# Distant metastases of melanoma exhibit varying extent of intrapatient proteogenomic heterogeneity

**DOI:** 10.1002/ctm2.70477

**Published:** 2025-09-28

**Authors:** Beata Szeitz, Yanick Paco Hagemeijer, Zoltan Gabor Pahi, Zsuzsanna Ujfaludi, Magdalena Kuras, Jimmy Rodriguez, Viktoria Doma, Reka Mohacsi, Magdolna Herold, Zoltan Herold, Zsolt Horvath, Indira Pla, Yutaka Sugihara, Bo Baldetorp, Henrik Lindberg, Henriett Oskolas, Melinda Rezeli, Jeovanis Gil, Roger Appelqvist, Lajos V. Kemeny, Jessica Guedes, Johan Malm, Aniel Sanchez, Imre Miklos Boros, Istvan Balazs Nemeth, Victor Guryev, Tibor Pankotai, Krzysztof Pawłowski, Elisabet Wieslander, Attila Marcell Szasz, David Fenyö, Peter Horvatovich, Jozsef Timar, György Marko‐Varga, Lazaro Hiram Betancourt

**Affiliations:** ^1^ Division of Oncology, Department of Internal Medicine and Oncology Semmelweis University Budapest Hungary; ^2^ Analytical Biochemistry, Groningen Research Institute of Pharmacy University of Groningen Groningen The Netherlands; ^3^ European Research Institute for the Biology of Ageing University of Groningen, University Medical Centre Groningen Groningen The Netherlands; ^4^ Department of Pathology, Albert Szent‐Györgyi Medical School University of Szeged Szeged Hungary; ^5^ Genome Integrity and DNA Repair Core Group, Hungarian Centre of Excellence for Molecular Medicine (HCEMM) University of Szeged Szeged Hungary; ^6^ Competence Centre of the Life Sciences Cluster of the Centre of Excellence for Interdisciplinary Research Development and Innovation University of Szeged Szeged Hungary; ^7^ Division of Clinical Protein Science & Imaging, Department of Biomedical Engineering Lund University Lund Sweden; ^8^ Department of Translational Medicine Skåne University Hospital Malmö, Lund University Malmö Sweden; ^9^ Department of Biochemistry and Biophysics Karolinska Institute Stockholm Sweden; ^10^ Department of Dermatology, Venerology and Dermatooncology, Faculty of Medicine Semmelweis University Budapest Hungary; ^11^ European Cancer Moonshot Lund Center Lund Sweden; ^12^ Section of Oncology, Department of Clinical Sciences Lund Lund University Lund Sweden; ^13^ BioMS−Swedish National Infrastructure for Biological Mass Spectrometry Lund University Lund Sweden; ^14^ HCEMM‐SU Translational Dermatology Research Group Semmelweis University Budapest Hungary; ^15^ Department of Physiology Semmelweis University Budapest Hungary; ^16^ Chemistry Institute Federal University of Rio de Janeiro Rio de Janeiro Brazil; ^17^ Department of Biochemistry and Molecular Biology University of Szeged Szeged Hungary; ^18^ Hungarian Research Network Biological Research Center Institute of Biochemistry Szeged Hungary; ^19^ Department of Dermatology and Allergology University of Szeged Szeged Hungary; ^20^ Department of Molecular Biology University of Texas Southwestern Medical Center Dallas Texas USA; ^21^ Howard Hughes Medical Institute Dallas Texas USA; ^22^ Institute for Systems Genetics NYU Grossman School of Medicine New York New York USA; ^23^ Department of Biochemistry and Molecular Pharmacology NYU Grossman School of Medicine New York New York USA; ^24^ Department of Pathology, Forensic and Insurance Medicine Semmelweis University Budapest Hungary

**Keywords:** distant metastasis, histopathology, mass spectrometry‐based proteomics, melanoma, post mortem, proteogenomics, RNA‐sequencing

## Abstract

**Background:**

Metastatic melanoma is a highly aggressive disease with poor survival rates despite recent therapeutic advancements with immunotherapy. The proteomic landscape of advanced melanoma remains poorly understood, especially regarding proteomic heterogeneity across metastases within patients.

**Methods:**

We collected 83 melanoma metastases from 19 different metastatic sites in 24 patients with advanced metastatic melanoma almost exclusively from the pre‐immunotherapy era, using semi‐rapid autopsies. The metastases were subjected to histopathological evaluation, RNA‐sequencing and mass spectrometry‐based proteomics for protein quantitation and non‐reference peptide (NRP) sequence detection using a proteogenomic data integration approach.

**Results:**

NRPs associated with mutations frequently occurred in proteins related to focal adhesion, vesicle‐mediated transport, MAPK signalling and immune response pathways across the cohort. Intrapatient heterogeneity was negligible when considering morphology and driver gene mutation status but was substantial at the proteogenomic level. This heterogeneity was not driven by metastasis location, albeit liver metastases exhibited distinct proteogenomic patterns, including upregulation of metabolic pathways. Cluster analysis outlined four proteomic clusters (C1–4) of the metastases, characterised by the upregulation of cell cycle and RNA‐splicing (C1), mitochondrial processes (C3), extracellular matrix (ECM) and immune pathways (C2) and ECM and vesicle‐mediated transport pathways (C4). Around two‐thirds of patients had metastases that had strongly distinct phenotypes. Patients in our cohort whose metastases were primarily assigned to clusters C1 and C3 exhibited shorter overall survival than patients whose metastases were categorised mainly into the C2 and C4 clusters.

**Conclusion:**

Our unique multi‐metastasis cohort captured the proteogenomic heterogeneity of immunotherapy‐naïve melanoma distant metastases, establishing a foundation for future studies aimed at identifying novel therapeutic targets to complement current immunotherapies.

**Key points:**

- Comprehensive proteogenomic profiling of post‐mortem melanoma metastases, collected primarily before the immunotherapy era.
- Description of 1177 protein sequence variants predicted by RNA‐Seq and validated via mass spectrometry‐based proteomics.
- Empirical evidence of prominent intrapatient heterogeneity, driven by heterogeneous protein expression related to cell cycle‐ and mitochondrial processes, immune system and extracellular matrix organization.

## Graphical abstract

Comprehensive proteogenomic profiling of post mortem melanoma metastases from patients with multiple lesions reveals the protein‐level mutation landscape and dominant phenotypes of distant metastases. The study uncovers varying levels of intrapatient heterogeneity, potentially associated with survival and therapeutic response.

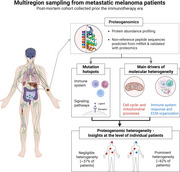

## 1 INTRODUCTION

Melanoma, one of the most lethal types of skin cancer, is linked to over 330 000 new cases and approximately 60 000 deaths annually worldwide.^1^ In contrast to other skin cancer types, melanoma can spread locally, regionally and to distant organs. Distant metastasis is associated with diminished survival rates.^2^ Melanoma also stands out with its high mutation rate compared to other malignant neoplasms^3^ and is among the most heterogeneous cancers.^4^

Notable advancements in melanoma treatment have occurred over the past few years, driven by a deeper understanding of cancer pathophysiology and the development of new therapeutic strategies.^5^ In particular, combination therapies can help delay the development of resistance, which can be caused by the substantial tumour heterogeneity.^4^ For instance, patients with unresectable stage IIIC or IV melanoma harbouring a BRAF V600E/K mutation can benefit from combination therapies targeting both BRAF and MEK. These combination therapies demonstrated improved survival rates and delayed resistance compared to monotherapies targeting BRAF alone.^6^, ^7^ In addition, immunotherapies, particularly immune checkpoint inhibitors targeting PD‐1/PD‐L1 and CTLA‐4 pathways, have revolutionised the treatment landscape of metastatic melanoma, offering durable responses in a subset of patients.^8^, ^9^ Nonetheless, the majority of metastatic melanoma patients still relapse on these therapies,^10^ underpinning an urgent need for novel therapeutic strategies. Ongoing research is therefore exploring multiple combinations of immunotherapies and their integration with targeted therapies to improve clinical outcomes.^11^, ^12^

Upon treatment, tumour cell subpopulations with distinct behaviour and altered antigenic landscapes emerge.^13^ This acquired intra‐tumoural heterogeneity results in the development of new resistance mechanisms, enabling tumour regrowth and disease relapse by evading drug cytotoxicity and/or immune system surveillance.^14^ Moreover, the aggressiveness of melanoma can be driven by alterations in the tumour microenvironment (TME) and the diversity of immune‐related cell populations.^15^, ^16^, ^17^ In this way, metastases can exhibit different aggressiveness patterns depending on the metastasis location and individual aspects of immune status. Molecular studies, and notably, studying the proteome of metastatic melanoma, can help us better understand this heterogeneity and treatment resistance,^18^, ^19^, ^20^ thereby contributing to the identification of novel drug targets and the development of therapeutic approaches that are more effective and offer long‐lasting benefits for patients.

There is an emerging number of studies based on research autopsy programs for metastatic cancer, which are essential yet underutilised in translational research. These programs are effective in collecting a large volume of diverse samples, leading to new insights into cancer progression, treatment resistance and more. Additionally, the challenges and strategies in post mortem sample collection must be emphasised and the establishment of new programs to advance cancer research are encouraged.^21^ The pronounced heterogeneity observed in multi‐omic and histopathological data underscores melanoma as a complex disease where such studies are urgently needed. From this perspective, we conducted large‐scale characterisation of metastases derived from 24 deceased melanoma patients after post mortem examination.^22^, ^23^ Notably, these patients were treated before the immunotherapy era except for one patient. The metastases underwent clinicopathological assessment and delicate tissue processing,^22^, ^23^ followed by RNA‐Seq and mass spectrometry (MS)‐based proteomics. The proteomic data are a part of the MM500 study,^20^, ^24^ and previously provided insights on copy number changes associated with metastatic sites and interferon therapy,^25^, ^26^ and was utilised in a recent study investigating the mitochondrial proteome landscape in melanoma.^27^ We hereby expanded on those efforts by incorporating RNA‐Seq data and integrating it with the proteomic data using an in‐house developed proteogenomic data integration approach, similarly to ref. 28 Through this advanced bioinformatic analysis, we aimed to explore the molecular complexity of melanoma in the pre‐immunotherapy era, establishing a baseline heterogeneity profile of distant metastases prior to the advent of more effective therapeutic strategies, and generating hypotheses on the connection between molecular profiles and patient outcomes.

## 2 MATERIALS AND METHODS

### 2.1 Metastasis sample collection

#### 2.1.1 Cohort collection

The research was conducted in compliance with the Declaration of Helsinki and was approved by the Semmelweis University Regional and Institutional Committee of Science and Research Ethics (IRB, SE TUKEB 114/2012). Distant metastasis samples from 24 melanoma patients were harvested during semi‐rapid autopsies at pathological departments across Hungary (Department of Pathology at Saint George Teaching Hospital of Fejér County, Székesfehérvár; Hospital of Zala County, Zalaegerszeg; Semmelweis University, Budapest). Post mortem examinations were conducted by pathologists, who identified metastatic sites and collected tissue samples from all affected areas. These harvested samples (*n* = 83, Table S1) were snap‐frozen in liquid nitrogen and stored at −80°C in ultra‐low temperature freezers. For further details on sample collection and post mortem procedures, refer to ref. 22 No samples were initially excluded from proteomic and RNA‐Seq analyses; however, nine and 11 metastases were not analysed via MS‐based proteomics and RNA‐Seq, respectively (see details in Materials and Methods sections ‘Sample preparation and analysis for quantitative proteomics’ and ‘Sample preparation and analysis for quantitative RNA‐Seq’).

#### 2.1.2 Tissue processing and histological characterisation

Frozen tissue samples were sliced into 10 µm sections, with 10–15 slices allocated for each omic analysis. The initial and final slices, along with (every 10th) intermediate slice, were utilised for haematoxylin and eosin (H&E) staining and histopathological analysis. The tissues underwent stepwise sectioning, with an average assessment of three sections per metastasis per setting (initial, intermediate, final). Frozen tissue sections were placed onto glass slides, stained with H&E, and subsequently transferred to an automated slide scanner system (Zeiss Mirax). Each metastasis sample underwent histopathological evaluation at multiple levels as detailed above. The H&E stained sections were imported into the QuPath software environment v.0.4.4, and regions of tumour cells, necrosis, surrounding (adjacent) normal tissue and lymphocytic infiltration were annotated by two investigators (A.M.S. and R.M.) independently. Surface areas on the complete slide and the selected region were calculated and the values (originally in µm^2^, and ratio of each component to the whole in %) of the selected areas for the given features were then averaged for all sections representing each metastasis sample. Cell morphology was grouped into either epithelioid, spindle or mixed morphology. Lymphocytic infiltration was scored as percentage (Table S1).

### 2.2 Sample preparation and analysis for quantitative proteomics

#### 2.2.1 Protein extraction

Metastasis samples were mixed with a lysis buffer consisting of 100 mM ammonium bicarbonate and 4 M urea in an ice bath for 30 min. The samples were placed into the tube holder of the Bioruptor, and sonication was performed using 40 cycles, consisting of 15 s at high power (on) with 15 s rest (off), at 4°C. Next, the samples were centrifuged at 10 000 × *g* for 10 min at 4°C, and the protein content was determined using the colorimetric micro BCA Protein Assay Kit according to the instructions supplied by the manufacturer (ThermoFisher Scientific).

#### 2.2.2 Automated in‐solution digestion

The AssayMAP Bravo platform (Agilent Technologies) was used to perform urea in‐solution digestion as published before^29^, ^30^ using the In‐Solution Digestion protocol: Single Plate v1.0 protocol in VWorks. Briefly, 40 µg of proteins were first reduced with 10 mM dithiothreitol for 1 h at 37°C and alkylated with 20 mM iodoacetamide for 30 min in the dark at room temperature (RT). This was followed by enzymatic digestion with endoproteinase Lys‐C in a 1:50 w/w ratio (enzyme/protein) in 1 M urea and incubated at RT for 7 h. Next, the samples were diluted with 100 mM ammonium bicarbonate to a concentration of .6 M urea, trypsin was added in a 1:50 w/w ratio (enzyme/protein) and the digestion was incubated overnight at RT. The peptides generated were acidified by adding 4 µL of 50% formic acid and peptide desalting was also performed in the AssayMAP Bravo using the Peptide Clean‐up v2.0 protocol. Here, C18 cartridges (Agilent, 5 µL bead volume, 150 µg capacity) were primed using 100 µL of 90% acetonitrile (ACN) and equilibrated with 70 µL of .1% trifluoroacetic acid (TFA) at 10 µL/min. Samples were loaded at 5 µL/min, followed by internal cartridge wash and cup wash with .1% TFA at 10 µL/min. The peptides were eluted using 30 µL 80% ACN with .1% TFA at 5 µL/min. This elution buffer was introduced on the deck of the instrument just prior to elution.

#### 2.2.3 High pH reversed‐phase high‐performance liquid chromatography fractionation

A pool of all digested samples was fractionated using an Aeris Widepore XB‐C8 (3.6 µm, 2.1 × 100 mm) column (Phenomenex) on an 1100 Series HPLC (Agilent) operating at 80 µL/min. The mobile phases were solvent A: 20 mM ammonium formate pH 10, and solvent B: 80% ACN and 20% water containing 20 mM ammonium formate pH 10. An estimated amount of 200 µg was separated using the following gradient: 0 min 5% B; 1 min 20% B; 60 min 40% B; 90 min 90% B; 120 min 90% B. The column was operated at RT and the detection wavelength was 220 nm. Then, 96 fractions were collected at 1 min intervals and further concatenated to 24 fractions and dried in a Speed‐Vac.

#### 2.2.4 Liquid chromatography–tandem mass spectrometry analysis

The liquid chromatography–tandem mass spectrometry (LC–MS/MS) analyses were done in an Ultimate 3000 nano LC (nLC; Thermo Scientific) coupled to a Q Exactive HF‐X mass spectrometer (Thermo Scientific). Peptide samples (1 mg) spiked‐in with iRT peptides (Biognosis AG) in a 1:10 dilution (iRT:peptides) were loaded in a trap column (Acclaim1 PepMap 100 pre‐column, 75 µm, 2 cm, C18, 3 m, 100 A, Thermo Scientific) and then separated on an analytical column (EASY‐Spray column 50 cm, 75 mm i.d., PepMap RSLC C18, 2 mm, 100 A, Thermo Scientific) using a flow rate of 300 nL/min and a water/ACN in .1% formic acid gradient of 2 h. The samples were measured in data‐independent acquisition (DIA) mode. The full scans were processed in the Orbitrap analyser with a resolution of 120 000 at (200 *m*/*z*), an injection time of 50 ms, and a target automatic gain control (AGC) value of 3 × 10^6^ in a range of 350–1410 *m*/*z*. Fragmentation was set to 54 variable isolation windows based on the density distribution of *m*/*z* precursors in the previously built spectral library (see next Materials and Methods section ‘Spectral library building’). MS2 scans were acquired with a resolution of 30 000 at 200 *m*/*z*, a normalised collision energy (NCE) of 25, a target AGC value of 1 × 10^6^, and 200 *m*/*z* as resolution reference mass.

#### 2.2.5 Spectral library building

The DIA–MS Spectral library was built from data‐dependent acquisition (DDA)–LC–MS/MS analyses using the same LC–MS/MS system as above. Here 24 high pH reversed‐phase high‐performance liquid chromatography (hpH‐RP‐HPLC) peptide fractions were dissolved in 2% ACN, .1% TFA and spiked‐in with iRT peptides (Biognosis AG) in a 1:10 dilution (iRT:peptides; Biognosis AG). The gradient used was the following: 0 min 4% B; 7 min 4% B; 139 min 30% B; 154 min 45% B; 155 min 98% B; 160 min 98% B. The MS parameters were set as follows: selection of the 40 most intense ions for fragmentation, full MS scans at *m*/*z* 375–1750 with a resolution of 120 000 at *m*/*z* 200, a target AGC value of 3 × 10^6^ and injection time of 100 ms, fragmentation in higher energy collision dissociation collision cell with NCE of 25 and MS/MS spectra acquisition in the Orbitrap analyser at a resolution of 60 000 (at *m*/*z* 200) with a maximum injection time of 120 ms and dynamic exclusion of 40 s.

#### 2.2.6 Proteomic data processing for protein quantification

For the peptide and protein identification and quantitation in DDA–MS experiments, raw DDA–LC–MS/MS files were analysed using ProteomeDiscoverer™ Software from Thermo Scientific™ (v2.3) employing the Uniprot Human protein sequence dataset with isoforms (downloaded 2018‐10‐01, 70 646 protein entries) using the common proteomics workflows (note: the variant protein sequences inferred based on RNA‐Seq were not utilised in these steps, see ‘Computational pipeline for proteogenomics’ in Materials and Methods section). The Sequest HT search engine was used for peptide identification, with carbamidomethylation specified as a static modification. Dynamic modifications included oxidation of methionine residues (+15.9949 Da) and acetylation at protein N‐termini (+42.0105 Da). Precursor and fragment mass tolerances were set at 20 ppm and .02 Da, respectively, with allowance for up to two missed cleavages for peptides.

Next, a spectral library was generated from the DDA experiments outlined above. Notably, nine of the 83 metastasis samples were excluded from subsequent proteomic analysis due to either >70% necrosis content or failure to yield results following LC–MS/MS (Table S1). Raw files underwent conversion to HTRMS files using a specialised converter provided by Biognosys AG. Subsequently, they were searched within Spectronaut v11 (Biognosys AG), employing the *Homo sapiens* database from Uniprot, including isoforms (downloaded 2018‐10‐01, 70 646 protein entries; note: the variant protein sequences inferred based on RNA‐Seq were not utilised in these steps either, see ‘Computational pipeline for proteogenomics’ in Materials and Methods section). Dynamic retention time prediction was chosen to facilitate non‐linear alignment of precursor retention times between the spectral library (with iRT‐normalised retention times) and the DIA–MS data through segmented regression. The following search parameters were employed: cysteine carbamidomethylation (+57.0215 Da) as a fixed modification, and methionine oxidation (+15.9949 Da) along with N‐terminal acetylation (+42.0105 Da) as dynamic modifications. Up to two missed cleavages were permitted. Precursor mass tolerance was set to 10 ppm, while for the MS/MS fragments, it was set to .02 Da. Between three and 25 fragments were used for peptide identification. Filtering was conducted at a 1% false discovery rate (FDR) for all peptides and proteins utilised in constructing the spectral library. The software calculated MS1 peptide abundance as the sum of the precursor extracted‐ion chromatogram (XIC) from the monoisotopic precursor ion and its isotopic envelope. Protein abundance was derived from the average of the top three most intense precursor ions corresponding to unique and razor peptides.

### 2.3 Sample preparation and analysis for quantitative RNA‐Seq

#### 2.3.1 RNA‐Seq sample processing

Total RNA was extracted from the metastasis samples allocated for RNA‐Seq using ReliaPrep RNA Miniprep Systems (Promega) according to the manufacturer's recommendations. RNA quantification and qualification were performed on Qubit 4 fluorometer (Thermo Fisher Scientific) and Agilent BioAnalyzer platforms (Agilent Biotechnologies), using Thermo Qubit RNA High Sensitivity Assay (Thermo Fisher Scientific) and RNA 6000 Nano Kit (Agilent Biotechnologies), respectively. Using a minimum of 100 ng input RNA per sample, sequencing libraries were prepared by TruSeq Stranded mRNA library preparation kit (Illumina) according to the manufacturer's instructions. Libraries were pooled based on the concentrations and fragment distributions determined previously by Qubit 4 fluorometer (Thermo Fisher Scientific) and Agilent BioAnalyzer platforms (Agilent Biotechnologies), using Thermo Qubit dsDNA High Sensitivity Assay (Thermo Fisher Scientific) and DNA1000 chip (Agilent Biotechnologies), respectively. Single‐read 75 bp read length sequencing was accomplished on NextSeq 550 Sequencing System (Illumina) using NextSeq 500 v2.5 high output kit.

RNA isolation was performed on all metastasis samples available within the cohort; however, 11 samples failed this initial RNA isolation step due to low or poor RNA quality and thus they were not sequenced (Table S1).

#### 2.3.2 Trimming, quality control and mapping of RNA‐sequencing data

The FASTQ files of the RNA‐sequencing were trimmed using Trimmomatic v0.39. It was run with default settings for single‐end data as stated on the tool homepage with an additional minimum adapter length of 2 for the palindrome mode. Then quality control was performed using FastQC v0.12.1 with the non‐standard nogroup parameter. Afterwards the reads were mapped to the human genome primary assembly (GRCh38.p13, gene build from Ensembl release 105) using Spliced Transcripts Alignment to a Reference (STAR) v2.7.11a, adjusting settings for read length and reference genome characteristics according to the manual. Two separate passes were performed to reduce the number of the novel splice junctions that get added per sample, according to the software author's guidelines (https://github.com/alexdobin/STAR/issues/733#issuecomment‐1499481954) before being fed into STAR for the second, more sensitive, mapping step. Additionally, to ensure compatibility with GATK, the outSAMmapqUnique parameter was set to 50. Unmapped reads were output as fastq files to be used in further downstream analysis.

#### 2.3.3 RNA‐seq quality assessment

To assess the quality of the RNA‐Seq data extracted from post mortem metastasis samples, we selected specific quality metrics to describe the RNA quality of each sample, namely, mRNA content in the samples (percentage of total bases that originate from mRNA), the percentage of reads assigned to genomic features (assigned reads), and the number of reads in millions (M assigned reads). We first determined confidence intervals (CIs) using Wilcoxon rank‐sum tests for each of these quality characteristics to define thresholds for each, and then categorised each into ‘good’, ‘medium’ or ‘poor’ quality groups. An mRNA content above 76.84%, more than 3.6 million assigned reads, and an assigned reads percentage exceeding 48.3% was classified as ‘good quality’. An mRNA content of 60% or less, assigned reads of 2.3 million or below, and an assigned reads percentage no higher than 38.9% was labelled as ‘poor quality’. Values falling between the upper limit of poor quality and the lower limit of good quality were considered ‘medium quality’. After defining the quality categories per individual sample characteristics (mRNA content, the percentage of assigned reads and the M assigned reads), the RNA quality per sample was determined the following manner: if all three sample characteristics were ‘poor’ for a sample, then the sample was labelled as ‘poor quality’; if all three sample characteristics were ‘good’ for a sample, then the sample was labelled as ‘good quality’. Remaining samples were labelled as ‘medium quality’. Importantly, we had sample replicates for five samples (MF13G, MF30E, MF55B, MF55E and MF56B). Using the quality metrics described above, we selected one replicate to be kept per sample based on the highest number of assigned reads and M assigned reads.

#### 2.3.4 Read quantification

After mapping the reads using STAR, the reads were quantified using FeatureCounts v2.0.1. The default settings of FeatureCounts were used, except for the ‘‐s 2’ option. Both reads per kilobase million (RPKM) and transcripts per kilobase million (TPM) values were calculated. A total of 61 541 transcripts were quantified. For downstream analyses (regardless of whether RPKM or TPM values were used), only transcripts that had raw counts above 9 in minimum 30% of the RNA‐Seq metastasis samples (22 samples out of 72) were retained (*n* = 11 757 transcripts).

### 2.4 Computational pipeline for proteogenomics

For the integrative processing of proteomics and RNA‐Seq, a custom in‐house developed computational pipeline was developed. This included processing the aligned RNA‐Seq data to predict protein sequence variants, which were subsequently searched and verified in the raw proteomics data. The special steps involved in this processing are detailed below.

#### 2.4.1 Variant calling

The Genome Analysis Toolkit v4.4.0.0 (GATK) was used according to the best practices ‘Data pre‐processing for variant discovery’ (https://gatk.broadinstitute.org/hc/en‐us/articles/360035535912‐Data‐pre‐processing‐for‐variant‐discovery) with additional RNA processing specific steps from the ‘RNAseq short variant discovery (SNPs + Indels)’ (https://gatk.broadinstitute.org/hc/en‐us/articles/360035531192‐RNAseq‐short‐variant‐discovery‐SNPs‐Indels) best practices. This included duplicate removal, splitting up reads with gapped alignments and performing base quality score recalibration. To detect as many variants in the RNA‐seq data as possible, we called variants in each sample with both HaplotypeCaller (a tool classically used to detect germline variants) and Mutect2 (a tool typically used to detect somatic variants in DNA sequencing data), as the non‐uniformity of RNA‐sequencing coverage might cause variants to be missed by HaplotypeCaller. These two tools were run according to the ‘Germline short variant discovery (SNPs + Indels)’ best practices (https://gatk.broadinstitute.org/hc/en‐us/articles/360035535932‐Germline‐short‐variant‐discovery‐SNPs‐Indels) and the ‘Somatic short variant discovery (SNVs + Indels)’ best practices (https://gatk.broadinstitute.org/hc/en‐us/articles/360035894731‐Somatic‐short‐variant‐discovery‐SNVs‐Indels) guides, respectively. As we had access to neither an RNA‐specific panel of normals, nor germline samples for the included patients, we relied on GATK's public panel of normals as described in their ‘Panel of Normals (PON)’ glossary (https://gatk.broadinstitute.org/hc/en‐us/articles/360035890631‐Panel‐of‐Normals‐PON) to run Mutect2 in tumour‐only mode for each individual sample. Additionally, sample‐specific DRAGstr models were used with both variant callers (https://gatk.broadinstitute.org/hc/en‐us/articles/360039984151‐DRAGEN‐GATK‐Update‐Let‐s‐get‐more‐specific), after which the sample‐specific VCF files from the HaplotypeCaller and Mutect2 tools were merged using the BCFtools merge command, providing the final list of called variants.

#### 2.4.2 Variant annotation

Variant Effect Predictor (VEP), Ensembl API v105, was run with the non‐standard ‘everything’ flag for additional annotation sources to annotate the called variants. A custom post‐processing script was used to exclude variants that would not affect protein sequences. Putative protein/RNA sequences were generated, encompassing all potential combinations of variants per 30 amino acid window, and categorised separately for in‐frame and frameshifting variants respectively. Genes with more than 10 000 generated variant combinations across all its transcripts were dropped on a per sample basis in order to avoid explosion of the protein variant search space.

#### 2.4.3 Alternative splicing

The deduplicated bam file created by GATK was analysed with StringTie v2.2.1 against a reference annotation GTF file (gene build from Ensembl release 105). The StringTie output was used to distinguish between novel and known pairings of known exons, or if one or both of the exons are not annotated. The sequence of transcripts without a reference id was retrieved from the primary assembly reference genome (GRCh38.p13, gene build from Ensembl release 105) using GffRead v0.12.7.

#### 2.4.4 De novo transcript assembly

The unmapped reads reported by STAR were used to run Trinity v2.15.1 for de novo assembly of unannotated transcripts.

#### 2.4.5 Open reading frame prediction

The RNA sequences resulting from de novo transcript assembly, alternative splicing events, and reading frame altering variants are more likely to contain spurious sequences. To enrich the real coding sequences, TransDecoder v5.7.1 (https://github.com/TransDecoder/TransDecoder) was used to predict open reading frames and the protein sequences therein. Homology searching, consisting of running BLASTp v2.15.0 against the genome annotation's protein FASTA file (Ensembl release 105), and running HMMER v3.4 against the Pfam‐A dataset (release 35.0), respectively, was performed according to the TransDecoder manual to filter out predicted protein sequences without sufficient homology.

#### 2.4.6 Creating a cohort‐specific protein sequence database

Since predicted sequences could be predicted in multiple samples, deduplication was performed by changing the FASTA headers into the MD5 checksum of the corresponding sequence. Sequences were only written to a ‘cohort wide’ protein FASTA file once, while the sample name, type of sequence prediction, original FASTA header and MD5 checksum were stored in a TSV file to aid in mapping back of peptide hits to their transcriptomic evidence. Using the ‘cohort wide’ FASTA file resulted in a larger search space but enabled the detection of variants at the peptide level that would otherwise be missed because of not being detected in some samples at the transcript level (i.e., as a result of RNA degradation). The resulting ‘cohort wide’ protein sequence database contained the Ensembl reference protein sequences (v105) as well as protein sequences reflecting mutations, indels, splicing variants and novel open reading frames that were detected in the RNA‐Seq data of any of the samples.

#### 2.4.7 Raw MS data processing for proteogenomics

Firstly, DIA raw files were converted to mzXML using the msConvert tool from ProteoWizard v3.0.21354 software (Linux version). The mzXML files were written in 32‐bit format and peak picking was enabled. Then, DIA‐Umpire v2.2.2 was used for signal extraction with exact parameters specified in Table S2A,B. All three pseudo MS/MS spectra files indicating different quality spectra (Q1, Q2, Q3) were subjected to database search. For the protein sequence database, the human reference proteome was fetched from UniProt (UP000005640, accessed on 21 October 2021) using FragPipe v20.0, downloading both canonical and isoform sequences from Swiss‐Prot as well as unreviewed sequences (TrEMBL). In addition, common contaminants and iRT sequences were added to the FASTA file. This reference FASTA file was then complemented with the sample‐specific protein sequences predicted with the proteogenomic pipeline to run the database search. Decoy sequences were added by Fragpipe. MSFragger v3.8 and Philosopher/PeptideProphet v5.0.0 were used for the database search and validation step. The full list of parameters for each software and example bash commands are listed in Table S2A,C. Importantly, only peptide‐spectrum matches (PSMs) passing the 1% FDR filter both at PSM, ion and peptide level were retained before further PSM quality filtering.

#### 2.4.8 PSM quality filtering

The PSMs that passed the 1% FDR filter and could not be mapped to the reference proteome (taking into account the indistinguishability of isoleucine and leucine amino acids by MS, as well as additionally checking if peptides could not be mapped to Ensembl v105 sequences) were subjected to downstream quality filtering using PepQuery v2.0.2. As the input spectra, the MGF files for all different quality spectra (Q1, Q2, Q3) created by DIA‐Umpire were used. The detailed PepQuery settings are listed in Table S2D. The individual PepQuery result files were merged into one summary table using custom R v4.2.0 scripts.

#### 2.4.9 Mapping back identified peptides to RNA evidence

The identified peptides were mapped back using custom scripts with the help of pyOpenMS v2.6.0 and Python 3.9 on a per sample basis, after which the results were aggregated to the cohort level to facilitate finding evidence shared across samples, as well as making sure these peptides were mapped uniquely to a single gene and could not have arisen from a reference protein sequence. This final list of peptides was labelled as ‘non‐reference peptides’ (NRPs). For each NRP, the pseudo MS/MS spectrum with the highest Hyperscore (derived from MSFragger outputs) is provided as File S1. For navigation within File S1, refer to Table S5A.

#### 2.4.10 Retrieval of single amino acid variant allele‐frequency information from public resources and scoring deleteriousness

To estimate gene‐specific mutation rates, NRPs mapping to multiple genes or to a stretch of sequence not predicted to contain single amino acid variants (SAAVs), and NRPs not confidently verified by PepQuery were excluded. The variants detected by the remaining peptides were looked up in the original unfiltered VCF files of all samples for maximum sensitivity. Next, all of these variants were looked up in Genome Aggregation Database (gnomAD) v3.1, to determine their allele frequency (AF) in the non‐cancer samples of non‐Finnish Europeans reference population, and additionally tallied distinct novel, or frequent mutations per gene.

The Combined Annotation‐Dependent Depletion (CADD) tool GRCh38‐v1.7 was used with default settings to score the deleteriousness of variants. When the NRP could be mapped to multiple locations on the genome, the scaled C‐scores calculated for the different locations were averaged.

### 2.5 Data post‐processing and statistics

#### 2.5.1 Proteomic data post‐processing

The raw proteomic data containing 10 121 unique protein groups for 143 sample replicates (corresponding to 74 unique metastasis samples) was log_2_‐transformed, which was followed by the removal of artefacts (values with log_2_ intensity <10 were replaced with *NA*s) and a median scale normalisation (i.e., centring the intensity values to the median of the distribution of each sample replicate, and then adding the global median to each intensity value). The sample replicates were averaged afterwards. For statistical analyses that required a protein intensity matrix without missing values, the protein intensity table was filtered for maximum 10% missing values across the metastases (5452 protein groups), followed by imputation of the remaining missing data using the stochastic minimal value approach implemented in the imputeLCMD R package v2.1 (‘impute.MinProb’ function, with settings *q* = .02, tune.sigma = 1).

#### 2.5.2 Proteogenomic data post‐processing

Binary tables were produced from the list of identified and validated PSMs, with cells containing ‘1’ if the NRP was identified and validated in a sample with at least one PSM and ‘0’ if the NRP was either not identified and/or not validated in a sample. Peptides that were only different due to missed cleavages were merged into one entry. To find associations between NRP presence and clinical/histopathological data, only peptides that were detected across minimum three patients (*n* = 364) were used.

#### 2.5.3 Data processing for previous studies

The normalised protein intensity table by Beck et al.^19^ was downloaded from the publication, containing 185 metastasis samples and 10 178 protein groups. Following their data processing workflow, the table was filtered for proteins with minimum 70% valid values across all samples (*n* = 4610). Using the stochastic minimal value approach from the imputeLCMD R package (impute.MinProb function), the missing values were imputed, with settings *q* = .01 and tune.sigma = 1, followed by batch effect removal using the removeBatchEffect function from the limma R package v3.62.2. Metastasis locations called ‘colon’, ‘small bowel’ and ‘rectum’ were merged into one category called ‘intestine’ to harmonise metastasis locations across our and their dataset.

The Cancer Genome Atlas (TCGA) melanoma dataset^31^ was downloaded from cBioPortal (https://www.cbioportal.org/study/summary?id=skcm_tcga_pan_can_atlas_2018) on 18 September, 2023.^32^, ^33^, ^34^ The clinical and expression data were extracted from the files ‘data_clinical_patient.txt’, ‘data_clinical_sample.txt’ and ‘data_mrna_seq_v2_rsem_zscores_ref_all_samples.txt’, and only metastasis samples from stages III and IV were further analysed to restrict the cohort to late‐stage metastases.

Three cohorts of advanced melanoma patients who have received modern anti‐cancer therapies before or after tumour sample collection (Van Allen, Gide and Liu cohorts)^35^, ^36^, ^37^ were also accessed. The clinical data were downloaded from the respective publications, while the processed RNA‐Seq data were available from https://github.com/ParkerICI/MORRISON‐1‐public.^38^ We downloaded the already post‐processed and normalised ‘RNA‐CancerCell‐MORRISON1‐no_batch_correction‐logcpm‐all_samples.tsv.zip’ file, ready for statistical analyses. In all three cohorts, we only retained samples that were both anti (a)‐PD1 and a‐CTLA‐4‐treatment naïve, to match the characteristics of our cohort.

#### 2.5.4 Statistics and bioinformatics

Refer to the Supporting Information for detailed information on the statistical and bioinformatics approaches used.

## 3 RESULTS

### 3.1 Clinical characteristics of the post mortem melanoma cohort

To explore intra‐ and inter‐tumoural molecular heterogeneity of melanoma metastases and the relation of this heterogeneity to clinical and histological metadata, we collected a post mortem cohort of 24 patients with 1–9 distant metastases (*n* = 83, Table S1). The main clinicopathological features of the cohort are summarised in Table 1, including details on the matched primary tumours which were removed during the patients’ life. Patients were originally diagnosed with stage IB, IIA/B/C and IIIA/B melanoma. The 5‐year overall survival (OS) rate was 33.3% (95% CI, 18.9%–58.7%). The patients had heterogeneous treatment histories, with two‐thirds of the patients receiving chemo‐, BRAF targeted‐ or interferon therapy. Only one patient had received immunotherapy (a‐CTLA‐4). In addition, four patients were untreated, and treatment history was missing for three other patients.

**TABLE 1:** Clinicopathological features of the study population.

Parameter	Value
Age at diagnosis (years; *n* = 24)	51.5 (32.0–78.0)
Sex (female:male; *n* = 24)	7 (29.2%):17 (70.8%)
Stage at initial diagnosis (AJCC8; *n* = 21)	
IB	2 (9.5%)
IIA:IIB:IIC	5 (23.8%):5 (23.8%):4 (19.0%)
IIIA:IIIB	1 (4.8%):4 (19.0%)
Anti‐tumour therapy (*n* = 21)	
Chemotherapy	13 (61.9%)
Interferon therapy	11 (52.4%)
Targeted therapy	9 (42.9%)
Immunotherapy	1 (4.8%)
No therapy	4 (19.0%)
Median survival time and its 95% CI (month; *n* = 24)	49.0 (26.0–69.0)
Post mortem interval (*n* = 24)	
Within 24 h	12 (50.0%)
Between 24–48 h	7 (29.2%)
After 48 h	5 (20.8)
Histology of the primary tumour (*n* = 23)	
SSM	10 (43.5%)
NM	6 (26.1%)
Other (ALM:mucosal:ocular)	1 (4.3%):1 (4.3%):1 (4.3%)
Unclassified	4 (17.4%)
Location of the primary tumour (*n* = 24)	
Head (eye)	4 (16.7%) (1)
Trunk	10 (41.7%)
Upper extremities	3 (12.5%)
Lower extremities (foot)	7 (29.2%) (1)
Number of metastatic sites (*n* = 24)	3.0 (1.0–9.0)

The number of patients without missing data in the individual categories in indicated in brackets. Numeric data are presented as median (range).

Abbreviations: AJCC8, 8th Edition of the American Joint Committee on Cancer; ALM, acral lentiginous melanoma; NM, nodular melanoma; SSM, superficial spreading melanoma; WT, wild type.

**FIGURE 1:**
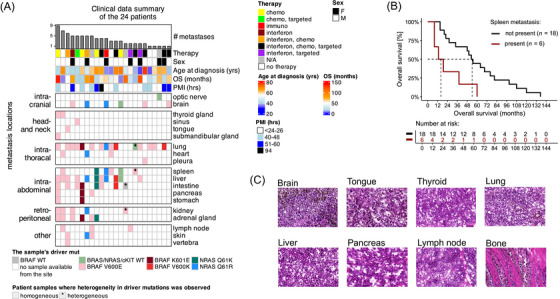
Overview of the metastatic melanoma post mortem cohort. (A) Overview of the patients' clinical data, including details on the number and localisation of their individual metastases, along with the specific driver mutations identified in each metastasis via targeted sequencing. The NRAS and cKIT mutation status is unknown for the metastasis labelled as BRAF WT. (B) Kaplan–Meier curves for patients with or without developing spleen metastases before their death. (C) Examples of histology images from distinct metastasis locations. The representative haematoxylin and eosin images of the melanoma metastases at selected regions were taken at 10× magnification with the QuPath v.4.0 software image viewer.

The distant metastases were snap‐frozen upon autopsy which was performed within an average of 38 h after death (post mortem interval [PMI] ranged from less than 24 to 96 h). The 83 metastases were located in 19 organ groups: with liver, brain, intestines, adrenal glands and spleen comprising 64% of metastases (Figure 1A). As expected clinically, the order of metastatic appearance was largely undeterminable according to the clinical reports: metastases appeared synchronously for 12 patients, the order was only partly known for four patients, and this information was missing for eight patients (Table S1).

To reveal the association between metastasis location(s) on OS in our cohort, Cox regression analyses were conducted (Supporting Information). Among the baseline clinical data, which included age at diagnosis, sex, staging, received therapies, driver mutation status and number of metastatic sites, only staging was statistical significantly associated with OS in a univariate setting (Figure S1). Interestingly, we found that the development of metastasis in the spleen was correlated with significantly reduced OS compared to patients not affected at this site in a univariate Cox regression analysis, with medians of 16 (95% CI, 8–*N/A*) and 54 (95% CI, 34–99) months, respectively (hazard ratio [HR]: 3.91, 95% CI, 1.40–10.95, *p* = .009; Figures 1B and S1). In a multivariate Cox model that accounted for the presence of metastases in other locations and stage, spleen metastases were again significantly associated with shorter patient survival (HR: 6.08; 95% CI, 1.55–23.81; *p* = .010; Figure S1). Importantly, the cohorts with and without spleen metastasis showed no variation in any of the clinicopathological parameters investigated besides OS.

Digital pathological assessment of the sectioned metastasis samples (Materials and Methods section) revealed that the metastatic melanocytes within each patient's metastases were strikingly uniform, predominantly on the spectrum of epithelioid to small cell appearance, with no spindle cell morphology notable in this cohort (Figure 1C). Additionally, the mutation status of BRAF, NRAS and CKIT (key proto‐oncogenes in melanoma; from the targeted sequencing analysis originally reported in ref. 22) was consistent across metastases within the same patient in 22 out of the 24 cases, indicating minimal proto‐oncogene heterogeneity (Figure 1A and Table S1).

### 3.2 Characterising post mortem melanoma metastases through proteomics and RNA‐Seq integrated with histopathology

**FIGURE 2:**
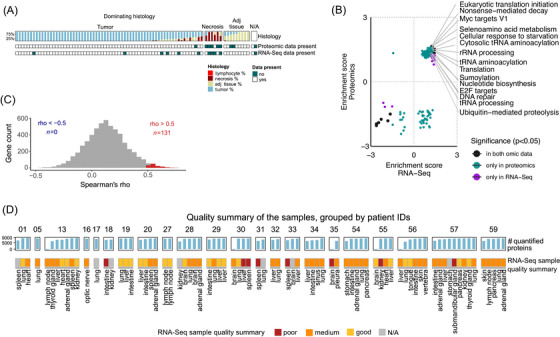
Multi‐omics analysis of the melanoma metastases. (A) Summary of the histological annotations (tumour, adjacent tissue, necrosis and lymphocyte percentage) for the individual metastasis samples, highlighting samples that were excluded from proteomic and/or RNA‐Seq analysis. (B) Pathways upregulated in samples with higher tumour percentages across both proteomic and RNA‐Seq data. The pre‐ranked GSEA results for the proteomic and RNA‐Seq data are displayed on the *x*‐ and *y*‐axis, respectively. Proteins and transcripts (both collapsed to gene IDs) were ranked according to the correlation between their abundance/expression levels and the tumour percentage of each sample. (C) Distribution of gene‐wise Spearman correlation coefficients (rho‐s) between transcript and protein abundance. Genes with a strong positive correlation are highlighted in red. (D) Summary of RNA‐Seq and proteomic data quality per metastasis sample. Metastases are grouped by patient.

The sectioned metastasis samples were subjected to RNA‐sequencing of the polyadenylated transcripts and quantitative MS‐based proteomics for a comprehensive molecular profiling, with only a few samples omitted from omics characterisation based on low quality (see exclusion details in Materials and Methods section). In addition, histological assessment of the tissues was performed on digitised slides collected directly adjacent to slices used for molecular analysis (Materials and Methods section) to assess the tumour, adjacent tissue, necrosis and lymphocyte content of the measured samples. The predominant component was tumour cell in 66 samples, necrosis in six samples and adjacent tissue in nine samples (Figure 2A). Histological assessment was only missing for two samples. Lymphocyte content was 0% in all samples, except for two samples with 3.2% and 14.0% lymphocyte content.

Quantitative proteomics and RNA‐Seq resulted in the quantification of 61 541 transcripts and 10 121 protein groups. Correlating protein abundance and gene expression with tumour content (Table S3A,B), followed by pre‐ranked gene set enrichment analysis (GSEA) separately performed on the proteomic and RNA‐Seq indicated the upregulation of cancer hallmark pathway members such as RNA metabolism, spliceosome, translation or MYC targets with higher tumour content, which was observable at both molecular layers with *p* < .05 (Table S3C and Figure 2B). This confirmed that the tissue slices assessed by histology accurately represent sample composition in the samples that were subjected to molecular profiling.

Gene‐wise correlation analyses for the 5619 genes robustly quantified both across the samples and across proteomics and RNA‐Seq (Supporting Information) indicated a general lack of concordance between transcript expression and corresponding protein abundance, with a mean Spearman rho of .128 ± .179 (Figure 2C). A total of 131 genes showed strong positive correlation across proteomics and RNA‐Seq with Spearman correlation coefficients >.5 and adjusted (adj.) *p* < .05 (Table S4A), including well‐known melanoma markers like MLANA, PMEL (HMB45) and multiple members of the S100 protein family. A significant enrichment (one‐sided Fisher's test, adj. *p* < .05) of three pathways, namely, ‘epithelial–mesenchymal transition’ (EMT), ‘hypoxia’ and ‘response to metal ions’ was observed for these 131 genes (Table S4B).

The low agreement between RNA‐Seq and proteomics warranted further investigation of molecular data quality, which may be affected by the post mortem nature of this cohort. Indeed, the quality of polyadenylated transcripts obtained from different patients and metastasis locations showed considerable variation. The samples showed differences in terms of mRNA content, the percentage of reads assigned to genomic features (assigned reads) and the number of reads in millions (M assigned reads), which reflect on differences in sequencing depth and, consequently, the quality of the sequences obtained. We used the aforementioned quality metrics to categorise the samples into ‘good’, ‘medium’ or ‘poor’ quality (Materials and Methods section, Table S1). The samples labelled as poor quality had low raw read counts and fewer protein‐coding genes were detected in them compared to better quality samples (Figure S2A). In general, the RNA‐Seq sample quality varied across and within patients (Figure 2D). In contrast to the RNA‐Seq data, 7794.5 ± 757.8 proteins were quantified per sample in the proteomics data (Figure 2D). Such variation in the number of quantified proteins is expected. Importantly, the RNA‐Seq quality differences and the number of quantified proteins were not significantly associated with metastasis location or PMI (Figure S2B–E).

To reduce technical confounders in the analysis of metastasis heterogeneity, two filtering criteria were applied in later analyses. First, RNA‐Seq gene expression data were excluded due to their low concordance with proteomic profiles and the greater reliability of the proteomics data. Second, metastasis samples with tumour content below 60% were removed from comparative analyses to minimise the influence of non‐tumour components on the results.

### 3.3 Advanced bioinformatic investigation provides insights into the proteogenome of melanoma metastases

**FIGURE 3:**
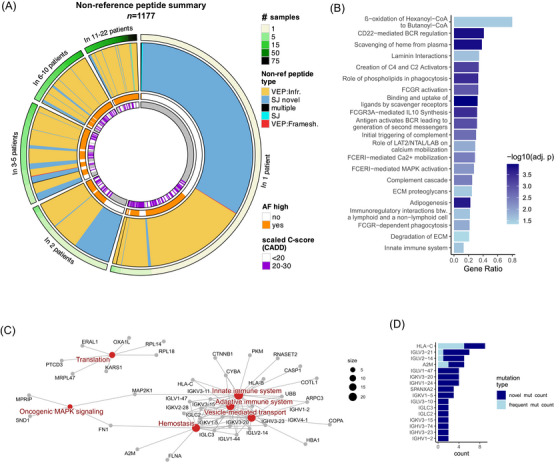
Insights into the proteogenome of melanoma metastases. (A) Summary of the non‐reference peptides (NRPs) on a circular plot. NRPs are grouped based on the number of patients in which they were detected. ‘AF high’ indicates AF >1% for an NRP. Infr, in‐frame; SJ, splice junction. (B) Enriched pathways for proteins with at least one identified NRP. (C) Enriched pathways (red nodes) for proteins with novel single amino acid mutations (grey nodes). (D) Proteins with significantly more novel single amino acid mutations detected than expected based on the reference population. The number of detected novel and frequent mutations are coloured separately.

To complement the proteomic data, we have utilised the RNA‐Seq data for predicting protein sequence variants with an in‐house developed workflow (Materials and Methods section). The predicted NRP sequences (i.e., peptides not present in the reference protein sequence databases UniProt/SwissProt, TrEMBL or NextProt) were then identified and validated through high‐quality PSMs in the proteomic data (Materials and Methods section, Table S2A–D). Importantly, to counterbalance the varying quality of RNA‐Seq within and across patients, protein sequence variants predicted in one sample were searched against all proteomic data from the entire cohort. Through these steps, a total of 1248 NRPs corresponding to 1177 unique sequence variants were confidently identified in at least one metastasis at the peptide level, which corresponded to 627 small variants (in‐frame and frameshift indels), 545 splice variants (known and novel splice junctions), and five peptides that may stem from multiple genomic alterations (Table S5A and Figure 3A). There was a slight bias towards detecting NRPs in proteins that we quantified with higher label‐free quantitation (LFQ) intensity, as expected (Wilcoxon test, *p* < .001, Figure S3A). Notably, the NRPs were highly variable across the patients, with the majority (69.1%) of the NRPs being detectable in one or two patients only (Figure 3A). On average, the NRPs were detectable only in the proteomes of 5.9 metastases, and 588 NRPs were only detected in one patient. For 533 in‐frame and frameshift indels, AF >1% was noted in either or both the 1000 genomes^39^ and gnomAD^40^ genetic variation databases (Materials and Methods section). A total of 149 NRPs were labelled as potentially deleterious using CADD predictions (scaled C‐score ≥20), 74 of which were only detected in one patient (Table S5A and Figure 3A).

The NRPs could be mapped to 975 genes. Compared to the list of proteins quantified in our quantitative proteomic data, these 975 genes showed an enrichment (Supporting Information, one‐sided Fisher's test, adj. *p* < .05) for pathways related to adipogenesis, innate and adaptive immune system (e.g., FCGR activation, complement cascade), vesicle‐mediated transport (scavenger receptors) and pathways associated with extracellular matrix (ECM) organisation (Figure 3B and Table S5C).

To provide an overview of pathways that are affected by SAAVs in our cohort, we turned our focus to NRPs corresponding to SAAVs and categorised them based on their previous observance in a reference population (non‐cancer samples from non‐Finnish European gnomAD subpopulation). SAAVs not present in the reference population were labelled as ‘novel mutations’, that is, likely causally linked to melanoma, while SAAVs detected in >1% of the reference population as ‘frequent mutations’, that is, likely not linked to melanoma. SAAVs detected in ≤1% of the reference population were removed due to uncertainty regarding their association with melanoma (Table S5B). A total of 69 proteins in our cohort harboured novel mutations (Table S5B), showing enrichment (one‐sided Fisher's test, adj. *p* < .05) for pathways including focal adhesion, innate and adaptive immune system including B‐cell receptor (BCR) signalling, vesicle‐mediated transport and oncogenic MAPK signalling. In addition, the translation pathway was marginally significant with adj. *p* = .20 (Figure 3C and Table S5C). Interestingly, oncogenic MAPK signalling was not enriched among the 975 genes containing any NRPs. Of note, mutations in neither BRAF nor NRAS were detected in our cohort, but other members of the MAPK signalling pathway were affected by mutations (MPRIP, SND1, MAP2K1, FN1) and contributed to the enrichment of this pathway in our data. There was no association between BRAF/NRAS driver mutation status for a metastasis and the presence or absence of NRPs in these four proteins (Figure S3B). Among the 69 proteins detected with novel mutations, 16 were more frequently mutated than expected in our cohort, which was assessed based on the average ratio of novel to frequent mutations in the reference population calculated for all genes (Figure 3D and Table S5B). This list of proteins includes A2M, SPANXA2 and multiple genes related to the immune system (HLA‐C and immunoglobulin genes).

### 3.4 Heterogeneity linked to metastasis location

**FIGURE 4:**
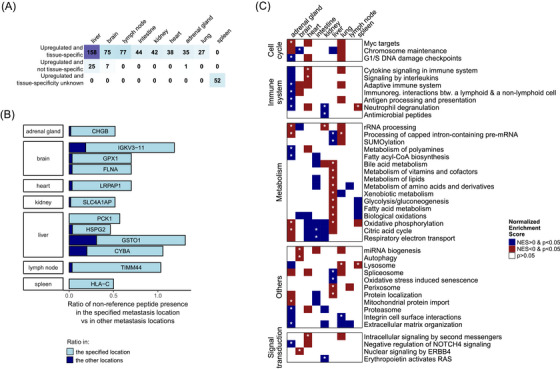
Proteogenomic differences associated with metastasis location. (A) Number of significantly upregulated proteins in distinct metastasis locations. Upregulated proteins that are tissue‐specific or not tissue‐specific according to the Human Protein Atlas (HPA) are displayed in separate rows. Information on spleen tissue specificity was not available in HPA. (B) Genes of non‐reference peptides more frequently detected in a metastasis location compared to other locations. (C) Pathway‐level signatures of metastasis locations as detected by pre‐ranked gene set enrichment analysis (GSEA) based on the global proteomics data. White cells depict results where GSEA *p* > .05, blue or red‐coloured cells without a star indicate *p* < .05 (normalised enrichment score [NES] lower than 0 or higher than 0, respectively) and cells with a star indicate adj. *p* < .05.

After the in‐depth bioinformatic analysis of our data, we aimed to study the heterogeneity in distant metastases of melanoma. Firstly, we investigated proteogenomic heterogeneity attributable to metastasis location. We used differential abundance analyses and Fisher tests to find upregulated proteins and enriched NRPs, respectively, for each metastasis location (Supporting Information). We only included samples with tumour content ≥60% and only compared metastasis locations that contained minimum three metastasis samples with tumour content ≥60%, namely, adrenal gland, brain, heart, intestine, kidney, liver, lung, lymph node and spleen (Table S6A). The number of uniquely upregulated proteins based on differential abundance analysis for each metastasis location was sparse, even with *p* < .01 (Figure 4A). The highest number of upregulated proteins was detected in the liver (*n* = 183) and brain (*n* = 82). Importantly, the number of upregulated proteins showing tissue specificity to the relevant tissue according to the Human Protein Atlas (HPA) database^41^ was minimal or not present (Figure S4A). When examining the significant enrichment (Fisher test, *p* < .01) of NRPs in the metastasis locations, we found only variants for which the corresponding protein was not upregulated in the specific metastasis location. In‐frame variants of CHGB, LRPAP1, SLC4A1AP, TIMM44 and HLA‐C (all with high AF in the reference population) were enriched in adrenal gland, heart, kidney, lymph node and spleen metastases, respectively (Table S6B and Figure 4B). Interestingly, in‐frame variant NRPs from genes GPX1, IGKV3‐11, FLNA were enriched in brain metastases, and only the GPX1 variant showed high AF in the reference population. In contrast, liver metastases showed enrichment for in‐frame variants of GSTO1, CYBA, PCK1 and HSPG2, all of which were highly frequent in the reference population. Considering the proteins of the above‐mentioned NRPs, the HPA database indicated that CHGB is upregulated in adrenal gland tissues alongside brain and pituitary gland tissues. In addition, both GSTO1 and PCK1 were noted with enhanced expression in liver according to the HPA database. No NRPs were enriched in the lung and intestine metastases. The observation that upregulated proteins and enriched NRPs for the metastasis locations were not strongly overlapping with the tissue‐specific proteins according to HPA underscores the precise histological annotations of the analysed samples; namely, that the metastasis samples with ≥60% tumour content had minimal (mean ± SD = 3.2 ± 7.4%) adjacent tissue presence.

We further investigated the metastasis location differences at pathway level with pre‐ranked GSEA (Table S6C). The representative pathways for the significantly (adj. *p* < .05) up‐ and downregulated processes in these metastasis locations, categorised into larger biological processes, are shown in Figure 4B. No pathways were significantly altered when comparing the lymph node metastases to other metastasis locations, and only a few pathways were significantly upregulated when comparing adrenal gland, brain, heart, intestine, kidney, lung and spleen to all other metastatic locations. The liver metastases showed the highest number of significant pathways. We noted the upregulation of various metabolic pathways (e.g., amino acids and lipids), peroxisome and protein localisation pathways in liver metastases, in parallel with the downregulation of RNA metabolism, SUMOylation, spliceosome and oxidative stress induced senescence.

We also utilised the proteomic data of a prospective melanoma distant metastasis cohort (Beck dataset),^19^ which includes sites such as brain, intestine, liver, lung, lymph node and spleen metastases, to verify our findings on metastasis location‐specific proteomic patterns. Importantly, only the upregulation of various metabolic processes and the protein localisation pathway in the liver, as well as the upregulation of neutrophil degranulation in the spleen was confirmed with the Beck dataset (Figure S4B). However, we found contrasting results for only one pathway, the mRNA‐splicing Reactome pathway, which showed significant enrichment with normalised enrichment score (NES) >0 in our dataset in the lung, but showed a significant opposite trend in the Beck dataset (*p* < .05). The short list of metastasis location‐associated proteins, NRPs and pathways, together with a lack of agreement with the Beck dataset may suggest that the molecular profile of a metastasis is not dominated by the metastasis location.

### 3.5 Proteogenomic heterogeneity of melanoma metastases

As the proteogenomic differences based on metastasis location were not strong, we also investigated what other drivers of heterogeneity were present for metastases within the same patients. Firstly, pairwise Pearson correlations and Jaccard similarities between the metastases with min. 60% tumour content were calculated based on the protein abundance and NRP presence, respectively, which indicated varying levels of intrapatient similarity (Figure S5A). Particularly at the proteomic level, metastases occasionally exhibited less similarity to other metastases from the same patient than to metastases from different patients.

**FIGURE 5:**
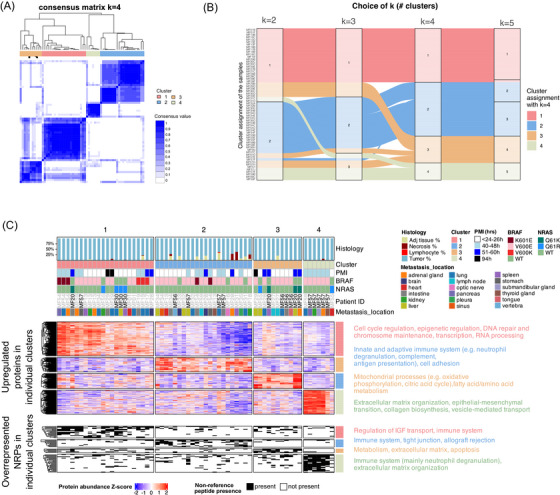
Proteogenomic heterogeneity of melanoma metastases. (A) Consensus matrix with number of clusters (*k*) = 4, as generated by the consensus clustering algorithm (partition around medoids algorithm with Pearson distance). (B) Alluvial diagram showing cluster assignment transitions for the samples across different values of *k*. Each vertical block represents the samples’ cluster assignment at *k* = 2, 3, 4 and 5. Samples (horizontal lines) are coloured based on their cluster membership at *k* = 4, and flows between the vertical blocks illustrate how samples are reassigned as the number of clusters changes. (C) Heatmap of metastasis clusters displaying upregulated proteins (top heatmap) and non‐reference peptides (NRPs) more frequently detected (bottom heatmap) in each cluster. The metastases, proteins and NRPs are clustered based on Euclidean distance and complete linkage. Metastases are annotated with their histological features, cluster assignment and various clinical data, while protein and NRP clusters are annotated with enriched pathways.

To investigate this varying intrapatient similarity in an unsupervised manner, the high tumour‐content (≥60%) metastasis samples were subjected to consensus clustering based on the abundance profile of 5542 proteins that were quantified across ≥90% of the metastases (Supporting Information). We identified four proteomic clusters, labelled as C1–4 (Figures 5A, S5B and Table S7A). The number of clusters (*k*) was determined based on the delta area plot, which indicated that with *k* > 4, the relative increase in consensus is small. In addition, a new cluster emerged with *k* = 4, collecting various samples from the different clusters into one cluster (cluster C3, Figure 5B). Importantly, clustering was not driven by PMI or the metastasis location (Supporting Information, Fisher tests *p* > .05, Table S7B), albeit seven out of 13 lung metastases were part of C1 (Figure S5C). Similarly, driver mutation status did not influence clustering. While C2 included only metastases without NRAS driver mutations (Fisher test, *p* = .008), NRAS driver mutation occurred only in three patients, thus this finding is limited by small sample size. Metastases from individual patients were distributed across multiple clusters, with a few exceptions (Supporting Information, Fisher tests *p* > .05, Figure 5C and Table S7B). Namely, only one patient ID was enriched in C1 (MF30, Fisher test, *p* = .041), three patient IDs were enriched in C3 (MF20, MF27 and MF56, Fisher tests, *p* = .016, .016 and .029, respectively). In addition, MF57 was enriched in C4 (Fisher test, *p* < .001) but notably, this patient had four additional metastases present in other clusters. This observation that metastases from the same patient did not necessarily cluster together indicate that prominent proteomic differences within patients exist.

We furthermore investigated how the clinical data relates to the clusters (Table S7B). Associations between clusters and the patient's treatment data were challenging to establish due to the high variability of treatment combinations across patients. We observed an enrichment for immunotherapy in C1 (this is due to the enrichment of patient MF30 in this C1 who solely received immunotherapy in this cohort, Fisher test, *p* = .041), enrichment of metastases from patients who were treated with interferon therapy in C3 (Fisher test, *p* = .014), and enrichment of metastases from patients who were not treated in C1 (Fisher test, *p* = .056). There were no significant differences across the clusters in terms of chemo‐ or targeted therapy.

To uncover the proteogenomic drivers of heterogeneity, we annotated the proteomic clusters with proteins showing uniquely upregulation in those clusters, followed by pathway enrichment analysis (adj. *p* < .05) to detect each clusters’ characteristic pathways. We also sought out significantly enriched (*p* < .01) NRPs for each cluster (Table S7C–F and Figure 5C). A total of 237, 100, 105 and 167 proteins showed specific upregulation in C1–4, respectively (differential abundance comparisons, adj. *p* < .05 for all relevant comparisons; Figure 5C), among which we also searched for druggable proteins (Table S7C and Figure S5D). Druggable proteins are defined as targets for Food and Drug Administration‐approved drugs, and are summarised in the HPA database, referencing DrugBank as its primary resource.^42^, ^43^ Additionally, 17, 11, 6 and 26 NRPs were enriched in C1–4, respectively (Fisher's tests, *p* < .01; Table S7E).

C1 was mainly distinguishable by the upregulation of proteins involved in RNA metabolism and processing, transcription and cell cycle regulation (adj. *p* < .05). In addition, C1 showed an enrichment of NRPs that were all in‐frame variants with AF >1% in external reference populations and multiple of their genes were associated with insulin growth‐like factor regulation (LAMC1, F2, SERPINA1) and the innate immune system (IFI16, F2, ARHGAP45, SERPINA1; Table S7E,F). Druggable proteins upregulated in C1 were CDK4, IMPDH2, PARP1, XPO1 and YES (Figure S5D), all of which are members of cell cycle‐, DNA repair‐ and transcription‐related pathways.

C2, the cluster with the lowest average tumour content (Kruskal–Wallis test, *p* = .018, Figure S5E), was characterised by the upregulation of proteins that are members of the immune system (such as neutrophil degranulation, complement, PD‐1 signalling, antigen presentation) and cell adhesion pathways (adj. *p* < .05). Next to NRPs that were all in‐frame variants with high AF in external reference populations, we also found enrichment for one peptide originating from alternative splicing on gene GPR146, as well as two in‐frame variants with low AF in the reference population (IGLV3‐21, IGHV3‐74). The NRPs were associated with the immune system (CYBA, PSMB4, IGLV3‐21), tight junction (MYH11, RAB13, HCLS1) and allograft rejection (HCLS1, CAPG; Table S7E,F). In total, 11 druggable proteins showed upregulation in C2 (Figure S5D), none of which were involved in the characteristic pathways of C2.

In C3, we observed upregulation for mitochondrial processes, oxidative phosphorylation (OXPHOS) and various metabolic pathways (fatty acid, amino acid and glucose; adj. *p* < .05). Concerning significantly enriched NRPs in C3, they were all in‐frame variants with high AF of multiple metabolism‐related genes (APIP, ESYT2, PYGB, QPRT), as well as the ECM‐related LAMC1 and apoptosis‐related APIP was present in this list (Table S7E,F). Nine of the upregulated proteins in C3 were druggable (Figure S5D), but none were involved in the characteristic pathways of this cluster.

Lastly, C4 contained metastases showing upregulation of proteins involved in ECM organisation, EMT and collagen biosynthesis (adj. *p* < .05). Enriched NRPs stem from immune system‐related genes (PKM, PGM1, CTSD, PRKCSH, PRCP, ITGAV, HLA‐C, IGLV1‐47, PSMB3; many of which are part of the neutrophil degranulation pathway) and ECM organisation‐related genes (CTSD, LAMA5, ITGAV). Interestingly, two NRPs were in‐frame variants with low AF (PKM, IGLV1‐47) and one NRP was a product of alternative splicing in gene TMEM242 (Table S7E,F). There were seven druggable proteins upregulated in C4 (Figure S5D), from which ATP1A1 was involved in ion homeostasis, and ITGB1 and ITGB3 were involved in ECM organisation and EMT processes.

Pre‐ranked GSEA applied on the differential protein abundance results further confirmed the distinct phenotypic characteristics associated with the clusters at a more granular level (Figure S5F and Table S7G). With significance set at adj. *p* < .05, both C1 and C3 clusters showed an upregulation of the translation pathway, however, C1 showed the upregulation of cell cycle, transcription and RNA‐splicing‐related pathways, while C3 showed the upregulation of mitochondrial processes, OXPHOS and various metabolism pathways. In contrast, both C2 and C4 showed an upregulation of the ECM and EMT pathways, accompanied by the upregulation of various immune pathways in C2, which was not present in C4. In contrast, C4 uniquely showed an upregulation of the vesicle‐mediated transport pathway.

### 3.6 Varying levels of heterogeneity across metastases within patients with prognostic implications

**FIGURE 6:**
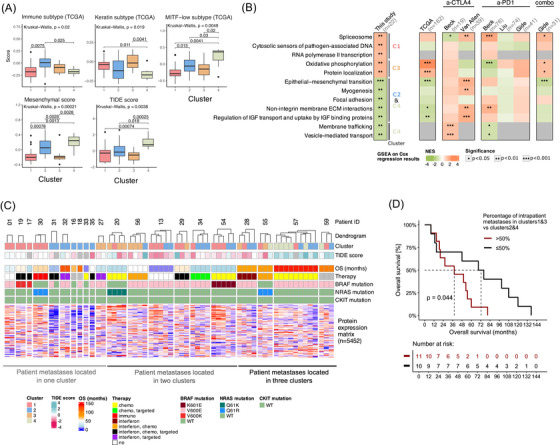
Prognostic implications of proteomic heterogeneity. (A) Boxplots showing differences in single‐sample scores across the proteomic clusters. From left to right: Immune/Keratin/MITF‐low TCGA subtype scores, mesenchymal scores and tumour immune dysfunction and exclusion (TIDE) scores. Kruskal‐Wallis and pairwise Wilcoxon test *p* values are shown on the top. (B) Pathways significantly associated with overall survival (OS) in our cohort, and their relationship to the proteomic clusters and survival relevance in external cohorts.^19^, ^31^, ^35^, ^36^, ^37^ Survival relevance for the pathways was assessed with Cox regression analyses followed by pre‐ranked gene set enrichment analysis (GSEA). The heatmaps show normalised enrichment scores from GSEA, with stars indicating significance. Normalised enrichment score (NES) >0 indicates that the pathway was upregulated in patients with shorter OS. The results on the external cohorts are grouped based on the applied therapy regimen, including anti (a)‐CTLA‐4, a‐PD‐1, combination therapy (combo). The TCGA cohort was not collected in the context of immunotherapy response. Missing NES values for a gene set indicate that <10 members of the gene set were quantified in the dataset. (C) Overview of cluster assignments, TIDE scores and patient‐level clinical data for each metastasis. Metastases are grouped by patient, and the dendrograms are shown to demonstrate the varying levels of intrapatient similarities based on their protein abundance profile. Both the proteins and metastases are clustered based on Euclidean distance and complete linkage. The protein abundance matrix is vertically compressed. Patients are ordered based on the number of clusters across which their metastases are distributed. (D) Kaplan–Meier curves for patients who have more than 50% of their metastases classified into C1 and C3 versus patients with a maximum of 50% of their metastases classified into C1 and C3.

We also annotated the metastasis samples with single‐sample scores derived from our RNA‐Seq data (Table S7A) and related those scores to the proteomic clusters. We investigated scores that describe the metastases’ relationship to the TCGA subtypes, which have been associated with patient survival; scores that describe mesenchymal characteristics (using melanocytic and mesenchymal markers published in ref. 44) as well as the tumour immune dysfunction and exclusion (TIDE) scores which predict immunotherapy response.^45^ The TCGA subtype scores, namely, Immune, Keratin and MITF‐low subtype scores, varied significantly across the clusters (Kruskal–Wallis tests, *p* < .05, Figure 6A), with C2 showing the highest Immune subtype scores (best prognosis subtype according to TCGA), C1 and C3 showing highest Keratin subtype scores (worse prognosis subtype according to TCGA) and C4 showing high MITF‐low subtype score. The mesenchymal and TIDE scores (where TIDE score <0 indicates improved response to immunotherapy) also differed across the clusters (Kruskal–Wallis test, *p* < .001 and *p* = .004, respectively, Figure 6A). C1 and C3 showed the lowest TIDE and mesenchymal scores, thereby indicating increased susceptibility to immunotherapies and a more pronounced melanocytic phenotype. These results signalled a potential prognostic and therapeutic relevance of the detected proteomic clusters.

To investigate how clusters are associated with OS in our cohort, univariate Cox regressions with patient ID included as random effect in the model were used to rank proteins based on their association with OS, with higher rank indicating upregulation in patients with shorter OS. These ranks were then used in a pre‐ranked GSEA to evaluate the significance of cluster‐specific pathways with OS (Materials and Methods section). We opted for this alternative approach instead of directly linking the clusters to OS because metastases from the same patient can fall into different clusters. Several cluster‐specific pathways were significantly associated with OS with *p* < .01 (Figure 6B and Table S7H). Specifically, pathways associated with C1 or C3 (such as RNA‐splicing and OXPHOS) were enriched in patients with shorter OS (NES >0). In contrast, the characteristic pathways of C2 and/or C4, such as EMT, focal adhesion and vesicle‐mediated transport, generally showed upregulation with longer OS (NES <0).

We analysed multiple external advanced melanoma cohorts to validate our survival analysis results. These included the TCGA RNA‐Seq dataset (collected prior to the immunotherapy era)^31^ as well as the Van Allen,^35^ Gide,^36^ and Liu^37^ RNA‐Seq cohorts, and the Beck proteomic dataset,^19^ all of which contained samples from patients who were subsequently treated with a‐PD‐1, a‐CTLA‐4 therapies or their combination (herein referred to as a‐PD‐1, a‐CTLA‐4 or combination therapy subcohorts). For each subcohort, Cox regression analysis was used to rank proteins or genes based on their association with OS, followed by pre‐ranked GSEA, mirroring the approach applied in our dataset (Materials and Methods section, Figure 6B). Interestingly, the Van Allen a‐CTLA‐4, Beck a‐PD‐1 and Gide combination therapy subcohorts supported that upregulation of RNA‐splicing‐related pathways was associated with shorter OS (pre‐ranked GSEA, *p* < .05). In addition, several findings from our cohort were corroborated by the TCGA cohort, including upregulation of mitochondria‐related pathways and downregulation of EMT and ECM‐related pathways in patients with shorter OS (pre‐ranked GSEA, *p* < .05, Figure 6B). However, the directionality of survival associations for other pathways varied across cohorts. In the Beck a‐PD‐1 subcohort, mitochondrial processes were associated with better outcomes. Additionally, the ECM and EMT pathways were associated with shorter OS in the Van Allen a‐CTLA‐4 and Beck a‐PD‐1 cohorts. Lastly, vesicle‐mediated transport pathways were linked to shorter OS in the Beck a‐CTLA‐4 cohort.

The heterogeneity of our patient cohort in terms of the clusters and further sample‐wise annotations is summarised in Figure 6C. We identified eight patients classified into both C1/C3 and C2/C4 clusters, out of which four patients’ metastases were spanning across three clusters and thus showing the strongest heterogeneity, namely, MF28 (C1, C2, C3), MF55 (C1, C3, C4), MF57 (C1, C2, C4) and MF59 (C1, C2, C4). Besides the different cluster assignment, the independently developed algorithm, TIDE also predicted immunotherapy response differences among the metastases of the same patient (Table S7A). In nine out of the 15 patients with multiple metastases annotated with a TIDE score, at least one metastasis had a TIDE score >0 and another had a TIDE score <0. For patients MF29 and MF56 these TIDE scores varied even more substantially, having at least one metastasis with TIDE score <1 and parallelly having another metastasis with TIDE score >1. Consistent with these findings, patients with more than 50% of their metastases classified into C1 or C3 had significantly poorer survival than those with a majority of C2 or C4‐type metastases (log‐rank test, *p* = .044; Figure 6D), These results show that the proteomic clusters, which we have demonstrated to have distinct prognostic and therapeutic implications, often co‐occur across the metastases within one patient.

## 4 DISCUSSION

The study of these unique post mortem clinical samples, collected primarily in the pre‐immunotherapy era, provided a detailed profile of multiple metastatic sites in melanoma patients. The high heterogeneity of melanoma was already evident in the clinical data of our cohort. While the number of metastases per patient did not correlate with OS, we found that spleen metastases were associated with lower OS, supported by previous studies showing that spleen metastases typically occur in cases of disseminated disease involving multiple organs.^46^, ^47^ As the spleen is a key immune organ, colonisation by tumour cells could represent a stage where the host immune system can no longer support an effective anti‐tumour response.

A previous analysis on this cohort^22^ showed that the metastases within patients did not show differences in their BRAF, NRAS and CKIT mutation status. Similarly, we found that the metastases displayed homogeneous morphologies within patients, emphasising the importance of our efforts in understanding melanoma heterogeneity utilising RNA‐Seq and proteomics. While the correlation between proteomics and RNA‐Seq in our study was lower than previously reported in large‐scale proteogenomic studies,^48^ we identified 131 genes with strong concordance at both levels. Notably, many of these genes, such as MLANA, PMEL (HMB45) and S100, correspond to well‐established immunohistochemical markers for cutaneous melanoma.^49^ The discrepancy between protein abundance and gene expression could stem from biological factors such as post‐transcriptional regulation, which can decouple mRNA levels from protein abundances.^50^ Technical considerations may also play a role, including differences in the post mortem stability of proteins and transcripts. RNA is known to degrade more rapidly than proteins after death due to the high rate of enzymatic degradation of transcripts, potentially affecting RNA‐Seq data quality.^51^ In addition, Ferreira et al. described that gene expression changes may occur in a tissue‐specific manner after death.^51^ We observed variable RNA‐Seq data quality in our study, which was not dependent on PMI or metastasis location, but showed differences across patients. To circumvent the technical issues with the RNA‐Seq data outlined above, we therefore opted to utilise the RNA‐Seq data to extract a new layer of information from our data, namely, to predict protein sequence variants with an in‐house developed proteogenomic data integration workflow, which identified variants were later confirmed at the peptide level. To mitigate potential gaps in RNA‐Seq prediction caused by variable RNA quality, we searched for all predicted sequence variants across all proteomic samples, regardless of whether they were initially predicted from those samples.

In total, we detected 1177 unique protein sequence variations mapping to 975 genes with this approach. Most identified NRPs were unique to individual patients. This was an expected finding, reflecting the high degree of patient‐specific genetic variability and the unique mutational landscapes of melanomas.^52^ Schiantarelli et al. recently reported the genomic analysis of matched biopsies from 25 melanoma patients before and after immunotherapy treatment and found no highly recurrent resistance‐associated mutations.^53^ In our study, NRPs were significantly enriched among members of the adipogenesis, immune system, vesicle‐mediated transport and ECM organisation pathways. In total, 45% of the NRPs were frequent (>1%) in the reference populations, strengthening the validity of our computational pipeline. A key advantage of our approach over genomics‐based mutation profiling is its focus on sequence variants that are detectable at the protein level, thereby confirming their translation and suggesting functional relevance. While not all of the identified NRPs may be directly related to oncogenesis, they could support tumour growth and survival by contributing to the heterogeneity of the metastases.^54^ This curated list of sequence variants therefore may be of interest for follow‐up investigations. To support future research, we provide this list of identified NRPs along with their frequency in our cohort, AF data from external databases and CADD deleteriousness predictions to help prioritise variants potentially relevant to melanoma. Interestingly, additional investigation of single amino acid mutations detected in our cohort that have not been previously described in the non‐cancer samples of non‐Finnish European gnomAD subpopulation (‘novel mutations’) revealed an enrichment of similar pathways mentioned above for all NRPs but was supplemented by an enrichment of the oncogenic MAPK signalling pathway. The MAPK pathway has a crucial role in melanoma pathogenesis and is activated by mutations in the key signalling pathway members, including BRAF, NRAS, NF1 and KIT.^55^ We did not detect mutations in the aforementioned driver genes at the proteomic level, likely due to sensitivity limitations, as previously reported in the MM500 study.^20^ However, we detected mutations in FN1, MAP2K1, MPRIP and SND1 genes, all of which are functionally linked to the MAPK pathway. Previous studies on BRAF‐ and NRAS‐mutated melanomas have identified the co‐occurrence of mutations affecting members of this critical pathway.^31^, ^56^, ^57^, ^58^ In addition, we detected A2M, SPANXA2, HLA‐C and multiple immunoglobulin genes more frequently mutated in our cohort than expected based on trends in larger reference populations. These mutated proteins therefore might have an important role in melanoma progression and treatment resistance. A2M has been shown to upregulate the tumour suppressor PTEN and inhibit cell proliferation in various cancer cell lines, including melanoma, suggesting a tumour‐inhibitory mechanism that could be therapeutically exploited.^59^ Furthermore, A2M was included in a transcript signature with prognostic capacity in melanoma, indicating its potential role in disease outcome prediction.^60^ On the other hand, the SPANX gene family, which is aberrantly expressed in aggressive metastatic melanomas, is implicated in cancer progression and immune evasion, making it a promising target for cancer immunotherapy.^61^ HLA‐C, a key immune system component, can undergo mutations or loss of heterozygosity, impairing immune surveillance and contributing to tumour escape. Such alterations in HLA genes, particularly HLA‐C, have been associated with poorer prognosis and resistance to immunotherapy in melanoma and other cancers where immune evasion is critical.^62^, ^63^, ^64^

The extent of proteogenomic differences associated with distinct metastasis locations, including adrenal gland, brain, heart, intestine, kidney, liver, lung, lymph node and spleen, varied substantially with the location. We also mapped only a short list of NRPs favourably appearing in the distinct metastasis locations. Interestingly, the liver metastases, the presence of which is known to significantly worsen patient prognosis,^65^ showed the most distinct protein abundance profile, with 183 uniquely upregulated proteins. At the pathway level, we observed the upregulation of various metabolic pathways and protein localisation in liver metastases, which was confirmed by the Beck dataset.^19^ We only detected a moderate number of upregulated proteins for the other examined metastasis locations (<100 proteins) and a handful of upregulated pathways. The Beck dataset^19^ neither supported nor contradicted our findings. Notably, both the Beck et al. study and the present study showed limited host tissue contamination and identified molecular heterogeneity within metastatic sites. In particular, Beck et al. previously defined three distinct proteomic clusters of lung metastases.^19^ Additionally, inherent differences between the cohorts (our study focusing on patients who succumbed to melanoma vs. Beck et al.’s biopsies from living patients) could contribute to the disparities, alongside with technical challenges. Specifically, both studies are underpowered regarding the number of metastases from each location, and the proteomics sample processing protocols and analytical pipelines were different across the cohorts. These findings highlight the need for analysing larger post mortem cohorts to understand the molecular heterogeneity associated with the different metastasis locations, and to establish location‐specific protein abundance patterns in the latest disease stages after accounting for this heterogeneity.

Unsupervised clustering of metastases based on protein abundance profiles revealed four clusters (C1–4) that formed independently of metastasis location, further indicating a lack of generalisability in the proteomic differences of melanoma metastases across locations. We identified four distinct clusters: one characterised by upregulation of cell cycle and RNA‐splicing pathways (C1), another enriched for mitochondrial and metabolic processes (C3) and two clusters showing higher abundance of ECM‐related proteins (C2 and C4), with C2 further distinguished by upregulation of immune‐related genes. Each cluster was associated with enriched NRPs and included potentially druggable proteins, which, together with the cluster‐associated pathway activations, may serve as candidates for follow‐up studies aimed at mapping therapeutic vulnerabilities specific to each cluster. Moreover, about two‐thirds of patients had metastases spanning across multiple proteomic clusters, thus indicating substantial intrapatient heterogeneity irrespective of metastasis location.

Follow‐up analyses for cluster and OS associations suggested that when the dominating fraction of the distant metastases showed upregulation of translation‐ and mitochondria‐associated pathways (characteristic of C1 and C3), the patients exhibited shorter OS compared to patients whose metastases largely showed upregulation of EMT and ECM organisation pathways (characteristic of C2 and C4). To validate our results, we analysed five publicly available RNA‐Seq and proteomic advanced melanoma cohorts, each with one sample per patient collected at a single timepoint. The TCGA melanoma dataset,^31^ which included patients not collected in the context of immunotherapy response, was most comparable to our cohort. Notably, the relationship between pathway activities and survival outcomes in the TCGA dataset was largely in agreement with our findings, reinforcing the validity of our survival analysis results despite the relatively small cohort size. In both this study and the TCGA dataset, upregulation of ECM and EMT‐related pathways was associated with longer survival, potentially reflecting a less proliferative yet more differentiated mesenchymal phenotype. Conversely, tumours enriched in mitochondrial and OXPHOS pathways were linked to poorer outcomes, consistent with a metabolically aggressive and adaptable tumour state. In line with this, a spatially resolved study conducted by our group previously on the tumour regions and TME of primary melanomas concluded that mitochondrial translation were upregulated both in the tumour and TME regions of recurrent melanomas^17^ compared to non‐recurrent melanomas, indicating a more aggressive phenotype. In contrast, validation of the identified prognostic trends for pathways in cohorts receiving modern anti‐cancer therapies (a‐PD‐1, a‐CTLA‐4 or their combination) did not yield uniform conclusions. Interestingly, across multiple external datasets, there was consistent evidence linking the upregulation of RNA‐splicing‐related pathways to shorter OS, highlighting this phenotype as a potential candidate for future targeted therapies as it may be more resistant to current standard‐of‐care treatments. Previous studies have already linked aberrant alternative splicing events to immunotherapy resistance.^66^ The relationship between other cluster‐specific pathways and patient survival was inconsistent across the immunotherapy cohorts. In the Van Allen cohort, activation of ECM and EMT pathways was associated with poor prognosis and resistance to immunotherapy, which may indicate an immunosuppressive TME that impairs T‐cell infiltration and antigen presentation.^67^, ^68^ Elevated mitochondrial activity has been linked to improved response to immunotherapy in the Beck proteomic dataset, possibly due to enhanced tumour immunogenicity, more efficient antigen processing and presentation, and a metabolic profile conducive to effective immune engagement.^18^ This was in line with the outputs from the TIDE algorithm^45^ which indicated that samples in C3 are the most susceptible to immunotherapies. In contrast, the Gide dataset associated enriched oxidative phosphorylation with immunotherapy non‐responders.^36^ Overall, this highlights that the prognostic value of these pathways is context‐dependent, emphasising the need to consider treatmentand the underlying tumour characteristics when interpreting molecular signatures.

To conclude, our work contributes to the ongoing efforts to understand and ultimately overcome intrinsic or acquired drug resistance in melanoma. We provide empirical evidence on molecular heterogeneity across metastases in a single patient, which heterogeneity was not linked to metastasis locations. This finding underscores a critical challenge: relying on a single metastasis sample to guide treatment decisions may be insufficient. To design effective targeted therapies, it is essential to study intrapatient heterogeneity in larger advanced melanoma patient cohorts, particularly to capture rare metastatic phenotypes that may have been missed in our study due to our limited sample size. Research autopsy programs are well‐positioned to facilitate this effort by providing comprehensive sampling across the metastatic sites and generating hypotheses for combination therapies involving surgery, immunotherapy and novel targeted therapies.^21^ Given that collecting samples from all metastatic sites in living patients is not feasible, future studies must also investigate predictive models that can infer treatment response from single tumour biopsies or other patient‐derived materials (e.g., blood).^69^

We also acknowledge the limitations of this study. Due to the relatively low number of patients and sample quality being affected by varying PMI, our results are primarily descriptive. While the proteomic clusters did not form based on PMI, the proteogenomic differences within patients may be affected by the received treatments and the order of metastasis appearance, and we could not extract the latter information from the clinical metadata. The limited cohort size also restricts our ability to capture rare melanoma phenotypes. In addition, no control tissues were collected from the metastasis locations, thereby we were unable to differentiate between germline and somatic mutations. For this reason and due to their largely patient‐specific occurrence, the NRPs could primarily serve as an additional layer of confirmation for proteomic heterogeneity. Further studies are required to confirm the somatic mutation status of these NRPs and to elucidate their potential mechanistic role in melanoma. While some of our findings from differential protein abundance analysis were supported by external melanoma datasets, further in vitro and in vivo validation is needed; particularly in the context of modern treatments such as immunotherapies. The introduction of immunotherapy may add further complexity to intrapatient heterogeneity in advanced melanoma that could not be addressed in this cohort.

## 5 CONCLUSIONS

In summary, we have performed a comprehensive characterisation of a post mortem melanoma cohort through histopathology, MS‐based proteomics and RNA‐Seq, generating an important dataset for enhancing our understanding of the molecular heterogeneity of metastatic melanoma before the influence of immunotherapy. Our data revealed that metastases within a single patient can have diverse molecular profiles, which may impact prognosis and treatment response. Future studies can leverage this dataset (1) to identify genetic variations directly associated with melanoma progression or therapy resistance by prioritising sequence alterations that are translated as evidenced by our MS/MS proteomics data, and (2) to leverage the phenotypes described here to explore novel targeted therapies, either in combination with immunotherapy or as second‐ or third‐line options when immunotherapy is no longer effective in advanced melanoma patients.

## AUTHOR CONTRIBUTIONS

*Conceptualisation*: Beata Szeitz, Zsuzsanna Ujfaludi, Magdolna Herold, Zoltan Herold, Tibor Pankotai, Krzysztof Pawłowski, György Marko‐Varga, Lazaro Hiram Betancourt. *Methodology*: Beata Szeitz, Yanick Paco Hagemeijer, Zsuzsanna Ujfaludi, Magdalena Kuras, Zoltan Herold, Melinda Rezeli, Tibor Pankotai, György Marko‐Varga, Lazaro Hiram Betancourt. *Software*: Beata Szeitz, Yanick Paco Hagemeijer. *Validation*: Beata Szeitz, Yanick Paco Hagemeijer. *Formal analysis*: Beata Szeitz, Yanick Paco Hagemeijer, Zoltan Gabor Pahi, Magdolna Herold, Zoltan Herold, Tibor Pankotai. *Investigation*: Zsuzsanna Ujfaludi, Magdalena Kuras, Jimmy Rodriguez, Viktoria Doma, Reka Mohacsi. *Resources*: Imre Miklos Boros, Tibor Pankotai, Peter Horvatovich, Jozsef Timar, György Marko‐Varga. *Data curation*: Beata Szeitz, Yanick Paco Hagemeijer, Viktoria Doma, Zsolt Horvath, Indira Pla, Istvan Balazs Nemeth, Tibor Pankotai, Jozsef Timar, Lazaro Hiram Betancourt. *Writing—original draft*: Beata Szeitz, Yanick Paco Hagemeijer, Zsuzsanna Ujfaludi, Zoltan Herold, Krzysztof Pawłowski. *Writing—review & editing*: Beata Szeitz, Yanick Paco Hagemeijer, Zoltan Gabor Pahi, Zsuzsanna Ujfaludi, Magdalena Kuras, Jimmy Rodriguez, Viktoria Doma, Reka Mohacsi, Magdolna Herold, Zoltan Herold, Zsolt Horvath, Indira Pla, Yutaka Sugihara, Bo Baldetorp, Henrik Lindberg, Henriett Oskolas, Melinda Rezeli, Jeovanis Gil, Roger Appelqvist, Lajos V. Kemeny, Jessica Guedes, Johan Malm, Aniel Sanchez, Imre Miklos Boros, Istvan Balazs Nemeth, Victor Guryev, Tibor Pankotai, Krzysztof Pawłowski, Elisabet Wieslander, Attila Marcell Szasz, David Fenyö, Peter Horvatovich, Jozsef Timar, György Marko‐Varga, Lazaro Hiram Betancourt. *Visualisation*: Beata Szeitz, Yanick Paco Hagemeijer, Zoltan Gabor Pahi, Zsuzsanna Ujfaludi, Tibor Pankotai. *Supervision*: Victor Guryev, Tibor Pankotai, Krzysztof Pawłowski, Elisabet Wieslander, Attila Marcell Szasz, David Fenyö, Peter Horvatovich, György Marko‐Varga, Lazaro Hiram Betancourt. *Project administration*: Tibor Pankotai, György Marko‐Varga, Lazaro Hiram Betancourt. *Funding acquisition*: Imre Miklos Boros, Tibor Pankotai, Jozsef Timar, György Marko‐Varga.

## CONFLICT OF INTEREST STATEMENT

The authors declare no conflicts of interest.

## ETHICS STATEMENT

The research was conducted in compliance with the Declaration of Helsinki and was approved by the Semmelweis University Regional and Institutional Committee of Science and Research Ethics (IRB, SE TUKEB 114/2012).

## Supporting information



Supporting Information

Supporting Information

Supporting Information

Supporting Information

Supporting Information

Supporting Information

Supporting Information

Supporting Information

Supporting Information

## Data Availability

The MS proteomic data have been deposited to the ProteomeXchange Consortium via the PRIDE partner repository with the dataset identifier PXD058546. RNA‐Seq data will be available upon acceptance on the HCEMM server. Histology images reported in this paper will be shared by the lead contact upon request. External datasets^19^, ^31^, ^35^, ^36^, ^37^ were accessed from their publication and from other data repositories (https://www.cbioportal.org/study/summary?id=skcm_tcga_pan_can_atlas_2018) and https://github.com/ParkerICI/MORRISON‐1‐public.^32^, ^33^, ^34^, ^38^
