# Supplementary material for: Distant metastases of melanoma exhibit varying extent of intrapatient proteogenomic heterogeneity
Source: Clin Transl Med. 2025 Sep 28;15(10):e70477. doi: 10.1002/ctm2.70477 (PMC12477065; doi:10.1002/ctm2.70477)
Supplement: Supplementary file 1 — Supporting Information [file CTM2-15-e70477-s005.docx]

**Distant metastases of melanoma exhibit varying extent of intrapatient proteogenomic heterogeneity**

**Supplementary material**

**Running title**: Proteogenomics on distant melanoma metastases

**Authors**:

Beata Szeitz^1^, Yanick Paco Hagemeijer^2,3^, Zoltan Gabor Pahi^4,5^, Zsuzsanna Ujfaludi^4,6^, Magdalena Kuras^7,8^, Jimmy Rodriguez^9^, Viktoria Doma^10^, Reka Mohacsi^1^, Magdolna Herold^1^, Zoltan Herold^1^, Zsolt Horvath^7^, Indira Pla^7,11^, Yutaka Sugihara^7,8^, Bo Baldetorp^12^, Henrik Lindberg^7^, Henriett Oskolas^8^, Melinda Rezeli^7,13^, Jeovanis Gil^8,11^, Roger Appelqvist^11,14^, Lajos V. Kemeny^10,15,16^, Jessica Guedes^7,11,17^, Johan Malm^8^, Aniel Sanchez^8,11^, Imre Miklos Boros^18,19^, Istvan Balazs Nemeth^20^, Victor Guryev^3^, Tibor Pankotai^4,5,6^, Krzysztof Pawłowski^21,22^, Elisabet Wieslander^8^, Attila Marcell Szasz^1^, David Fenyö^23,24^, Peter Horvatovich^2^, Jozsef Timar^25^, György Marko-Varga^7,11*^, Lazaro Hiram Betancourt^8*^

**Affiliations**:
^1^Division of Oncology, Department of Internal Medicine and Oncology, Semmelweis University, Budapest, Hungary
^2^Analytical Biochemistry, Groningen Research Institute of Pharmacy, University of Groningen, Groningen, The Netherlands
^3^European Research Institute for the Biology of Ageing, University of Groningen, University Medical Centre Groningen, Groningen, The Netherlands
^4^Department of Pathology, Albert Szent-Györgyi Medical School, University of Szeged, Szeged, Hungary
^5^Hungarian Centre of Excellence for Molecular Medicine (HCEMM), Genome Integrity and DNA Repair Core Group, University of Szeged, Szeged, Hungary
^6^Competence Centre of the Life Sciences Cluster of the Centre of Excellence for Interdisciplinary Research, Development and Innovation, University of Szeged, Szeged, Hungary
^7^Department of Biomedical Engineering, Lund University, Lund, Sweden
^8^Department of Translational Medicine, Lund University, Skåne University Hospital Malmö, Malmö, Sweden
^9^Department of Biochemistry and Biophysics, Karolinska Institute, Stockholm, Sweden
^10^Department of Dermatology, Venerology and Dermatooncology, Faculty of Medicine, Semmelweis University, Budapest, Hungary
^11^European Cancer Moonshot Lund Center, Sweden
^12^Section of Oncology, Department of Clinical Sciences Lund, Lund University, Lund, Sweden
^13^BioMS−Swedish National Infrastructure for Biological Mass Spectrometry, Lund University, Lund, Sweden
^14^Clinical Protein Science & Imaging, Biomedical Centre, Department of Biomedical Engineering, Lund University, Lund, Sweden
^15^HCEMM-SU Translational Dermatology Research Group, Semmelweis University, Budapest, Hungary
^16^Department of Physiology, Semmelweis University, Budapest, Hungary
^17^Chemistry Institute, Federal University of Rio de Janeiro, Rio de Janeiro, Brazil
^18^Department of Biochemistry and Molecular Biology, University of Szeged, Szeged, Hungary
^19^Hungarian Research Network Biological Research Center, Institute of Biochemistry, Szeged, Hungary
^20^Department of Dermatology and Allergology, University of Szeged, Szeged, Hungary
^21^Department of Molecular Biology, University of Texas Southwestern Medical Center, Dallas, Texas, USA
^22^Howard Hughes Medical Institute, Dallas, Texas, USA
^23^Institute for Systems Genetics, NYU Grossman School of Medicine, New York, NY, USA
^24^Department of Biochemistry and Molecular Pharmacology, NYU Grossman School of Medicine, New York, NY, USA
^25^Department of Pathology, Forensic and Insurance Medicine Semmelweis University, Budapest, Hungary

Correspondence:

Lazaro Hiram Betancourt*

Department of Translational Medicine,

Lund University,

Skåne University Hospital Malmö,

Malmö, Sweden

lazaro_hiram.betancourt_nunez@med.lu.se

Gyorgy Marko-Varga*

Department of Biomedical Engineering,

Div. Clinical Protein Science & Imaging

Lund University, Lund, Sweden

gyorgy.marko-varga@bme.lth.se

**Supplementary Methods**

*Statistics on the clinical data*

Descriptive and comparative statistics of clinicopathological data was performed in R v4.4.2 (R Foundation for Statistical Computing, Vienna, Austria). Wilcoxon rank sum test with continuity correction and Fisher's exact test was used to compare numeric and count data of cohorts, respectively. To assess survival data, Cox regression models were applied, using the survival R package v3.8-3. Naïve Kaplan-Meier-type plots were drawn with the survminer v0.5.0 R package. Proportional hazards assumption of all Cox regression models was verified.

*Correlation analyses with expression data*

For correlation analyses, the filtered TPM-normalized RNA-Seq dataset (n=11,757 transcripts) and the proteins that were quantified across minimum 50% of the metastasis samples (n=8,028 proteins) were considered. The calculations were performed using the log_2_-transformed TPM values for the retained transcripts and the log_2_-transformed median scale-normalized protein abundances.

In addition, transcript and protein IDs were first mapped to gene names before comparing protein abundance and transcript expression across metastases. Whenever gene names were assigned to multiple protein groups / transcripts, only the row with the largest sum label-free quantitation (LFQ) intensity / TPM value for each gene was kept. In total, 5,619 genes were present in both RNA-Seq and proteome data and were used for the correlation analysis.

Correlation was calculated by Spearman’s correlation method using the cor.test function (stats R package v4.4.2). In both cases, adjusted (adj.) p-values were calculated using the Benjamini-Hochberg (BH) procedure.

*Mutation hot spot regions*

Following the mapping back process, a subset of peptides was created that solely overlapped SAAV sequences, which were confidently verified by PepQuery, and originated from genes encoded in the nuclear genome. Additionally, variants observed in the non-cancer non-Finnish European gnomAD subpopulation reference (v3.1), at an allele frequency (AF) below 1% were filtered out, considering their higher probability of having unknown effects on cancer-related traits (the lack of germline data for these patients means we cannot classify variants as being germline or somatic). The remaining alleles were therefore classified as either gnomAD variants (>1% in the reference subpopulation, later referred to as “frequent mutations”) or “novel mutations” (not annotated in the gnomAD subpopulation). Next, the resulting set of genes for which a mutation hotspot score could be calculated was then ordered by the fraction of non-gnomAD variants as an approximation of how many mutations accumulated in the metastasis compared to the population background. Genes with confirmed mutations at the proteome level were subjected to a binomial test to check if the ratio of novel to frequent mutations in each gene was different from the average background ratio calculated over all genes with confirmed mutations.

During construction of the protein database, all protein variants detected in the RNA-Seq data of the whole dataset were added regardless of their expression level to create one protein sequence database containing the sequence of all protein variants in the analyzed dataset. Genes of which all transcripts were detected at levels below one TPM while searching for evidence of alternative splicing using StringTie as described above across all metastases were removed from the background gene set used for the enrichment analysis.

Additionally, the total number of novel and frequent mutations was determined and the ratio thereof used as the reference value in genewise binomial tests to see which genes were significantly more or less mutated than expected.

*Intrapatient similarity assessment*

Both the Z-score normalized protein intensity table and the binary table indicating the presence (=1) or absence (=0) of a NRP in the metastasis were used to calculate the intrapatient similarity metrics. Only high tumor content (≥60%) metastasis samples were considered (63 samples from 21 patients). Pairwise Pearson and Jaccard similarities were calculated between the metastases using the proteomic and proteogenomic data respectively, via R packages stats v4.4.2 and vegan v2.6-10.

*Defining and annotating metastasis clusters*

The ConsensusClusterPlus function from the ConsensusClusterPlus R package v1.70.0 was used to define the metastasis clusters. As an input, only the metastasis samples with ≥60% tumor content were used (for detailed description of tumor content calculation, see Materials and Methods section “Metastasis sample collection”). This resulted in analyzing the filtered and imputed, Z-score normalized protein expression table of 63 metastases from 21 patients. The following settings were specified: maxK=10, reps=1000, pItem=0.8, pFeature=1, clusterAlg=”pam”, distance=”pearson”. Four clusters (k=4) were selected based on the ConsensusClusterPlus outputs.

Differences in any metastasis characteristics across the metastasis clusters were determined using one-sided Fisher’s exact test for categorical data and Kruskal-Wallis supplemented with pairwise Wilcoxon tests for numerical data. P-values less than 0.05 were considered significant.

*Overall survival-associated proteins and genes*

We utilized a Cox proportional hazards model with mixed effects to evaluate the impact of protein expression on overall survival. The filtered and imputed protein expression table was used as an input, and only metastasis samples with ≥60% tumor content were considered (63 metastases from 21 patients). The model was fitted using the coxme function from the coxme R package v2.2-22, and the protein expression was used as the fixed effect, while the patient IDs were used as the random effect.

In case of external datasets (TCGA, Beck, Van Allen, Gide, Liu) (19,31,35–37), Cox regressions were also performed but without mixed effects to evaluate the associations between protein/transcript expression and overall survival. The Beck and Gide cohorts were further subdivided into distinct cohorts based on what therapies they were subjected to (anti (a)-CTLA4, a-PD1, or their combination). For the TCGA, Gide and Liu datasets, a stage-stratified model was used. In case of the Beck and Van Allen dataset, staging information was not accessible from the publications’ supplementary material.

*Group comparisons*

Differential protein expression analyses were performed using the empirical Bayes statistic implemented in the limma R package v3.62.2.

Considering differential expression analysis of the proteome-based metastasis clusters, the filtered and imputed protein expression table was used as an input, only using metastasis samples with ≥60% tumor content (63 metastases from 21 patients), and all pairwise comparisons between the clusters were tested. To find uniquely overexpressed proteins for each cluster, all pairwise comparisons between a cluster needed to be significant with adj. p<0.05.

In the case of metastasis location comparisons, only the unique expression patterns of locations with minimum 3 metastases (with ≥60% tumor content) were investigated, namely the adrenal gland (n=6), brain (n=3), heart (n=3), intestine (n=7), kidney (n=4), liver (n=7), lung (n=13), lymph node (n=3) and spleen (n=4) locations. The differential expression analysis was separately performed for each aforementioned metastasis site, by comparing the metastases in the selected metastasis site to all other metastases from various metastasis sites, including those metastasis sites as well that did not have minimum 3 metastases. As the input, the protein expression table was filtered for proteins with <50% missing values across the metastases. The same methodology was applied to calculate differential expression on the filtered and imputed Beck protein expression dataset.

For both cluster and metastasis location comparisons, NRPs enriched within these metastasis groups were detected using one-sided Fisher’s exact tests, and p<0.05 was considered significant. Only those NRPs were considered that were present in the samples with ≥60% tumor content and in minimum three patients.

*Pathway analysis*

Overrepresentation analysis for pathways among the selected proteins or NRPs was performed using the fgsea R package v1.32.2. Protein accession numbers were always converted to gene symbols and NRPs were collapsed to gene IDs, and the gene background was defined as all unique gene IDs matching to proteins that were quantified in at least one metastasis in our quantitative proteomic data. Enrichments for Hallmark, Reactome and Kyoto Encyclopedia of Genes and Genomes (KEGG) gene sets downloaded from the MSigDB database v.7.5.1 were tested. P-value adjustments were made with the BH procedure.

For pre-ranked gene set enrichment analysis (GSEA), the proteins were ranked by multiplying the correlation coefficient, log_2_(fold change, FC) or Cox regression coefficient values with the -log_10_(p-values) derived from the relevant statistical test, respectively. In the case of defining cluster-specific pathways, the log_2_FC and p-values for the relevant comparisons were averaged, while ensuring the correct directionality of the FC values for each individual cluster. The same list of gene sets were tested as in the overrepresentation analysis. The BH procedure was used for p-value adjustment.

*Calculation of single-sample scores*

For the single-sample enrichment analysis, the filtered TPM-normalized RNA-Seq dataset (n=11,757 transcripts) was used. The Ensembl gene IDs (ENSG IDs) were then matched to HUGO Gene Nomenclature Committee (HGNC) symbols, and in case of multiple transcripts matching to the same gene, the transcript with the highest sum TPM value across the metastasis samples was kept (n=11,573). The KEGG, Reactome and Hallmark gene sets (MSigDB v.7.5.1; 1,851 gene sets in total) were accessed for this analysis. In addition, we defined custom gene sets to annotate the metastases. Firstly, we extracted lists of genes that define the melanoma TCGA subtypes (upregulated in Keratin subtype, upregulated in Immune subtype, upregulated in MITF-low subtype, downregulated in MITF-low subtype, denoted as Keratin-UP, Immune-UP, MITF-low-UP and MITF-low-DOWN respectively). In addition, we collected lists of genes that define the melanocytic and undifferentiated mesenchymal state. “Singscore”, a single-sample gene-set scoring method was employed to derive single-sample enrichment scores. This algorithm has the advantage that it scores each sample independently without relying on the other samples in the dataset, i.e., potential low-quality or outlier metastasis samples do not affect the scores in other samples. The single-sample scores were only calculated if a minimum of 10 genes were shared between the dataset and the gene set (which criteria was lowered to a minimum of 5 genes for the gene sets defining the TCGA subtypes). Otherwise, the default settings in the simpleScore function from the singscore R package v1.26.0 were used. At the last step, a combined single-sample score for the MITF-low subtype was calculated by subtracting the MITF-low-DOWN single-sample scores from the MITF-low-UP single-sample scores. In addition, a combined mesenchymal score was derived by subtracting the melanocytic state single-sample scores from the undifferentiated mesenchymal state single-sample scores.

*Prediction of immune checkpoint inhibitor therapy response*

The TPM-normalized RNA-Seq dataset collapsed to gene names (n = 11,573) used for the “Singscore” approach was exported and utilized as input in the Tumor Immune Dysfunction and Exclusion (TIDE) algorithm. Only RNA-Seq samples with ≥60% tumor content were considered (57 metastases from 18 patients). The TIDE python package v.1.3.8 (<https://pypi.org/project/tidepy/>) was used to run the TIDE prediction in the command line with a single command: “tidepy -o <output filename> -c Melanoma --force_normalize --vthres 0.0 <input filename>”. After running the TIDE algorithm, each metastasis received a TIDE prediction score. Metastases with scores higher than 0 were categorized as non-responders whereas metastases with scores under 0 were categorized as responders (in alignment with the default setting of TIDE).

*Visualizations*

Plots were created in R v4.4.2 and Inkscape v1.3.2, using mainly R packages clusterProfiler v4.14.4, ComplexHeatmap v2.22.0, ggplot2 v3.5.1, ggbiplot v0.6.2 and survminer v0.5.0.

**Supplementary Tables**

***Table S1. Clinical and histopathological data of the cohort.***

***Table S2. Proteogenomics data processing settings.*** **A)** Example bash commands for the DIA-Umpire conversion and database search. **B)** DIA-Umpire parameters. **C)** MSFragger parameters. **D)** PepQuery parameters.

***Table S3. Correlation analysis results between tumor content and gene/protein expression.*** **A)** Correlation analysis results between protein expression and tumor content. P<0.05 filter. **B)** Correlation analysis results between gene expression and tumor content. P<0.05 filter. **C)** Pre-ranked GSEA results based on the correlation analysis results. P<0.05 filter.

***Table S4. Gene-wise correlation analyses.* A)** Correlation analysis results between protein and gene expression. Adj.p<0.05 filter. **B)** Pathway overrepresentation analysis (ORA) for the genes with strong positive correlation (rho>0.5). Adj.p<0.05 filter.

***Table S5. Non-reference peptide summary and analyses.* A)** NRP annotations. **B)** Hotspot analysis results. **C)** Pathway overrepresentation analysis (ORA) for genes with any NRPs or only with novel mutations. Adj.p<0.20 filter.

***Table S6. Metastasis location-differences.* A)** Differential expression (DE) results for all metastasis locations. P<0.01 filter. **B)** Pre-ranked GSEA results based on the DE analysis results. P<0.01 filter. **C)** Enriched NRPs at the metastasis locations. P<0.01 filter.

***Table S7.*** ***Proteomic cluster characterization.*** **A)** Cluster assignments and the metastases' single-sample scores calculated from RNA-Seq data. **B)** Enriched metastasis annotations in the clusters. P<0.05 filter. **C)** Overexpressed proteins in each cluster. Adj.p<0.05 filter. **D)** Enriched pathways (overrepresentation analysis, ORA) for the overexpressed proteins. Adj.p<0.05 filter. **E)** Enriched NRPs in the clusters. P<0.05 filter. **F)** Pathway involvement for the enriched NRPs in the clusters. **G)** Proteins ranked based on differential expression results between clusters, followed by pre-ranked GSEA. Adj.p<0.05 filter. **H)** Pre-ranked GSEA results based on mixed effects Cox regression analysis results. P<0.01 filter.

**Supplementary File**

***File S1. Pseudo MS/MS spectra for each NRP.*** The best spectra for each NRP with the highest Hyperscore was visualized. Refer to Table S5A for easy navigation within this file; it provides the exact page numbers for each NRP.

**Supplementary Figures**


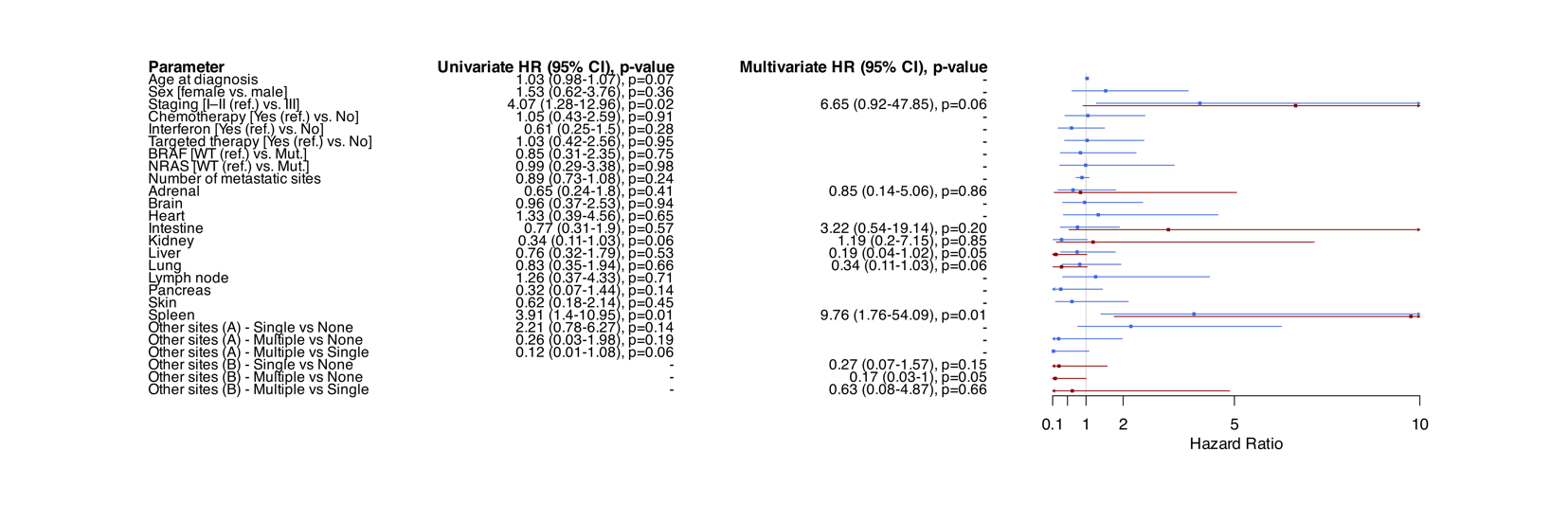


***Figure S1. Associations between metastasis locations and overall survival.*** *Univariate and multivariate Cox regression analysis results for baseline clinical variables, and the metastasis locations are shown on this forest plot. There were a few metastasis locations, which occurred only sporadically. We combined those in the "Other sites (A)" group. In the multivariate analysis, Staging was included alongside the various metastasis locations. Furthermore, in order not to lose any data, in the multivariate survival model, the count data about brain-, heart-, lymph node-, pancreas- and skin metastases were incorporated into the “Other sites (B)” variable. In both "Other" groups, metastases were grouped into the ‘none - single - multiple’ cohorts instead of the ‘none - present’ dummy variable. In the “Other sites (A)” and “Other sites (B)” groups 17 - 5 - 2 and 9 - 8 - 7 patients had no, single or multiple “other-location metastasis", respectively. Other-metastasis locations included the thyroid gland, optic nerve, sinuses, pleura, stomach, tongue, vertebra and the submandibular glands. CI: confidence interval.*


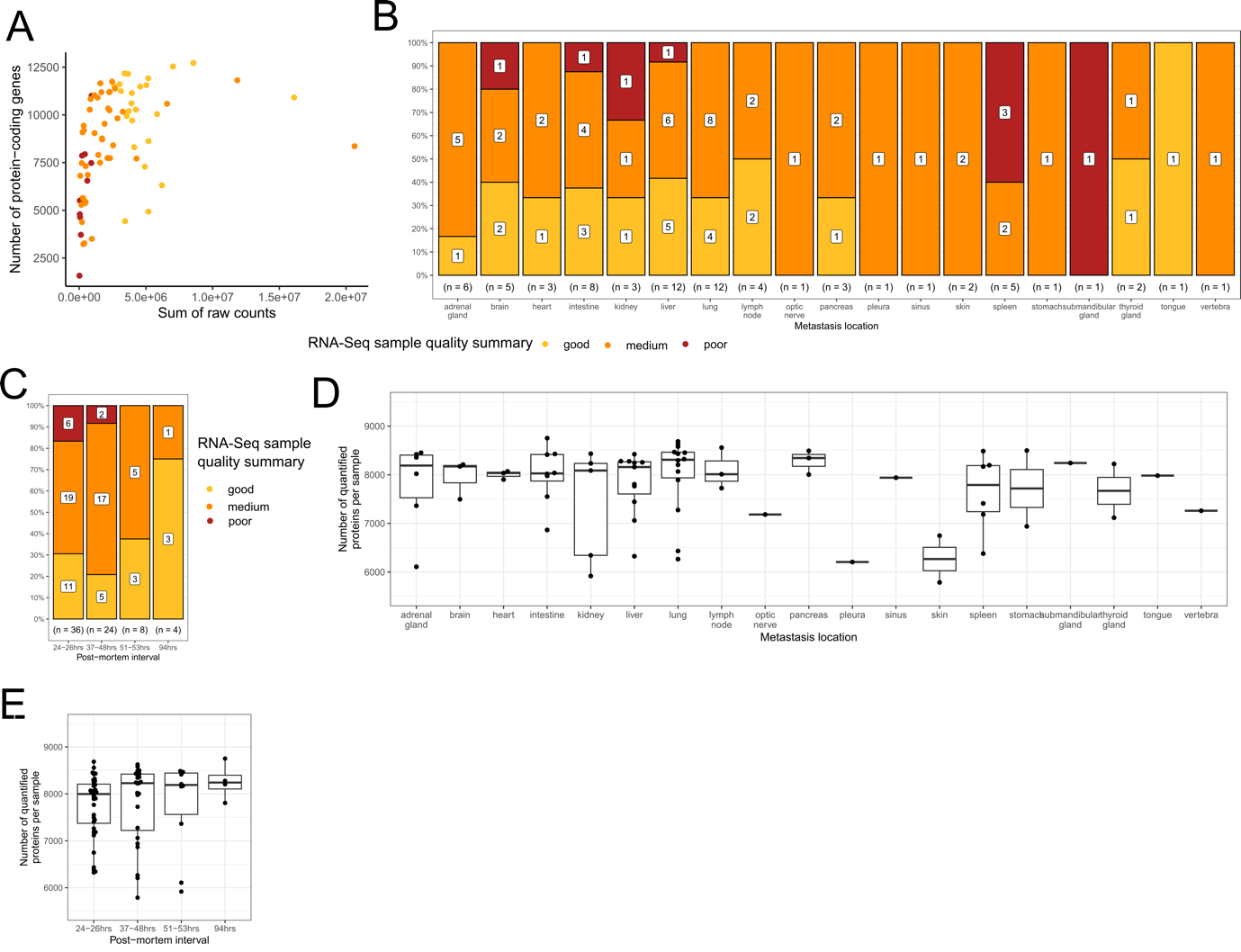


***Figure S2. RNA-Seq and proteomic data quality. A)*** *Number of detected protein-coding genes (y-axis) vs total raw read count (x-axis) per RNA-Seq sample. The samples are colored based on the RNA-Seq sample quality, which was created by summing up the “mRNA content” (percentage of total bases that originate from mRNA), “percentage of Assigned reads”, and “M Assigned reads” (Assigned reads in millions) parameters, as described in the Materials and Methods.* ***B)*** *Distribution of RNA-Seq samples of varying quality (poor, medium, good) across the different metastasis locations.* ***C)*** *Distribution of RNA-Seq samples of varying quality (poor, medium, good) across the different post-mortem interval (PMI) categories.* ***D)*** *Number of quantified proteins vs metastasis locations in the proteomic samples.* ***G)*** *Number of quantified proteins vs PMI categories in the proteomic samples.*

*
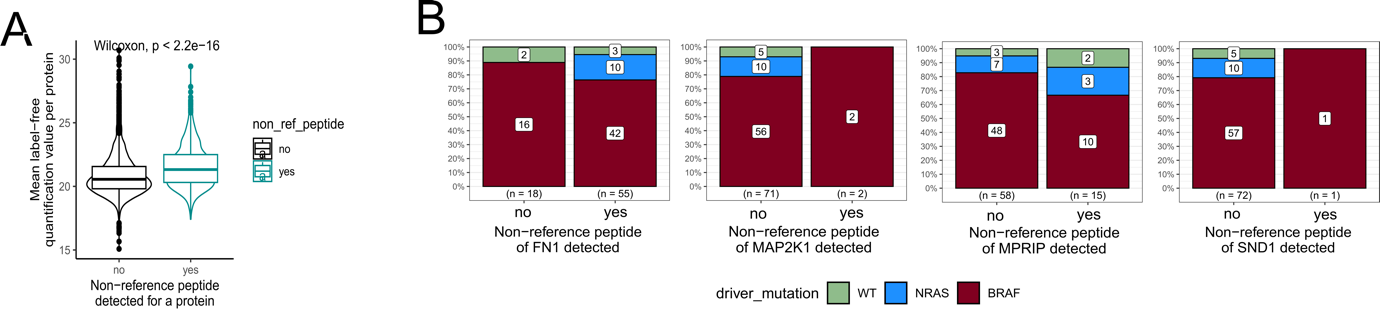
*

***Figure S3. Proteogenomic data of melanoma metastases. A)*** *Distribution of label-free quantities for proteins for which we did not detect NRPs vs for proteins with detected NRPs. The Wilcoxon test p-value is shown on the top.* ***B)*** *Distribution of BRAF/NRAS driver mutations across metastases in which NRPs from FN1, MAP2K1, MPRIP and SND1 were detected vs not detected.*


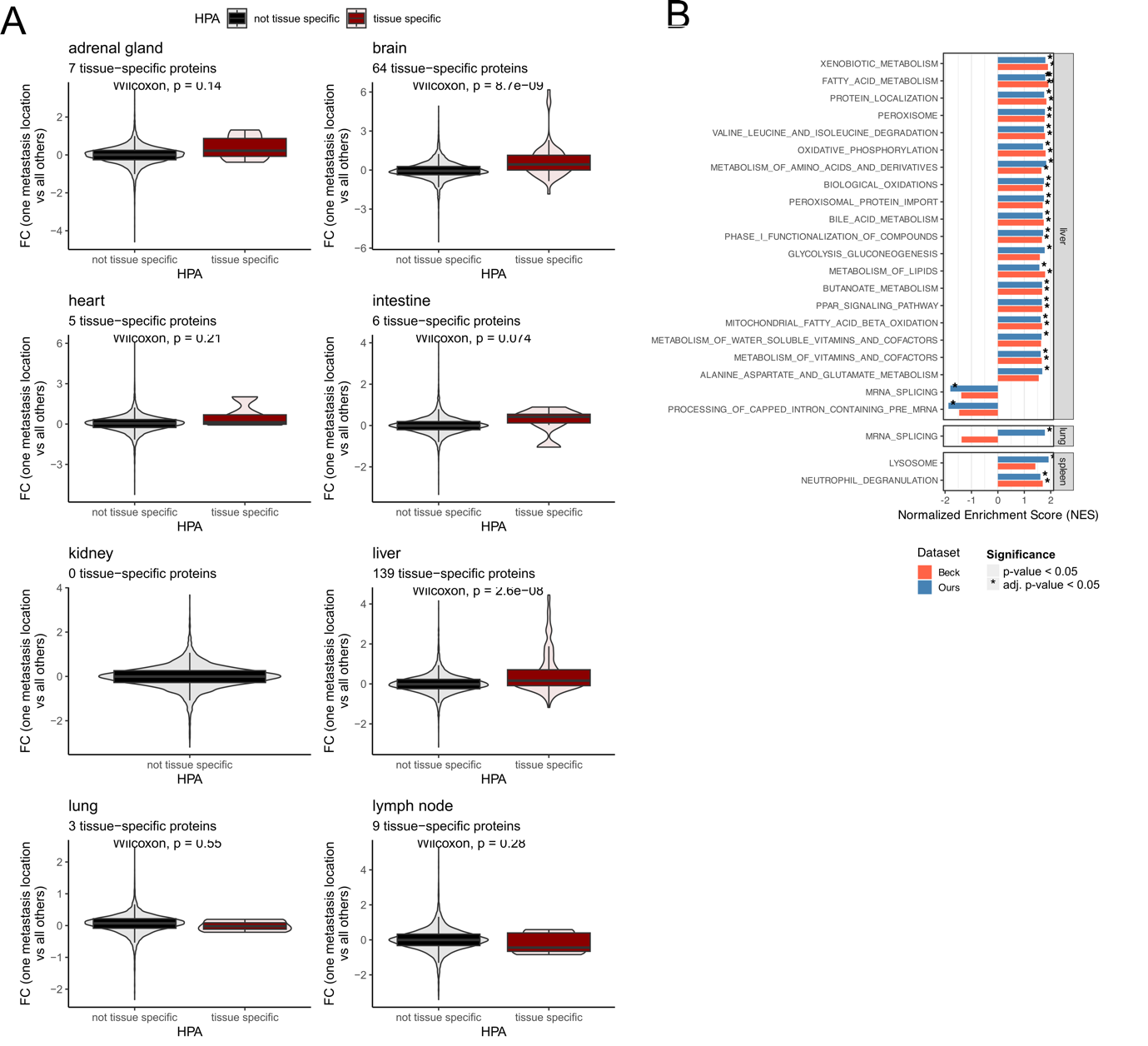


***Figure S4. Metastasis location-specific differences across melanoma metastases. A)*** *Boxplots showing the log_2_FC distribution of all proteins for one metastasis location vs all other metastasis location comparisons. The log_2_FC distributions of proteins with tissue-specific expression according to the HPA database vs proteins with no such specificity are compared. Wilcoxon test p-values are shown on the top.* ***B)*** *Pathways that were significant (adj. p<0.05) in our study for the comparisons related to one metastasis location vs all other locations and were also significant (p<0.05) in the Beck dataset. The bars are labeled with a star when results were significant with adj. p<0.05.*


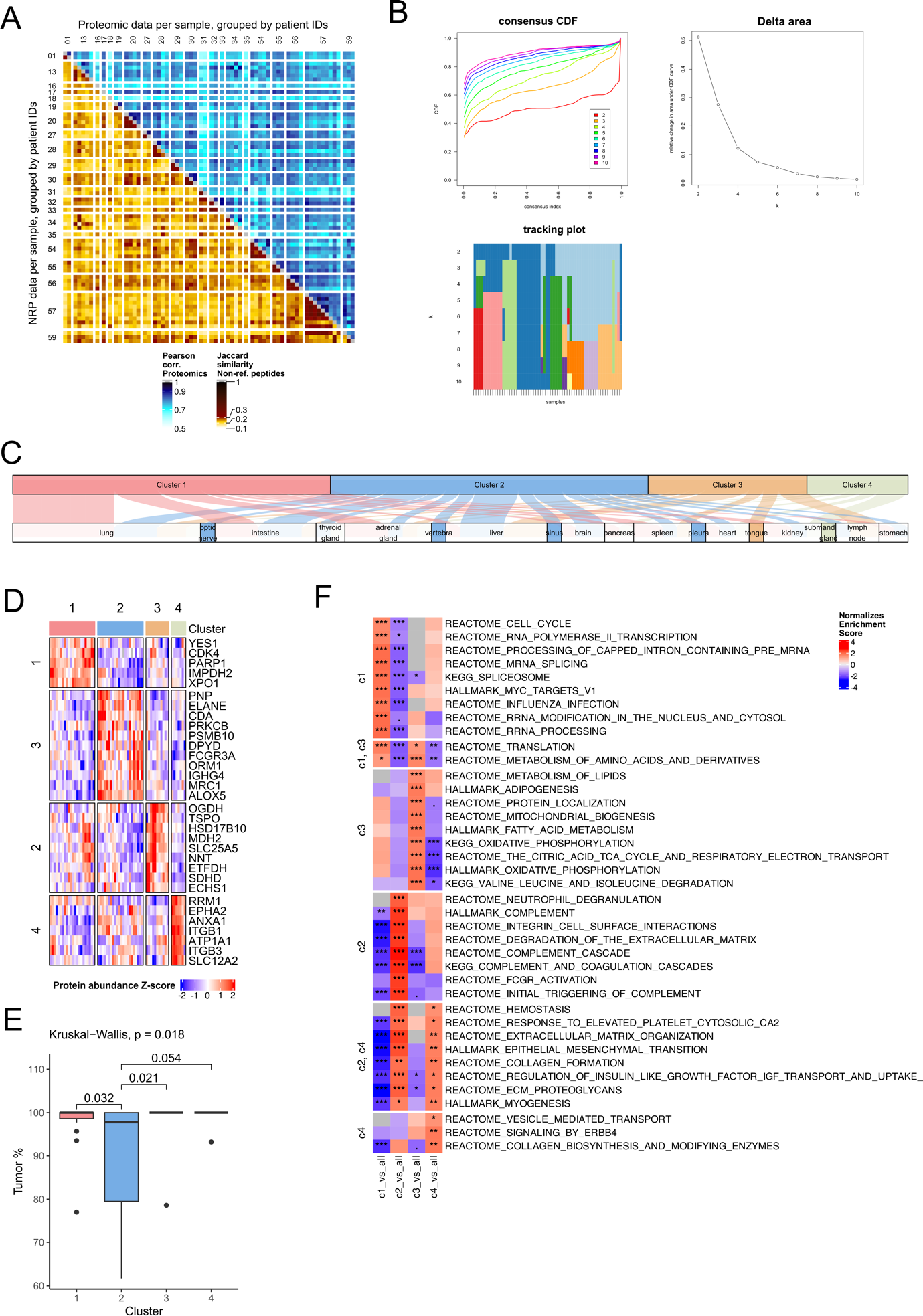


***Figure S5. Proteogenomic heterogeneity of melanoma metastases. A)*** *Pairwise similarities between metastases, grouped by patients. Upper triangle shows pairwise Pearson correlation coefficients calculated based on protein expression data, while the lower triangle shows pairwise Jaccard similarities based on the overlap of detected non-reference peptides. The gray diagonal line separates the two data types.* ***B)*** *Consensus clustering outputs, including consensus cumulative distribution function (CFD), delta area, and tracking plot for number of clusters (k) ranging between 2-10.* ***C)*** *Distribution of metastasis locations across the four proteomic clusters shown with an alluvial plot.* ***D)*** *Heatmap displaying the expression of proteins that were labeled as druggable based on the HPA database and also showed overexpression in one of the proteomic clusters vs the other proteomic clusters. E) Boxplot displaying differences in tumor content across the proteomic clusters. Kruskal-Wallis and pairwise Wilcoxon test p-values are shown on the top. F) Pre-ranked gene set enrichment analysis (GSEA) results for C1, C2, C3 or C4 vs all. For each comparison, the top 10 upregulated pathways (NES>0, adj. p<0.05) are shown, and are grouped based on their cluster-specificity.*
