# Supplementary material for: Distant metastases of melanoma exhibit varying extent of intrapatient proteogenomic heterogeneity
Source: Clin Transl Med. 2025 Sep 28;15(10):e70477. doi: 10.1002/ctm2.70477 (PMC12477065; doi:10.1002/ctm2.70477)
Supplement: Supplementary file 2 — Supporting Information [file CTM2-15-e70477-s003.pdf]

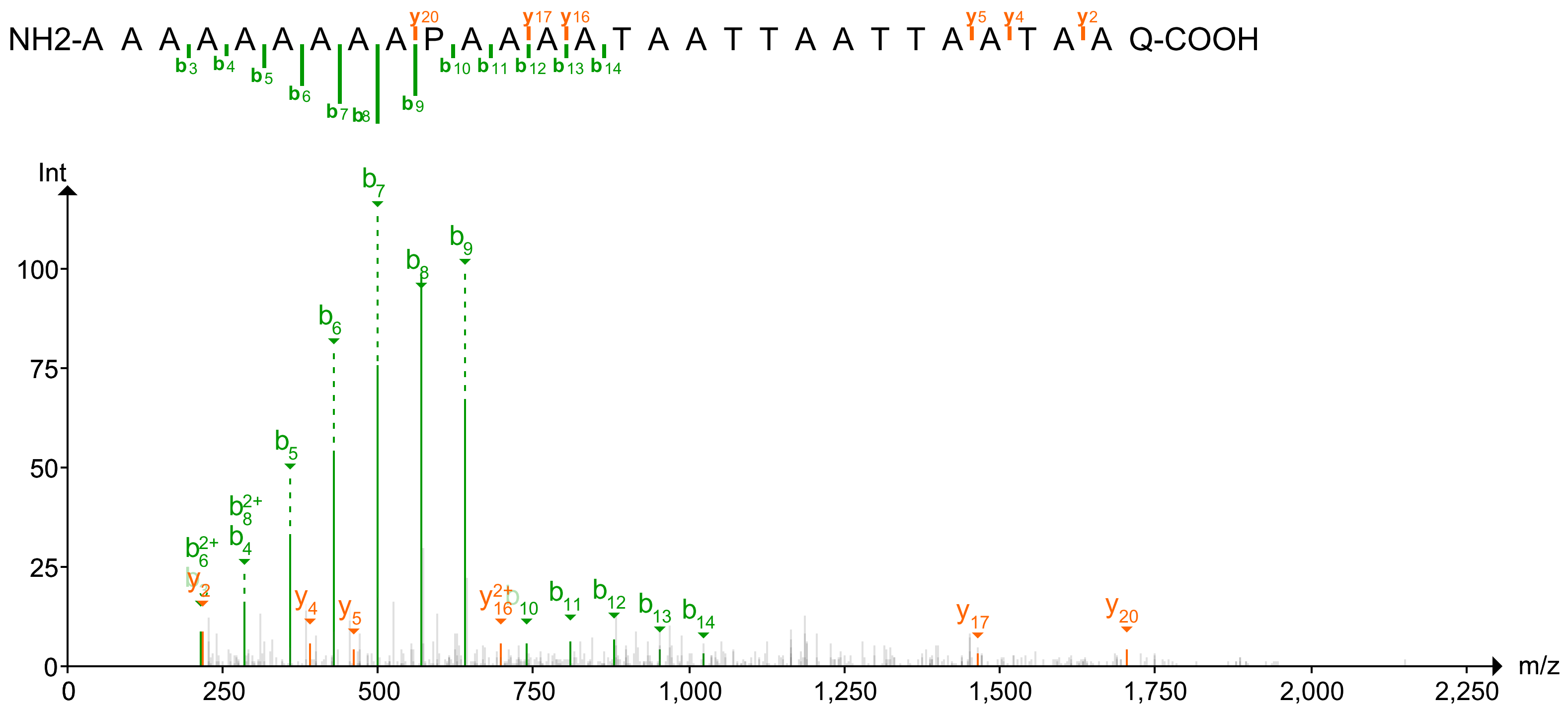

NH2-A A A A A A A A A P A A A A T A A T T A A T T A A T A A Q-COOH
y20 y17 y16 y5 y4 y2
b3 b4 b5 b6 b7 b8 b9 b10 b11 b12 b13 b14
Int
m/z
0 25 50 75 100
0 250 500 750 1,000 1,250 1,500 1,750 2,000 2,250
b7 b9 b8 b6 b5 b8 2+ b4 b6 2+ y2 y4 y5 y16 2+ b10 b11 b12 b13 b14 y17 y20

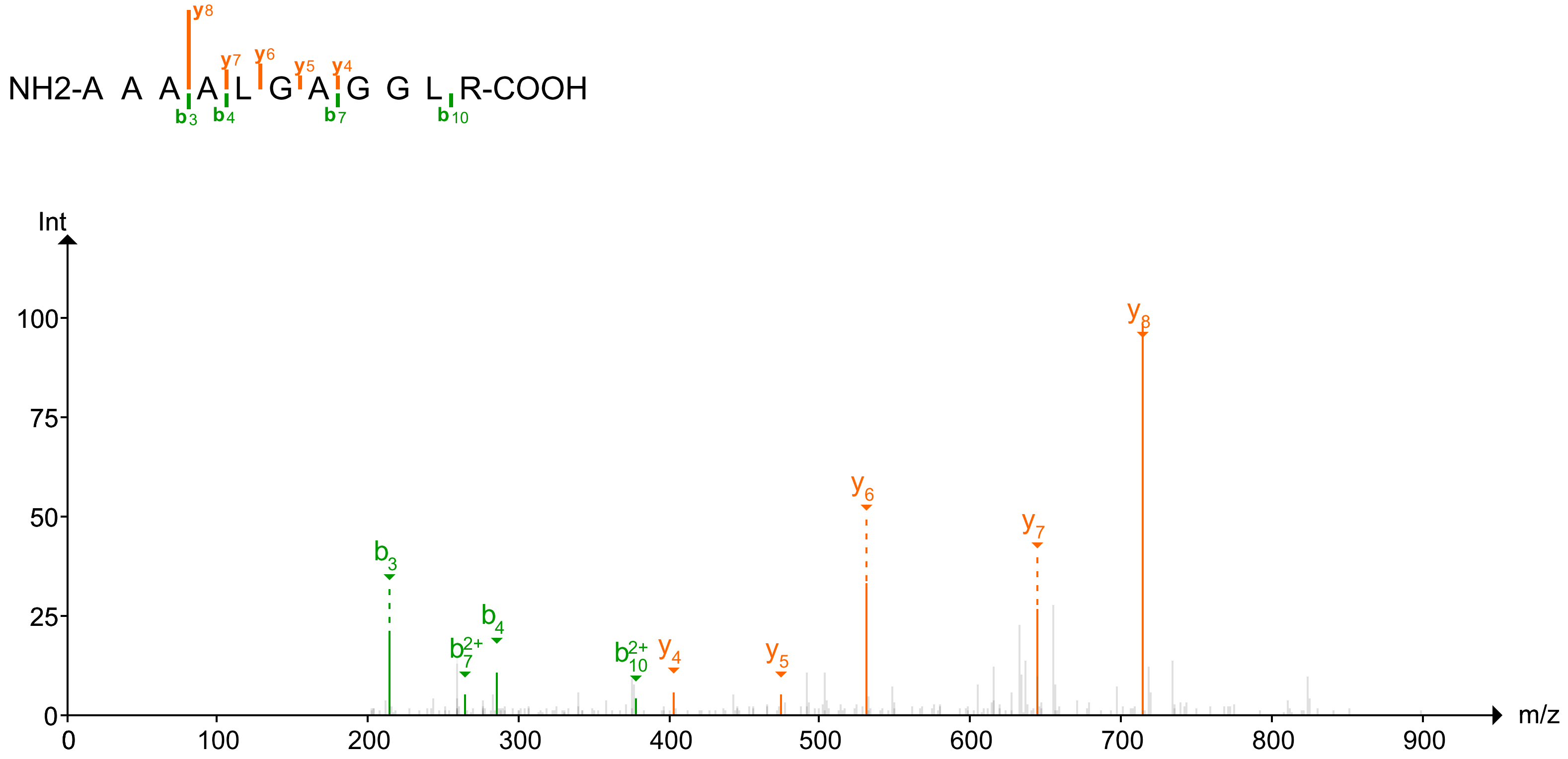

NH2-A A A A L G A G G L R-COOH
y8
y7
y6
y5
y4
b3
b4
b7
b10
Int
100
75
50
25
0
m/z
0
100
200
300
400
500
600
700
800
900
b3
b4
b7 2+
b10 2+
y4
y5
y6
y7
y8

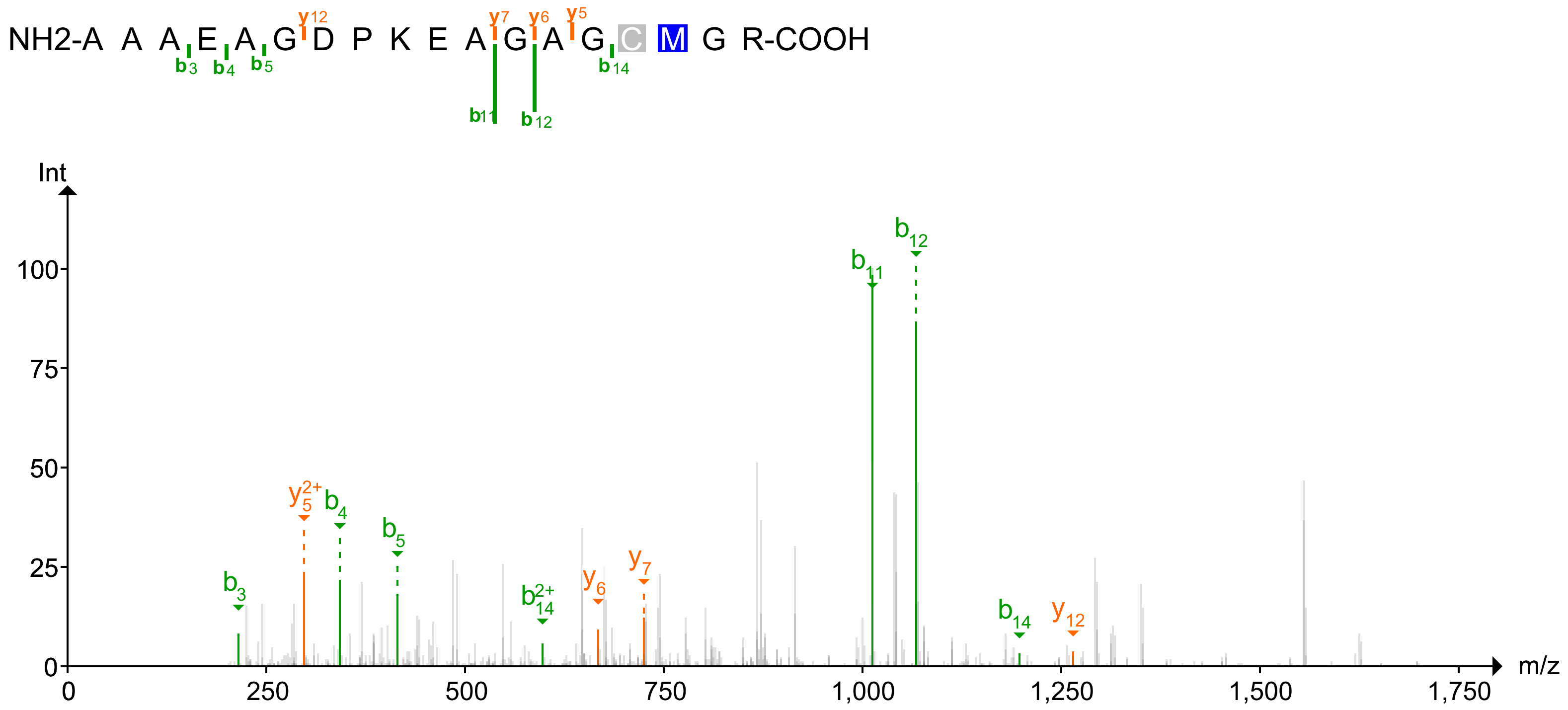
NH2-A A A E A G D P K E A G A G C M G R-COOH
y12
y7
y6
y5
b3
b4
b5
b11
b12
b14
Int
100
75
50
25
0
m/z
0
250
500
750
1,000
1,250
1,500
1,750
$y_5^{2+}$
$b_3$
$b_4$
$b_5$
$b_{14}^{2+}$
$y_6$
$y_7$
$b_{11}$
$b_{12}$
$b_{14}$
$y_{12}$

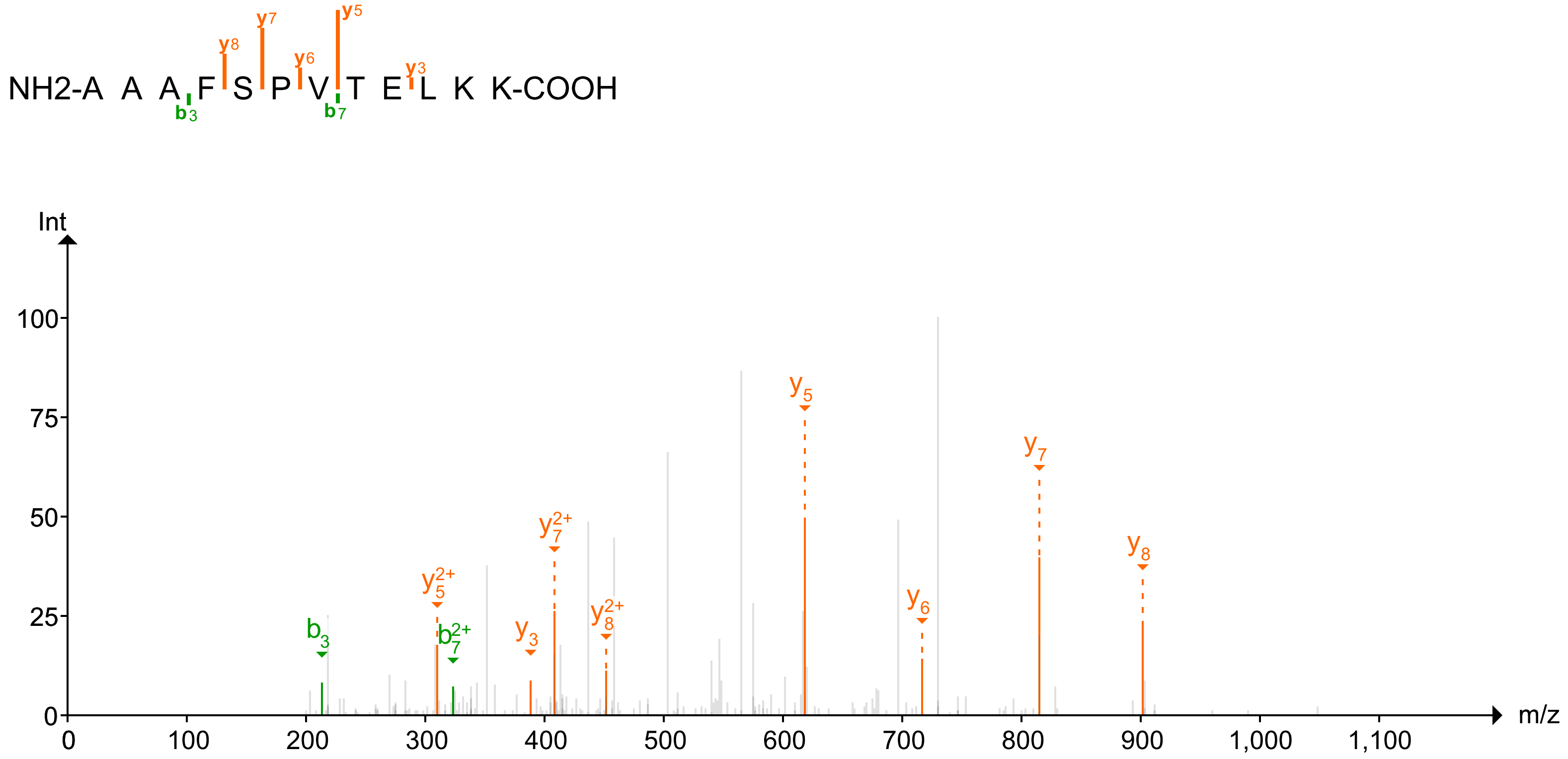

NH2-A A A F S P V T E L K K-COOH
y8 y7 y6 y5 y3
b3 b7
Int
100
75
50
25
0
m/z
0 100 200 300 400 500 600 700 800 900 1,000 1,100
y5
y7
y8
y6
y7 2+
y5 2+
y8 2+
y3
b3
b7 2+

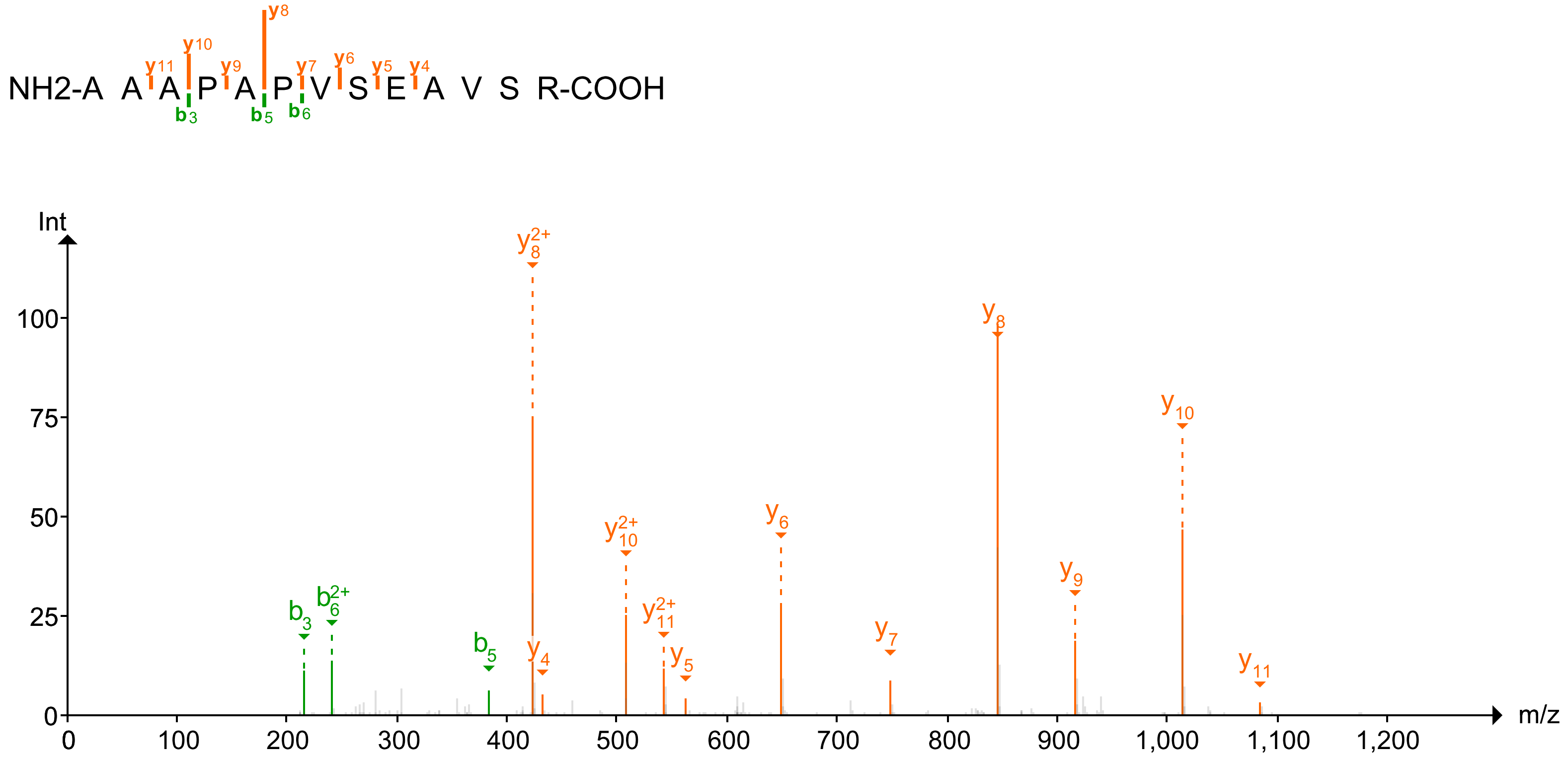

NH2-A A A P A P V S E A V S R-COOH
y11 y10 y9 y8 y7 y6 y5 y4
b3 b5 b6
Int
m/z
0 25 50 75 100
0 100 200 300 400 500 600 700 800 900 1,000 1,100 1,200
b3
b6 2+
b5
y8 2+
y4
y10 2+
y11 2+
y5
y6
y7
y8
y9
y10
y11

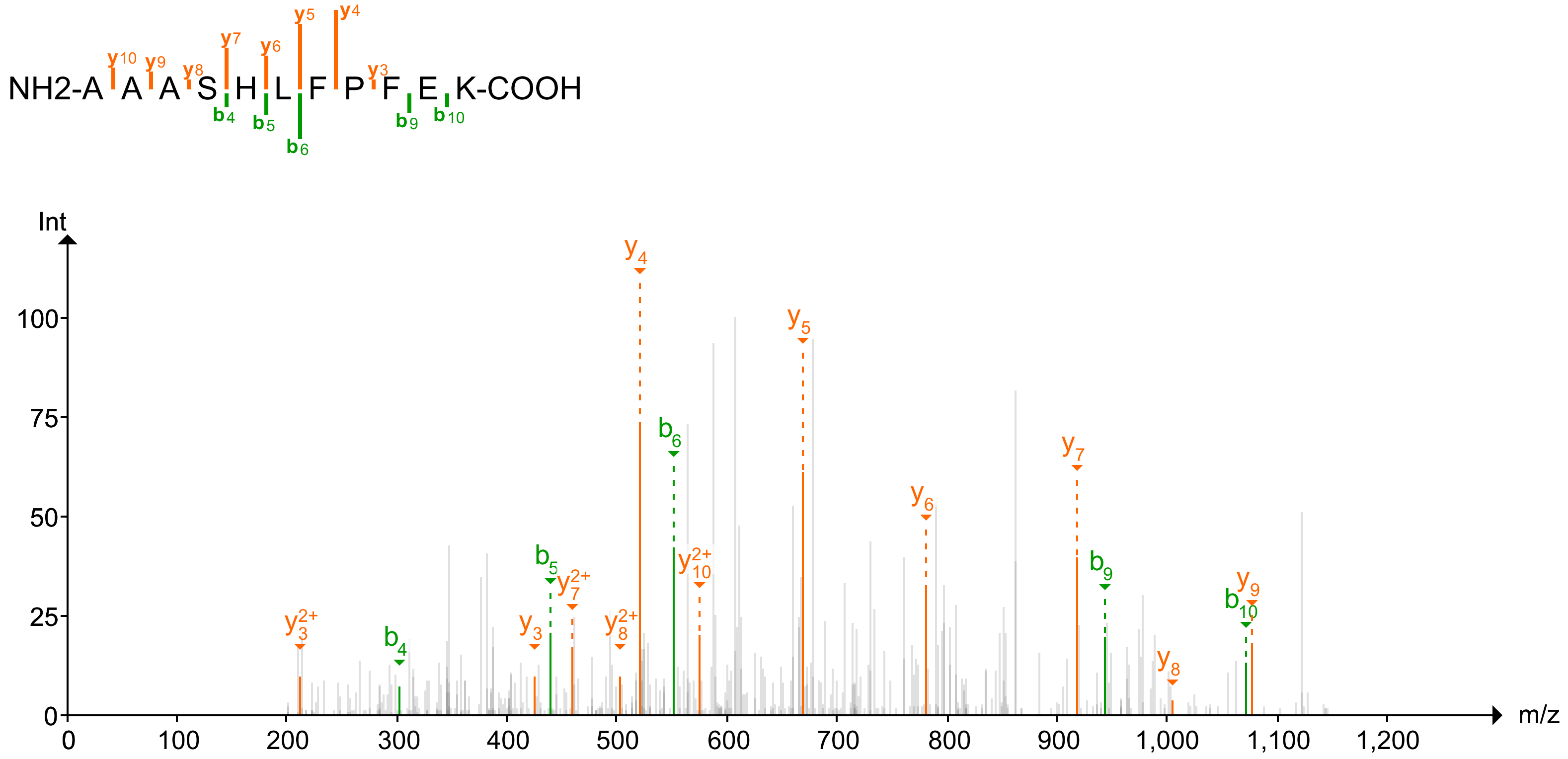

NH2-A A A S H L F P F E K-COOH
y10 y9 y8 y7 y6 y5 y4 y3
b4 b5 b6 b9 b10
Int
100
75
50
25
0
0 100 200 300 400 500 600 700 800 900 1,000 1,100 1,200
m/z
y4 y5 b6 y7 y6 b5 y7 2+ y10 2+ b9 y9 b10 y3 2+ b4 y3 y8 2+ y8

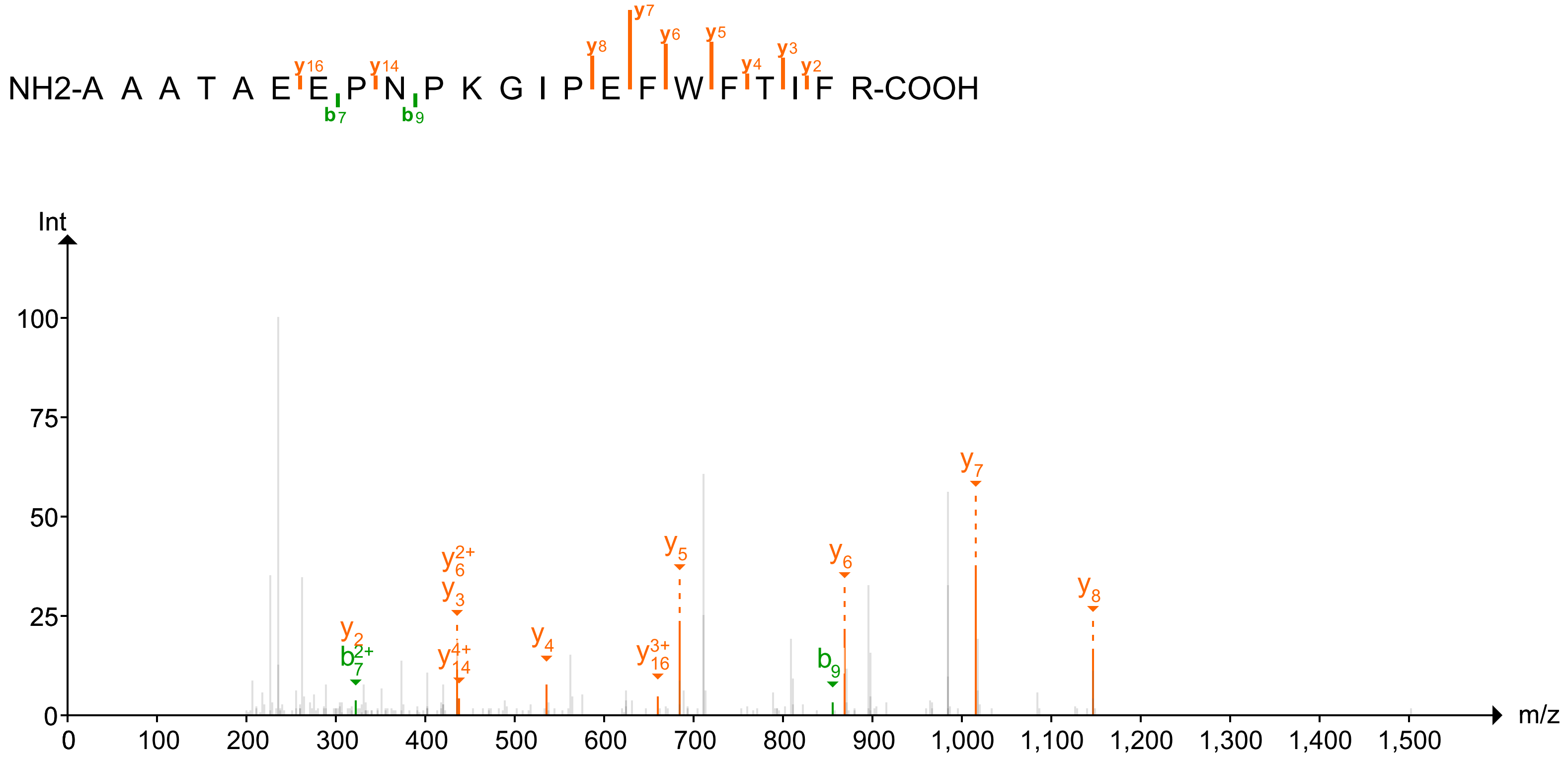

NH2-A A A T A E E P N P K G I P E F W F T I F R-COOH
y16 y14 y8 y7 y6 y5 y4 y3 y2
b7 b9
Int
100
75
50
25
0
y2
b7 2+
y6 2+
y3
y14 4+
y4
y16 3+
y5
b9
y6
y7
y8
m/z
0 100 200 300 400 500 600 700 800 900 1,000 1,100 1,200 1,300 1,400 1,500

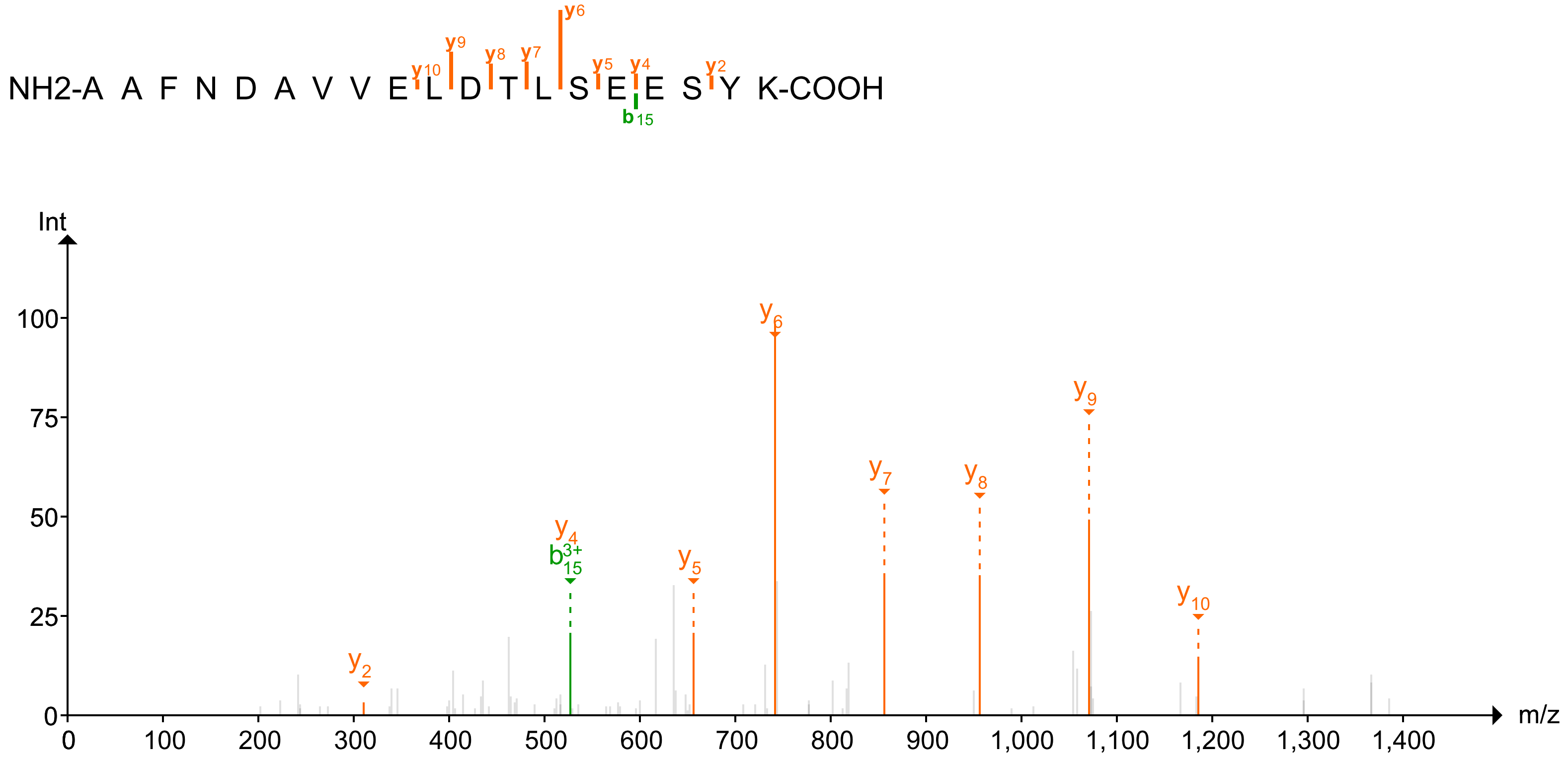

NH2-A A F N D A V V E L D T L S E E S Y K-COOH
y10 y9 y8 y7 y6 y5 y4 y2
b15
Int
100
75
50
25
0
y2
y4
b3+ 15
y5
y6
y7
y8
y9
y10
0 100 200 300 400 500 600 700 800 900 1,000 1,100 1,200 1,300 1,400
m/z

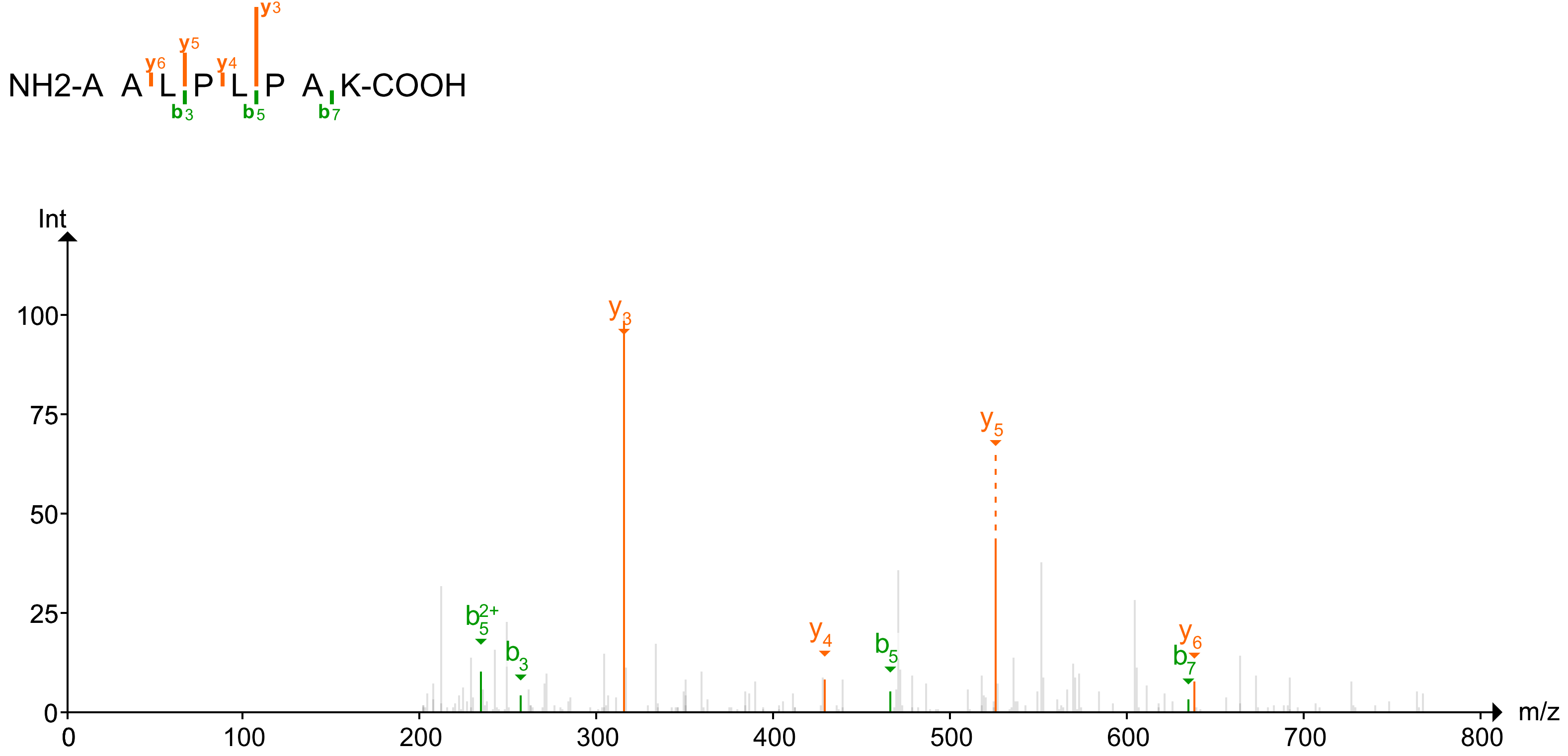

NH2-A A L P L P A K-COOH
y6 y5 y4 y3
b3 b5 b7
Int
100
75
50
25
0
m/z
0
100
200
300
400
500
600
700
800
y3
y5
y4
y6
b5 2+
b3
b5
b7

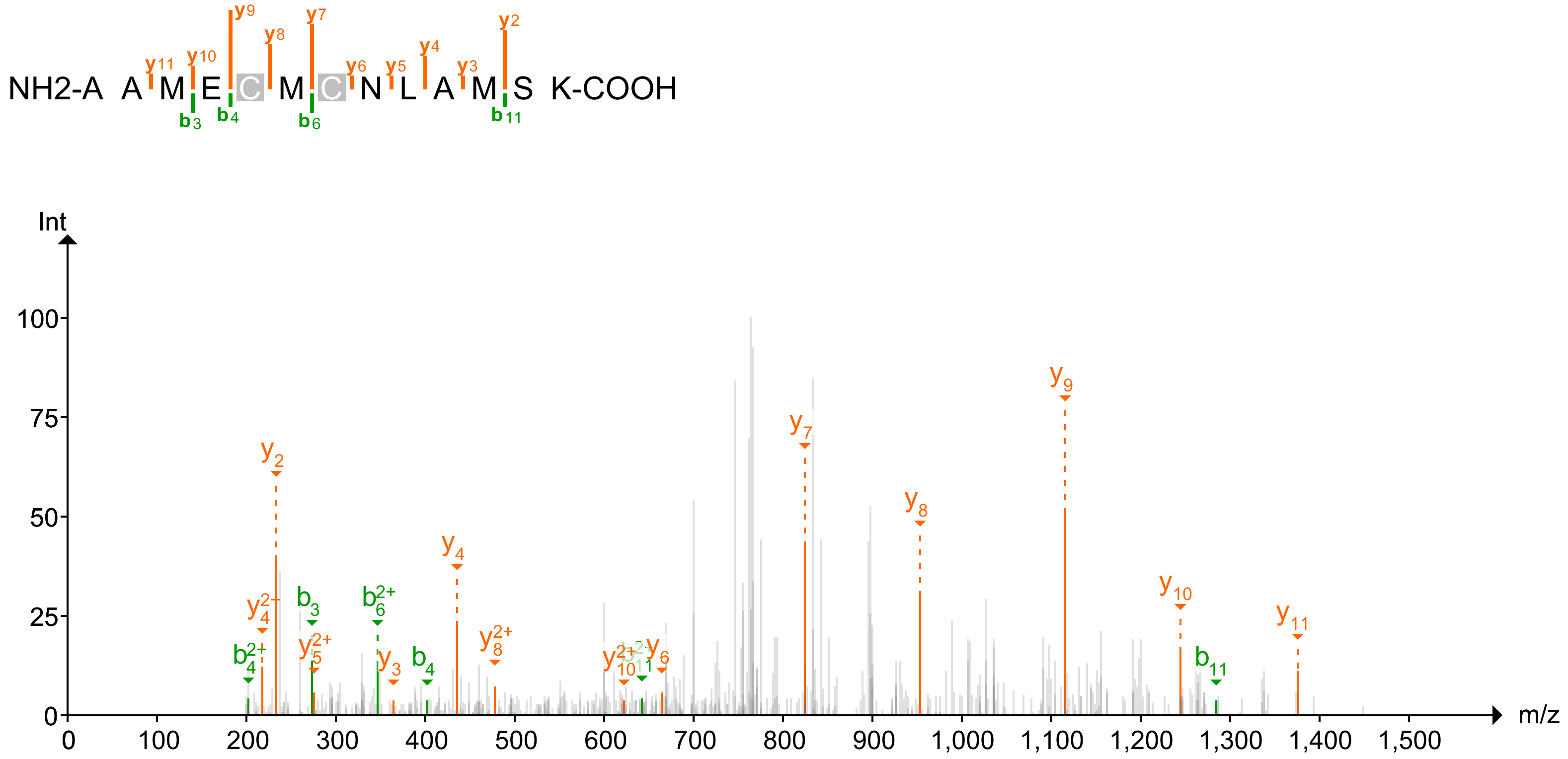

NH2-A A M E C M C N L A M S K-COOH
y11 y10 y9 y8 y7 y6 y5 y4 y3 y2
b3 b4 b6 b11
Int
100
75
50
25
0
m/z
0 100 200 300 400 500 600 700 800 900 1,000 1,100 1,200 1,300 1,400 1,500
y2 y4 y7 y8 y9 y10 y11 y3 y6
$y_4^{2+}$ $y_5^{2+}$ $y_8^{2+}$ $y_{10}^{2+}$
b3 b4 b11 $b_4^{2+}$ $b_6^{2+}$ $b_{11}^{2+}$

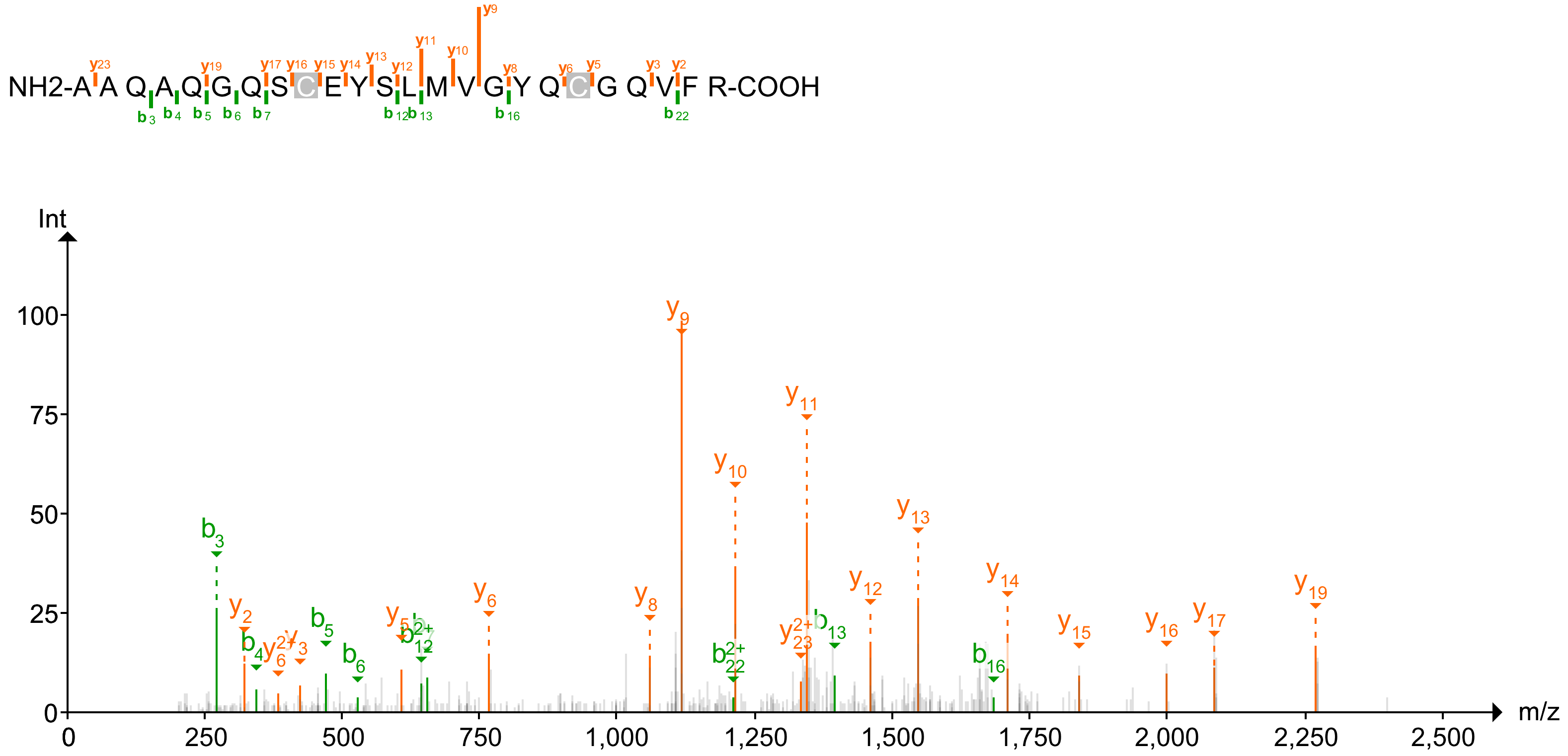

NH2-A A Q A Q G Q S C E Y S L M V G Y Q C G Q V F R-COOH
y23 y19 y17 y16 y15 y14 y13 y12 y11 y10 y9 y8 y6 y5 y3 y2
b3 b4 b5 b6 b7 b12 b13 b16 b22
Int
100
75
50
25
0
m/z
0
250
500
750
1,000
1,250
1,500
1,750
2,000
2,250
2,500
y9
y11
y10
y13
y14
y12
y19
y17
y16
y15
b3
y2
b4
y6 2+
y3
b5
b6
y5
b12 2+
y6
y8
b22 2+
y23 2+
b13
b16

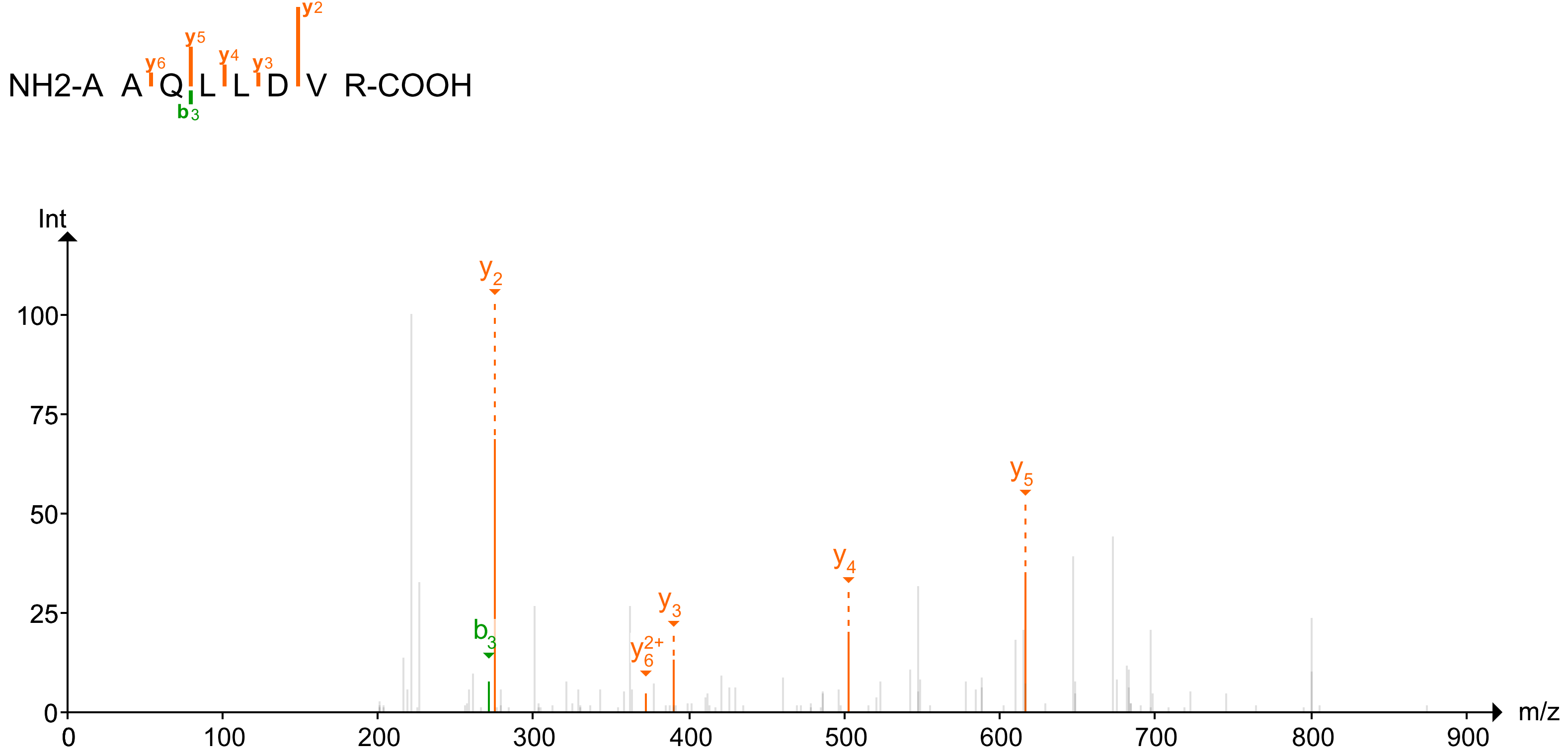

NH2-A A Q L L D V R-COOH
y2
y5
y6
y4
y3
b3
Int
100
75
50
25
0
y2
y5
y4
y3
y6 2+
b3
0
100
200
300
400
500
600
700
800
900
m/z

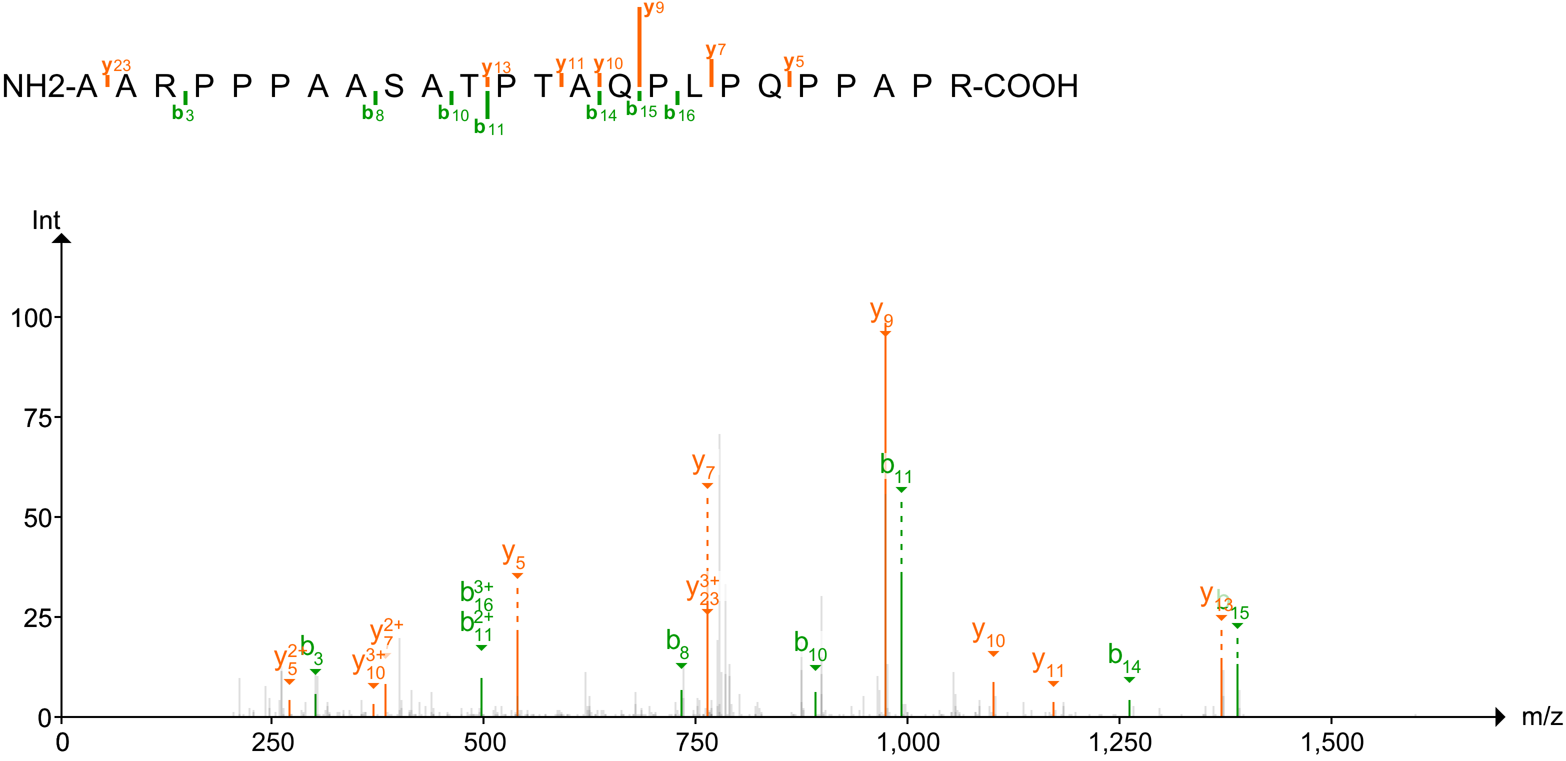

NH2-A A R P P P A A S A T P T A Q P L P Q P P A P R-COOH
y23 y13 y11 y10 y9 y7 y5
b3 b8 b10 b11 b14 b15 b16
Int
100
75
50
25
0
250
500
750
1,000
1,250
1,500
m/z
y9
b11
y7
y5
y23 3+
b16 3+
b11 2+
y7 2+
y5 2+
b3
y10 3+
b8
b10
y10
y11
b14
y13
b15

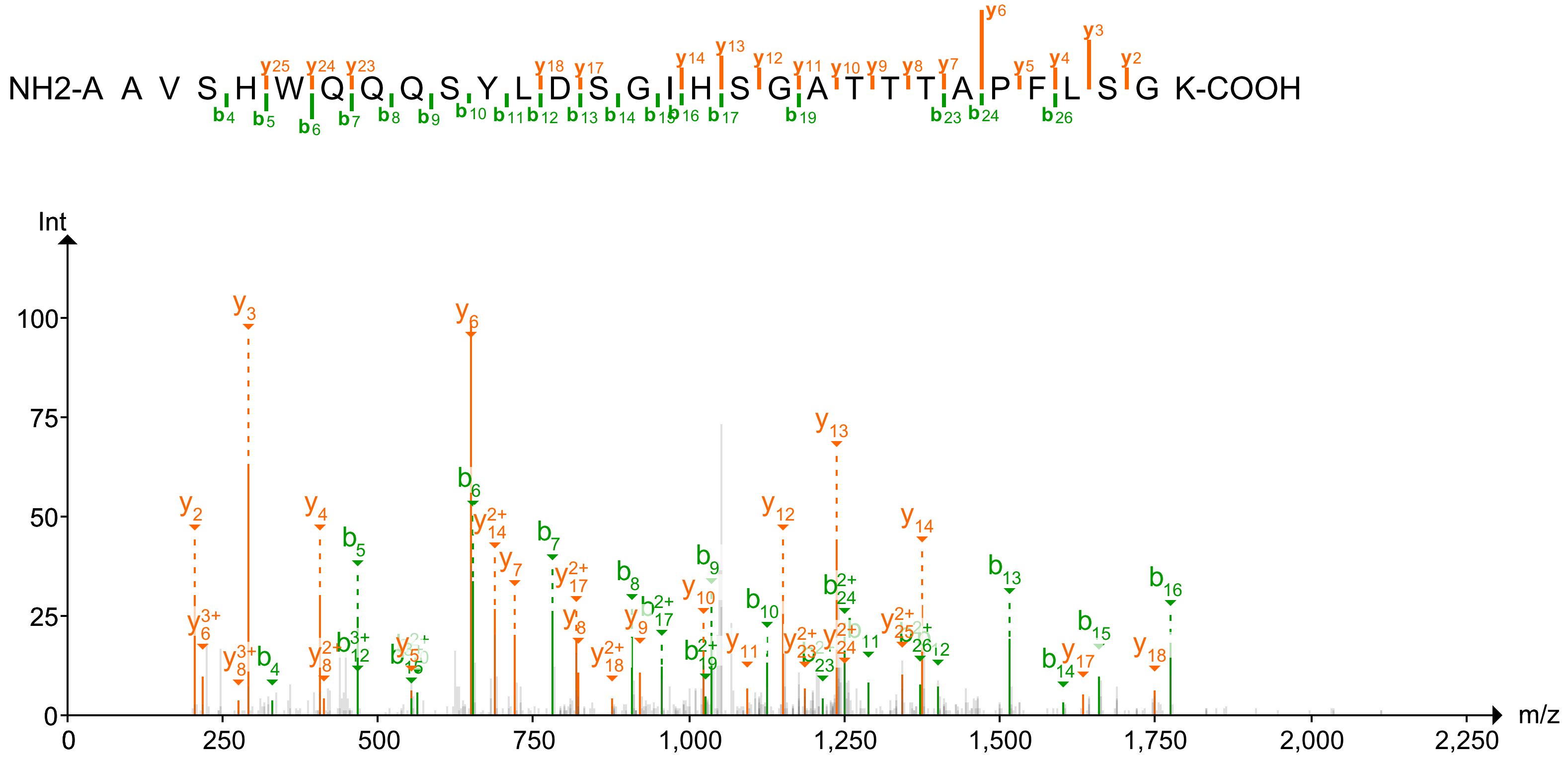

NH2-A A V S H W Q Q Q S Y L D S G I H S G A T T T A P F L S G K-COOH
Int
100
75
50
25
0
m/z
0
250
500
750
1,000
1,250
1,500
1,750
2,000
2,250

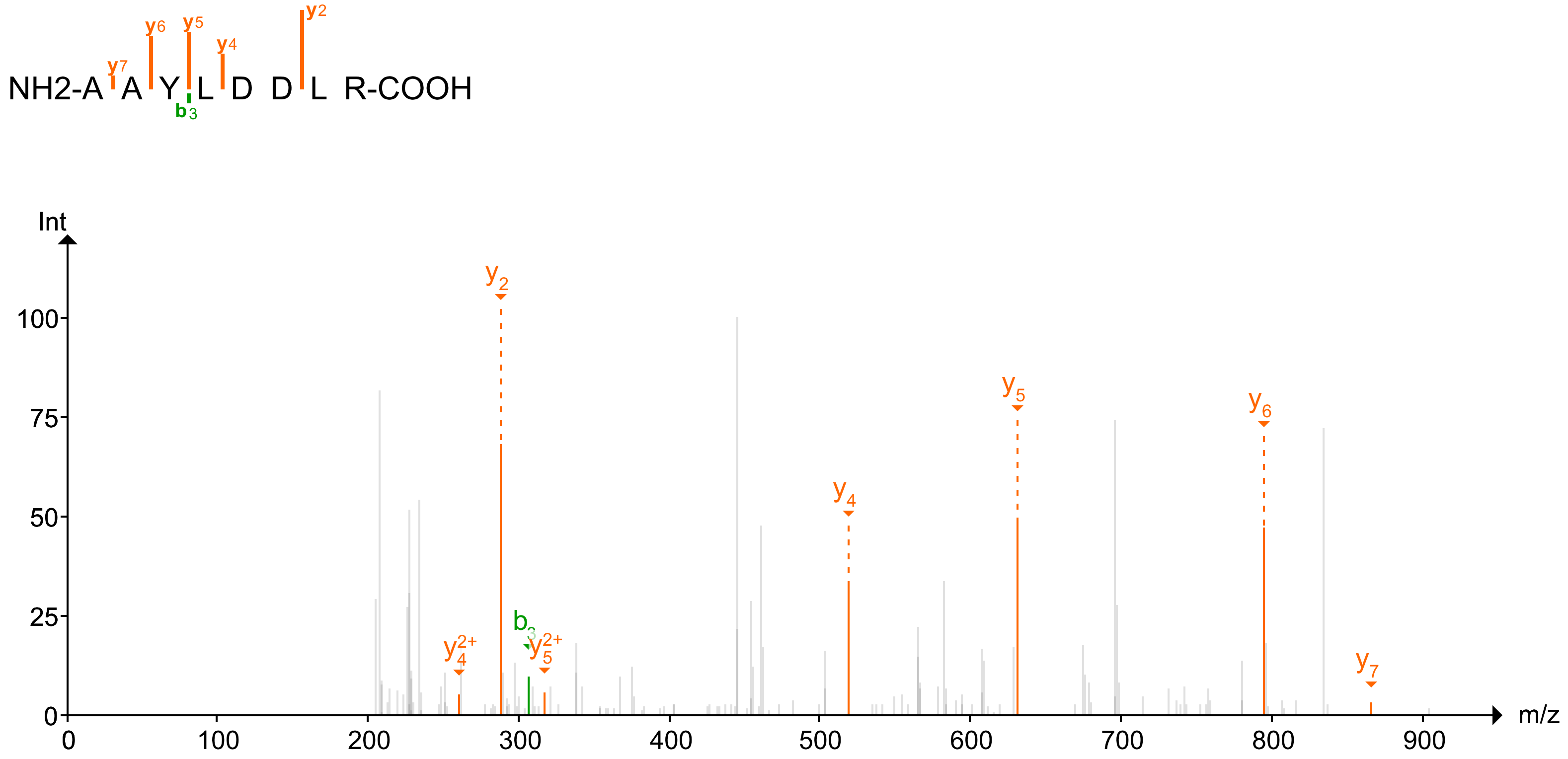

NH2-A A Y L D D L R-COOH
y7 y6 y5 y4 y2 b3
Int
100
75
50
25
0
y2
y5
y6
y4
y7
$y_4^{2+}$
$y_5^{2+}$
b3
0 100 200 300 400 500 600 700 800 900
m/z

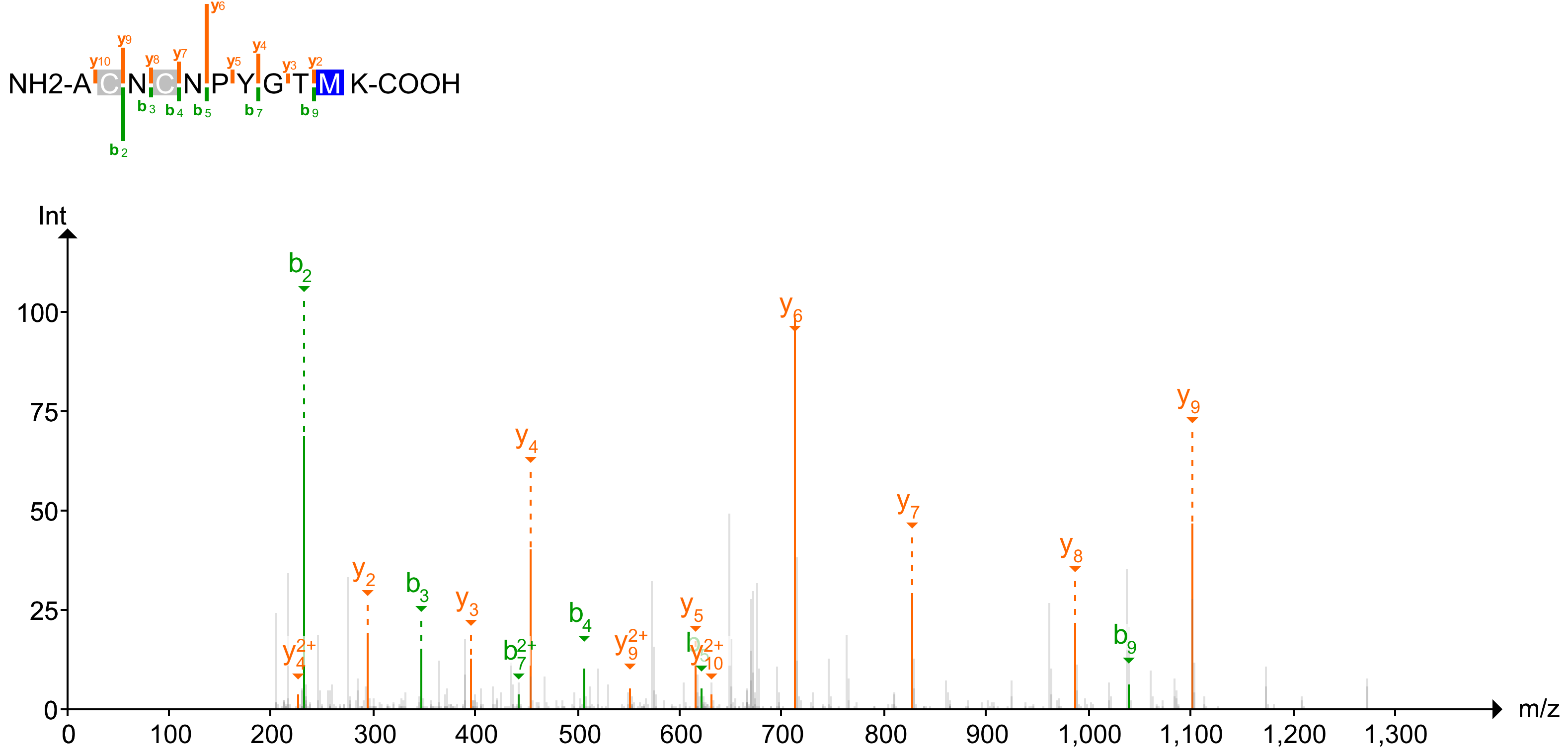

NH2-ACNCNPYGTMK-COOH
Int
m/z
b2
y6
y9
y4
y7
y8
y2
b3
y3
b4
y5
b9
0
25
50
75
100
0
100
200
300
400
500
600
700
800
900
1,000
1,100
1,200
1,300

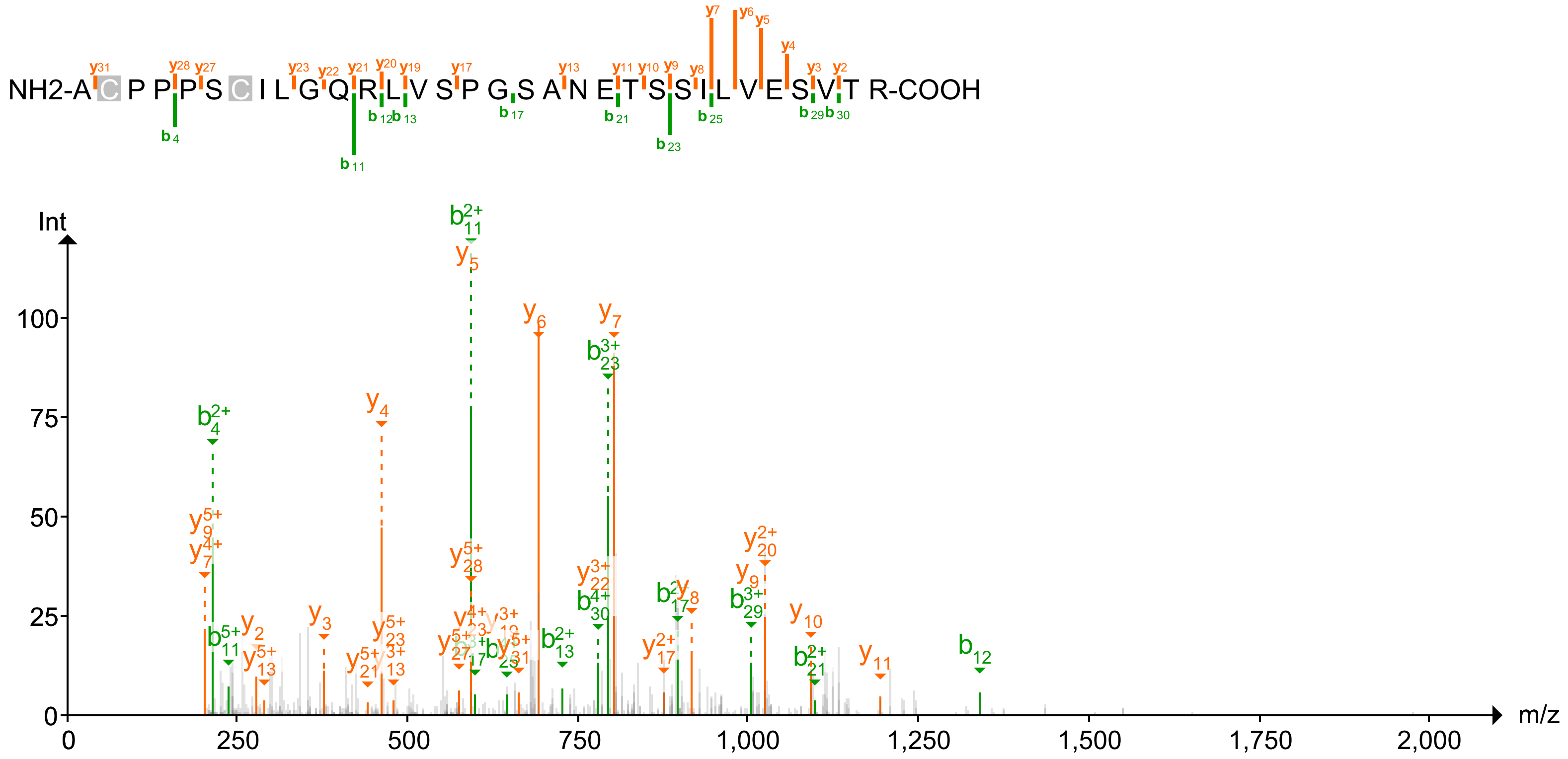

NH2-A C P P P S C I L G Q R L V S P G S A N E T S S I L V E S V T R-COOH
Int
m/z
0
25
50
75
100
0
250
500
750
1,000
1,250
1,500
1,750
2,000

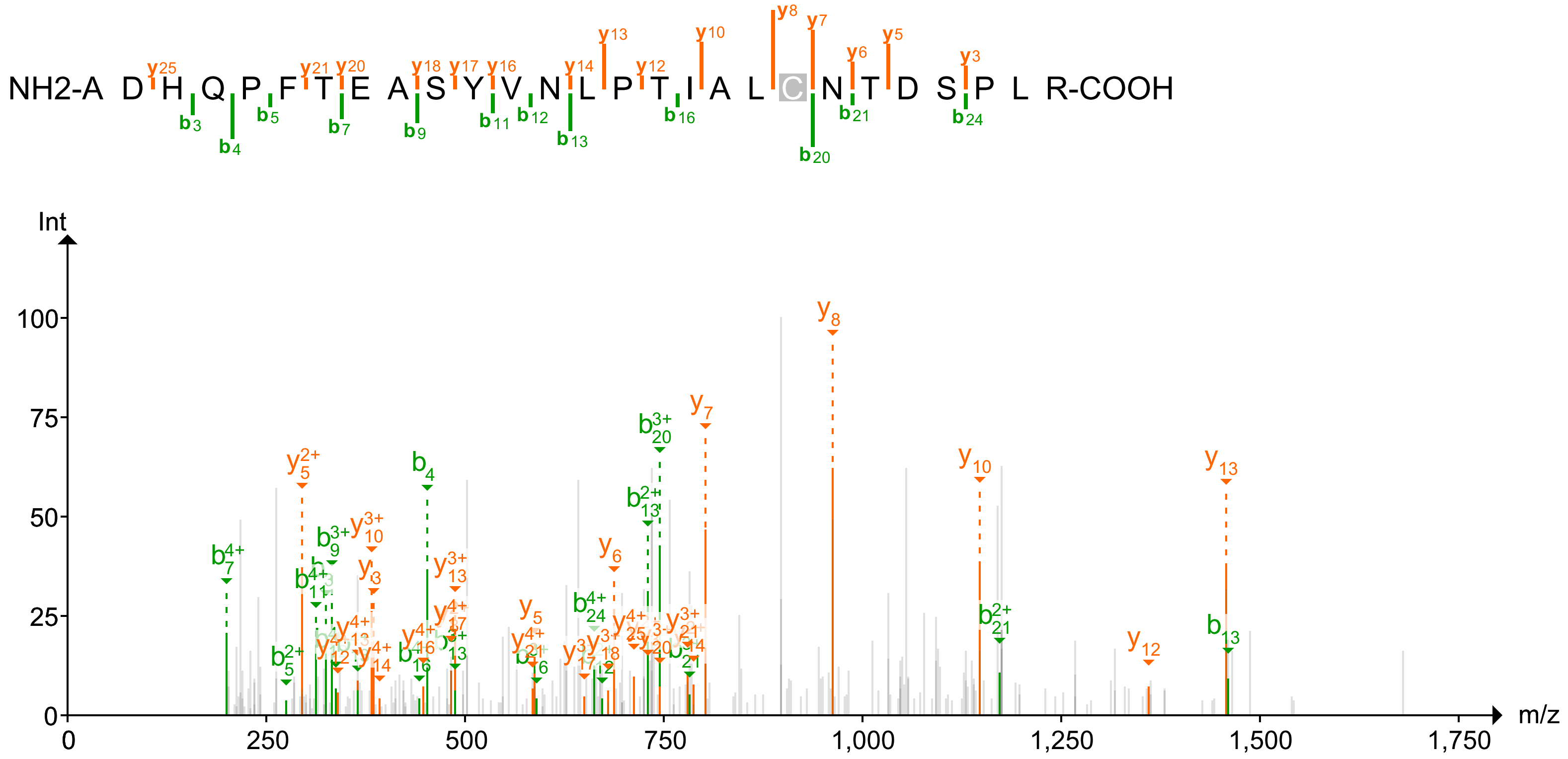

NH2-A D H Q P F T E A S Y V N L P T I A L C N T D S P L R-COOH
Int
m/z
0
25
50
75
100
250
500
750
1,000
1,250
1,500
1,750

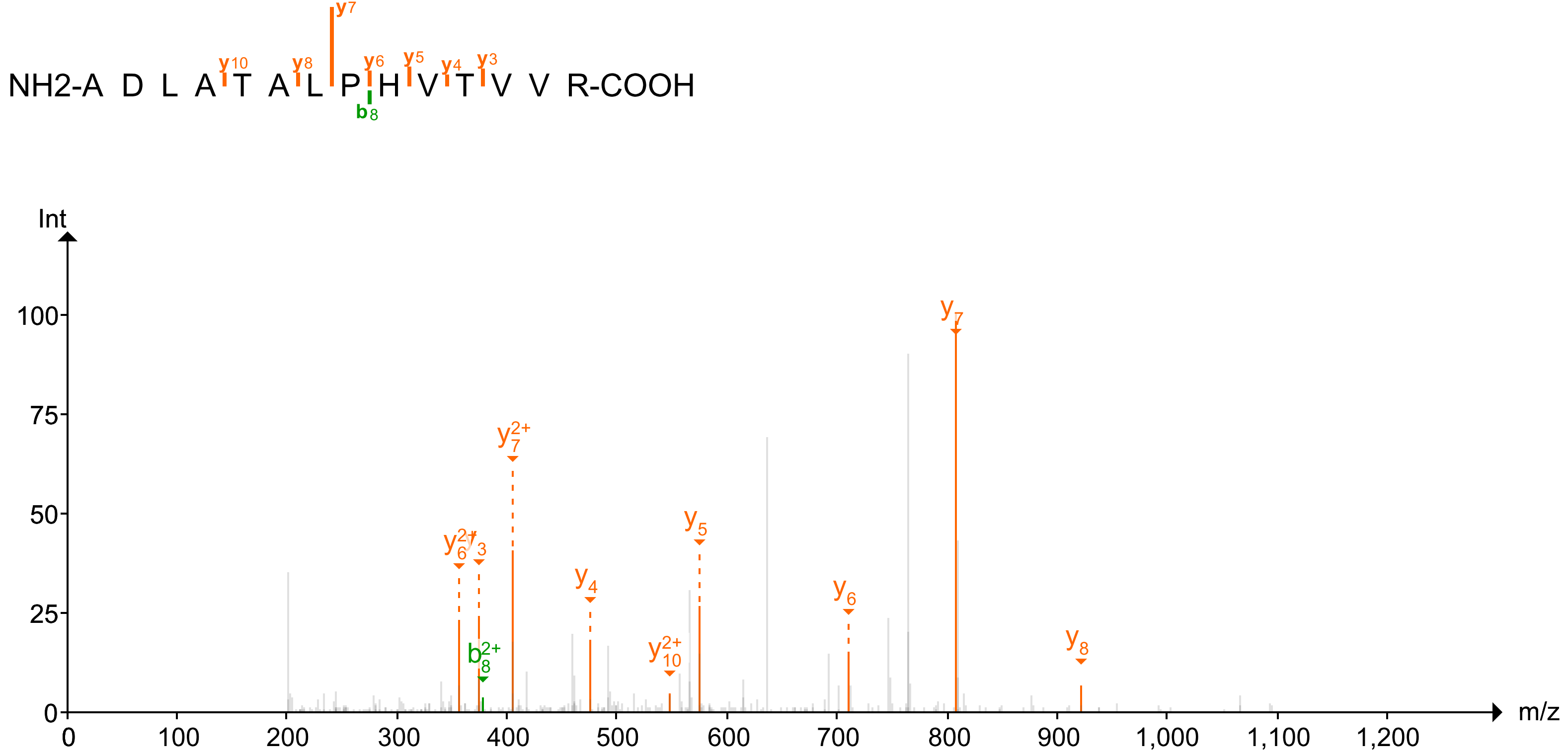

NH2-A D L A T A L P H V T V V R-COOH
y10 y8 y7 y6 y5 y4 y3
b8
Int
100
75
50
25
0
m/z
0 100 200 300 400 500 600 700 800 900 1,000 1,100 1,200
y7
y7 2+
y6 2+
y3
y4
y5
y6
y8
y10 2+
b8 2+

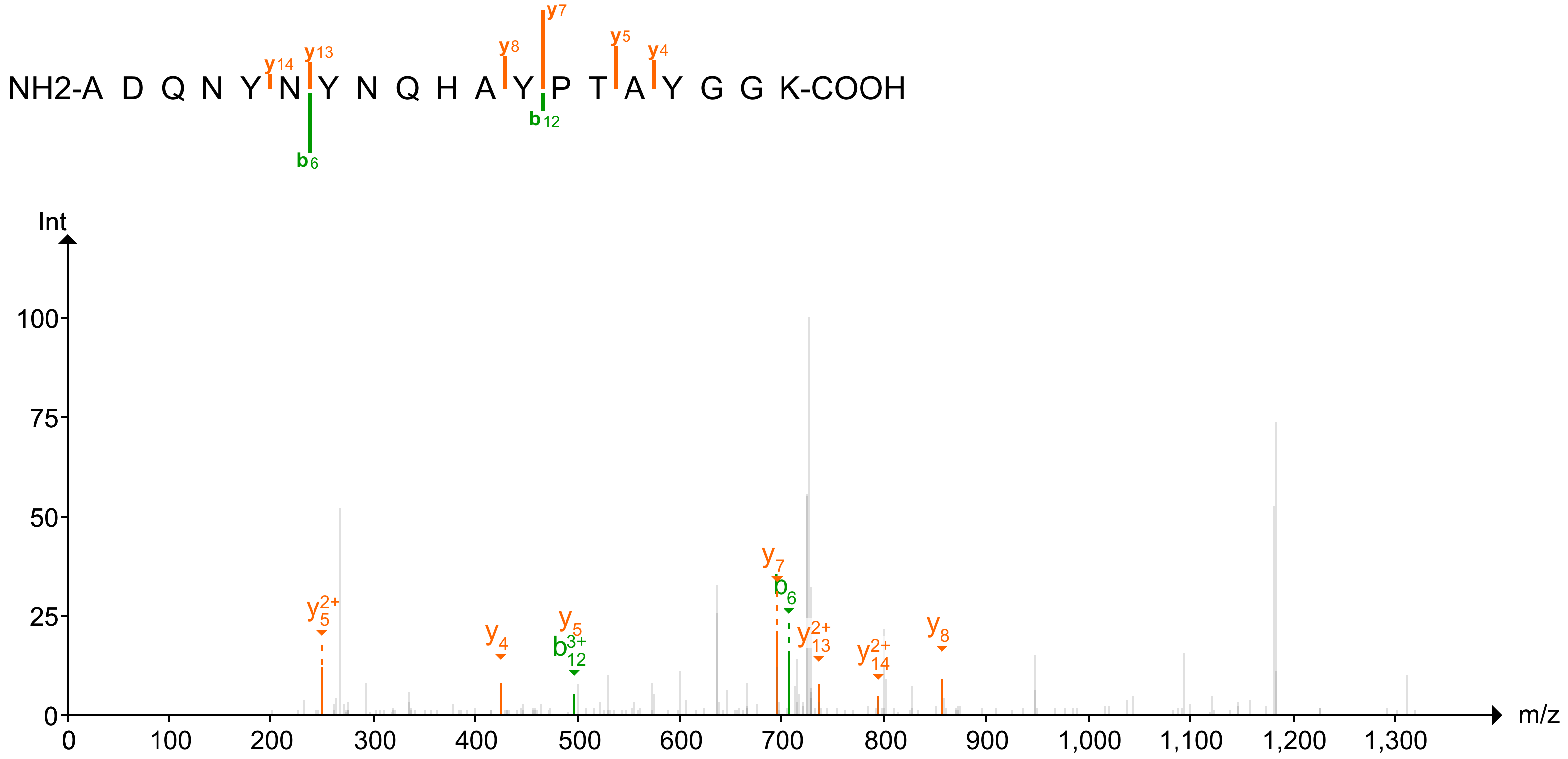

NH2-A D Q N Y N Y N Q H A Y P T A Y G G K-COOH
y14
y13
y8
y7
y5
y4
b6
b12
Int
100
75
50
25
0
m/z
0
100
200
300
400
500
600
700
800
900
1,000
1,100
1,200
1,300
y5 2+
y4
y5
b12 3+
y7
b6
y13 2+
y14 2+
y8

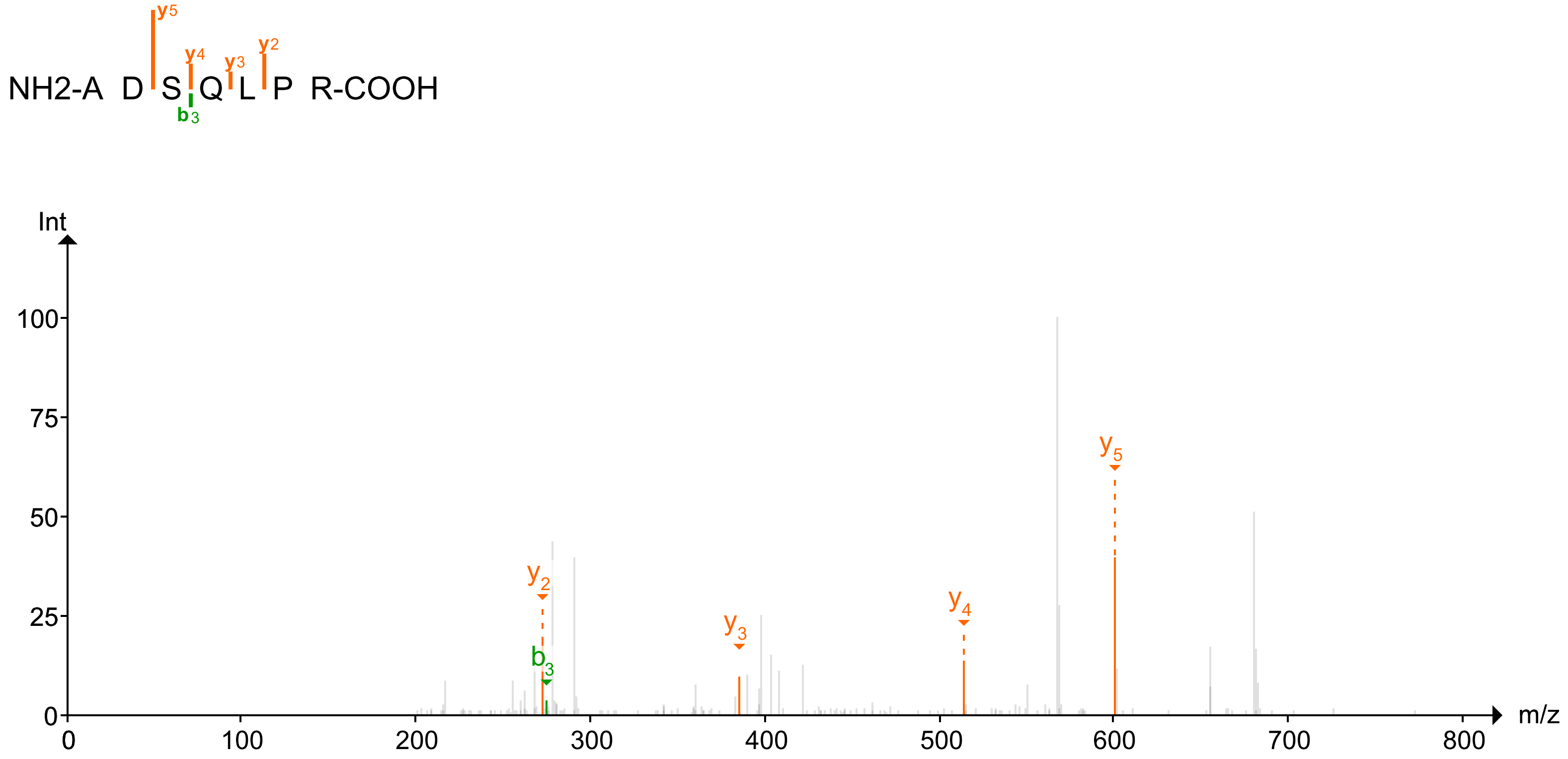

y5
y4
y3
y2
NH2-A D S Q L P R-COOH
b3
Int
100
75
50
25
0
y2
b3
y3
y4
y5
0
100
200
300
400
500
600
700
800
m/z

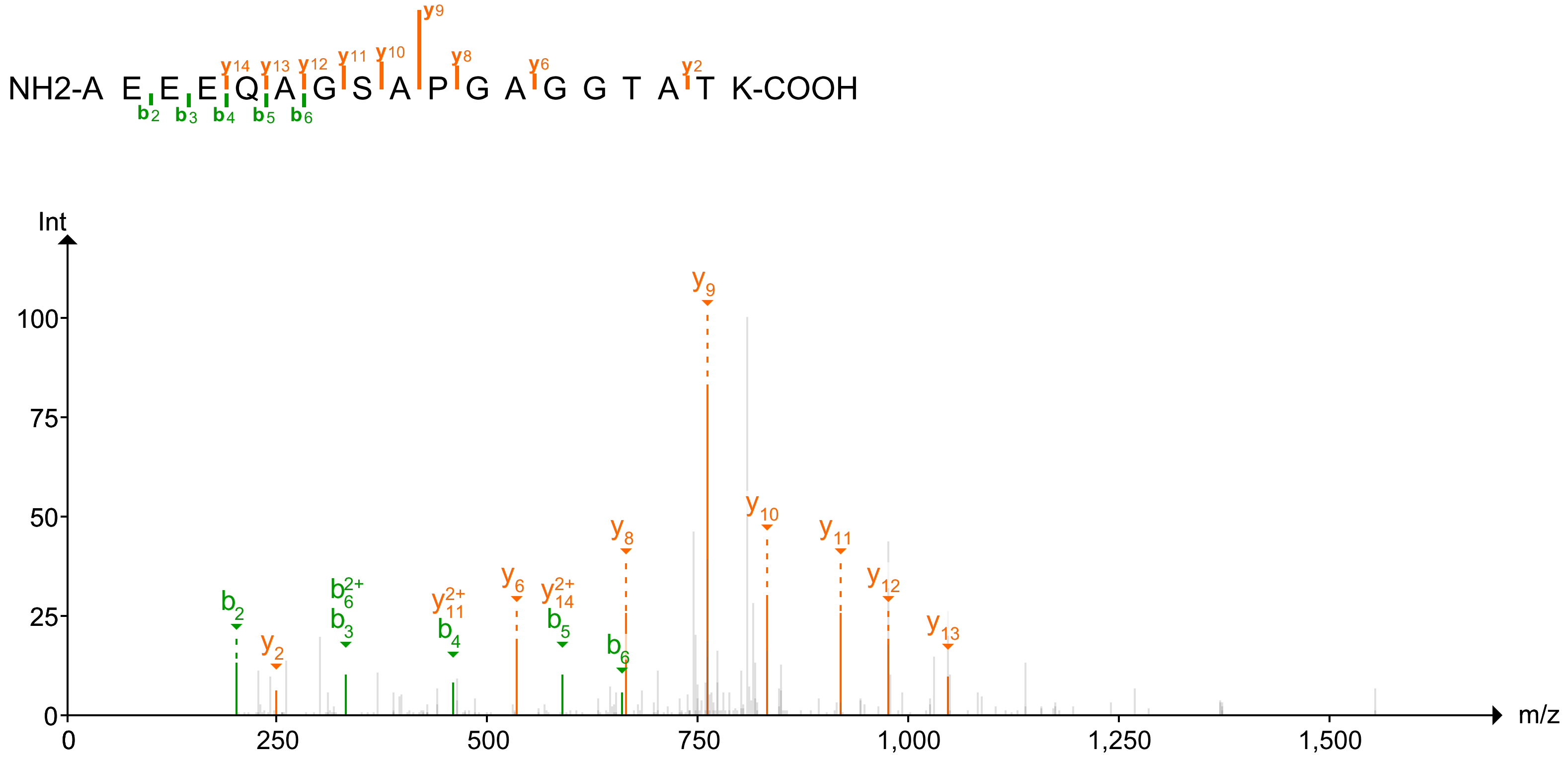
NH2-A E E E Q A G S A P G A G G T A T K-COOH
y14 y13 y12 y11 y10 y9 y8 y6 y2
b2 b3 b4 b5 b6
Int
100
75
50
25
0
m/z
0
250
500
750
1,000
1,250
1,500
b2
y2
b6 2+
b3
y11 2+
b4
y6
y14 2+
b5
b6
y8
y9
y10
y11
y12
y13

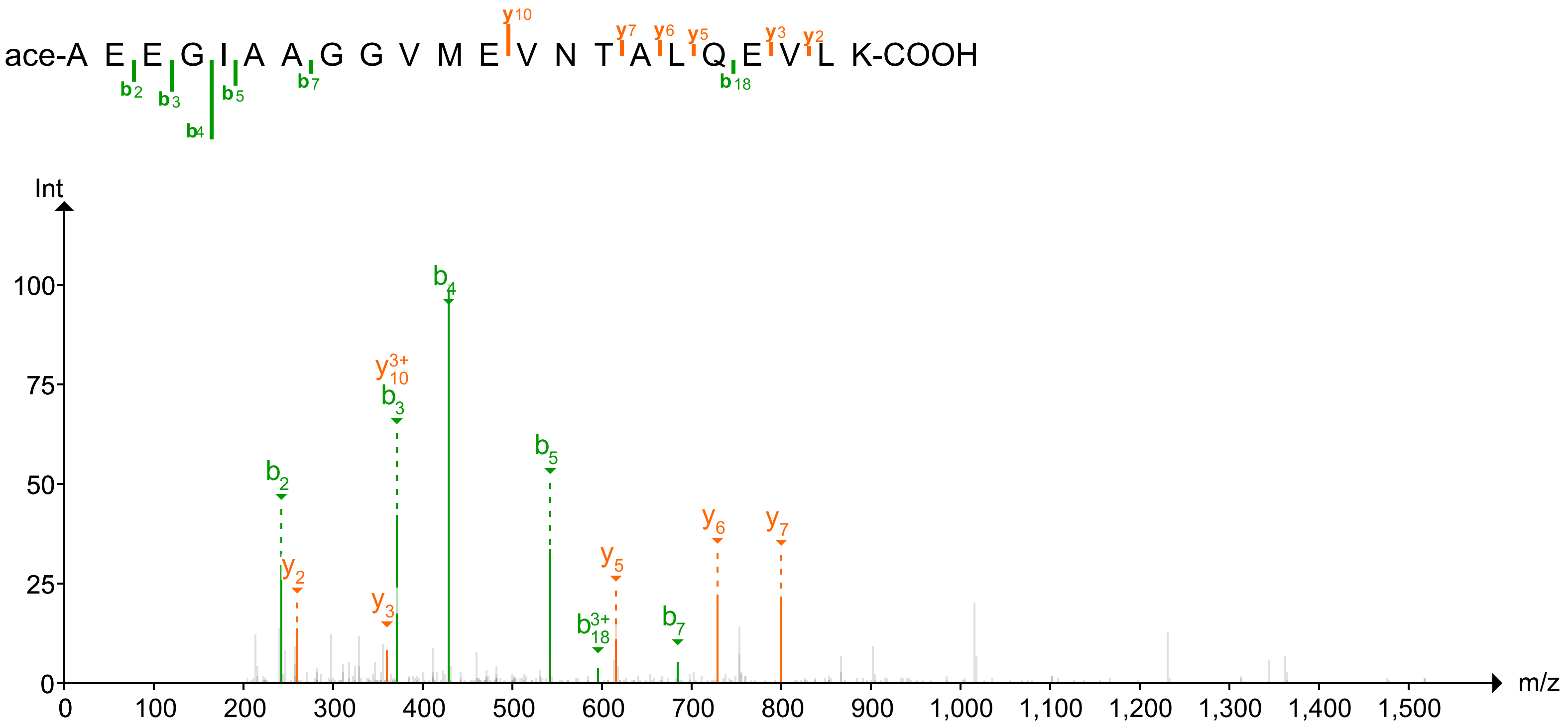
ace-A E E G I A A G G V M E V N T A L Q E V L K-COOH
b2 b3 b4 b5 b7 b18
y10 y7 y6 y5 y3 y2
Int
100
75
50
25
0
b4
y10 3+
b3
b2
b5
y2
y3
b18 3+
y5
y6
y7
b7
0
100
200
300
400
500
600
700
800
900
1,000
1,100
1,200
1,300
1,400
1,500
m/z

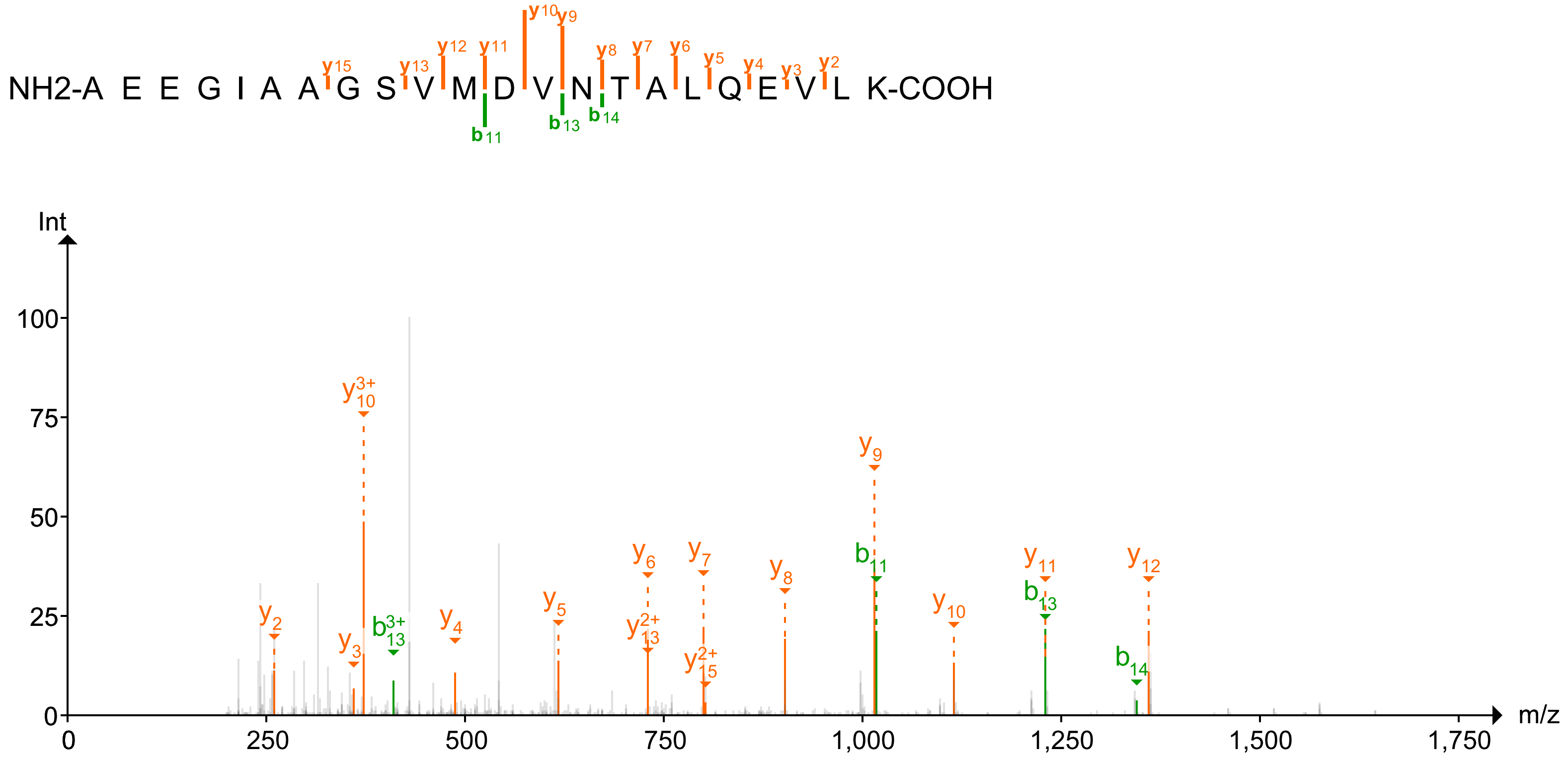
NH2-A E E G I A A G S V M D V N T A L Q E V L K-COOH
y15 y13 y12 y11 y10 y9 y8 y7 y6 y5 y4 y3 y2
b11 b13 b14
Int
100
75
50
25
0
0
250
500
750
1,000
1,250
1,500
1,750
m/z
$y_{10}^{3+}$
$y_9$
$b_{11}$
$y_6$
$y_7$
$y_8$
$y_{11}$
$y_{12}$
$b_{13}$
$y_{10}$
$y_5$
$y_2$
$y_3$
$b_{13}^{3+}$
$y_4$
$y_{13}^{2+}$
$y_{15}^{2+}$
$b_{14}$

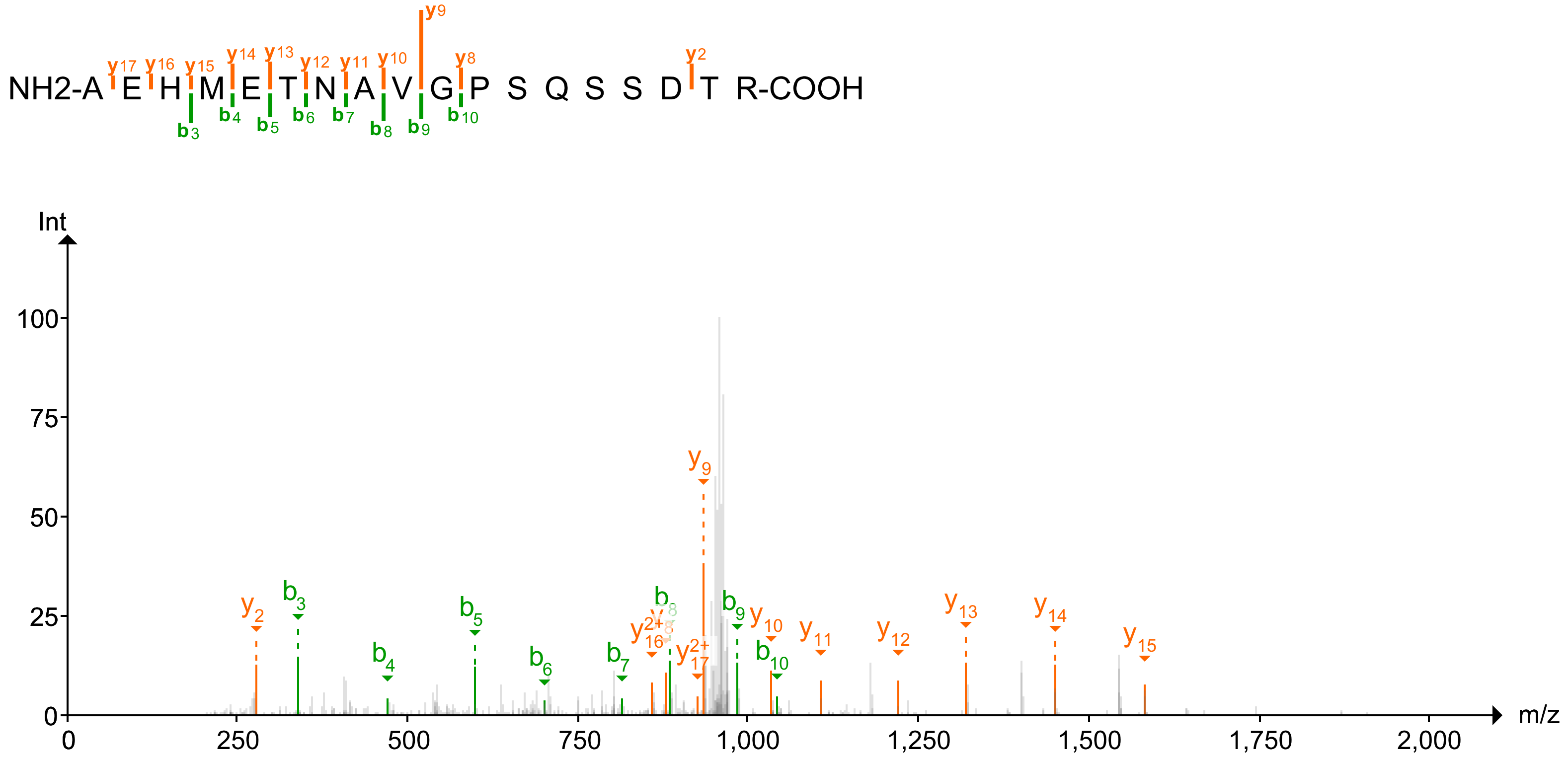

NH2-A E H M E T N A V G P S Q S S D T R-COOH
y17 y16 y15 y14 y13 y12 y11 y10 y9 y8 y2
b3 b4 b5 b6 b7 b8 b9 b10
Int
100
75
50
25
0
m/z
0
250
500
750
1,000
1,250
1,500
1,750
2,000

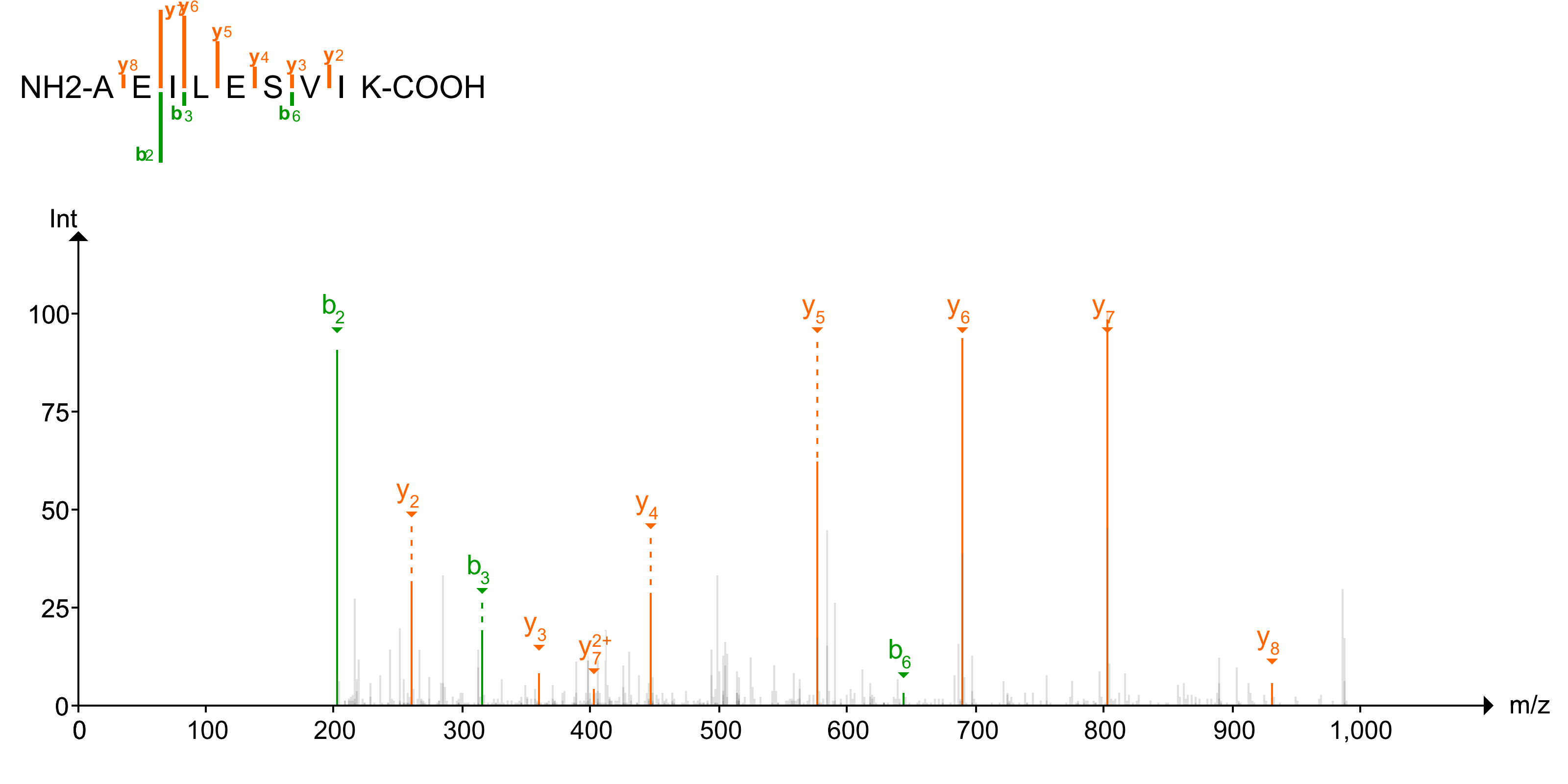

NH2-A E I L E S V I K-COOH
y8
y7
y6
y5
y4
y3
y2
b2
b3
b6
Int
100
75
50
25
0
m/z
0
100
200
300
400
500
600
700
800
900
1,000

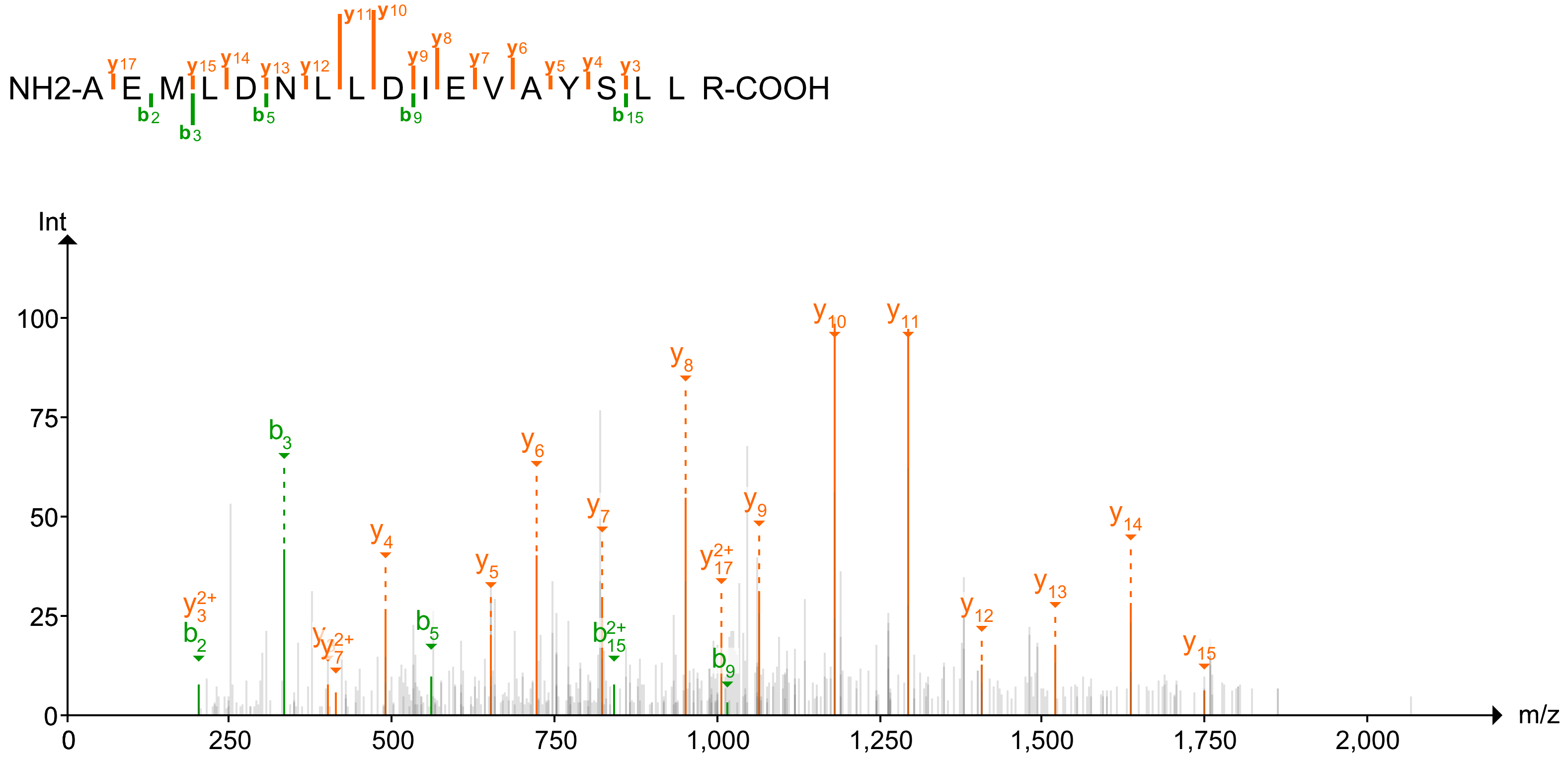

NH2-A E M L D N L L D I E V A Y S L L R-COOH
y17 y15 y14 y13 y12 y11 y10 y9 y8 y7 y6 y5 y4 y3
b2 b3 b5 b9 b15
Int
m/z
0
25
50
75
100
250
500
750
1,000
1,250
1,500
1,750
2,000

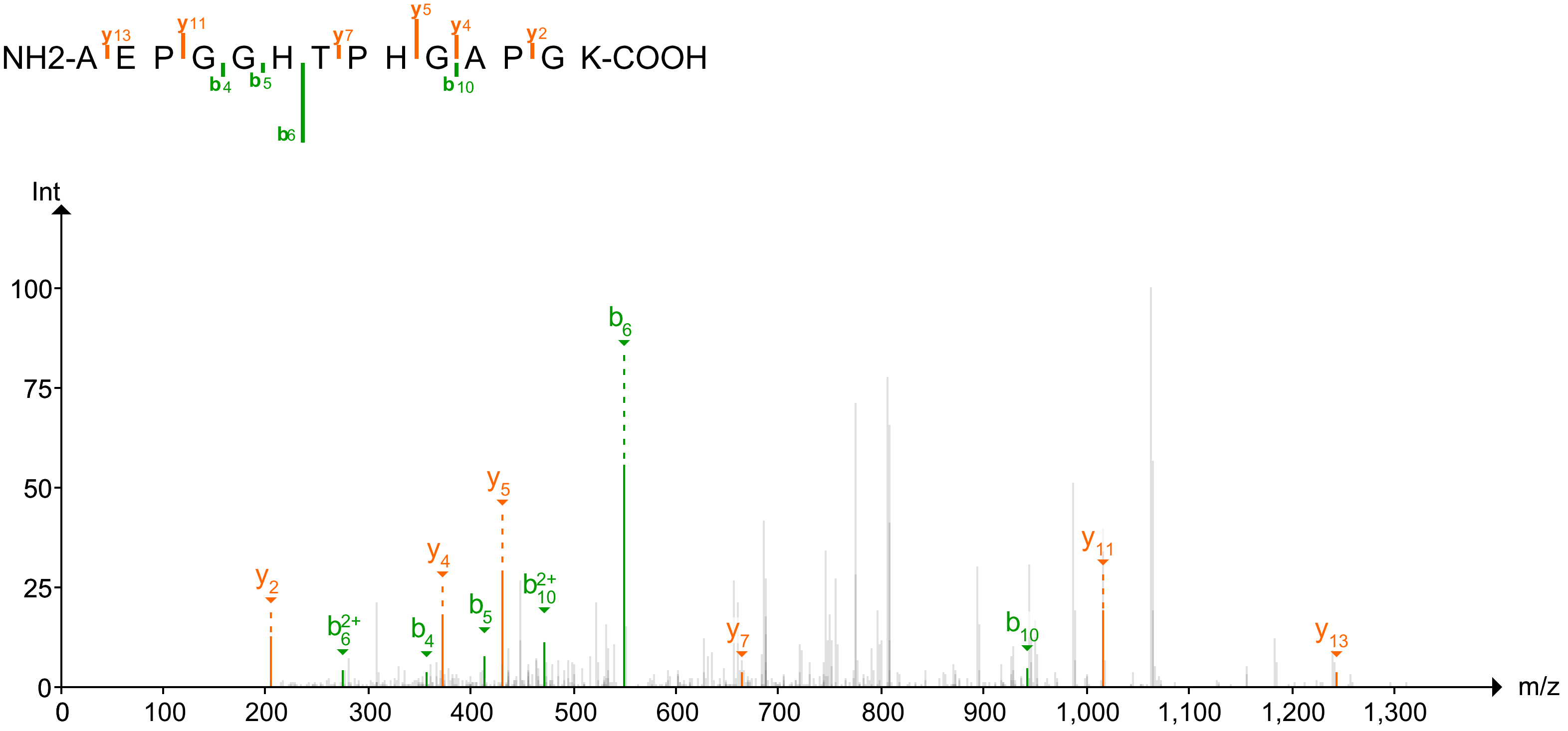

NH2-A E P G G H T P H G A P G K-COOH
y13
y11
y7
y5
y4
y2
b4
b5
b6
b10
Int
100
75
50
25
0
m/z
0
100
200
300
400
500
600
700
800
900
1,000
1,100
1,200
1,300

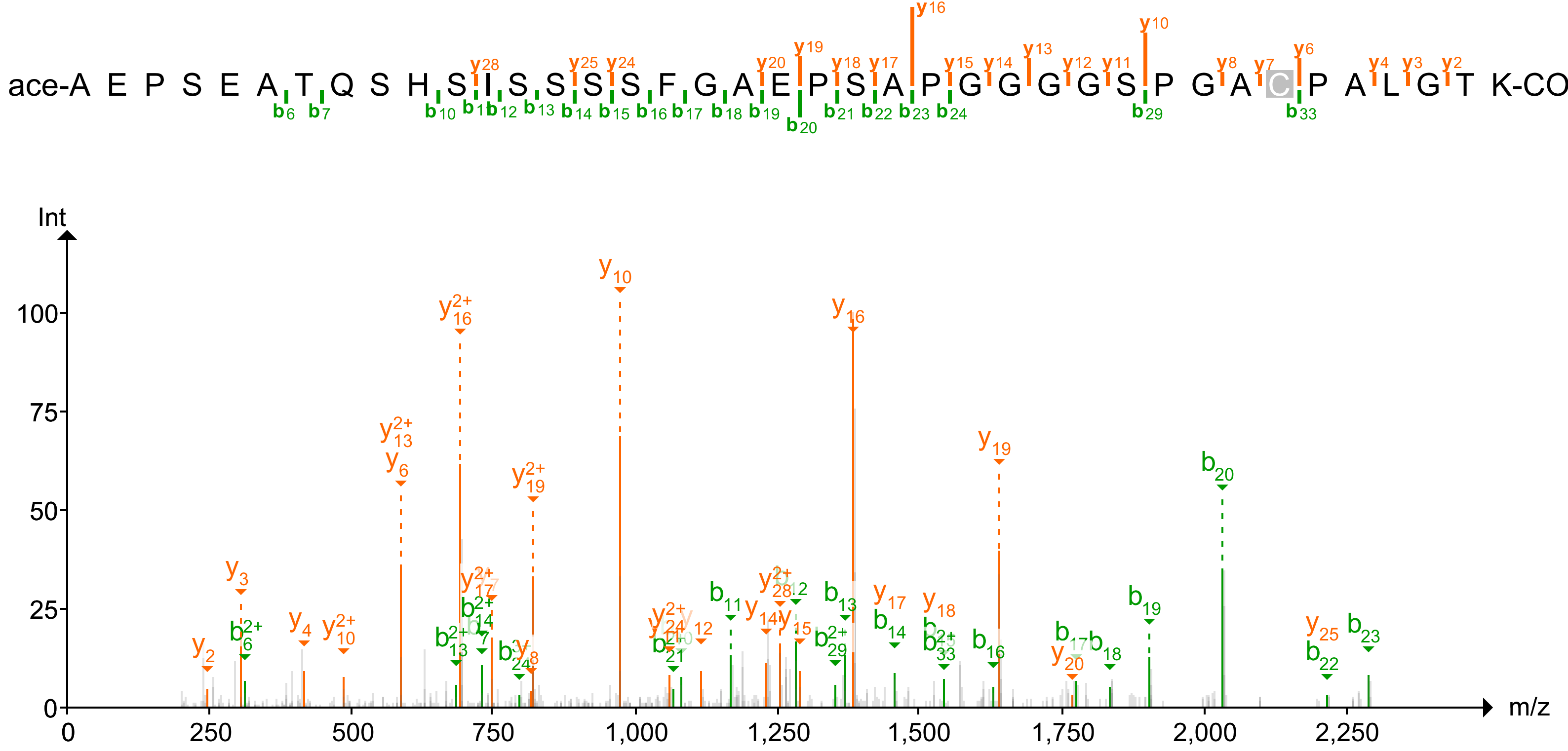

ace-A E P S E A T Q S H S I S S S S F G A E P S A P G G G S P G A C P A L G T K-CO
Int
100
75
50
25
0
0
250
500
750
1,000
1,250
1,500
1,750
2,000
2,250
m/z
y10
y16
y19
b20
y3
y2
y4
b11
b13
y17
y18
b14
b16
b18
b19
y25
b22
b23
y20

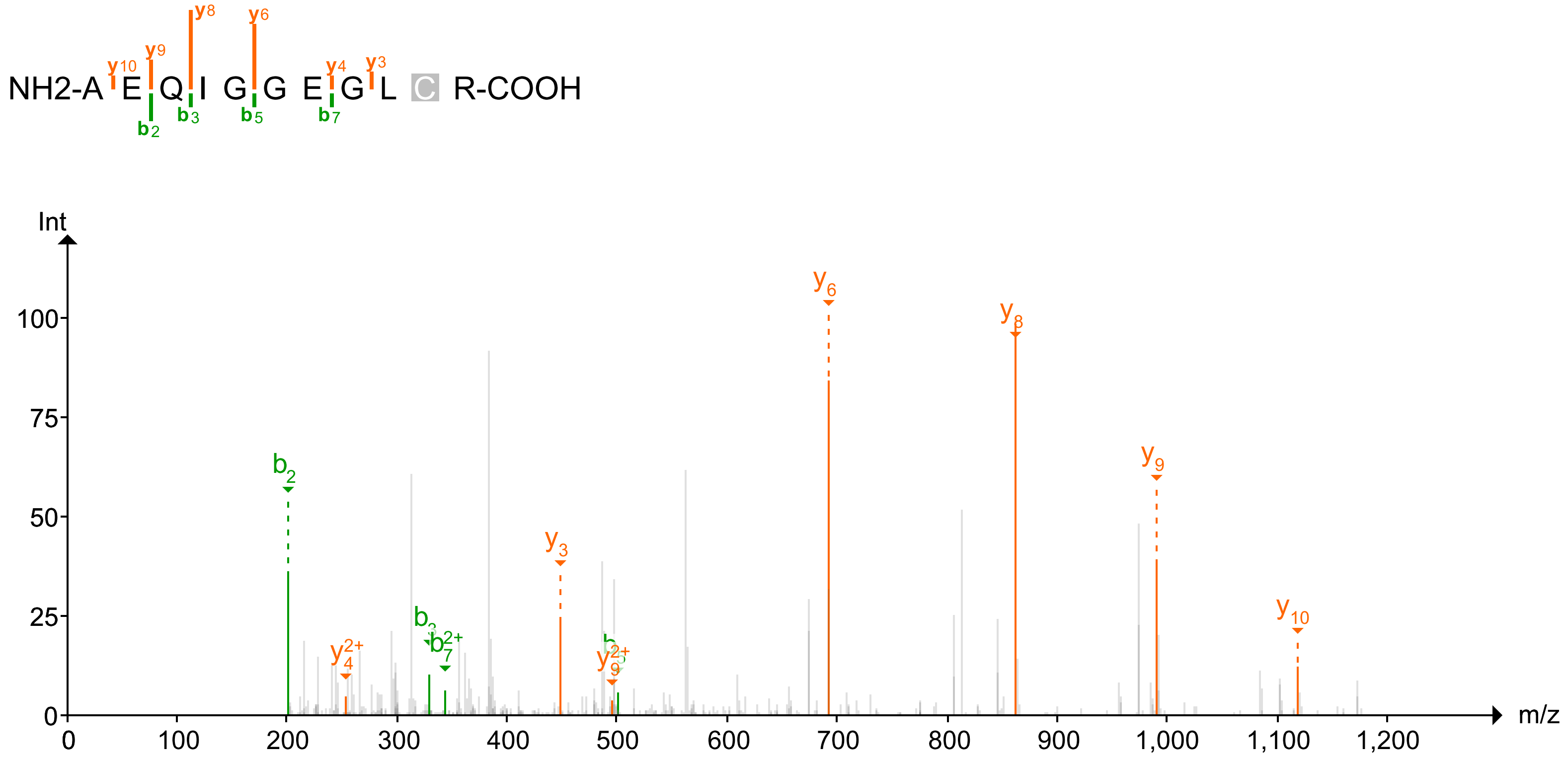

NH2-A E Q I G G E G L C R-COOH
y10
y9
y8
y6
y4
y3
b2
b3
b5
b7
Int
m/z
b2
y6
y8
y9
y3
y10

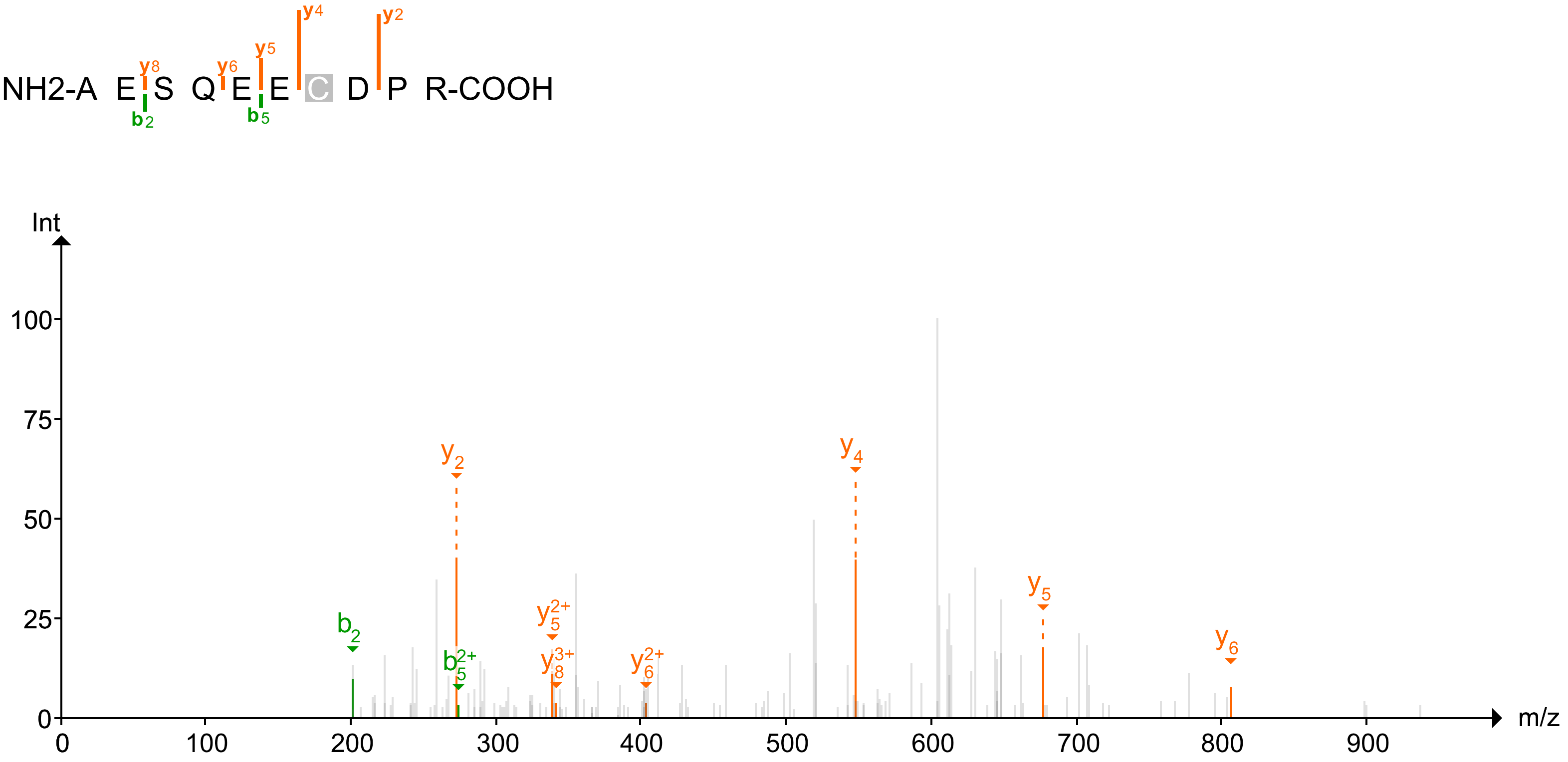
NH2-A E S Q E E C D P R-COOH
y8 y6 y5 y4 y2
b2 b5
Int
100
75
50
25
0
m/z
0 100 200 300 400 500 600 700 800 900
y2
y4
y5
y6
b2
b5 2+
y5 2+
y8 3+
y6 2+

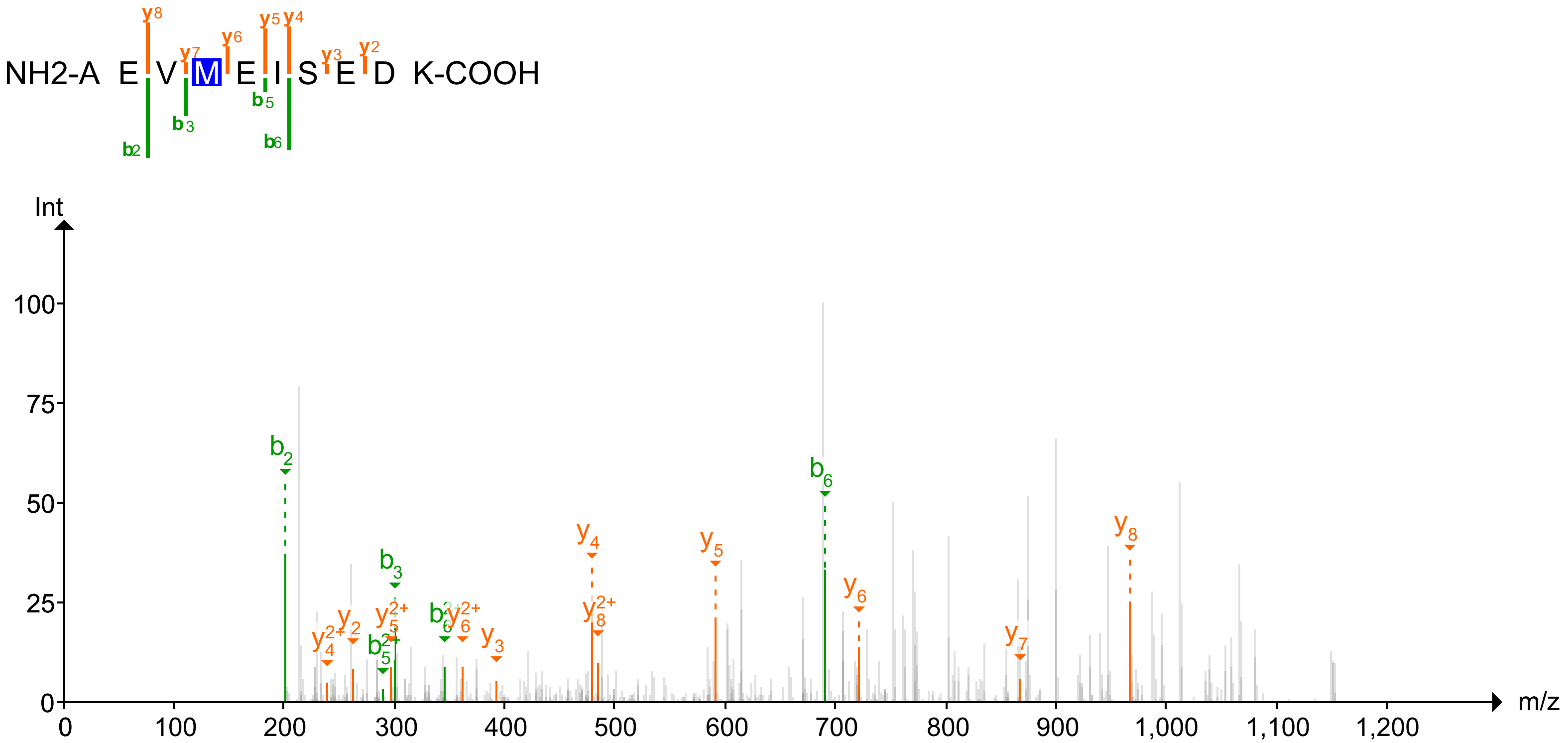

NH2-A E V M E I S E D K-COOH
y8
y7
y6
y5
y4
y3
y2
b2
b3
b5
b6
Int
100
75
50
25
0
m/z
0
100
200
300
400
500
600
700
800
900
1,000
1,100
1,200

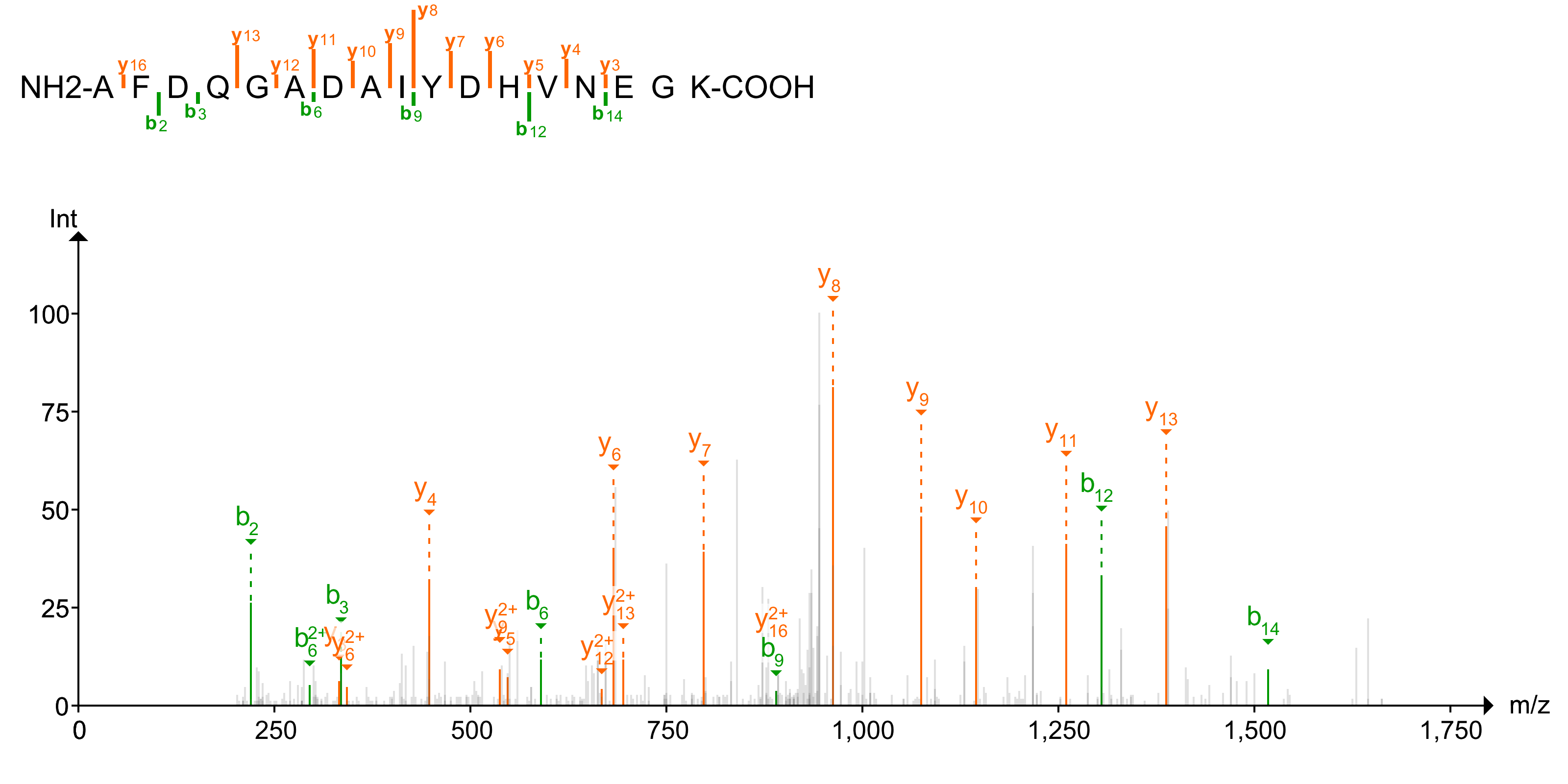

NH2-A F D Q G A D A I Y D H V N E G K-COOH
Int
m/z

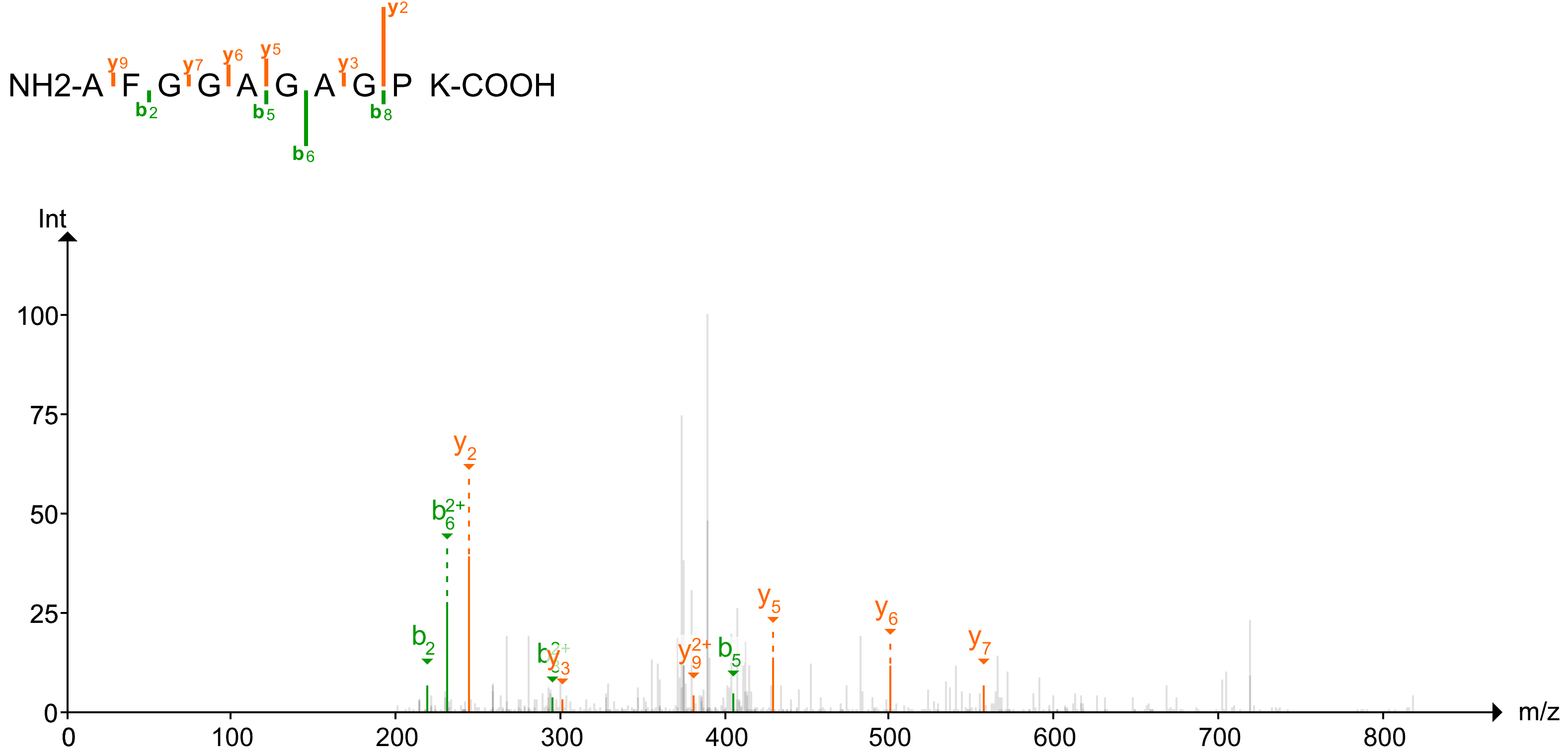

NH2-A F G G A G A G P K-COOH
y9 y7 y6 y5 y3 y2
b2 b5 b6 b8
Int
m/z
0
25
50
75
100
0
100
200
300
400
500
600
700
800
y2
b6 2+
b2
b3 2+
y3
y9 2+
b5
y5
y6
y7

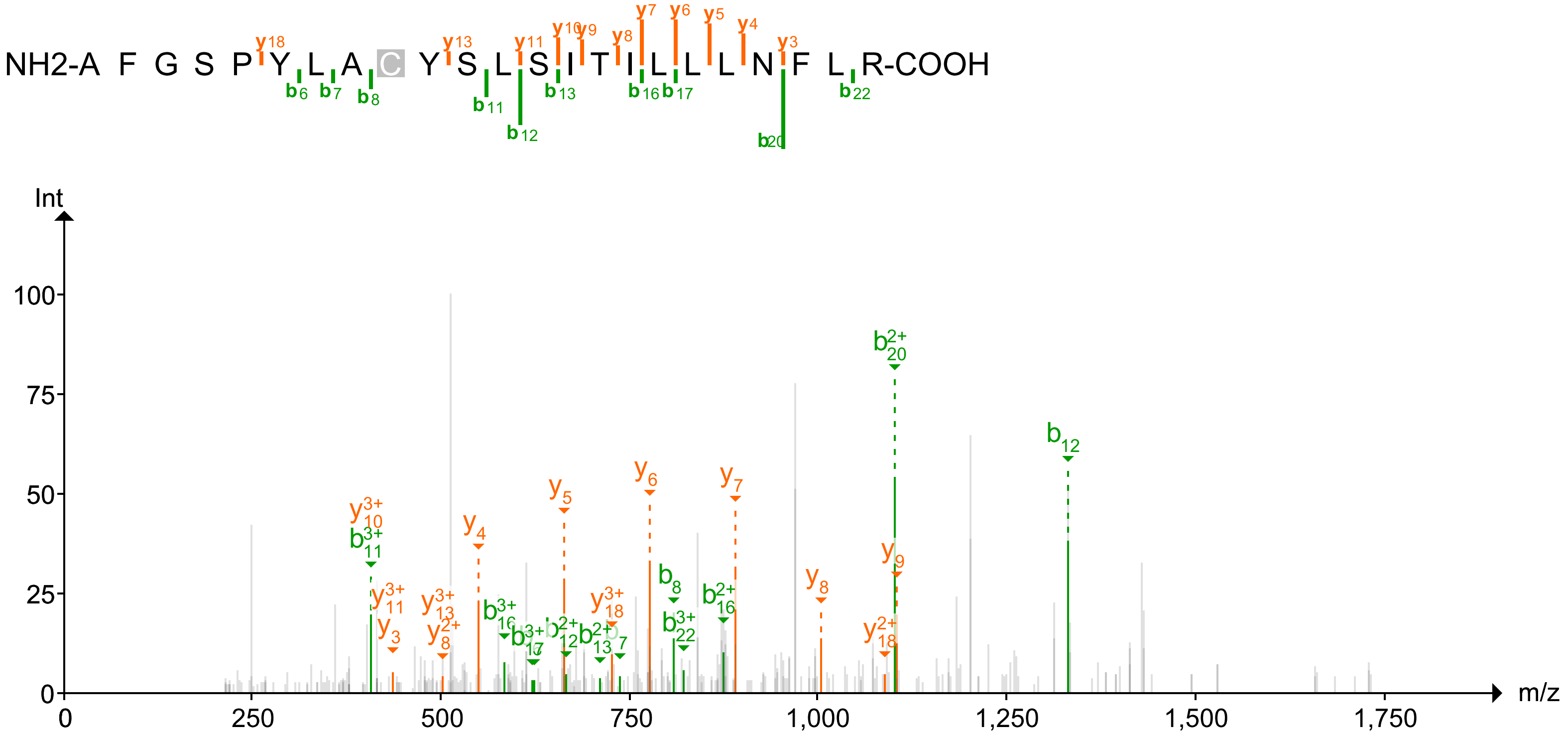

NH2-A F G S P Y L A C Y S L S I T I L L L N F L R-COOH
Int
m/z
0
25
50
75
100
250
500
750
1,000
1,250
1,500
1,750

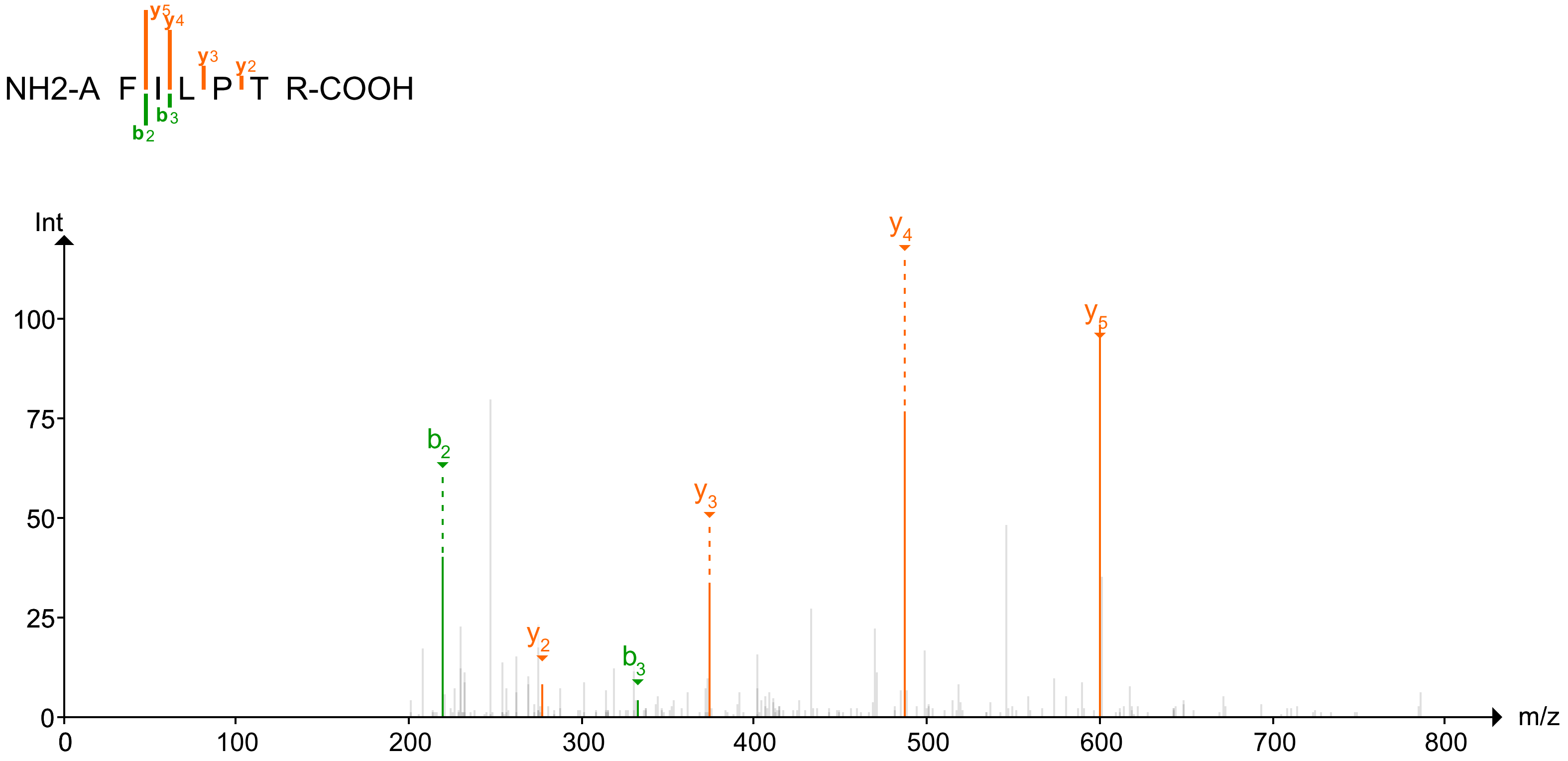

NH2-A F I L P T R-COOH
y5 y4 y3 y2
b2 b3
Int
100
75
50
25
0
0 100 200 300 400 500 600 700 800
m/z
b2
y2
b3
y3
y4
y5

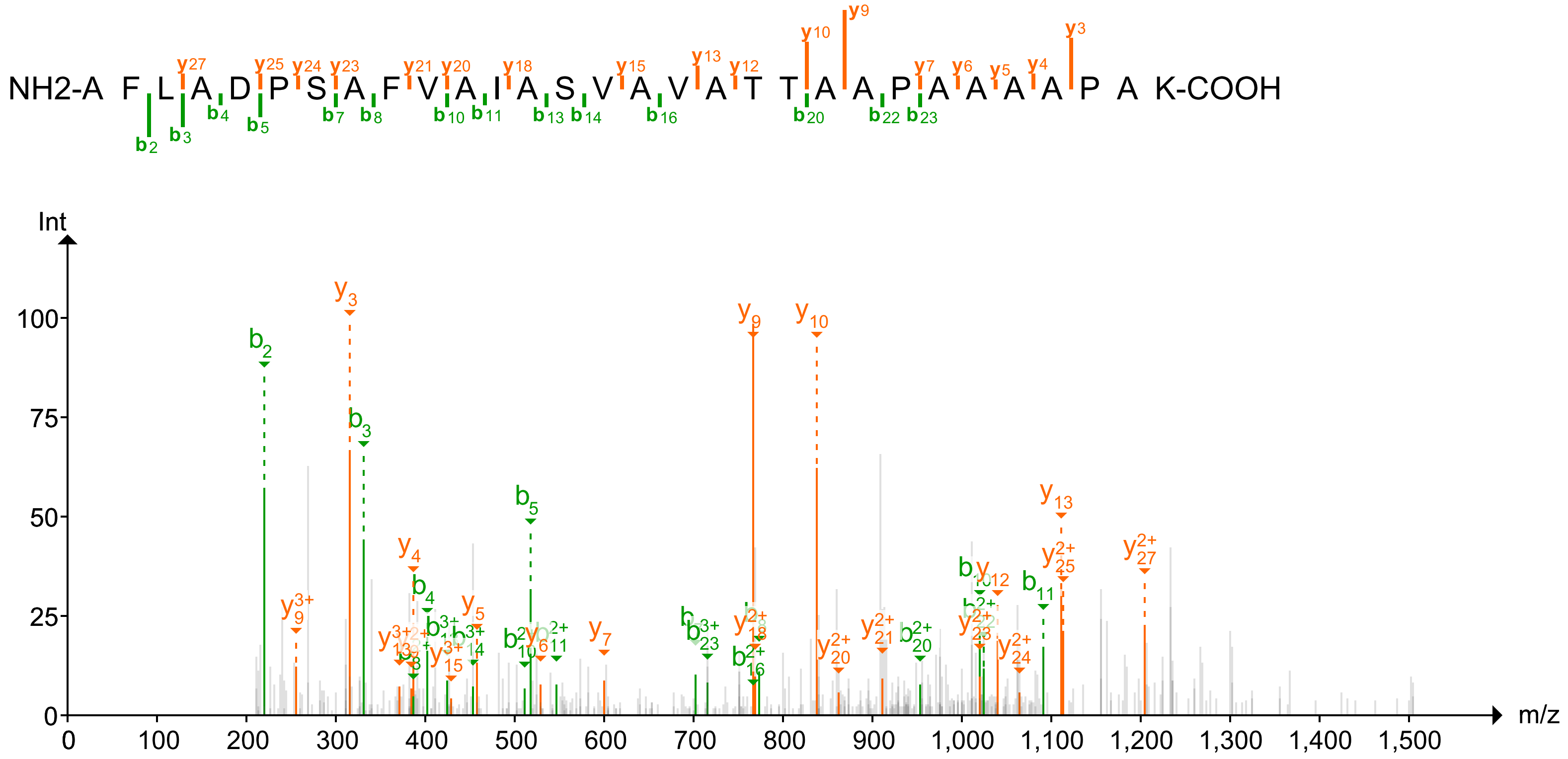
NH2-A F L A D P S A F V A I A S V A V A T T A A P A A A A P A K-COOH
Int
m/z
0
25
50
75
100
0
100
200
300
400
500
600
700
800
900
1,000
1,100
1,200
1,300
1,400
1,500

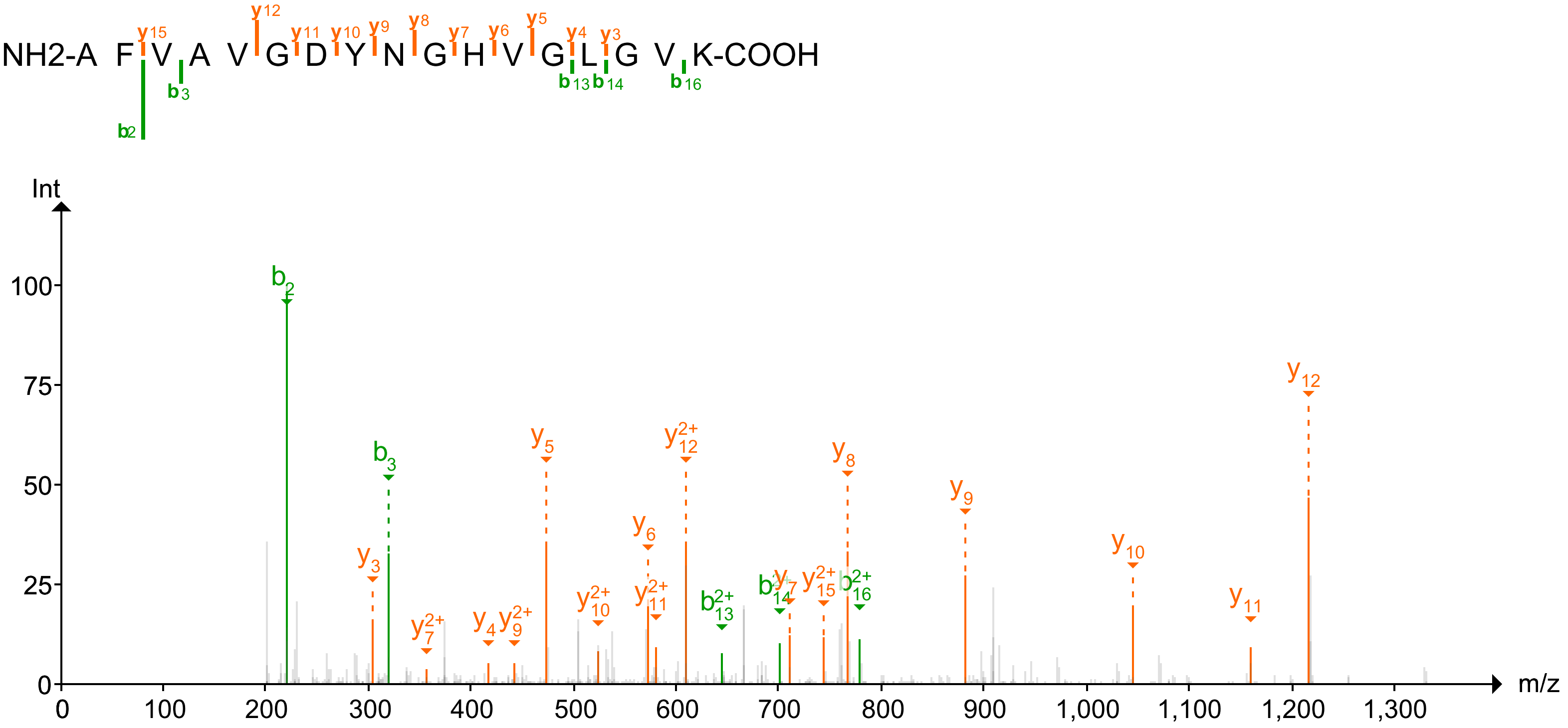

NH2-A F V A V G D Y N G H V G L G V K-COOH
y15 y12 y11 y10 y9 y8 y7 y6 y5 y4 y3
b2 b3 b13 b14 b16
Int
100
75
50
25
0
b2
b3
y3
y7 2+
y4
y9 2+
y5
y10 2+
y6
y11 2+
y12 2+
b13 2+
b14 2+
y7
y15 2+
y8
b16 2+
y9
y10
y11
y12
0 100 200 300 400 500 600 700 800 900 1,000 1,100 1,200 1,300
m/z

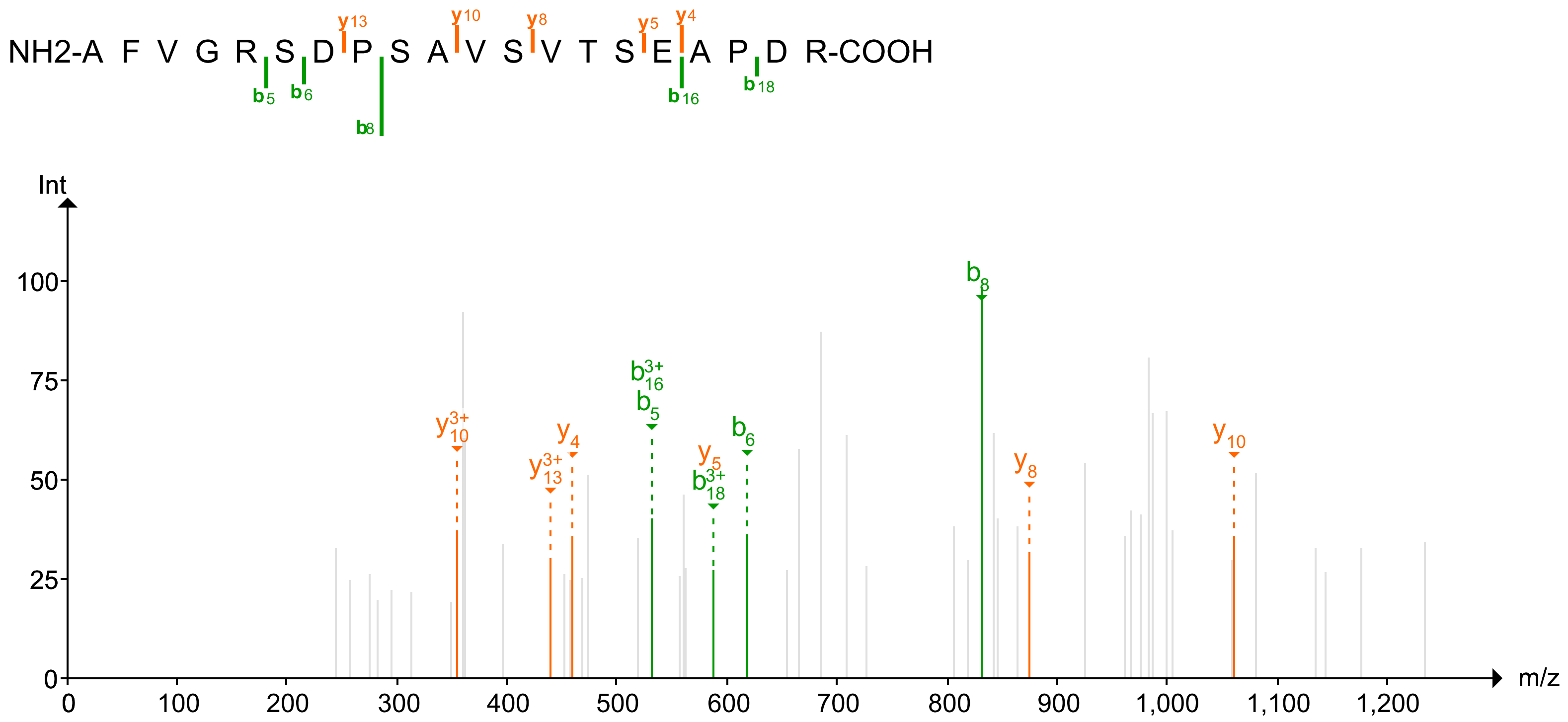

NH2-A F V G R S D P S A V S V T S E A P D R-COOH
y13 y10 y8 y5 y4
b5 b6 b8 b16 b18
Int
m/z
b8
b16 3+
b5
y10 3+
y4
y13 3+
b6
y5
b18 3+
y8
y10
0
25
50
75
100
0
100
200
300
400
500
600
700
800
900
1,000
1,100
1,200

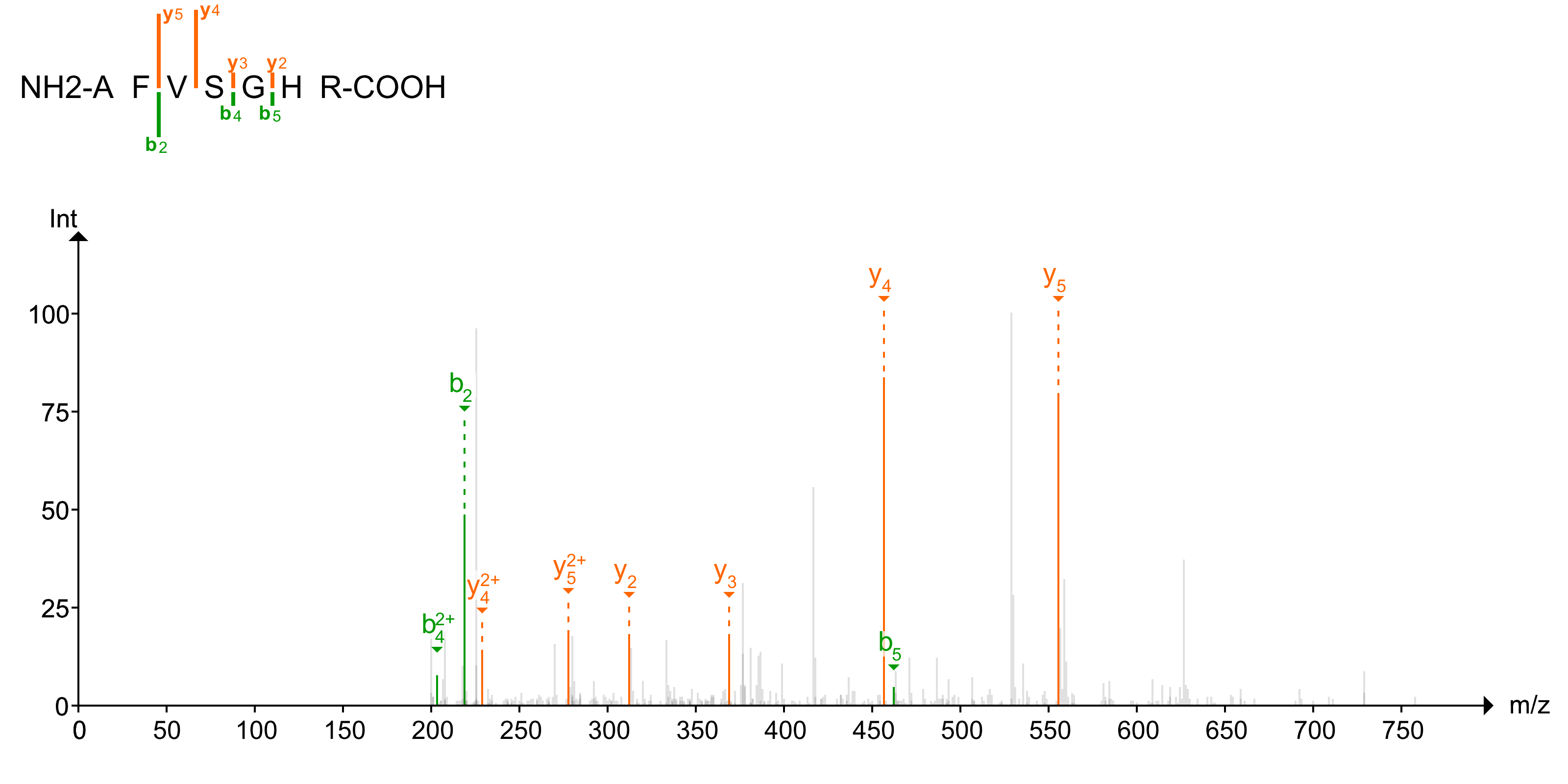

NH2-A F V S G H R-COOH
y5 y4 y3 y2
b2 b4 b5
Int
100
75
50
25
0
b2
y4
y5
b4 2+
y4 2+
y5 2+
y2
y3
b5
0 50 100 150 200 250 300 350 400 450 500 550 600 650 700 750
m/z

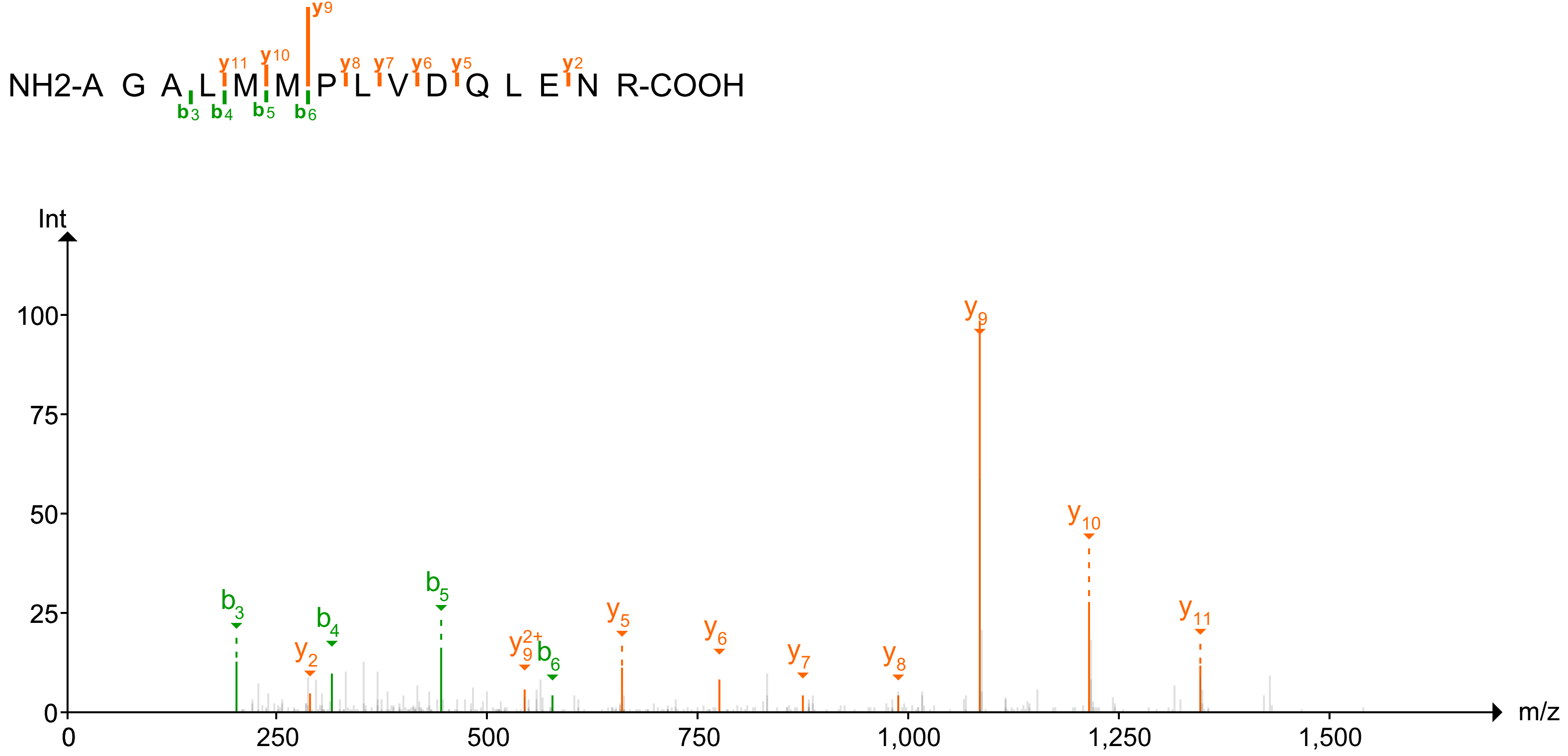

NH2-A G A L M M P L V D Q L E N R-COOH
y11 y10 y9 y8 y7 y6 y5 y2
b3 b4 b5 b6
Int
100
75
50
25
0
m/z
0
250
500
750
1,000
1,250
1,500
b3
y2
b4
b5
y9 2+
b6
y5
y6
y7
y8
y9
y10
y11

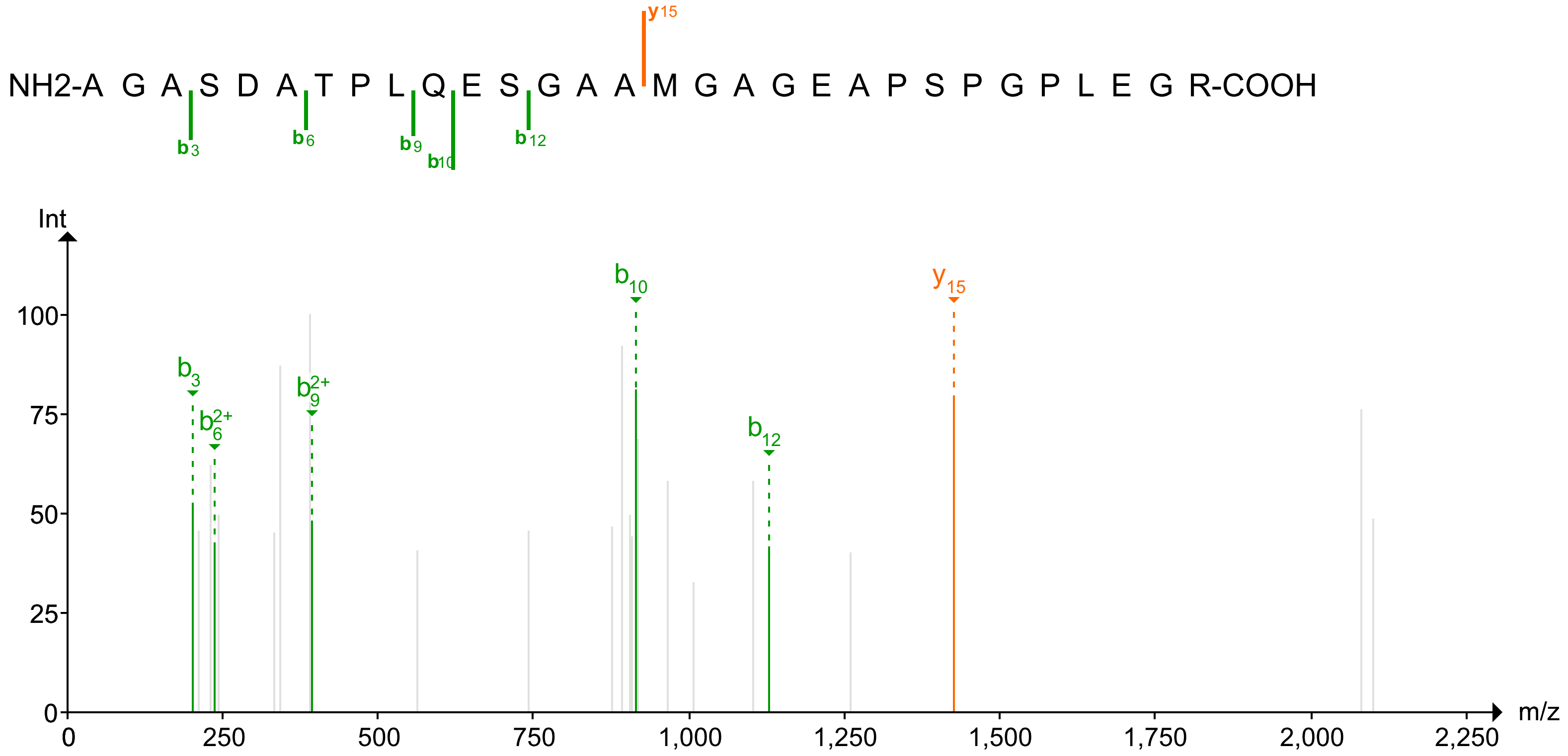

NH2-A G A S D A T P L Q E S G A A M G A G E A P S P G P L E G R-COOH
y15
b3
b6
b9
b10
b12
Int
m/z
0
25
50
75
100
250
500
750
1,000
1,250
1,500
1,750
2,000
2,250
b3
b6 2+
b9 2+
b10
b12
y15

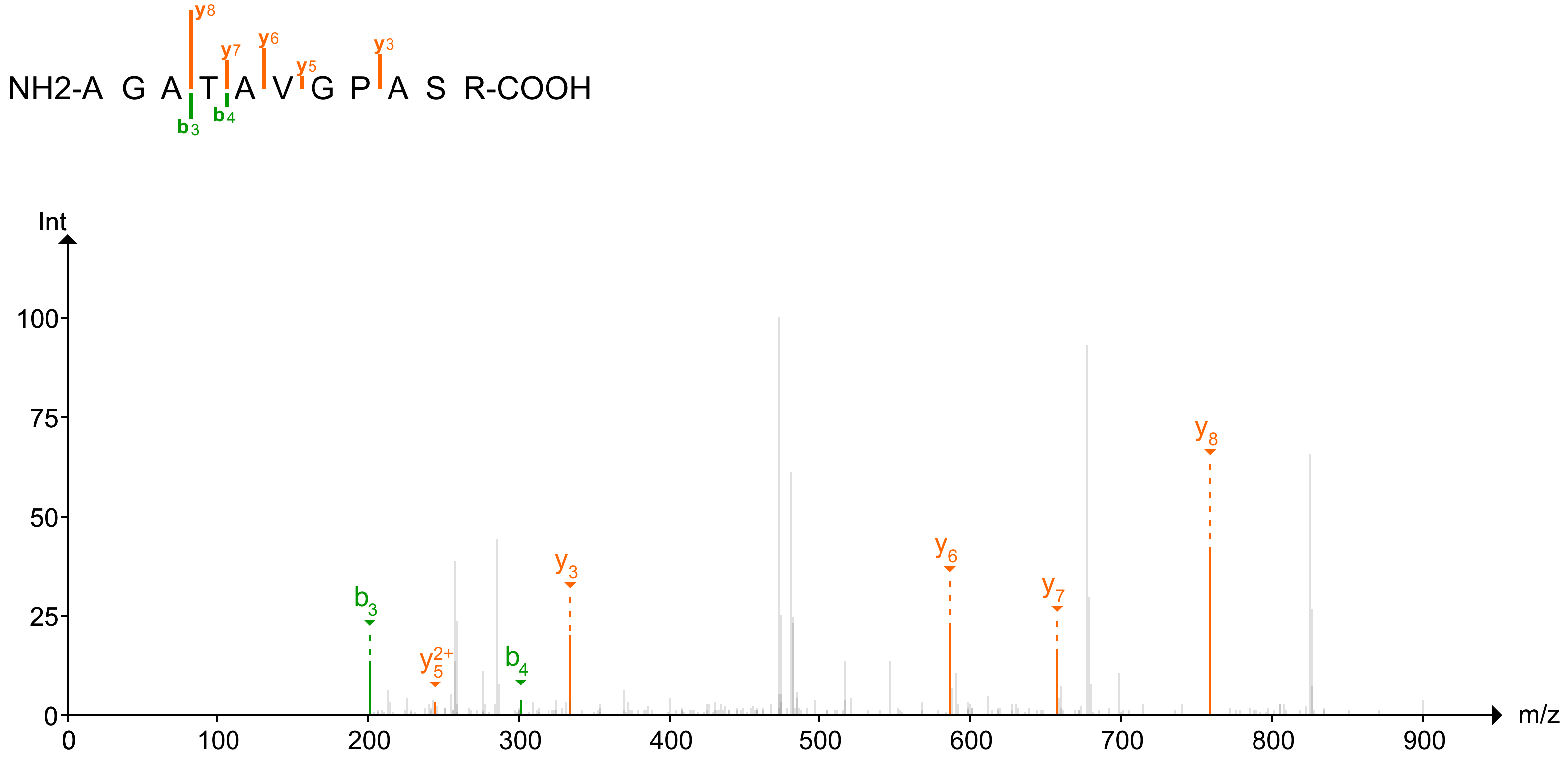

NH2-A G A T A V G P A S R-COOH
y8
y7
y6
y5
y3
b3
b4
Int
100
75
50
25
0
b3
y5 2+
b4
y3
y6
y7
y8
0
100
200
300
400
500
600
700
800
900
m/z

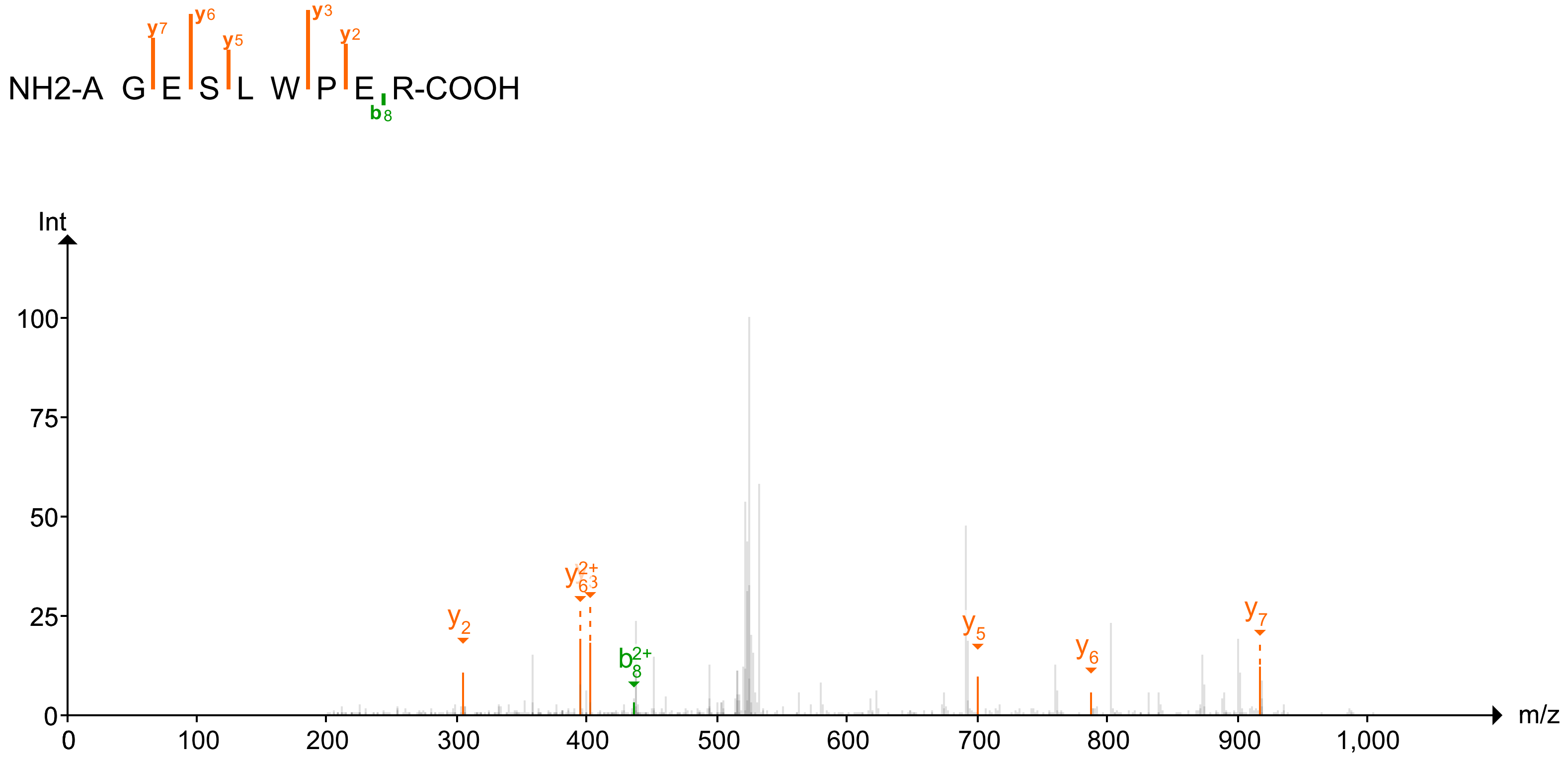
NH2-A G E S L W P E R-COOH
y7
y6
y5
y3
y2
b8
Int
100
75
50
25
0
y2
y6 2+
y3
b8 2+
y5
y6
y7
m/z
0
100
200
300
400
500
600
700
800
900
1,000

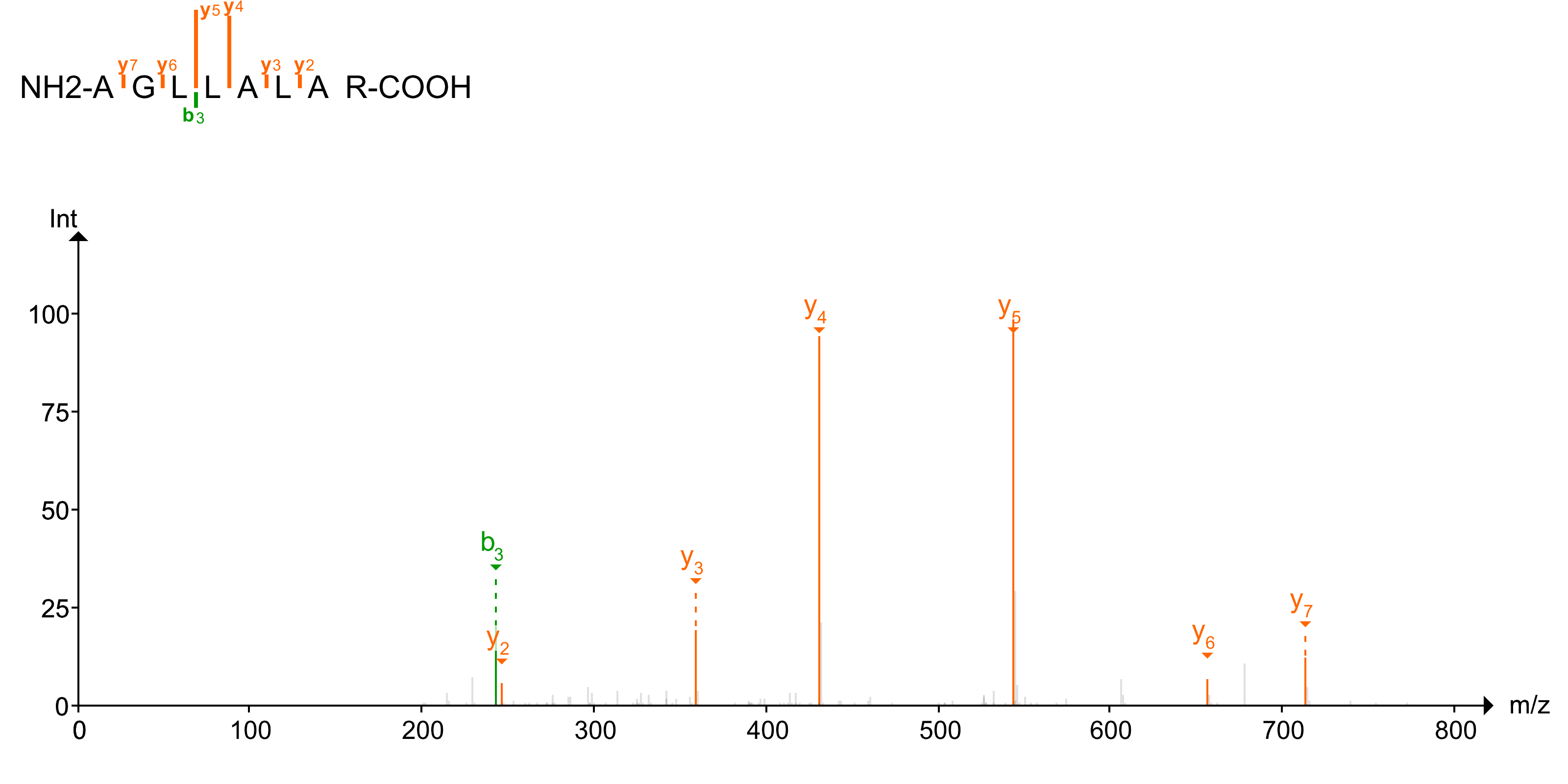
NH2-A G L L A L A R-COOH
y7 y6 y5 y4 y3 y2
b3
Int
100
75
50
25
0
y4
y5
b3
y3
y2
y6
y7
0
100
200
300
400
500
600
700
800
m/z

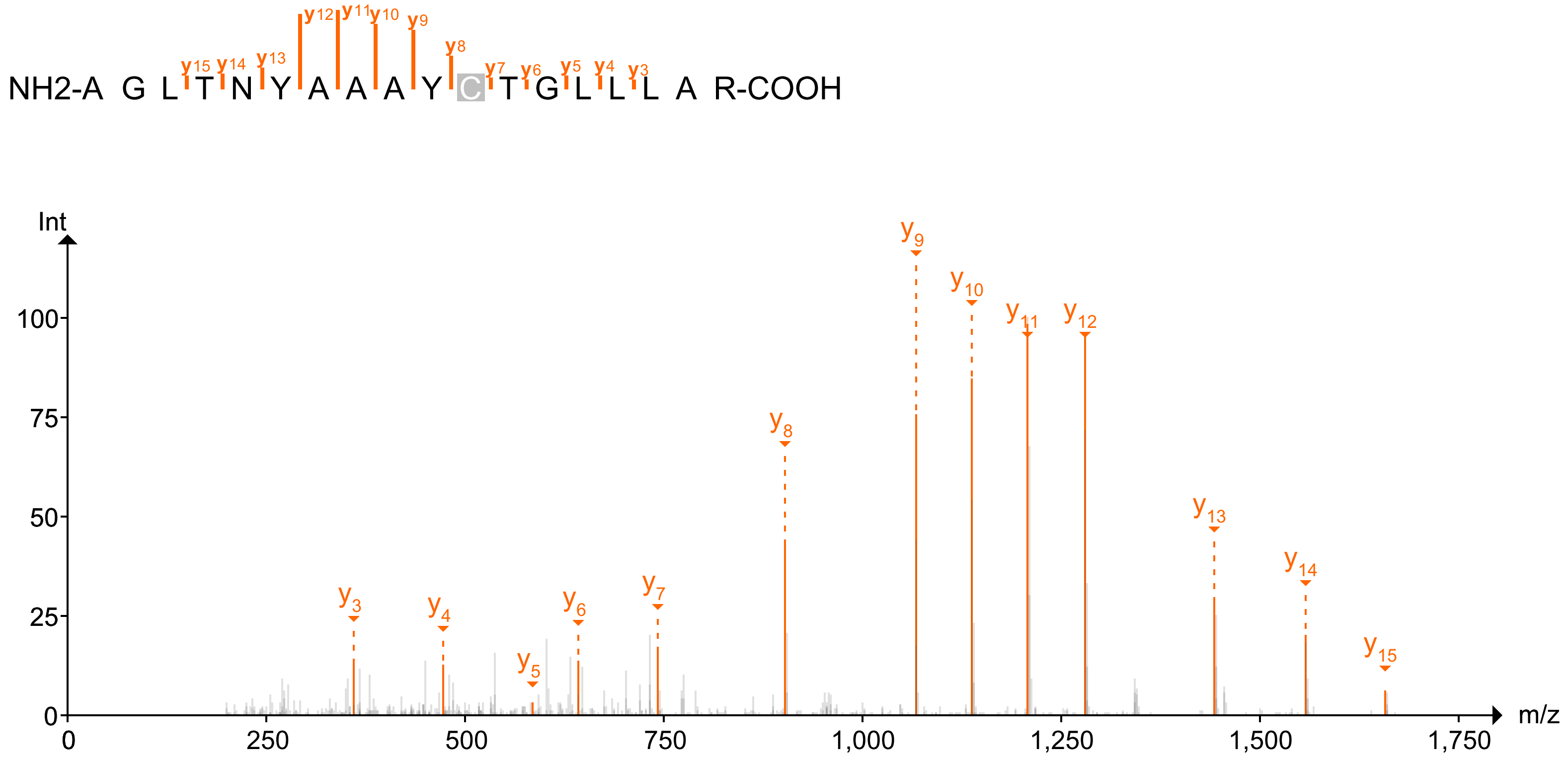

NH2-A G L T N Y A A A Y C T G L L L A R-COOH
y15 y14 y13 y12 y11 y10 y9 y8 y7 y6 y5 y4 y3
Int
100
75
50
25
0
m/z
0
250
500
750
1,000
1,250
1,500
1,750
y3
y4
y5
y6
y7
y8
y9
y10
y11
y12
y13
y14
y15

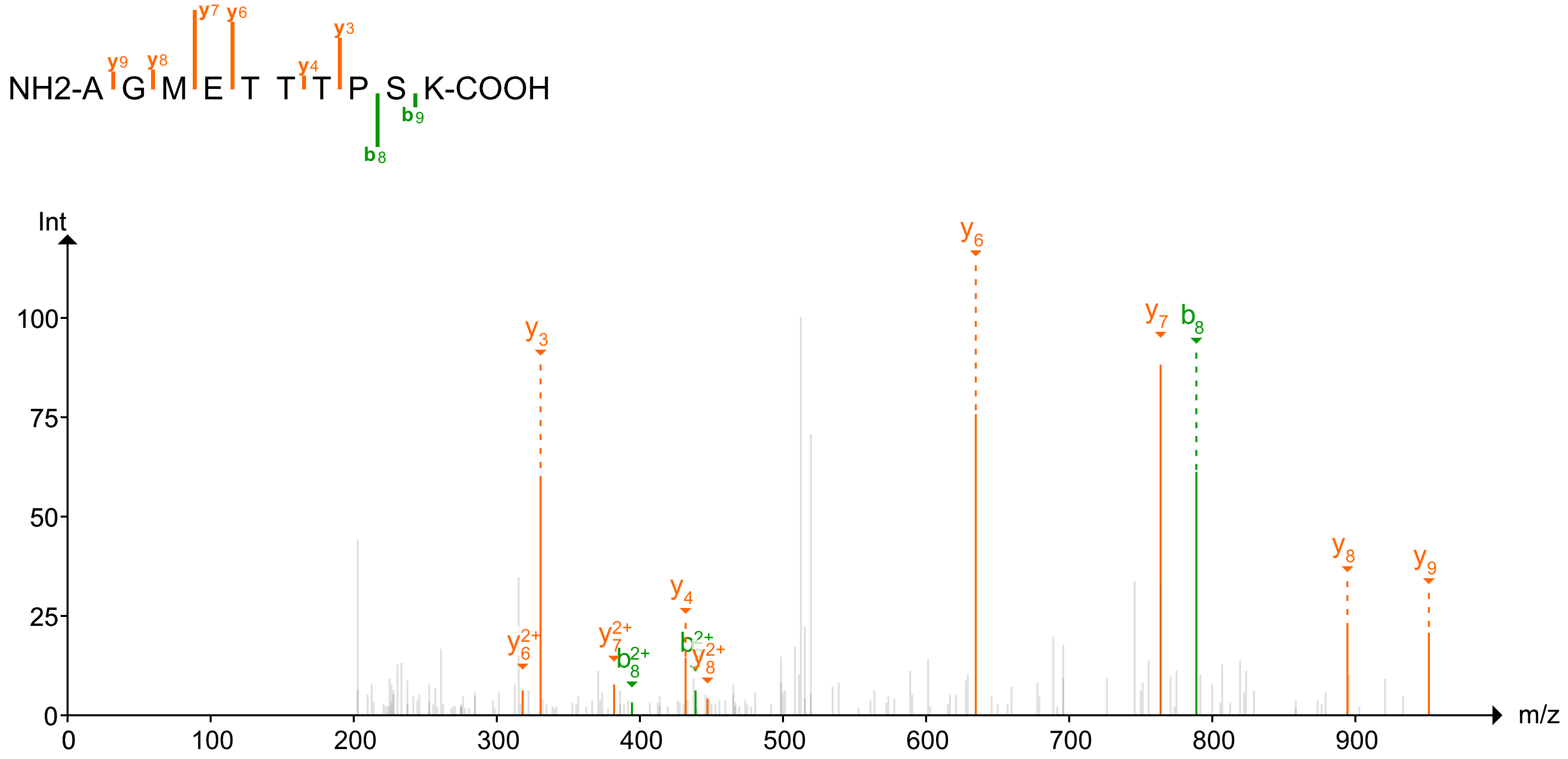
NH2-A G M E T T T P S K-COOH
y9 y8 y7 y6 y4 y3
b8 b9
Int
m/z
0
25
50
75
100
0
100
200
300
400
500
600
700
800
900
y3
y6
y7
b8
y8
y9
y4

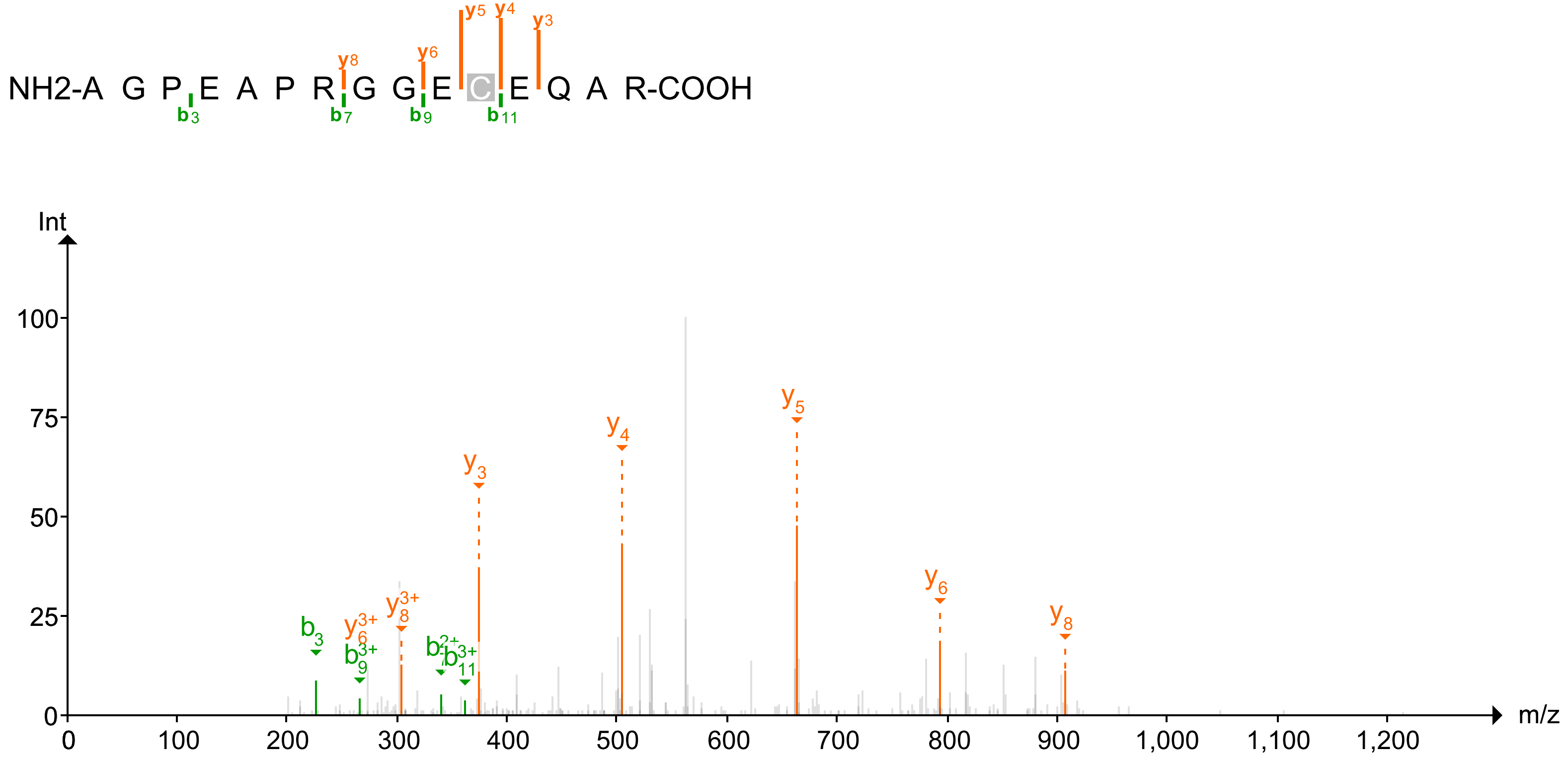

NH2-A G P E A P R G G E C E Q A R-COOH
b3
b7
b9
b11
y8
y6
y5
y4
y3
Int
100
75
50
25
0
m/z
0
100
200
300
400
500
600
700
800
900
1,000
1,100
1,200

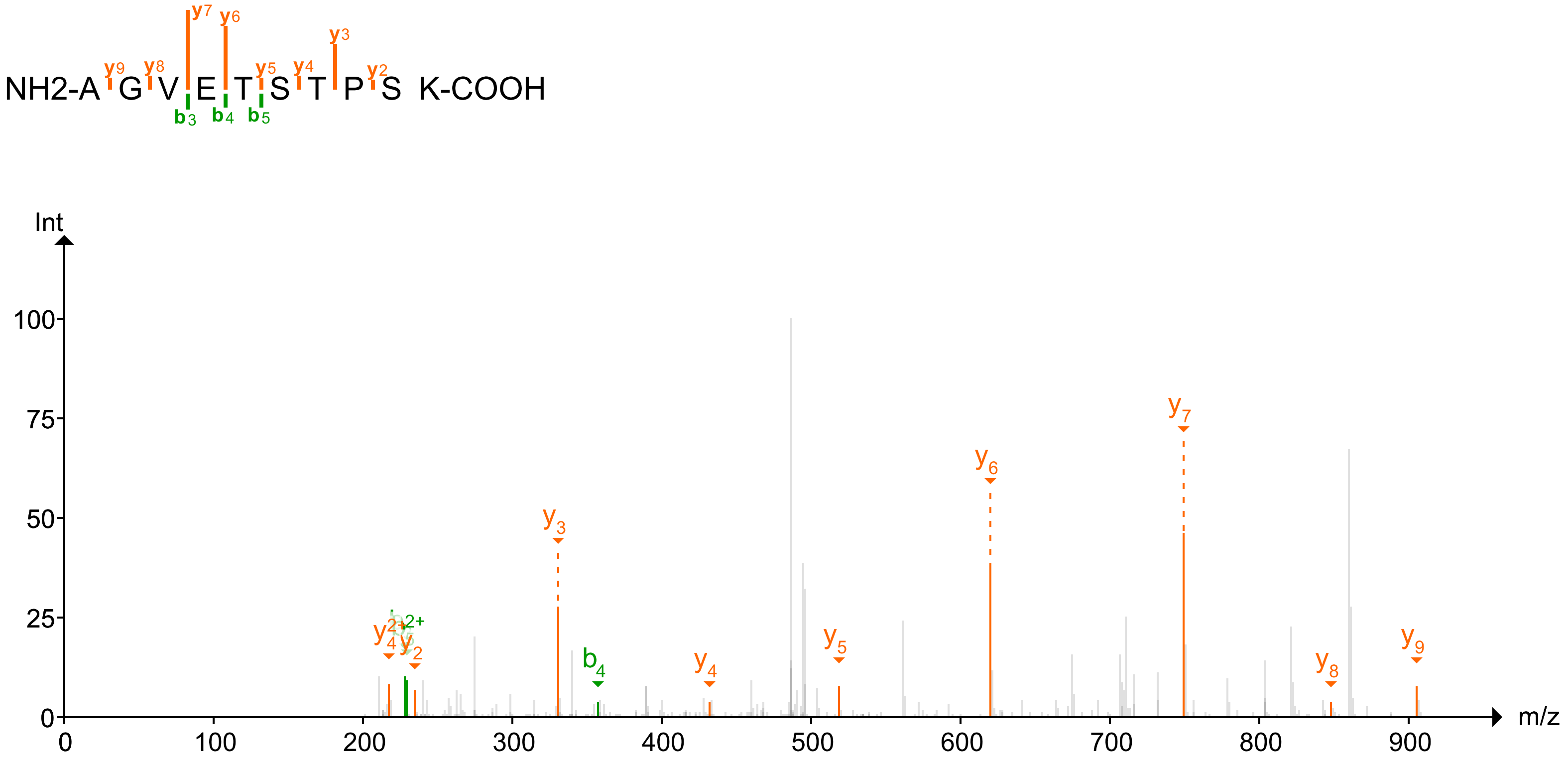

NH2-A G V E T S T P S K-COOH
y9 y8 y7 y6 y5 y4 y3 y2
b3 b4 b5
Int
100
75
50
25
0
m/z
0
100
200
300
400
500
600
700
800
900
y7
y6
y3
y5
y4
y8
y9
b4
y2

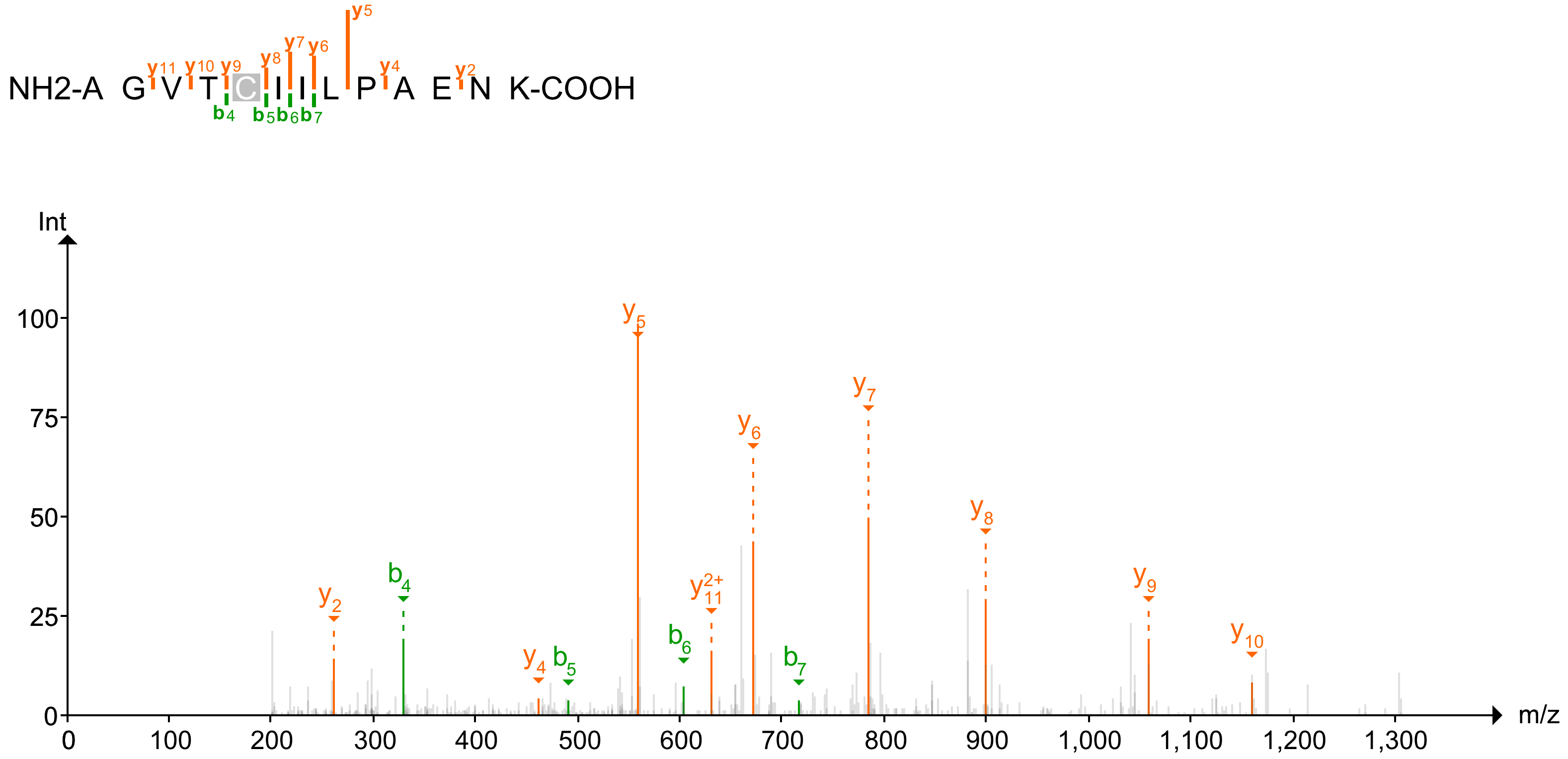
NH2-A G V T C I I L P A E N K-COOH
y11 y10 y9 y8 y7 y6 y5 y4 y2
b4 b5 b6 b7
Int
100
75
50
25
0
y5
y7
y6
y8
y2
b4
$y_{11}^{2+}$
y9
y10
y4 b5
b6
b7
0 100 200 300 400 500 600 700 800 900 1,000 1,100 1,200 1,300
m/z

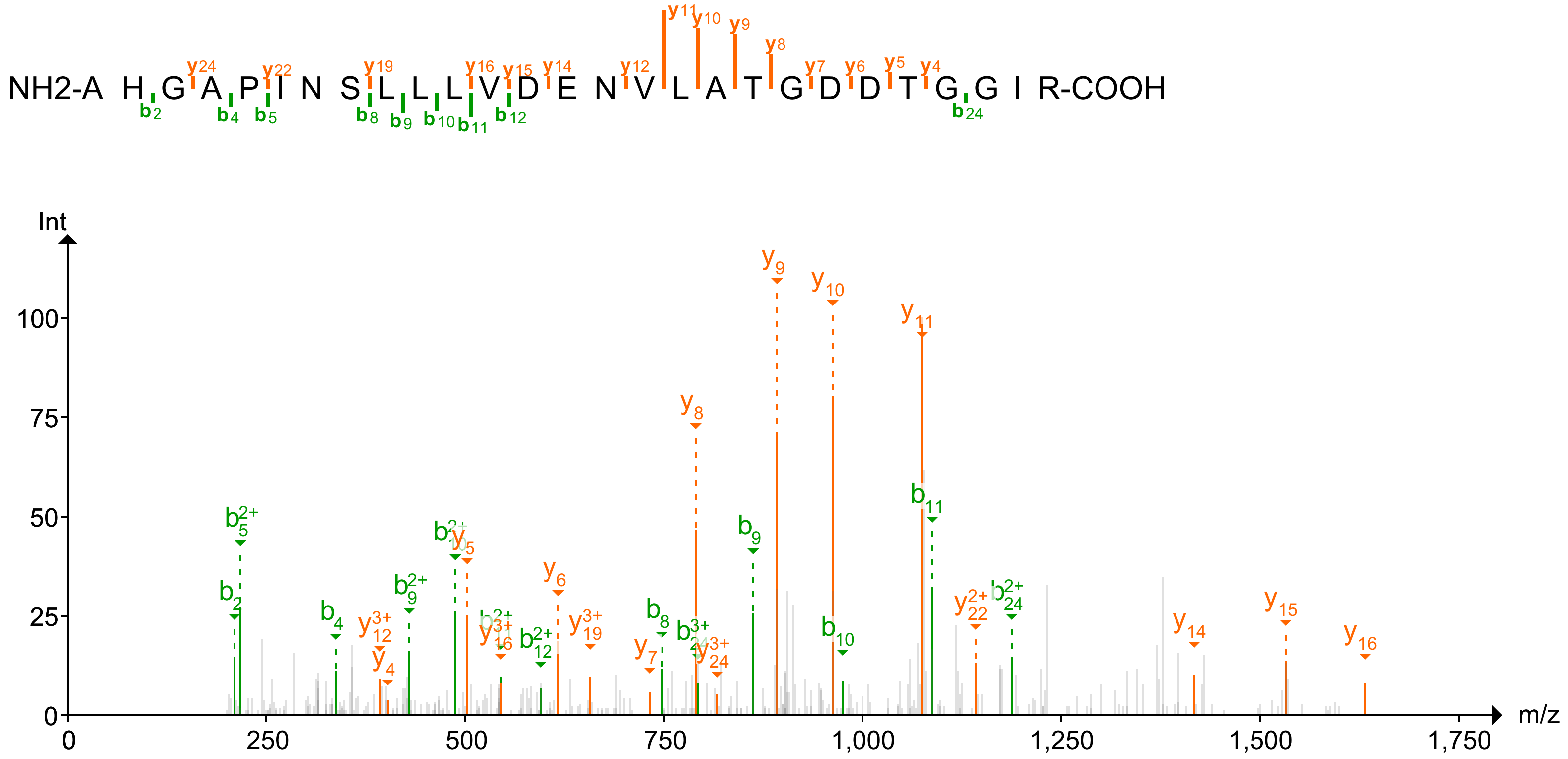

NH2-A H G A P I N S L L L V D E N V L A T G D D T G G I R-COOH
y24 y22 y19 y16 y15 y14 y12 y11 y10 y9 y8 y7 y6 y5 y4
b2 b4 b5 b8 b9 b10 b11 b12 b24
Int
100
75
50
25
0
250
500
750
1,000
1,250
1,500
1,750
m/z

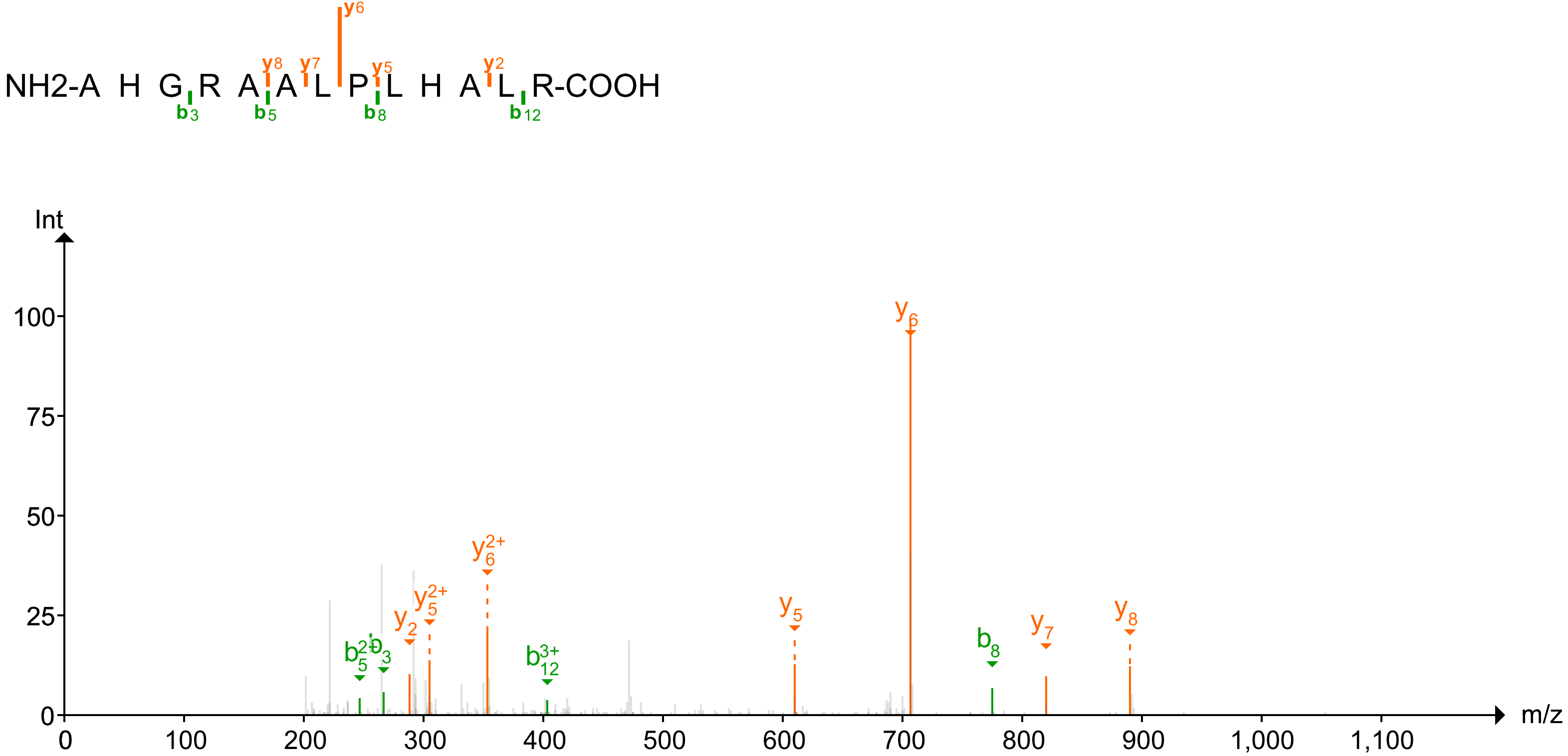

NH2-A H G R A A L P L H A L R-COOH
y6 y8 y7 y5 y2
b3 b5 b8 b12
Int
m/z
0 25 50 75 100
0 100 200 300 400 500 600 700 800 900 1,000 1,100
y6
y6 2+
y5 2+
y2
b5 2+
b3
b12 3+
y5
b8
y7
y8

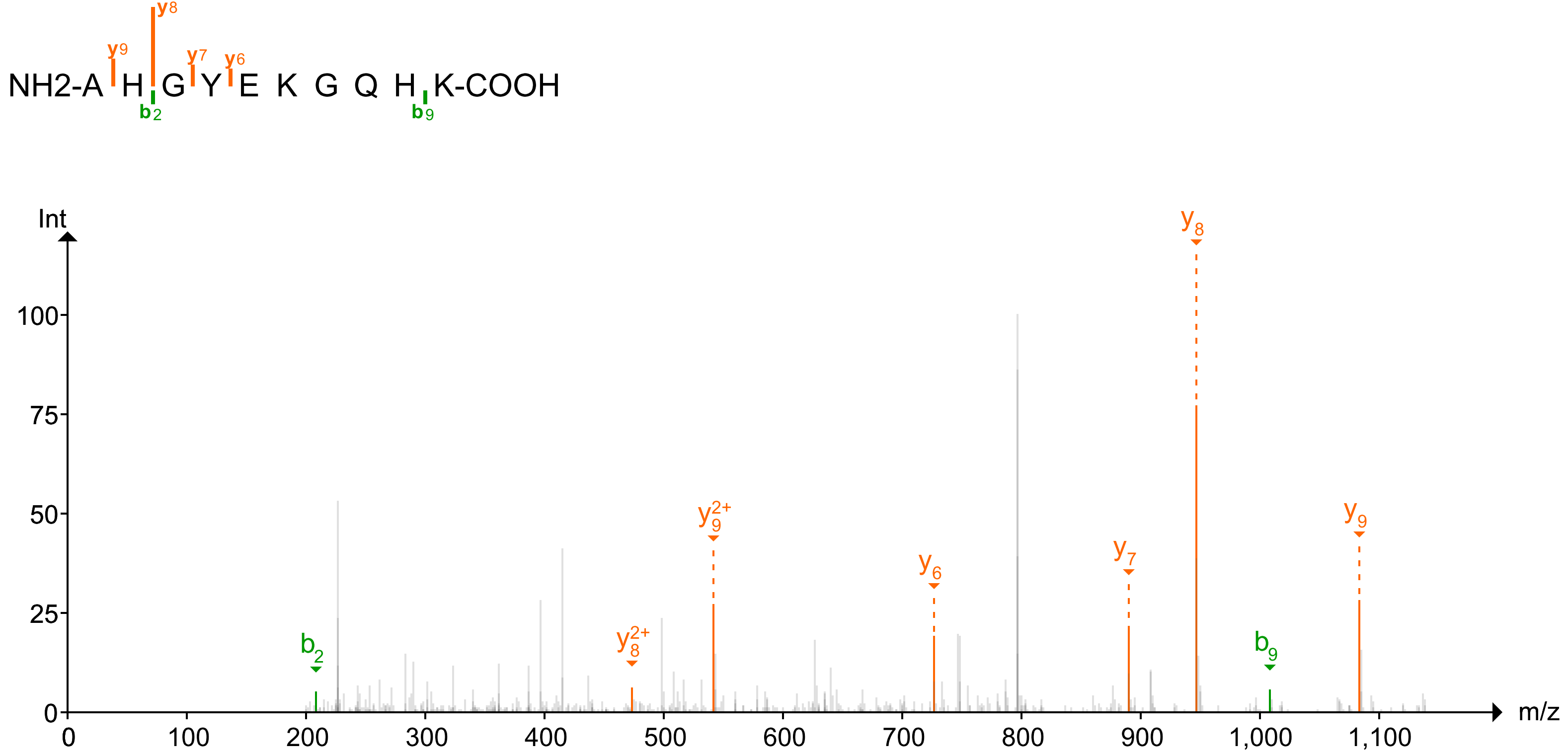

y8
y9
y7
y6
NH2-A H G Y E K G Q H K-COOH
b2
b9
Int
100
75
50
25
0
y8
y9 2+
y9
y6
y7
b2
y8 2+
b9
0
100
200
300
400
500
600
700
800
900
1,000
1,100
m/z

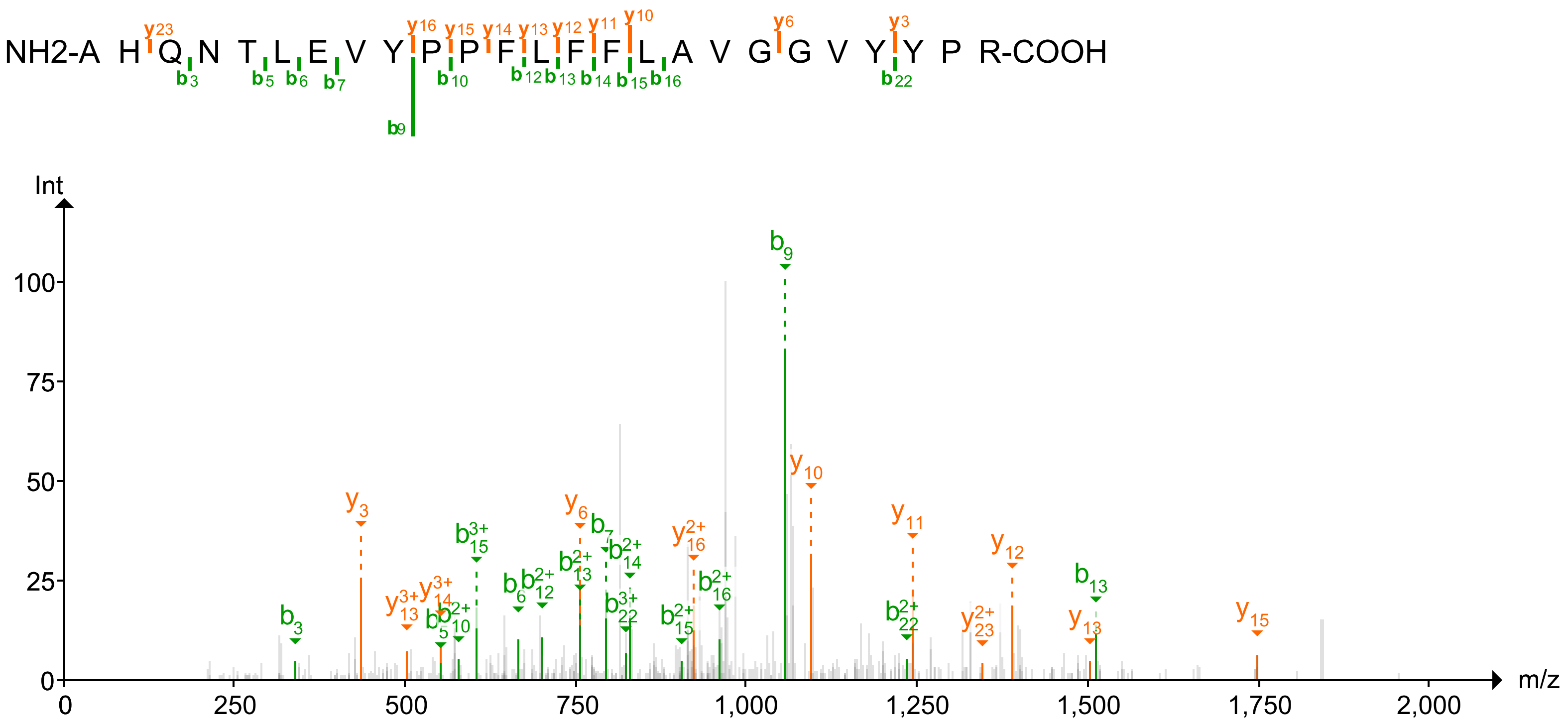
NH2-A H Q N T L E V Y P P F L F F L A V G G V Y Y P R-COOH
y23 y16 y15 y14 y13 y12 y11 y10 y6 y3
b3 b5 b6 b7 b9 b10 b12 b13 b14 b15 b16 b22
Int
100
75
50
25
0
m/z
0
250
500
750
1,000
1,250
1,500
1,750
2,000
b9
y10
y3
y6
b7
y11
y12
b13
y15
b3
y13
b22
b5
b6

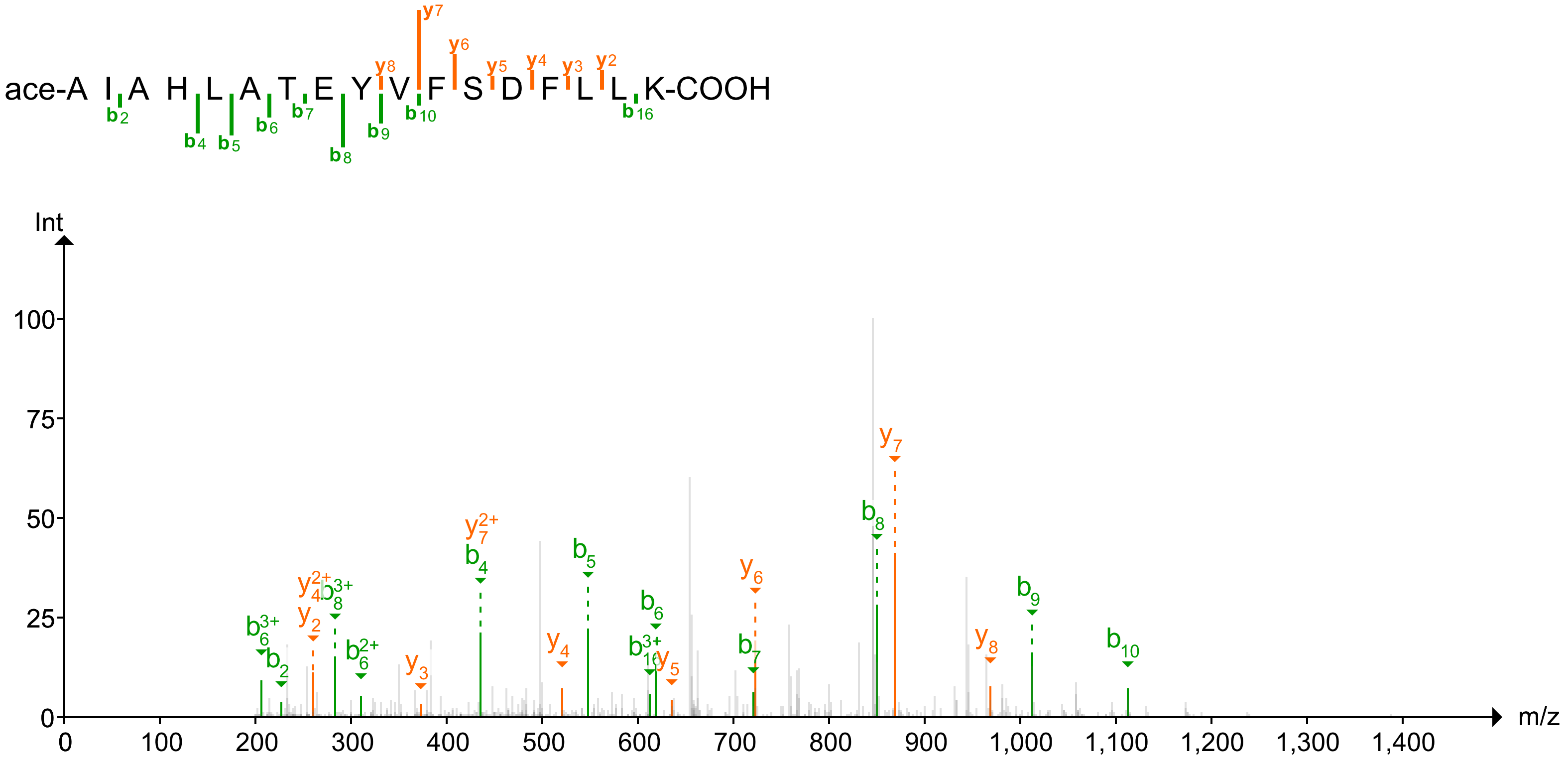

ace-A I A H L A T E Y V F S D F L L K-COOH
Int
m/z

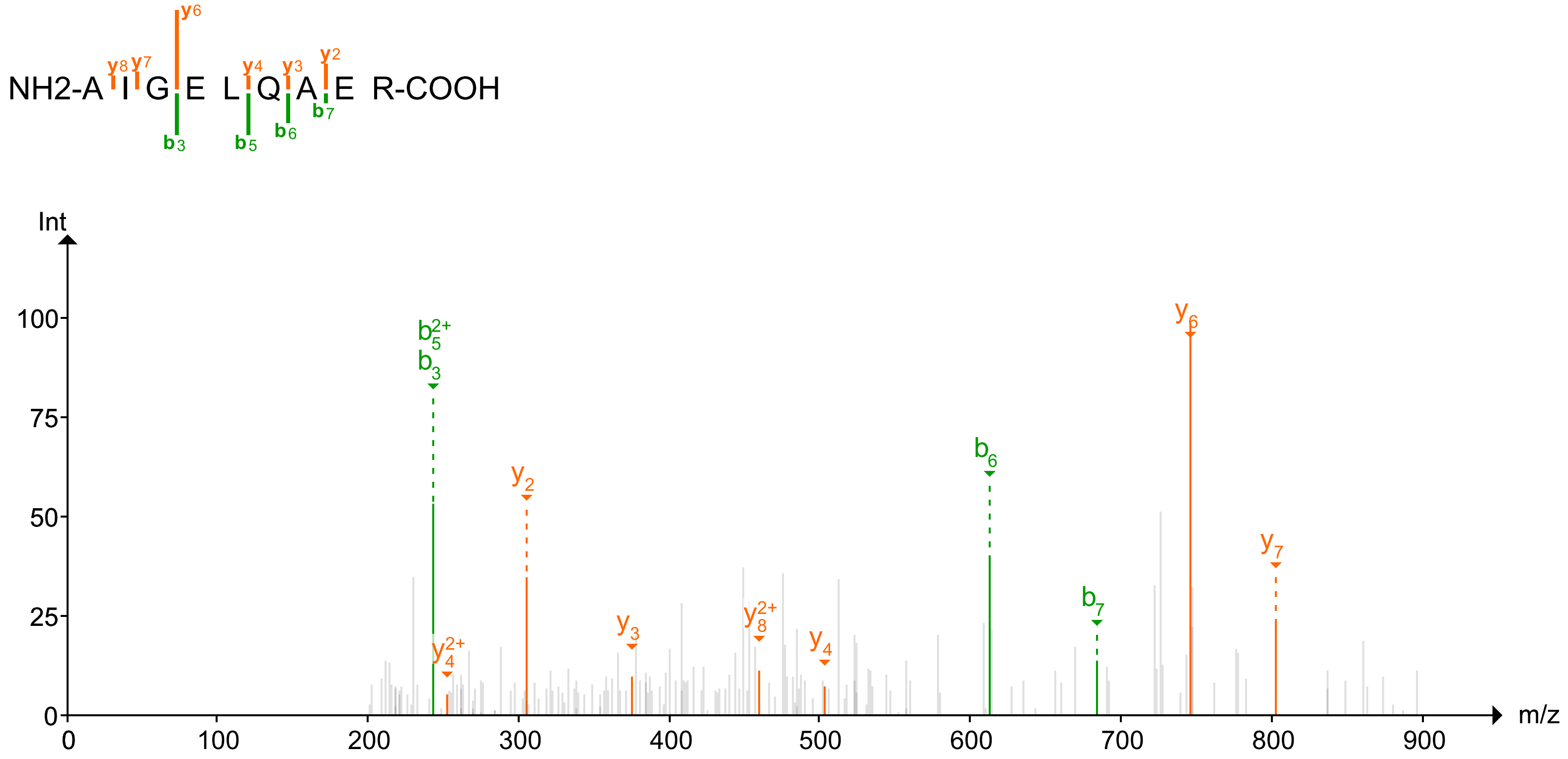

NH2-A I G E L Q A E R-COOH
y8 y7 y6 y4 y3 y2
b3 b5 b6 b7
Int
m/z
0
25
50
75
100
0
100
200
300
400
500
600
700
800
900
b5 2+
b3
y2
y4 2+
y3
y8 2+
y4
b6
b7
y6
y7

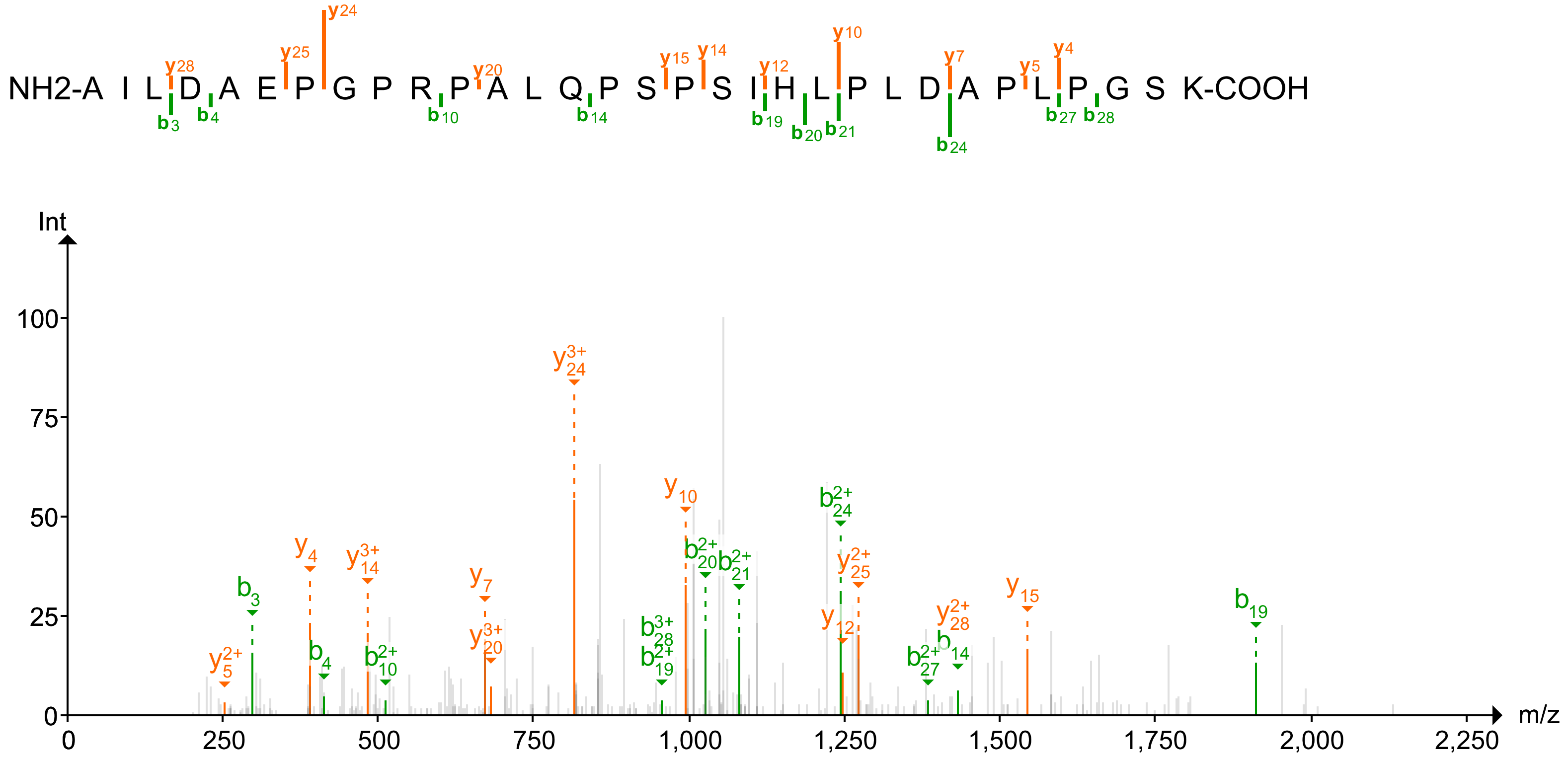

NH2-A I L D A E P G P R P A L Q P S P S I H L P L D A P L P G S K-COOH
Int
m/z

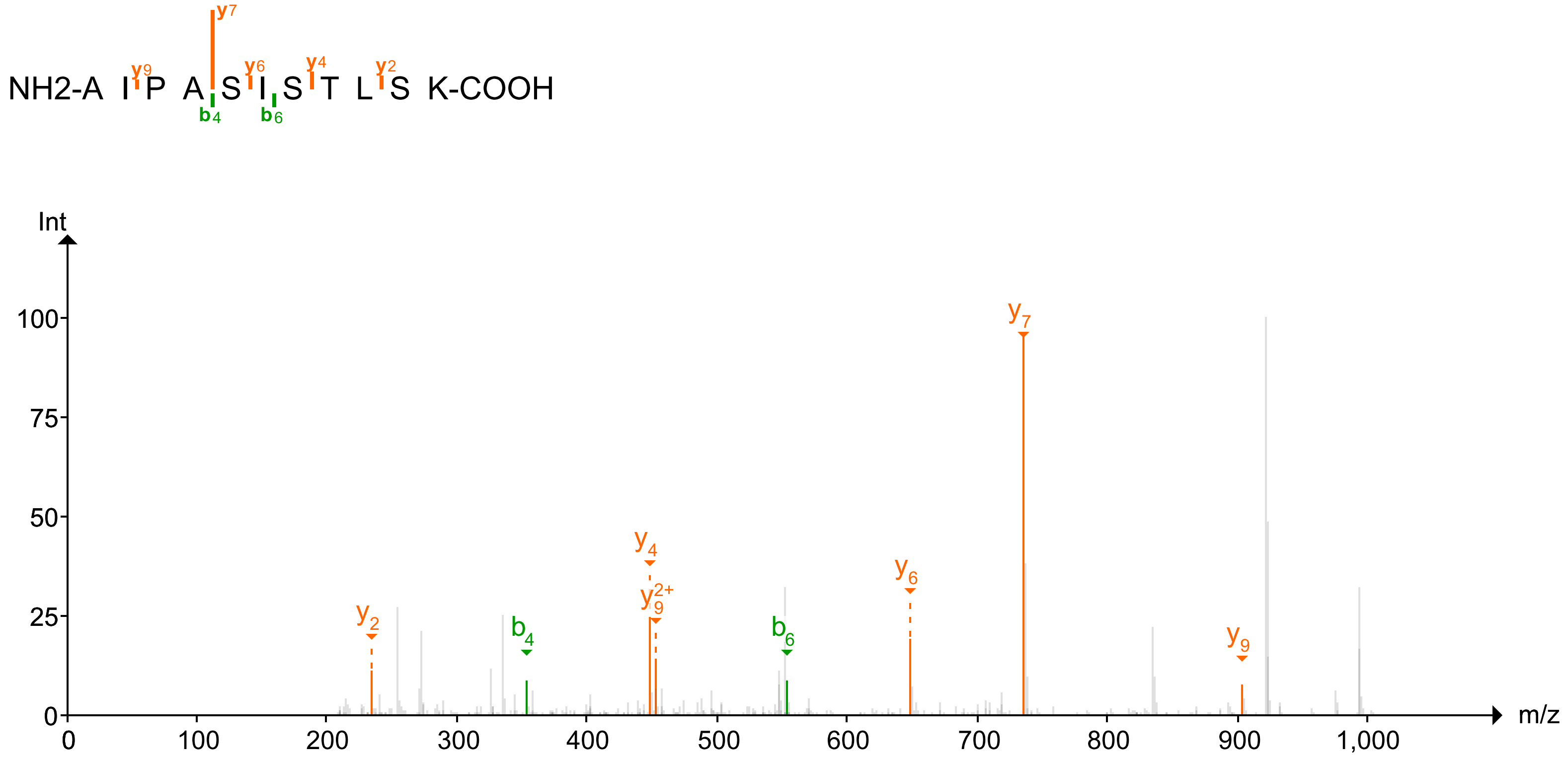

NH2-A I P A S I S T L S K-COOH
y7
y9
y6
y4
y2
b4
b6
Int
100
75
50
25
0
m/z
0
100
200
300
400
500
600
700
800
900
1,000
$y_2$
$b_4$
$y_4$
$y_9^{2+}$
$b_6$
$y_6$
$y_7$
$y_9$

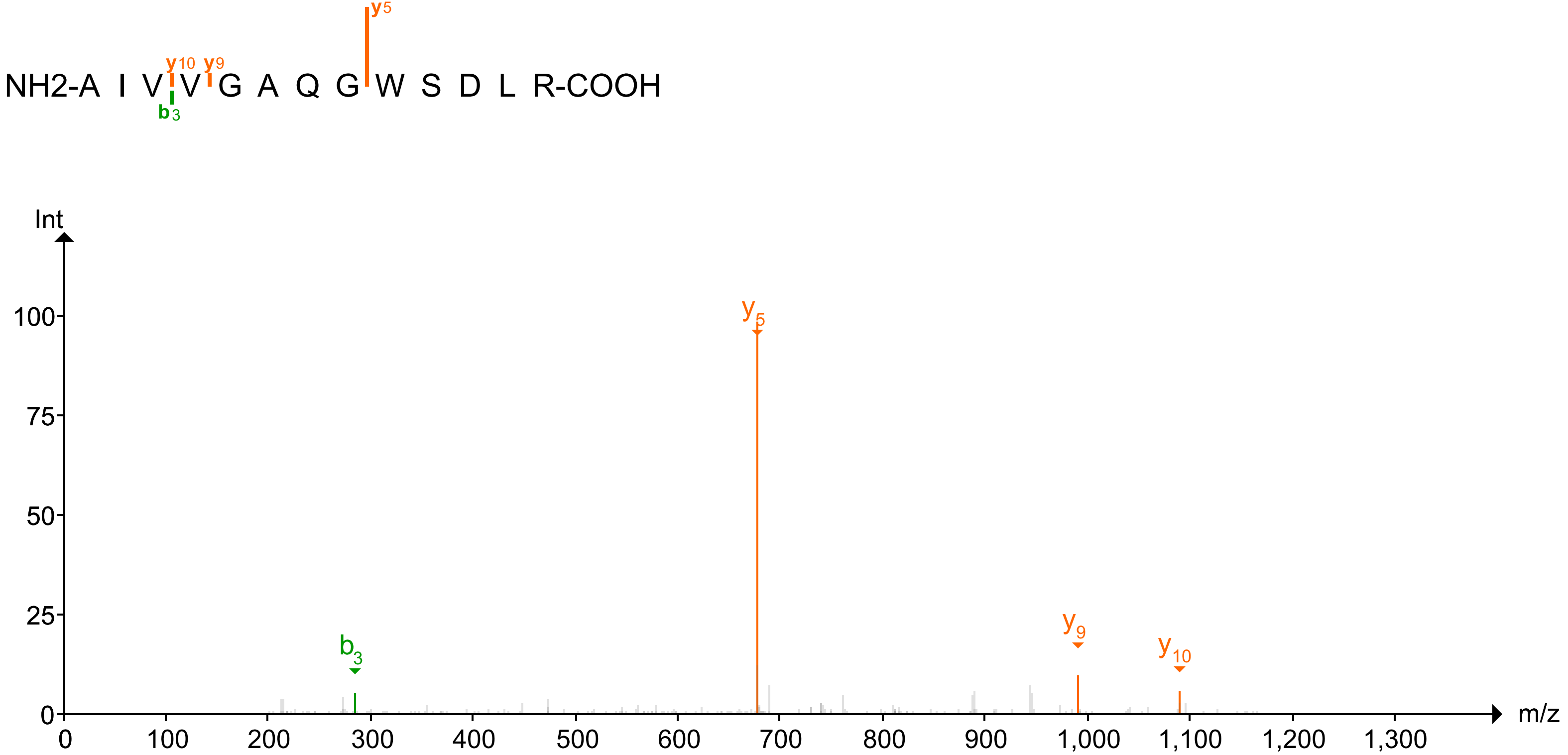

y5
y10 y9
NH2-A I V V G A Q G W S D L R-COOH
b3
Int
100
75
50
25
0
y5
b3
y9
y10
0
100
200
300
400
500
600
700
800
900
1,000
1,100
1,200
1,300
m/z

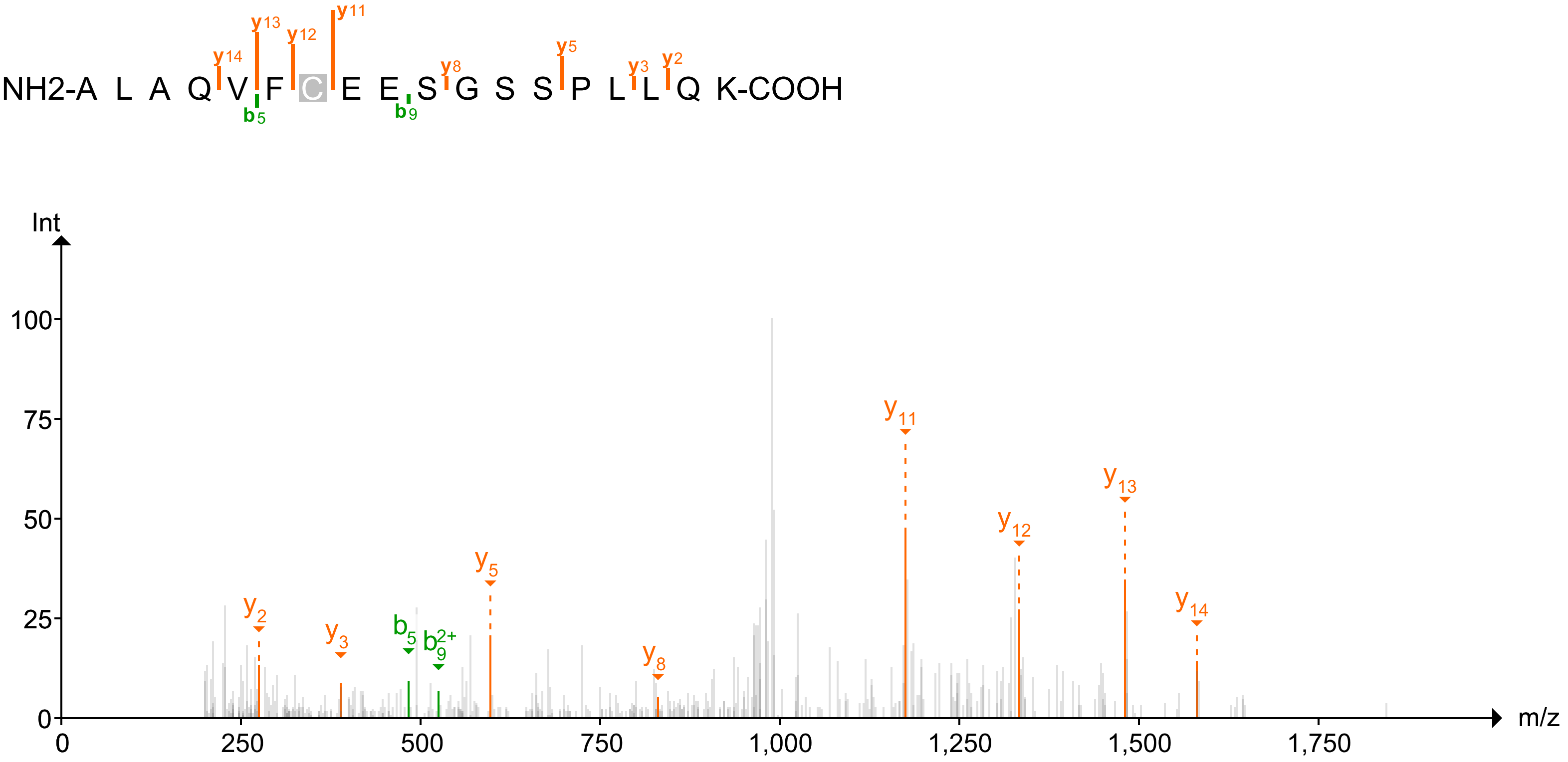

NH2-A L A Q V F C E E S G S S P L L Q K-COOH
y14 y13 y12 y11 y8 y5 y3 y2
b5 b9
Int
100
75
50
25
0
m/z
0
250
500
750
1,000
1,250
1,500
1,750
y2 y3 b5 b9 2+ y5 y8 y11 y12 y13 y14

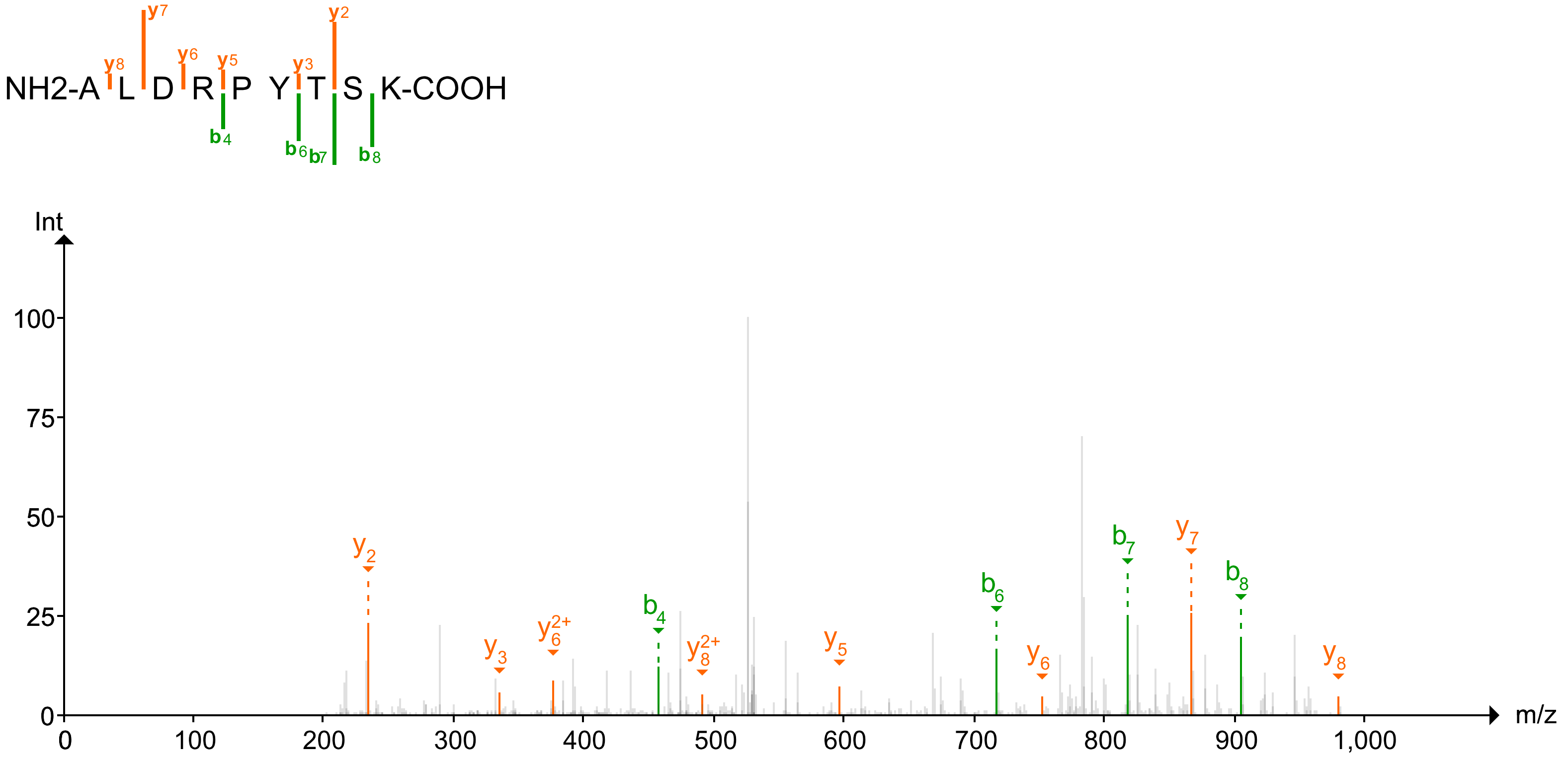

NH2-A L D R P Y T S K-COOH
y8 y7 y6 y5 y3 y2
b4 b6 b7 b8
Int
100
75
50
25
0
y2
y3
y6 2+
b4
y8 2+
y5
b6
y6
b7
y7
b8
y8
m/z
0
100
200
300
400
500
600
700
800
900
1,000

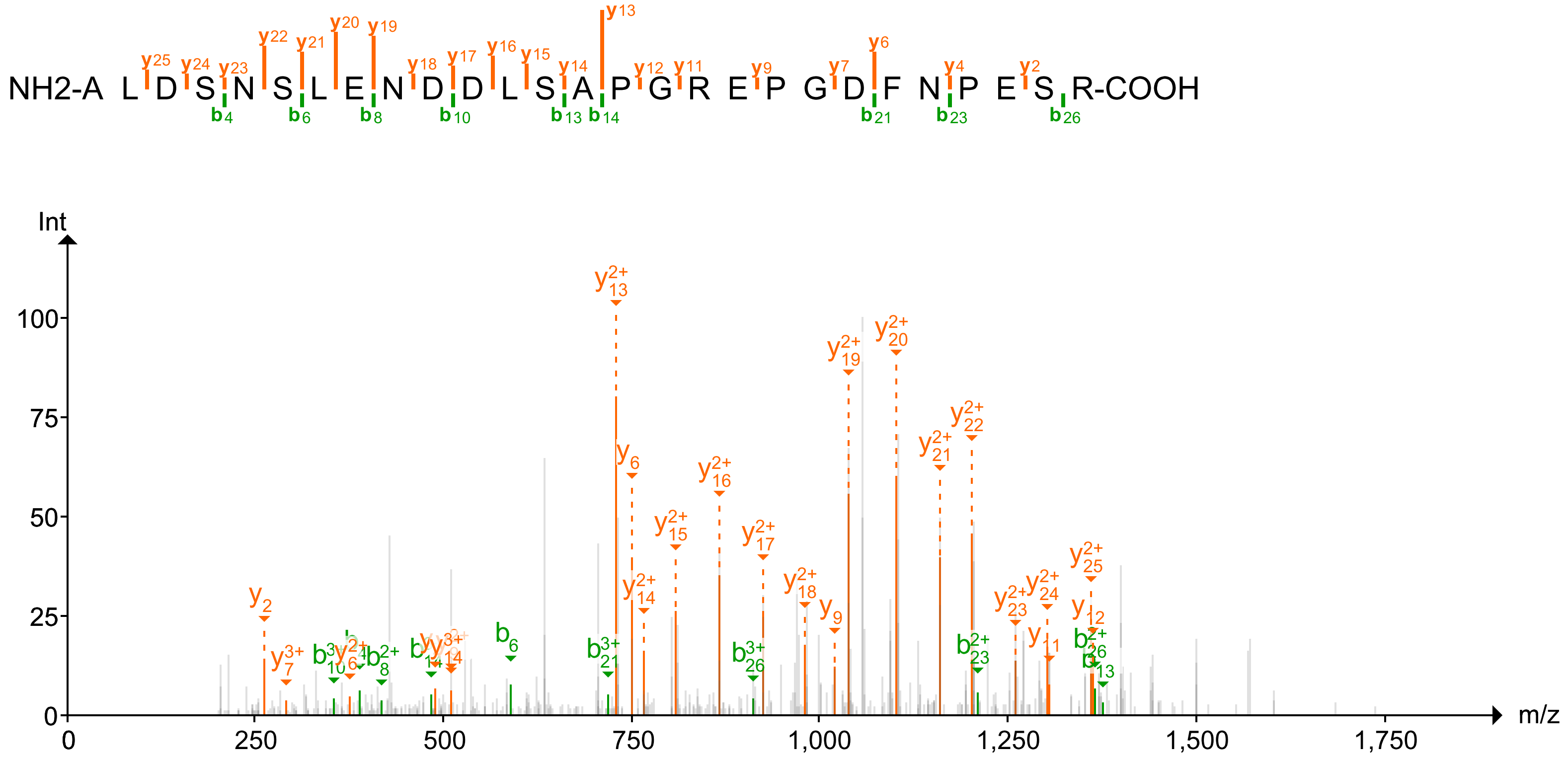

NH2-A L D S N S L E N D D L S A P G R E P G D F N P E S R-COOH
Int
m/z
0
25
50
75
100
250
500
750
1,000
1,250
1,500
1,750

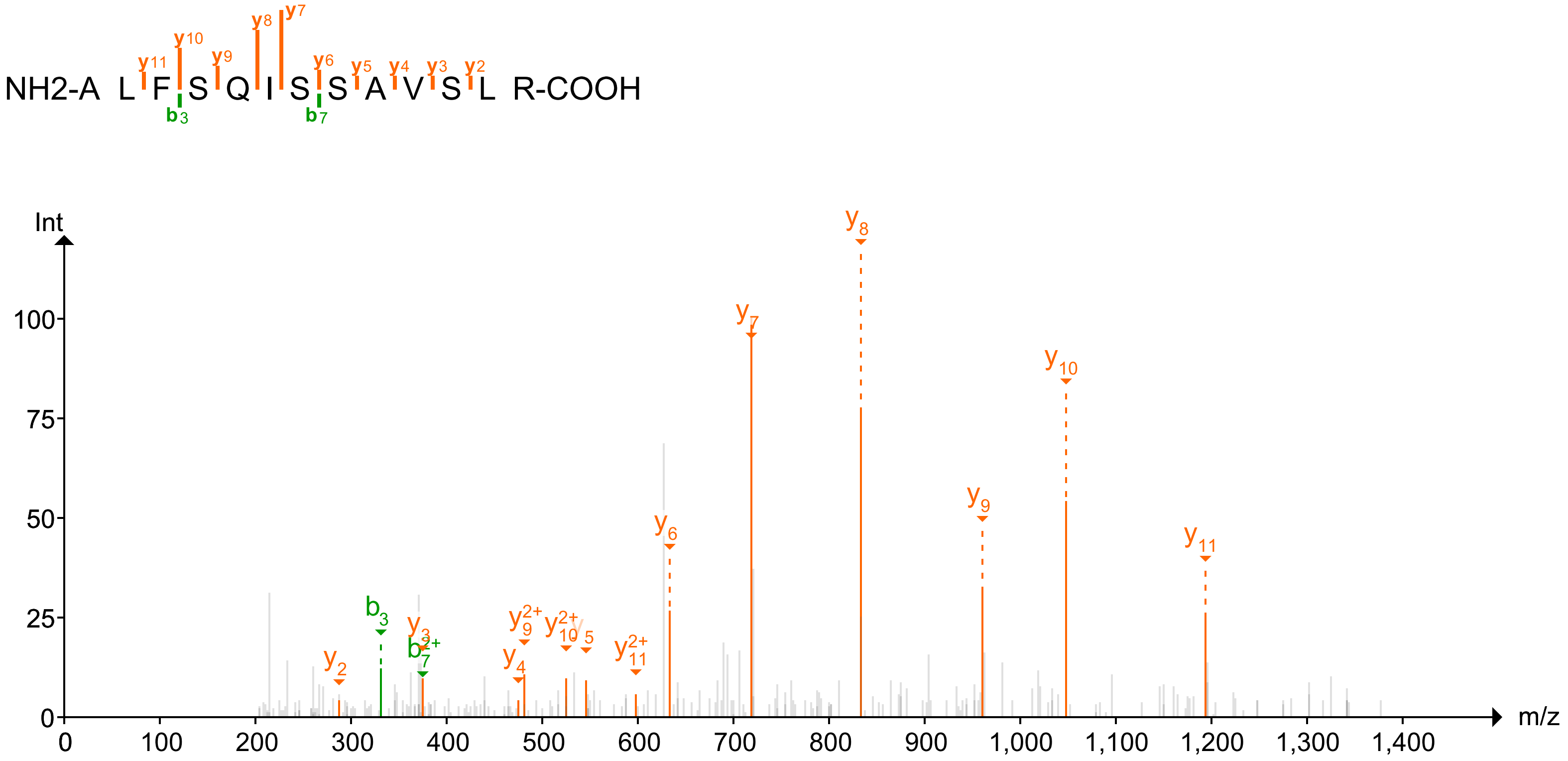

NH2-A L F S Q I S S A V S L R-COOH
y11 y10 y9 y8 y7 y6 y5 y4 y3 y2
b3 b7
Int
100
75
50
25
0
m/z
0 100 200 300 400 500 600 700 800 900 1,000 1,100 1,200 1,300 1,400
y8 y7 y10 y9 y6 y11
b3 y2 y3 $b_7^{2+}$ y4 $y_9^{2+}$ $y_{10}^{2+}$ y5 $y_{11}^{2+}$

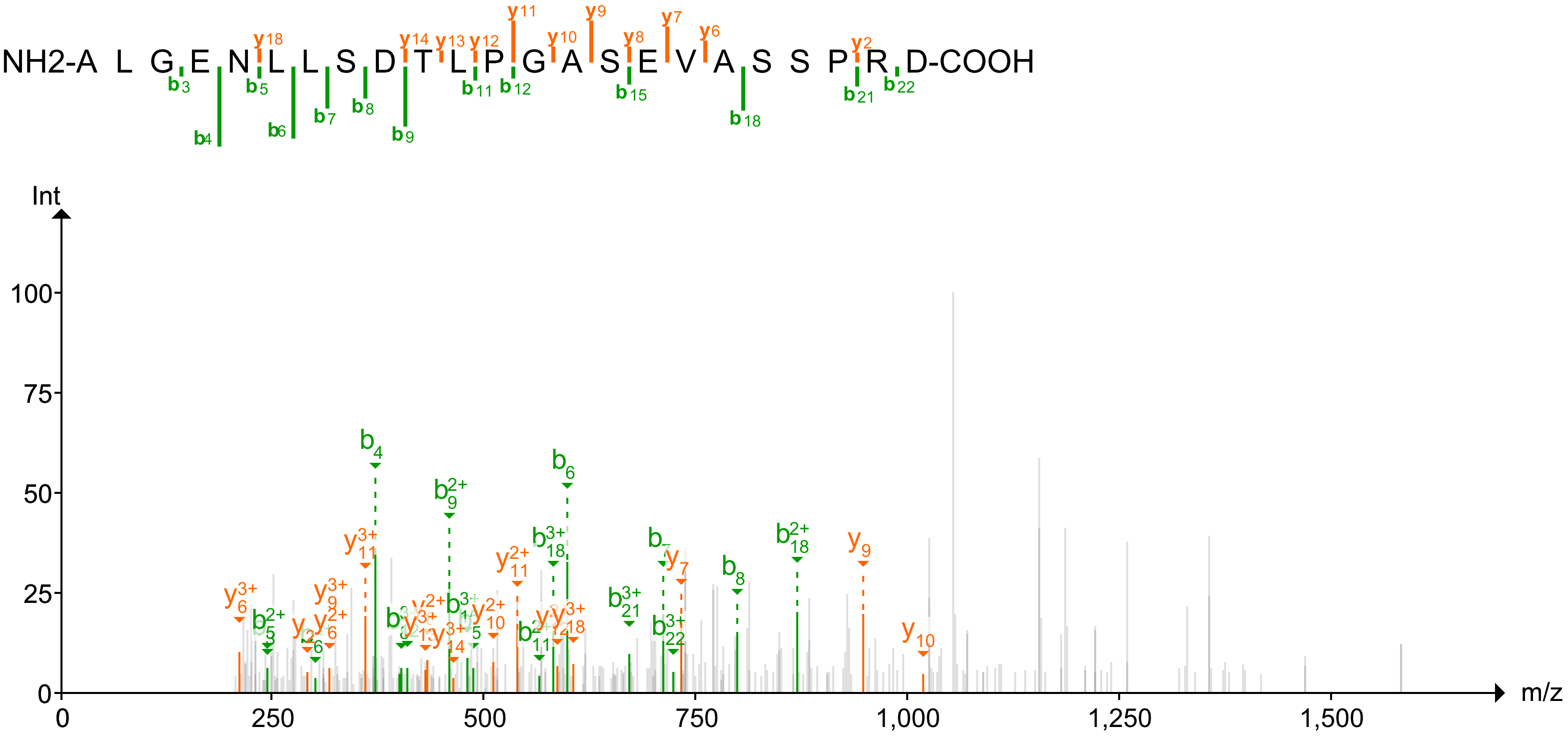

NH2-A L G E N L L S D T L P G A S E V A S S P R D-COOH
y18 y14 y13 y12 y11 y10 y9 y8 y7 y6 y2
b3 b4 b5 b6 b7 b8 b9 b11 b12 b15 b18 b21 b22
Int
100
75
50
25
0
0
250
500
750
1,000
1,250
1,500
m/z

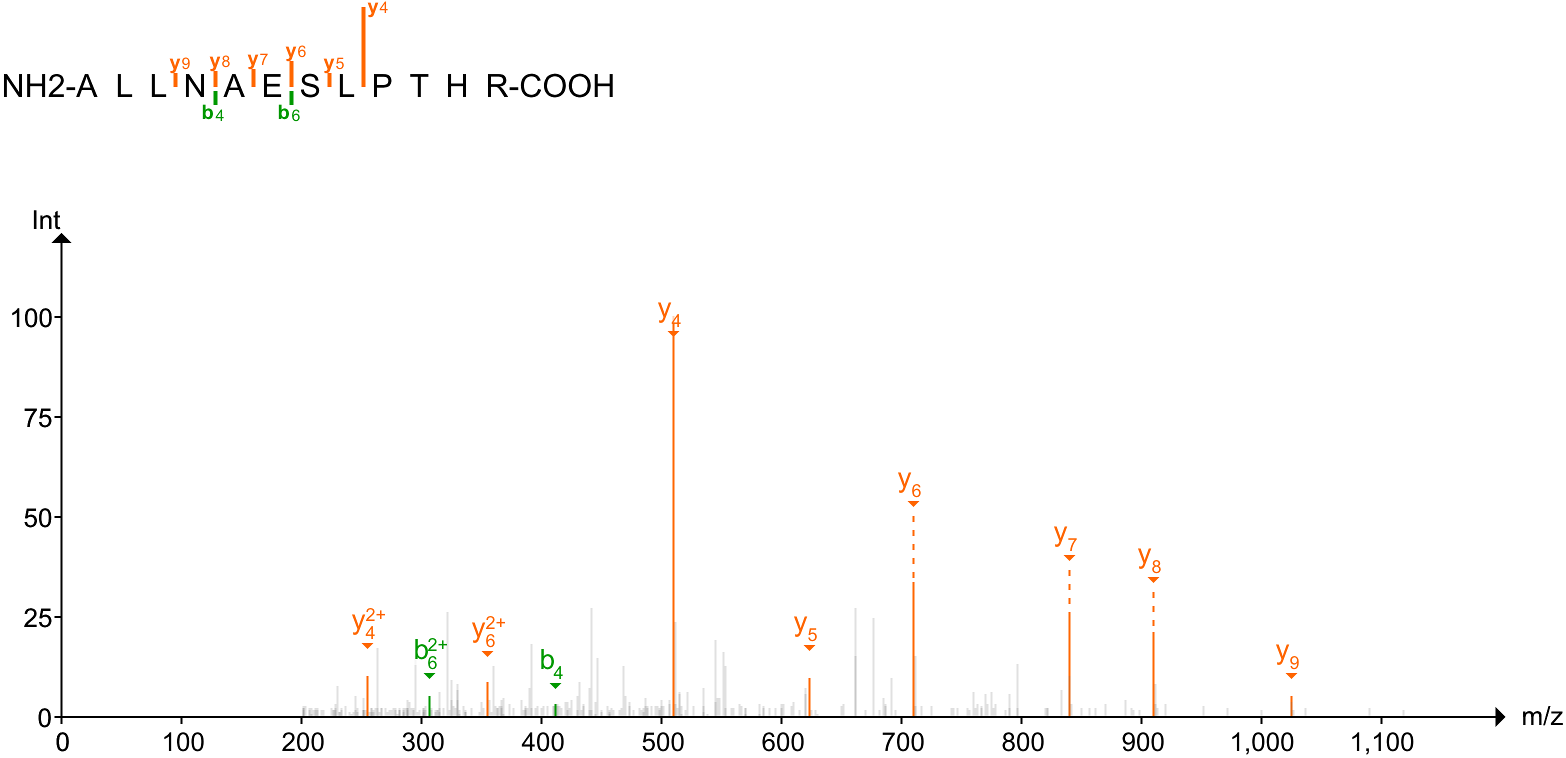

NH2-A L L N A E S L P T H R-COOH
y9 y8 y7 y6 y5 y4
b4 b6
Int
100
75
50
25
0
m/z
0 100 200 300 400 500 600 700 800 900 1,000 1,100
y4
y6
y7
y8
y9
y5
y4 2+
y6 2+
b6 2+
b4

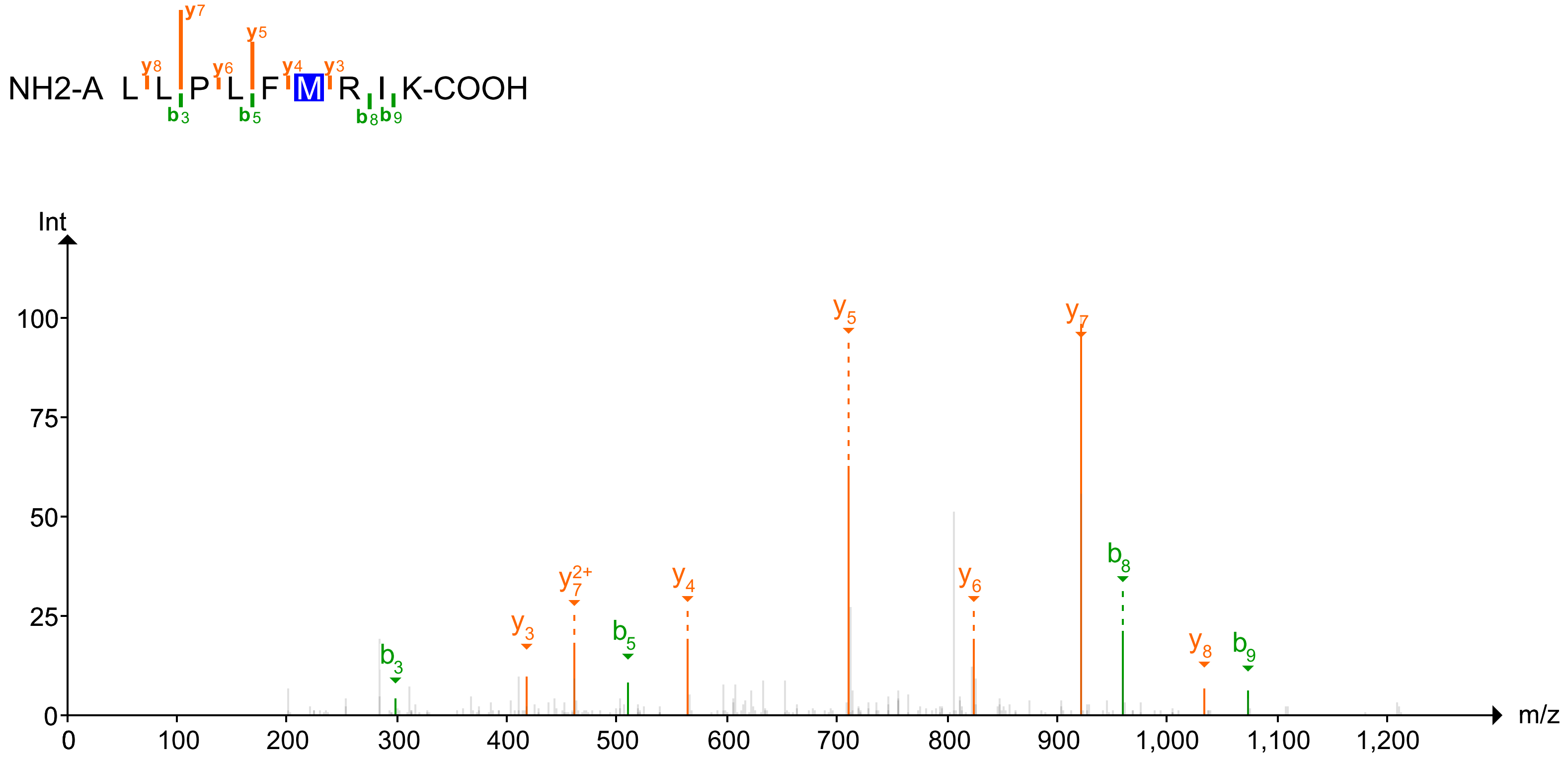

NH2-A L L P L F M R I K-COOH
y7 y5 y8 y6 y4 y3
b3 b5 b8 b9
Int
100
75
50
25
0
y5
y7
b8
y$_7^{2+}$
y4
y6
y3
b5
b3
y8
b9
0 100 200 300 400 500 600 700 800 900 1,000 1,100 1,200
m/z

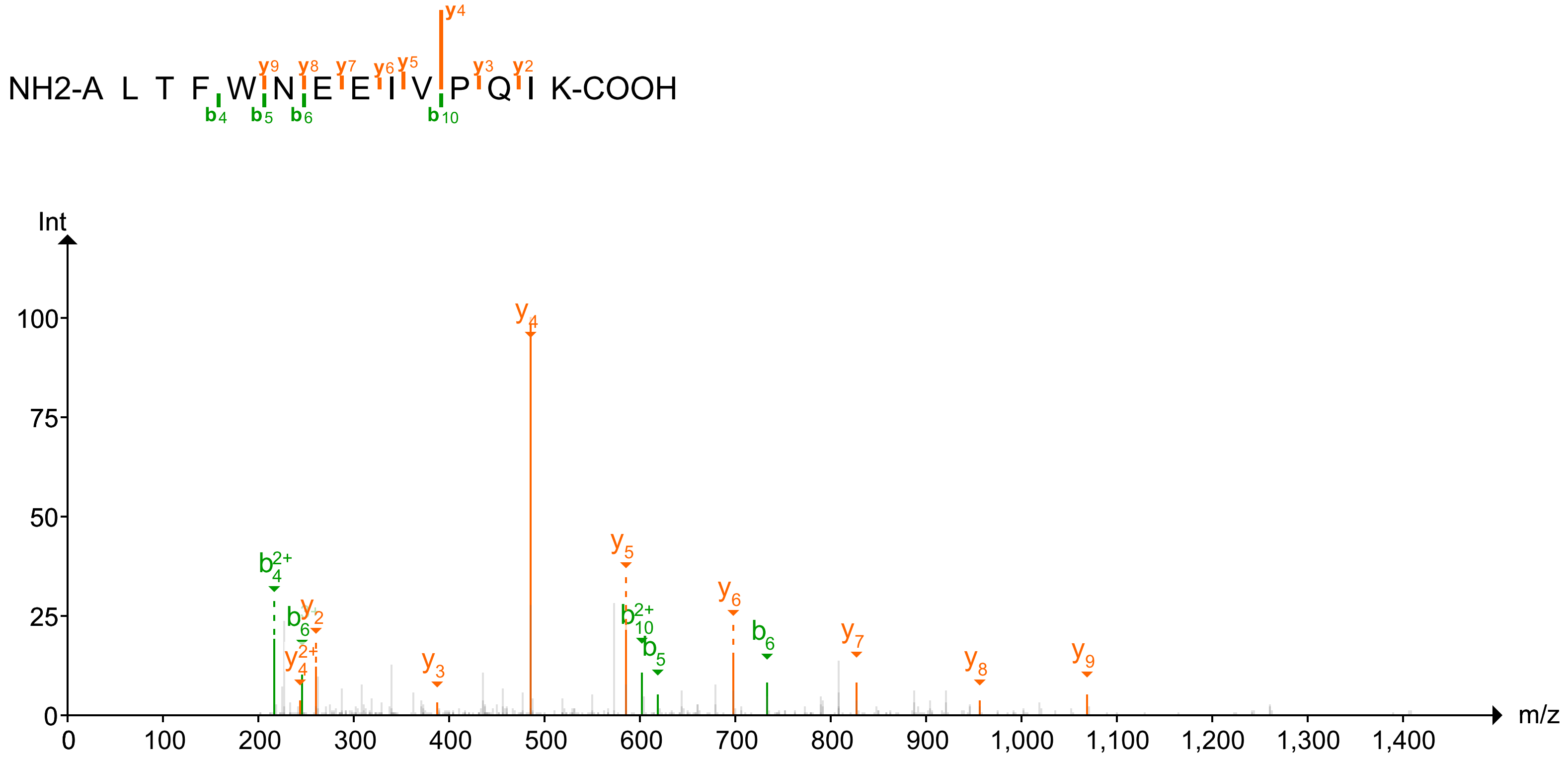
NH2-A L T F W N E E I V P Q I K-COOH
y4
y9
y8
y7
y6
y5
y3
y2
b4
b5
b6
b10
Int
100
75
50
25
0
m/z
0
100
200
300
400
500
600
700
800
900
1,000
1,100
1,200
1,300
1,400
y4
y5
y6
y7
y8
y9
y3
y2
b4 2+
b6 2+
y4 2+
b10 2+
b5
b6

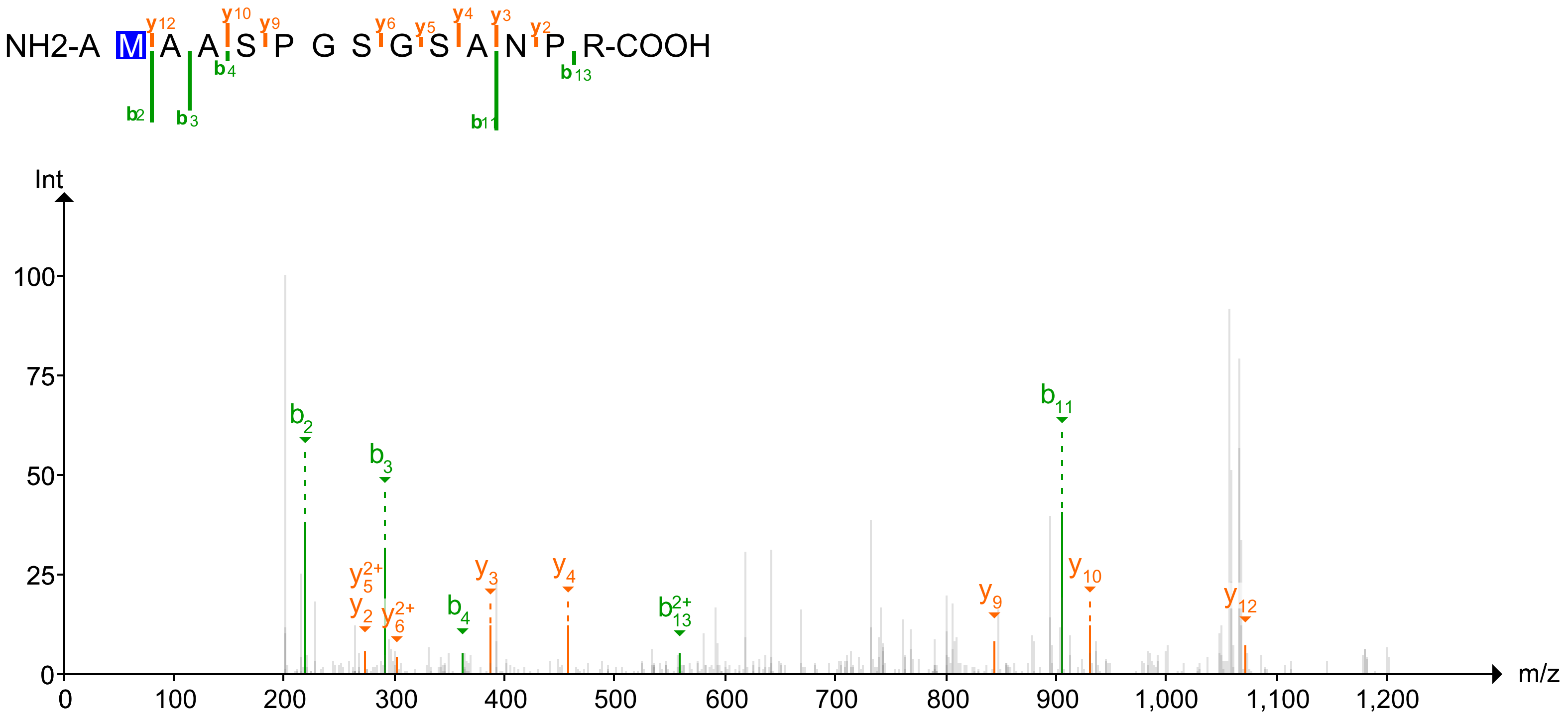

NH2-A M A A S P G S G S A N P R-COOH
y12 y10 y9 y6 y5 y4 y3 y2
b2 b3 b4 b11 b13
Int
m/z
b2
b3
b4
b11
b13 2+
y2
y3
y4
y5 2+
y6 2+
y9
y10
y12

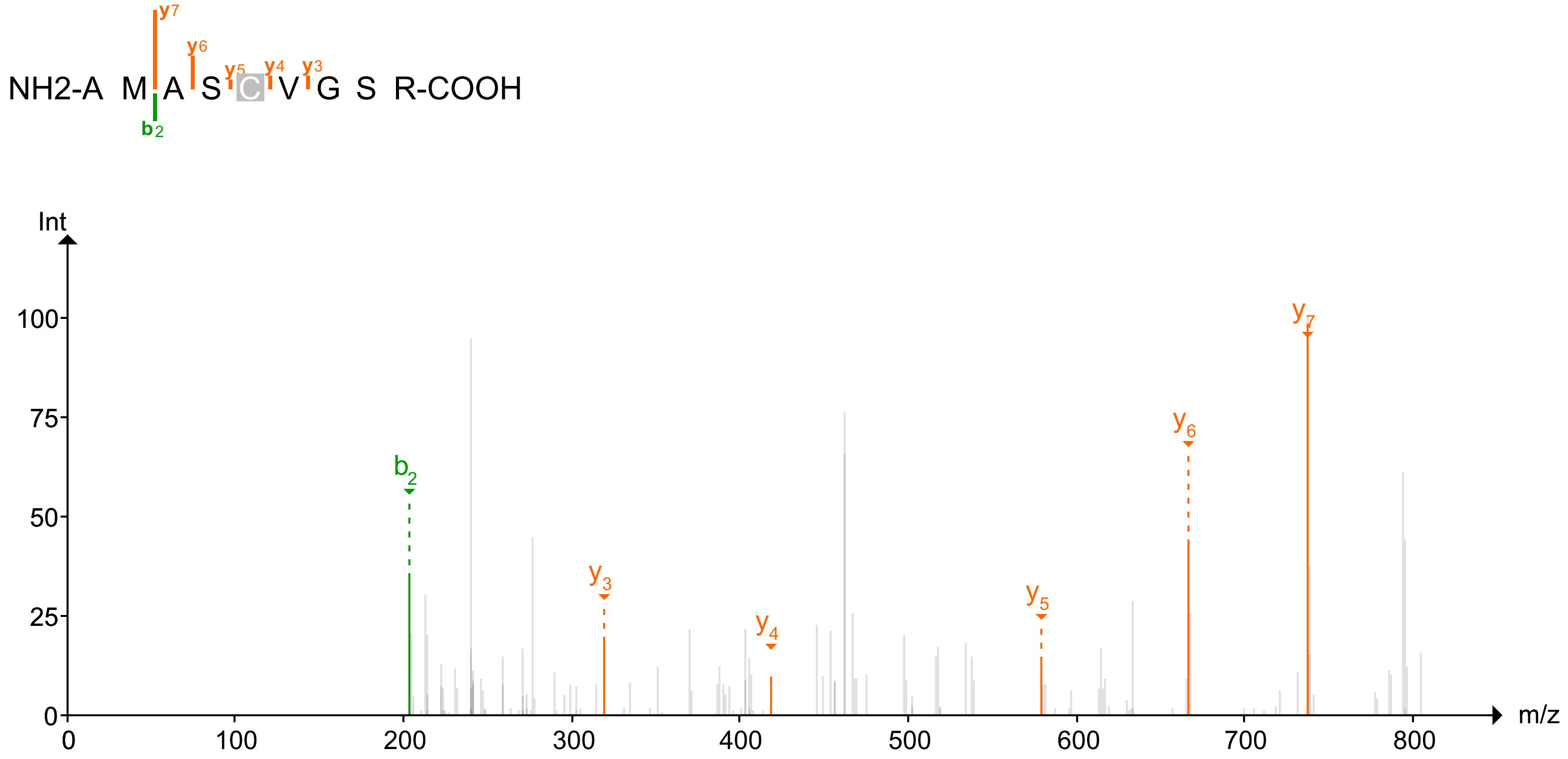

NH2-A M A S C V G S R-COOH
y7
y6
y5
y4
y3
b2
Int
100
75
50
25
0
m/z
0
100
200
300
400
500
600
700
800

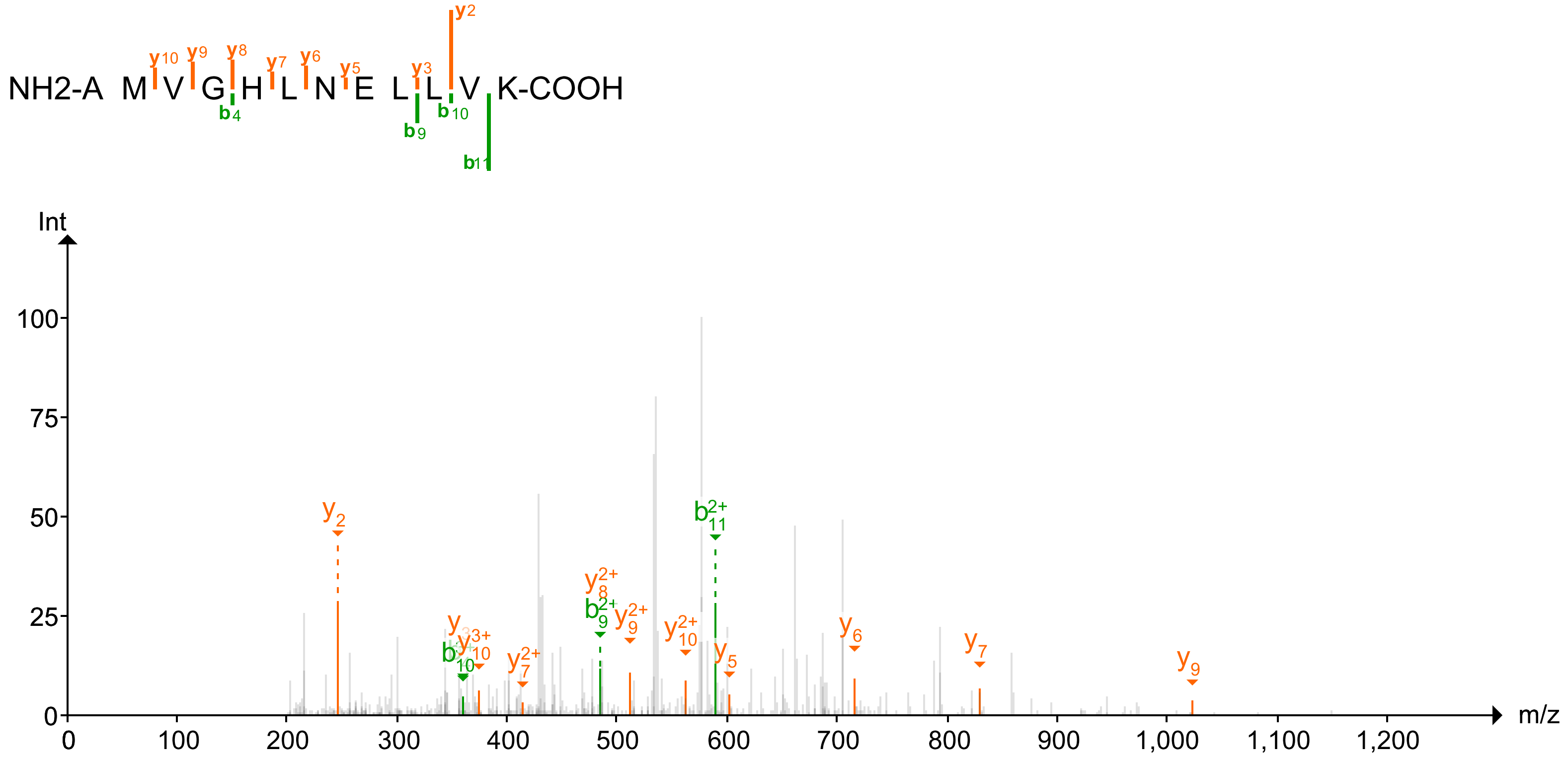

NH2-A M V G H L N E L L V K-COOH
y10 y9 y8 y7 y6 y5 y3 y2
b4 b9 b10 b11
Int
100
75
50
25
0
m/z
0 100 200 300 400 500 600 700 800 900 1,000 1,100 1,200
y2
b11 2+
y8 2+
b9 2+
y9 2+
y10 2+
y10 3+
b10 2+
y7 2+
y5
y6
y7
y9

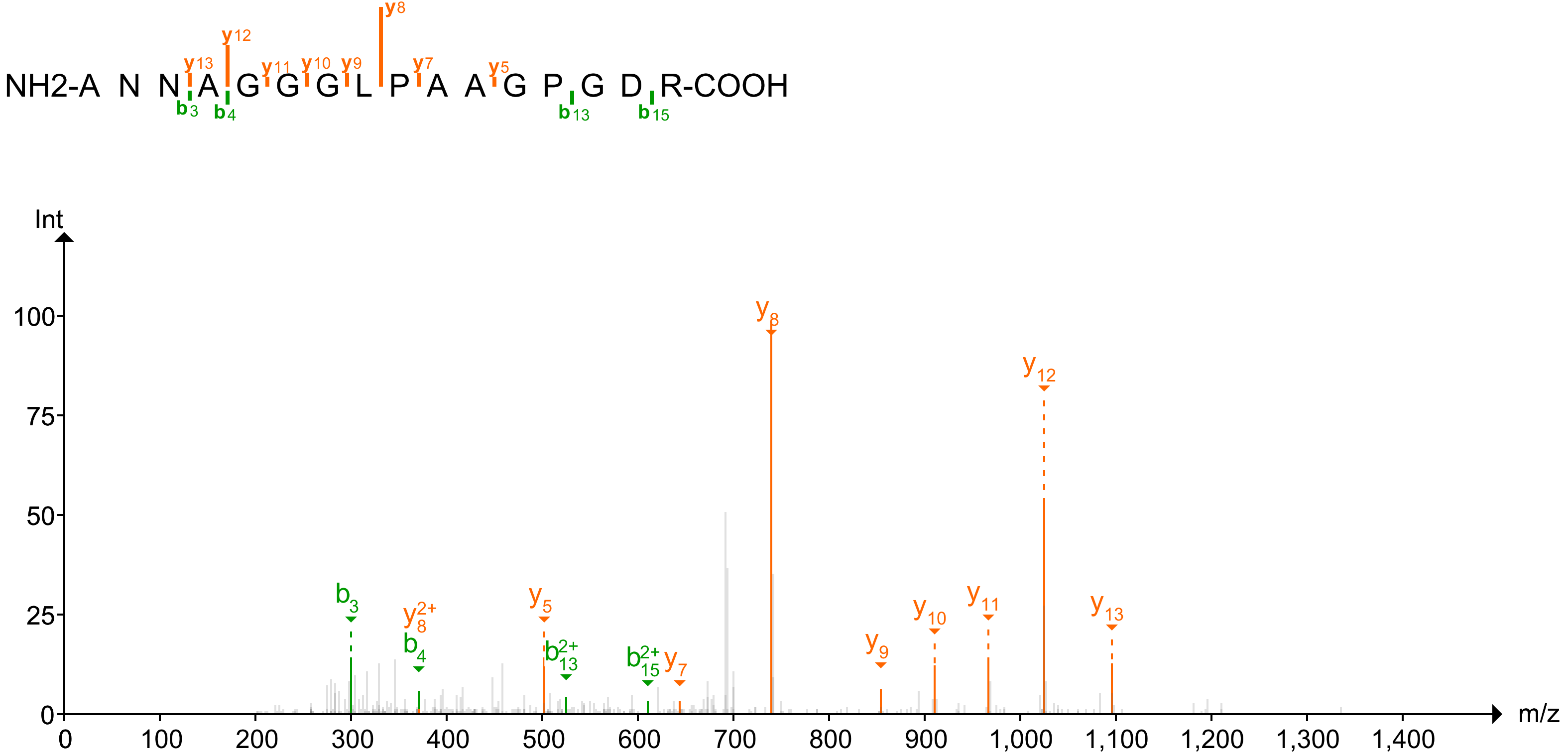

NH2-A N N A G G G L P A A G P G D R-COOH
y13 y12 y11 y10 y9 y8 y7 y5
b3 b4 b13 b15
Int
m/z
y8
y12
b3
y8 2+
b4
y5
b13 2+
b15 2+
y7
y9
y10
y11
y13

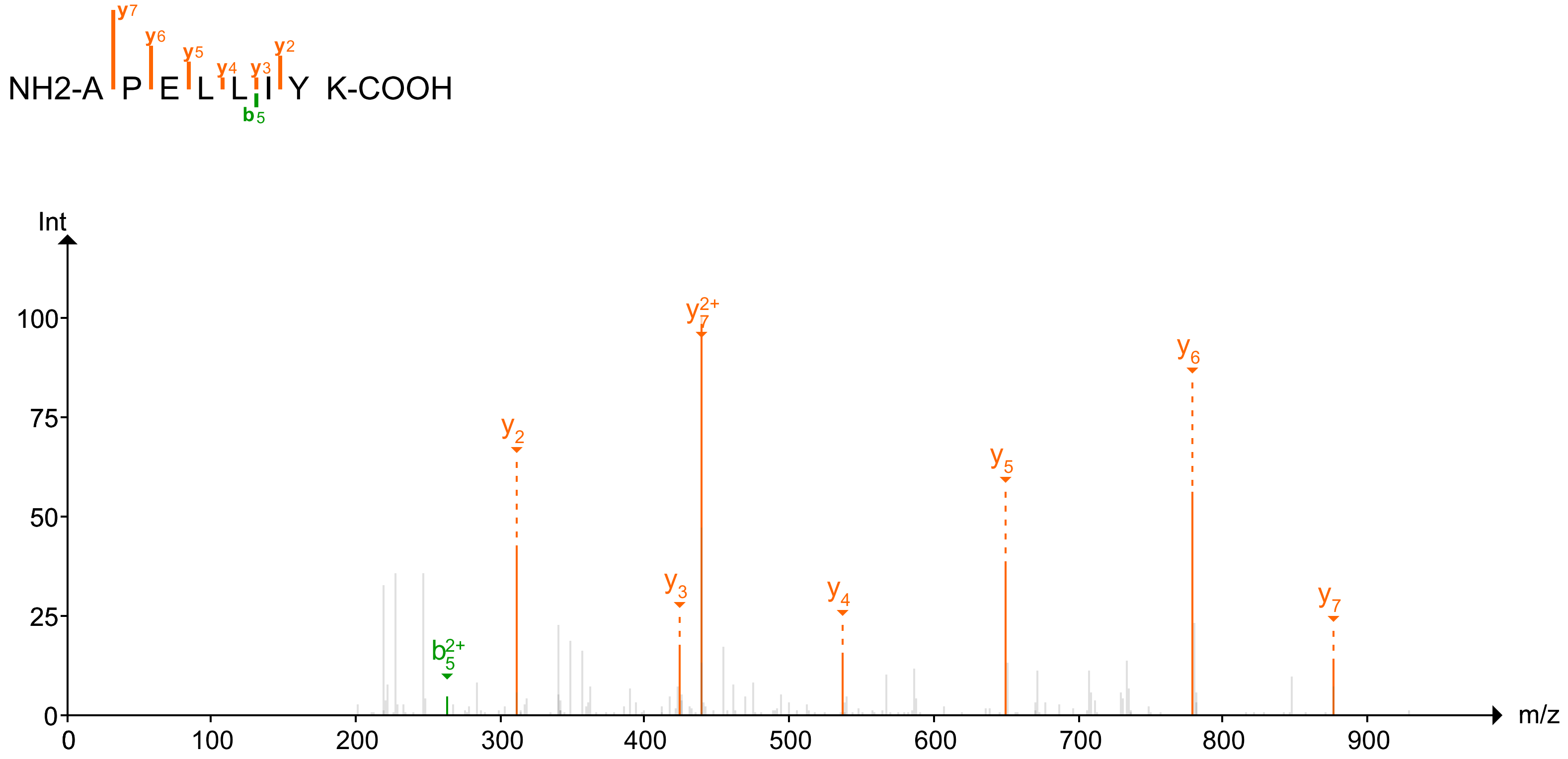

NH2-A P E L L I Y K-COOH
y7
y6
y5
y4
y3
y2
b5
Int
100
75
50
25
0
0
100
200
300
400
500
600
700
800
900
m/z
$y_7^{2+}$
$y_6$
$y_2$
$y_5$
$y_3$
$y_4$
$y_7$
$b_5^{2+}$

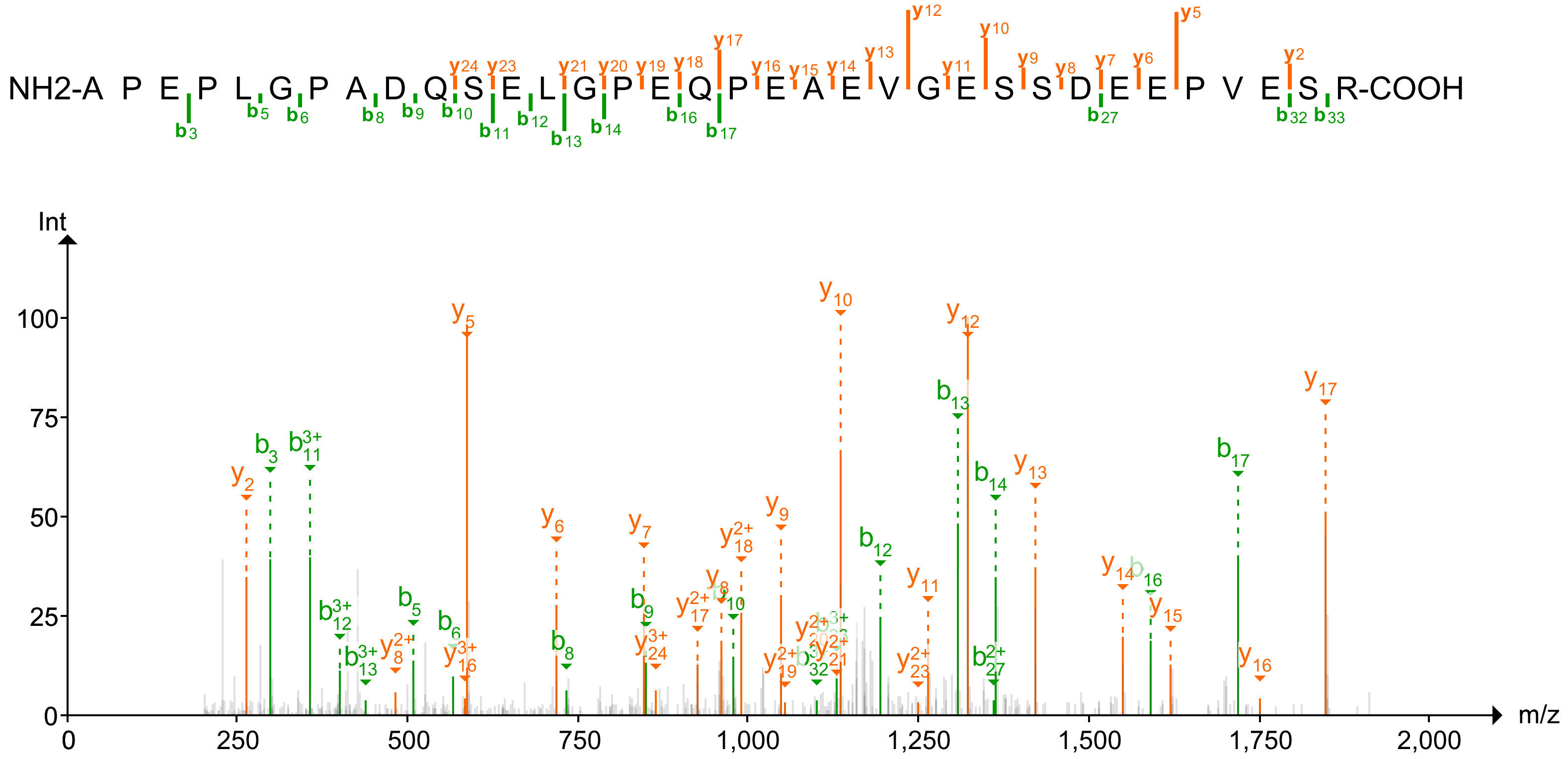

NH2-A P E P L G P A D Q S E L G P E Q P E A E V G E S S D E E P V E S R-COOH
Int
100
75
50
25
0
m/z
0
250
500
750
1,000
1,250
1,500
1,750
2,000

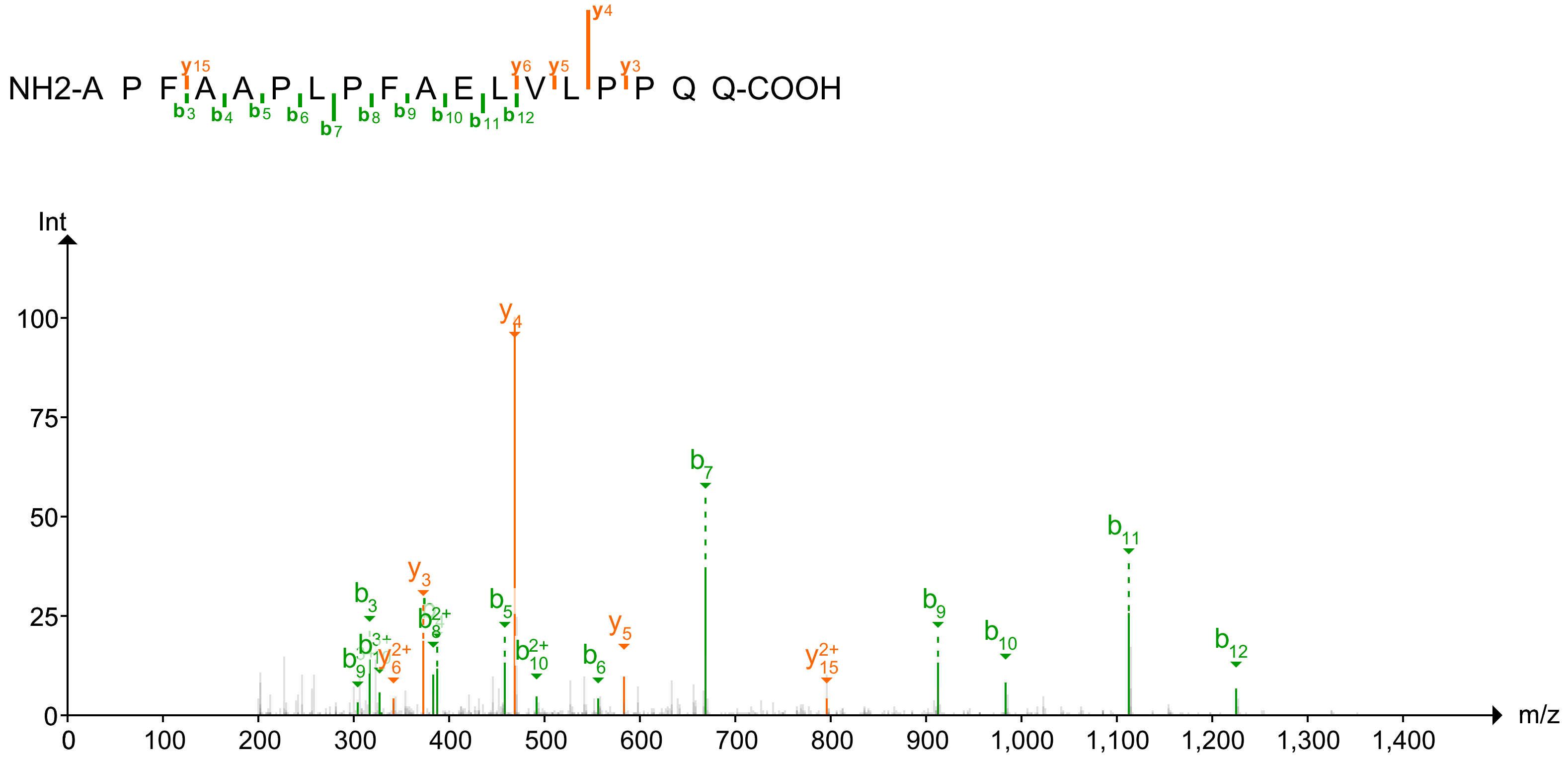
NH2-A P F A A P L P F A E L V L P P Q Q-COOH
Int
m/z

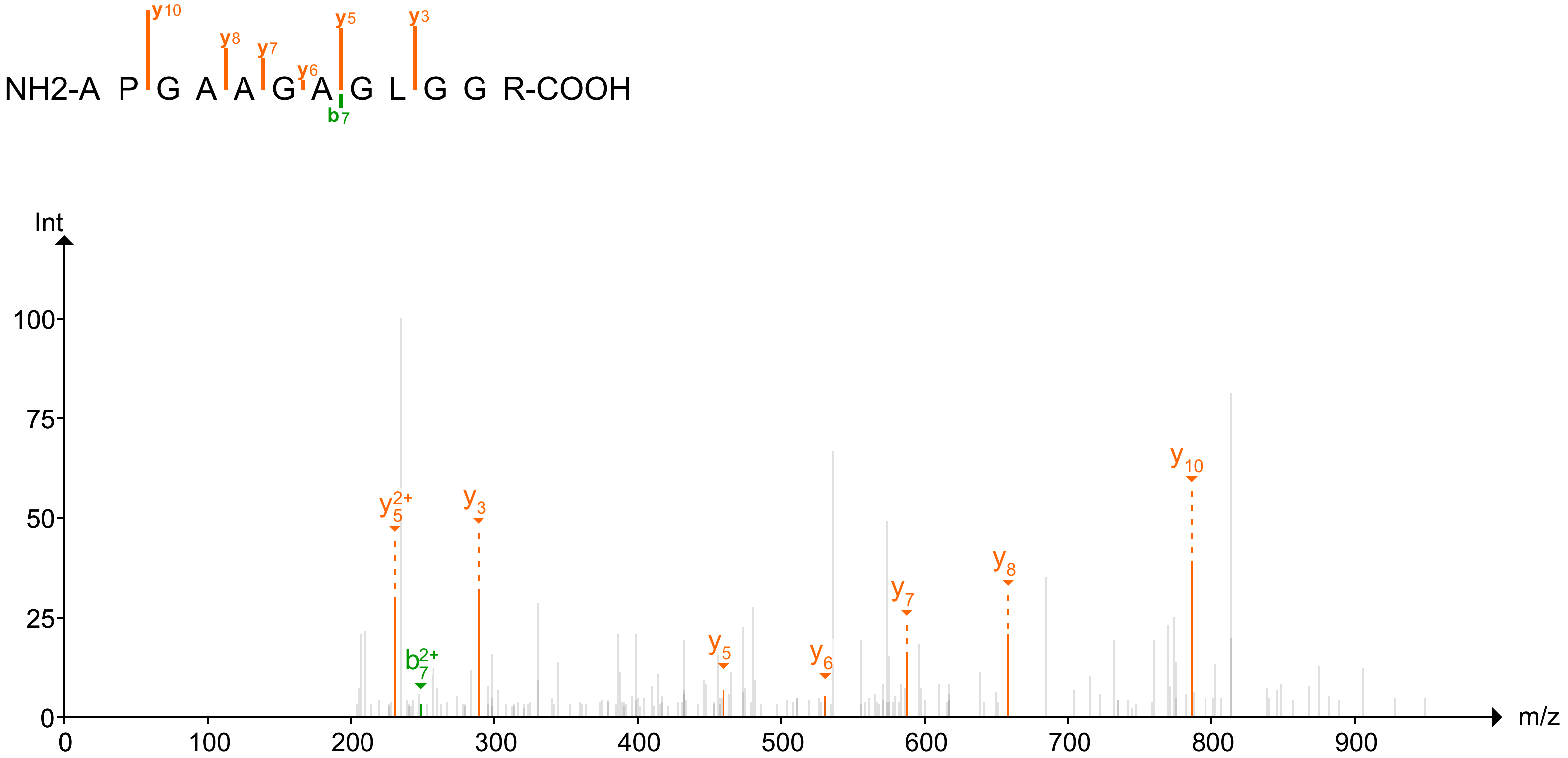

NH2-A P G A A G A G L G G R-COOH
y10
y8
y7
y6
y5
y3
b7
Int
100
75
50
25
0
m/z
0
100
200
300
400
500
600
700
800
900
y5 2+
b7 2+
y3
y5
y6
y7
y8
y10

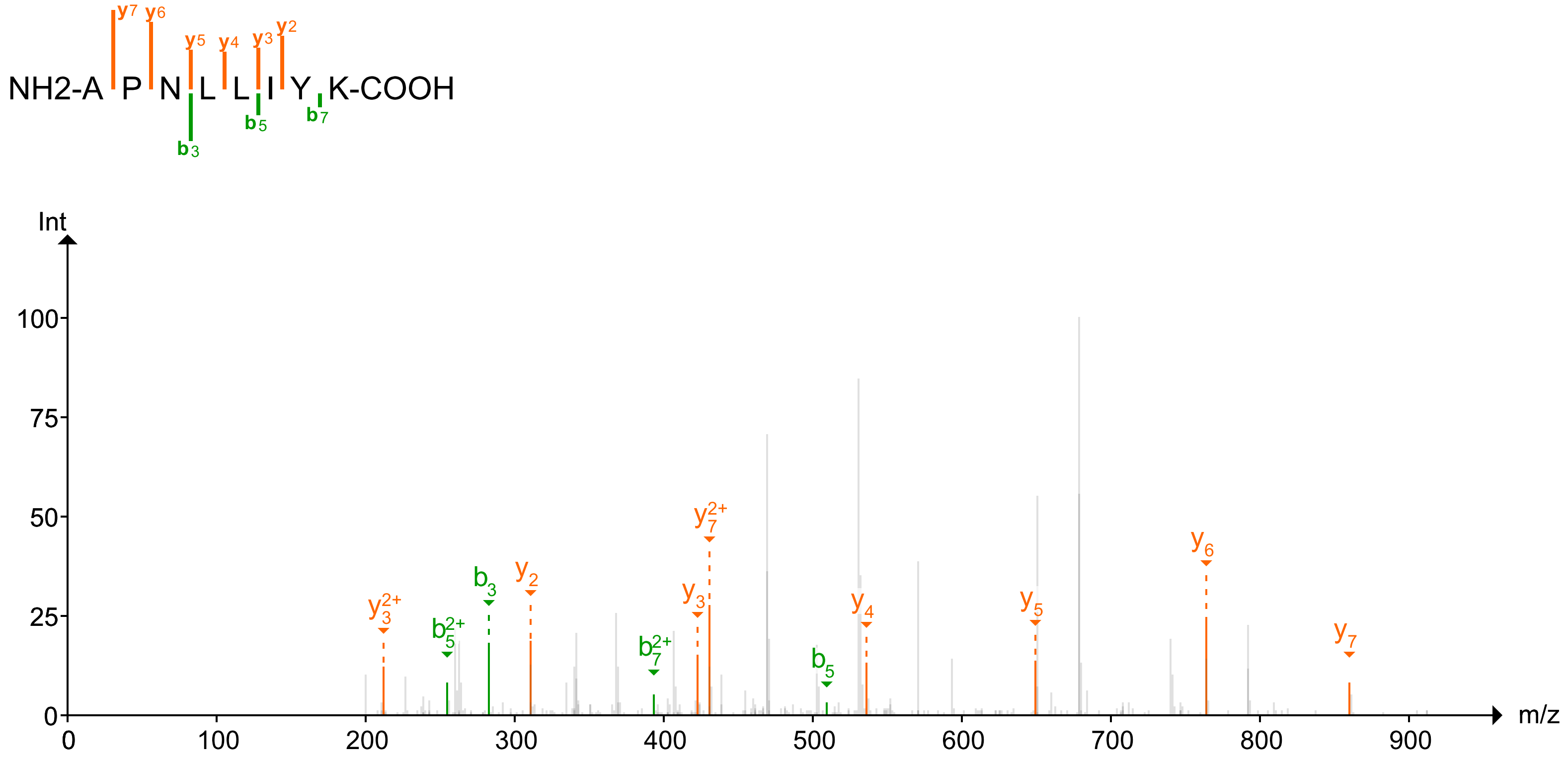

NH2-A P N L L I Y K-COOH
y7 y6 y5 y4 y3 y2
b3 b5 b7
Int
100
75
50
25
0
m/z
0 100 200 300 400 500 600 700 800 900
$y_3^{2+}$
$b_5^{2+}$
$b_3$
$y_2$
$b_7^{2+}$
$y_3$
$y_7^{2+}$
$b_5$
$y_4$
$y_5$
$y_6$
$y_7$

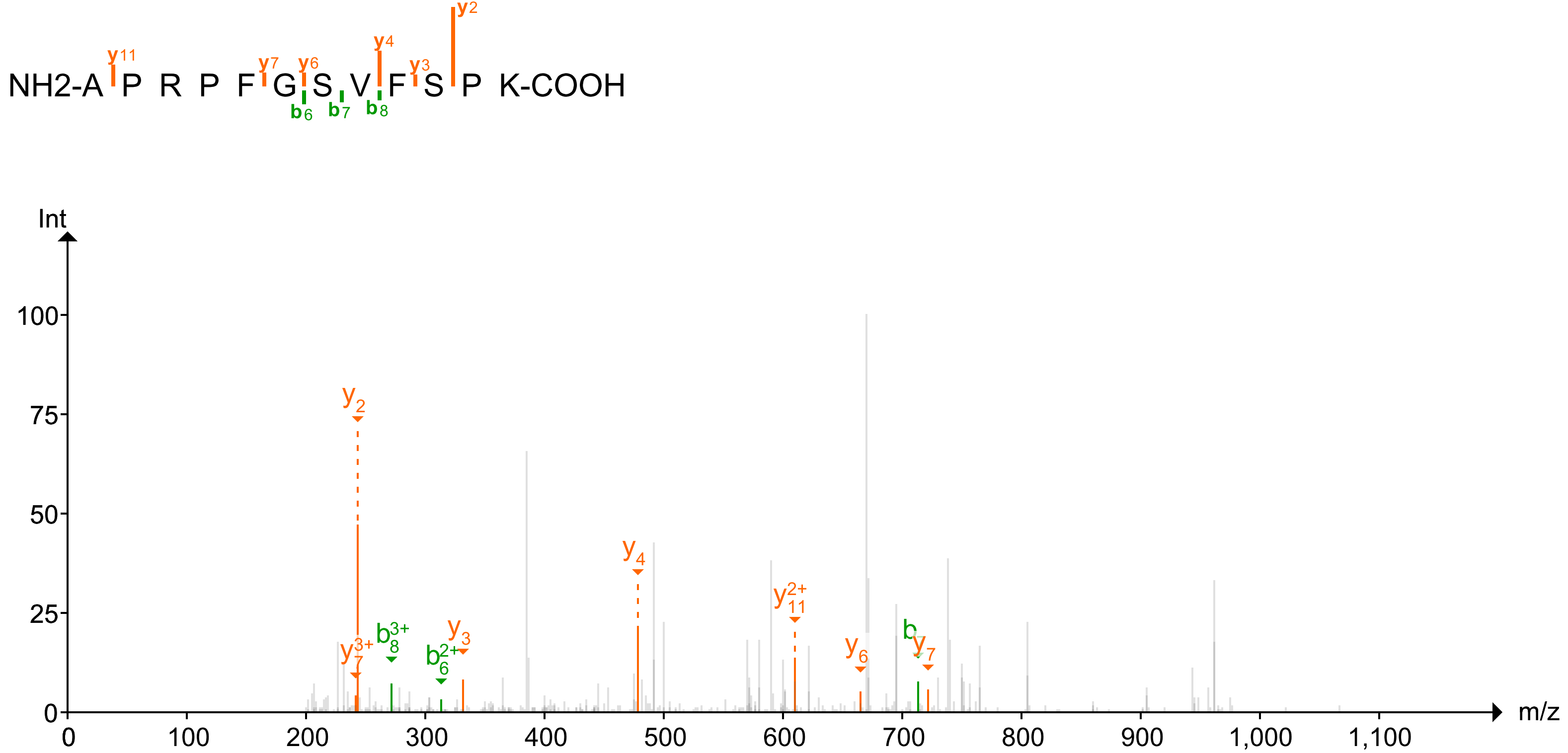

NH2-A P R P F G S V F S P K-COOH
y11
y7
y6
y4
y3
y2
b6
b7
b8
Int
m/z
y2
y4
y7 3+
b8 3+
b6 2+
y3
y11 2+
y6
y7
0
25
50
75
100
0
100
200
300
400
500
600
700
800
900
1,000
1,100

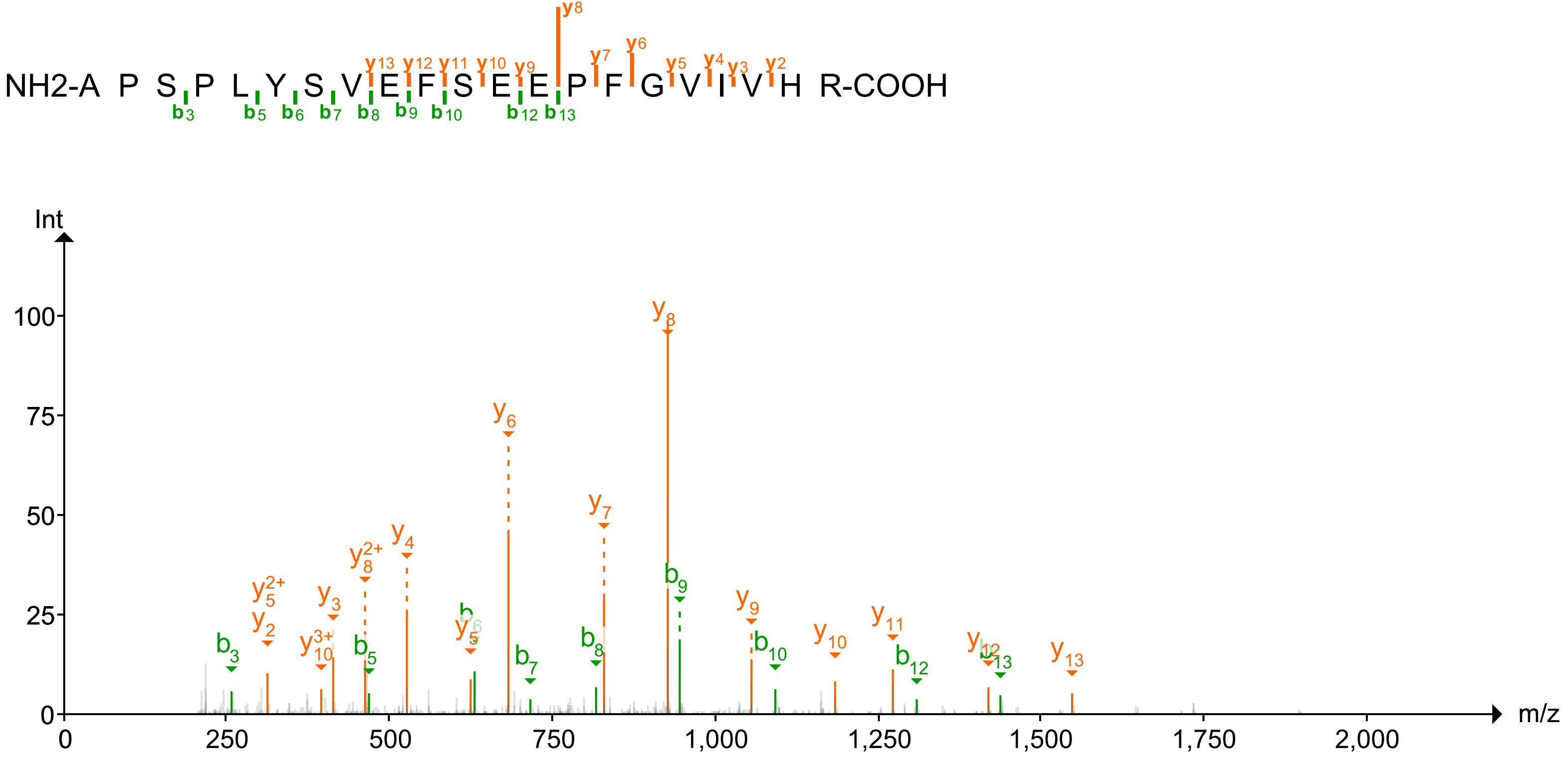

NH2-A P S P L Y S V E F S E E P F G V I V H R-COOH
y13 y12 y11 y10 y9 y8 y7 y6 y5 y4 y3 y2
b3 b5 b6 b7 b8 b9 b10 b12 b13
Int
100
75
50
25
0
m/z
0
250
500
750
1,000
1,250
1,500
1,750
2,000
y8
y6
y7
y4
y8 2+
y5 2+
y3
y2
y10 3+
b3
b5
b6
y5
b7
b8
b9
y9
b10
y10
y11
b12
y12
b13
y13

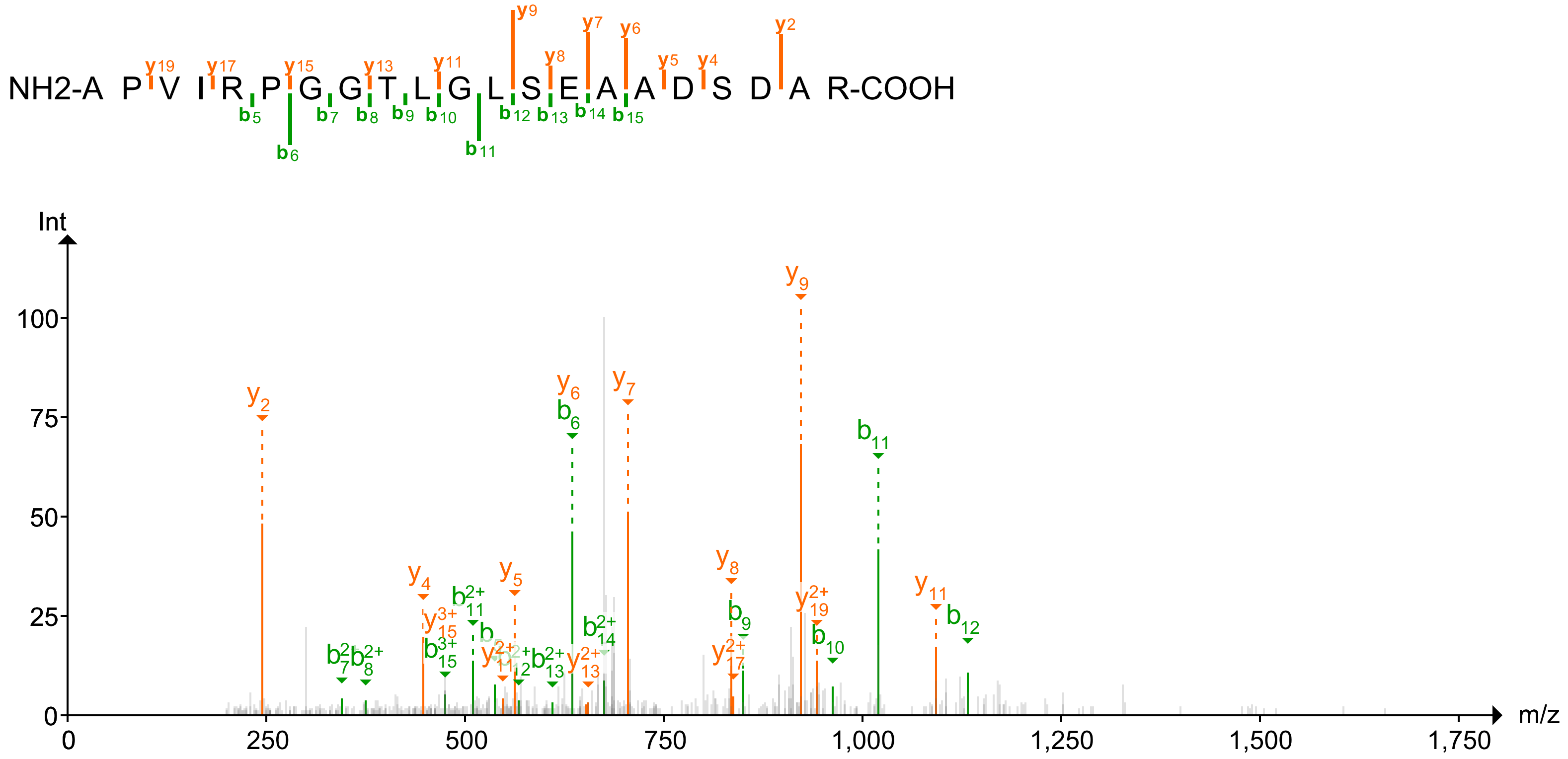

NH2-A P V I R P G G T L G L S E A A D S D A R-COOH
y19 y17 y15 y13 y11 y9 y8 y7 y6 y5 y4 y2
b5 b6 b7 b8 b9 b10 b11 b12 b13 b14 b15
Int
100
75
50
25
0
m/z
0
250
500
750
1,000
1,250
1,500
1,750

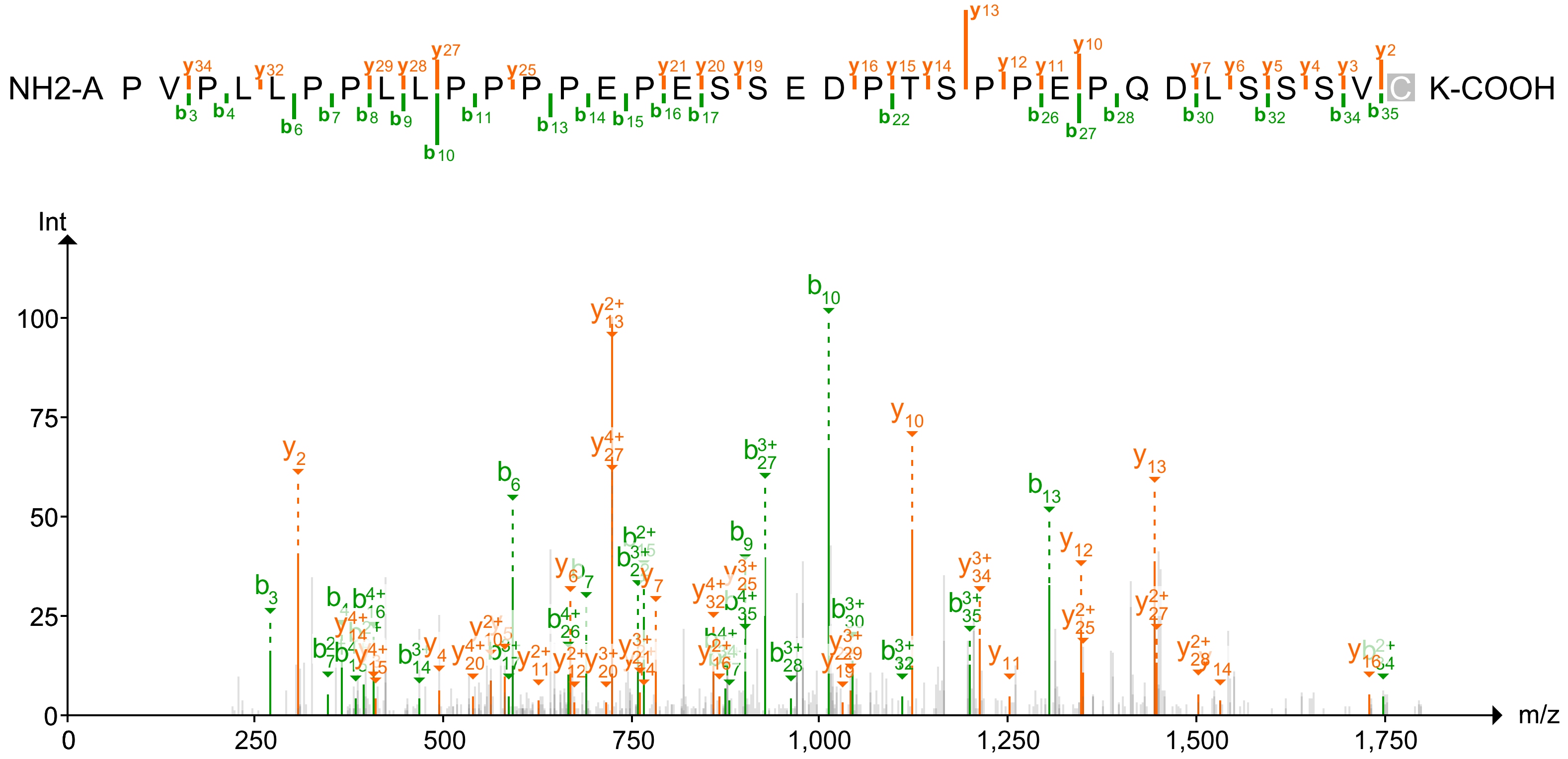

NH2-A P V P L L P P L L P P P P E P E S S E D P T S P P E P Q D L S S S V C K-COOH
Int
100
75
50
25
0
m/z
0
250
500
750
1,000
1,250
1,500
1,750
y2
b3
b6
b10
y10
b13
y13
y12
y11
b9

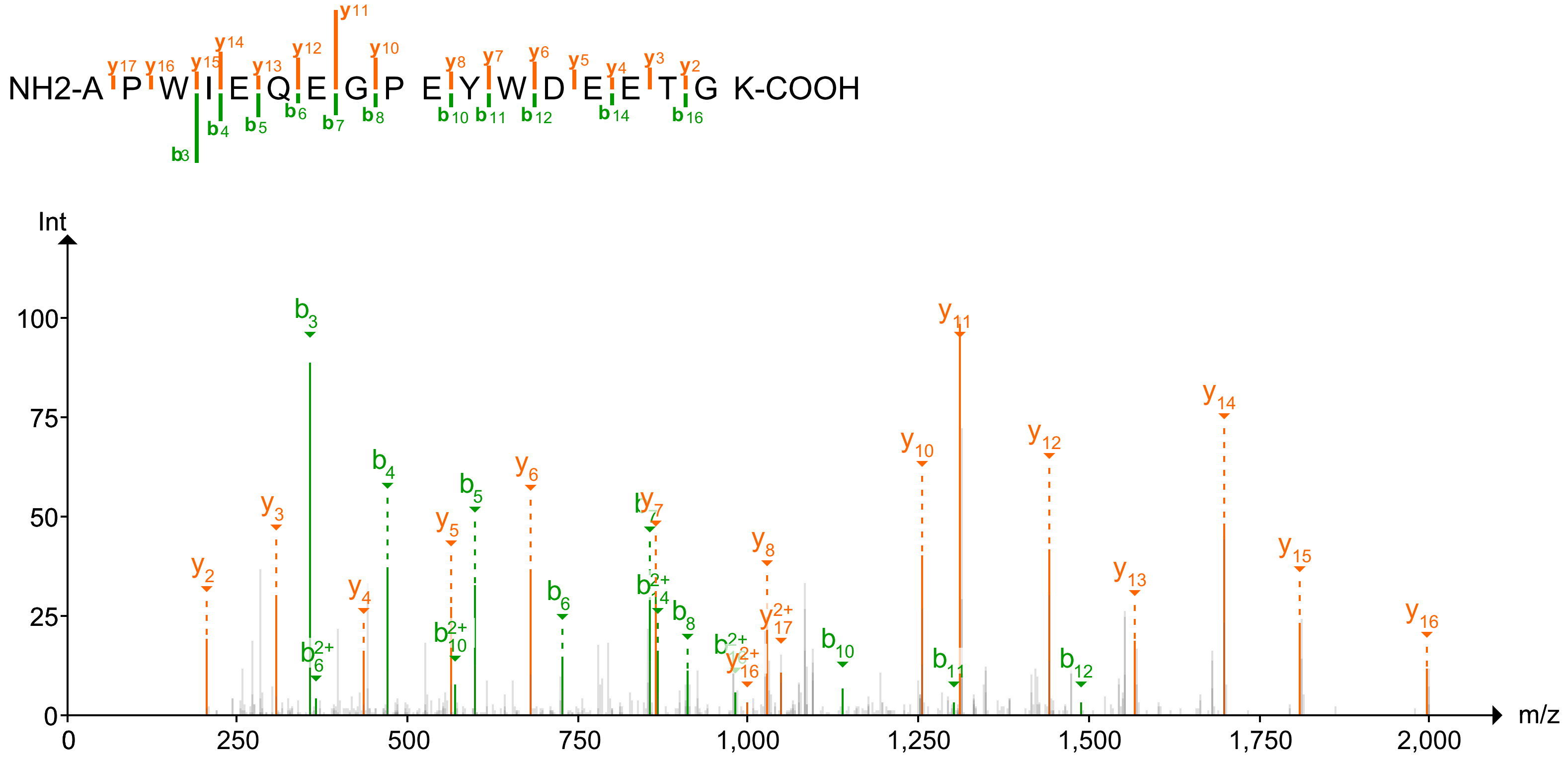

NH2-A P W I E Q E G P E Y W D E E T G K-COOH
y17 y16 y15 y14 y13 y12 y11 y10 y8 y7 y6 y5 y4 y3 y2
b3 b4 b5 b6 b7 b8 b10 b11 b12 b14 b16
Int
100
75
50
25
0
m/z
0
250
500
750
1,000
1,250
1,500
1,750
2,000
y2 y3 b3 y4 b4 b5 y5 y6 b6 y7 b7 y8 b8 b10 y10 y11 b11 y12 b12 y13 y14 y15 y16

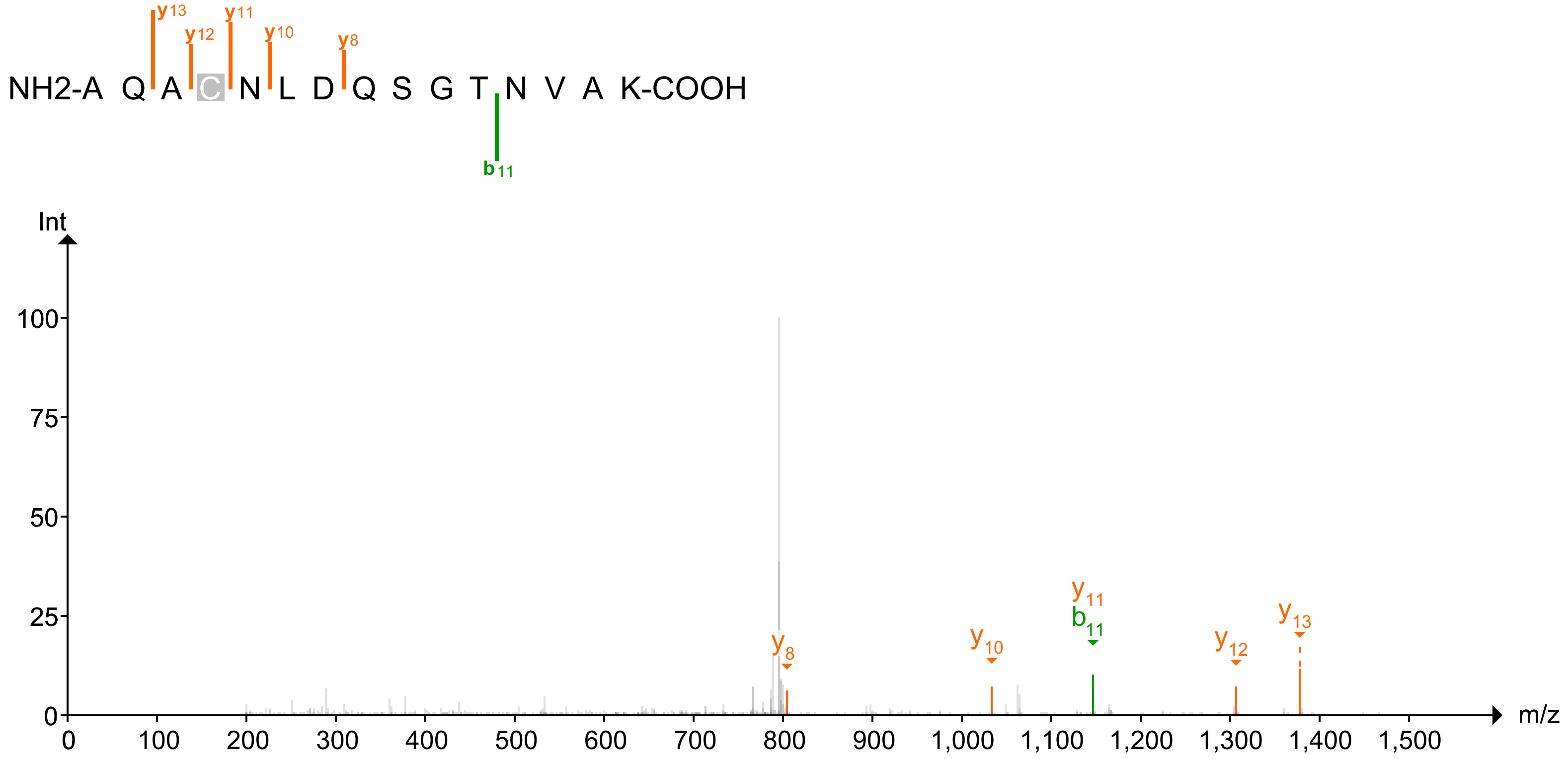

NH2-A Q A C N L D Q S G T N V A K-COOH
y13
y12
y11
y10
y8
b11
Int
100
75
50
25
0
m/z
0
100
200
300
400
500
600
700
800
900
1,000
1,100
1,200
1,300
1,400
1,500
y8
y10
y11
b11
y12
y13

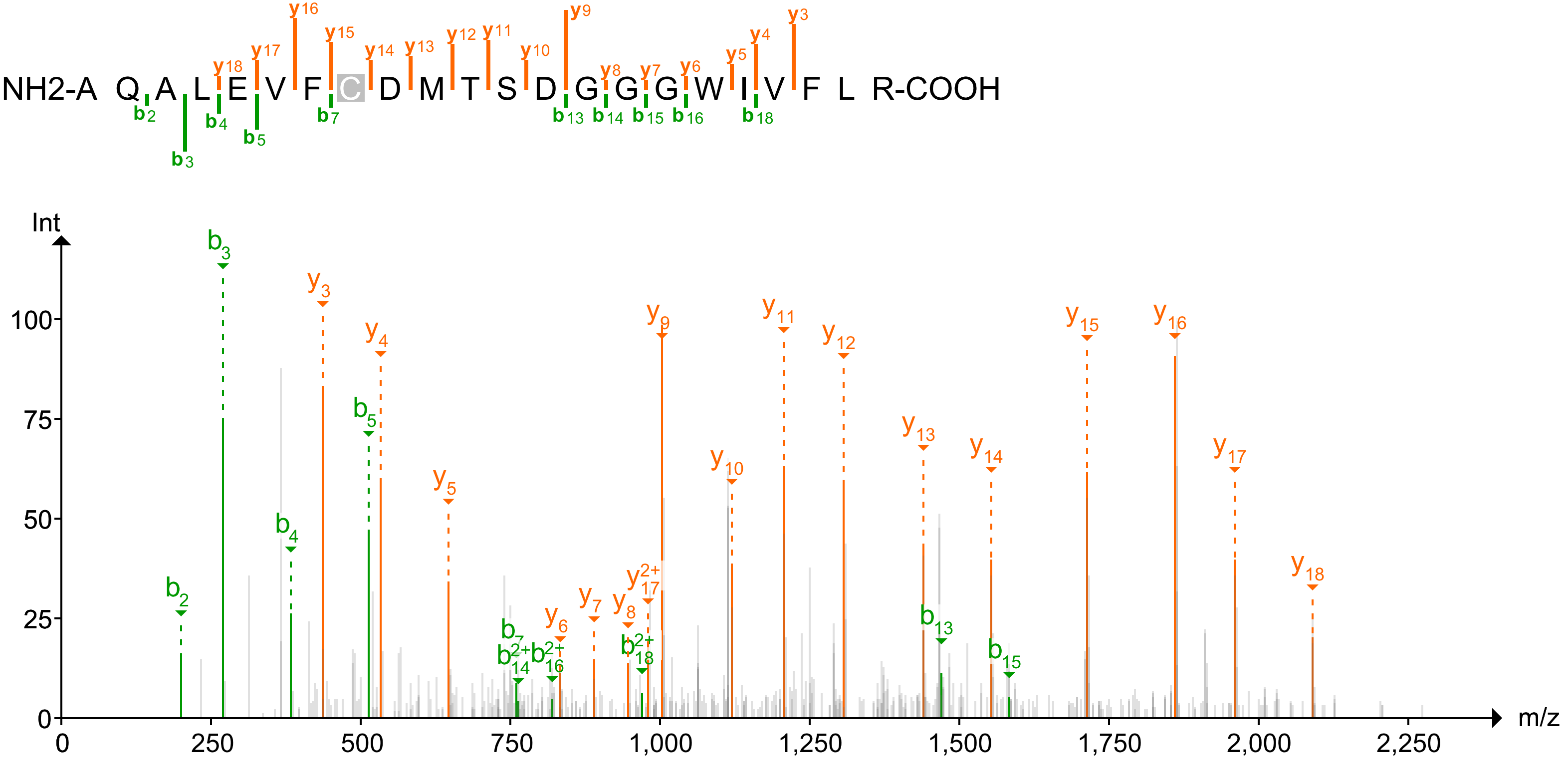

NH2-A Q A L E V F C D M T S D G G G W I V F L R-COOH
Int
m/z

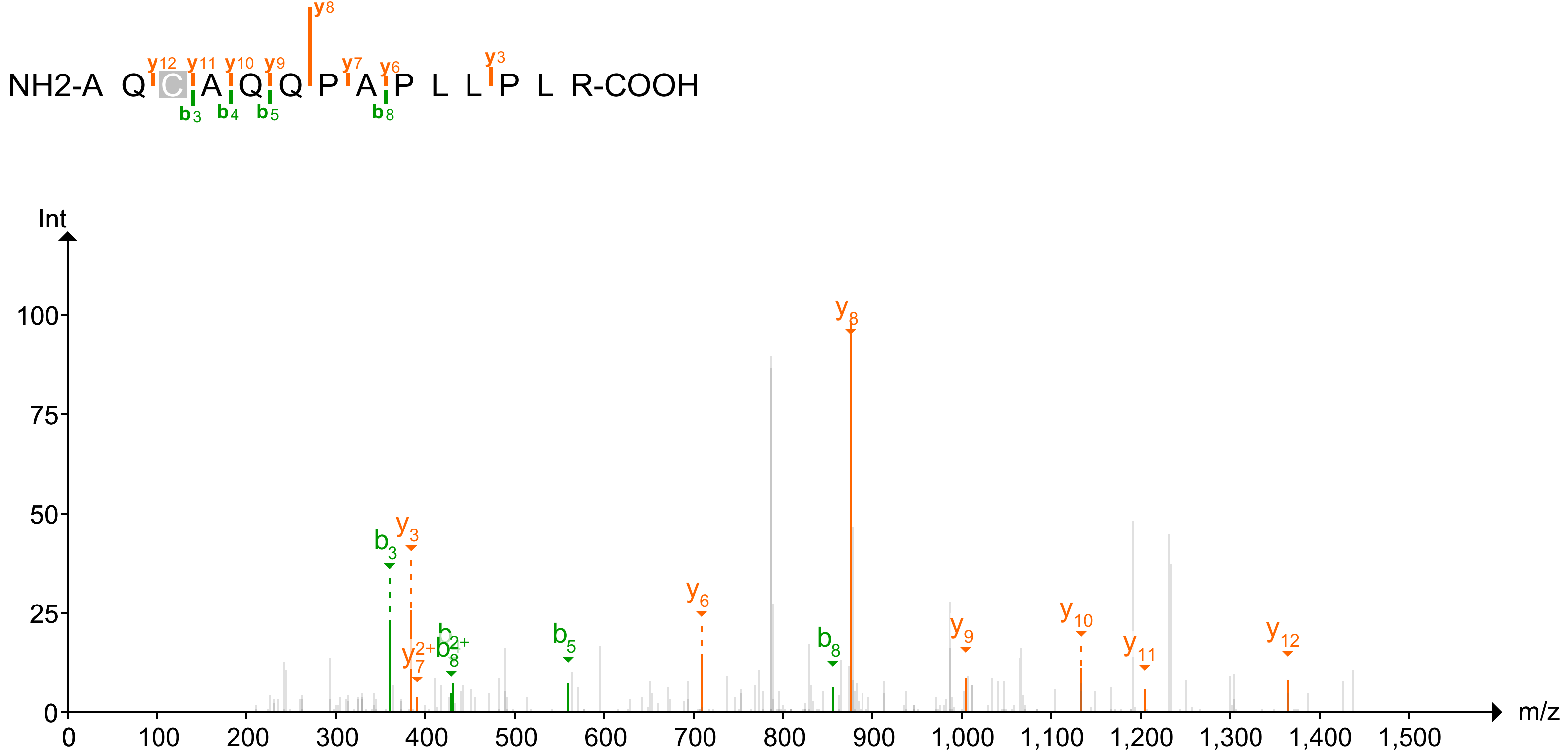

NH2-A Q C A Q Q P A P L L P L R-COOH
y12 y11 y10 y9 y8 y7 y6 y3
b3 b4 b5 b8
Int
100
75
50
25
0
m/z
0 100 200 300 400 500 600 700 800 900 1,000 1,100 1,200 1,300 1,400 1,500
y8
b3
y3
y6
y7 2+
b8 2+
b5
b8
y9
y10
y11
y12

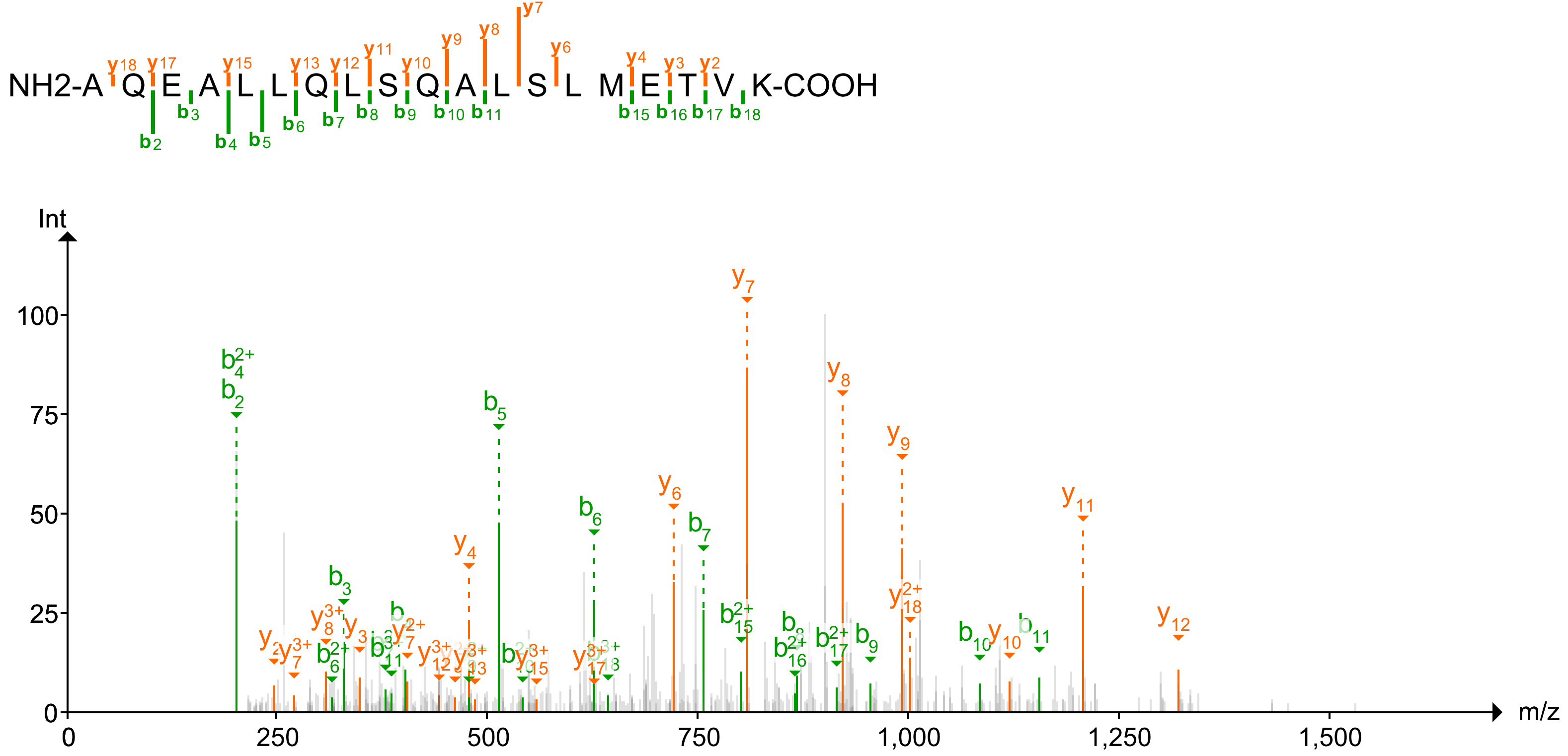

NH2-A Q E A L L Q L S Q A L S L M E T V K-COOH
y18 y17 y15 y13 y12 y11 y10 y9 y8 y7 y6 y4 y3 y2
b2 b3 b4 b5 b6 b7 b8 b9 b10 b11 b15 b16 b17 b18
Int
100
75
50
25
0
m/z
0
250
500
750
1,000
1,250
1,500

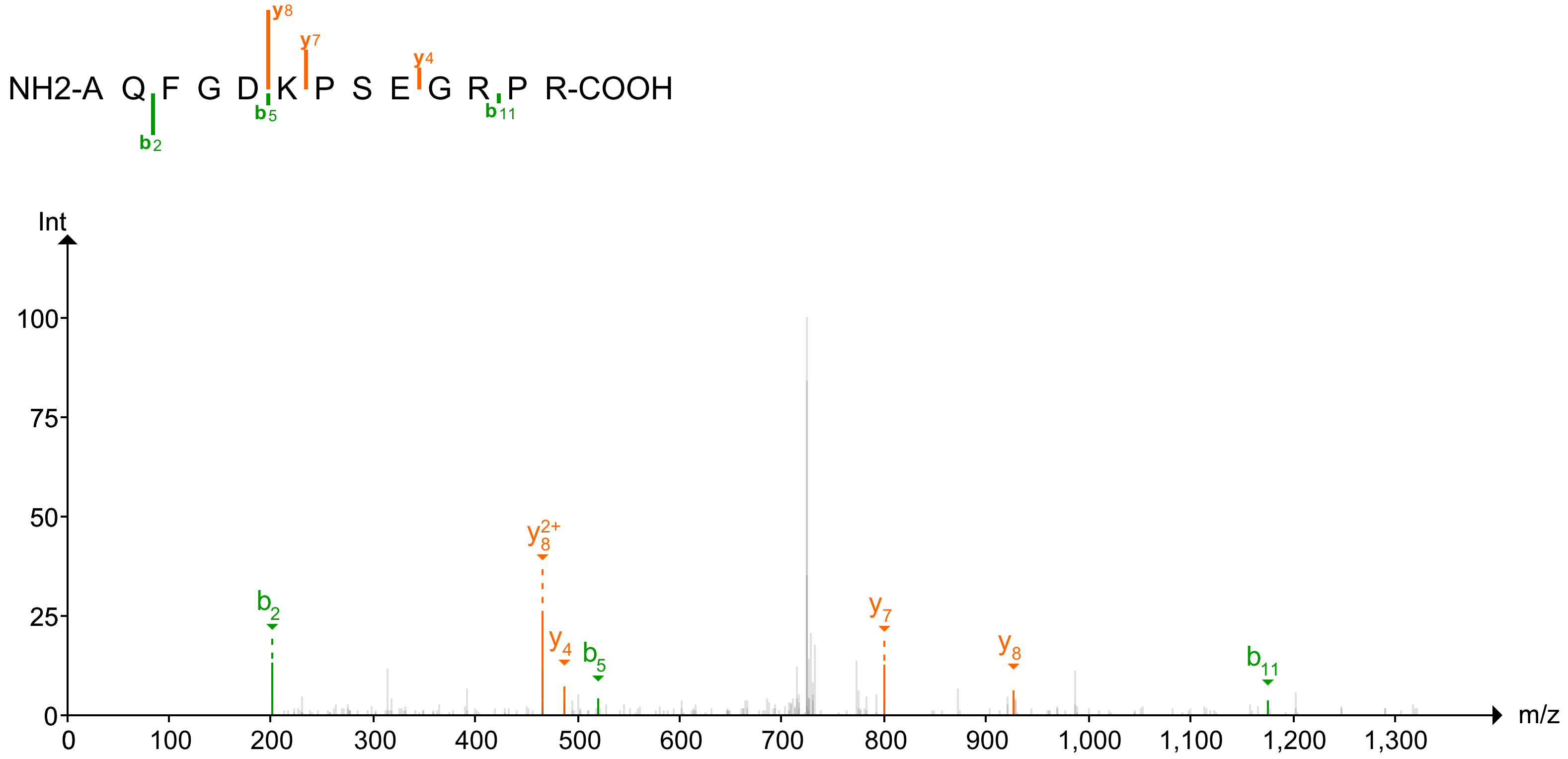

y8
y7
y4
NH2-A Q F G D K P S E G R P R-COOH
b2
b5
b11
Int
100
75
50
25
0
$y_8^{2+}$
$b_2$
$y_4$
$b_5$
$y_7$
$y_8$
$b_{11}$
0
100
200
300
400
500
600
700
800
900
1,000
1,100
1,200
1,300
m/z

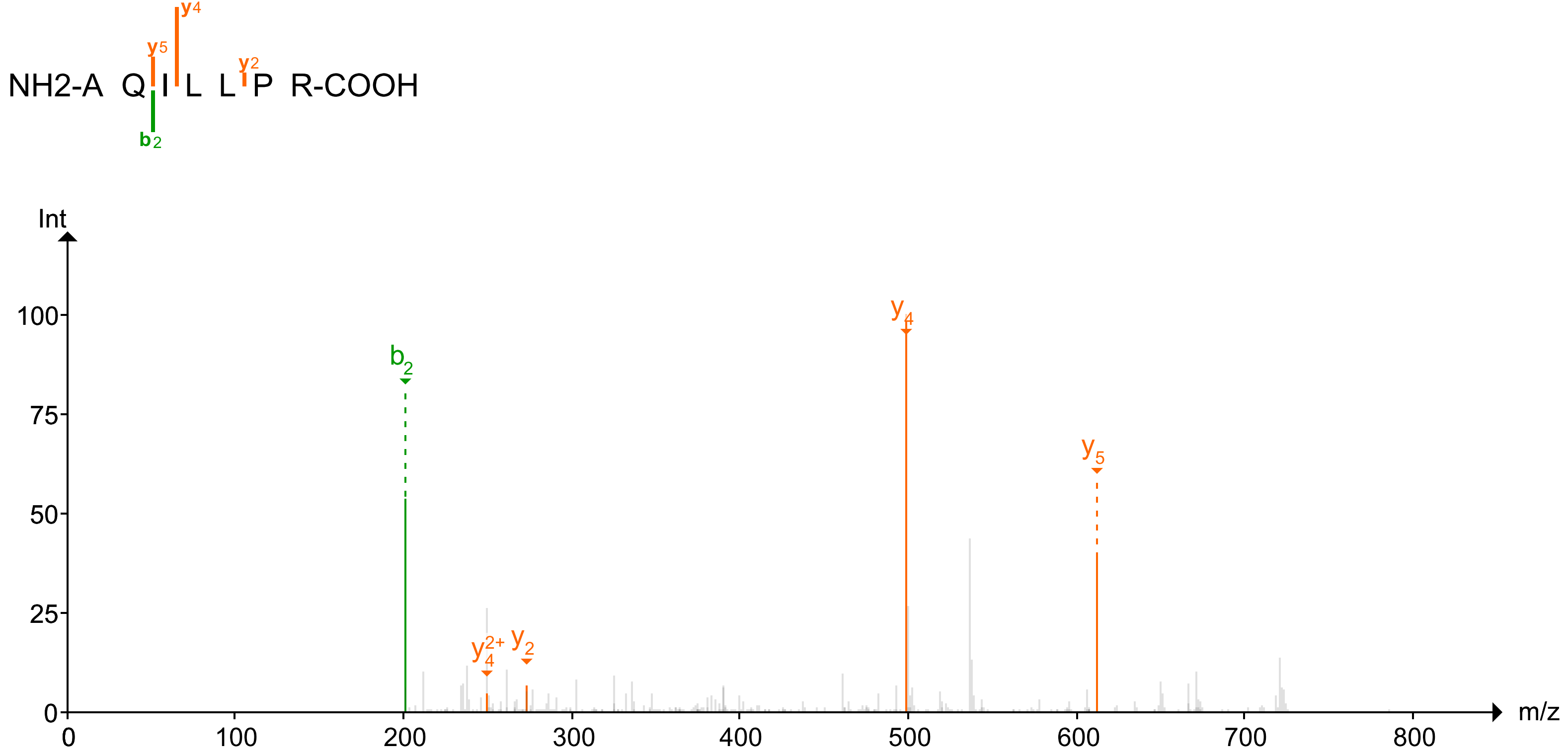

NH2-A Q I L L P R-COOH
y4
y5
y2
b2
Int
m/z
100
75
50
25
0
0
100
200
300
400
500
600
700
800
b2
y4
y5
y4 2+
y2

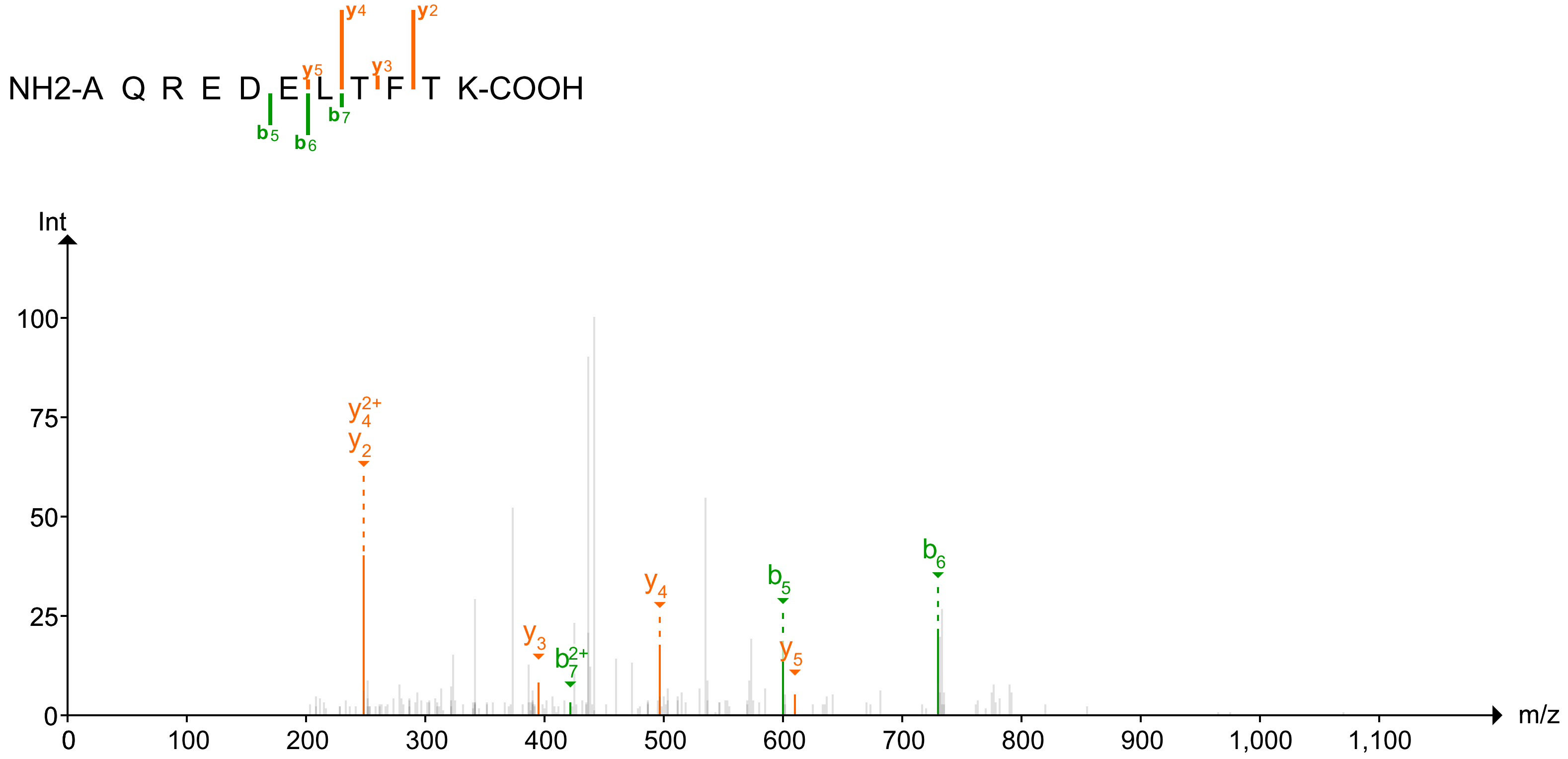

NH2-A Q R E D E L T F T K-COOH
y4
y2
y5
y3
b5
b6
b7
Int
100
75
50
25
0
m/z
0
100
200
300
400
500
600
700
800
900
1,000
1,100

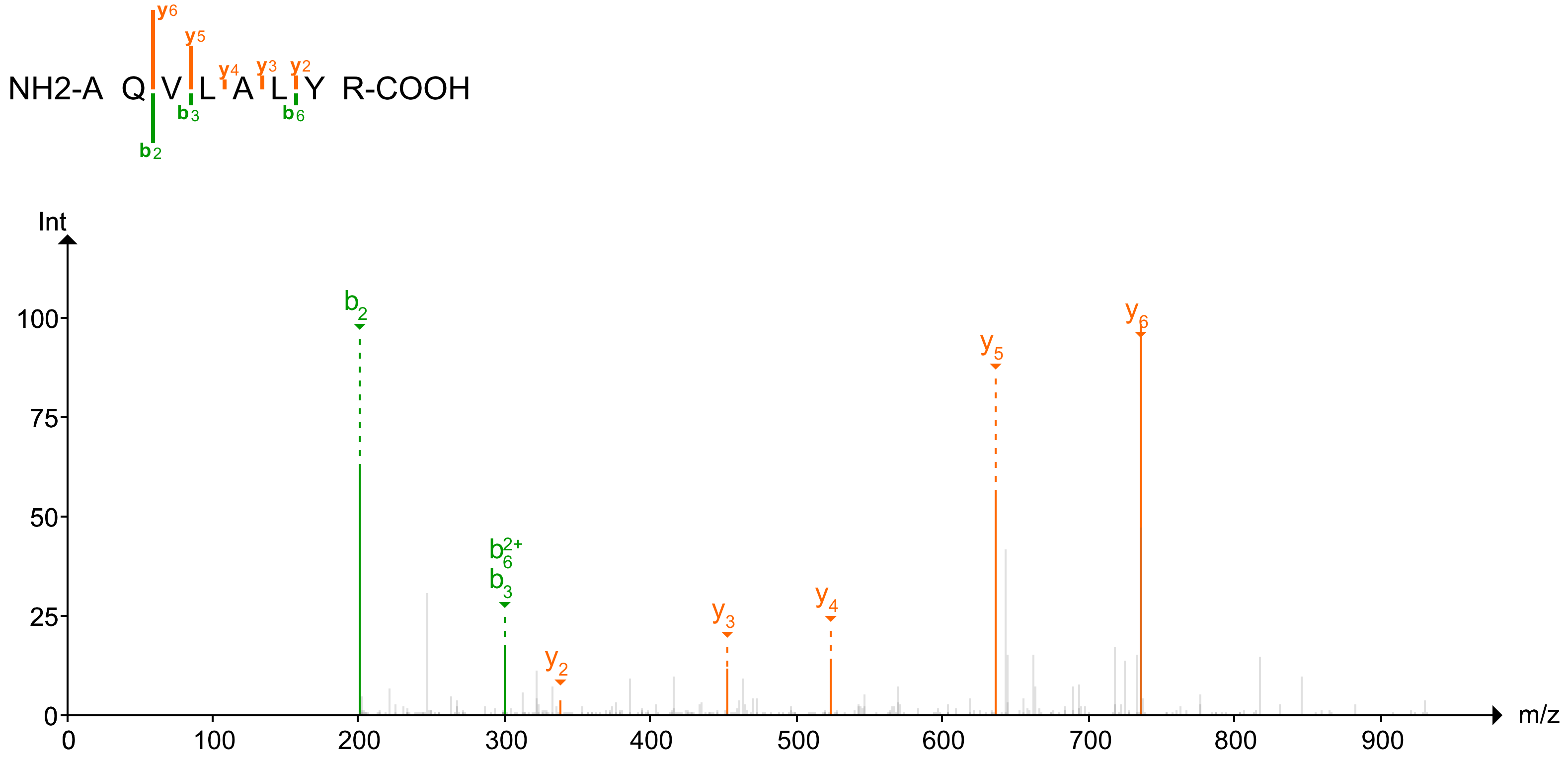

NH2-A Q V L A L Y R-COOH
y6
y5
y4
y3
y2
b2
b3
b6
Int
100
75
50
25
0
b2
b6 2+
b3
y2
y3
y4
y5
y6
0
100
200
300
400
500
600
700
800
900
m/z

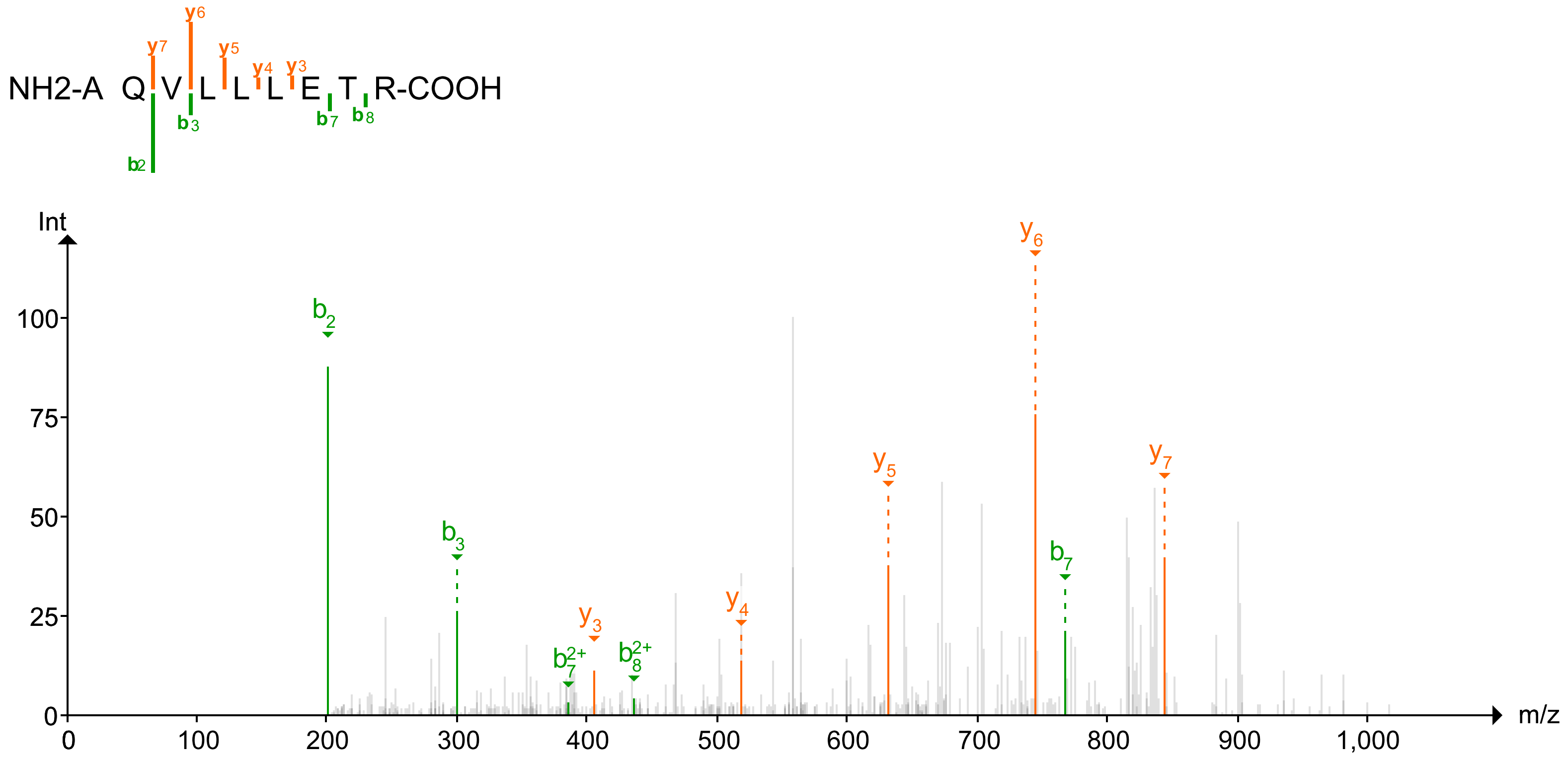

NH2-A Q V L L L E T R-COOH
y7 y6 y5 y4 y3
b2 b3 b7 b8
Int
m/z
100
75
50
25
0
100
200
300
400
500
600
700
800
900
1,000
$b_2$
$b_3$
$b_7^{2+}$
$y_3$
$b_8^{2+}$
$y_4$
$y_5$
$y_6$
$b_7$
$y_7$

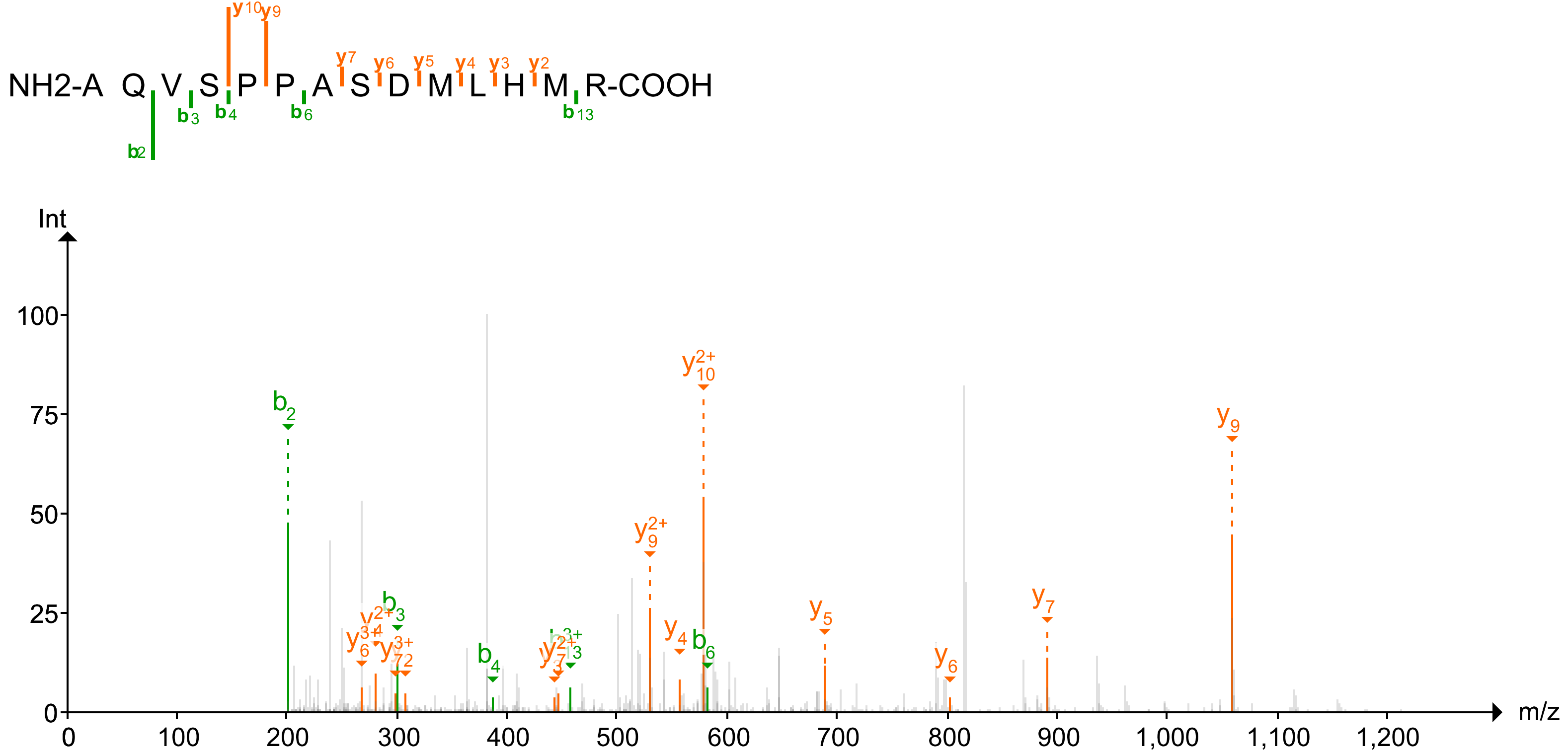

NH2-A Q V S P P A S D M L H M R-COOH
y10 y9 y7 y6 y5 y4 y3 y2
b2 b3 b4 b6 b13
Int
m/z
0
25
50
75
100
0
100
200
300
400
500
600
700
800
900
1,000
1,100
1,200
b2
b3
b4
b6
y10 2+
y9 2+
y9
y7
y6
y5
y4

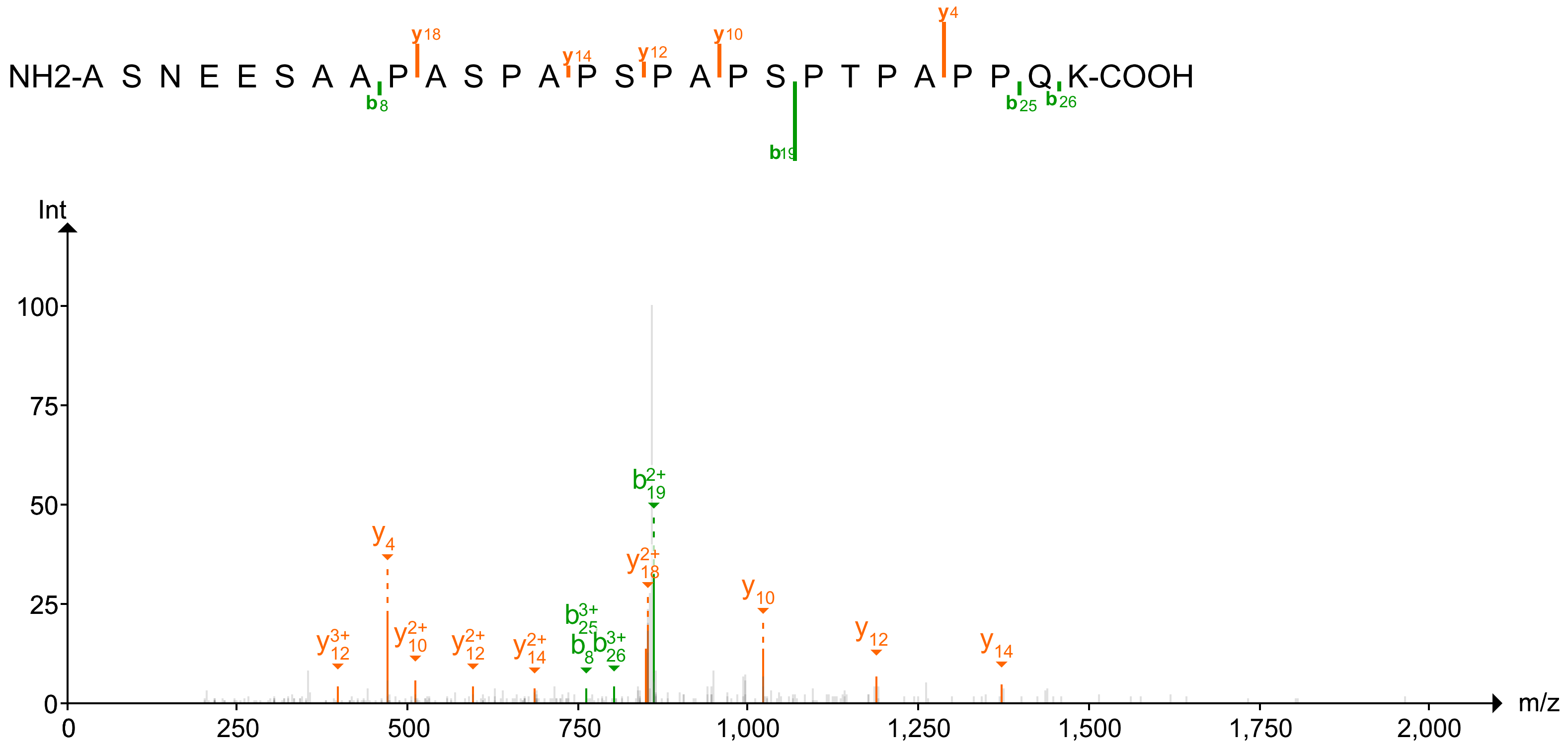

NH2-A S N E E S A A P A S P A P S P A P S P T P A P P Q K-COOH
y18 y14 y12 y10 y4
b8 b19 b25 b26
Int
100
75
50
25
0
m/z
0
250
500
750
1,000
1,250
1,500
1,750
2,000
y4
y10
y12
y14
y3+ 12
y2+ 10
y2+ 12
y2+ 14
b3+ 25
b8
b3+ 26
y2+ 18
b2+ 19

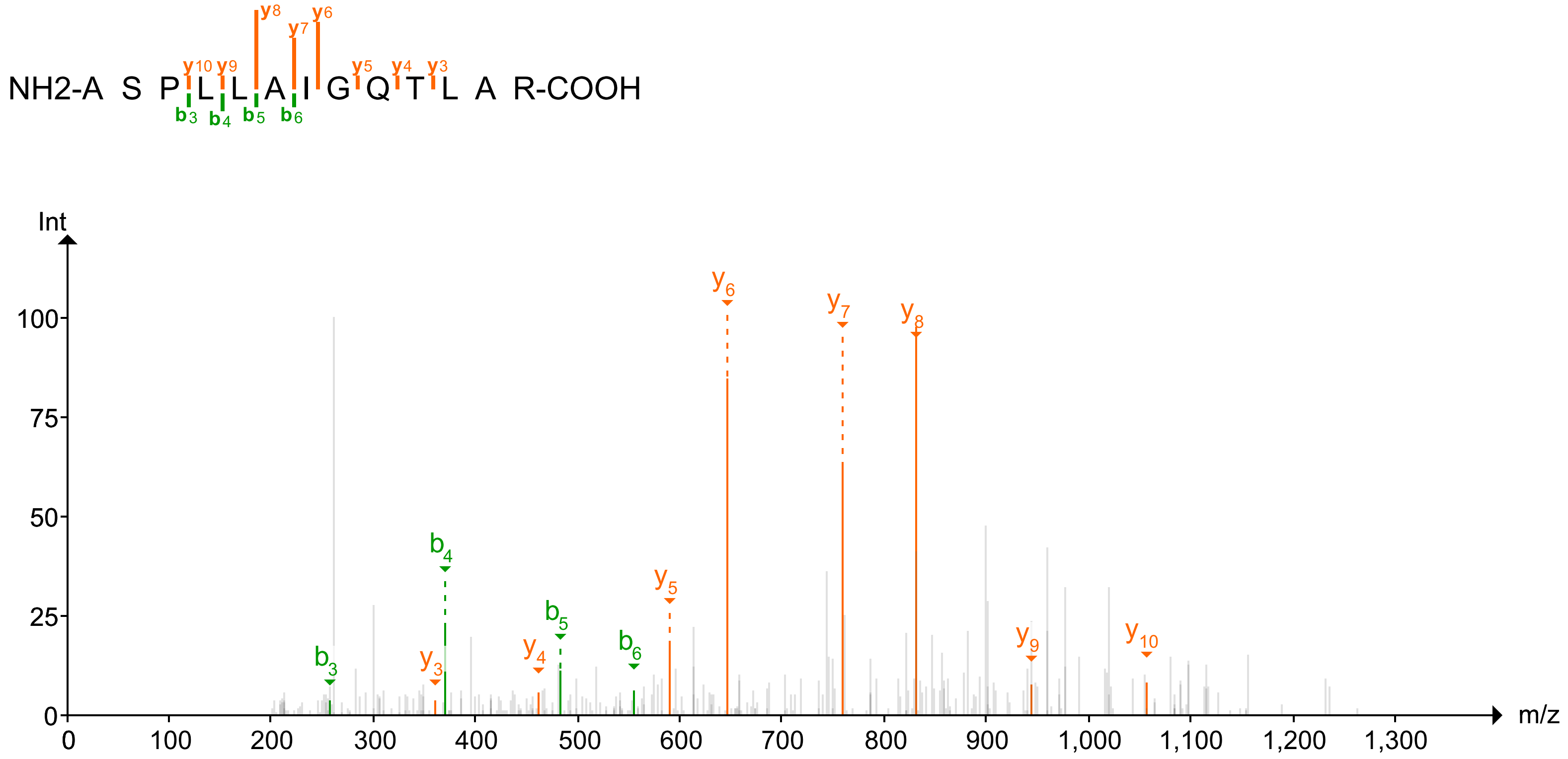

NH2-A S P L L A I G Q T L A R-COOH
y10 y9 y8 y7 y6 y5 y4 y3
b3 b4 b5 b6
Int
m/z
0
25
50
75
100
0
100
200
300
400
500
600
700
800
900
1,000
1,100
1,200
1,300
b3
y3
b4
y4
b5
b6
y5
y6
y7
y8
y9
y10

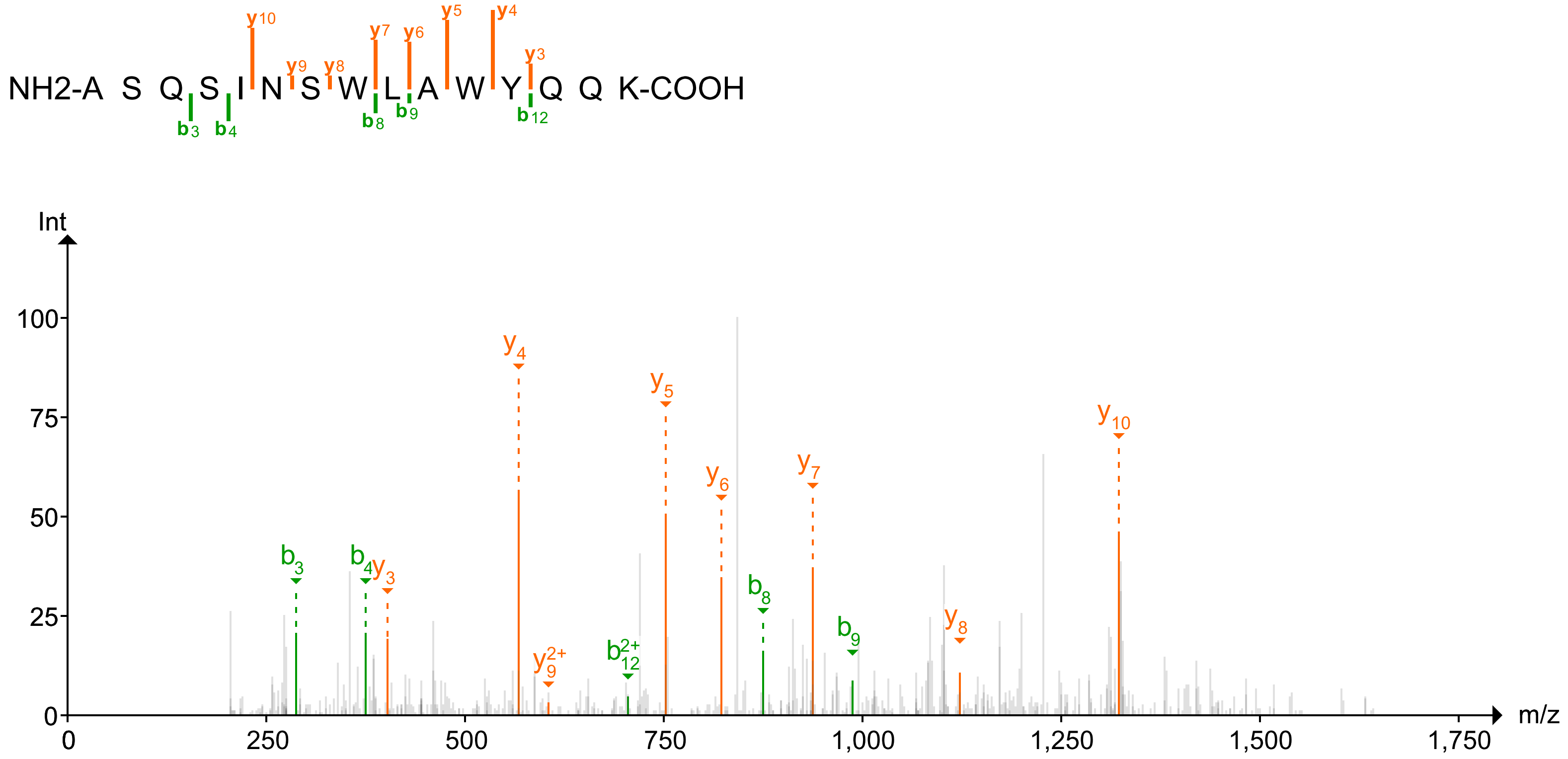

NH2-A S Q S I N S W L A W Y Q Q K-COOH
y10 y9 y8 y7 y6 y5 y4 y3
b3 b4 b8 b9 b12
Int
100
75
50
25
0
m/z
0
250
500
750
1,000
1,250
1,500
1,750
b3
b4
y3
y4
y9 2+
b12 2+
y5
y6
y7
b8
b9
y8
y10

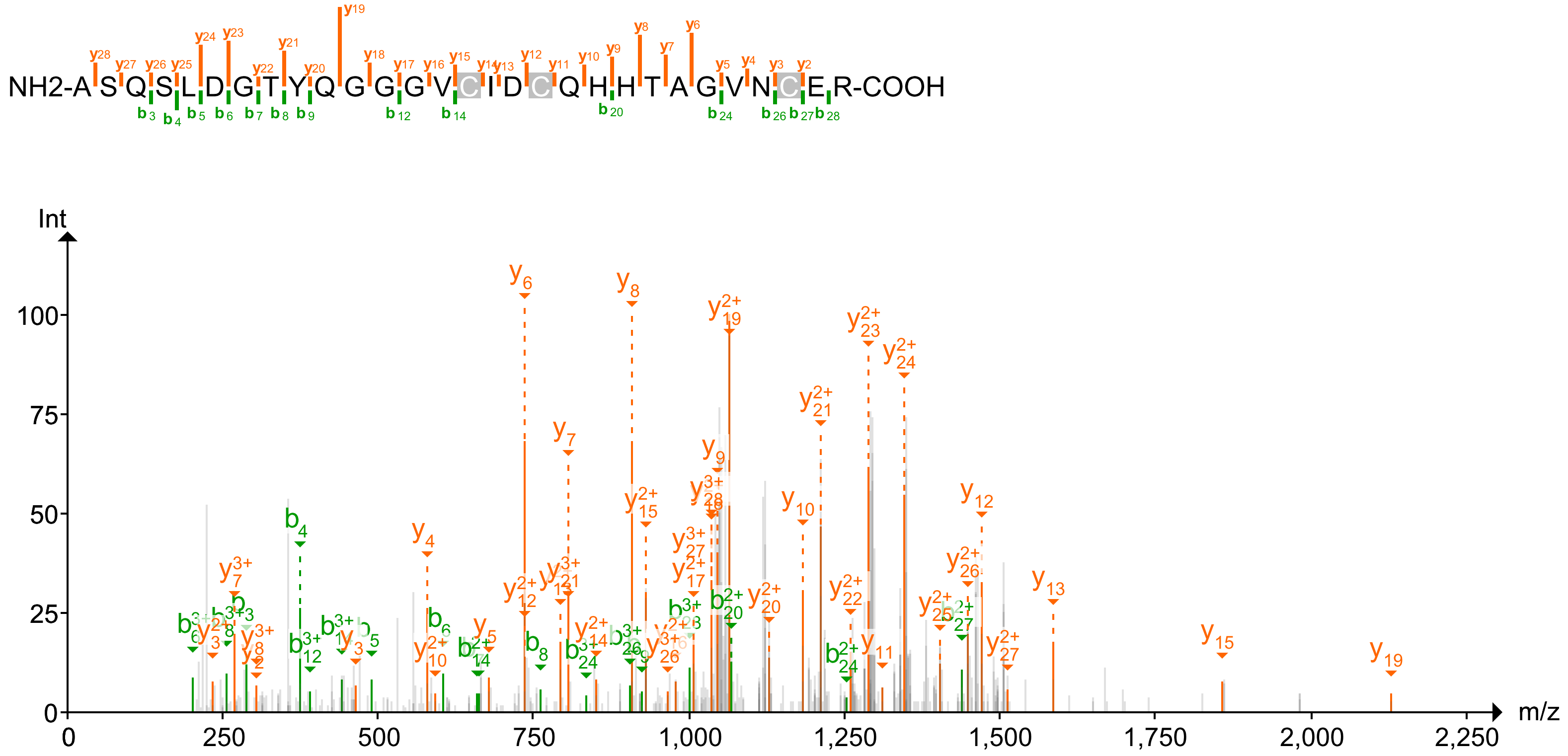
NH2-ASQSLDGTYQGGGVCIDCQHHTAGVNCER-COOH
Int
100
75
50
25
0
m/z
0
250
500
750
1,000
1,250
1,500
1,750
2,000
2,250

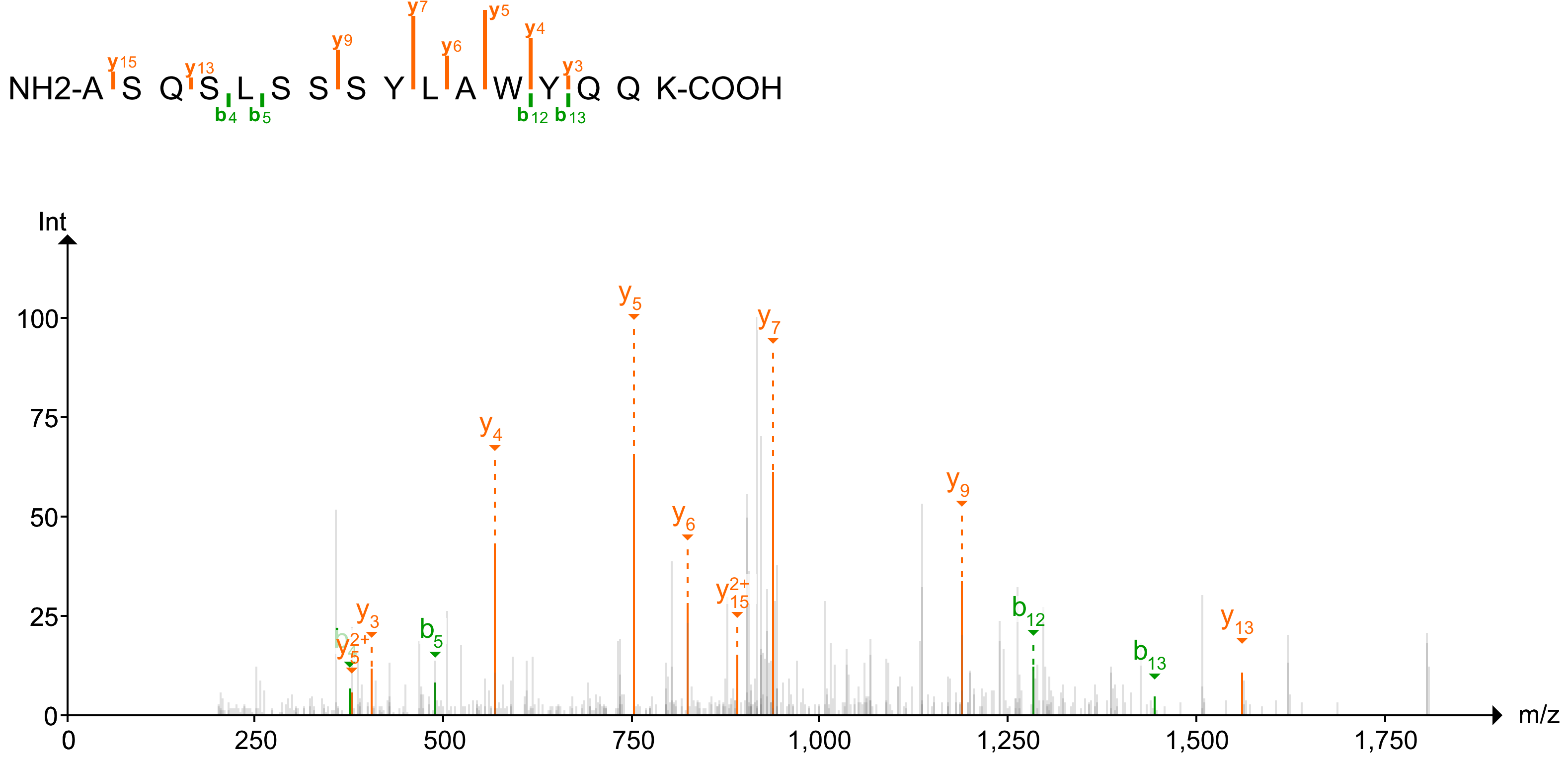

NH2-A S Q S L S S S Y L A W Y Q Q K-COOH
y15 y13 y9 y7 y6 y5 y4 y3
b4 b5 b12 b13
Int
100
75
50
25
0
y5
y7
y4
y9
y6
y15 2+
b12
y13
b13
y3
b5
y5 2+
0
250
500
750
1,000
1,250
1,500
1,750
m/z

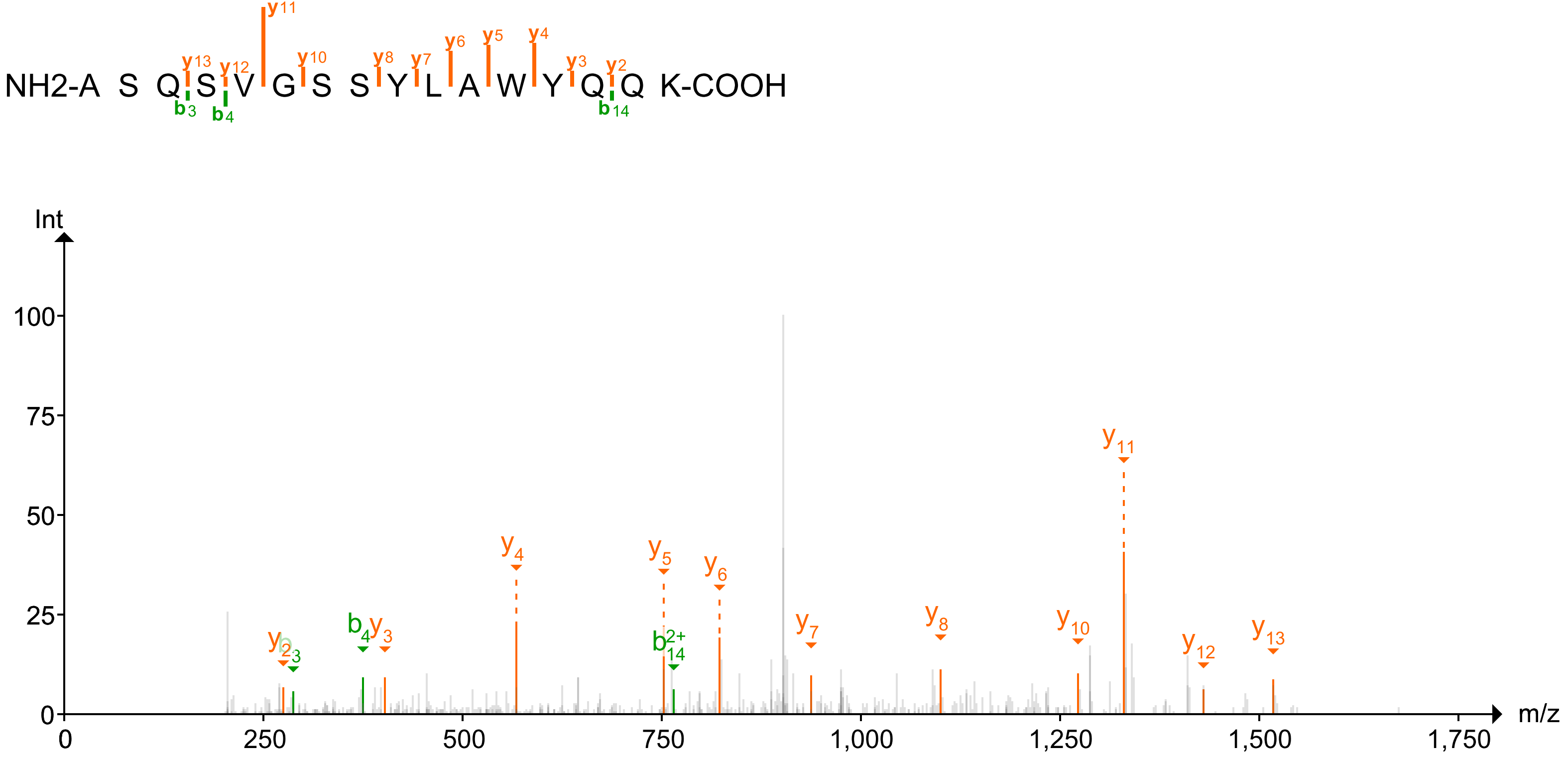
NH2-A S Q S V G S S Y L A W Y Q Q K-COOH
y11
y13
y12
y10
y8
y7
y6
y5
y4
y3
y2
b3
b4
b14
Int
100
75
50
25
0
m/z
0
250
500
750
1,000
1,250
1,500
1,750
y4
y5
y6
y7
y8
y10
y11
y12
y13
y2
b3
b4
y3
b2+14

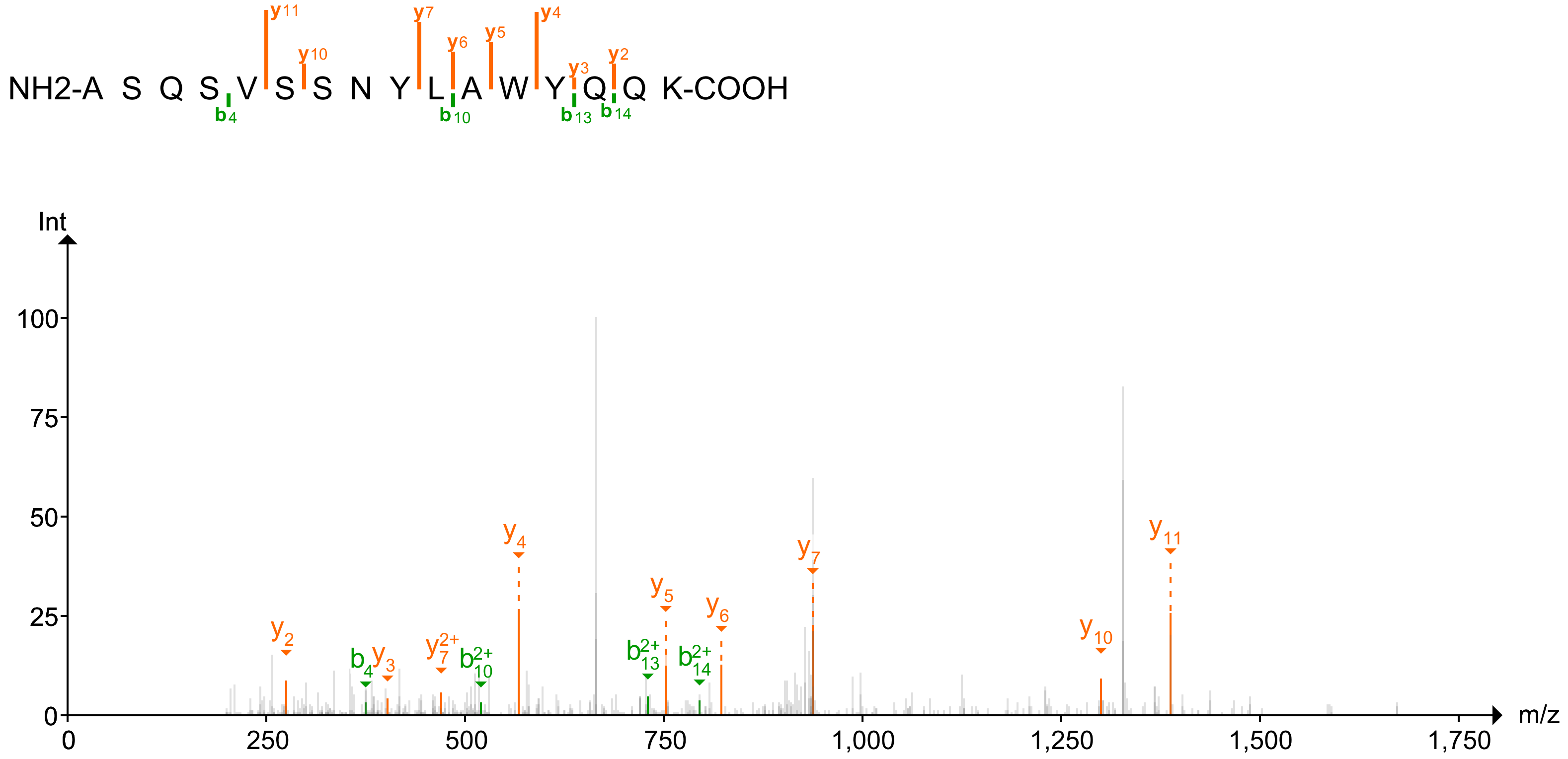

NH2-A S Q S V S S N Y L A W Y Q Q K-COOH
y11 y10 y7 y6 y5 y4 y3 y2
b4 b10 b13 b14
Int
100
75
50
25
0
y2 b4 y3 y7 2+ b10 2+ y4 b13 2+ y5 b14 2+ y6 y7 y10 y11
0 250 500 750 1,000 1,250 1,500 1,750
m/z

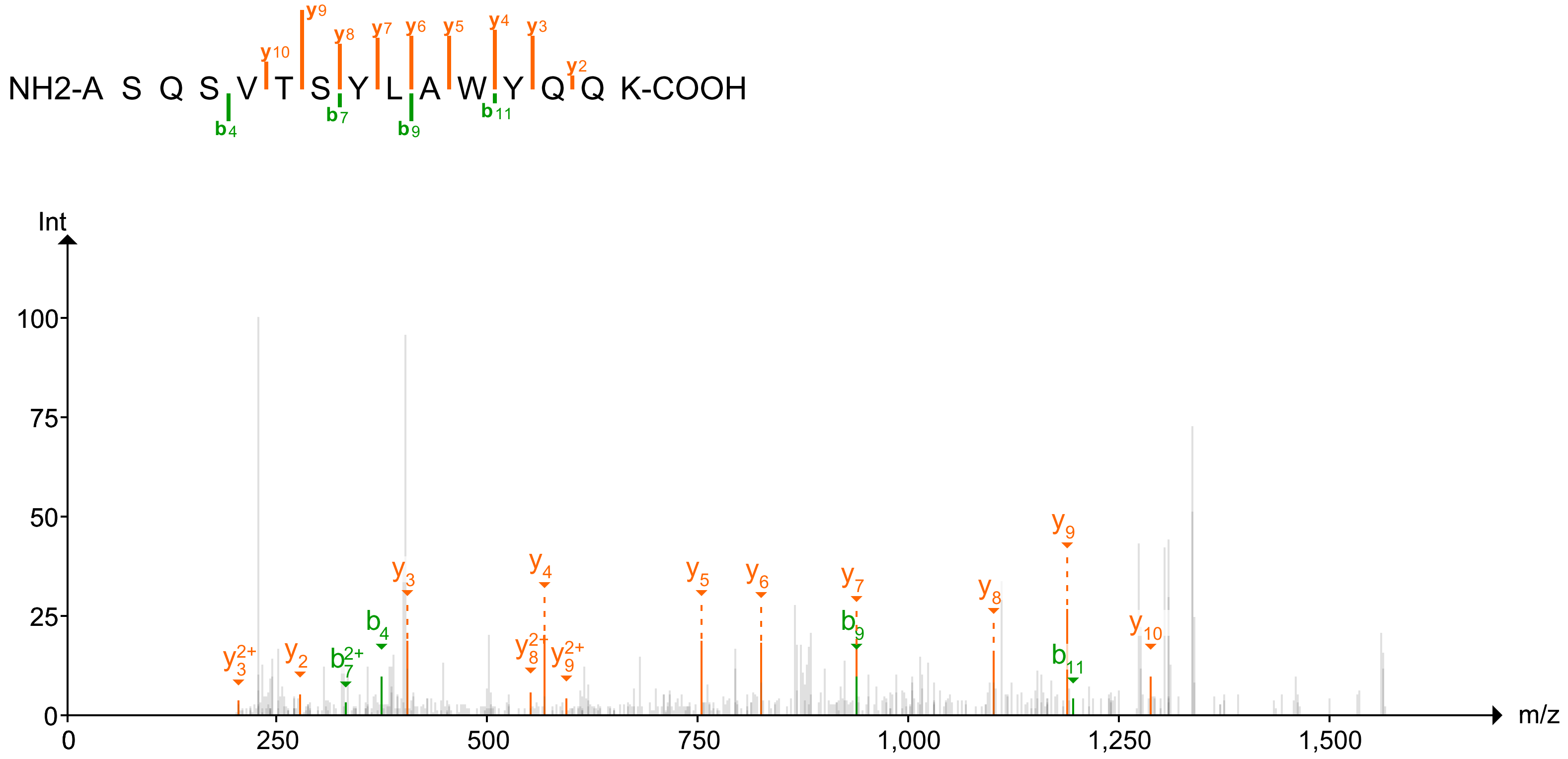

NH2-A S Q S V T S Y L A W Y Q Q K-COOH
y10 y9 y8 y7 y6 y5 y4 y3 y2
b4 b7 b9 b11
Int
100
75
50
25
0
0
250
500
750
1,000
1,250
1,500
m/z

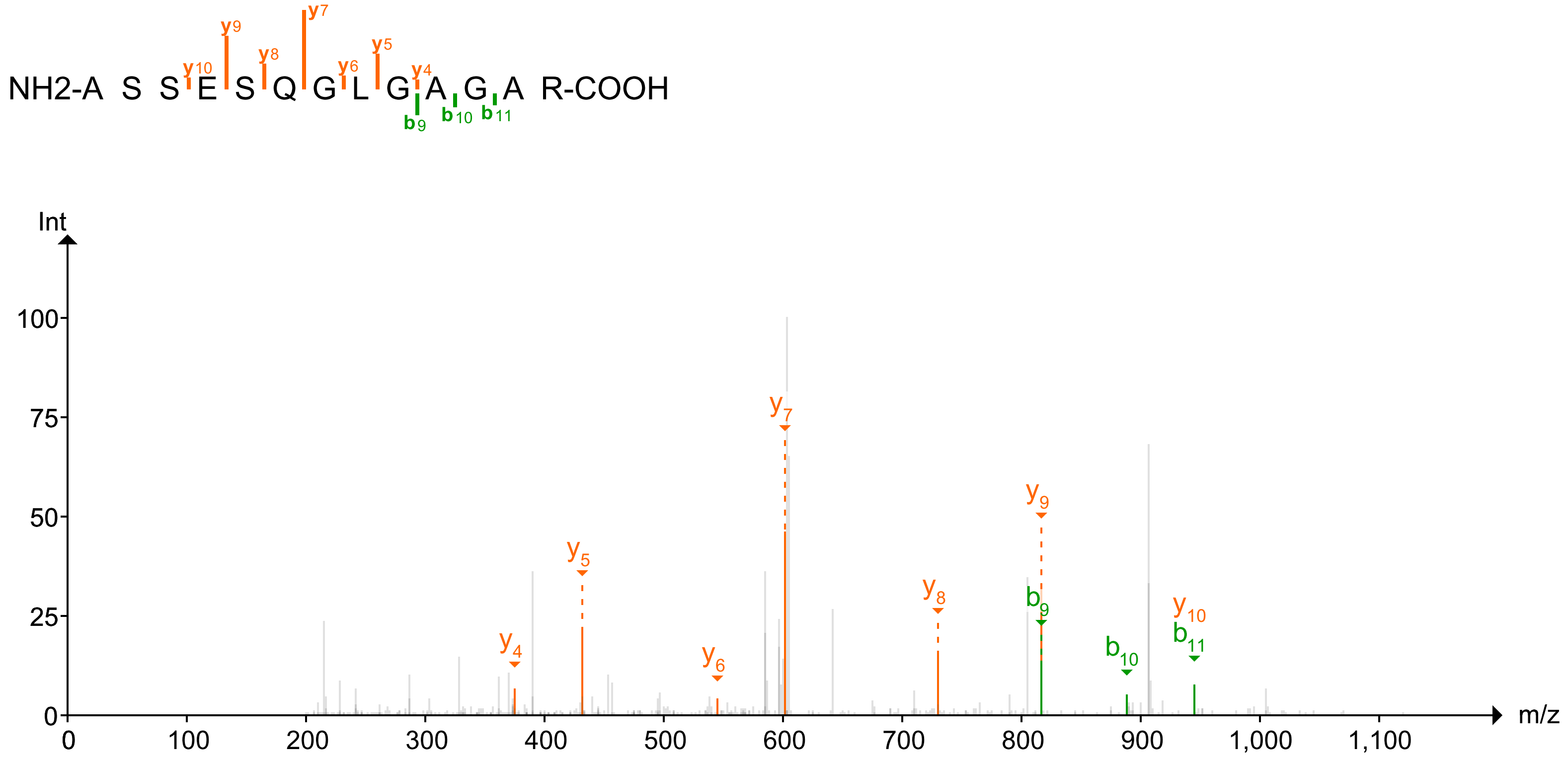

NH2-A S S E S Q G L G A G A R-COOH
y10 y9 y8 y7 y6 y5 y4
b9 b10 b11
Int
100
75
50
25
0
0 100 200 300 400 500 600 700 800 900 1,000 1,100
m/z
y4 y5 y6 y7 y8 y9 y10
b9 b10 b11

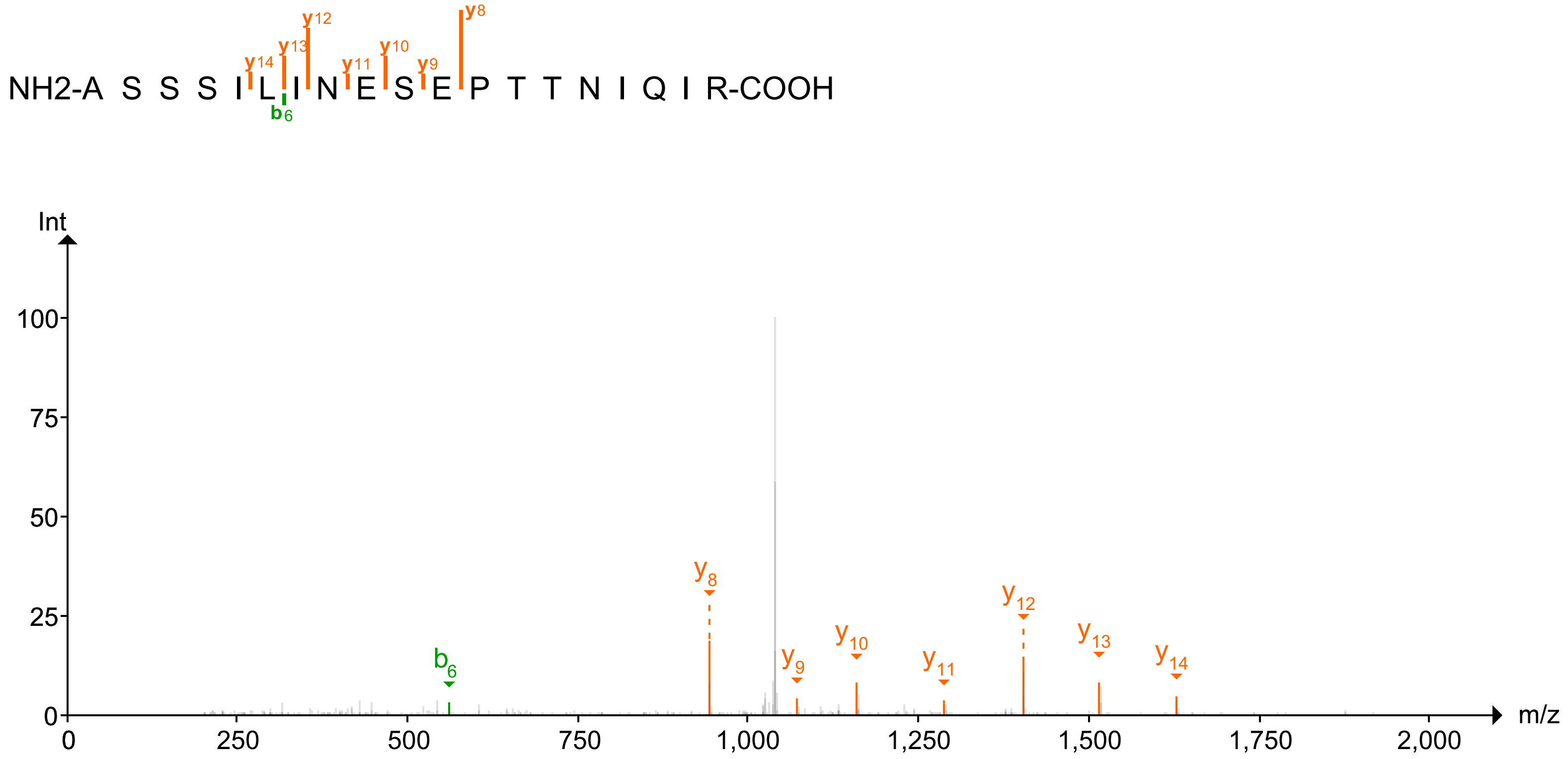

NH2-A S S S I L I N E S E P T T N I Q I R-COOH
y14 y13 y12 y11 y10 y9 y8
b6
Int
100
75
50
25
0
m/z
0
250
500
750
1,000
1,250
1,500
1,750
2,000
b6
y8
y9
y10
y11
y12
y13
y14

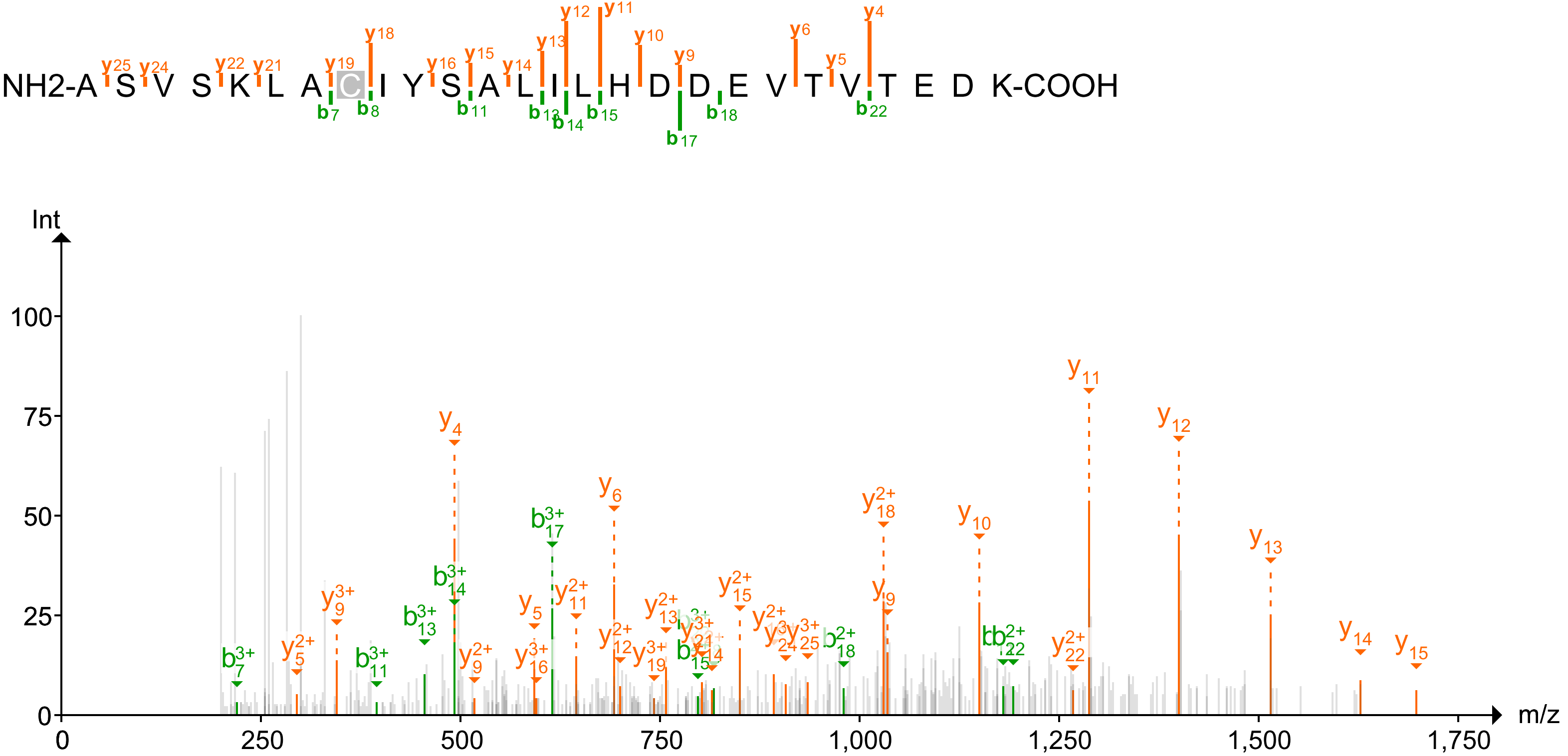

NH2-A S V S K L A C I Y S A L I L H D D E V T V T E D K-COOH
Int
m/z
0
25
50
75
100
250
500
750
1,000
1,250
1,500
1,750

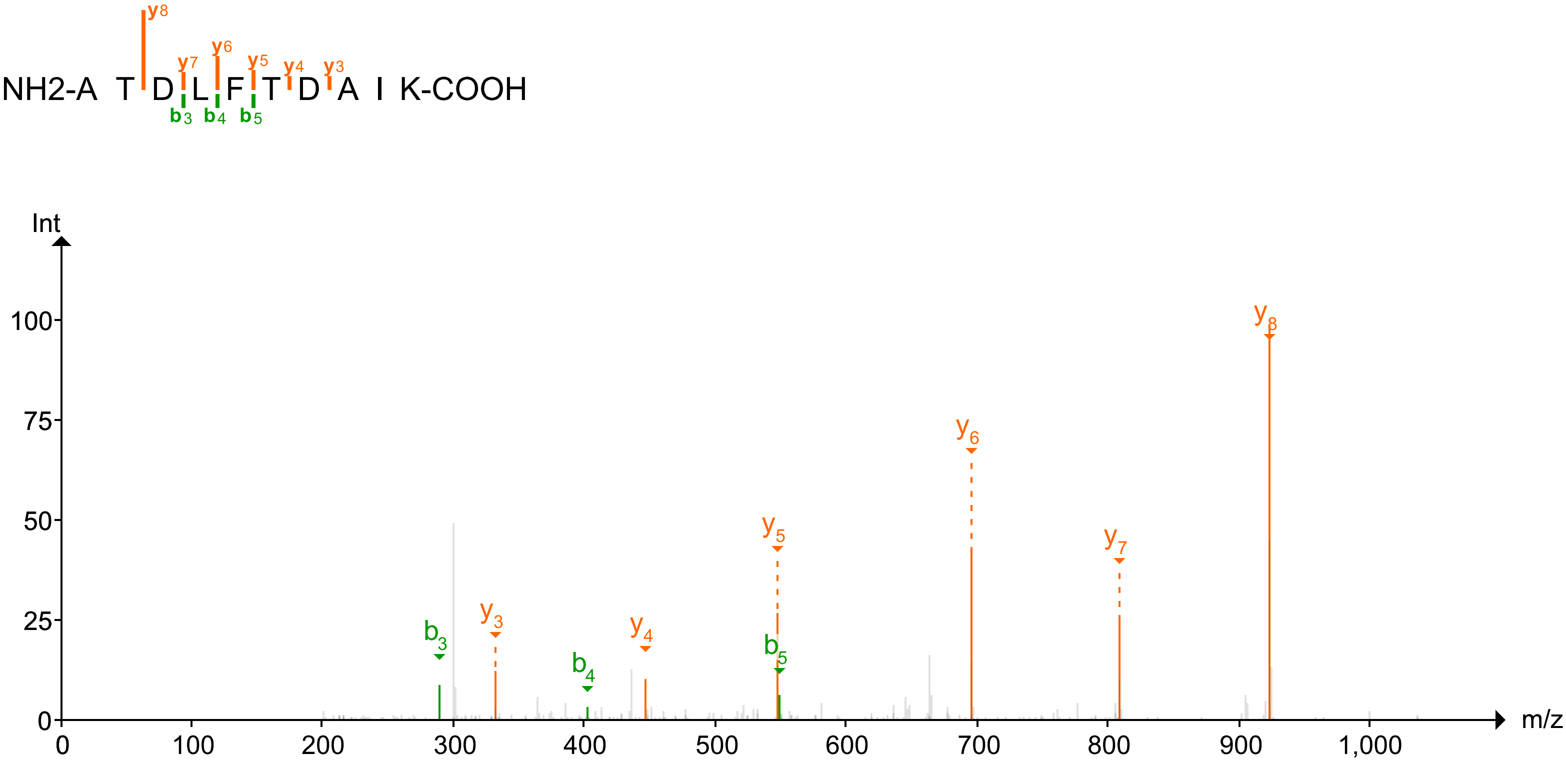
NH2-A T D L F T D A I K-COOH
y8
y7
y6
y5
y4
y3
b3
b4
b5
Int
m/z
0
25
50
75
100
0
100
200
300
400
500
600
700
800
900
1,000

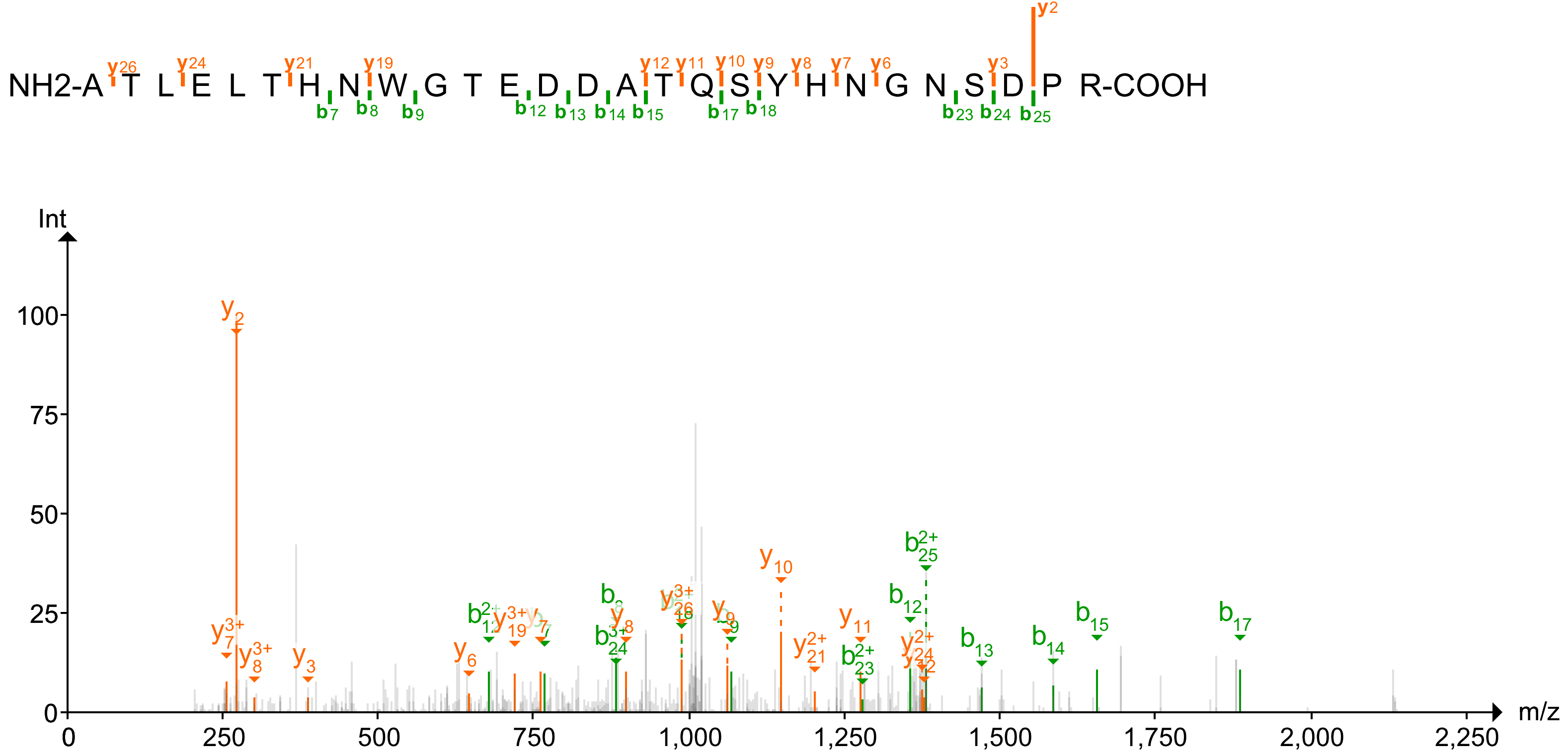

NH2-A T L E L T H N W G T E D D A T Q S Y H N G N S D P R-COOH
y26 y24 y21 y19 y12 y11 y10 y9 y8 y7 y6 y3 y2
b7 b8 b9 b12 b13 b14 b15 b17 b18 b23 b24 b25
Int
100
75
50
25
0
m/z
0
250
500
750
1,000
1,250
1,500
1,750
2,000
2,250

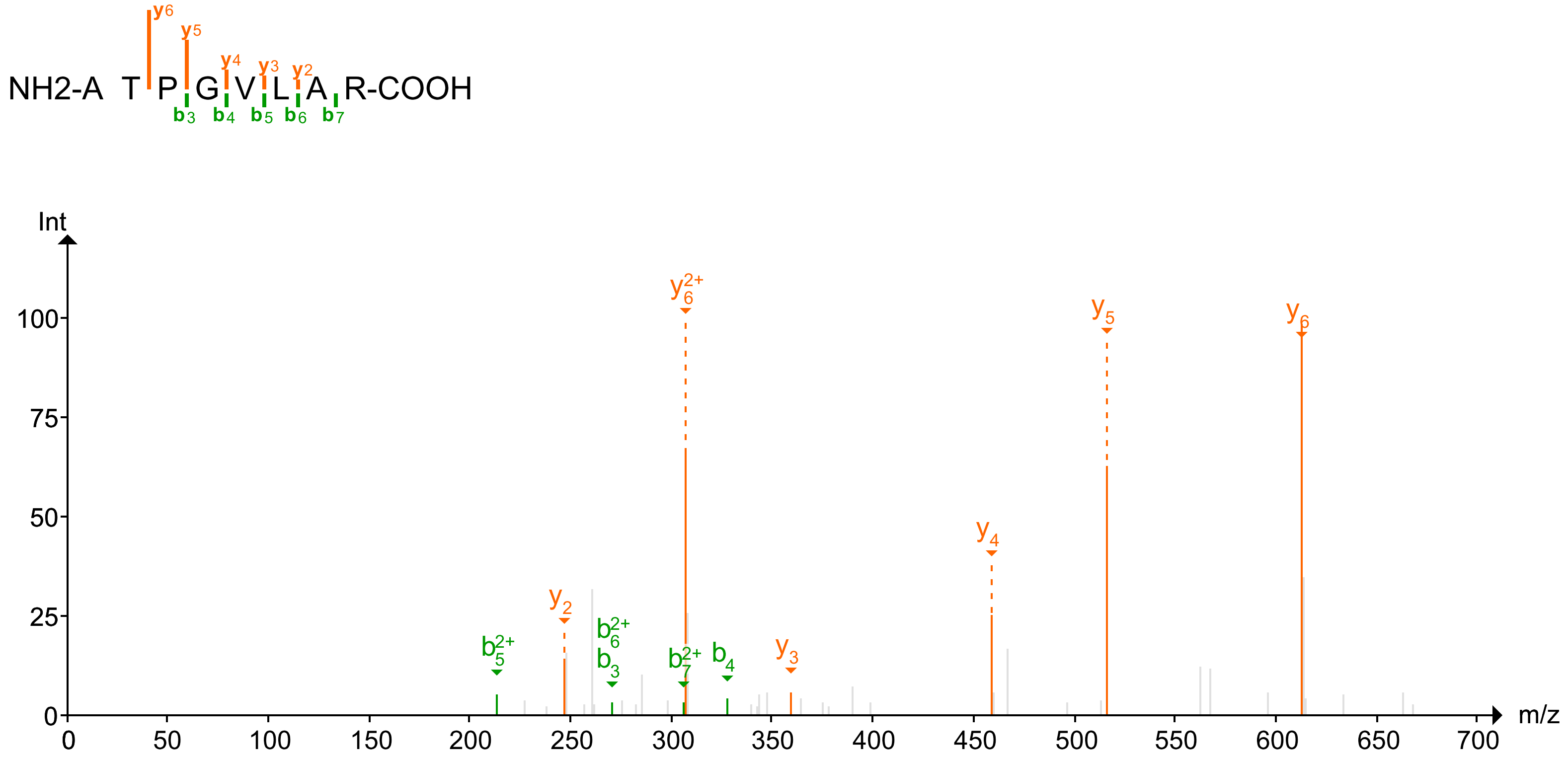
NH2-A T P G V L A R-COOH
y6 y5 y4 y3 y2
b3 b4 b5 b6 b7
Int
m/z
0 25 50 75 100
0 50 100 150 200 250 300 350 400 450 500 550 600 650 700
y6 2+
y5
y6
y4
y2
b5 2+
b6 2+
b3
b7 2+
b4
y3

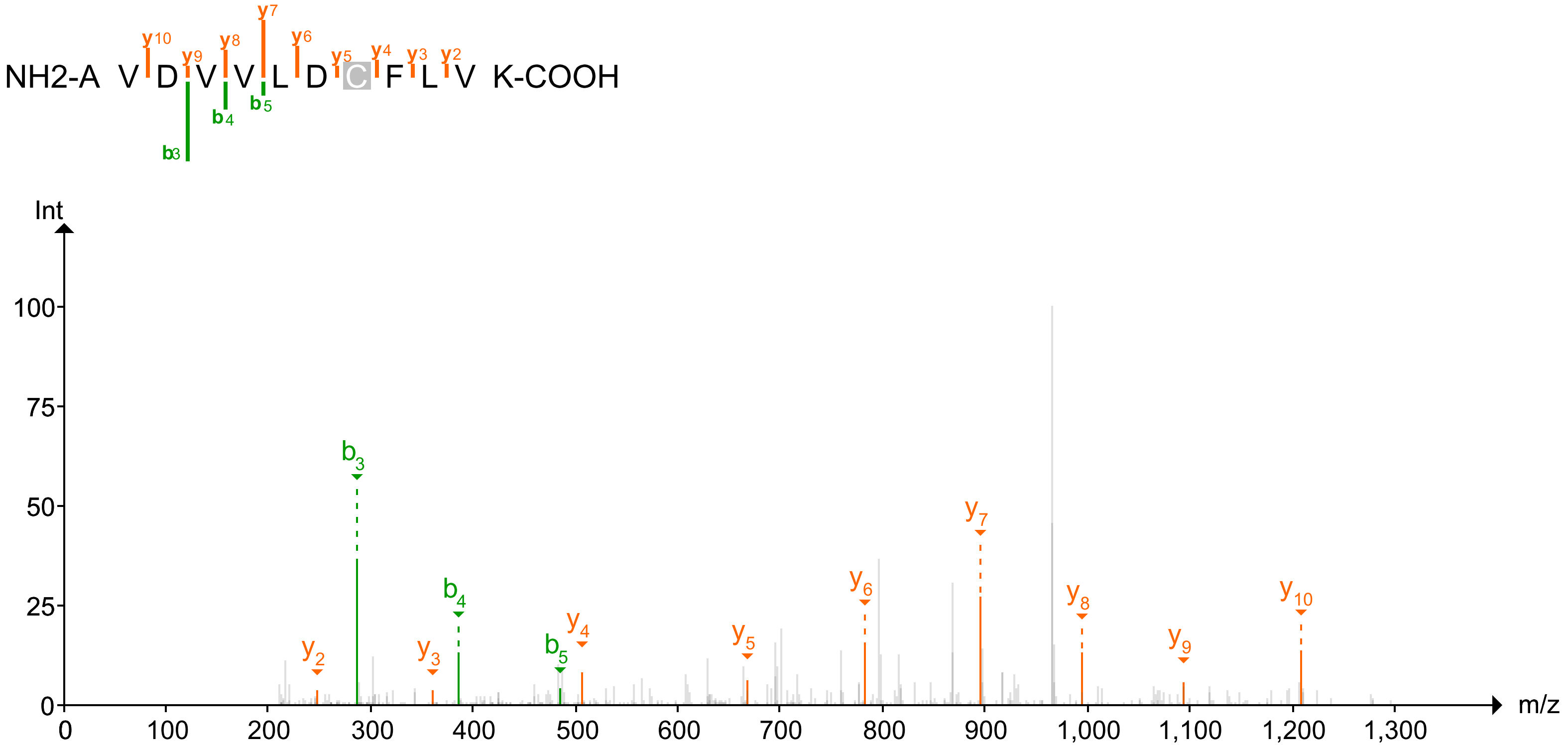

NH2-A V D V V L D C F L V K-COOH
y10 y9 y8 y7 y6 y5 y4 y3 y2
b3 b4 b5
Int
100
75
50
25
0
m/z
0 100 200 300 400 500 600 700 800 900 1,000 1,100 1,200 1,300
b3 b4 b5
y2 y3 y4 y5 y6 y7 y8 y9 y10

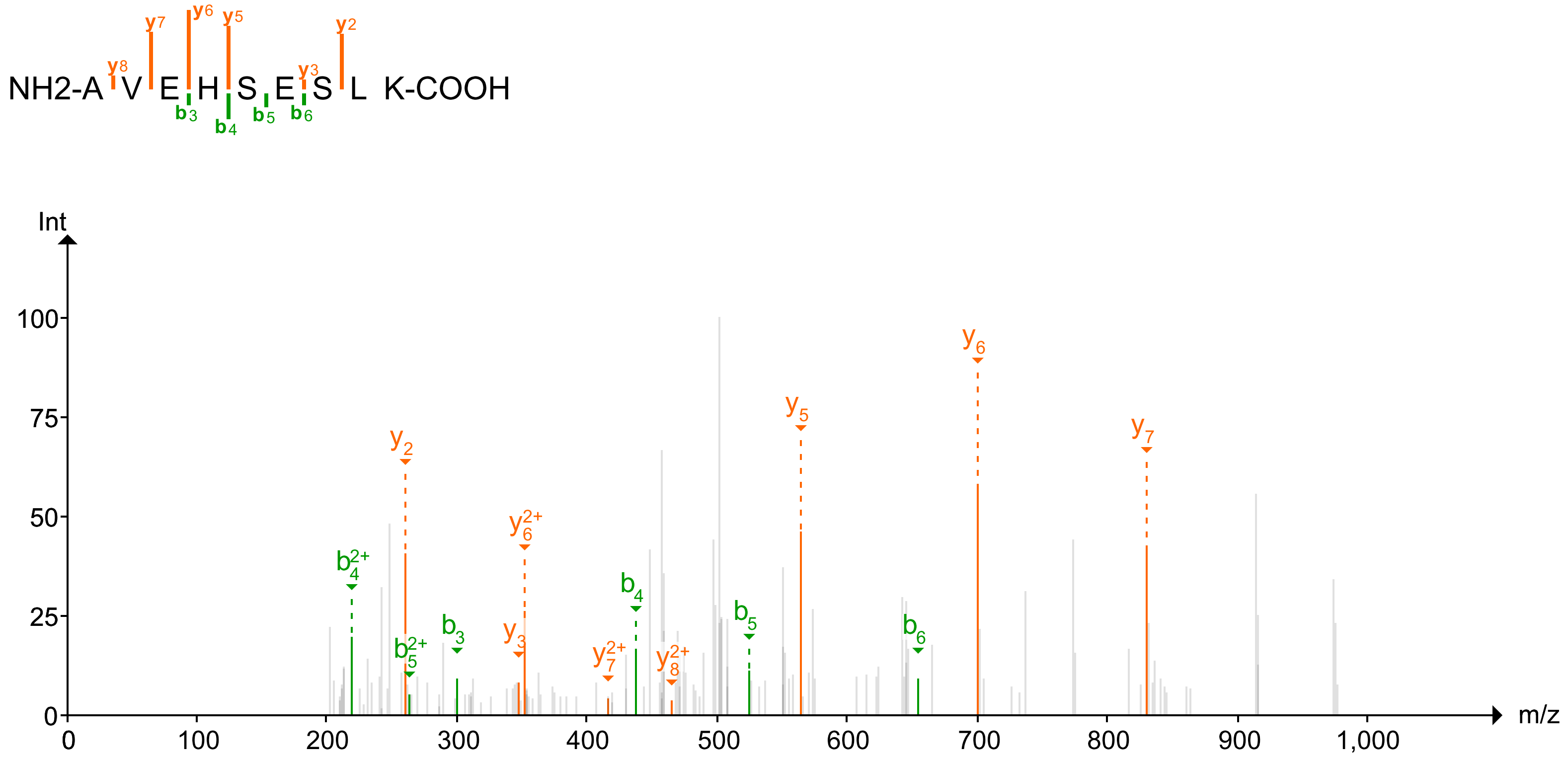

NH2-A V E H S E S L K-COOH
y8 y7 y6 y5 y3 y2
b3 b4 b5 b6
Int
100
75
50
25
0
m/z
0
100
200
300
400
500
600
700
800
900
1,000
$b_4^{2+}$
$y_2$
$b_5^{2+}$
$b_3$
$y_3$
$y_6^{2+}$
$y_7^{2+}$
$b_4$
$y_8^{2+}$
$b_5$
$y_5$
$b_6$
$y_6$
$y_7$

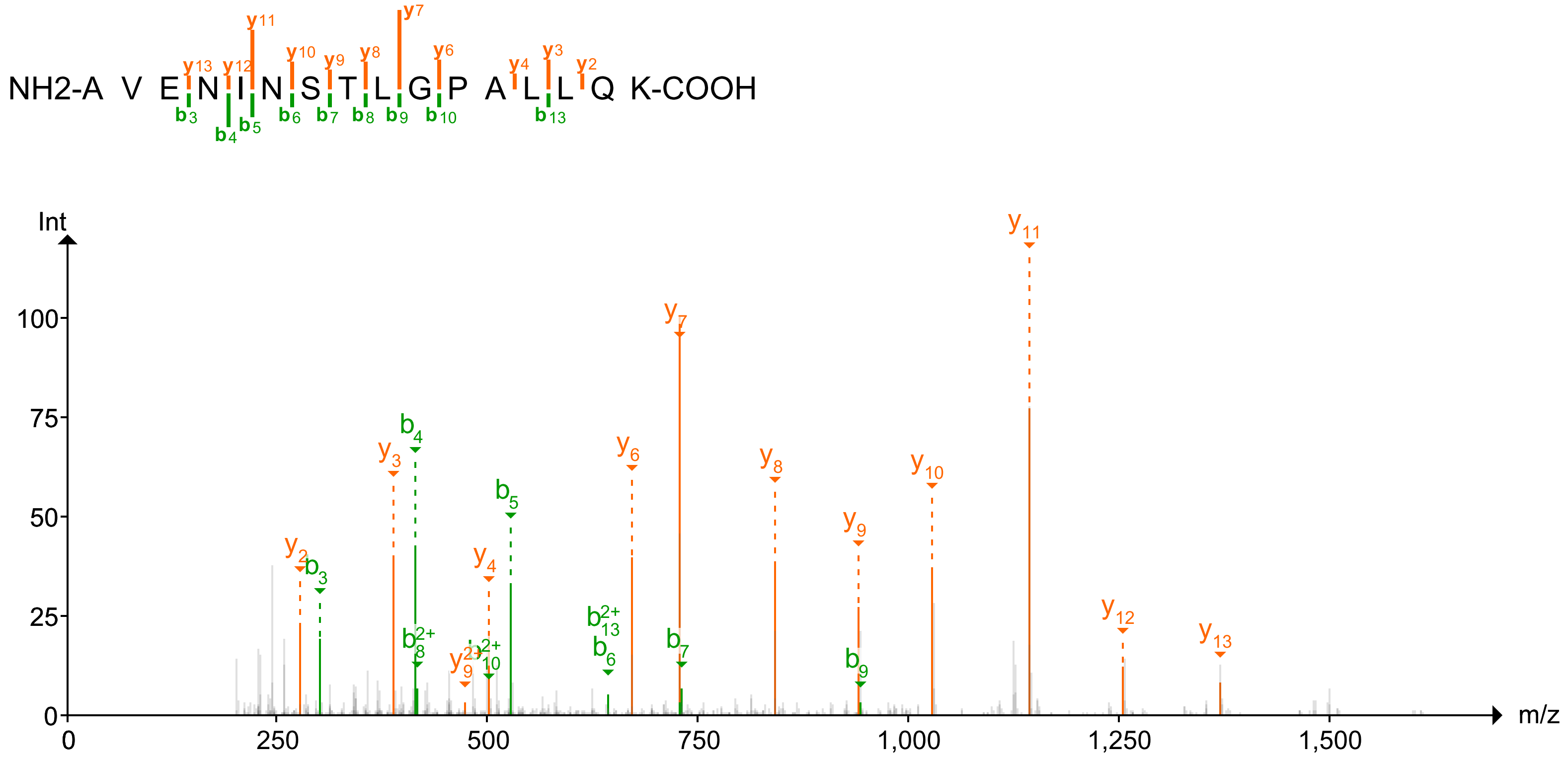
NH2-A V E N I N S T L G P A L L Q K-COOH
y13 y12 y11 y10 y9 y8 y7 y6 y4 y3 y2
b3 b4 b5 b6 b7 b8 b9 b10 b13
Int
m/z
0
25
50
75
100
250
500
750
1,000
1,250
1,500
y11
y7
b4
y3
y6
y8
y10
b5
y9
y2
b3
y4
y12
y13
b8 2+
y9 2+
b10 2+
b13 2+
b6
b7
b9

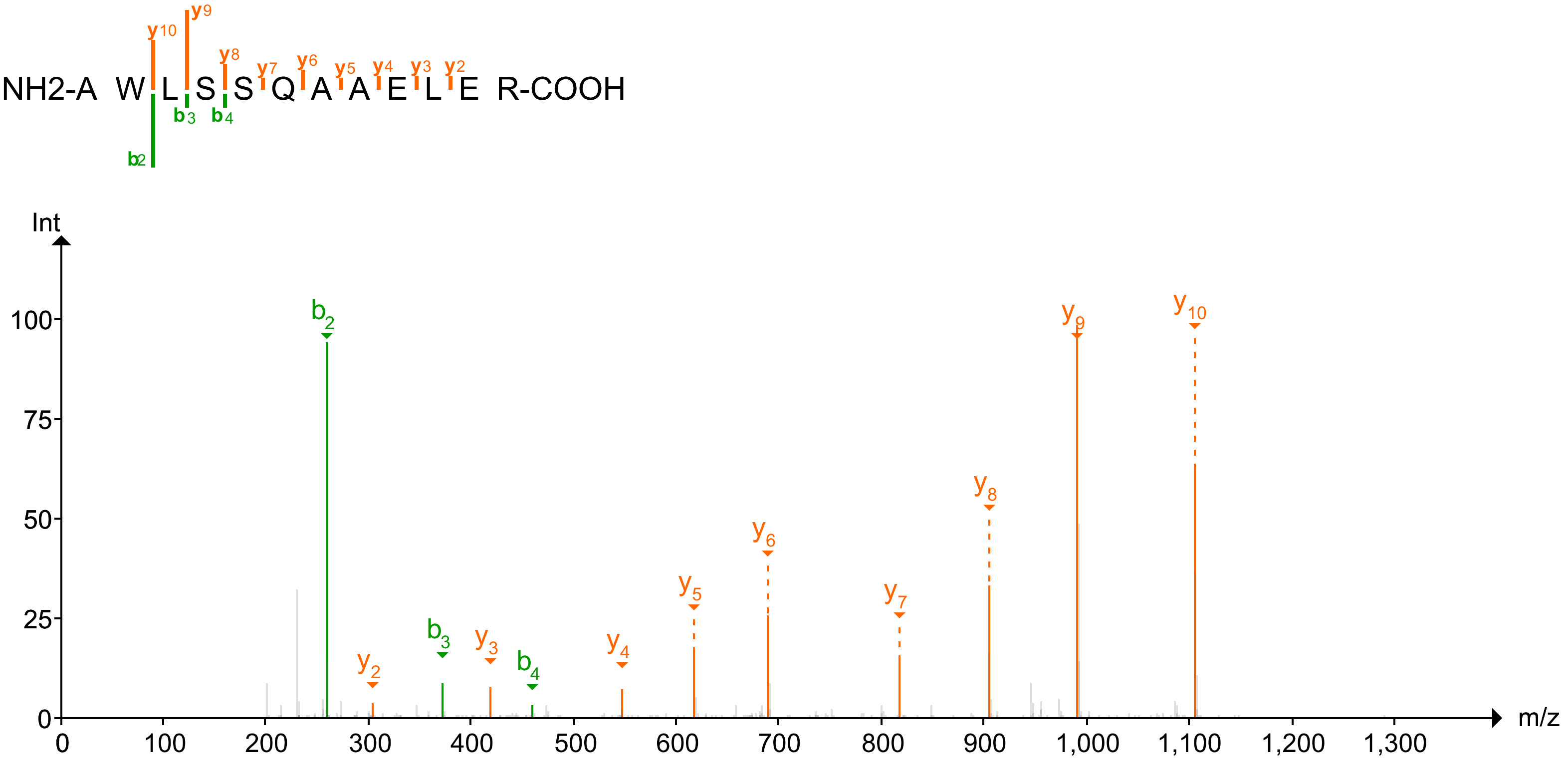

NH2-A W L S S Q A A E L E R-COOH
y10 y9 y8 y7 y6 y5 y4 y3 y2
b2 b3 b4
Int
100
75
50
25
0
m/z
0 100 200 300 400 500 600 700 800 900 1,000 1,100 1,200 1,300
b2 y2 b3 y3 b4 y4 y5 y6 y7 y8 y9 y10

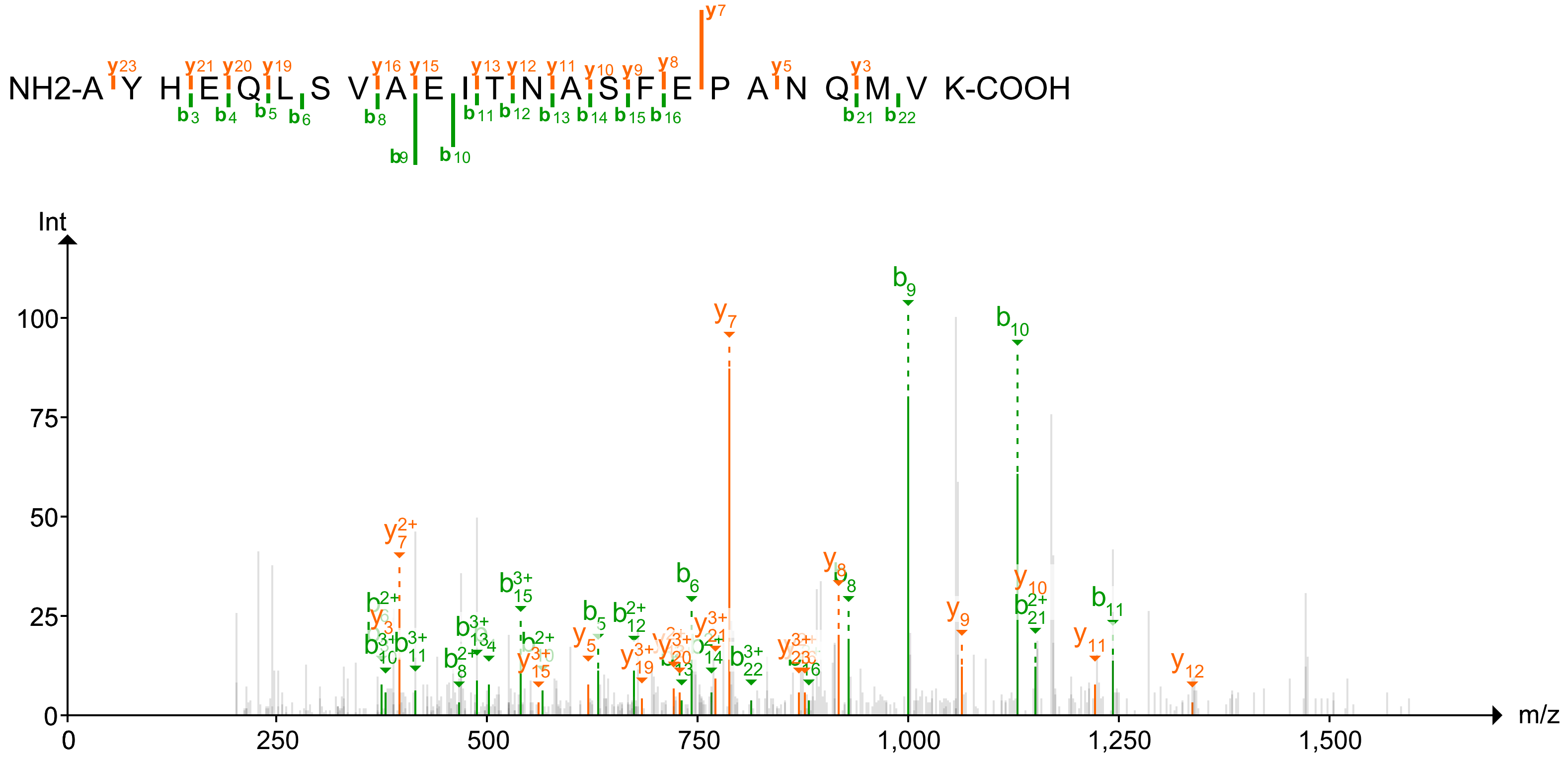

NH2-A Y H E Q L S V A E I T N A S F E P A N Q M V K-COOH
y23 y21 y20 y19 y16 y15 y13 y12 y11 y10 y9 y8 y7 y5 y3
b3 b4 b5 b6 b8 b9 b10 b11 b12 b13 b14 b15 b16 b21 b22
Int
100
75
50
25
0
m/z
0
250
500
750
1,000
1,250
1,500

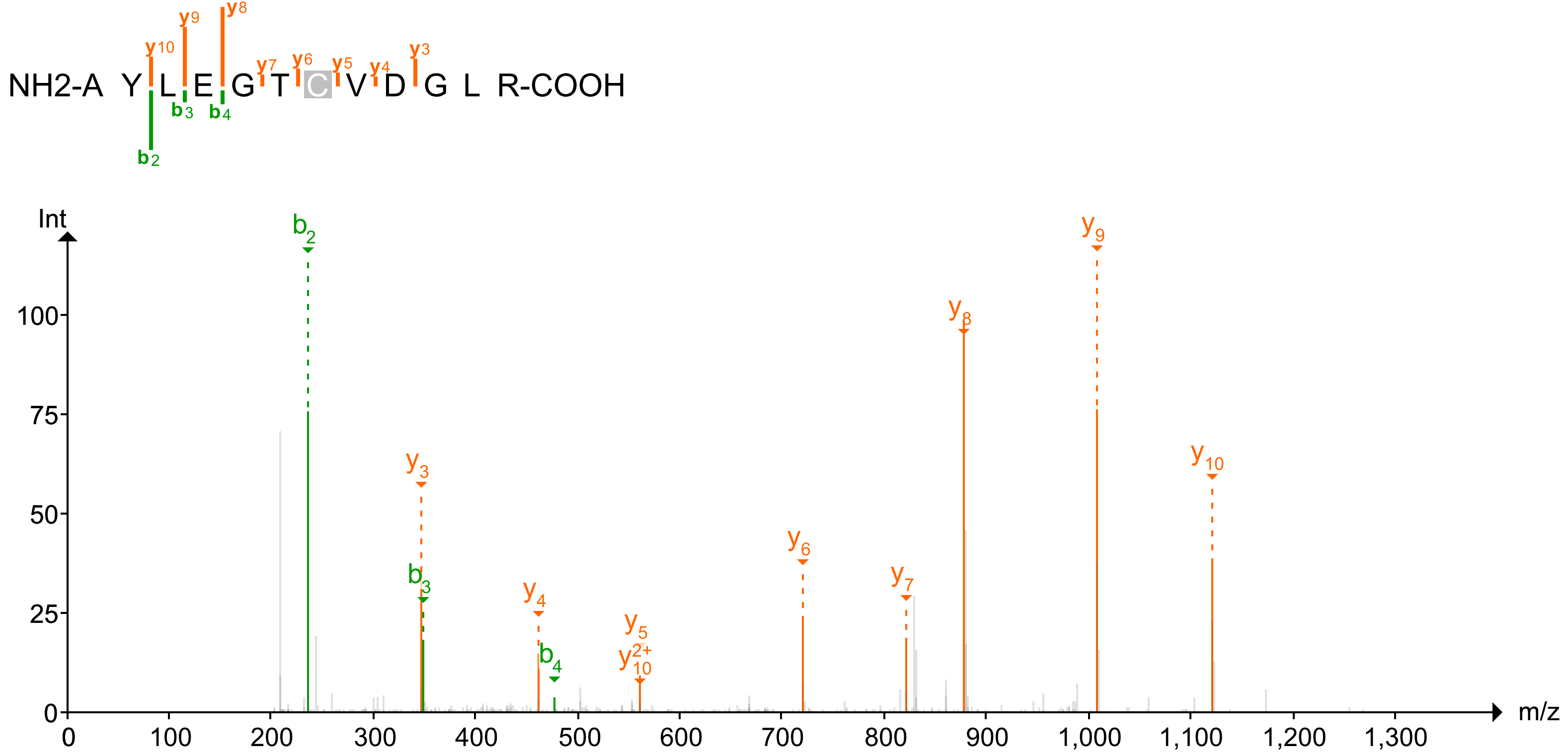

NH2-A Y L E G T C V D G L R-COOH
y10 y9 y8 y7 y6 y5 y4 y3
b2 b3 b4
Int
m/z
0 25 50 75 100
0 100 200 300 400 500 600 700 800 900 1,000 1,100 1,200 1,300
b2 b3 b4
y3 y4 y5 y10 2+ y6 y7 y8 y9 y10

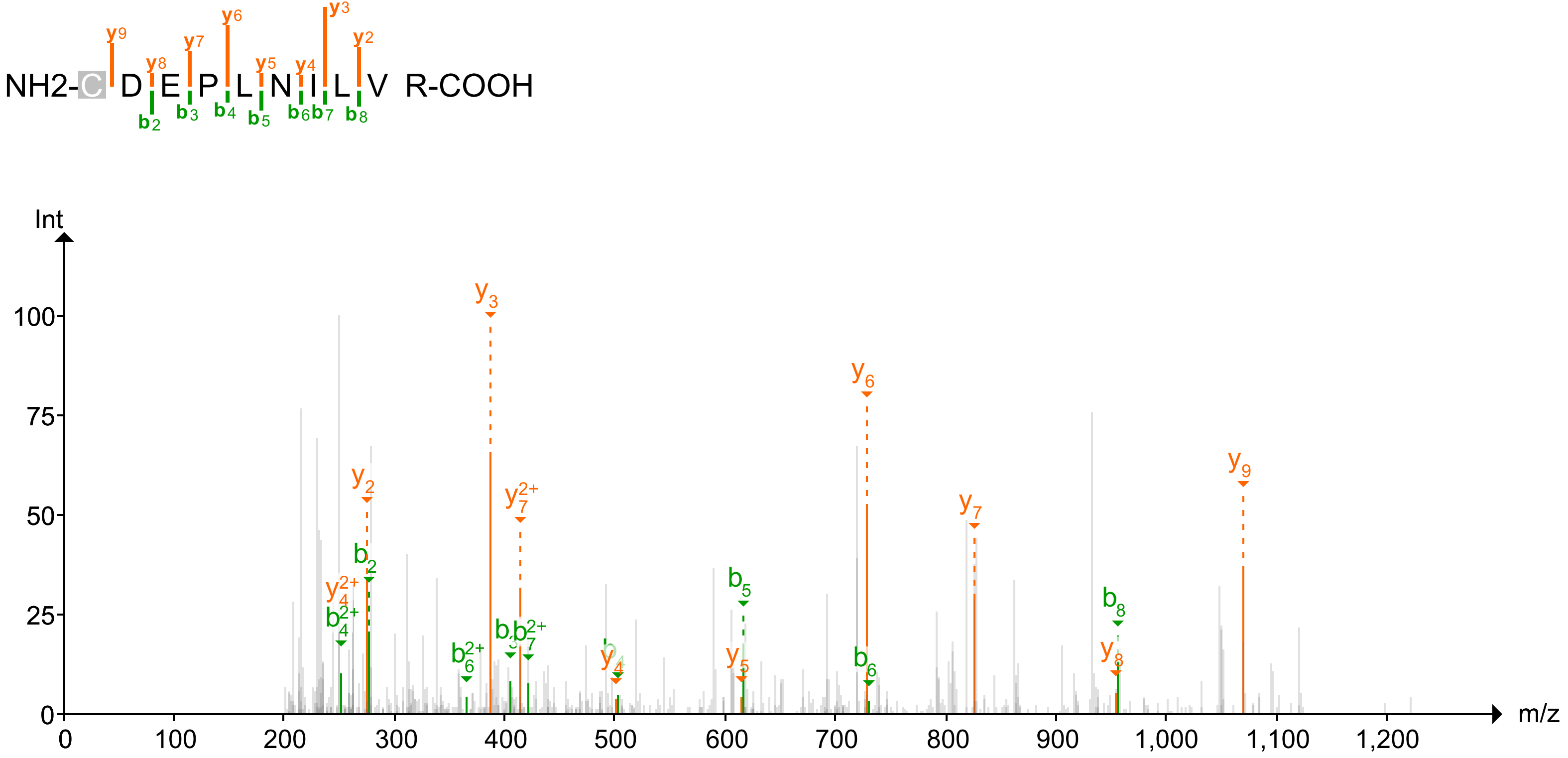

NH2-C D E P L N I L V R-COOH
y9 y8 y7 y6 y5 y4 y3 y2
b2 b3 b4 b5 b6 b7 b8
Int
100
75
50
25
0
m/z
0 100 200 300 400 500 600 700 800 900 1,000 1,100 1,200
y3
y6
y9
y2
y7
y7 2+
b2
y4 2+
b4 2+
b5
b8
b6 2+
b3
b7 2+
y4
y5
b6
y8

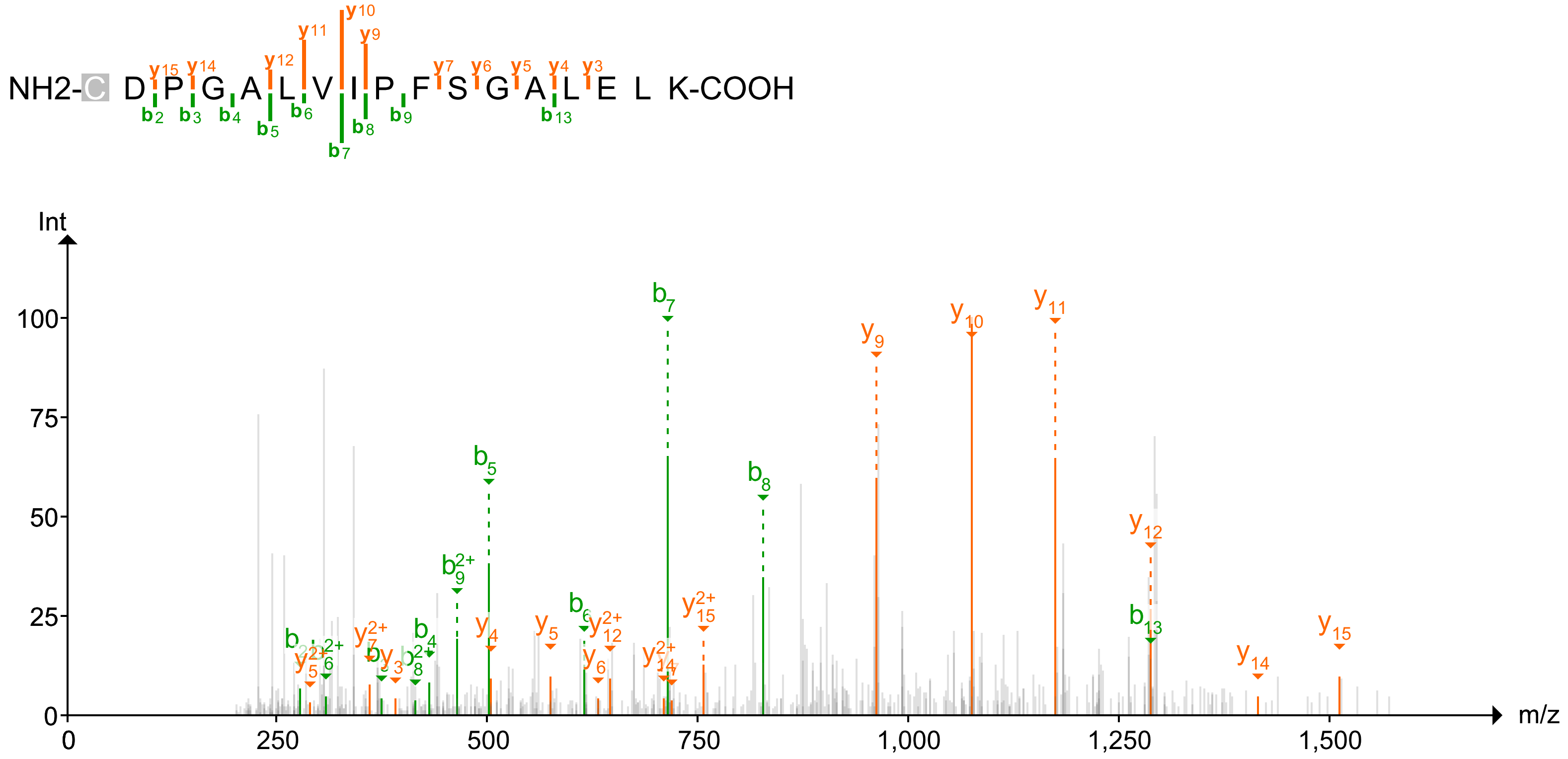

NH2-C D P G A L V I P F S G A L E L K-COOH
y15 y14 y12 y11 y10 y9 y7 y6 y5 y4 y3
b2 b3 b4 b5 b6 b7 b8 b9 b13
Int
100
75
50
25
0
m/z
0
250
500
750
1,000
1,250
1,500
b7
y9
y10
y11
b5
b8
y12
b13
y15
y14

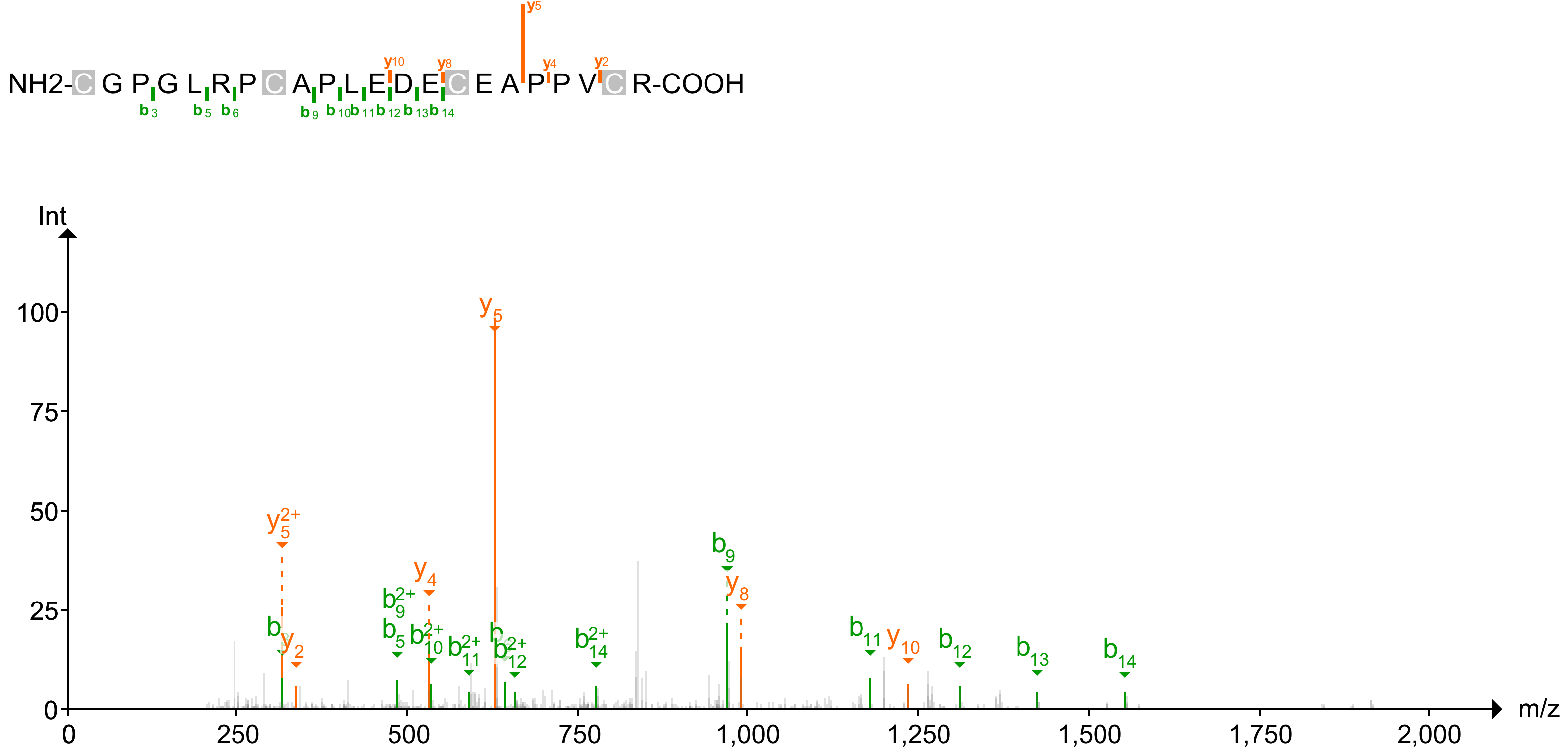

NH2-C G P G L R P C A P L E D E C E A P P V C R-COOH
y5
y10
y8
y4
y2
b3
b5
b6
b9
b10
b11
b12
b13
b14
Int
100
75
50
25
0
m/z
0
250
500
750
1,000
1,250
1,500
1,750
2,000
y5
y5 2+
y4
y2
b9
y8
b9 2+
b5
b10 2+
b11 2+
b12 2+
b14 2+
b11
y10
b12
b13
b14

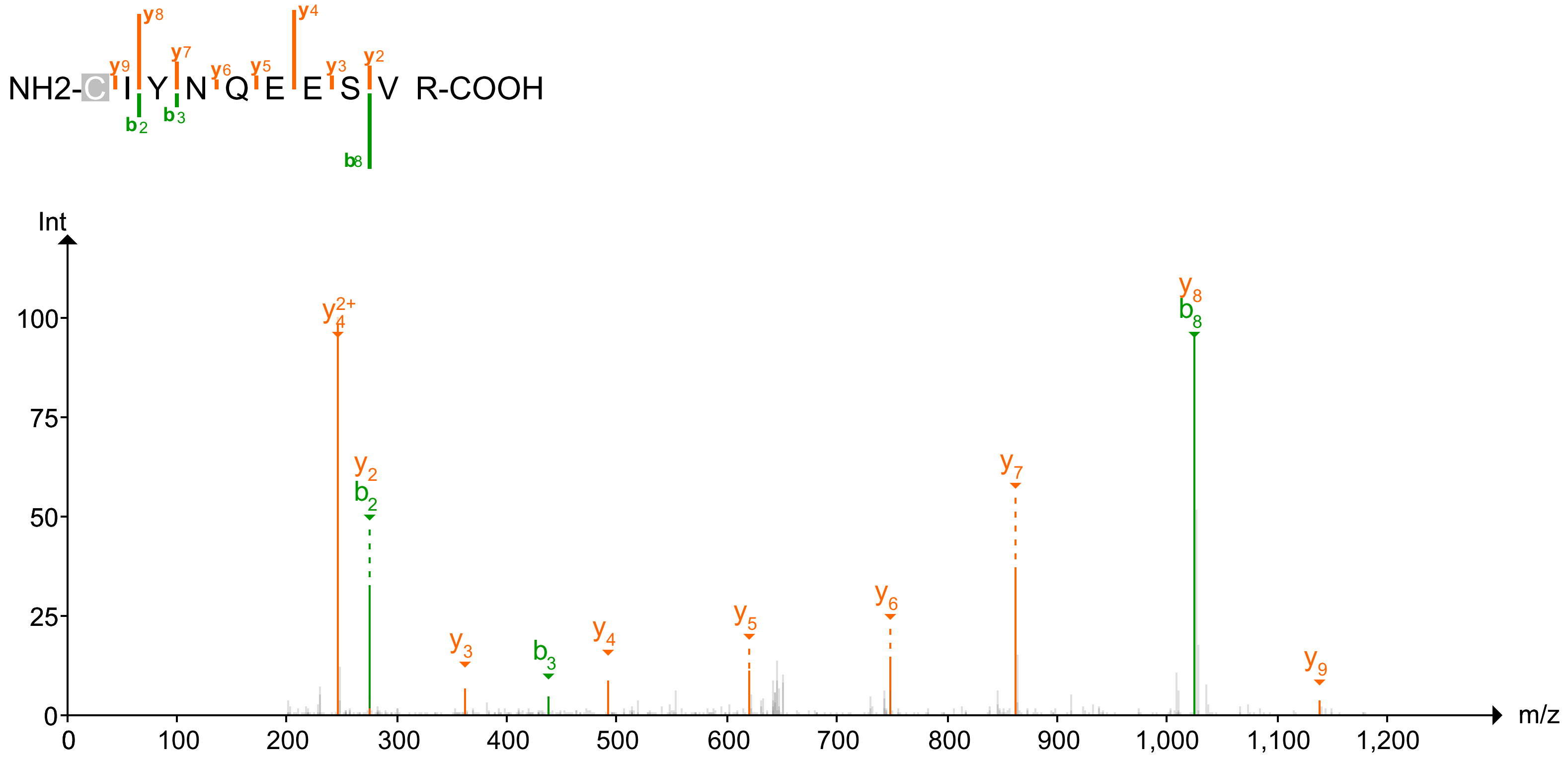

NH2-C I Y N Q E E S V R-COOH
y9 y8 y7 y6 y5 y4 y3 y2
b2 b3 b8
Int
100
75
50
25
0
m/z
0 100 200 300 400 500 600 700 800 900 1,000 1,100 1,200
y4 2+
y2 b2
y3
b3
y4
y5
y6
y7
y8 b8
y9

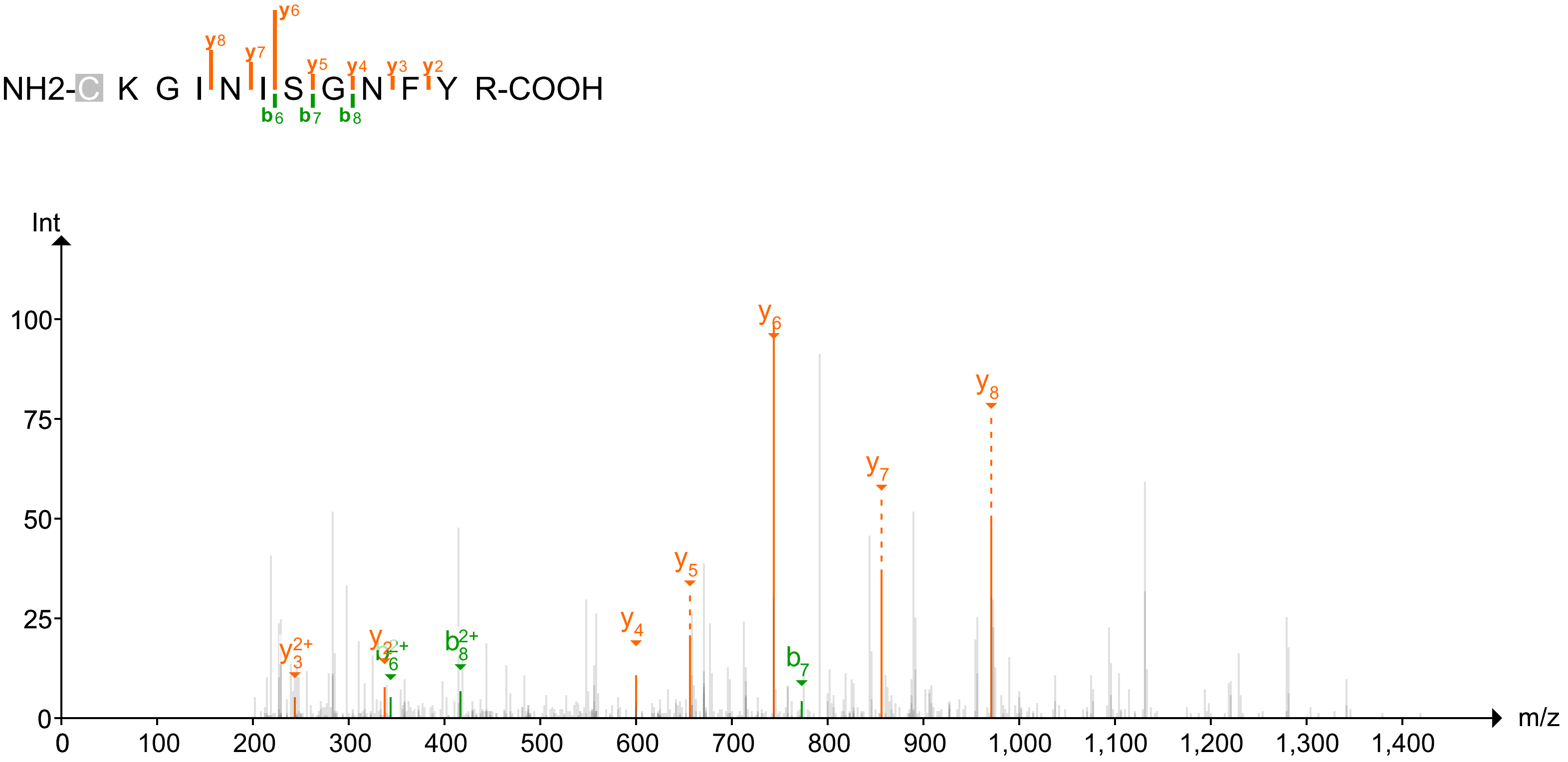

NH2-C K G I N I S G N F Y R-COOH
y8 y7 y6 y5 y4 y3 y2
b6 b7 b8
Int
100
75
50
25
0
y6
y8
y7
y5
y4
y3 2+
b6 2+
b8 2+
b7
m/z
0
100
200
300
400
500
600
700
800
900
1,000
1,100
1,200
1,300
1,400

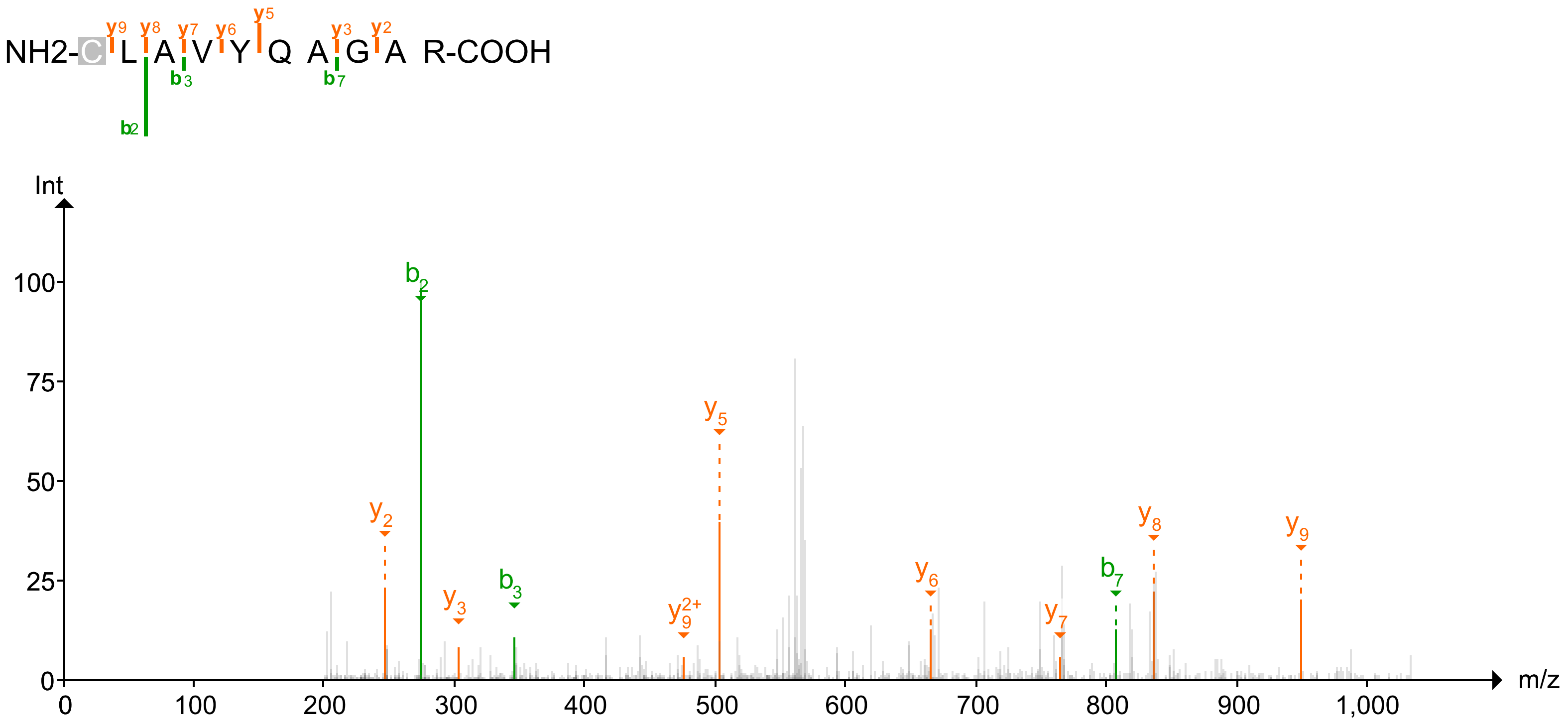

NH2-C L A V Y Q A G A R-COOH
y9 y8 y7 y6 y5 y3 y2
b2 b3 b7
Int
100
75
50
25
0
0 100 200 300 400 500 600 700 800 900 1,000
m/z
b2
y5
y2
y3
b3
y9 2+
y6
y7
b7
y8
y9

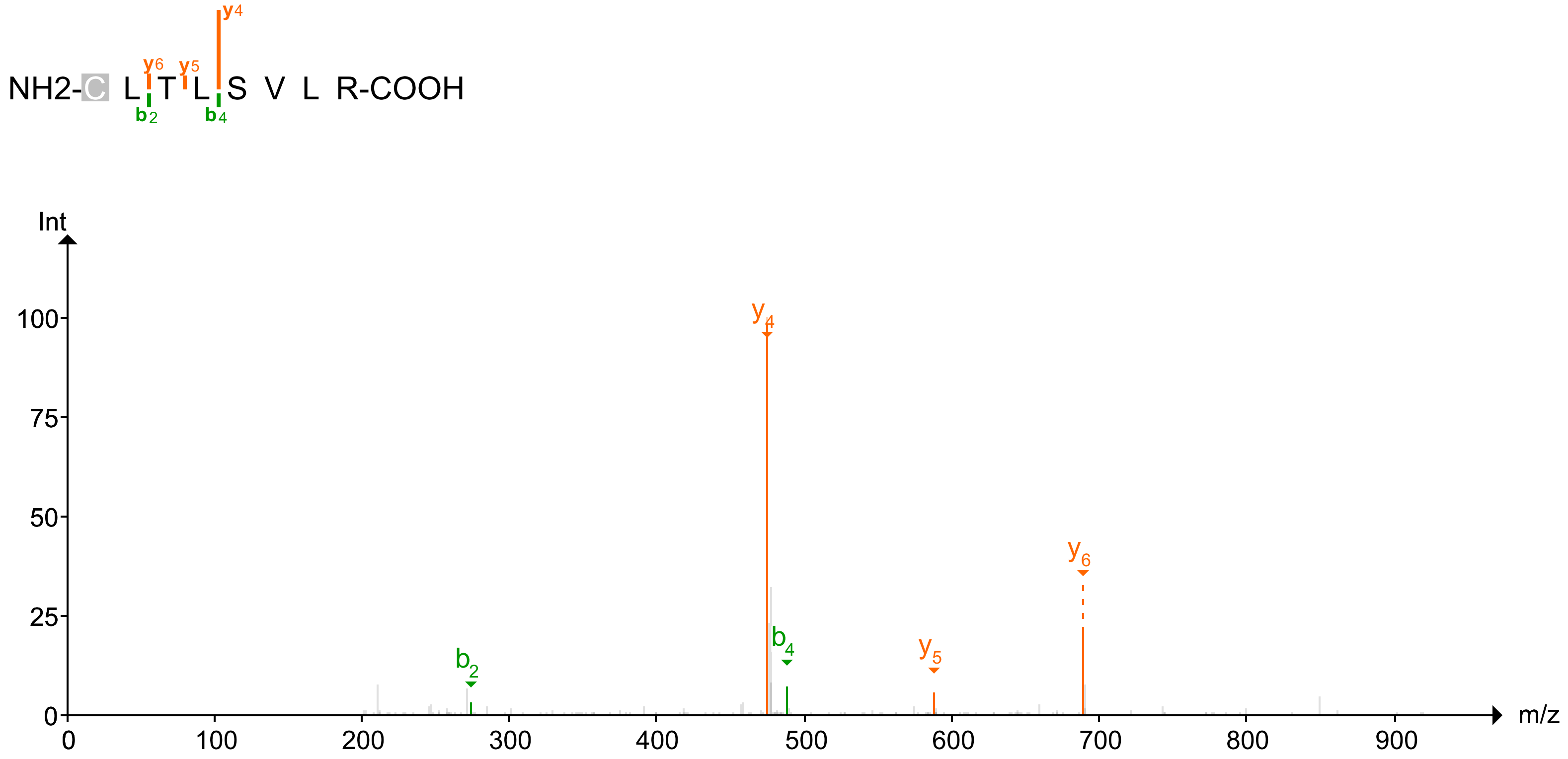
NH2-C L T L S V L R-COOH
y4
y6
y5
b2
b4
Int
100
75
50
25
0
0
100
200
300
400
500
600
700
800
900
m/z
y4
y6
b4
y5
b2

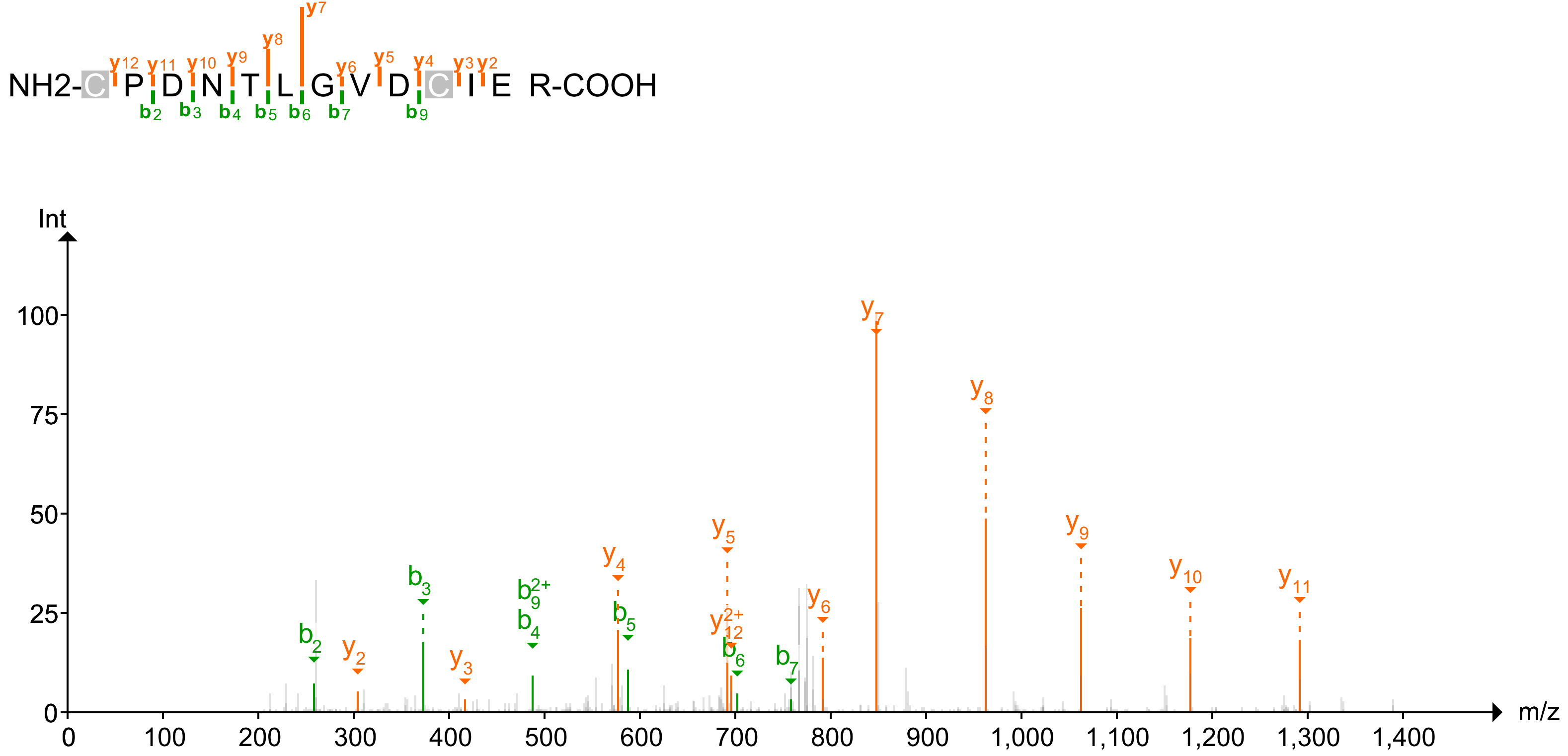

NH2-C P D N T L G V D C I E R-COOH
y12 y11 y10 y9 y8 y7 y6 y5 y4 y3 y2
b2 b3 b4 b5 b6 b7 b9
Int
100
75
50
25
0
m/z
0
100
200
300
400
500
600
700
800
900
1,000
1,100
1,200
1,300
1,400
b2
y2
b3
y3
b9 2+
b4
y4
b5
y5
y12 2+
b6
b7
y6
y7
y8
y9
y10
y11

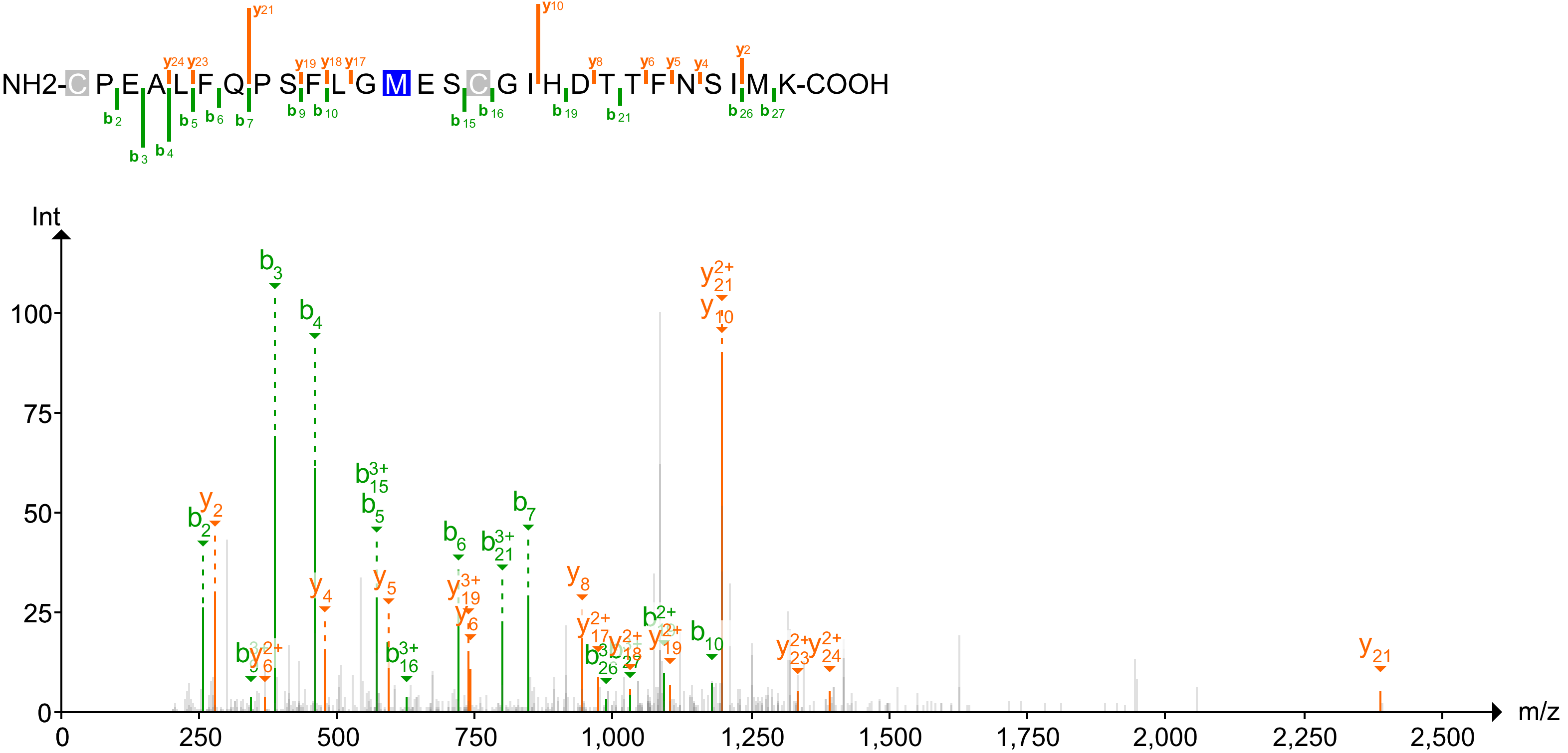
NH2-C P E A L F Q P S F L G M E S C G I H D T T F N S I M K-COOH
y24 y23 y21 y19 y18 y17 y10 y8 y6 y5 y4 y2
b2 b3 b4 b5 b6 b7 b9 b10 b15 b16 b19 b21 b26 b27
Int
m/z
0
25
50
75
100
0
250
500
750
1,000
1,250
1,500
1,750
2,000
2,250
2,500
b3
b4
b2
y2
b5
y4
y5
b6
b7
y8
y10
y21
b10

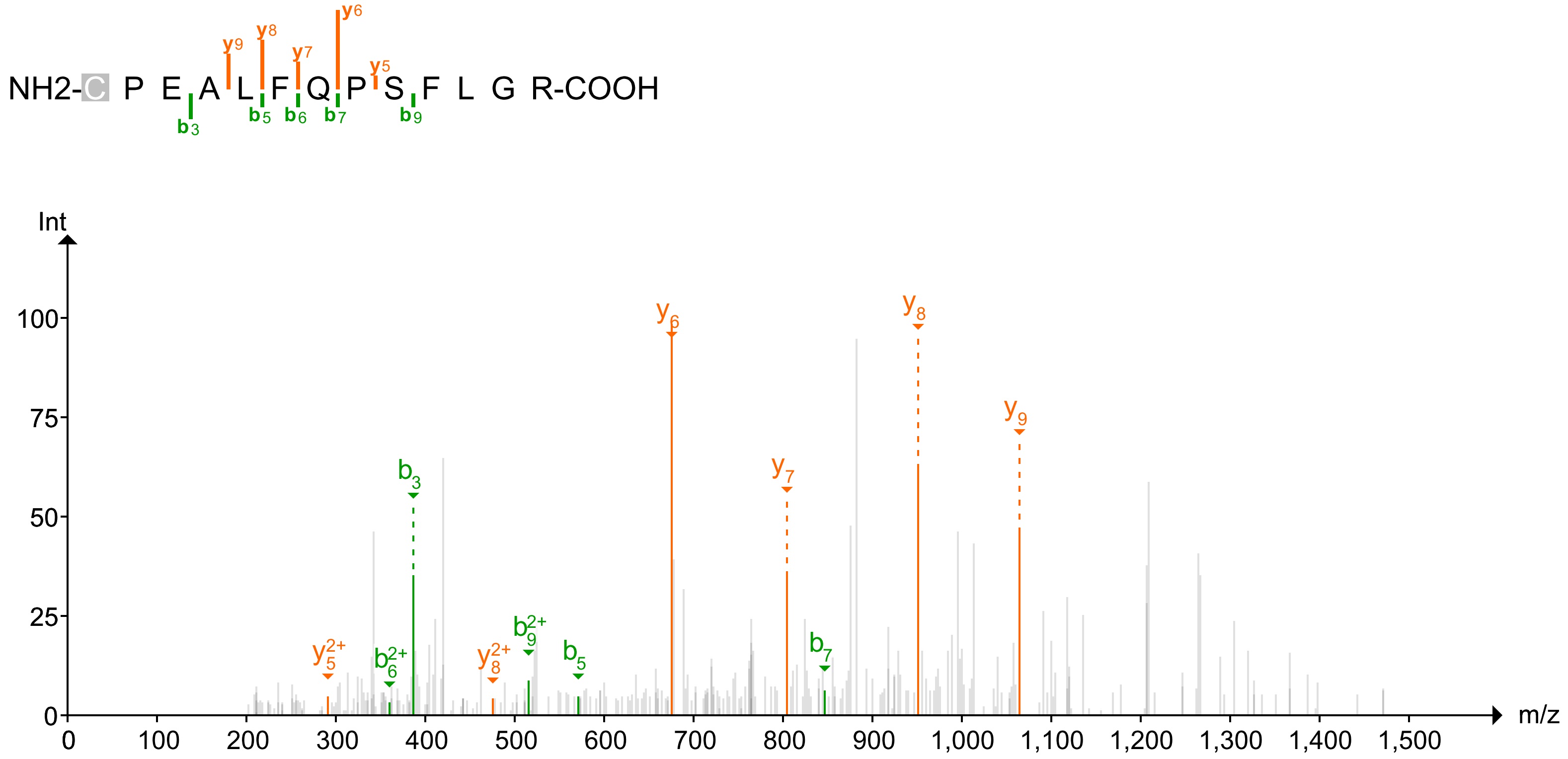

NH2-C P E A L F Q P S F L G R-COOH
y9 y8 y7 y6 y5
b3 b5 b6 b7 b9
Int
100
75
50
25
0
m/z
0 100 200 300 400 500 600 700 800 900 1,000 1,100 1,200 1,300 1,400 1,500
$y_6$ $y_8$ $y_9$ $y_7$ $b_3$ $b_7$ $b_5$ $b_9^{2+}$ $b_6^{2+}$ $y_5^{2+}$ $y_8^{2+}$

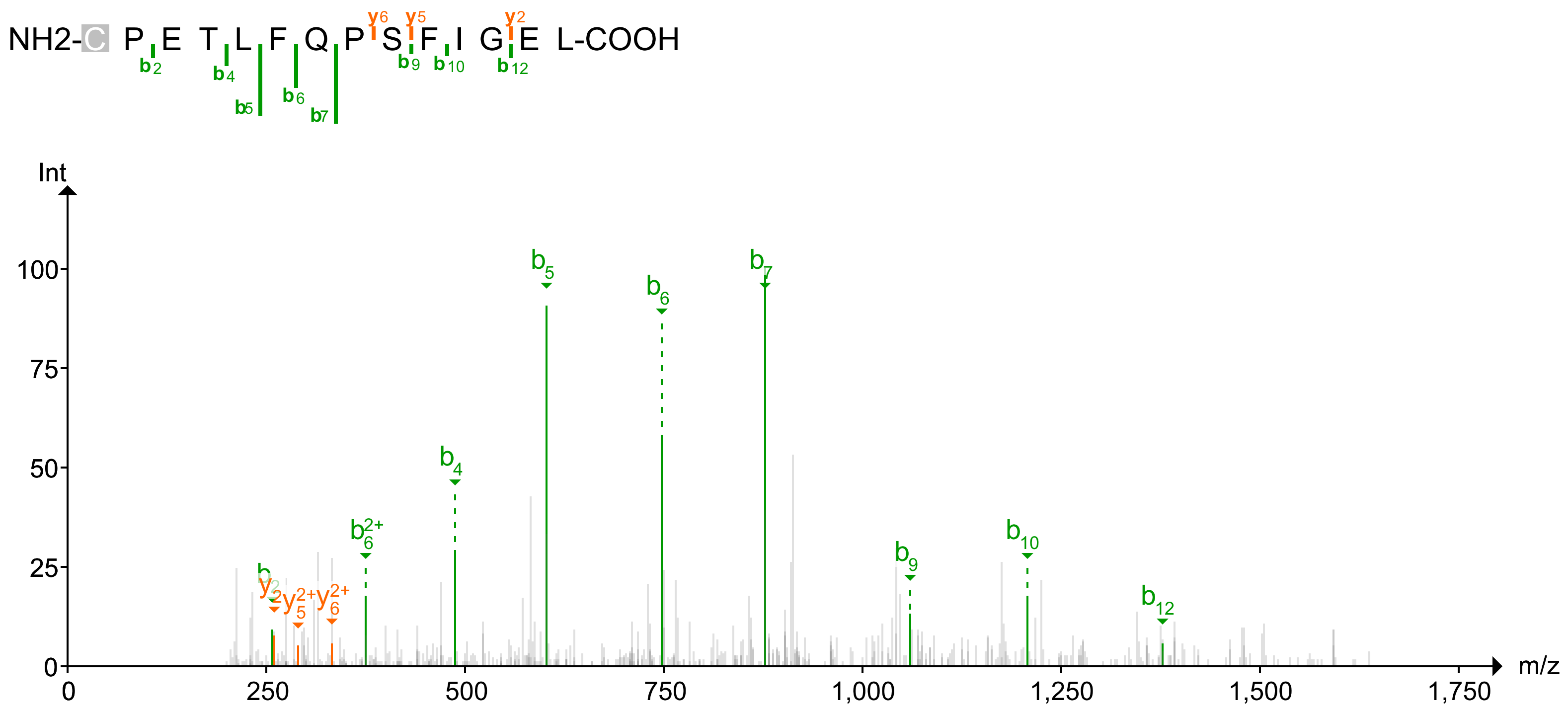

NH2-C P E T L F Q P S F I G E L-COOH
y6 y5 y2
b2 b4 b5 b6 b7 b9 b10 b12
Int
100
75
50
25
0
m/z
0
250
500
750
1,000
1,250
1,500
1,750
b5
b6
b7
b4
b6 2+
b9
b10
b12
b2
y2
y5 2+
y6 2+

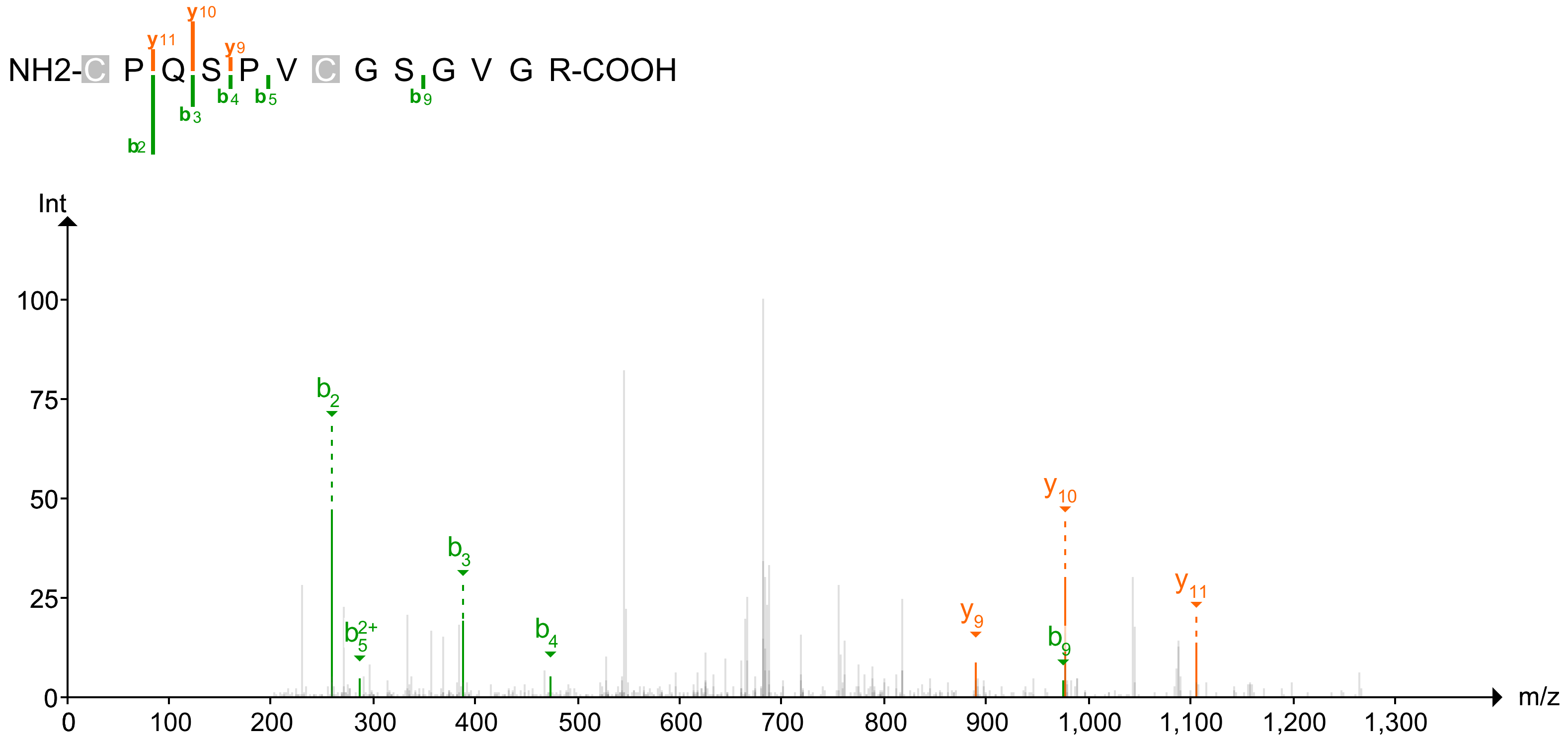

NH2-C P Q S P V C G S G V G R-COOH
y11 y10 y9
b2 b3 b4 b5 b9
Int
100
75
50
25
0
m/z
0 100 200 300 400 500 600 700 800 900 1,000 1,100 1,200 1,300
b2
b3
b5 2+
b4
y9
y10
b9
y11

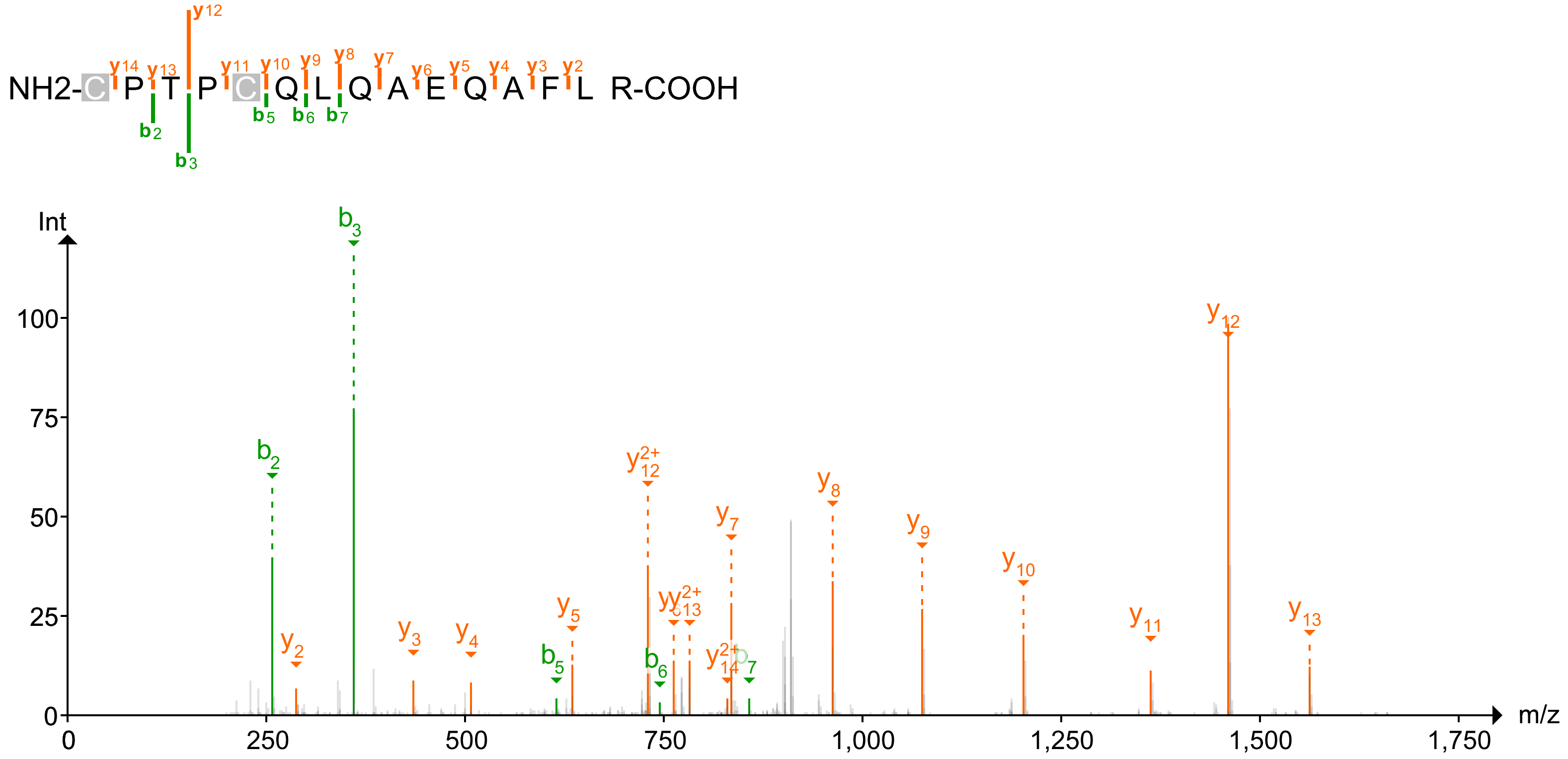

NH2-C P T P C Q L Q A E Q A F L R-COOH
y14 y13 y12 y11 y10 y9 y8 y7 y6 y5 y4 y3 y2
b2 b3 b5 b6 b7
Int
m/z
b3
b2
y12
y8
y7
y9
y10
y11
y13
y5
y4
y3
y2
b5
b6
b7
0
25
50
75
100
250
500
750
1,000
1,250
1,500
1,750

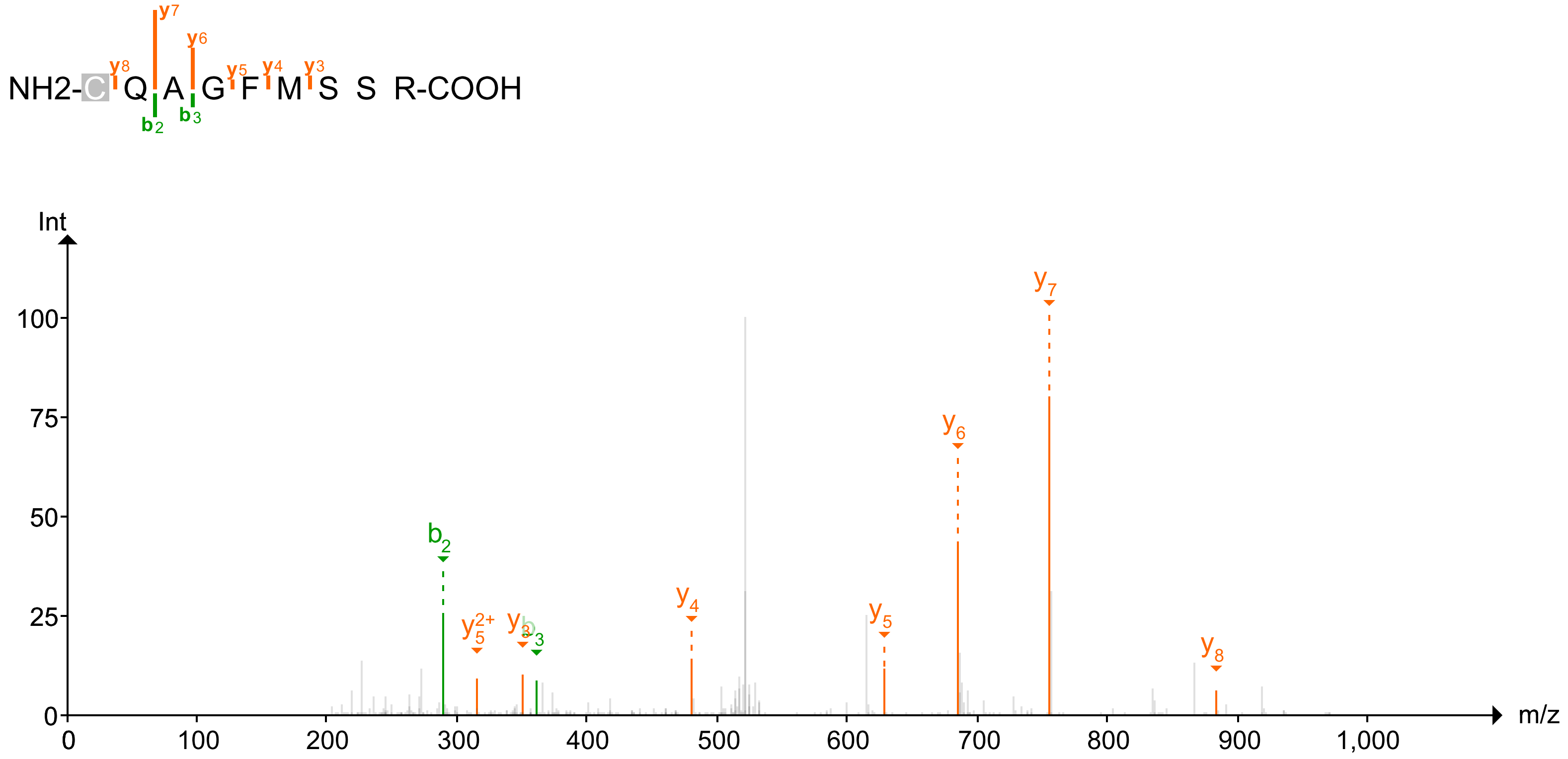

NH2-C Q A G F M S S R-COOH
y7
y6
y8
y5
y4
y3
b2
b3
Int
100
75
50
25
0
b2
y5 2+
y3
b3
y4
y5
y6
y7
y8
0
100
200
300
400
500
600
700
800
900
1,000
m/z

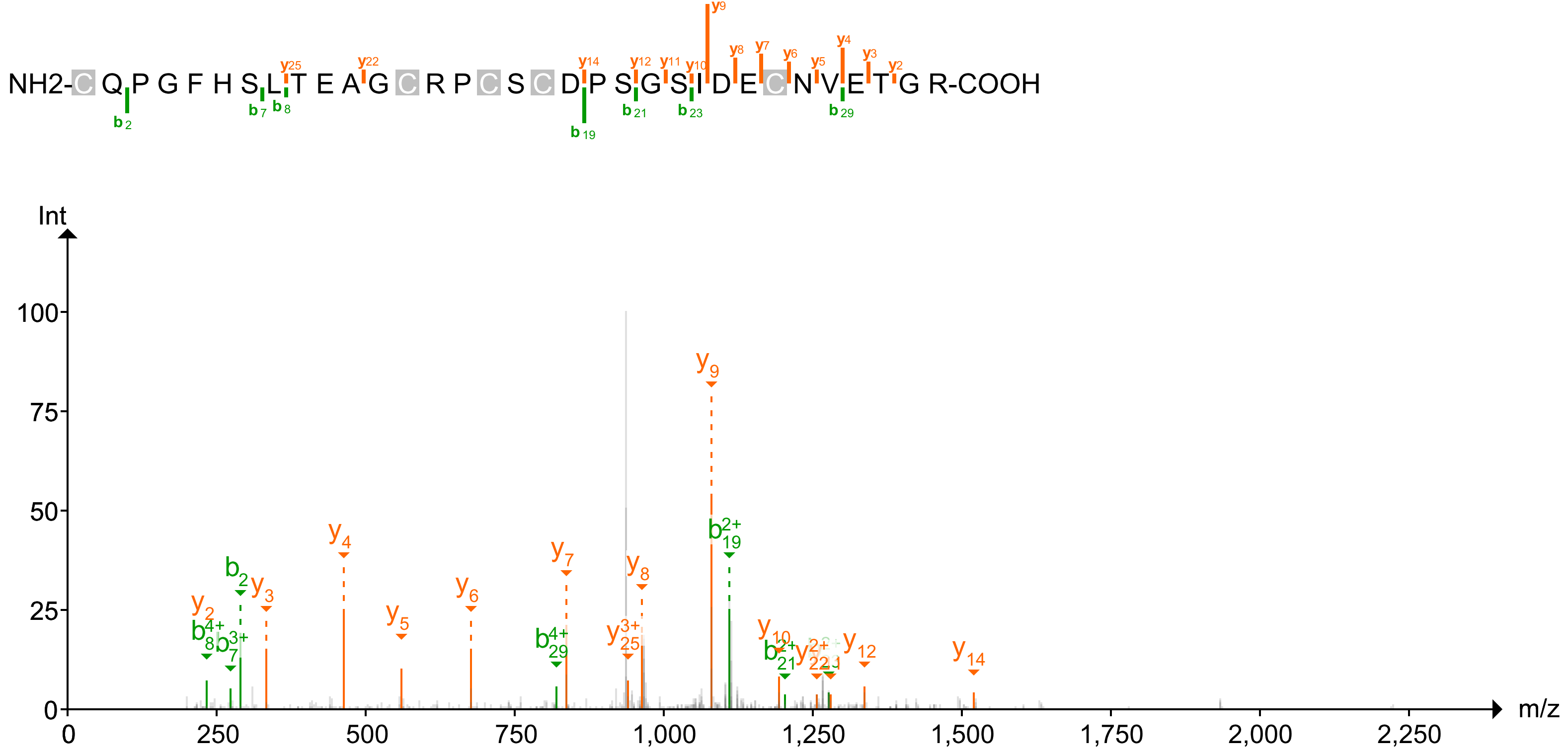
NH2-C Q P G F H S L T E A G C R P C S C D P S G S I D E C N V E T G R-COOH
Int
m/z
100
75
50
25
0
250
500
750
1,000
1,250
1,500
1,750
2,000
2,250

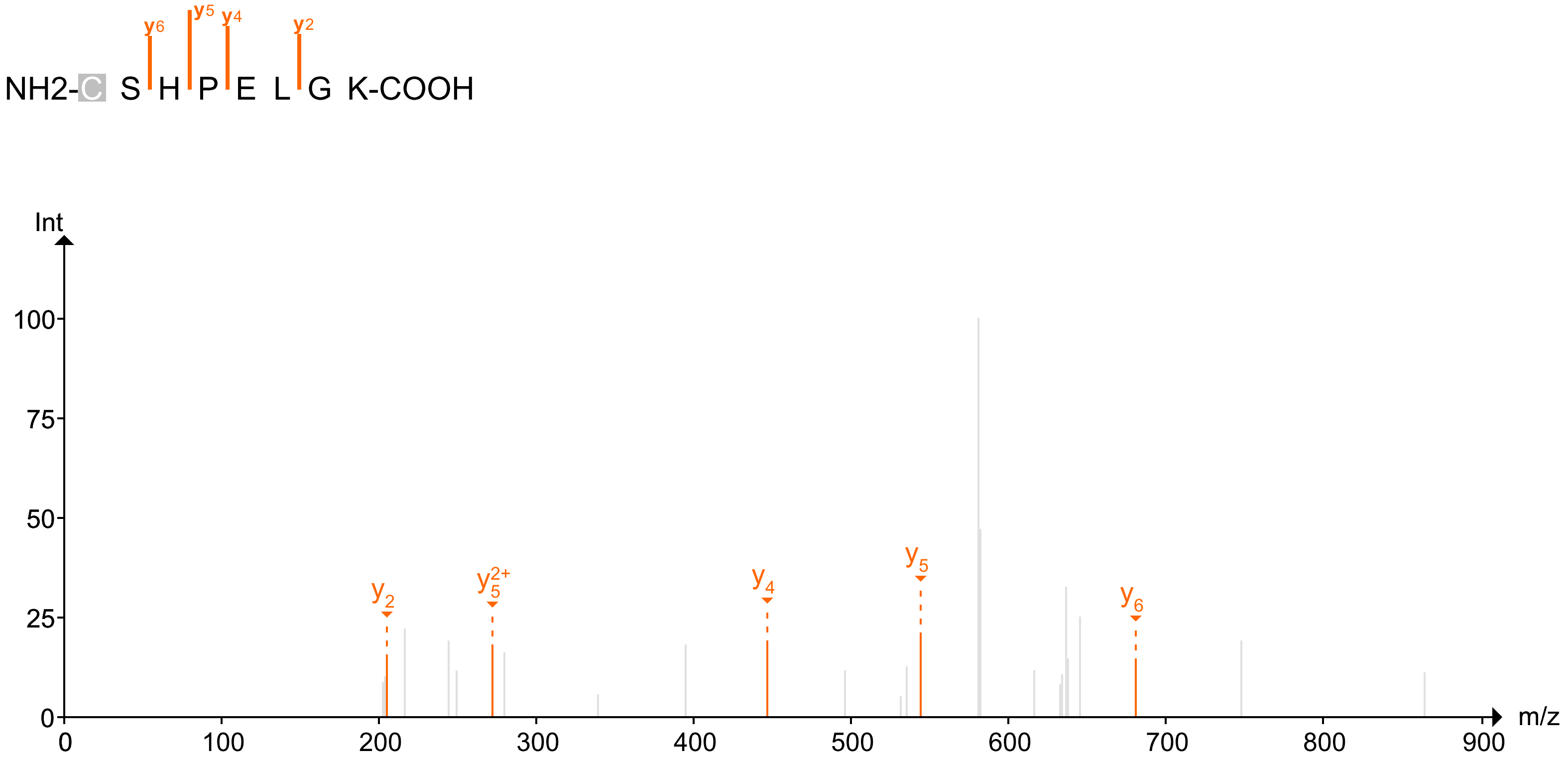
NH2-C S H P E L G K-COOH
y6
y5
y4
y2
Int
m/z
y2
y5 2+
y4
y5
y6

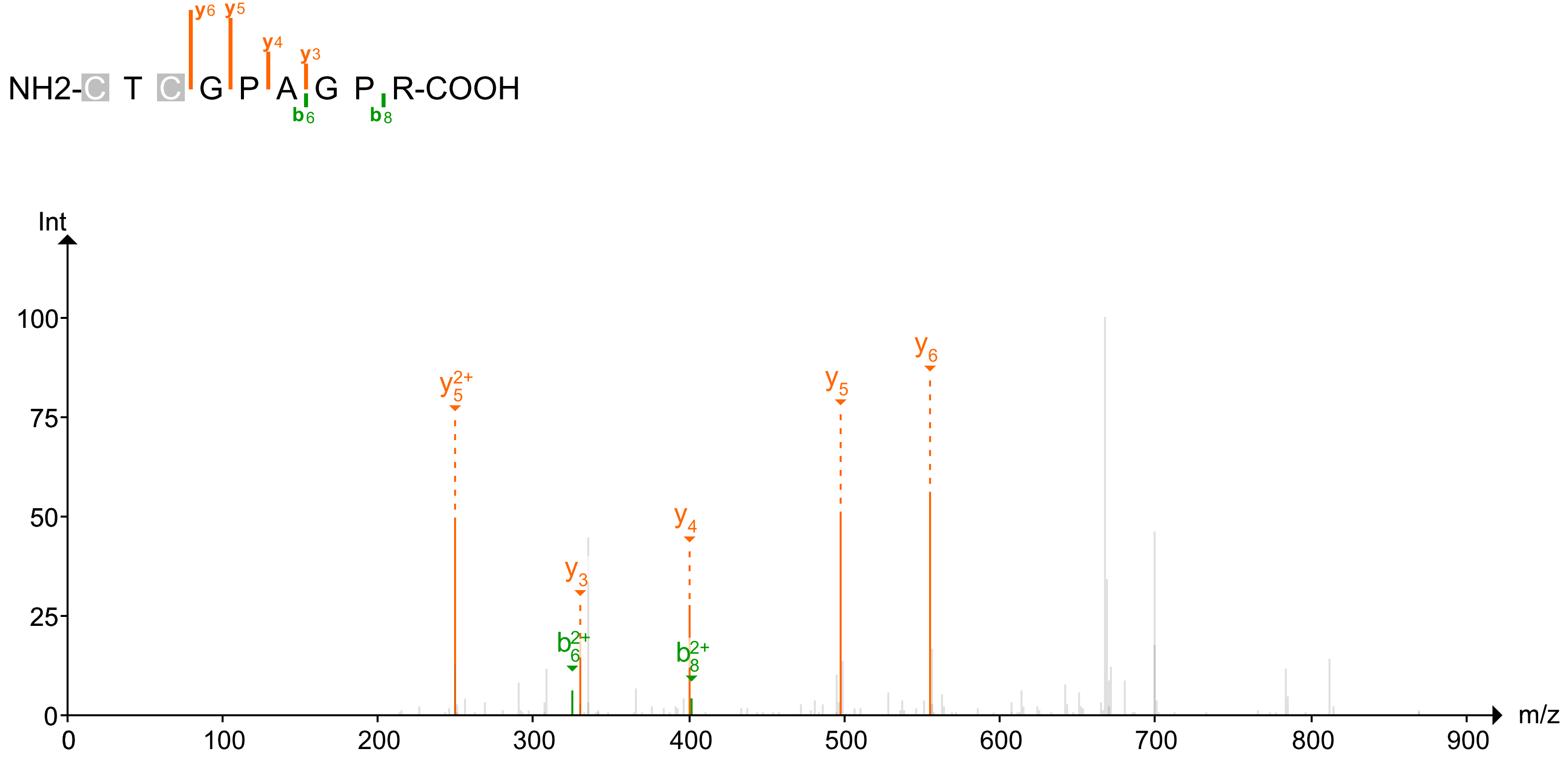

NH2-C T C G P A G P R-COOH
y6 y5 y4 y3
b6 b8
Int
100
75
50
25
0
m/z
0
100
200
300
400
500
600
700
800
900
y5 2+
y3
b6 2+
y4
b8 2+
y5
y6

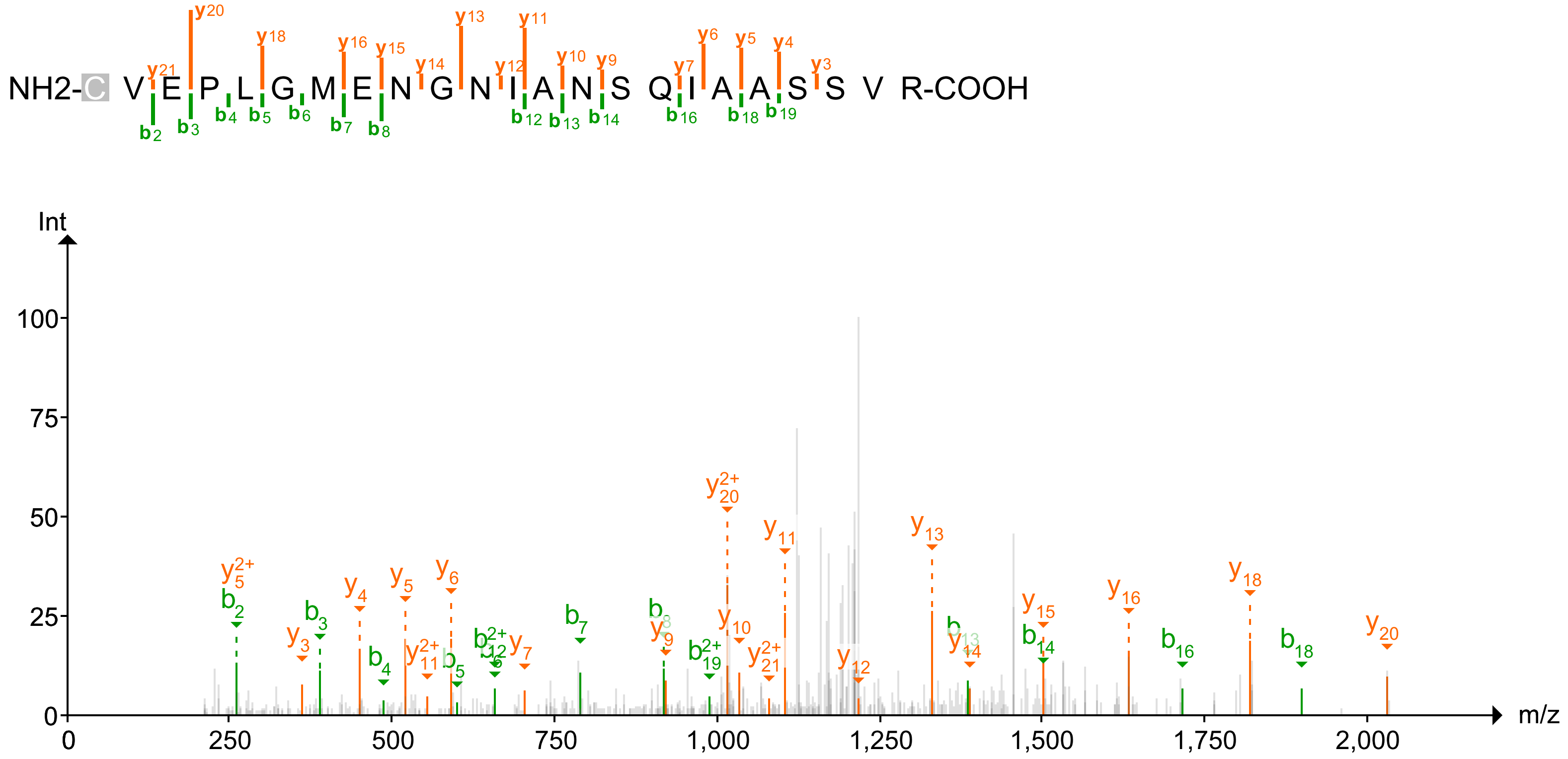

NH2-C V E P L G M E N G N I A N S Q I A A S S V R-COOH
Int
m/z
0
25
50
75
100
250
500
750
1,000
1,250
1,500
1,750
2,000

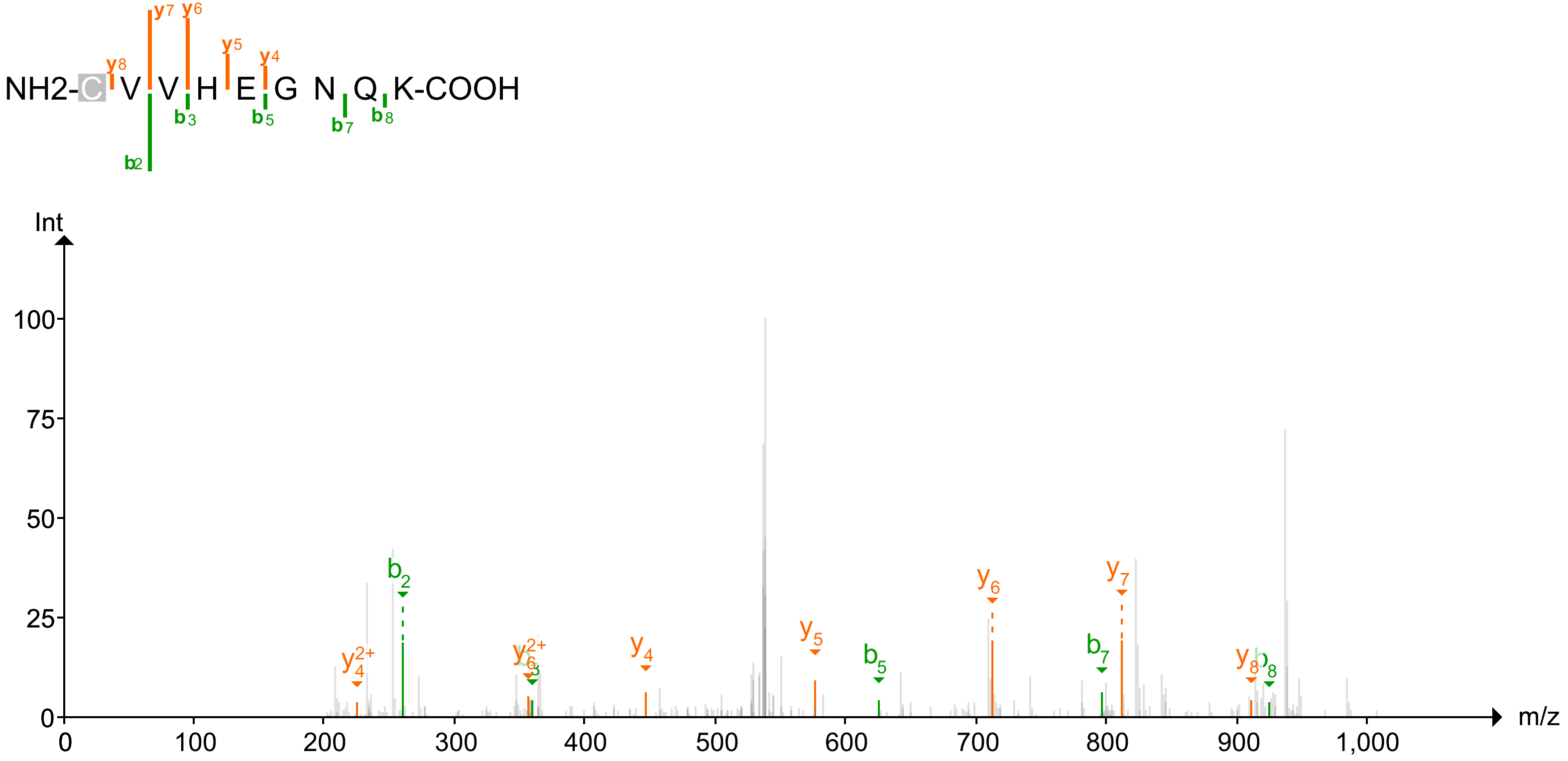

NH2-C V V H E G N Q K-COOH
y8 y7 y6 y5 y4
b2 b3 b5 b7 b8
Int
100
75
50
25
0
0 100 200 300 400 500 600 700 800 900 1,000
m/z
b2
y4 2+
y6 2+
b3
y4
y5
b5
y6
b7
y7
y8
b8

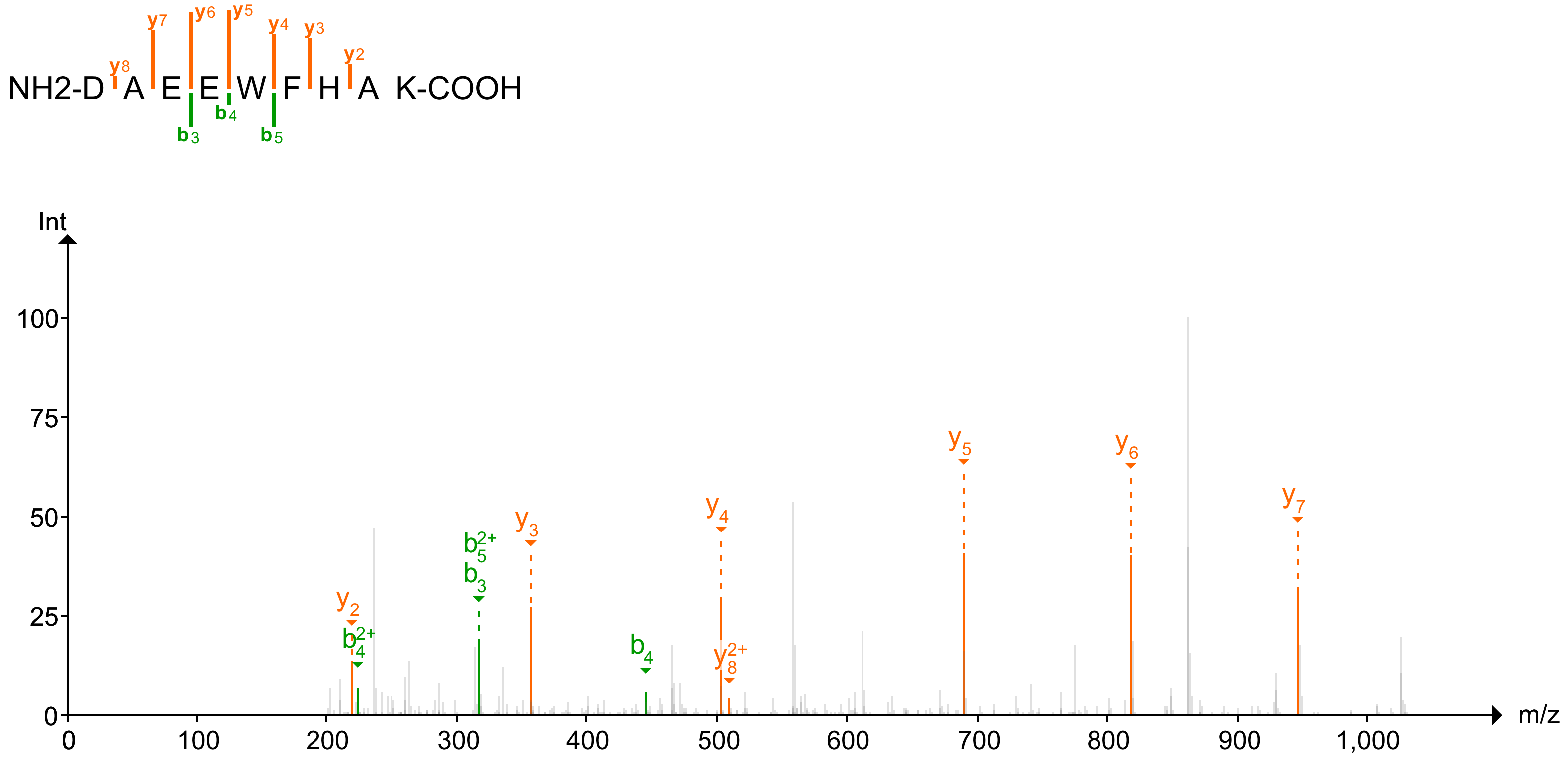

NH2-D A E E W F H A K-COOH
y8 y7 y6 y5 y4 y3 y2
b3 b4 b5
Int
m/z
y2
b4 2+
b5 2+
b3
y3
b4
y4
y8 2+
y5
y6
y7

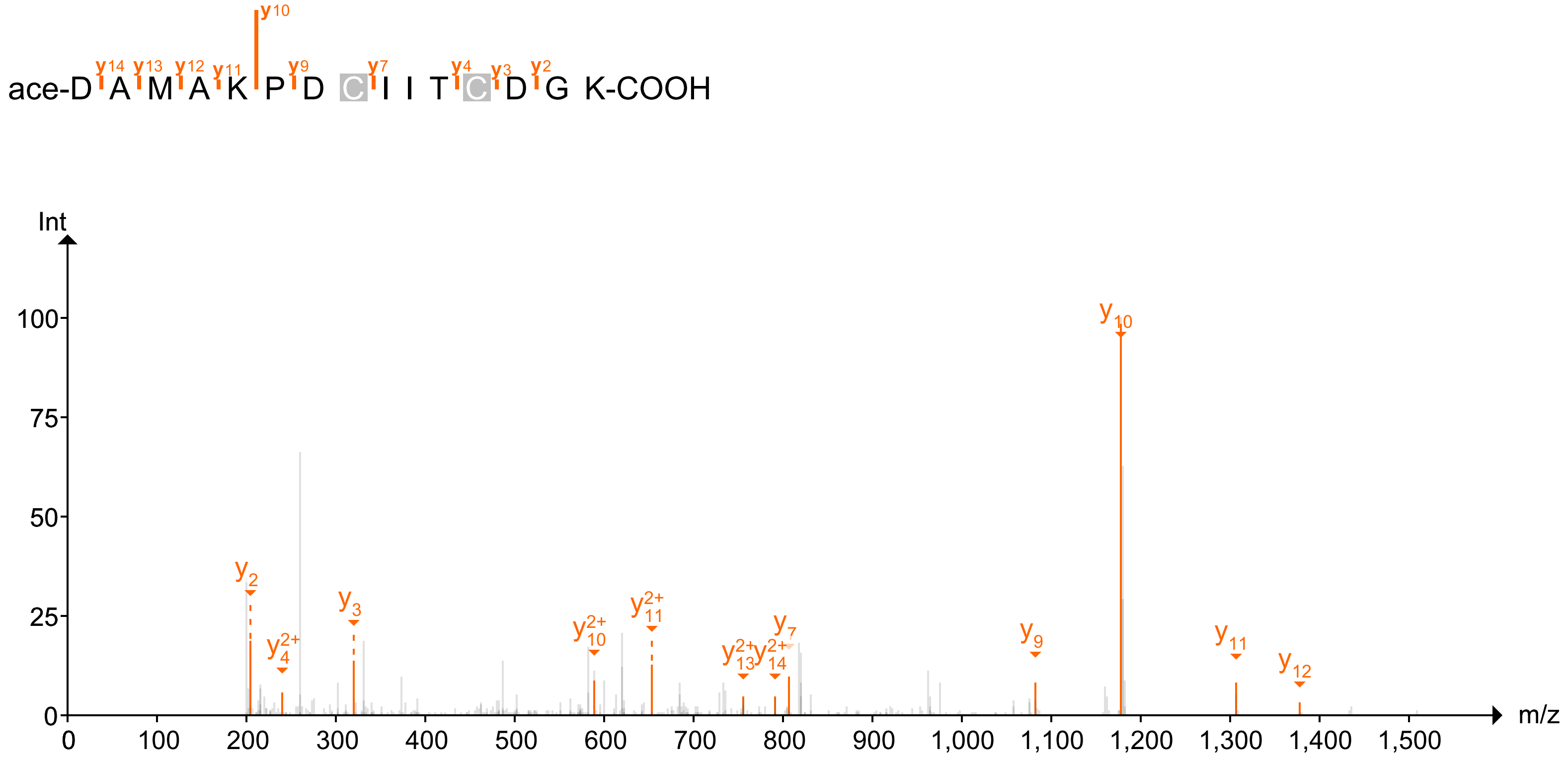

ace-D A M A K P D C I I T C D G K-COOH
y14 y13 y12 y11 y10 y9 y7 y4 y3 y2
Int
100
75
50
25
0
y10
y2
y3
y4 2+
y10 2+
y11 2+
y13 2+
y14 2+
y7
y9
y11
y12
0
100
200
300
400
500
600
700
800
900
1,000
1,100
1,200
1,300
1,400
1,500
m/z

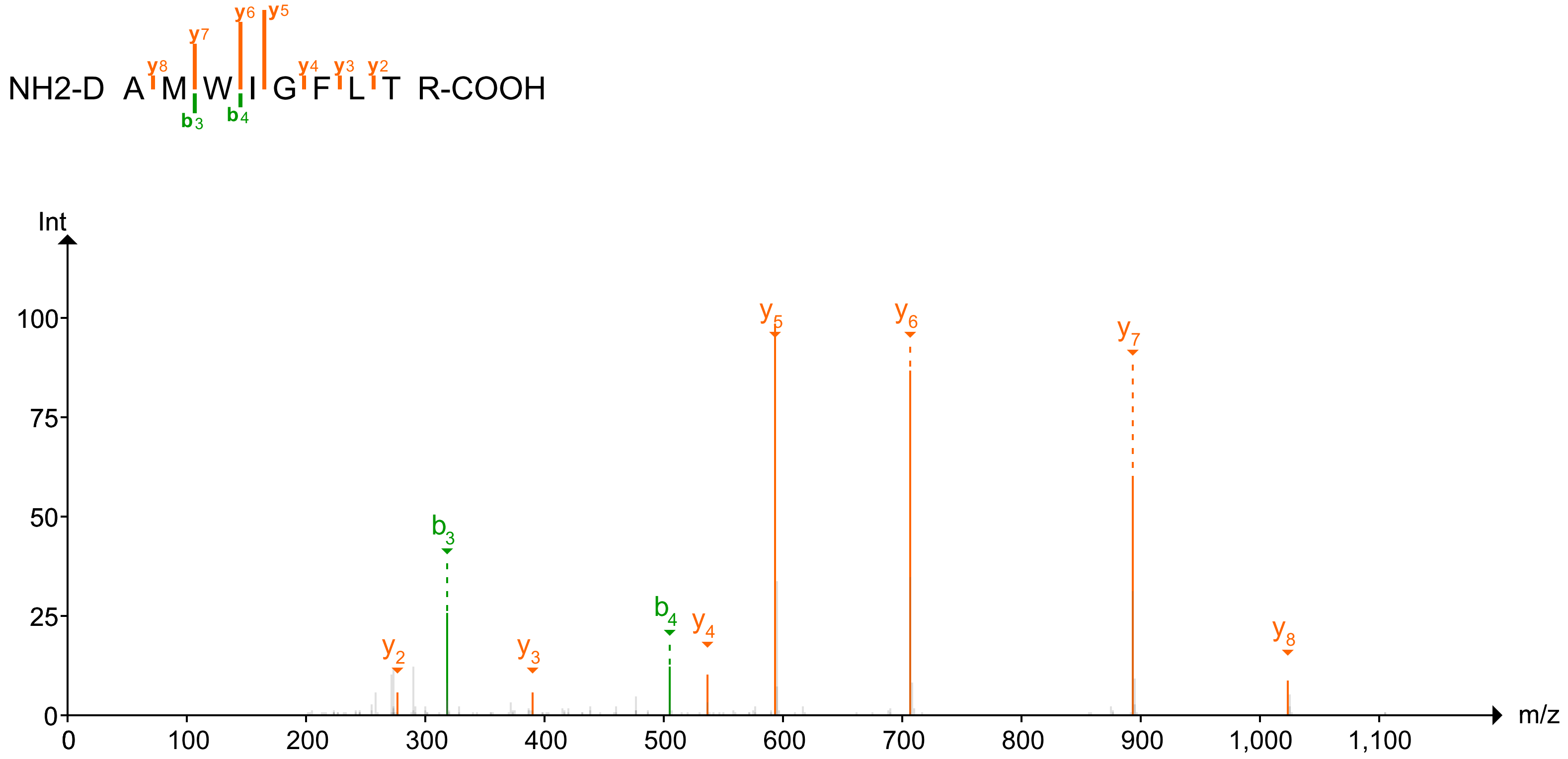

NH2-D A M W I G F L T R-COOH
y8 y7 y6 y5 y4 y3 y2
b3 b4
Int
100
75
50
25
0
m/z
0 100 200 300 400 500 600 700 800 900 1,000 1,100
y2 y3 y4 y5 y6 y7 y8
b3 b4

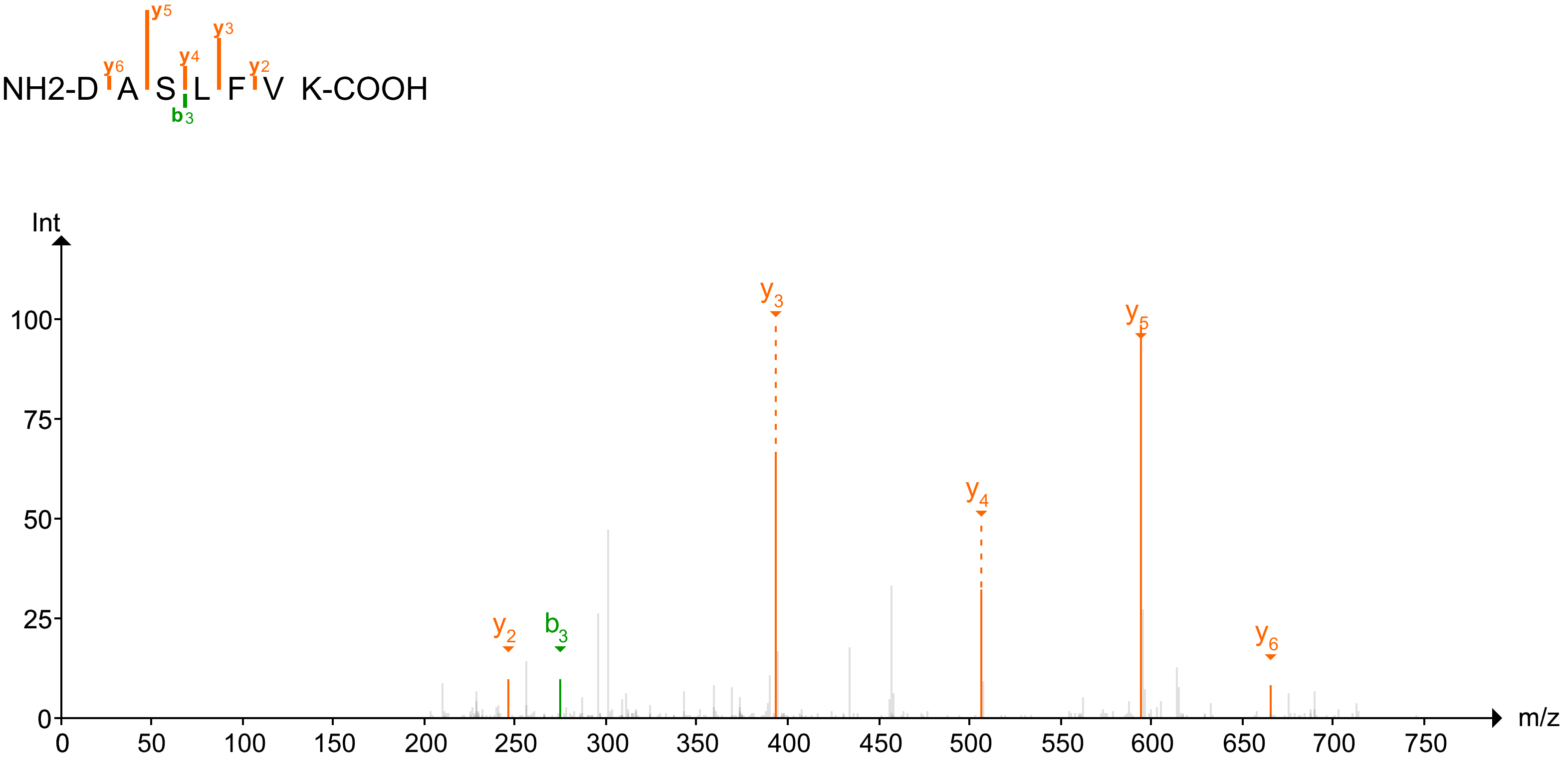

NH2-D A S L F V K-COOH
y6 y5 y4 y3 y2 b3
Int
100
75
50
25
0
m/z
0 50 100 150 200 250 300 350 400 450 500 550 600 650 700 750

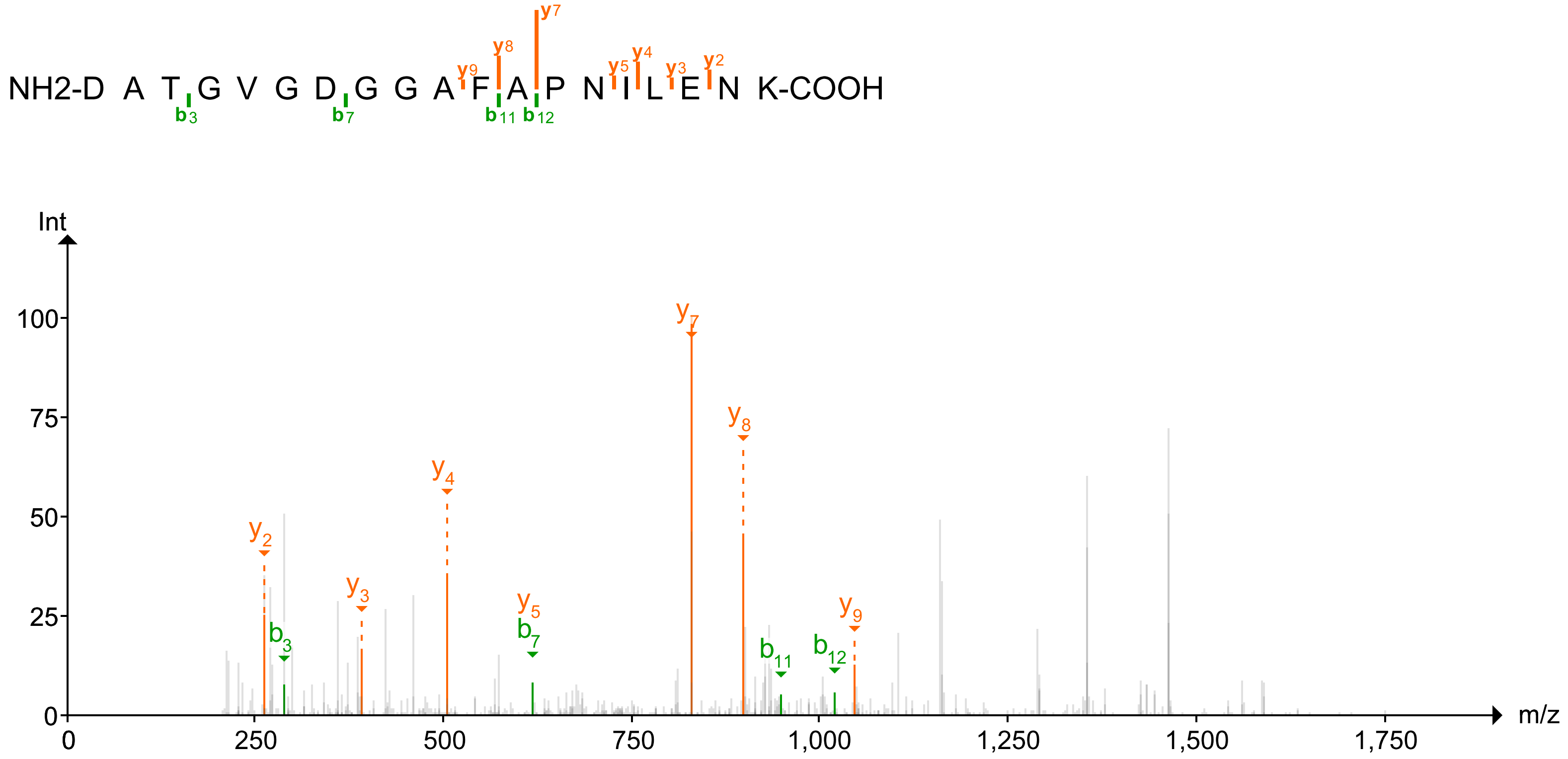
NH2-D A T G V G D G G A F A P N I L E N K-COOH
y7 y8 y9 y5 y4 y3 y2
b3 b7 b11 b12
Int
100
75
50
25
0
250
500
750
1,000
1,250
1,500
1,750
m/z

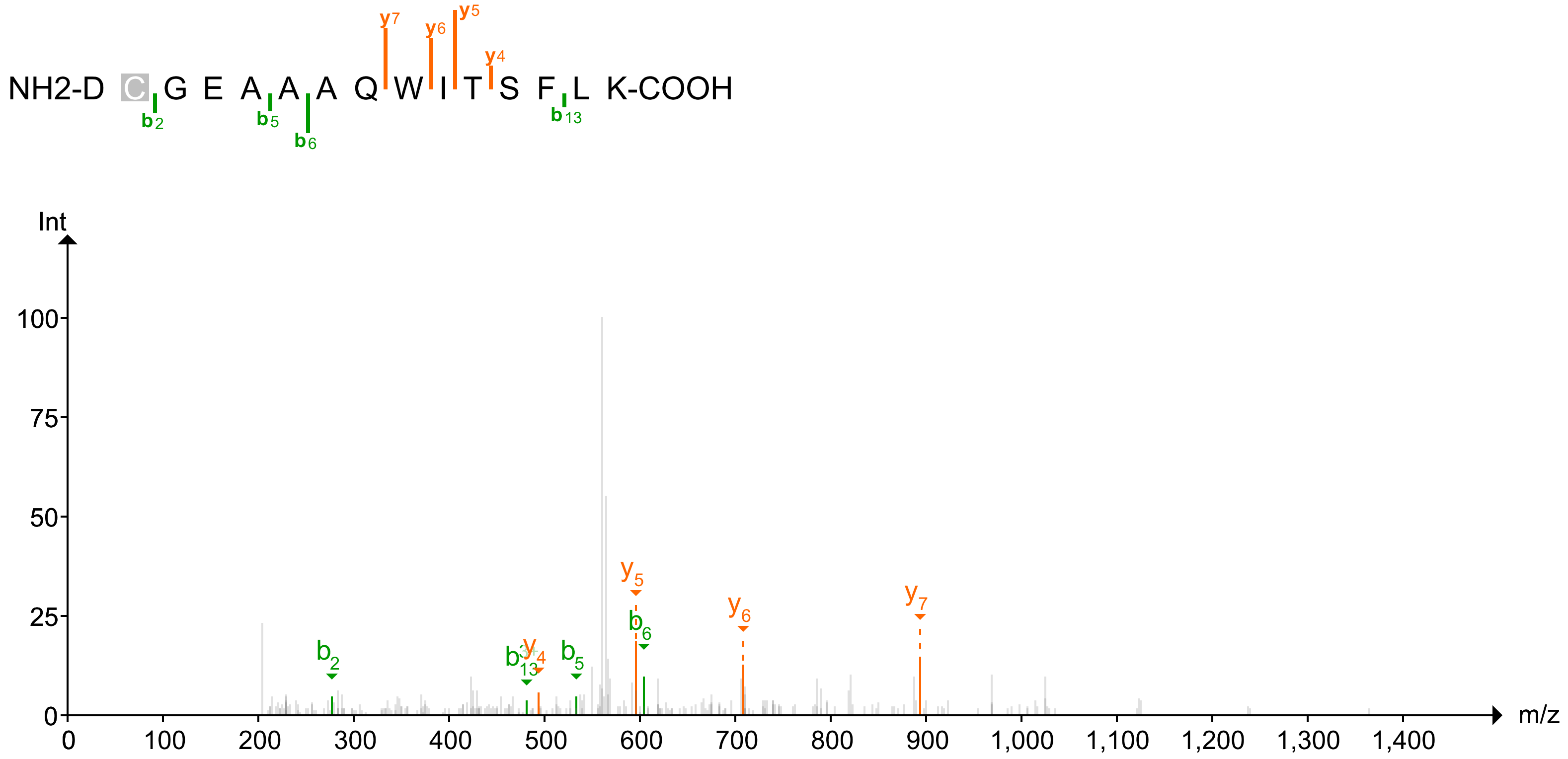

NH2-D C G E A A A Q W I T S F L K-COOH
y7 y6 y5 y4
b2 b5 b6 b13
Int
100
75
50
25
0
m/z
0 100 200 300 400 500 600 700 800 900 1,000 1,100 1,200 1,300 1,400
y5 y6 y7 b6 b2 b5 y4 b13

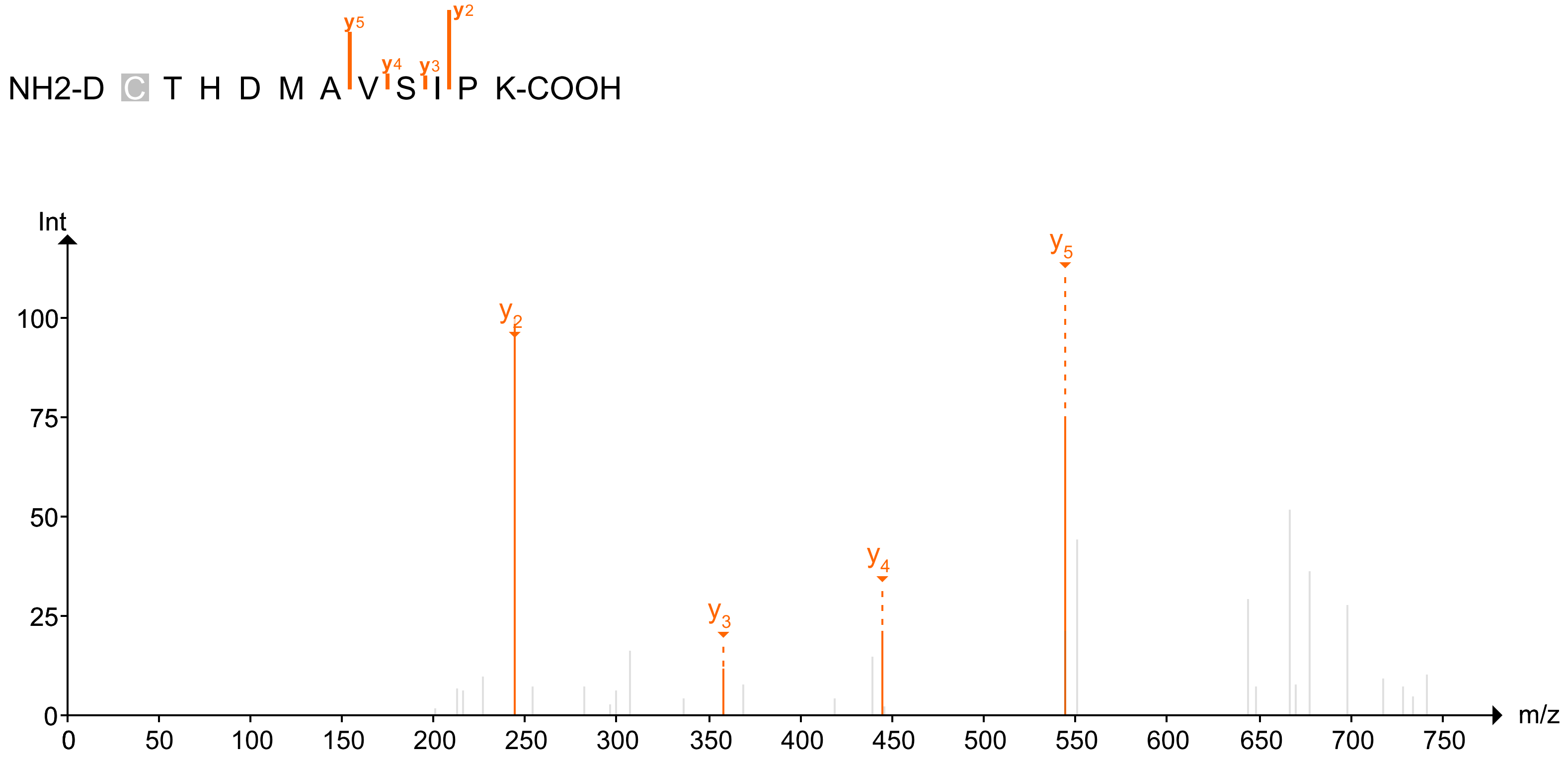

NH2-D C T H D M A V S I P K-COOH
y5
y4
y3
y2
Int
100
75
50
25
0
m/z
0
50
100
150
200
250
300
350
400
450
500
550
600
650
700
750
y2
y3
y4
y5

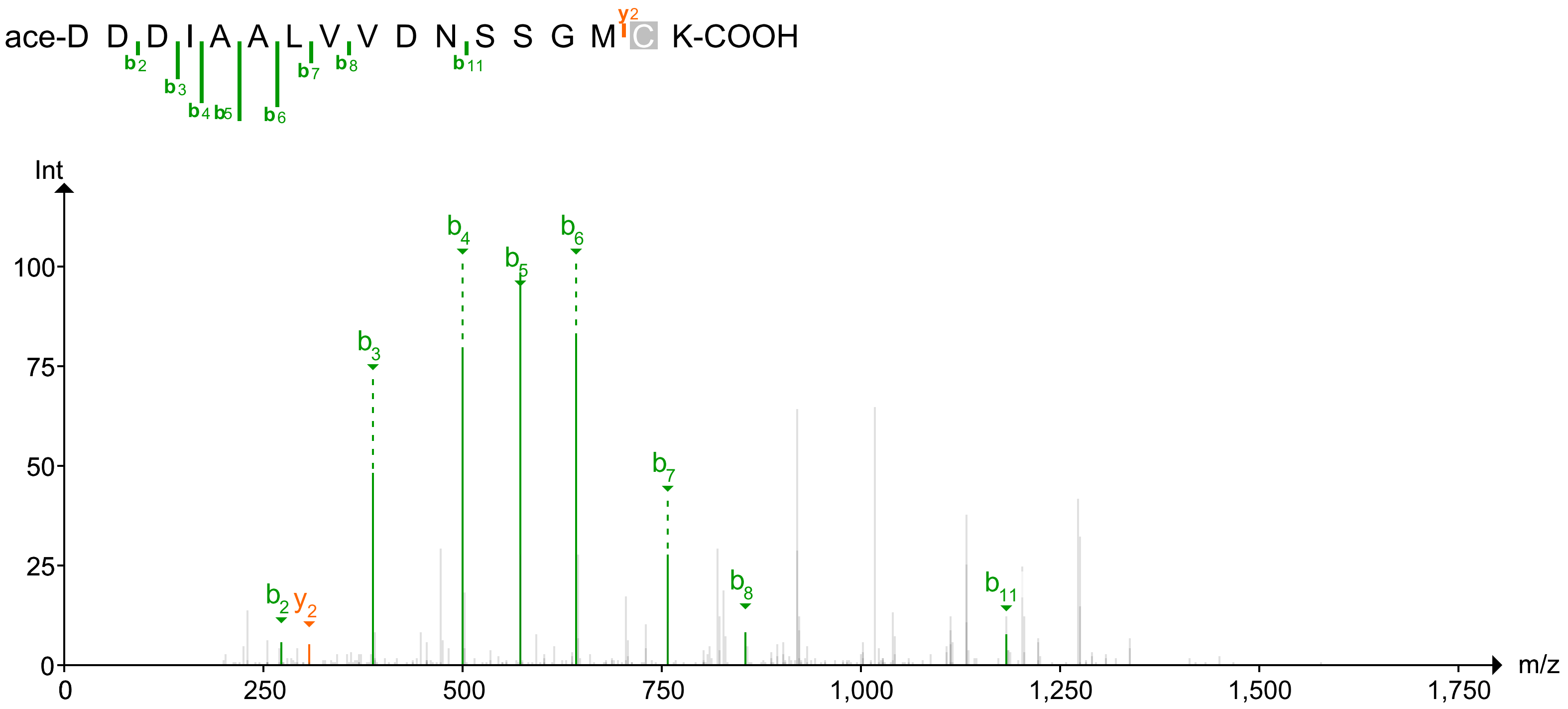

ace-D D D I A A L V V D N S S G M C K-COOH
y2
b2 b3 b4 b5 b6 b7 b8 b11
Int
100
75
50
25
0
b4
b5
b6
b3
b7
b8
b11
b2
y2
0
250
500
750
1,000
1,250
1,500
1,750
m/z

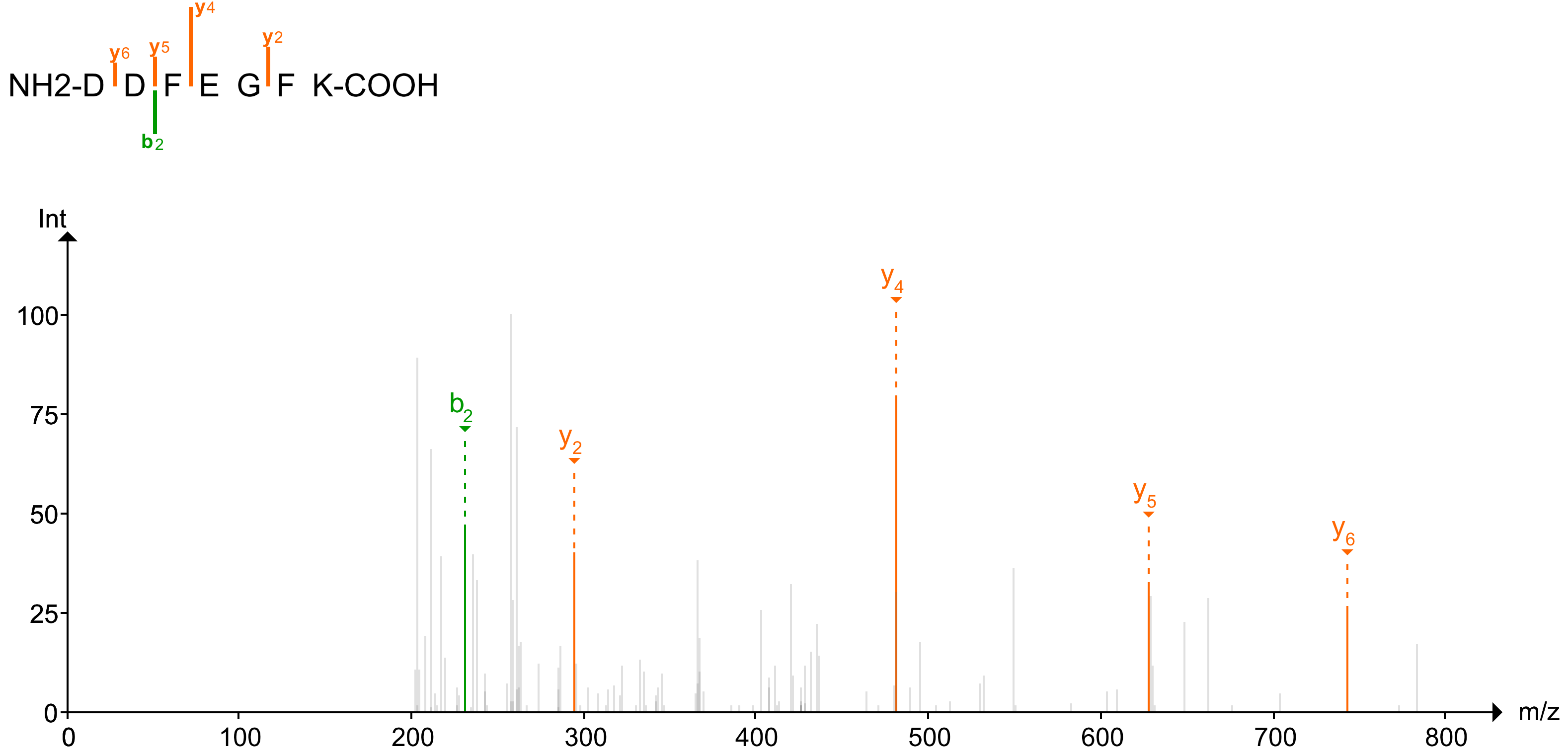

y4
y6
y5
y2
NH2-D D F E G F K-COOH
b2
Int
100
75
50
25
0
y4
b2
y2
y5
y6
0
100
200
300
400
500
600
700
800
m/z

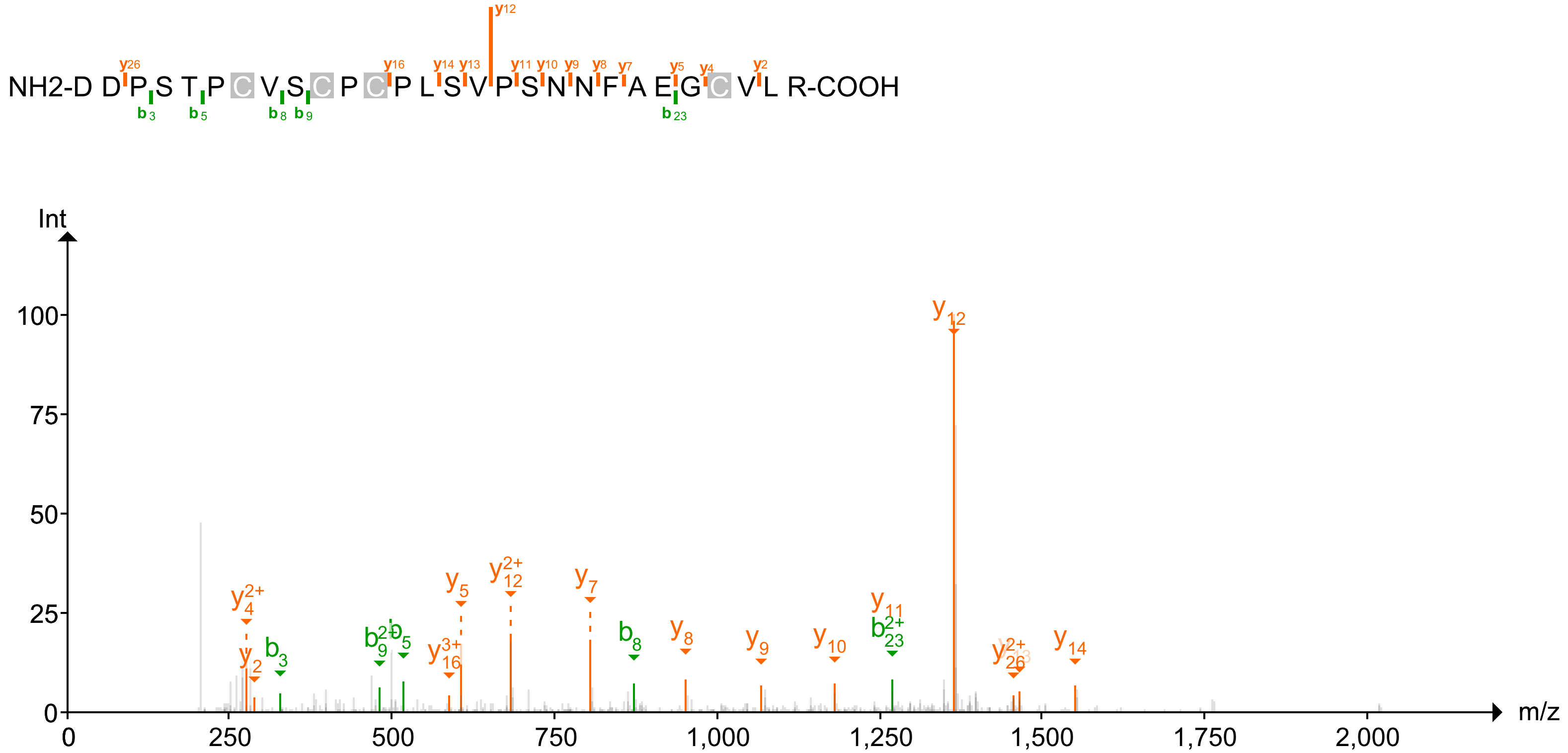

NH2-D D P S T P C V S C P C P L S V P S N N F A E G C V L R-COOH
y26 y16 y14 y13 y12 y11 y10 y9 y8 y7 y5 y4 y2
b3 b5 b8 b9 b23
Int
100
75
50
25
0
m/z
0
250
500
750
1,000
1,250
1,500
1,750
2,000
y12
y4 2+
y2
b3
b9 2+
b5
y16 3+
y5
y12 2+
y7
b8
y8
y9
y10
y11
b23 2+
y26 2+
y14

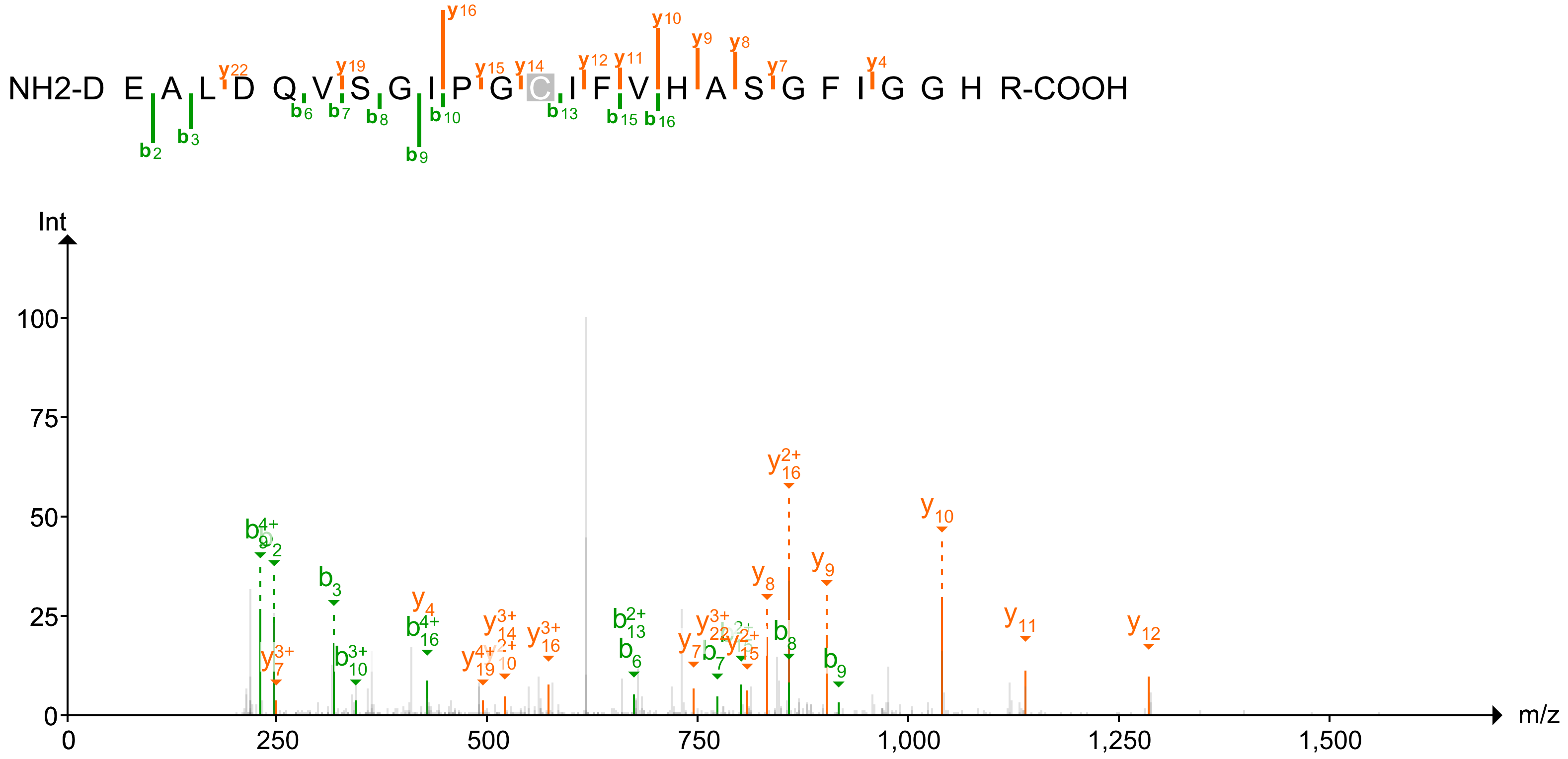

NH2-D E A L D Q V S G I P G C I F V H A S G F I G G H R-COOH
Int
100
75
50
25
0
m/z
0
250
500
750
1,000
1,250
1,500

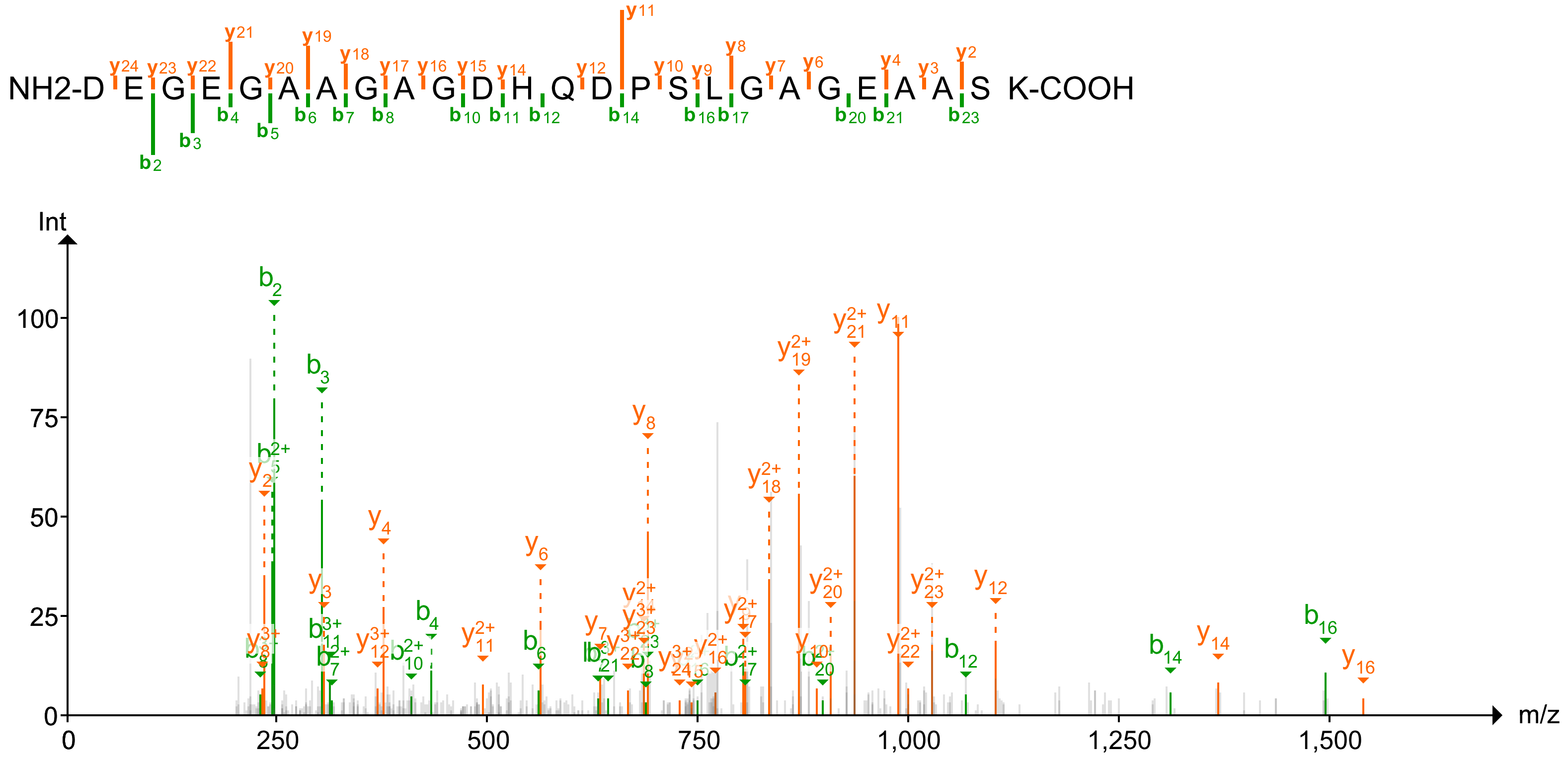

NH2-D E G E G A A G A G D H Q D P S L G A G E A A S K-COOH
y24 y23 y22 y21 y20 y19 y18 y17 y16 y15 y14 y12 y11 y10 y9 y8 y7 y6 y4 y3 y2
b2 b3 b4 b5 b6 b7 b8 b10 b11 b12 b14 b16 b17 b20 b21 b23
Int
m/z
0
25
50
75
100
0
250
500
750
1,000
1,250
1,500
b2
b3
y8
y2+19
y2+21
y11
y2+18
y4
y6
y3
b4
y2+11
y2+20
y2+23
y12
y2+22
b12
b14
y14
b16
y16

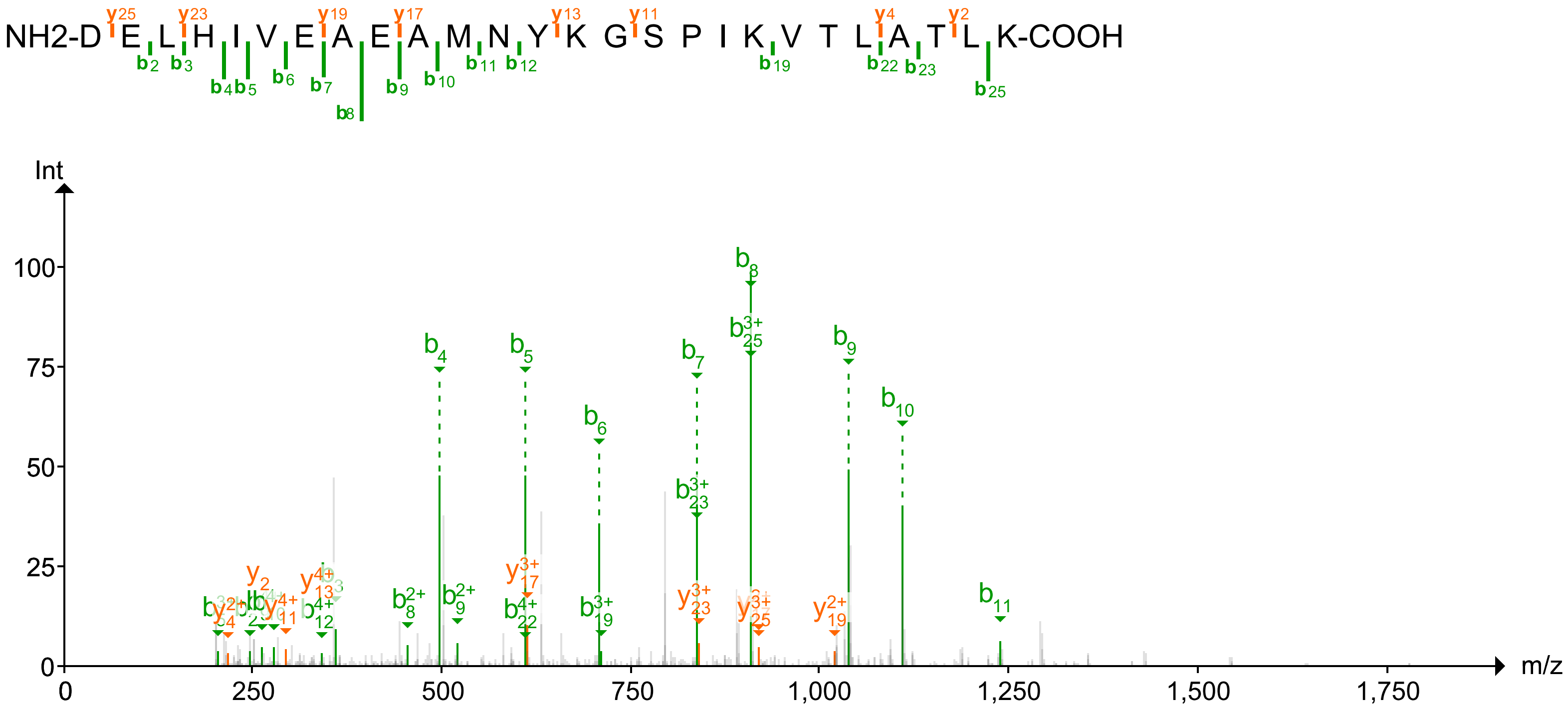

NH2-D E L H I V E A E A M N Y K G S P I K V T L A T L K-COOH
y25 y23 y19 y17 y13 y11 y4 y2
b2 b3 b4 b5 b6 b7 b8 b9 b10 b11 b12 b19 b22 b23 b25
Int
100
75
50
25
0
m/z
0
250
500
750
1,000
1,250
1,500
1,750
b8
b4
b5
b7
b9
b6
b10
b11
y2

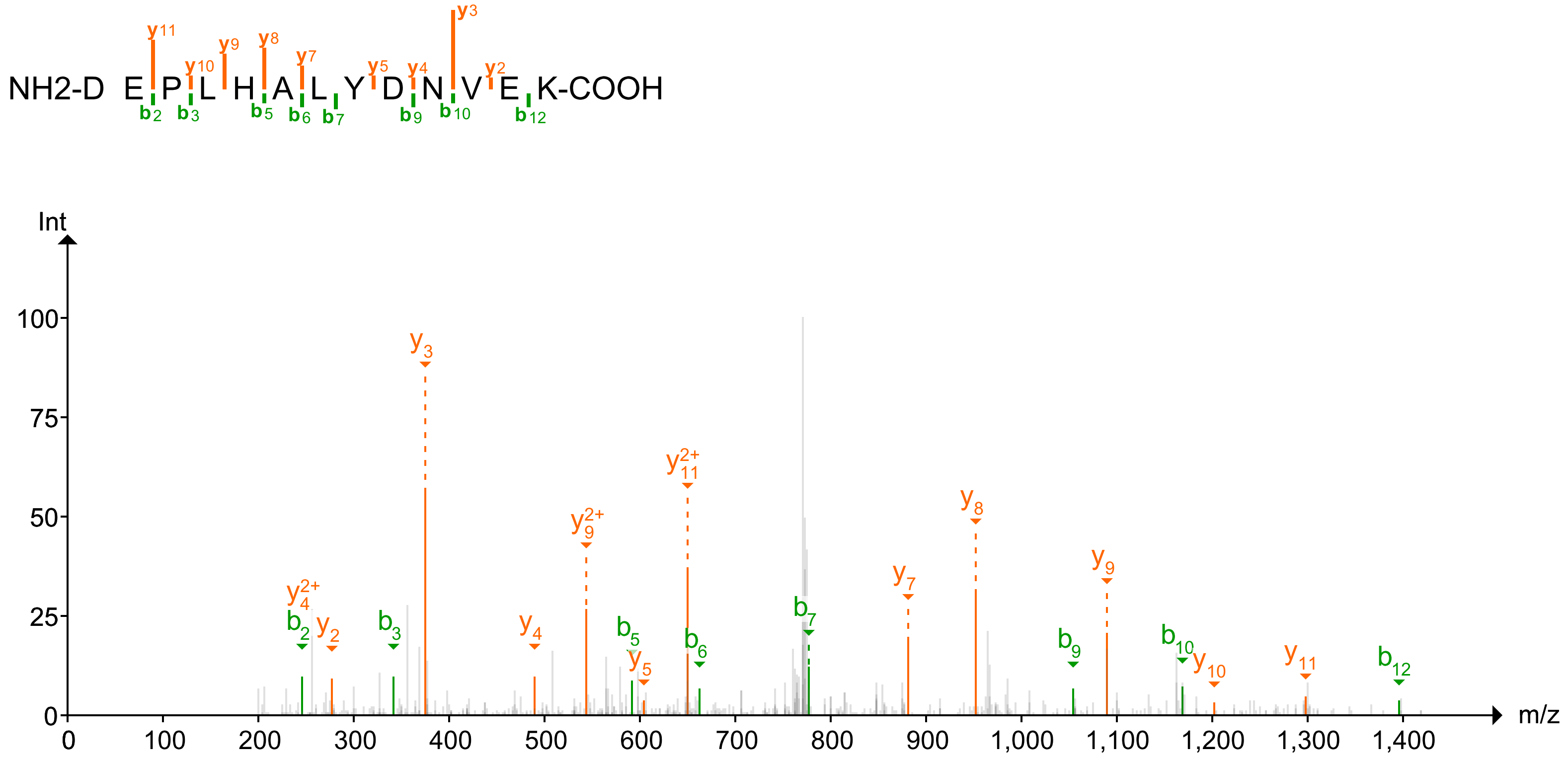

NH2-D E P L H A L Y D N V E K-COOH
y11 y10 y9 y8 y7 y5 y4 y3 y2
b2 b3 b5 b6 b7 b9 b10 b12
Int
100
75
50
25
0
m/z
0 100 200 300 400 500 600 700 800 900 1,000 1,100 1,200 1,300 1,400
y3
y11 2+
y9 2+
y8
y9
y7
y4 2+
b2 y2
b3
y4
b5
y5
b6
b7
b9
b10
y10
y11
b12

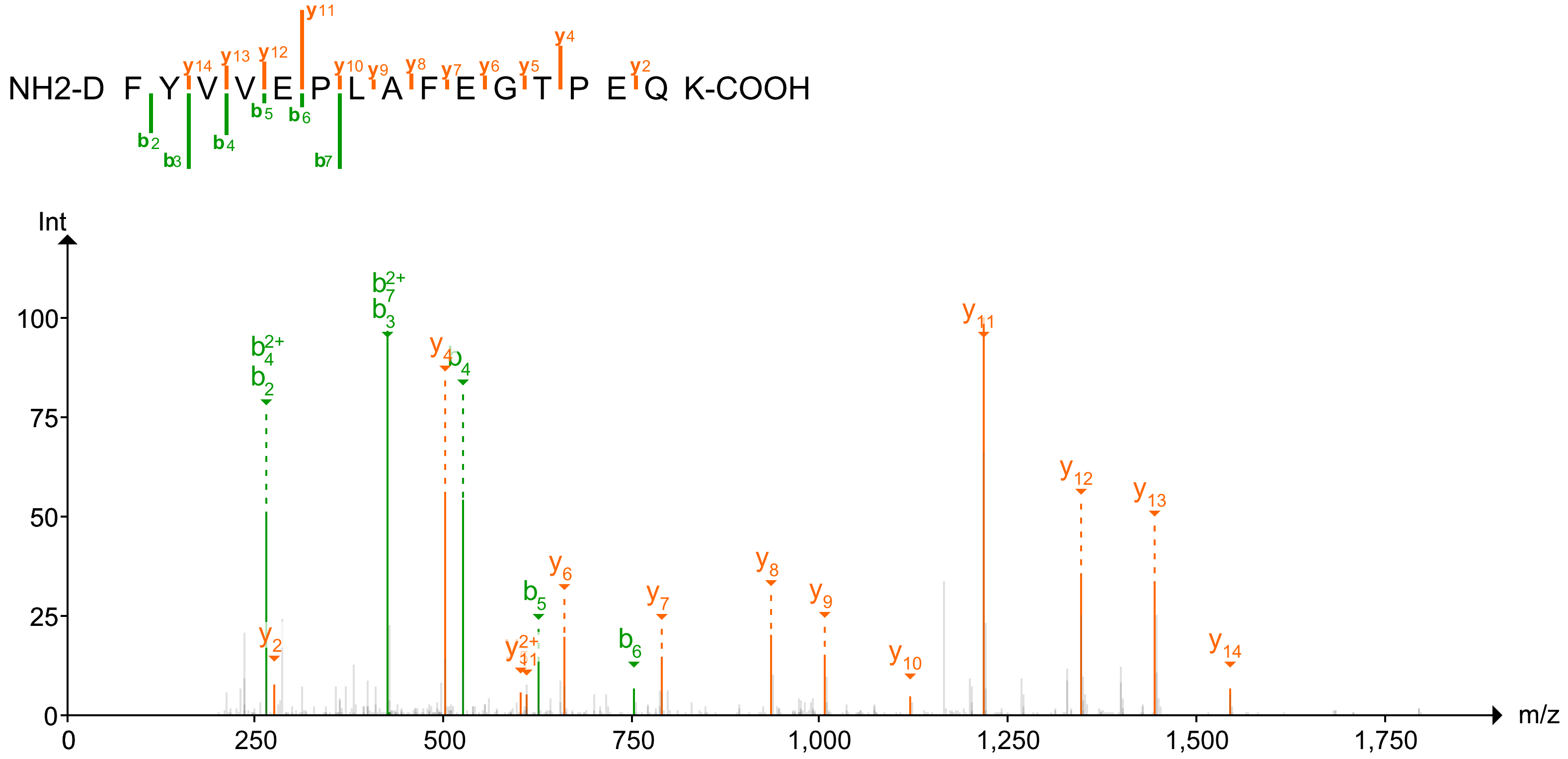

NH2-D F Y V V E P L A F E G T P E Q K-COOH
y11 y4 y14 y13 y12 y10 y9 y8 y7 y6 y5 y2
b2 b3 b4 b5 b6 b7
Int
100
75
50
25
0
m/z
0
250
500
750
1,000
1,250
1,500
1,750
b7 2+
b3
b4 2+
b2
y4
b4
y11
y12
y13
y6
b5
y7
y8
y9
y2
y11 2+
b6
y10
y14

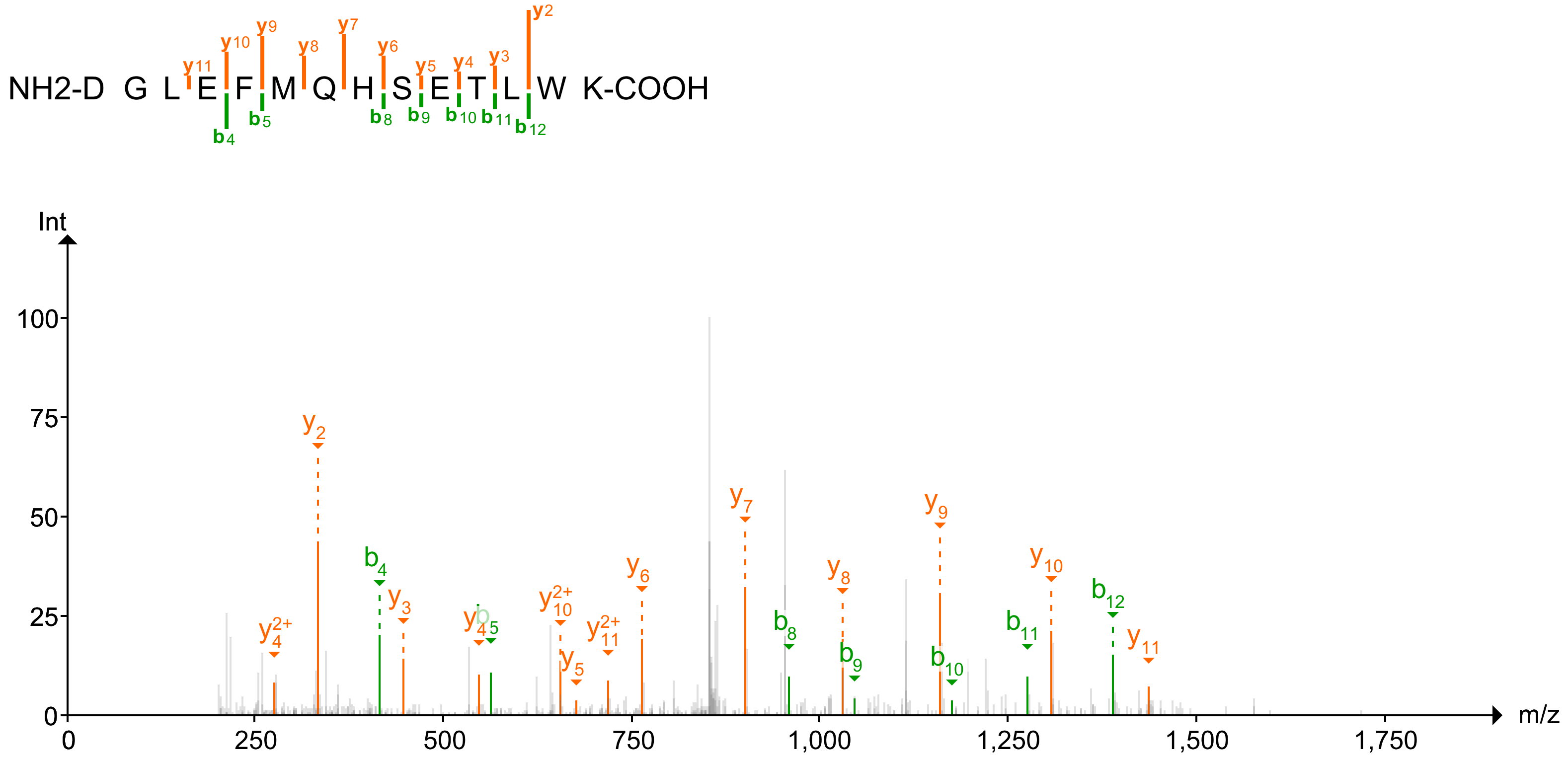

NH2-D G L E F M Q H S E T L W K-COOH
y11 y10 y9 y8 y7 y6 y5 y4 y3 y2
b4 b5 b8 b9 b10 b11 b12
Int
100
75
50
25
0
250
500
750
1,000
1,250
1,500
1,750
m/z

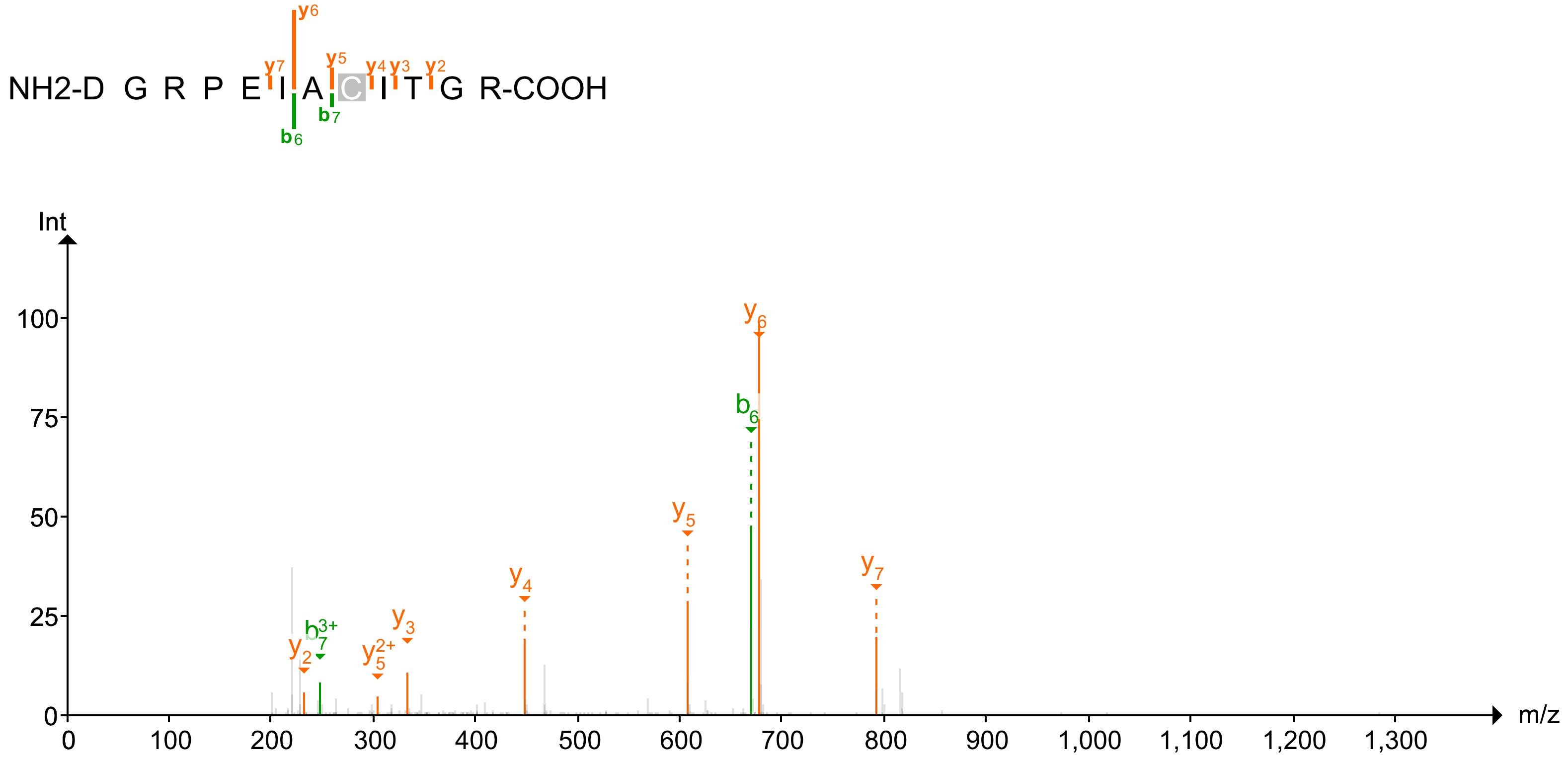

NH2-D G R P E I A C I T G R-COOH
y6
y7
y5
y4
y3
y2
b6
b7
Int
100
75
50
25
0
y6
b6
y5
y4
y7
y3
y2
b7 3+
y5 2+
m/z
0
100
200
300
400
500
600
700
800
900
1,000
1,100
1,200
1,300

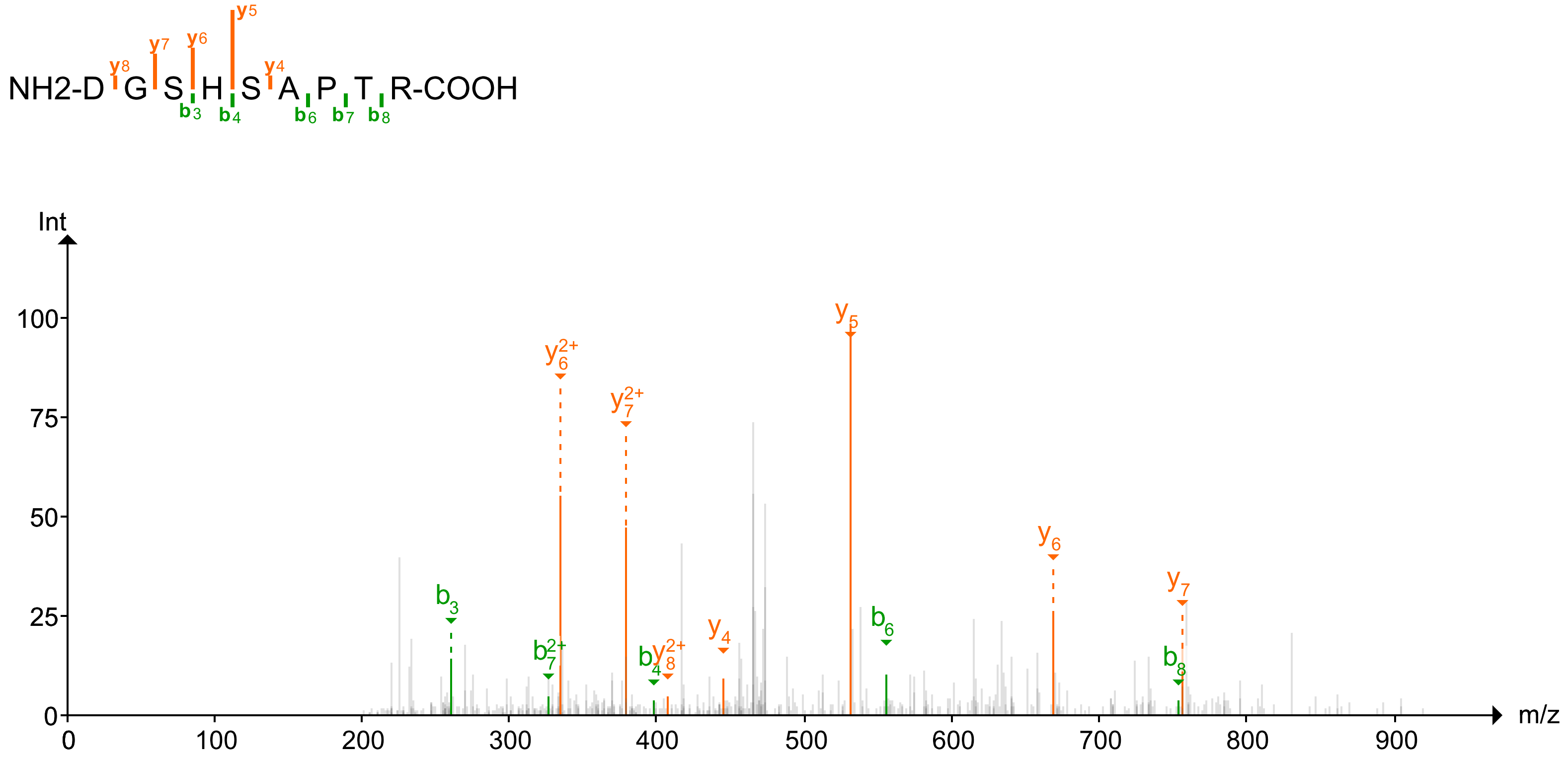

NH2-D G S H S A P T R-COOH
y8 y7 y6 y5 y4
b3 b4 b6 b7 b8
Int
m/z
0
25
50
75
100
0
100
200
300
400
500
600
700
800
900
b3
y6 2+
b7 2+
y7 2+
b4
y8 2+
y4
y5
b6
y6
b8
y7

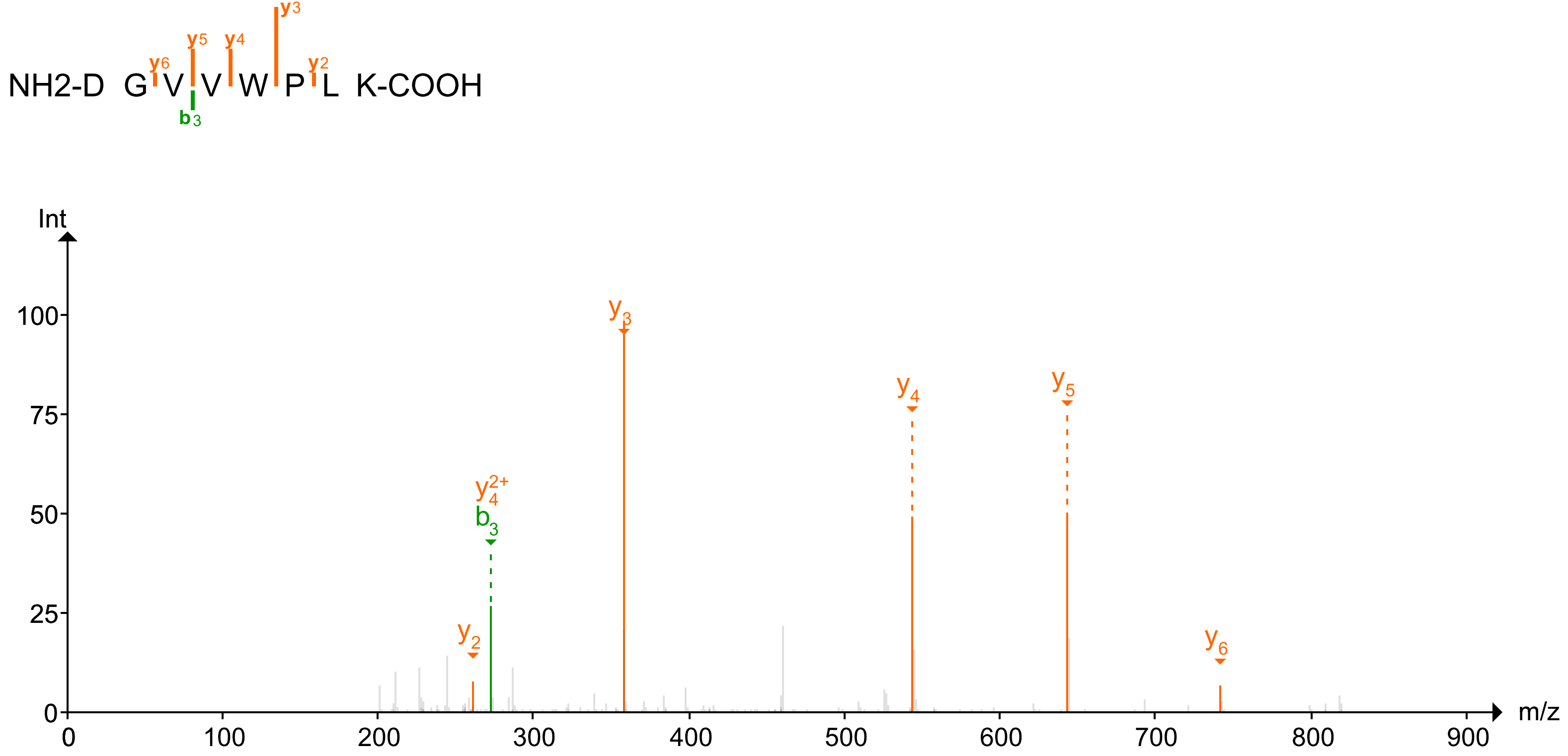

NH2-D G V V W P L K-COOH
y6 y5 y4 y3 y2
b3
Int
100
75
50
25
0
y3
y4
y5
y4 2+
b3
y2
y6
m/z
0
100
200
300
400
500
600
700
800
900

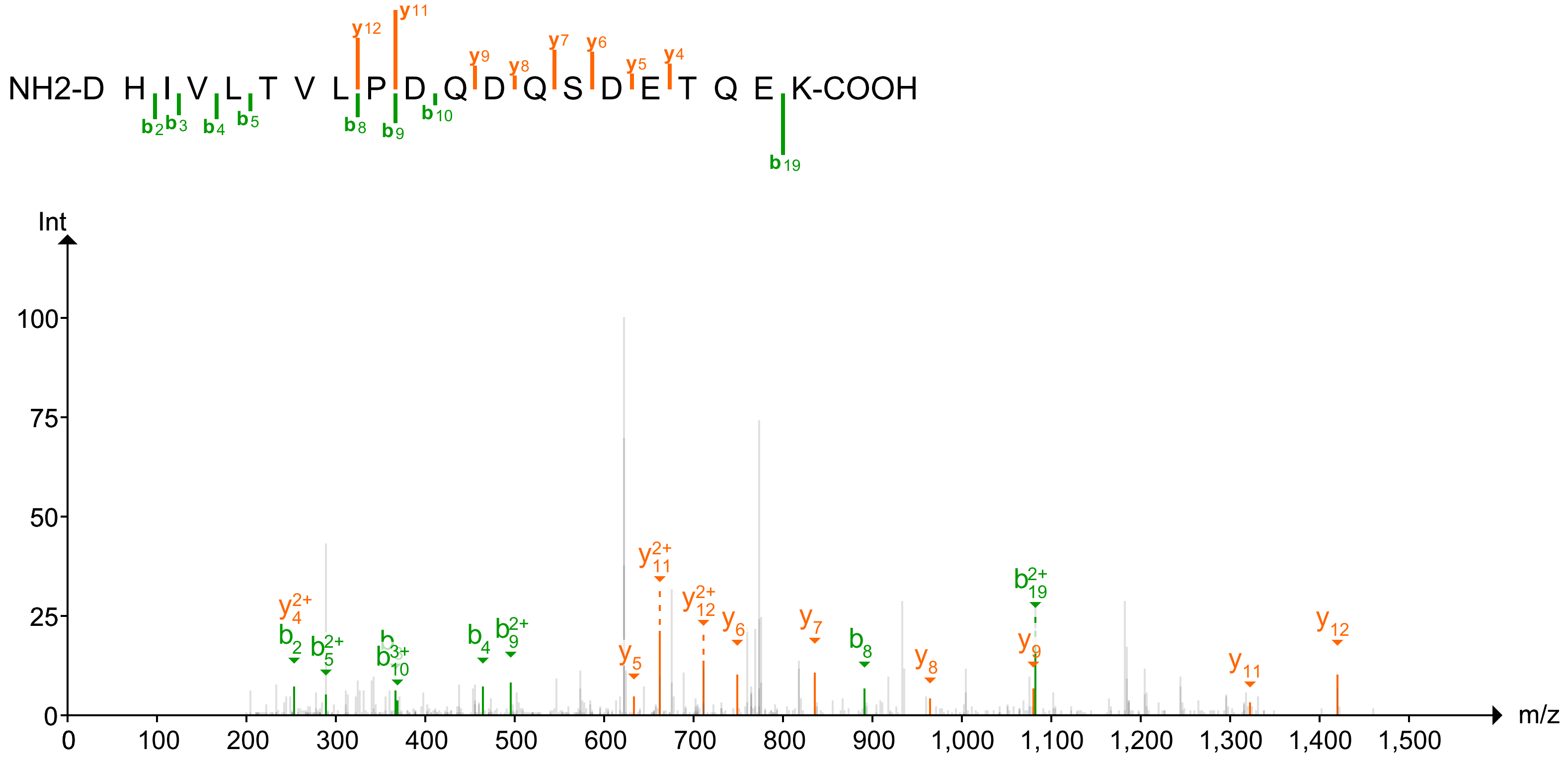

NH2-D H I V L T V L P D Q D Q S D E T Q E K-COOH
y12 y11 y9 y8 y7 y6 y5 y4
b2 b3 b4 b5 b8 b9 b10 b19
Int
100
75
50
25
0
$y_{11}^{2+}$
$y_{12}^{2+}$
$b_{19}^{2+}$
$y_4^{2+}$
$b_2$
$b_5^{2+}$
$b_{10}^{3+}$
$b_4$
$b_9^{2+}$
$y_5$
$y_6$
$y_7$
$b_8$
$y_8$
$y_9$
$y_{11}$
$y_{12}$
0 100 200 300 400 500 600 700 800 900 1,000 1,100 1,200 1,300 1,400 1,500
m/z

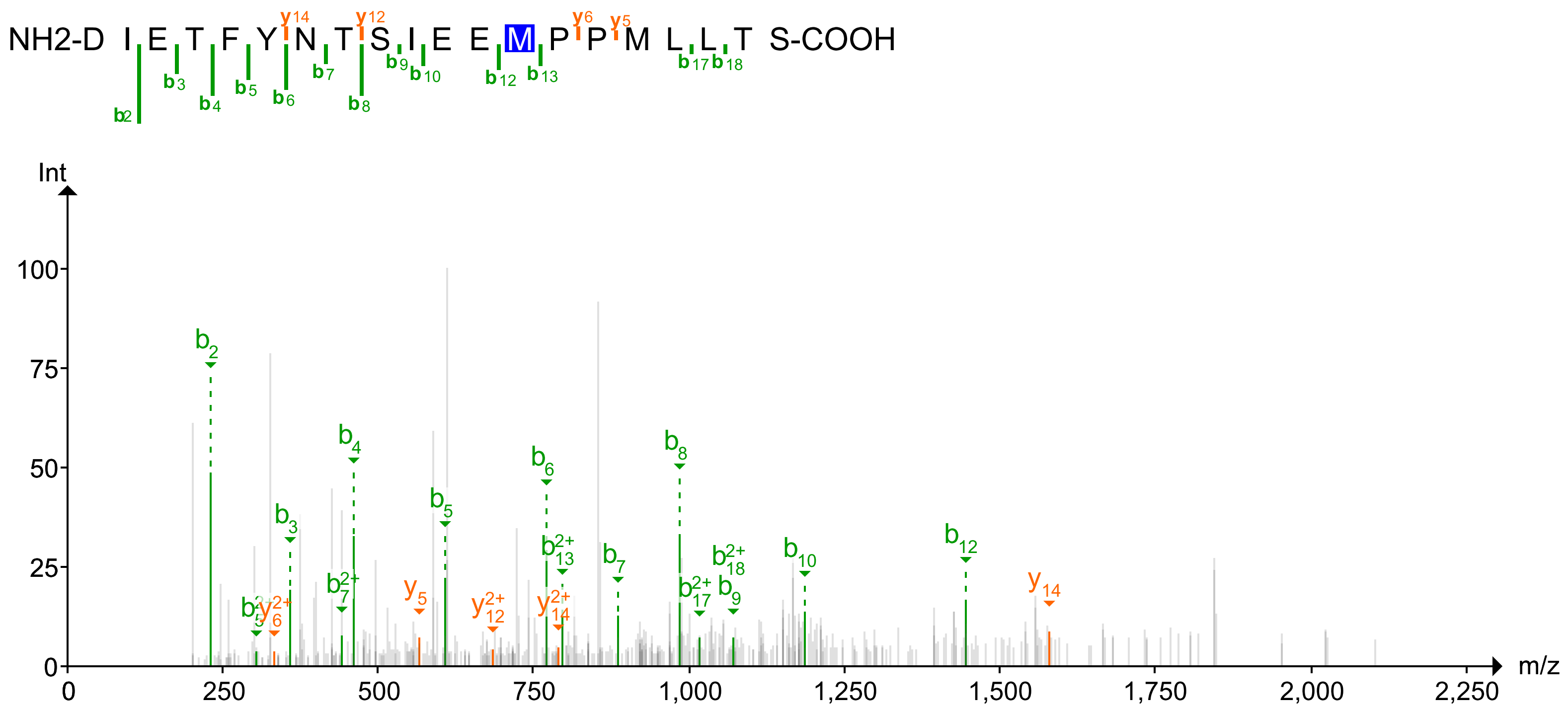
NH2-D I E T F Y N T S I E E M P P M L L T S-COOH
Int
m/z
0
250
500
750
1,000
1,250
1,500
1,750
2,000
2,250
25
50
75
100

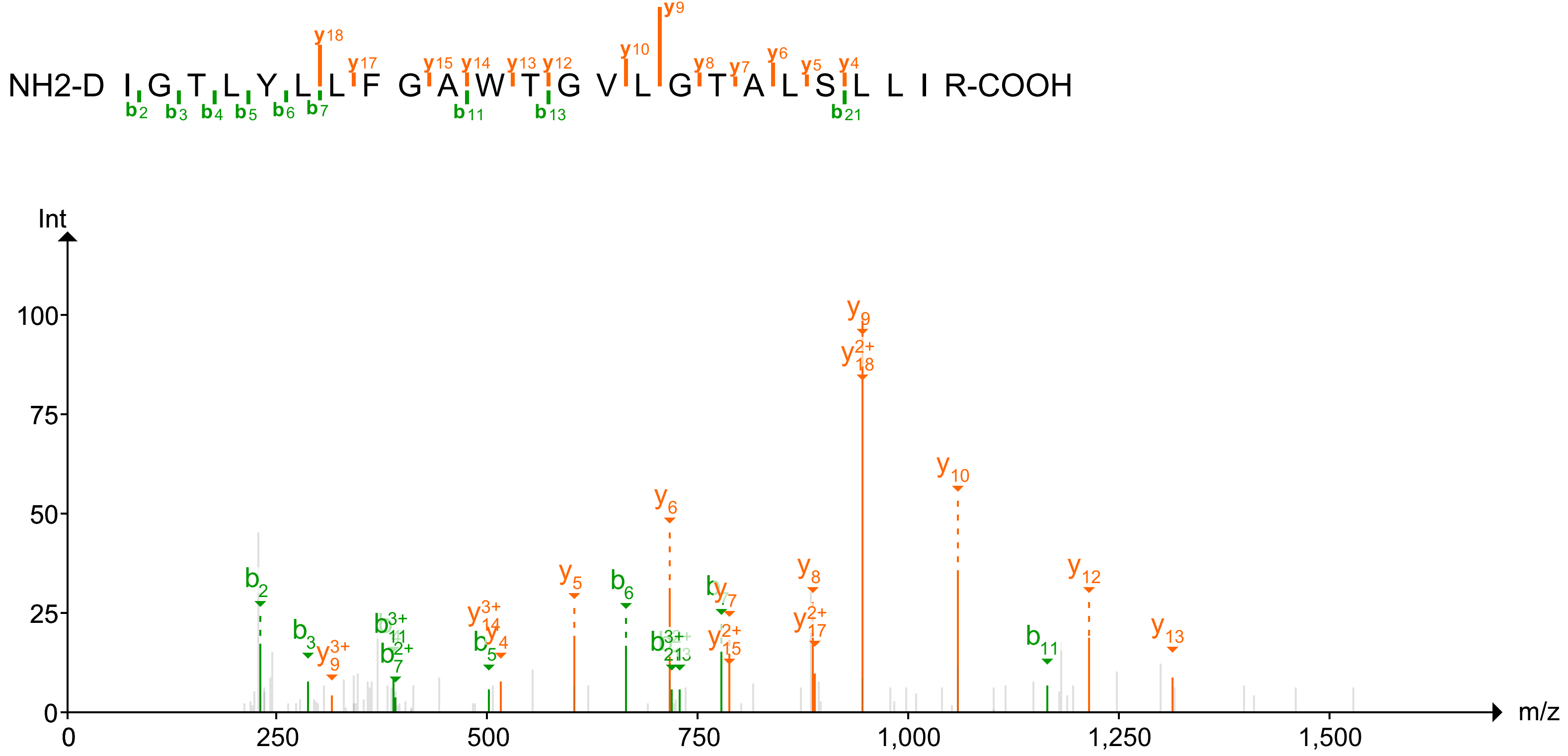
NH2-D I G T L Y L L F G A W T G V L G T A L S L L I R-COOH
y18 y17 y15 y14 y13 y12 y10 y9 y8 y7 y6 y5 y4
b2 b3 b4 b5 b6 b7 b11 b13 b21
Int
100
75
50
25
0
m/z
0
250
500
750
1,000
1,250
1,500
b2
b3
y9 3+
b11 3+
b7 2+
y14 3+
y4
b5
y5
b6
y6
b21 3+
b7
y7
y15 2+
y8
y17 2+
y9
y18 2+
y10
b11
y12
y13

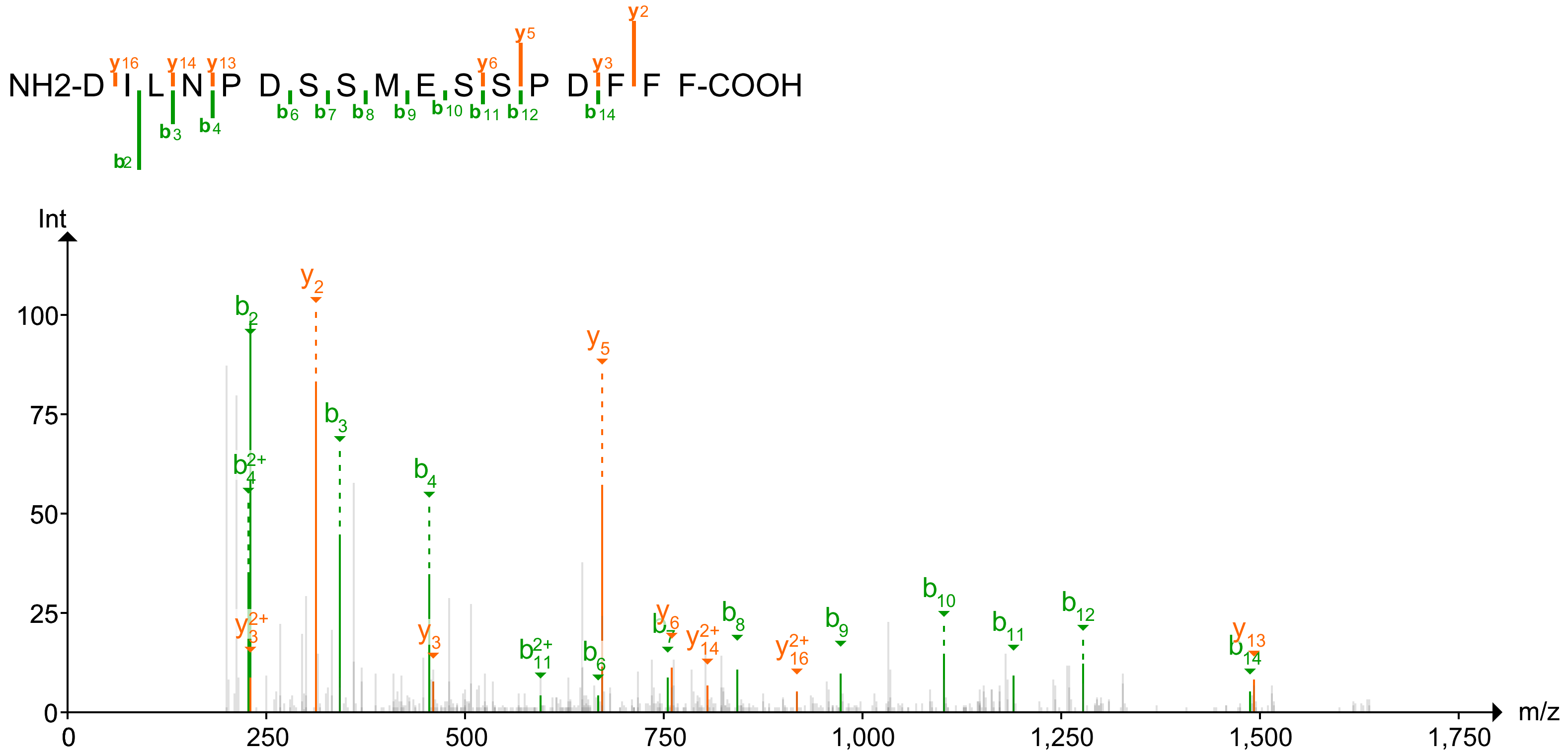

NH2-D I L N P D S S M E S S P D F F F-COOH
y16 y14 y13 y6 y5 y3 y2
b2 b3 b4 b6 b7 b8 b9 b10 b11 b12 b14
Int
100
75
50
25
0
m/z
0
250
500
750
1,000
1,250
1,500
1,750

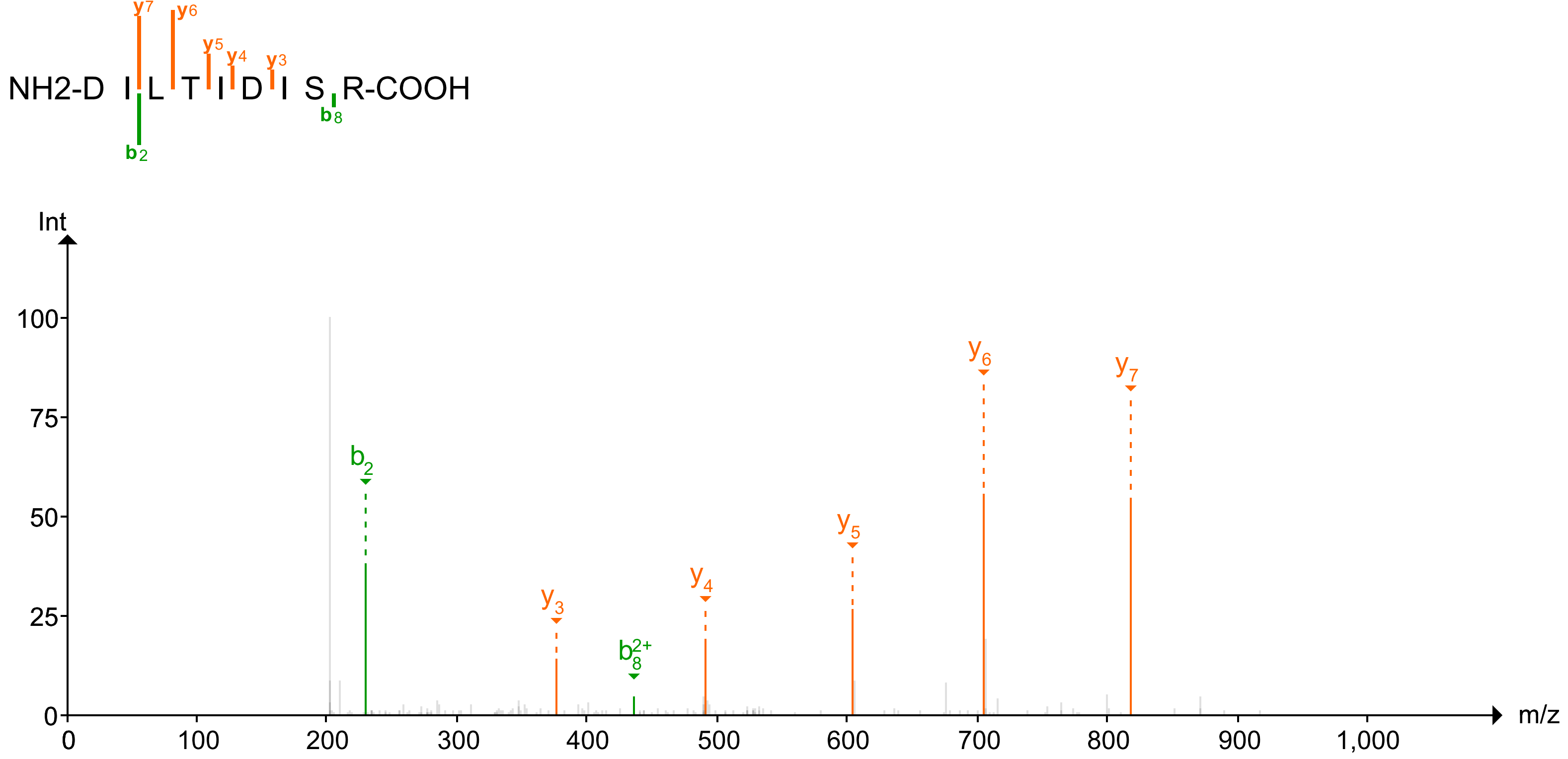

NH2-D I L T I D I S R-COOH
y7
y6
y5
y4
y3
b2
b8
Int
100
75
50
25
0
m/z
0
100
200
300
400
500
600
700
800
900
1,000
b2
y3
b8 2+
y4
y5
y6
y7

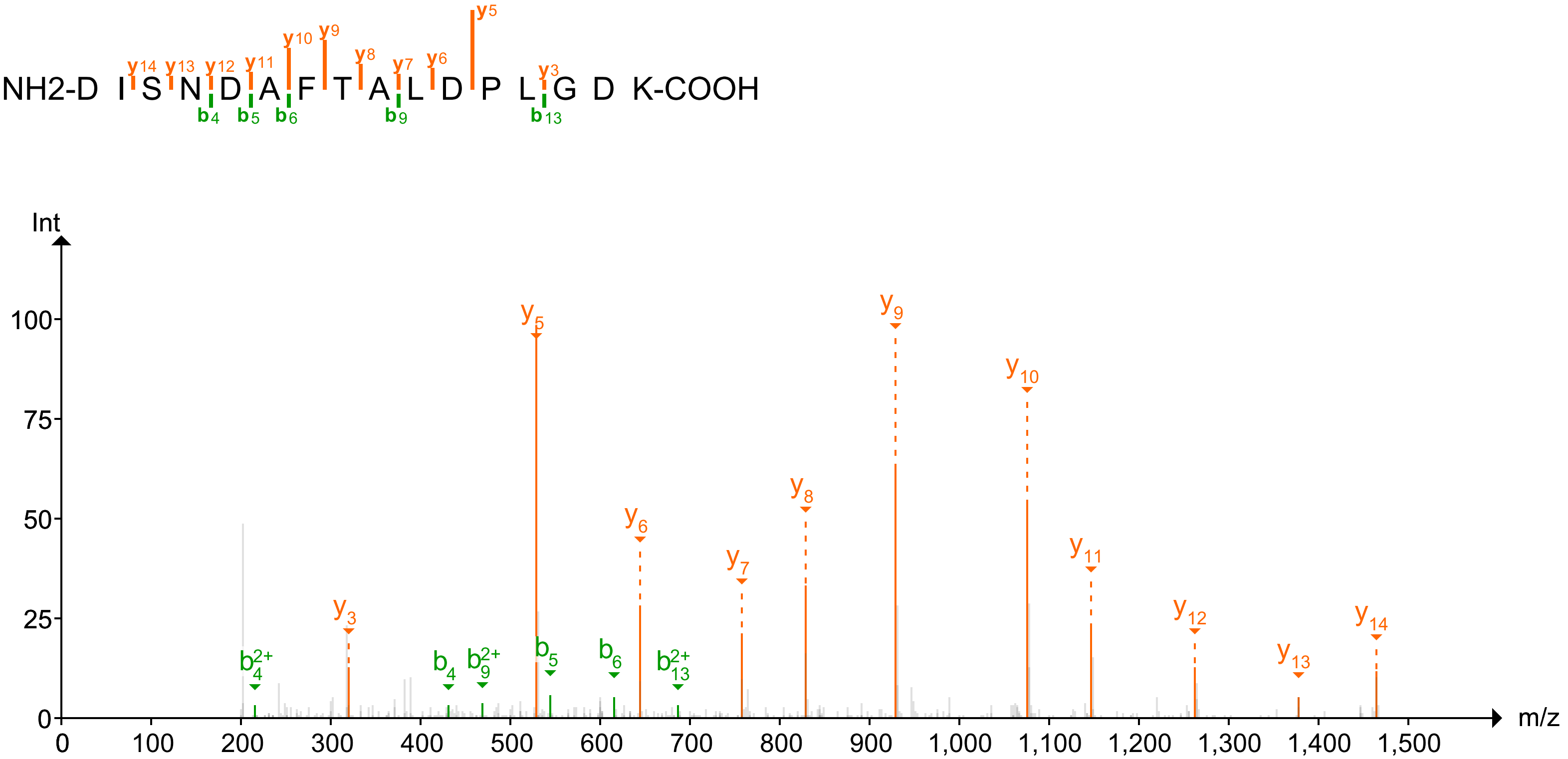

NH2-D I S N D A F T A L D P L G D K-COOH
y14 y13 y12 y11 y10 y9 y8 y7 y6 y5 y3
b4 b5 b6 b9 b13
Int
100
75
50
25
0
m/z
0 100 200 300 400 500 600 700 800 900 1,000 1,100 1,200 1,300 1,400 1,500
y5 y9 y10 y8 y6 y7 y11 y12 y14 y13 y3
b4 2+ b4 b9 2+ b5 b6 b13 2+

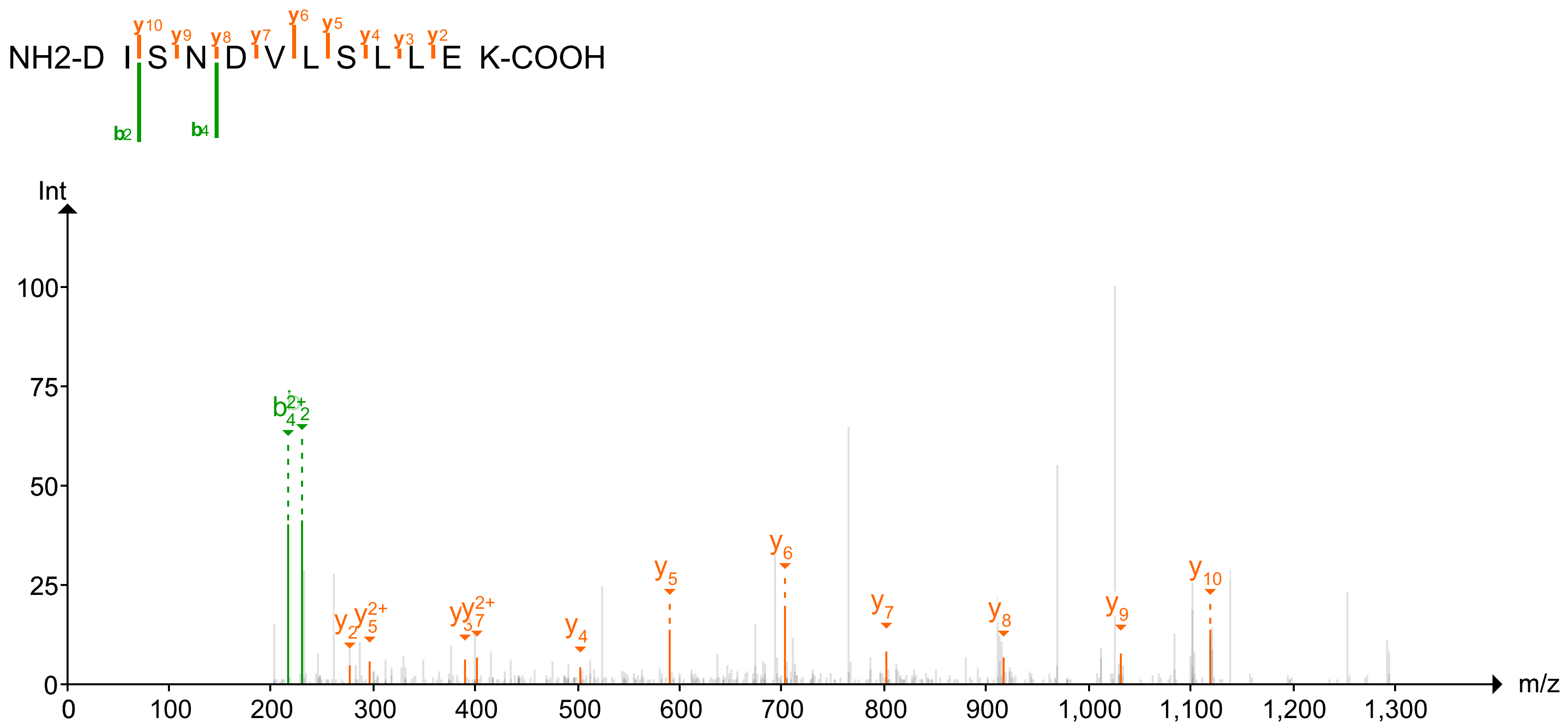

NH2-D I S N D V L S L L E K-COOH
y10 y9 y8 y7 y6 y5 y4 y3 y2
b2 b4
Int
m/z
0 25 50 75 100
0 100 200 300 400 500 600 700 800 900 1,000 1,100 1,200 1,300
b$_4^{2+}$ b$_2$
y$_2$ y$_5^{2+}$ y$_3$ y$_7^{2+}$ y$_4$ y$_5$ y$_6$ y$_7$ y$_8$ y$_9$ y$_{10}$

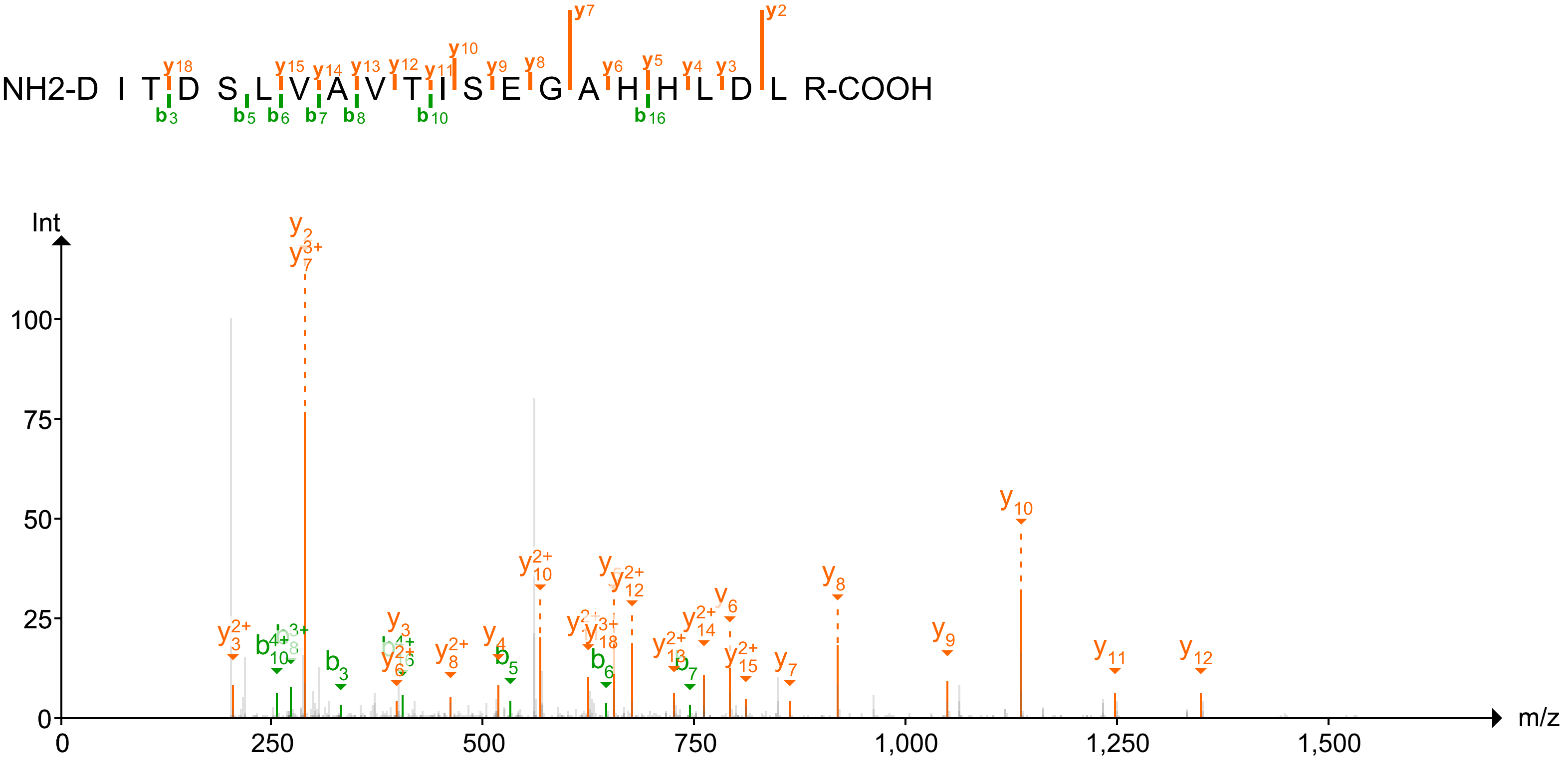

NH2-D I T D S L V A V T I S E G A H H L D L R-COOH
Int
m/z
0
25
50
75
100
0
250
500
750
1,000
1,250
1,500

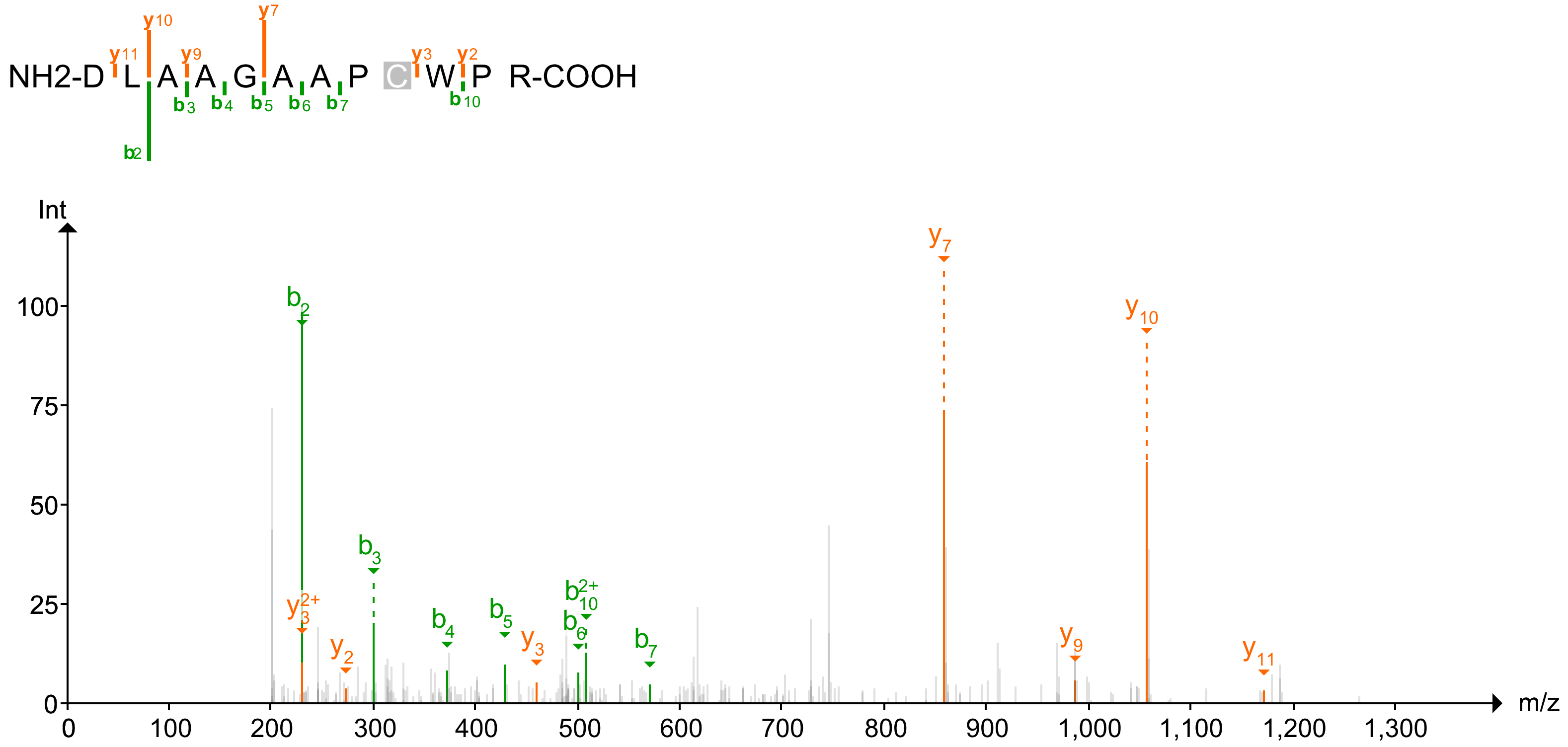

NH2-D L A A G A A P C W P R-COOH
y11 y10 y9 y7 y3 y2
b2 b3 b4 b5 b6 b7 b10
Int
100
75
50
25
0
m/z
0 100 200 300 400 500 600 700 800 900 1,000 1,100 1,200 1,300
y7 y10 b2 b3 y3 2+ y2 b4 b5 y3 b10 2+ b6 b7 y9 y11

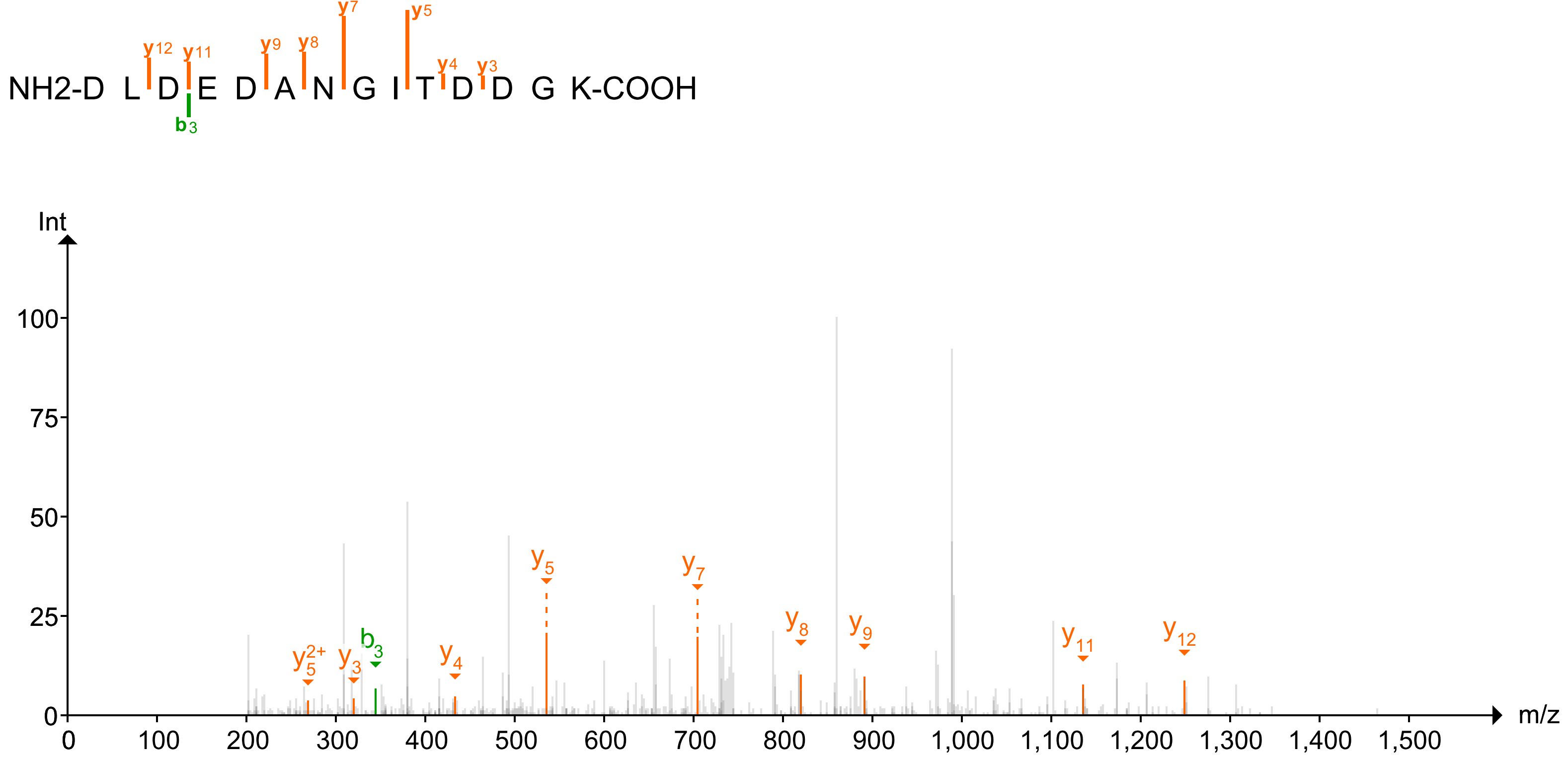

NH2-D L D E D A N G I T D D G K-COOH
y12 y11 y9 y8 y7 y5 y4 y3
b3
Int
100
75
50
25
0
m/z
0 100 200 300 400 500 600 700 800 900 1,000 1,100 1,200 1,300 1,400 1,500
y5 2+
y3
b3
y4
y5
y7
y8
y9
y11
y12

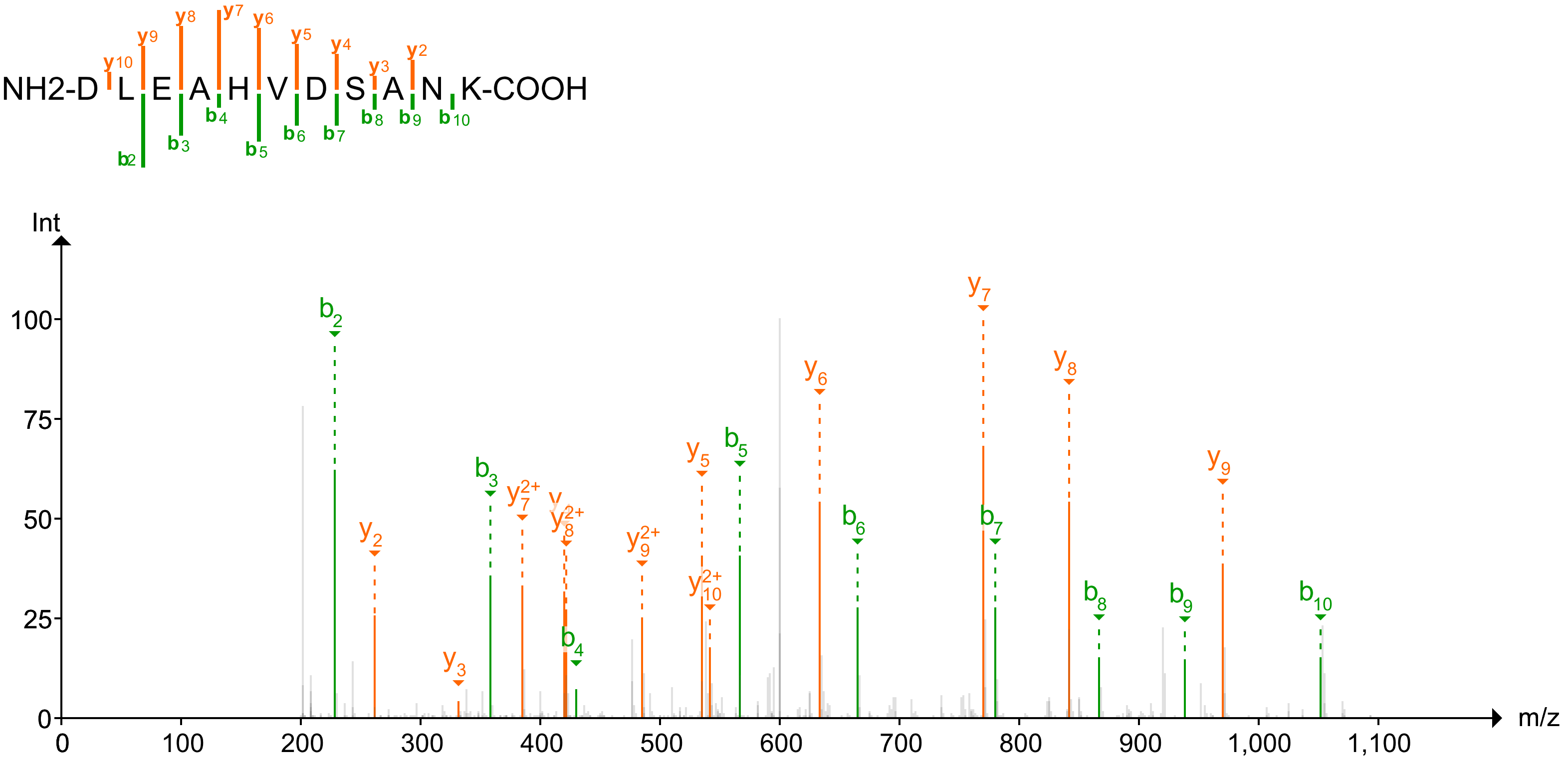
NH2-D L E A H V D S A N K-COOH
Int
m/z
0
25
50
75
100
0
100
200
300
400
500
600
700
800
900
1,000
1,100

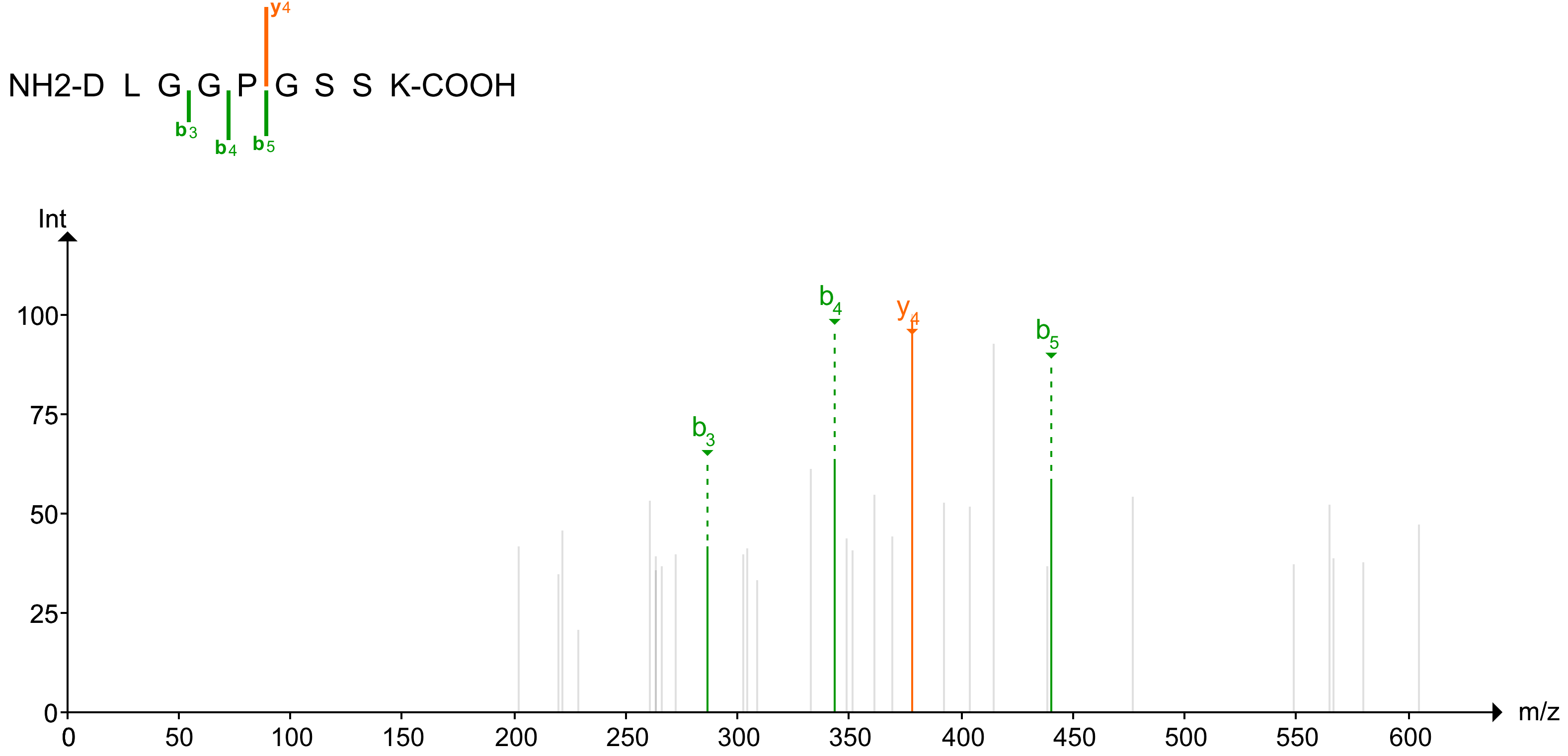
NH2-D L G G P G S S K-COOH
y4
b3
b4
b5
Int
100
75
50
25
0
0
50
100
150
200
250
300
350
400
450
500
550
600
m/z

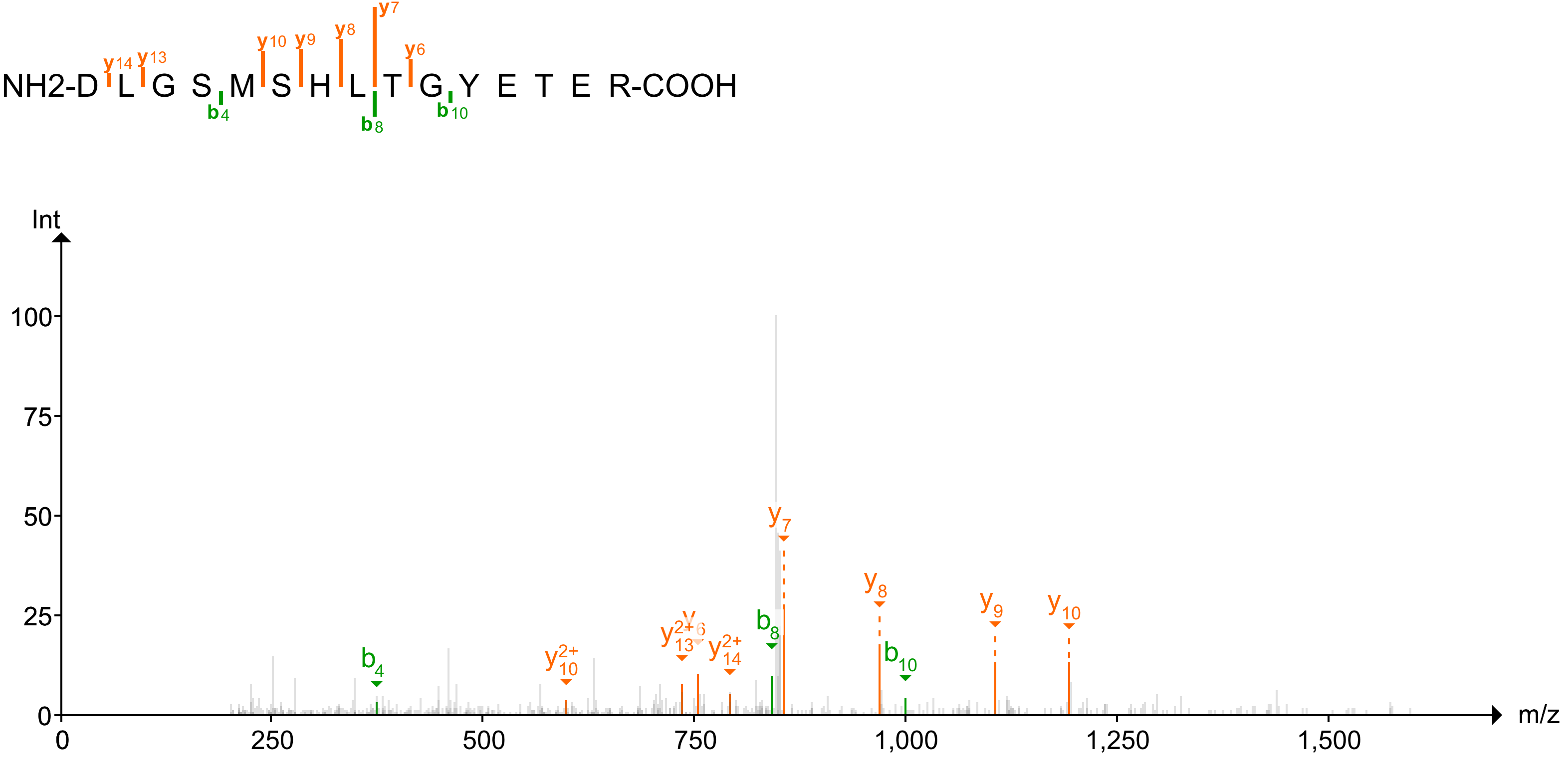

NH2-D L G S M S H L T G Y E T E R-COOH
y14 y13 y10 y9 y8 y7 y6
b4 b8 b10
Int
100
75
50
25
0
250
500
750
1,000
1,250
1,500
m/z
b4
y2+ 10
y2+ 13
y6
y2+ 14
b8
y7
y8
b10
y9
y10

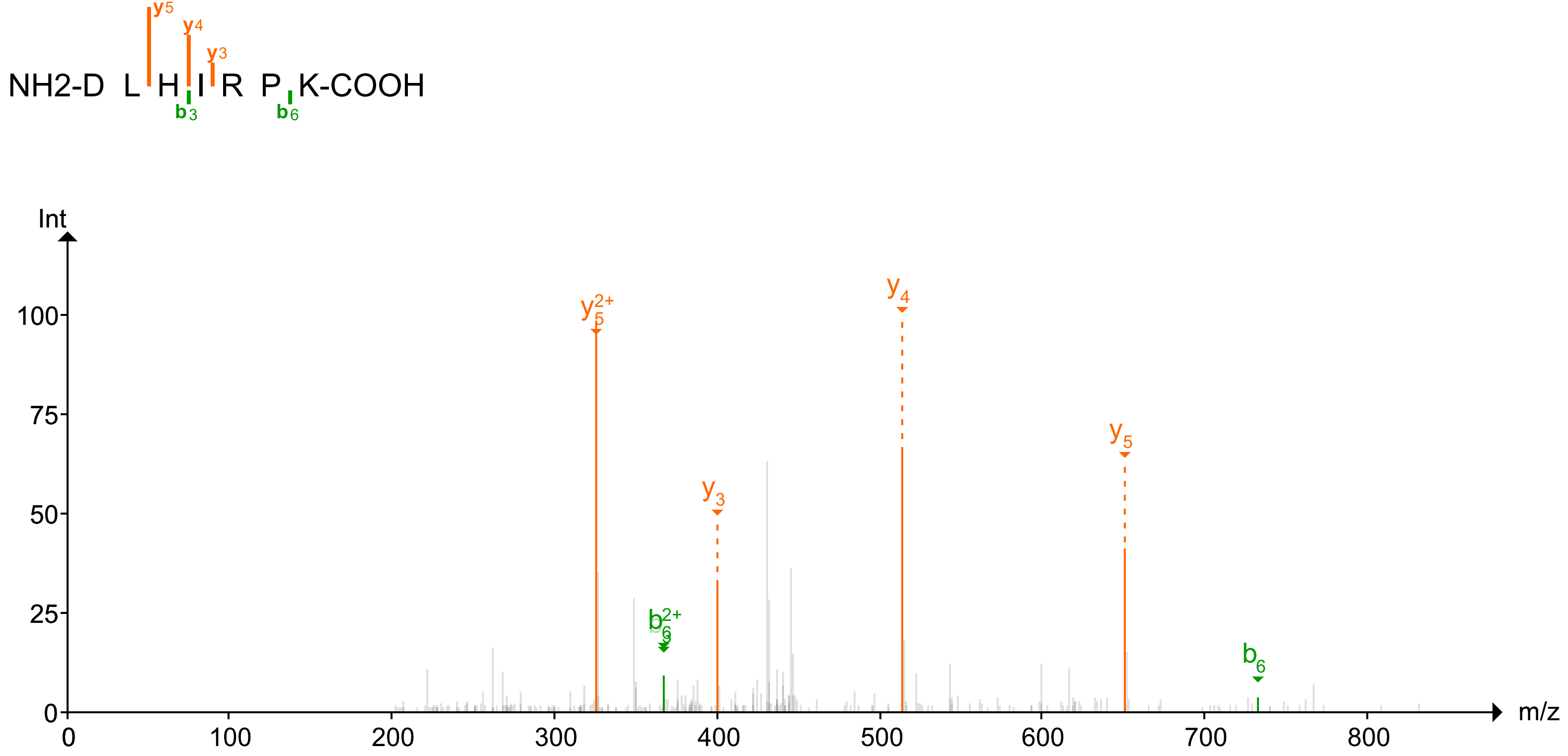

NH2-D L H I R P K-COOH
y5
y4
y3
b3
b6
Int
100
75
50
25
0
m/z
0
100
200
300
400
500
600
700
800
$y_5^{2+}$
$y_4$
$y_3$
$y_5$
$b_6^{2+}$
$b_6$

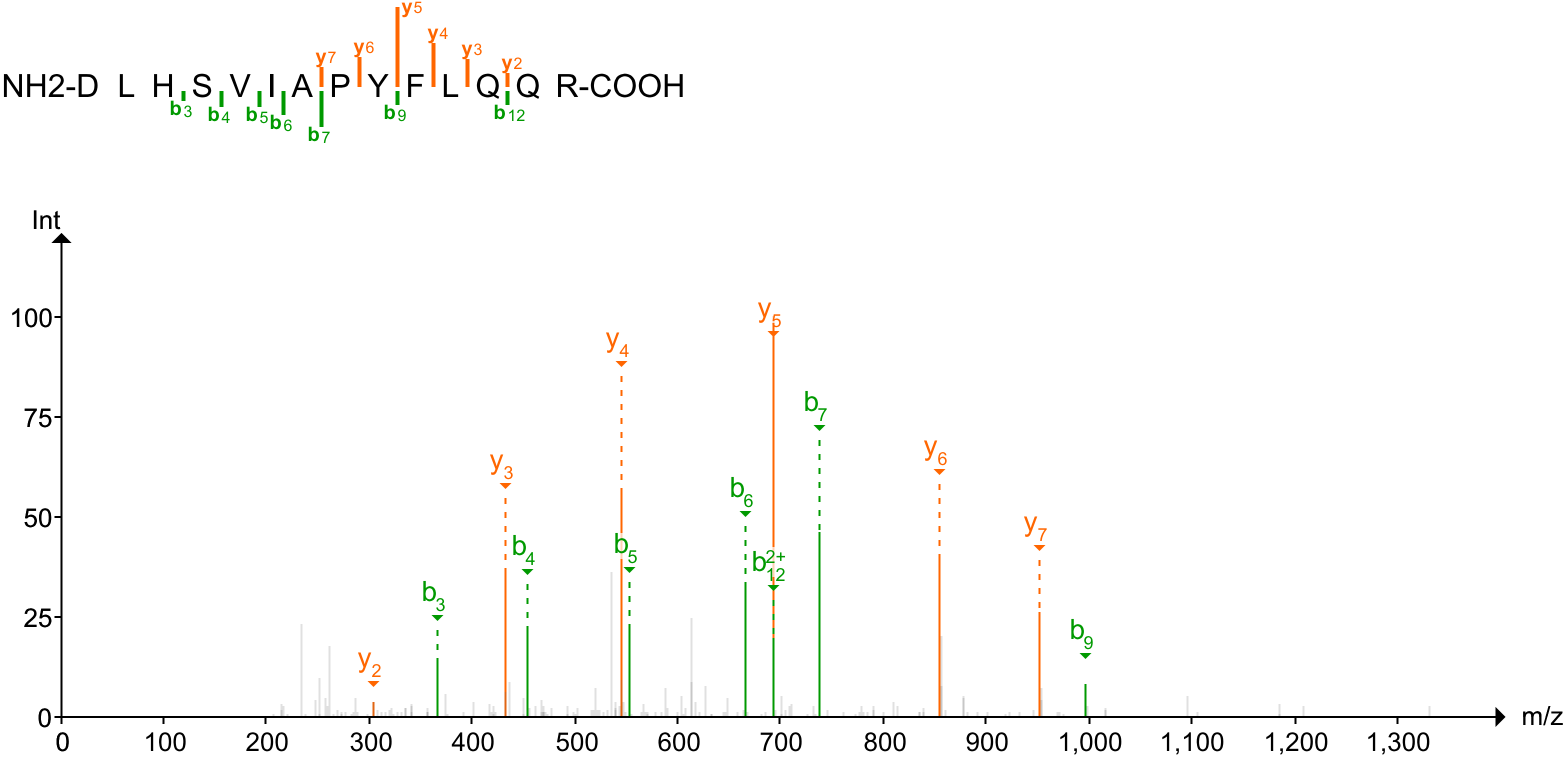
NH2-D L H S V I A P Y F L Q Q R-COOH
y5 y4 y3 y2 y7 y6
b3 b4 b5 b6 b7 b9 b12
Int
m/z
y2 y3 y4 y5 y6 y7
b3 b4 b5 b6 b7 b9 b12 2+

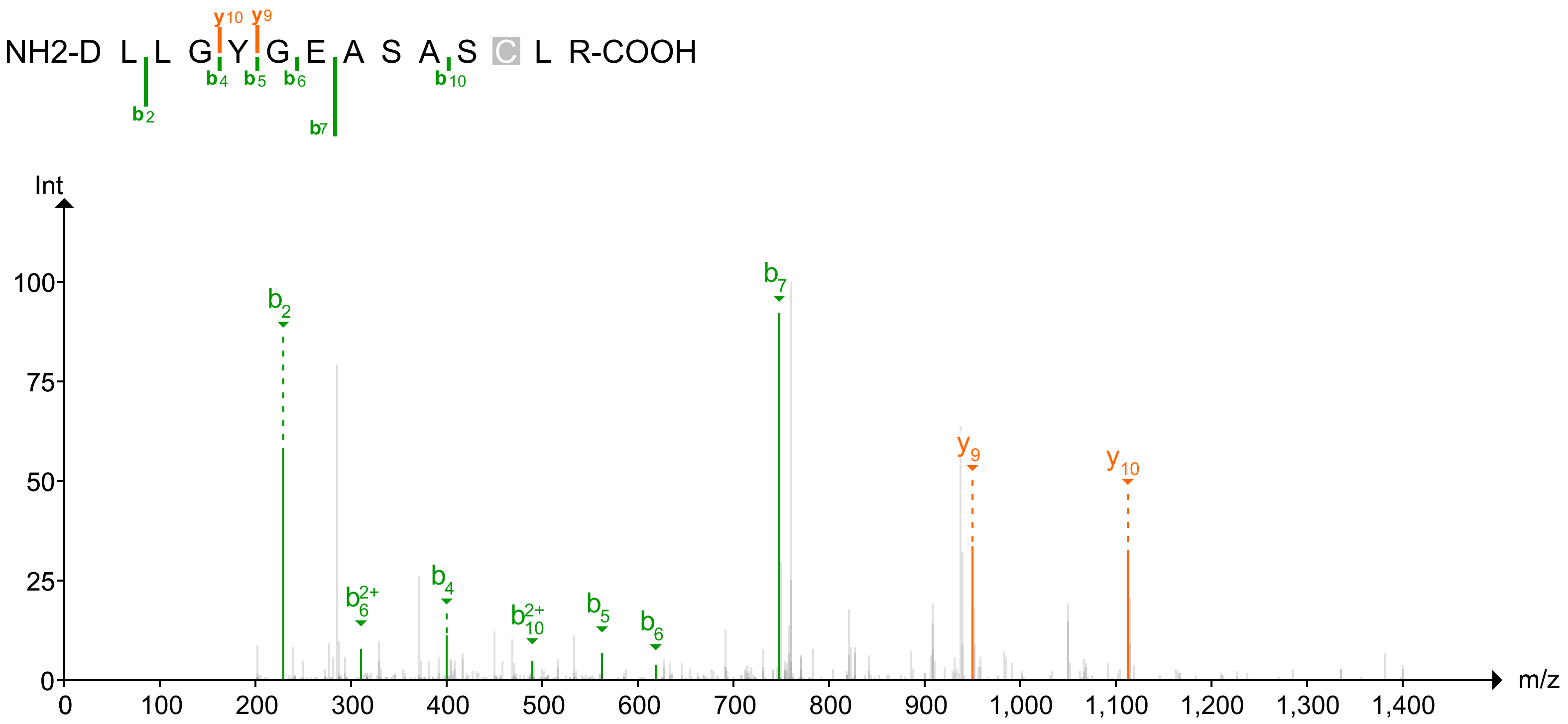

NH2-D L L G Y G E A S A S C L R-COOH
y10 y9
b2 b4 b5 b6 b7 b10
Int
100
75
50
25
0
b2
b7
y9
y10
b4
b6 2+
b10 2+
b5
b6
0 100 200 300 400 500 600 700 800 900 1,000 1,100 1,200 1,300 1,400
m/z

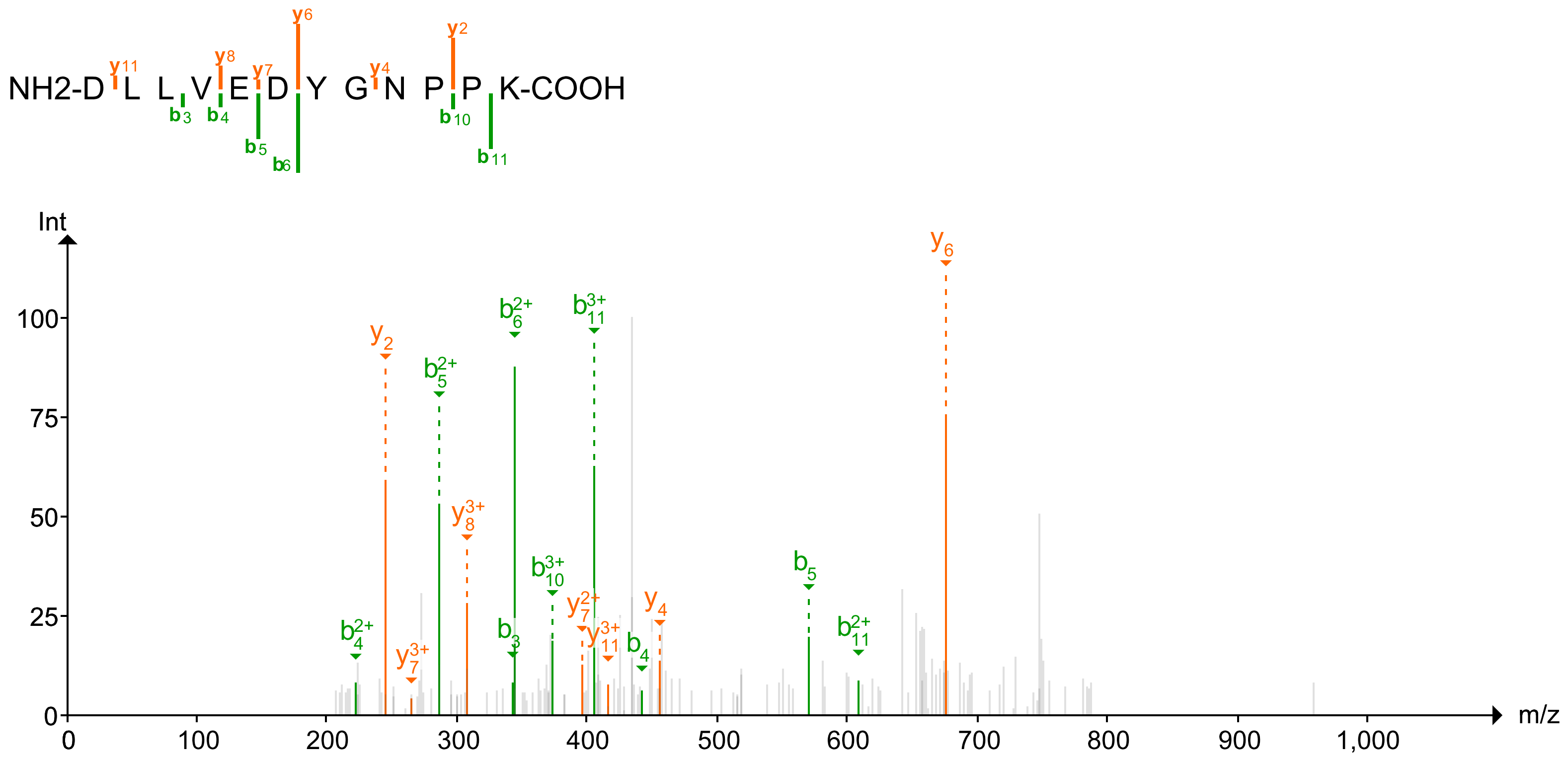

NH2-D L L V E D Y G N P P K-COOH
y11 y8 y7 y6 y4 y2
b3 b4 b5 b6 b10 b11
Int
100
75
50
25
0
m/z
0
100
200
300
400
500
600
700
800
900
1,000
y6
y2
b6 2+
b11 3+
b5 2+
y8 3+
b10 3+
b5
y7 2+
y11 3+
y4
b4 2+
y7 3+
b3
b4
b11 2+

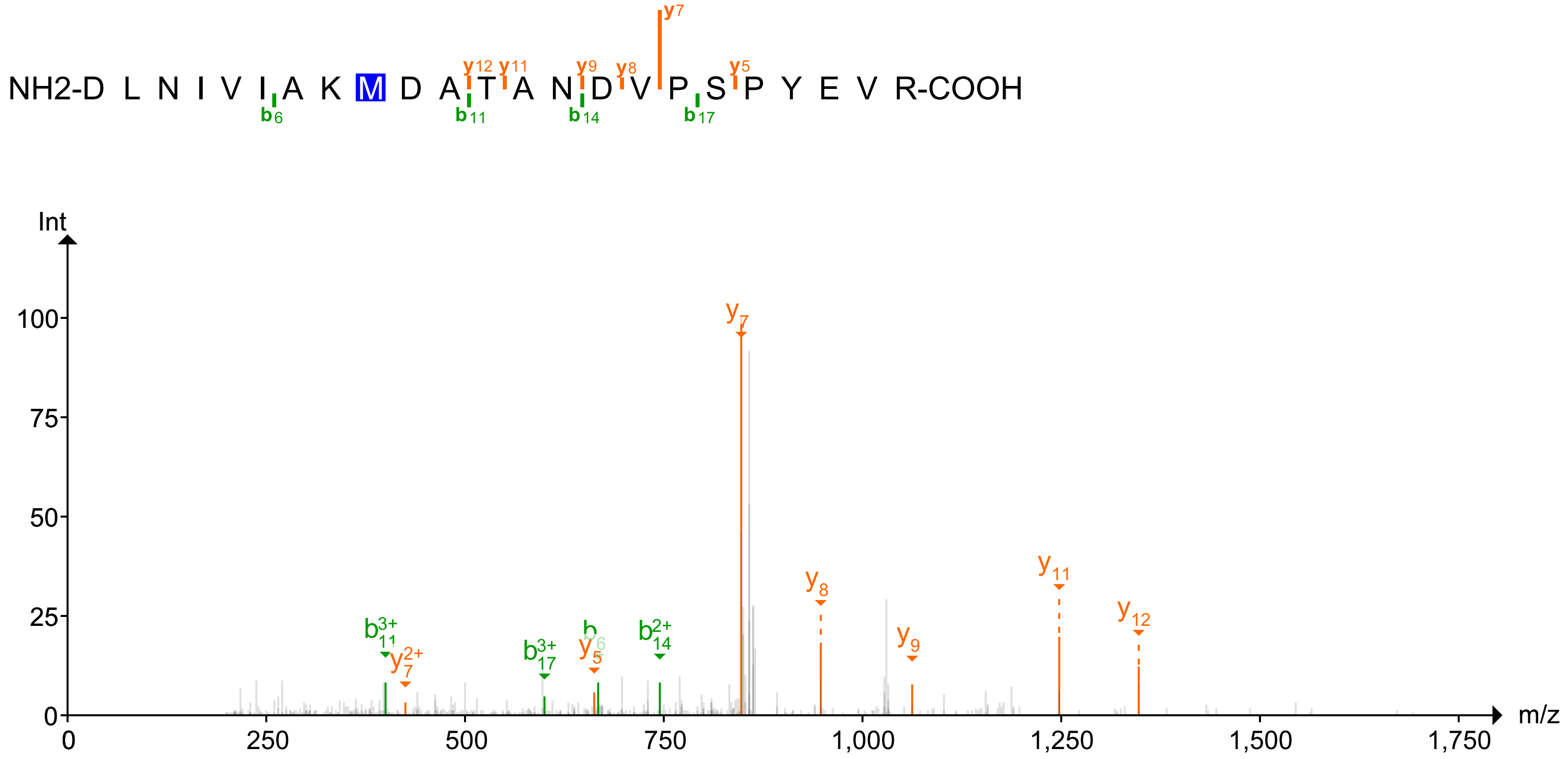

NH2-D L N I V I A K M D A T A N D V P S P Y E V R-COOH
b6 b11 b14 b17
y12 y11 y9 y8 y7 y5
Int
100
75
50
25
0
0
250
500
750
1,000
1,250
1,500
1,750
m/z
y7
y8
y11
y12
y9
b3+ 11
y2+ 7
b3+ 17
b6
y5
b2+ 14

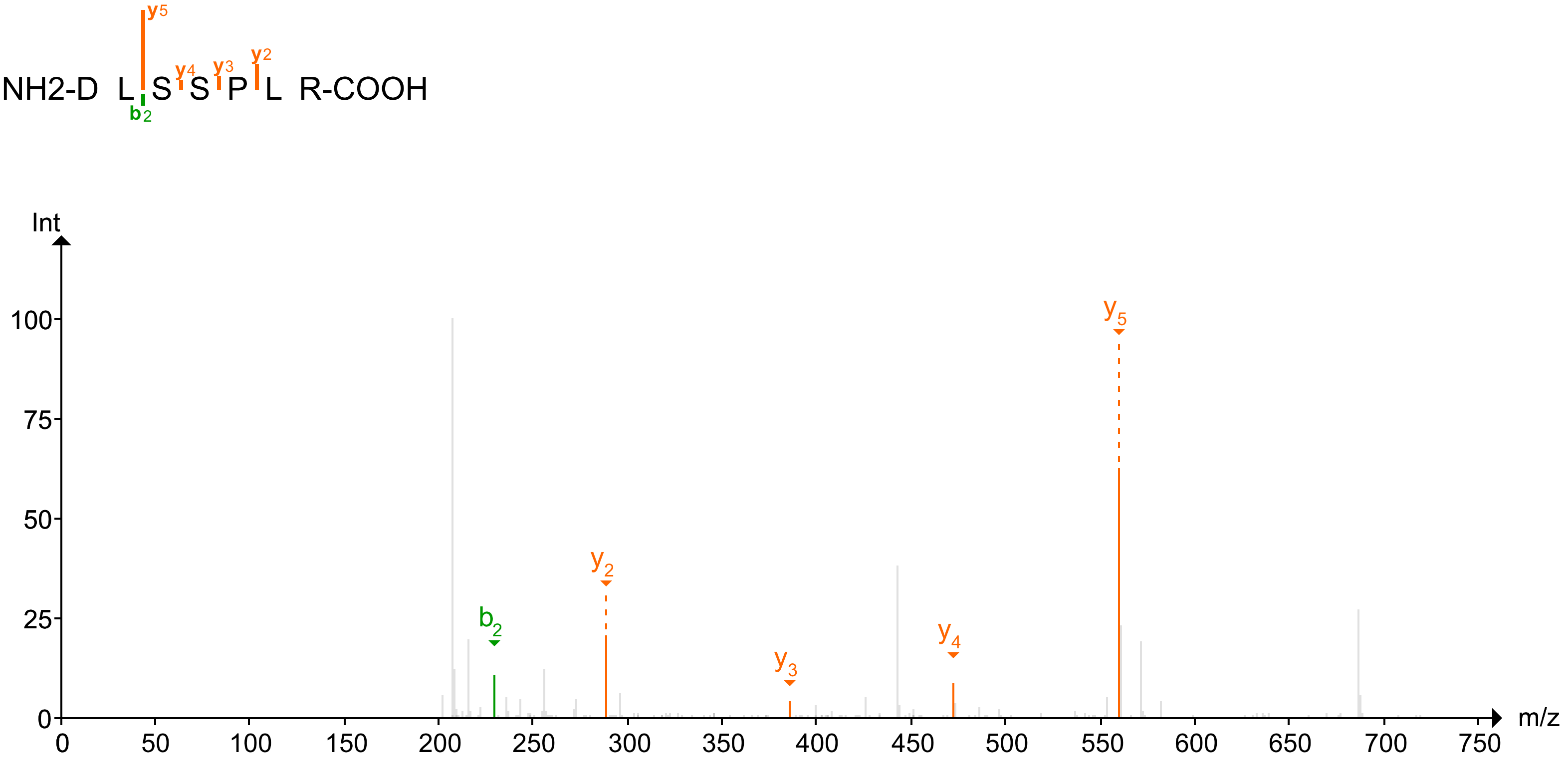
NH2-D L S S P L R-COOH
y5
y4
y3
y2
b2
Int
100
75
50
25
0
m/z
0
50
100
150
200
250
300
350
400
450
500
550
600
650
700
750
b2
y2
y3
y4
y5

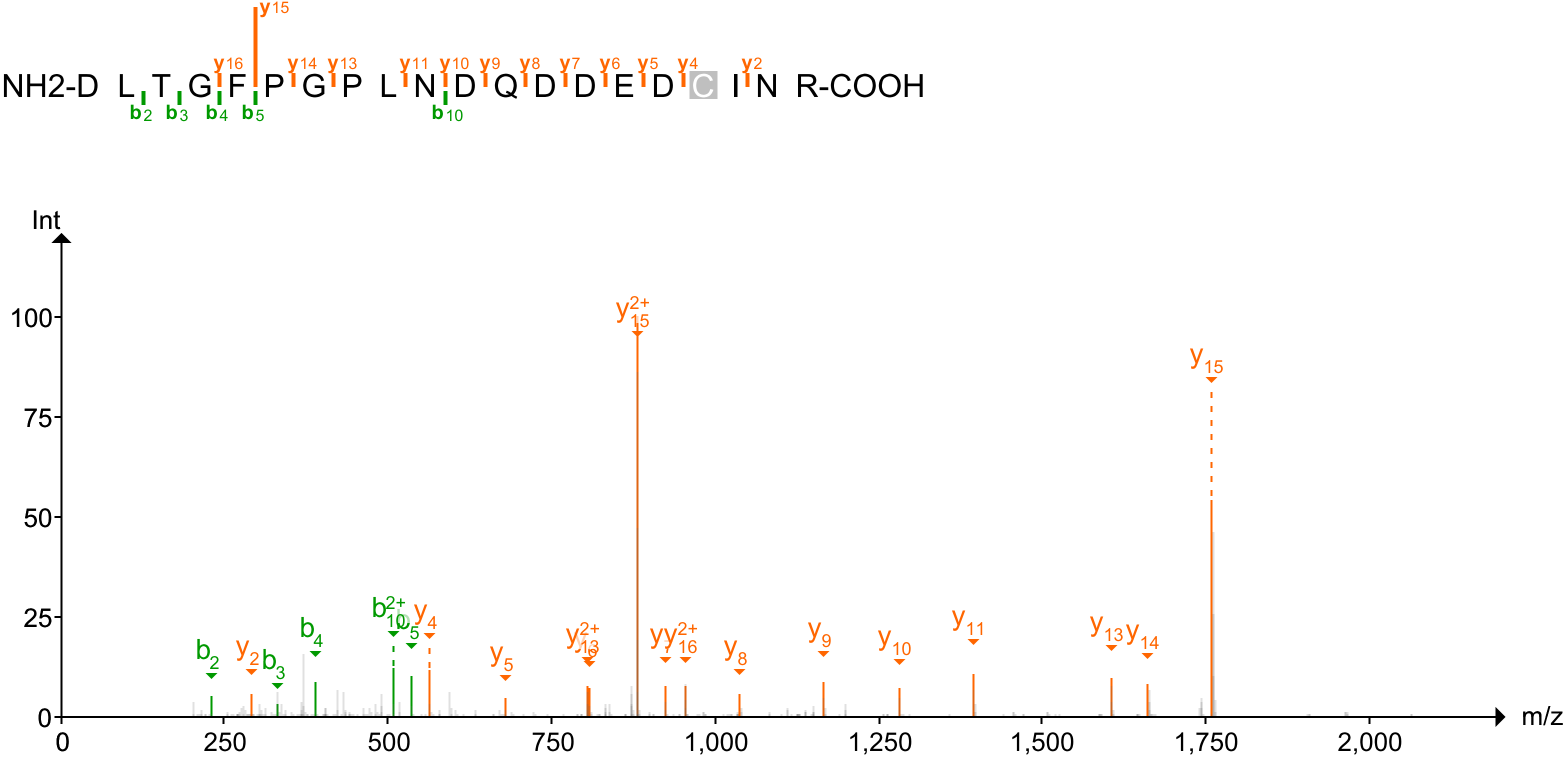

NH2-D L T G F P G P L N D Q D D E D C I N R-COOH
y15
y16 y14 y13 y11 y10 y9 y8 y7 y6 y5 y4 y2
b2 b3 b4 b5 b10
Int
100
75
50
25
0
m/z
0
250
500
750
1,000
1,250
1,500
1,750
2,000

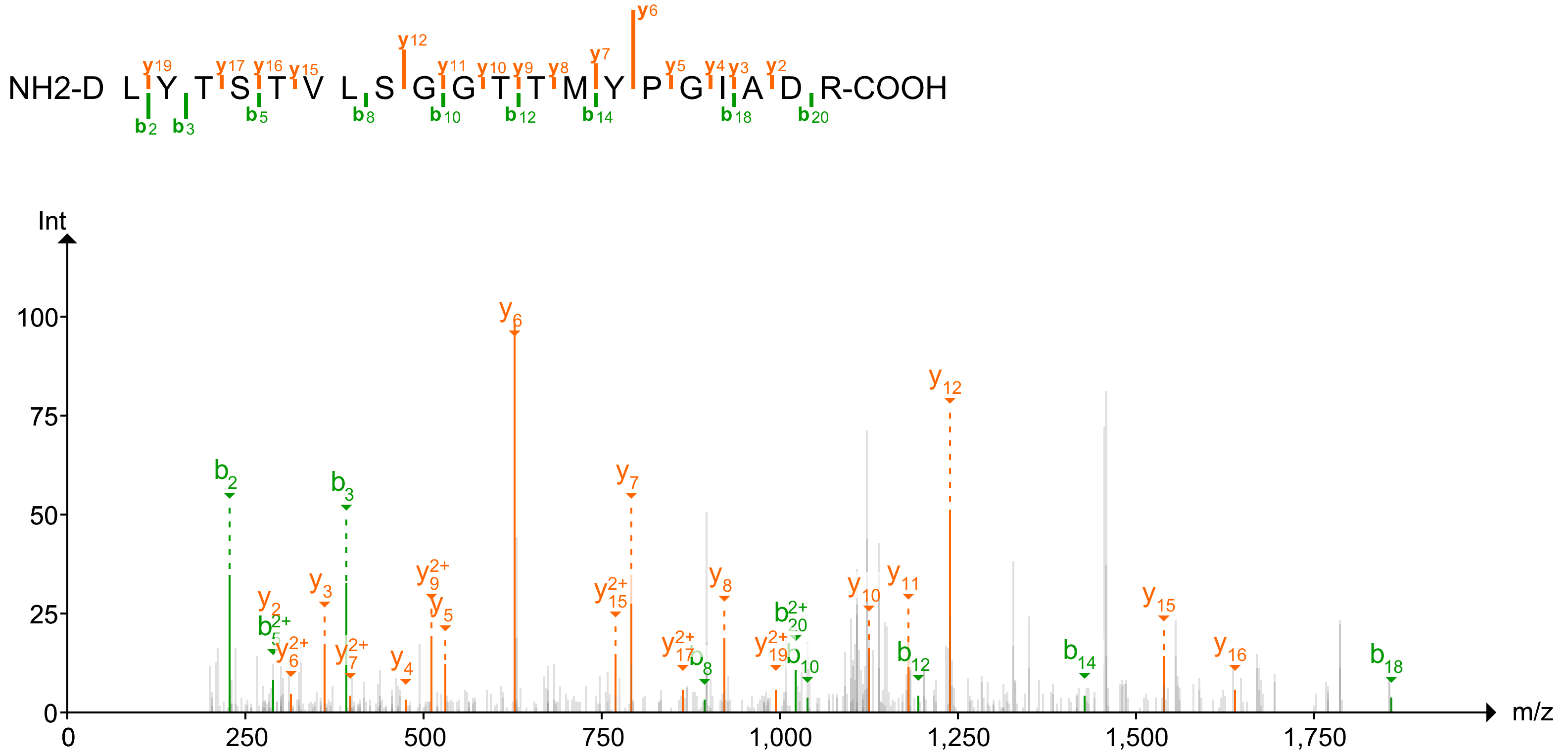

NH2-D L Y T S T V L S G G T T M Y P G I A D R-COOH
y19 y17 y16 y15 y12 y11 y10 y9 y8 y7 y6 y5 y4 y3 y2
b2 b3 b5 b8 b10 b12 b14 b18 b20
Int
100
75
50
25
0
m/z
0
250
500
750
1,000
1,250
1,500
1,750

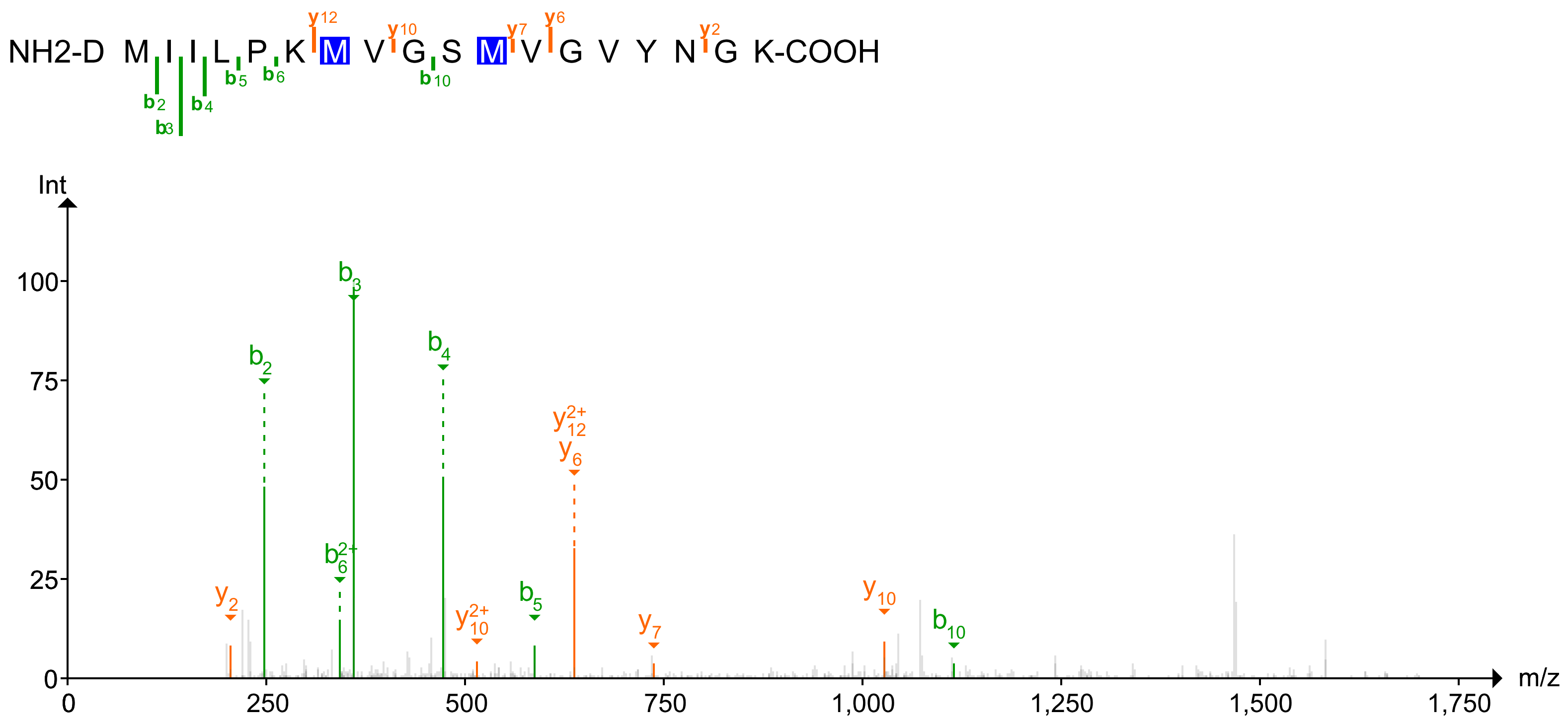

NH2-D M I I L P K M V G S M V G V Y N G K-COOH
b2 b3 b4 b5 b6 b10
y12 y10 y7 y6 y2
Int
100
75
50
25
0
b3
b2
b4
$y_{12}^{2+}$
$y_6$
$b_6^{2+}$
$y_2$
$y_{10}^{2+}$
b5
y7
y10
b10
0
250
500
750
1,000
1,250
1,500
1,750
m/z

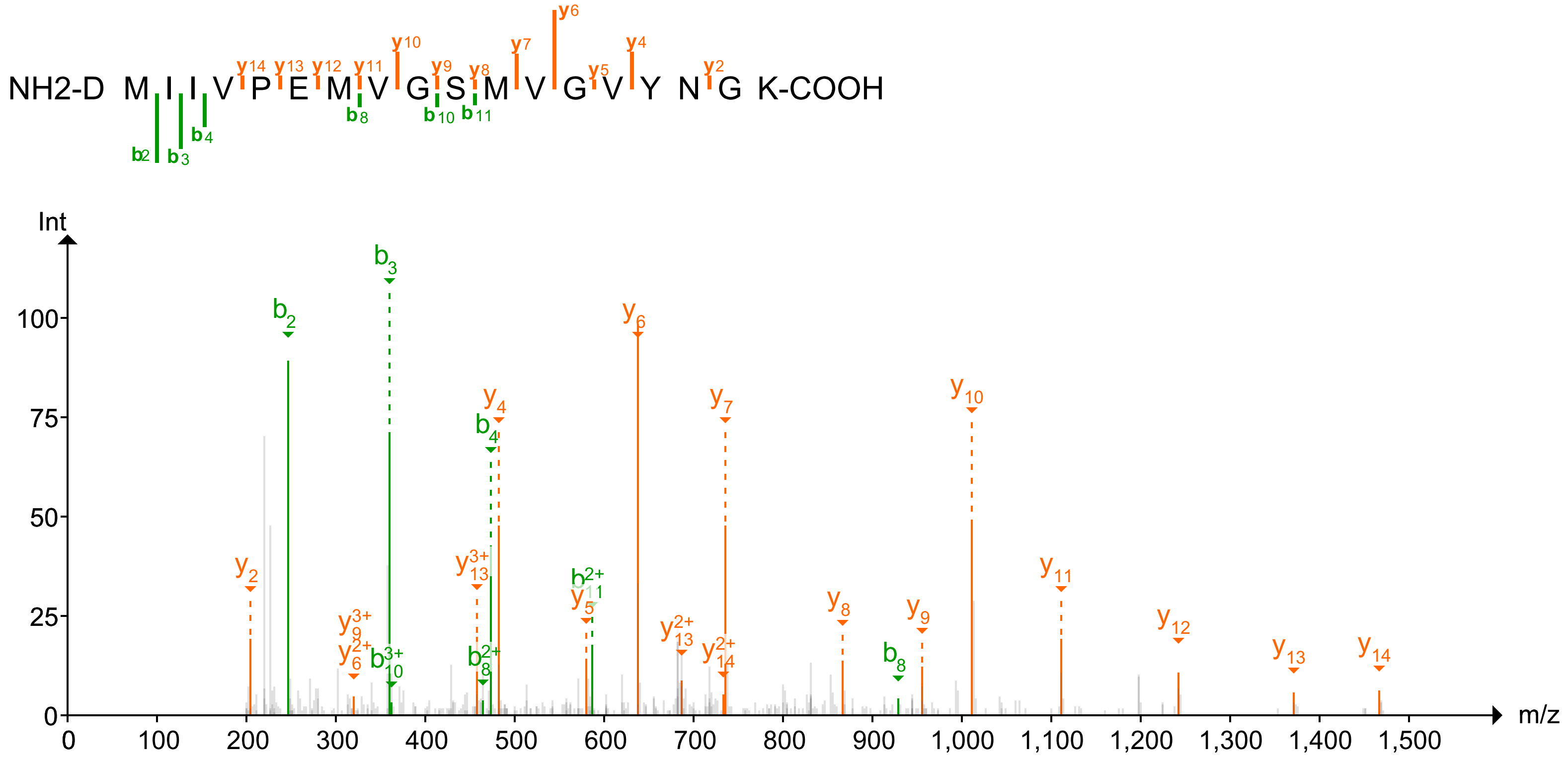

NH2-D M I I V P E M V G S M V G V Y N G K-COOH
Int
m/z

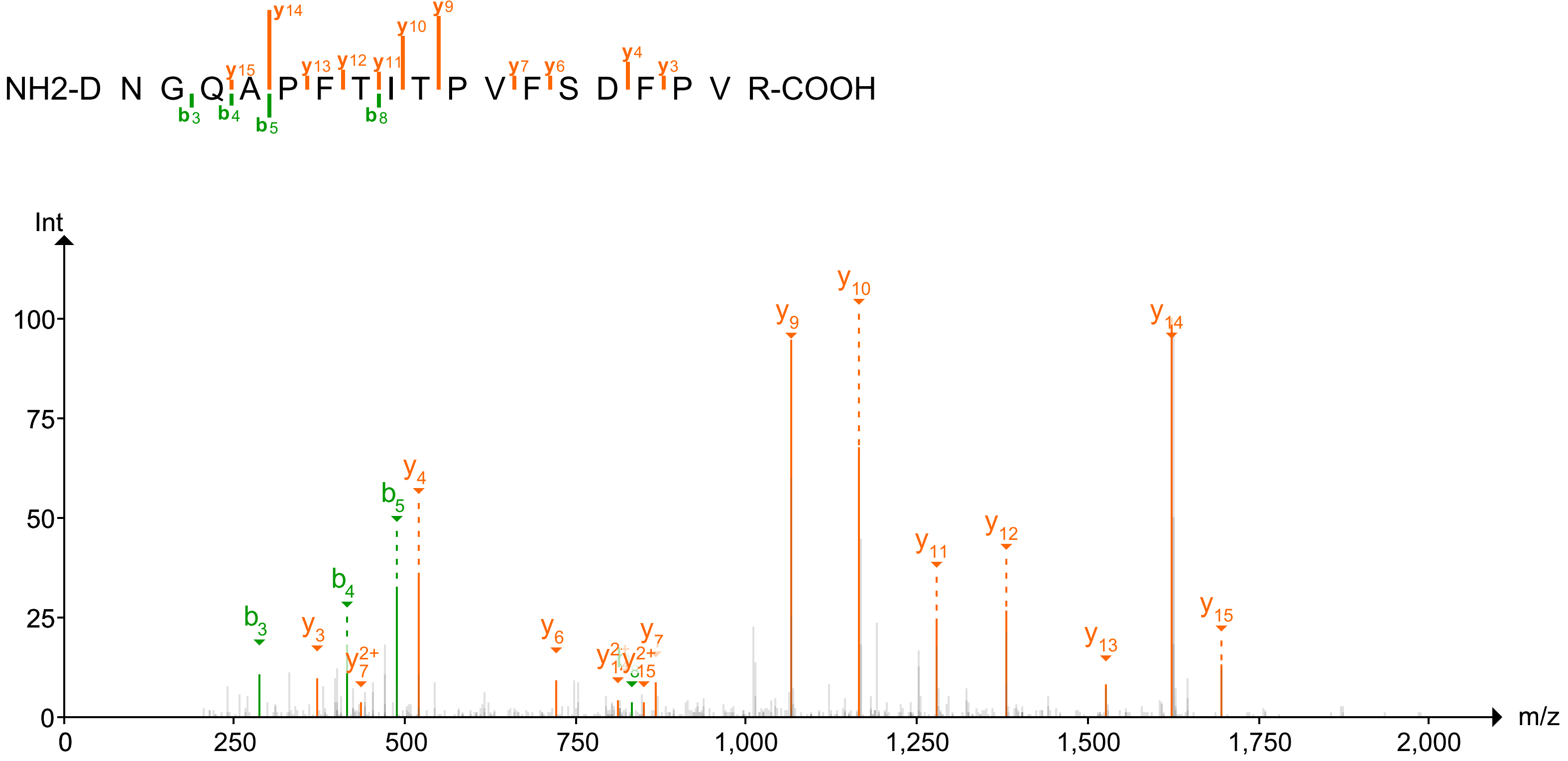
NH2-D N G Q A P F T I T P V F S D F P V R-COOH
Int
m/z
100
75
50
25
0
250
500
750
1,000
1,250
1,500
1,750
2,000
y9
y10
y14
y4
b5
b4
b3
y3
y11
y12
y13
y15
y6
y7

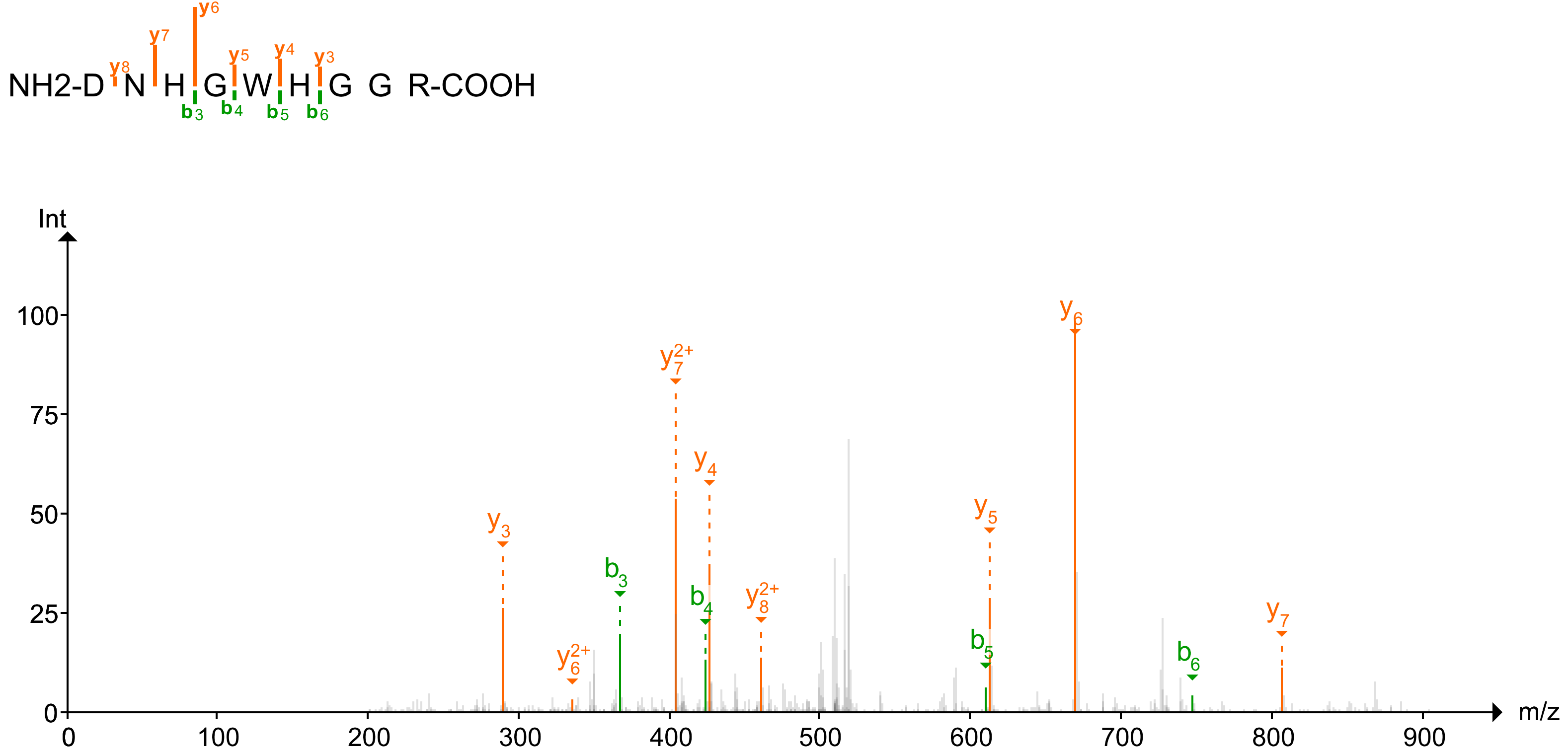

NH2-D N H G W H G G R-COOH
y8 y7 y6 y5 y4 y3
b3 b4 b5 b6
Int
100
75
50
25
0
m/z
0 100 200 300 400 500 600 700 800 900
y3
y6 2+
b3
y7 2+
b4
y4
y8 2+
b5
y5
y6
b6
y7

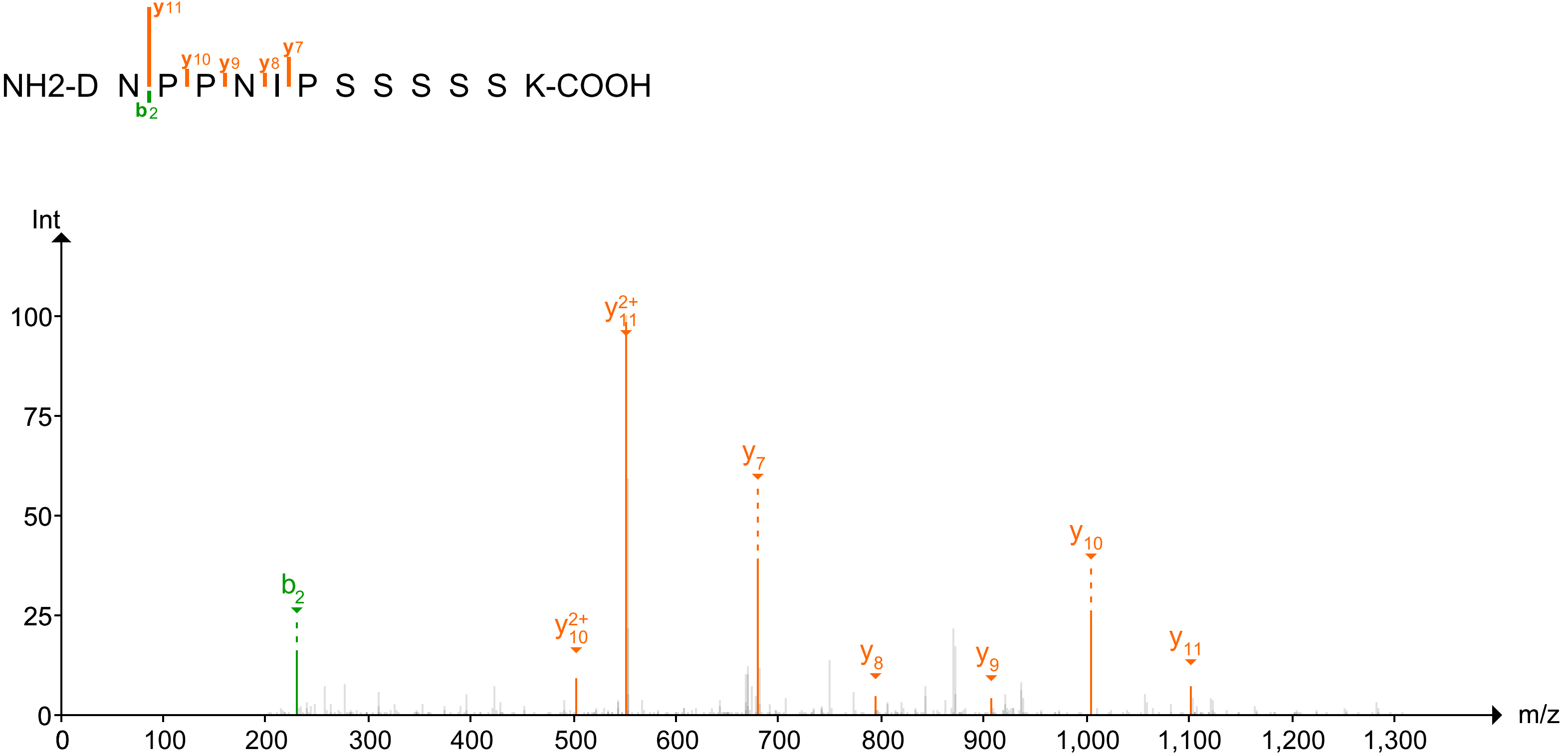

NH2-D N P P N I P S S S S S K-COOH
y11
y10
y9
y8
y7
b2
Int
100
75
50
25
0
0
100
200
300
400
500
600
700
800
900
1,000
1,100
1,200
1,300
m/z
$y_{11}^{2+}$
$y_7$
$y_{10}$
$b_2$
$y_{10}^{2+}$
$y_8$
$y_9$
$y_{11}$

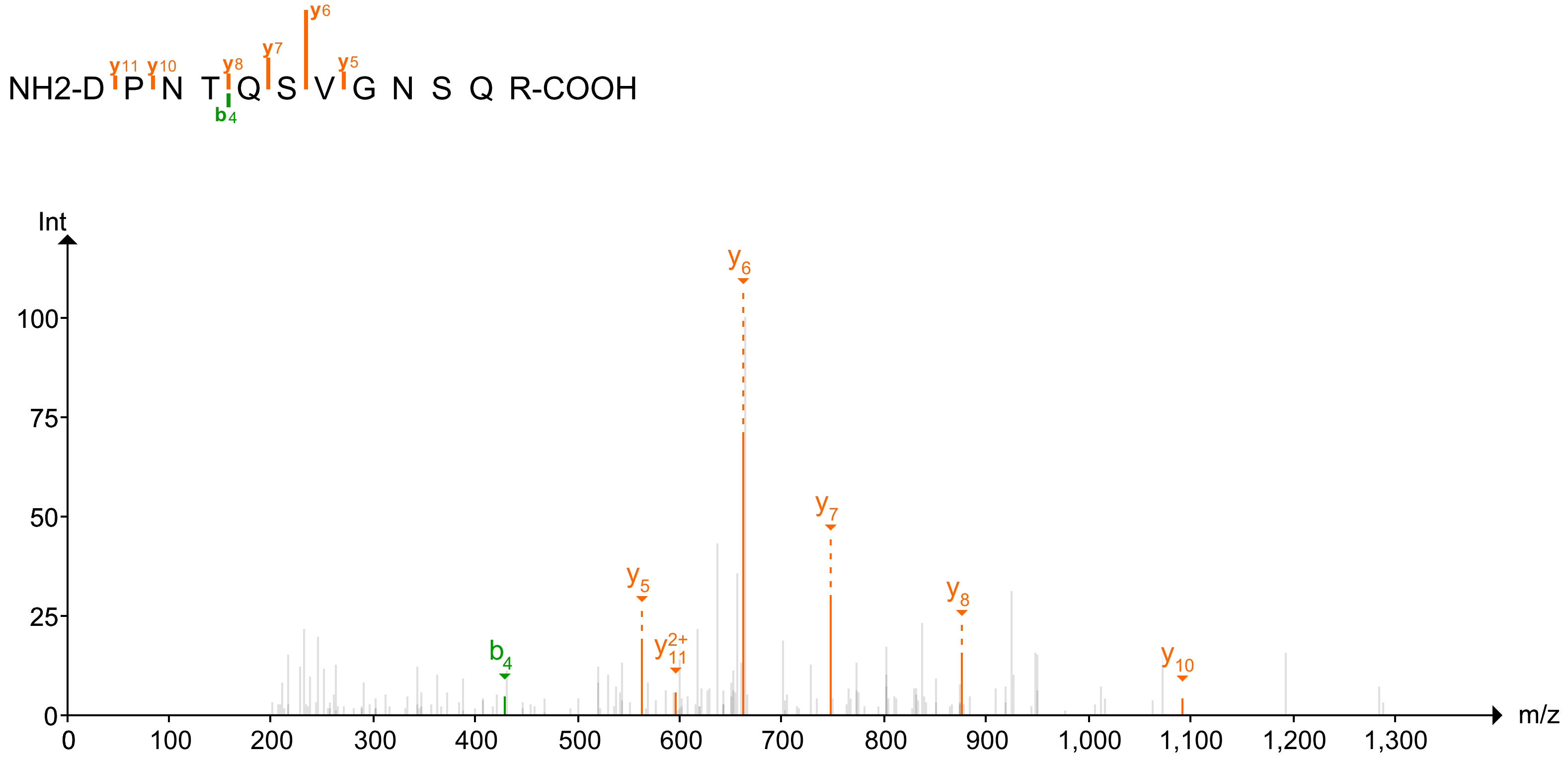

NH2-D P N T Q S V G N S Q R-COOH
y11 y10 y8 y7 y6 y5
b4
Int
100
75
50
25
0
y6
y7
y5
y8
b4
y10
y11 2+
m/z
0
100
200
300
400
500
600
700
800
900
1,000
1,100
1,200
1,300

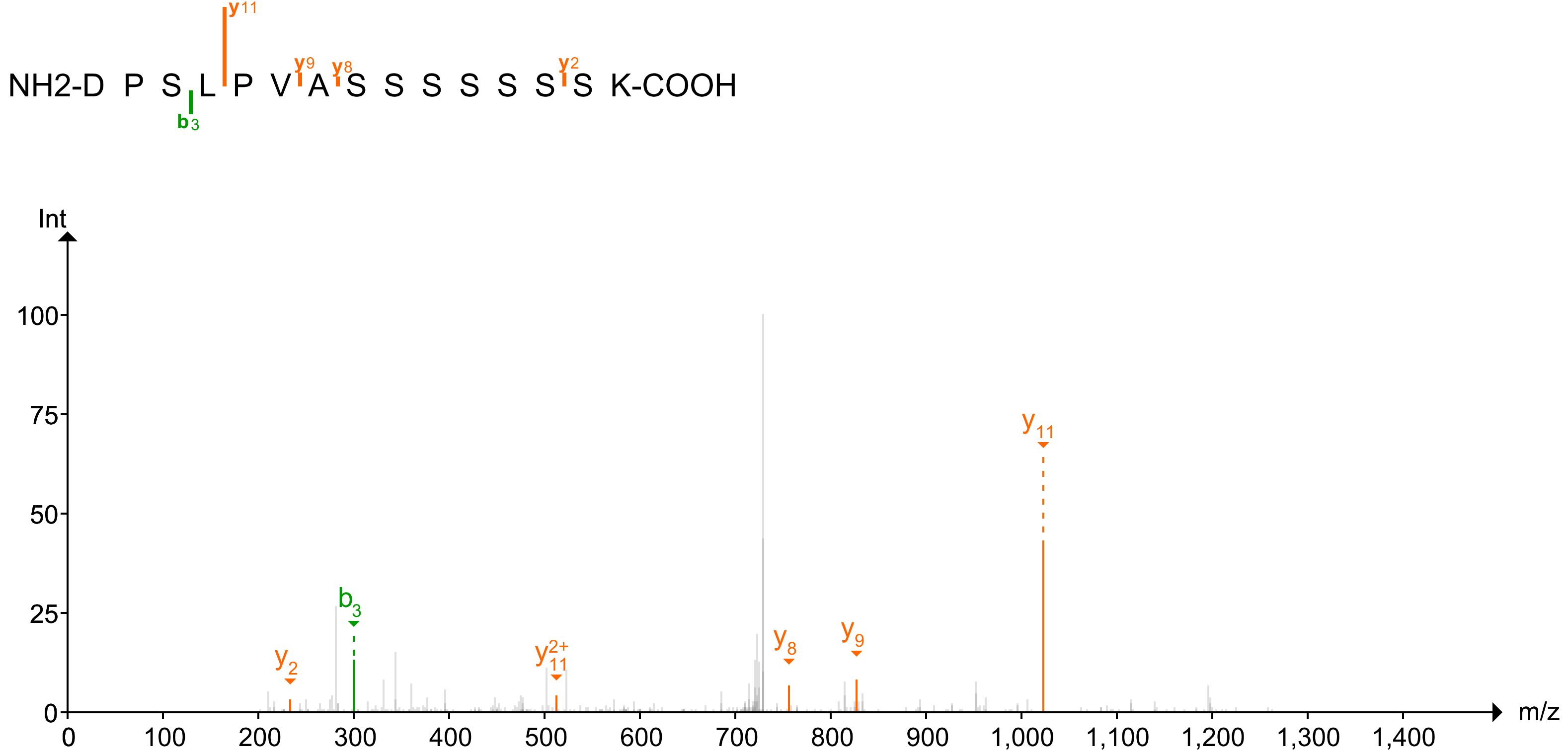

NH2-D P S L P V A S S S S S S S K-COOH
b3
y11
y9
y8
y2
Int
100
75
50
25
0
m/z
0
100
200
300
400
500
600
700
800
900
1,000
1,100
1,200
1,300
1,400
y2
b3
y11 2+
y8
y9
y11

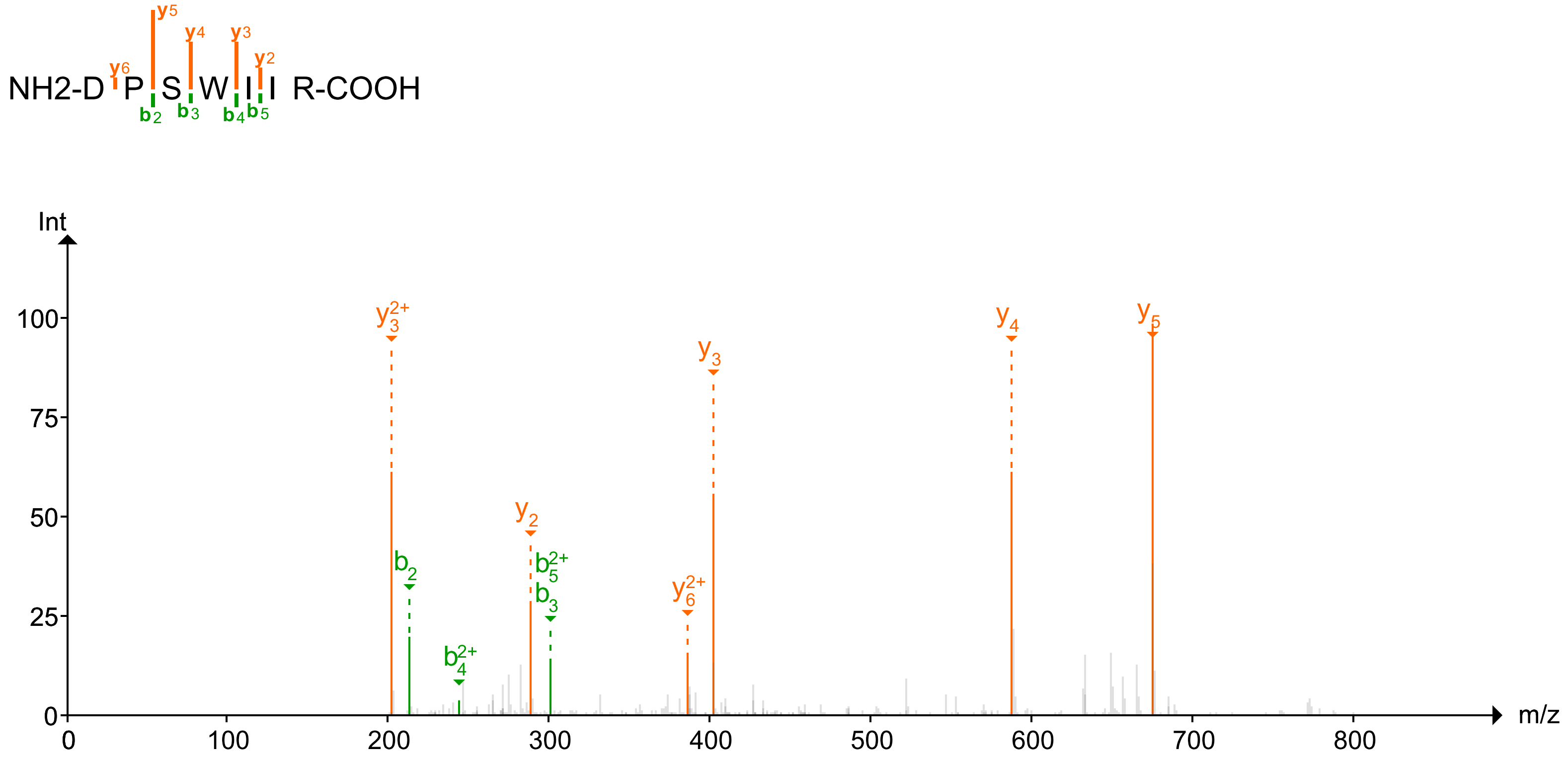

NH2-D P S W I I R-COOH
y6 y5 y4 y3 y2
b2 b3 b4 b5
Int
100
75
50
25
0
m/z
0
100
200
300
400
500
600
700
800
y3 2+
b2
b4 2+
y2
b5 2+
b3
y6 2+
y3
y4
y5

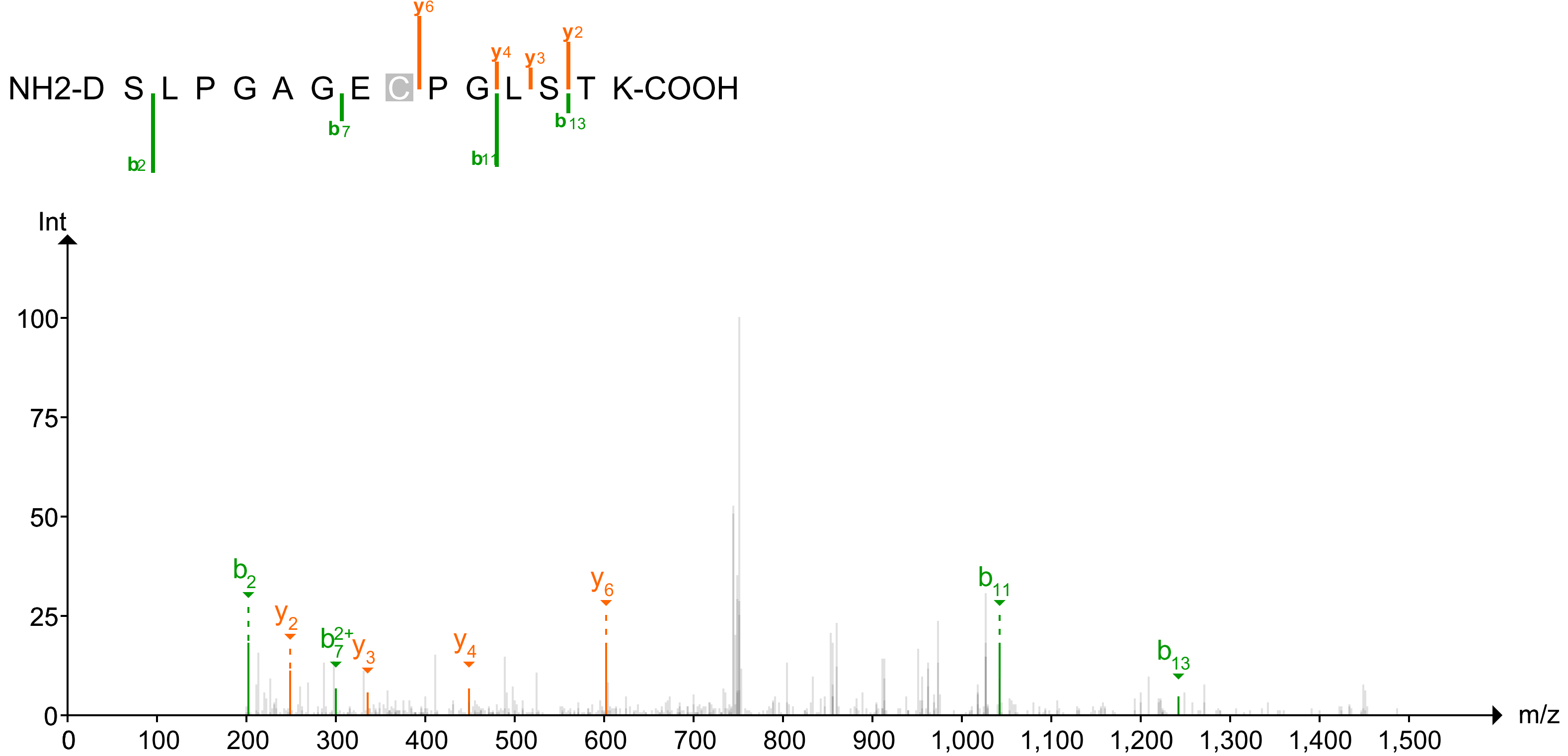

NH2-D S L P G A G E C P G L S T K-COOH
y6 y4 y3 y2
b2 b7 b11 b13
Int
100
75
50
25
0
b2
y2
b7 2+
y3
y4
y6
b11
b13
0 100 200 300 400 500 600 700 800 900 1,000 1,100 1,200 1,300 1,400 1,500
m/z

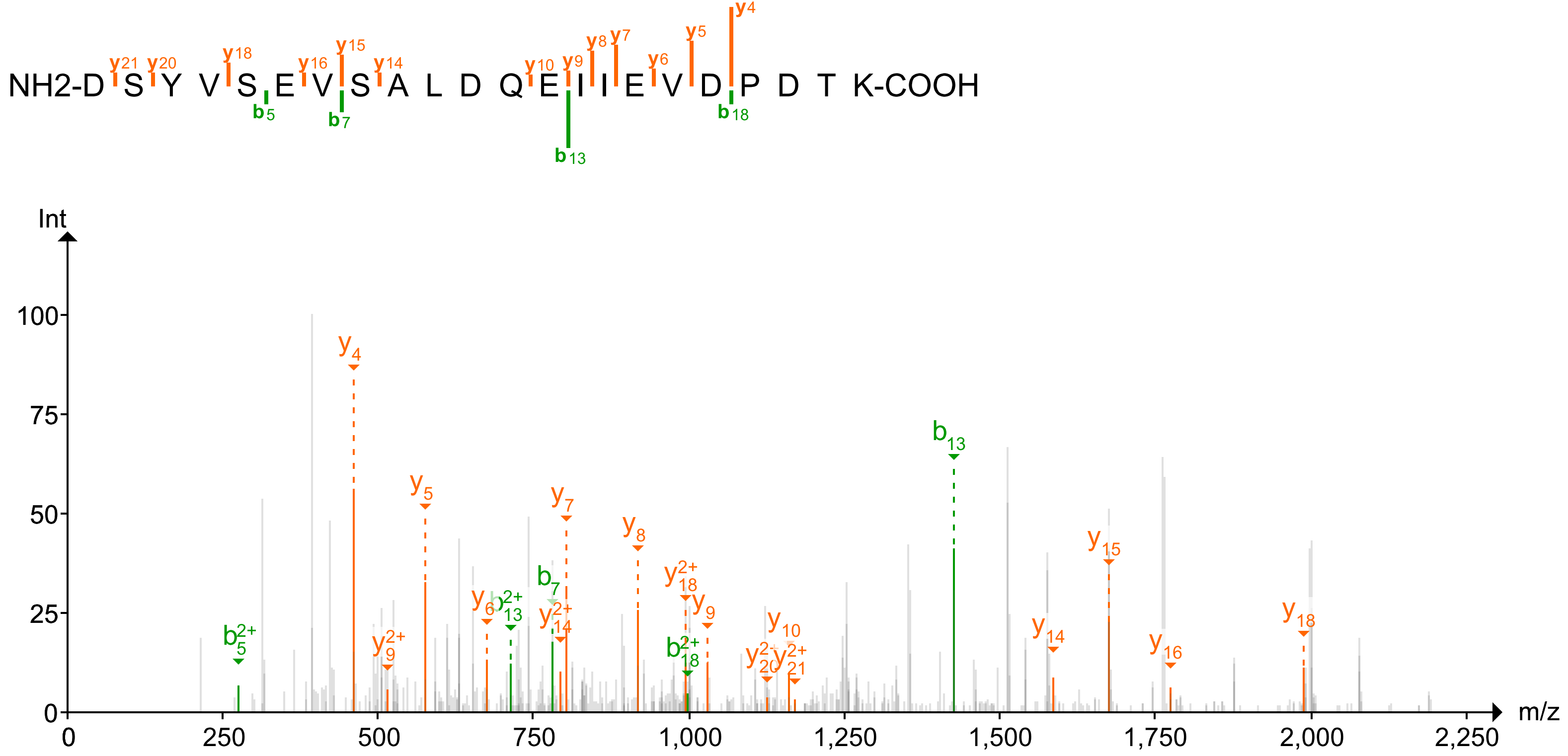
NH2-D S Y V S E V S A L D Q E I I E V D P D T K-COOH
y21 y20 y18 y16 y15 y14 y10 y9 y8 y7 y6 y5 y4
b5 b7 b13 b18
Int
100
75
50
25
0
m/z
0
250
500
750
1,000
1,250
1,500
1,750
2,000
2,250

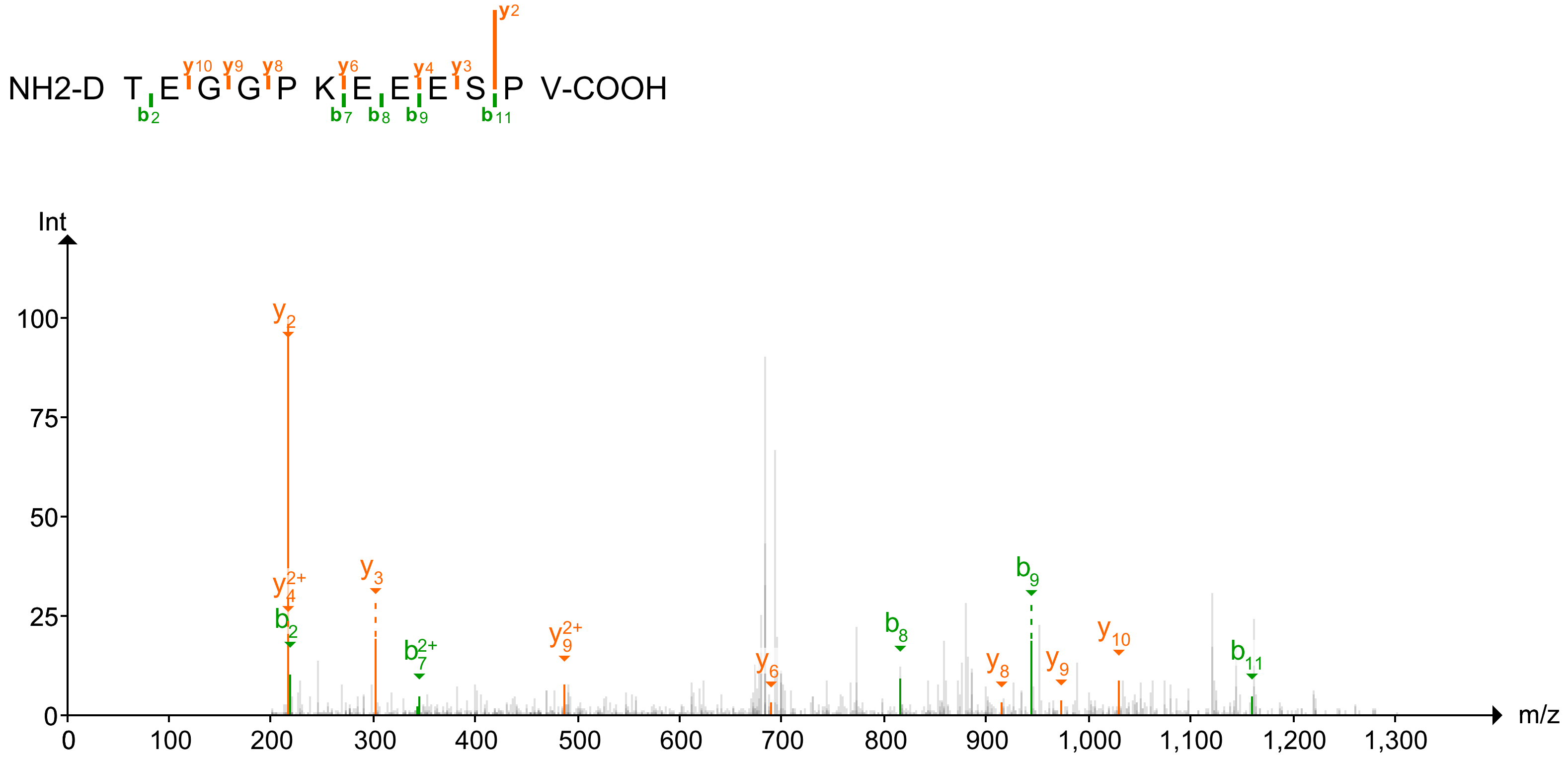
NH2-D T E G G P K E E E S P V-COOH
y10 y9 y8 y6 y4 y3 y2
b2 b7 b8 b9 b11
Int
100
75
50
25
0
m/z
0 100 200 300 400 500 600 700 800 900 1,000 1,100 1,200 1,300
y2
y4 2+
b2
y3
b7 2+
y9 2+
y6
b8
y8
b9
y9
y10
b11

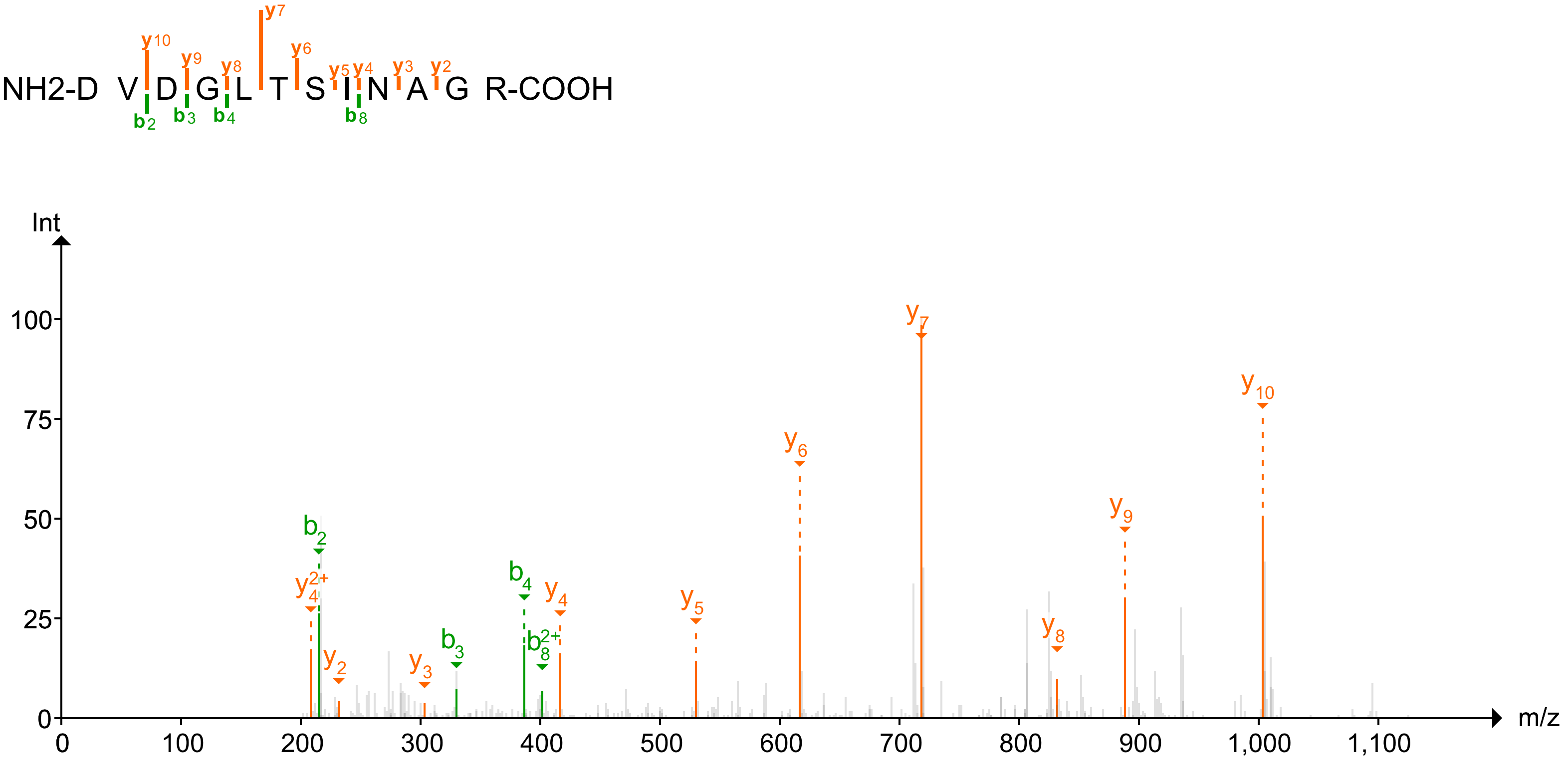

NH2-D V D G L T S I N A G R-COOH
y10 y9 y8 y7 y6 y5 y4 y3 y2
b2 b3 b4 b8
Int
m/z
0
25
50
75
100
0
100
200
300
400
500
600
700
800
900
1,000
1,100

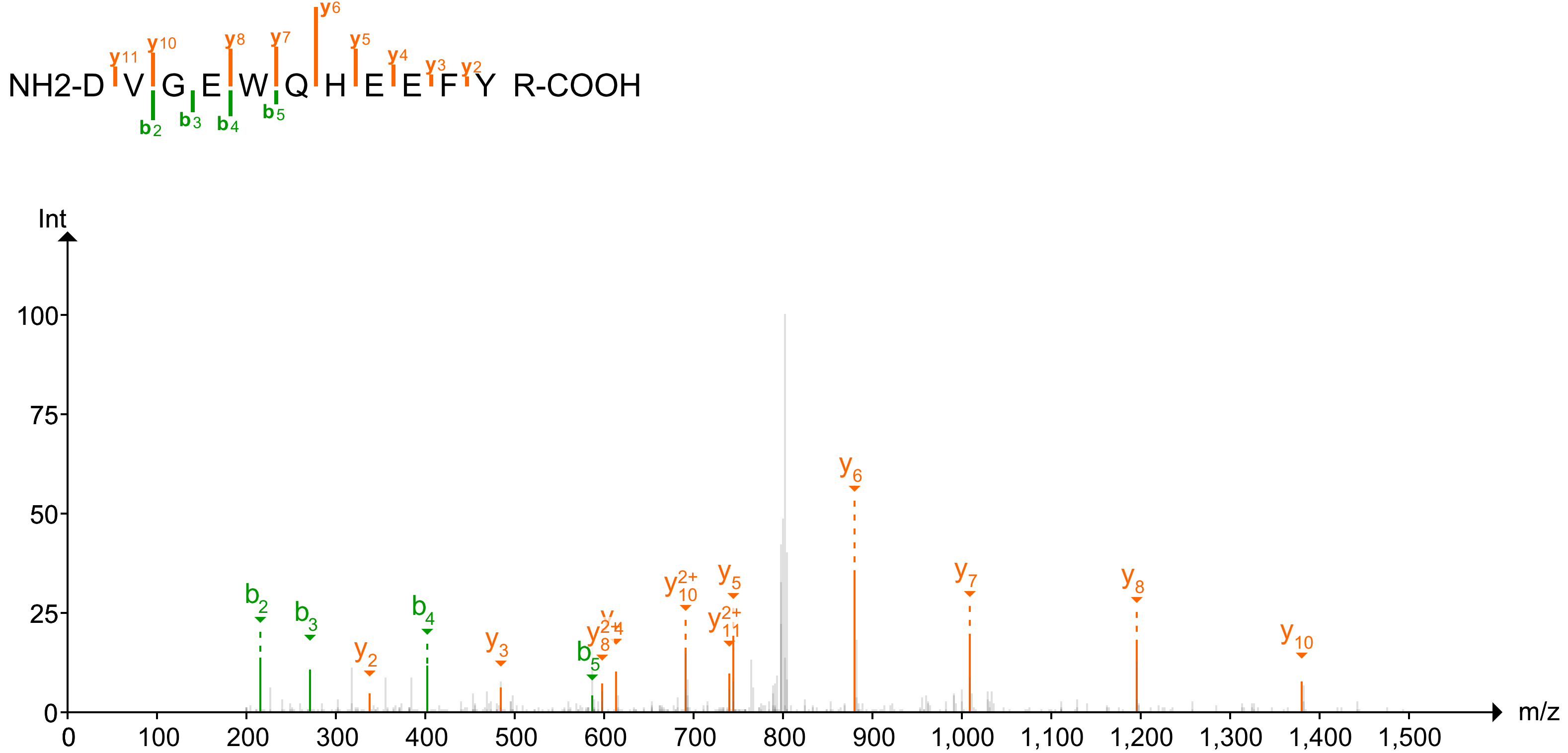
NH2-D V G E W Q H E E F Y R-COOH
y11 y10 y8 y7 y6 y5 y4 y3 y2
b2 b3 b4 b5
Int
100
75
50
25
0
m/z
0 100 200 300 400 500 600 700 800 900 1,000 1,100 1,200 1,300 1,400 1,500
b2 b3 b4 b5
y2 y3 y5 y6 y7 y8 y10
y8 2+
y10 2+
y11 2+

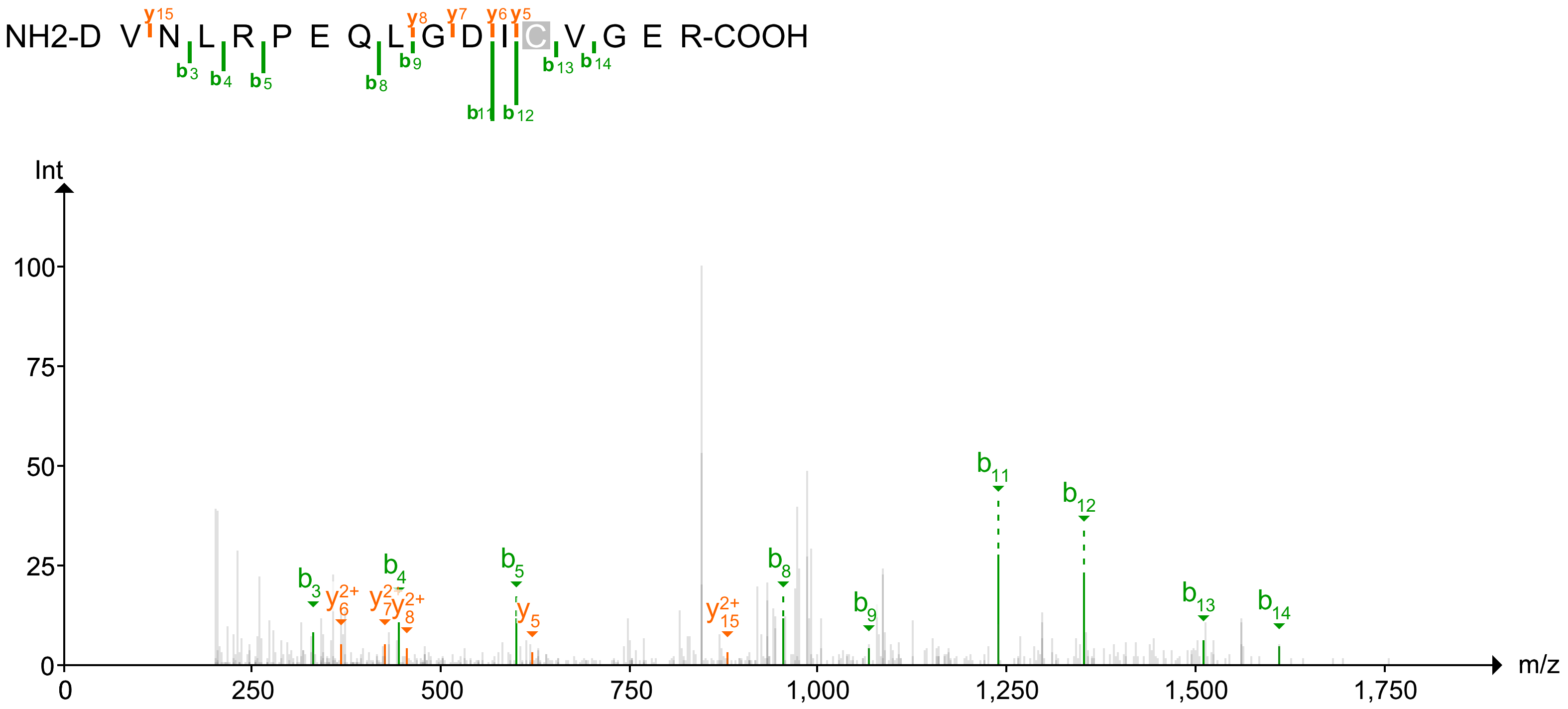

NH2-D V N L R P E Q L G D I C V G E R-COOH
y15 y8 y7 y6 y5
b3 b4 b5 b8 b9 b11 b12 b13 b14
Int
100
75
50
25
0
0 250 500 750 1,000 1,250 1,500 1,750
m/z
b3 y6 2+ y7 2+ b4 y8 2+ b5 y5 y15 2+ b8 b9 b11 b12 b13 b14

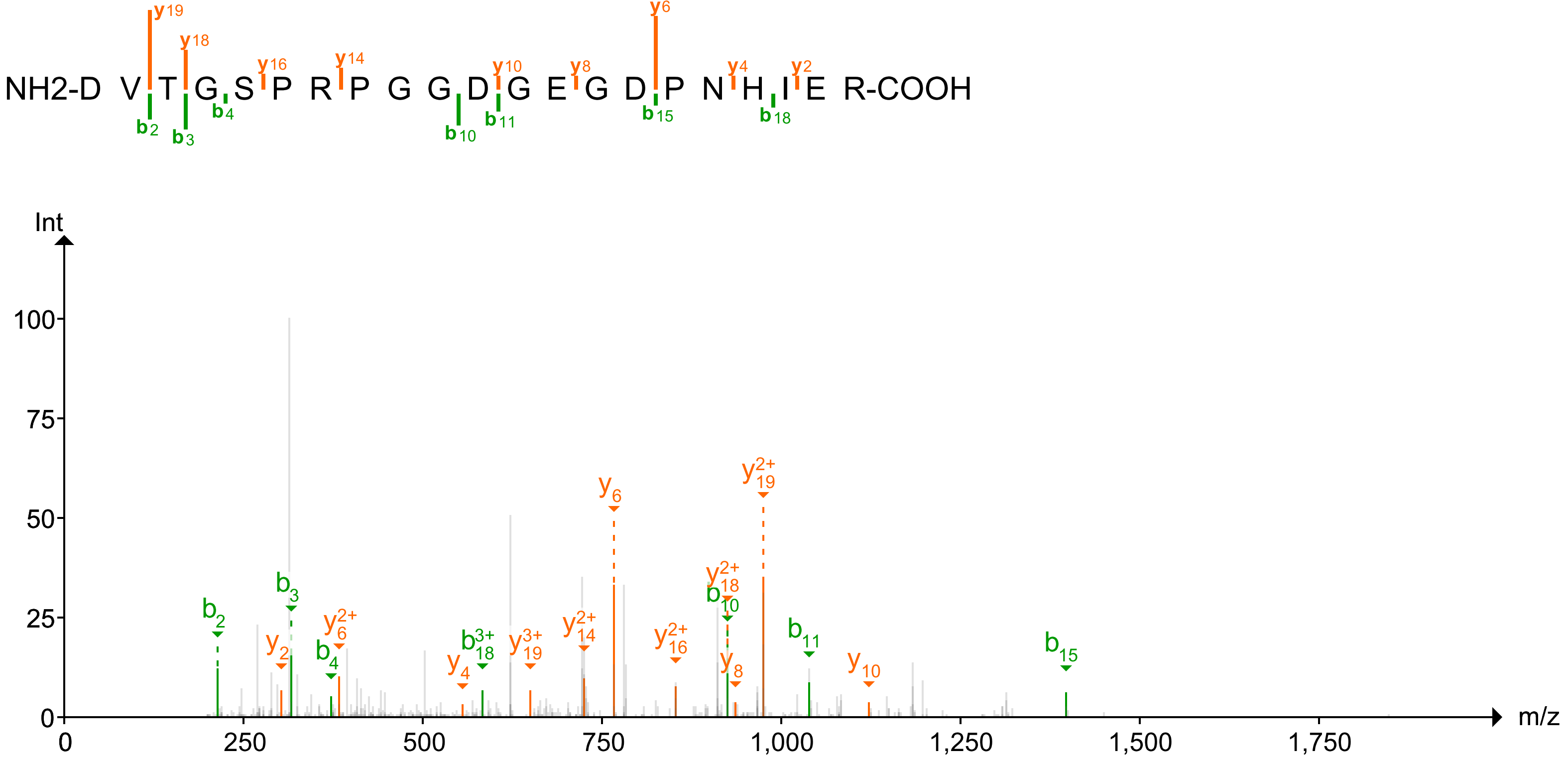

NH2-D V T G S P R P G G D G E G D P N H I E R-COOH
y19 y18 y16 y14 y10 y8 y6 y4 y2
b2 b3 b4 b10 b11 b15 b18
Int
100
75
50
25
0
m/z
0
250
500
750
1,000
1,250
1,500
1,750
b2
b3
y2
b4
y6 2+
y4
b18 3+
y19 3+
y14 2+
y6
y16 2+
y18 2+
b10
y8
y19 2+
b11
y10
b15

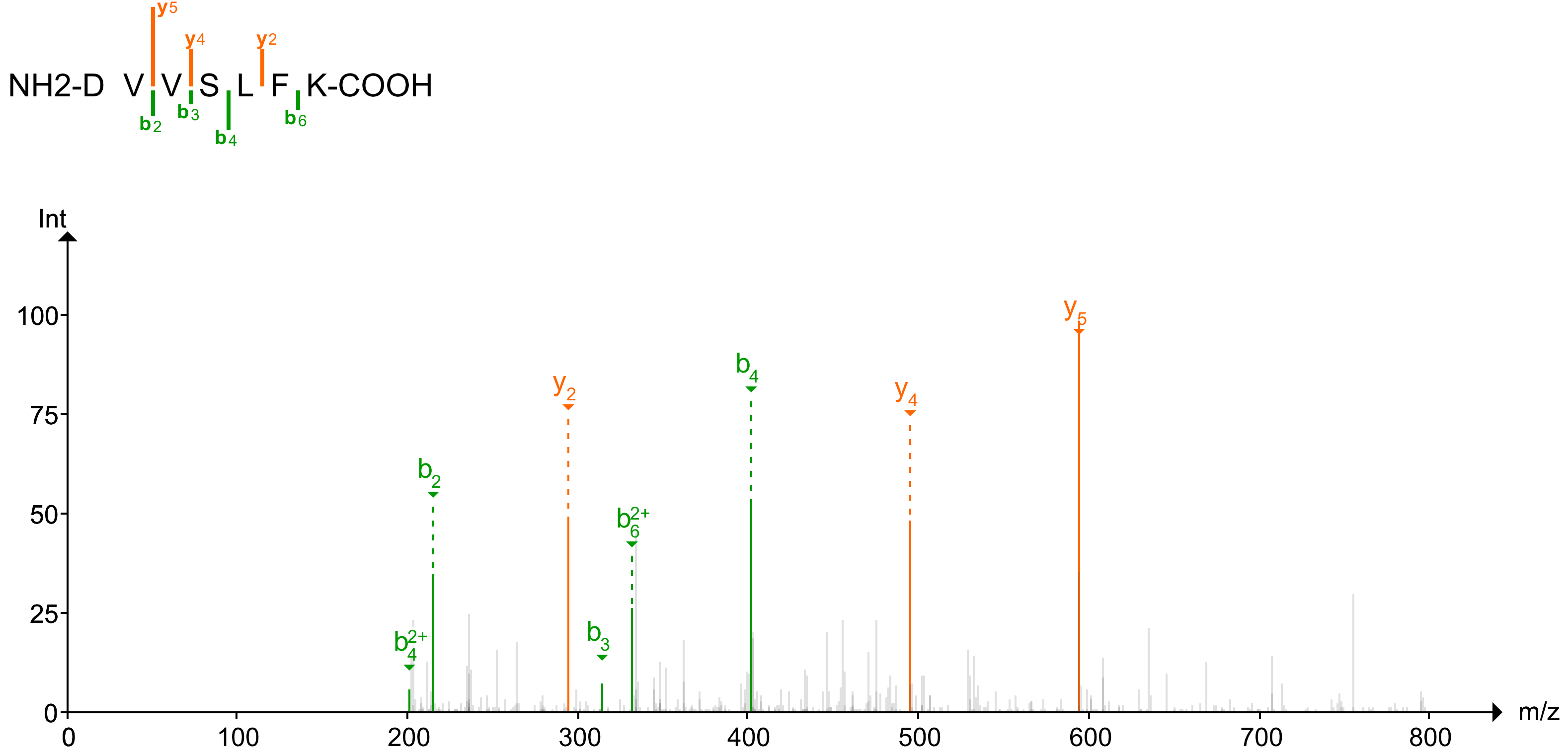

NH2-D V V S L F K-COOH
y5
y4
y2
b2
b3
b4
b6
Int
m/z
0
25
50
75
100
0
100
200
300
400
500
600
700
800
$b_4^{2+}$
$b_2$
$y_2$
$b_3$
$b_6^{2+}$
$b_4$
$y_4$
$y_5$

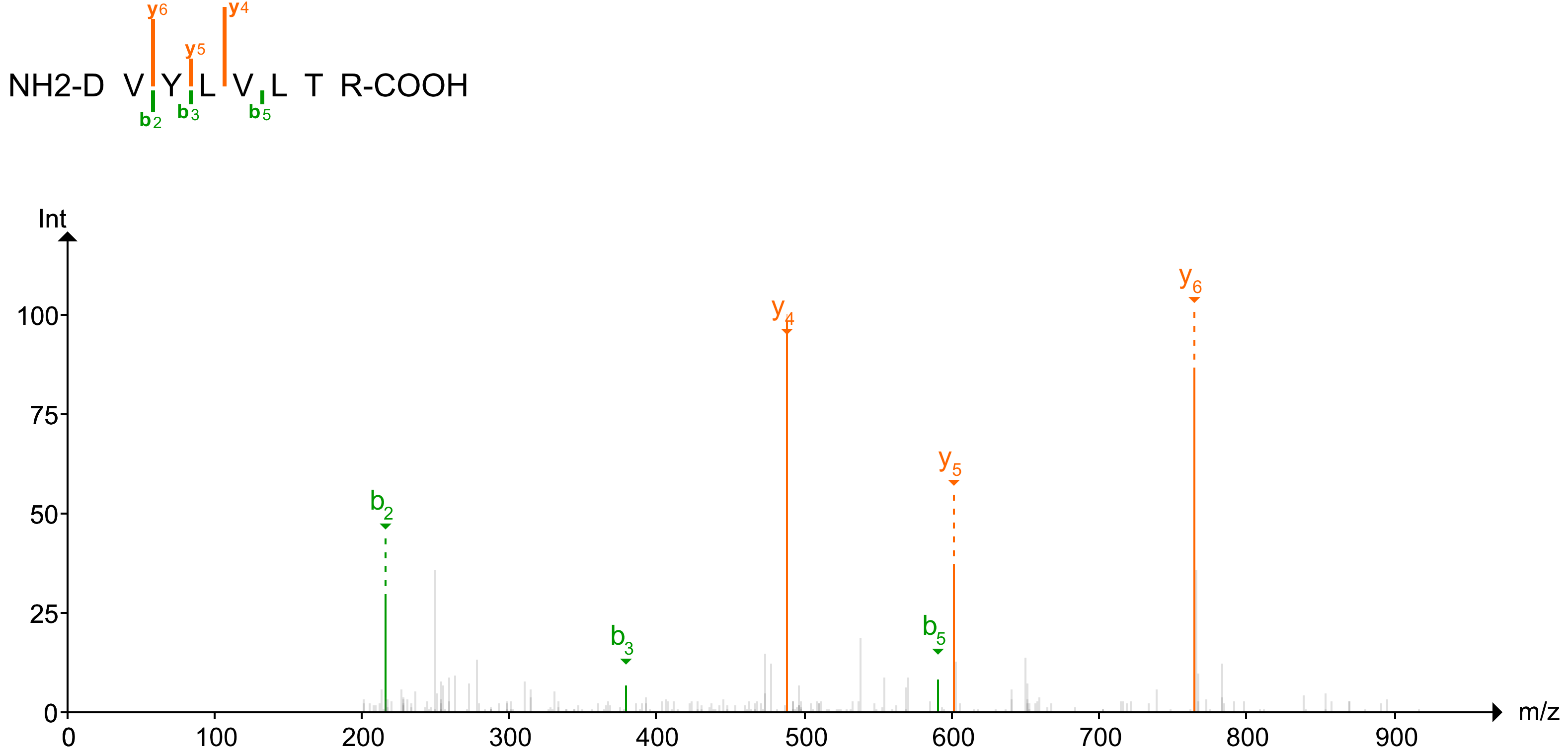

NH2-D V Y L V L T R-COOH
y6
y5
y4
b2
b3
b5
Int
100
75
50
25
0
m/z
0
100
200
300
400
500
600
700
800
900
b2
b3
y4
b5
y5
y6

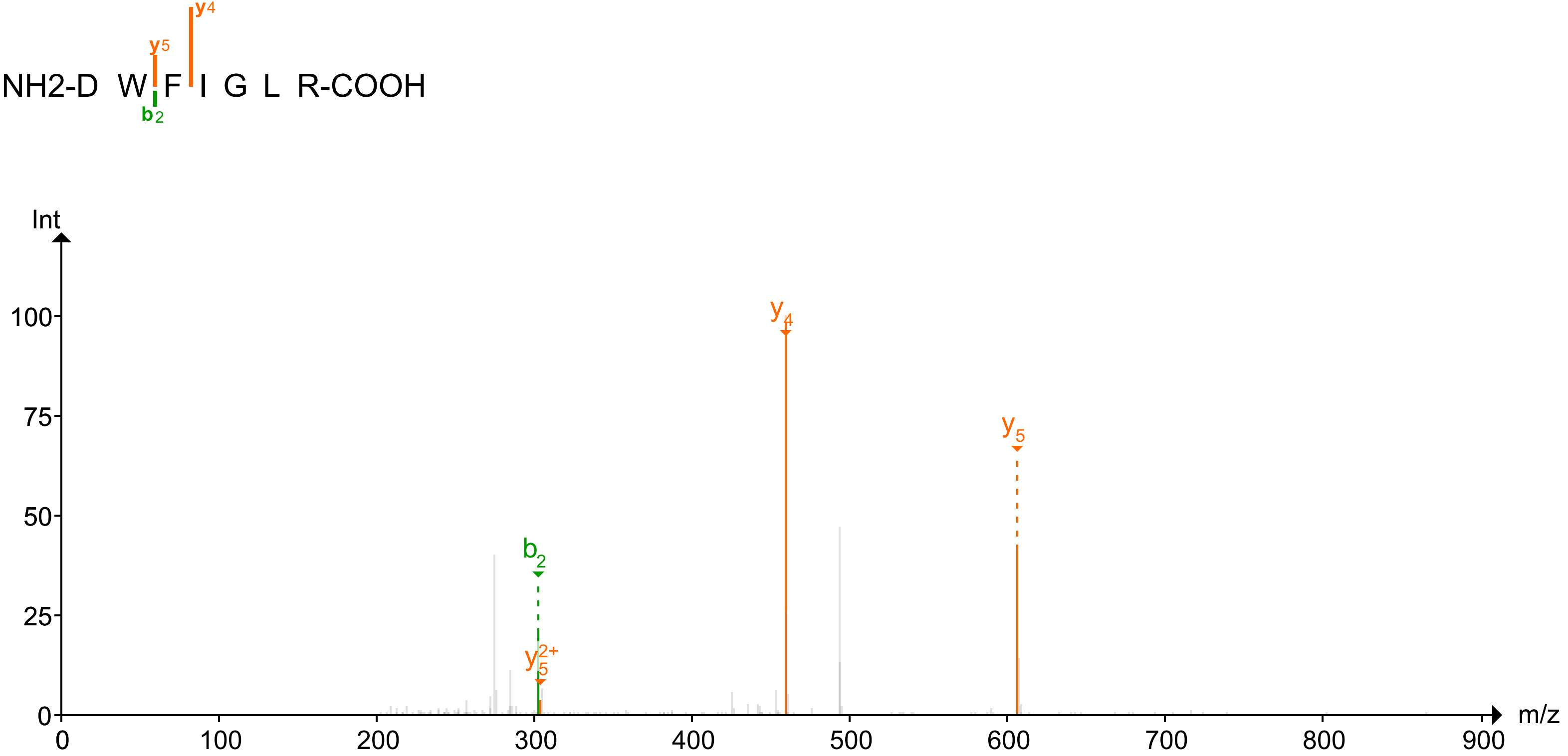

y4
y5
NH2-D W F I G L R-COOH
b2
Int
100
75
50
25
0
b2
y5 2+
y4
y5
0
100
200
300
400
500
600
700
800
900
m/z

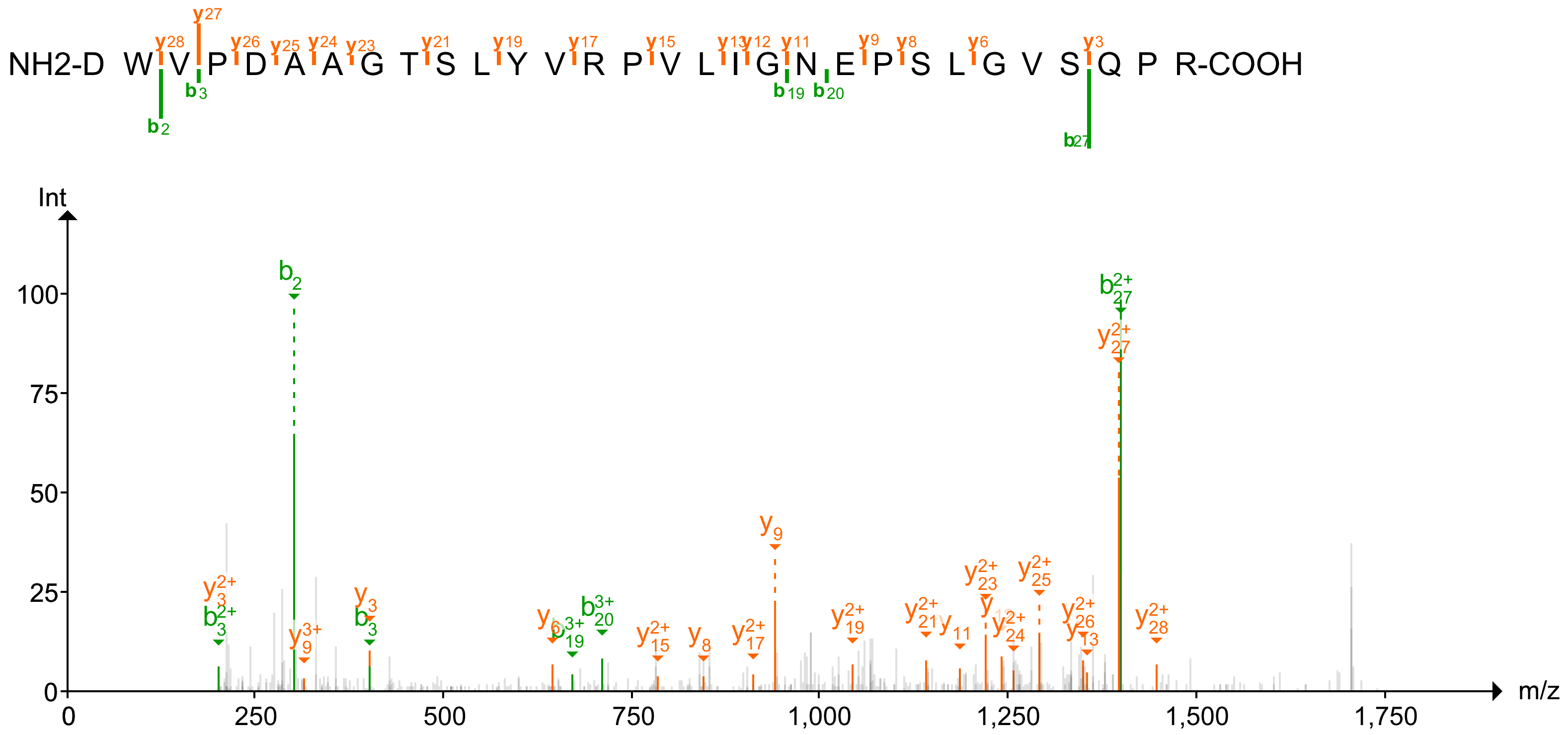
NH2-D W V P D A A G T S L Y V R P V L I G N E P S L G V S Q P R-COOH
y28 y27 y26 y25 y24 y23 y21 y19 y17 y15 y13 y12 y11 y9 y8 y6 y3
b2 b3 b19 b20 b27
Int
100
75
50
25
0
0
250
500
750
1,000
1,250
1,500
1,750
m/z
b2
b27 2+
y27 2+
y9
y3 2+
b3 2+
y9 3+
y3
b3
b19 3+
b20 3+
y15 2+
y8
y17 2+
y19 2+
y21 2+
y11
y23 2+
y24 2+
y25 2+
y26 2+
y13
y28 2+

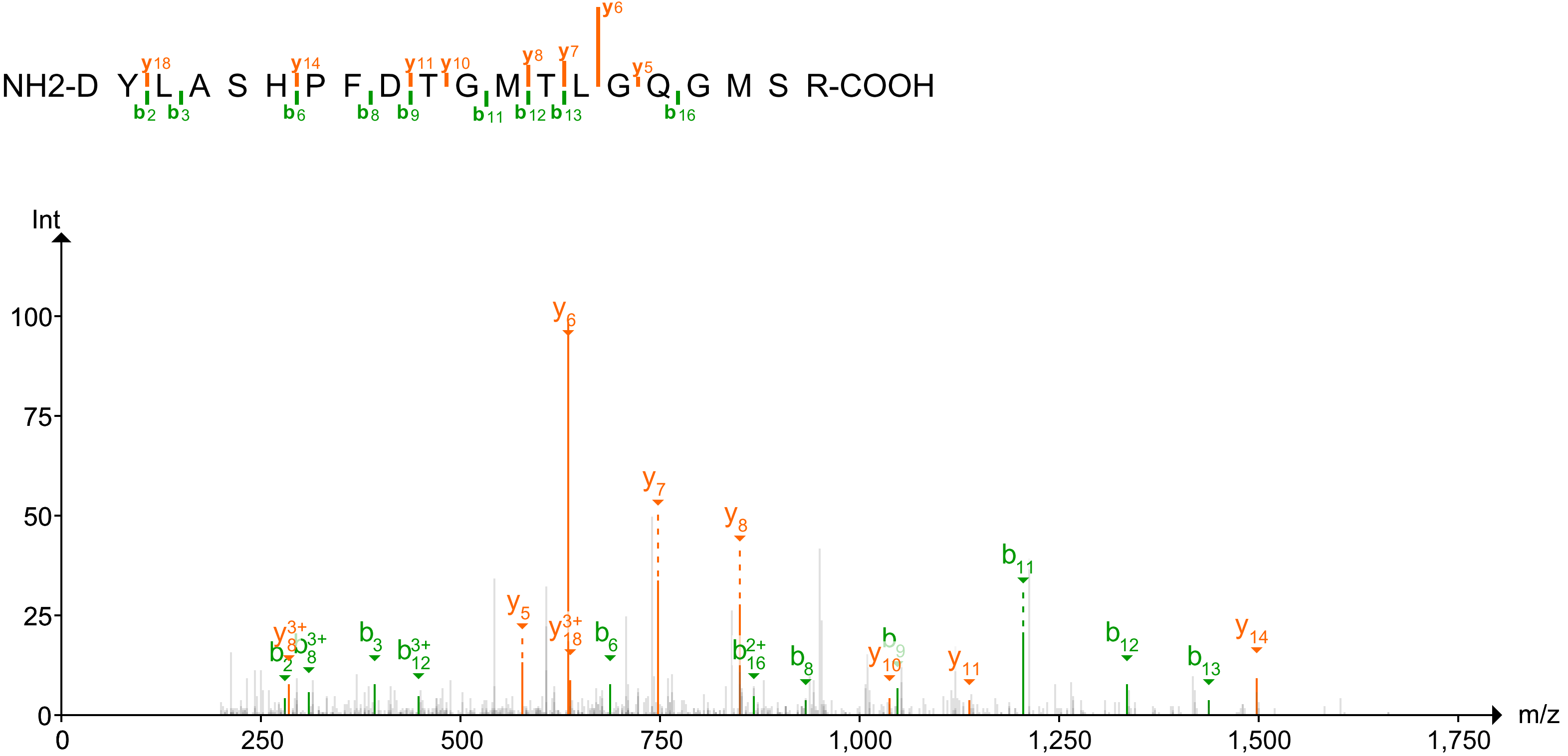

NH2-D Y L A S H P F D T G M T L G Q G M S R-COOH
y18 y14 y11 y10 y8 y7 y6 y5
b2 b3 b6 b8 b9 b11 b12 b13 b16
Int
100
75
50
25
0
m/z
0
250
500
750
1,000
1,250
1,500
1,750
$y_6$
$y_7$
$y_8$
$b_{11}$
$y_5$
$y_{18}^{3+}$
$b_6$
$b_3$
$b_{12}^{3+}$
$y_8^{3+}$
$b_2$
$b_8^{3+}$
$b_{16}^{2+}$
$b_8$
$y_{10}$
$b_9$
$y_{11}$
$b_{12}$
$b_{13}$
$y_{14}$

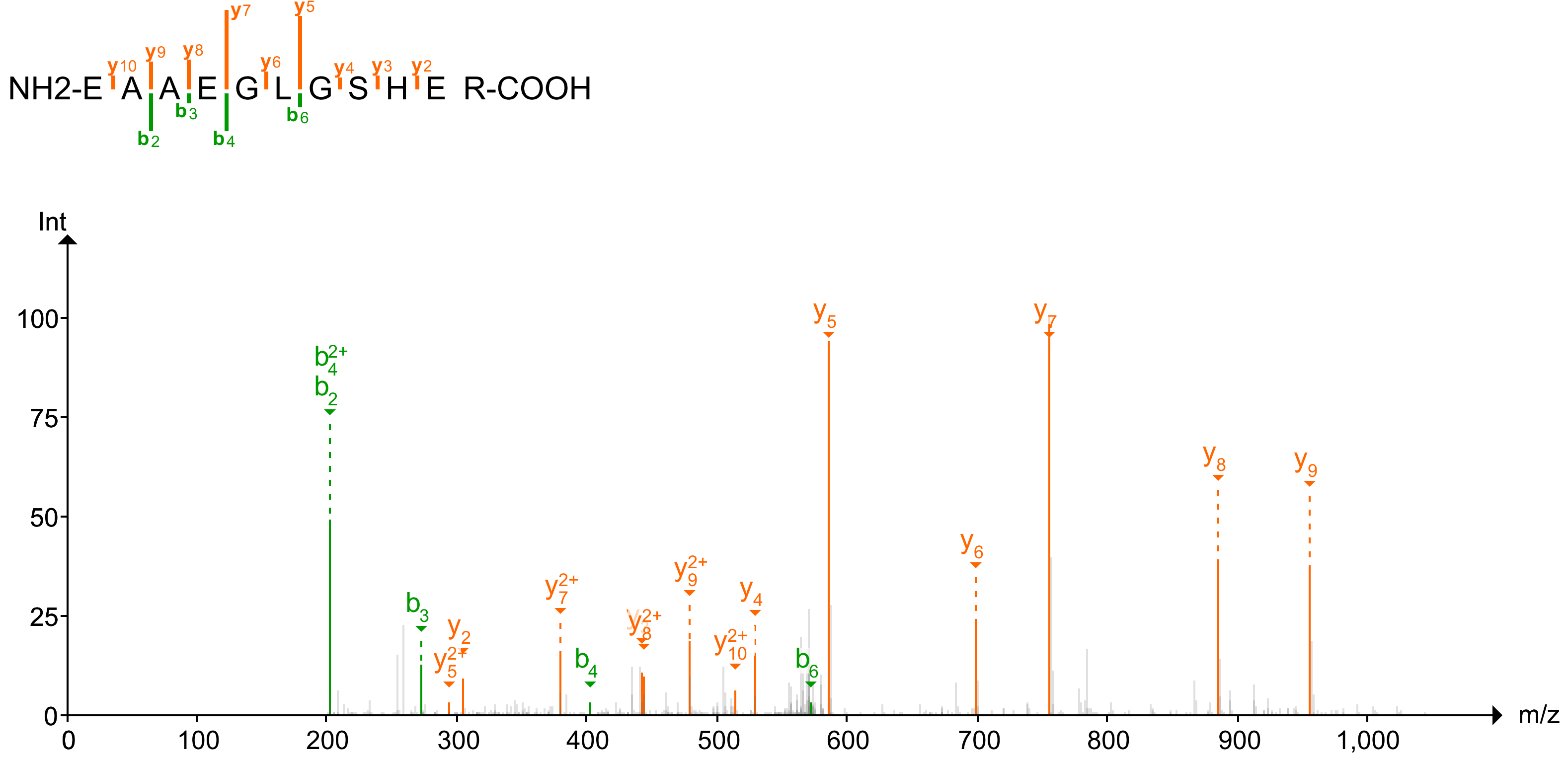

NH2-E A A E G L G S H E R-COOH
y10 y9 y8 y7 y6 y5 y4 y3 y2
b2 b3 b4 b6
Int
100
75
50
25
0
m/z
0 100 200 300 400 500 600 700 800 900 1,000
b4 2+
b2
b3
y2
y5 2+
y7 2+
b4
y8 2+
y9 2+
y10 2+
y4
b6
y5
y6
y7
y8
y9

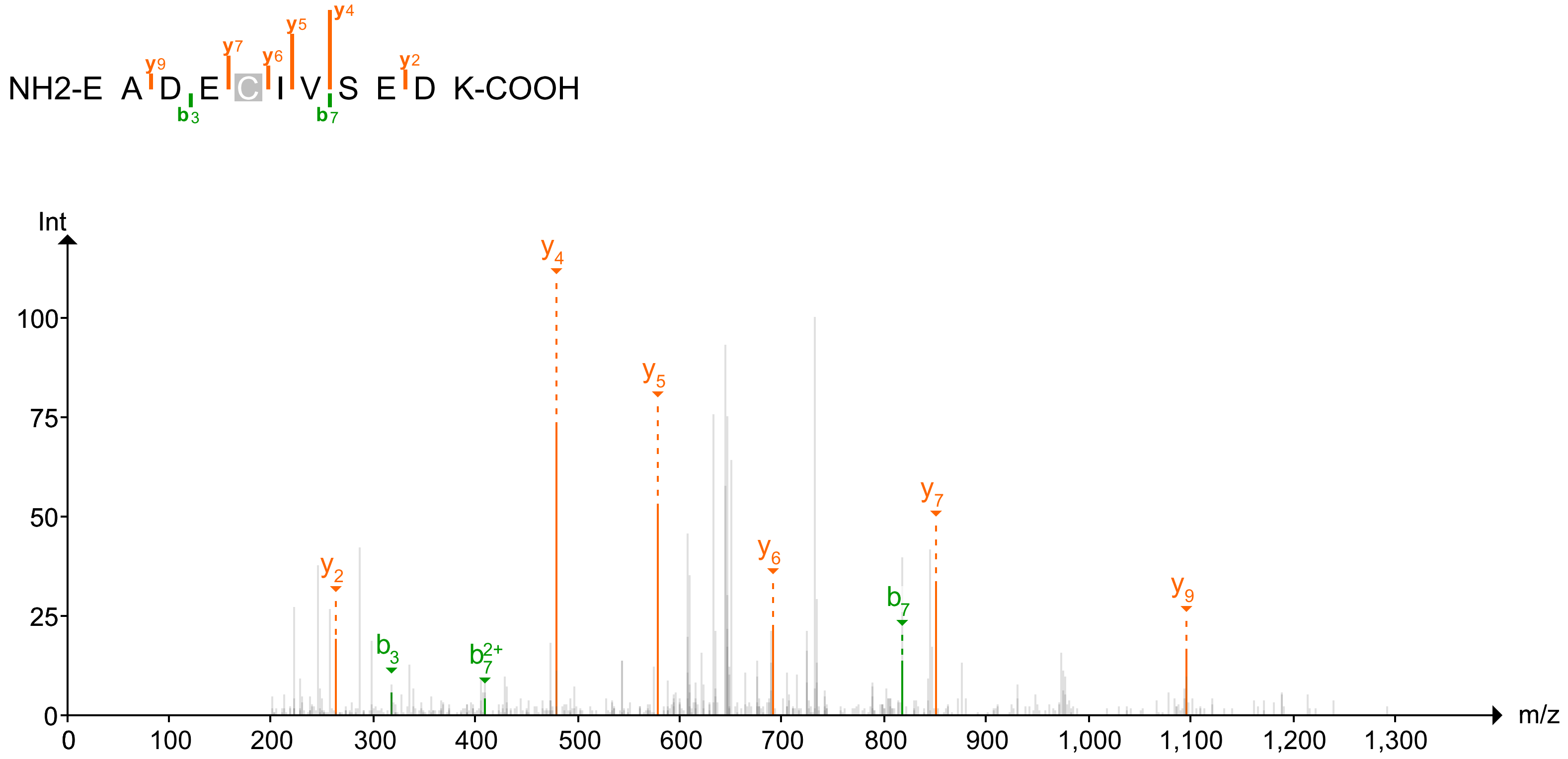

NH2-E A D E C I V S E D K-COOH
y9 y7 y6 y5 y4 y2
b3 b7
Int
100
75
50
25
0
y4 y5 y2 y6 y7 y9
b3 b7 b7 2+
0 100 200 300 400 500 600 700 800 900 1,000 1,100 1,200 1,300
m/z

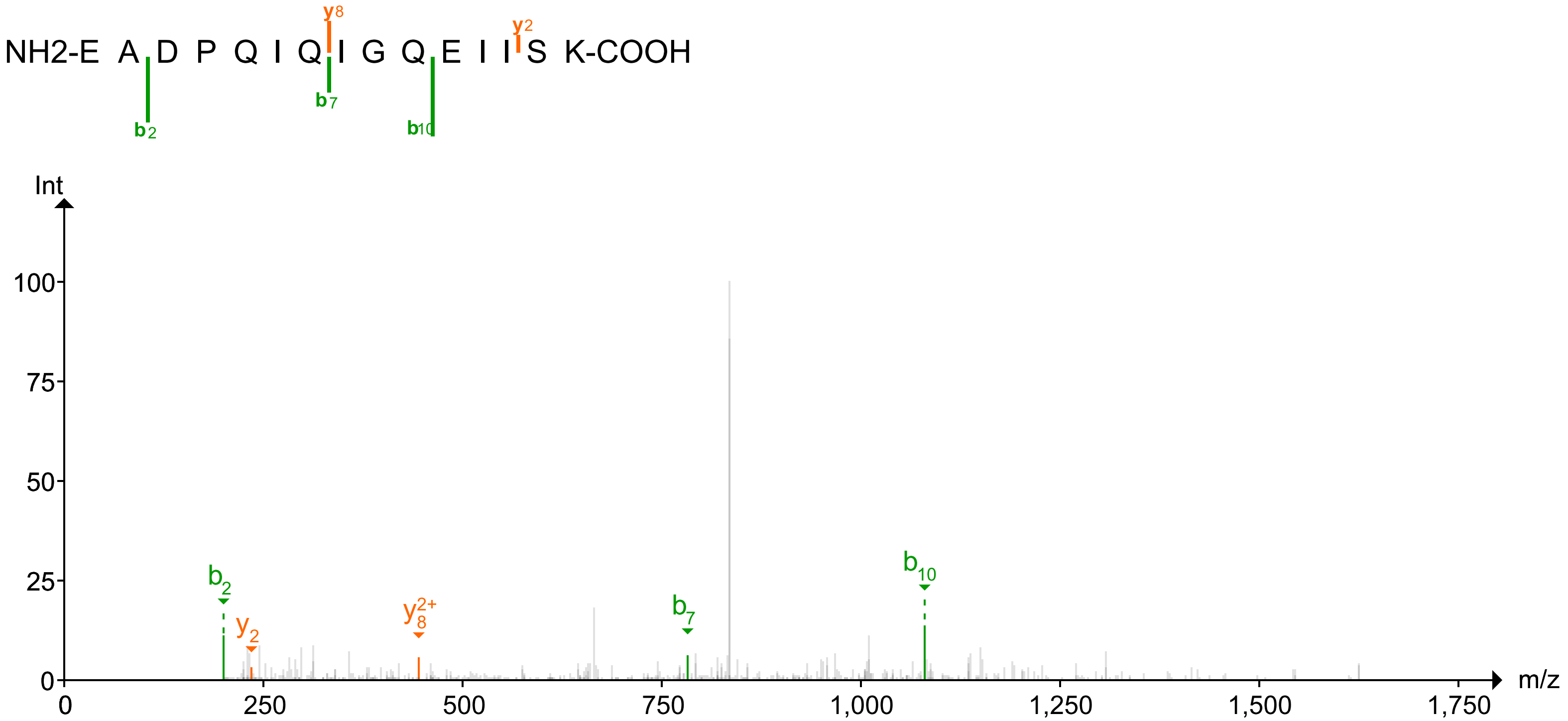

NH2-E A D P Q I Q I G Q E I I S K-COOH
y8
y2
b2
b7
b10
Int
100
75
50
25
0
b2
y2
y8 2+
b7
b10
0
250
500
750
1,000
1,250
1,500
1,750
m/z

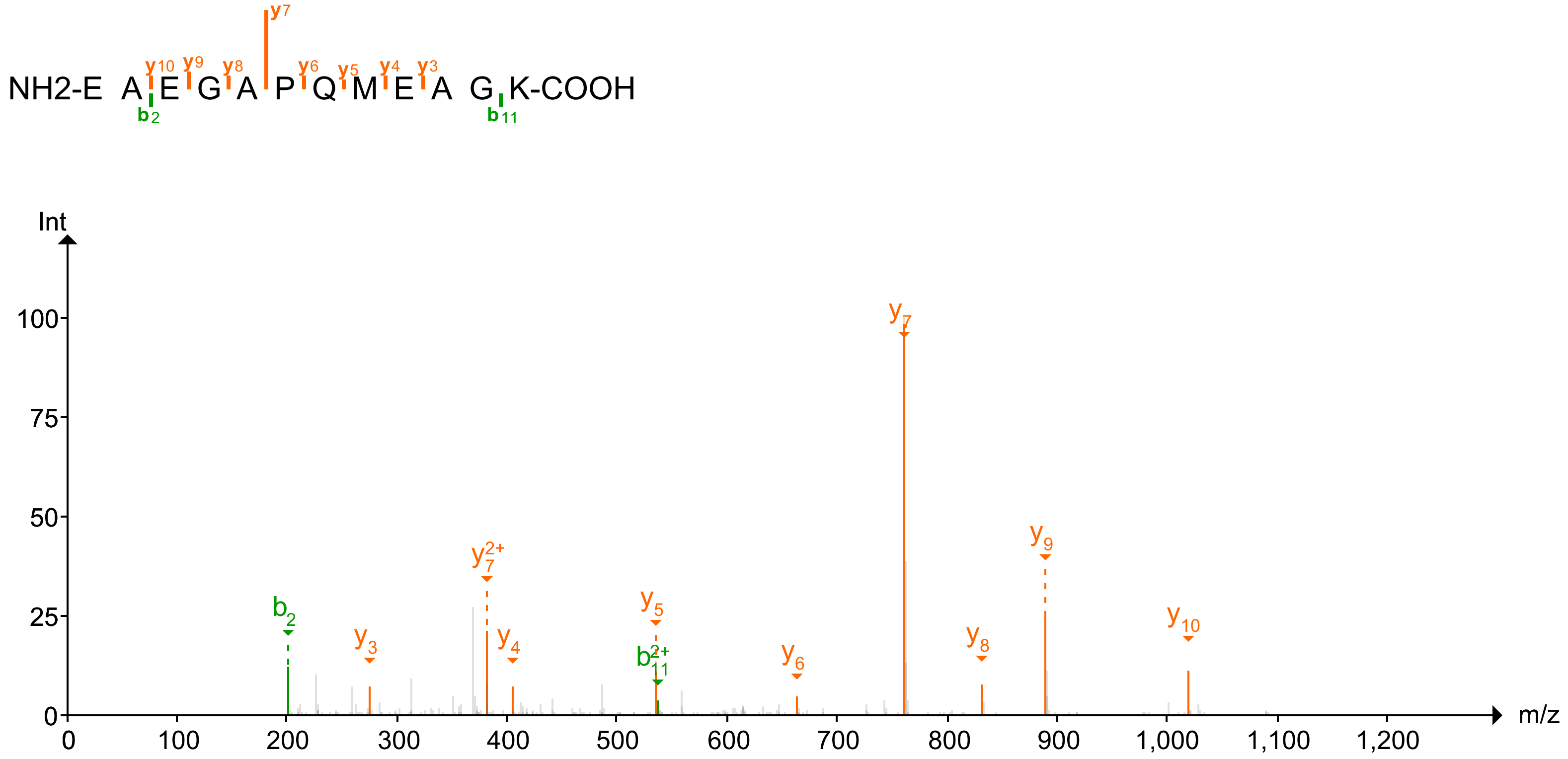

NH2-E A E G A P Q M E A G K-COOH
y10 y9 y8 y7 y6 y5 y4 y3
b2 b11
Int
100
75
50
25
0
m/z
0
100
200
300
400
500
600
700
800
900
1,000
1,100
1,200
y7
y9
y7 2+
y5
b2
y3
y4
b11 2+
y6
y8
y10

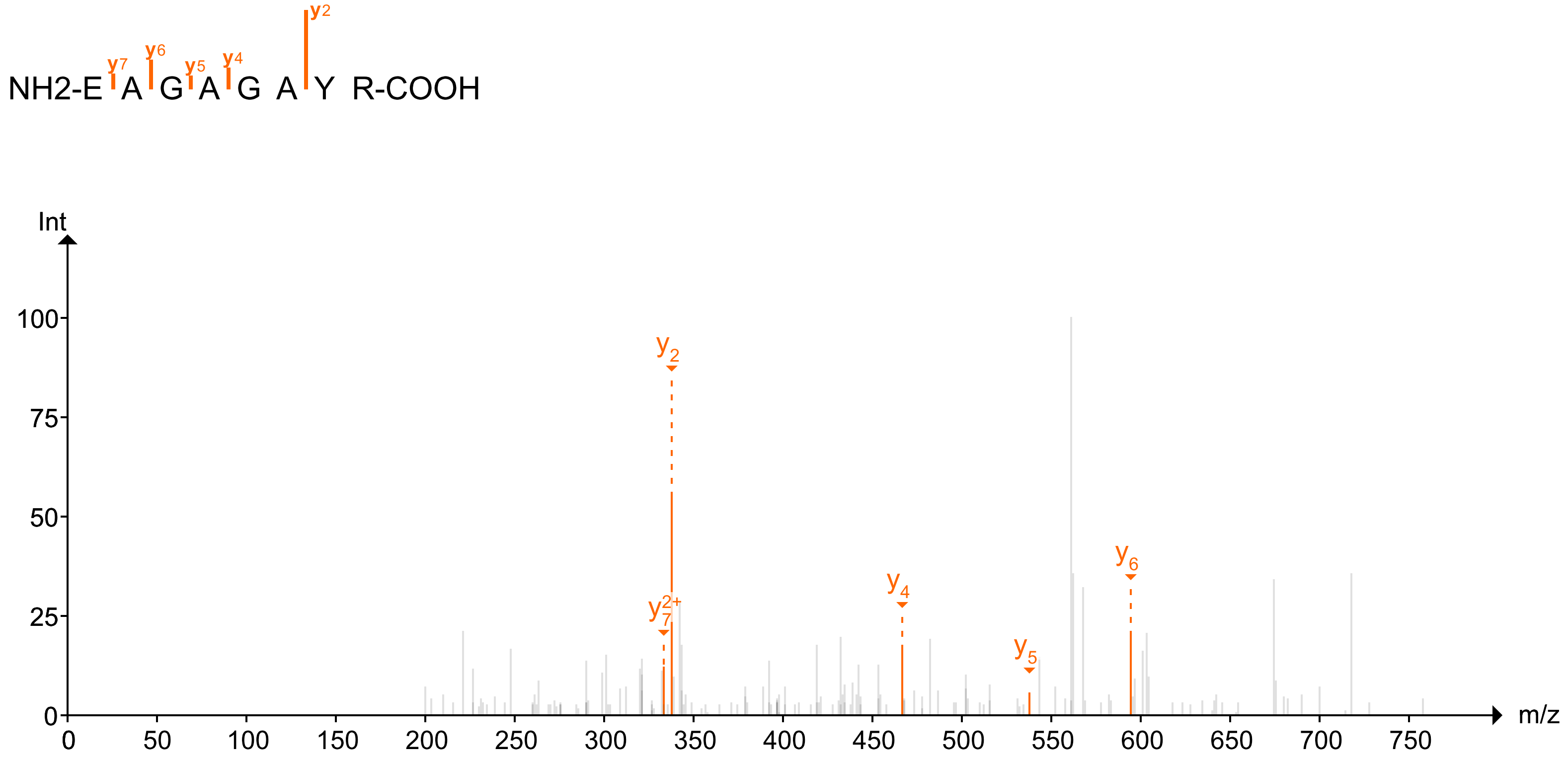

NH2-E A G A G A Y R-COOH
y7
y6
y5
y4
y2
Int
100
75
50
25
0
y2
y4
y5
y6
y7 2+
0
50
100
150
200
250
300
350
400
450
500
550
600
650
700
750
m/z

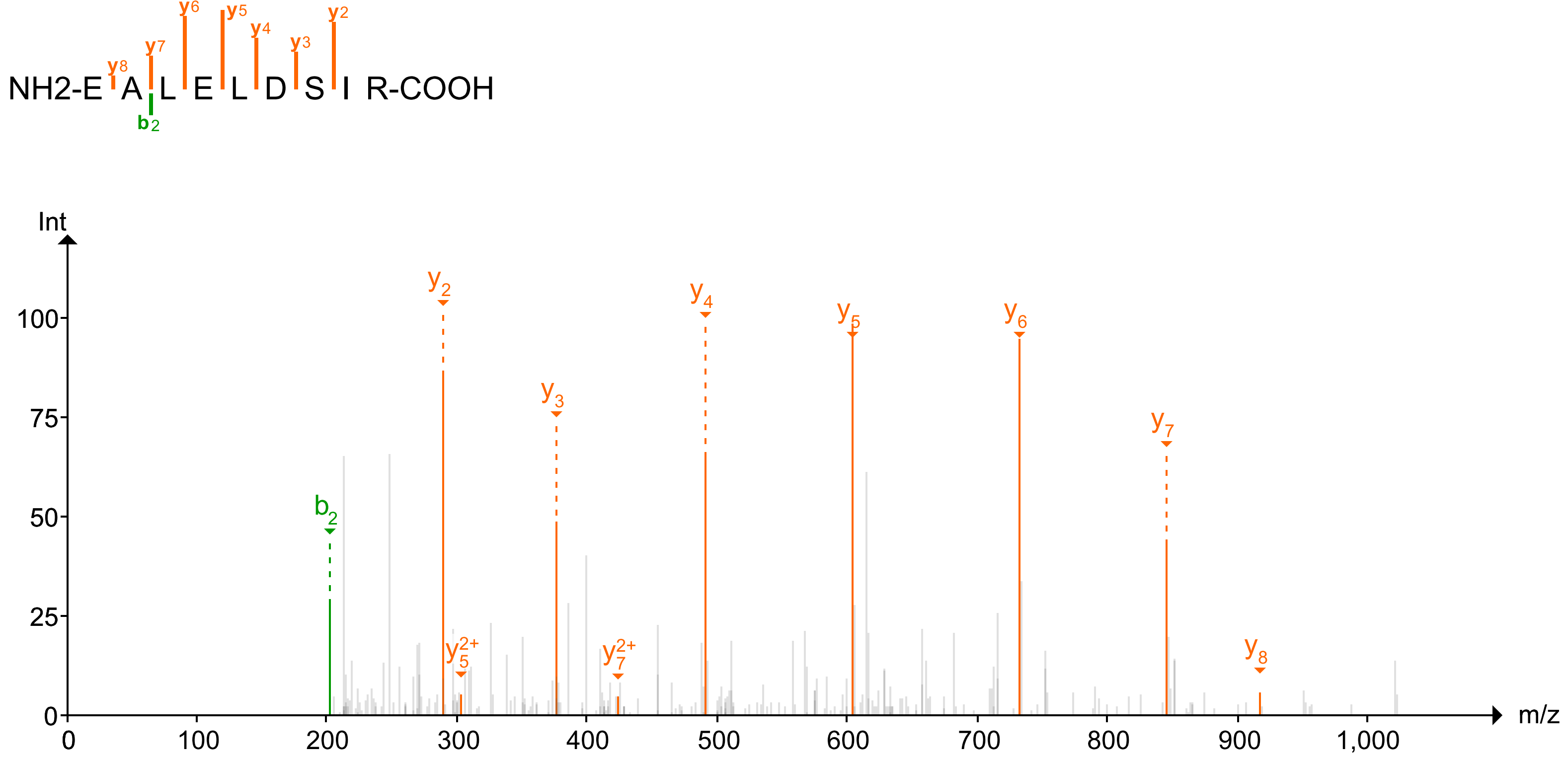
NH2-E A L E L D S I R-COOH
y8 y7 y6 y5 y4 y3 y2
b2
Int
100
75
50
25
0
m/z
0
100
200
300
400
500
600
700
800
900
1,000
y2
y3
y4
y5
y6
y7
y8
b2
y5 2+
y7 2+

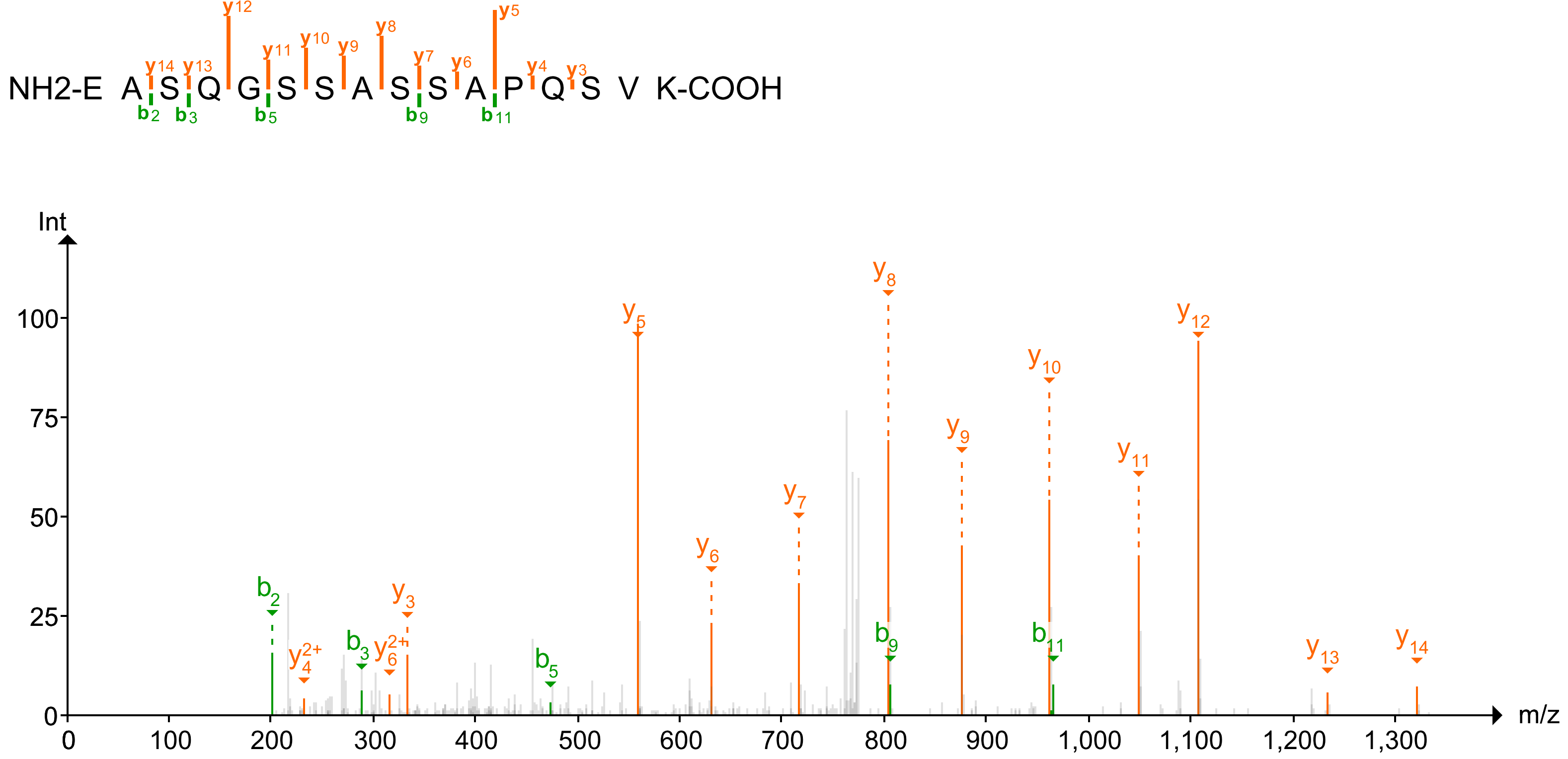

NH2-E A S Q G S S A S S A P Q S V K-COOH
y12 y14 y13 y11 y10 y9 y8 y7 y6 y5 y4 y3
b2 b3 b5 b9 b11
Int
100
75
50
25
0
m/z
0 100 200 300 400 500 600 700 800 900 1,000 1,100 1,200 1,300

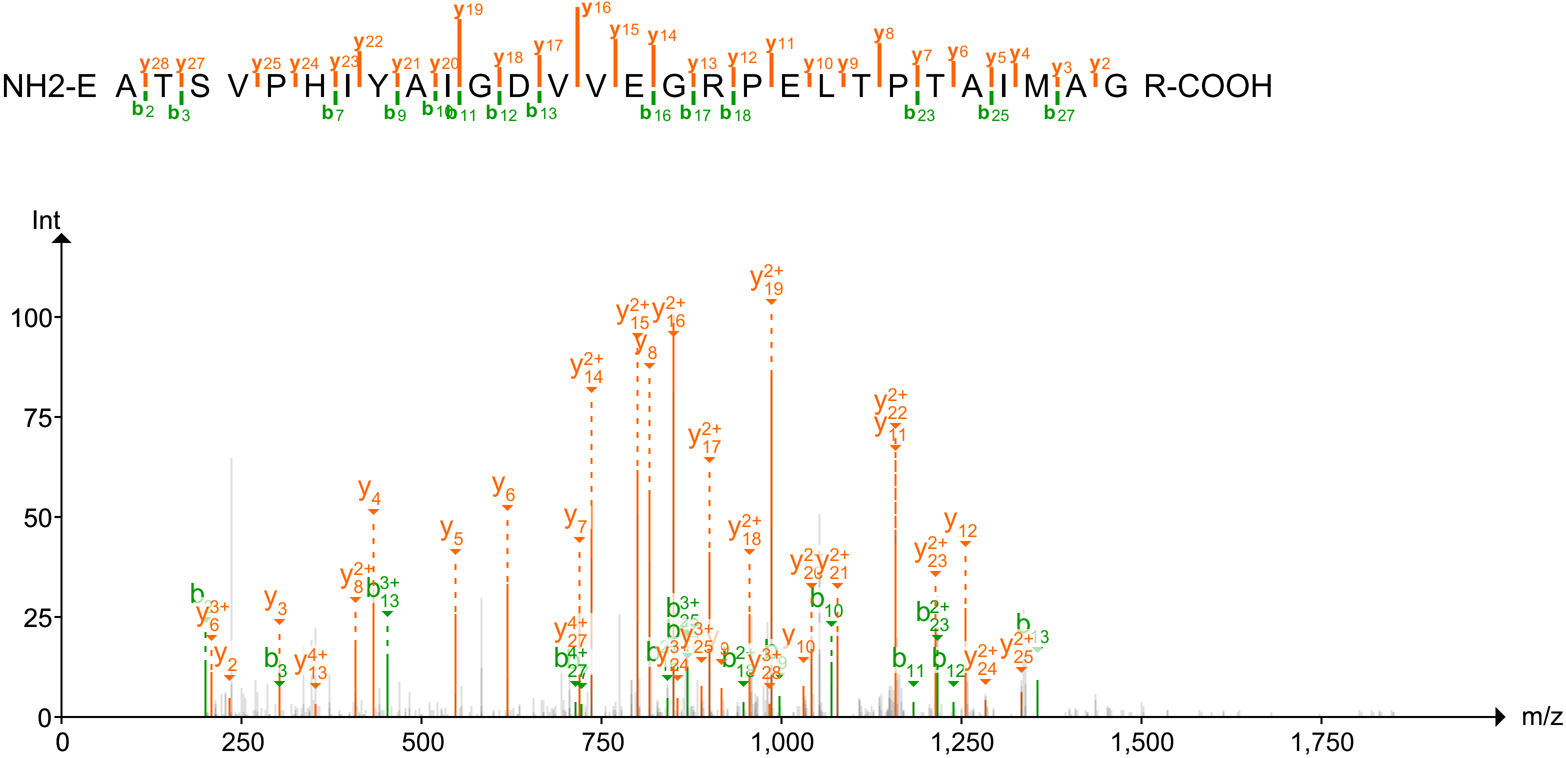

NH2-E A T S V P H I Y A I G D V V E G R P E L T P T A I M A G R-COOH
Int
m/z
0
25
50
75
100
250
500
750
1,000
1,250
1,500
1,750

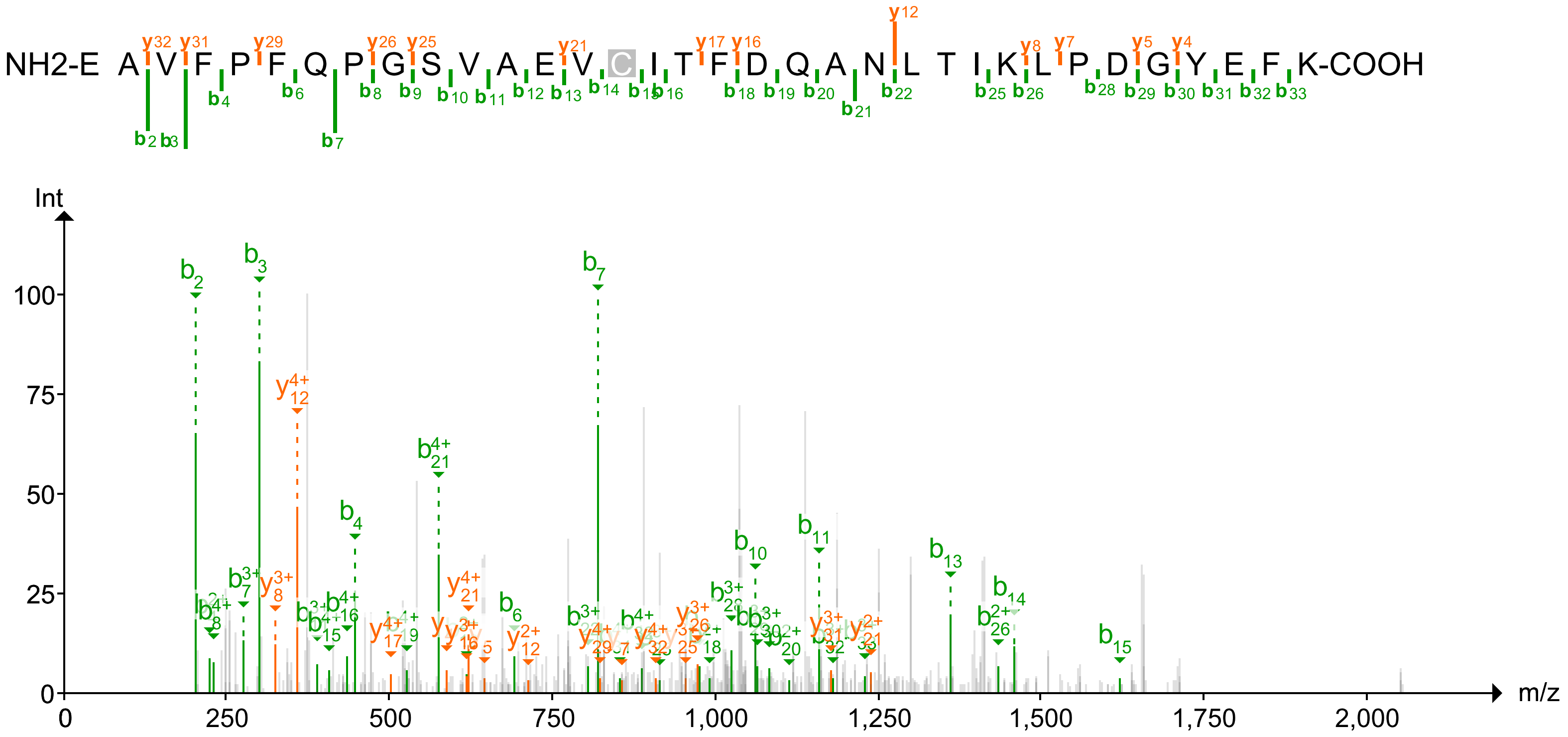

NH2-E A V F P F Q P G S V A E V C I T F D Q A N L T I K L P D G Y E F K-COOH
Int
m/z
0
25
50
75
100
250
500
750
1,000
1,250
1,500
1,750
2,000

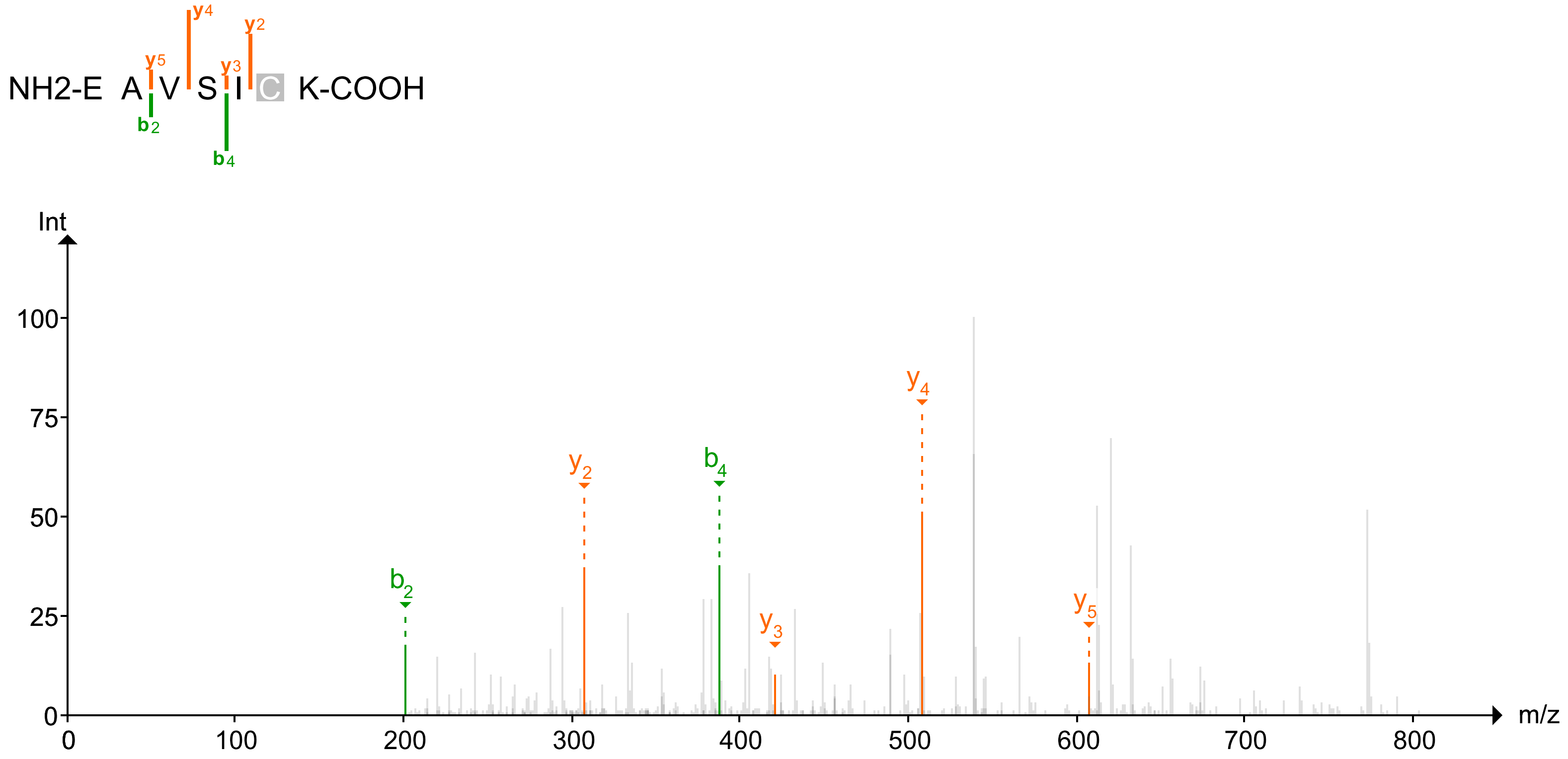

NH2-E A V S I C K-COOH
y4
y2
y5
y3
b2
b4
Int
100
75
50
25
0
m/z
0
100
200
300
400
500
600
700
800

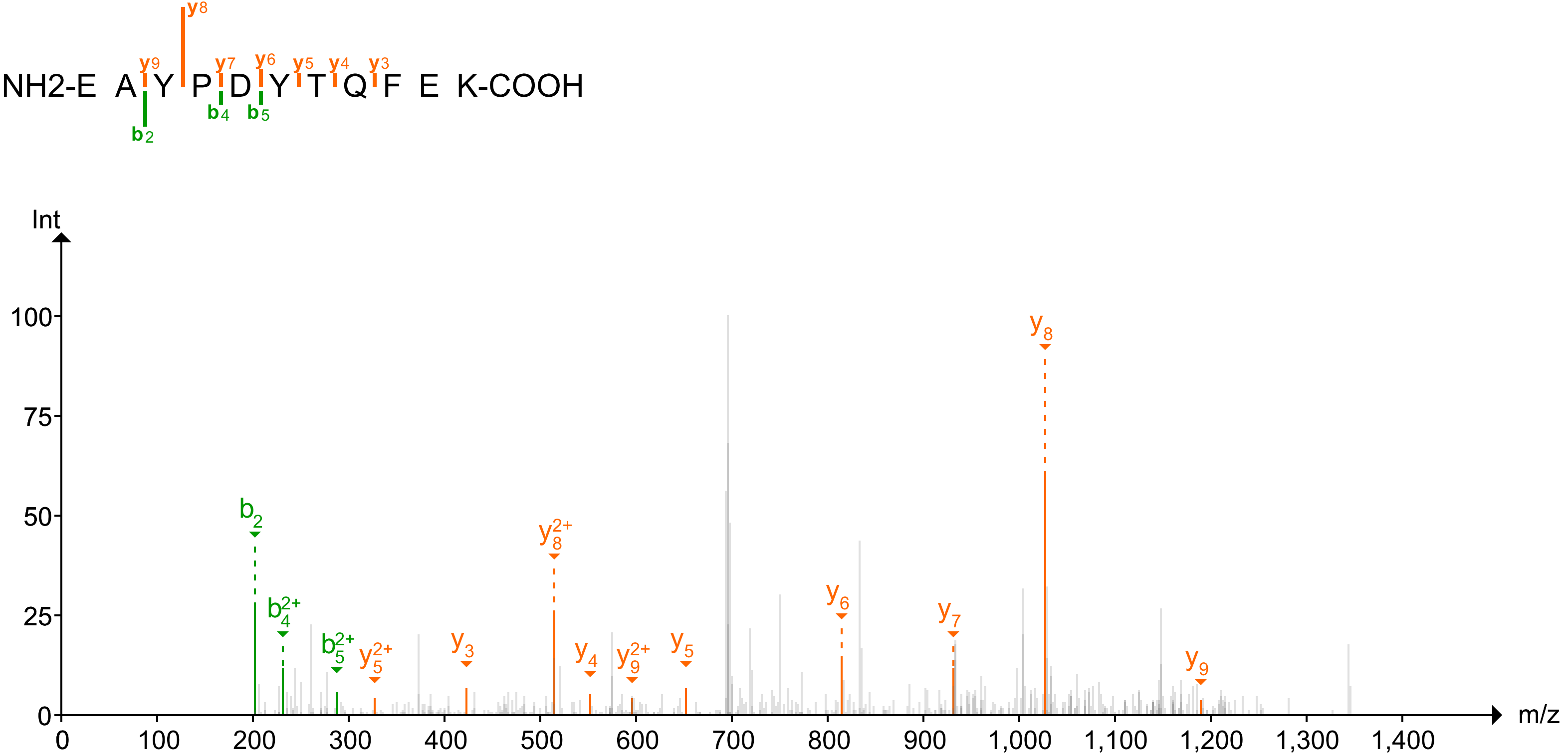
NH2-E A Y P D Y T Q F E K-COOH
y9 y8 y7 y6 y5 y4 y3
b2 b4 b5
Int
100
75
50
25
0
m/z
0 100 200 300 400 500 600 700 800 900 1,000 1,100 1,200 1,300 1,400
b2
b4 2+
b5 2+
y5 2+
y3
y8 2+
y4
y9 2+
y5
y6
y7
y8
y9

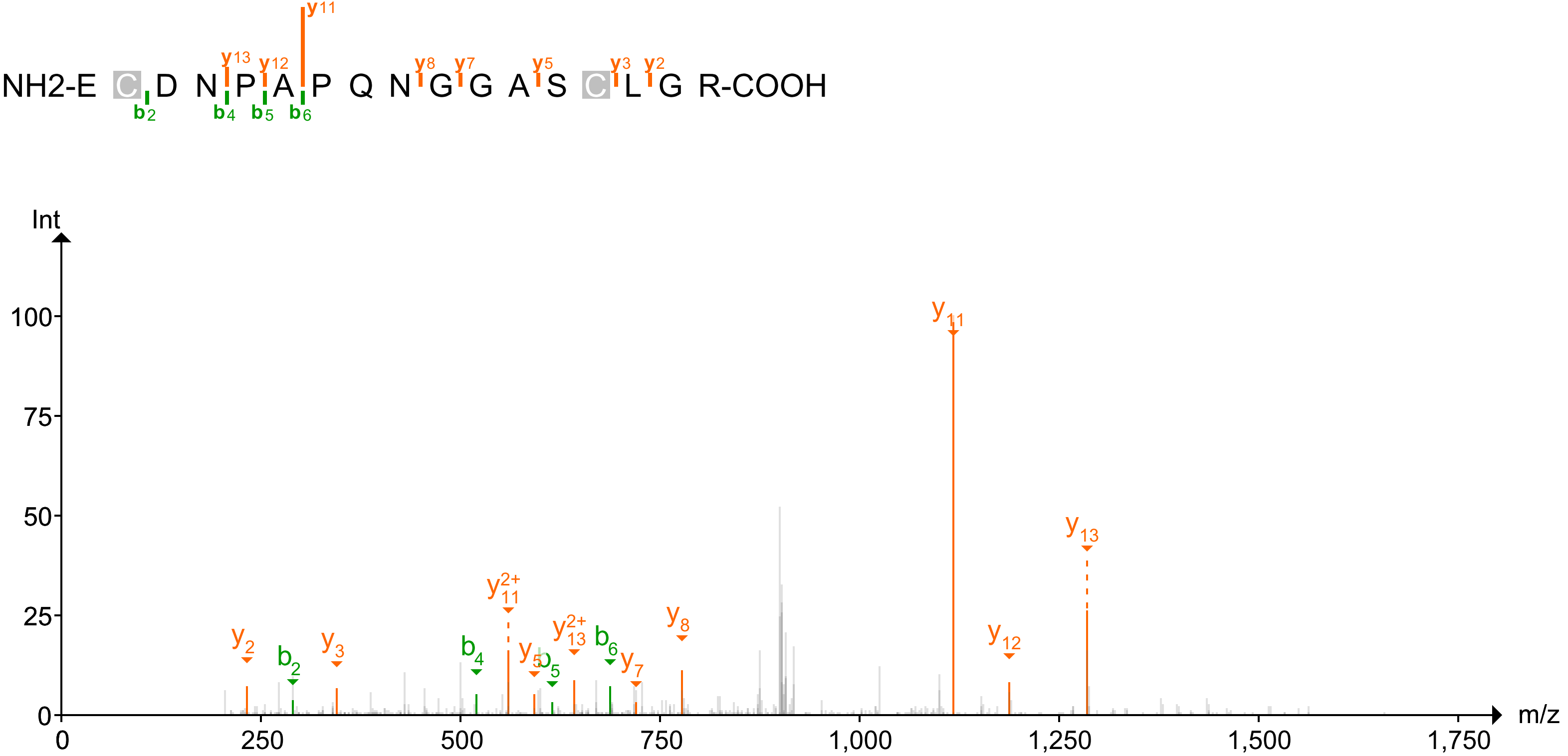

NH2-E C D N P A P Q N G G A S C L G R-COOH
y11
y13
y12
y8
y7
y5
y3
y2
b2
b4
b5
b6
Int
100
75
50
25
0
m/z
0
250
500
750
1,000
1,250
1,500
1,750
y11
y13
y12
y8
y7
y5
y3
y2
b2
b4
b5
b6
y11 2+
y13 2+

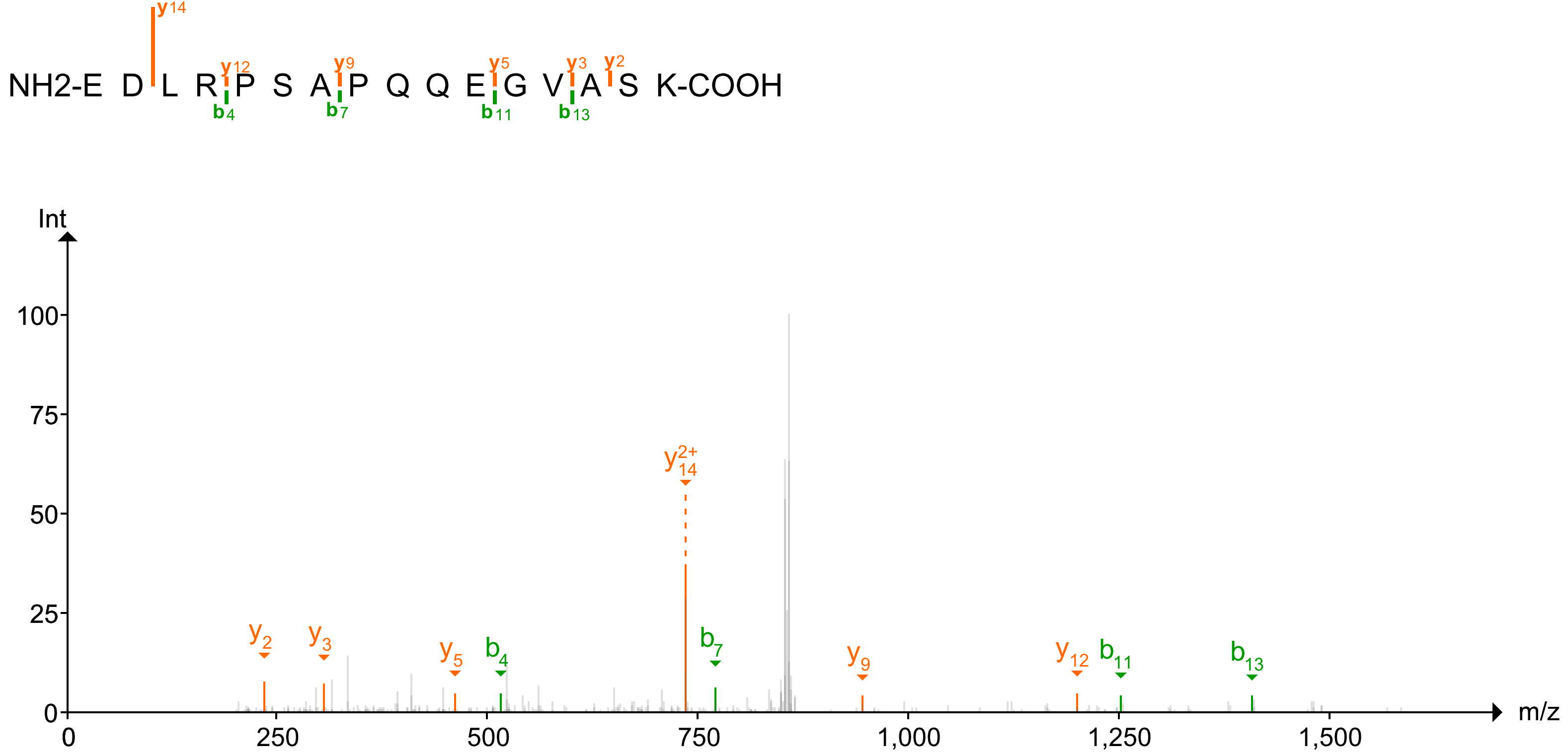

NH2-E D L R P S A P Q Q E G V A S K-COOH
y14
y12
y9
y5
y3
y2
b4
b7
b11
b13
Int
100
75
50
25
0
m/z
0
250
500
750
1,000
1,250
1,500
y2
y3
y5
b4
y14 2+
b7
y9
y12
b11
b13

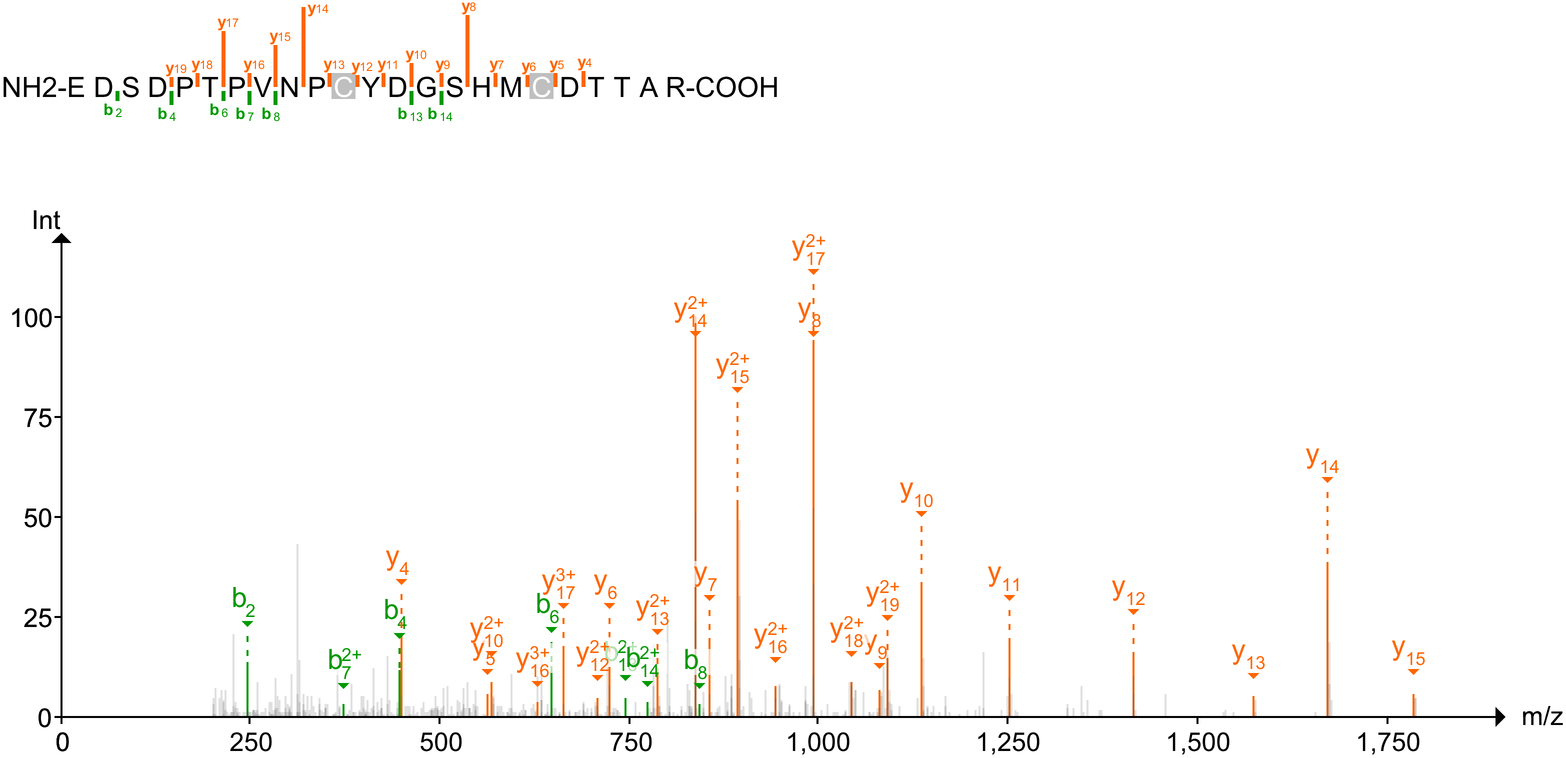

NH2-E D S D P T P V N P C Y D G S H M C D T T A R-COOH
y19 y18 y17 y16 y15 y14 y13 y12 y11 y10 y9 y8 y7 y6 y5 y4
b2 b4 b6 b7 b8 b13 b14
Int
100
75
50
25
0
m/z
0
250
500
750
1,000
1,250
1,500
1,750

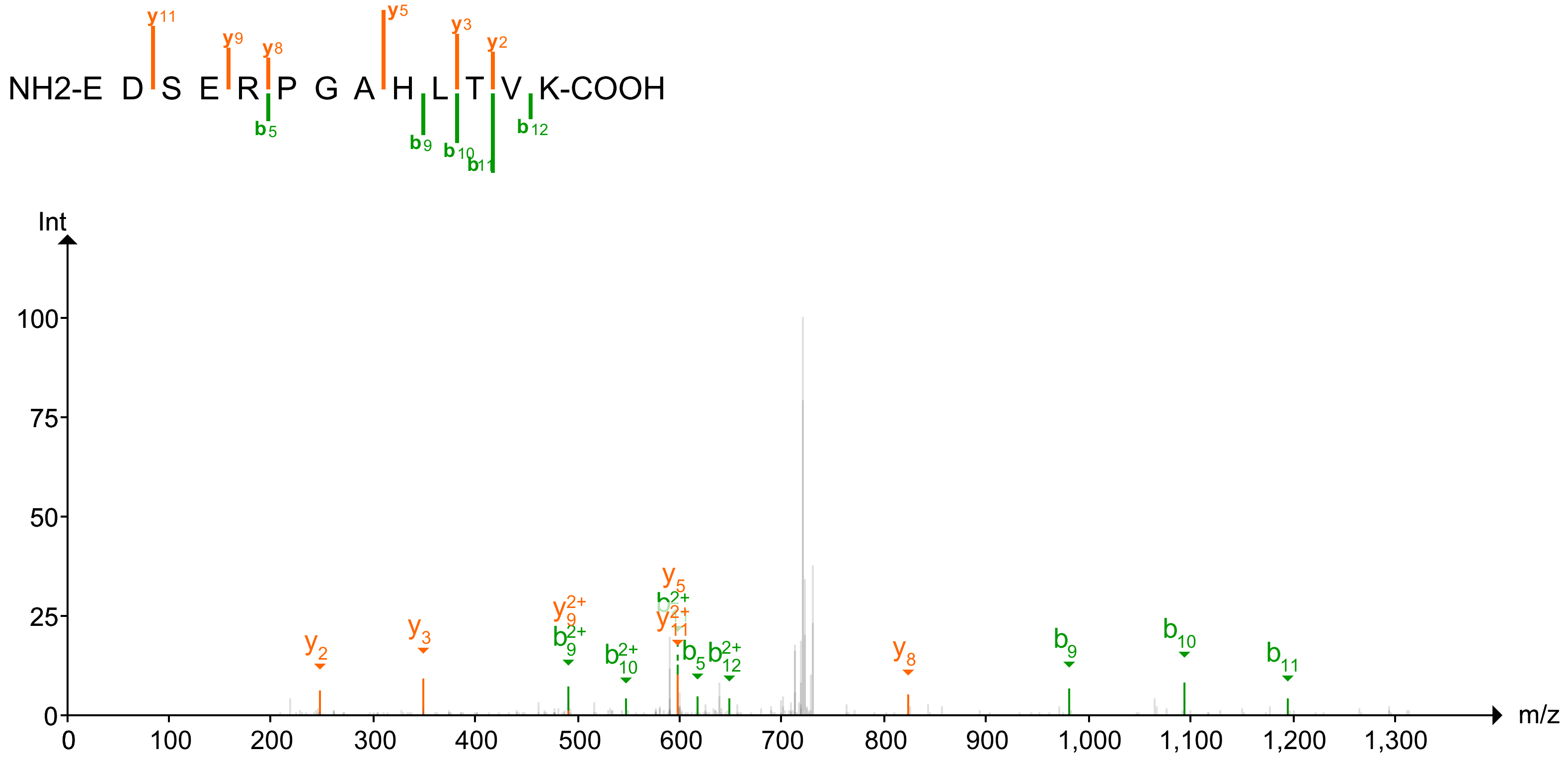

NH2-E D S E R P G A H L T V K-COOH
y11
y9
y8
y5
y3
y2
b5
b9
b10
b11
b12
Int
100
75
50
25
0
m/z
0
100
200
300
400
500
600
700
800
900
1,000
1,100
1,200
1,300
y2
y3
y9 2+
b9 2+
b10 2+
y5
b2+
y11 2+
b5
b12 2+
y8
b9
b10
b11

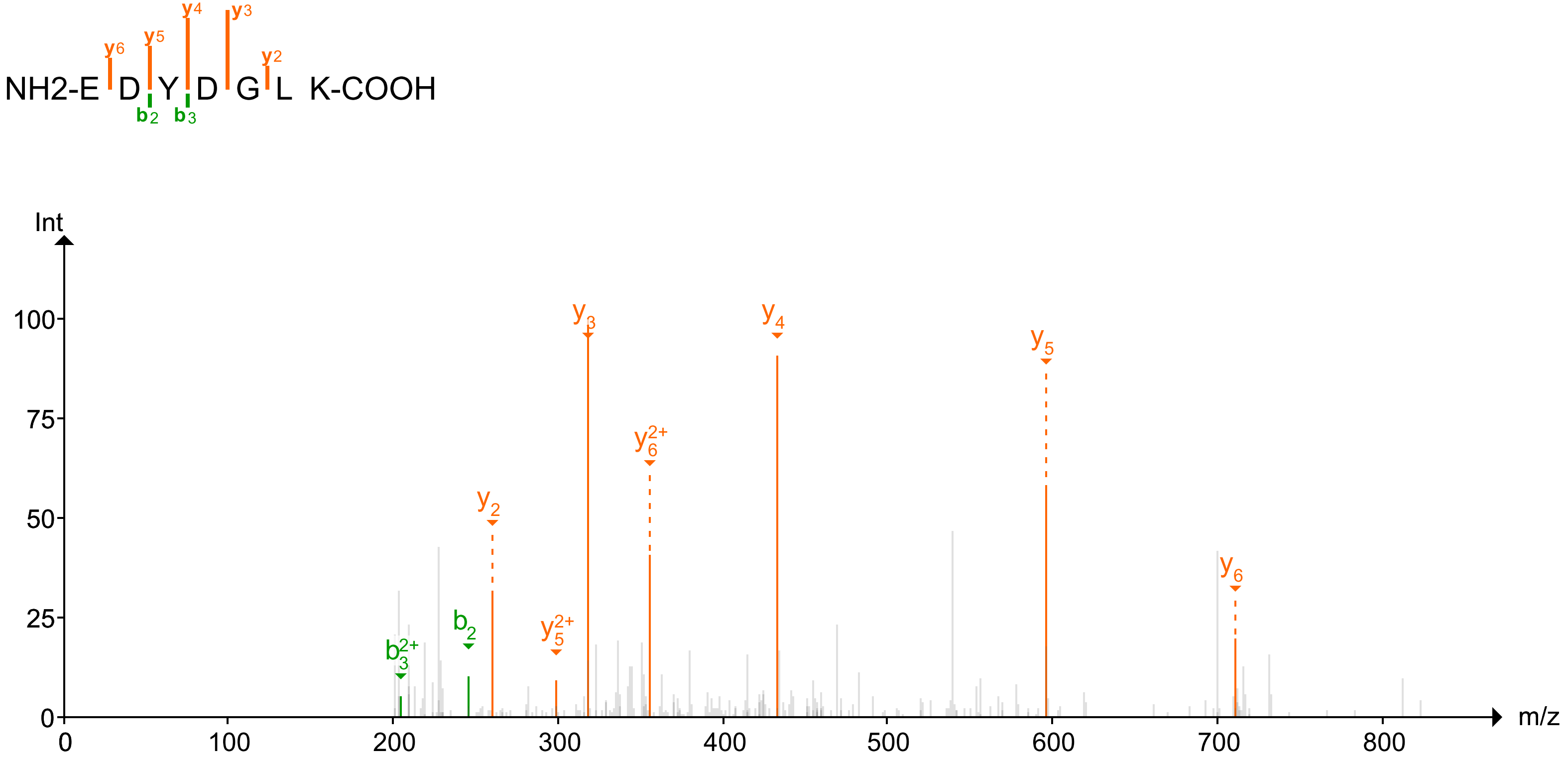

NH2-E D Y D G L K-COOH
y6 y5 y4 y3 y2
b2 b3
Int
100
75
50
25
0
y3
y4
y5
y6 2+
y2
y5 2+
b2
b3 2+
y6
0
100
200
300
400
500
600
700
800
m/z

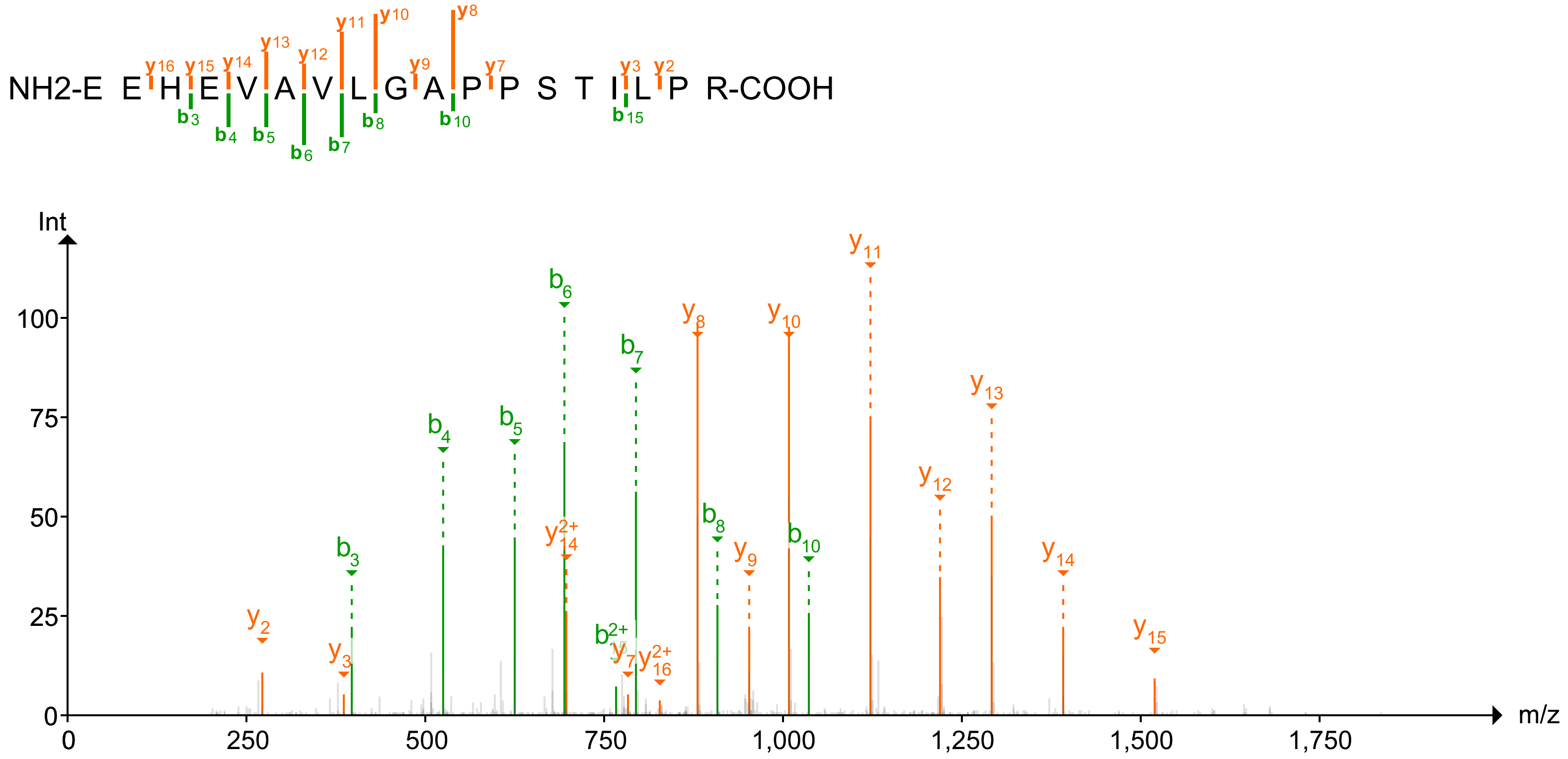

NH2-E E H E V A V L G A P P S T I L P R-COOH
y16 y15 y14 y13 y12 y11 y10 y9 y8 y7 y3 y2
b3 b4 b5 b6 b7 b8 b10 b15
Int
100
75
50
25
0
0
250
500
750
1,000
1,250
1,500
1,750
m/z
y11
b6
y8
y10
b7
y13
b4
b5
y12
b8
y14 2+
b3
y9
b10
y14
y15
y2
y3
b2+ 15
y7
y2+ 16

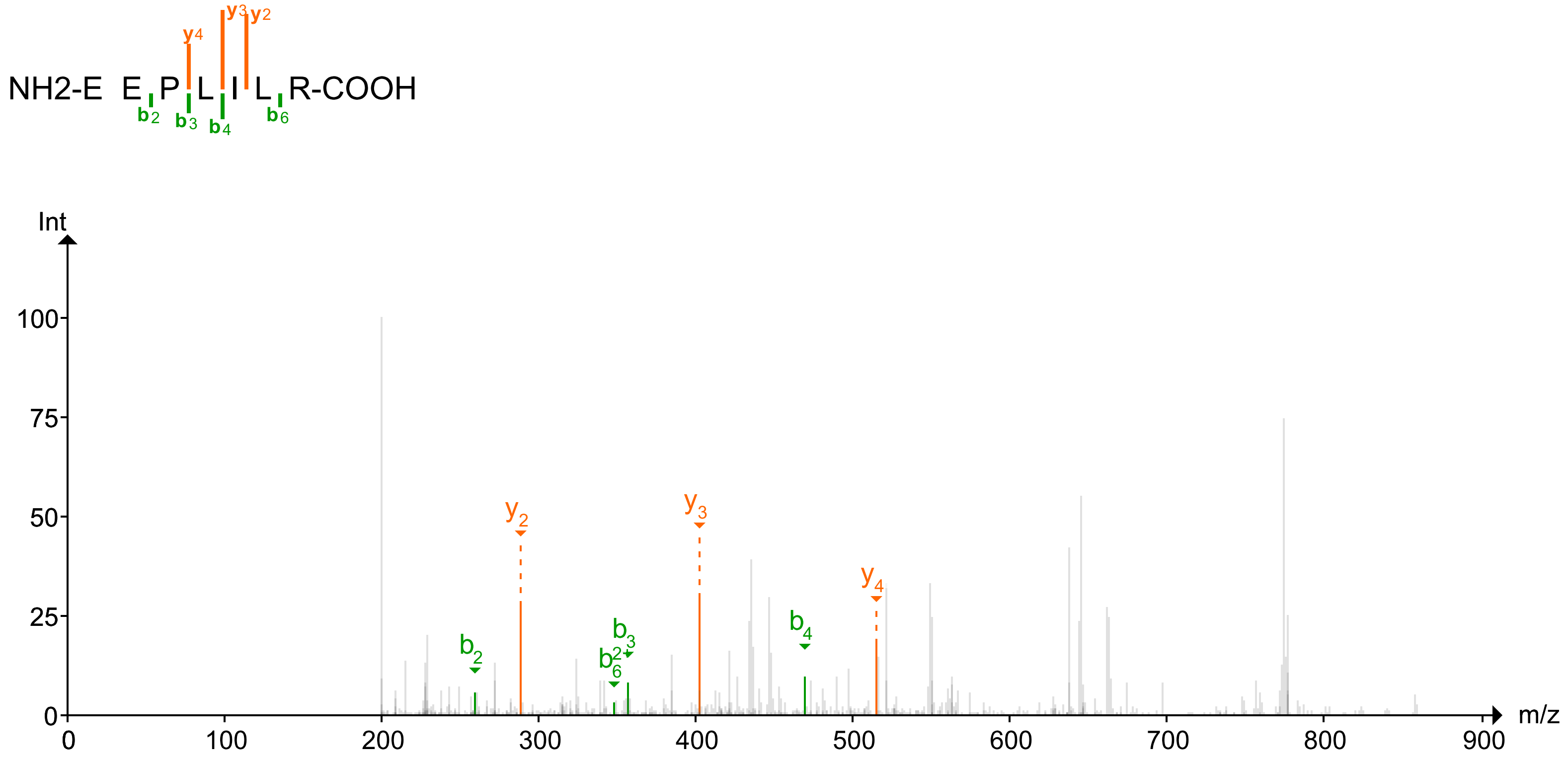

NH2-E E P L I L R-COOH
y4 y3 y2
b2 b3 b4 b6
Int
100
75
50
25
0
0 100 200 300 400 500 600 700 800 900
m/z
y2
y3
y4
b2
b3
b4
b6 2+

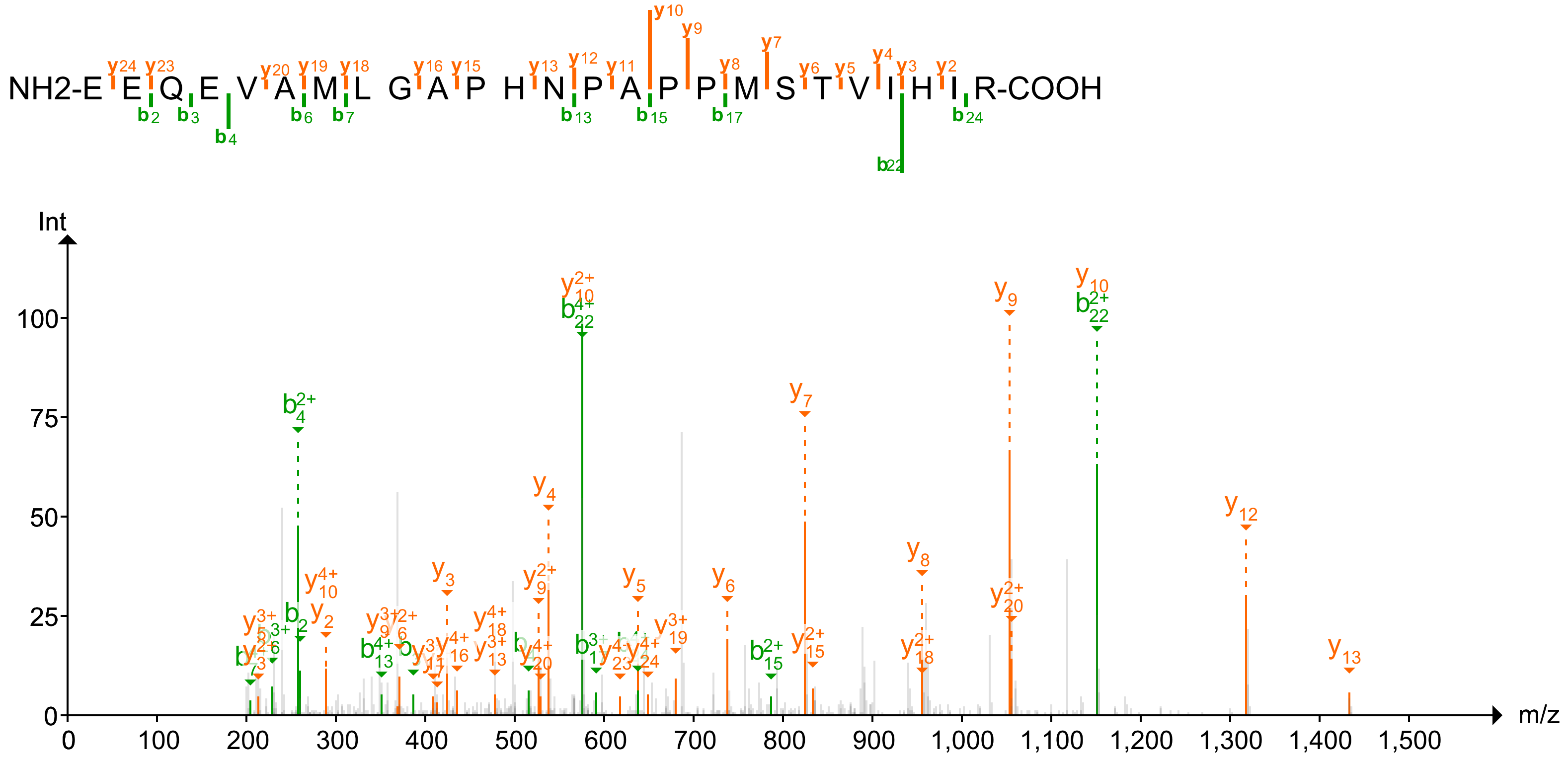

NH2-E E Q E V A M L G A P H N P A P P M S T V I H I R-COOH
Int
m/z
0
25
50
75
100
0
100
200
300
400
500
600
700
800
900
1,000
1,100
1,200
1,300
1,400
1,500

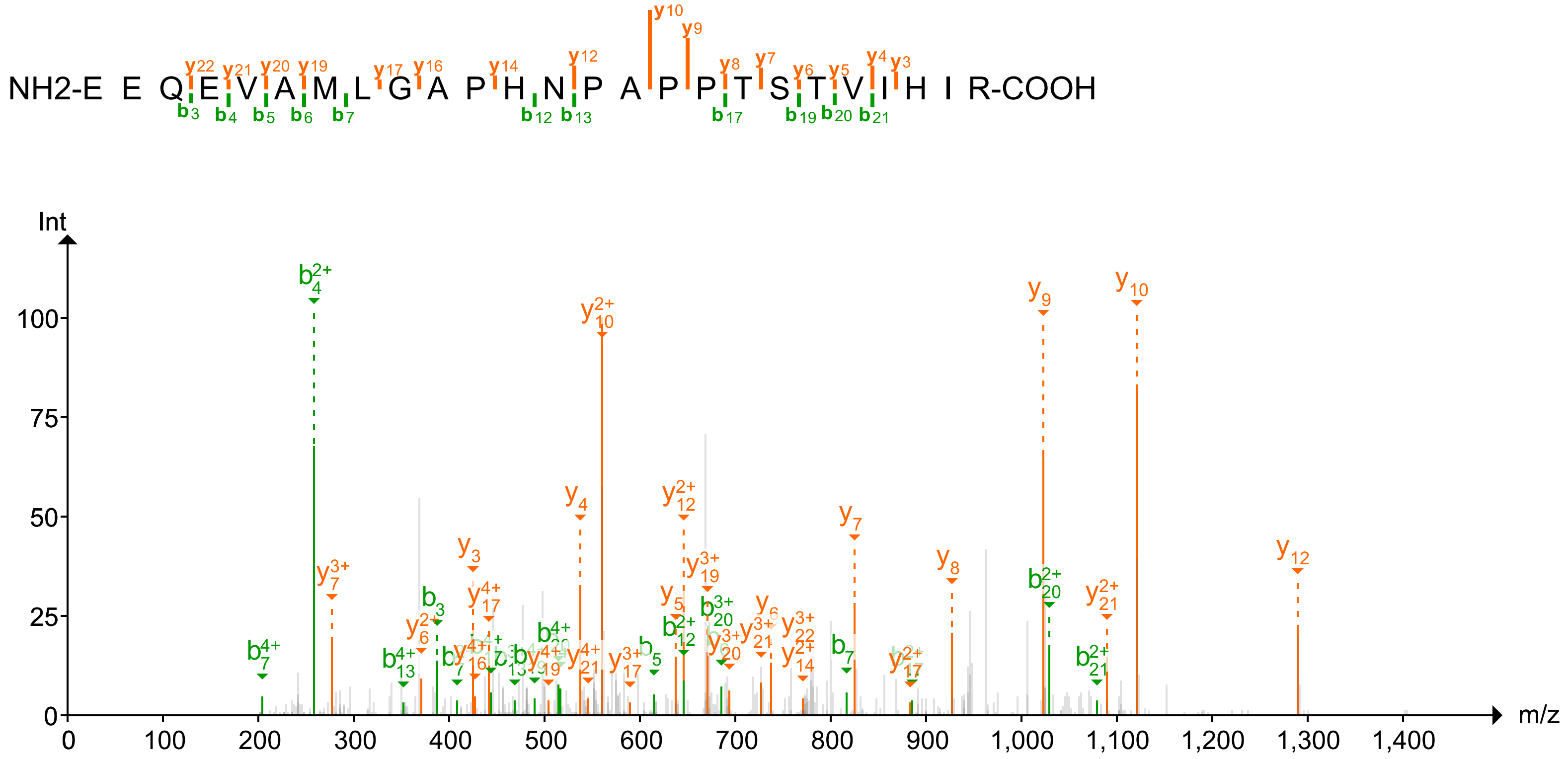
NH2-E E Q E V A M L G A P H N P A P P T S T V I H I R-COOH
y22 y21 y20 y19 y17 y16 y14 y12 y10 y9 y8 y7 y6 y5 y4 y3
b3 b4 b5 b6 b7 b12 b13 b17 b19 b20 b21
Int
m/z
0
25
50
75
100
0
100
200
300
400
500
600
700
800
900
1,000
1,100
1,200
1,300
1,400

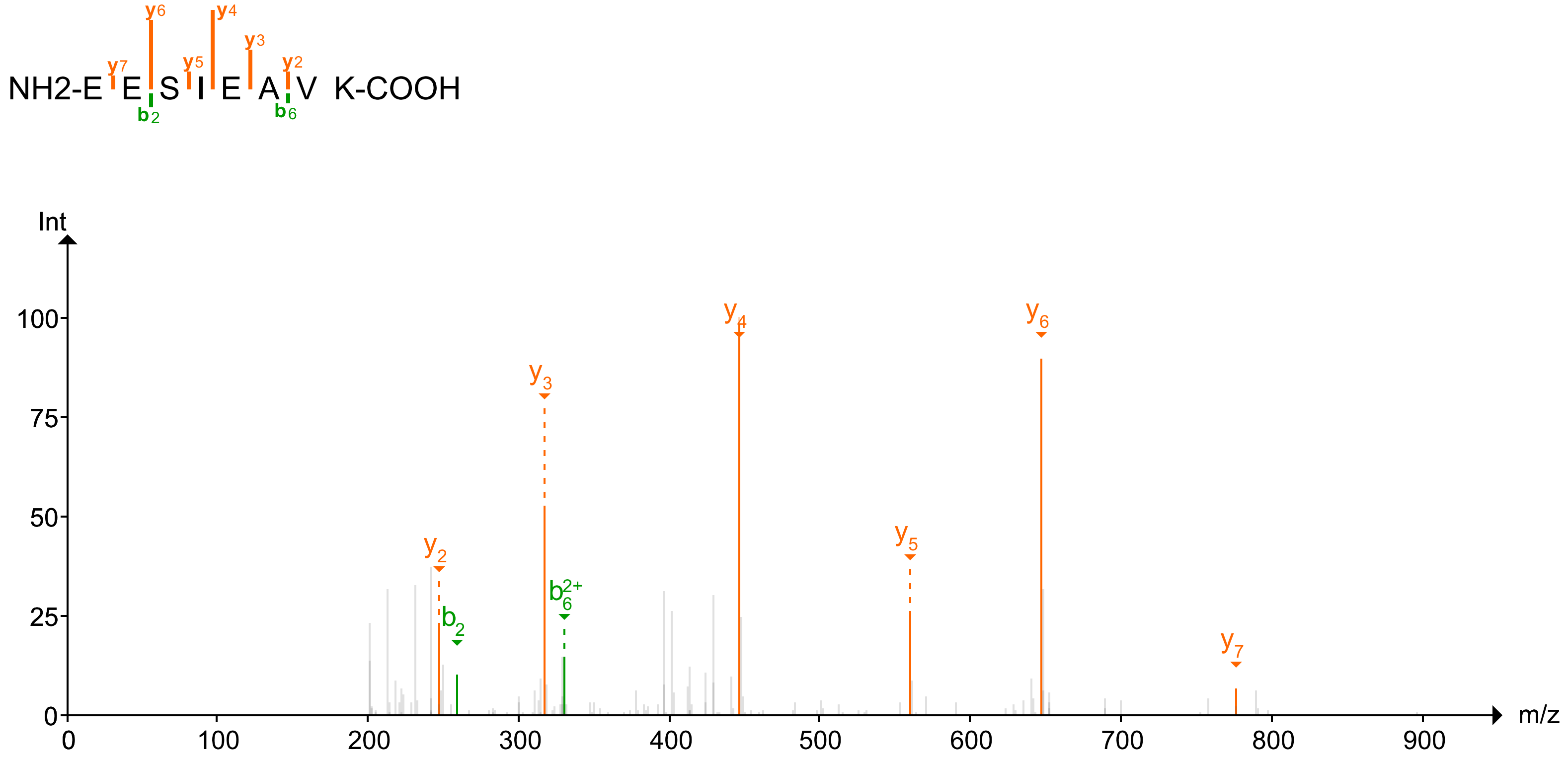

NH2-E E S I E A V K-COOH
y7 y6 y5 y4 y3 y2
b2 b6
Int
100
75
50
25
0
y2
b2
y3
b6 2+
y4
y5
y6
y7
0 100 200 300 400 500 600 700 800 900
m/z

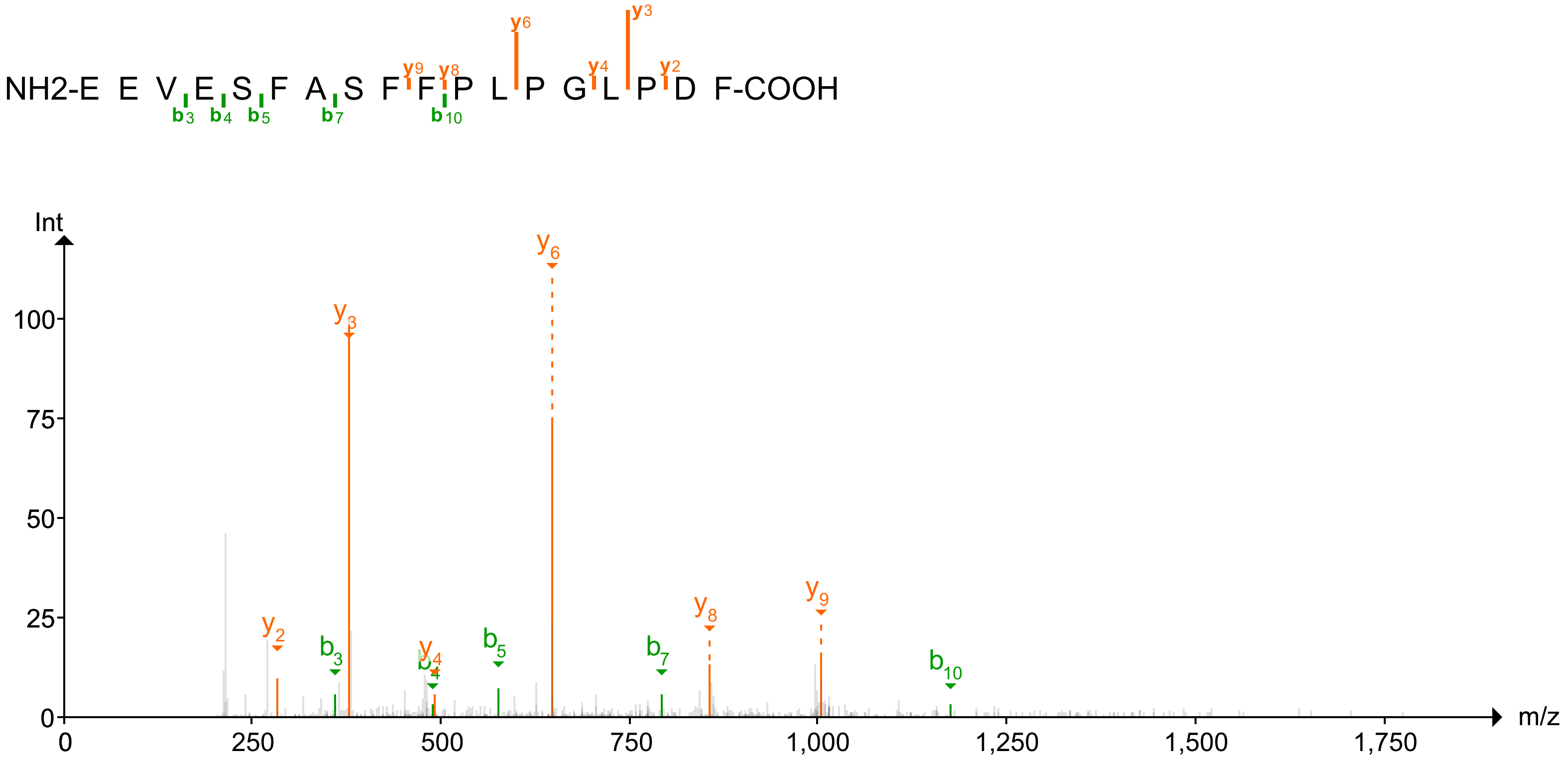

NH2-E E V E S F A S F F P L P G L P D F-COOH
y6
y3
y9
y8
y4
y2
b3
b4
b5
b7
b10
Int
100
75
50
25
0
y3
y6
y2
b3
y4
b4
b5
b7
y8
y9
b10
0
250
500
750
1,000
1,250
1,500
1,750
m/z

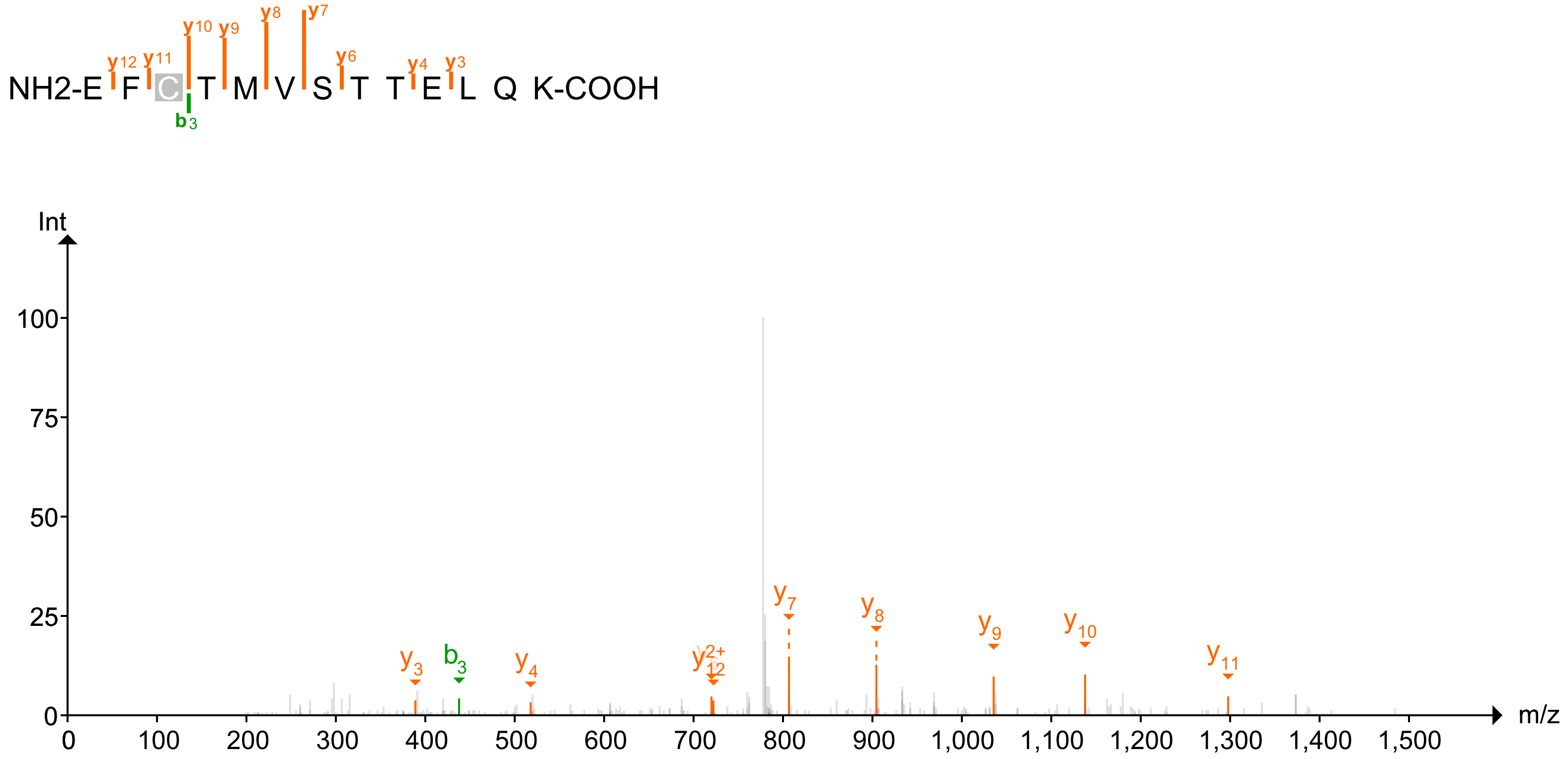

NH2-E F C T M V S T T E L Q K-COOH
y12 y11 y10 y9 y8 y7 y6 y4 y3
b3
Int
100
75
50
25
0
m/z
0 100 200 300 400 500 600 700 800 900 1,000 1,100 1,200 1,300 1,400 1,500
y3 b3 y4 y12 2+ y7 y8 y9 y10 y11

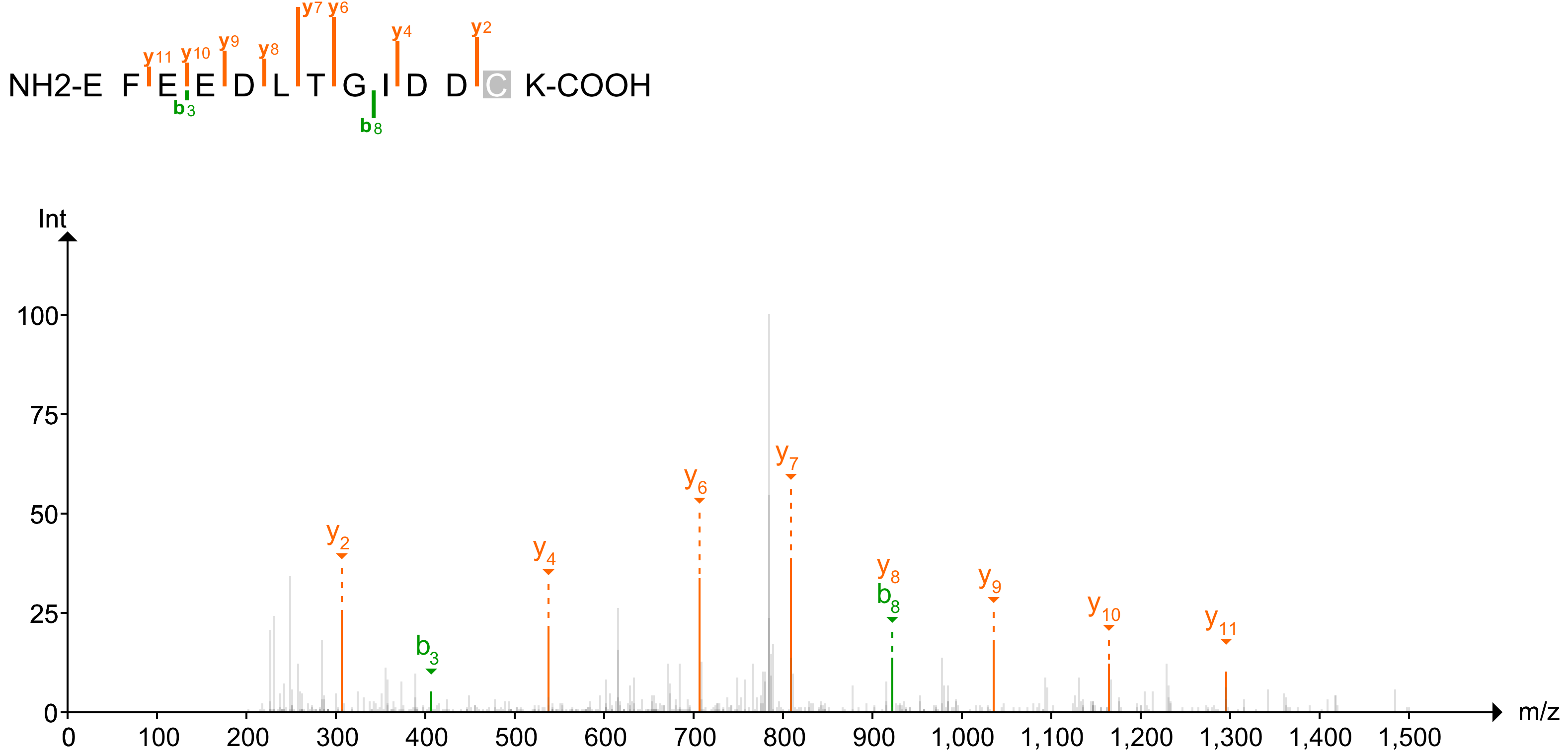

NH2-E F E E D L T G I D D C K-COOH
y11 y10 y9 y8 y7 y6 y4 y2
b3 b8
Int
100
75
50
25
0
y2
b3
y4
y6
y7
y8
b8
y9
y10
y11
0 100 200 300 400 500 600 700 800 900 1,000 1,100 1,200 1,300 1,400 1,500
m/z

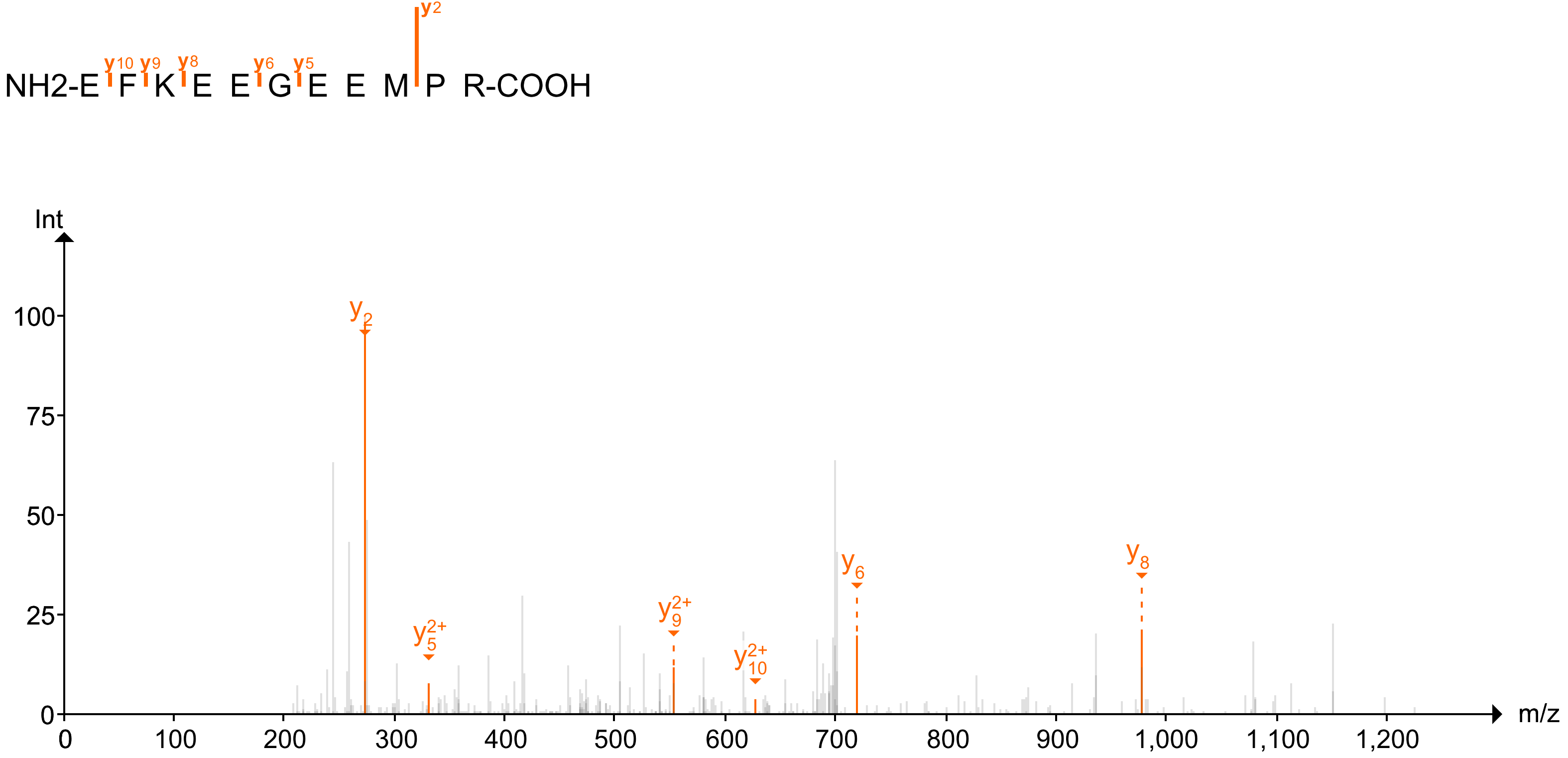

y2
y10 y9 y8 y6 y5
NH2-E F K E E G E E M P R-COOH
Int
100
75
50
25
0
y2
y5 2+
y9 2+
y10 2+
y6
y8
0
100
200
300
400
500
600
700
800
900
1,000
1,100
1,200
m/z

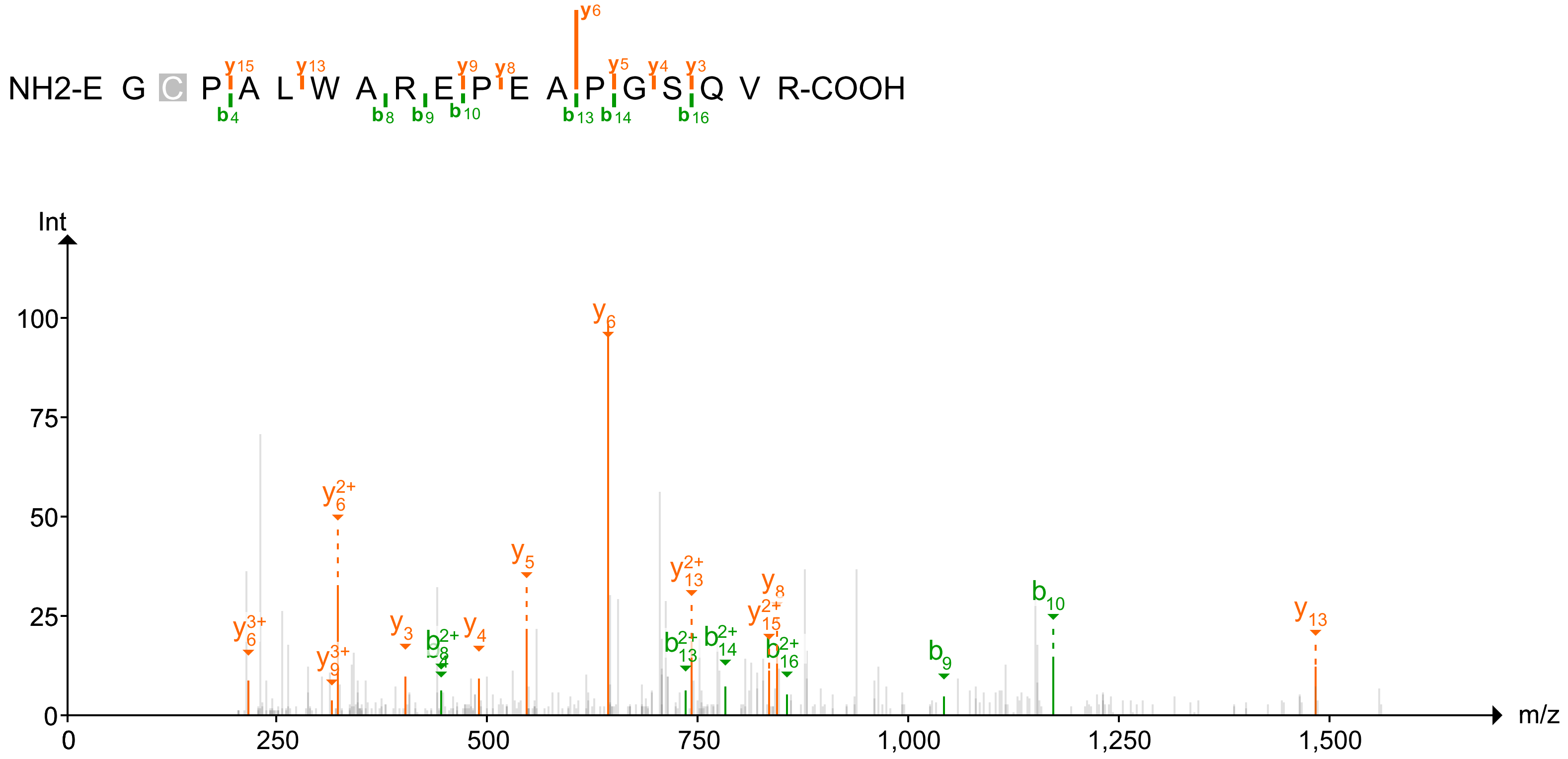

NH2-E G C P A L W A R E P E A P G S Q V R-COOH
y15 y13 y9 y8 y6 y5 y4 y3
b4 b8 b9 b10 b13 b14 b16
Int
100
75
50
25
0
250
500
750
1,000
1,250
1,500
m/z
y6
y6 2+
y5
y13 2+
y8
y15 2+
b10
y13
y6 3+
y9 3+
y3
b8 2+
y4
b13 2+
b14 2+
b16 2+
b9

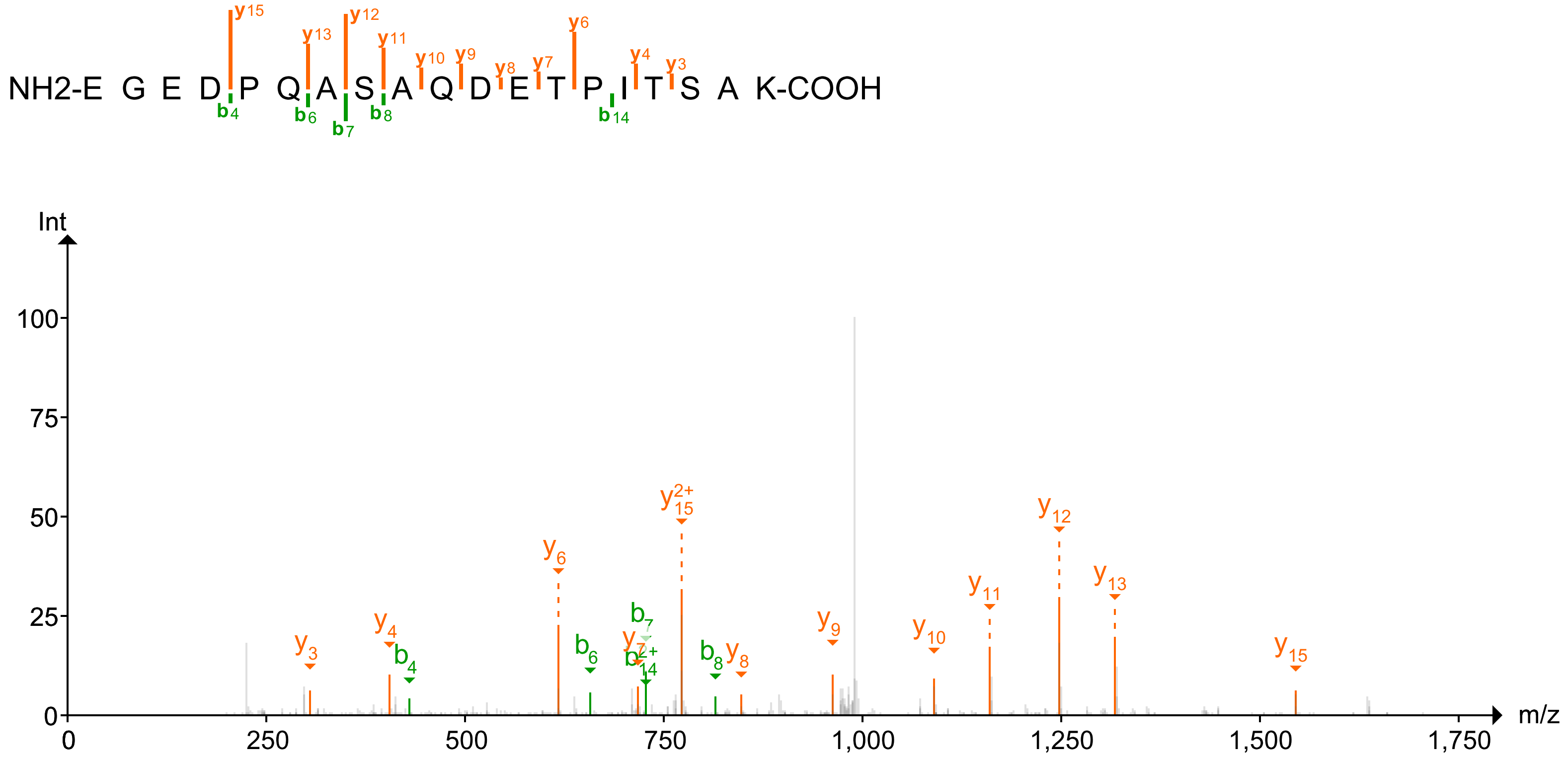

NH2-E G E D P Q A S A Q D E T P I T S A K-COOH
y15
y13
y12
y11
y10
y9
y8
y7
y6
y4
y3
b4
b6
b7
b8
b14
Int
m/z

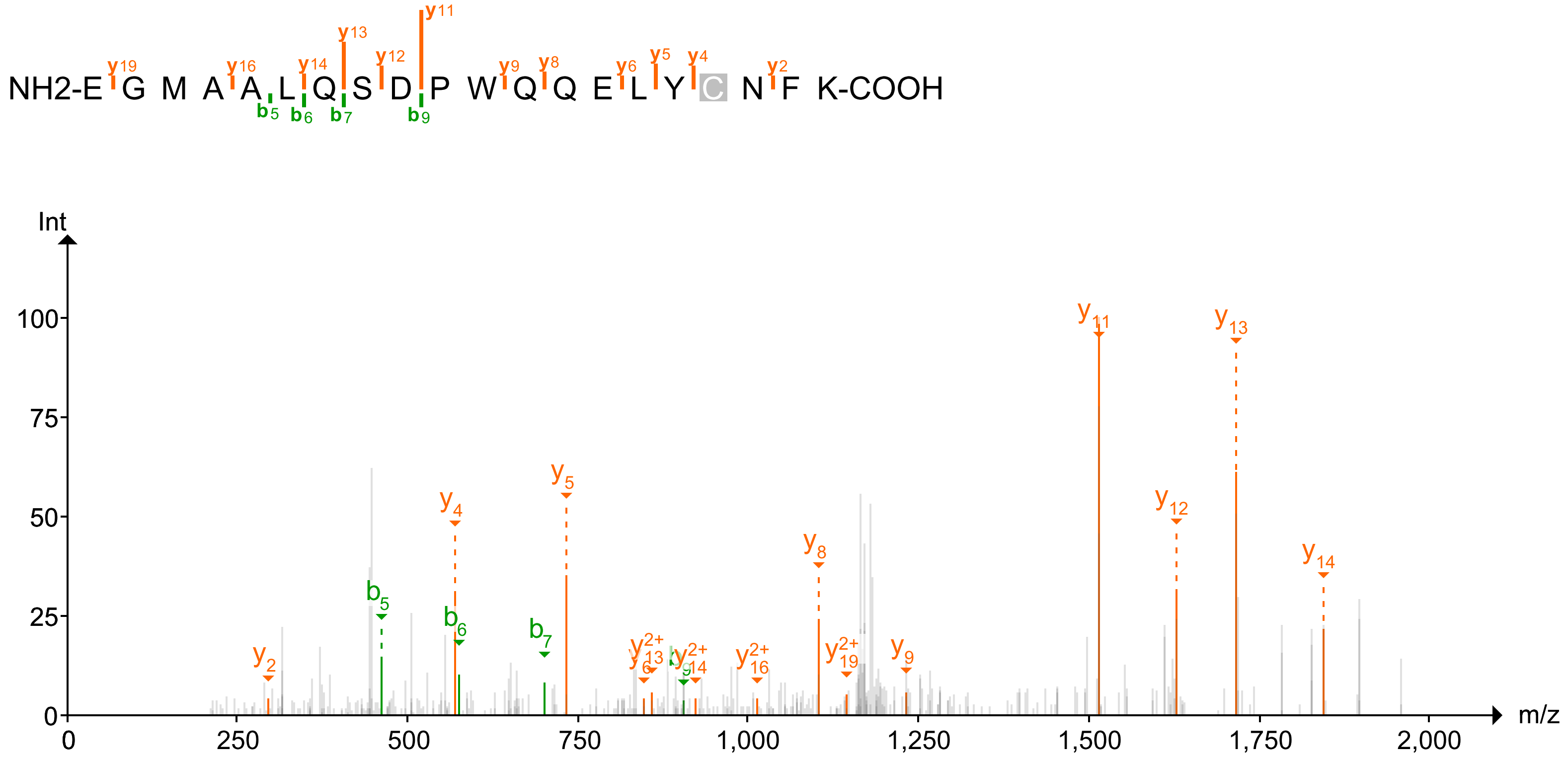
NH2-E G M A A L Q S D P W Q Q E L Y C N F K-COOH
Int
m/z
0
25
50
75
100
250
500
750
1,000
1,250
1,500
1,750
2,000

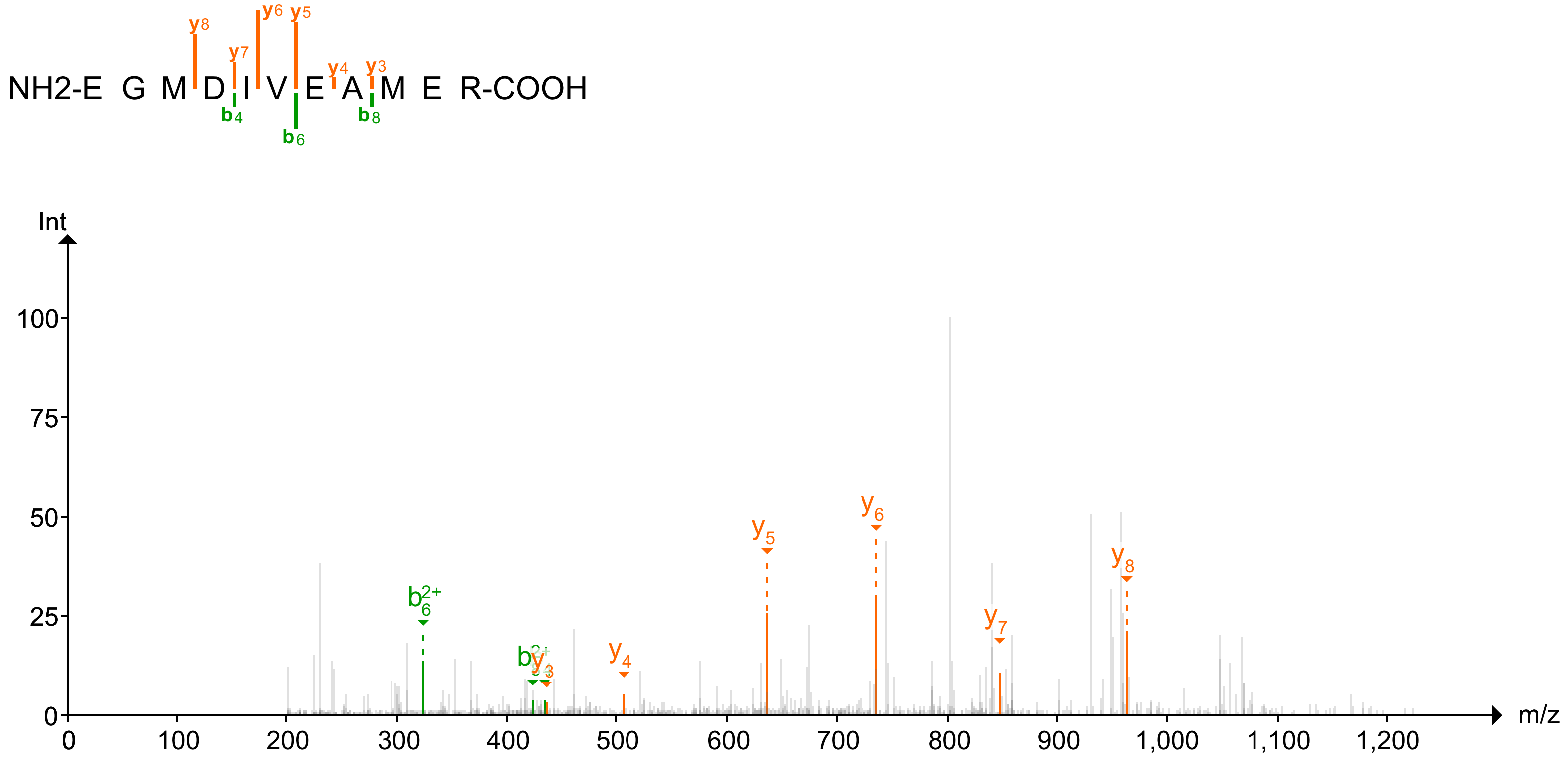

NH2-E G M D I V E A M E R-COOH
y8 y7 y6 y5 y4 y3
b4 b6 b8
Int
100
75
50
25
0
m/z
0 100 200 300 400 500 600 700 800 900 1,000 1,100 1,200
b6 2+
y5
y6
y7
y8
y4
y3

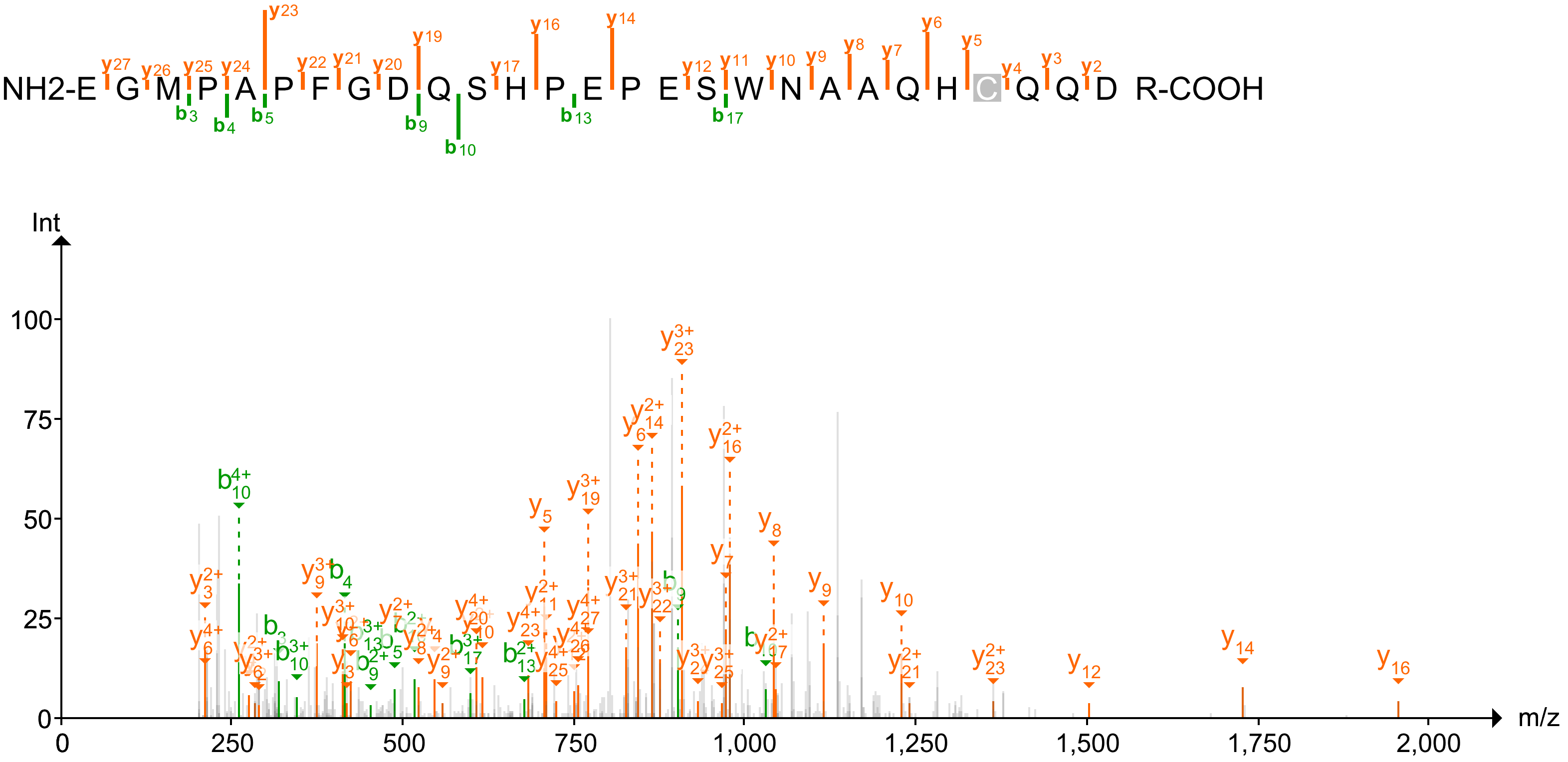

NH2-E G M P A P F G D Q S H P E P E S W N A A Q H C Q Q D R-COOH
Int
100
75
50
25
0
m/z
0
250
500
750
1,000
1,250
1,500
1,750
2,000

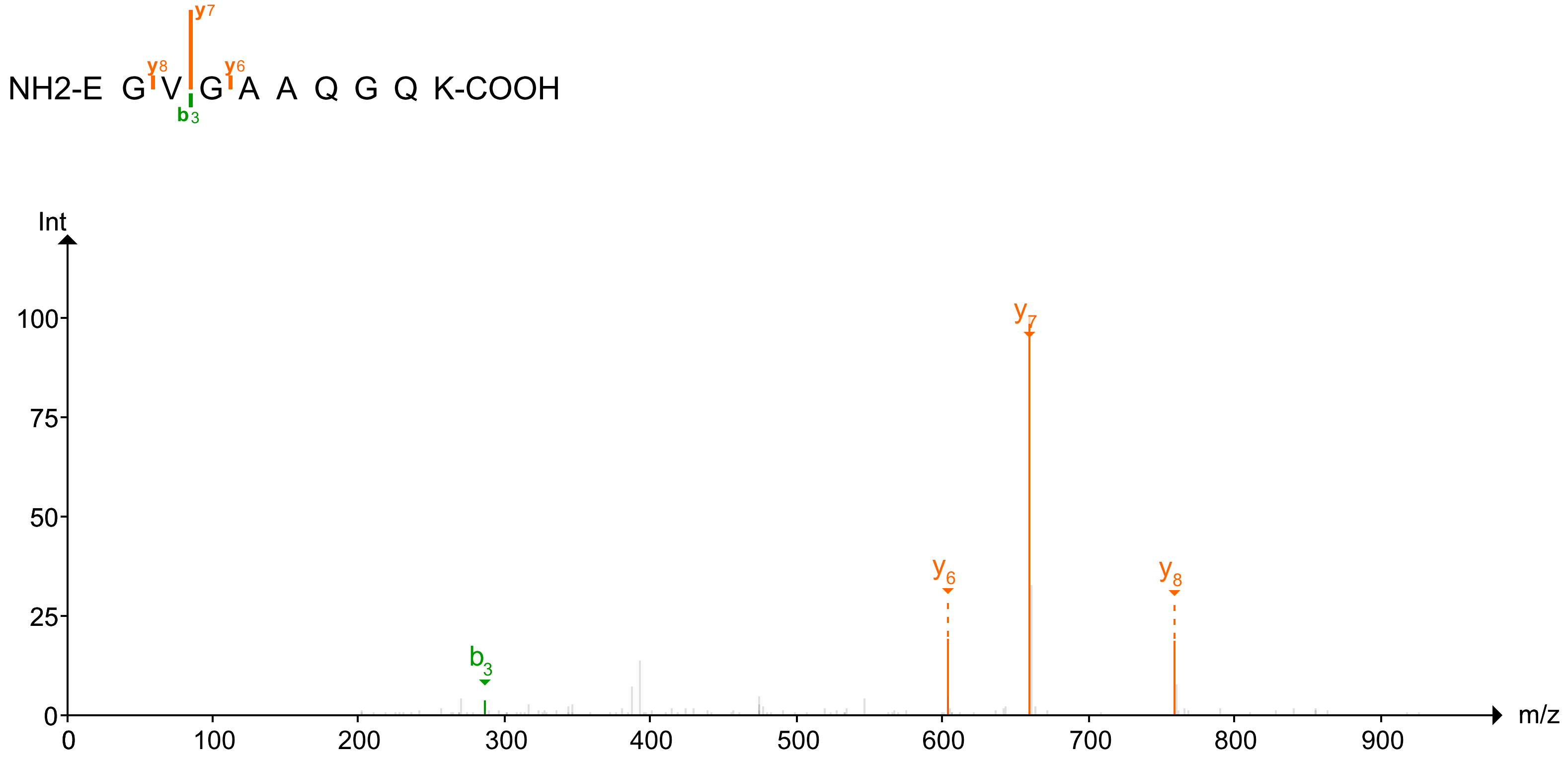

NH2-E G V G A A Q G Q K-COOH
y7
y8
y6
b3
Int
100
75
50
25
0
0
100
200
300
400
500
600
700
800
900
m/z
b3
y6
y7
y8

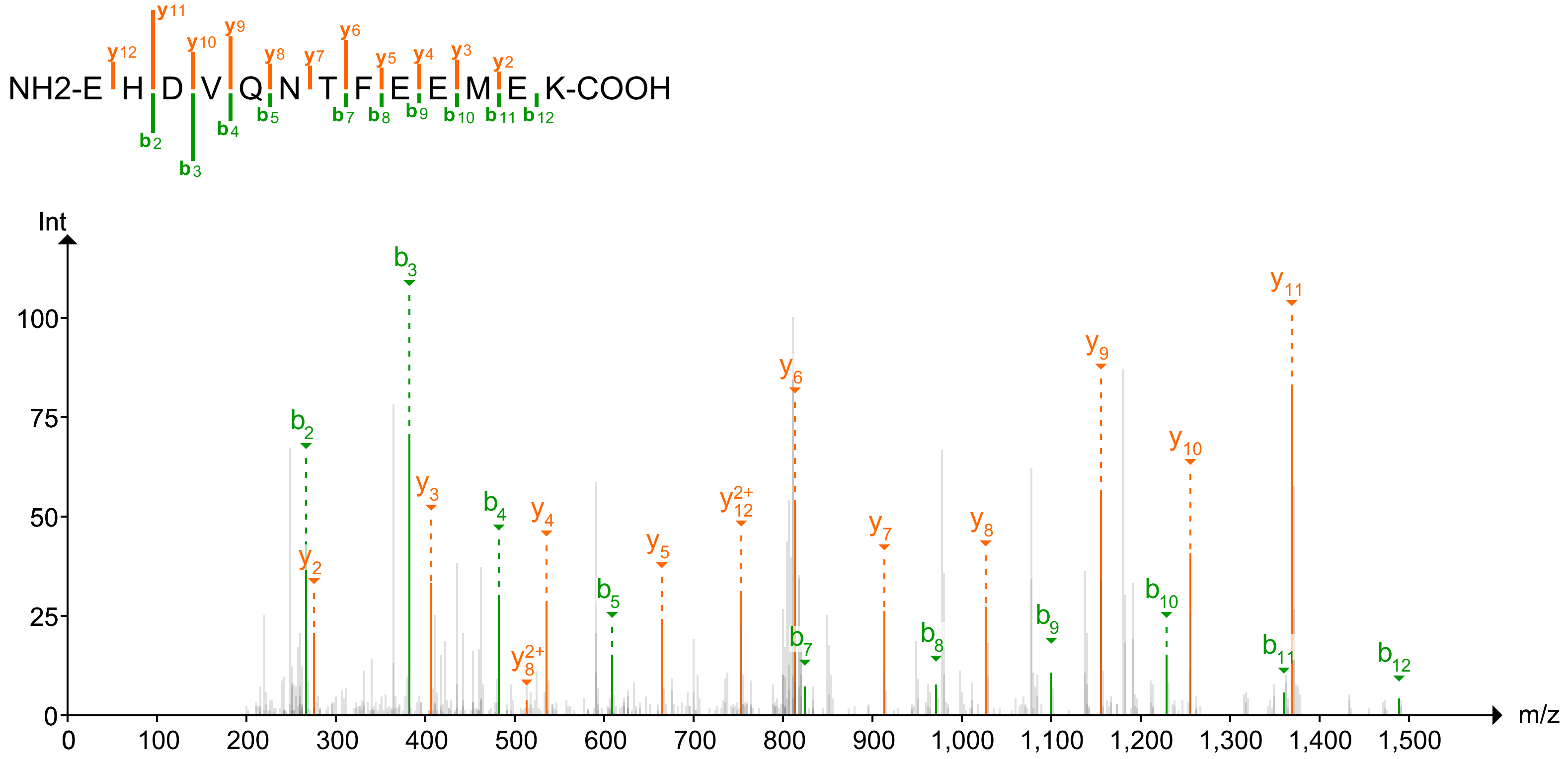
NH2-E H D V Q N T F E E M E K-COOH
y12 y11 y10 y9 y8 y7 y6 y5 y4 y3 y2
b2 b3 b4 b5 b7 b8 b9 b10 b11 b12
Int
m/z
0 25 50 75 100
0 100 200 300 400 500 600 700 800 900 1,000 1,100 1,200 1,300 1,400 1,500
b3 b2 y2 y3 b4 y4 $y_8^{2+}$ b5 y5 $y_{12}^{2+}$ y6 b7 y7 b8 y8 b9 y9 b10 y10 b11 y11 b12

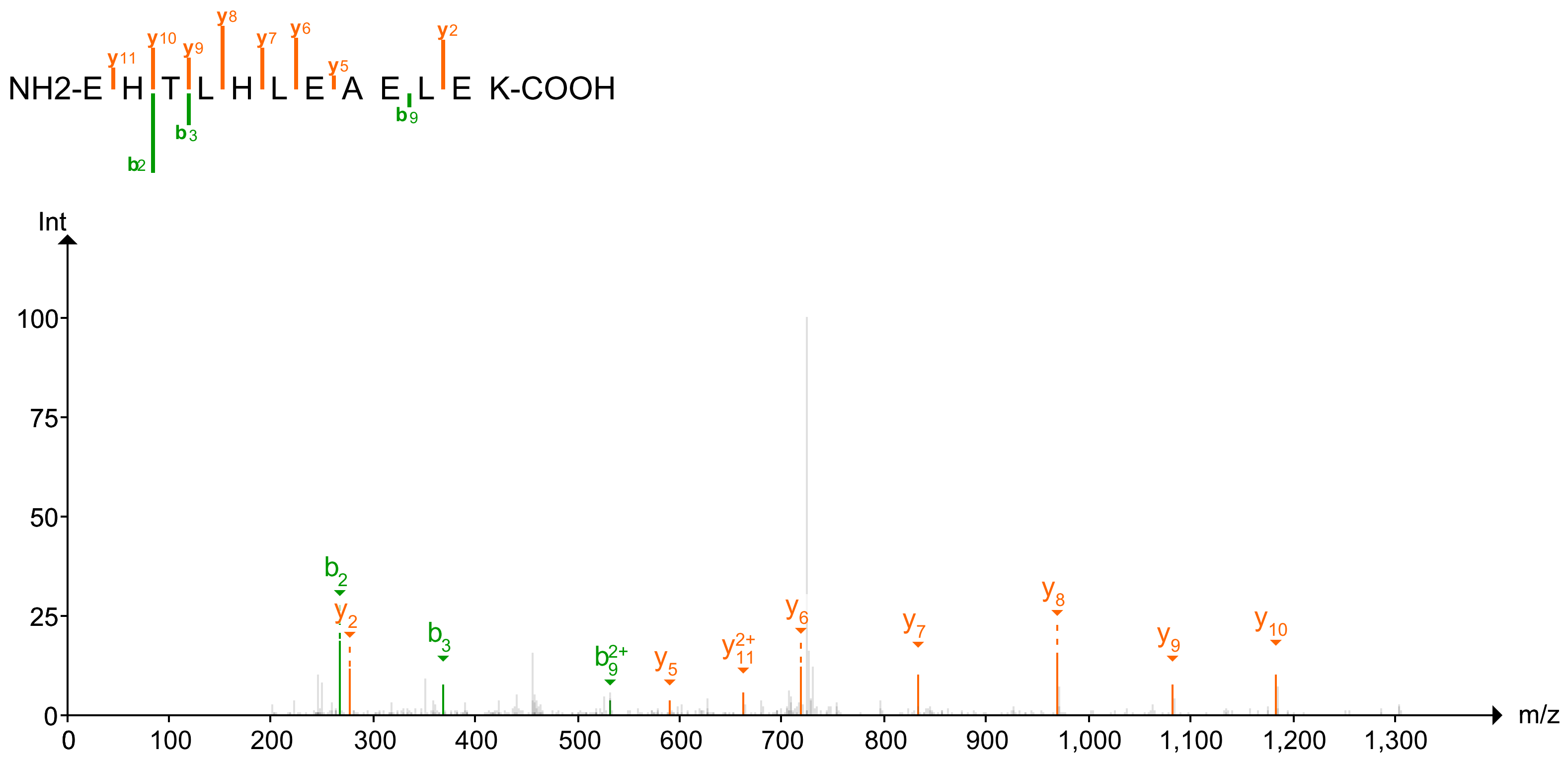

NH2-E H T L H L E A E L E K-COOH
y11 y10 y9 y8 y7 y6 y5 y2
b2 b3 b9
Int
100
75
50
25
0
b2 y2 b3 $b_9^{2+}$ y5 $y_{11}^{2+}$ y6 y7 y8 y9 y10
0 100 200 300 400 500 600 700 800 900 1,000 1,100 1,200 1,300
m/z

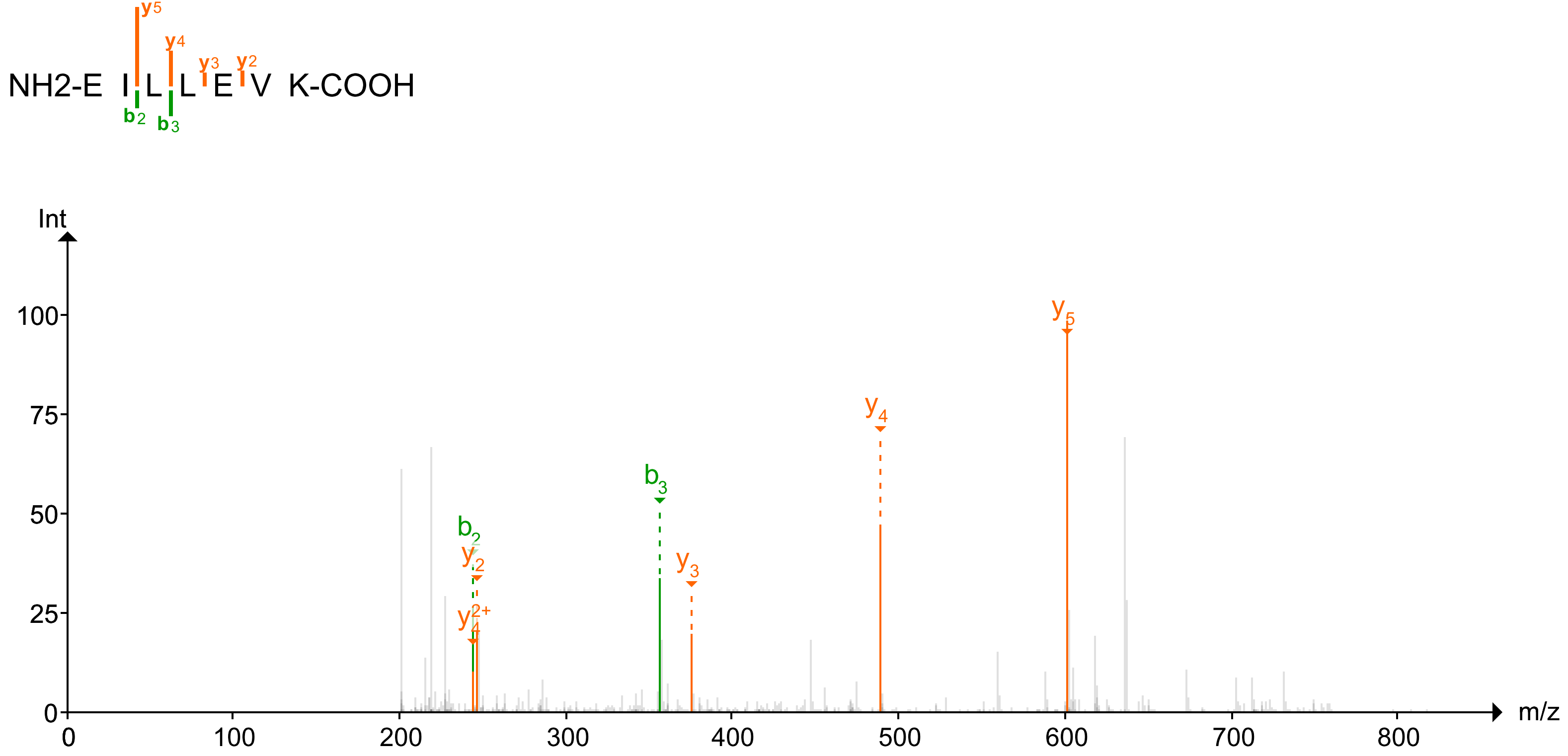

NH2-E I L L E V K-COOH
y5
y4
y3
y2
b2
b3
Int
100
75
50
25
0
m/z
0
100
200
300
400
500
600
700
800

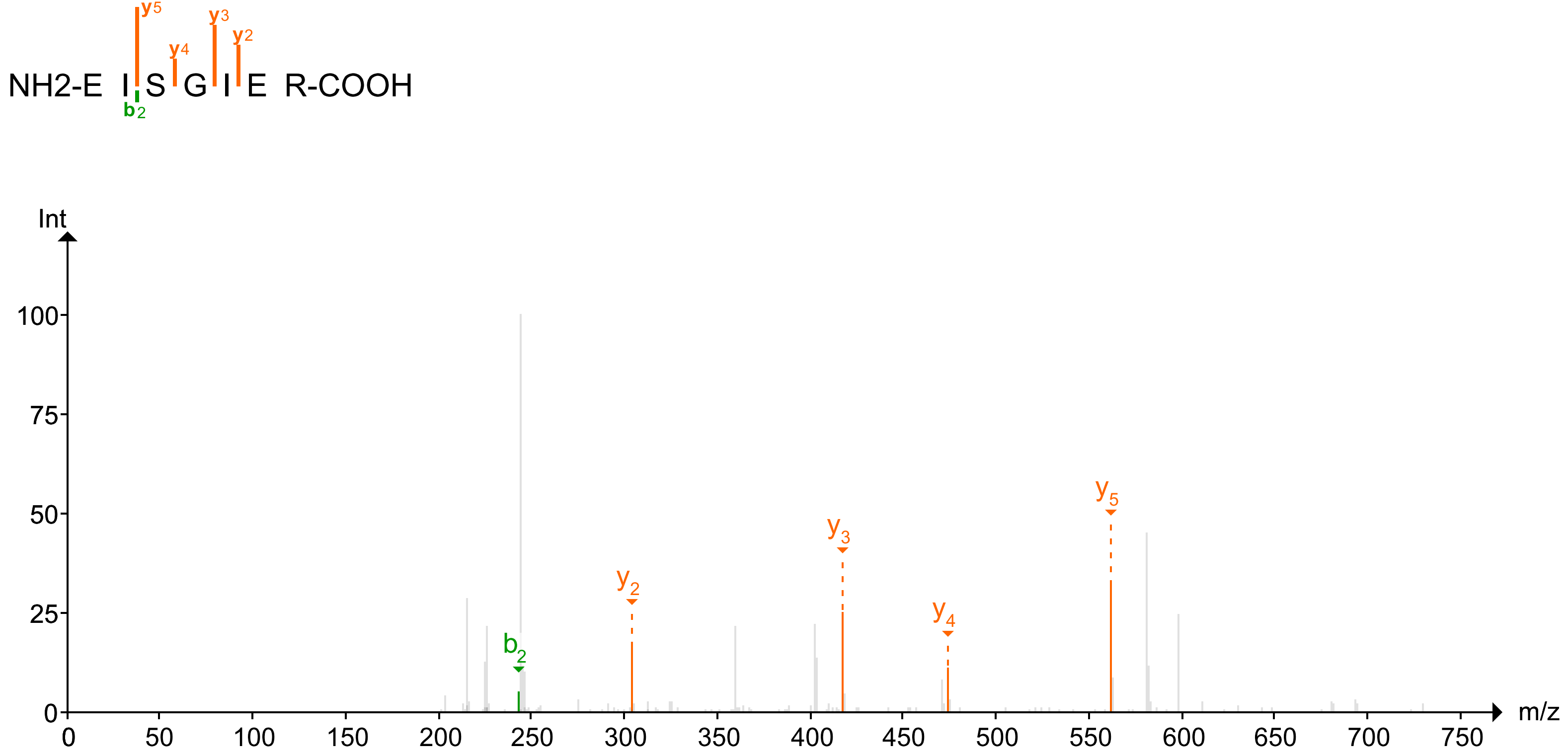

NH2-E I S G I E R-COOH
y5
y4
y3
y2
b2
Int
100
75
50
25
0
m/z
0
50
100
150
200
250
300
350
400
450
500
550
600
650
700
750

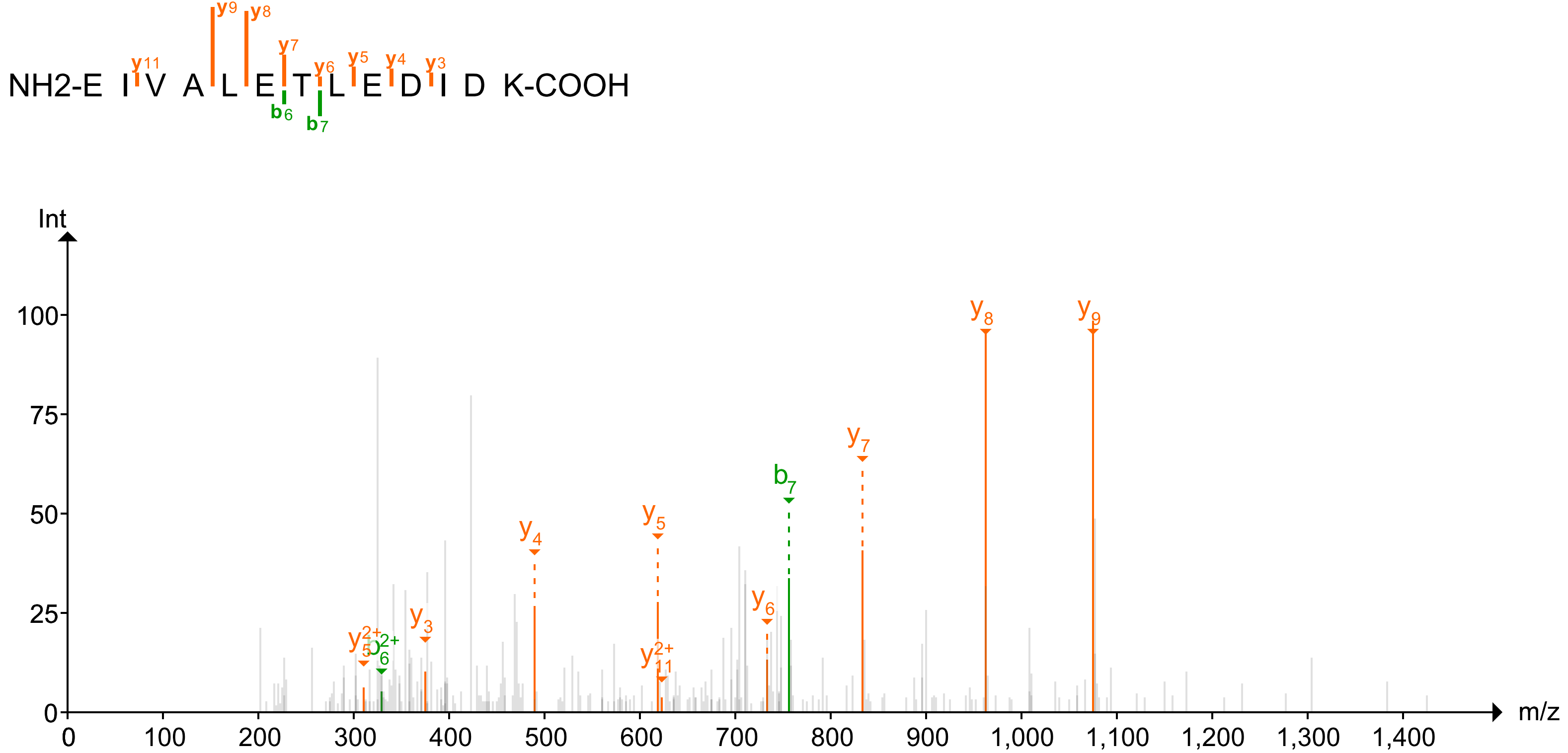

NH2-E I V A L E T L E D I D K-COOH
y9 y8 y7 y11 y6 y5 y4 y3
b6 b7
Int
m/z
y8 y9 y7 b7 y5 y4 y6 y3
100
75
50
25
0
0 100 200 300 400 500 600 700 800 900 1,000 1,100 1,200 1,300 1,400

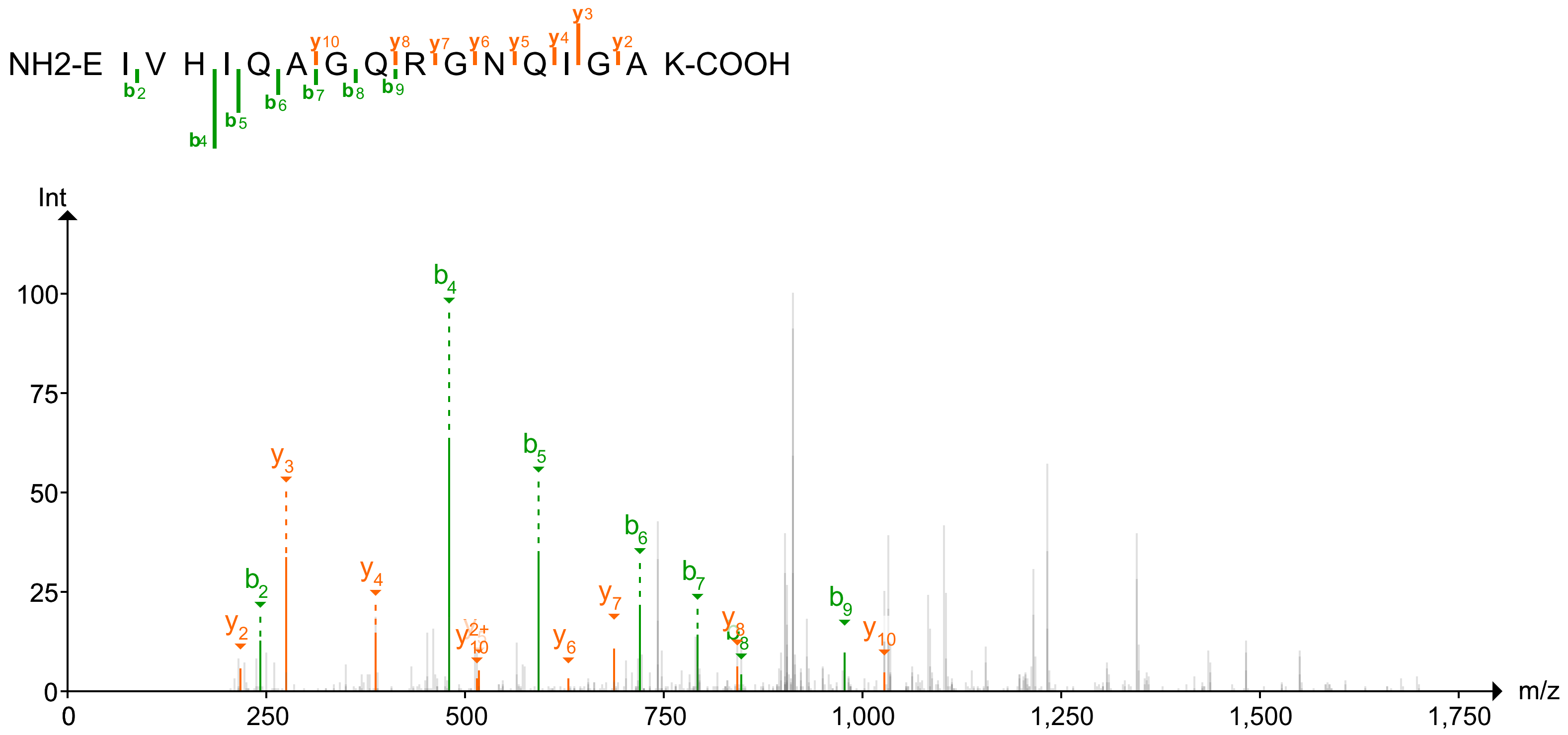

NH2-E I V H I Q A G Q R G N Q I G A K-COOH
b2 b4 b5 b6 b7 b8 b9
y10 y8 y7 y6 y5 y4 y3 y2
Int
100
75
50
25
0
m/z
0
250
500
750
1,000
1,250
1,500
1,750
b4
b5
y3
b6
b7
b2
y4
y7
b9
y2
y6
y10

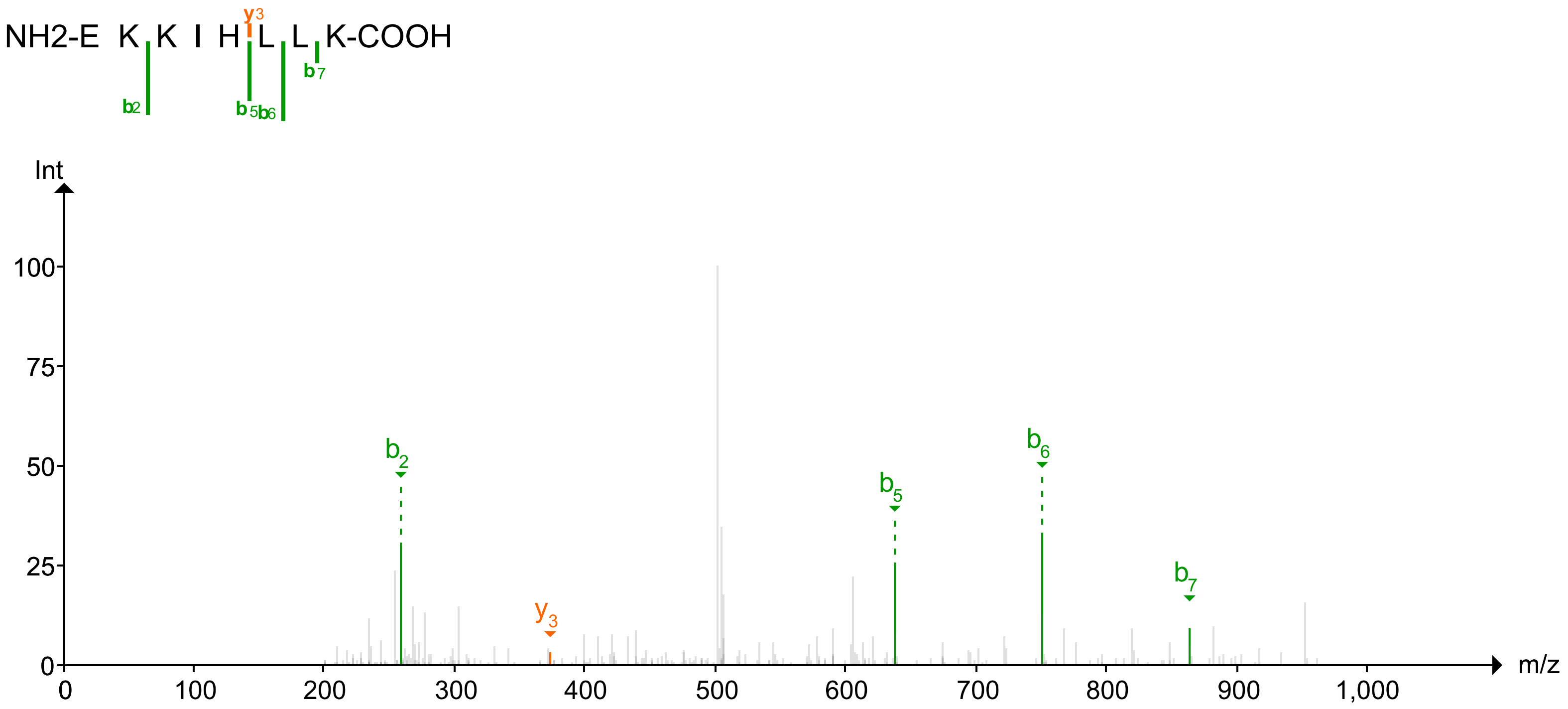

NH2-E K K I H L L K-COOH
y3
b2
b5
b6
b7
Int
100
75
50
25
0
m/z
0
100
200
300
400
500
600
700
800
900
1,000

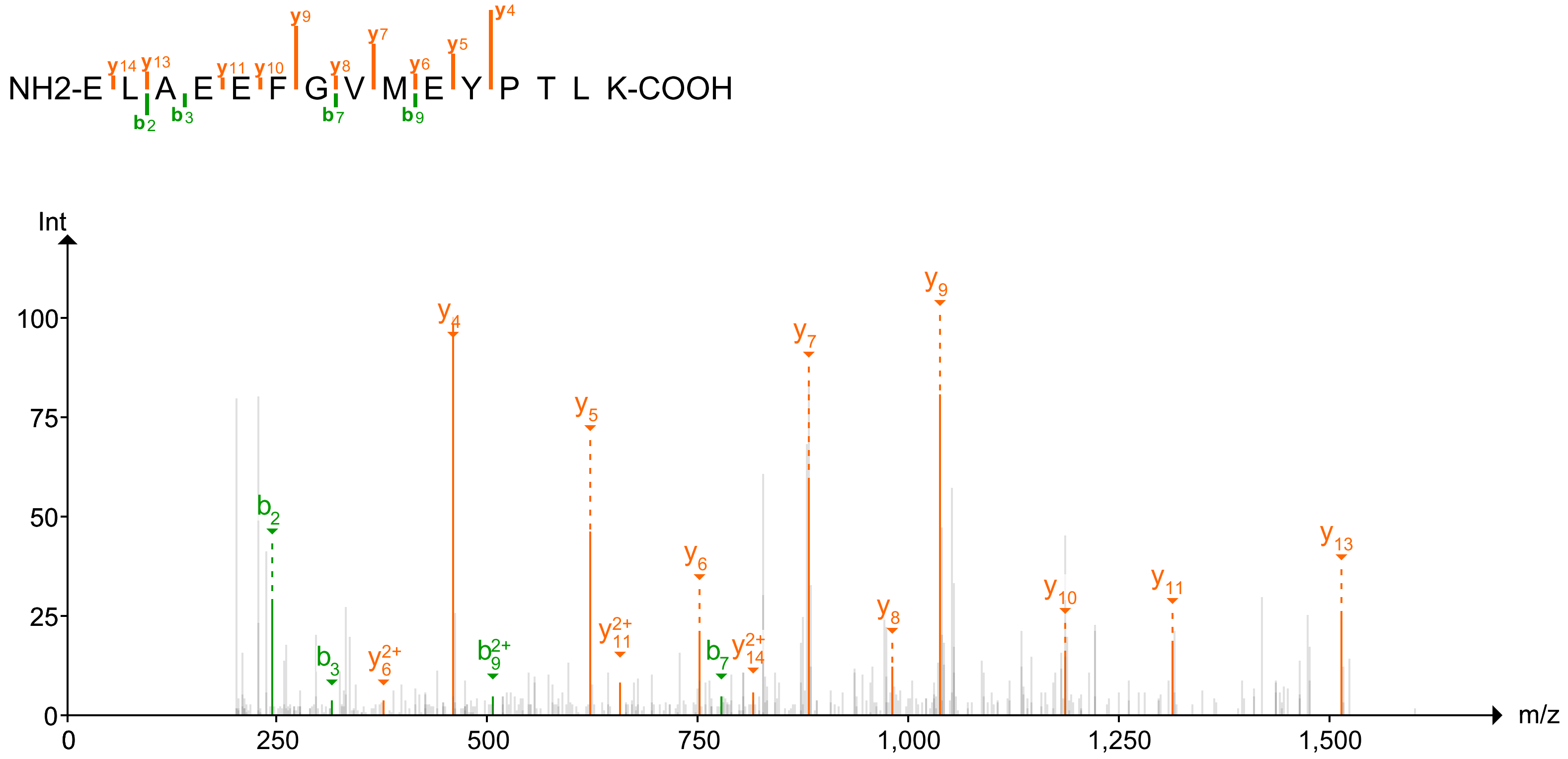

NH2-E L A E E F G V M E Y P T L K-COOH
y14 y13 y11 y10 y9 y8 y7 y6 y5 y4
b2 b3 b7 b9
Int
100
75
50
25
0
m/z
0
250
500
750
1,000
1,250
1,500
y4
y9
y7
y5
b2
y6
y13
y11
y10
y8
b3
y6 2+
b9 2+
y11 2+
b7
y14 2+

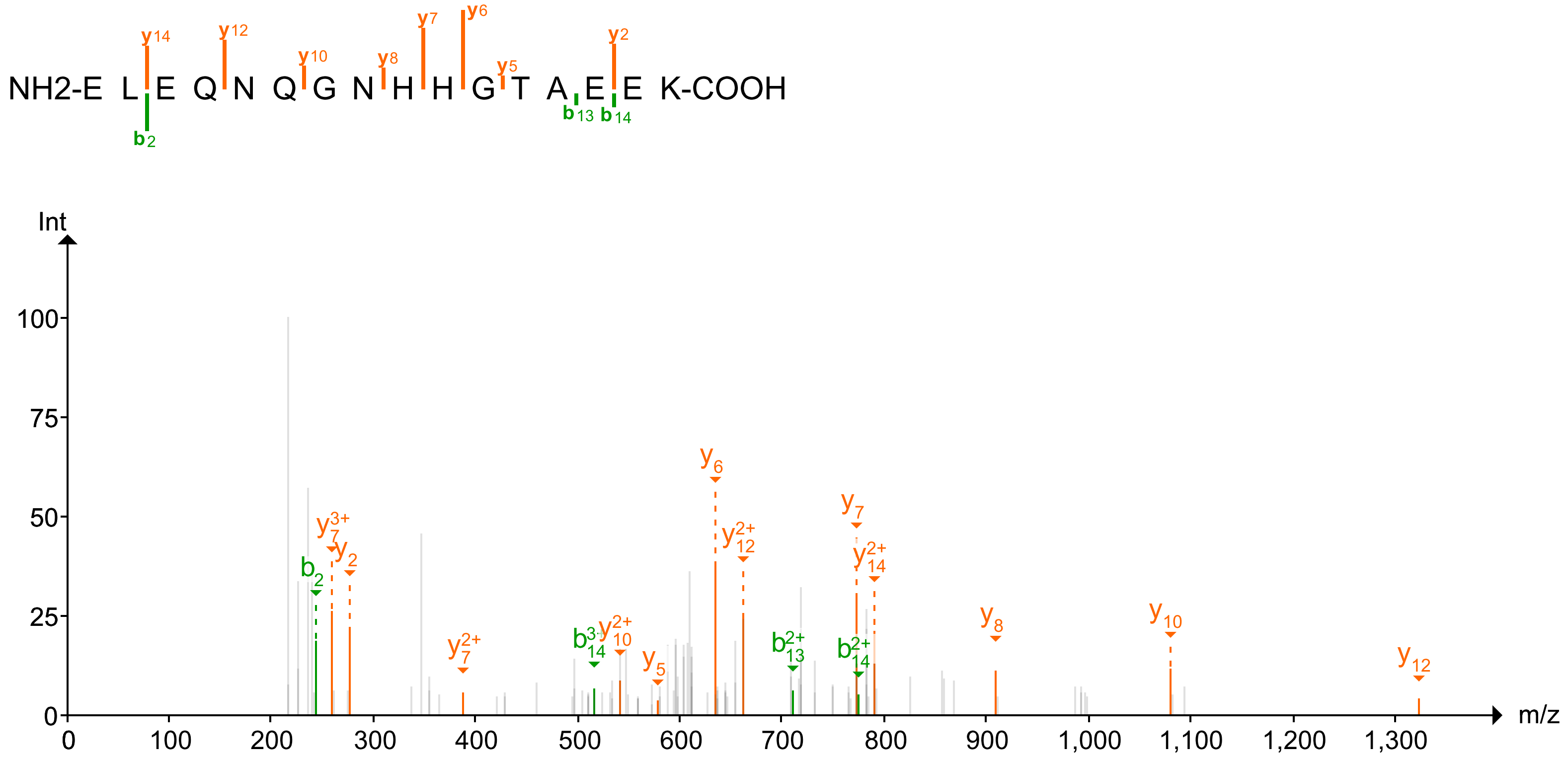

NH2-E L E Q N Q G N H H G T A E E K-COOH
Int
m/z

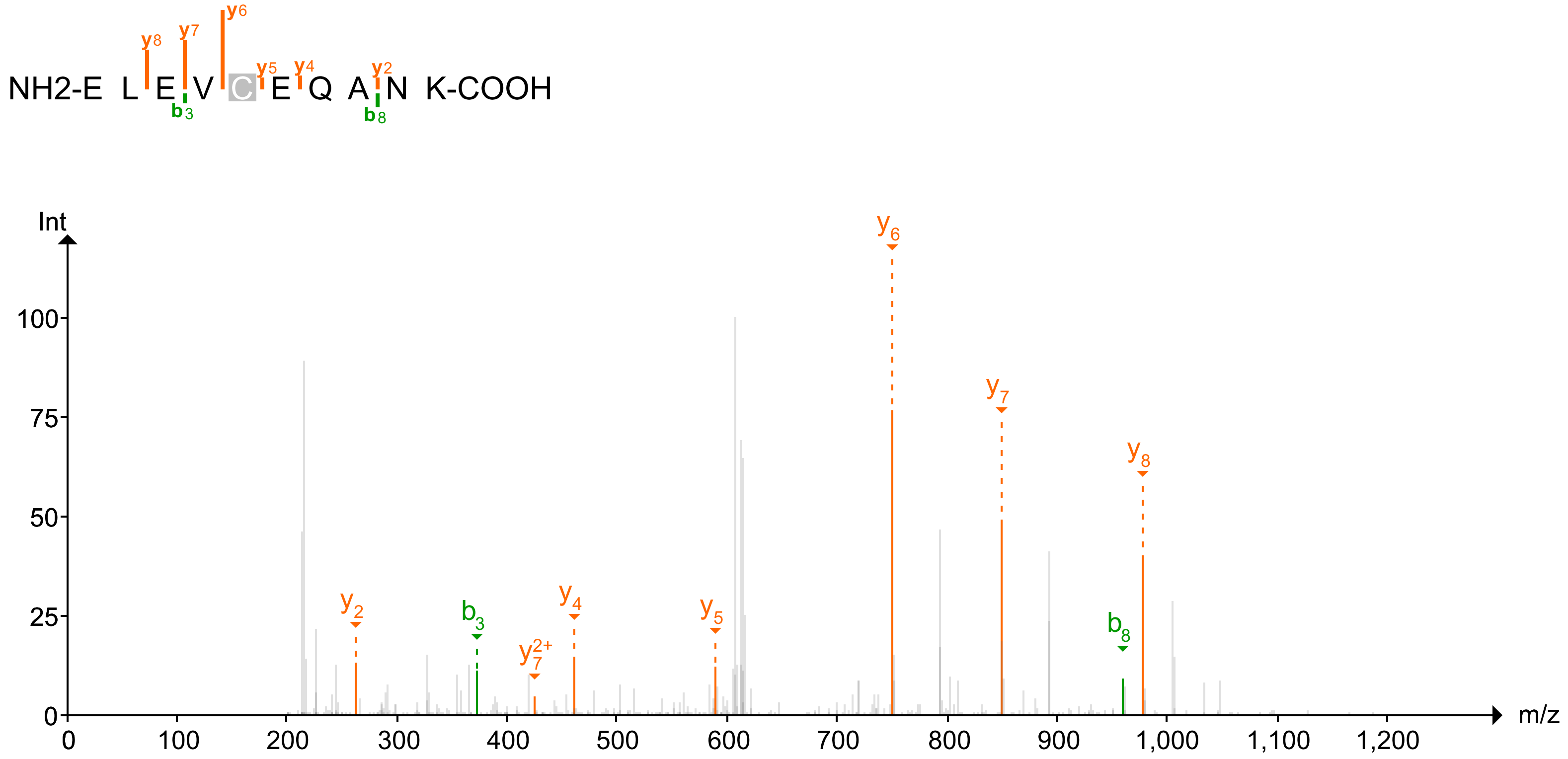

NH2-E L E V C E Q A N K-COOH
y8 y7 y6 y5 y4 y2
b3 b8
Int
100
75
50
25
0
0 100 200 300 400 500 600 700 800 900 1,000 1,100 1,200
m/z
y6 y7 y8 y2 b3 y7 2+ y4 y5 b8

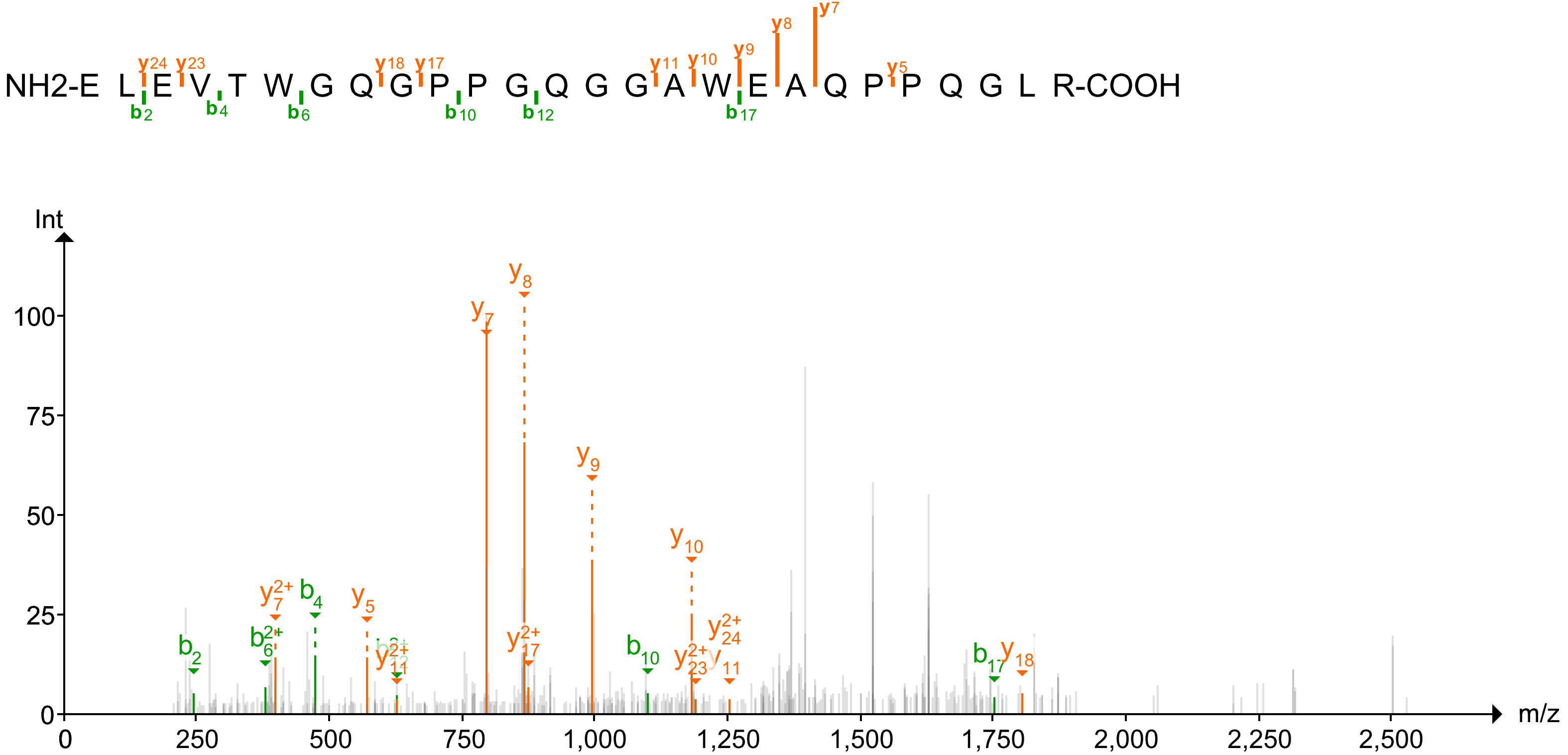

NH2-E L E V T W G Q G P P G Q G G A W E A Q P P Q G L R-COOH
y24 y23 y18 y17 y11 y10 y9 y8 y7 y5
b2 b4 b6 b10 b12 b17
Int
m/z
0
25
50
75
100
250
500
750
1,000
1,250
1,500
1,750
2,000
2,250
2,500

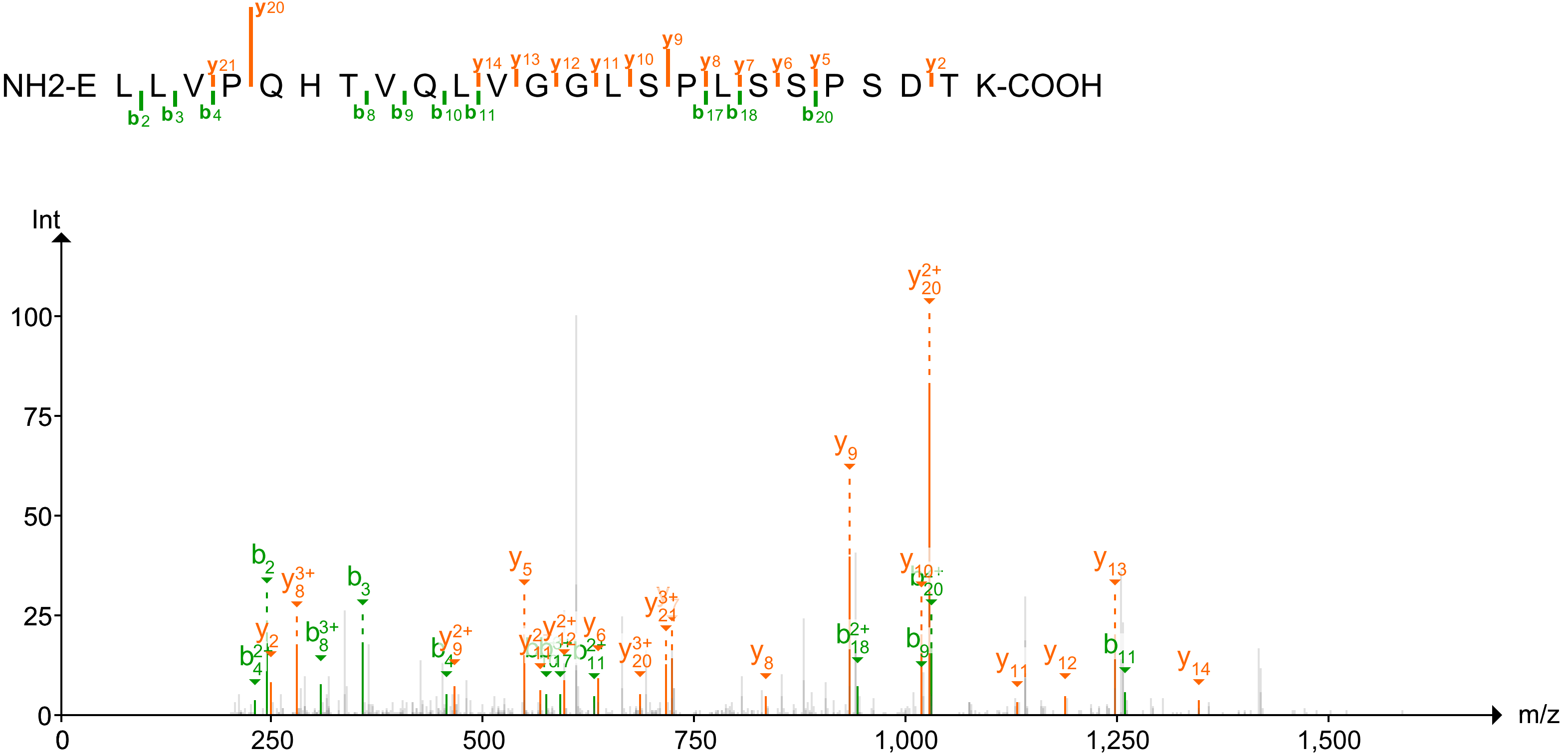

NH2-E L L V P Q H T V Q L V G G L S P L S S P S D T K-COOH
y20 y21 y14 y13 y12 y11 y10 y9 y8 y7 y6 y5 y2
b2 b3 b4 b8 b9 b10 b11 b17 b18 b20
Int
100
75
50
25
0
m/z
0
250
500
750
1,000
1,250
1,500
$y_{20}^{2+}$
$y_9$
$b_2$
$y_8^{3+}$
$b_3$
$y_5$
$y_{13}$
$y_2$
$b_4^{2+}$
$b_8^{3+}$
$y_9^{2+}$
$y_{12}^{2+}$
$y_6$
$b_{11}^{2+}$
$y_{20}^{3+}$
$y_{21}^{3+}$
$y_8$
$b_{18}^{2+}$
$b_{20}^{2+}$
$b_9$
$y_{11}$
$y_{12}$
$b_{11}$
$y_{14}$

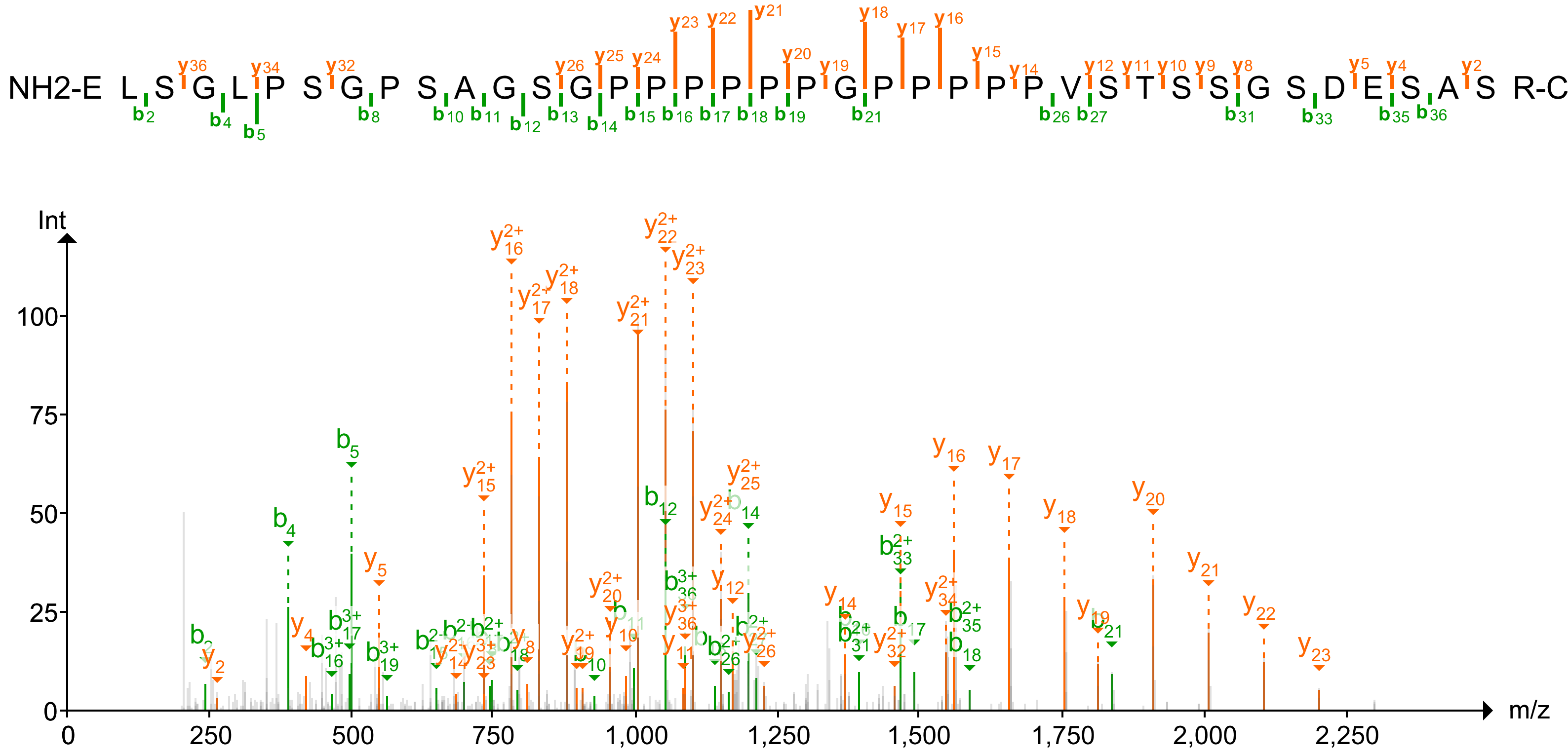

NH2-E L S G L P S G P S A G S G P P P P P P G P P P P P V S T S S G S D E S A S R-C
Int
100
75
50
25
0
m/z
0
250
500
750
1,000
1,250
1,500
1,750
2,000
2,250

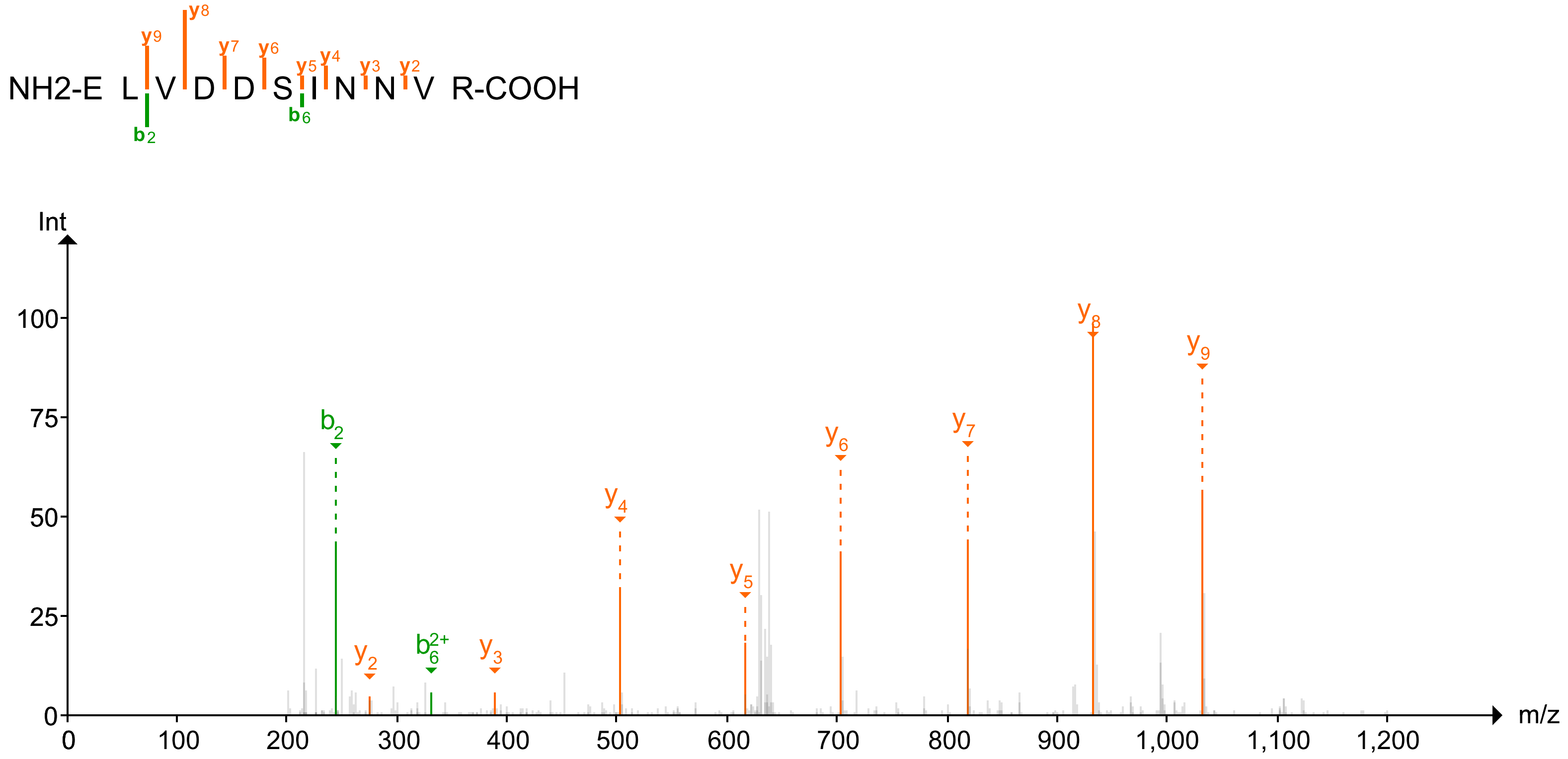
NH2-E L V D D S I N N V R-COOH
y9 y8 y7 y6 y5 y4 y3 y2
b2 b6
Int
100
75
50
25
0
b2
y2
b6 2+
y3
y4
y5
y6
y7
y8
y9
0 100 200 300 400 500 600 700 800 900 1,000 1,100 1,200
m/z

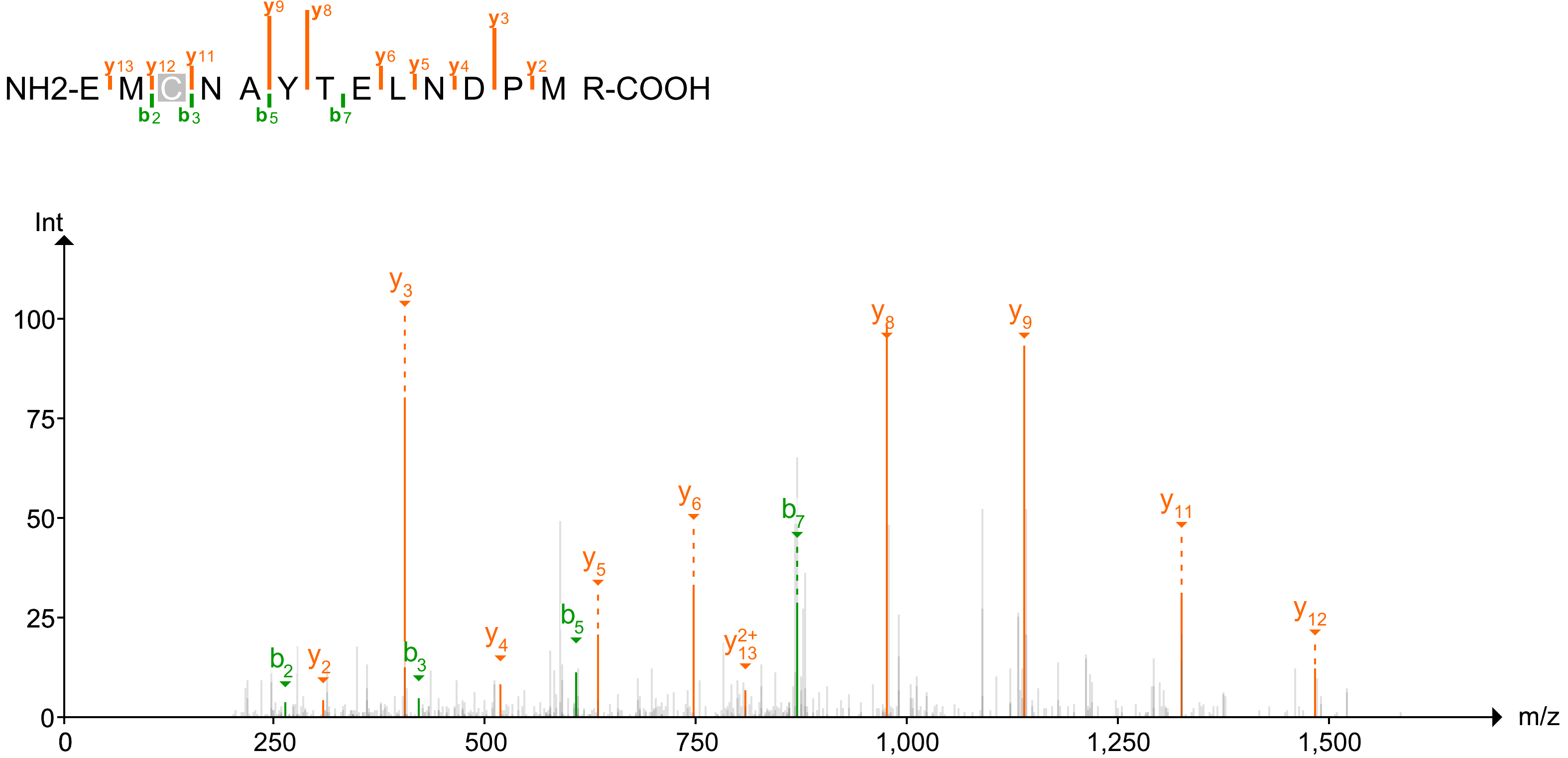

NH2-E M C N A Y T E L N D P M R-COOH
y13 y12 y11 y9 y8 y6 y5 y4 y3 y2
b2 b3 b5 b7
Int
m/z
y3
y8
y9
y6
b7
y11
y5
b5
y4
y12
b2
y2
b3
y13 2+
100
75
50
25
0
250
500
750
1,000
1,250
1,500

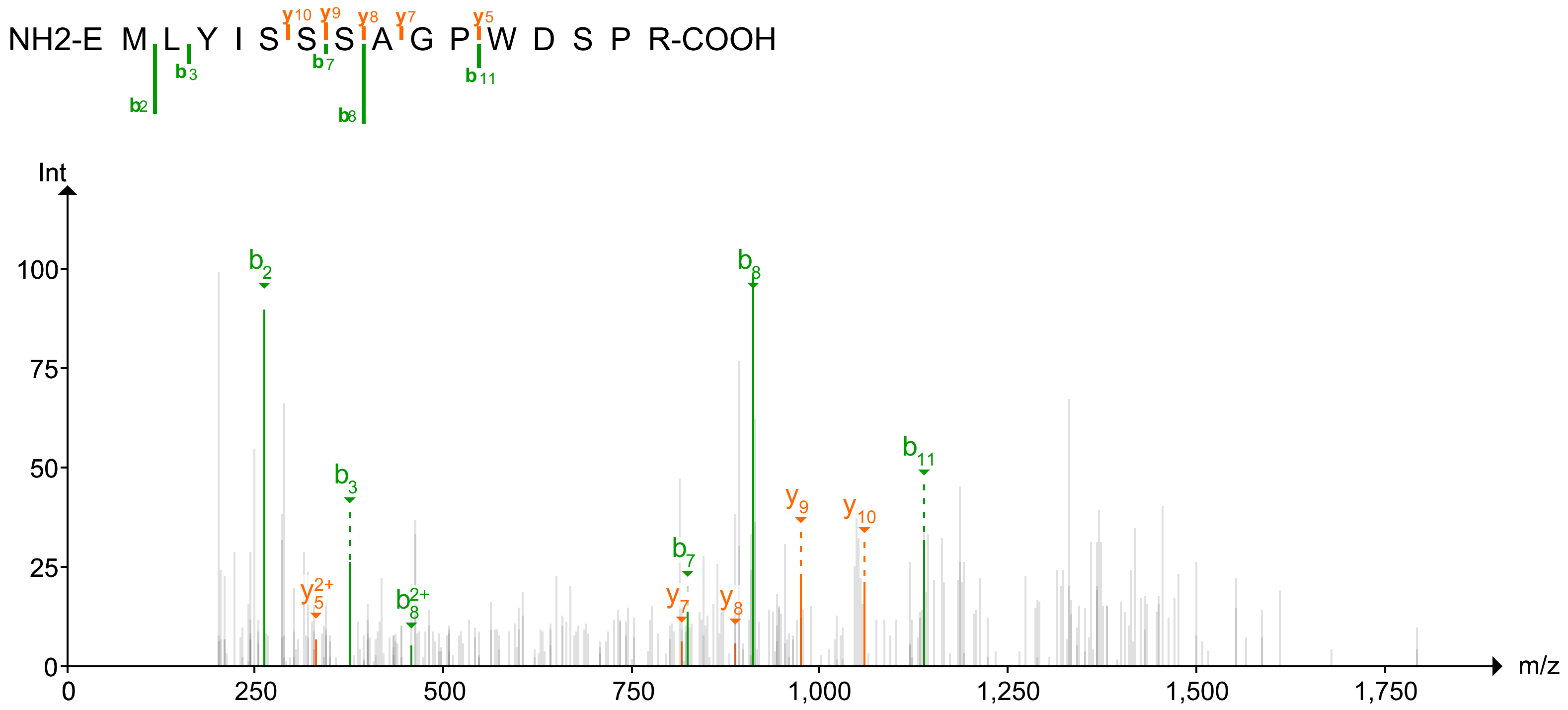

NH2-E M L Y I S S S A G P W D S P R-COOH
y10 y9 y8 y7 y5
b2 b3 b7 b8 b11
Int
100
75
50
25
0
m/z
0
250
500
750
1,000
1,250
1,500
1,750
b2
b8
b11
b3
y9
y10
b7
y5 2+
b8 2+
y7
y8

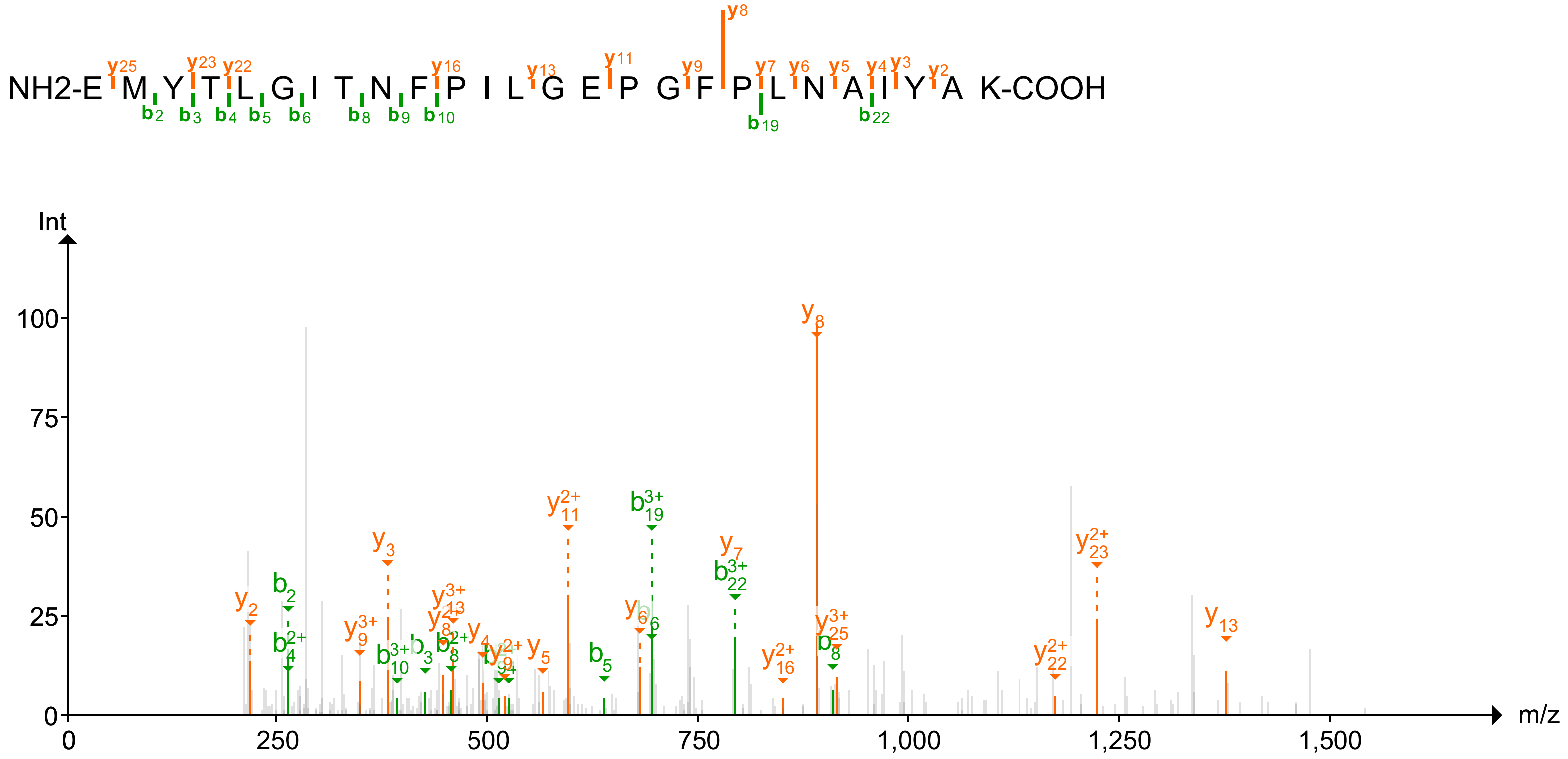
NH2-E M Y T L G I T N F P I L G E P G F P L N A I Y A K-COOH
Int
m/z
0
25
50
75
100
250
500
750
1,000
1,250
1,500

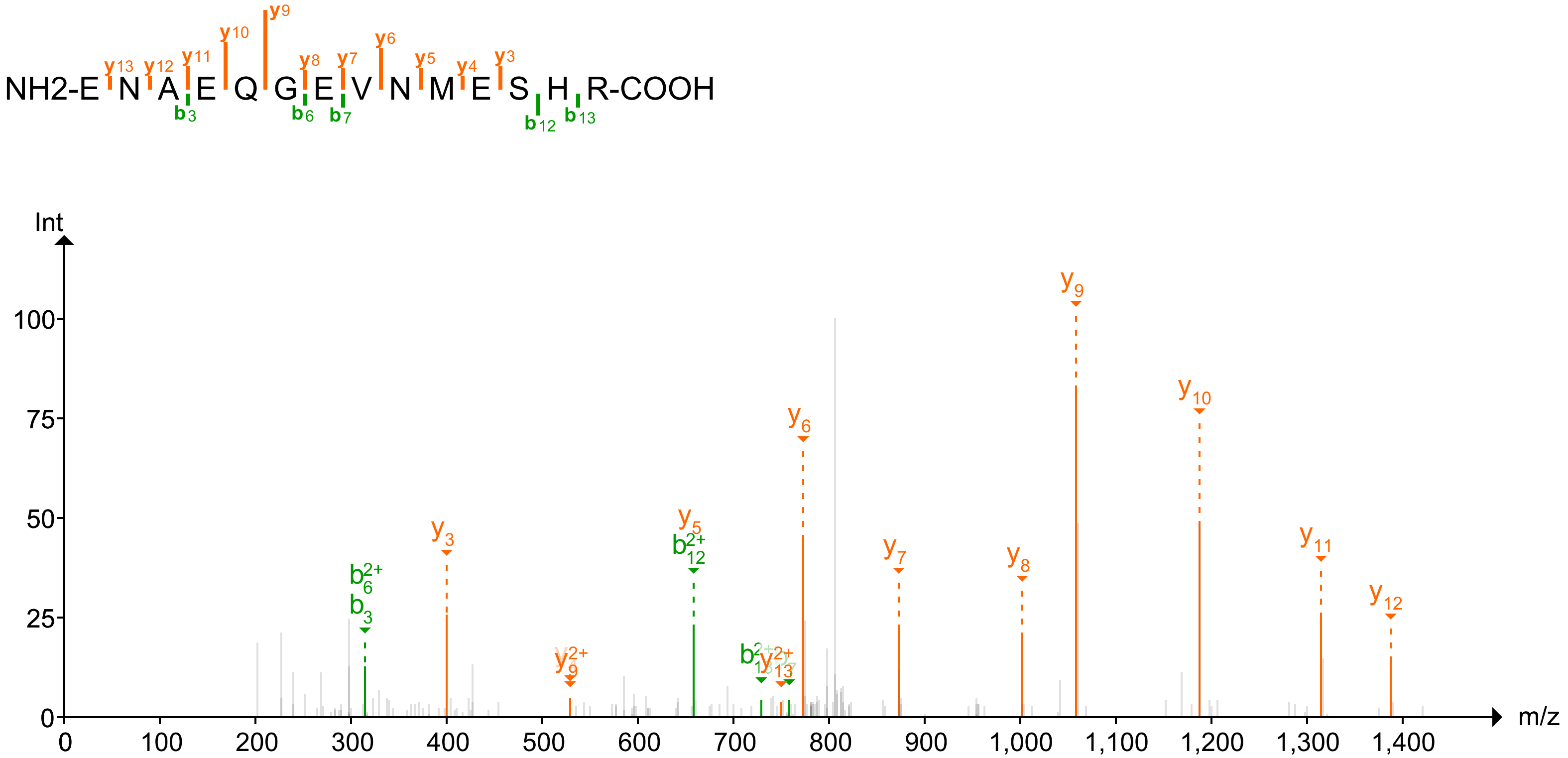

NH2-E N A E Q G E V N M E S H R-COOH
y13 y12 y11 y10 y9 y8 y7 y6 y5 y4 y3
b3 b6 b7 b12 b13
Int
m/z
0
25
50
75
100
0
100
200
300
400
500
600
700
800
900
1,000
1,100
1,200
1,300
1,400
y9
y10
y6
y5
y3
y11
y7
y8
y12

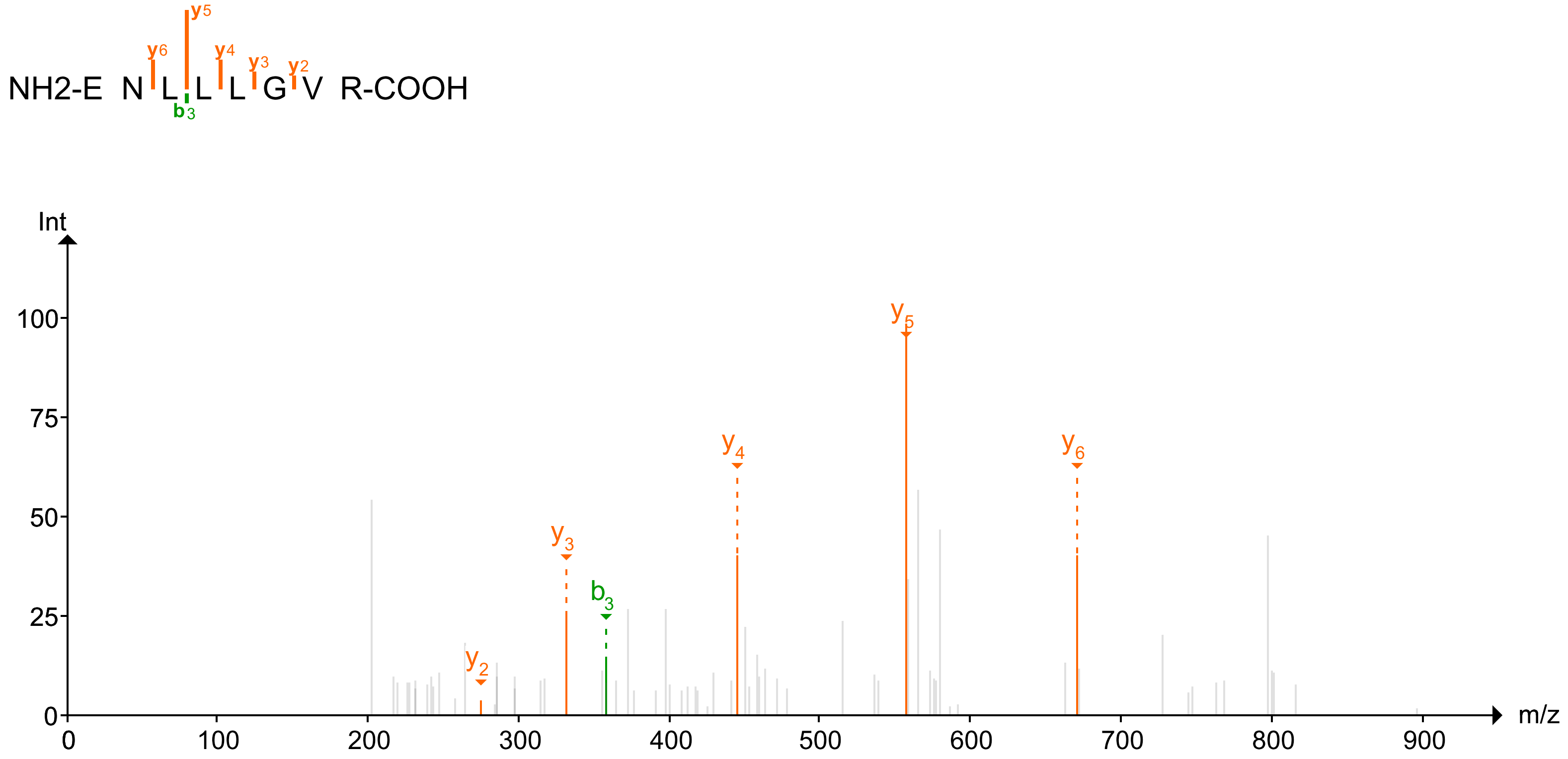

NH2-E N L L L G V R-COOH
y6
y5
y4
y3
y2
b3
Int
100
75
50
25
0
y5
y4
y6
y3
b3
y2
0
100
200
300
400
500
600
700
800
900
m/z

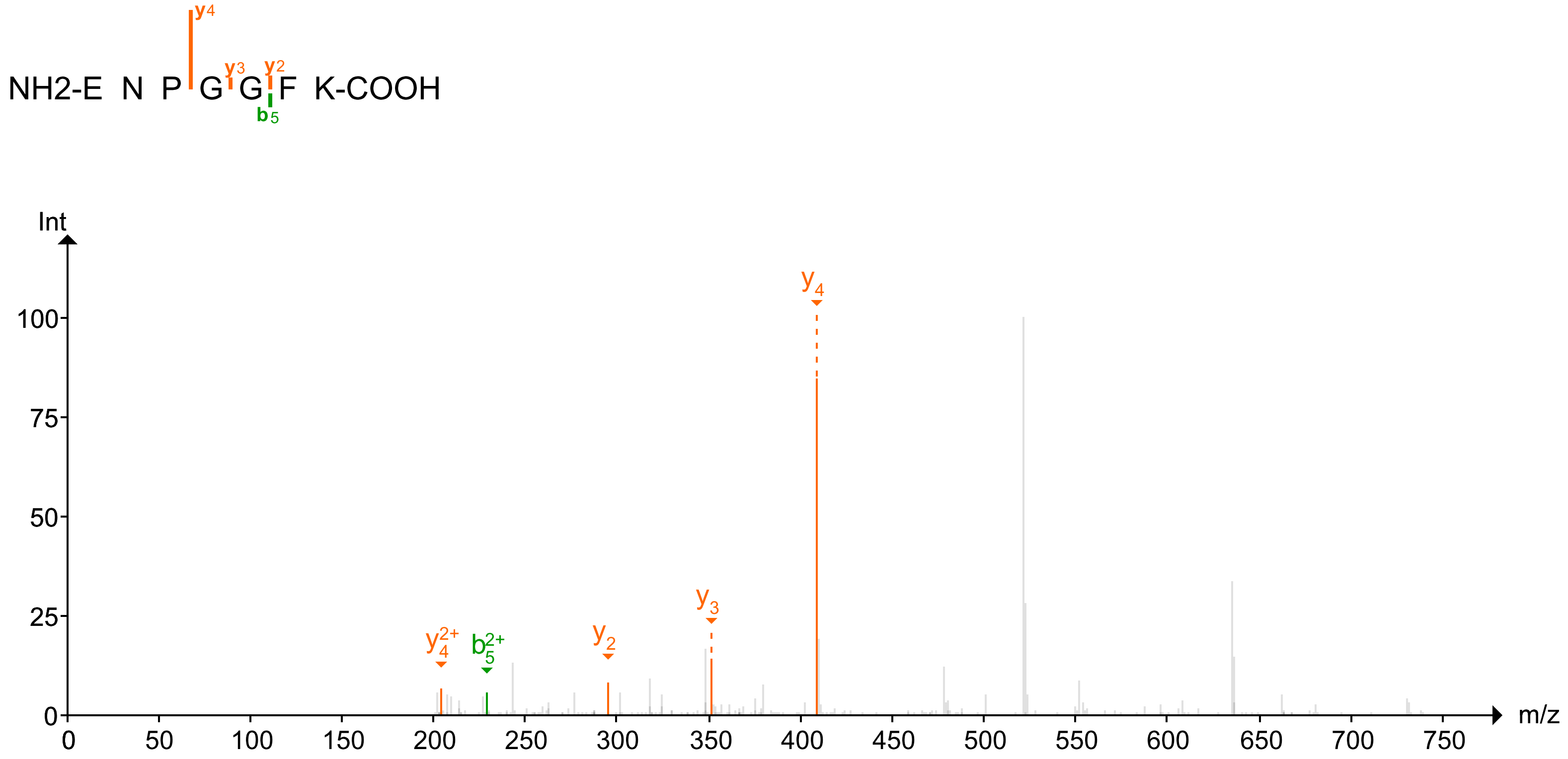

NH2-E N P G G F K-COOH
y4
y3
y2
b5
Int
m/z
y4
y3
y2
y4 2+
b5 2+
0
25
50
75
100
0
50
100
150
200
250
300
350
400
450
500
550
600
650
700
750

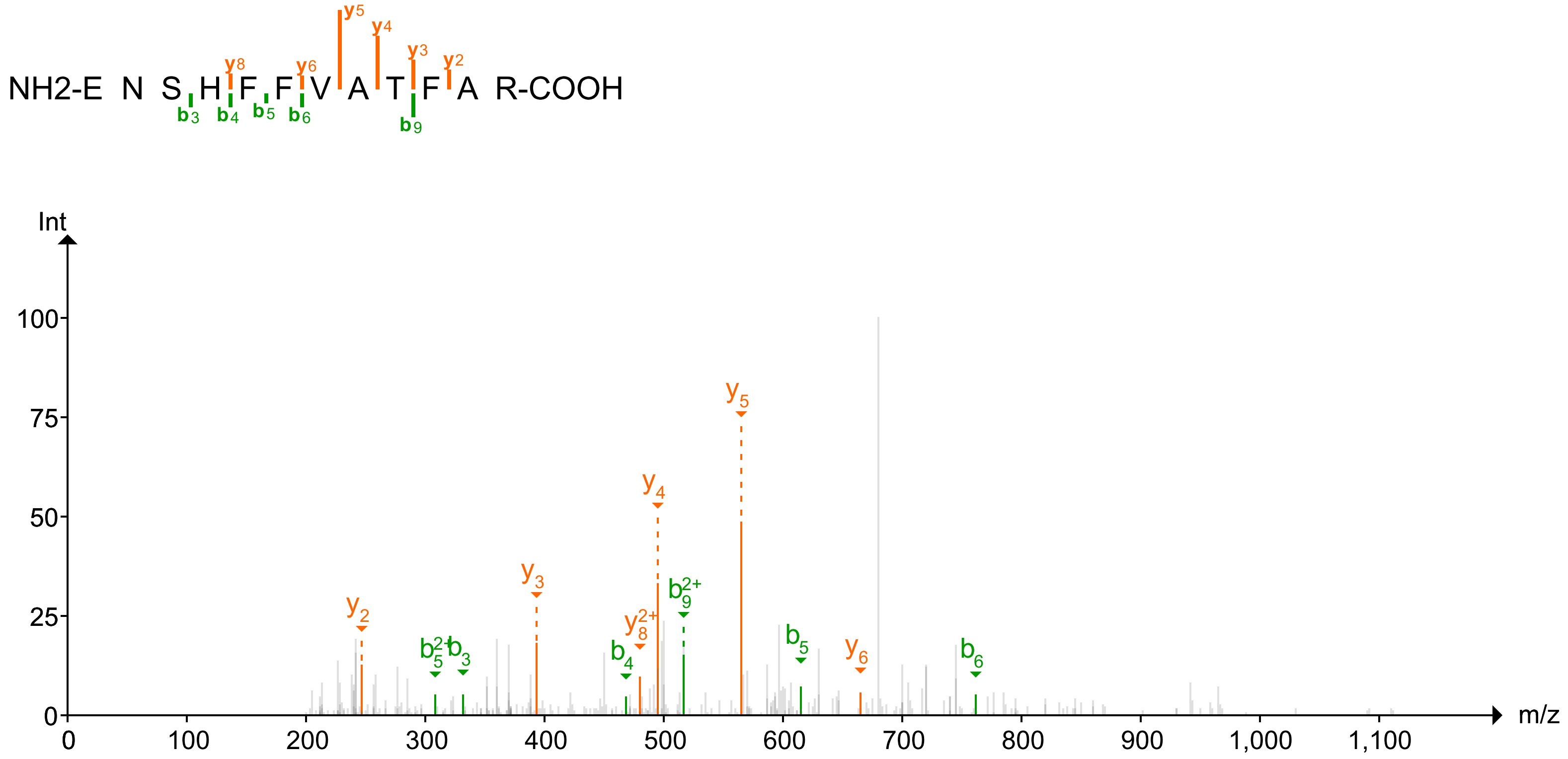

NH2-E N S H F F V A T F A R-COOH
y5 y4 y3 y2 y8 y6
b3 b4 b5 b6 b9
Int
100
75
50
25
0
y5
y4
y3
y2
b9 2+
y8 2+
b5 2+
b3
b4
b5
y6
b6
0 100 200 300 400 500 600 700 800 900 1,000 1,100
m/z

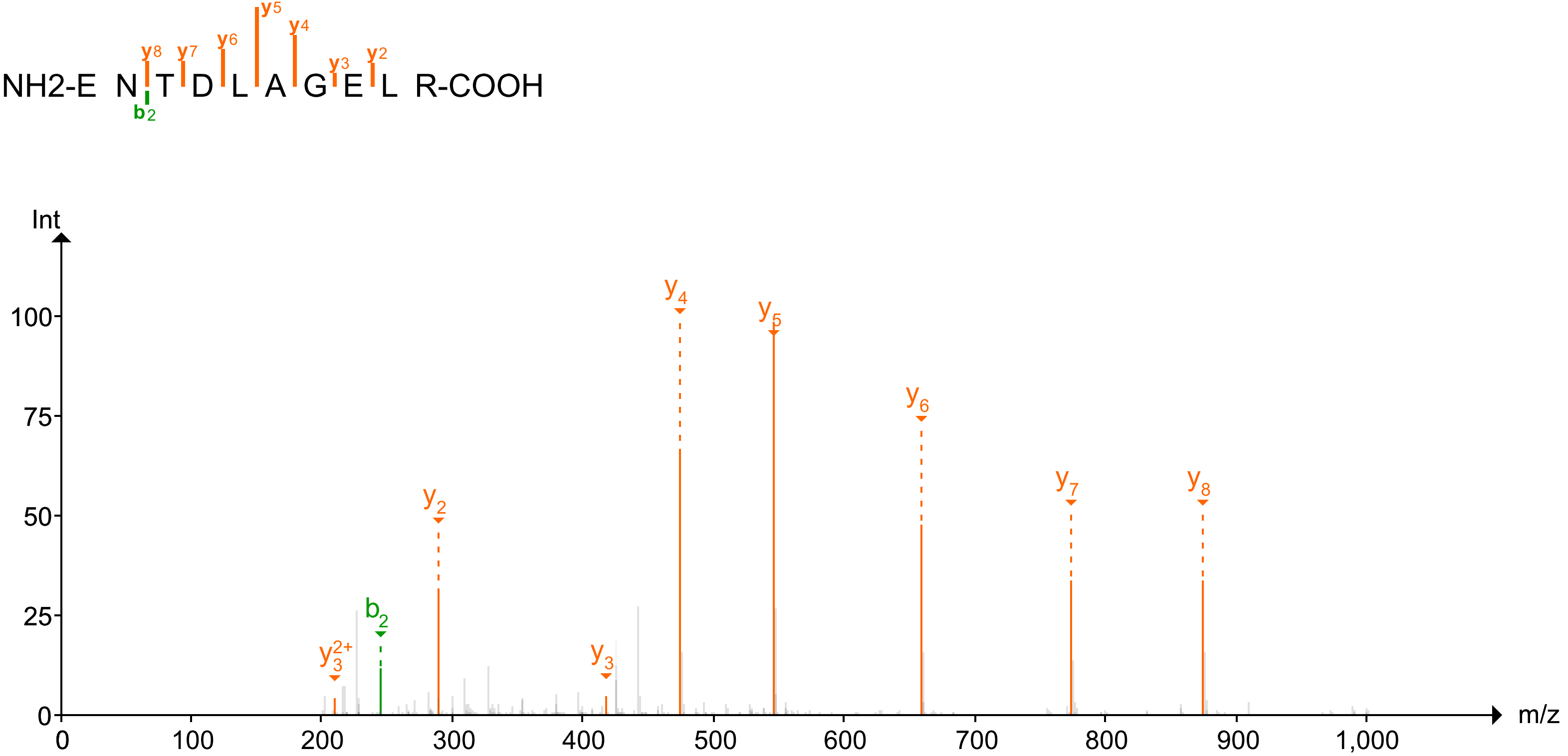

NH2-E N T D L A G E L R-COOH
y8 y7 y6 y5 y4 y3 y2
b2
Int
100
75
50
25
0
m/z
0
100
200
300
400
500
600
700
800
900
1,000
$y_3^{2+}$
$b_2$
$y_2$
$y_3$
$y_4$
$y_5$
$y_6$
$y_7$
$y_8$

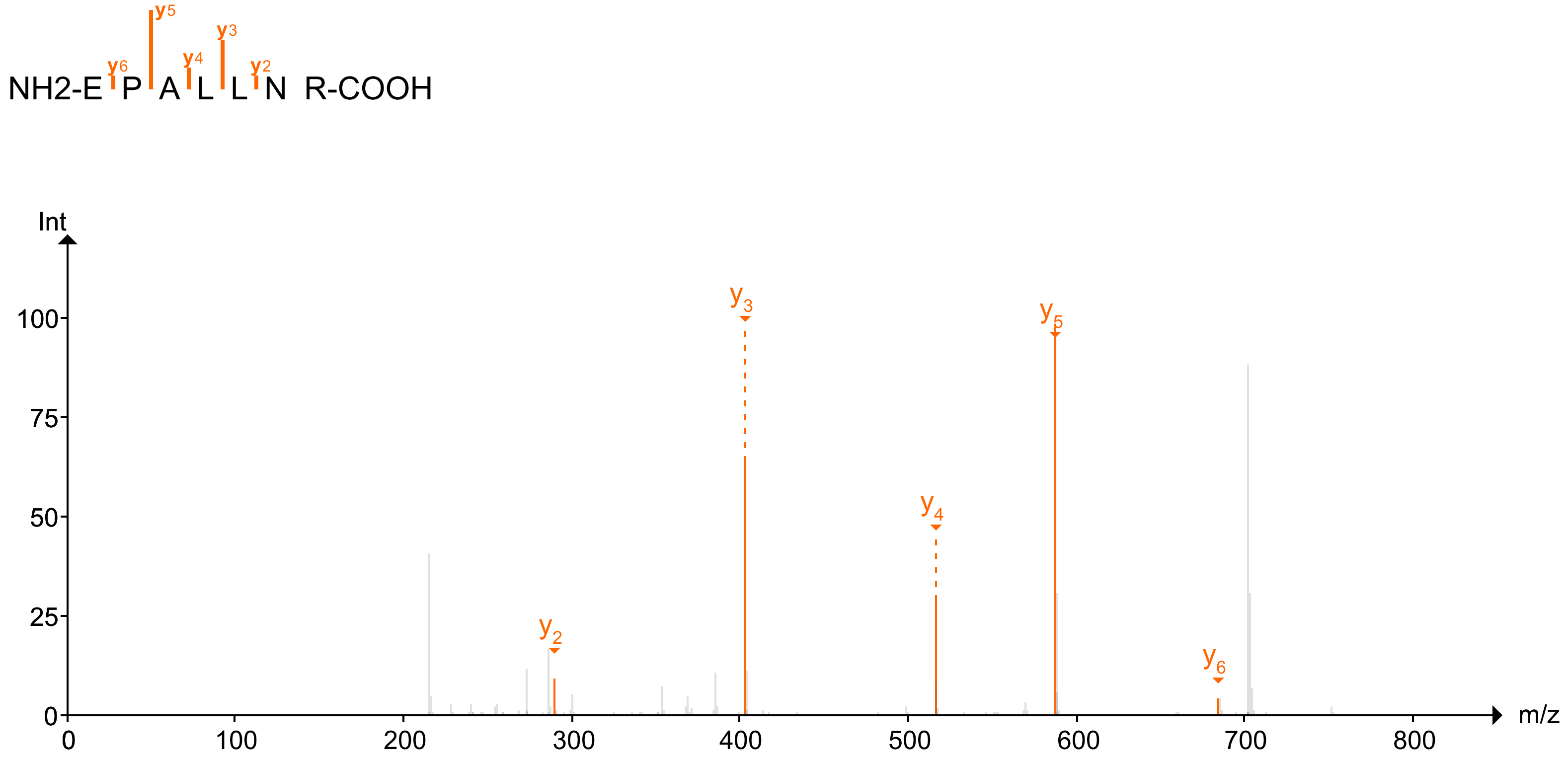

NH2-E P A L L N R-COOH
y6
y5
y4
y3
y2
Int
100
75
50
25
0
m/z
0
100
200
300
400
500
600
700
800

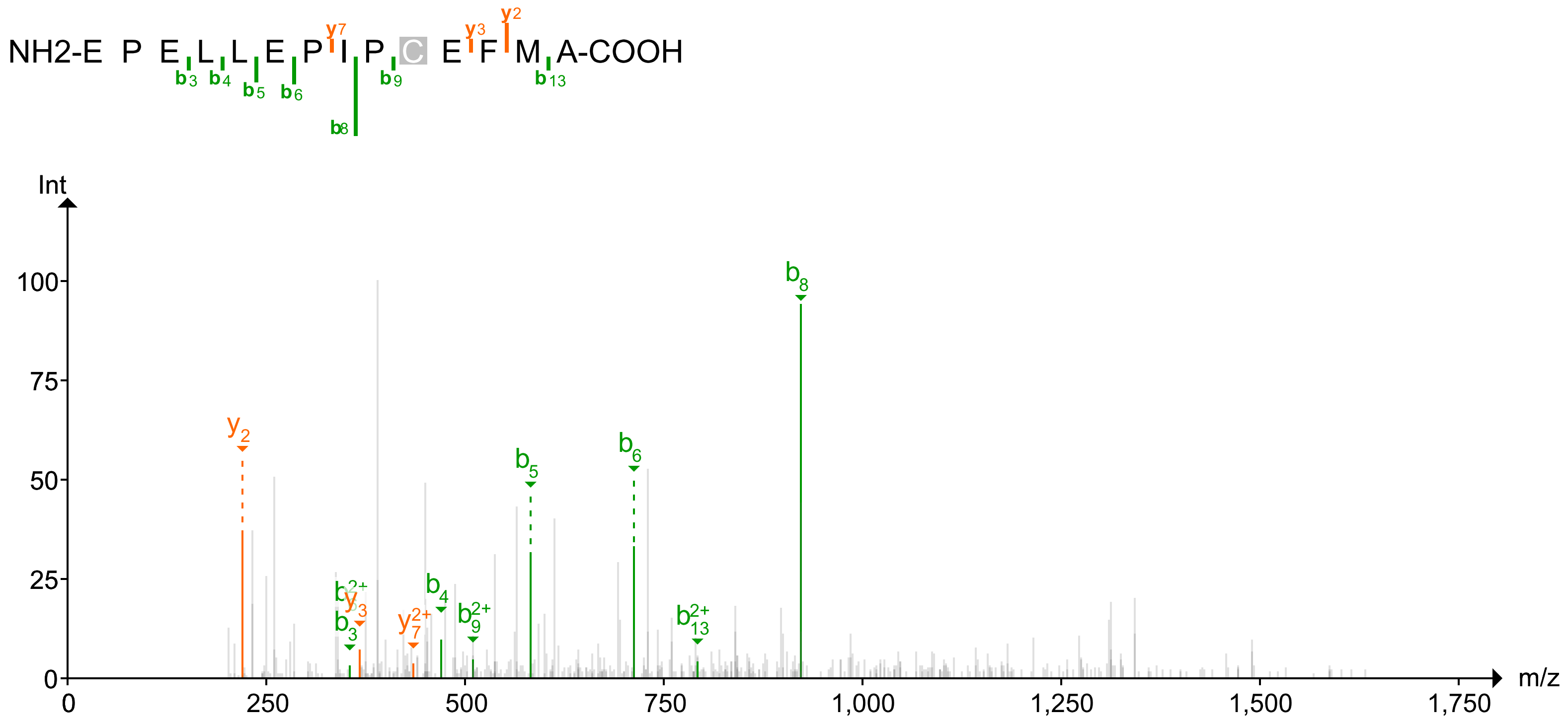

NH2-E P E L L E P I P C E F M A-COOH
y7 y3 y2
b3 b4 b5 b6 b8 b9 b13
Int
100
75
50
25
0
y2
b8
b5
b6
b4
b9 2+
b13 2+
y7 2+
y3
b3
b3 2+
0
250
500
750
1,000
1,250
1,500
1,750
m/z

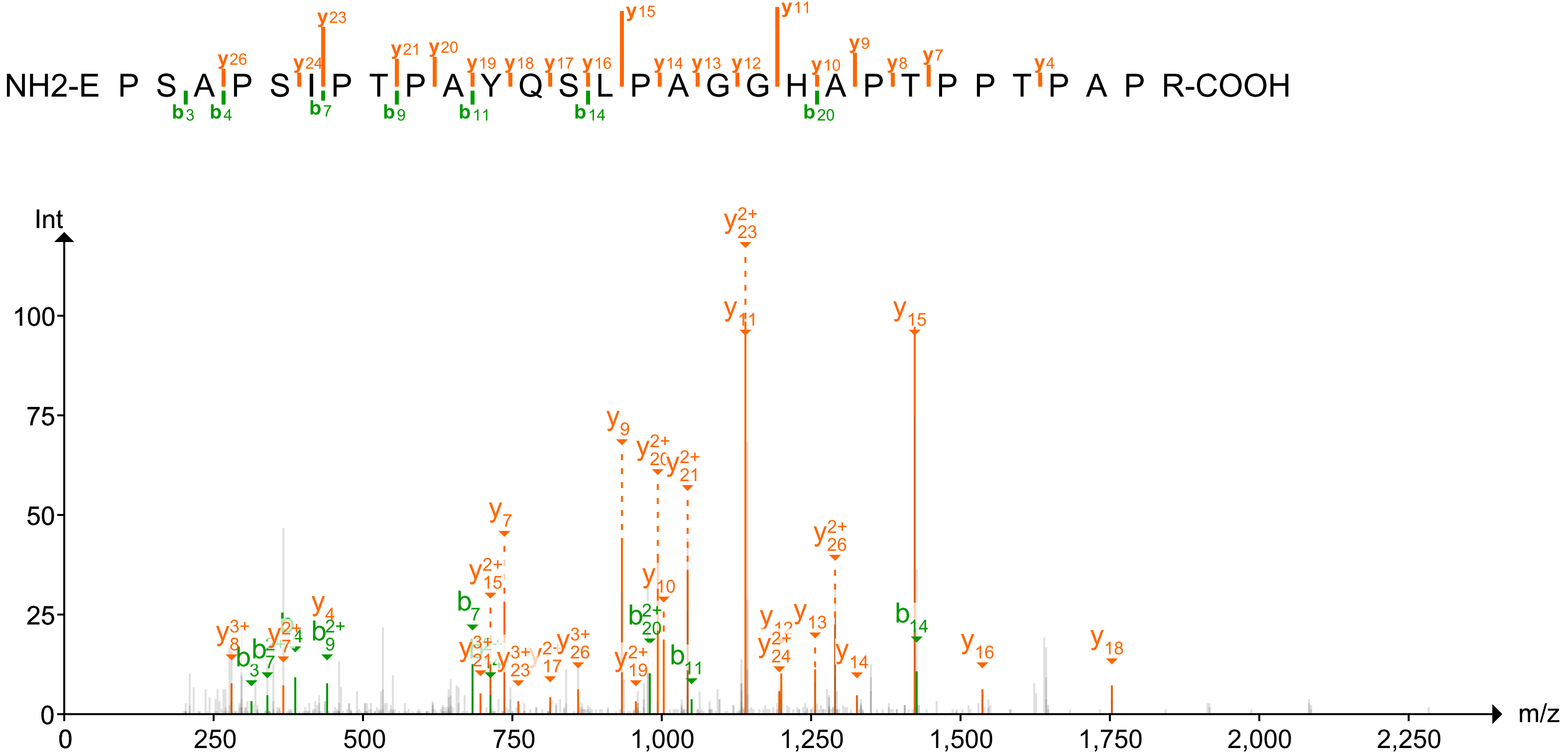

NH2-E P S A P S I P T P A Y Q S L P A G G H A P T P P T P A P R-COOH
y26 y24 y23 y21 y20 y19 y18 y17 y16 y15 y14 y13 y12 y11 y10 y9 y8 y7 y4
b3 b4 b7 b9 b11 b14 b20
Int
m/z
0
25
50
75
100
250
500
750
1,000
1,250
1,500
1,750
2,000
2,250

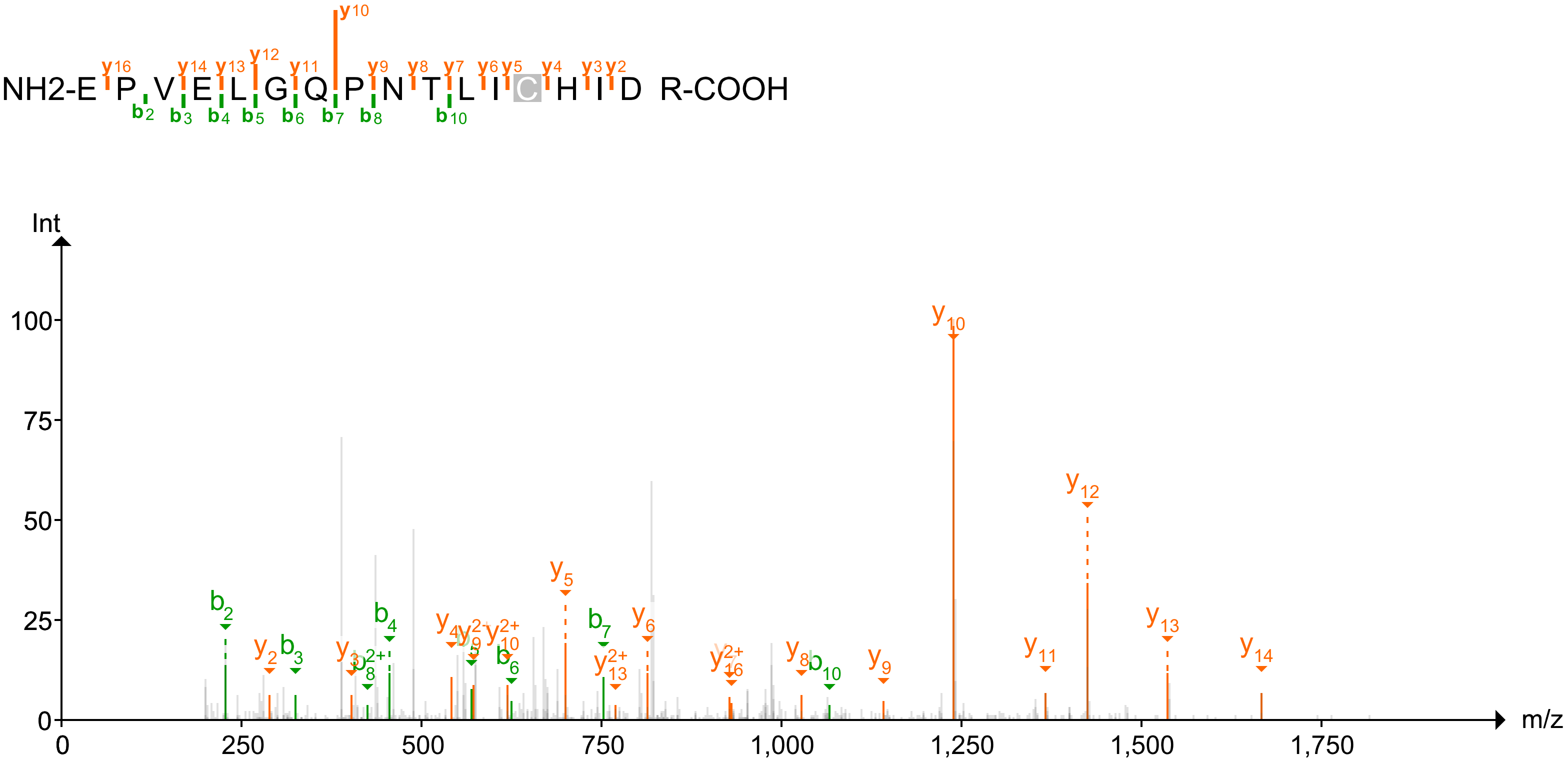

NH2-E P V E L G Q P N T L I C H I D R-COOH
y16 y14 y13 y12 y11 y10 y9 y8 y7 y6 y5 y4 y3 y2
b2 b3 b4 b5 b6 b7 b8 b10
Int
100
75
50
25
0
m/z
0
250
500
750
1,000
1,250
1,500
1,750
y10
y12
y5
y13
y11
y14
y9
y8
b10
y6
b7
b2
b4
b3
y2
y3
b8 2+
y4
y9 2+
y10 2+
b6
y13 2+
y16 2+

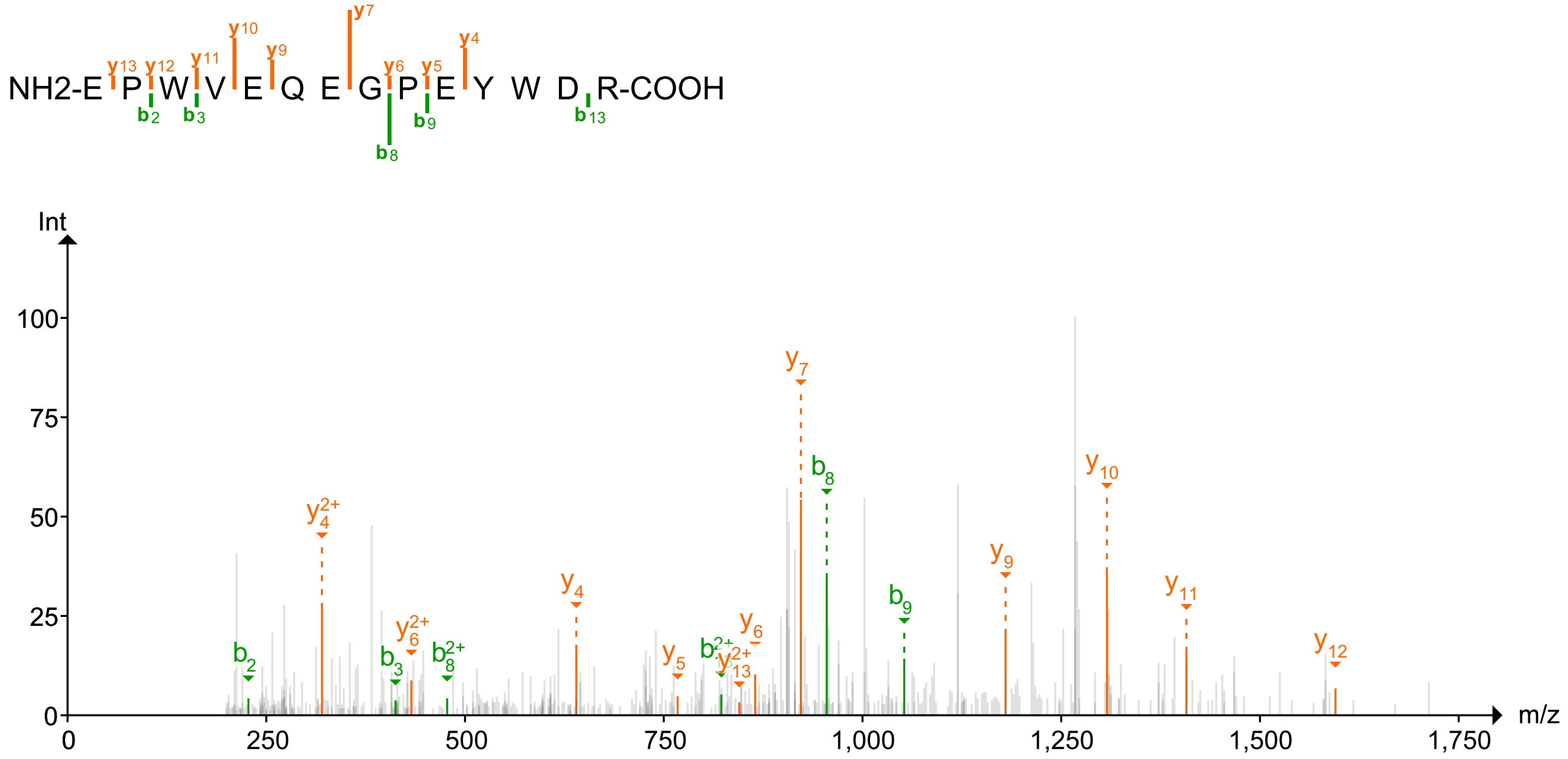

NH2-E P W V E Q E G P E Y W D R-COOH
Int
m/z

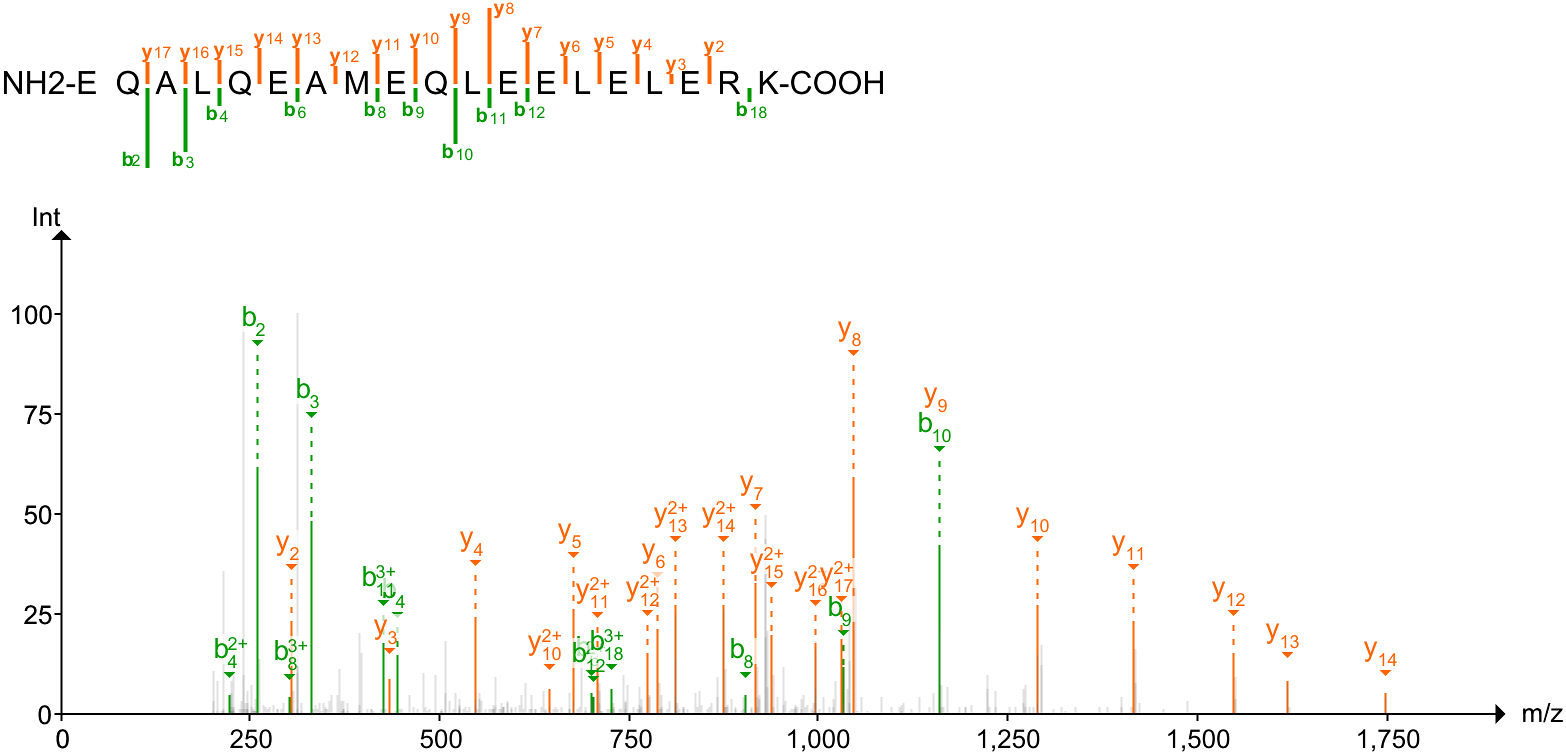

NH2-E Q A L Q E A M E Q L E E L E L E R K-COOH
y17 y16 y15 y14 y13 y12 y11 y10 y9 y8 y7 y6 y5 y4 y3 y2
b2 b3 b4 b6 b8 b9 b10 b11 b12 b18
Int
100
75
50
25
0
m/z
0
250
500
750
1,000
1,250
1,500
1,750
b2
b3
y2
y8
y9
b10
y7
y4
y5
y6
y10
y11
y12
y13
y14
b8
b9
y3

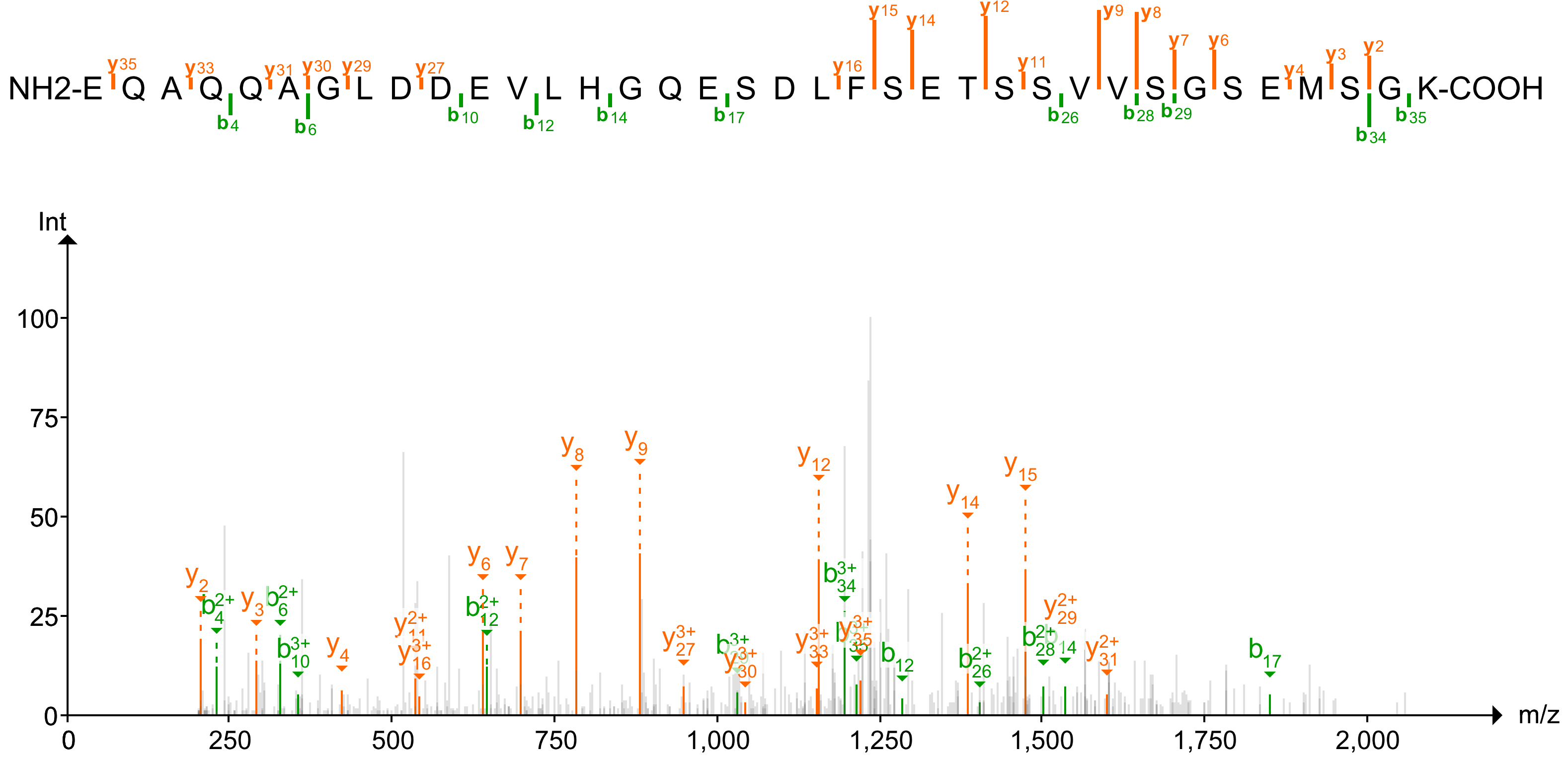

NH2-E Q A Q Q A G L D D E V L H G Q E S D L F S E T S S V V S G S E M S G K-COOH
y35 y33 y31 y30 y29 y27 y16 y15 y14 y12 y11 y9 y8 y7 y6 y4 y3 y2
b4 b6 b10 b12 b14 b17 b26 b28 b29 b34 b35
Int
100
75
50
25
0
m/z
0
250
500
750
1,000
1,250
1,500
1,750
2,000
y2 y3 y4 y6 y7 y8 y9 y12 y14 y15
b4 2+
b6 2+
b10 3+
y11 2+
y16 3+
b12 2+
y27 3+
y30 3+
y33 3+
b34 3+
y35 3+
b12
b26 2+
b28 2+
y29 2+
y31 2+
b17

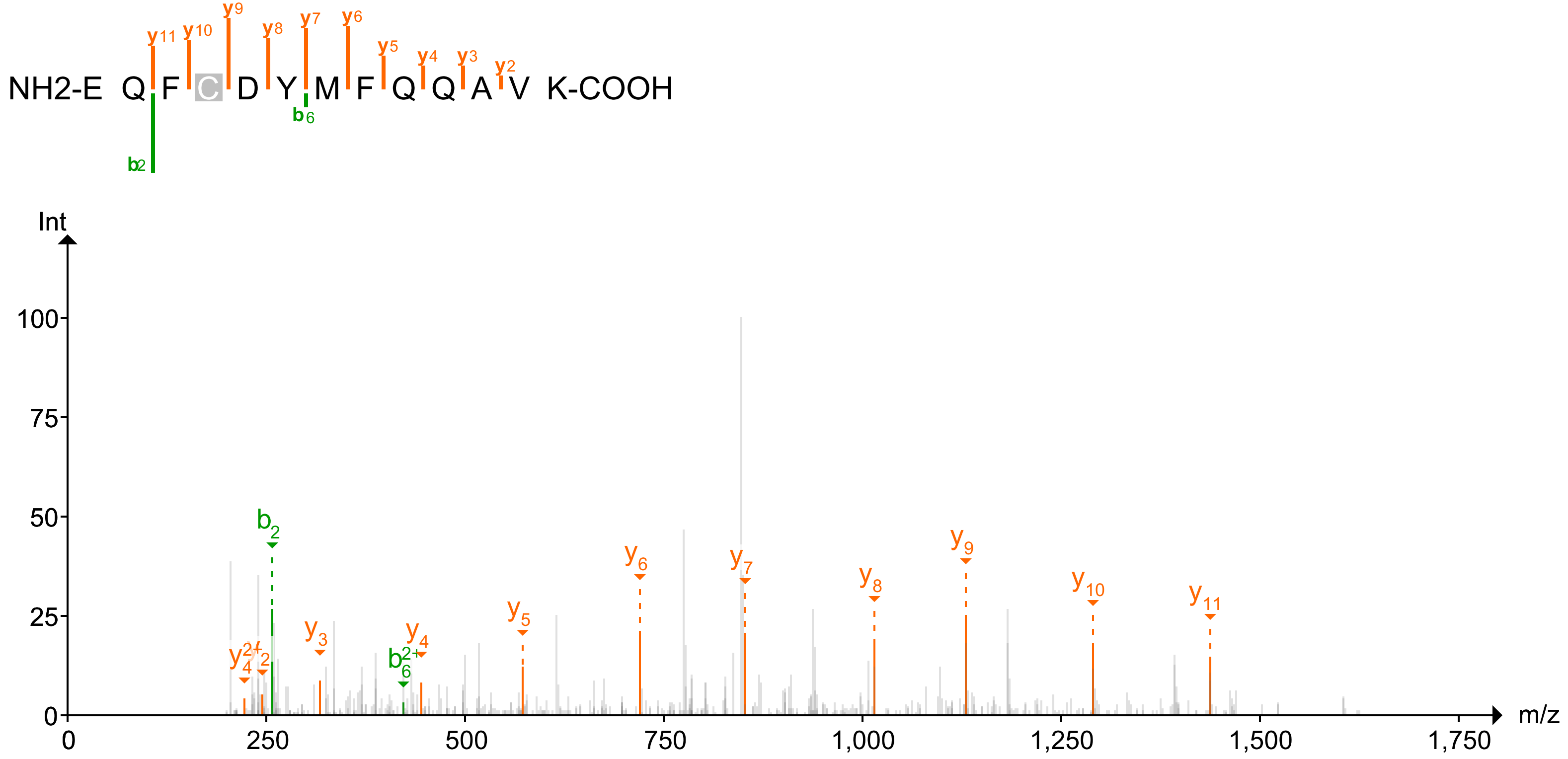

NH2-E Q F C D Y M F Q Q A V K-COOH
y11 y10 y9 y8 y7 y6 y5 y4 y3 y2
b2 b6
Int
100
75
50
25
0
m/z
0
250
500
750
1,000
1,250
1,500
1,750
b2
y6
y7
y8
y9
y10
y11
y5
y3
y4
b6 2+
y4 2+
y2

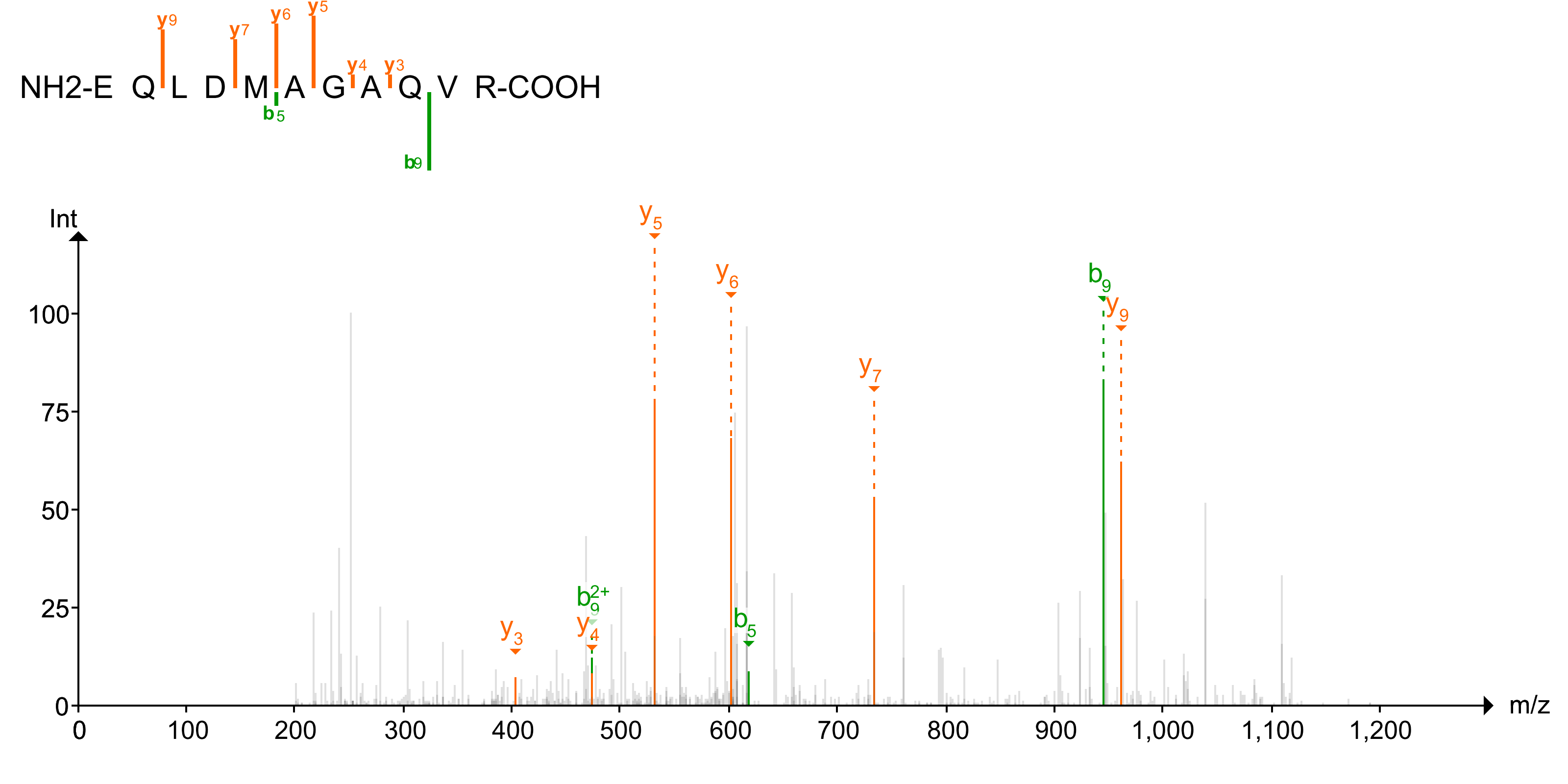

NH2-E Q L D M A G A Q V R-COOH
y9 y7 y6 y5 y4 y3
b5 b9
Int
m/z
y5 y6 b9 y9 y7 y3 y4 b5
0 25 50 75 100
0 100 200 300 400 500 600 700 800 900 1,000 1,100 1,200

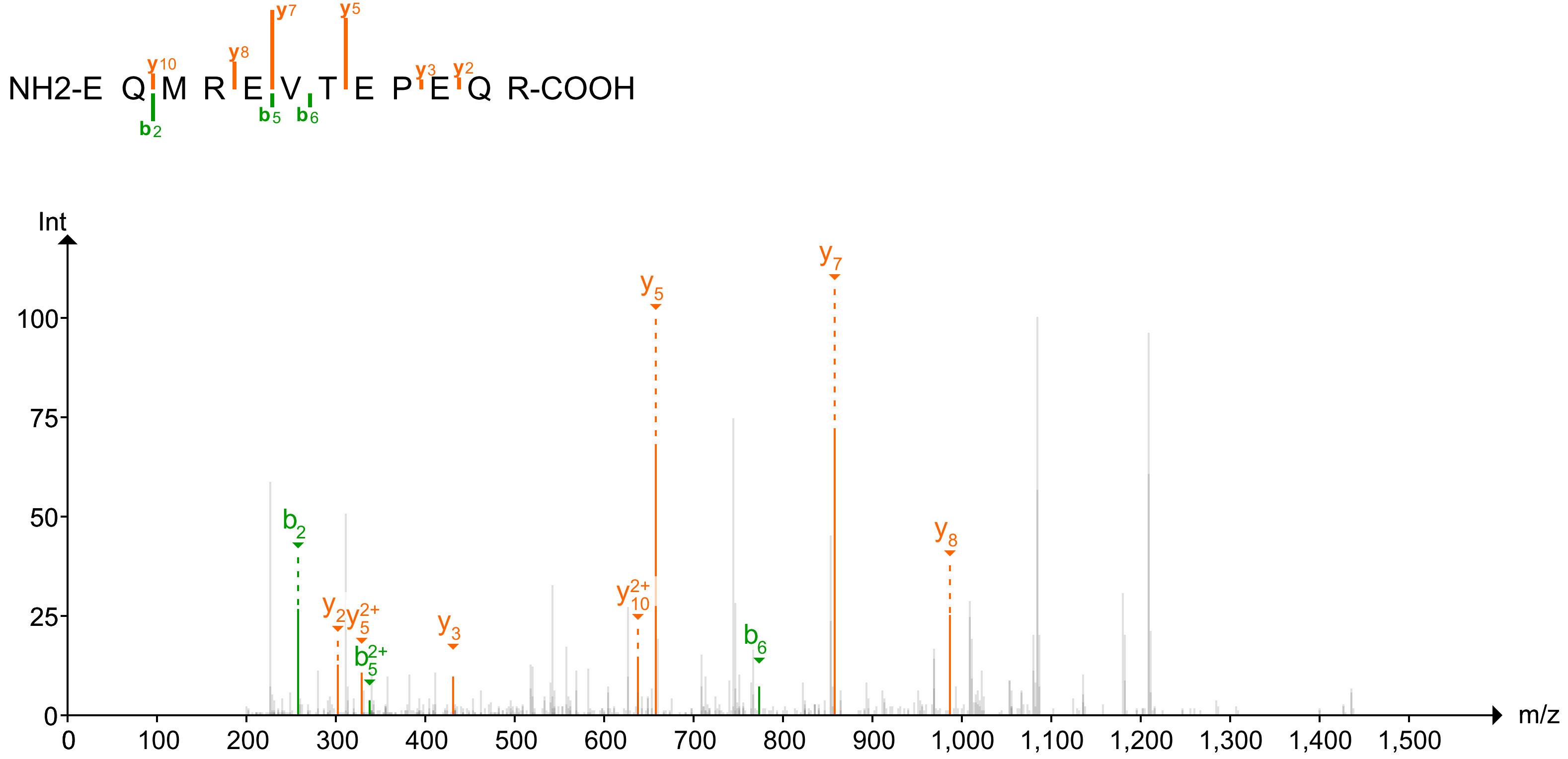
NH2-E Q M R E V T E P E Q R-COOH
y10 y8 y7 y5 y3 y2
b2 b5 b6
Int
m/z
0
25
50
75
100
0
100
200
300
400
500
600
700
800
900
1,000
1,100
1,200
1,300
1,400
1,500
b2
y2
y5 2+
b5 2+
y3
y10 2+
y5
b6
y7
y8

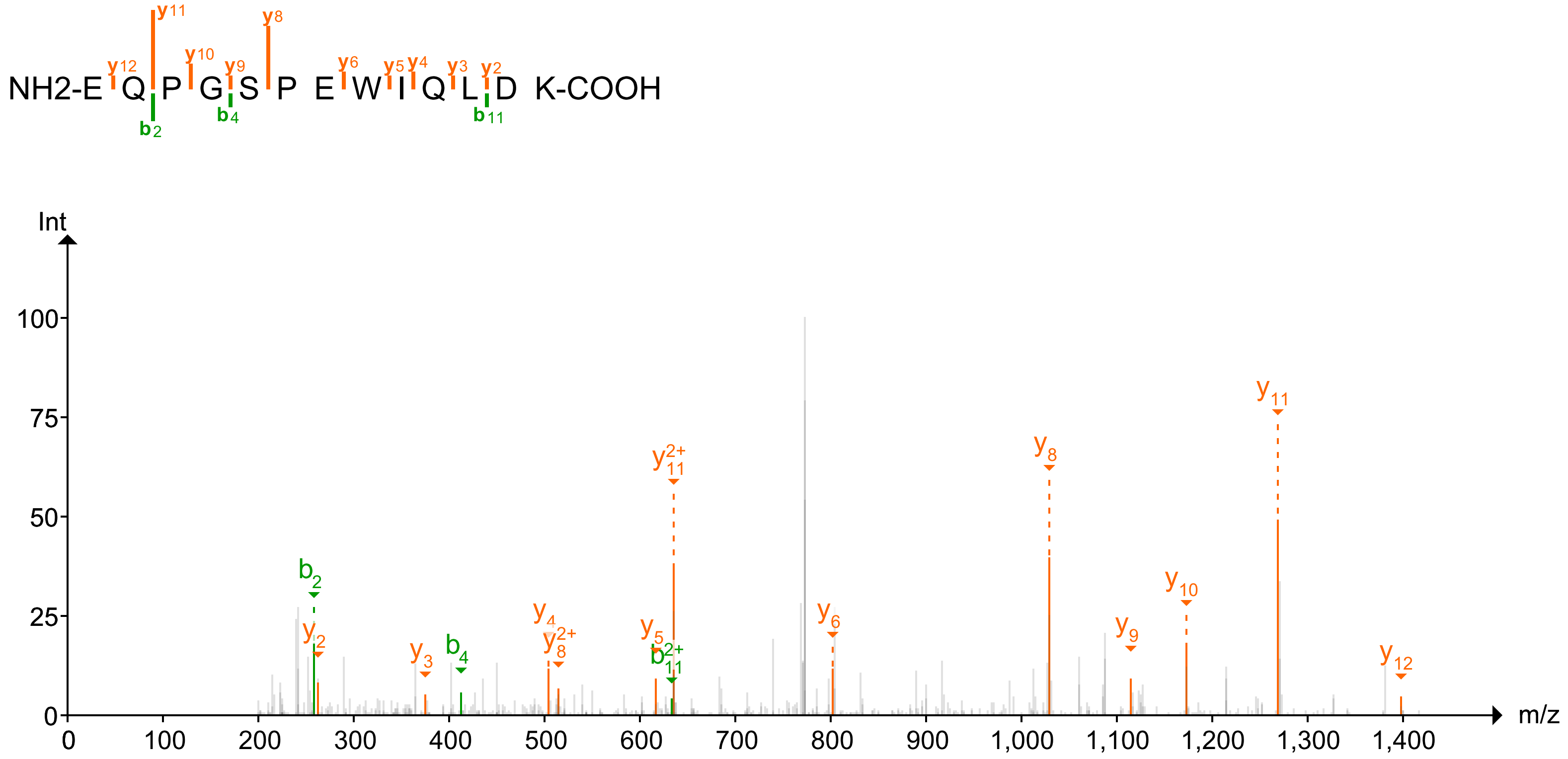

NH2-E Q P G S P E W I Q L D K-COOH
y12 y11 y10 y9 y8 y6 y5 y4 y3 y2
b2 b4 b11
Int
100
75
50
25
0
m/z
0
100
200
300
400
500
600
700
800
900
1,000
1,100
1,200
1,300
1,400

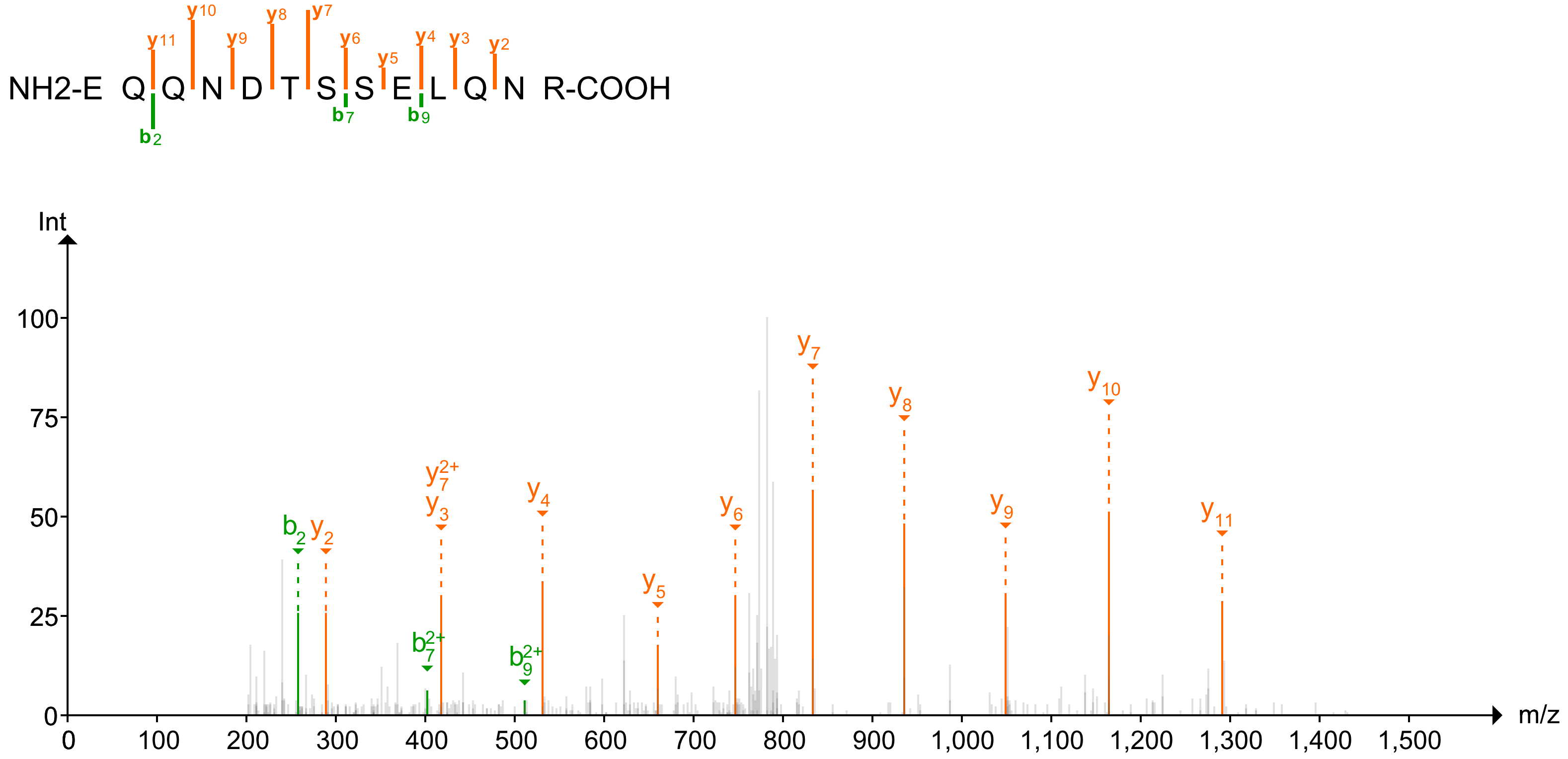

NH2-E Q Q N D T S S E L Q N R-COOH
y11 y10 y9 y8 y7 y6 y5 y4 y3 y2
b2 b7 b9
Int
100
75
50
25
0
b2 y2
b7 2+
y7 2+
y3
b9 2+
y4
y5
y6
y7
y8
y9
y10
y11
0 100 200 300 400 500 600 700 800 900 1,000 1,100 1,200 1,300 1,400 1,500
m/z

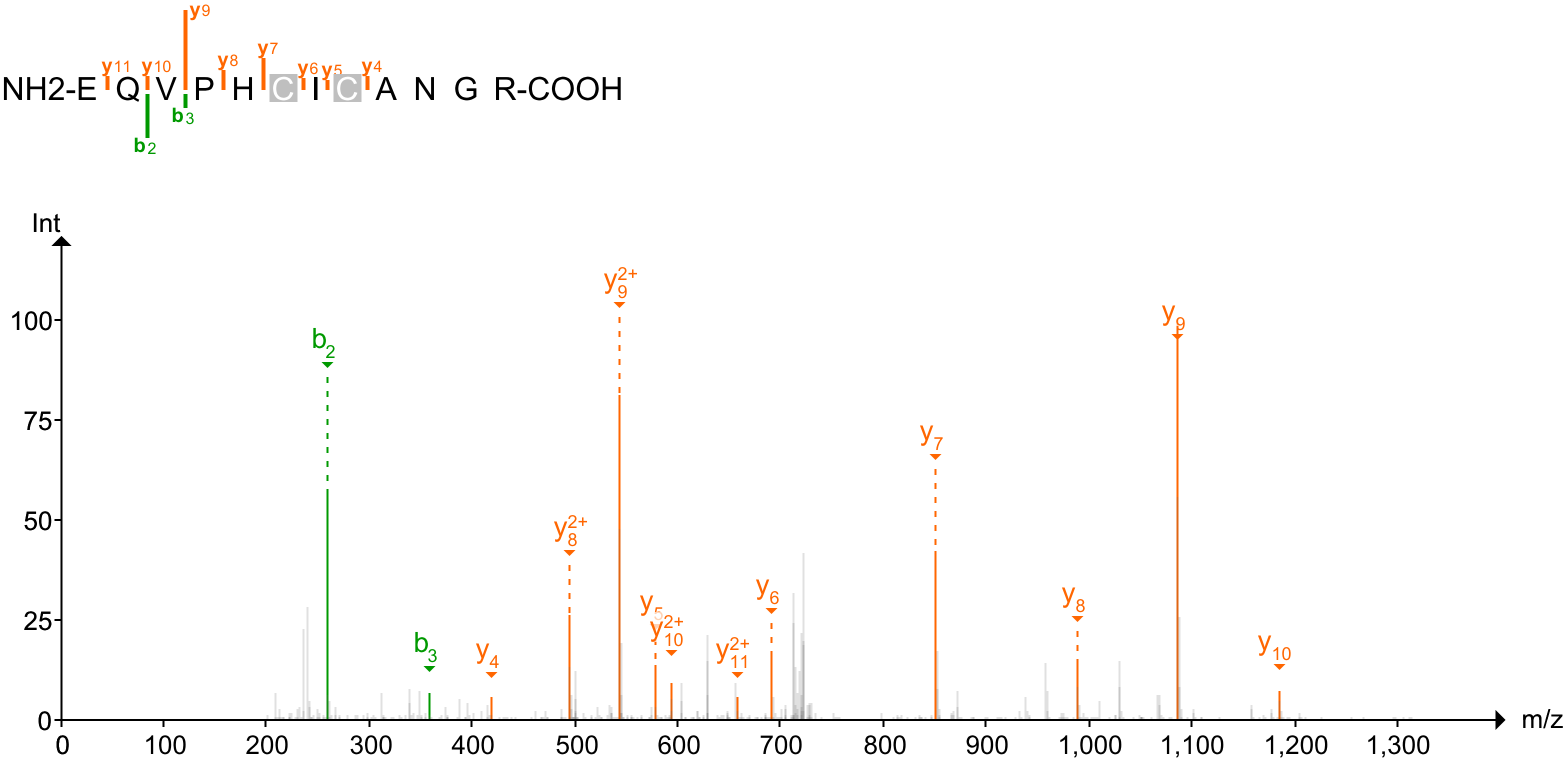

NH2-E Q V P H C I C A N G R-COOH
y11 y10 y9 y8 y7 y6 y5 y4
b2 b3
Int
100
75
50
25
0
m/z
0 100 200 300 400 500 600 700 800 900 1,000 1,100 1,200 1,300
b2
b3
y4
y8 2+
y9 2+
y5 2+
y10
y11 2+
y6
y7
y8
y9
y10

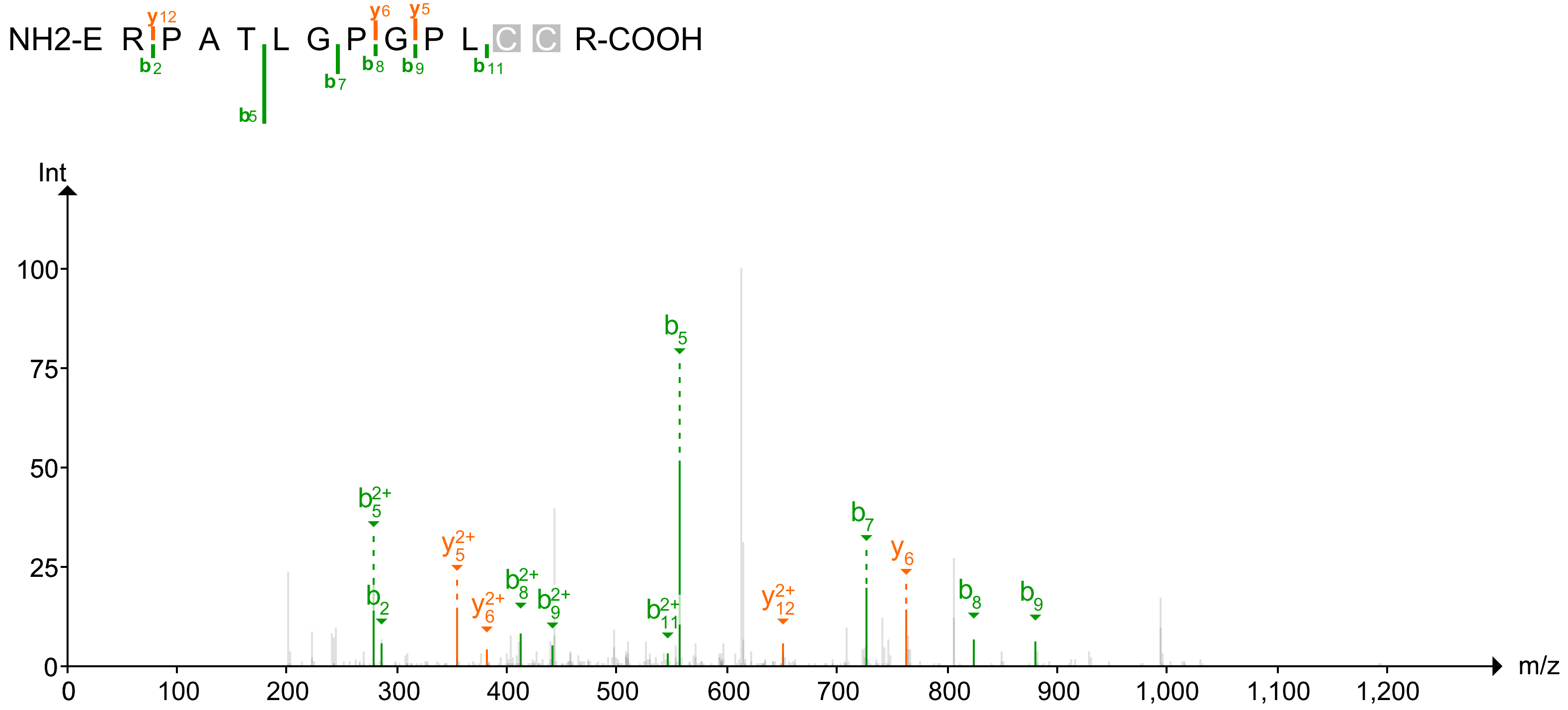

NH2-E R P A T L G P G P L C C R-COOH
y12 y6 y5
b2 b5 b7 b8 b9 b11
Int
100
75
50
25
0
b5
b5 2+
y5 2+
b2
b8 2+
b9 2+
y6 2+
b11 2+
y12 2+
b7
y6
b8
b9
0
100
200
300
400
500
600
700
800
900
1,000
1,100
1,200
m/z

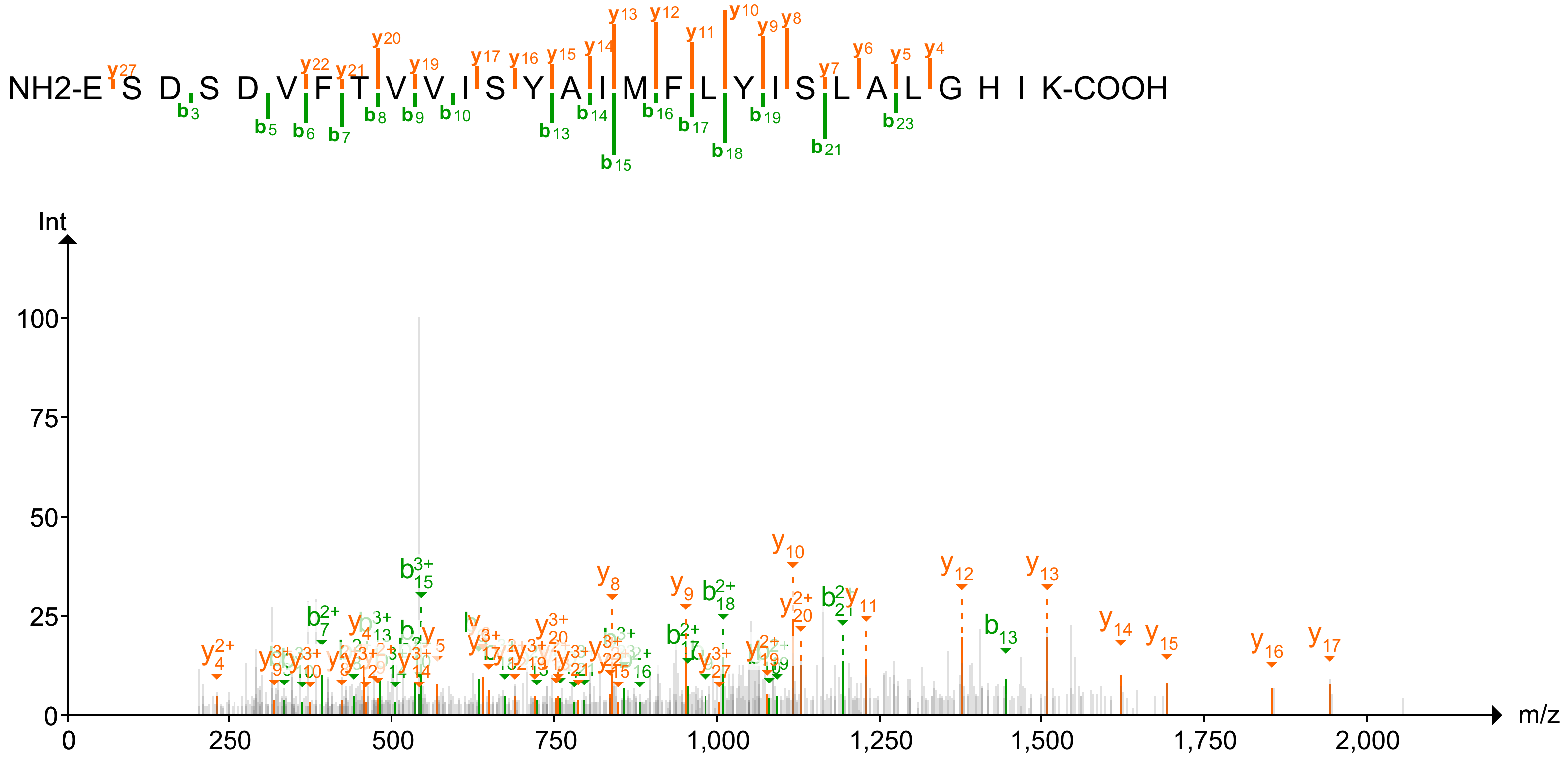

NH2-E S D S D V F T V V I S Y A I M F L Y I S L A L G H I K-COOH
Int
100
75
50
25
0
0
250
500
750
1,000
1,250
1,500
1,750
2,000
m/z

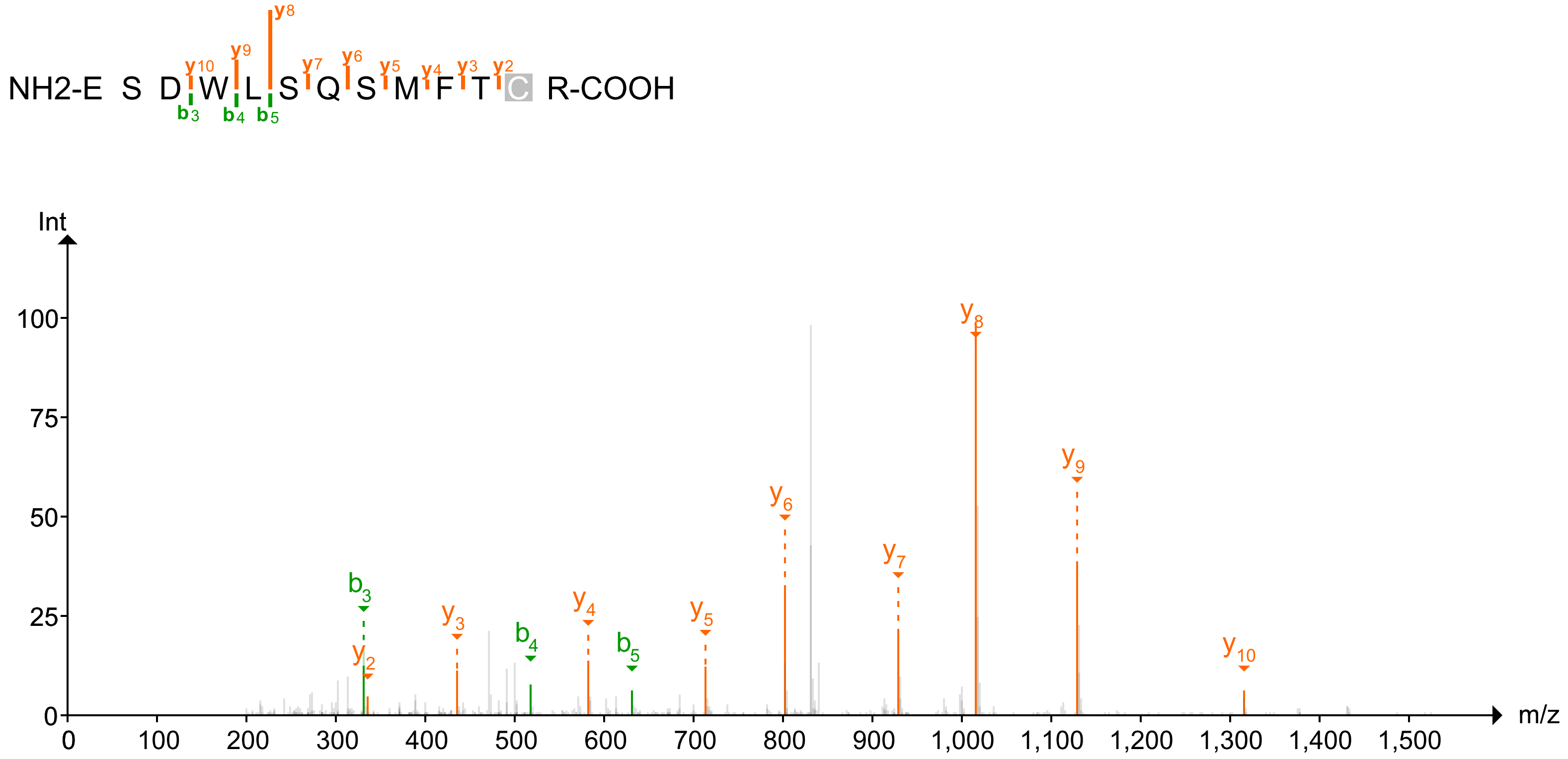

NH2-E S D W L S Q S M F T C R-COOH
y10 y9 y8 y7 y6 y5 y4 y3 y2
b3 b4 b5
Int
100
75
50
25
0
m/z
0 100 200 300 400 500 600 700 800 900 1,000 1,100 1,200 1,300 1,400 1,500
b3 y2 y3 b4 y4 b5 y5 y6 y7 y8 y9 y10

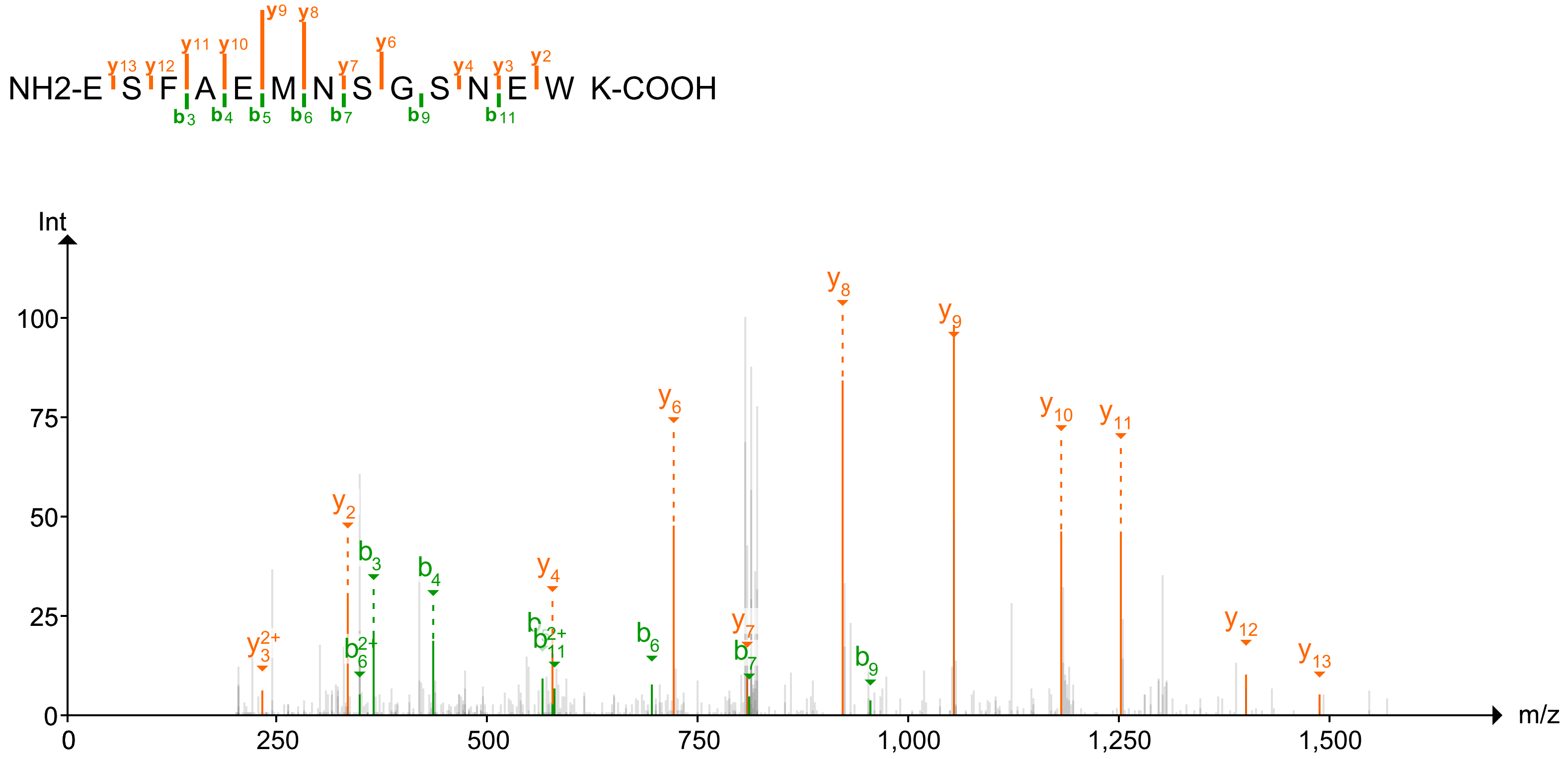
NH2-E S F A E M N S G S N E W K-COOH
y13 y12 y11 y10 y9 y8 y7 y6 y4 y3 y2
b3 b4 b5 b6 b7 b9 b11
Int
100
75
50
25
0
0
250
500
750
1,000
1,250
1,500
m/z
y8
y9
y6
y10
y11
y2
b3
b4
y4
y3 2+
b6 2+
b11 2+
b6
y7
b7
b9
y12
y13

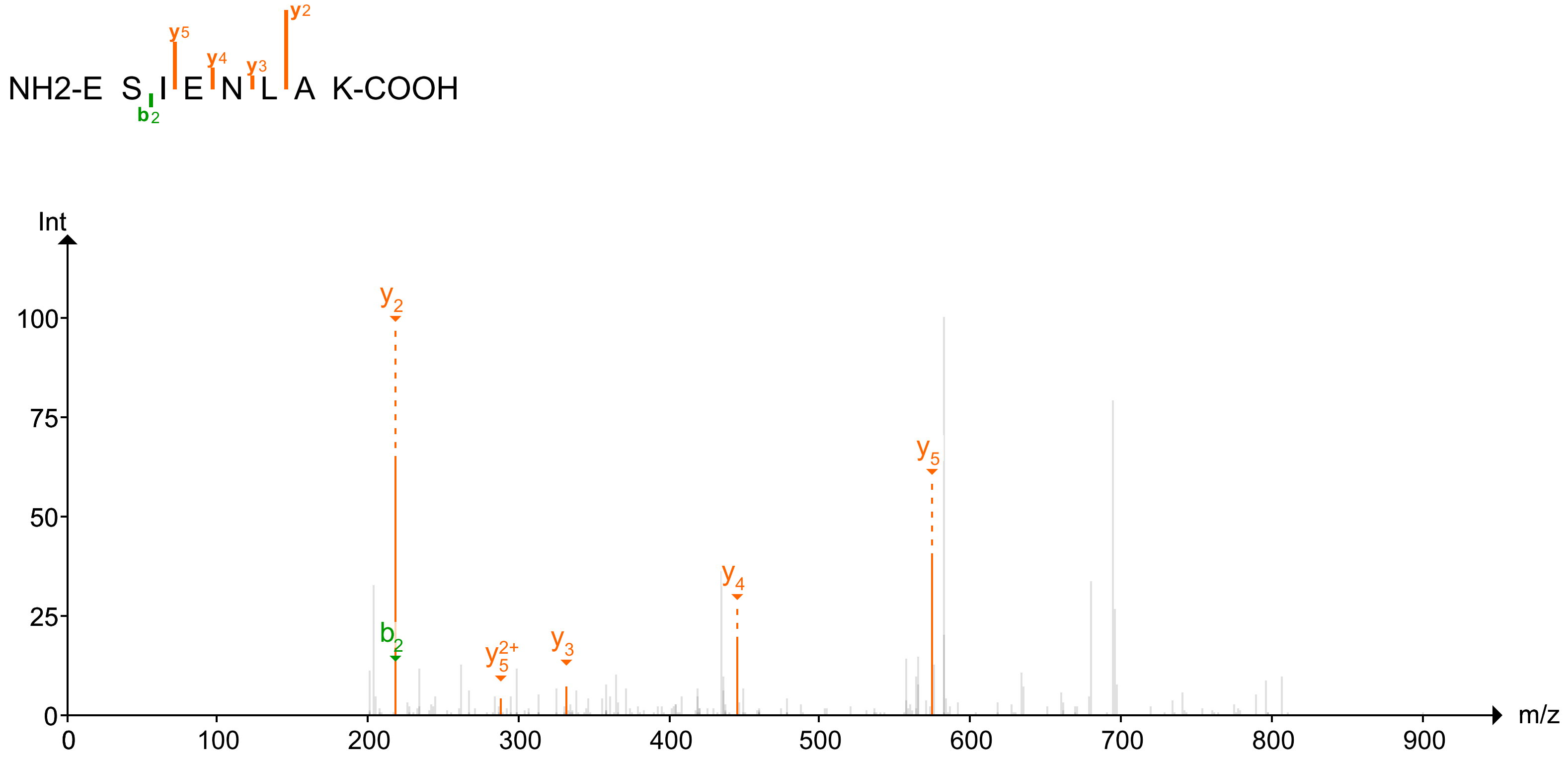

NH2-E S I E N L A K-COOH
y5
y4
y3
y2
b2
Int
100
75
50
25
0
y2
b2
y5 2+
y3
y4
y5
0
100
200
300
400
500
600
700
800
900
m/z

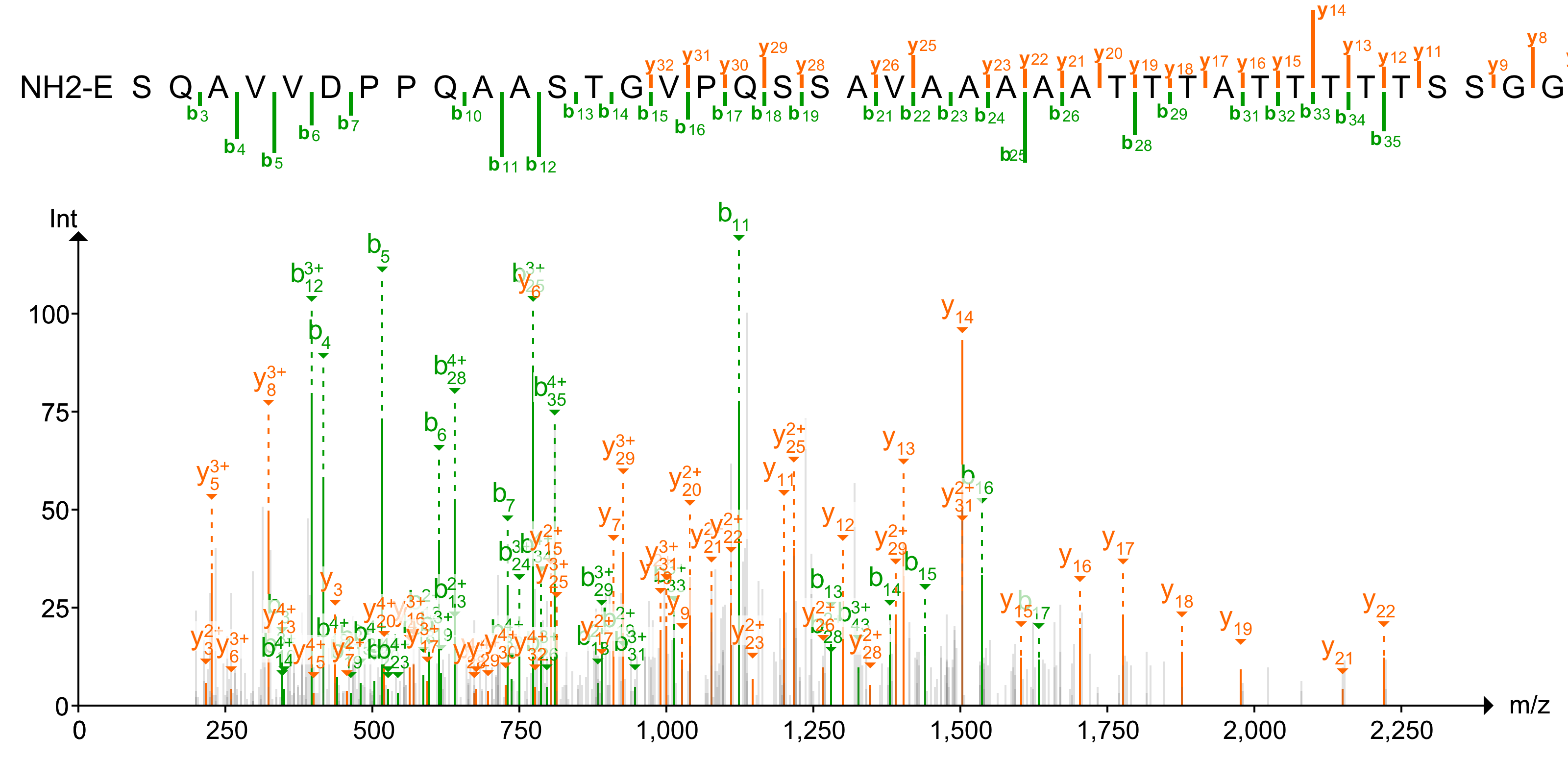

NH2-E S Q A V V D P P Q A A S T G V P Q S S A V A A A A A T T T A T T T T T S S G G
Int
0
25
50
75
100
m/z
0
250
500
750
1,000
1,250
1,500
1,750
2,000
2,250

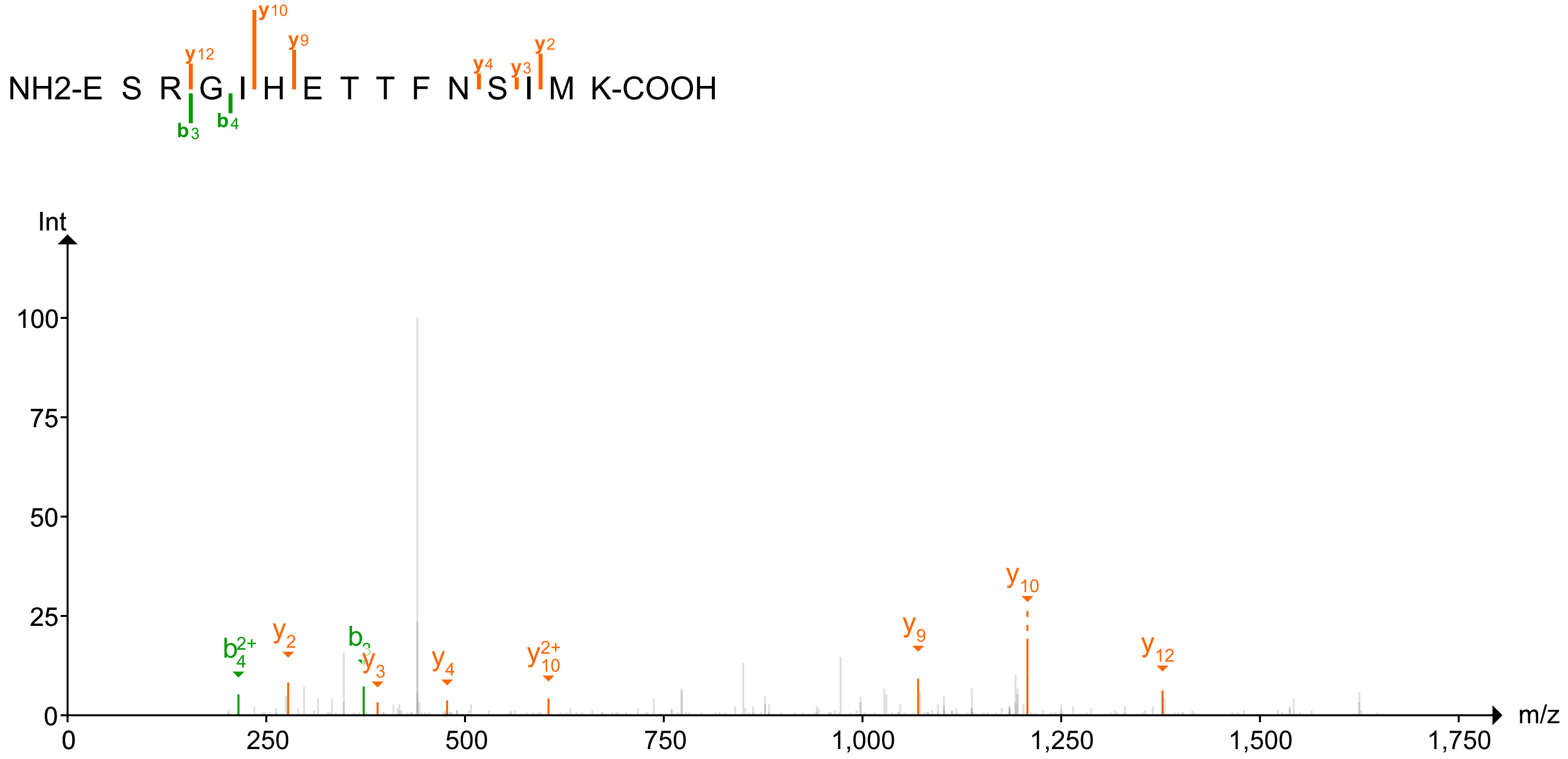

NH2-E S R G I H E T T F N S I M K-COOH
y10
y9
y12
y4
y3
y2
b3
b4
Int
100
75
50
25
0
m/z
250
500
750
1,000
1,250
1,500
1,750
$b_4^{2+}$
$y_2$
$b_3$
$y_3$
$y_4$
$y_{10}^{2+}$
$y_9$
$y_{10}$
$y_{12}$

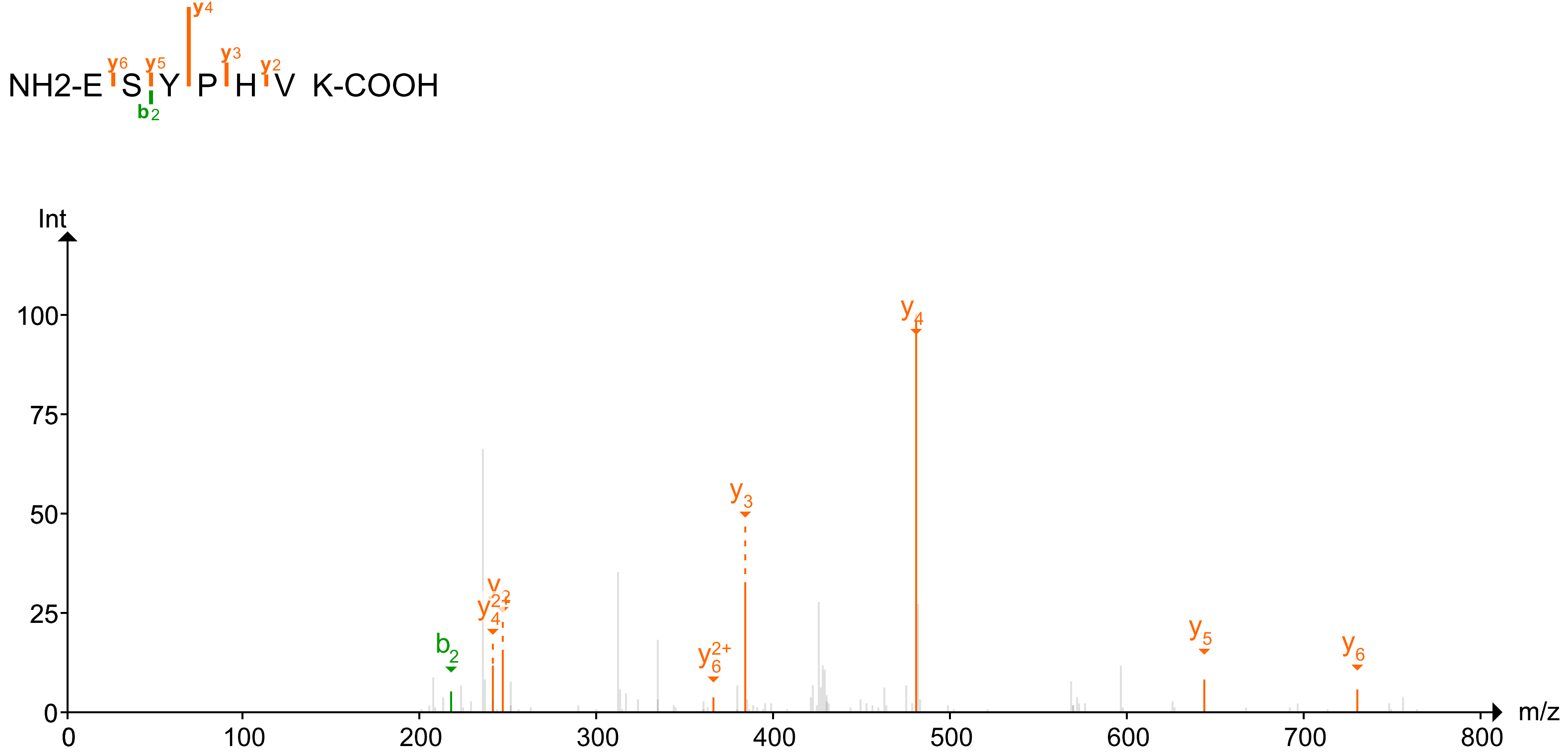

NH2-E S Y P H V K-COOH
y6
y5
y4
y3
y2
b2
Int
100
75
50
25
0
m/z
0
100
200
300
400
500
600
700
800

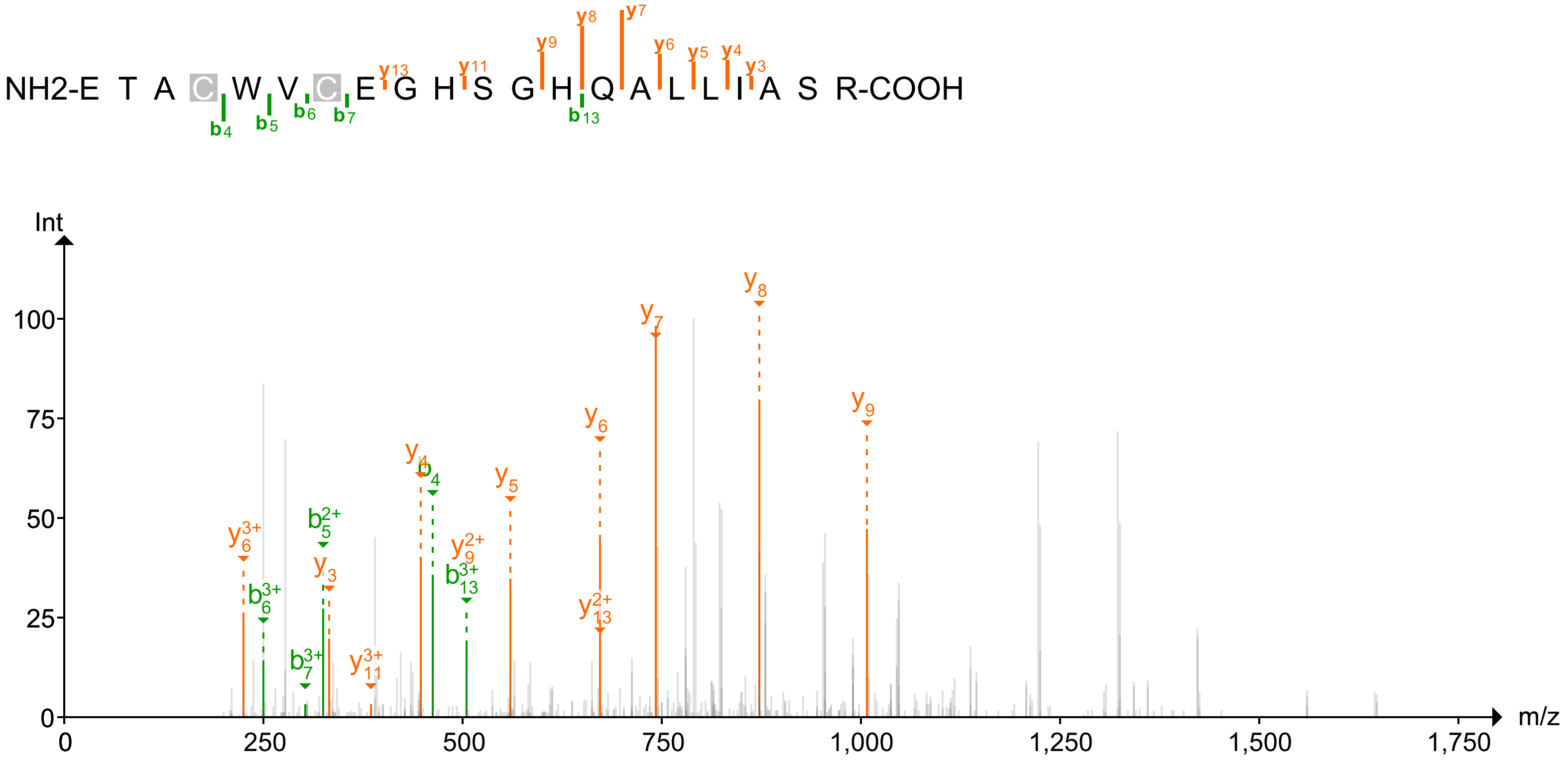

NH2-E T A C W V C E G H S G H Q A L L I A S R-COOH
b4 b5 b6 b7 b13
y13 y11 y9 y8 y7 y6 y5 y4 y3
Int
m/z
0
25
50
75
100
250
500
750
1,000
1,250
1,500
1,750

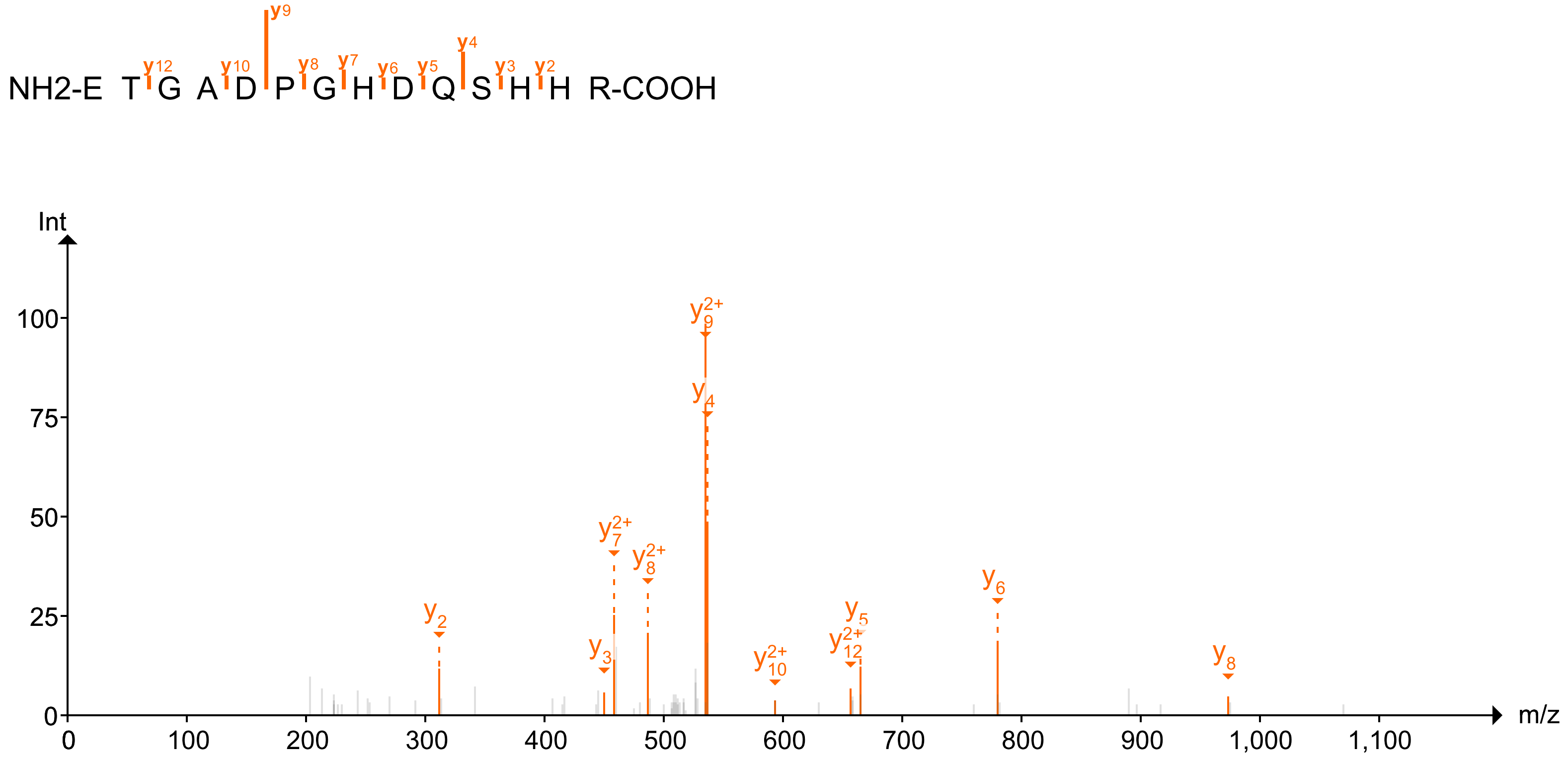
NH2-E T G A D P G H D Q S H H R-COOH
y12 y10 y9 y8 y7 y6 y5 y4 y3 y2
Int
100
75
50
25
0
m/z
0 100 200 300 400 500 600 700 800 900 1,000 1,100
$y_9^{2+}$
$y_4$
$y_7^{2+}$
$y_8^{2+}$
$y_2$
$y_3$
$y_{10}^{2+}$
$y_5$
$y_{12}^{2+}$
$y_6$
$y_8$

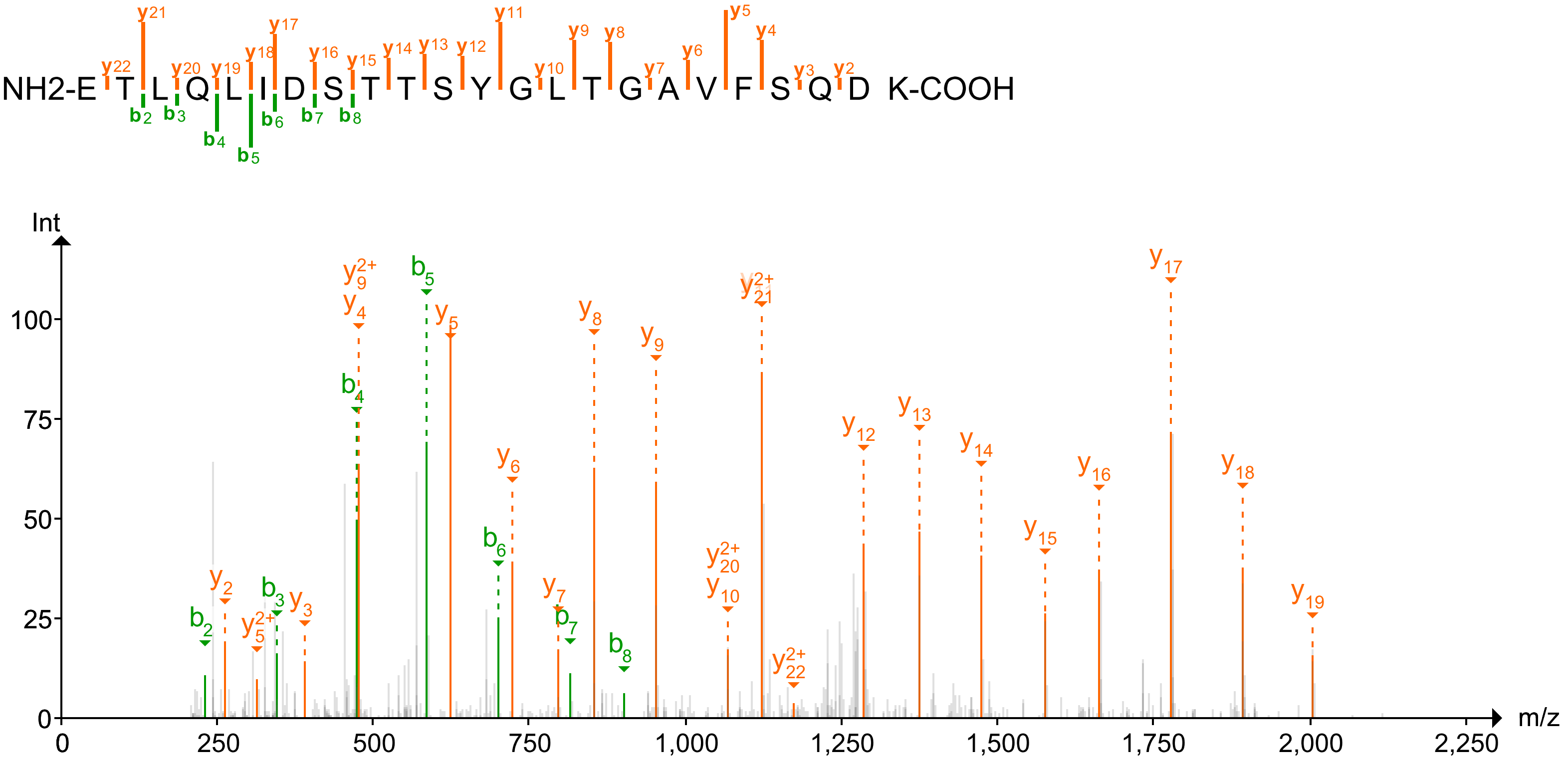

NH2-E T L Q L I D S T T S Y G L T G A V F S Q D K-COOH
y22 y21 y20 y19 y18 y17 y16 y15 y14 y13 y12 y11 y10 y9 y8 y7 y6 y5 y4 y3 y2
b2 b3 b4 b5 b6 b7 b8
Int
100
75
50
25
0
m/z
0
250
500
750
1,000
1,250
1,500
1,750
2,000
2,250
b2 y2 y5 2+ b3 y3 y9 2+ y4 b4 b5 y5 y6 b6 y7 b7 y8 b8 y9 y20 2+ y10 y21 2+ y22 2+ y12 y13 y14 y15 y16 y17 y18 y19

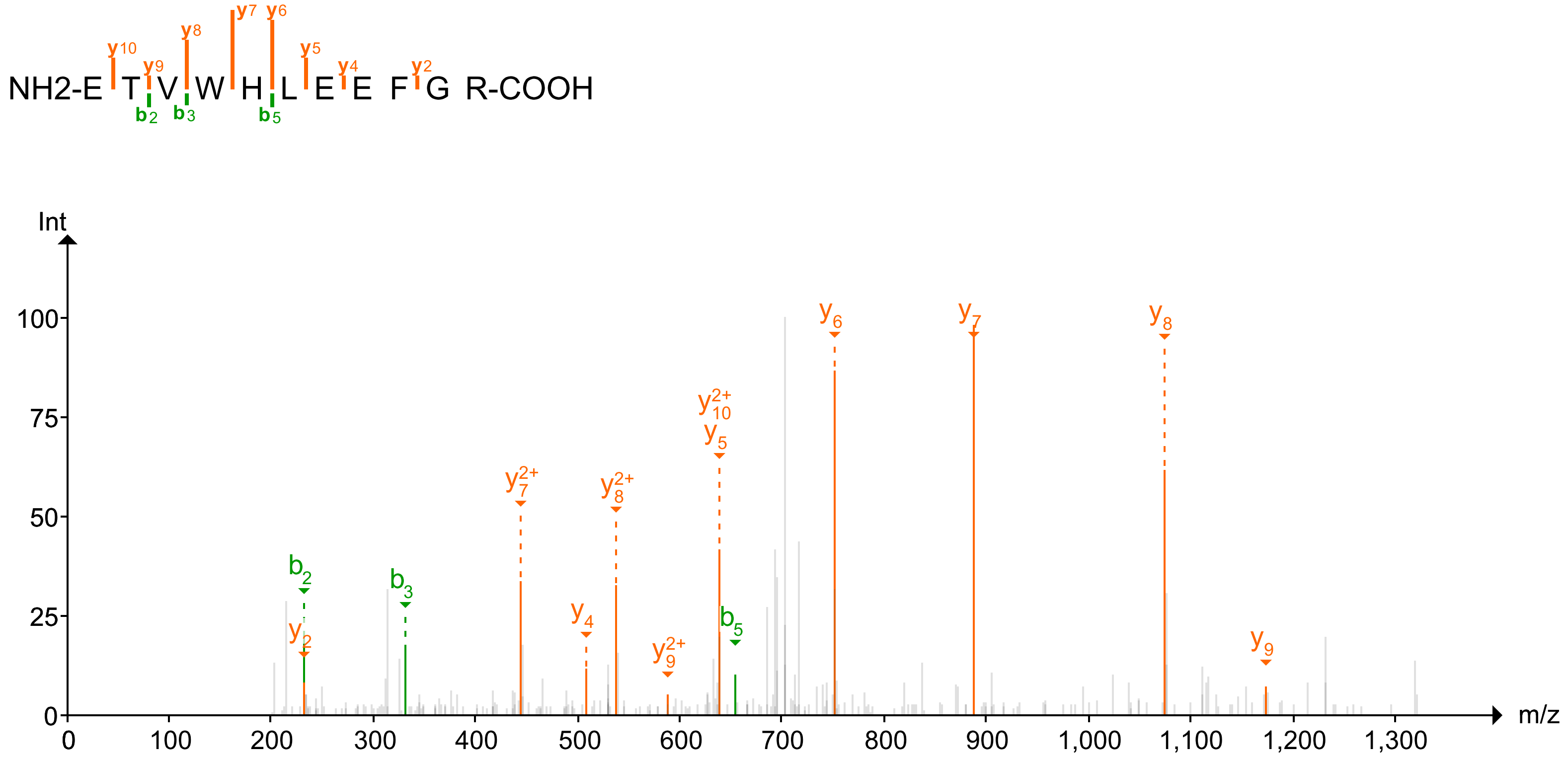

NH2-E T V W H L E E F G R-COOH
y10 y9 y8 y7 y6 y5 y4 y2
b2 b3 b5
Int
100
75
50
25
0
m/z
0 100 200 300 400 500 600 700 800 900 1,000 1,100 1,200 1,300
y6 y7 y8
y10 2+
y5
y7 2+
y8 2+
b2 b3
y2
y4
y9 2+
b5
y9

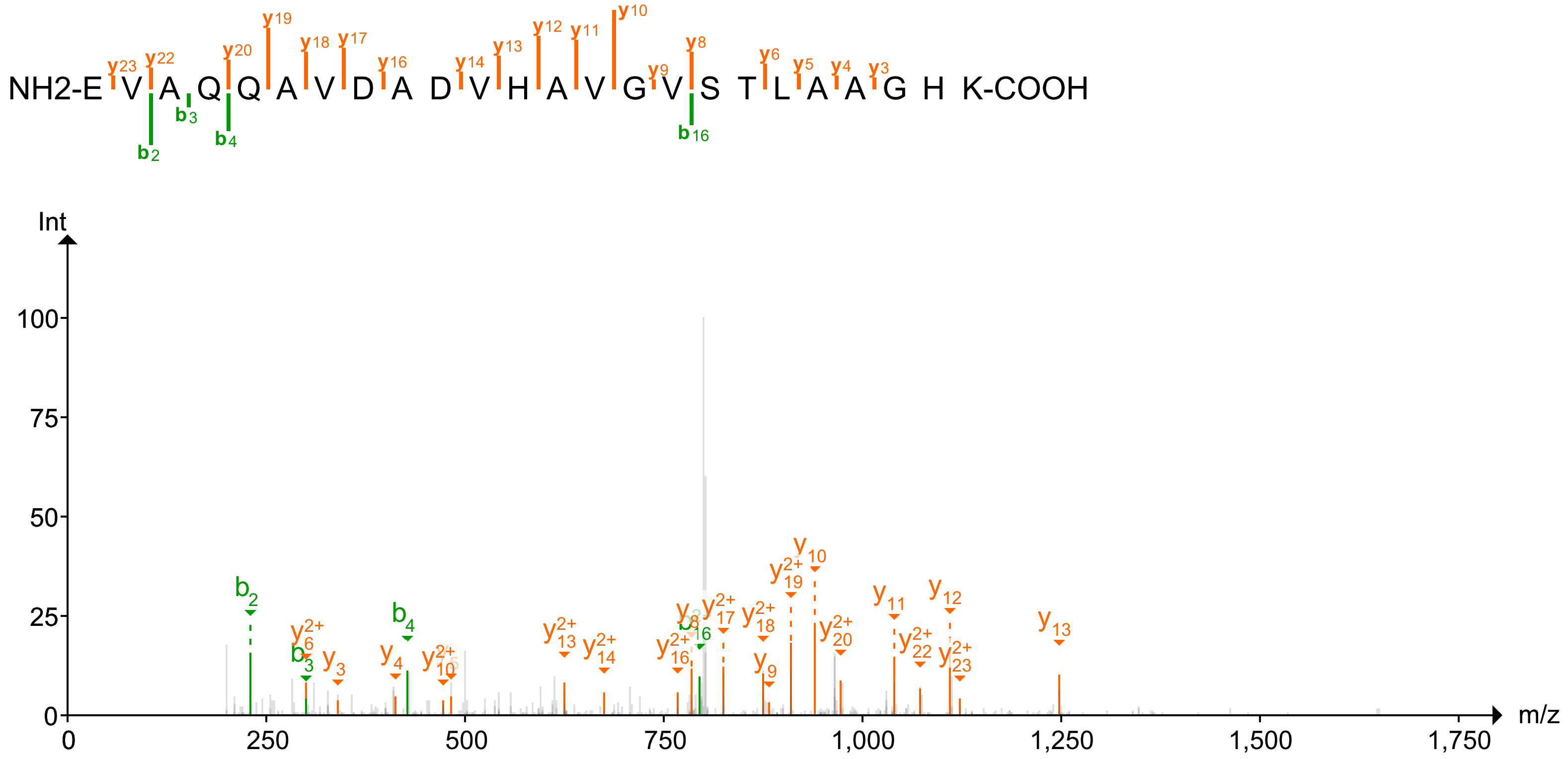

NH2-E V A Q Q A V D A D V H A V G V S T L A A G H K-COOH
Int
100
75
50
25
0
m/z
0
250
500
750
1,000
1,250
1,500
1,750

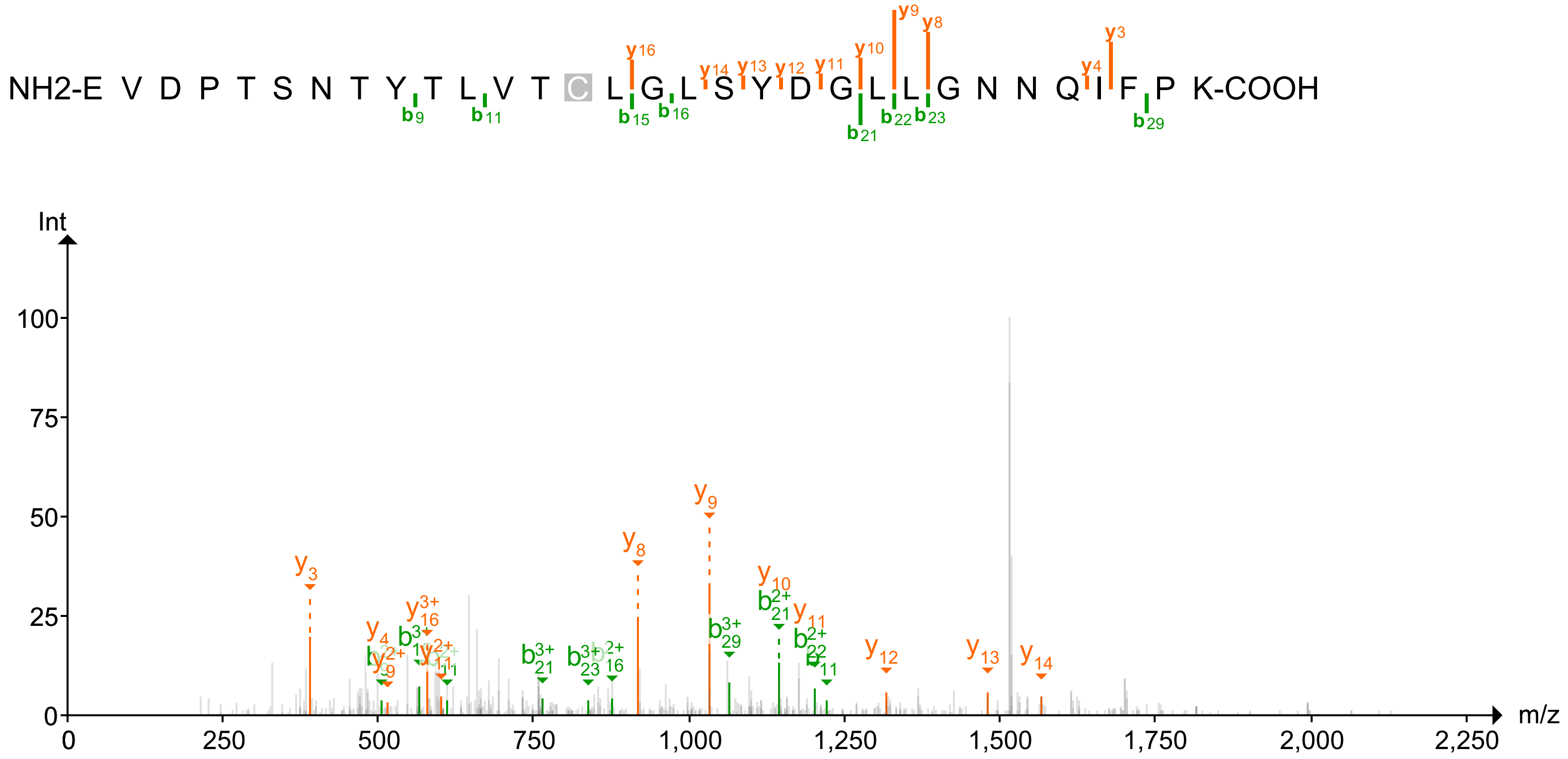

NH2-E V D P T S N T Y T L V T C L G L S Y D G L L G N N Q I F P K-COOH
Int
m/z
0
25
50
75
100
0
250
500
750
1,000
1,250
1,500
1,750
2,000
2,250

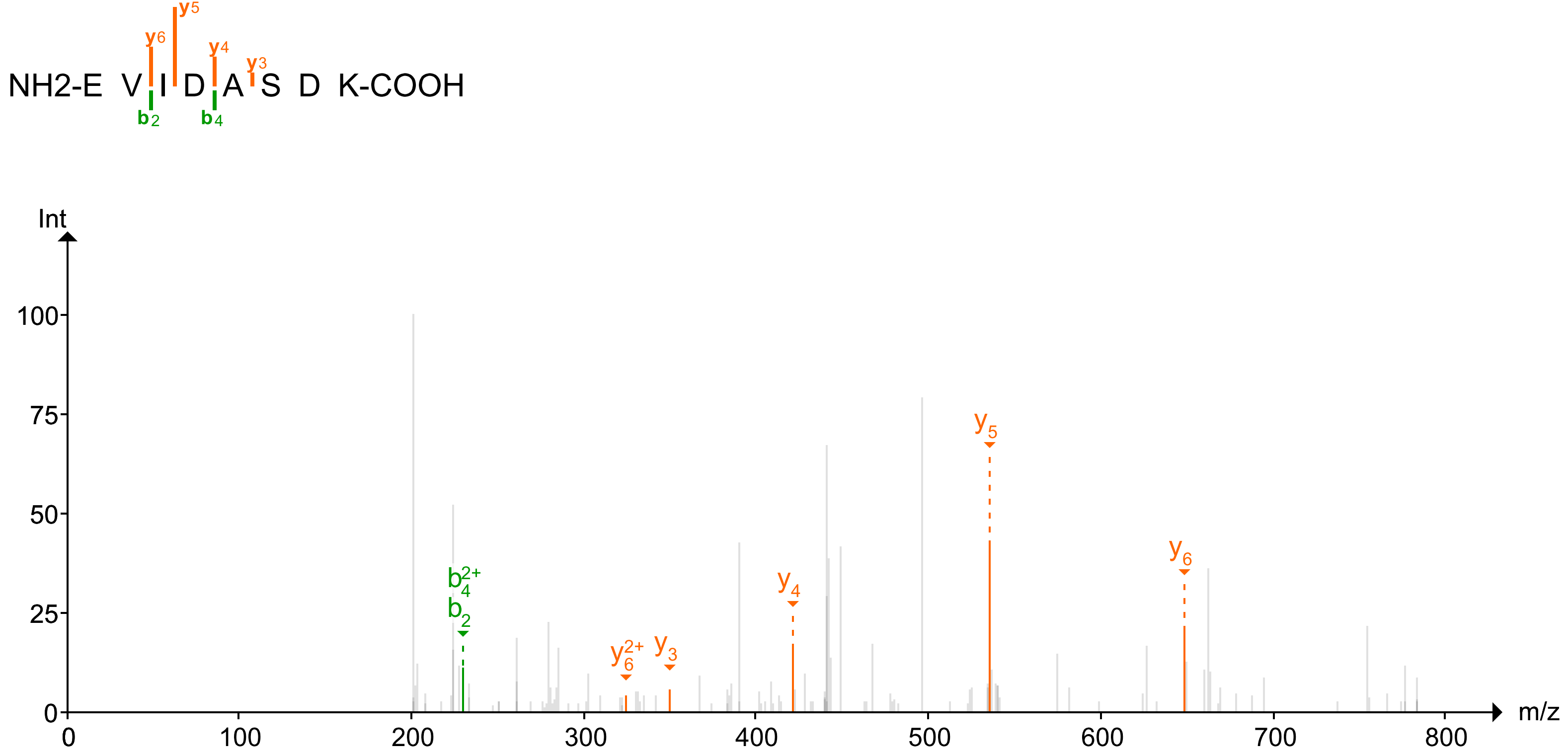

NH2-E V I D A S D K-COOH
y5
y6
y4
y3
b2
b4
Int
100
75
50
25
0
$b_4^{2+}$
$b_2$
$y_6^{2+}$
$y_3$
$y_4$
$y_5$
$y_6$
0
100
200
300
400
500
600
700
800
m/z

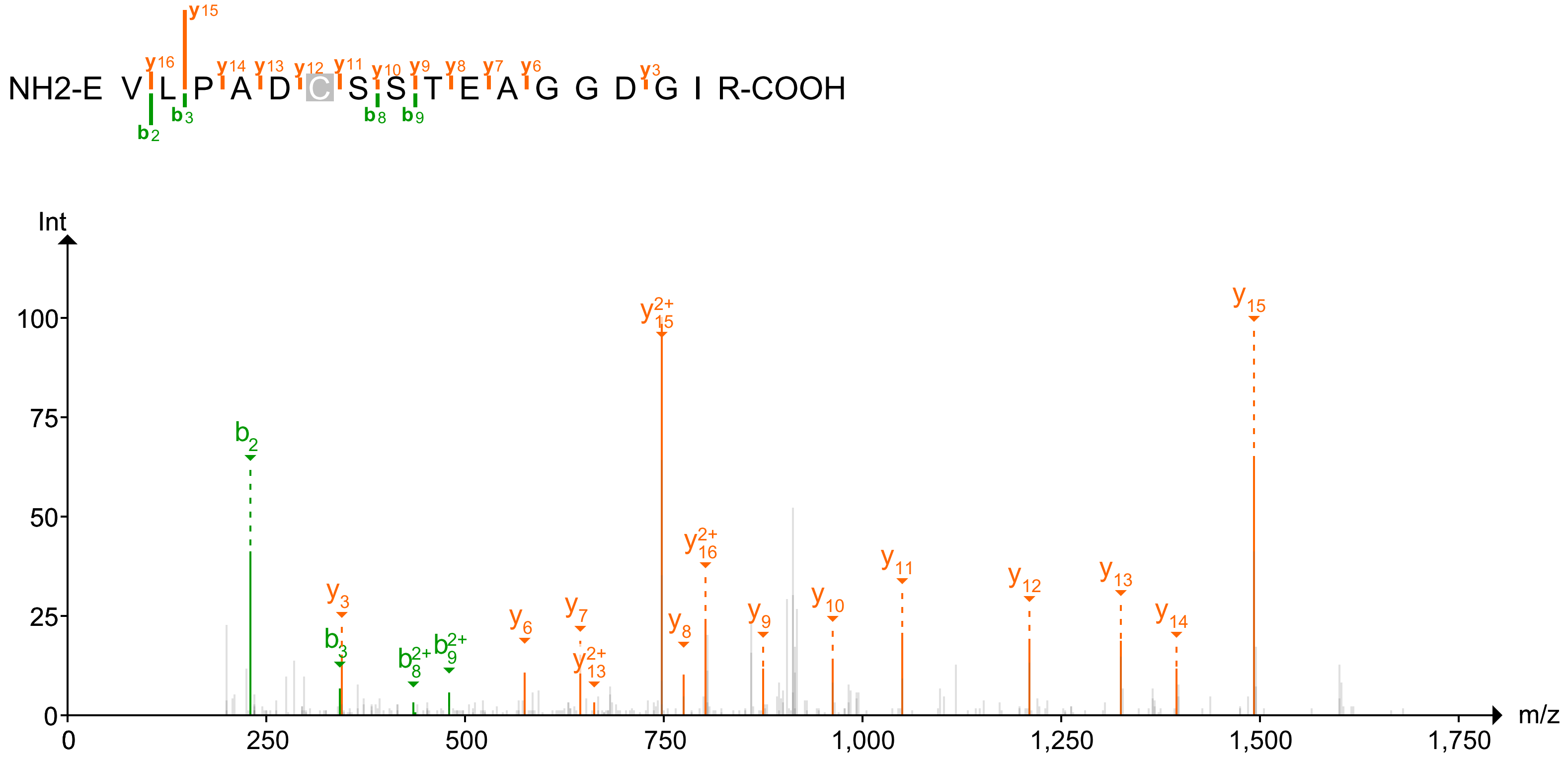

NH2-E V L P A D C S S T E A G G D G I R-COOH
y15 y16 y14 y13 y12 y11 y10 y9 y8 y7 y6 y3
b2 b3 b8 b9
Int
100
75
50
25
0
m/z
0
250
500
750
1,000
1,250
1,500
1,750
b2
b3
b8 2+
b9 2+
y3
y6
y7
y13 2+
y15 2+
y8
y16 2+
y9
y10
y11
y12
y13
y14
y15

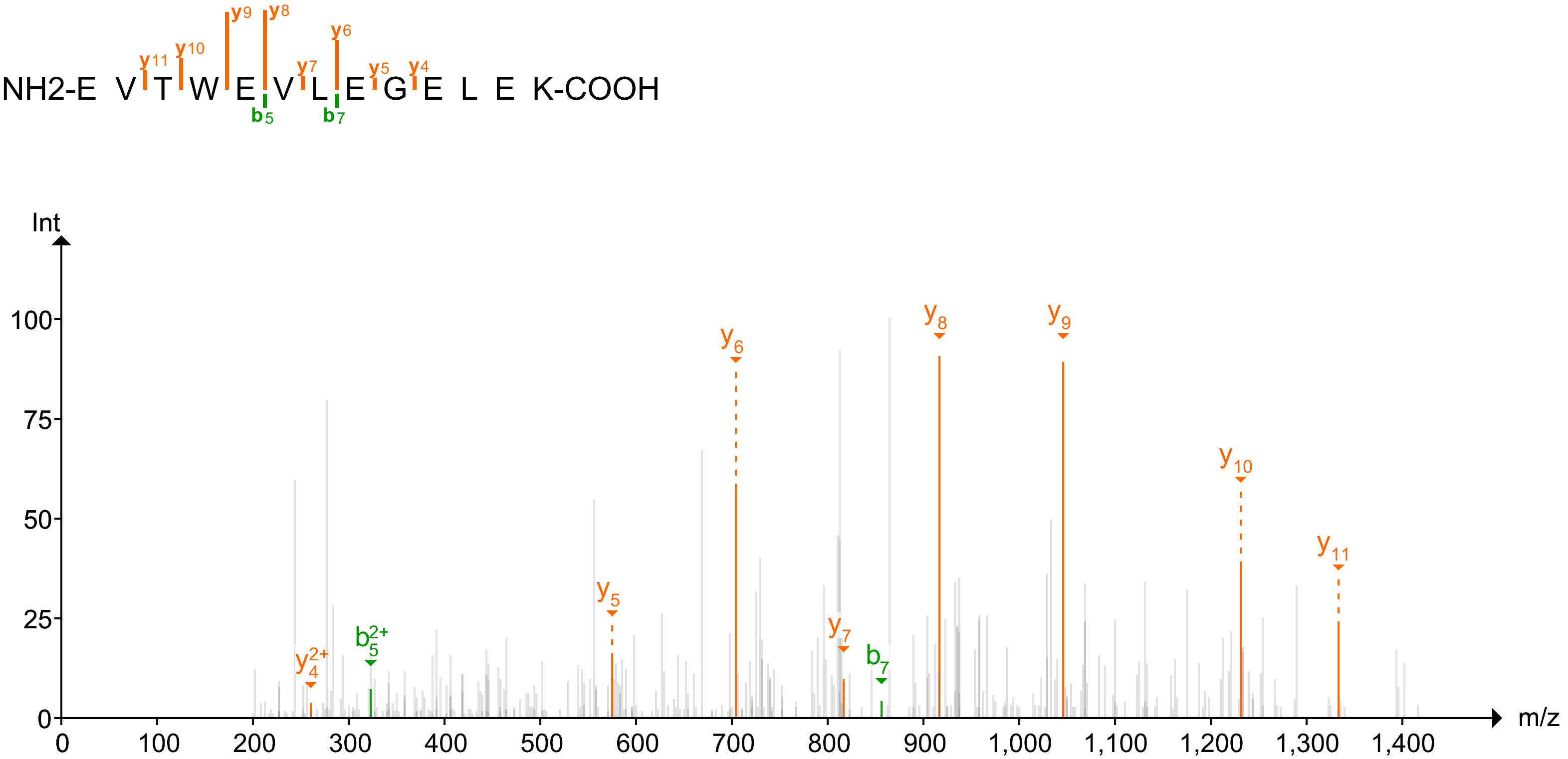

NH2-E V T W E V L E G E L E K-COOH
y11 y10 y9 y8 y7 y6 y5 y4
b5 b7
Int
100
75
50
25
0
m/z
0 100 200 300 400 500 600 700 800 900 1,000 1,100 1,200 1,300 1,400
y6 y8 y9 y10 y11 y5 y7 b7

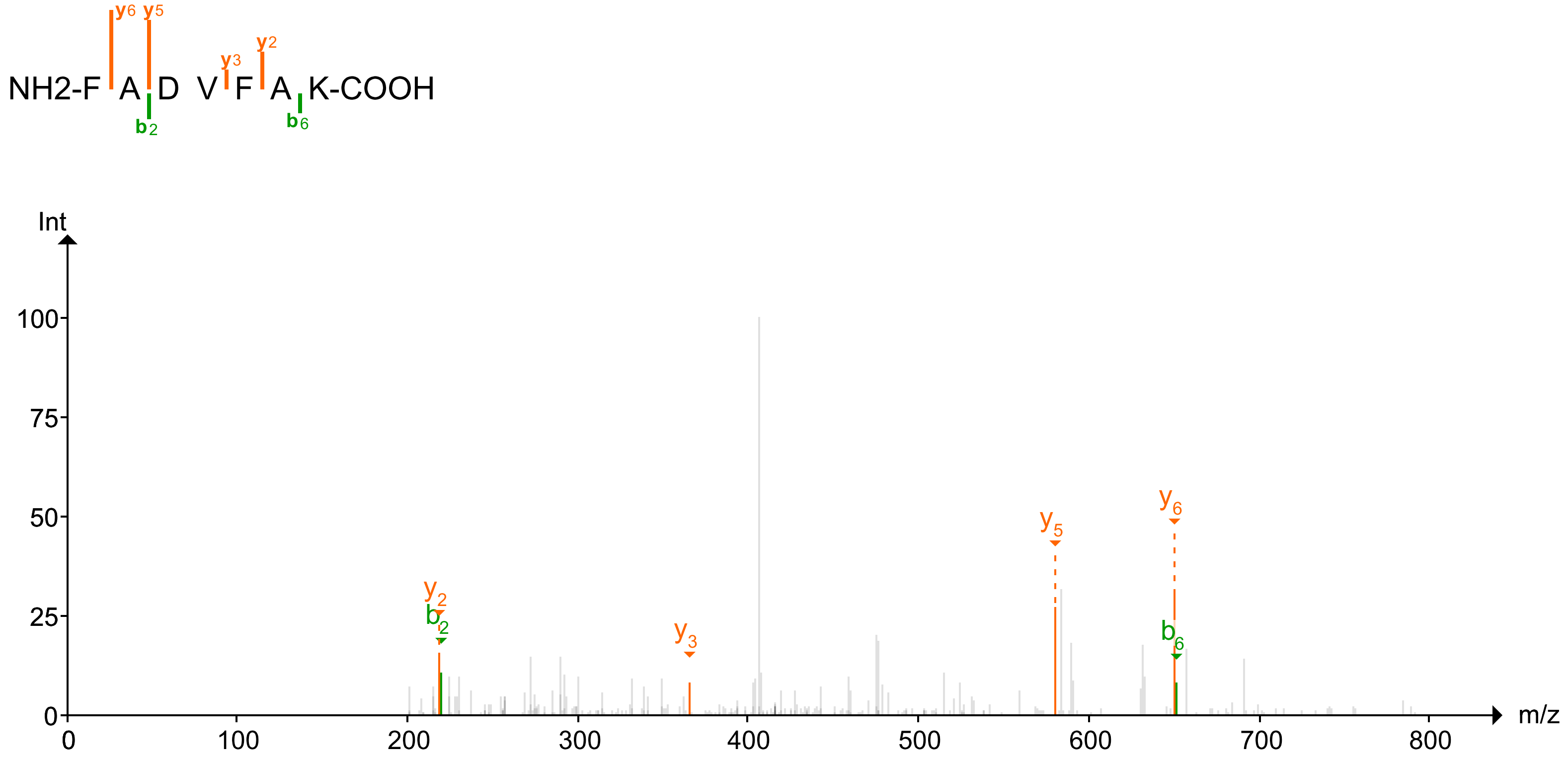
NH2-F A D V F A K-COOH
y6 y5 y3 y2
b2 b6
Int
100
75
50
25
0
0 100 200 300 400 500 600 700 800
m/z
y2 b2 y3 y5 y6 b6

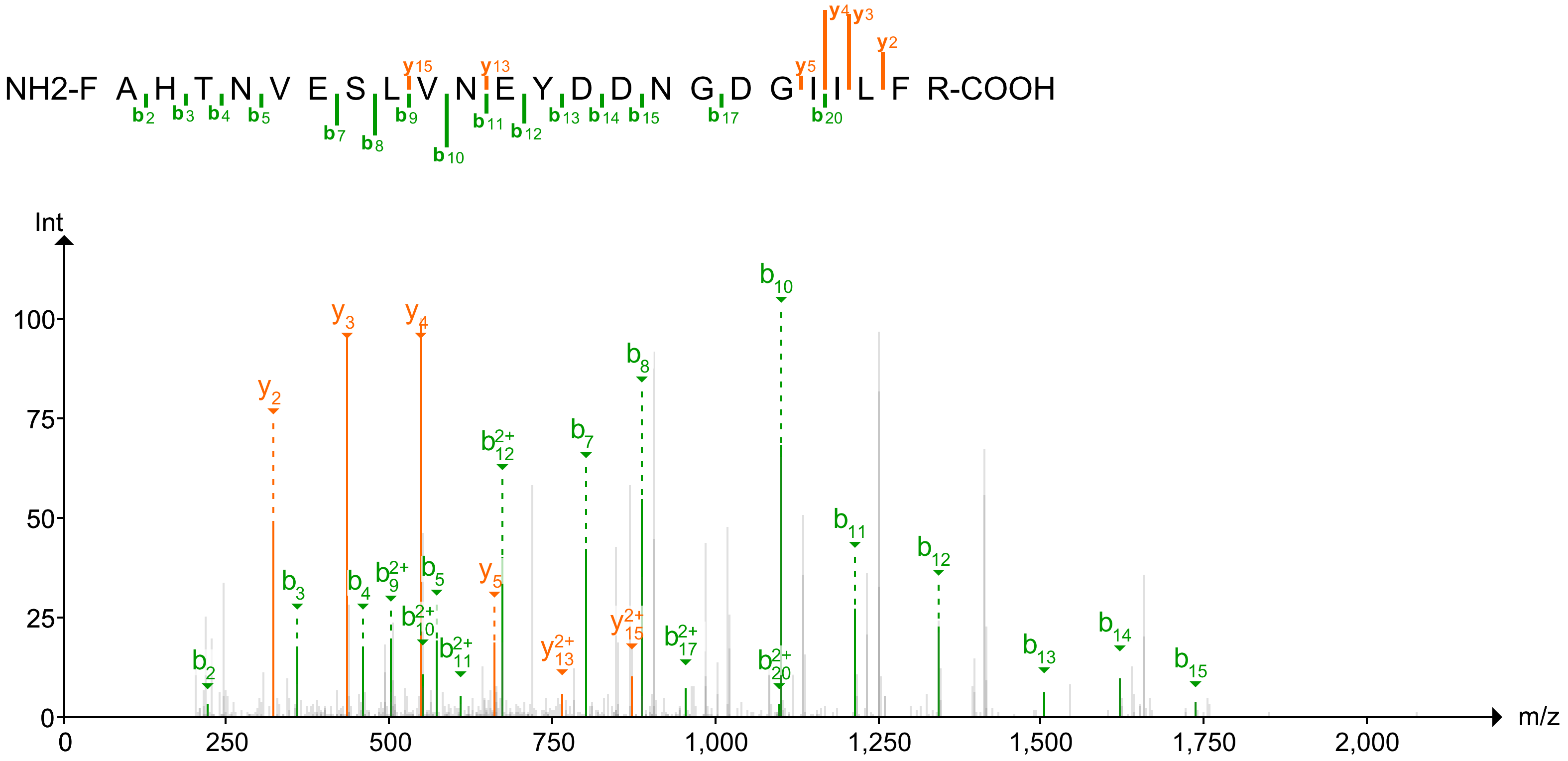

NH2-F A H T N V E S L V N E Y D D N G D G I I L F R-COOH
Int
m/z
y2
y3
y4
y5
b3
b4
b5
b7
b8
b10
b11
b12
b13
b14
b15

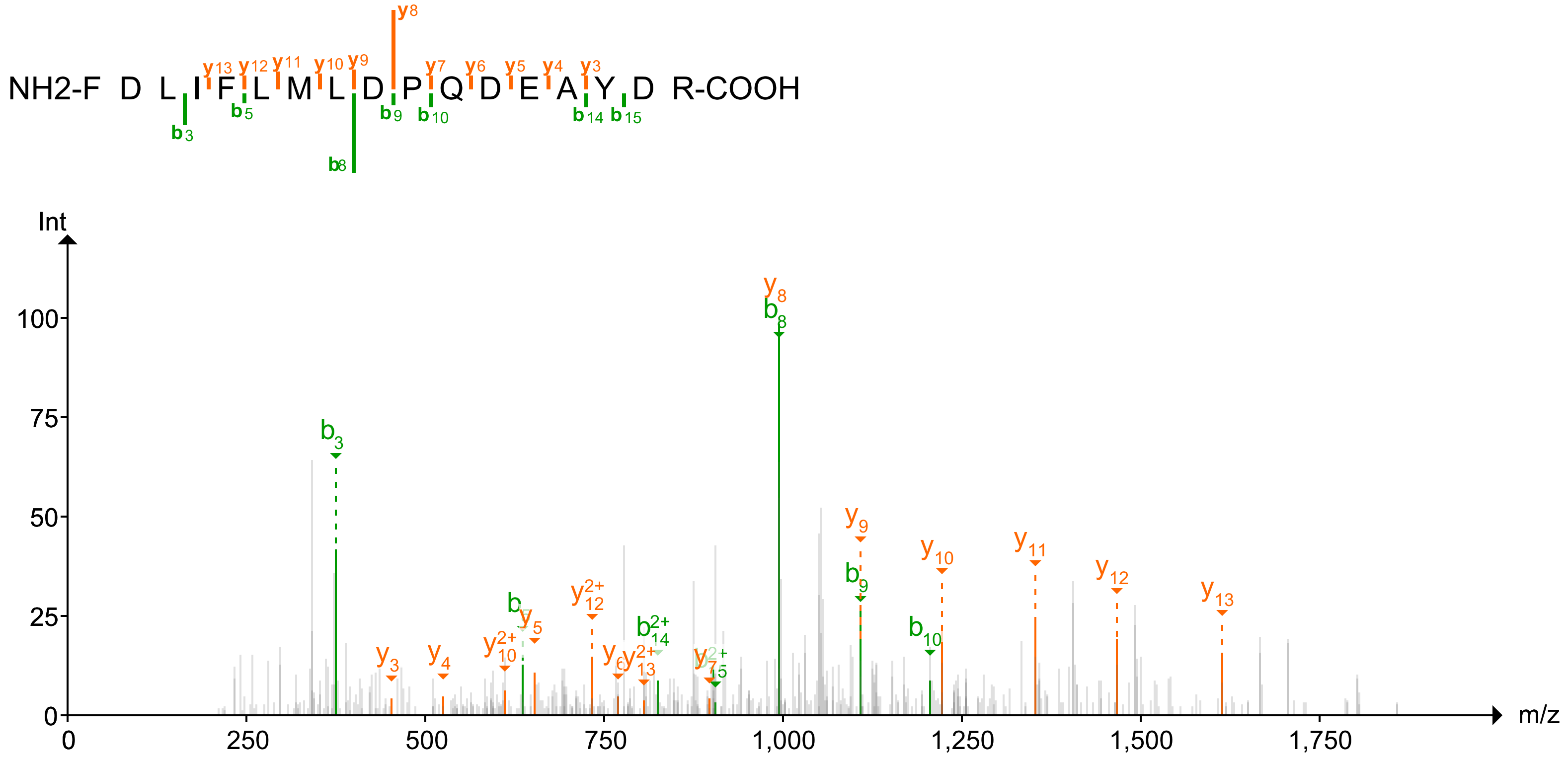
NH2-F D L I F L M L D P Q D E A Y D R-COOH
y13 y12 y11 y10 y9 y8 y7 y6 y5 y4 y3
b3 b5 b8 b9 b10 b14 b15
Int
100
75
50
25
0
m/z
0
250
500
750
1,000
1,250
1,500
1,750

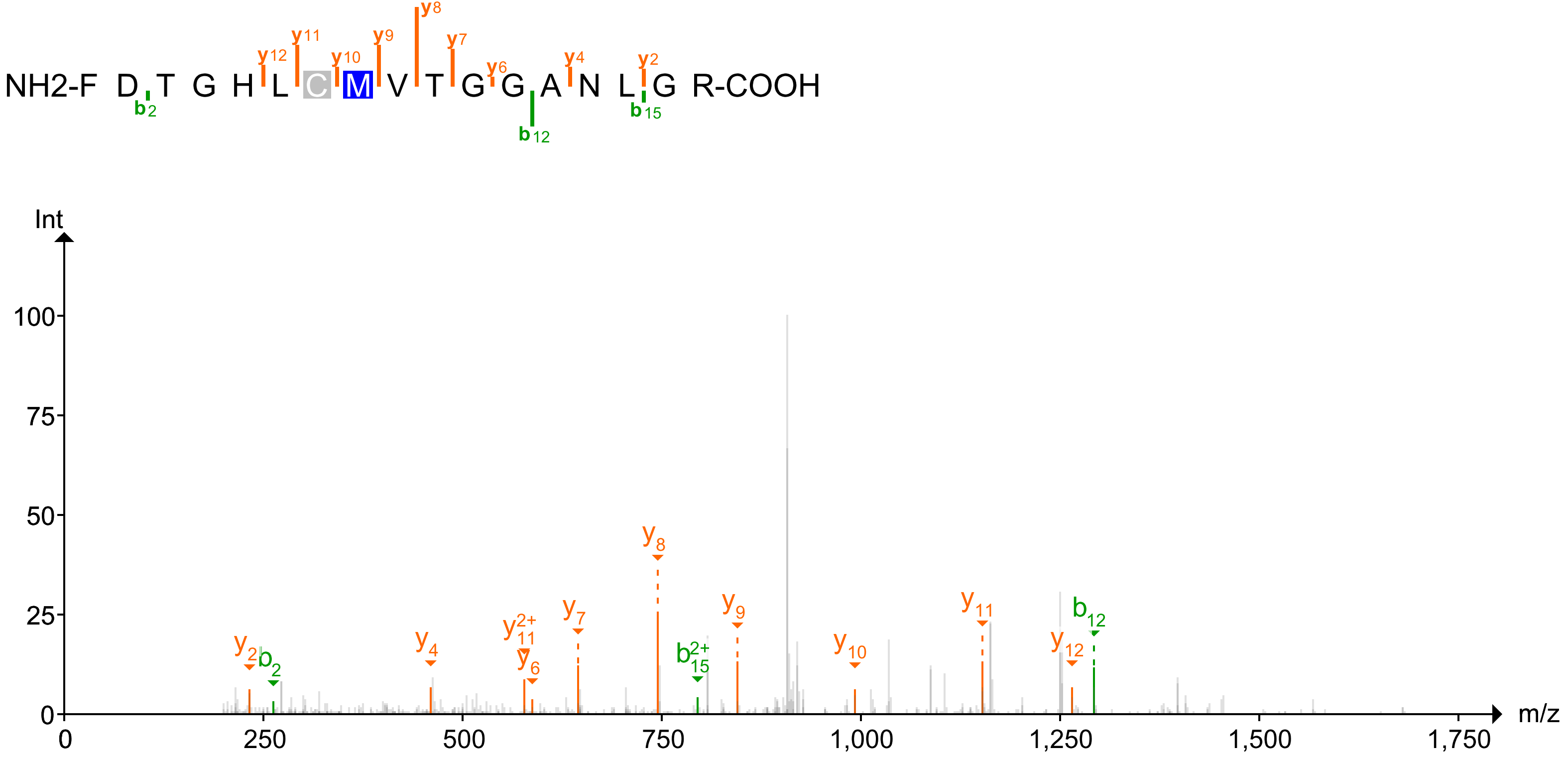

NH2-F D T G H L C M V T G G A N L G R-COOH
y12 y11 y10 y9 y8 y7 y6 y4 y2
b2 b12 b15
Int
100
75
50
25
0
m/z
0
250
500
750
1,000
1,250
1,500
1,750
y2 b2 y4 y11 2+ y6 y7 y8 b15 2+ y9 y10 y11 y12 b12

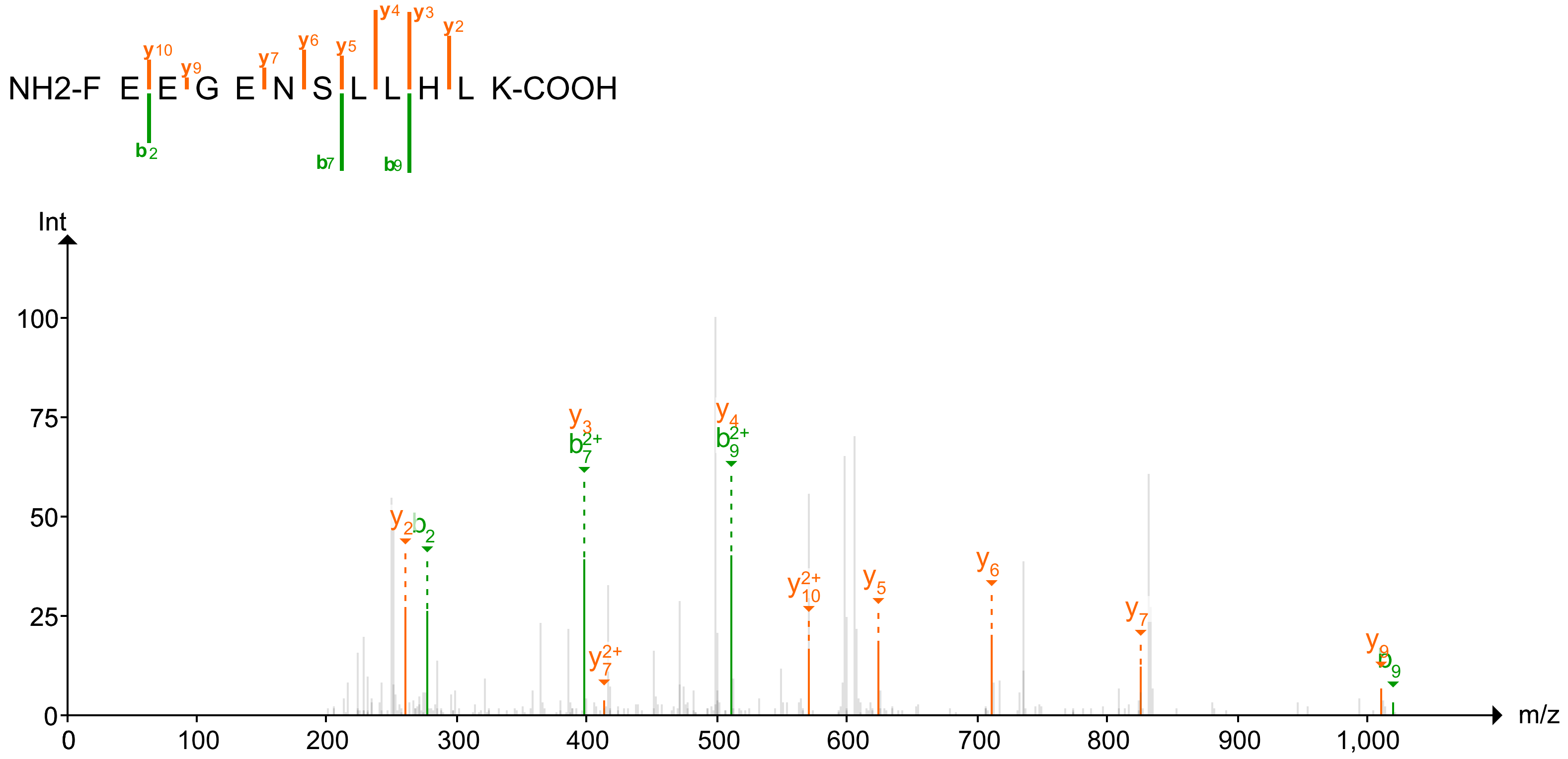

NH2-F E E G E N S L L H L K-COOH
y10
y9
y7
y6
y5
y4
y3
y2
b2
b7
b9
Int
100
75
50
25
0
m/z
0
100
200
300
400
500
600
700
800
900
1,000
y2
b2
y3
b7 2+
y7 2+
y4
b9 2+
y10 2+
y5
y6
y7
y9
b9

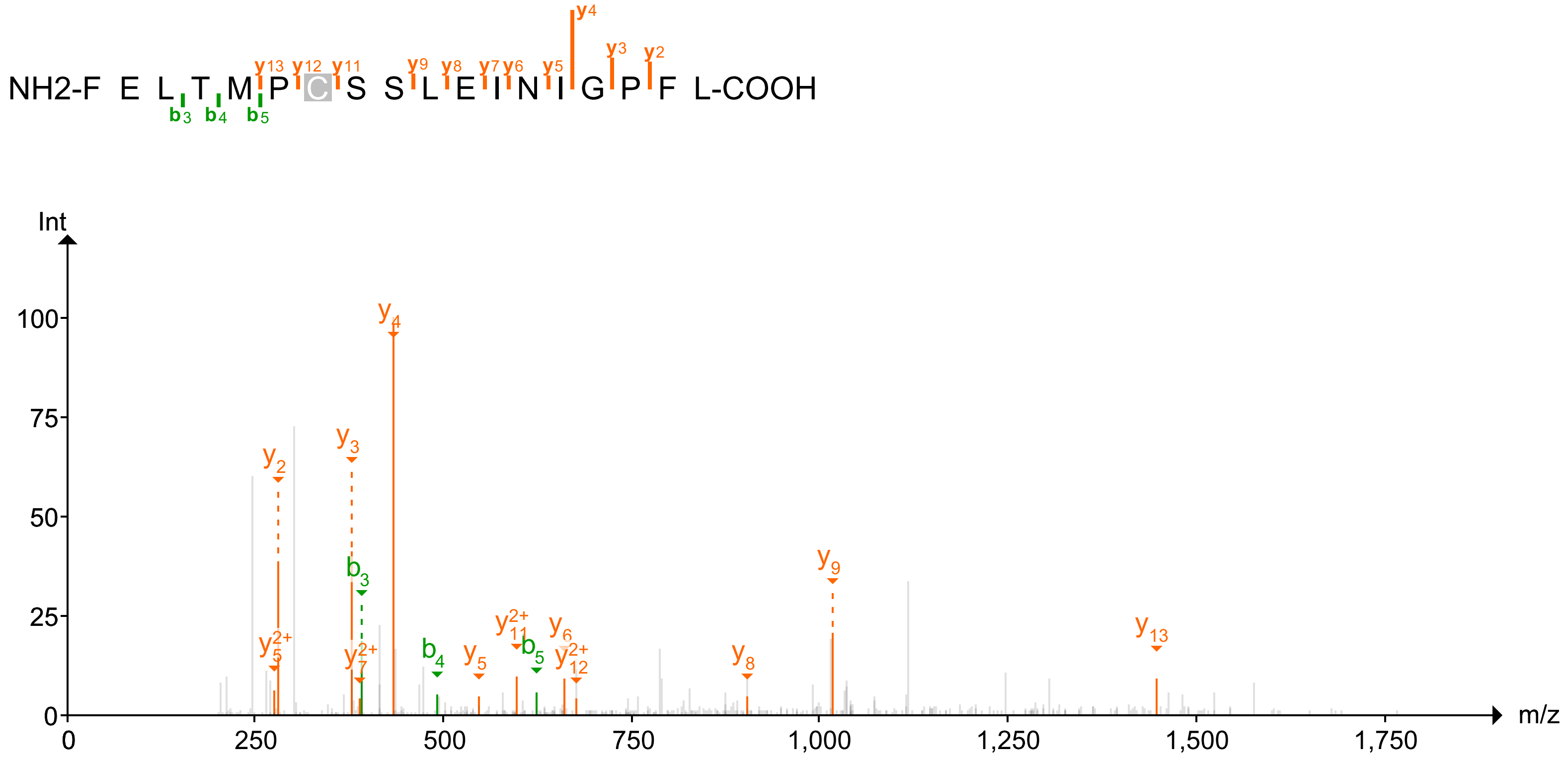

NH2-F E L T M P C S S L E I N I G P F L-COOH
b3 b4 b5
y13 y12 y11 y9 y8 y7 y6 y5 y4 y3 y2
Int
100
75
50
25
0
m/z
0
250
500
750
1,000
1,250
1,500
1,750

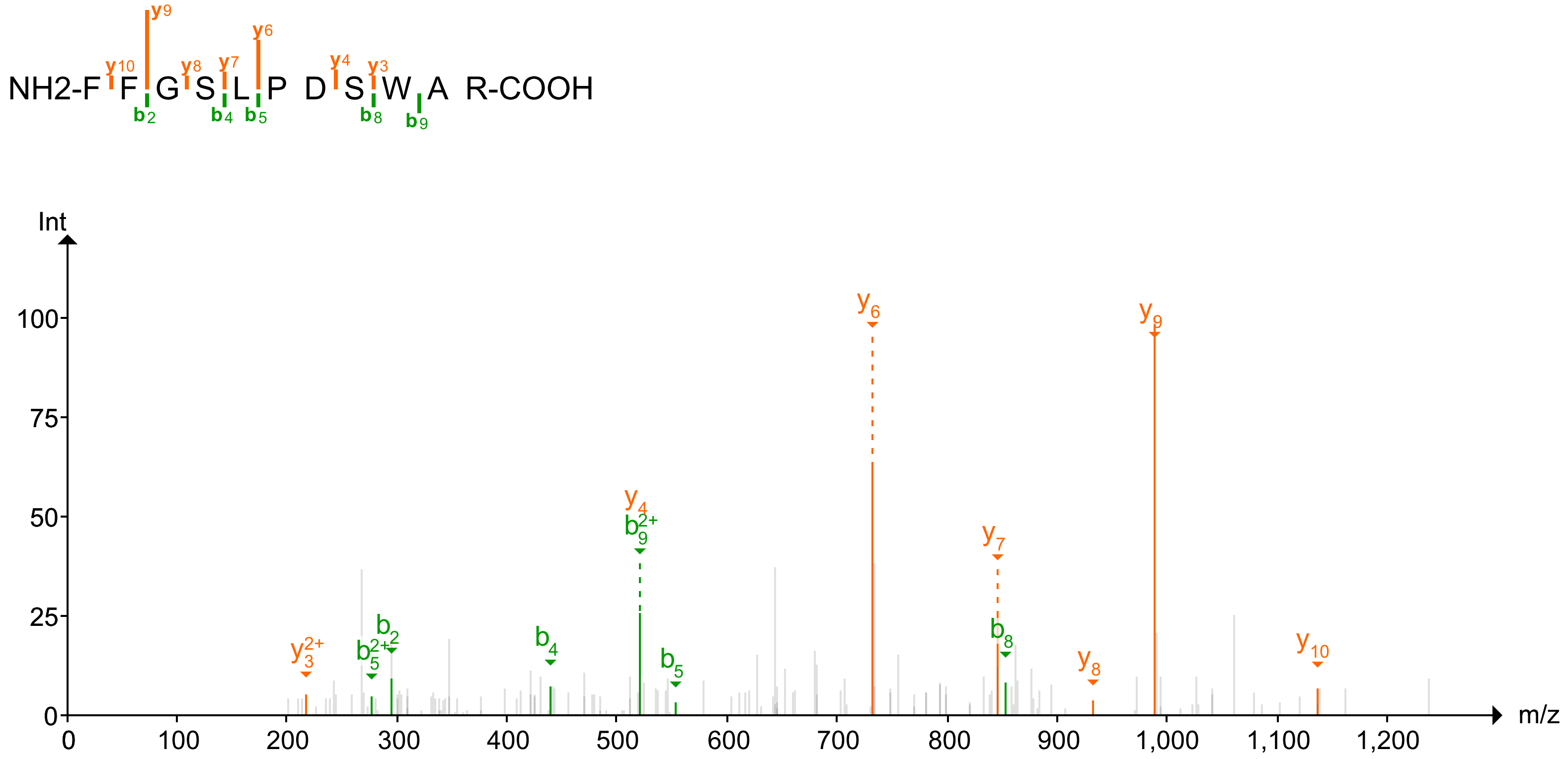

NH2-F F G S L P D S W A R-COOH
y10 y9 y8 y7 y6 y4 y3
b2 b4 b5 b8 b9
Int
100
75
50
25
0
m/z
0
100
200
300
400
500
600
700
800
900
1,000
1,100
1,200
y6
y9
y4
b9 2+
y7
b8
y3 2+
b5 2+
b2
b4
b5
y8
y10

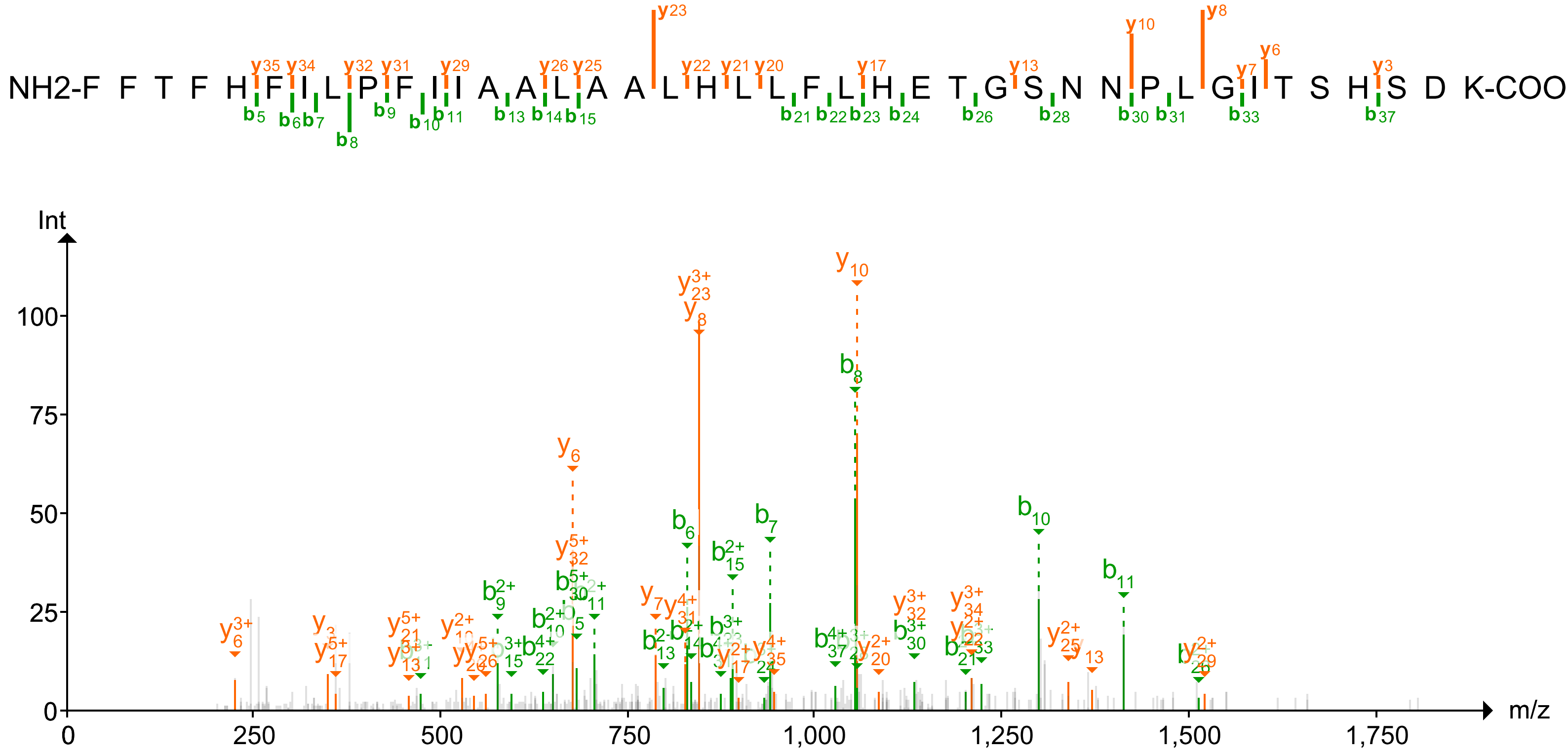

NH2-F F T F H F I L P F I I A A L A A L H L L F L H E T G S N N P L G I T S H S D K-COO
Int
m/z
0
25
50
75
100
0
250
500
750
1,000
1,250
1,500
1,750

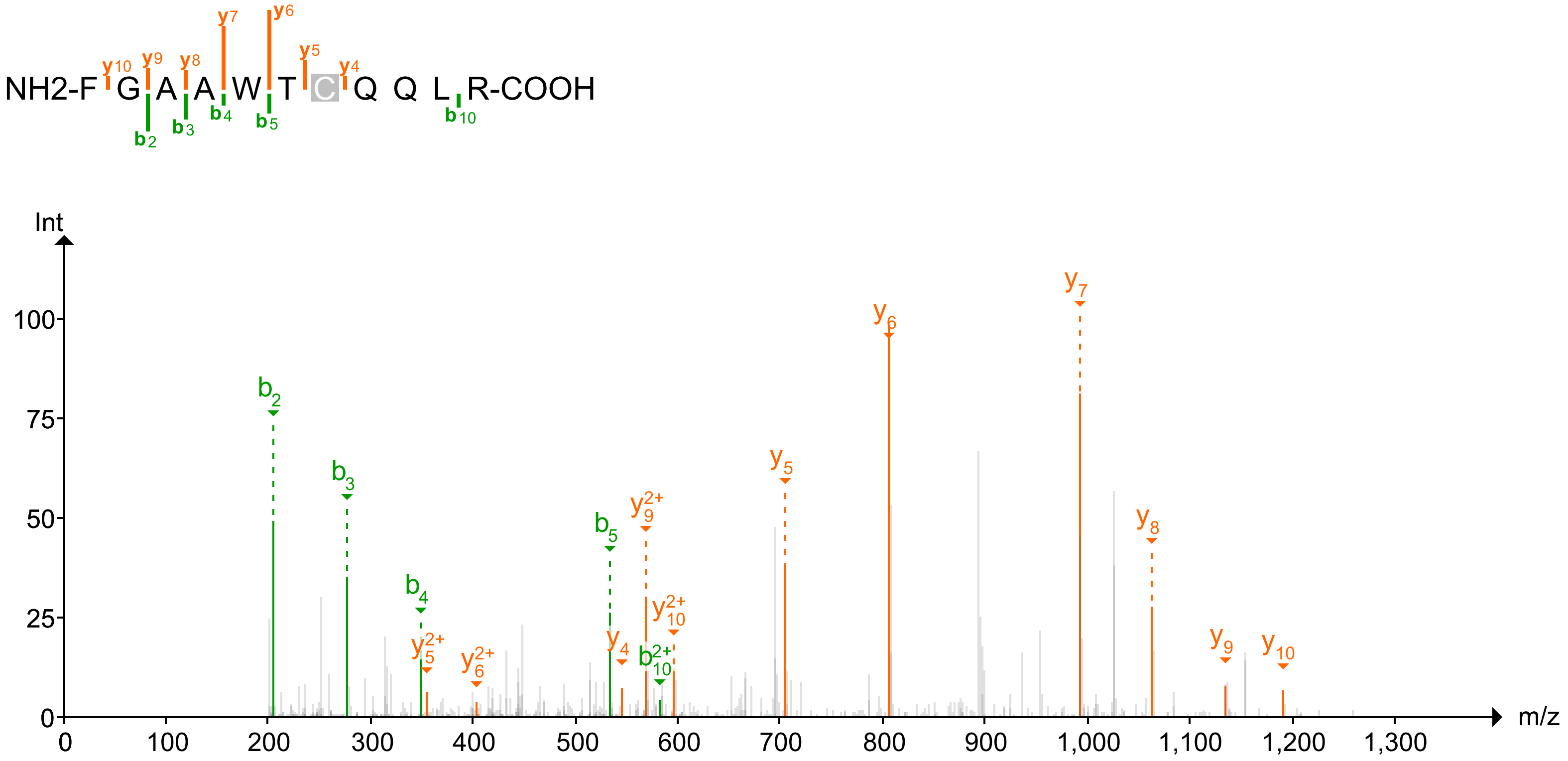

NH2-F G A A W T C Q Q L R-COOH
y10 y9 y8 y7 y6 y5 y4
b2 b3 b4 b5 b10
Int
100
75
50
25
0
b2 b3 b4 b5 y5 2+ y6 2+ y4 y9 2+ b10 2+ y10 2+ y5 y6 y7 y8 y9 y10
0 100 200 300 400 500 600 700 800 900 1,000 1,100 1,200 1,300
m/z

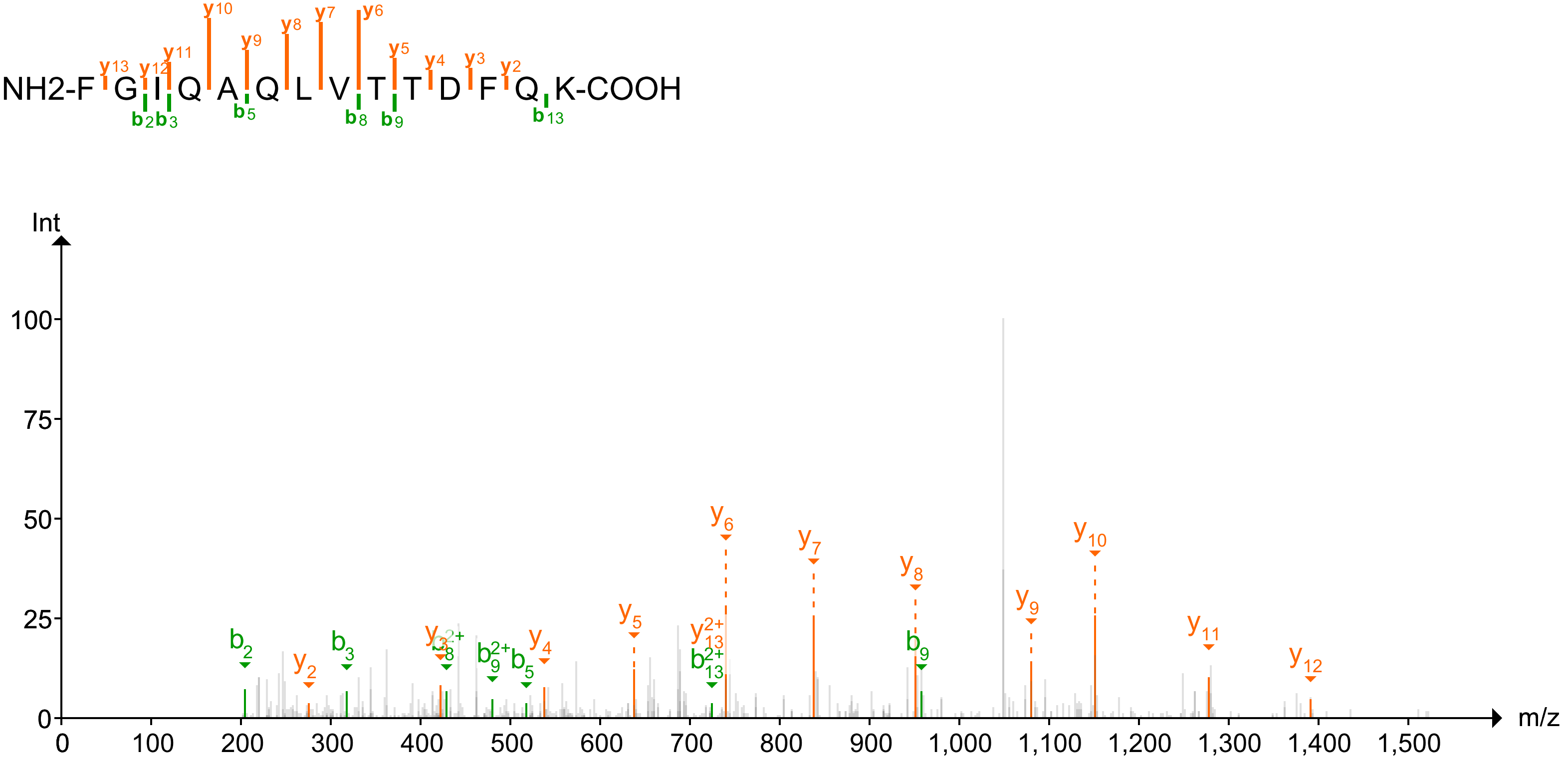

NH2-F G I Q A Q L V T T D F Q K-COOH
Int
m/z
y2
y4
y5
y6
y7
y8
y9
y10
y11
y12
b2
b3
b5
b9

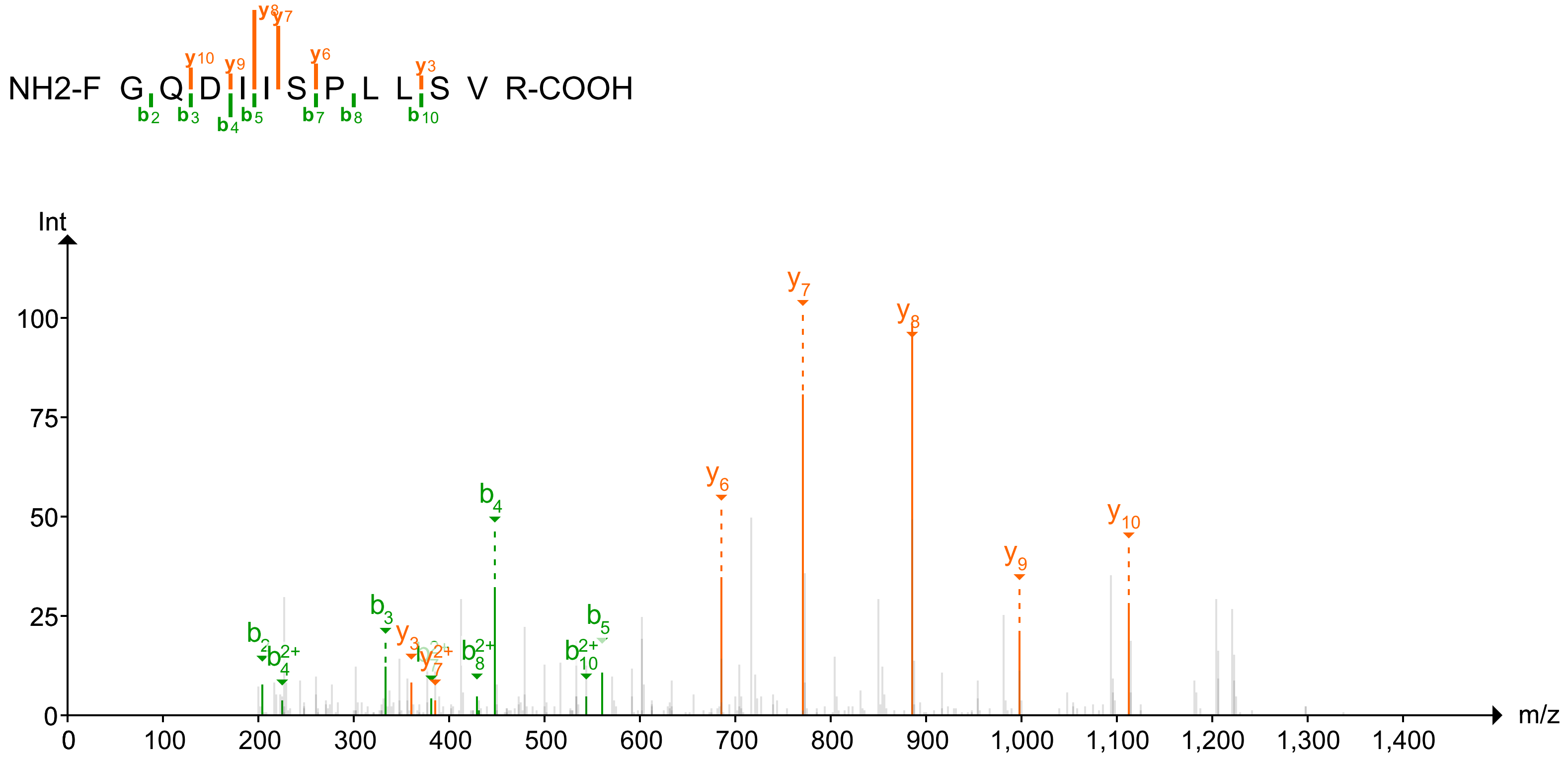
NH2-F G Q D I I S P L L S V R-COOH
y10 y9 y8 y7 y6 y3
b2 b3 b4 b5 b7 b8 b10
Int
100
75
50
25
0
y7
y8
y6
b4
y10
y9
b3
y3
b2
b5
b2+ 4
b2+ 8
b2+ 10
m/z
0
100
200
300
400
500
600
700
800
900
1,000
1,100
1,200
1,300
1,400

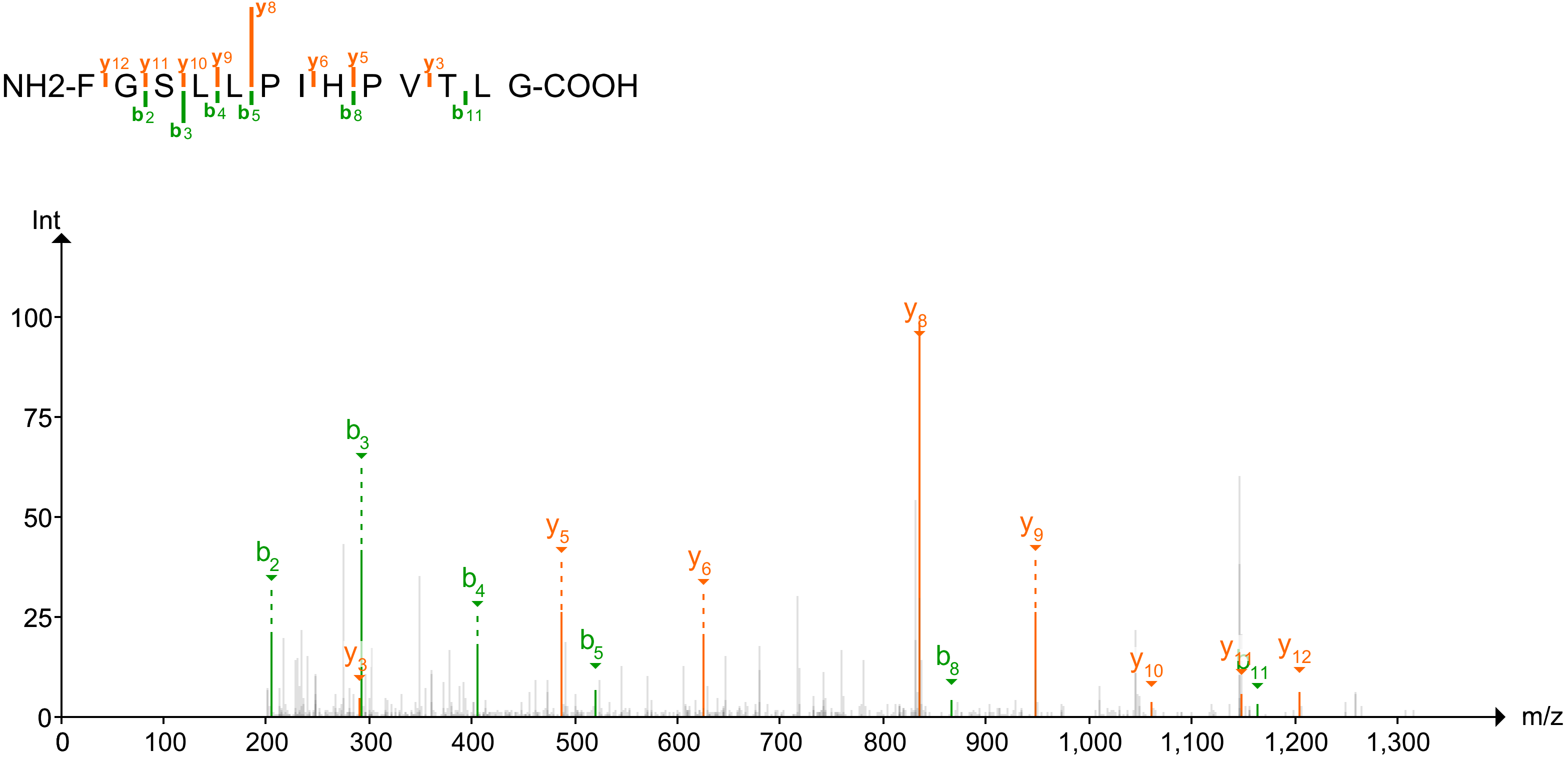

NH2-F G S L L P I H P V T L G-COOH
y12 y11 y10 y9 y8 y6 y5 y3
b2 b3 b4 b5 b8 b11
Int
100
75
50
25
0
m/z
0
100
200
300
400
500
600
700
800
900
1,000
1,100
1,200
1,300

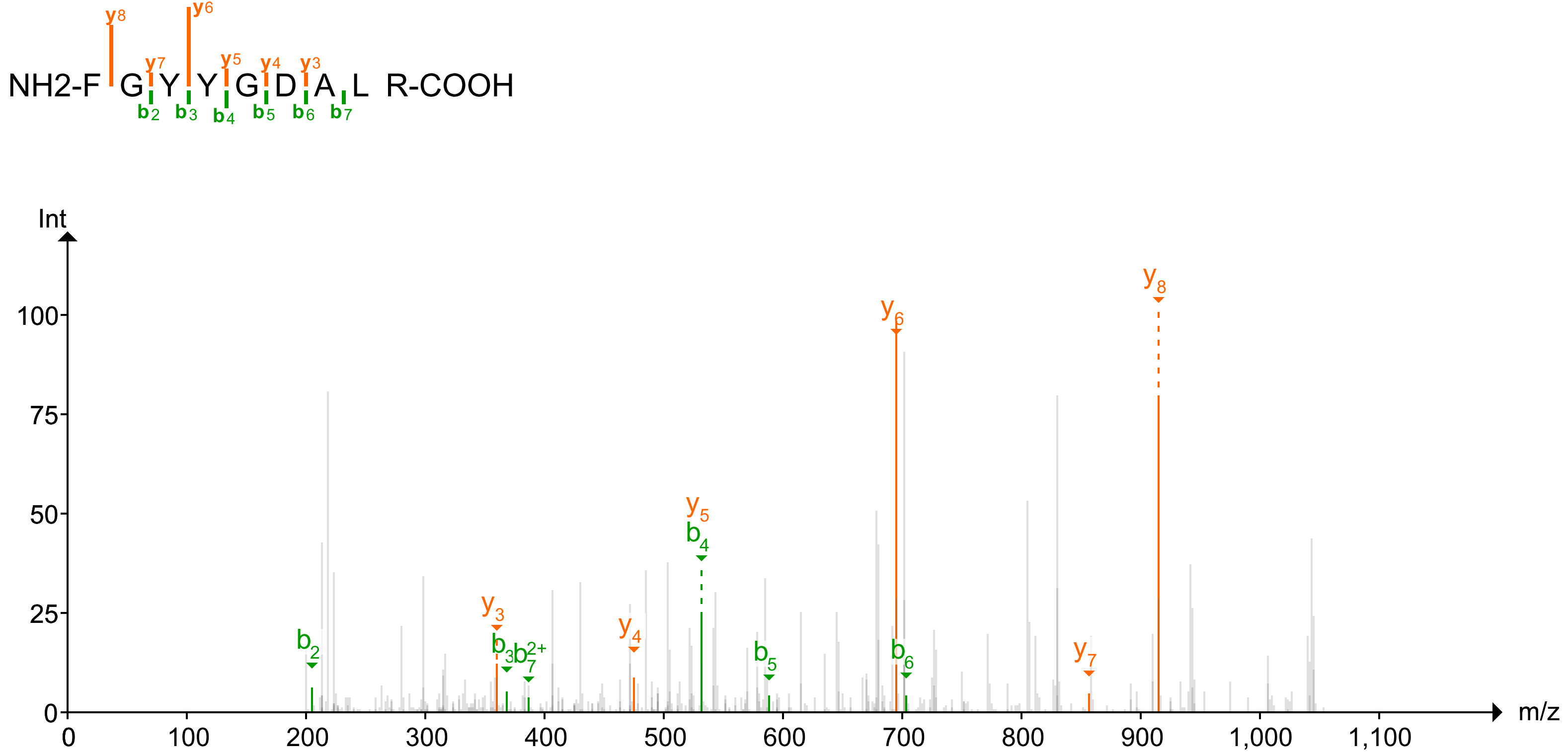

NH2-F G Y Y G D A L R-COOH
y8 y7 y6 y5 y4 y3
b2 b3 b4 b5 b6 b7
Int
100
75
50
25
0
m/z
0
100
200
300
400
500
600
700
800
900
1,000
1,100

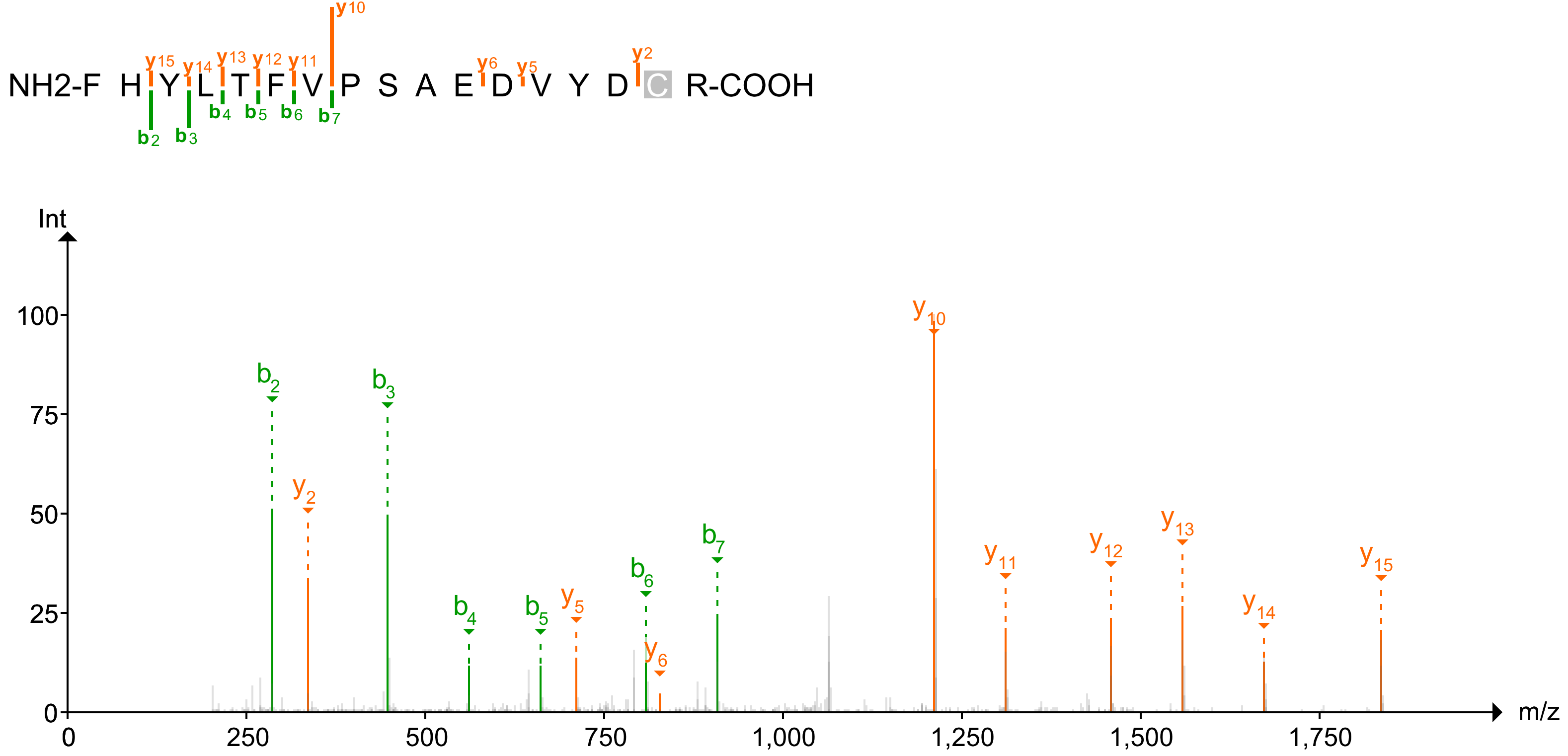

NH2-F H Y L T F V P S A E D V Y D C R-COOH
Int
m/z

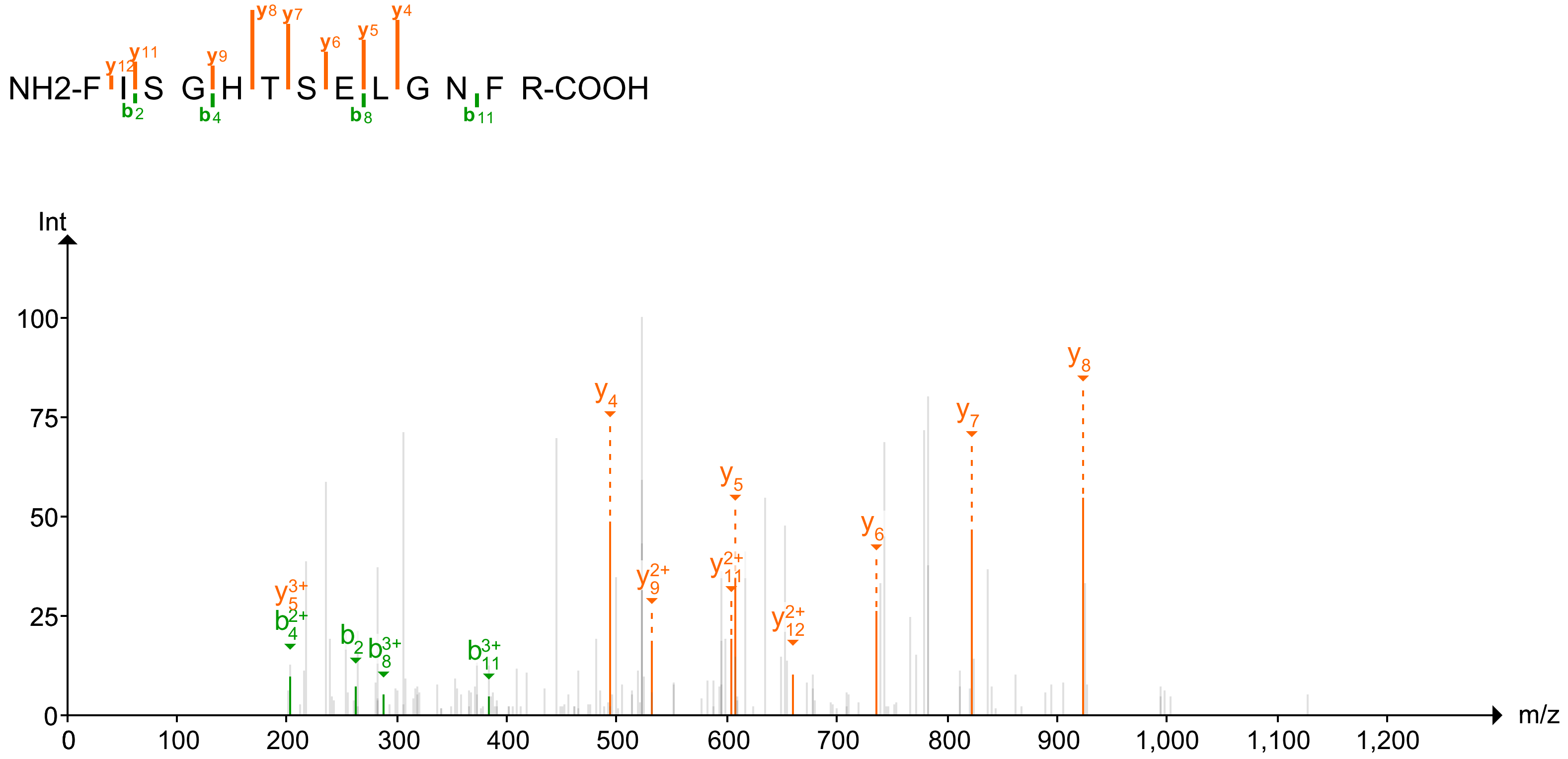

NH2-F I S G H T S E L G N F R-COOH
y12 y11 y9 y8 y7 y6 y5 y4
b2 b4 b8 b11
Int
100
75
50
25
0
m/z
0 100 200 300 400 500 600 700 800 900 1,000 1,100 1,200
y4
y8
y7
y5
y6
y9 2+
y11 2+
y12 2+
y5 3+
b4 2+
b2
b8 3+
b11 3+

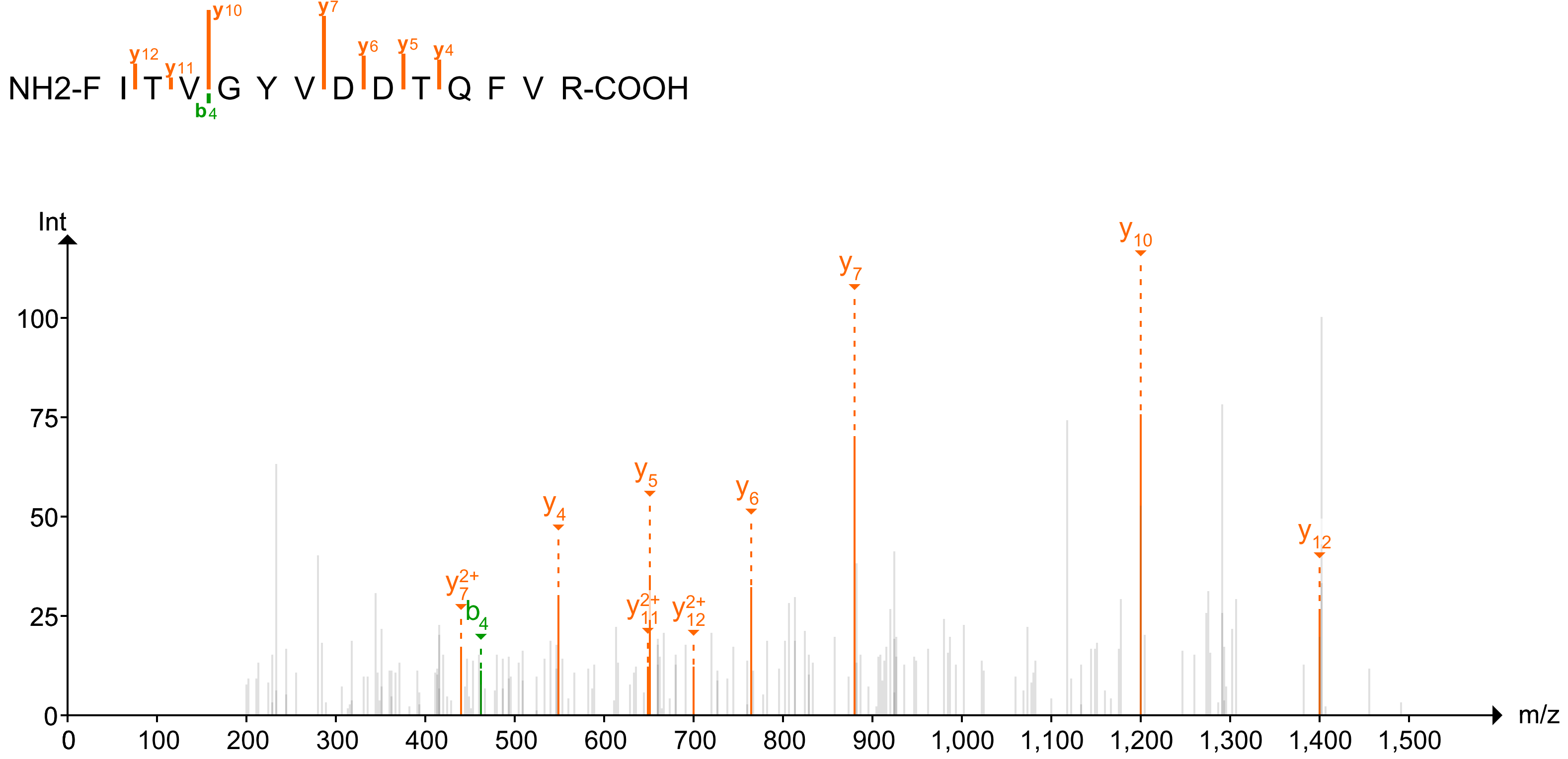

NH2-F I T V G Y V D D T Q F V R-COOH
y12 y11 y10 y7 y6 y5 y4
b4
Int
100
75
50
25
0
y10
y7
y12
y6
y5
y4
y7 2+
b4
y11 2+
y12 2+
0 100 200 300 400 500 600 700 800 900 1,000 1,100 1,200 1,300 1,400 1,500
m/z

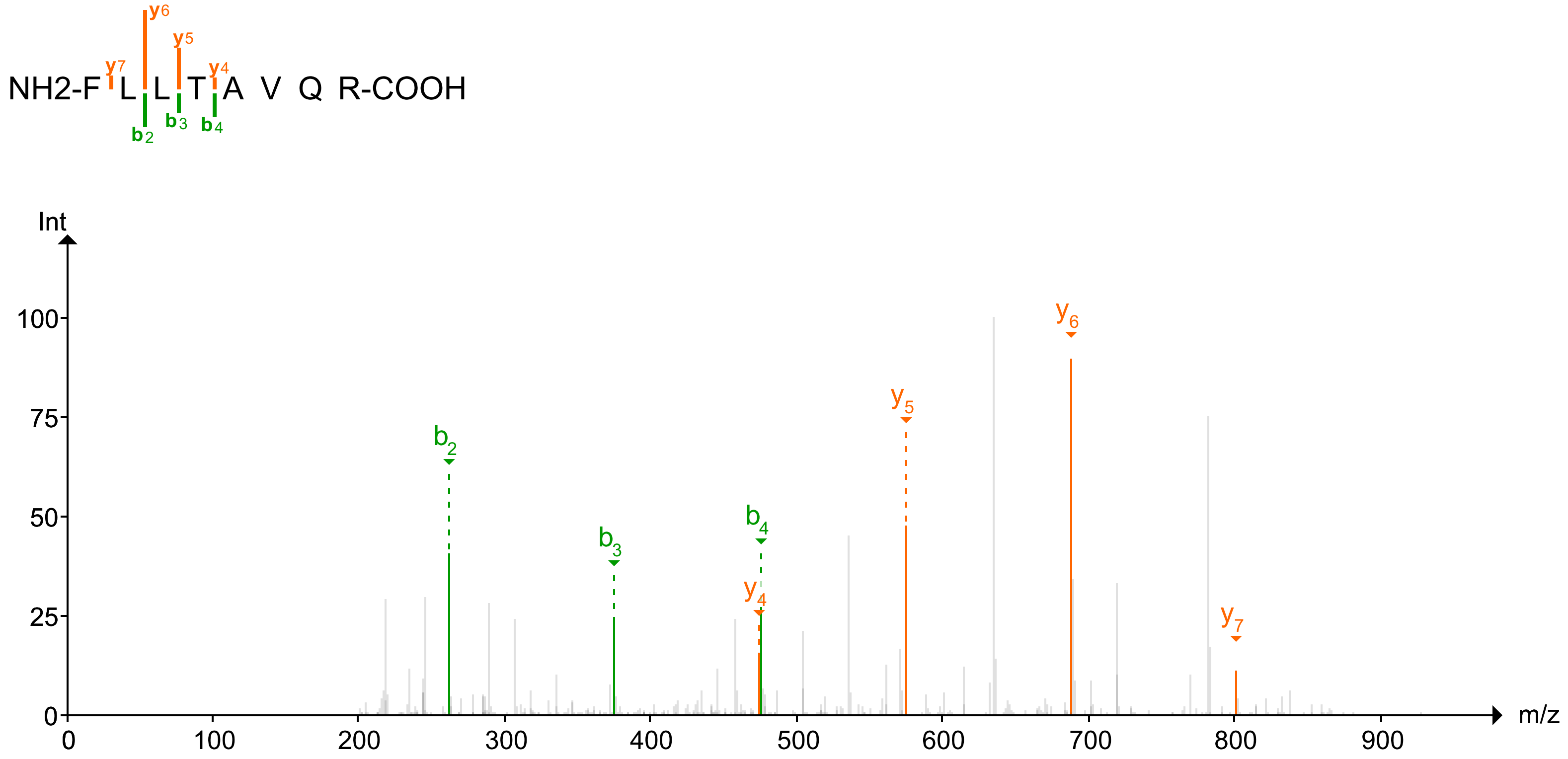

NH2-F L L T A V Q R-COOH
y7
y6
y5
y4
b2
b3
b4
Int
100
75
50
25
0
m/z
0
100
200
300
400
500
600
700
800
900

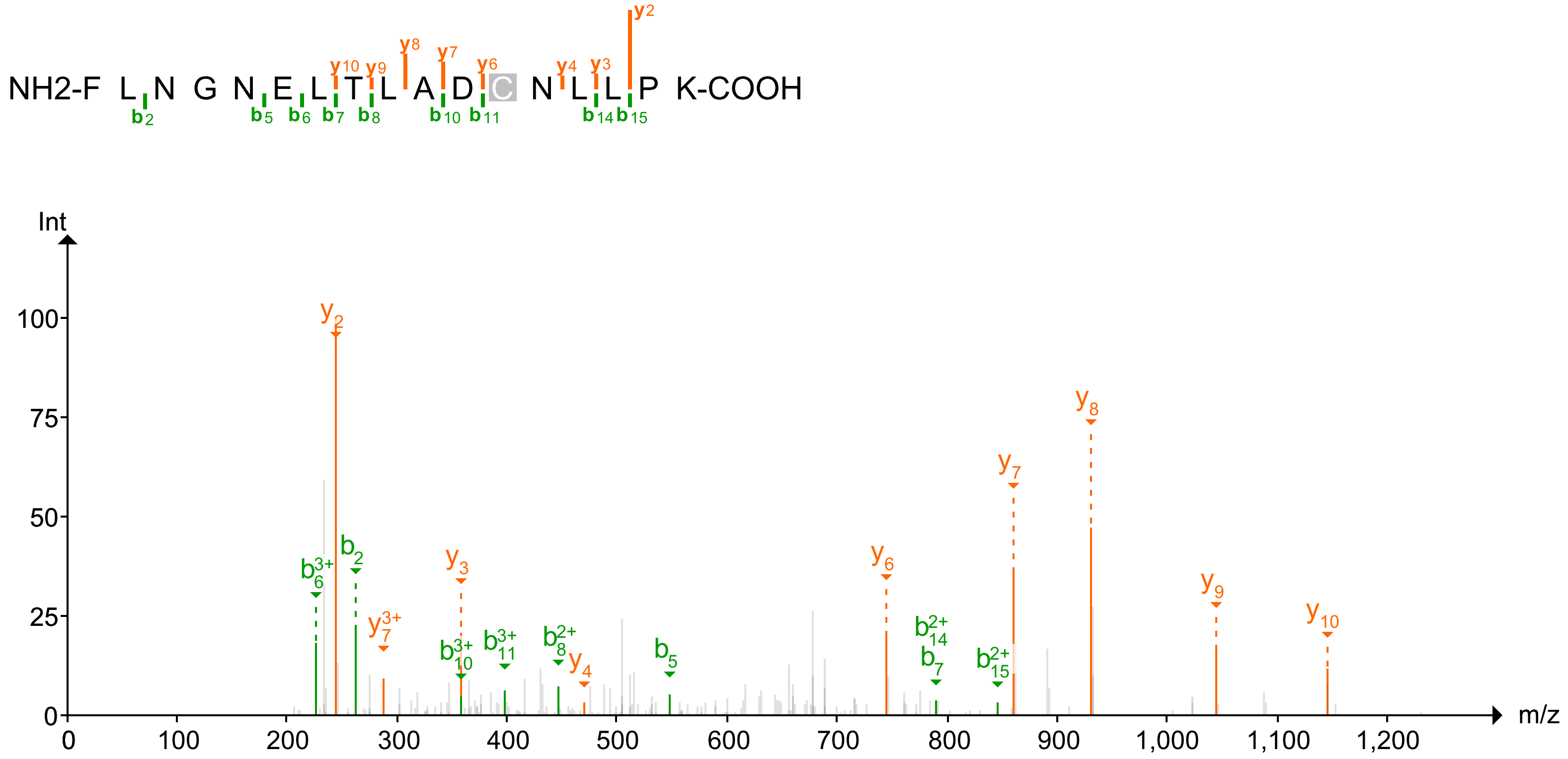

NH2-F L N G N E L T L A D C N L L P K-COOH
Int
m/z

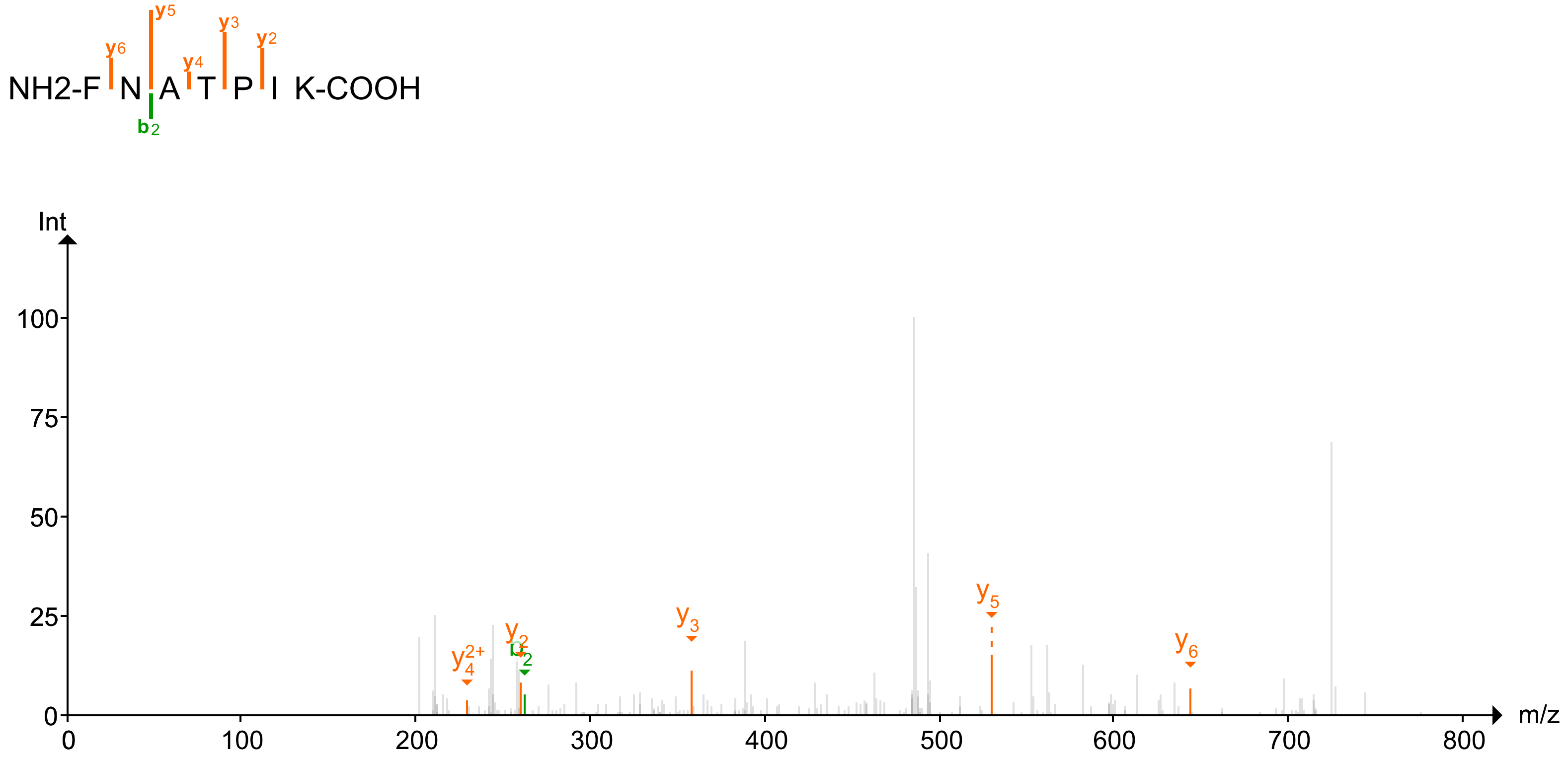
NH2-F N A T P I K-COOH
y6 y5 y4 y3 y2
b2
Int
100
75
50
25
0
m/z
0
100
200
300
400
500
600
700
800
y4 2+
y2
b2
y3
y5
y6

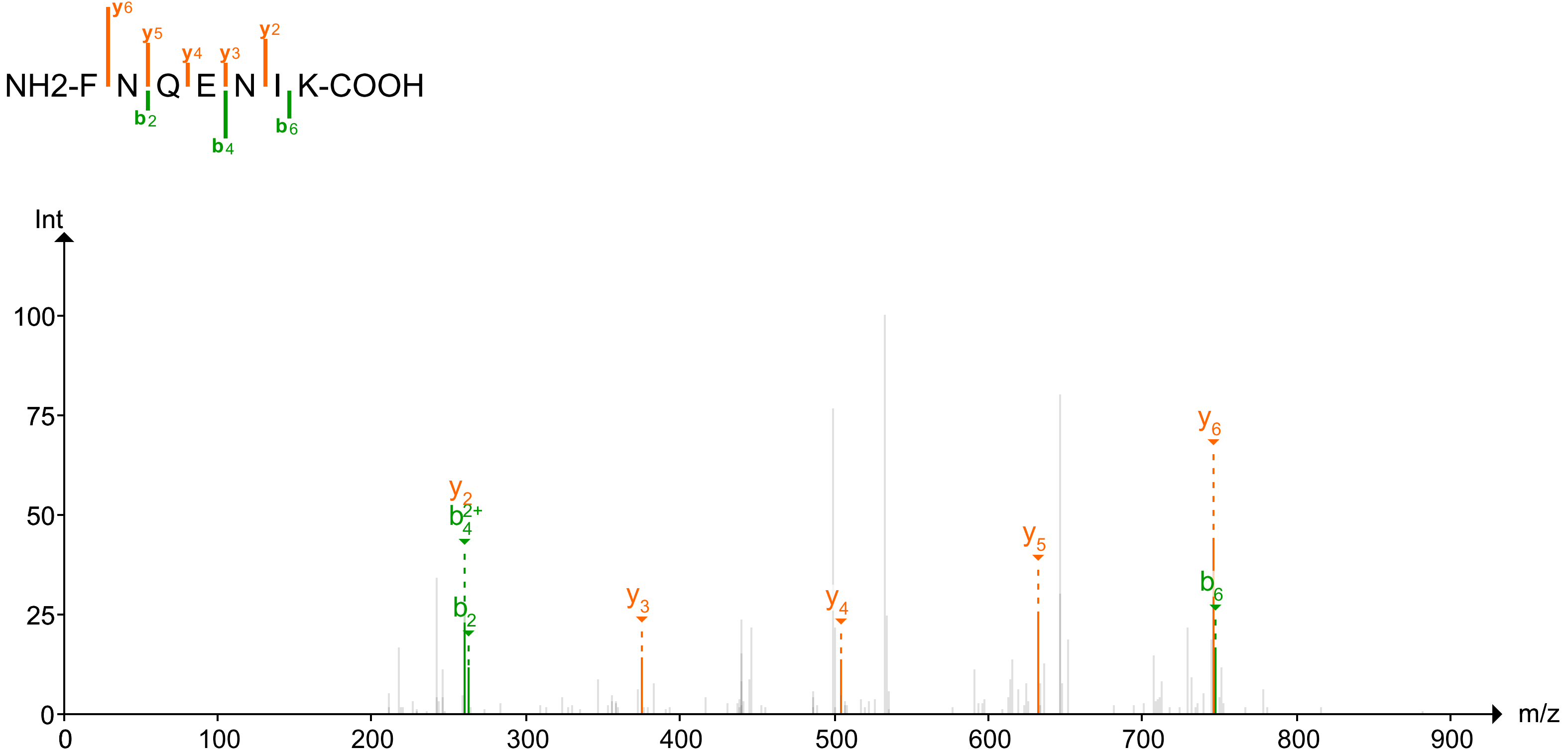

NH2-F N Q E N I K-COOH
y6
y5
y4
y3
y2
b2
b4
b6
Int
100
75
50
25
0
m/z
0
100
200
300
400
500
600
700
800
900
y2
b4 2+
b2
y3
y4
y5
y6
b6

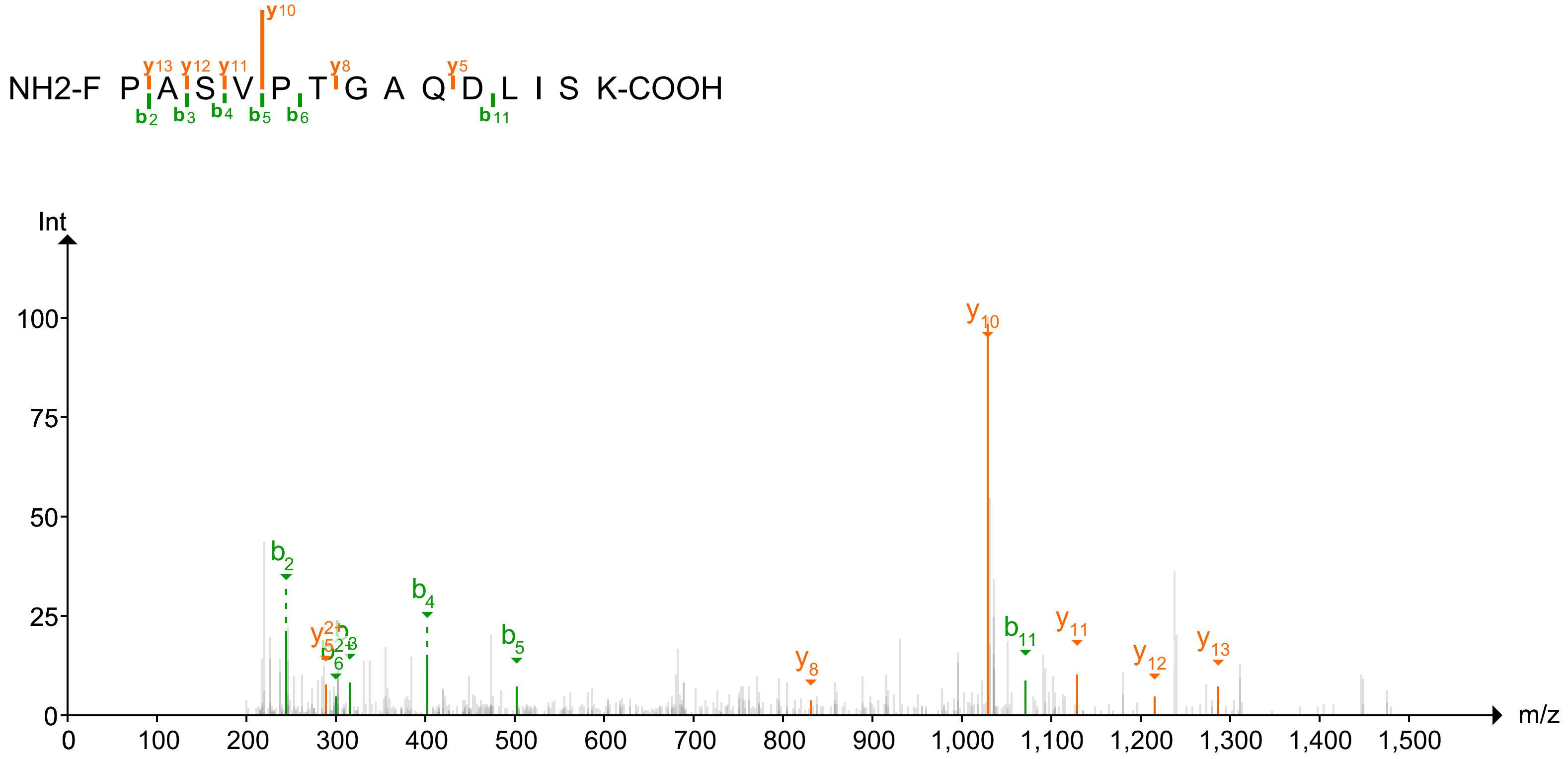
NH2-F P A S V P T G A Q D L I S K-COOH
y10
y13 y12 y11 y8 y5
b2 b3 b4 b5 b6 b11
Int
100
75
50
25
0
m/z
0 100 200 300 400 500 600 700 800 900 1,000 1,100 1,200 1,300 1,400 1,500
y10
b2
b4
b5
b11
y11
y12
y13
y8
b6

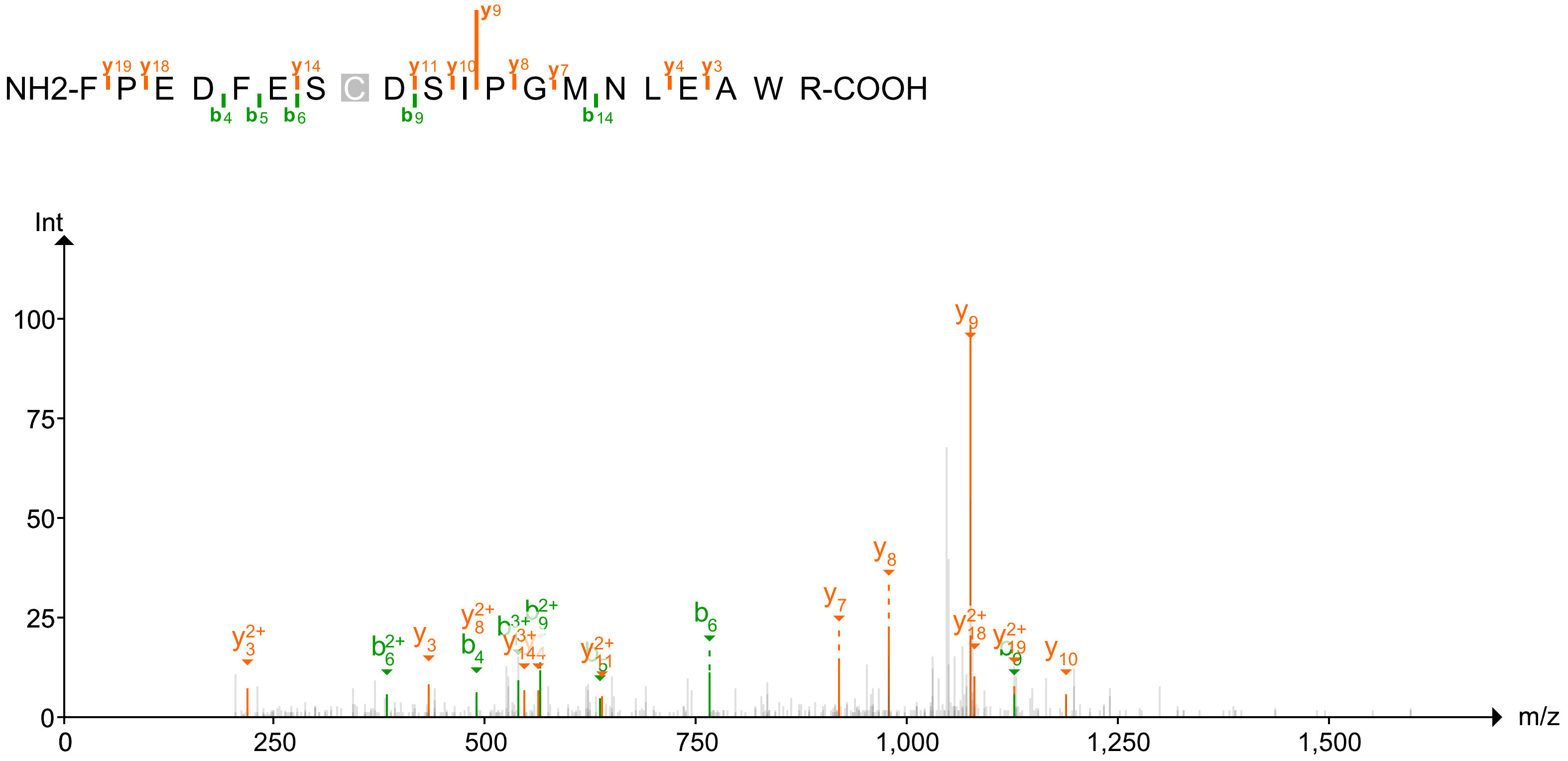
NH2-F P E D F E S C D S I P G M N L E A W R-COOH
y19 y18 y14 y11 y10 y9 y8 y7 y4 y3
b4 b5 b6 b9 b14
Int
100
75
50
25
0
250
500
750
1,000
1,250
1,500
m/z
y9
y8
y7
b6
y10
y3
y3 2+
b6 2+
b4
y8 2+
b9 2+
y11 2+
y18 2+
y19 2+

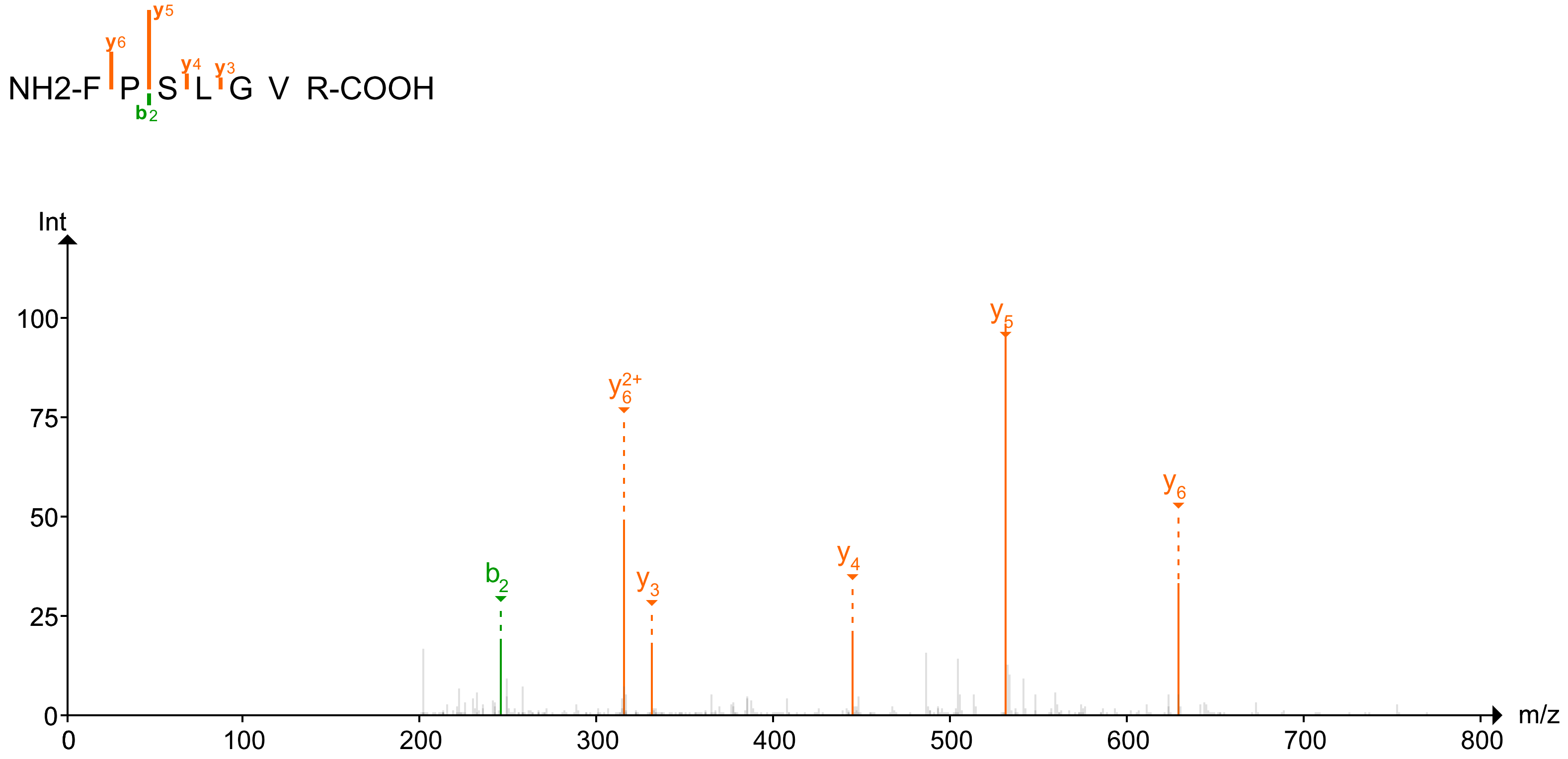

NH2-F P S L G V R-COOH
y6
y5
y4
y3
b2
Int
100
75
50
25
0
m/z
0
100
200
300
400
500
600
700
800
b2
y6 2+
y3
y4
y5
y6

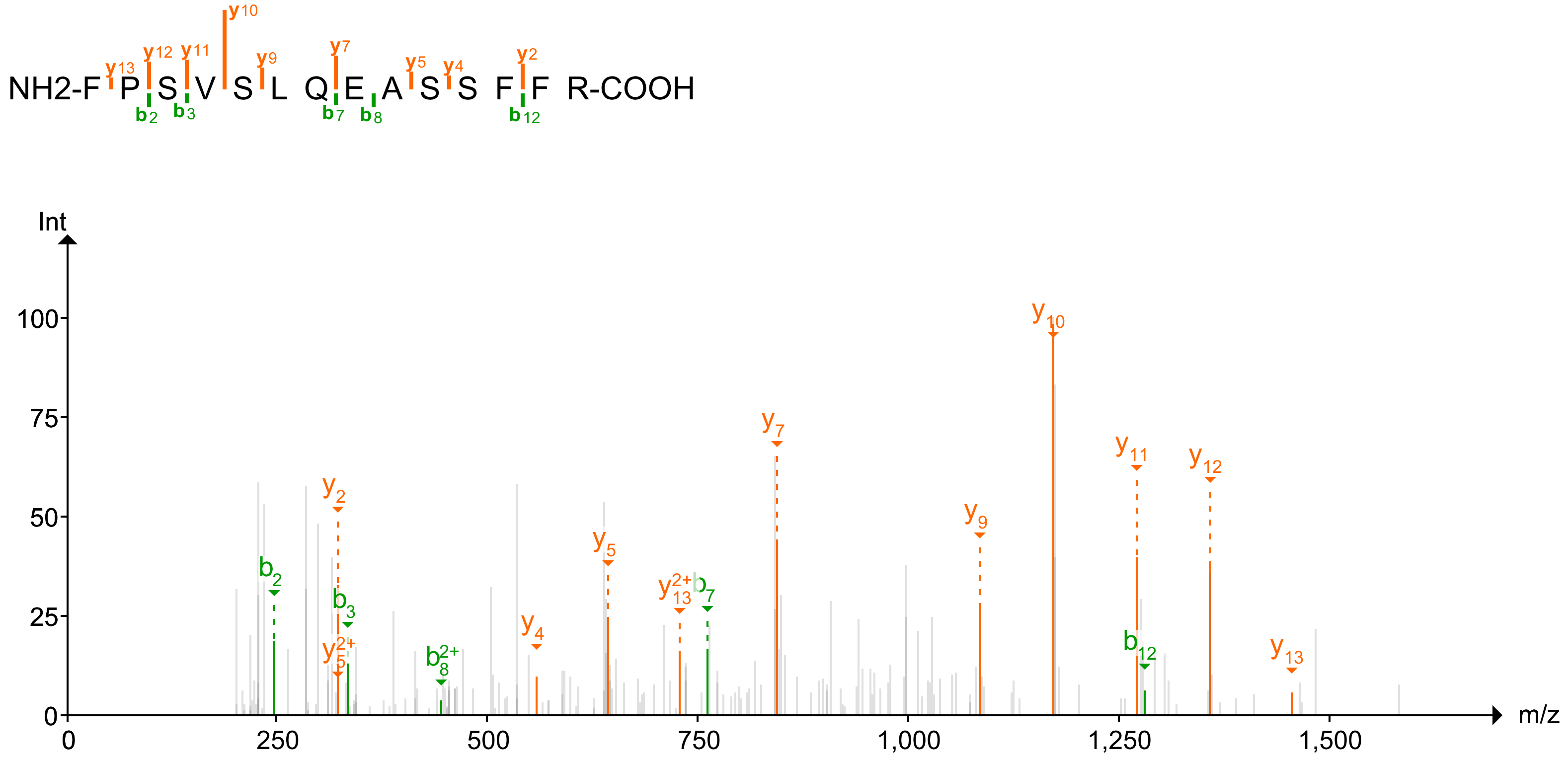

NH2-F P S V S L Q E A S S F F R-COOH
y13 y12 y11 y10 y9 y7 y5 y4 y2
b2 b3 b7 b8 b12
Int
100
75
50
25
0
250
500
750
1,000
1,250
1,500
m/z

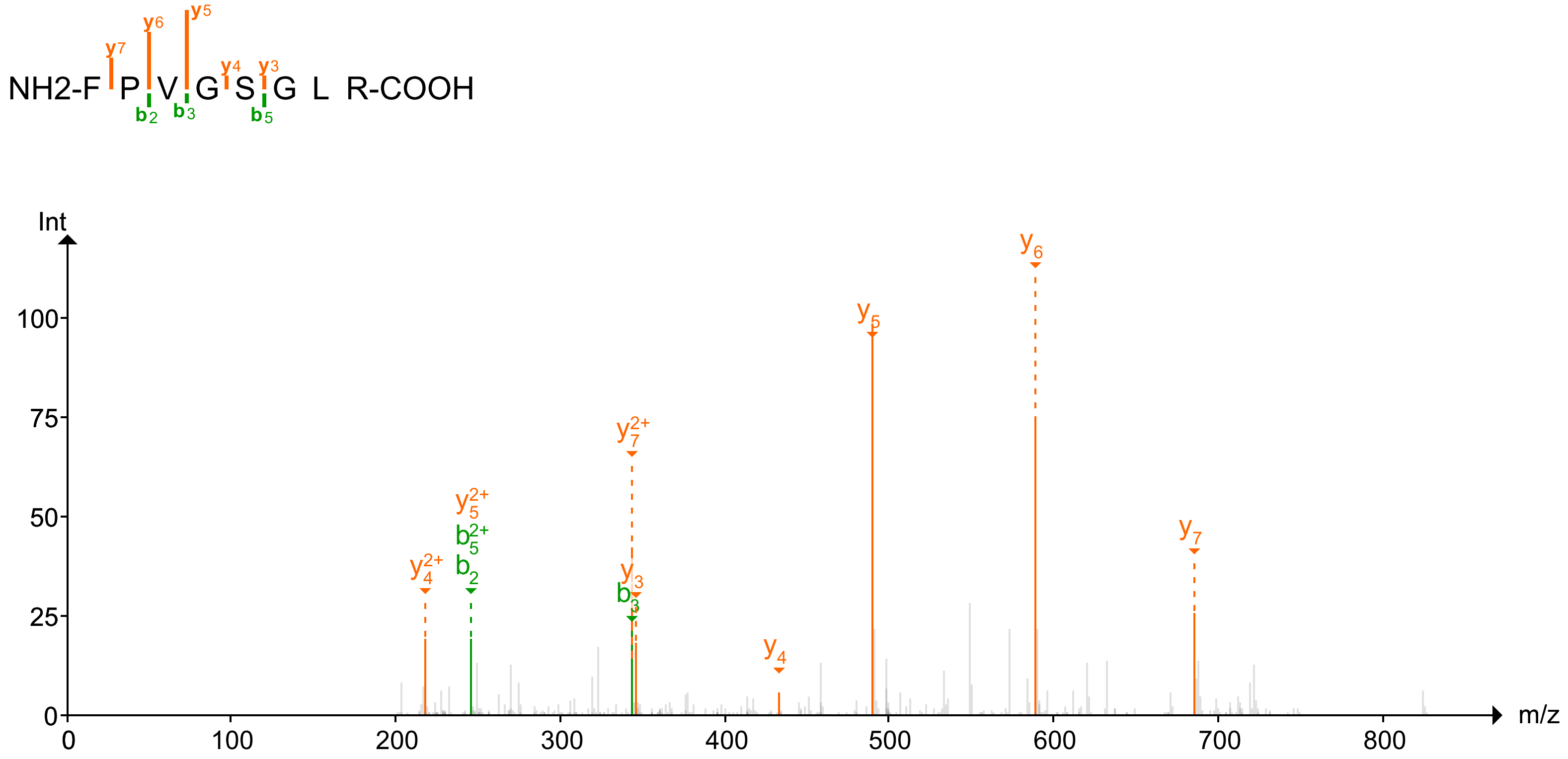

NH2-F P V G S G L R-COOH
y7
y6
y5
y4
y3
b2
b3
b5
Int
m/z
0
25
50
75
100
0
100
200
300
400
500
600
700
800
y6
y5
y7 2+
y5 2+
b5 2+
b2
y4 2+
y3
b3
y4
y7

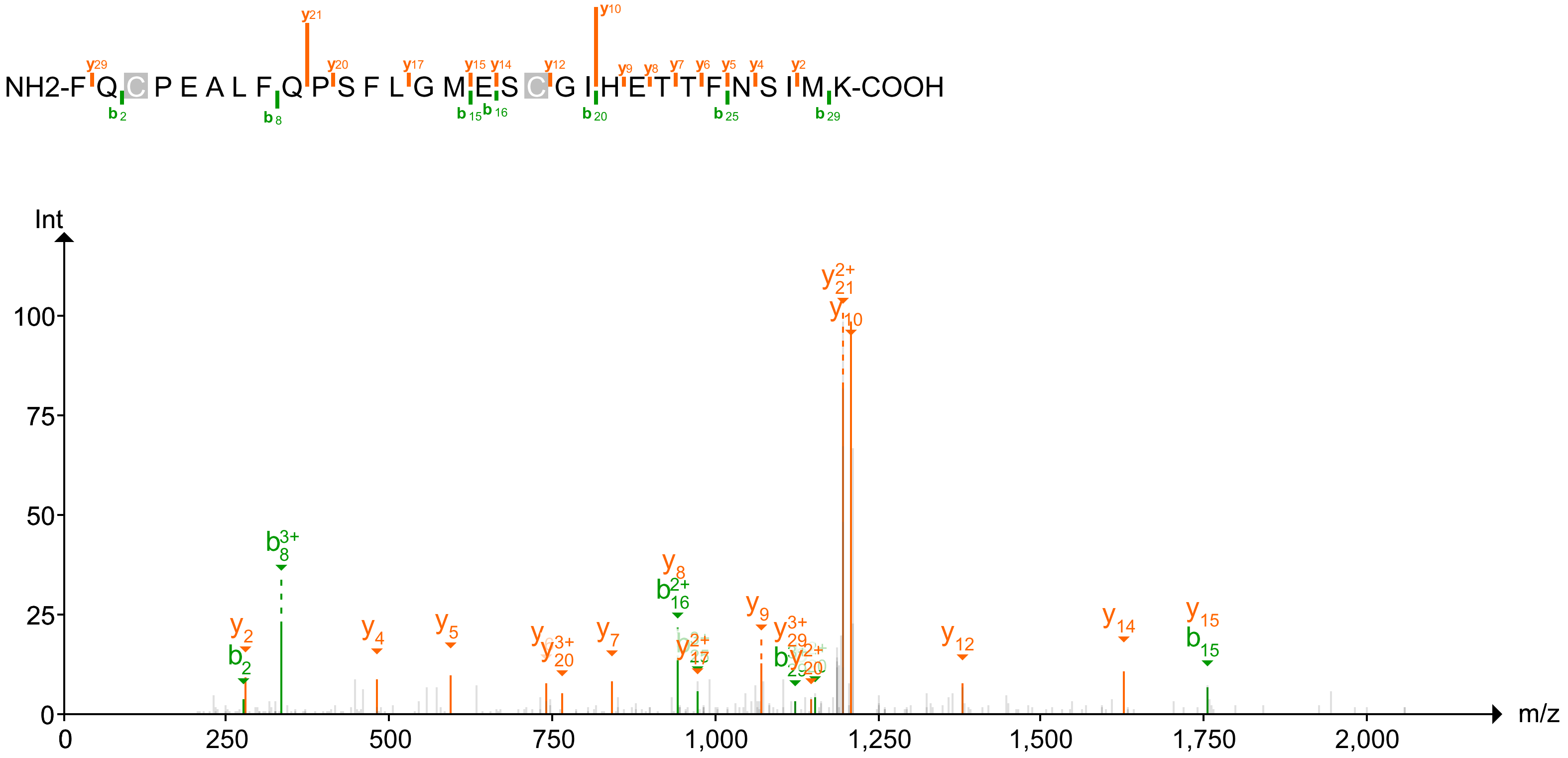
NH2-F Q C P E A L F Q P S F L G M E S C G I H E T T F N S I M K-COOH
y29 y21 y20 y17 y15 y14 y12 y10 y9 y8 y7 y6 y5 y4 y2
b 2 b 8 b 15 b 16 b 20 b 25 b 29
Int
100
75
50
25
0
m/z
0
250
500
750
1,000
1,250
1,500
1,750
2,000
y21 2+
y10
b8 3+
y8
b16 2+
y2
b2
y4
y5
y20 3+
y7
y9
y29 3+
y12
y14
y15
b15

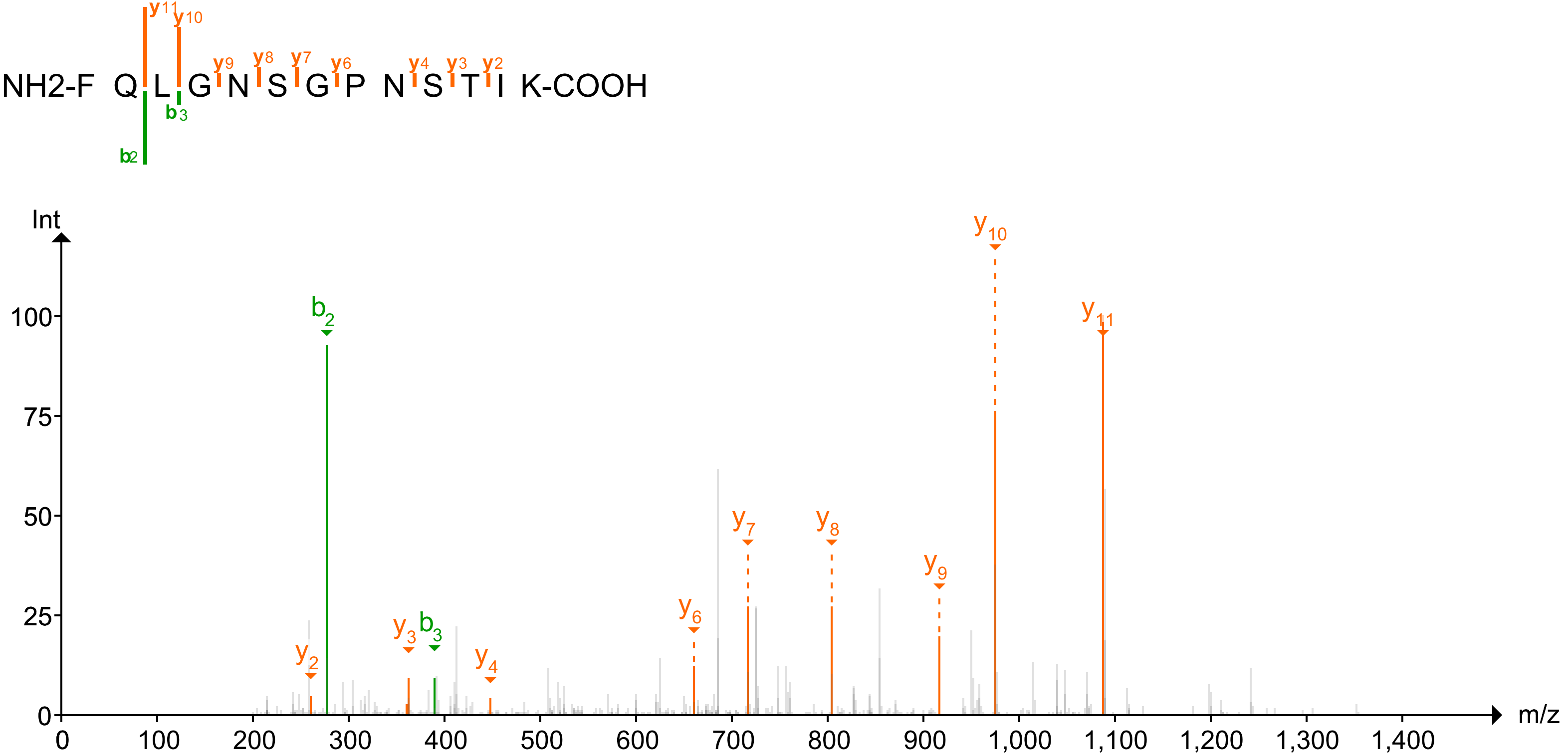

NH2-F Q L G N S G P N S T I K-COOH
y11 y10 y9 y8 y7 y6 y4 y3 y2
b2 b3
Int
m/z
y10 y11 b2 y7 y8 y9 y6 y3 b3 y2 y4
0 25 50 75 100
0 100 200 300 400 500 600 700 800 900 1,000 1,100 1,200 1,300 1,400

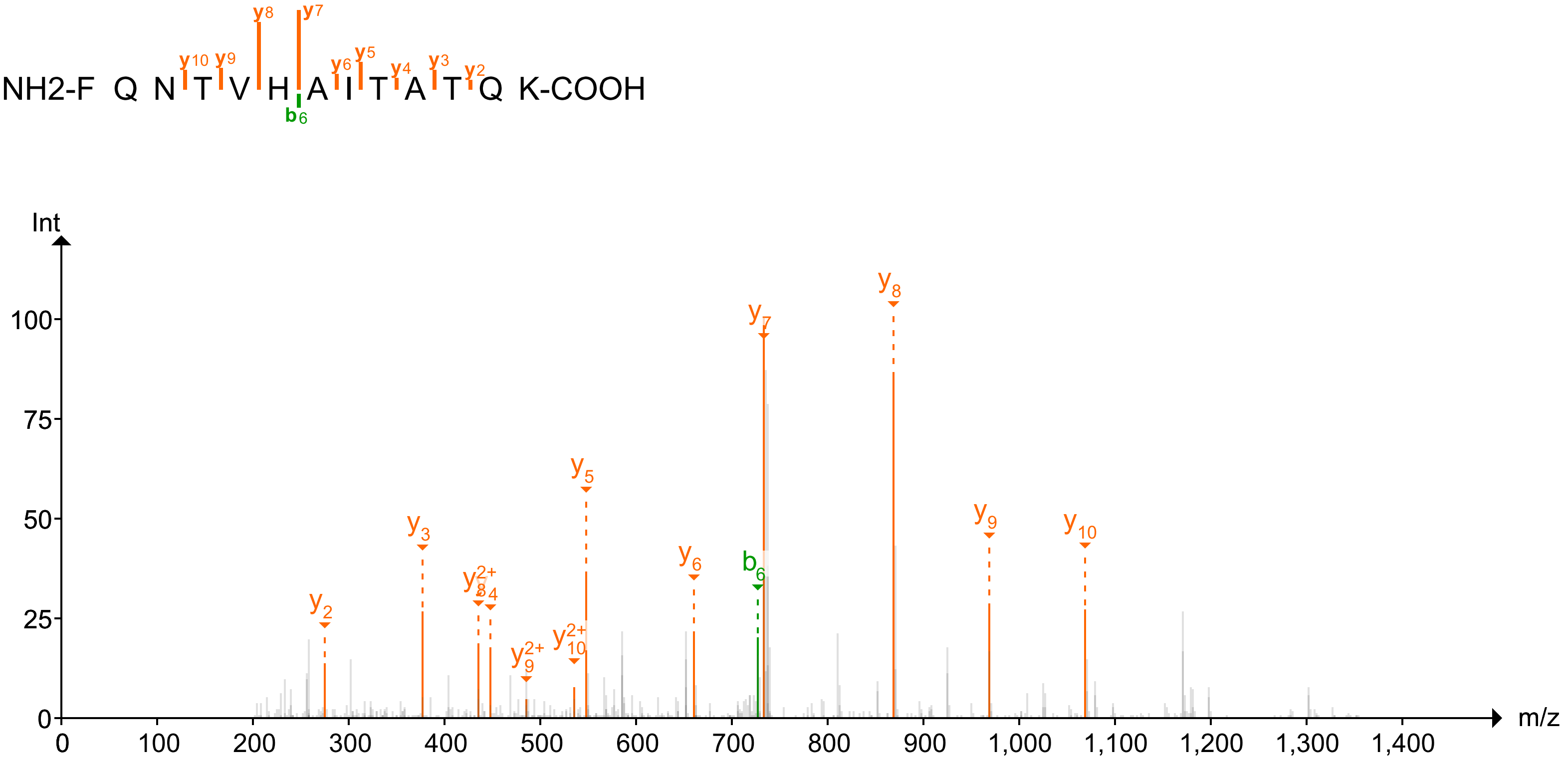

NH2-F Q N T V H A I T A T Q K-COOH
y10 y9 y8 y7 y6 y5 y4 y3 y2
b6
Int
m/z
0
25
50
75
100
0
100
200
300
400
500
600
700
800
900
1,000
1,100
1,200
1,300
1,400

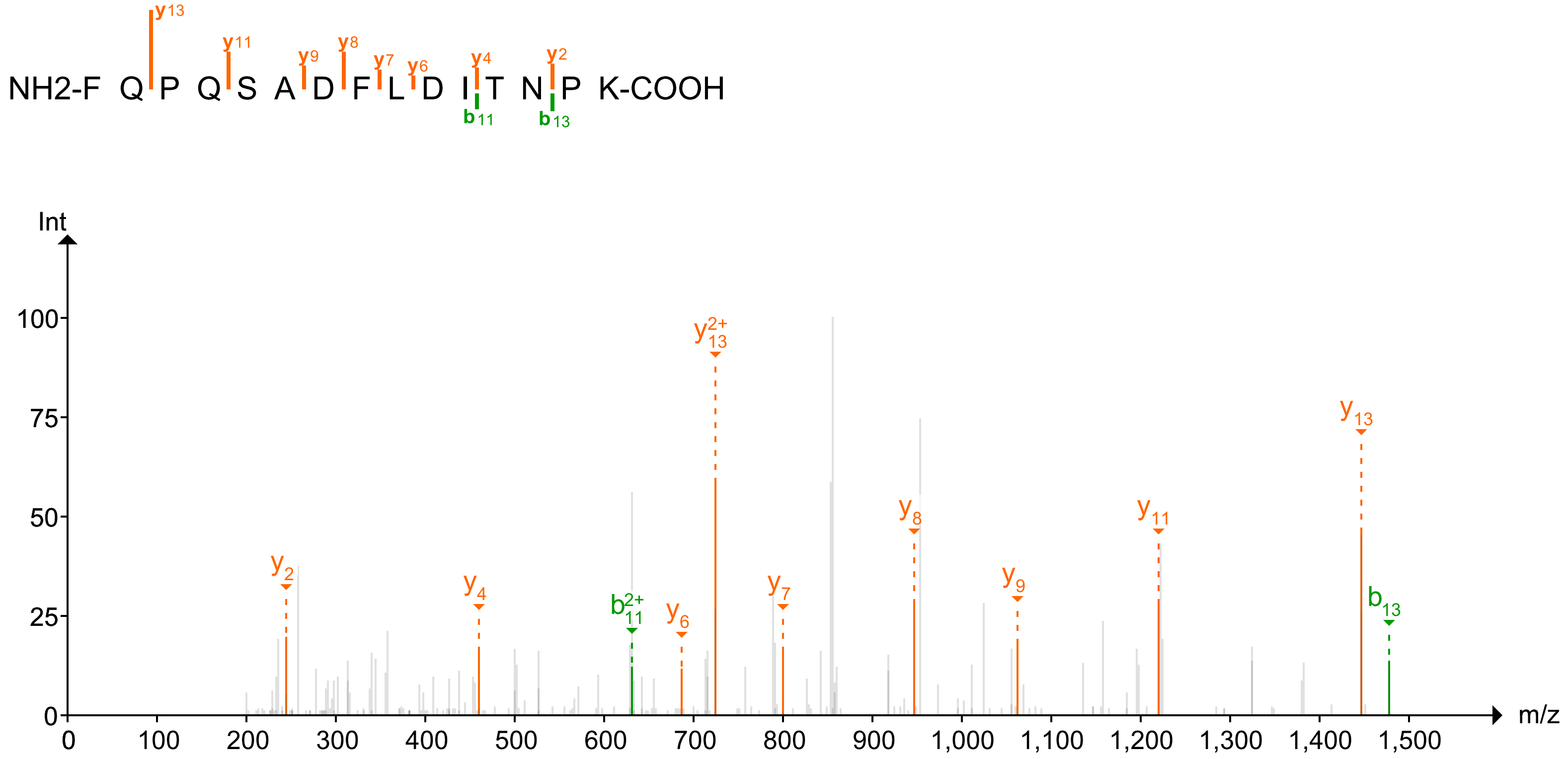

NH2-F Q P Q S A D F L D I T N P K-COOH
y13
y11
y9
y8
y7
y6
y4
y2
b11
b13
Int
100
75
50
25
0
m/z
0
100
200
300
400
500
600
700
800
900
1,000
1,100
1,200
1,300
1,400
1,500
$y_{13}^{2+}$
$y_{13}$
$y_8$
$y_{11}$
$y_2$
$y_4$
$b_{11}^{2+}$
$y_6$
$y_7$
$y_9$
$b_{13}$

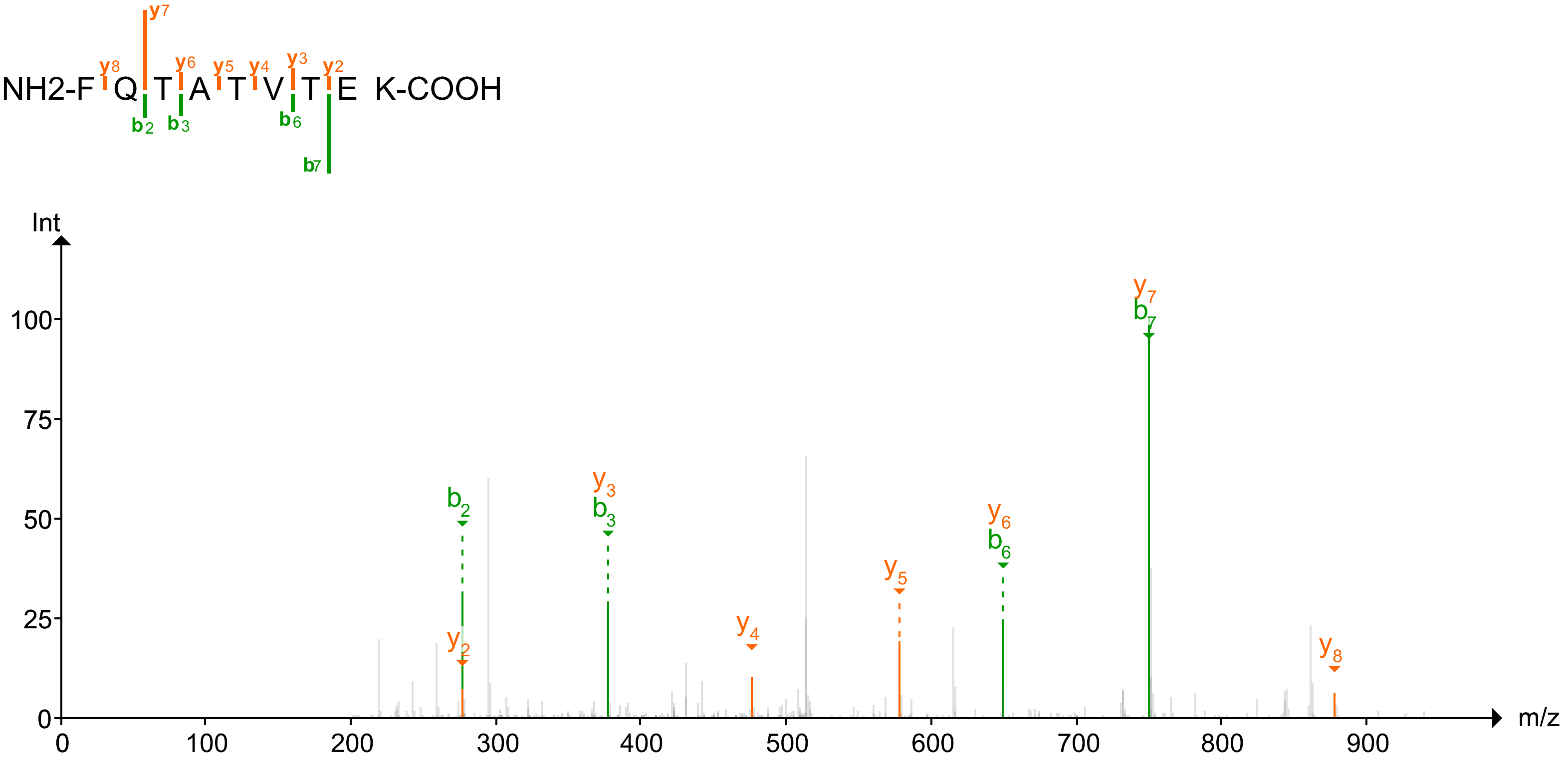

NH2-F Q T A T V T E K-COOH
y7
y8
y6
y5
y4
y3
y2
b2
b3
b6
b7
Int
100
75
50
25
0
m/z
0
100
200
300
400
500
600
700
800
900

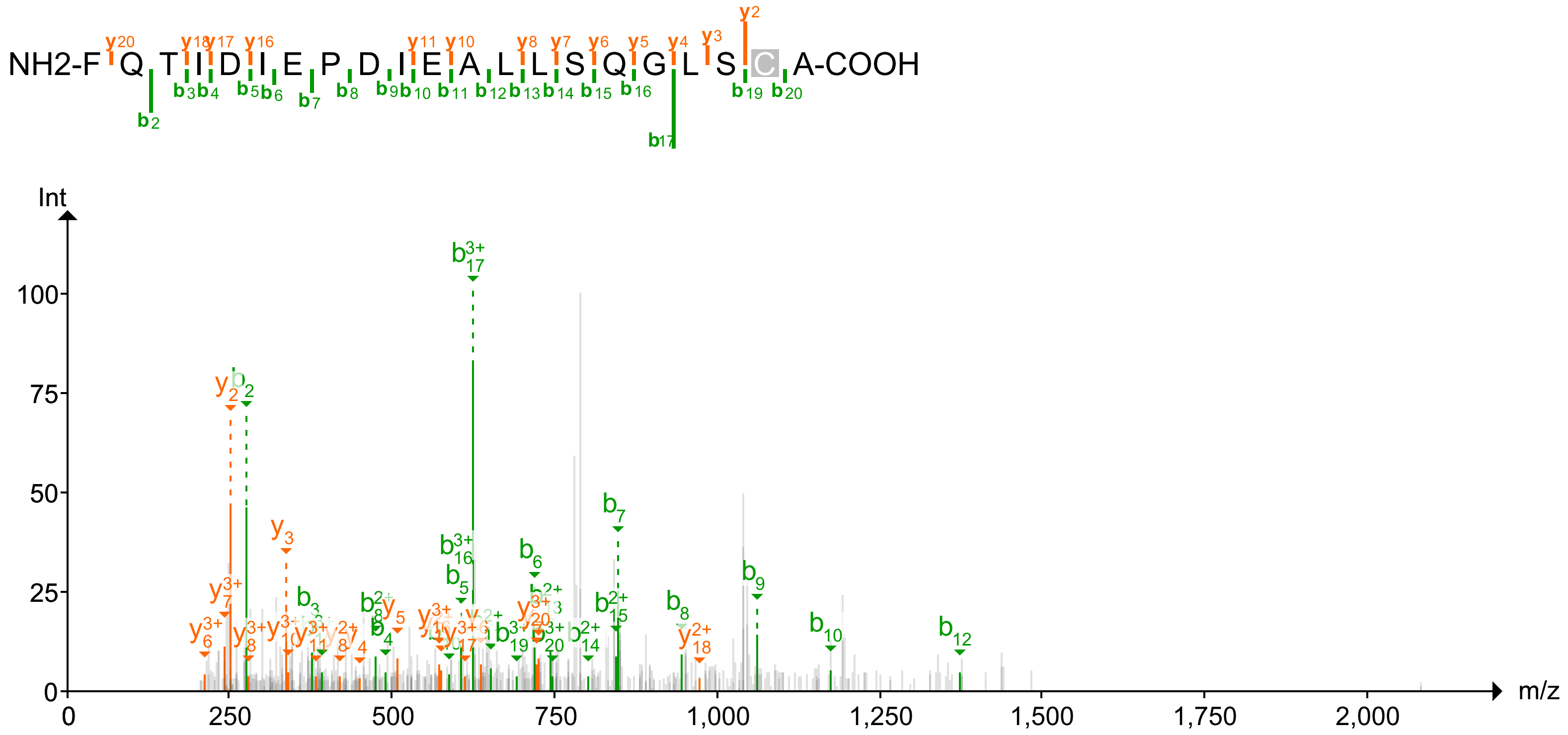

NH2-F Q T I I D I E P D I E A L L S Q G L S C A-COOH
y20 y18 y17 y16 y11 y10 y8 y7 y6 y5 y4 y3 y2
b2 b3 b4 b5 b6 b7 b8 b9 b10 b11 b12 b13 b14 b15 b16 b17 b19 b20
Int
m/z

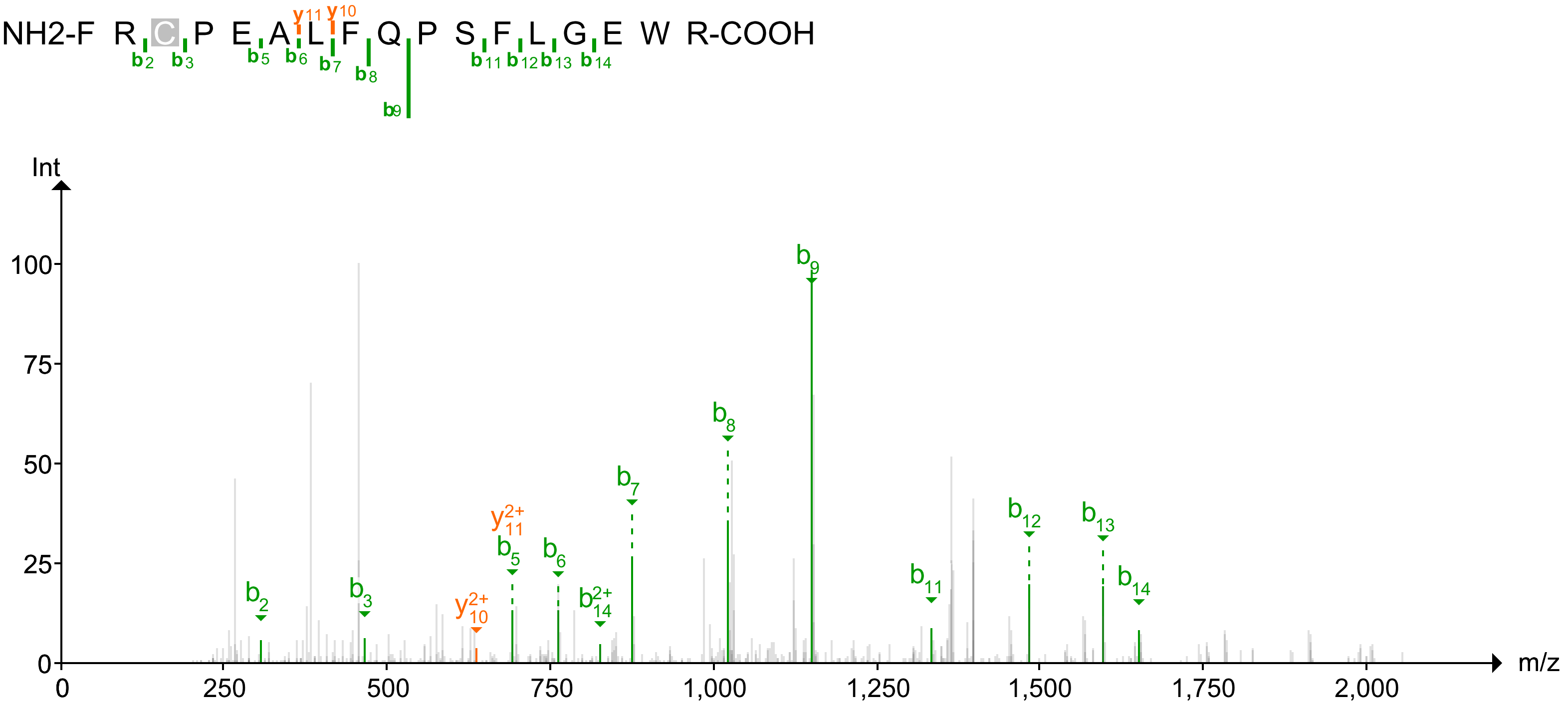

NH2-F R C P E A L F Q P S F L G E W R-COOH
y11 y10
b2 b3 b5 b6 b7 b8 b9 b11 b12 b13 b14
Int
100
75
50
25
0
b9
b8
b7
$y_{11}^{2+}$
b5
b6
b12
b13
b2
b3
$y_{10}^{2+}$
$b_{14}^{2+}$
b11
b14
0
250
500
750
1,000
1,250
1,500
1,750
2,000
m/z

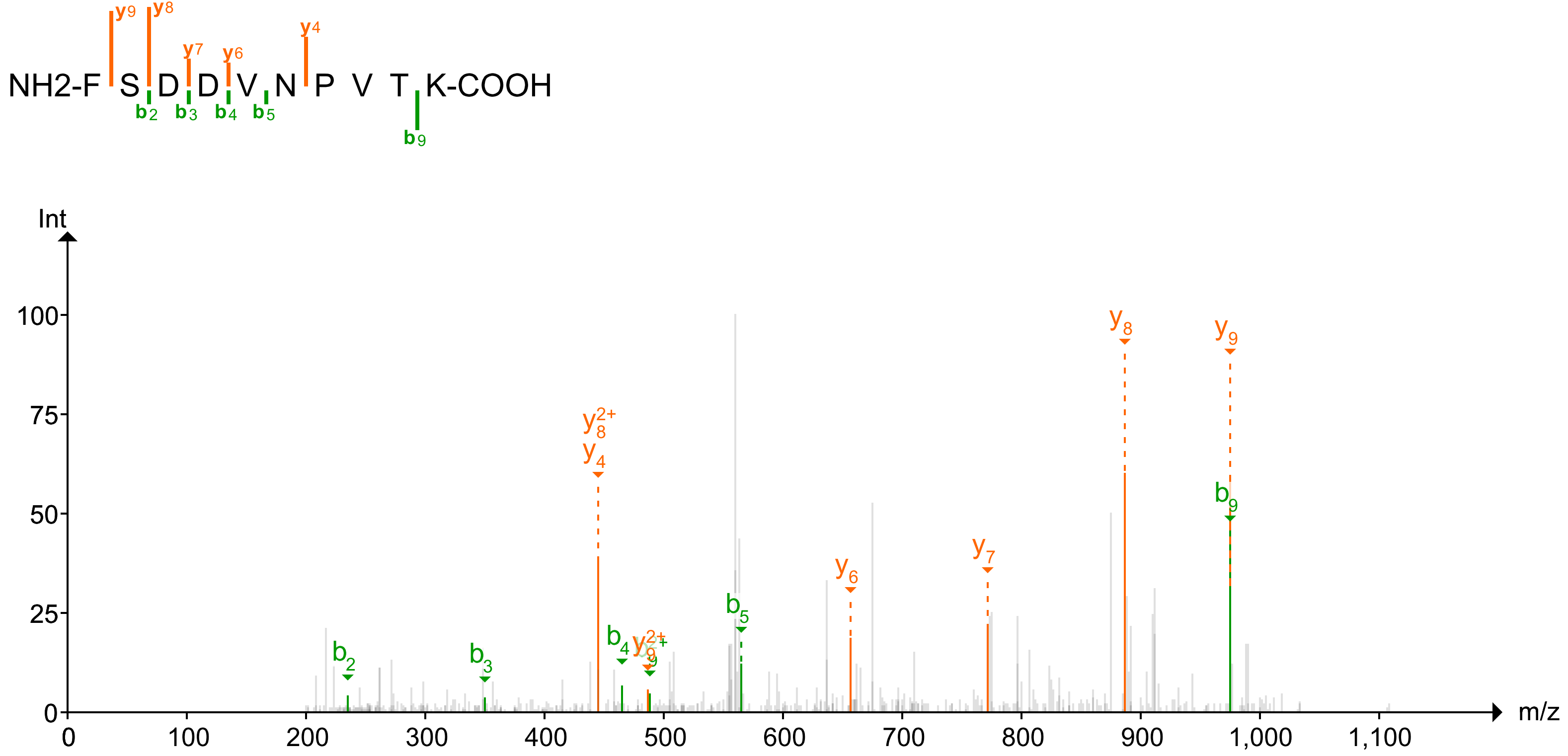

NH2-F S D D V N P V T K-COOH
y9 y8 y7 y6 y4
b2 b3 b4 b5 b9
Int
100
75
50
25
0
m/z
0 100 200 300 400 500 600 700 800 900 1,000 1,100

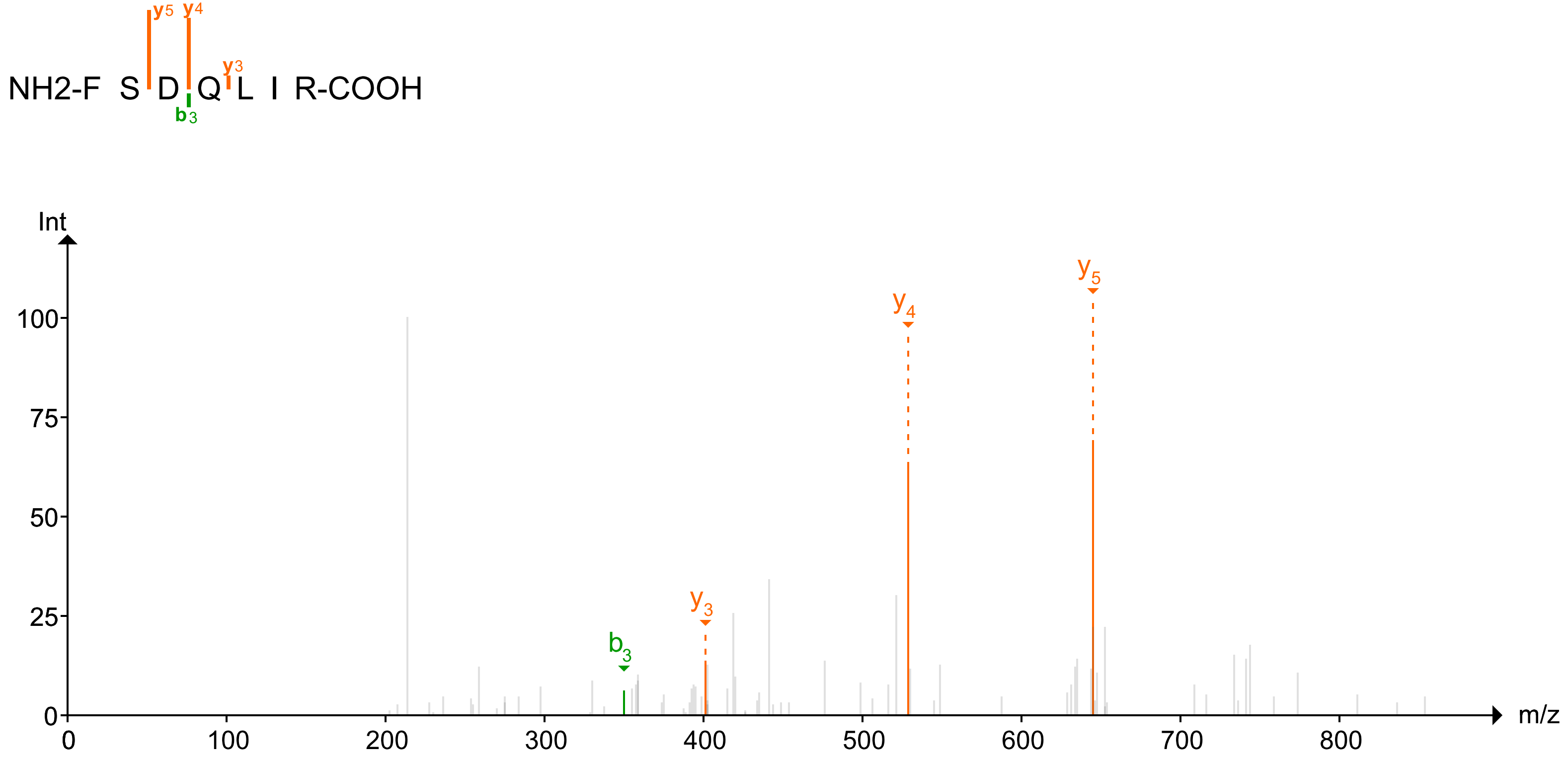

NH2-F S D Q L I R-COOH
y5
y4
y3
b3
Int
100
75
50
25
0
m/z
0
100
200
300
400
500
600
700
800

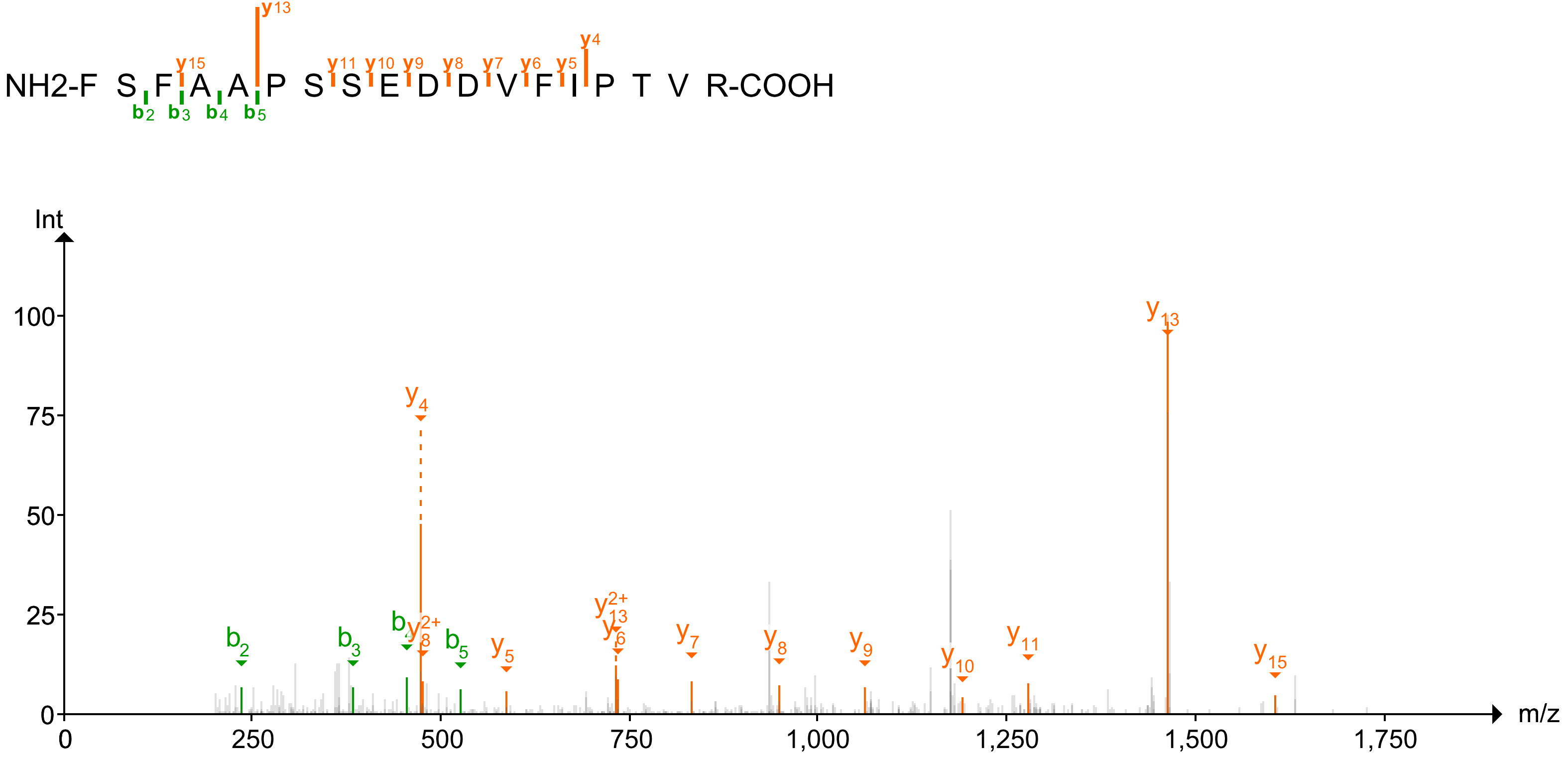

NH2-F S F A A P S S E D D V F I P T V R-COOH
y13
y15
y11
y10
y9
y8
y7
y6
y5
y4
b2
b3
b4
b5
Int
100
75
50
25
0
m/z
250
500
750
1,000
1,250
1,500
1,750

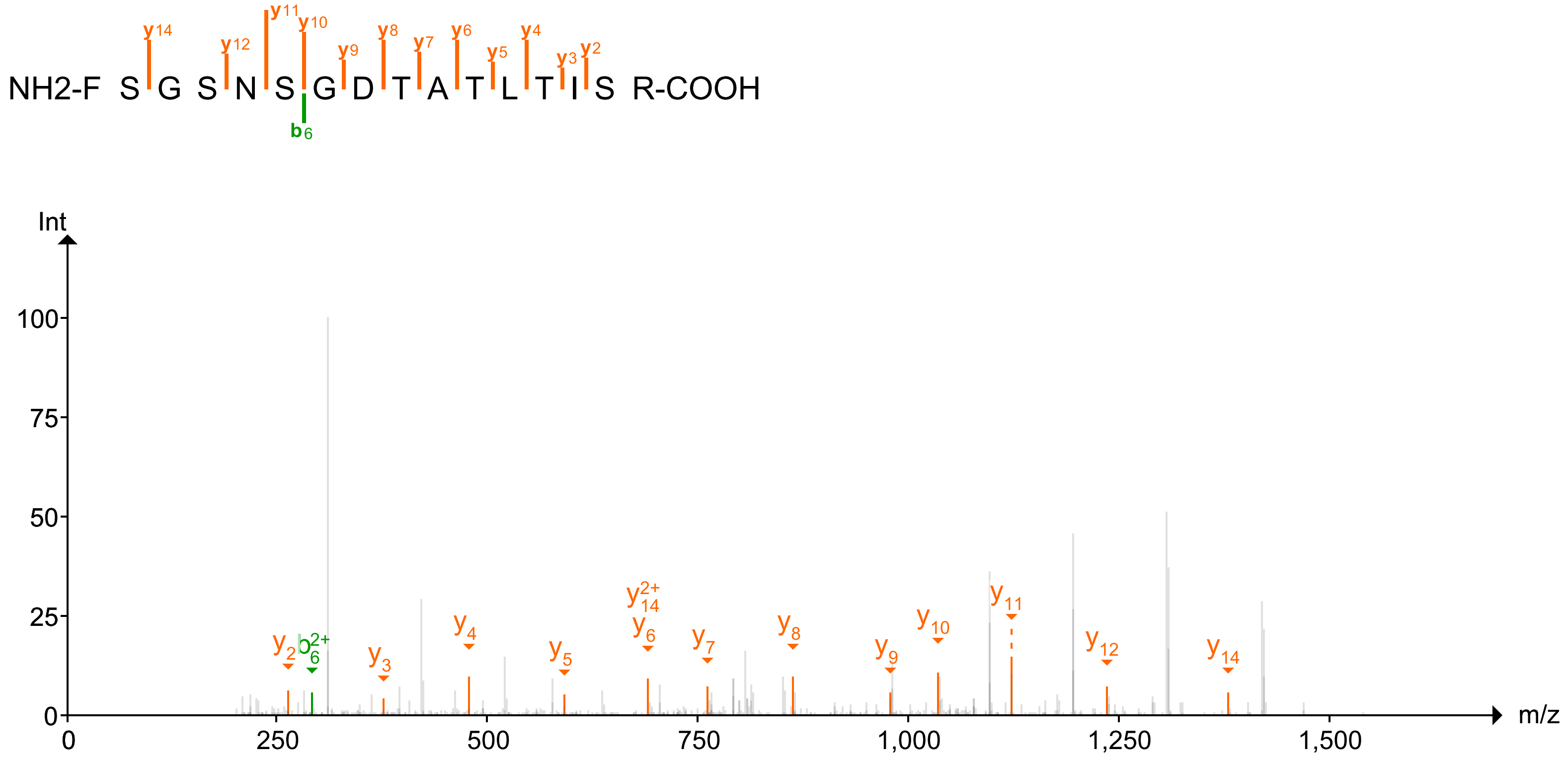

NH2-F S G S N S G D T A T L T I S R-COOH
y14 y12 y11 y10 y9 y8 y7 y6 y5 y4 y3 y2
b6
Int
100
75
50
25
0
y2
b6 2+
y3
y4
y14 2+
y6
y5
y7
y8
y9
y10
y11
y12
y14
0
250
500
750
1,000
1,250
1,500
m/z

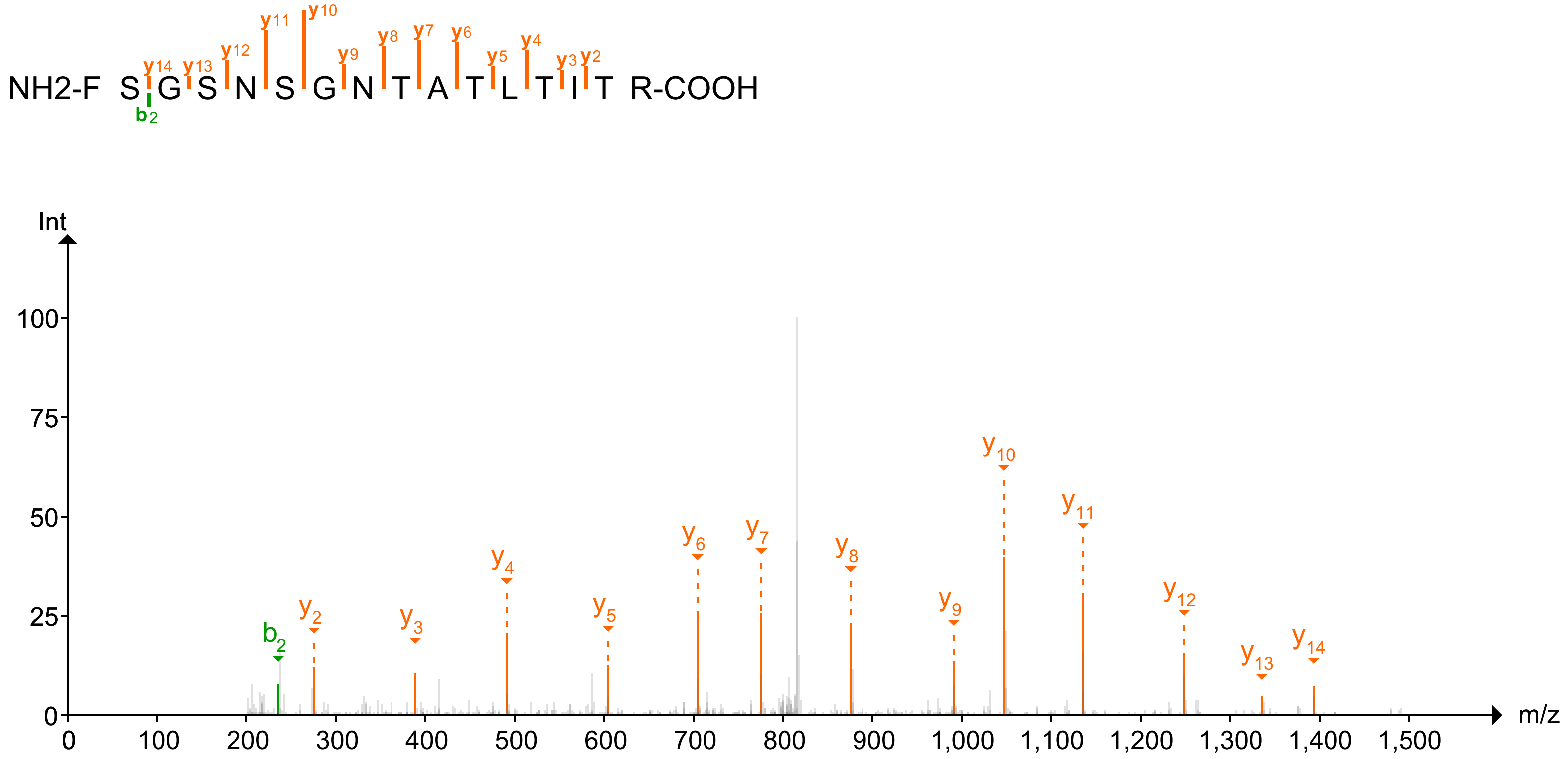

NH2-F S G S N S G N T A T L T I T R-COOH
y14 y13 y12 y11 y10 y9 y8 y7 y6 y5 y4 y3 y2
b2
Int
100
75
50
25
0
m/z
0 100 200 300 400 500 600 700 800 900 1,000 1,100 1,200 1,300 1,400 1,500
b2 y2 y3 y4 y5 y6 y7 y8 y9 y10 y11 y12 y13 y14

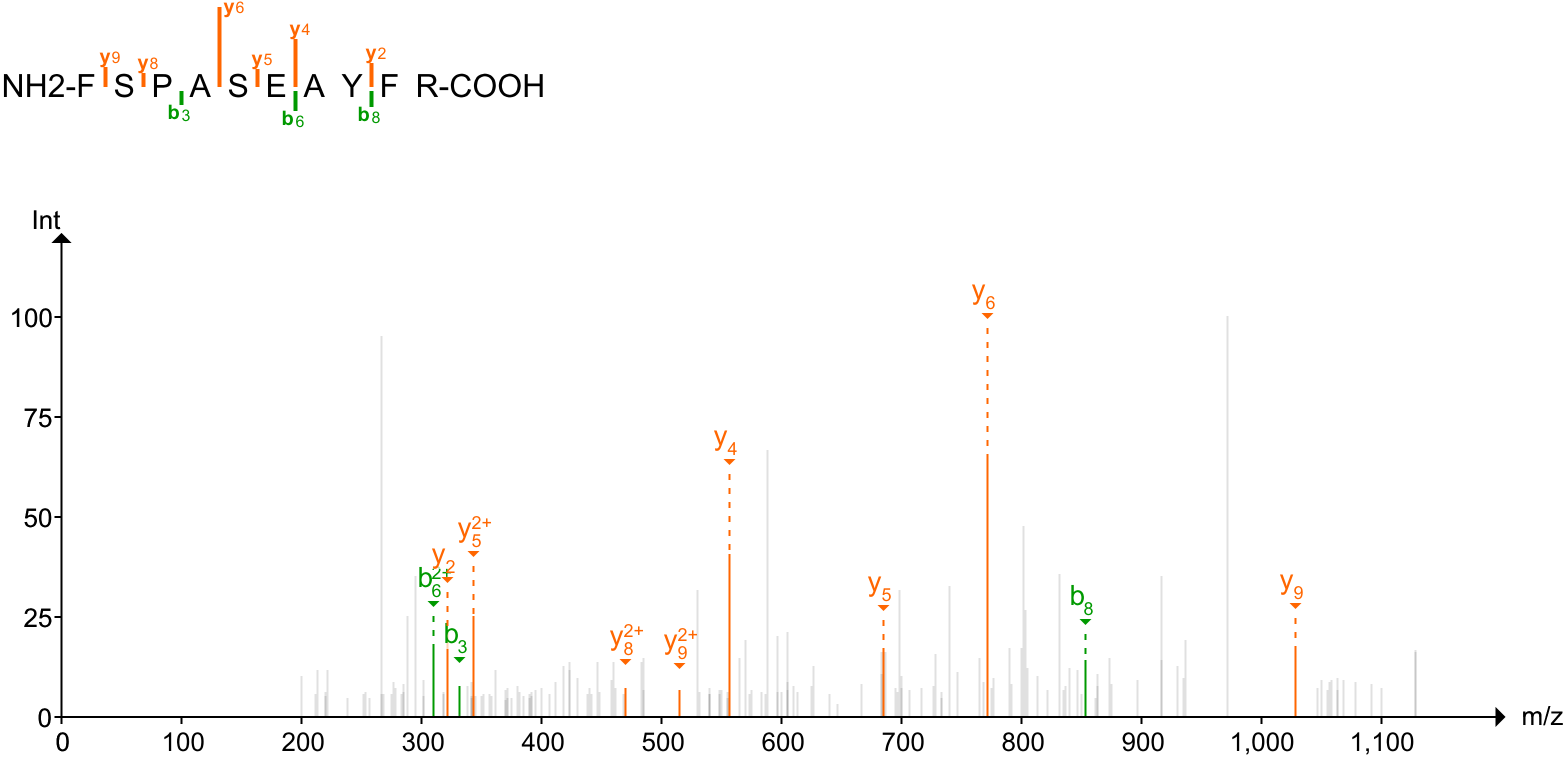

NH2-F S P A S E A Y F R-COOH
y9 y8 y6 y5 y4 y2
b3 b6 b8
Int
100
75
50
25
0
m/z
0
100
200
300
400
500
600
700
800
900
1,000
1,100
y6
y4
y5 2+
y2
b6 2+
b3
y8 2+
y9 2+
y5
b8
y9

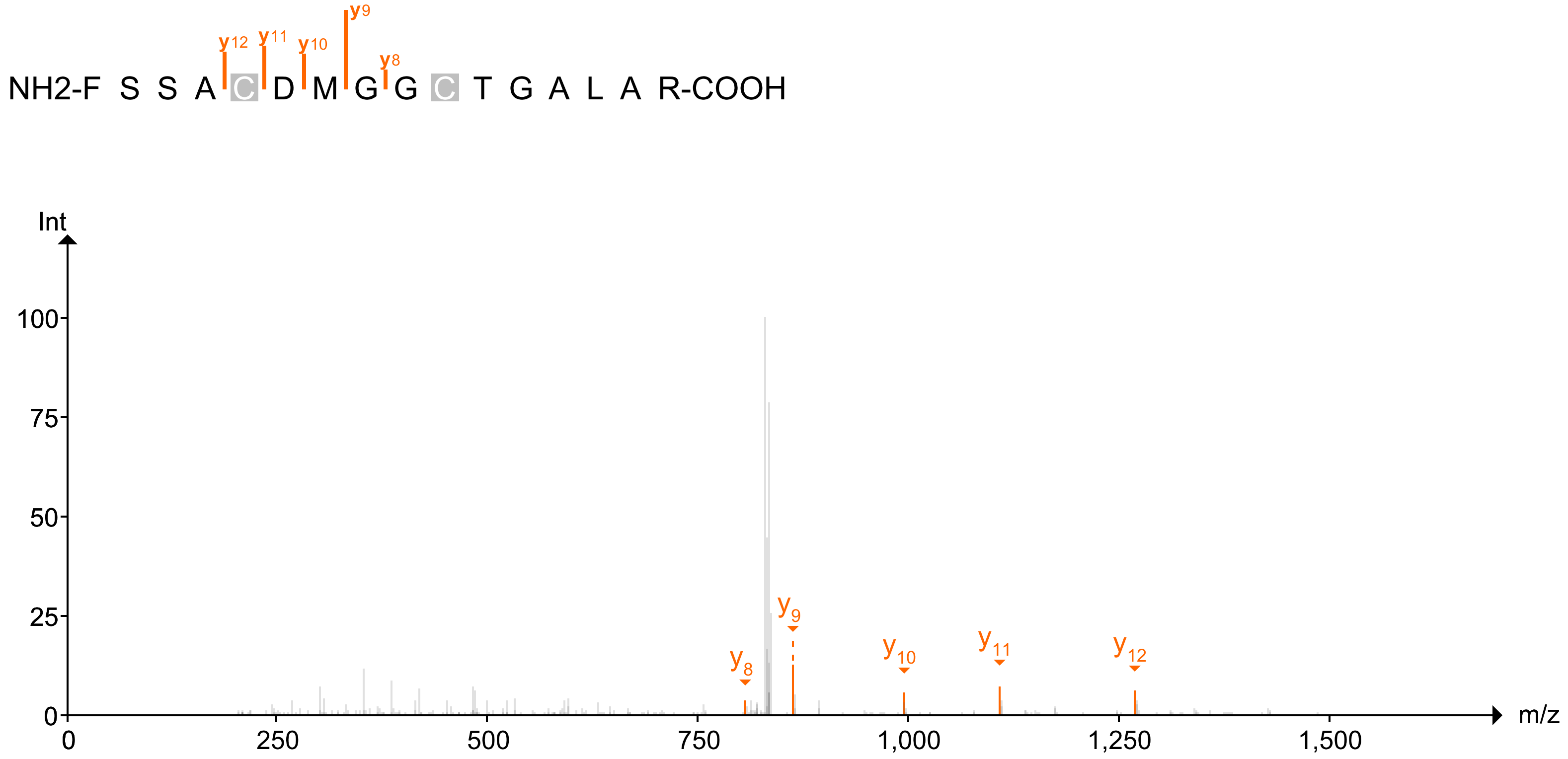

y12
y11
y10
y9
y8
NH2-F S S A C D M G G C T G A L A R-COOH
Int
100
75
50
25
0
y9
y8
y10
y11
y12
0
250
500
750
1,000
1,250
1,500
m/z

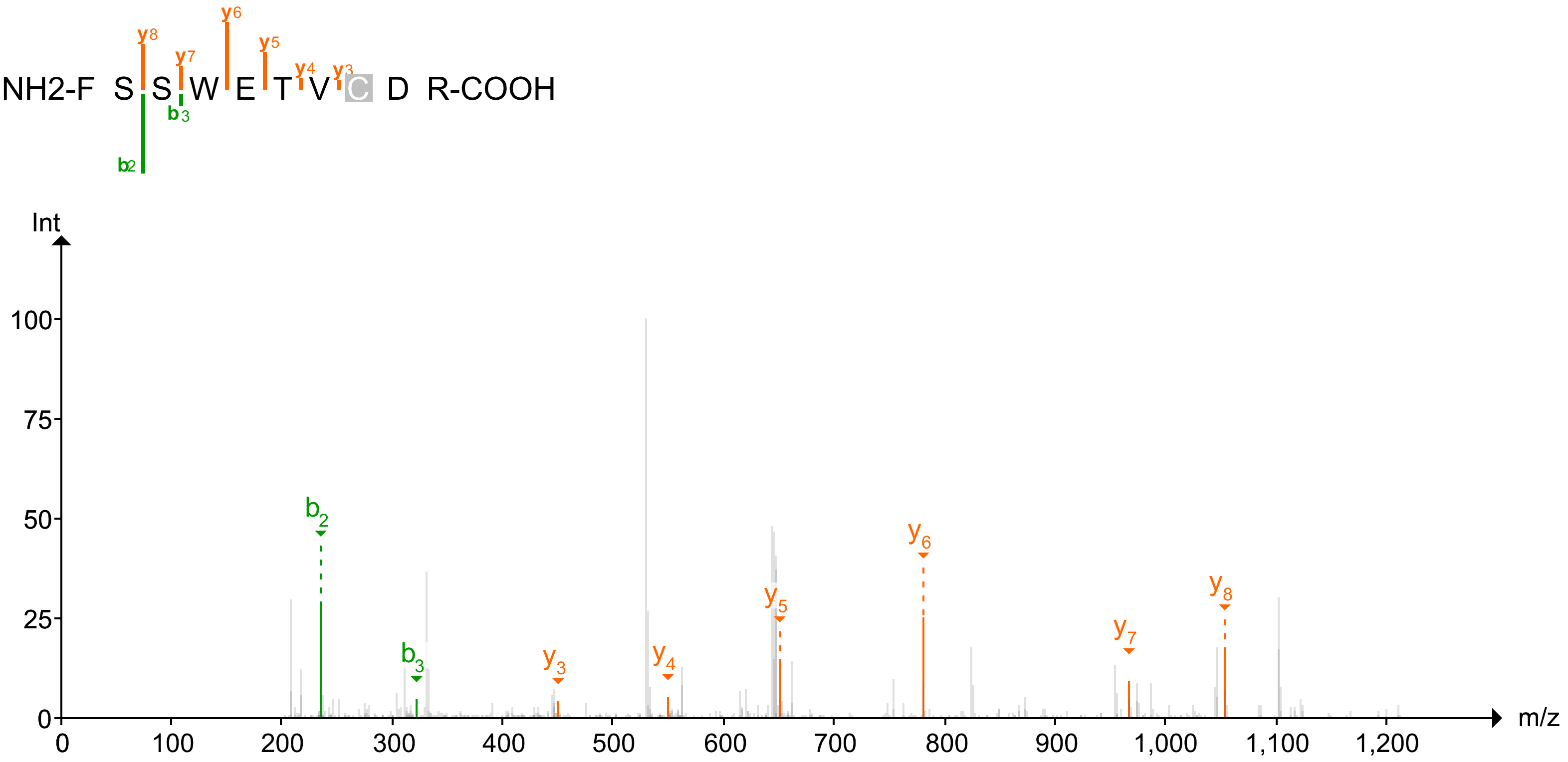

NH2-F S S W E T V C D R-COOH
y8
y7
y6
y5
y4
y3
b2
b3
Int
100
75
50
25
0
m/z
0
100
200
300
400
500
600
700
800
900
1,000
1,100
1,200

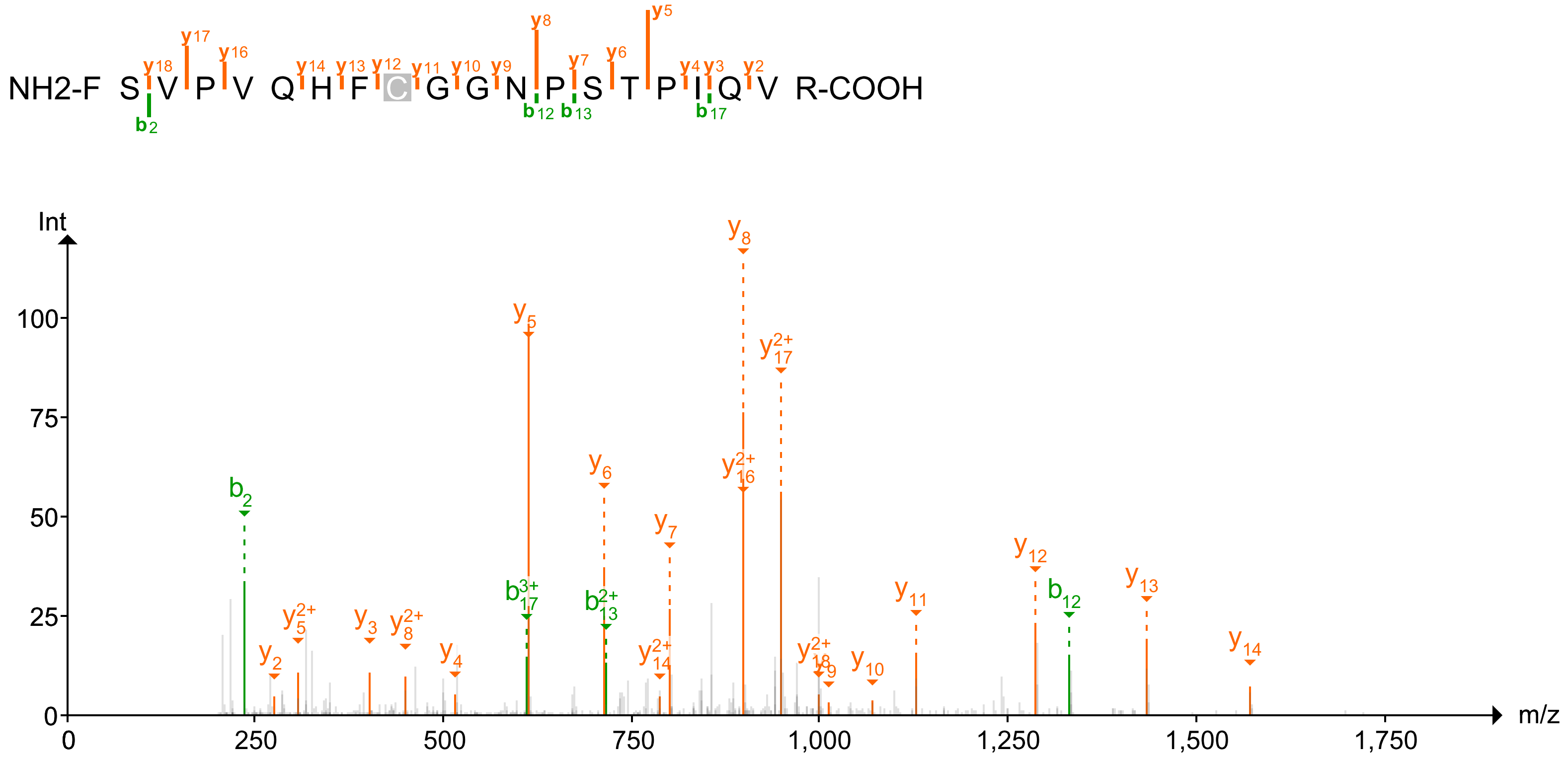

NH2-F S V P V Q H F C G G N P S T P I Q V R-COOH
y18 y17 y16 y14 y13 y12 y11 y10 y9 y8 y7 y6 y5 y4 y3 y2
b2 b12 b13 b17
Int
100
75
50
25
0
0
250
500
750
1,000
1,250
1,500
1,750
m/z
$b_2$
$y_2$
$y_5^{2+}$
$y_3$
$y_8^{2+}$
$y_4$
$b_{17}^{3+}$
$y_5$
$b_{13}^{2+}$
$y_6$
$y_{14}^{2+}$
$y_7$
$y_{16}^{2+}$
$y_8$
$y_{17}^{2+}$
$y_{18}^{2+}$
$y_9$
$y_{10}$
$y_{11}$
$y_{12}$
$b_{12}$
$y_{13}$
$y_{14}$

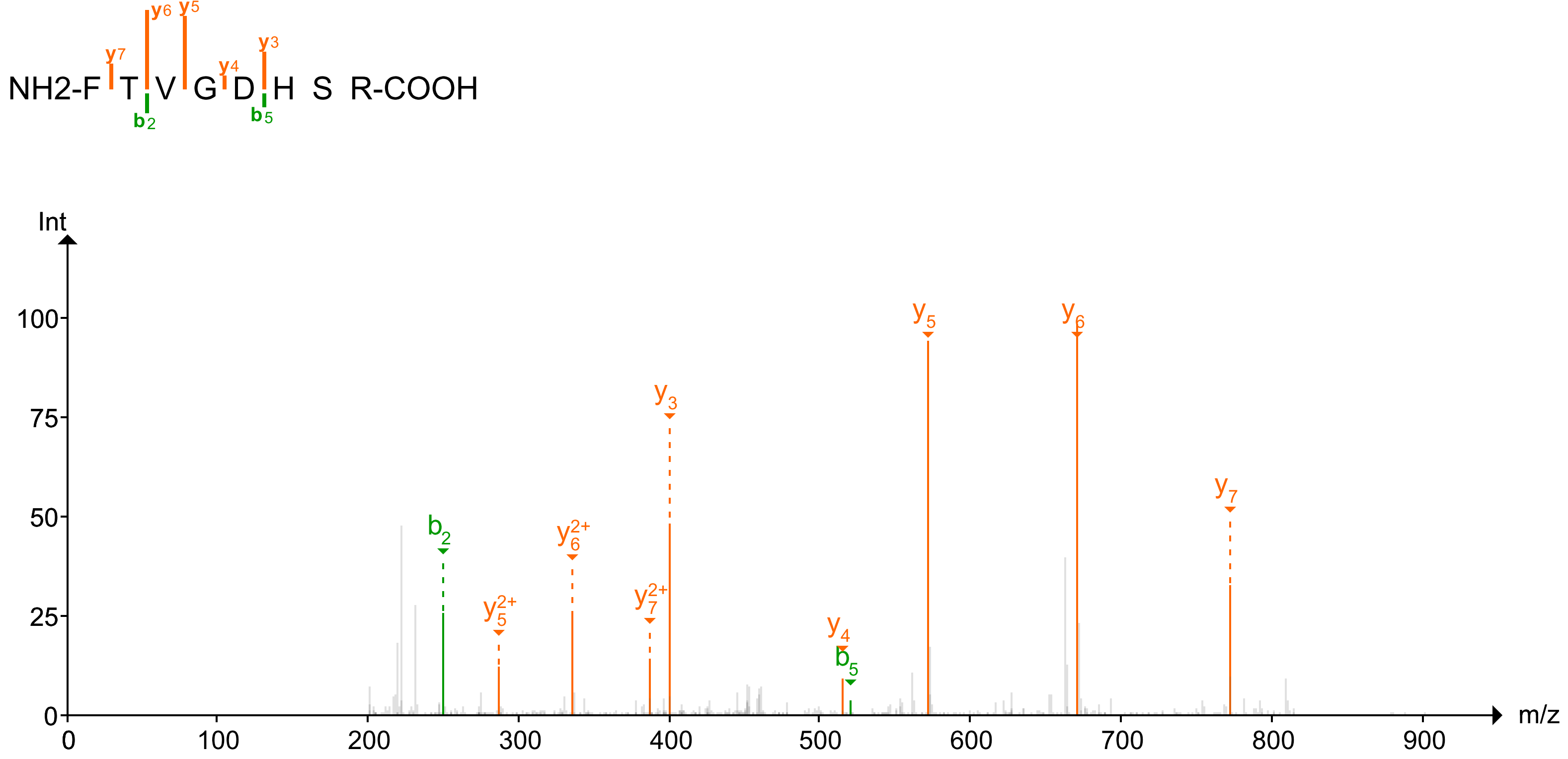

NH2-F T V G D H S R-COOH
y7 y6 y5 y4 y3
b2 b5
Int
100
75
50
25
0
m/z
0 100 200 300 400 500 600 700 800 900
b2
y5 2+
y6 2+
y7 2+
y3
y4
b5
y5
y6
y7

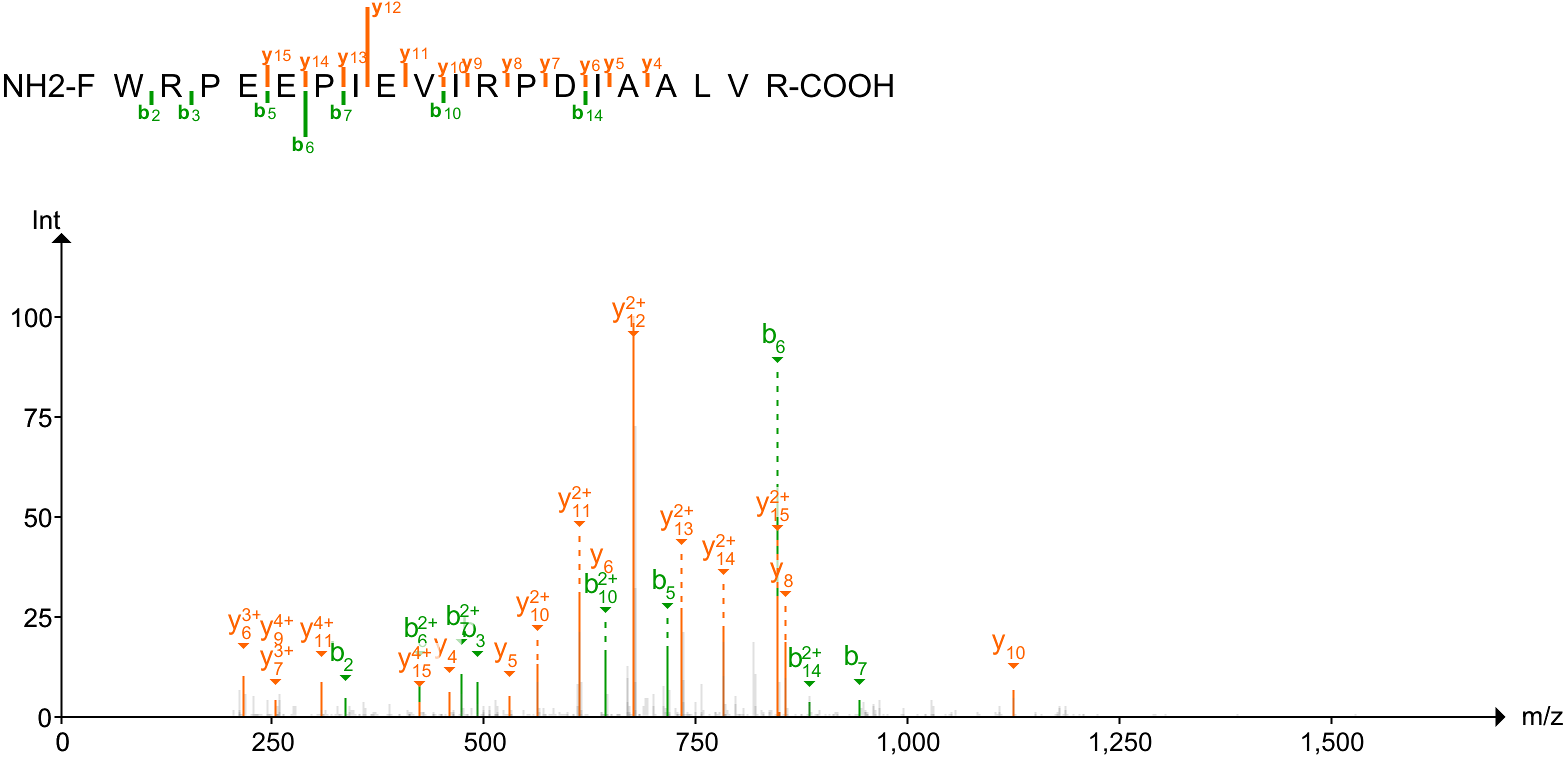

NH2-F W R P E E P I E V I R P D I A A L V R-COOH
y15 y14 y13 y12 y11 y10 y9 y8 y7 y6 y5 y4
b2 b3 b5 b6 b7 b10 b14
Int
100
75
50
25
0
m/z
0
250
500
750
1,000
1,250
1,500
y12 2+
b6
y11 2+
y13 2+
y15 2+
y14 2+
y6
b10 2+
b5
y8
y10 2+
y6 3+
y9 4+
y7 3+
y11 4+
b2
b6 2+
y15 4+
y4
b7 2+
b3
y5
b14 2+
b7
y10

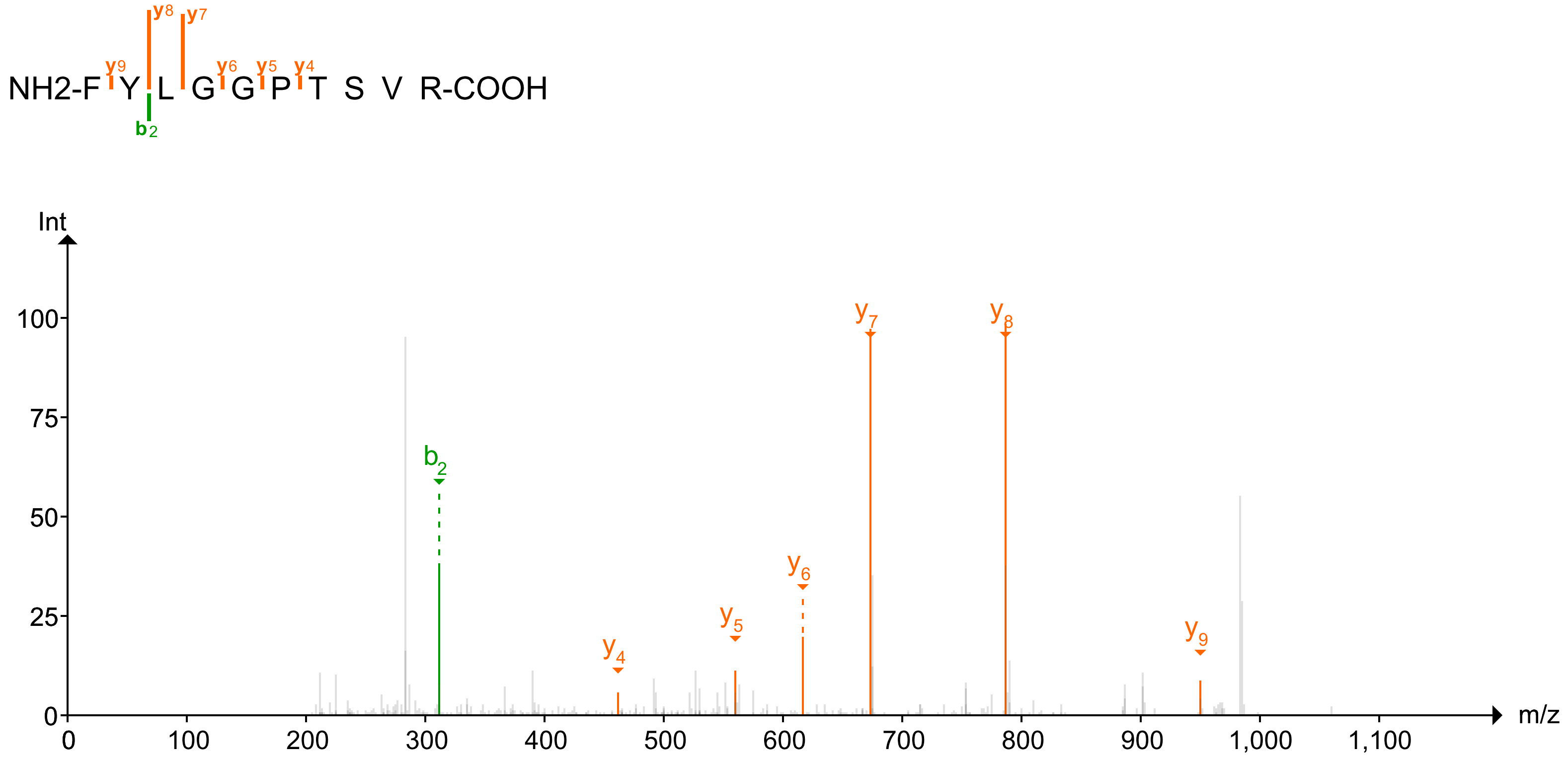

NH2-F Y L G G P T S V R-COOH
y9 y8 y7 y6 y5 y4
b2
Int
100
75
50
25
0
m/z
0
100
200
300
400
500
600
700
800
900
1,000
1,100
b2
y4
y5
y6
y7
y8
y9

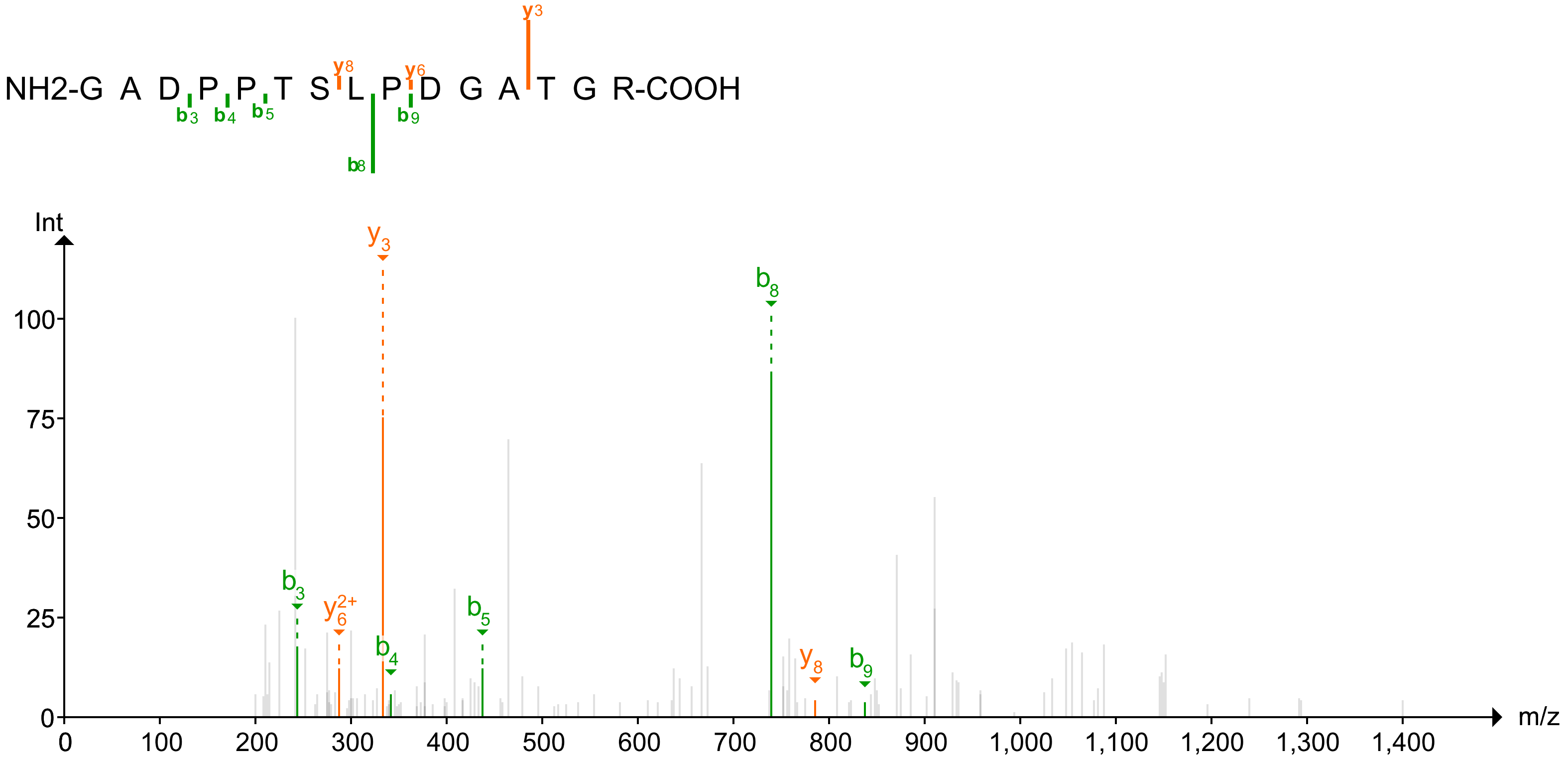
NH2-G A D P P T S L P D G A T G R-COOH
y3
y8
y6
b3
b4
b5
b8
b9
Int
m/z
y3
b8
b3
y6 2+
b4
b5
y8
b9
0
25
50
75
100
0
100
200
300
400
500
600
700
800
900
1,000
1,100
1,200
1,300
1,400

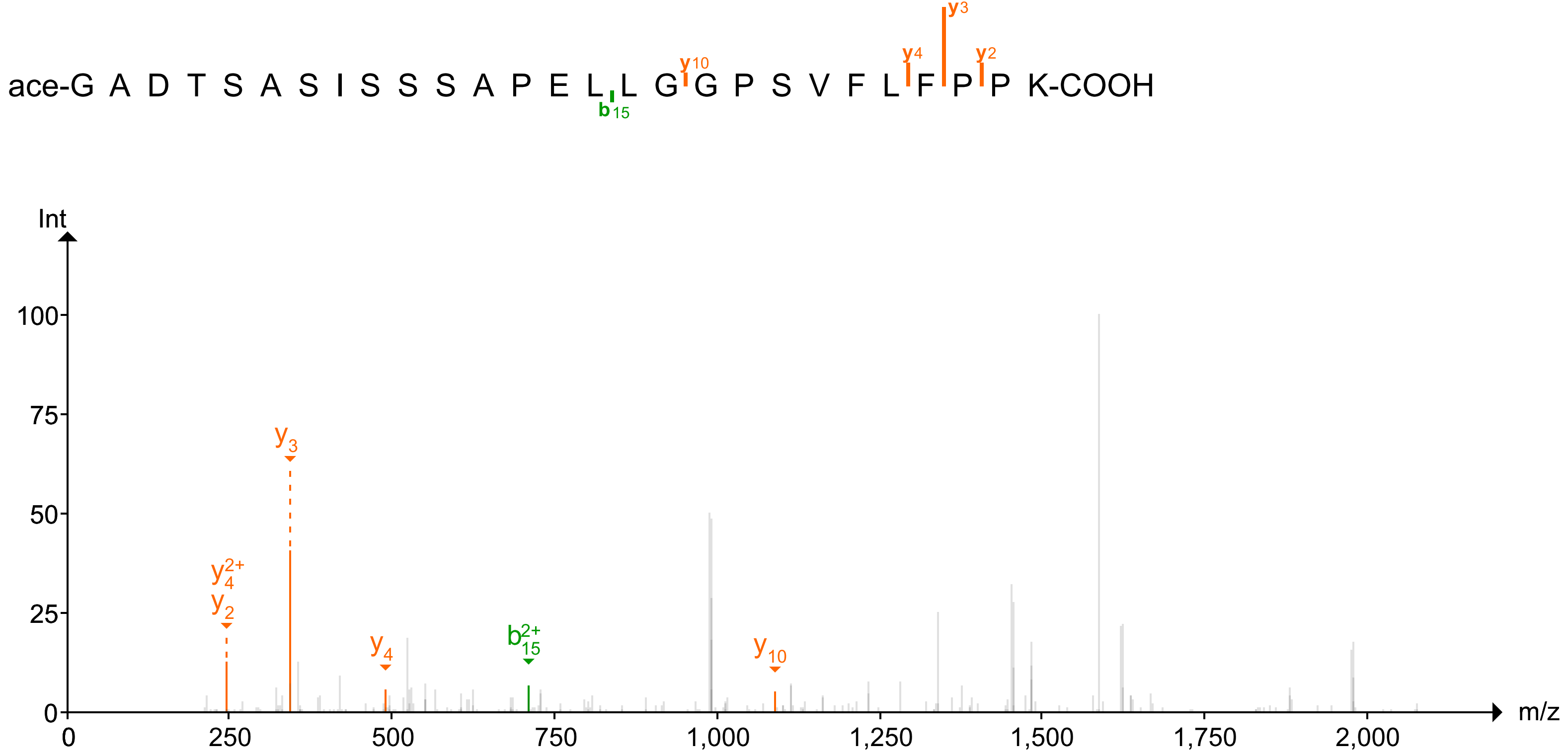

ace-G A D T S A S I S S S A P E L L G G P S V F L F P P K-COOH
b15
y10
y4
y3
y2
Int
100
75
50
25
0
y3
y4 2+
y2
y4
b15 2+
y10
0
250
500
750
1,000
1,250
1,500
1,750
2,000
m/z

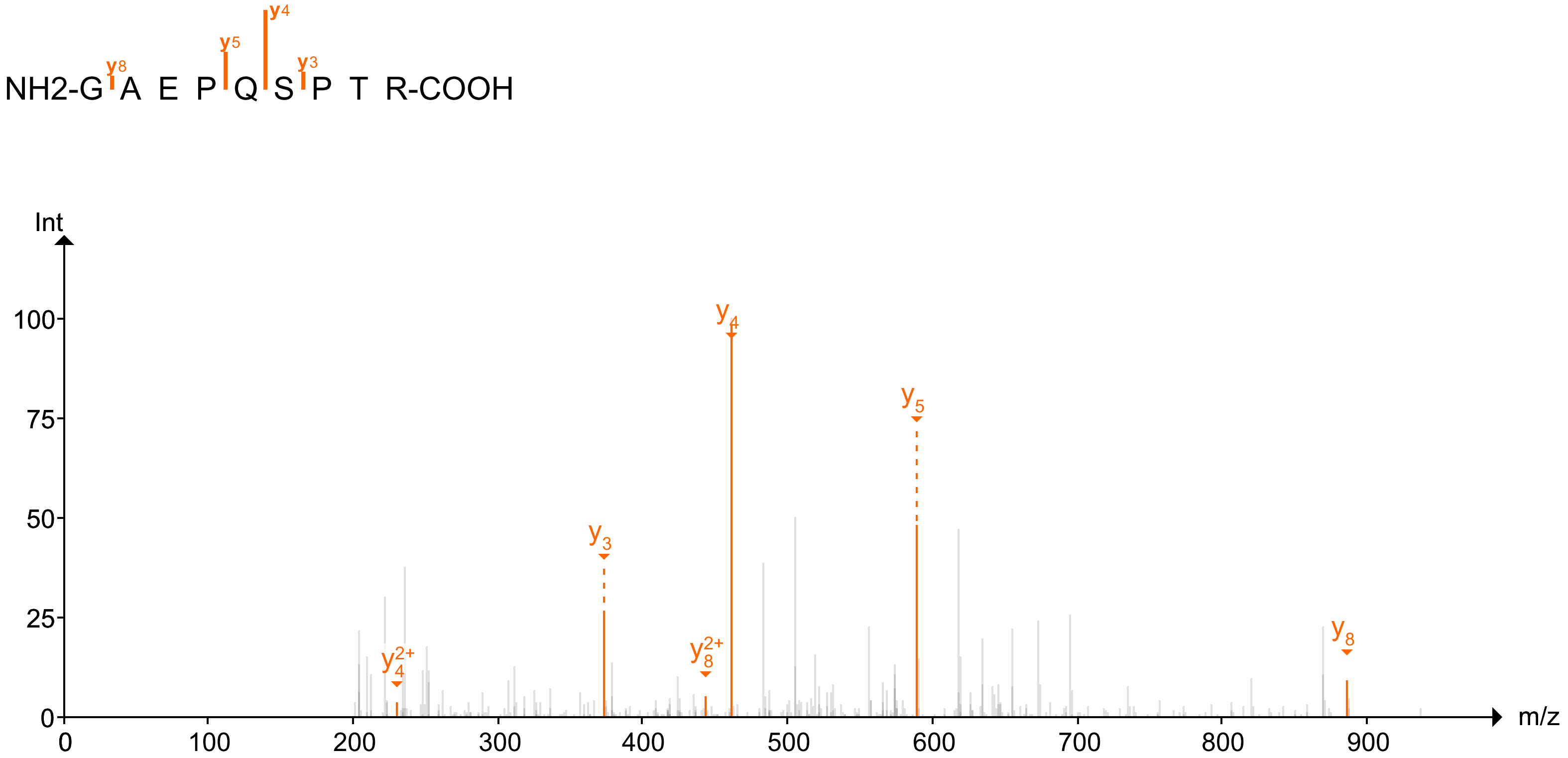

NH2-G A E P Q S P T R-COOH
y8
y5
y4
y3
Int
100
75
50
25
0
y4
y5
y3
y4 2+
y8 2+
y8
0
100
200
300
400
500
600
700
800
900
m/z

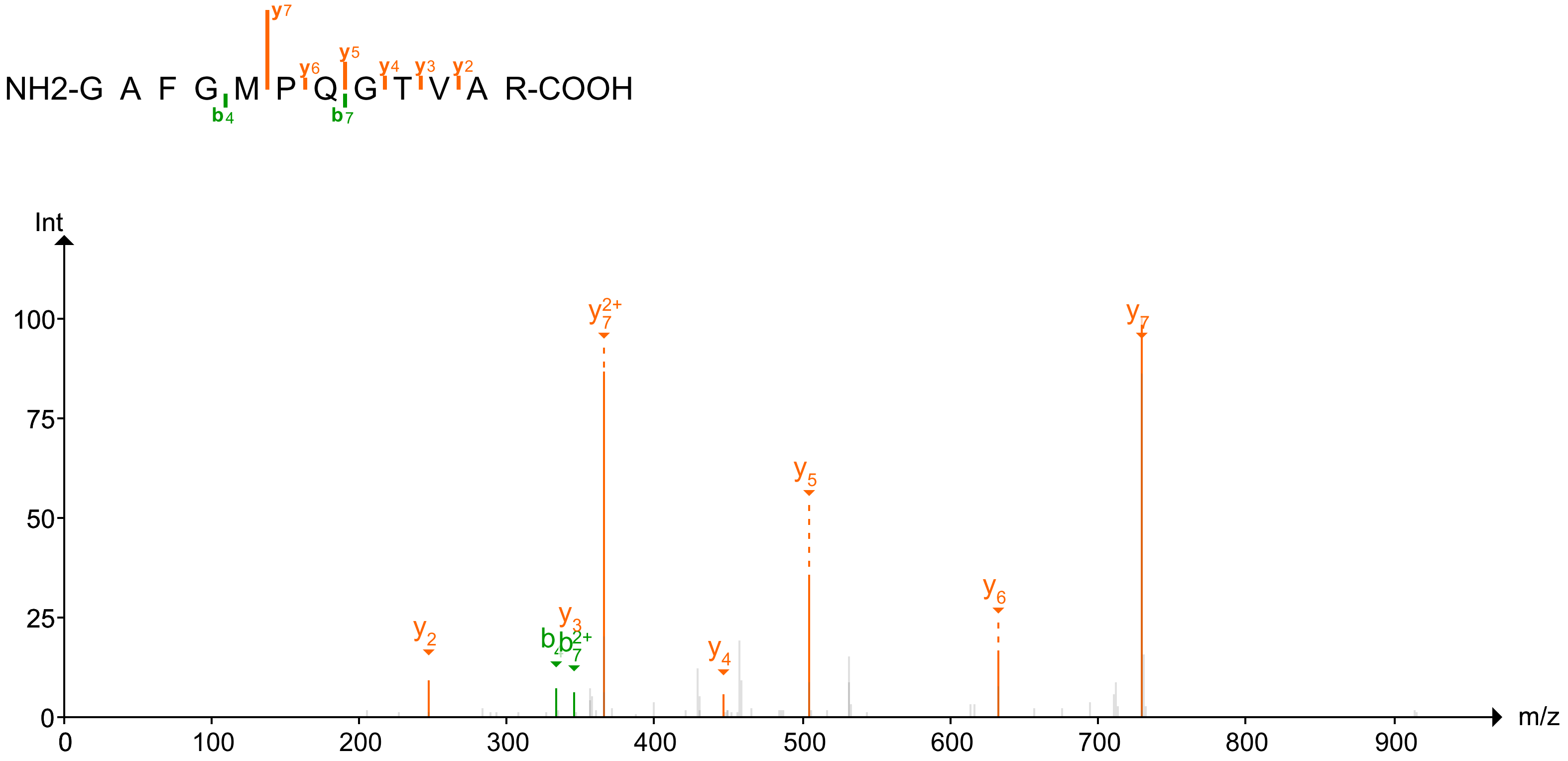

NH2-G A F G M P Q G T V A R-COOH
y7
y6
y5
y4
y3
y2
b4
b7
Int
100
75
50
25
0
m/z
0
100
200
300
400
500
600
700
800
900
y2
b4
y3
b7 2+
y7 2+
y4
y5
y6
y7

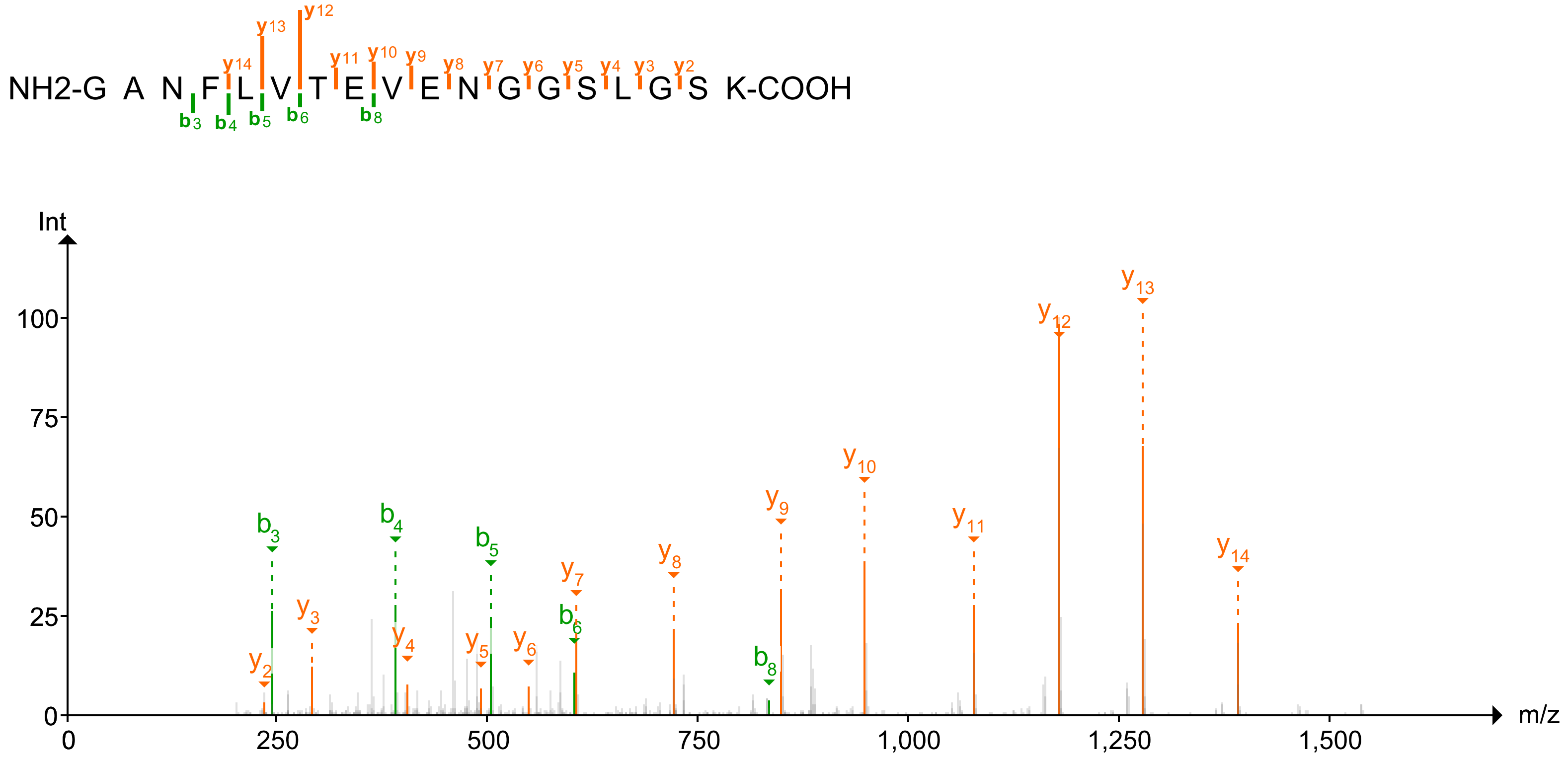

NH2-G A N F L V T E V E N G G S L G S K-COOH
y14 y13 y12 y11 y10 y9 y8 y7 y6 y5 y4 y3 y2
b3 b4 b5 b6 b8
Int
100
75
50
25
0
0
250
500
750
1,000
1,250
1,500
m/z

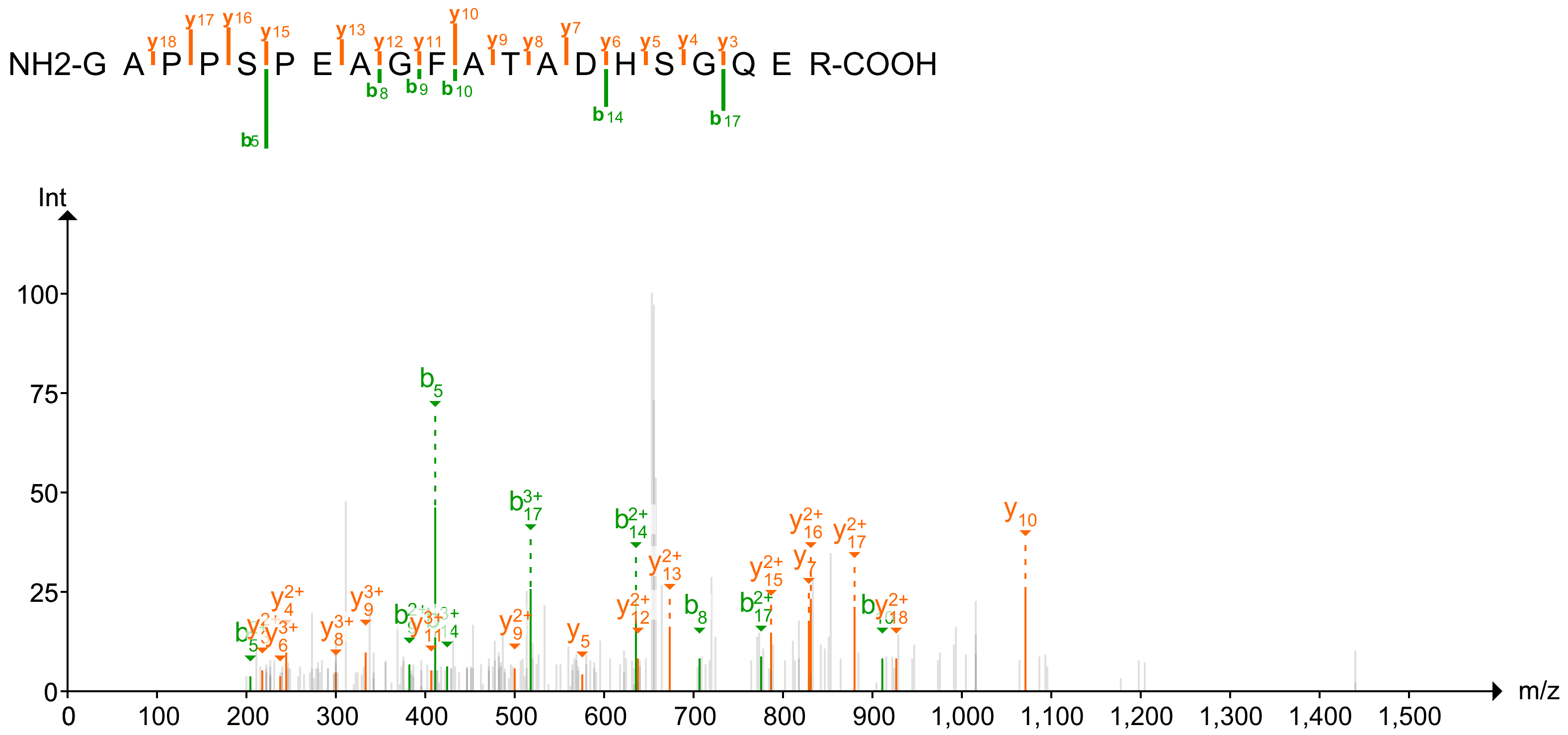

NH2-G A P P S P E A G F A T A D H S G Q E R-COOH
y18 y17 y16 y15 y13 y12 y11 y10 y9 y8 y7 y6 y5 y4 y3
b5 b8 b9 b10 b14 b17
Int
100
75
50
25
0
m/z
0
100
200
300
400
500
600
700
800
900
1,000
1,100
1,200
1,300
1,400
1,500
b5
b3+17
b2+14
y10
y2+16
y2+17
y2+13
y2+15
y7
b8
b2+17
y2+12
y2+18
y2+4
y3+9
y3+8
y2+9
y5
y3+6
y3+11

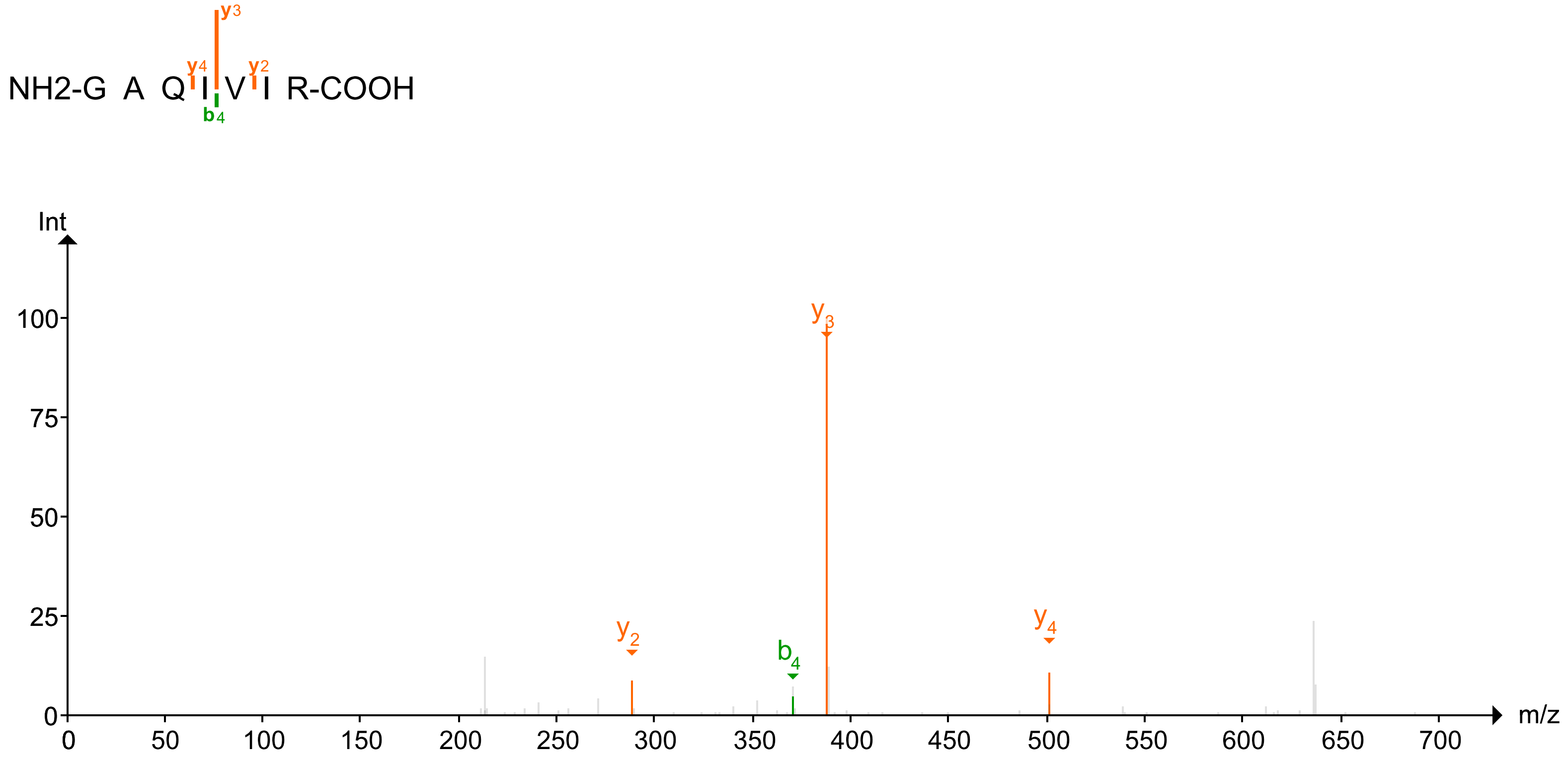

NH2-G A Q I V I R-COOH
y3
y4
y2
b4
Int
100
75
50
25
0
m/z
0
50
100
150
200
250
300
350
400
450
500
550
600
650
700

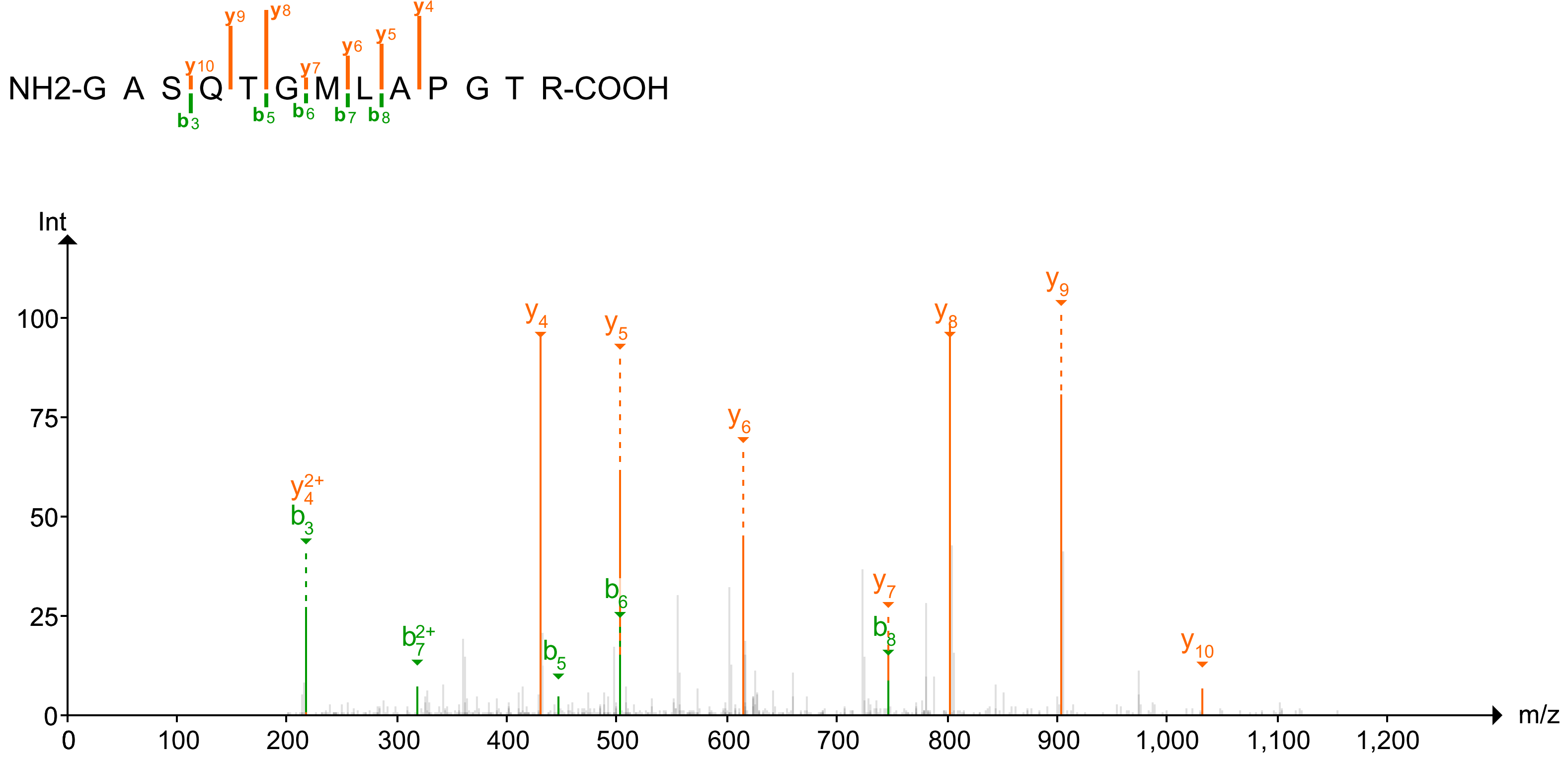

NH2-G A S Q T G M L A P G T R-COOH
y10 y9 y8 y7 y6 y5 y4
b3 b5 b6 b7 b8
Int
100
75
50
25
0
m/z
0
100
200
300
400
500
600
700
800
900
1,000
1,100
1,200
$y_4^{2+}$
$b_3$
$b_7^{2+}$
$y_4$
$b_5$
$y_5$
$b_6$
$y_6$
$y_7$
$b_8$
$y_8$
$y_9$
$y_{10}$

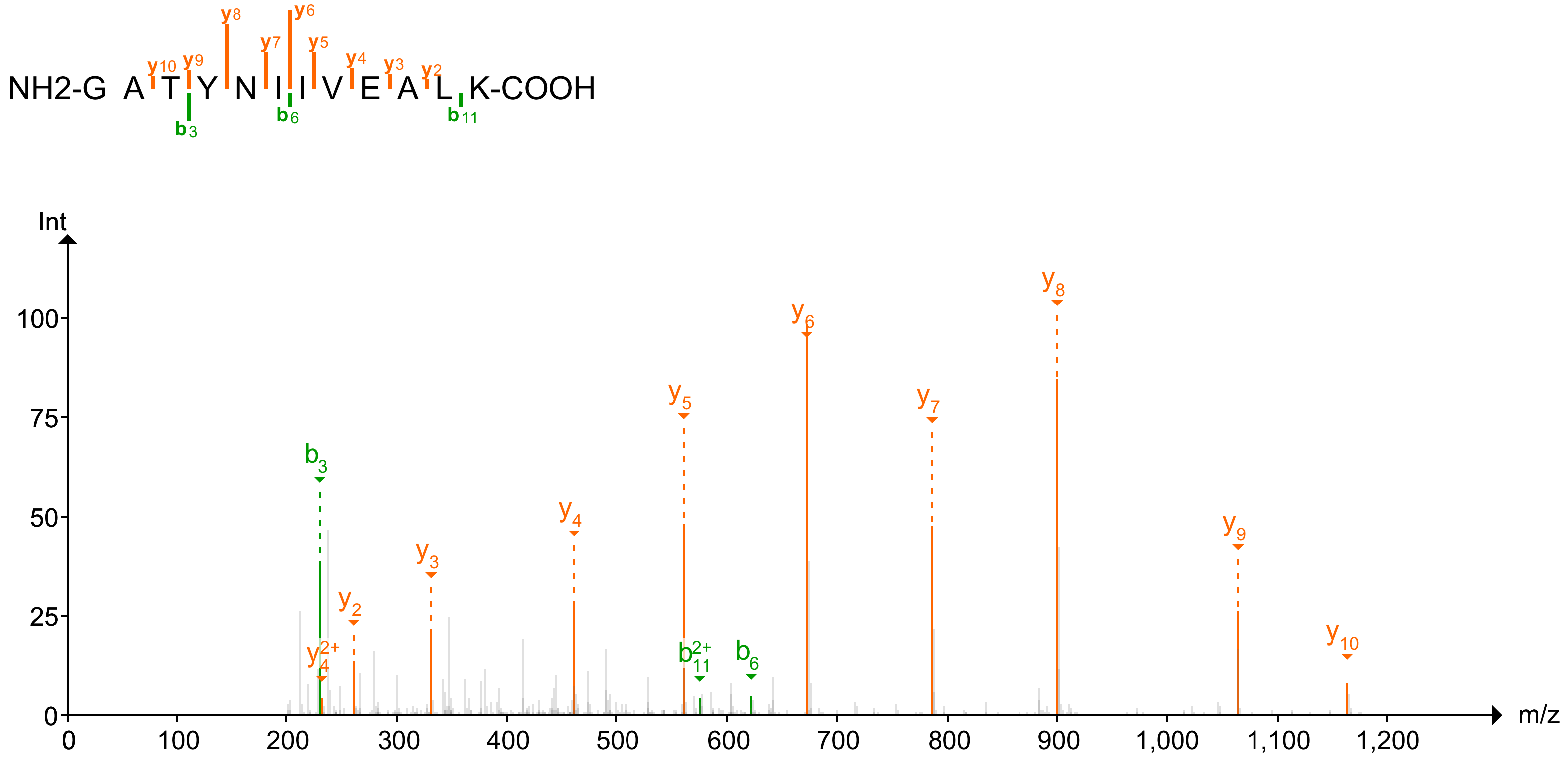

NH2-G A T Y N I I V E A L K-COOH
y10 y9 y8 y7 y6 y5 y4 y3 y2
b3 b6 b11
Int
100
75
50
25
0
m/z
0 100 200 300 400 500 600 700 800 900 1,000 1,100 1,200
b3
y2
y3
y4
y5
y6
y7
y8
y9
y10
y4 2+
b11 2+
b6

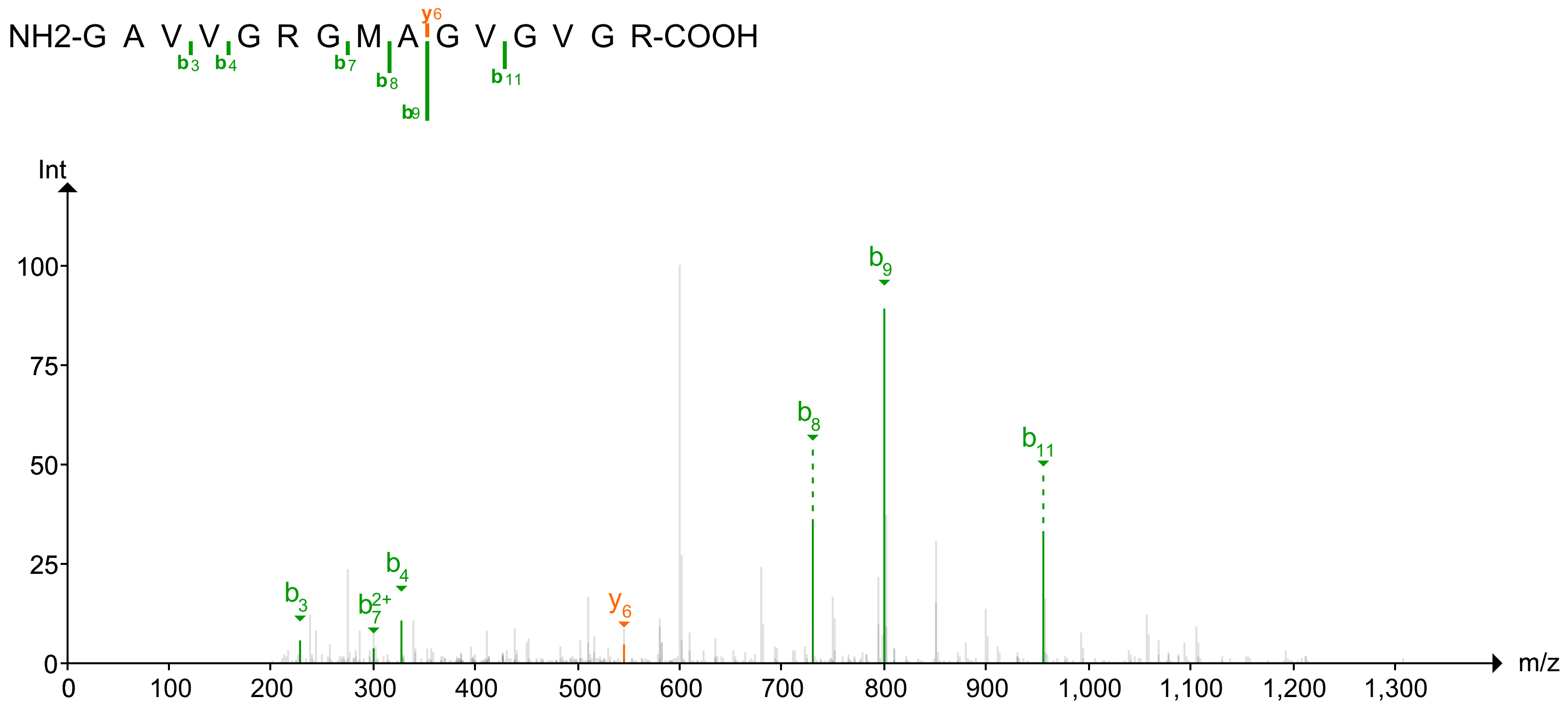
NH2-G A V V G R G M A G V G V G R-COOH
y6
b3
b4
b7
b8
b9
b11
Int
100
75
50
25
0
b9
b8
b11
b3
b7 2+
b4
y6
0
100
200
300
400
500
600
700
800
900
1,000
1,100
1,200
1,300
m/z

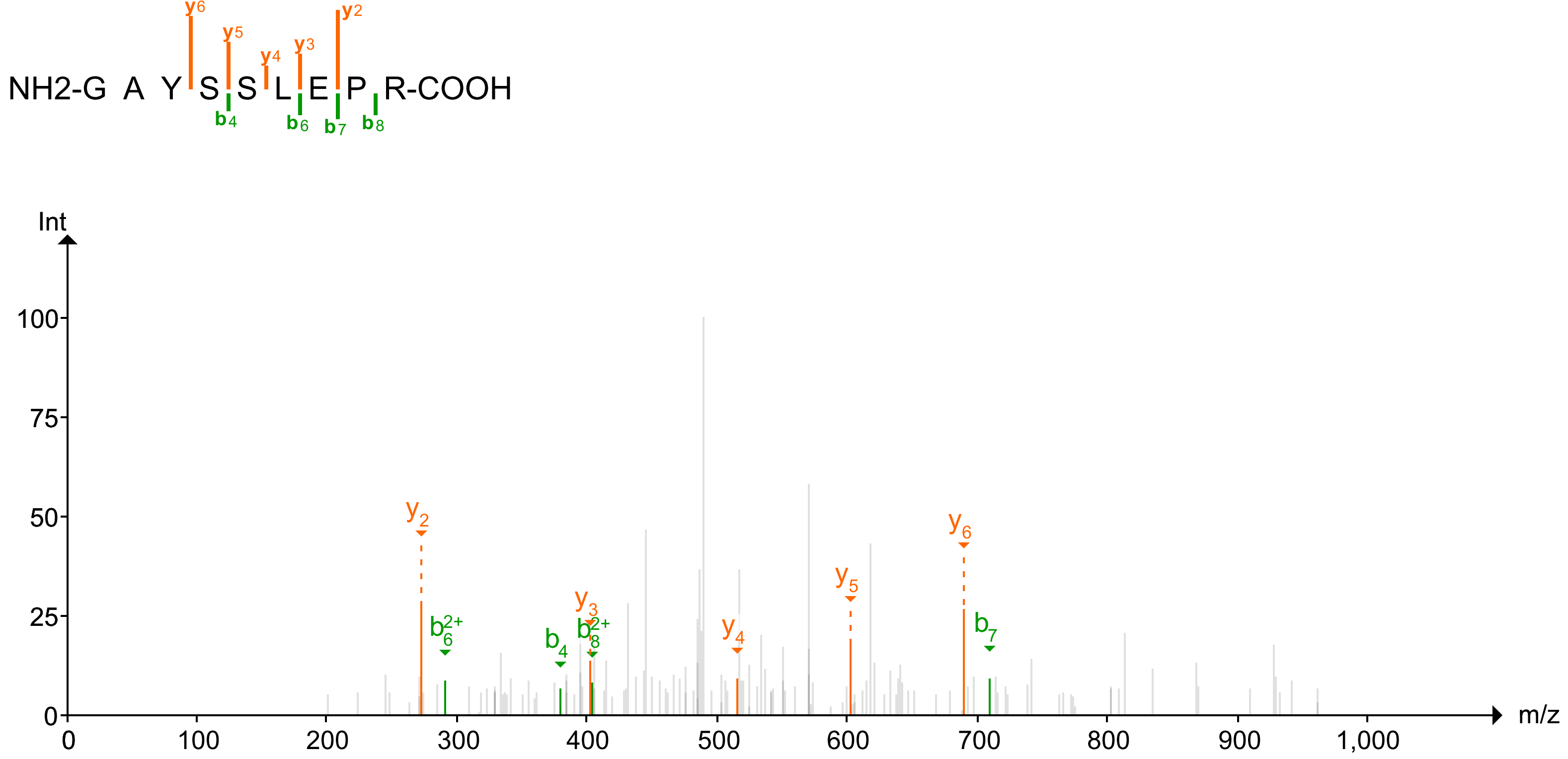

NH2-G A Y S S L E P R-COOH
y6
y5
y4
y3
y2
b4
b6
b7
b8
Int
100
75
50
25
0
m/z
0
100
200
300
400
500
600
700
800
900
1,000
y2
b6 2+
b4
y3
b8 2+
y4
y5
y6
b7

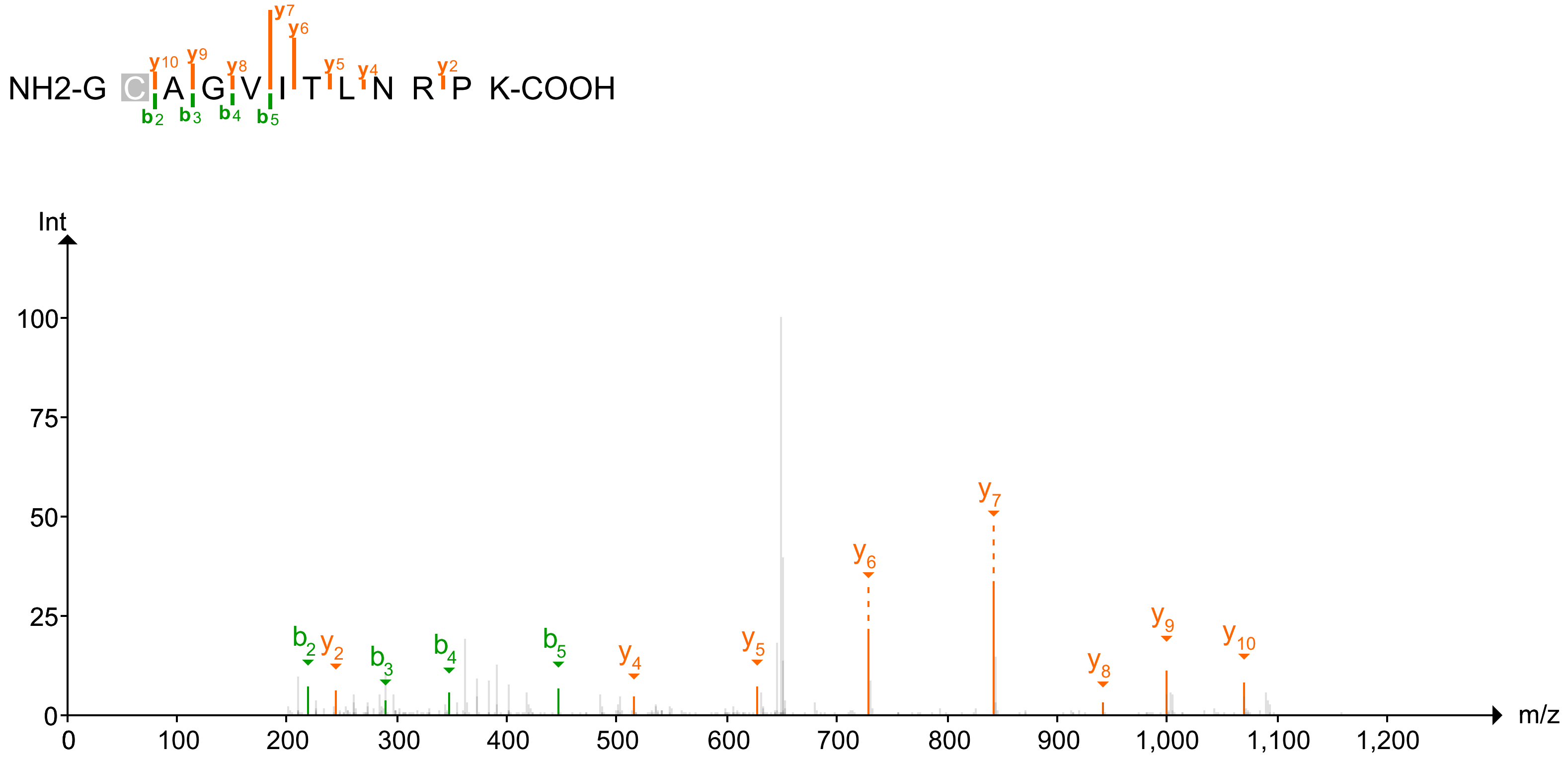

NH2-G C A G V I T L N R P K-COOH
y10 y9 y8 y7 y6 y5 y4 y2
b2 b3 b4 b5
Int
100
75
50
25
0
m/z
0 100 200 300 400 500 600 700 800 900 1,000 1,100 1,200

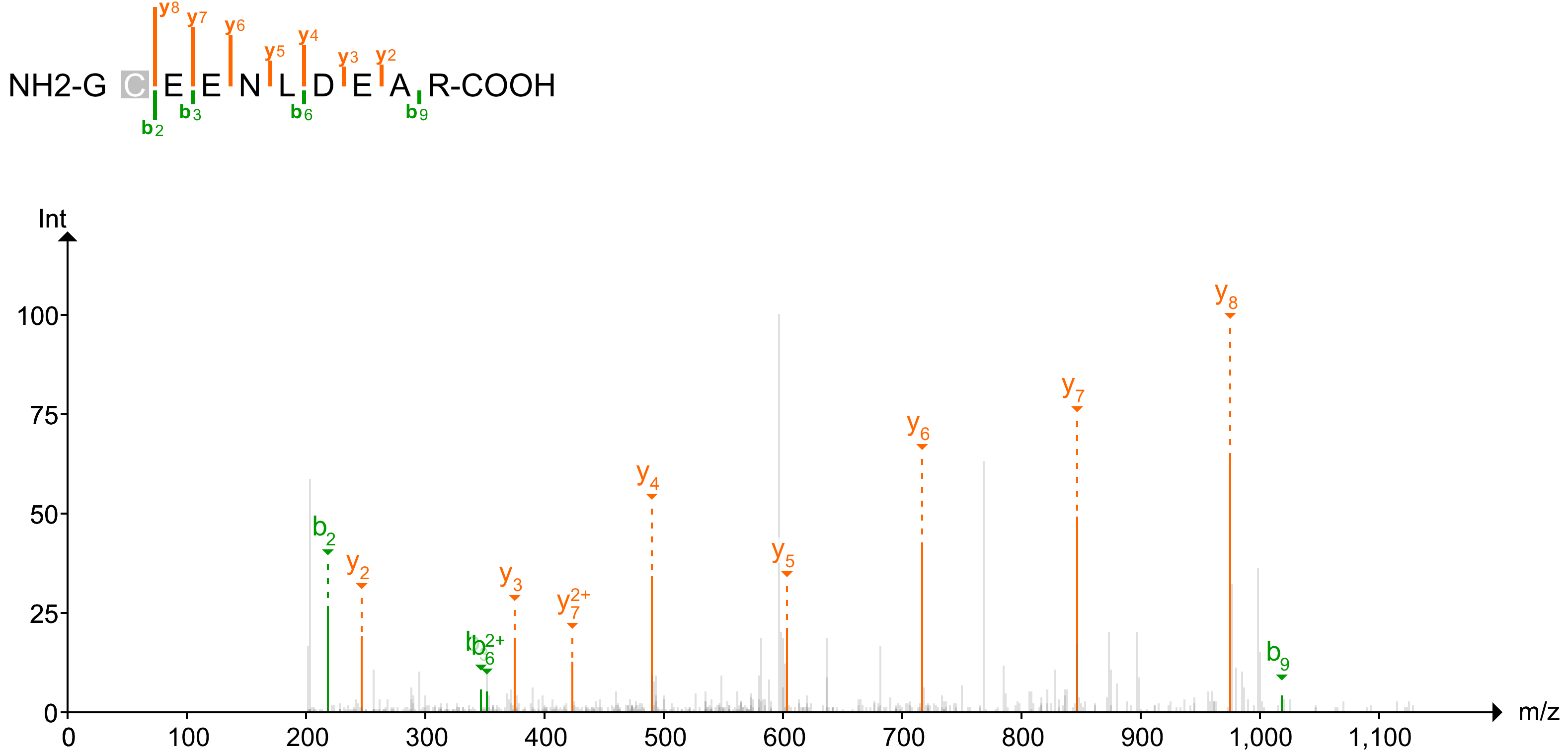

NH2-G C E E N L D E A R-COOH
y8 y7 y6 y5 y4 y3 y2
b2 b3 b6 b9
Int
100
75
50
25
0
0 100 200 300 400 500 600 700 800 900 1,000 1,100
m/z

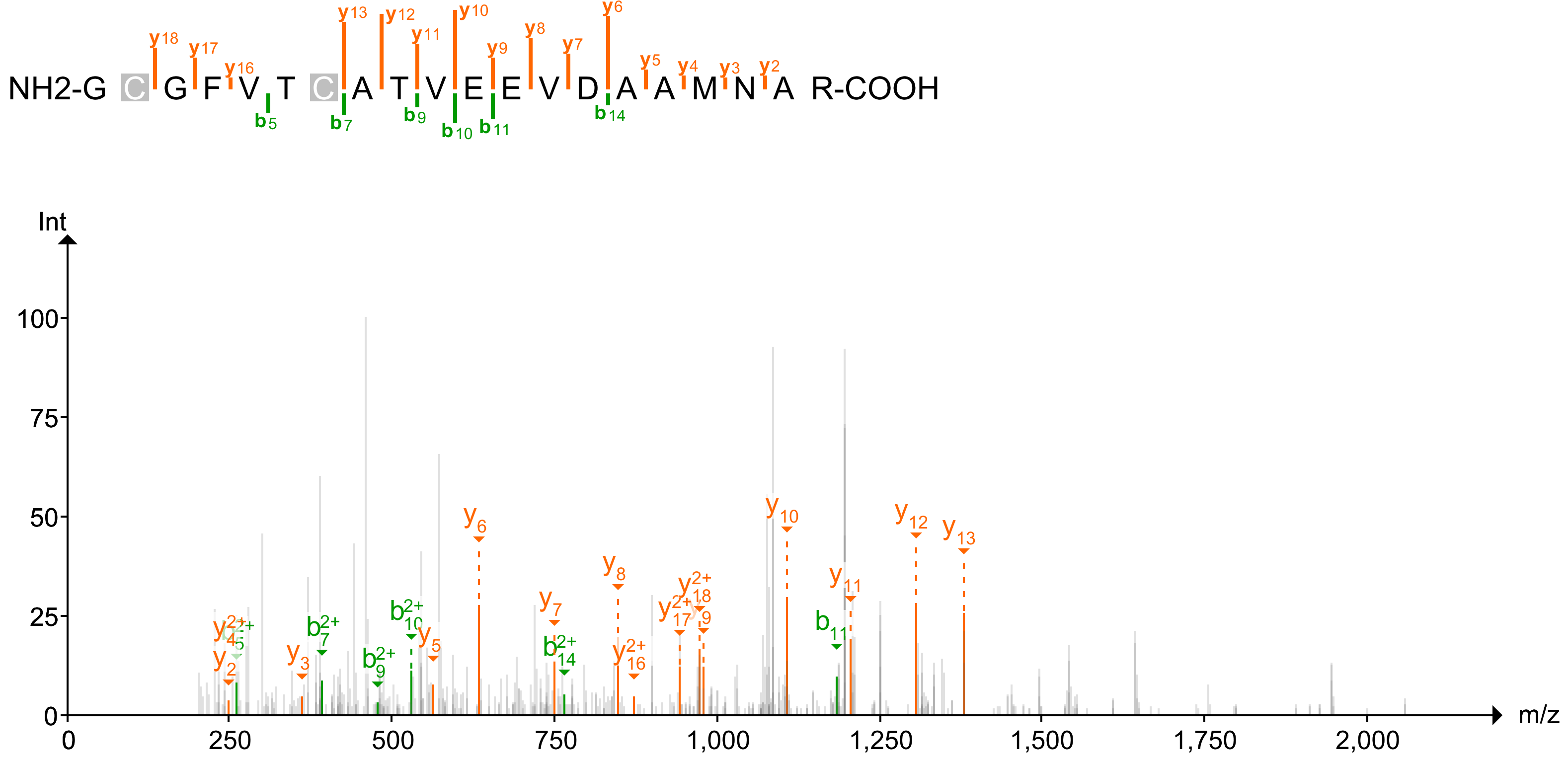

NH2-G C G F V T C A T V E E V D A A M N A R-COOH
y18 y17 y16 y13 y12 y11 y10 y9 y8 y7 y6 y5 y4 y3 y2
b5 b7 b9 b10 b11 b14
Int
100
75
50
25
0
m/z
0
250
500
750
1,000
1,250
1,500
1,750
2,000

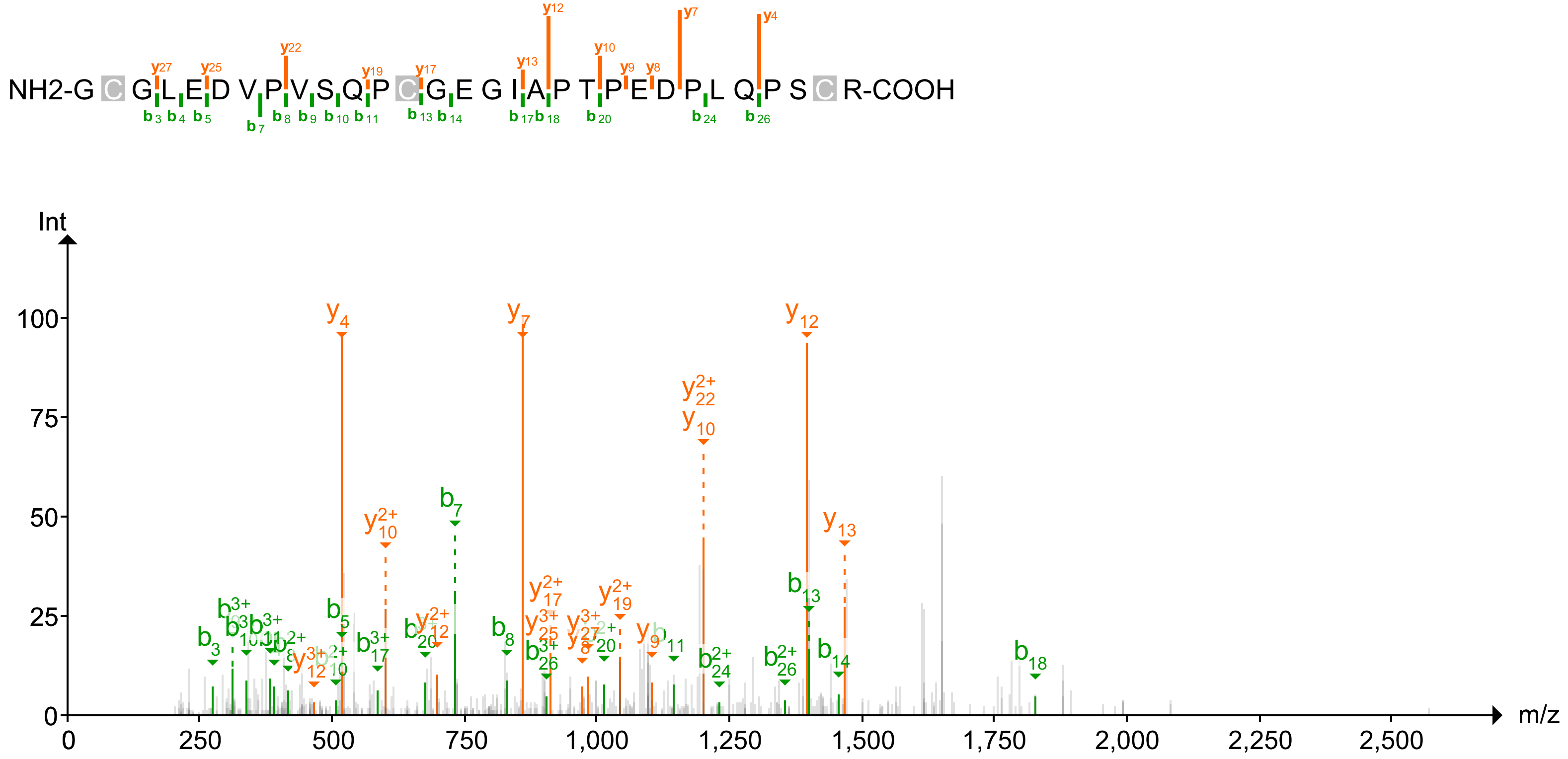

NH2-G C G L E D V P V S Q P C G E G I A P T P E D P L Q P S C R-COOH
Int
100
75
50
25
0
m/z
0
250
500
750
1,000
1,250
1,500
1,750
2,000
2,250
2,500

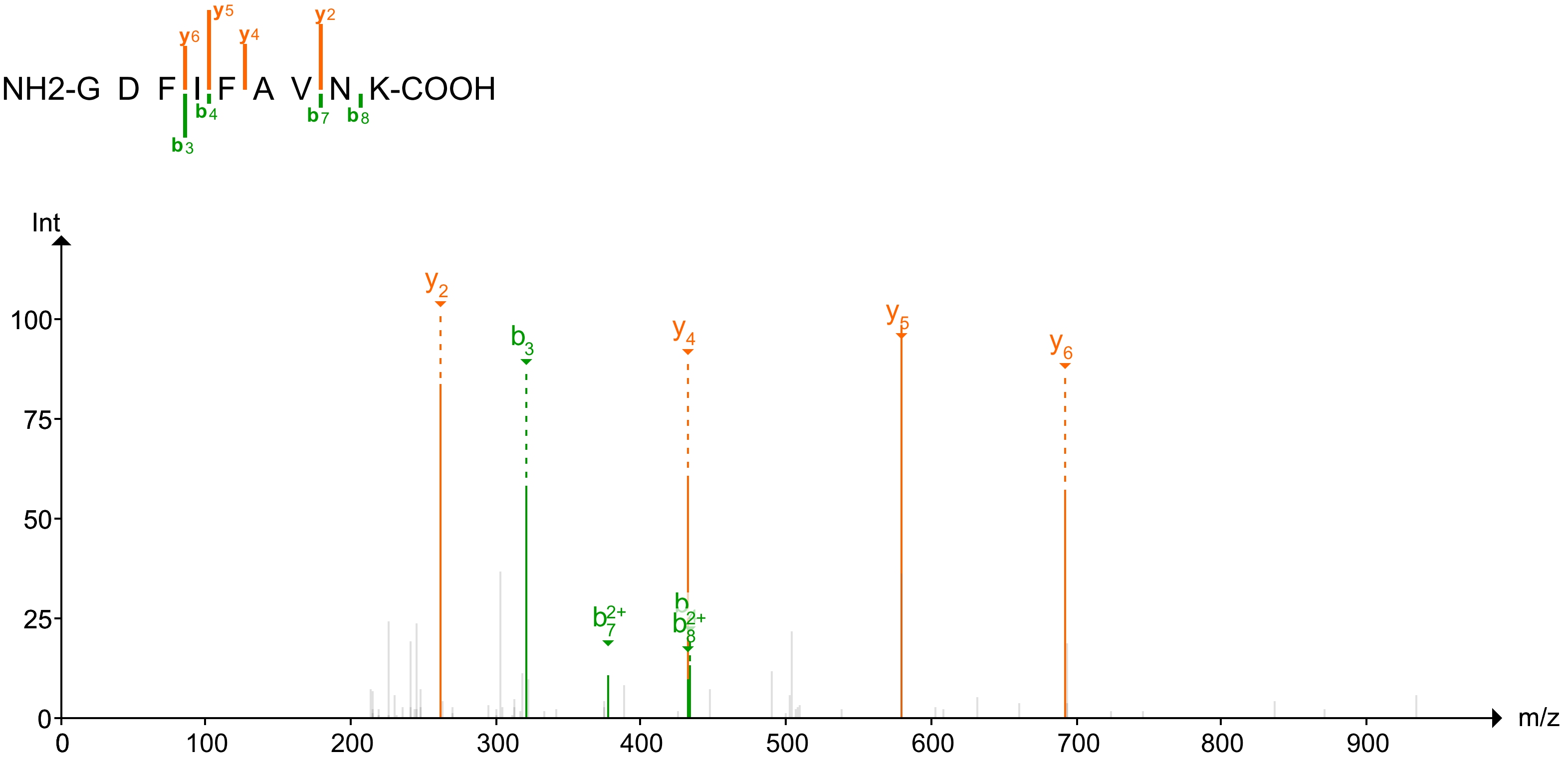

NH2-G D F I F A V N K-COOH
y6
y5
y4
y2
b3
b4
b7
b8
Int
m/z
100
75
50
25
0
0
100
200
300
400
500
600
700
800
900

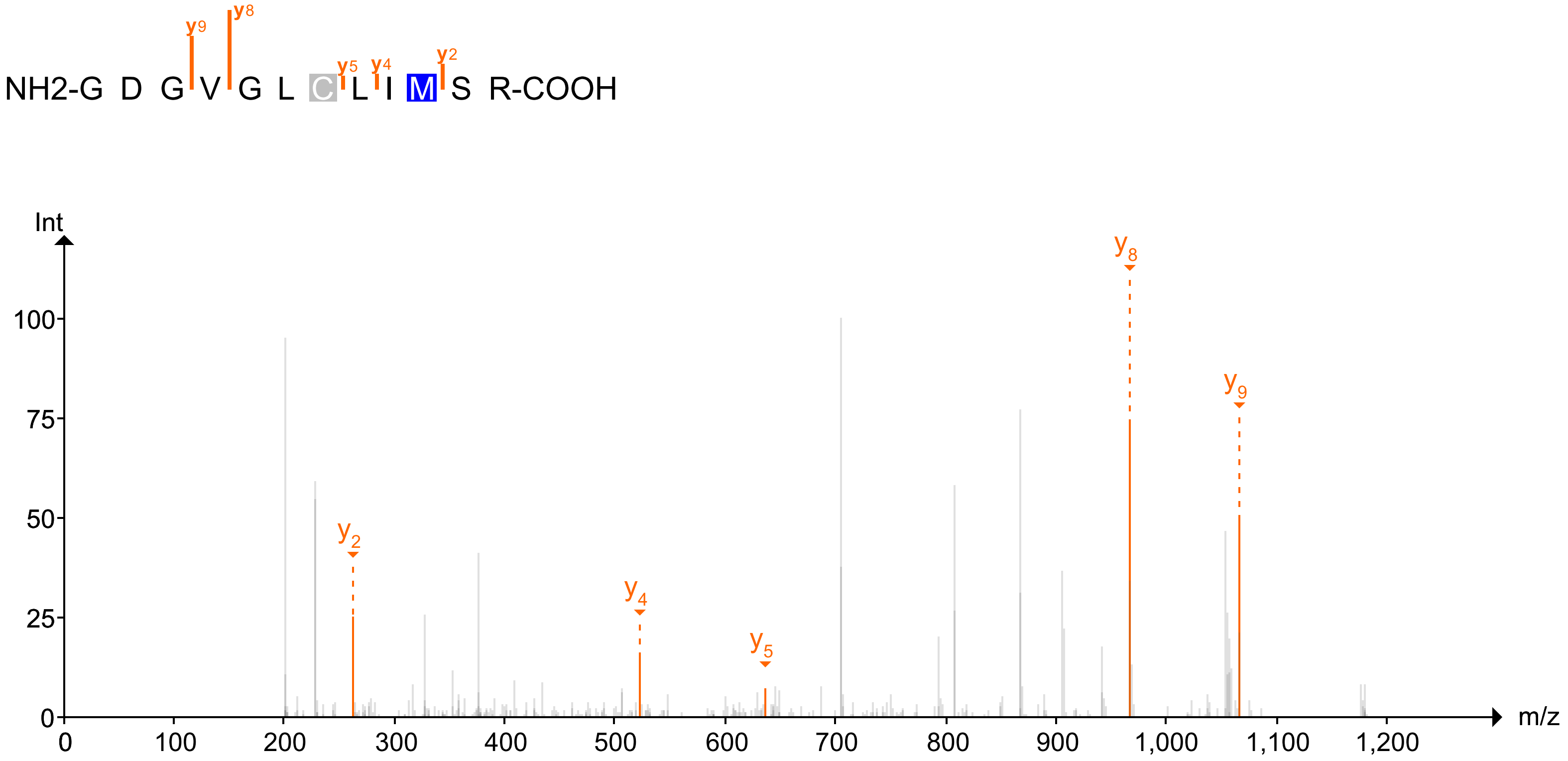

y9
y8
y5
y4
y2
NH2-G D G V G L C L I M S R-COOH
Int
100
75
50
25
0
y8
y9
y2
y4
y5
0
100
200
300
400
500
600
700
800
900
1,000
1,100
1,200
m/z

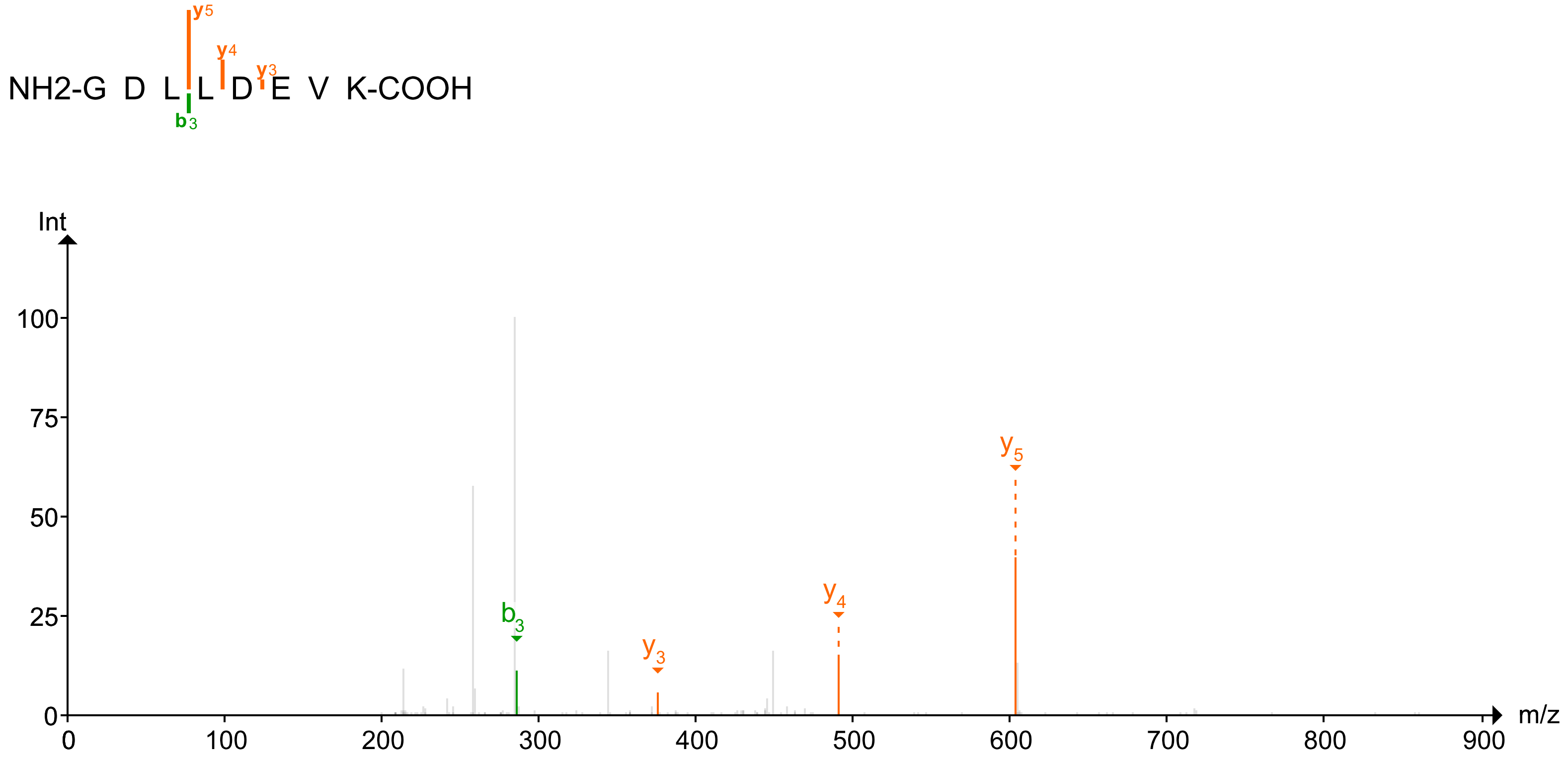

NH2-G D L L D E V K-COOH
y5
y4
y3
b3
Int
100
75
50
25
0
m/z
0
100
200
300
400
500
600
700
800
900

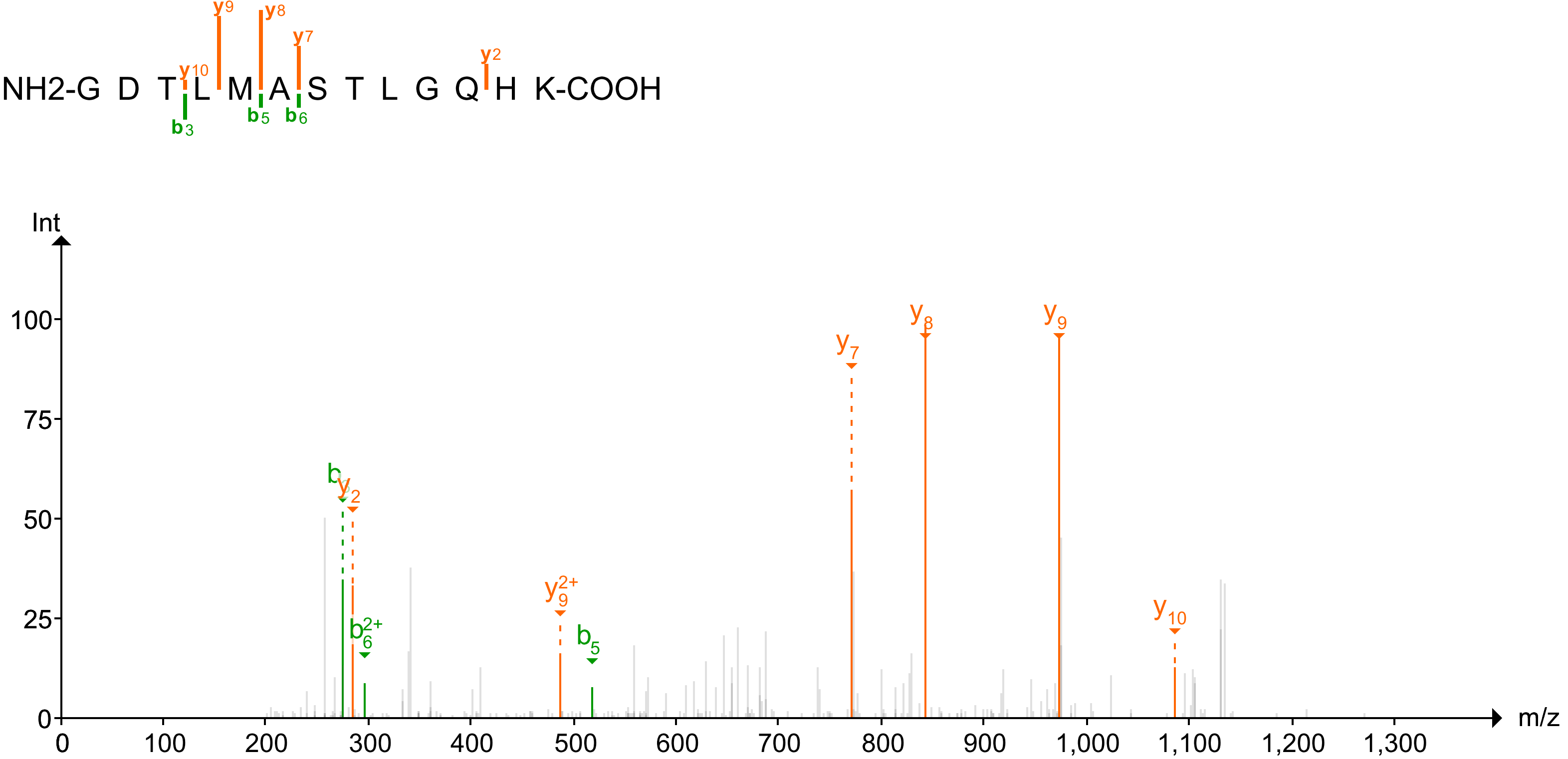

NH2-G D T L M A S T L G Q H K-COOH
y9 y8 y7 y10 y2
b3 b5 b6
Int
100
75
50
25
0
m/z
0 100 200 300 400 500 600 700 800 900 1,000 1,100 1,200 1,300
y7 y8 y9 y10
y2 b6
y9 2+
b6 2+
b5

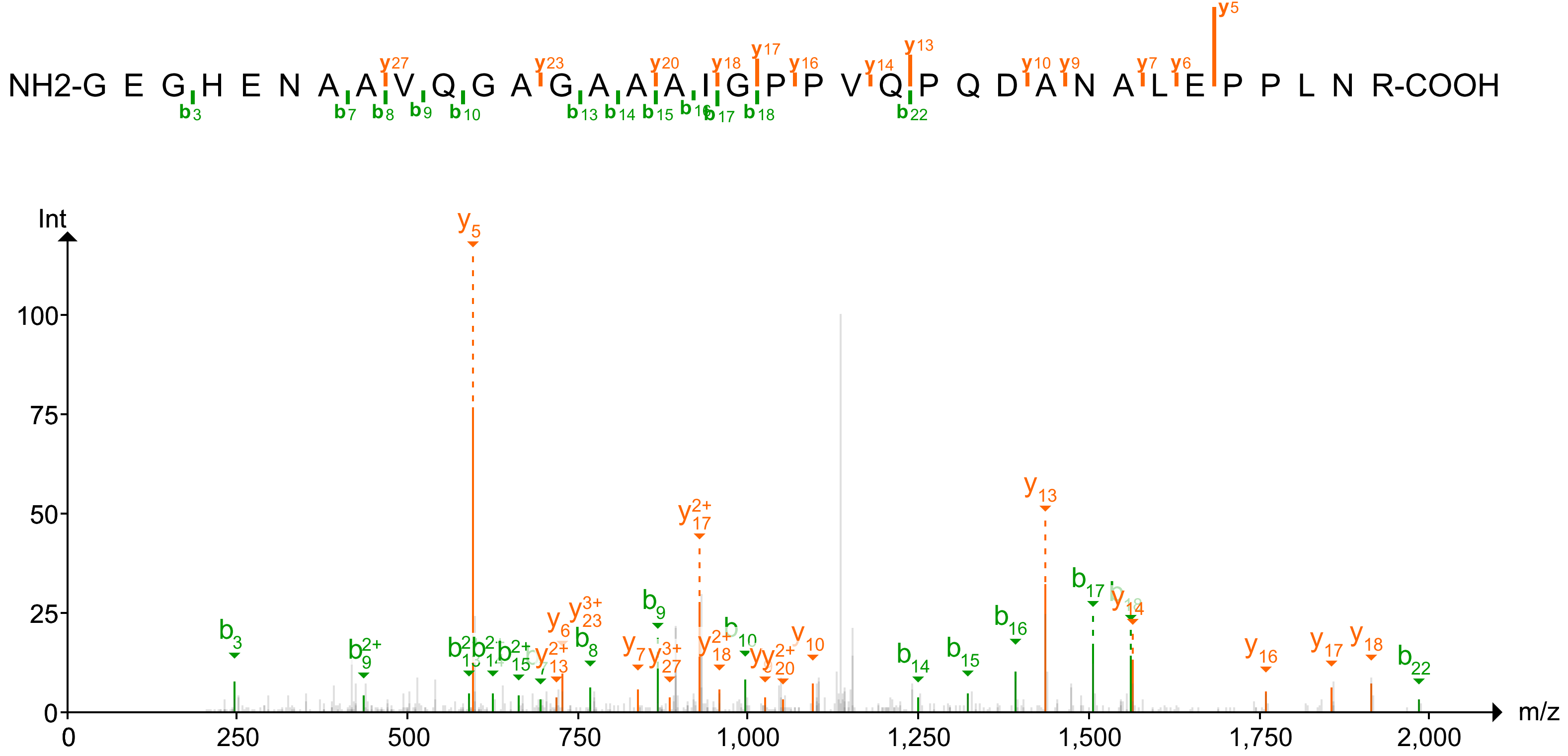

NH2-G E G H E N A A V Q G A G A A A I G P P V Q P Q D A N A L E P P L N R-COOH
Int
m/z

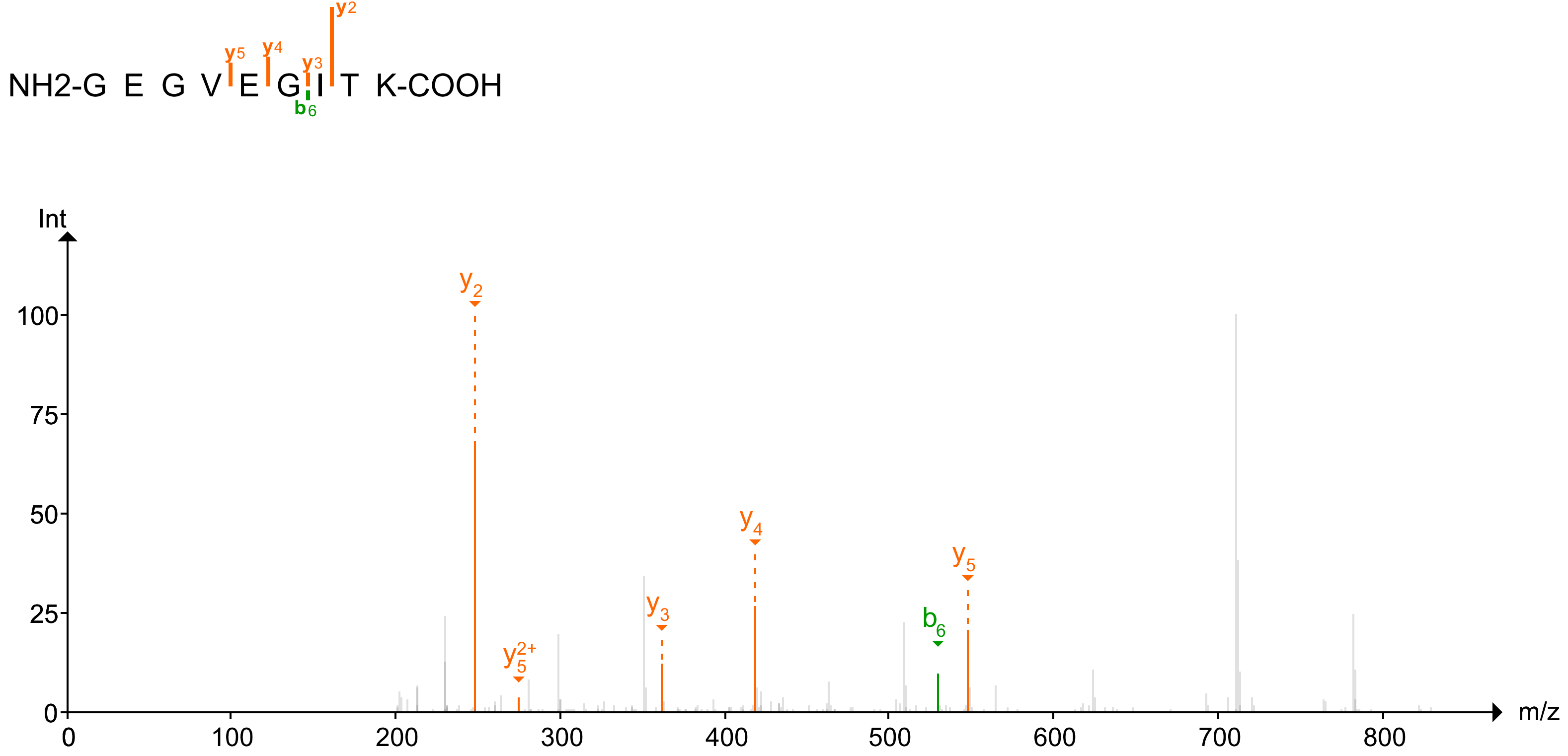

NH2-G E G V E G I T K-COOH
y5
y4
y3
y2
b6
Int
100
75
50
25
0
y2
y5 2+
y3
y4
b6
y5
0
100
200
300
400
500
600
700
800
m/z

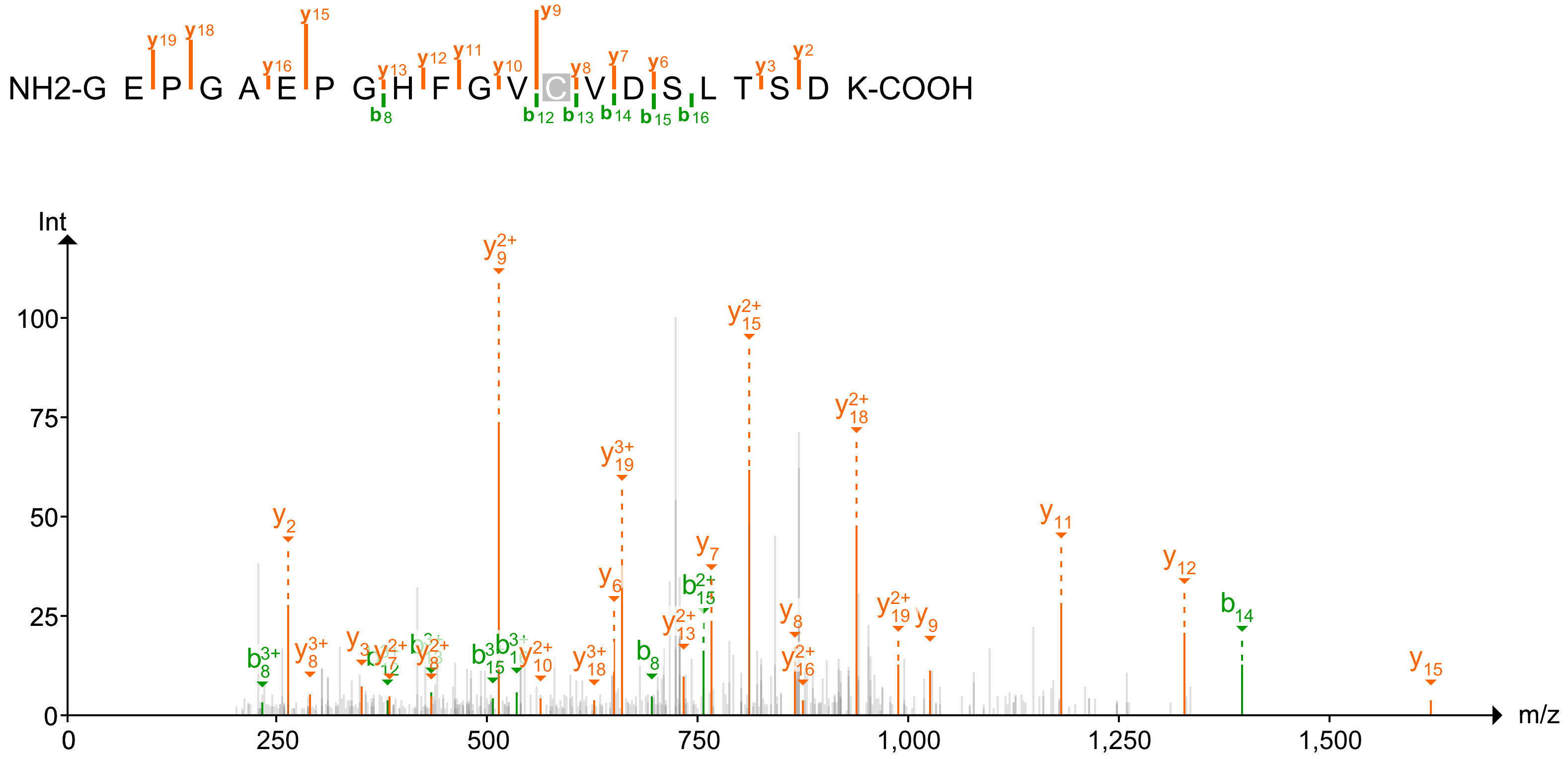
NH2-G E P G A E P G H F G V C V D S L T S D K-COOH
Int
m/z
0
25
50
75
100
250
500
750
1,000
1,250
1,500

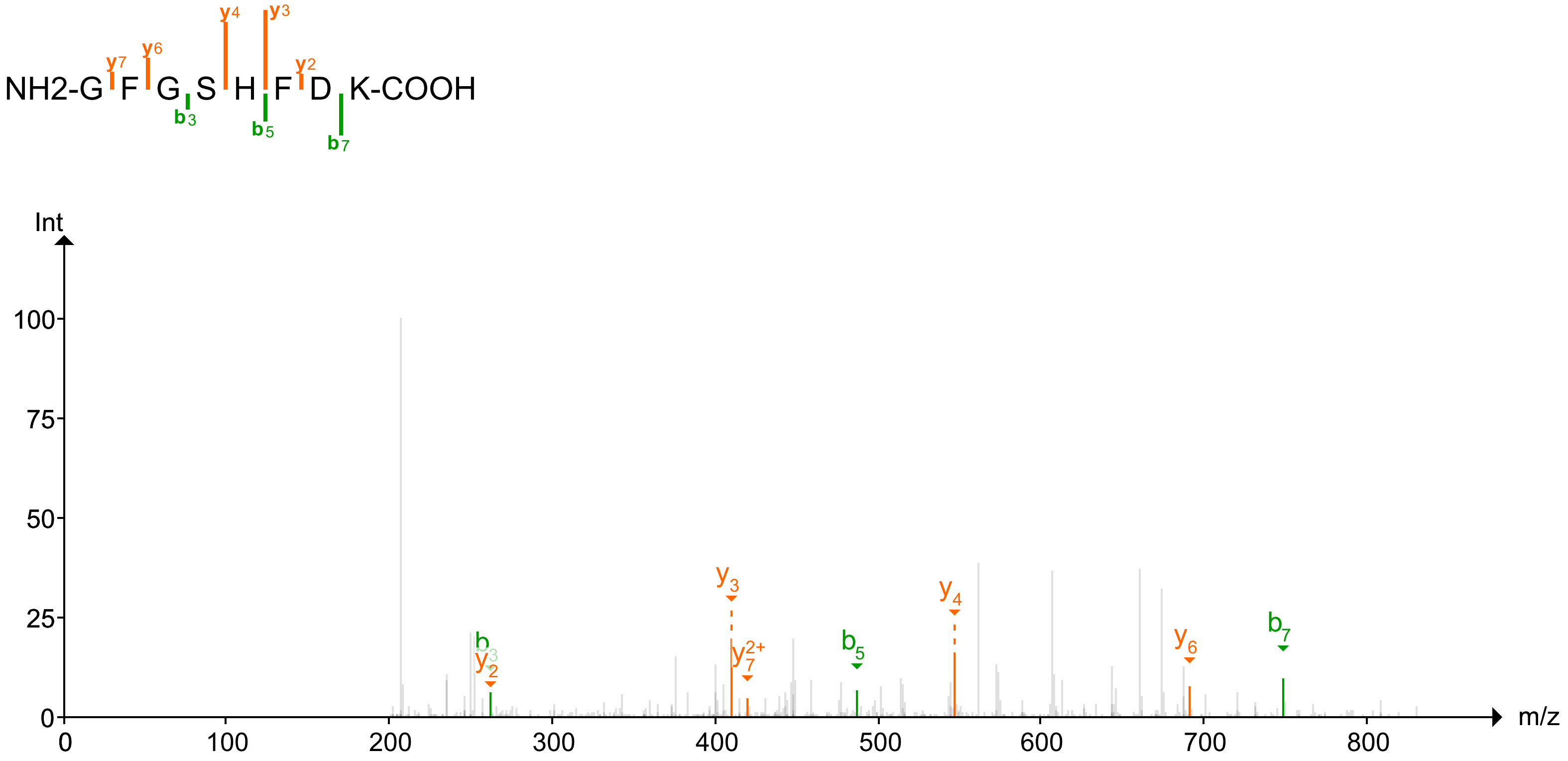

NH2-G F G S H F D K-COOH
y7
y6
y4
y3
y2
b3
b5
b7
Int
100
75
50
25
0
m/z
0
100
200
300
400
500
600
700
800
b3
y2
y3
y7 2+
b5
y4
y6
b7

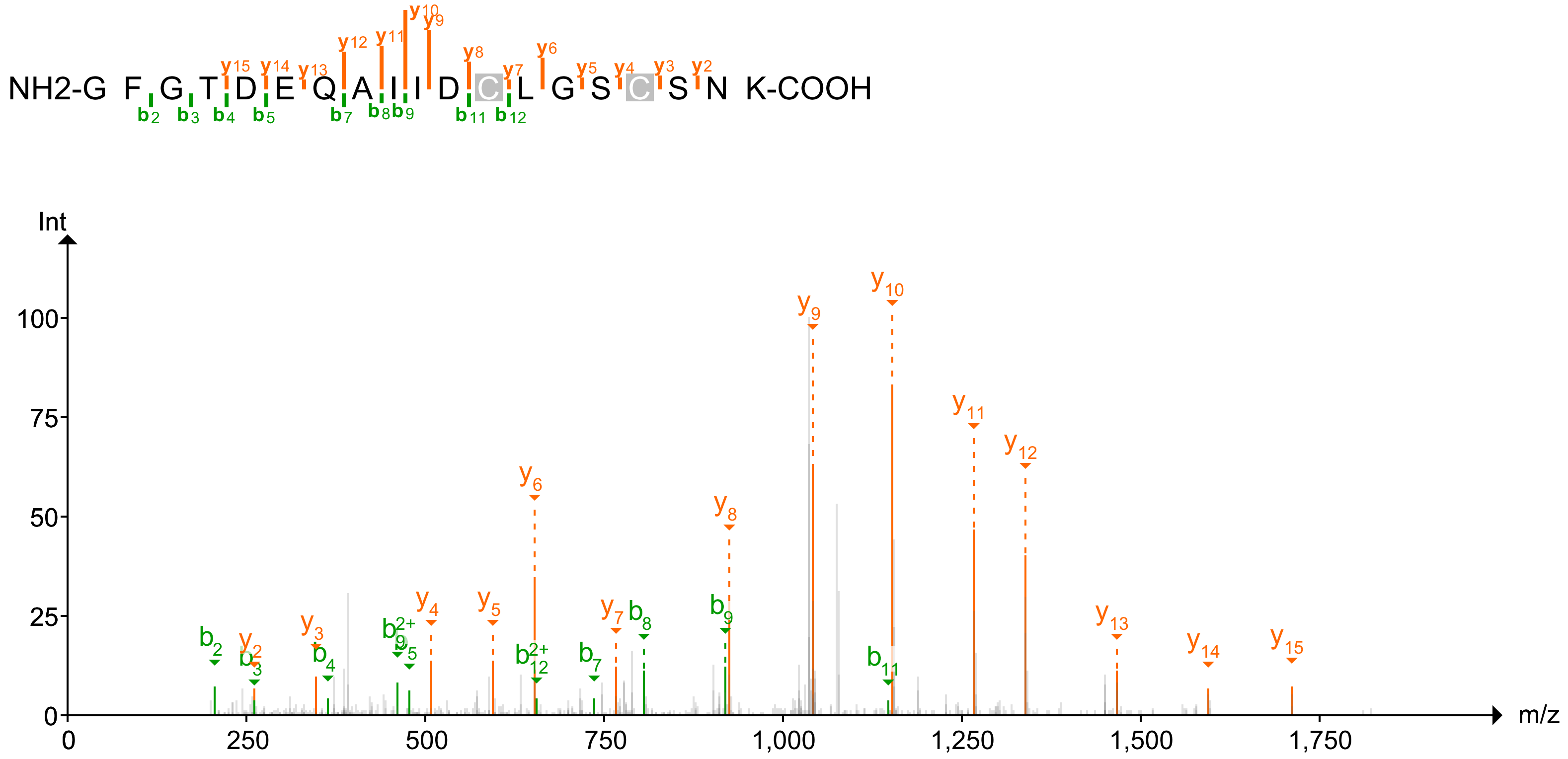

NH2-G F G T D E Q A I I D C L G S C S N K-COOH
Int
100
75
50
25
0
m/z
0
250
500
750
1,000
1,250
1,500
1,750

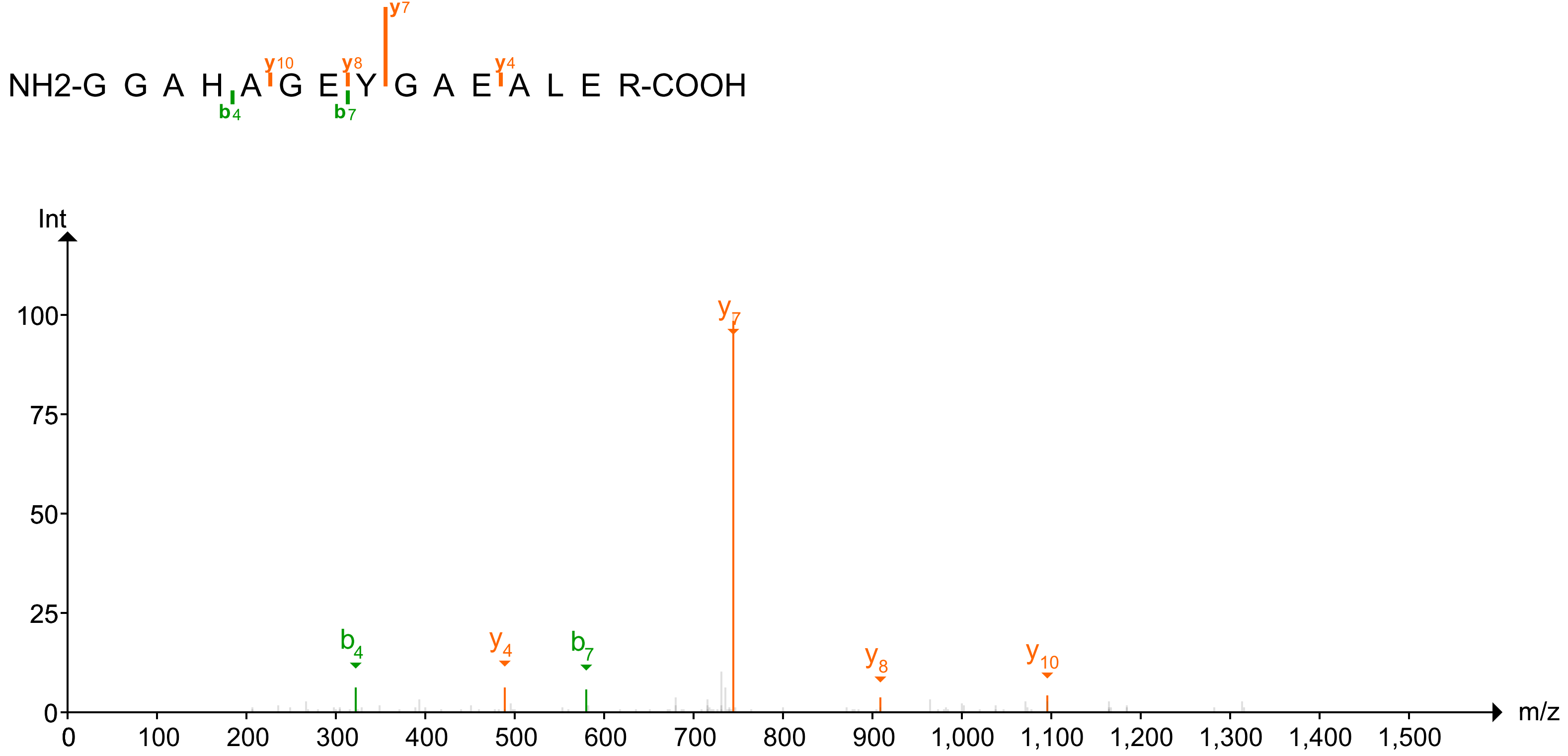

NH2-G G A H A G E Y G A E A L E R-COOH
y7
y10
y8
y4
b4
b7
Int
100
75
50
25
0
y7
b4
y4
b7
y8
y10
m/z
0
100
200
300
400
500
600
700
800
900
1,000
1,100
1,200
1,300
1,400
1,500

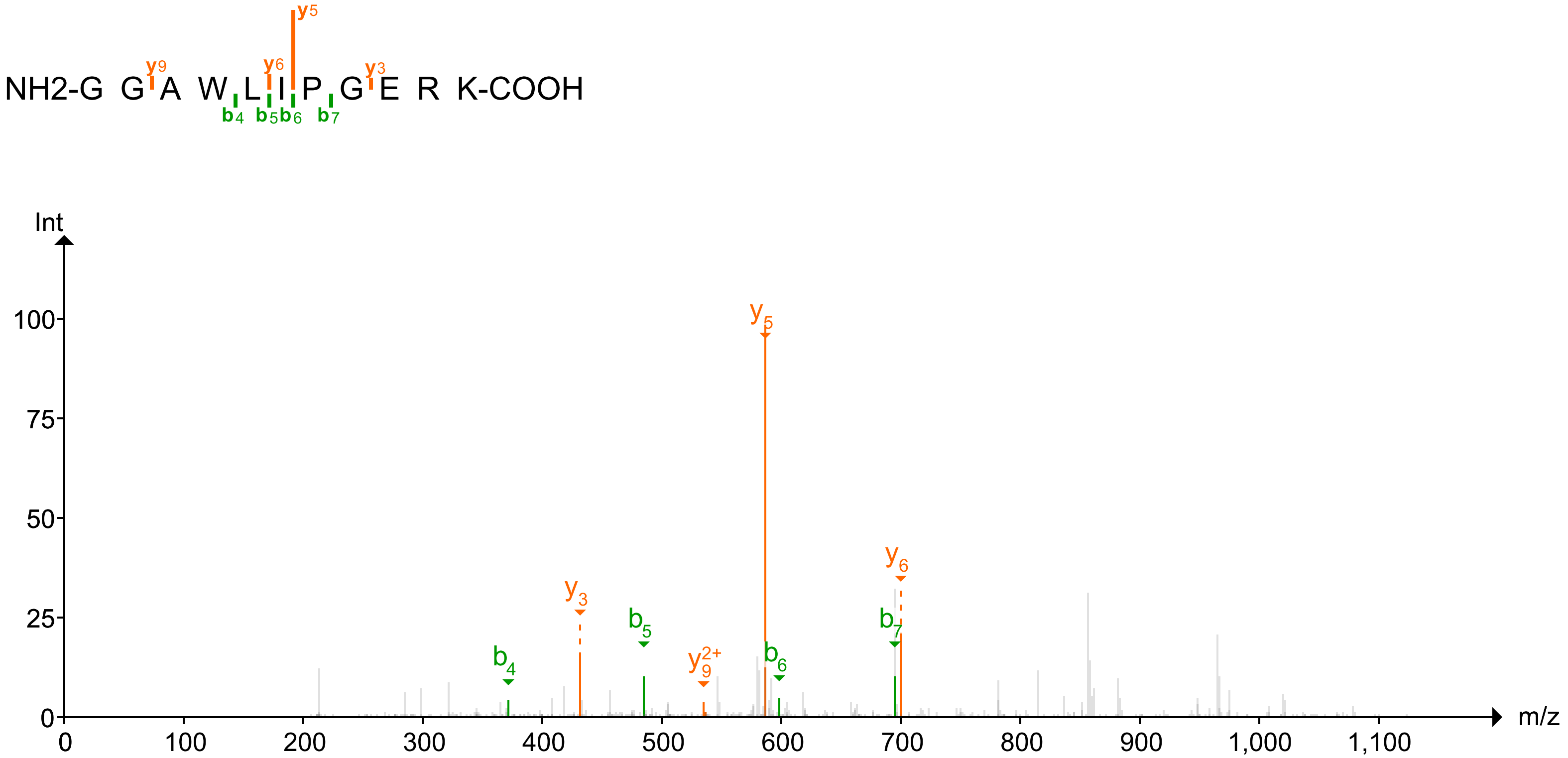
NH2-G G A W L I P G E R K-COOH
y9 y6 y5 y3
b4 b5 b6 b7
Int
100
75
50
25
0
y5
y6
y3
b5
b4
$y_9^{2+}$
b6
b7
0 100 200 300 400 500 600 700 800 900 1,000 1,100
m/z

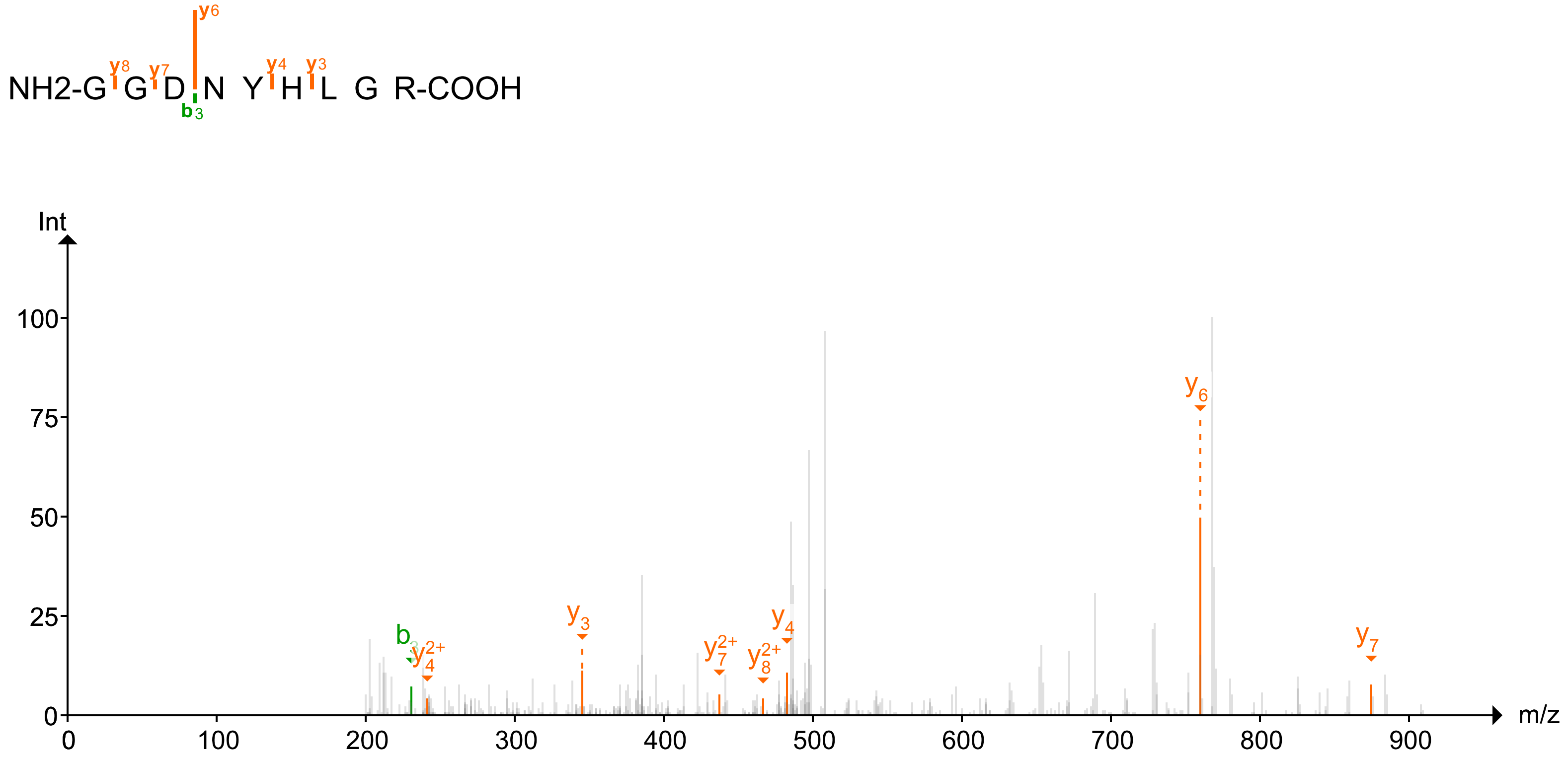

NH2-G G D N Y H L G R-COOH
y8 y7 y6 y4 y3
b3
Int
100
75
50
25
0
b3
y4 2+
y3
y7 2+
y8 2+
y4
y6
y7
0 100 200 300 400 500 600 700 800 900
m/z

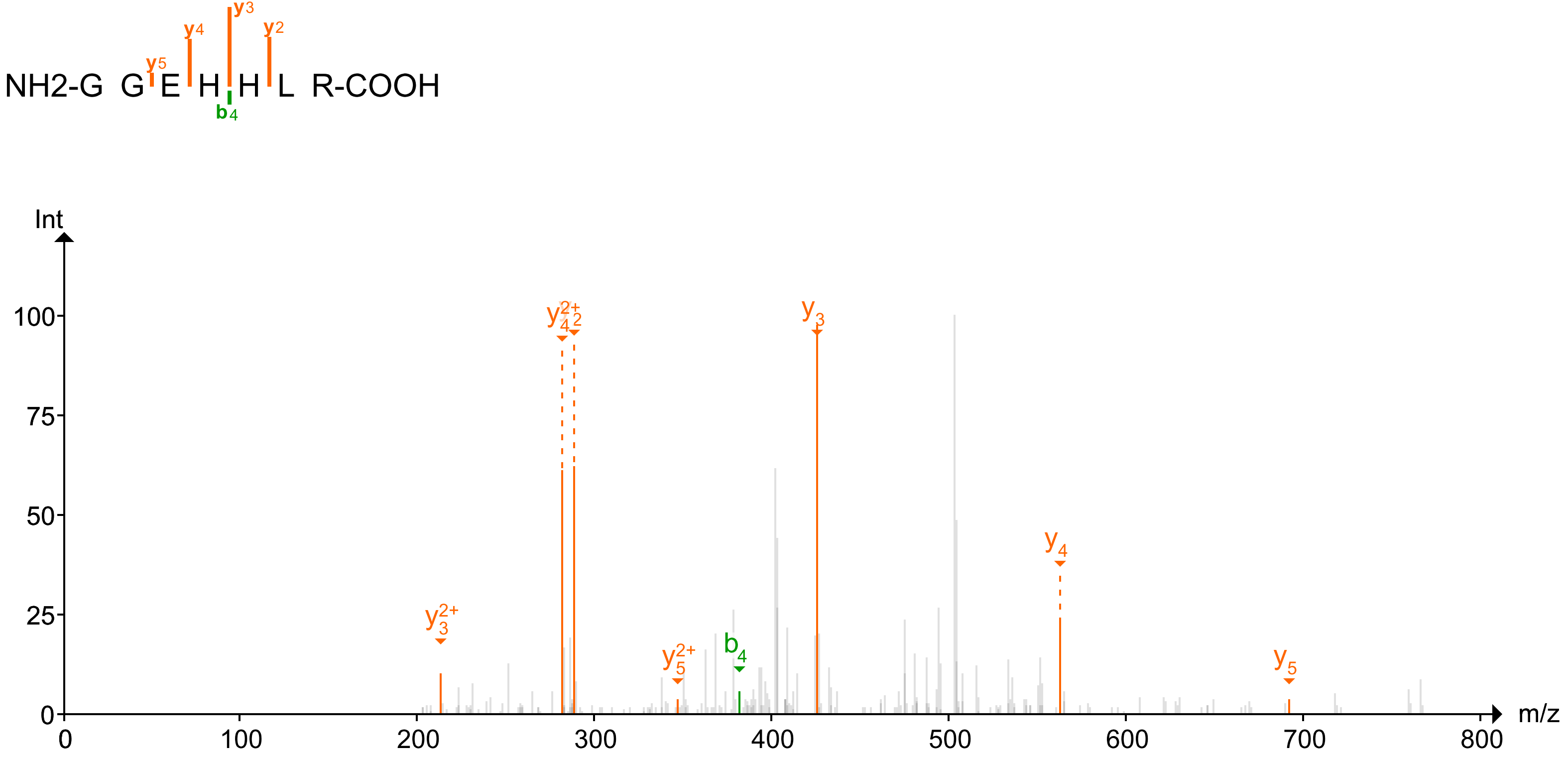

NH2-G G E H H L R-COOH
y5 y4 y3 y2
b4
Int
100
75
50
25
0
m/z
0 100 200 300 400 500 600 700 800
$y_4^{2+}$ $y_2$ $y_3$ $y_4$ $y_3^{2+}$ $y_5^{2+}$ $b_4$ $y_5$

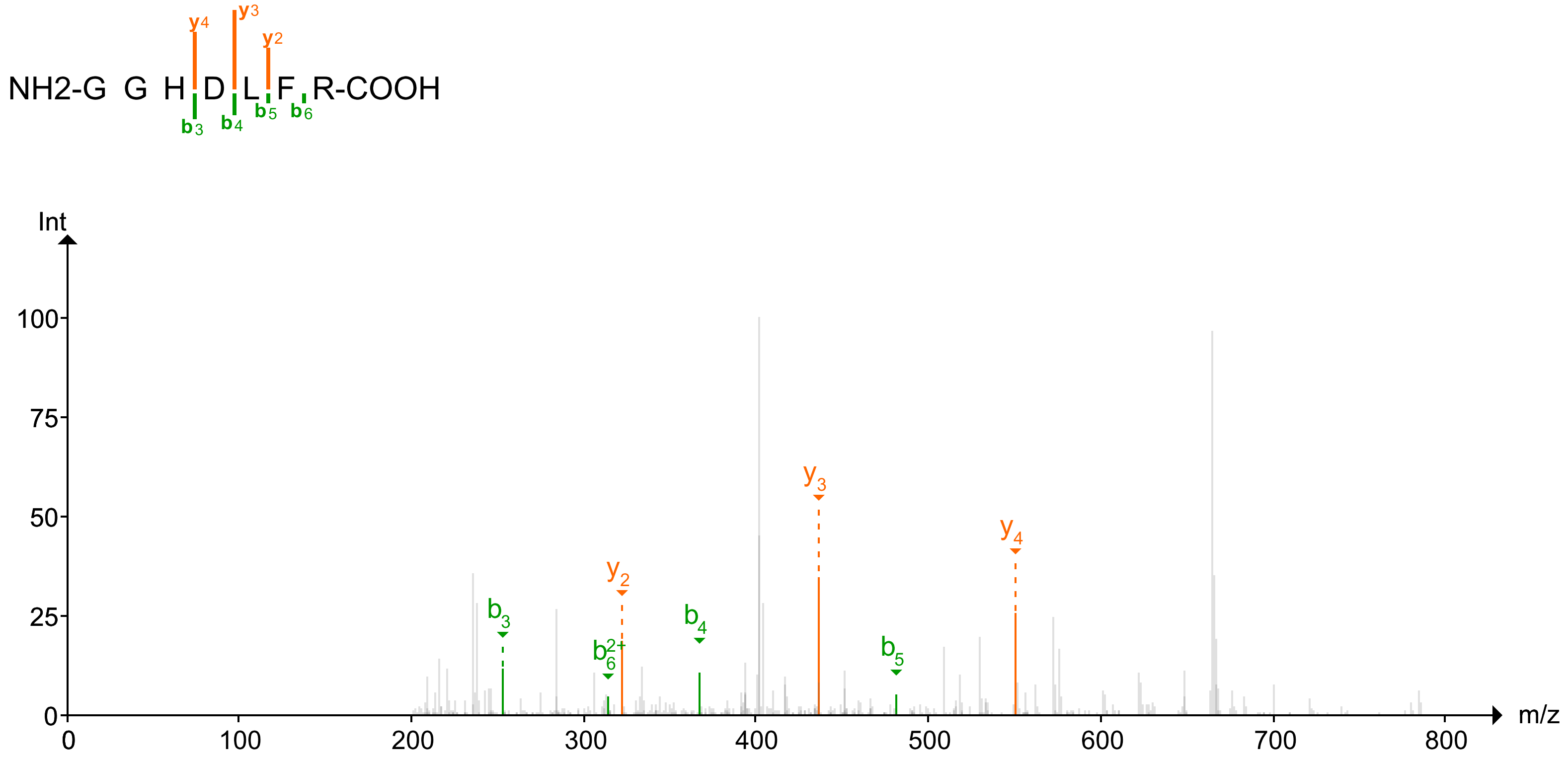

NH2-G G H D L F R-COOH
y4 y3 y2
b3 b4 b5 b6
Int
100
75
50
25
0
y3
y4
y2
b3
b4
b5
b6 2+
0
100
200
300
400
500
600
700
800
m/z

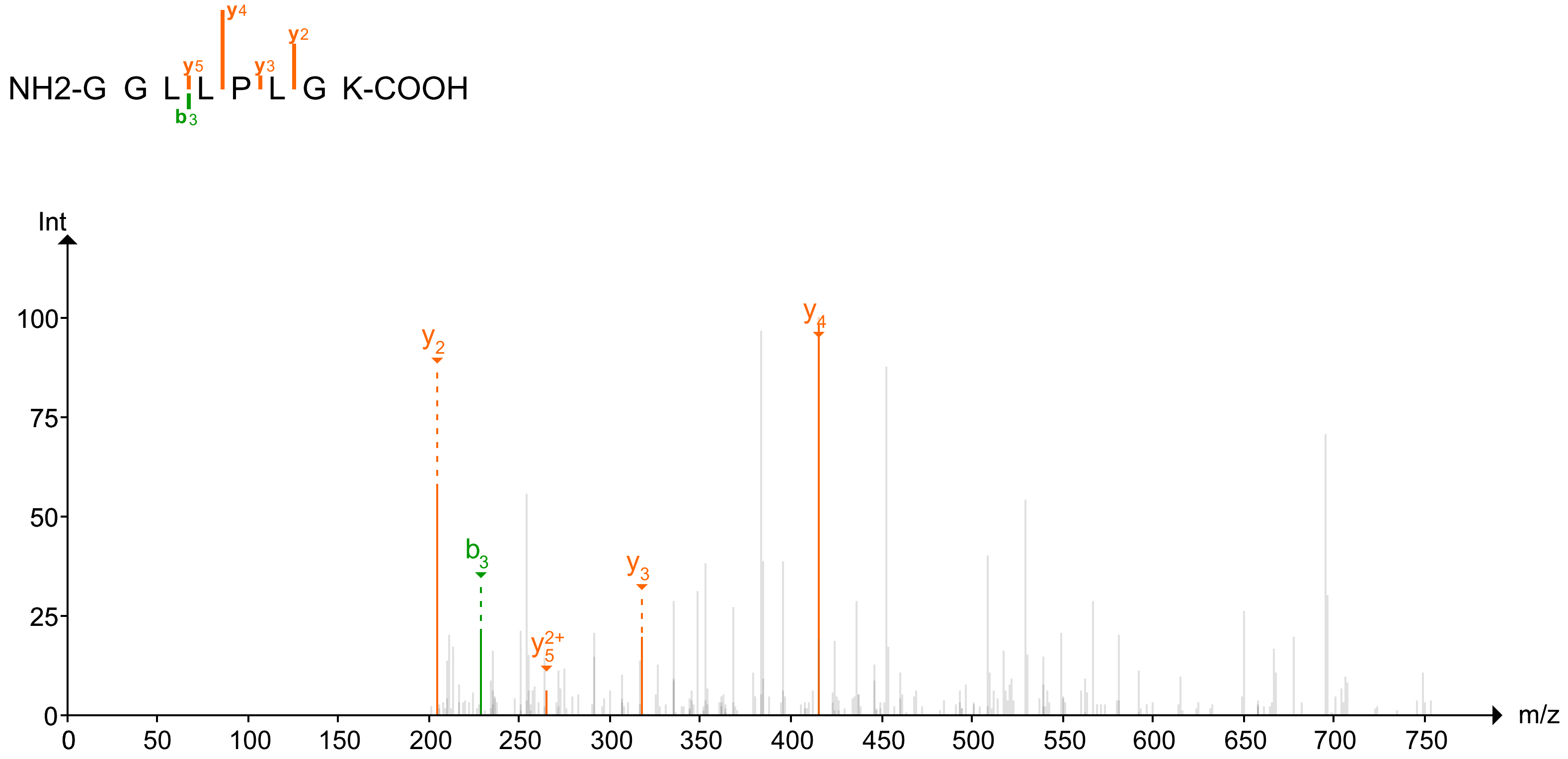

NH2-G G L L P L G K-COOH
y4
y2
y5
y3
b3
Int
m/z
y2
y4
b3
y3
y5 2+
0
25
50
75
100
0 50 100 150 200 250 300 350 400 450 500 550 600 650 700 750

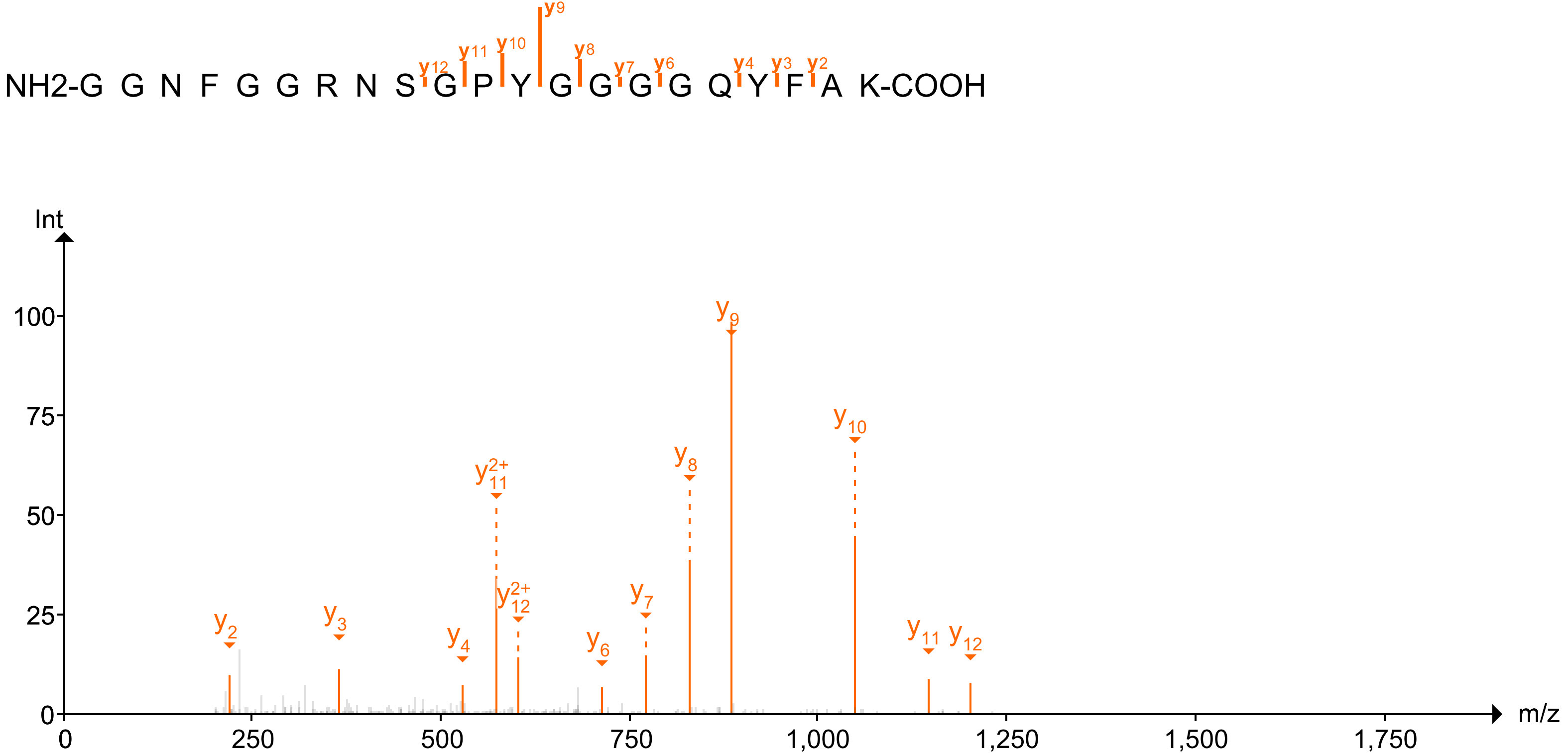

NH2-G G N F G G R N S G P Y G G G G Q Y F A K-COOH
y12 y11 y10 y9 y8 y7 y6 y4 y3 y2
Int
100
75
50
25
0
0 250 500 750 1,000 1,250 1,500 1,750
m/z
y2 y3 y4 y11 2+ y12 2+ y6 y7 y8 y9 y10 y11 y12

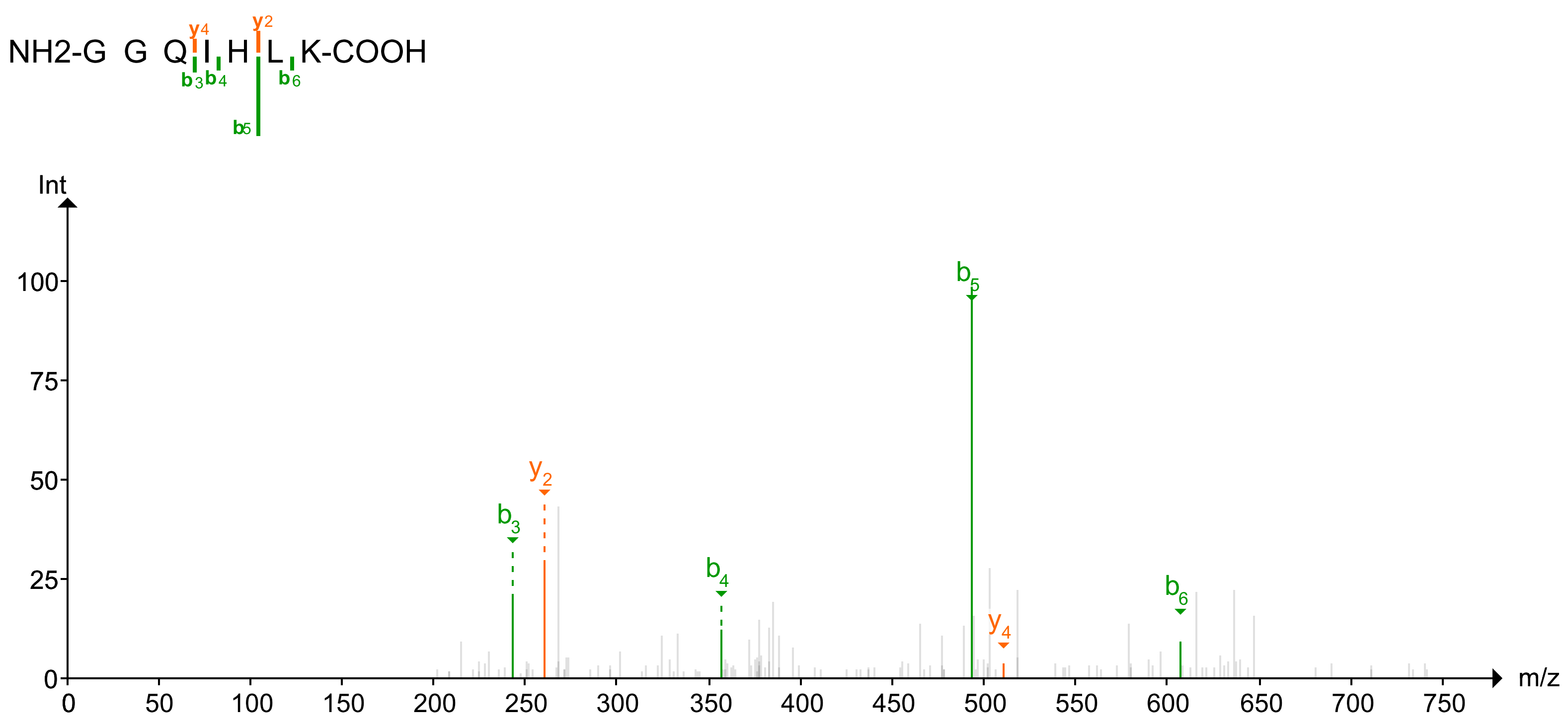

NH2-G G Q I H L K-COOH
y4
y2
b3
b4
b5
b6
Int
100
75
50
25
0
0
50
100
150
200
250
300
350
400
450
500
550
600
650
700
750
m/z

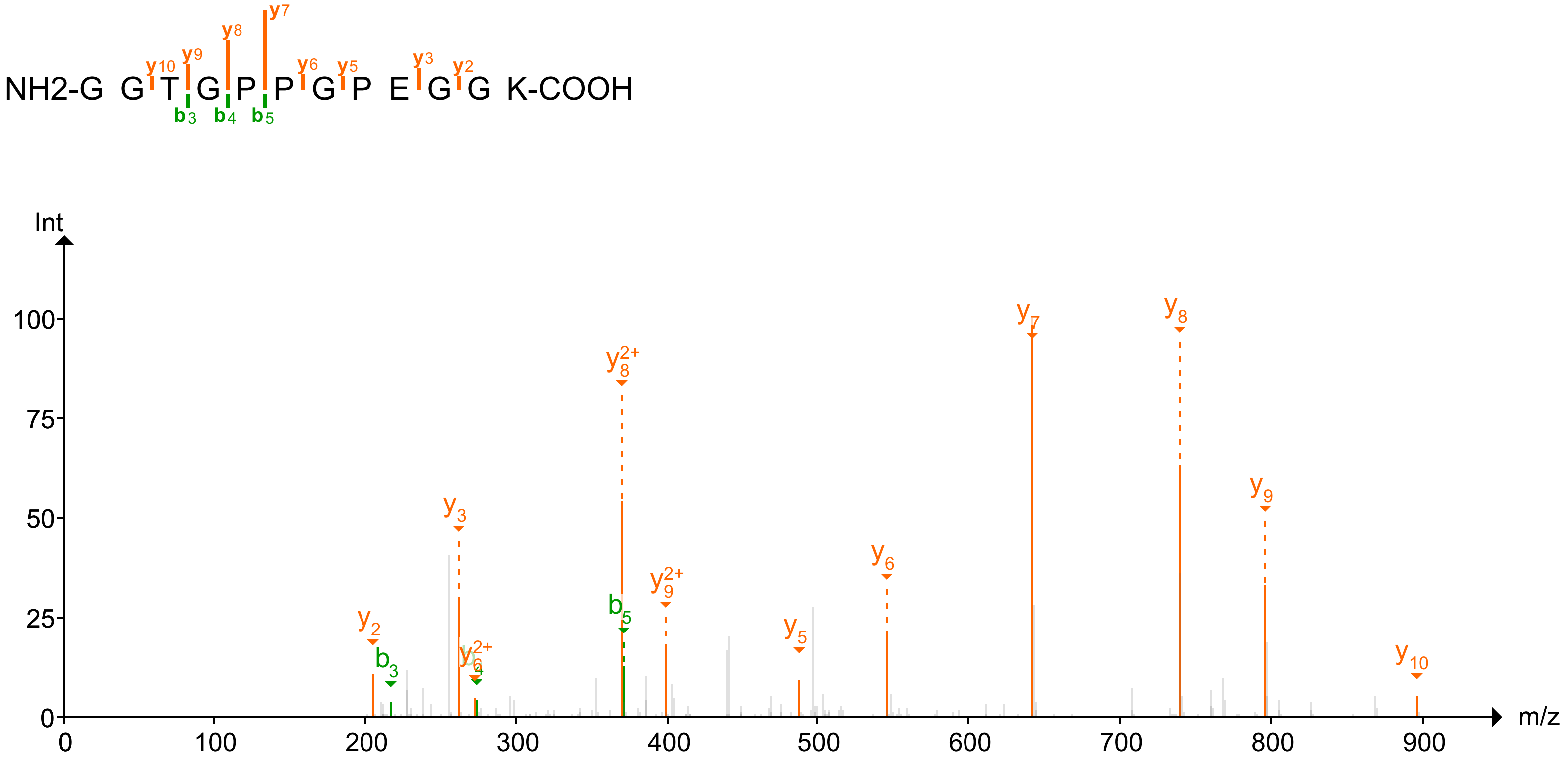

NH2-G G T G P P G P E G G K-COOH
y10 y9 y8 y7 y6 y5 y3 y2
b3 b4 b5
Int
m/z
0
25
50
75
100
0 100 200 300 400 500 600 700 800 900
y2
b3
y3
y6 2+
b4
y8 2+
b5
y9 2+
y5
y6
y7
y8
y9
y10

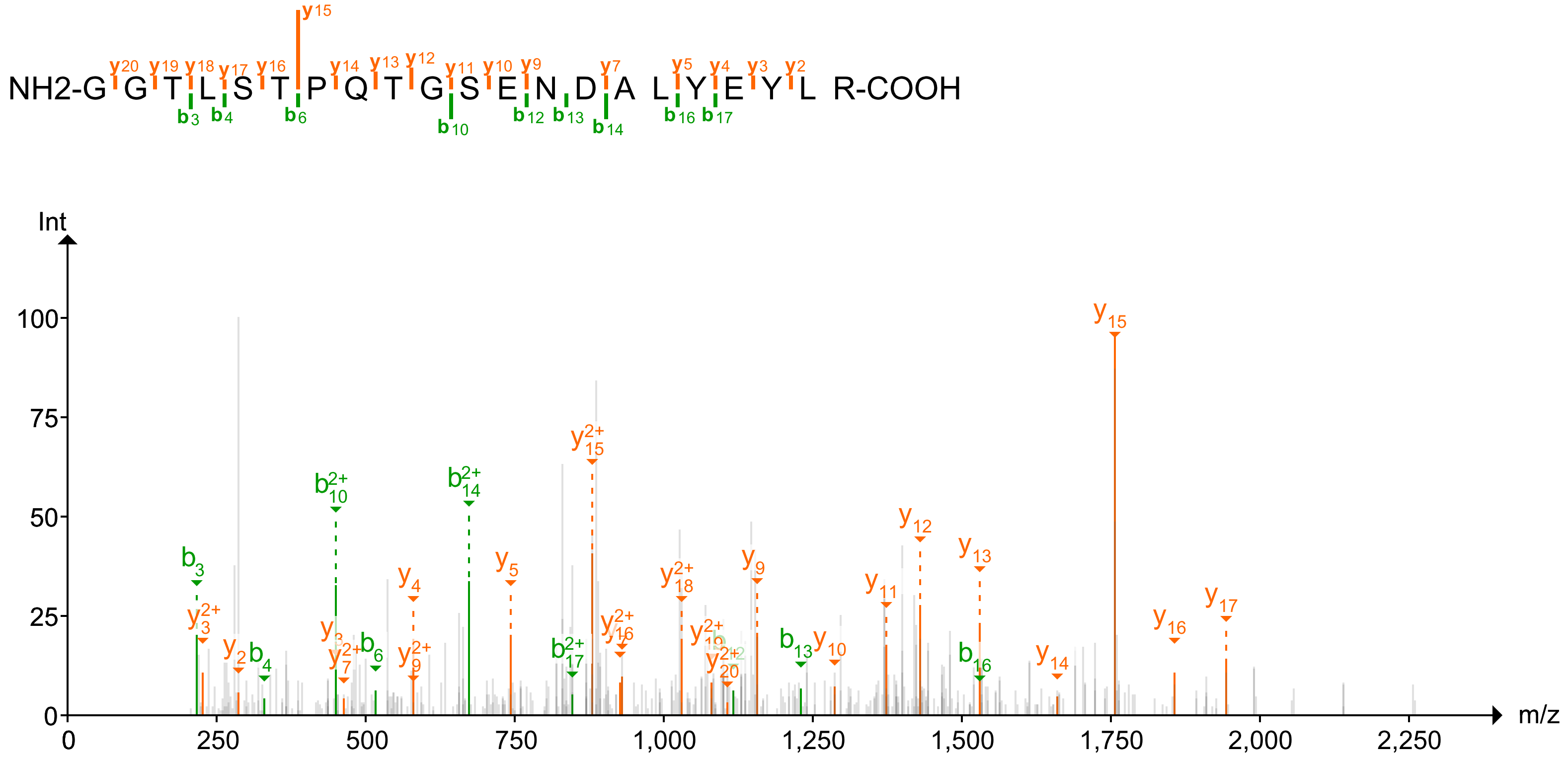
NH2-G G T L S T P Q T G S E N D A L Y E Y L R-COOH
Int
m/z
0
25
50
75
100
250
500
750
1,000
1,250
1,500
1,750
2,000
2,250

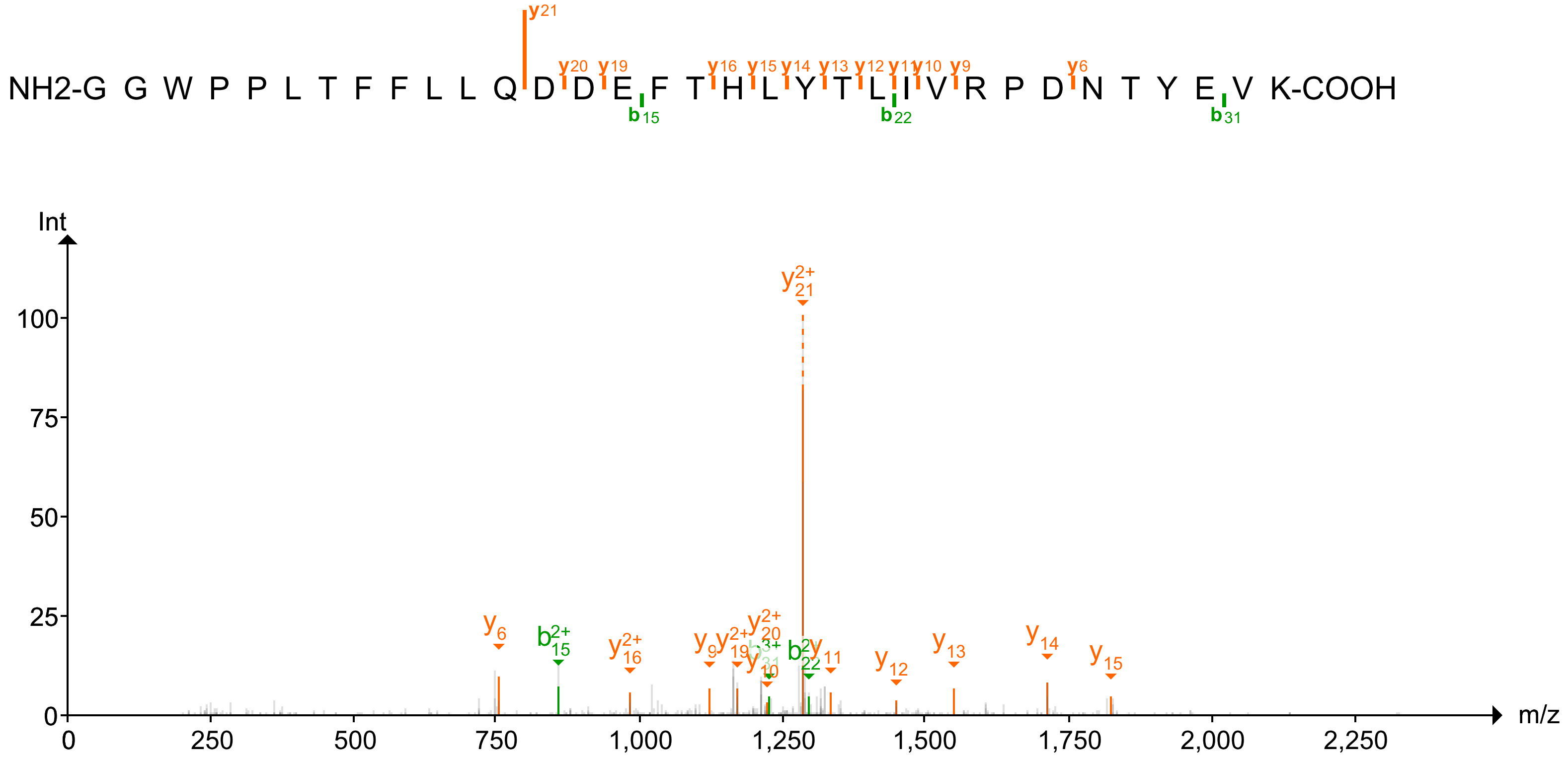
NH2-G G W P P L T F F L L Q D D E F T H L Y T L I V R P D N T Y E V K-COOH
y21 y20 y19 y16 y15 y14 y13 y12 y11 y10 y9 y6
b15 b22 b31
Int
m/z
0 25 50 75 100
0 250 500 750 1,000 1,250 1,500 1,750 2,000 2,250
y21 2+
y6
b15 2+
y16 2+
y9
y19 2+
y20 2+
b31 3+
y10
b22 2+
y11
y12
y13
y14
y15

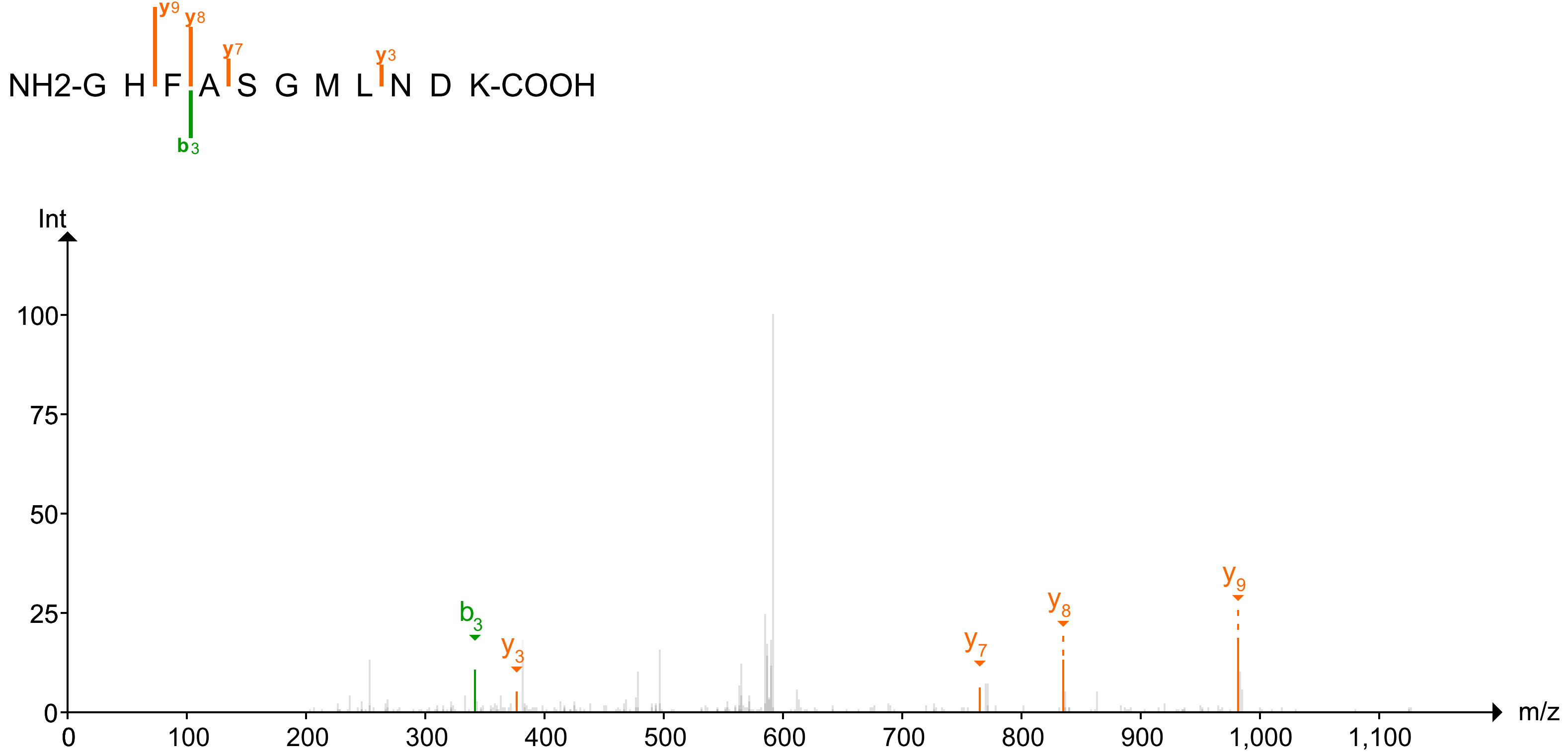

NH2-G H F A S G M L N D K-COOH
y9
y8
y7
y3
b3
Int
100
75
50
25
0
m/z
0
100
200
300
400
500
600
700
800
900
1,000
1,100

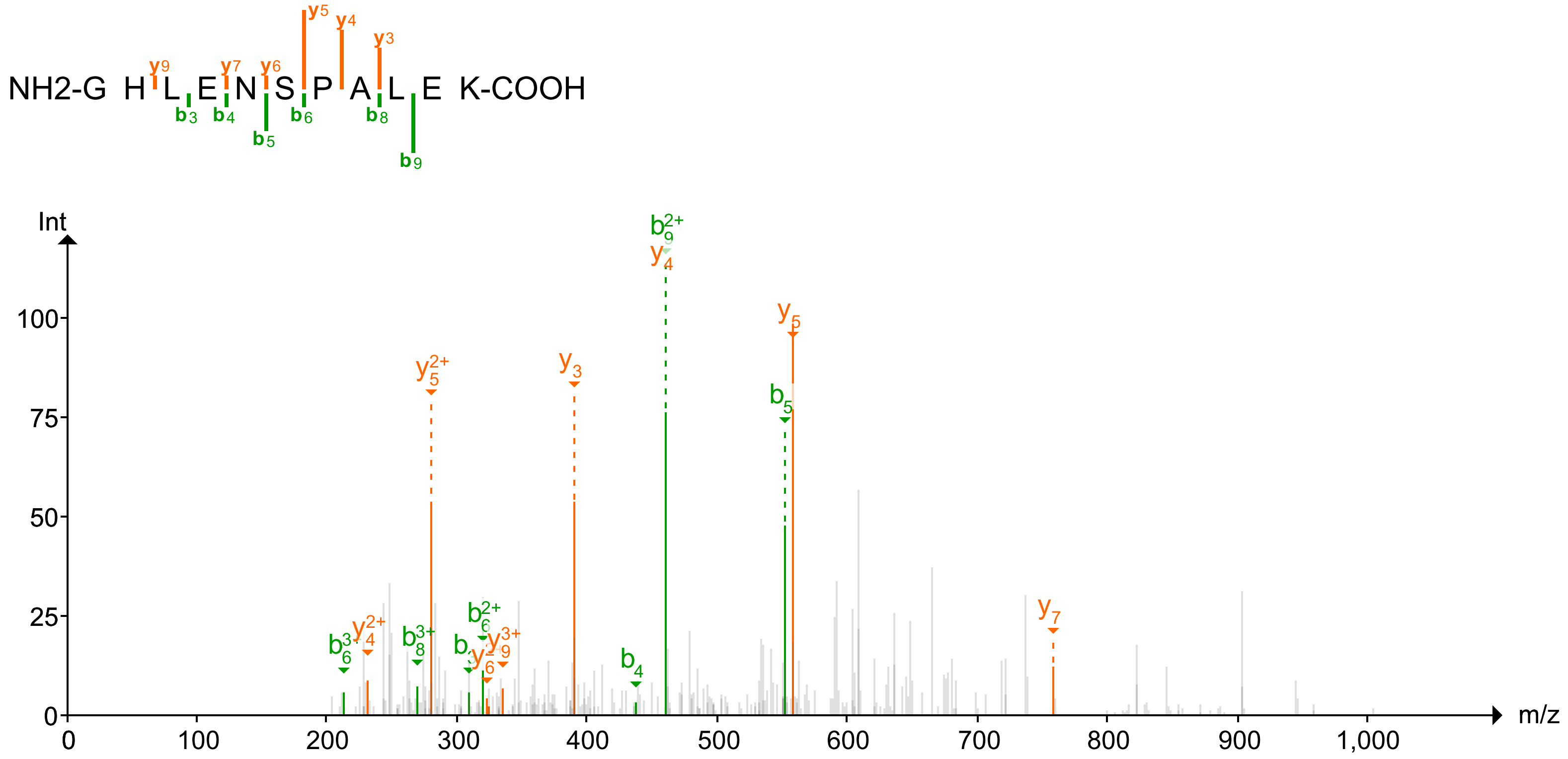

NH2-G H L E N S P A L E K-COOH
y9 y7 y6 y5 y4 y3
b3 b4 b5 b6 b8 b9
Int
m/z
0
25
50
75
100
0
100
200
300
400
500
600
700
800
900
1,000
$b_9^{2+}$
$y_4$
$y_5$
$y_5^{2+}$
$y_3$
$b_5$
$y_7$
$b_4$
$b_6^{3+}$
$y_4^{2+}$
$b_8^{3+}$
$b_6^{2+}$
$y_9^{3+}$

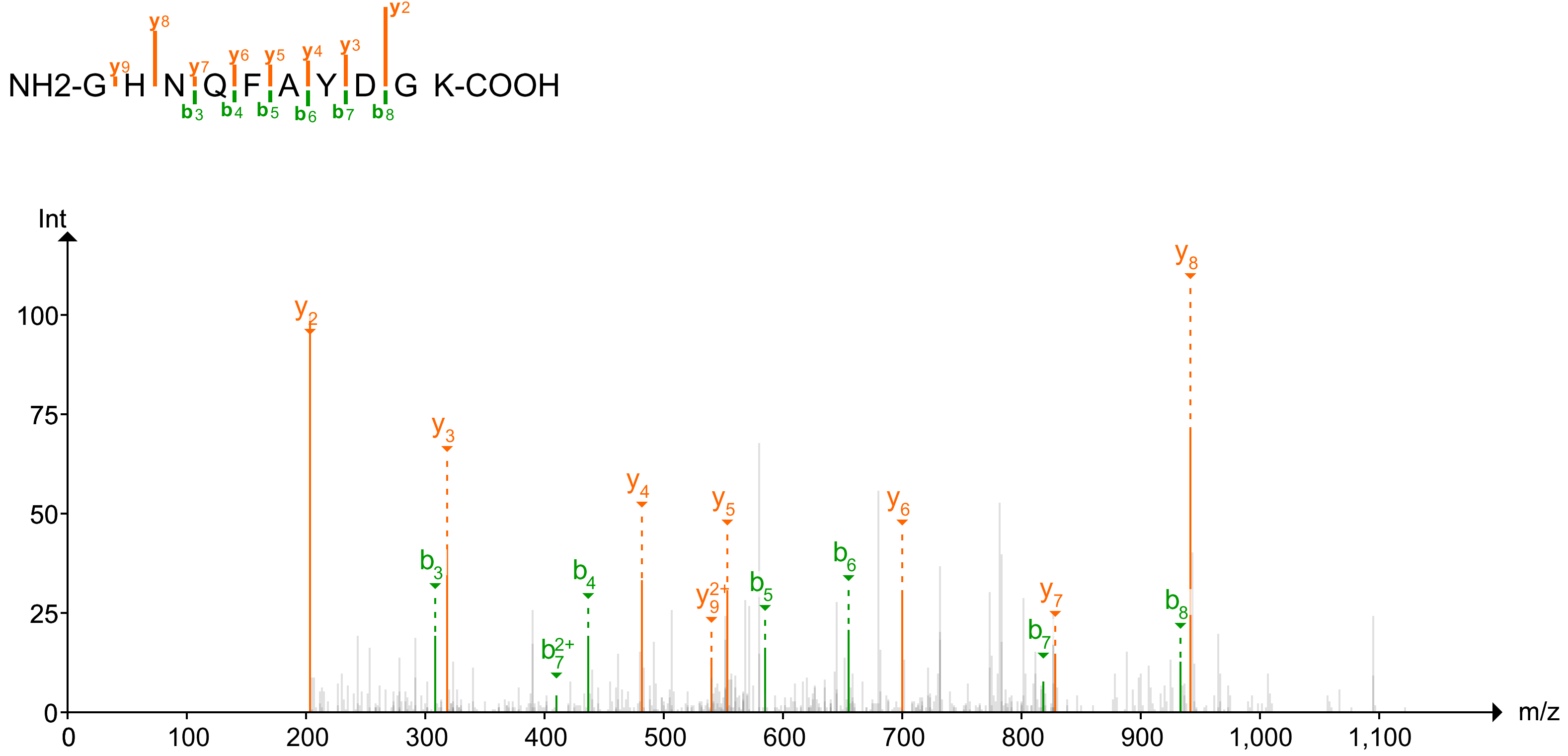

NH2-G H N Q F A Y D G K-COOH
y9 y8 y7 y6 y5 y4 y3 y2
b3 b4 b5 b6 b7 b8
Int
m/z
0
25
50
75
100
0
100
200
300
400
500
600
700
800
900
1,000
1,100
y2
y3
y4
y5
y6
y7
y8
y9 2+
b3
b4
b5
b6
b7
b8
b7 2+

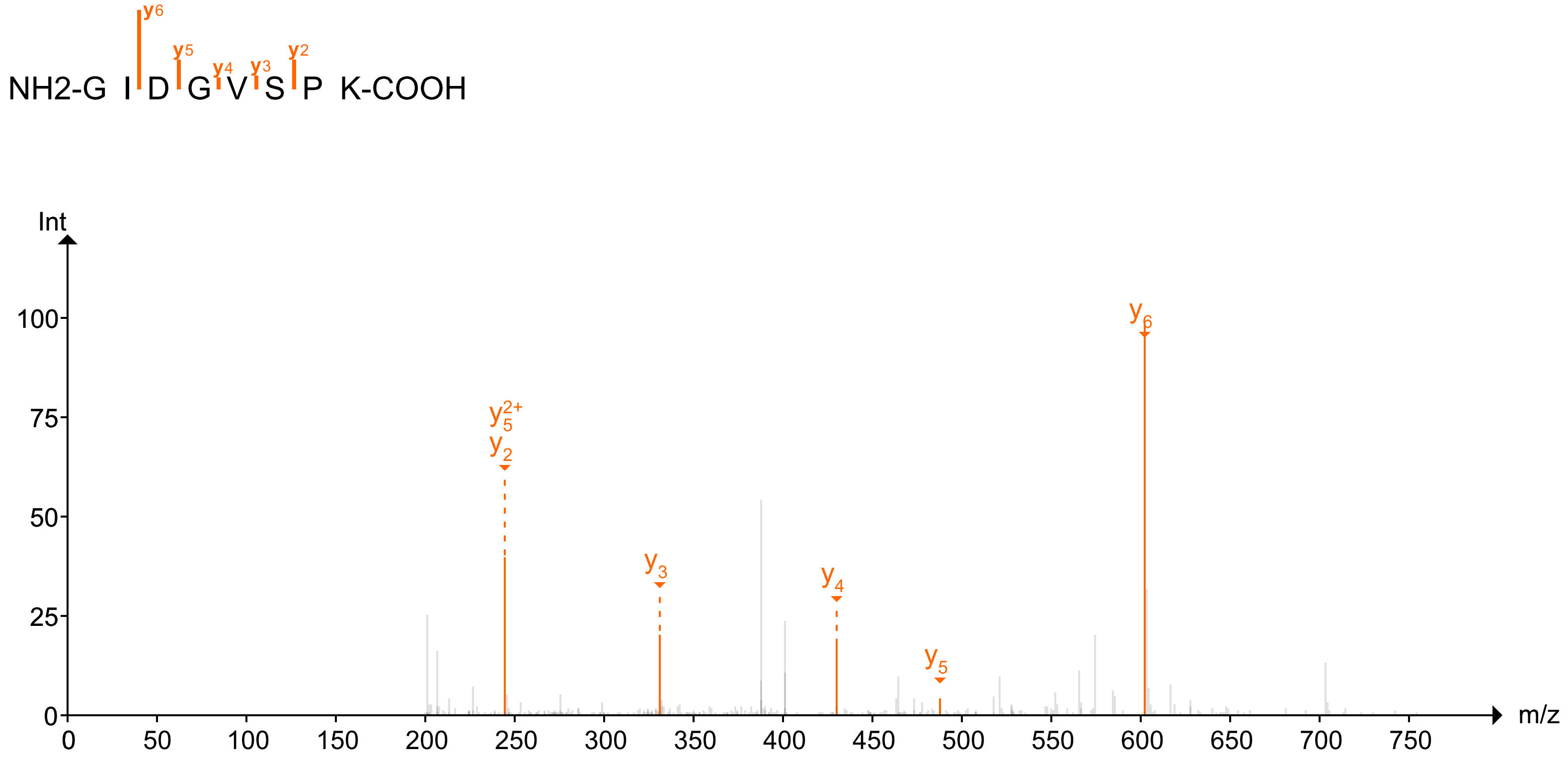

NH2-G I D G V S P K-COOH
y6
y5
y4
y3
y2
Int
100
75
50
25
0
$y_5^{2+}$
$y_2$
$y_3$
$y_4$
$y_5$
$y_6$
0
50
100
150
200
250
300
350
400
450
500
550
600
650
700
750
m/z

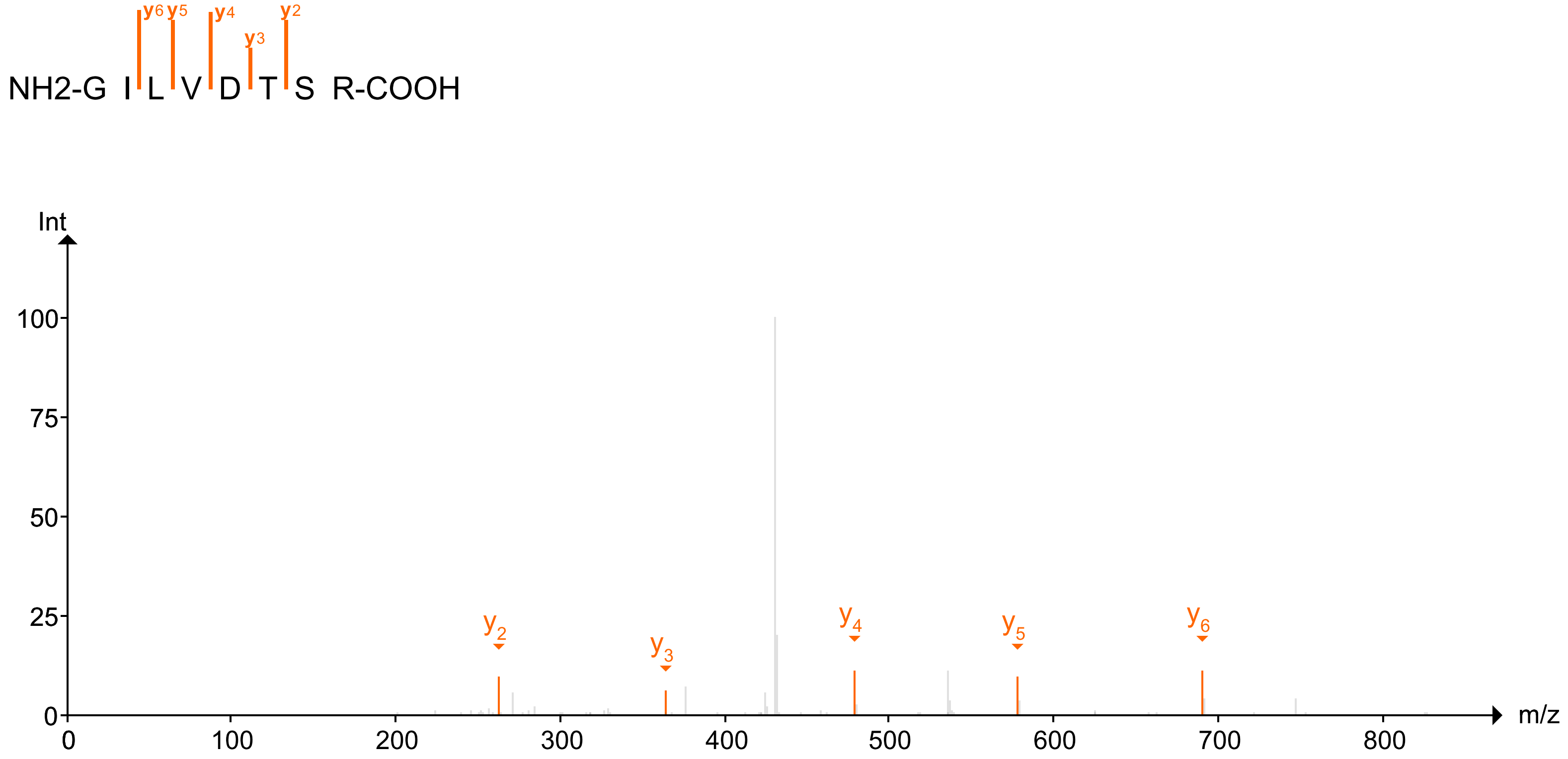

NH2-G I L V D T S R-COOH
y6 y5 y4 y3 y2
Int
100
75
50
25
0
y2
y3
y4
y5
y6
0
100
200
300
400
500
600
700
800
m/z

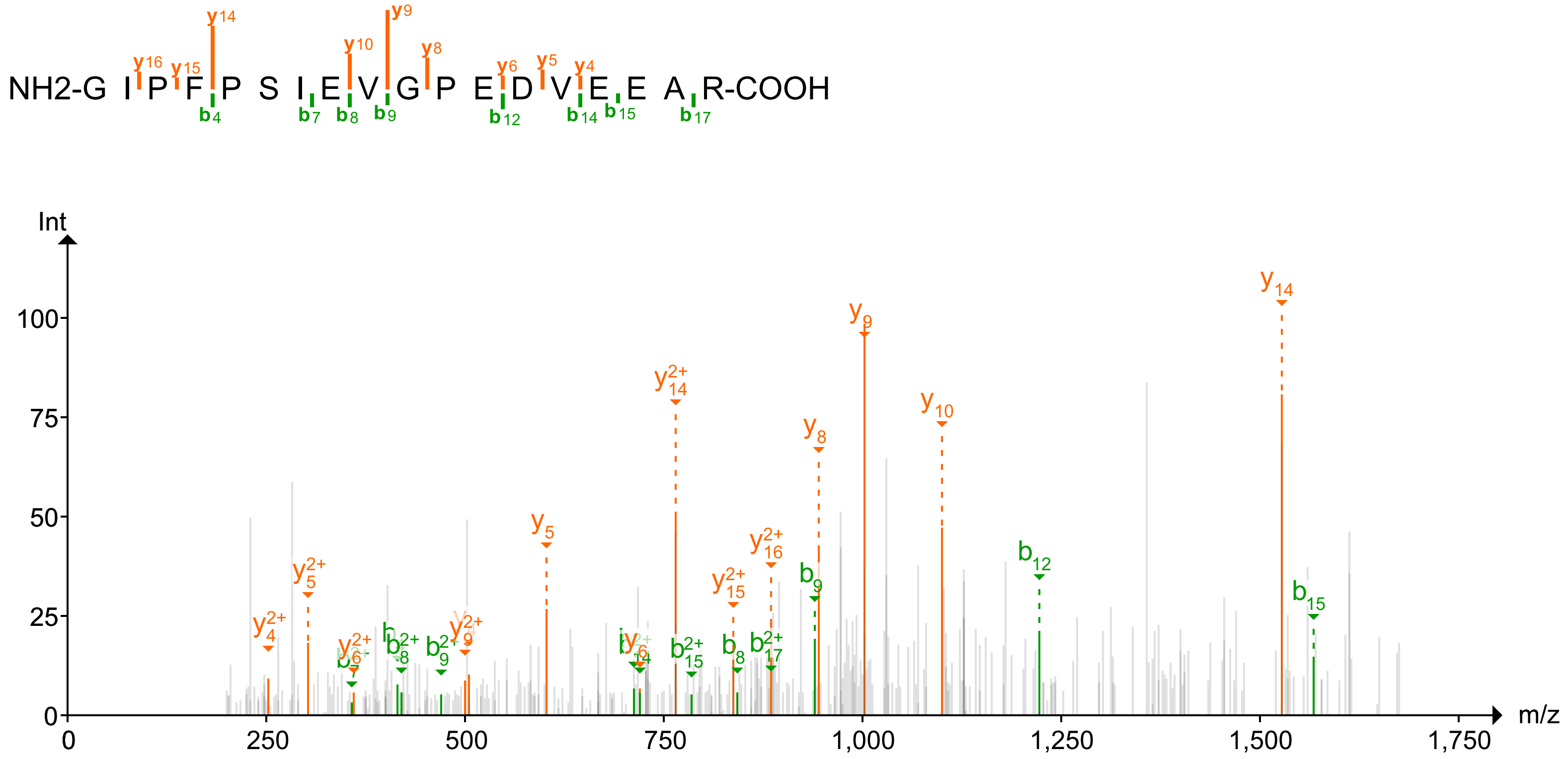

NH2-G I P F P S I E V G P E D V E E A R-COOH
y14 y16 y15 y10 y9 y8 y6 y5 y4
b4 b7 b8 b9 b12 b14 b15 b17
Int
m/z
0
25
50
75
100
0
250
500
750
1,000
1,250
1,500
1,750

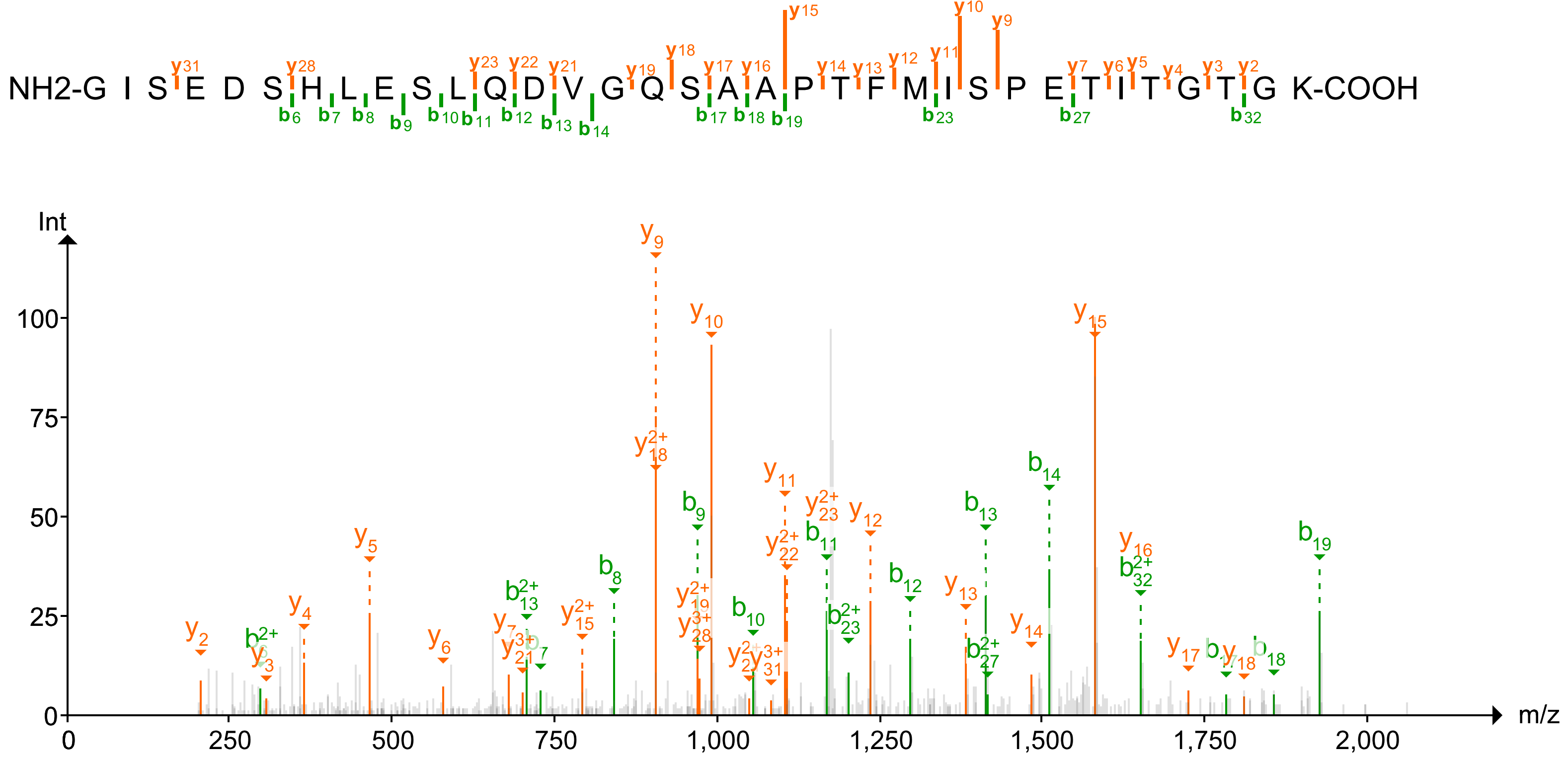

NH2-G I S E D S H L E S L Q D V G Q S A A P T F M I S P E T I T G T G K-COOH
y31 y28 y23 y22 y21 y19 y18 y17 y16 y15 y14 y13 y12 y11 y10 y9 y7 y6 y5 y4 y3 y2
b6 b7 b8 b9 b10 b11 b12 b13 b14 b17 b18 b19 b23 b27 b32
Int
100
75
50
25
0
250
500
750
1,000
1,250
1,500
1,750
2,000
m/z

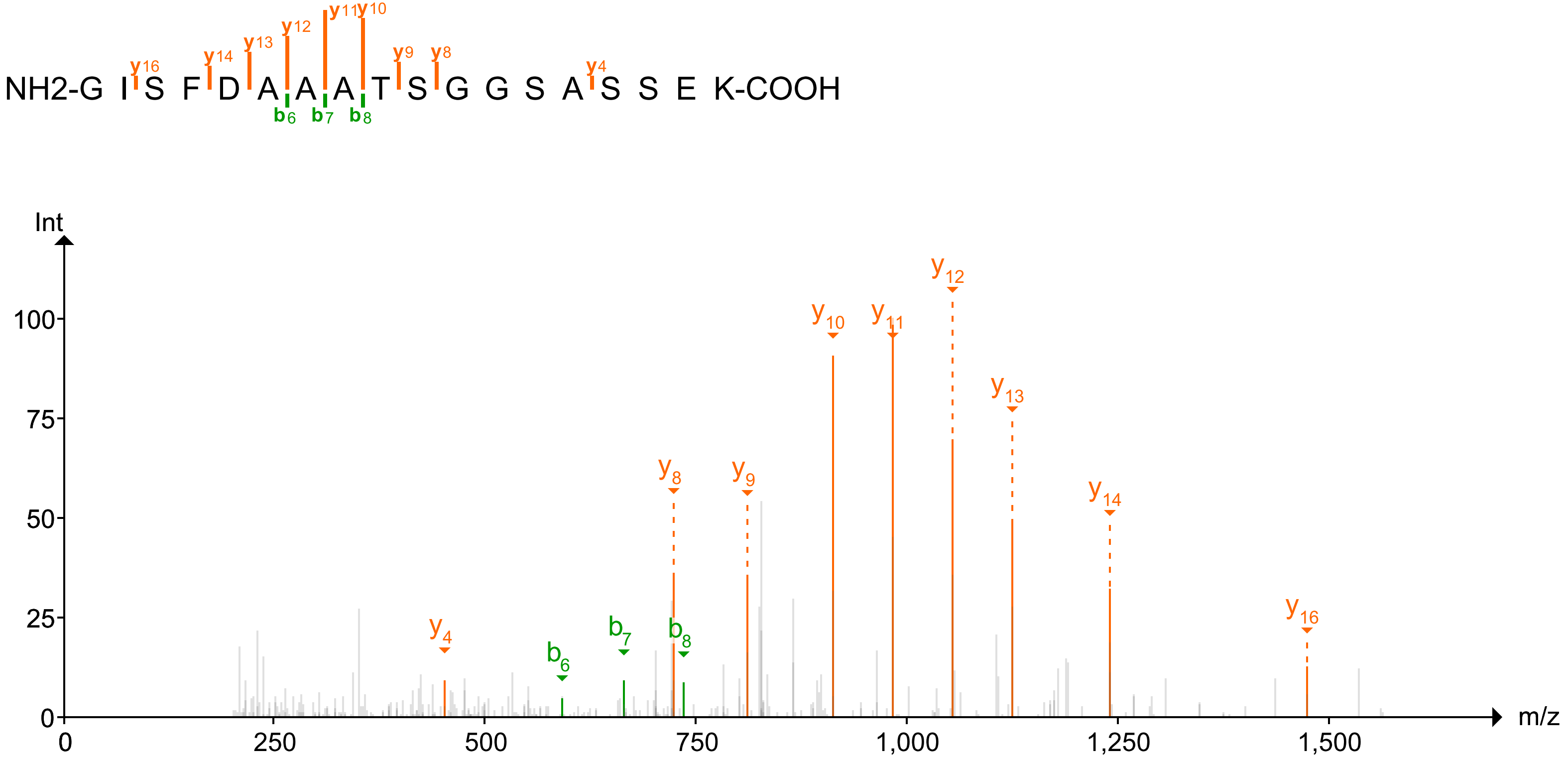

NH2-G I S F D A A A T S G G S A S S E K-COOH
y16 y14 y13 y12 y11 y10 y9 y8 y4
b6 b7 b8
Int
100
75
50
25
0
m/z
250
500
750
1,000
1,250
1,500
y4
b6
b7
b8
y8
y9
y10
y11
y12
y13
y14
y16

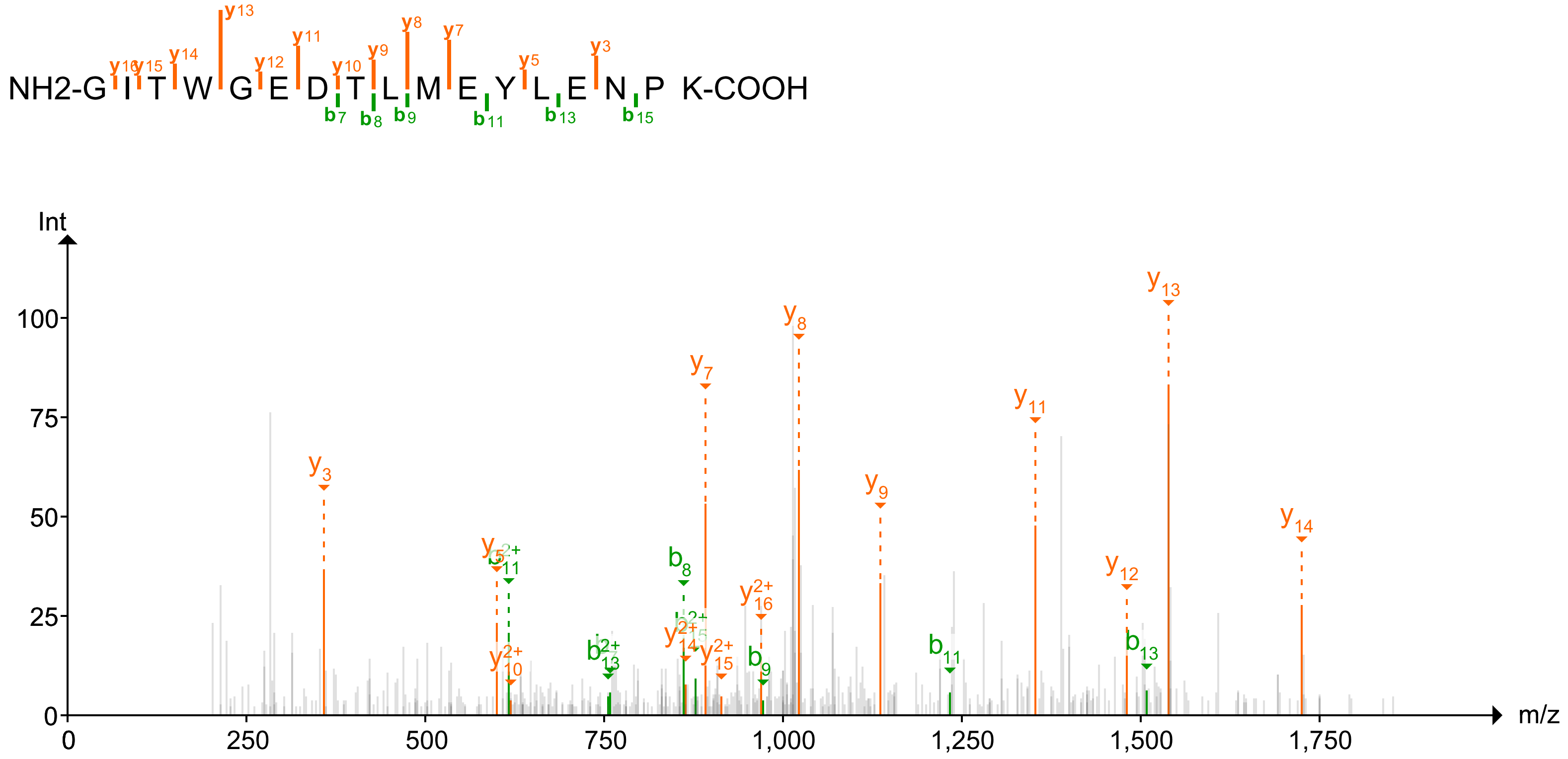

NH2-G I T W G E D T L M E Y L E N P K-COOH
y16 y15 y14 y13 y12 y11 y10 y9 y8 y7 y5 y3
b7 b8 b9 b11 b13 b15
Int
100
75
50
25
0
m/z
0
250
500
750
1,000
1,250
1,500
1,750
y3
y5
b2+ 11
y2+ 10
b2+ 13
b8
y2+ 14
y2+ 15
y7
y8
y2+ 16
b9
y9
b11
y11
y12
b13
y13
y14

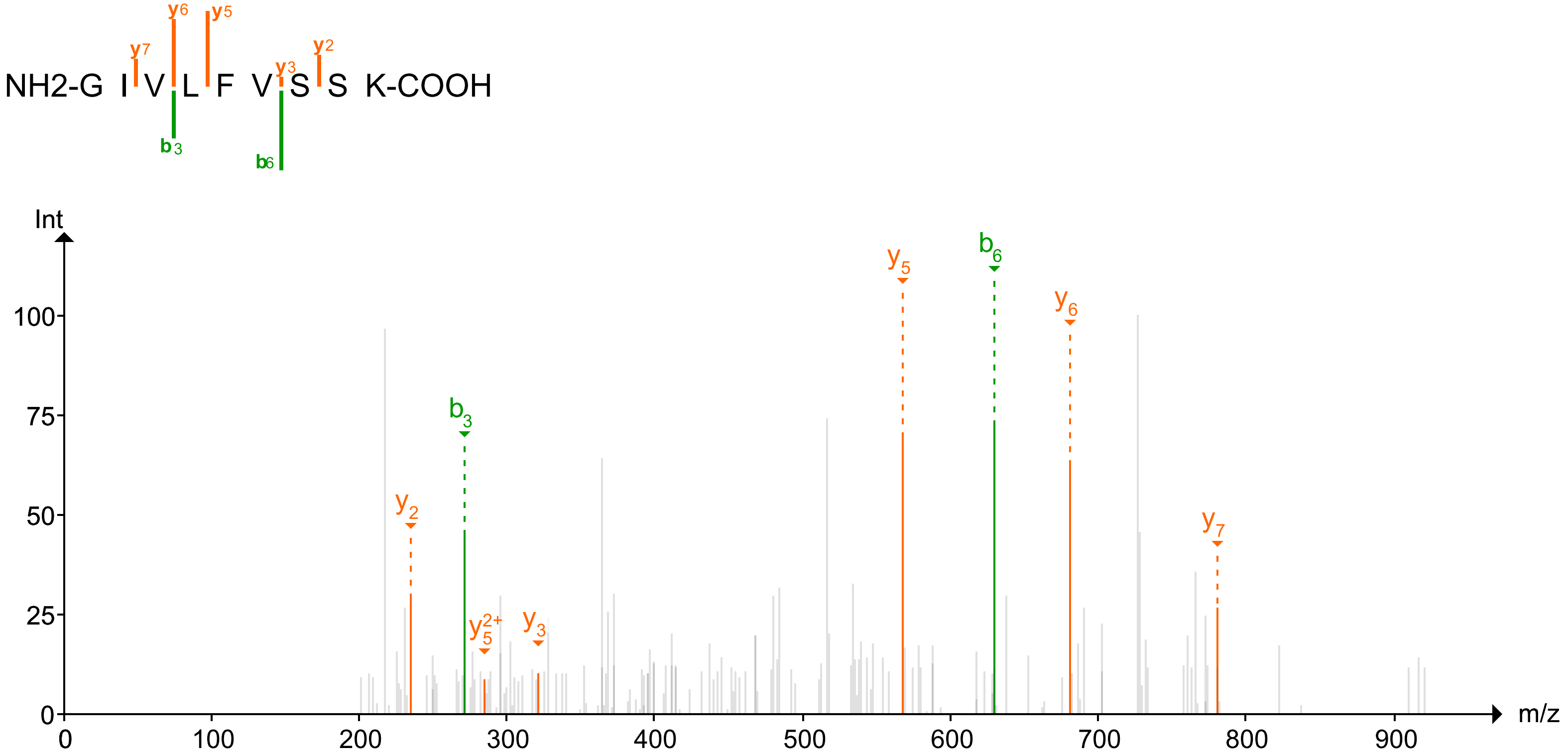

NH2-G I V L F V S S K-COOH
y7
y6
y5
y3
y2
b3
b6
Int
100
75
50
25
0
y2
b3
y5 2+
y3
y5
b6
y6
y7
0
100
200
300
400
500
600
700
800
900
m/z

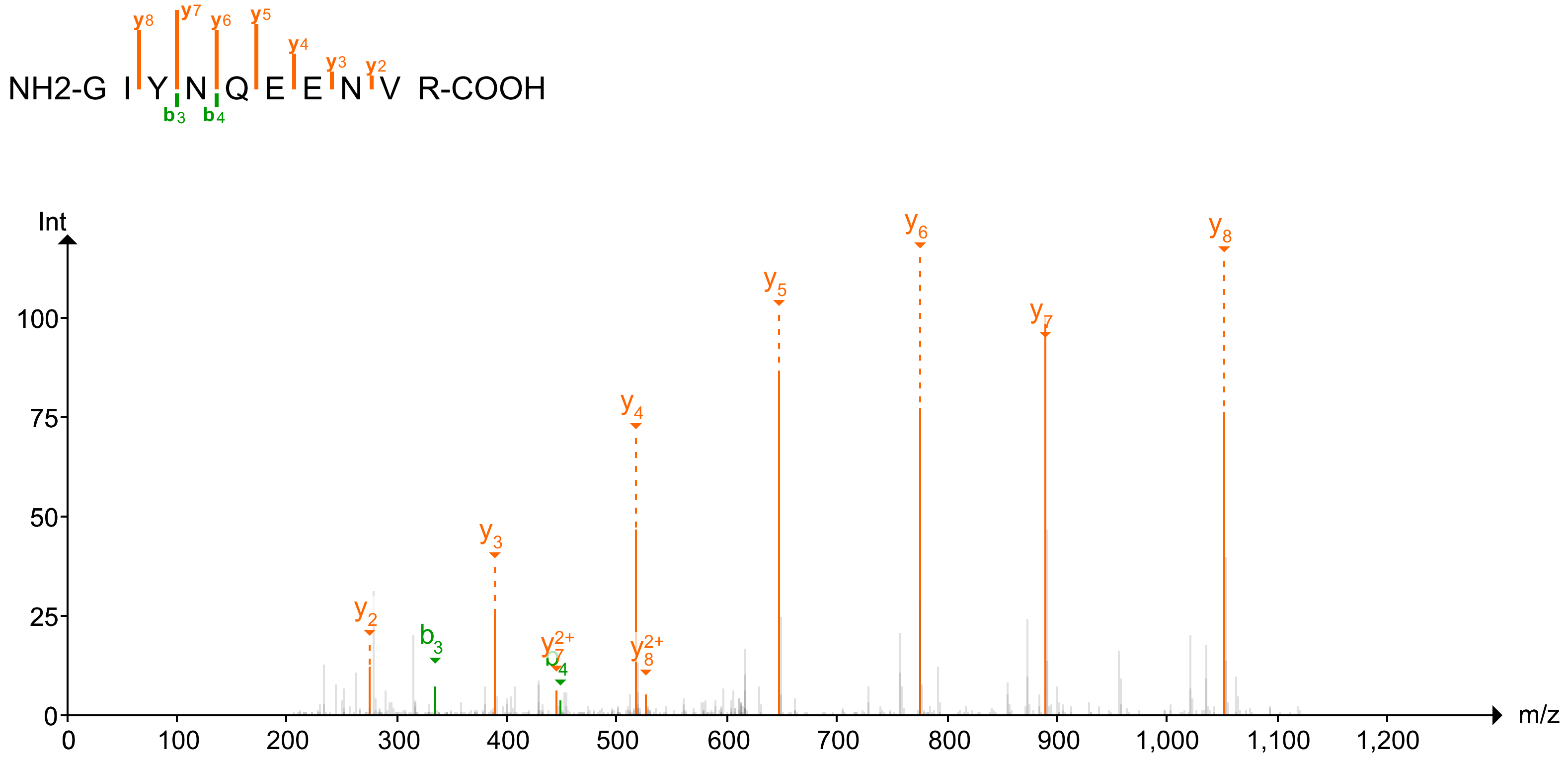

NH2-G I Y N Q E E N V R-COOH
y8 y7 y6 y5 y4 y3 y2
b3 b4
Int
m/z
0
25
50
75
100
0
100
200
300
400
500
600
700
800
900
1,000
1,100
1,200
y2
b3
y3
y7 2+
b4
y4
y8 2+
y5
y6
y7
y8

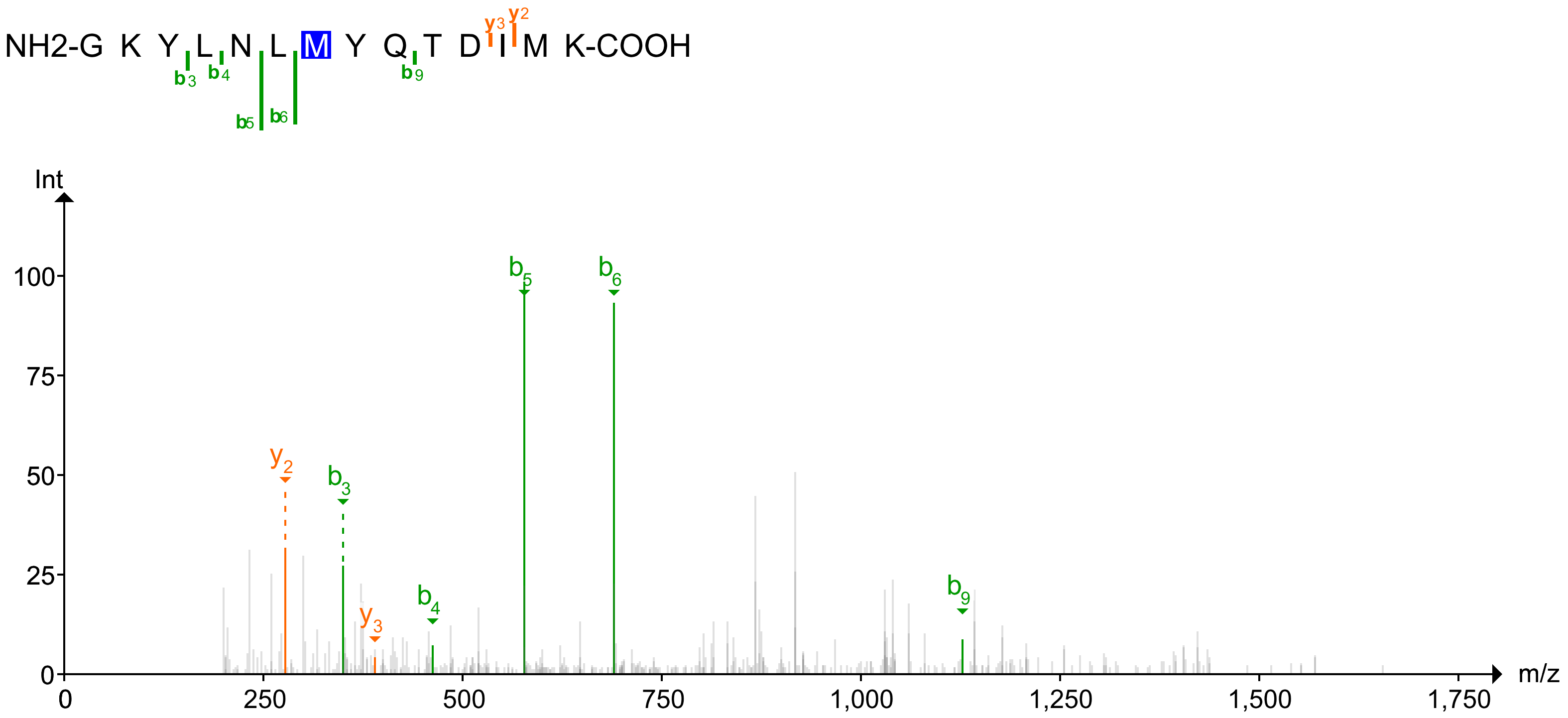

NH2-G K Y L N L M Y Q T D I M K-COOH
b3 b4 b5 b6 b9 y3 y2
Int
100
75
50
25
0
b5 b6 y2 b3 y3 b4 b9
0 250 500 750 1,000 1,250 1,500 1,750
m/z

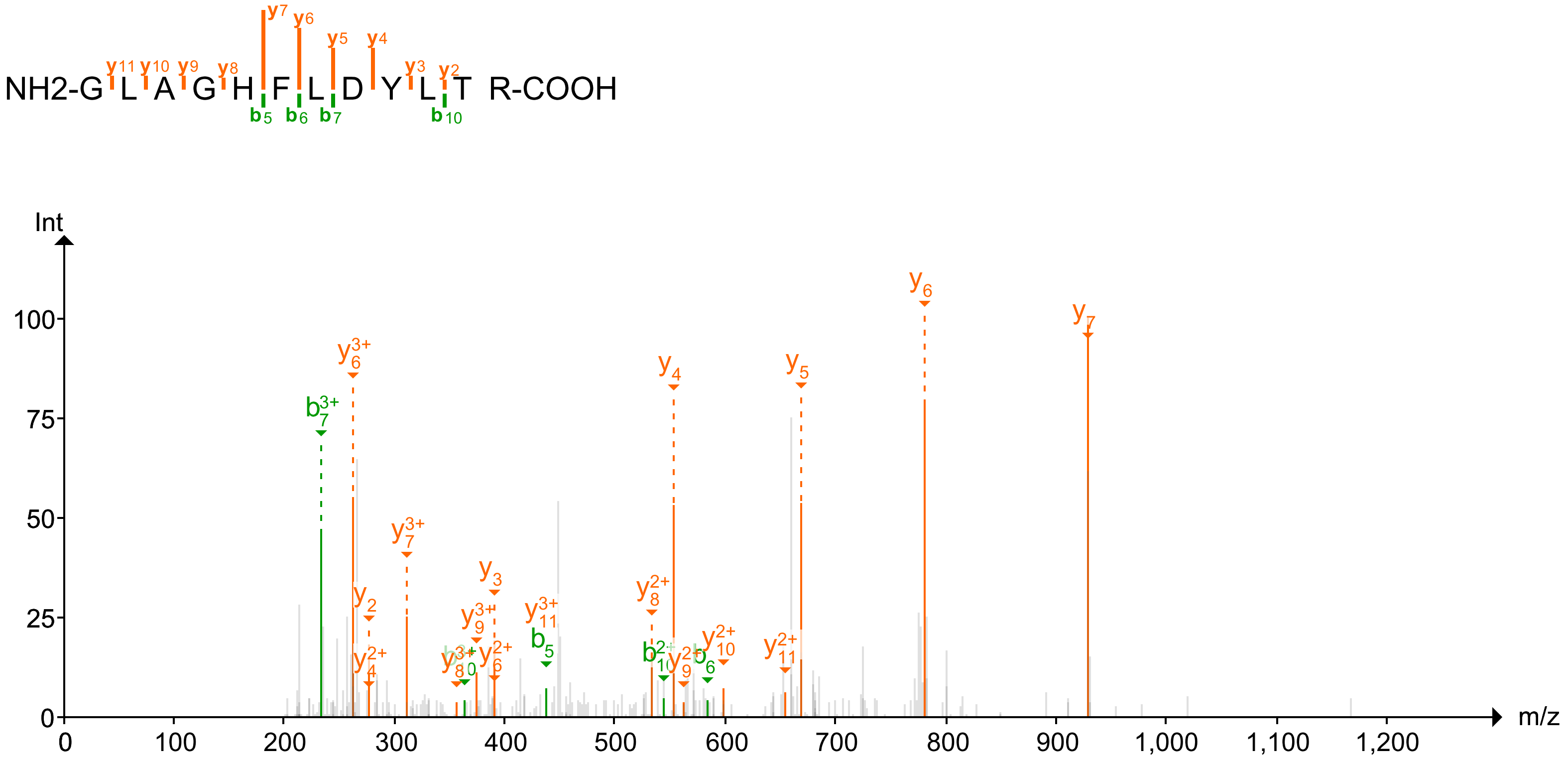
NH2-G L A G H F L D Y L T R-COOH
Int
m/z

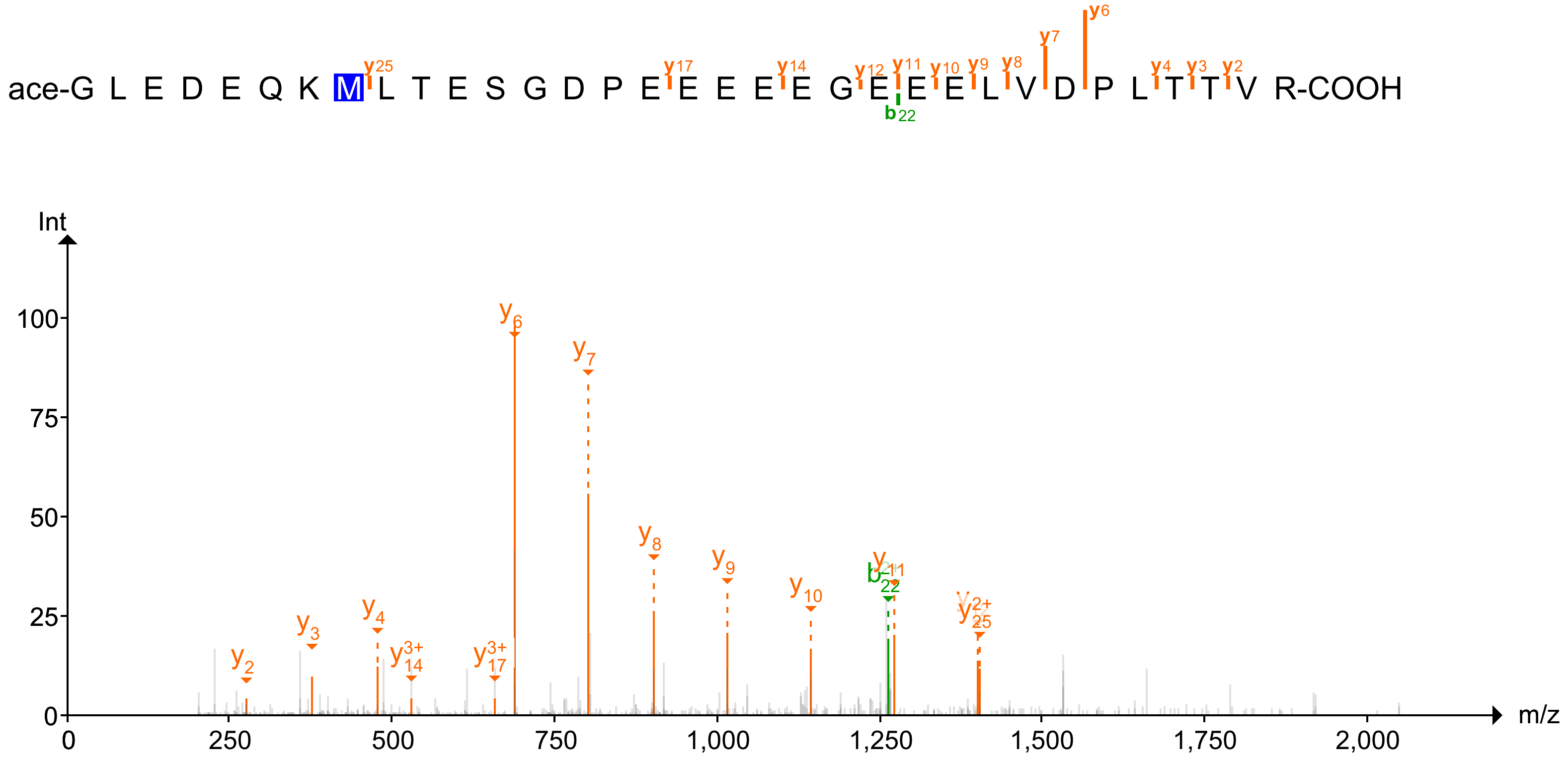

ace-G L E D E Q K M L T E S G D P E E E E E G E E E L V D P L T T V R-COOH
y25 y17 y14 y12 y11 y10 y9 y8 y7 y6 y4 y3 y2
b22
Int
100
75
50
25
0
m/z
0
250
500
750
1,000
1,250
1,500
1,750
2,000
$y_2$ $y_3$ $y_4$ $y_{14}^{3+}$ $y_{17}^{3+}$ $y_6$ $y_7$ $y_8$ $y_9$ $y_{10}$ $b_{22}$ $y_{11}$ $y_{25}^{2+}$

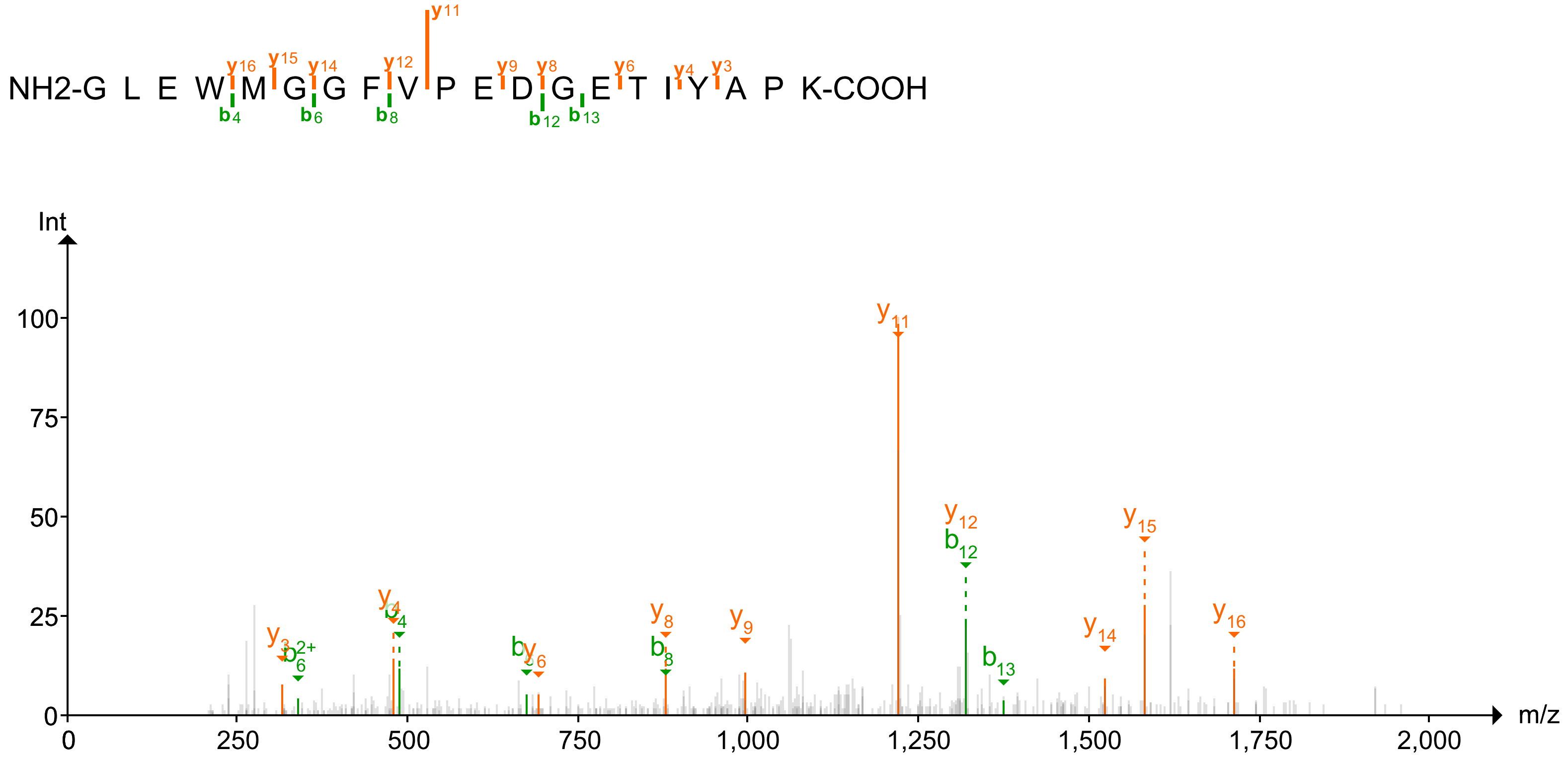
NH2-G L E W M G G F V P E D G E T I Y A P K-COOH
y11
y16
y15
y14
y12
y9
y8
y6
y4
y3
b4
b6
b8
b12
b13
Int
100
75
50
25
0
m/z
0
250
500
750
1,000
1,250
1,500
1,750
2,000

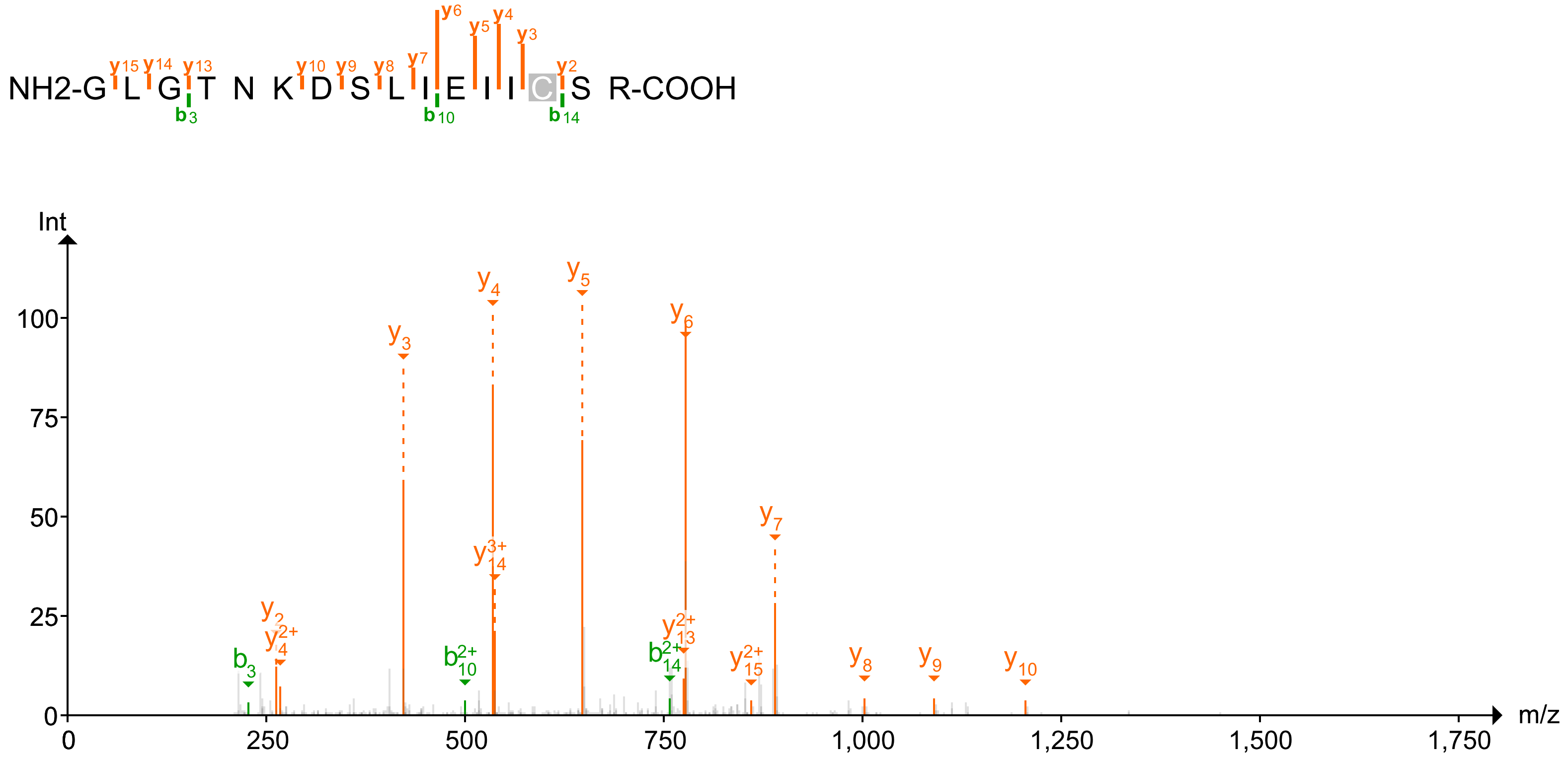

NH2-G L G T N K D S L I E I I C S R-COOH
y15 y14 y13 y10 y9 y8 y7 y6 y5 y4 y3 y2
b3 b10 b14
Int
100
75
50
25
0
y3
y4
y5
y6
y7
y2
b3
b10 2+
b14 2+
y8
y9
y10
m/z
0
250
500
750
1,000
1,250
1,500
1,750

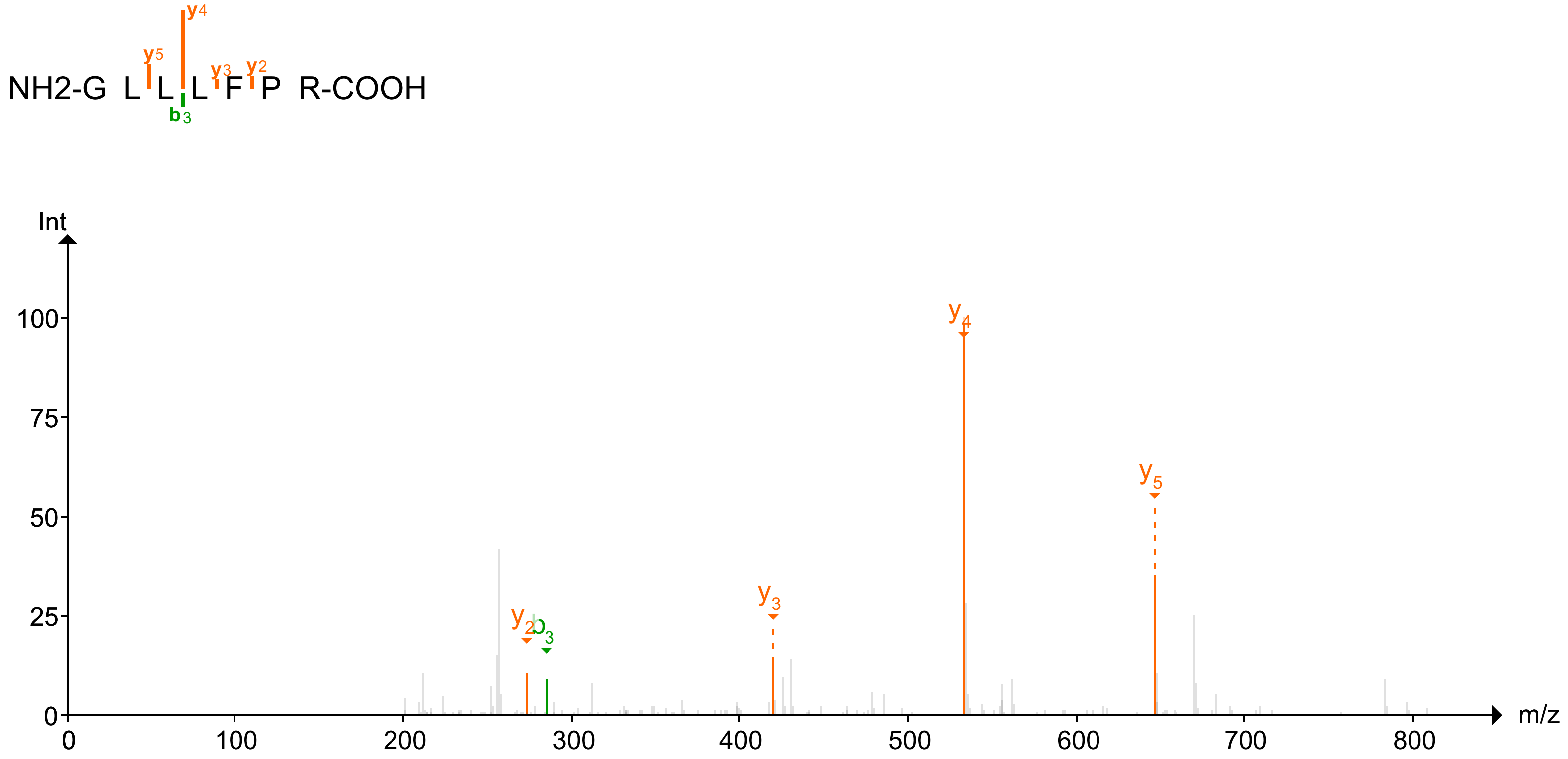

NH2-G L L L F P R-COOH
y5 y4 y3 y2
b3
Int
100
75
50
25
0
0 100 200 300 400 500 600 700 800
m/z
y4
y5
y3
y2
b3

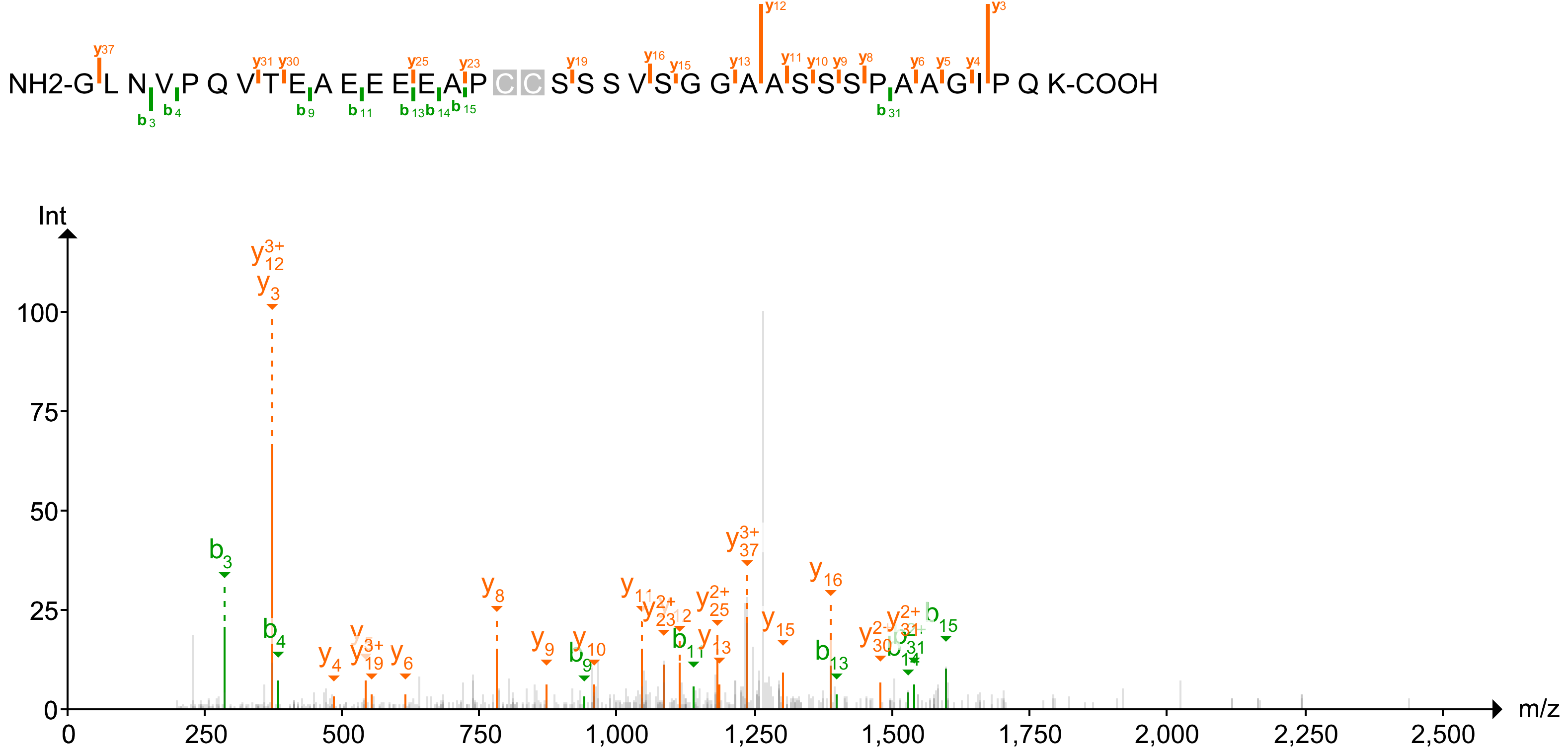

NH2-G L N V P Q V T E A E E E E A P C C S S S V S G G A A S S S P A A G I P Q K-COOH
y37 y31 y30 y25 y23 y19 y16 y15 y13 y12 y11 y10 y9 y8 y6 y5 y4 y3
b3 b4 b9 b11 b13 b14 b15 b31
Int
m/z
0
25
50
75
100
250
500
750
1,000
1,250
1,500
1,750
2,000
2,250
2,500

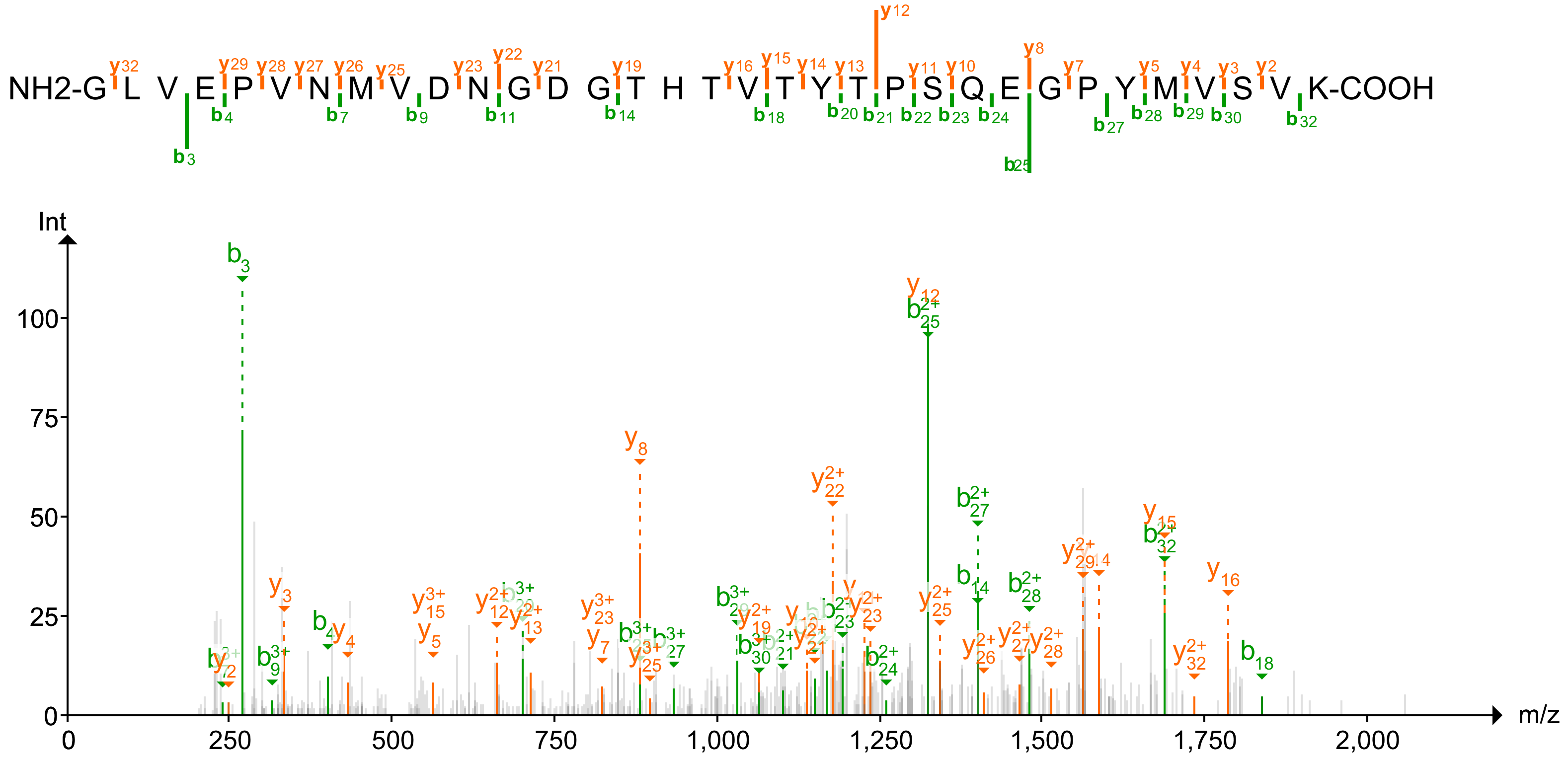

NH2-G L V E P V N M V D N G D G T H T V T Y T P S Q E G P Y M V S V K-COOH
Int
m/z
0
25
50
75
100
0
250
500
750
1,000
1,250
1,500
1,750
2,000

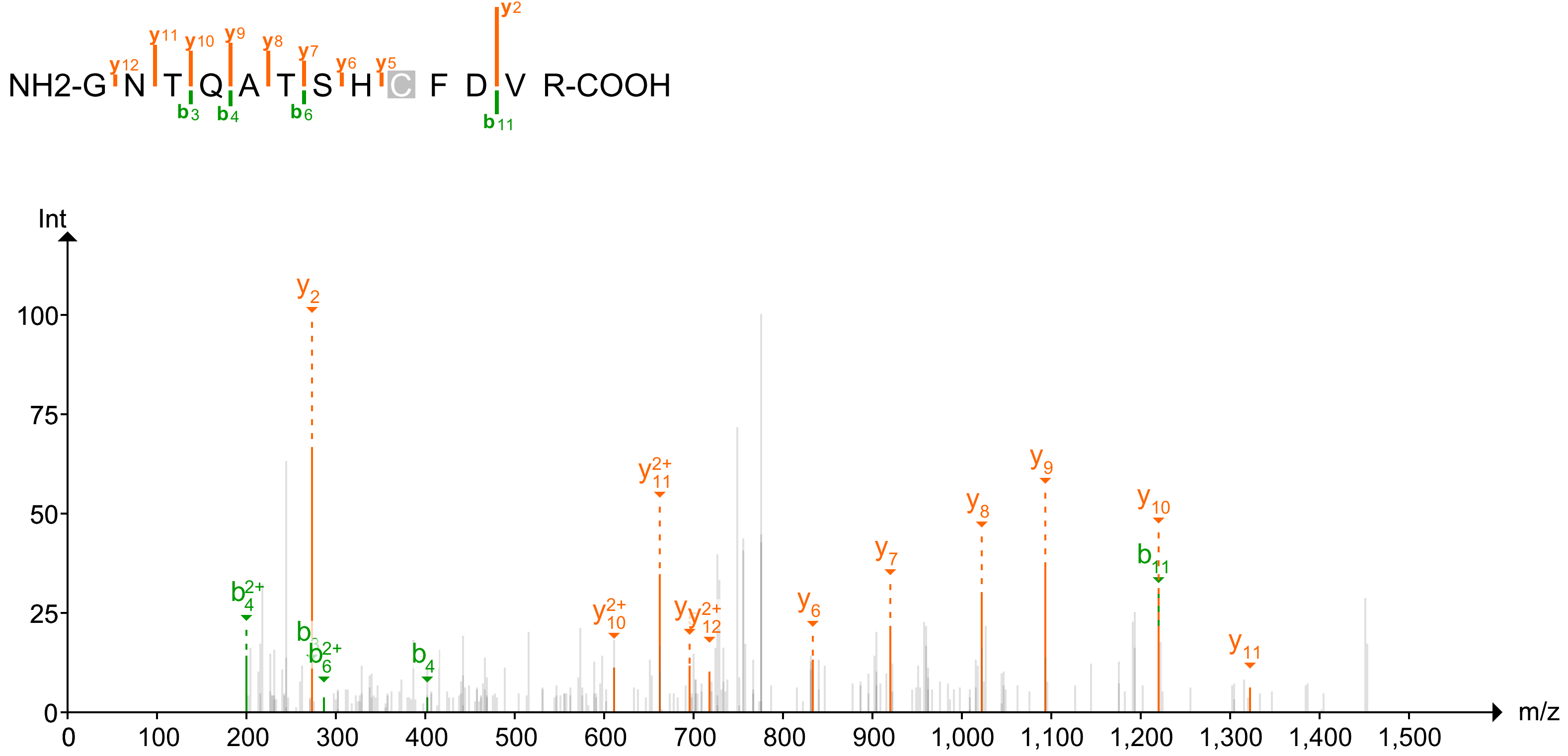

NH2-G N T Q A T S H C F D V R-COOH
y12 y11 y10 y9 y8 y7 y6 y5 y2
b3 b4 b6 b11
Int
100
75
50
25
0
m/z
0 100 200 300 400 500 600 700 800 900 1,000 1,100 1,200 1,300 1,400 1,500

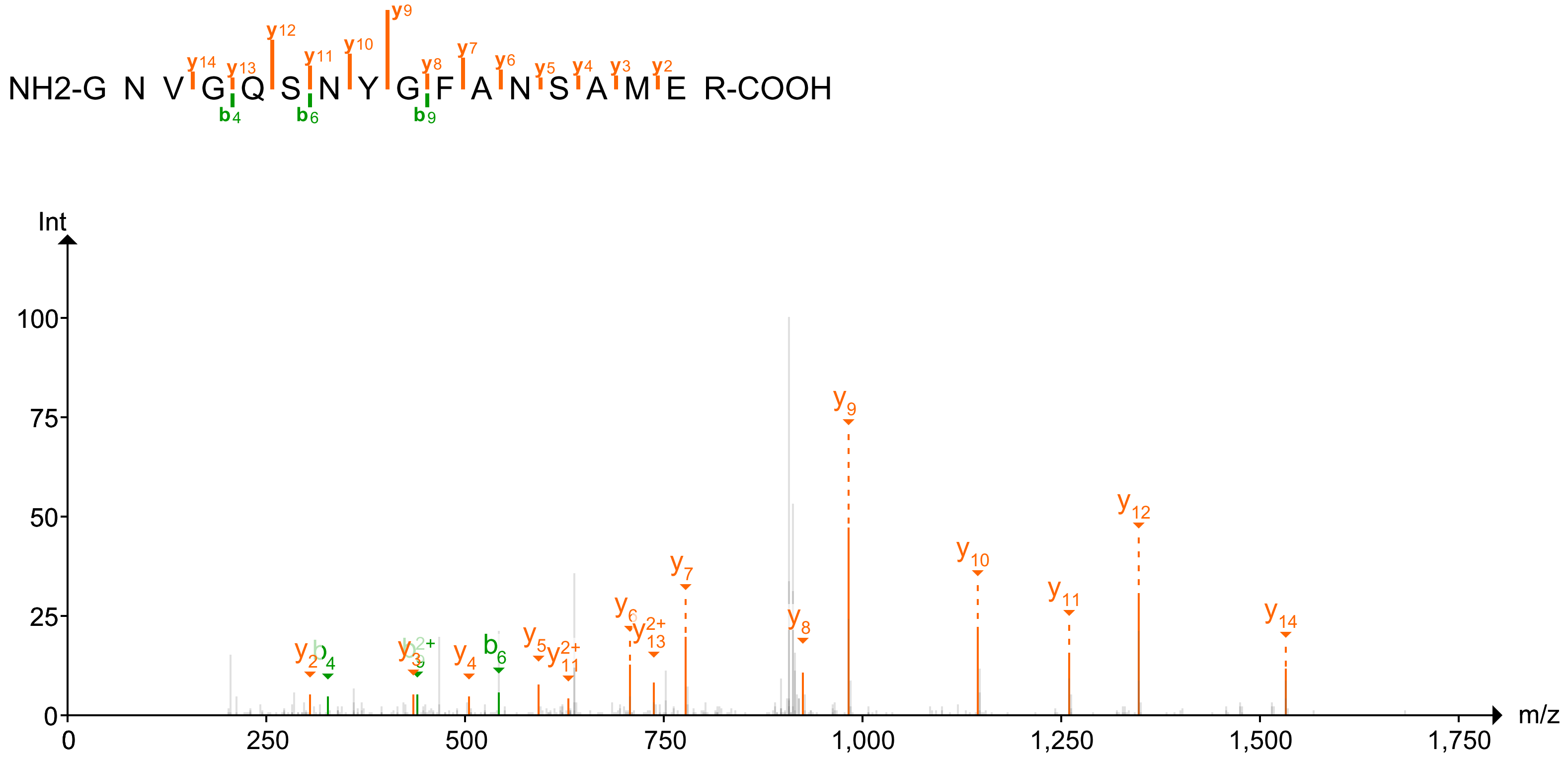

NH2-G N V G Q S N Y G F A N S A M E R-COOH
y14 y13 y12 y11 y10 y9 y8 y7 y6 y5 y4 y3 y2
b4 b6 b9
Int
100
75
50
25
0
m/z
0
250
500
750
1,000
1,250
1,500
1,750

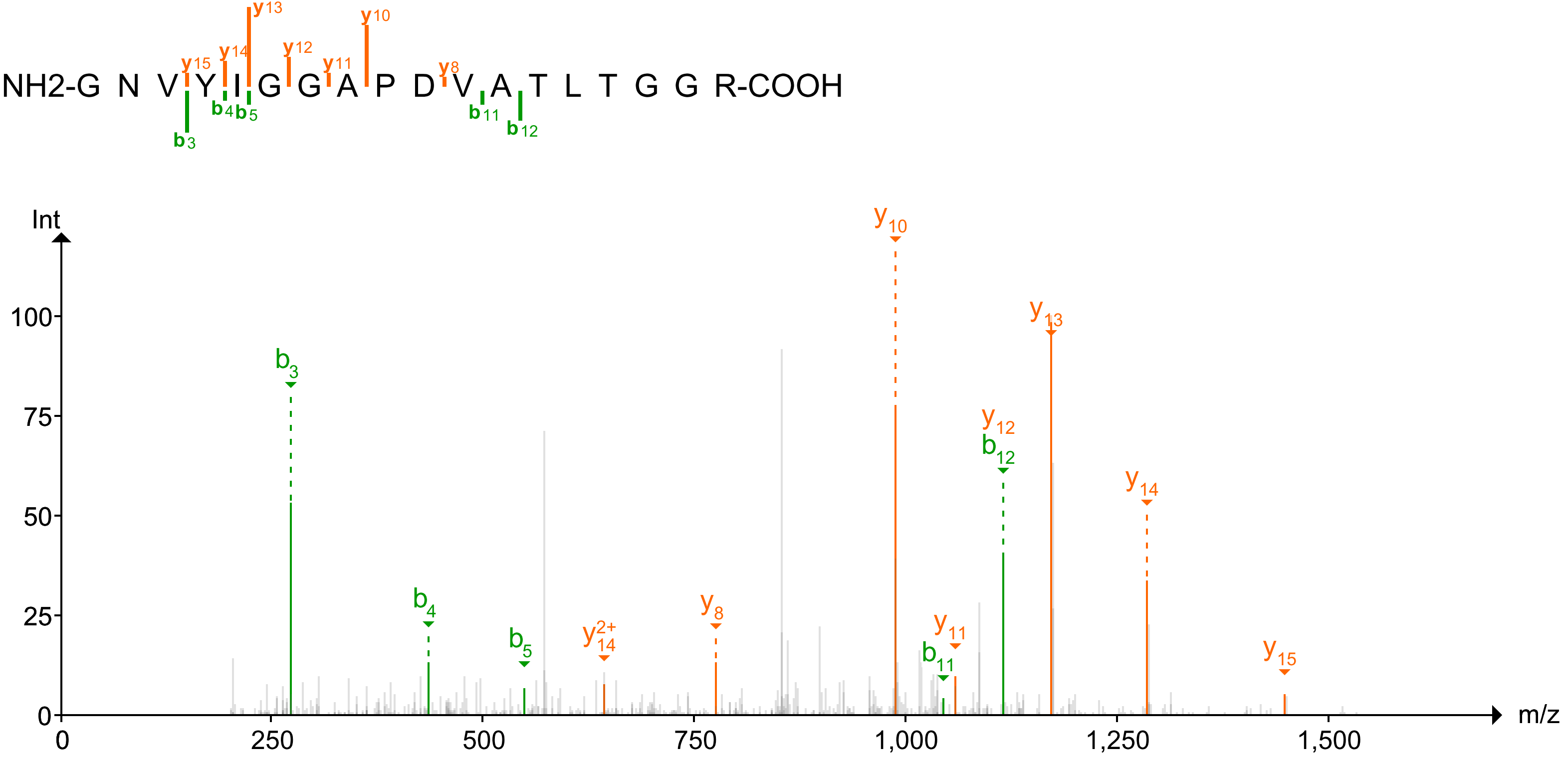

NH2-G N V Y I G G A P D V A T L T G G R-COOH
y15 y14 y13 y12 y11 y10 y8
b3 b4 b5 b11 b12
Int
m/z
0
25
50
75
100
250
500
750
1,000
1,250
1,500
b3
b4
b5
y14 2+
y8
y10
b11
y11
y12
b12
y13
y14
y15

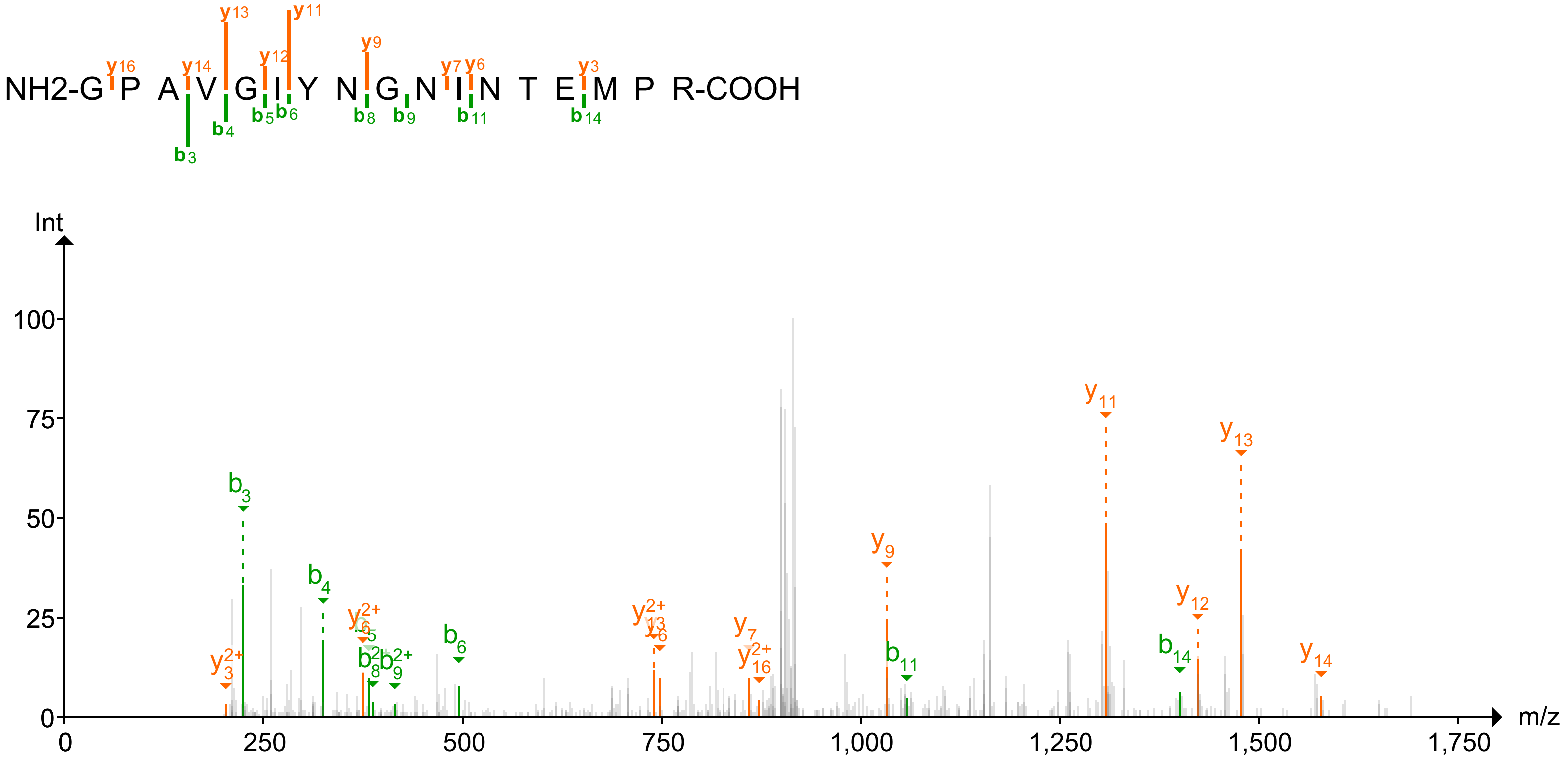

NH2-G P A V G I Y N G N I N T E M P R-COOH
y16 y14 y13 y12 y11 y9 y7 y6 y3
b3 b4 b5 b6 b8 b9 b11 b14
Int
100
75
50
25
0
m/z
0
250
500
750
1,000
1,250
1,500
1,750
b3
b4
b6
b11
b14
y9
y11
y13
y12
y14
y7

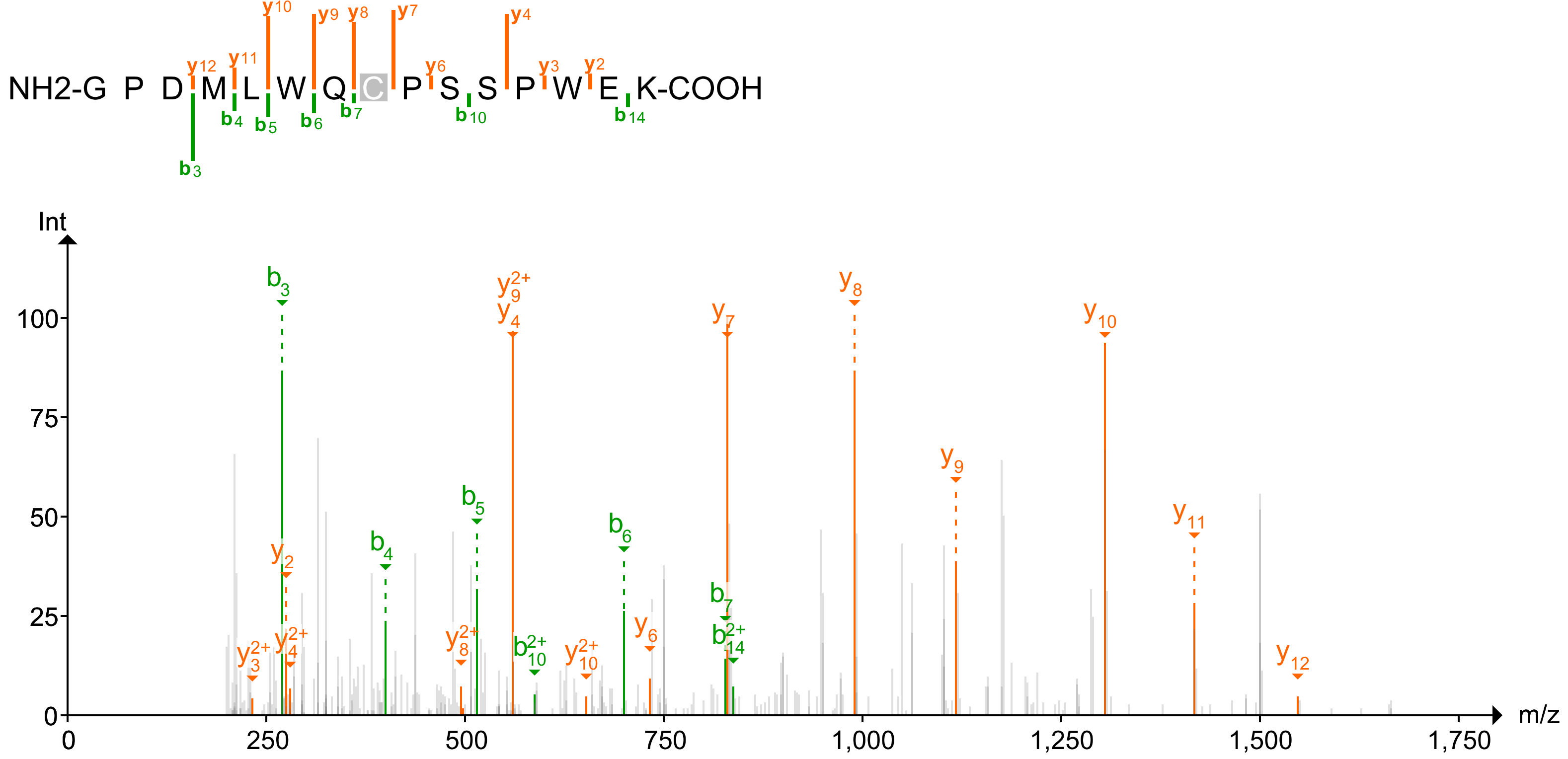

NH2-G P D M L W Q C P S S P W E K-COOH
y12 y11 y10 y9 y8 y7 y6 y4 y3 y2
b3 b4 b5 b6 b7 b10 b14
Int
100
75
50
25
0
m/z
0
250
500
750
1,000
1,250
1,500
1,750
b3
y9 2+
y4
y7
y8
y10
y9
y11
y12
y2
b4
b5
b6
b7
b14 2+
b10 2+
y3 2+
y4 2+
y8 2+
y10 2+
y6

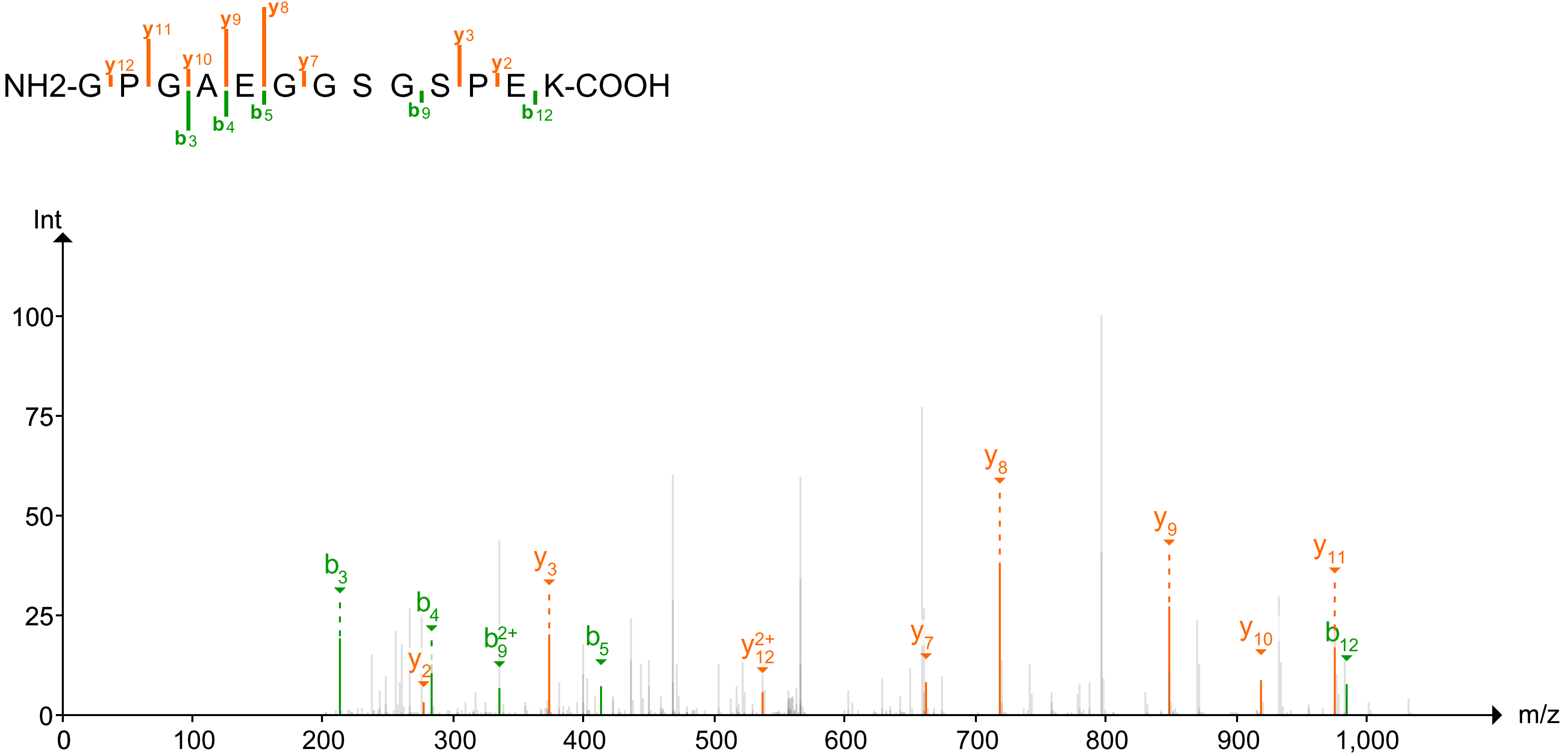

NH2-G P G A E G G S G S P E K-COOH
y12 y11 y10 y9 y8 y7 y3 y2
b3 b4 b5 b9 b12
Int
100
75
50
25
0
m/z
0 100 200 300 400 500 600 700 800 900 1,000
b3
b4
$b_9^{2+}$
b5
y2
y3
$y_{12}^{2+}$
y7
y8
y9
y10
y11
b12

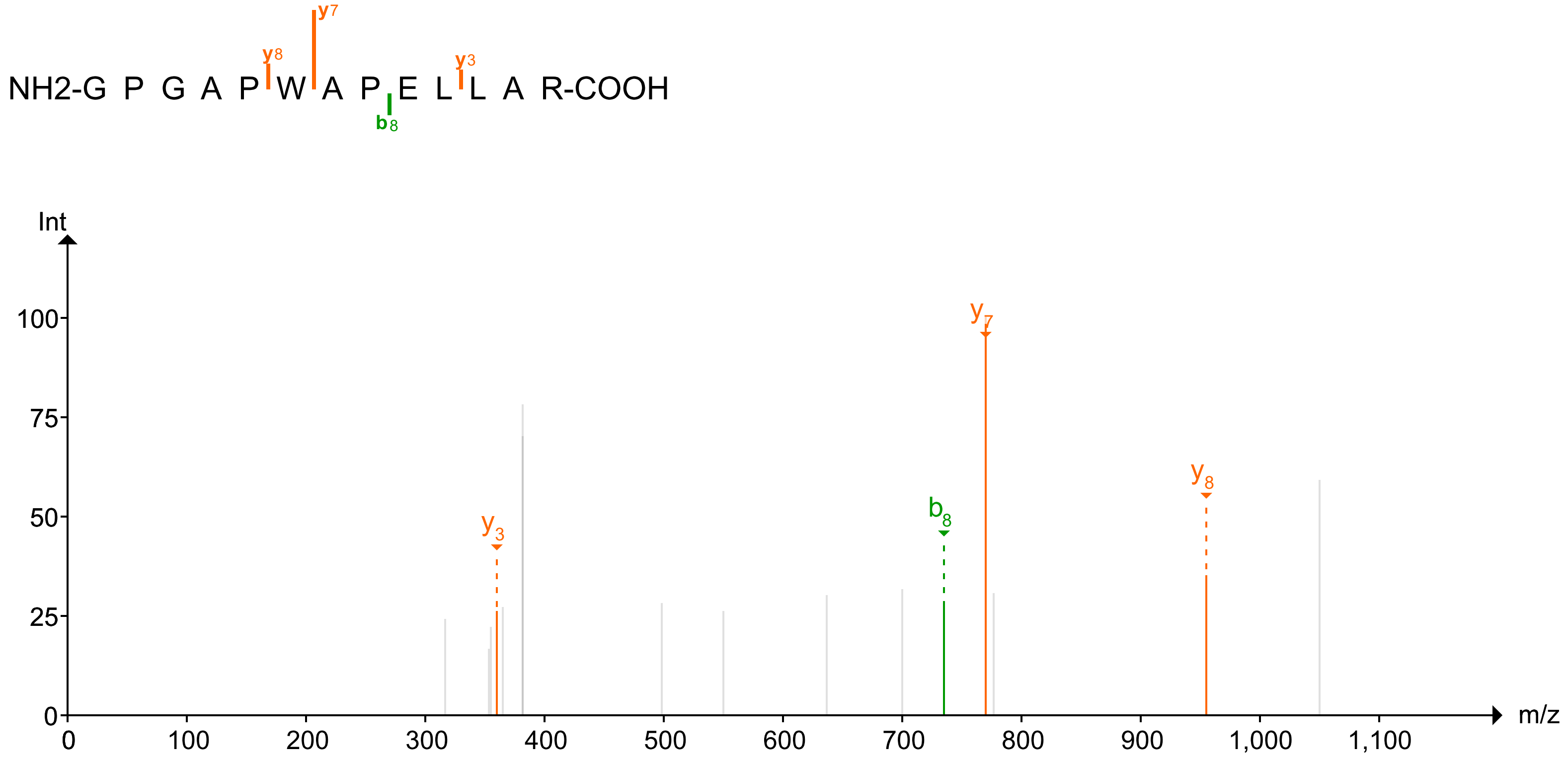

NH2-G P G A P W A P E L L A R-COOH
y7
y8
y3
b8
Int
100
75
50
25
0
0
100
200
300
400
500
600
700
800
900
1,000
1,100
m/z

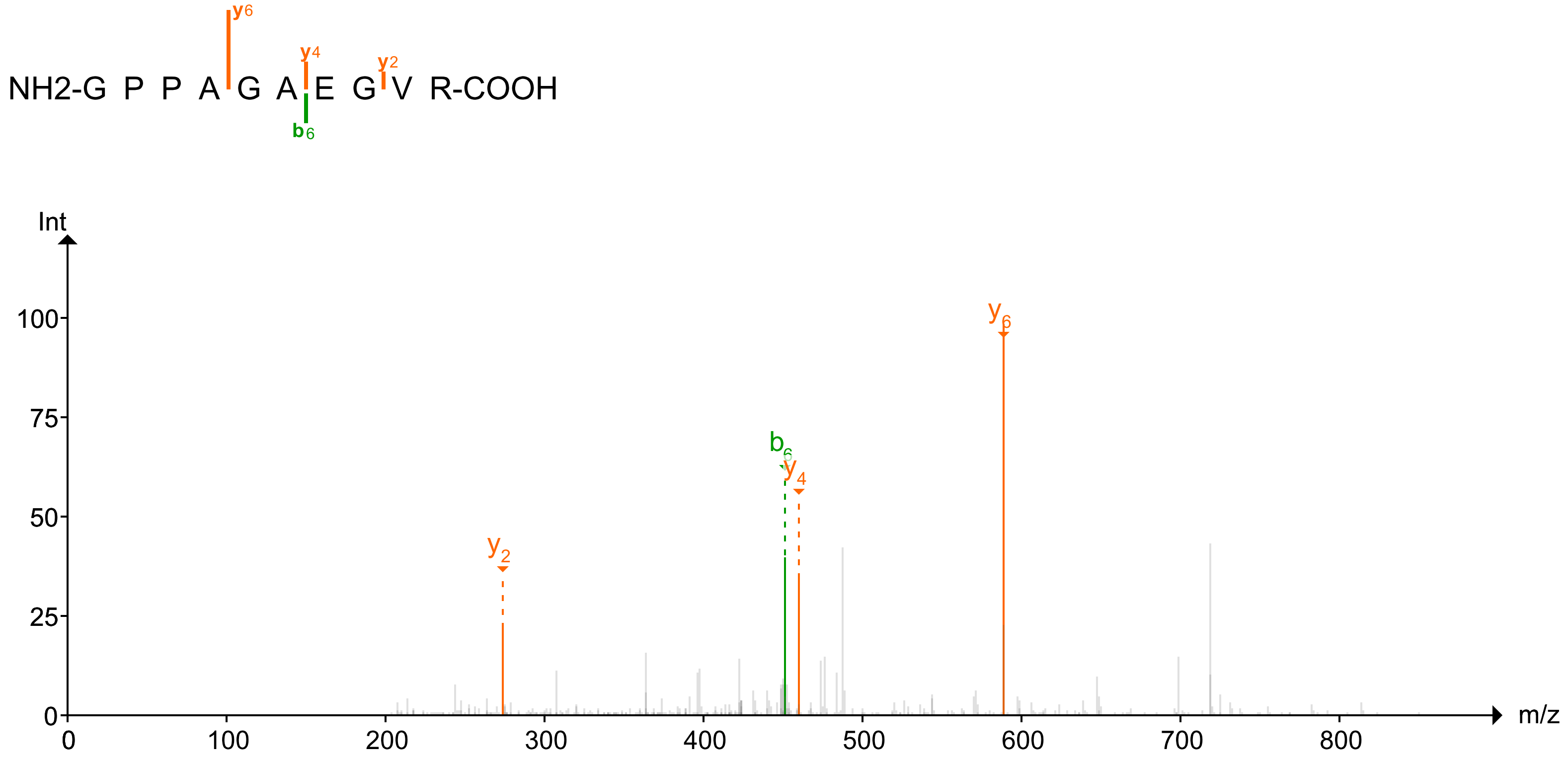

NH2-G P P A G A E G V R-COOH
y6
y4
y2
b6
Int
m/z
0
25
50
75
100
0
100
200
300
400
500
600
700
800

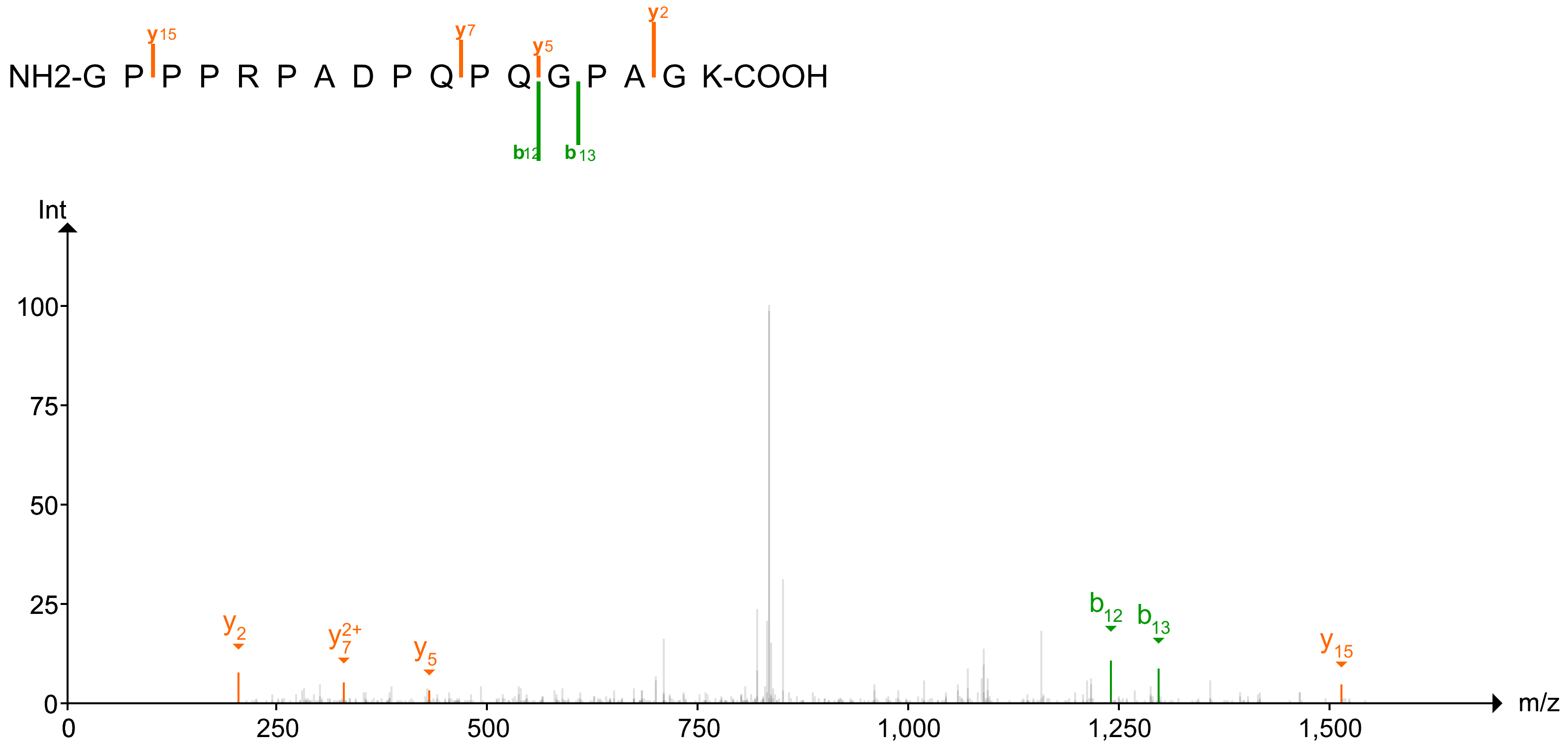

NH2-G P P P R P A D P Q P Q G P A G K-COOH
y15
y7
y5
y2
b12
b13
Int
100
75
50
25
0
y2
y7 2+
y5
b12
b13
y15
0
250
500
750
1,000
1,250
1,500
m/z

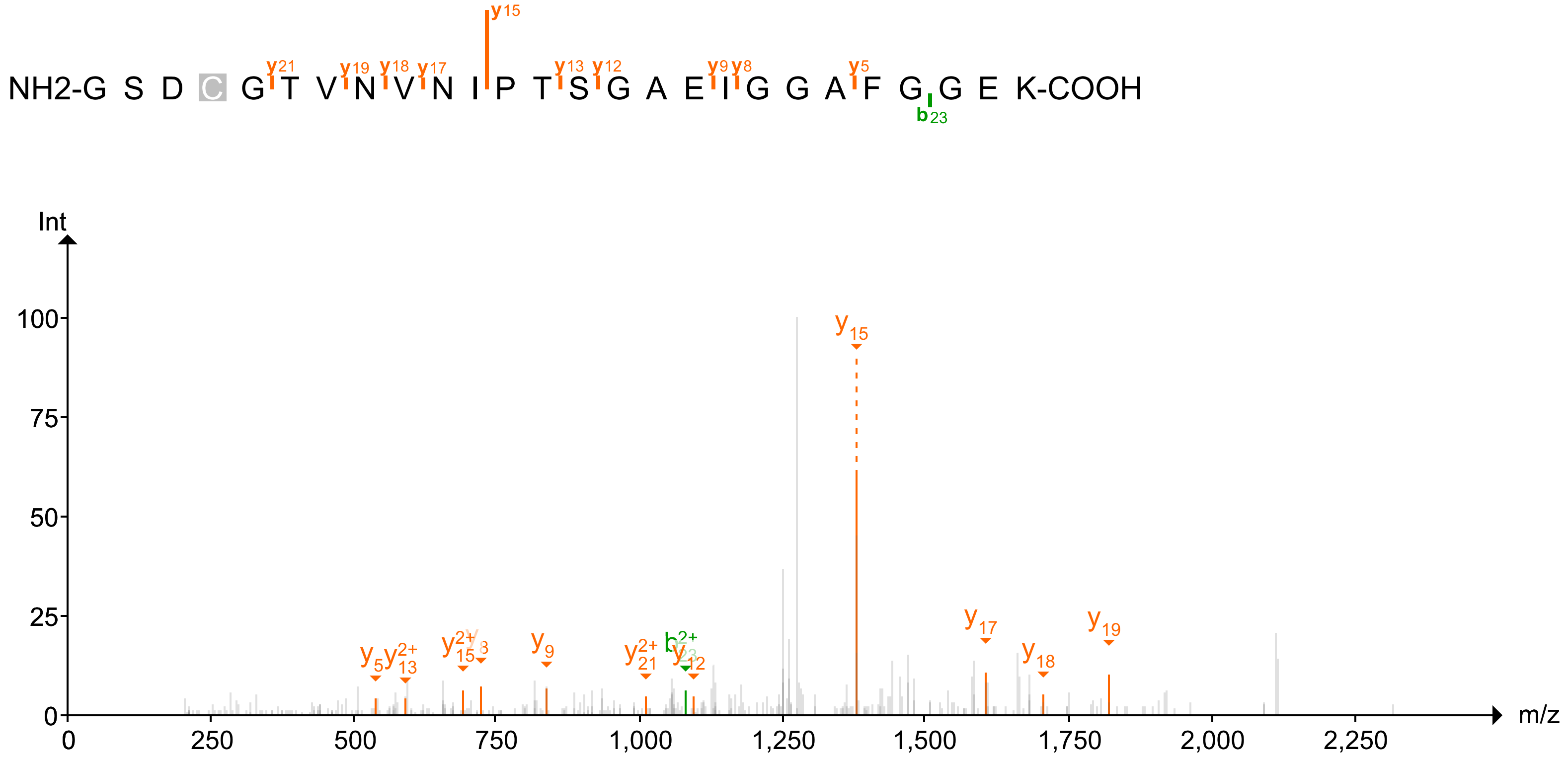

NH2-G S D C G T V N V N I P T S G A E I G G A F G G E K-COOH
y21 y19 y18 y17 y15 y13 y12 y9 y8 y5
b23
Int
m/z
0
25
50
75
100
0
250
500
750
1,000
1,250
1,500
1,750
2,000
2,250
y15
y17
y19
y18
y5
y13 2+
y15 2+
y9
y21 2+
b23 2+
y12

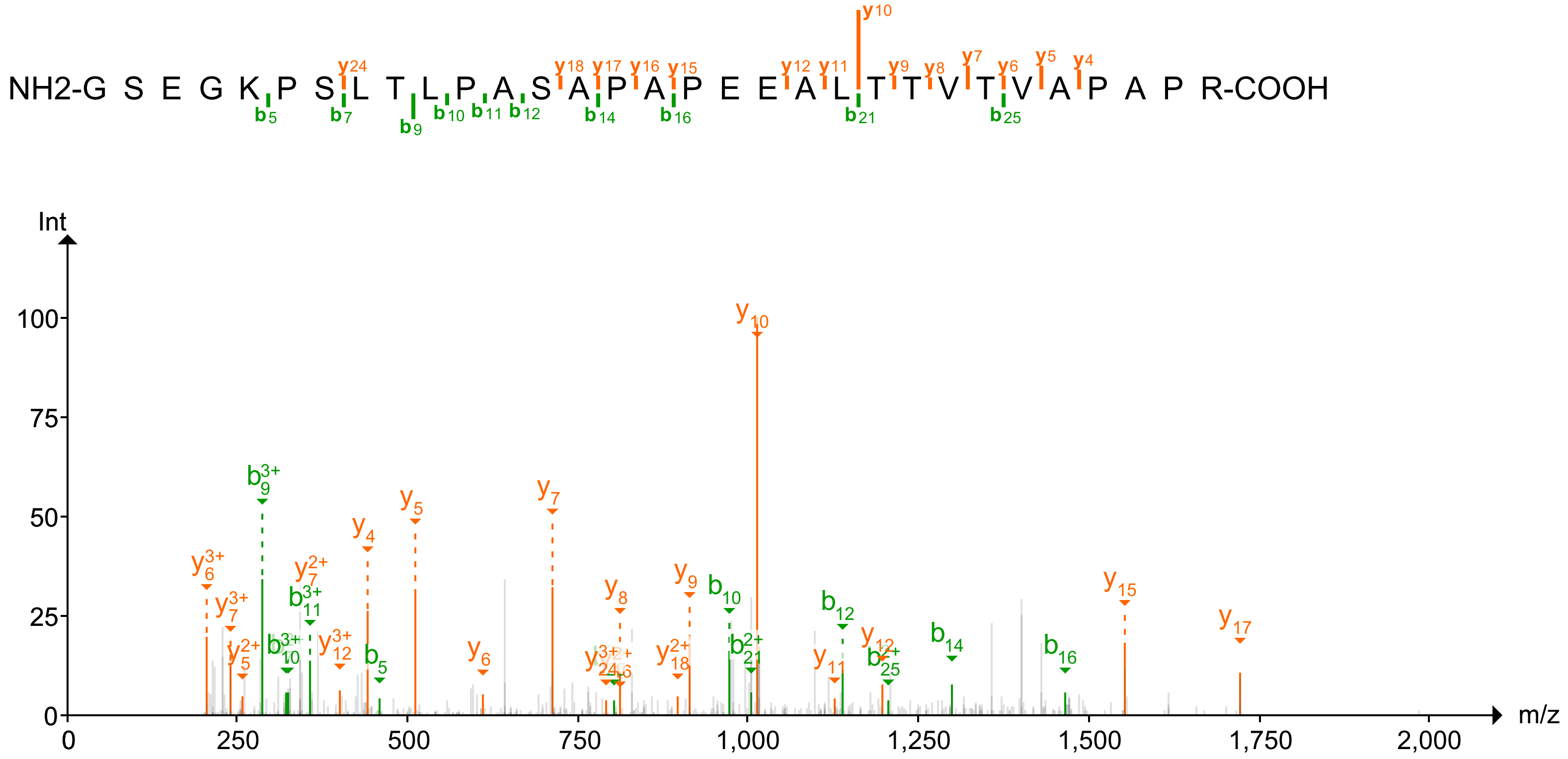

NH2-G S E G K P S L T L P A S A P A P E E A L T T V T V A P A P R-COOH
y24 y18 y17 y16 y15 y12 y11 y10 y9 y8 y7 y6 y5 y4
b5 b7 b9 b10 b11 b12 b14 b16 b21 b25
Int
100
75
50
25
0
m/z
0
250
500
750
1,000
1,250
1,500
1,750
2,000
y10
b9 3+
y5
y7
y4
y6 3+
y7 2+
b11 3+
y7 3+
y5 2+
b10 3+
y12 3+
b5
y6
y8
y9
b10
y18 2+
b12
b21 2+
y11
y12
b25 2+
b14
b16
y15
y17

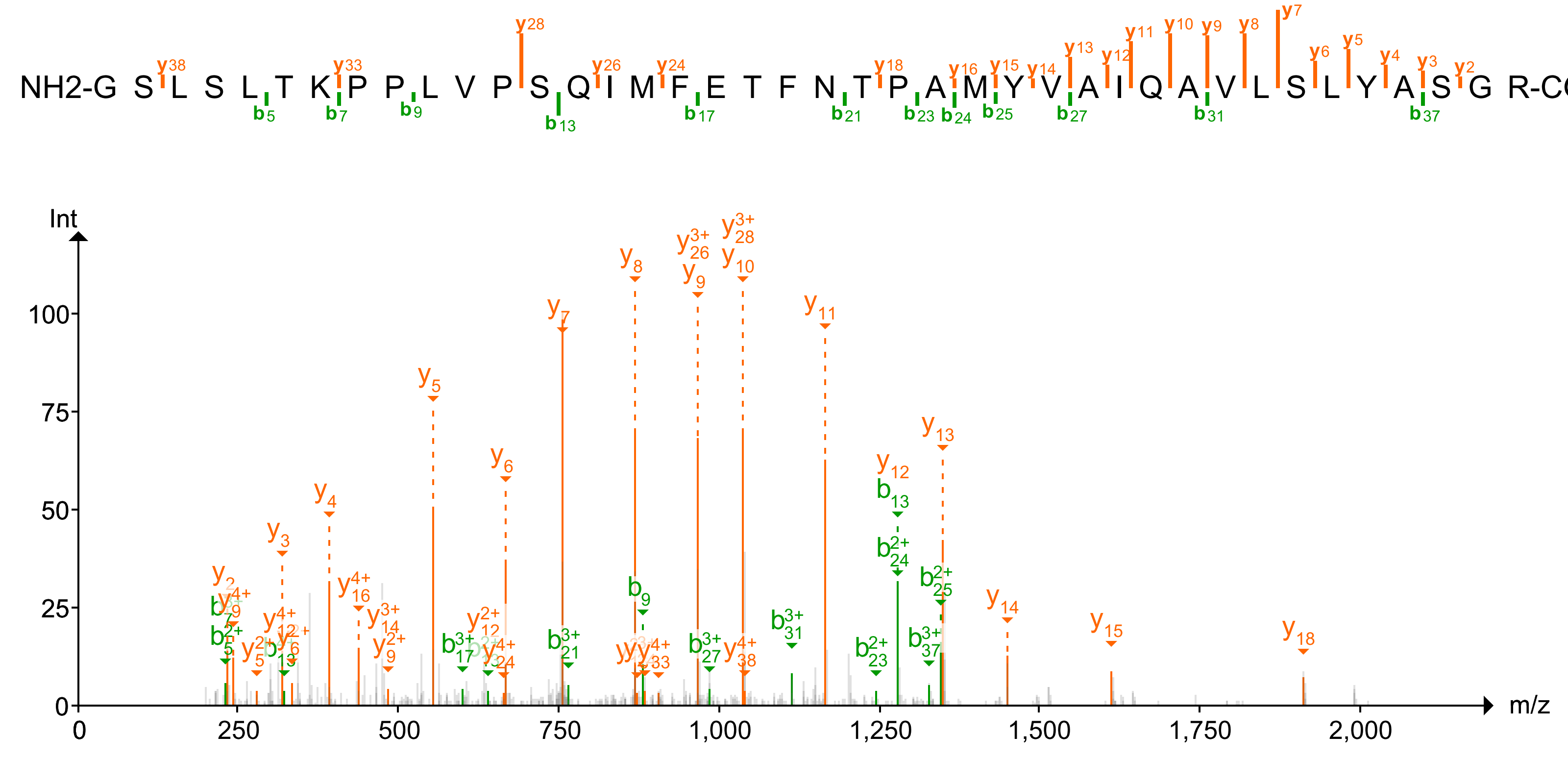

NH2-G S L S L T K P P L V P S Q I M F E T F N T P A M Y V A I Q A V L S L Y A S G R-C[illegible]
y38 y33 y28 y26 y24 y18 y16 y15 y14 y13 y12 y11 y10 y9 y8 y7 y6 y5 y4 y3 y2
b5 b7 b9 b13 b17 b21 b23 b24 b25 b27 b31 b37
Int
m/z
0
25
50
75
100
0
250
500
750
1,000
1,250
1,500
1,750
2,000
y8
y9
y10
y11
y7
y5
y6
y4
y3
y13
y12
b13
y14
y15
y18
b9

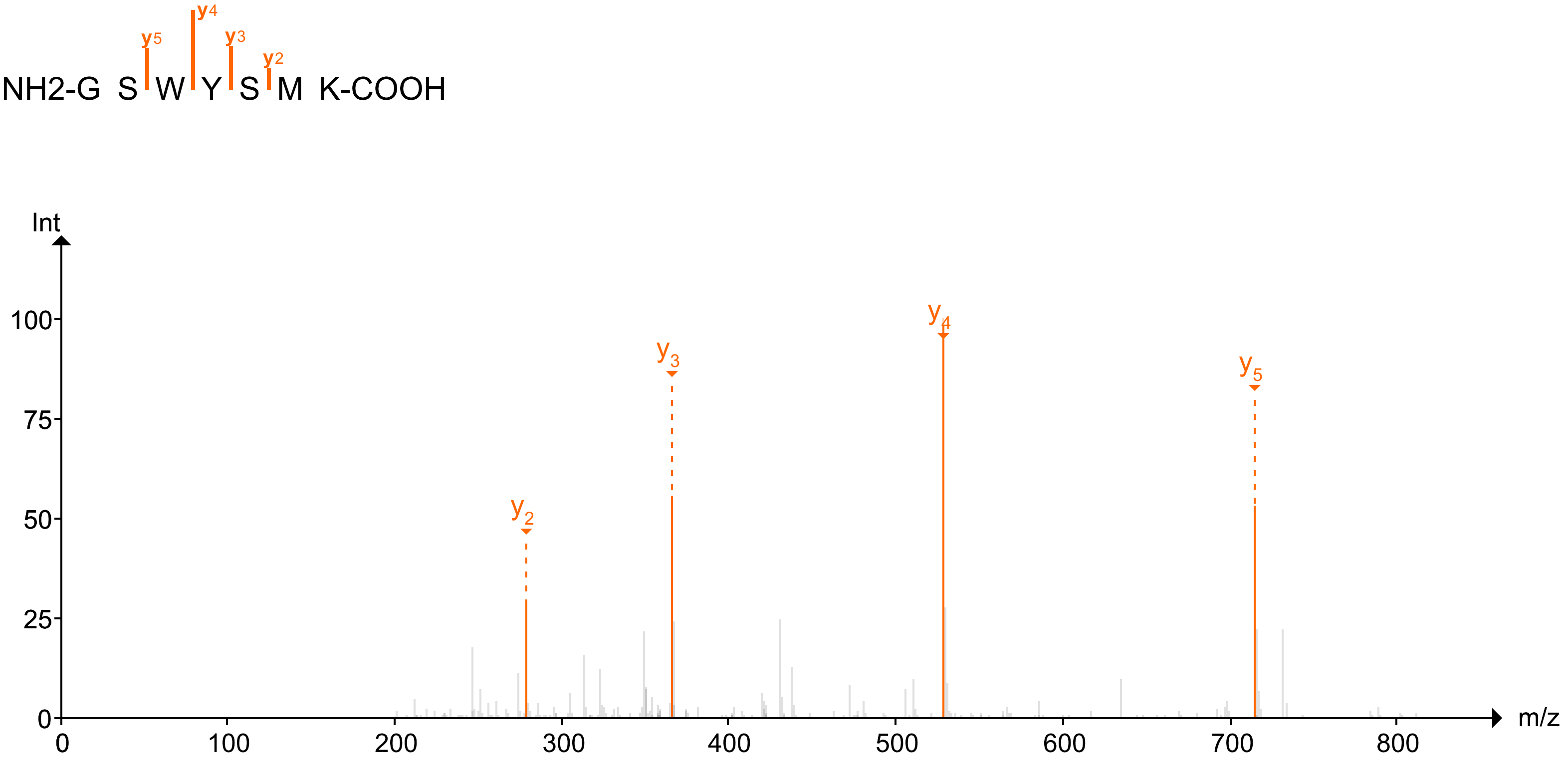

y5
y4
y3
y2
NH2-G S W Y S M K-COOH
Int
100
75
50
25
0
y2
y3
y4
y5
0
100
200
300
400
500
600
700
800
m/z

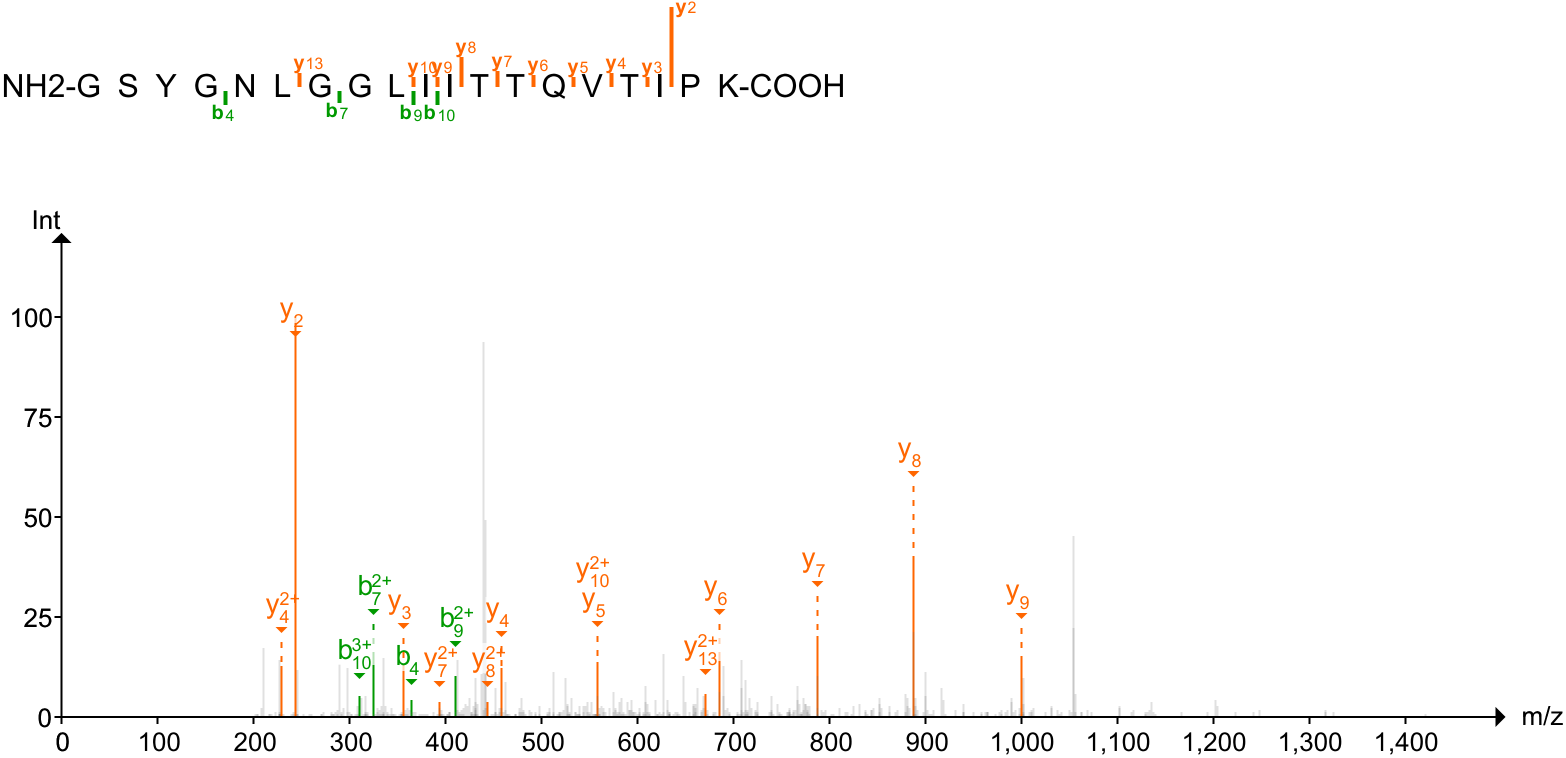
NH2-G S Y G N L G G L I I T T Q V T I P K-COOH
y13 y10 y9 y8 y7 y6 y5 y4 y3 y2
b4 b7 b9 b10
Int
100
75
50
25
0
m/z
0 100 200 300 400 500 600 700 800 900 1,000 1,100 1,200 1,300 1,400
y2
y8
y7
y9
y6
y5
y10 2+
y4
y3
y4 2+
b7 2+
b9 2+
b10 3+
b4
y7 2+
y8 2+
y13 2+

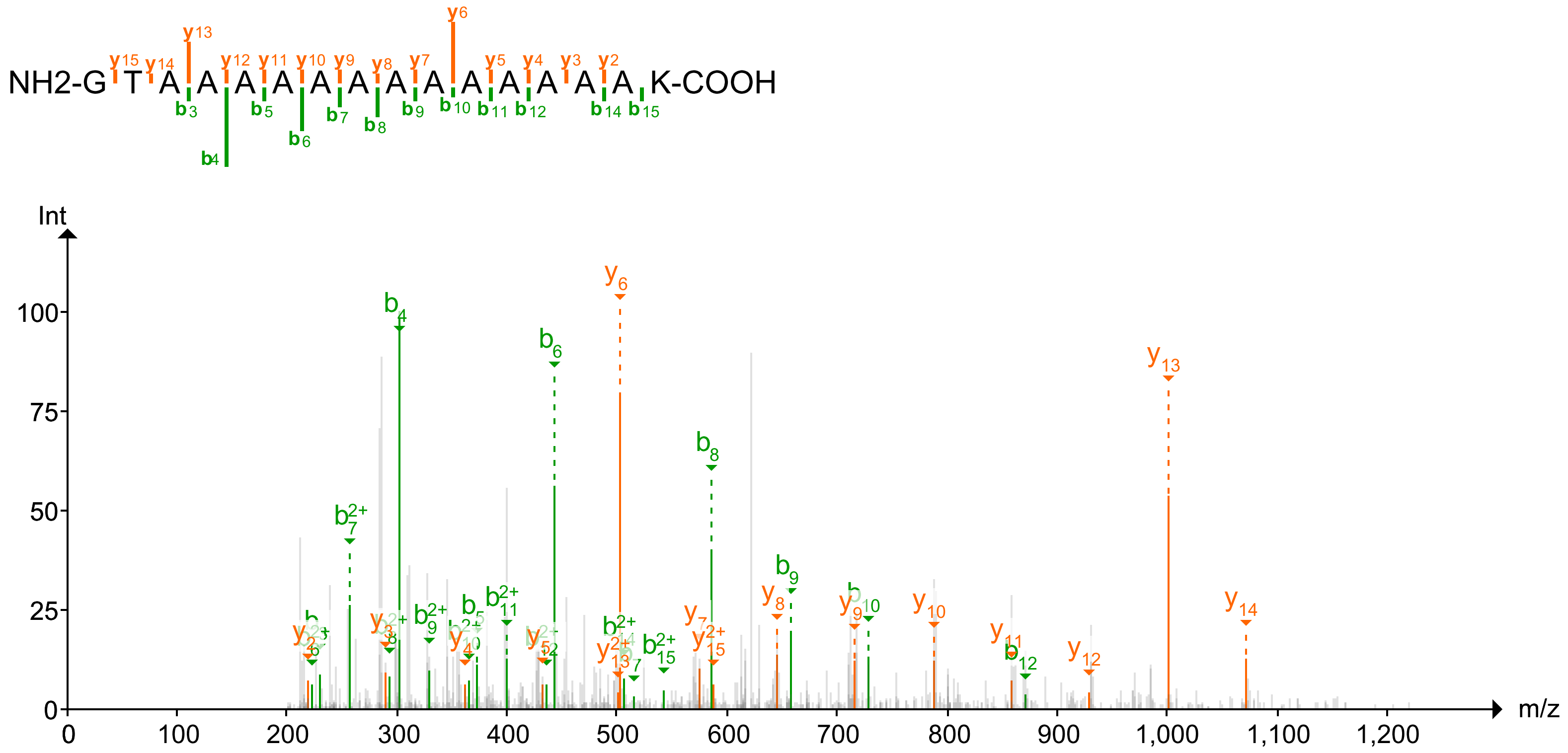

NH2-G T A A A A A A A A A A A A A A K-COOH
Int
m/z

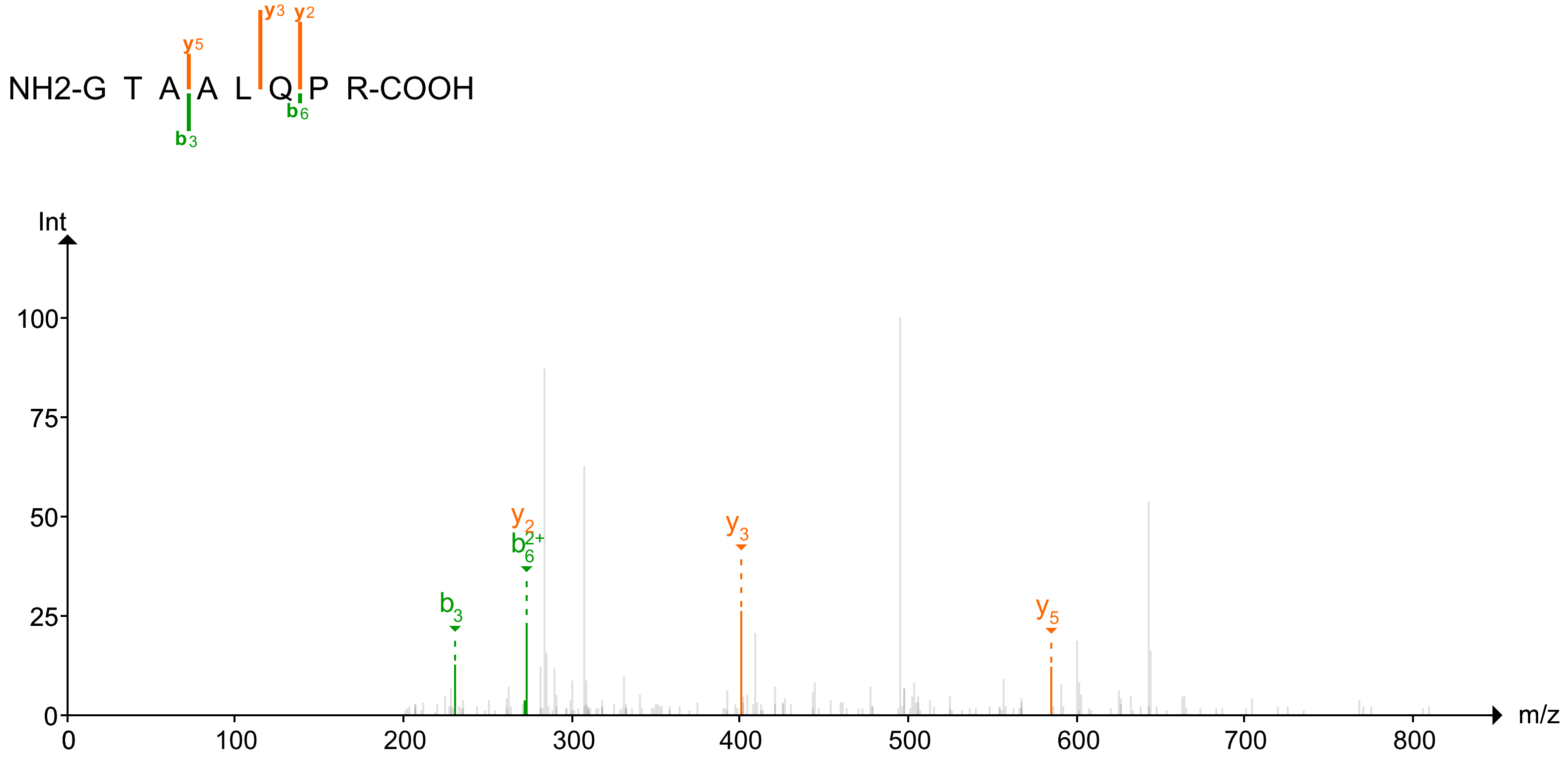

NH2-G T A A L Q P R-COOH
y5
y3
y2
b3
b6
Int
100
75
50
25
0
m/z
0
100
200
300
400
500
600
700
800
b3
y2
b6 2+
y3
y5

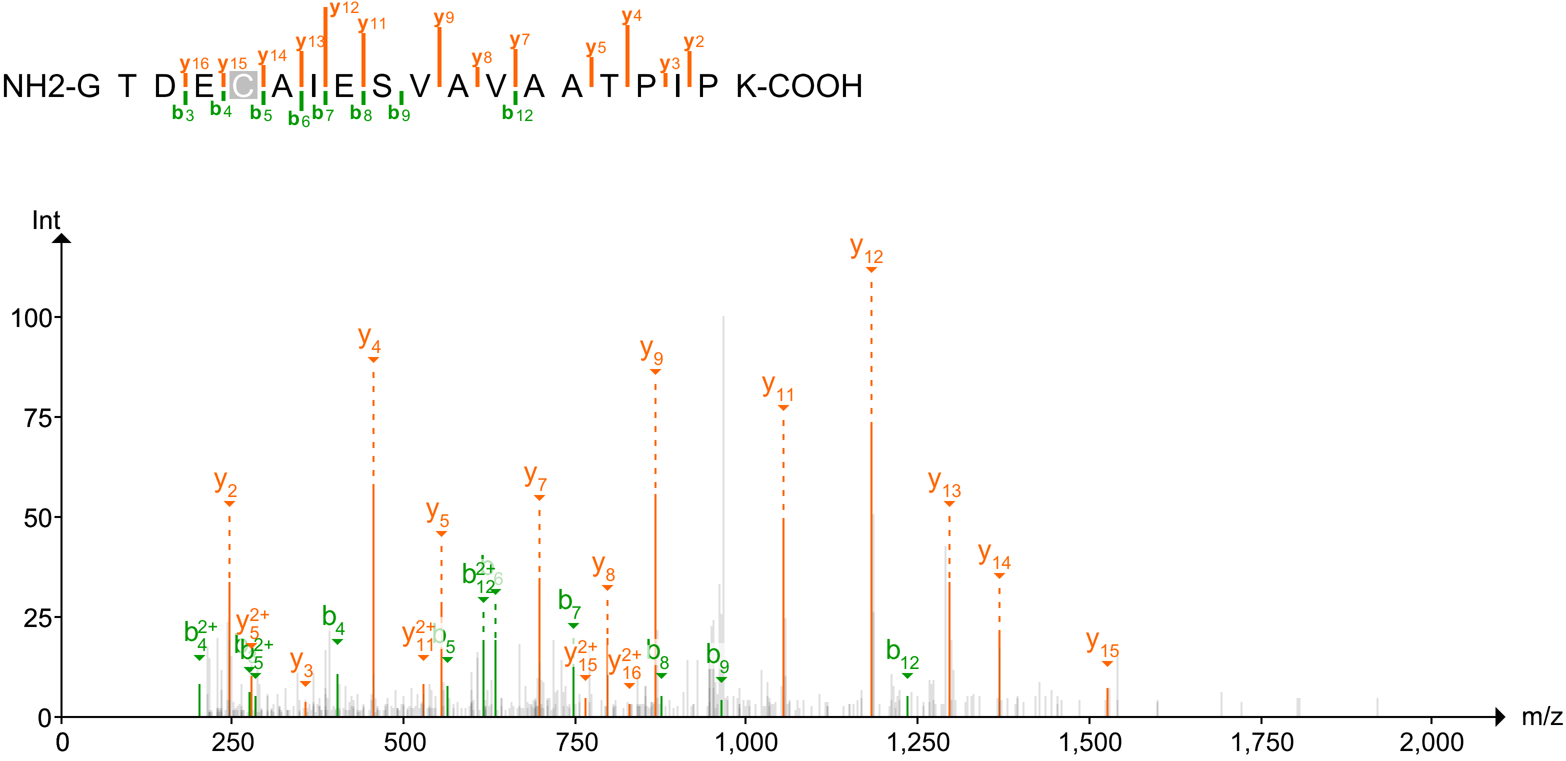

NH2-G T D E C A I E S V A V A A T P I P K-COOH
y16 y15 y14 y13 y12 y11 y9 y8 y7 y5 y4 y3 y2
b3 b4 b5 b6 b7 b8 b9 b12
Int
100
75
50
25
0
0
250
500
750
1,000
1,250
1,500
1,750
2,000
m/z

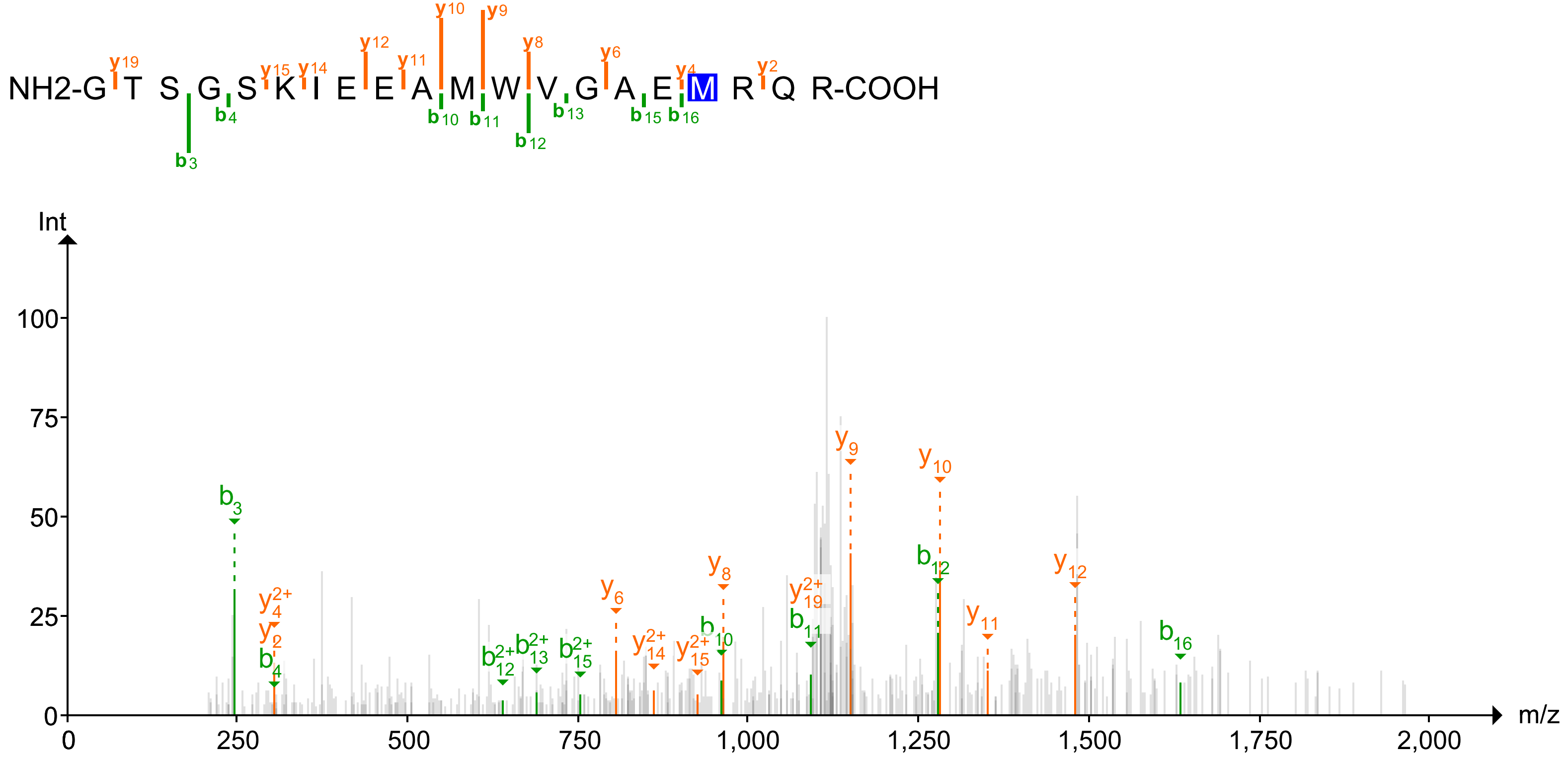

NH2-G T S G S K I E E A M W V G A E M R Q R-COOH
y19 y15 y14 y12 y11 y10 y9 y8 y6 y4 y2
b3 b4 b10 b11 b12 b13 b15 b16
Int
100
75
50
25
0
m/z
0
250
500
750
1,000
1,250
1,500
1,750
2,000
b3
y4 2+
y2
b4
b12 2+
b13 2+
b15 2+
y6
y14 2+
y15 2+
b10
y8
y19 2+
b11
y9
y10
b12
y11
y12
b16

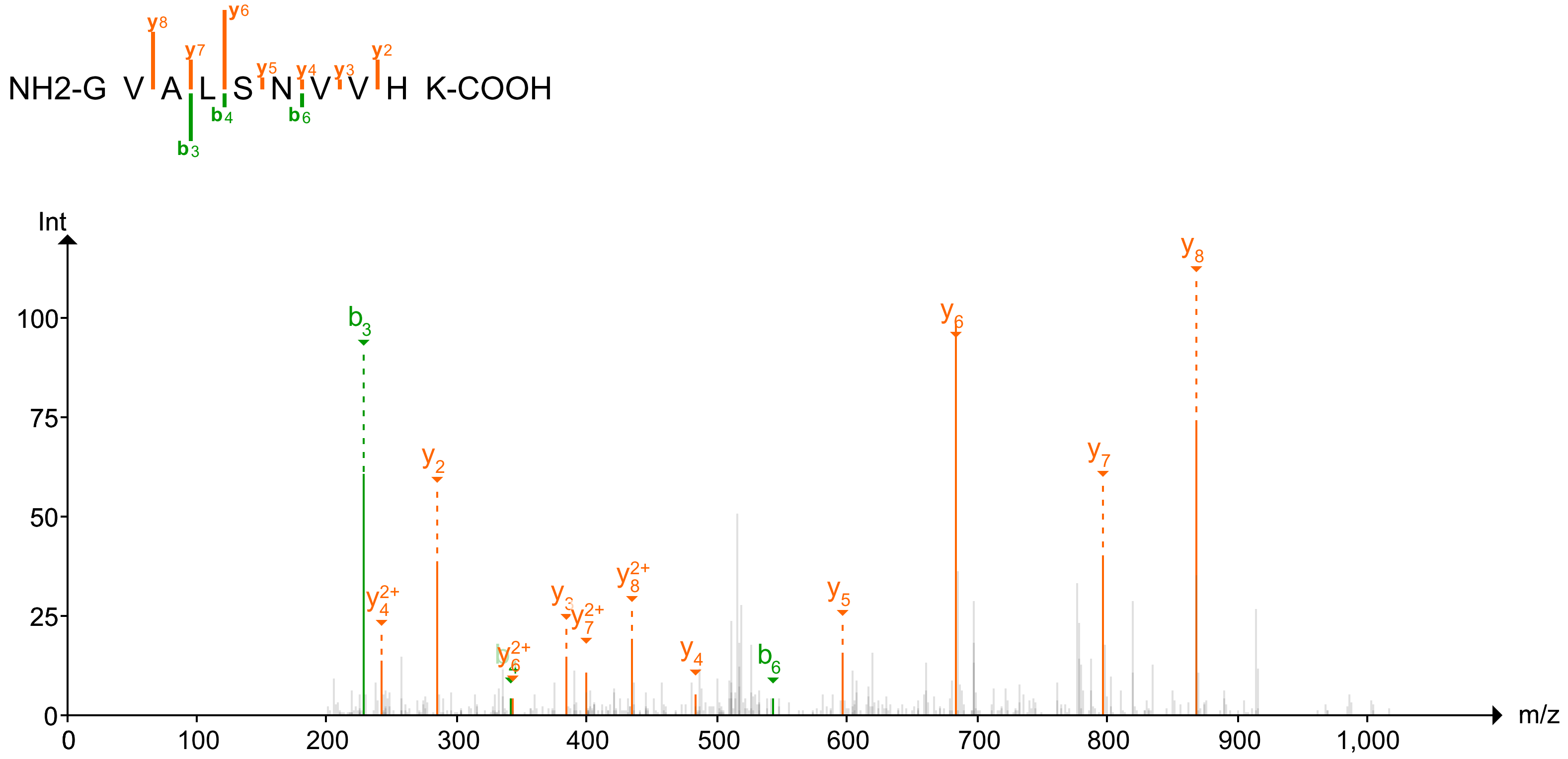

NH2-G V A L S N V V H K-COOH
y8 y7 y6 y5 y4 y3 y2
b3 b4 b6
Int
100
75
50
25
0
b3
y2
y6
y7
y8
y4 2+
y6 2+
y3
y7 2+
y8 2+
y4
b6
y5
m/z
0
100
200
300
400
500
600
700
800
900
1,000

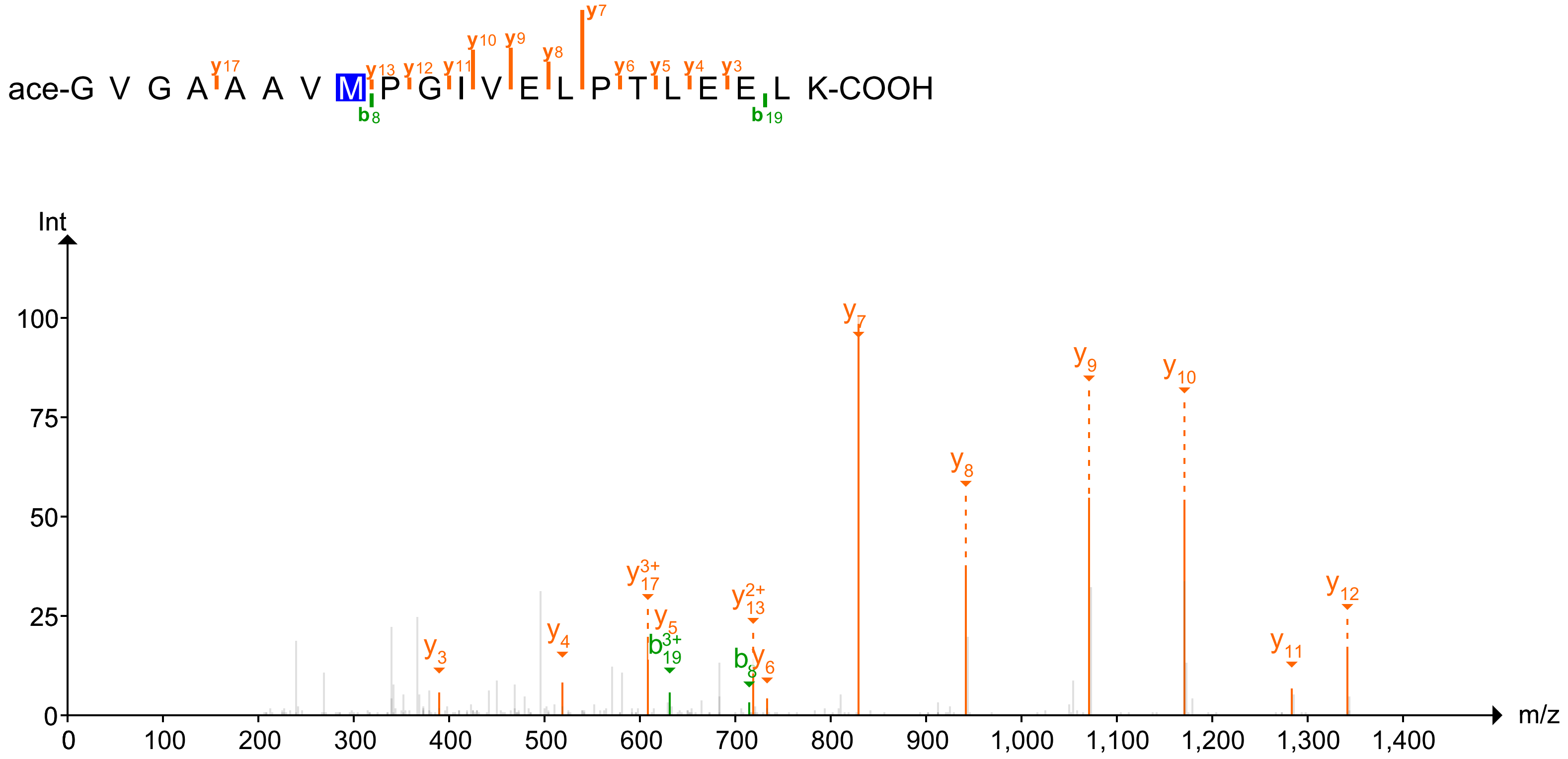
ace-G V G A A A V M P G I V E L P T L E E L K-COOH
y17 y13 y12 y11 y10 y9 y8 y7 y6 y5 y4 y3
b8 b19
Int
100
75
50
25
0
m/z
0 100 200 300 400 500 600 700 800 900 1,000 1,100 1,200 1,300 1,400
y7
y9
y10
y8
y12
y11
y3
y4
y17 3+
y5
b19 3+
y13 2+
b8
y6

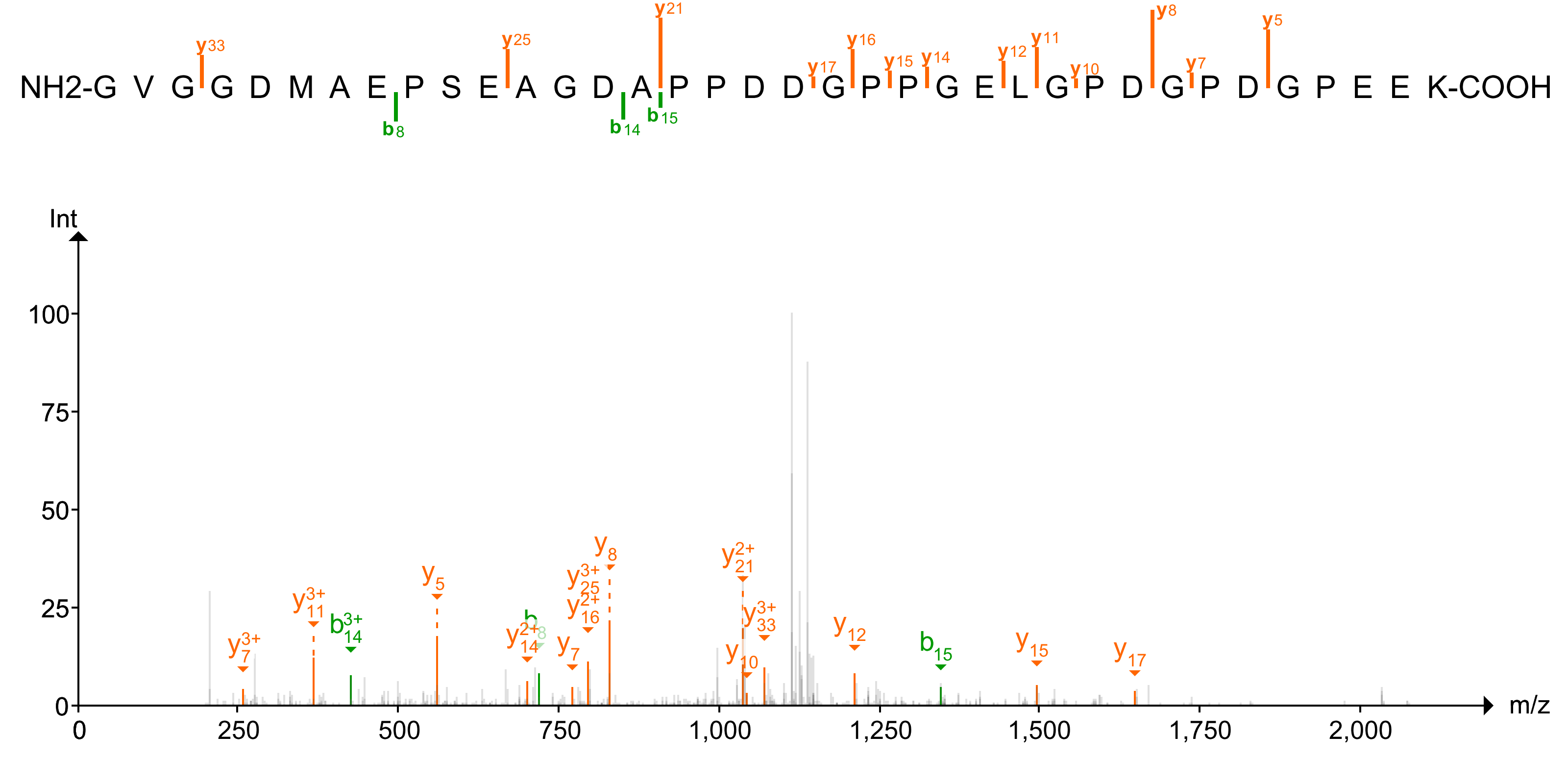

NH2-G V G G D M A E P S E A G D A P P D D G P P G E L G P D G P D G P E E K-COOH
y33
y25
y21
y17
y16
y15
y14
y12
y11
y10
y8
y7
y5
b8
b14
b15
Int
m/z
0
25
50
75
100
250
500
750
1,000
1,250
1,500
1,750
2,000

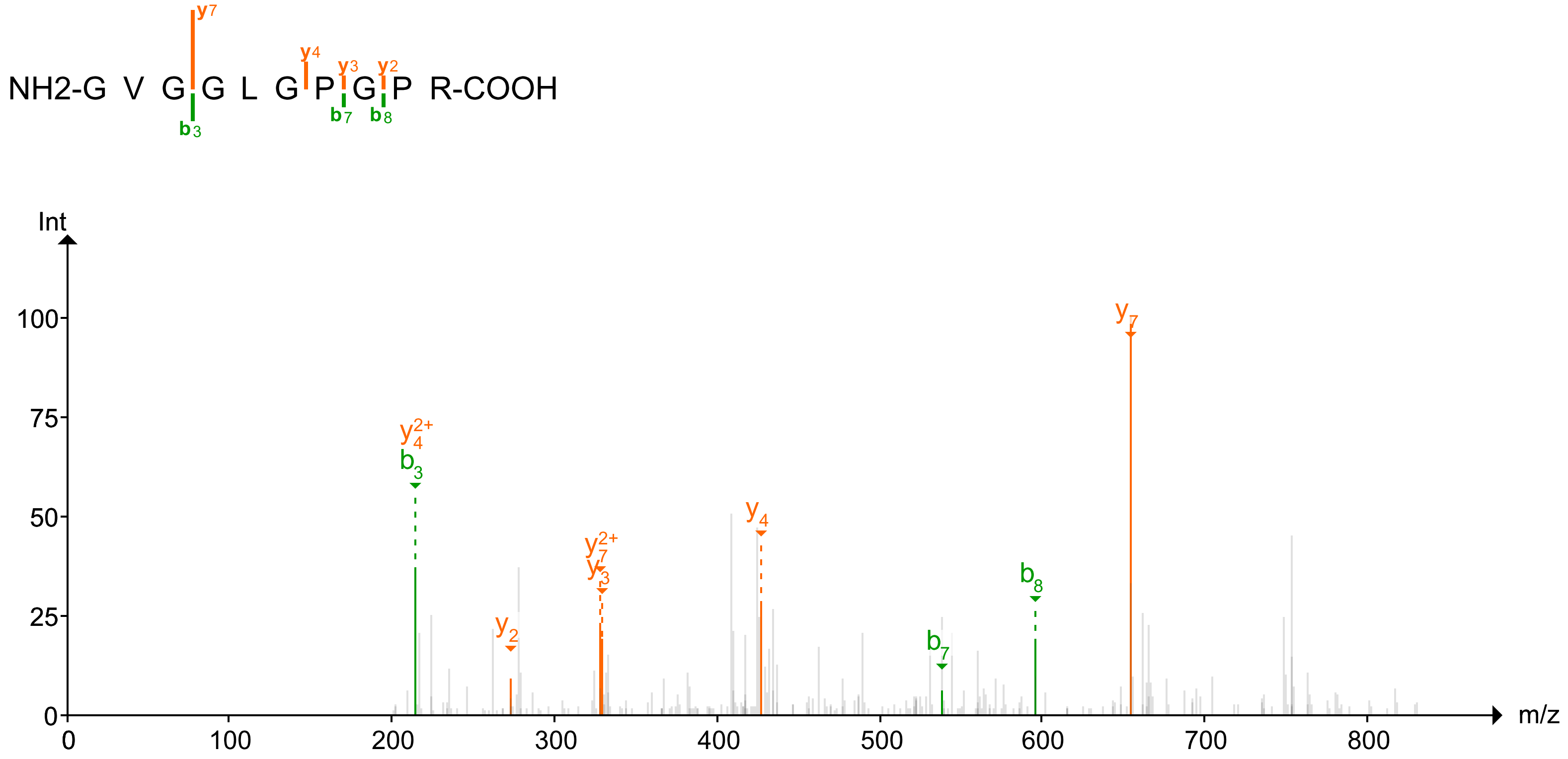

NH2-G V G G L G P G P R-COOH
y7
y4
y3
y2
b3
b7
b8
Int
100
75
50
25
0
0
100
200
300
400
500
600
700
800
m/z
y4 2+
b3
y2
y7 2+
y3
y4
b7
b8
y7

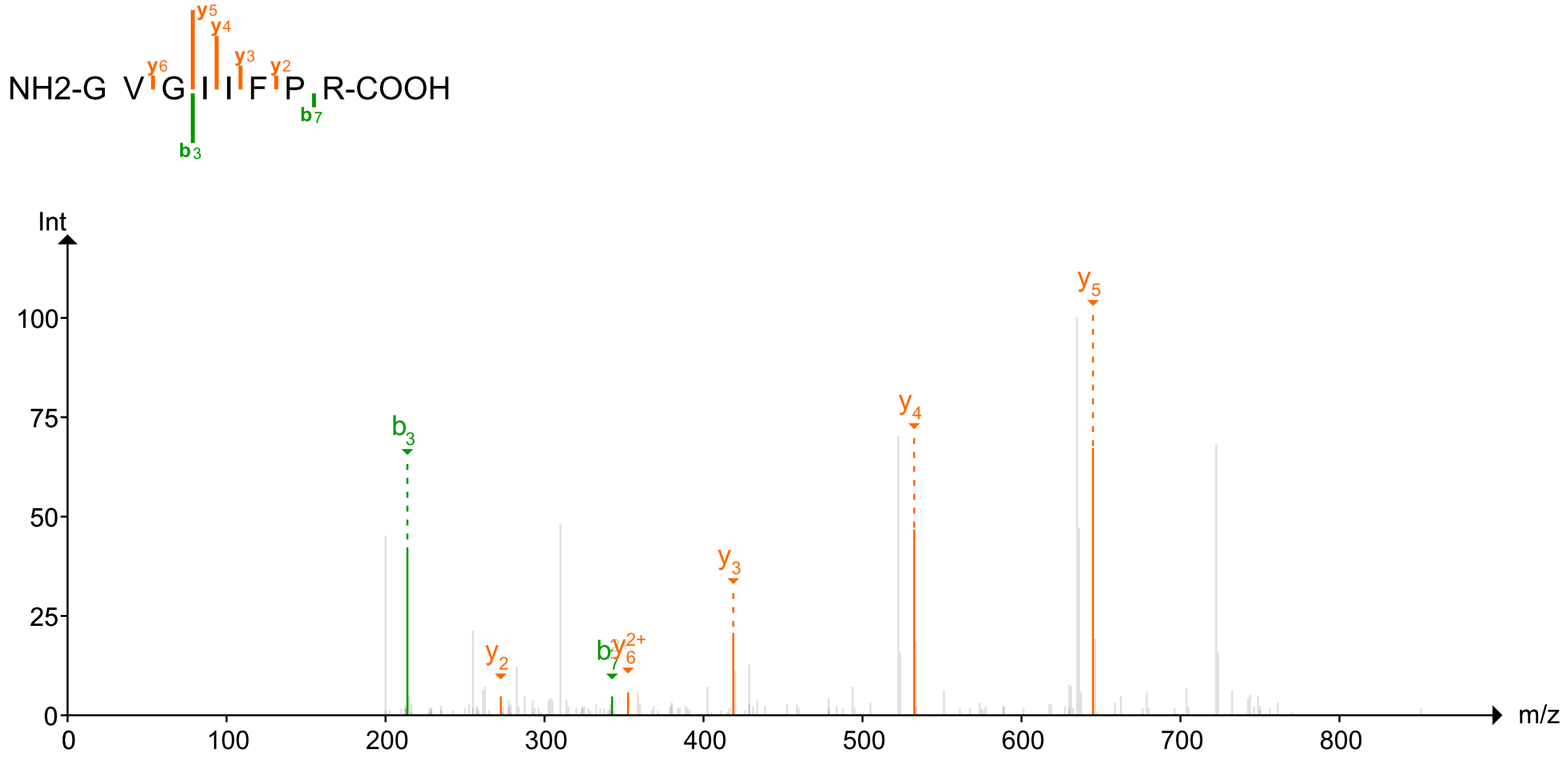

NH2-G V G I I F P R-COOH
y5
y4
y6
y3
y2
b3
b7
Int
100
75
50
25
0
b3
y2
b7
y6 2+
y3
y4
y5
0
100
200
300
400
500
600
700
800
m/z

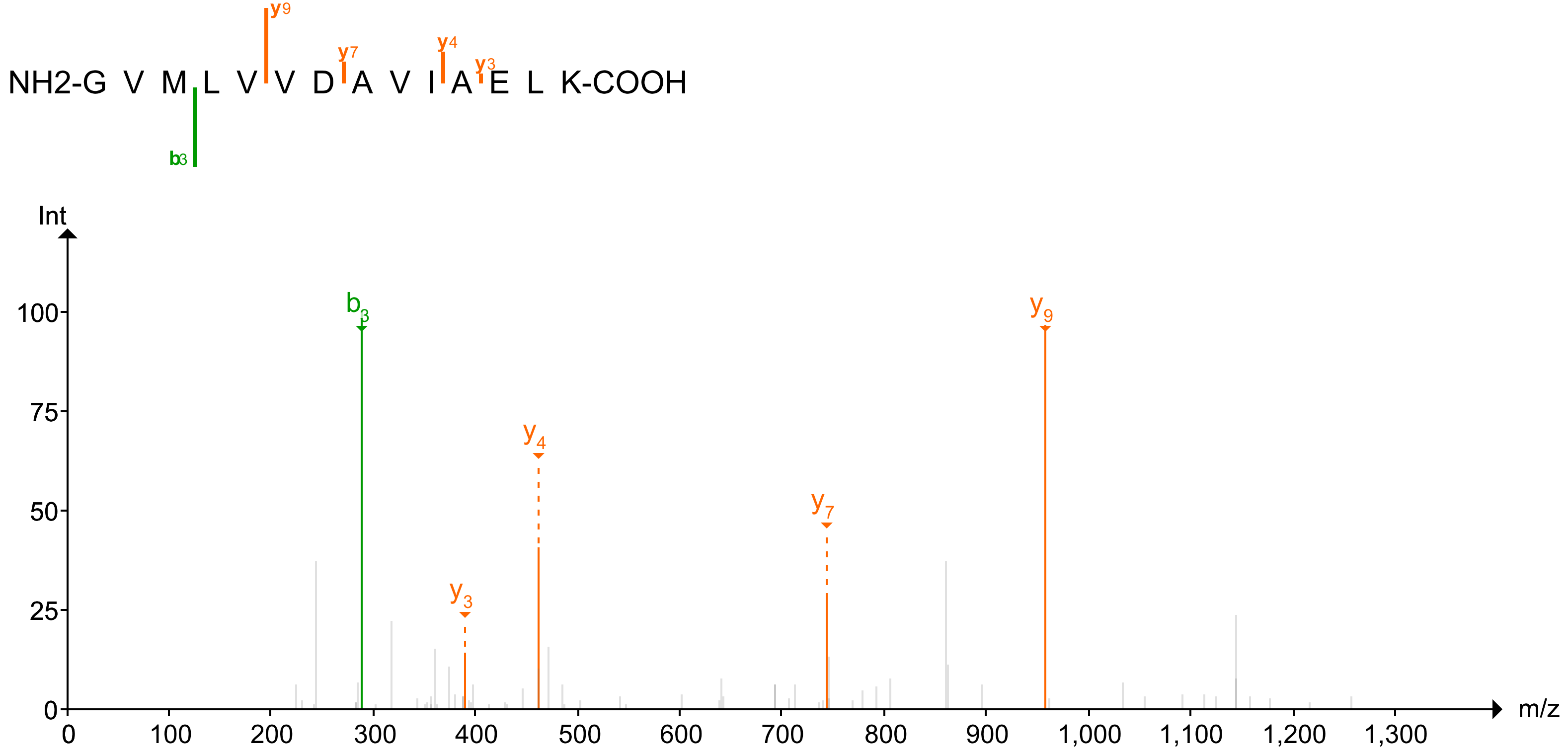

NH2-G V M L V V D A V I A E L K-COOH
y9
y7
y4
y3
b3
Int
100
75
50
25
0
$b_3$
$y_9$
$y_4$
$y_7$
$y_3$
0
100
200
300
400
500
600
700
800
900
1,000
1,100
1,200
1,300
m/z

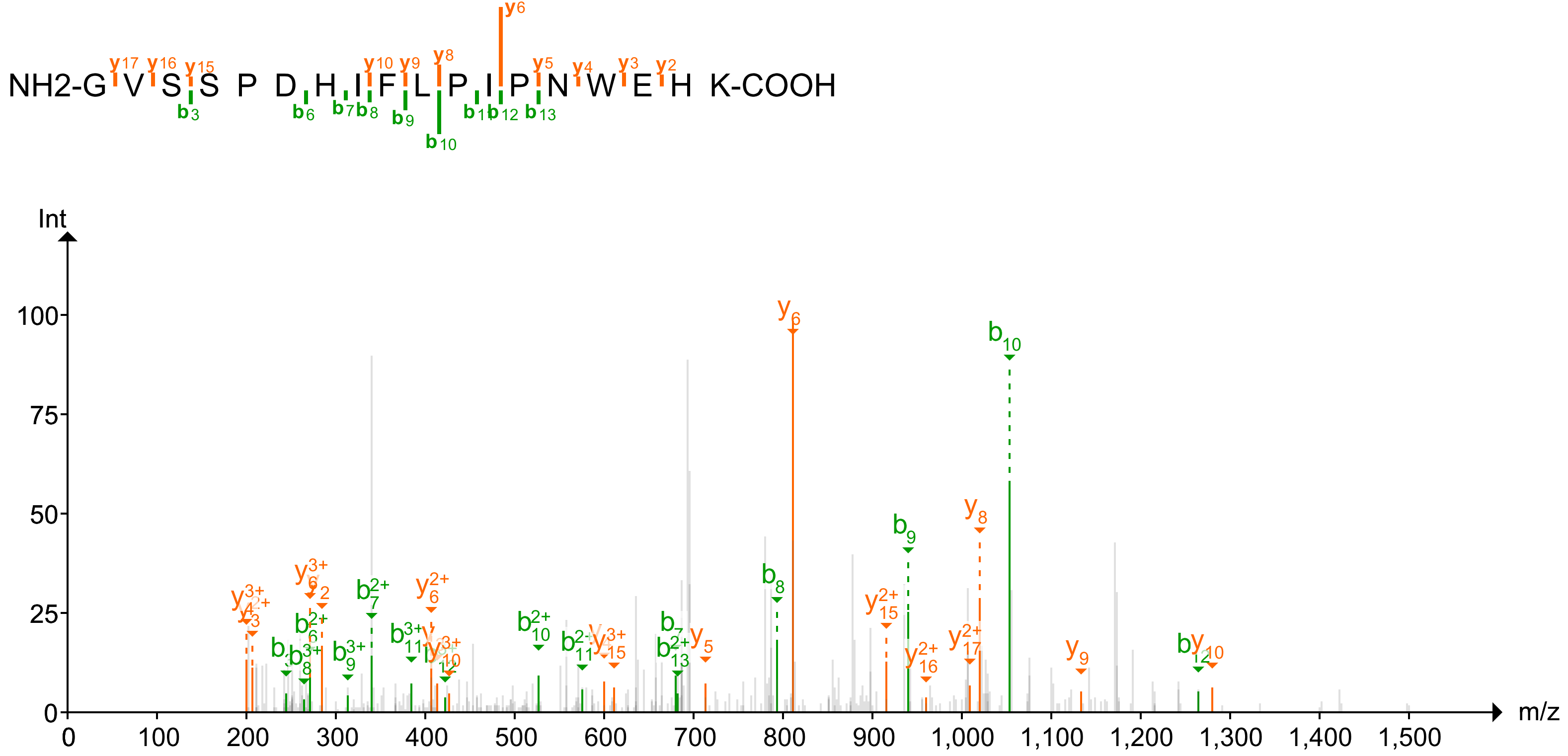
NH2-G V S S P D H I F L P I P N W E H K-COOH
y17 y16 y15 y10 y9 y8 y6 y5 y4 y3 y2
b3 b6 b7 b8 b9 b10 b12 b13
Int
100
75
50
25
0
m/z
0
100
200
300
400
500
600
700
800
900
1,000
1,100
1,200
1,300
1,400
1,500

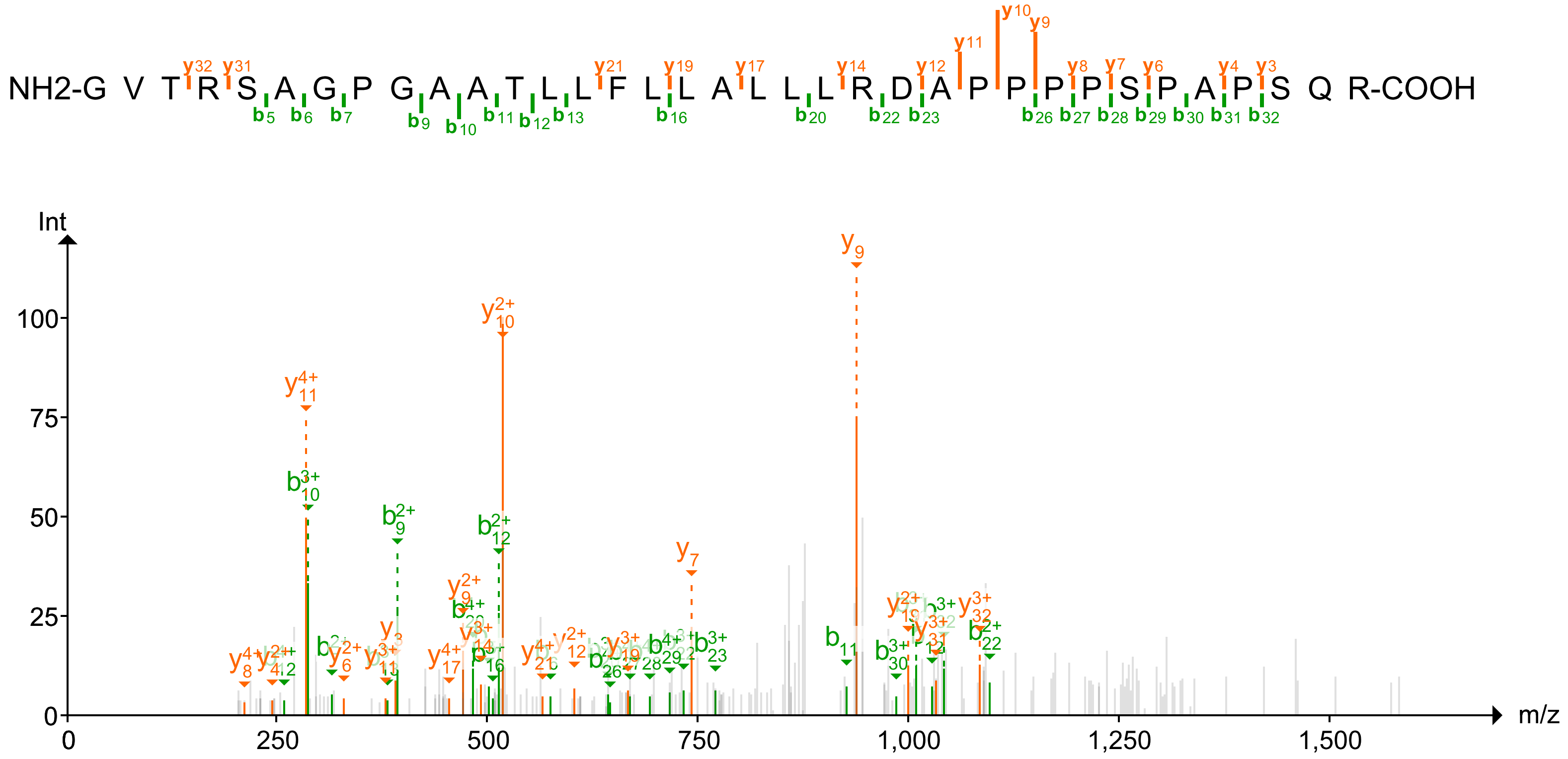

NH2-G V T R S A G P G A A T L L F L L A L L L R D A P P P P S P A P S Q R-COOH
y32 y31 y21 y19 y17 y14 y12 y11 y10 y9 y8 y7 y6 y4 y3
b5 b6 b7 b9 b10 b11 b12 b13 b16 b20 b22 b23 b26 b27 b28 b29 b30 b31 b32
Int
100
75
50
25
0
m/z
250
500
750
1,000
1,250
1,500

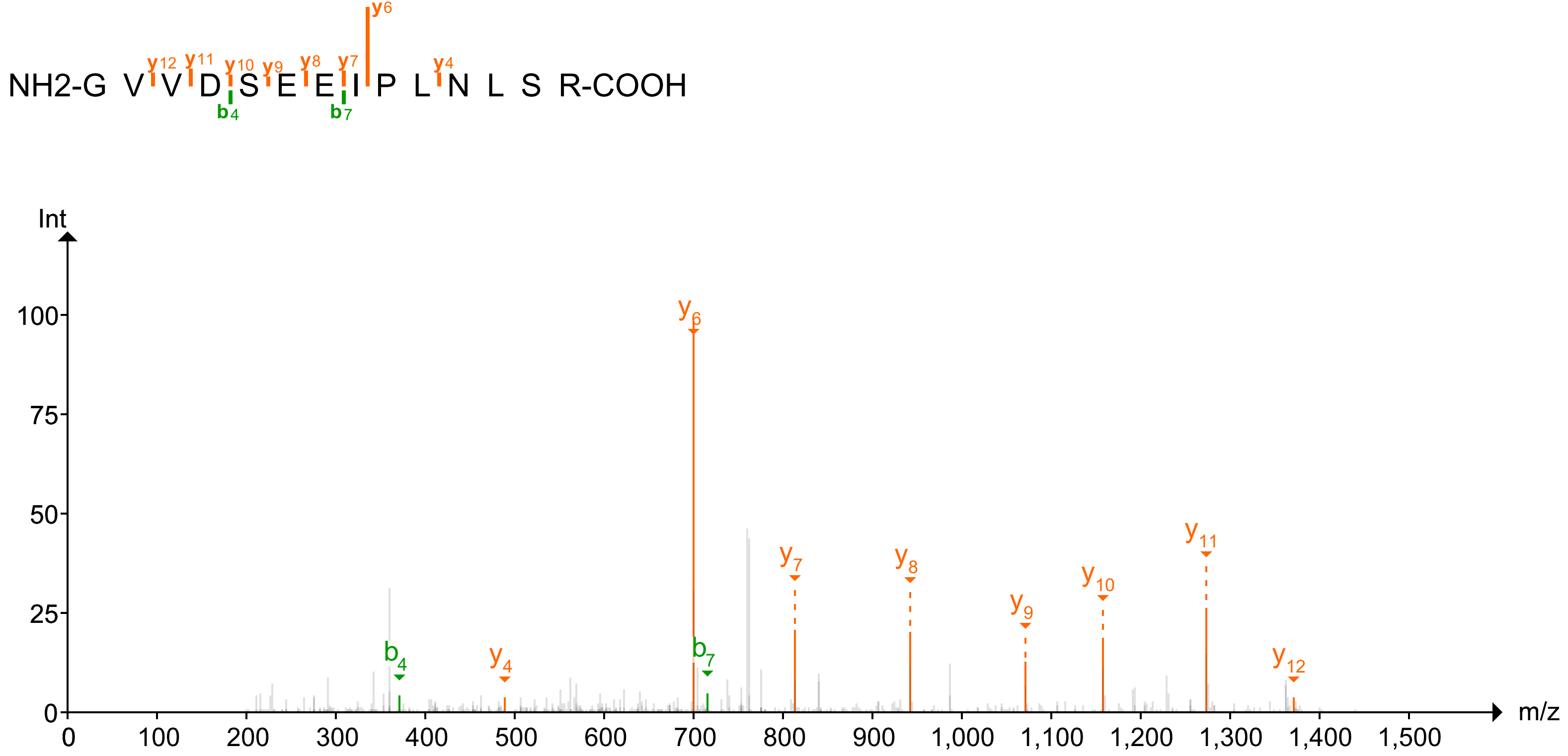

NH2-G V V D S E E I P L N L S R-COOH
y12 y11 y10 y9 y8 y7 y6 y4
b4 b7
Int
100
75
50
25
0
0 100 200 300 400 500 600 700 800 900 1,000 1,100 1,200 1,300 1,400 1,500
m/z
y6 y7 y8 y9 y10 y11 y12 y4 b4 b7

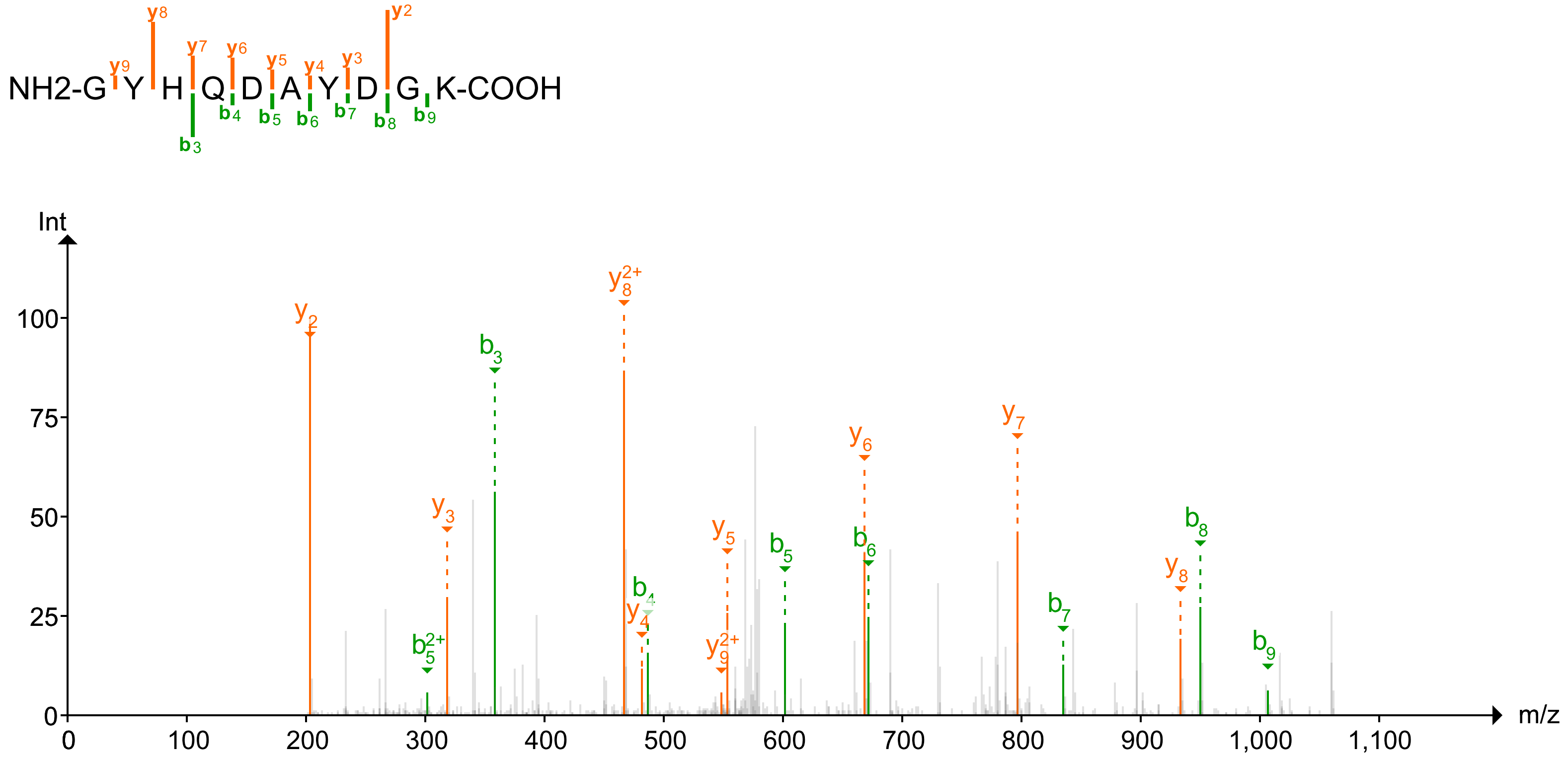

NH2-G Y H Q D A Y D G K-COOH
y9 y8 y7 y6 y5 y4 y3 y2
b3 b4 b5 b6 b7 b8 b9
Int
m/z
0
25
50
75
100
0
100
200
300
400
500
600
700
800
900
1,000
1,100
$y_2$
$y_8^{2+}$
$b_3$
$y_3$
$b_5^{2+}$
$b_4$
$y_4$
$y_5$
$y_9^{2+}$
$b_5$
$y_6$
$b_6$
$y_7$
$b_7$
$y_8$
$b_8$
$b_9$

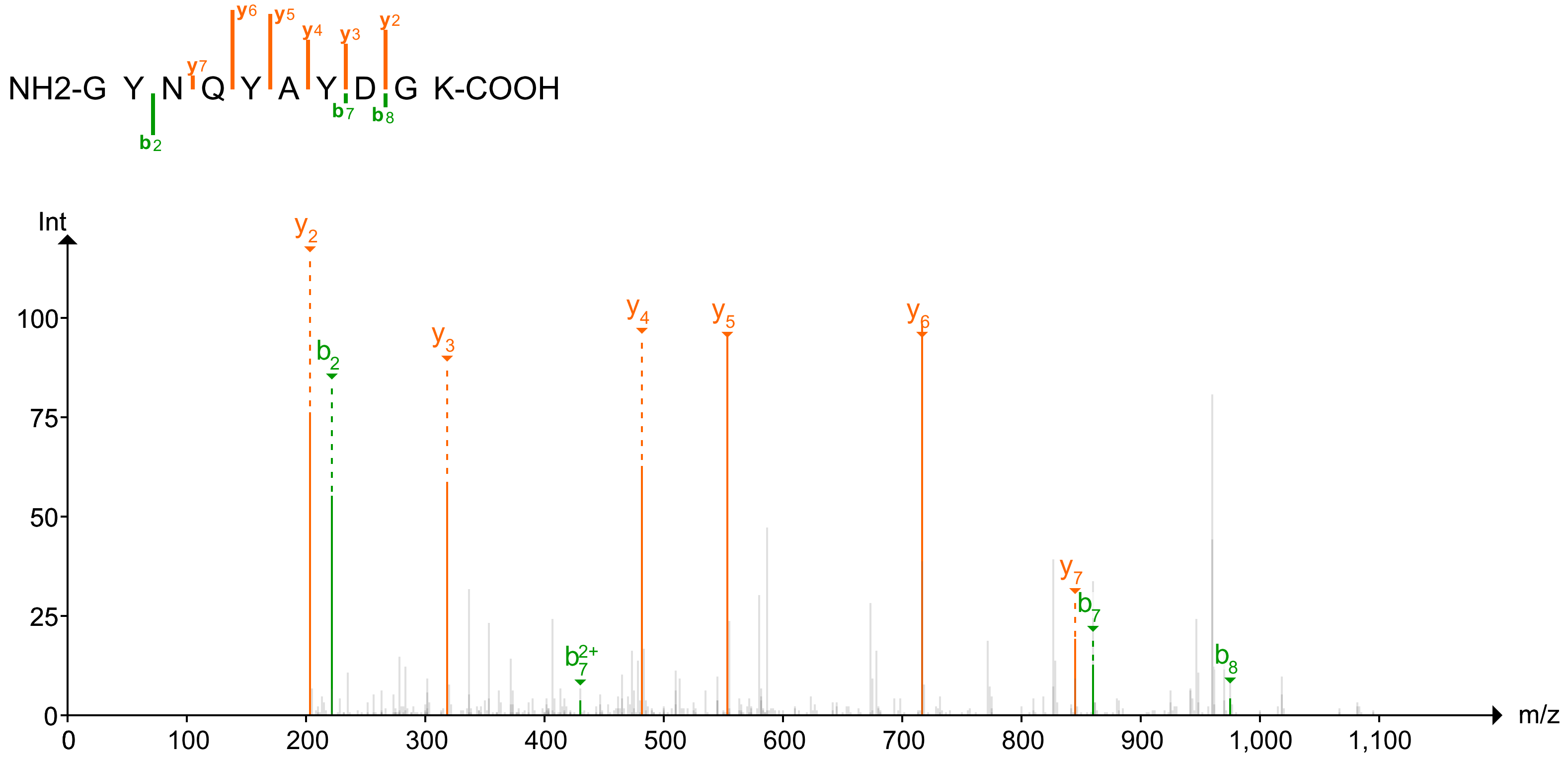

NH2-G Y N Q Y A Y D G K-COOH
y6 y5 y4 y3 y2 y7
b2 b7 b8
Int
m/z
y2 b2 y3 y4 y5 y6 y7 b7 b8 b7 2+
0 25 50 75 100
0 100 200 300 400 500 600 700 800 900 1,000 1,100

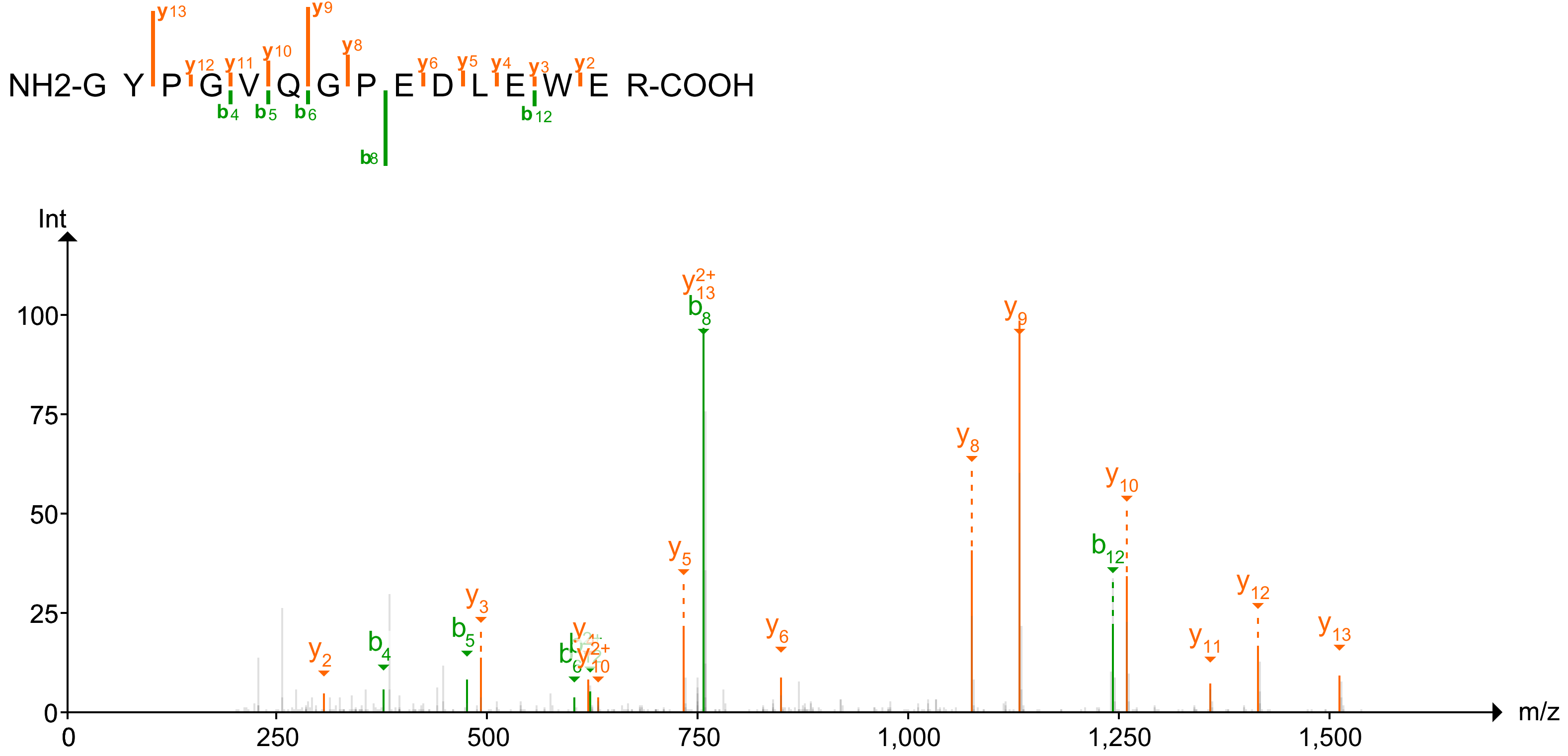

NH2-G Y P G V Q G P E D L E W E R-COOH
y13 y12 y11 y10 y9 y8 y6 y5 y4 y3 y2
b4 b5 b6 b8 b12
Int
m/z
0
25
50
75
100
250
500
750
1,000
1,250
1,500
y2
b4
b5
y3
y5
y13 2+
b8
y6
y8
y9
y10
b12
y11
y12
y13

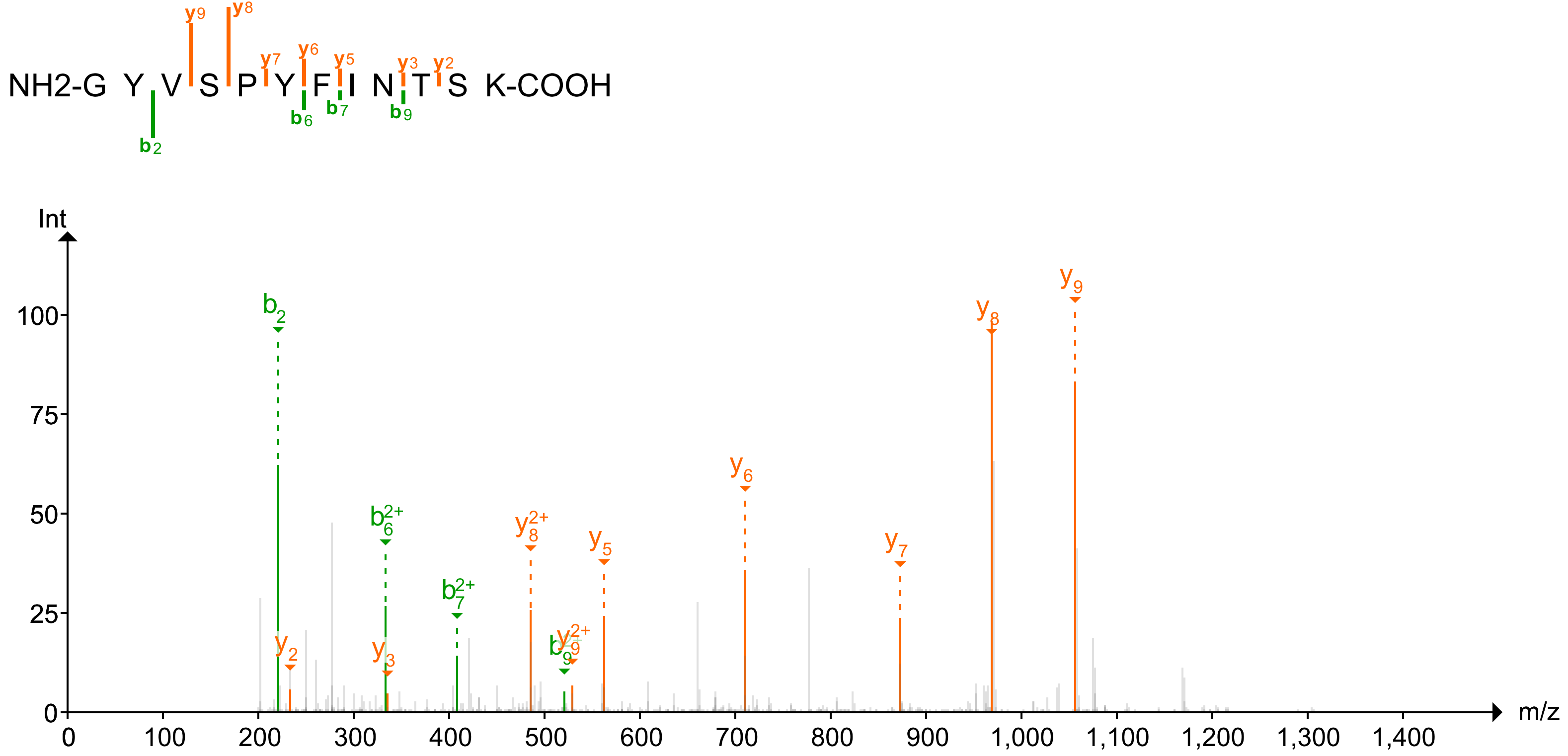

NH2-G Y V S P Y F I N T S K-COOH
y9 y8 y7 y6 y5 y3 y2
b2 b6 b7 b9
Int
100
75
50
25
0
$b_2$
$b_6^{2+}$
$b_7^{2+}$
$b_9$
$y_2$
$y_3$
$y_8^{2+}$
$y_9^{2+}$
$y_5$
$y_6$
$y_7$
$y_8$
$y_9$
0 100 200 300 400 500 600 700 800 900 1,000 1,100 1,200 1,300 1,400
m/z

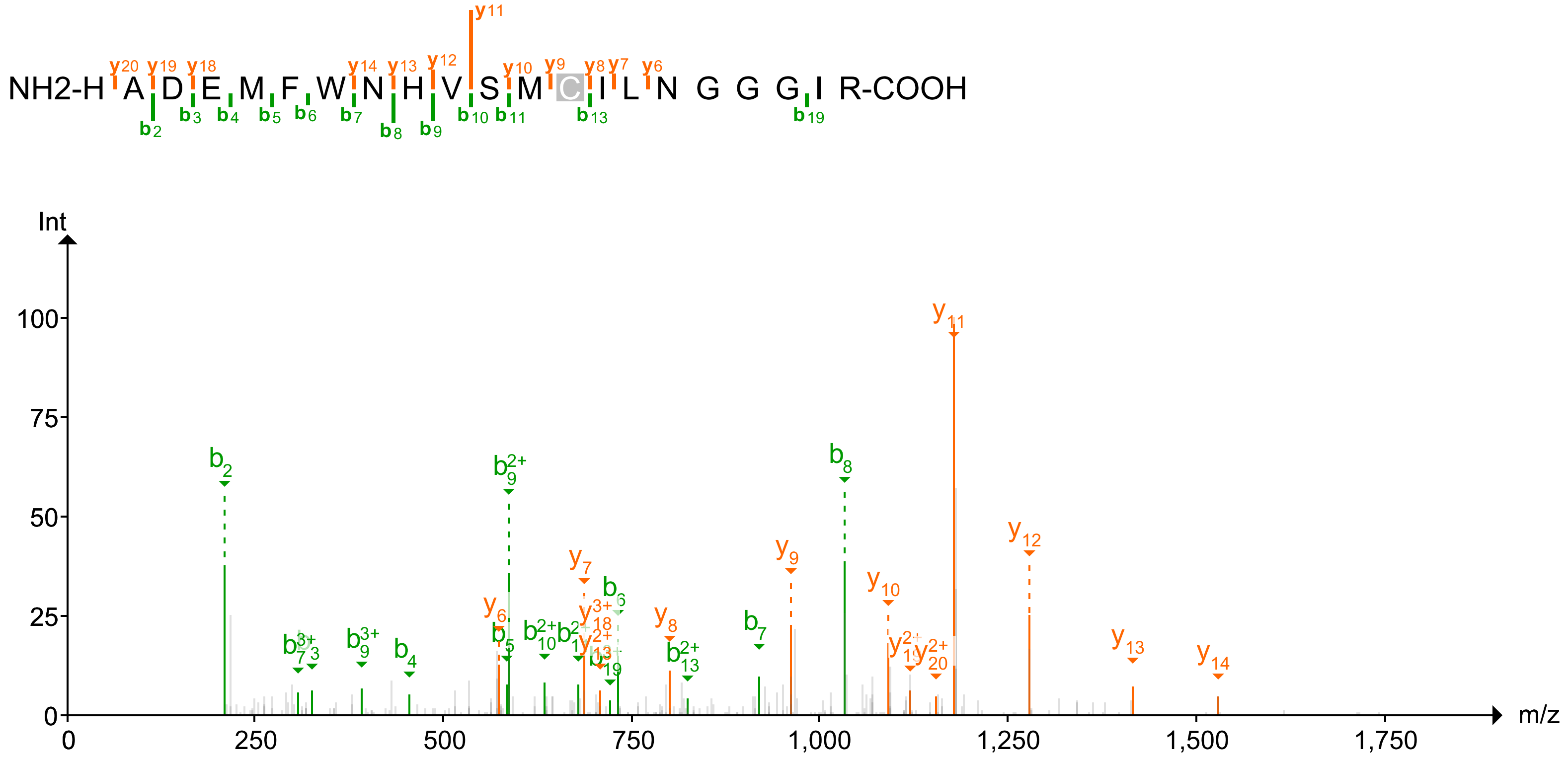

NH2-H A D E M F W N H V S M C I L N G G G I R-COOH
Int
100
75
50
25
0
0
250
500
750
1,000
1,250
1,500
1,750
m/z

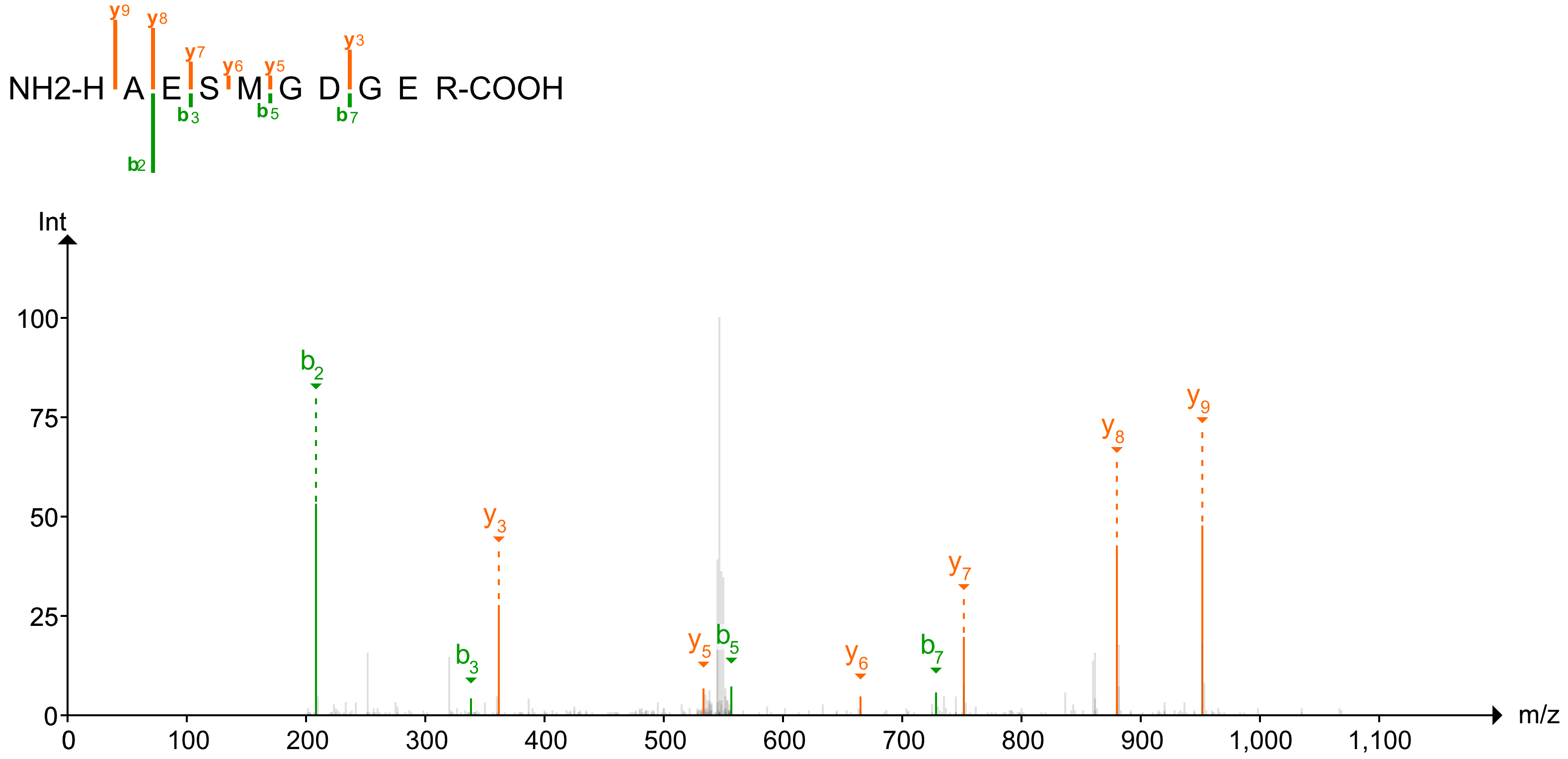

NH2-H A E S M G D G E R-COOH
y9 y8 y7 y6 y5 y3
b2 b3 b5 b7
Int
100
75
50
25
0
m/z
0 100 200 300 400 500 600 700 800 900 1,000 1,100

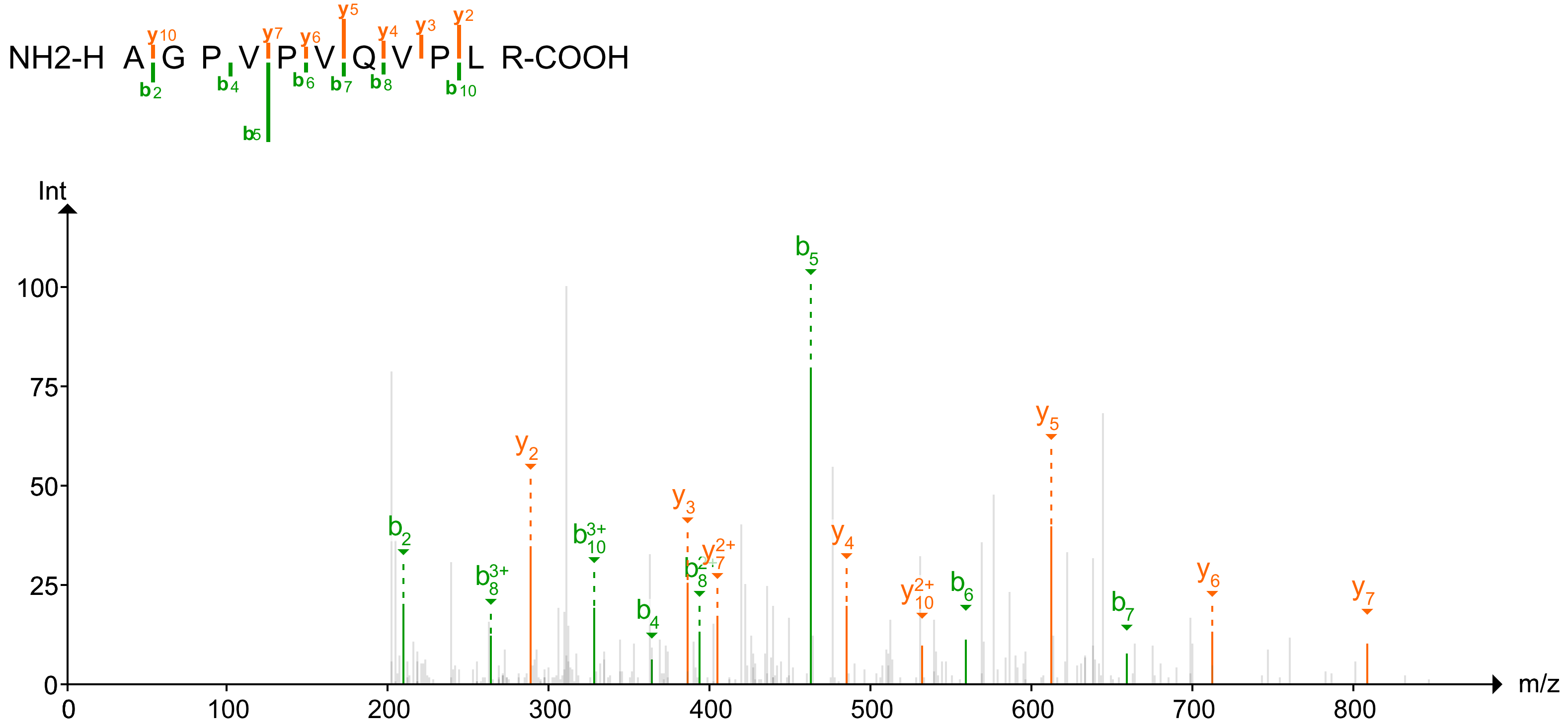

NH2-H A G P V P V Q V P L R-COOH
y10 y7 y6 y5 y4 y3 y2
b2 b4 b5 b6 b7 b8 b10
Int
100
75
50
25
0
m/z
0
100
200
300
400
500
600
700
800
b5
y5
y2
b2
y3
b3+10
y4
y2+7
b2+8
b3+8
b4
y2+10
b6
b7
y6
y7

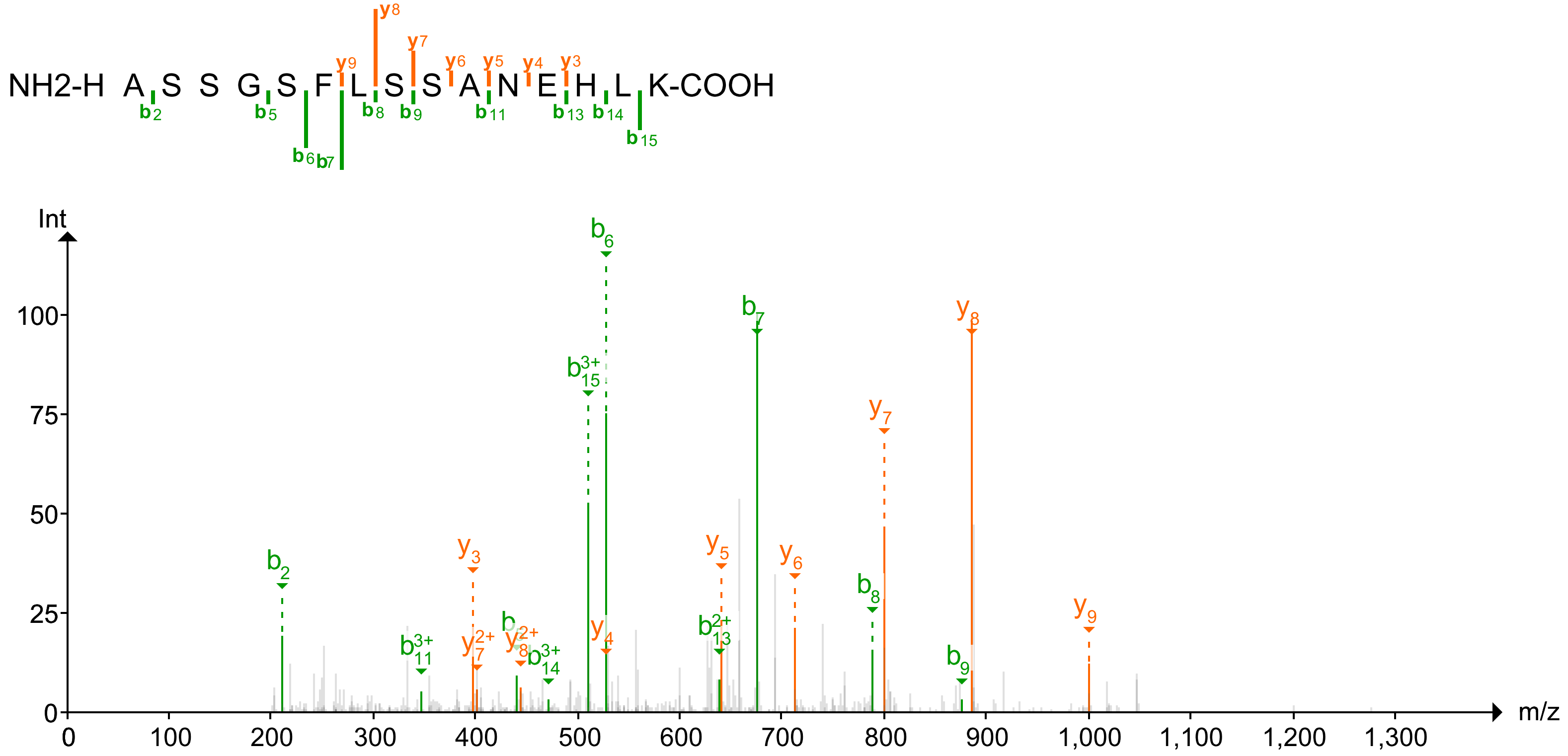

NH2-H A S S G S F L S S A N E H L K-COOH
y8 y7 y9 y6 y5 y4 y3
b2 b5 b6 b7 b8 b9 b11 b13 b14 b15
Int
m/z
0
25
50
75
100
0 100 200 300 400 500 600 700 800 900 1,000 1,100 1,200 1,300

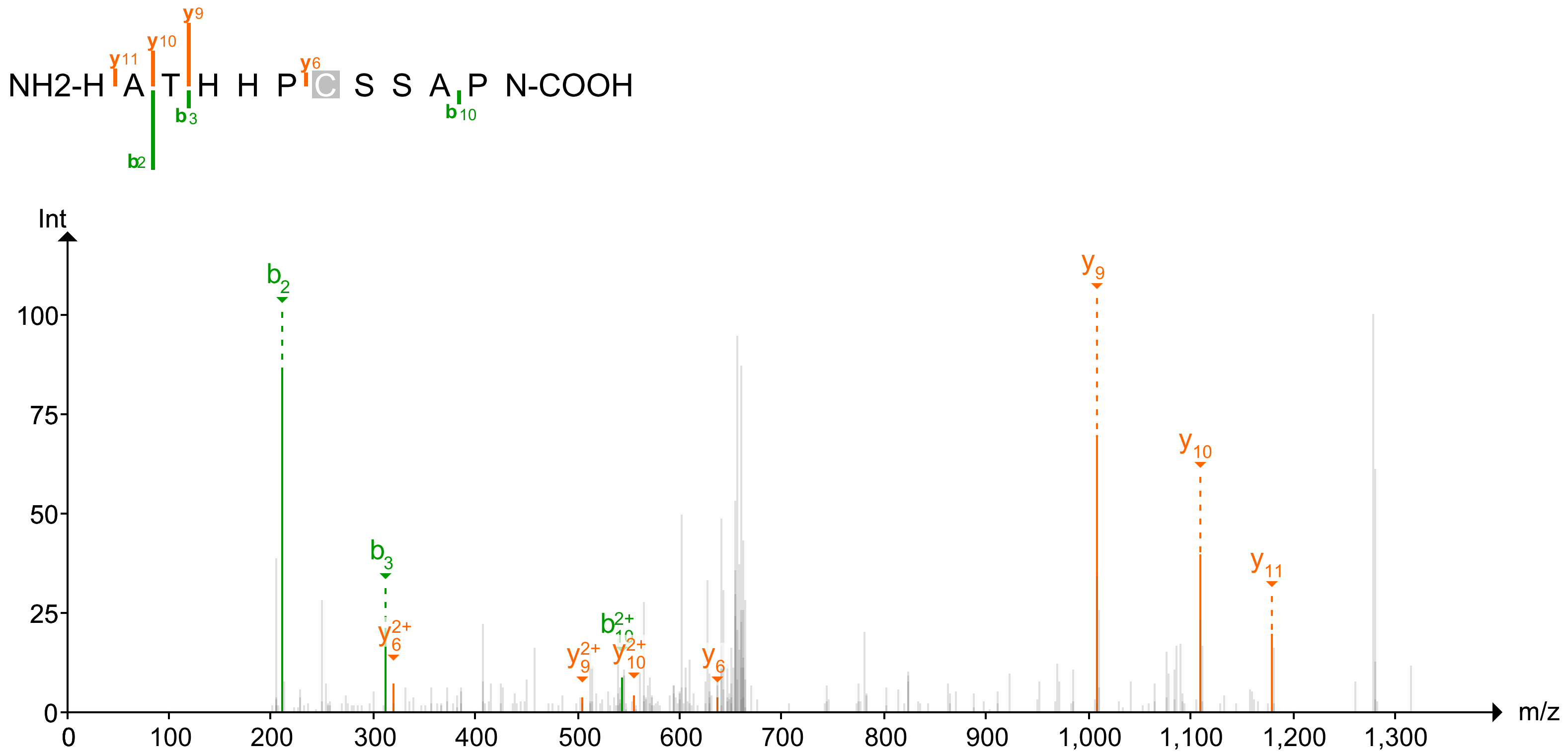

NH2-H A T H H P C S S A P N-COOH
y11 y10 y9 y6
b2 b3 b10
Int
m/z
b2
b3
y9
y10
y11
y6

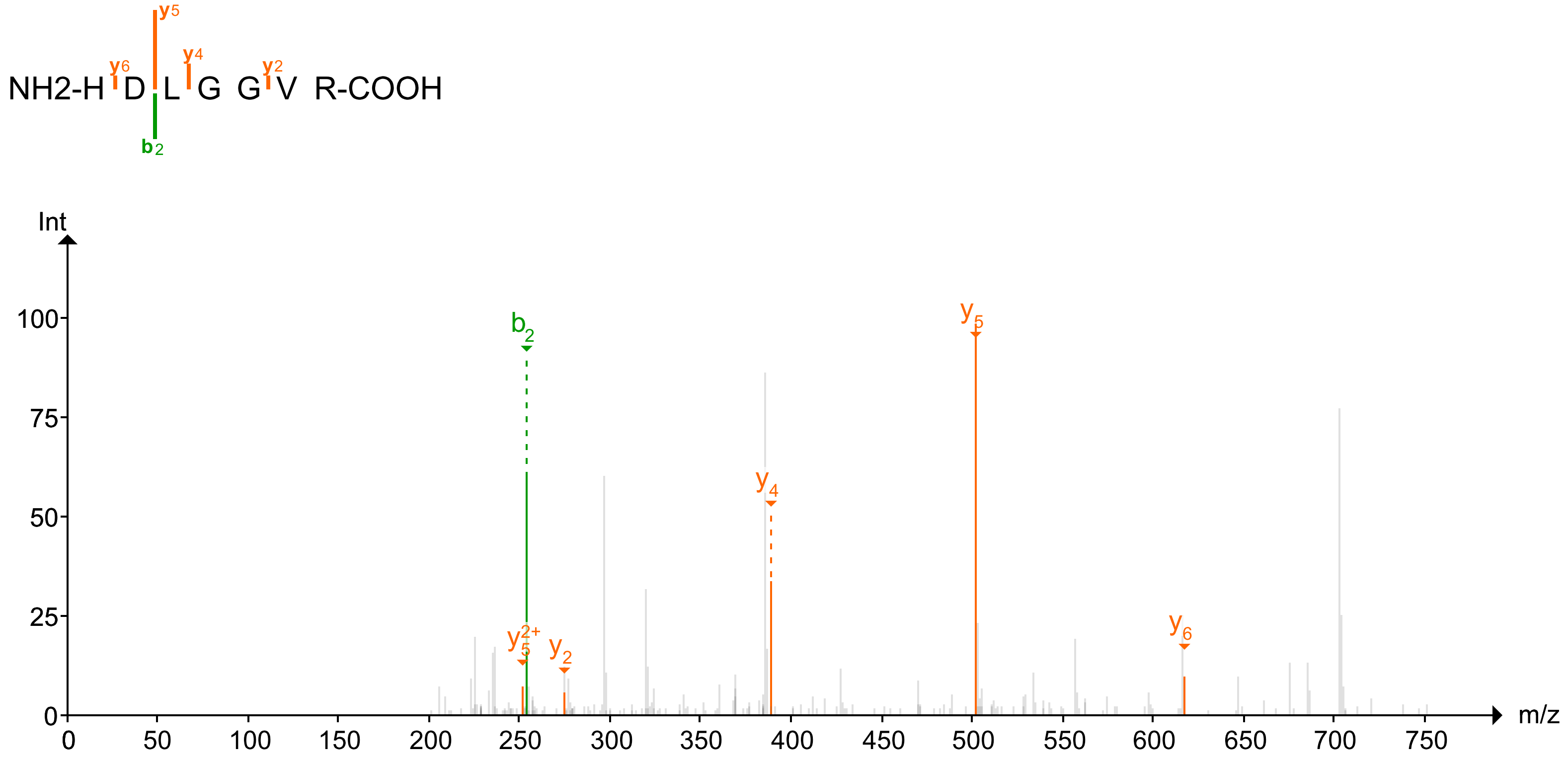

NH2-H D L G G V R-COOH
y6
y5
y4
y2
b2
Int
100
75
50
25
0
b2
y5
y4
y5 2+
y2
y6
0
50
100
150
200
250
300
350
400
450
500
550
600
650
700
750
m/z

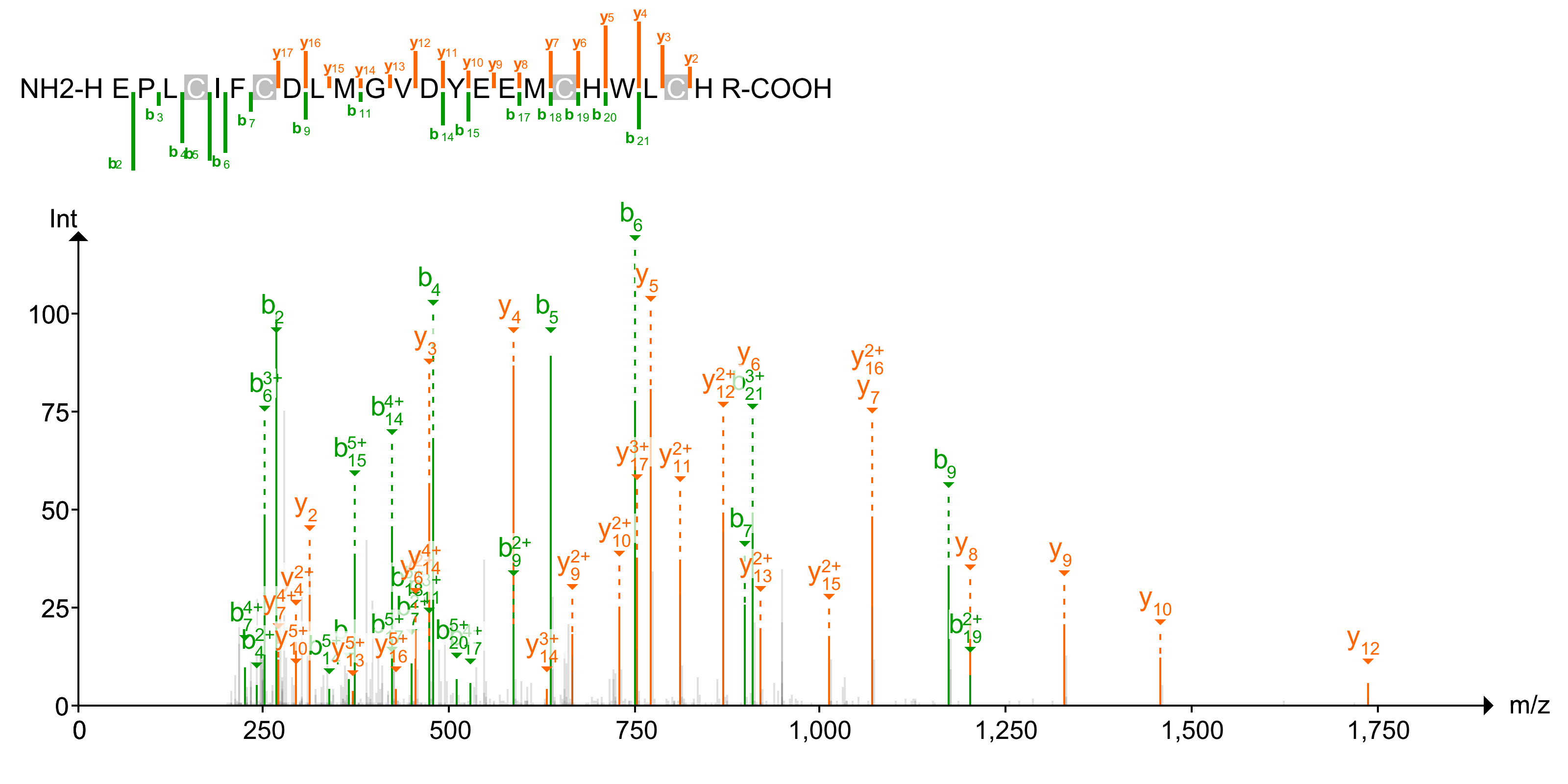

NH2-H E P L C I F C D L M G V D Y E E M C H W L C H R-COOH
Int
m/z
0
25
50
75
100
0
250
500
750
1,000
1,250
1,500
1,750

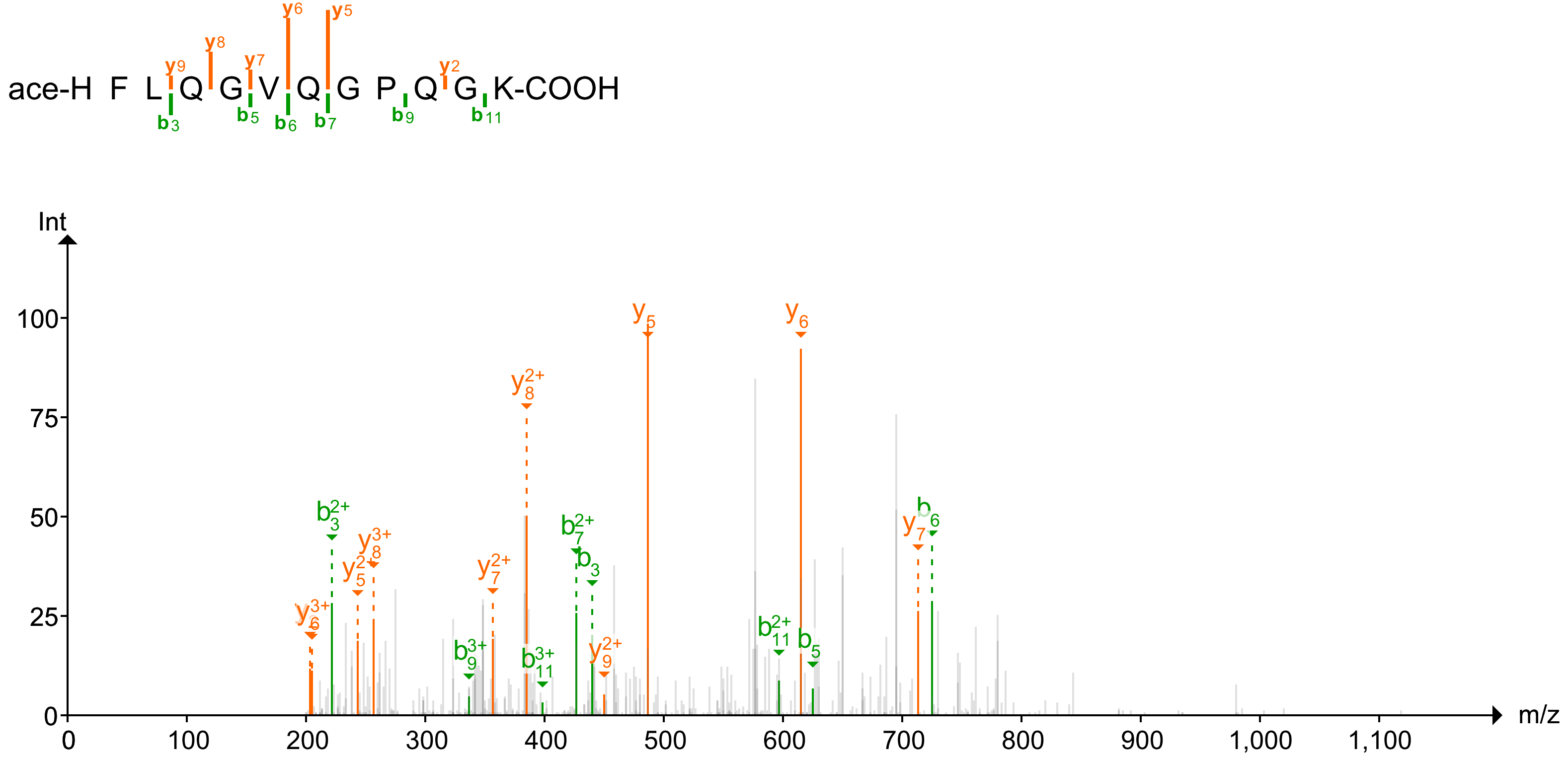

ace-H F L Q G V Q G P Q G K-COOH
y9 y8 y7 y6 y5 y2
b3 b5 b6 b7 b9 b11
Int
100
75
50
25
0
m/z
0
100
200
300
400
500
600
700
800
900
1,000
1,100

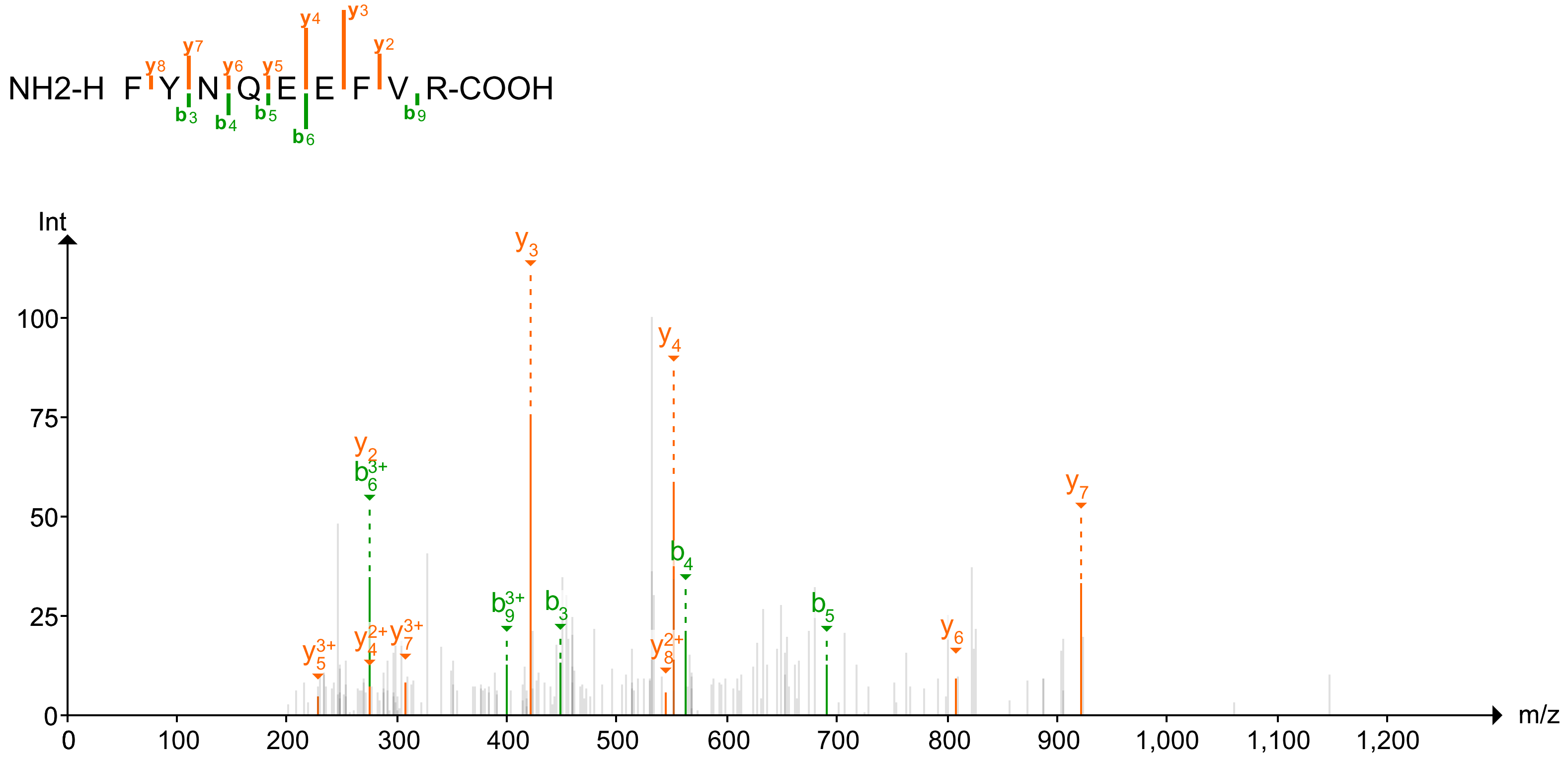

NH2-H F Y N Q E E F V R-COOH
y8 y7 y6 y5 y4 y3 y2
b3 b4 b5 b6 b9
Int
m/z
y3
y4
y2
b6 3+
y7
b4
b9 3+
b3
b5
y6
y5 3+
y4 2+
y7 3+
y8 2+

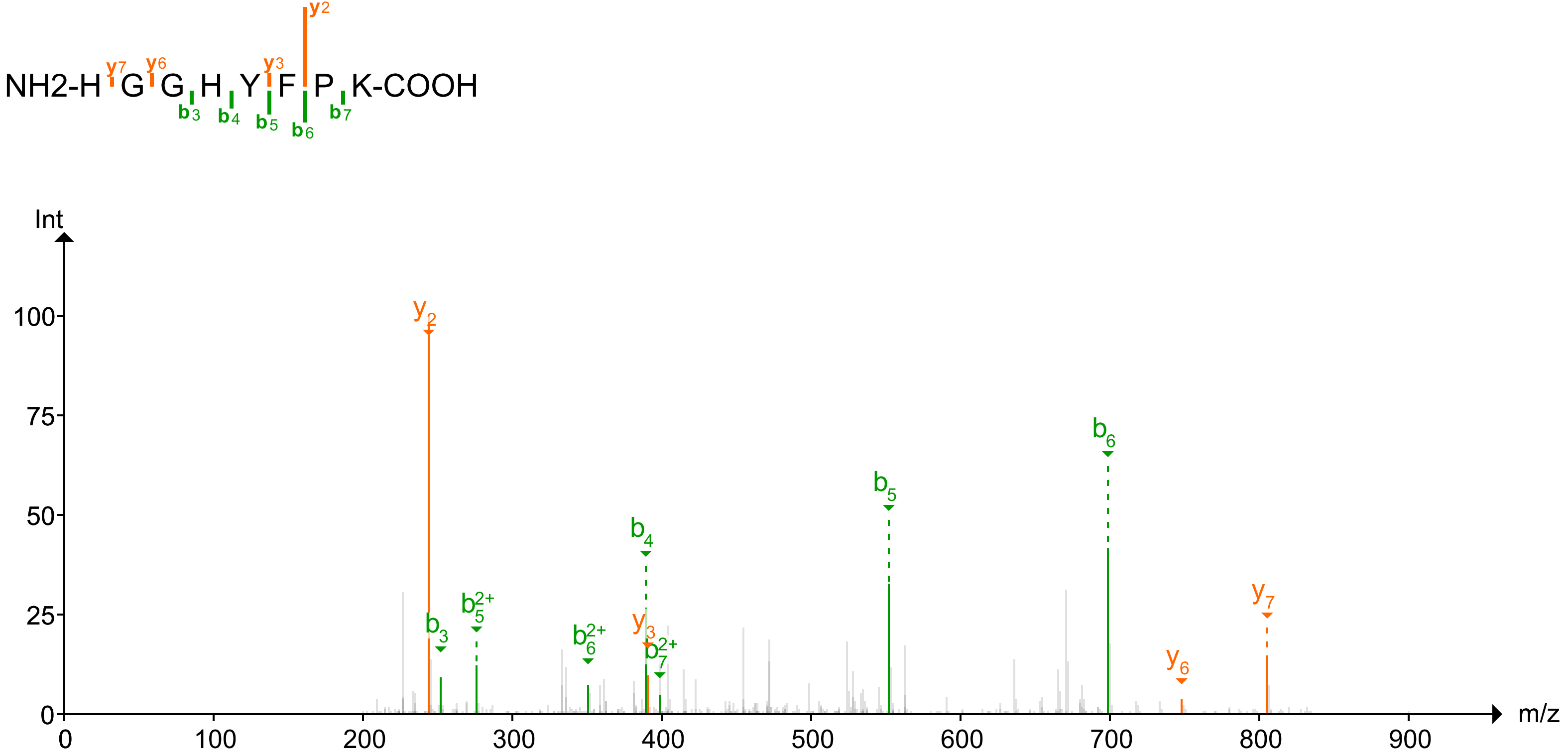

NH2-H G G H Y F P K-COOH
y7 y6 y3 y2
b3 b4 b5 b6 b7
Int
100
75
50
25
0
y2
b3
b5 2+
b6 2+
b4
y3
b7 2+
b5
b6
y6
y7
0 100 200 300 400 500 600 700 800 900
m/z

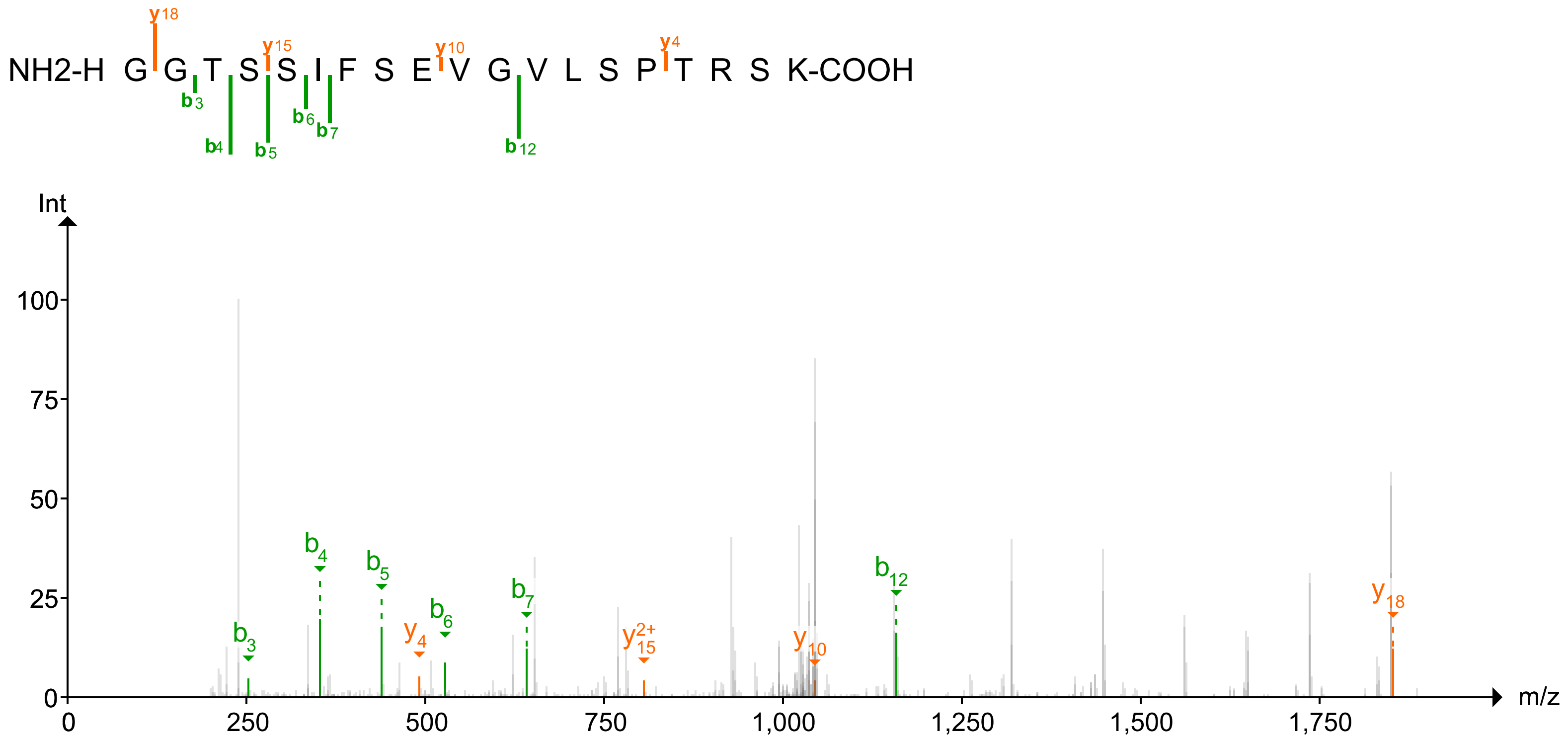

NH2-H G G T S S I F S E V G V L S P T R S K-COOH
y18 y15 y10 y4
b3 b4 b5 b6 b7 b12
Int
100
75
50
25
0
0 250 500 750 1,000 1,250 1,500 1,750
m/z
b3 b4 b5 y4 b6 b7 y15 2+ y10 b12 y18

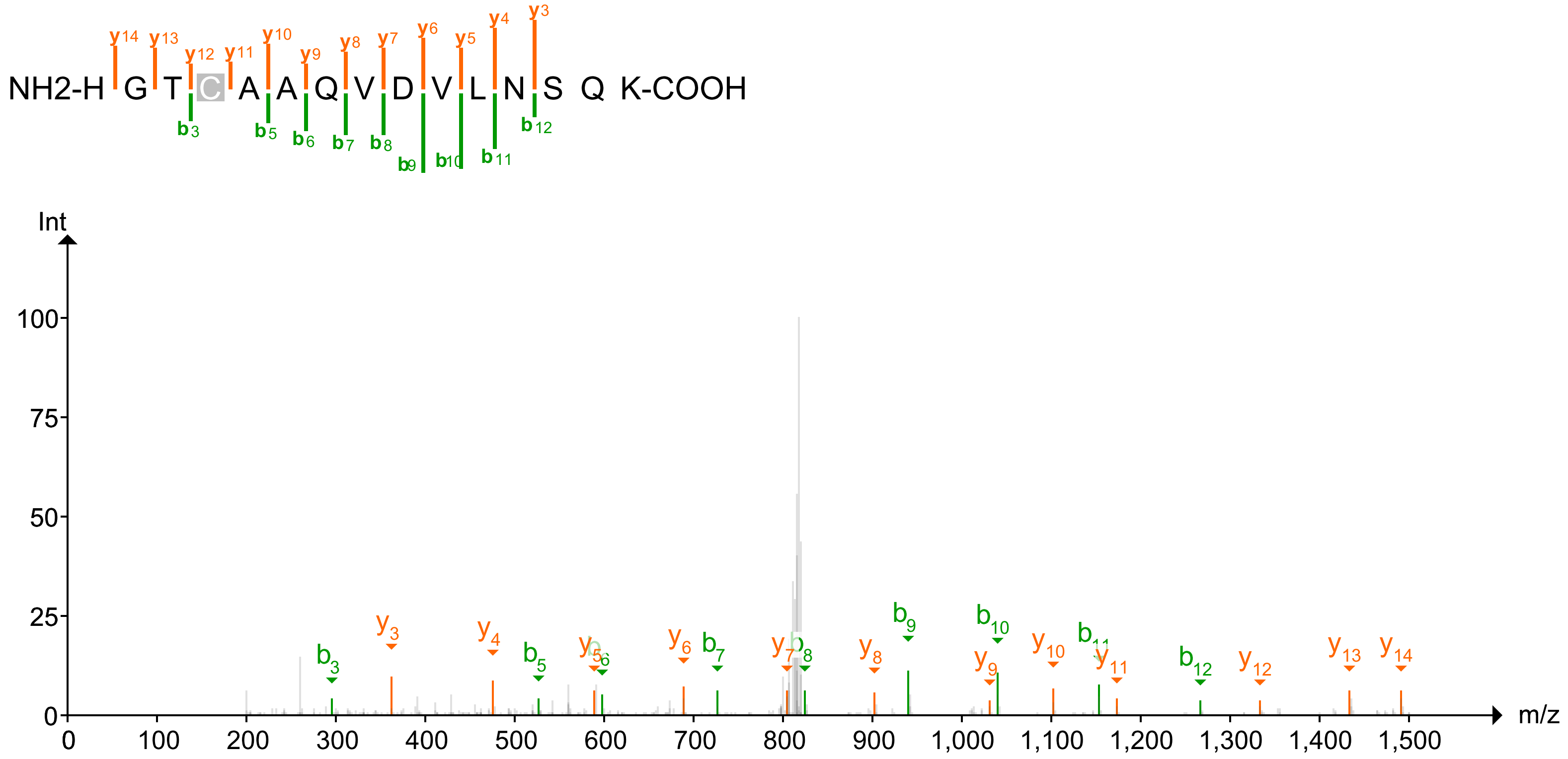

NH2-H G T C A A Q V D V L N S Q K-COOH
y14 y13 y12 y11 y10 y9 y8 y7 y6 y5 y4 y3
b3 b5 b6 b7 b8 b9 b10 b11 b12
Int
100
75
50
25
0
m/z
0 100 200 300 400 500 600 700 800 900 1,000 1,100 1,200 1,300 1,400 1,500

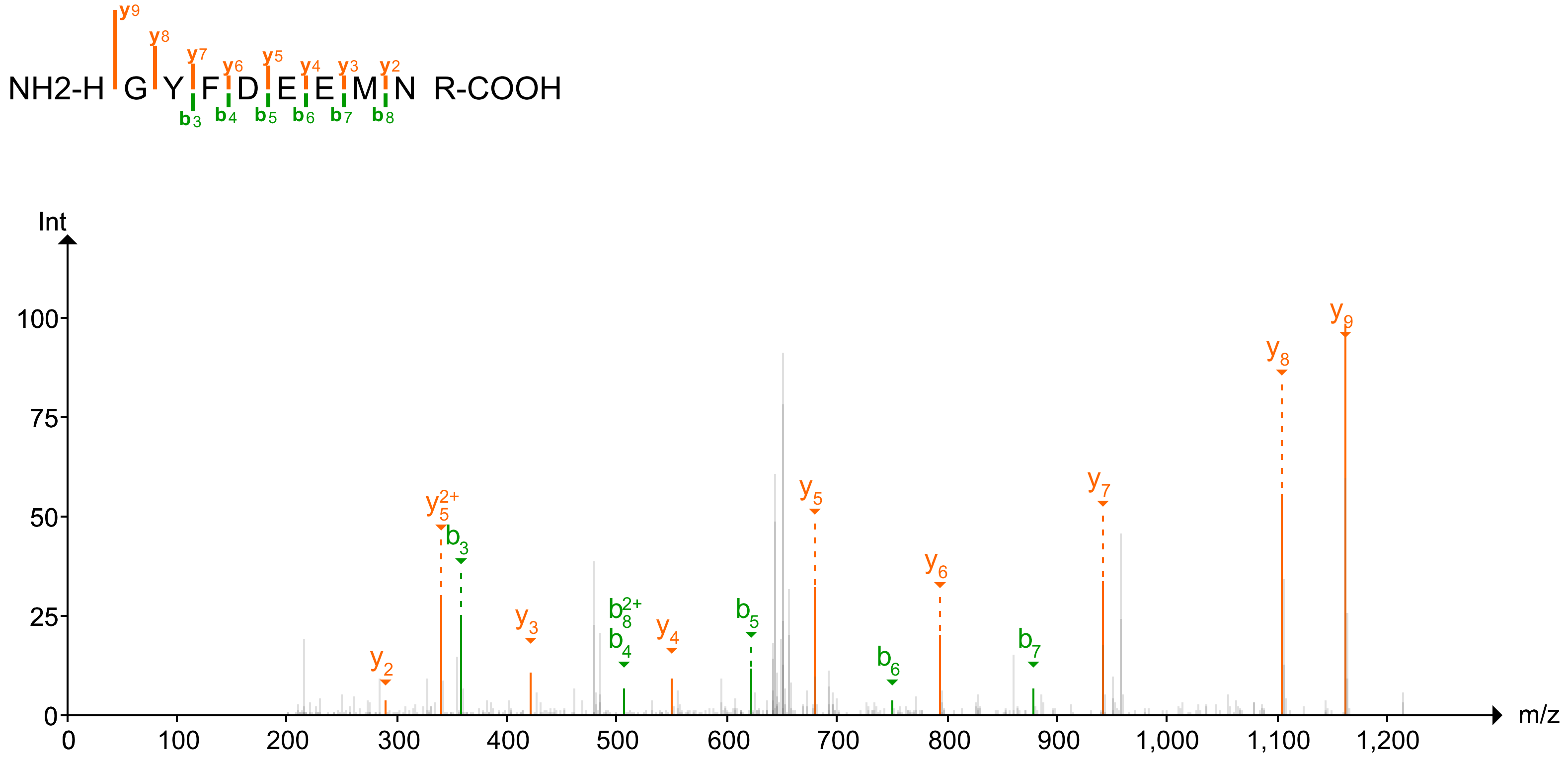

NH2-H G Y F D E E M N R-COOH
y9 y8 y7 y6 y5 y4 y3 y2
b3 b4 b5 b6 b7 b8
Int
100
75
50
25
0
$y_9$
$y_8$
$y_7$
$y_5$
$y_5^{2+}$
$b_3$
$y_6$
$y_3$
$b_8^{2+}$
$b_4$
$y_4$
$b_5$
$b_7$
$b_6$
$y_2$
0 100 200 300 400 500 600 700 800 900 1,000 1,100 1,200
m/z

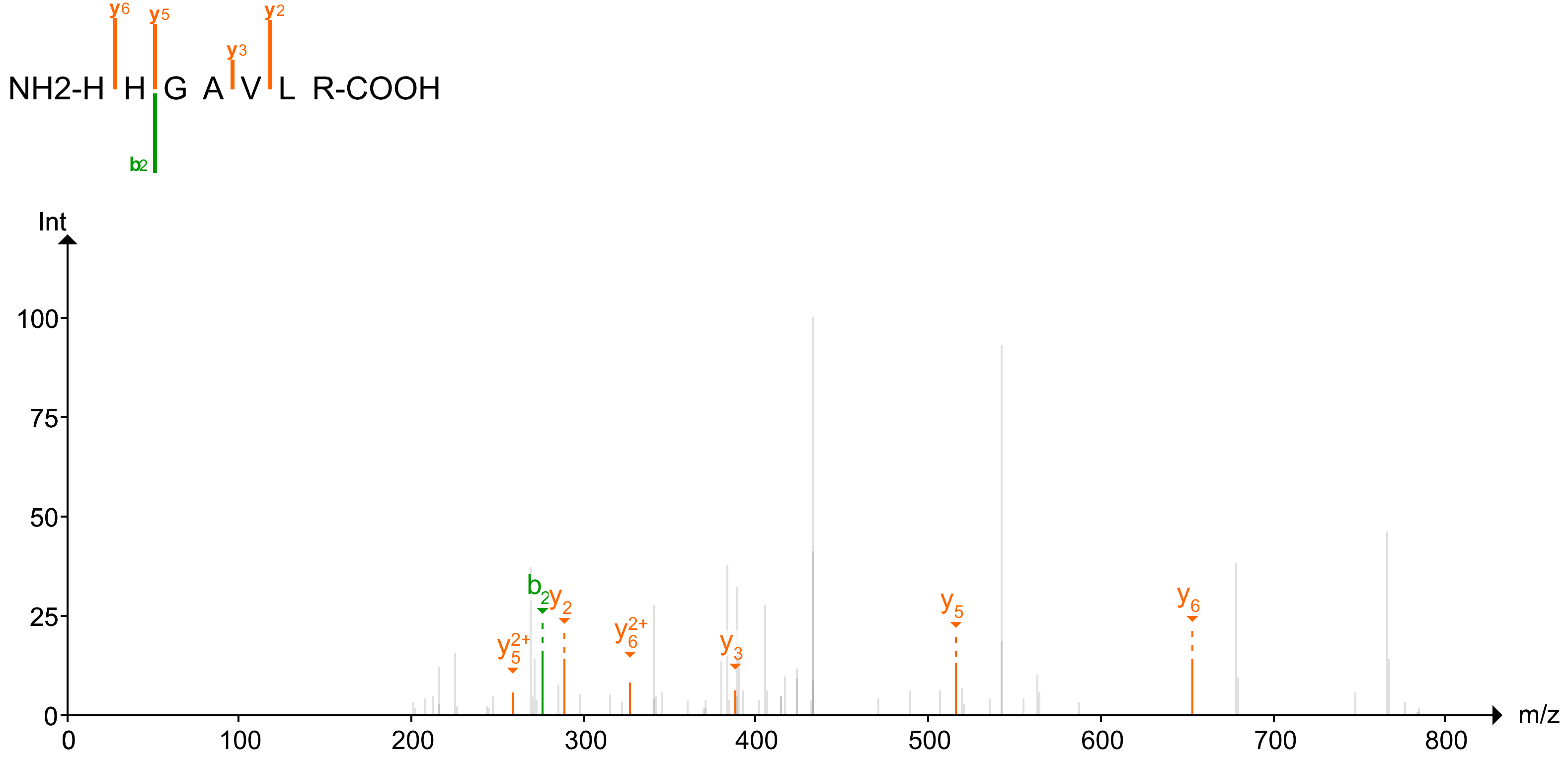

NH2-H H G A V L R-COOH
y6
y5
y3
y2
b2
Int
100
75
50
25
0
b2
y2
y5 2+
y6 2+
y3
y5
y6
0
100
200
300
400
500
600
700
800
m/z

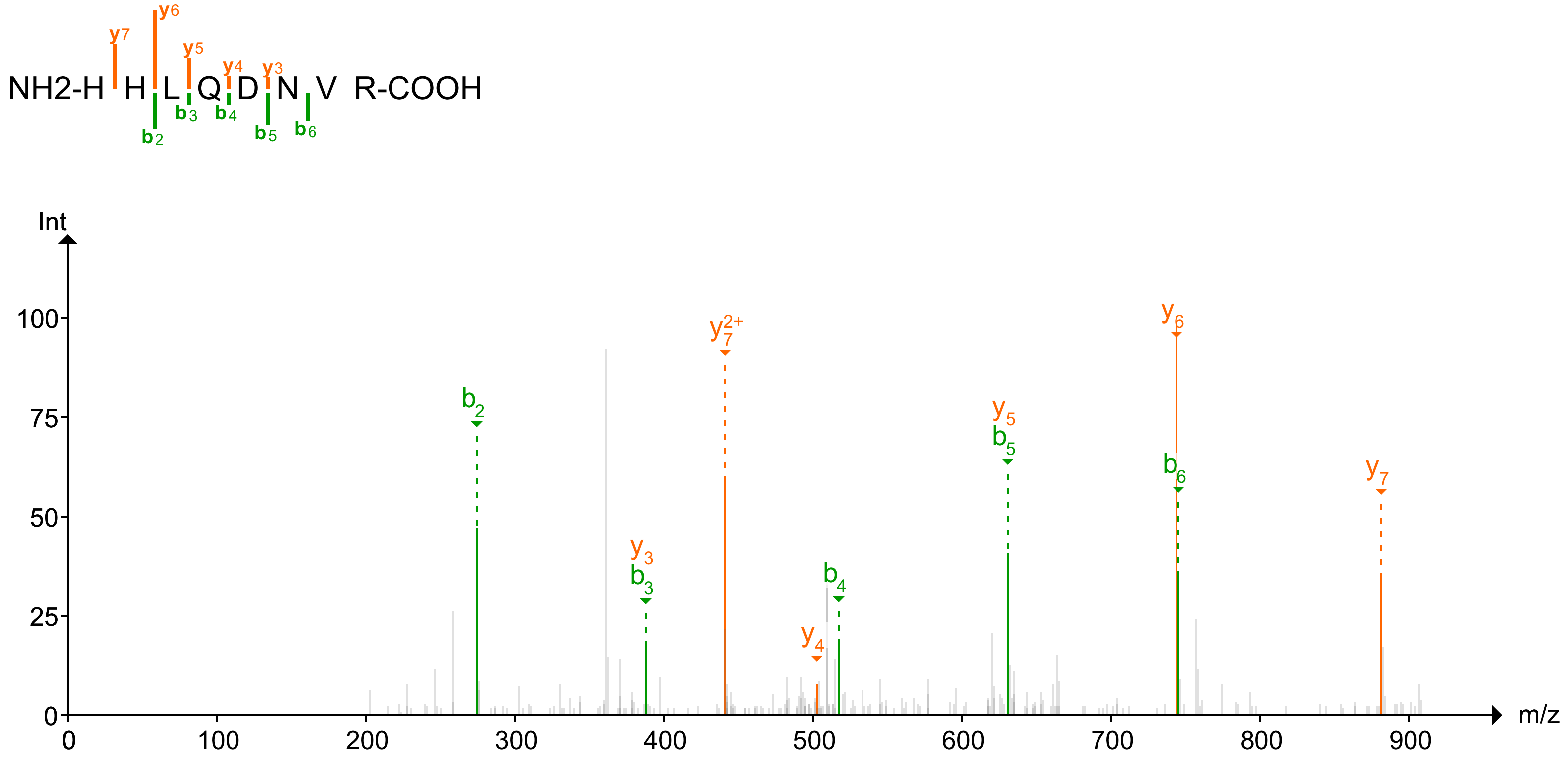

NH2-H H L Q D N V R-COOH
y7 y6 y5 y4 y3
b2 b3 b4 b5 b6
Int
100
75
50
25
0
m/z
0
100
200
300
400
500
600
700
800
900
b2
y3
b3
y7 2+
y4
b4
y5
b5
y6
b6
y7

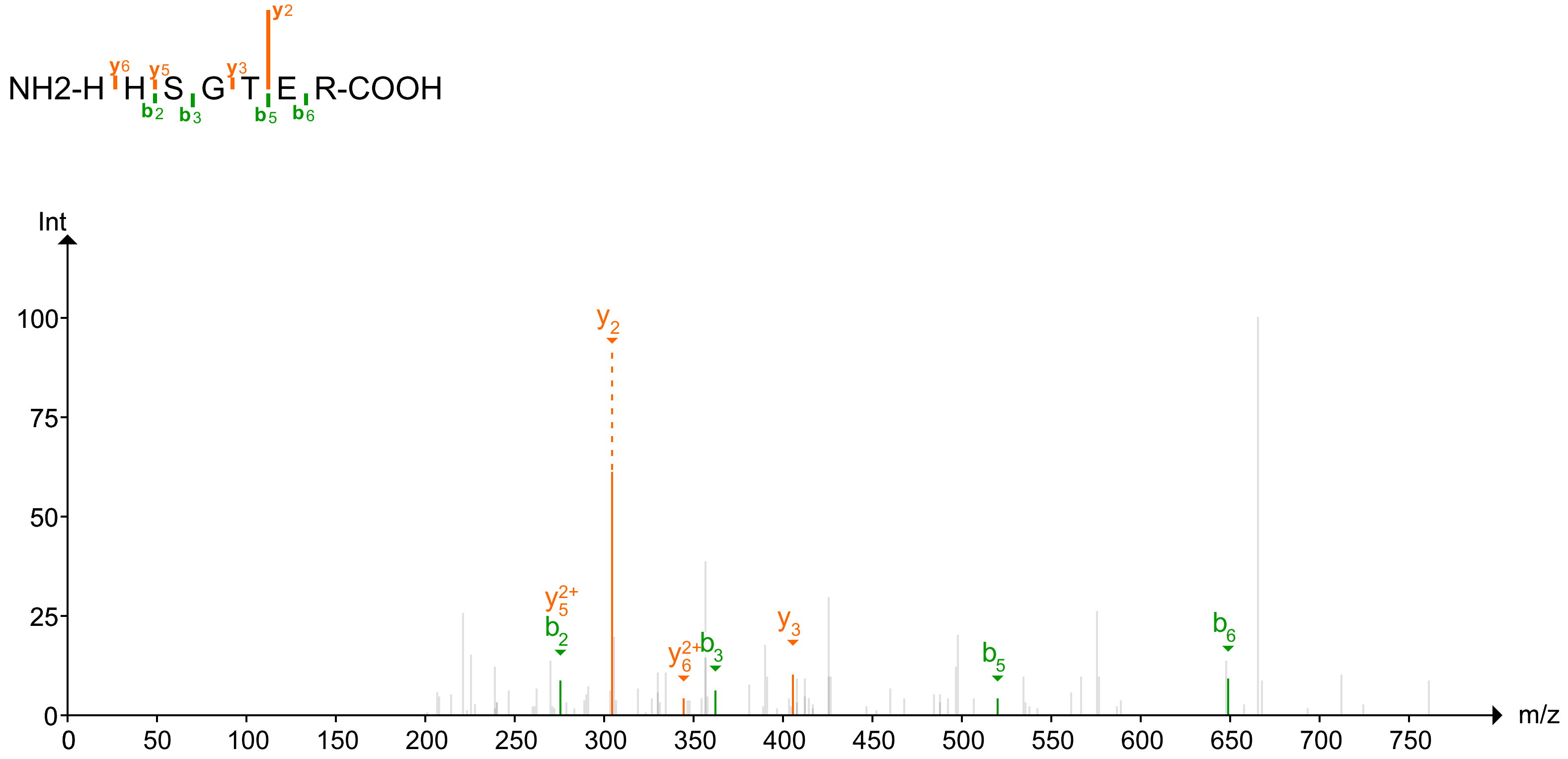

NH2-H H S G T E R-COOH
y6 y5 y3 y2
b2 b3 b5 b6
Int
m/z
0
25
50
75
100
0 50 100 150 200 250 300 350 400 450 500 550 600 650 700 750
y2
y5 2+
b2
y6 2+
b3
y3
b5
b6

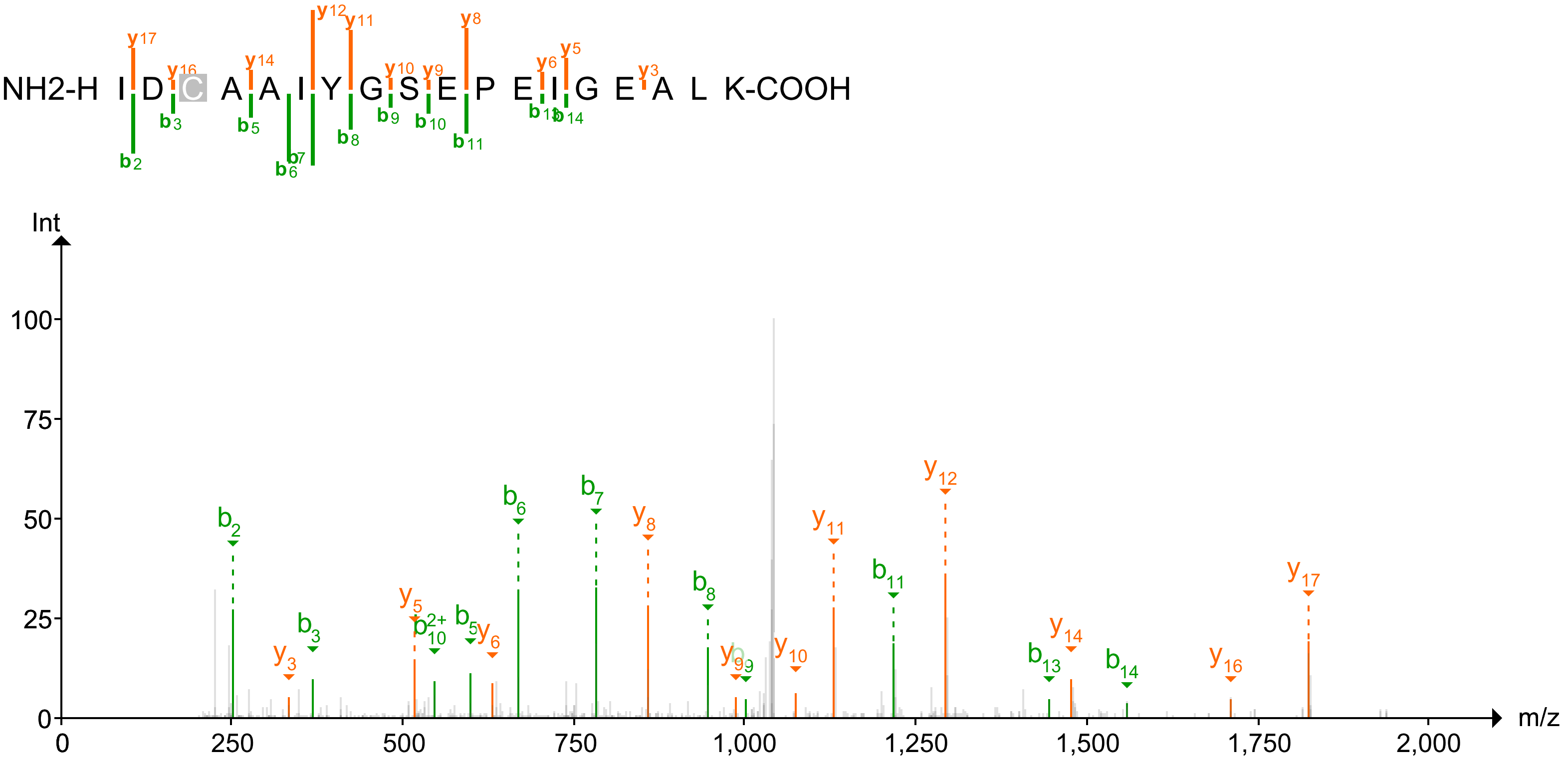
NH2-H I D C A A I Y G S E P E I G E A L K-COOH
y17 y16 y14 y12 y11 y10 y9 y8 y6 y5 y3
b2 b3 b5 b6 b7 b8 b9 b10 b11 b13 b14
Int
100
75
50
25
0
m/z
0
250
500
750
1,000
1,250
1,500
1,750
2,000
b2 y3 b3 y5 b10 2+ b5 y6 b6 b7 y8 b8 y9 b9 y10 y11 b11 y12 b13 y14 b14 y16 y17

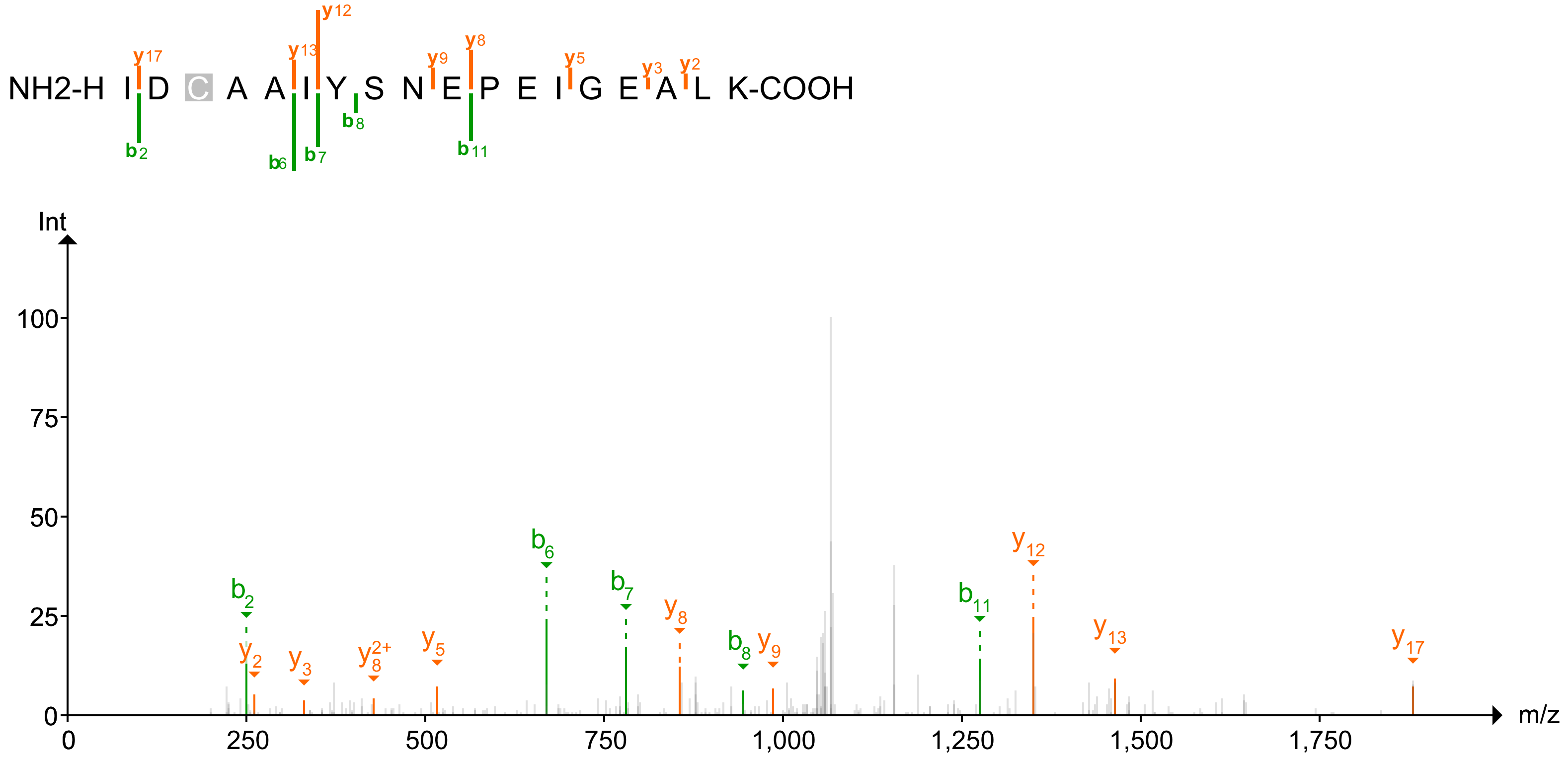
NH2-H I D C A A I Y S N E P E I G E A L K-COOH
y17 y13 y12 y9 y8 y5 y3 y2
b2 b6 b7 b8 b11
Int
100
75
50
25
0
b2 y2 y3 y8 2+ y5 b6 b7 y8 b8 y9 b11 y12 y13 y17
0
250
500
750
1,000
1,250
1,500
1,750
m/z

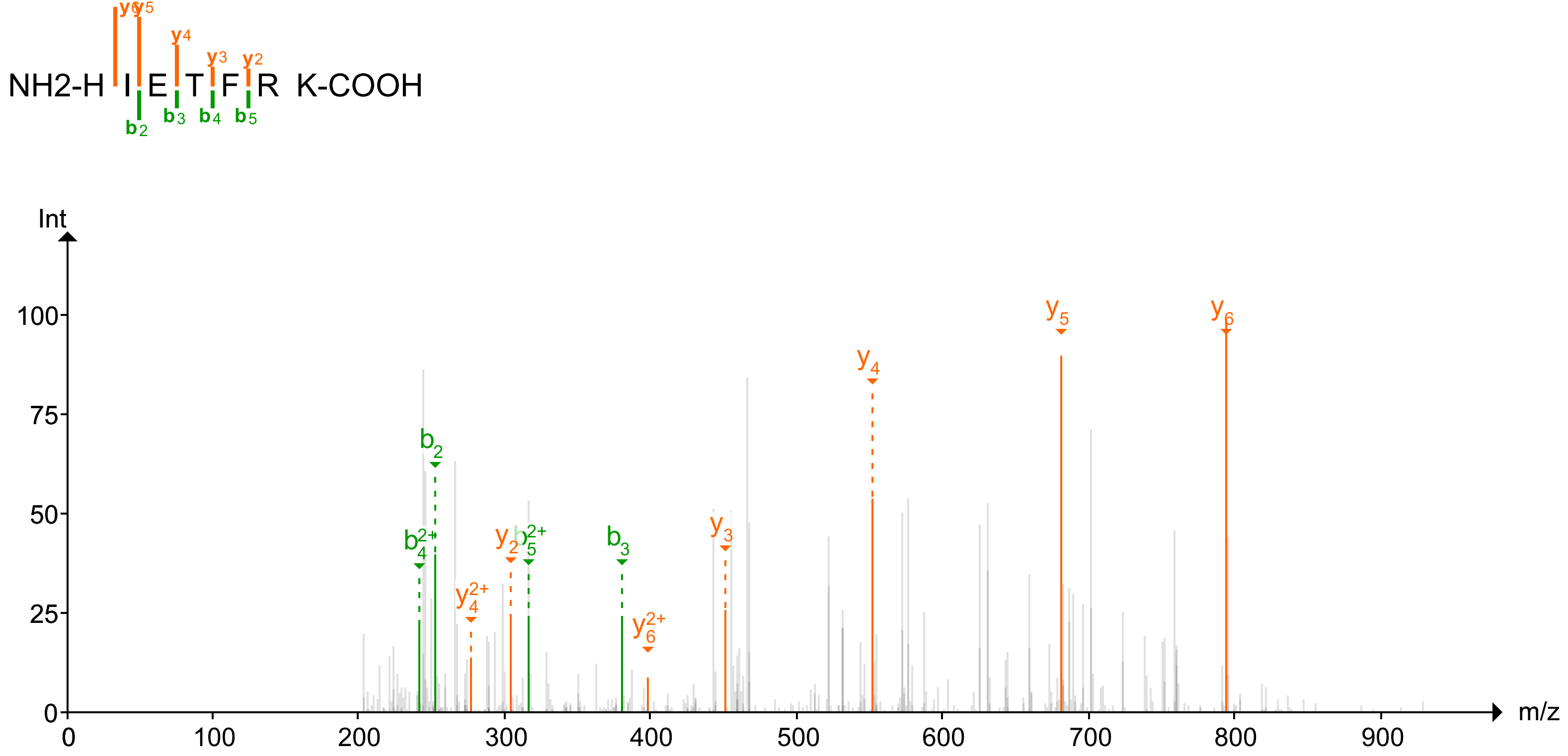

NH2-H I E T F R K-COOH
y6 y5 y4 y3 y2
b2 b3 b4 b5
Int
m/z
y5
y6
y4
b2
y3
b4 2+
y2
b5 2+
b3
y4 2+
y6 2+

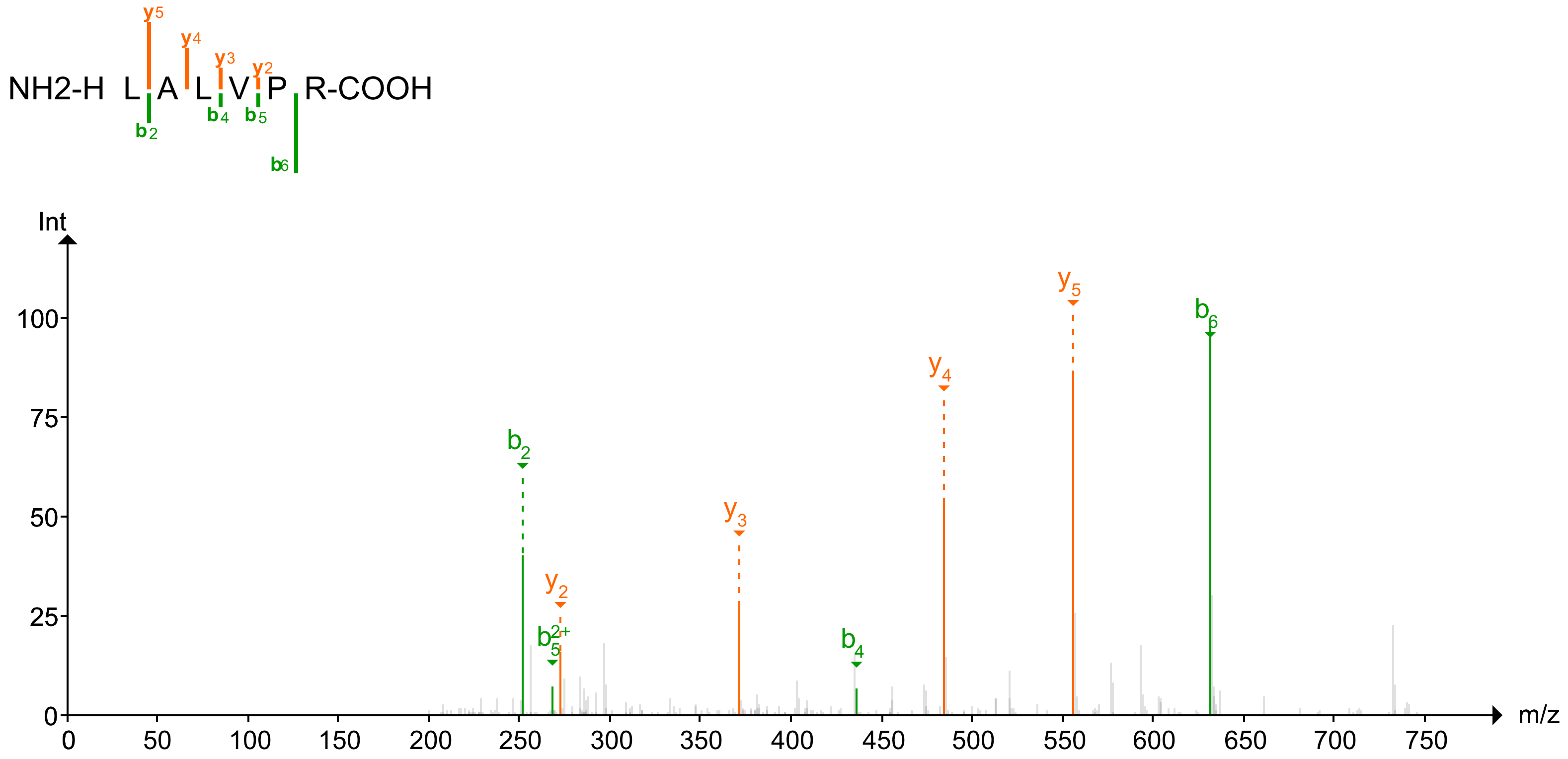

NH2-H L A L V P R-COOH
y5
y4
y3
y2
b2
b4
b5
b6
Int
100
75
50
25
0
m/z
0
50
100
150
200
250
300
350
400
450
500
550
600
650
700
750
$b_2$
$y_2$
$b_5^{2+}$
$y_3$
$b_4$
$y_4$
$y_5$
$b_6$

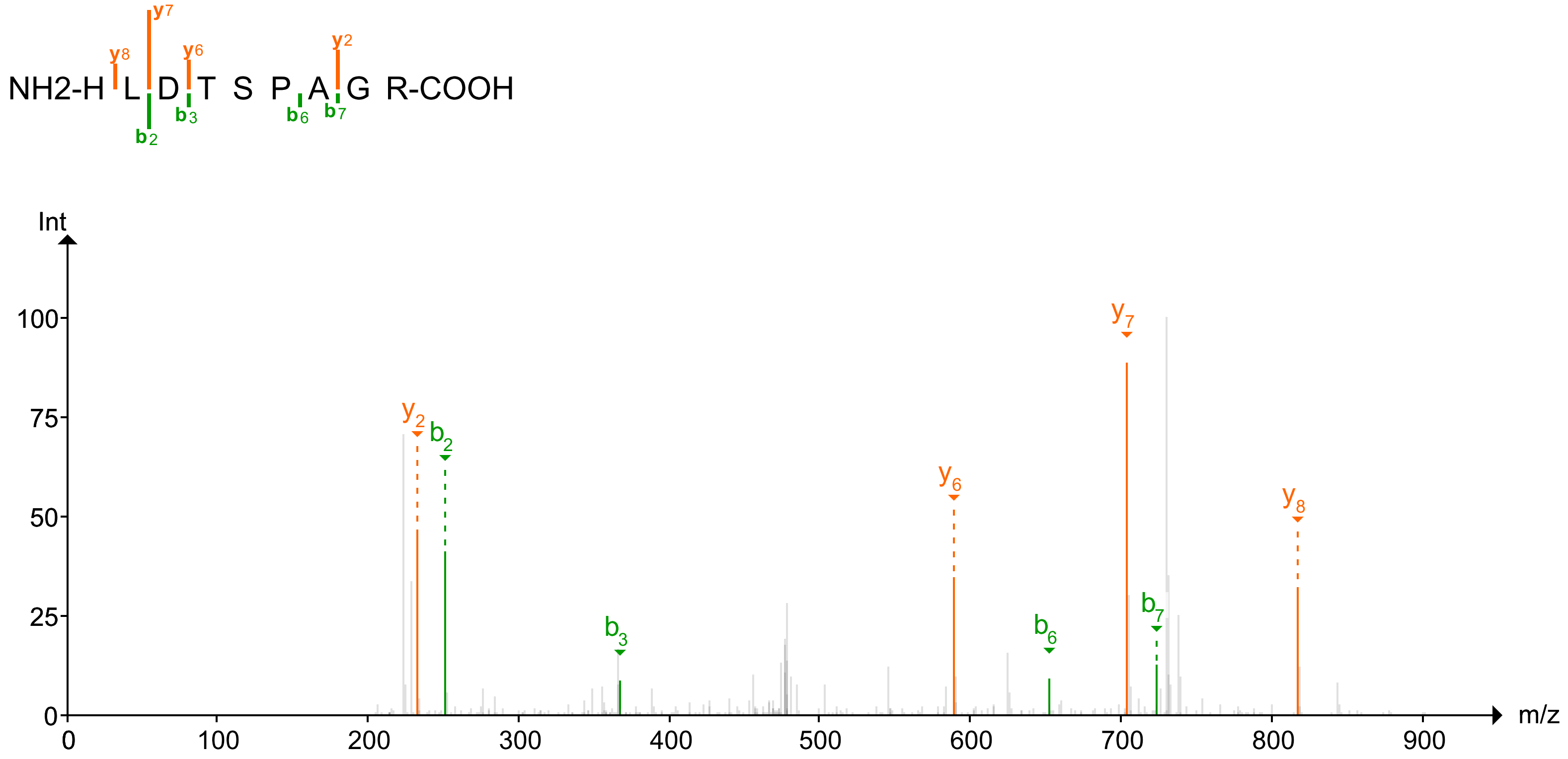

NH2-H L D T S P A G R-COOH
y7
y8
y6
y2
b2
b3
b6
b7
Int
m/z
100
75
50
25
0
0
100
200
300
400
500
600
700
800
900

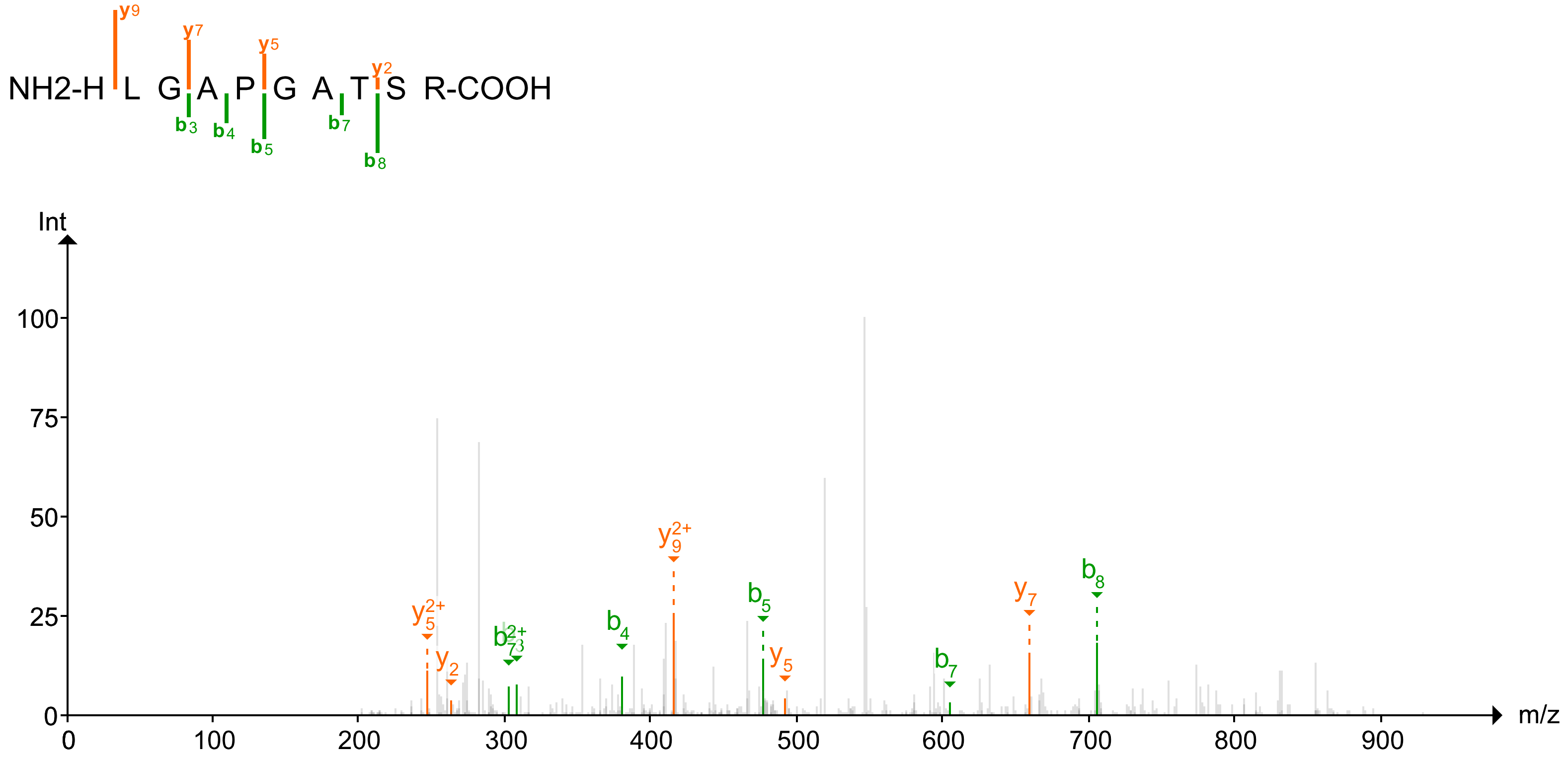

NH2-H L G A P G A T S R-COOH
y9
y7
y5
y2
b3
b4
b5
b7
b8
Int
100
75
50
25
0
m/z
0
100
200
300
400
500
600
700
800
900

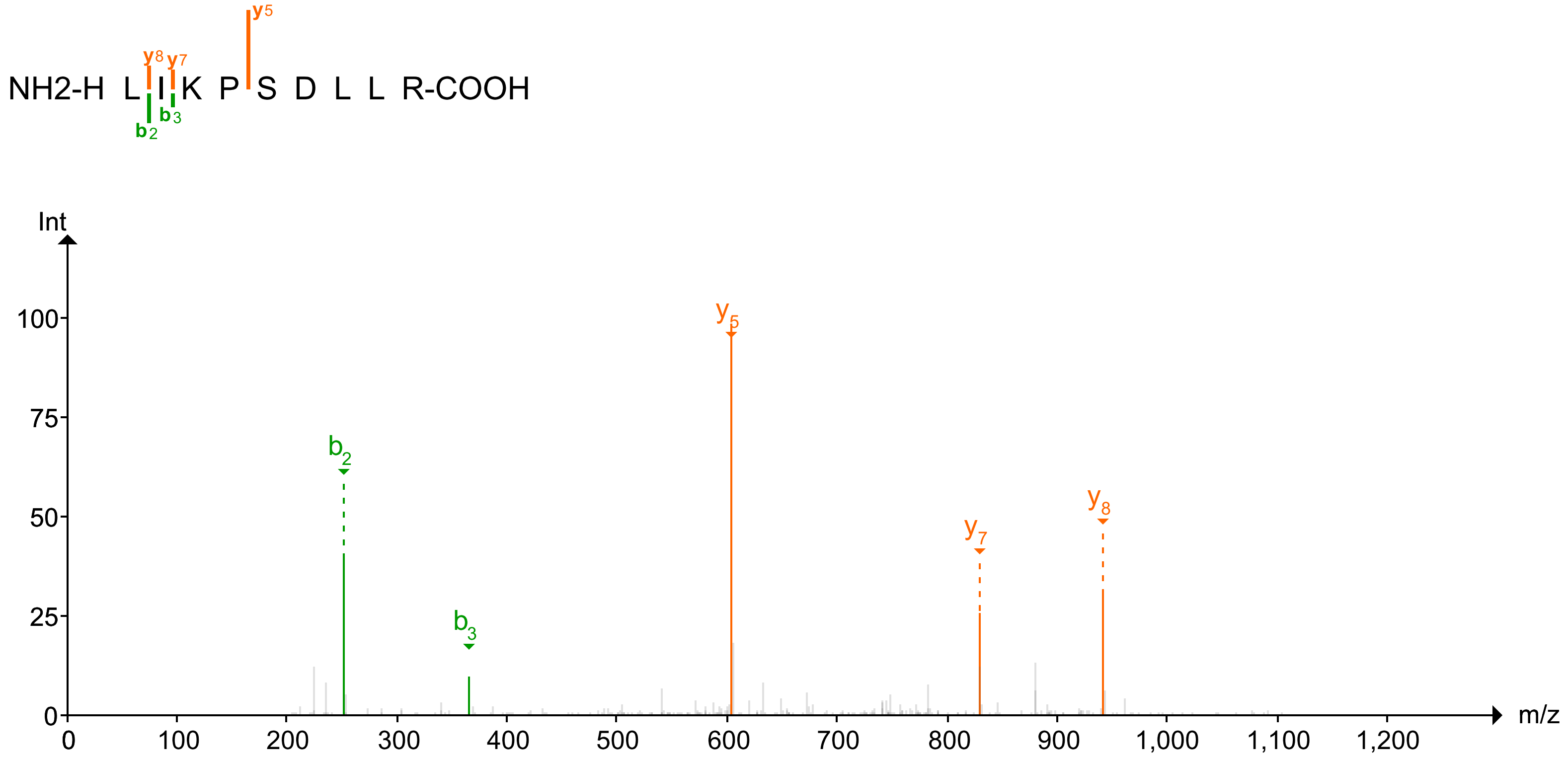

NH2-H L I K P S D L L R-COOH
y5
y8
y7
b2
b3
Int
100
75
50
25
0
m/z
0
100
200
300
400
500
600
700
800
900
1,000
1,100
1,200

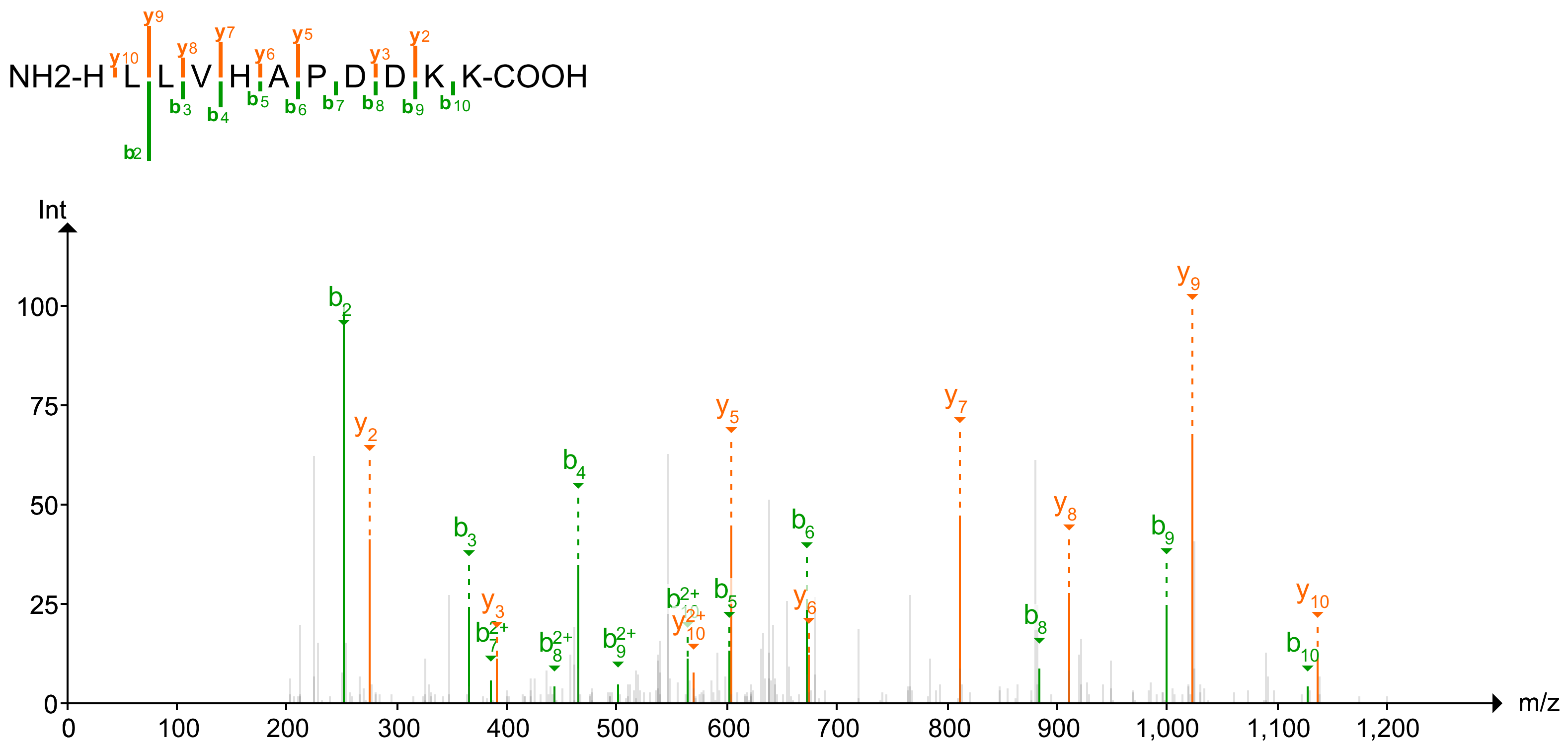

NH2-H L L V H A P D D K K-COOH
Int
m/z

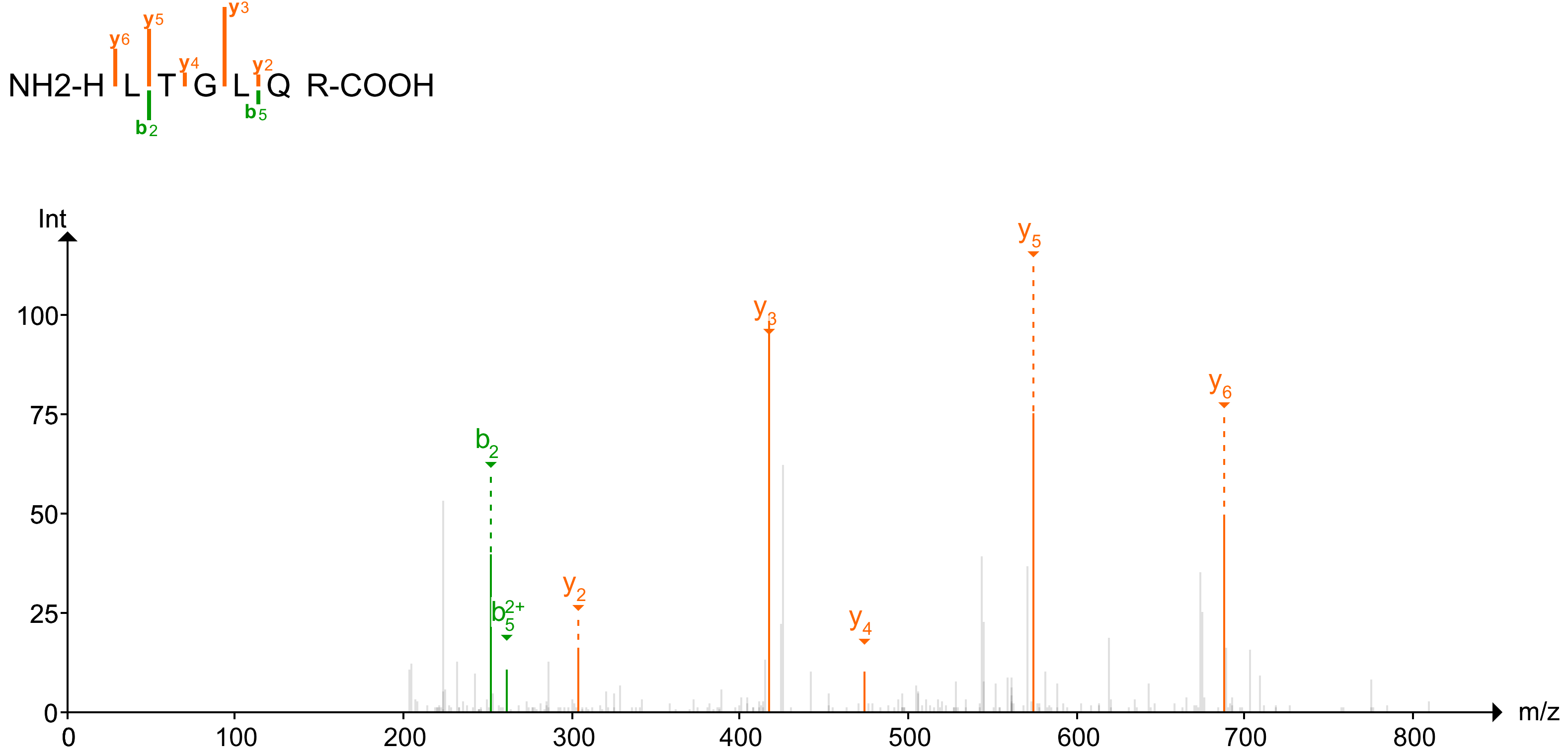

NH2-H L T G L Q R-COOH
y6 y5 y4 y3 y2
b2 b5
Int
100
75
50
25
0
m/z
0
100
200
300
400
500
600
700
800
b2
b5 2+
y2
y3
y4
y5
y6

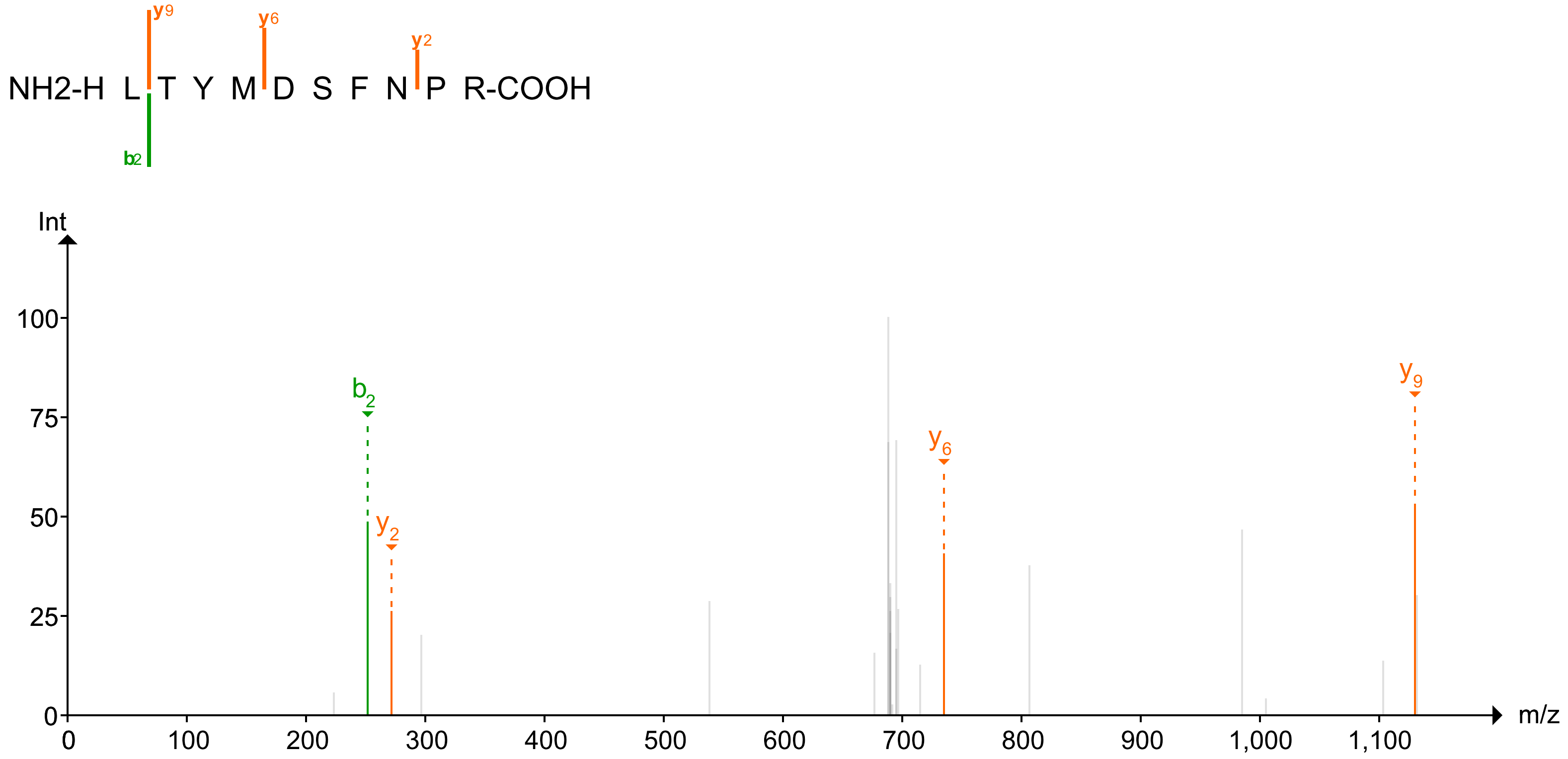

NH2-H L T Y M D S F N P R-COOH
y9
y6
y2
b2
Int
100
75
50
25
0
m/z
0
100
200
300
400
500
600
700
800
900
1,000
1,100
b2
y2
y6
y9

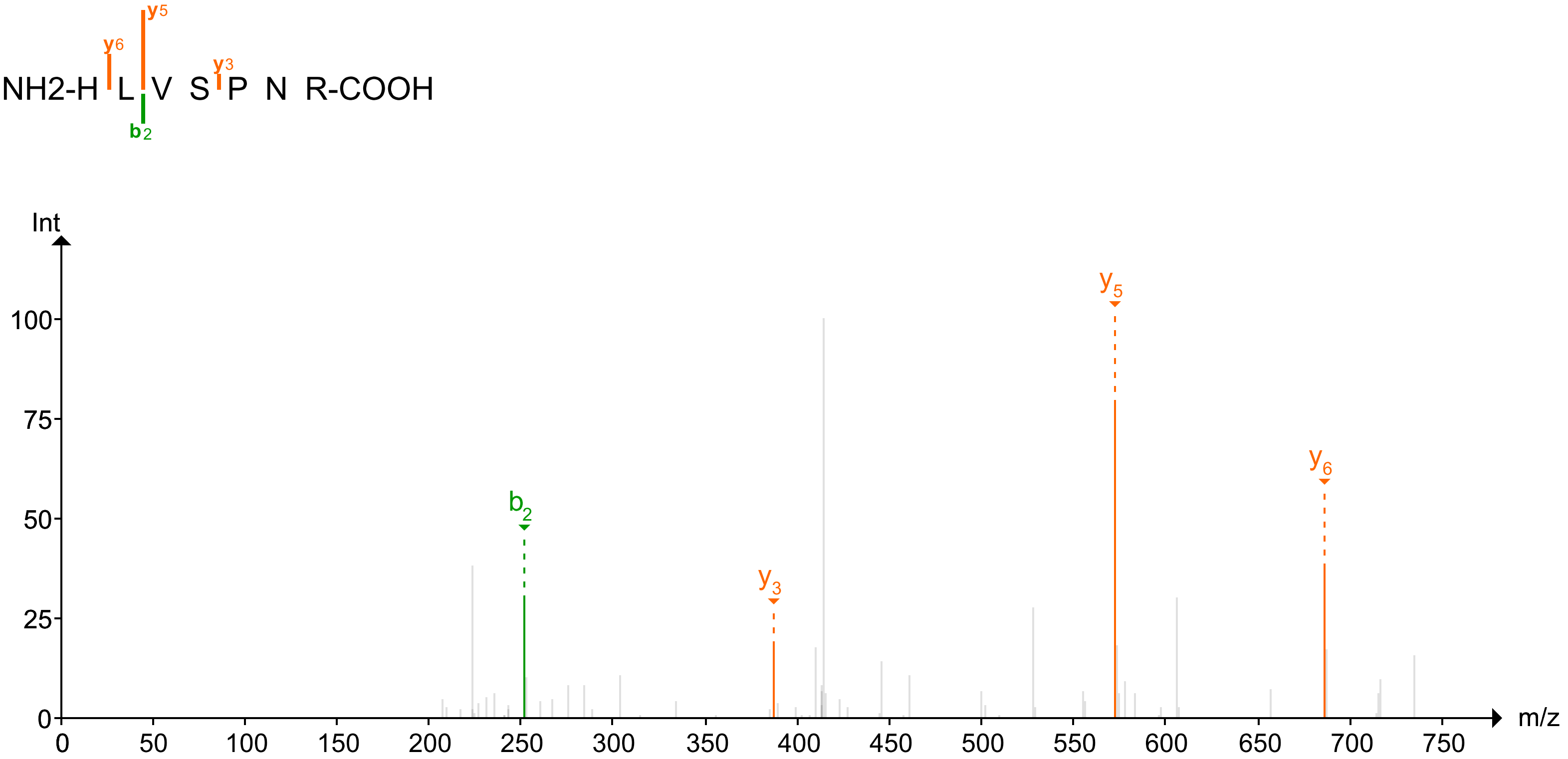

NH2-H L V S P N R-COOH
y6
y5
y3
b2
Int
m/z
0
25
50
75
100
0
50
100
150
200
250
300
350
400
450
500
550
600
650
700
750

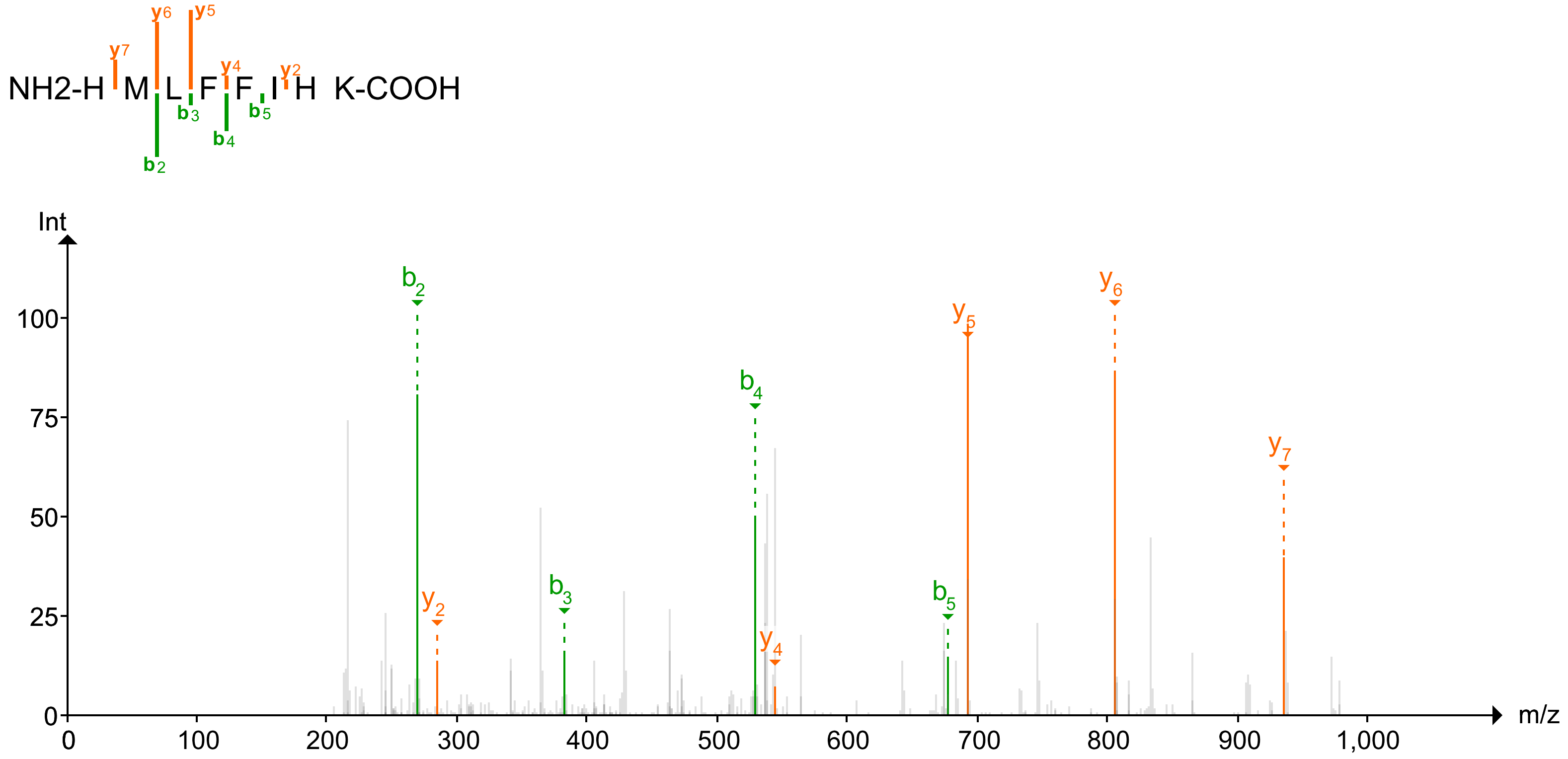

NH2-H M L F F I H K-COOH
y7
y6
y5
y4
y2
b2
b3
b4
b5
Int
m/z
100
75
50
25
0
0
100
200
300
400
500
600
700
800
900
1,000

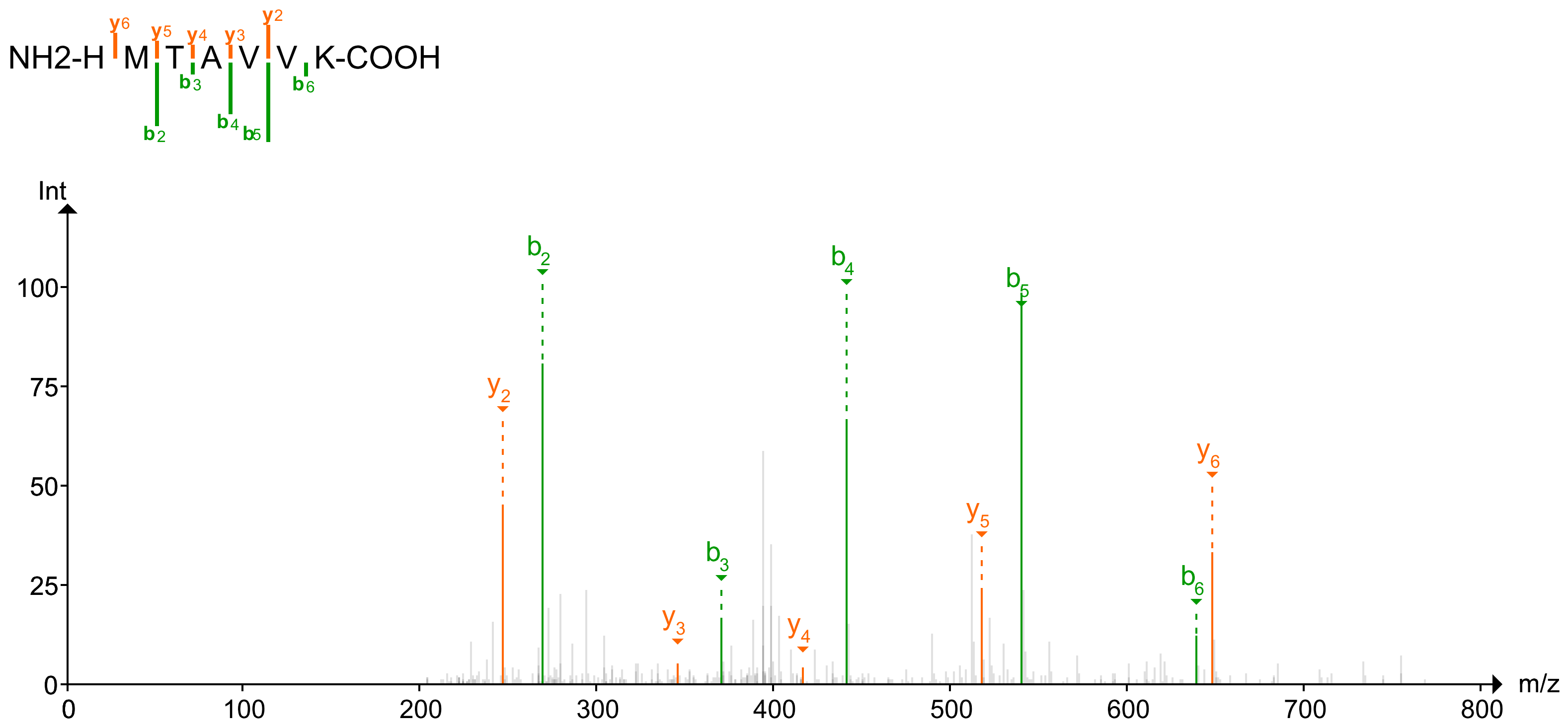

NH2-H M T A V V K-COOH
y6 y5 y4 y3 y2
b2 b3 b4 b5 b6
Int
100
75
50
25
0
b2
b4
b5
y2
y6
y5
b3
y3
y4
b6
0
100
200
300
400
500
600
700
800
m/z

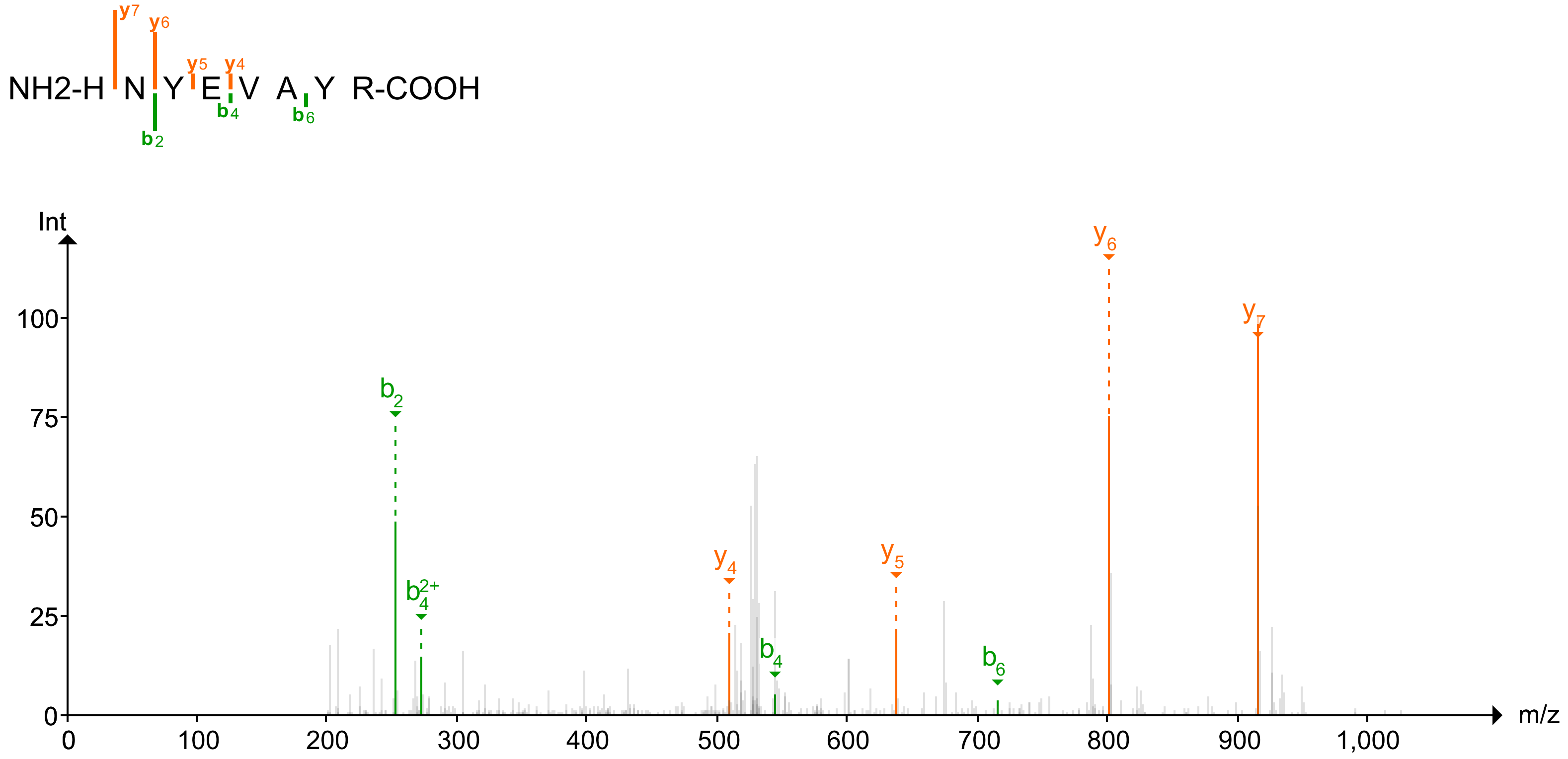

NH2-H N Y E V A Y R-COOH
y7
y6
y5
y4
b2
b4
b6
Int
100
75
50
25
0
0
100
200
300
400
500
600
700
800
900
1,000
m/z
b2
b4 2+
y4
b4
y5
b6
y6
y7

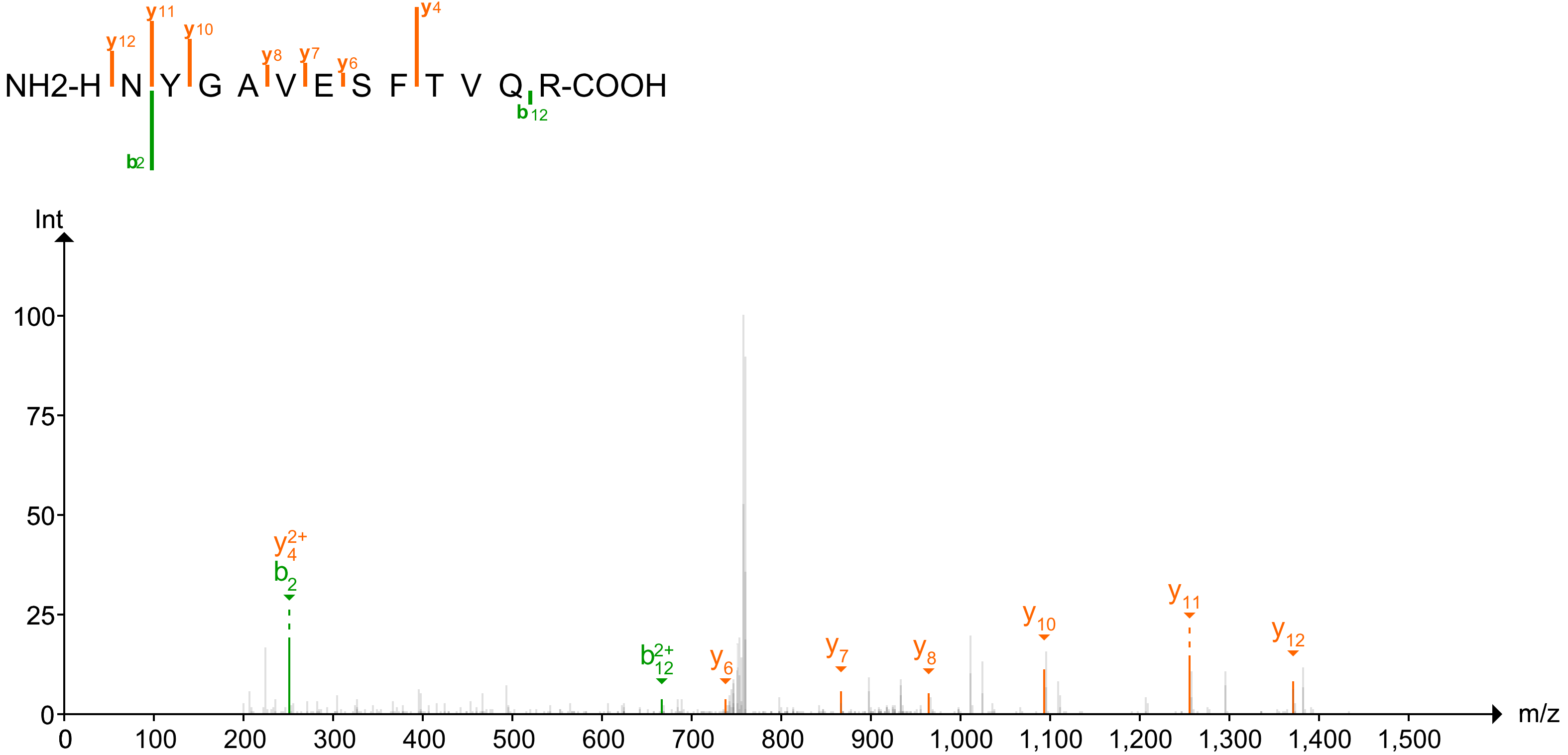

NH2-H N Y G A V E S F T V Q R-COOH
y12 y11 y10 y8 y7 y6 y4
b2 b12
Int
100
75
50
25
0
$y_4^{2+}$
$b_2$
$b_{12}^{2+}$
$y_6$
$y_7$
$y_8$
$y_{10}$
$y_{11}$
$y_{12}$
0 100 200 300 400 500 600 700 800 900 1,000 1,100 1,200 1,300 1,400 1,500
m/z

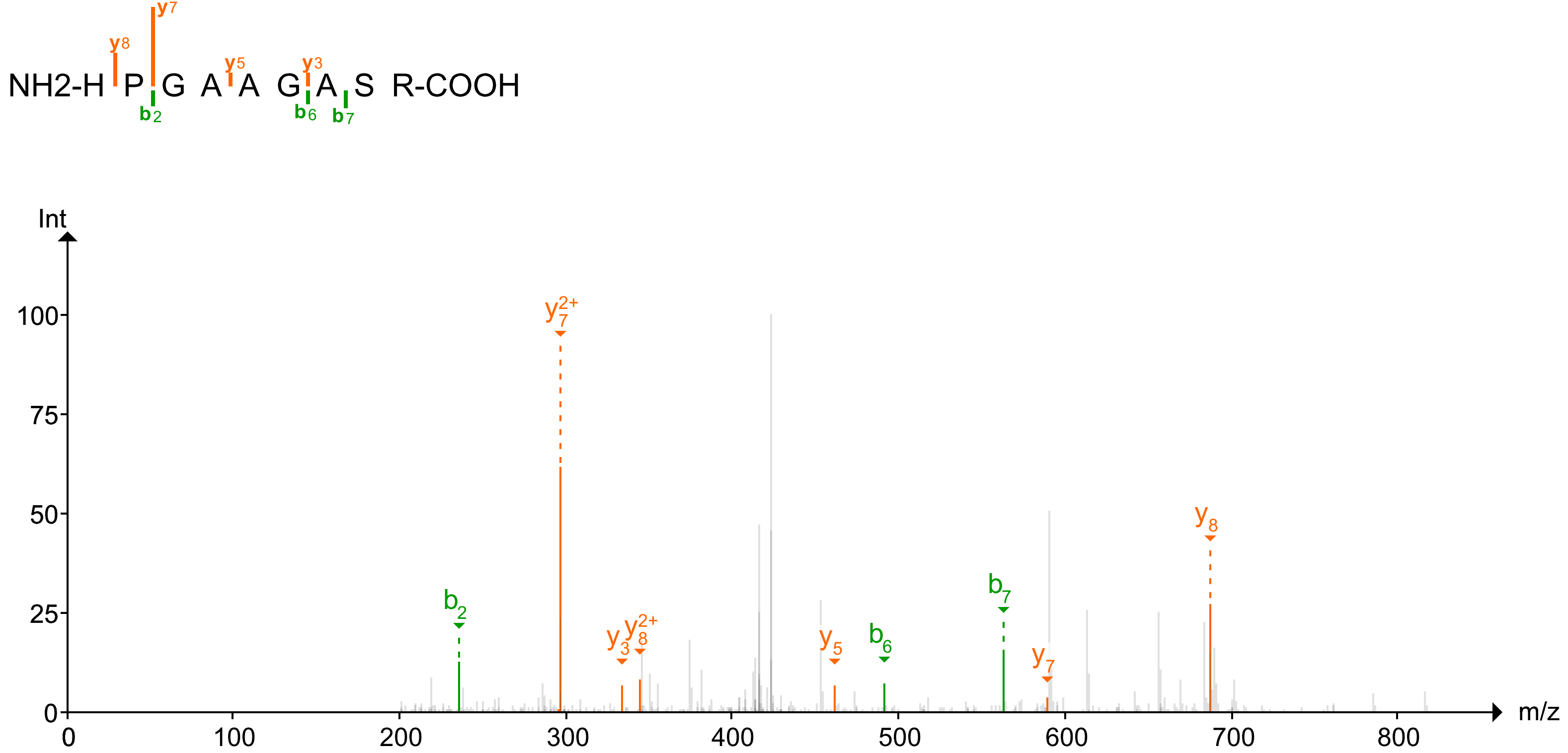

NH2-H P G A A G A S R-COOH
y8 y7 y5 y3
b2 b6 b7
Int
100
75
50
25
0
0 100 200 300 400 500 600 700 800
m/z
$y_7^{2+}$
$b_2$
$y_3$
$y_8^{2+}$
$y_5$
$b_6$
$b_7$
$y_7$
$y_8$

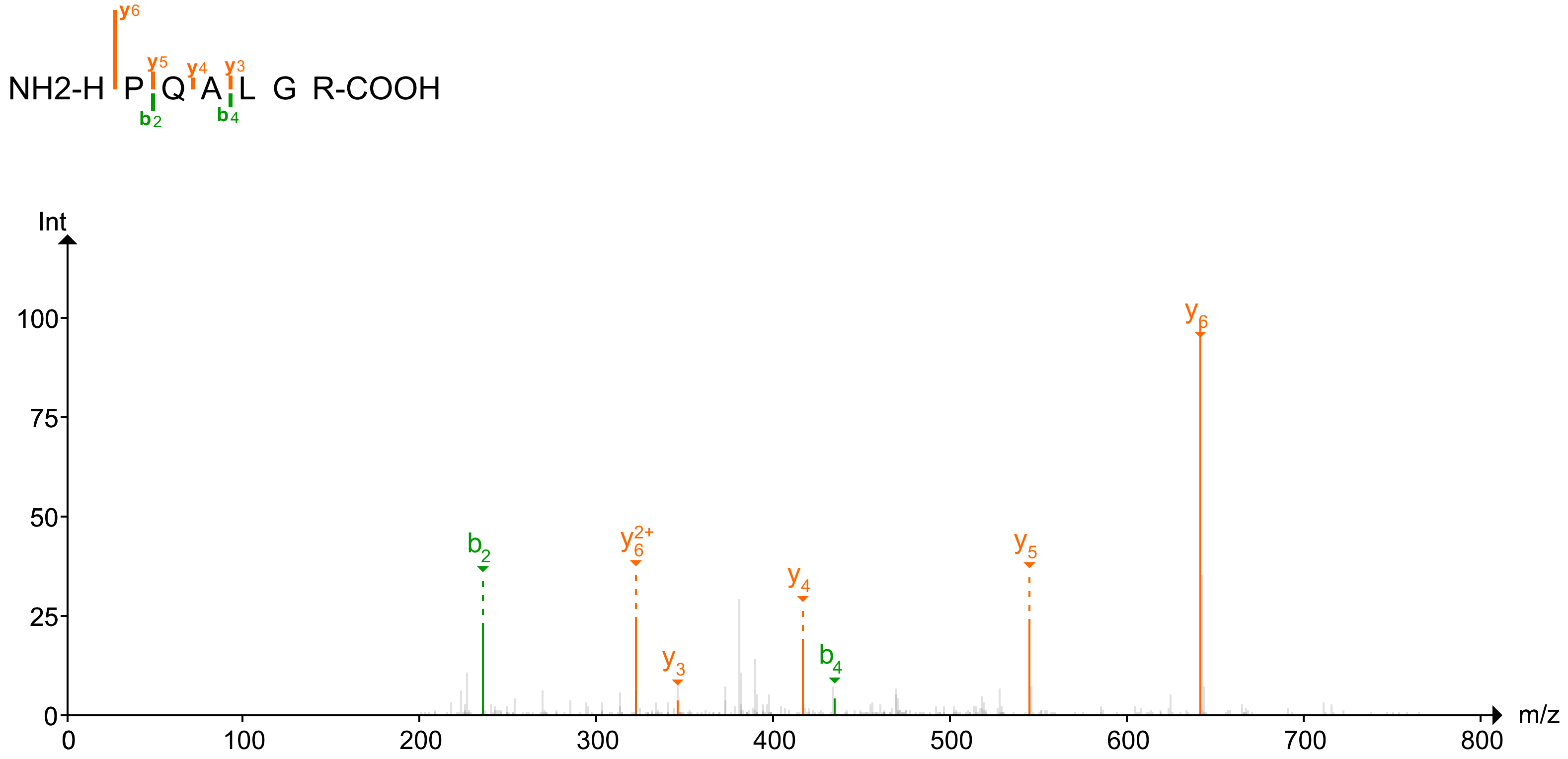

NH2-H P Q A L G R-COOH
y6
y5
y4
y3
b2
b4
Int
100
75
50
25
0
0
100
200
300
400
500
600
700
800
m/z
b2
y6 2+
y3
y4
b4
y5
y6

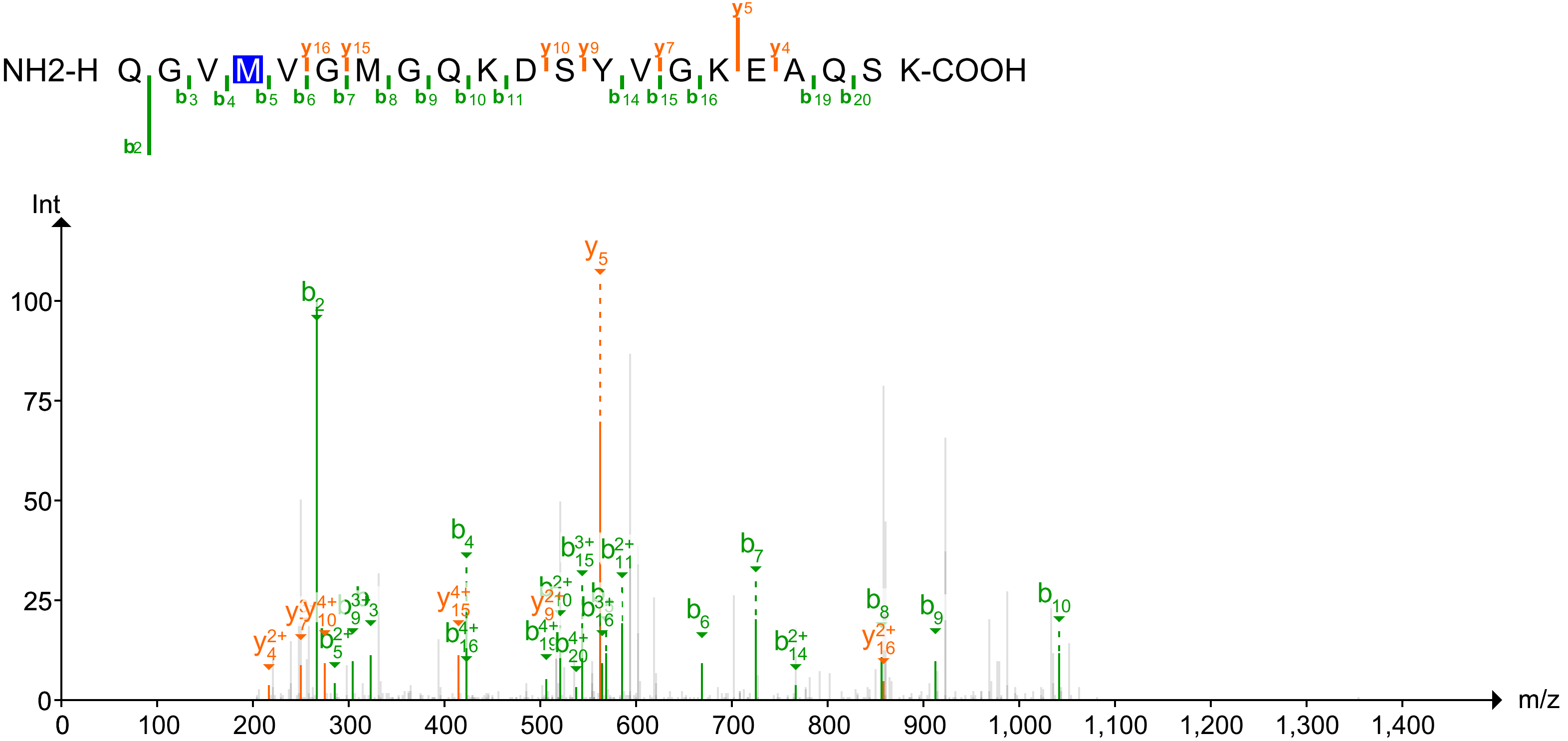

NH2-H Q G V M V G M G Q K D S Y V G K E A Q S K-COOH
Int
m/z
0
25
50
75
100
0
100
200
300
400
500
600
700
800
900
1,000
1,100
1,200
1,300
1,400

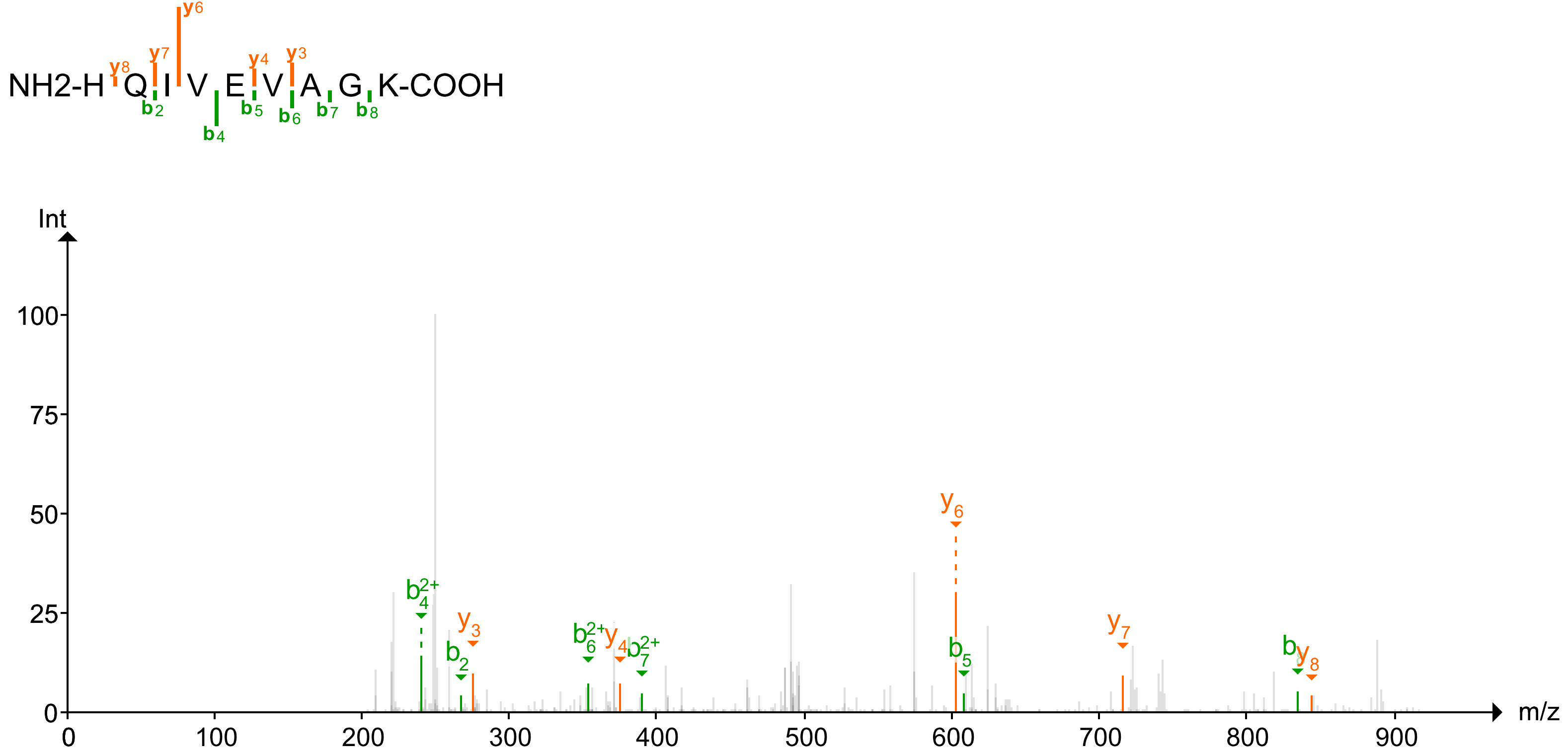
NH2-HQIVEVAGK-COOH
Int
m/z
y6
y7
y8
y4
y3
b2
b4
b5
b6
b7
b8
0
25
50
75
100
0
100
200
300
400
500
600
700
800
900

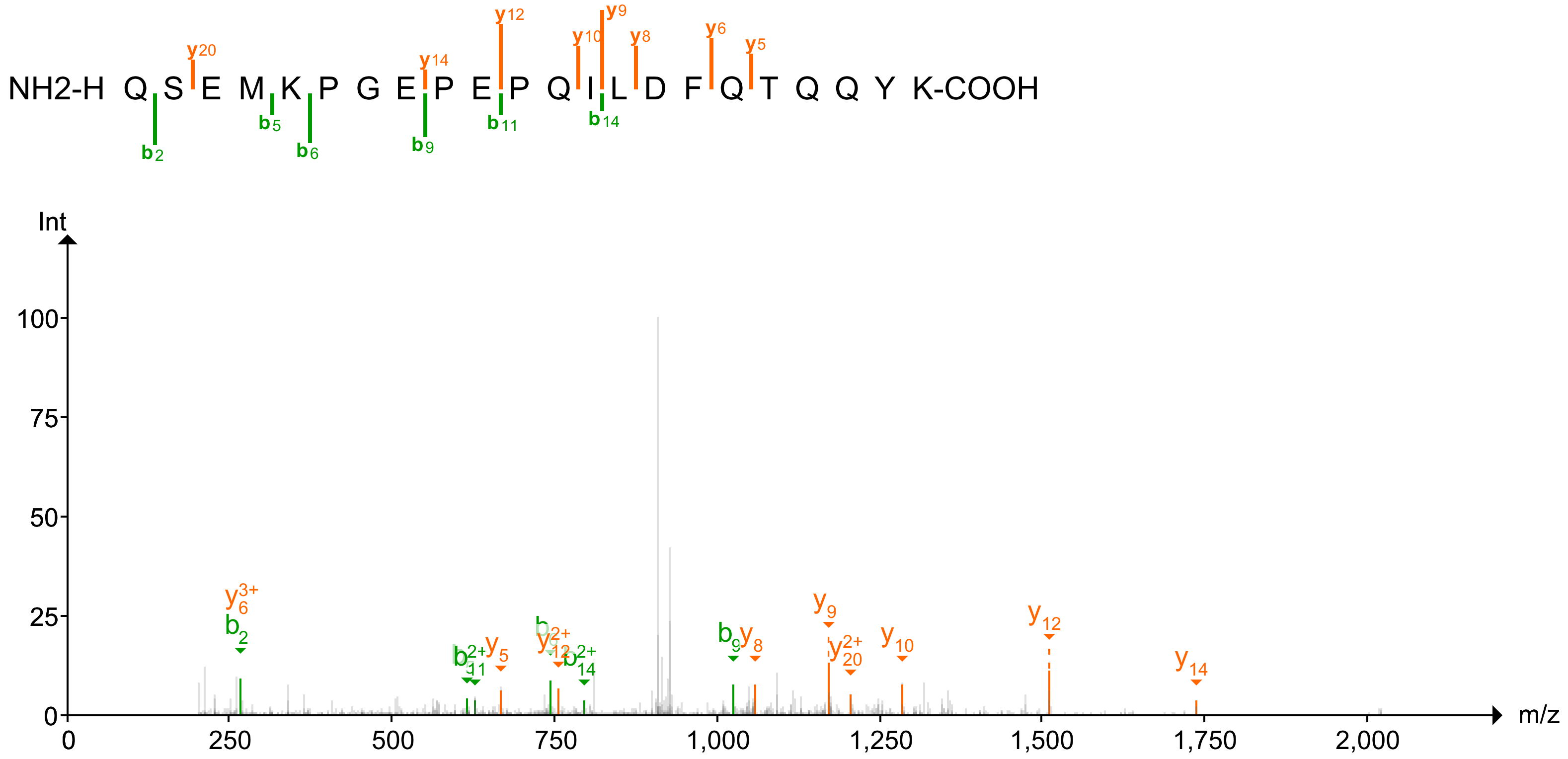

NH2-H Q S E M K P G E P E P Q I L D F Q T Q Q Y K-COOH
y20 y14 y12 y10 y9 y8 y6 y5
b2 b5 b6 b9 b11 b14
Int
100
75
50
25
0
m/z
0 250 500 750 1,000 1,250 1,500 1,750 2,000

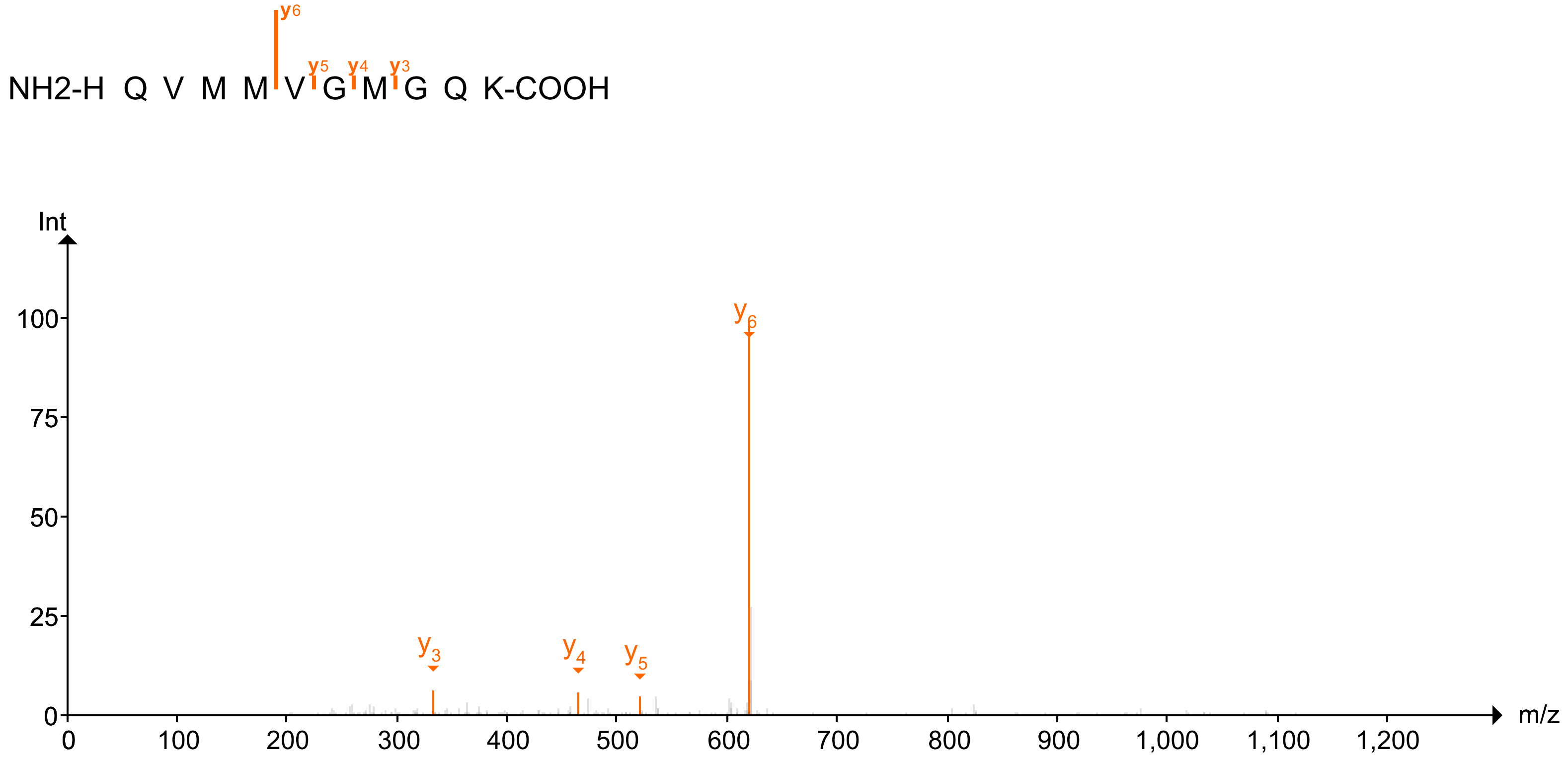

NH2-H Q V M M V G M G Q K-COOH
y6
y5
y4
y3
Int
100
75
50
25
0
m/z
0
100
200
300
400
500
600
700
800
900
1,000
1,100
1,200

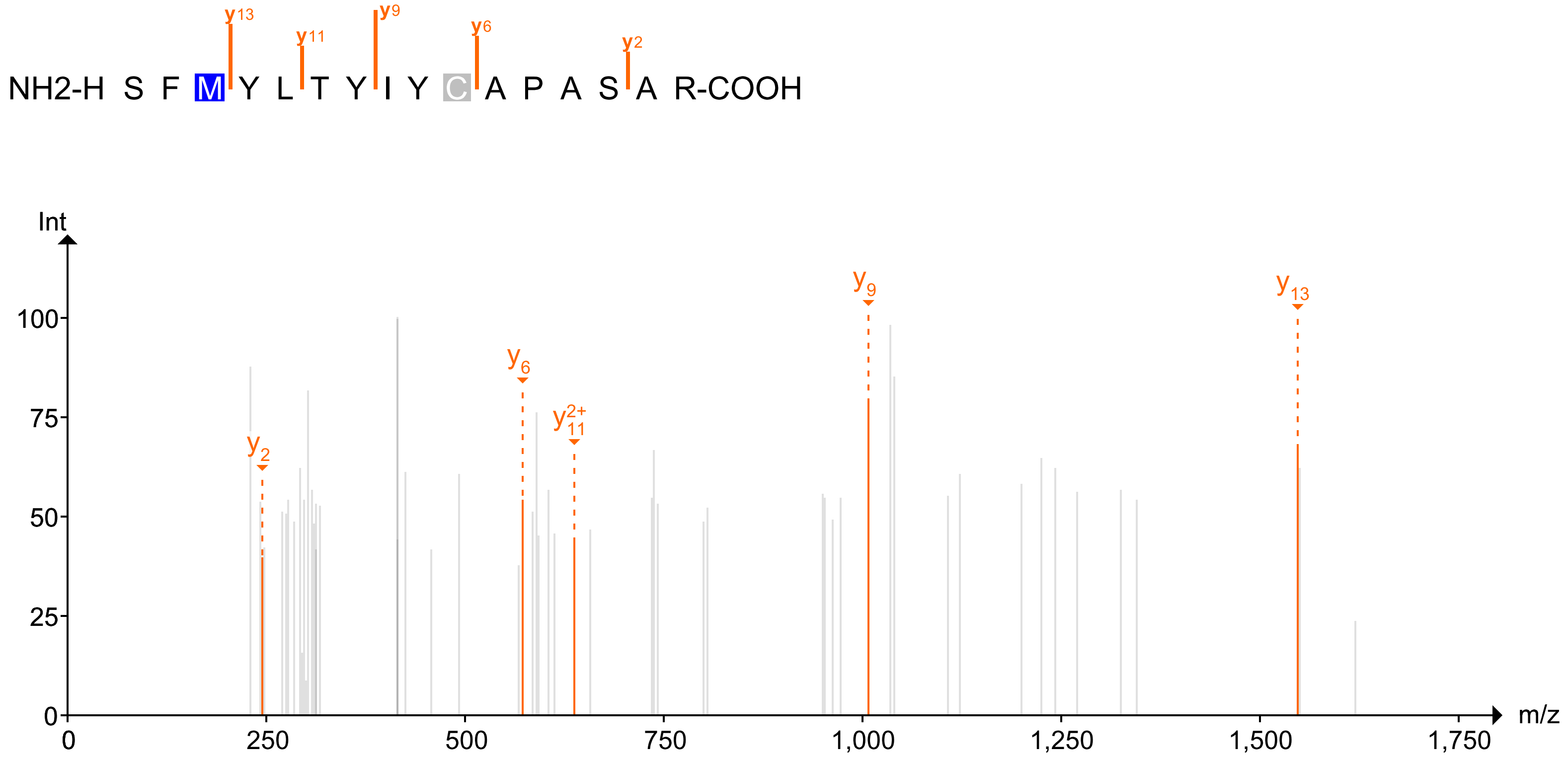

NH2-H S F M Y L T Y I Y C A P A S A R-COOH
y13
y11
y9
y6
y2
Int
m/z
100
75
50
25
0
0
250
500
750
1,000
1,250
1,500
1,750
y2
y6
y2+11
y9
y13

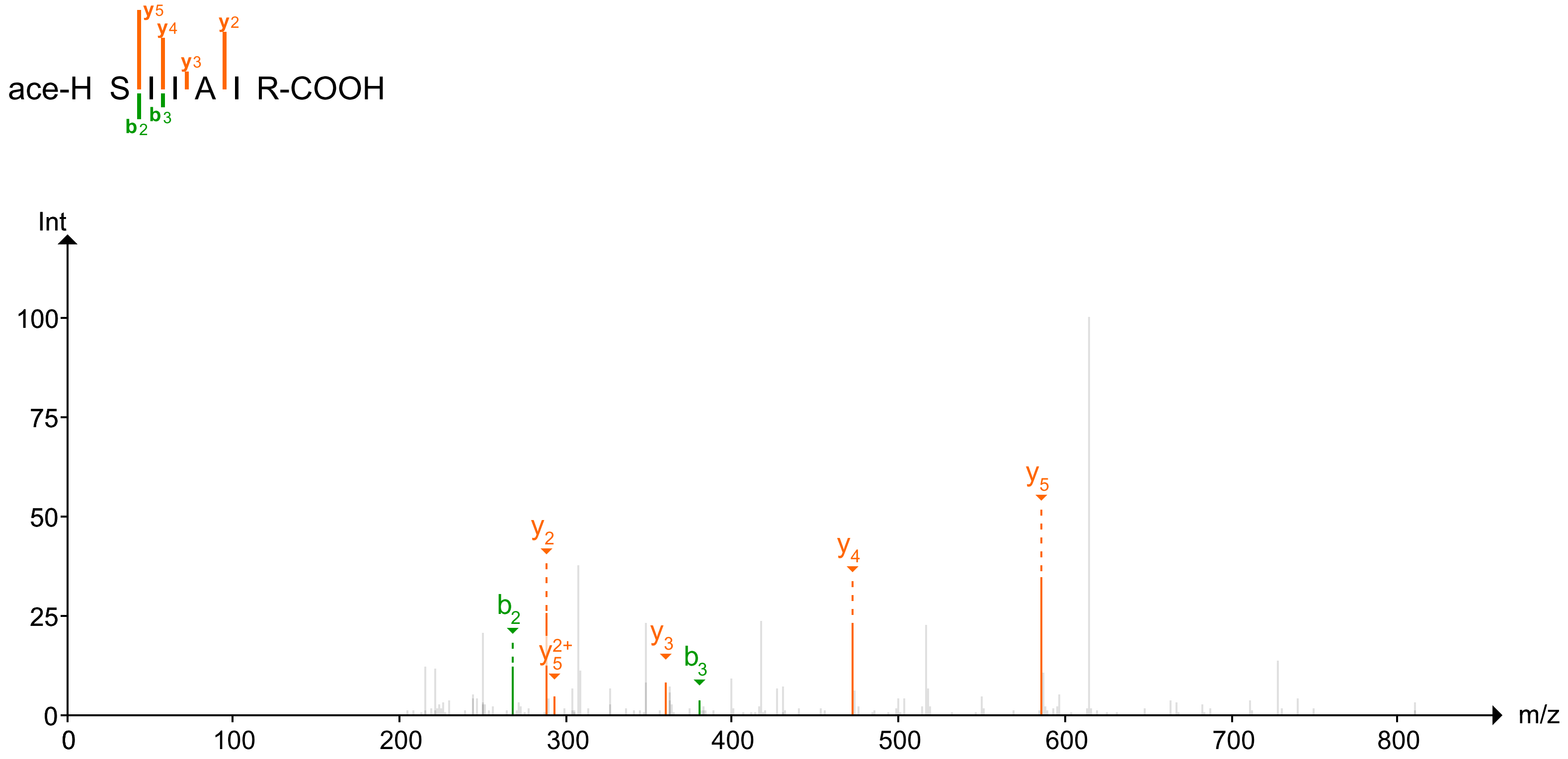

ace-H S I I A I R-COOH
y5
y4
y3
y2
b2
b3
Int
100
75
50
25
0
m/z
0
100
200
300
400
500
600
700
800
y2
y5 2+
y3
y4
y5
b2
b3

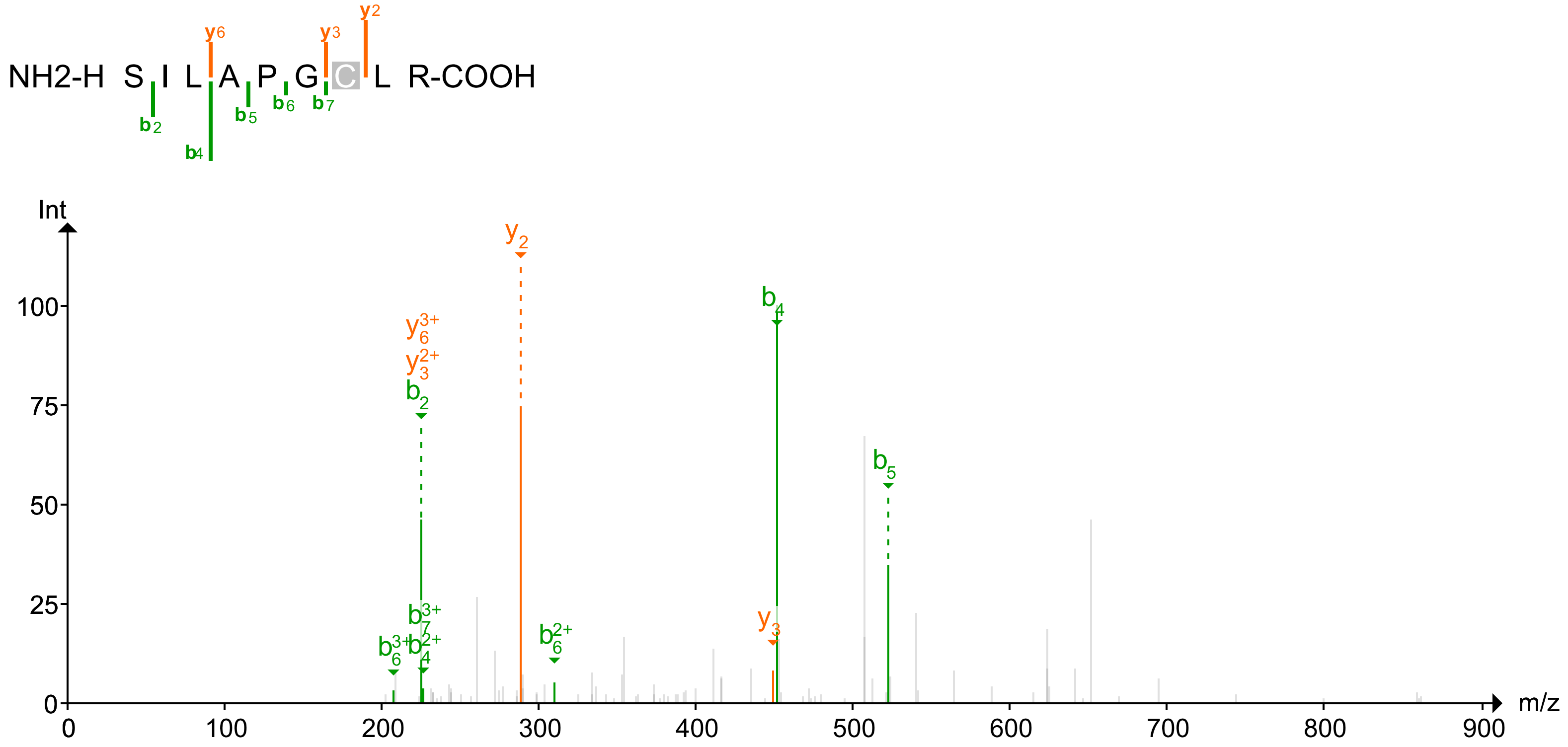

NH2-H S I L A P G C L R-COOH
y6
y3
y2
b2
b4
b5
b6
b7
Int
m/z
y2
b4
y6 3+
y3 2+
b2
b5
b7 3+
b6 3+
b4 2+
b6 2+
y3
0
25
50
75
100
0
100
200
300
400
500
600
700
800
900

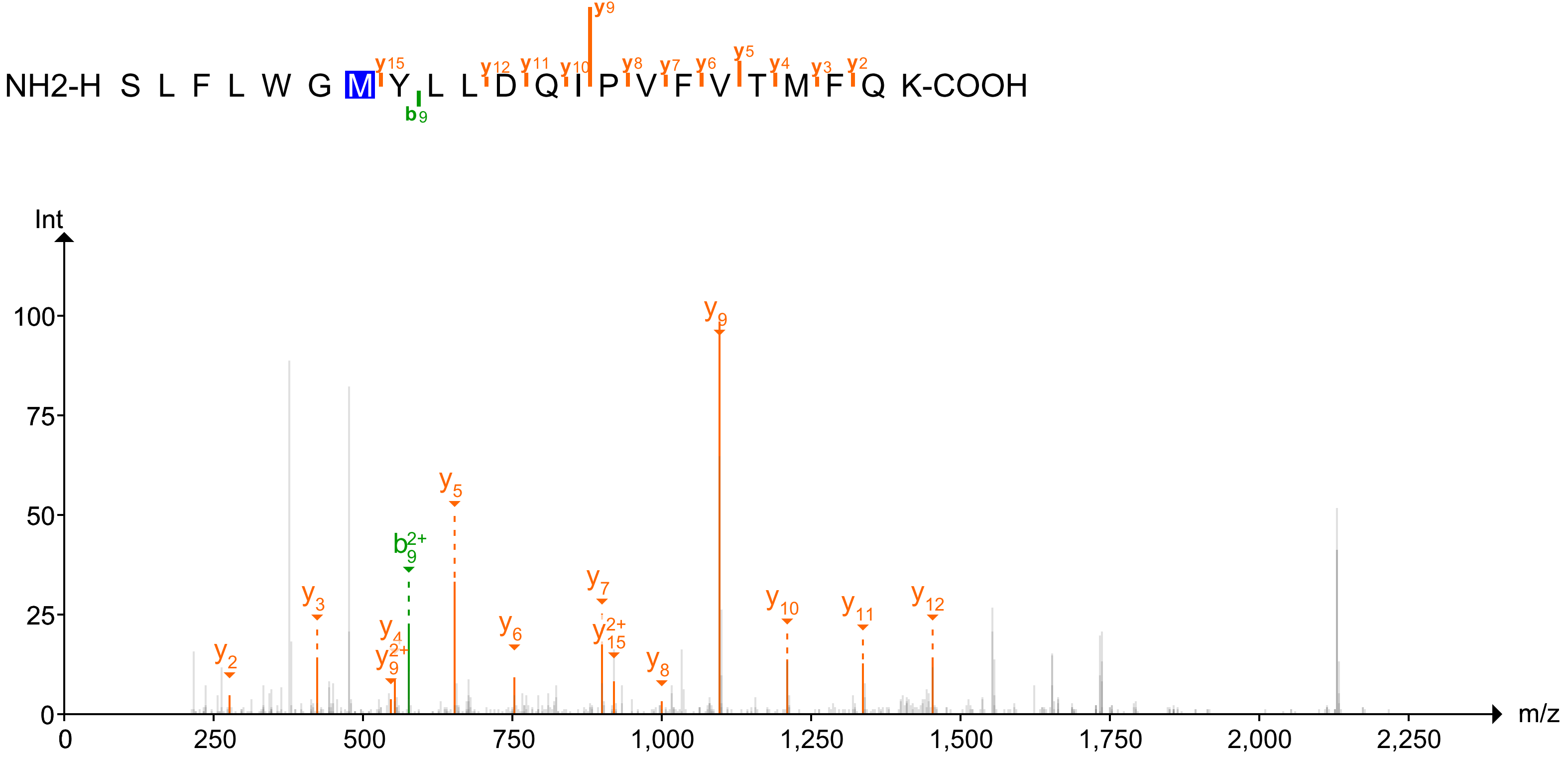

NH2-H S L F L W G M Y L L D Q I P V F V T M F Q K-COOH
y15 y12 y11 y10 y9 y8 y7 y6 y5 y4 y3 y2
b9
Int
100
75
50
25
0
m/z
0
250
500
750
1,000
1,250
1,500
1,750
2,000
2,250
y2 y3 y4 y9 2+ b9 2+ y5 y6 y7 y15 2+ y8 y9 y10 y11 y12

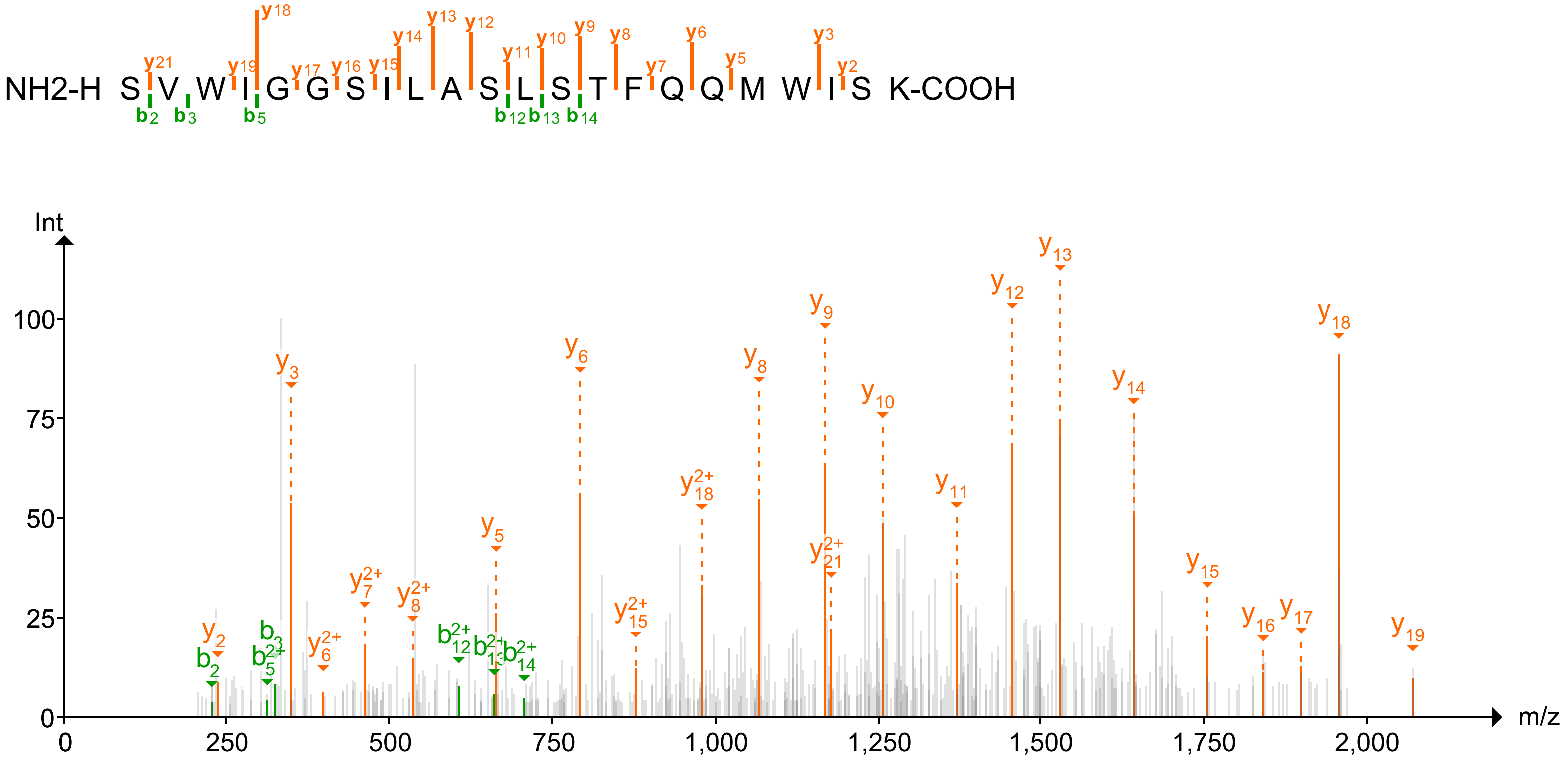

NH2-H S V W I G G S I L A S L S T F Q Q M W I S K-COOH
Int
m/z
0
25
50
75
100
0
250
500
750
1,000
1,250
1,500
1,750
2,000

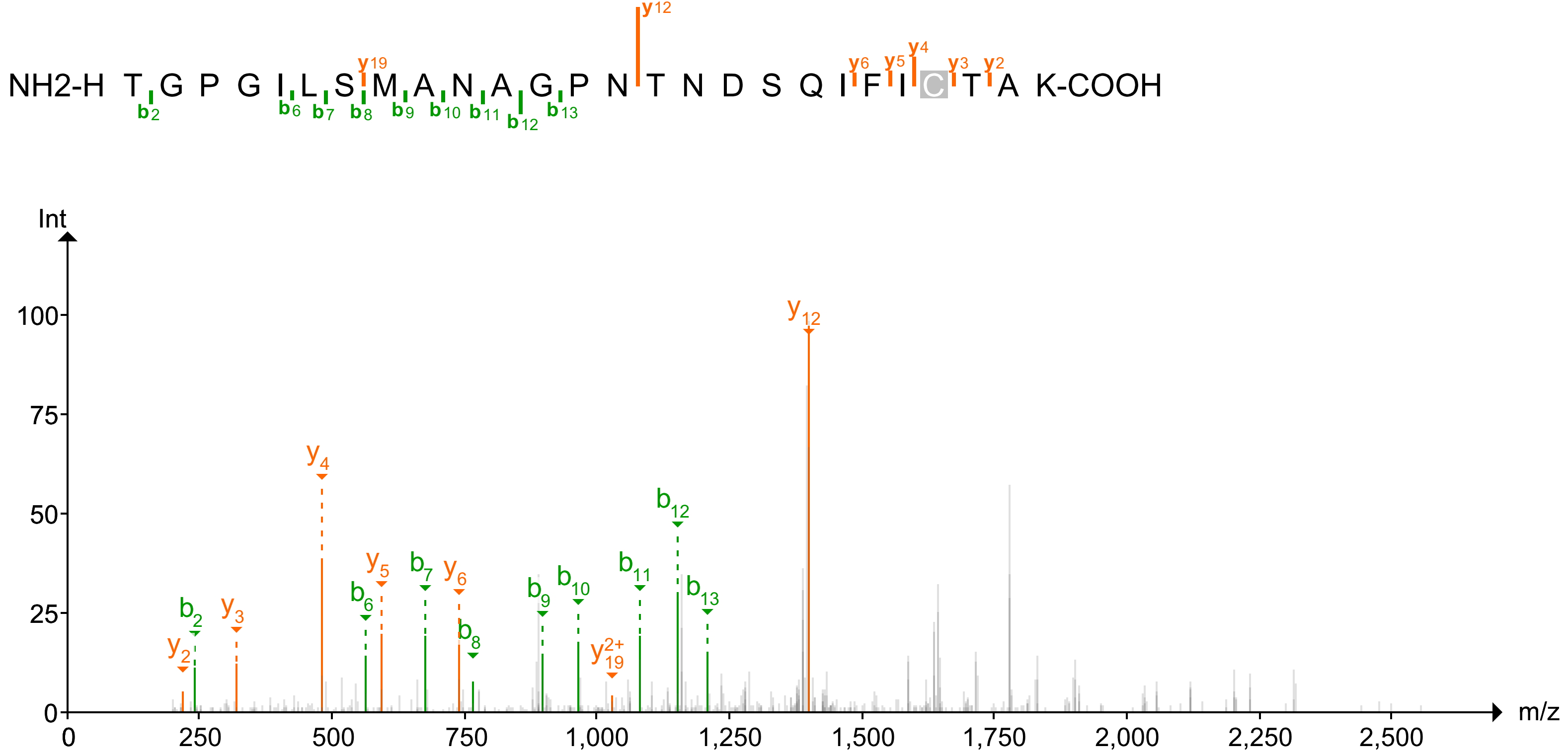

NH2-H T G P G I L S M A N A G P N T N D S Q I F I C T A K-COOH
b2 b6 b7 b8 b9 b10 b11 b12 b13
y19 y12 y6 y5 y4 y3 y2
Int
100
75
50
25
0
m/z
0
250
500
750
1,000
1,250
1,500
1,750
2,000
2,250
2,500
$y_{12}$
$y_4$
$b_{12}$
$y_5$
$b_7$
$y_6$
$b_6$
$b_{11}$
$b_9$
$b_{10}$
$b_{13}$
$b_2$
$y_3$
$b_8$
$y_2$
$y^{2+}_{19}$

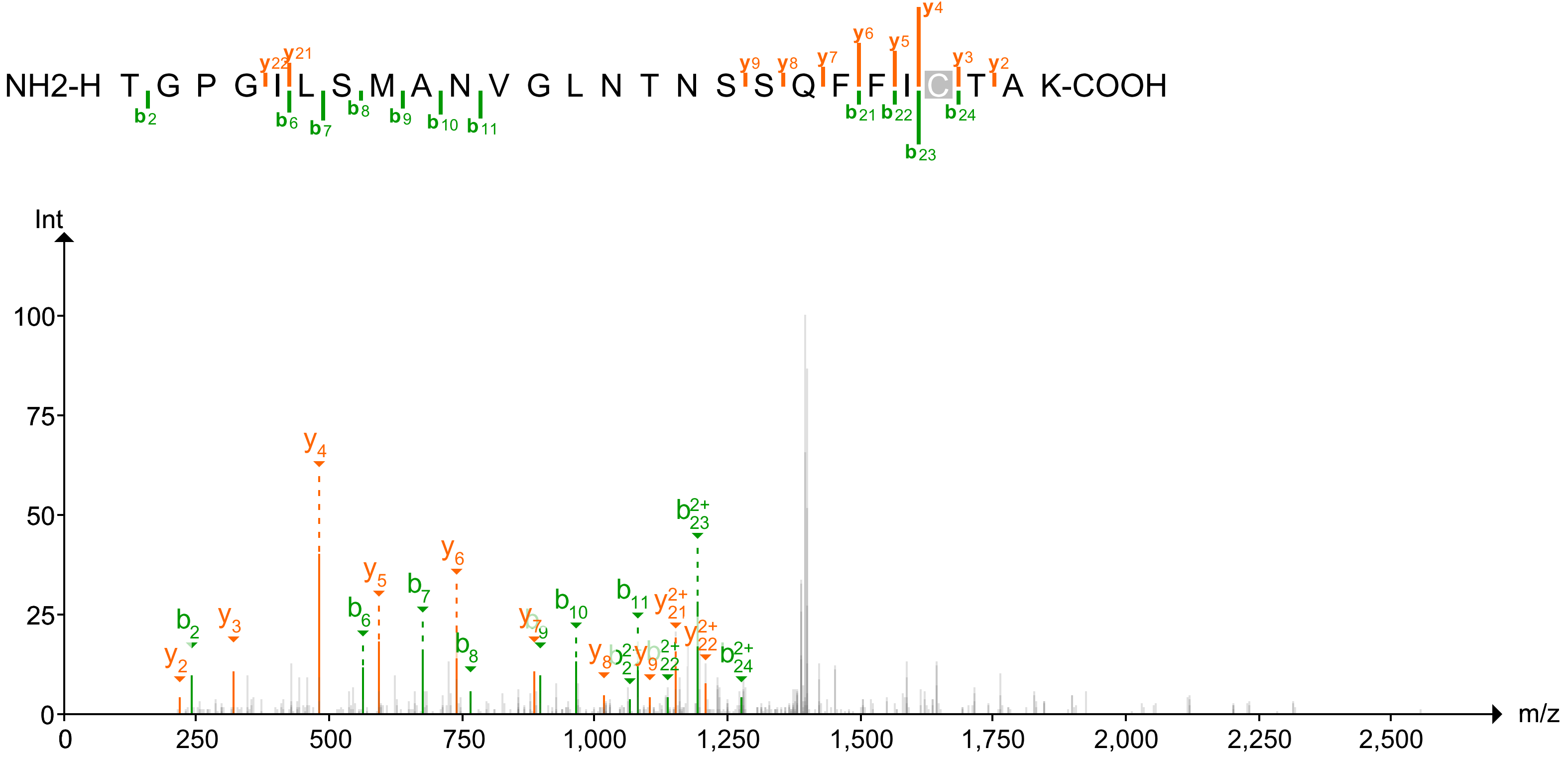

NH2-H T G P G I L S M A N V G L N T N S S Q F F I C T A K-COOH
y22 y21 y9 y8 y7 y6 y5 y4 y3 y2
b2 b6 b7 b8 b9 b10 b11 b21 b22 b23 b24
Int
100
75
50
25
0
m/z
0
250
500
750
1,000
1,250
1,500
1,750
2,000
2,250
2,500
$y_2$
$b_2$
$y_3$
$y_4$
$b_6$
$y_5$
$b_7$
$y_6$
$b_8$
$y_7$
$b_9$
$b_{10}$
$y_8$
$b_{11}$
$y_{21}^{2+}$
$y_9$
$b_{22}^{2+}$
$b_{23}^{2+}$
$y_{22}^{2+}$
$b_{24}^{2+}$

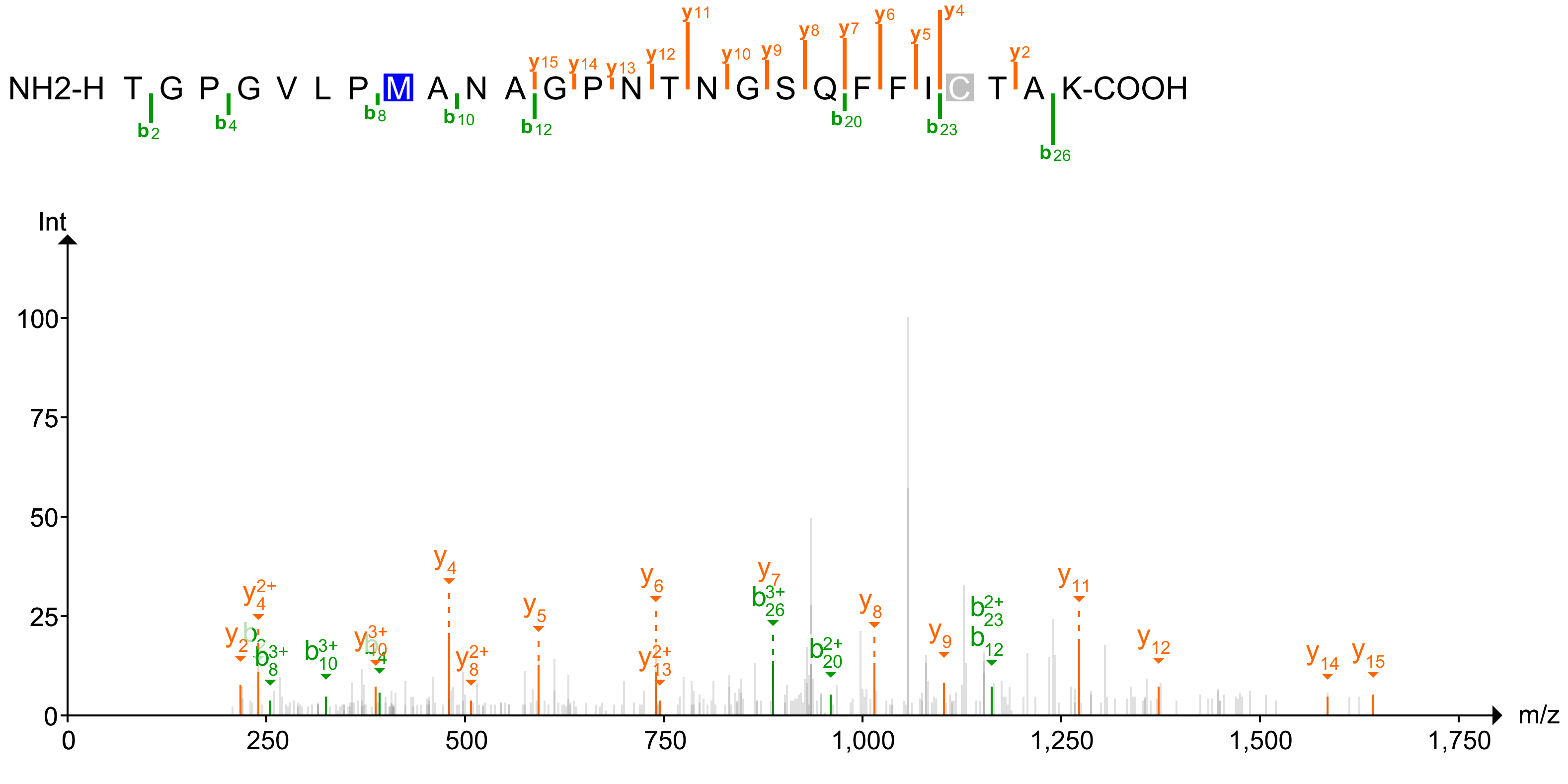

NH2-H T G P G V L P M A N A G P N T N G S Q F F I C T A K-COOH
b2 b4 b8 b10 b12 b20 b23 b26
y15 y14 y13 y12 y11 y10 y9 y8 y7 y6 y5 y4 y2
Int
100
75
50
25
0
m/z
0
250
500
750
1,000
1,250
1,500
1,750
y2
y4 2+
b8 3+
b10 3+
y10 3+
y4
y8 2+
y5
y6
y13 2+
y7
b26 3+
b20 2+
y8
y9
b23 2+
b12
y11
y12
y14
y15

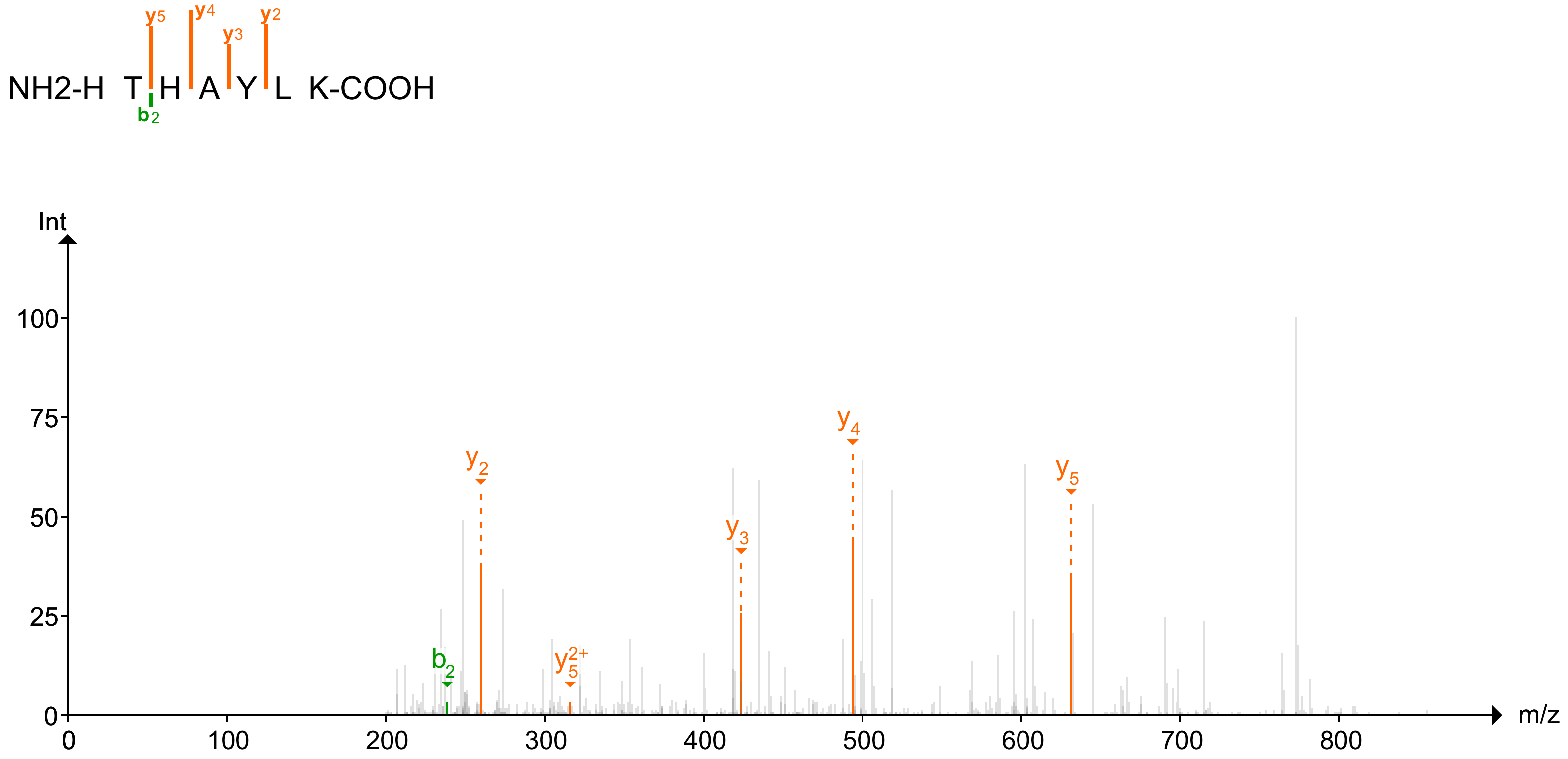

NH2-H T H A Y L K-COOH
y5
y4
y3
y2
b2
Int
100
75
50
25
0
m/z
0
100
200
300
400
500
600
700
800
b2
y2
y2+5
y3
y4
y5

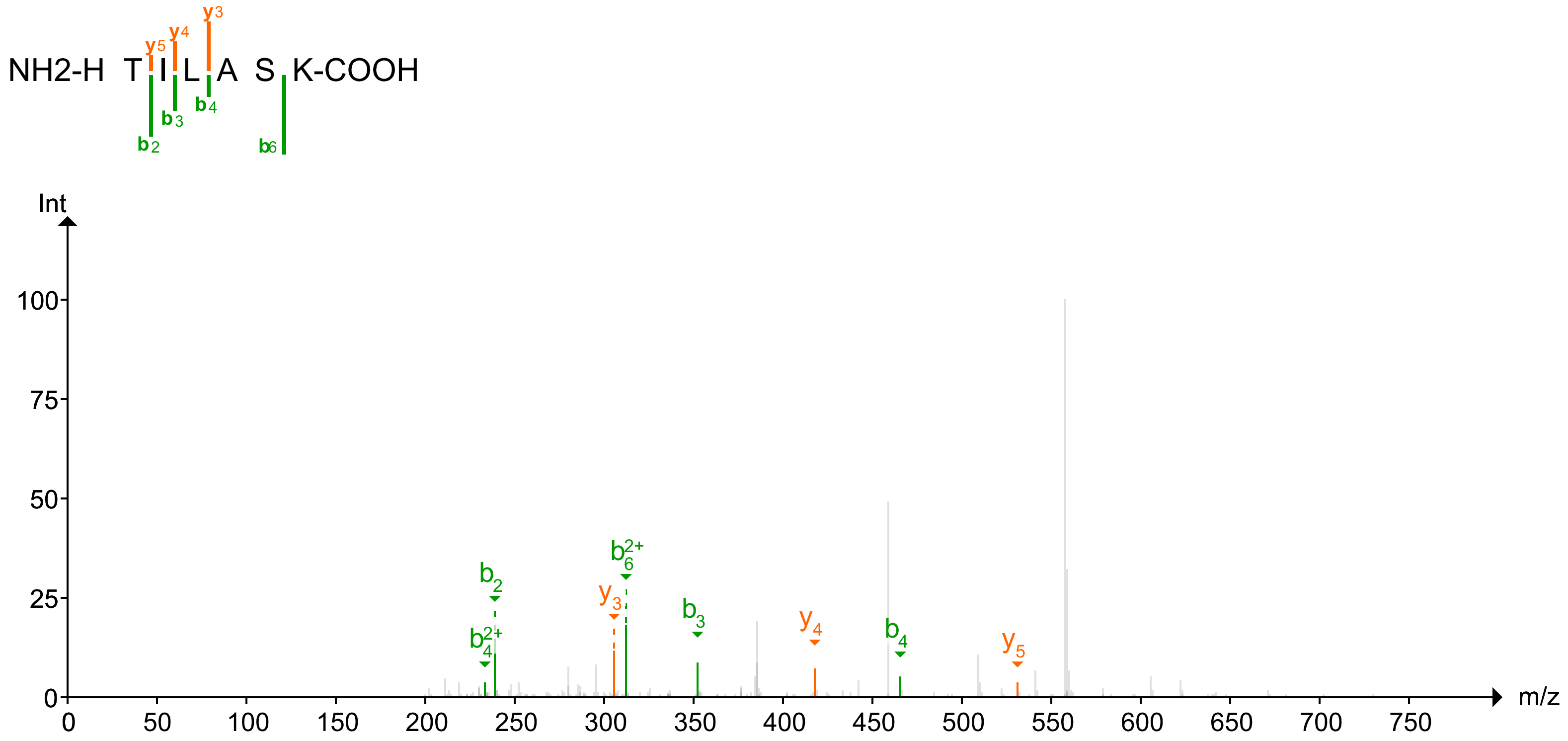

NH2-H T I L A S K-COOH
y3
y4
y5
b2
b3
b4
b6
Int
m/z
100
75
50
25
0
0
50
100
150
200
250
300
350
400
450
500
550
600
650
700
750
b2
b42+
b62+
y3
b3
y4
b4
y5

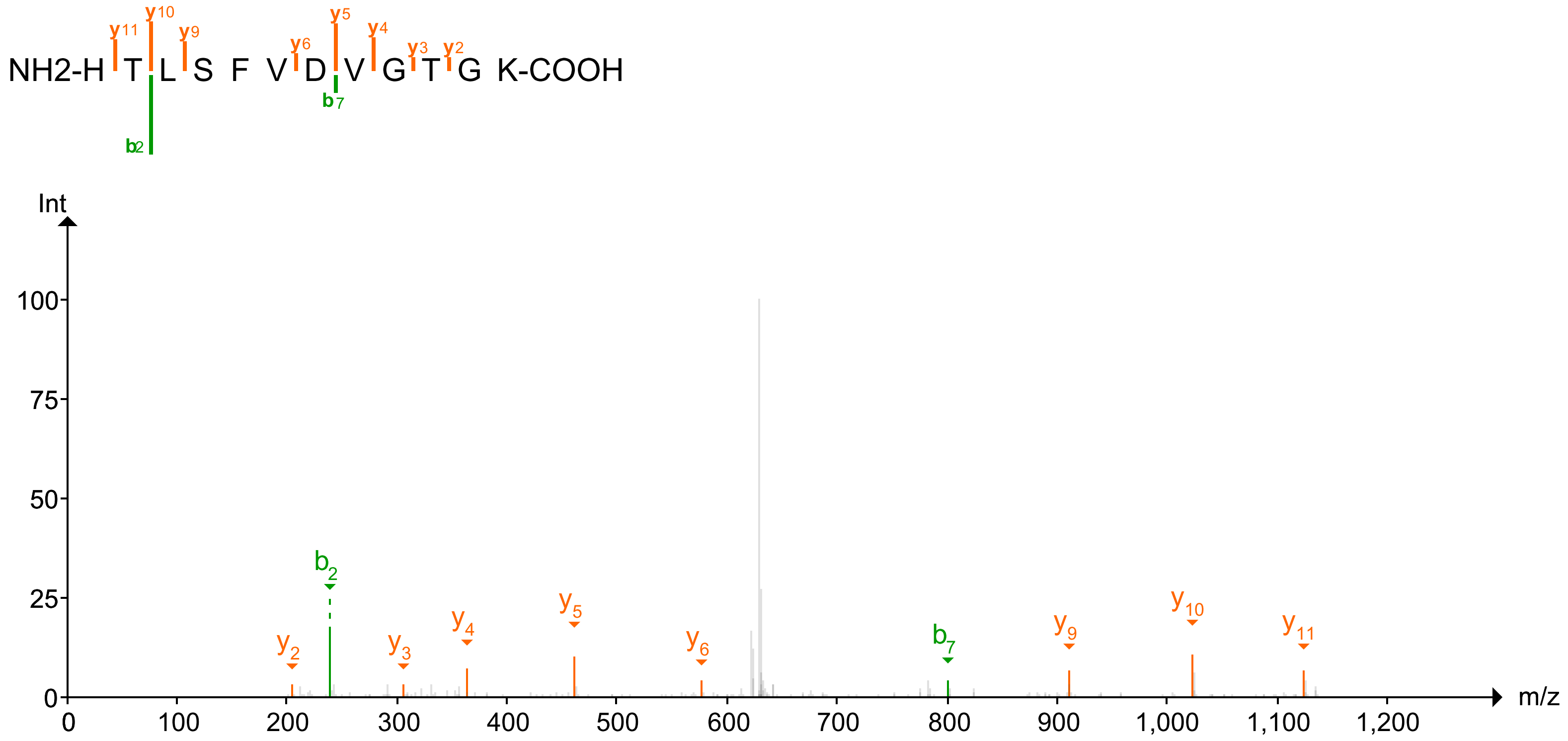

NH2-H T L S F V D V G T G K-COOH
y11 y10 y9 y6 y5 y4 y3 y2
b2 b7
Int
100
75
50
25
0
b2
y2 y3 y4 y5 y6 b7 y9 y10 y11
0 100 200 300 400 500 600 700 800 900 1,000 1,100 1,200
m/z

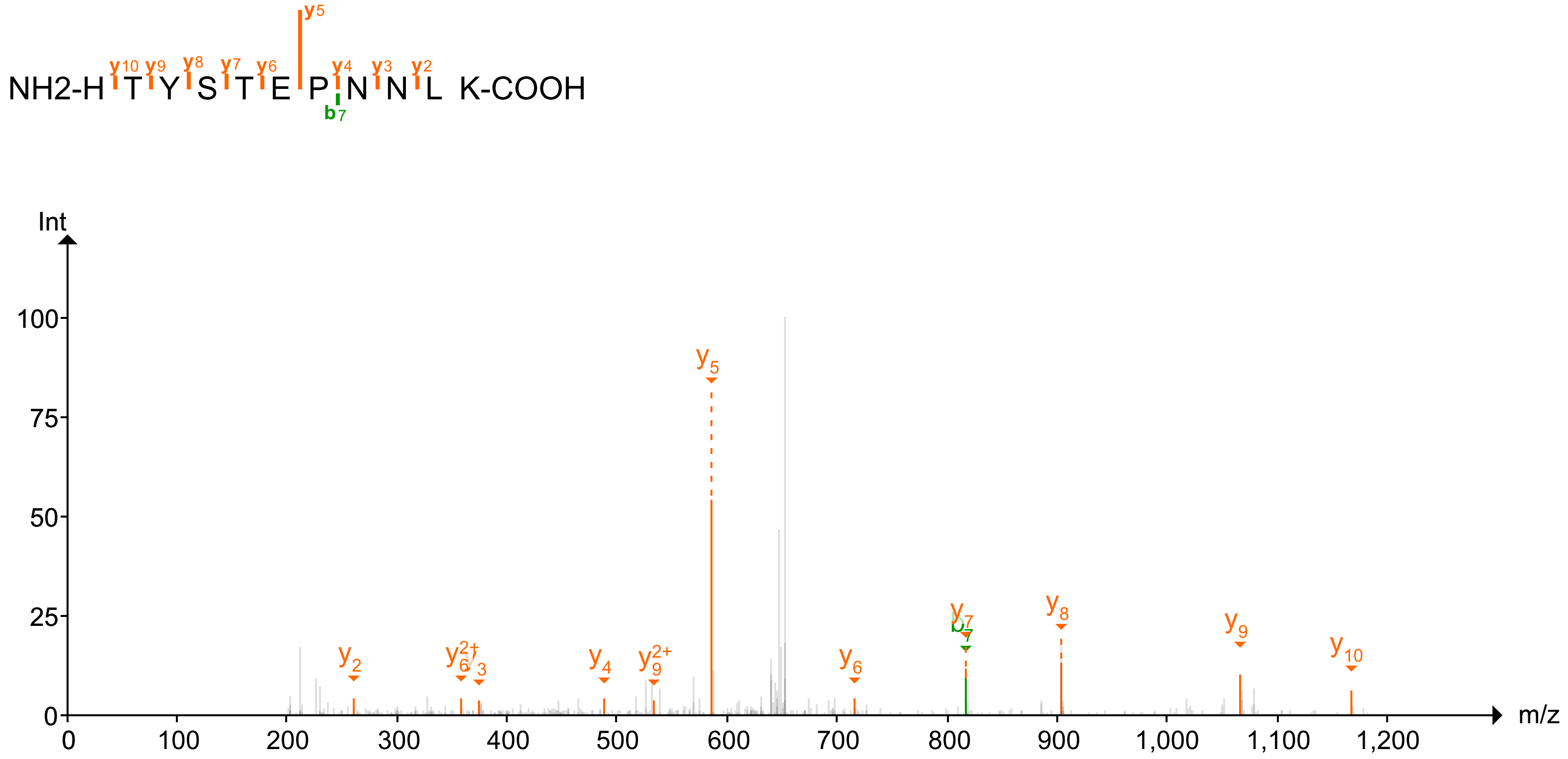

NH2-H T Y S T E P N N L K-COOH
y10 y9 y8 y7 y6 y5 y4 y3 y2
b7
Int
100
75
50
25
0
m/z
0
100
200
300
400
500
600
700
800
900
1,000
1,100
1,200
y5
y2
y6 2+
y3
y4
y9 2+
y6
y7
b7
y8
y9
y10

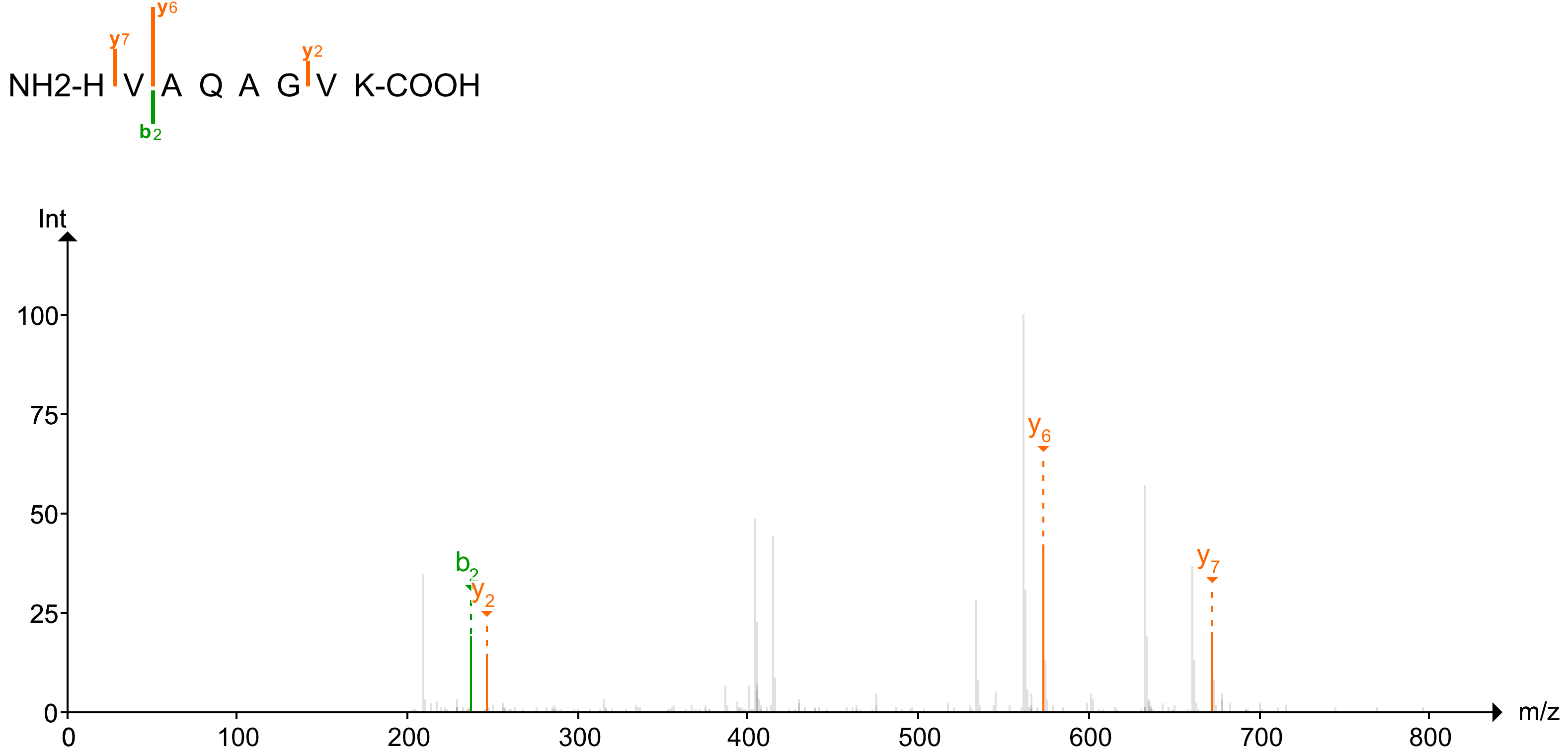

NH2-H V A Q A G V K-COOH
y6
y7
y2
b2
Int
100
75
50
25
0
m/z
0
100
200
300
400
500
600
700
800
b2
y2
y6
y7

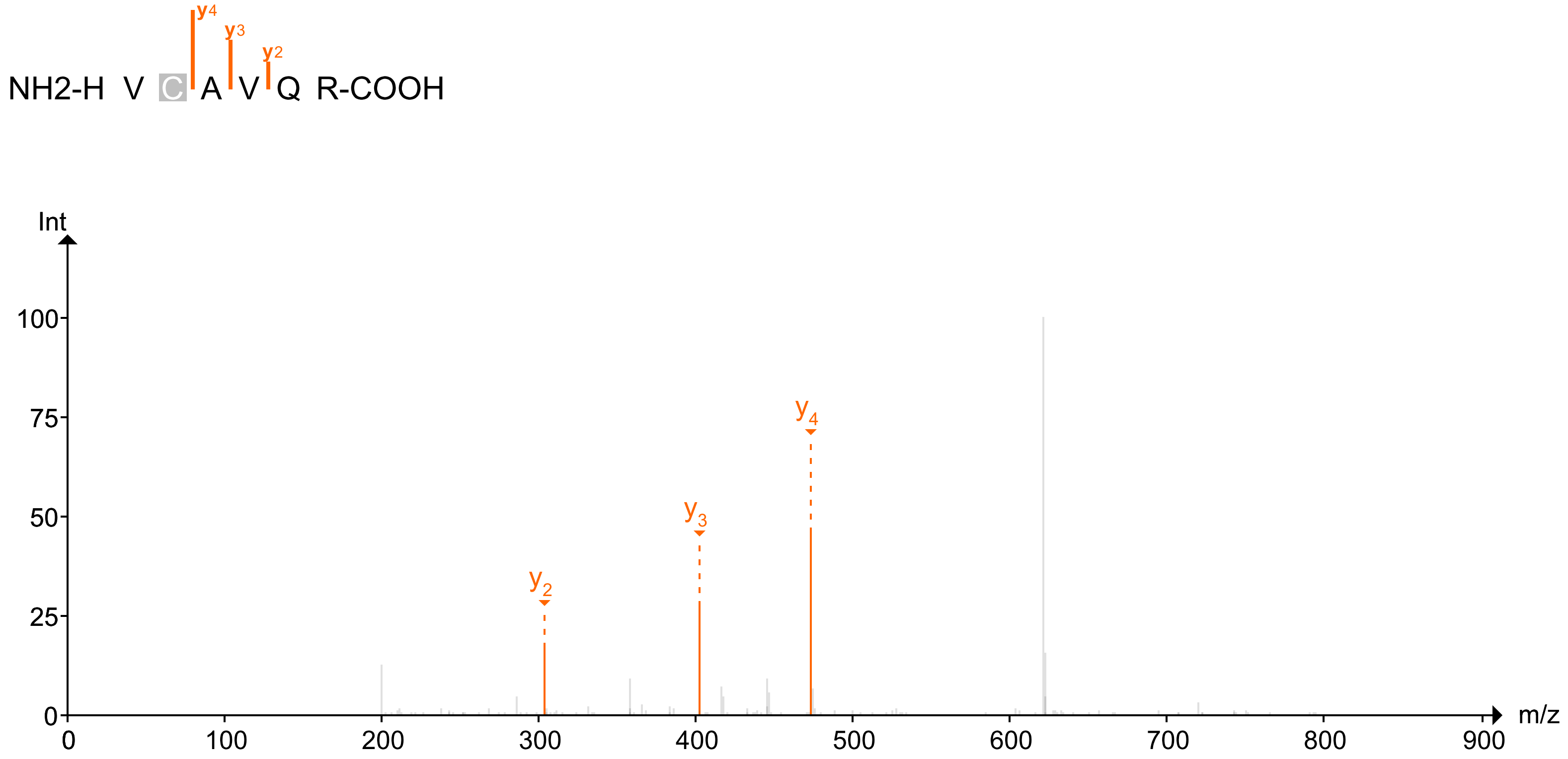
NH2-H V C A V Q R-COOH
y4
y3
y2
Int
100
75
50
25
0
m/z
0
100
200
300
400
500
600
700
800
900
y2
y3
y4

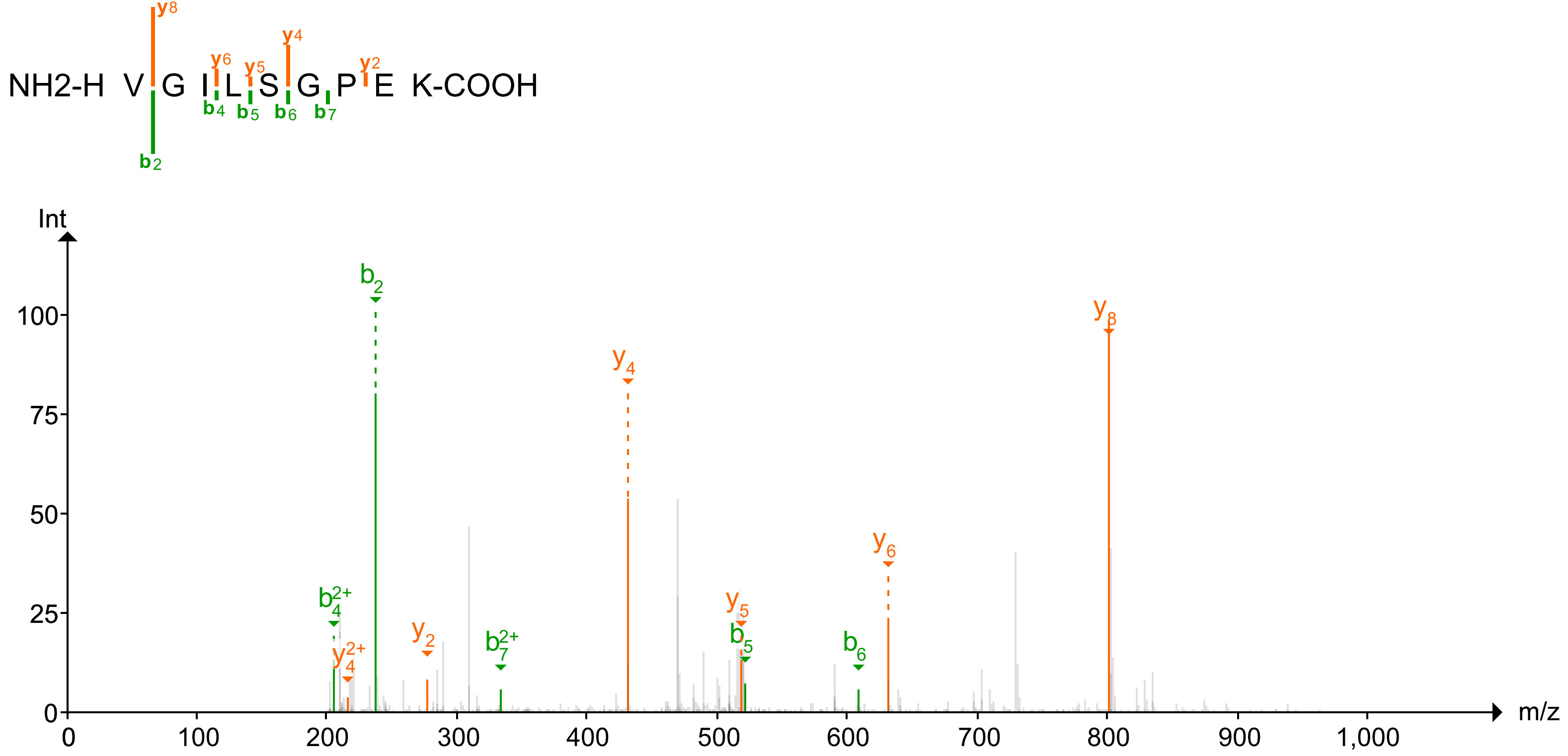

NH2-H V G I L S G P E K-COOH
y8
y6
y5
y4
y2
b2
b4
b5
b6
b7
Int
100
75
50
25
0
m/z
0
100
200
300
400
500
600
700
800
900
1,000
b2
y4
y8
y6
y5
b5
b6
y2
b7 2+
b4 2+
y4 2+

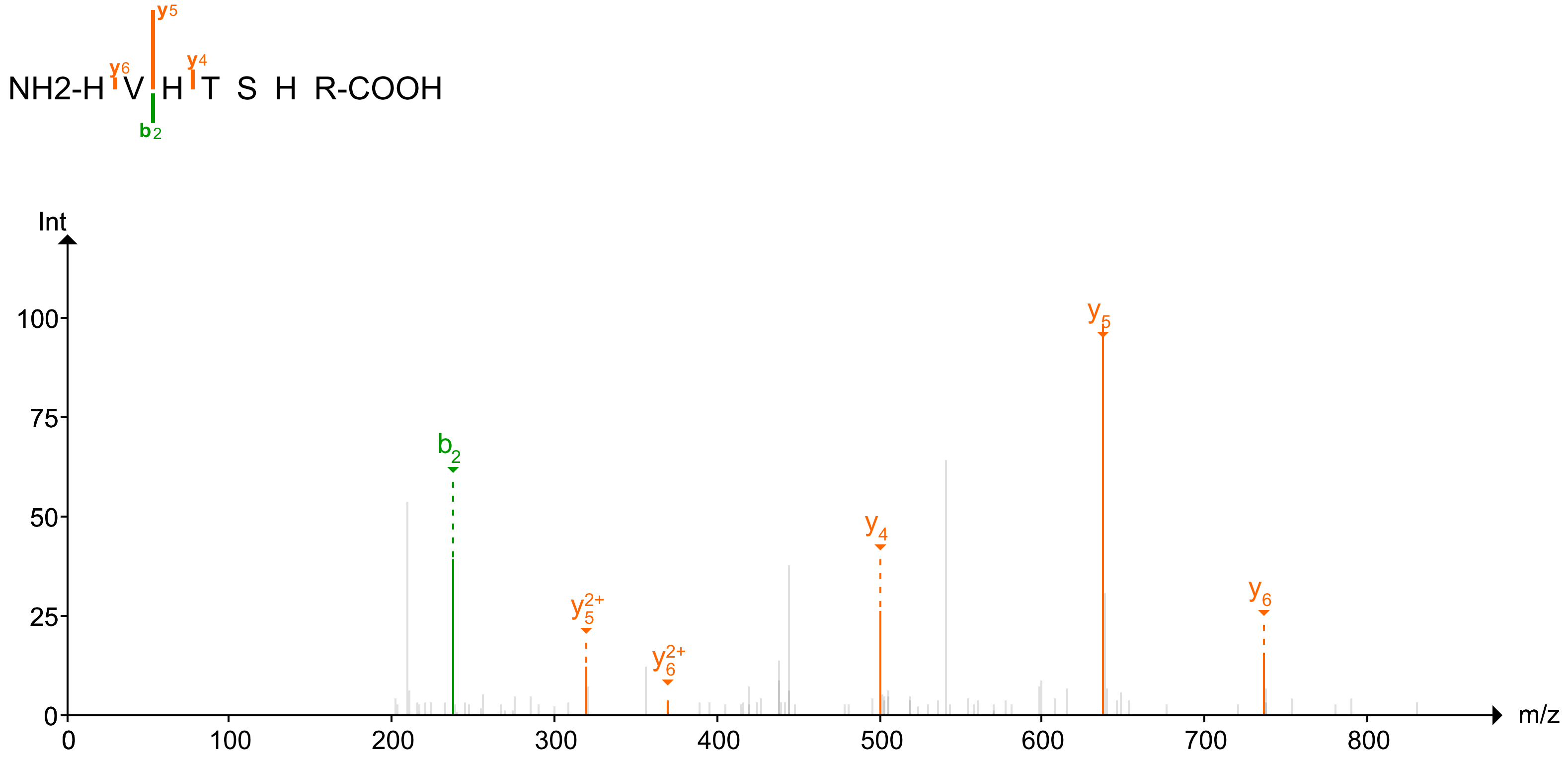

y5
y6
y4
NH2-H V H T S H R-COOH
b2
Int
100
75
50
25
0
b2
y5 2+
y6 2+
y4
y5
y6
0
100
200
300
400
500
600
700
800
m/z

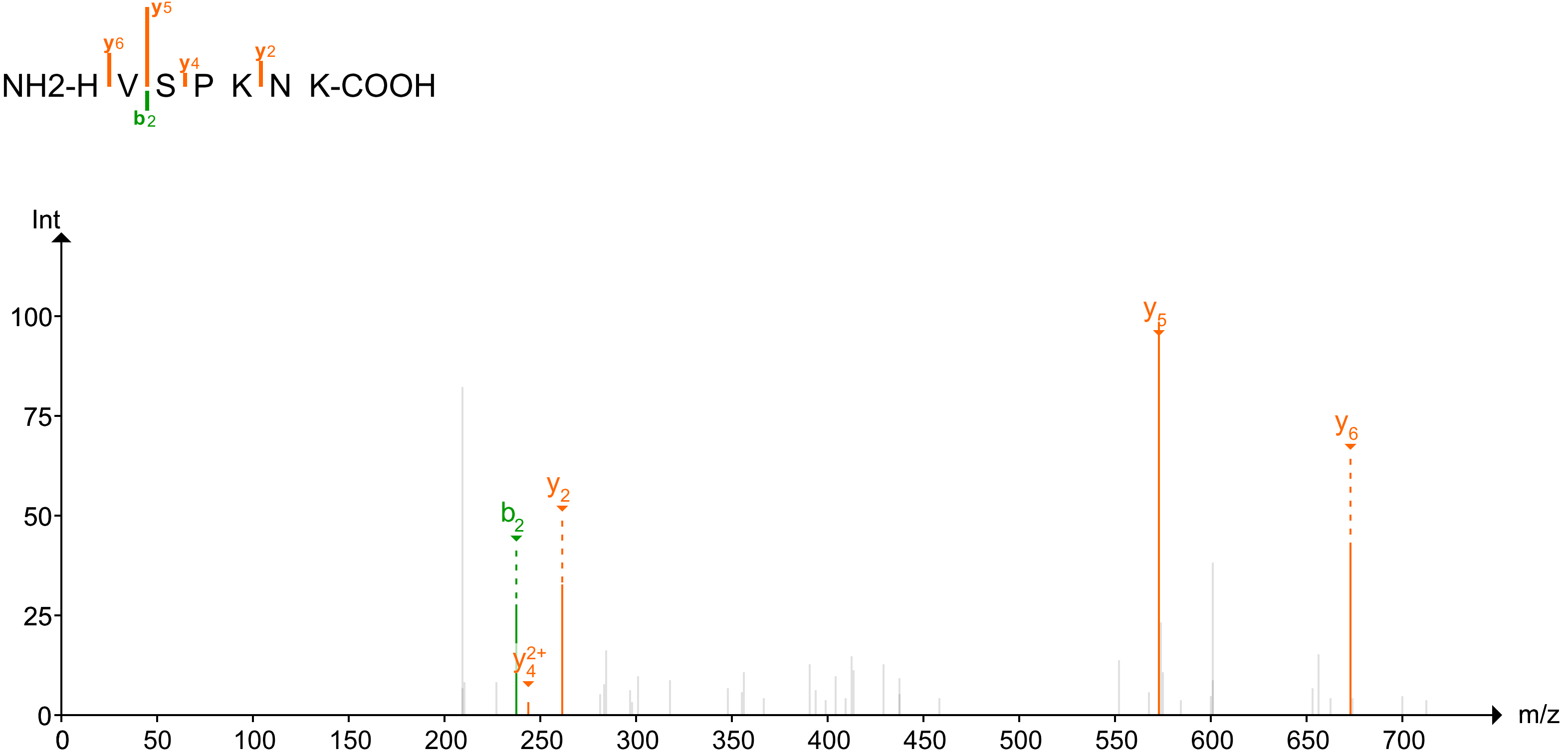

NH2-H V S P K N K-COOH
y5
y6
y4
y2
b2
Int
100
75
50
25
0
0
50
100
150
200
250
300
350
400
450
500
550
600
650
700
m/z
y5
y6
y2
b2
y4 2+

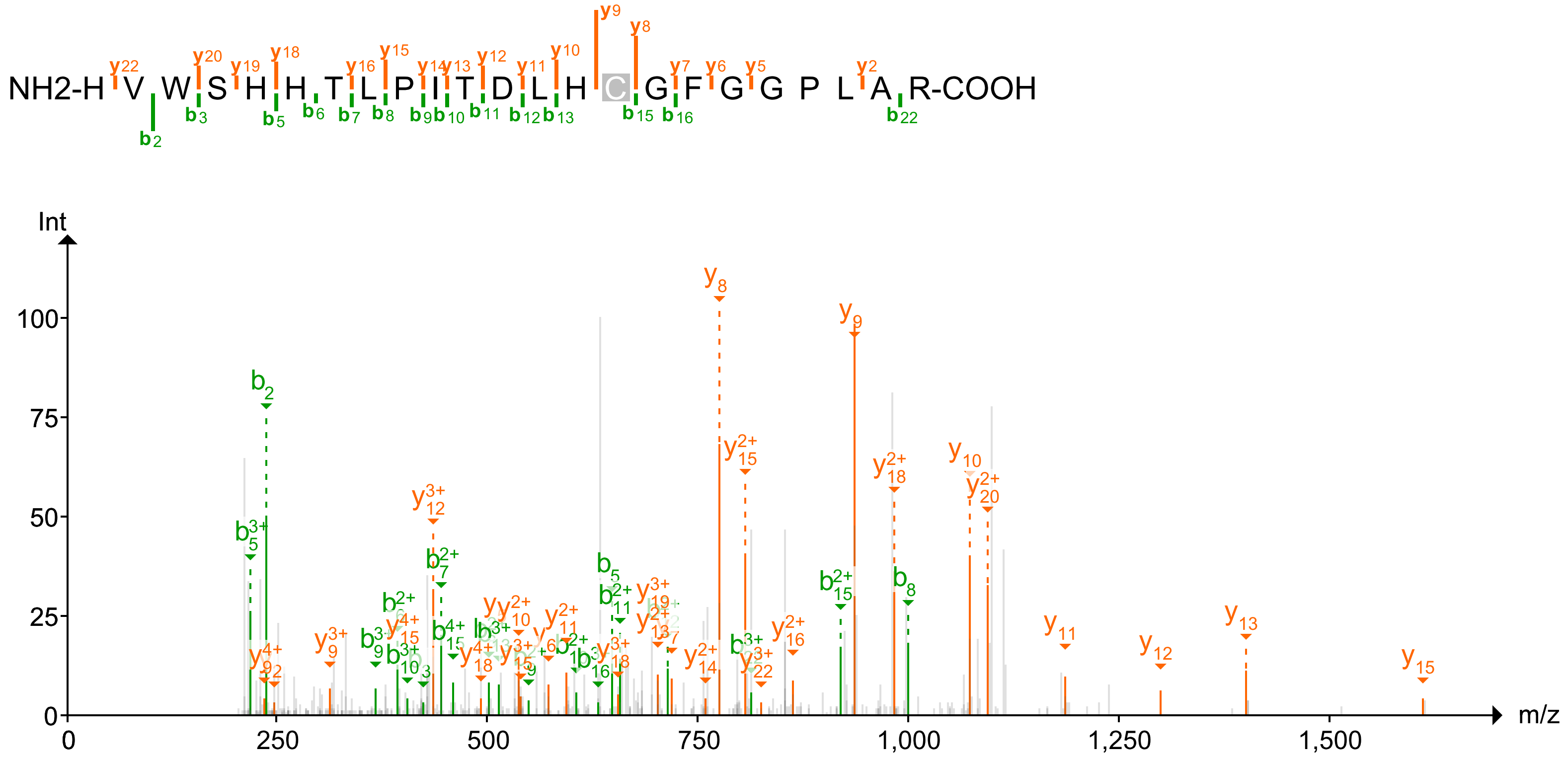
NH2-H V W S H H T L P I T D L H C G F G G P L A R-COOH
Int
m/z
0
25
50
75
100
250
500
750
1,000
1,250
1,500

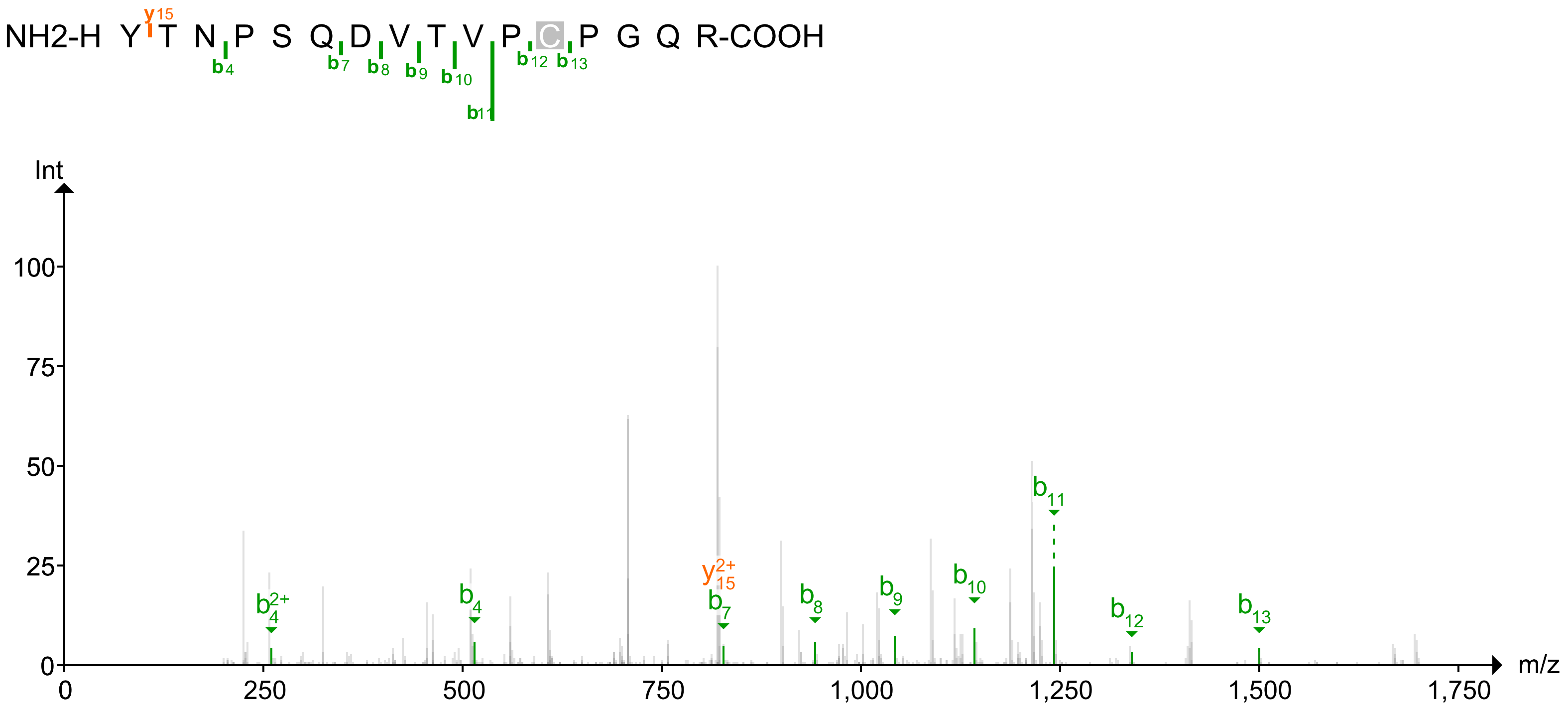

NH2-H Y T N P S Q D V T V P C P G Q R-COOH
y15
b4 b7 b8 b9 b10 b11 b12 b13
Int
100
75
50
25
0
m/z
0
250
500
750
1,000
1,250
1,500
1,750
b4 2+
b4
y15 2+
b7
b8
b9
b10
b11
b12
b13

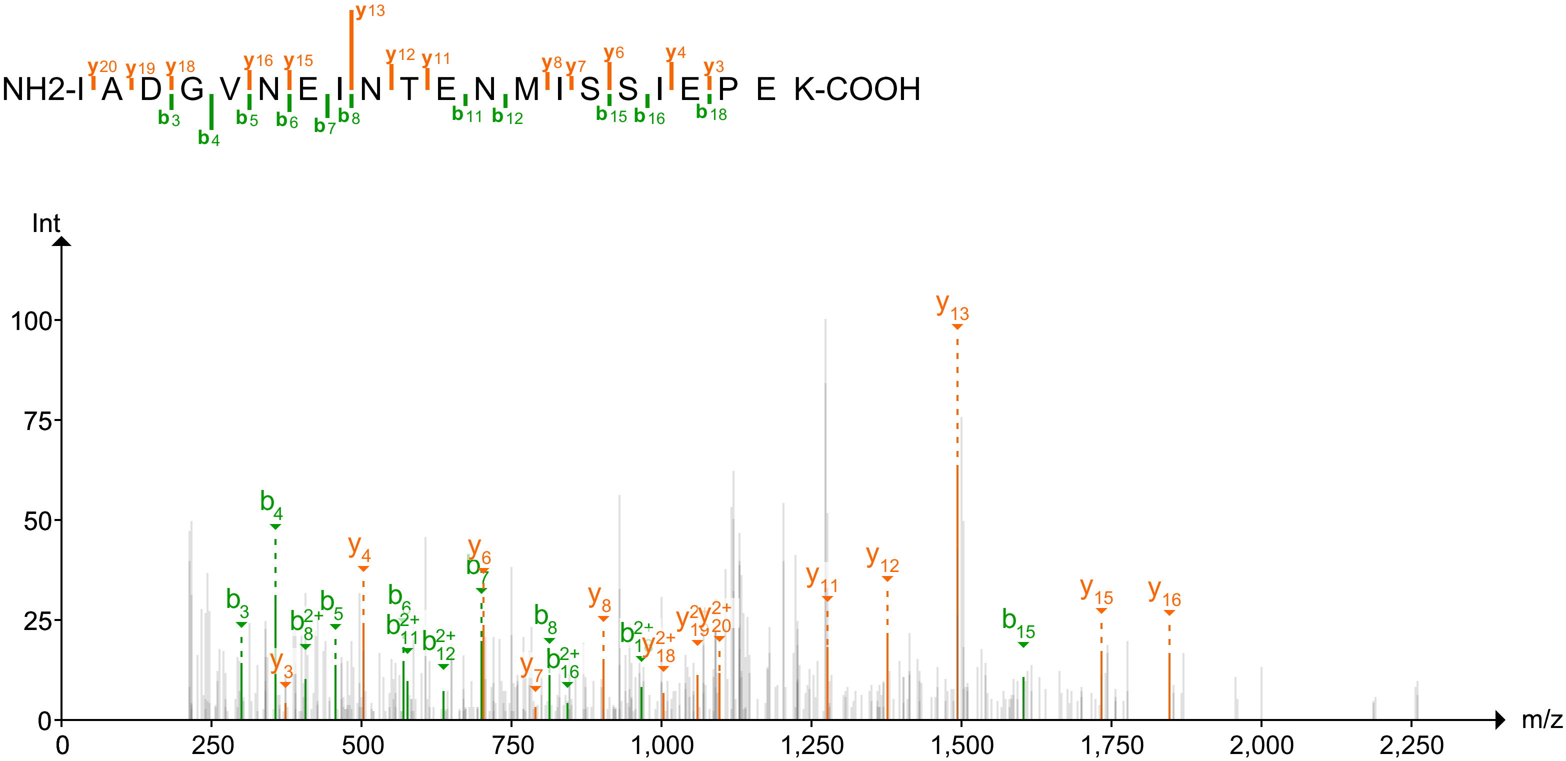

NH2-I A D G V N E I N T E N M I S S I E P E K-COOH
y20 y19 y18 y16 y15 y13 y12 y11 y8 y7 y6 y4 y3
b3 b4 b5 b6 b7 b8 b11 b12 b15 b16 b18
Int
m/z
0
25
50
75
100
0
250
500
750
1,000
1,250
1,500
1,750
2,000
2,250

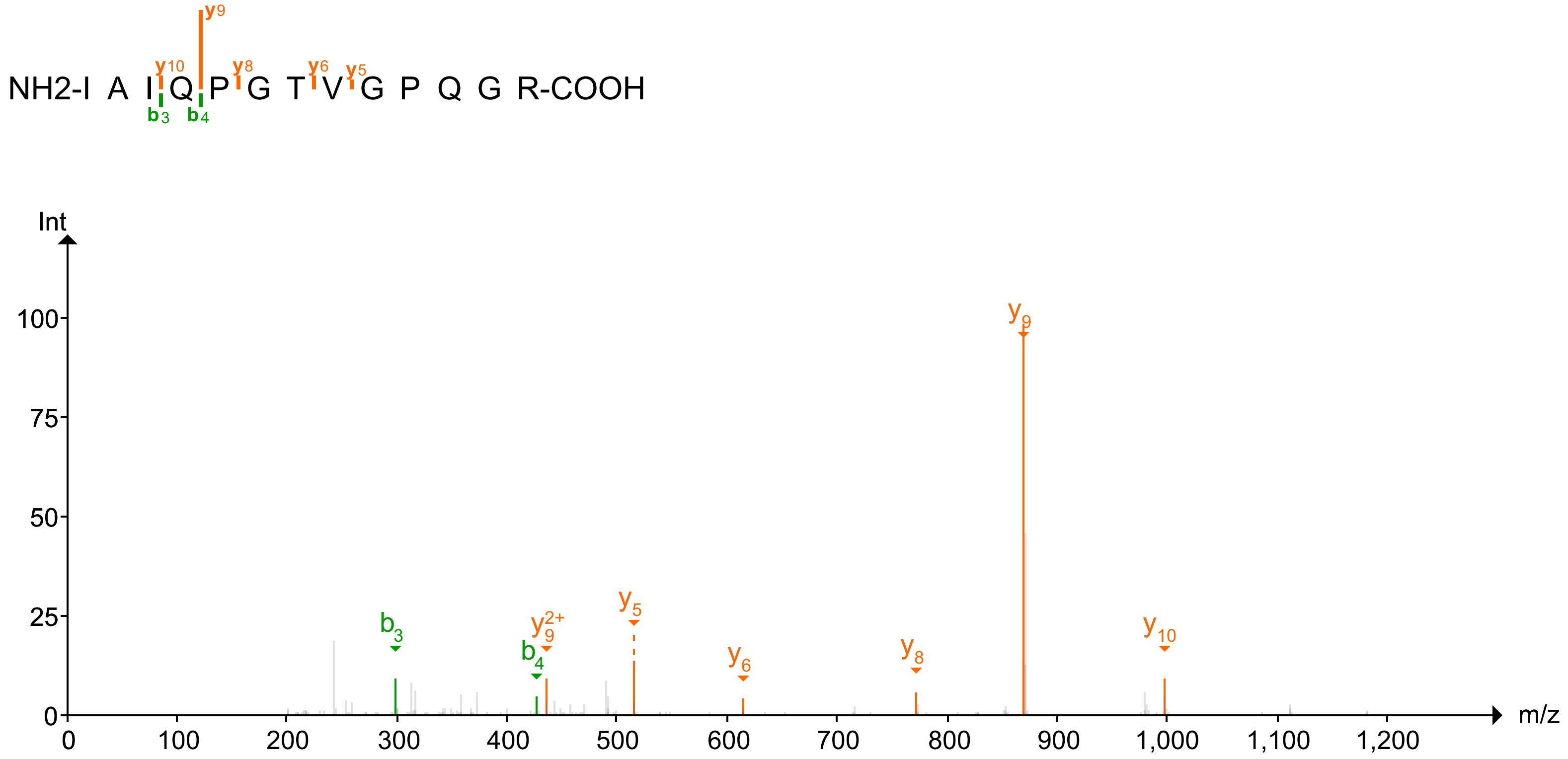

NH2-I A I Q P G T V G P Q G R-COOH
y9
y10
y8
y6
y5
b3
b4
Int
100
75
50
25
0
y9
y2+ 9
y5
b3
b4
y6
y8
y10
0
100
200
300
400
500
600
700
800
900
1,000
1,100
1,200
m/z

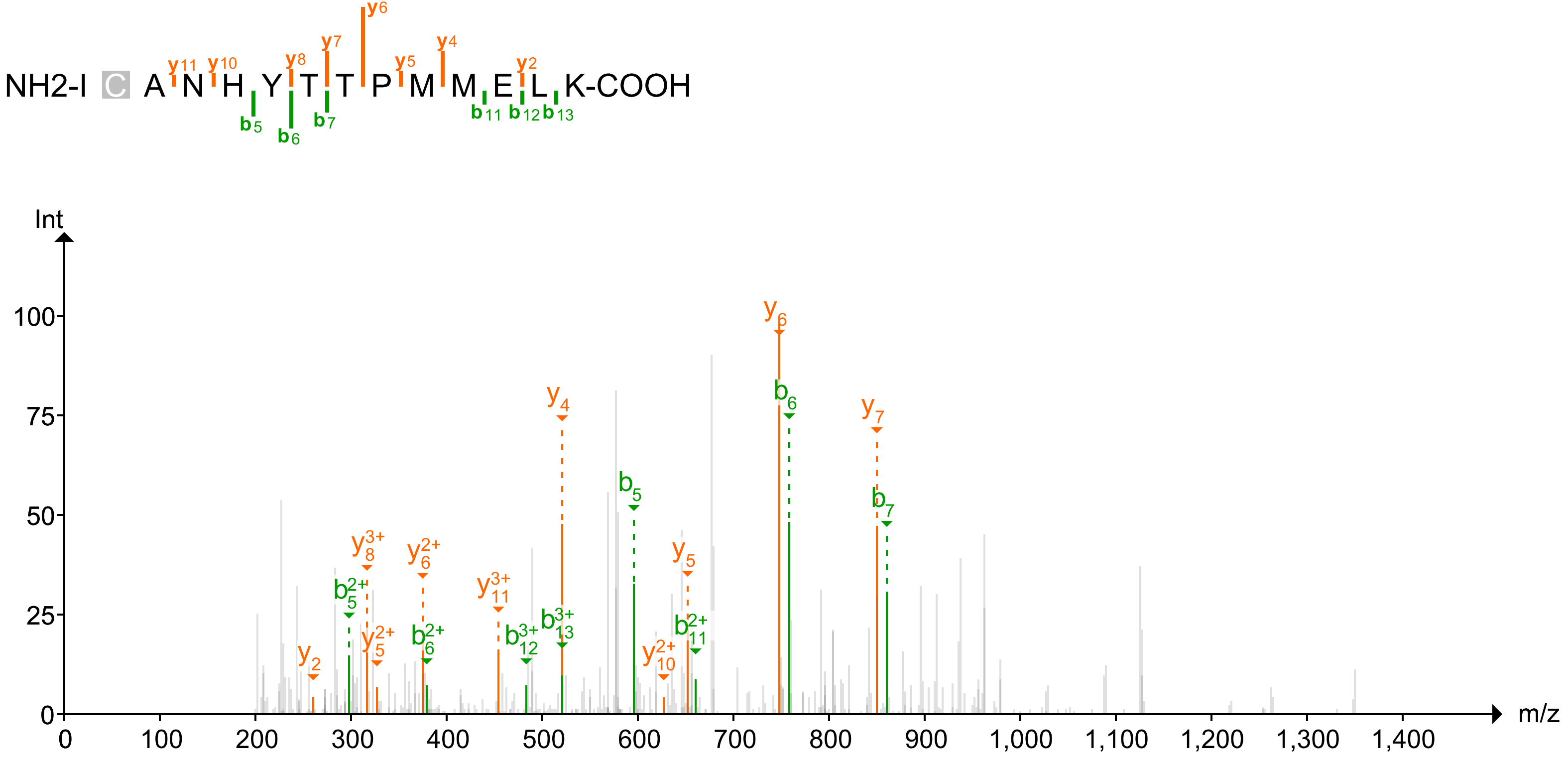
NH2-I C A N H Y T T P M M E L K-COOH
y11 y10 y8 y7 y6 y5 y4 y2
b5 b6 b7 b11 b12 b13
Int
m/z
y6
y4
b6
y7
b5
b7
y5
y8 3+
y6 2+
b5 2+
y11 3+
b13 3+
b12 3+
b6 2+
y5 2+
b11 2+
y10 2+
y2

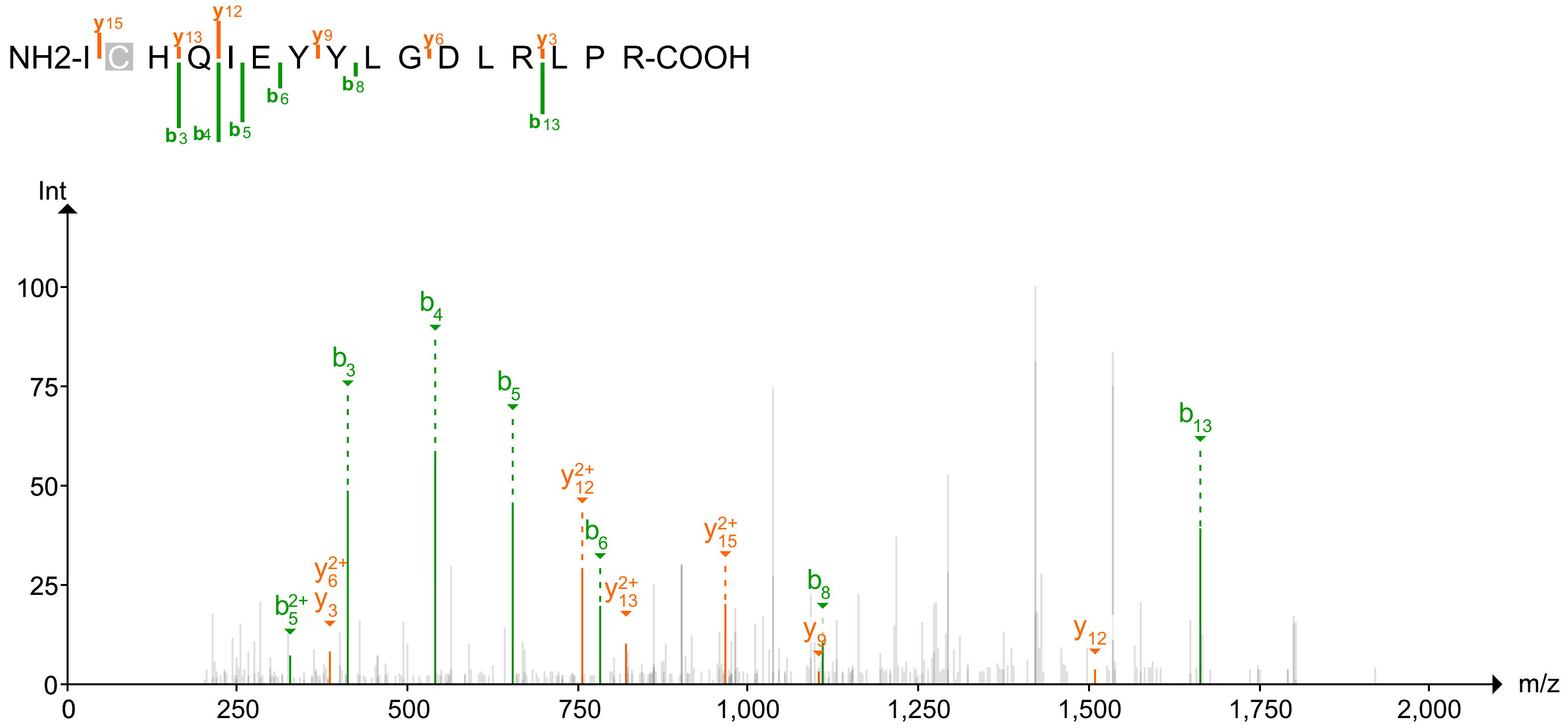

NH2-I C H Q I E Y Y L G D L R L P R-COOH
y15 y13 y12 y9 y6 y3
b3 b4 b5 b6 b8 b13
Int
100
75
50
25
0
b4
b3
b5
y12 2+
b6
y15 2+
b13
b8
y6 2+
b5 2+
y3
y13 2+
y9
y12
0
250
500
750
1,000
1,250
1,500
1,750
2,000
m/z

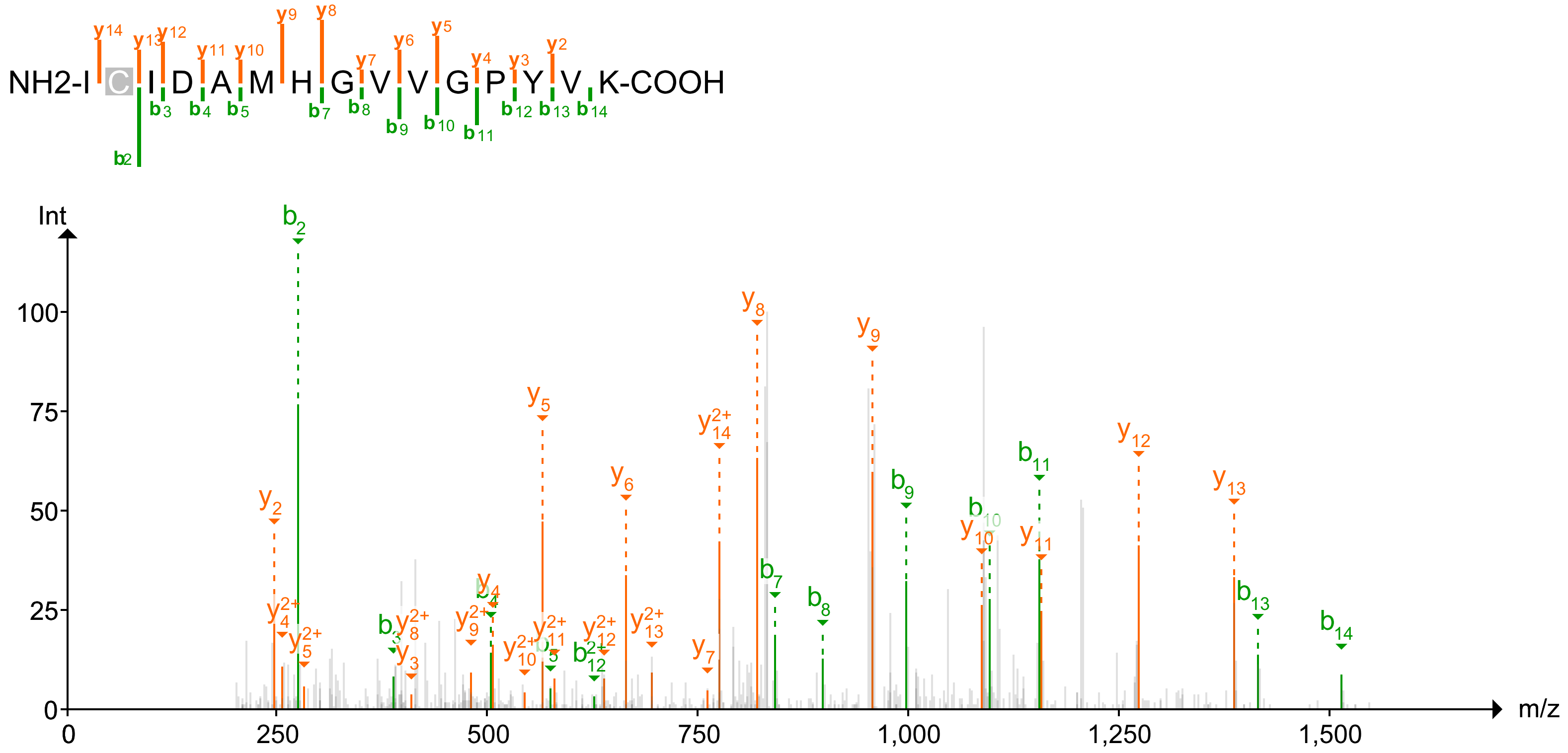

NH2-I C I D A M H G V V G P Y V K-COOH
Int
m/z
0
250
500
750
1,000
1,250
1,500
25
50
75
100

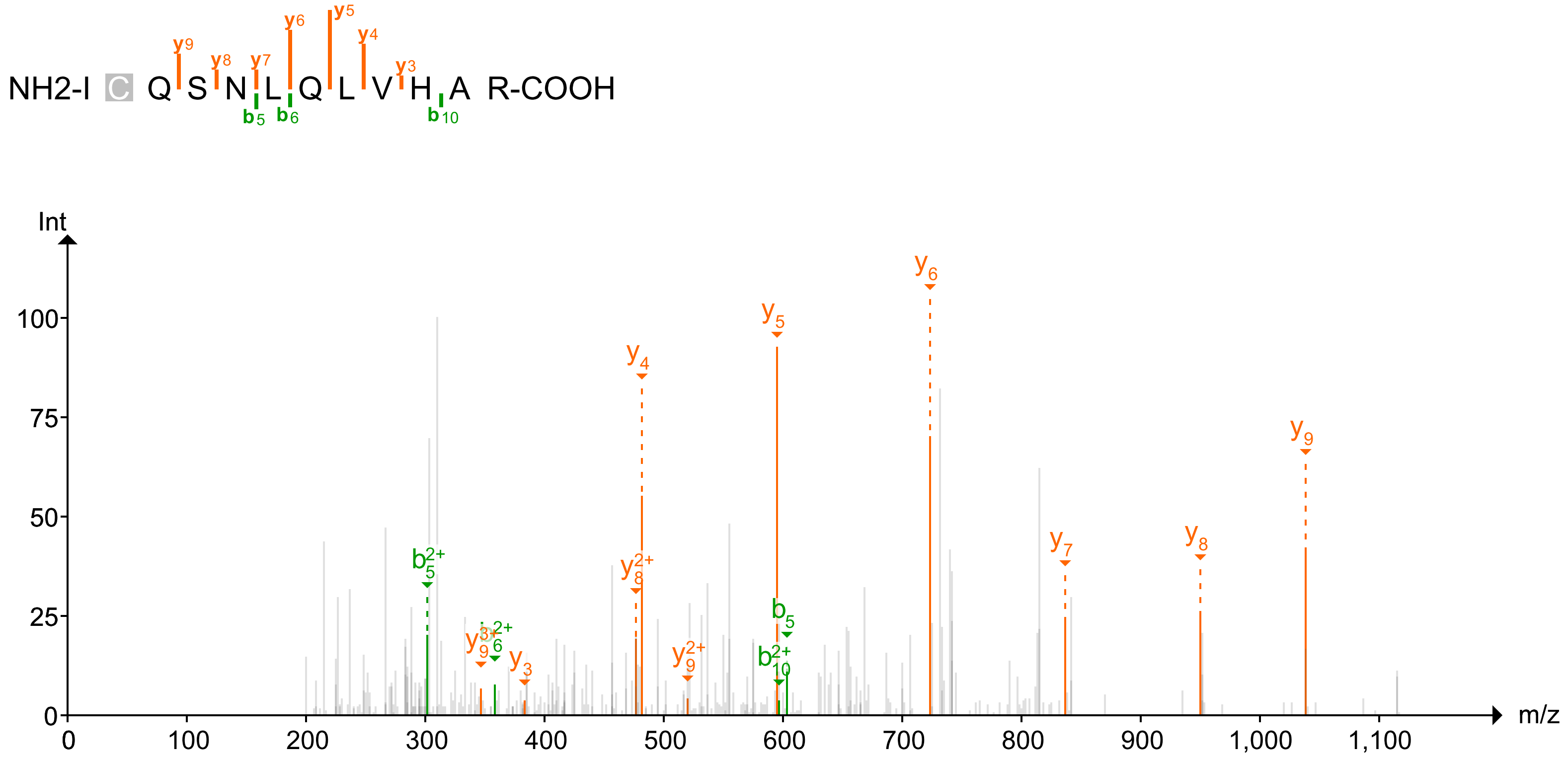

NH2-I C Q S N L Q L V H A R-COOH
y9 y8 y7 y6 y5 y4 y3
b5 b6 b10
Int
m/z
100
75
50
25
0
0 100 200 300 400 500 600 700 800 900 1,000 1,100
y6
y5
y4
y9
y7
y8
b5 2+
y8 2+
y9 3+
b6 2+
y3
y9 2+
b5
b10 2+

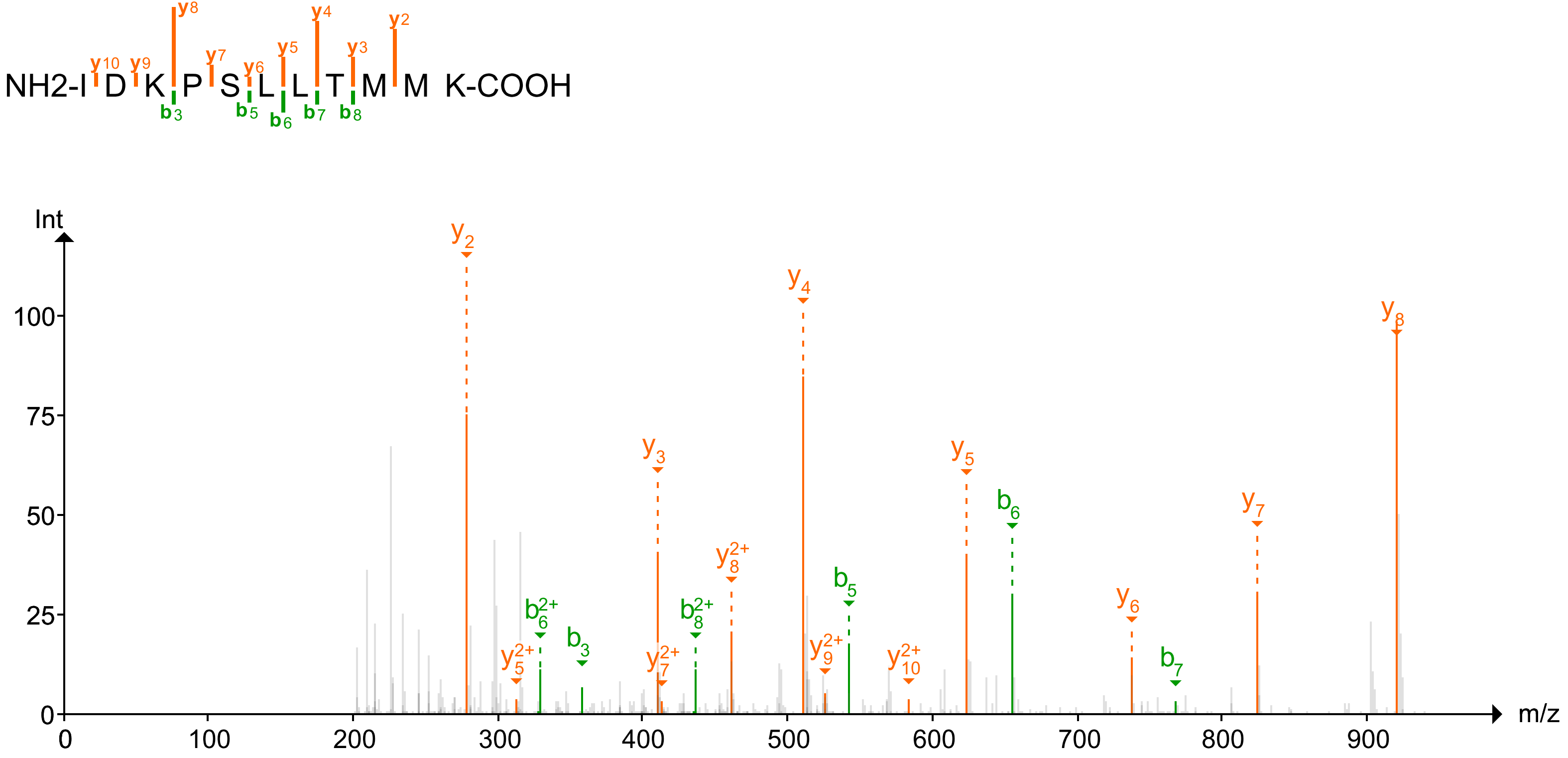

NH2-I D K P S L L T M M K-COOH
y10 y9 y8 y7 y6 y5 y4 y3 y2
b3 b5 b6 b7 b8
Int
100
75
50
25
0
m/z
0
100
200
300
400
500
600
700
800
900
y2
y4
y8
y3
y5
b6
y7
y8 2+
b5
b6 2+
b3
b8 2+
y5 2+
y7 2+
y9 2+
y10 2+
y6
b7

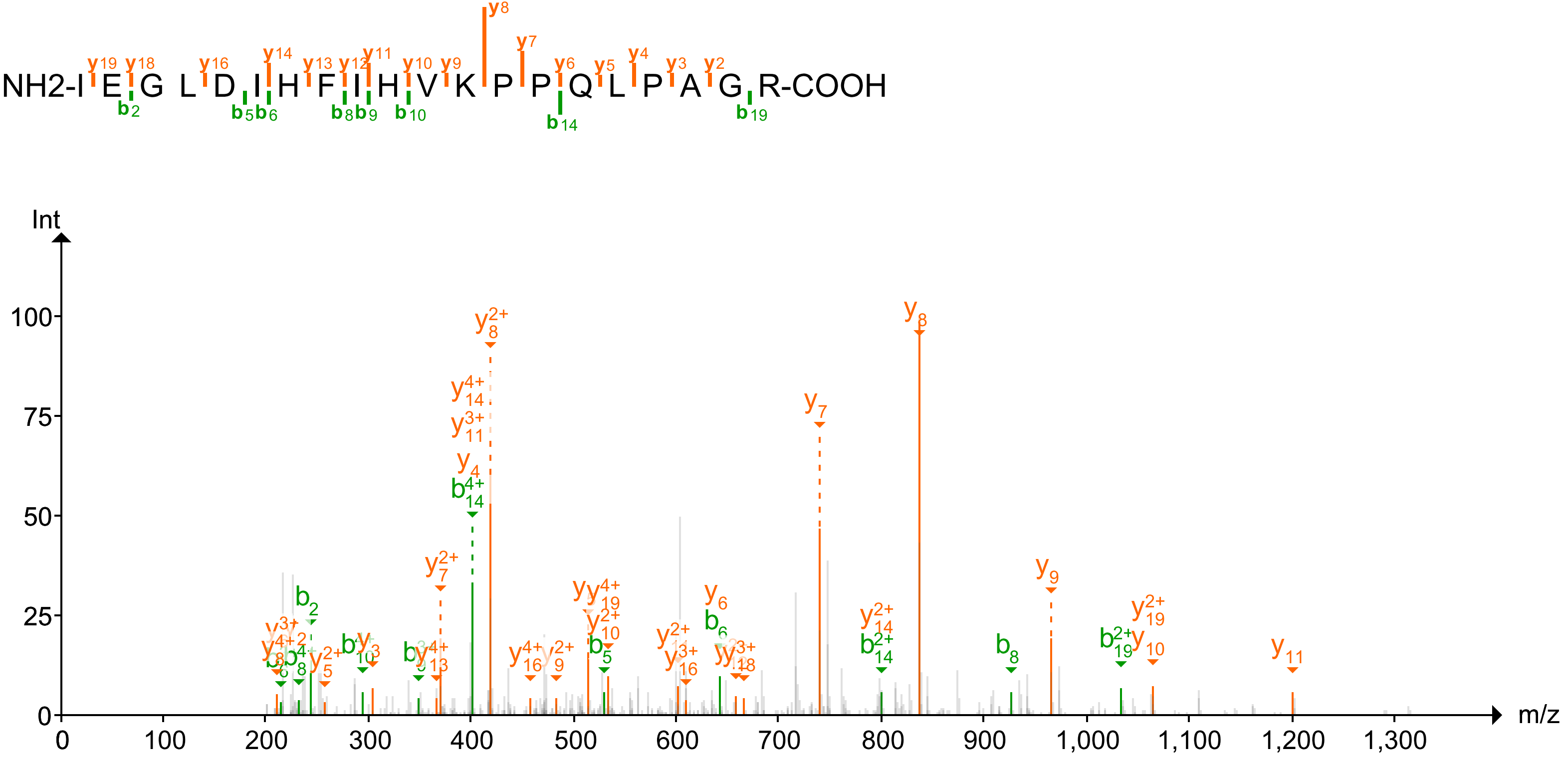

NH2-I E G L D I H F I H V K P P Q L P A G R-COOH
y19 y18 y16 y14 y13 y12 y11 y10 y9 y8 y7 y6 y5 y4 y3 y2
b2 b5 b6 b8 b9 b10 b14 b19
Int
100
75
50
25
0
m/z
0
100
200
300
400
500
600
700
800
900
1,000
1,100
1,200
1,300

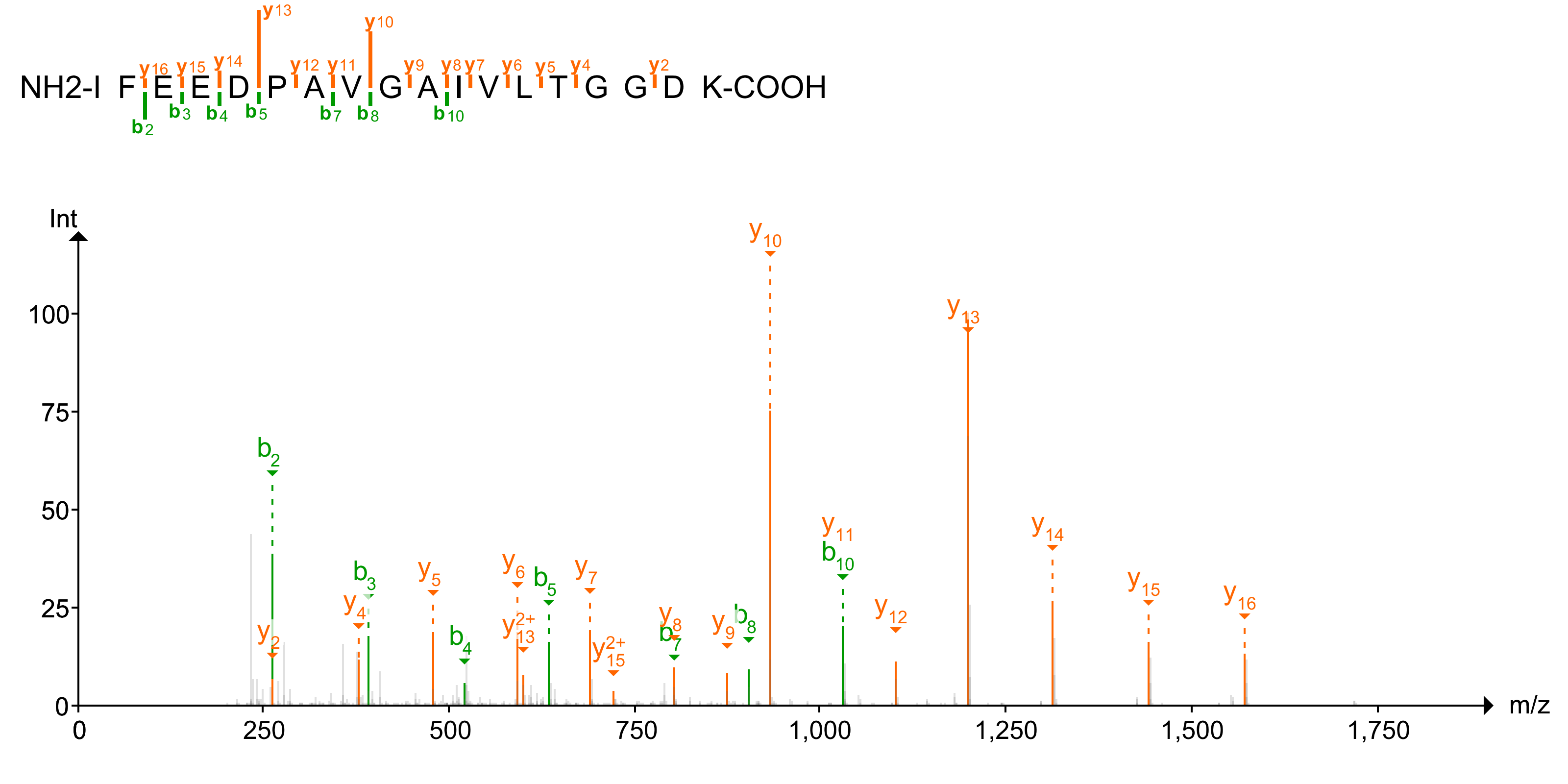

NH2-I F E E D P A V G A I V L T G G D K-COOH
Int
m/z
0
25
50
75
100
250
500
750
1,000
1,250
1,500
1,750

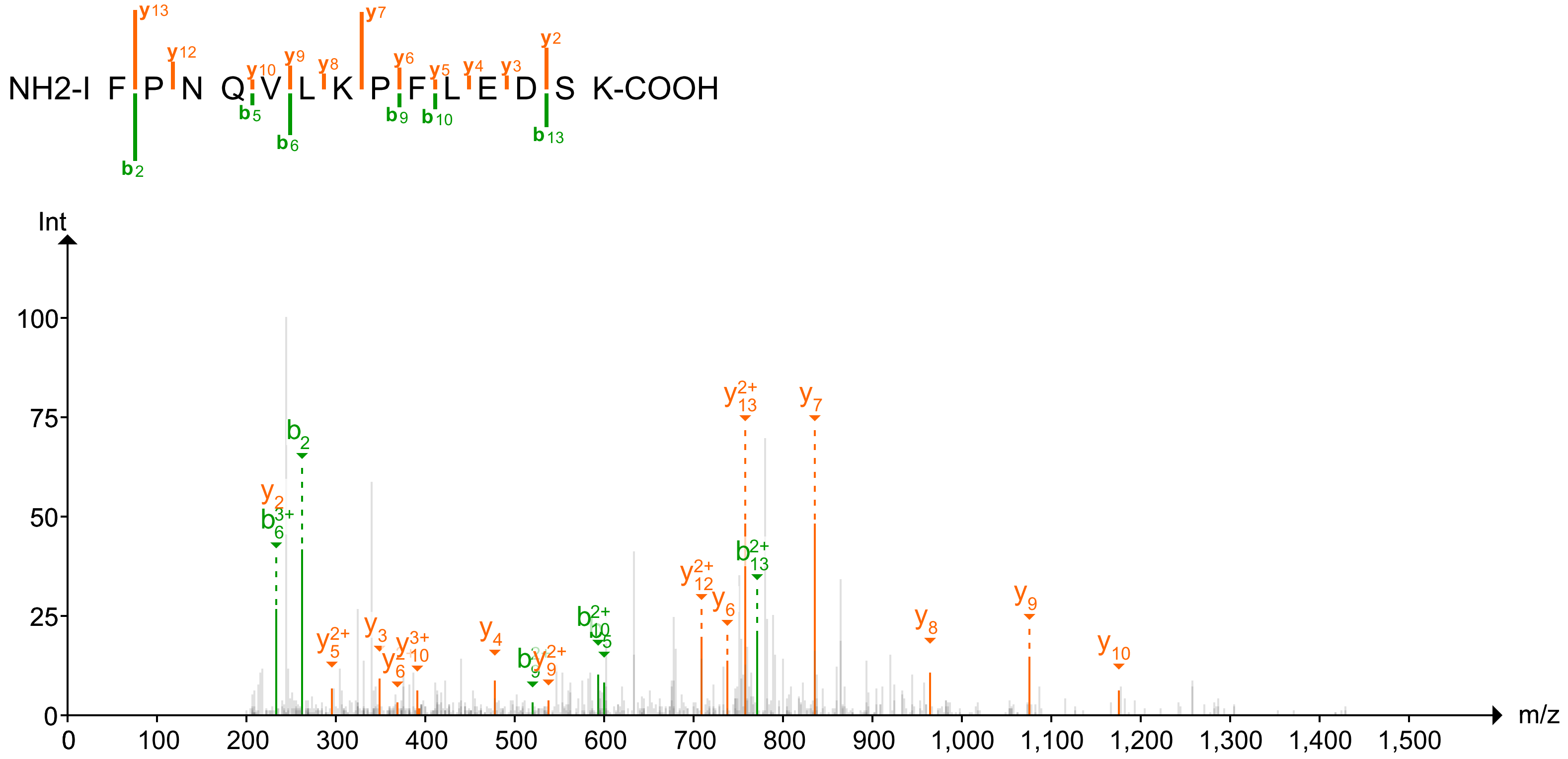

NH2-I F P N Q V L K P F L E D S K-COOH
y13 y12 y10 y9 y8 y7 y6 y5 y4 y3 y2
b2 b5 b6 b9 b10 b13
Int
100
75
50
25
0
m/z
0 100 200 300 400 500 600 700 800 900 1,000 1,100 1,200 1,300 1,400 1,500

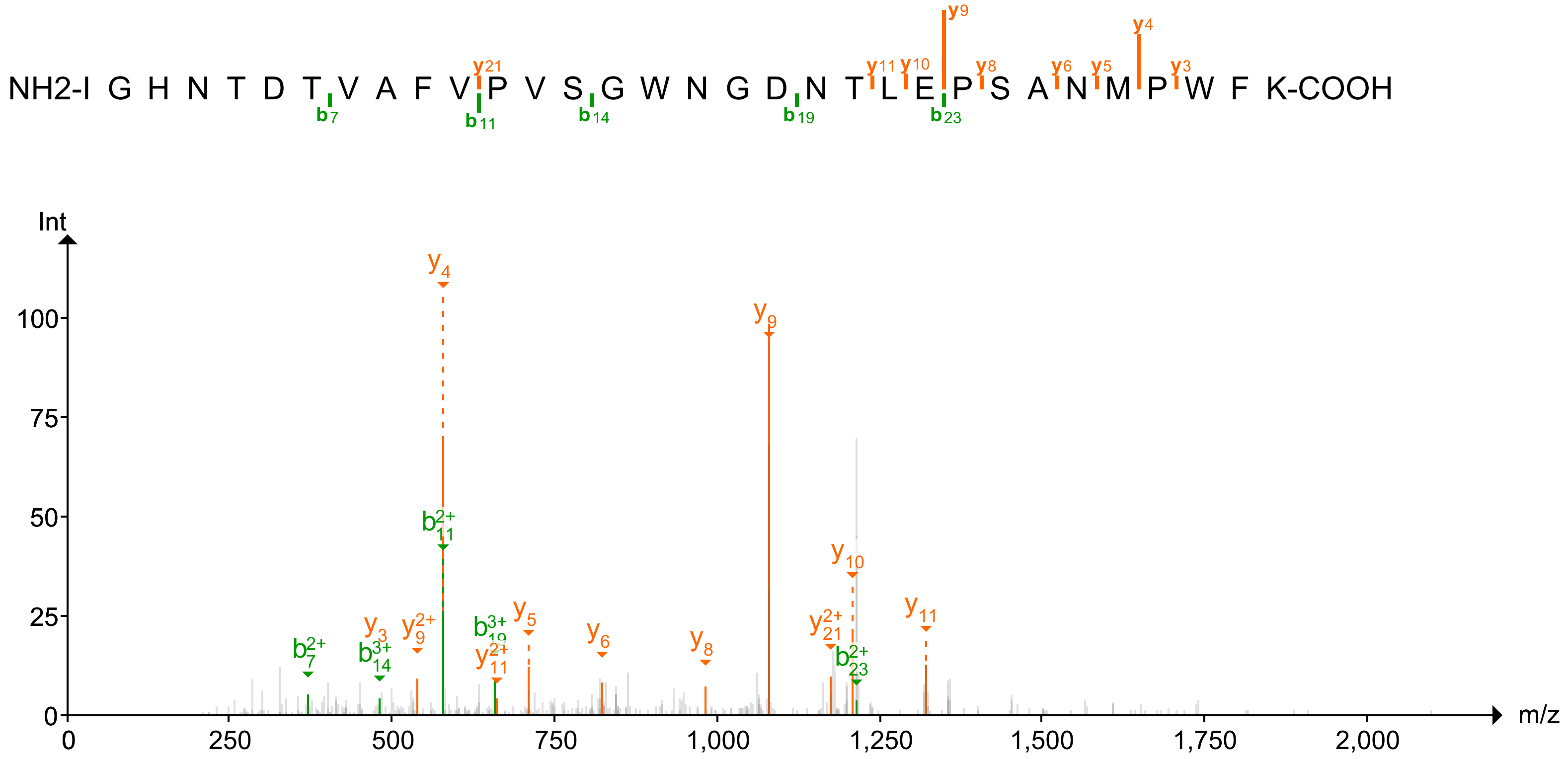

NH2-I G H N T D T V A F V P V S G W N G D N T L E P S A N M P W F K-COOH
b7 b11 b14 b19 b23
y21 y11 y10 y9 y8 y6 y5 y4 y3
Int
100
75
50
25
0
m/z
0 250 500 750 1,000 1,250 1,500 1,750 2,000

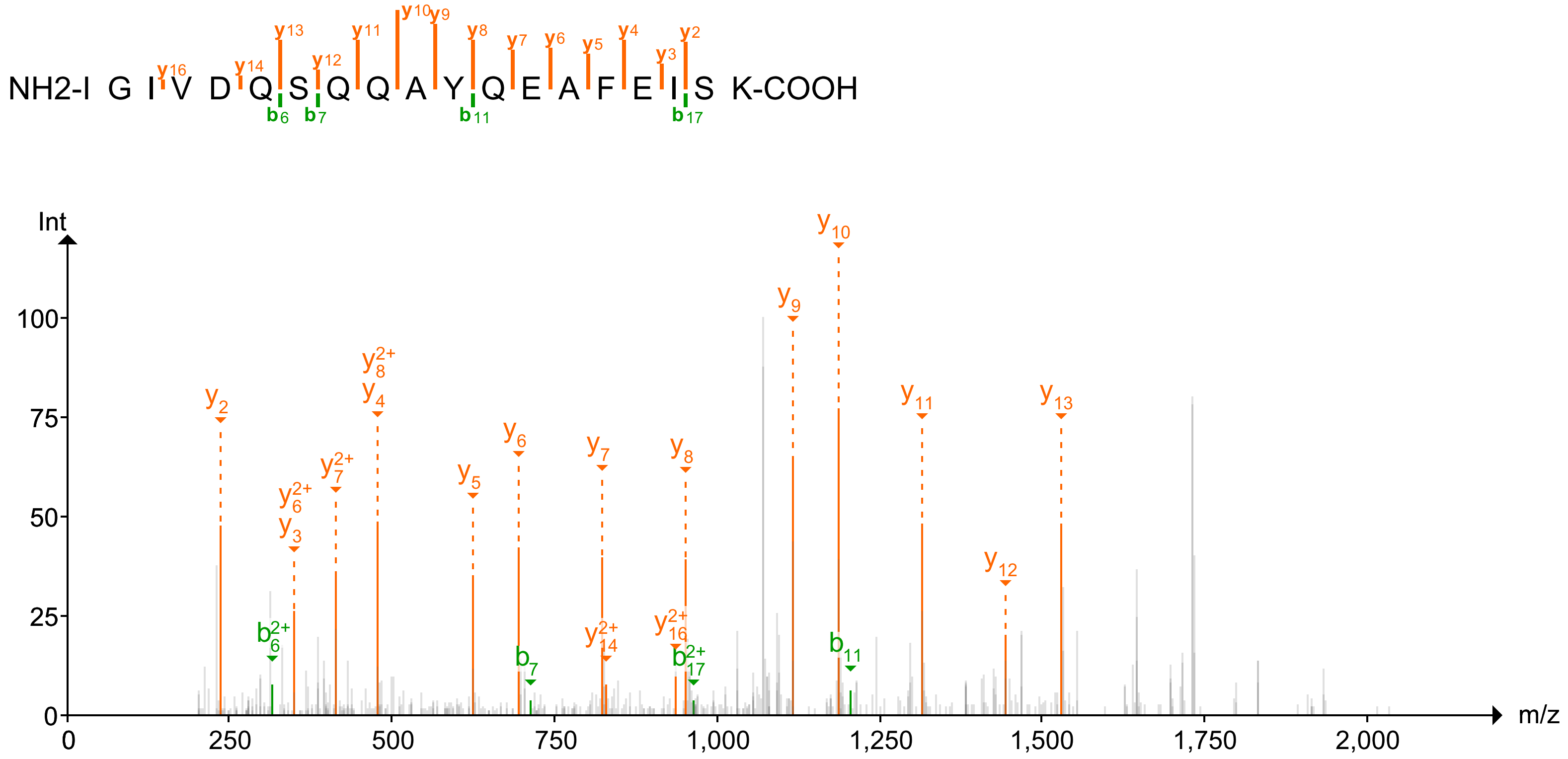

NH2-I G I V D Q S Q Q A Y Q E A F E I S K-COOH
y16 y14 y13 y12 y11 y10 y9 y8 y7 y6 y5 y4 y3 y2
b6 b7 b11 b17
Int
m/z
0
25
50
75
100
250
500
750
1,000
1,250
1,500
1,750
2,000
$y_2$
$y_3$
$y_6^{2+}$
$y_7^{2+}$
$y_8^{2+}$
$y_4$
$y_5$
$y_6$
$y_7$
$y_8$
$y_9$
$y_{10}$
$y_{11}$
$y_{12}$
$y_{13}$
$y_{14}^{2+}$
$y_{16}^{2+}$
$b_6^{2+}$
$b_7$
$b_{17}^{2+}$
$b_{11}$

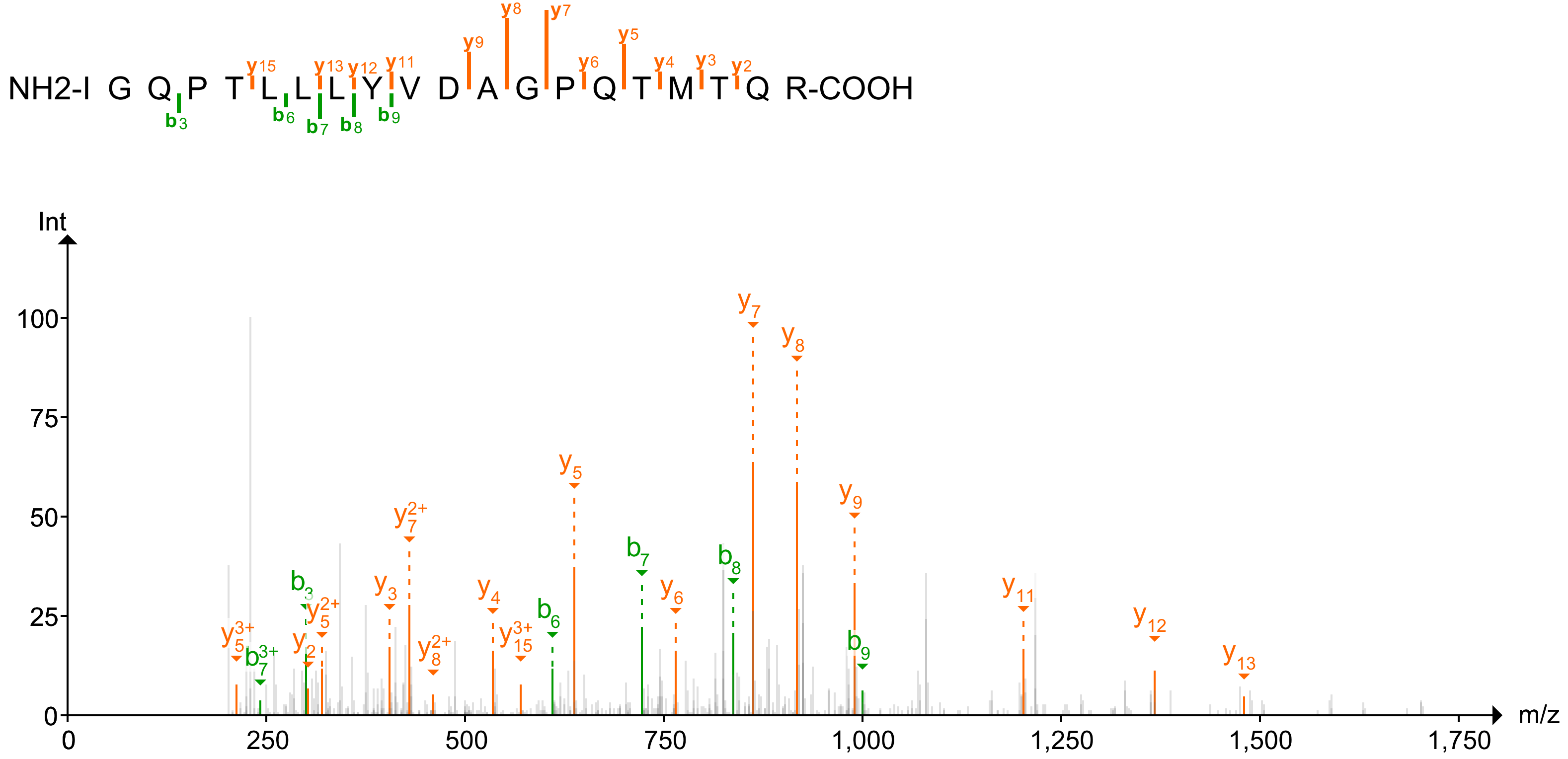

NH2-I G Q P T L L L Y V D A G P Q T M T Q R-COOH
Int
m/z

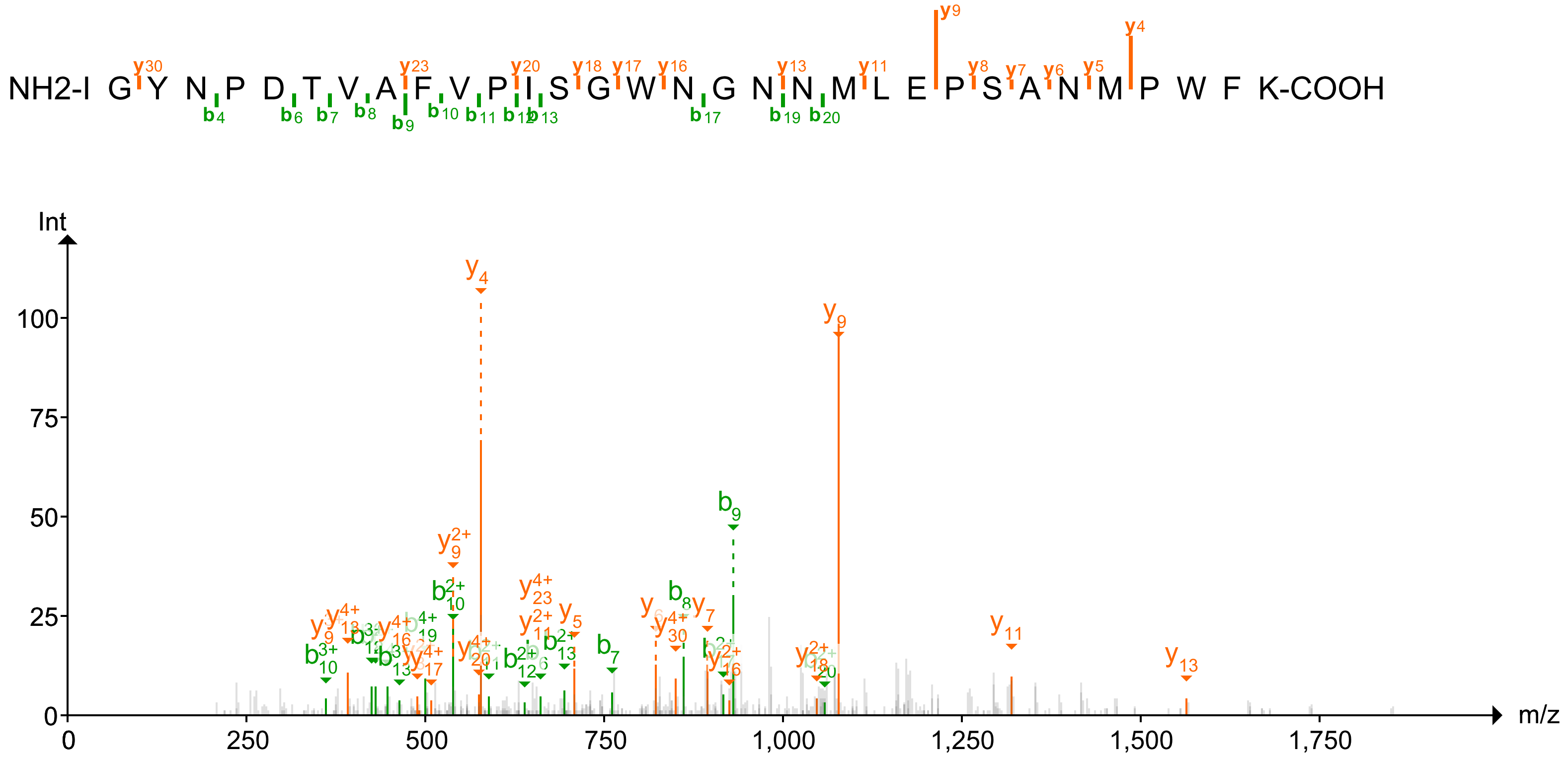

NH2-I G Y N P D T V A F V P I S G W N G N N M L E P S A N M P W F K-COOH
y30 y23 y20 y18 y17 y16 y13 y11 y9 y8 y7 y6 y5 y4
b4 b6 b7 b8 b9 b10 b11 b12 b13 b17 b19 b20
Int
100
75
50
25
0
m/z
0
250
500
750
1,000
1,250
1,500
1,750
y4
y9
b9
y11
y13
y5
y7
b7

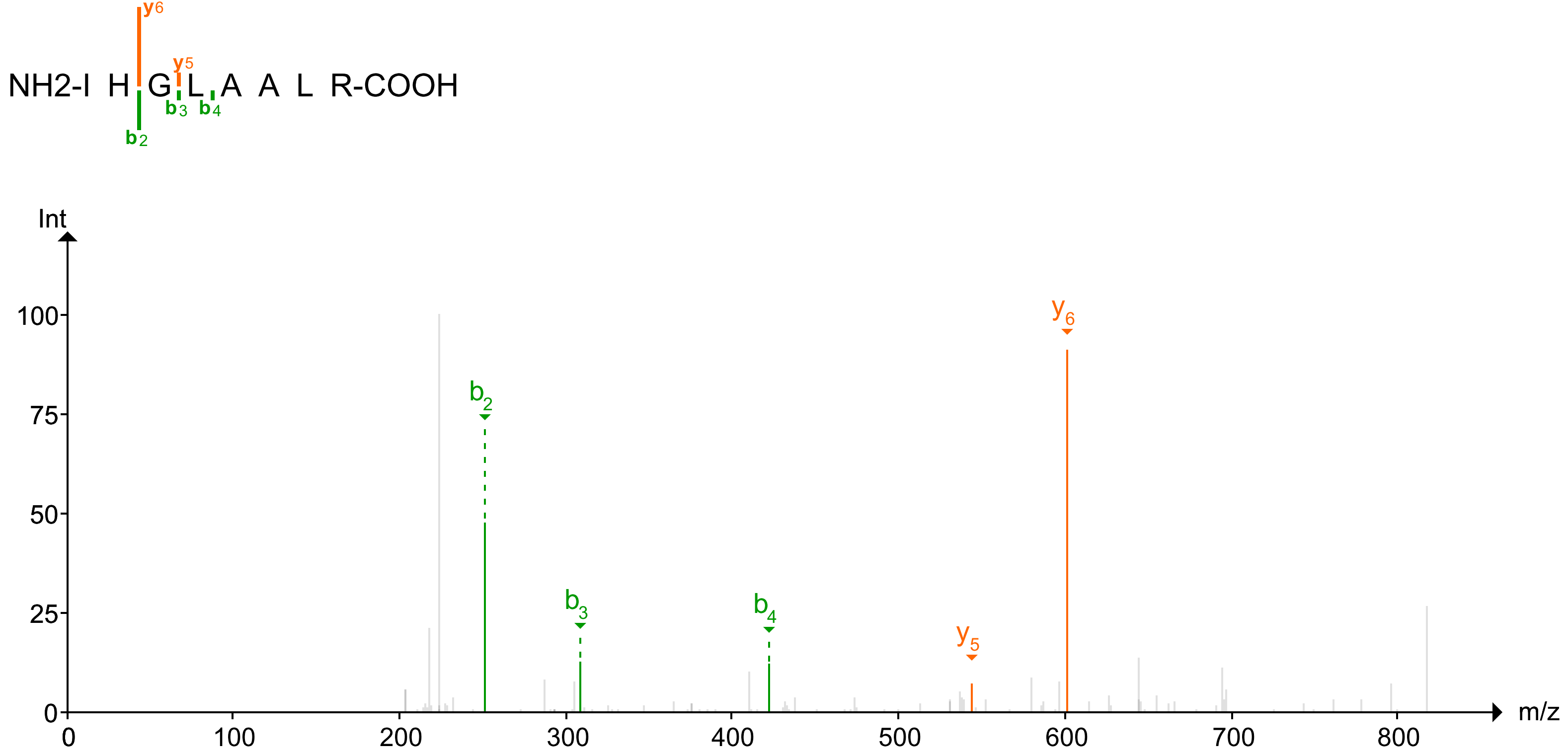

NH2-I H G L A A L R-COOH
y6
y5
b2
b3
b4
Int
100
75
50
25
0
m/z
0
100
200
300
400
500
600
700
800

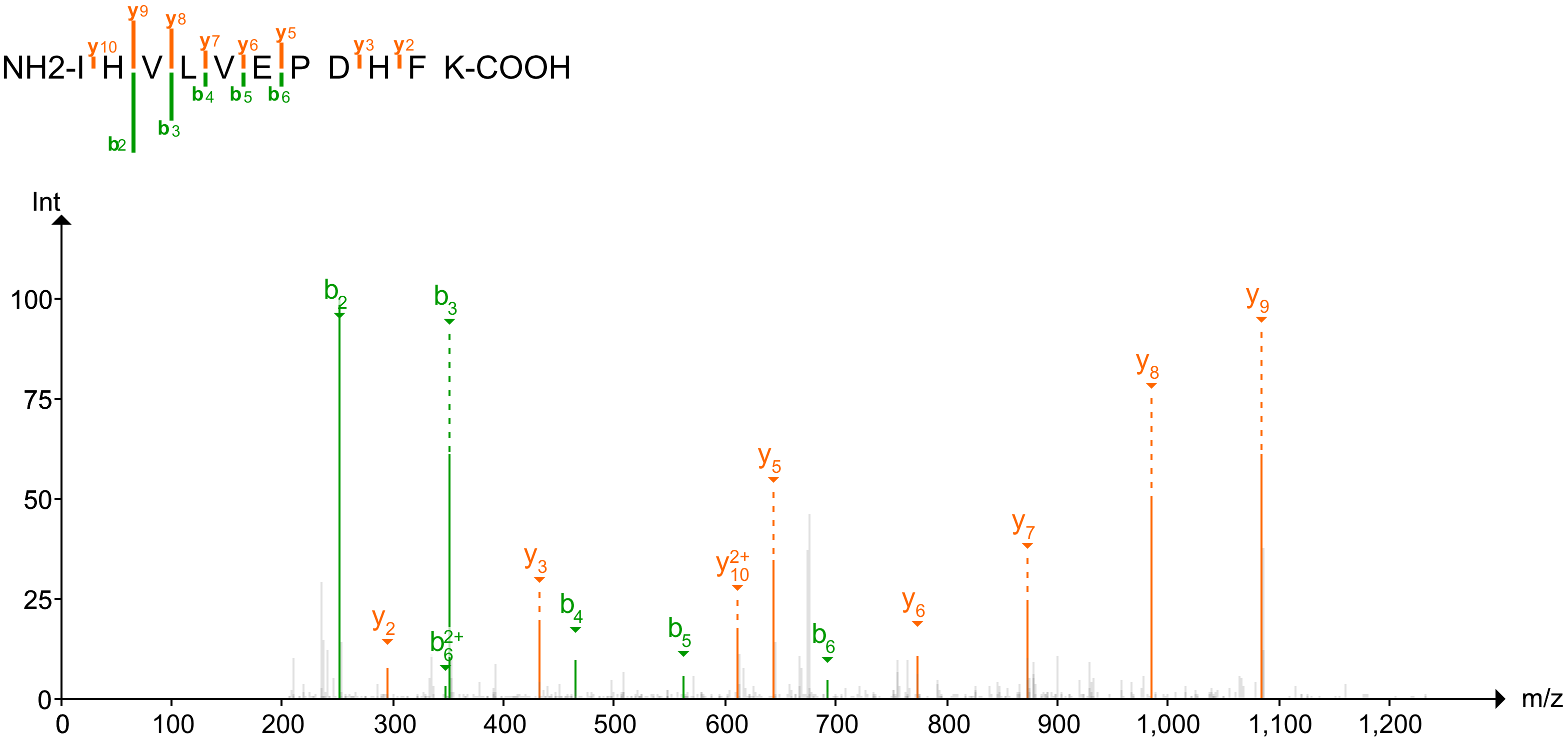
NH2-I H V L V E P D H F K-COOH
y10 y9 y8 y7 y6 y5 y3 y2
b2 b3 b4 b5 b6
Int
100
75
50
25
0
m/z
0
100
200
300
400
500
600
700
800
900
1,000
1,100
1,200
b2 b3 y2 b6 2+ y3 b4 b5 y10 2+ y5 b6 y6 y7 y8 y9

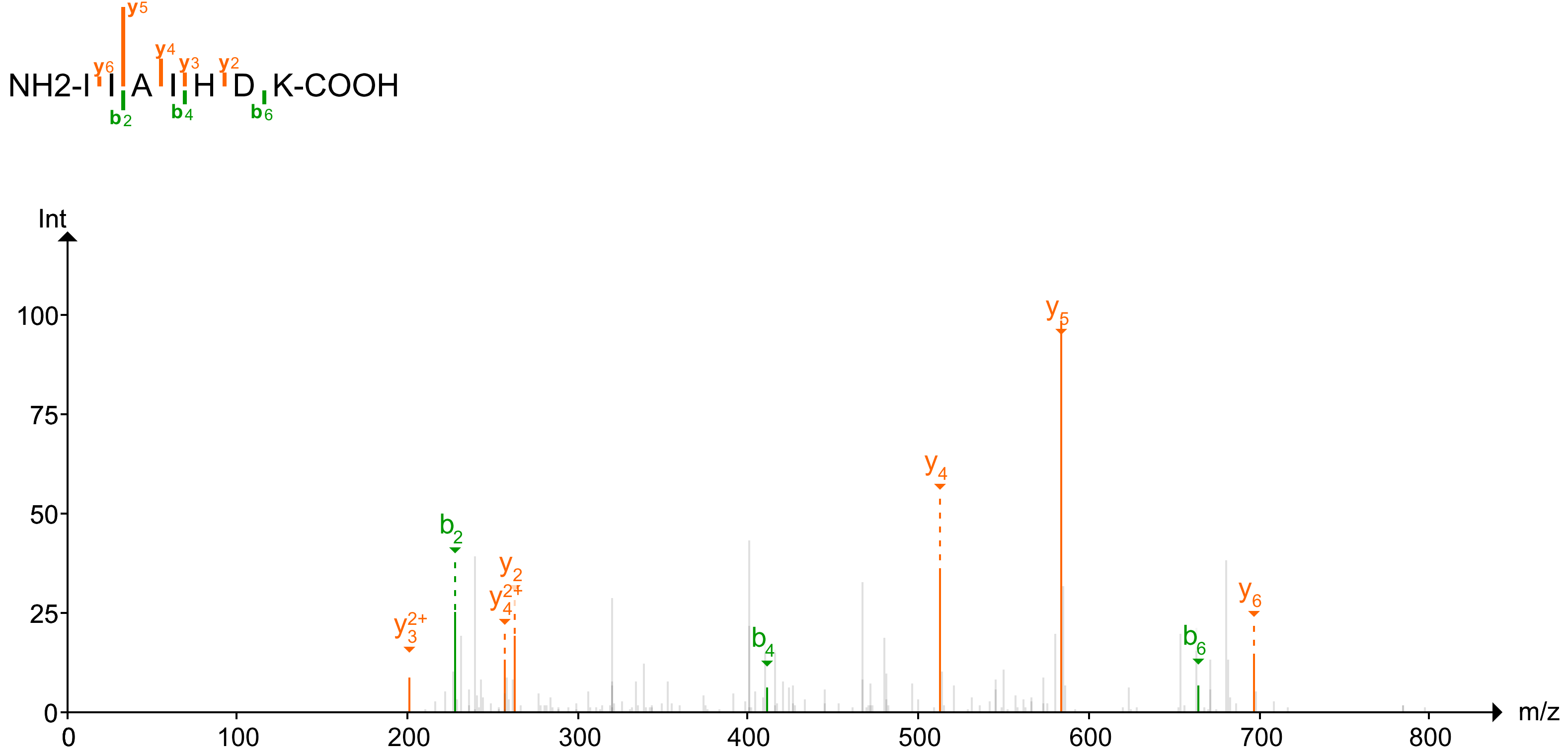

NH2-I I A I H D K-COOH
y5
y6
y4
y3
y2
b2
b4
b6
Int
100
75
50
25
0
0
100
200
300
400
500
600
700
800
m/z
$y_5$
$y_4$
$b_2$
$y_2$
$y_4^{2+}$
$y_3^{2+}$
$b_4$
$b_6$
$y_6$

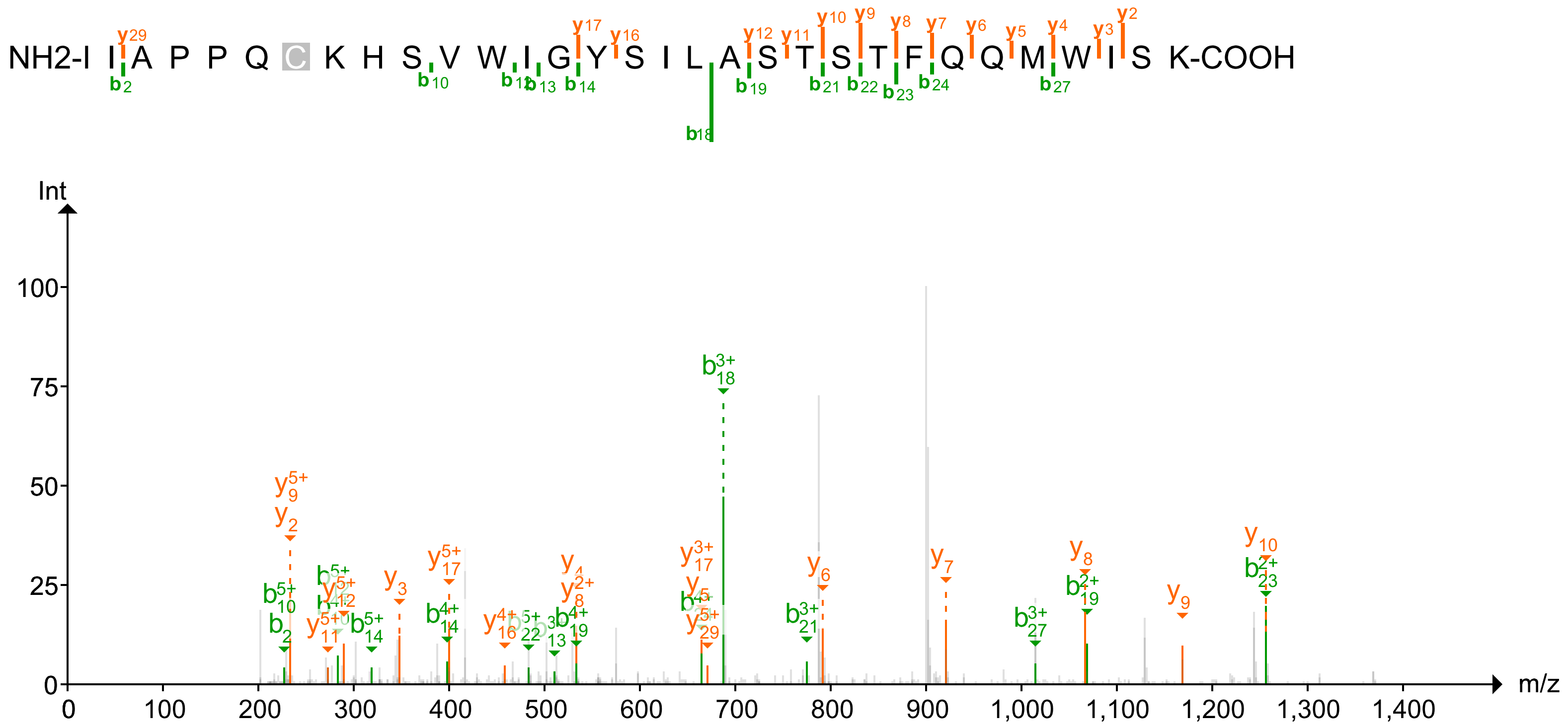

NH2-I I A P P Q C K H S V W I G Y S I L A S T S T F Q Q M W I S K-COOH
y29
y17
y16
y12
y11
y10
y9
y8
y7
y6
y5
y4
y3
y2
b2
b10
b13
b14
b18
b19
b21
b22
b23
b24
b27
Int
m/z
0
25
50
75
100
0
100
200
300
400
500
600
700
800
900
1,000
1,100
1,200
1,300
1,400

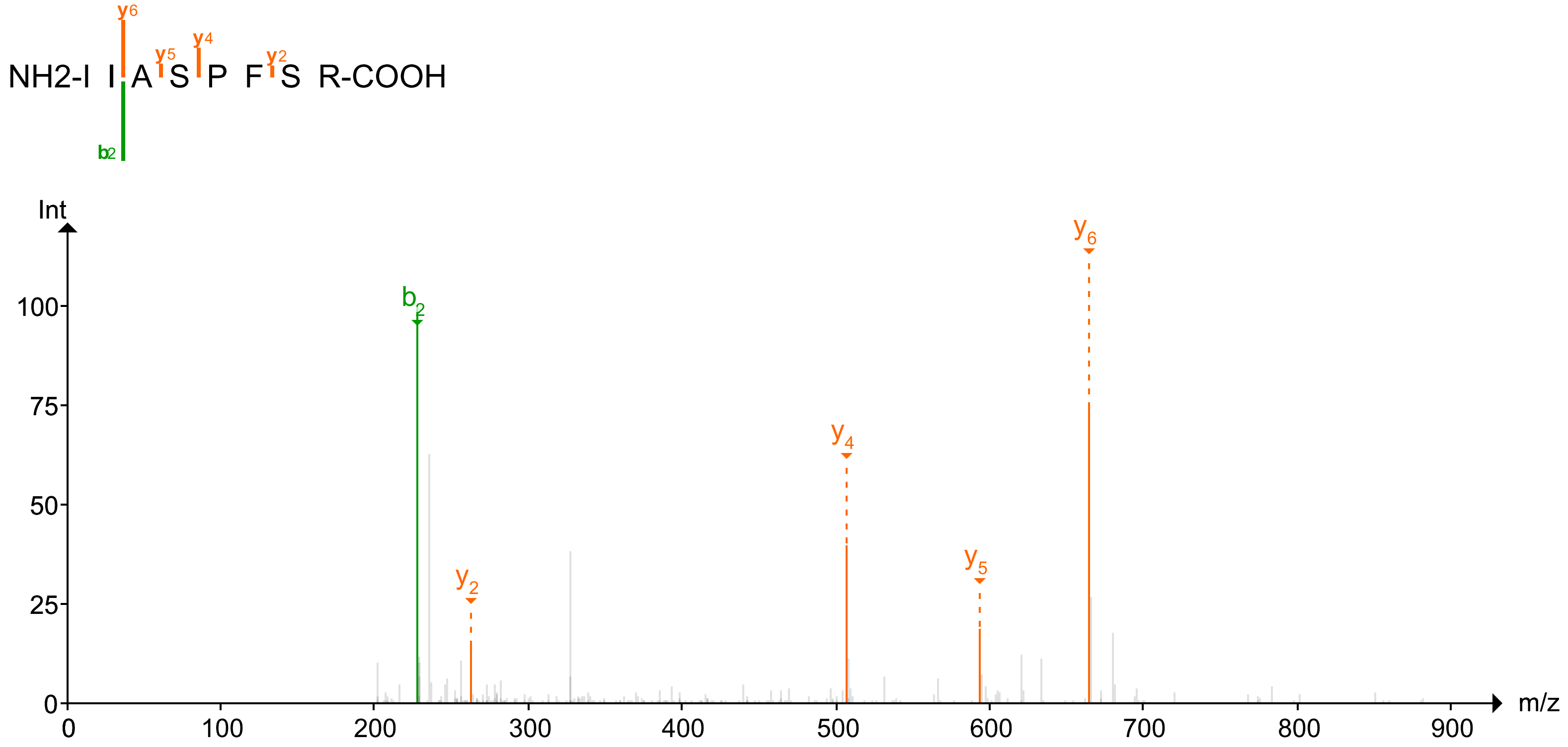

NH2-I I A S P F S R-COOH
y6
y5
y4
y2
b2
Int
m/z
0
25
50
75
100
0
100
200
300
400
500
600
700
800
900
b2
y2
y4
y5
y6

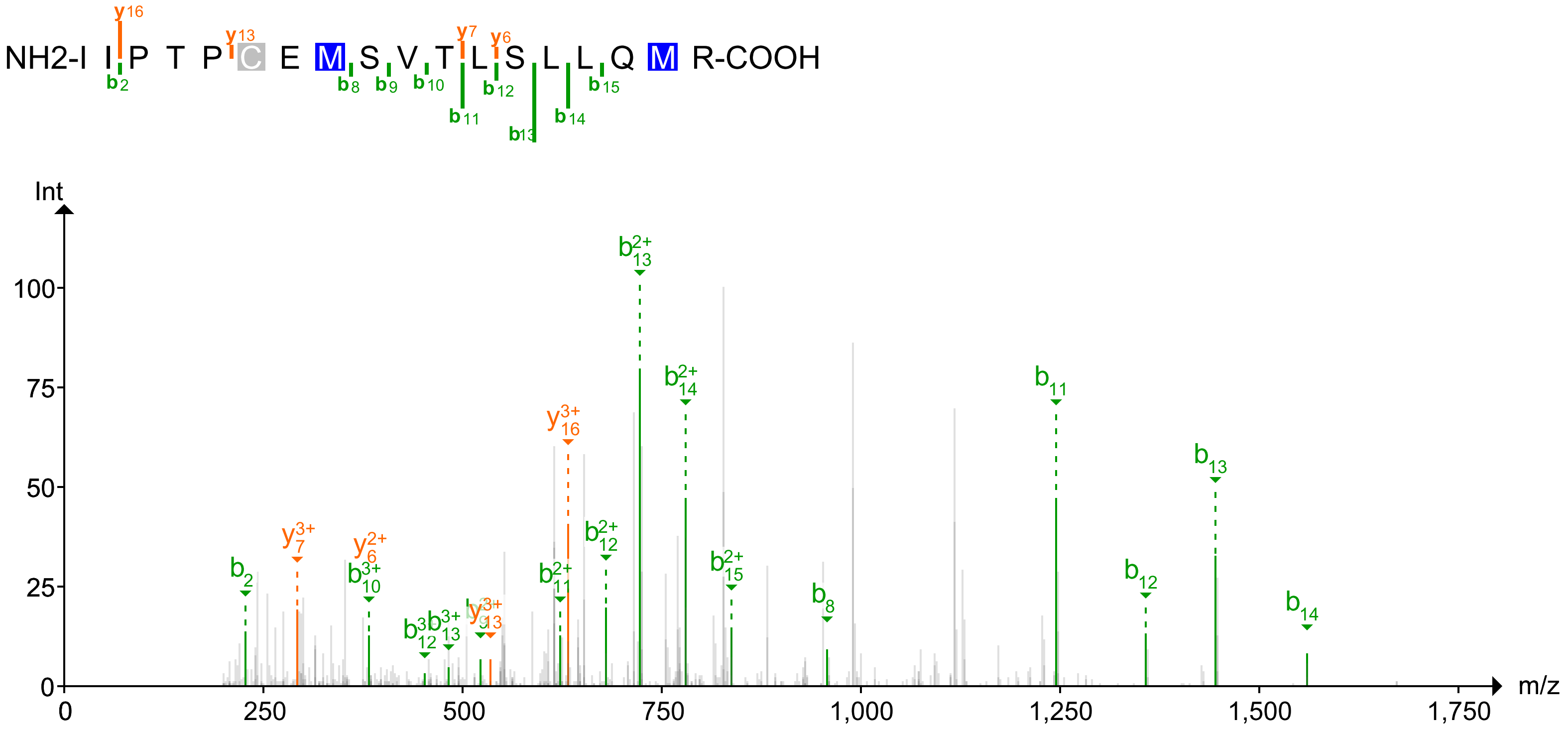

NH2-I I P T P C E M S V T L S L L Q M R-COOH
y16
y13
y7
y6
b2
b8
b9
b10
b11
b12
b13
b14
b15
Int
m/z
0
25
50
75
100
250
500
750
1,000
1,250
1,500
1,750

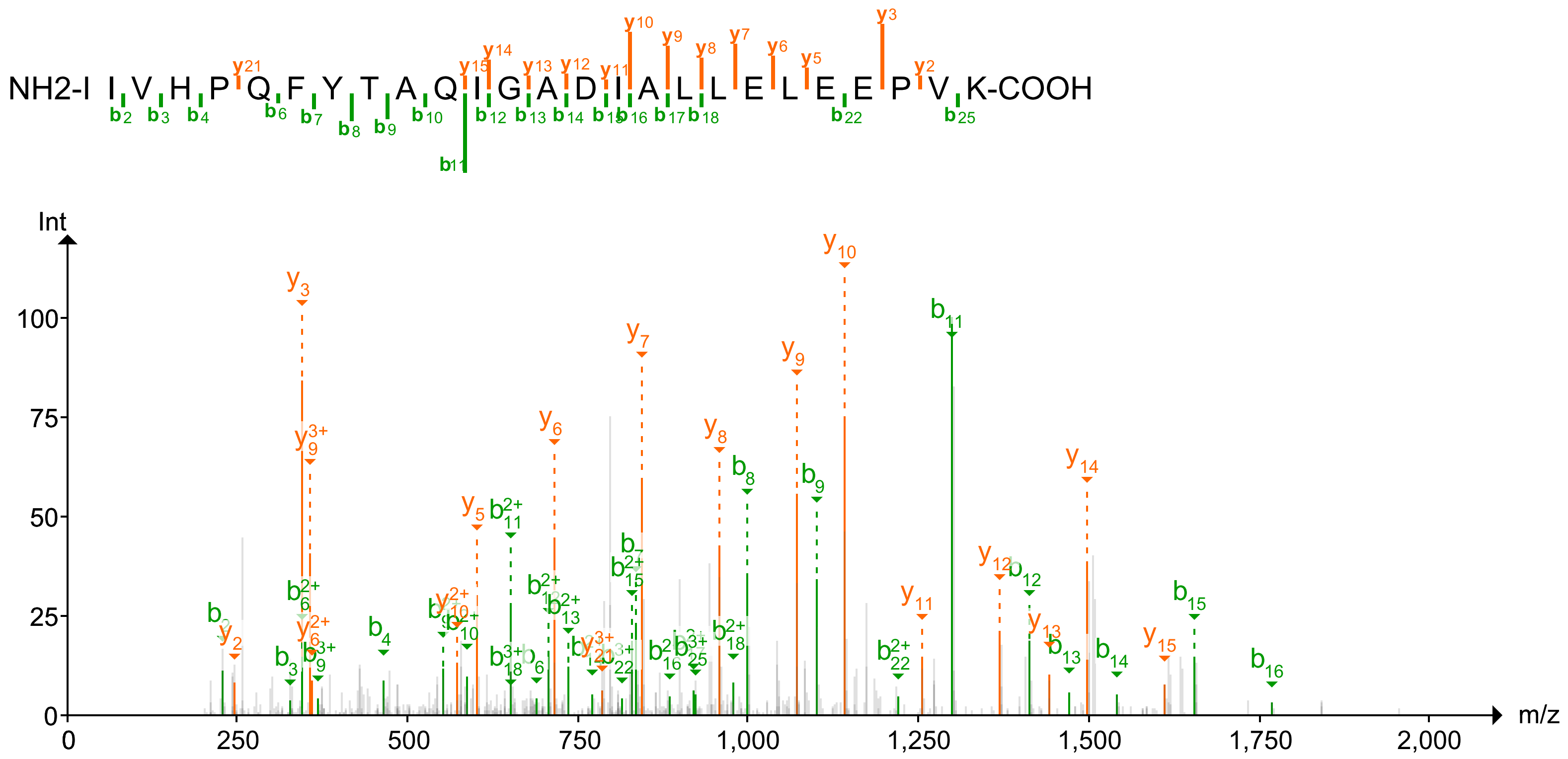
NH2-I I V H P Q F Y T A Q I G A D I A L L E L E E P V K-COOH
Int
m/z
0
25
50
75
100
250
500
750
1,000
1,250
1,500
1,750
2,000

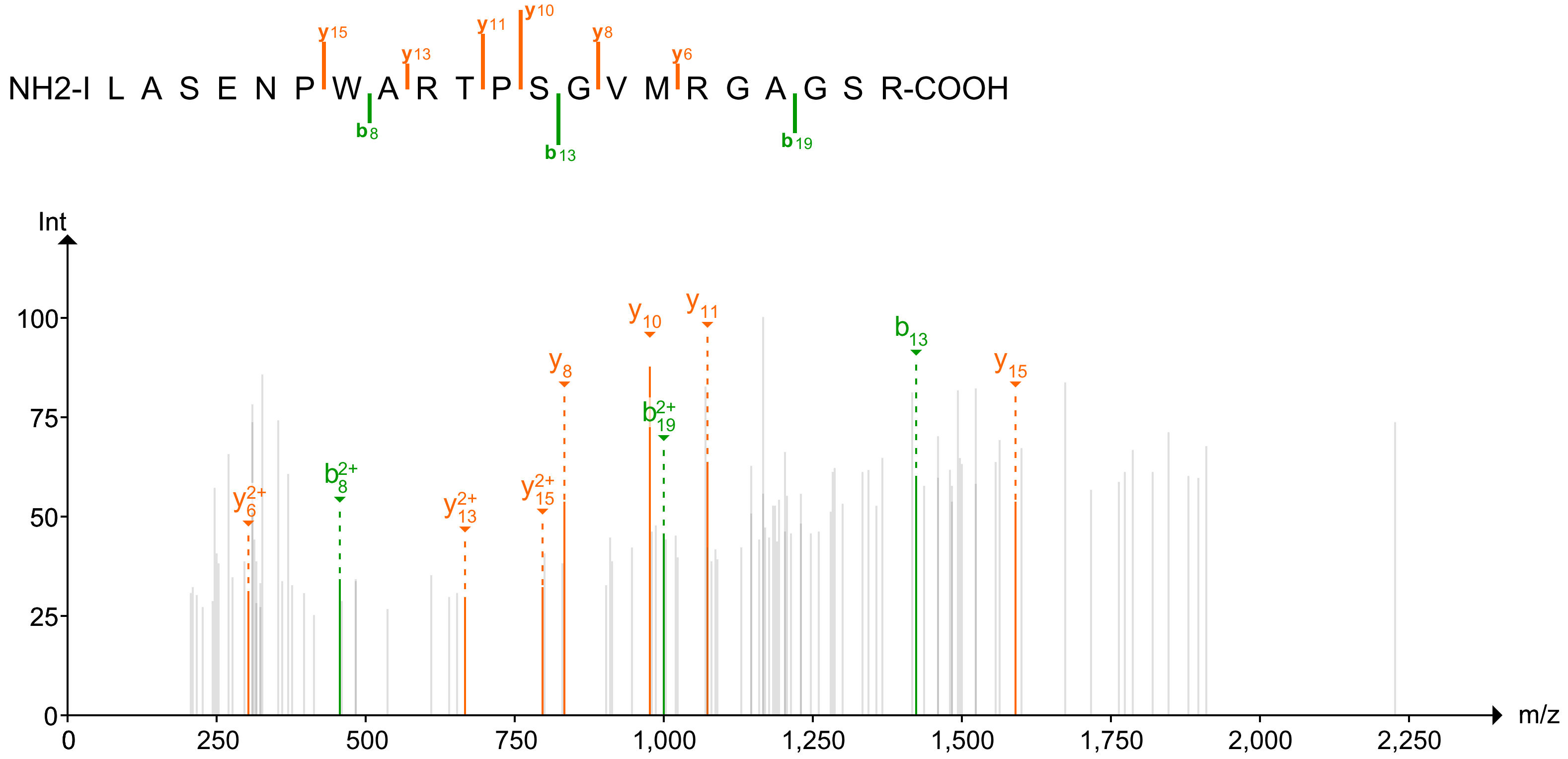

NH2-I L A S E N P W A R T P S G V M R G A G S R-COOH
y15
y13
y11
y10
y8
y6
b8
b13
b19
Int
m/z
y6 2+
b8 2+
y13 2+
y15 2+
y8
y10
y11
b19 2+
b13
y15
0
25
50
75
100
0
250
500
750
1,000
1,250
1,500
1,750
2,000
2,250

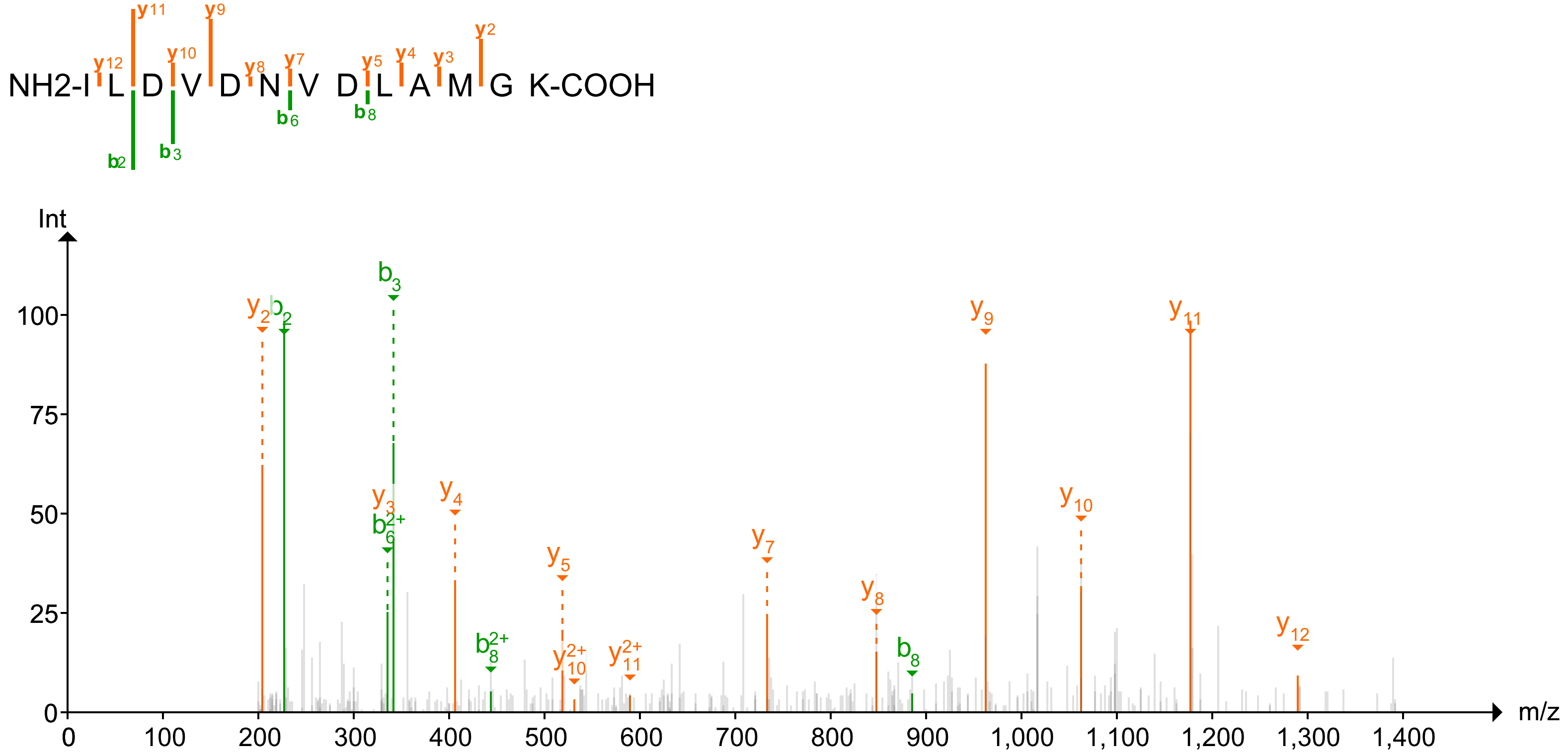

NH2-I L D V D N V D L A M G K-COOH
y12 y11 y10 y9 y8 y7 y5 y4 y3 y2
b2 b3 b6 b8
Int
100
75
50
25
0
m/z
0 100 200 300 400 500 600 700 800 900 1,000 1,100 1,200 1,300 1,400
y2 b2 b3 y3 b6 2+ y4 b8 2+ y5 y10 2+ y11 2+ y7 y8 b8 y9 y10 y11 y12

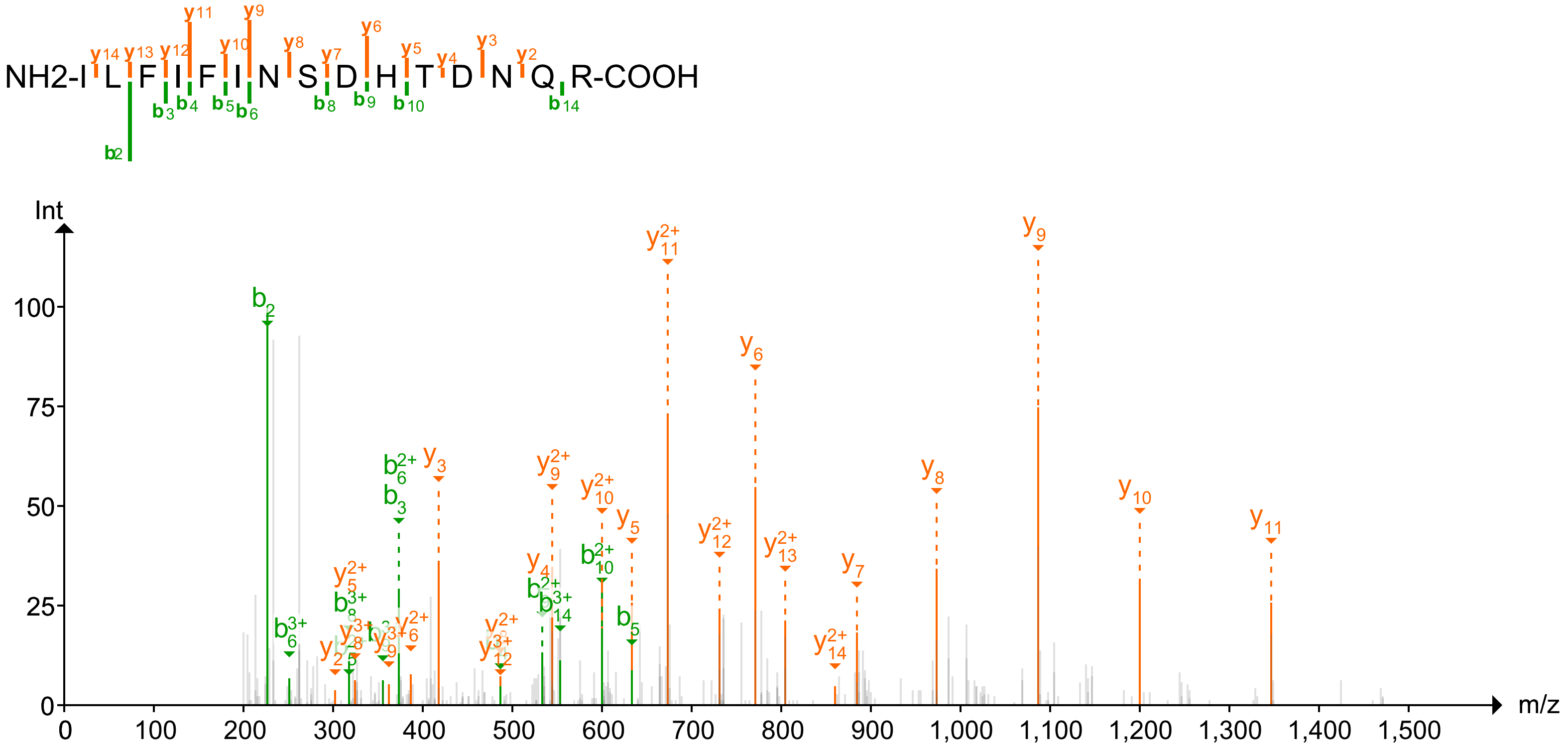

NH2-I L F I F I N S D H T D N Q R-COOH
Int
m/z
0
25
50
75
100
0
100
200
300
400
500
600
700
800
900
1,000
1,100
1,200
1,300
1,400
1,500

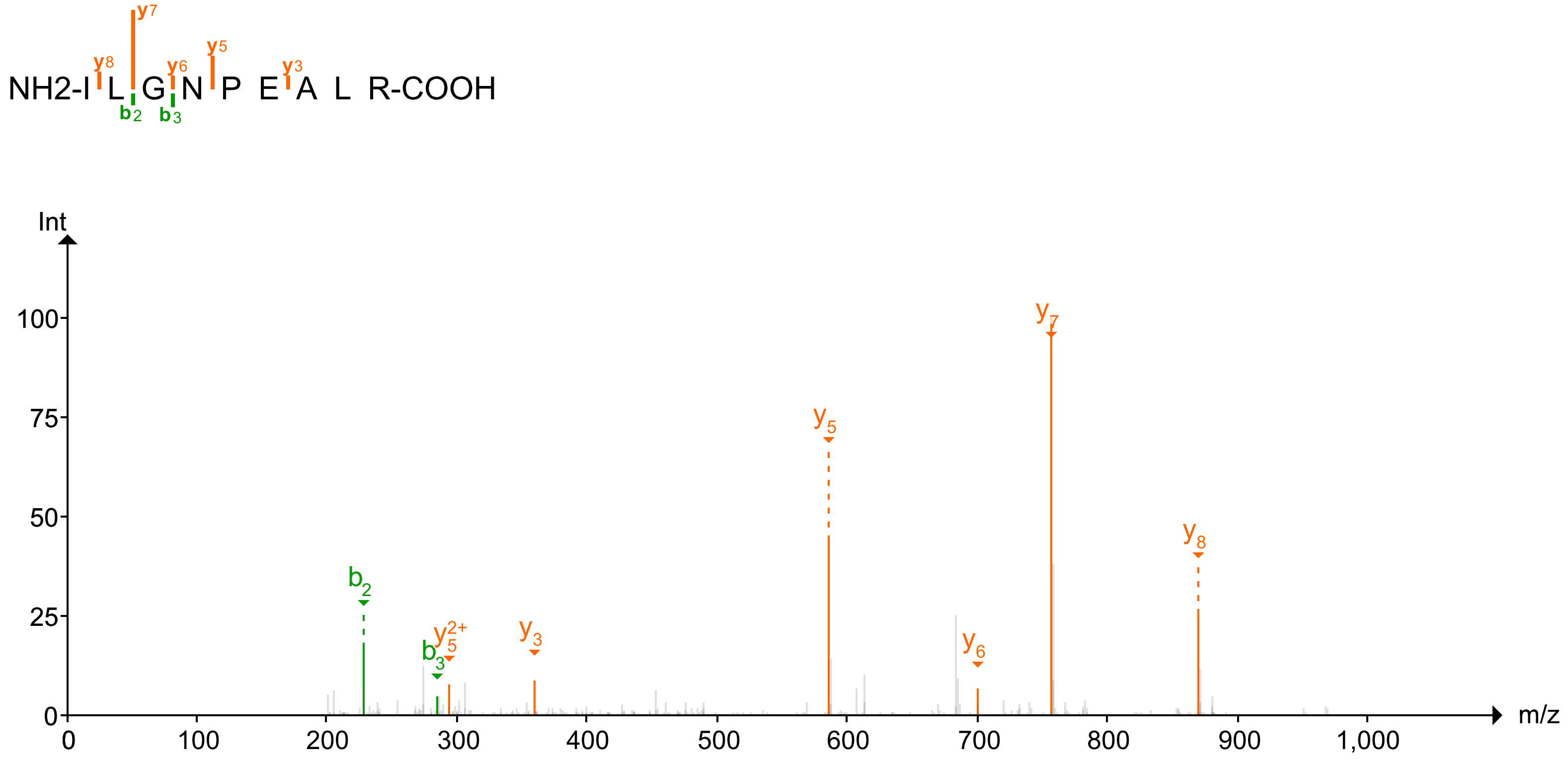

NH2-I L G N P E A L R-COOH
y8 y7 y6 y5 y3
b2 b3
Int
m/z
0 25 50 75 100
0 100 200 300 400 500 600 700 800 900 1,000
b2 b3 y5 2+ y3 y5 y6 y7 y8

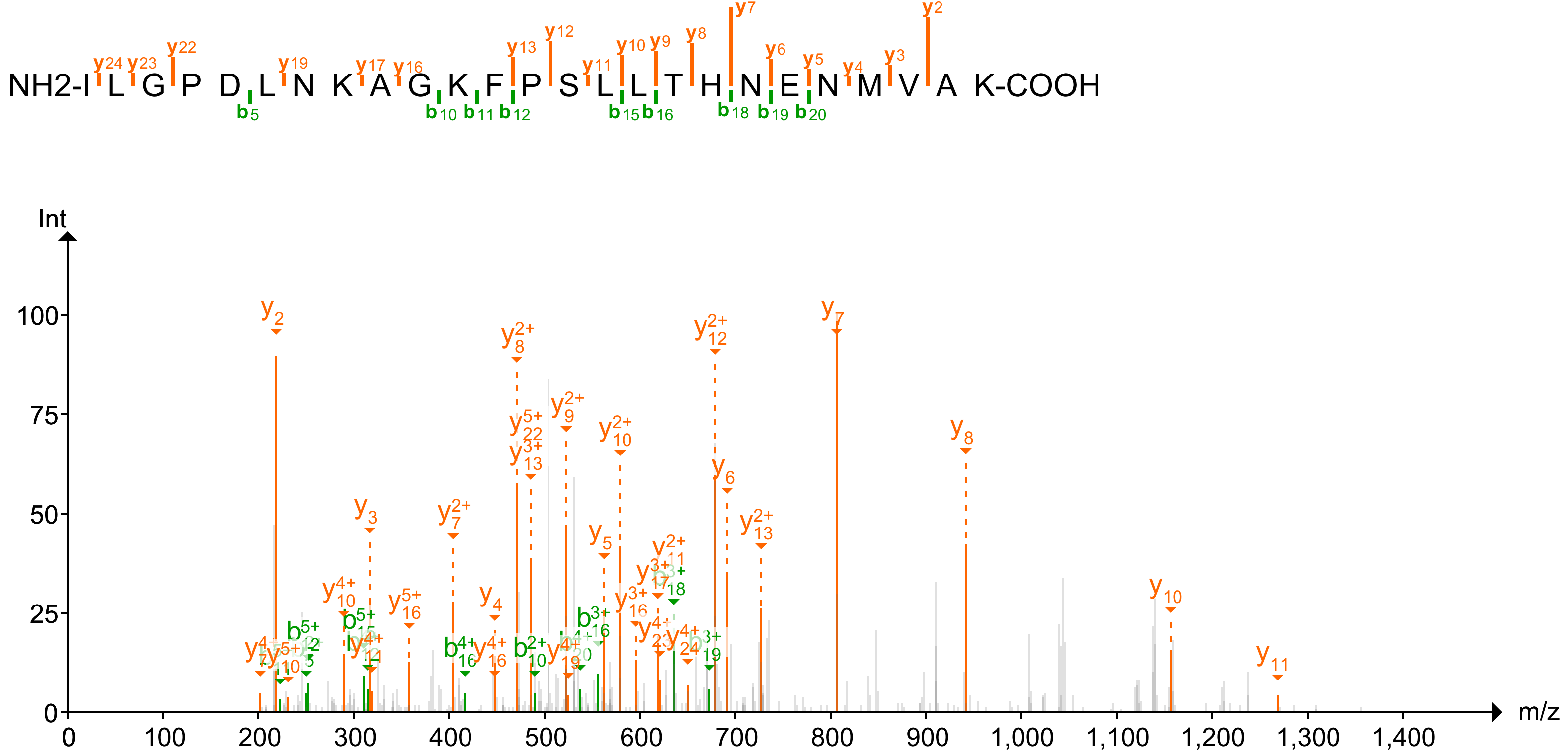
NH2-I L G P D L N K A G K F P S L L T H N E N M V A K-COOH
y24 y23 y22 y19 y17 y16 y13 y12 y11 y10 y9 y8 y7 y6 y5 y4 y3 y2
b5 b10 b11 b12 b15 b16 b18 b19 b20
Int
100
75
50
25
0
m/z
0
100
200
300
400
500
600
700
800
900
1,000
1,100
1,200
1,300
1,400

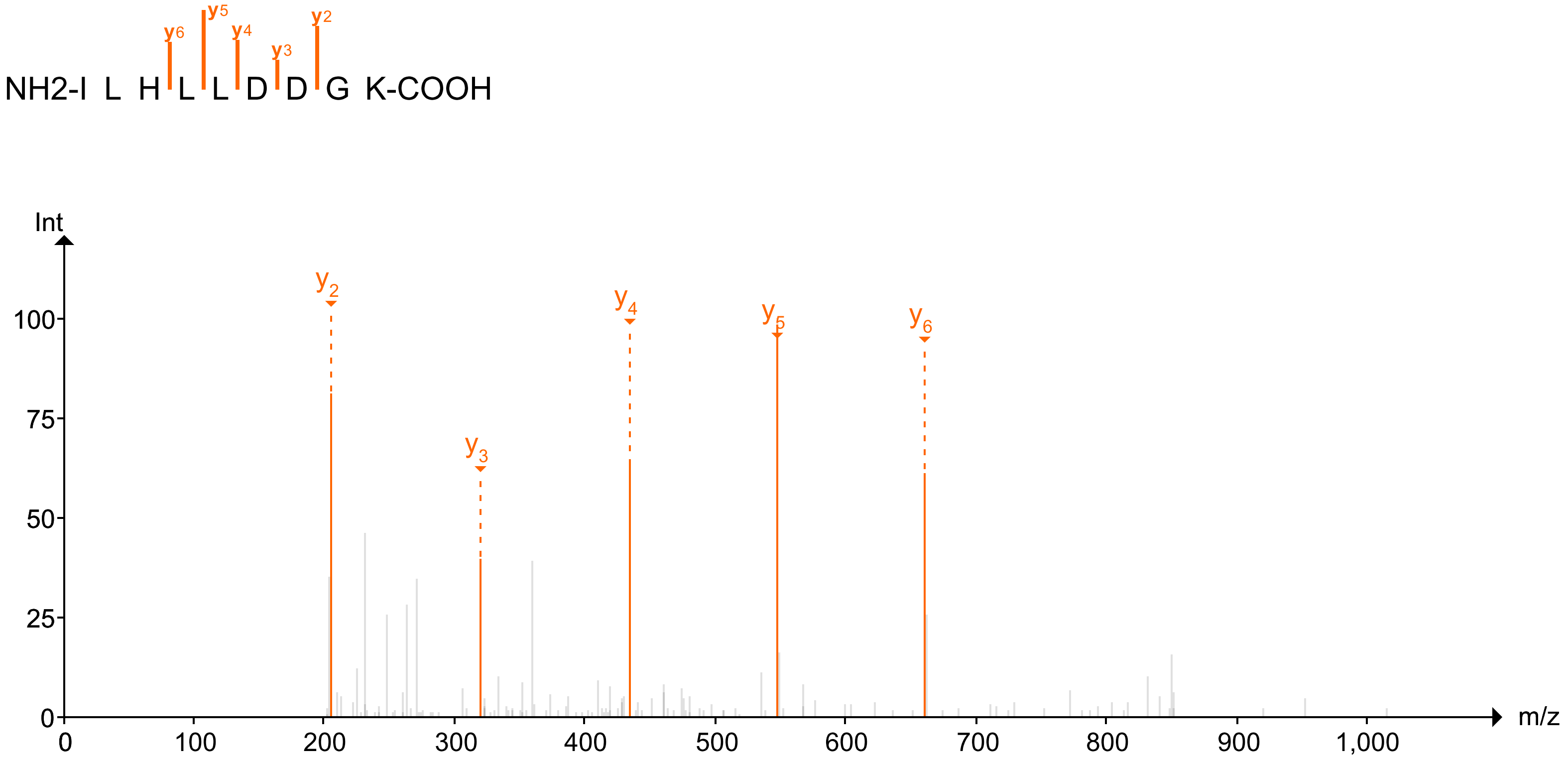

NH2-I L H L L D D G K-COOH
y6
y5
y4
y3
y2
Int
100
75
50
25
0
y2
y3
y4
y5
y6
0
100
200
300
400
500
600
700
800
900
1,000
m/z

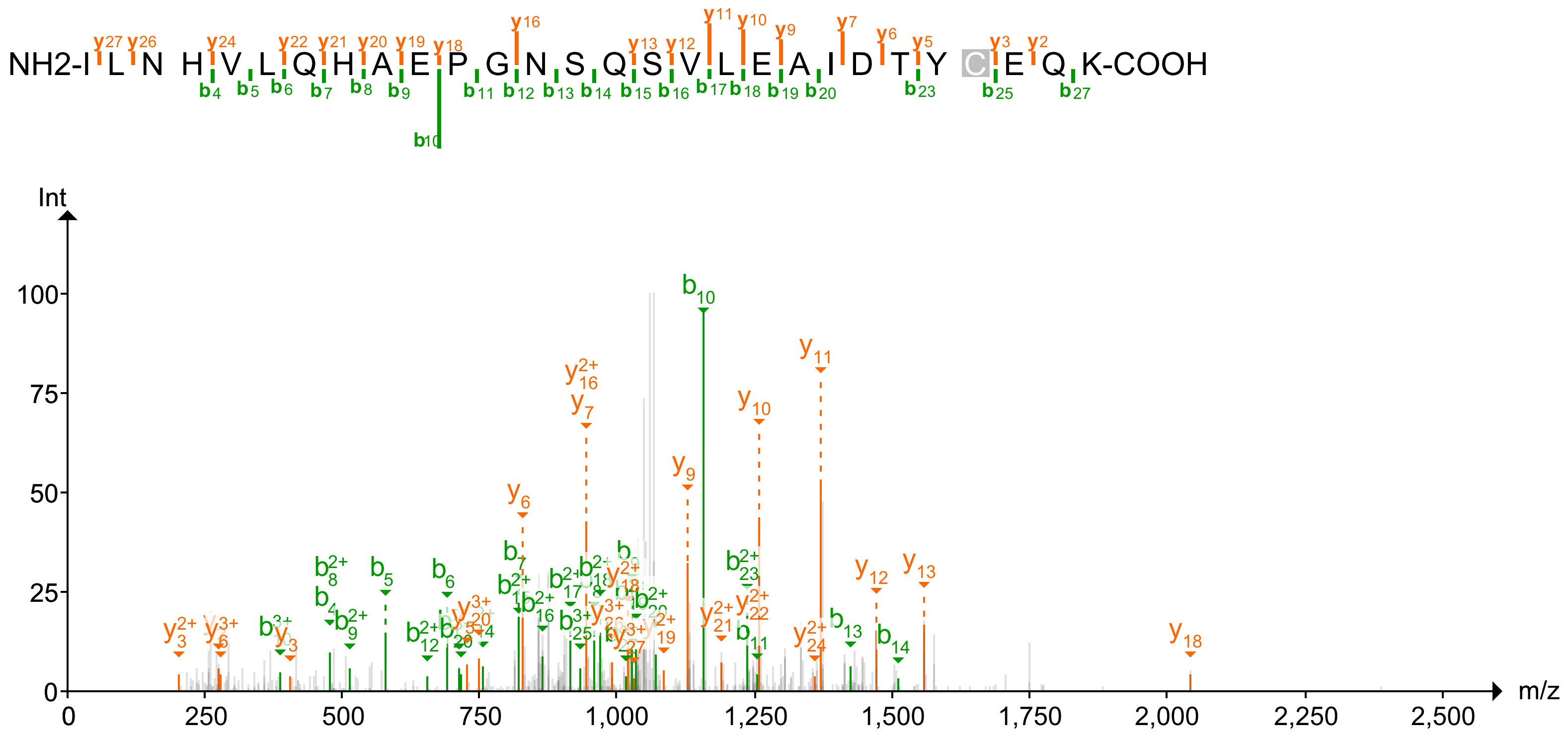

NH2-I L N H V L Q H A E P G N S Q S V L E A I D T Y C E Q K-COOH
Int
100
75
50
25
0
0
250
500
750
1,000
1,250
1,500
1,750
2,000
2,250
2,500
m/z
b10
y11
y10
y9
y7
y6
y12
y13
y18
b5
b6
b7
b13
b14

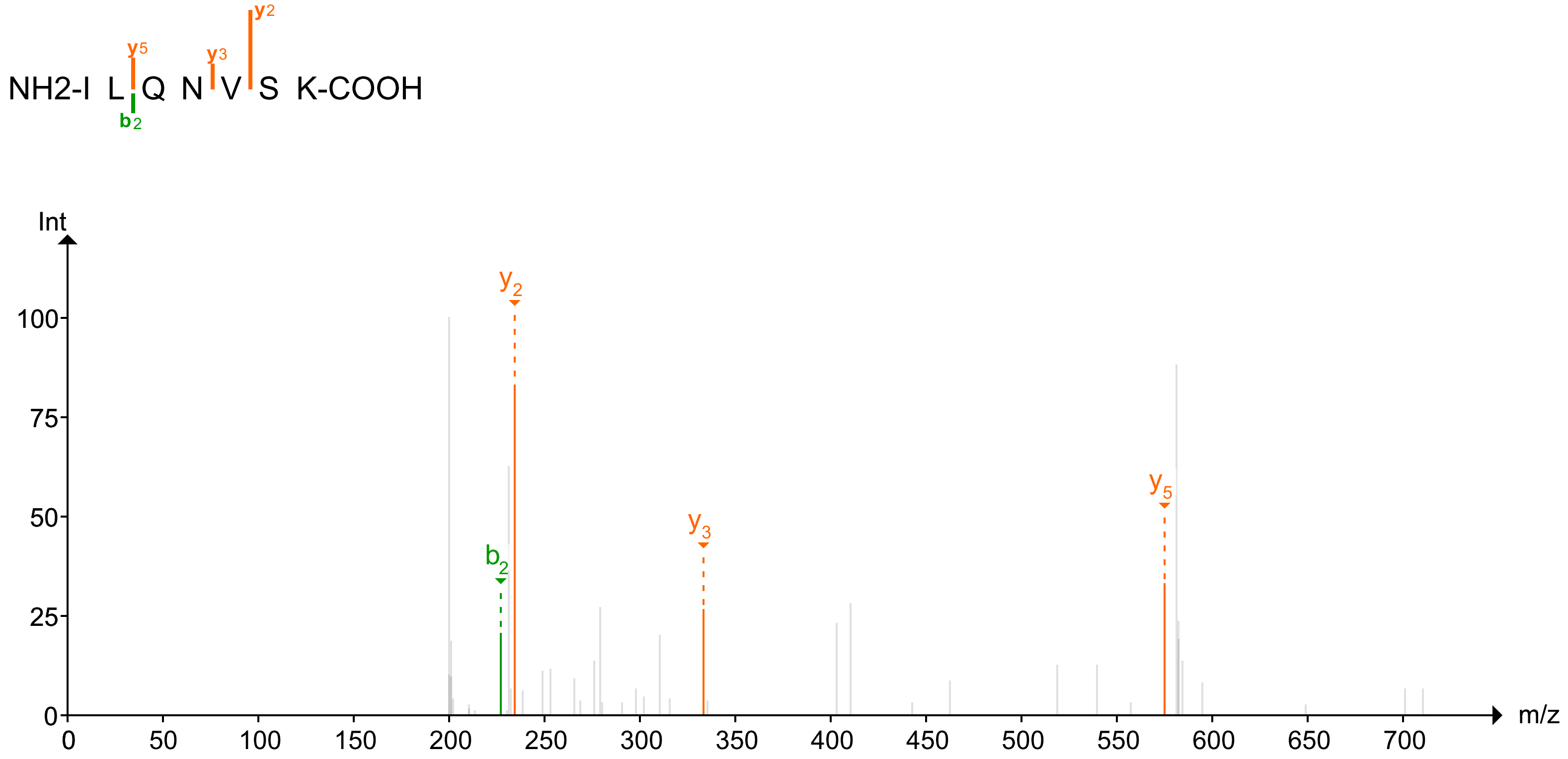

NH2-I L Q N V S K-COOH
y5
y3
y2
b2
Int
100
75
50
25
0
y2
y3
y5
b2
0
50
100
150
200
250
300
350
400
450
500
550
600
650
700
m/z

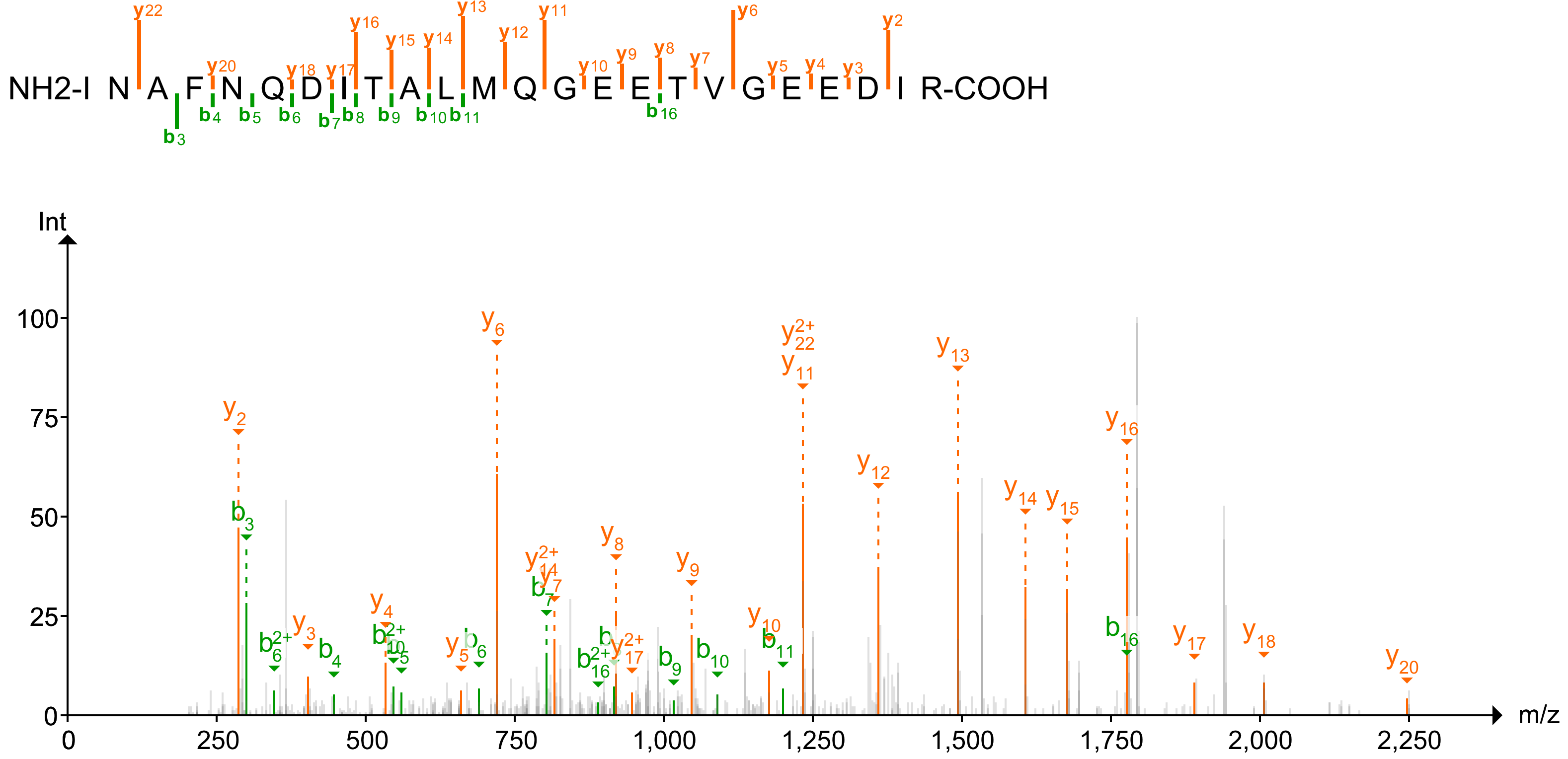

NH2-I N A F N Q D I T A L M Q G E E T V G E E D I R-COOH
y22 y20 y18 y17 y16 y15 y14 y13 y12 y11 y10 y9 y8 y7 y6 y5 y4 y3 y2
b3 b4 b5 b6 b7 b8 b9 b10 b11 b16
Int
100
75
50
25
0
m/z
0
250
500
750
1,000
1,250
1,500
1,750
2,000
2,250

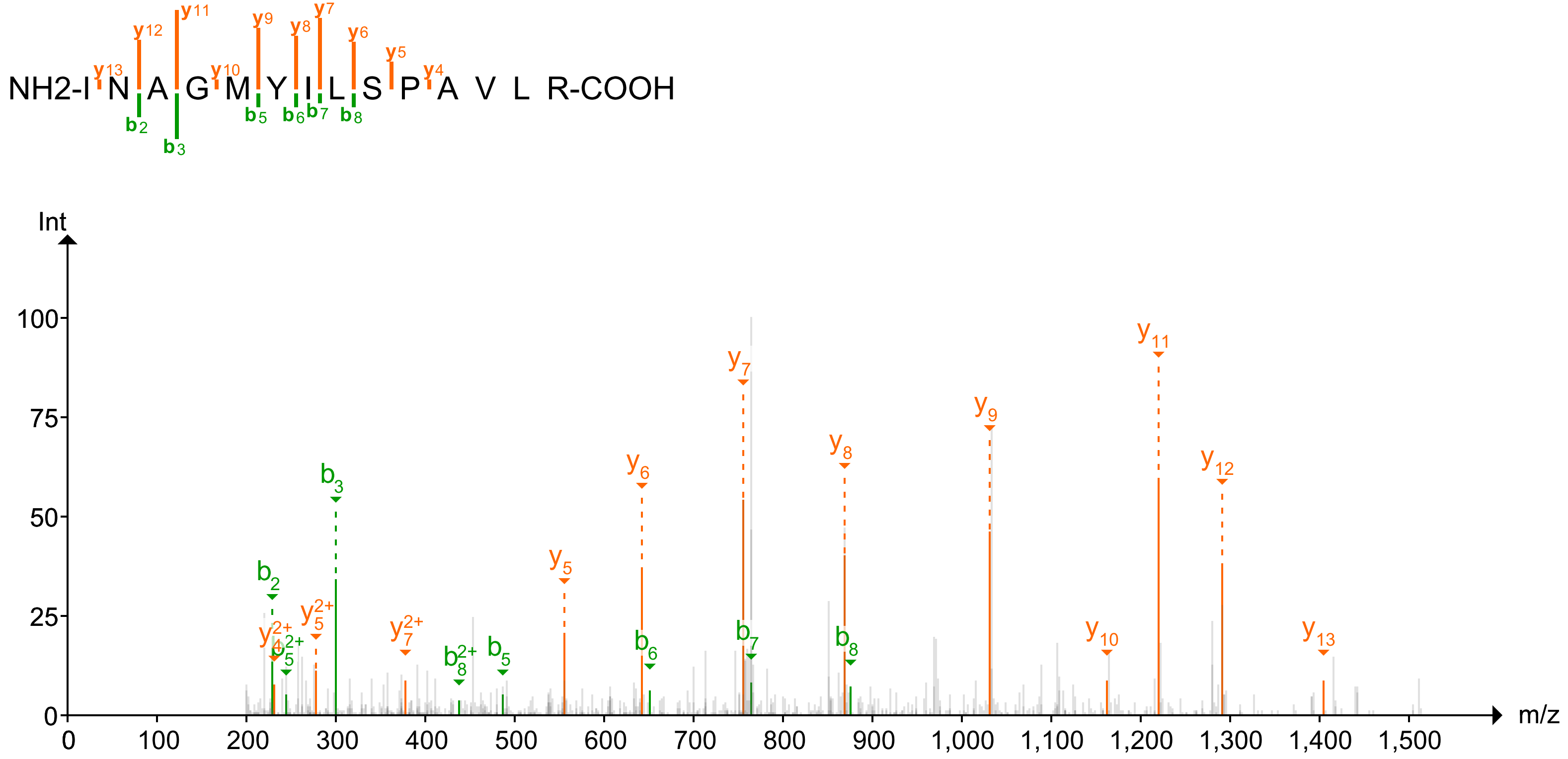

NH2-I N A G M Y I L S P A V L R-COOH
y13 y12 y11 y10 y9 y8 y7 y6 y5 y4
b2 b3 b5 b6 b7 b8
Int
100
75
50
25
0
m/z
0 100 200 300 400 500 600 700 800 900 1,000 1,100 1,200 1,300 1,400 1,500
b2 b3 b5 b6 b7 b8
y5 y6 y7 y8 y9 y10 y11 y12 y13
y4 2+ y5 2+ y7 2+ b5 2+ b8 2+

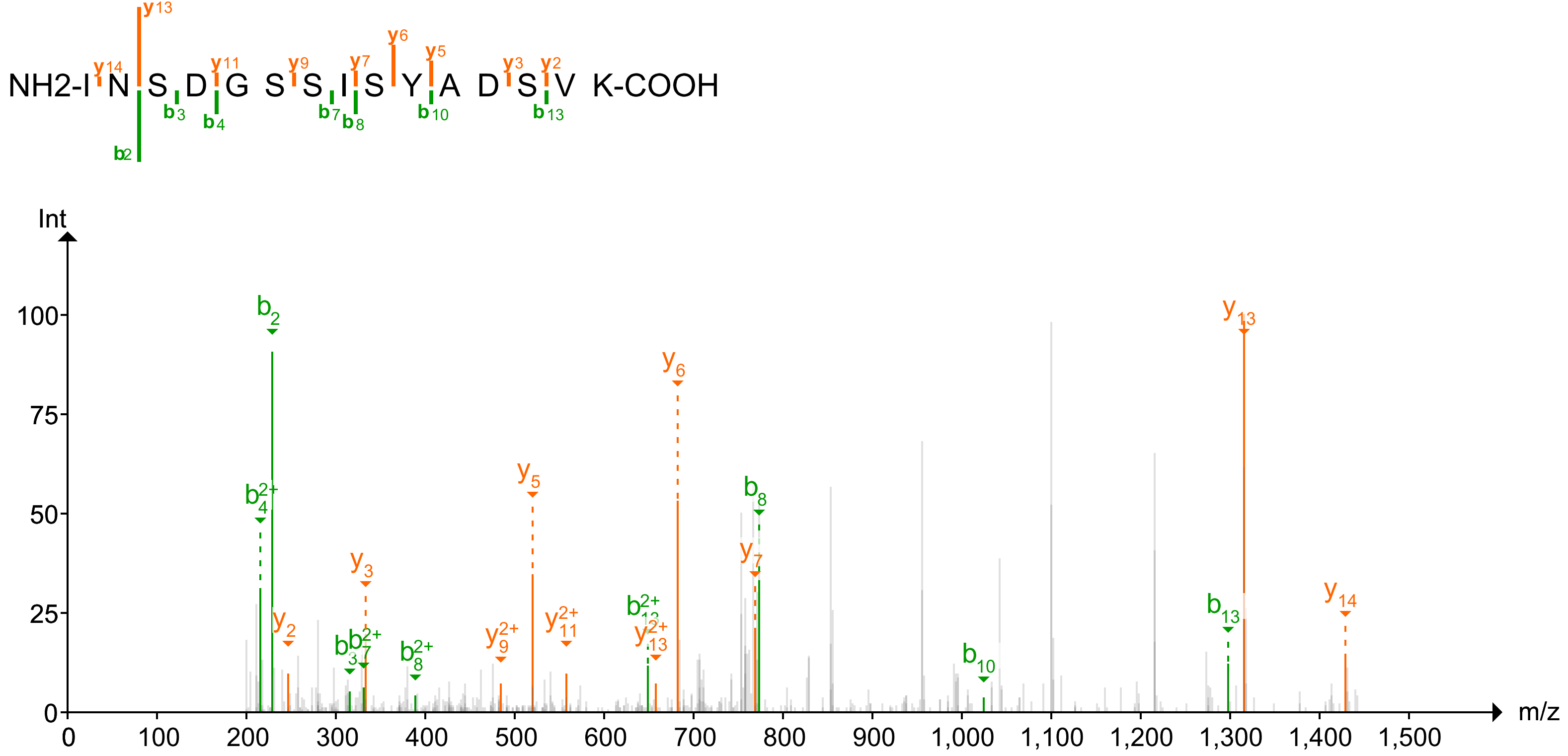

NH2-I N S D G S S I S Y A D S V K-COOH
Int
m/z

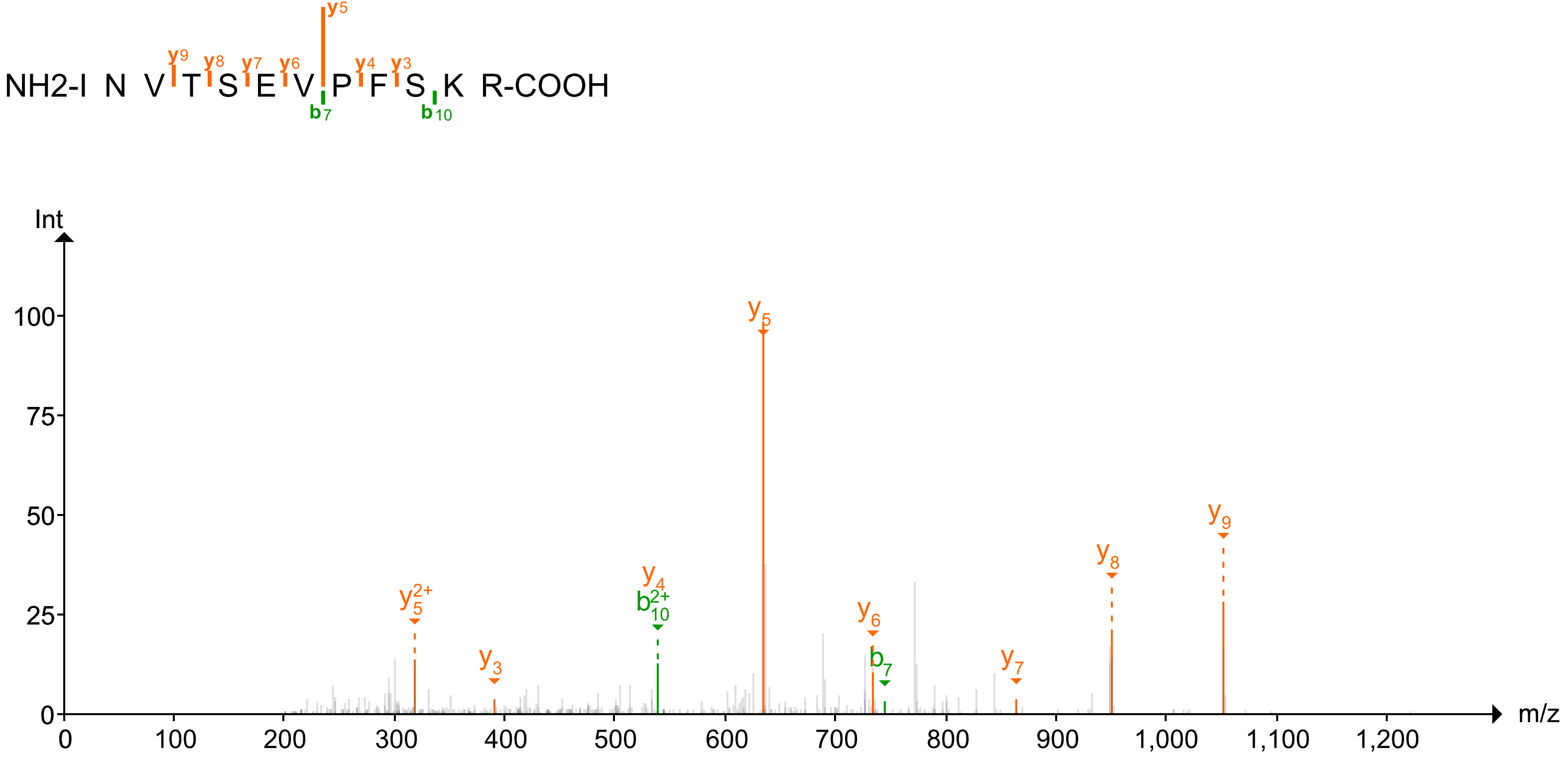
NH2-I N V T S E V P F S K R-COOH
y9 y8 y7 y6 y5 y4 y3
b7 b10
Int
m/z
0 25 50 75 100
0 100 200 300 400 500 600 700 800 900 1,000 1,100 1,200
y5
y9
y8
y4
b10 2+
y5 2+
y6
b7
y3
y7

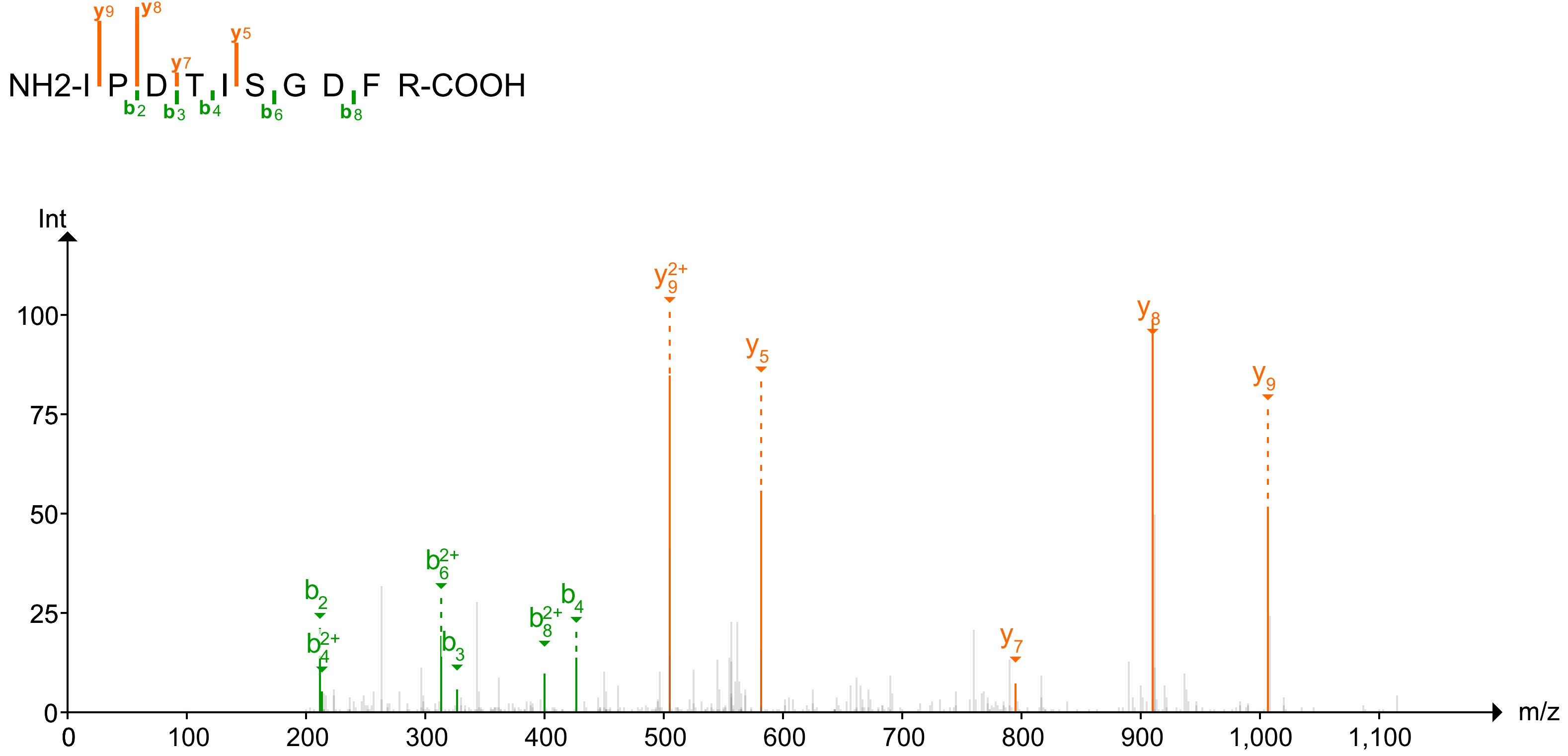
NH2-I P D T I S G D F R-COOH
y9
y8
y7
y5
b2
b3
b4
b6
b8
Int
100
75
50
25
0
m/z
0
100
200
300
400
500
600
700
800
900
1,000
1,100
y9 2+
y5
y8
y9
y7
b2
b4 2+
b6 2+
b3
b8 2+
b4

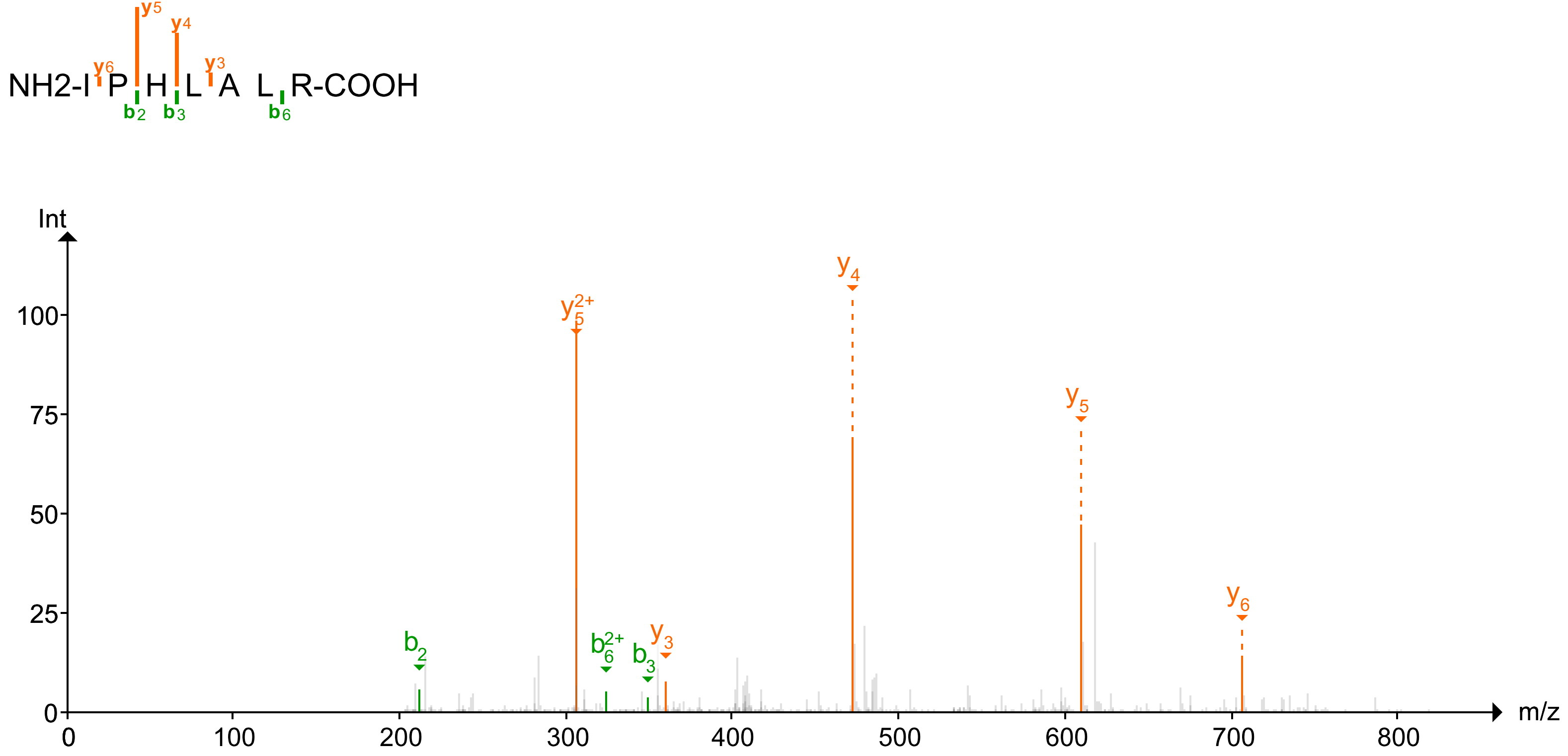

NH2-I P H L A L R-COOH
y5
y4
y6
y3
b2
b3
b6
Int
100
75
50
25
0
y4
y2+5
y5
y6
b2
b2+6
b3
y3
0
100
200
300
400
500
600
700
800
m/z

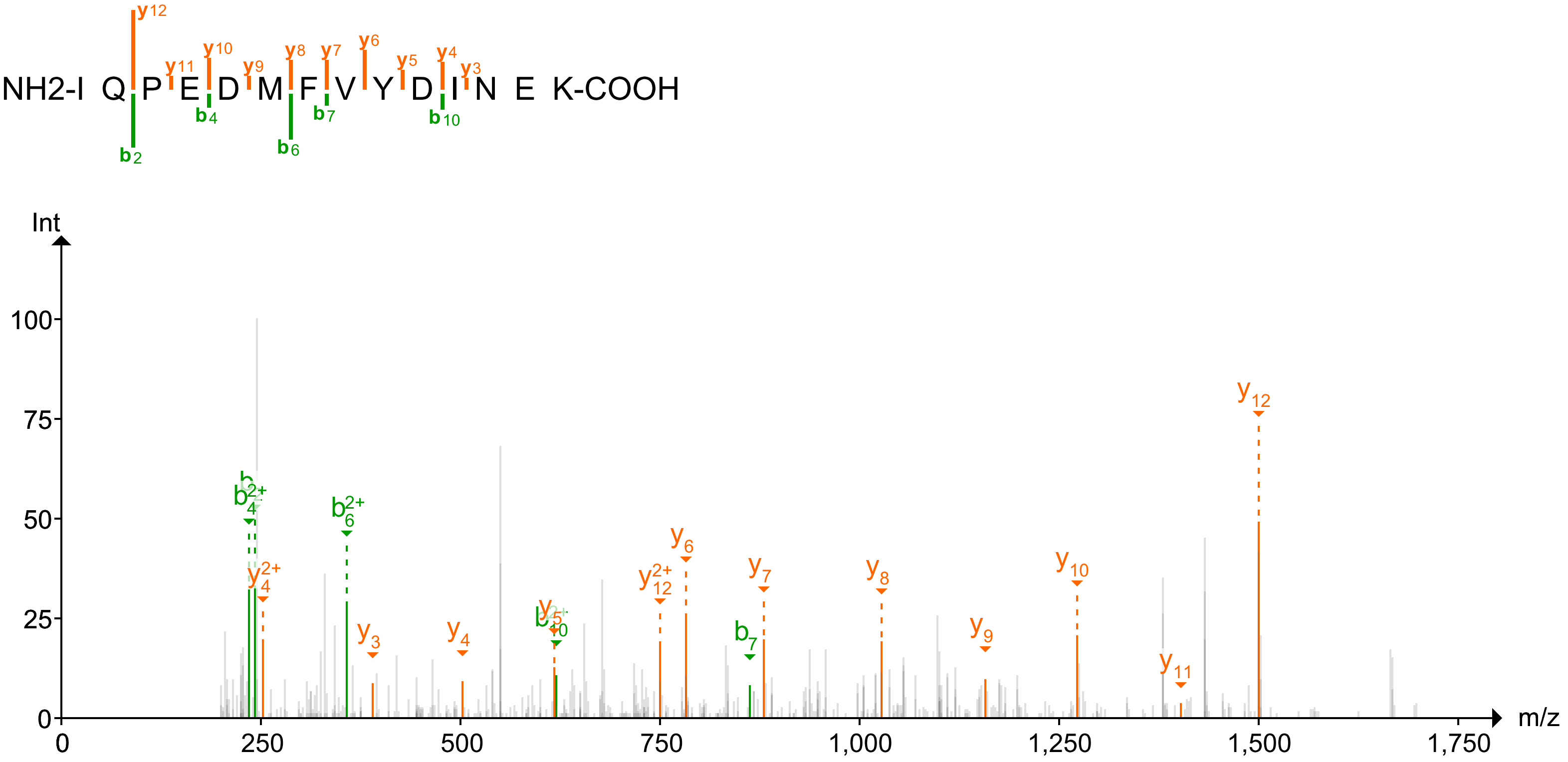

NH2-I Q P E D M F V Y D I N E K-COOH
y12 y11 y10 y9 y8 y7 y6 y5 y4 y3
b2 b4 b6 b7 b10
Int
100
75
50
25
0
m/z
0
250
500
750
1,000
1,250
1,500
1,750
y12
y6
y7
y8
y10
y9
y11
y3
y4
b7

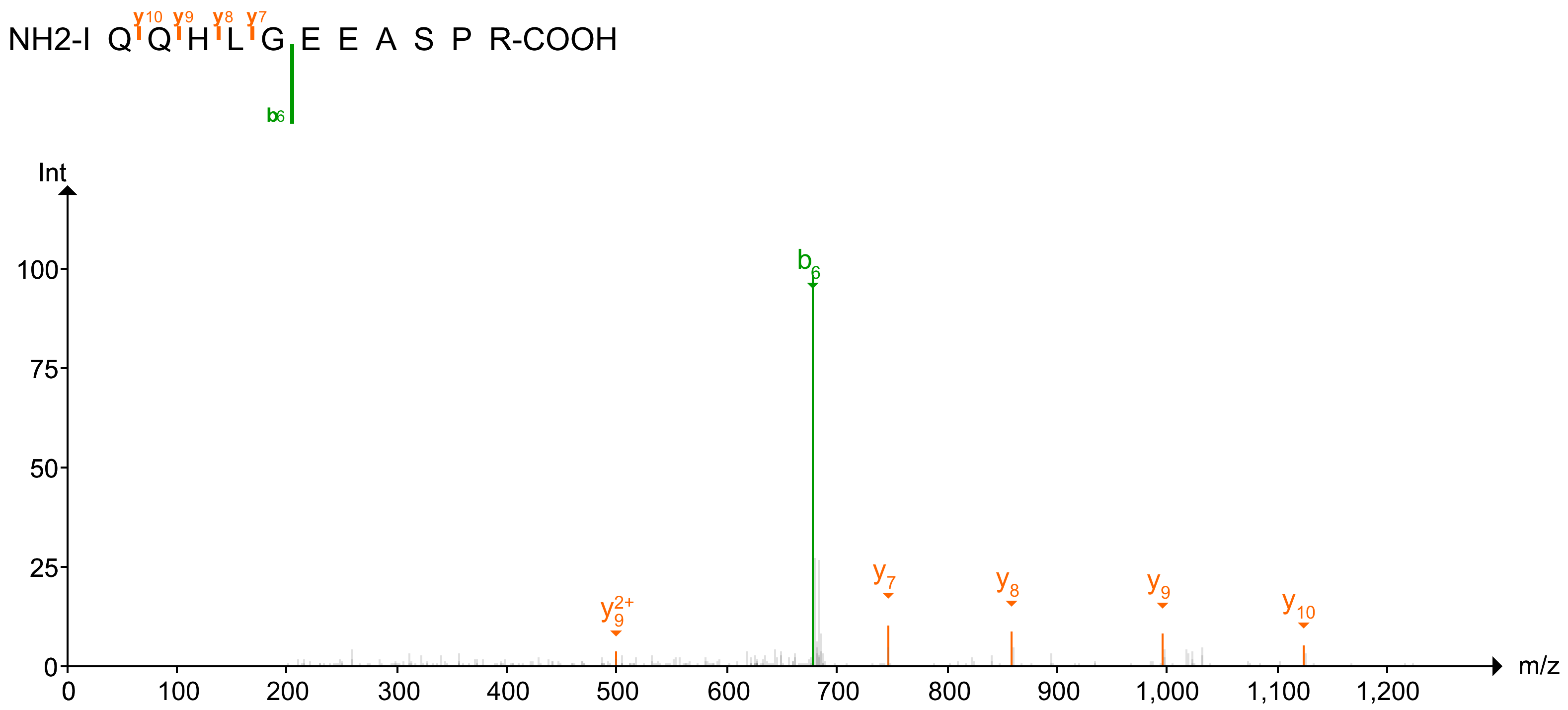

NH2-I Q Q H L G E E A S P R-COOH
y10 y9 y8 y7
b6
Int
100
75
50
25
0
m/z
0 100 200 300 400 500 600 700 800 900 1,000 1,100 1,200
b6
y9 2+
y7
y8
y9
y10

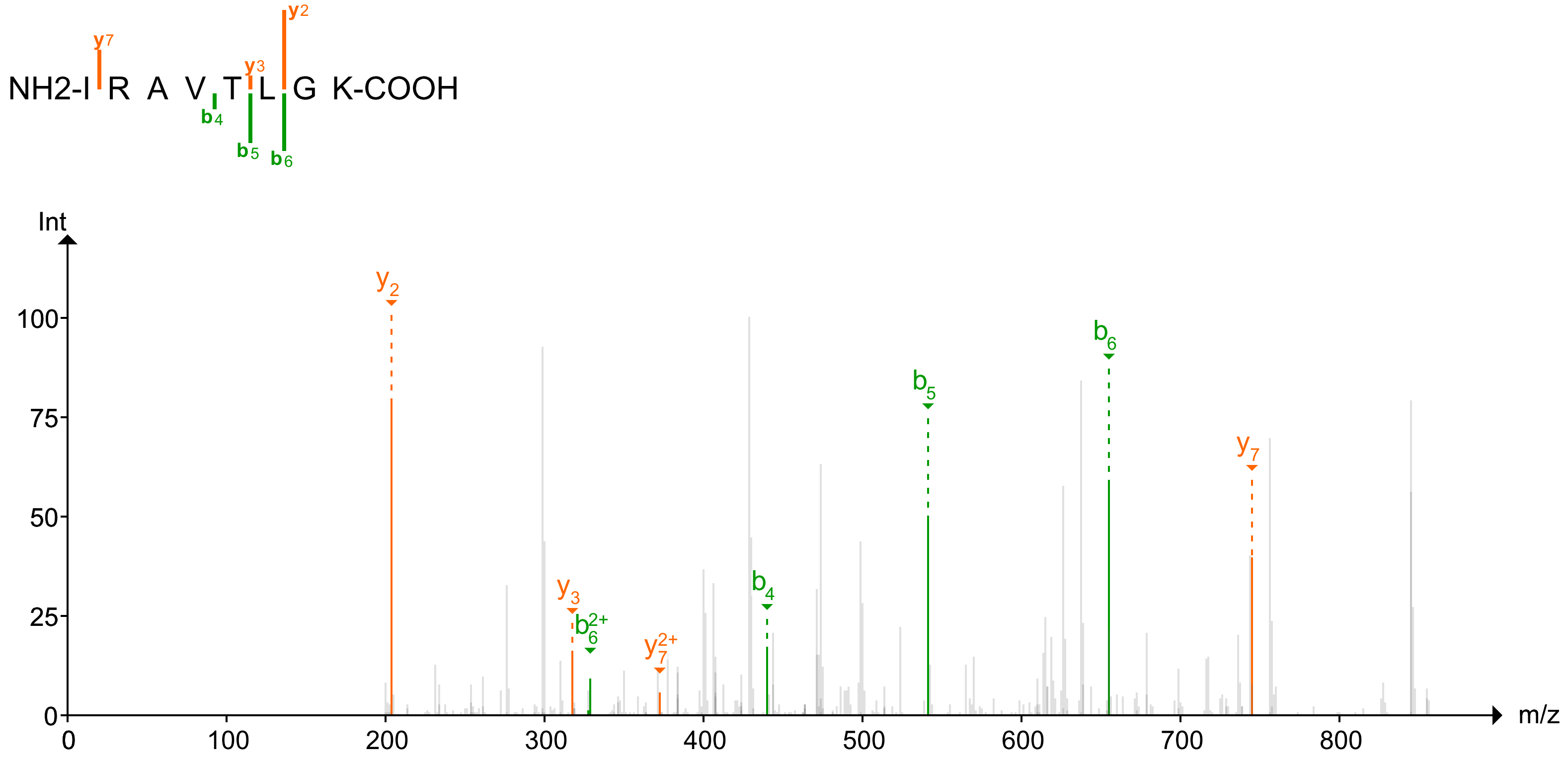

NH2-I R A V T L G K-COOH
y7
y3
y2
b4
b5
b6
Int
100
75
50
25
0
y2
y3
b6 2+
y7 2+
b4
b5
b6
y7
0
100
200
300
400
500
600
700
800
m/z

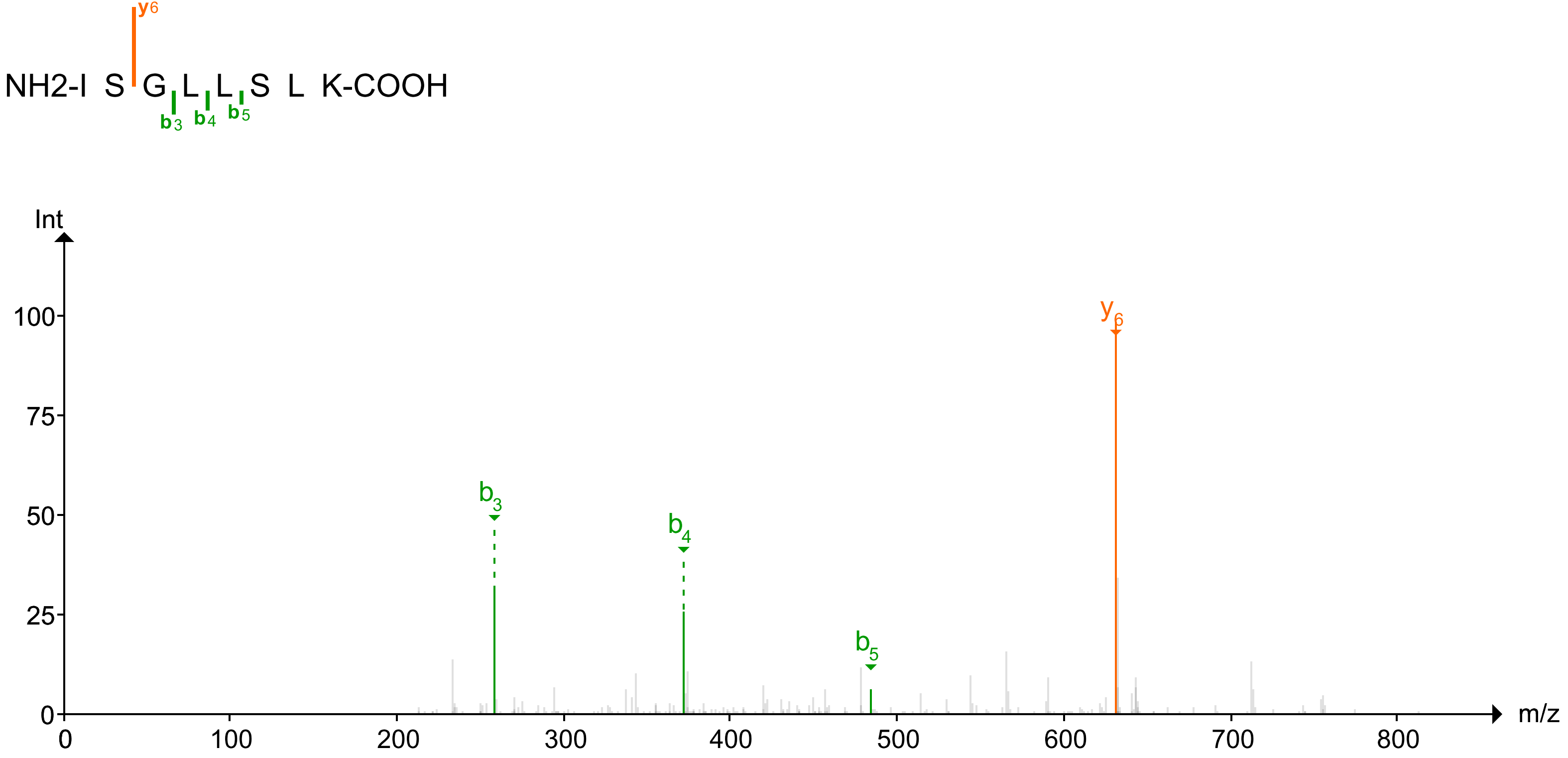

y6
NH2-I S G L L S L K-COOH
b3
b4
b5
Int
100
75
50
25
0
b3
b4
b5
y6
0
100
200
300
400
500
600
700
800
m/z

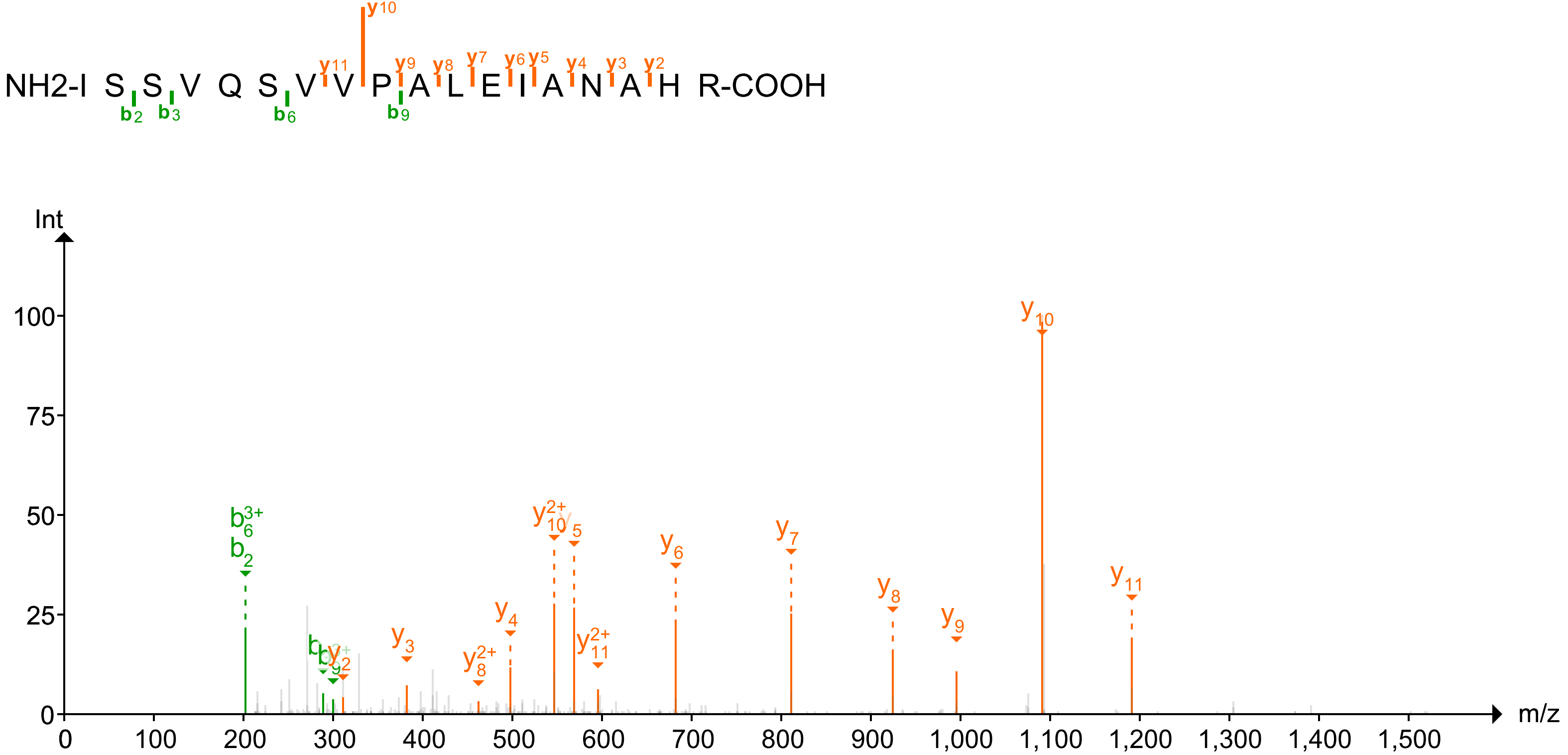

NH2-I S S V Q S V V P A L E I A N A H R-COOH
b2 b3 b6 b9
y11 y10 y9 y8 y7 y6 y5 y4 y3 y2
Int
100
75
50
25
0
m/z
0 100 200 300 400 500 600 700 800 900 1,000 1,100 1,200 1,300 1,400 1,500

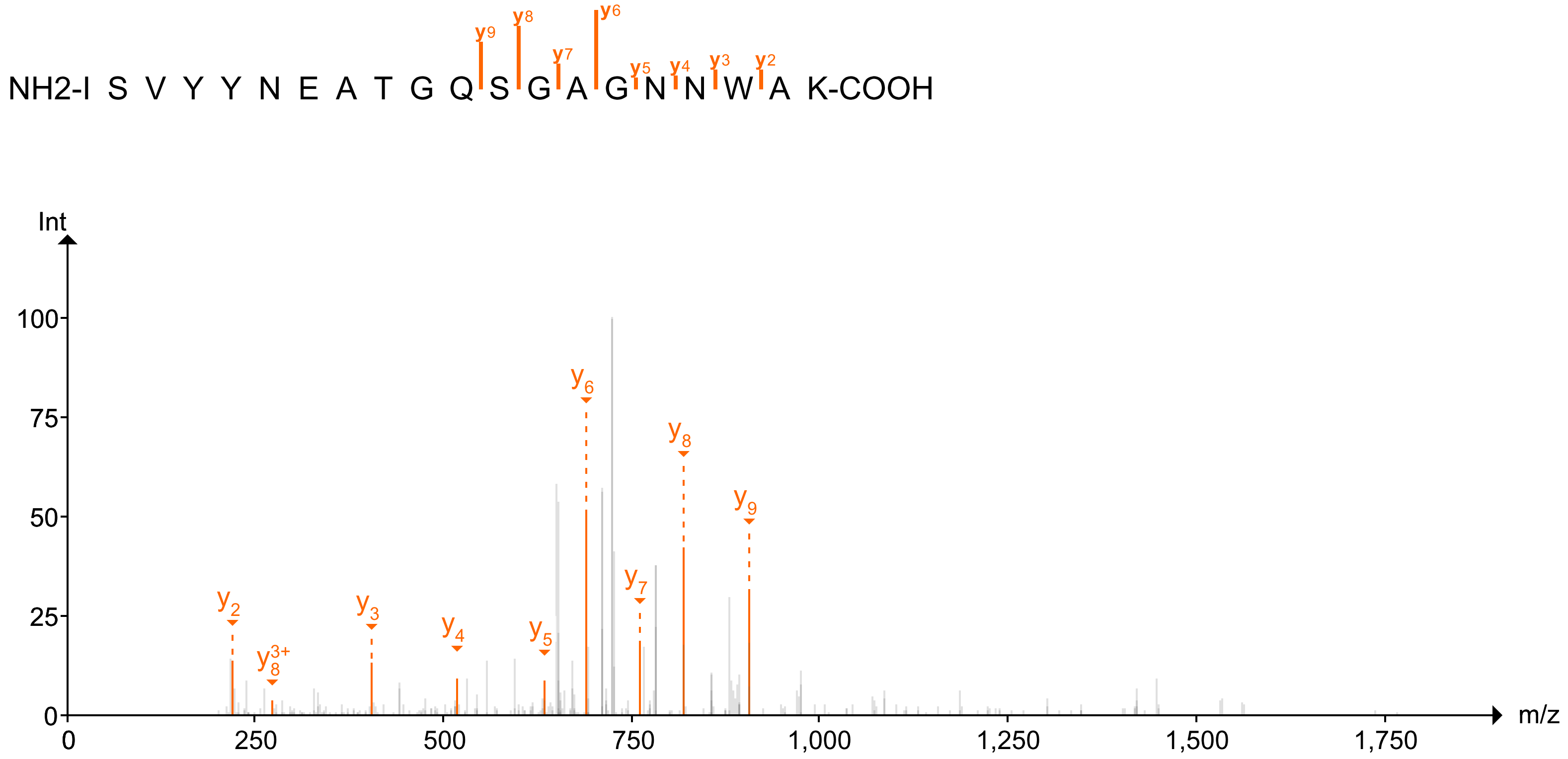

NH2-I S V Y Y N E A T G Q S G A G N N W A K-COOH
y9
y8
y7
y6
y5
y4
y3
y2
Int
100
75
50
25
0
y2
y8 3+
y3
y4
y5
y6
y7
y8
y9
0
250
500
750
1,000
1,250
1,500
1,750
m/z

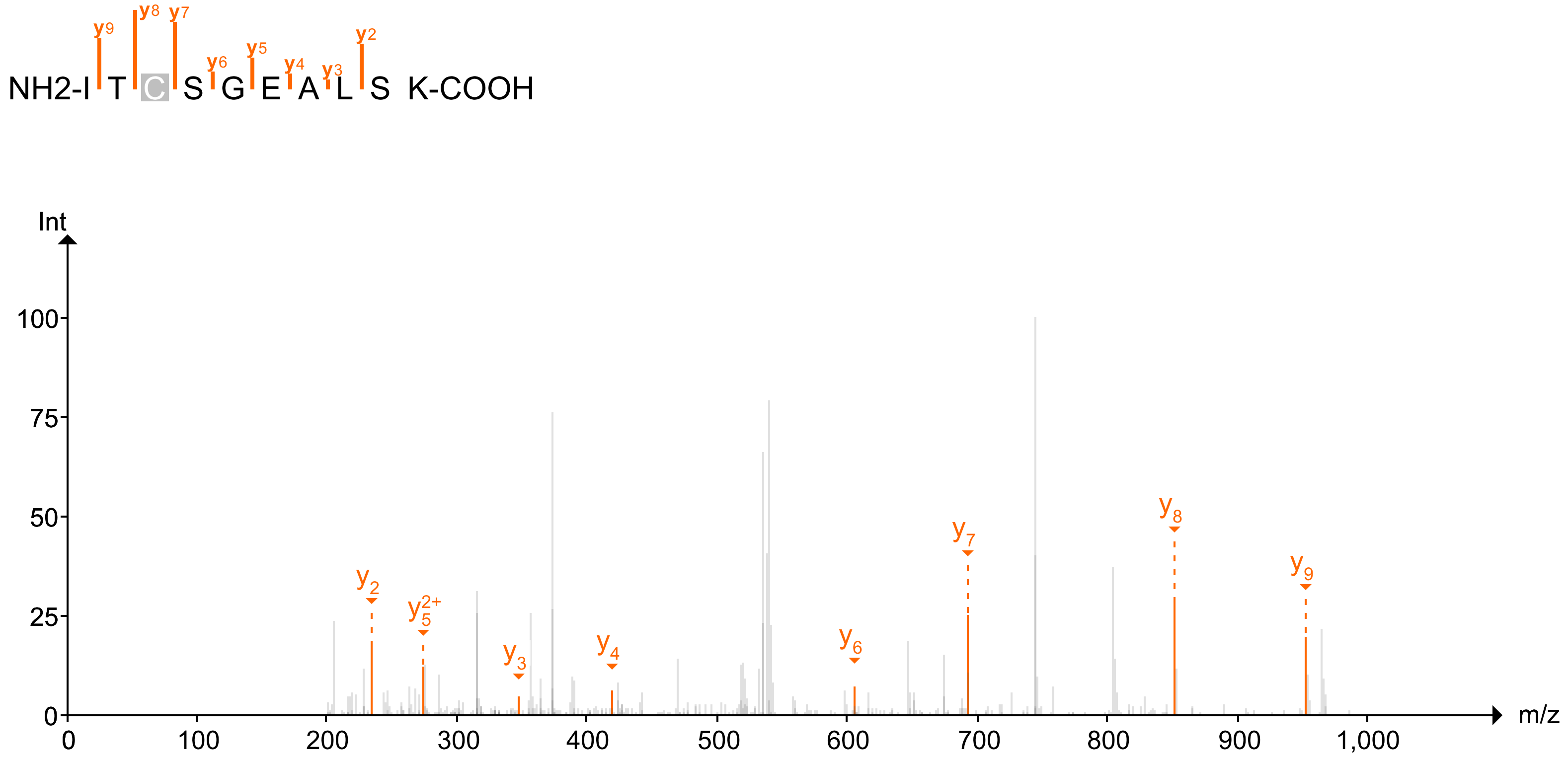

NH2-I T C S G E A L S K-COOH
y9 y8 y7 y6 y5 y4 y3 y2
Int
100
75
50
25
0
y2
$y_5^{2+}$
y3
y4
y6
y7
y8
y9
0 100 200 300 400 500 600 700 800 900 1,000
m/z

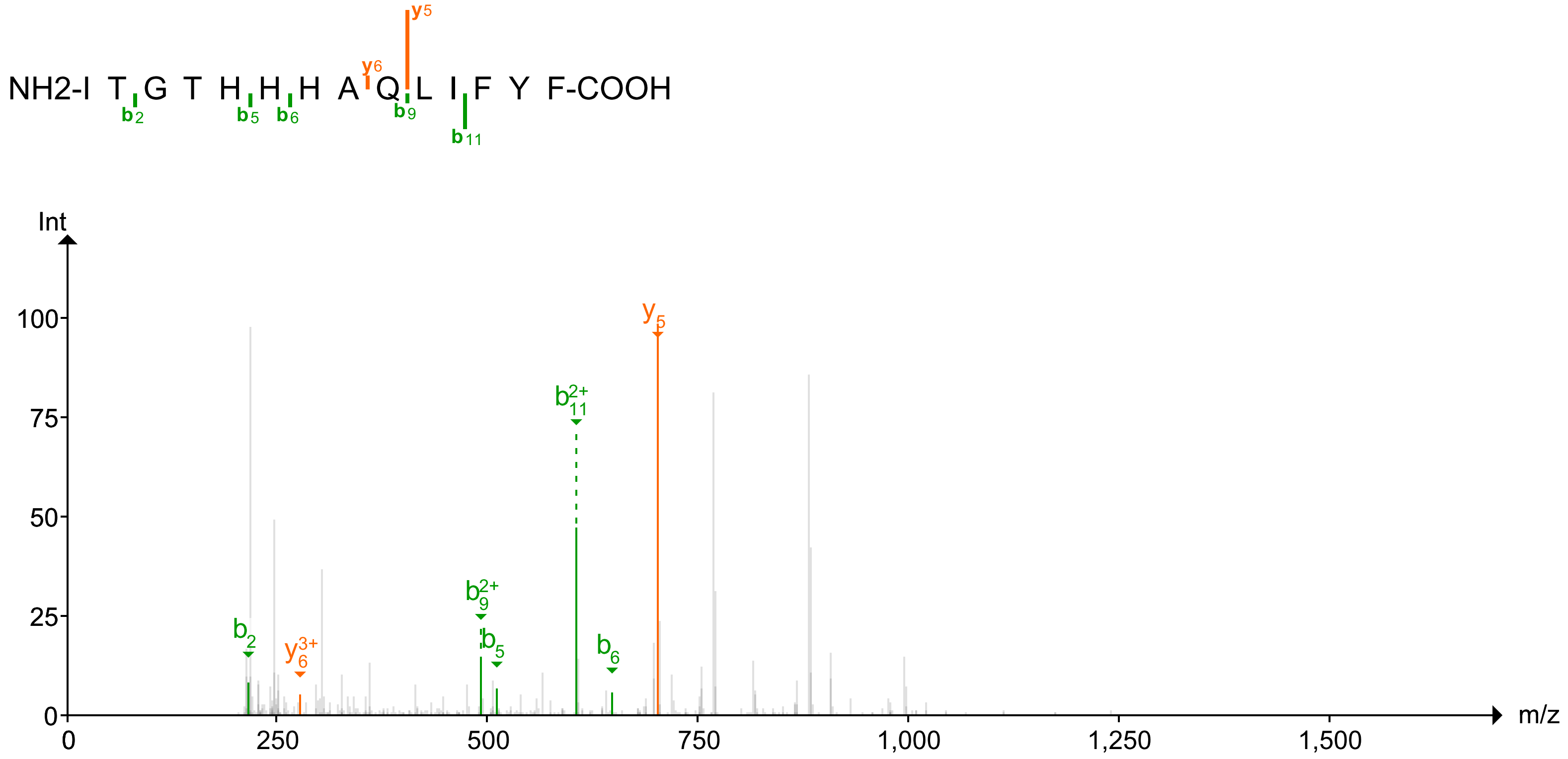
NH2-I T G T H H H A Q L I F Y F-COOH
b2
b5
b6
b9
b11
y6
y5
Int
100
75
50
25
0
m/z
0
250
500
750
1,000
1,250
1,500
b2
y6 3+
b9 2+
b5
b11 2+
b6
y5

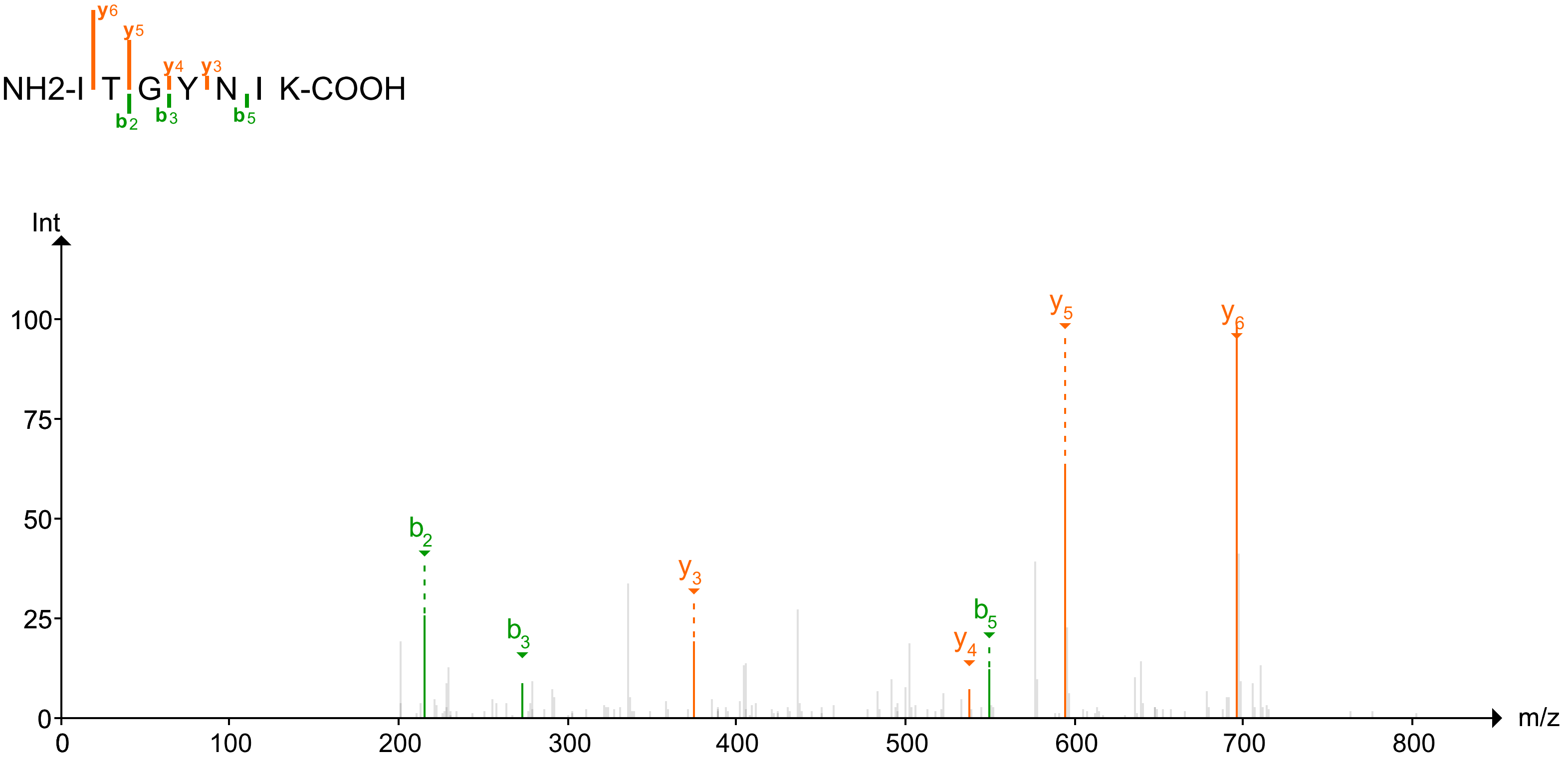

NH2-I T G Y N I K-COOH
y6
y5
y4
y3
b2
b3
b5
Int
100
75
50
25
0
m/z
0
100
200
300
400
500
600
700
800
$b_2$
$b_3$
$y_3$
$y_4$
$b_5$
$y_5$
$y_6$

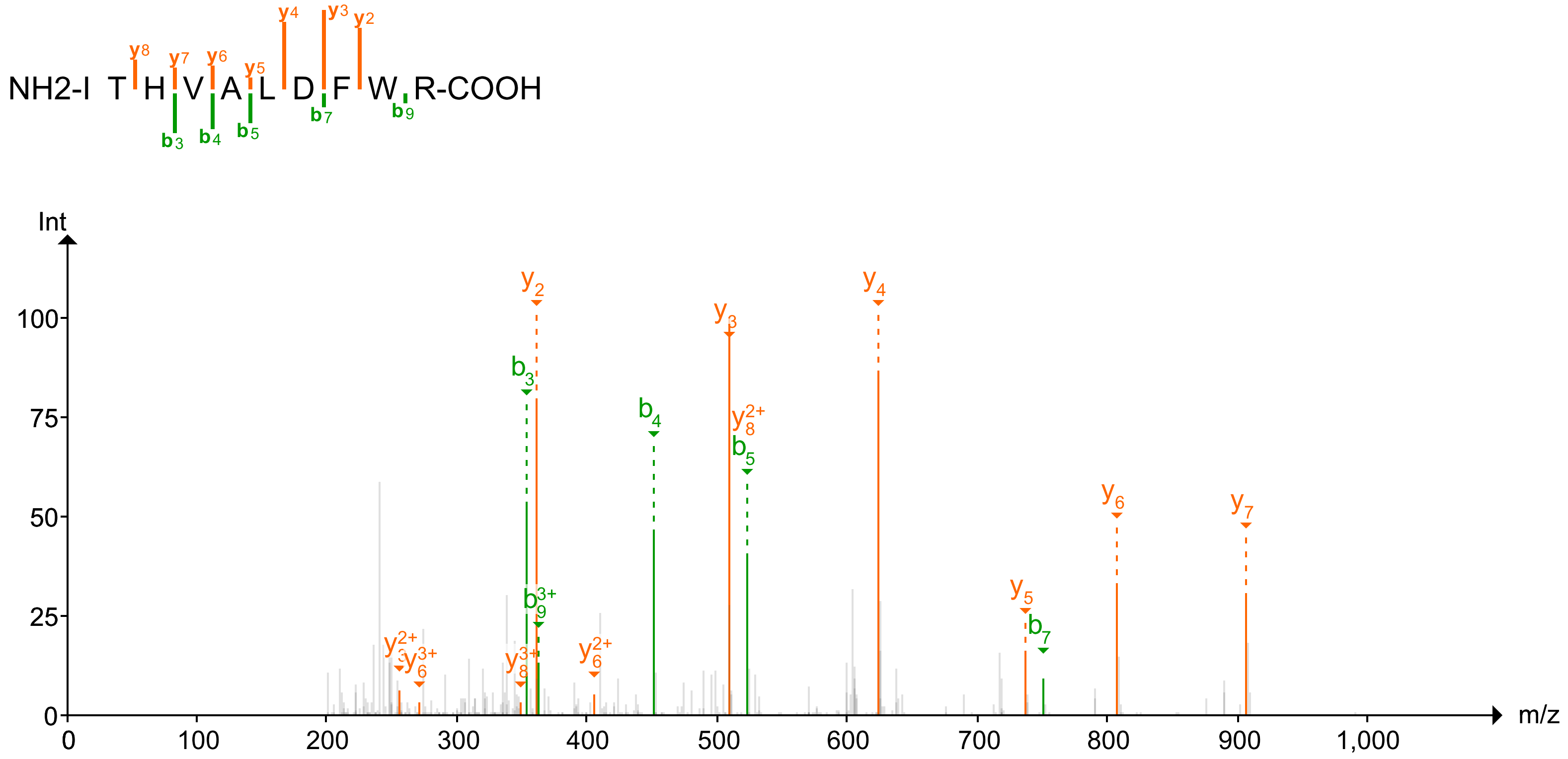

NH2-I T H V A L D F W R-COOH
y8 y7 y6 y5 y4 y3 y2
b3 b4 b5 b7 b9
Int
m/z
0
25
50
75
100
0
100
200
300
400
500
600
700
800
900
1,000
y2
y3
y4
b3
b4
b5
y8 2+
y6
y7
y5
b7
b9 3+
y3 2+
y6 3+
y8 3+
y6 2+

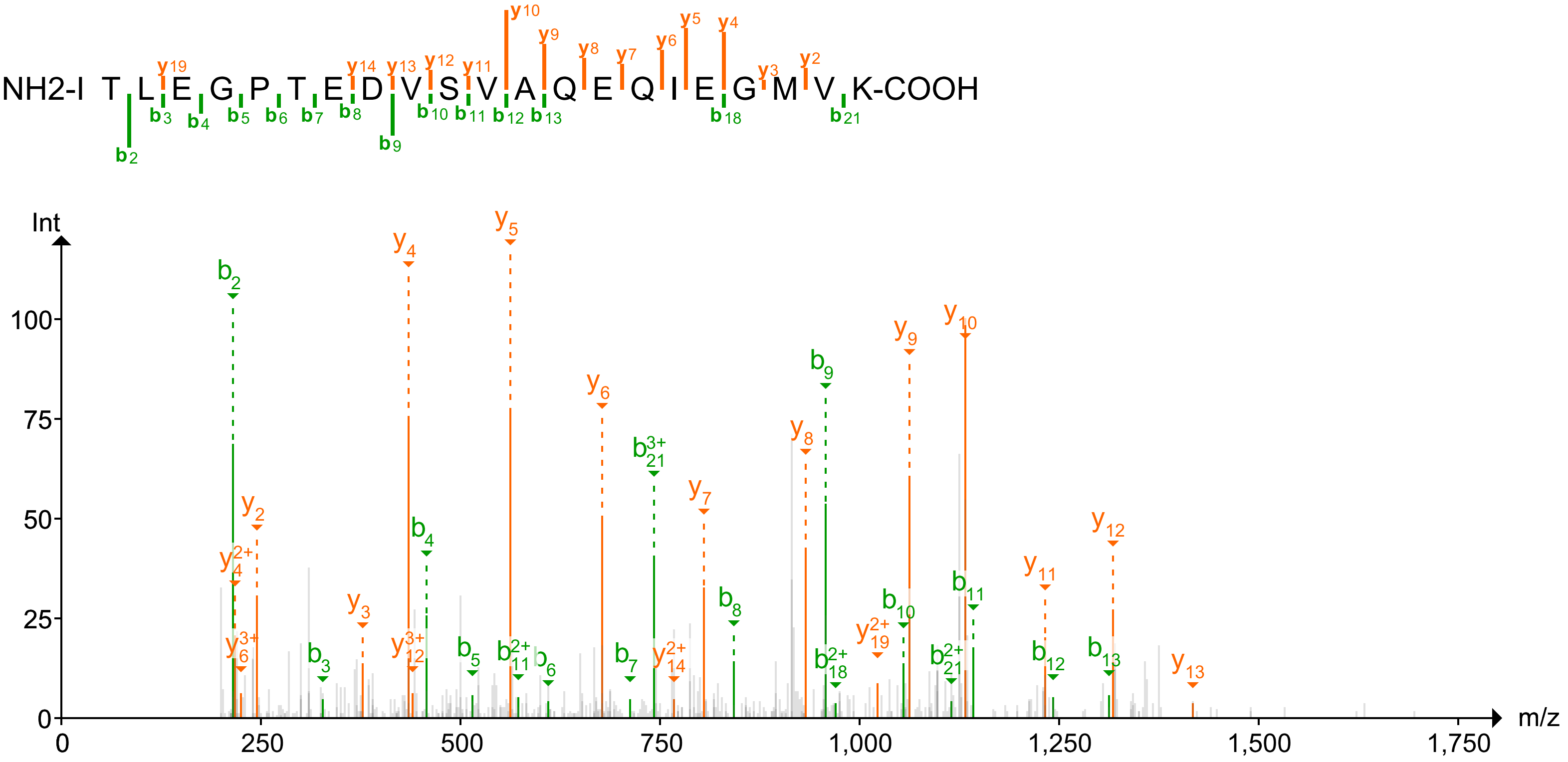

NH2-I T L E G P T E D V S V A Q E Q I E G M V K-COOH
Int
m/z
0
25
50
75
100
250
500
750
1,000
1,250
1,500
1,750

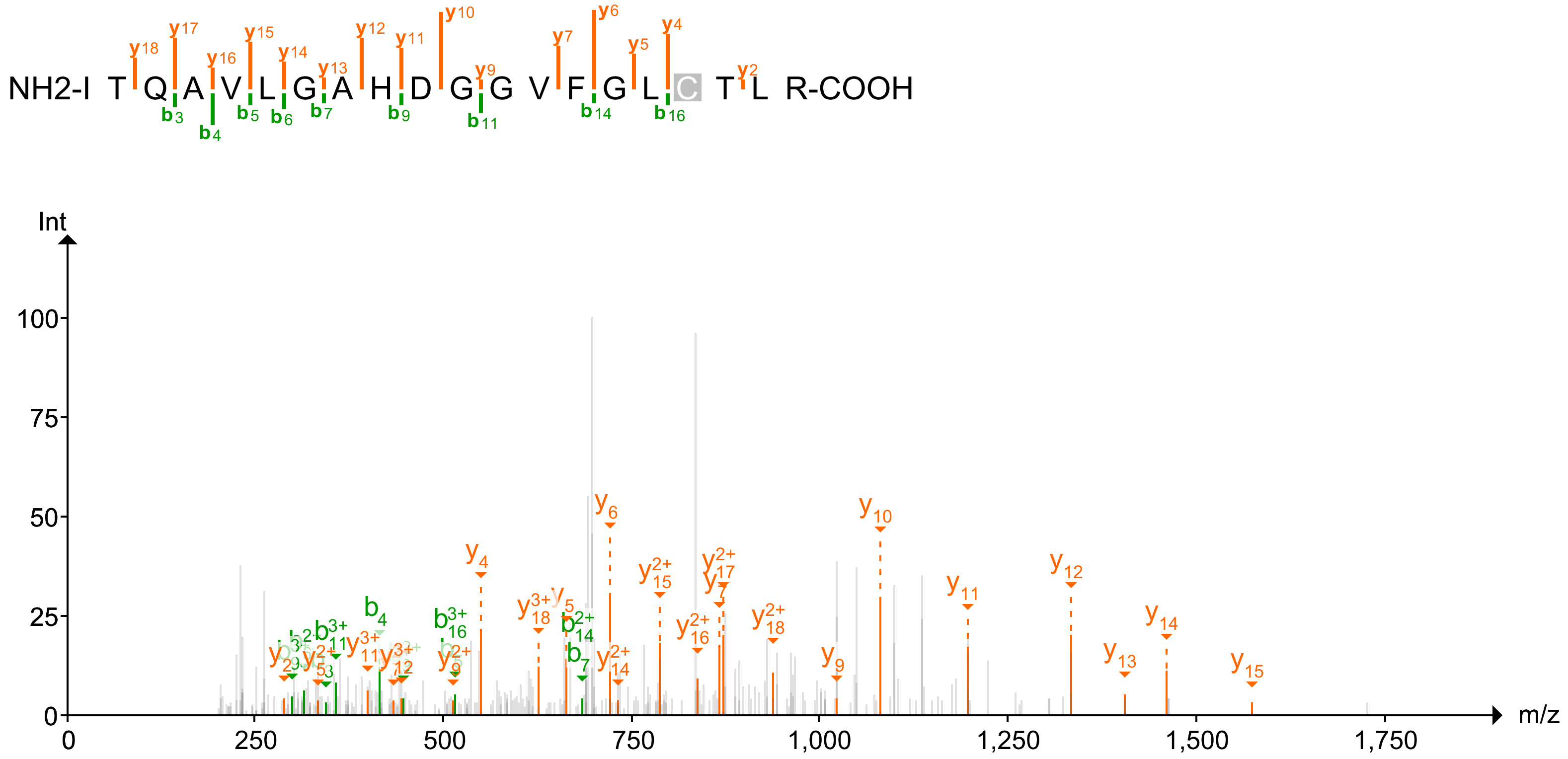

NH2-I T Q A V L G A H D G G V F G L C T L R-COOH
y18 y17 y16 y15 y14 y13 y12 y11 y10 y9 y7 y6 y5 y4 y2
b3 b4 b5 b6 b7 b9 b11 b14 b16
Int
100
75
50
25
0
250
500
750
1,000
1,250
1,500
1,750
m/z
y4
y6
y10
y12
y11
y14
y13
y15
y9
y2+15
y2+17
y7
y2+16
y2+18
y2+14
y3+18
y5
b2+14
b7
b3+16
y2+9
b4
y3+11
y3+12

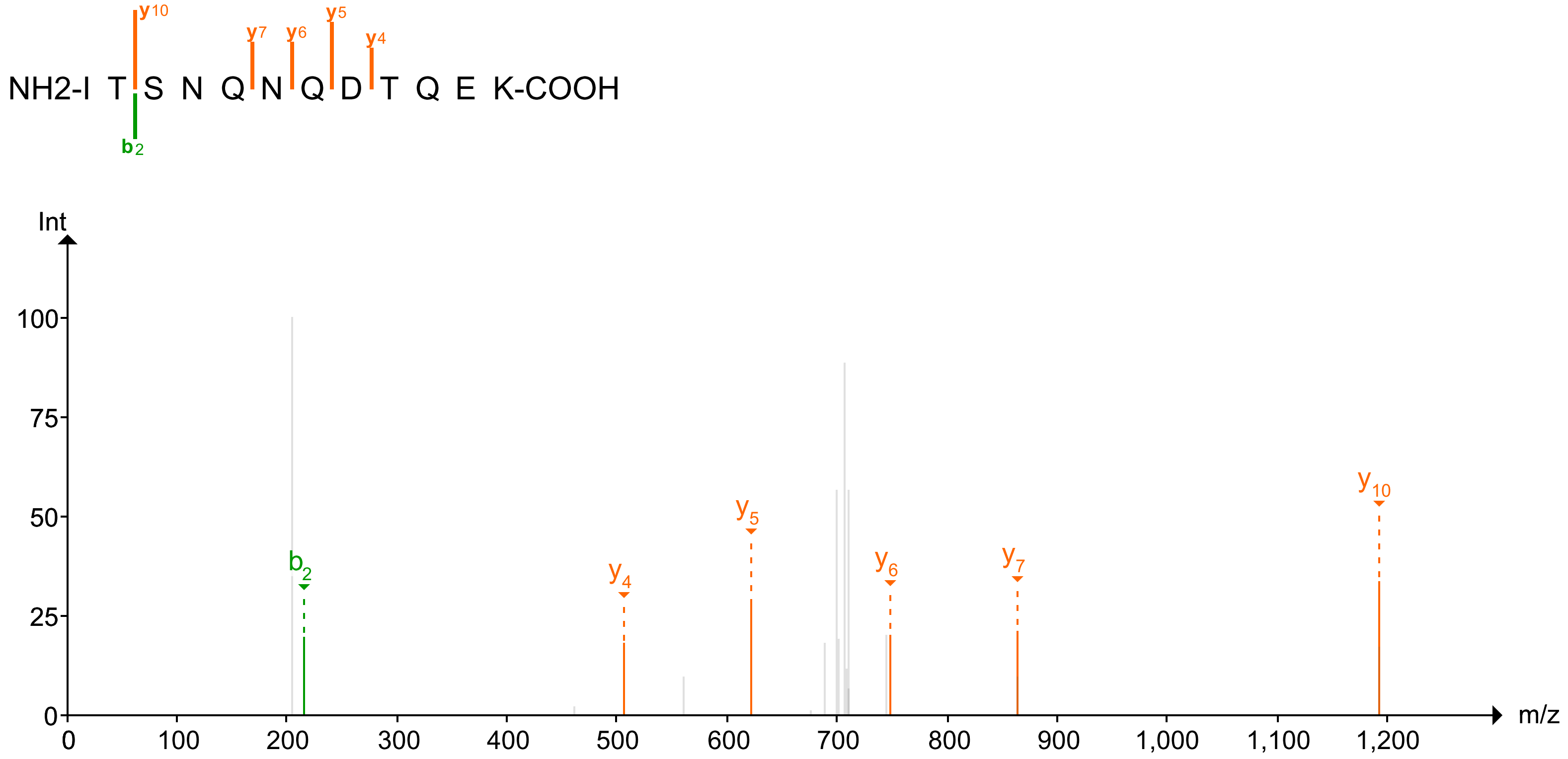

NH2-I T S N Q N Q D T Q E K-COOH
y10
y7
y6
y5
y4
b2
Int
100
75
50
25
0
m/z
0
100
200
300
400
500
600
700
800
900
1,000
1,100
1,200

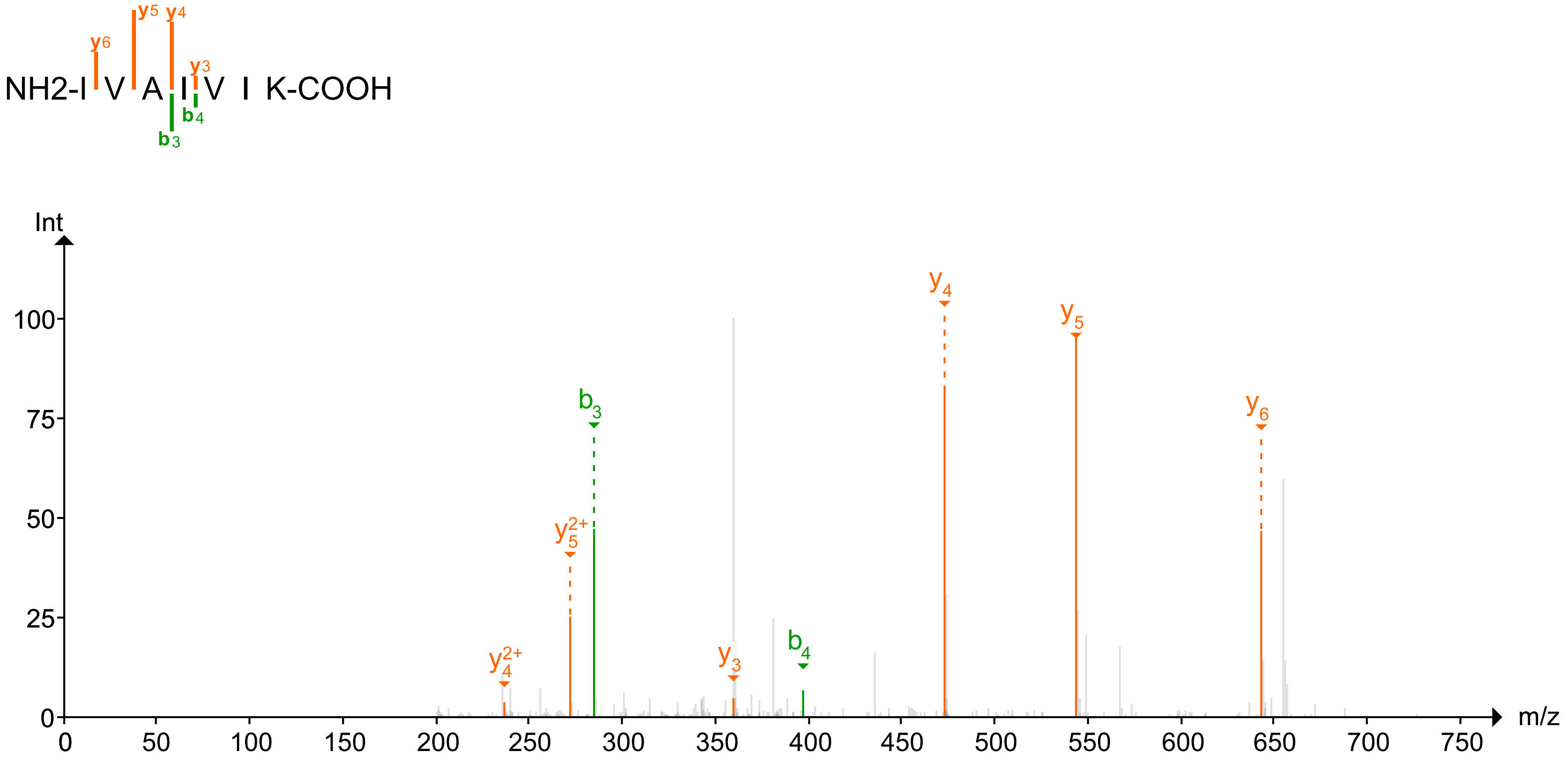

y6
y5
y4
y3
NH2-I V A I V I K-COOH
b3
b4
Int
100
75
50
25
0
b3
y5 2+
y4
y5
y6
y4 2+
y3
b4
0
50
100
150
200
250
300
350
400
450
500
550
600
650
700
750
m/z

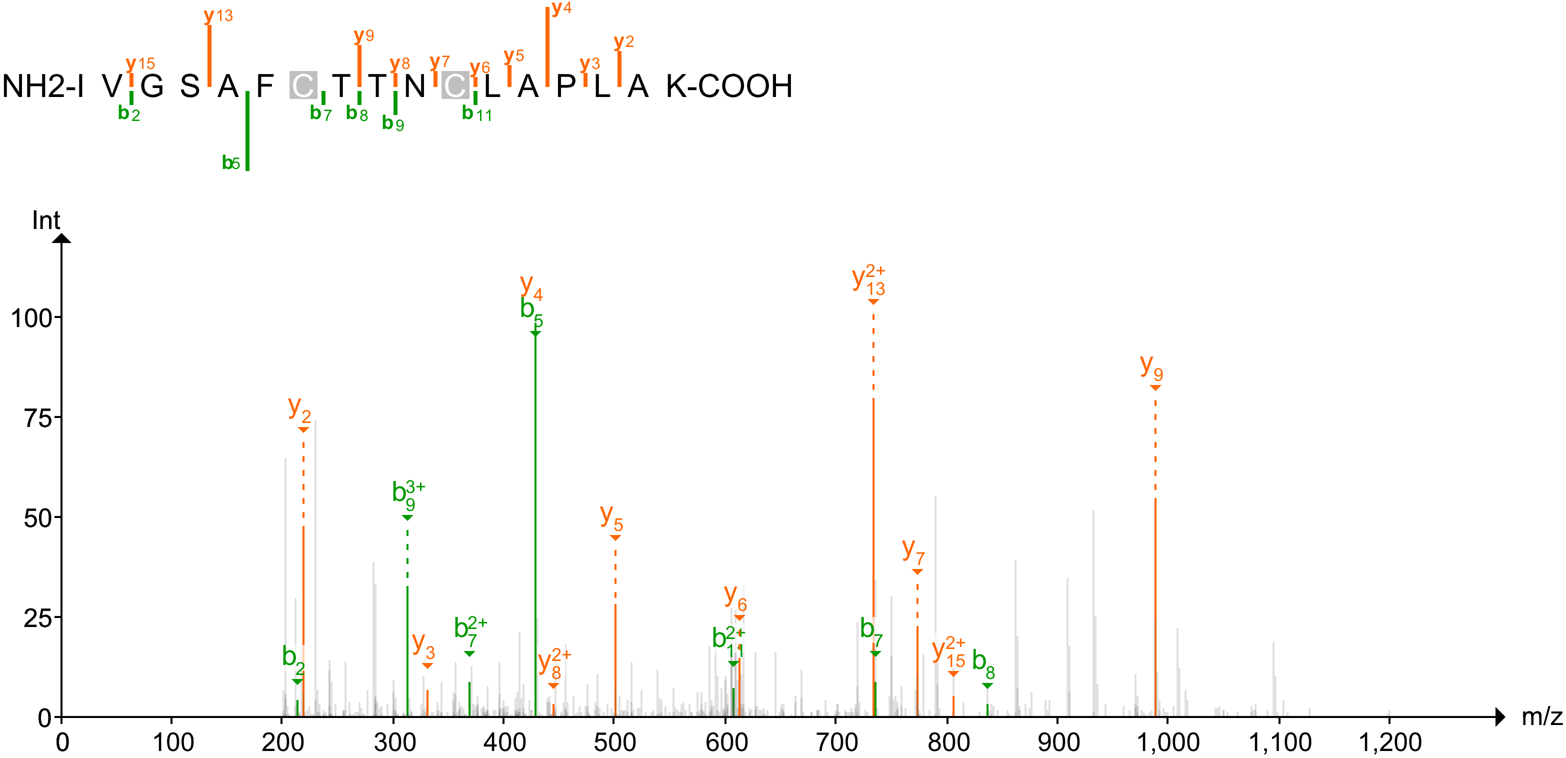

NH2-I V G S A F C T T N C L A P L A K-COOH
y15 y13 y9 y8 y7 y6 y5 y4 y3 y2
b2 b5 b7 b8 b9 b11
Int
100
75
50
25
0
m/z
0
100
200
300
400
500
600
700
800
900
1,000
1,100
1,200

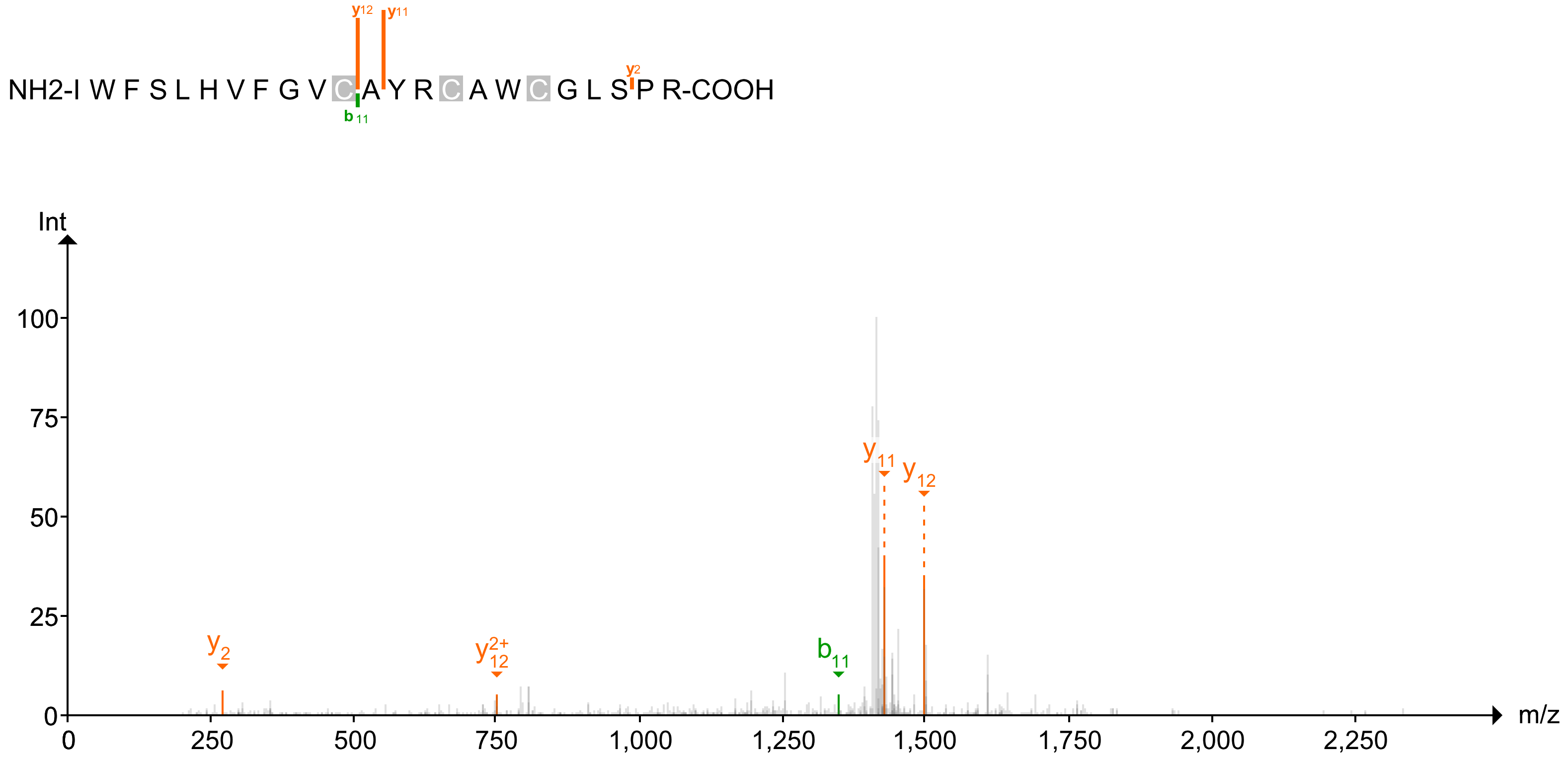

NH2-I W F S L H V F G V C A Y R C A W C G L S P R-COOH
y12
y11
b11
y2
Int
100
75
50
25
0
y11
y12
y2
y12 2+
b11
m/z
0
250
500
750
1,000
1,250
1,500
1,750
2,000
2,250

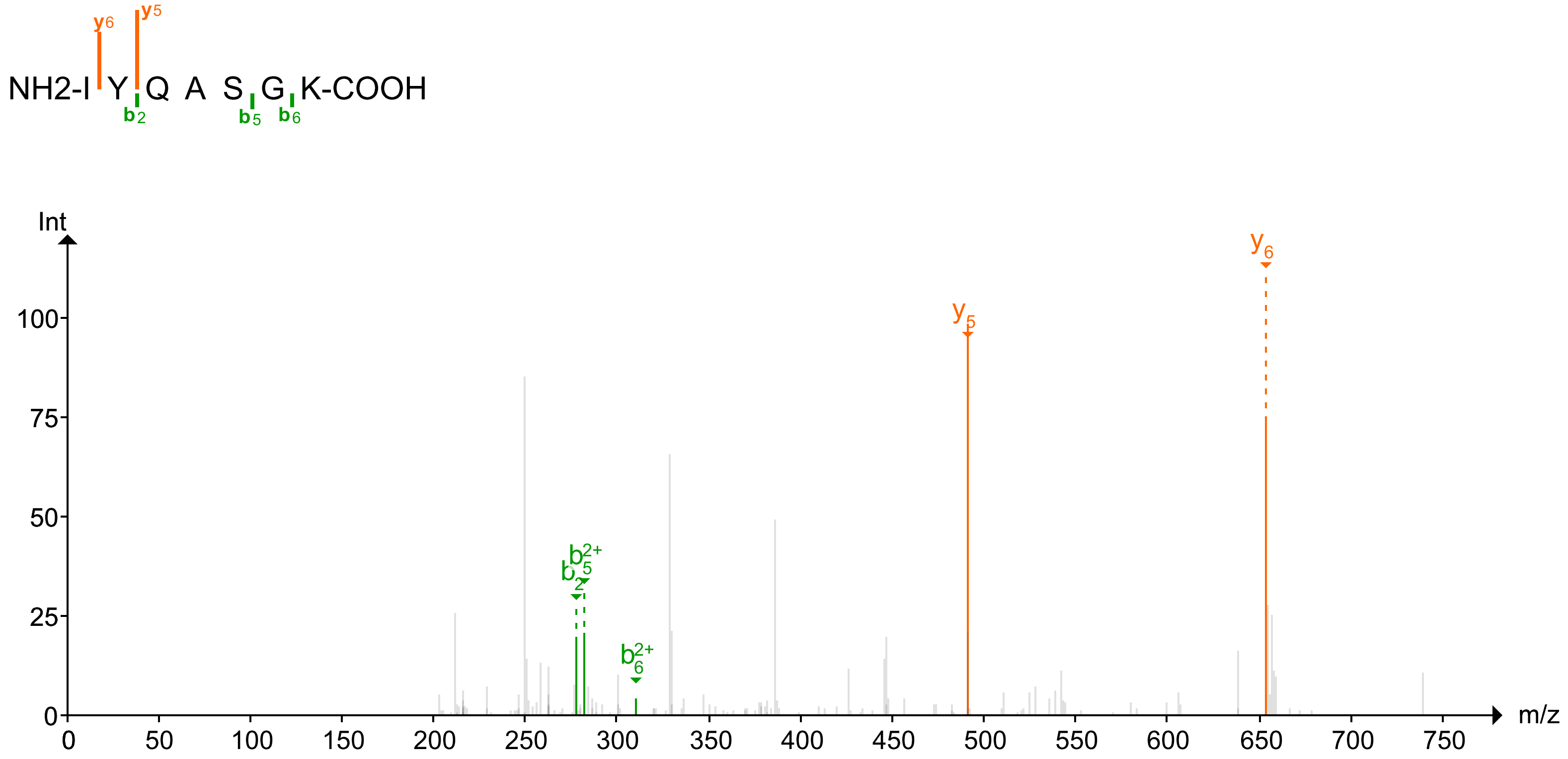

NH2-I Y Q A S G K-COOH
y6
y5
b2
b5
b6
Int
m/z
0
25
50
75
100
0
50
100
150
200
250
300
350
400
450
500
550
600
650
700
750
y6
y5
b5 2+
b2
b6 2+

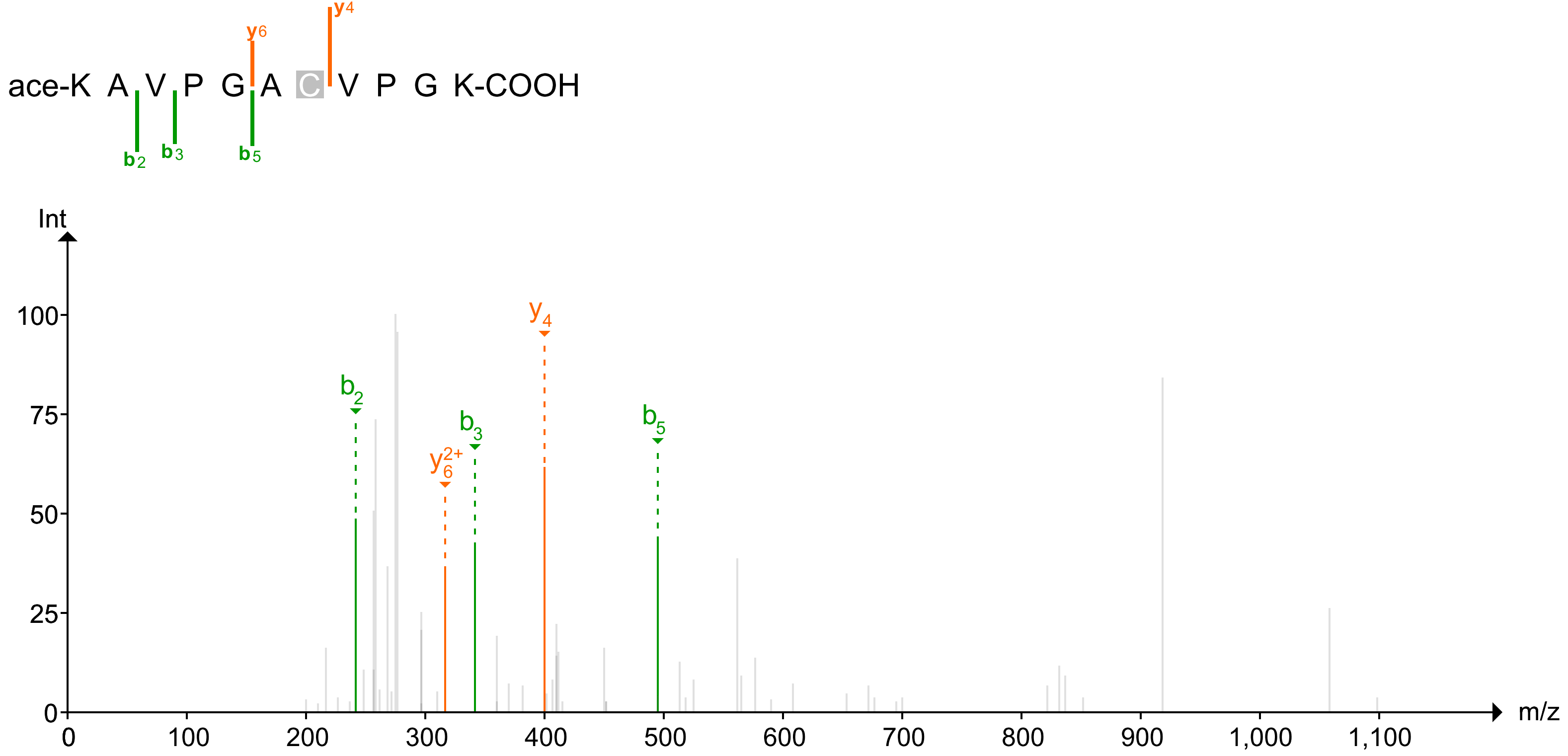

ace-K A V P G A C V P G K-COOH
y6
y4
b2
b3
b5
Int
100
75
50
25
0
b2
y6 2+
b3
y4
b5
0
100
200
300
400
500
600
700
800
900
1,000
1,100
m/z

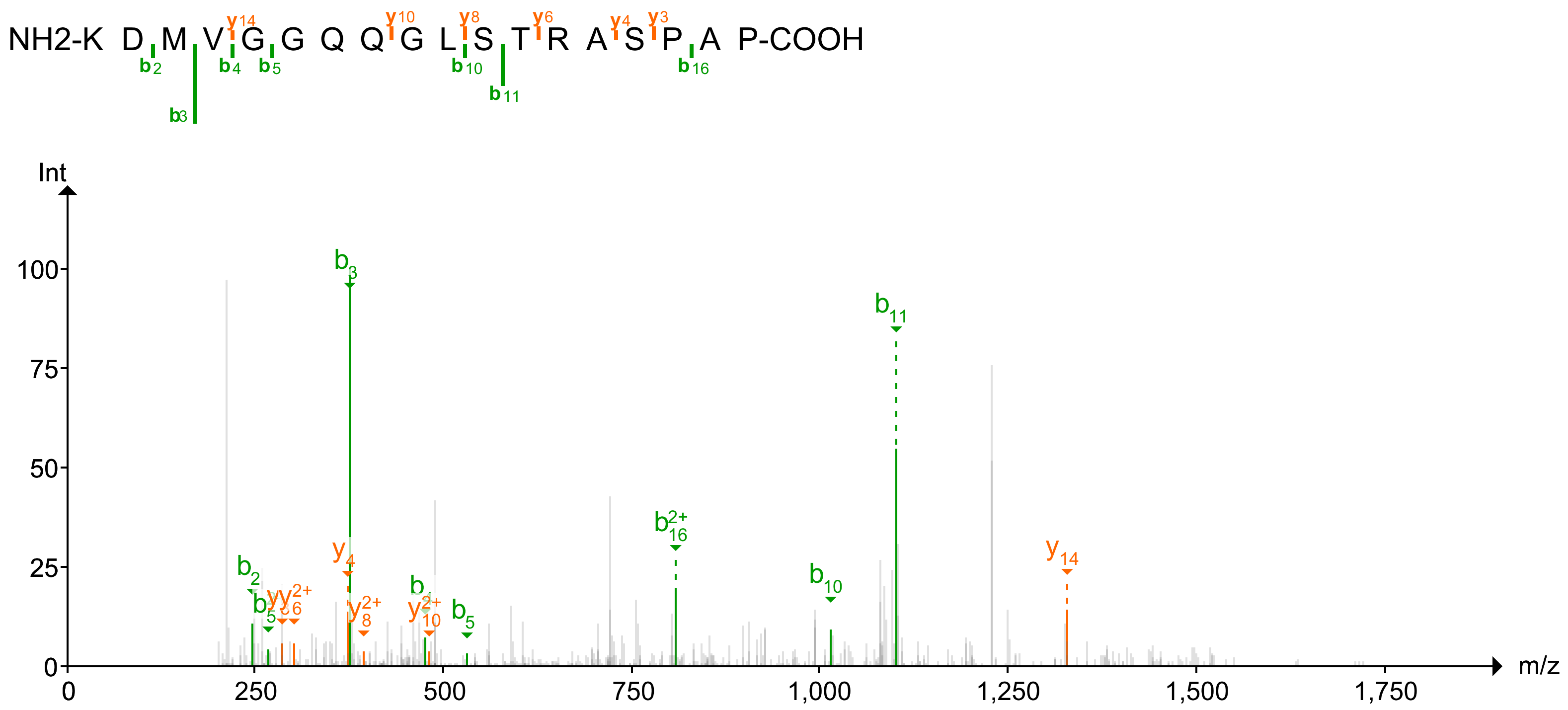

NH2-K D M V G G Q Q G L S T R A S P A P-COOH
y14 y10 y8 y6 y4 y3
b2 b3 b4 b5 b10 b11 b16
Int
100
75
50
25
0
m/z
0
250
500
750
1,000
1,250
1,500
1,750
b3
b11
b16 2+
y14
b10
b2
y4
b5
y8 2+
y10 2+

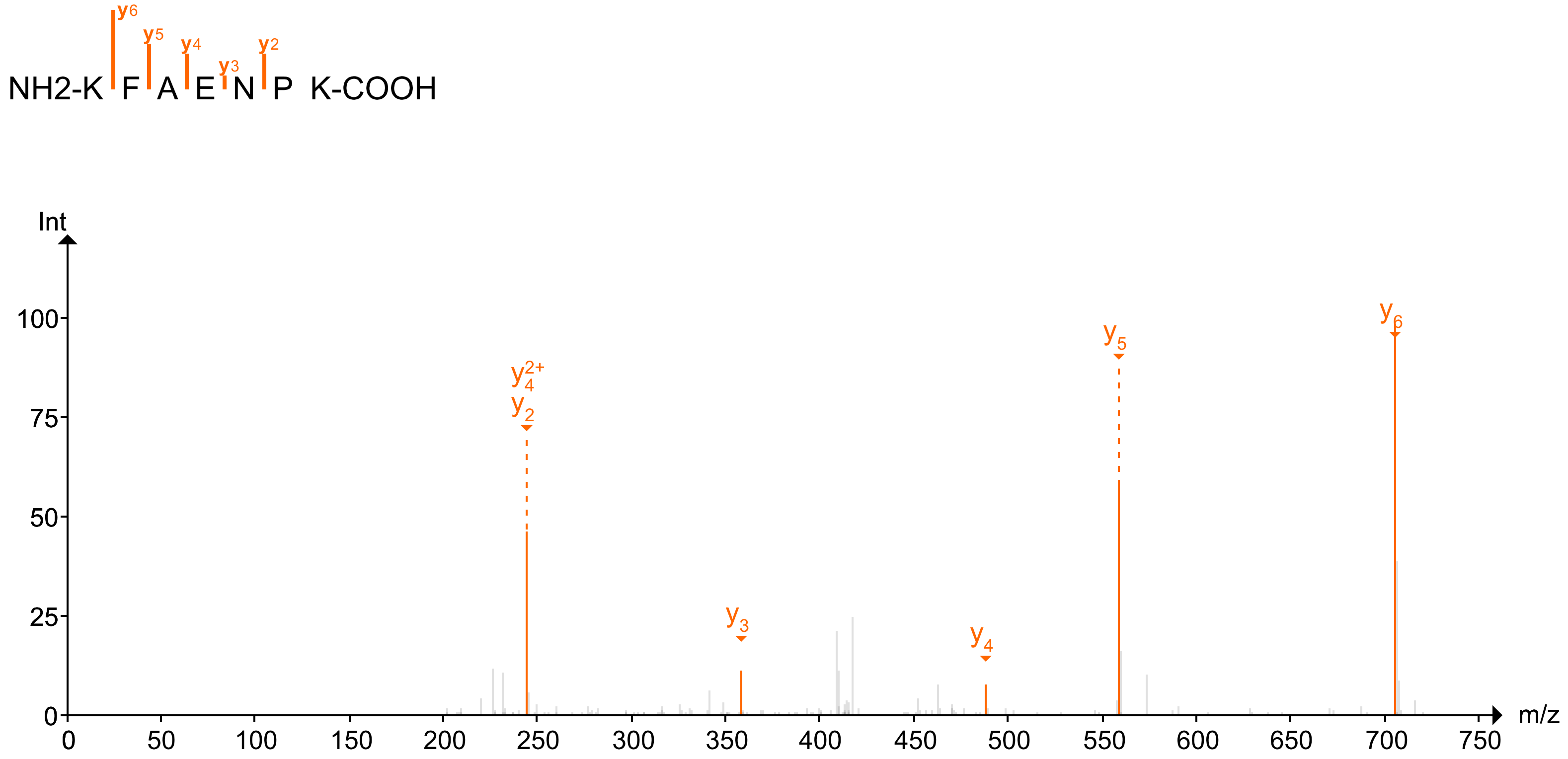

y6
y5
y4
y3
y2
NH2-K F A E N P K-COOH
Int
100
75
50
25
0
$y_4^{2+}$
$y_2$
$y_3$
$y_4$
$y_5$
$y_6$
0
50
100
150
200
250
300
350
400
450
500
550
600
650
700
750
m/z

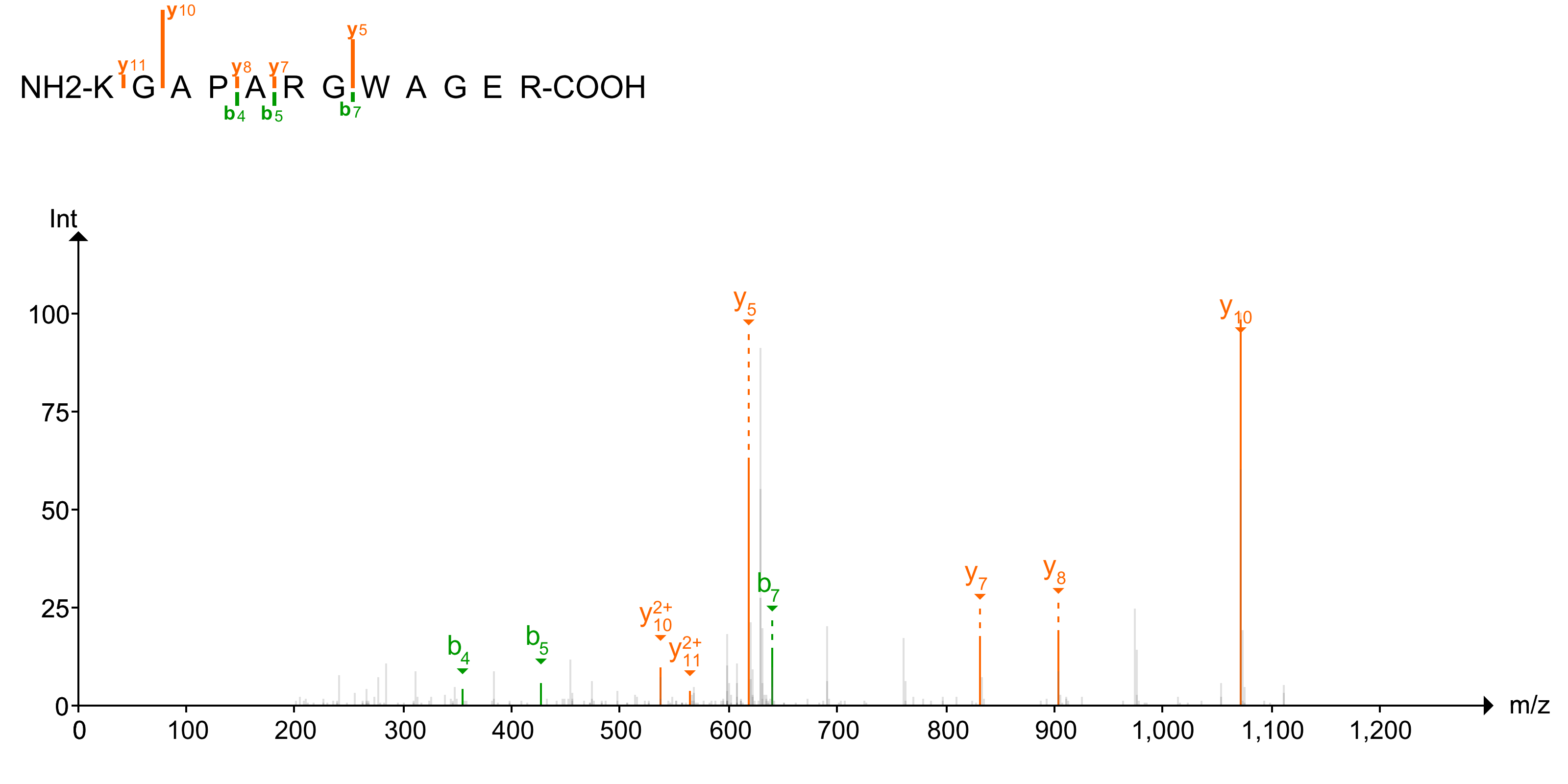
NH2-K G A P A R G W A G E R-COOH
y11
y10
y8
y7
y5
b4
b5
b7
Int
m/z
100
75
50
25
0
0
100
200
300
400
500
600
700
800
900
1,000
1,100
1,200

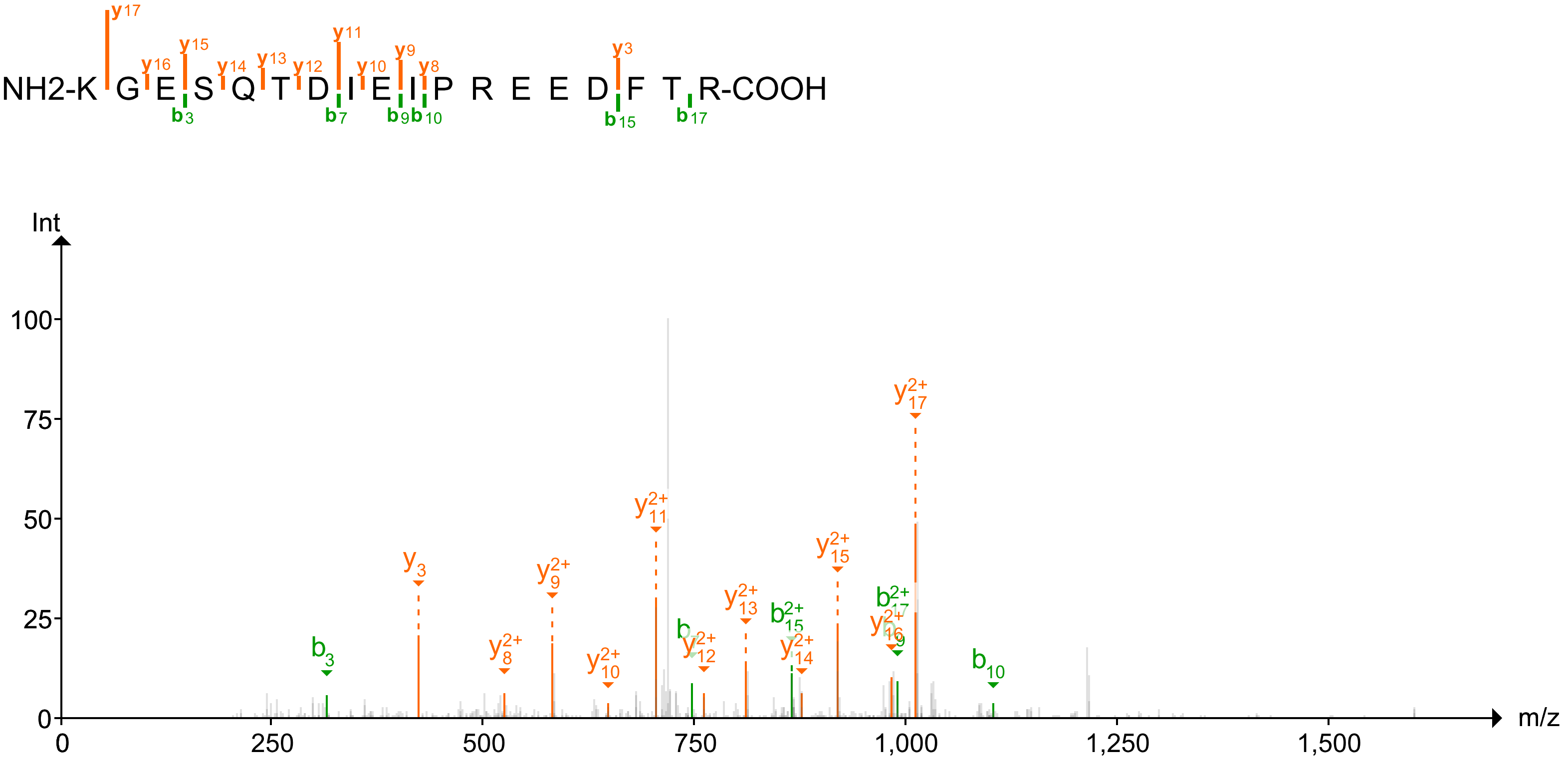

NH2-K G E S Q T D I E I P R E E D F T R-COOH
y17 y16 y15 y14 y13 y12 y11 y10 y9 y8 y3
b3 b7 b9 b10 b15 b17
Int
100
75
50
25
0
0 250 500 750 1,000 1,250 1,500
m/z

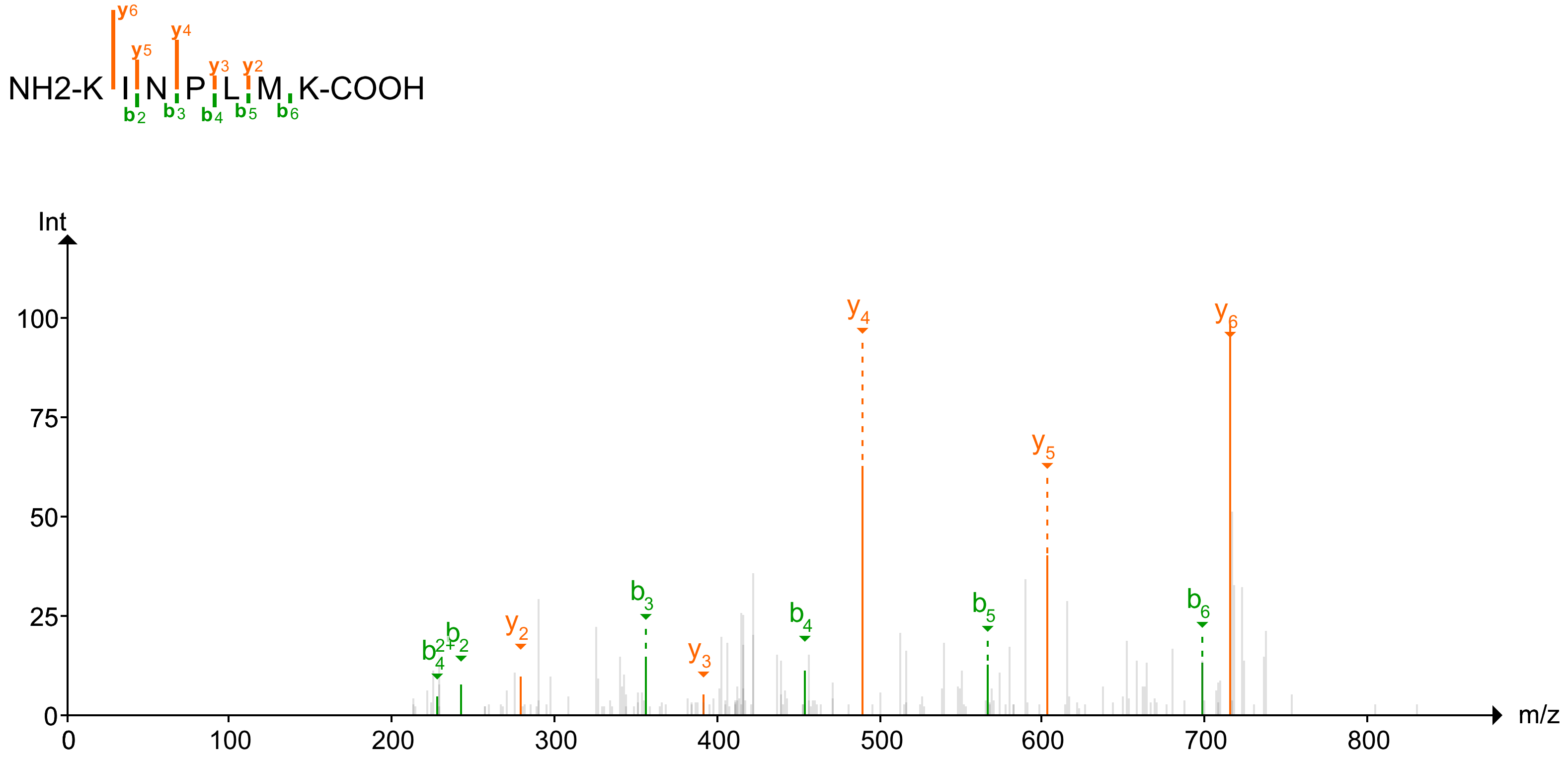

NH2-K I N P L M K-COOH
y6 y5 y4 y3 y2
b2 b3 b4 b5 b6
Int
m/z
0
25
50
75
100
0
100
200
300
400
500
600
700
800
y4
y6
y5
b3
b4
b5
b6
y2
y3
b2
b4 2+

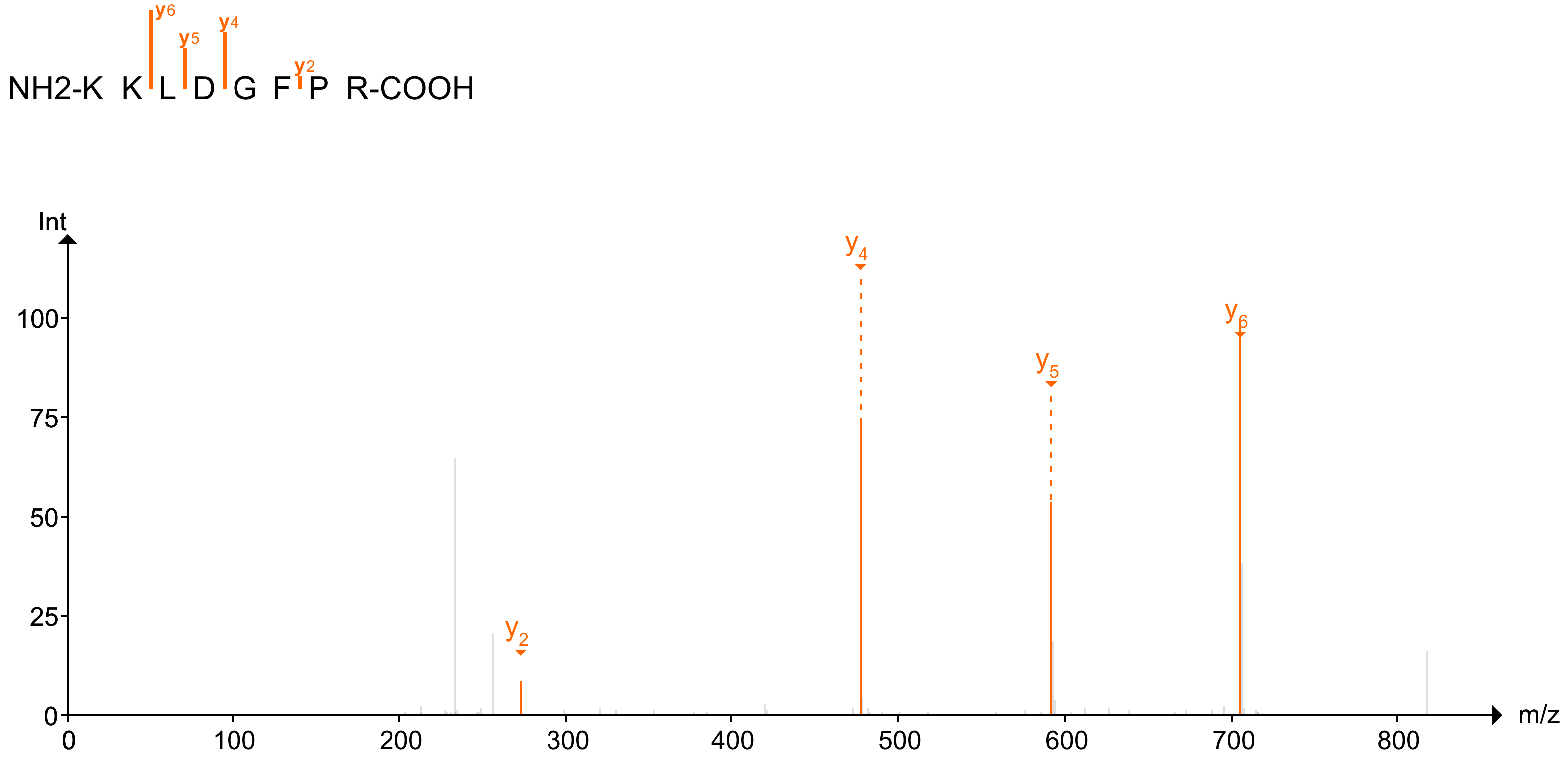

NH2-K K L D G F P R-COOH
y6
y5
y4
y2
Int
100
75
50
25
0
m/z
0
100
200
300
400
500
600
700
800

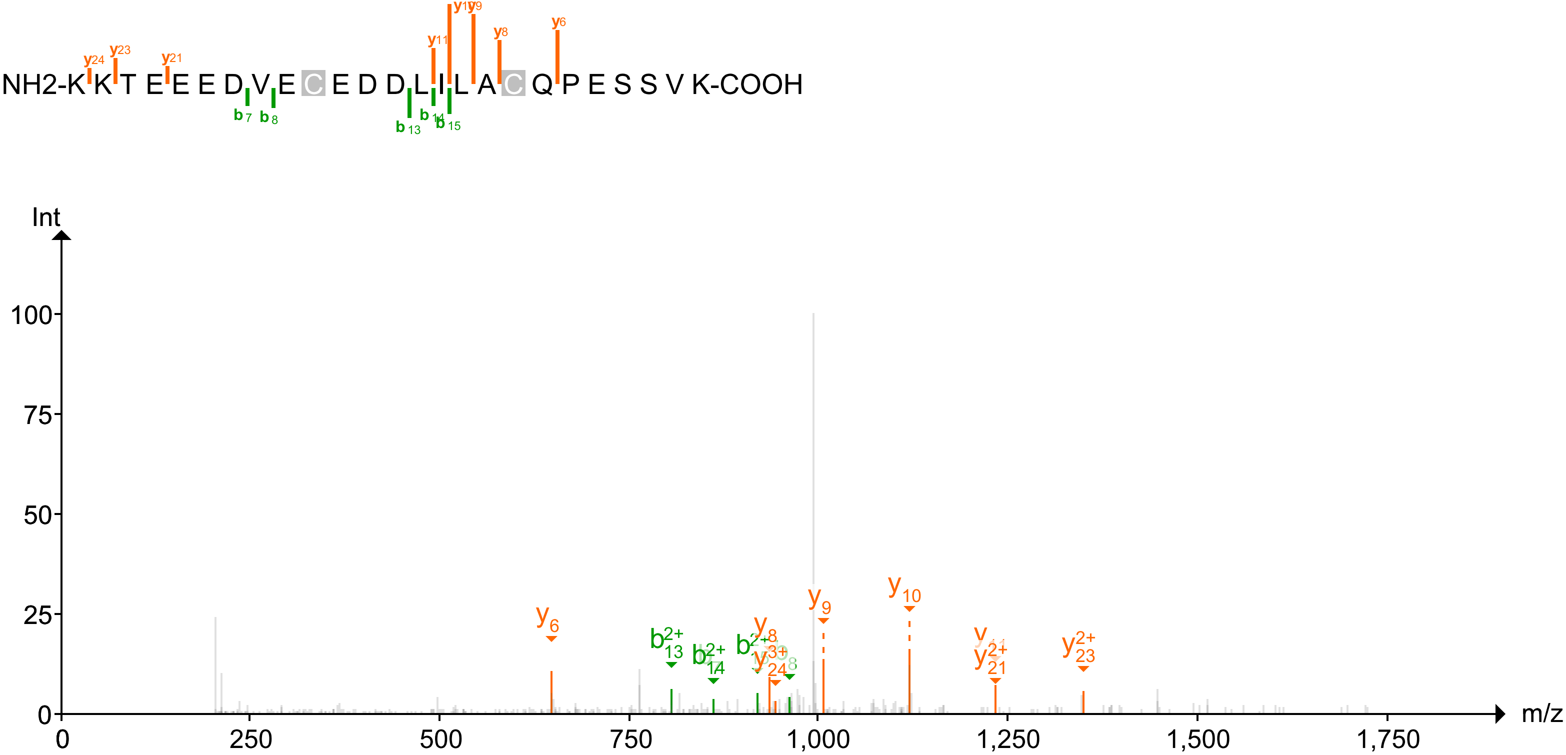

NH2-K K T E E E D V E C E D D L I L A C Q P E S S V K-COOH
y24
y23
y21
y11
y8
y6
b7
b8
b13
b15
Int
100
75
50
25
0
m/z
0
250
500
750
1,000
1,250
1,500
1,750
y6
y9
y10
y8
y23 2+
y21 2+
b13 2+
b14 2+
y24 3+

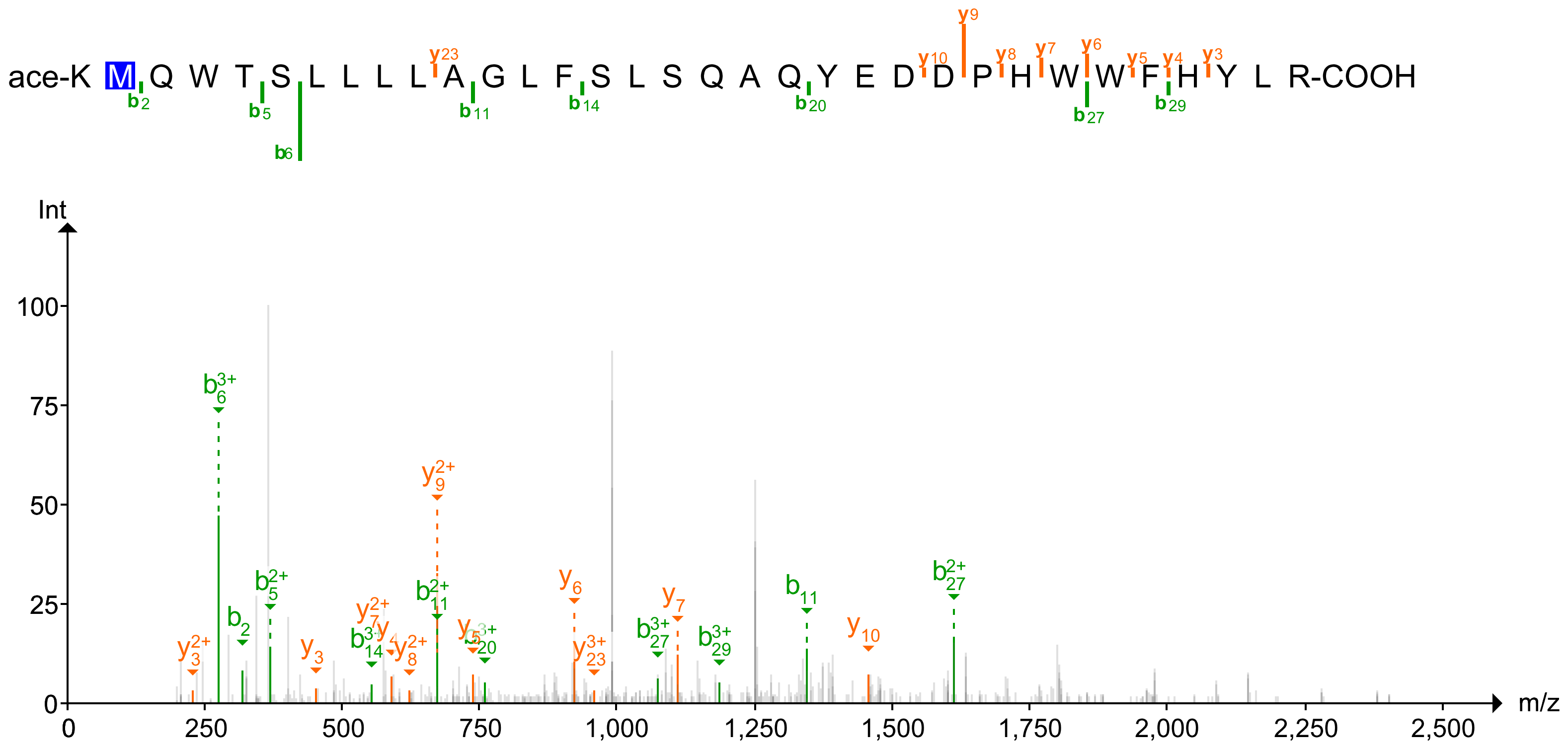

ace-K M Q W T S L L L L A G L F S L S Q A Q Y E D D P H W W F H Y L R-COOH
Int
100
75
50
25
0
m/z
0
250
500
750
1,000
1,250
1,500
1,750
2,000
2,250
2,500

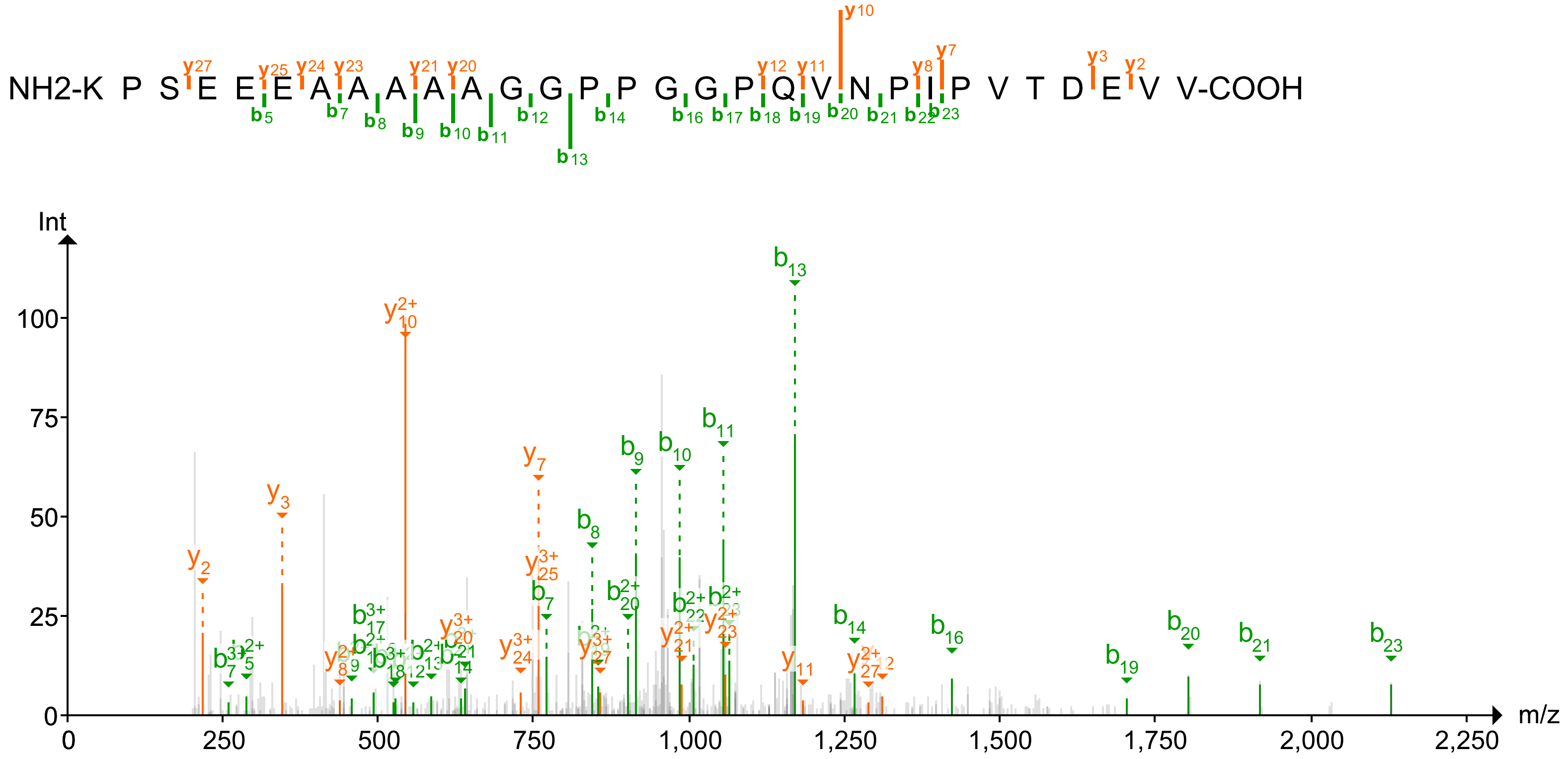

NH2-K P S E E E A A A A A G G P P G G P Q V N P I P V T D E V V-COOH
y27 y25 y24 y23 y21 y20 y12 y11 y10 y8 y7 y3 y2
b5 b7 b8 b9 b10 b11 b12 b13 b14 b16 b17 b18 b19 b20 b21 b22 b23
Int
m/z
0
25
50
75
100
0
250
500
750
1,000
1,250
1,500
1,750
2,000
2,250
$y_2$
$y_3$
$y^{2+}_{10}$
$y_7$
$y^{3+}_{25}$
$b_7$
$b_8$
$b_9$
$b_{10}$
$b_{11}$
$b_{13}$
$b^{2+}_{20}$
$b^{2+}_{22}$
$y^{2+}_{21}$
$y^{2+}_{23}$
$y^{3+}_{24}$
$y^{3+}_{27}$
$y^{2+}_{8}$
$b^{3+}_{17}$
$b^{3+}_{18}$
$y_{11}$
$b_{14}$
$y^{2+}_{27}$
$b_{16}$
$b_{19}$
$b_{20}$
$b_{21}$
$b_{23}$

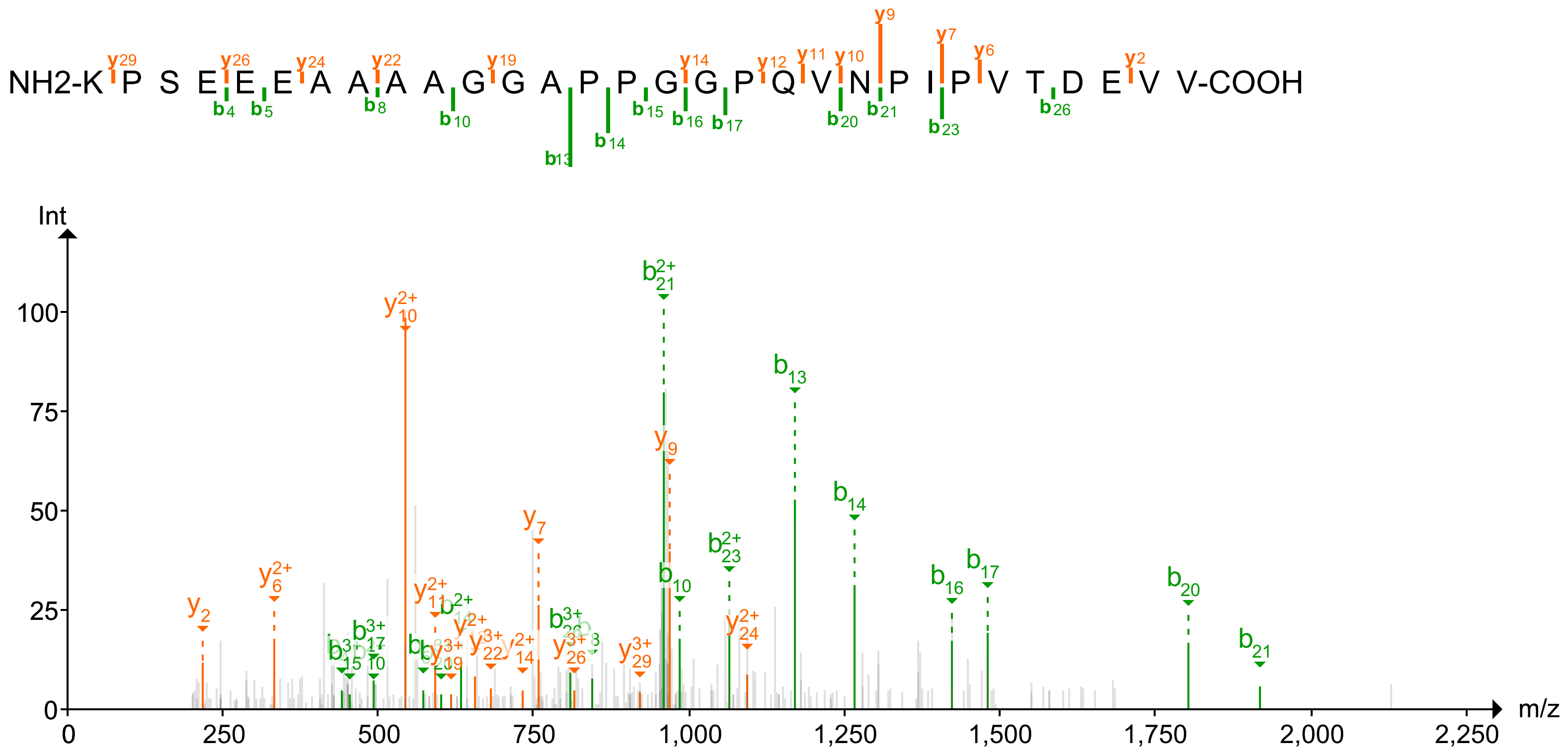

NH2-K P S E E E A A A A G G A P P G G P Q V N P I P V T D E V V-COOH
y29 y26 y24 y22 y19 y14 y12 y11 y10 y9 y7 y6 y2
b4 b5 b8 b10 b13 b14 b15 b16 b17 b20 b21 b23 b26
Int
m/z
0
25
50
75
100
0
250
500
750
1,000
1,250
1,500
1,750
2,000
2,250
y2
y6 2+
y10 2+
y11 2+
y7
y9
y24 2+
y29 3+
b21 2+
b13
b14
b23 2+
b10
b16
b17
b20
b21

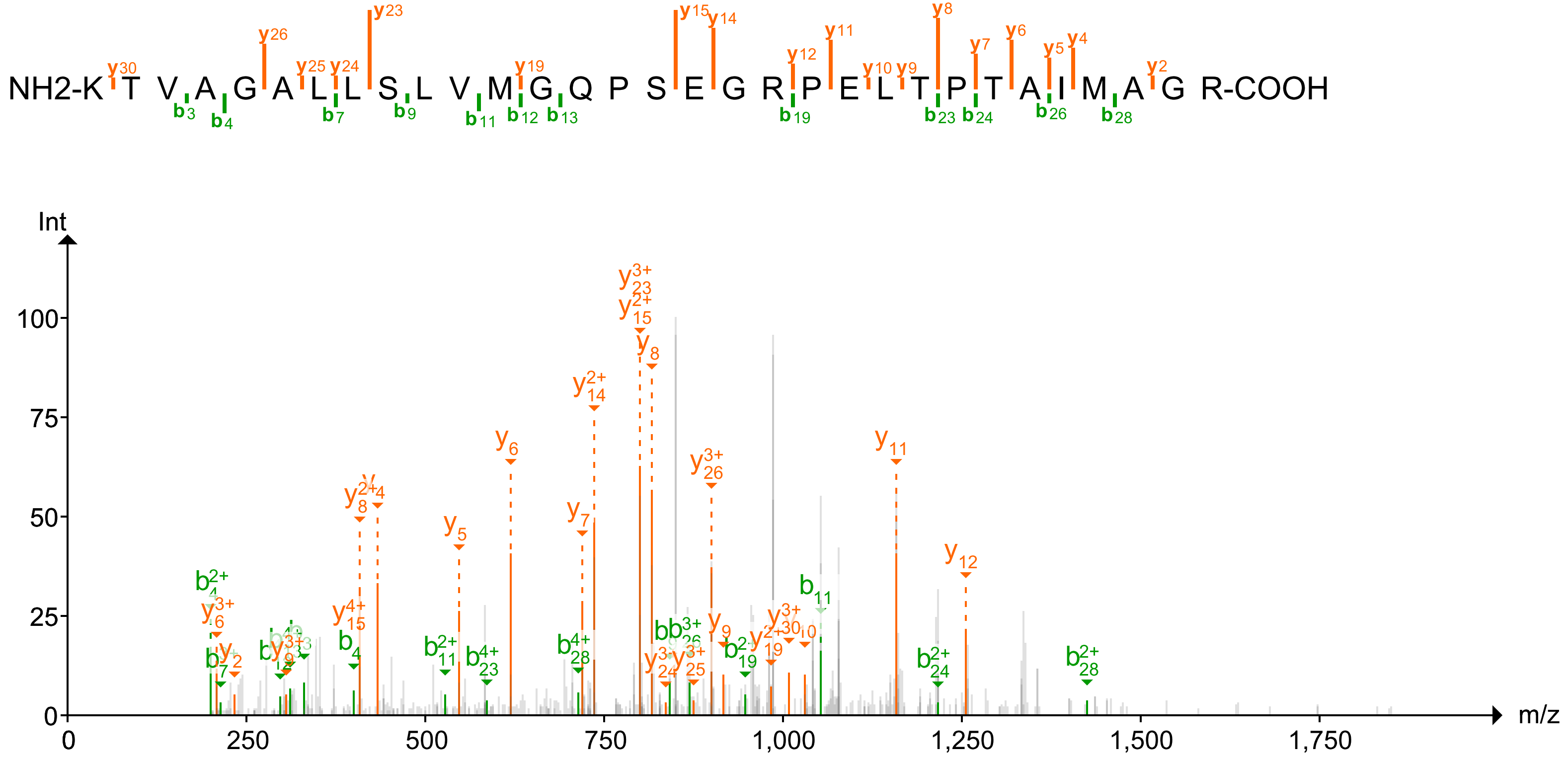

NH2-K T V A G A L L S L V M G Q P S E G R P E L T P T A I M A G R-COOH
y30 y26 y25 y24 y23 y19 y15 y14 y12 y11 y10 y9 y8 y7 y6 y5 y4 y2
b3 b4 b7 b9 b11 b12 b13 b19 b23 b24 b26 b28
Int
0
25
50
75
100
m/z
0
250
500
750
1,000
1,250
1,500
1,750

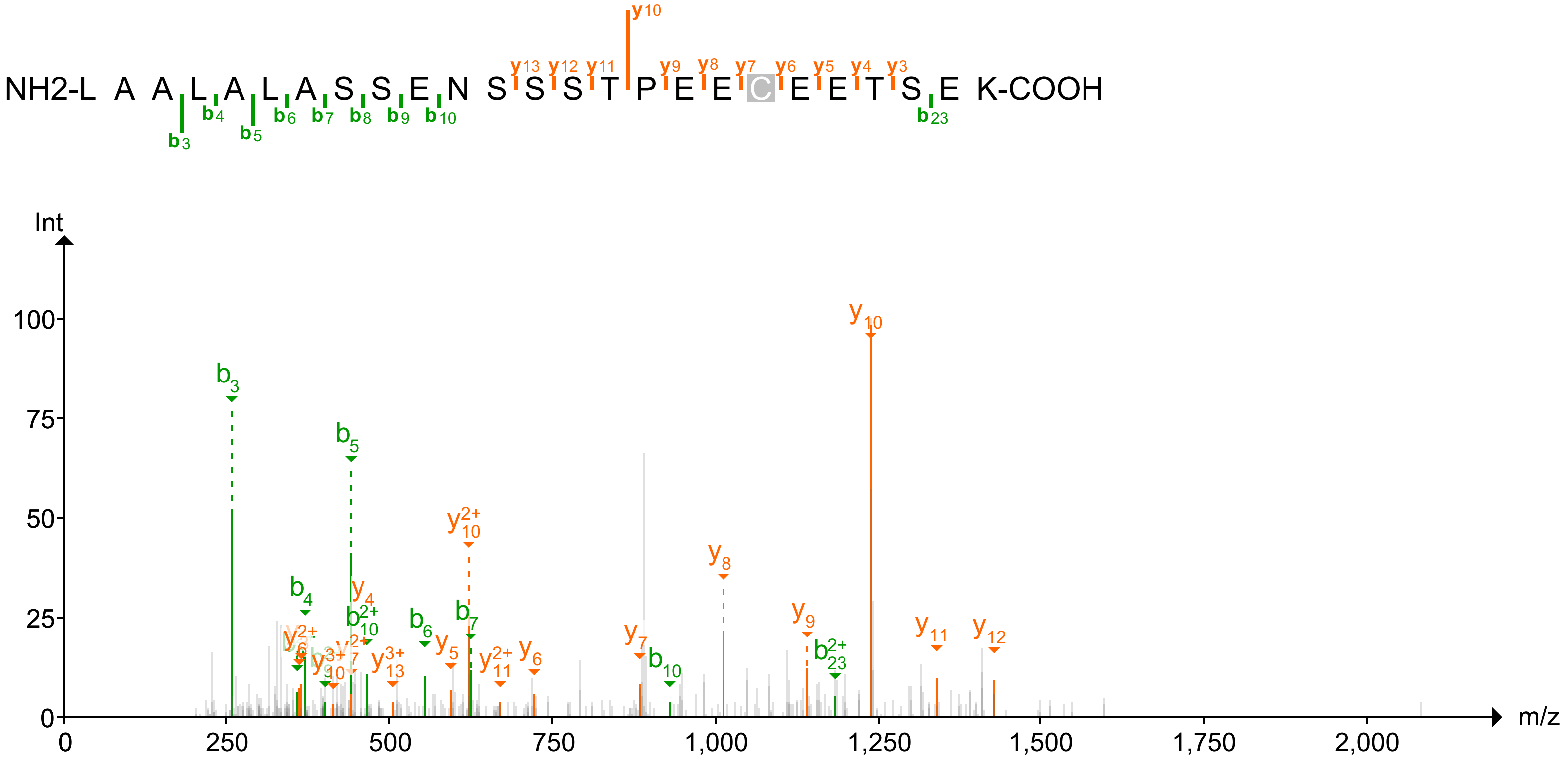

NH2-L A A L A L A S S E N S S S T P E E C E E T S E K-COOH
Int
m/z

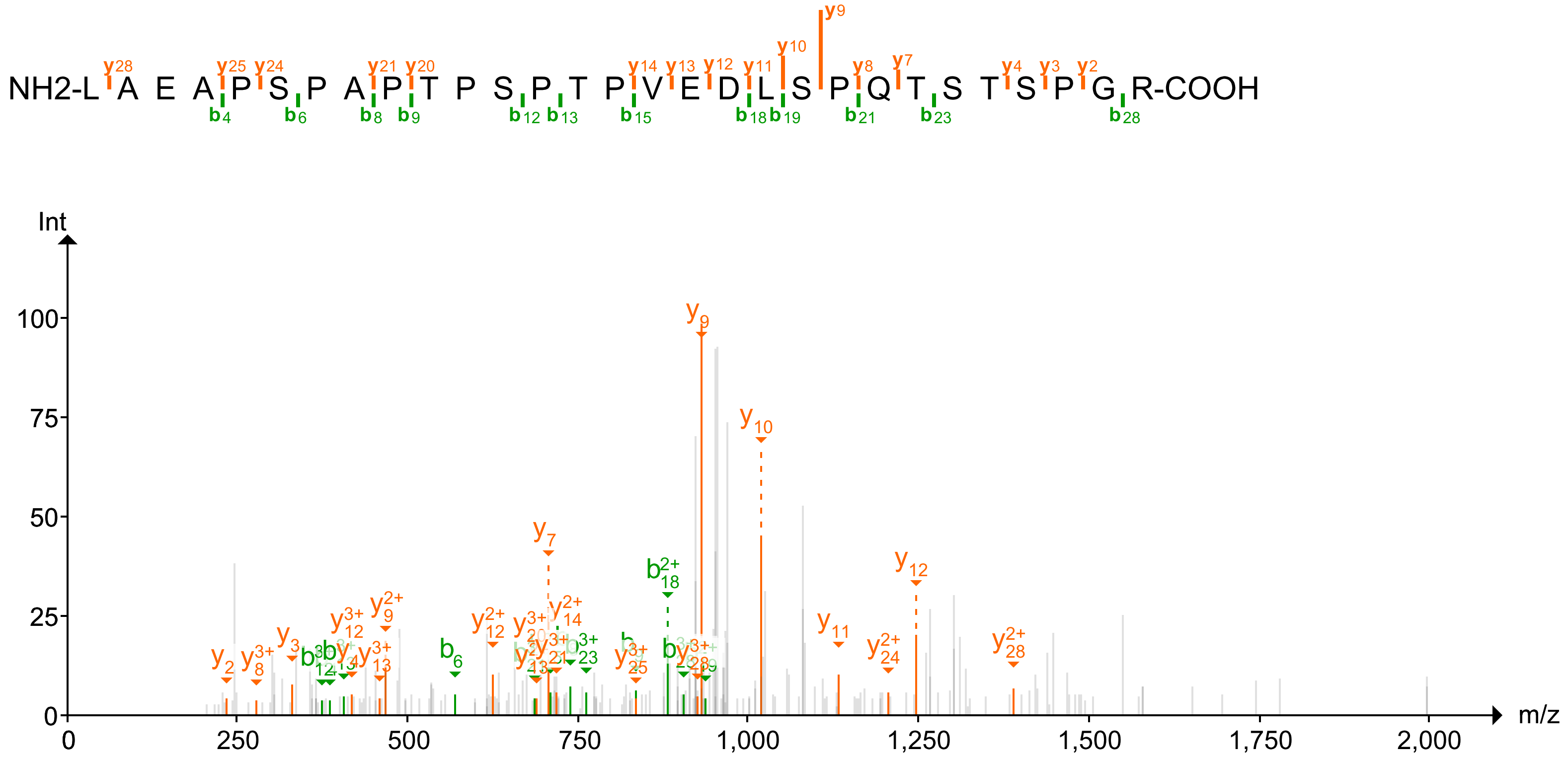

NH2-L A E A P S P A P T P S P T P V E D L S P Q T S T S P G R-COOH
y28 y25 y24 y21 y20 y14 y13 y12 y11 y10 y9 y8 y7 y4 y3 y2
b4 b6 b8 b9 b12 b13 b15 b18 b19 b21 b23 b28
Int
100
75
50
25
0
m/z
0
250
500
750
1,000
1,250
1,500
1,750
2,000

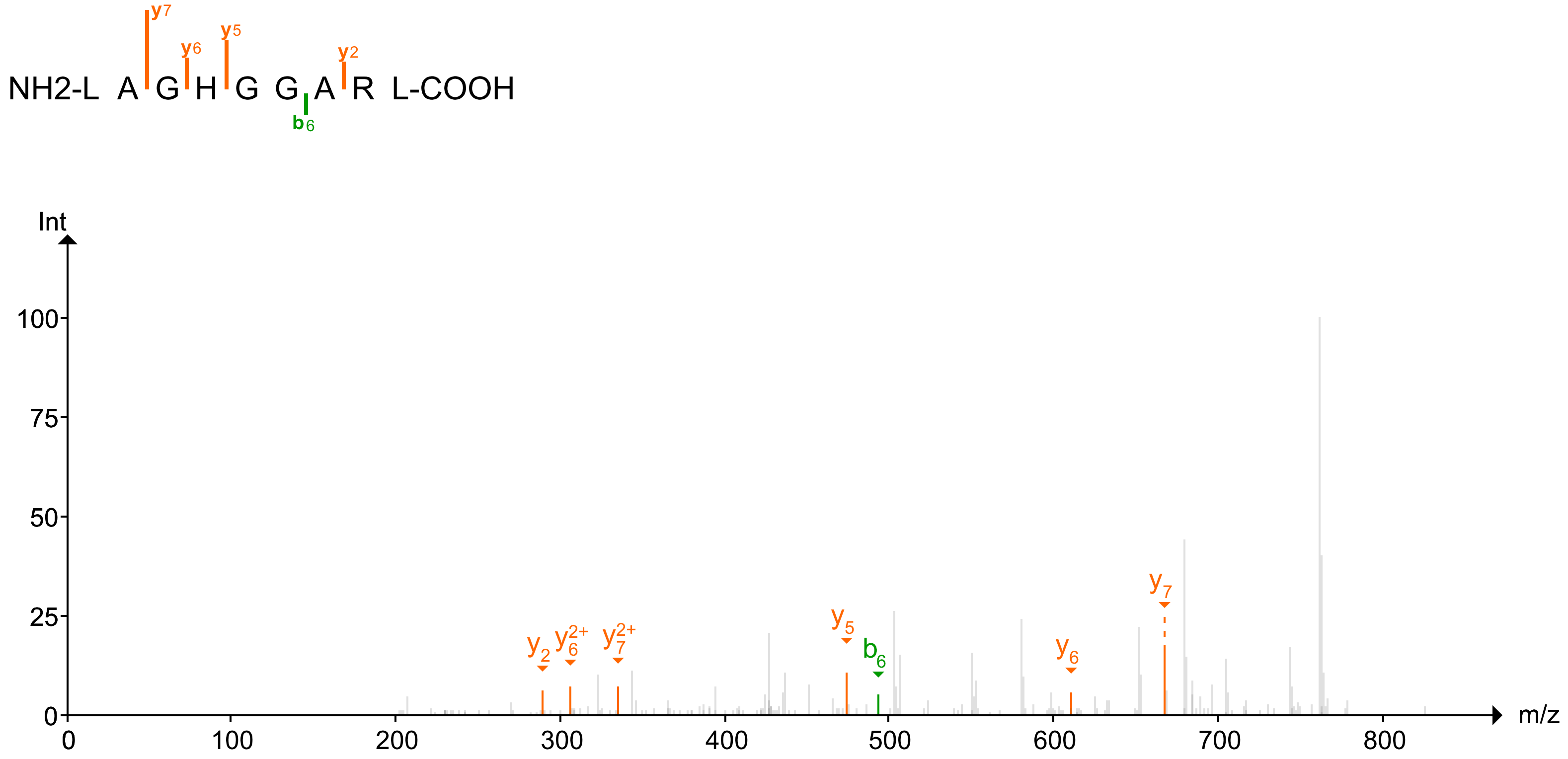
NH2-L A G H G G A R L-COOH
y7
y6
y5
y2
b6
Int
100
75
50
25
0
m/z
0
100
200
300
400
500
600
700
800
y2
y6 2+
y7 2+
y5
b6
y6
y7

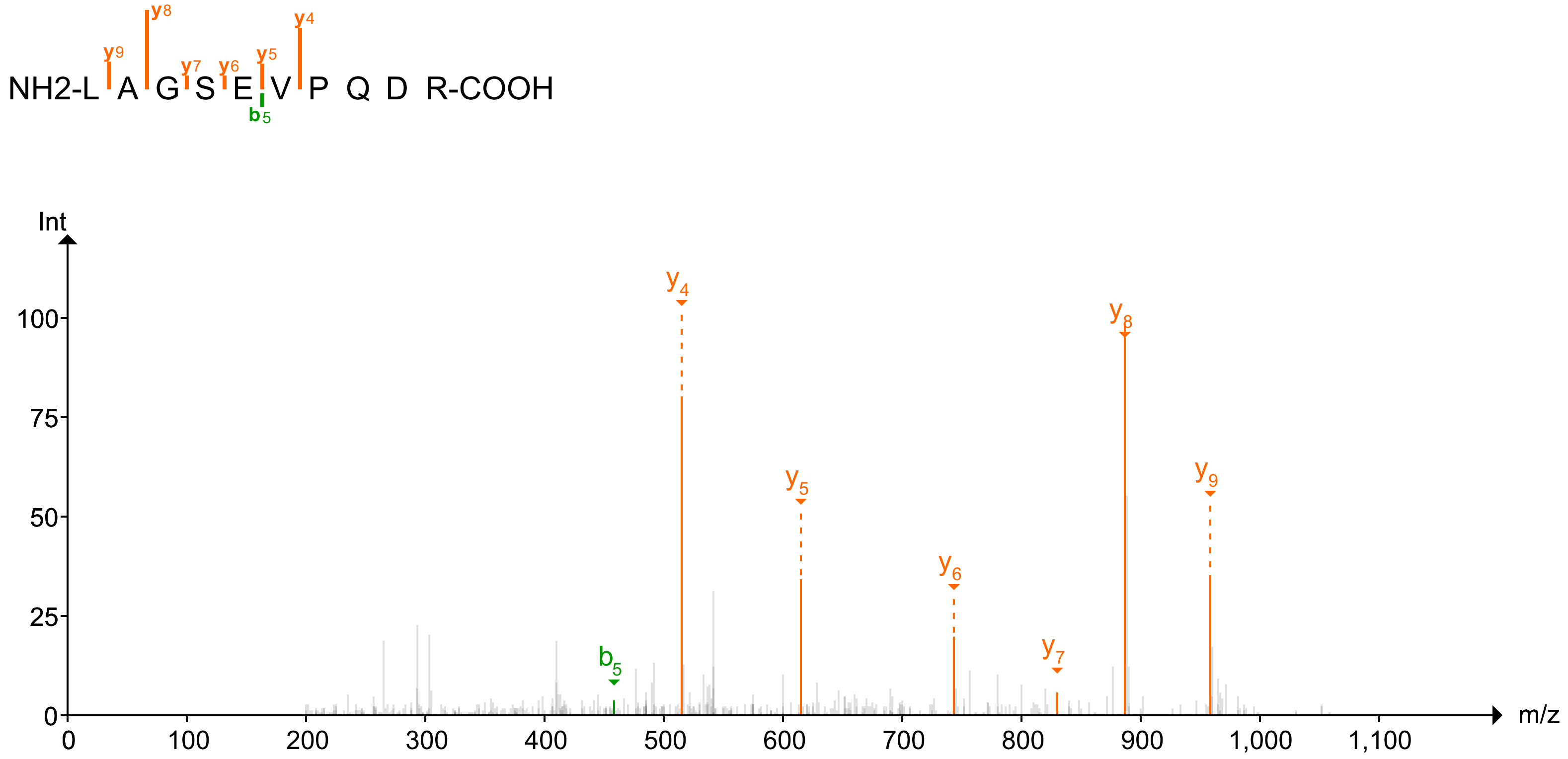

NH2-L A G S E V P Q D R-COOH
y9
y8
y7
y6
y5
y4
b5
Int
m/z
100
75
50
25
0
100
200
300
400
500
600
700
800
900
1,000
1,100
y4
y5
y6
y7
y8
y9
b5

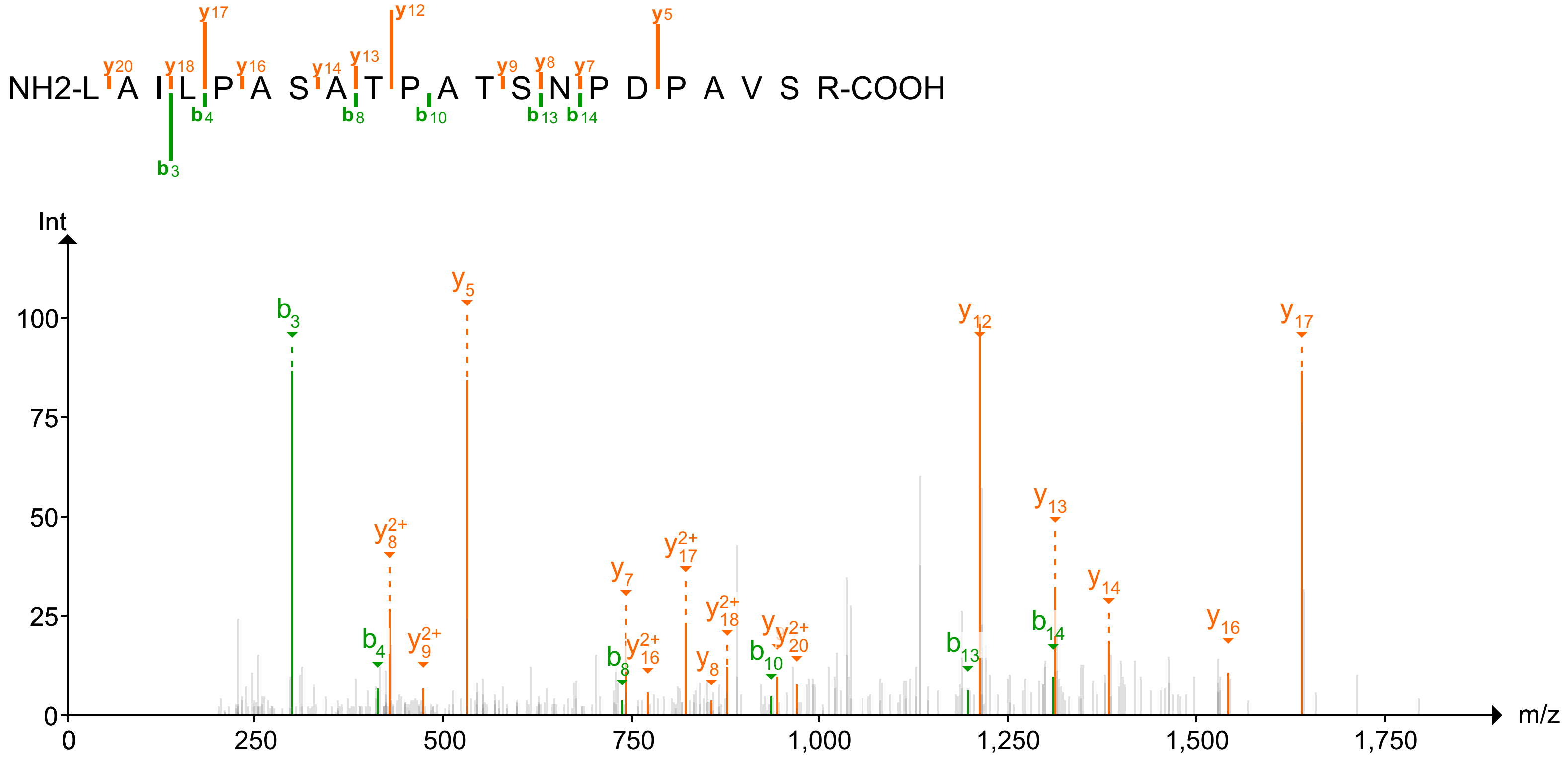
NH2-L A I L P A S A T P A T S N P D P A V S R-COOH
y20 y18 y17 y16 y14 y13 y12 y9 y8 y7 y5
b3 b4 b8 b10 b13 b14
Int
m/z
0
25
50
75
100
250
500
750
1,000
1,250
1,500
1,750

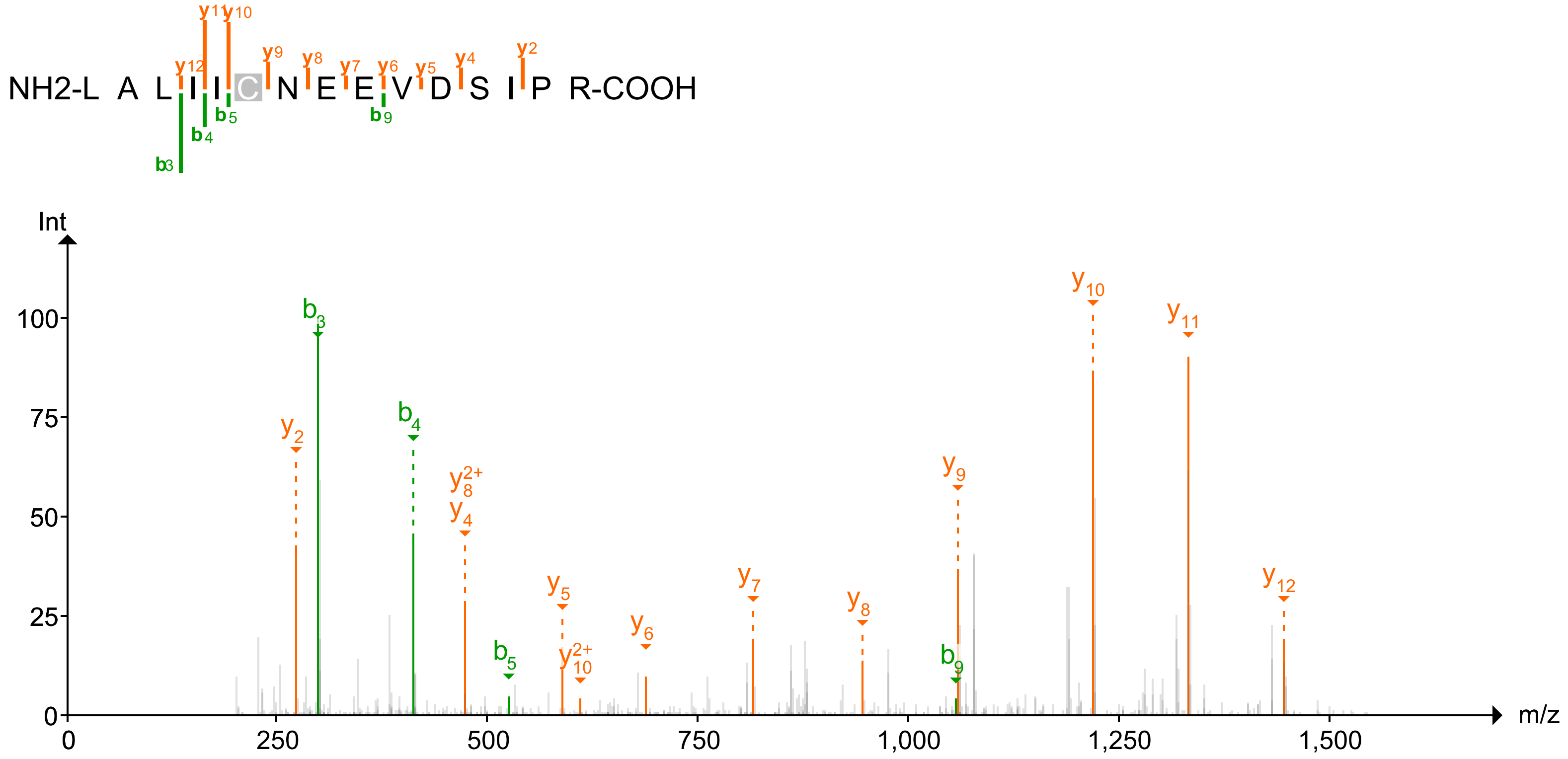

NH2-L A L I I C N E E V D S I P R-COOH
y11 y10 y12 y9 y8 y7 y6 y5 y4 y2
b3 b4 b5 b9
Int
m/z

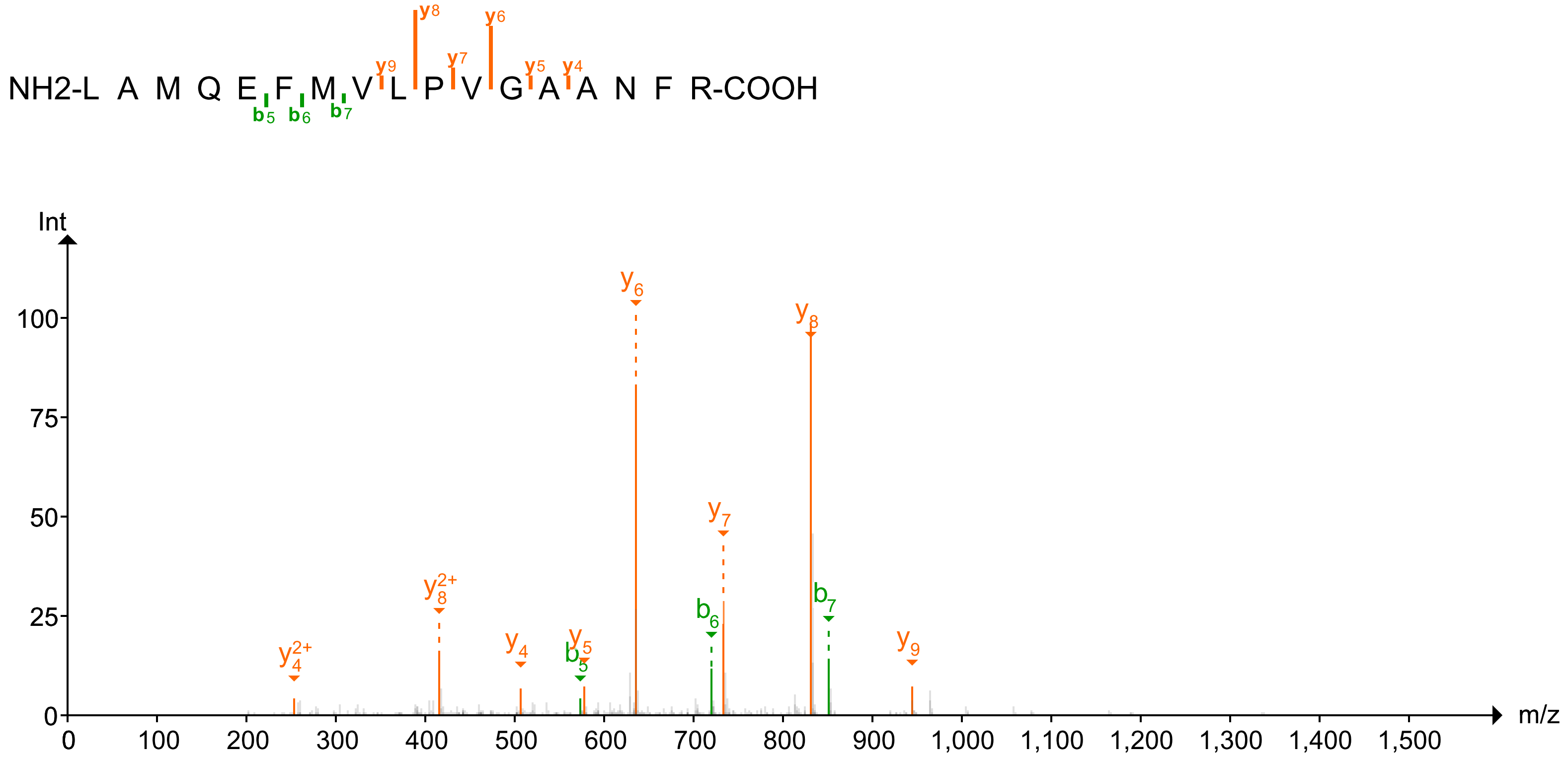

NH2-L A M Q E F M V L P V G A A N F R-COOH
b5 b6 b7
y9 y8 y7 y6 y5 y4
Int
m/z
0
25
50
75
100
0
100
200
300
400
500
600
700
800
900
1,000
1,100
1,200
1,300
1,400
1,500
y6
y8
y7
y8 2+
y4 2+
y4
y5
b5
b6
b7
y9

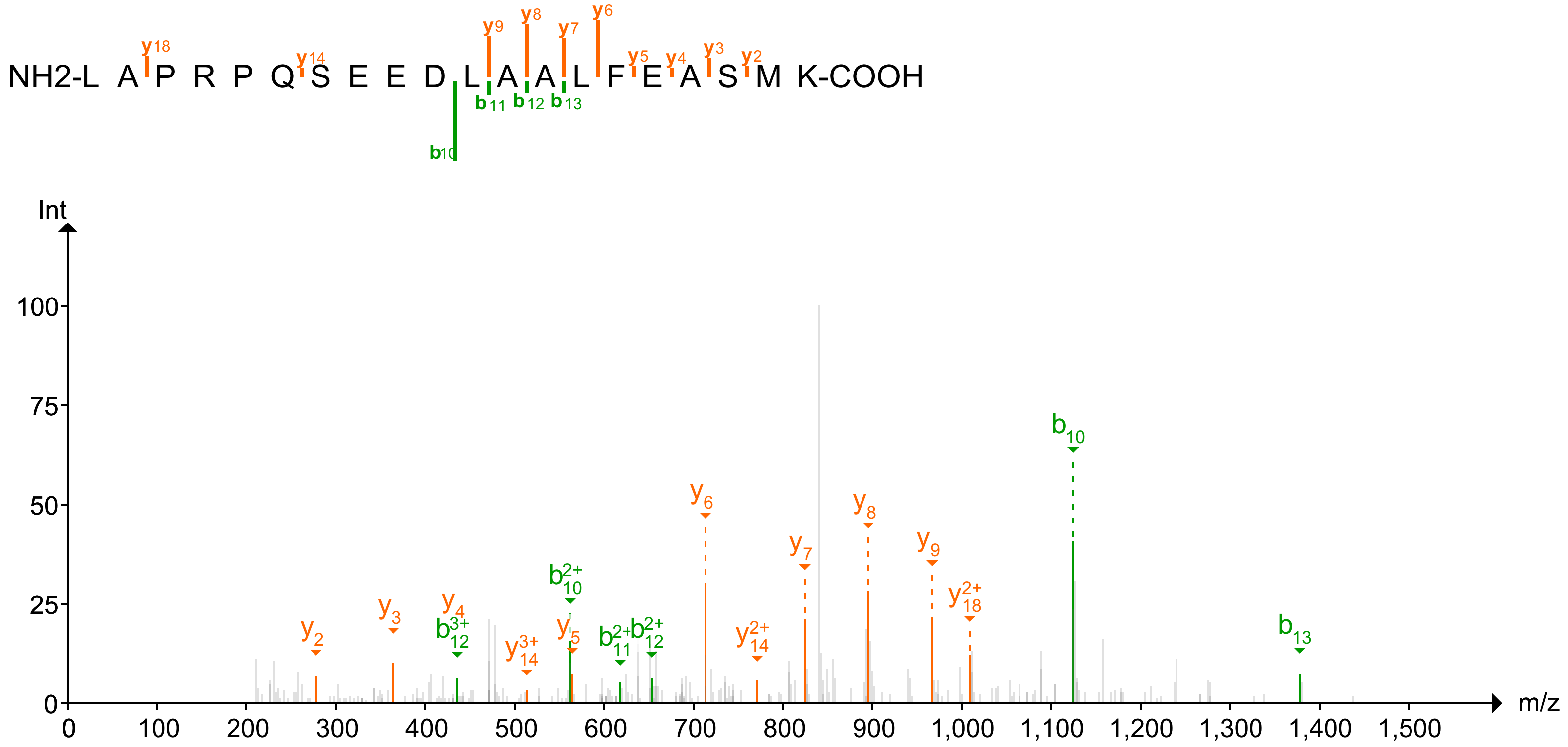

NH2-L A P R P Q S E E D L A A L F E A S M K-COOH
y18 y14 y9 y8 y7 y6 y5 y4 y3 y2
b10 b11 b12 b13
Int
m/z
$y_2$
$y_3$
$y_4$
$b_{12}^{3+}$
$y_{14}^{3+}$
$b_{10}^{2+}$
$y_5$
$b_{11}^{2+}$
$b_{12}^{2+}$
$y_6$
$y_{14}^{2+}$
$y_7$
$y_8$
$y_9$
$y_{18}^{2+}$
$b_{10}$
$b_{13}$

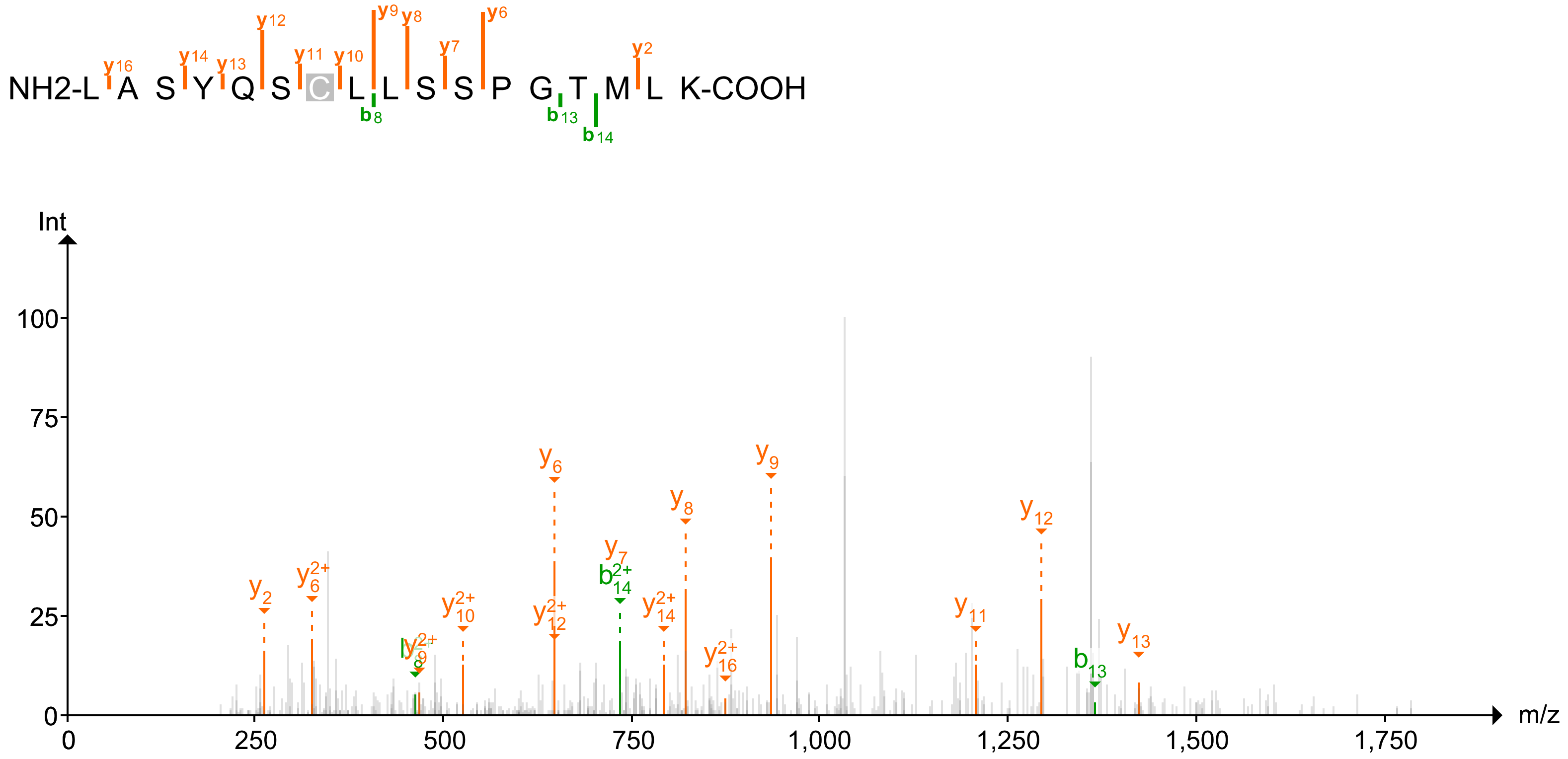

NH2-L A S Y Q S C L L S S P G T M L K-COOH
y16 y14 y13 y12 y11 y10 y9 y8 y7 y6 y2
b8 b13 b14
Int
100
75
50
25
0
y6
y9
y8
y12
y7
b2+ 14
y2
y2+ 6
y2+ 10
y2+ 12
y2+ 14
y2+ 16
y2+ 9
y11
y13
b13
0
250
500
750
1,000
1,250
1,500
1,750
m/z

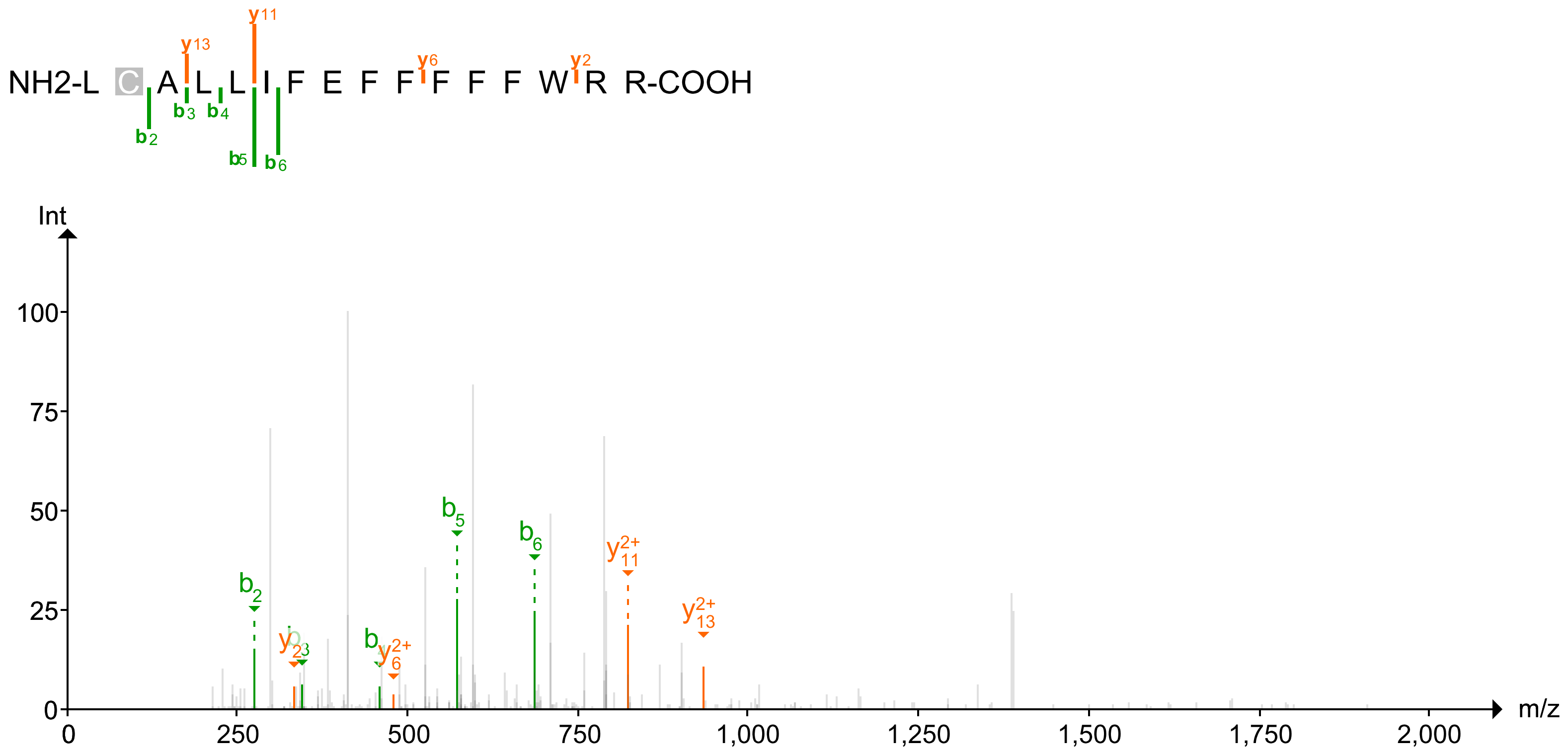

NH2-L C A L L I F E F F F F F W R R-COOH
y11
y13
y6
y2
b2
b3
b4
b5
b6
Int
100
75
50
25
0
b2
y2
b5
b6
y11 2+
y13 2+
y6 2+
m/z
0
250
500
750
1,000
1,250
1,500
1,750
2,000

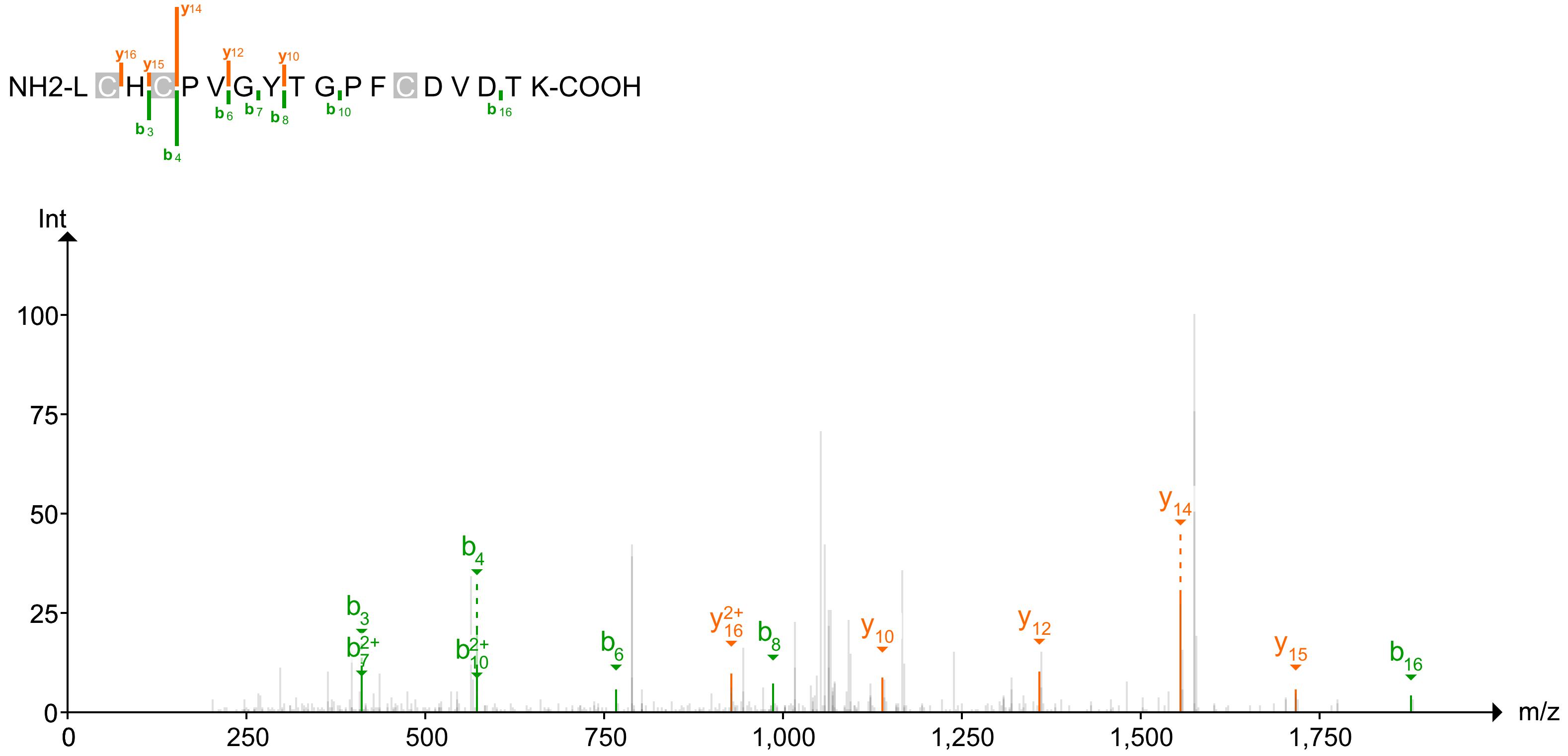

NH2-L C H C P V G Y T G P F C D V D T K-COOH
y16 y15 y14 y12 y10
b3 b4 b6 b7 b8 b10 b16
Int
100
75
50
25
0
0
250
500
750
1,000
1,250
1,500
1,750
m/z
b3
b7 2+
b4
b10 2+
b6
y16 2+
b8
y10
y12
y14
y15
b16

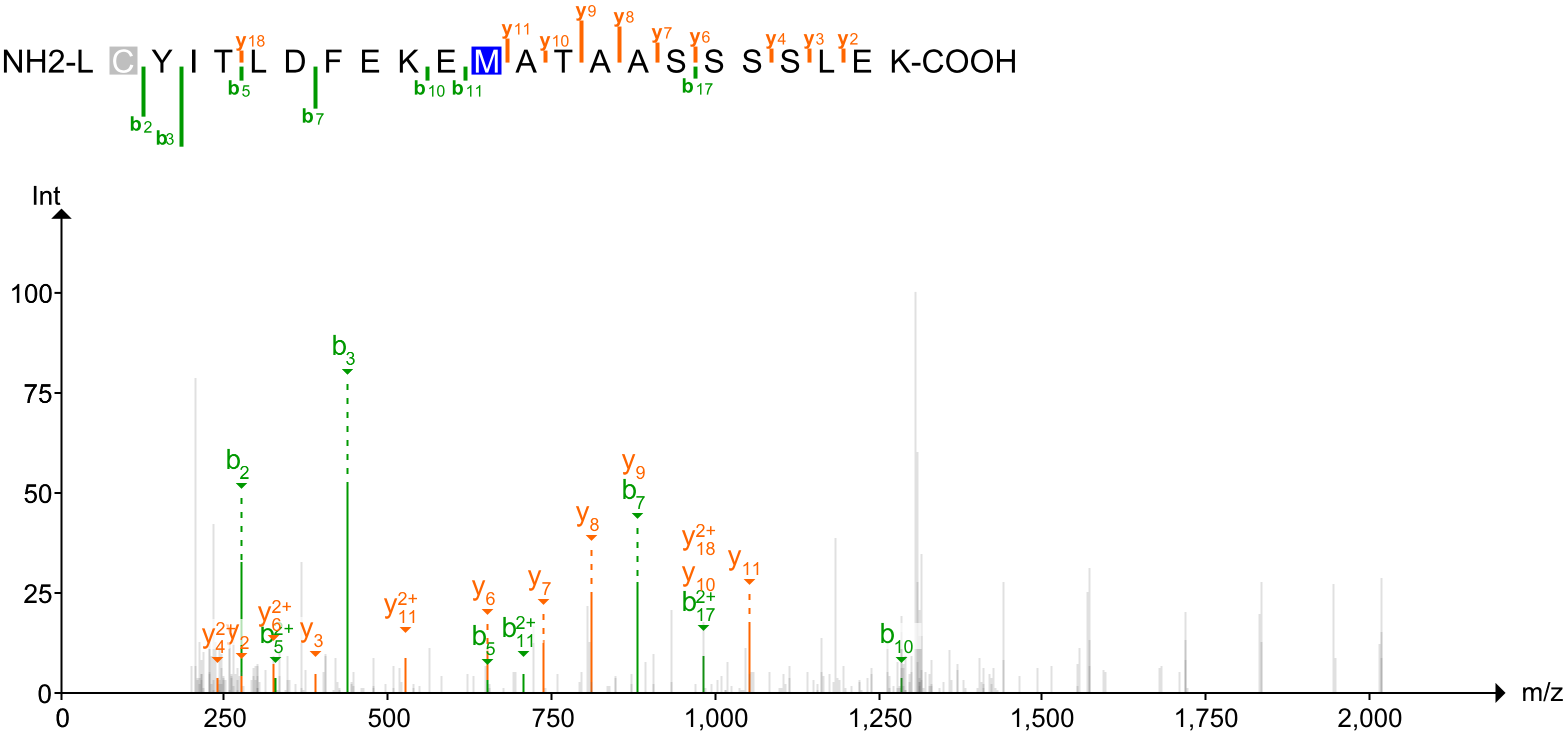

NH2-L C Y I T L D F E K E M A T A A S S S S L E K-COOH
y18 y11 y10 y9 y8 y7 y6 y4 y3 y2
b2 b3 b5 b7 b10 b11 b17
Int
100
75
50
25
0
b3
b2
y9
b7
y8
y2+18
y11
y10
b2+17
y7
y6
b5
b2+11
y2+11
y2+6
b2+5
y3
y2+4
y2
b10
0
250
500
750
1,000
1,250
1,500
1,750
2,000
m/z

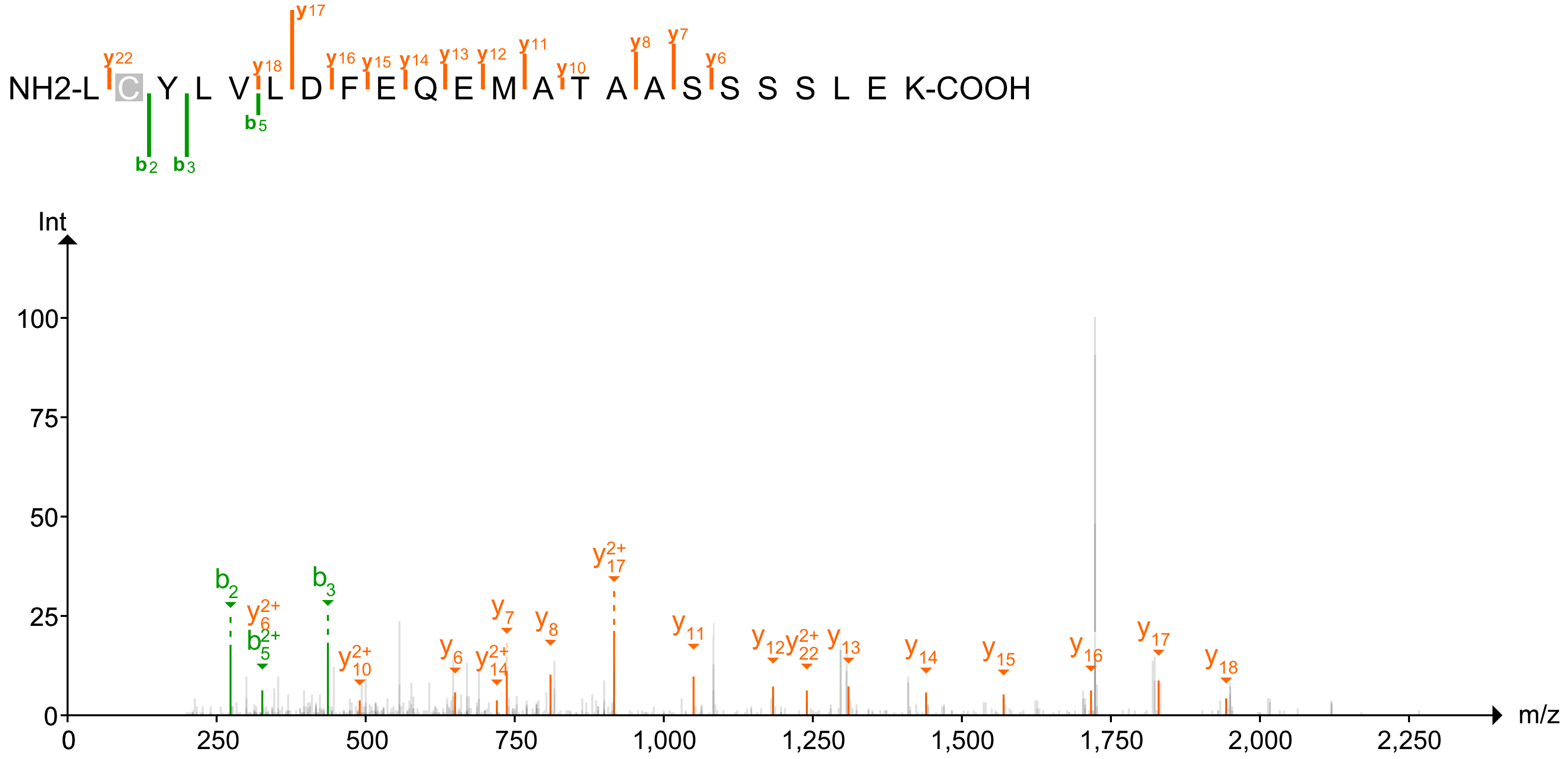

NH2-L C Y L V L D F E Q E M A T A A S S S S L E K-COOH
y22 y18 y17 y16 y15 y14 y13 y12 y11 y10 y8 y7 y6
b2 b3 b5
Int
100
75
50
25
0
m/z
0 250 500 750 1,000 1,250 1,500 1,750 2,000 2,250
b2 b3 y6 2+ b5 2+ y10 2+ y6 y14 2+ y7 y8 y17 2+ y11 y12 y22 2+ y13 y14 y15 y16 y17 y18

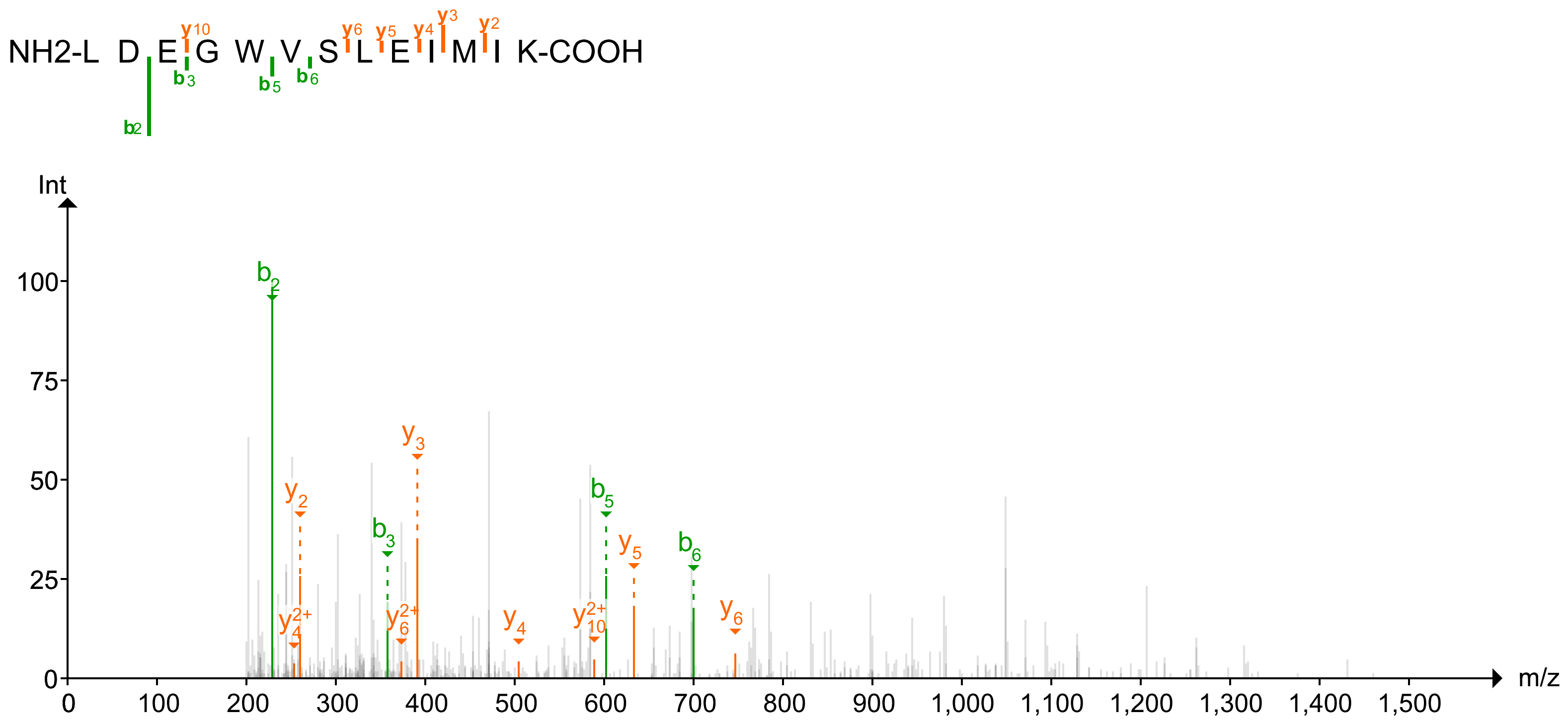

NH2-L D E G W V S L E I M I K-COOH
y10 y6 y5 y4 y3 y2
b2 b3 b5 b6
Int
100
75
50
25
0
b2
y3
y2
b3
b5
y5
b6
y6
y4 2+
y6 2+
y4
y10 2+
0 100 200 300 400 500 600 700 800 900 1,000 1,100 1,200 1,300 1,400 1,500
m/z

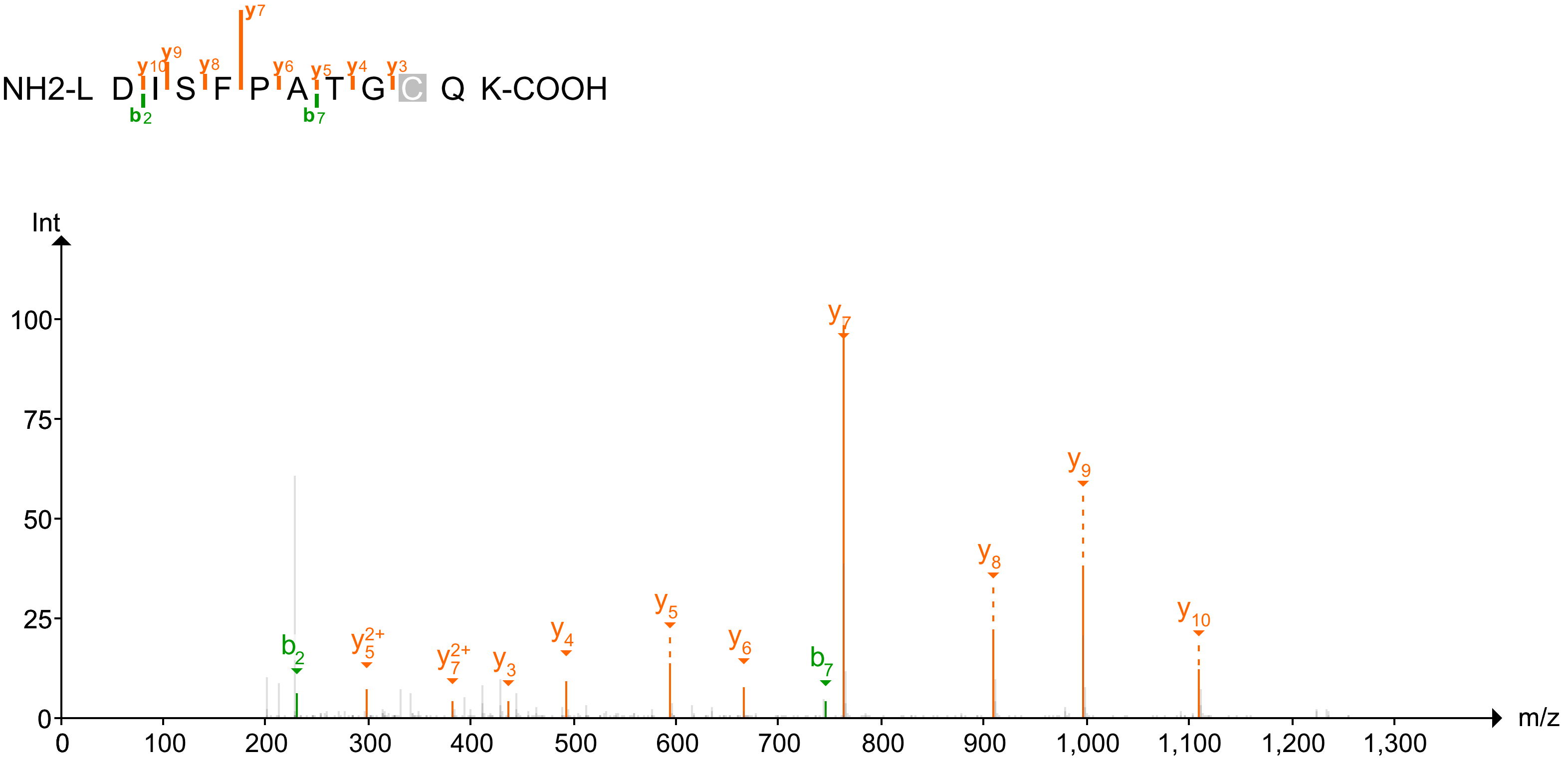

NH2-L D I S F P A T G C Q K-COOH
y7
y9
y10
y8
y6
y5
y4
y3
b2
b7
Int
100
75
50
25
0
m/z
0
100
200
300
400
500
600
700
800
900
1,000
1,100
1,200
1,300
y7
y9
y8
y10
y5
y6
b7
y4
y3
y7 2+
y5 2+
b2

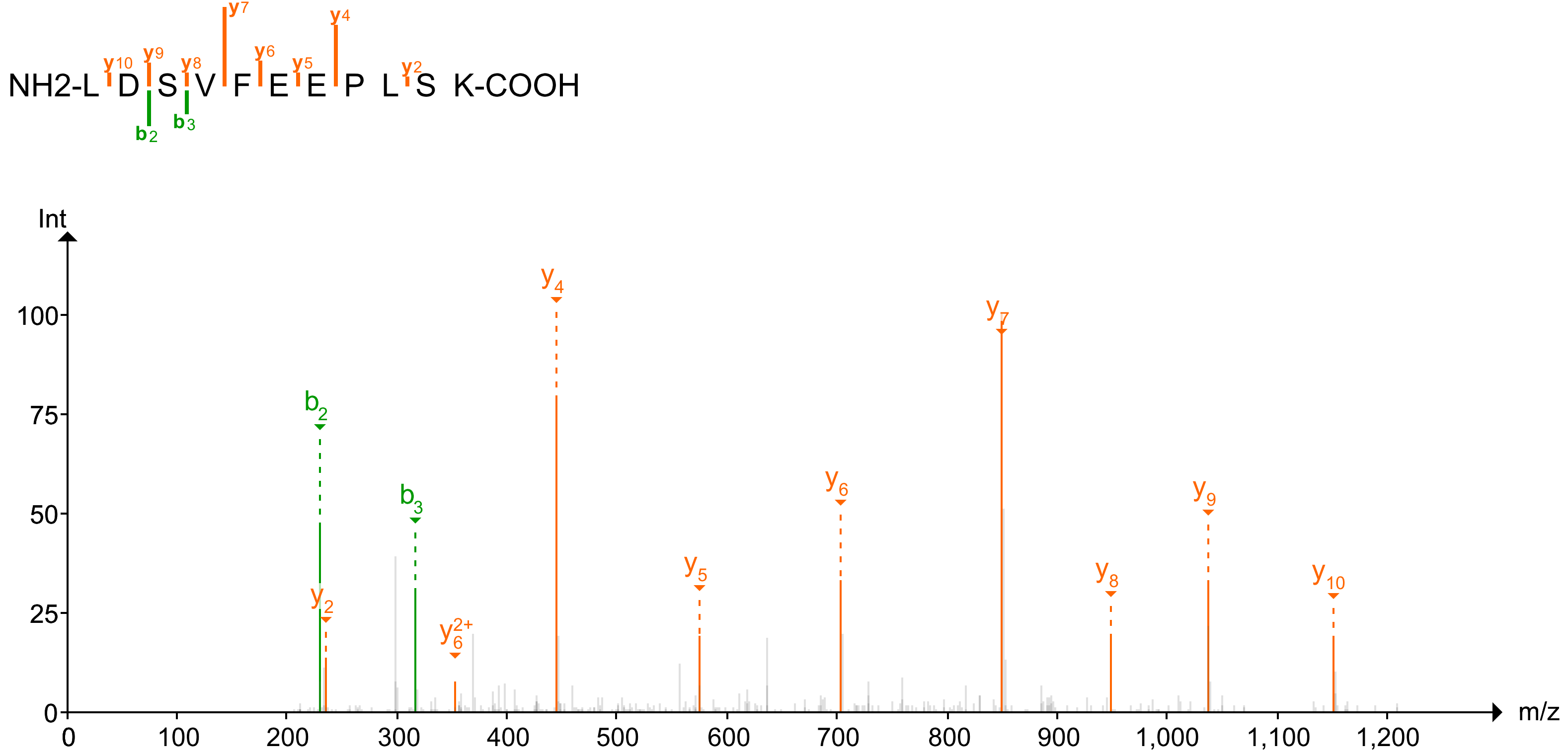

NH2-L D S V F E E P L S K-COOH
y10 y9 y8 y7 y6 y5 y4 y2
b2 b3
Int
m/z
0
25
50
75
100
0
100
200
300
400
500
600
700
800
900
1,000
1,100
1,200
b2
b3
y2
y6 2+
y4
y5
y6
y7
y8
y9
y10

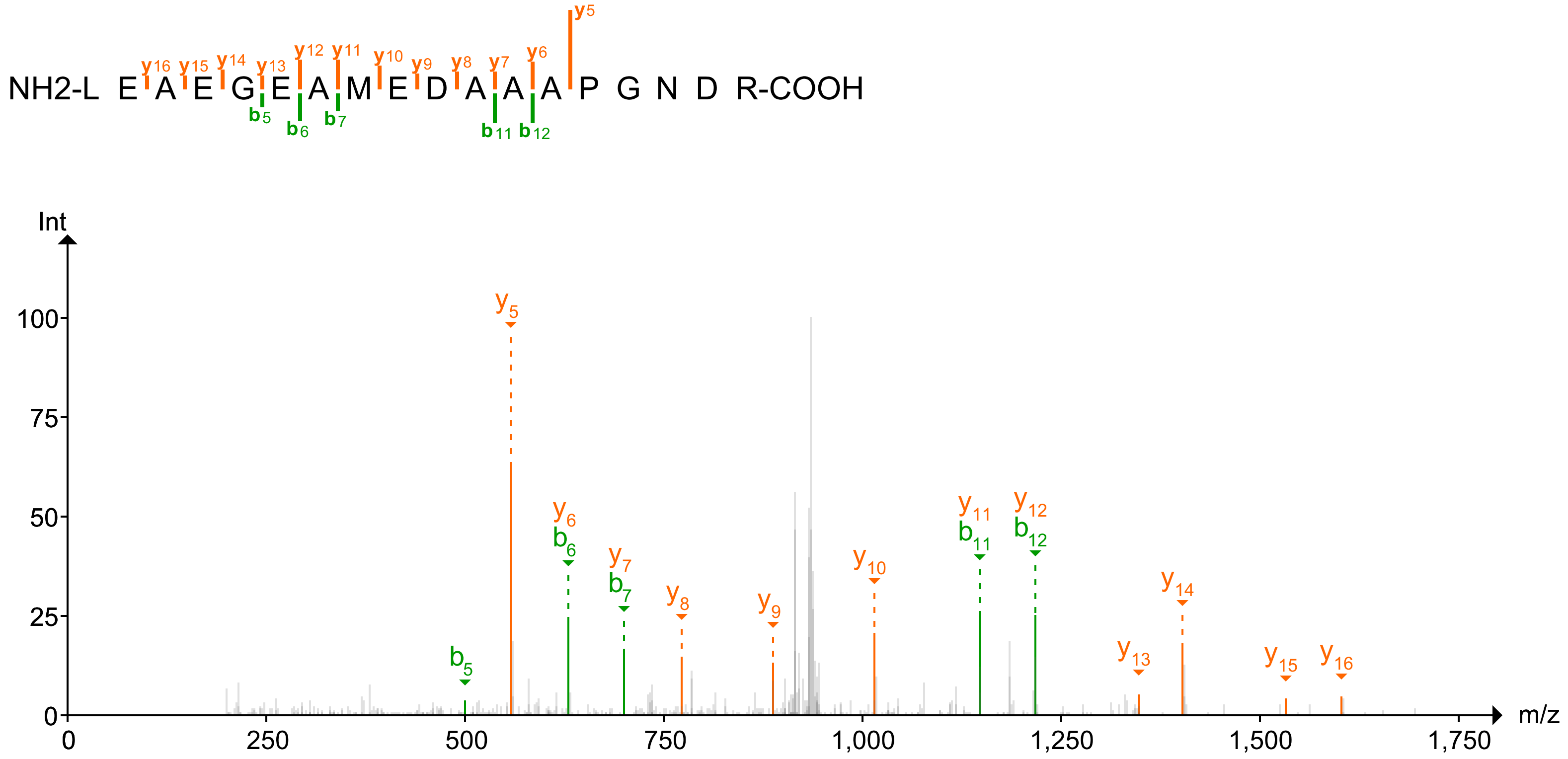
NH2-L E A E G E A M E D A A A P G N D R-COOH
y16 y15 y14 y13 y12 y11 y10 y9 y8 y7 y6 y5
b5 b6 b7 b11 b12
Int
100
75
50
25
0
0
250
500
750
1,000
1,250
1,500
1,750
m/z
y5
y6
b6
y7
b7
b5
y8
y9
y10
y11
b11
y12
b12
y13
y14
y15
y16

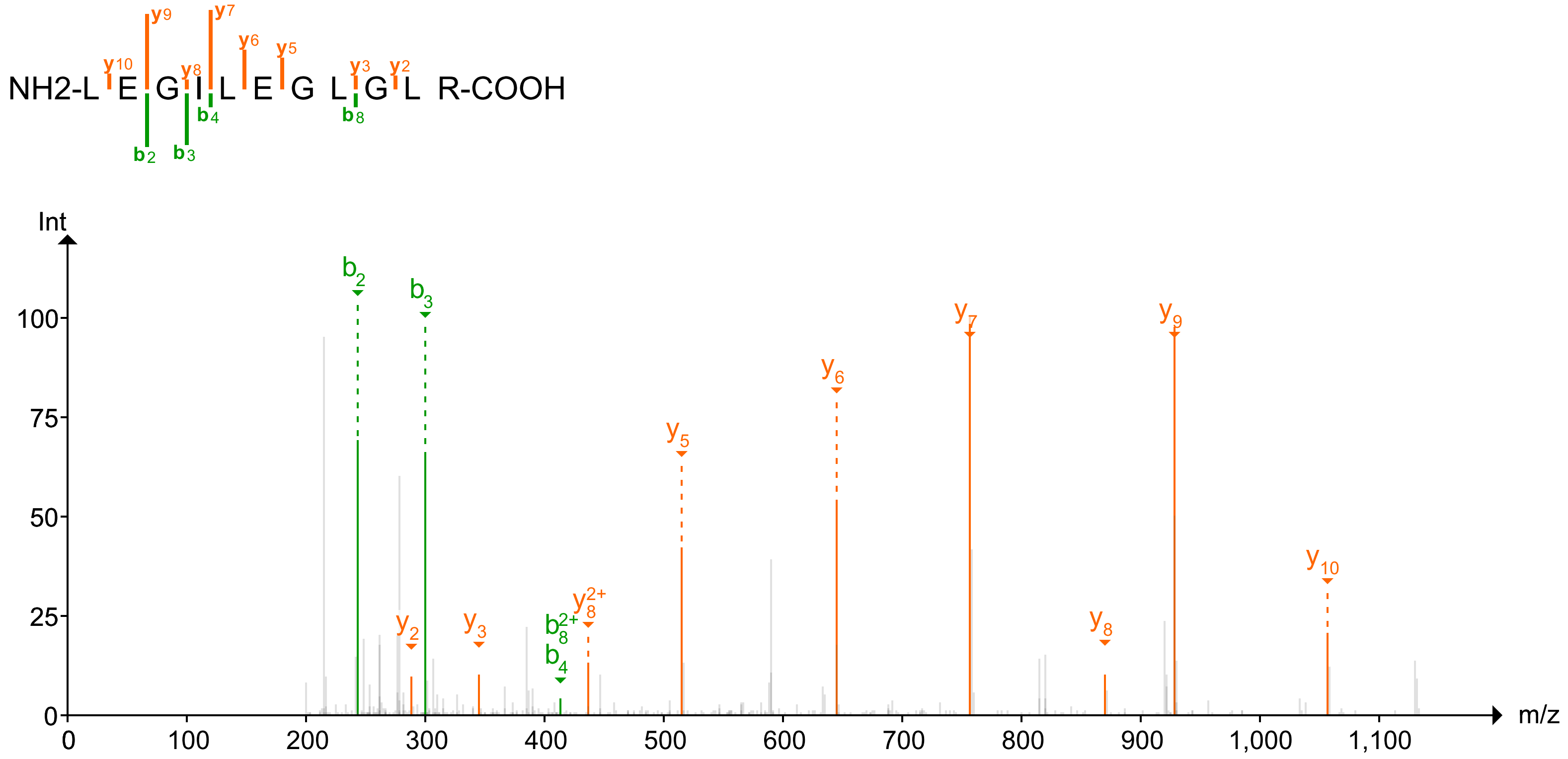

NH2-L E G I L E G L G L R-COOH
y10 y9 y8 y7 y6 y5 y3 y2
b2 b3 b4 b8
Int
m/z
0
25
50
75
100
0 100 200 300 400 500 600 700 800 900 1,000 1,100
$b_2$
$b_3$
$y_2$
$y_3$
$b_8^{2+}$
$b_4$
$y_8^{2+}$
$y_5$
$y_6$
$y_7$
$y_8$
$y_9$
$y_{10}$

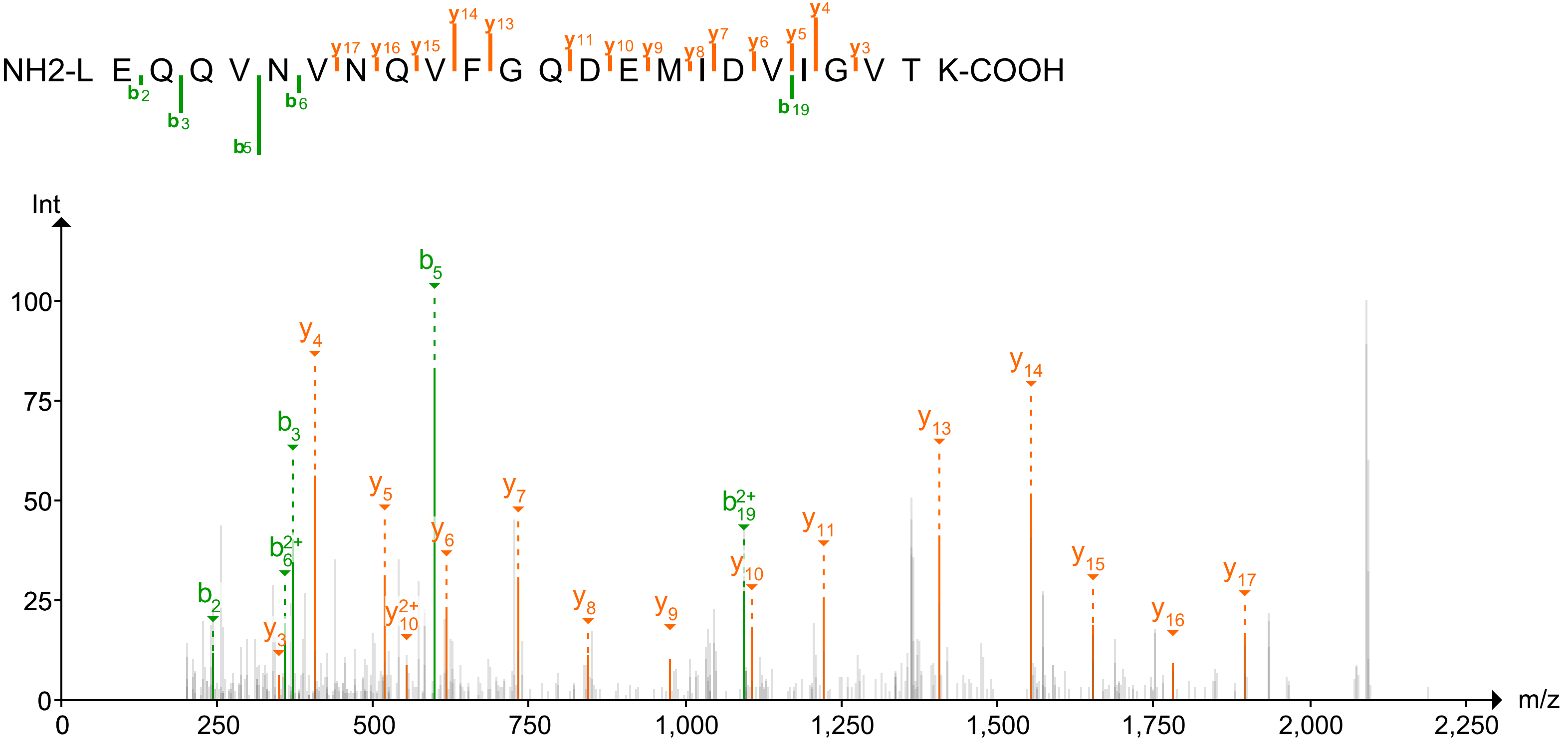

NH2-L E Q Q V N V N Q V F G Q D E M I D V I G V T K-COOH
Int
m/z
0
25
50
75
100
250
500
750
1,000
1,250
1,500
1,750
2,000
2,250

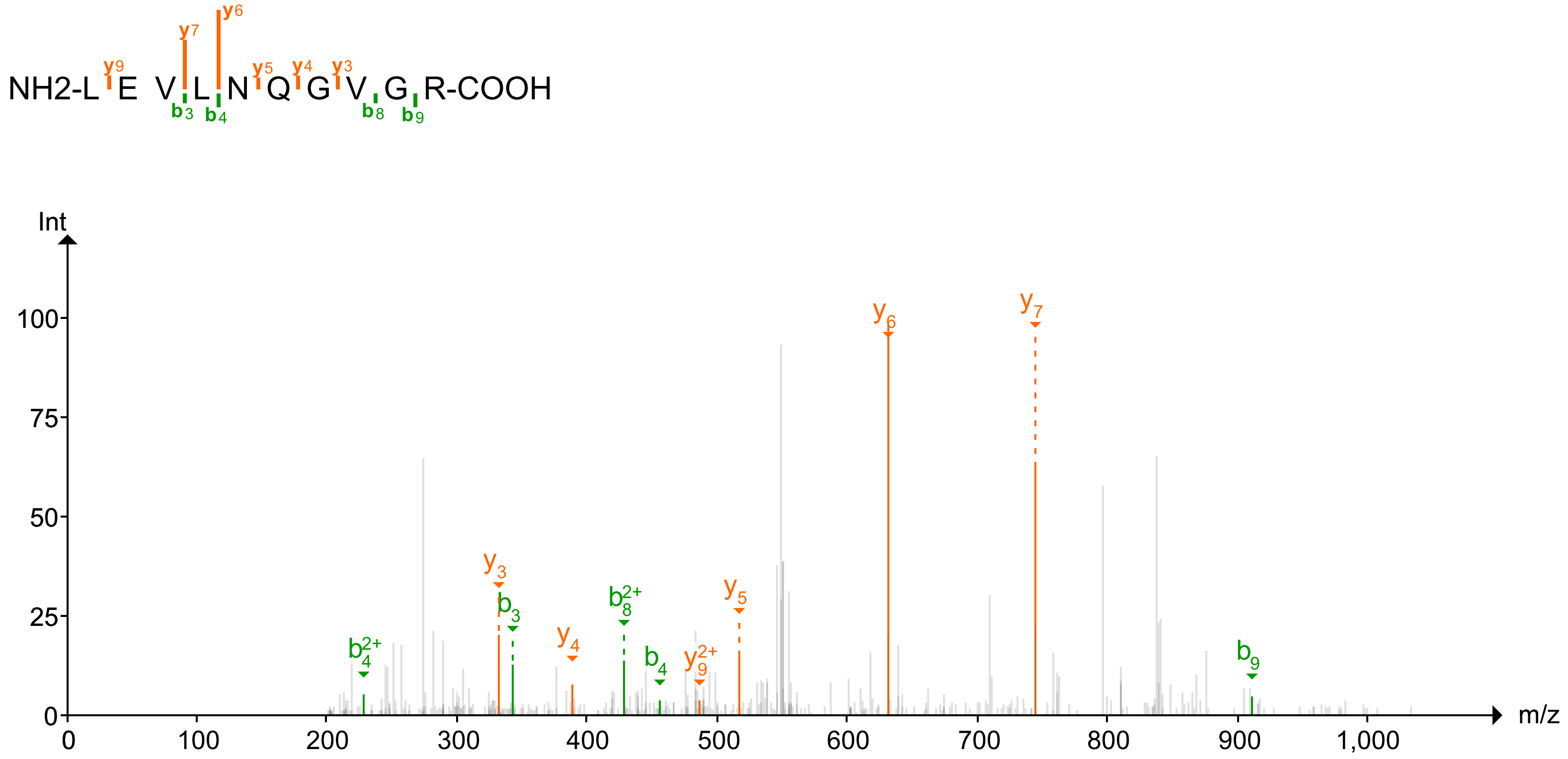
NH2-L E V L N Q G V G R-COOH
y9 y7 y6 y5 y4 y3
b3 b4 b8 b9
Int
100
75
50
25
0
0
100
200
300
400
500
600
700
800
900
1,000
m/z
y6
y7
y3
b3
b4 2+
b8 2+
y4
b4
y9 2+
y5
b9

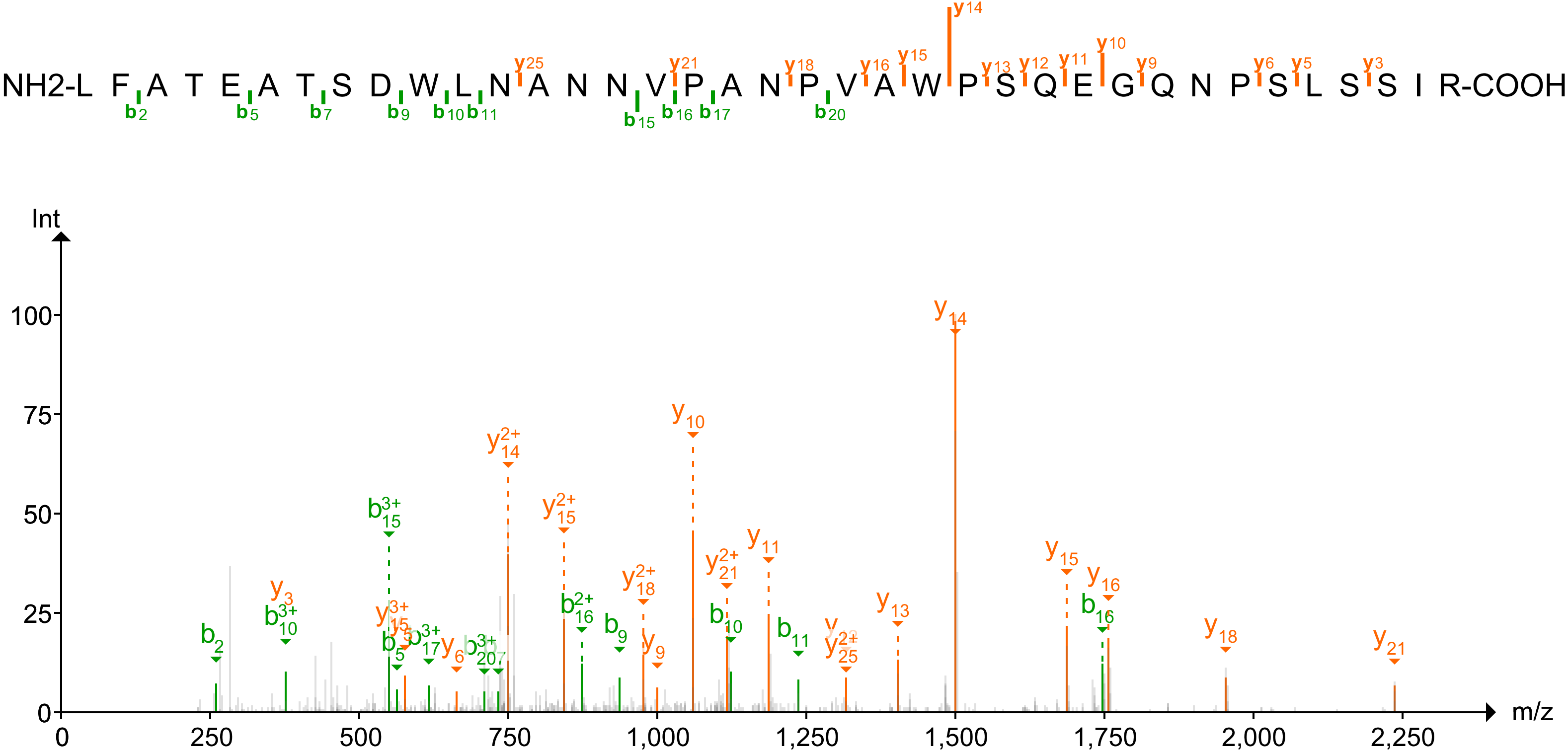

NH2-L F A T E A T S D W L N A N N V P A N P V A W P S Q E G Q N P S L S S I R-COOH
b2 b5 b7 b9 b10 b11 b15 b16 b17 b20
y25 y21 y18 y16 y15 y14 y13 y12 y11 y10 y9 y6 y5 y3
Int
m/z
0
25
50
75
100
250
500
750
1,000
1,250
1,500
1,750
2,000
2,250

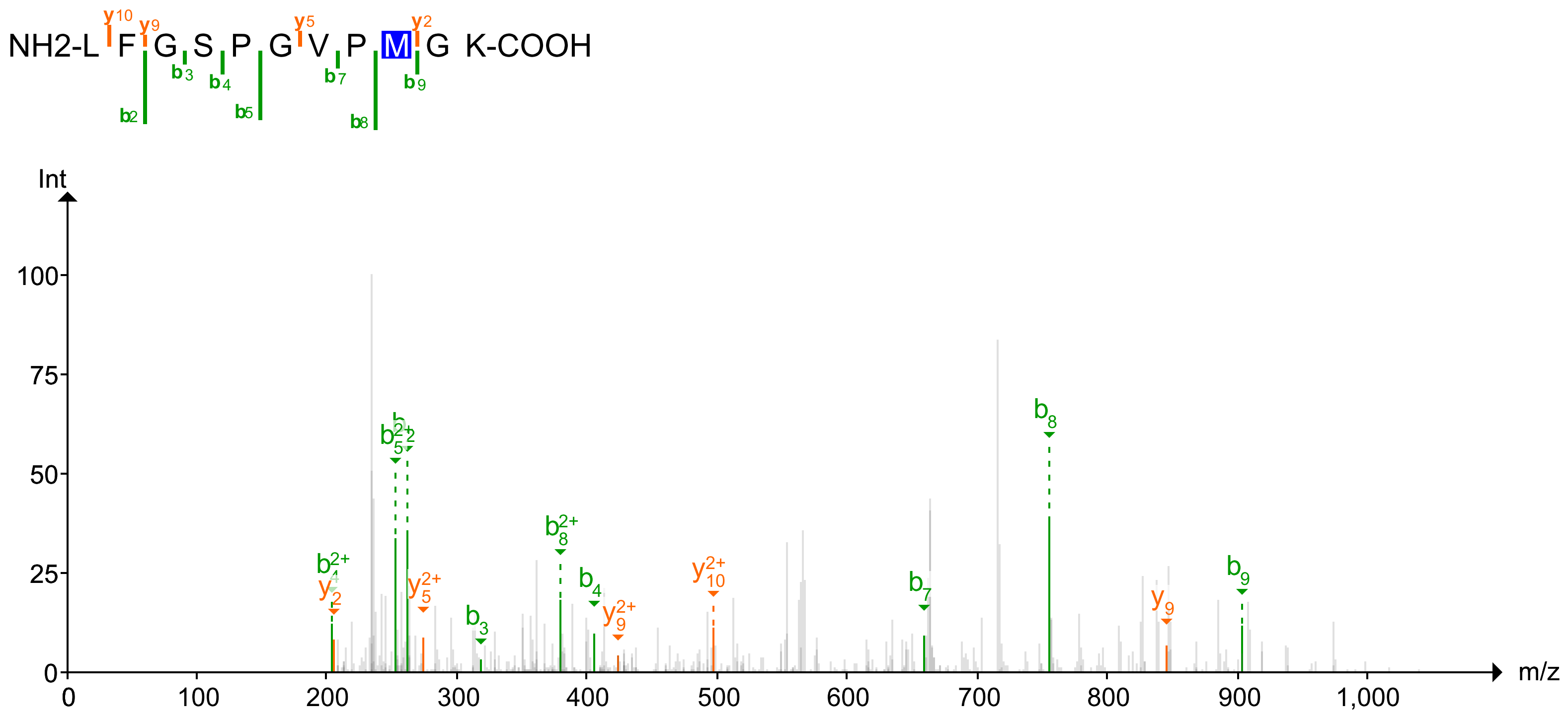

NH2-L F G S P G V P M G K-COOH
y10 y9 y5 y2
b2 b3 b4 b5 b7 b8 b9
Int
100
75
50
25
0
m/z
0
100
200
300
400
500
600
700
800
900
1,000

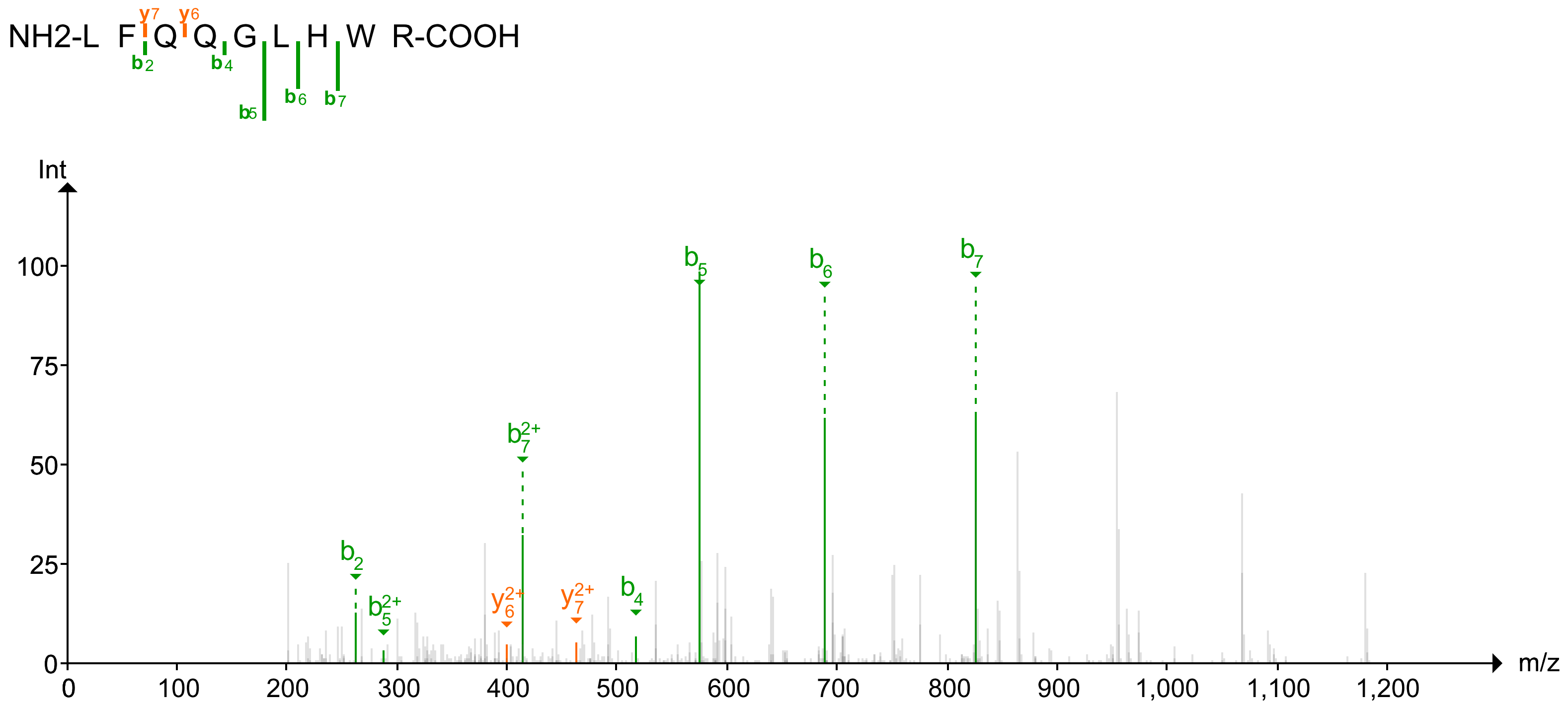

NH2-L F Q Q G L H W R-COOH
y7
y6
b2
b4
b5
b6
b7
Int
100
75
50
25
0
m/z
0
100
200
300
400
500
600
700
800
900
1,000
1,100
1,200
b5
b6
b7
b7 2+
b2
b5 2+
y6 2+
y7 2+
b4

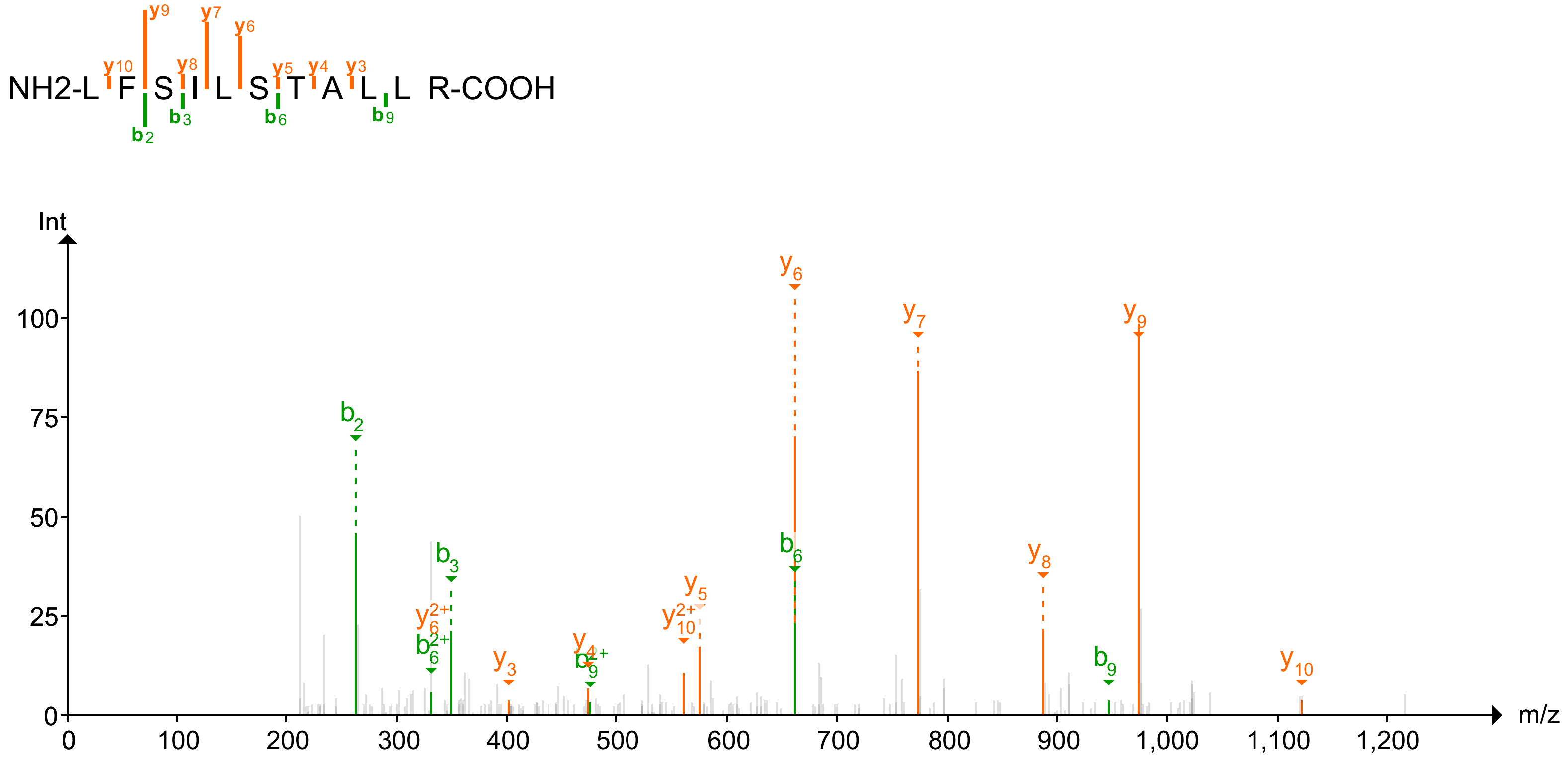
NH2-L F S I L S T A L L R-COOH
y10
y9
y8
y7
y6
y5
y4
y3
b2
b3
b6
b9
Int
100
75
50
25
0
m/z
0
100
200
300
400
500
600
700
800
900
1,000
1,100
1,200
y6
y7
y9
b2
b3
y6 2+
b6 2+
y3
y4
b9 2+
y10 2+
y5
b6
y8
b9
y10

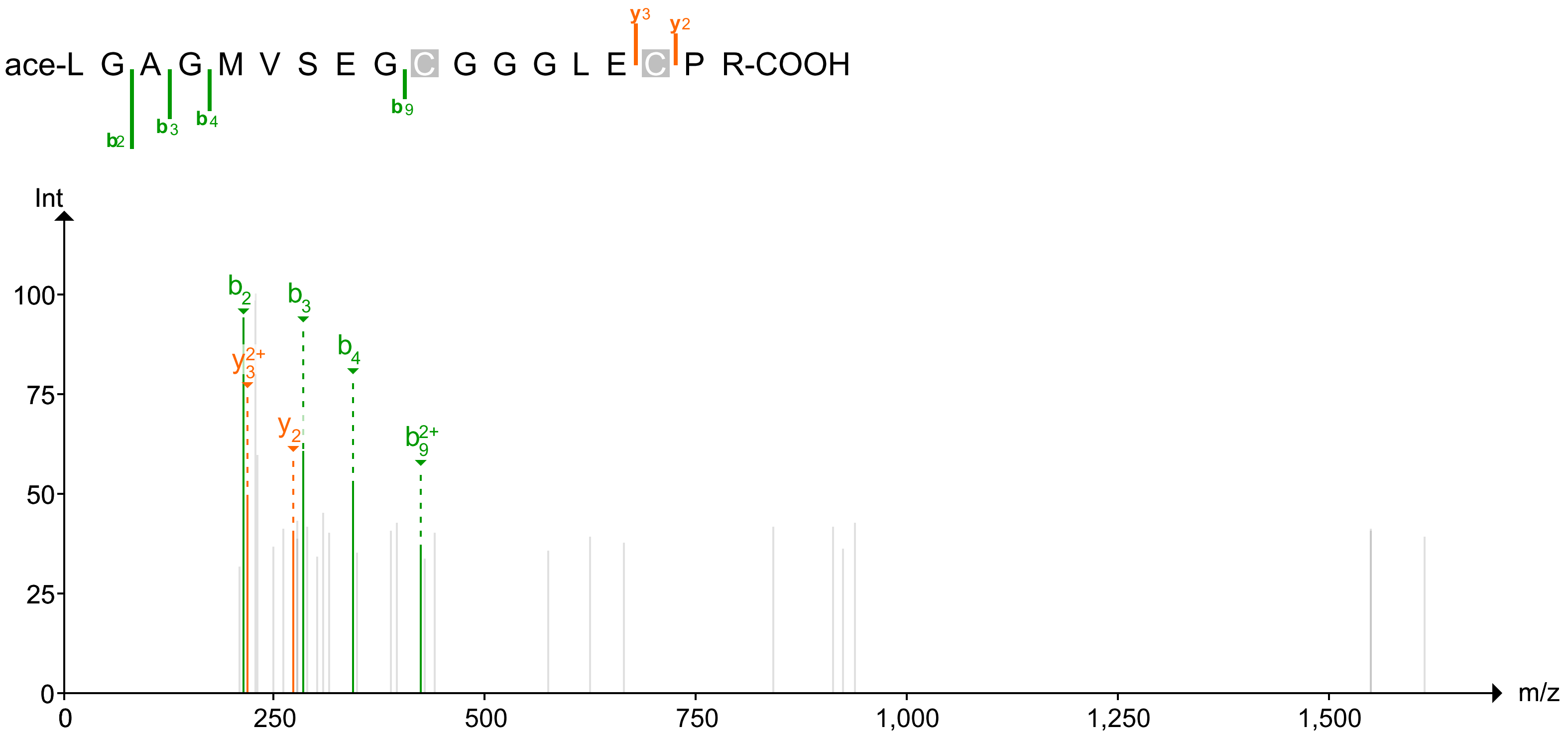
ace-L G A G M V S E G C G G G L E C P R-COOH
b2 b3 b4 b9 y3 y2
Int
m/z
0
25
50
75
100
250
500
750
1,000
1,250
1,500
b2
b3
y3 2+
b4
y2
b9 2+

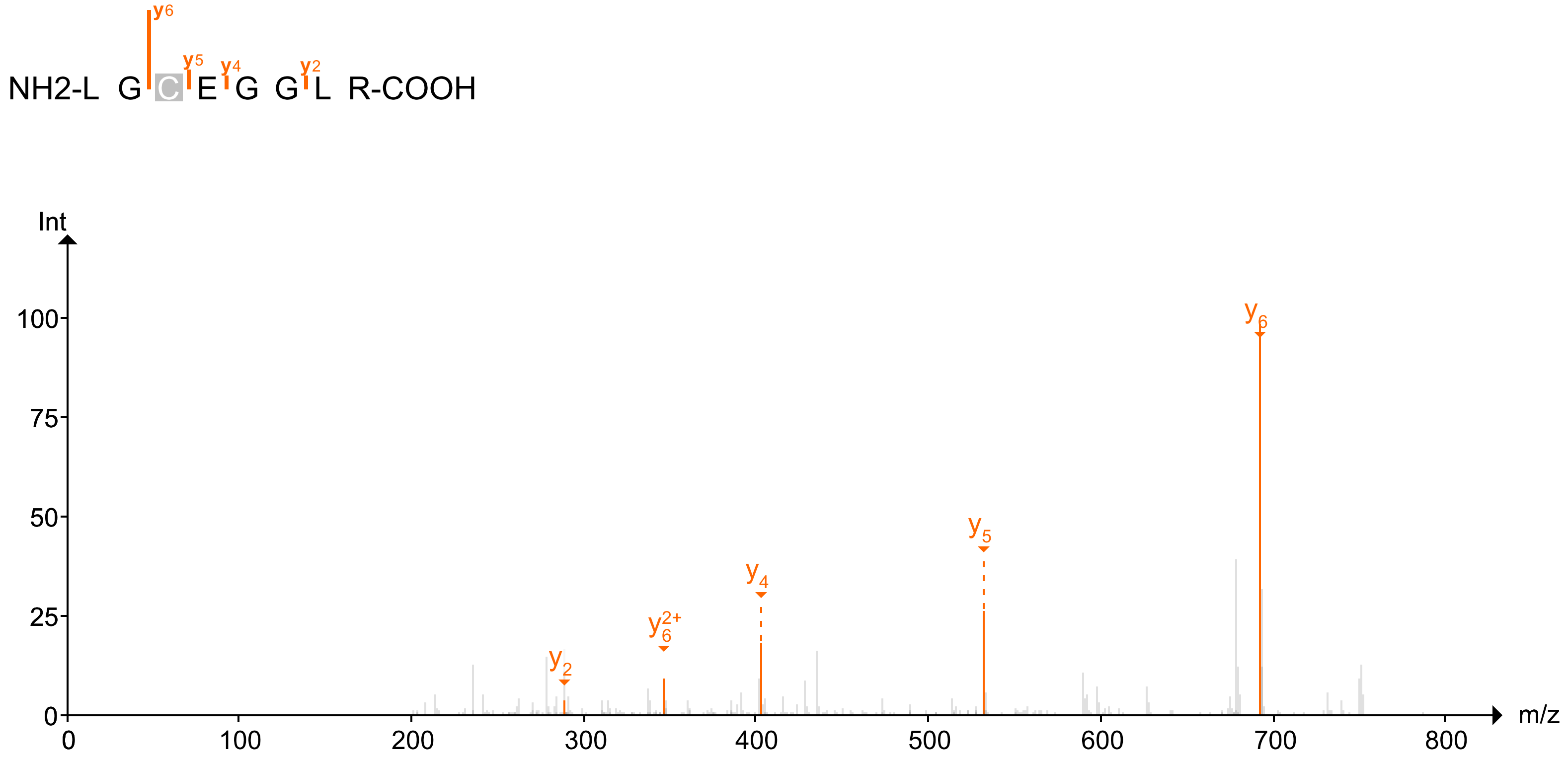

NH2-L G C E G G L R-COOH
y6
y5
y4
y2
Int
100
75
50
25
0
y6
y5
y4
y6 2+
y2
0
100
200
300
400
500
600
700
800
m/z

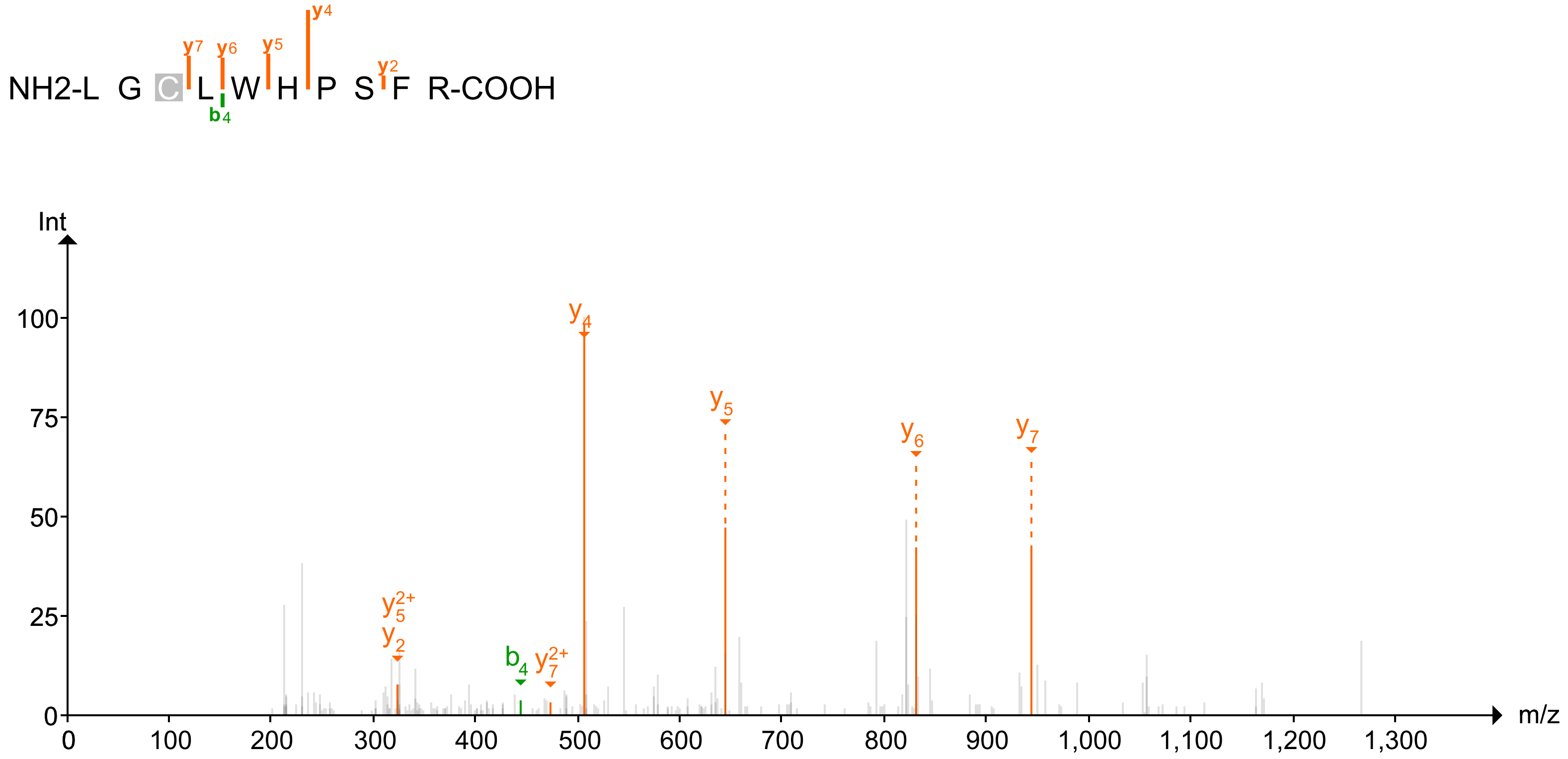

NH2-L G C L W H P S F R-COOH
y7 y6 y5 y4 y2
b4
Int
100
75
50
25
0
m/z
0 100 200 300 400 500 600 700 800 900 1,000 1,100 1,200 1,300
y4
y5
y6
y7
y5 2+
y2
b4
y7 2+

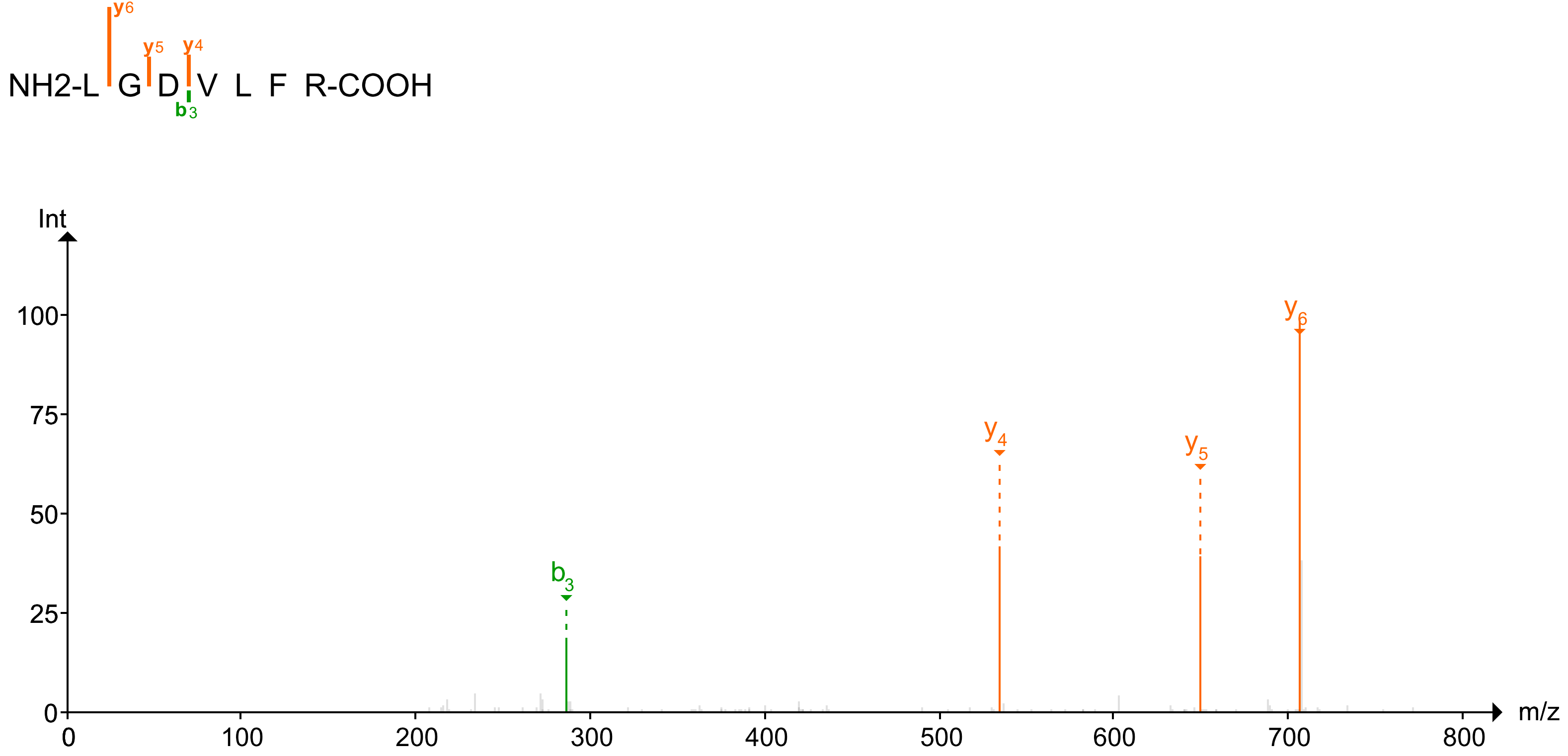

NH2-L G D V L F R-COOH
y6
y5
y4
b3
Int
100
75
50
25
0
m/z
0
100
200
300
400
500
600
700
800

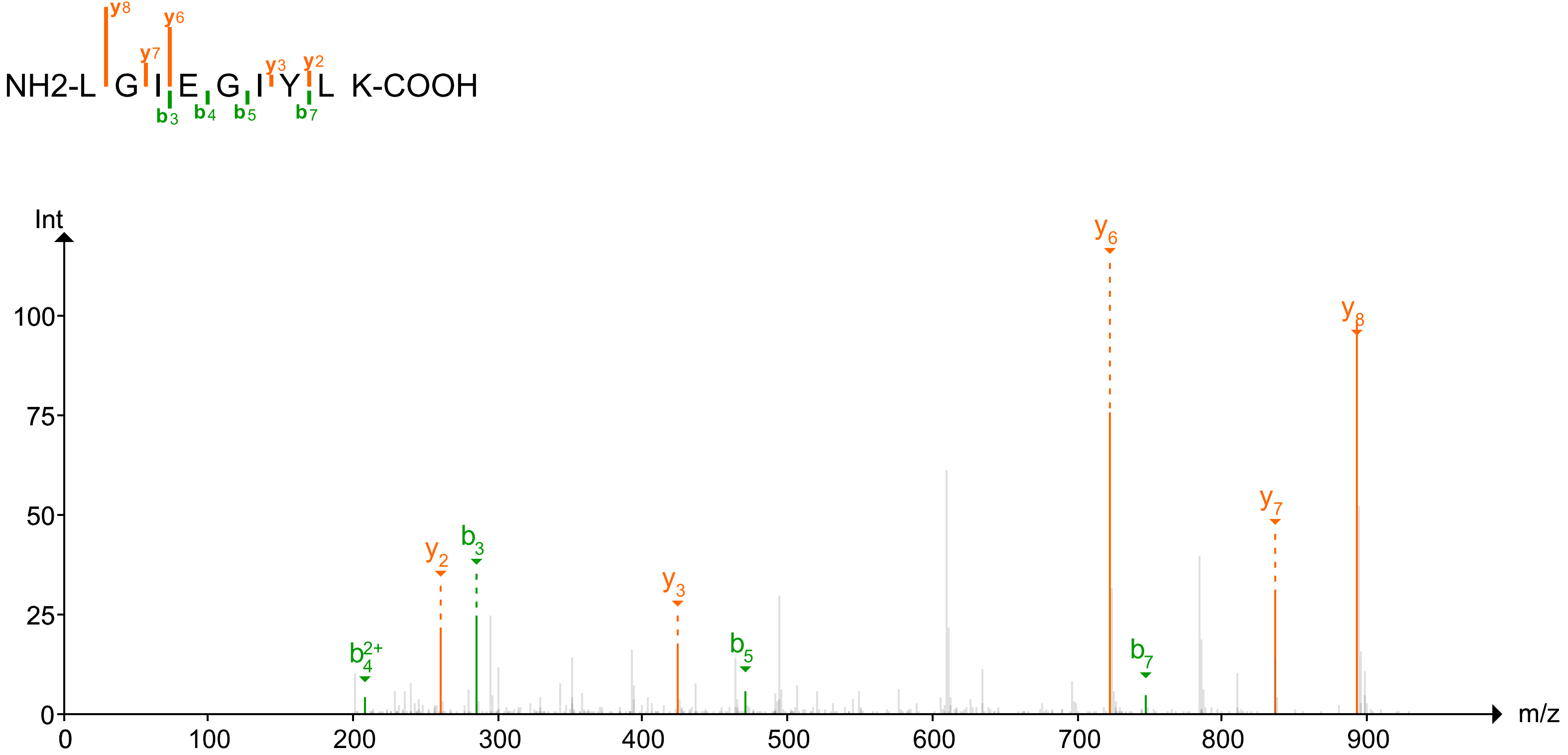

NH2-L G I E G I Y L K-COOH
y8
y7
y6
y3
y2
b3
b4
b5
b7
Int
100
75
50
25
0
m/z
0
100
200
300
400
500
600
700
800
900
$b_4^{2+}$
$y_2$
$b_3$
$y_3$
$b_5$
$y_6$
$b_7$
$y_7$
$y_8$

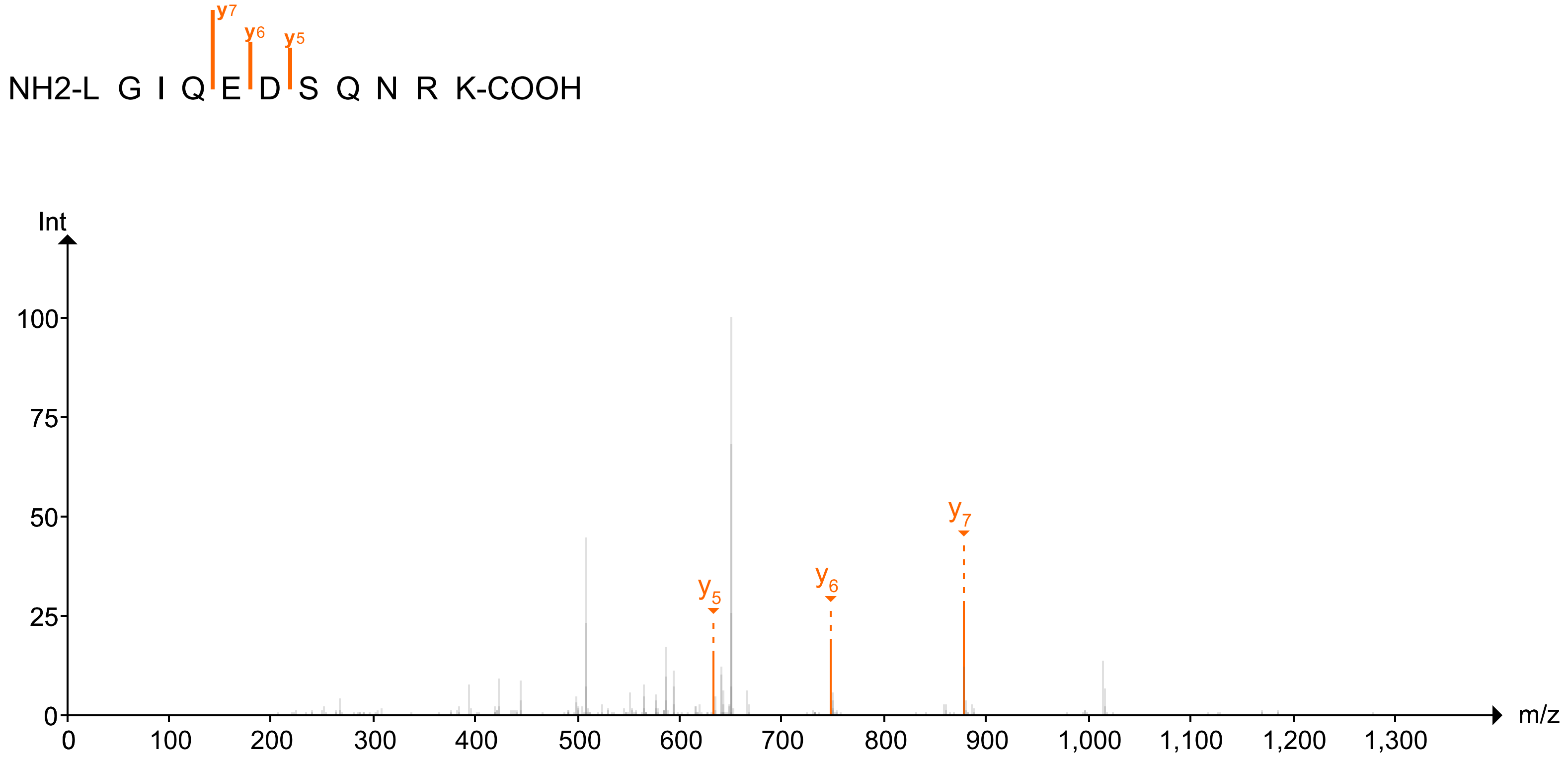

NH2-L G I Q E D S Q N R K-COOH
y7
y6
y5
Int
100
75
50
25
0
m/z
0
100
200
300
400
500
600
700
800
900
1,000
1,100
1,200
1,300

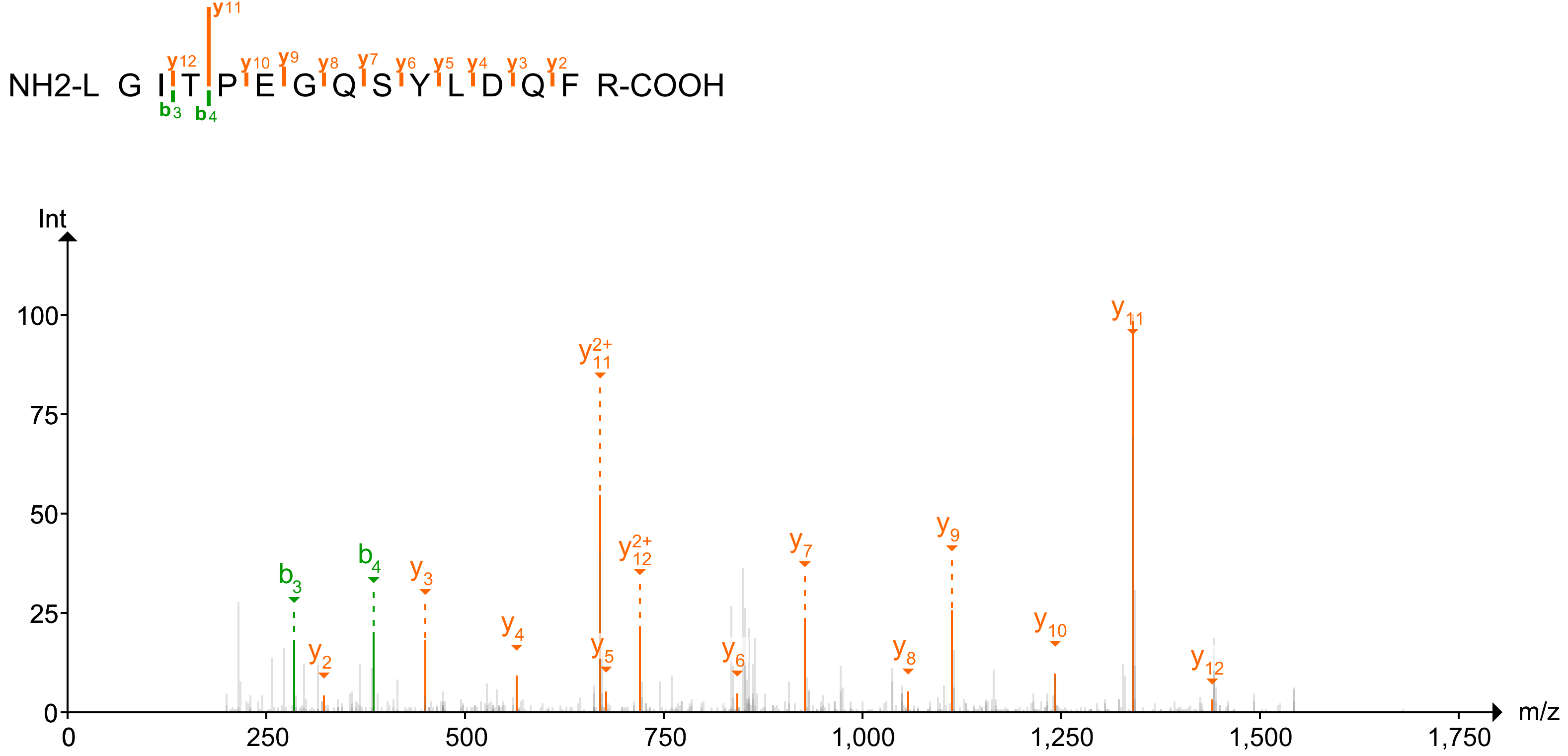

NH2-L G I T P E G Q S Y L D Q F R-COOH
y11
y12
y10
y9
y8
y7
y6
y5
y4
y3
y2
b3
b4
Int
100
75
50
25
0
m/z
0
250
500
750
1,000
1,250
1,500
1,750
b3
b4
y3
y2
y4
y11 2+
y5
y12 2+
y6
y7
y8
y9
y10
y11
y12

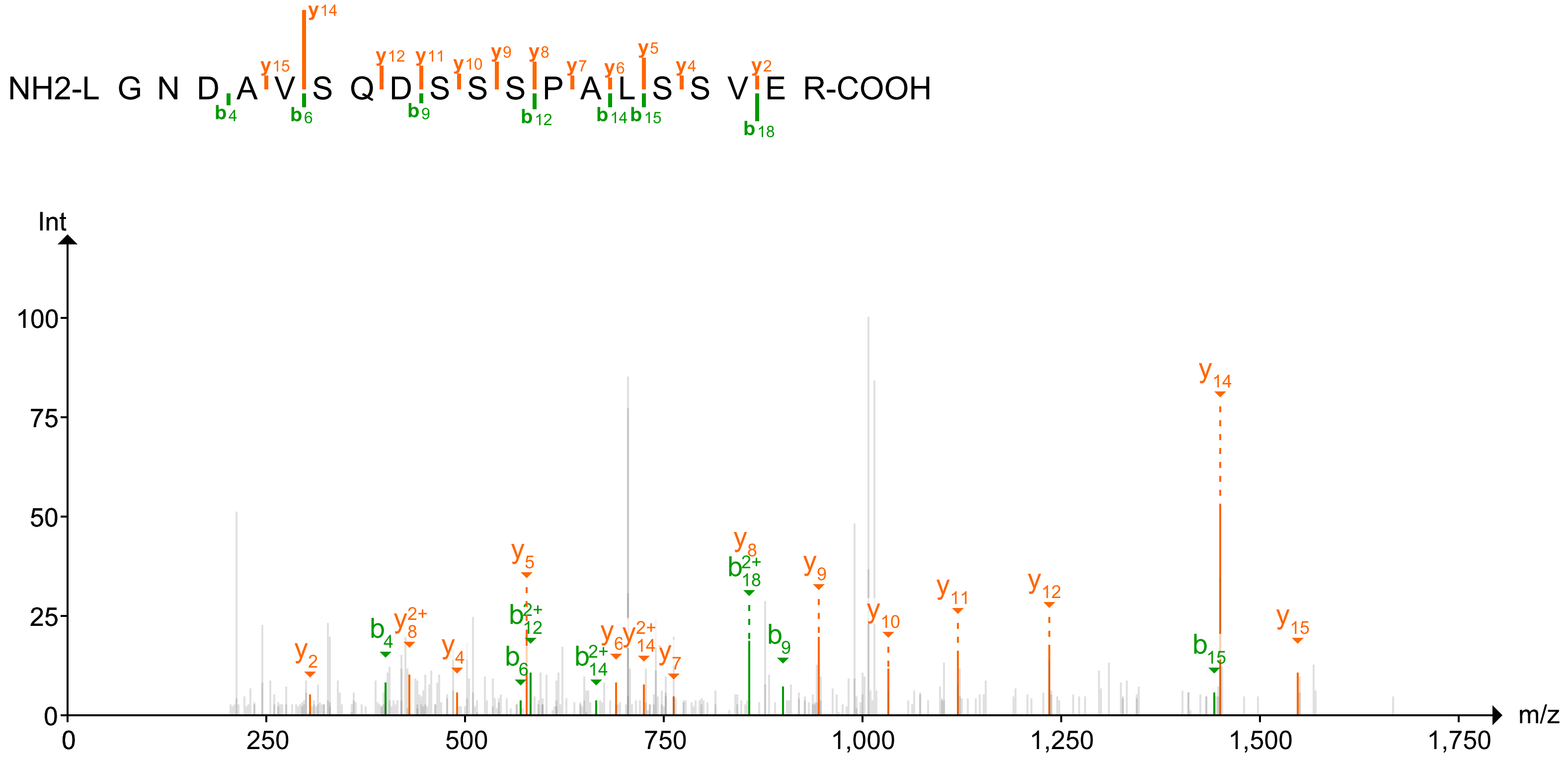

NH2-L G N D A V S Q D S S S P A L S S V E R-COOH
y14 y15 y12 y11 y10 y9 y8 y7 y6 y5 y4 y2
b4 b6 b9 b12 b14 b15 b18
Int
100
75
50
25
0
m/z
0
250
500
750
1,000
1,250
1,500
1,750
y14
y15
y12
y11
y10
y9
y8
b18 2+
y5
b4
y8 2+
y2
y4
b12 2+
b6
b14 2+
y6
y14 2+
y7
b9
b15

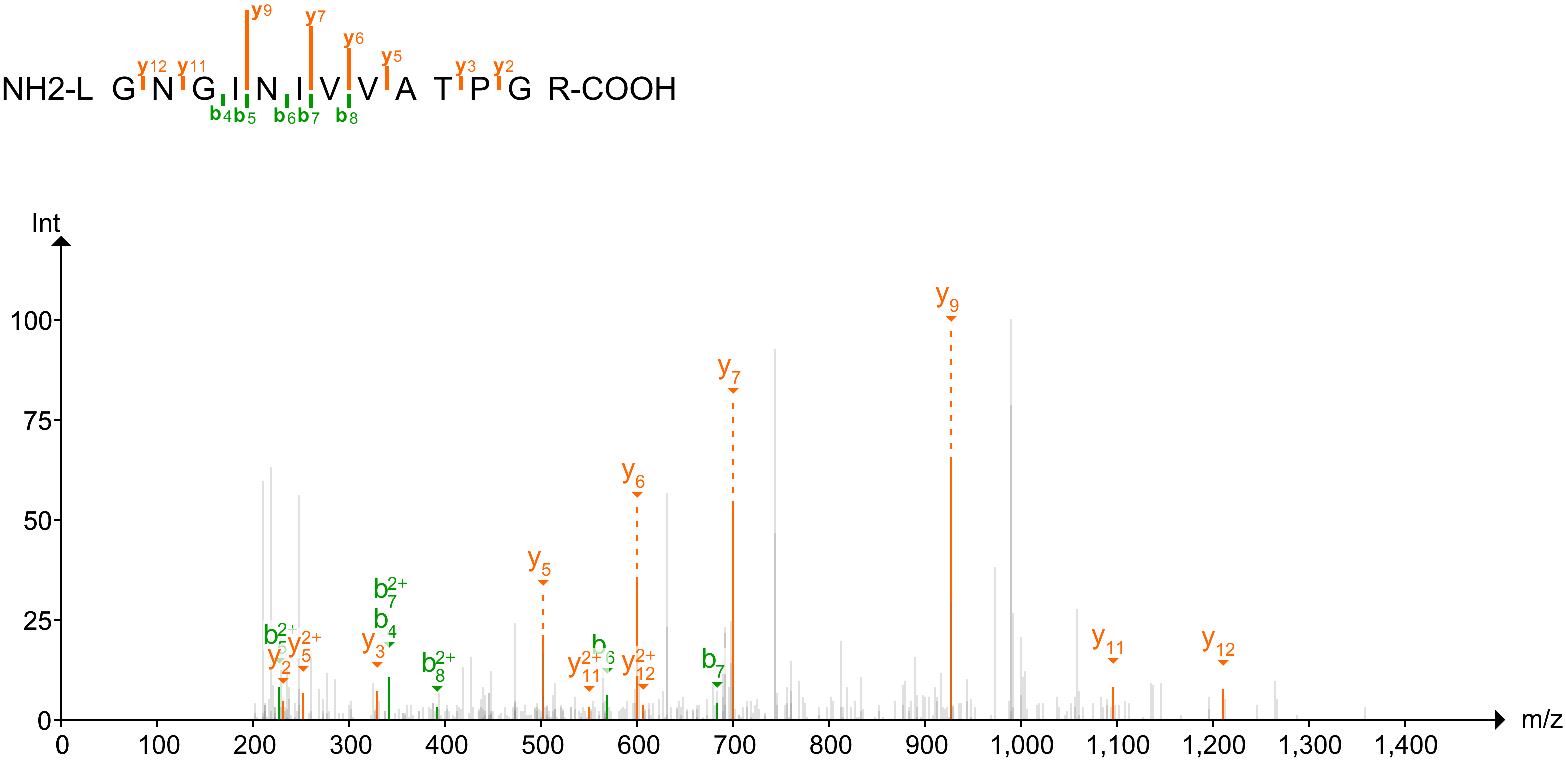

NH2-L G N G I N I V V A T P G R-COOH
y12 y11 y9 y7 y6 y5 y3 y2
b4 b5 b6 b7 b8
Int
100
75
50
25
0
m/z
0
100
200
300
400
500
600
700
800
900
1,000
1,100
1,200
1,300
1,400

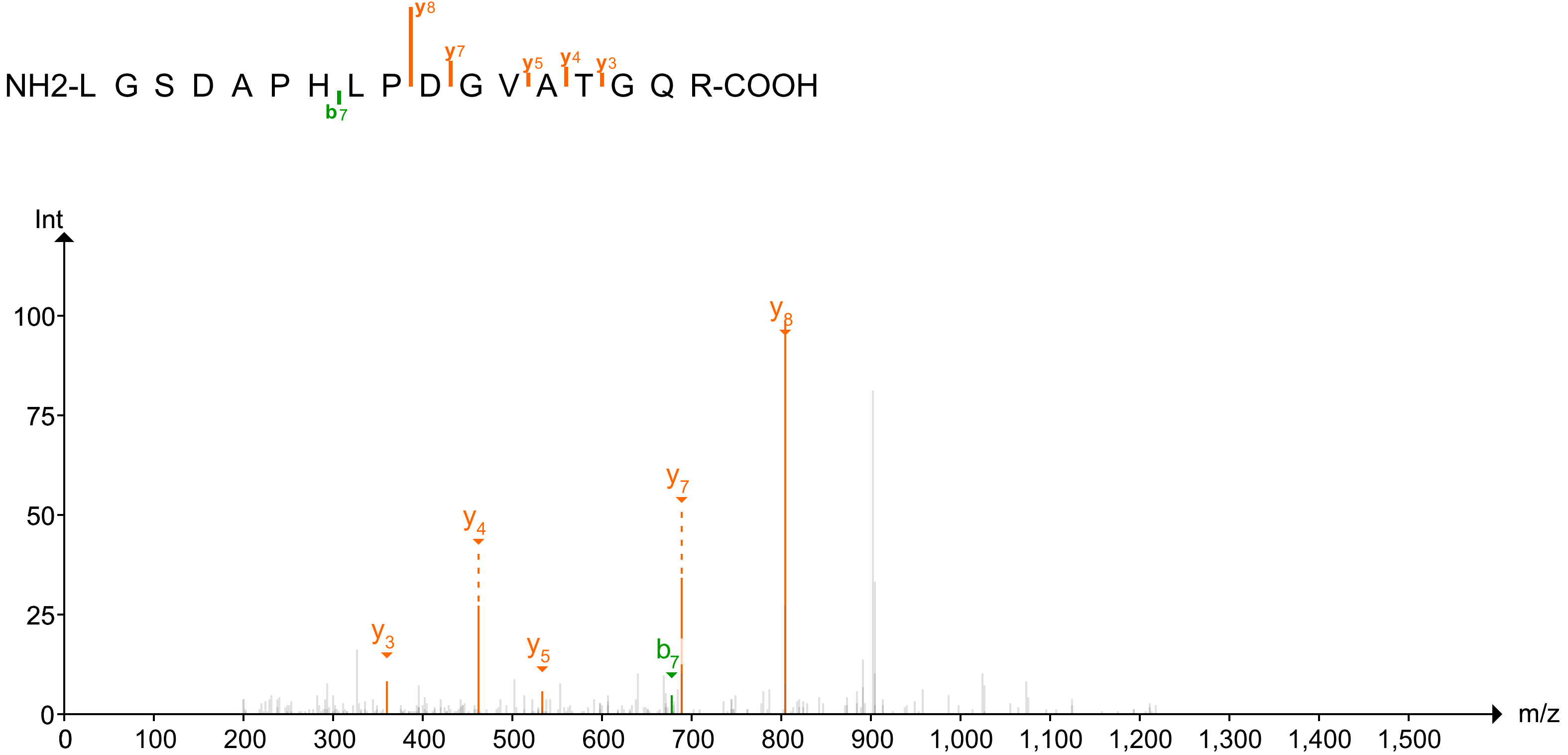

NH2-L G S D A P H L P D G V A T G Q R-COOH
b7
y8
y7
y5
y4
y3
Int
m/z
0
25
50
75
100
0
100
200
300
400
500
600
700
800
900
1,000
1,100
1,200
1,300
1,400
1,500

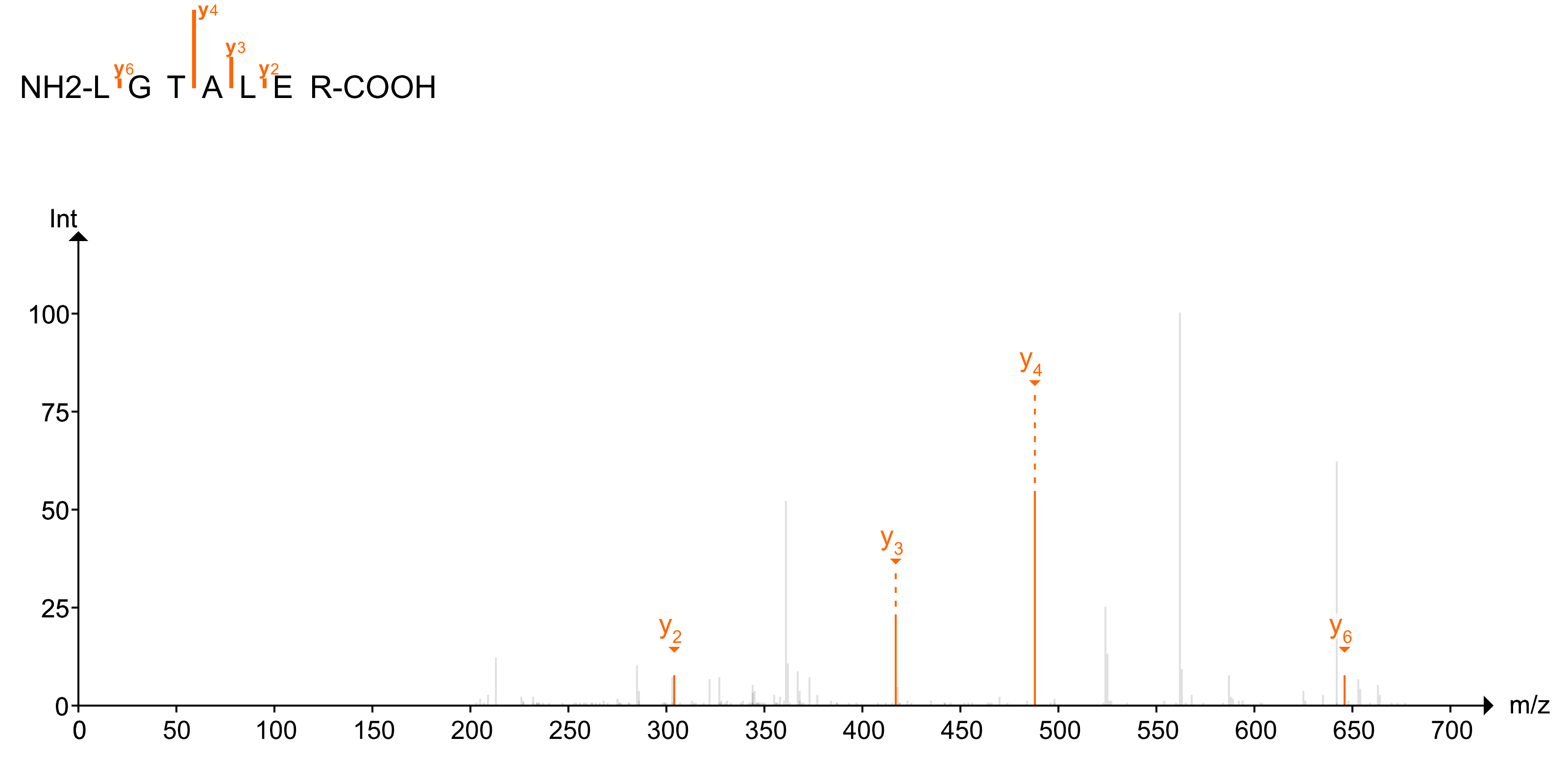

y4
y3
y6
y2
NH2-L G T A L E R-COOH
Int
100
75
50
25
0
y4
y3
y2
y6
0
50
100
150
200
250
300
350
400
450
500
550
600
650
700
m/z

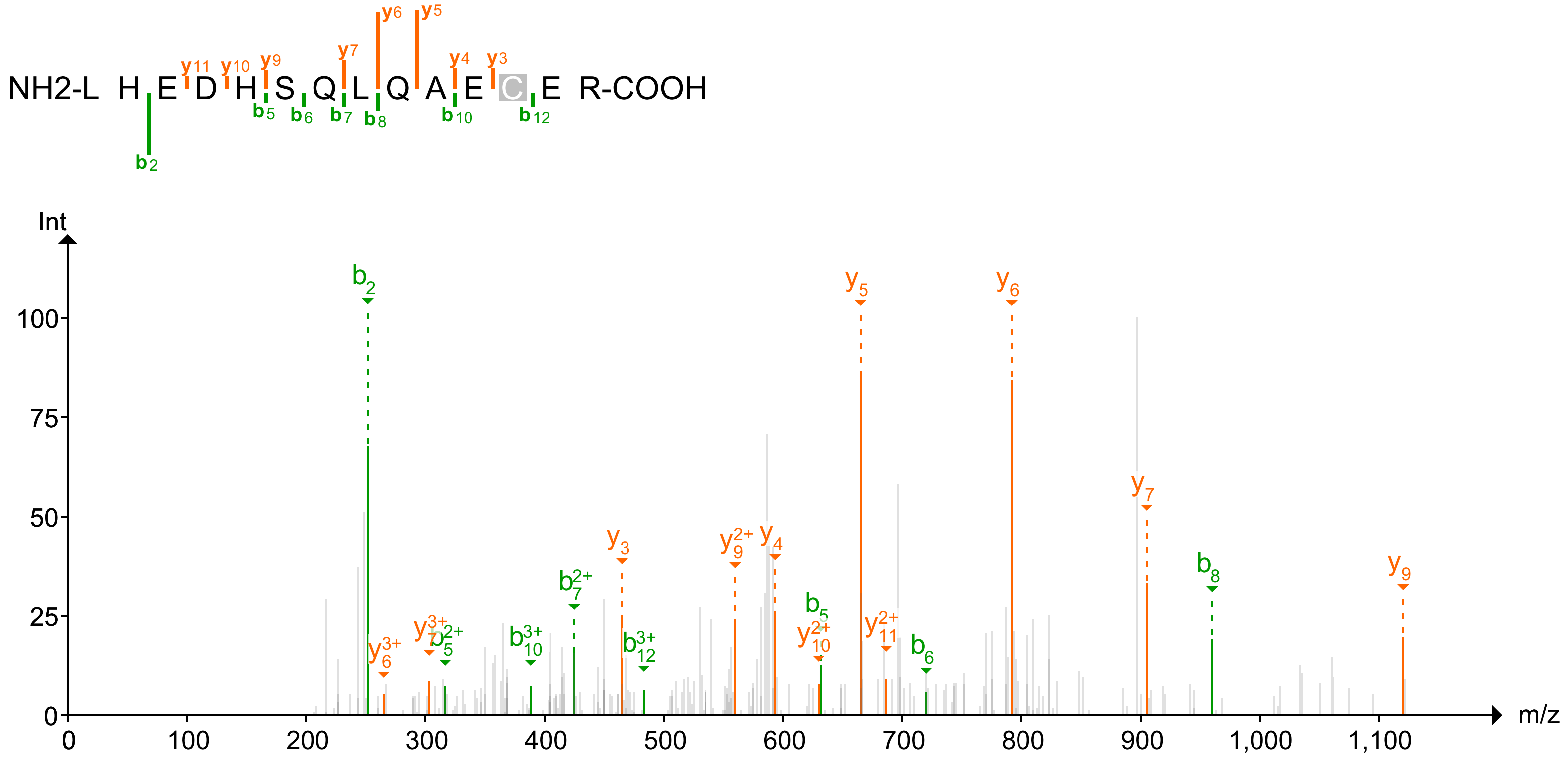

NH2-L H E D H S Q L Q A E C E R-COOH
Int
m/z

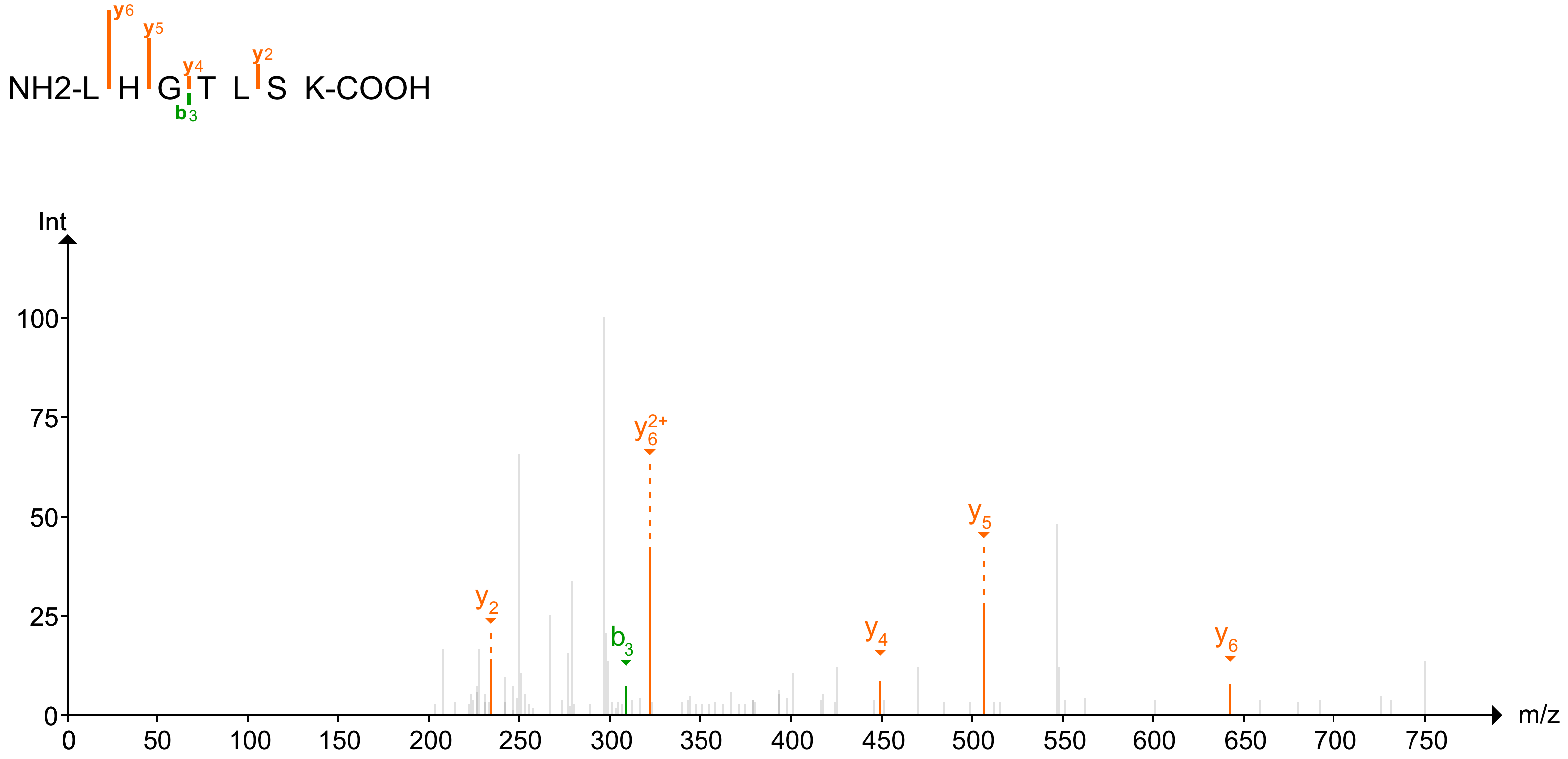

NH2-L H G T L S K-COOH
y6
y5
y4
y2
b3
Int
100
75
50
25
0
m/z
0
50
100
150
200
250
300
350
400
450
500
550
600
650
700
750
y2
b3
$y_6^{2+}$
y4
y5
y6

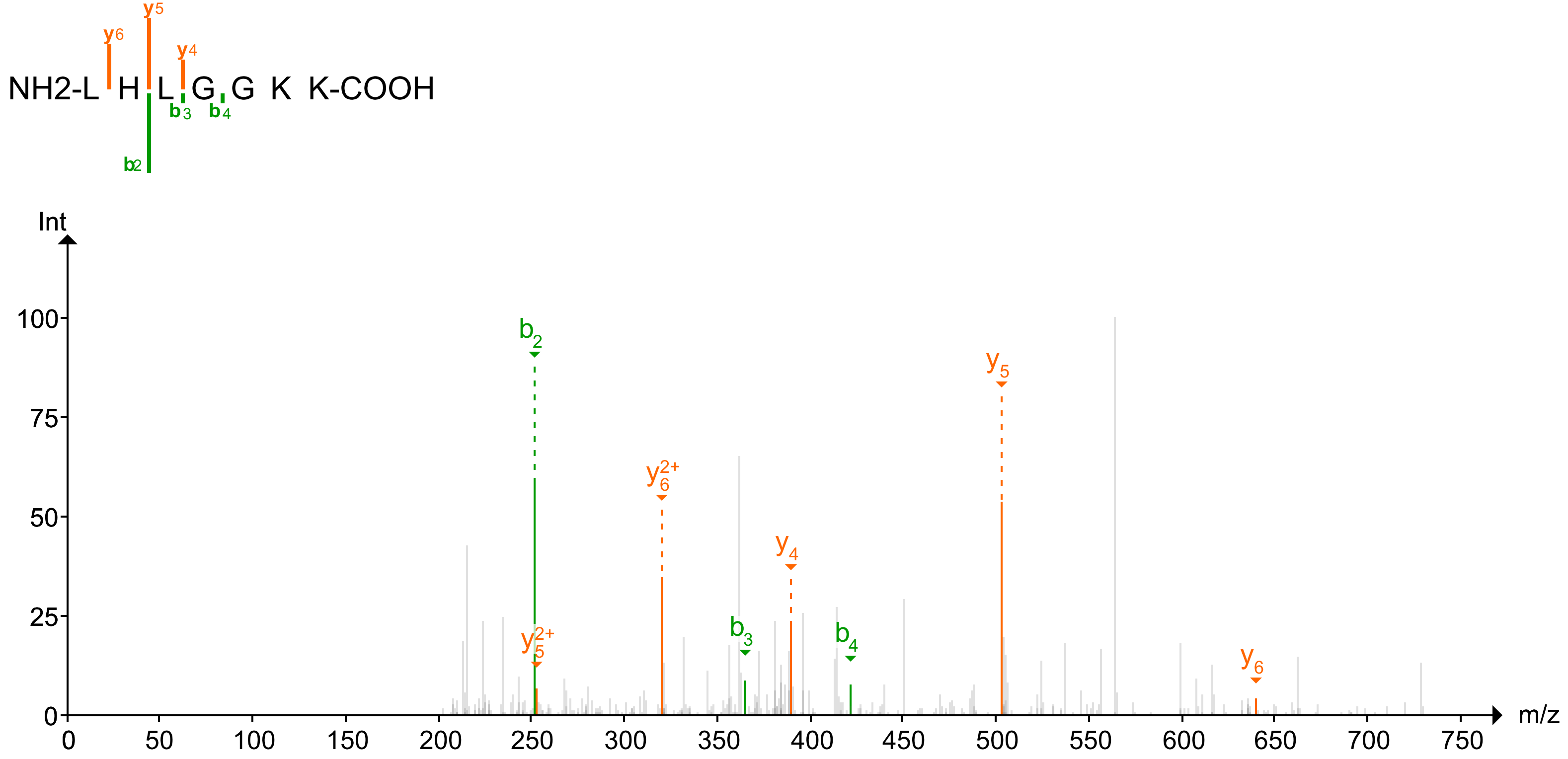

NH2-L H L G G K K-COOH
y6 y5 y4
b2 b3 b4
Int
m/z
0 25 50 75 100
0 50 100 150 200 250 300 350 400 450 500 550 600 650 700 750
$b_2$
$y_5^{2+}$
$y_6^{2+}$
$b_3$
$y_4$
$b_4$
$y_5$
$y_6$

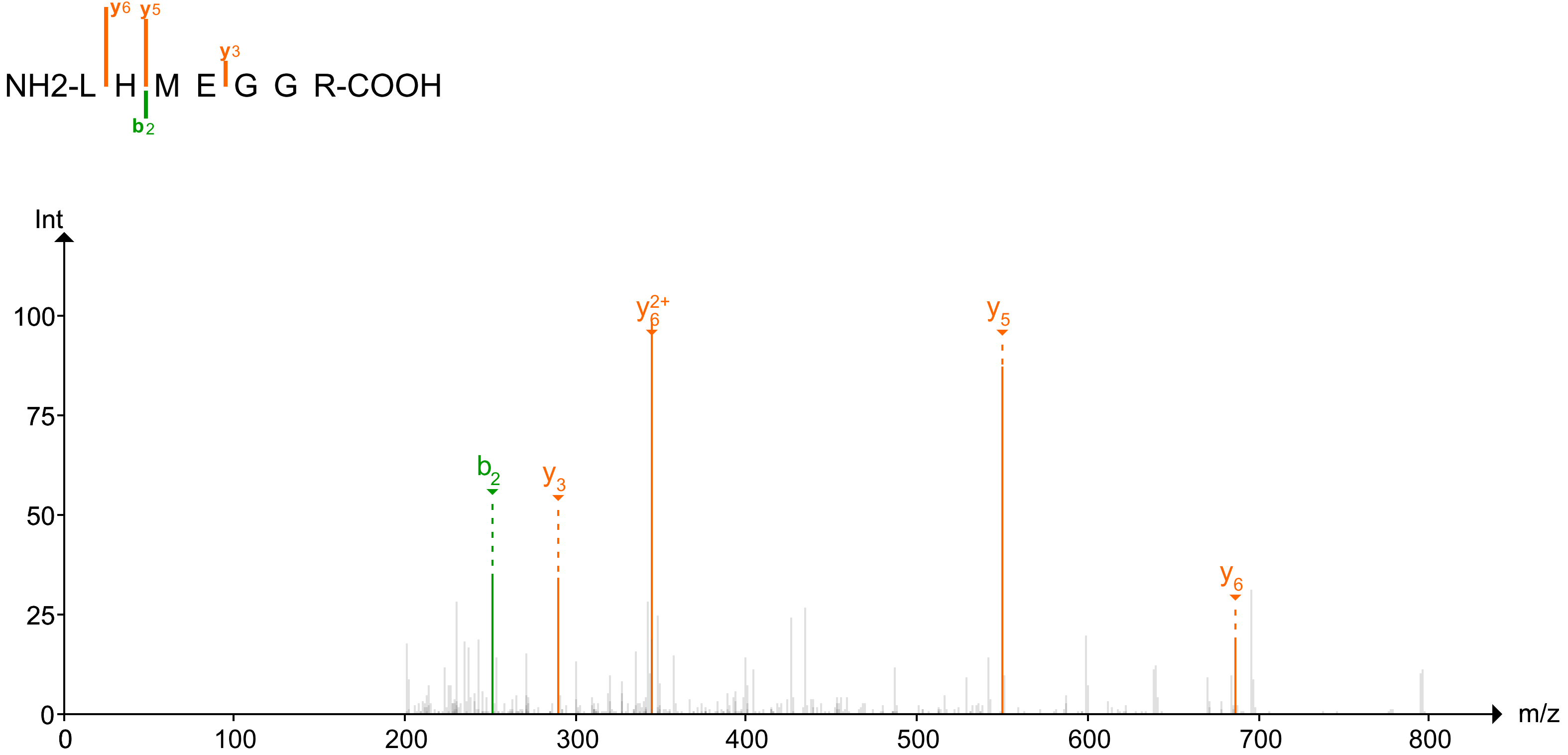

NH2-L H M E G G R-COOH
y6
y5
y3
b2
Int
100
75
50
25
0
m/z
0
100
200
300
400
500
600
700
800
$y_6^{2+}$
$y_5$
$b_2$
$y_3$
$y_6$

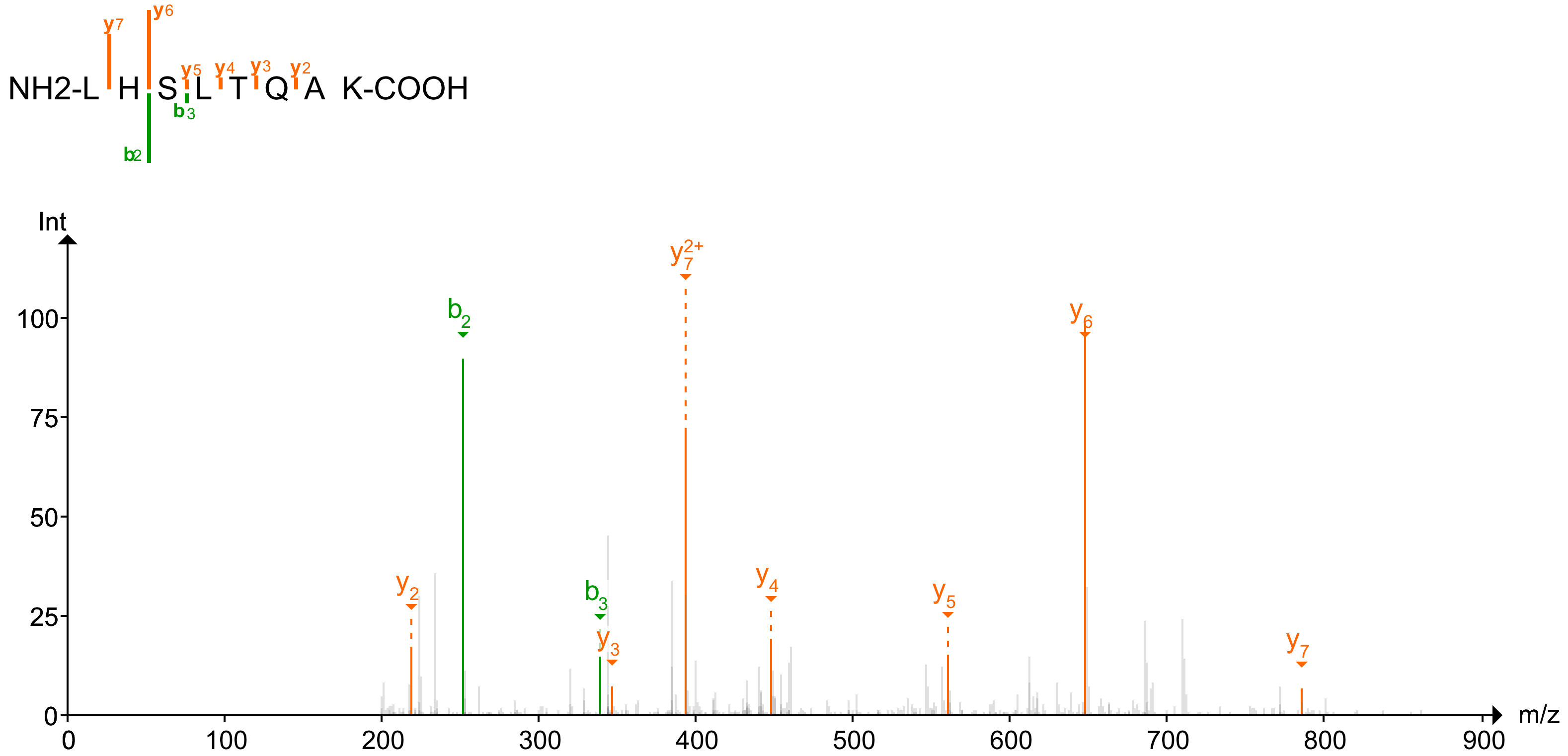

NH2-L H S L T Q A K-COOH
y7
y6
y5
y4
y3
y2
b3
b2
Int
100
75
50
25
0
m/z
0
100
200
300
400
500
600
700
800
900
b2
y7 2+
y6
y2
b3
y3
y4
y5
y7

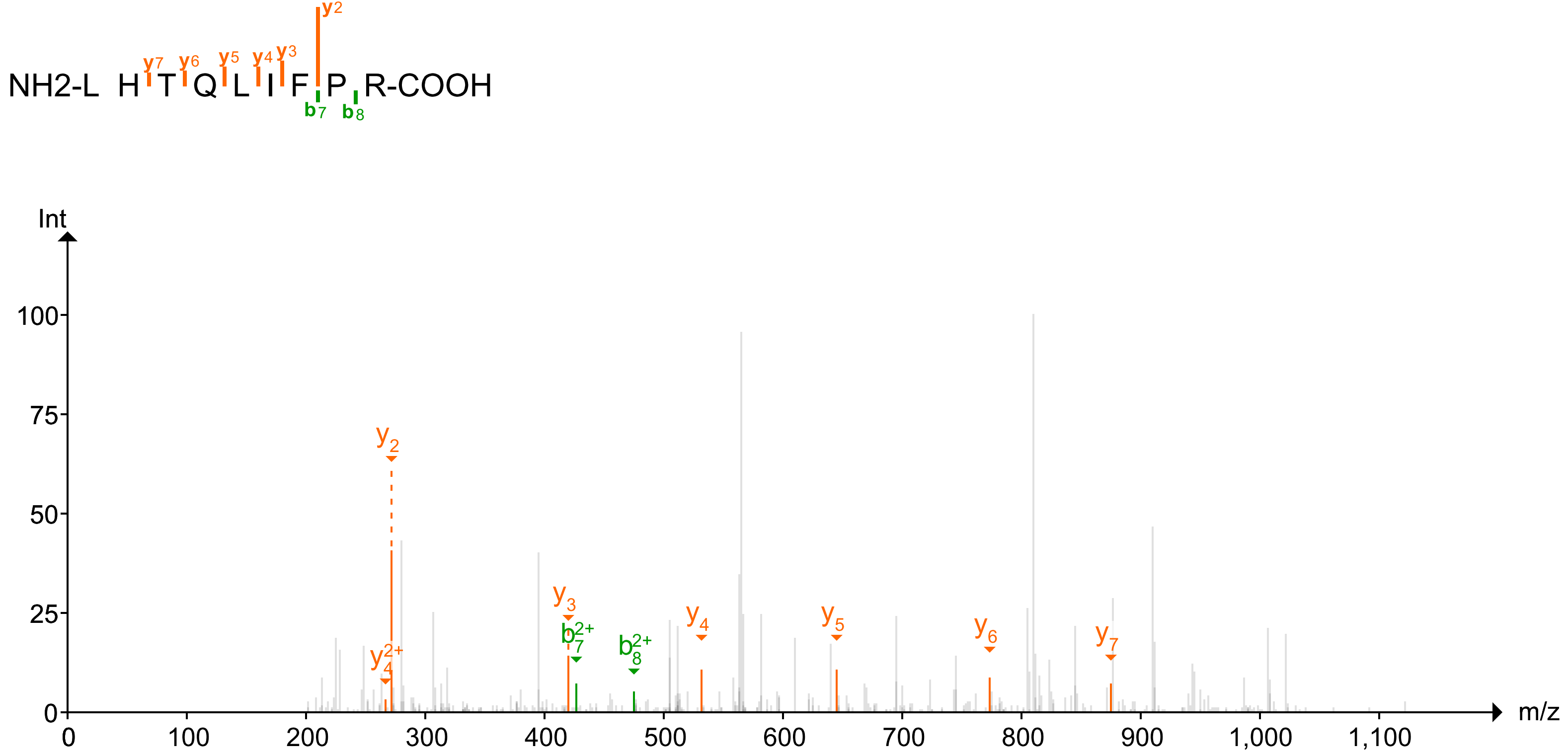

NH2-L H T Q L I F P R-COOH
y2
y7
y6
y5
y4
y3
b7
b8
Int
100
75
50
25
0
y2
y3
$b_7^{2+}$
$b_8^{2+}$
y4
y5
y6
y7
$y_4^{2+}$
m/z
0
100
200
300
400
500
600
700
800
900
1,000
1,100

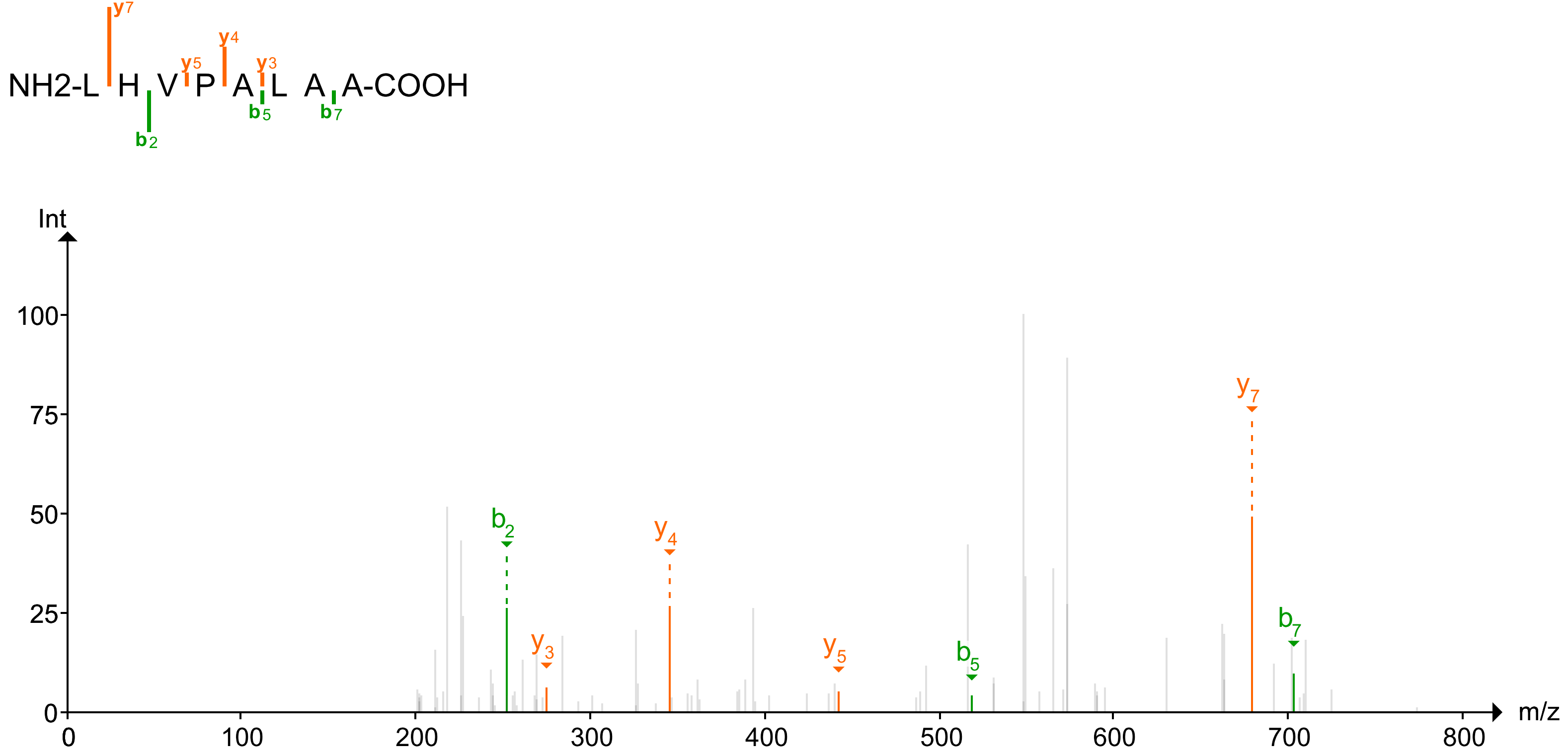
NH2-L H V P A L A A-COOH
y7
y5
y4
y3
b2
b5
b7
Int
100
75
50
25
0
0
100
200
300
400
500
600
700
800
m/z
b2
y3
y4
y5
b5
y7
b7

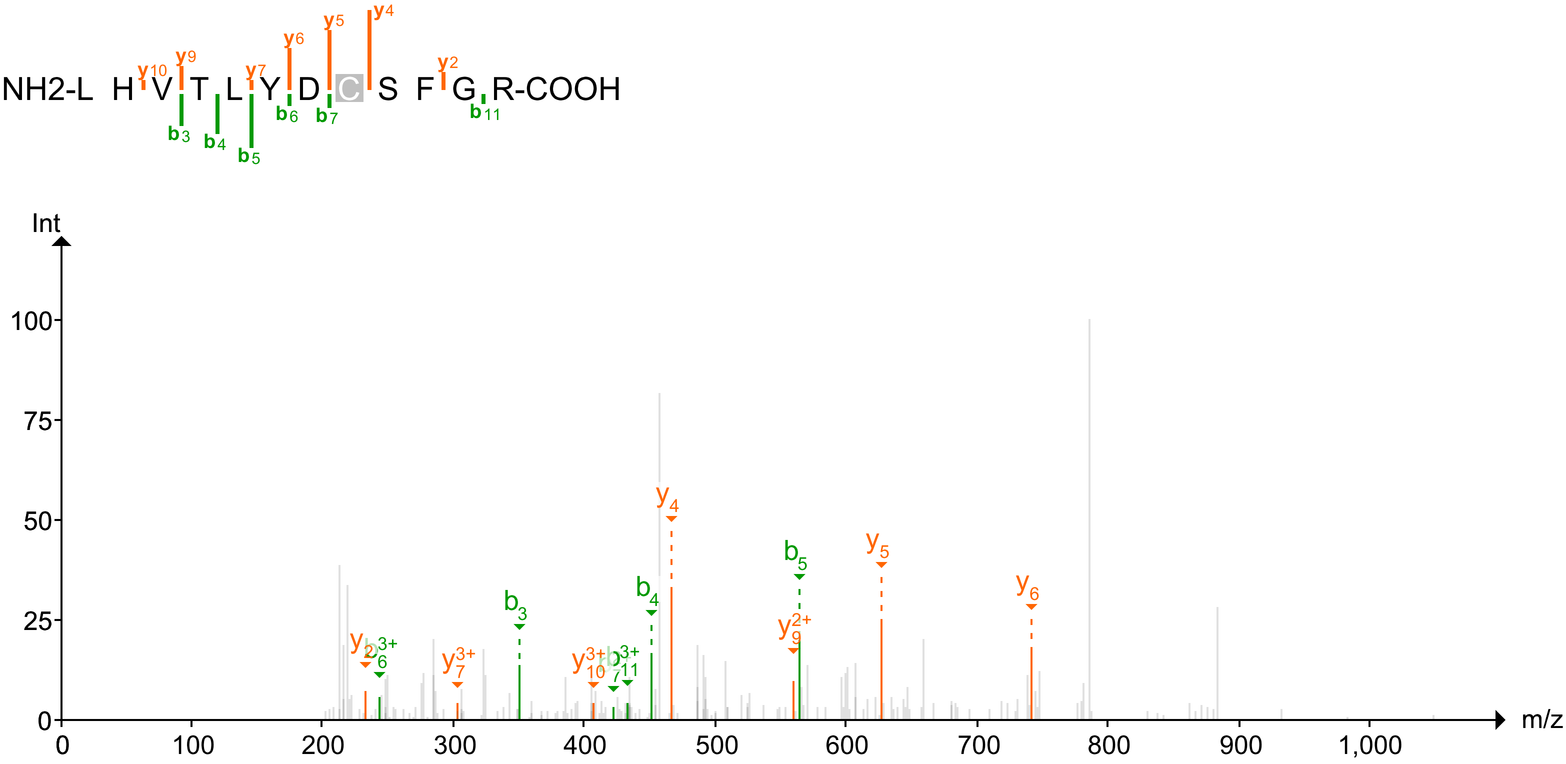

NH2-L H V T L Y D C S F G R-COOH
Int
m/z

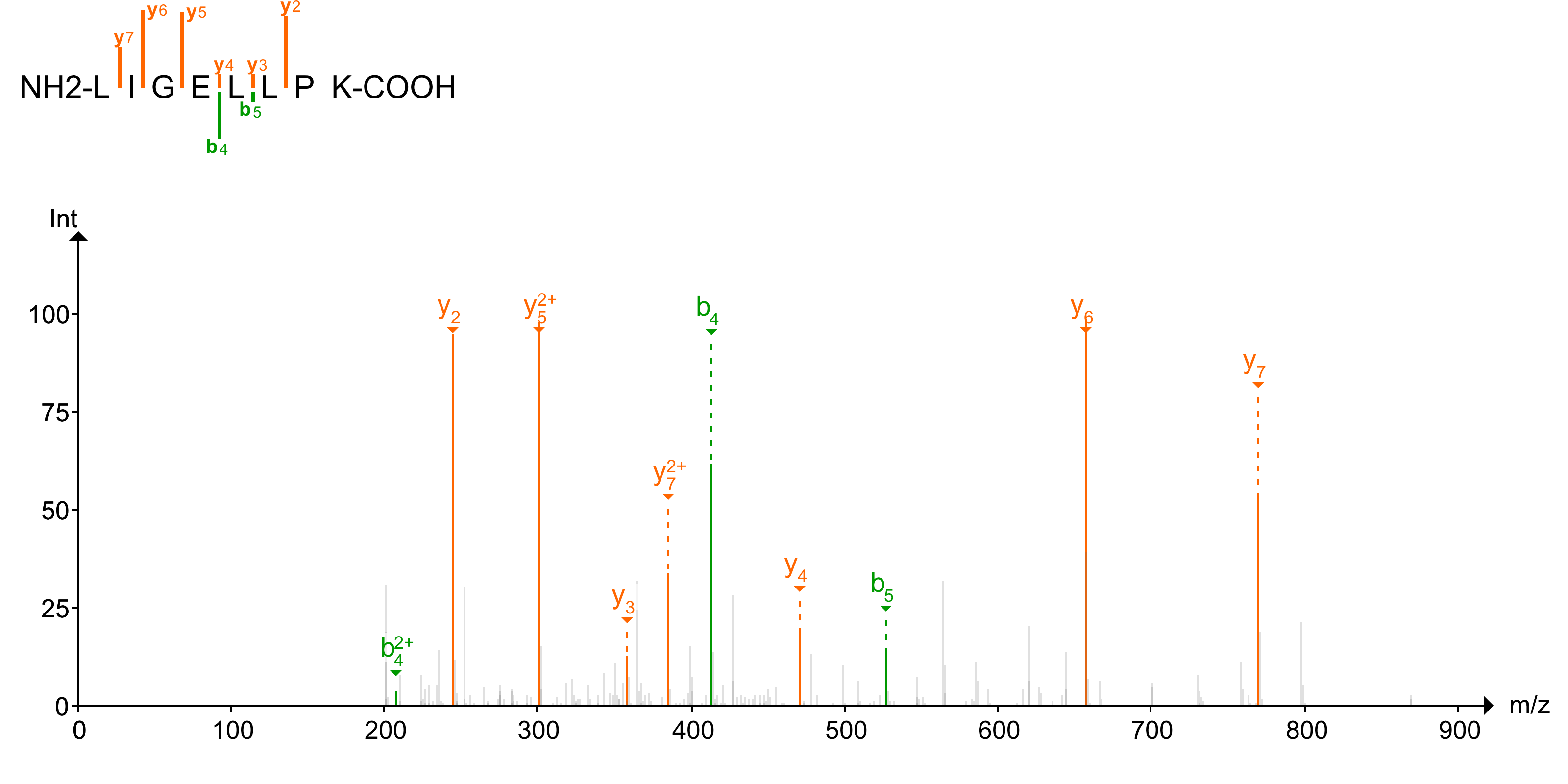

NH2-L I G E L L P K-COOH
y7 y6 y5 y4 y3 y2
b4 b5
Int
100
75
50
25
0
0 100 200 300 400 500 600 700 800 900
m/z
y2
y5 2+
b4
y6
y7
y7 2+
y3
y4
b5
b4 2+

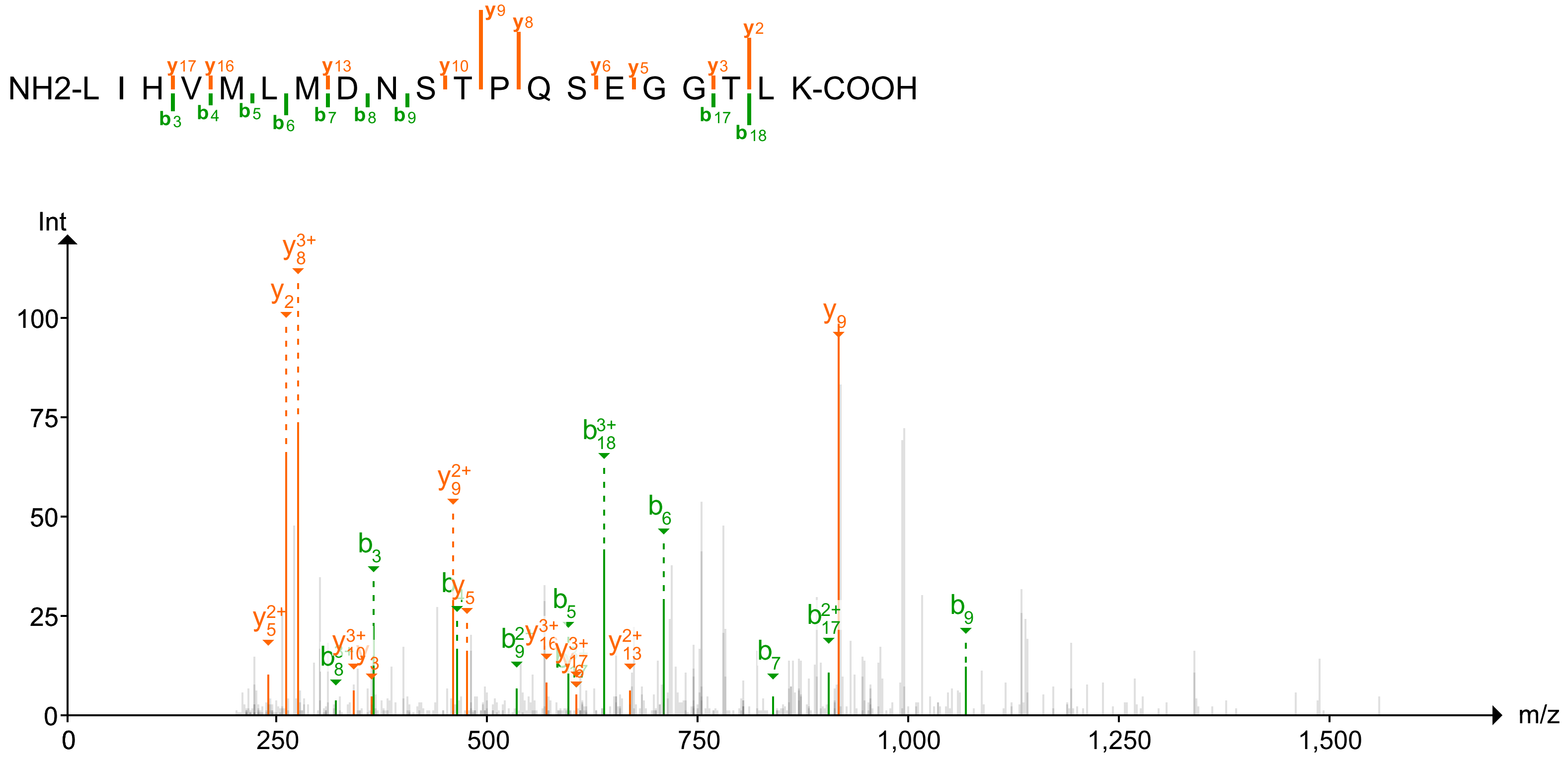

NH2-L I H V M L M D N S T P Q S E G G T L K-COOH
y17 y16 y13 y10 y9 y8 y6 y5 y3 y2
b3 b4 b5 b6 b7 b8 b9 b17 b18
Int
100
75
50
25
0
0
250
500
750
1,000
1,250
1,500
m/z
$y_8^{3+}$
$y_2$
$y_9$
$y_9^{2+}$
$b_{18}^{3+}$
$b_6$
$b_3$
$y_5^{2+}$
$y_{10}^{3+}$
$b_8$
$y_3$
$y_5$
$b_5$
$b_9^{2+}$
$y_{16}^{3+}$
$y_{17}^{3+}$
$y_{13}^{2+}$
$b_7$
$b_{17}^{2+}$
$b_9$

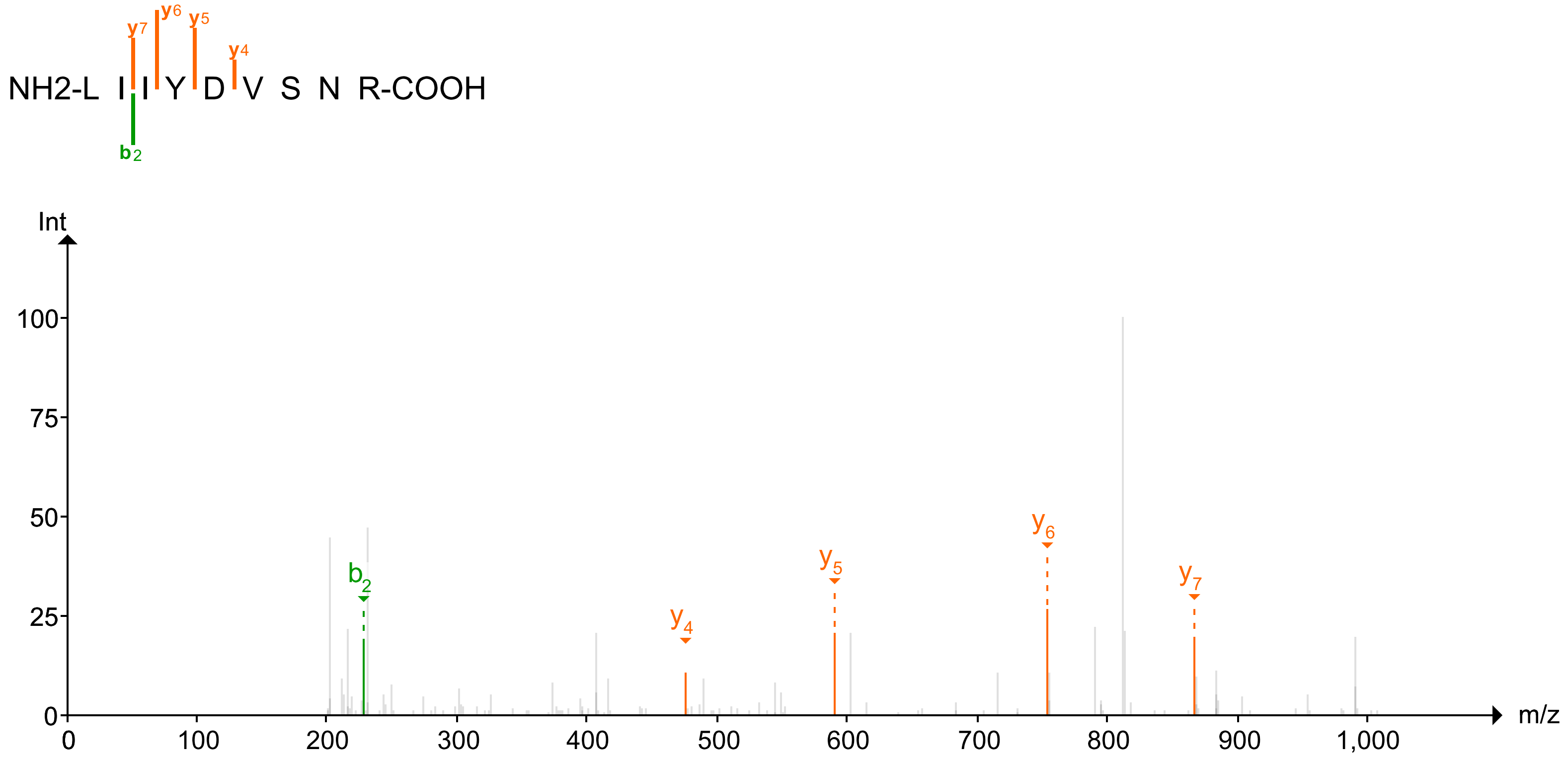

y7 y6 y5 y4
NH2-L I I Y D V S N R-COOH
b2
Int
100
75
50
25
0
b2
y4
y5
y6
y7
0 100 200 300 400 500 600 700 800 900 1,000
m/z

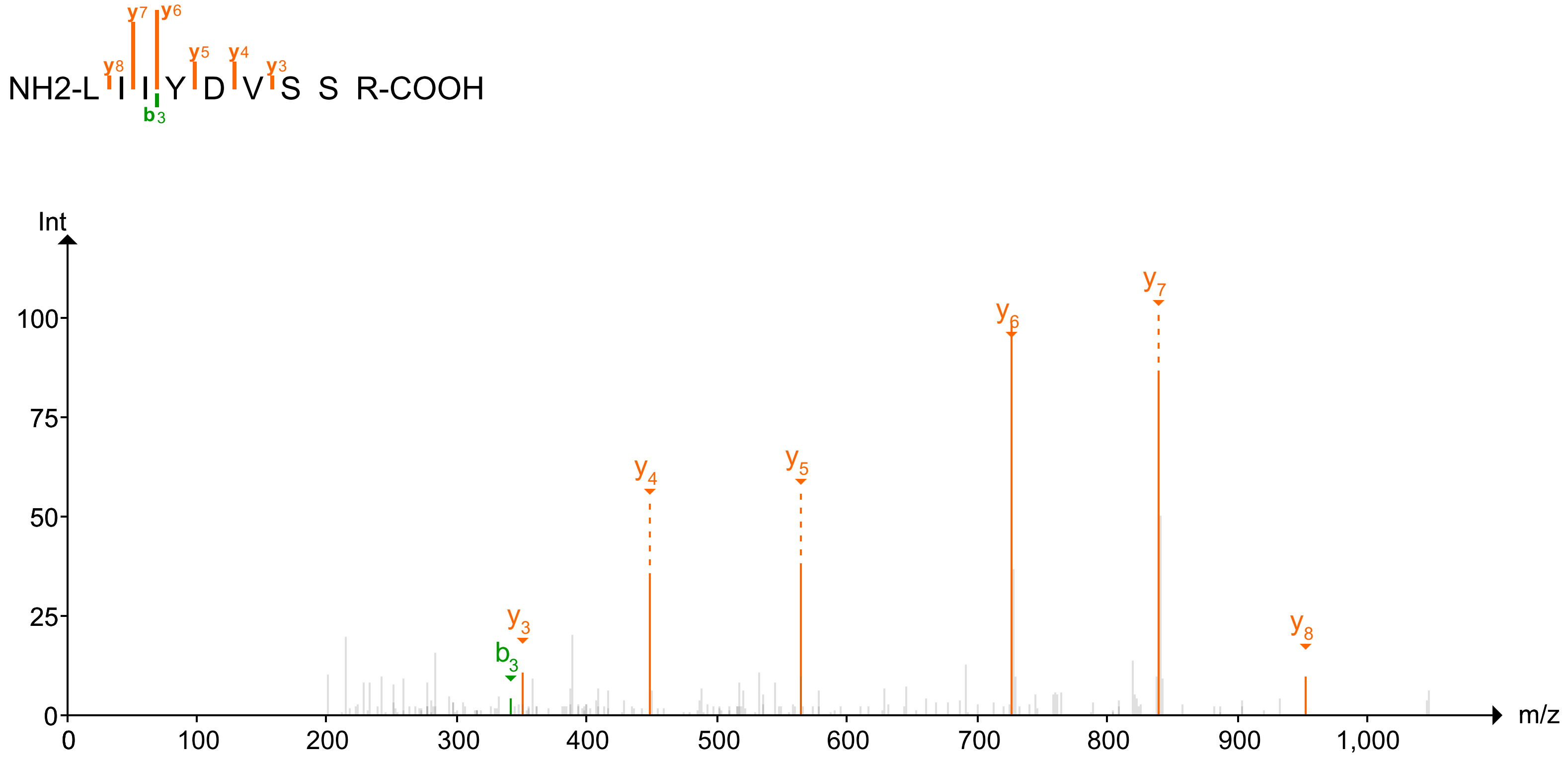

NH2-L I I Y D V S S R-COOH
y7 y6 y8 y5 y4 y3 b3
Int
100
75
50
25
0
y7
y6
y5
y4
y3
b3
y8
0 100 200 300 400 500 600 700 800 900 1,000
m/z

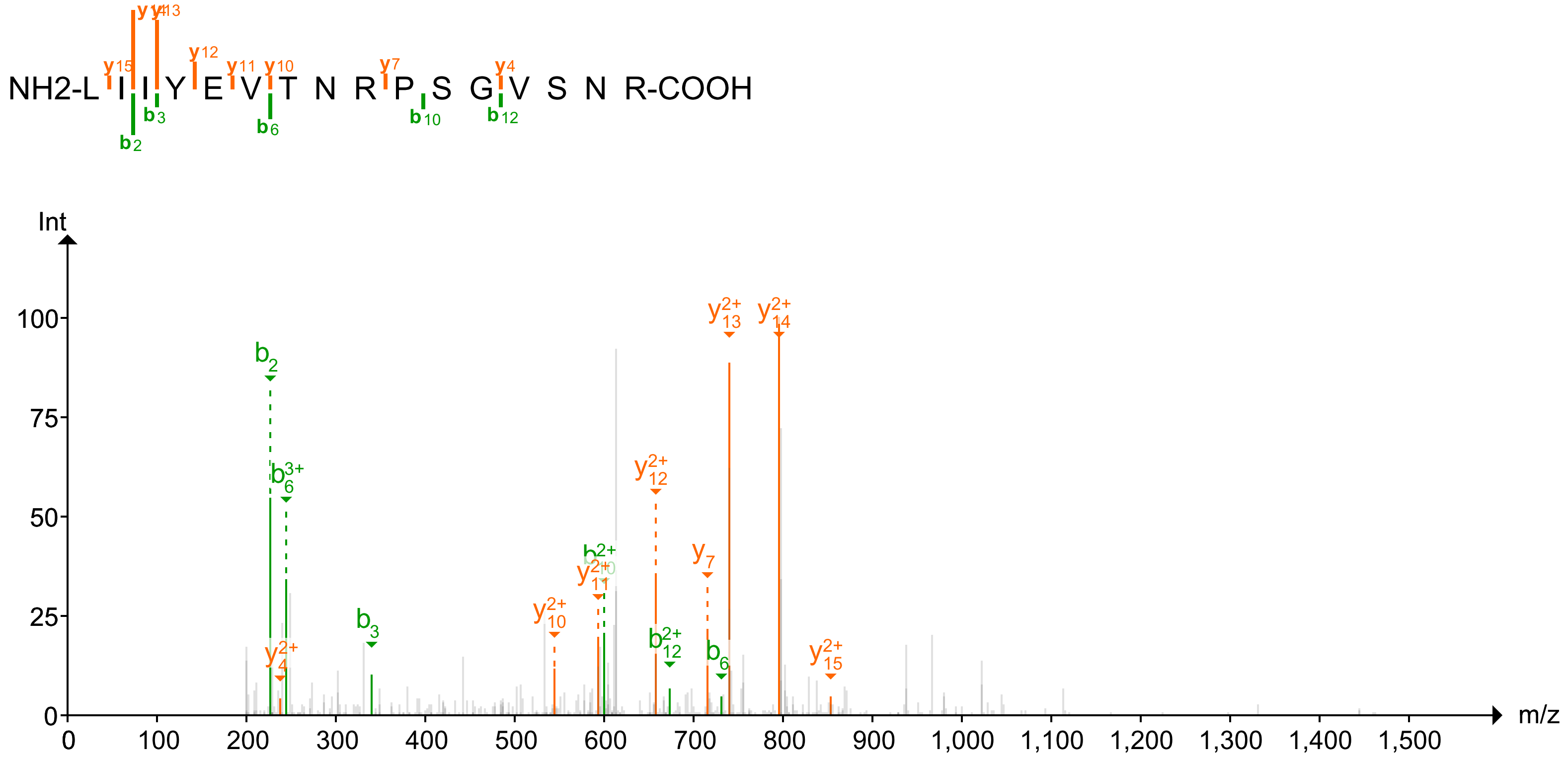

NH2-L I I Y E V T N R P S G V S N R-COOH
y15 y y13 y12 y11 y10 y7 y4
b2 b3 b6 b10 b12
Int
m/z
0 25 50 75 100
0 100 200 300 400 500 600 700 800 900 1,000 1,100 1,200 1,300 1,400 1,500

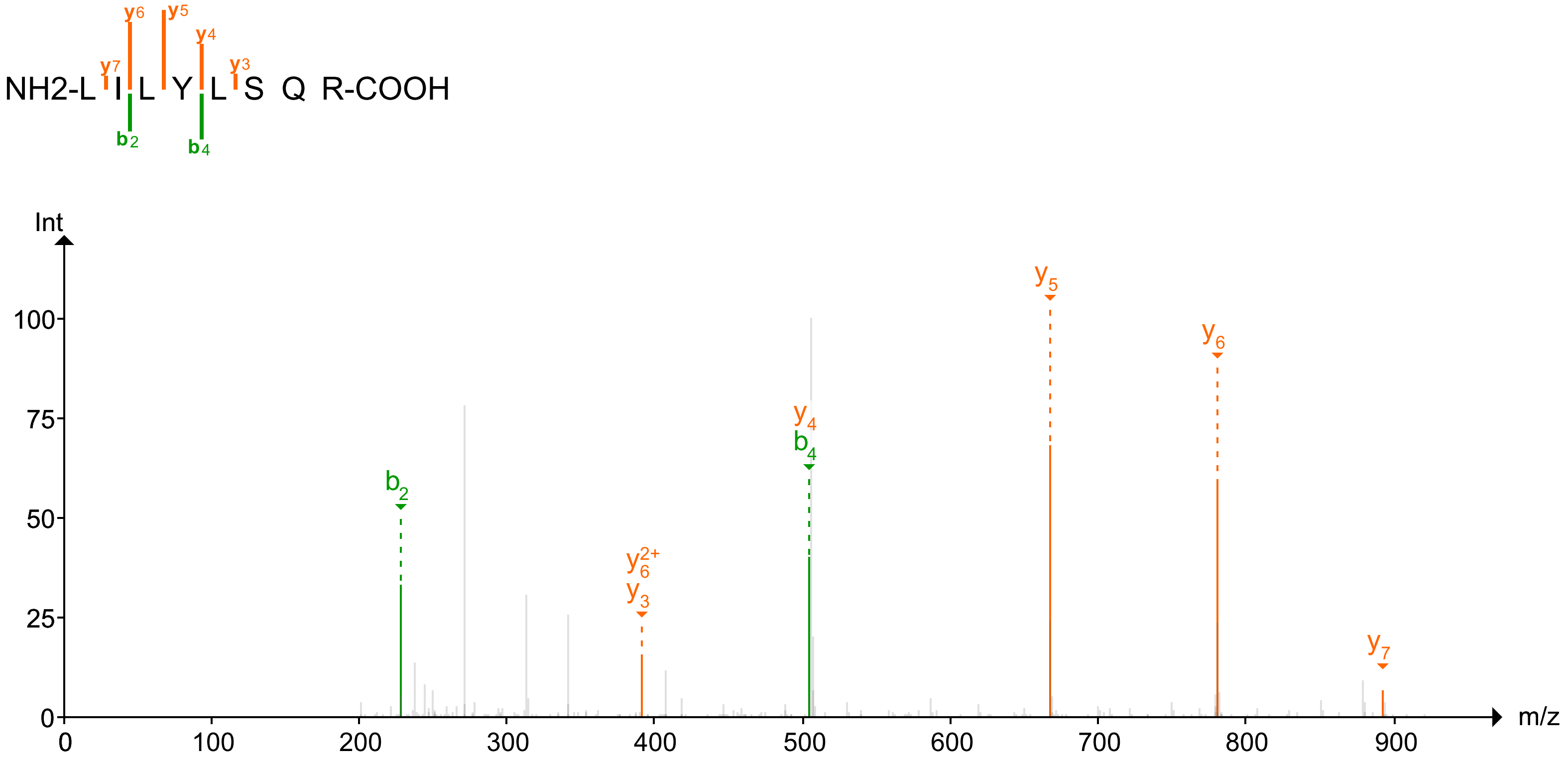

NH2-L I L Y L S Q R-COOH
y6 y5 y4 y7 y3
b2 b4
Int
m/z
0
25
50
75
100
0
100
200
300
400
500
600
700
800
900
b2
y6 2+
y3
y4
b4
y5
y6
y7

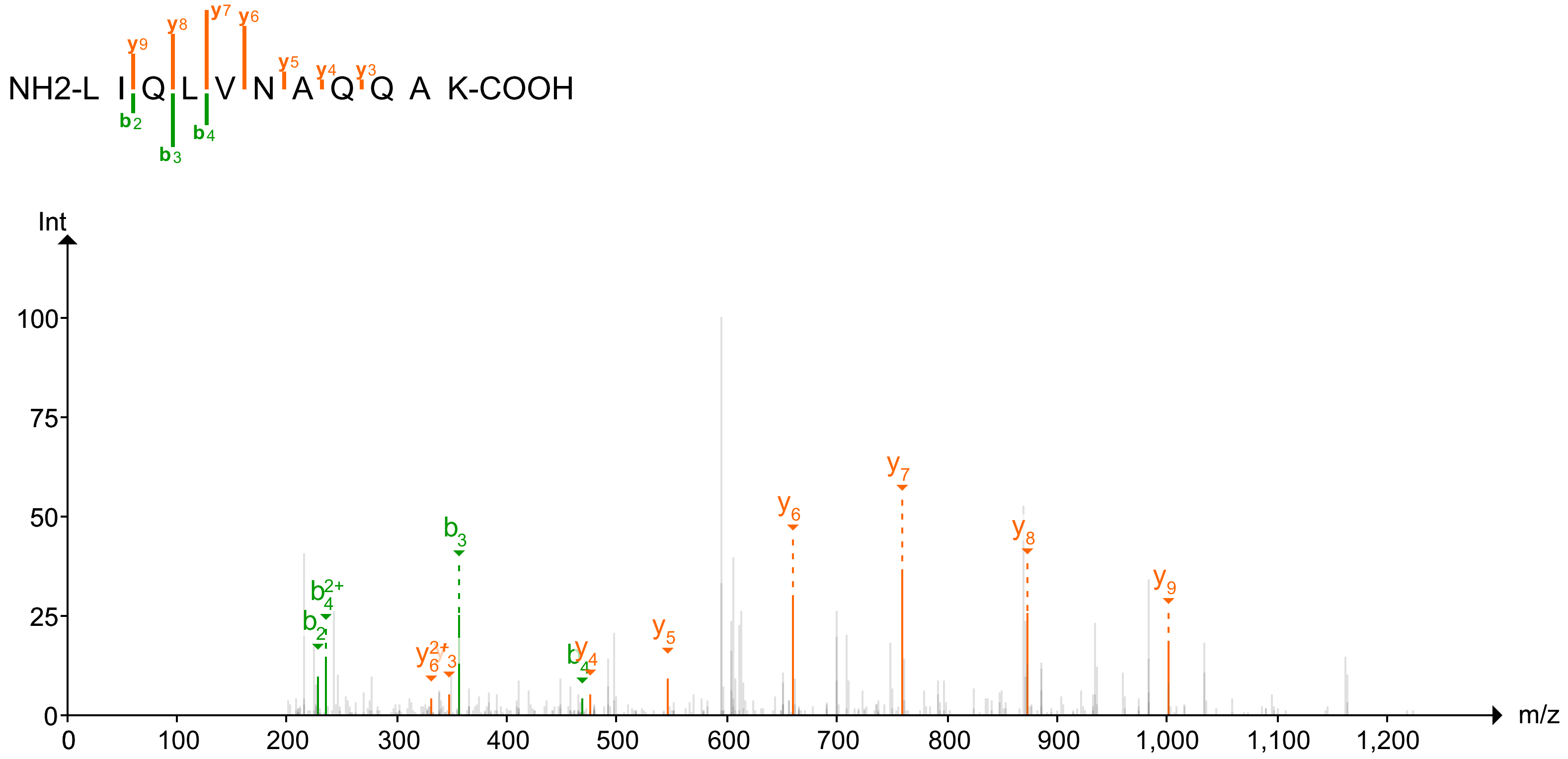

NH2-L I Q L V N A Q Q A K-COOH
y9 y8 y7 y6 y5 y4 y3
b2 b3 b4
Int
100
75
50
25
0
m/z
0 100 200 300 400 500 600 700 800 900 1,000 1,100 1,200
b2
b4 2+
b3
y6 2+
y3
b4
y4
y5
y6
y7
y8
y9

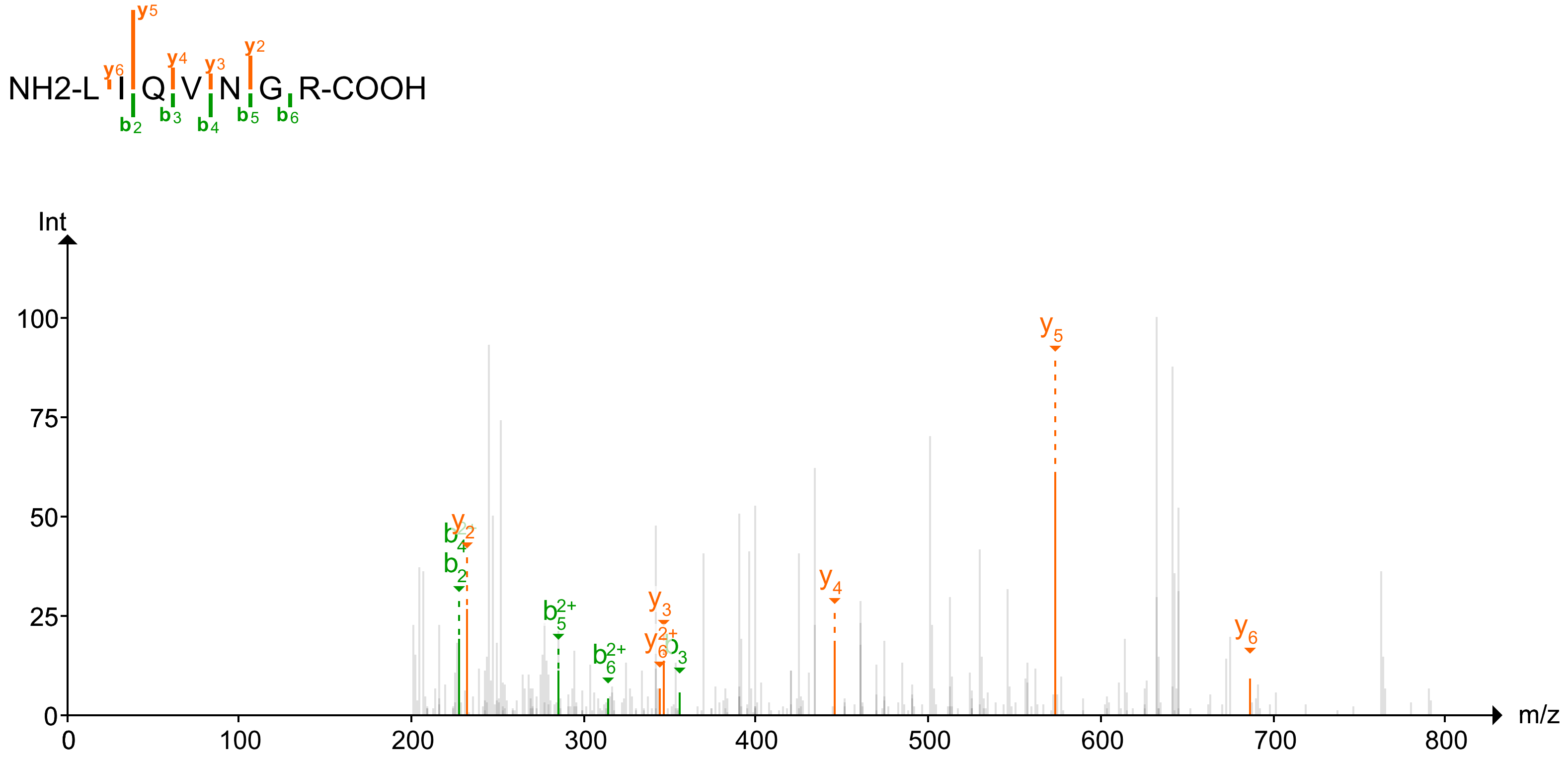

NH2-L I Q V N G R-COOH
y5
y6
y4
y3
y2
b2
b3
b4
b5
b6
Int
100
75
50
25
0
0
100
200
300
400
500
600
700
800
m/z

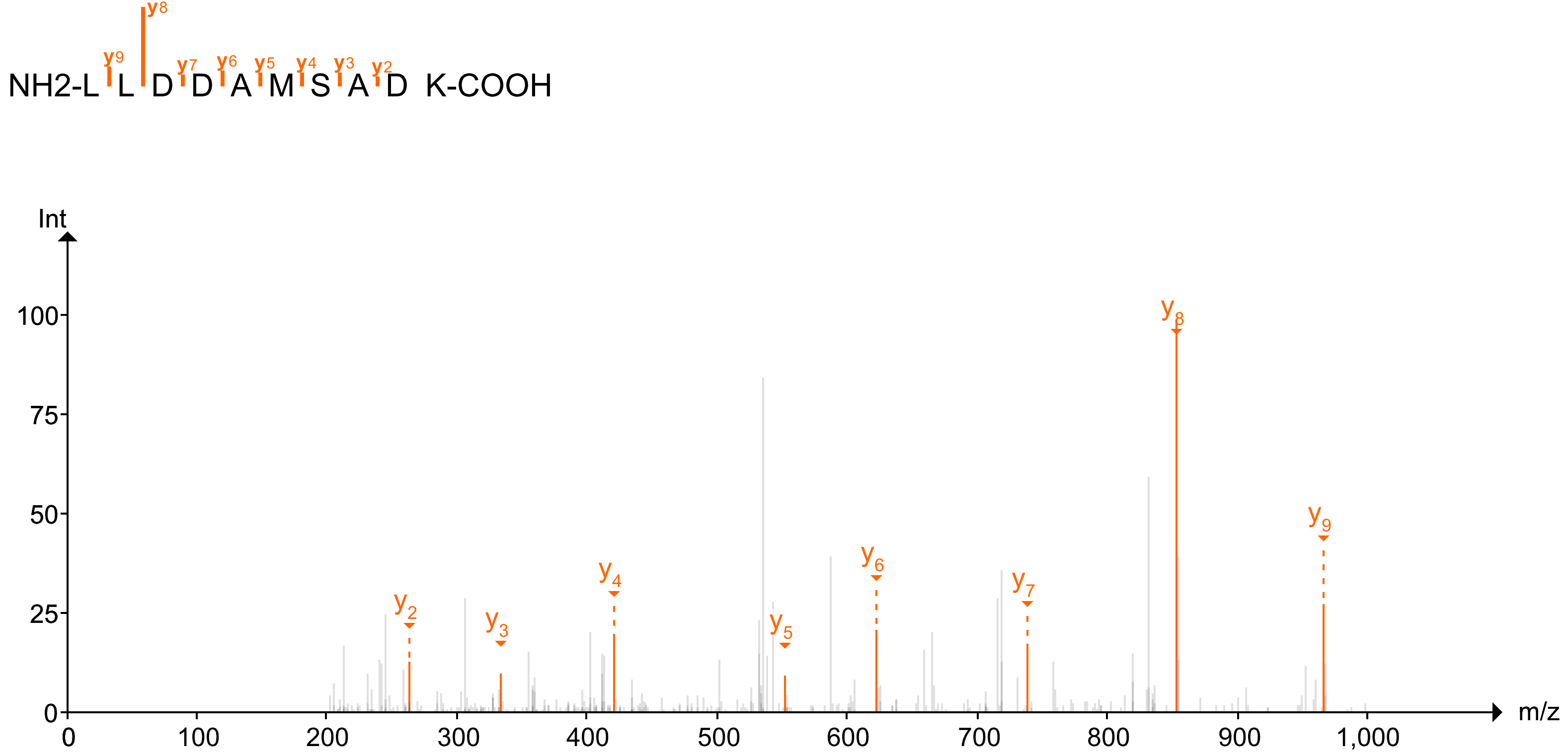

NH2-L L D D A M S A D K-COOH
y9
y8
y7
y6
y5
y4
y3
y2
Int
100
75
50
25
0
m/z
0
100
200
300
400
500
600
700
800
900
1,000

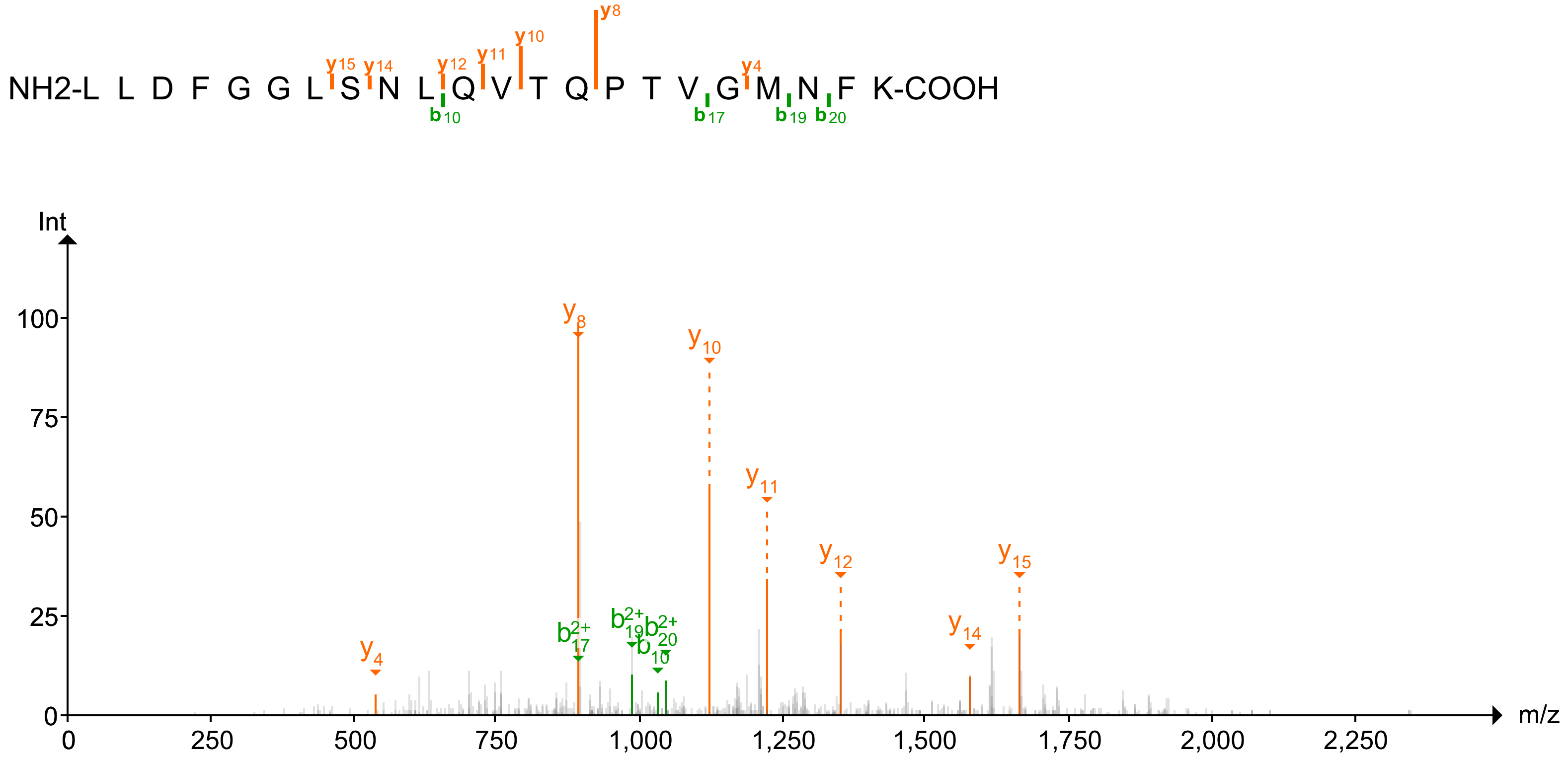

NH2-L L D F G G L S N L Q V T Q P T V G M N F K-COOH
y15 y14 y12 y11 y10 y8 y4
b10 b17 b19 b20
Int
100
75
50
25
0
m/z
0
250
500
750
1,000
1,250
1,500
1,750
2,000
2,250

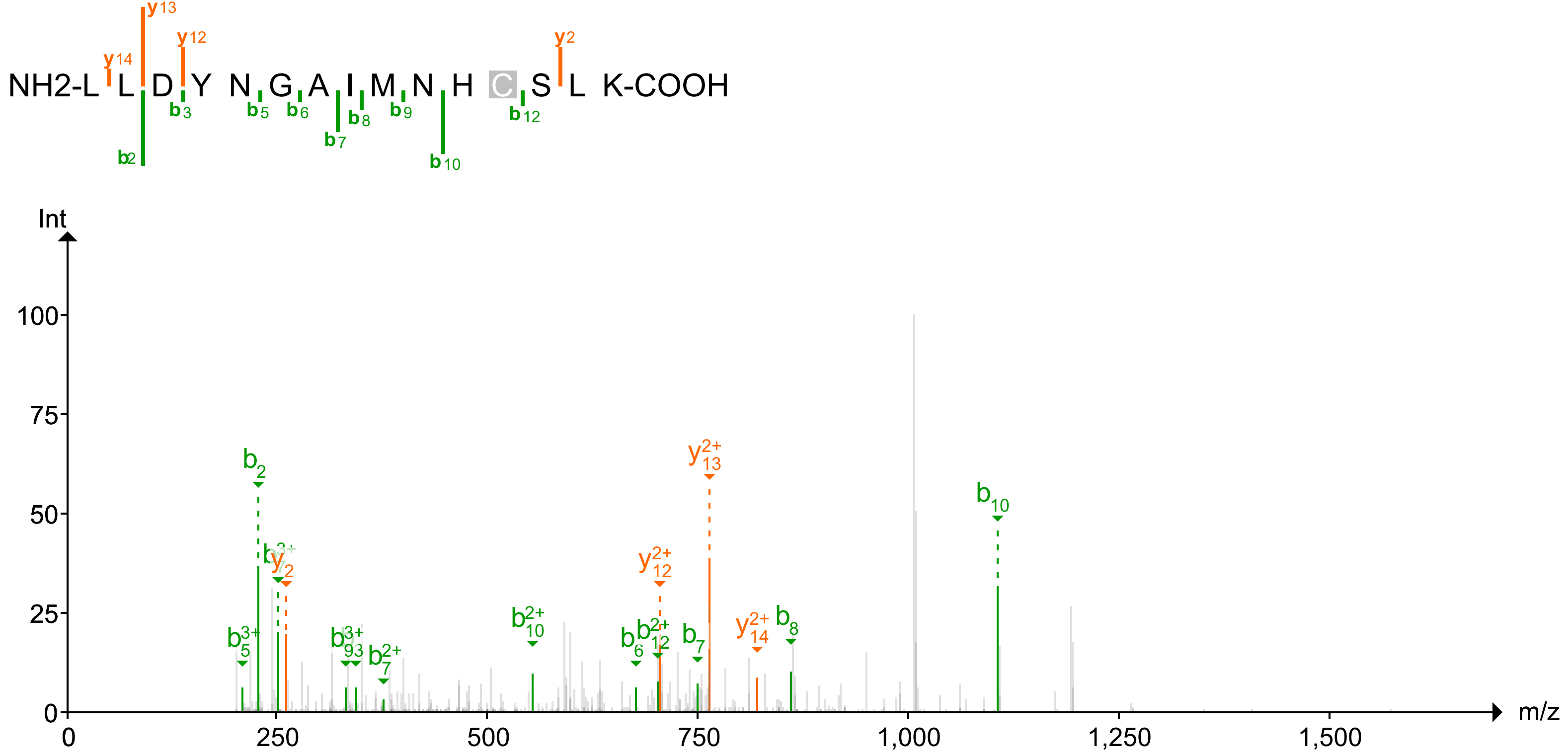

NH2-L L D Y N G A I M N H C S L K-COOH
y14 y13 y12 y2
b2 b3 b5 b6 b7 b8 b9 b10 b12
Int
100
75
50
25
0
0
250
500
750
1,000
1,250
1,500
m/z

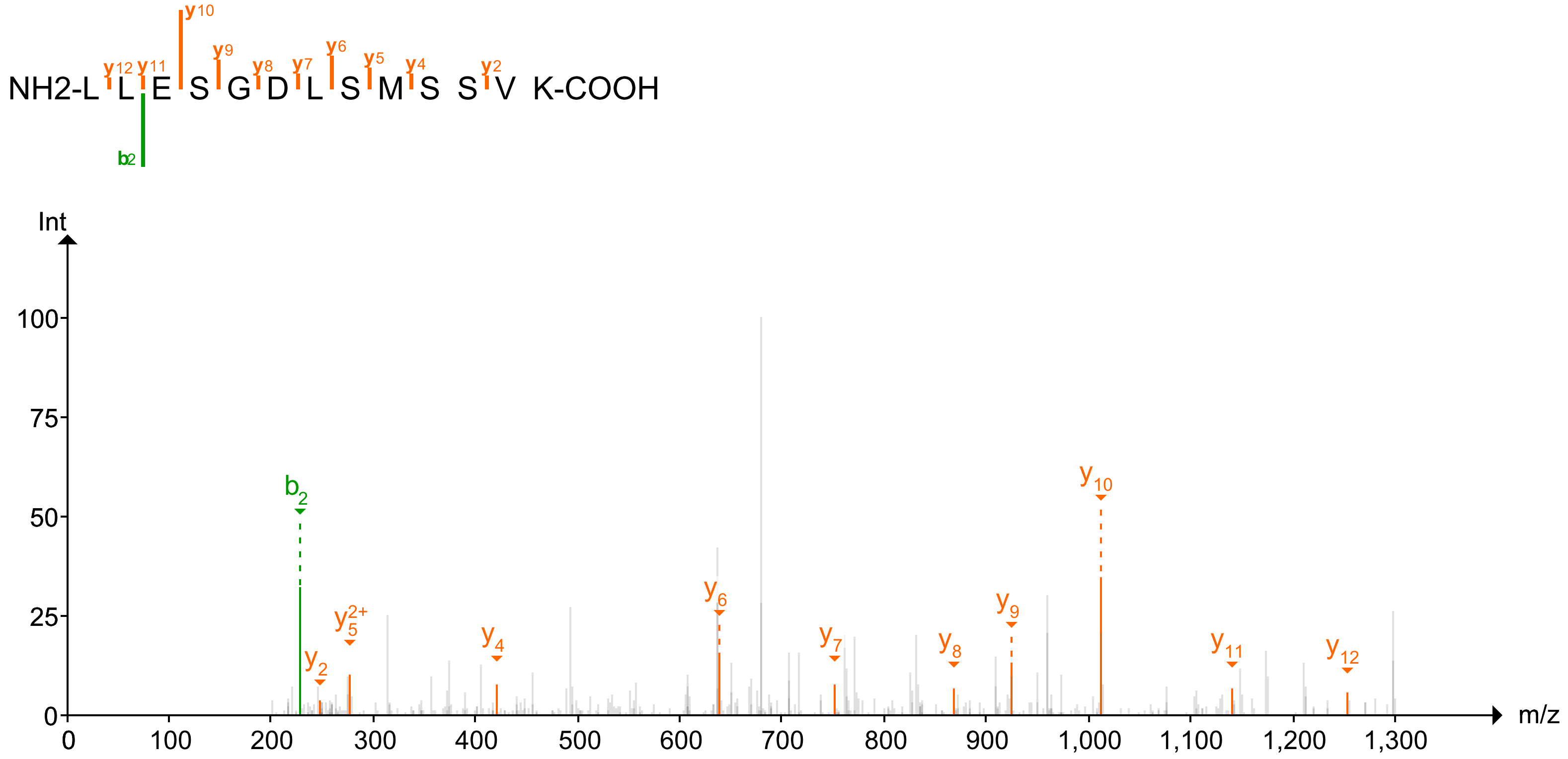

NH2-L L E S G D L S M S S V K-COOH
y12 y11 y10 y9 y8 y7 y6 y5 y4 y2
b2
Int
100
75
50
25
0
m/z
0 100 200 300 400 500 600 700 800 900 1,000 1,100 1,200 1,300
b2
y2
y5 2+
y4
y6
y7
y8
y9
y10
y11
y12

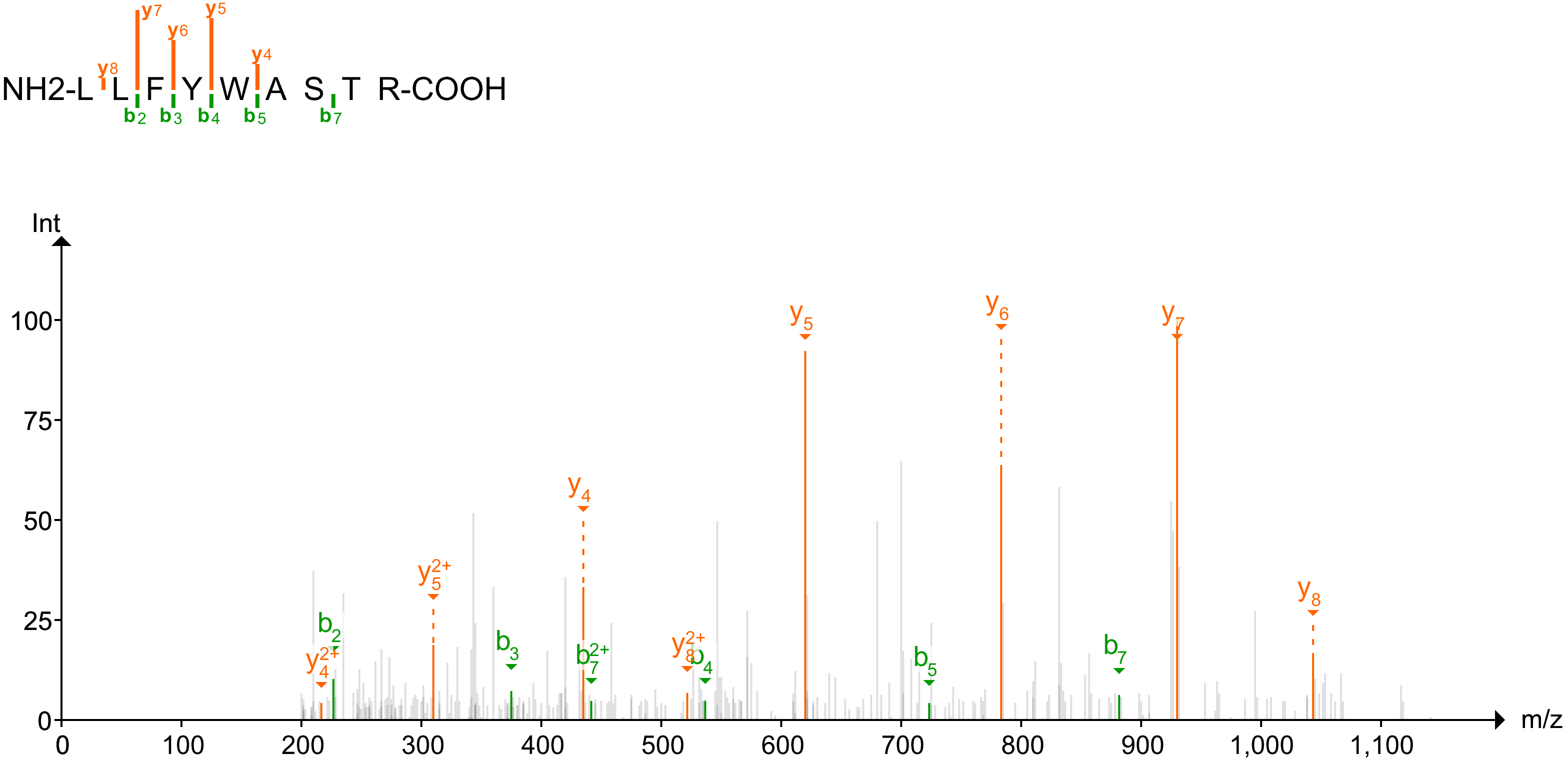

NH2-L L F Y W A S T R-COOH
y8 y7 y6 y5 y4
b2 b3 b4 b5 b7
Int
100
75
50
25
0
0 100 200 300 400 500 600 700 800 900 1,000 1,100
m/z
y5
y6
y7
y4
y8
y5 2+
y4 2+
b2
b3
b7 2+
y8 2+
b4
b5
b7

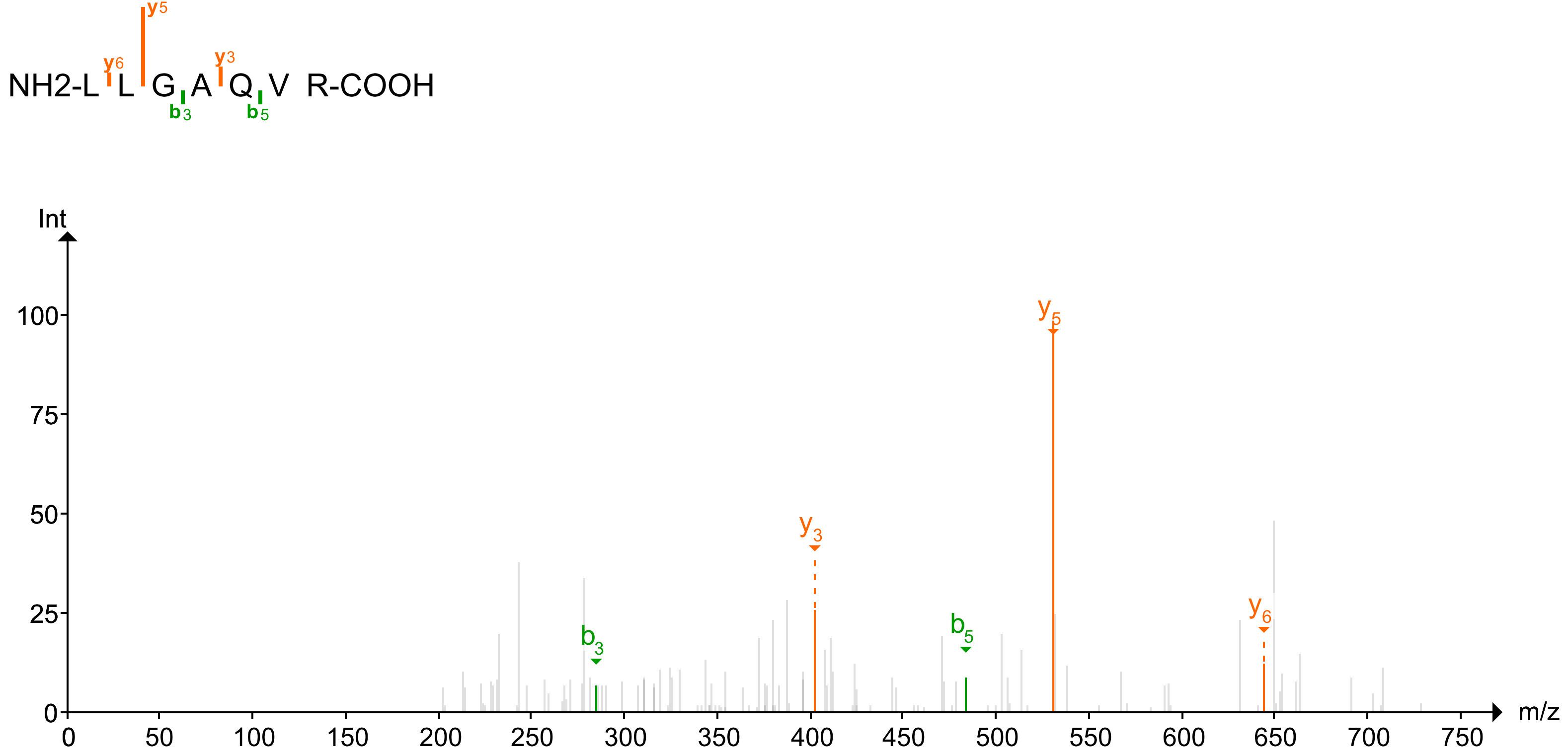

NH2-L L G A Q V R-COOH
y6
y5
y3
b3
b5
Int
100
75
50
25
0
m/z
0
50
100
150
200
250
300
350
400
450
500
550
600
650
700
750

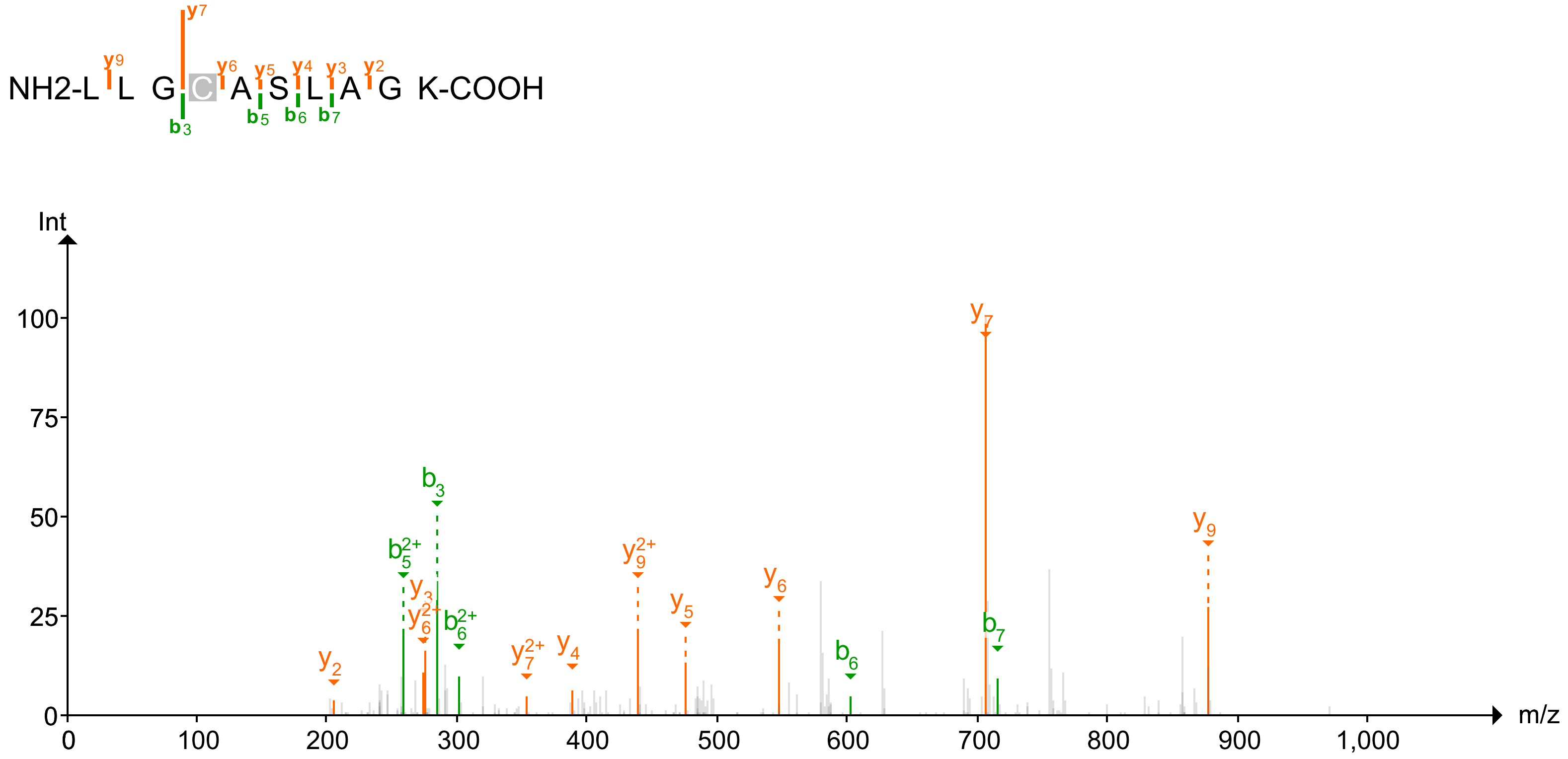

NH2-L L G C A S L A G K-COOH
y9 y7 y6 y5 y4 y3 y2
b3 b5 b6 b7
Int
m/z
0
25
50
75
100
0
100
200
300
400
500
600
700
800
900
1,000
y2
b5 2+
b3
y3
y6 2+
b6 2+
y7 2+
y4
y9 2+
y5
y6
b6
y7
b7
y9

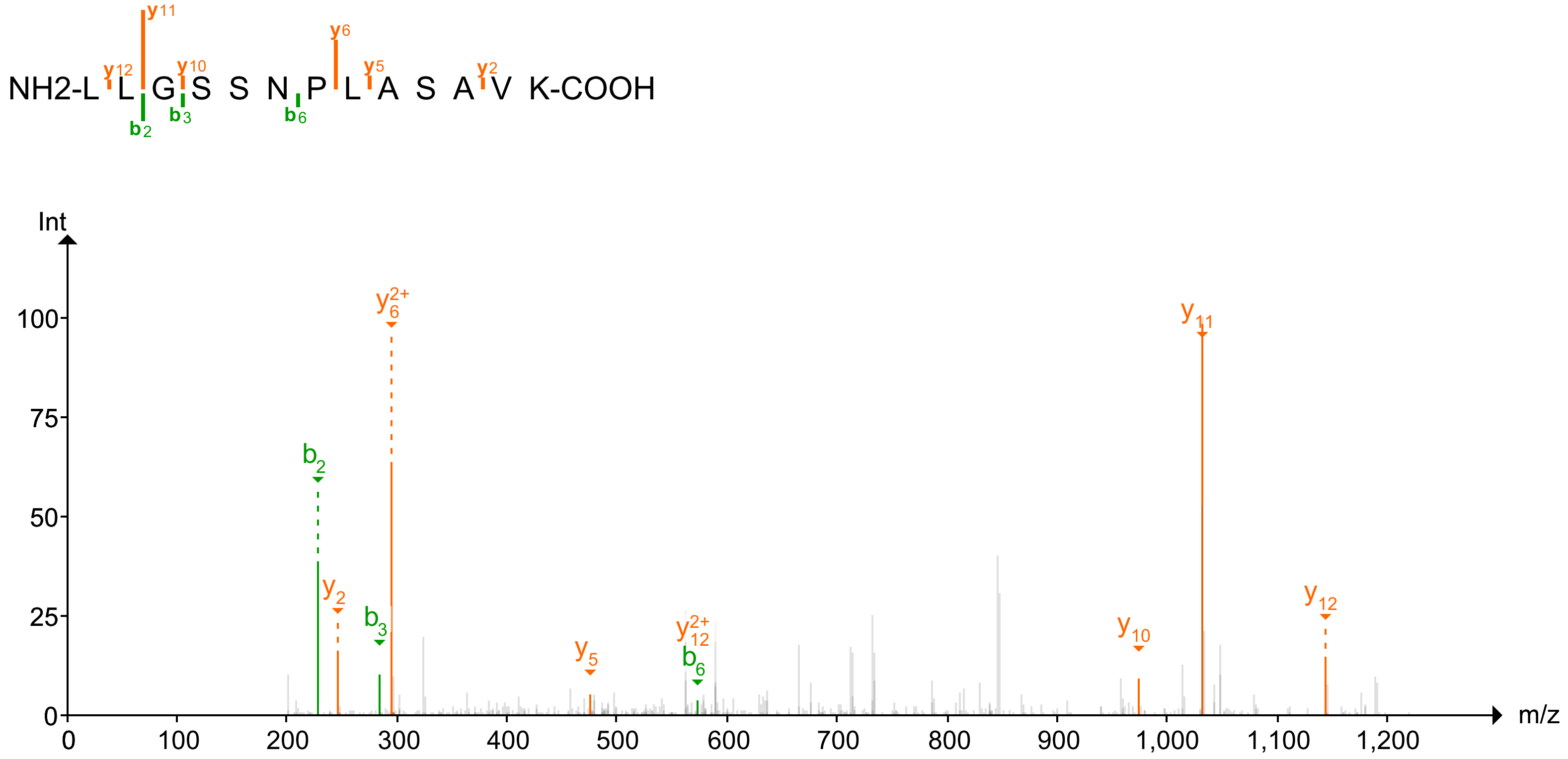

NH2-L L G S S N P L A S A V K-COOH
y11
y12
y10
y6
y5
y2
b2
b3
b6
Int
100
75
50
25
0
m/z
0
100
200
300
400
500
600
700
800
900
1,000
1,100
1,200

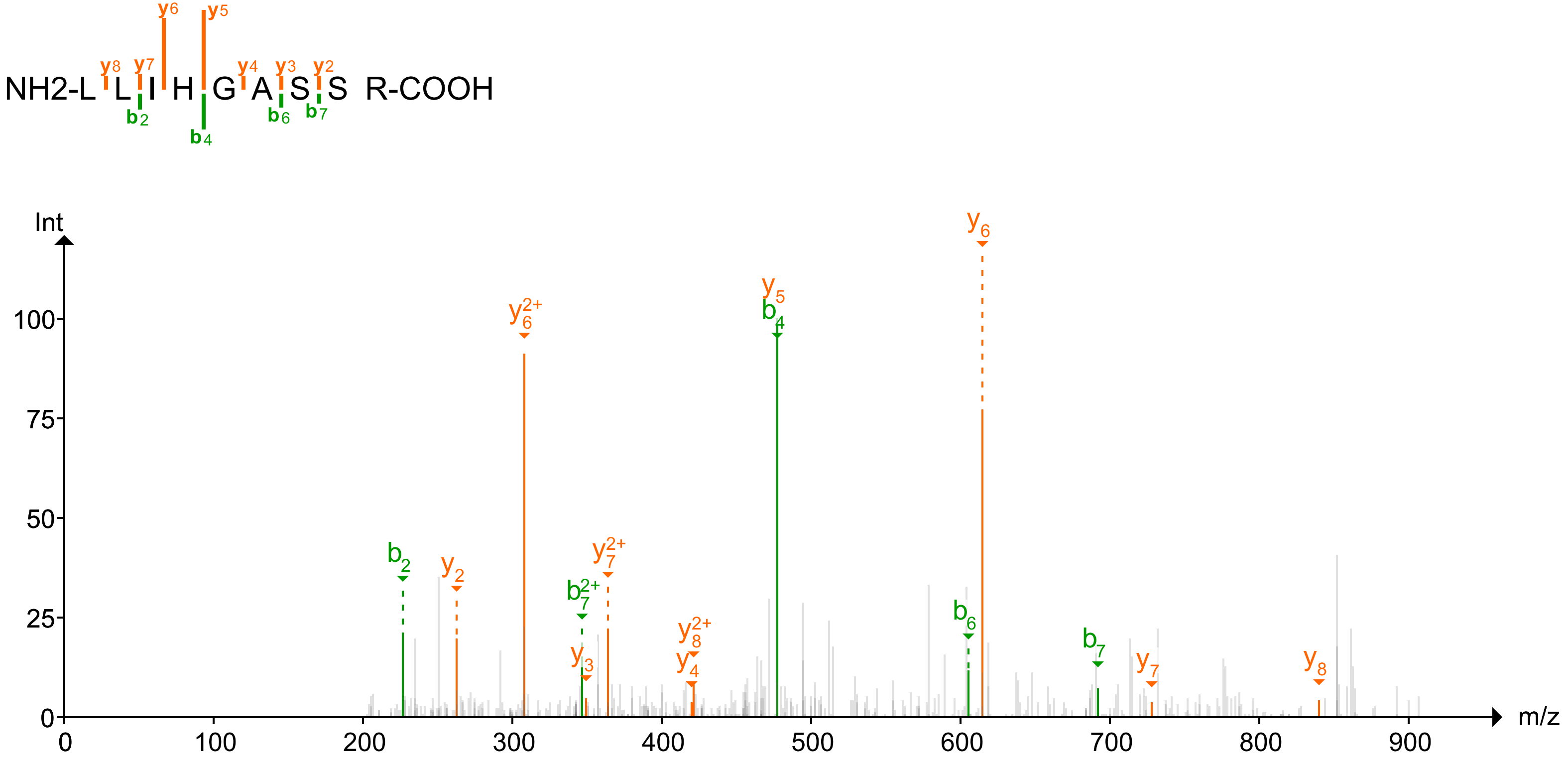

NH2-L L I H G A S S R-COOH
y8 y7 y6 y5 y4 y3 y2
b2 b4 b6 b7
Int
m/z
0
25
50
75
100
0
100
200
300
400
500
600
700
800
900

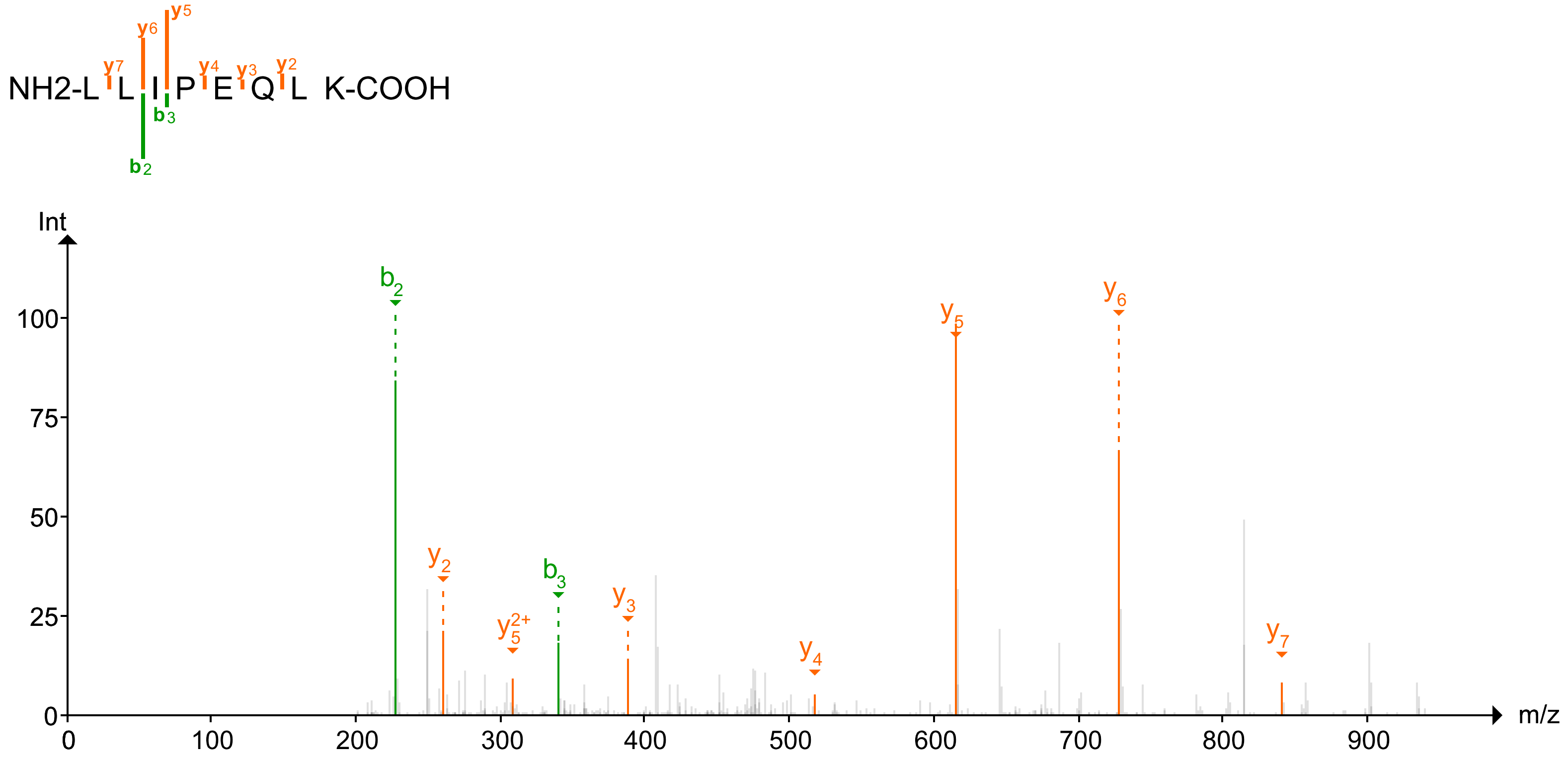

NH2-L L I P E Q L K-COOH
y7 y6 y5 y4 y3 y2
b2 b3
Int
m/z
b2
y2
y5 2+
b3
y3
y4
y5
y6
y7
0
25
50
75
100
0
100
200
300
400
500
600
700
800
900

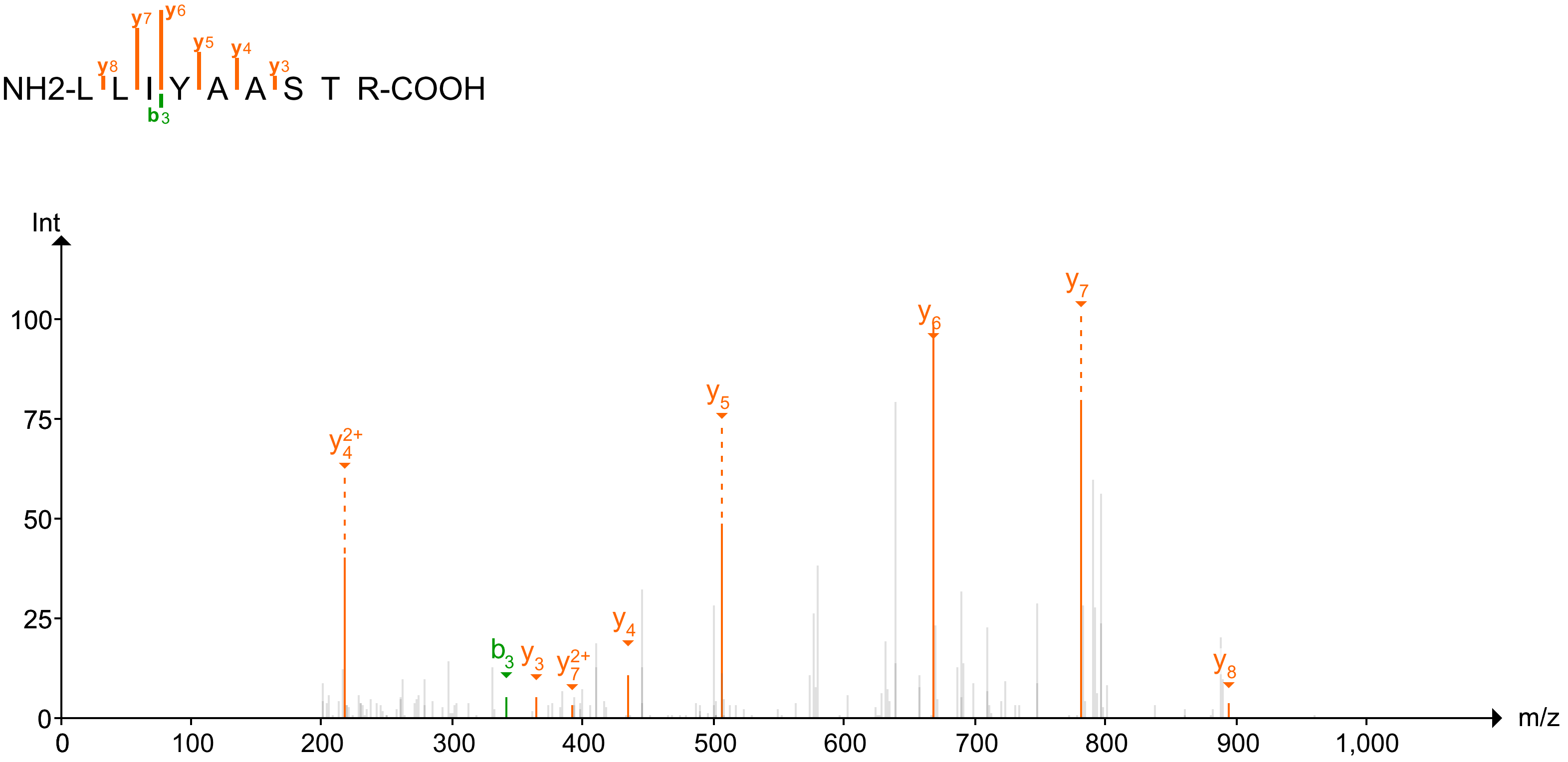

NH2-L L I Y A A S T R-COOH
y8 y7 y6 y5 y4 y3
b3
Int
100
75
50
25
0
0 100 200 300 400 500 600 700 800 900 1,000
m/z
y4 2+
b3 y3 y7 2+ y4 y5 y6 y7 y8

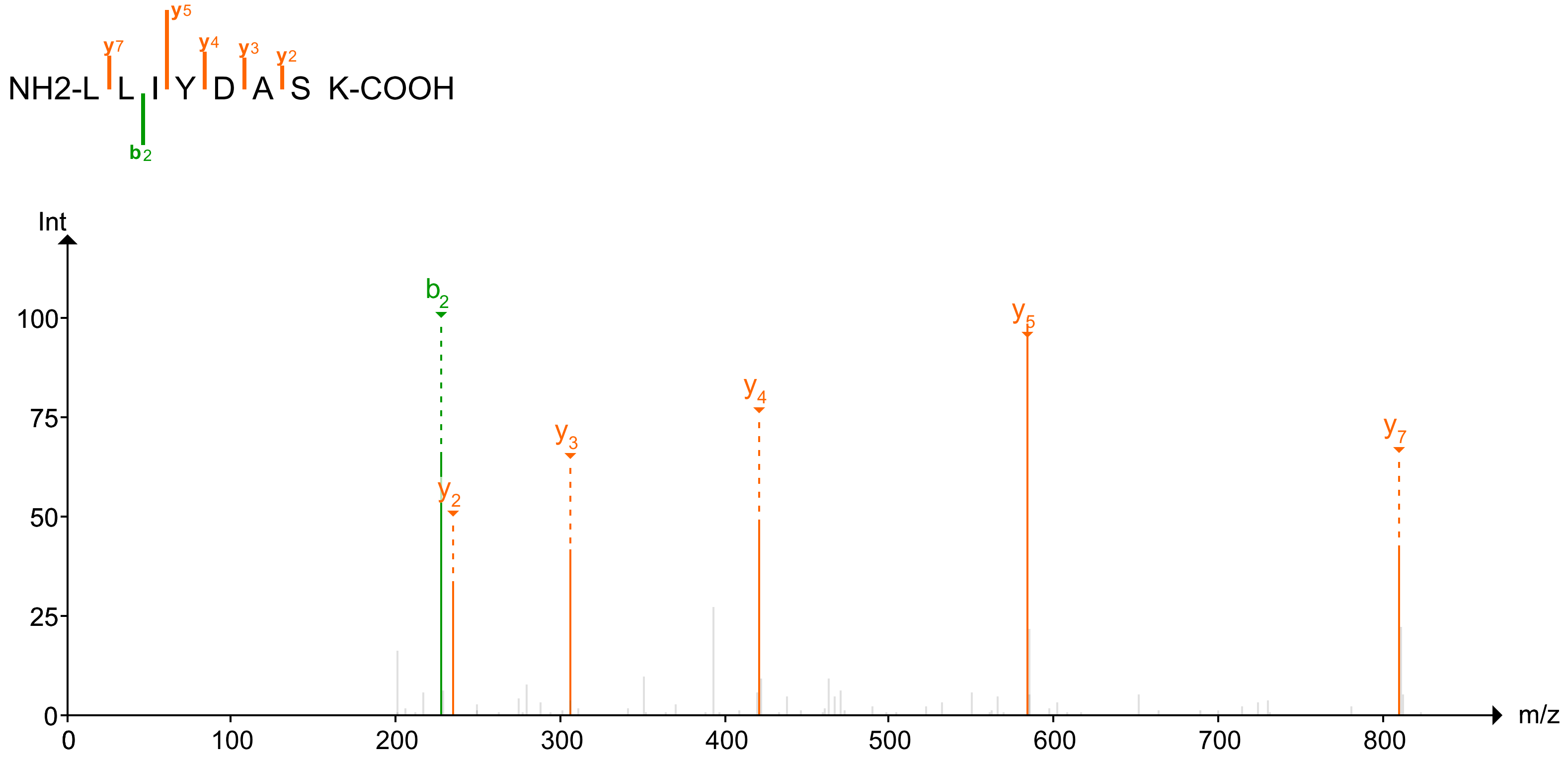

NH2-L L I Y D A S K-COOH
y7 y5 y4 y3 y2
b2
Int
m/z
0 25 50 75 100
0 100 200 300 400 500 600 700 800
b2 y2 y3 y4 y5 y7

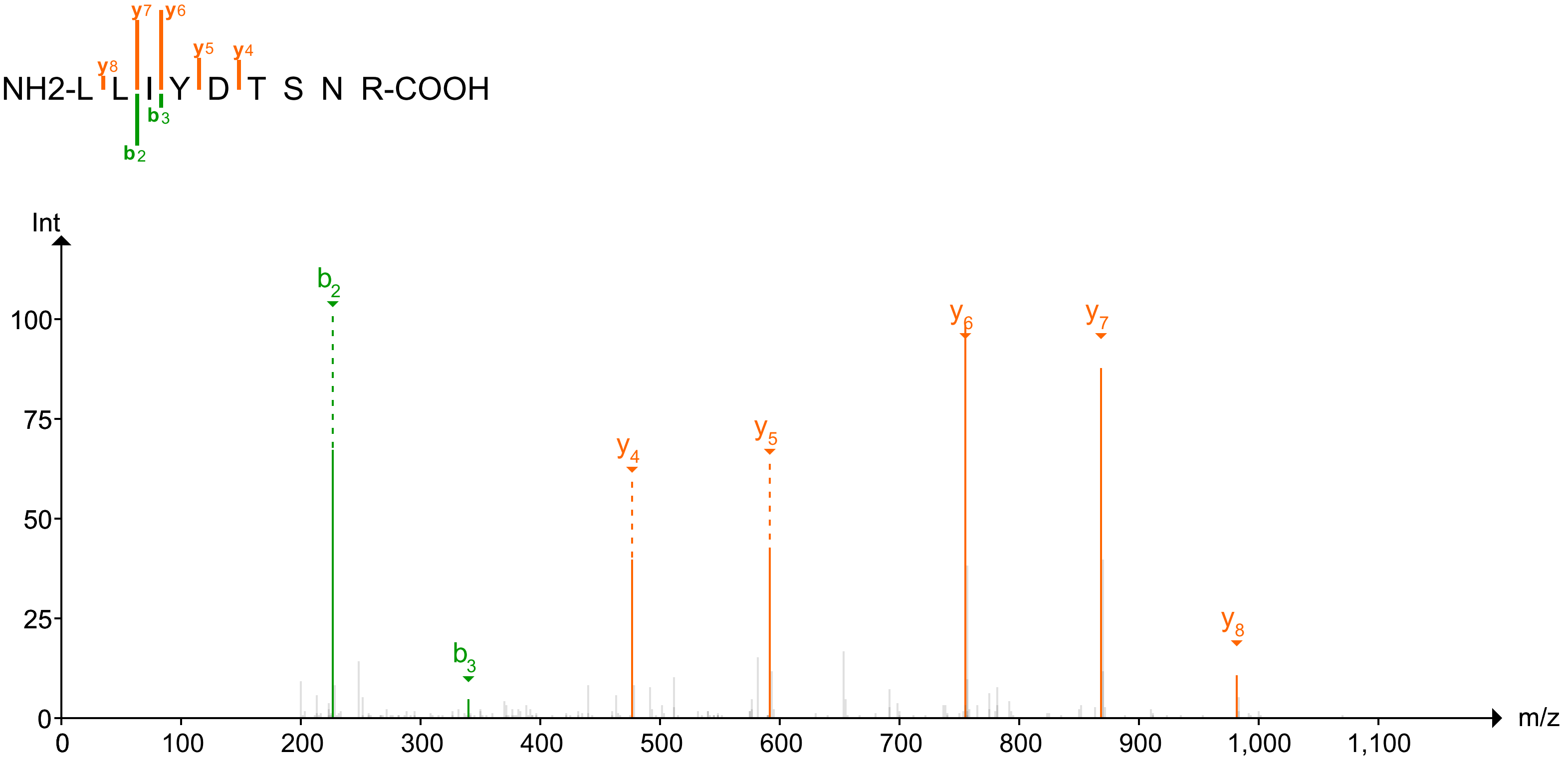

NH2-L L I Y D T S N R-COOH
y8
y7
y6
y5
y4
b2
b3
Int
100
75
50
25
0
b2
b3
y4
y5
y6
y7
y8
0
100
200
300
400
500
600
700
800
900
1,000
1,100
m/z

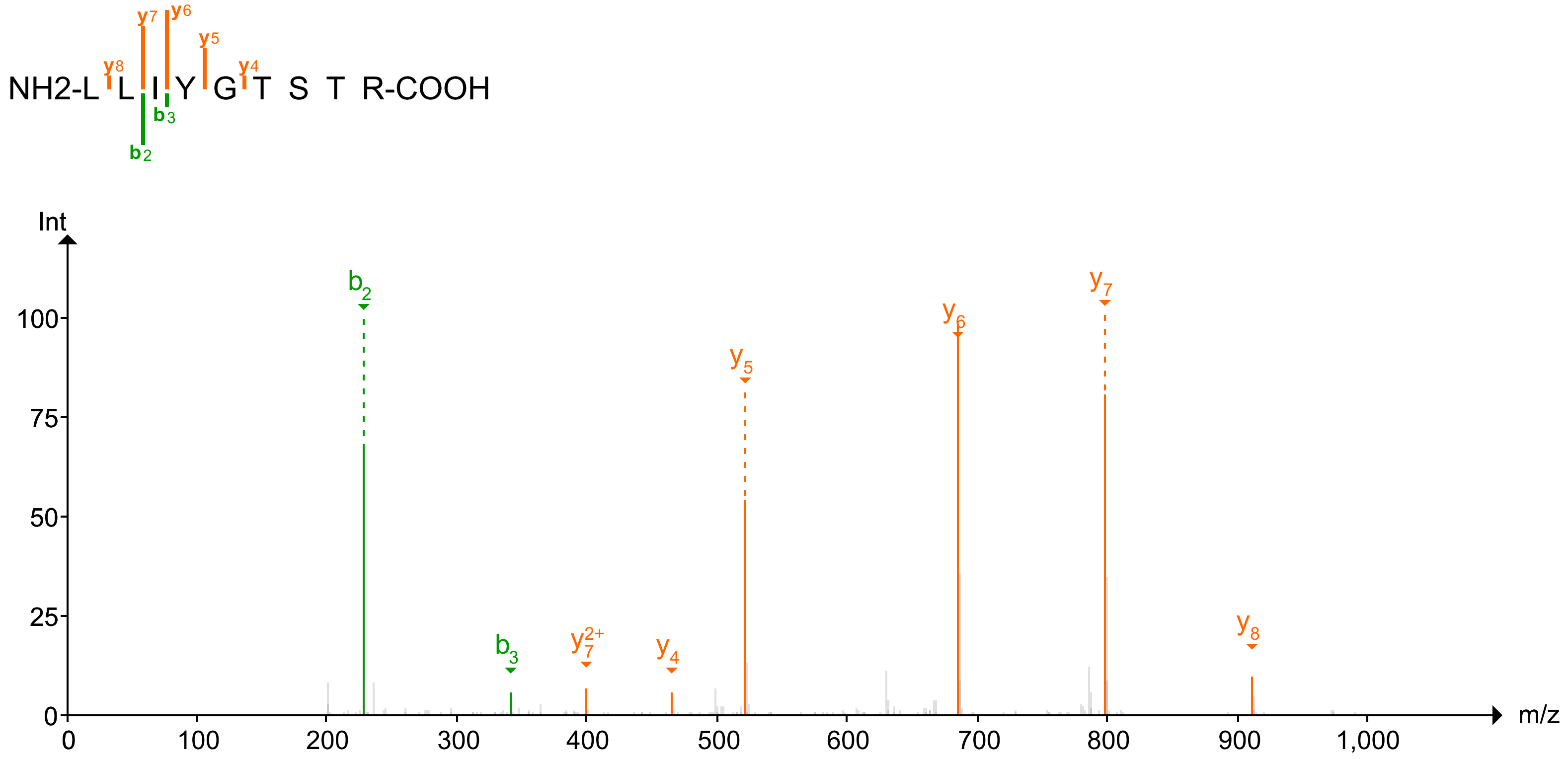

NH2-L L I Y G T S T R-COOH
y8 y7 y6 y5 y4
b2 b3
Int
m/z
100
75
50
25
0
0
100
200
300
400
500
600
700
800
900
1,000
b2
b3
y7 2+
y4
y5
y6
y7
y8

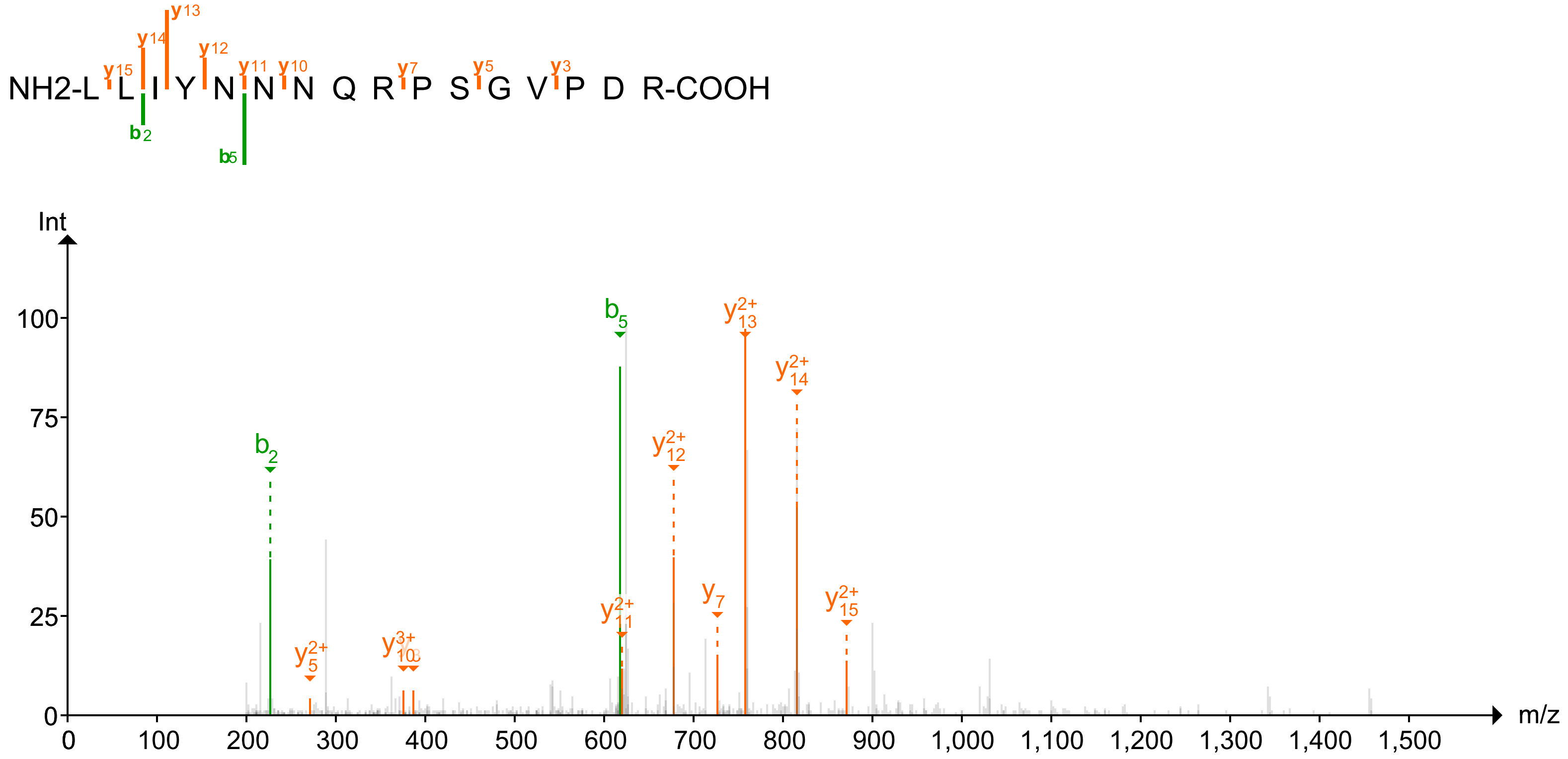

NH2-L L I Y N N N Q R P S G V P D R-COOH
y15 y14 y13 y12 y11 y10 y7 y5 y3
b2 b5
Int
m/z
b2
b5
$y_{13}^{2+}$
$y_{14}^{2+}$
$y_{12}^{2+}$
$y_{11}^{2+}$
$y_7$
$y_{15}^{2+}$
$y_5^{2+}$
$y_{10}^{3+}$
0 25 50 75 100
0 100 200 300 400 500 600 700 800 900 1,000 1,100 1,200 1,300 1,400 1,500

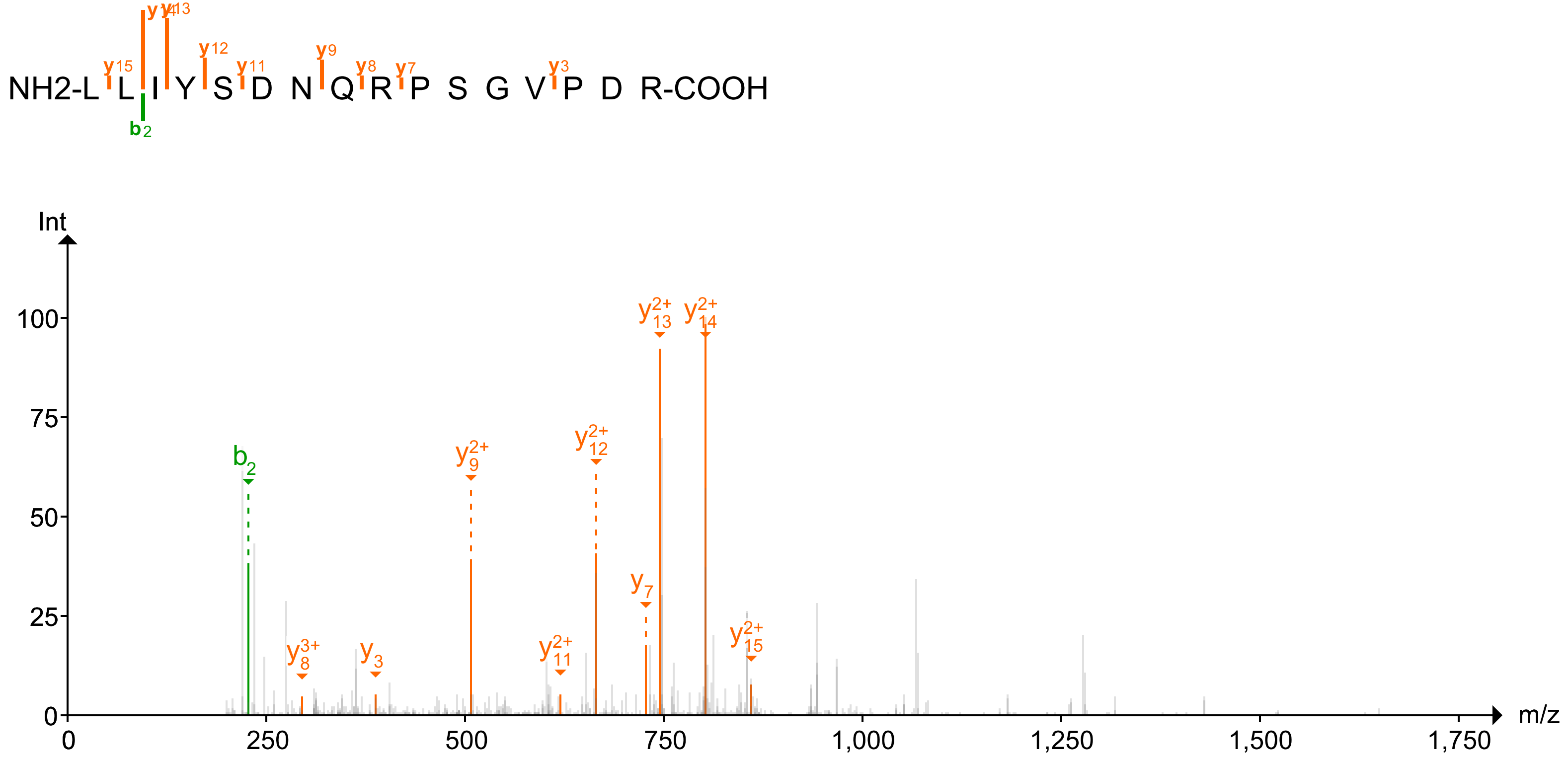

NH2-L L I Y S D N Q R P S G V P D R-COOH
y15 y14 y13 y12 y11 y9 y8 y7 y3
b2
Int
100
75
50
25
0
b2
y8 3+
y3
y9 2+
y11 2+
y12 2+
y7
y13 2+
y14 2+
y15 2+
0
250
500
750
1,000
1,250
1,500
1,750
m/z

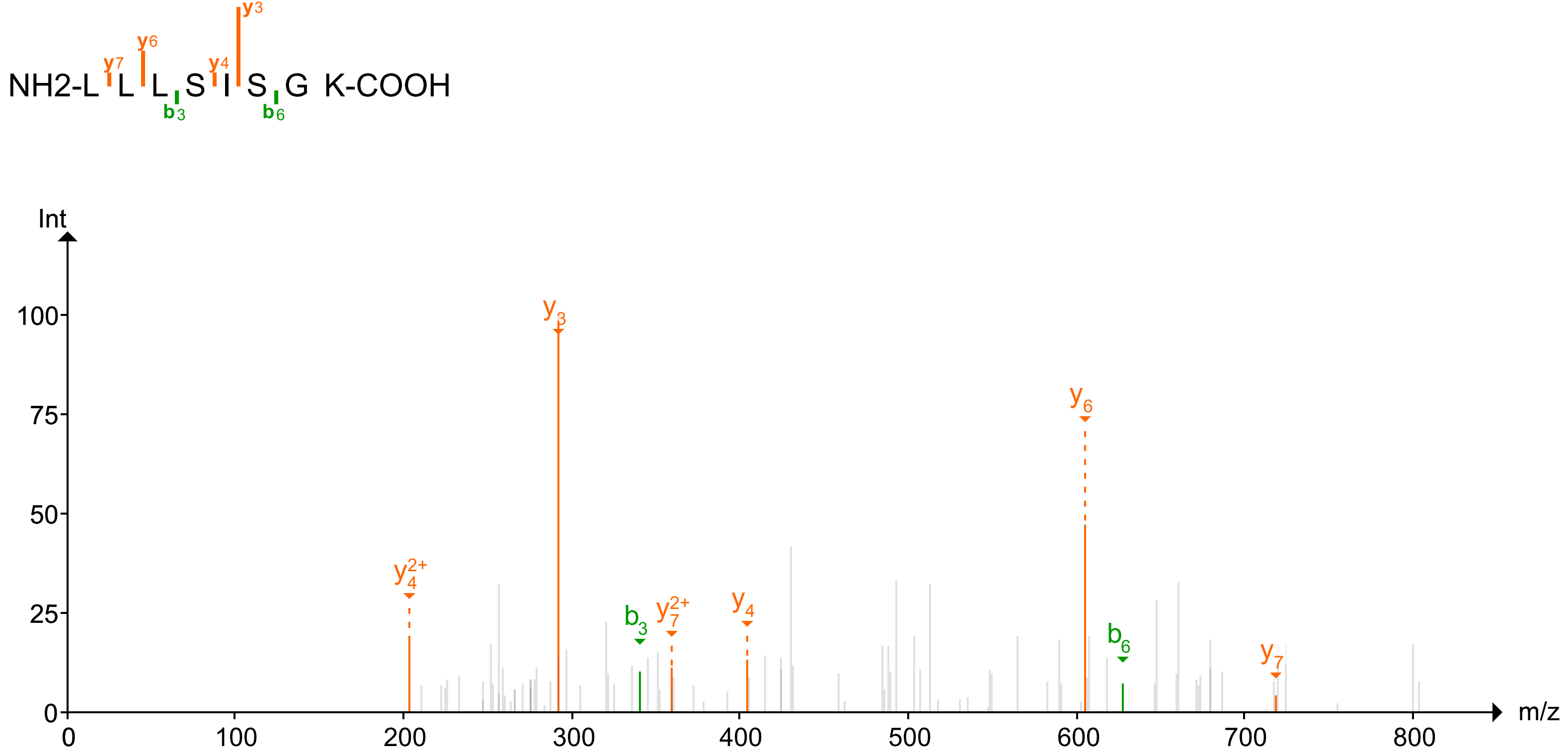

NH2-L L L S I S G K-COOH
y7
y6
y4
y3
b3
b6
Int
100
75
50
25
0
y3
y6
y4 2+
b3
y7 2+
y4
b6
y7
0
100
200
300
400
500
600
700
800
m/z

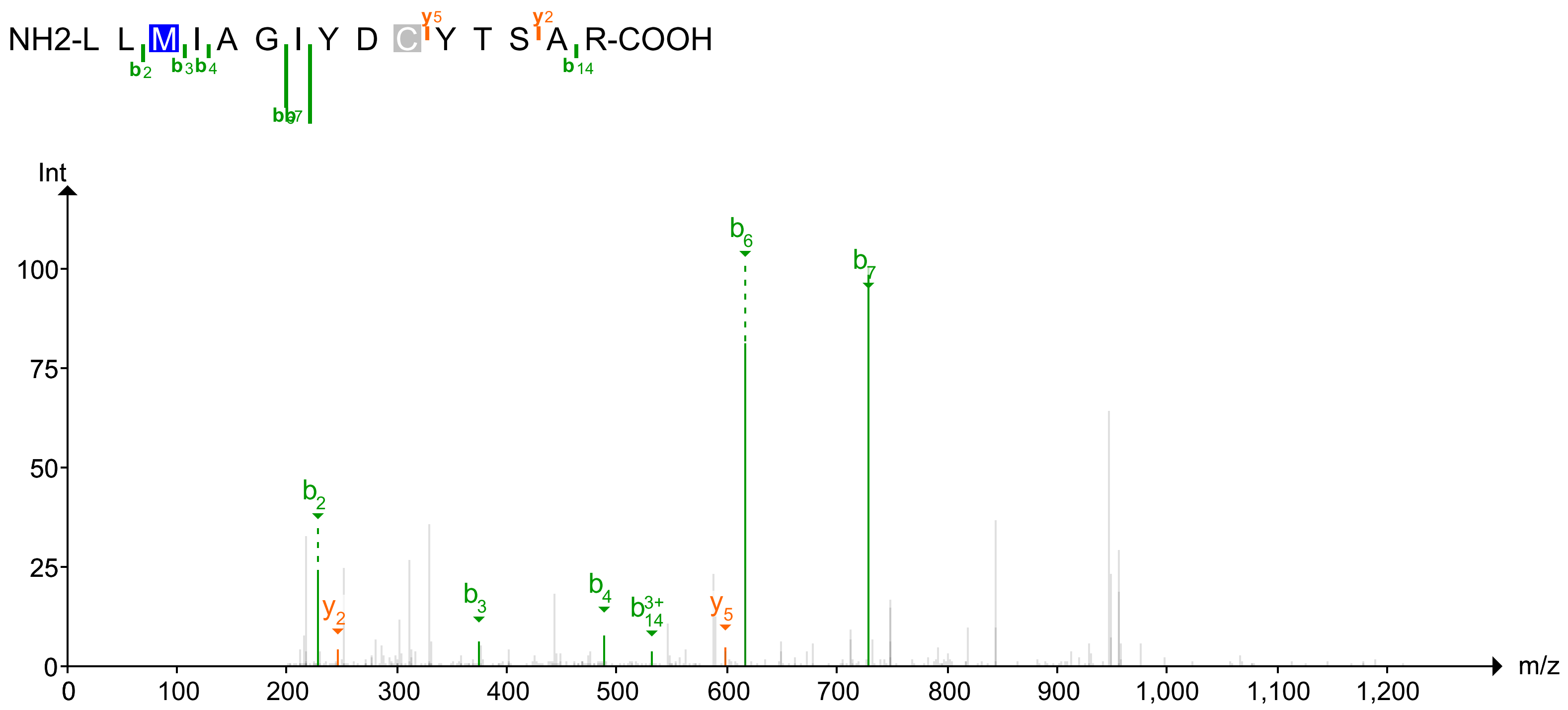

NH2-L L M I A G I Y D C Y T S A R-COOH
y5
y2
b2
b3
b4
b14
Int
100
75
50
25
0
b6
b7
b2
y2
b3
b4
b3+14
y5
0
100
200
300
400
500
600
700
800
900
1,000
1,100
1,200
m/z

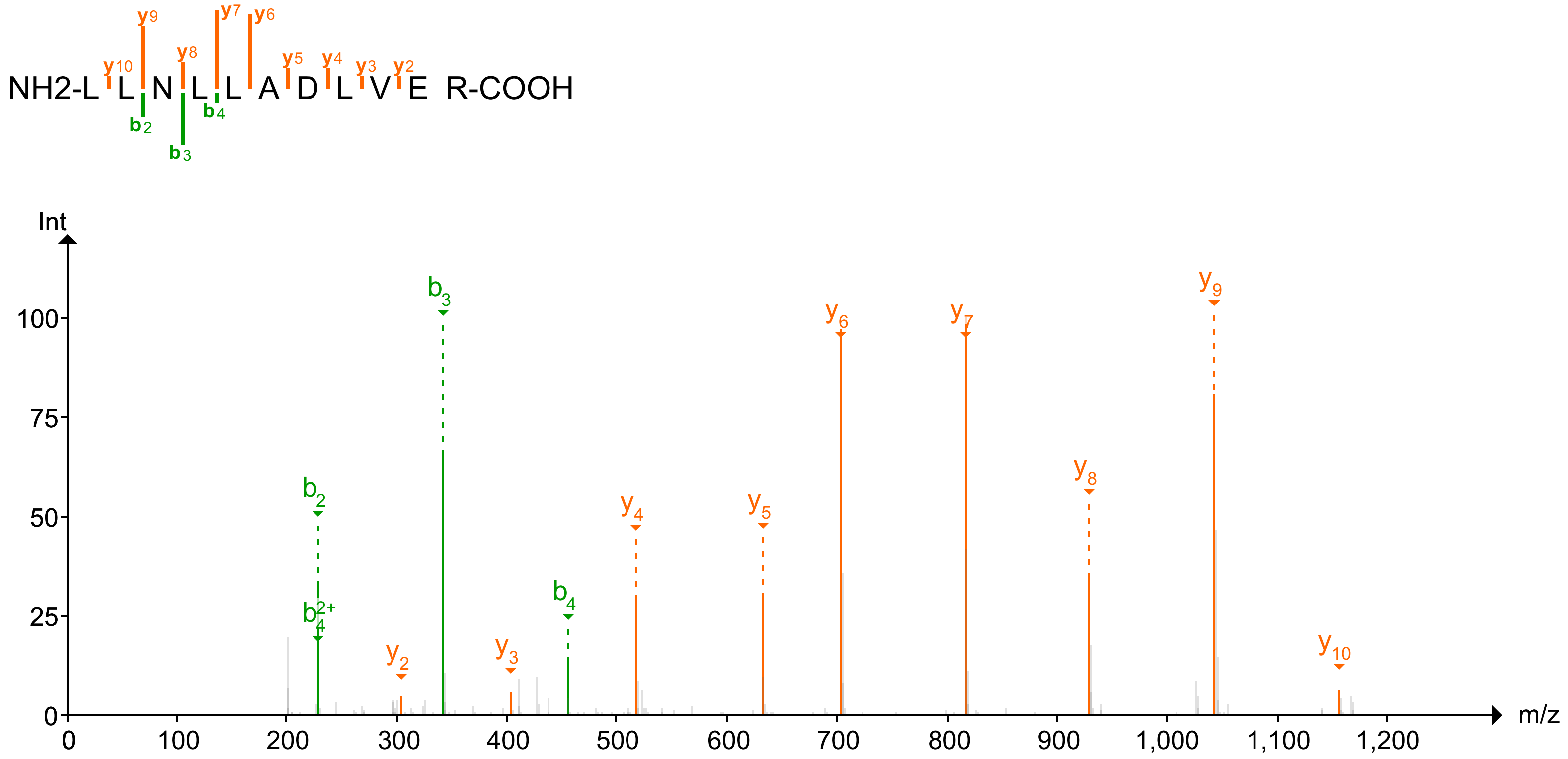

NH2-L L N L L A D L V E R-COOH
y10 y9 y8 y7 y6 y5 y4 y3 y2
b2 b3 b4
Int
m/z
b3 y6 y7 y9 y8 b2 y4 y5 b4 b4 2+ y2 y3 y10

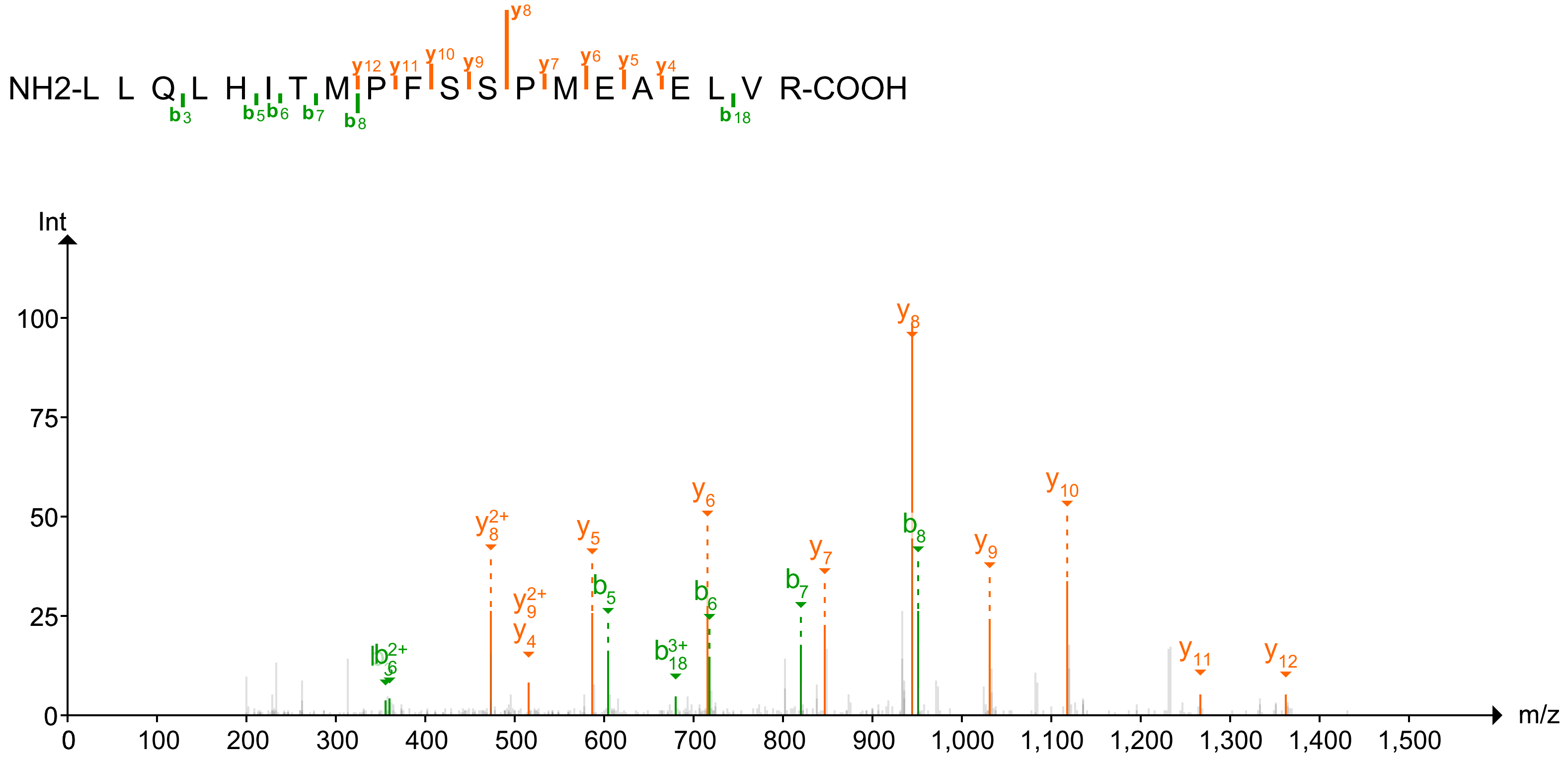

NH2-L L Q L H I T M P F S S P M E A E L V R-COOH
b3 b5 b6 b7 b8 b18
y12 y11 y10 y9 y8 y7 y6 y5 y4
Int
m/z
100
75
50
25
0
0 100 200 300 400 500 600 700 800 900 1,000 1,100 1,200 1,300 1,400 1,500

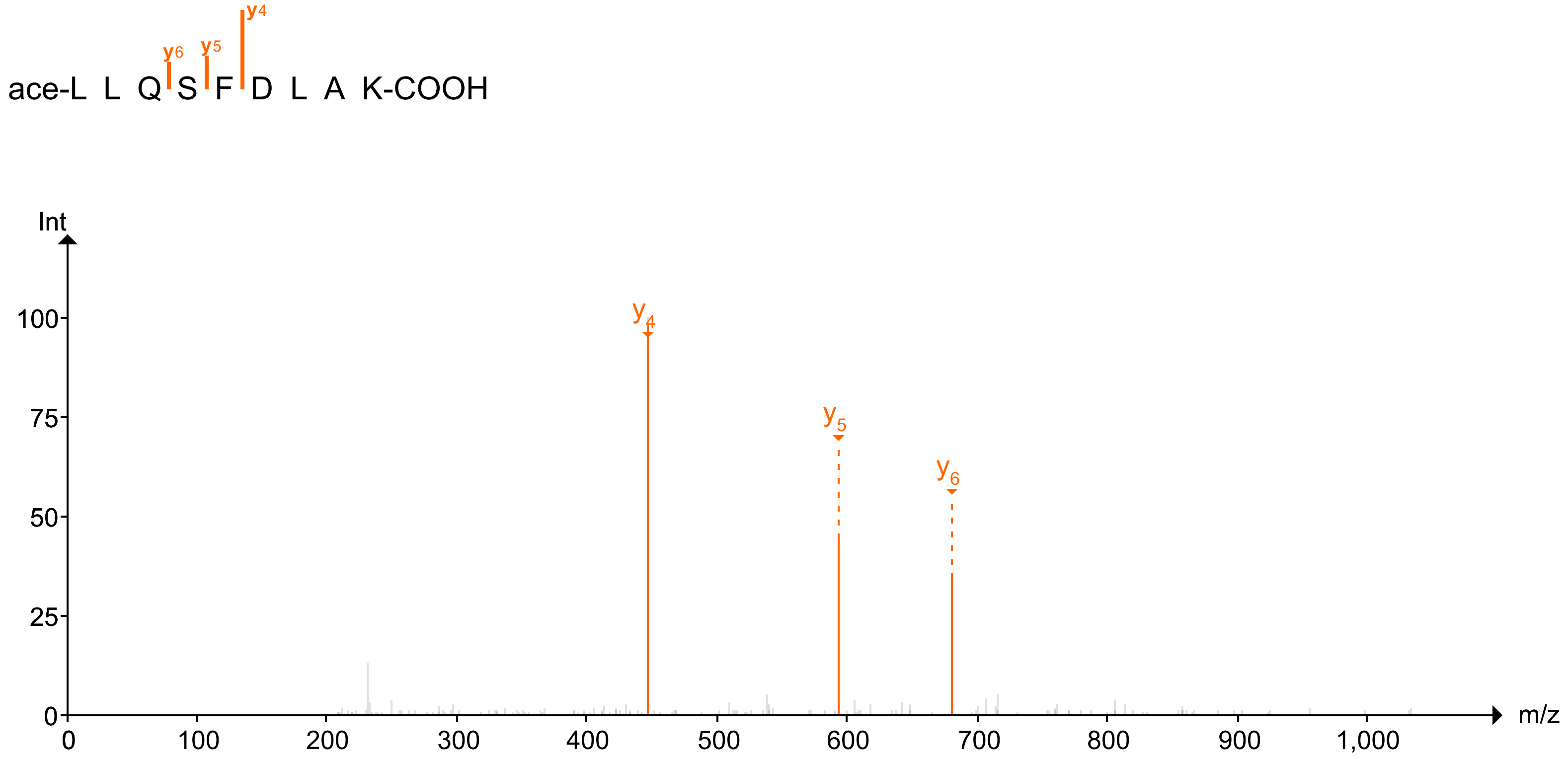

y4
y6
y5
ace-L L Q S F D L A K-COOH
Int
100
75
50
25
0
y4
y5
y6
0
100
200
300
400
500
600
700
800
900
1,000
m/z

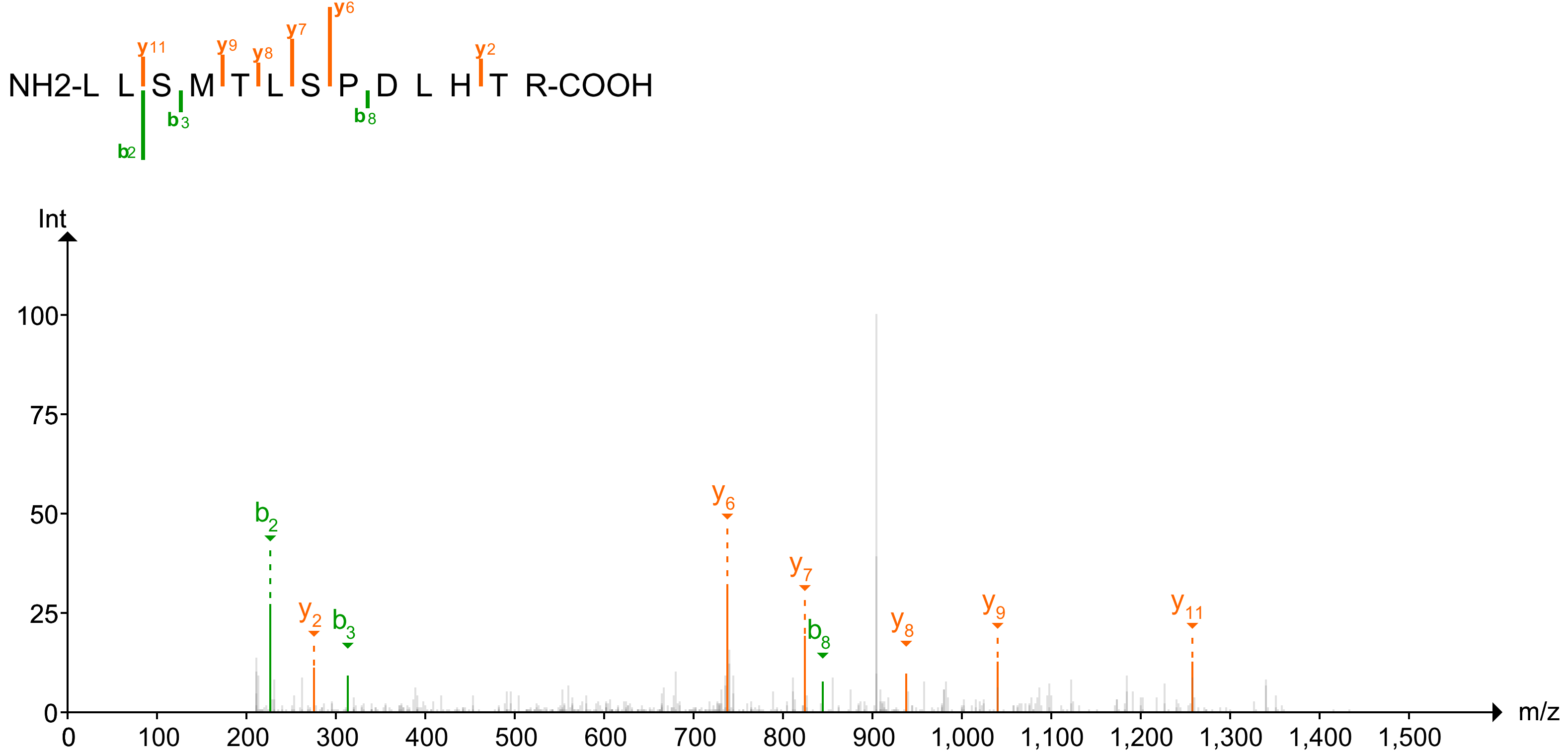

NH2-L L S M T L S P D L H T R-COOH
y11 y9 y8 y7 y6 y2
b2 b3 b8
Int
100
75
50
25
0
m/z
0 100 200 300 400 500 600 700 800 900 1,000 1,100 1,200 1,300 1,400 1,500
b2 y2 b3 y6 y7 b8 y8 y9 y11

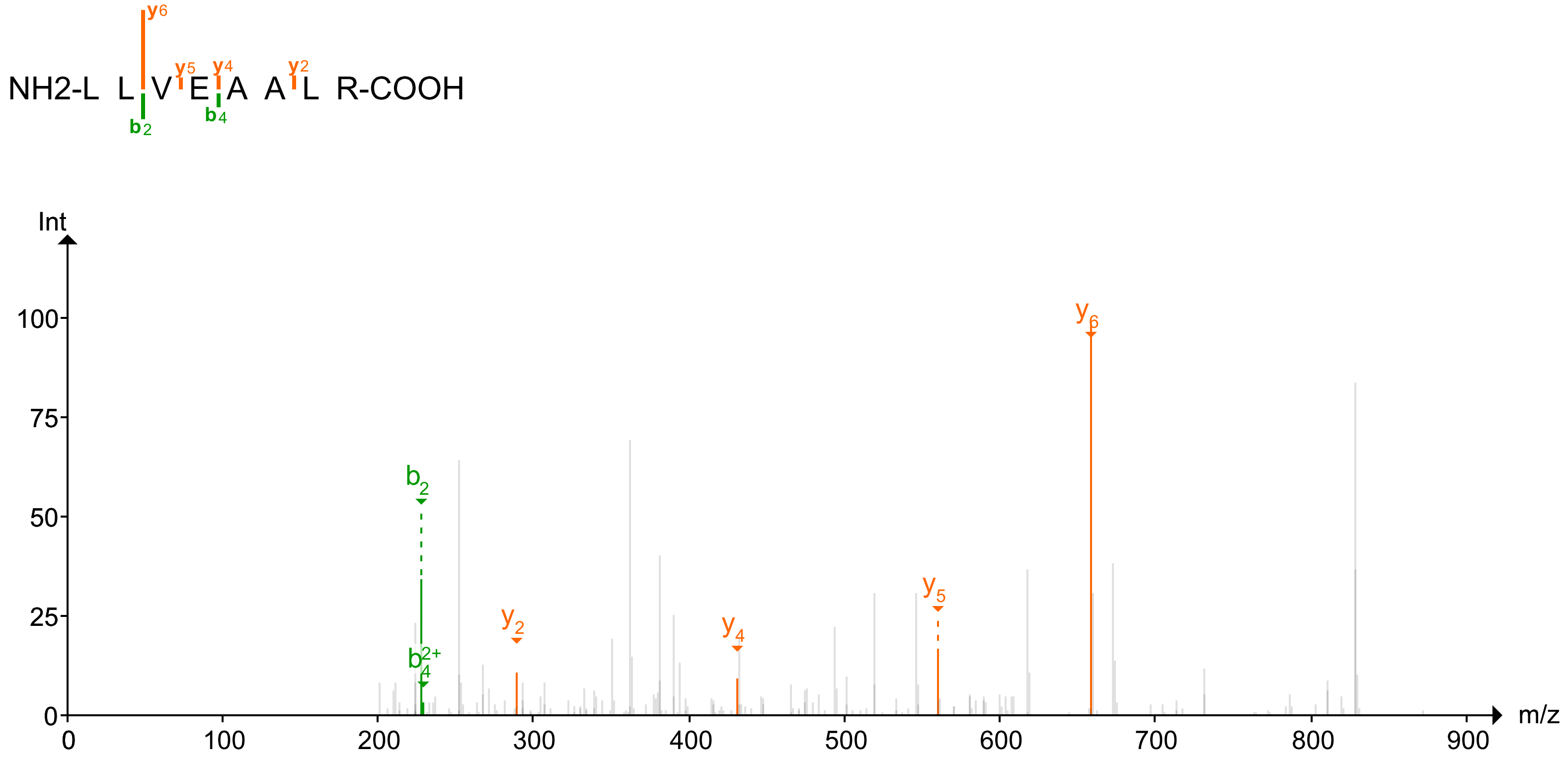
NH2-L L V E A A L R-COOH
y6
y5
y4
y2
b2
b4
Int
100
75
50
25
0
m/z
0
100
200
300
400
500
600
700
800
900
b2
y2
y4
y5
y6
b4 2+

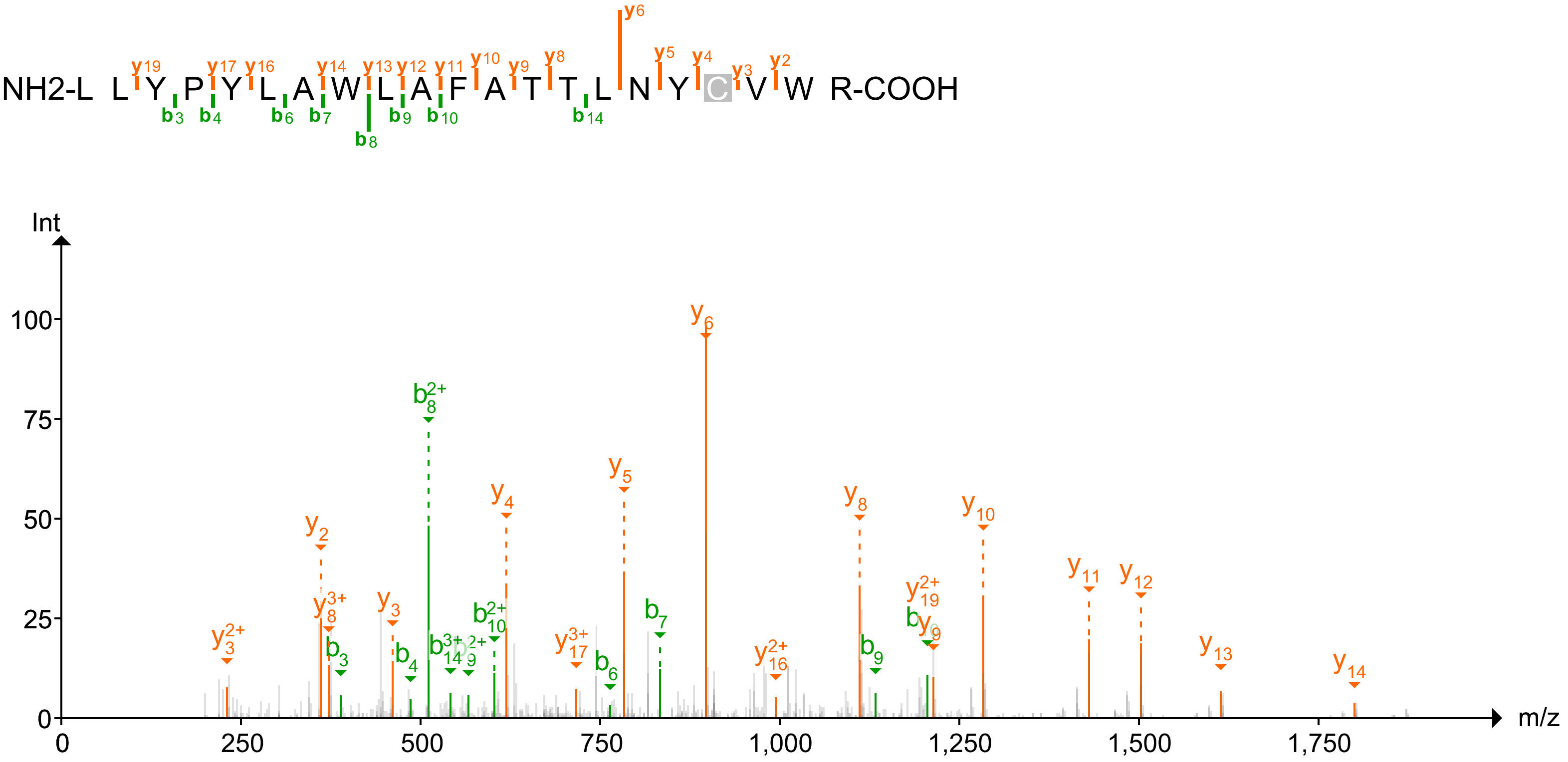

NH2-L L Y P Y L A W L A F A T T L N Y C V W R-COOH
Int
m/z

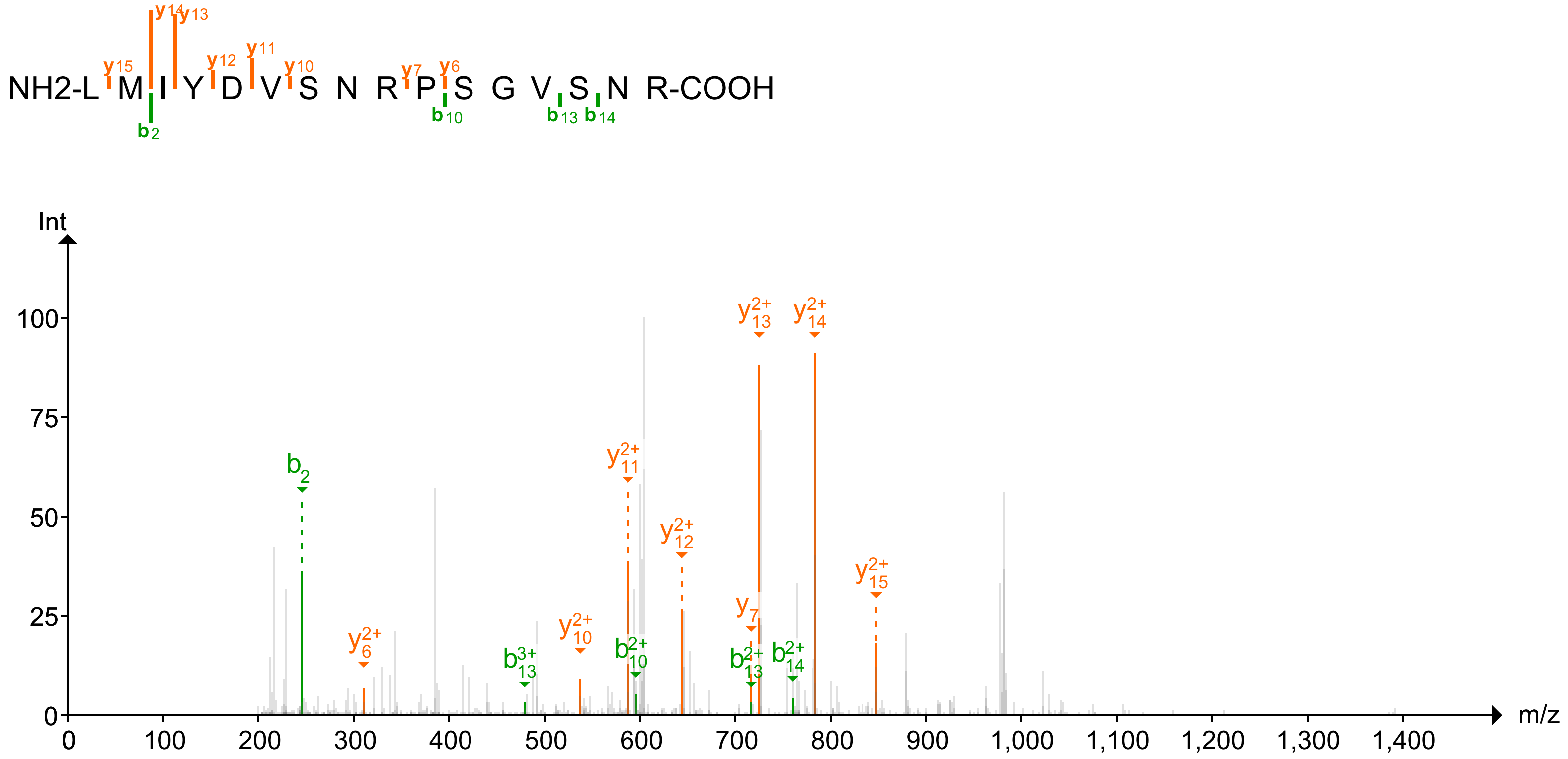

NH2-L M I Y D V S N R P S G V S N R-COOH
y15 y14 y13 y12 y11 y10 y7 y6
b2 b10 b13 b14
Int
100
75
50
25
0
b2
$y_6^{2+}$
$b_{13}^{3+}$
$y_{10}^{2+}$
$y_{11}^{2+}$
$b_{10}^{2+}$
$y_{12}^{2+}$
$y_7$
$b_{13}^{2+}$
$y_{13}^{2+}$
$y_{14}^{2+}$
$b_{14}^{2+}$
$y_{15}^{2+}$
0 100 200 300 400 500 600 700 800 900 1,000 1,100 1,200 1,300 1,400
m/z

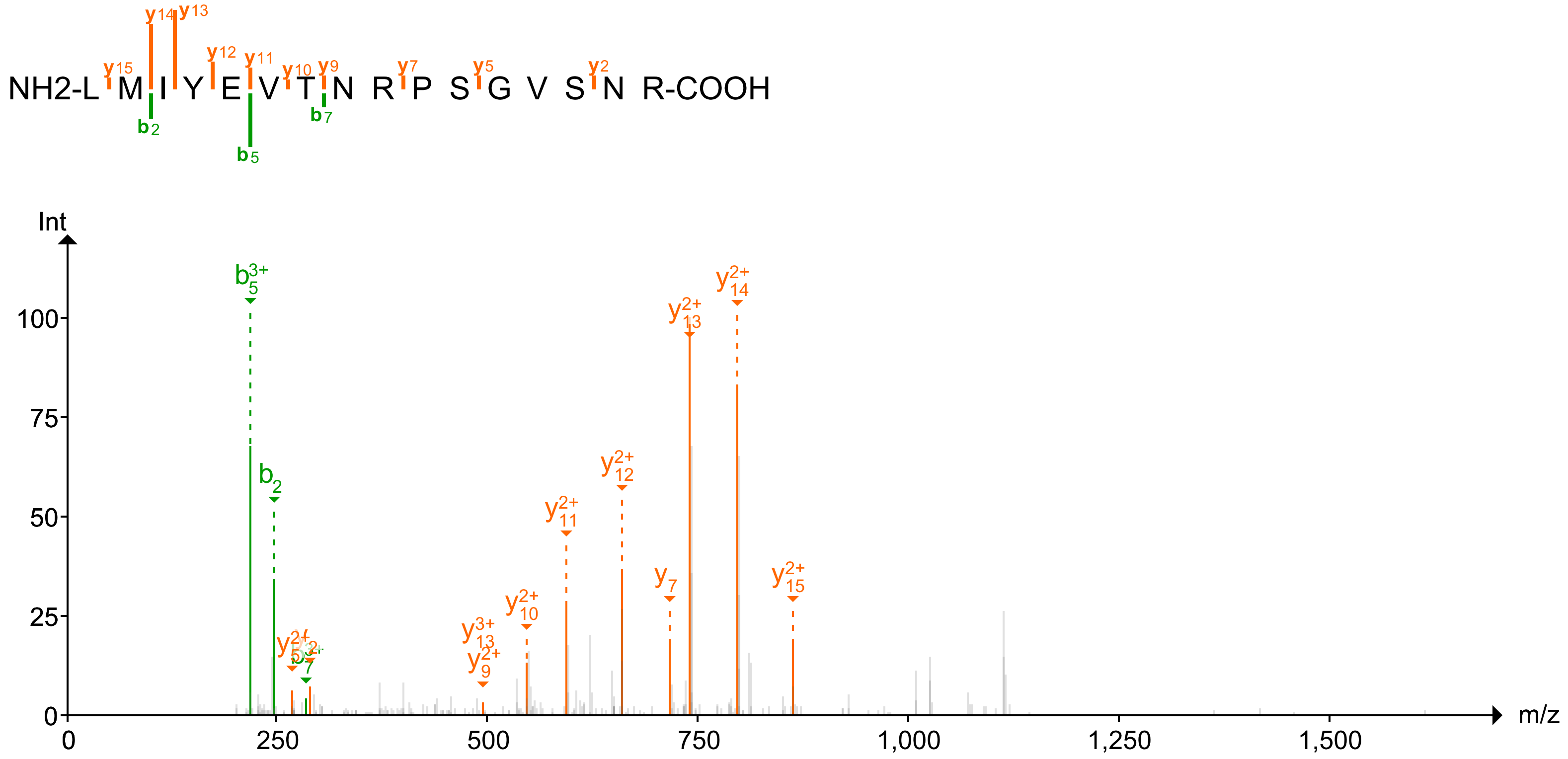

NH2-L M I Y E V T N R P S G V S N R-COOH
y15 y14 y13 y12 y11 y10 y9 y7 y5 y2
b2 b5 b7
Int
100
75
50
25
0
0
250
500
750
1,000
1,250
1,500
m/z

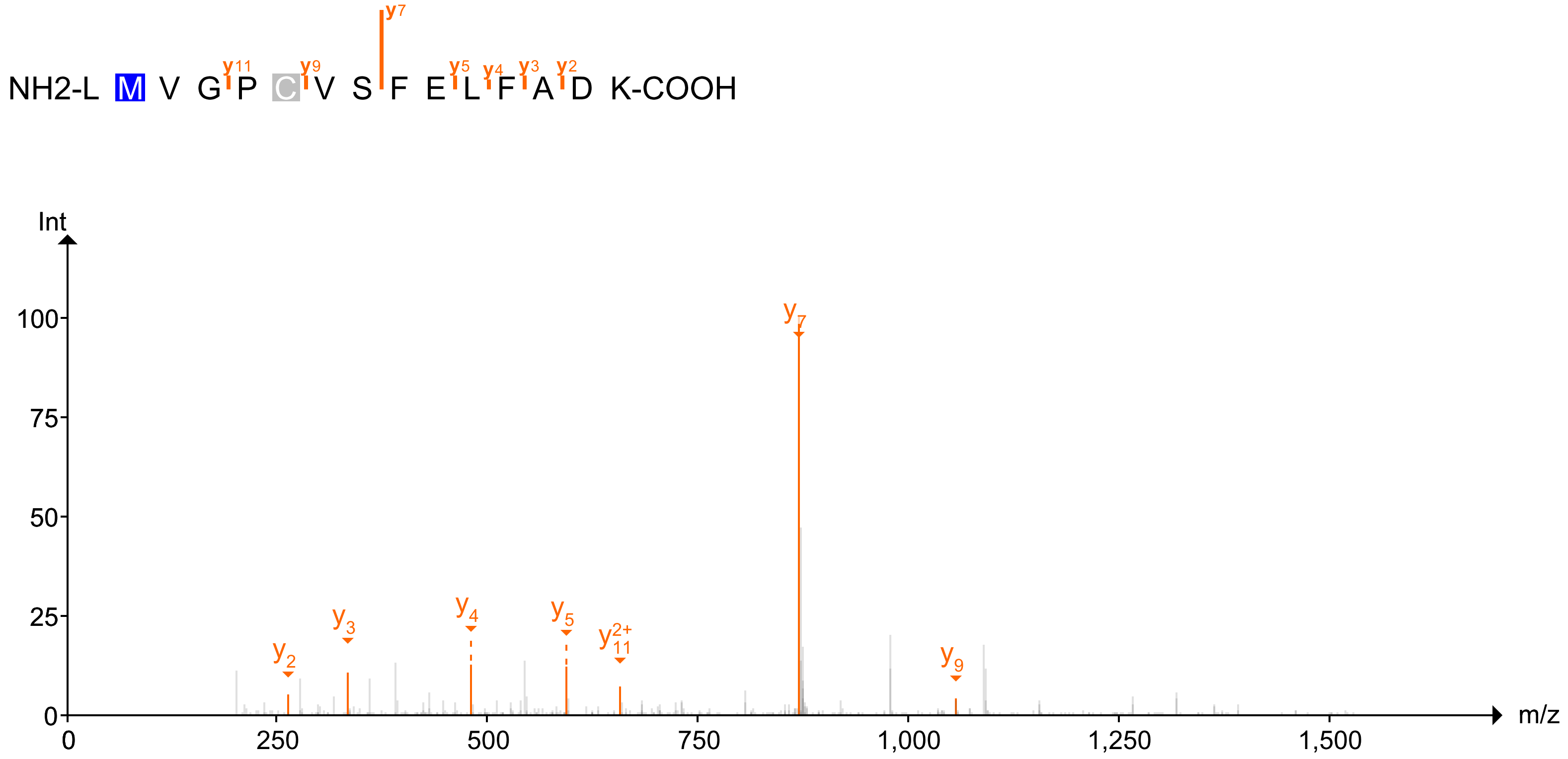

NH2-L M V G P C V S F E L F A D K-COOH
y11 y9 y7 y5 y4 y3 y2
Int
100
75
50
25
0
y2
y3
y4
y5
y11 2+
y7
y9
0
250
500
750
1,000
1,250
1,500
m/z

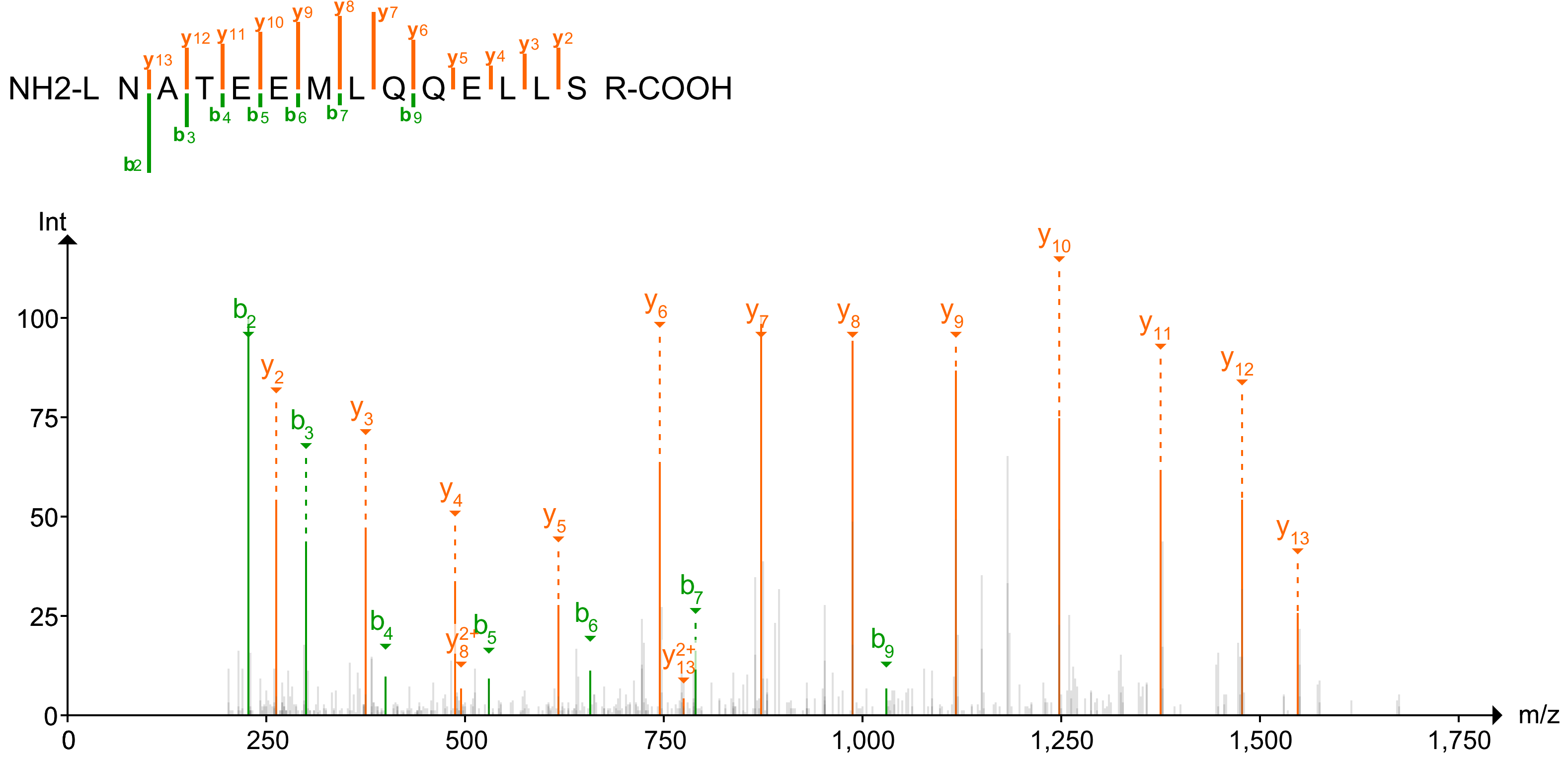

NH2-L N A T E E M L Q Q E L L S R-COOH
y13 y12 y11 y10 y9 y8 y7 y6 y5 y4 y3 y2
b2 b3 b4 b5 b6 b7 b9
Int
m/z
0
25
50
75
100
250
500
750
1,000
1,250
1,500
1,750

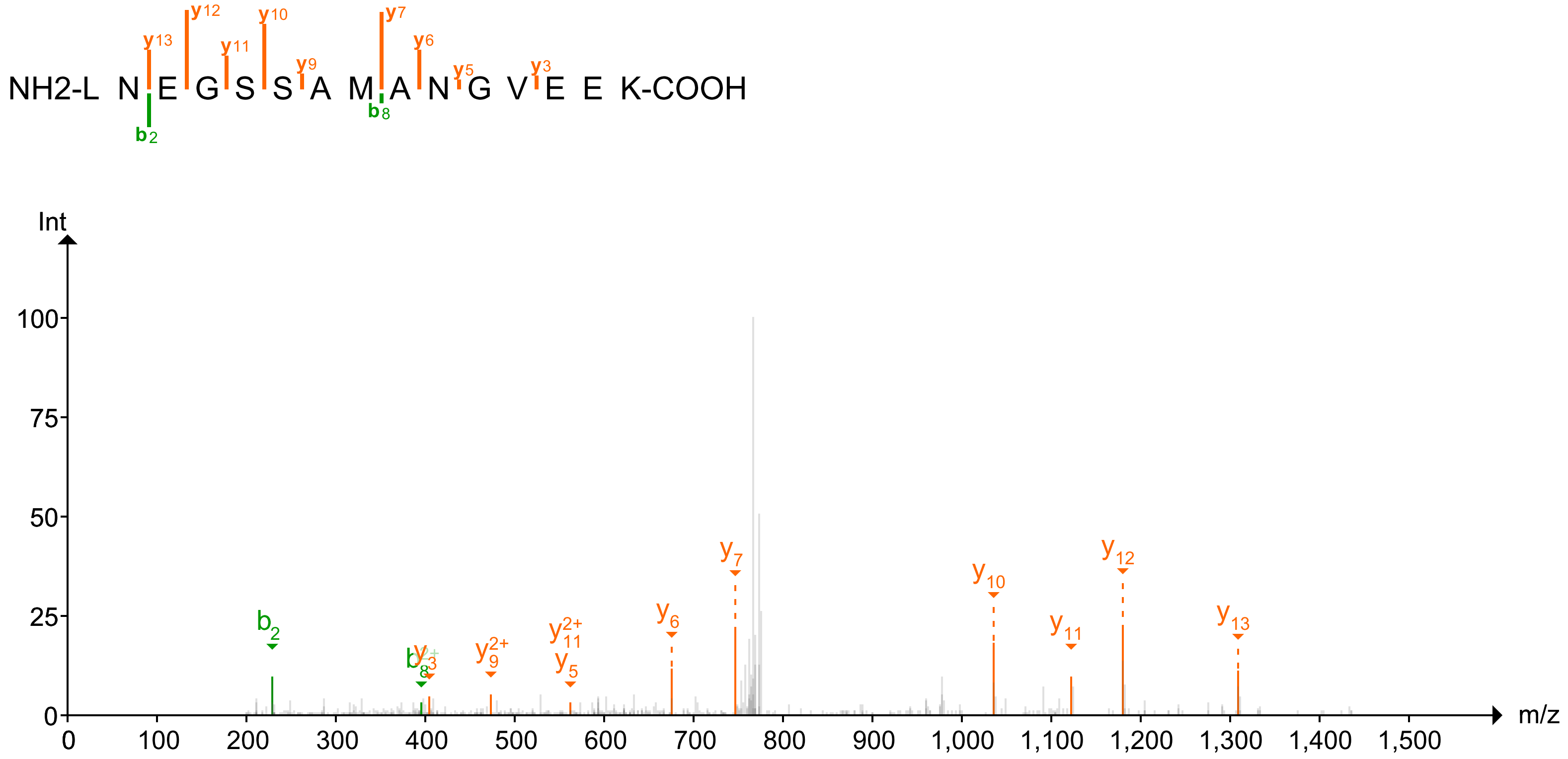
NH2-L N E G S S A M A N G V E E K-COOH
y13 y12 y11 y10 y9 y7 y6 y5 y3
b2 b8
Int
100
75
50
25
0
b2
b8
y3
y9 2+
y11 2+
y5
y6
y7
y10
y11
y12
y13
0 100 200 300 400 500 600 700 800 900 1,000 1,100 1,200 1,300 1,400 1,500
m/z

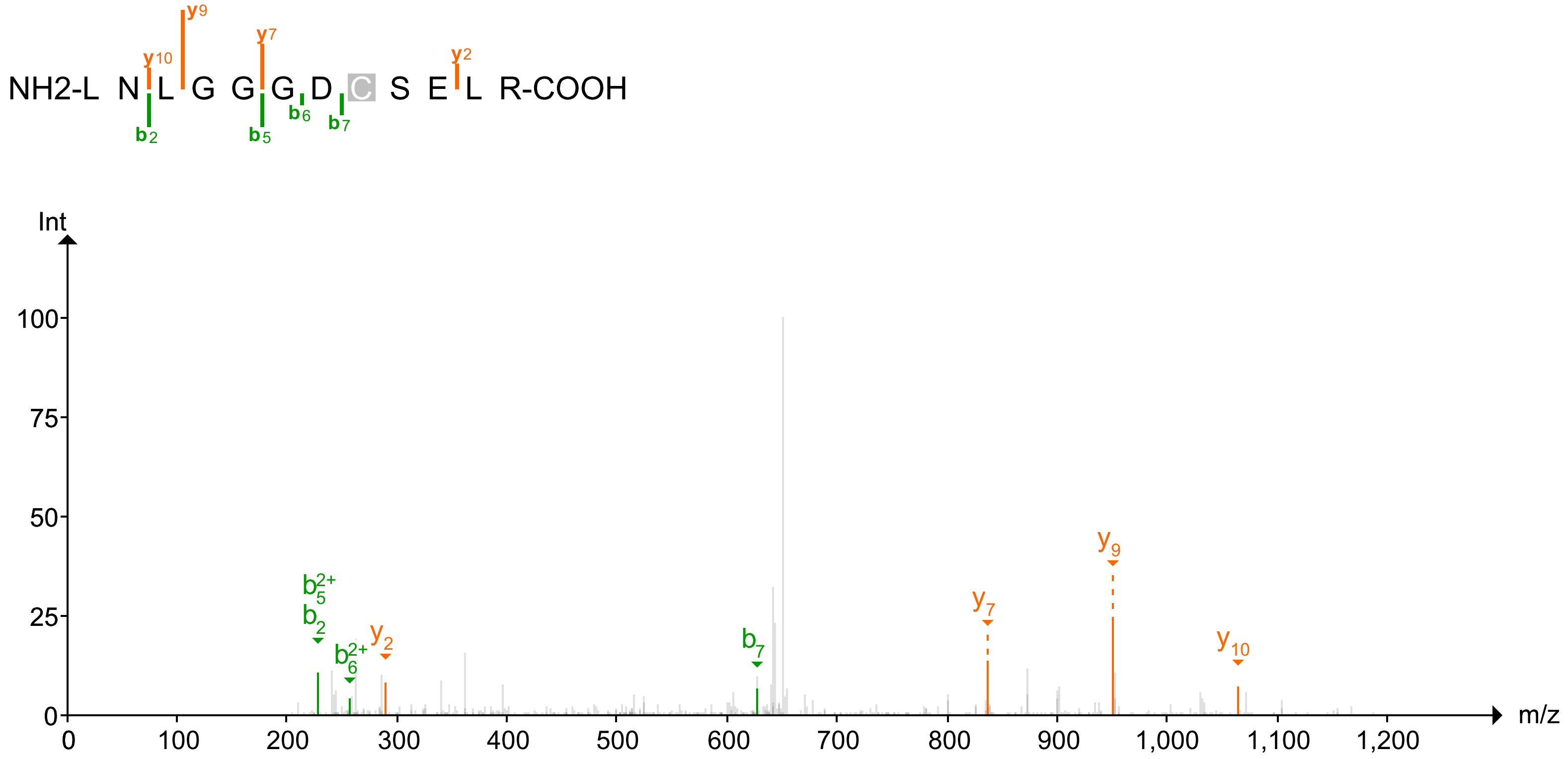

NH2-L N L G G G D C S E L R-COOH
y10
y9
y7
y2
b2
b5
b6
b7
Int
100
75
50
25
0
m/z
0
100
200
300
400
500
600
700
800
900
1,000
1,100
1,200
b5 2+
b2
b6 2+
y2
b7
y7
y9
y10

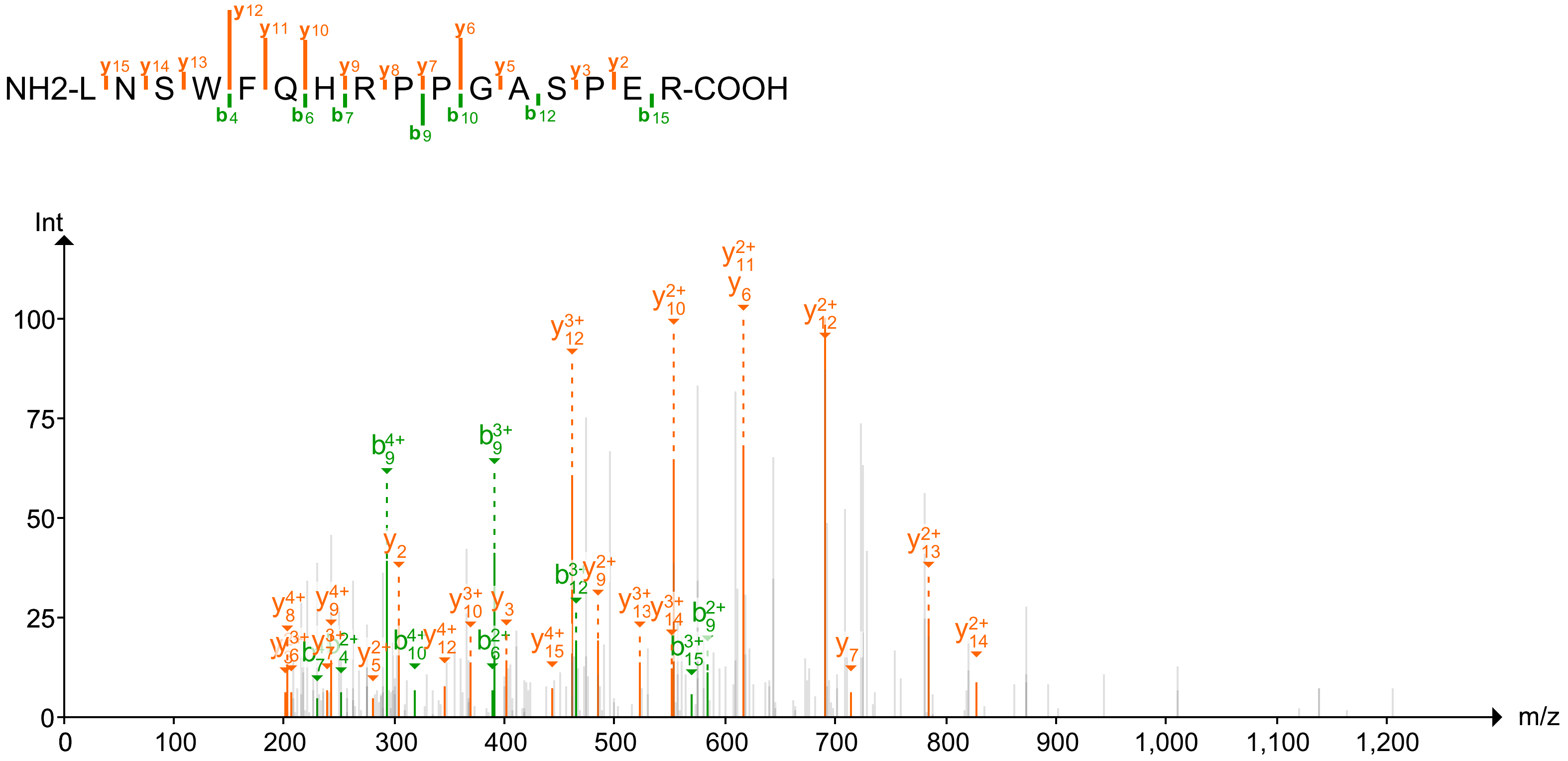
NH2-L N S W F Q H R P P G A S P E R-COOH
y15 y14 y13 y12 y11 y10 y9 y8 y7 y6 y5 y3 y2
b4 b6 b7 b9 b10 b12 b15
Int
100
75
50
25
0
m/z
0
100
200
300
400
500
600
700
800
900
1,000
1,100
1,200

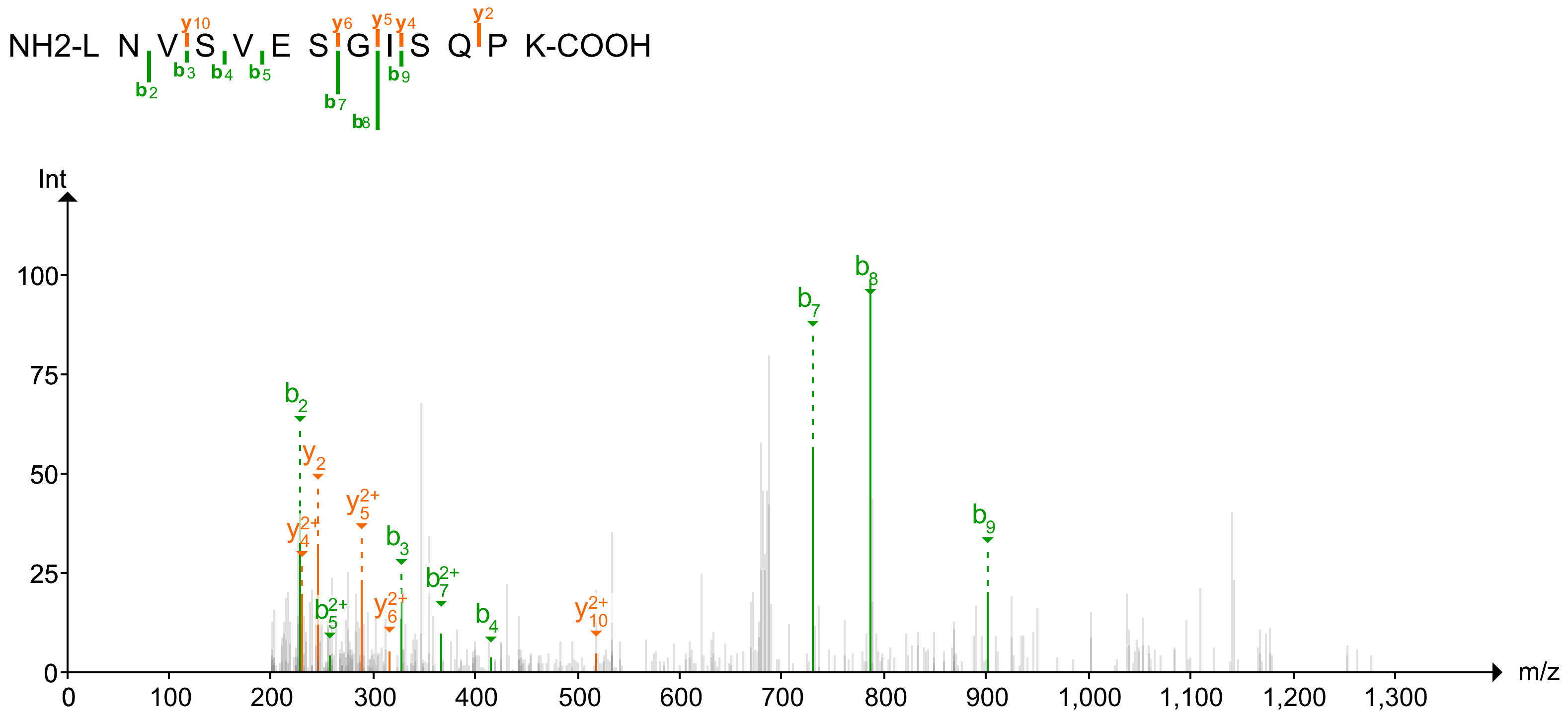

NH2-L N V S V E S G I S Q P K-COOH
y10 y6 y5 y4 y2
b2 b3 b4 b5 b7 b8 b9
Int
m/z
b8
b7
b2
y2
y5 2+
y4 2+
b3
b7 2+
b5 2+
y6 2+
b4
y10 2+
b9

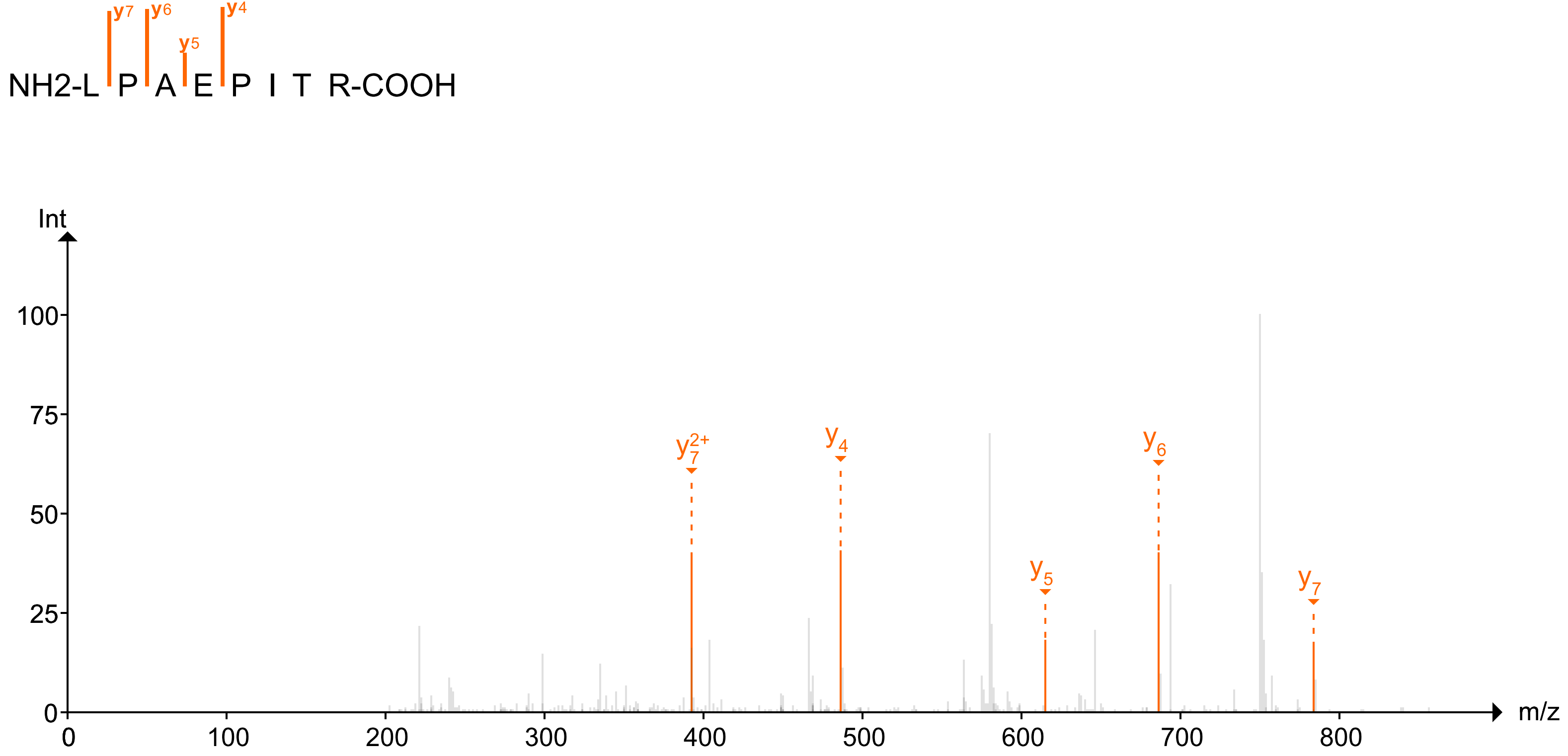

y7
y6
y5
y4
NH2-L P A E P I T R-COOH
Int
100
75
50
25
0
$y_7^{2+}$
$y_4$
$y_5$
$y_6$
$y_7$
0
100
200
300
400
500
600
700
800
m/z

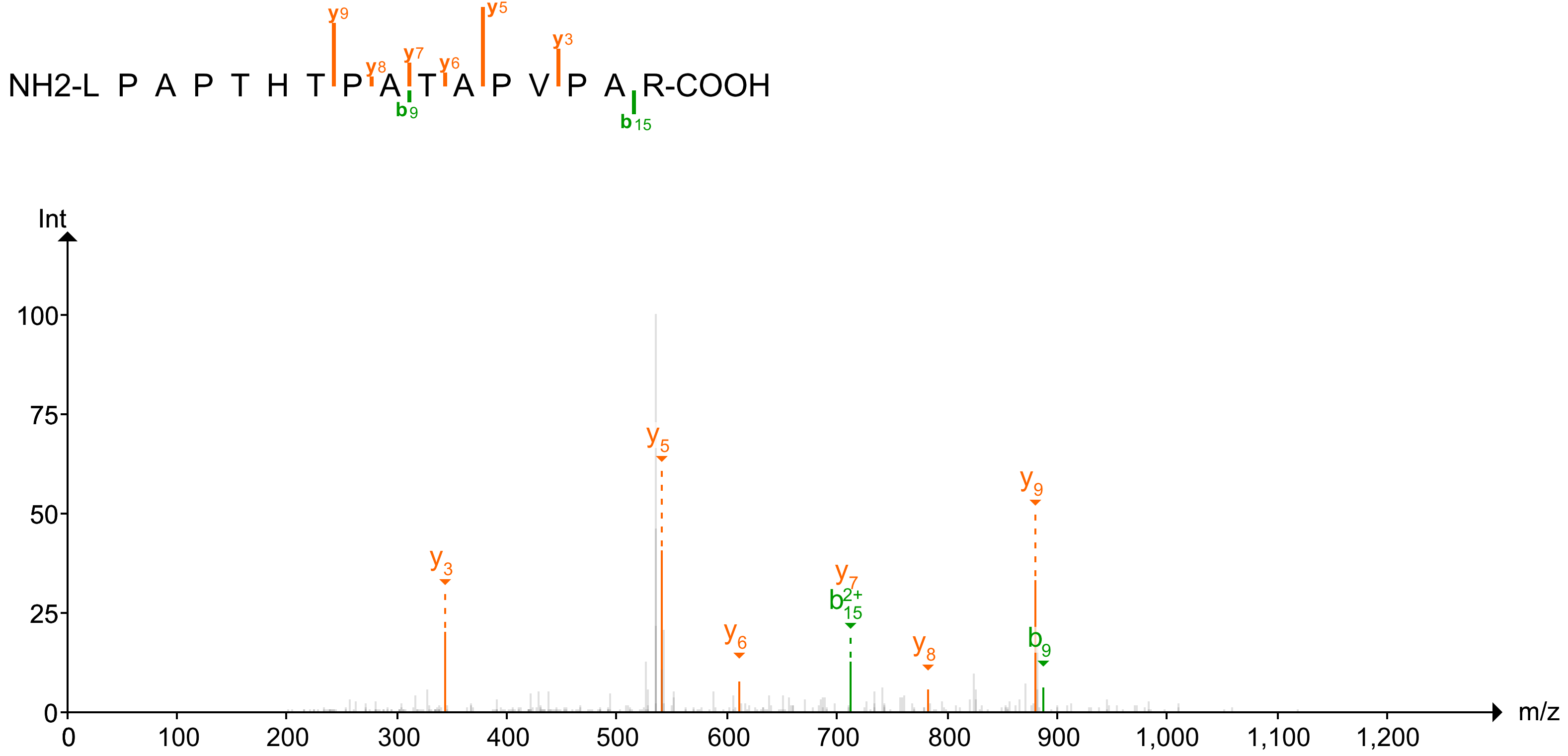

NH2-L P A P T H T P A T A P V P A R-COOH
y9
y8
y7
y6
y5
y3
b9
b15
Int
100
75
50
25
0
y3
y5
y6
y7
b2+15
y8
y9
b9
0
100
200
300
400
500
600
700
800
900
1,000
1,100
1,200
m/z

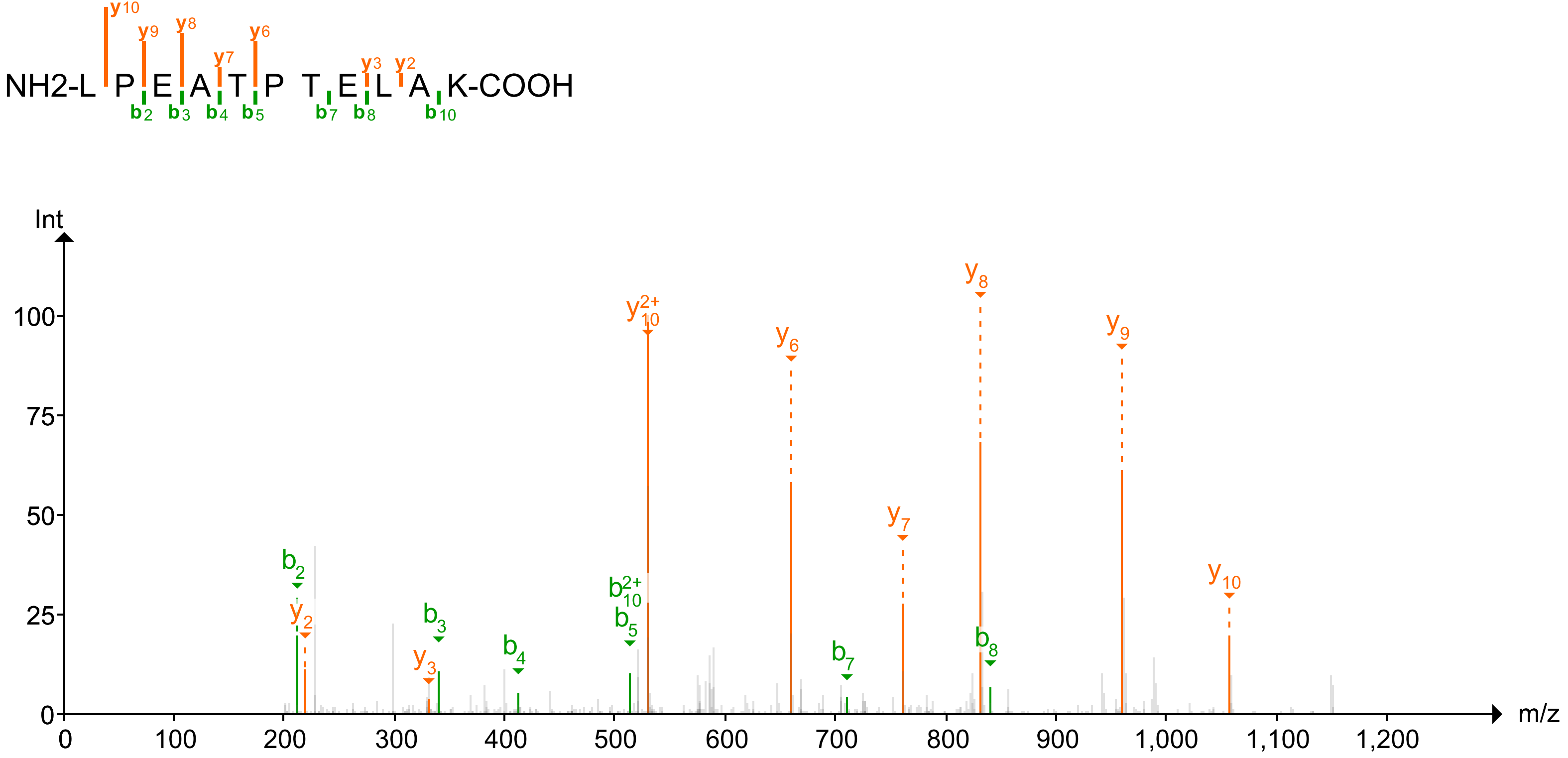

NH2-L P E A T P T E L A K-COOH
y10 y9 y8 y7 y6 y3 y2
b2 b3 b4 b5 b7 b8 b10
Int
100
75
50
25
0
m/z
0
100
200
300
400
500
600
700
800
900
1,000
1,100
1,200
y8
y10 2+
y6
y9
y7
y10
b2
y2
b3
y3
b4
b10 2+
b5
b7
b8

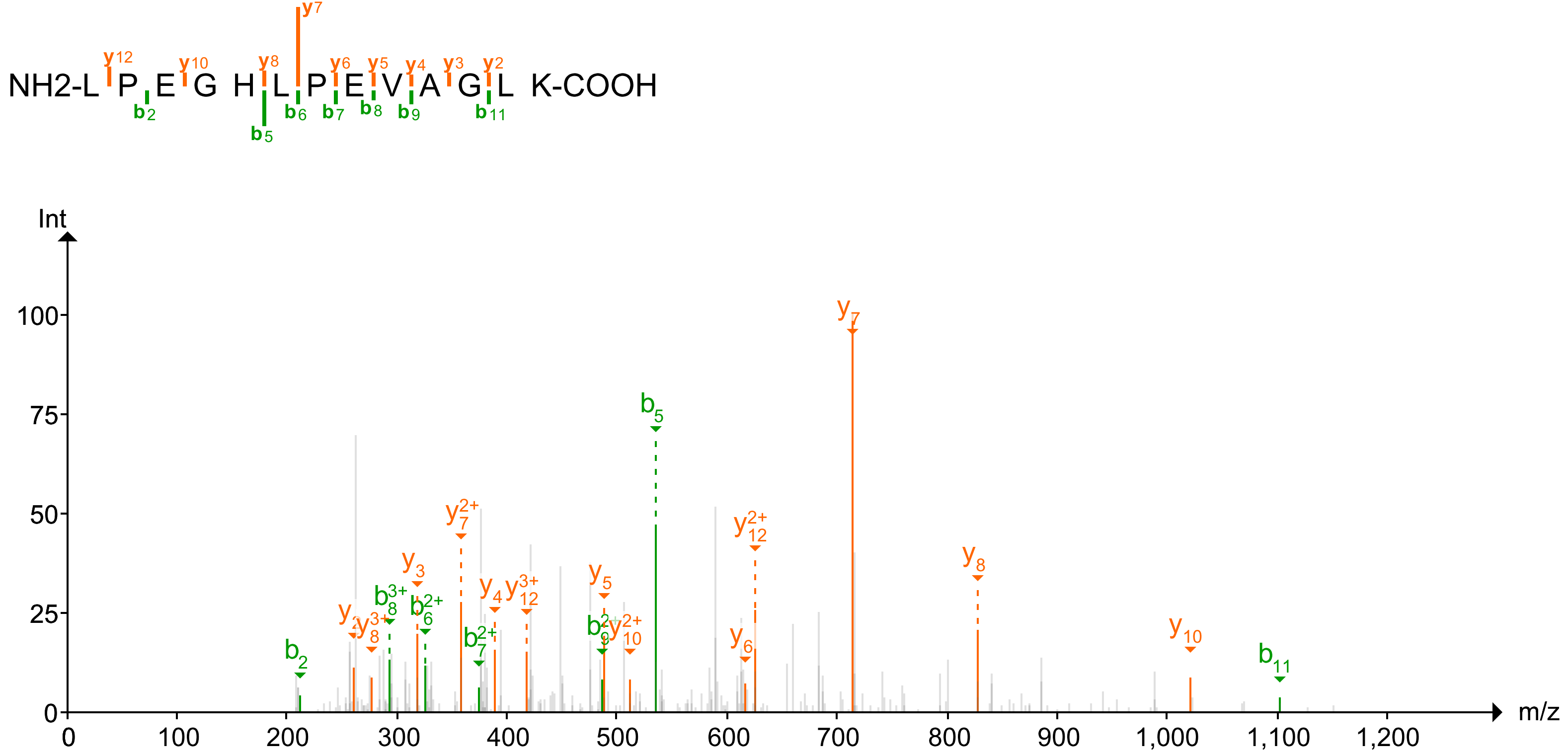

NH2-L P E G H L P E V A G L K-COOH
y12 y10 y8 y7 y6 y5 y4 y3 y2
b2 b5 b6 b7 b8 b9 b11
Int
100
75
50
25
0
m/z
0 100 200 300 400 500 600 700 800 900 1,000 1,100 1,200

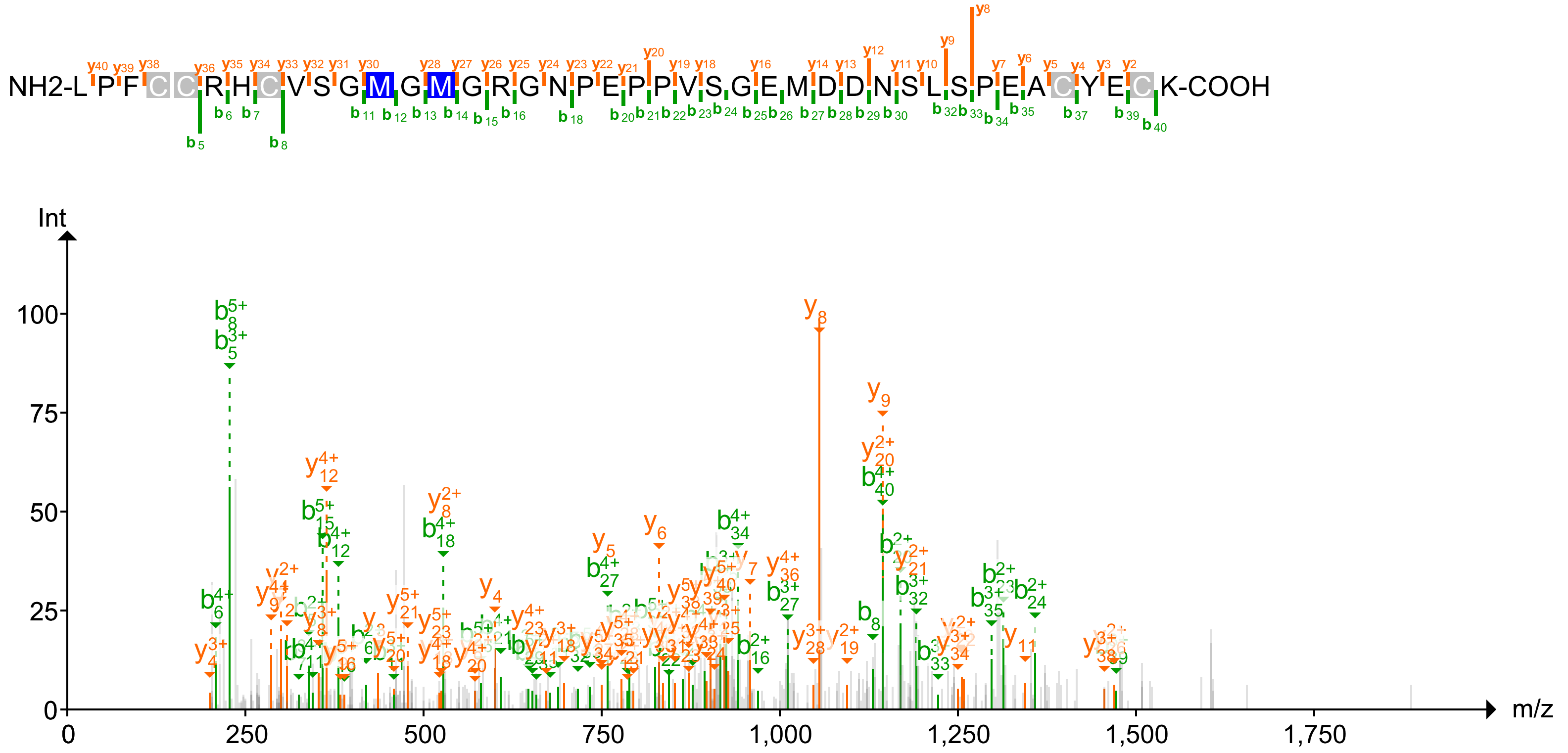

NH2-L P F C C R H C V S G M G M G R G N P E P P V S G E M D D N S L S P E A C Y E C K-COOH
Int
m/z
0
25
50
75
100
0
250
500
750
1,000
1,250
1,500
1,750

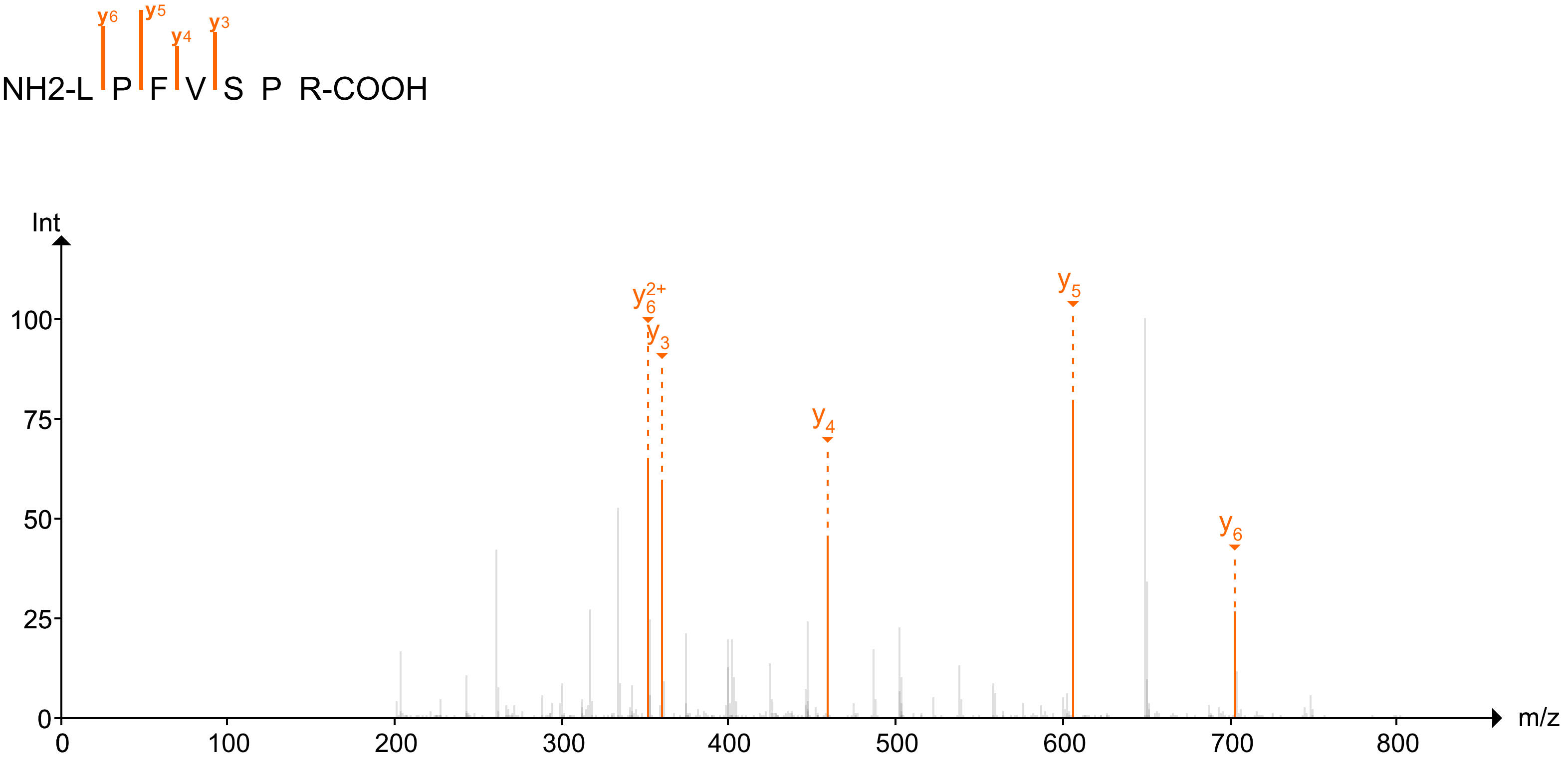

y6 y5 y4 y3
NH2-L P F V S P R-COOH
Int
100
75
50
25
0
$y_6^{2+}$
$y_3$
$y_4$
$y_5$
$y_6$
0
100
200
300
400
500
600
700
800
m/z

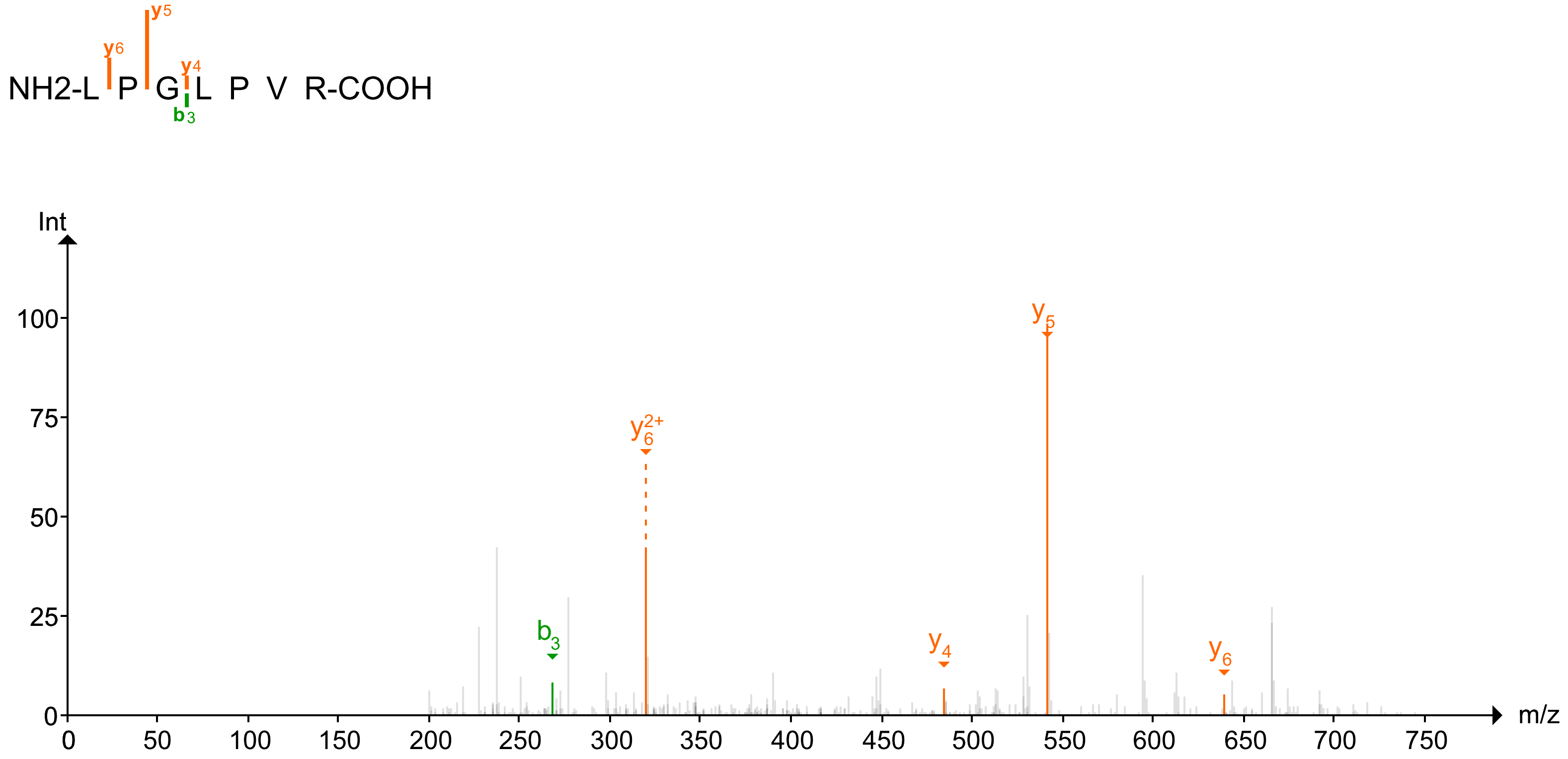
NH2-L P G L P V R-COOH
y6
y5
y4
b3
Int
100
75
50
25
0
y5
y6 2+
b3
y4
y6
0
50
100
150
200
250
300
350
400
450
500
550
600
650
700
750
m/z

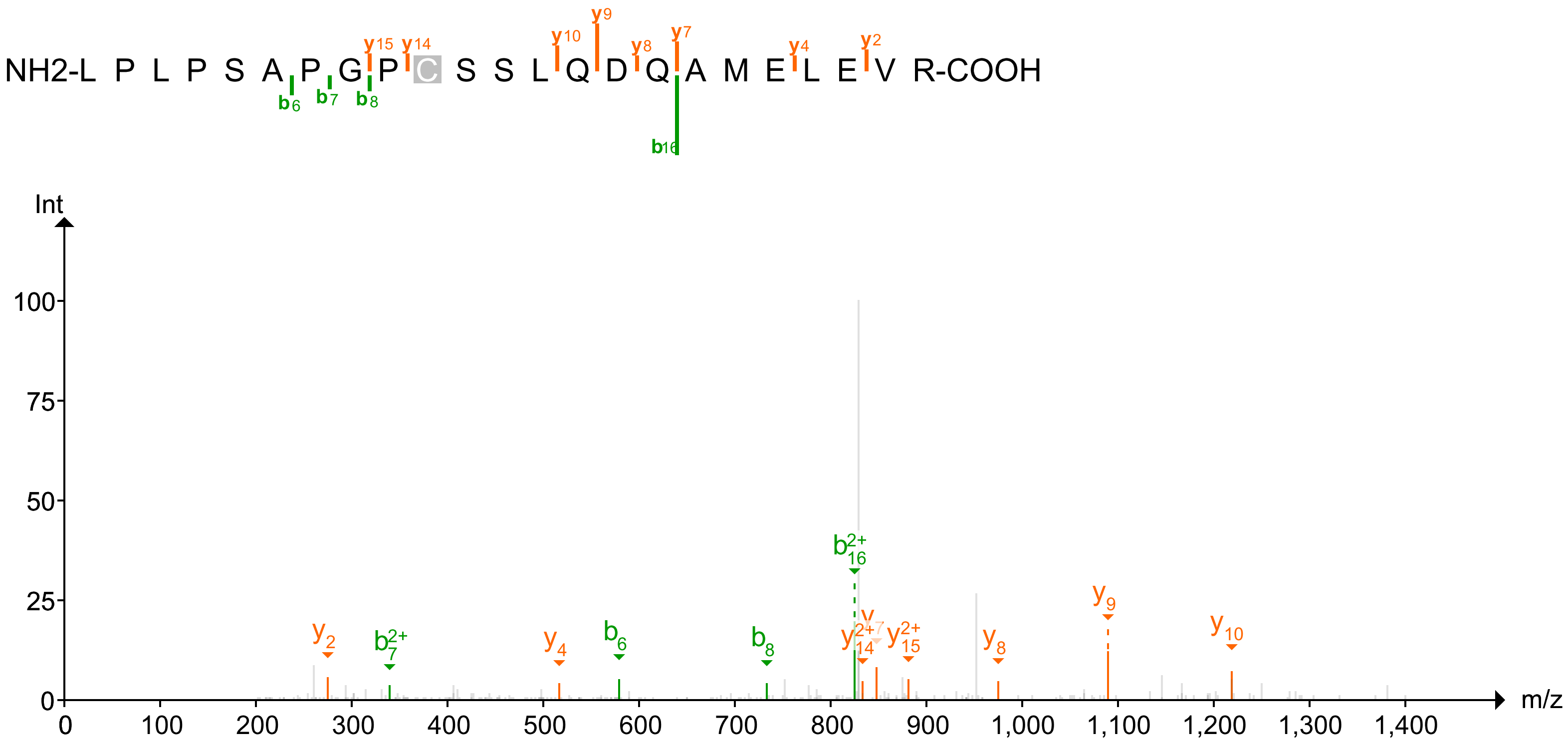

NH2-L P L P S A P G P C S S L Q D Q A M E L E V R-COOH
y15 y14 y10 y9 y8 y7 y4 y2
b6 b7 b8 b16
Int
100
75
50
25
0
m/z
0 100 200 300 400 500 600 700 800 900 1,000 1,100 1,200 1,300 1,400
y2
b7 2+
y4
b6
b8
b16 2+
y14 2+
y15 2+
y8
y9
y10

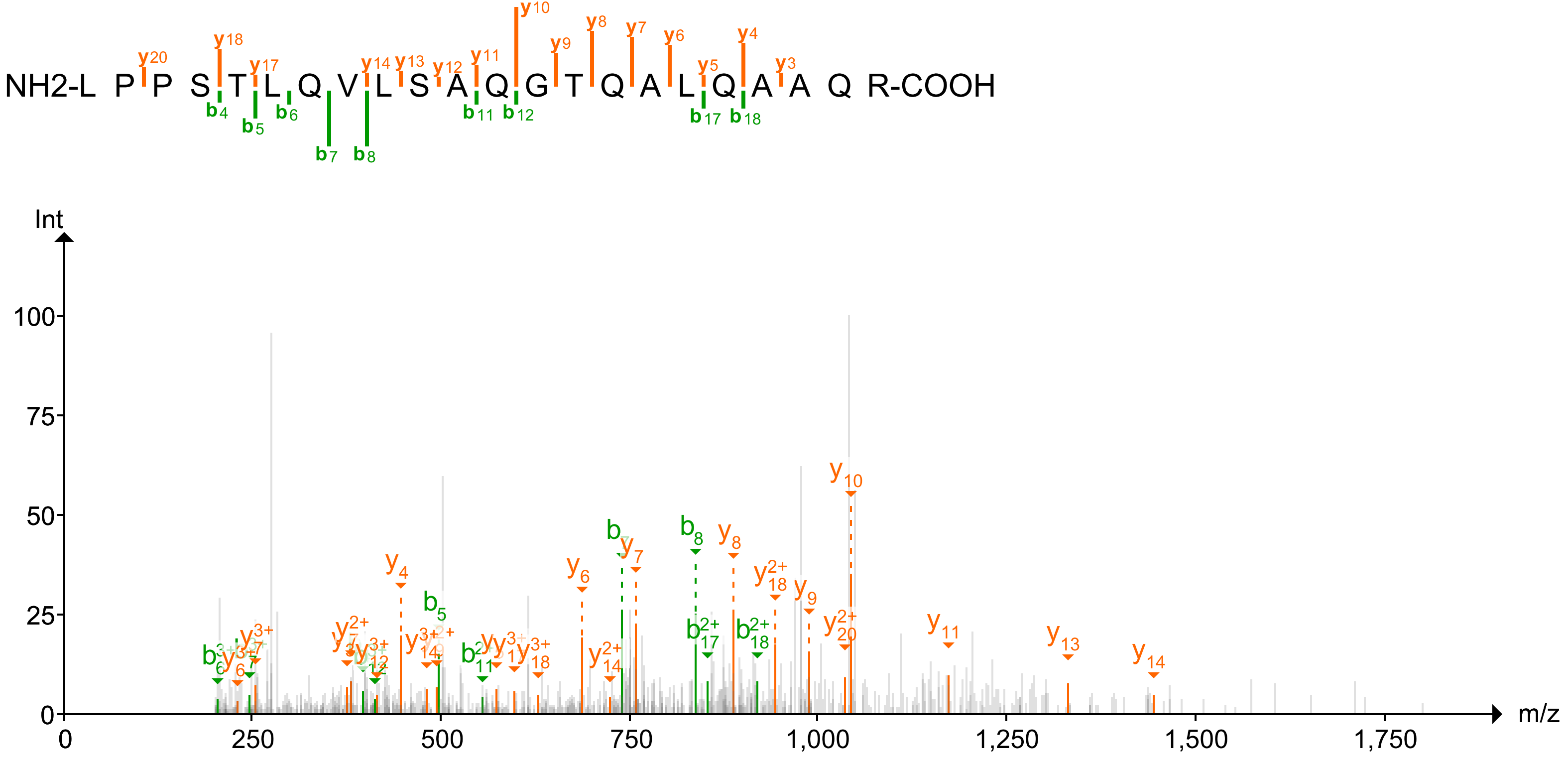

NH2-L P P S T L Q V L S A Q G T Q A L Q A A Q R-COOH
y20 y18 y17 y14 y13 y12 y11 y10 y9 y8 y7 y6 y5 y4 y3
b4 b5 b6 b7 b8 b11 b12 b17 b18
Int
100
75
50
25
0
0
250
500
750
1,000
1,250
1,500
1,750
m/z
y10
b7
b8
y8
y7
y4
y6
b5
y2+ 18
y9
b2+ 17
b2+ 18
y2+ 20
y11
y13
y14
y2+ 14

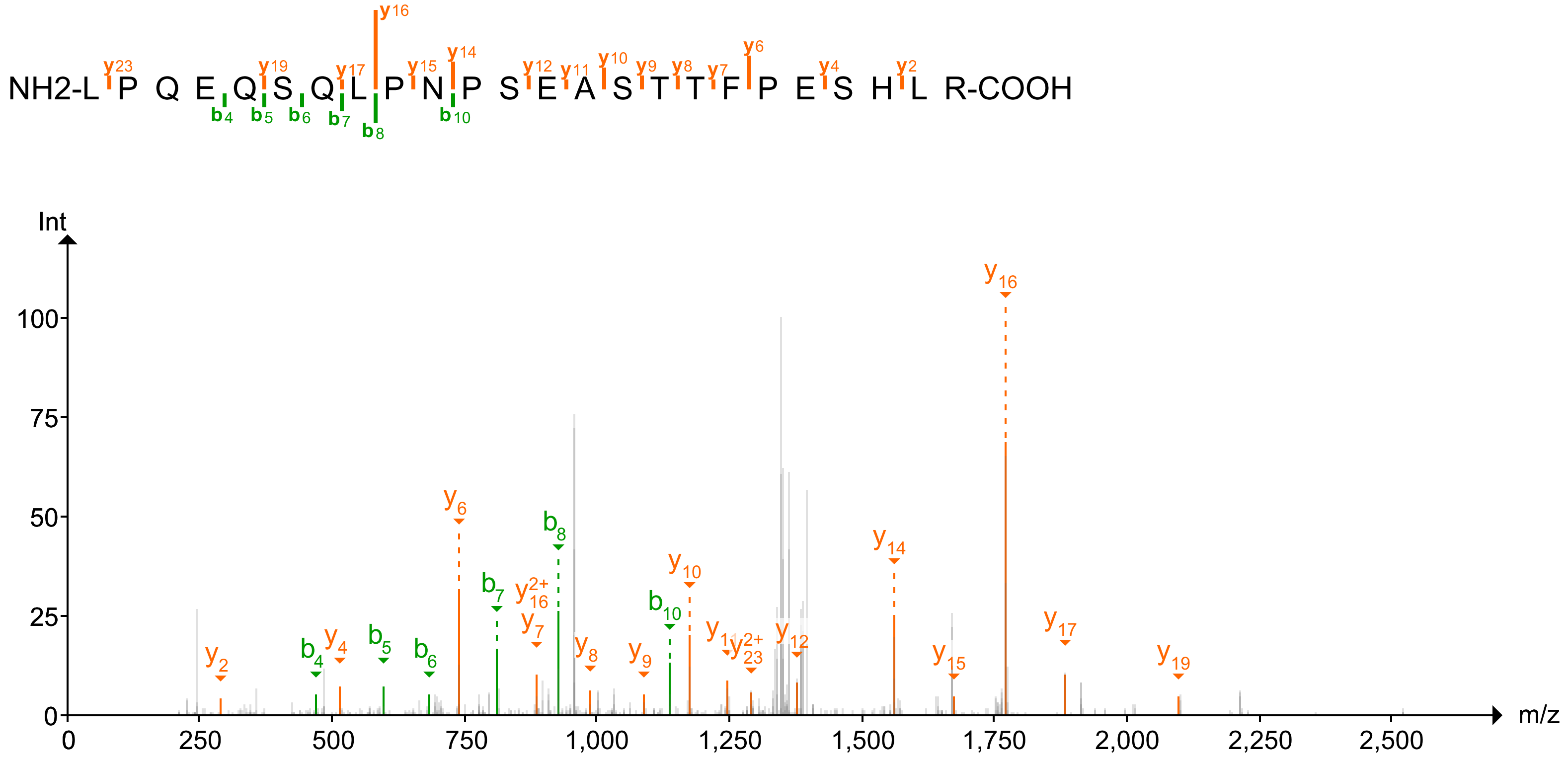
NH2-L P Q E Q S Q L P N P S E A S T T F P E S H L R-COOH
y23 y19 y17 y16 y15 y14 y12 y11 y10 y9 y8 y7 y6 y4 y2
b4 b5 b6 b7 b8 b10
Int
100
75
50
25
0
m/z
0
250
500
750
1,000
1,250
1,500
1,750
2,000
2,250
2,500
y16
y6
b8
y10
y14
b7
$y_{16}^{2+}$
y7
b10
y17
y2
b4
y4
b5
b6
y8
y9
y11
$y_{23}^{2+}$
y12
y15
y19

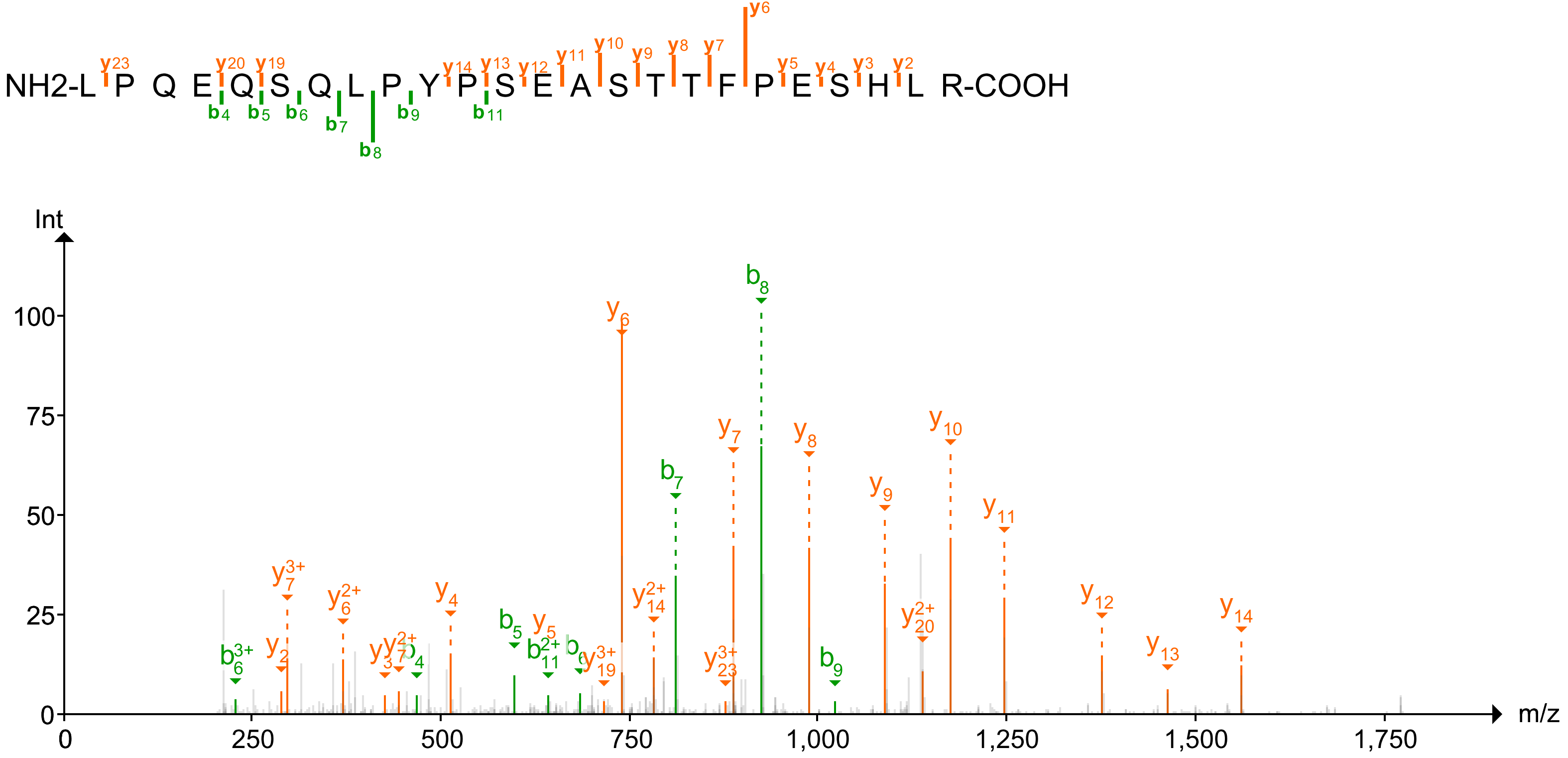

NH2-L P Q E Q S Q L P Y P S E A S T T F P E S H L R-COOH
y23 y20 y19 y14 y13 y12 y11 y10 y9 y8 y7 y6 y5 y4 y3 y2
b4 b5 b6 b7 b8 b9 b11
Int
100
75
50
25
0
m/z
0
250
500
750
1,000
1,250
1,500
1,750
b8
y6
y7
b7
y8
y10
y9
y11
y12
y13
y14
y20 2+
y14 2+
y7 3+
y6 2+
y4
b5
y5
b11 2+
y19 3+
y23 3+
b9
b6 3+
y2
y3
b4

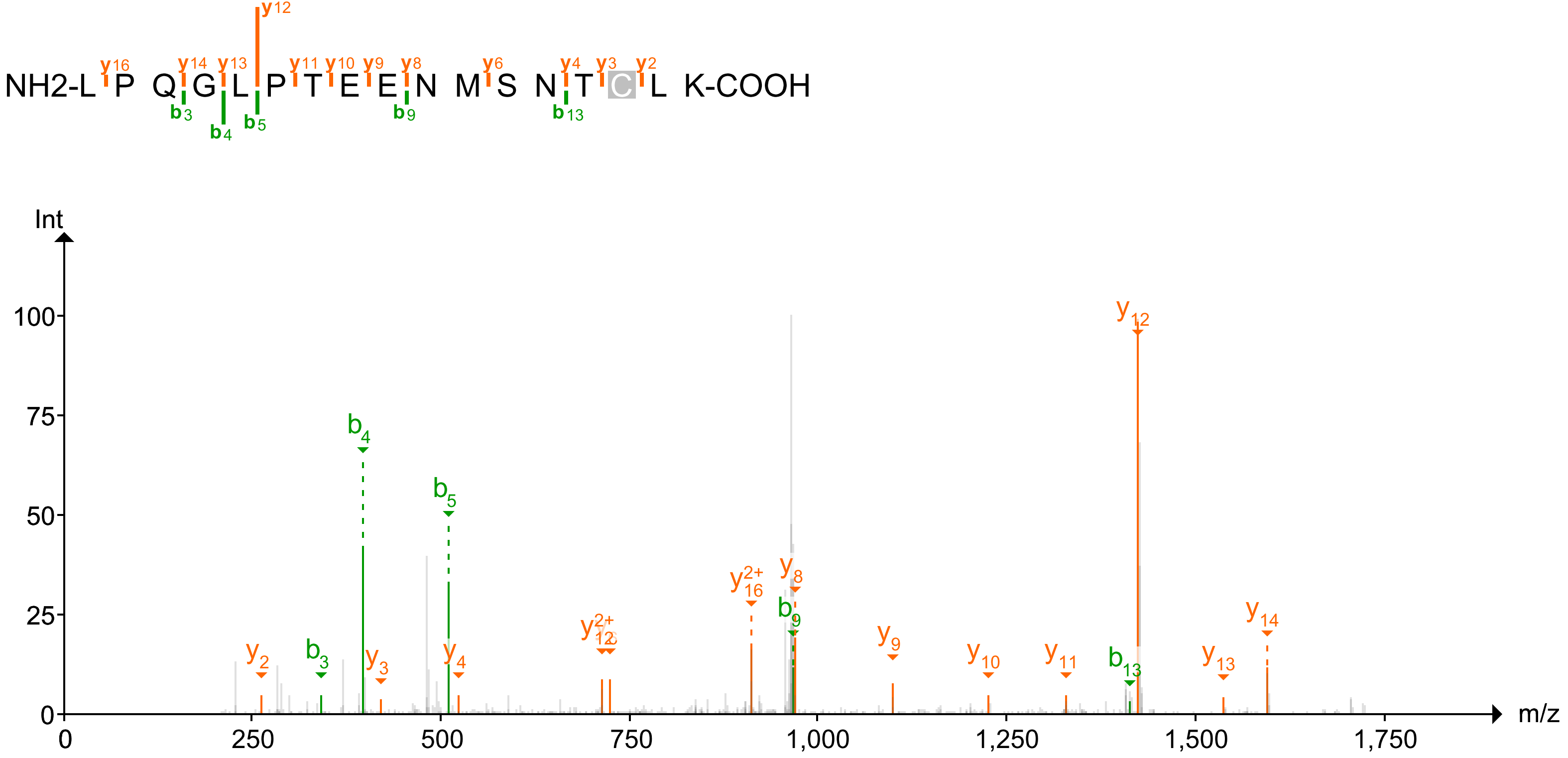

NH2-L P Q G L P T E E N M S N T C L K-COOH
y16 y14 y13 y12 y11 y10 y9 y8 y6 y4 y3 y2
b3 b4 b5 b9 b13
Int
100
75
50
25
0
m/z
0
250
500
750
1,000
1,250
1,500
1,750
y12
b4
b5
y16 2+
y8
b9
y12 2+
y2
b3
y3
y4
y9
y10
y11
b13
y13
y14

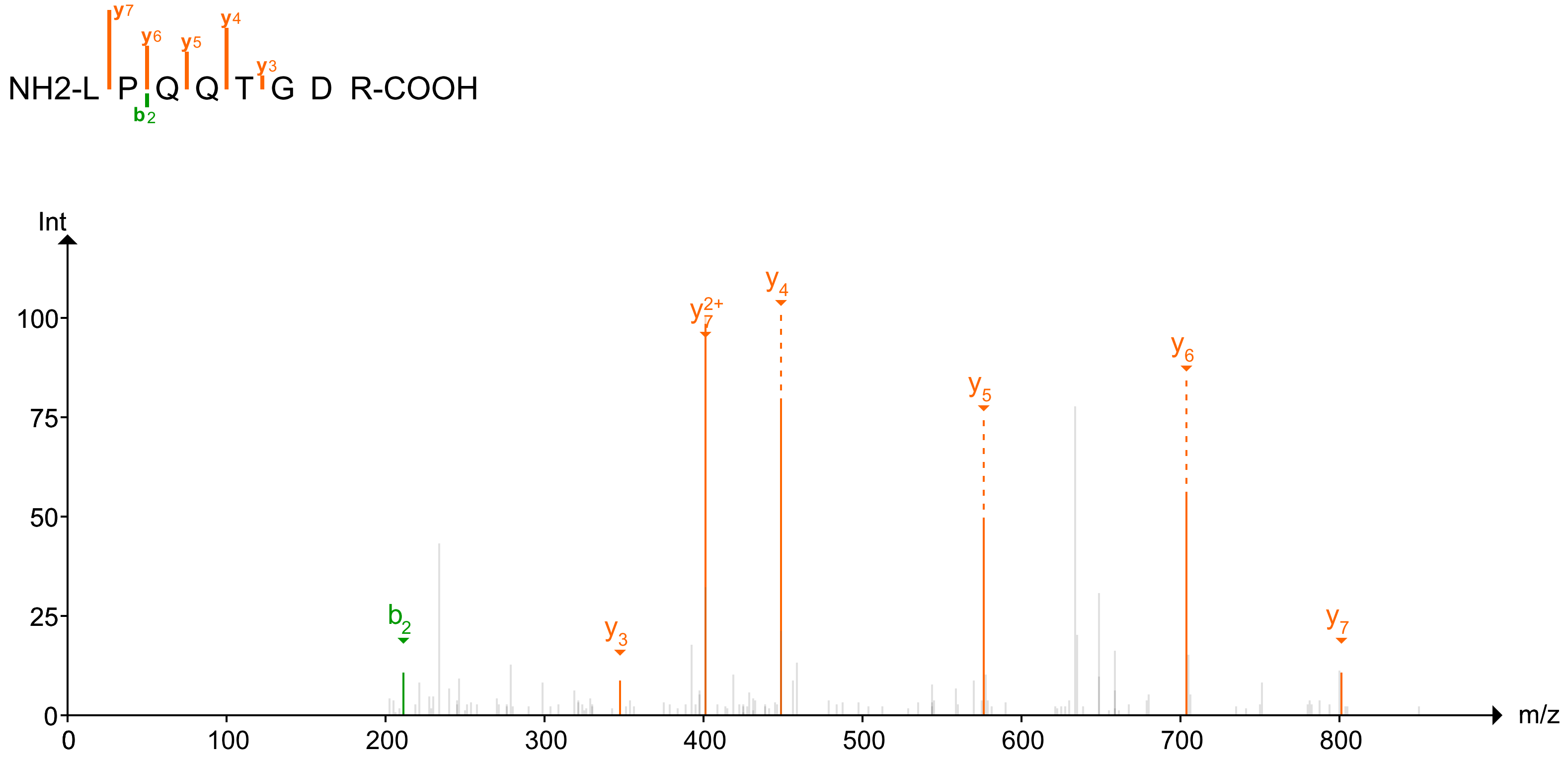

NH2-L P Q Q T G D R-COOH
y7
y6
y5
y4
y3
b2
Int
m/z
0
25
50
75
100
0
100
200
300
400
500
600
700
800
b2
y3
y7 2+
y4
y5
y6
y7

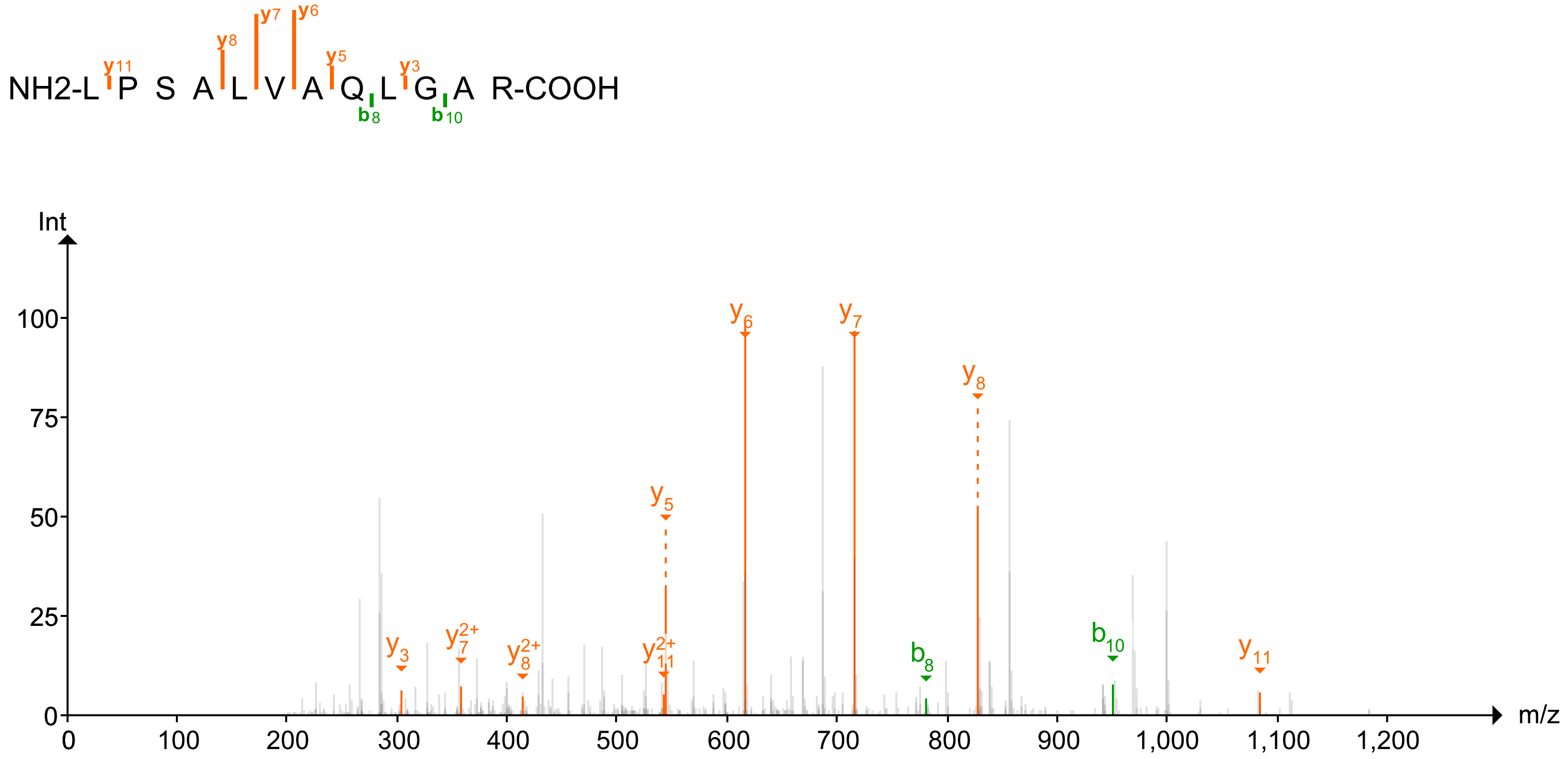

NH2-L P S A L V A Q L G A R-COOH
y11 y8 y7 y6 y5 y3
b8 b10
Int
100
75
50
25
0
y6 y7 y8 y5 y3 y7 2+ y8 2+ y11 2+ b8 b10 y11
0 100 200 300 400 500 600 700 800 900 1,000 1,100 1,200
m/z

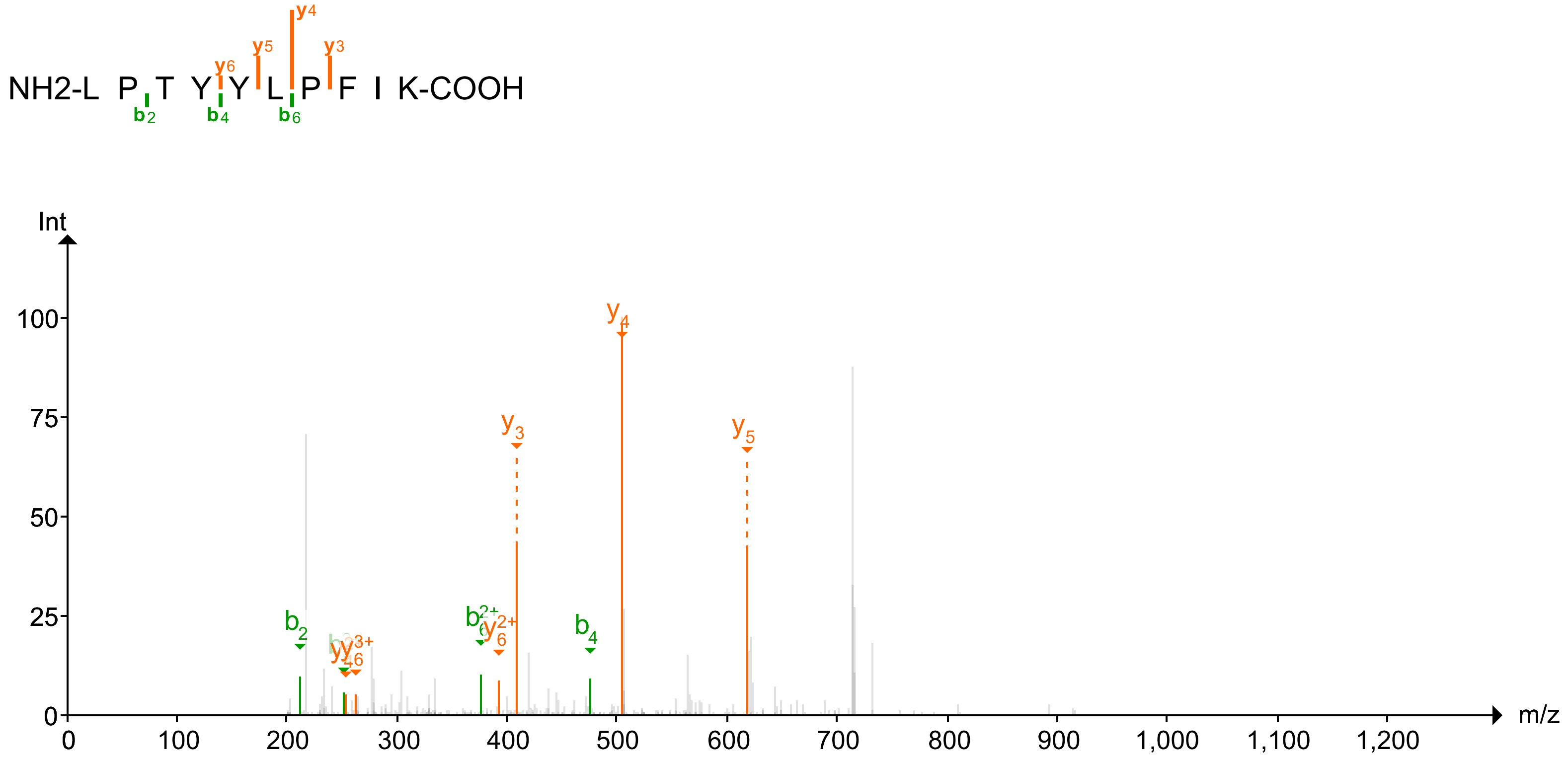

NH2-L P T Y Y L P F I K-COOH
y4
y5
y3
y6
b2
b4
b6
Int
100
75
50
25
0
m/z
0
100
200
300
400
500
600
700
800
900
1,000
1,100
1,200

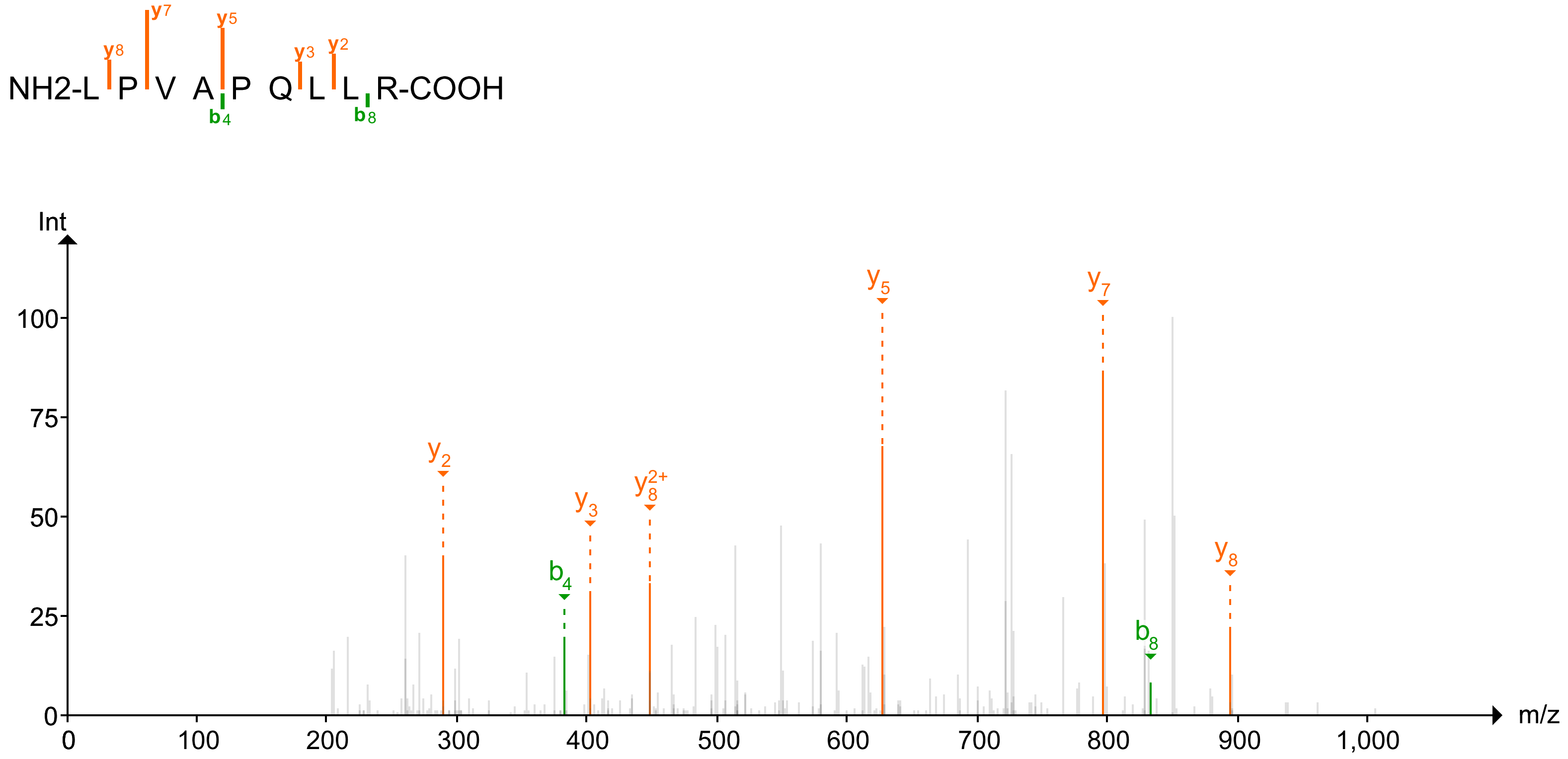

NH2-L P V A P Q L L R-COOH
y8
y7
y5
y3
y2
b4
b8
Int
100
75
50
25
0
y5
y7
y2
y3
y8 2+
b4
y8
b8
0
100
200
300
400
500
600
700
800
900
1,000
m/z

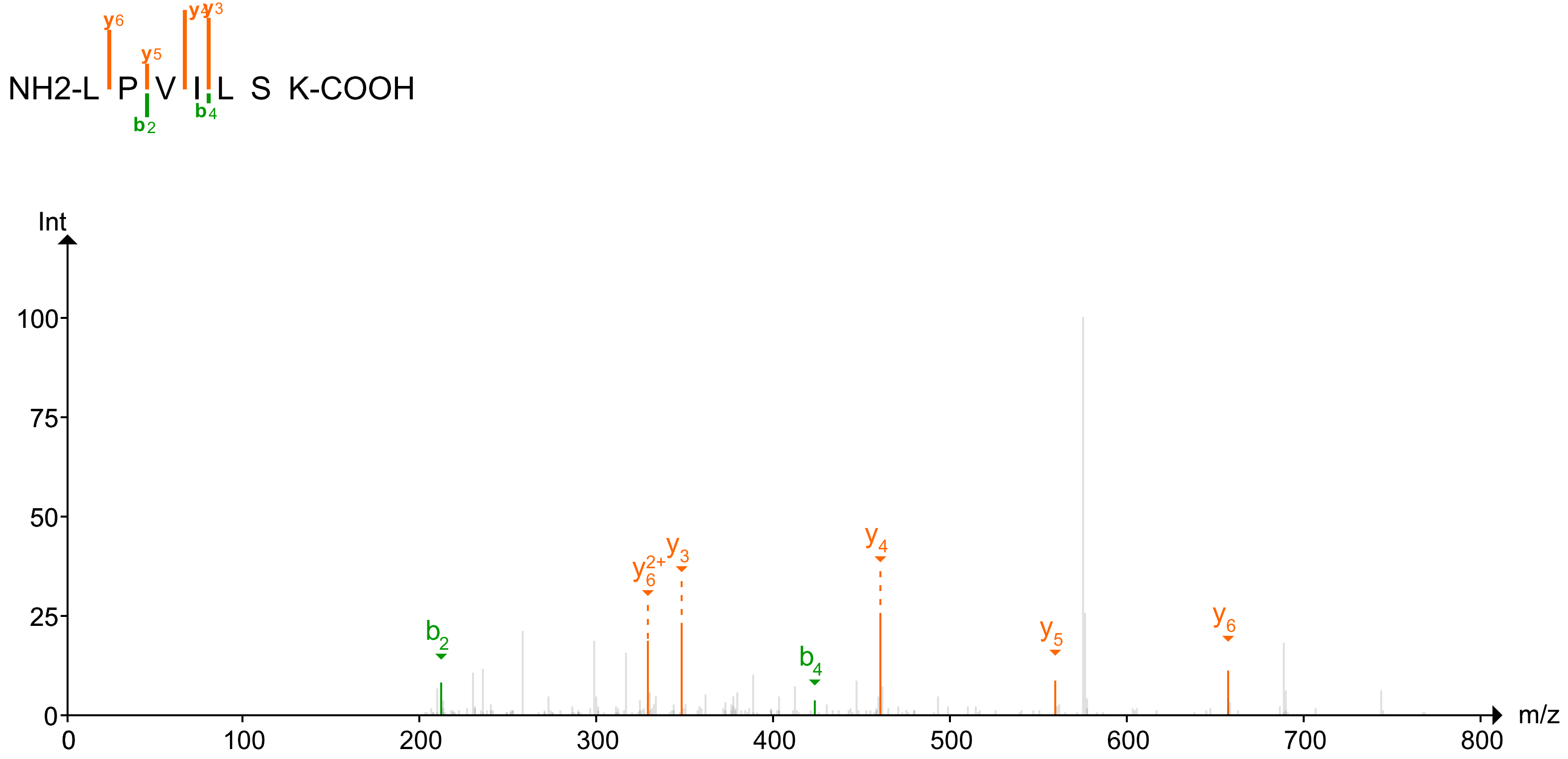

NH2-L P V I L S K-COOH
y6
y5
y4
y3
b2
b4
Int
100
75
50
25
0
m/z
0
100
200
300
400
500
600
700
800
$b_2$
$y_6^{2+}$
$y_3$
$b_4$
$y_4$
$y_5$
$y_6$

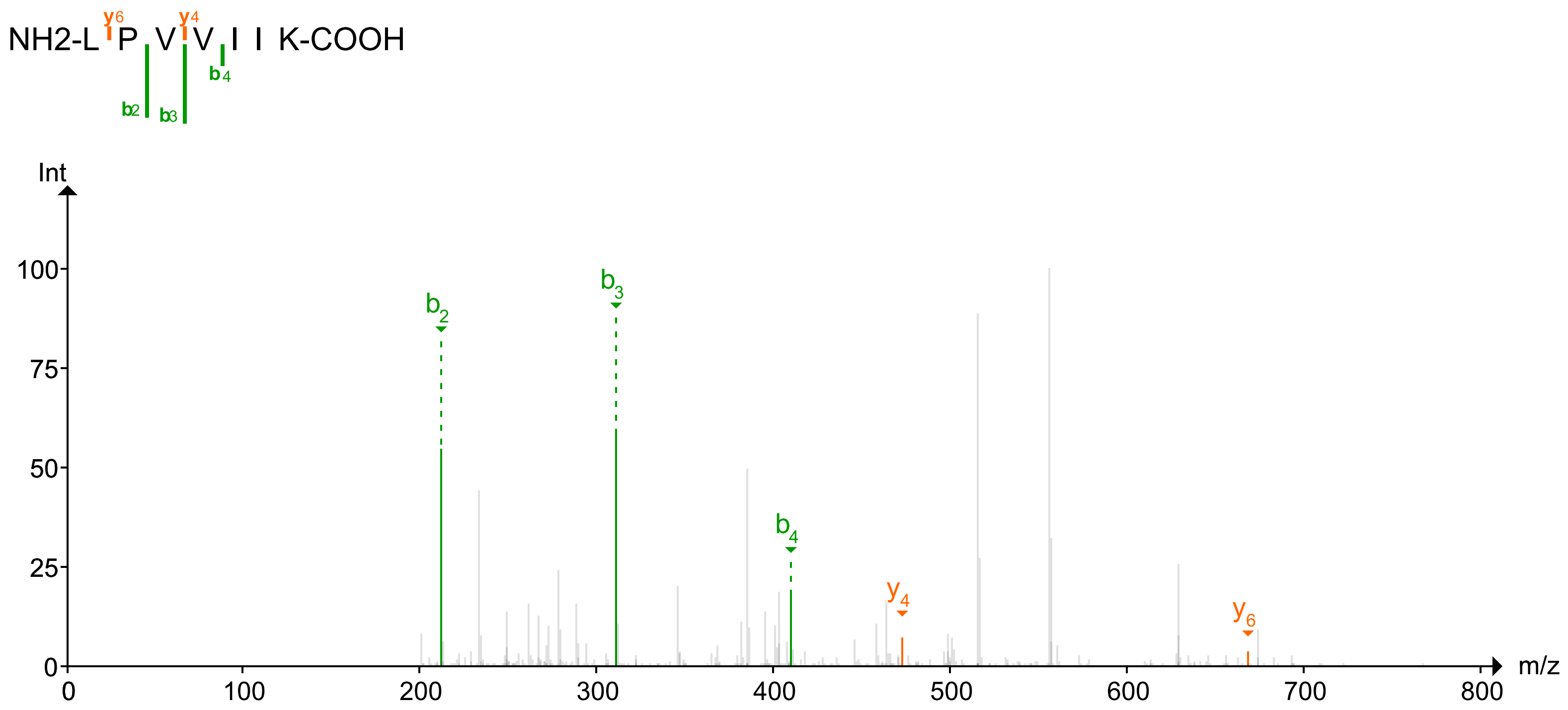

NH2-L P V V I I K-COOH
y6
y4
b2
b3
b4
Int
100
75
50
25
0
m/z
0
100
200
300
400
500
600
700
800
b2
b3
b4
y4
y6

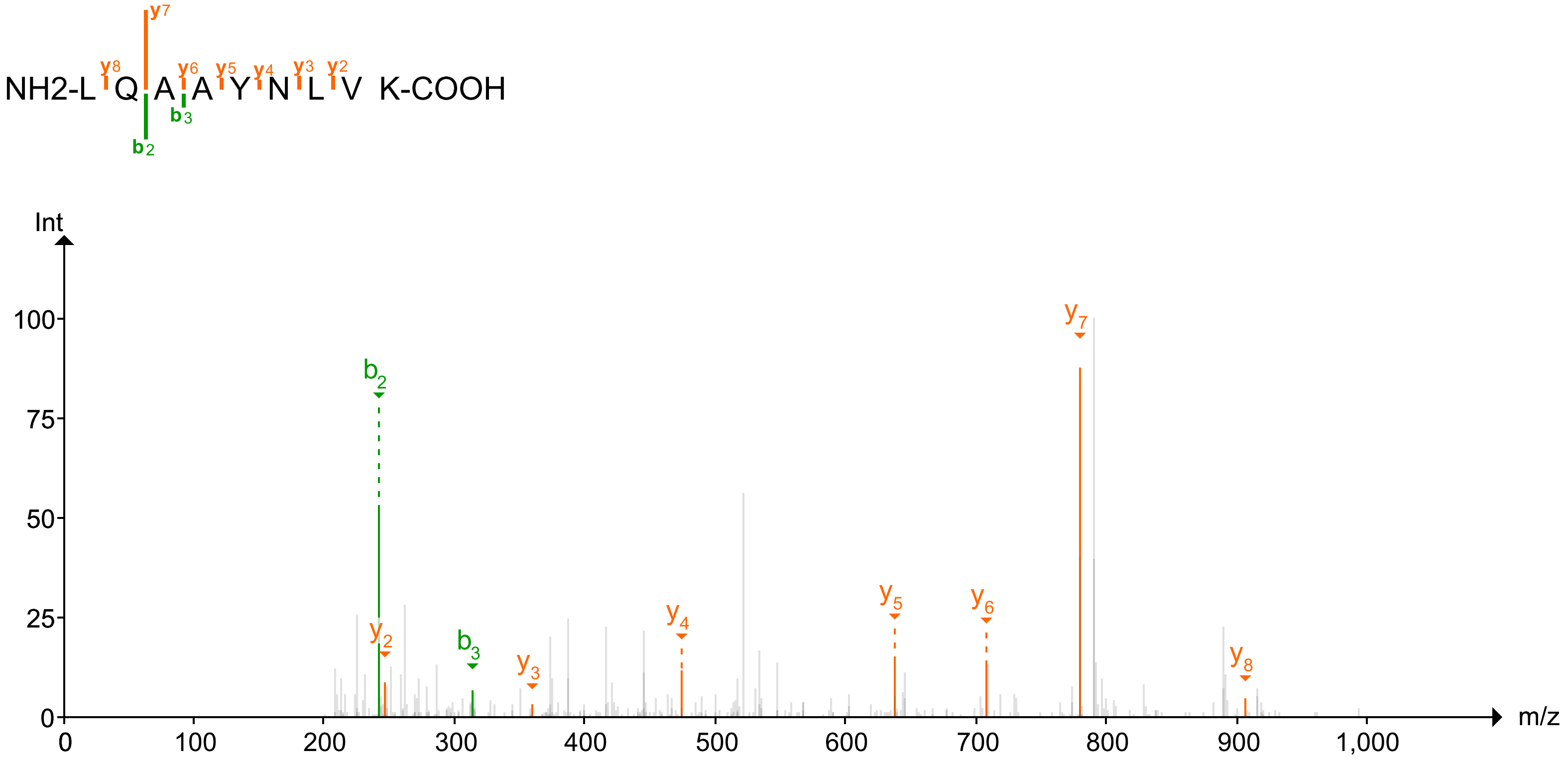
NH2-L Q A A Y N L V K-COOH
y7
y8
y6
y5
y4
y3
y2
b2
b3
Int
100
75
50
25
0
m/z
0
100
200
300
400
500
600
700
800
900
1,000
b2
y2
b3
y3
y4
y5
y6
y7
y8

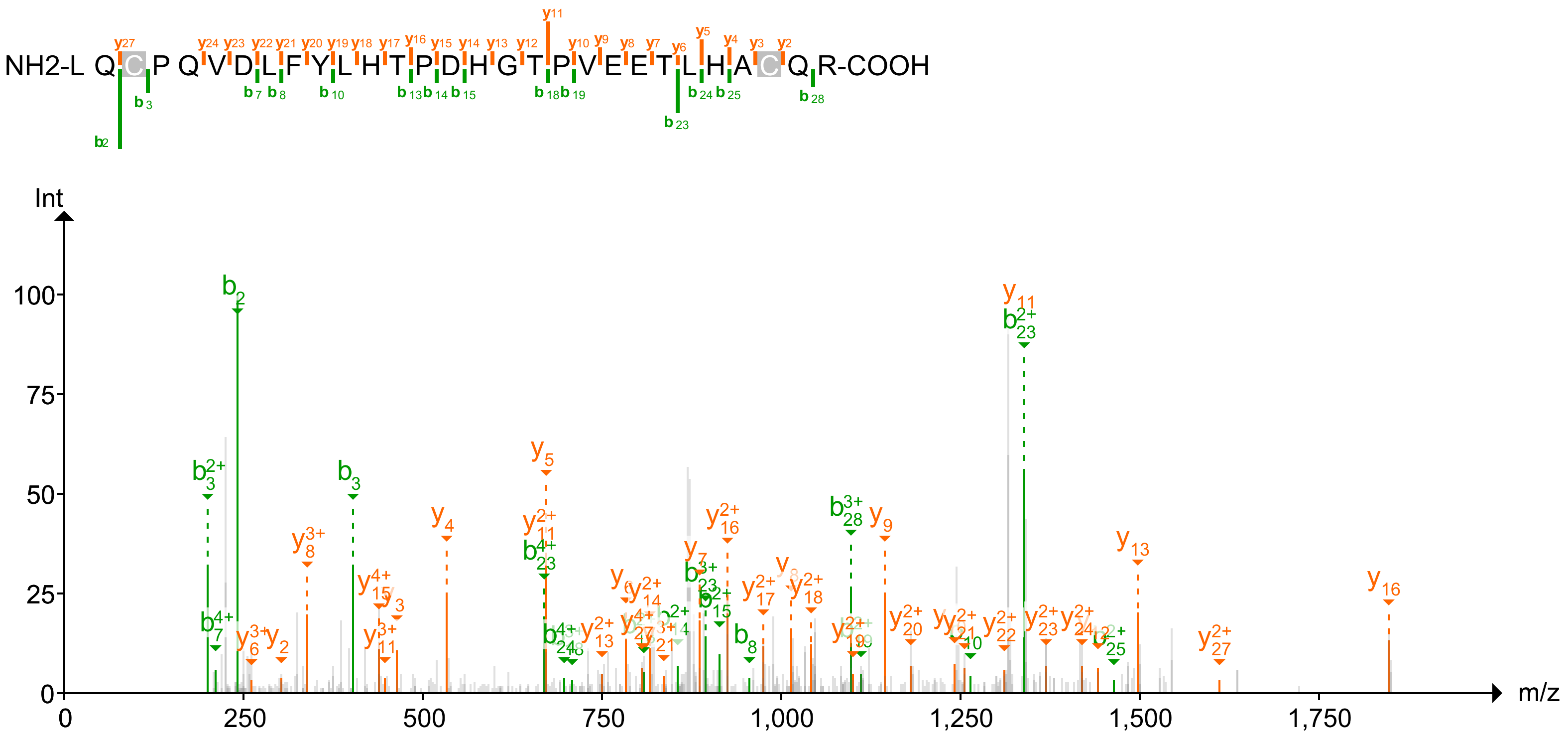

NH2-L Q C P Q V D L F Y L H T P D H G T P V E E T L H A C Q R-COOH
Int
100
75
50
25
0
m/z
0
250
500
750
1,000
1,250
1,500
1,750

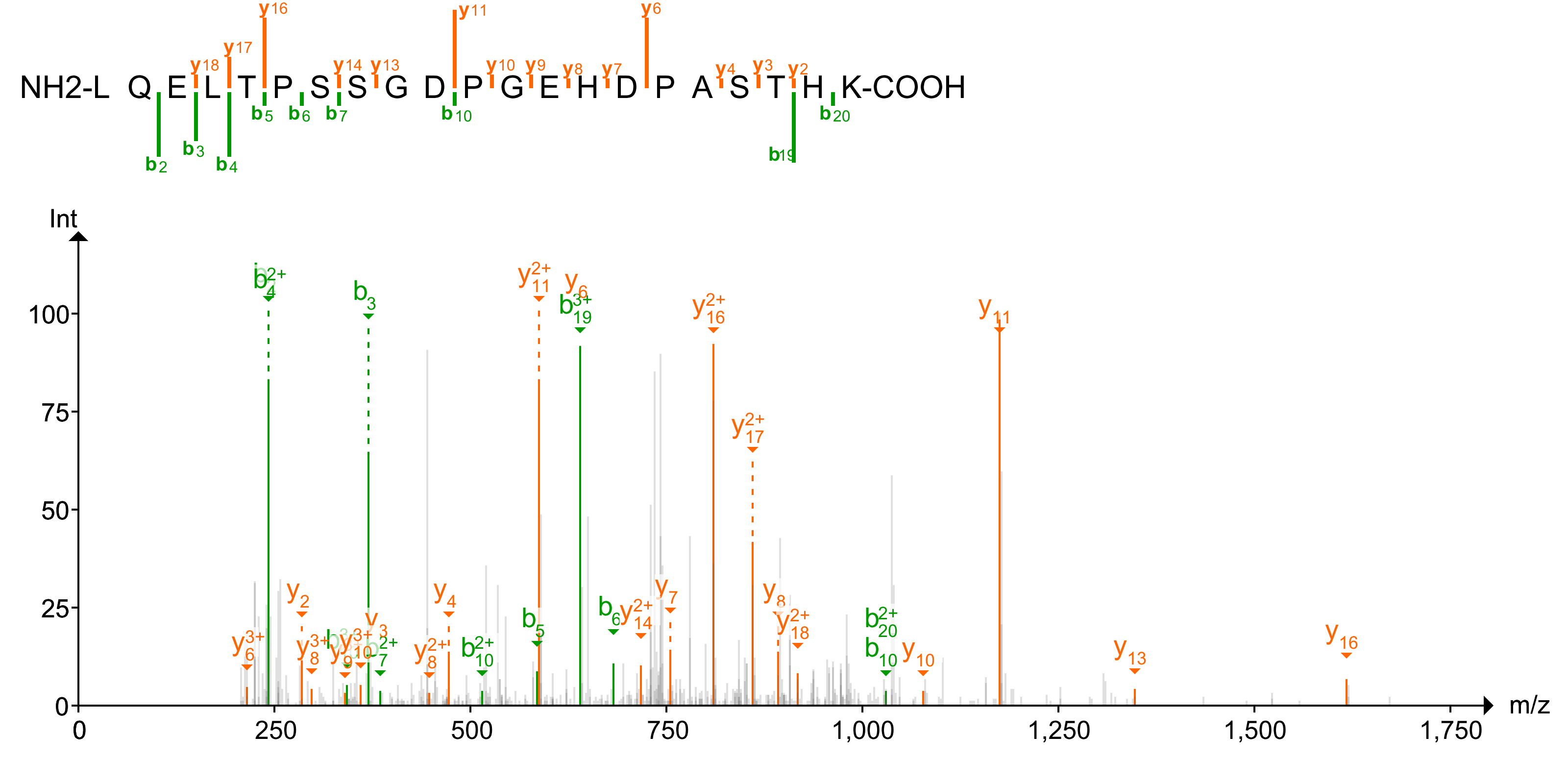

NH2-L Q E L T P S S G D P G E H D P A S T H K-COOH
Int
m/z
0
25
50
75
100
250
500
750
1,000
1,250
1,500
1,750

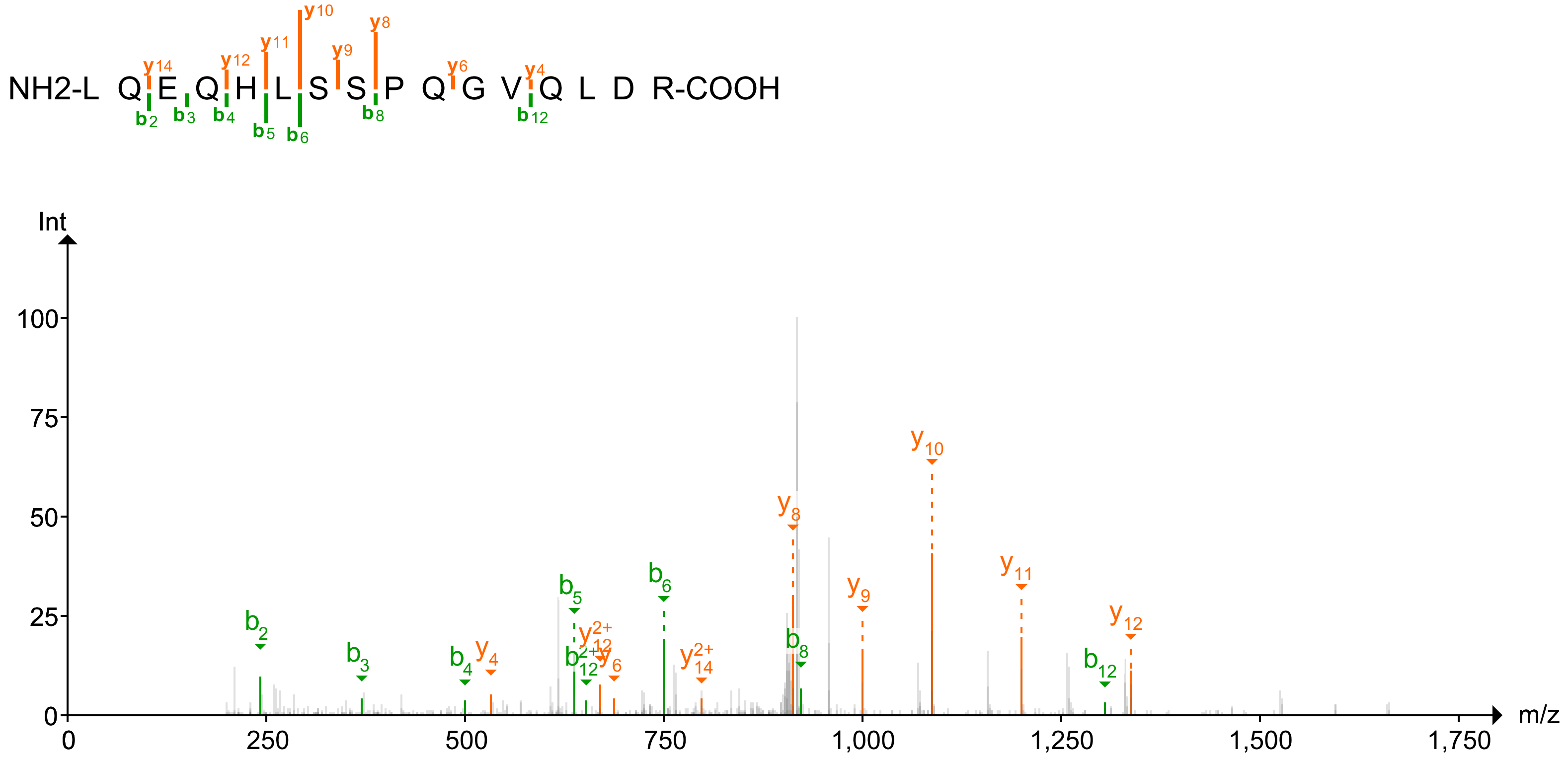

NH2-L Q E Q H L S S P Q G V Q L D R-COOH
y14 y12 y11 y10 y9 y8 y6 y4
b2 b3 b4 b5 b6 b8 b12
Int
100
75
50
25
0
250
500
750
1,000
1,250
1,500
1,750
m/z

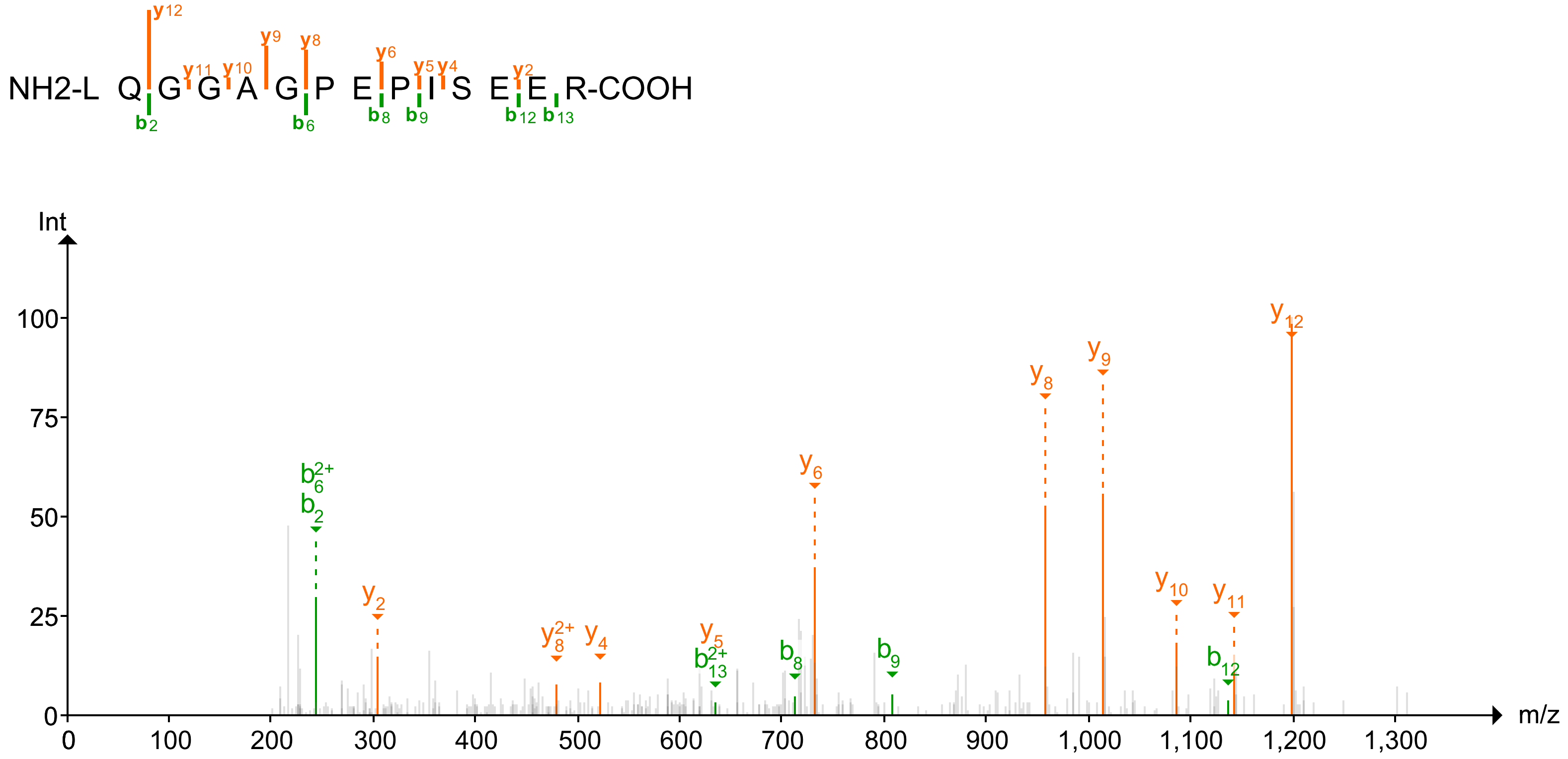

NH2-L Q G G A G P E P I S E E R-COOH
Int
m/z

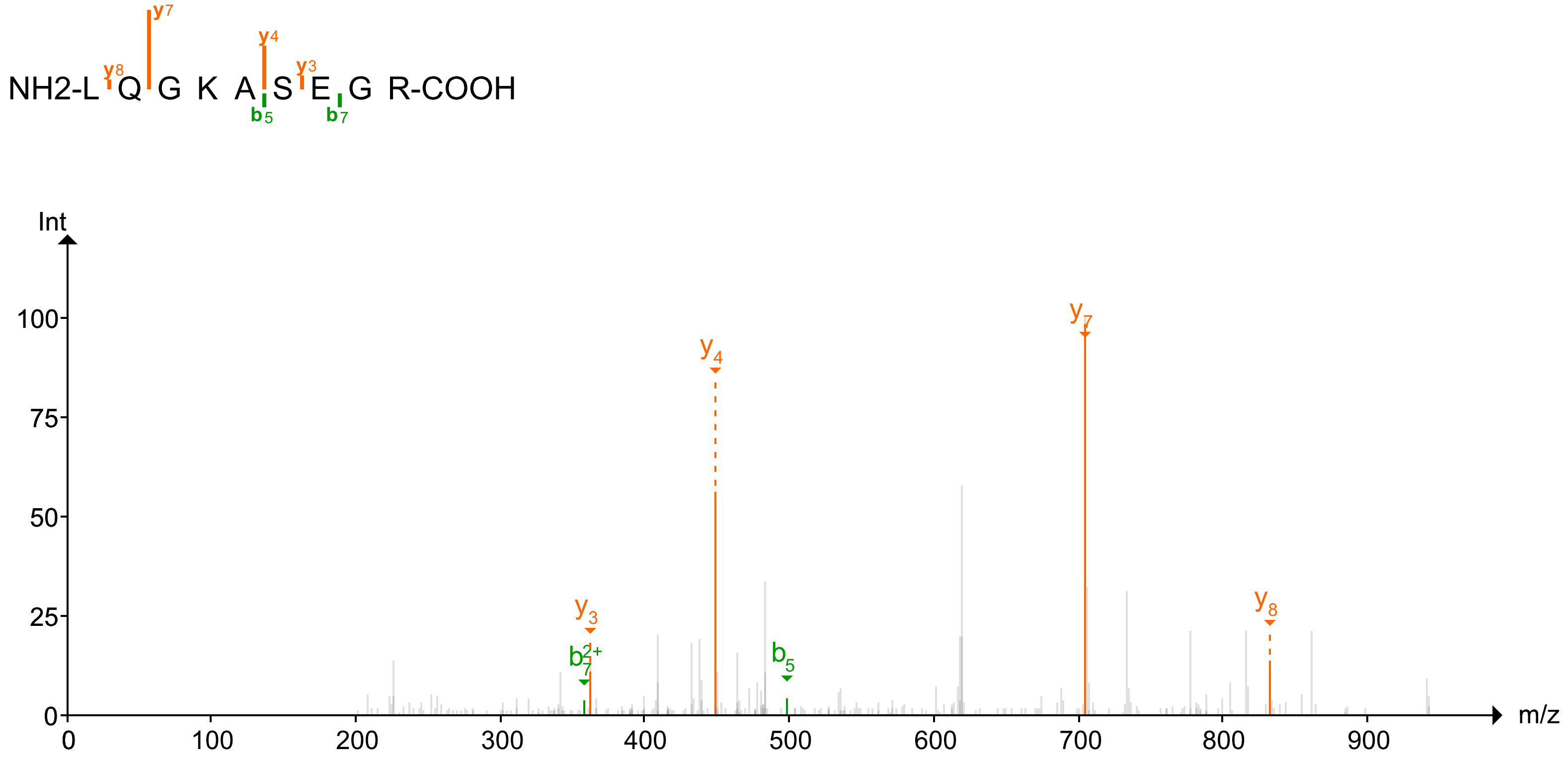

NH2-L Q G K A S E G R-COOH
y8
y7
y4
y3
b5
b7
Int
100
75
50
25
0
m/z
0
100
200
300
400
500
600
700
800
900
y7
y4
y3
$b_7^{2+}$
b5
y8

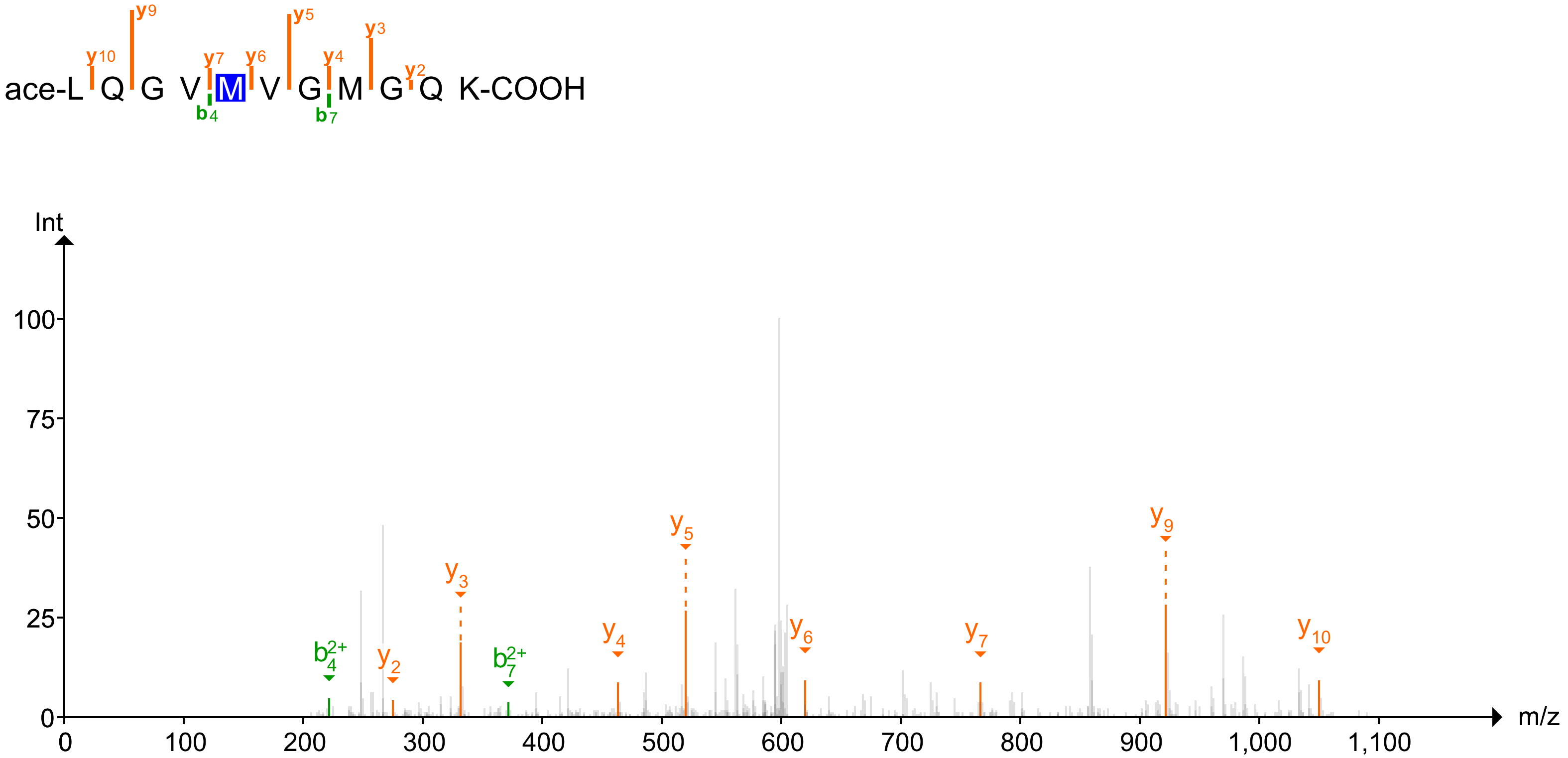

ace-L Q G V M V G M G Q K-COOH
y10 y9 y7 y6 y5 y4 y3 y2
b4 b7
Int
100
75
50
25
0
m/z
0 100 200 300 400 500 600 700 800 900 1,000 1,100
$b_4^{2+}$ $y_2$ $y_3$ $b_7^{2+}$ $y_4$ $y_5$ $y_6$ $y_7$ $y_9$ $y_{10}$

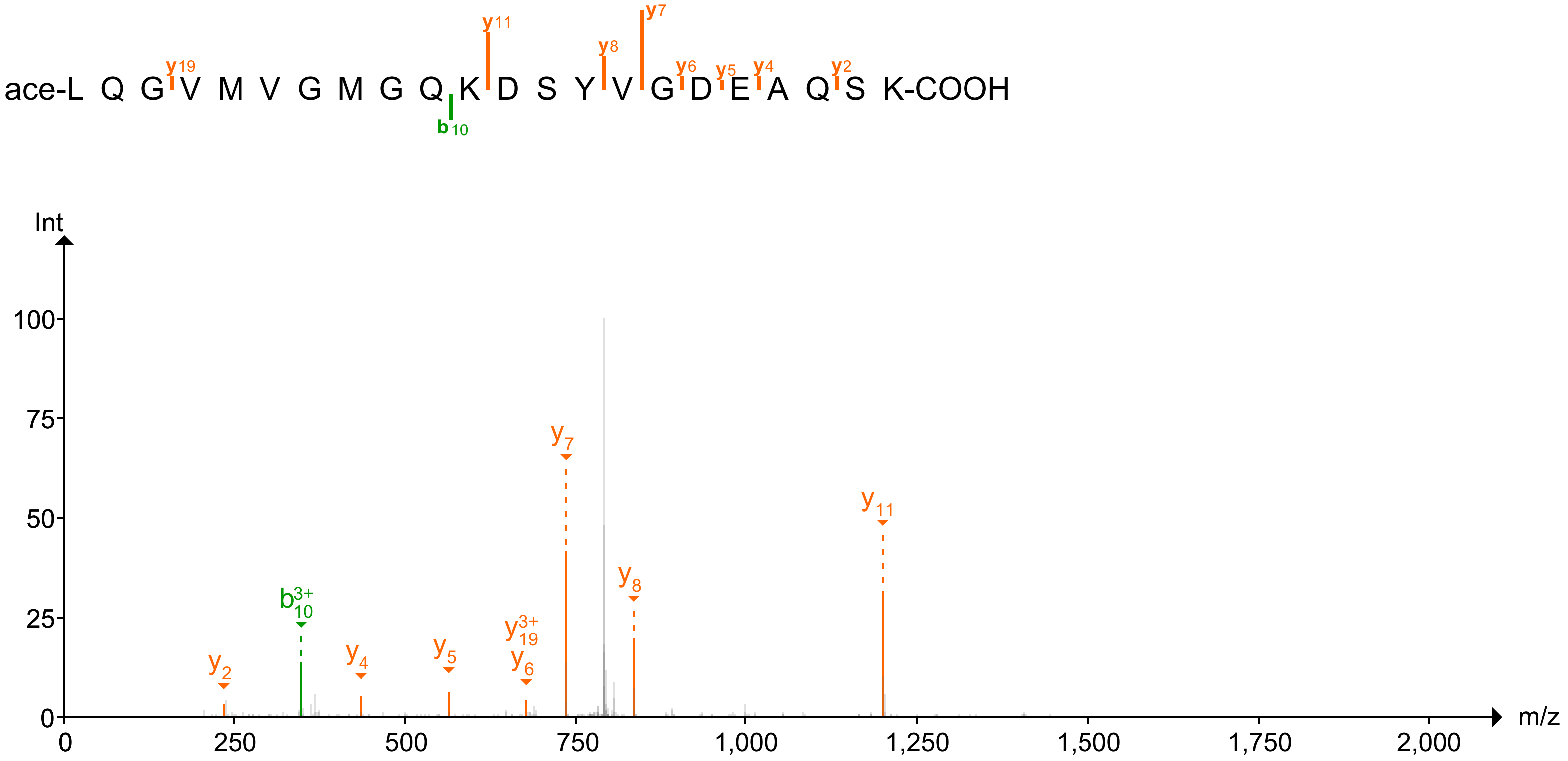
ace-L Q G V M V G M G Q K D S Y V G D E A Q S K-COOH
y19
y11
y8
y7
y6
y5
y4
y2
b10
Int
100
75
50
25
0
m/z
0
250
500
750
1,000
1,250
1,500
1,750
2,000
y7
y11
y8
b10 3+
y2
y4
y5
y19 3+
y6

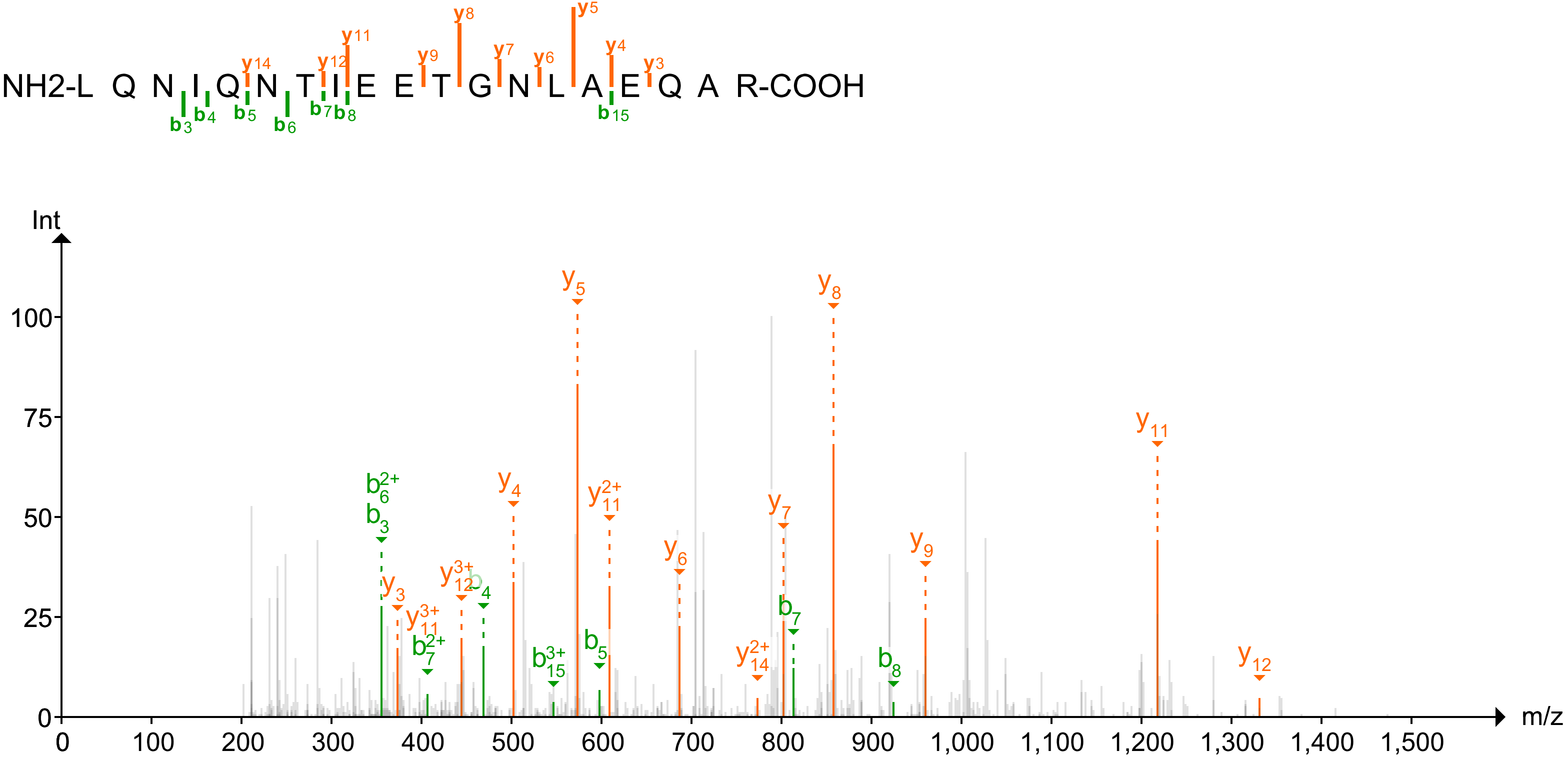

NH2-L Q N I Q N T I E E T G N L A E Q A R-COOH
y14 y12 y11 y9 y8 y7 y6 y5 y4 y3
b3 b4 b5 b6 b7 b8 b15
Int
100
75
50
25
0
m/z
0
100
200
300
400
500
600
700
800
900
1,000
1,100
1,200
1,300
1,400
1,500

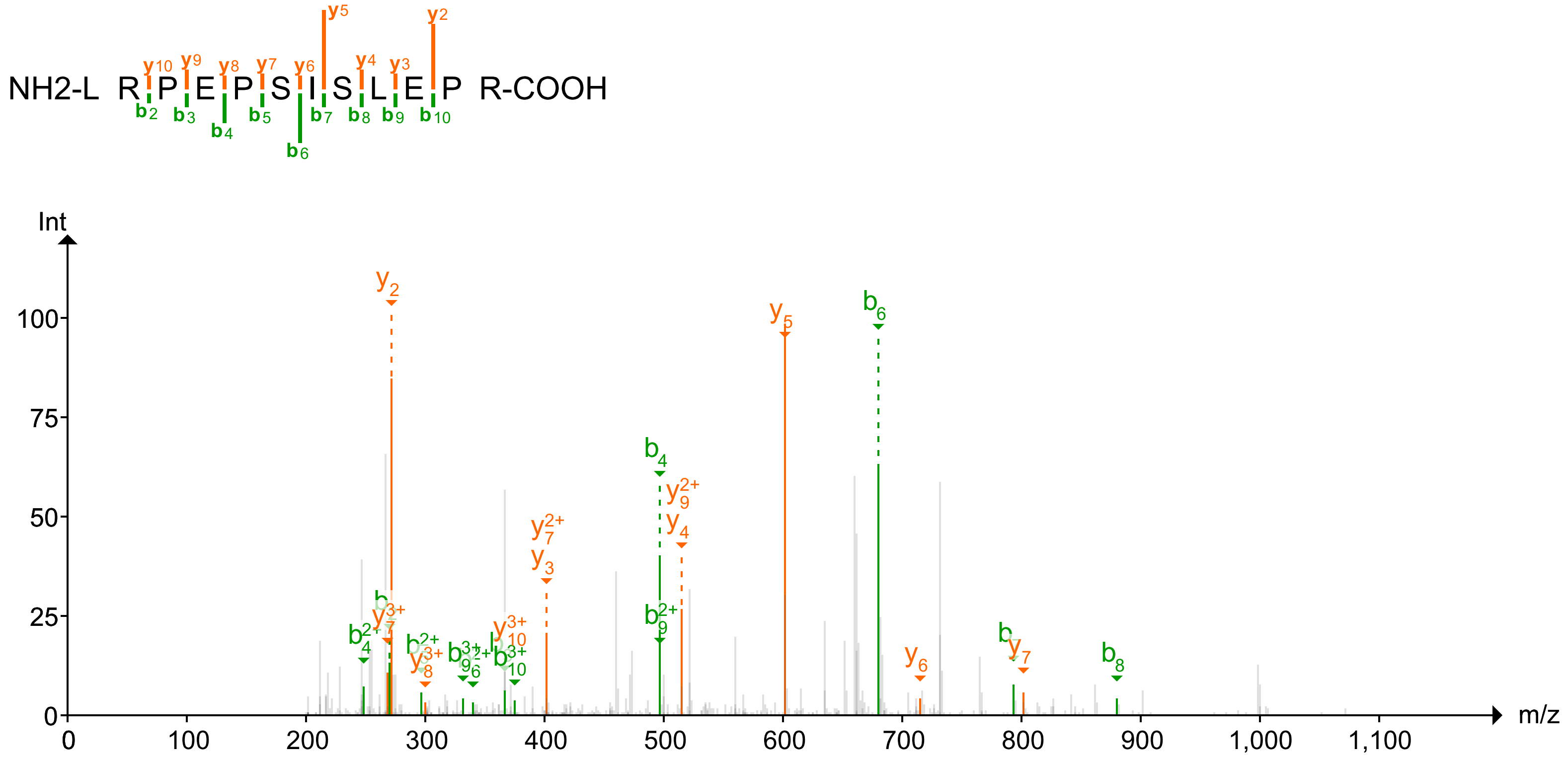

NH2-L R P E P S I S L E P R-COOH
y10 y9 y8 y7 y6 y5 y4 y3 y2
b2 b3 b4 b5 b6 b7 b8 b9 b10
Int
100
75
50
25
0
m/z
0 100 200 300 400 500 600 700 800 900 1,000 1,100
y2
y5
b6
b4
y9 2+
y4
y7 2+
y3
b9 2+
y6
y7
b8

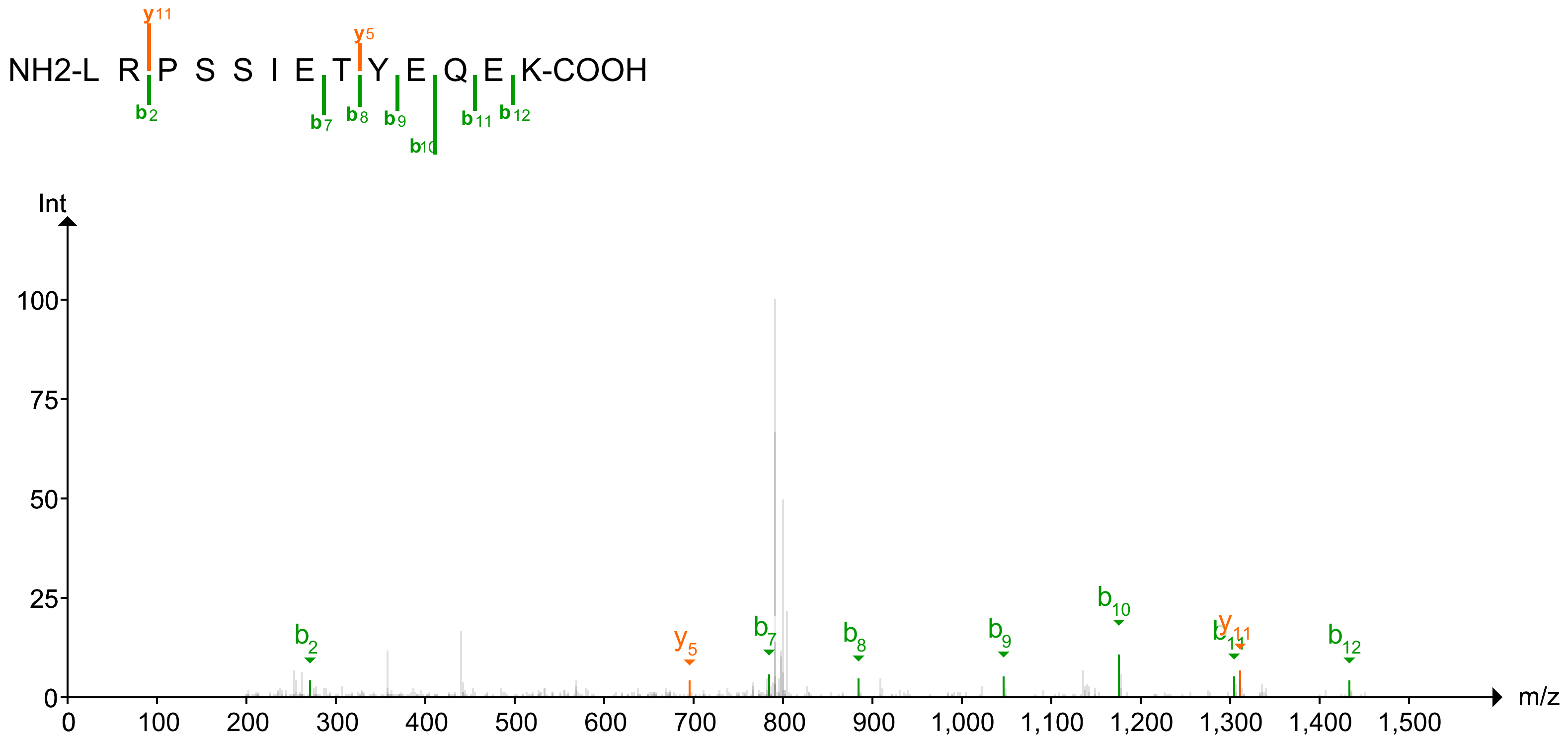

NH2-L R P S S I E T Y E Q E K-COOH
y11
y5
b2
b7
b8
b9
b10
b11
b12
Int
100
75
50
25
0
m/z
0
100
200
300
400
500
600
700
800
900
1,000
1,100
1,200
1,300
1,400
1,500

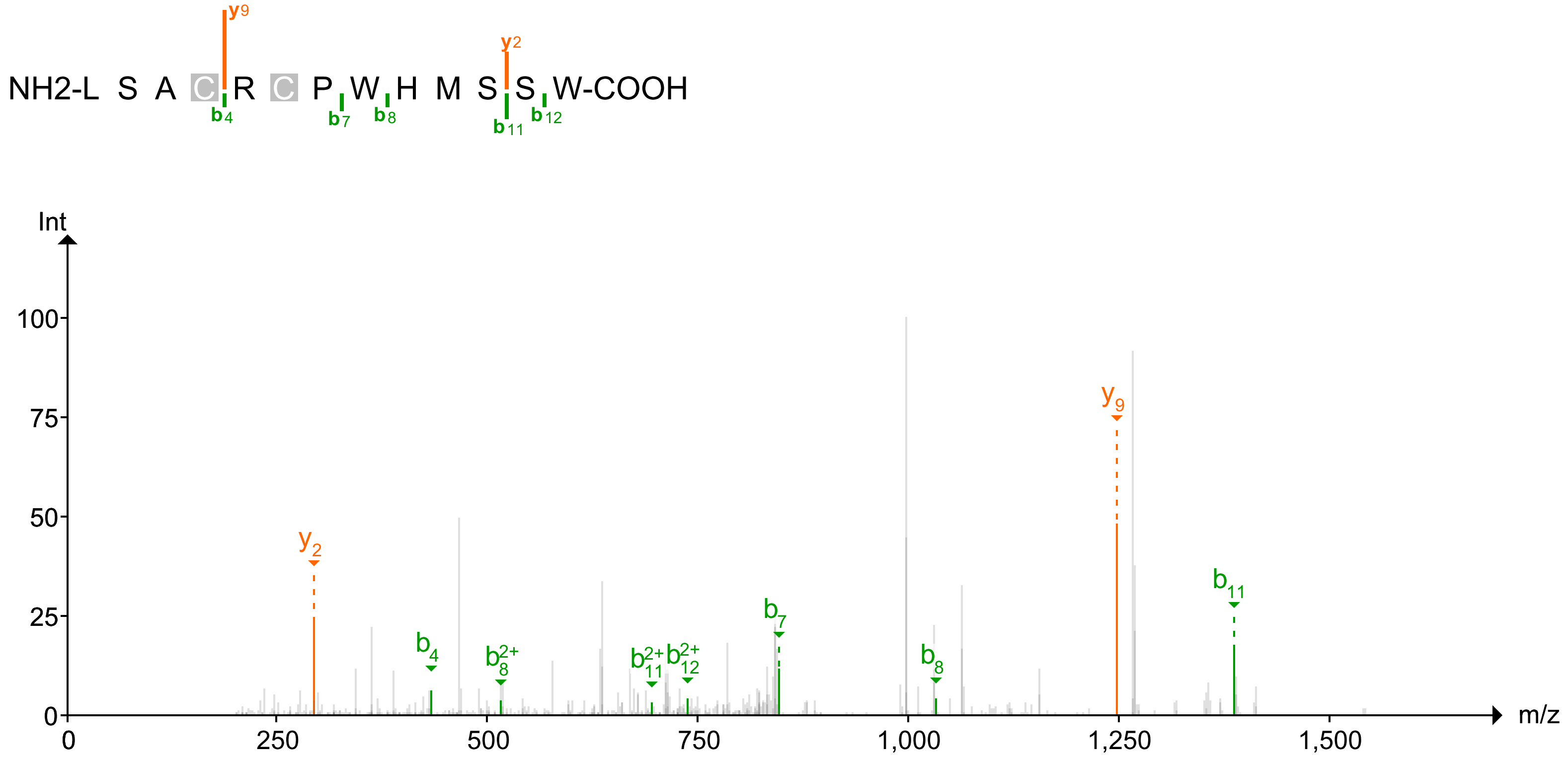

NH2-L S A C R C P W H M S S W-COOH
y9
y2
b4
b7
b8
b11
b12
Int
100
75
50
25
0
m/z
0
250
500
750
1,000
1,250
1,500
$y_2$
$b_4$
$b_8^{2+}$
$b_{11}^{2+}$
$b_{12}^{2+}$
$b_7$
$b_8$
$y_9$
$b_{11}$

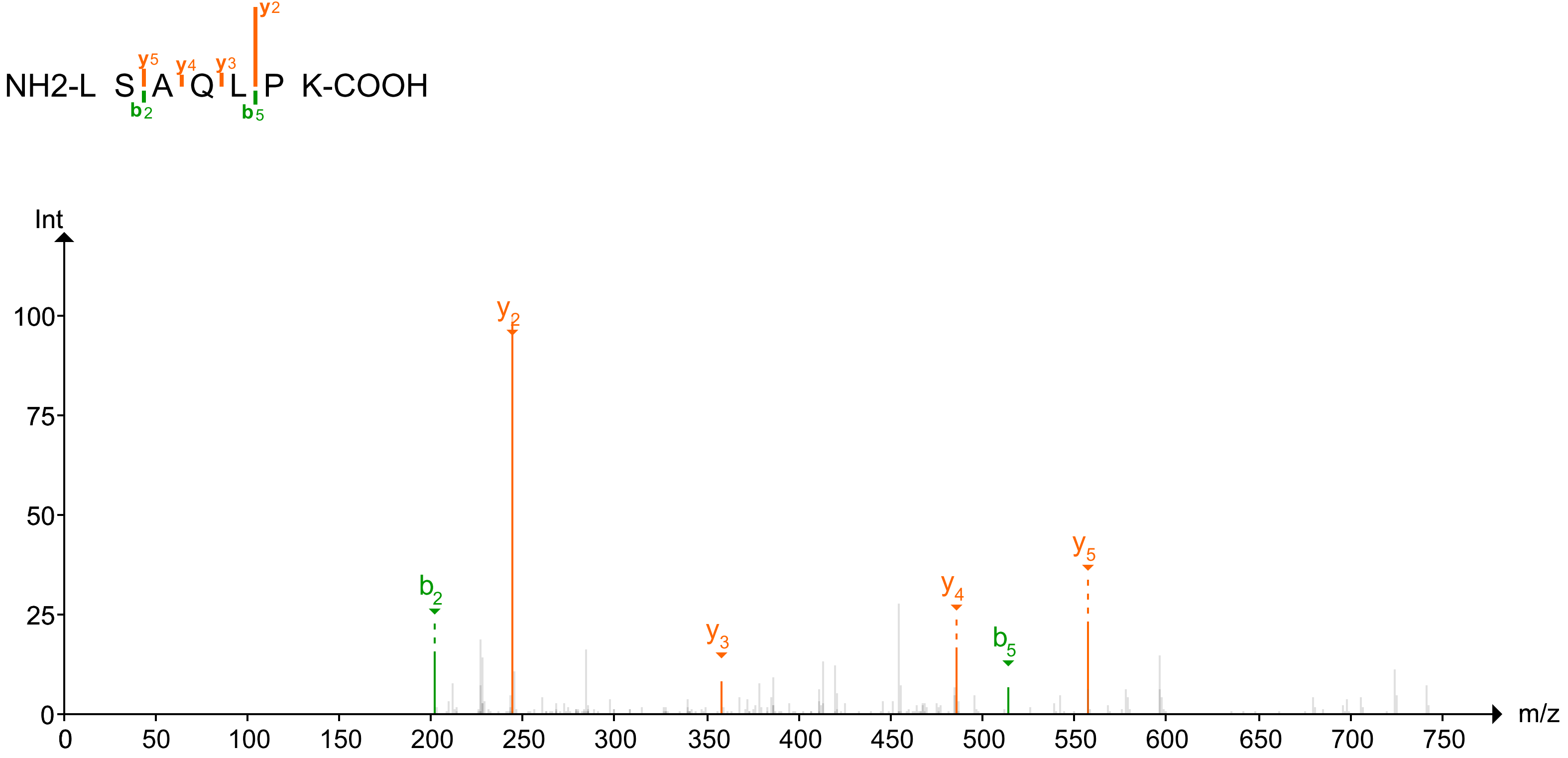

NH2-L S A Q L P K-COOH
y2
y5
y4
y3
b2
b5
Int
100
75
50
25
0
m/z
0
50
100
150
200
250
300
350
400
450
500
550
600
650
700
750

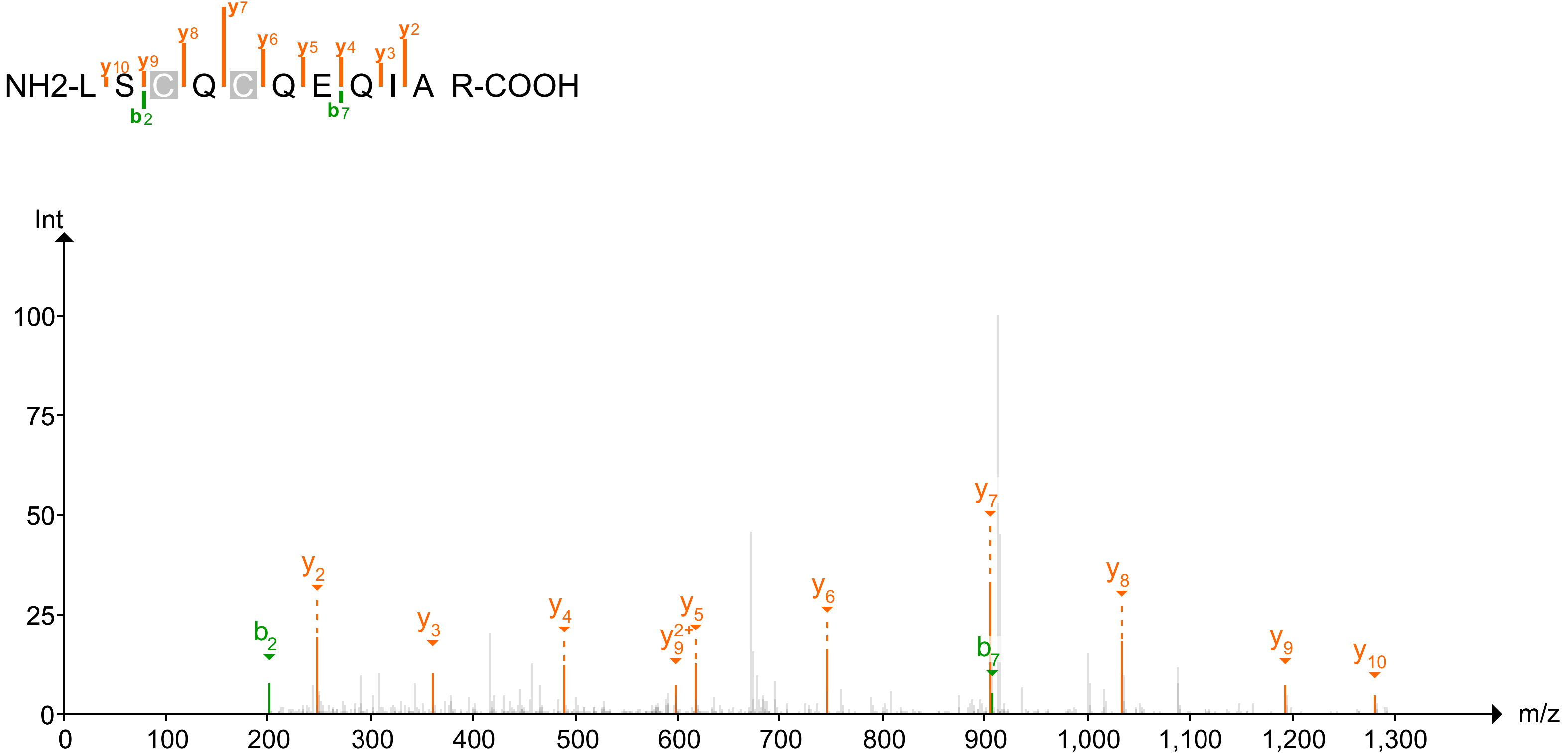

NH2-L S C Q C Q E Q I A R-COOH
y10 y9 y8 y7 y6 y5 y4 y3 y2
b2 b7
Int
100
75
50
25
0
m/z
0 100 200 300 400 500 600 700 800 900 1,000 1,100 1,200 1,300
b2
y2
y3
y4
y9 2+
y5
y6
y7
b7
y8
y9
y10

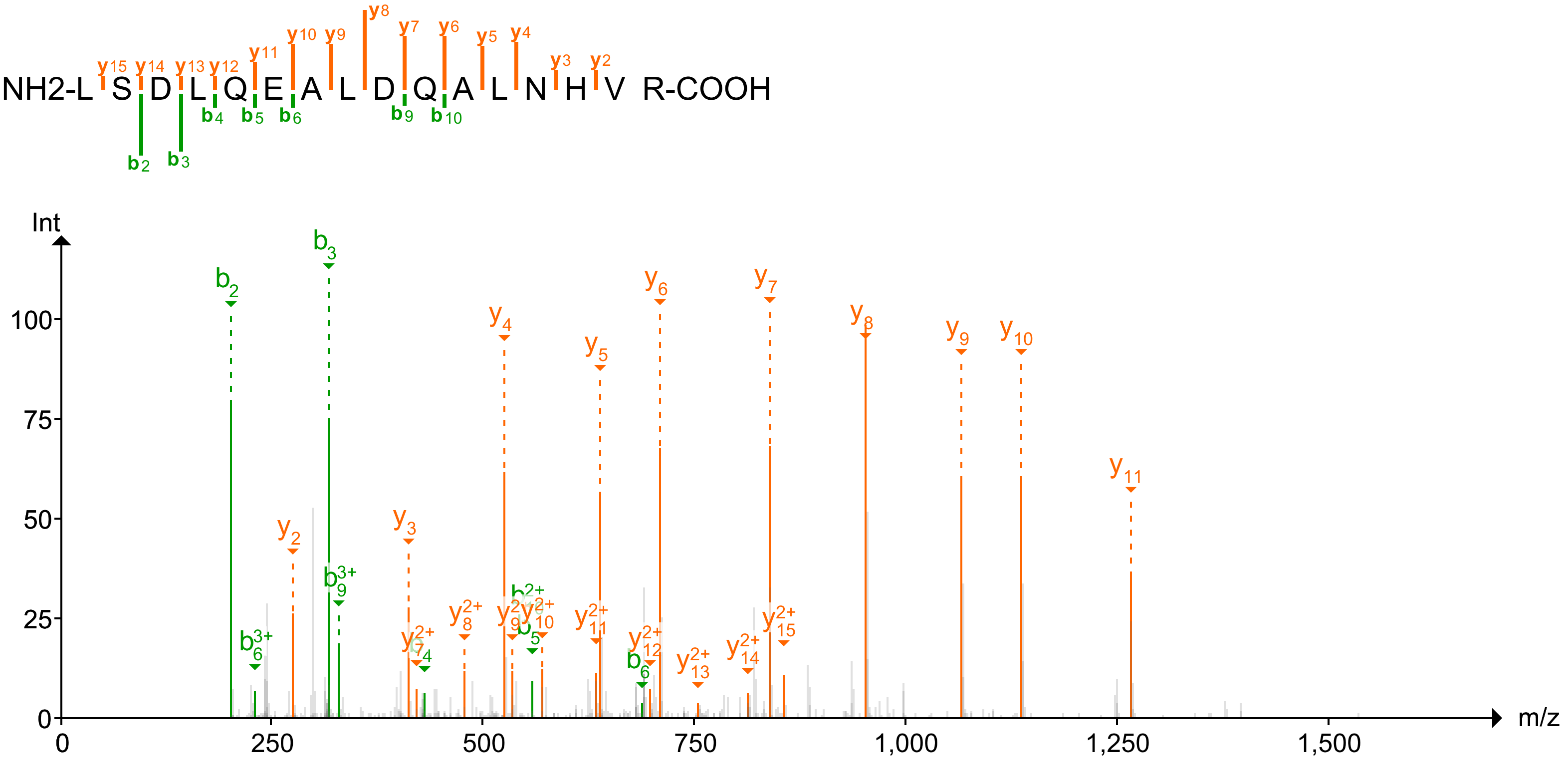
NH2-L S D L Q E A L D Q A L N H V R-COOH
y15 y14 y13 y12 y11 y10 y9 y8 y7 y6 y5 y4 y3 y2
b2 b3 b4 b5 b6 b9 b10
Int
m/z
0
25
50
75
100
250
500
750
1,000
1,250
1,500
b2
b3
y2
y3
y4
y5
y6
y7
y8
y9
y10
y11
b6 3+
b9 3+
b5
b6
y7 2+
y8 2+
y9 2+
y10 2+
y11 2+
y12 2+
y13 2+
y14 2+
y15 2+

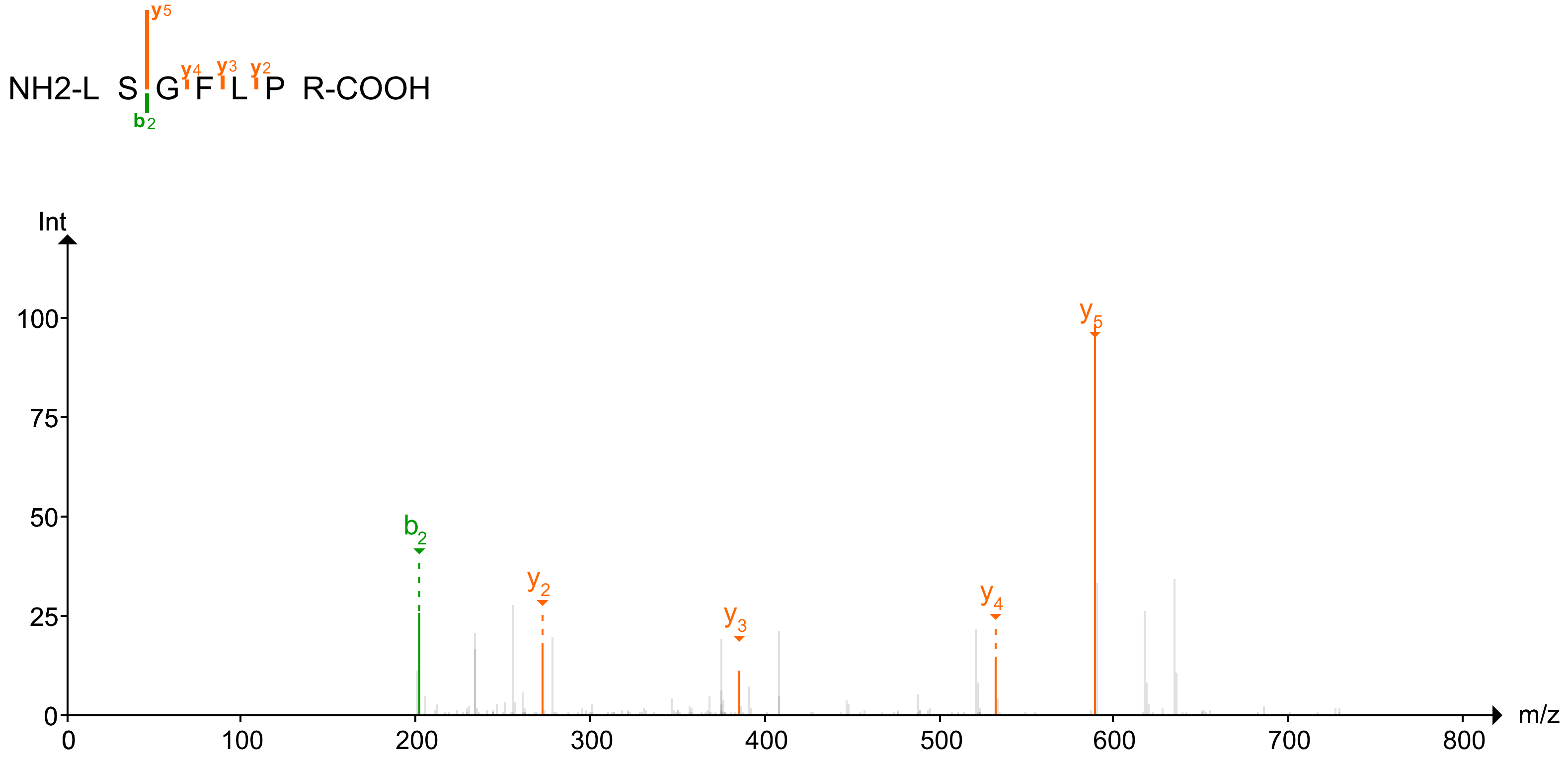
NH2-L S G F L P R-COOH
y5
y4
y3
y2
b2
Int
100
75
50
25
0
m/z
0
100
200
300
400
500
600
700
800
b2
y2
y3
y4
y5

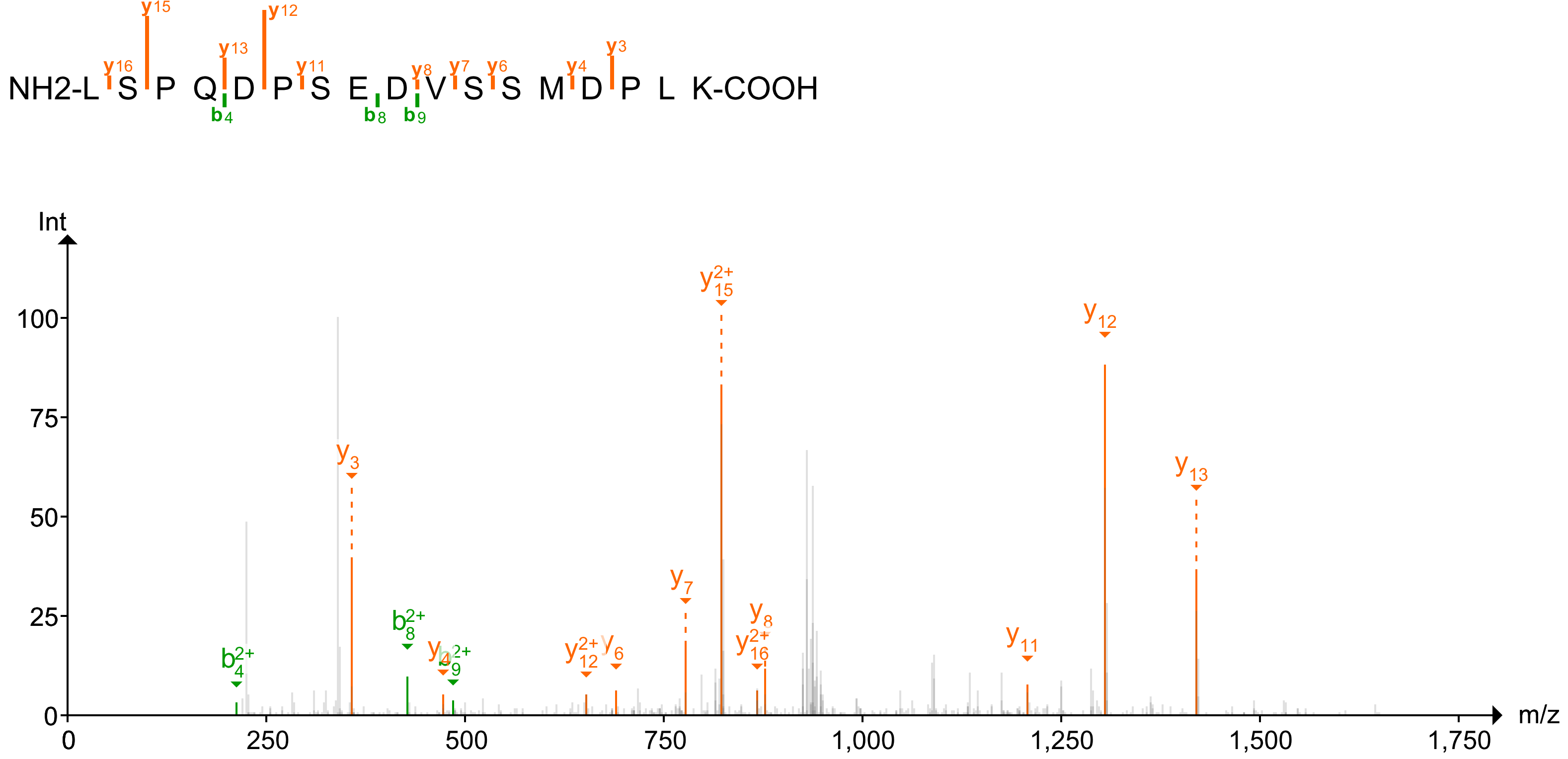

NH2-L S P Q D P S E D V S S M D P L K-COOH
y15 y12 y13 y16 y11 y8 y7 y6 y4 y3
b4 b8 b9
Int
100
75
50
25
0
m/z
0
250
500
750
1,000
1,250
1,500
1,750

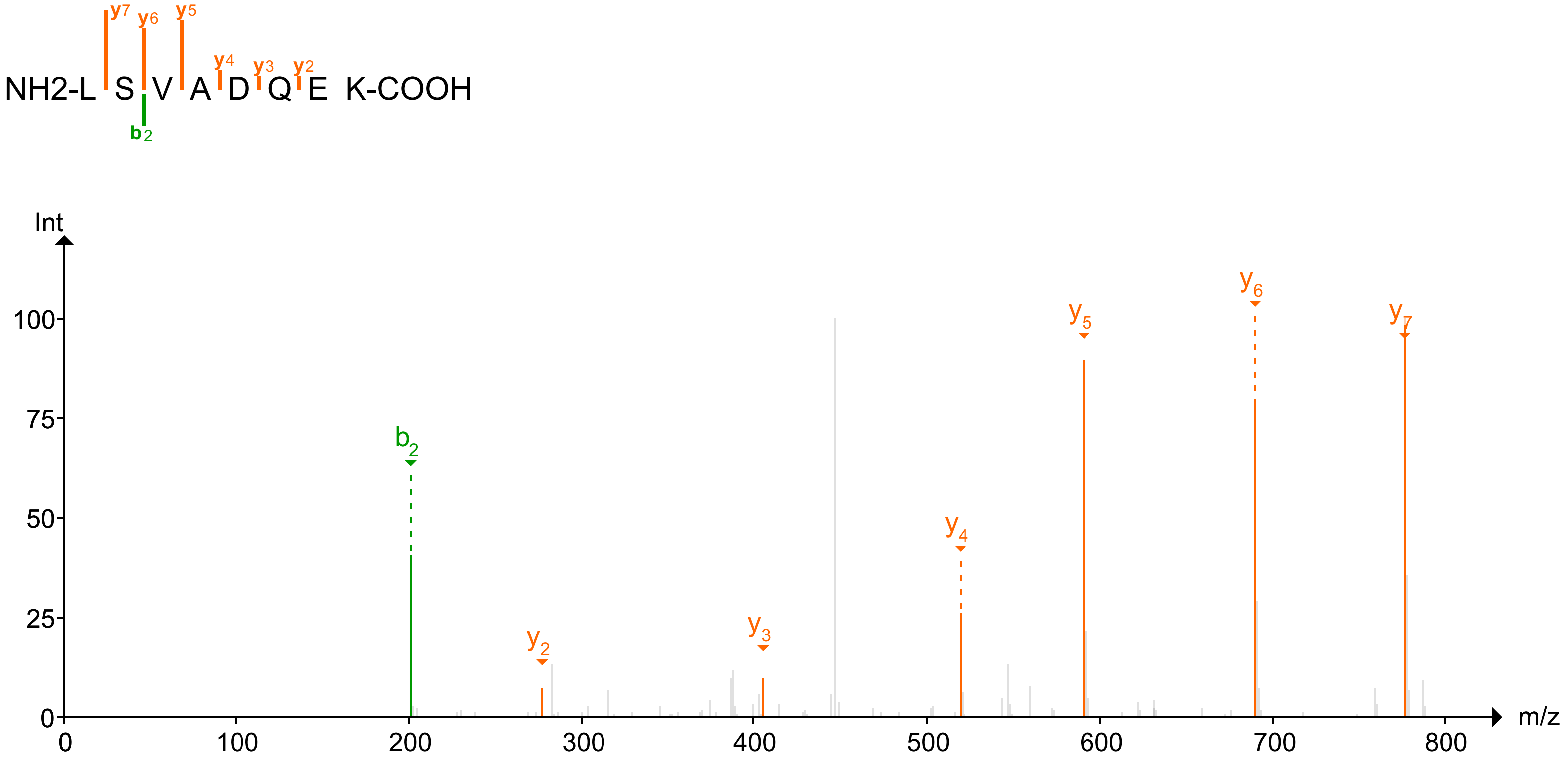

NH2-L S V A D Q E K-COOH
y7 y6 y5 y4 y3 y2
b2
Int
100
75
50
25
0
m/z
0
100
200
300
400
500
600
700
800
b2
y2
y3
y4
y5
y6
y7

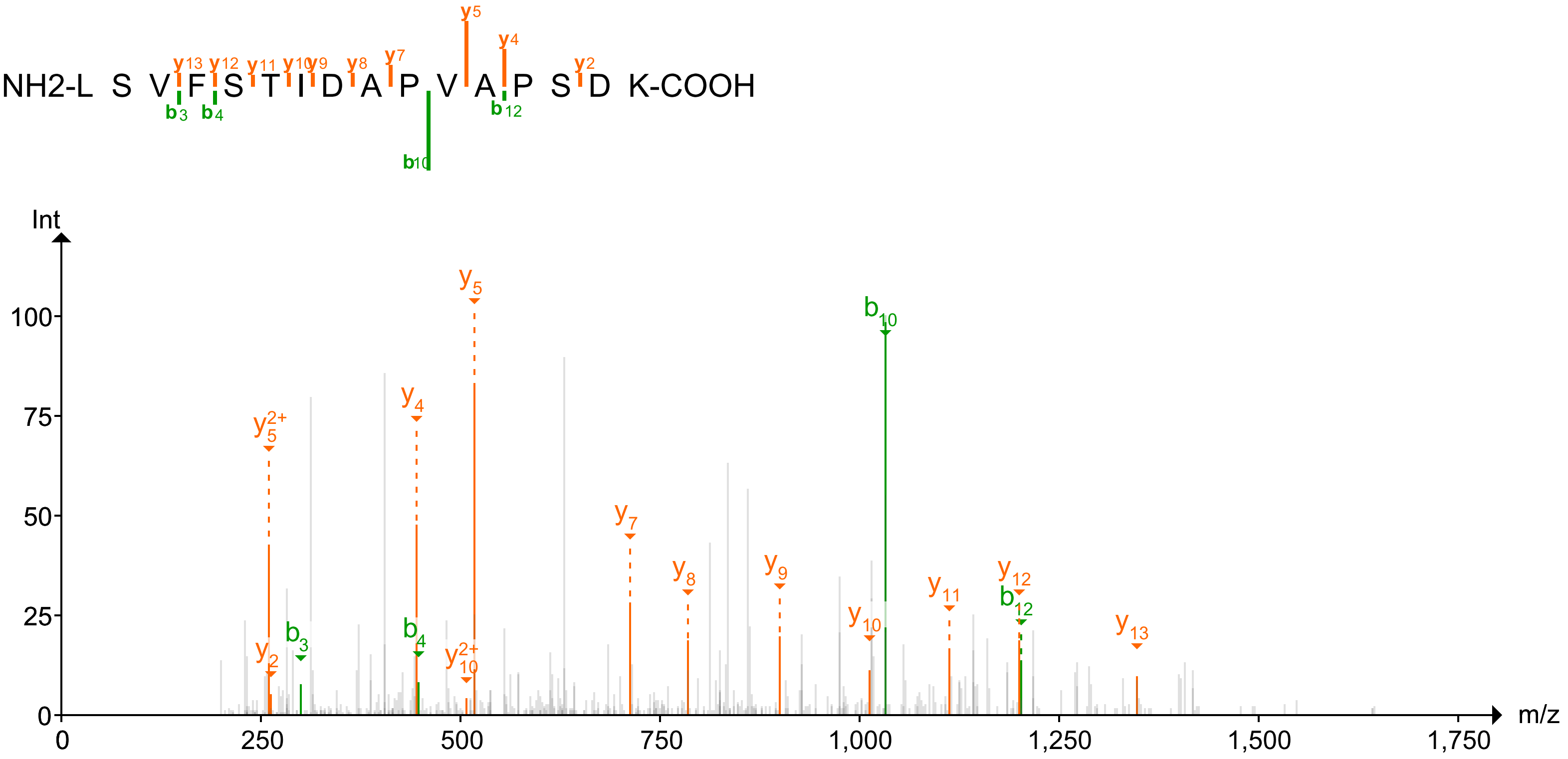

NH2-L S V F S T I D A P V A P S D K-COOH
y13 y12 y11 y10 y9 y8 y7 y5 y4 y2
b3 b4 b10 b12
Int
m/z
0
25
50
75
100
0
250
500
750
1,000
1,250
1,500
1,750

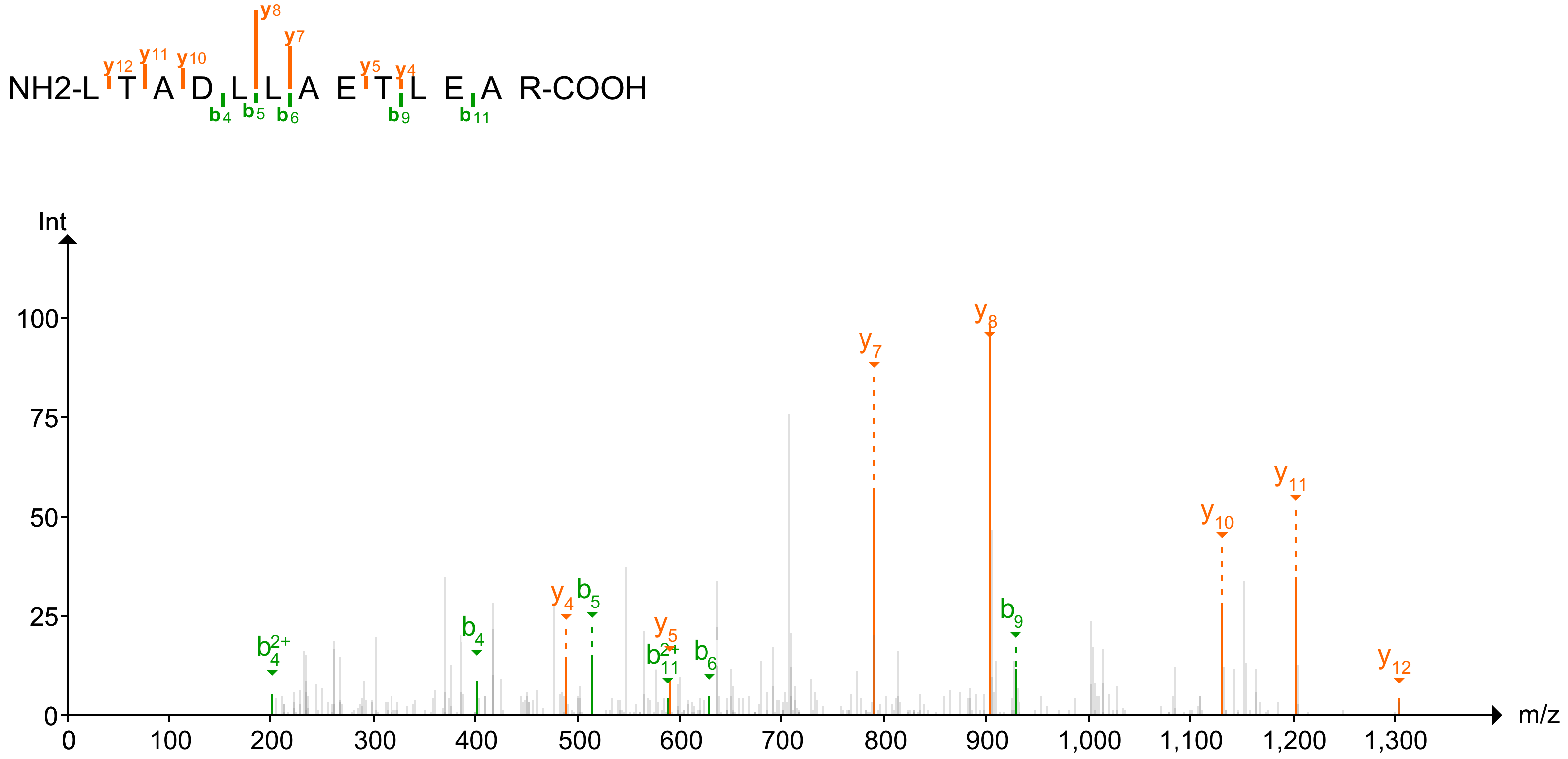

NH2-L T A D L L A E T L E A R-COOH
y12 y11 y10 y8 y7 y5 y4
b4 b5 b6 b9 b11
Int
m/z
0
25
50
75
100
0
100
200
300
400
500
600
700
800
900
1,000
1,100
1,200
1,300
b4 2+
b4
y4
b5
y5
b11 2+
b6
y7
y8
b9
y10
y11
y12

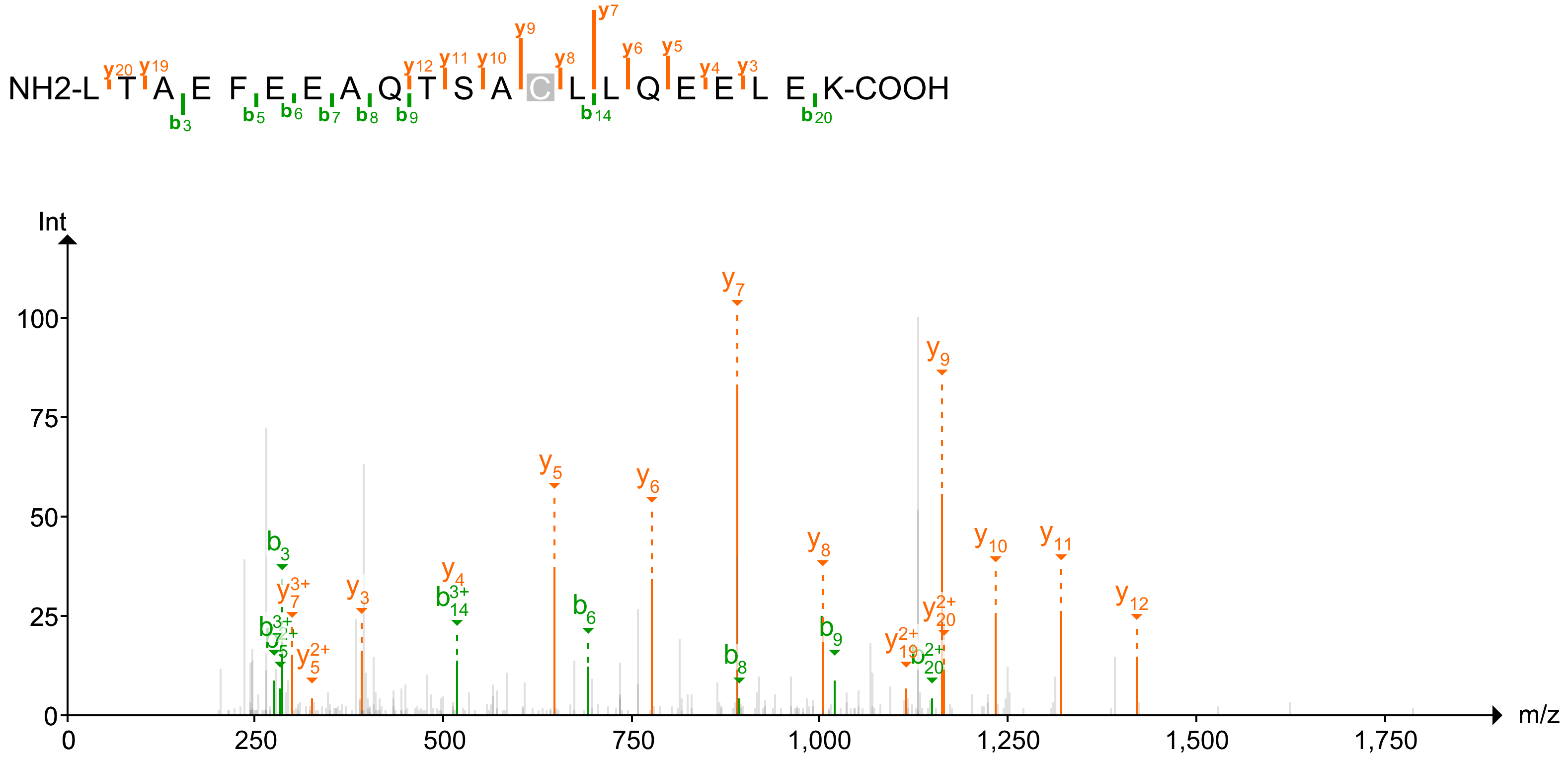

NH2-L T A E F E E A Q T S A C L L Q E E L E K-COOH
Int
m/z
0
25
50
75
100
250
500
750
1,000
1,250
1,500
1,750

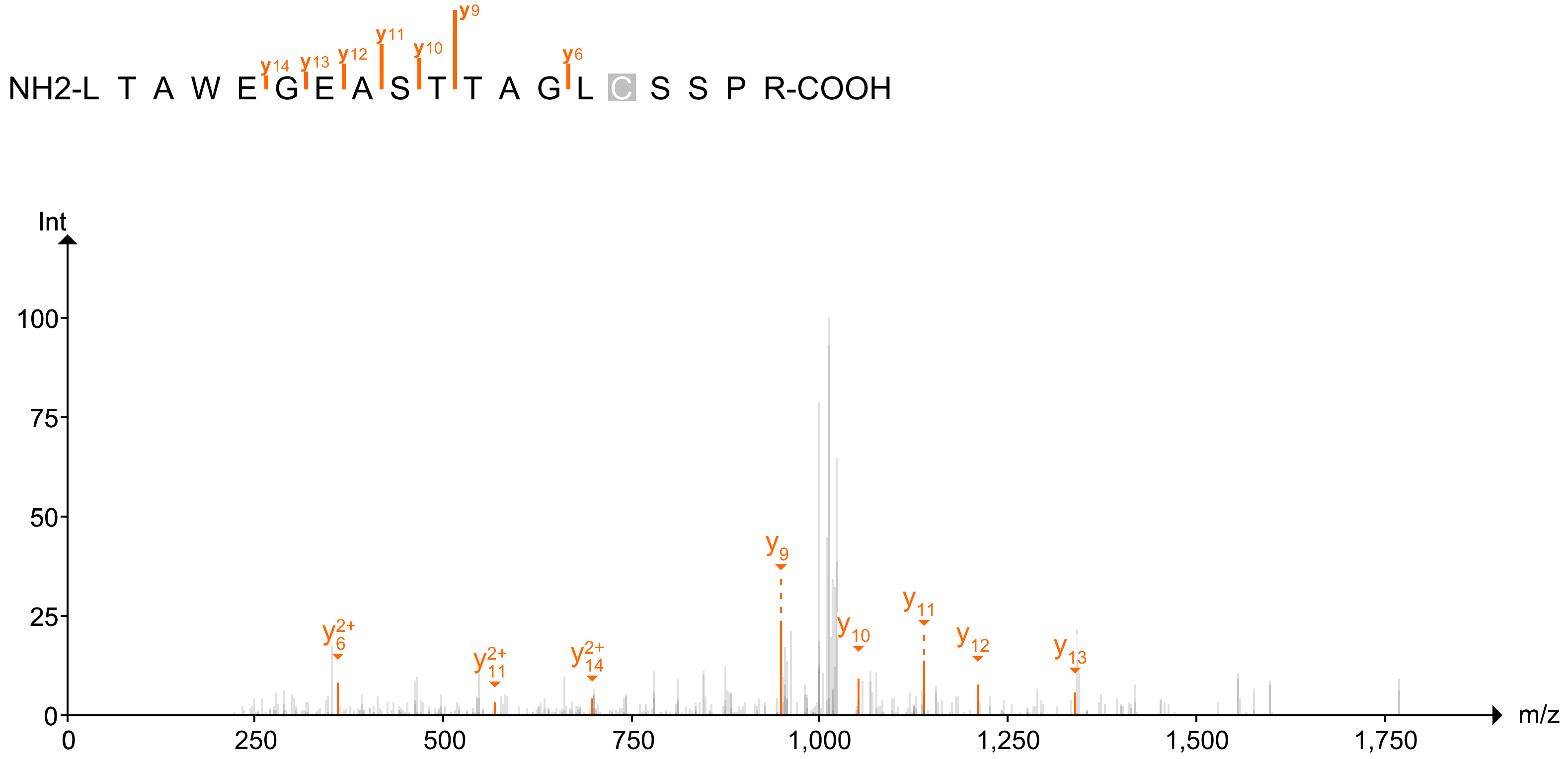

NH2-L T A W E G E A S T T A G L C S S P R-COOH
y14
y13
y12
y11
y10
y9
y6
Int
100
75
50
25
0
m/z
0
250
500
750
1,000
1,250
1,500
1,750
y6 2+
y11 2+
y14 2+
y9
y10
y11
y12
y13

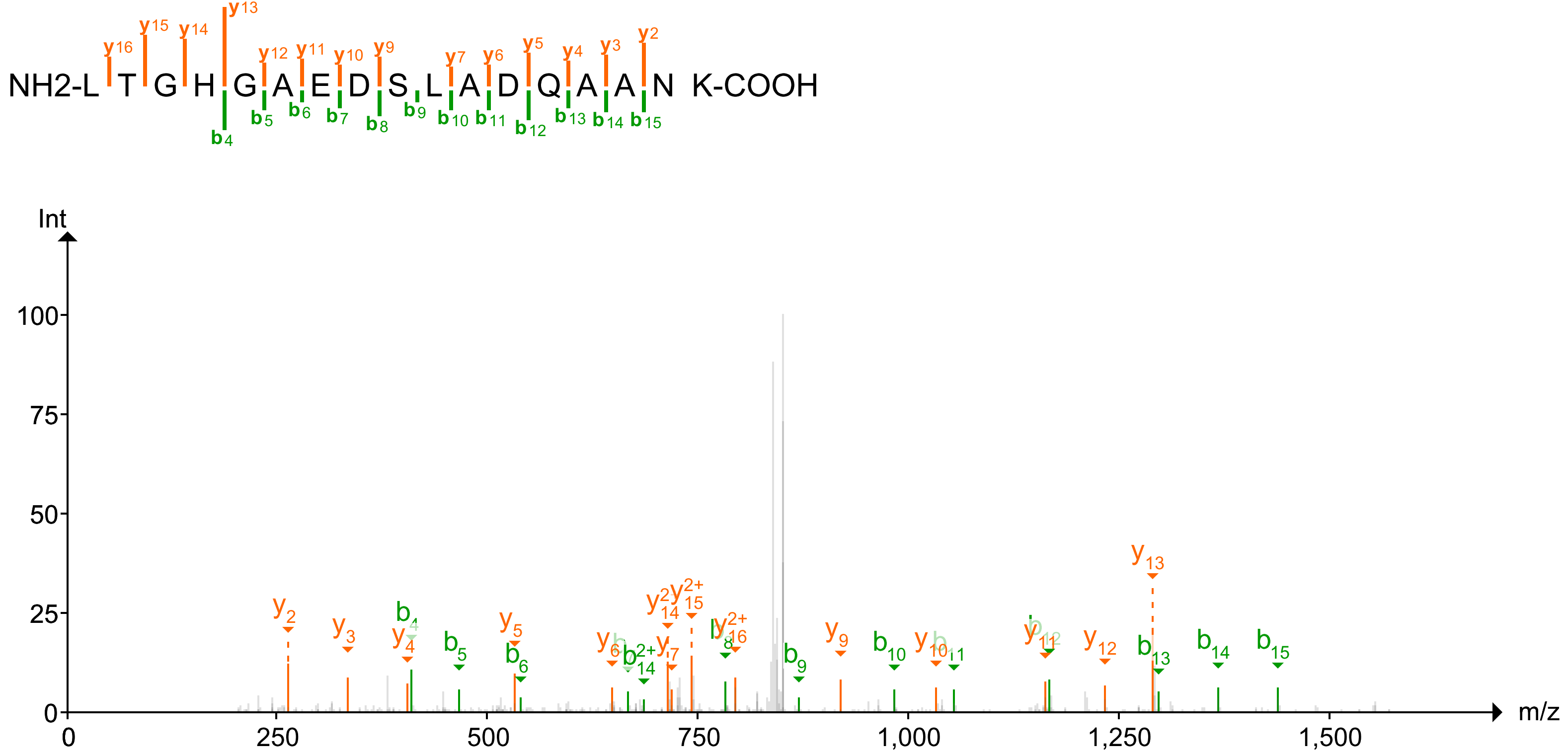
NH2-L T G H G A E D S L A D Q A A N K-COOH
Int
m/z
0
25
50
75
100
250
500
750
1,000
1,250
1,500

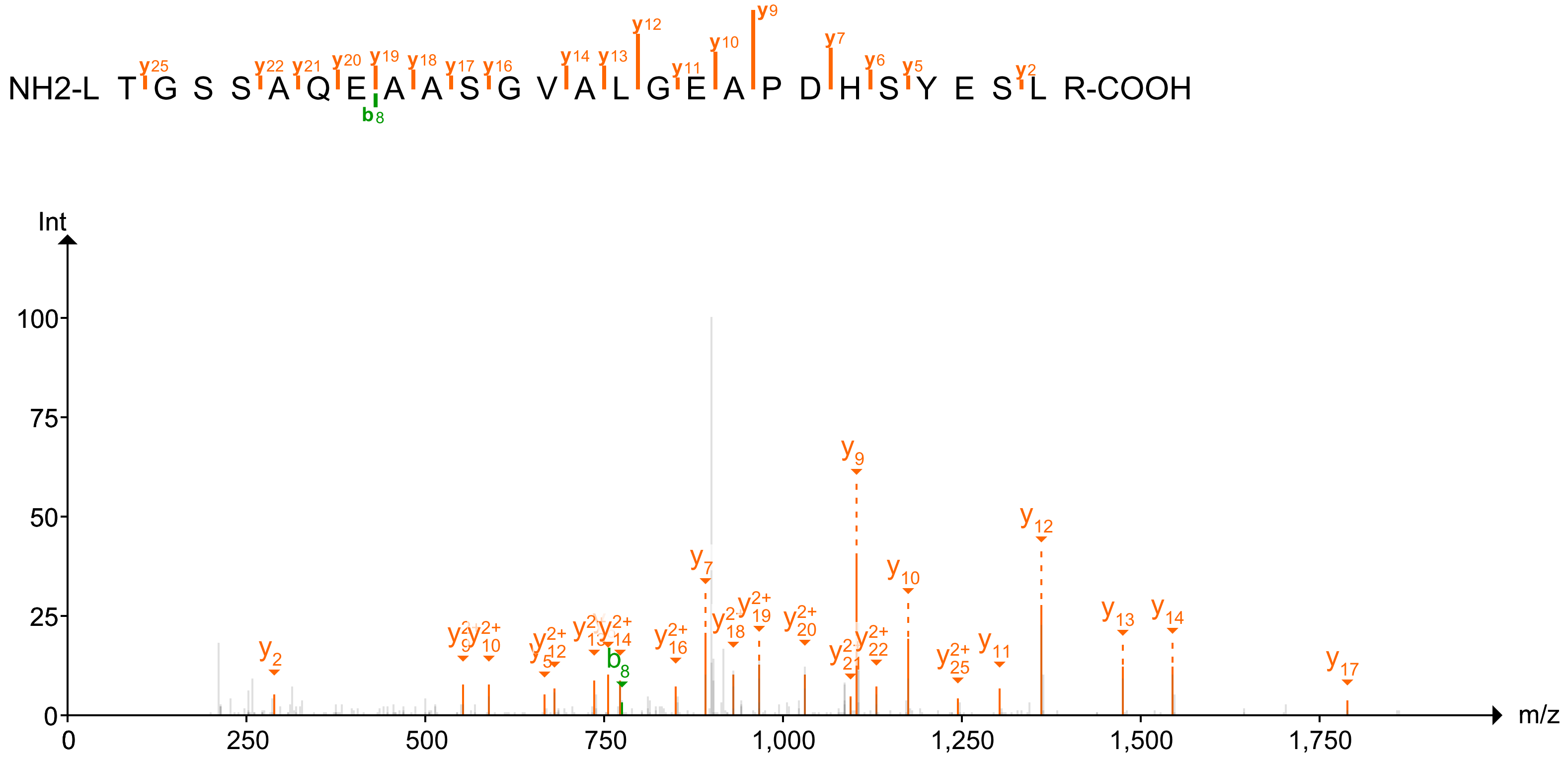

NH2-L T G S S A Q E A A S G V A L G E A P D H S Y E S L R-COOH
y25 y22 y21 y20 y19 y18 y17 y16 y14 y13 y12 y11 y10 y9 y7 y6 y5 y2
b8
Int
100
75
50
25
0
m/z
0
250
500
750
1,000
1,250
1,500
1,750

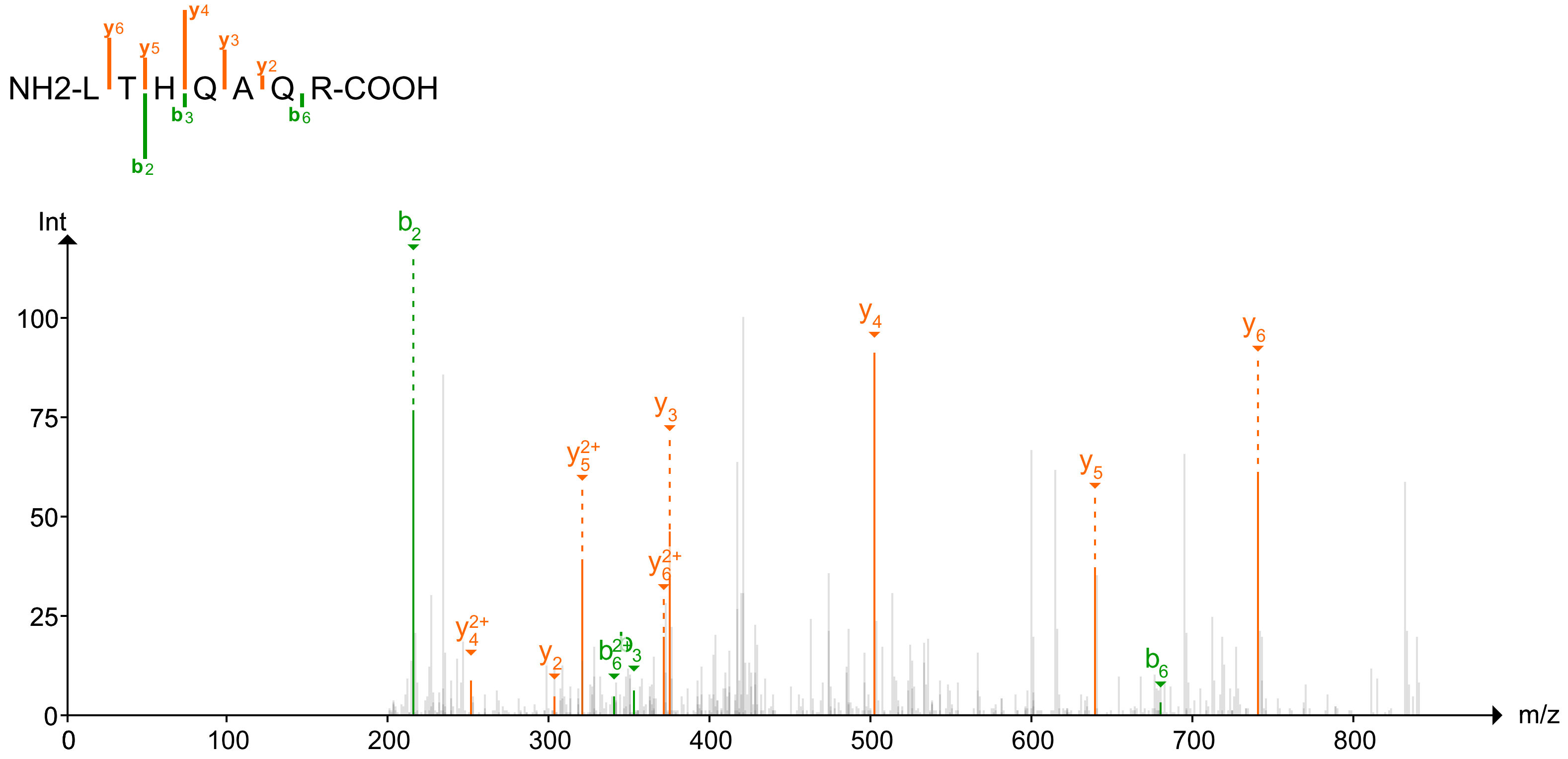

NH2-L T H Q A Q R-COOH
y6 y5 y4 y3 y2
b2 b3 b6
Int
m/z
0 25 50 75 100
0 100 200 300 400 500 600 700 800

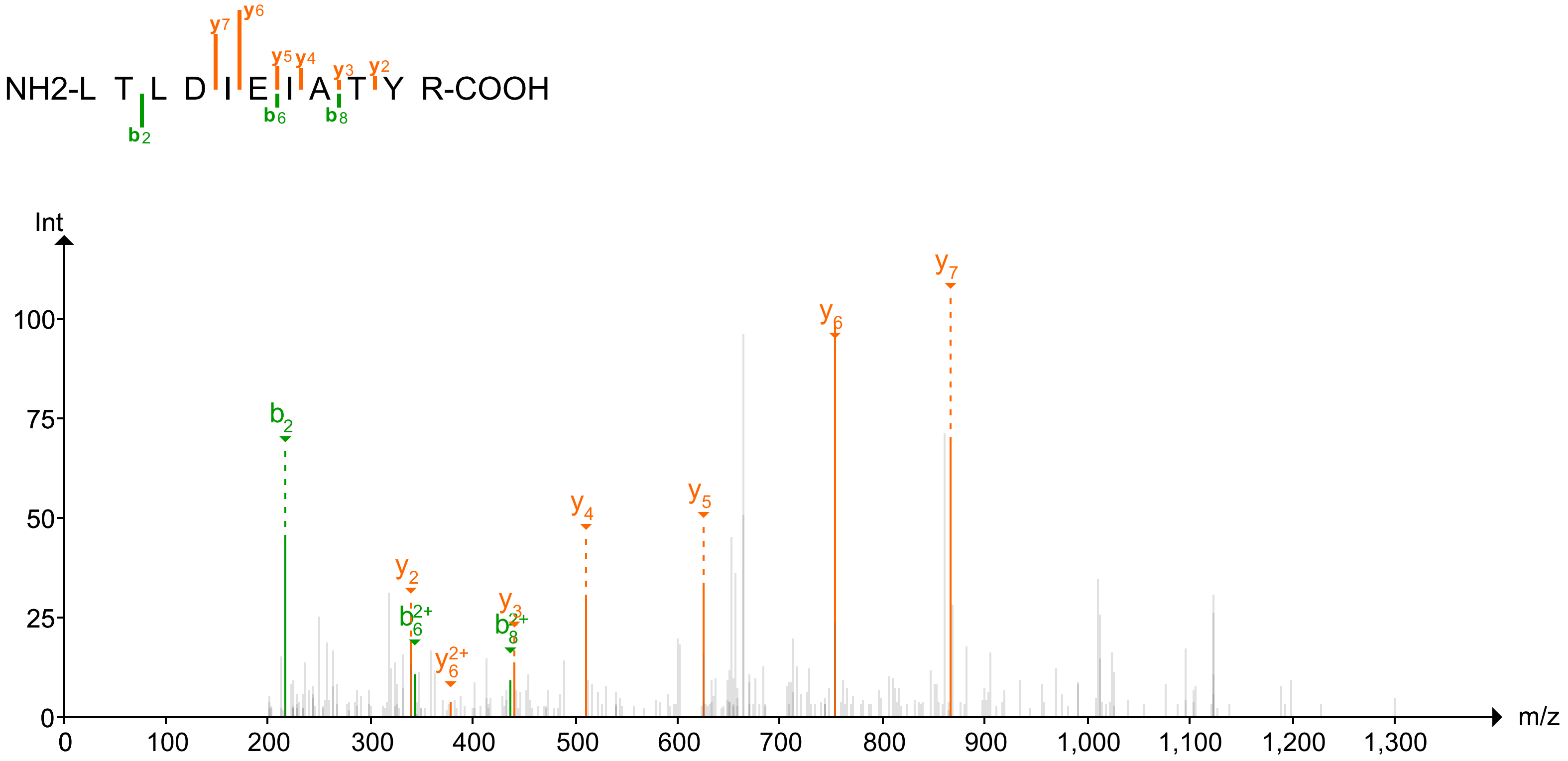

NH2-L T L D I E I A T Y R-COOH
y7 y6 y5 y4 y3 y2
b2 b6 b8
Int
100
75
50
25
0
m/z
0
100
200
300
400
500
600
700
800
900
1,000
1,100
1,200
1,300

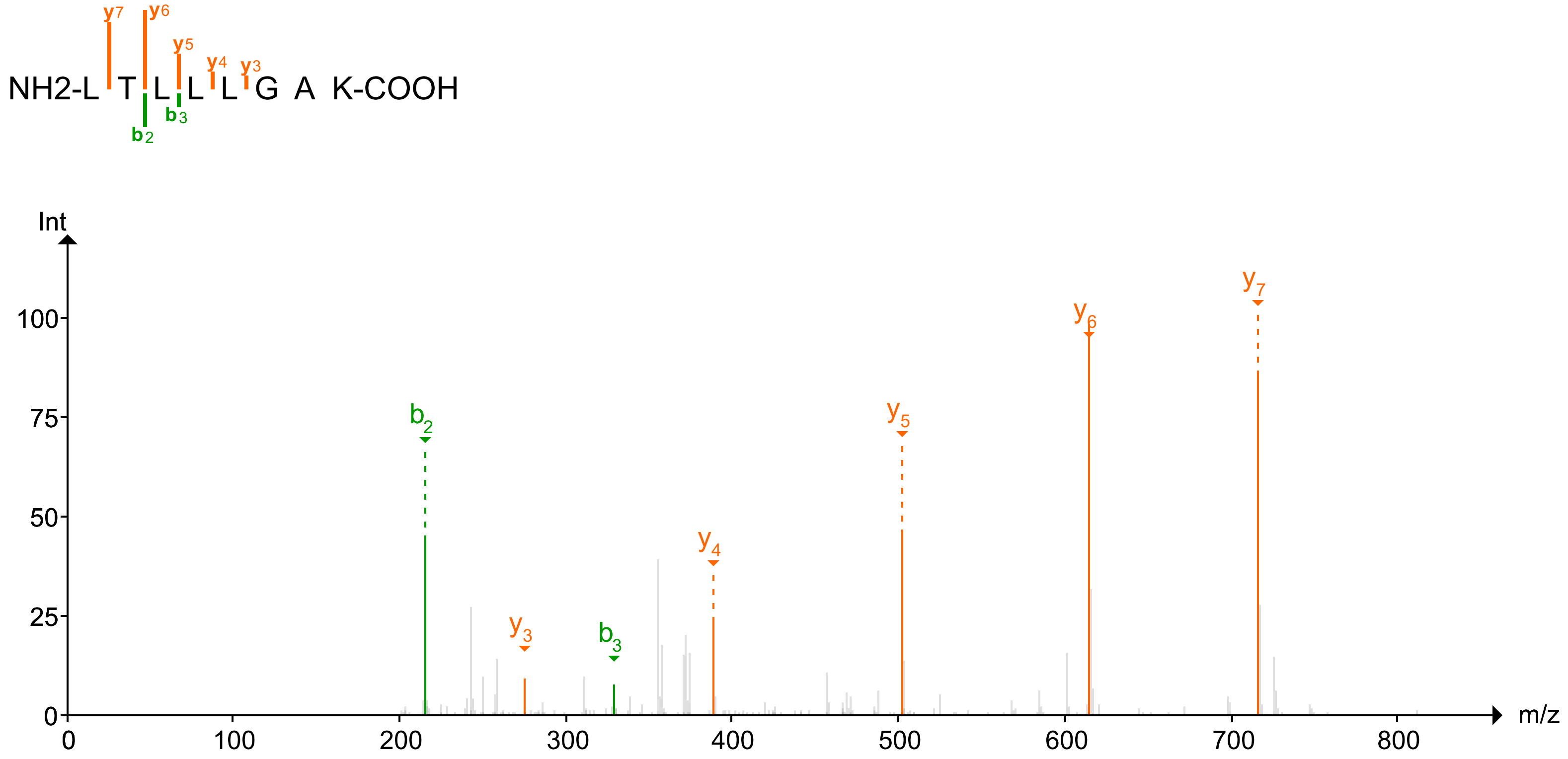

NH2-L T L L L G A K-COOH
y7 y6 y5 y4 y3
b2 b3
Int
100
75
50
25
0
m/z
0
100
200
300
400
500
600
700
800
b2
y3
b3
y4
y5
y6
y7

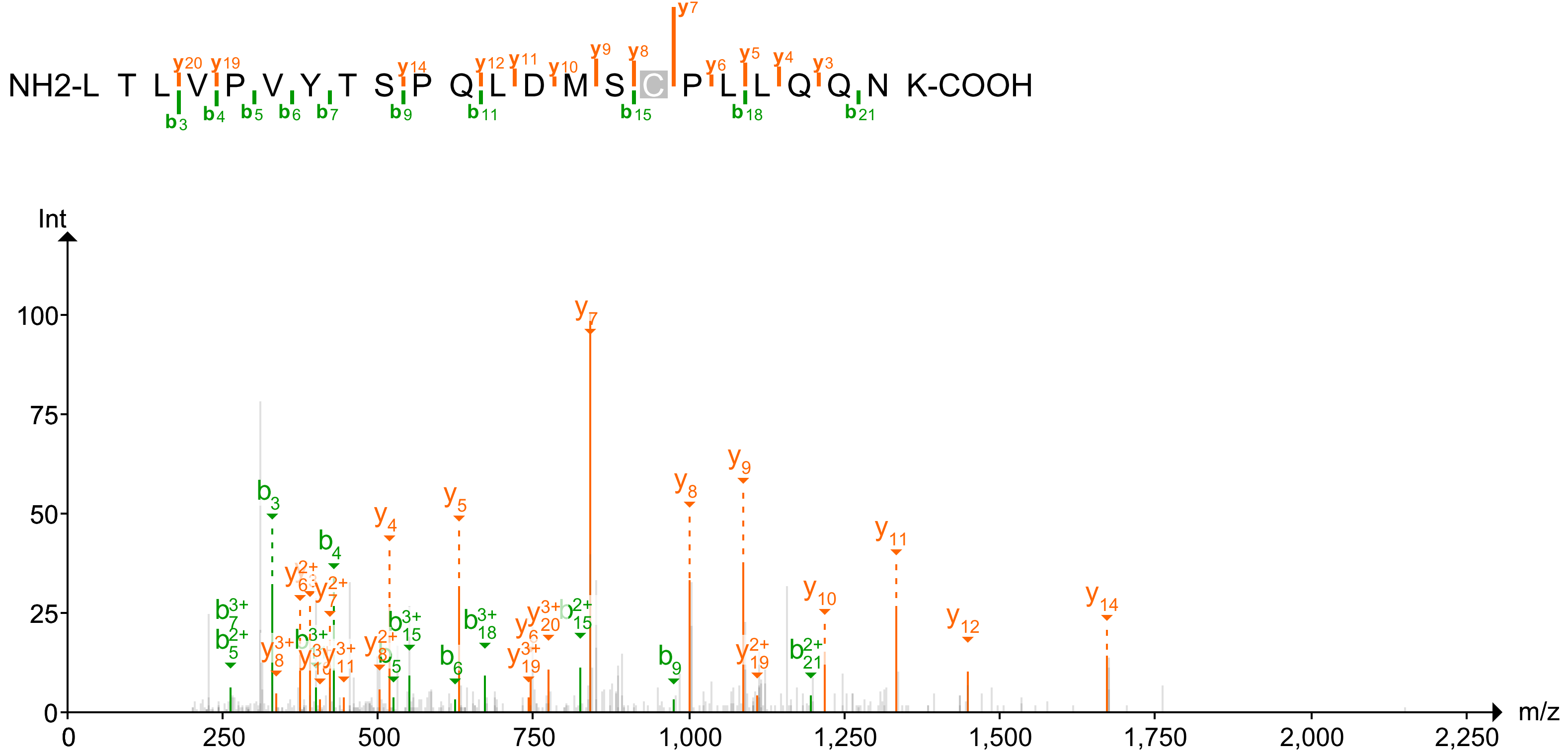

NH2-L T L V P V Y T S P Q L D M S C P L L Q Q N K-COOH
y20 y19 y14 y12 y11 y10 y9 y8 y7 y6 y5 y4 y3
b3 b4 b5 b6 b7 b9 b11 b15 b18 b21
Int
100
75
50
25
0
m/z
0
250
500
750
1,000
1,250
1,500
1,750
2,000
2,250
y7
y8
y9
y11
y10
y12
y14
y4
y5
b3
b4
b6
b9

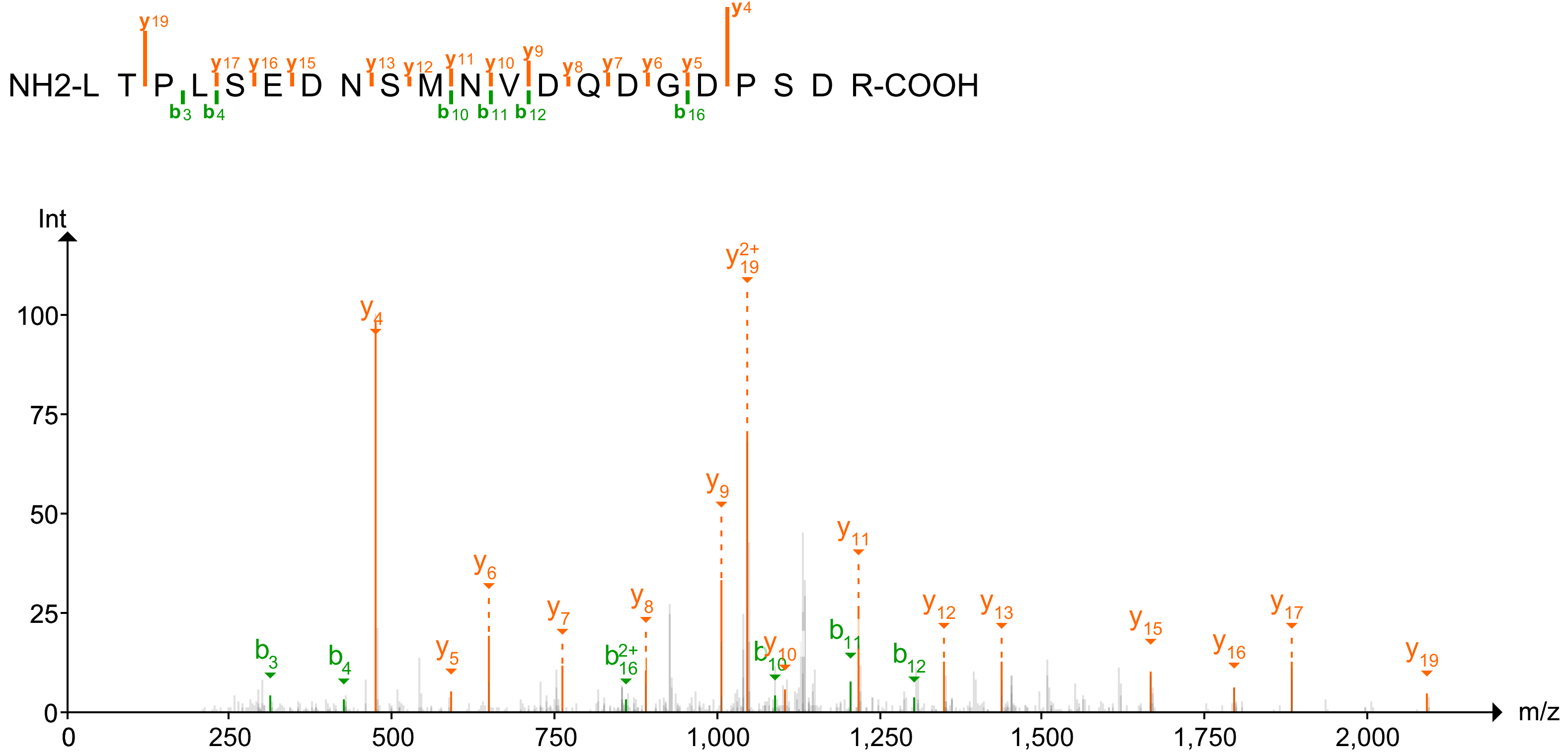

NH2-L T P L S E D N S M N V D Q D G D P S D R-COOH
y19 y17 y16 y15 y13 y12 y11 y10 y9 y8 y7 y6 y5 y4
b3 b4 b10 b11 b12 b16
Int
100
75
50
25
0
y4
y19 2+
y9
y11
y6
y7
y8
y5
y12
y13
y15
y16
y17
y19
y10
b3
b4
b16 2+
b10
b11
b12
0
250
500
750
1,000
1,250
1,500
1,750
2,000
m/z

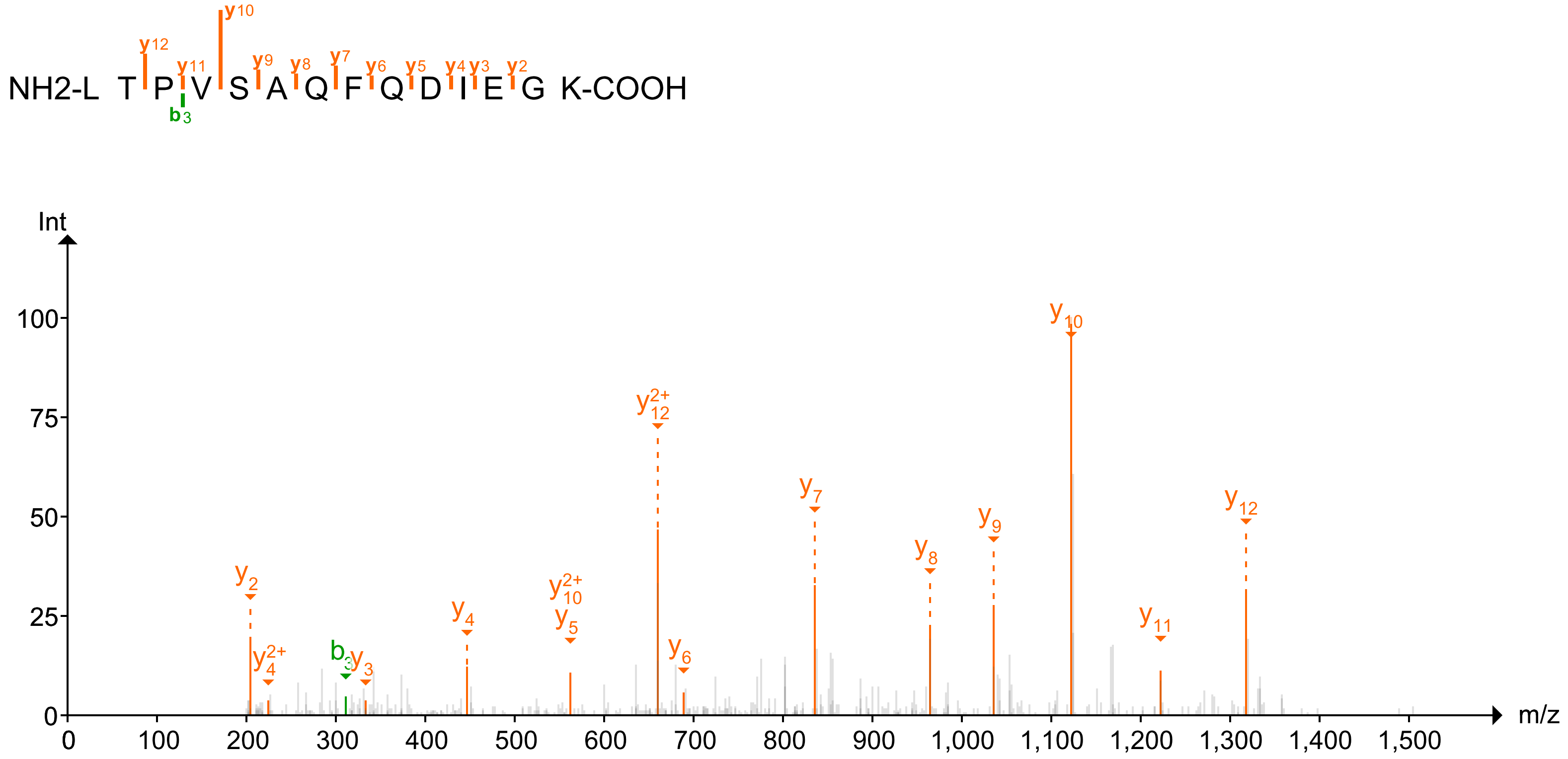

NH2-L T P V S A Q F Q D I E G K-COOH
y12 y11 y10 y9 y8 y7 y6 y5 y4 y3 y2
b3
Int
100
75
50
25
0
m/z
0 100 200 300 400 500 600 700 800 900 1,000 1,100 1,200 1,300 1,400 1,500
y2
y4 2+
b3
y3
y4
y10 2+
y5
y12 2+
y6
y7
y8
y9
y10
y11
y12

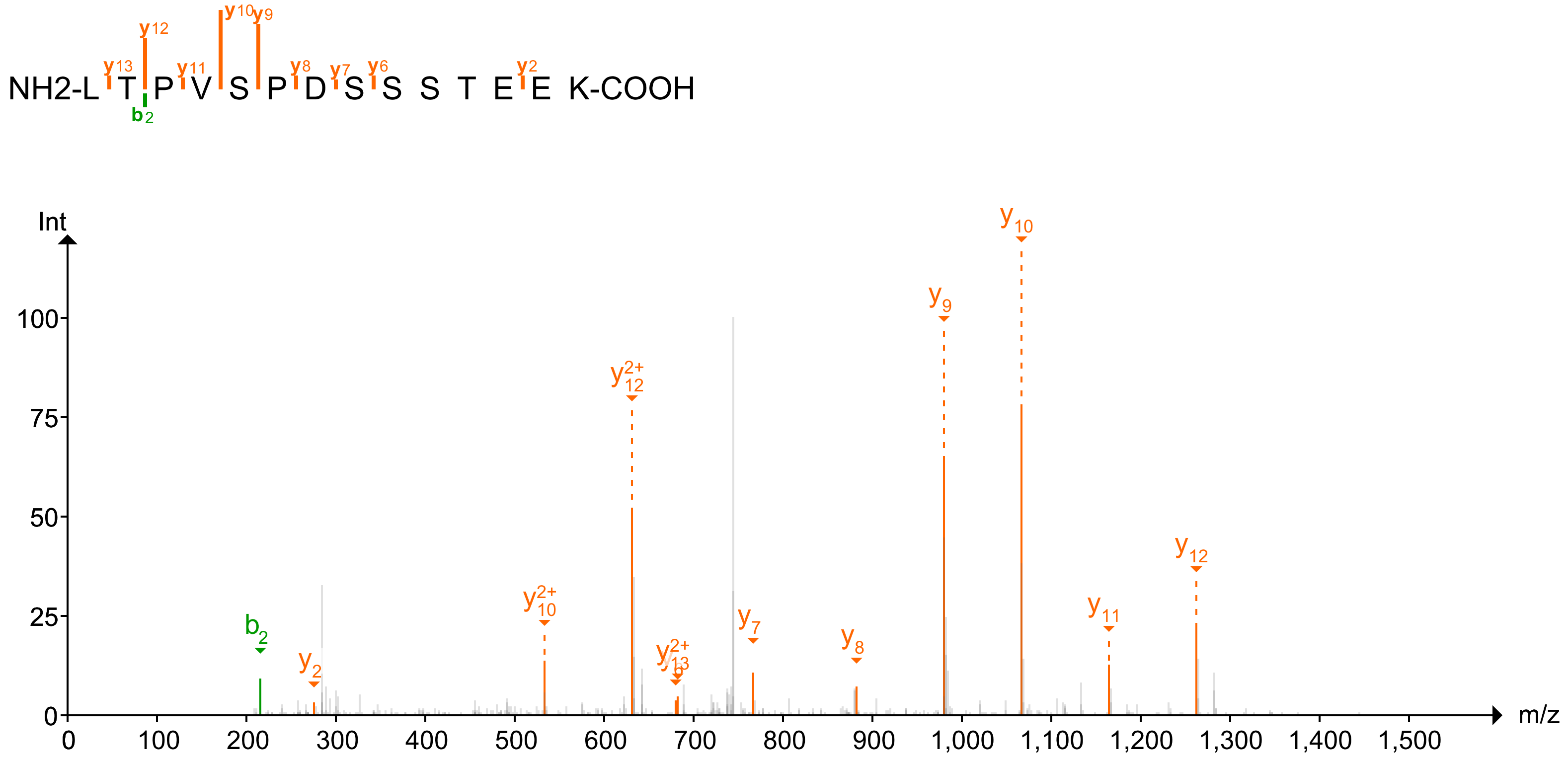

NH2-L T P V S P D S S S T E E K-COOH
y13 y12 y11 y10 y9 y8 y7 y6 y2
b2
Int
m/z
0
25
50
75
100
0 100 200 300 400 500 600 700 800 900 1,000 1,100 1,200 1,300 1,400 1,500
b2
y2
y10 2+
y12 2+
y13 2+
y7
y8
y9
y10
y11
y12

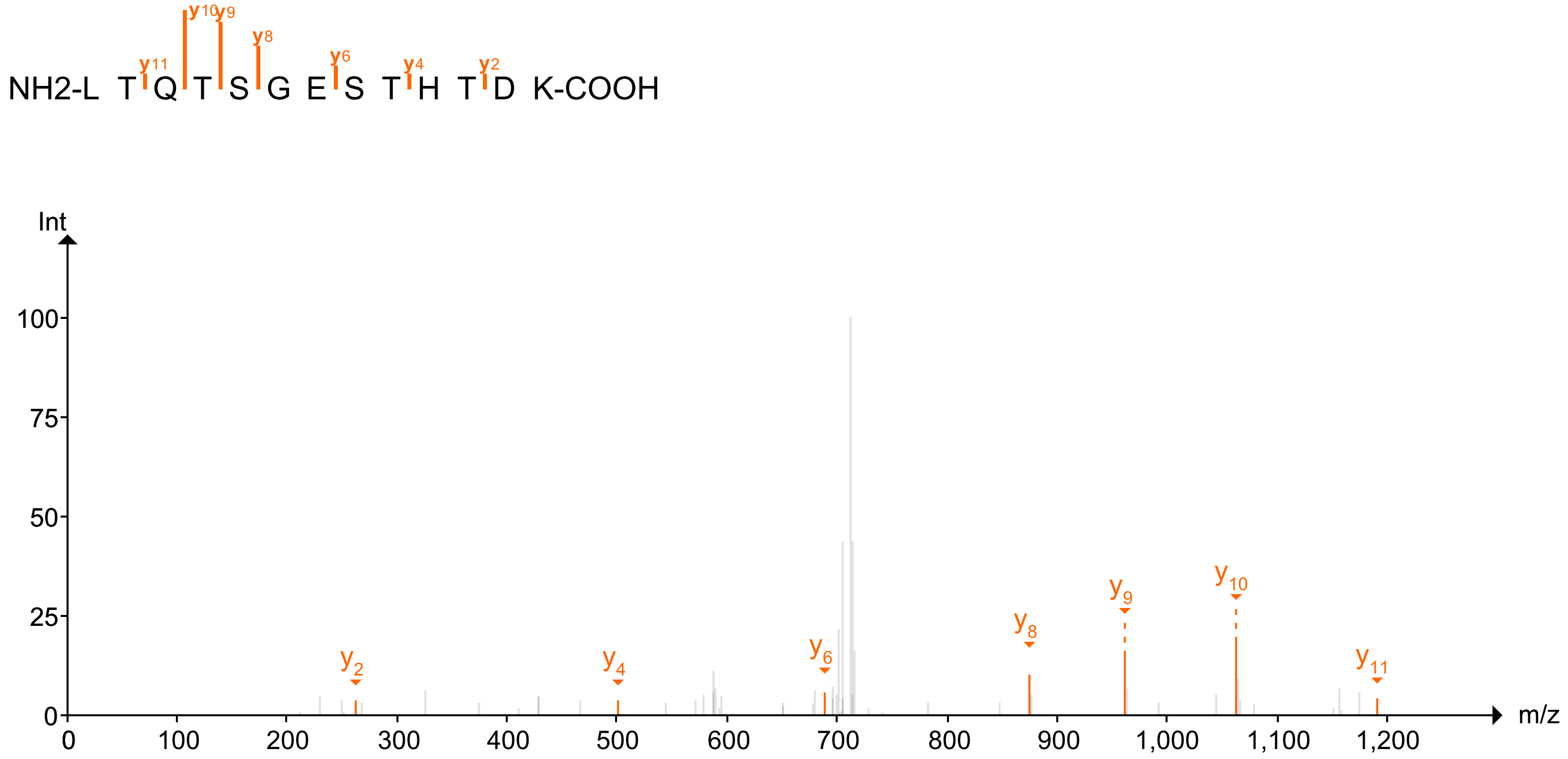

NH2-L T Q T S G E S T H T D K-COOH
y11 y10 y9 y8 y6 y4 y2
Int
100
75
50
25
0
y2
y4
y6
y8
y9
y10
y11
0 100 200 300 400 500 600 700 800 900 1,000 1,100 1,200
m/z

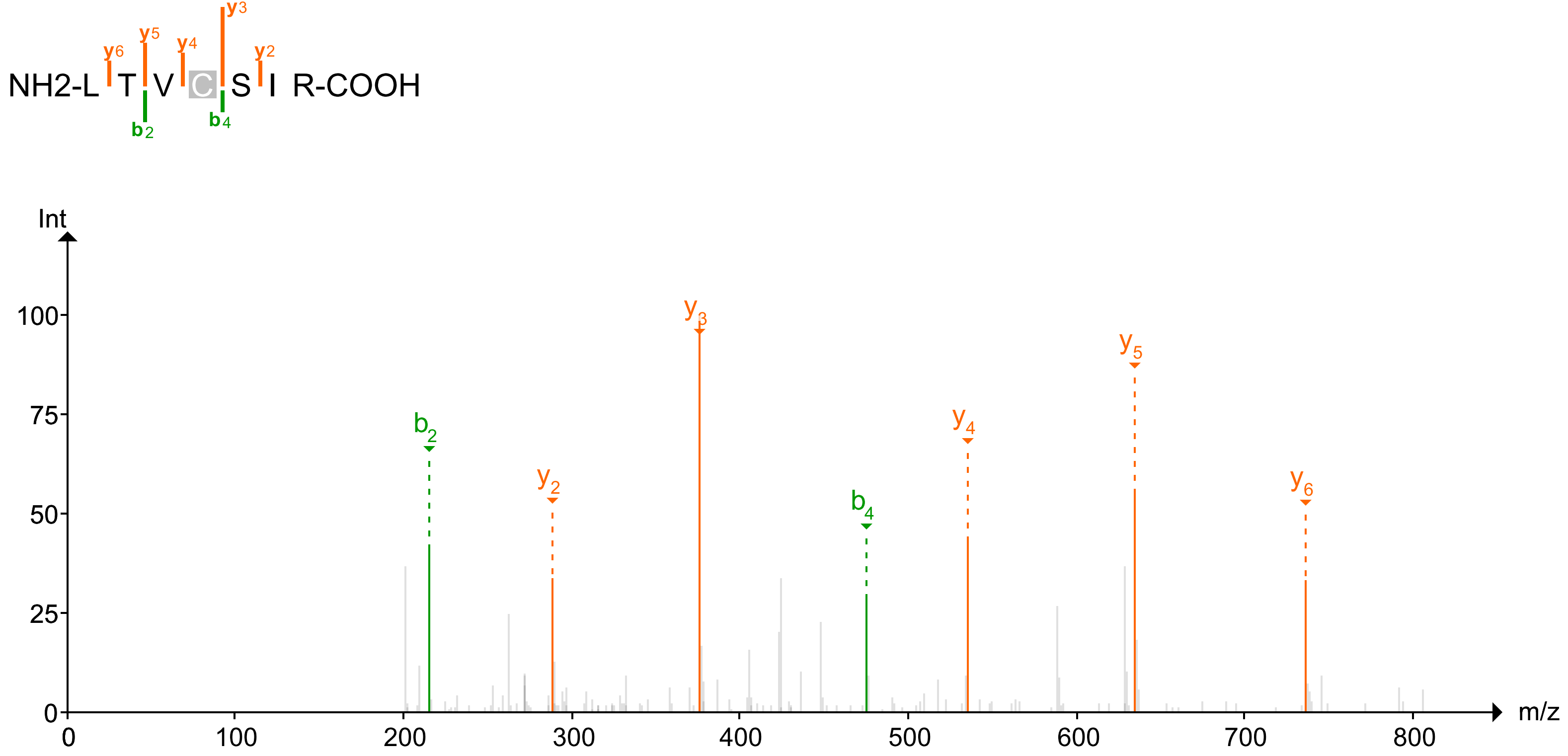

NH2-L T V C S I R-COOH
y6 y5 y4 y3 y2
b2 b4
Int
100
75
50
25
0
m/z
0
100
200
300
400
500
600
700
800
$b_2$
$y_2$
$y_3$
$b_4$
$y_4$
$y_5$
$y_6$

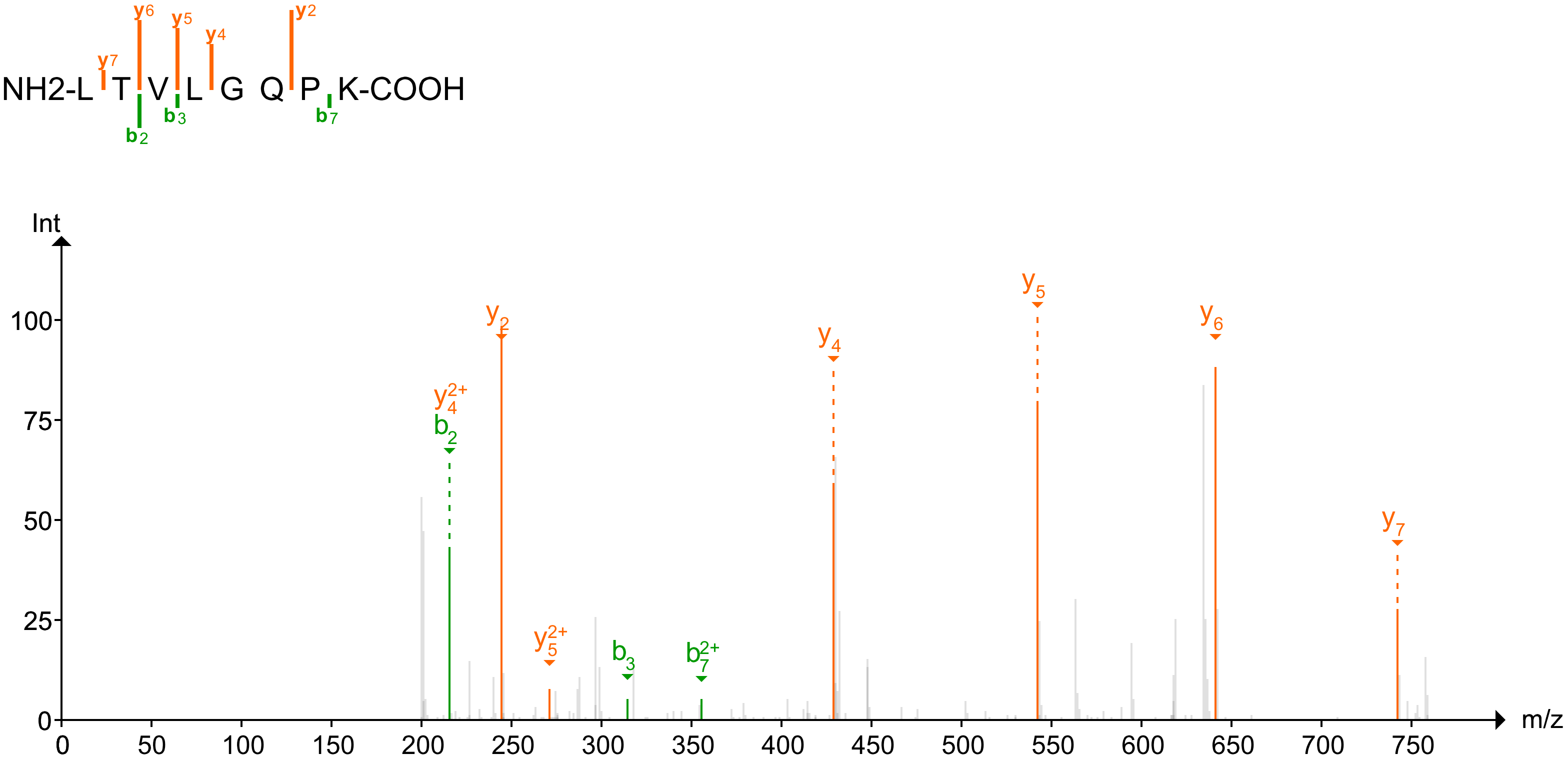

NH2-L T V L G Q P K-COOH
y7 y6 y5 y4 y2
b2 b3 b7
Int
m/z
y2
y4
y5
y6
y7
y4 2+
b2
y5 2+
b3
b7 2+
0 25 50 75 100
0 50 100 150 200 250 300 350 400 450 500 550 600 650 700 750

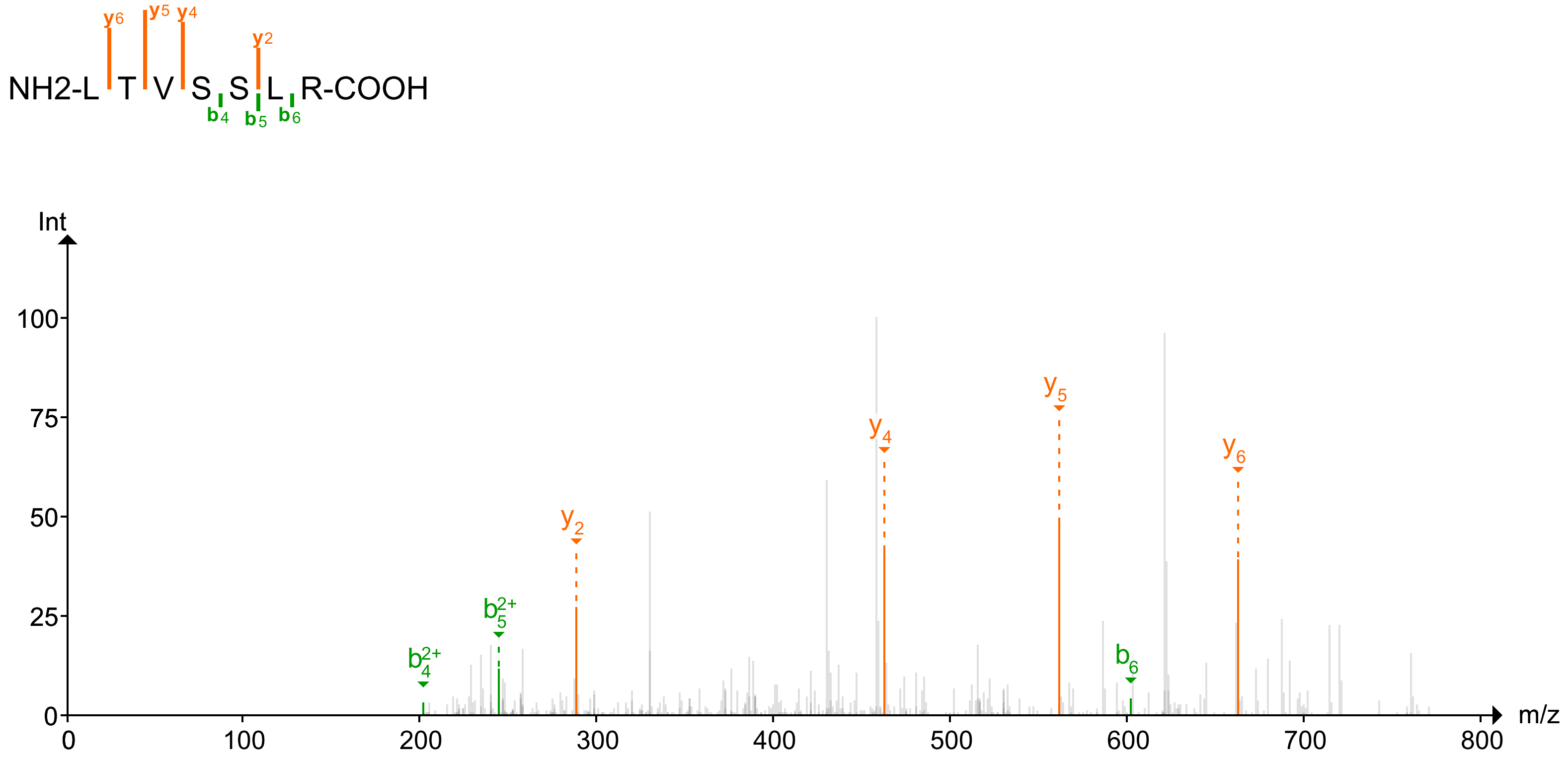

NH2-L T V S S L R-COOH
y6 y5 y4 y2
b4 b5 b6
Int
100
75
50
25
0
0 100 200 300 400 500 600 700 800
m/z
$y_2$
$y_4$
$y_5$
$y_6$
$b_4^{2+}$
$b_5^{2+}$
$b_6$

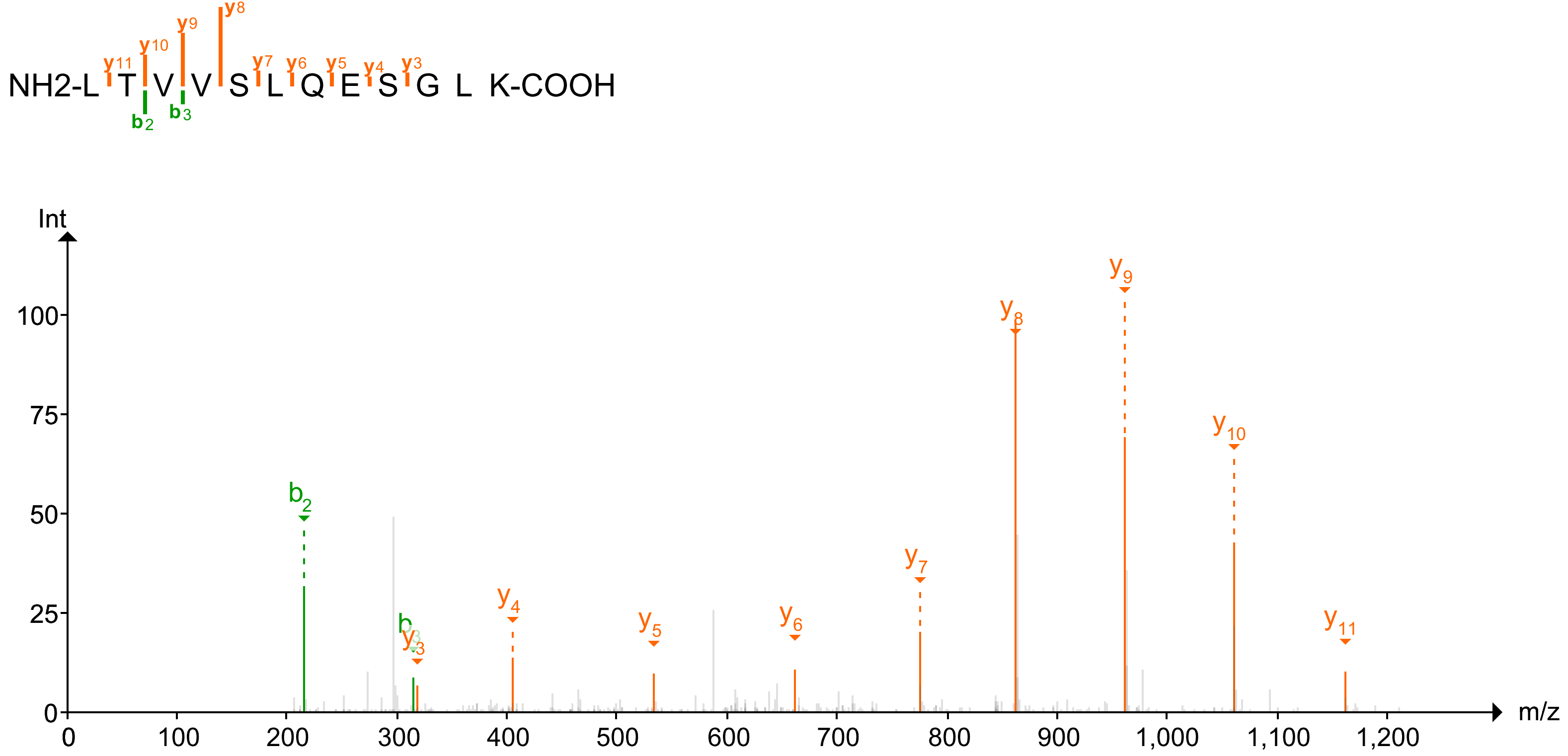

NH2-L T V V S L Q E S G L K-COOH
y11 y10 y9 y8 y7 y6 y5 y4 y3
b2 b3
Int
100
75
50
25
0
m/z
0
100
200
300
400
500
600
700
800
900
1,000
1,100
1,200
b2
b3
y3
y4
y5
y6
y7
y8
y9
y10
y11

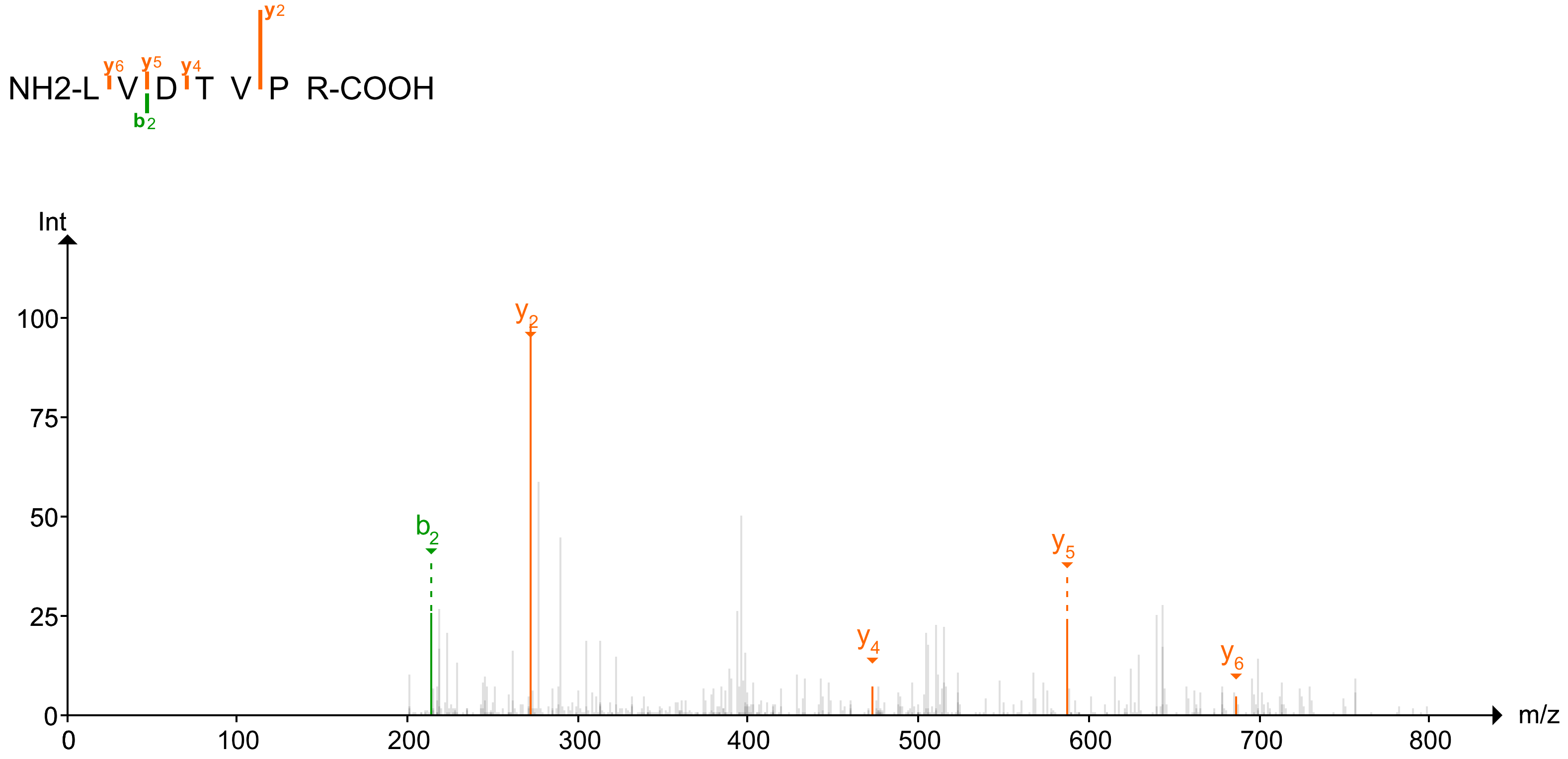

y2
y6
y5
y4
NH2-L V D T V P R-COOH
b2
Int
100
75
50
25
0
y2
b2
y5
y4
y6
0
100
200
300
400
500
600
700
800
m/z

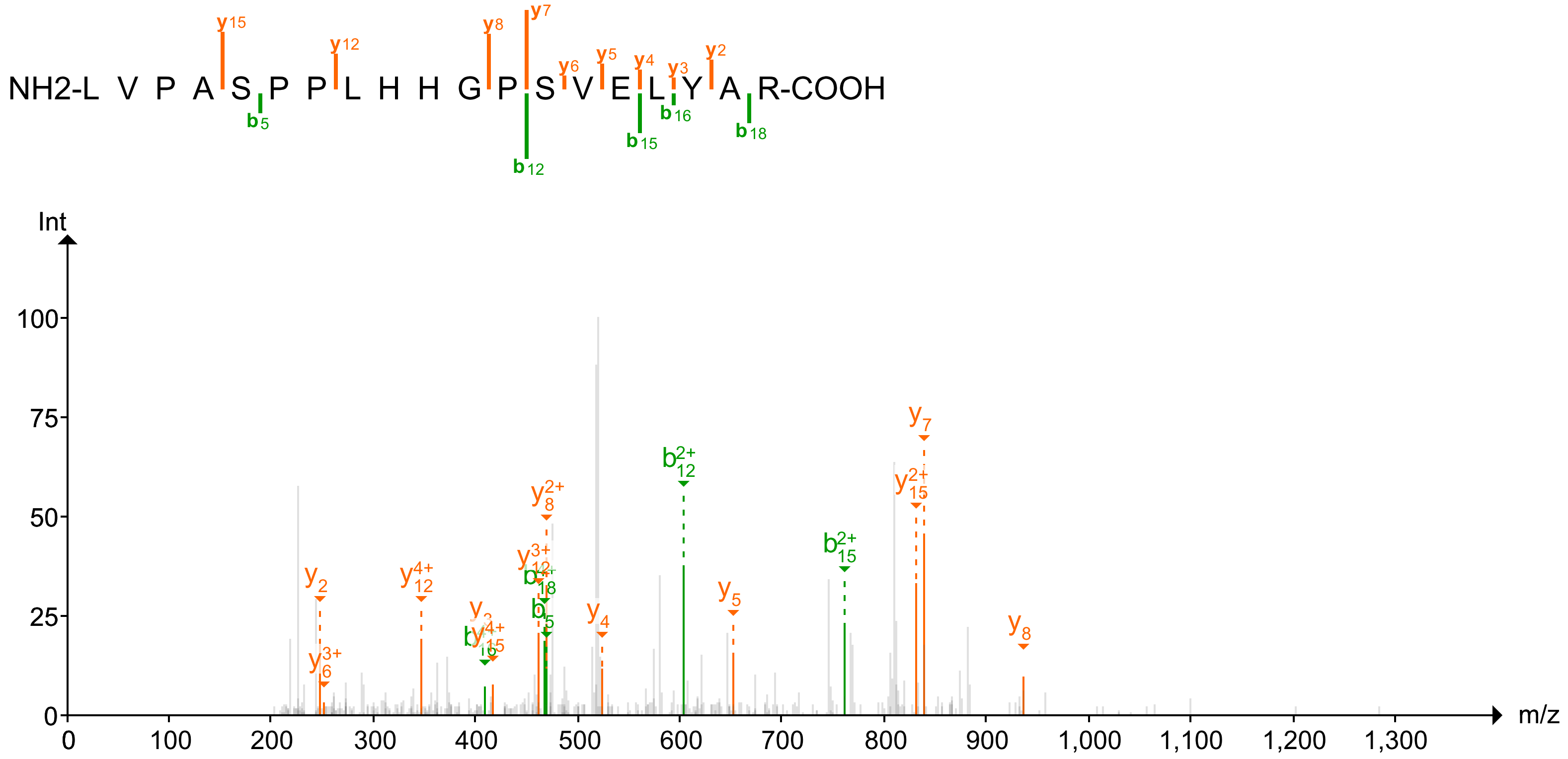

NH2-L V P A S P P L H H G P S V E L Y A R-COOH
y15 y12 y8 y7 y6 y5 y4 y3 y2
b5 b12 b15 b16 b18
Int
m/z
0 25 50 75 100
0 100 200 300 400 500 600 700 800 900 1,000 1,100 1,200 1,300

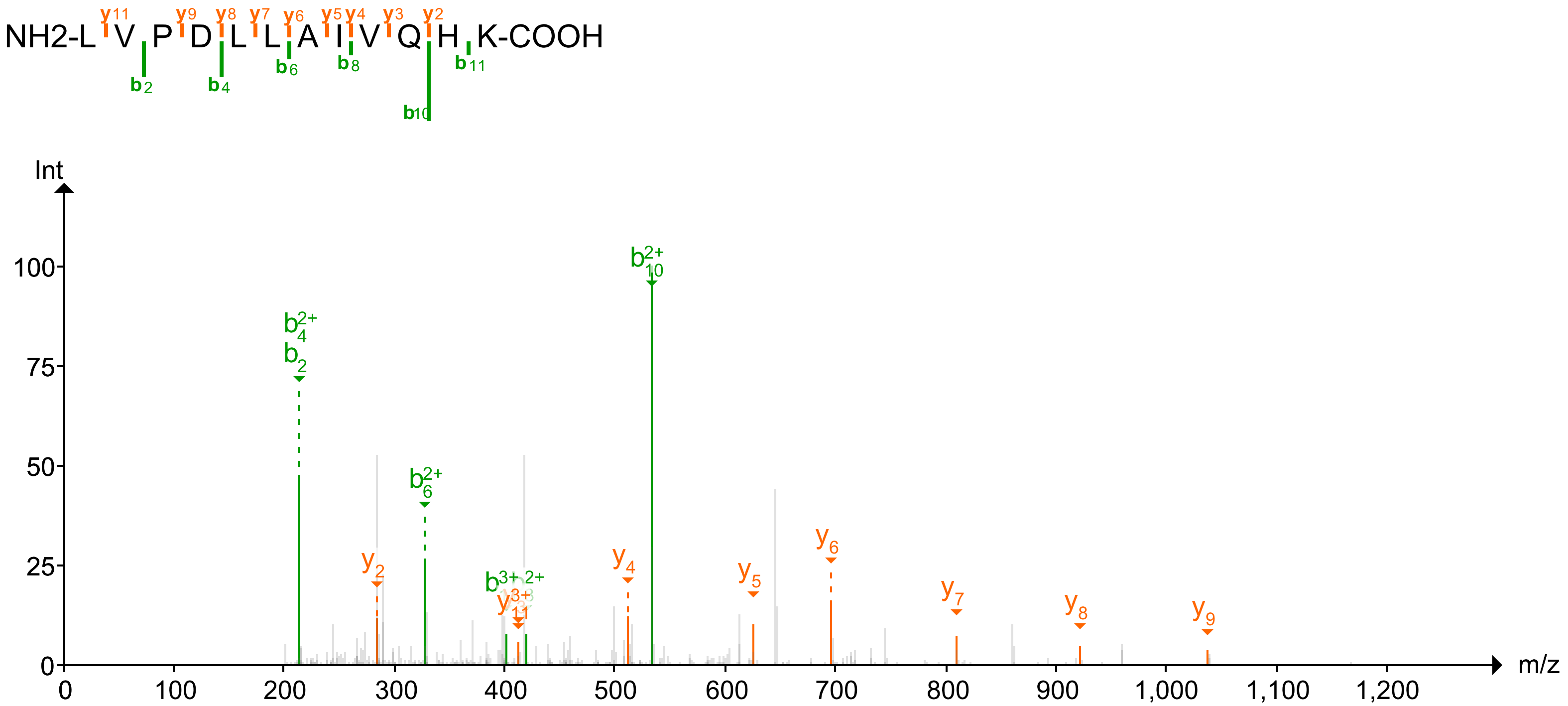

NH2-L V P D L L A I V Q H K-COOH
y11 y9 y8 y7 y6 y5 y4 y3 y2
b2 b4 b6 b8 b10 b11
Int
100
75
50
25
0
$b_{10}^{2+}$
$b_4^{2+}$
$b_2$
$b_6^{2+}$
$y_2$
$y_4$
$y_5$
$y_6$
$y_7$
$y_8$
$y_9$
$y_{11}^{3+}$
m/z
0 100 200 300 400 500 600 700 800 900 1,000 1,100 1,200

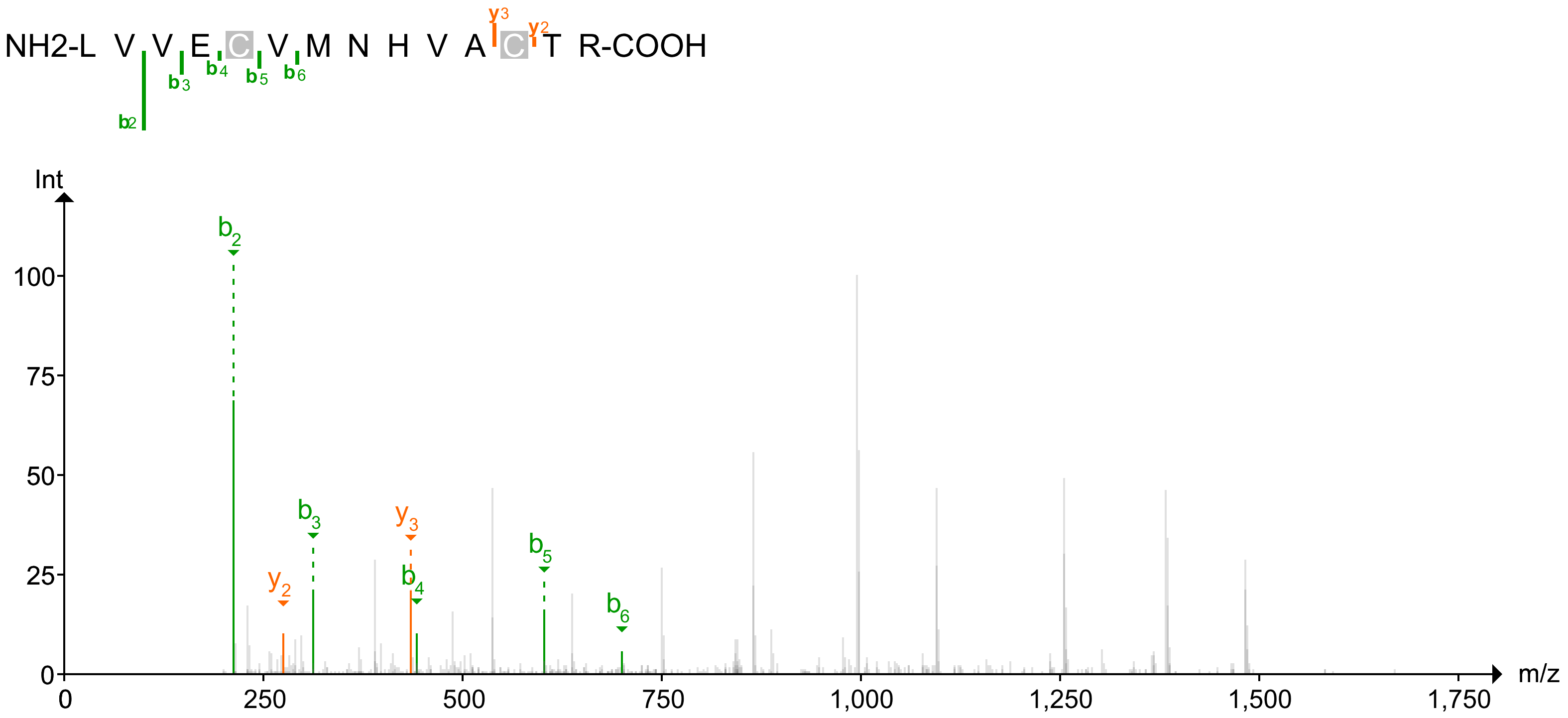

NH2-L V V E C V M N H V A C T R-COOH
b2 b3 b4 b5 b6 y3 y2
Int
100
75
50
25
0
m/z
0
250
500
750
1,000
1,250
1,500
1,750
b2
b3
y2
y3
b4
b5
b6

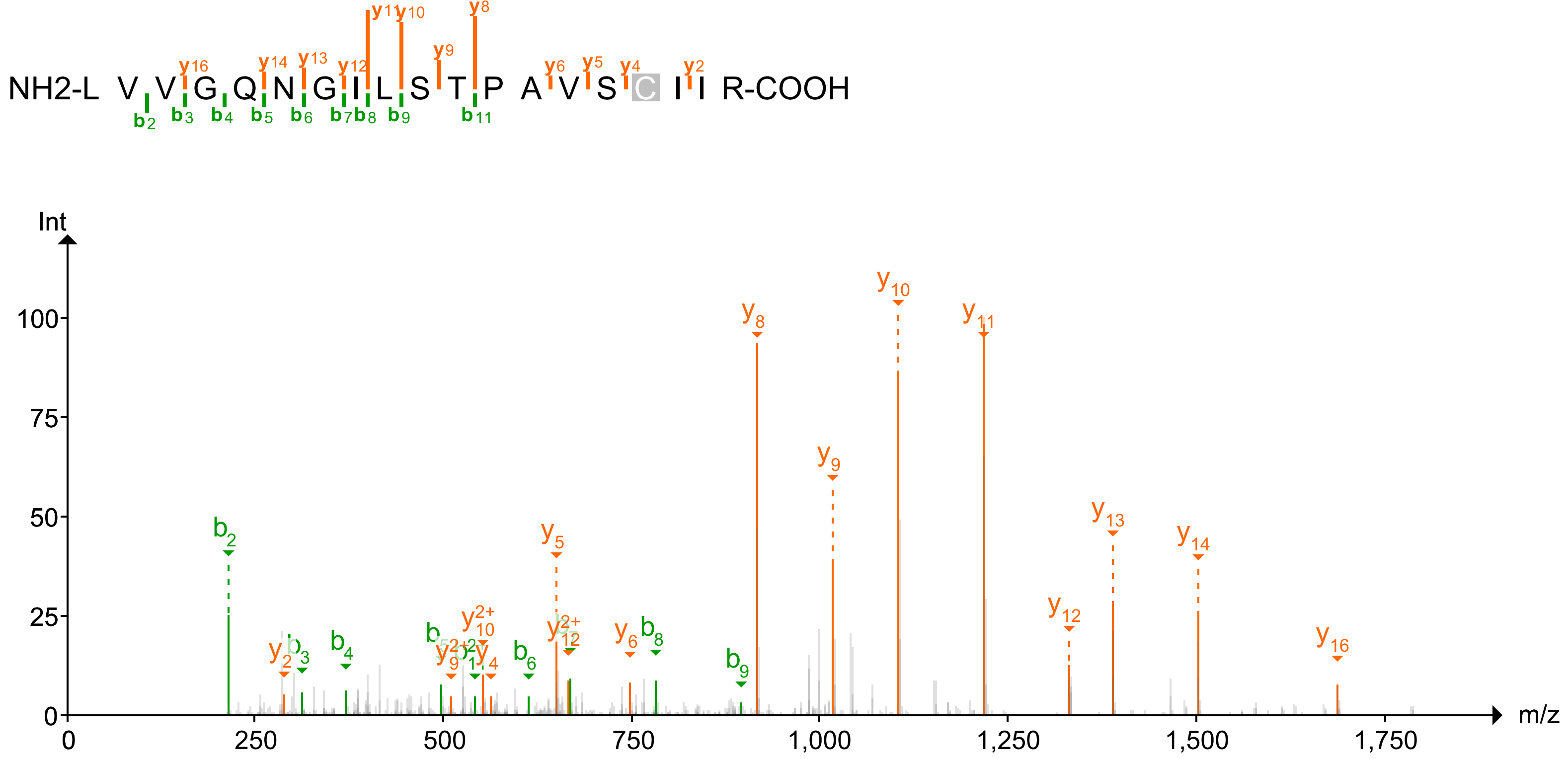

NH2-L V V G Q N G I L S T P A V S C I I R-COOH
y16 y14 y13 y12 y11 y10 y9 y8 y6 y5 y4 y2
b2 b3 b4 b5 b6 b7 b8 b9 b11
Int
m/z
0
25
50
75
100
250
500
750
1,000
1,250
1,500
1,750

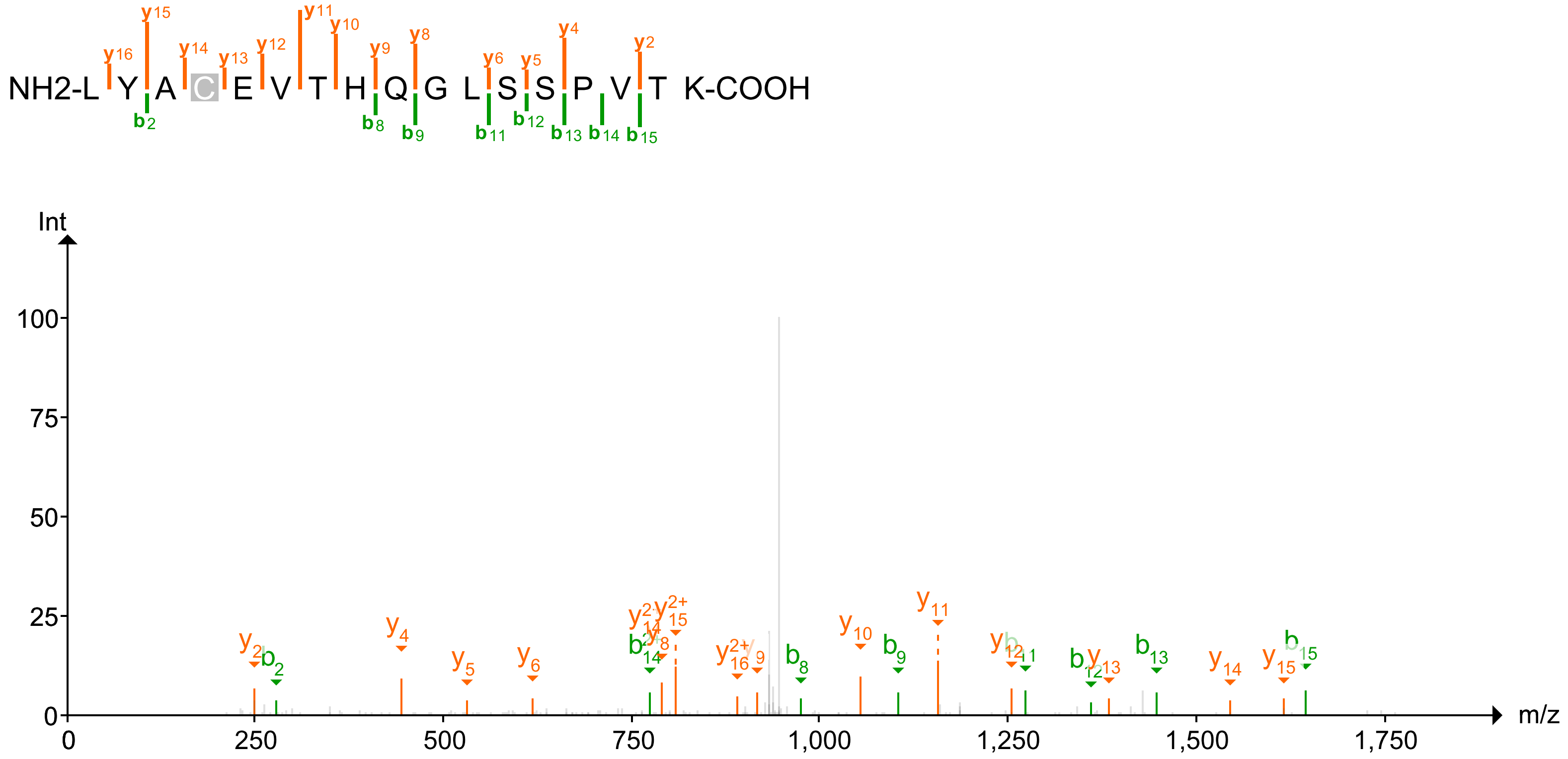

NH2-LYACEVTHQGLSSPVTK-COOH
Int
100
75
50
25
0
m/z
0
250
500
750
1,000
1,250
1,500
1,750

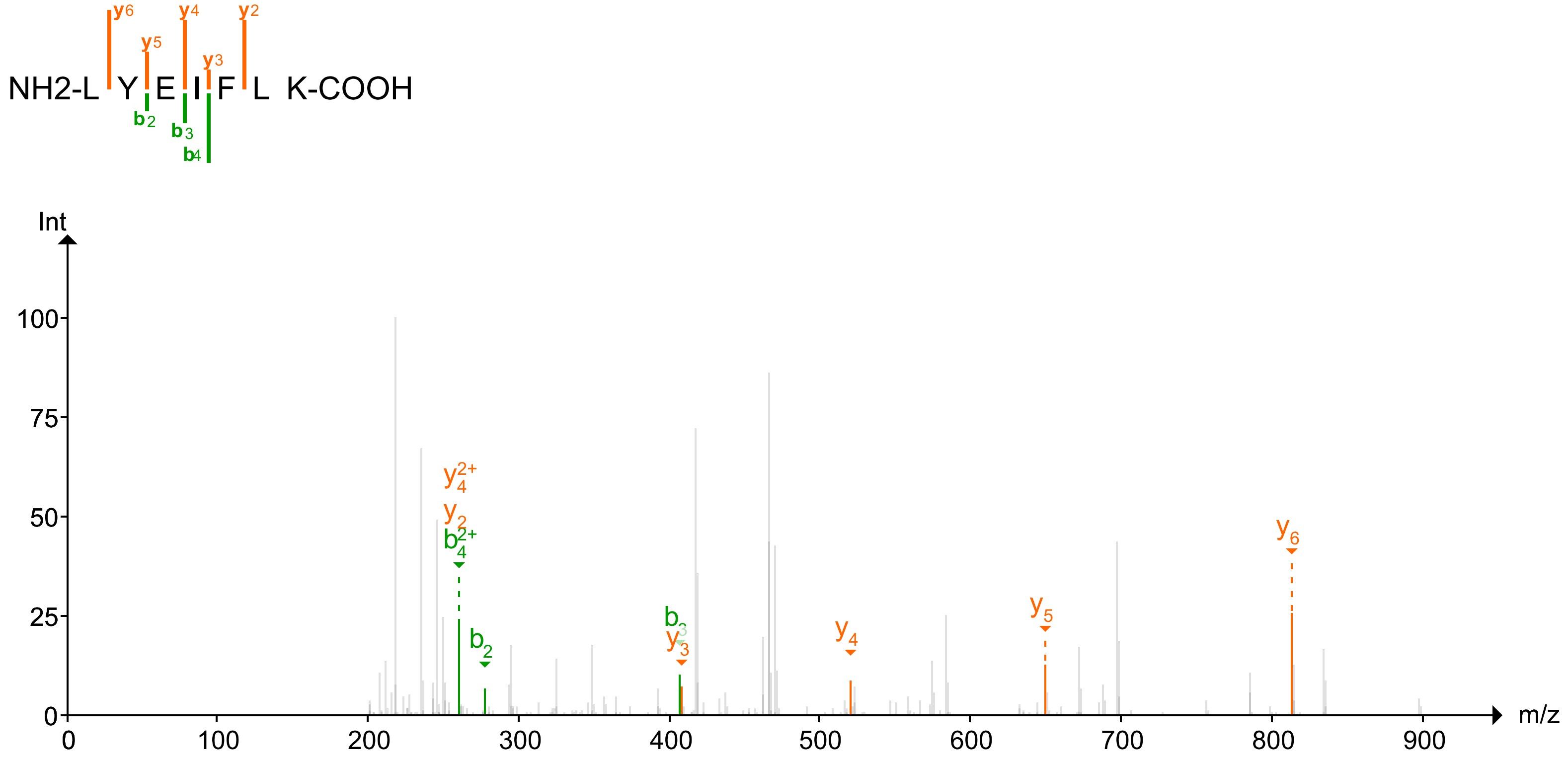

NH2-L Y E I F L K-COOH
y6 y5 y4 y3 y2
b2 b3 b4
Int
100
75
50
25
0
m/z
0
100
200
300
400
500
600
700
800
900
y4 2+
y2
b4 2+
b2
b3
y3
y4
y5
y6

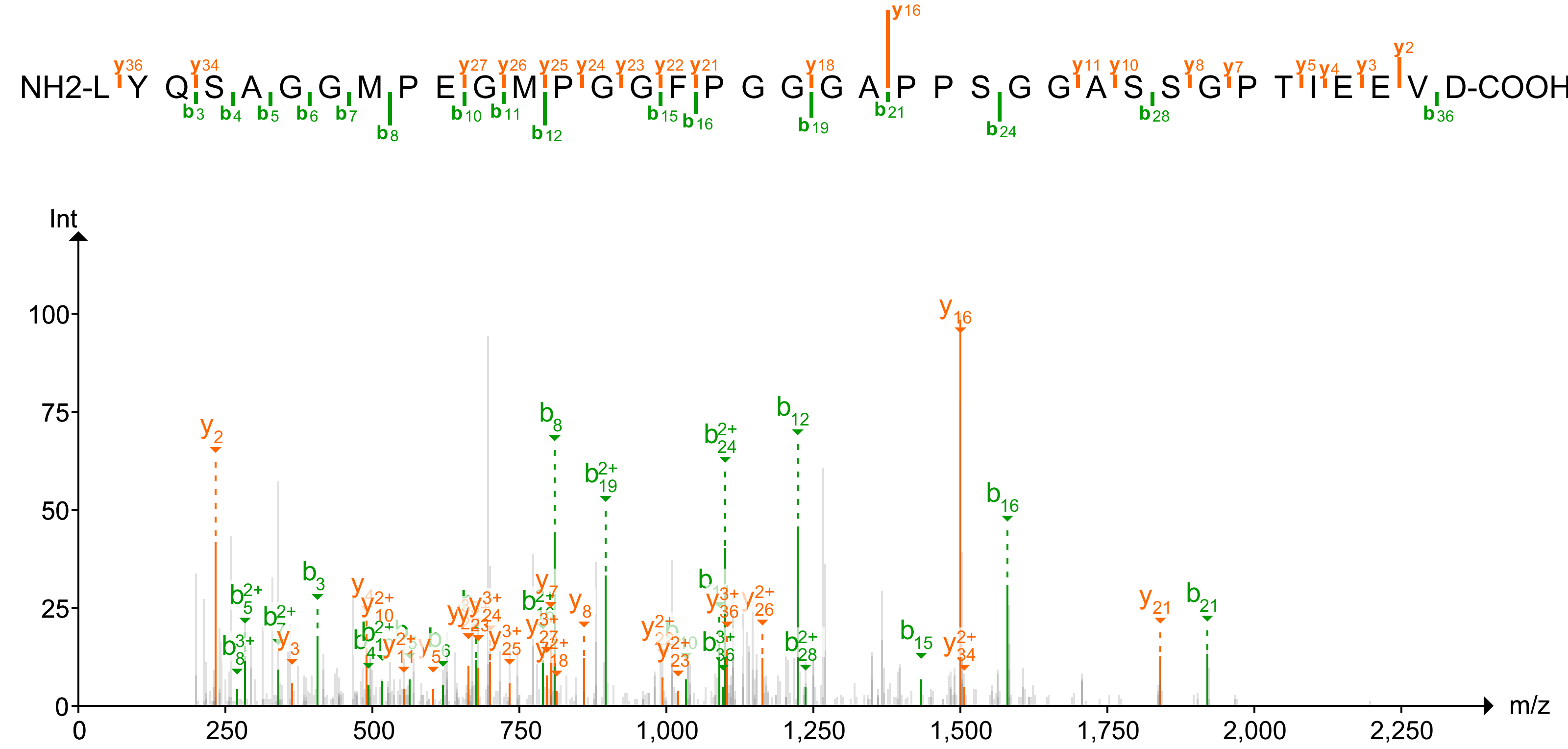

NH2-L Y Q S A G G M P E G M P G G F P G G G A P P S G G A S S G P T I E E V D-COOH
y36 y34 y27 y26 y25 y24 y23 y22 y21 y18 y16 y11 y10 y8 y7 y5 y4 y3 y2
b3 b4 b5 b6 b7 b8 b10 b11 b12 b15 b16 b19 b21 b24 b28 b36
Int
100
75
50
25
0
m/z
0
250
500
750
1,000
1,250
1,500
1,750
2,000
2,250
y2
b8
b12
y16
b16
y21
b21
b15
b3
y3
y8

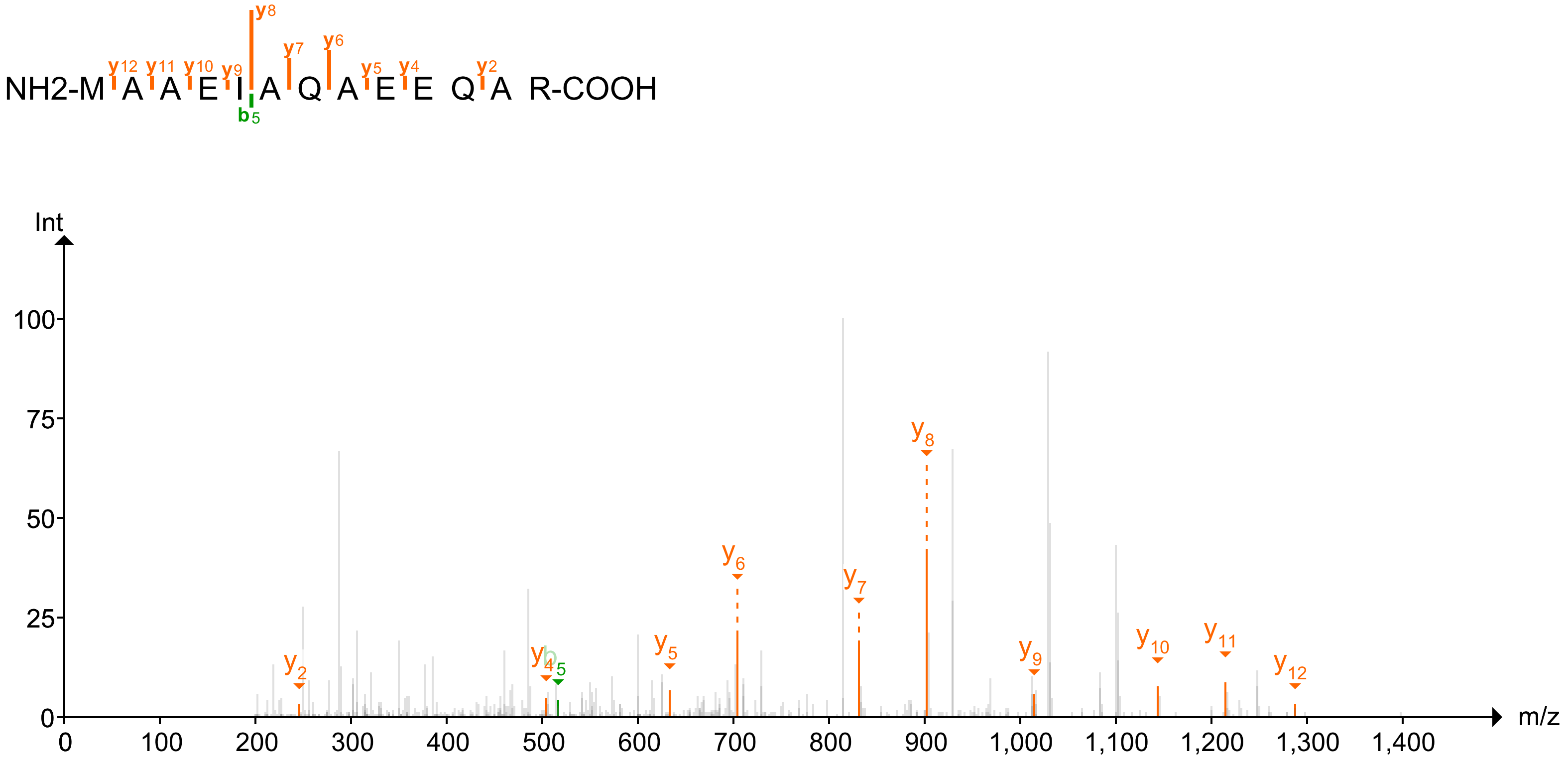

NH2-M A A E I A Q A E E Q A R-COOH
y12 y11 y10 y9 y8 y7 y6 y5 y4 y2
b5
Int
100
75
50
25
0
y2 y4 b5 y5 y6 y7 y8 y9 y10 y11 y12
0 100 200 300 400 500 600 700 800 900 1,000 1,100 1,200 1,300 1,400
m/z

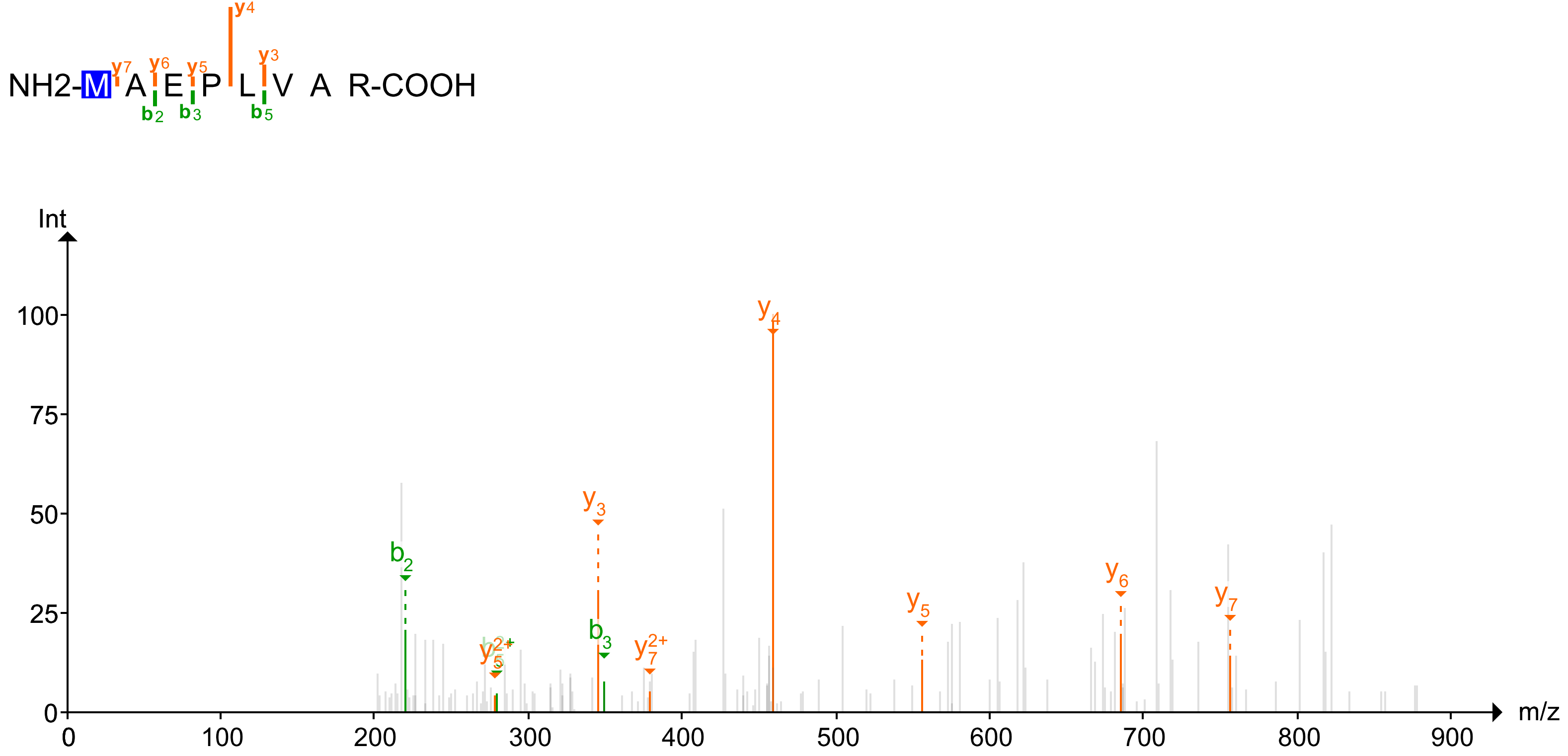

NH2-M A E P L V A R-COOH
y7 y6 y5 y4 y3
b2 b3 b5
Int
100
75
50
25
0
m/z
0
100
200
300
400
500
600
700
800
900
b2
y3
y4
y5
y6
y7
b3
y7 2+
y5 2+

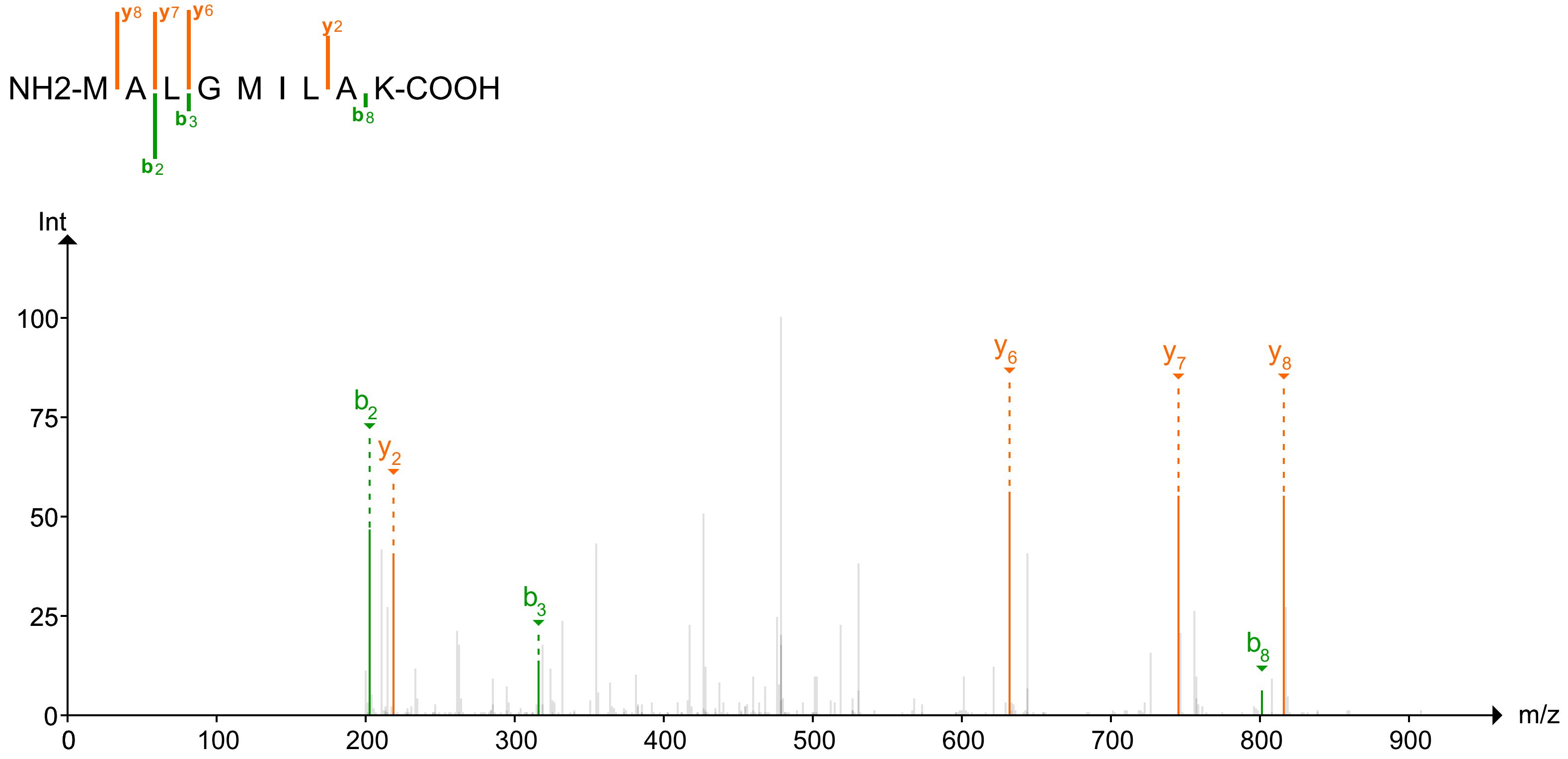

NH2-M A L G M I L A K-COOH
y8 y7 y6 y2
b2 b3 b8
Int
100
75
50
25
0
0 100 200 300 400 500 600 700 800 900
m/z
b2 y2 b3 y6 y7 b8 y8

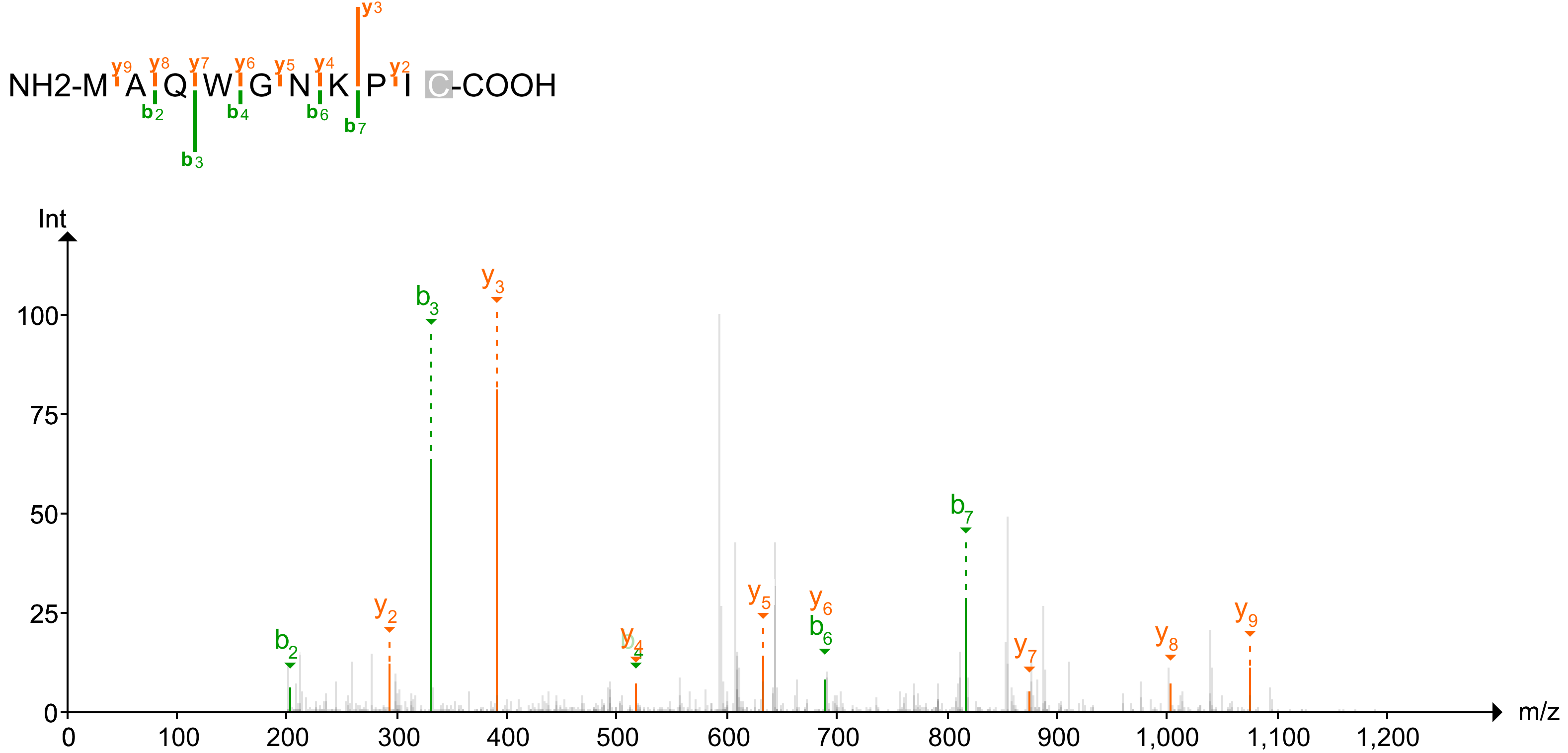

NH2-M A Q W G N K P I C-COOH
y9 y8 y7 y6 y5 y4 y3 y2
b2 b3 b4 b6 b7
Int
m/z
0
25
50
75
100
0
100
200
300
400
500
600
700
800
900
1,000
1,100
1,200
b2
y2
b3
y3
y4
y5
y6
b6
b7
y7
y8
y9

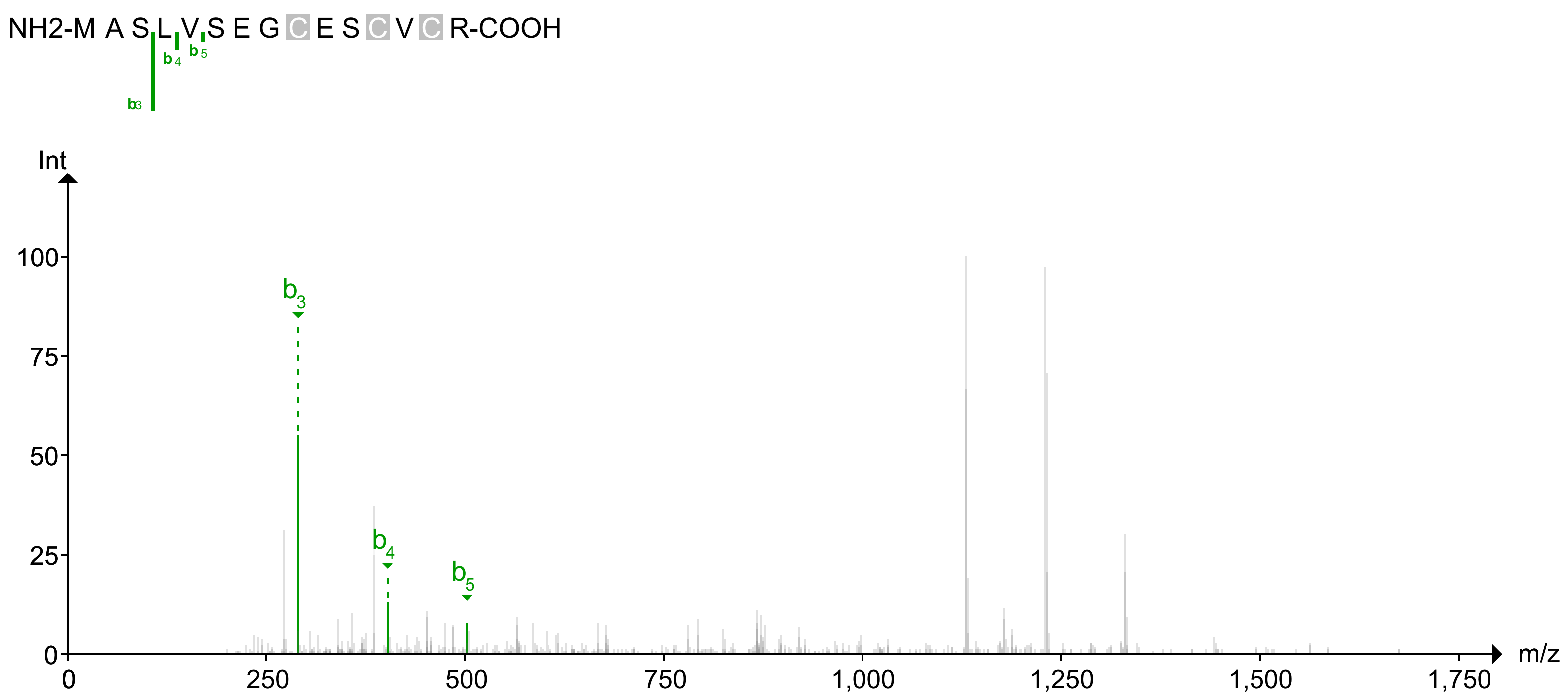

NH2-M A S L V S E G C E S C V C R-COOH
b3
b4
b5
Int
100
75
50
25
0
250
500
750
1,000
1,250
1,500
1,750
m/z

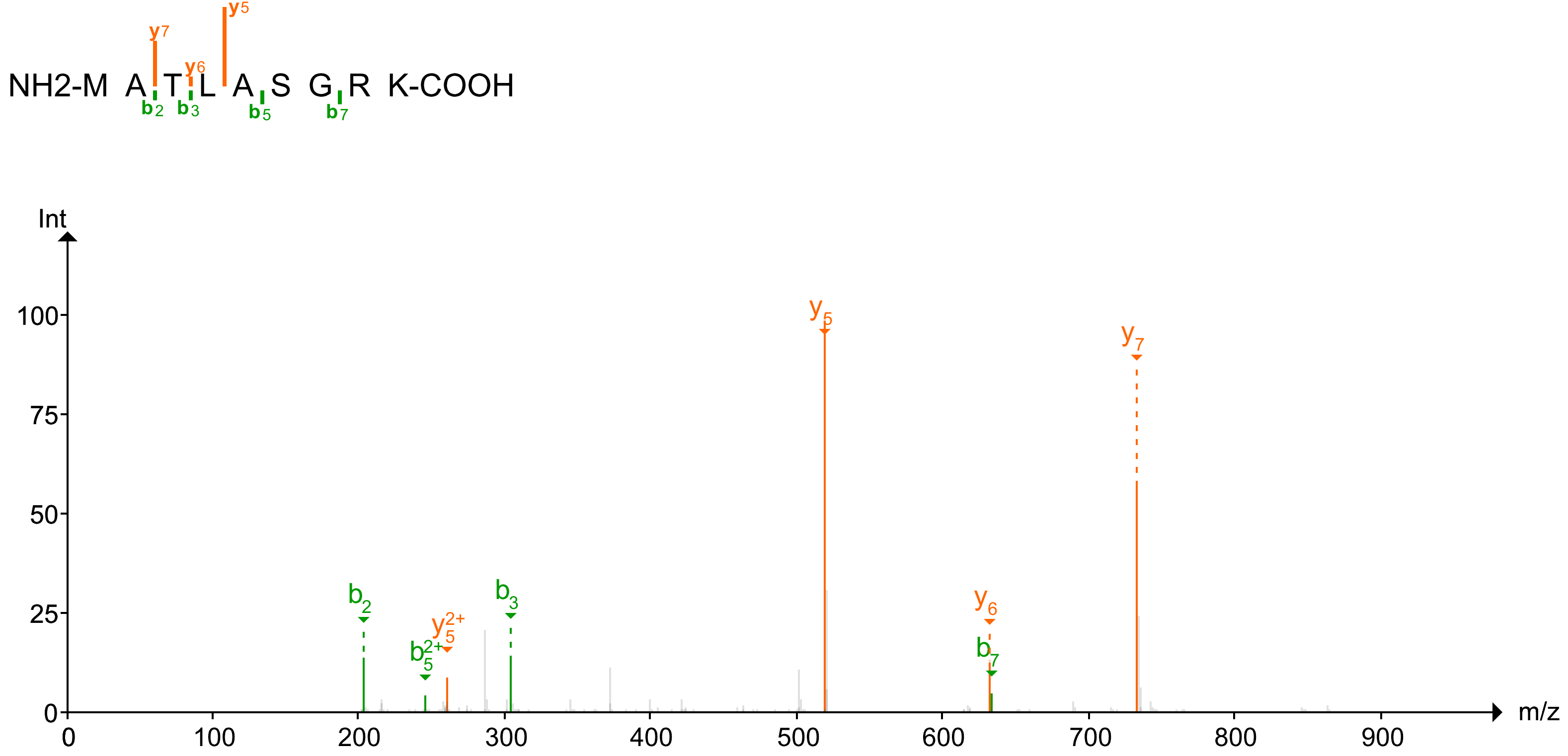

y7
y6
y5
NH2-M A T L A S G R K-COOH
b2
b3
b5
b7
Int
100
75
50
25
0
y5
y7
b2
b3
y6
b7
0
100
200
300
400
500
600
700
800
900
m/z

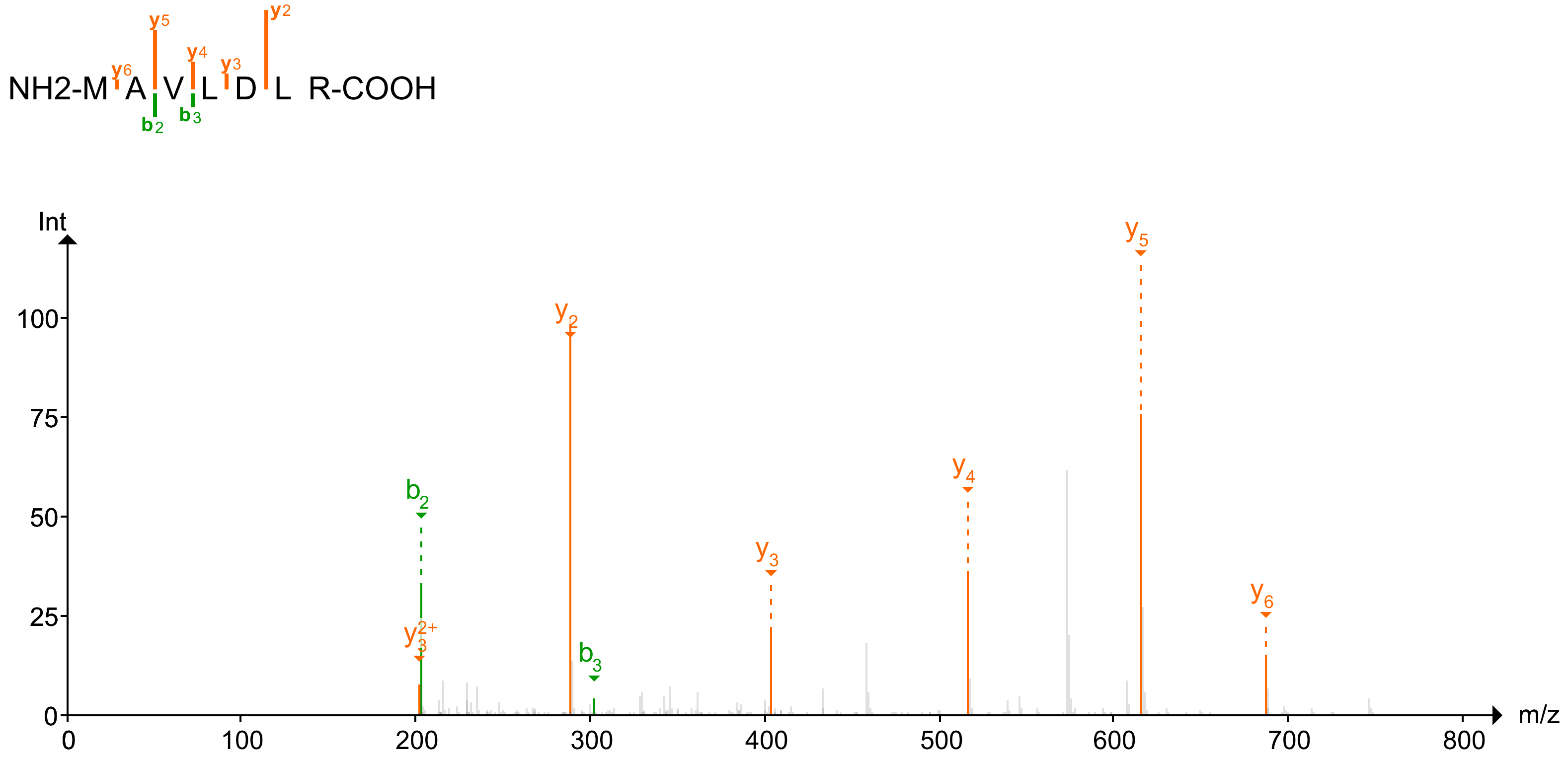

NH2-M A V L D L R-COOH
y6 y5 y4 y3 y2
b2 b3
Int
m/z
0
25
50
75
100
0
100
200
300
400
500
600
700
800
y5
y2
y4
y3
y6
b2
b3
y3 2+

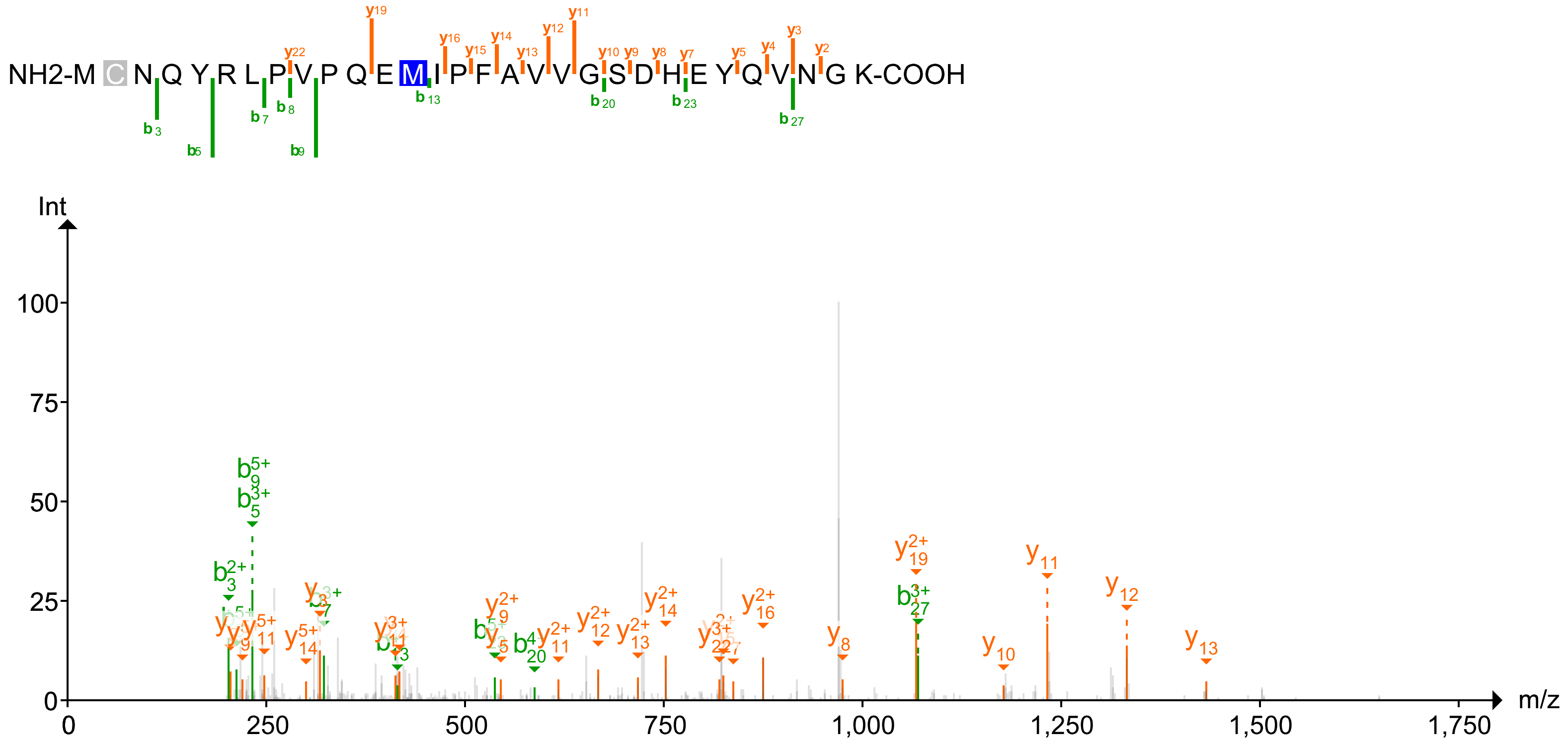
NH2-M C N Q Y R L P V P Q E M I P F A V V G S D H E Y Q V N G K-COOH
y19
y11
y16
y15
y14
y13
y12
y22
y10
y9
y8
y7
y5
y4
y3
y2
b3
b5
b7
b8
b9
b13
b20
b23
b27
Int
100
75
50
25
0
m/z
0
250
500
750
1,000
1,250
1,500
1,750

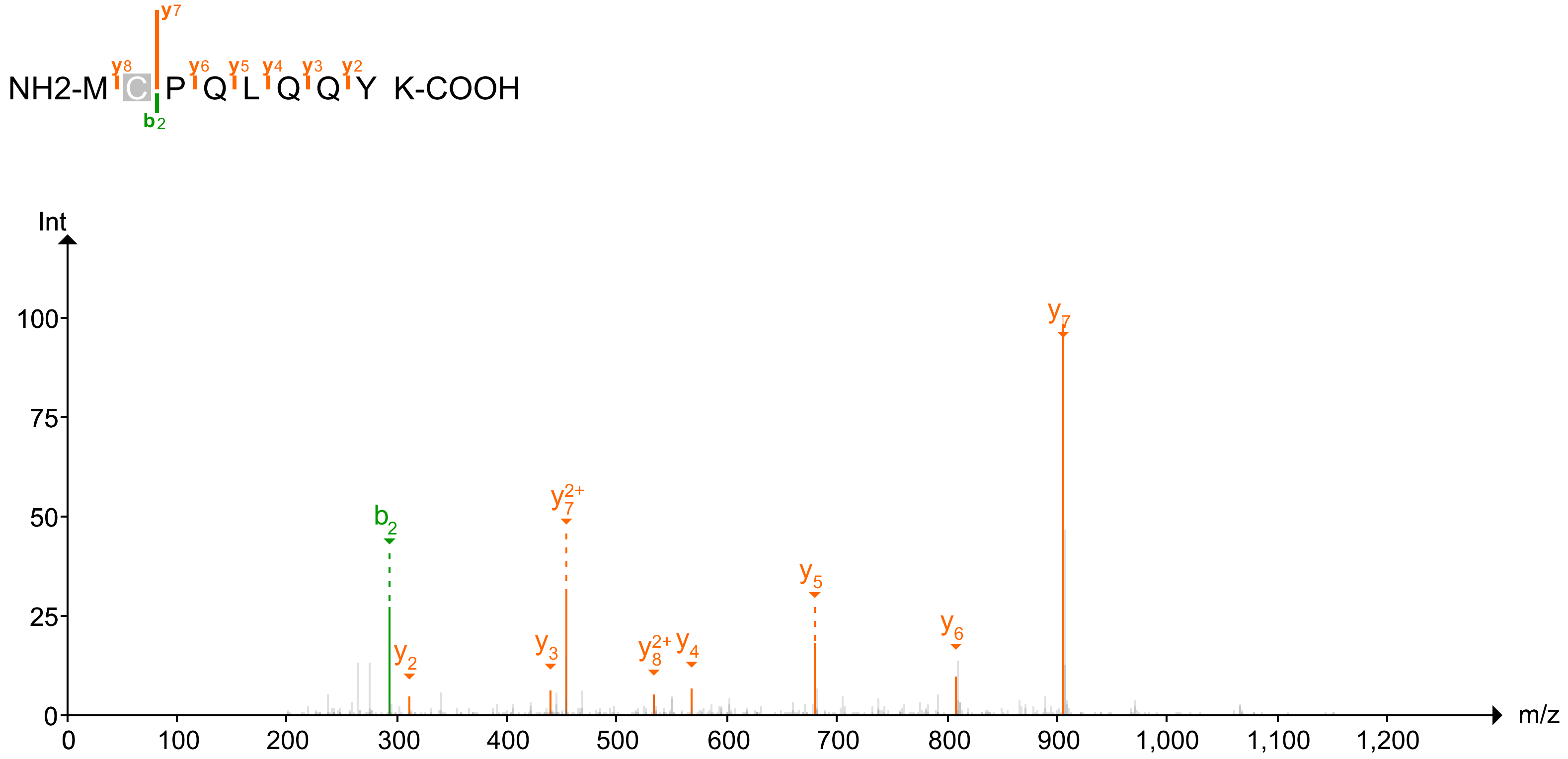

NH2-M C P Q L Q Q Y K-COOH
y7
y8
y6
y5
y4
y3
y2
b2
Int
100
75
50
25
0
m/z
0
100
200
300
400
500
600
700
800
900
1,000
1,100
1,200
b2
y2
y3
y7 2+
y8 2+
y4
y5
y6
y7

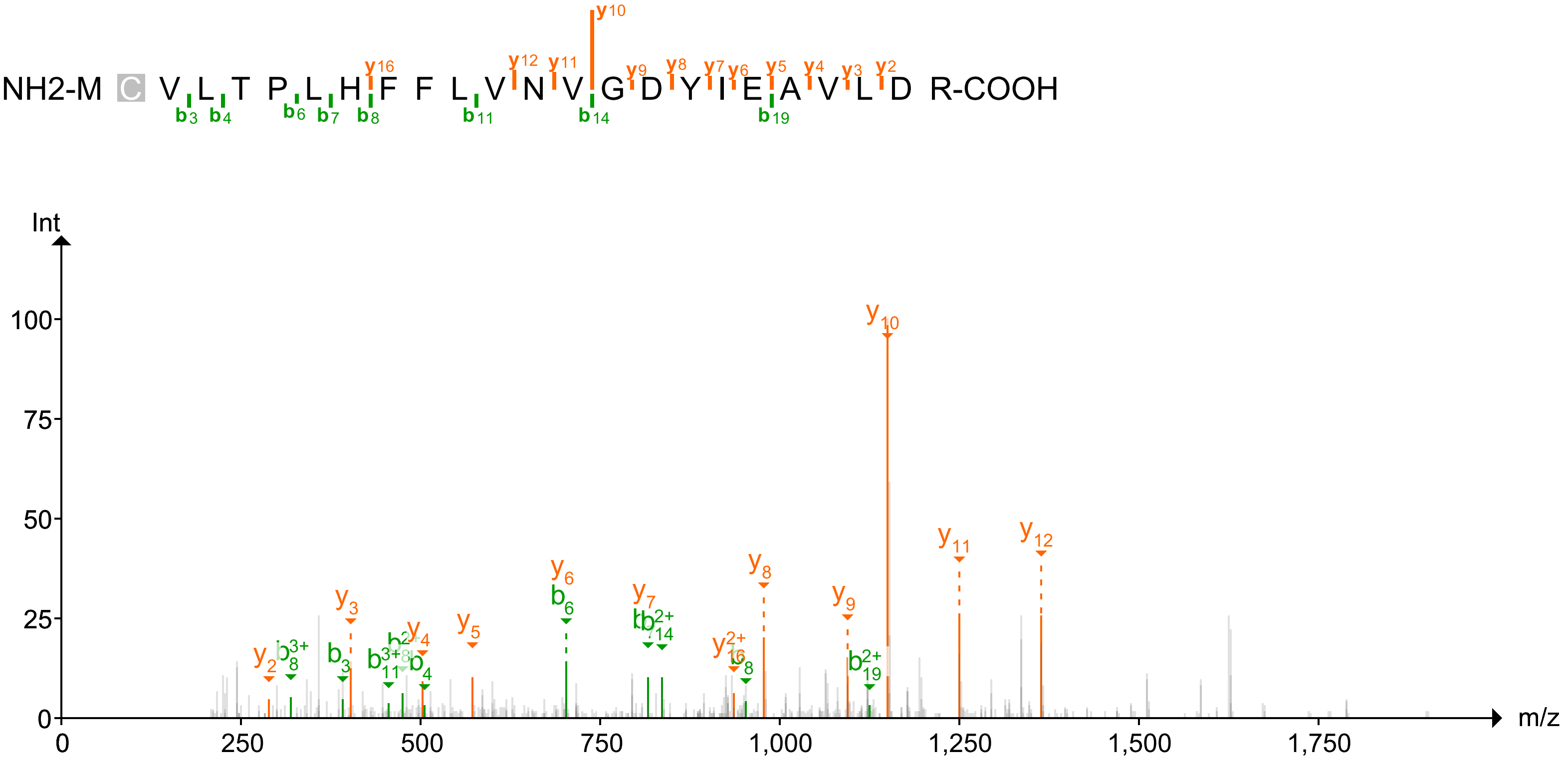

NH2-M C V L T P L H F F L V N V G D Y I E A V L D R-COOH
y16 y12 y11 y10 y9 y8 y7 y6 y5 y4 y3 y2
b3 b4 b6 b7 b8 b11 b14 b19
Int
100
75
50
25
0
250
500
750
1,000
1,250
1,500
1,750
m/z
y10
y11
y12
y8
y9
y7
y6
b6
y3
y5
y2
b3
y4
b4

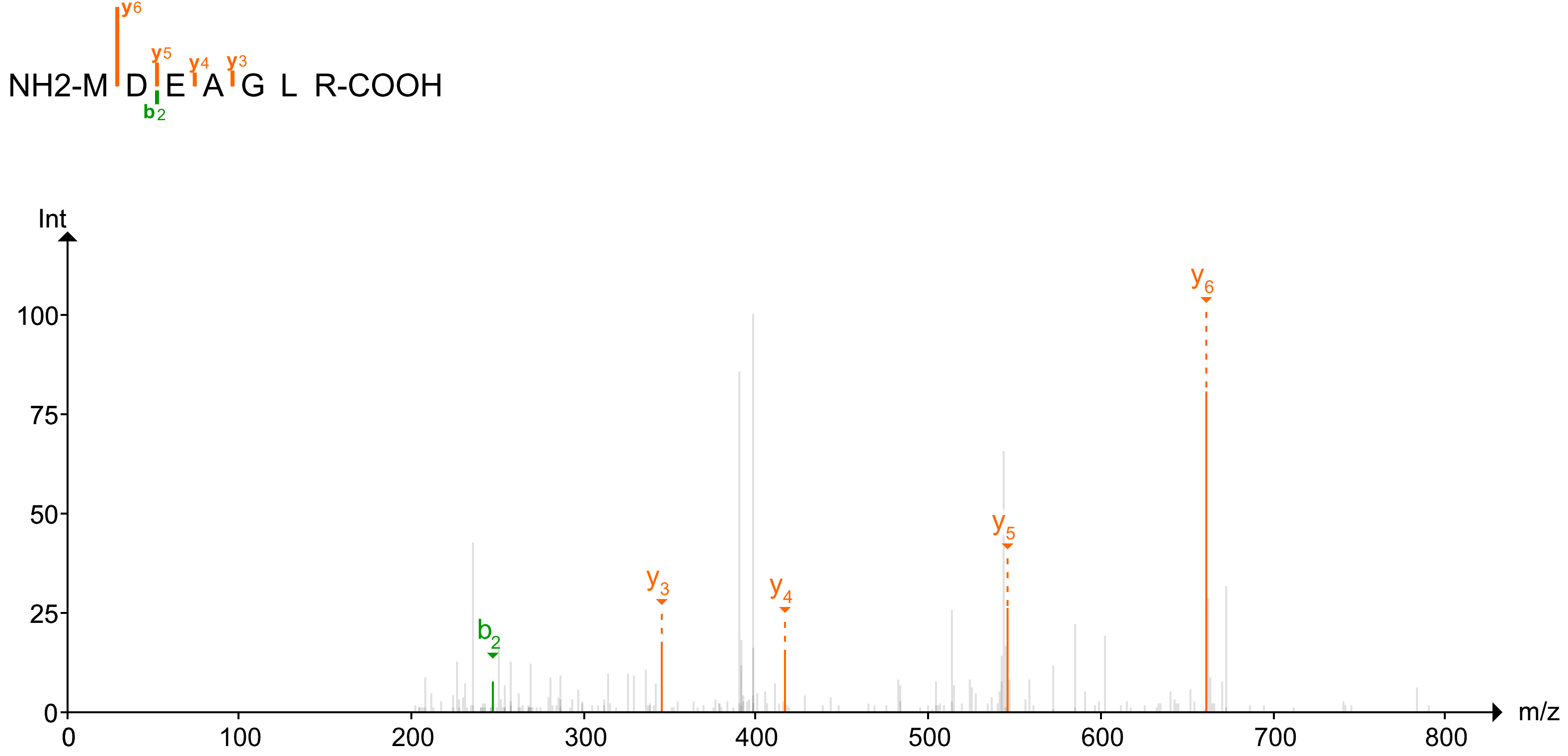

NH2-M D E A G L R-COOH
y6
y5
y4
y3
b2
Int
100
75
50
25
0
m/z
0
100
200
300
400
500
600
700
800
y6
y5
y4
y3
b2

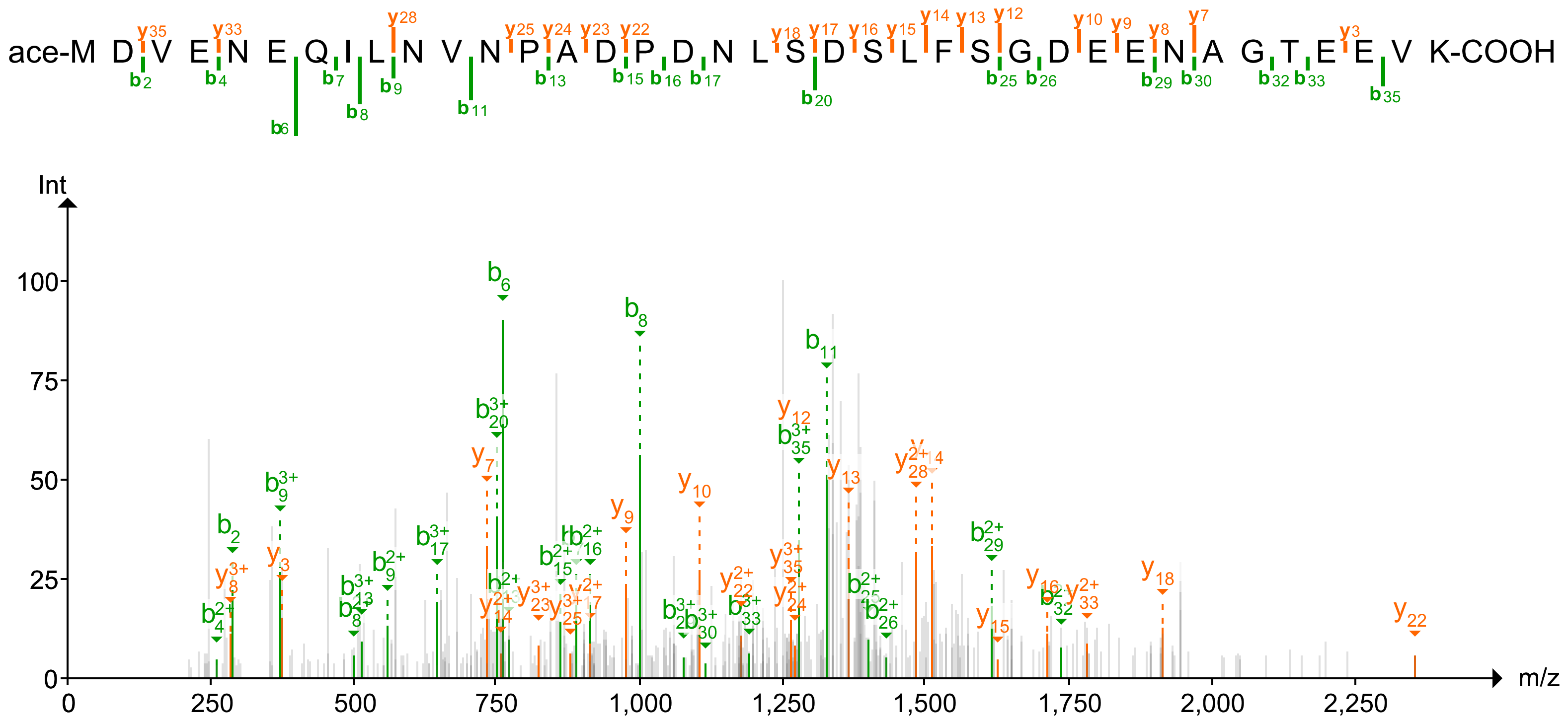
ace-M D V E N E Q I L N V N P A D P D N L S D S L F S G D E E N A G T E E V K-COOH
Int
m/z
0
25
50
75
100
0
250
500
750
1,000
1,250
1,500
1,750
2,000
2,250

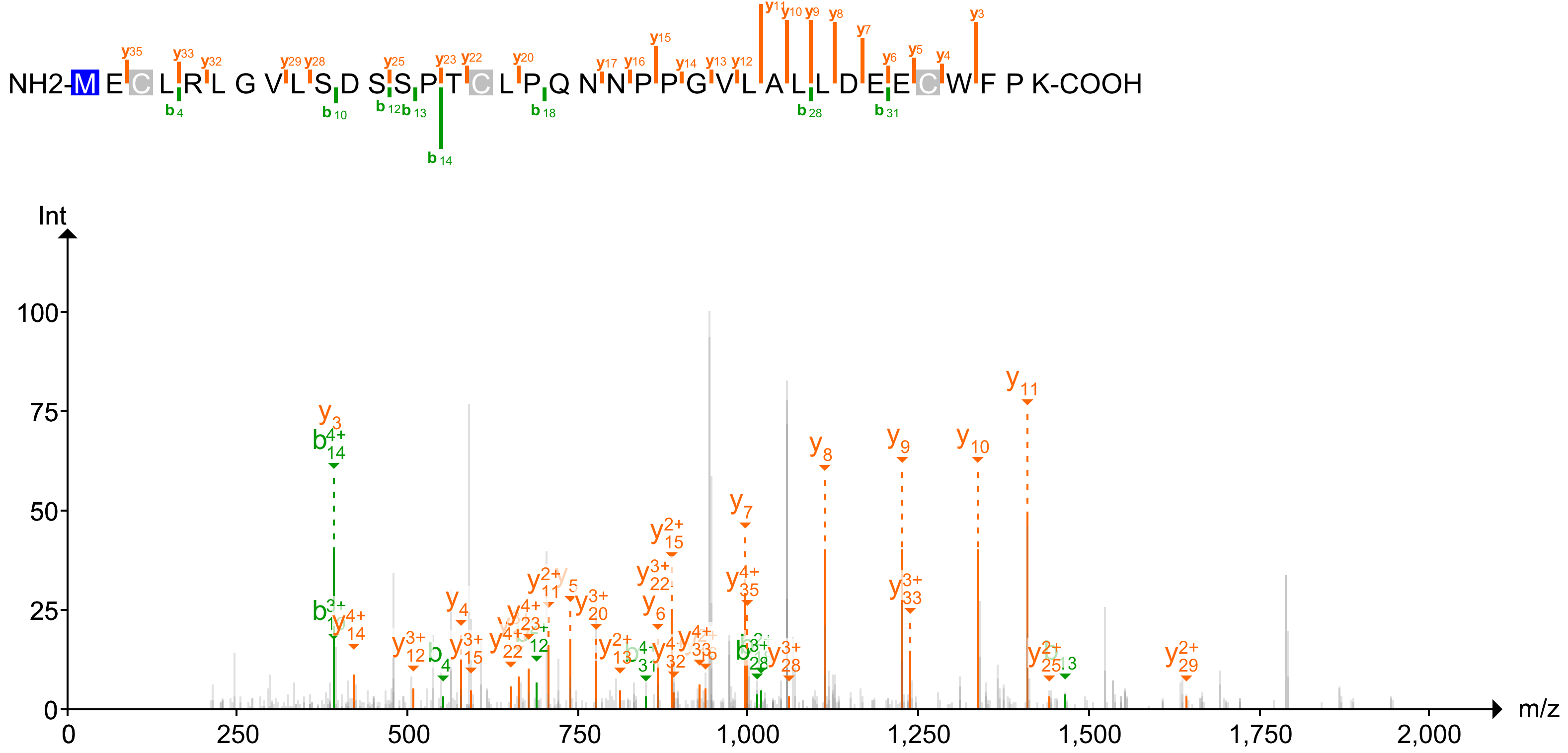

NH2-M E C L R L G V L S D S S P T C L P Q N N P P G V L A L L D E E C W F P K-COOH
Int
100
75
50
25
0
m/z
0
250
500
750
1,000
1,250
1,500
1,750
2,000

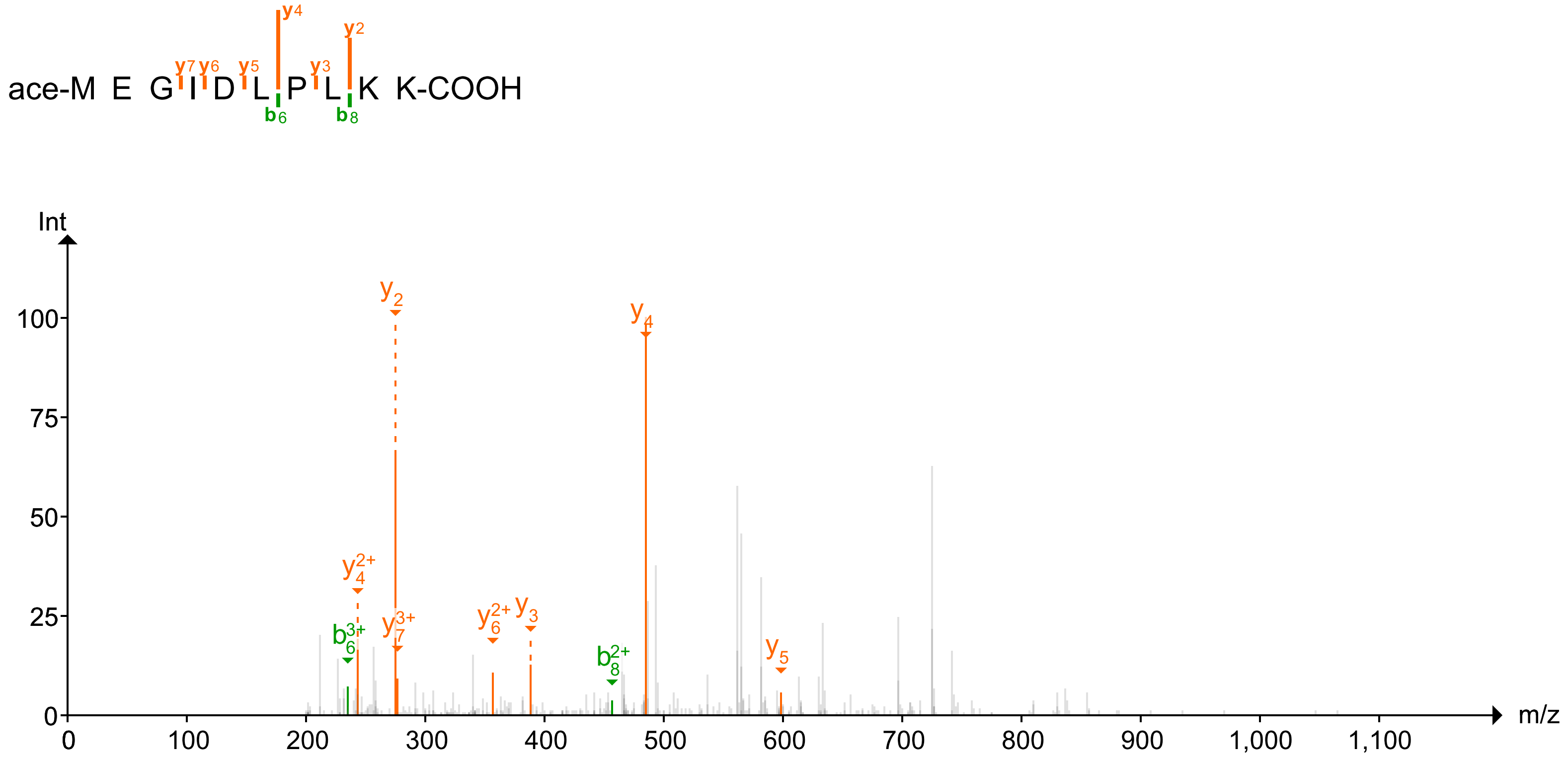

ace-M E G I D L P L K K-COOH
y7 y6 y5 y4 y3 y2
b6 b8
Int
m/z
y2
y4
y4 2+
b6 3+
y7 3+
y6 2+
y3
b8 2+
y5
0
25
50
75
100
0
100
200
300
400
500
600
700
800
900
1,000
1,100

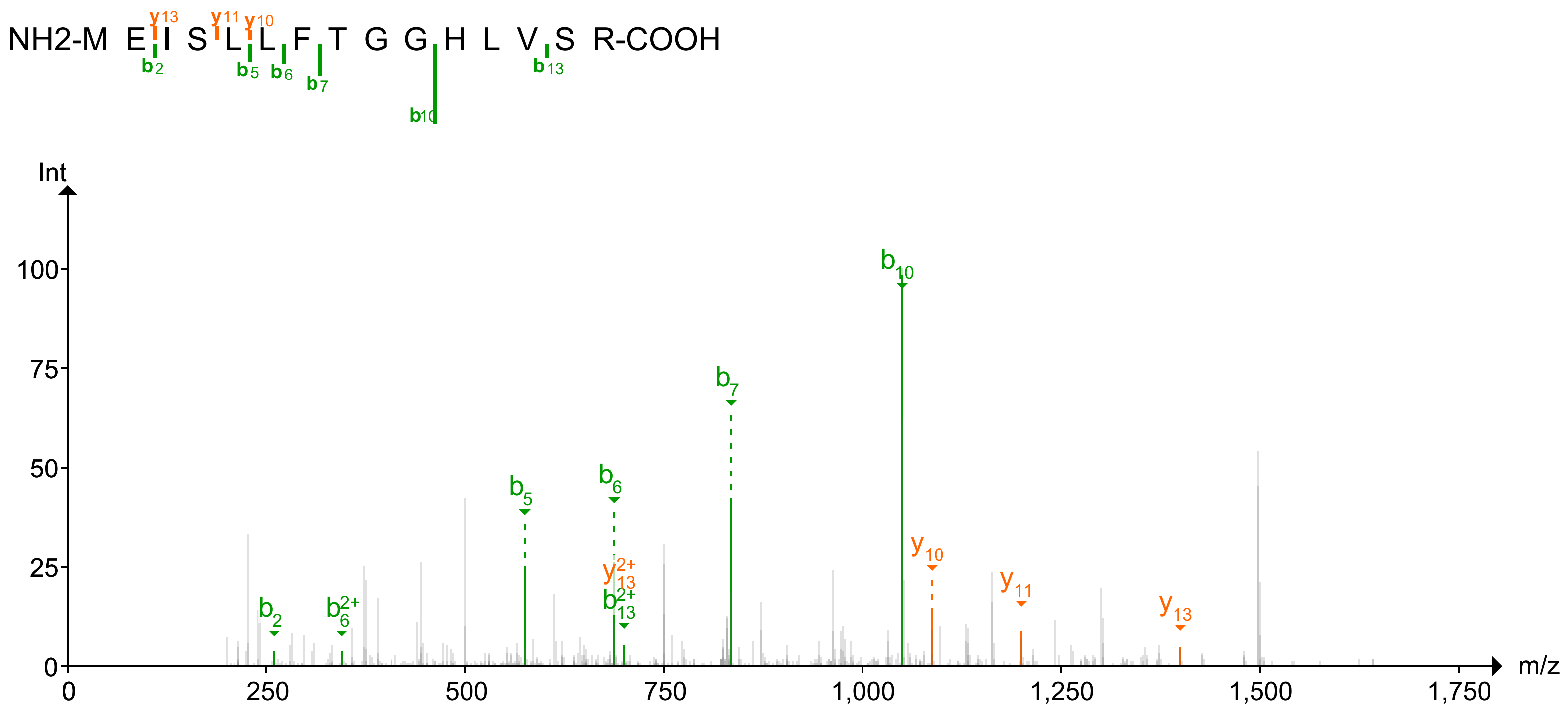

NH2-M E I S L L F T G G H L V S R-COOH
y13 y11 y10
b2 b5 b6 b7 b10 b13
Int
100
75
50
25
0
b10
b7
b5
b6
y10
y11
y13
b2
b6 2+
y13 2+
b13 2+
0
250
500
750
1,000
1,250
1,500
1,750
m/z

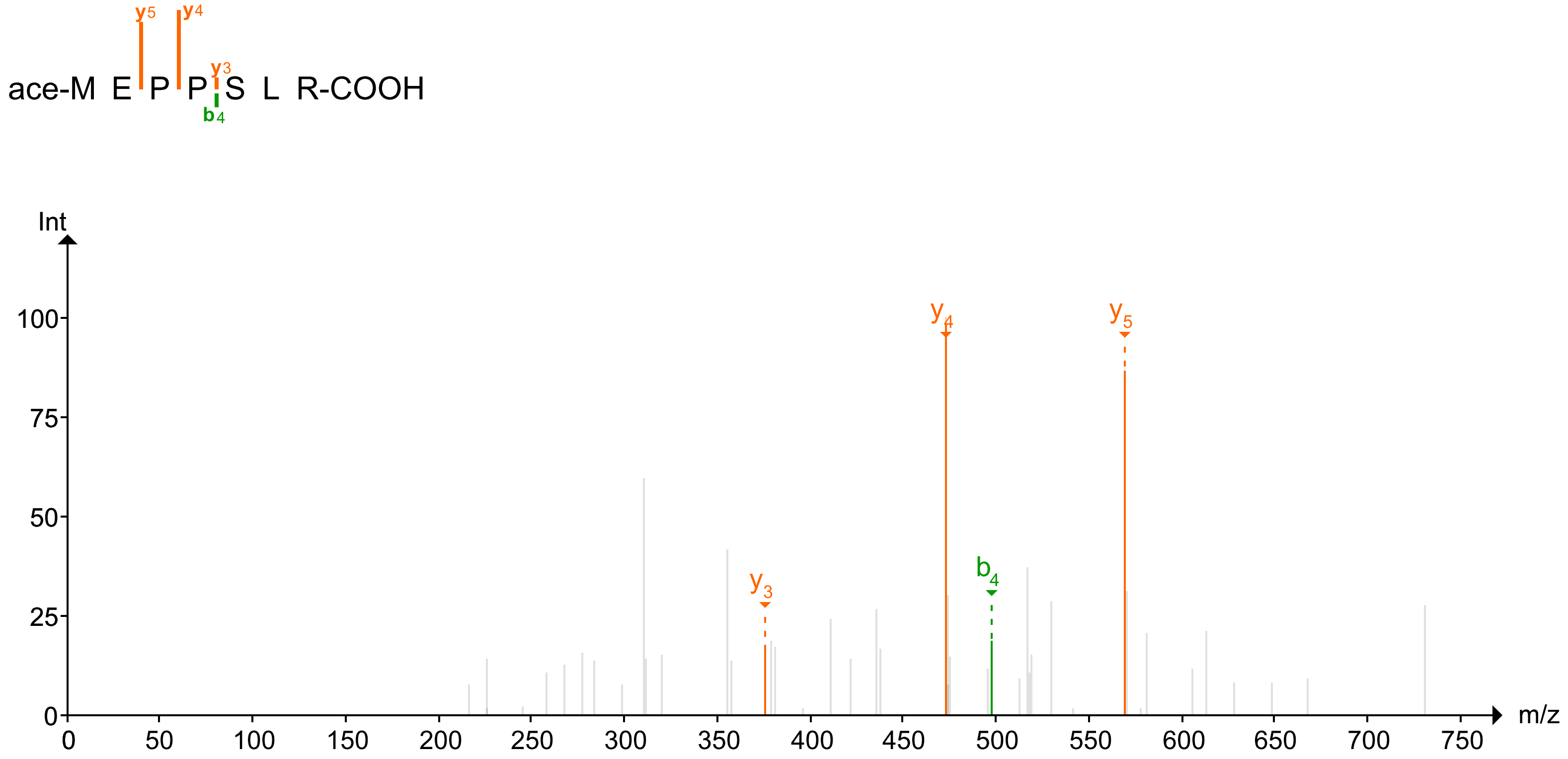

ace-M E P P S L R-COOH
y5
y4
y3
b4
Int
m/z
100
75
50
25
0
0
50
100
150
200
250
300
350
400
450
500
550
600
650
700
750

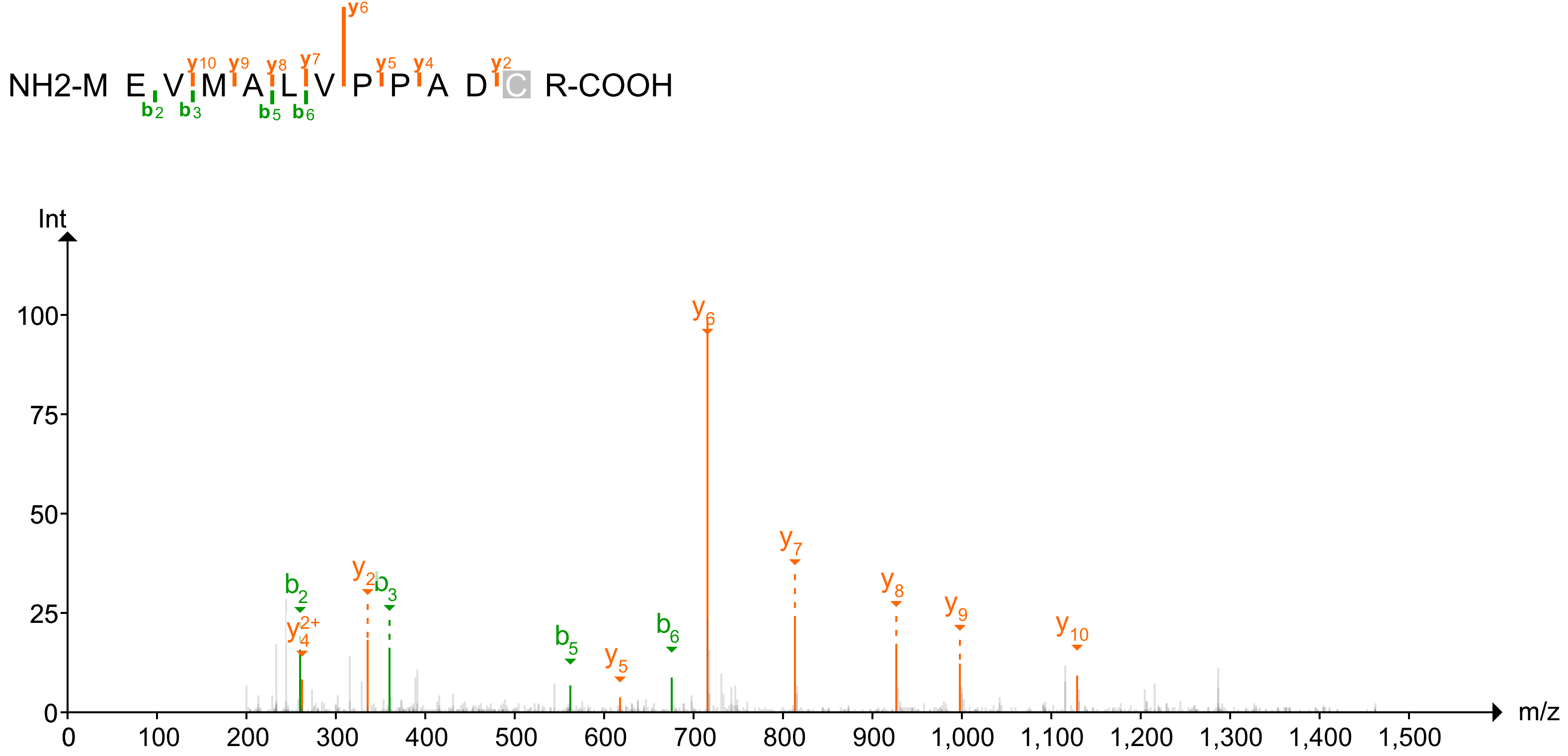

NH2-M E V M A L V P P A D C R-COOH
y10 y9 y8 y7 y6 y5 y4 y2
b2 b3 b5 b6
Int
m/z
0
25
50
75
100
0 100 200 300 400 500 600 700 800 900 1,000 1,100 1,200 1,300 1,400 1,500
b2
y4 2+
y2
b3
b5
y5
b6
y6
y7
y8
y9
y10

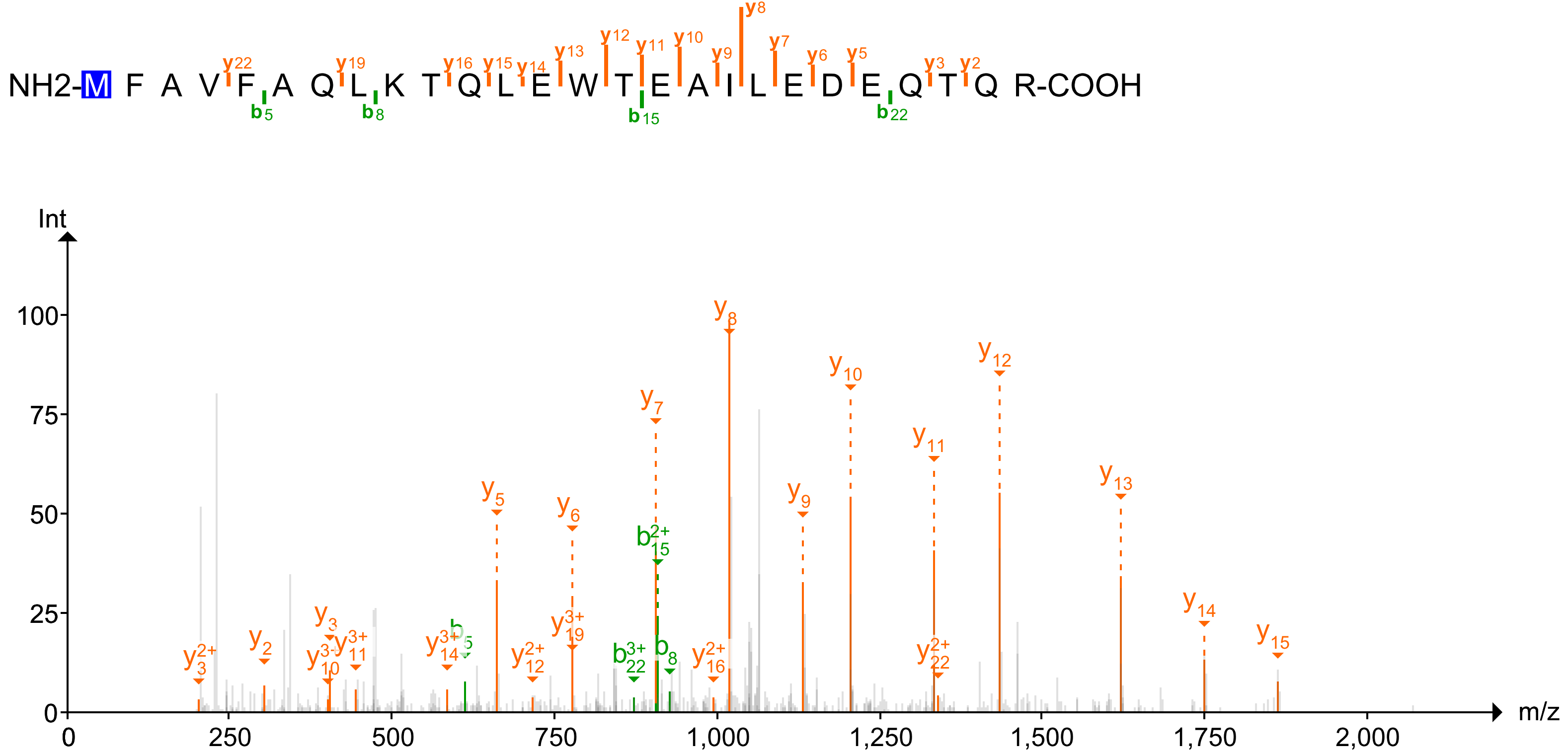

NH2-M F A V F A Q L K T Q L E W T E A I L E D E Q T Q R-COOH
y22 y19 y16 y15 y14 y13 y12 y11 y10 y9 y8 y7 y6 y5 y3 y2
b5 b8 b15 b22
Int
100
75
50
25
0
m/z
0
250
500
750
1,000
1,250
1,500
1,750
2,000
y8
y10
y12
y7
y11
y13
y5
y6
y9
b15 2+
y14
y15
y3 2+
y2
y3
y10 3+
y11 3+
y14 3+
b5
y12 2+
y19 3+
b22 3+
b8
y16 2+
y22 2+

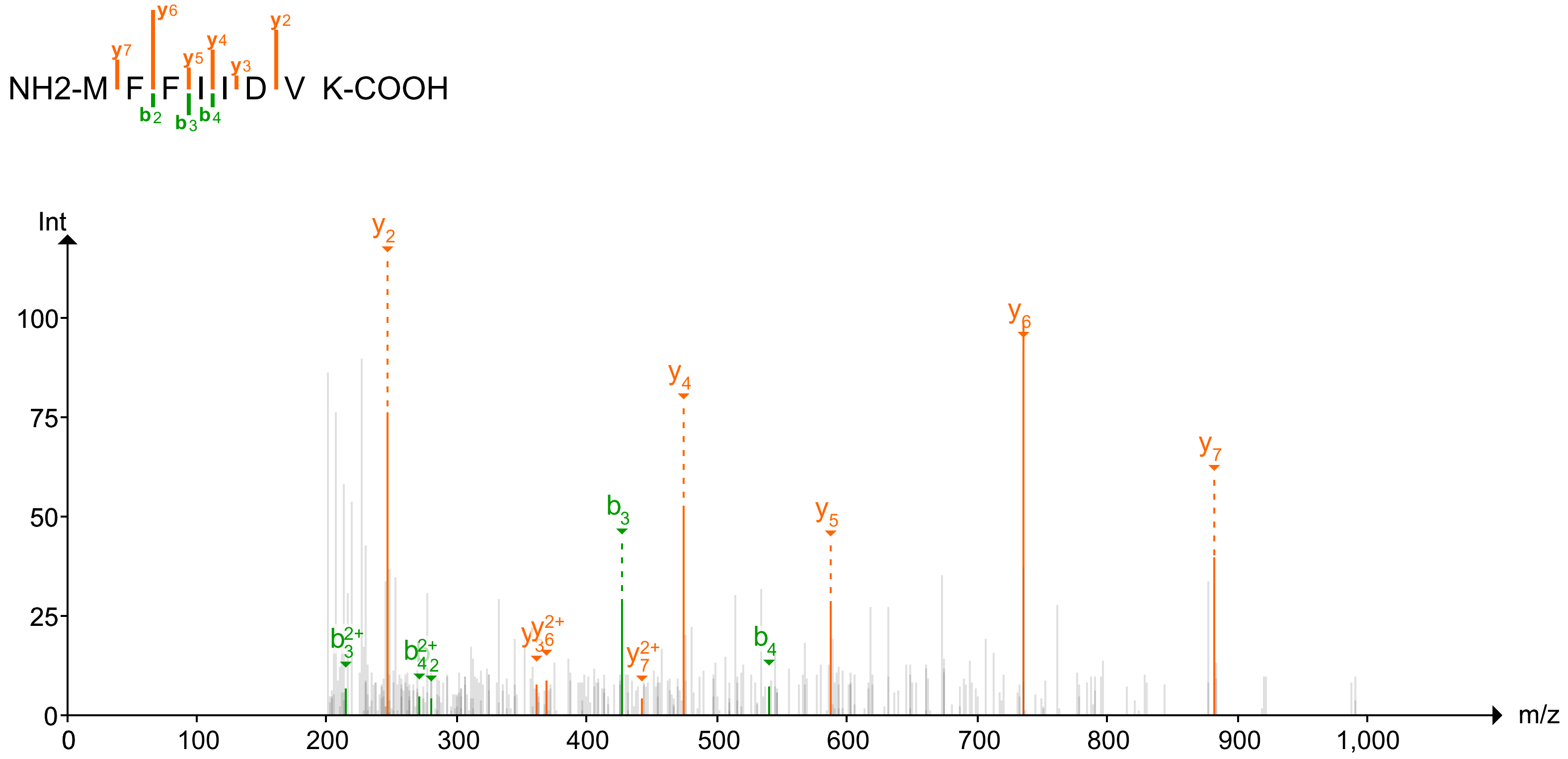

NH2-M F F I I D V K-COOH
y7 y6 y5 y4 y3 y2
b2 b3 b4
Int
m/z
0
25
50
75
100
0
100
200
300
400
500
600
700
800
900
1,000
y2
y4
y6
y7
y5
b3
b4

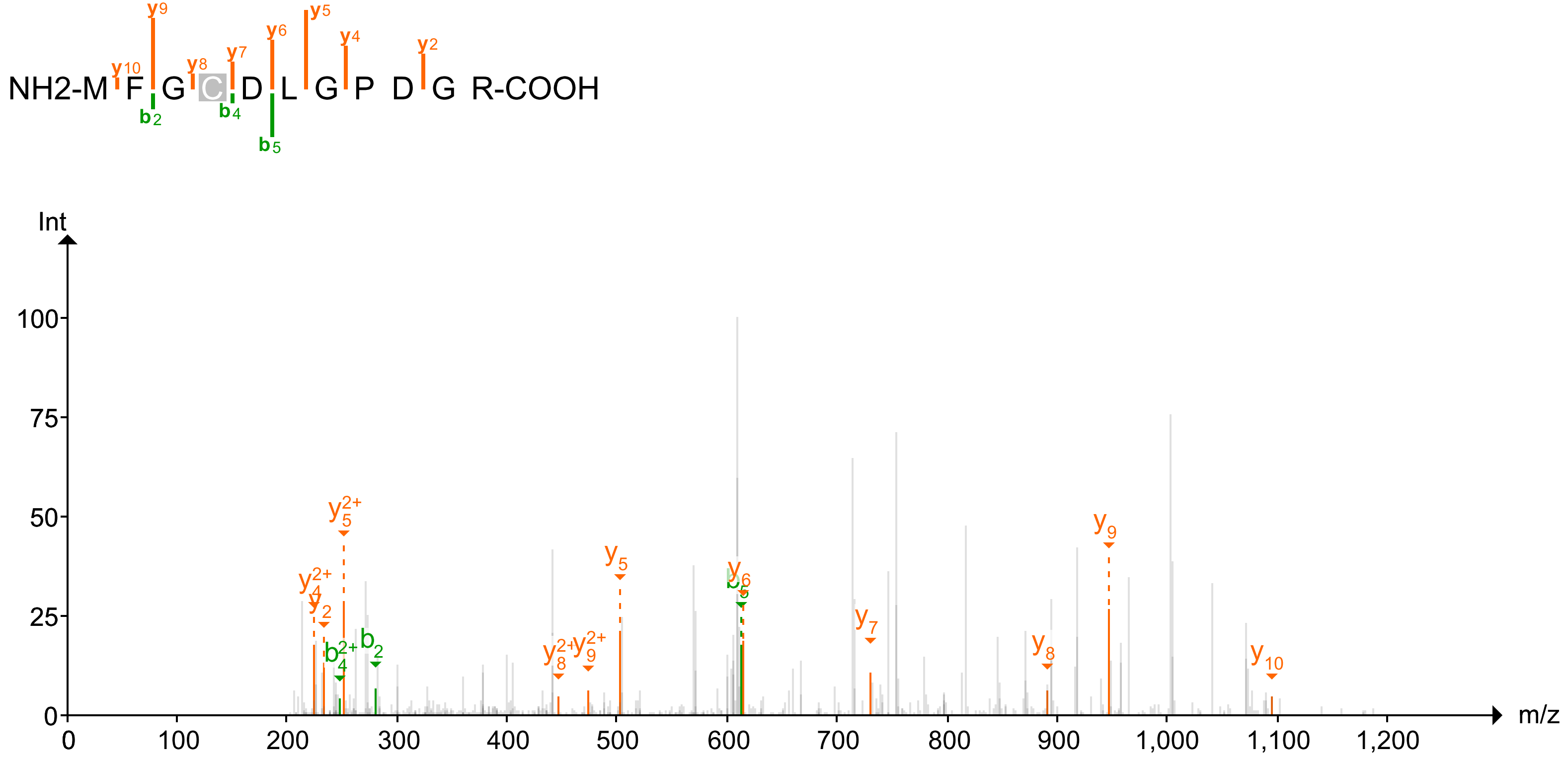

NH2-M F G C D L G P D G R-COOH
y10 y9 y8 y7 y6 y5 y4 y2
b2 b4 b5
Int
100
75
50
25
0
m/z
0 100 200 300 400 500 600 700 800 900 1,000 1,100 1,200

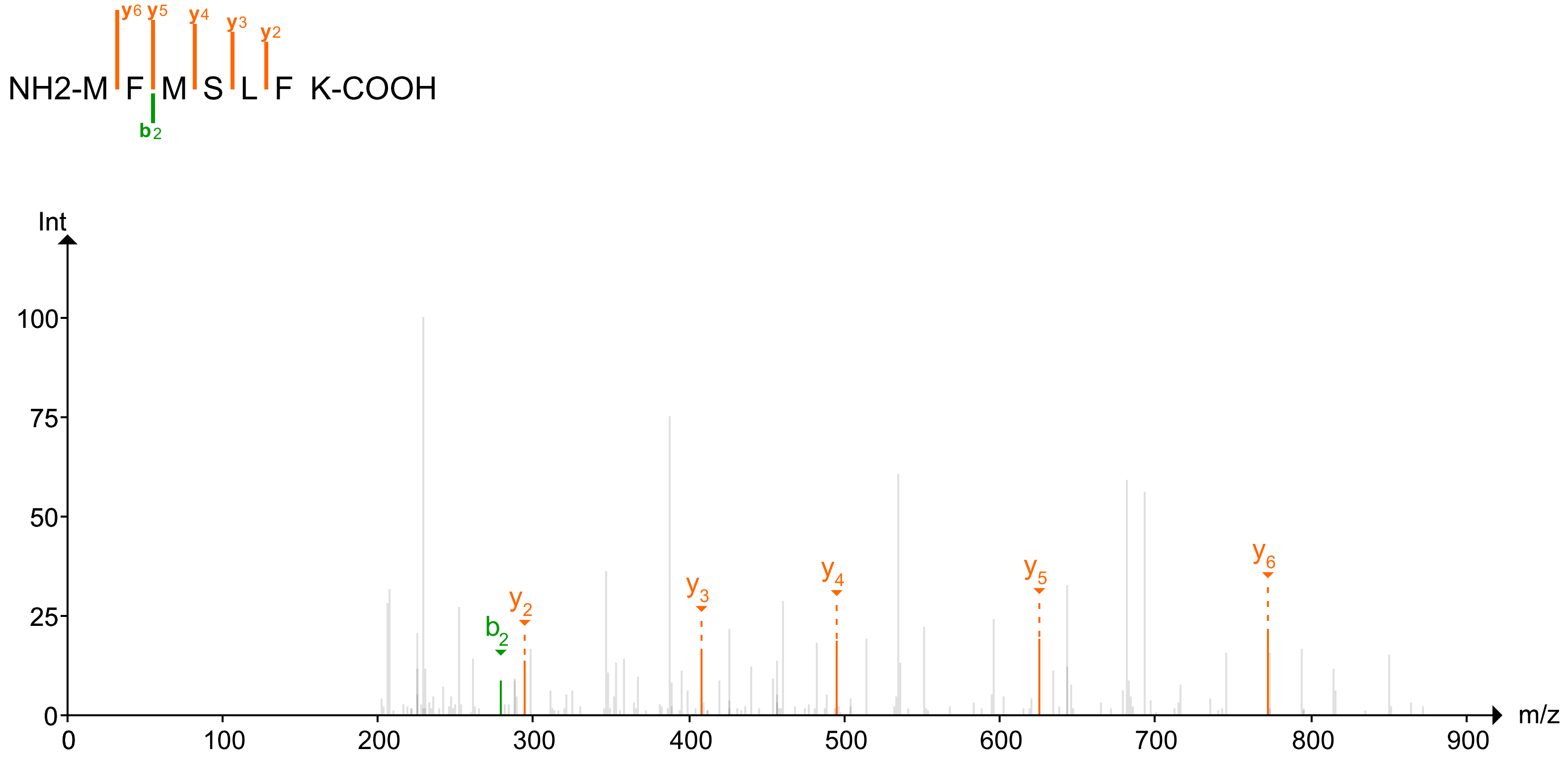

y6 y5 y4 y3 y2
NH2-M F M S L F K-COOH
b2
Int
100
75
50
25
0
0
100
200
300
400
500
600
700
800
900
m/z
b2
y2
y3
y4
y5
y6

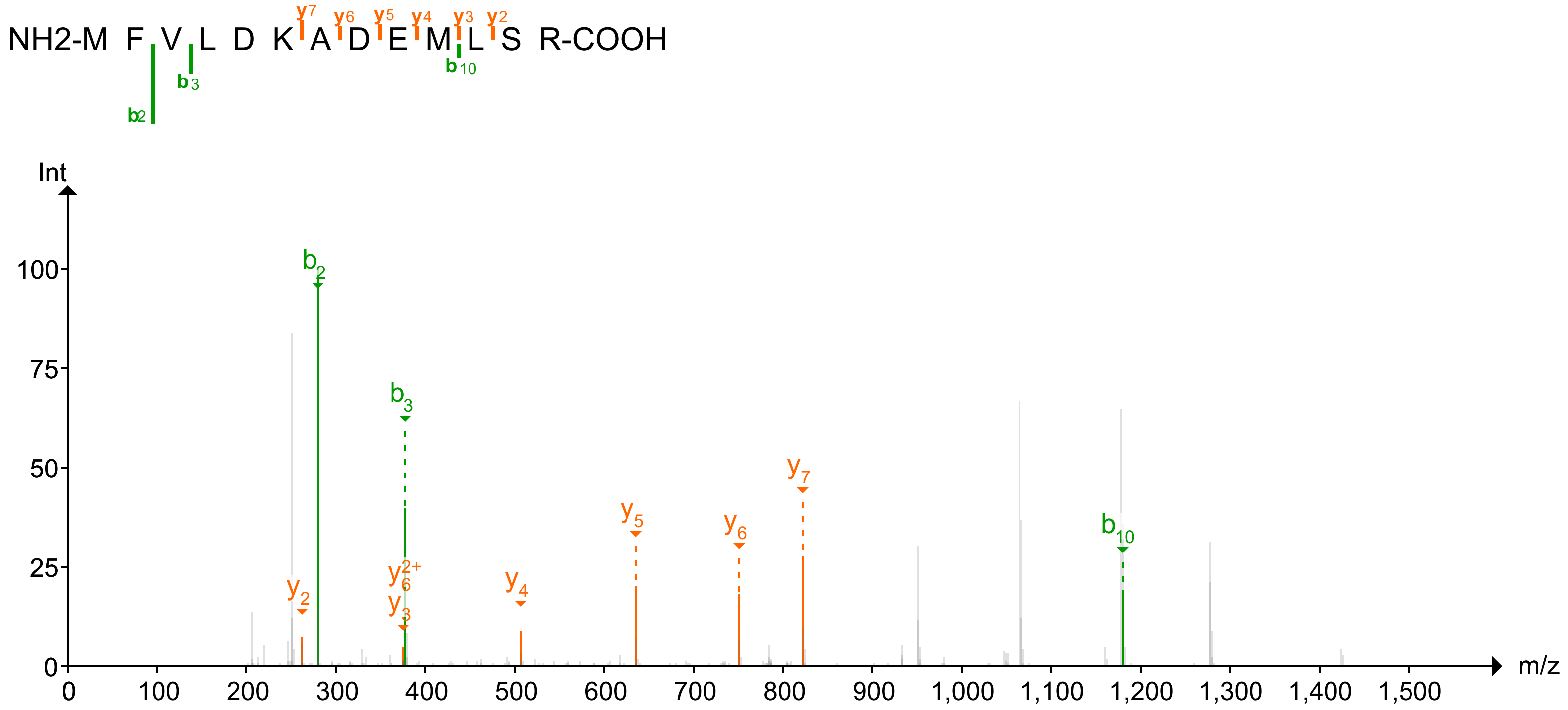

NH2-M F V L D K A D E M L S R-COOH
y7 y6 y5 y4 y3 y2
b2 b3 b10
Int
100
75
50
25
0
b2
b3
y7
y5
y6
b10
y2
y6 2+
y3
y4
m/z
0
100
200
300
400
500
600
700
800
900
1,000
1,100
1,200
1,300
1,400
1,500

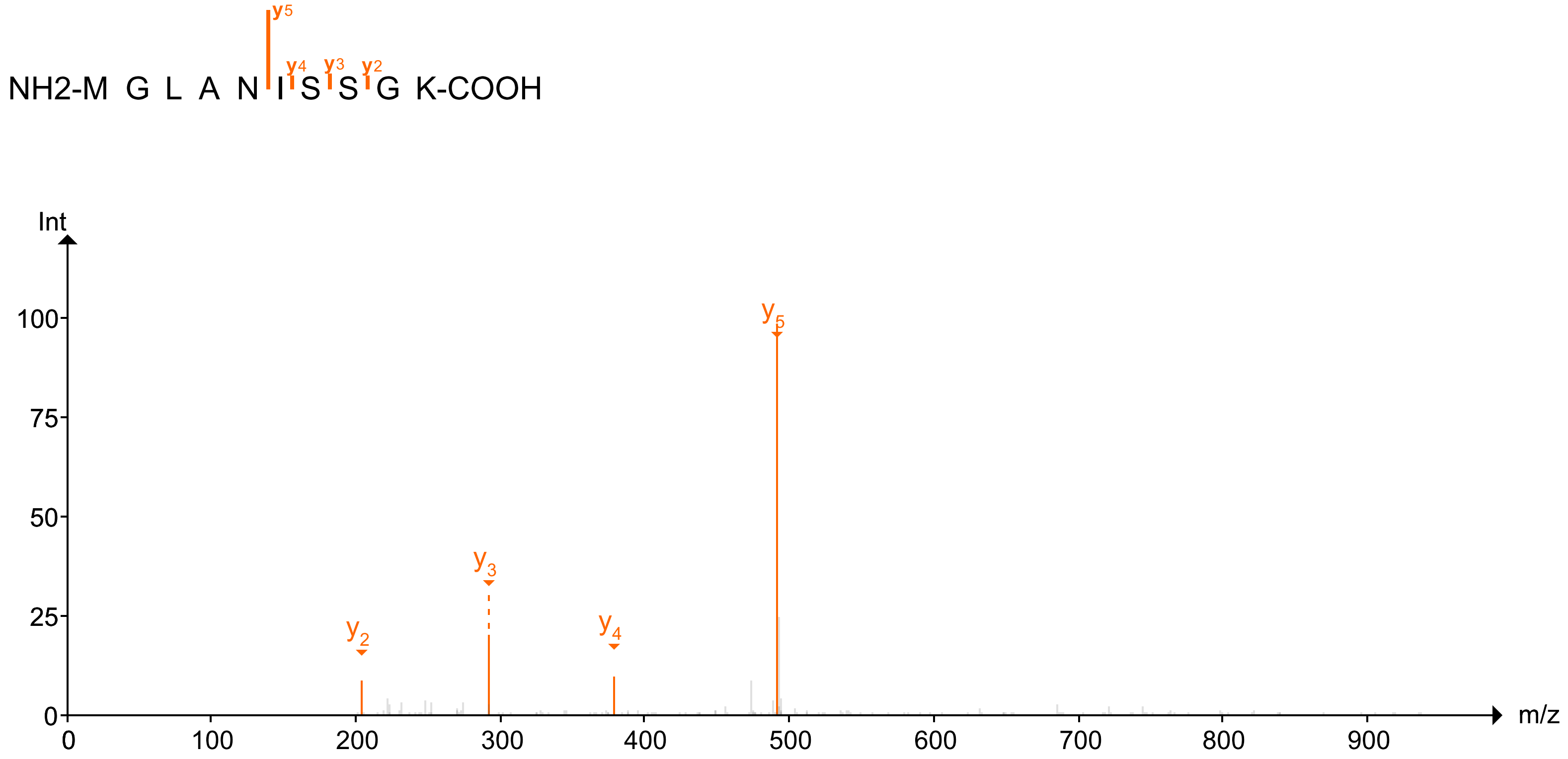

NH2-M G L A N I S S G K-COOH
y5
y4
y3
y2
Int
100
75
50
25
0
m/z
0
100
200
300
400
500
600
700
800
900

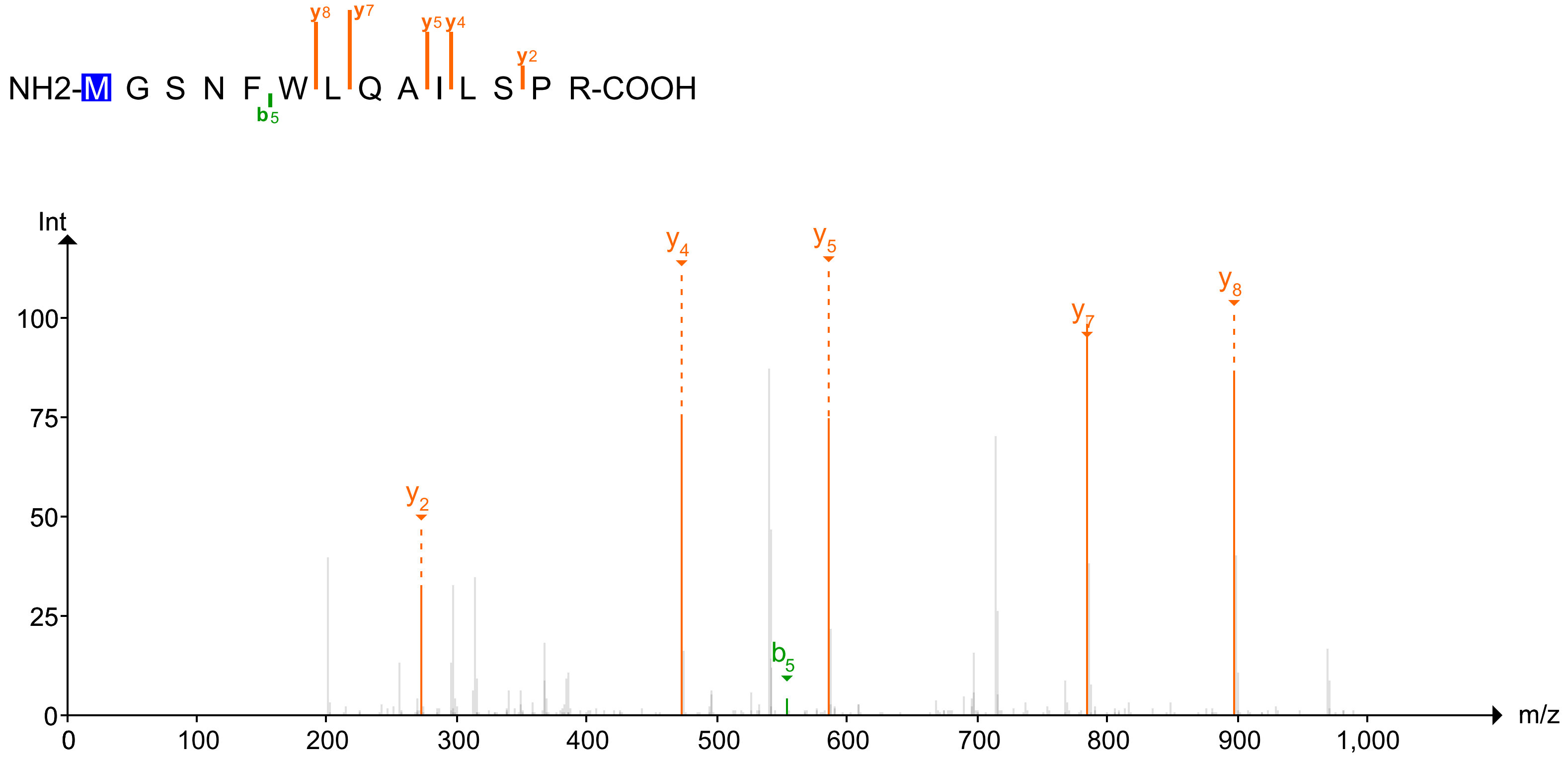

NH2-M G S N F W L Q A I L S P R-COOH
y8 y7 y5 y4 y2 b5
Int
100
75
50
25
0
y4 y5 y8 y7 y2 b5
0 100 200 300 400 500 600 700 800 900 1,000
m/z

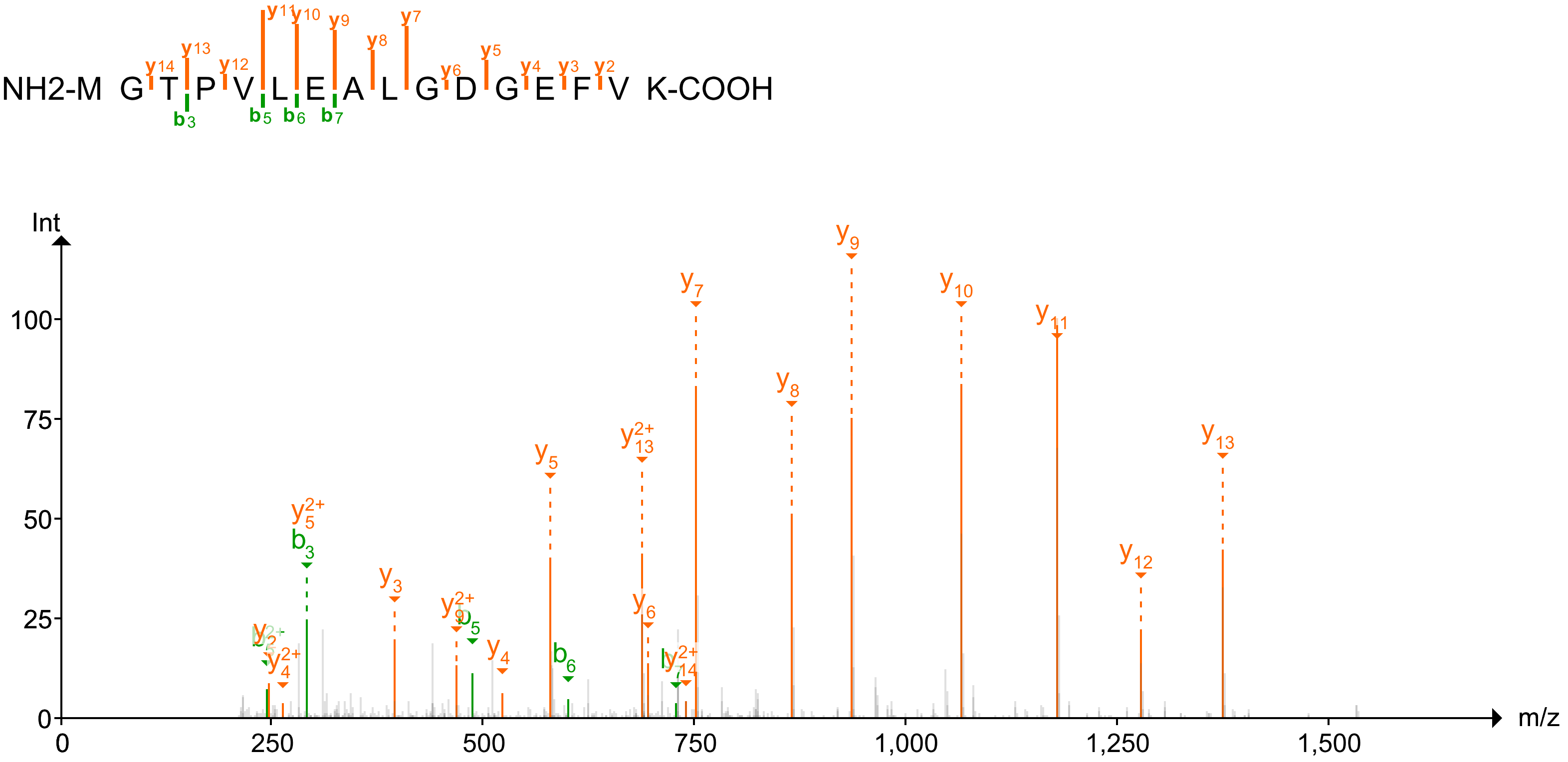

NH2-M G T P V L E A L G D G E F V K-COOH
Int
m/z
0
25
50
75
100
250
500
750
1,000
1,250
1,500

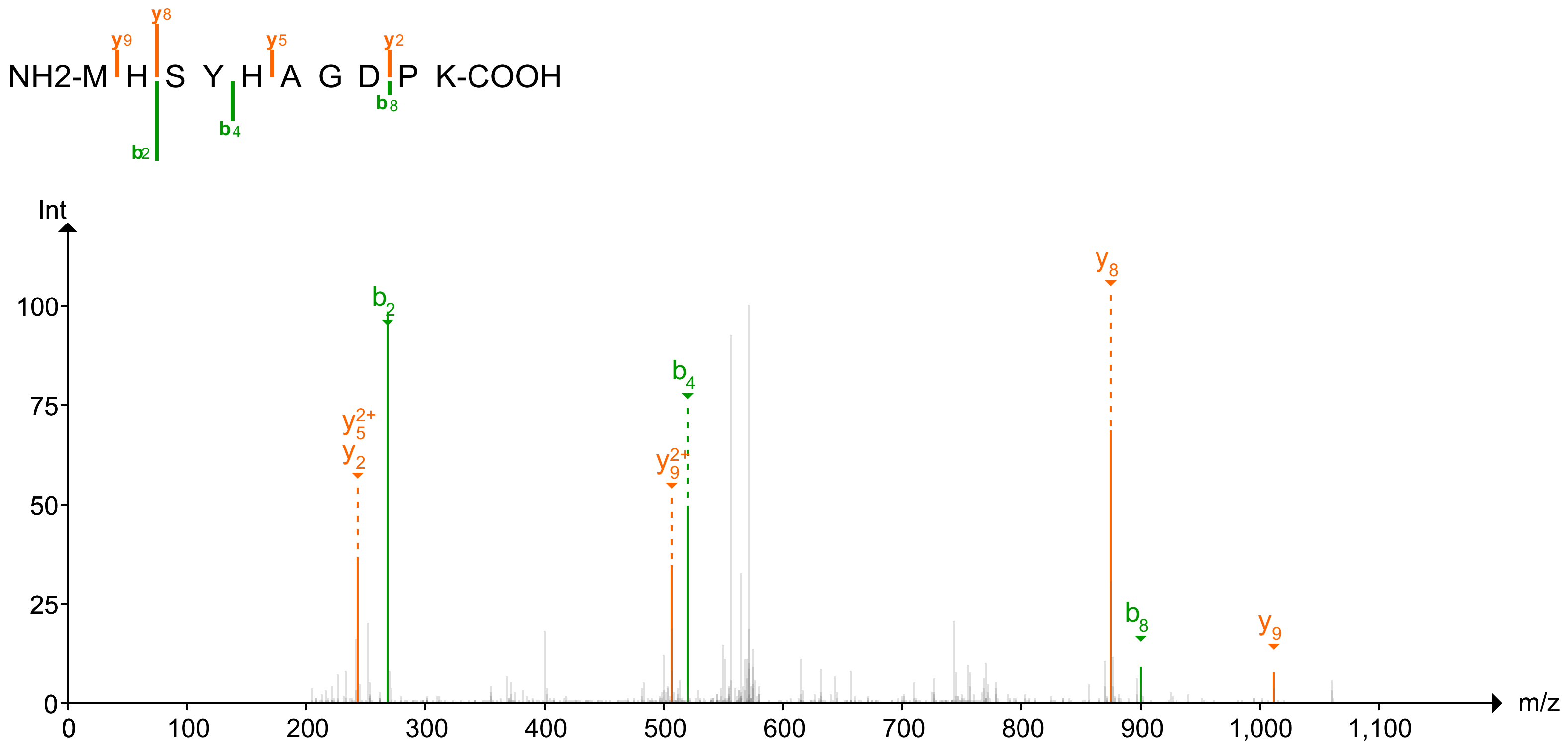

NH2-M H S Y H A G D P K-COOH
y9
y8
y5
y2
b2
b4
b8
Int
100
75
50
25
0
m/z
0
100
200
300
400
500
600
700
800
900
1,000
1,100
b2
y5 2+
y2
b4
y9 2+
y8
b8
y9

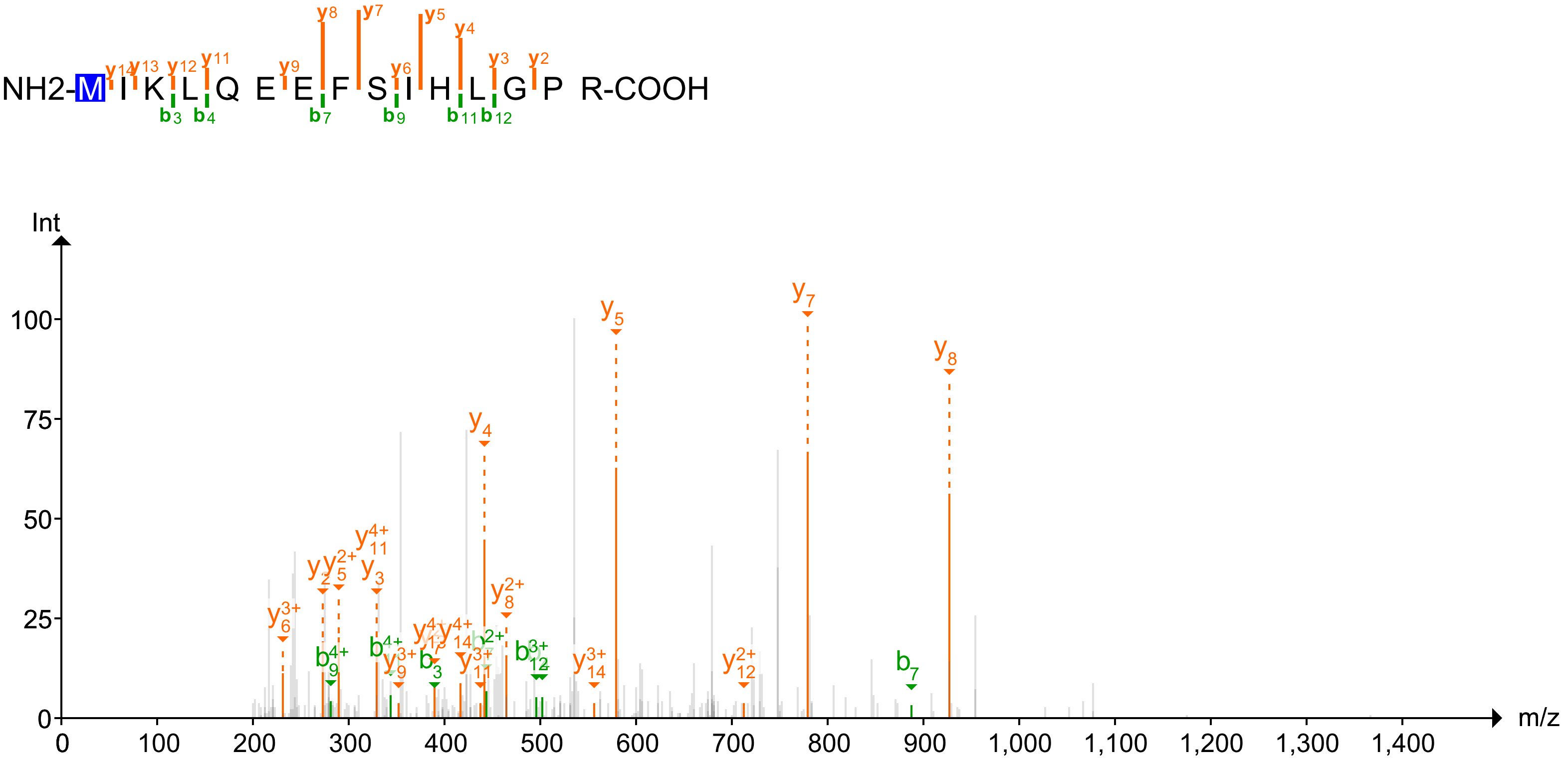
NH2-M I K L Q E E F S I H L G P R-COOH
y14 y13 y12 y11 y9 y8 y7 y6 y5 y4 y3 y2
b3 b4 b7 b9 b11 b12
Int
100
75
50
25
0
m/z
0 100 200 300 400 500 600 700 800 900 1,000 1,100 1,200 1,300 1,400
y5
y7
y8
y4
y2
y5 2+
y3
y11 4+
y6 3+
y8 2+
b9 4+
y9 3+
b3
y11 3+
y14 3+
y12 2+
b7

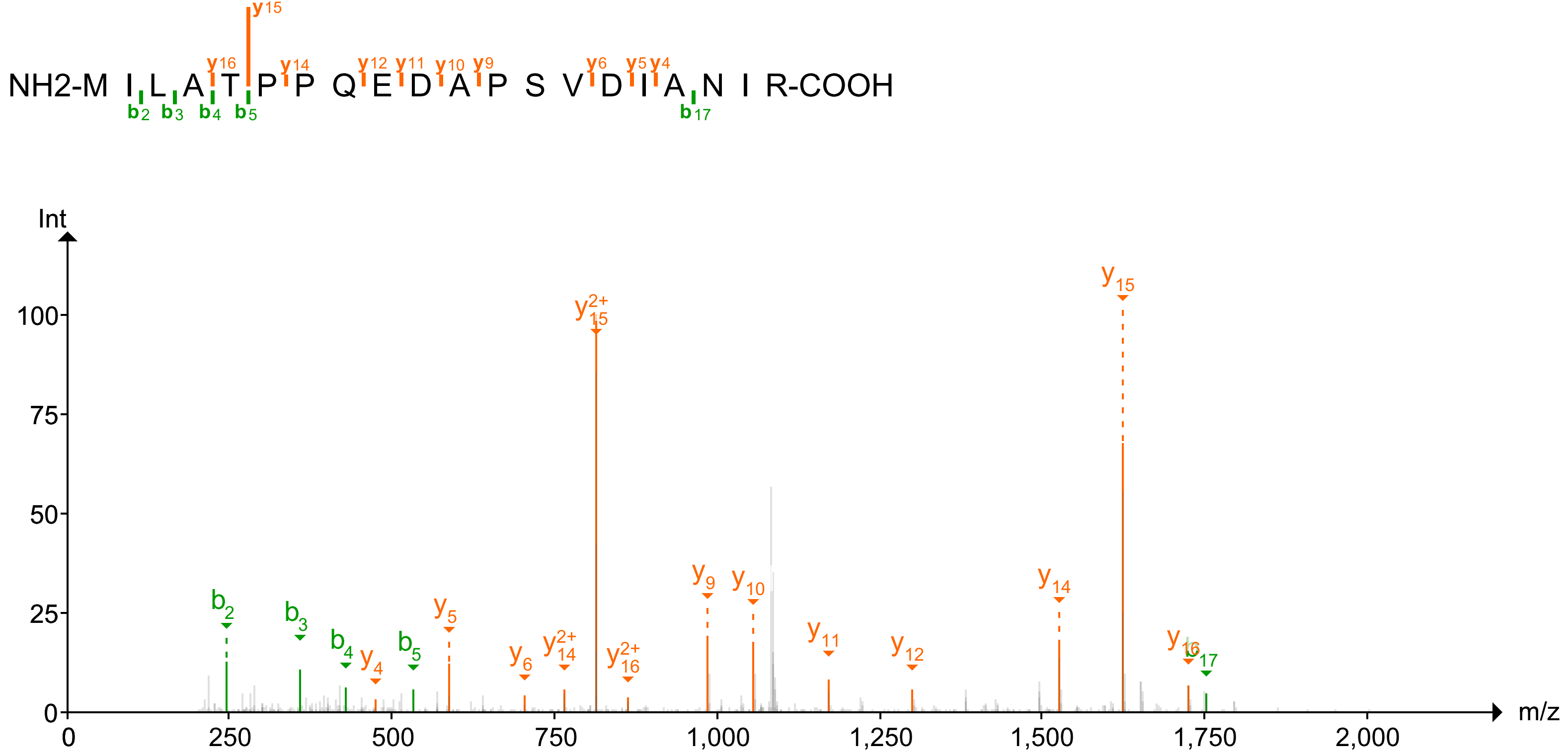

NH2-M I L A T P P Q E D A P S V D I A N I R-COOH
y15 y16 y14 y12 y11 y10 y9 y6 y5 y4
b2 b3 b4 b5 b17
Int
100
75
50
25
0
m/z
0 250 500 750 1,000 1,250 1,500 1,750 2,000
b2 b3 b4 b5 y4 y5 y6 y9 y10 y11 y12 y14 y15 y16 b17

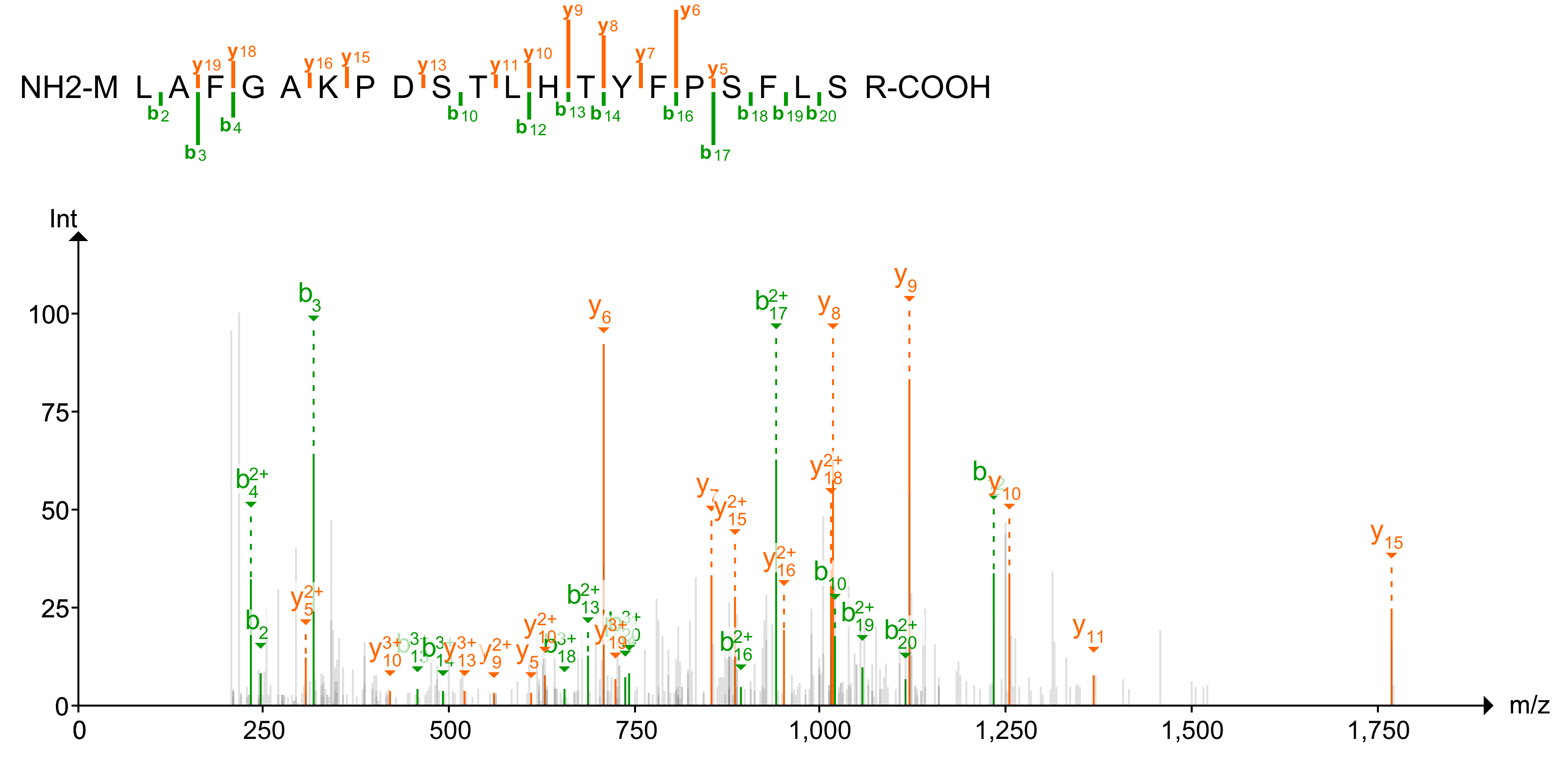

NH2-M L A F G A K P D S T L H T Y F P S F L S R-COOH
Int
m/z
0
25
50
75
100
250
500
750
1,000
1,250
1,500
1,750

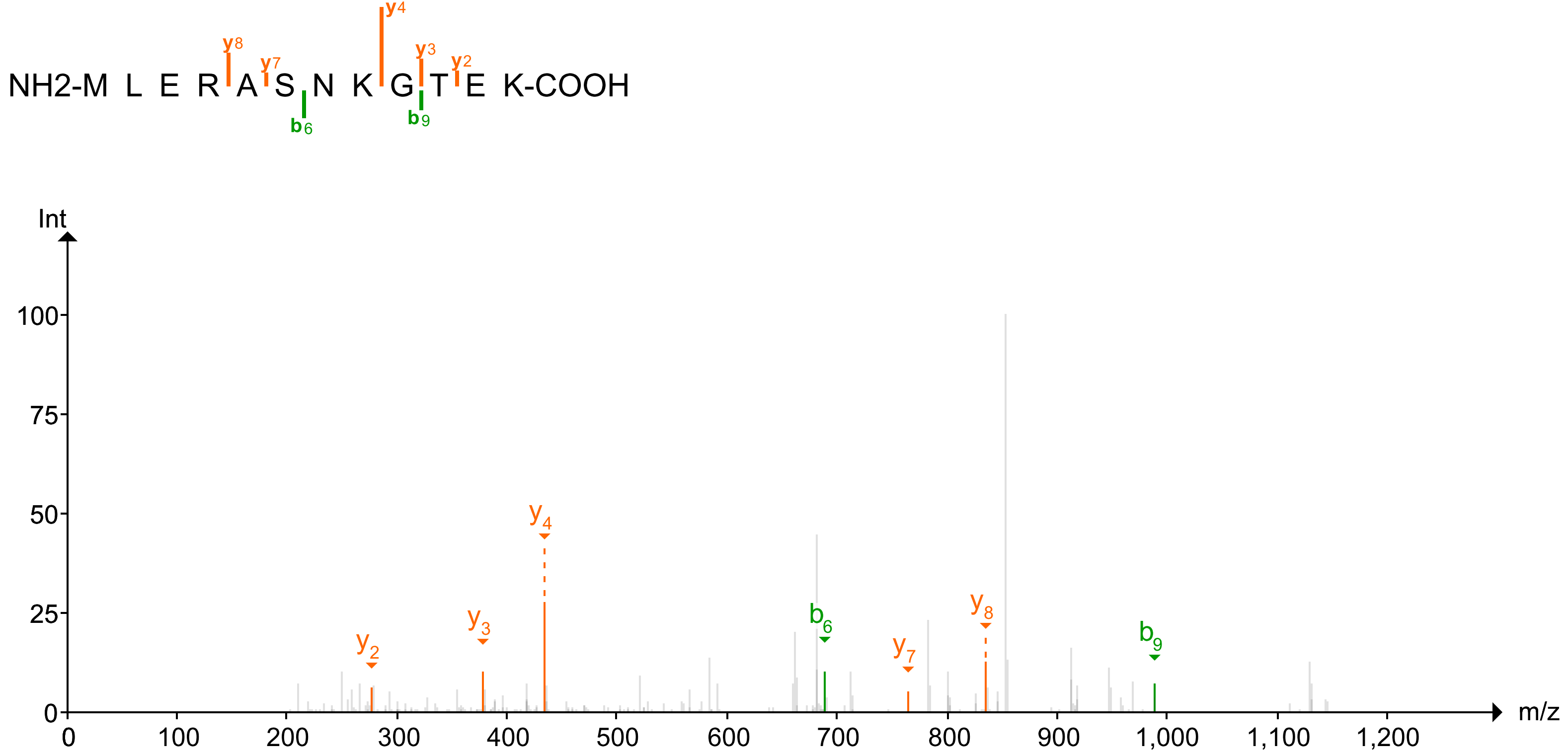

NH2-M L E R A S N K G T E K-COOH
y8
y7
y4
y3
y2
b6
b9
Int
100
75
50
25
0
y2
y3
y4
b6
y7
y8
b9
0
100
200
300
400
500
600
700
800
900
1,000
1,100
1,200
m/z

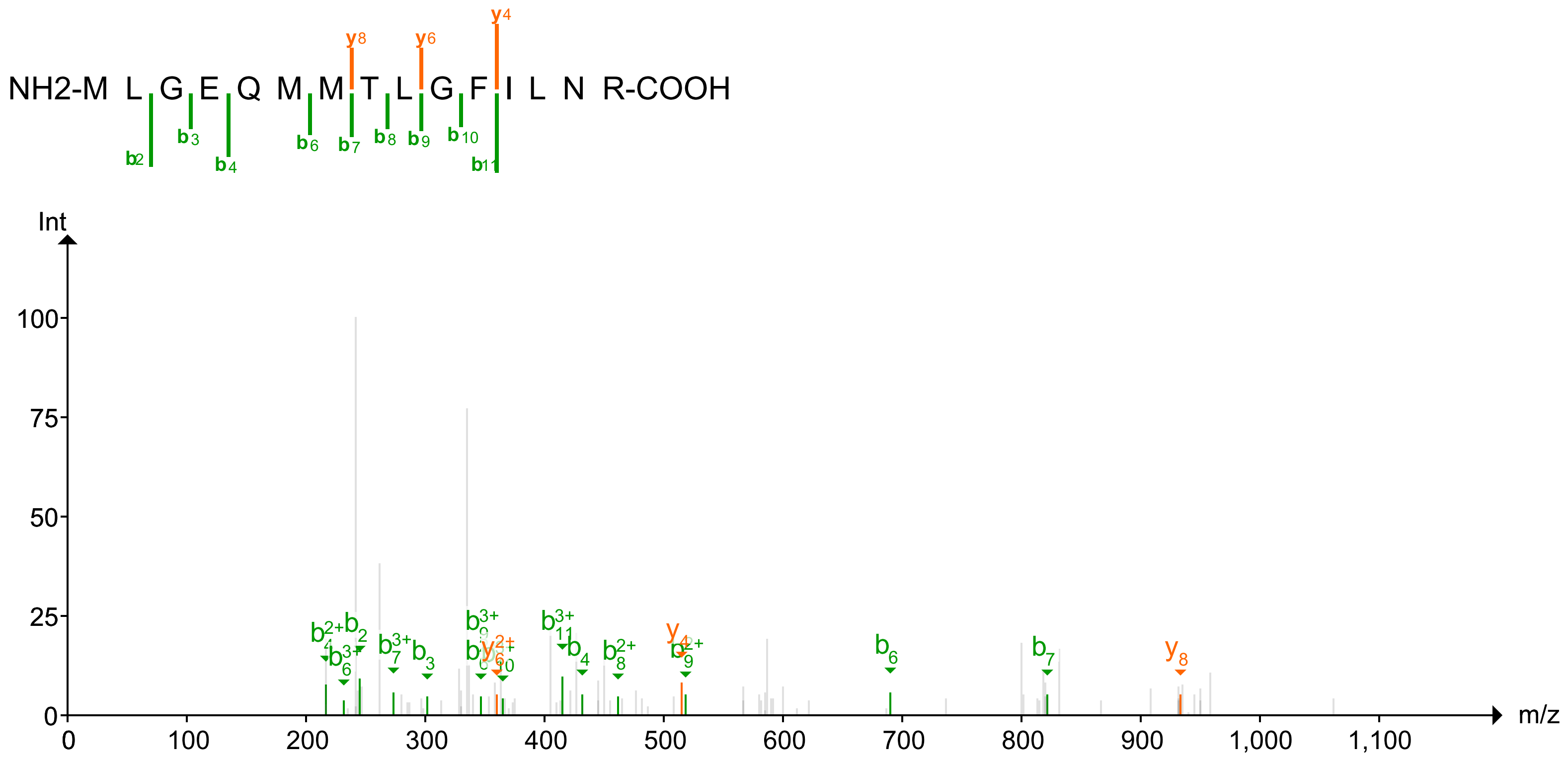

NH2-M L G E Q M M T L G F I L N R-COOH
y8
y6
y4
b2
b3
b4
b6
b7
b8
b9
b10
b11
Int
100
75
50
25
0
m/z
0
100
200
300
400
500
600
700
800
900
1,000
1,100
b6
b7
y8

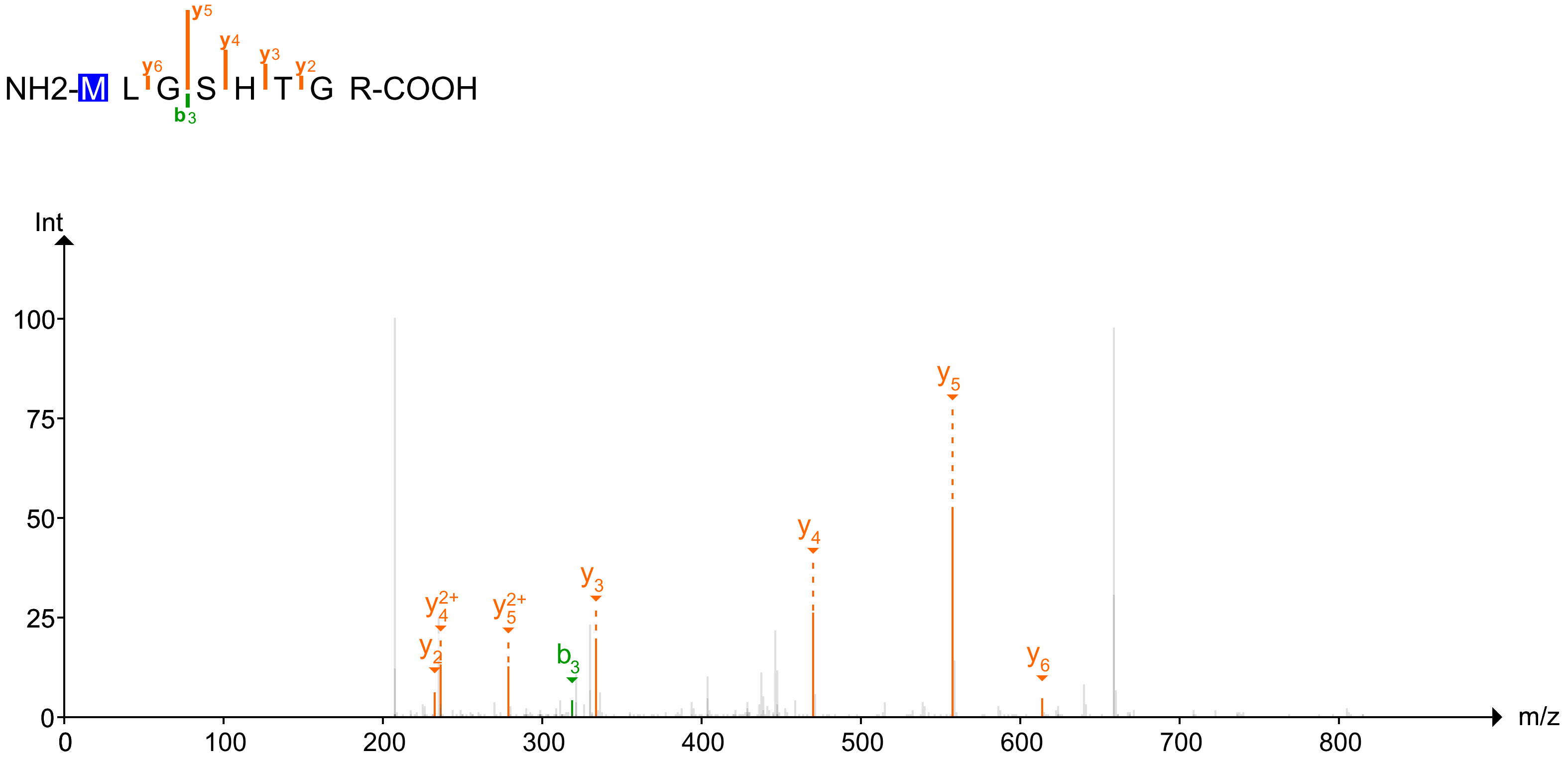

NH2-M L G S H T G R-COOH
y6 y5 y4 y3 y2
b3
Int
100
75
50
25
0
y5
y4
y3
y4 2+
y5 2+
y2
b3
y6
0 100 200 300 400 500 600 700 800
m/z

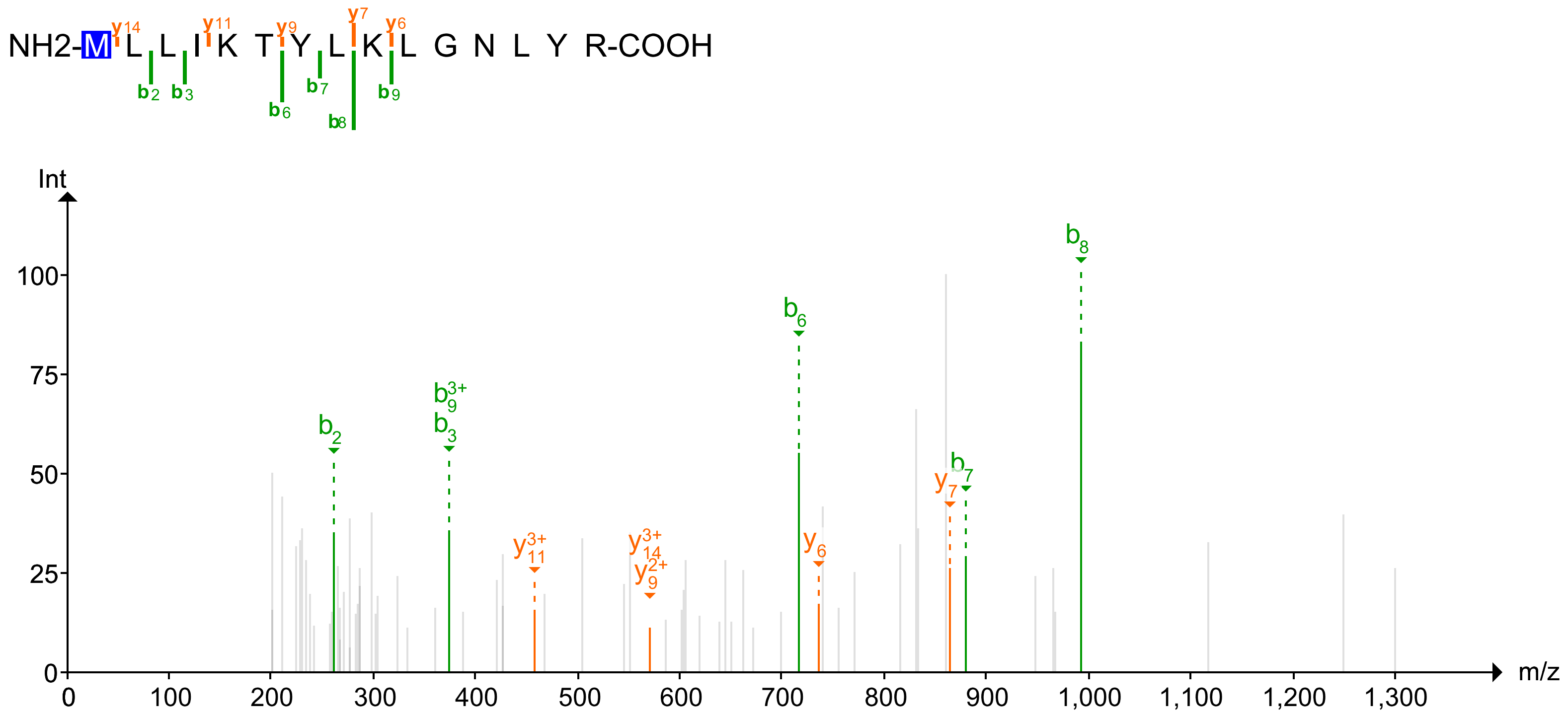

NH2-M L L I K T Y L K L G N L Y R-COOH
y14 y11 y9 y7 y6
b2 b3 b6 b7 b8 b9
Int
100
75
50
25
0
b8
b6
b2
b9 3+
b3
y11 3+
y14 3+
y9 2+
y6
y7
b7
0 100 200 300 400 500 600 700 800 900 1,000 1,100 1,200 1,300
m/z

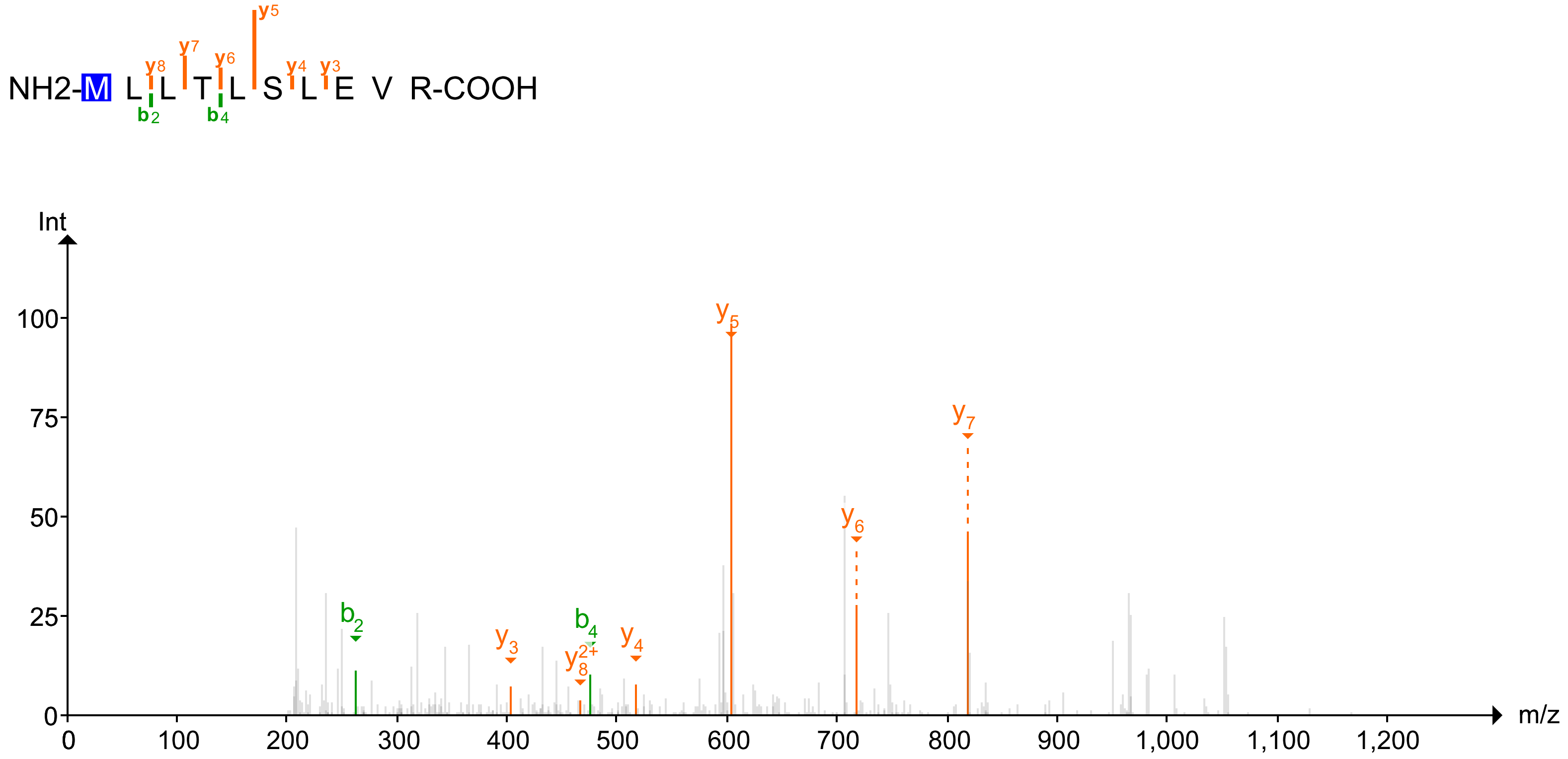

NH2-M L L T L S L E V R-COOH
y5
y7
y8
y6
y4
y3
b2
b4
Int
100
75
50
25
0
m/z
0
100
200
300
400
500
600
700
800
900
1,000
1,100
1,200

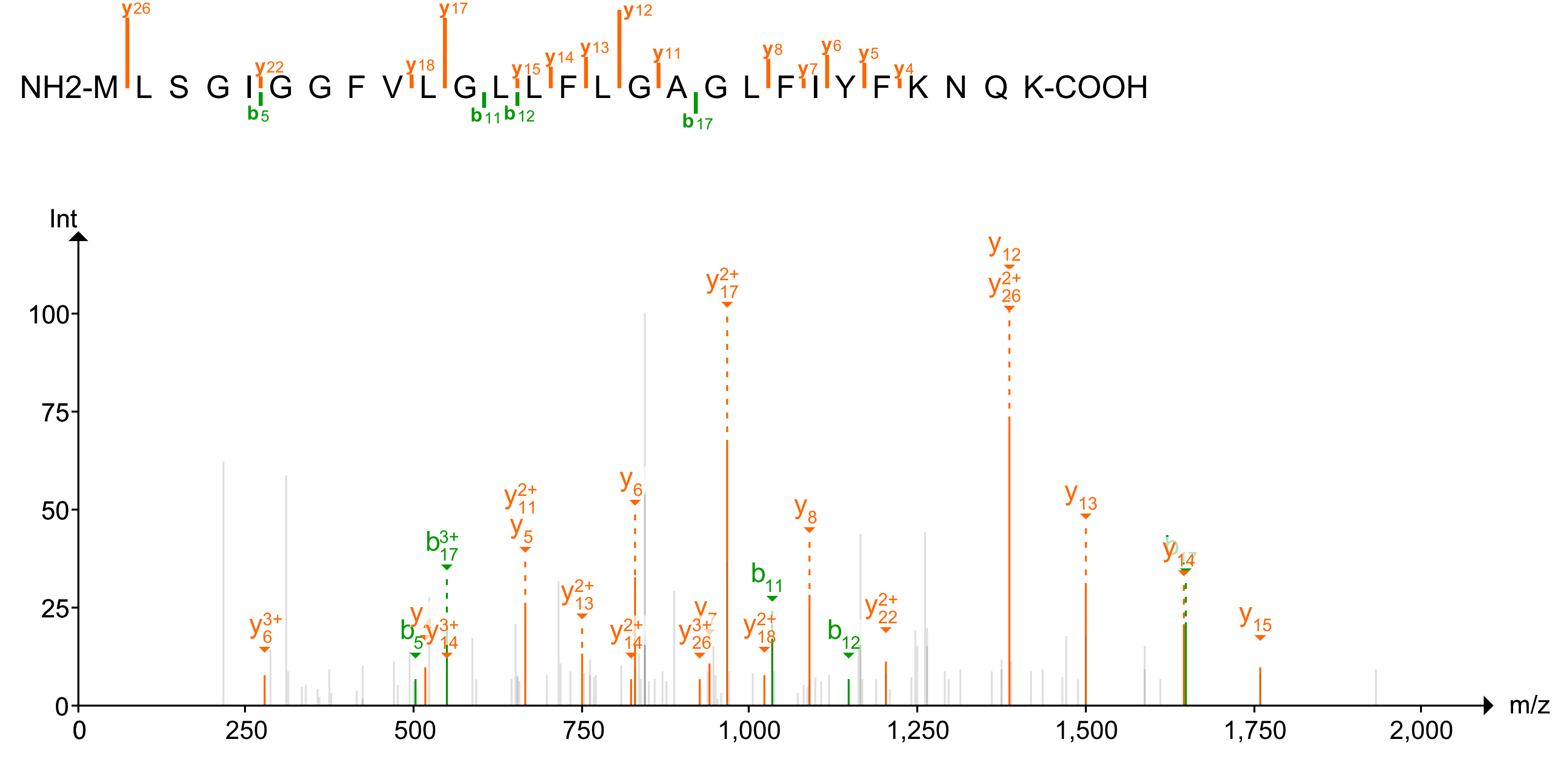

NH2-M L S G I G G F V L G L L F L G A G L F I Y F K N Q K-COOH
y26 y22 y18 y17 y15 y14 y13 y12 y11 y8 y7 y6 y5 y4
b5 b11 b12 b17
Int
m/z
y12
y2+ 26
y2+ 17
y6
y2+ 11
y5
y8
y13
b3+ 17
y2+ 13
b11
y14
y3+ 6
b5
y3+ 14
y2+ 14
y3+ 26
y2+ 18
b12
y2+ 22
y15

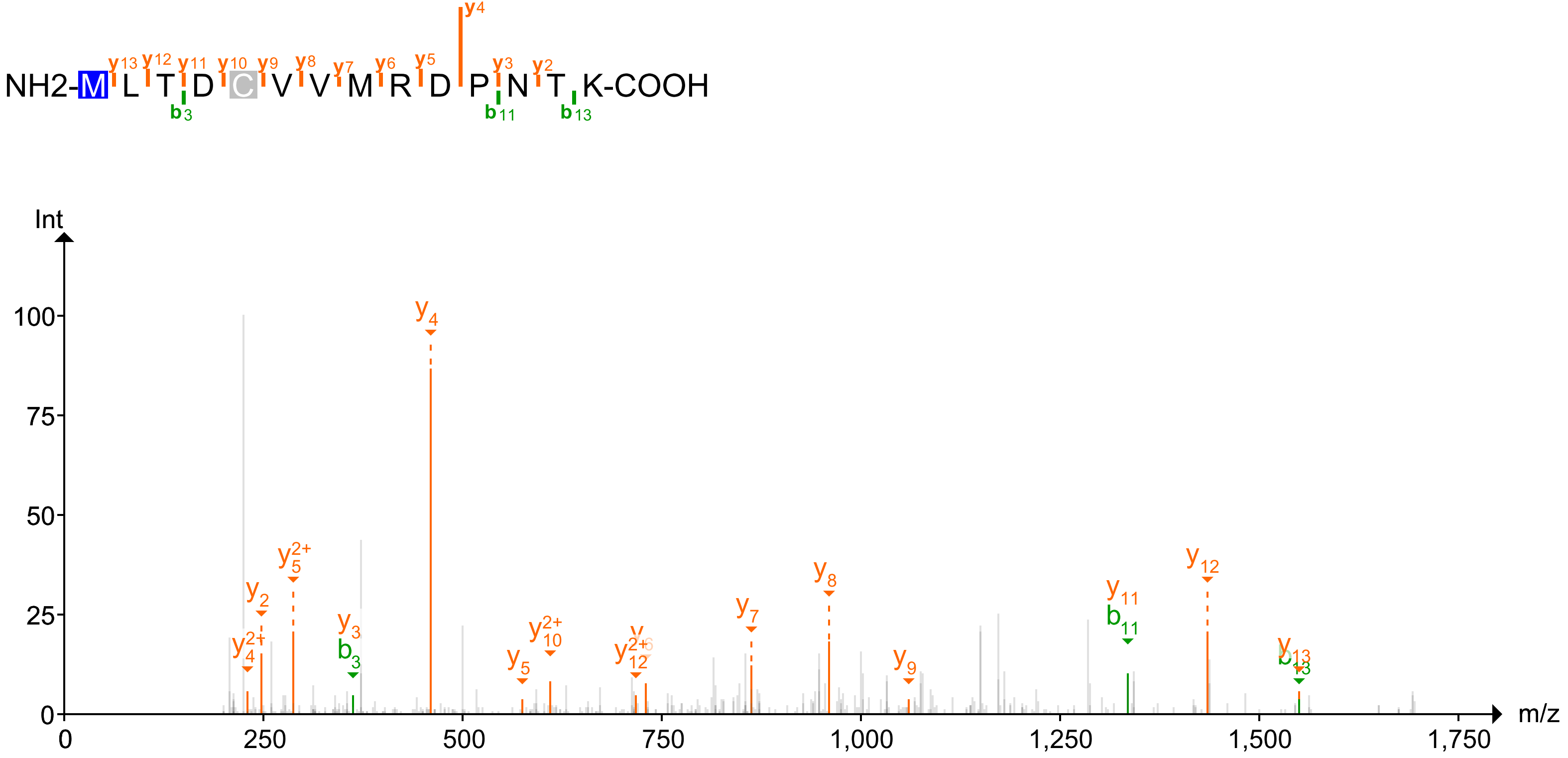

NH2-M L T D C V V M R D P N T K-COOH
y13 y12 y11 y10 y9 y8 y7 y6 y5 y4 y3 y2
b3 b11 b13
Int
100
75
50
25
0
250
500
750
1,000
1,250
1,500
1,750
m/z
y4
y2
y5 2+
y4 2+
y3
b3
y5
y10 2+
y12 2+
y7
y8
y9
y11
b11
y12
y13
b13

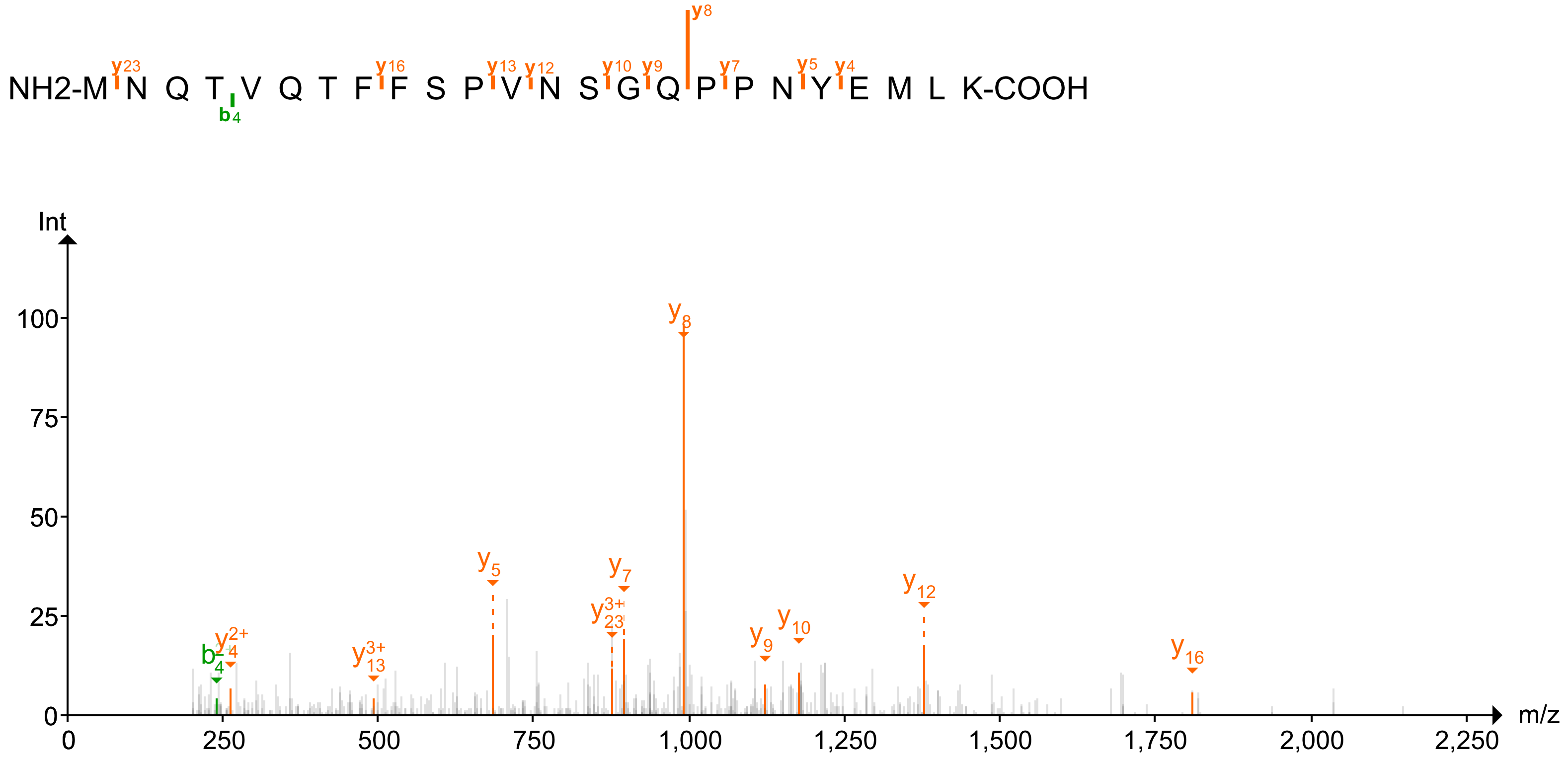

NH2-M N Q T V Q T F F S P V N S G Q P P N Y E M L K-COOH
y23 y16 y13 y12 y10 y9 y8 y7 y5 y4
b4
Int
100
75
50
25
0
y8
y5
y7
y23 3+
y12
y10
y9
y16
y4 2+
b4
y13 3+
0
250
500
750
1,000
1,250
1,500
1,750
2,000
2,250
m/z

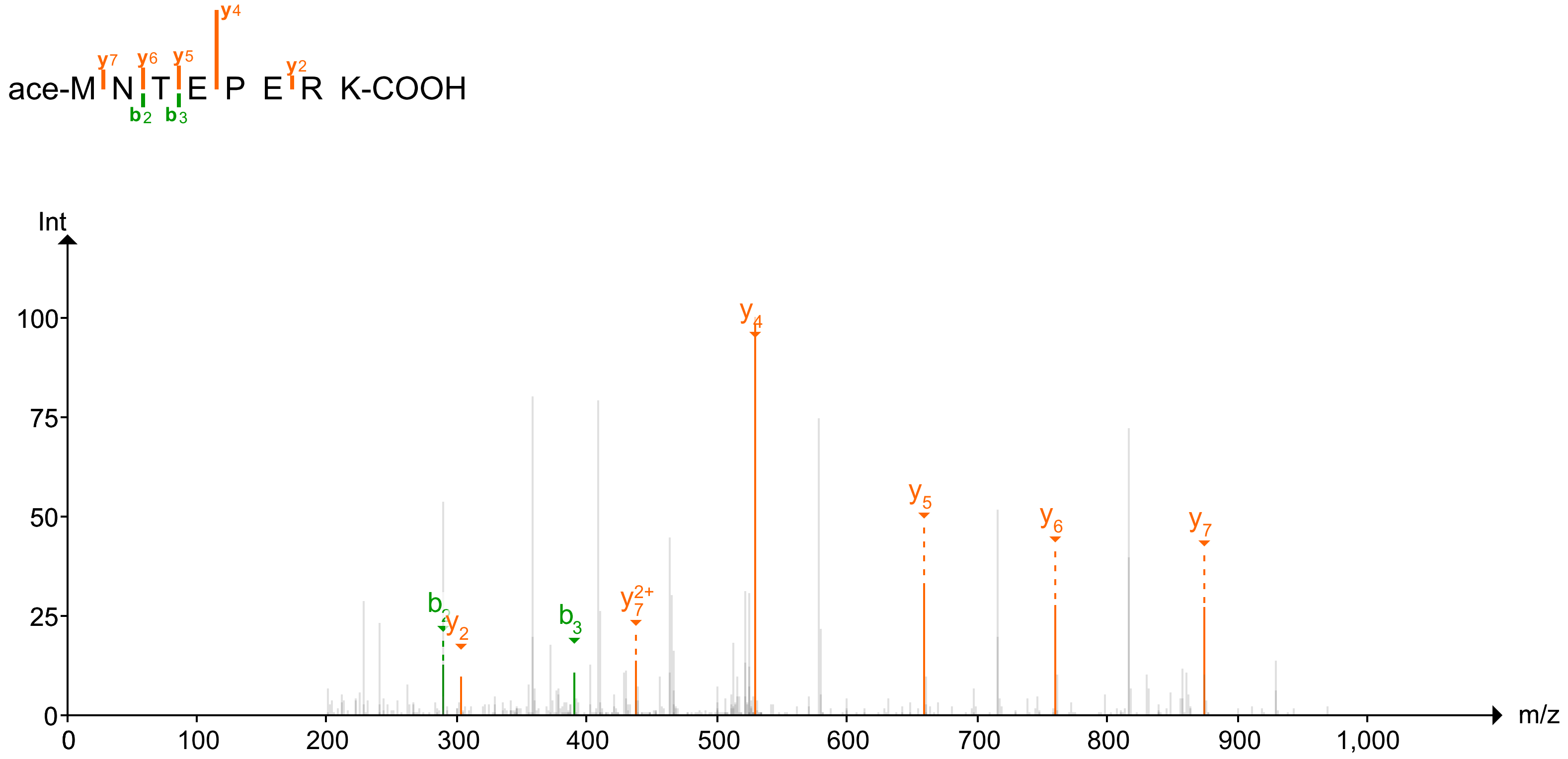

ace-M N T E P E R K-COOH
y7
y6
y5
y4
y2
b2
b3
Int
100
75
50
25
0
m/z
0
100
200
300
400
500
600
700
800
900
1,000
b2
y2
b3
y7 2+
y4
y5
y6
y7

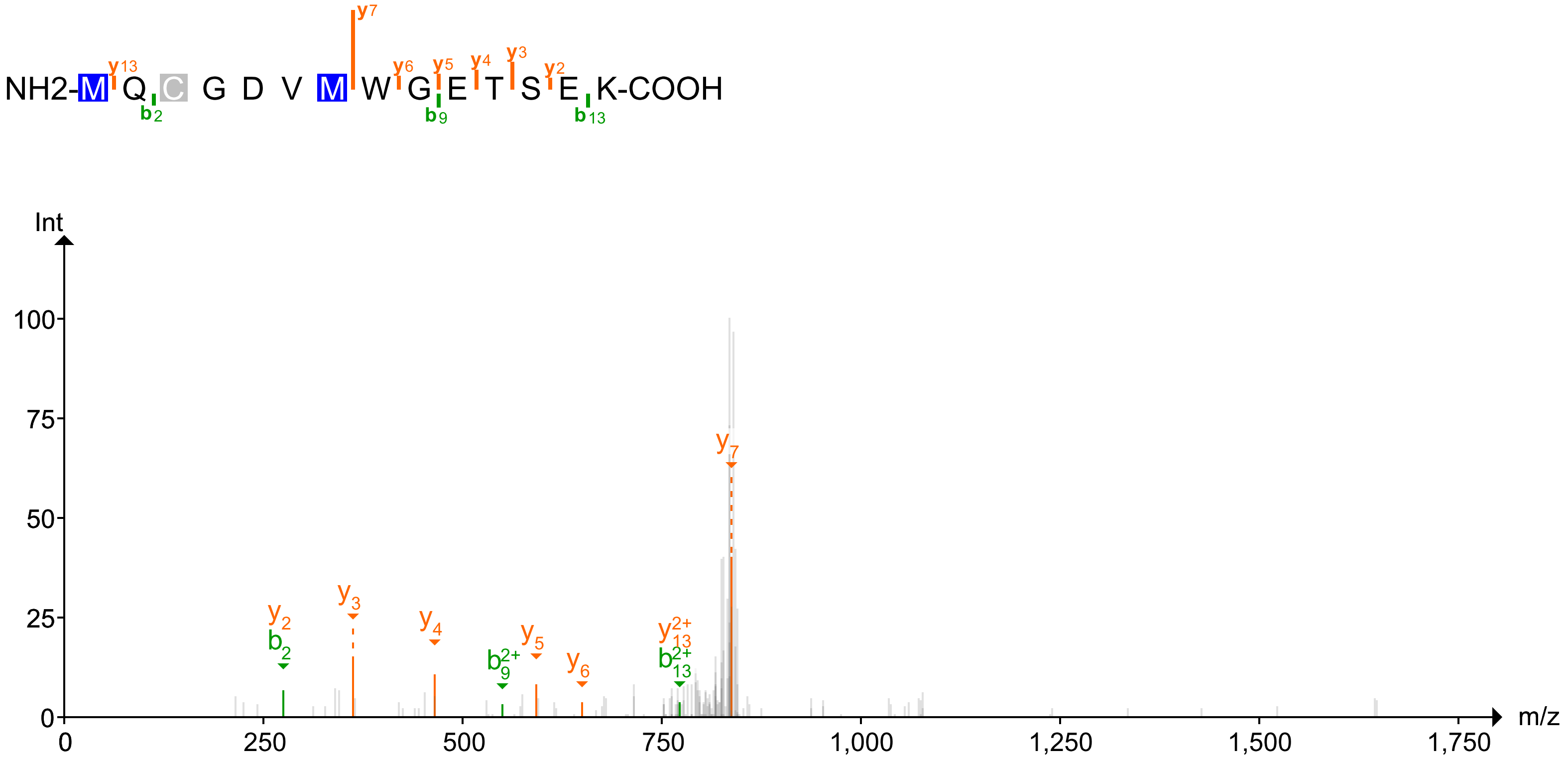

NH2-M Q C G D V M W G E T S E K-COOH
y7
y13
y6
y5
y4
y3
y2
b2
b9
b13
Int
100
75
50
25
0
m/z
0
250
500
750
1,000
1,250
1,500
1,750

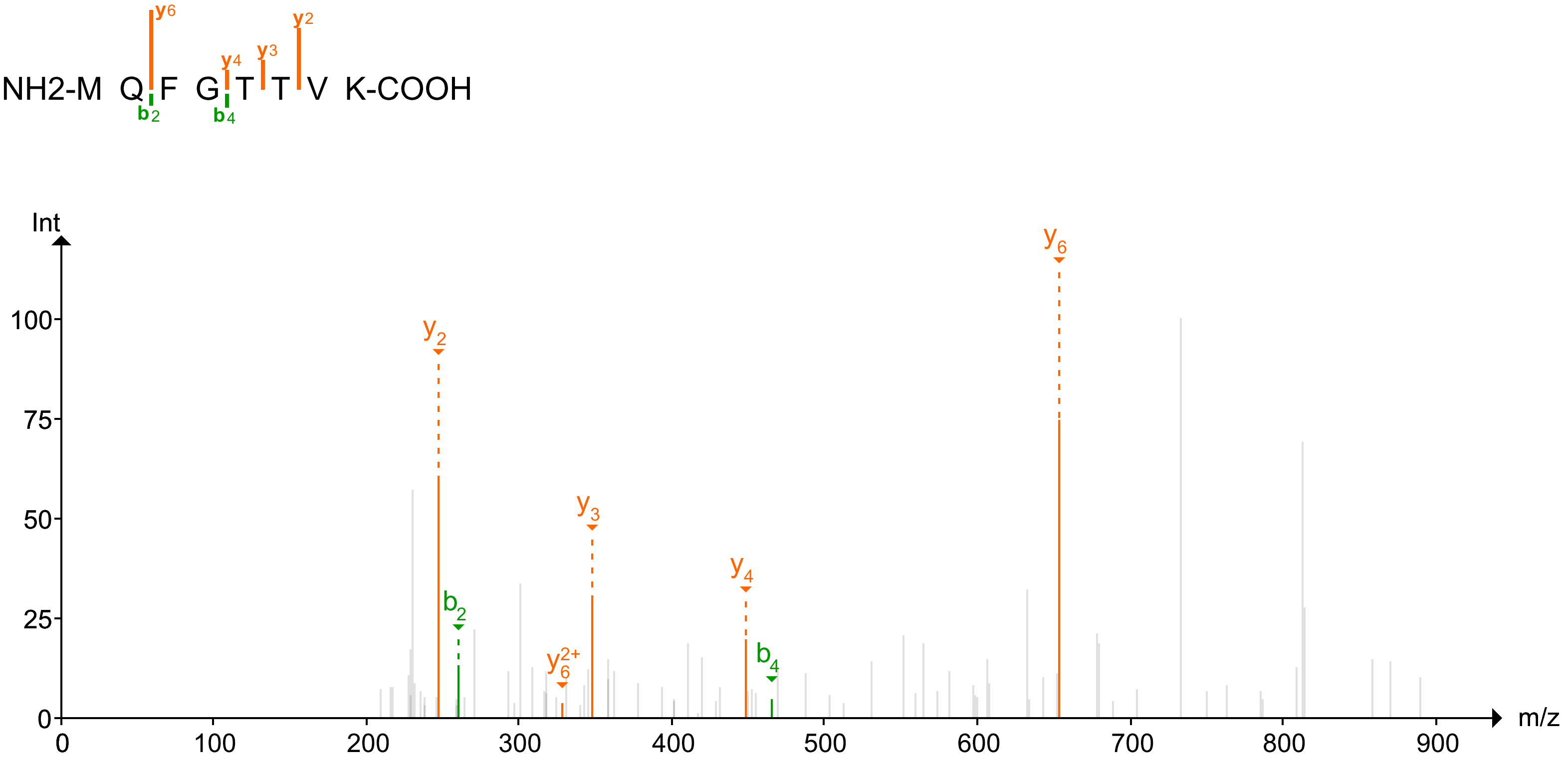

NH2-M Q F G T T V K-COOH
y6
y4
y3
y2
b2
b4
Int
100
75
50
25
0
m/z
0
100
200
300
400
500
600
700
800
900
y2
b2
y6 2+
y3
y4
b4
y6

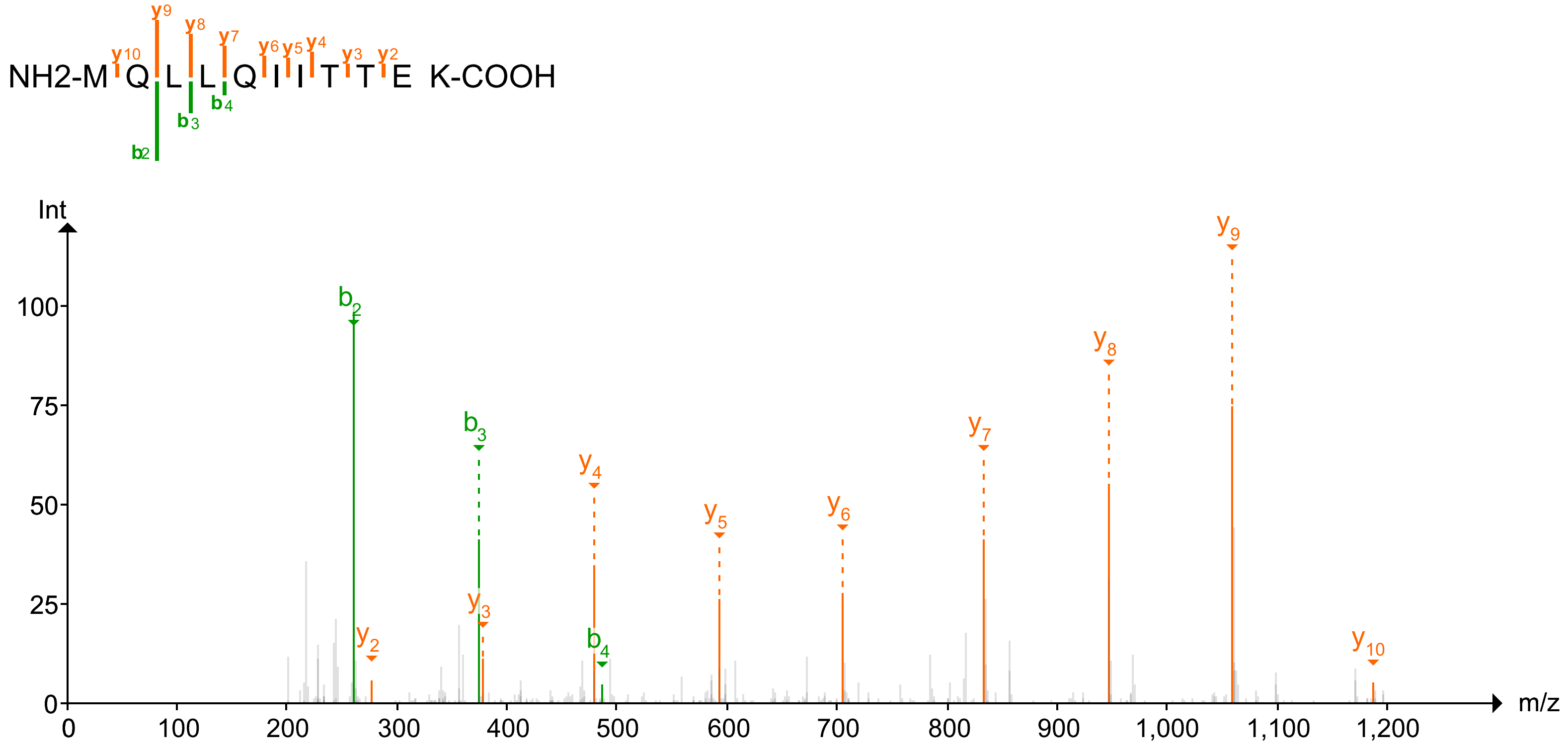

NH2-M Q L L Q I I T T E K-COOH
y10 y9 y8 y7 y6 y5 y4 y3 y2
b2 b3 b4
Int
100
75
50
25
0
m/z
0 100 200 300 400 500 600 700 800 900 1,000 1,100 1,200

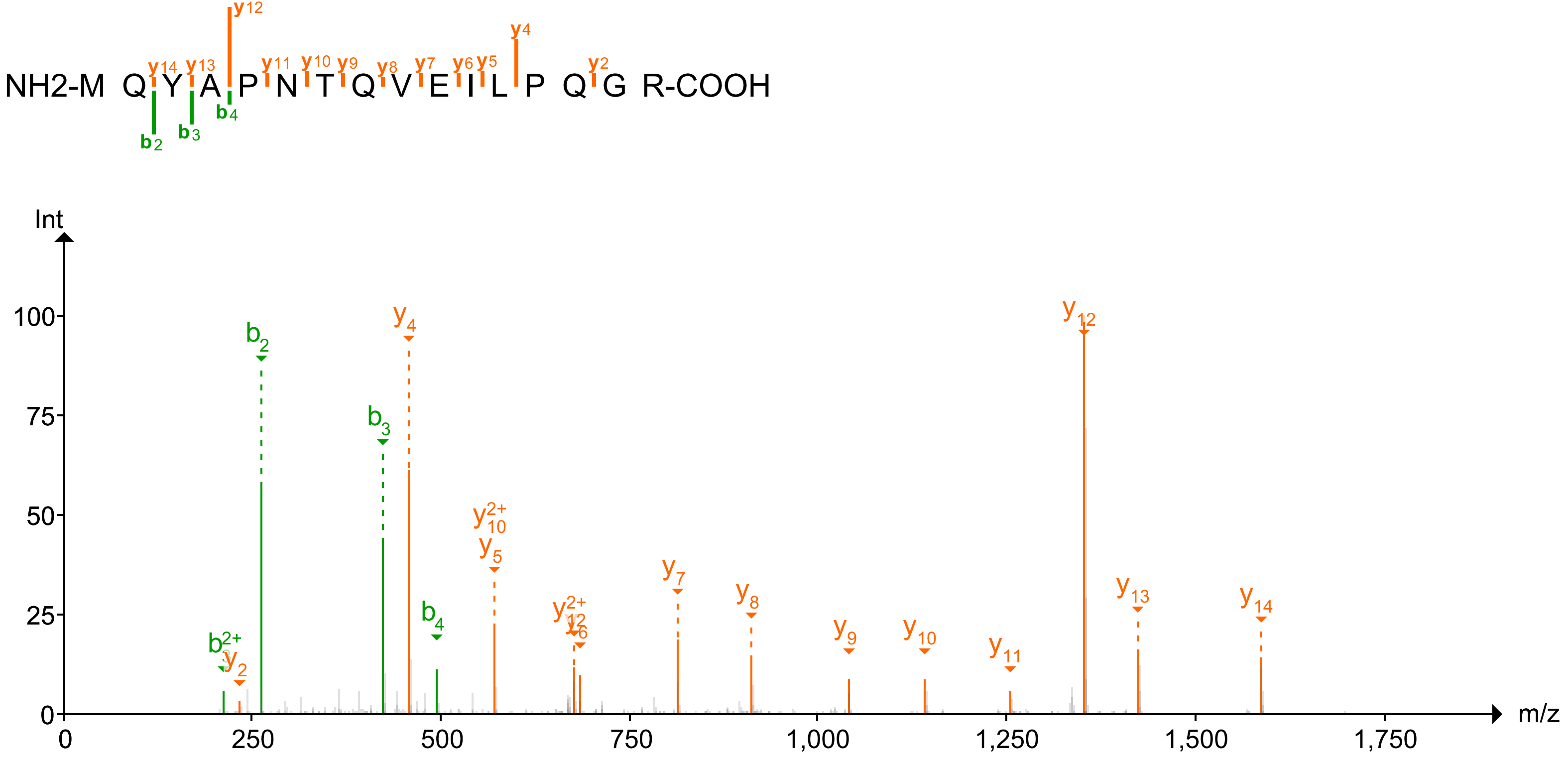

NH2-M Q Y A P N T Q V E I L P Q G R-COOH
y14 y13 y12 y11 y10 y9 y8 y7 y6 y5 y4 y2
b2 b3 b4
Int
100
75
50
25
0
0
250
500
750
1,000
1,250
1,500
1,750
m/z

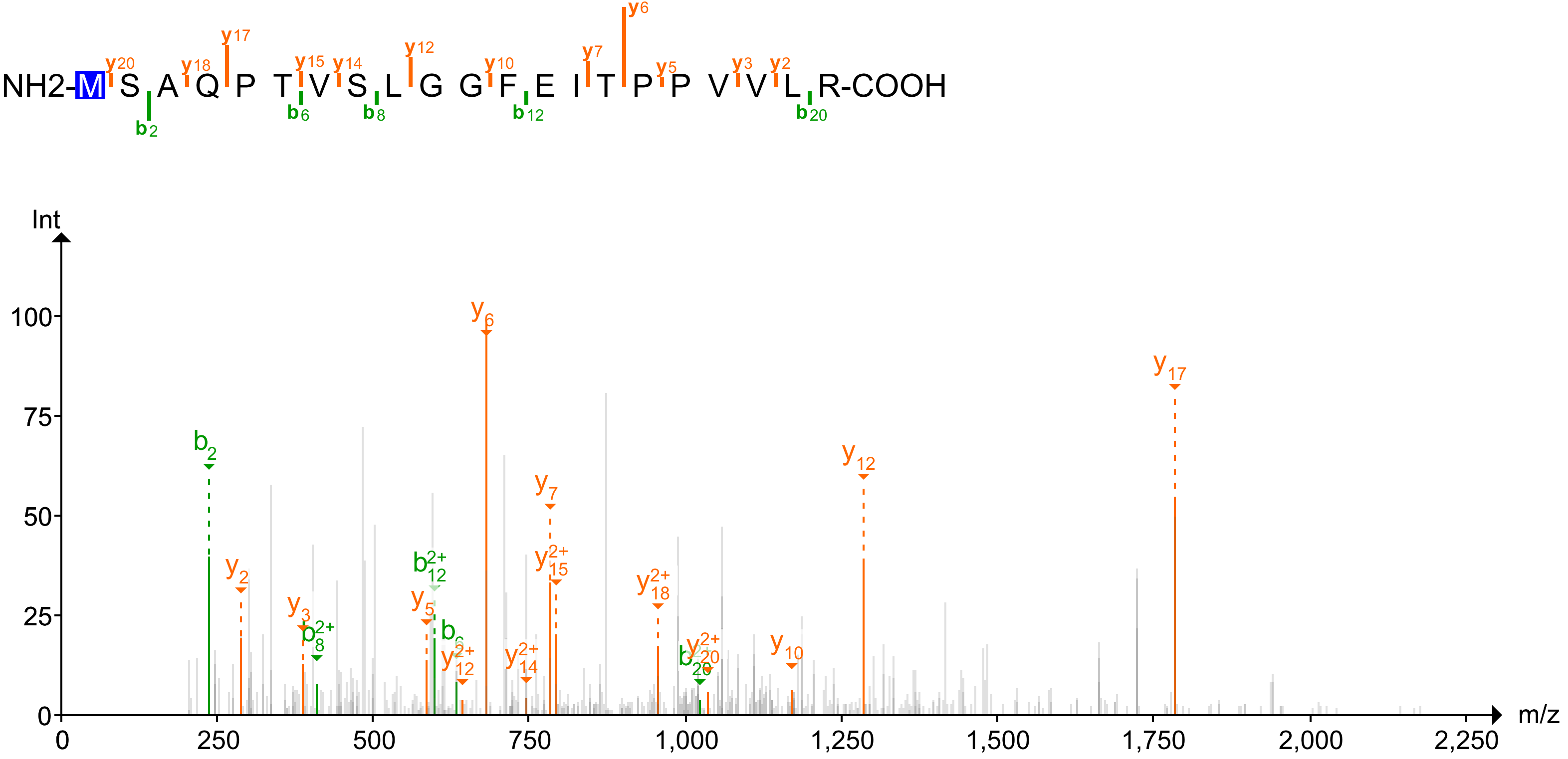

NH2-M S A Q P T V S L G G F E I T P P V V L R-COOH
y20 y18 y17 y15 y14 y12 y10 y7 y6 y5 y3 y2
b2 b6 b8 b12 b20
Int
m/z
0
25
50
75
100
0
250
500
750
1,000
1,250
1,500
1,750
2,000
2,250
b2
y2
y3
b8 2+
b12 2+
y5
b6
y12 2+
y6
y14 2+
y7
y15 2+
y18 2+
b20
y20 2+
y10
y12
y17

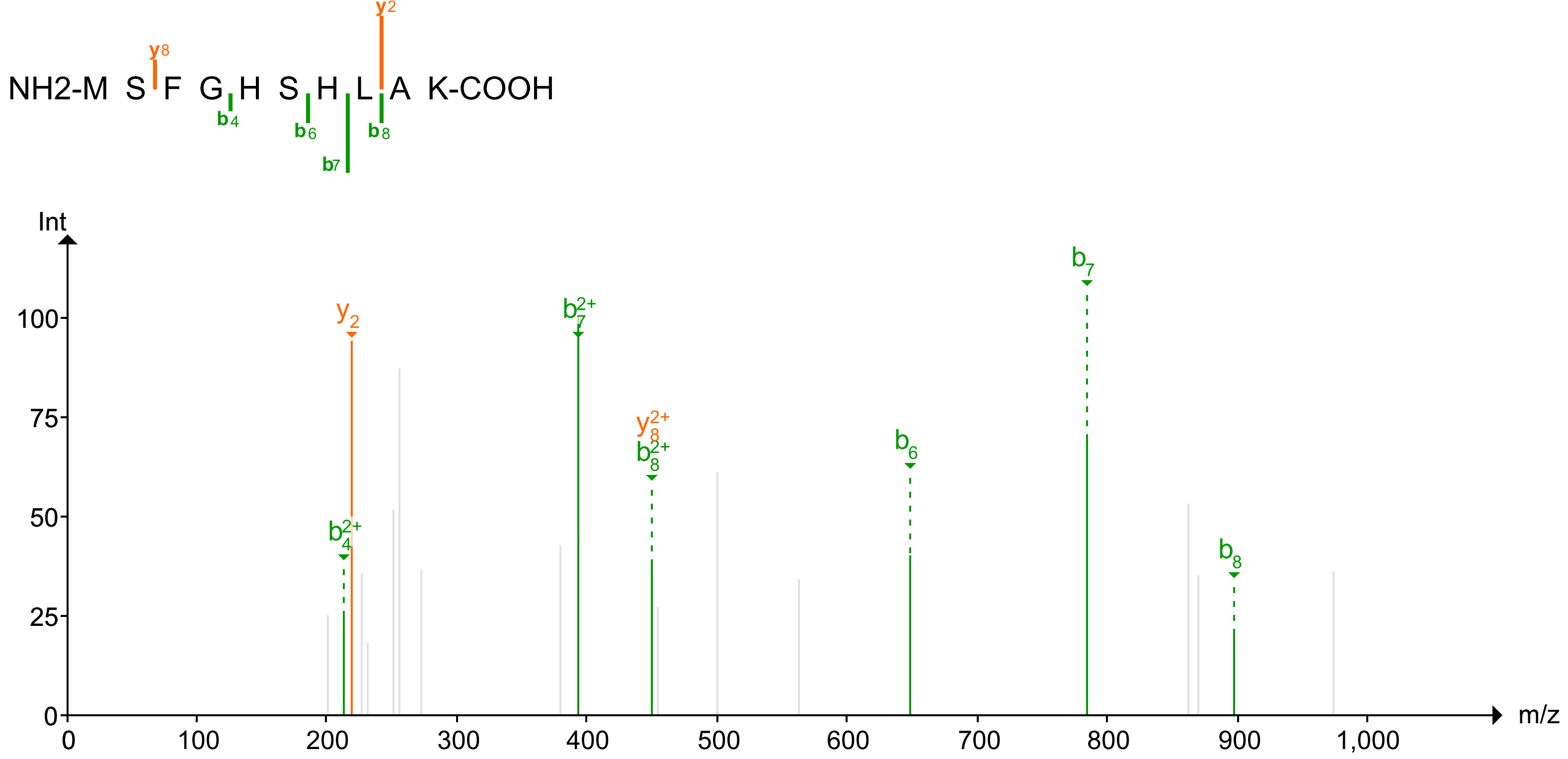

NH2-M S F G H S H L A K-COOH
y8
y2
b4
b6
b7
b8
Int
100
75
50
25
0
y2
b7 2+
y8 2+
b8 2+
b6
b7
b4 2+
b8
0
100
200
300
400
500
600
700
800
900
1,000
m/z

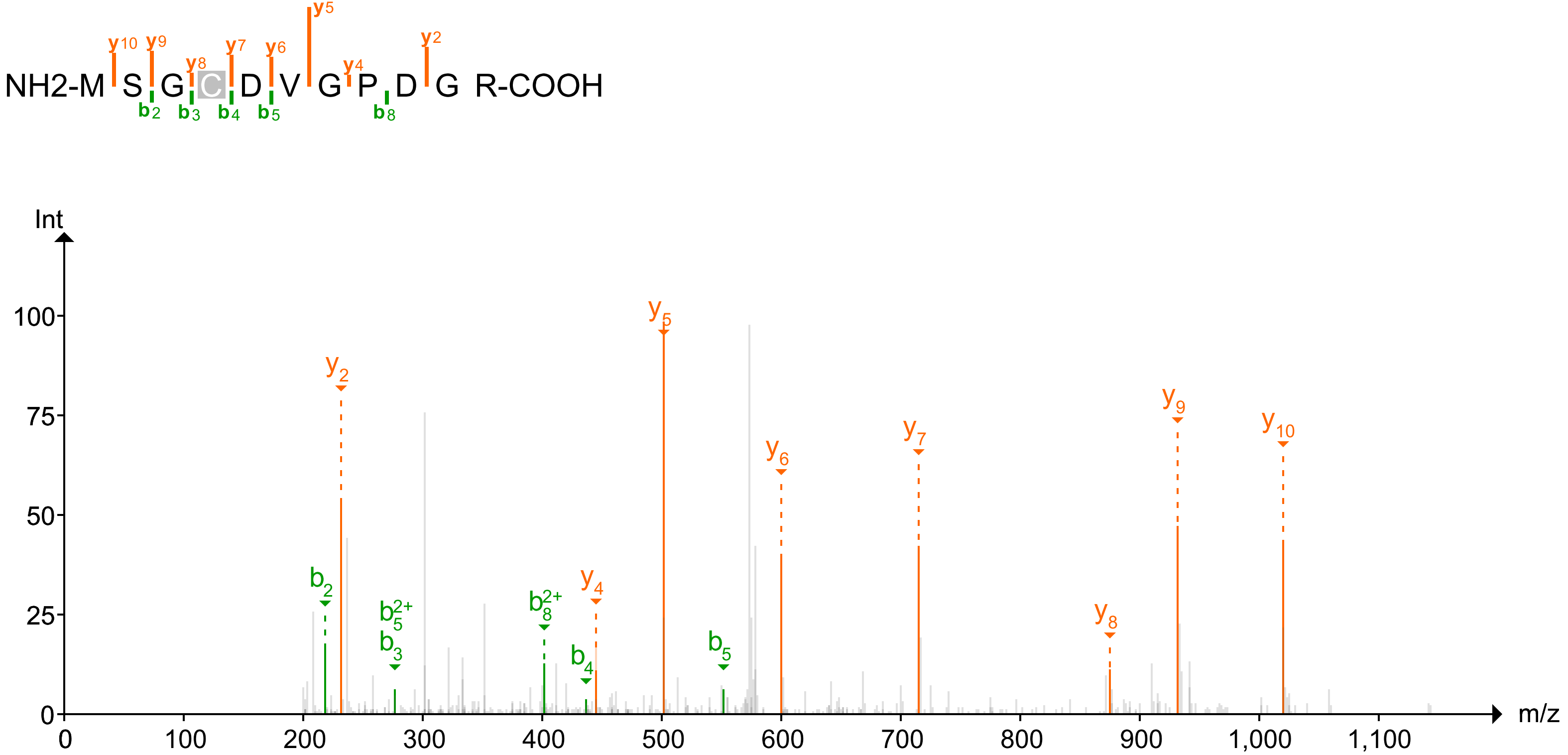
NH2-M S G C D V G P D G R-COOH
y10 y9 y8 y7 y6 y5 y4 y2
b2 b3 b4 b5 b8
Int
100
75
50
25
0
y5
y2
y9
y10
y7
y6
b2
b5 2+
b3
b8 2+
b4
y4
b5
y8
0 100 200 300 400 500 600 700 800 900 1,000 1,100
m/z

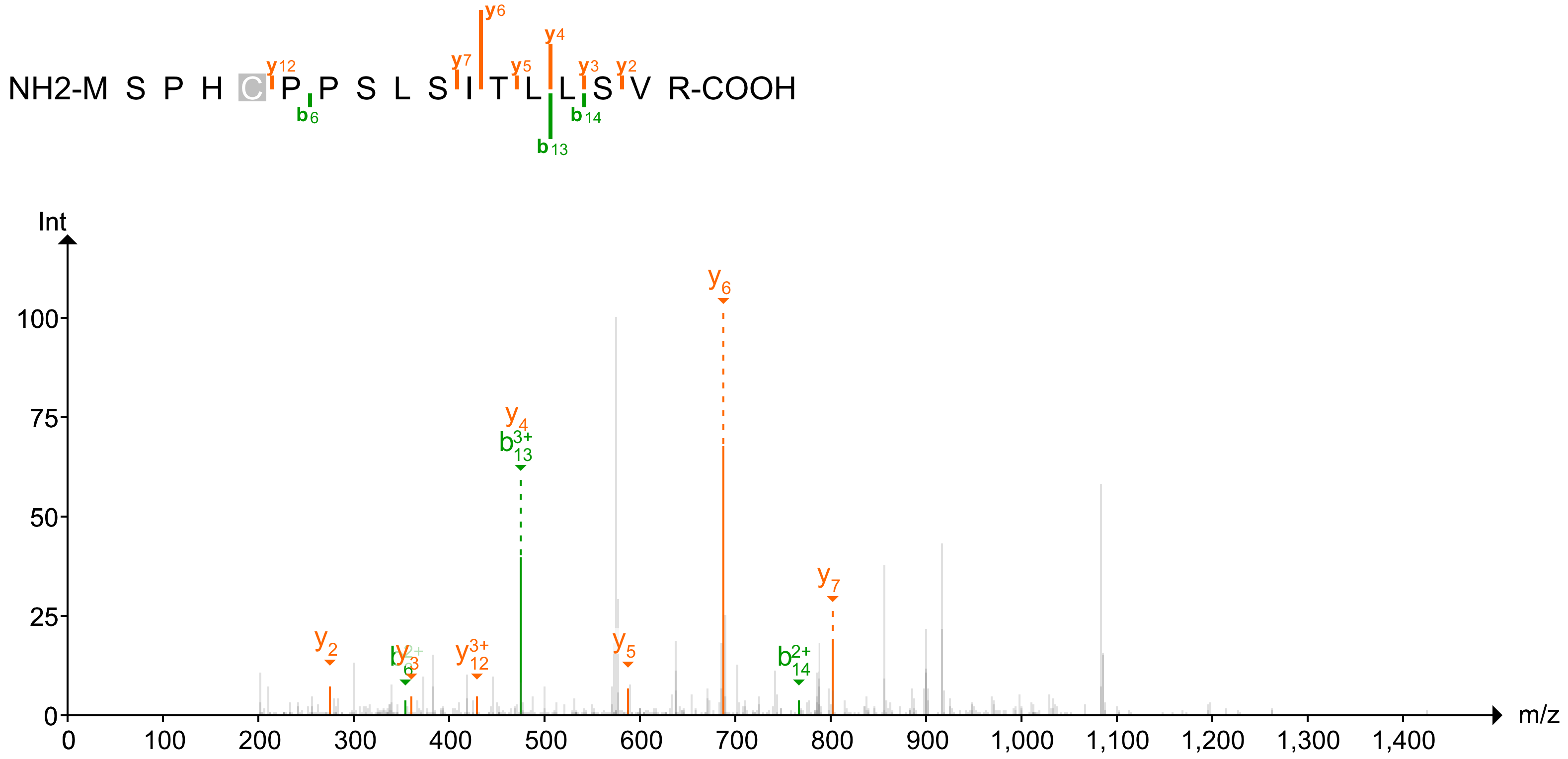

NH2-M S P H C P P S L S I T L L S V R-COOH
y12 y7 y6 y5 y4 y3 y2
b6 b13 b14
Int
100
75
50
25
0
y6
y4
b13 3+
y7
y2
b6 2+
y3
y12 3+
y5
b14 2+
0 100 200 300 400 500 600 700 800 900 1,000 1,100 1,200 1,300 1,400
m/z

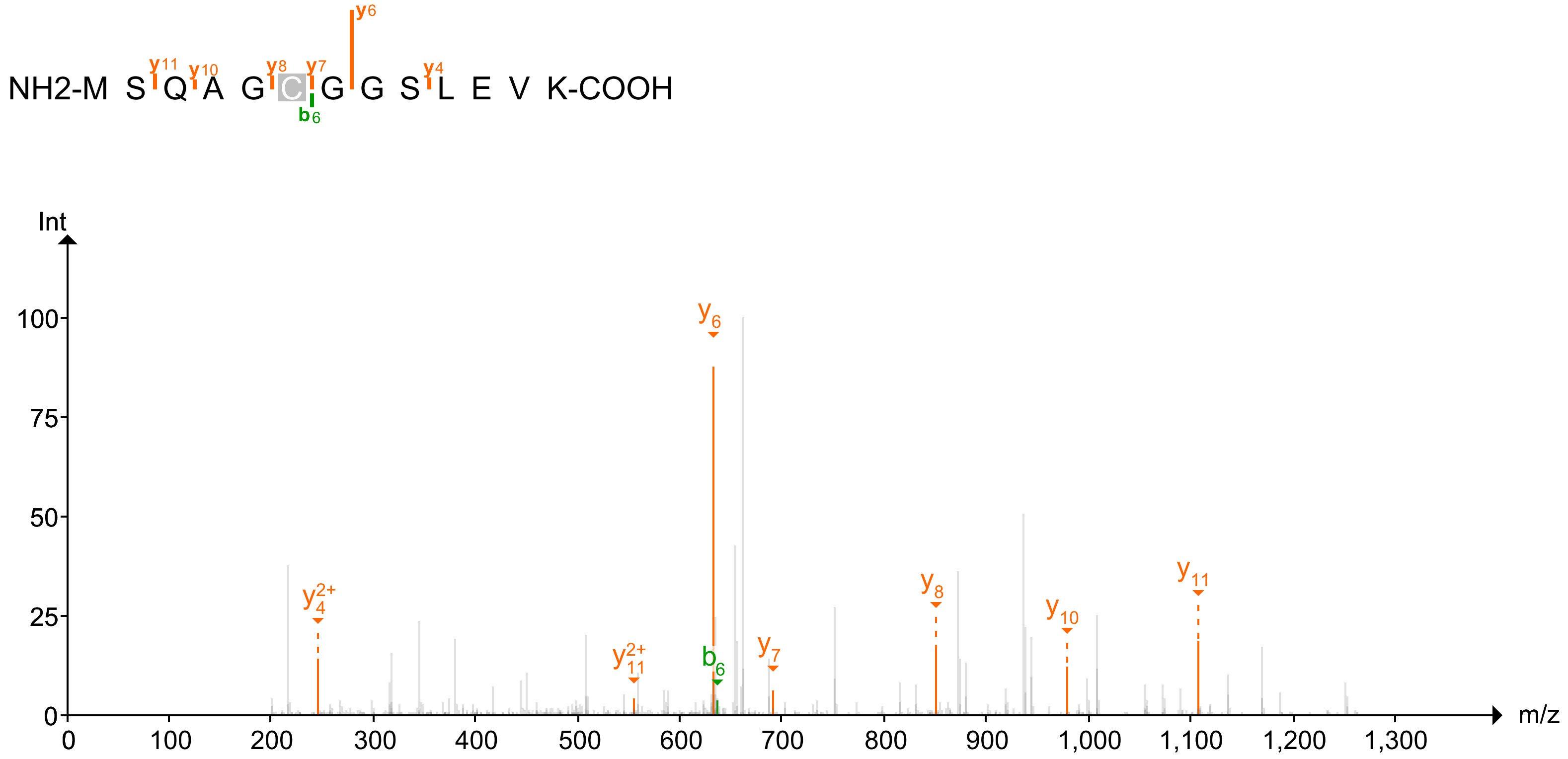

NH2-M S Q A G C G G S L E V K-COOH
y11 y10 y8 y7 y6 y4
b6
Int
100
75
50
25
0
y6
y4 2+
y11 2+
b6
y7
y8
y10
y11
0 100 200 300 400 500 600 700 800 900 1,000 1,100 1,200 1,300
m/z

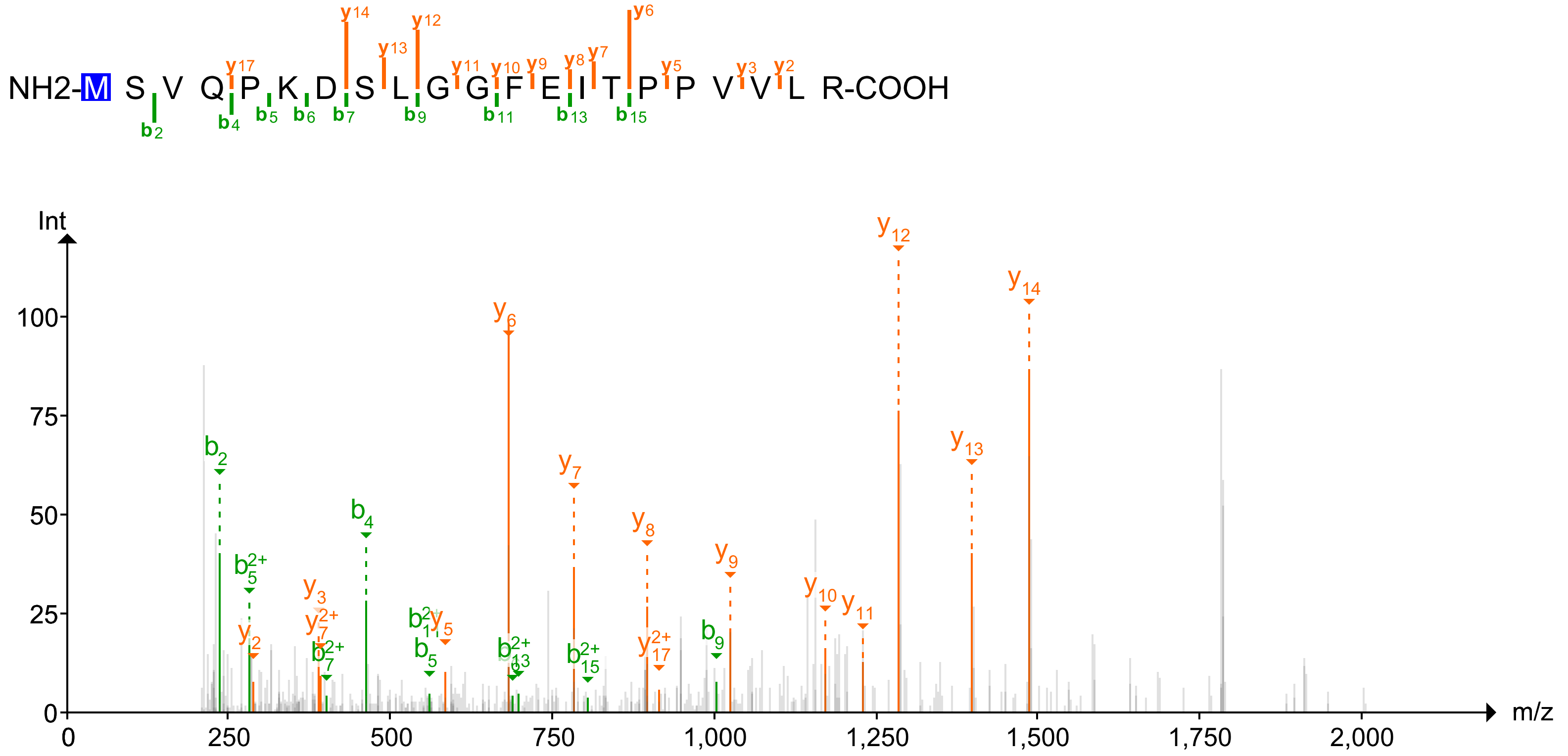
NH2-M S V Q P K D S L G G F E I T P P V V L R-COOH
Int
m/z
0
25
50
75
100
250
500
750
1,000
1,250
1,500
1,750
2,000

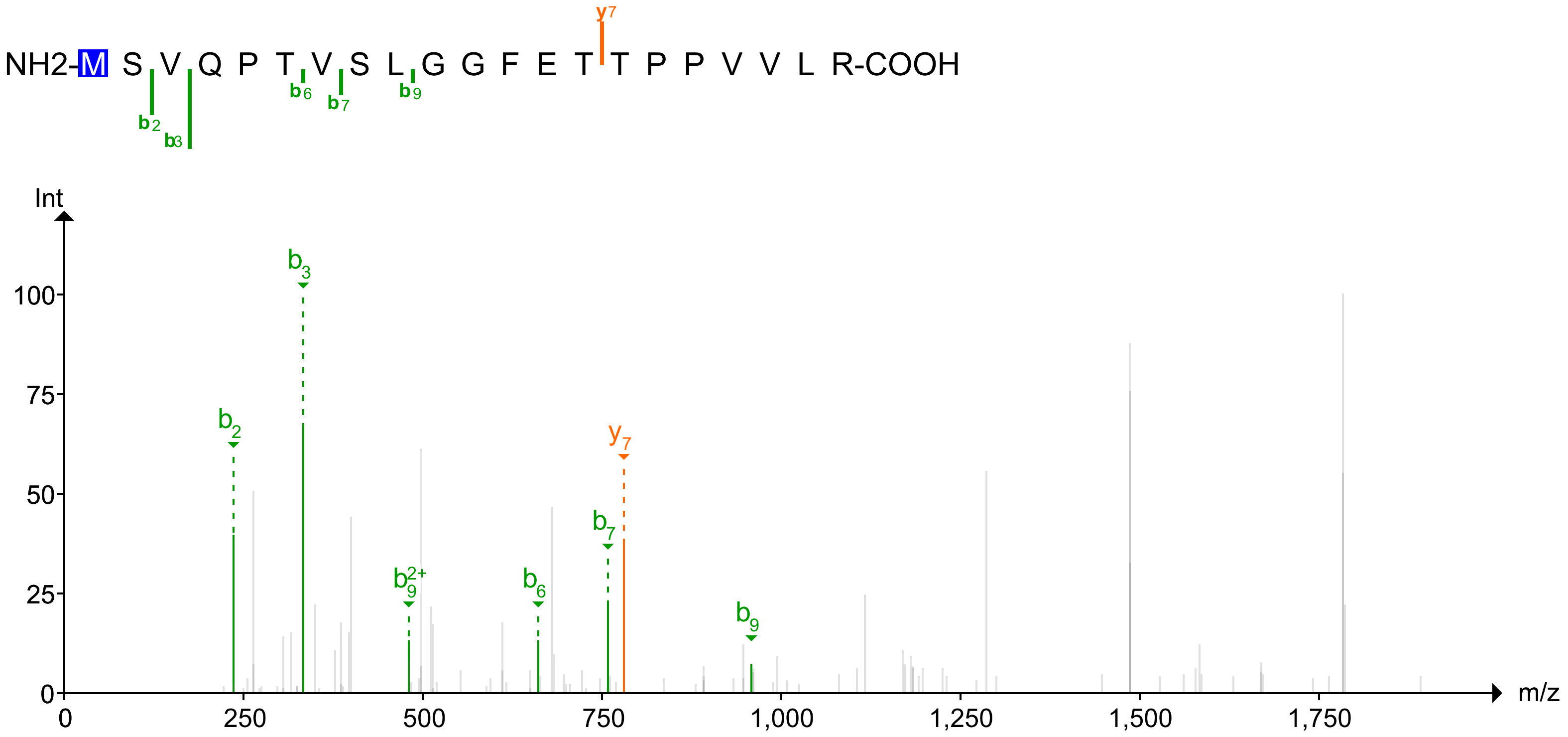

NH2-M S V Q P T V S L G G F E T T P P V V L R-COOH
y7
b2
b3
b6
b7
b9
Int
100
75
50
25
0
b3
b2
y7
b7
b9 2+
b6
b9
0
250
500
750
1,000
1,250
1,500
1,750
m/z

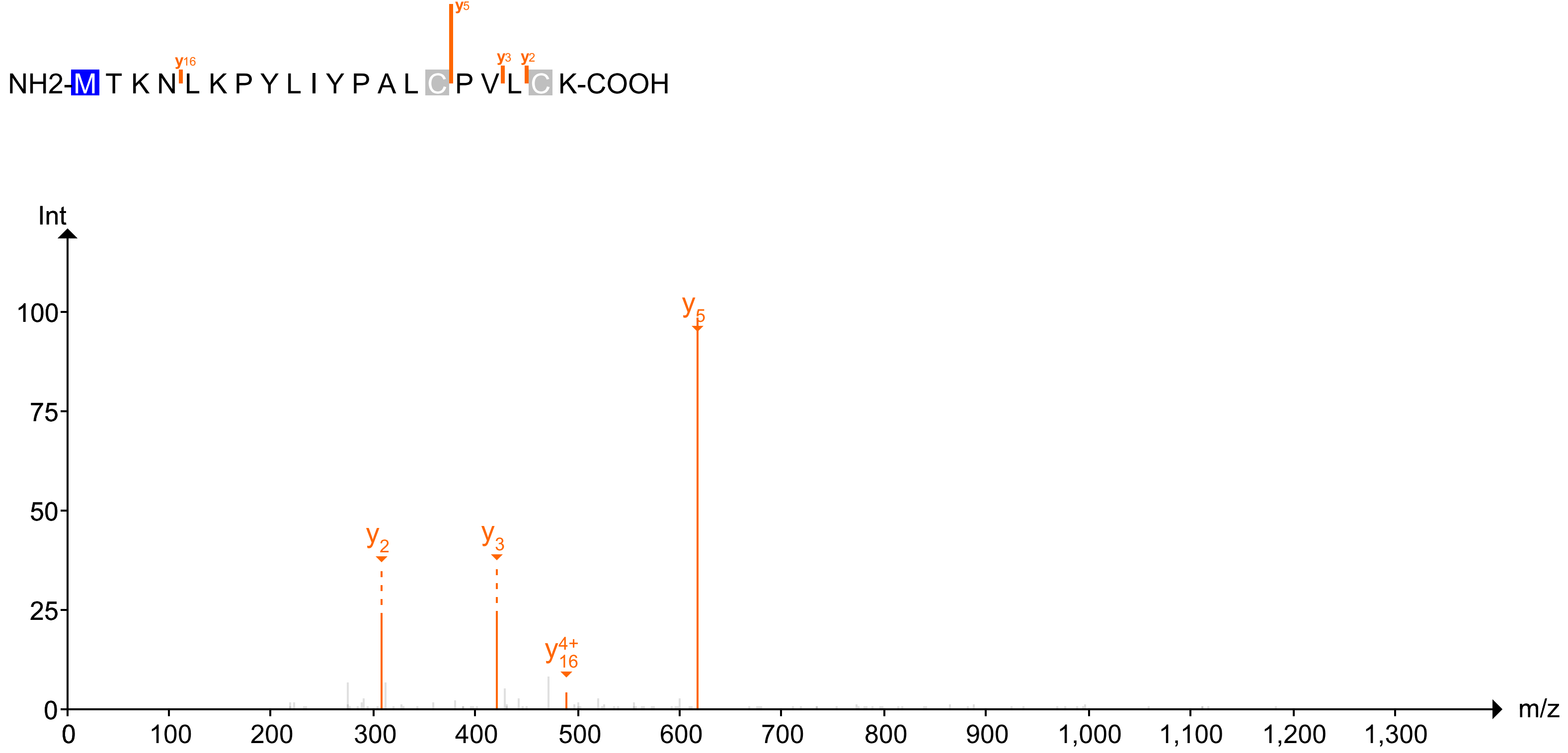
NH2-M T K N L K P Y L I Y P A L C P V L C K-COOH
y16
y5
y3
y2
Int
100
75
50
25
0
y5
y2
y3
y16 4+
m/z
0
100
200
300
400
500
600
700
800
900
1,000
1,100
1,200
1,300

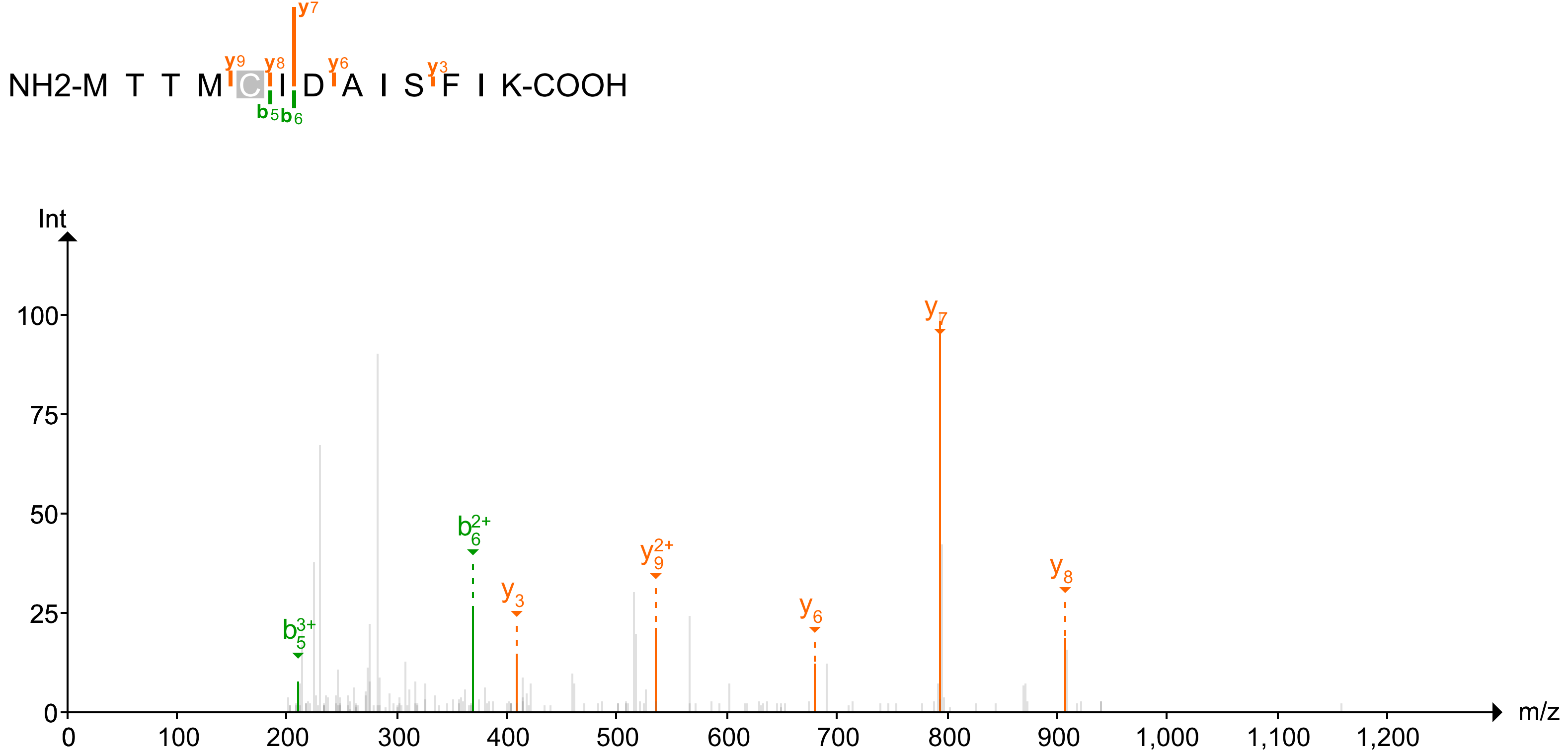

NH2-M T T M C I D A I S F I K-COOH
y9 y8 y7 y6 y3
b5 b6
Int
100
75
50
25
0
m/z
0 100 200 300 400 500 600 700 800 900 1,000 1,100 1,200
b5 3+
b6 2+
y3
y9 2+
y6
y7
y8

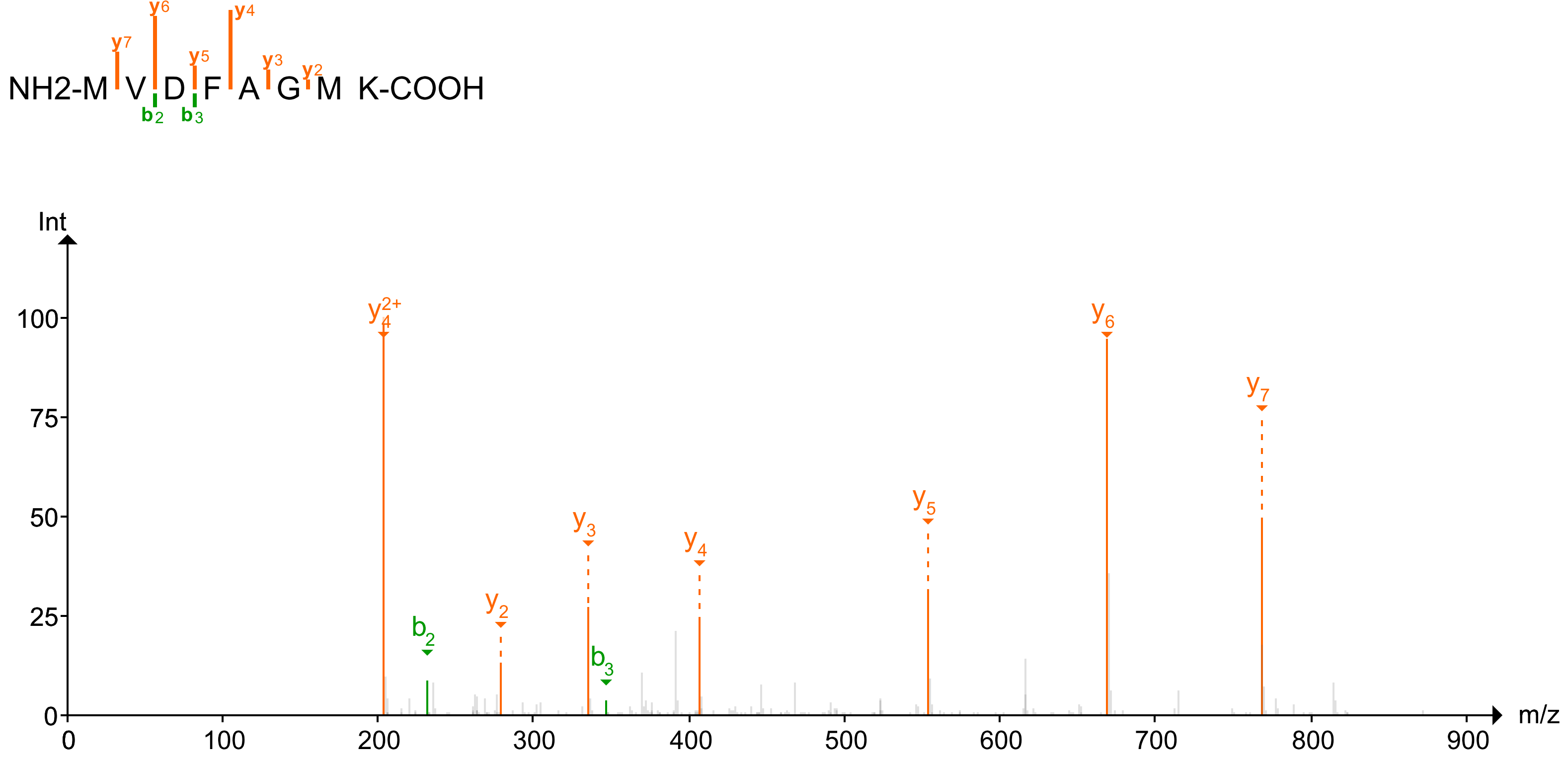

NH2-M V D F A G M K-COOH
y7
y6
y5
y4
y3
y2
b2
b3
Int
m/z
0
25
50
75
100
0
100
200
300
400
500
600
700
800
900
y4 2+
b2
y2
y3
b3
y4
y5
y6
y7

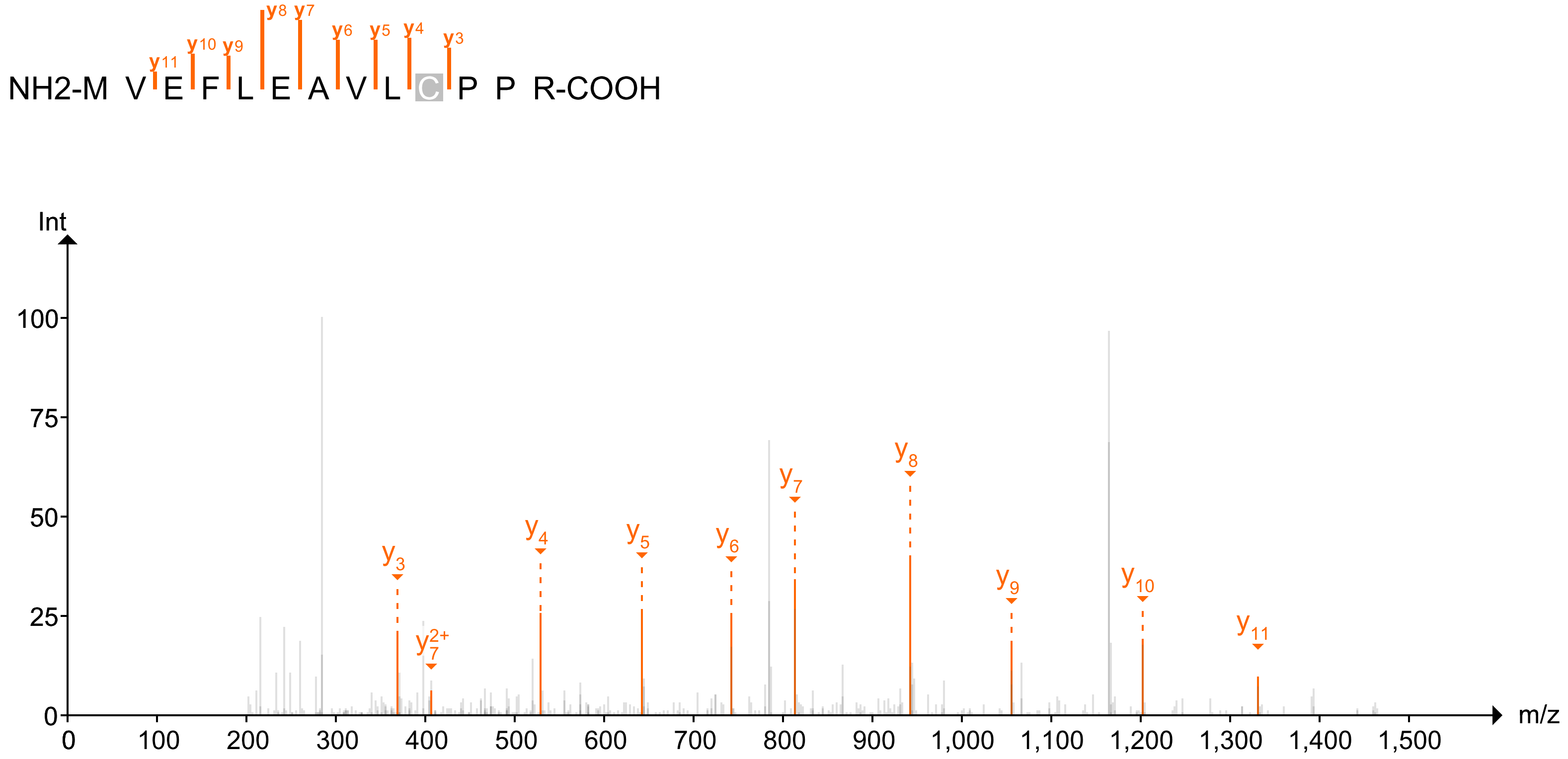

NH2-M V E F L E A V L C P P R-COOH
y11 y10 y9 y8 y7 y6 y5 y4 y3
Int
100
75
50
25
0
y3
y7 2+
y4
y5
y6
y7
y8
y9
y10
y11
0 100 200 300 400 500 600 700 800 900 1,000 1,100 1,200 1,300 1,400 1,500
m/z

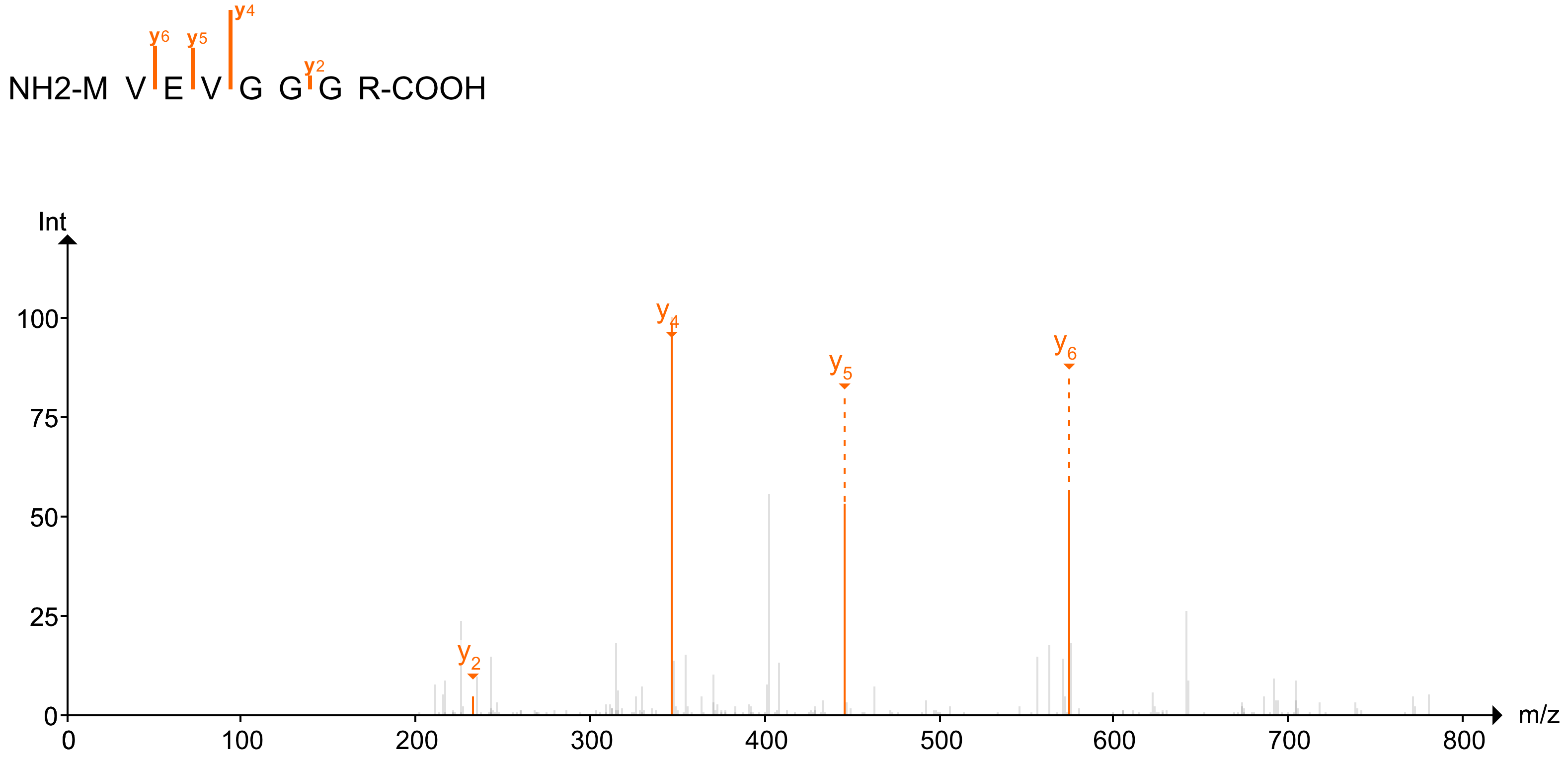

NH2-M V E V G G G R-COOH
y6
y5
y4
y2
Int
100
75
50
25
0
0
100
200
300
400
500
600
700
800
m/z

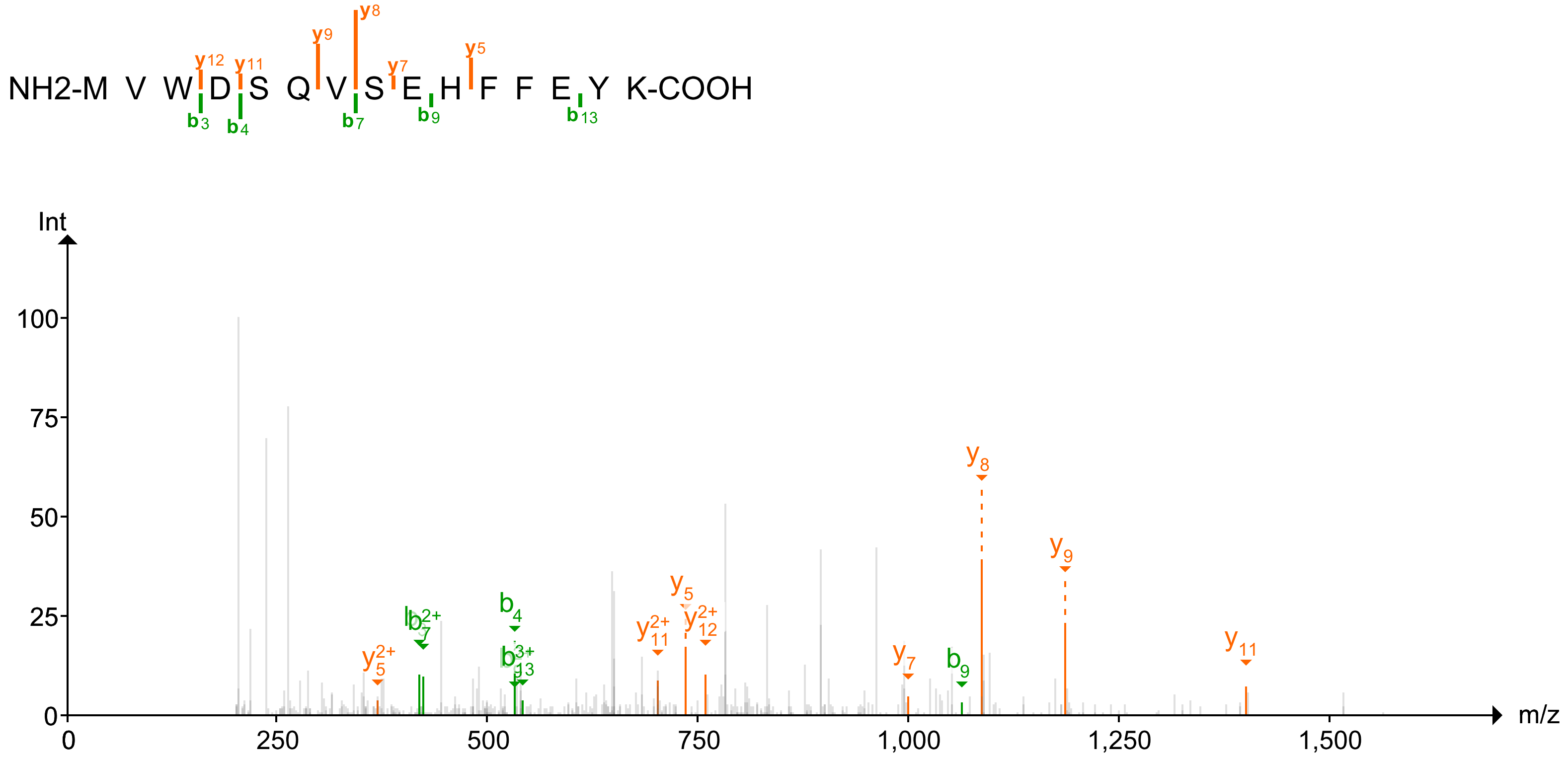

NH2-M V W D S Q V S E H F F E Y K-COOH
y12 y11 y9 y8 y7 y5
b3 b4 b7 b9 b13
Int
100
75
50
25
0
250
500
750
1,000
1,250
1,500
m/z
y8
y9
y5
y11
y7
b9
b4

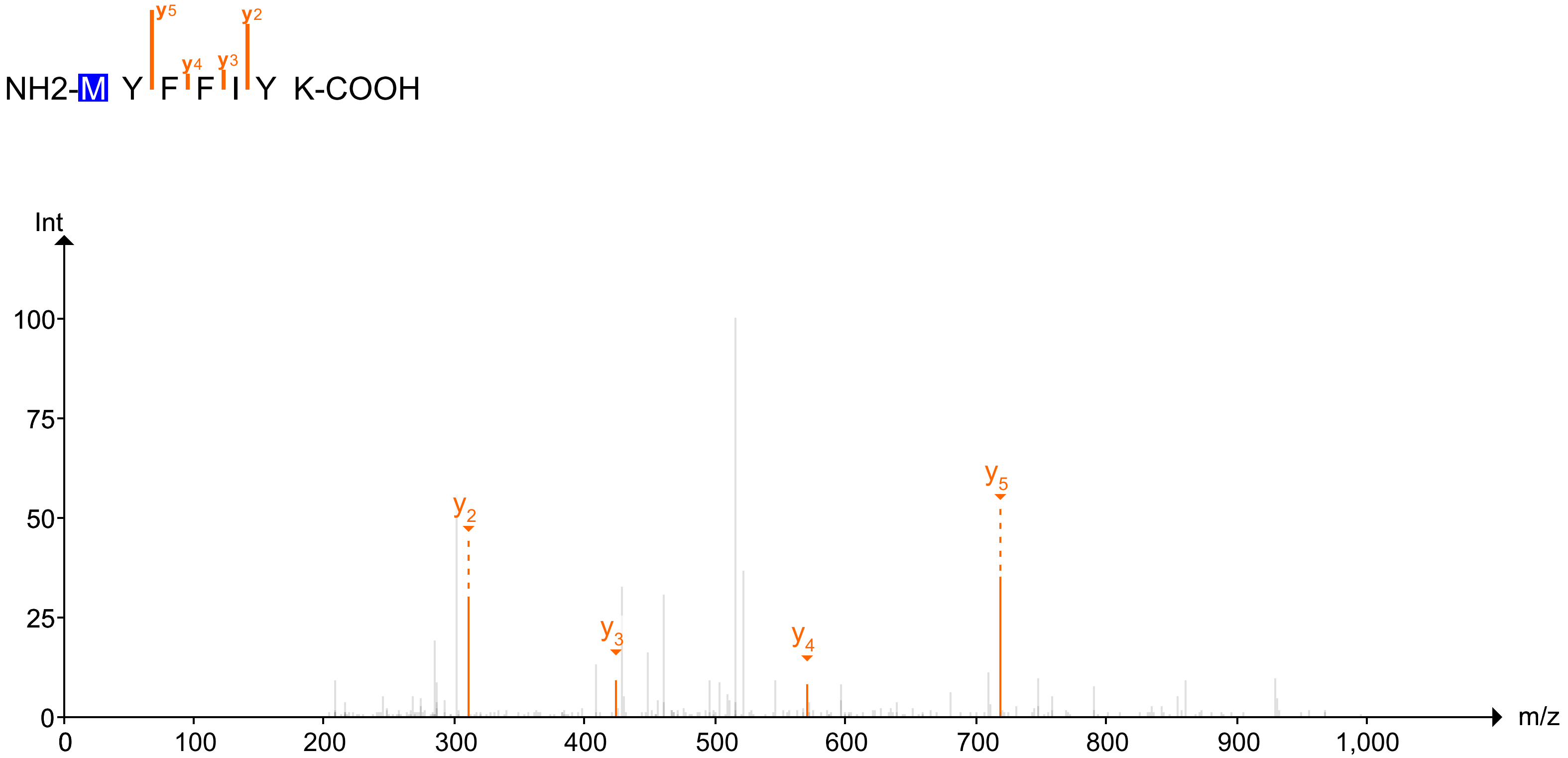

NH2-M Y F F I Y K-COOH
y5
y4
y3
y2
Int
100
75
50
25
0
m/z
0
100
200
300
400
500
600
700
800
900
1,000

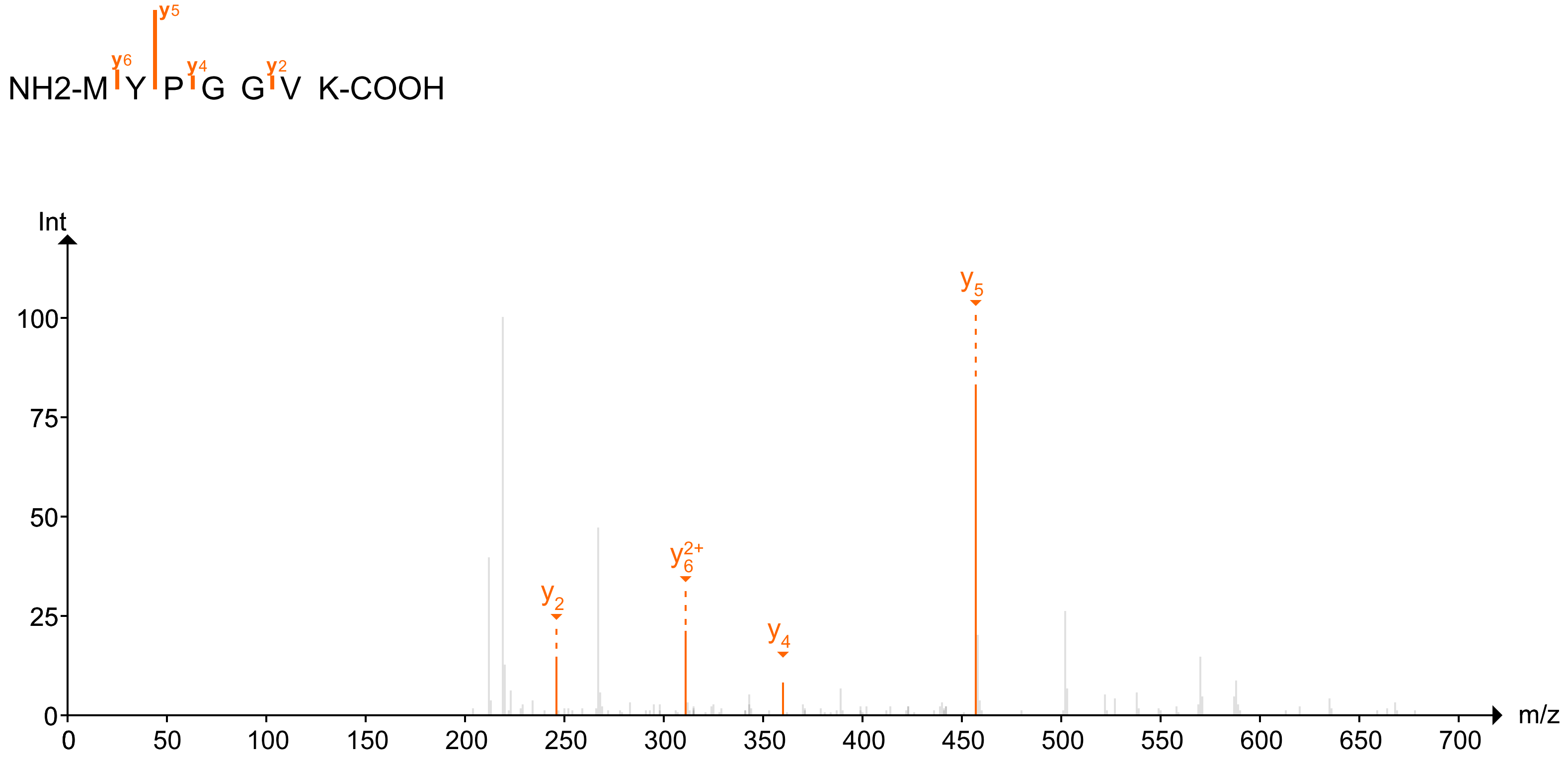
NH2-M Y P G G V K-COOH
y6
y5
y4
y2
Int
100
75
50
25
0
0
50
100
150
200
250
300
350
400
450
500
550
600
650
700
m/z
y2
y6 2+
y4
y5

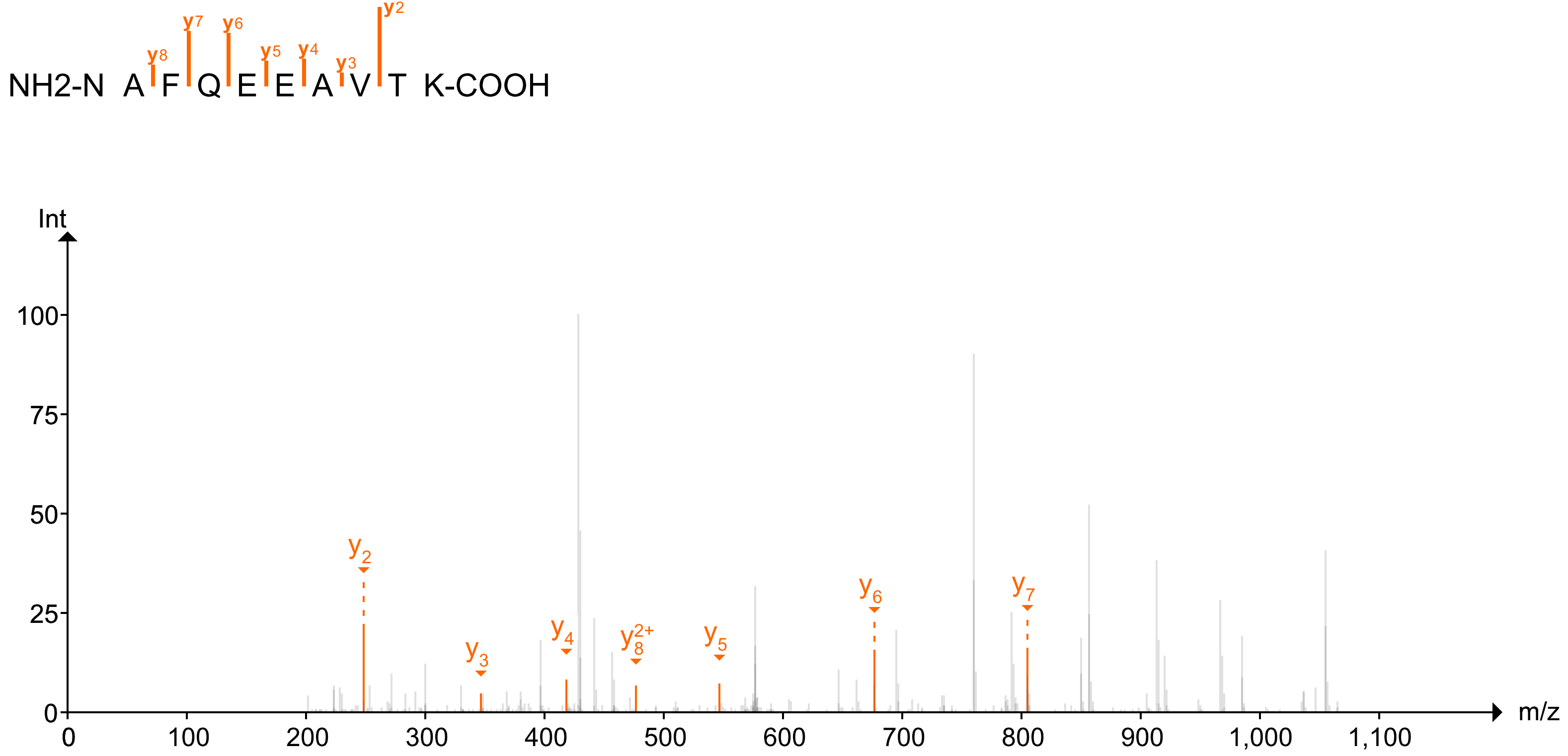

NH2-N A F Q E E A V T K-COOH
y8
y7
y6
y5
y4
y3
y2
Int
100
75
50
25
0
y2
y3
y4
y8 2+
y5
y6
y7
0
100
200
300
400
500
600
700
800
900
1,000
1,100
m/z

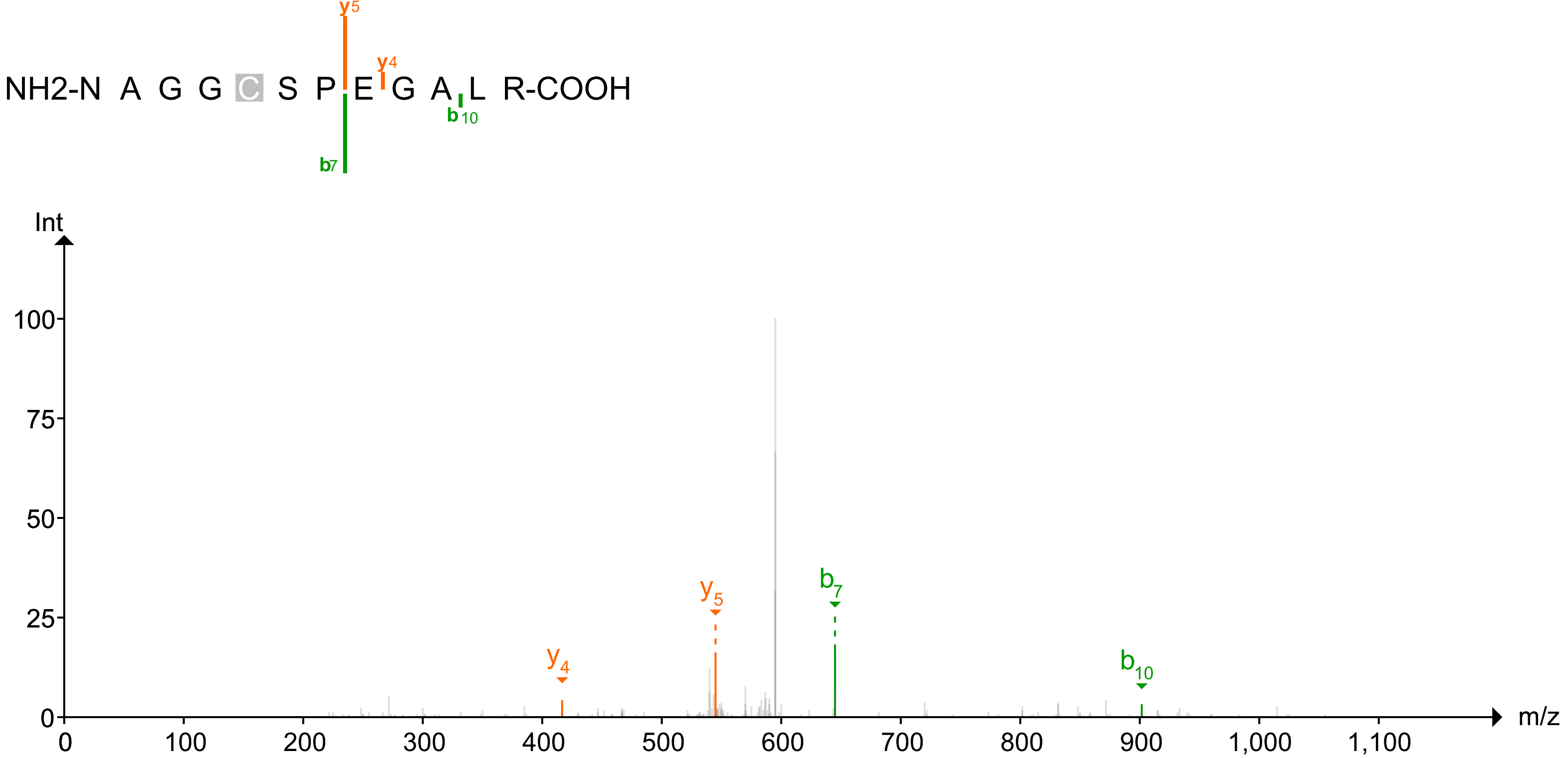

NH2-N A G G C S P E G A L R-COOH
y5
y4
b7
b10
Int
100
75
50
25
0
m/z
0
100
200
300
400
500
600
700
800
900
1,000
1,100
y4
y5
b7
b10

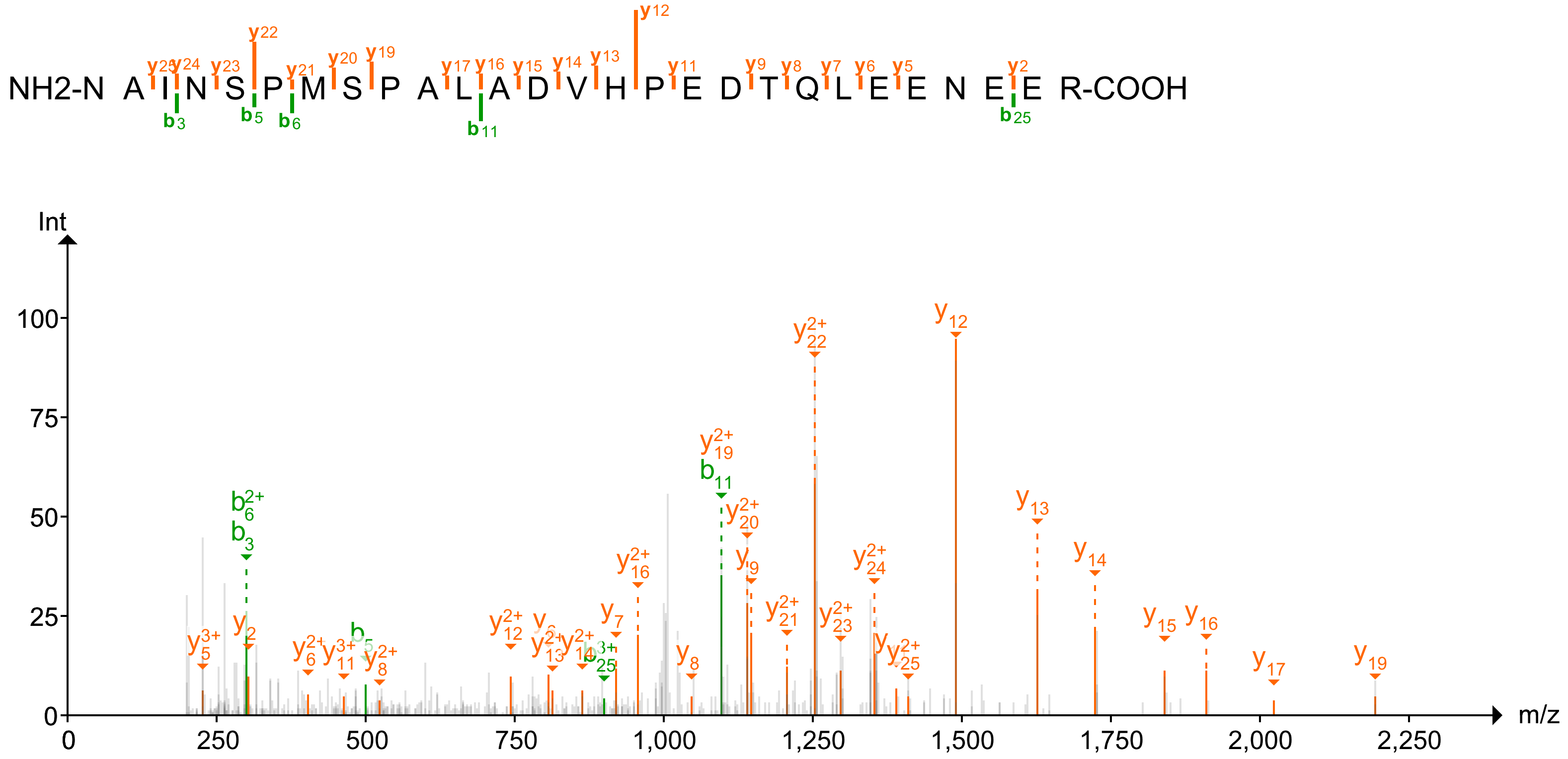

NH2-N A I N S P M S P A L A D V H P E D T Q L E E N E E R-COOH
Int
m/z
0
25
50
75
100
250
500
750
1,000
1,250
1,500
1,750
2,000
2,250

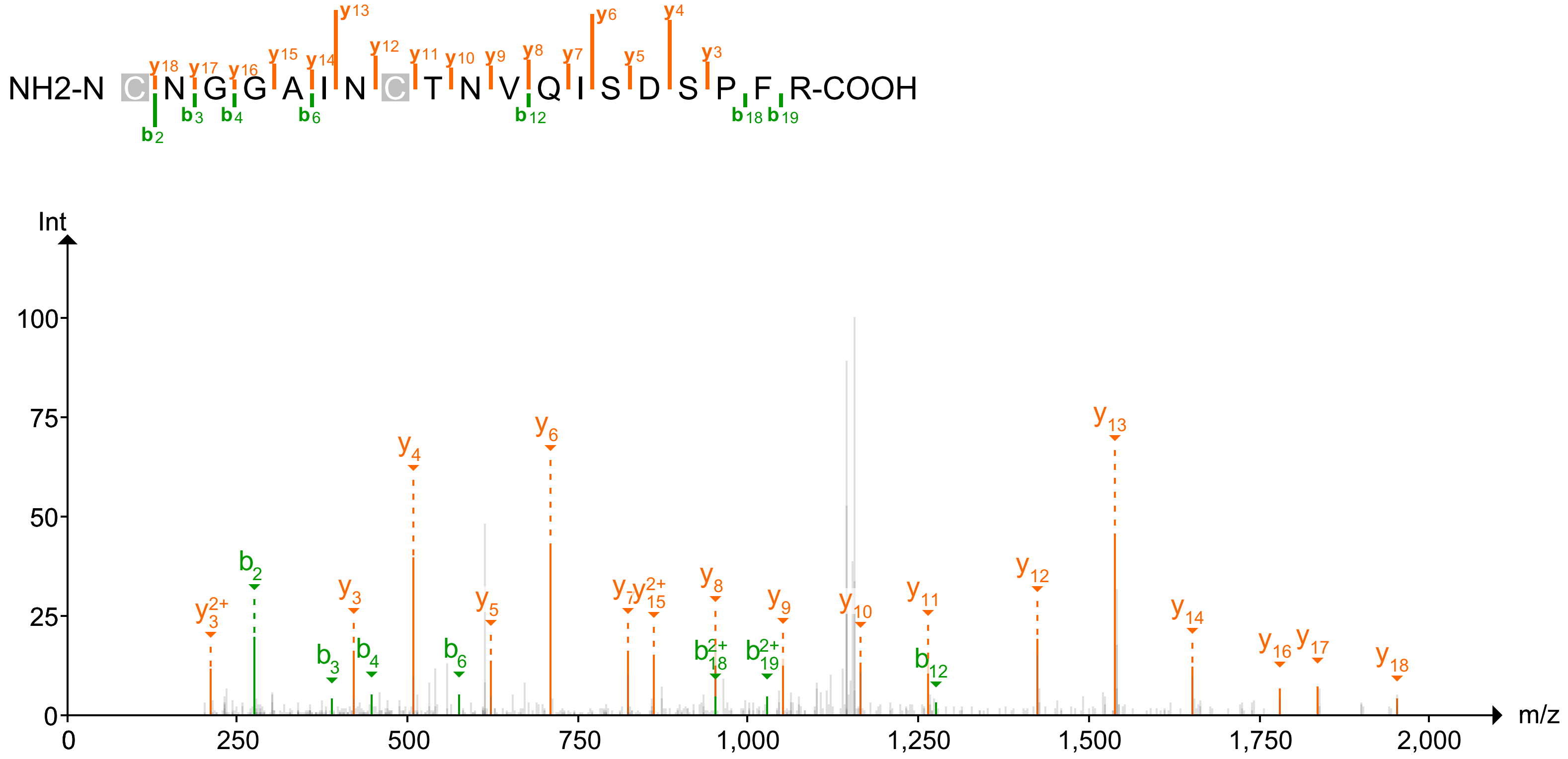

NH2-N C N G G A I N C T N V Q I S D S P F R-COOH
y18 y17 y16 y15 y14 y13 y12 y11 y10 y9 y8 y7 y6 y5 y4 y3
b2 b3 b4 b6 b12 b18 b19
Int
100
75
50
25
0
0
250
500
750
1,000
1,250
1,500
1,750
2,000
m/z
y3 2+
b2
b3
y3
b4
y4
b6
y5
y6
y7
y15 2+
y8
b18 2+
y9
b19 2+
y10
y11
b12
y12
y13
y14
y16
y17
y18

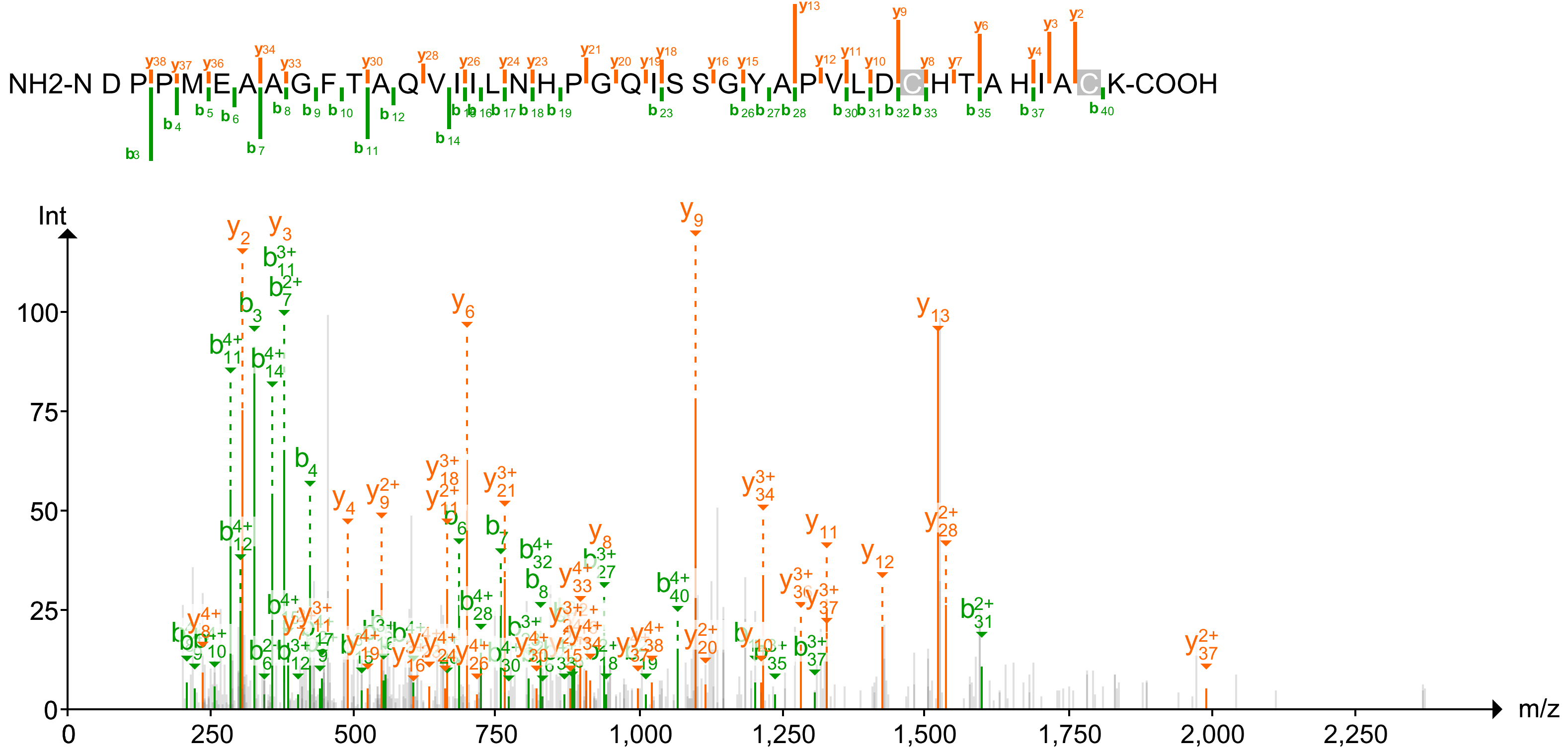

NH2-N D P P M E A A G F T A Q V I I L N H P G Q I S S G Y A P V L D C H T A H I A C K-COOH
Int
100
75
50
25
0
m/z
0
250
500
750
1,000
1,250
1,500
1,750
2,000
2,250

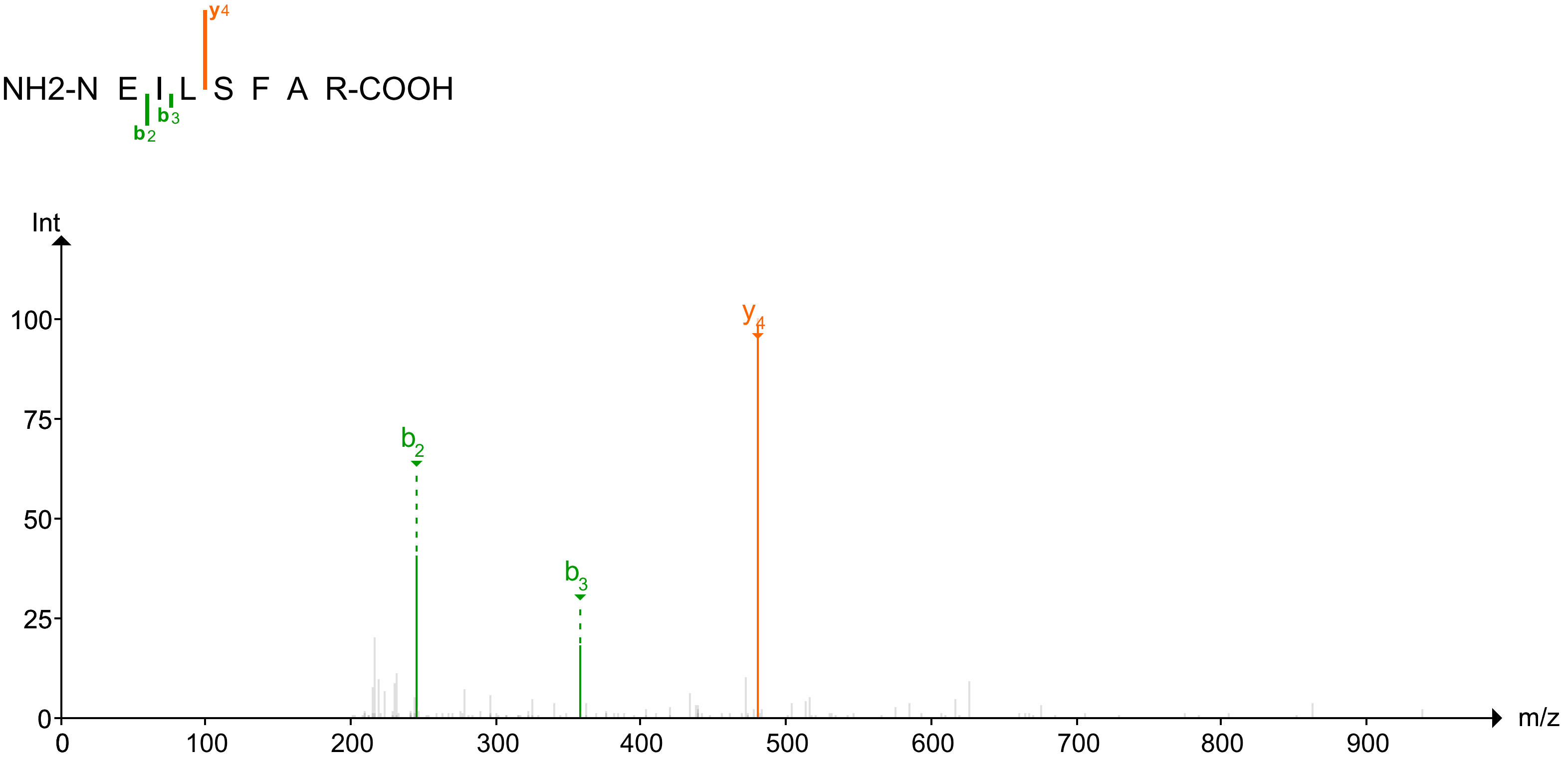

y4
NH2-N E I L S F A R-COOH
b2
b3
Int
100
75
50
25
0
b2
b3
y4
0
100
200
300
400
500
600
700
800
900
m/z

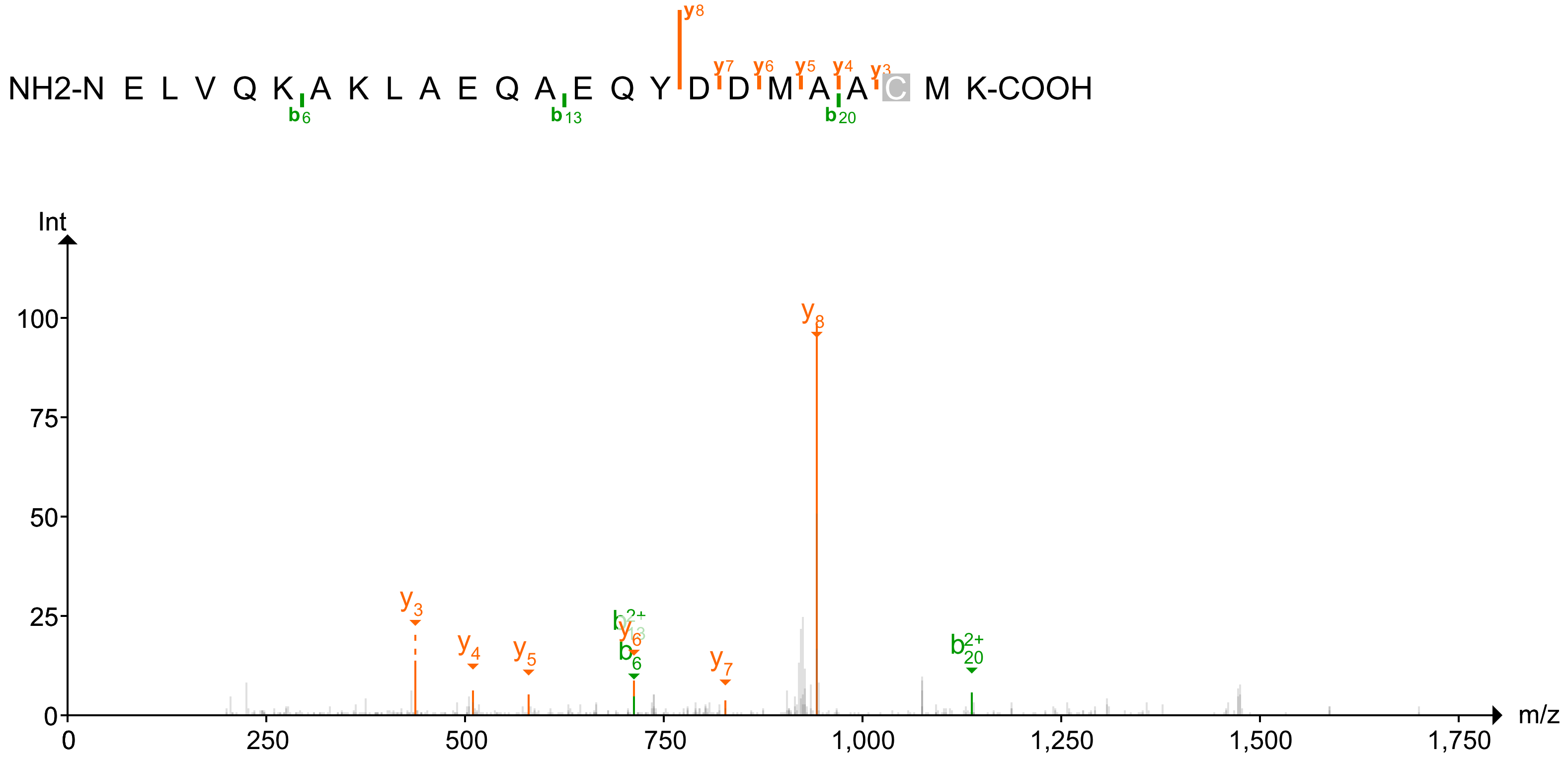

NH2-N E L V Q K A K L A E Q A E Q Y D D M A A C M K-COOH
y8
y7
y6
y5
y4
y3
b6
b13
b20
Int
100
75
50
25
0
m/z
0
250
500
750
1,000
1,250
1,500
1,750
y3
y4
y5
y6
b6
y7
y8
b2+ 20

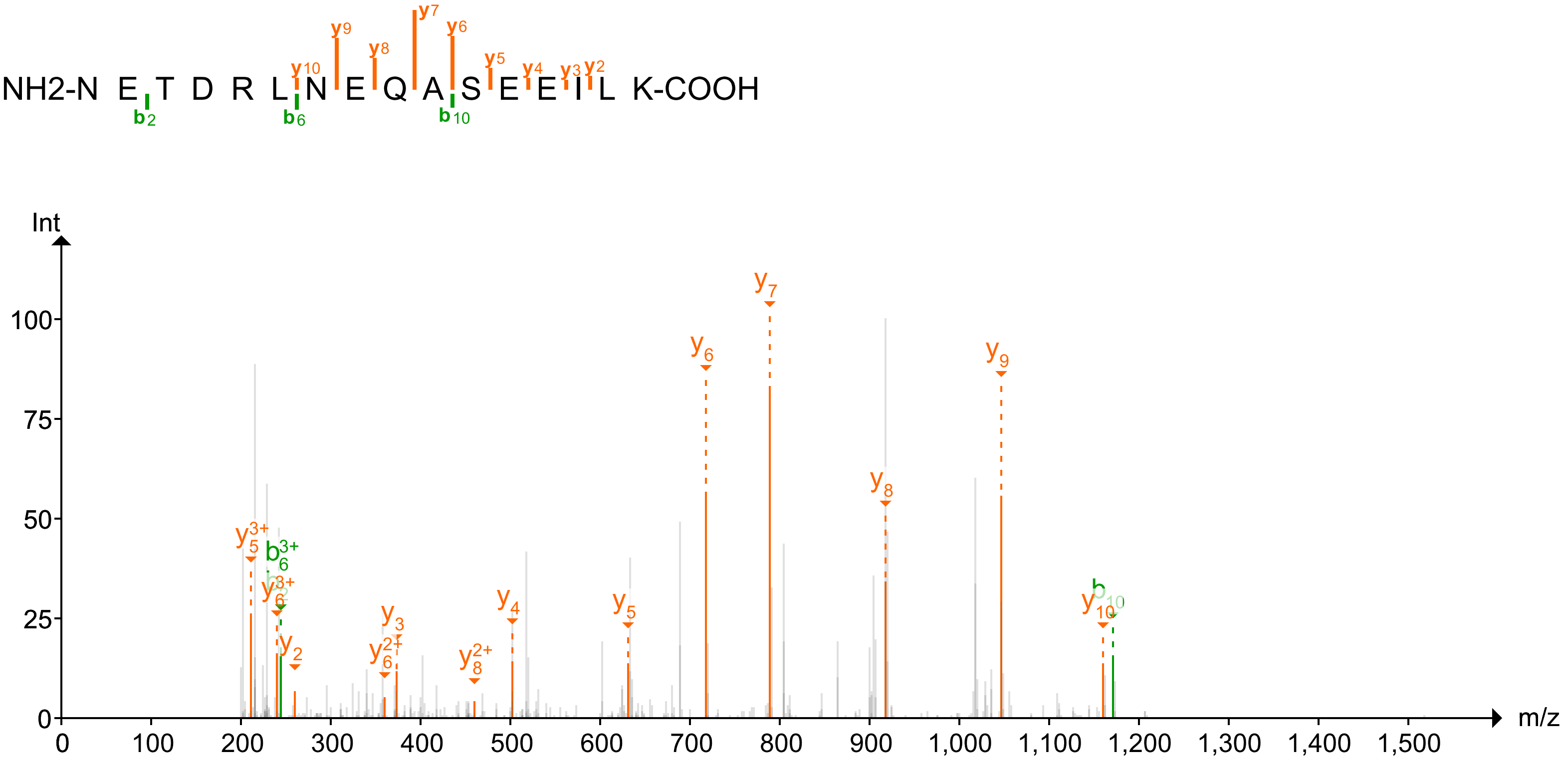

NH2-N E T D R L N E Q A S E E I L K-COOH
y10 y9 y8 y7 y6 y5 y4 y3 y2
b2 b6 b10
Int
100
75
50
25
0
m/z
0 100 200 300 400 500 600 700 800 900 1,000 1,100 1,200 1,300 1,400 1,500
y7
y6
y9
y8
y5 3+
b6 3+
y6 3+
y2
y3
y6 2+
y8 2+
y4
y5
y10
b10

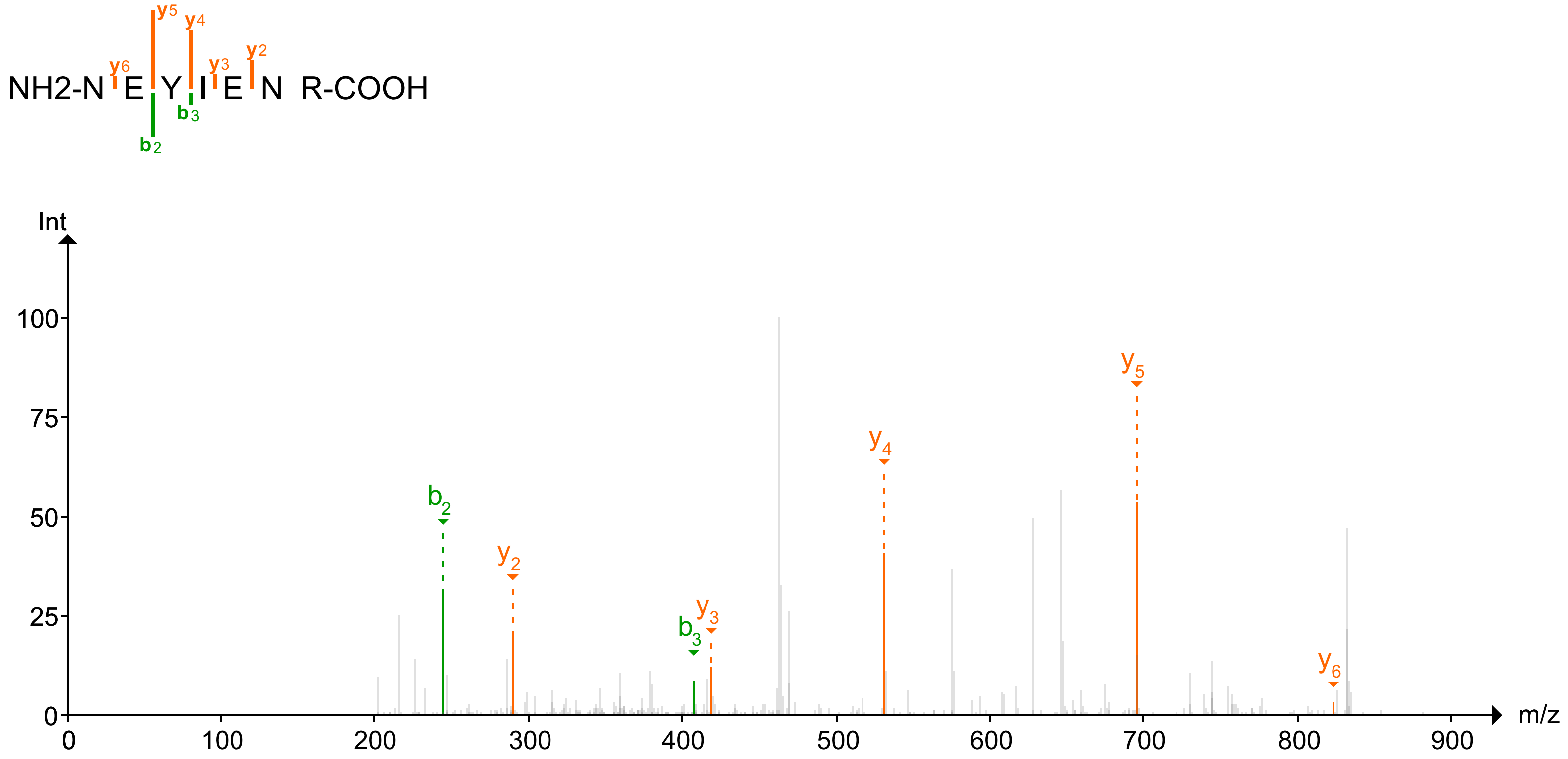

NH2-N E Y I E N R-COOH
y5 y4 y6 y3 y2
b2 b3
Int
m/z
0
25
50
75
100
0 100 200 300 400 500 600 700 800 900
b2
y2
b3
y3
y4
y5
y6

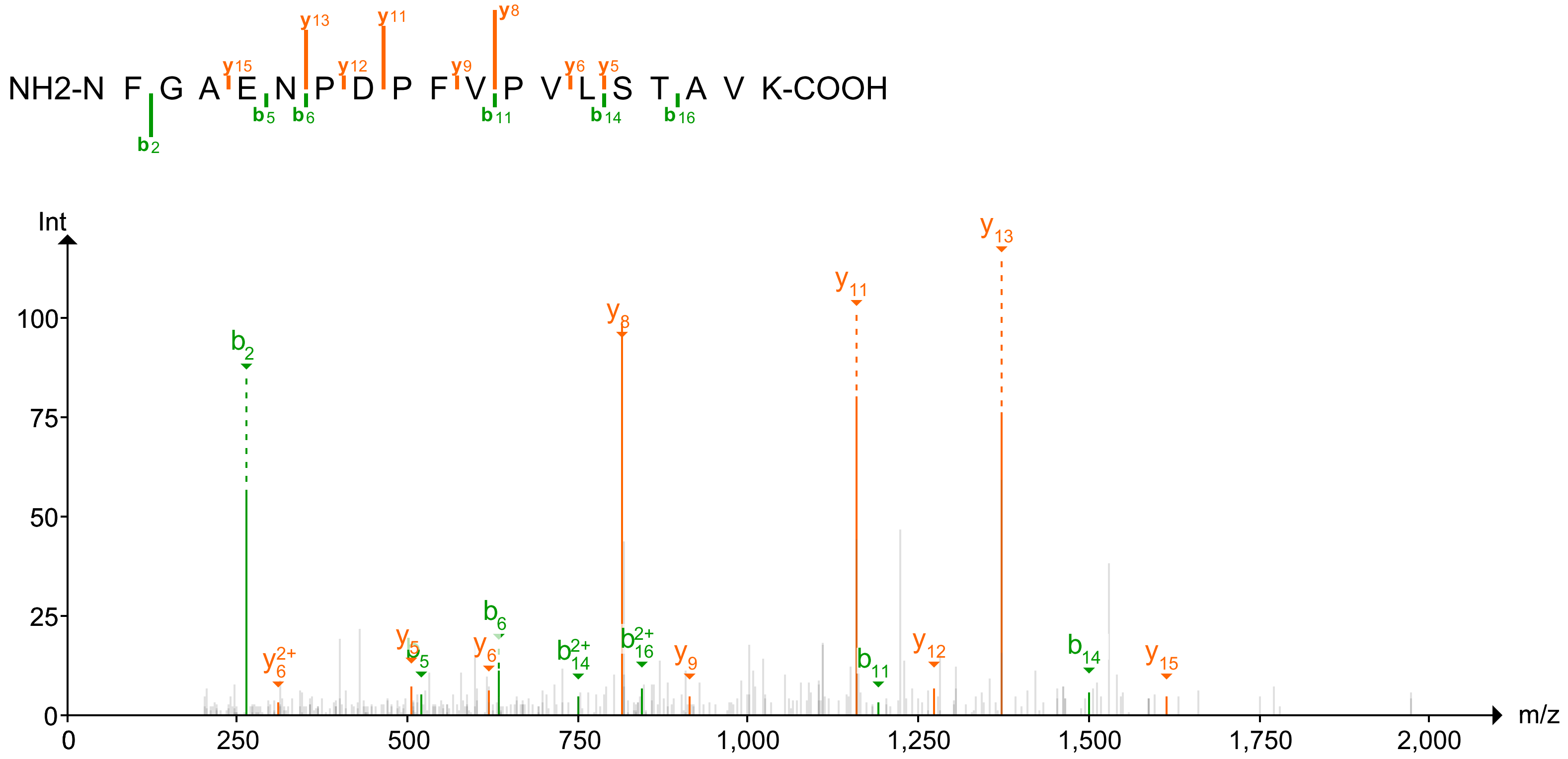

NH2-N F G A E N P D P F V P V L S T A V K-COOH
y15 y13 y12 y11 y9 y8 y6 y5
b2 b5 b6 b11 b14 b16
Int
m/z
b2
y8
y11
y13
$y_6^{2+}$
y5
b5
b6
y6
$b_{14}^{2+}$
$b_{16}^{2+}$
y9
b11
y12
b14
y15

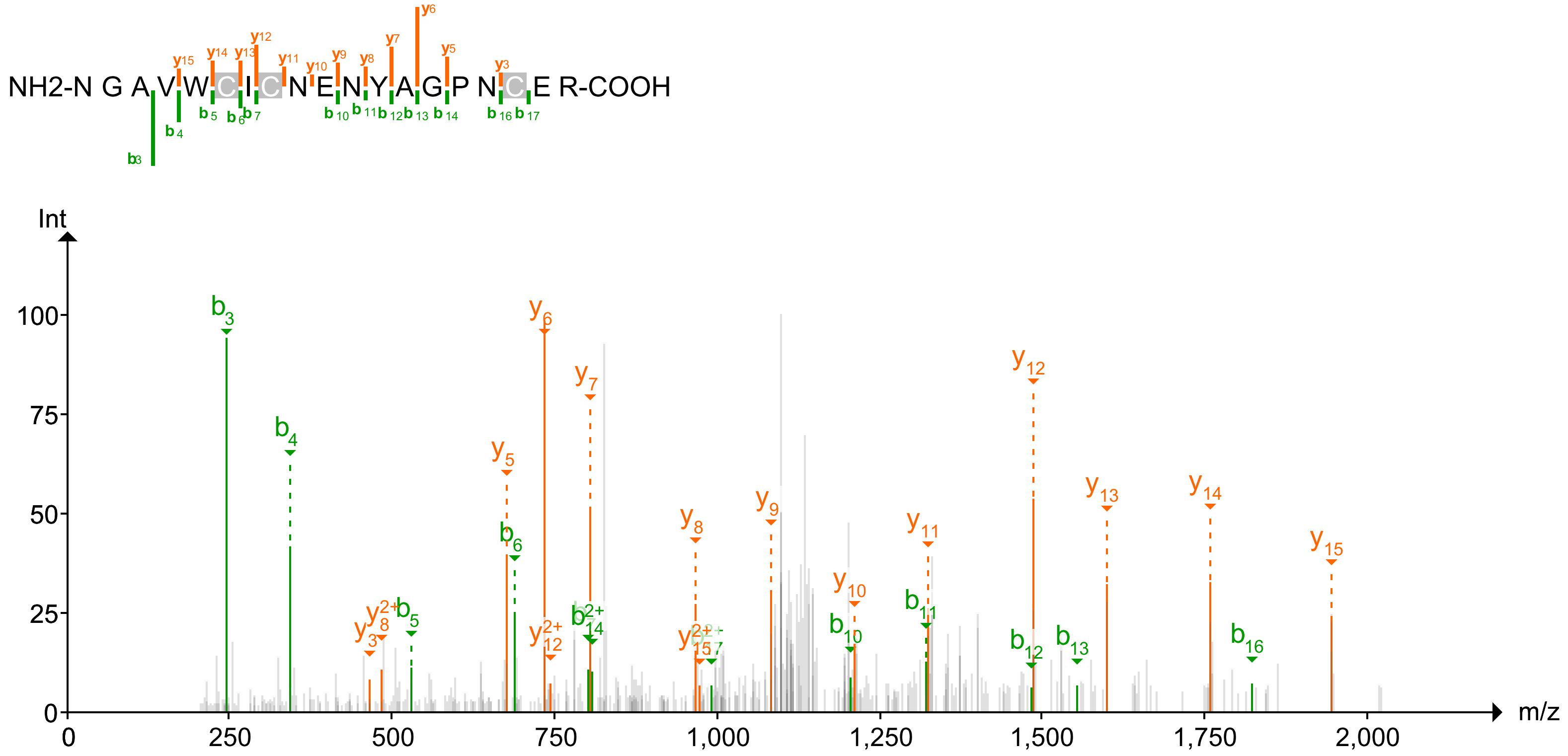

NH2-N G A V W C I C N E N Y A G P N C E R-COOH
y15 y14 y13 y12 y11 y10 y9 y8 y7 y6 y5 y3
b3 b4 b5 b6 b7 b10 b11 b12 b13 b14 b16 b17
Int
100
75
50
25
0
m/z
0
250
500
750
1,000
1,250
1,500
1,750
2,000
b3
b4
y3
y8 2+
b5
y5
b6
y6
y12 2+
y7
b14 2+
y8
y15 2+
y9
y10
b10
y11
b11
y12
b12
b13
y13
y14
b16
y15

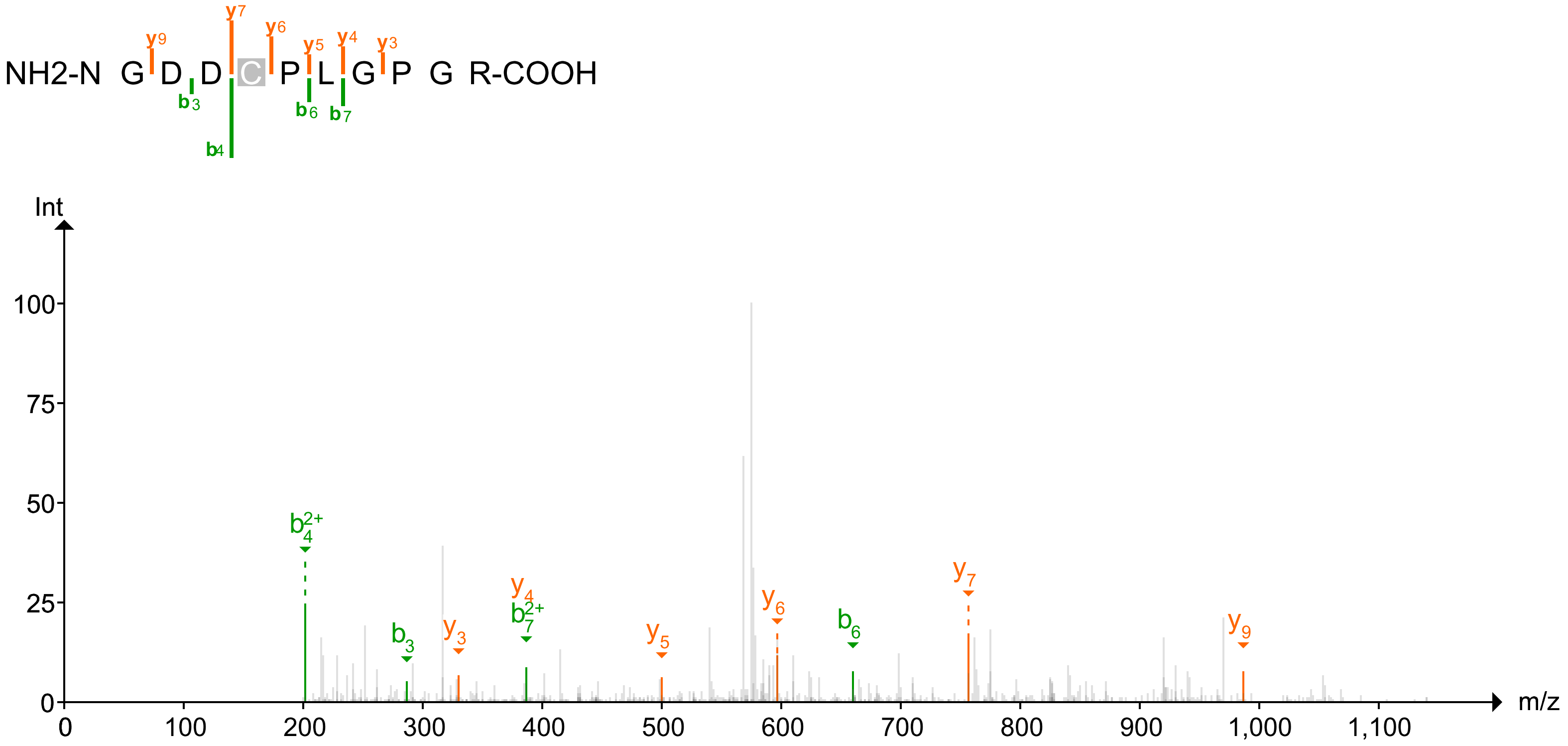

NH2-N G D D C P L G P G R-COOH
y9 y7 y6 y5 y4 y3
b3 b4 b6 b7
Int
100
75
50
25
0
$b_4^{2+}$
$b_3$
$y_3$
$y_4$
$b_7^{2+}$
$y_5$
$y_6$
$b_6$
$y_7$
$y_9$
0
100
200
300
400
500
600
700
800
900
1,000
1,100
m/z

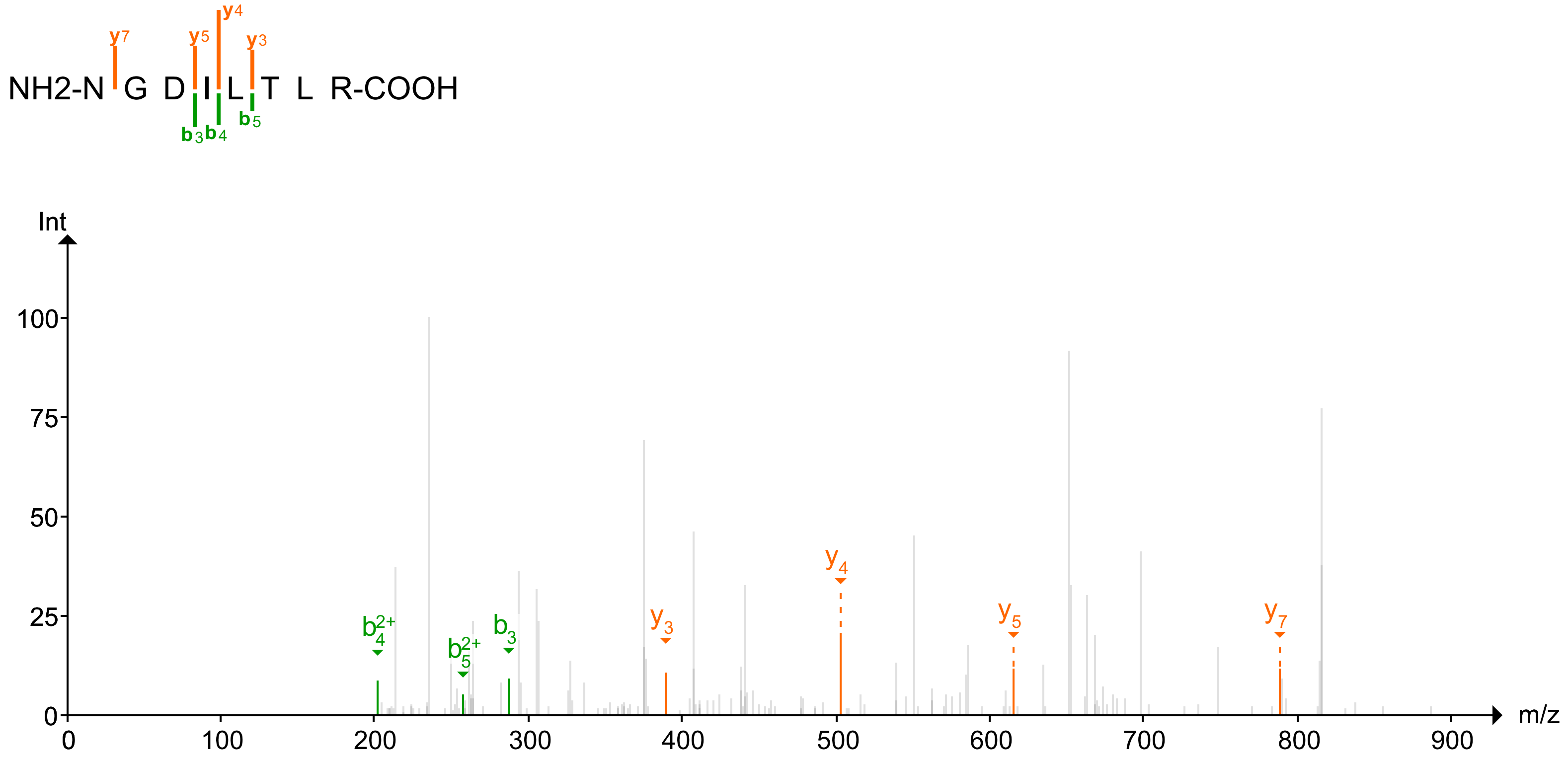

NH2-N G D I L T L R-COOH
y7
y5
y4
y3
b3
b4
b5
Int
100
75
50
25
0
0
100
200
300
400
500
600
700
800
900
m/z
$b_4^{2+}$
$b_5^{2+}$
$b_3$
$y_3$
$y_4$
$y_5$
$y_7$

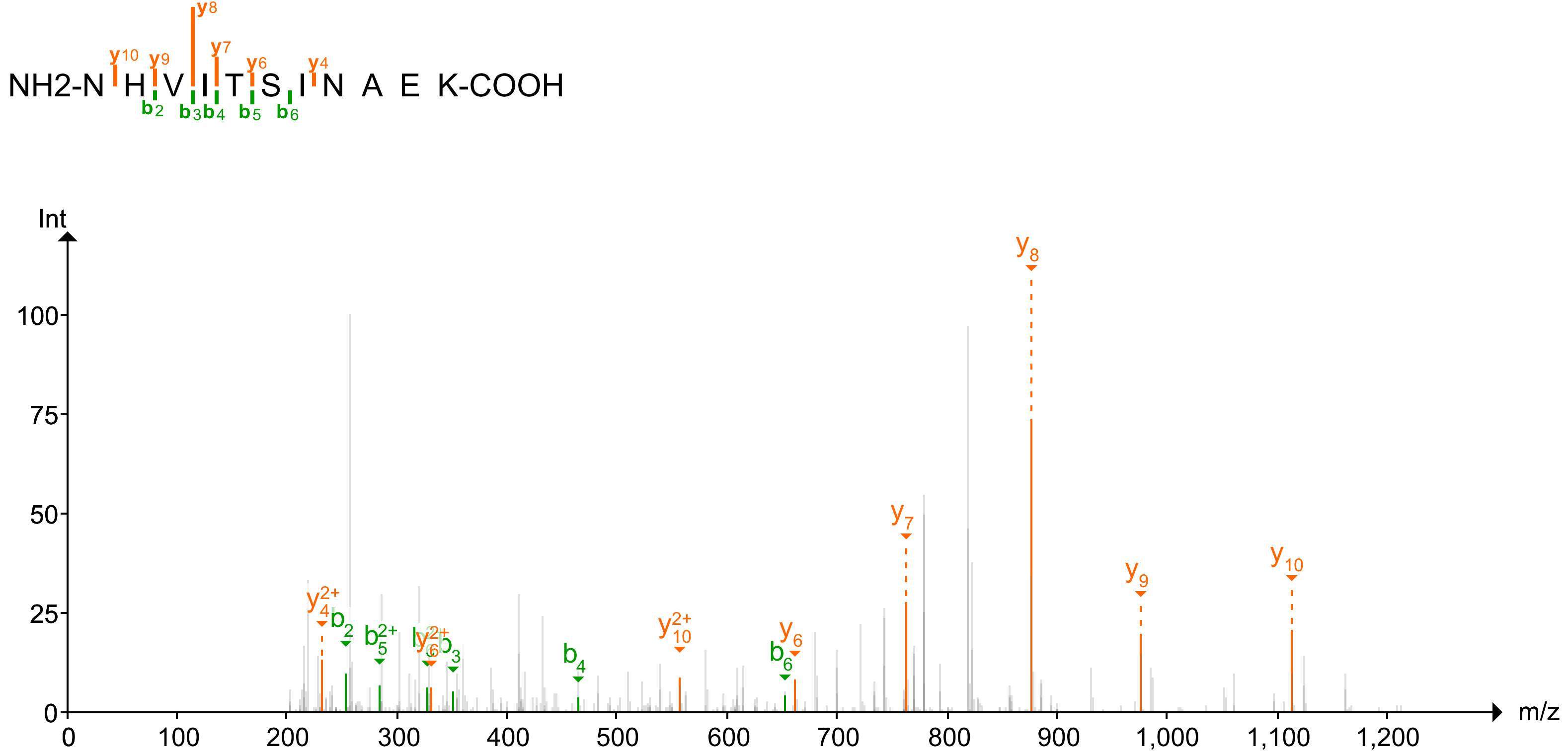

NH2-N H V I T S I N A E K-COOH
y10 y9 y8 y7 y6 y4
b2 b3 b4 b5 b6
Int
m/z
0
25
50
75
100
0
100
200
300
400
500
600
700
800
900
1,000
1,100
1,200
y8
y7
y9
y10
y6
b6
b4
b3
b2
b5 2+
y4 2+
y10 2+
y6 2+

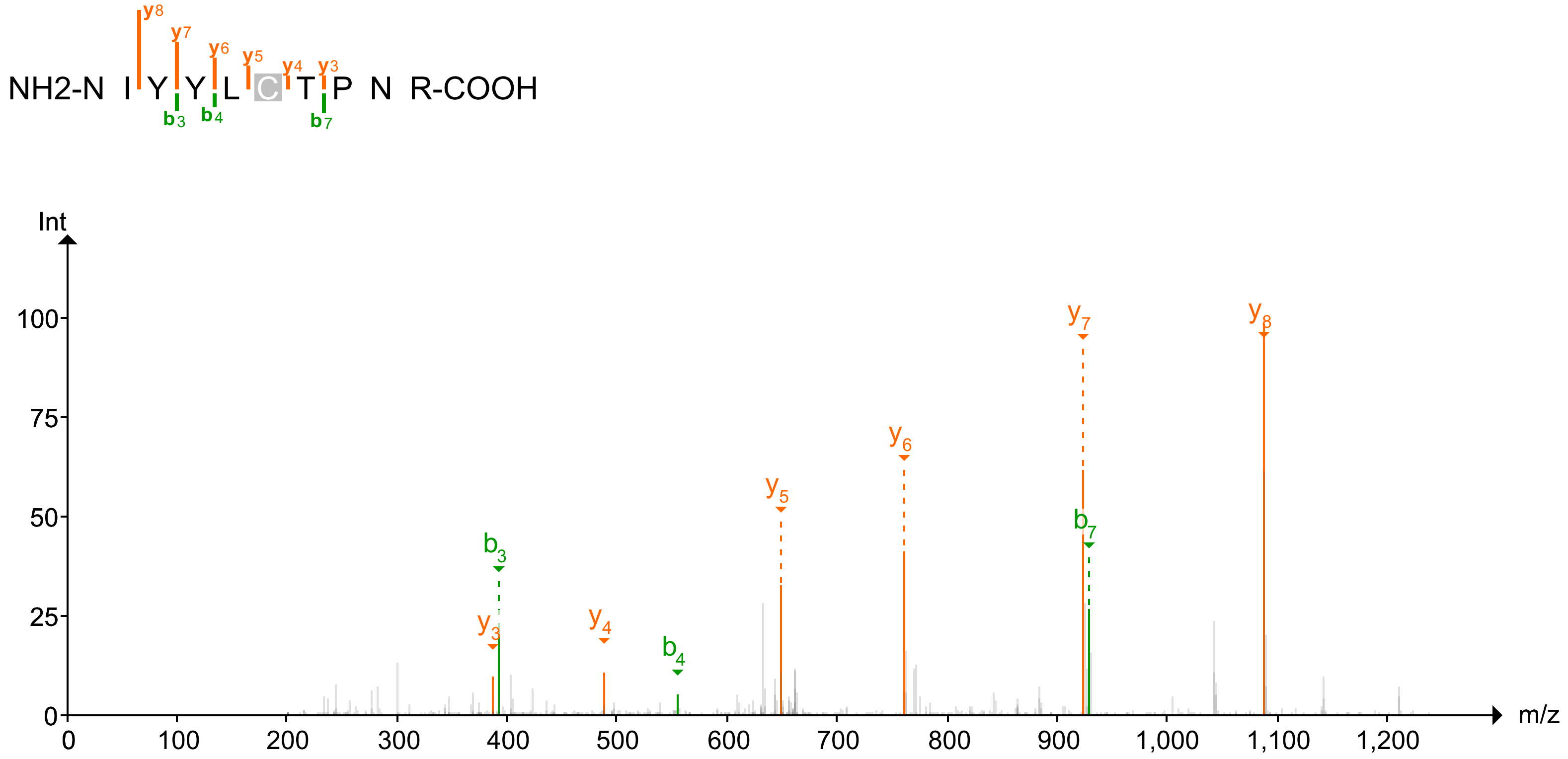

NH2-N I Y Y L C T P N R-COOH
y8
y7
y6
y5
y4
y3
b3
b4
b7
Int
100
75
50
25
0
m/z
0
100
200
300
400
500
600
700
800
900
1,000
1,100
1,200

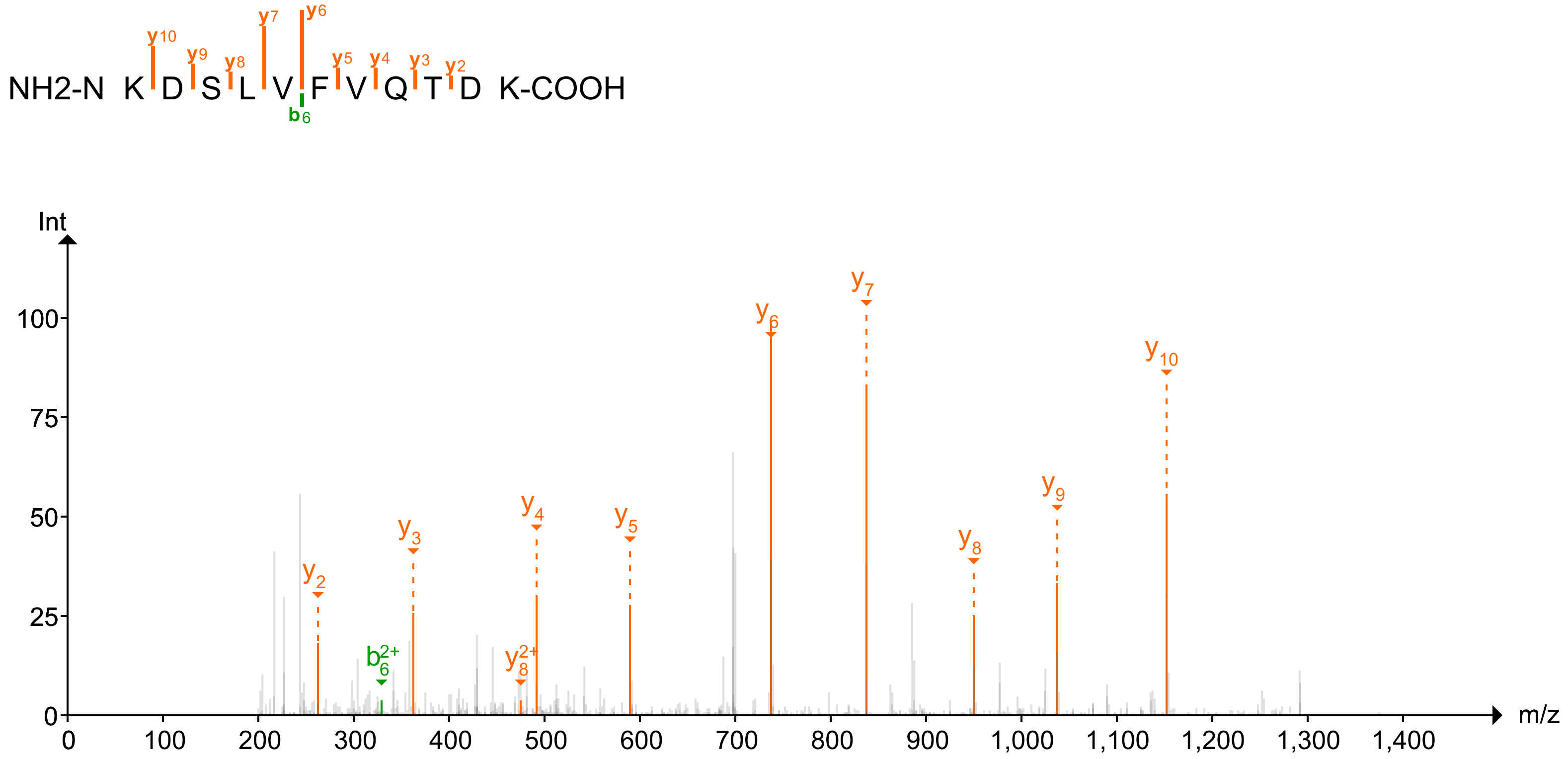

NH2-N K D S L V F V Q T D K-COOH
y10 y9 y8 y7 y6 y5 y4 y3 y2
b6
Int
100
75
50
25
0
y6
y7
y10
y9
y8
y5
y4
y3
y2
b6 2+
y8 2+
0 100 200 300 400 500 600 700 800 900 1,000 1,100 1,200 1,300 1,400
m/z

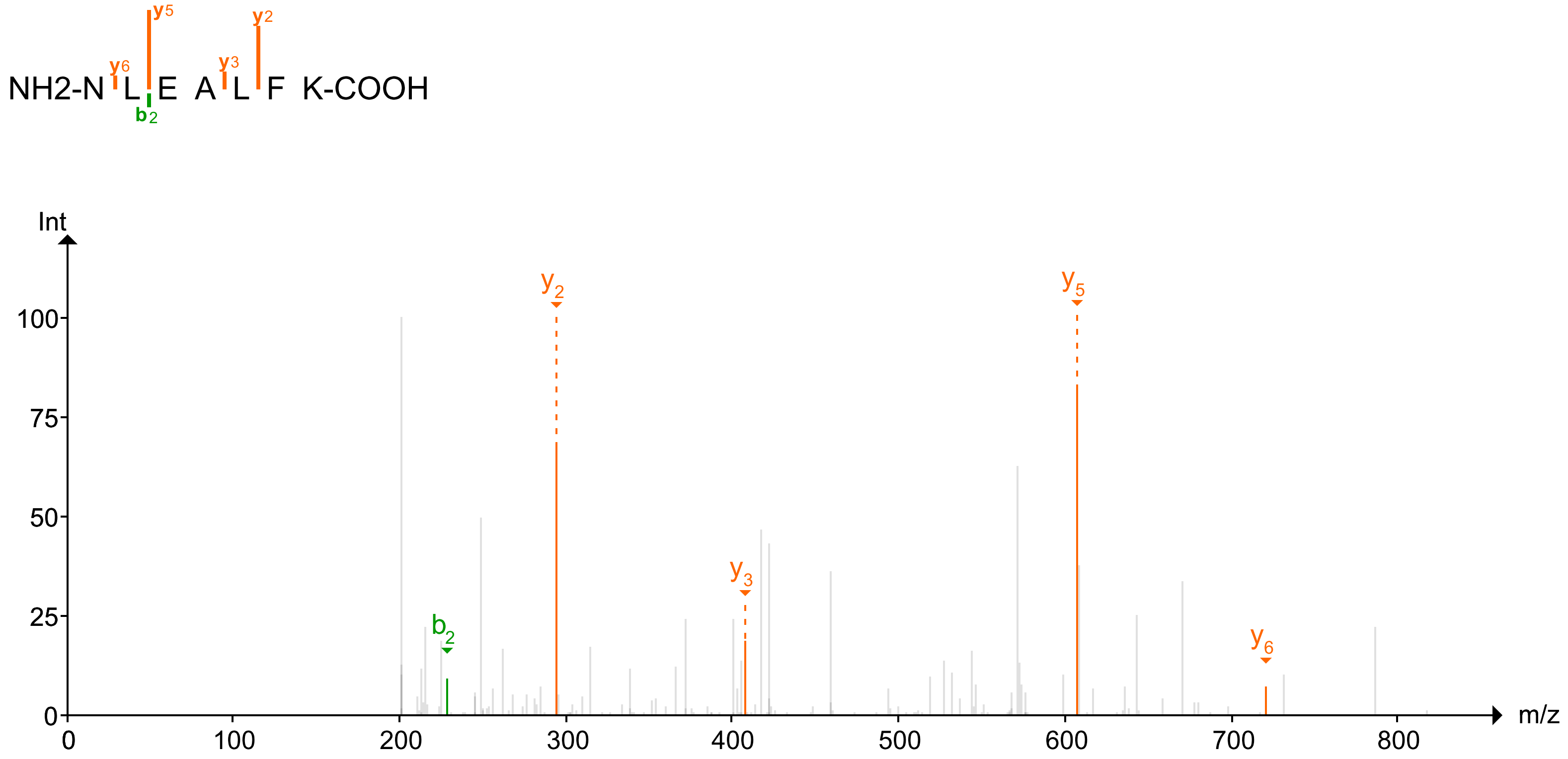

NH2-N L E A L F K-COOH
y5
y2
y6
y3
b2
Int
100
75
50
25
0
m/z
0
100
200
300
400
500
600
700
800

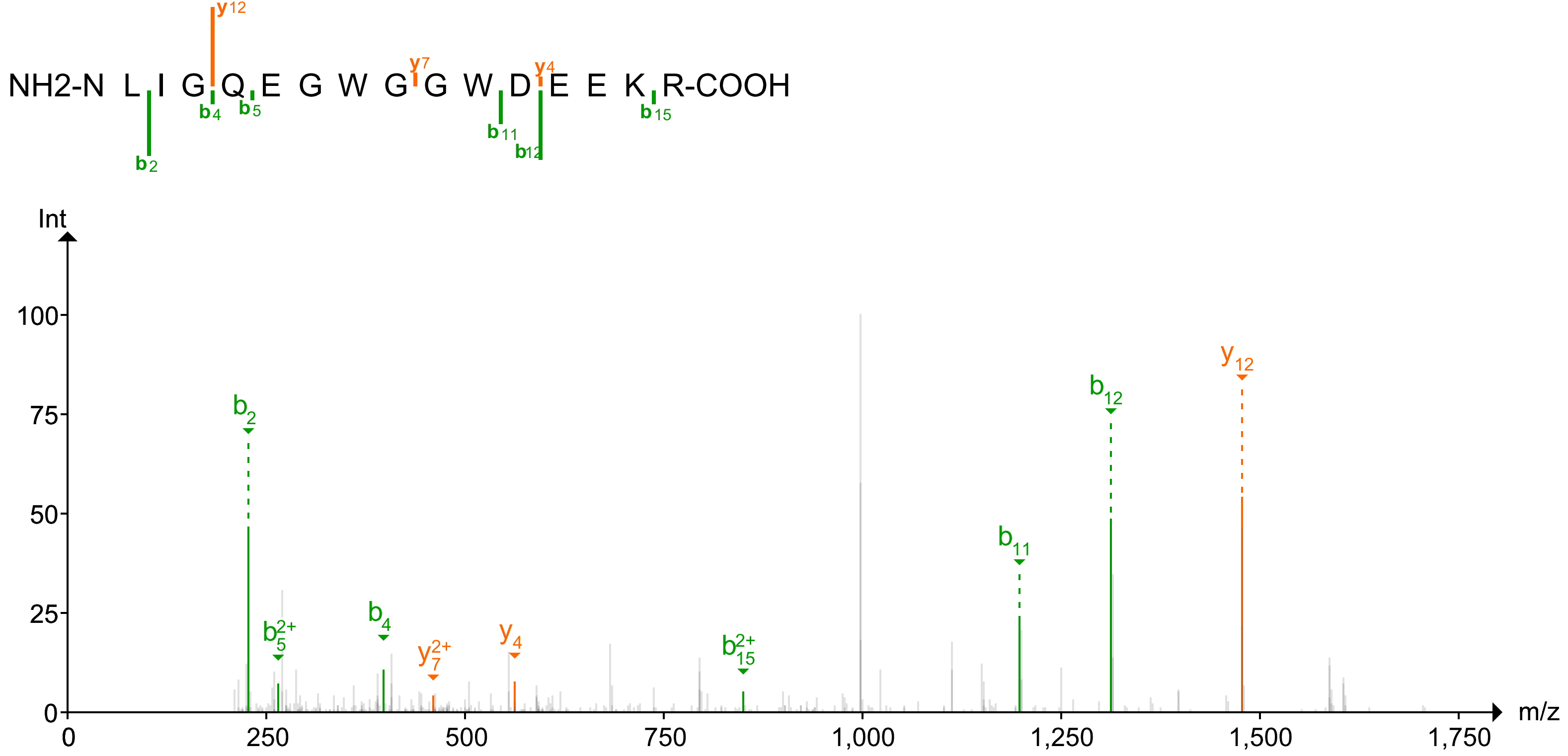

NH2-N L I G Q E G W G G W D E E K R-COOH
y12
y7
y4
b2
b4
b5
b11
b12
b15
Int
100
75
50
25
0
b2
b5 2+
b4
y7 2+
y4
b15 2+
b11
b12
y12
0
250
500
750
1,000
1,250
1,500
1,750
m/z

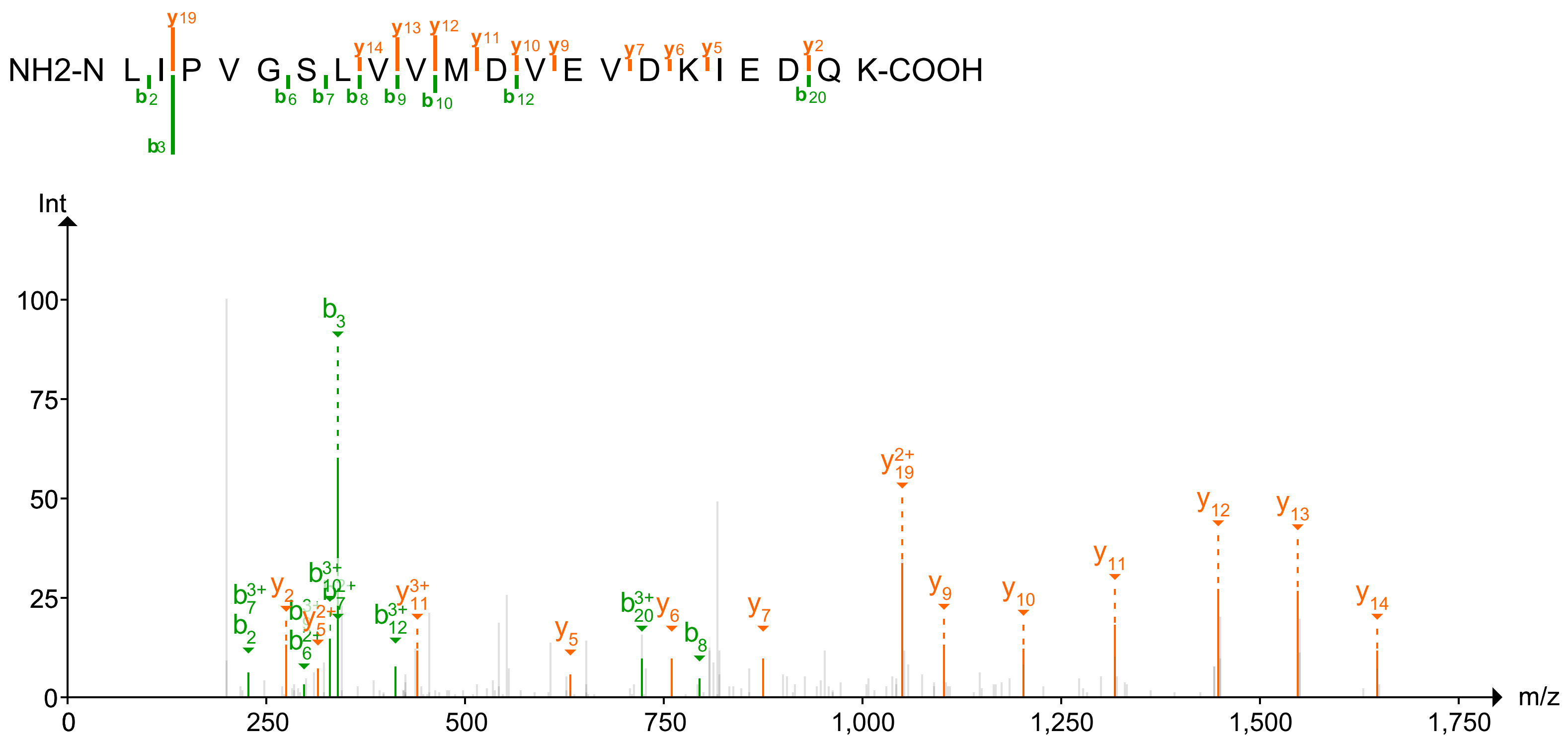

NH2-N L I P V G S L V V M D V E V D K I E D Q K-COOH
Int
m/z
0
25
50
75
100
250
500
750
1,000
1,250
1,500
1,750

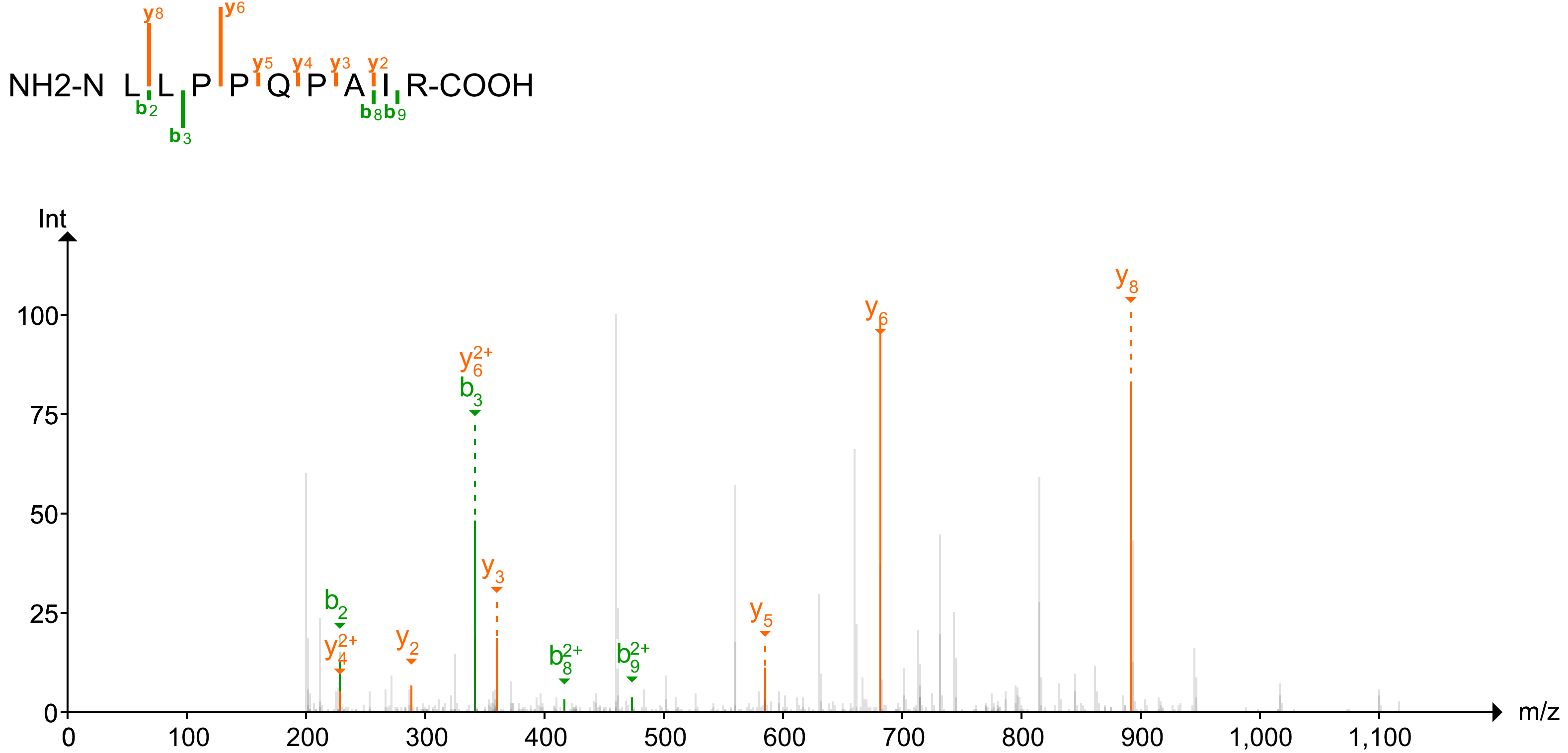

NH2-N L L P P Q P A I R-COOH
y8 y6 y5 y4 y3 y2
b2 b3 b8 b9
Int
m/z
0
25
50
75
100
0
100
200
300
400
500
600
700
800
900
1,000
1,100
$y_6^{2+}$
$b_3$
$y_3$
$b_2$
$y_4^{2+}$
$y_2$
$b_8^{2+}$
$b_9^{2+}$
$y_5$
$y_6$
$y_8$

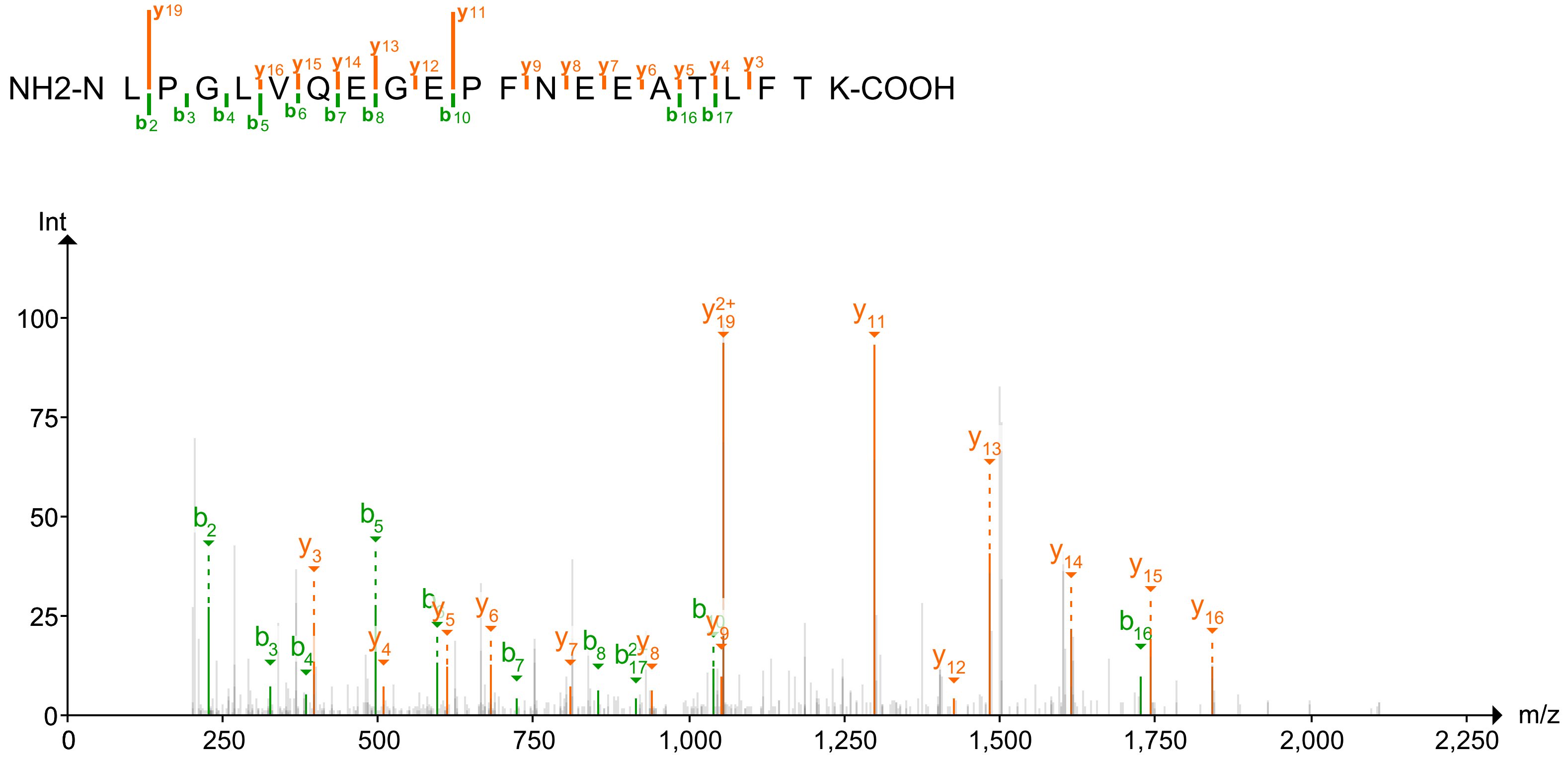
NH2-N L P G L V Q E G E P F N E E A T L F T K-COOH
y19 y16 y15 y14 y13 y12 y11 y9 y8 y7 y6 y5 y4 y3
b2 b3 b4 b5 b6 b7 b8 b10 b16 b17
Int
100
75
50
25
0
m/z
0
250
500
750
1,000
1,250
1,500
1,750
2,000
2,250
y19 2+
y11
y13
y14
y15
y16
y12
y3
y4
y5
y6
y7
y8
y9
b2
b3
b4
b5
b7
b8
b16
b17 2+

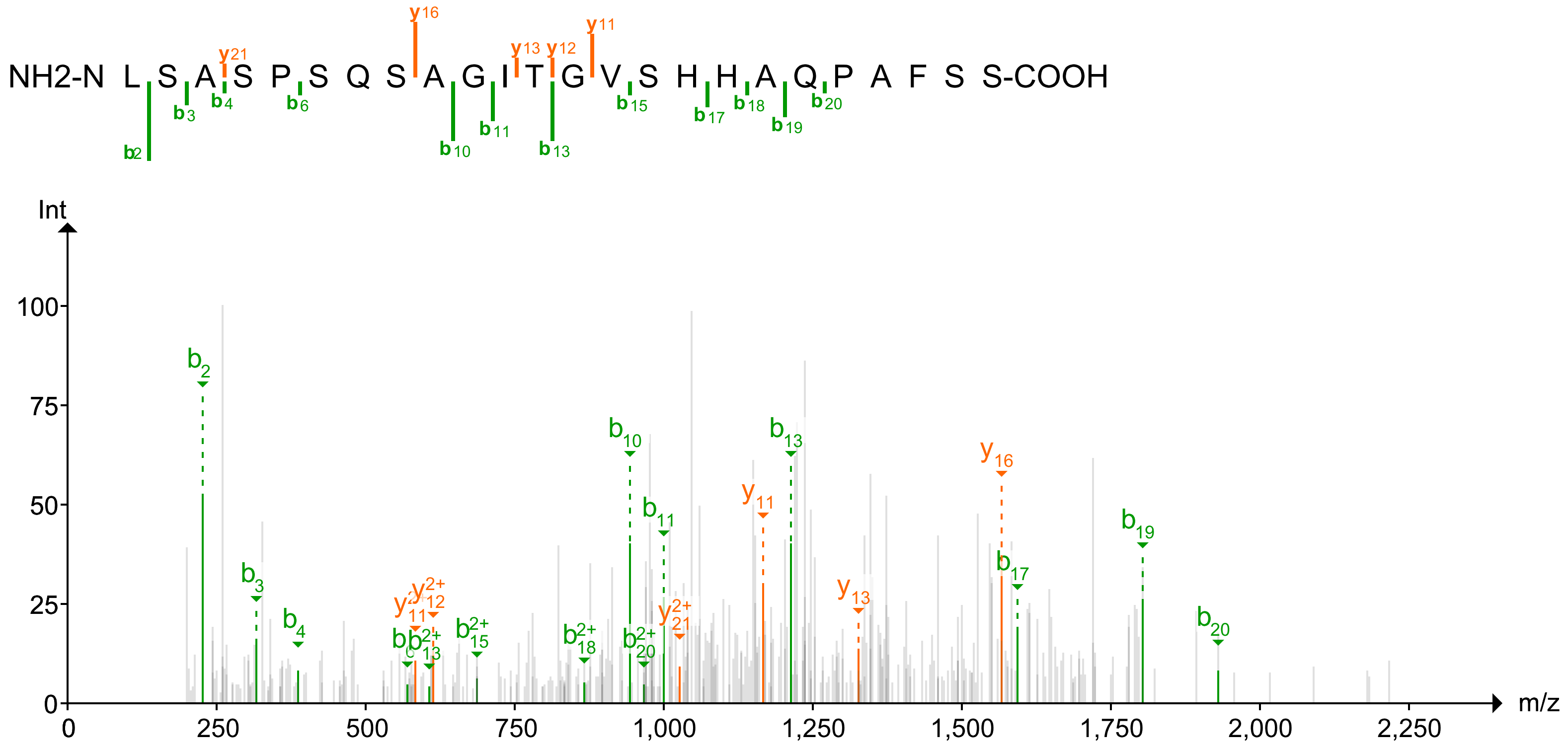

NH2-N L S A S P S Q S A G I T G V S H H A Q P A F S S-COOH
y16
y21
y13
y12
y11
b2
b3
b4
b6
b10
b11
b13
b15
b17
b18
b19
b20
Int
100
75
50
25
0
m/z
0
250
500
750
1,000
1,250
1,500
1,750
2,000
2,250

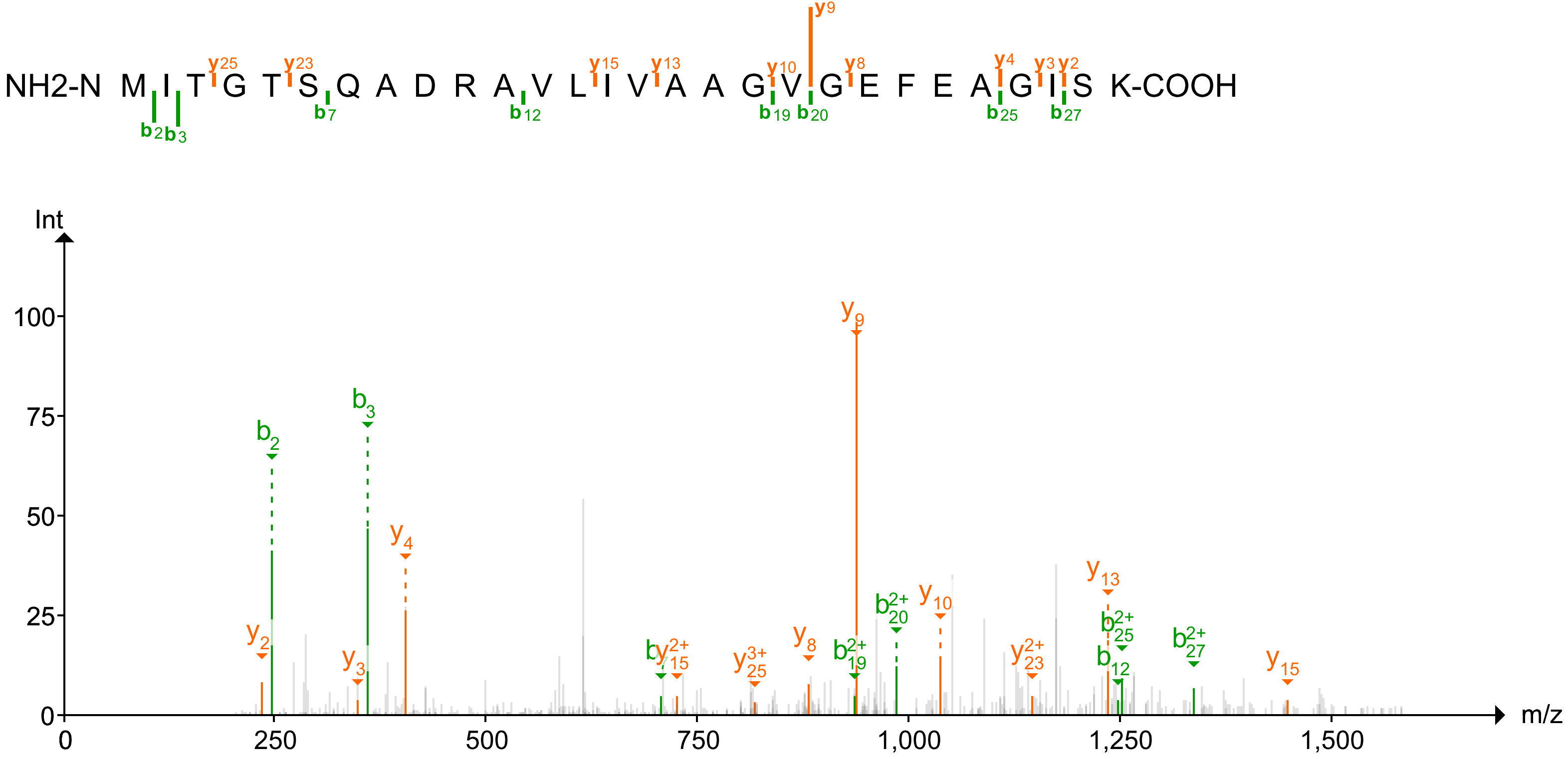

NH2-N M I T G T S Q A D R A V L I V A A G V G E F E A G I S K-COOH
y25 y23 y15 y13 y10 y9 y8 y4 y3 y2
b2 b3 b7 b12 b19 b20 b25 b27
Int
100
75
50
25
0
m/z
0
250
500
750
1,000
1,250
1,500
b2
b3
y2
y3
y4
y9
y10
y13
y15
y8
y3+ 25
y2+ 15
y2+ 23
b2+ 19
b2+ 20
b2+ 25
b2+ 27
b12

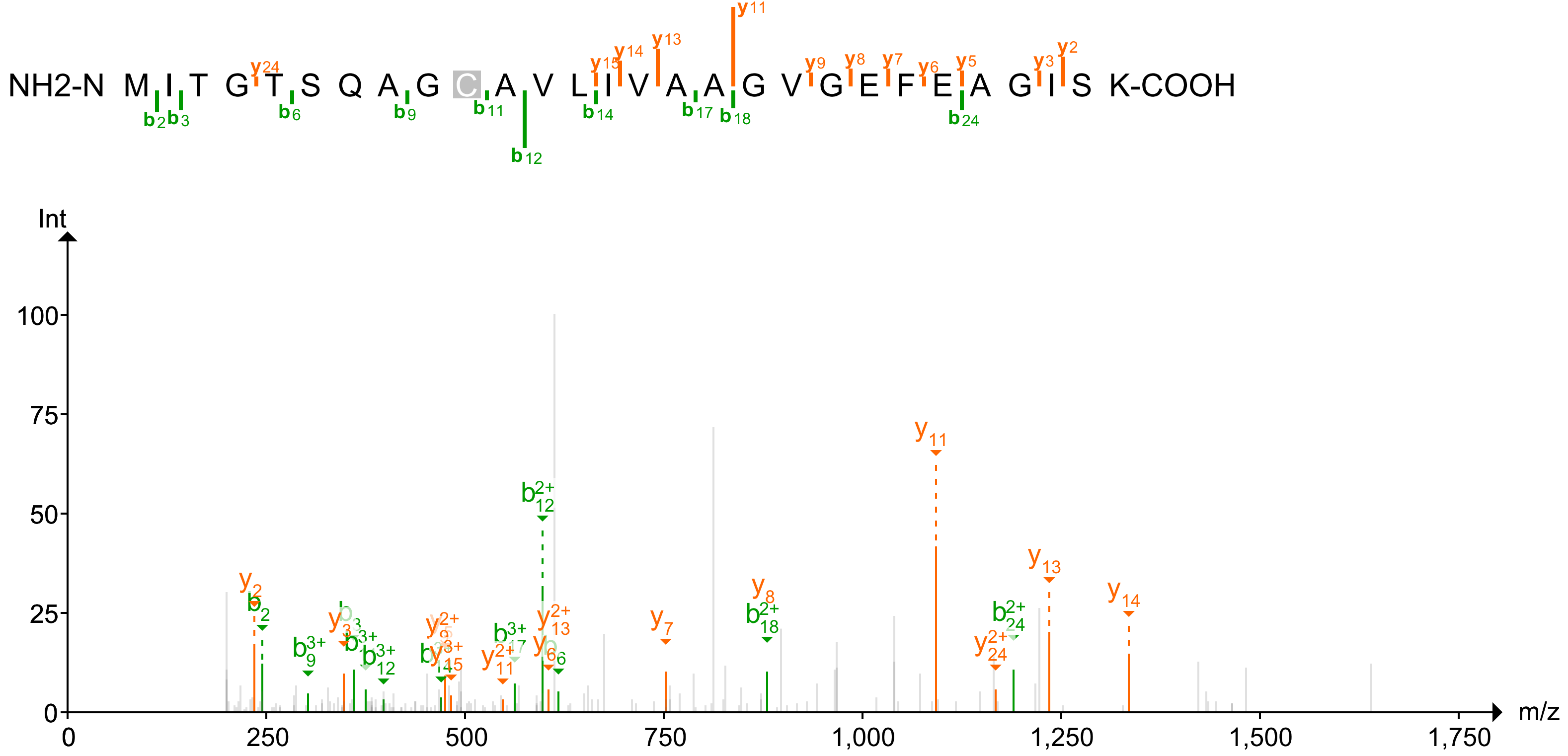

NH2-N M I T G T S Q A G C A V L I V A A G V G E F E A G I S K-COOH
Int
m/z
100
75
50
25
0
250
500
750
1,000
1,250
1,500
1,750

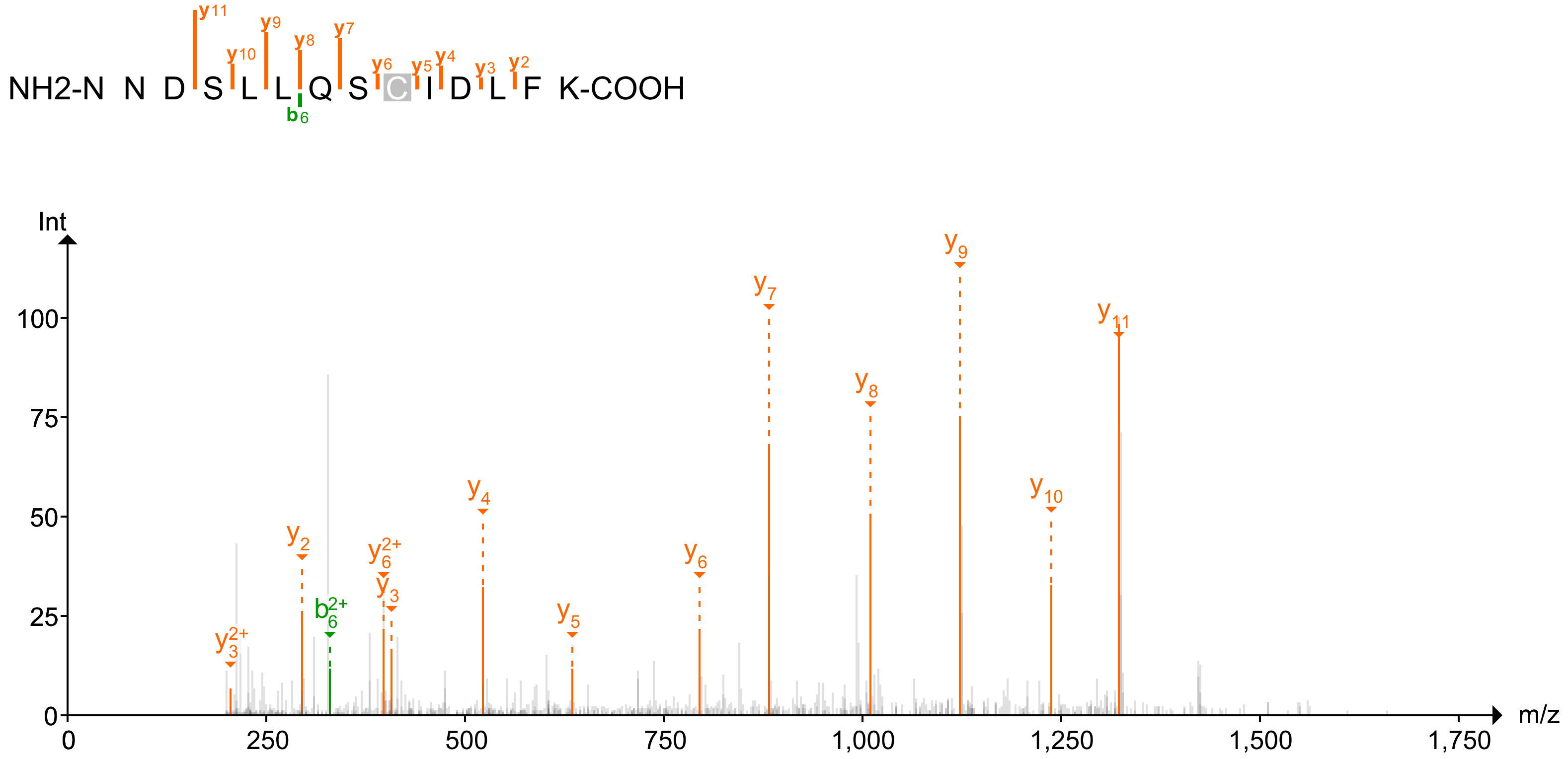

NH2-N N D S L L Q S C I D L F K-COOH
y11 y10 y9 y8 y7 y6 y5 y4 y3 y2
b6
Int
m/z
y9
y7
y11
y8
y4
y10
y2
y6 2+
y3
b6 2+
y6
y5
y3 2+

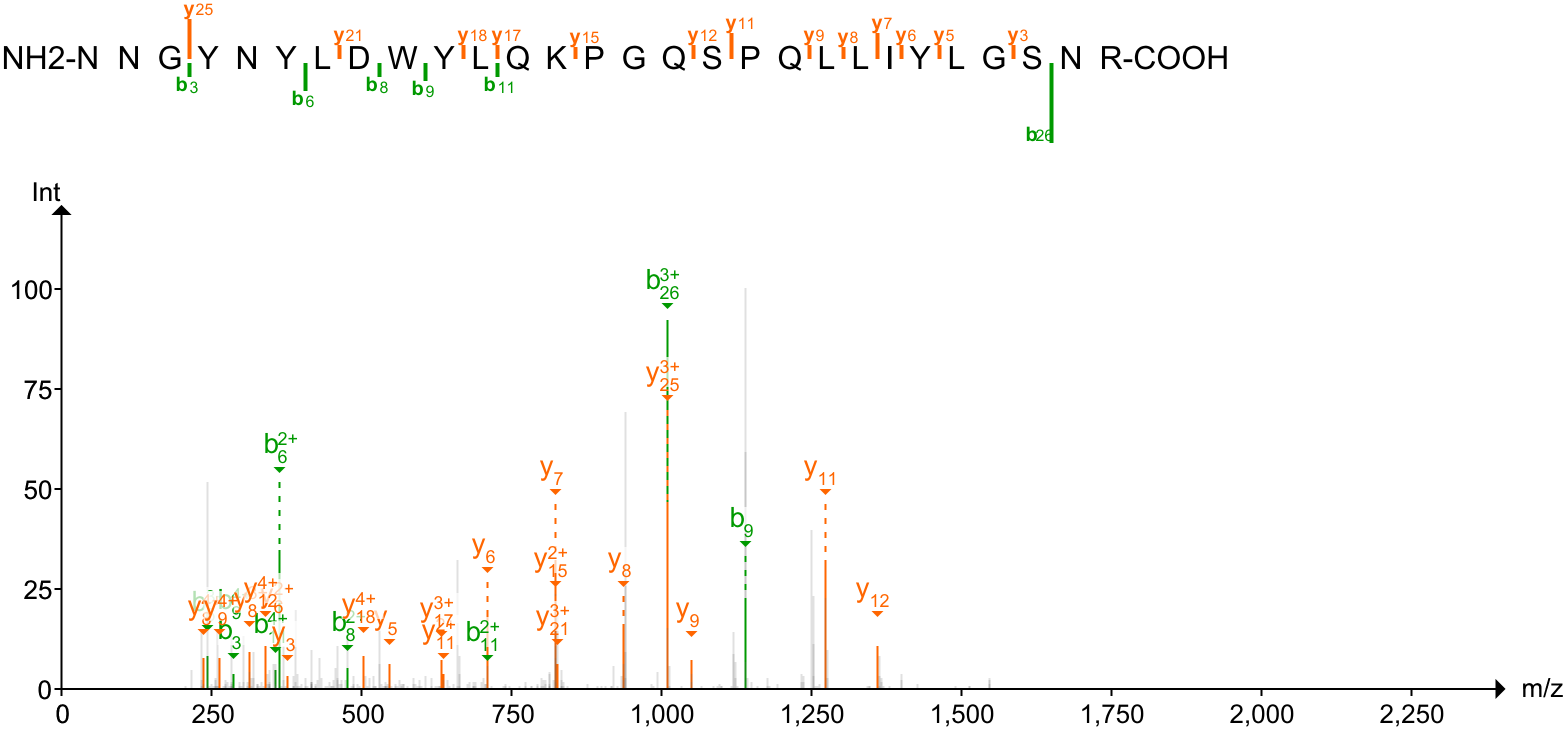

NH2-N N G Y N Y L D W Y L Q K P G Q S P Q L L I Y L G S N R-COOH
y25 y21 y18 y17 y15 y12 y11 y9 y8 y7 y6 y5 y3
b3 b6 b8 b9 b11 b26
Int
100
75
50
25
0
m/z
0
250
500
750
1,000
1,250
1,500
1,750
2,000
2,250

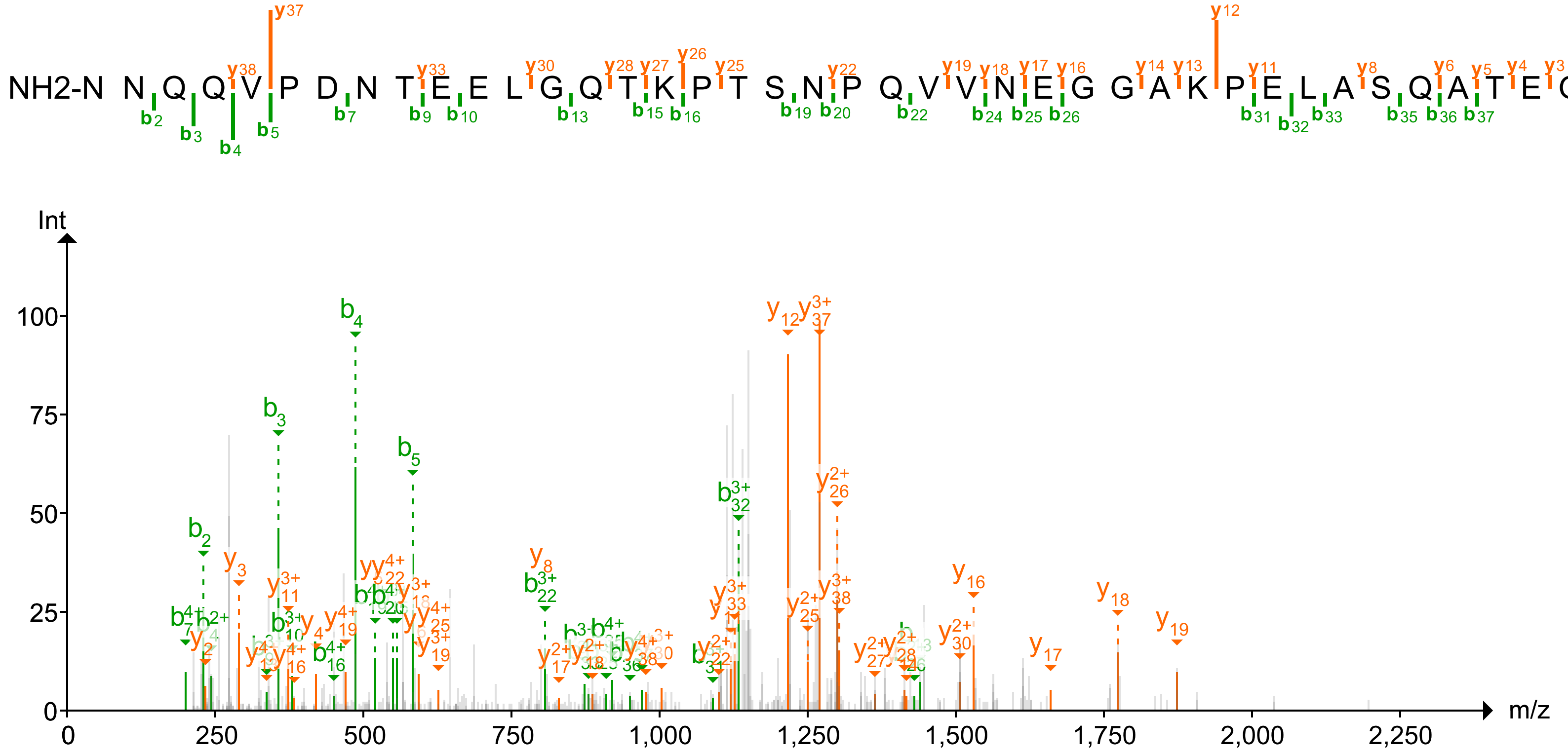

NH2-N N Q Q V P D N T E E L G Q T K P T S N P Q V V N E G G A K P E L A S Q A T E
Int
100
75
50
25
0
m/z
0
250
500
750
1,000
1,250
1,500
1,750
2,000
2,250

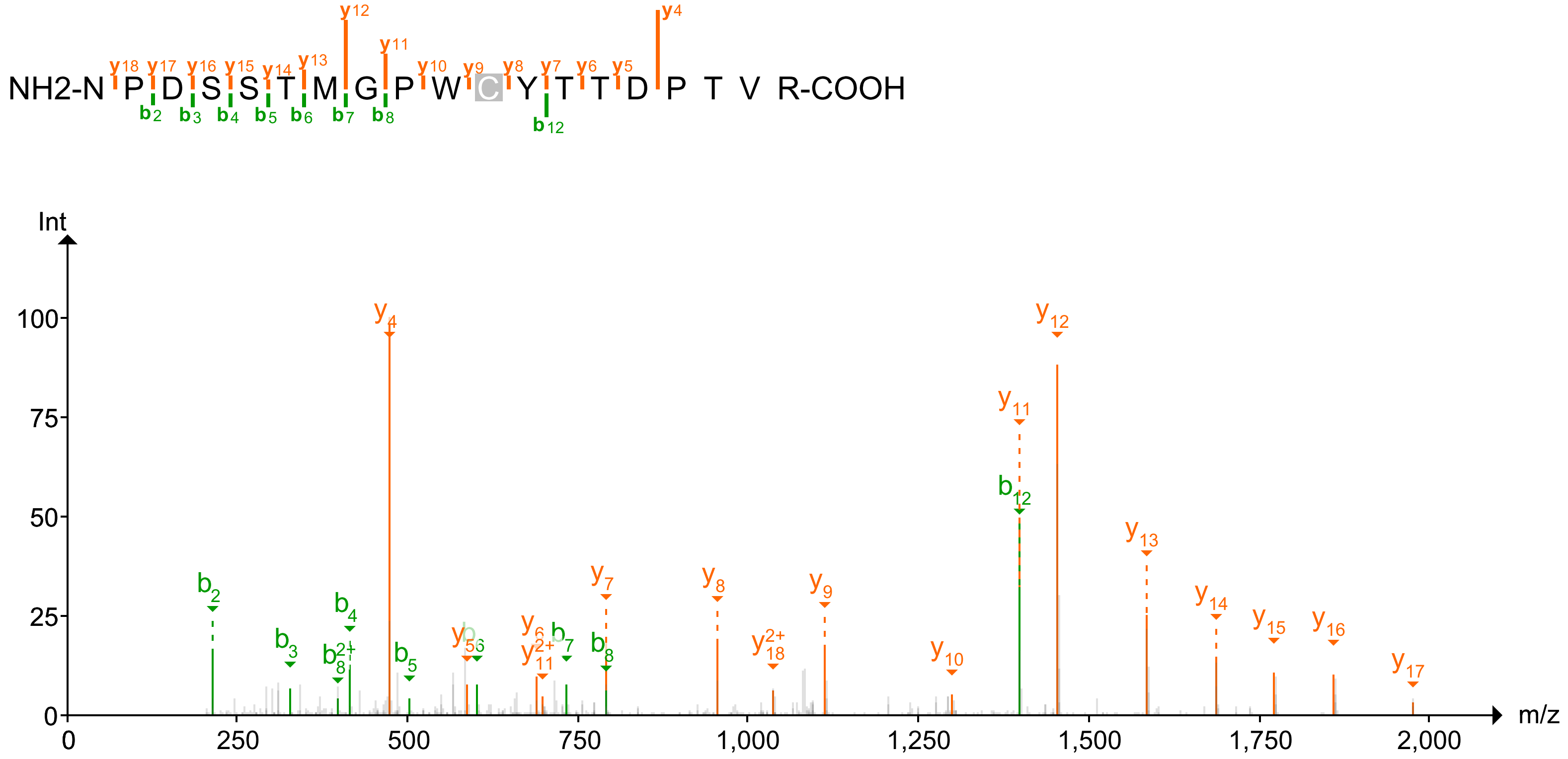

NH2-N P D S S T M G P W C Y T T D P T V R-COOH
y18 y17 y16 y15 y14 y13 y12 y11 y10 y9 y8 y7 y6 y5 y4
b2 b3 b4 b5 b6 b7 b8 b12
Int
m/z
0
25
50
75
100
250
500
750
1,000
1,250
1,500
1,750
2,000

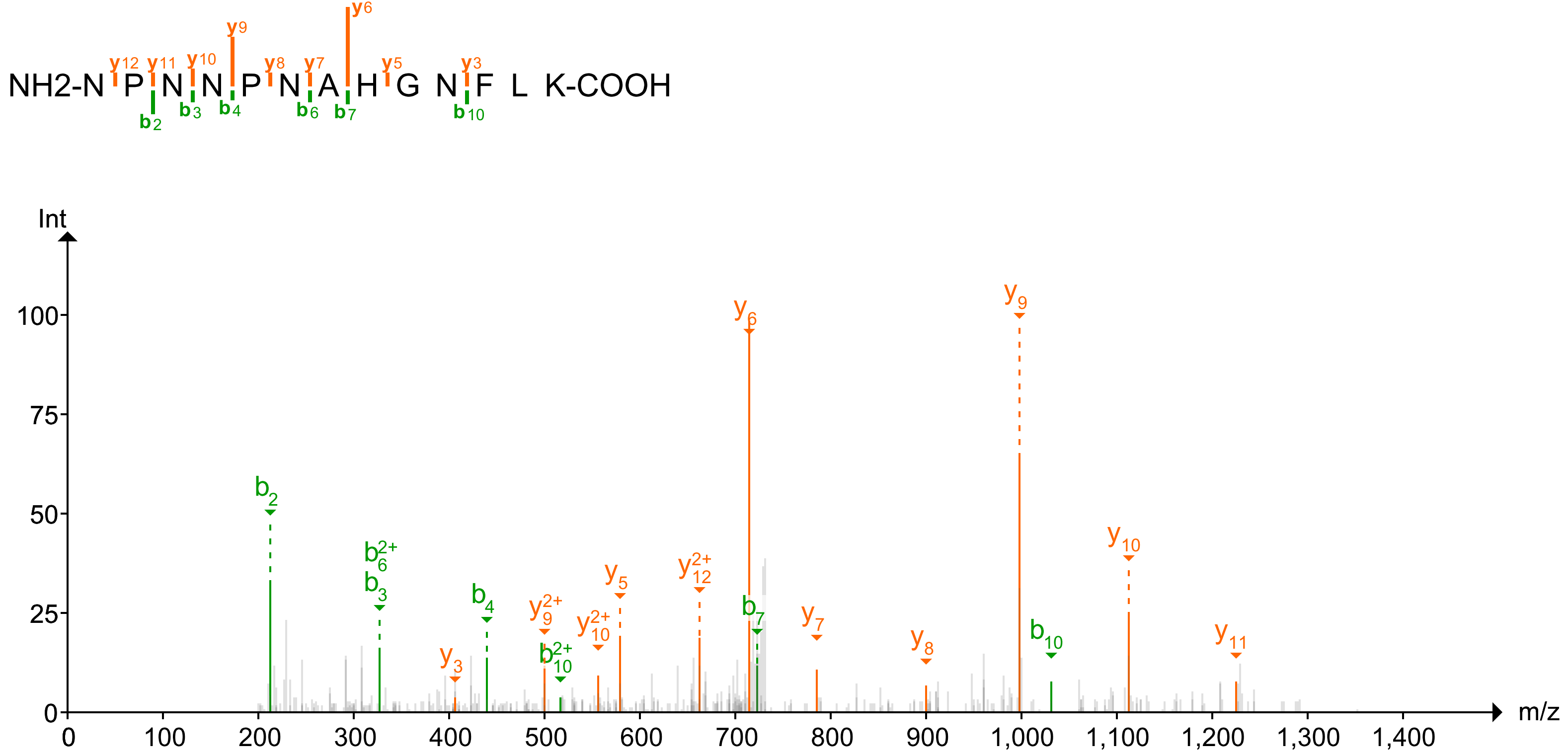

NH2-N P N N P N A H G N F L K-COOH
y12 y11 y10 y9 y8 y7 y6 y5 y3
b2 b3 b4 b6 b7 b10
Int
m/z
0
25
50
75
100
0
100
200
300
400
500
600
700
800
900
1,000
1,100
1,200
1,300
1,400
b2
b6 2+
b3
b4
y3
y9 2+
b10 2+
y10 2+
y5
y12 2+
y6
b7
y7
y8
y9
b10
y10
y11

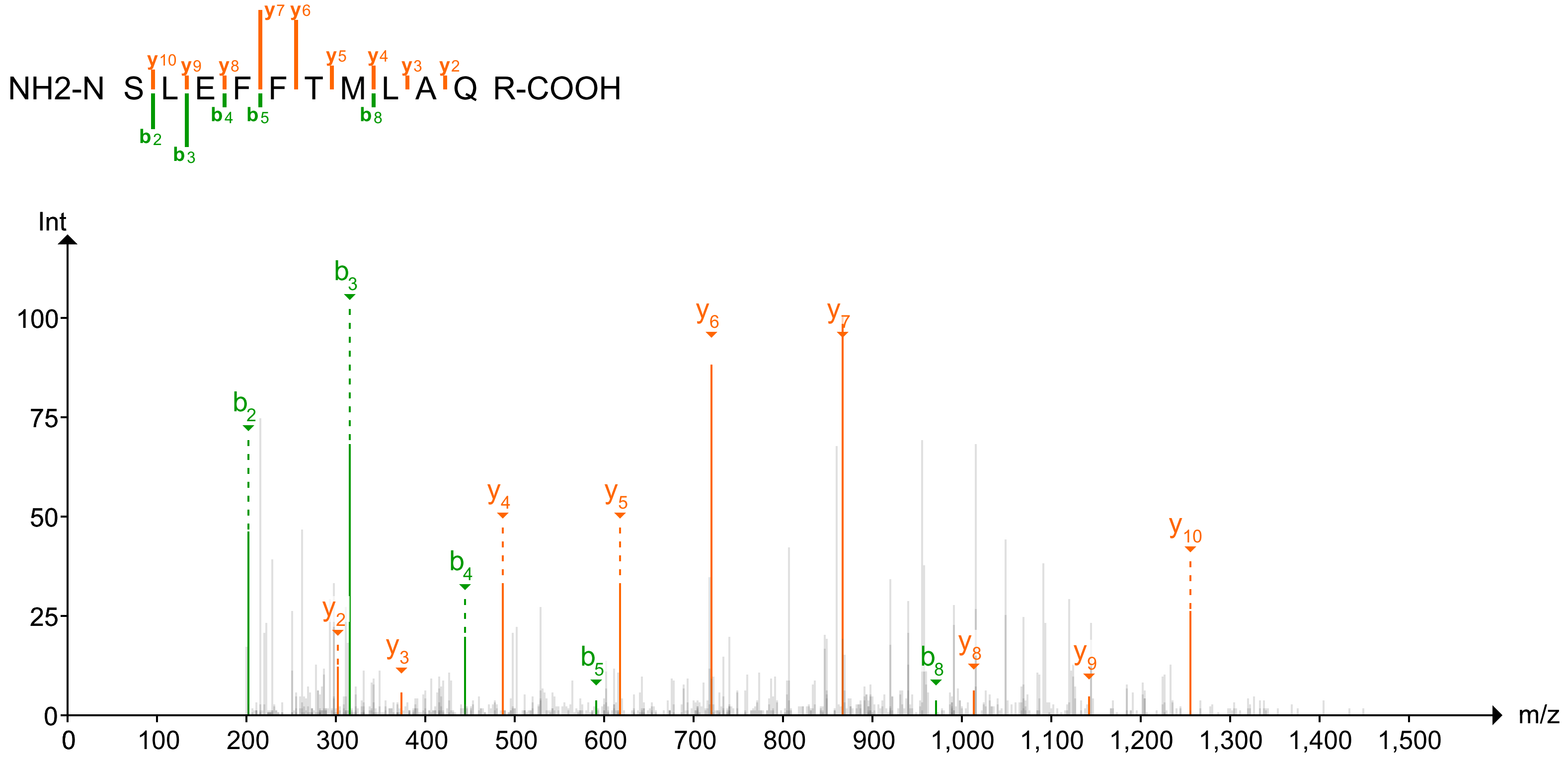

NH2-N S L E F F T M L A Q R-COOH
y10 y9 y8 y7 y6 y5 y4 y3 y2
b2 b3 b4 b5 b8
Int
m/z

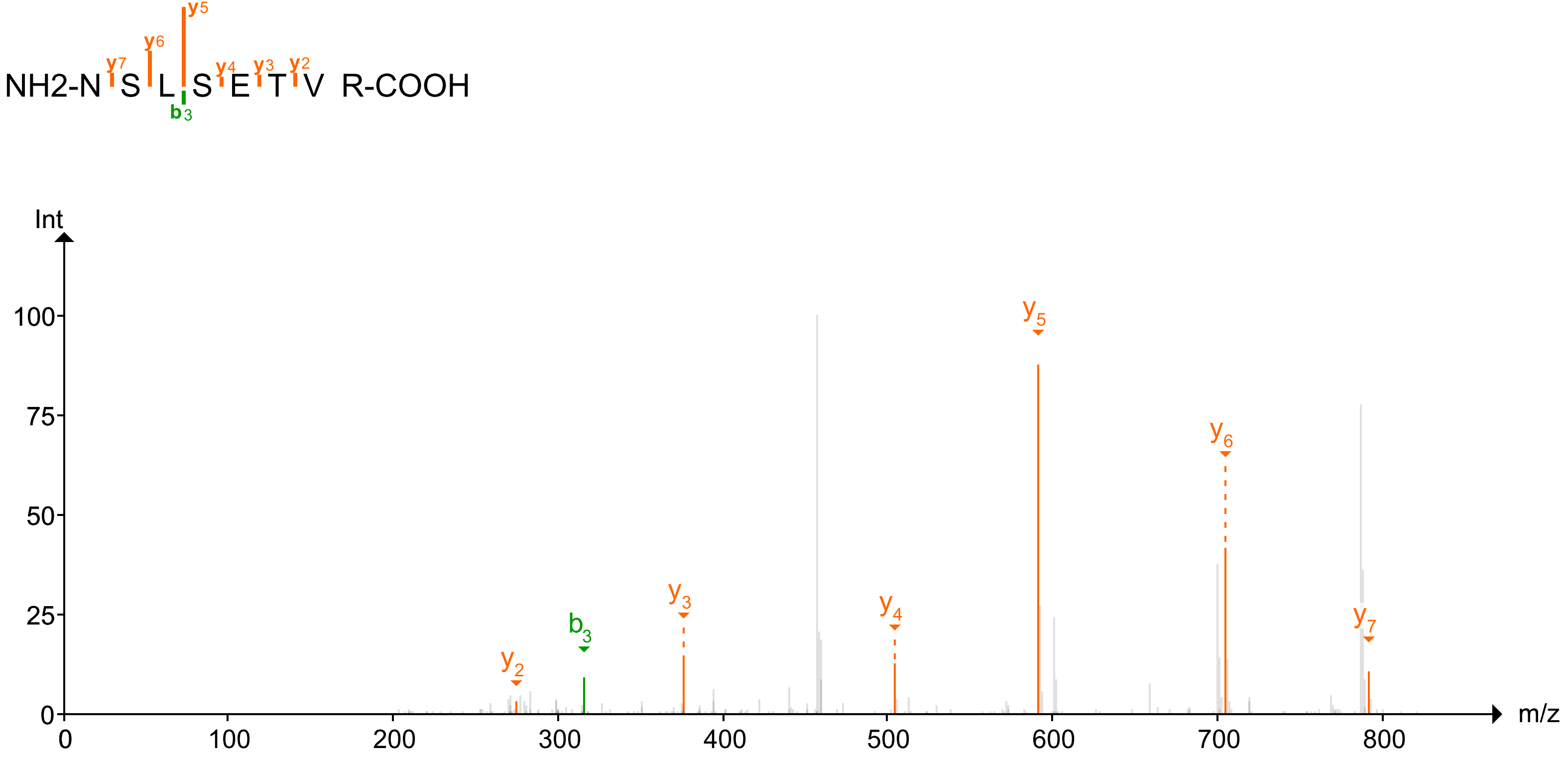

NH2-N S L S E T V R-COOH
y7
y6
y5
y4
y3
y2
b3
Int
100
75
50
25
0
m/z
0
100
200
300
400
500
600
700
800
y5
y6
y3
y4
y7
y2
b3

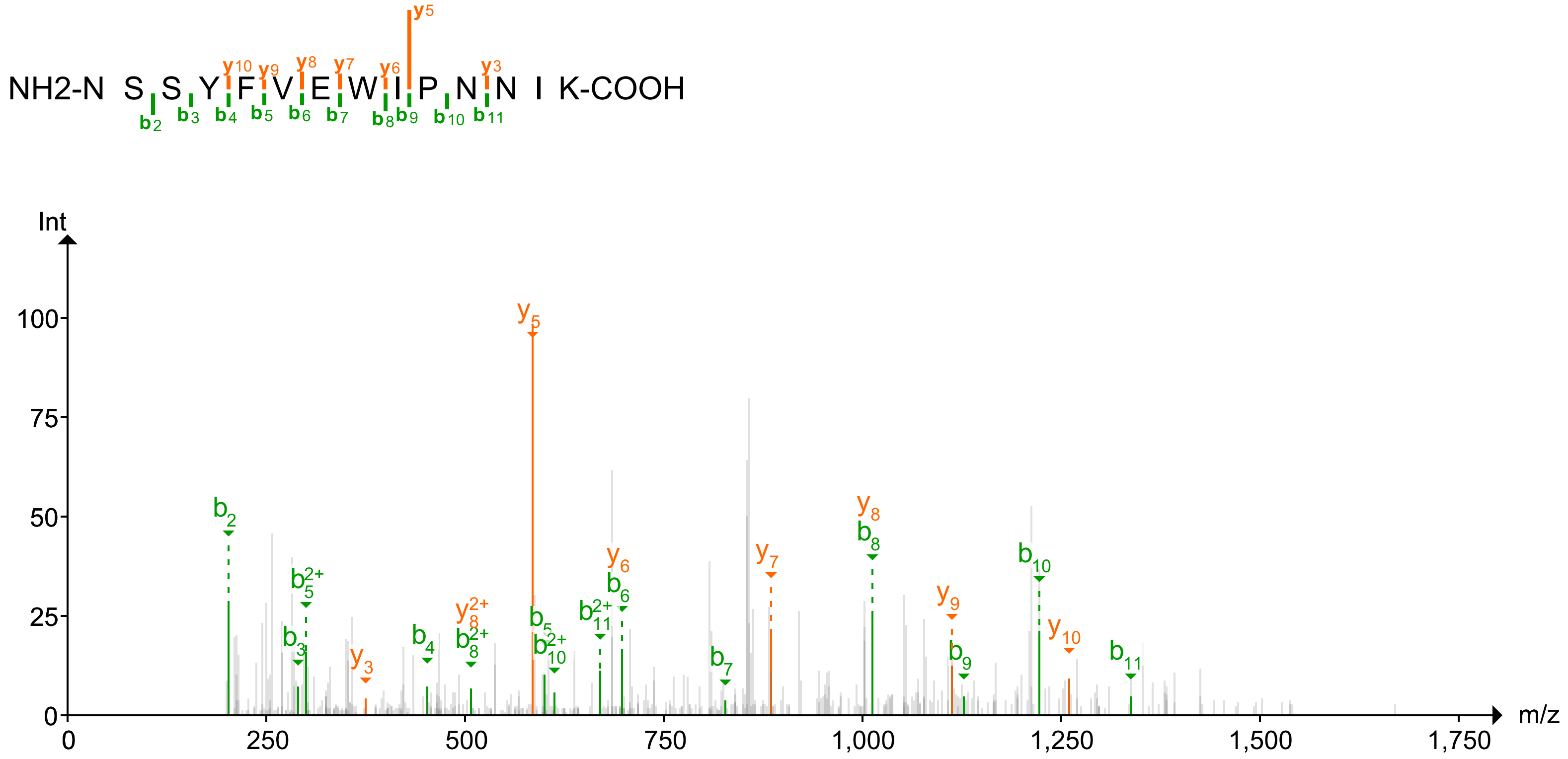

NH2-N S S Y F V E W I P N N I K-COOH
y10 y9 y8 y7 y6 y5 y3
b2 b3 b4 b5 b6 b7 b8 b9 b10 b11
Int
100
75
50
25
0
250
500
750
1,000
1,250
1,500
1,750
m/z

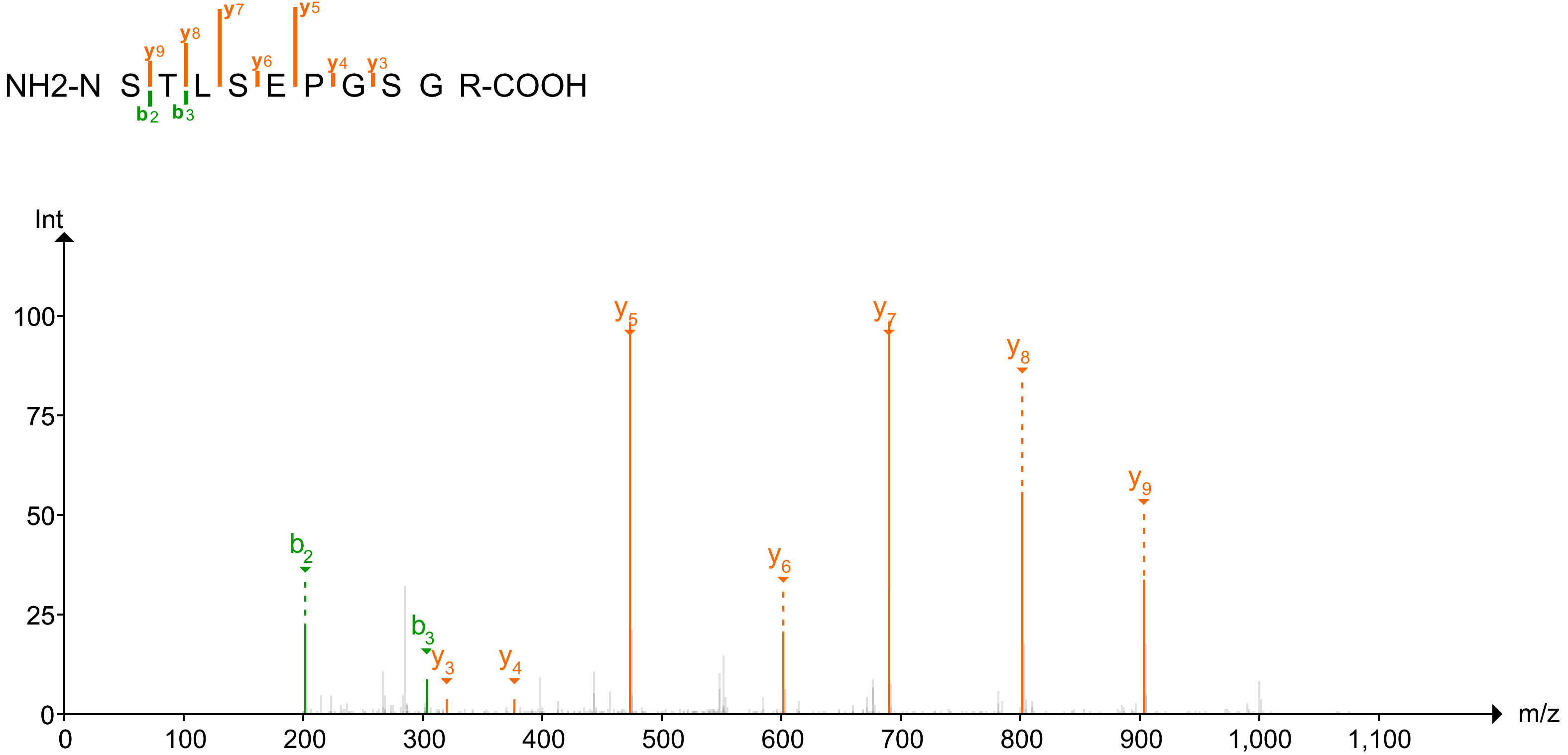
NH2-N S T L S E P G S G R-COOH
y9 y8 y7 y6 y5 y4 y3
b2 b3
Int
100
75
50
25
0
m/z
0
100
200
300
400
500
600
700
800
900
1,000
1,100
b2 b3 y3 y4 y5 y6 y7 y8 y9

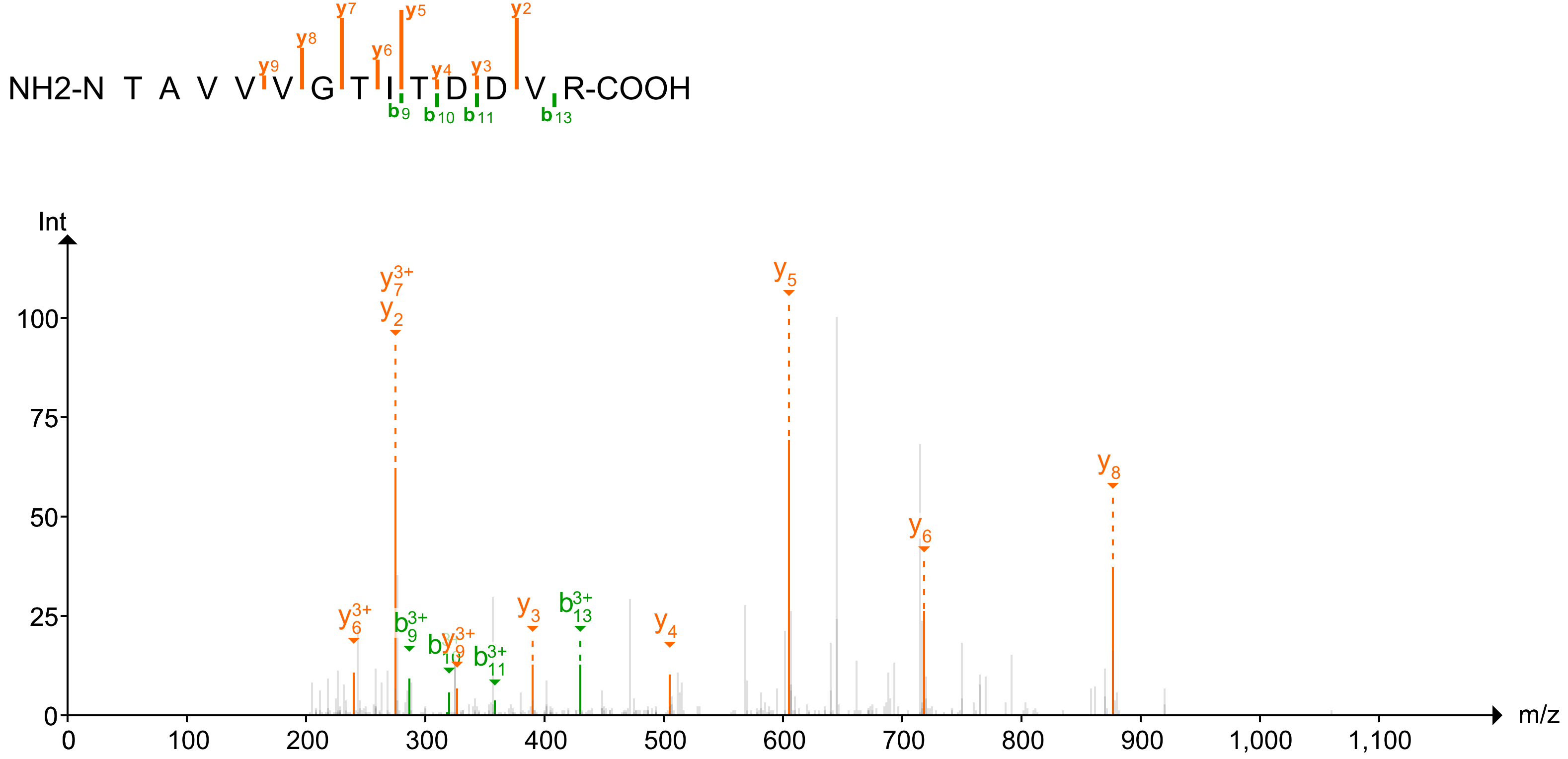

NH2-N T A V V V G T I T D D V R-COOH
y9 y8 y7 y6 y5 y4 y3 y2
b9 b10 b11 b13
Int
100
75
50
25
0
$y_7^{3+}$
$y_2$
$y_5$
$y_8$
$y_6$
$y_6^{3+}$
$b_9^{3+}$
$b_{10}$
$y_9^{3+}$
$b_{11}^{3+}$
$y_3$
$b_{13}^{3+}$
$y_4$
0
100
200
300
400
500
600
700
800
900
1,000
1,100
m/z

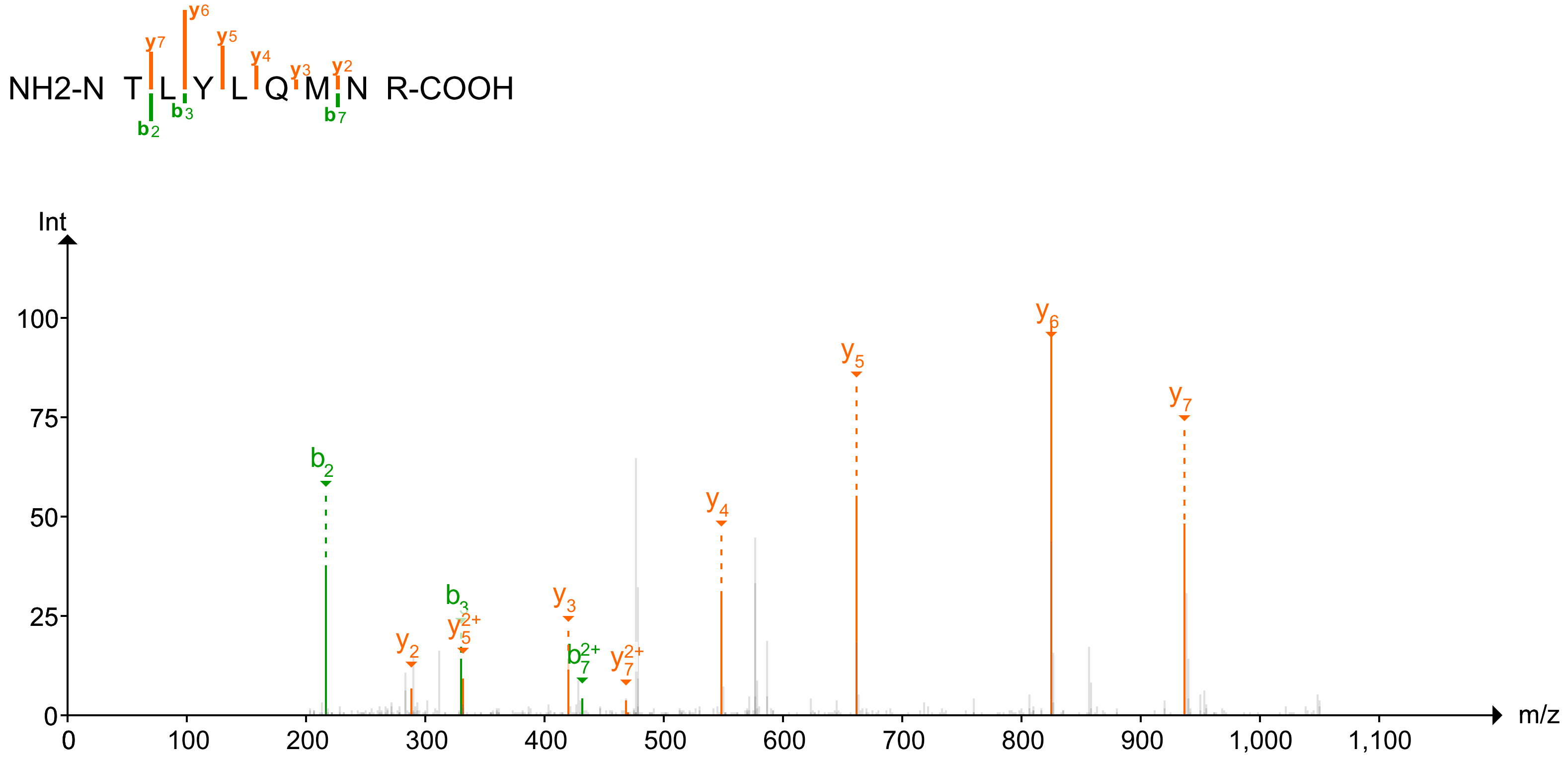

NH2-N T L Y L Q M N R-COOH
y7 y6 y5 y4 y3 y2
b2 b3 b7
Int
100
75
50
25
0
m/z
0 100 200 300 400 500 600 700 800 900 1,000 1,100
b2
y2
b3
y5 2+
y3
b7 2+
y7 2+
y4
y5
y6
y7

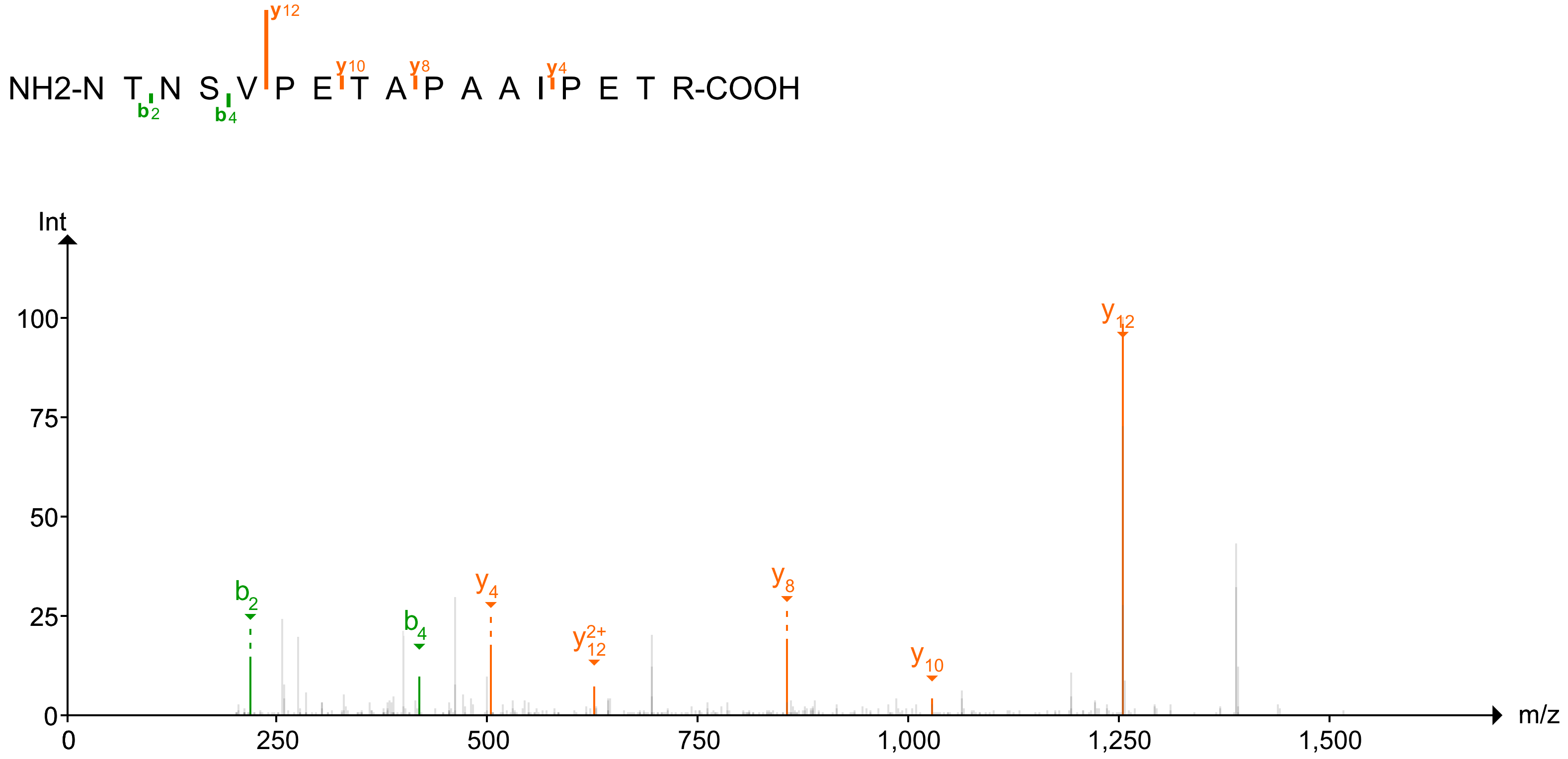

NH2-N T N S V P E T A P A A I P E T R-COOH
y12
y10
y8
y4
b2
b4
Int
100
75
50
25
0
m/z
0
250
500
750
1,000
1,250
1,500
b2
b4
y4
y12 2+
y8
y10
y12

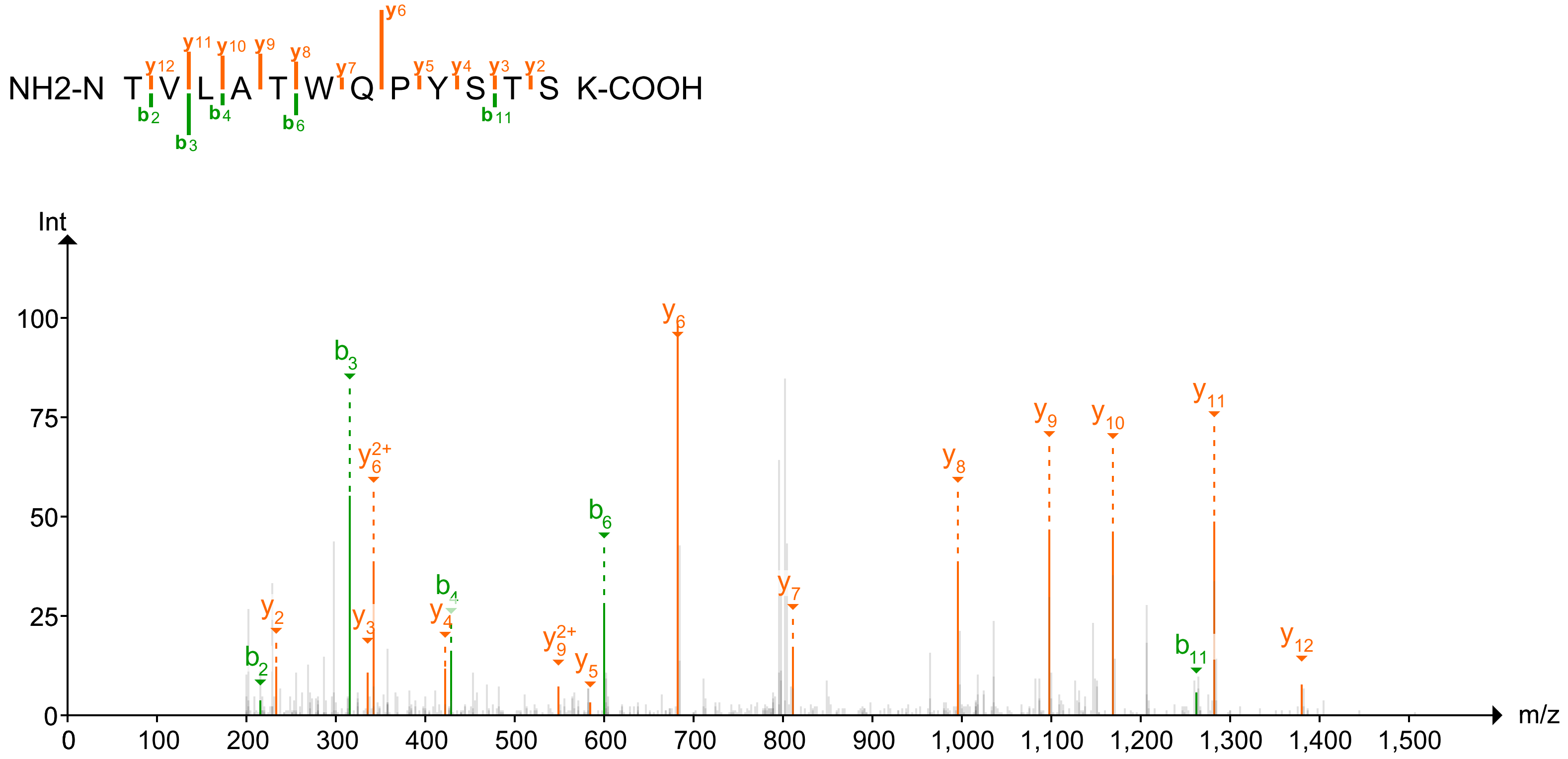

NH2-N T V L A T W Q P Y S T S K-COOH
y12 y11 y10 y9 y8 y7 y6 y5 y4 y3 y2
b2 b3 b4 b6 b11
Int
100
75
50
25
0
m/z
0
100
200
300
400
500
600
700
800
900
1,000
1,100
1,200
1,300
1,400
1,500
y6
b3
y6 2+
b6
y7
y8
y9
y10
y11
y12
b11
y2
b2
y3
y4
b4
y9 2+
y5

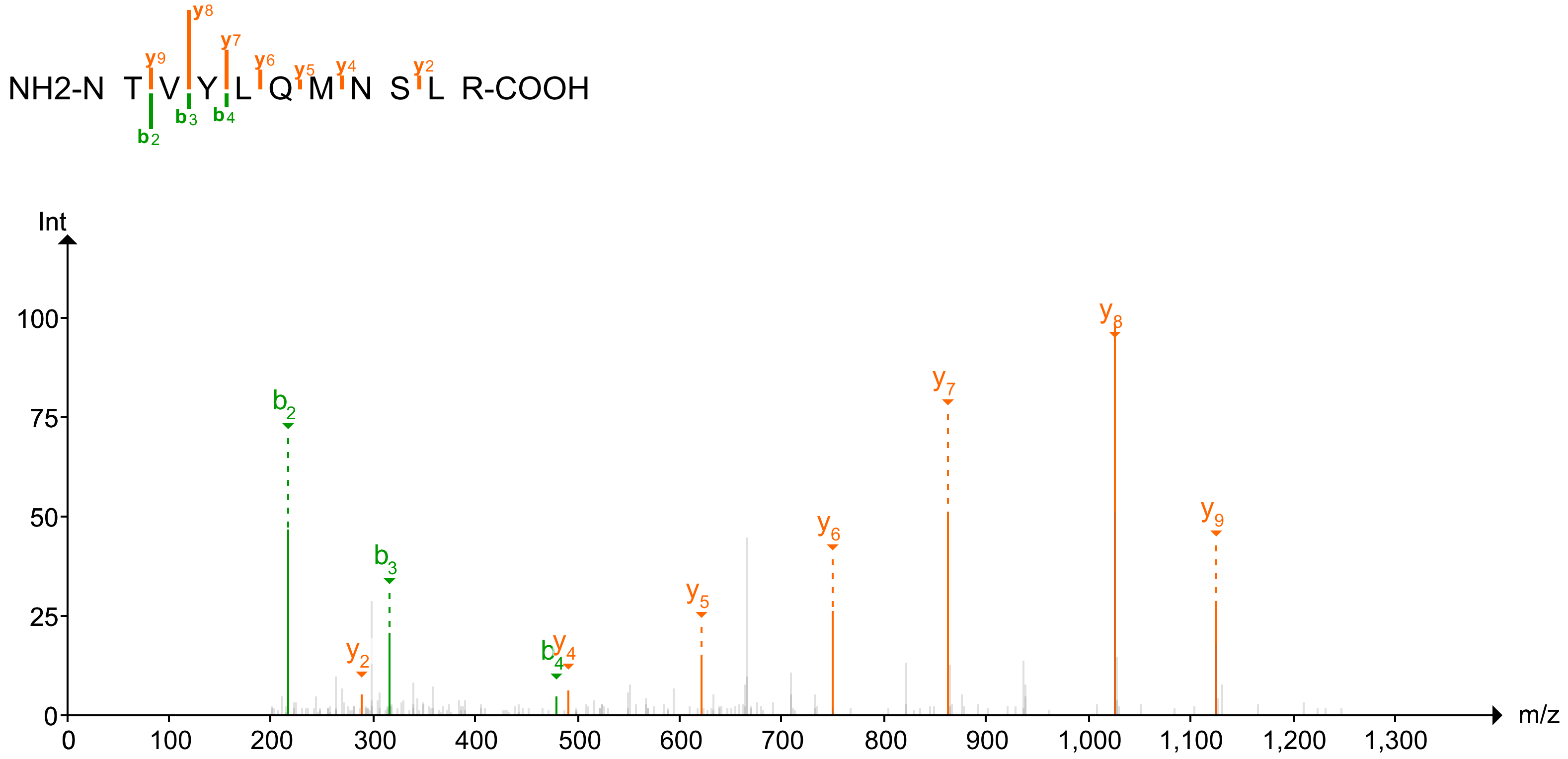

NH2-N T V Y L Q M N S L R-COOH
y9 y8 y7 y6 y5 y4 y2
b2 b3 b4
Int
100
75
50
25
0
b2
b3
y2
b4
y4
y5
y6
y7
y8
y9
0 100 200 300 400 500 600 700 800 900 1,000 1,100 1,200 1,300
m/z

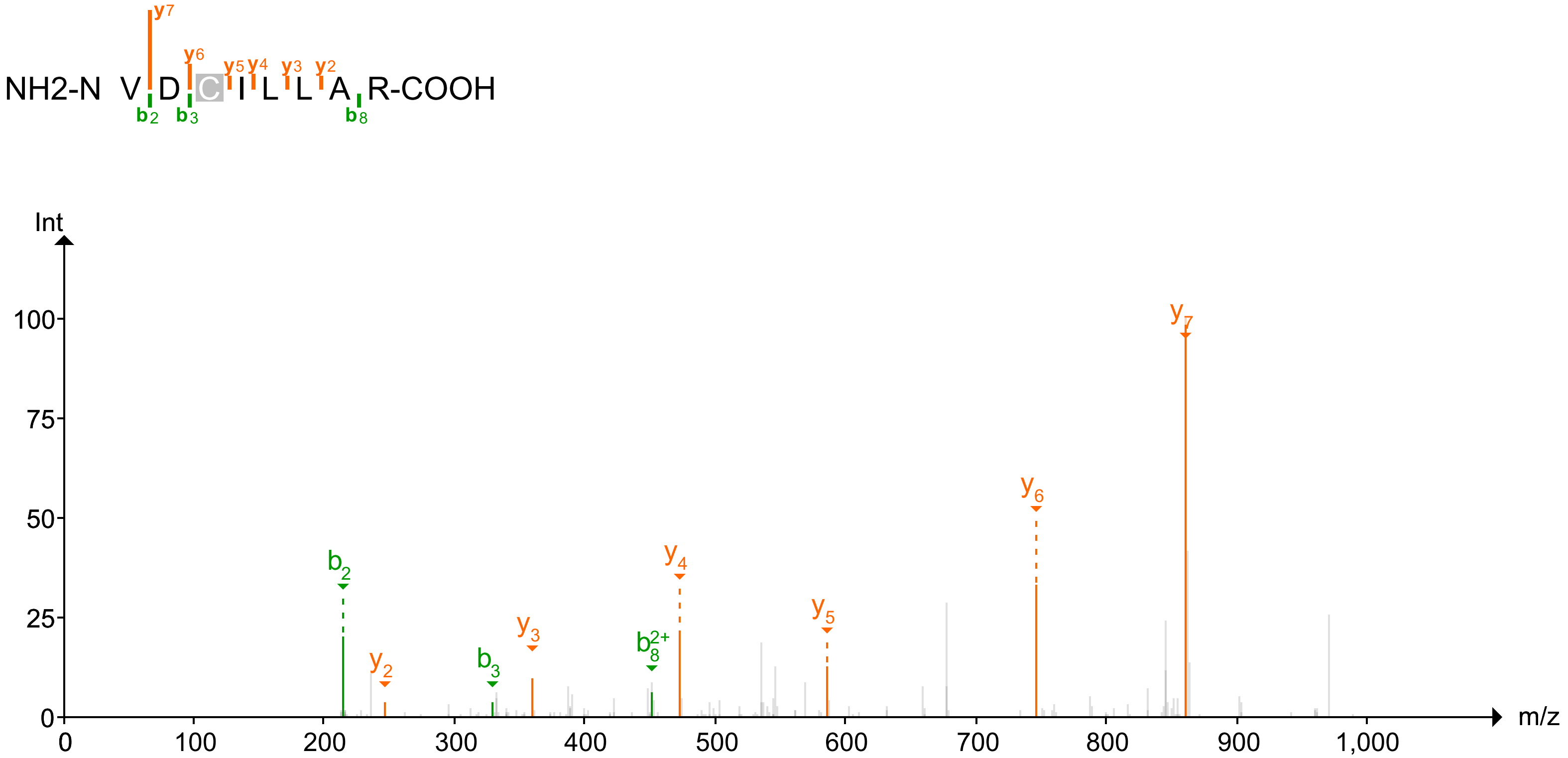

NH2-N V D C I L L A R-COOH
y7 y6 y5 y4 y3 y2
b2 b3 b8
Int
100
75
50
25
0
0 100 200 300 400 500 600 700 800 900 1,000
m/z
b2 y2 b3 y3 b8 2+ y4 y5 y6 y7

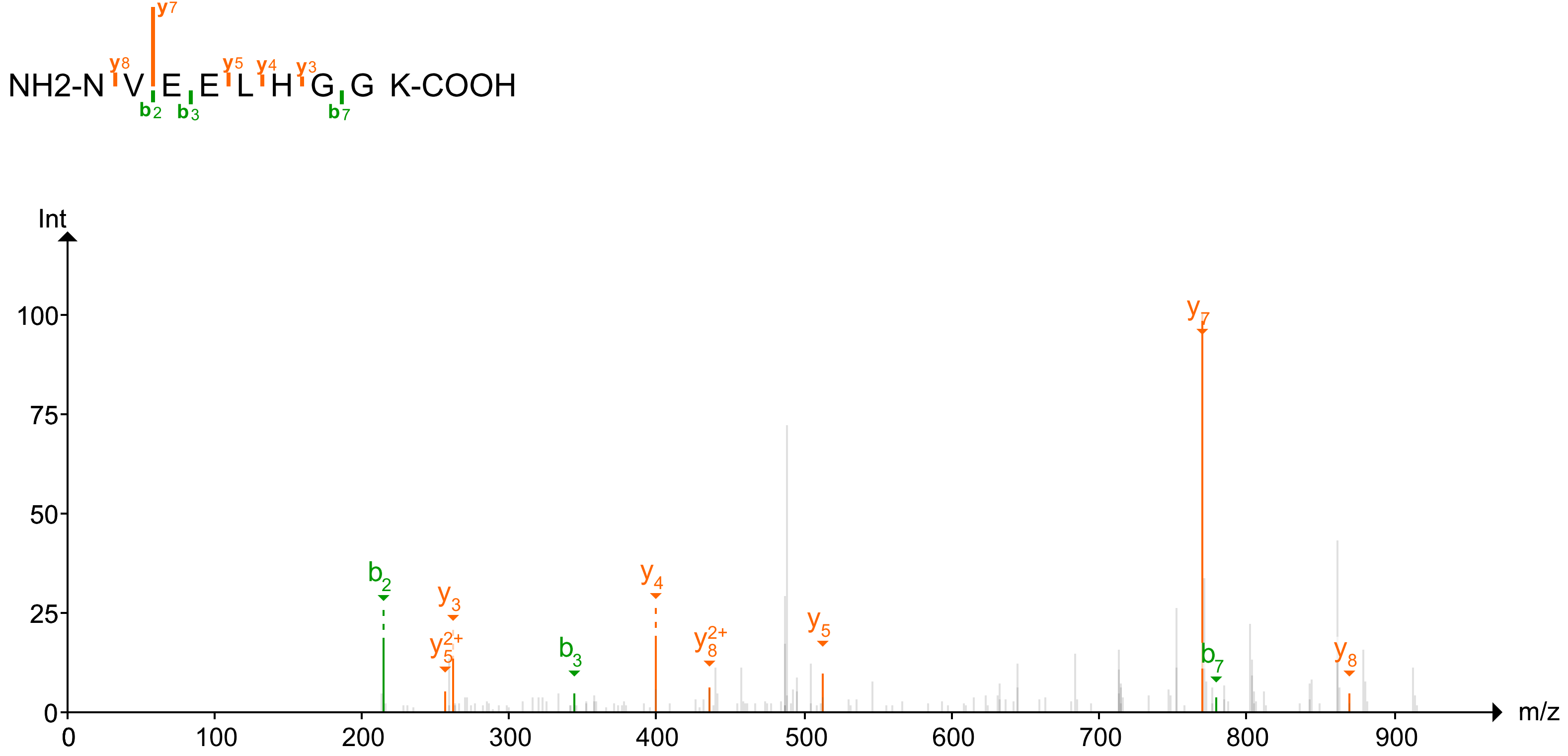

NH2-N V E E L H G G K-COOH
y7
y8
y5
y4
y3
b2
b3
b7
Int
100
75
50
25
0
m/z
0
100
200
300
400
500
600
700
800
900
b2
y3
y5 2+
b3
y4
y8 2+
y5
y7
b7
y8

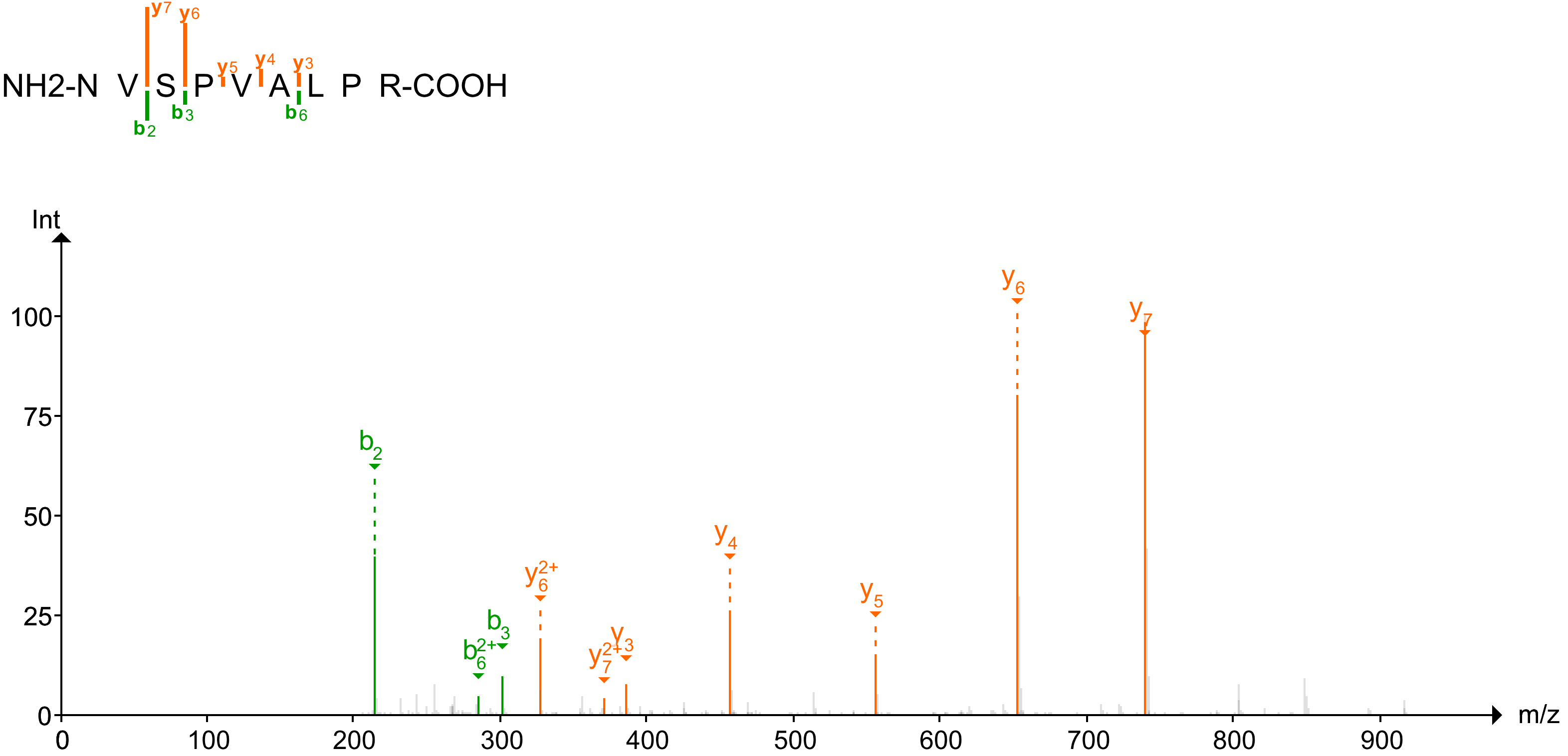
NH2-N V S P V A L P R-COOH
y7 y6 y5 y4 y3
b2 b3 b6
Int
100
75
50
25
0
m/z
0 100 200 300 400 500 600 700 800 900
b2
b3
b6 2+
y6 2+
y7 2+
y3
y4
y5
y6
y7

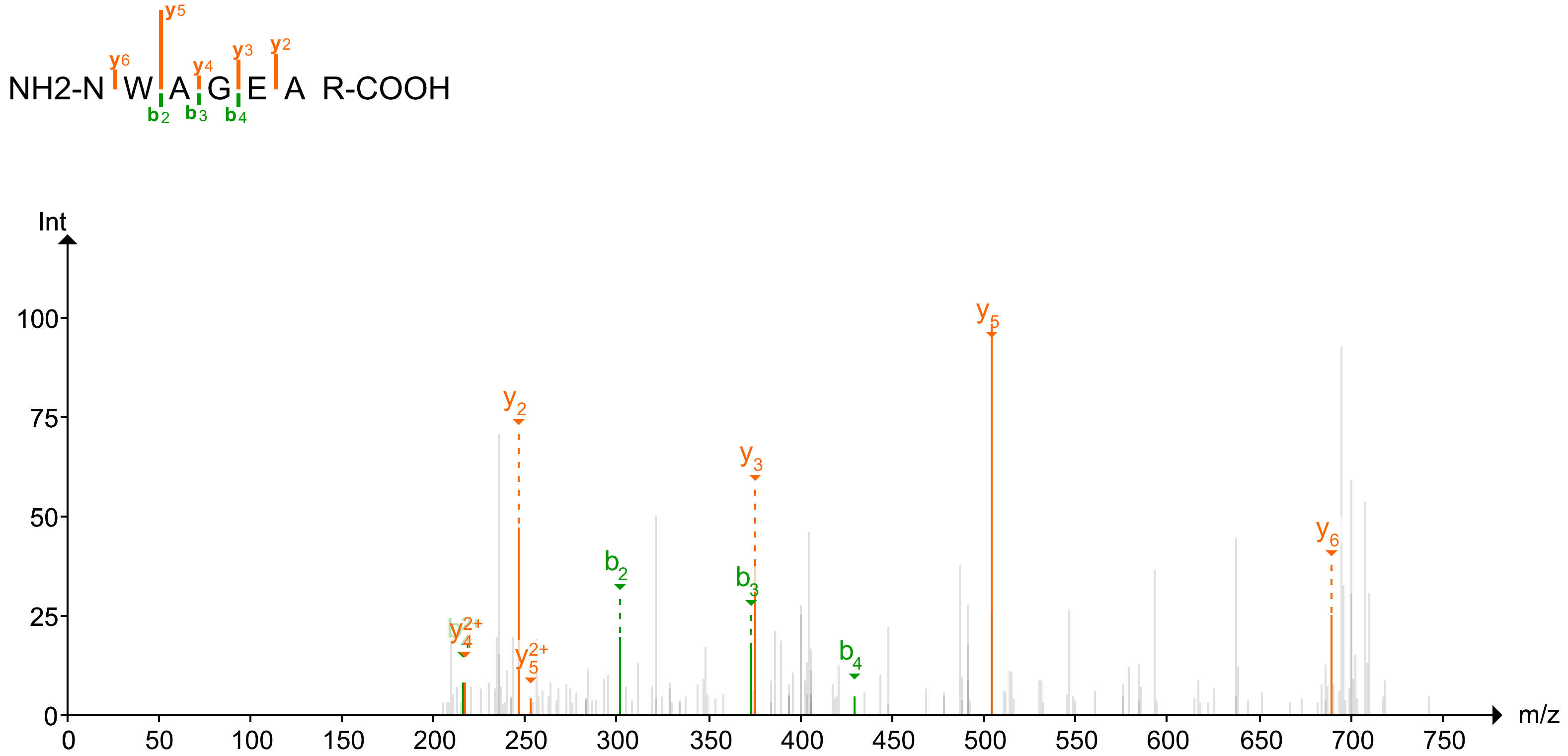

NH2-N W A G E A R-COOH
y6 y5 y4 y3 y2
b2 b3 b4
Int
100
75
50
25
0
y5
y2
y3
y6
b2
b3
b4
y4 2+
y5 2+
0 50 100 150 200 250 300 350 400 450 500 550 600 650 700 750
m/z

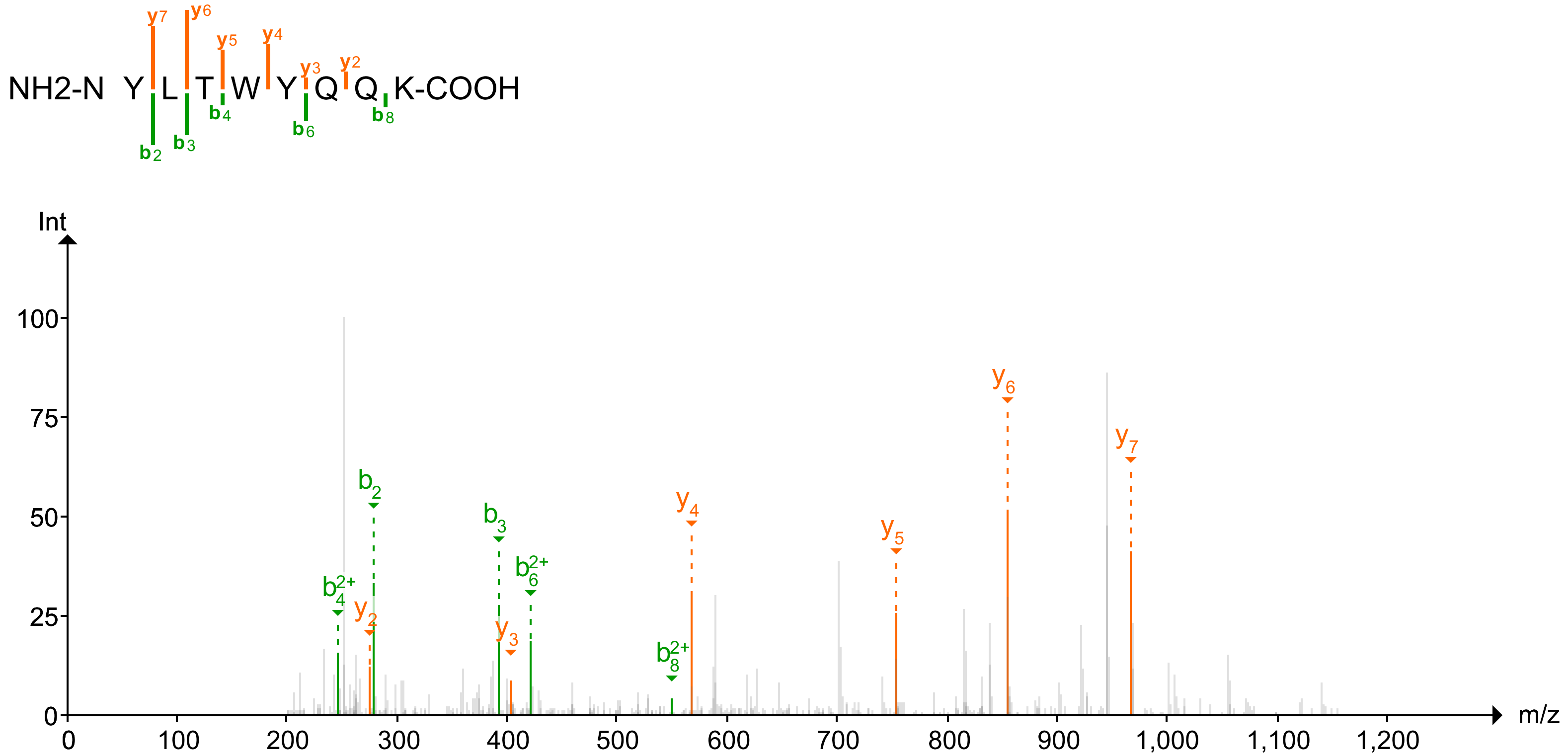

NH2-N Y L T W Y Q Q K-COOH
y7 y6 y5 y4 y3 y2
b2 b3 b4 b6 b8
Int
100
75
50
25
0
m/z
0
100
200
300
400
500
600
700
800
900
1,000
1,100
1,200
$b_2$
$b_3$
$b_4^{2+}$
$b_6^{2+}$
$b_8^{2+}$
$y_2$
$y_3$
$y_4$
$y_5$
$y_6$
$y_7$

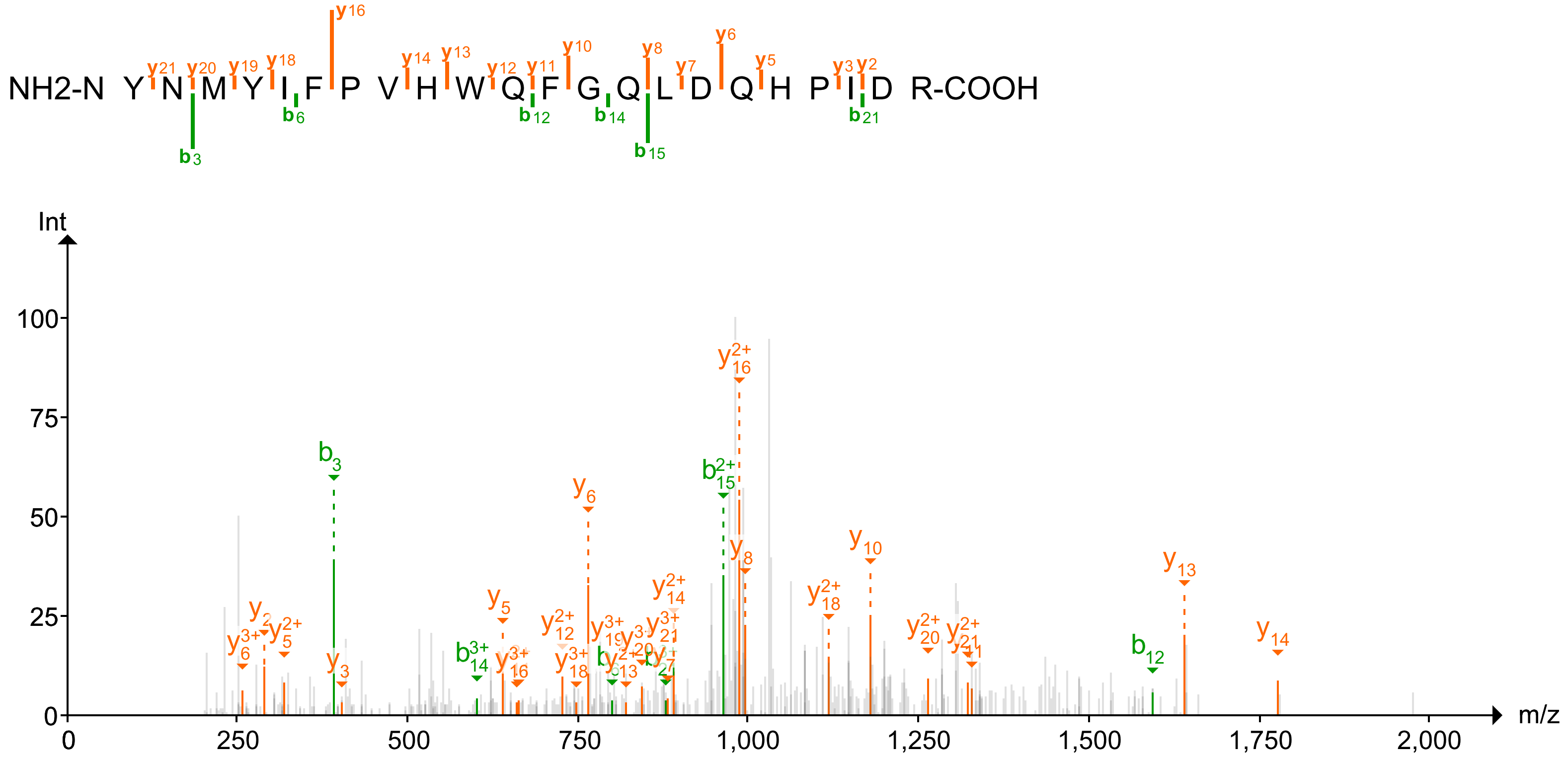

NH2-N Y N M Y I F P V H W Q F G Q L D Q H P I D R-COOH
y21 y20 y19 y18 y16 y14 y13 y12 y11 y10 y8 y7 y6 y5 y3 y2
b3 b6 b12 b14 b15 b21
Int
100
75
50
25
0
m/z
0
250
500
750
1,000
1,250
1,500
1,750
2,000

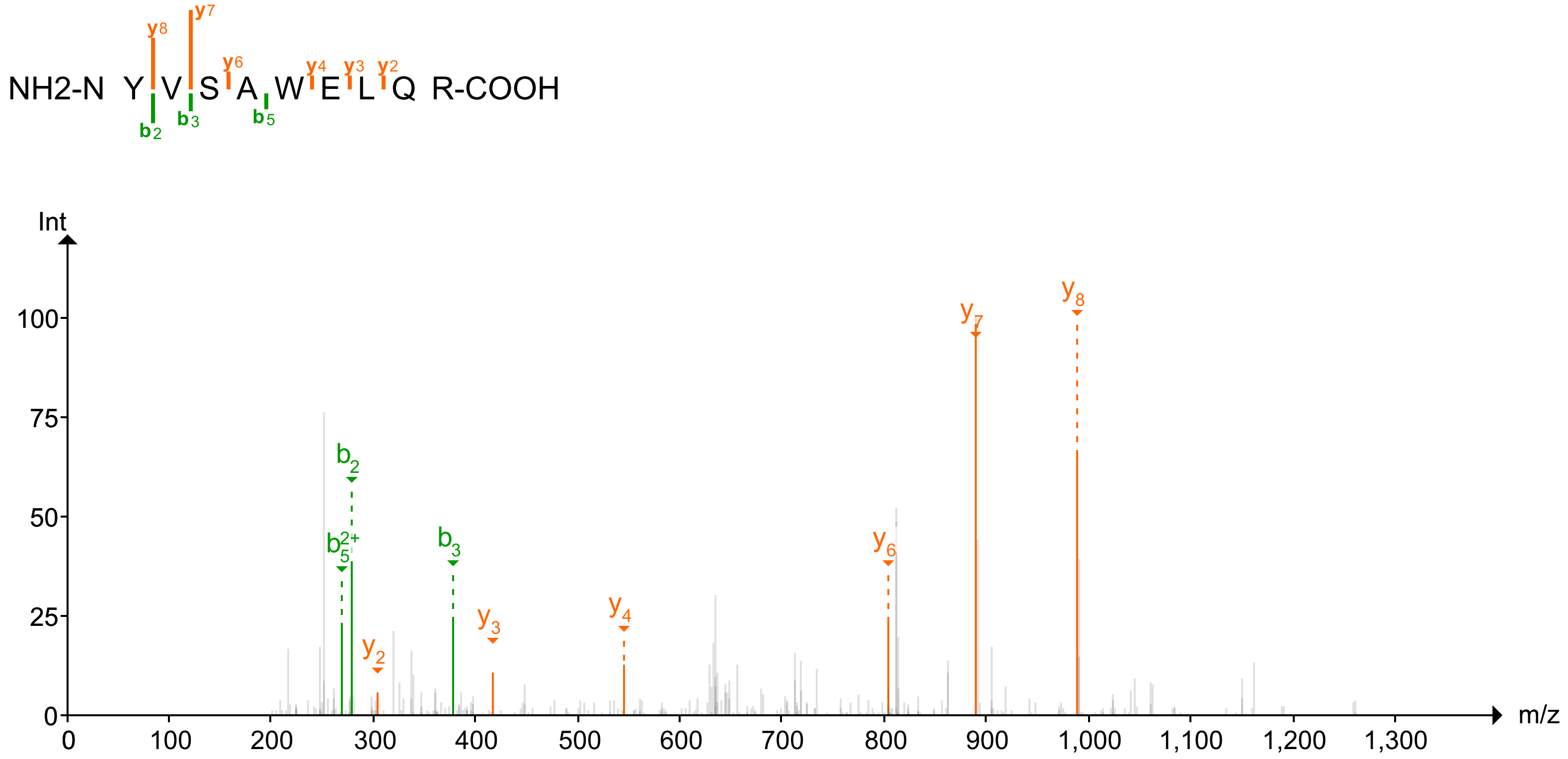

NH2-N Y V S A W E L Q R-COOH
y8
y7
y6
y4
y3
y2
b2
b3
b5
Int
100
75
50
25
0
m/z
0
100
200
300
400
500
600
700
800
900
1,000
1,100
1,200
1,300
b2
b5 2+
b3
y2
y3
y4
y6
y7
y8

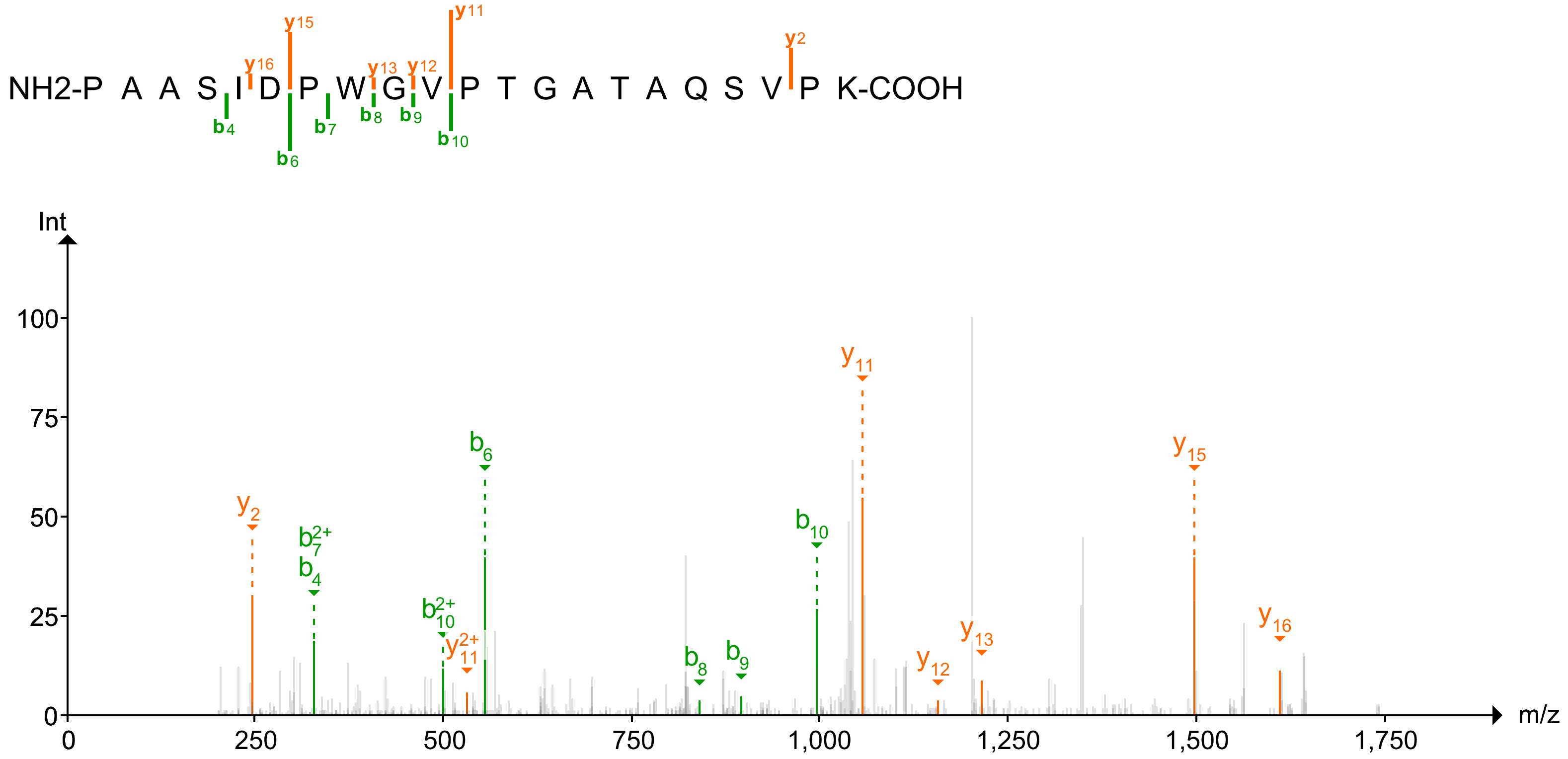

NH2-P A A S I D P W G V P T G A T A Q S V P K-COOH
y15
y11
y16
y13
y12
y2
b4
b6
b7
b8
b9
b10
Int
100
75
50
25
0
m/z
0
250
500
750
1,000
1,250
1,500
1,750

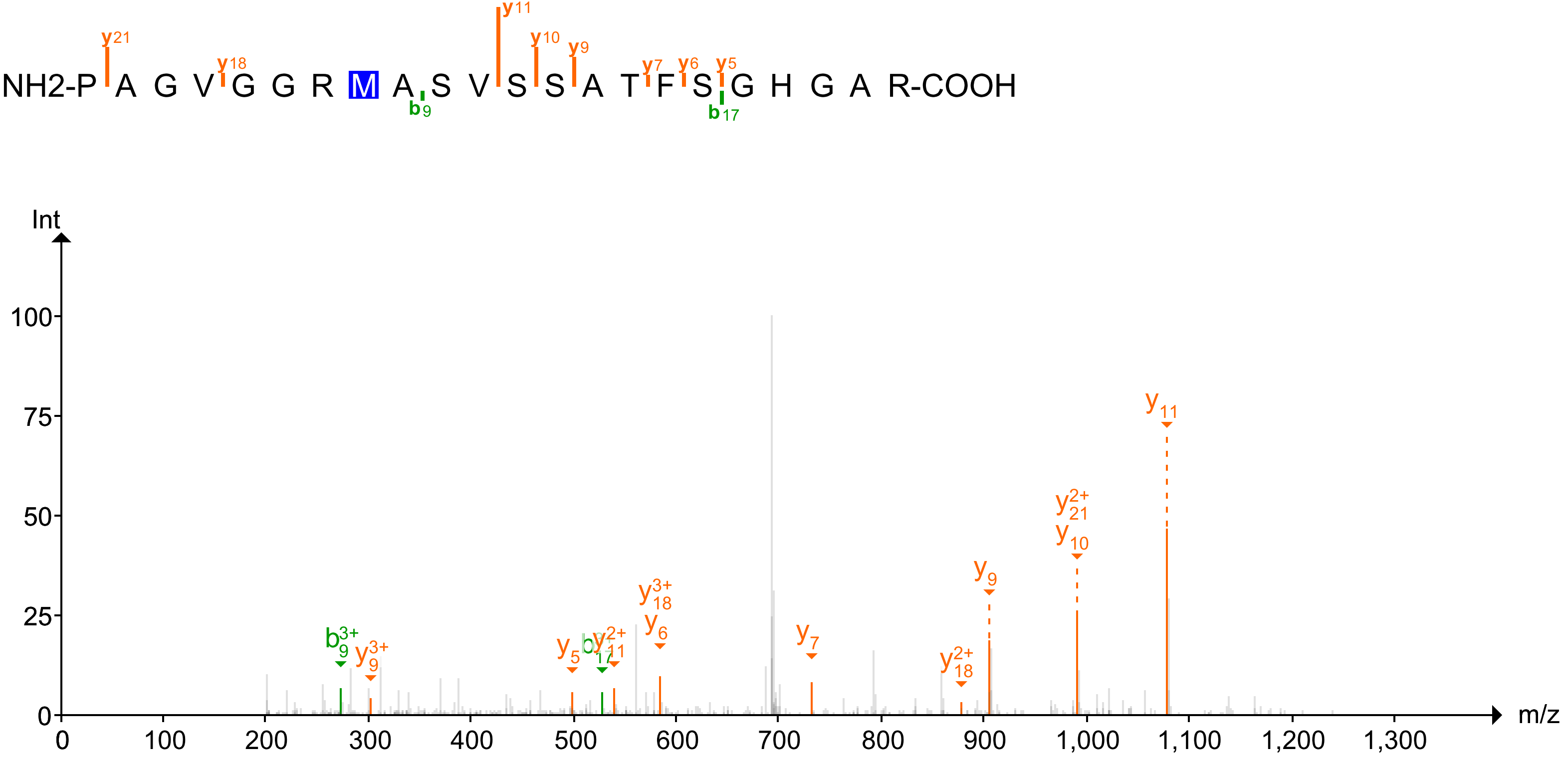

NH2-P A G V G G R M A S V S S A T F S G H G A R-COOH
y21 y18 y11 y10 y9 y7 y6 y5
b9 b17
Int
100
75
50
25
0
m/z
0 100 200 300 400 500 600 700 800 900 1,000 1,100 1,200 1,300
y11
y21 2+
y10
y9
y18 3+
y6
y7
y18 2+
b9 3+
y9 3+
y5
b17
y11 2+

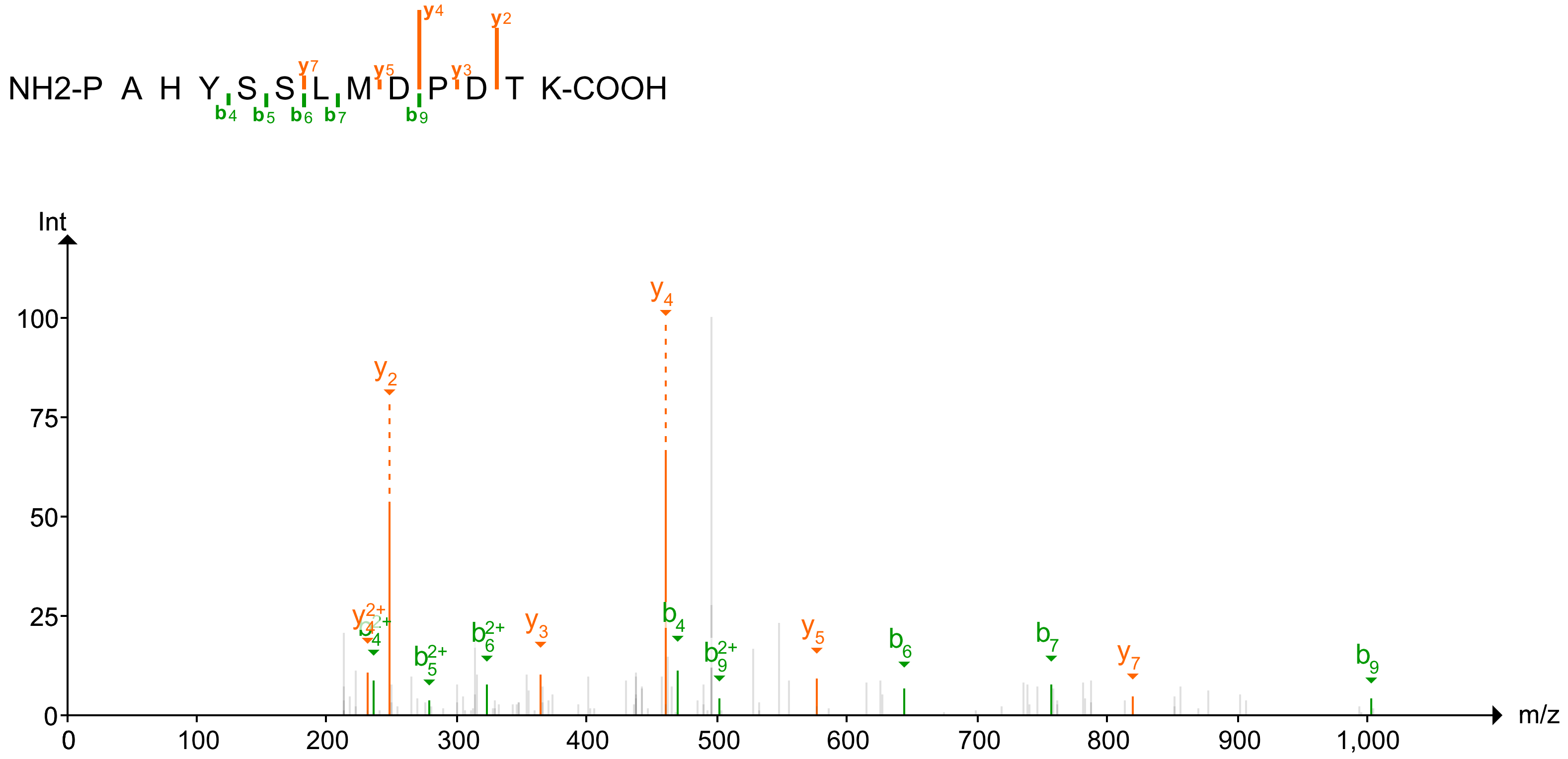

NH2-P A H Y S S L M D P D T K-COOH
Int
m/z
y4
y2
y2+4
b2+4
b2+5
b2+6
y3
b4
b2+9
y5
b6
b7
y7
b9

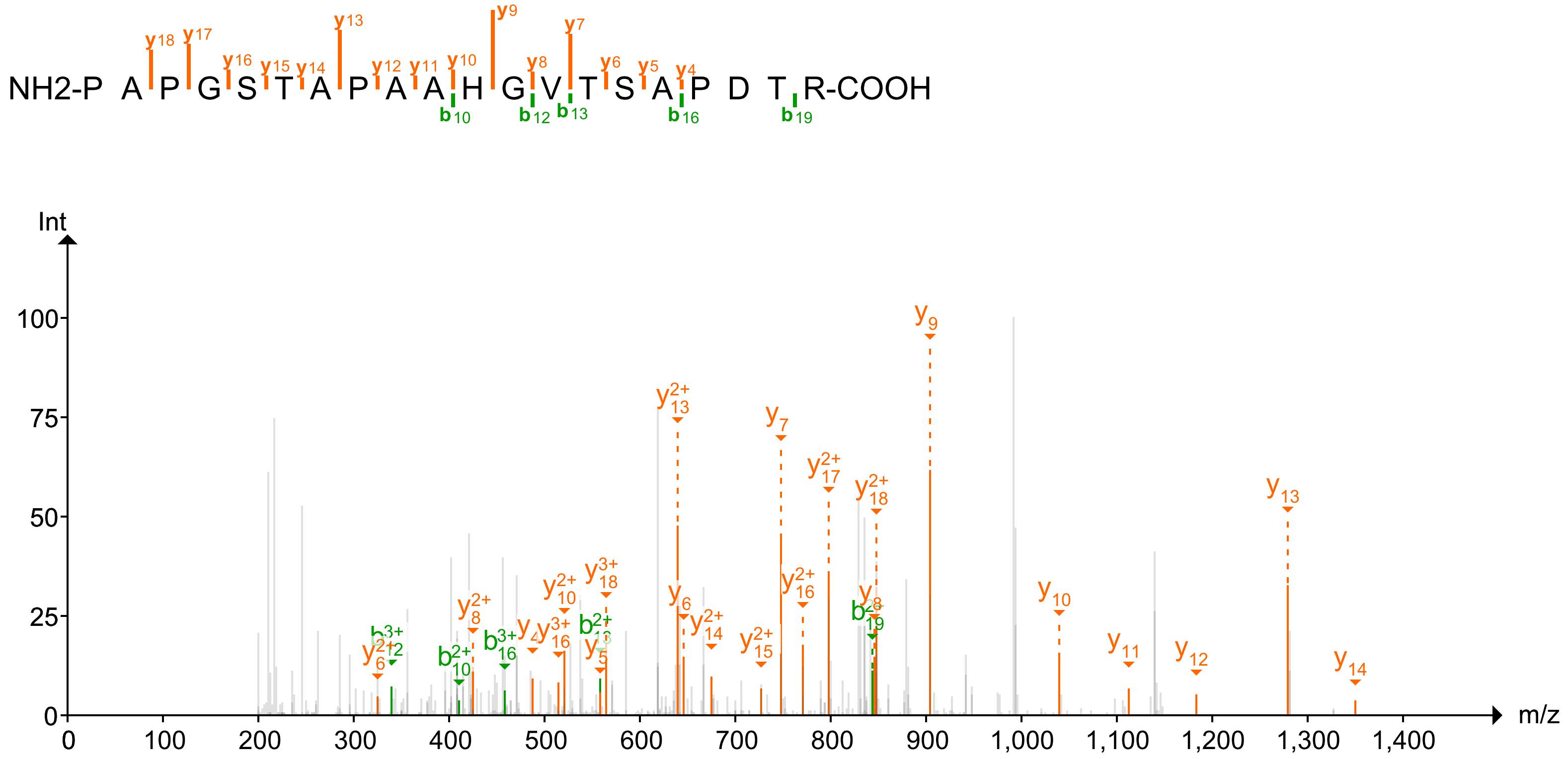

NH2-P A P G S T A P A A H G V T S A P D T R-COOH
y18 y17 y16 y15 y14 y13 y12 y11 y10 y9 y8 y7 y6 y5 y4
b10 b12 b13 b16 b19
Int
m/z
0 25 50 75 100
0 100 200 300 400 500 600 700 800 900 1,000 1,100 1,200 1,300 1,400
y9
y2+13
y7
y2+17
y2+18
y13
y10
y11
y12
y14
y6
y2+14
y2+15
y2+16
y3+18
y2+10
y3+16
y4
y5
y2+8
y2+6
b3+12
b2+10
b3+16
b2+19

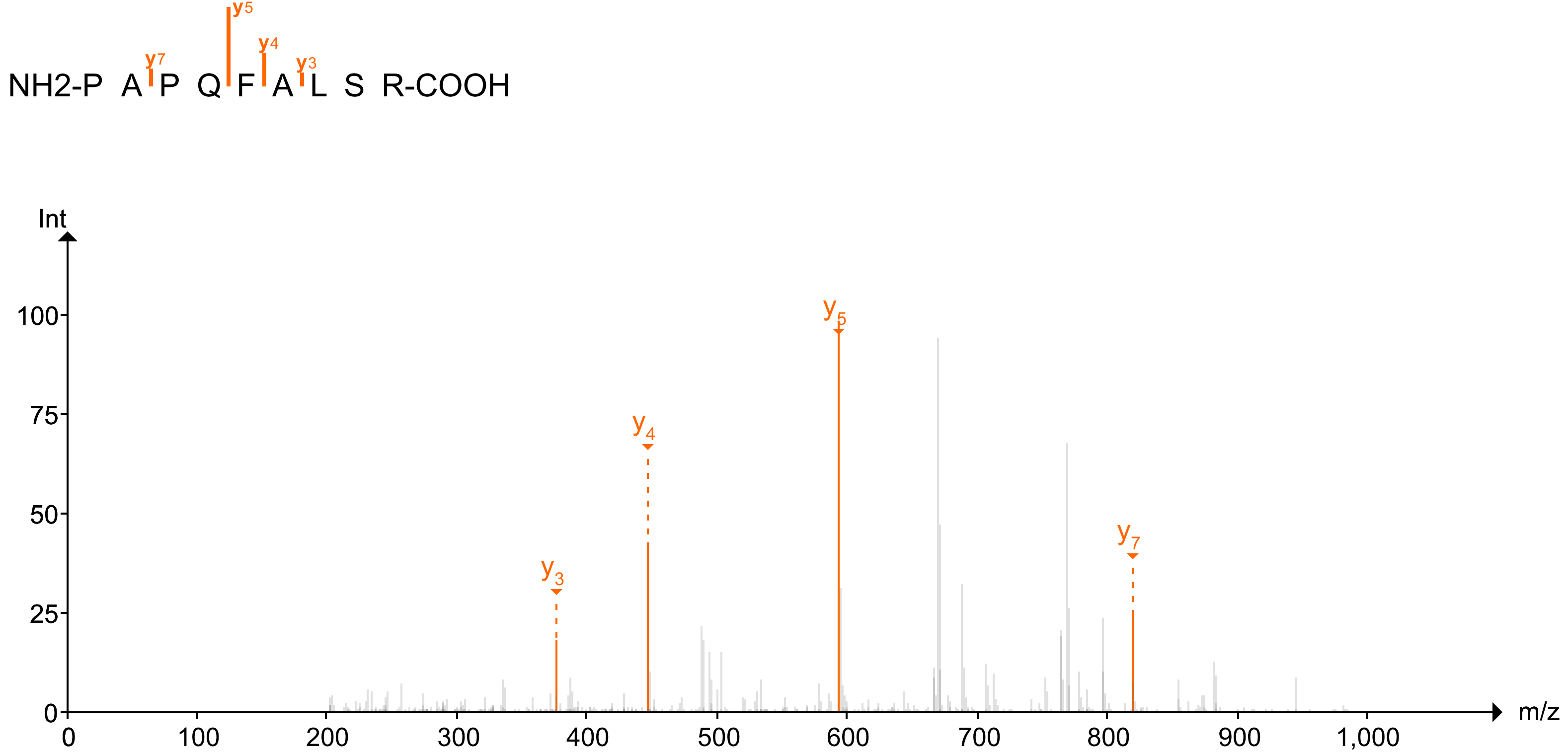

NH2-P A P Q F A L S R-COOH
y7
y5
y4
y3
Int
100
75
50
25
0
y3
y4
y5
y7
0
100
200
300
400
500
600
700
800
900
1,000
m/z

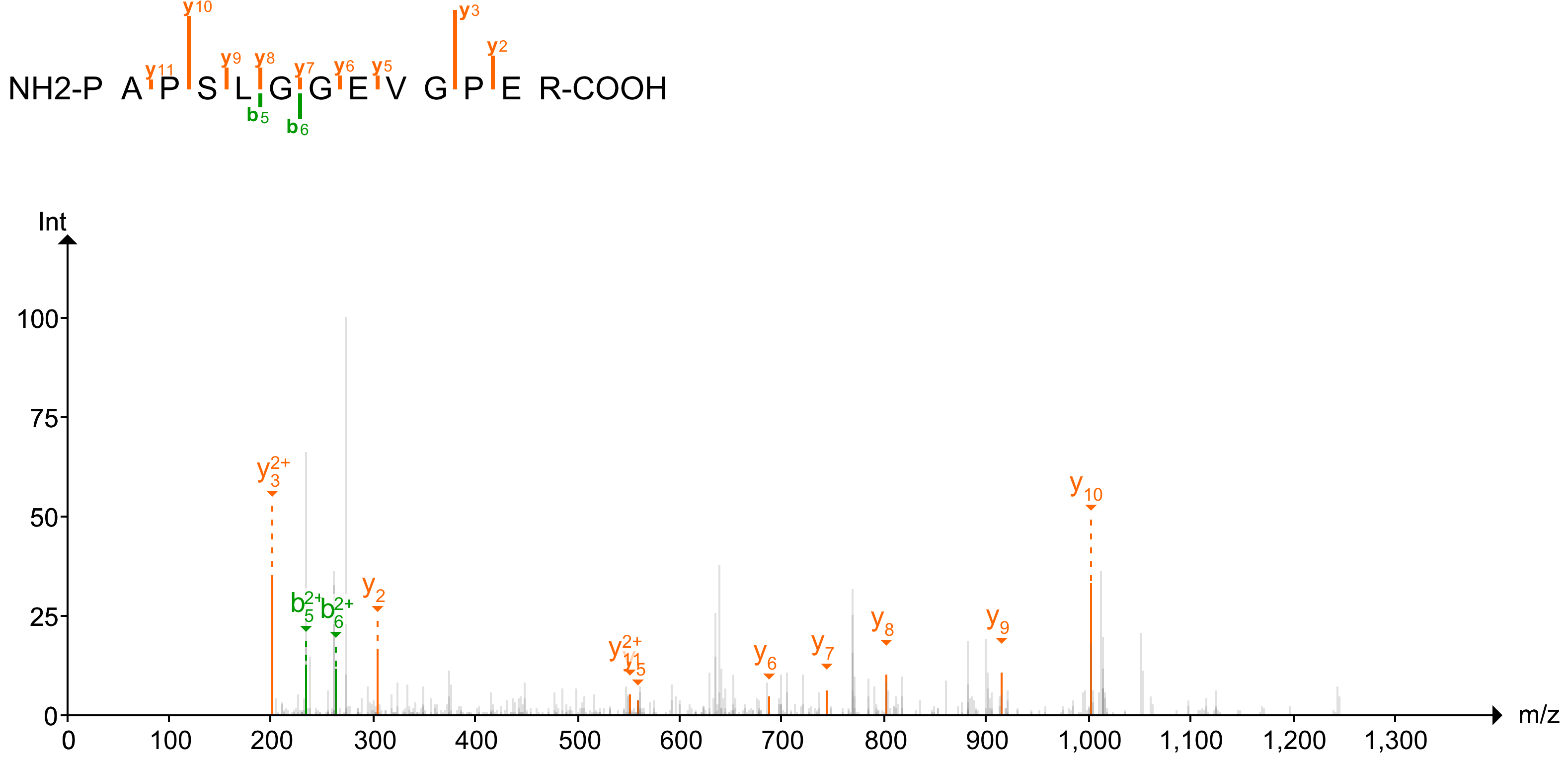

NH2-P A P S L G G E V G P E R-COOH
y11 y10 y9 y8 y7 y6 y5 y3 y2
b5 b6
Int
100
75
50
25
0
m/z
0 100 200 300 400 500 600 700 800 900 1,000 1,100 1,200 1,300
y3 2+
b5 2+
b6 2+
y2
y11 2+
y5
y6
y7
y8
y9
y10

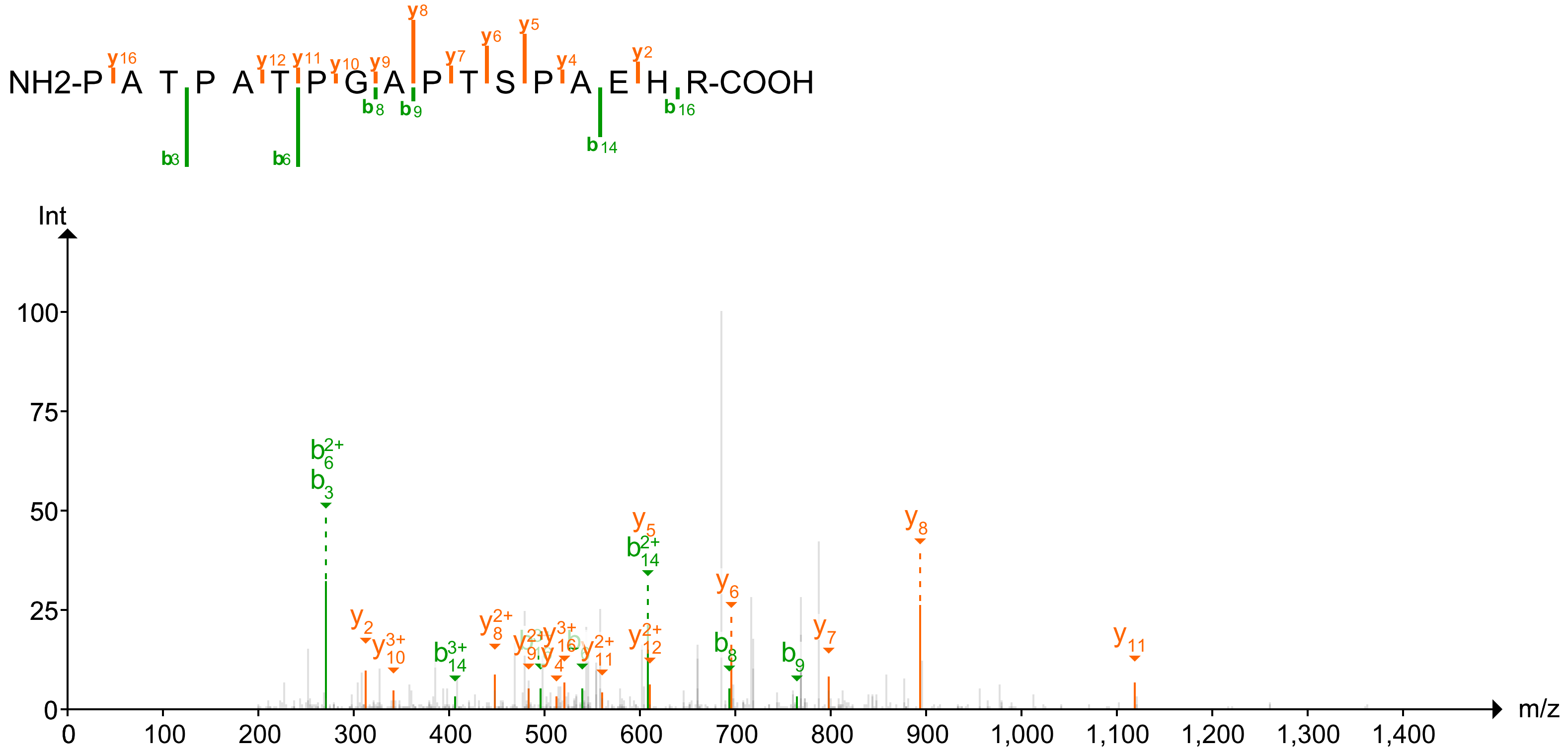
NH2-P A T P A T P G A P T S P A E H R-COOH
y16 y12 y11 y10 y9 y8 y7 y6 y5 y4 y2
b3 b6 b8 b9 b14 b16
Int
m/z
0 25 50 75 100
0 100 200 300 400 500 600 700 800 900 1,000 1,100 1,200 1,300 1,400
$b_6^{2+}$ $b_3$
$y_2$ $y_{10}^{3+}$ $b_{14}^{3+}$ $y_8^{2+}$ $y_9^{2+}$ $y_{16}^{3+}$ $y_4$ $y_{11}^{2+}$
$y_5$ $b_{14}^{2+}$ $y_{12}^{2+}$
$y_6$ $b_8$ $b_9$ $y_7$ $y_8$ $y_{11}$

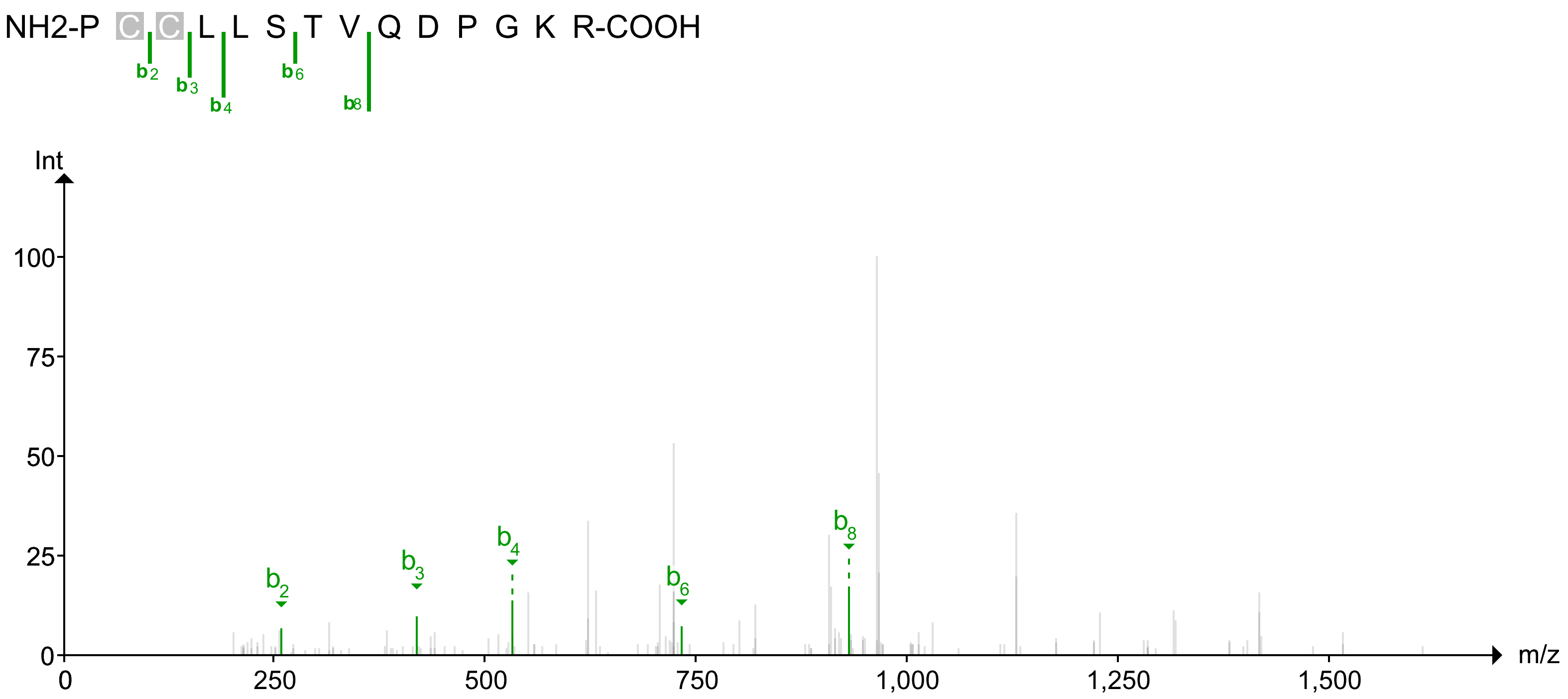
NH2-P C C L L S T V Q D P G K R-COOH
b2
b3
b4
b6
b8
Int
100
75
50
25
0
0
250
500
750
1,000
1,250
1,500
m/z
b2
b3
b4
b6
b8

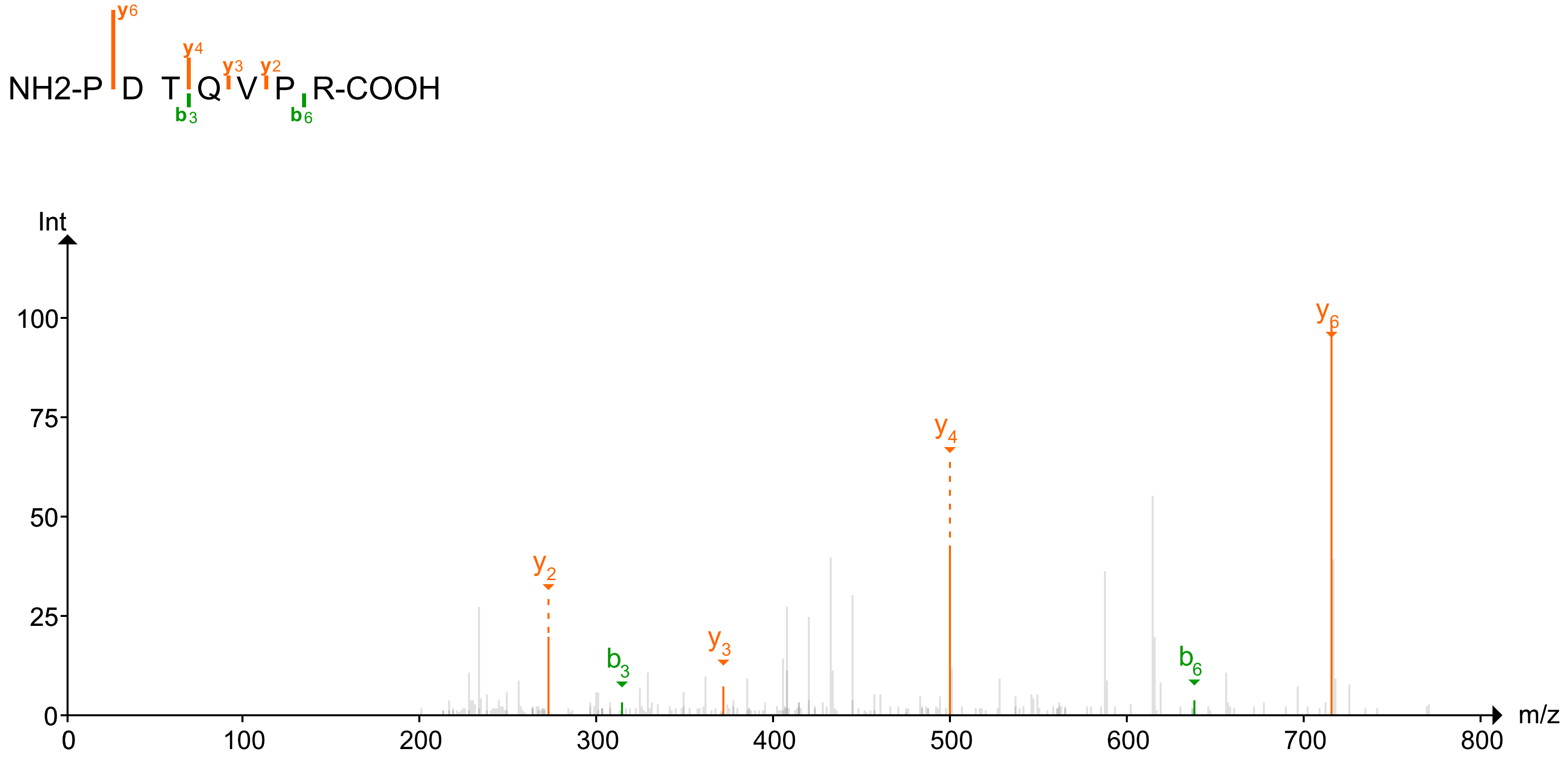

NH2-P D T Q V P R-COOH
y6
y4
y3
y2
b3
b6
Int
100
75
50
25
0
0
100
200
300
400
500
600
700
800
m/z
y2
b3
y3
y4
b6
y6

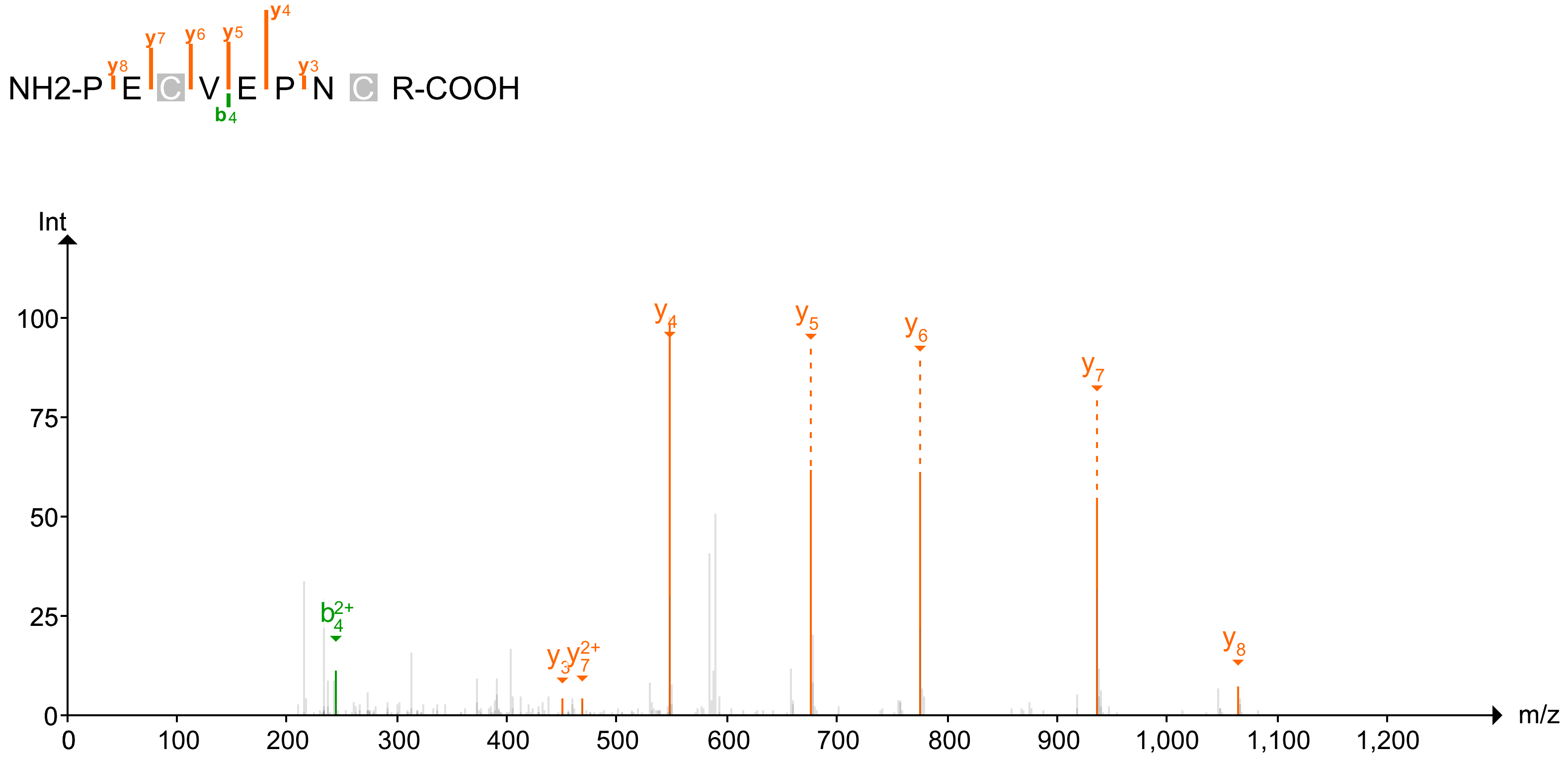

NH2-P E C V E P N C R-COOH
y8
y7
y6
y5
y4
y3
b4
Int
100
75
50
25
0
m/z
0
100
200
300
400
500
600
700
800
900
1,000
1,100
1,200
$b_4^{2+}$
$y_3$
$y_7^{2+}$
$y_4$
$y_5$
$y_6$
$y_7$
$y_8$

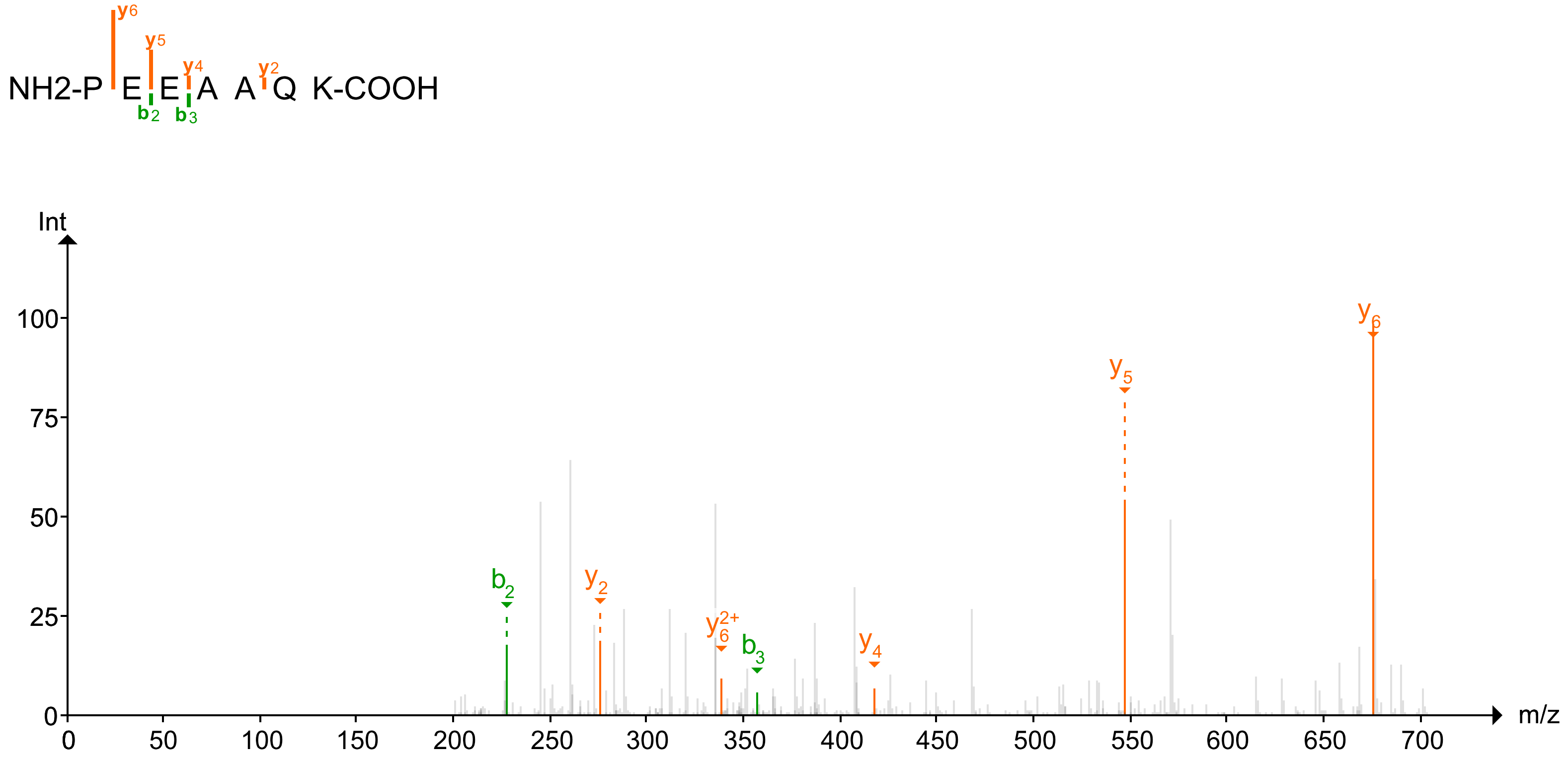

NH2-P E E A A Q K-COOH
y6
y5
y4
y2
b2
b3
Int
m/z
100
75
50
25
0
0
50
100
150
200
250
300
350
400
450
500
550
600
650
700
b2
y2
y6 2+
b3
y4
y5
y6

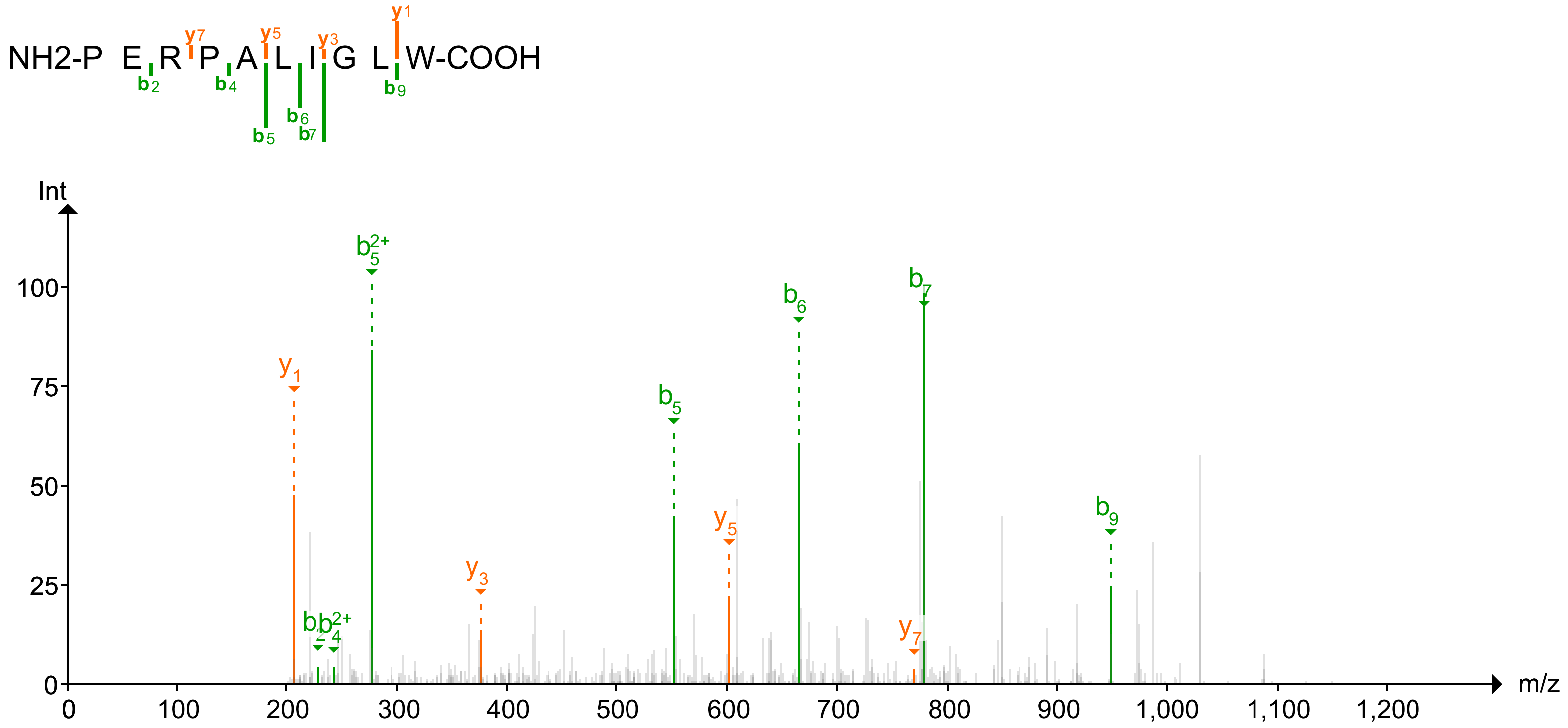

NH2-P E R P A L I G L W-COOH
y7 y5 y3 y1
b2 b4 b5 b6 b7 b9
Int
100
75
50
25
0
m/z
0
100
200
300
400
500
600
700
800
900
1,000
1,100
1,200
b5 2+
y1
b5
b6
b7
y5
y3
b9
y7
b2
b4 2+

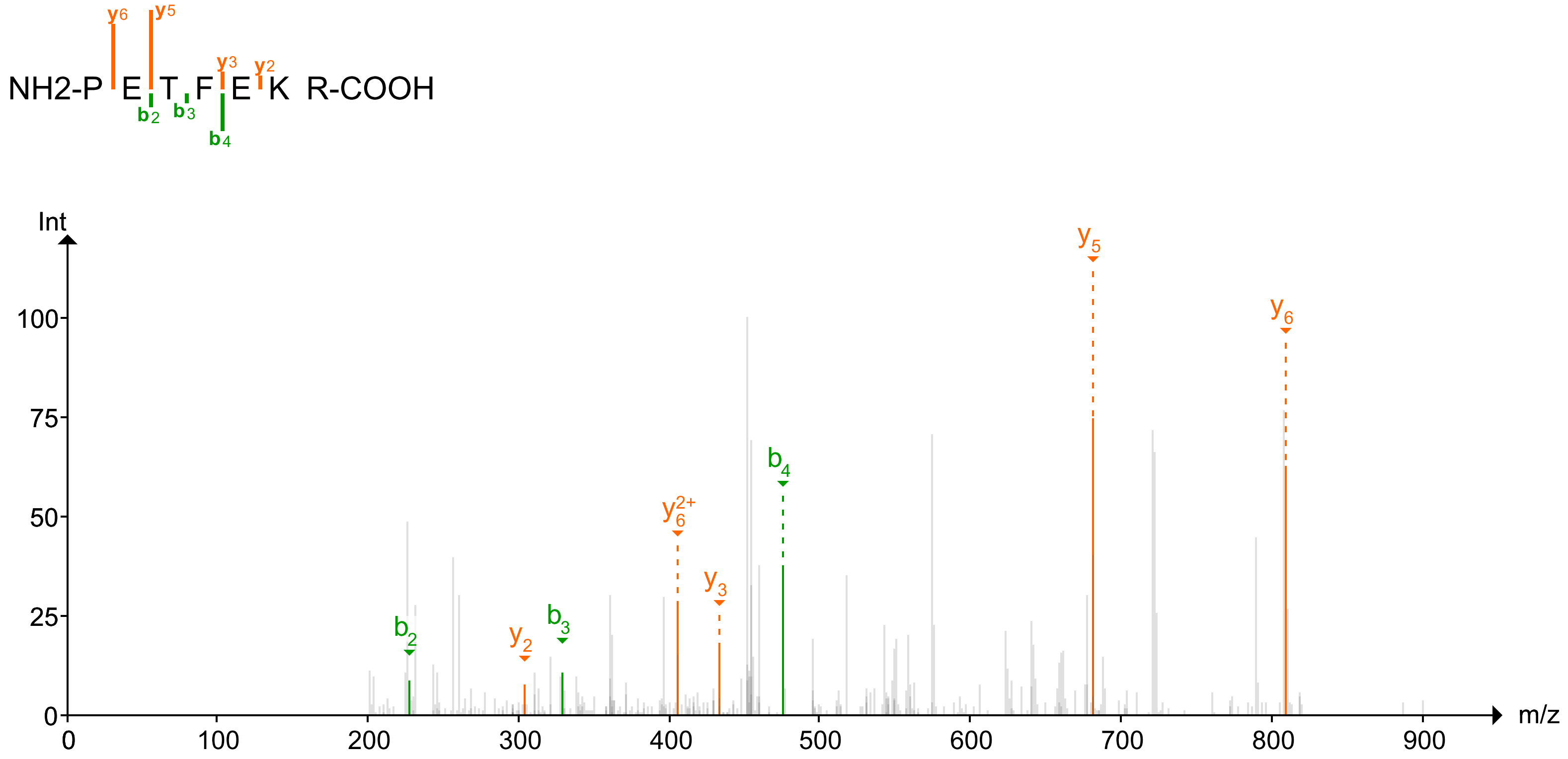

y6
y5
y3
y2
NH2-P E T F E K R-COOH
b2
b3
b4
Int
100
75
50
25
0
y5
y6
b4
y6 2+
y3
b2
y2
b3
0
100
200
300
400
500
600
700
800
900
m/z

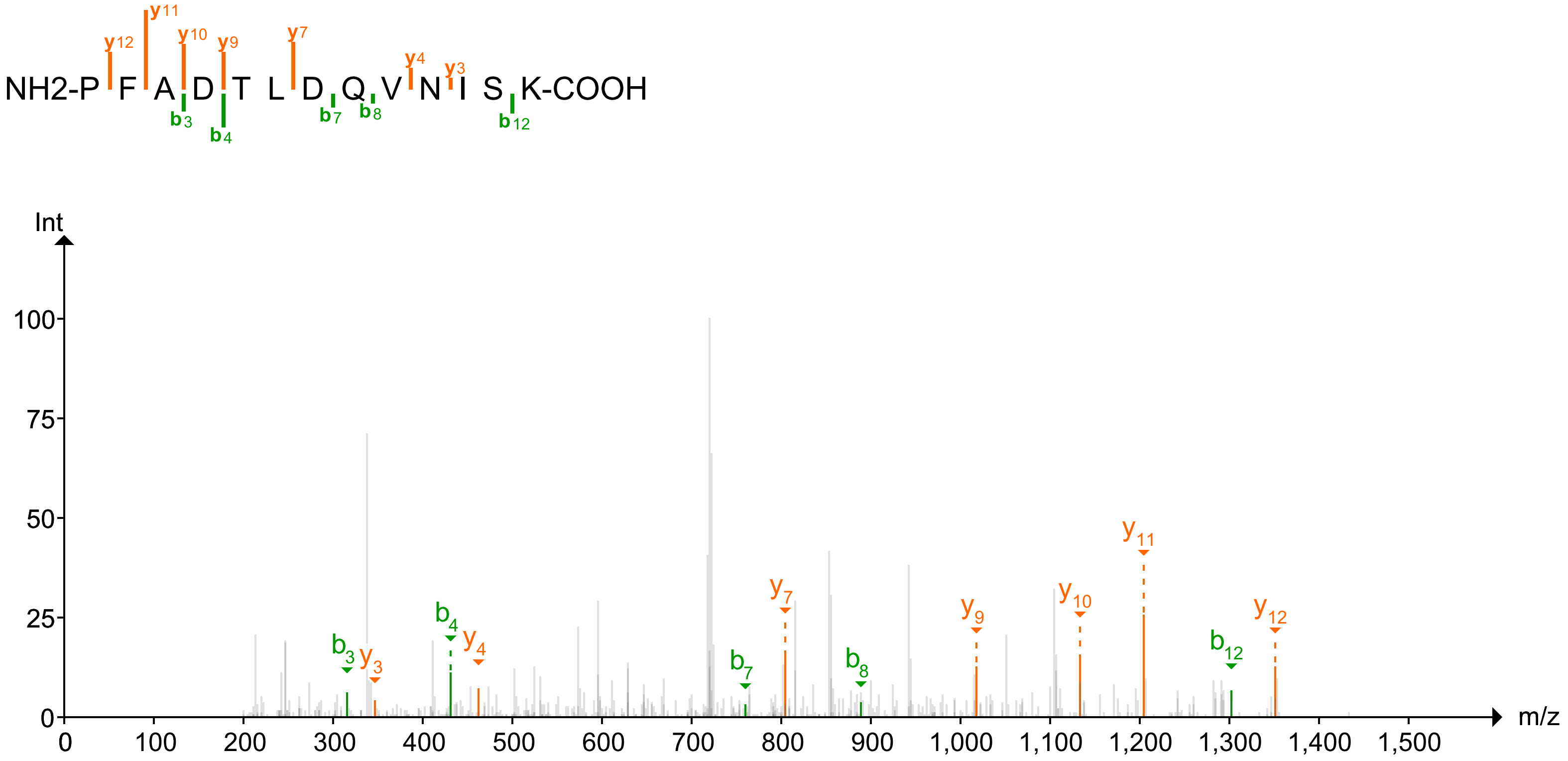
NH2-P F A D T L D Q V N I S K-COOH
y12 y11 y10 y9 y7 y4 y3
b3 b4 b7 b8 b12
Int
100
75
50
25
0
m/z
0 100 200 300 400 500 600 700 800 900 1,000 1,100 1,200 1,300 1,400 1,500
b3 y3 b4 y4 b7 y7 b8 y9 y10 y11 b12 y12

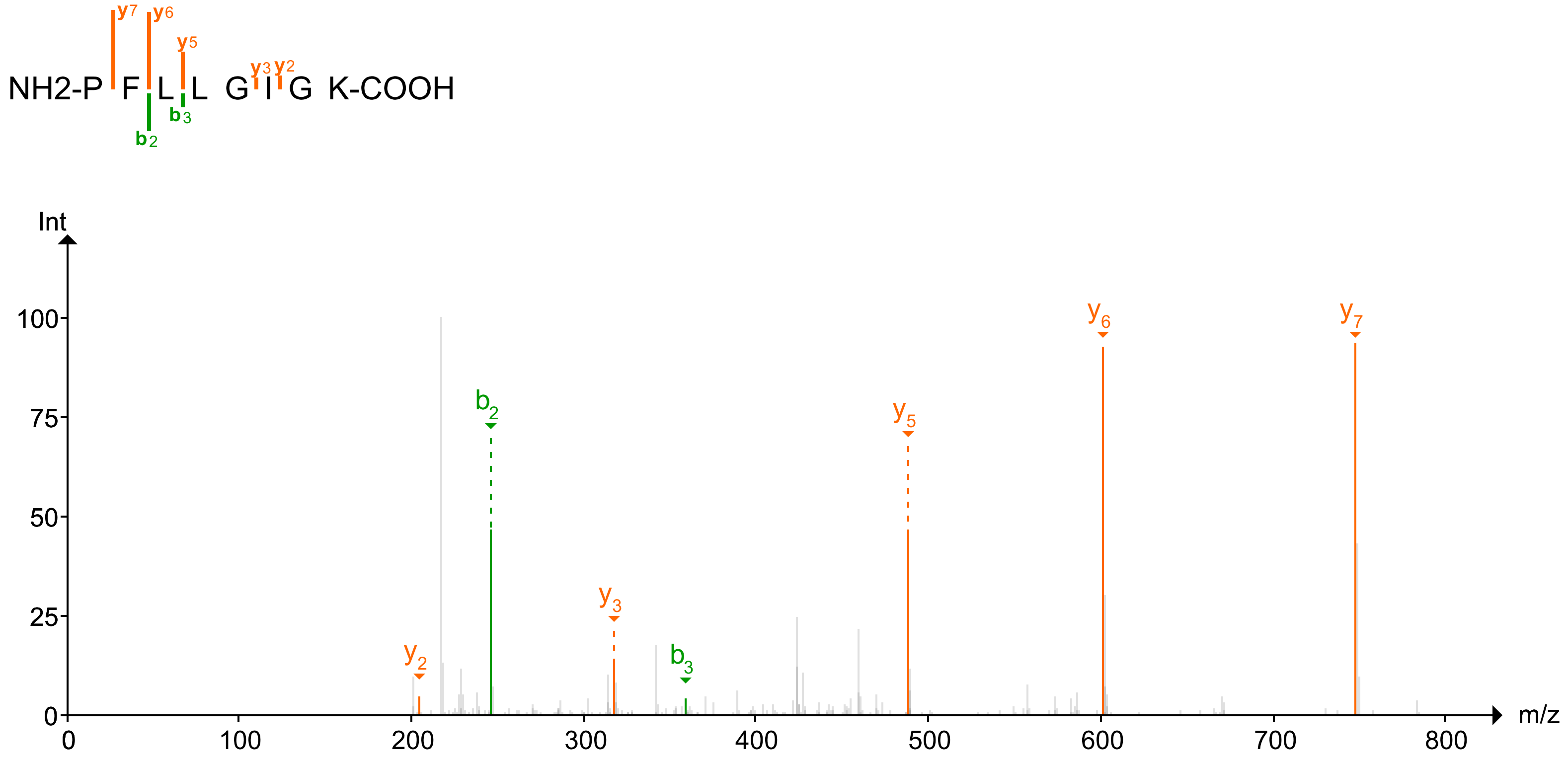

NH2-P F L L G I G K-COOH
y7 y6 y5 y3 y2
b2 b3
Int
100
75
50
25
0
m/z
0
100
200
300
400
500
600
700
800
y2
b2
y3
b3
y5
y6
y7

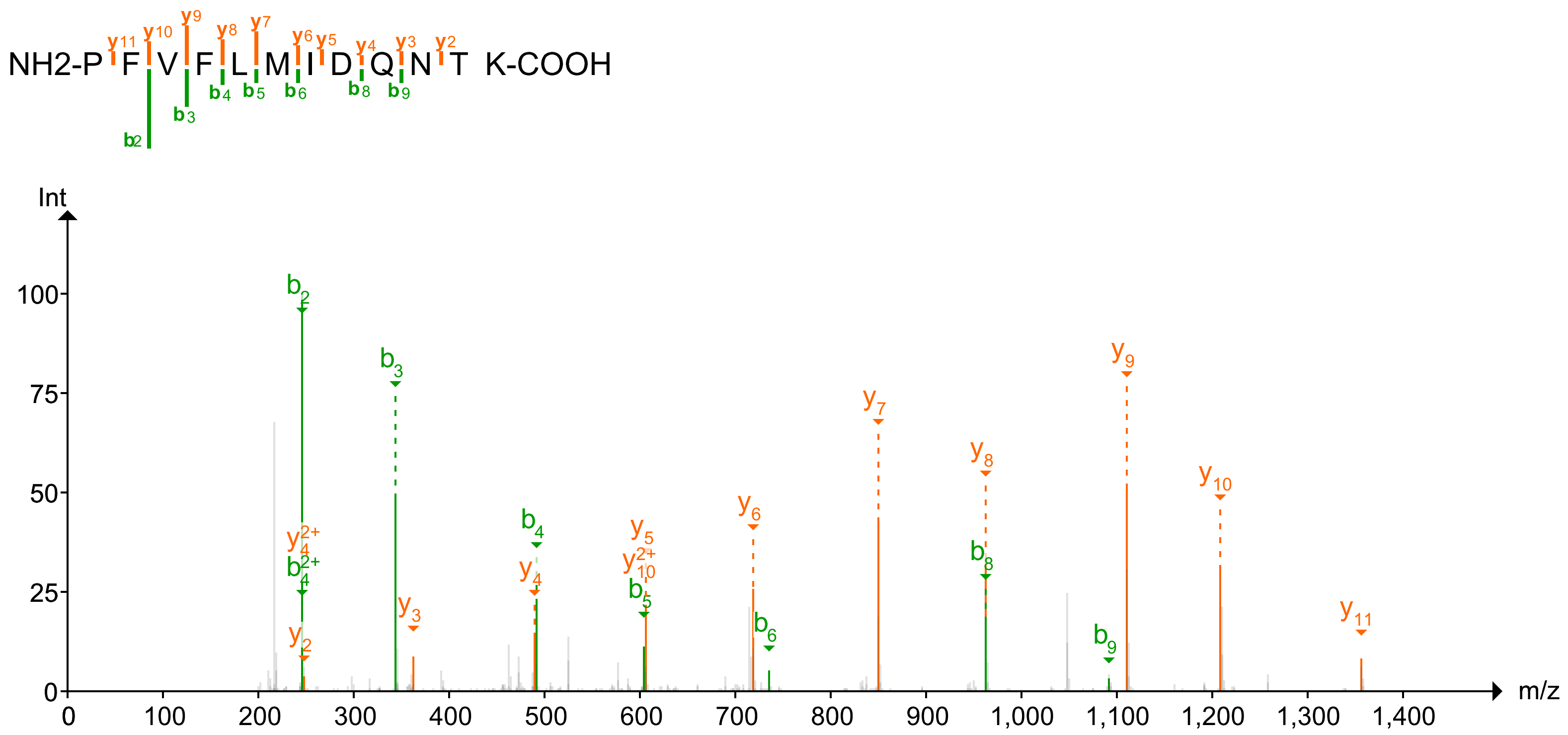

NH2-P F V F L M I D Q N T K-COOH
Int
m/z
b2
b3
b4
b5
b6
b8
b9
y2
y3
y4
y5
y6
y7
y8
y9
y10
y11
0
25
50
75
100
0
100
200
300
400
500
600
700
800
900
1,000
1,100
1,200
1,300
1,400

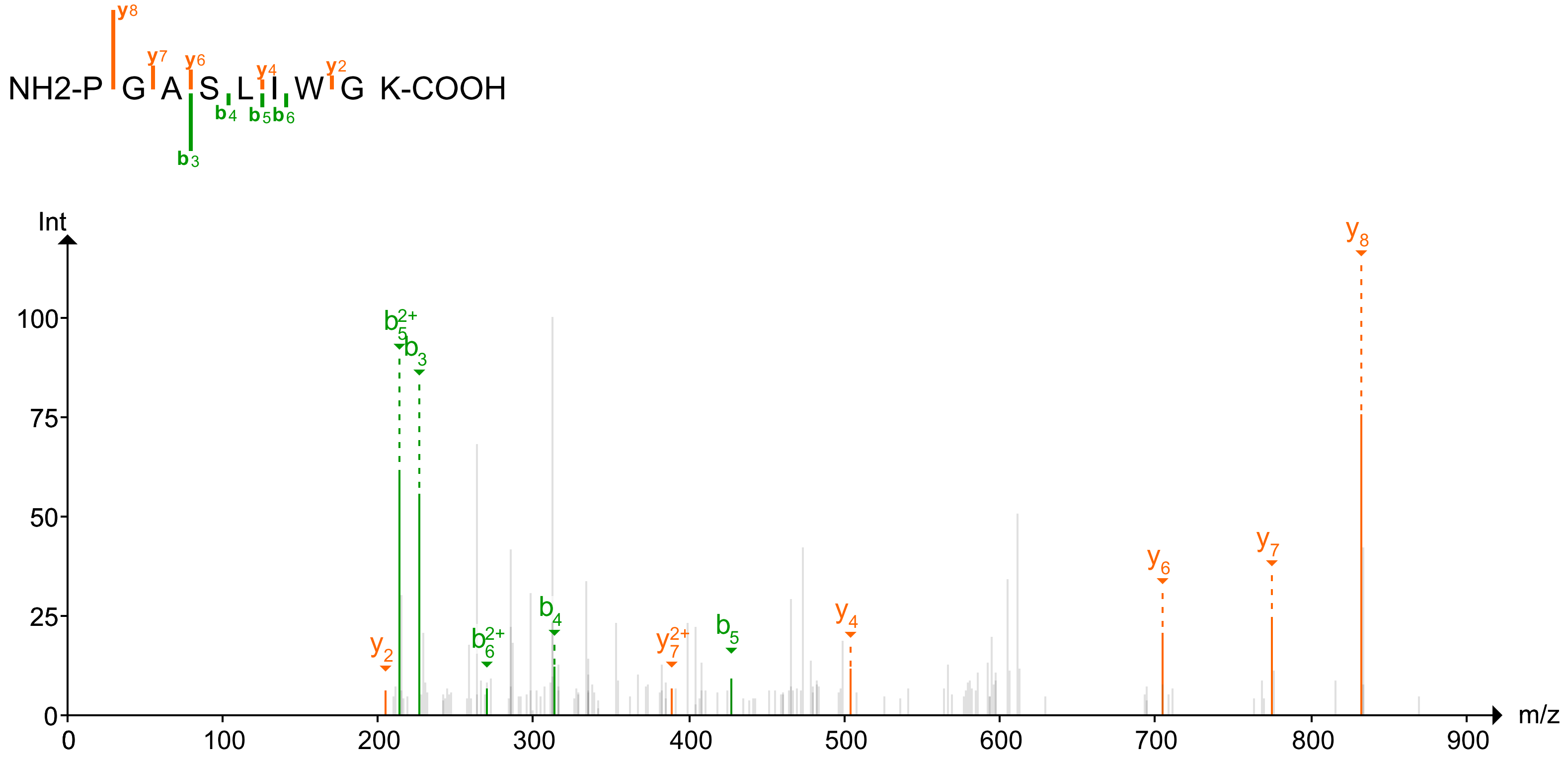

NH2-P G A S L I W G K-COOH
y8 y7 y6 y4 y2
b3 b4 b5 b6
Int
100
75
50
25
0
m/z
0 100 200 300 400 500 600 700 800 900
b5 2+
b3
b6 2+
b4
y2
y7 2+
b5
y4
y6
y7
y8

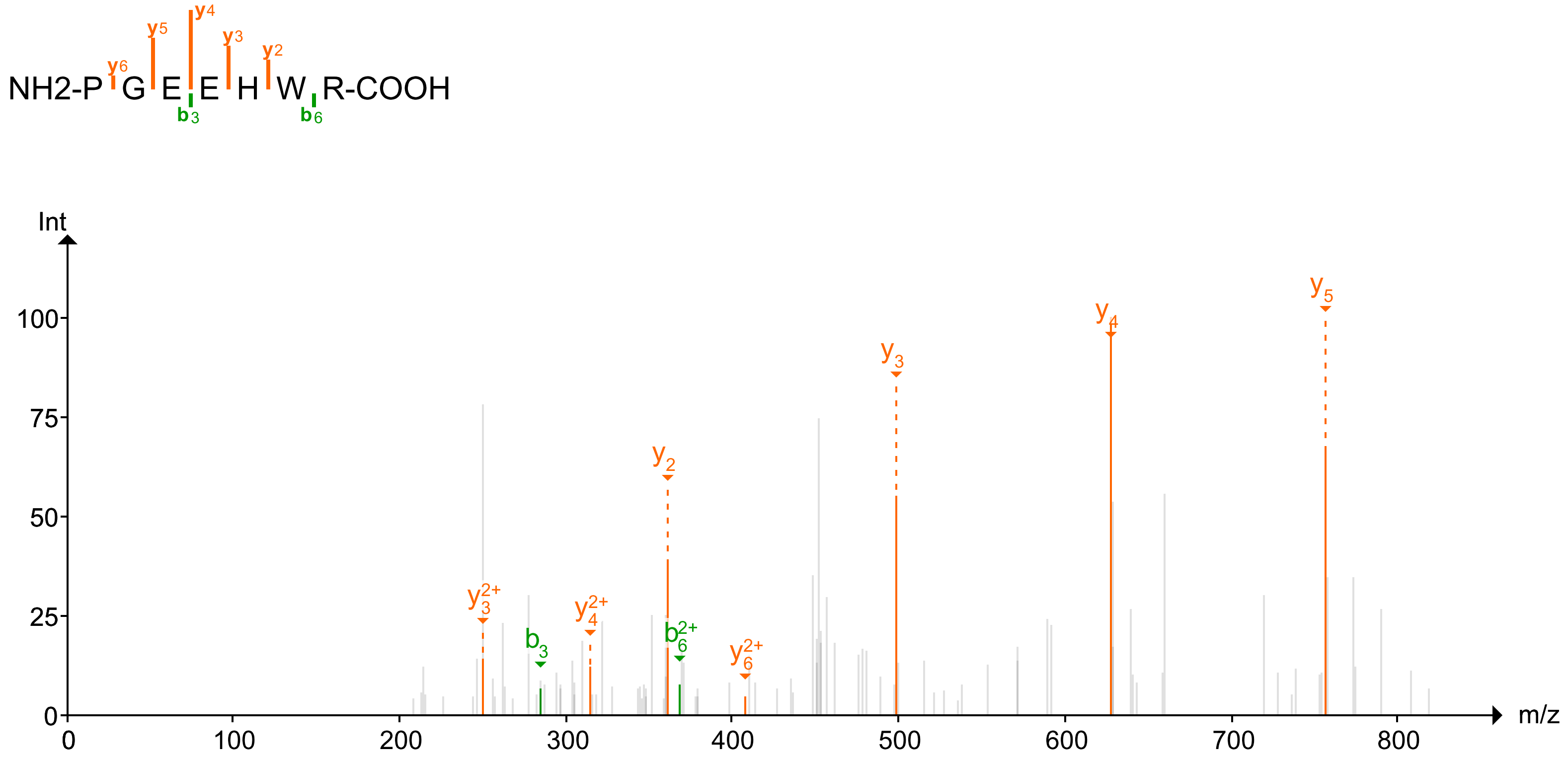

NH2-P G E E H W R-COOH
y6 y5 y4 y3 y2
b3 b6
Int
m/z
0 25 50 75 100
0 100 200 300 400 500 600 700 800
y5 y4 y3 y2
y3 2+ b3 y4 2+ b6 2+ y6 2+

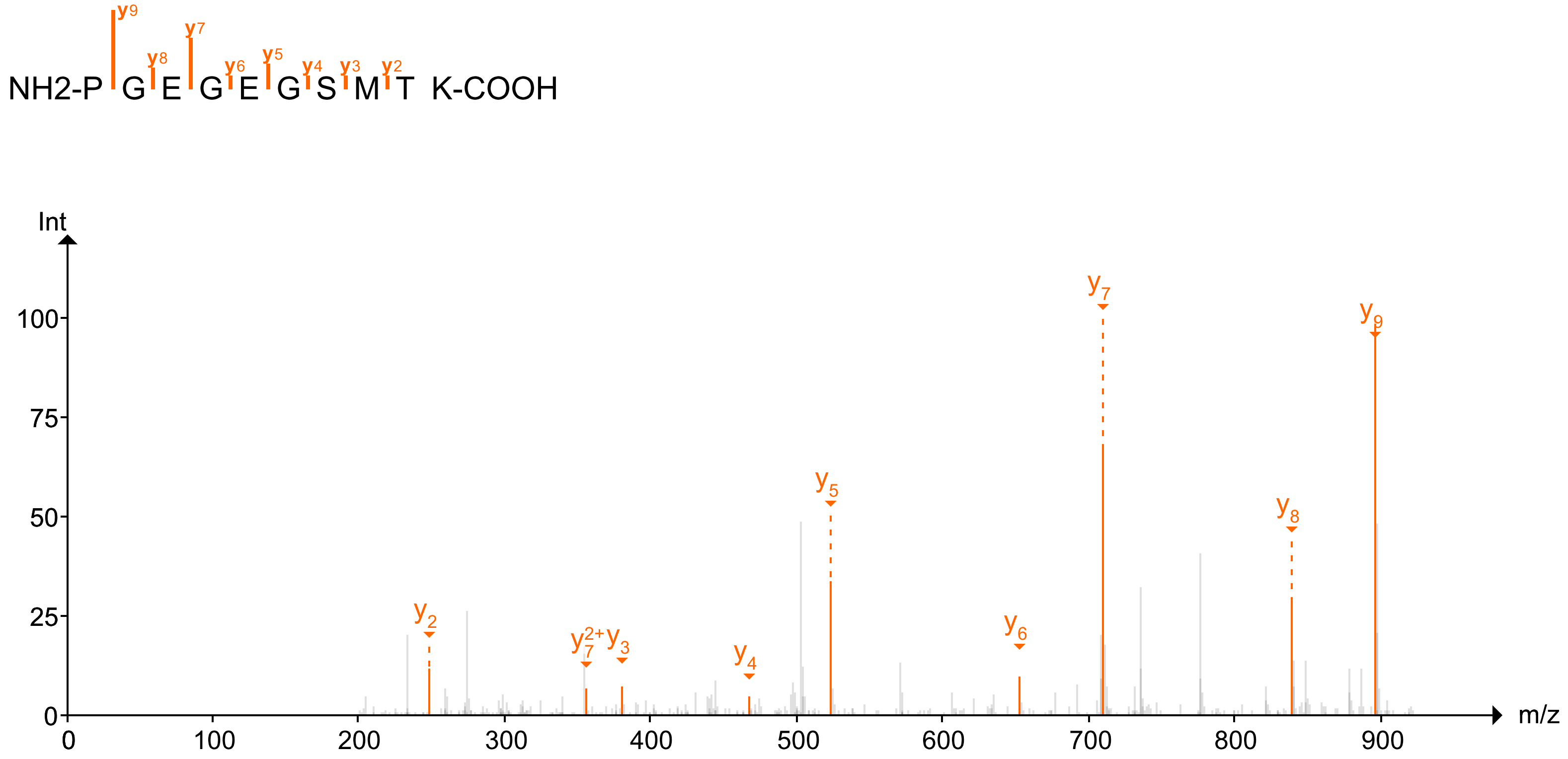

NH2-P G E G E G S M T K-COOH
y9 y8 y7 y6 y5 y4 y3 y2
Int
m/z
0
25
50
75
100
0
100
200
300
400
500
600
700
800
900
y2
y7 2+ y3
y4
y5
y6
y7
y8
y9

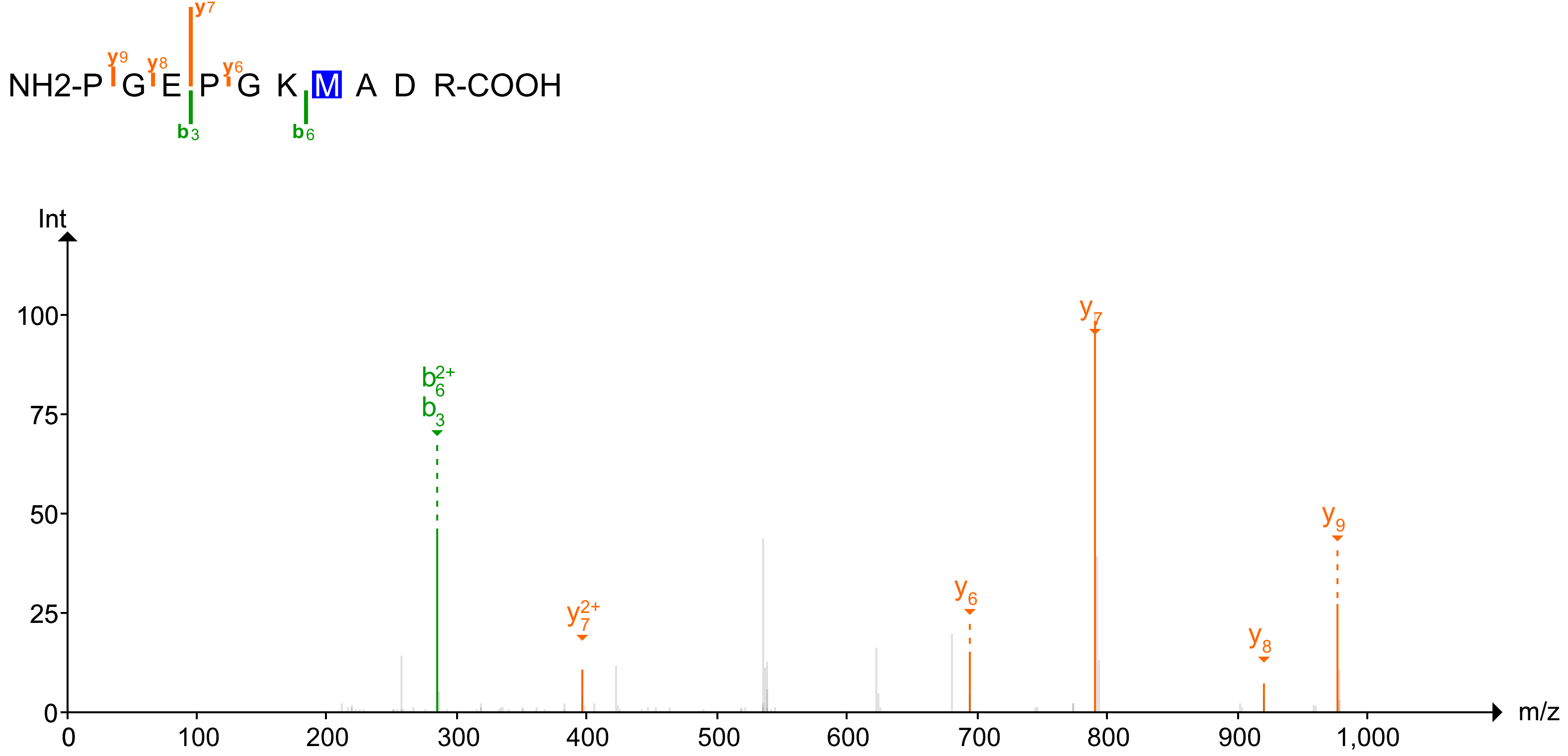

NH2-P G E P G K M A D R-COOH
y9
y8
y7
y6
b3
b6
Int
100
75
50
25
0
$b_6^{2+}$
$b_3$
$y_7^{2+}$
$y_6$
$y_7$
$y_8$
$y_9$
0
100
200
300
400
500
600
700
800
900
1,000
m/z

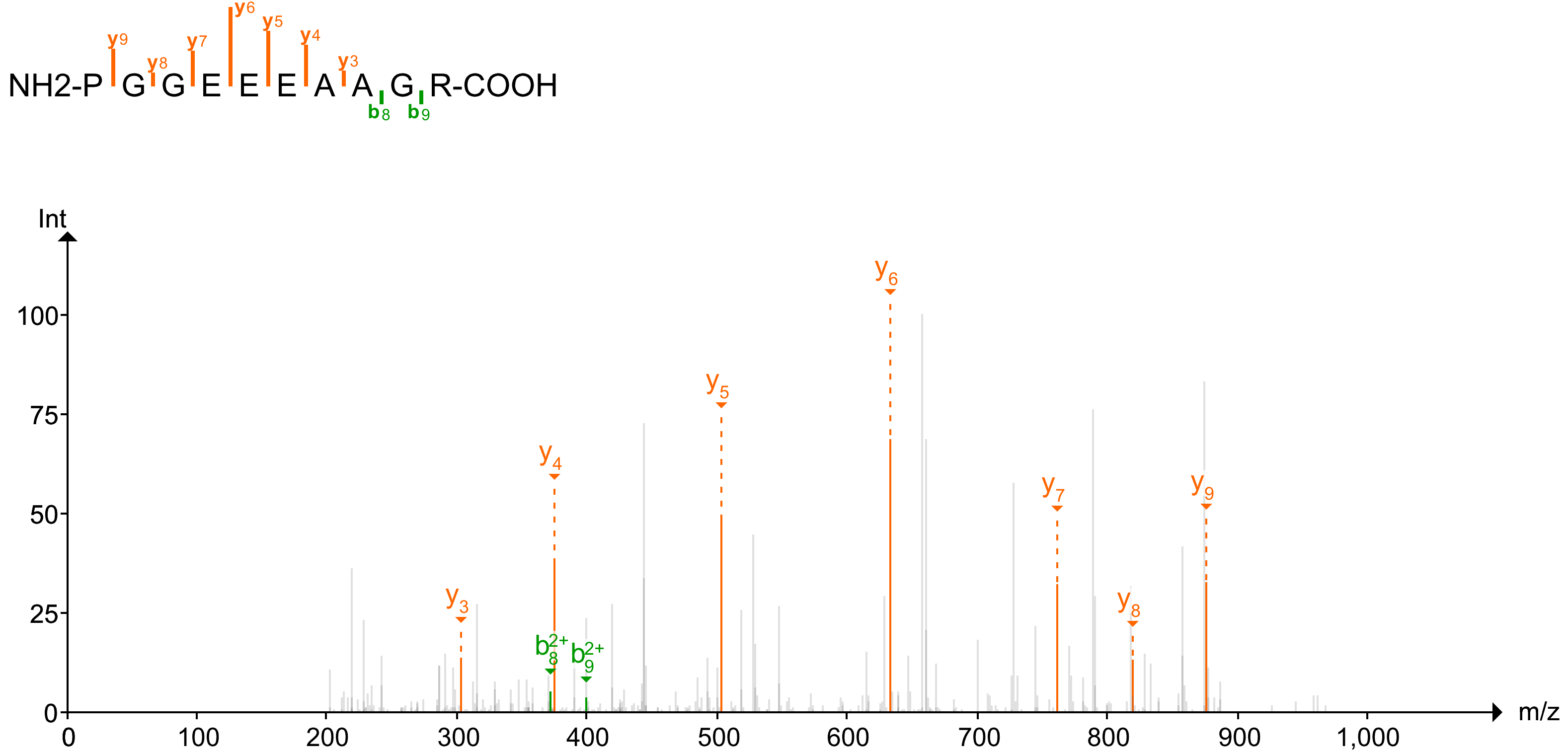

NH2-P G G E E E A A G R-COOH
y9 y8 y7 y6 y5 y4 y3
b8 b9
Int
m/z
y6
y5
y4
y7
y9
y3
y8
$b_8^{2+}$
$b_9^{2+}$

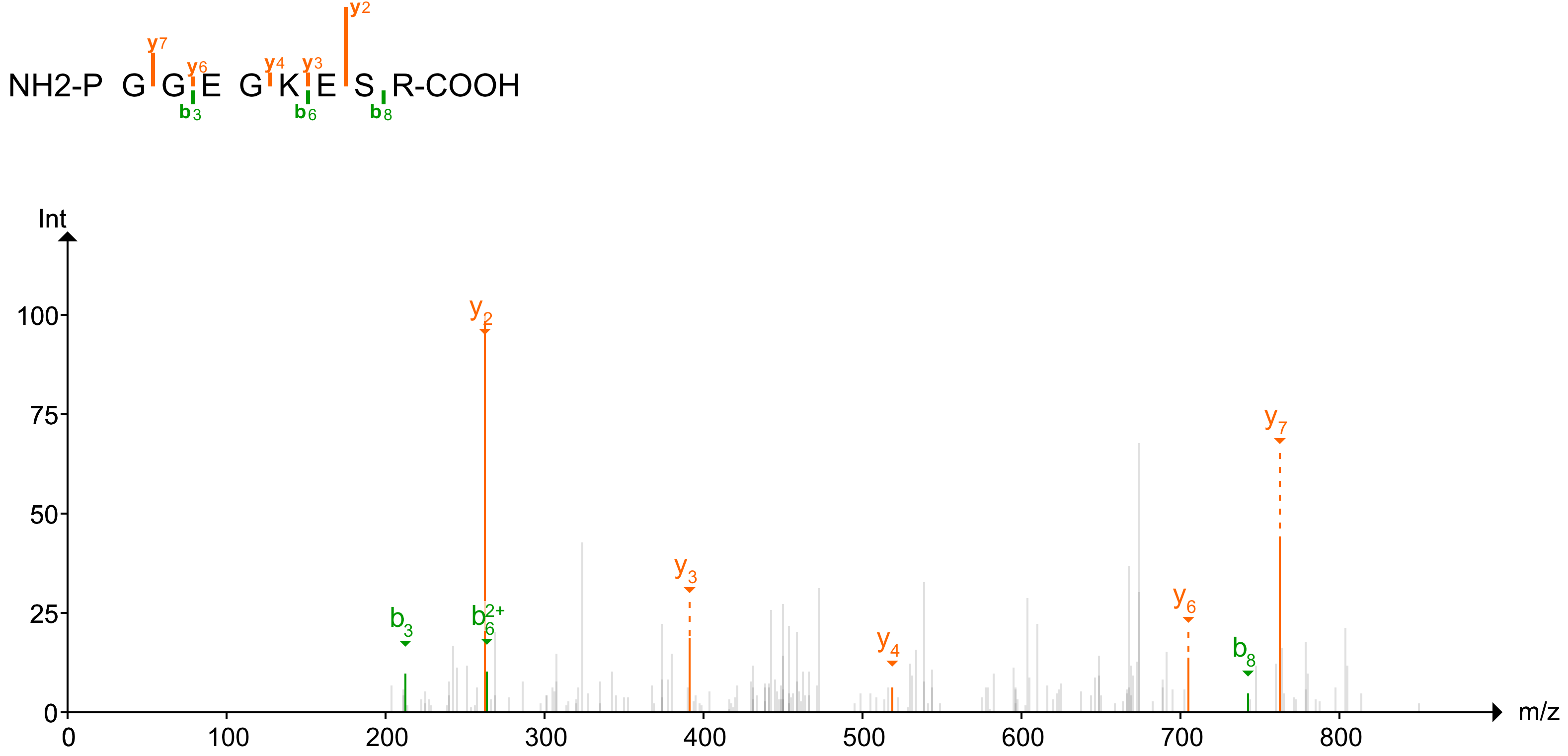

NH2-P G G E G K E S R-COOH
y7
y6
y4
y3
y2
b3
b6
b8
Int
100
75
50
25
0
m/z
0
100
200
300
400
500
600
700
800
y2
b3
b6 2+
y3
y4
y6
b8
y7

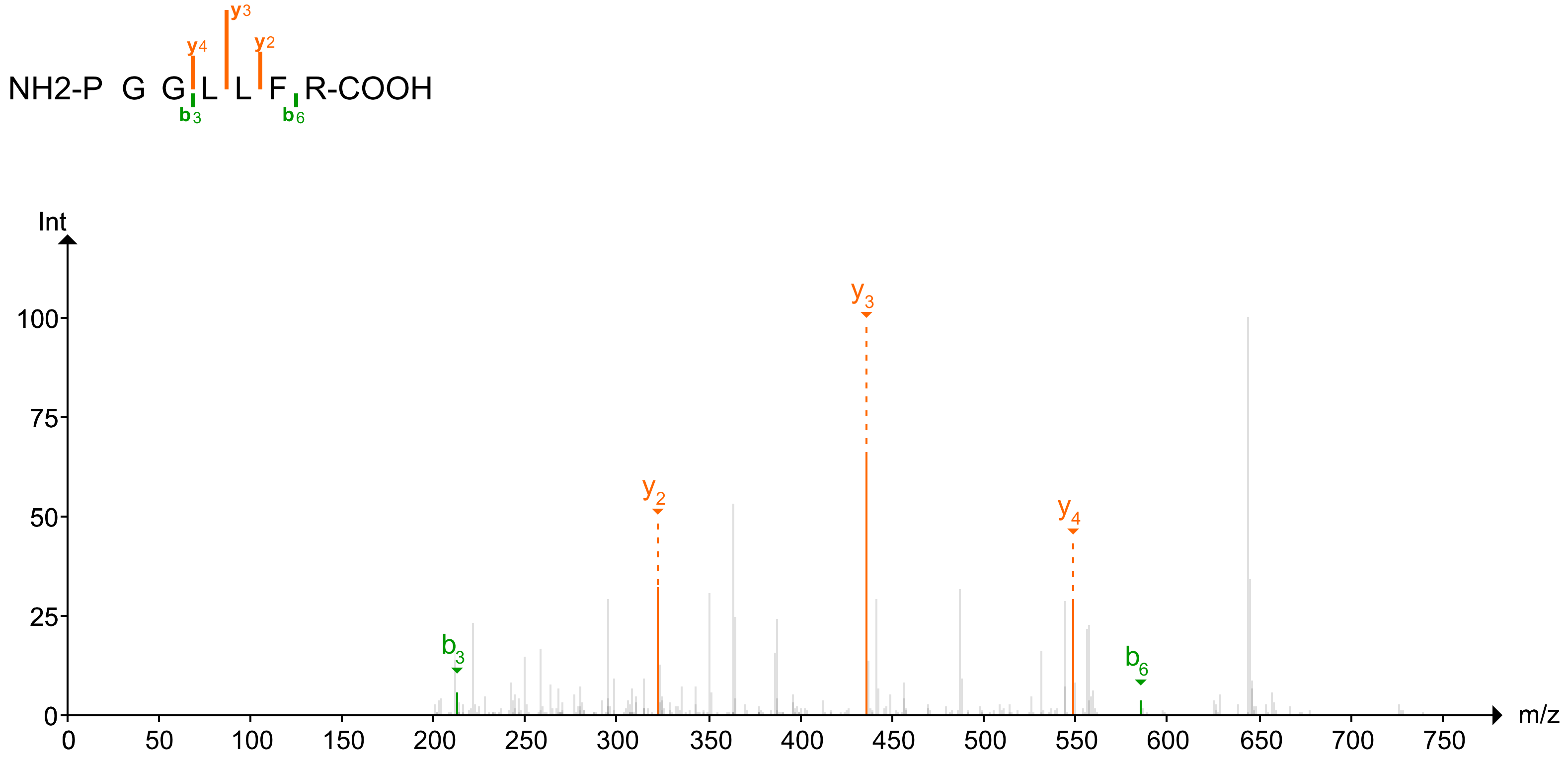
NH2-P G G L L F R-COOH
y4
y3
y2
b3
b6
Int
100
75
50
25
0
y3
y2
y4
b3
b6
0 50 100 150 200 250 300 350 400 450 500 550 600 650 700 750
m/z

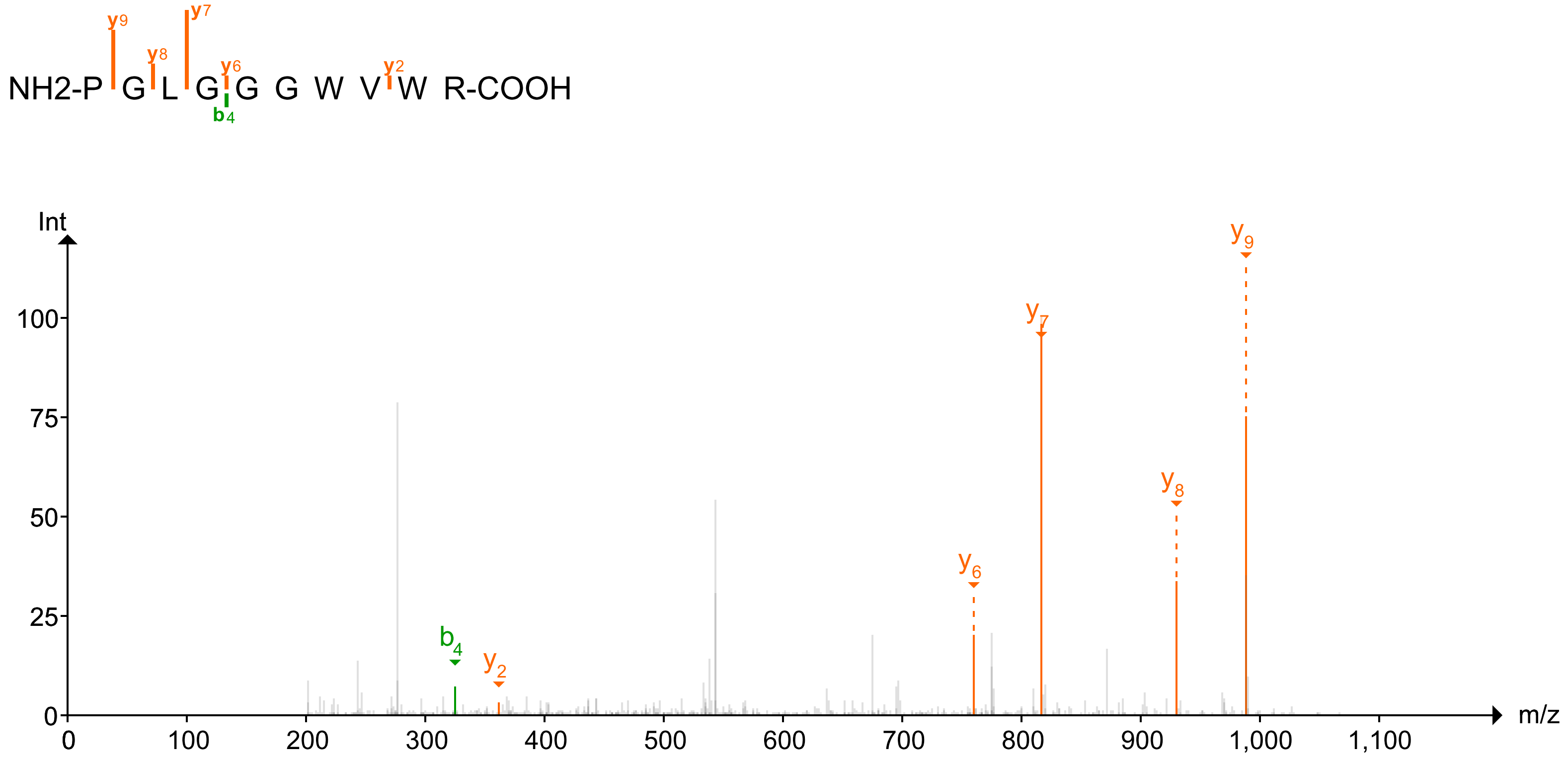

NH2-P G L G G G W V W R-COOH
y9
y8
y7
y6
y2
b4
Int
100
75
50
25
0
m/z
0
100
200
300
400
500
600
700
800
900
1,000
1,100

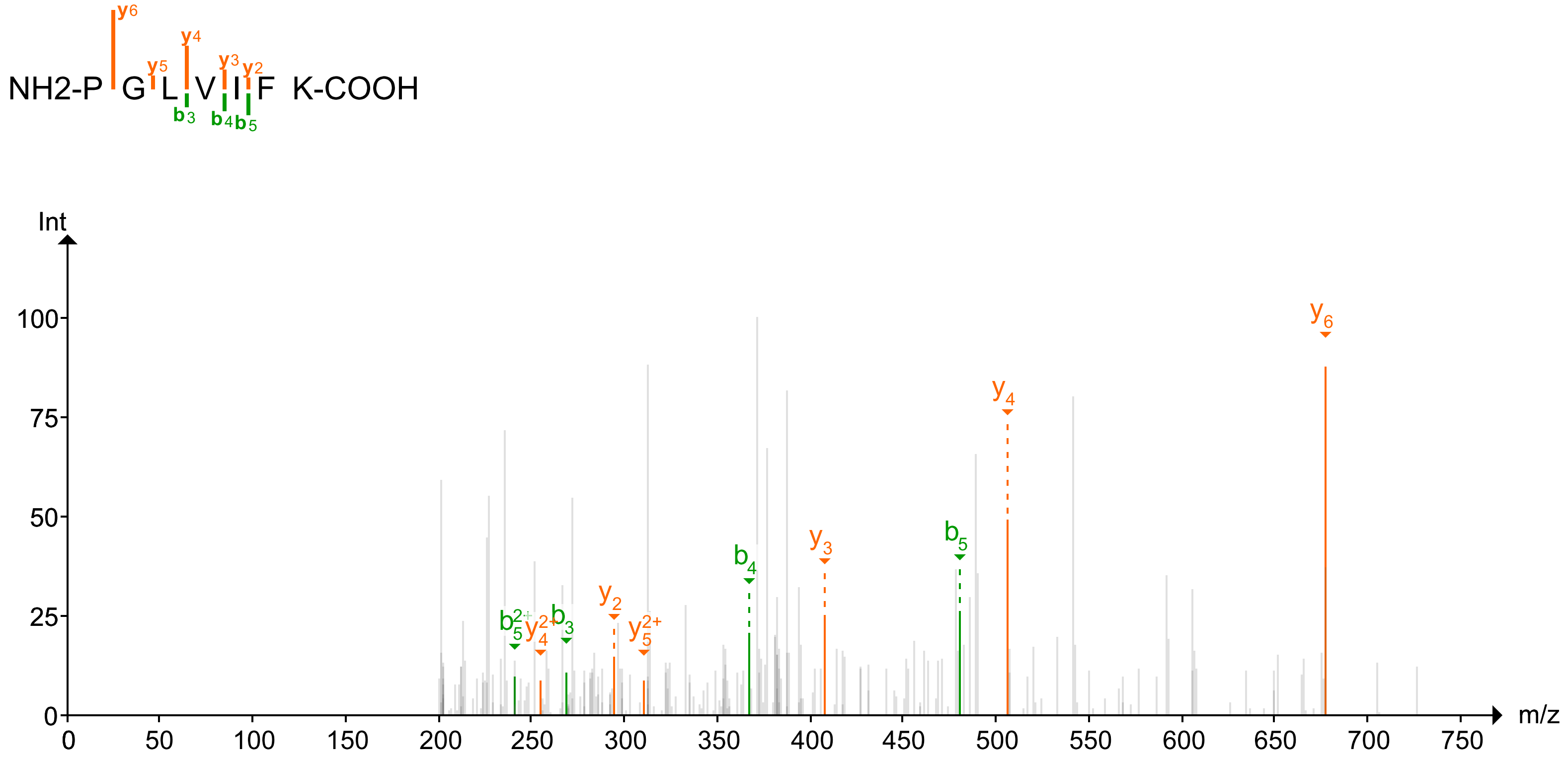

NH2-P G L V I F K-COOH
y6 y5 y4 y3 y2
b3 b4 b5
Int
100
75
50
25
0
0 50 100 150 200 250 300 350 400 450 500 550 600 650 700 750
m/z
y6
y4
y3
b5
b4
y2
b5 2+
y4 2+
b3
y5 2+

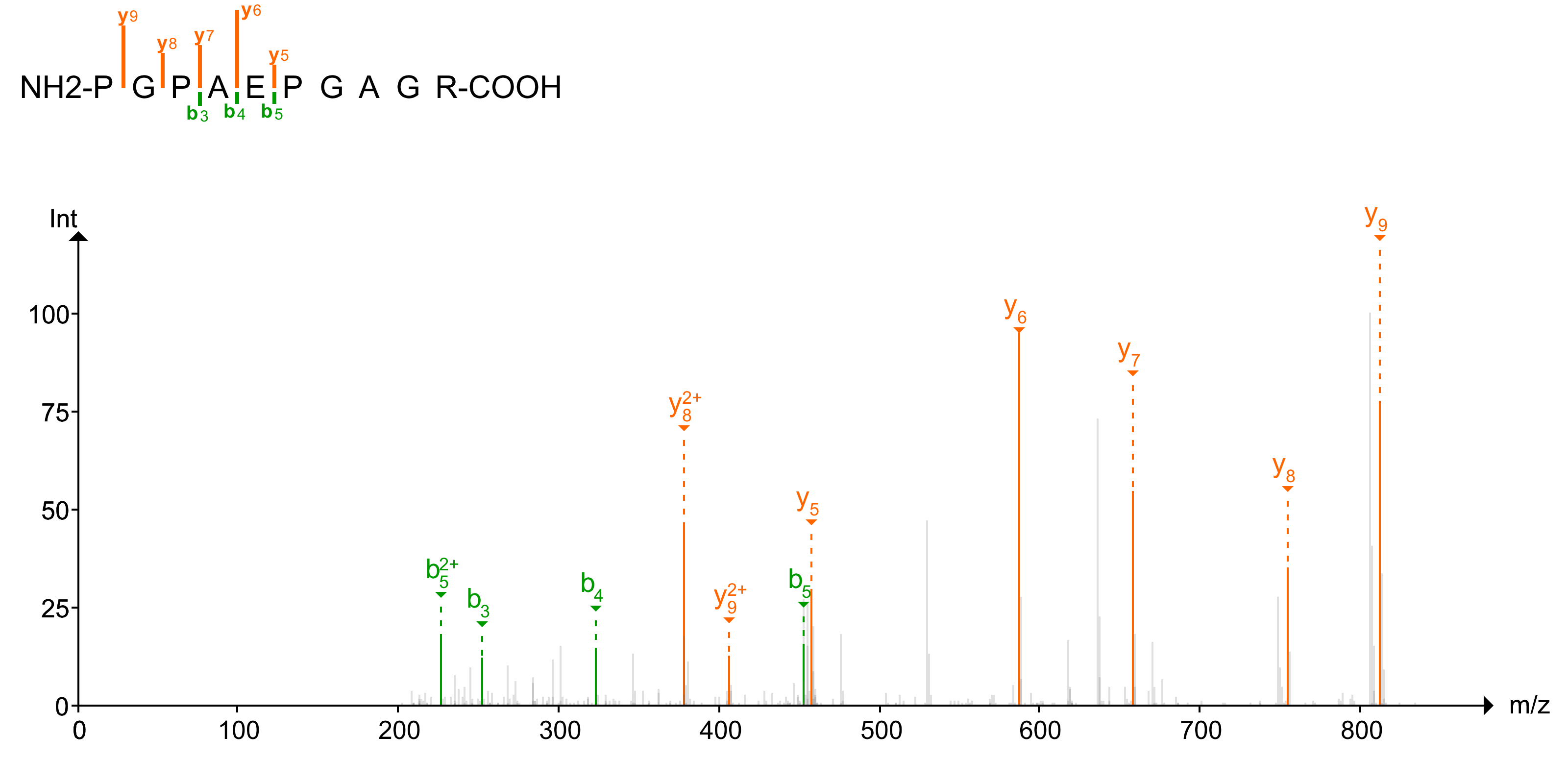

NH2-P G P A E P G A G R-COOH
y9 y8 y7 y6 y5
b3 b4 b5
Int
100
75
50
25
0
m/z
0
100
200
300
400
500
600
700
800
$b_5^{2+}$
$b_3$
$b_4$
$y_8^{2+}$
$y_9^{2+}$
$b_5$
$y_5$
$y_6$
$y_7$
$y_8$
$y_9$

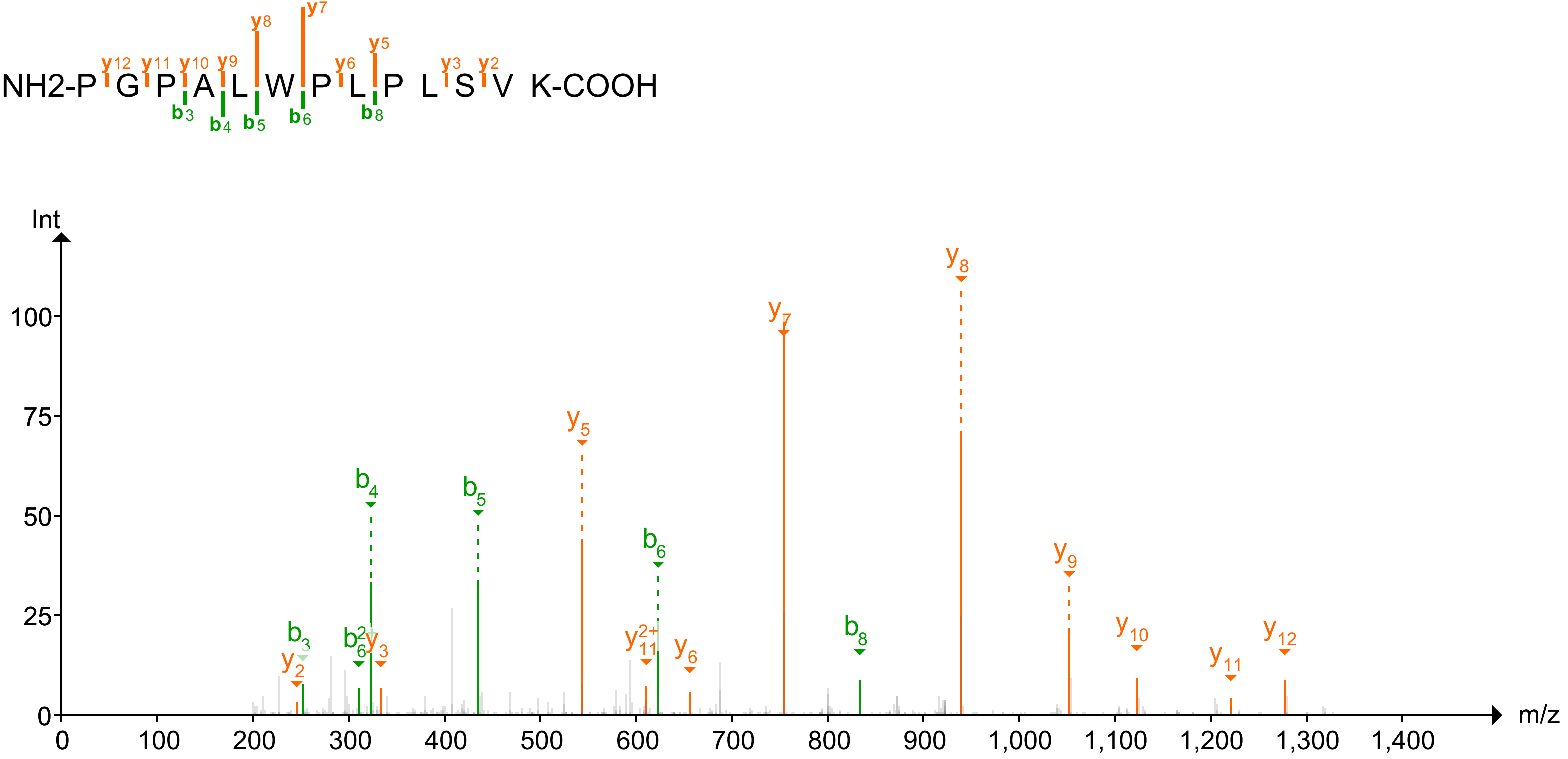

NH2-P G P A L W P L P L S V K-COOH
y12 y11 y10 y9 y8 y7 y6 y5 y3 y2
b3 b4 b5 b6 b8
Int
m/z
0
25
50
75
100
0 100 200 300 400 500 600 700 800 900 1,000 1,100 1,200 1,300 1,400
y2 b3 b6 2+ y3 b4 b5 y5 y11 2+ b6 y6 y7 b8 y8 y9 y10 y11 y12

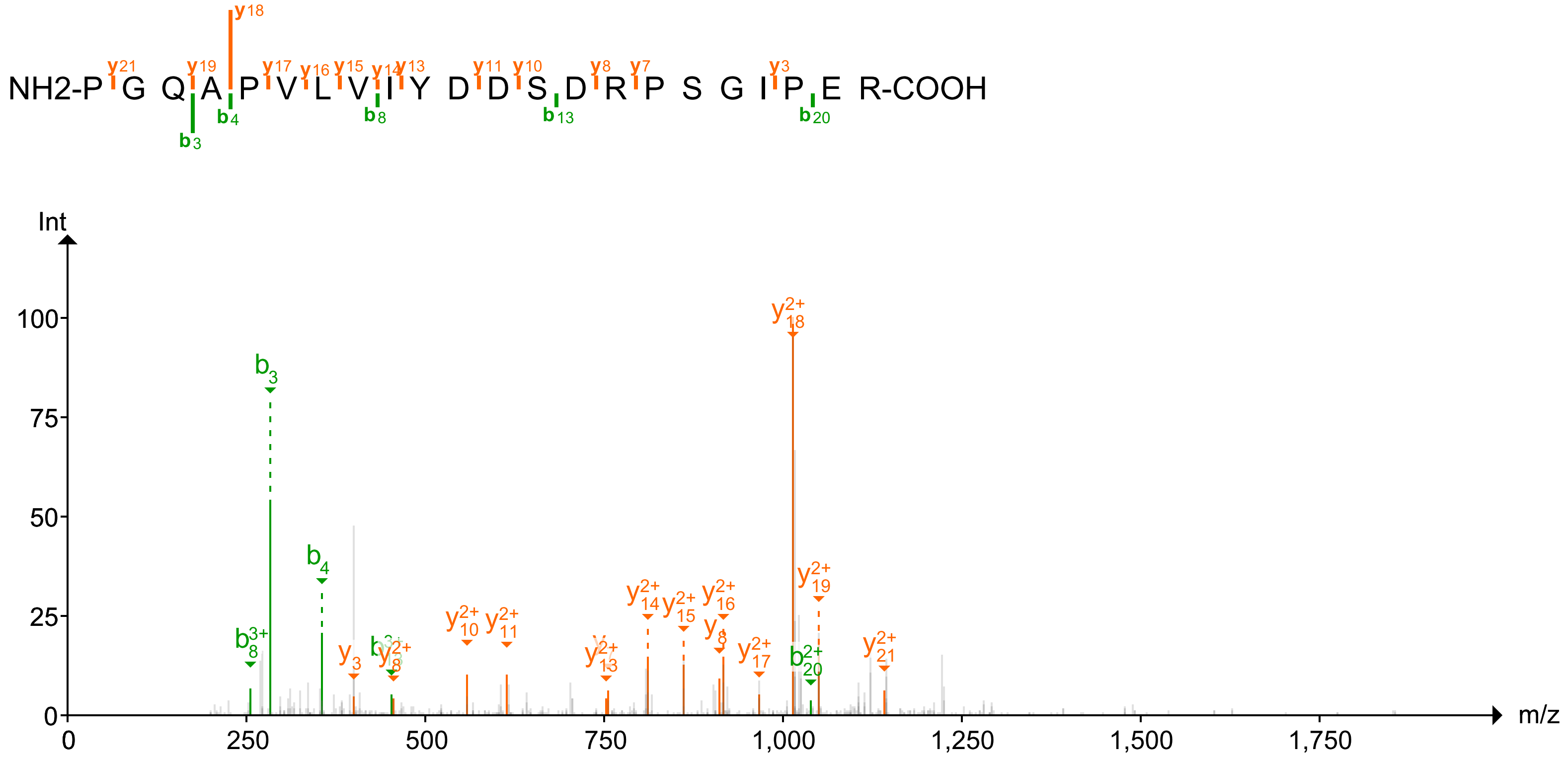
NH2-P G Q A P V L V I Y D D S D R P S G I P E R-COOH
y21 y19 y18 y17 y16 y15 y14 y13 y11 y10 y8 y7 y3
b3 b4 b8 b13 b20
Int
100
75
50
25
0
m/z
0
250
500
750
1,000
1,250
1,500
1,750
b3
b4
b8 3+
y3
y10 2+
y11 2+
y13 2+
y14 2+
y15 2+
y16 2+
y8
y17 2+
y18 2+
y19 2+
b20 2+
y21 2+

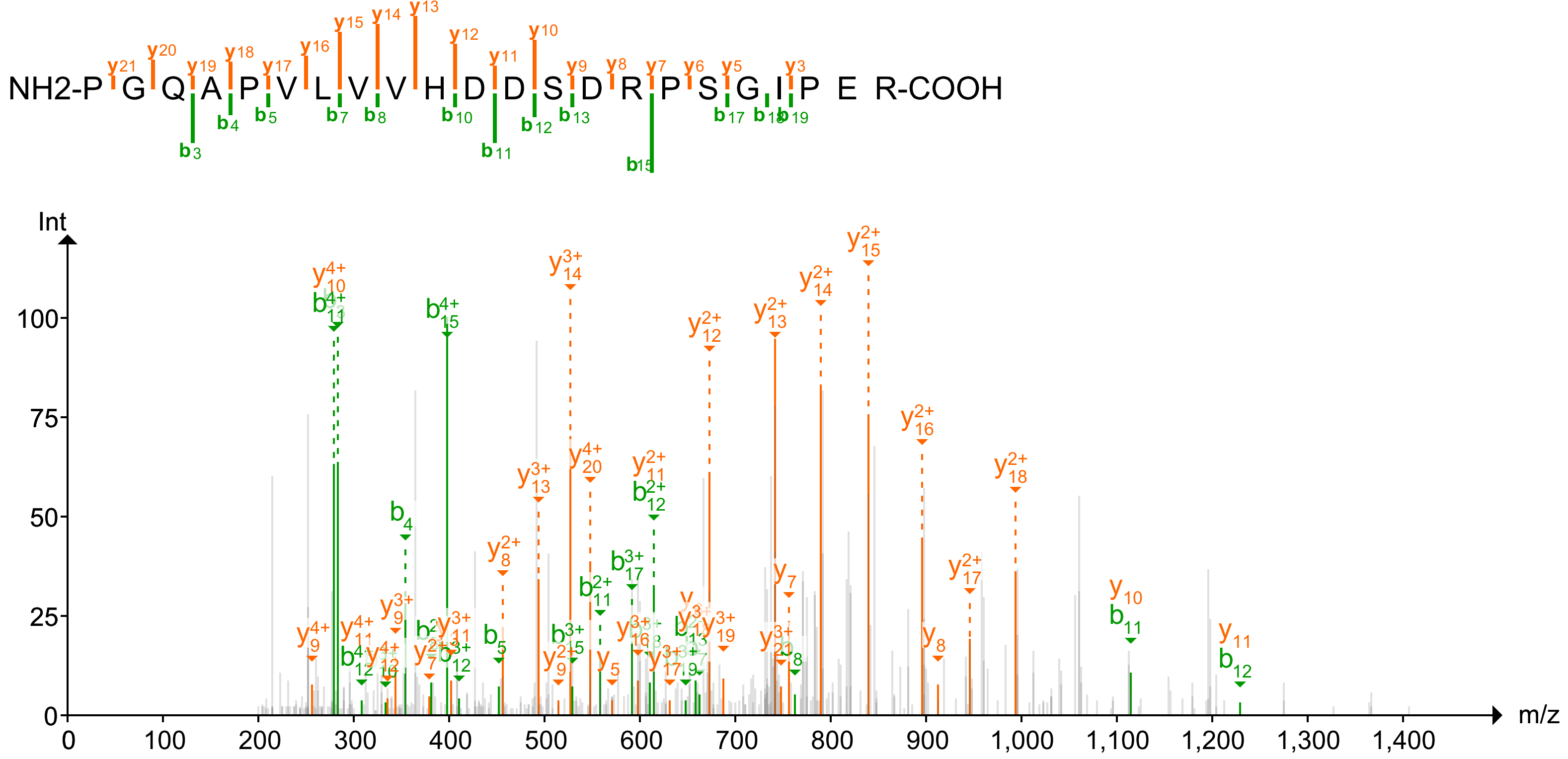

NH2-P G Q A P V L V V H D D S D R P S G I P E R-COOH
Int
100
75
50
25
0
0
100
200
300
400
500
600
700
800
900
1,000
1,100
1,200
1,300
1,400
m/z

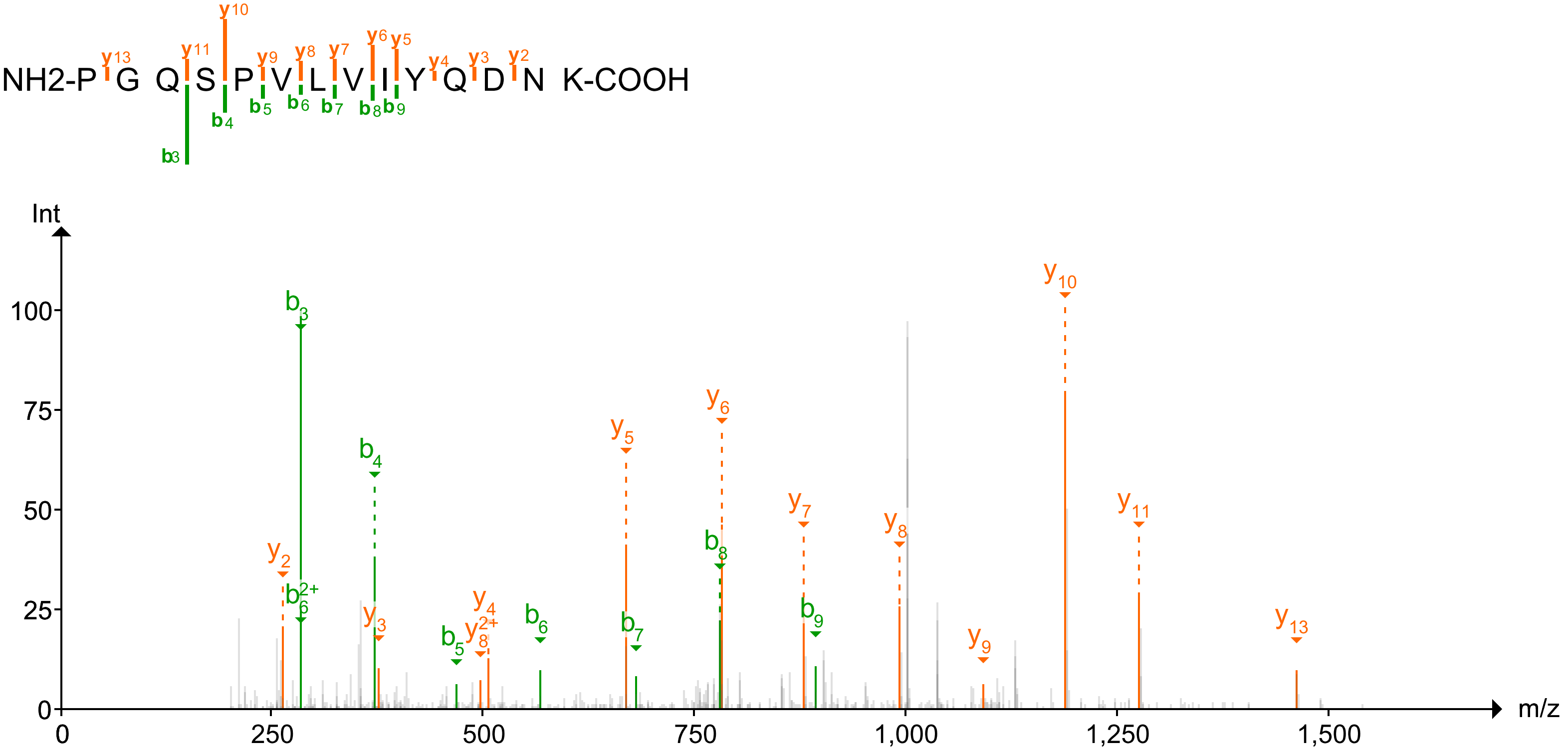

NH2-P G Q S P V L V I Y Q D N K-COOH
y13 y11 y10 y9 y8 y7 y6 y5 y4 y3 y2
b3 b4 b5 b6 b7 b8 b9
Int
100
75
50
25
0
0
250
500
750
1,000
1,250
1,500
m/z
b3
b4
y2
b6 2+
y3
b5
y4
y8 2+
b6
y5
b7
y6
b8
y7
b9
y8
y9
y10
y11
y13

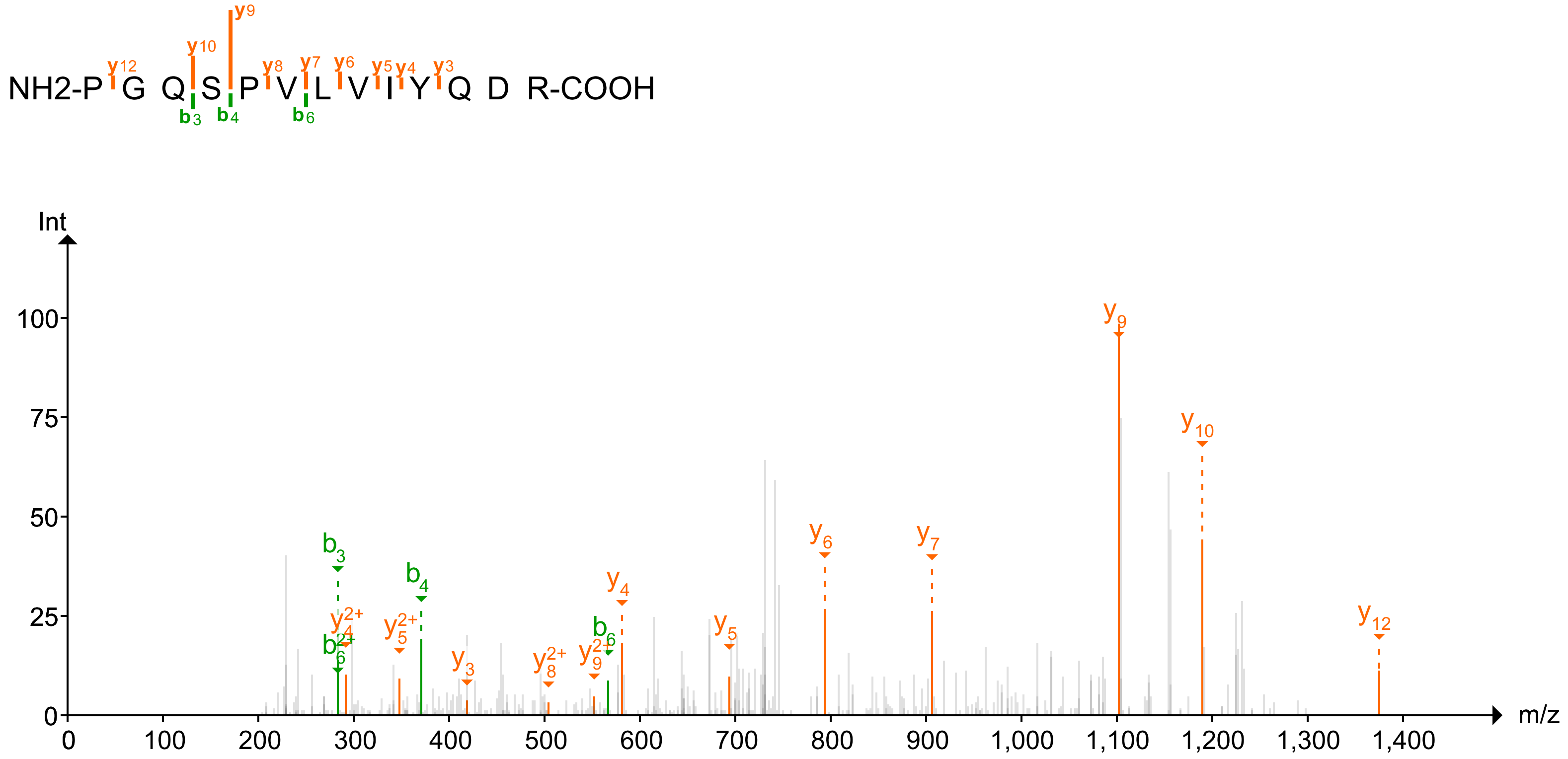

NH2-P G Q S P V L V I Y Q D R-COOH
y12 y10 y9 y8 y7 y6 y5 y4 y3
b3 b4 b6
Int
100
75
50
25
0
0 100 200 300 400 500 600 700 800 900 1,000 1,100 1,200 1,300 1,400
m/z
y9
y10
y6
y7
y12
b3
b4
y4
y5
y3
y4 2+
y5 2+
b6 2+
y8 2+
y9 2+
b6

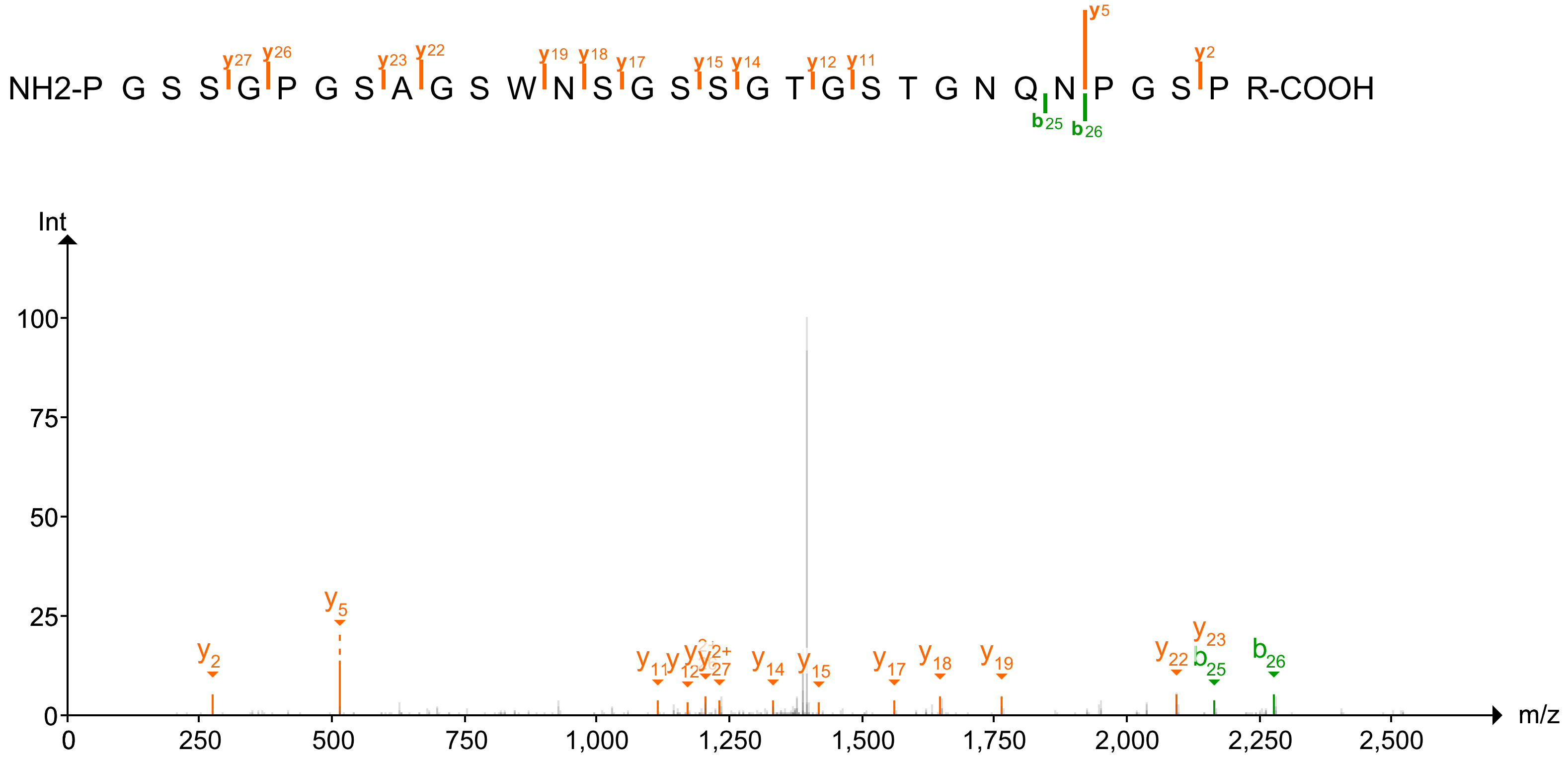

NH2-P G S S G P G S A G S W N S G S S G T G S T G N Q N P G S P R-COOH
y27 y26 y23 y22 y19 y18 y17 y15 y14 y12 y11 y5 y2
b25 b26
Int
100
75
50
25
0
y2
y5
y11
y12
y27
y14
y15
y17
y18
y19
y22
y23
b25
b26
0
250
500
750
1,000
1,250
1,500
1,750
2,000
2,250
2,500
m/z

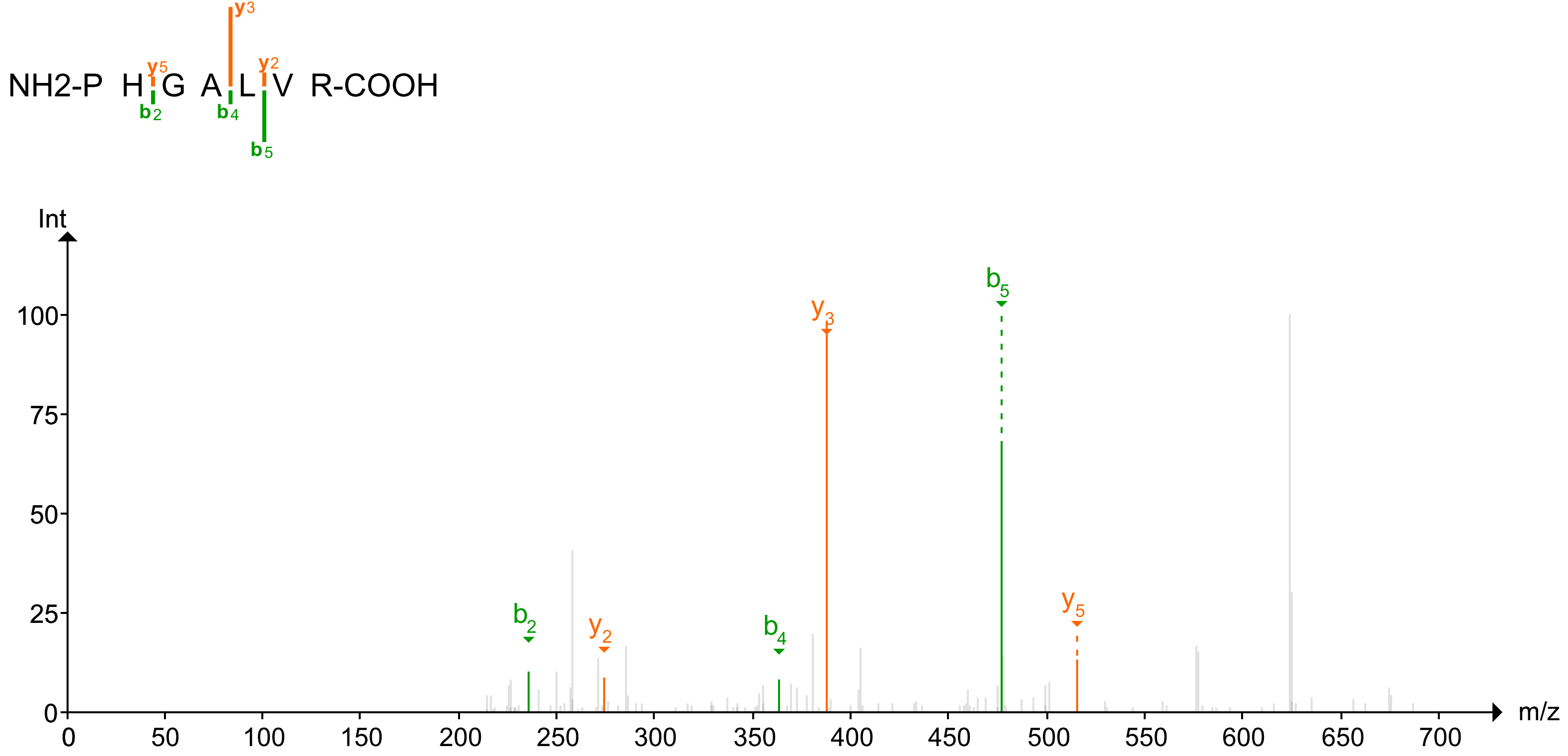

NH2-P H G A L V R-COOH
y5 y3 y2
b2 b4 b5
Int
100
75
50
25
0
y3
b5
b2
y2
b4
y5
0 50 100 150 200 250 300 350 400 450 500 550 600 650 700
m/z

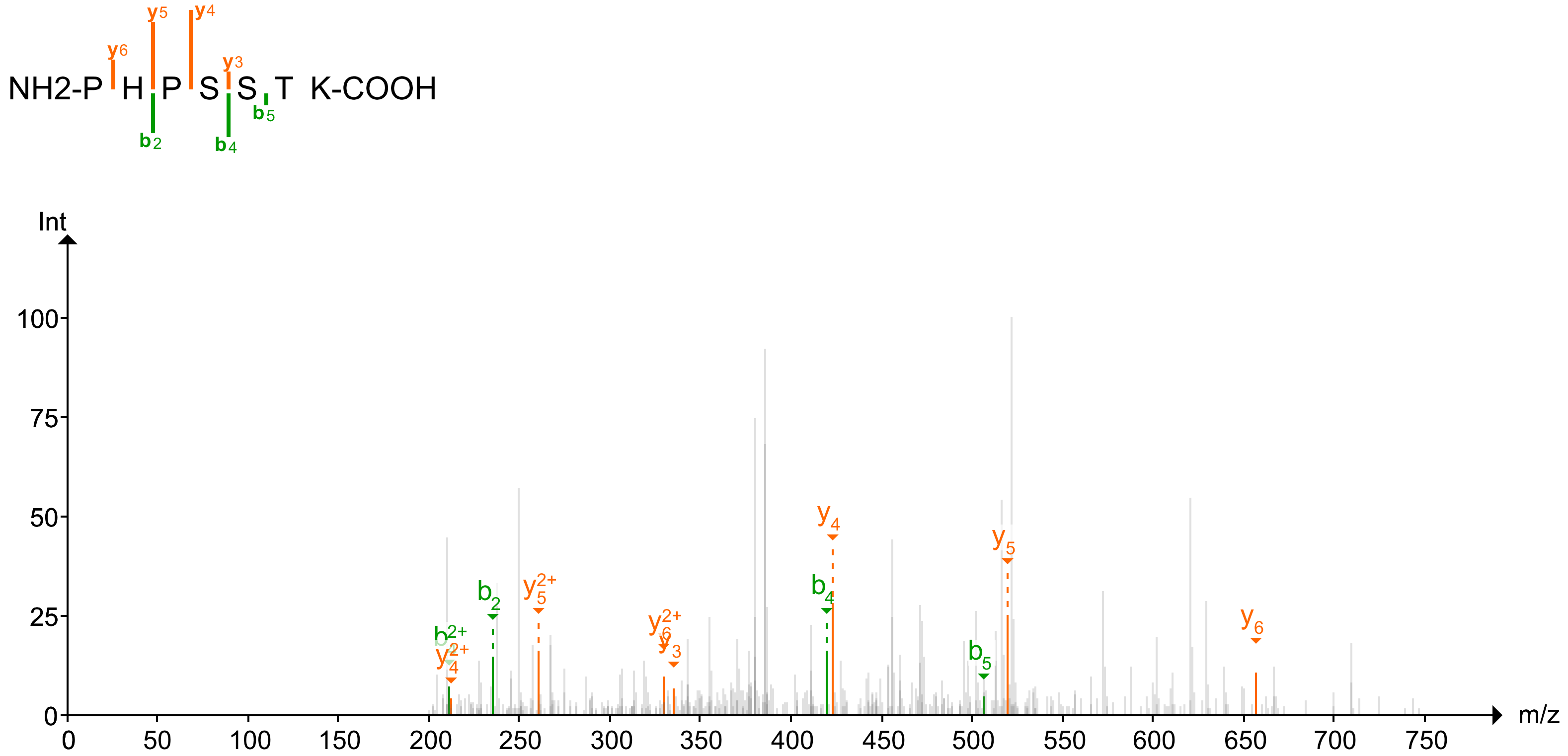

NH2-P H P S S T K-COOH
y6 y5 y4 y3
b2 b4 b5
Int
m/z
0
25
50
75
100
0 50 100 150 200 250 300 350 400 450 500 550 600 650 700 750
b2
y5 2+
y6 2+
y3
b4
y4
y5
b5
y6
y4 2+

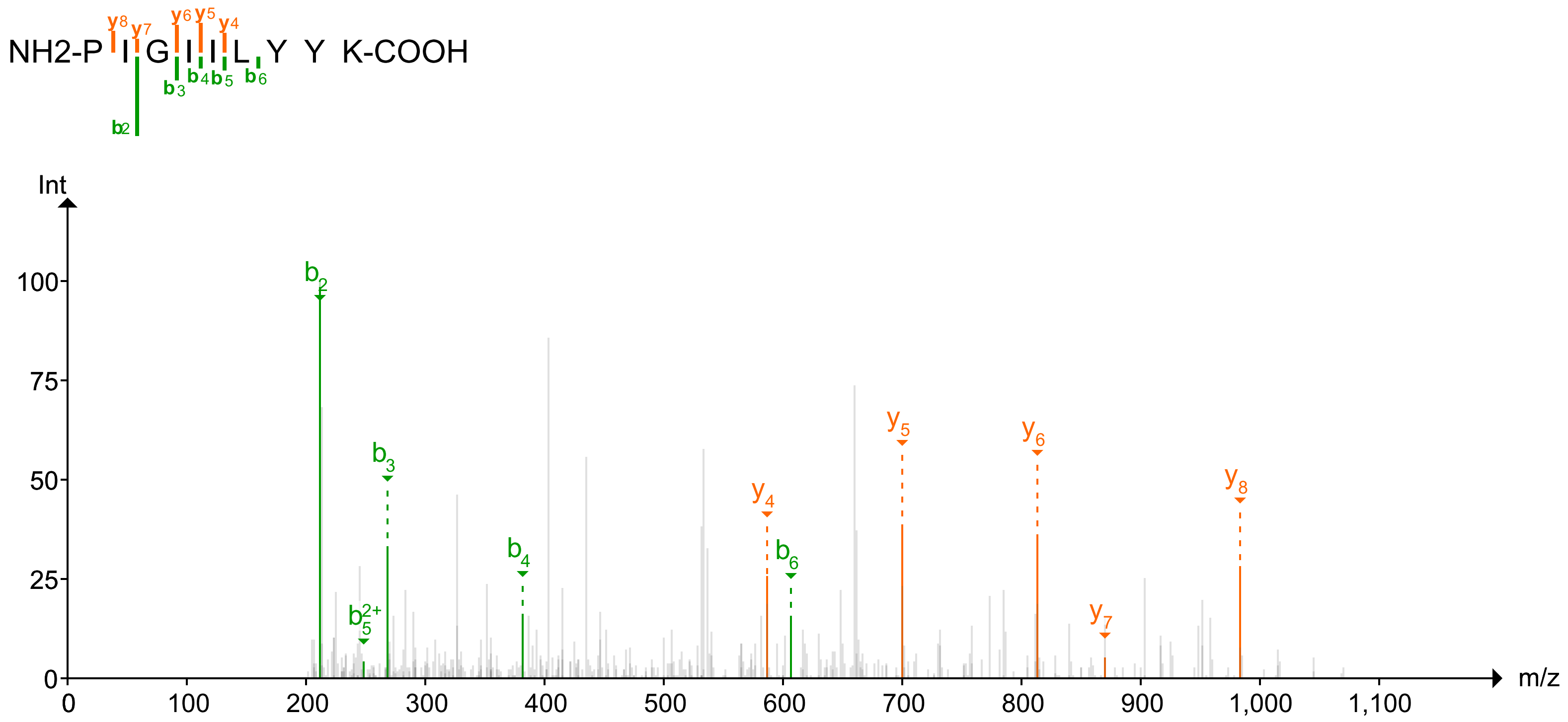

NH2-P I G I I L Y Y K-COOH
y8 y7 y6 y5 y4
b2 b3 b4 b5 b6
Int
100
75
50
25
0
b2
b3
b5 2+
b4
y4
b6
y5
y6
y7
y8
0
100
200
300
400
500
600
700
800
900
1,000
1,100
m/z

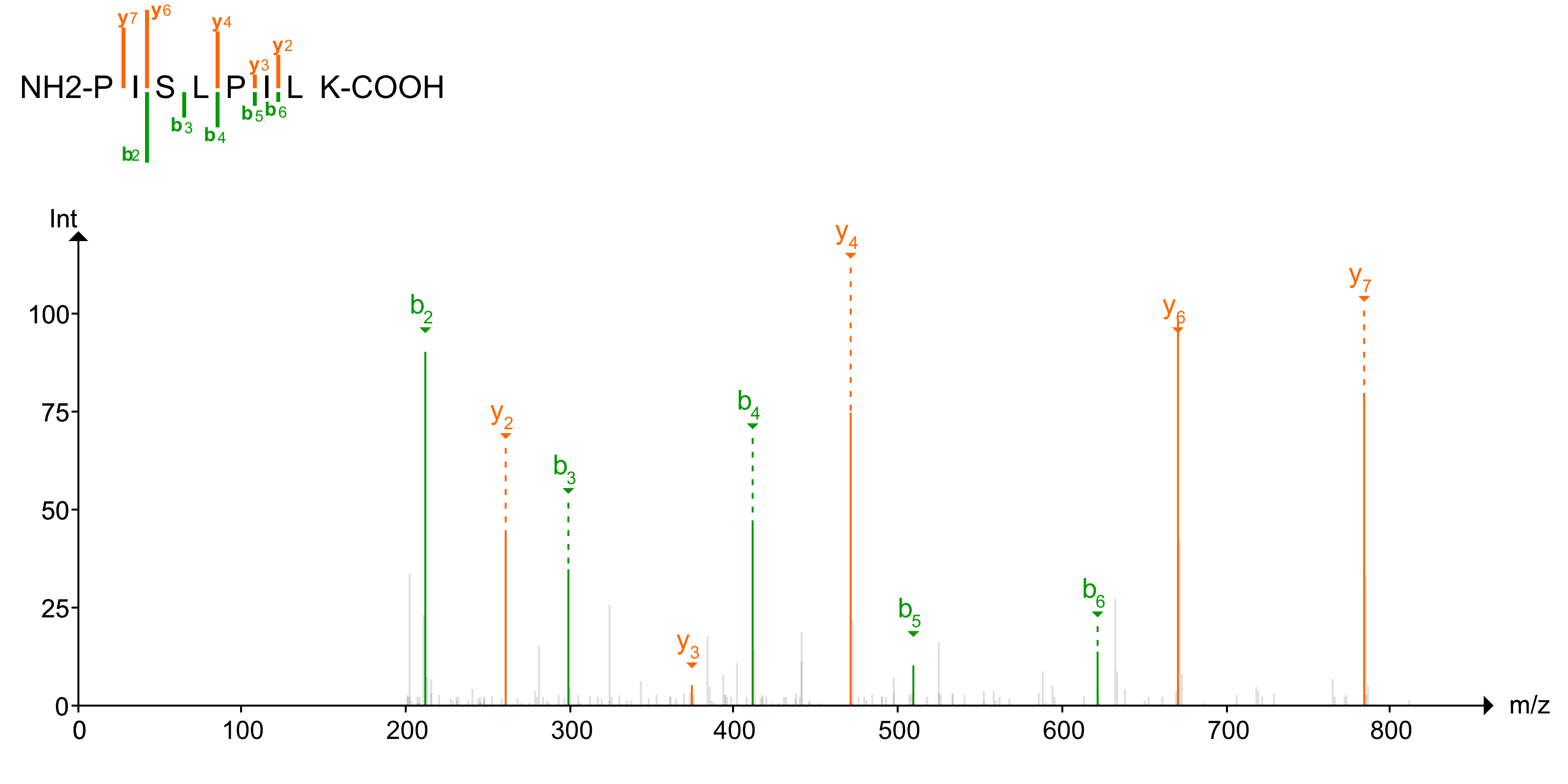

NH2-P I S L P I L K-COOH
y7
y6
y4
y3
y2
b2
b3
b4
b5
b6
Int
100
75
50
25
0
m/z
0
100
200
300
400
500
600
700
800
b2
y2
b3
y3
b4
y4
b5
b6
y6
y7

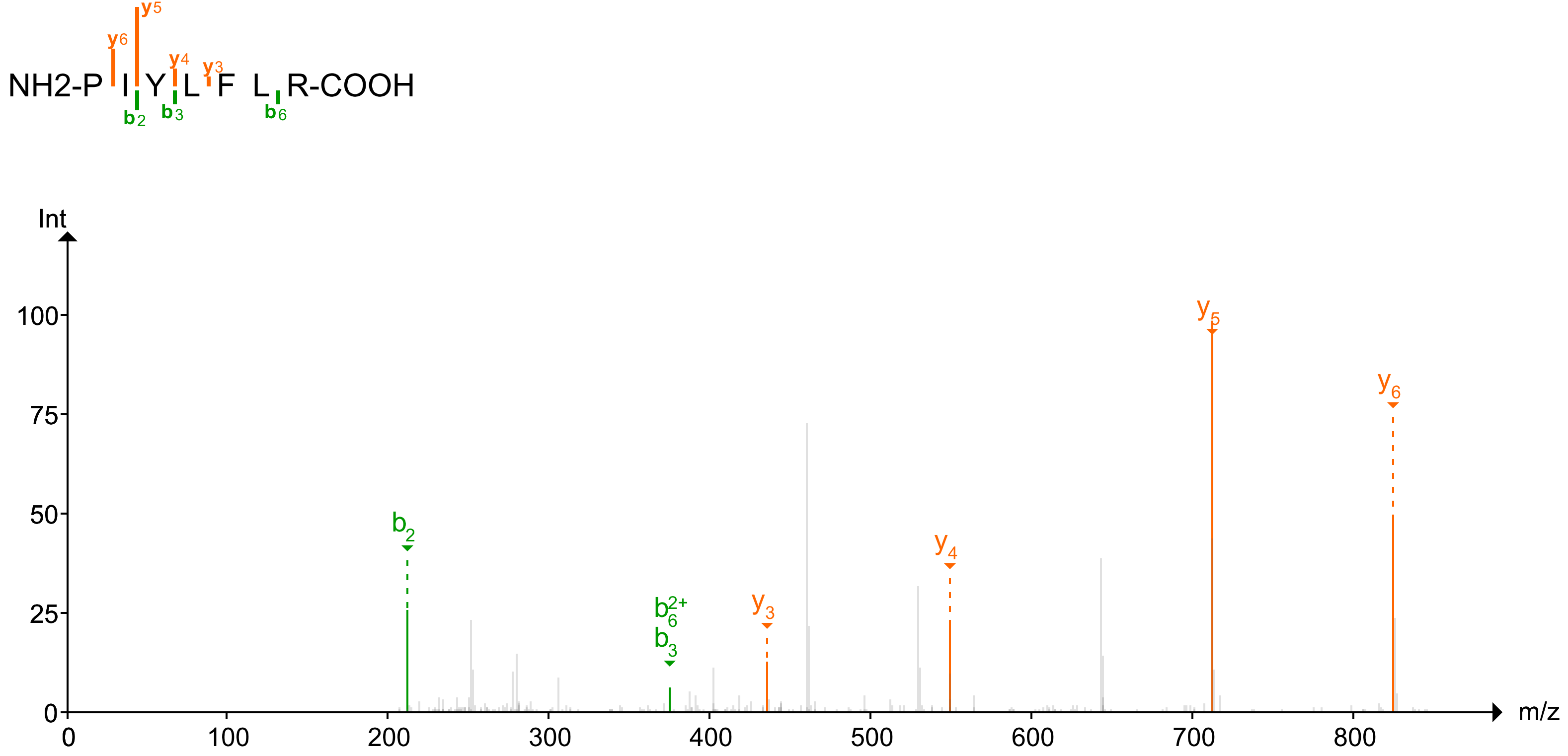

NH2-P I Y L F L R-COOH
y5
y6
y4
y3
b2
b3
b6
Int
100
75
50
25
0
0
100
200
300
400
500
600
700
800
m/z
b2
b6 2+
b3
y3
y4
y5
y6

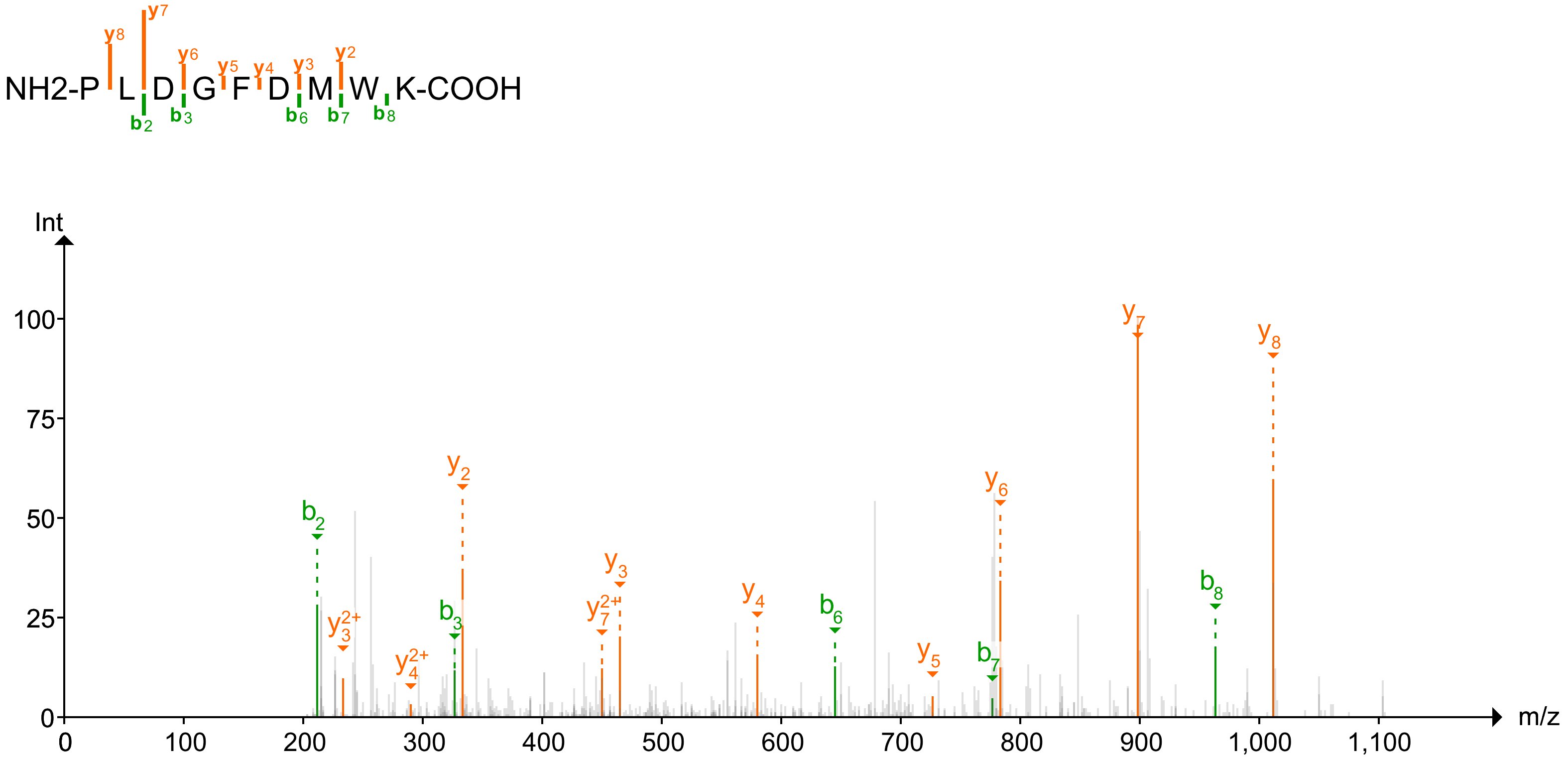

NH2-P L D G F D M W K-COOH
y8 y7 y6 y5 y4 y3 y2
b2 b3 b6 b7 b8
Int
100
75
50
25
0
m/z
0
100
200
300
400
500
600
700
800
900
1,000
1,100
b2
y3 2+
y4 2+
b3
y2
y7 2+
y3
y4
b6
y5
b7
y6
y7
b8
y8

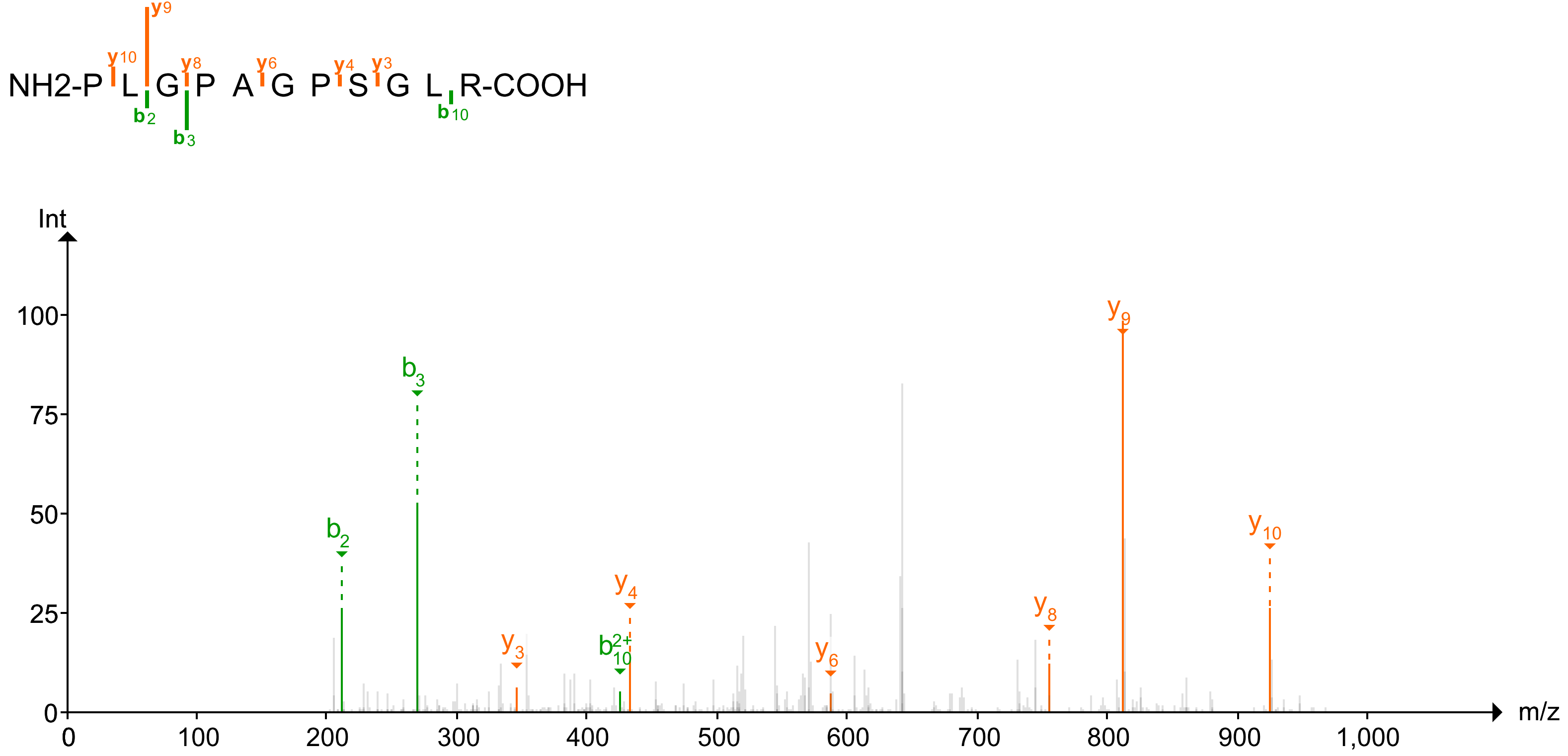

NH2-P L G P A G P S G L R-COOH
y9
y10
y8
y6
y4
y3
b2
b3
b10
Int
100
75
50
25
0
b3
b2
y3
$b_{10}^{2+}$
y4
y6
y8
y9
y10
m/z
0
100
200
300
400
500
600
700
800
900
1,000

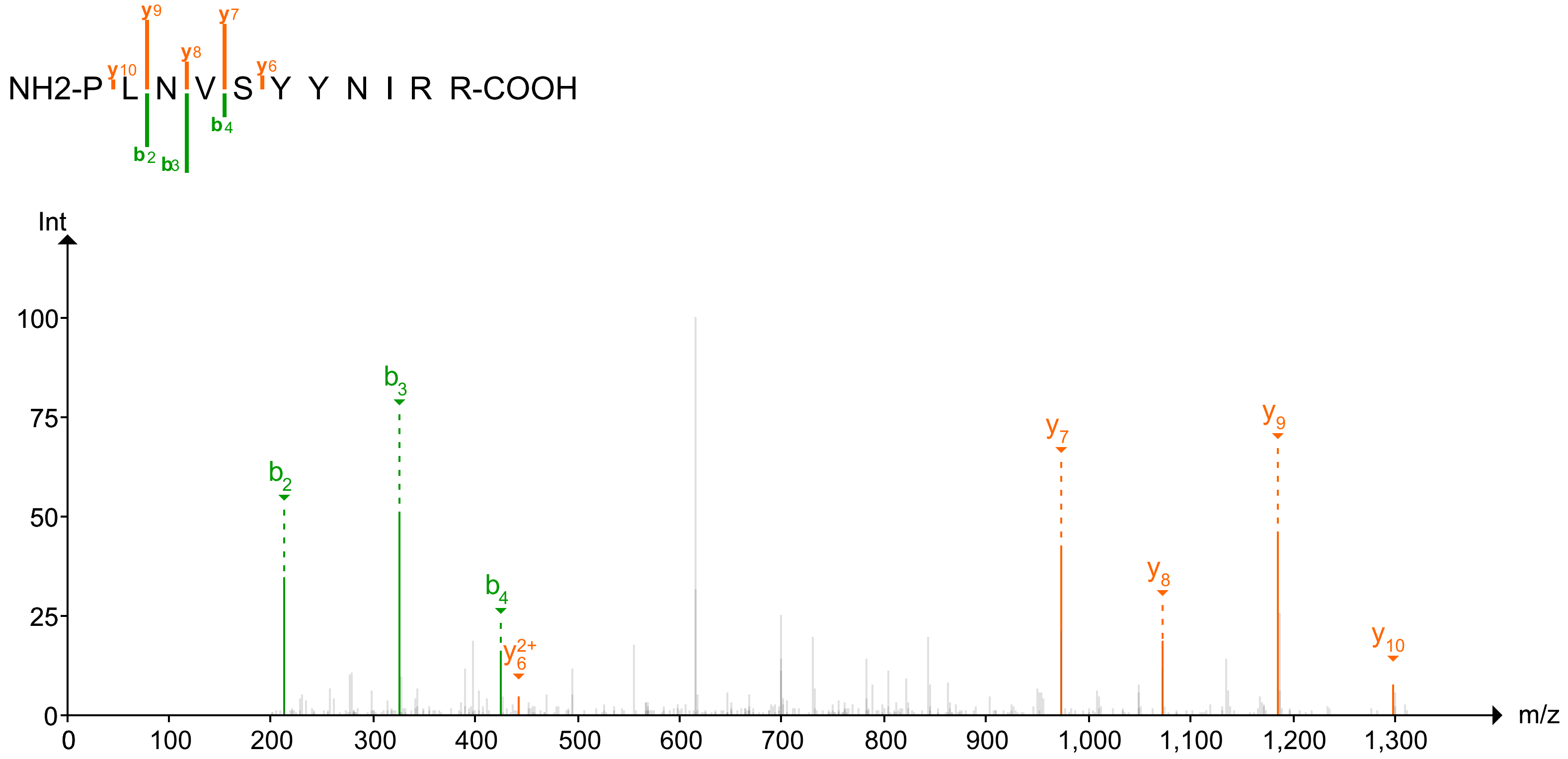

NH2-P L N V S Y Y N I R R-COOH
y10
y9
y8
y7
y6
b2
b3
b4
Int
100
75
50
25
0
b2
b3
b4
y6 2+
y7
y8
y9
y10
0
100
200
300
400
500
600
700
800
900
1,000
1,100
1,200
1,300
m/z

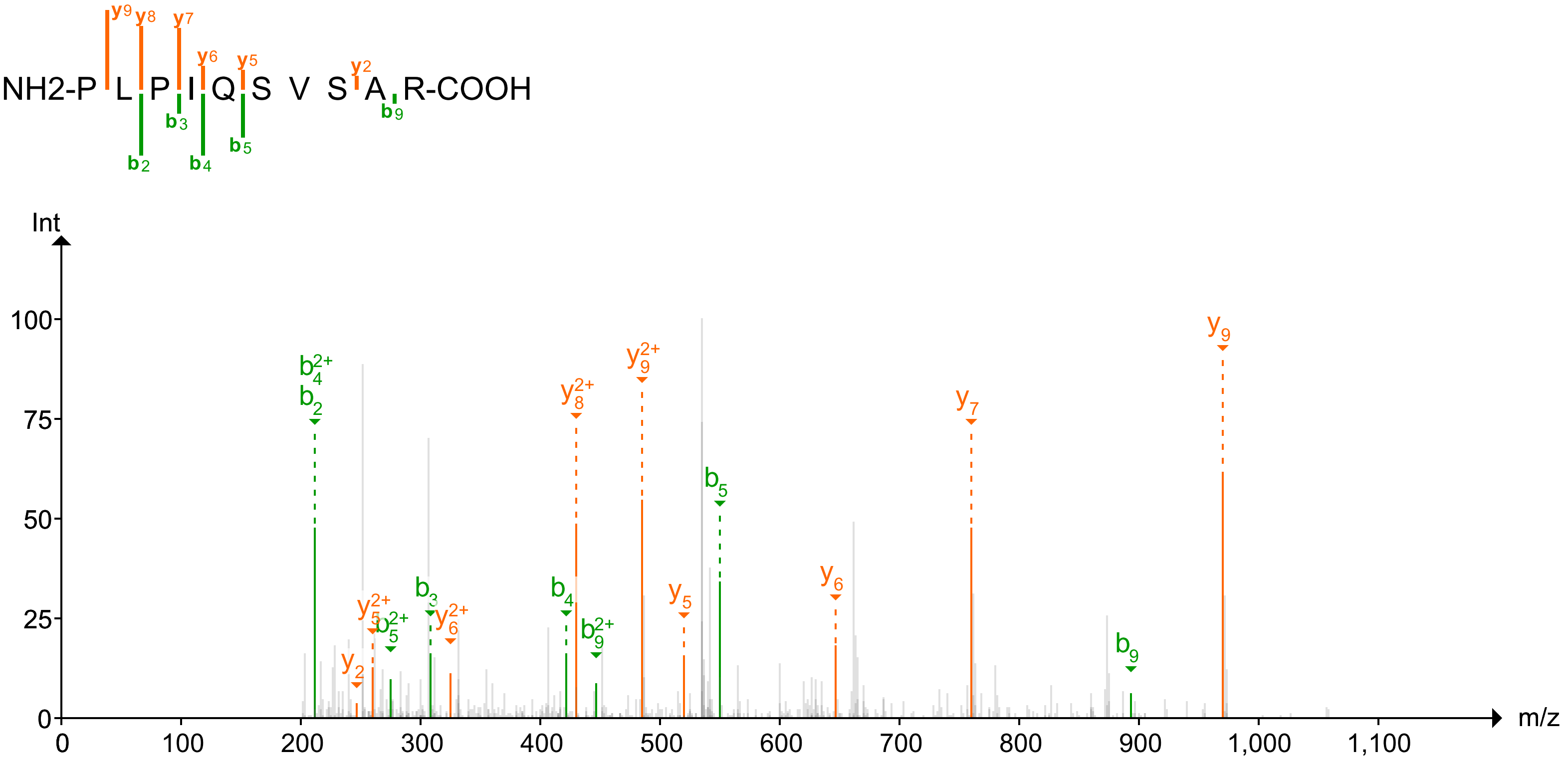

NH2-P L P I Q S V S A R-COOH
y9 y8 y7 y6 y5 y2
b2 b3 b4 b5 b9
Int
100
75
50
25
0
m/z
0 100 200 300 400 500 600 700 800 900 1,000 1,100
$b_4^{2+}$
$b_2$
$y_2$
$y_5^{2+}$
$b_5^{2+}$
$b_3$
$y_6^{2+}$
$y_8^{2+}$
$b_4$
$b_9^{2+}$
$y_9^{2+}$
$y_5$
$b_5$
$y_6$
$y_7$
$b_9$
$y_9$

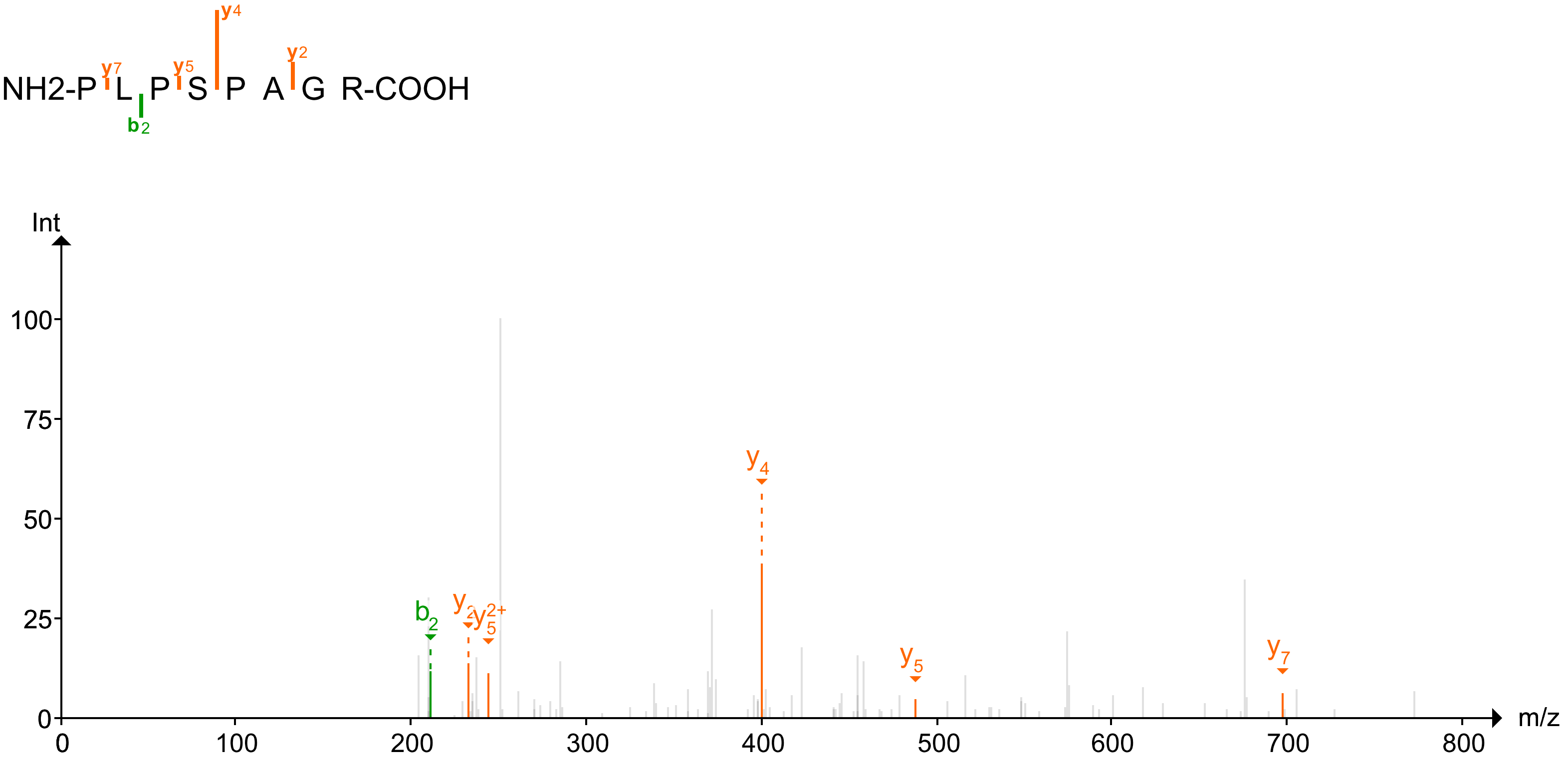

NH2-P L P S P A G R-COOH
y7
y5
y4
y2
b2
Int
m/z
0
25
50
75
100
0
100
200
300
400
500
600
700
800
b2
y2
y5 2+
y4
y5
y7

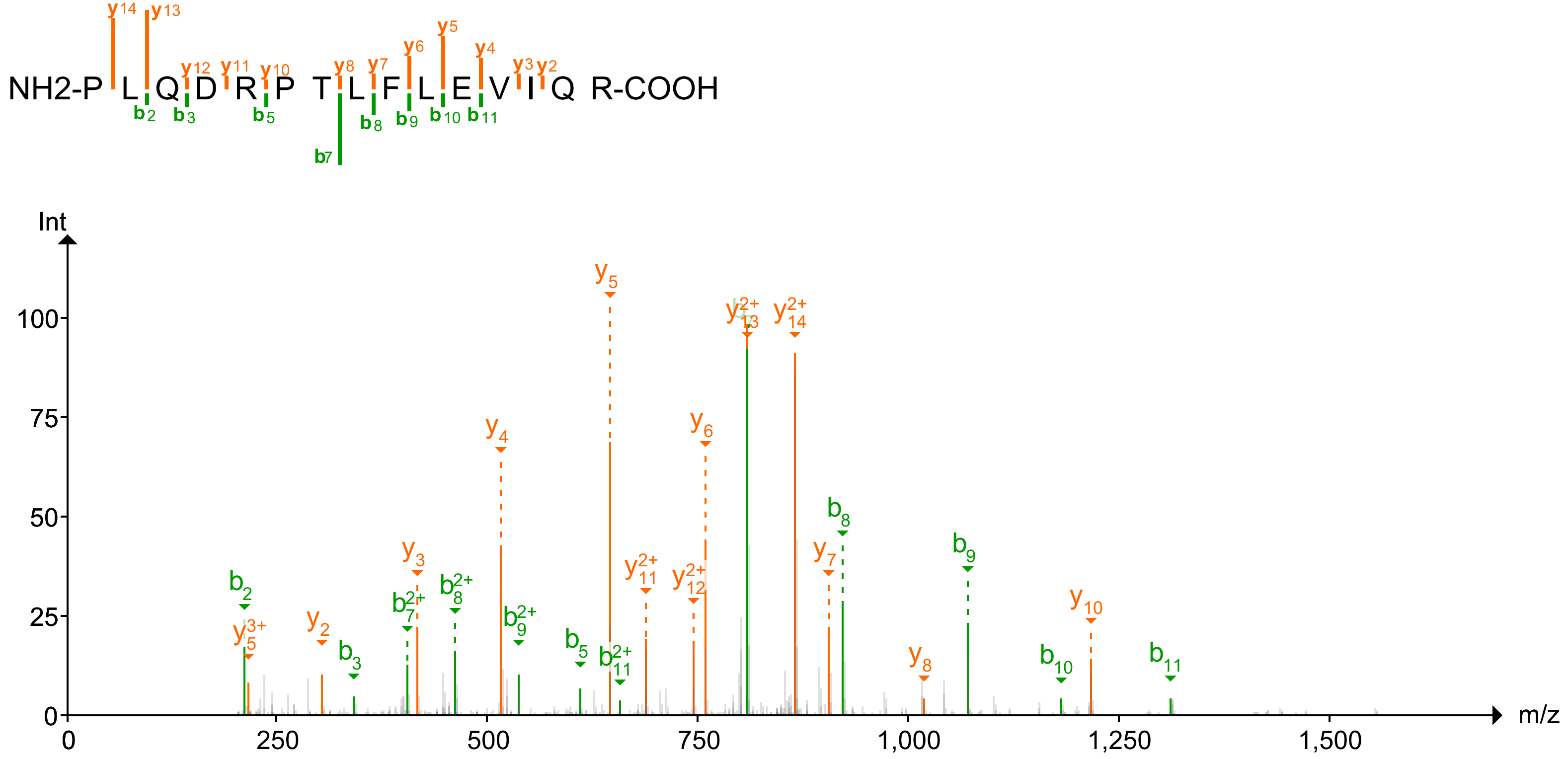

NH2-P L Q D R P T L F L E V I Q R-COOH
y14 y13 y12 y11 y10 y8 y7 y6 y5 y4 y3 y2
b2 b3 b5 b7 b8 b9 b10 b11
Int
m/z
0
25
50
75
100
250
500
750
1,000
1,250
1,500
y5
y2+13
y2+14
y4
y6
b8
y3
y7
b9
y2+11
y2+12
b2
b2+8
b2+7
b2+9
y3+5
y2
b3
b5
b2+11
y10
y8
b10
b11

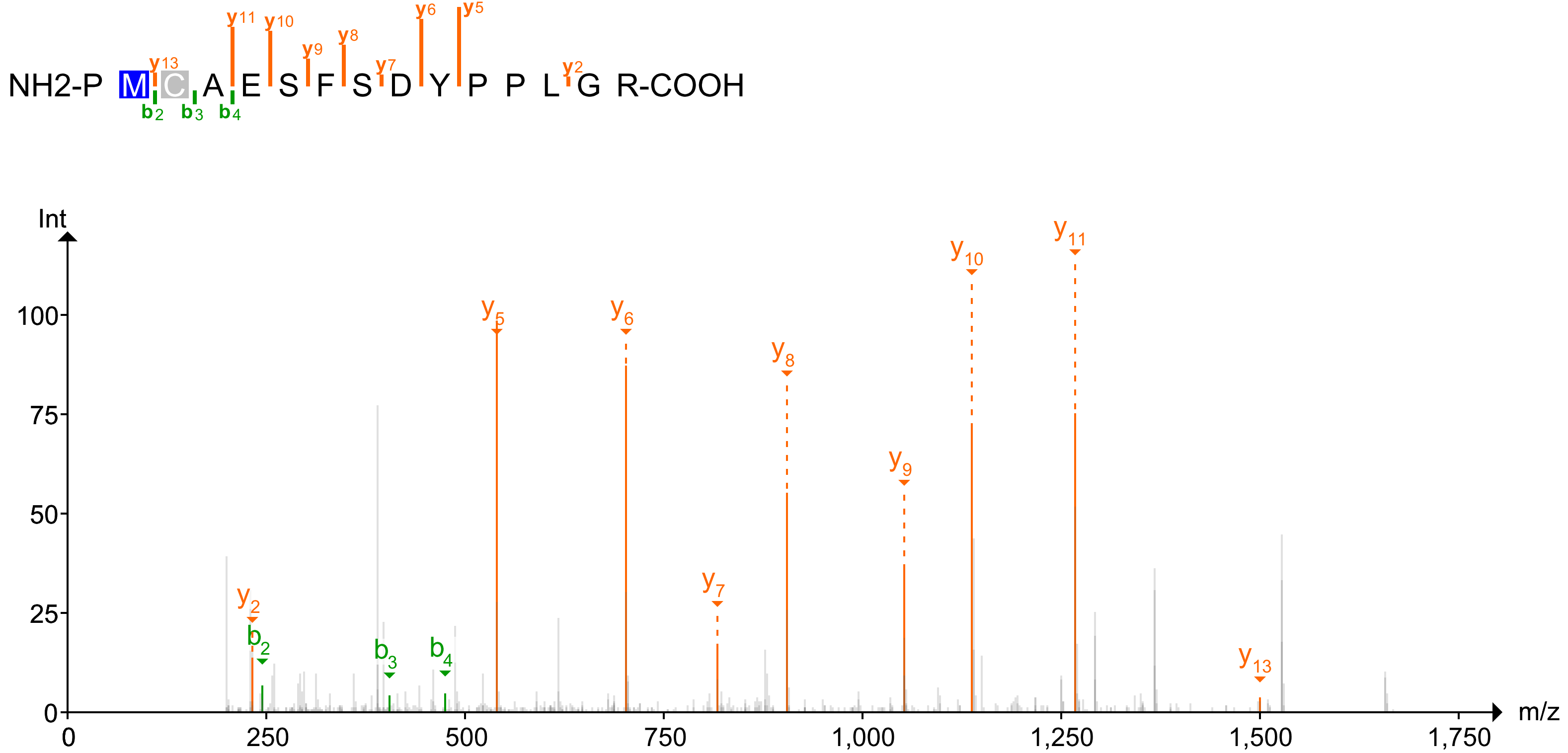

NH2-P M C A E S F S D Y P P L G R-COOH
y13 y11 y10 y9 y8 y7 y6 y5 y2
b2 b3 b4
Int
100
75
50
25
0
m/z
0
250
500
750
1,000
1,250
1,500
1,750

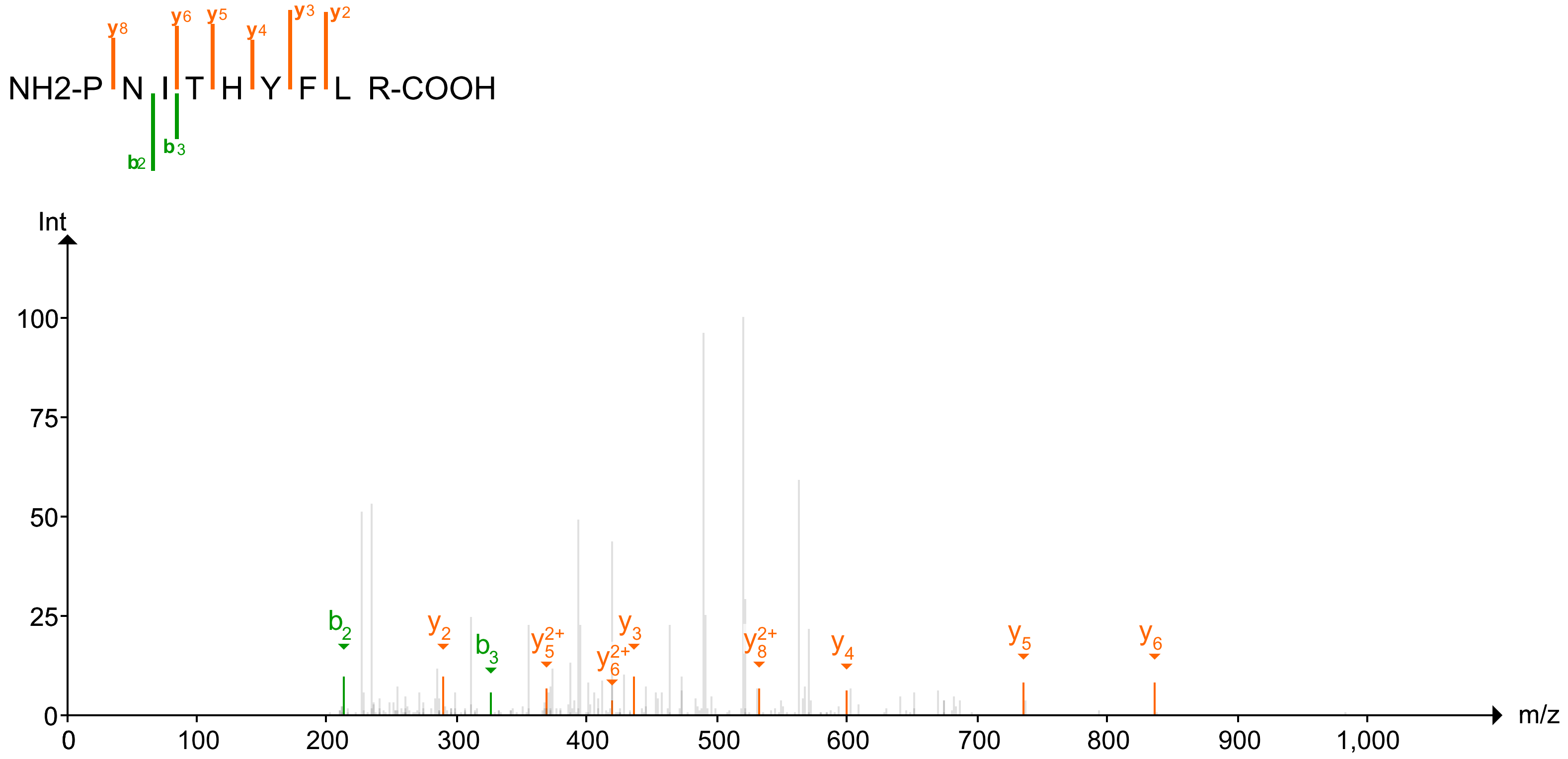

NH2-P N I T H Y F L R-COOH
y8 y6 y5 y4 y3 y2
b2 b3
Int
100
75
50
25
0
0 100 200 300 400 500 600 700 800 900 1,000
m/z
b2 y2 b3 y5 2+ y6 2+ y3 y8 2+ y4 y5 y6

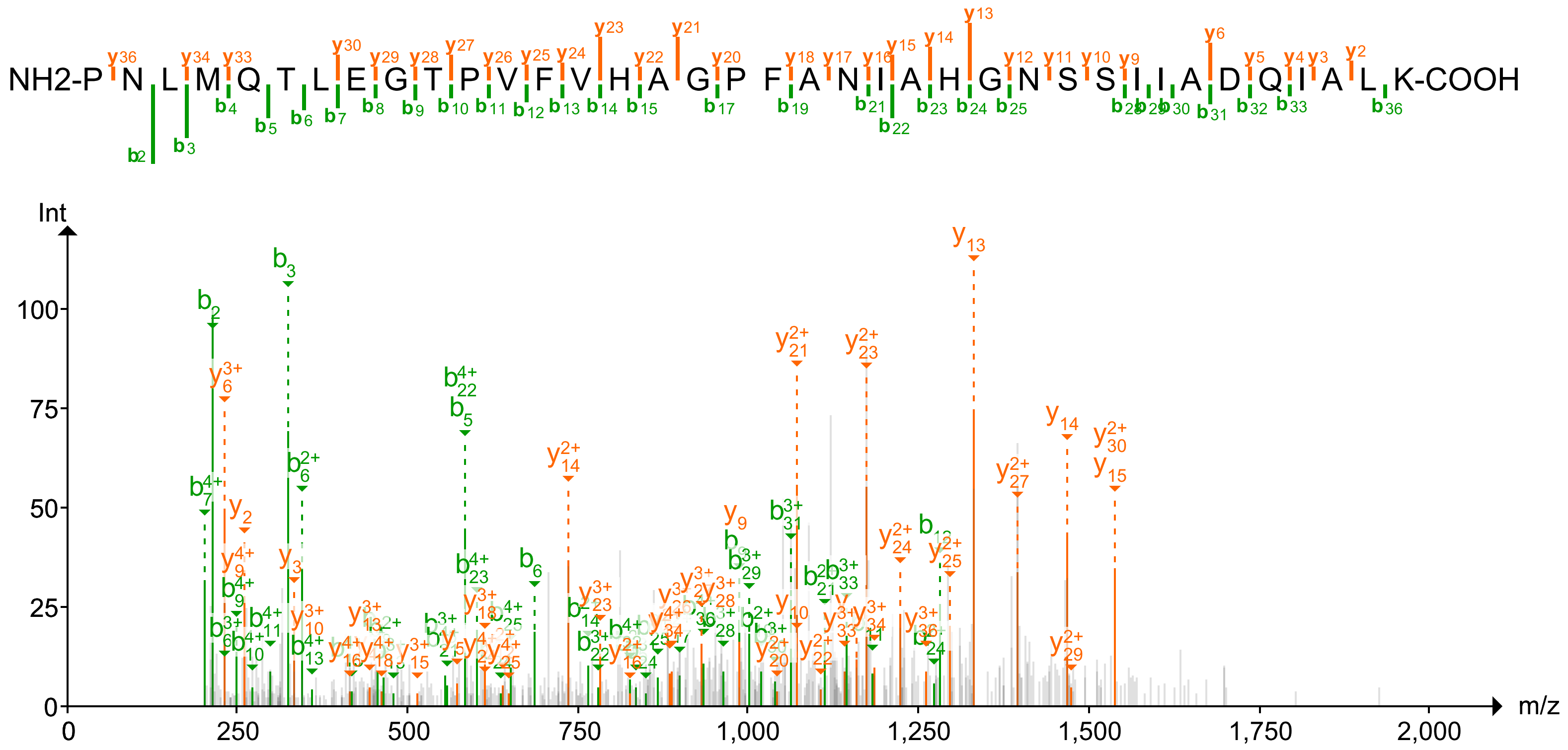

NH2-PNLMQTLEGTPVFVHAGPFANIAHGNSSIIADQIALK-COOH
Int
m/z
0
25
50
75
100
250
500
750
1,000
1,250
1,500
1,750
2,000

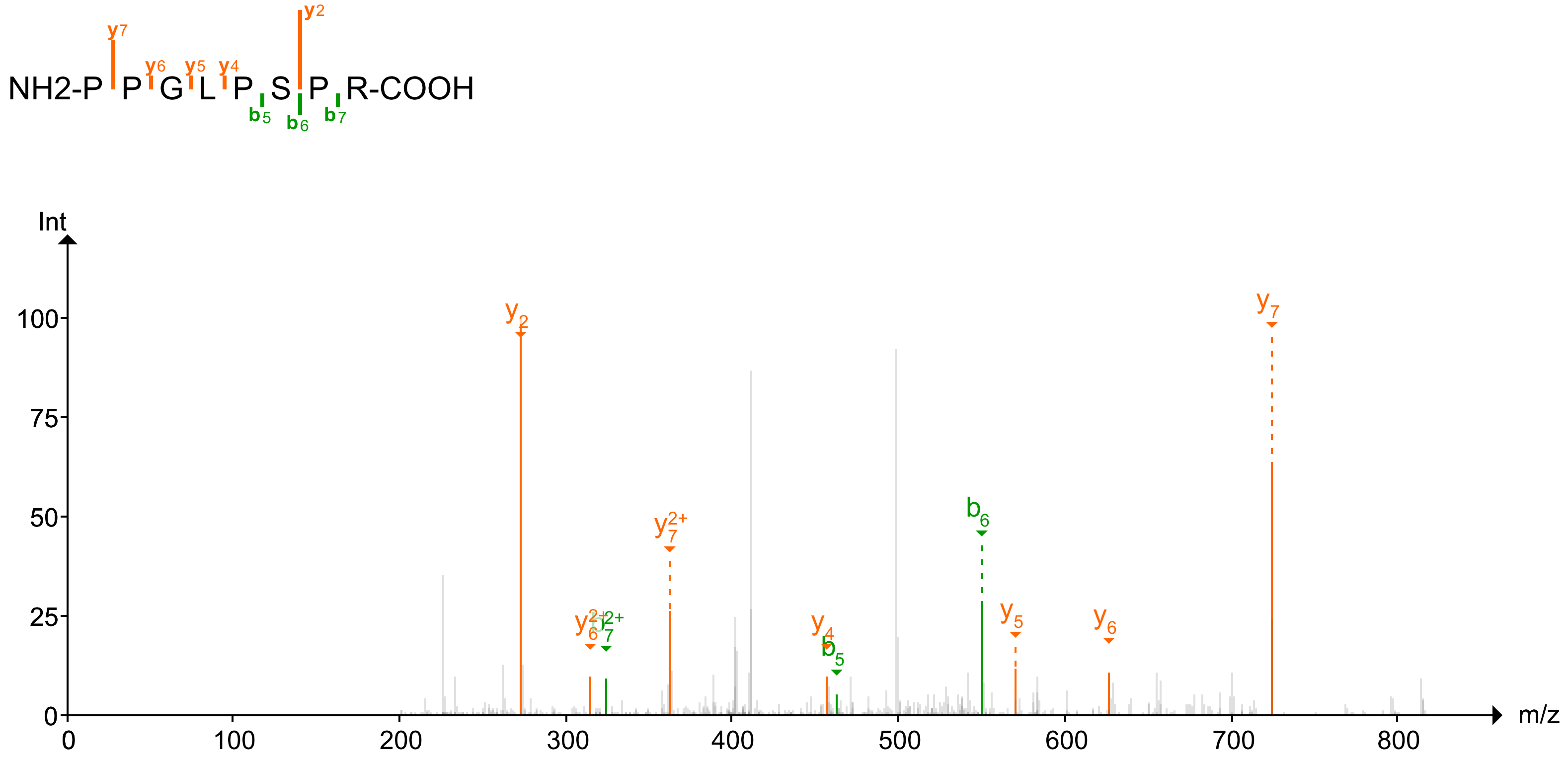

NH2-P P G L P S P R-COOH
y7 y6 y5 y4 y2
b5 b6 b7
Int
100
75
50
25
0
0
100
200
300
400
500
600
700
800
m/z
y2
y7
y7 2+
b6
y6 2+
b7 2+
y4
b5
y5
y6

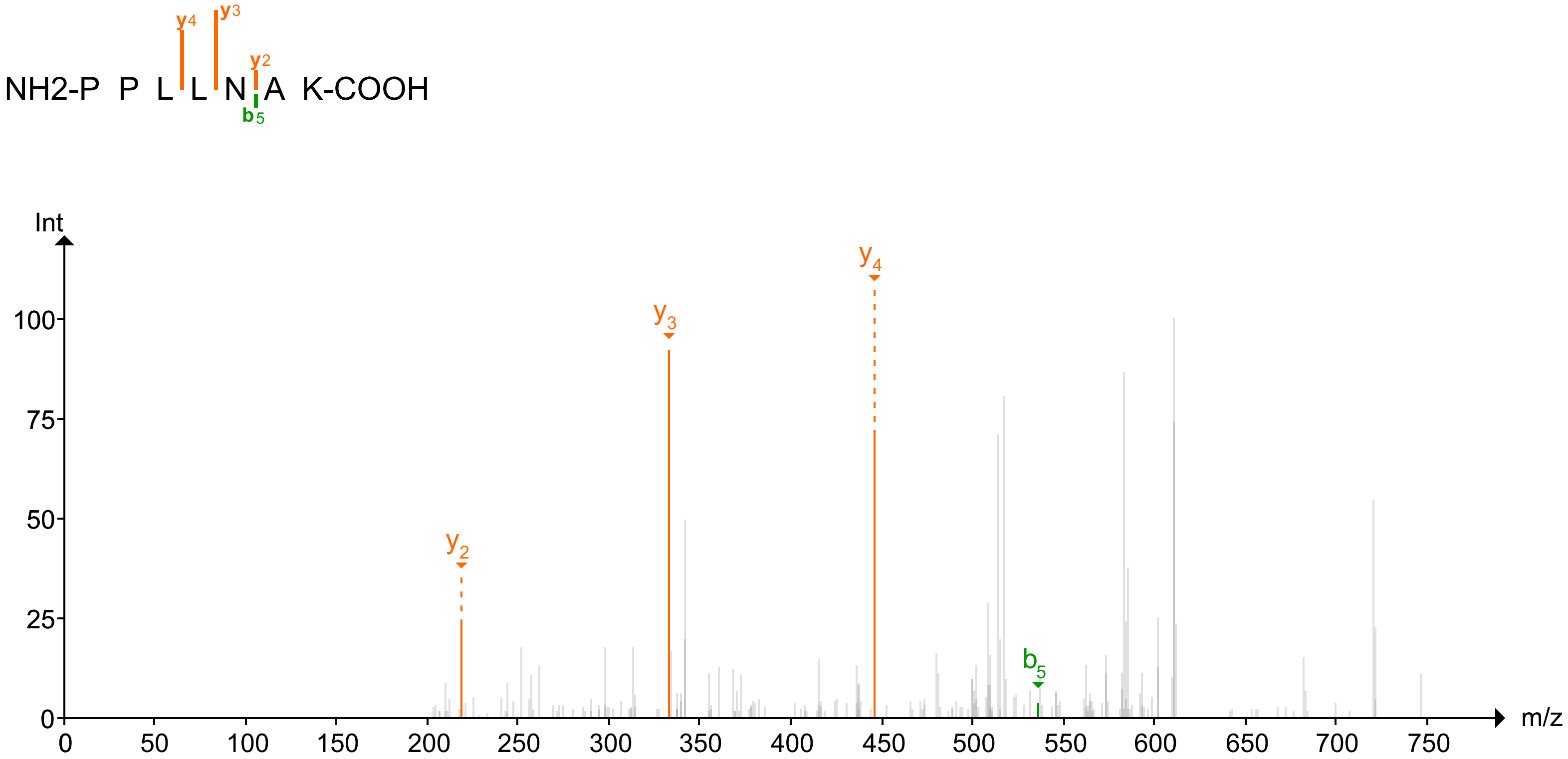

NH2-P P L L N A K-COOH
y4
y3
y2
b5
Int
100
75
50
25
0
0 50 100 150 200 250 300 350 400 450 500 550 600 650 700 750
m/z

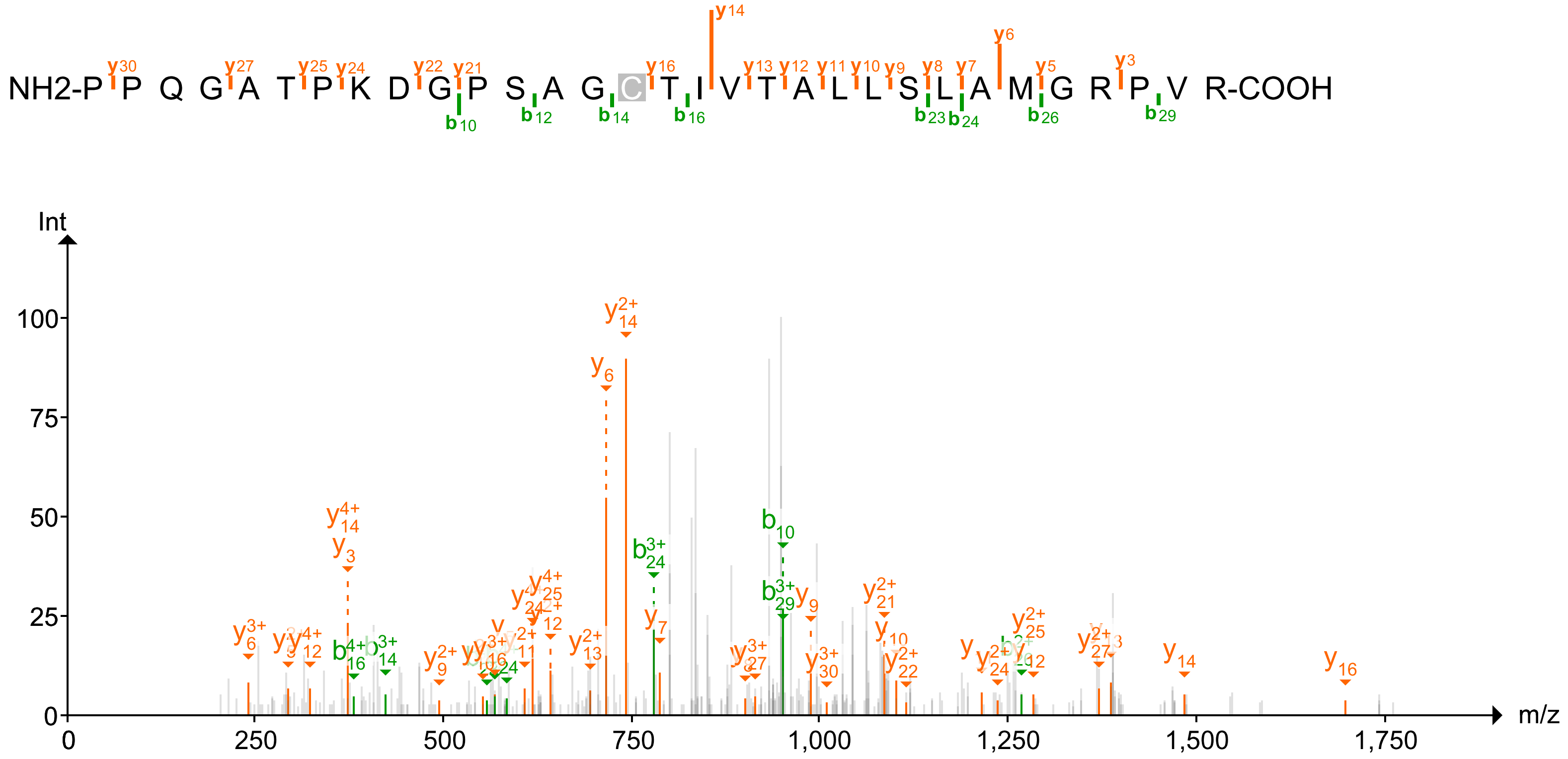

NH2-P P Q G A T P K D G P S A G C T I V T A L L S L A M G R P V R-COOH
y30 y27 y25 y24 y22 y21 y16 y14 y13 y12 y11 y10 y9 y8 y7 y6 y5 y3
b10 b12 b14 b16 b23 b24 b26 b29
Int
100
75
50
25
0
m/z
0
250
500
750
1,000
1,250
1,500
1,750
y14 2+
y6
y14 4+
y3
b10
b24 3+
b29 3+
y7
y9
y21 2+
y10
y22 2+
y25 2+
y27 2+
y14
y16
y6 3+
y12 4+
b16 4+
b14 3+
y9 2+
y13 2+
y25 4+
y30 3+
y27 3+
y24 2+

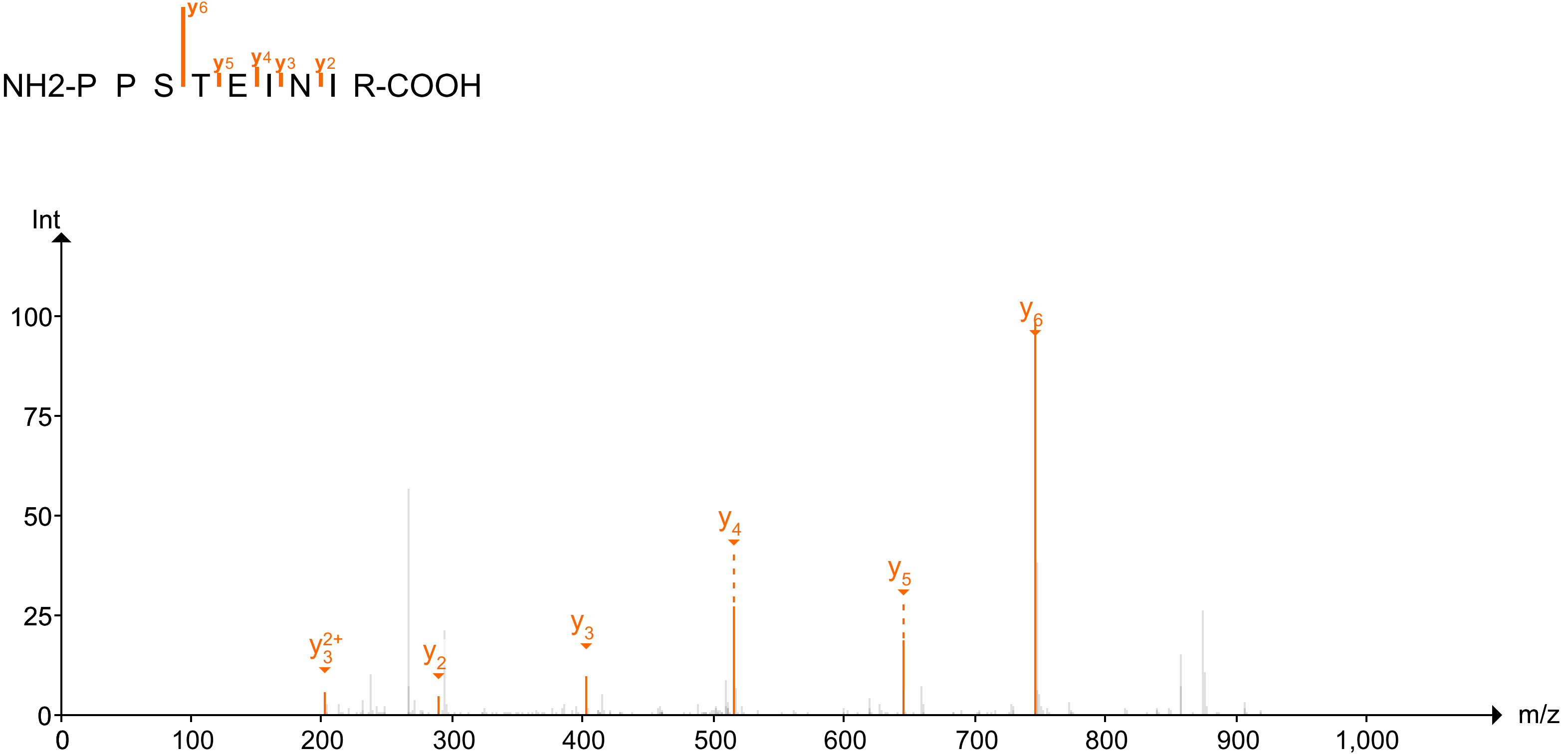

NH2-P P S T E I N I R-COOH
y6
y5
y4
y3
y2
Int
100
75
50
25
0
m/z
0
100
200
300
400
500
600
700
800
900
1,000
$y_3^{2+}$
$y_2$
$y_3$
$y_4$
$y_5$
$y_6$

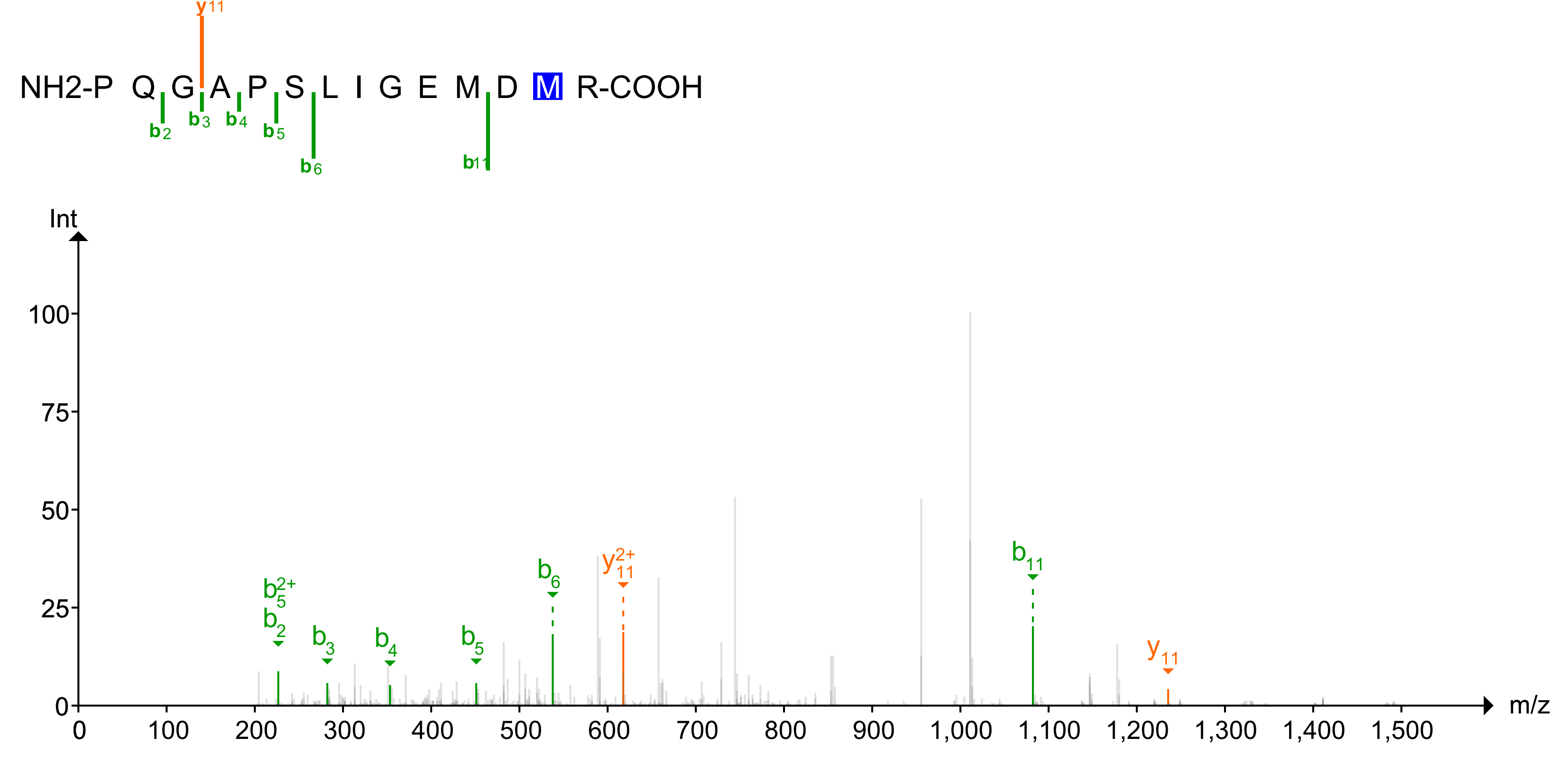

y11
NH2-P Q G A P S L I G E M D M R-COOH
b2
b3
b4
b5
b6
b11
Int
100
75
50
25
0
b5 2+
b2
b3
b4
b5
b6
y11 2+
b11
y11
m/z
0
100
200
300
400
500
600
700
800
900
1,000
1,100
1,200
1,300
1,400
1,500

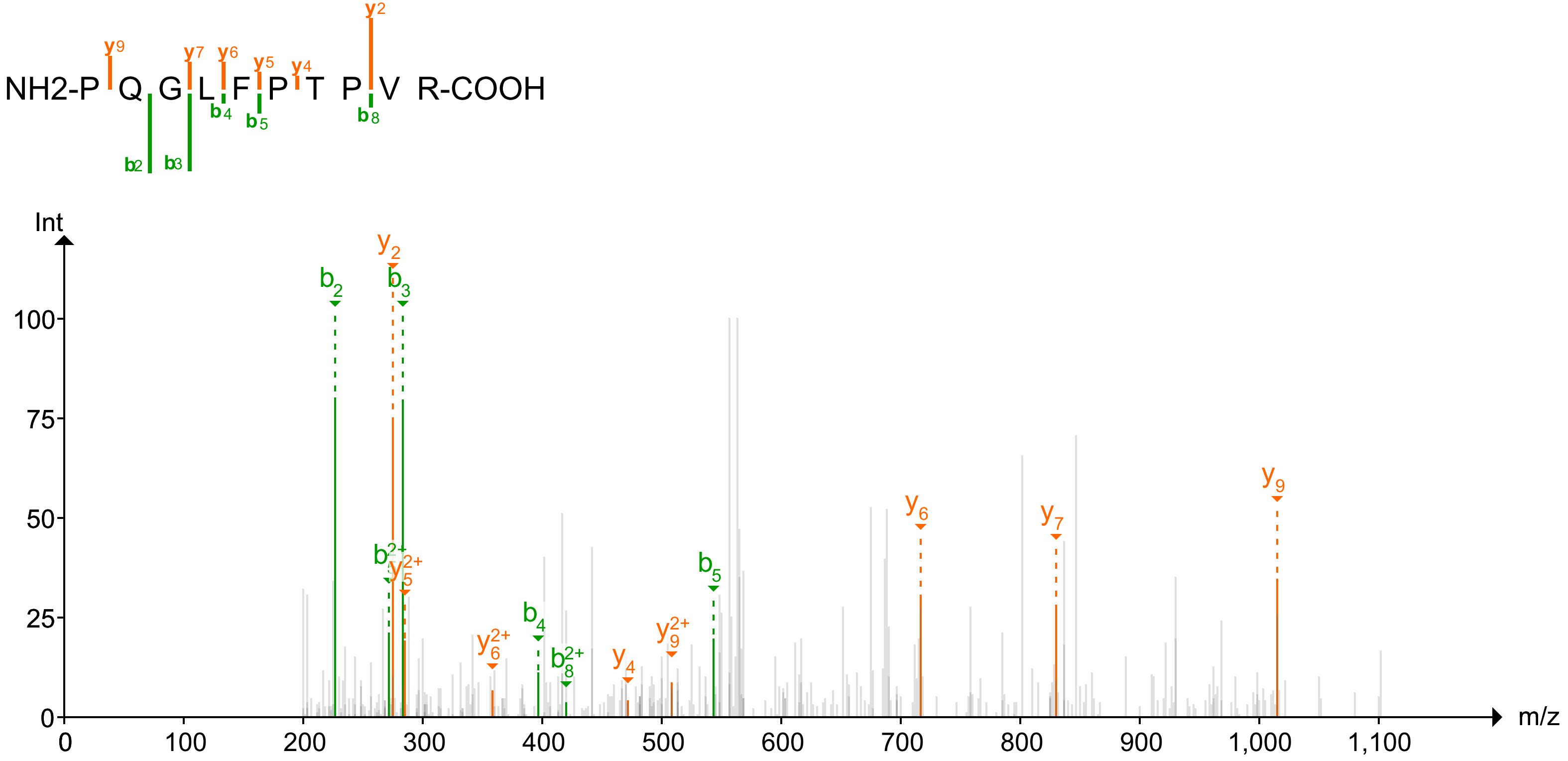
NH2-P Q G L F P T P V R-COOH
y9 y7 y6 y5 y4 y2
b2 b3 b4 b5 b8
Int
m/z
0
25
50
75
100
0 100 200 300 400 500 600 700 800 900 1,000 1,100
b2
y2
b3
b8 2+
y5 2+
y6 2+
b4
b8 2+
y4
y9 2+
b5
y6
y7
y9

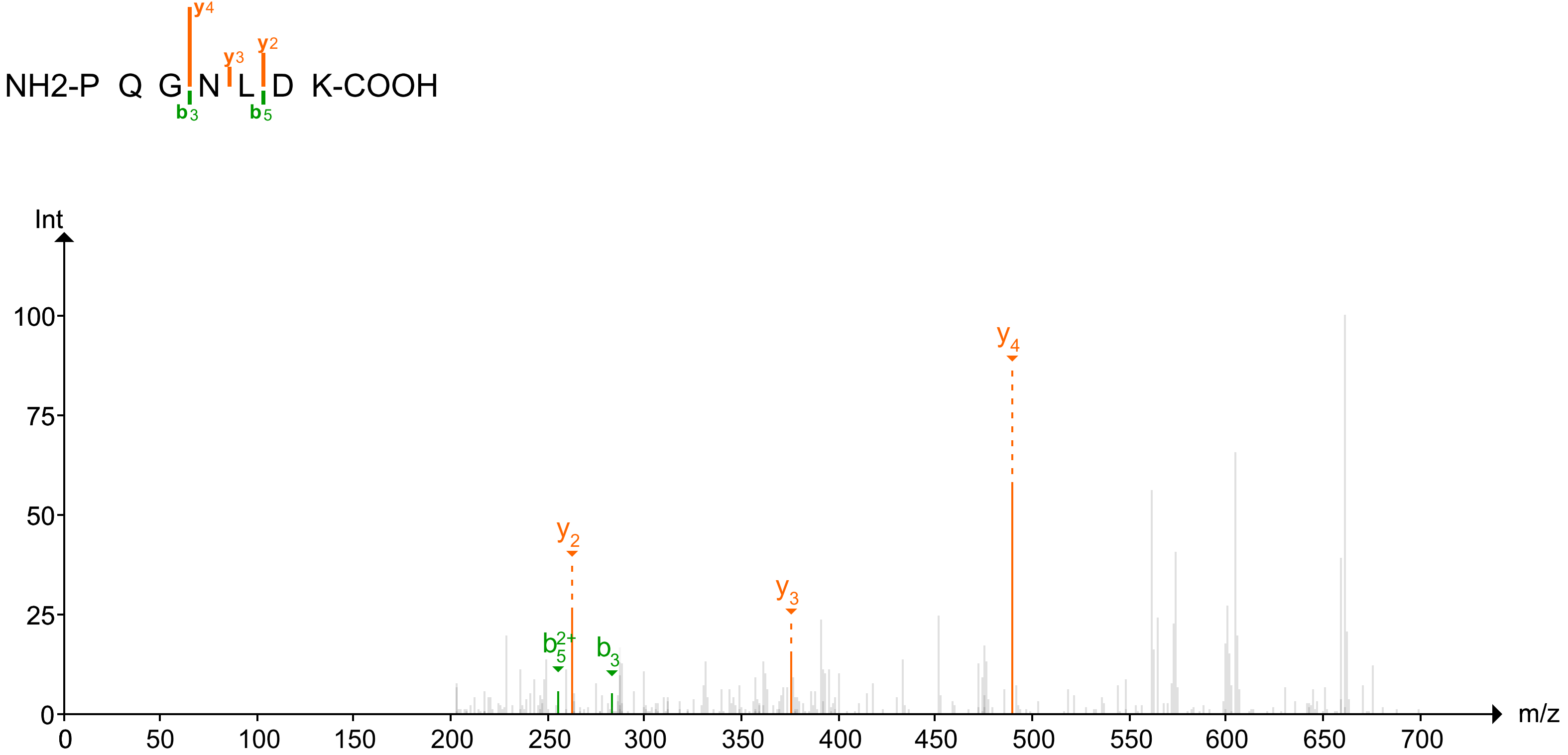

NH2-P Q G N L D K-COOH
y4
y3
y2
b3
b5
Int
100
75
50
25
0
y4
y2
y3
b5 2+
b3
0 50 100 150 200 250 300 350 400 450 500 550 600 650 700
m/z

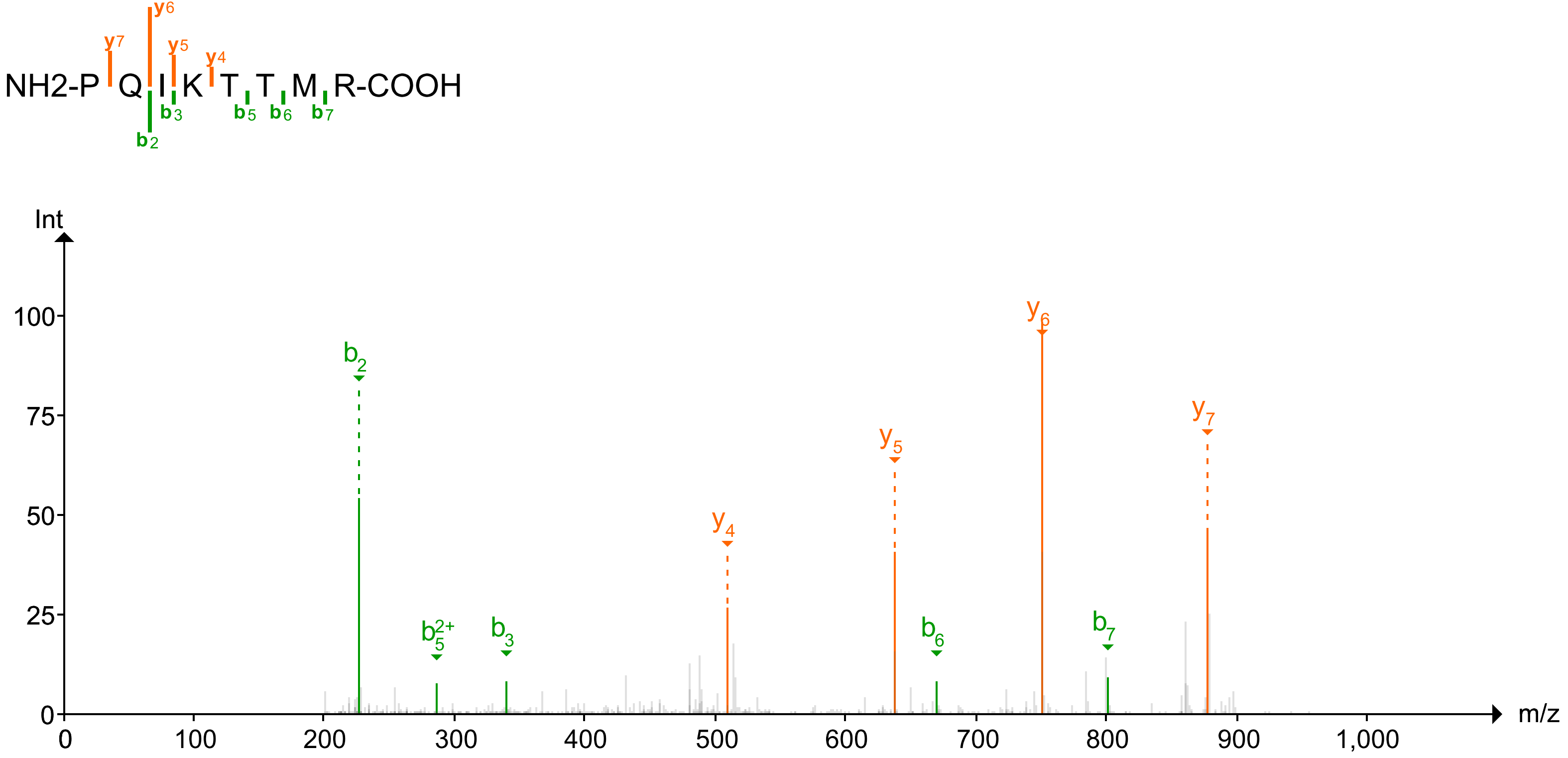

NH2-P Q I K T T M R-COOH
y7 y6 y5 y4
b2 b3 b5 b6 b7
Int
100
75
50
25
0
m/z
0
100
200
300
400
500
600
700
800
900
1,000
b2
b5 2+
b3
y4
y5
b6
y6
b7
y7

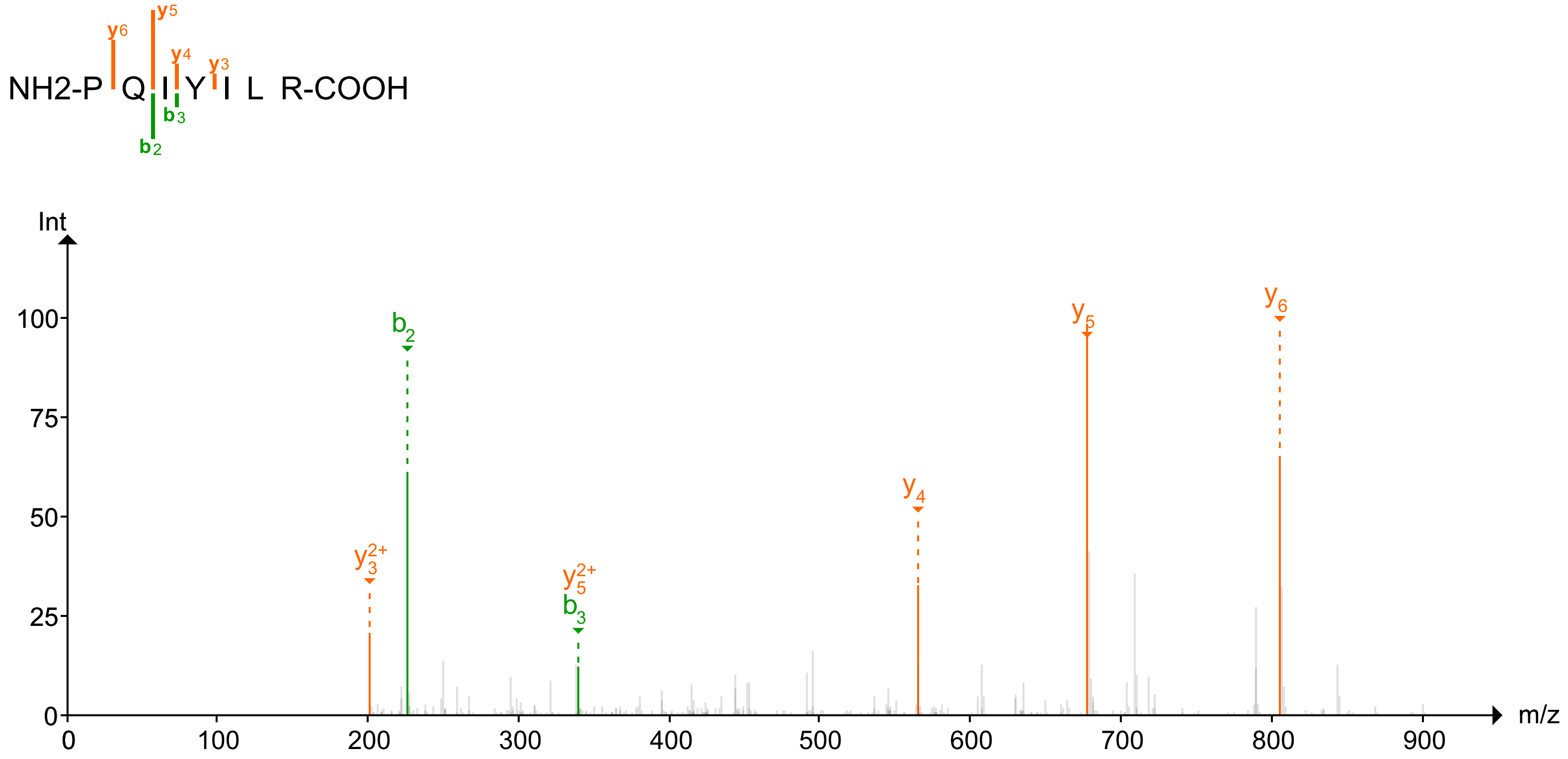
NH2-P Q I Y I L R-COOH
y6
y5
y4
y3
b2
b3
Int
100
75
50
25
0
m/z
0
100
200
300
400
500
600
700
800
900
$y_3^{2+}$
$b_2$
$y_5^{2+}$
$b_3$
$y_4$
$y_5$
$y_6$

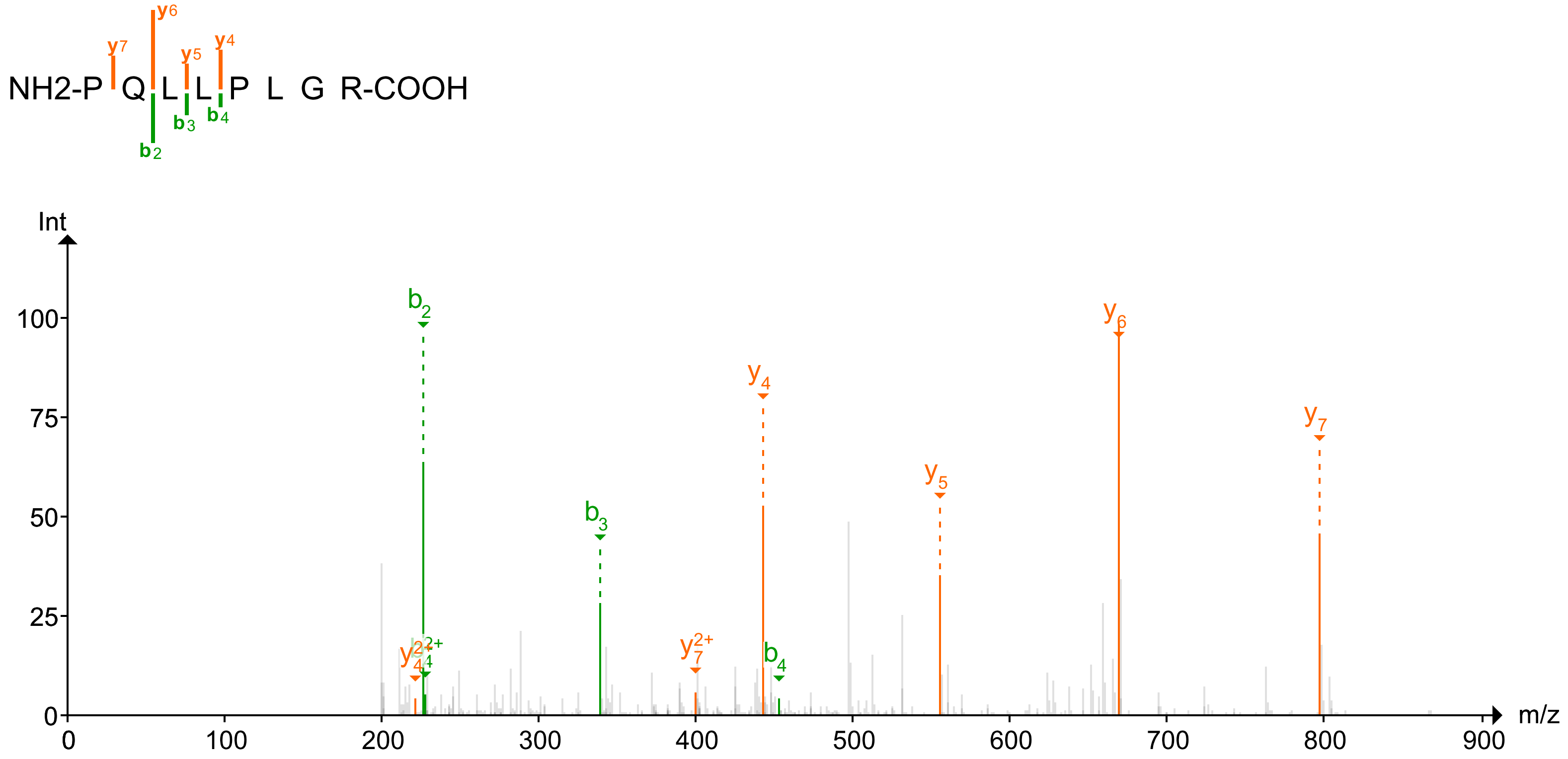

NH2-P Q L L P L G R-COOH
y7 y6 y5 y4
b2 b3 b4
Int
100
75
50
25
0
m/z
0 100 200 300 400 500 600 700 800 900
b2
y6
y4
y7
y5
b3
y7 2+
b4
y4 2+
b4 2+

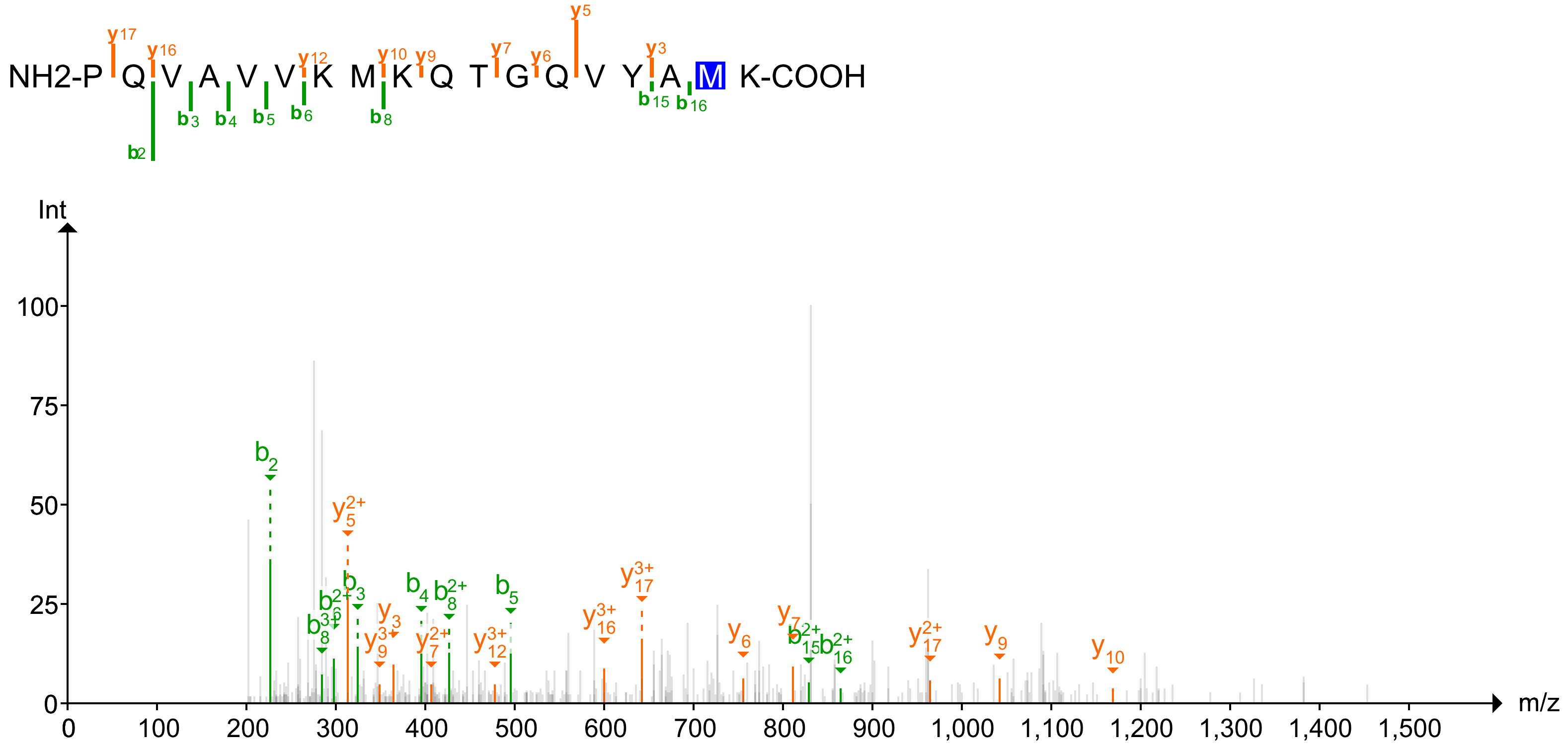

NH2-P Q V A V V K M K Q T G Q V Y A M K-COOH
y17 y16 y12 y10 y9 y7 y6 y5 y3
b2 b3 b4 b5 b6 b8 b15 b16
Int
100
75
50
25
0
m/z
0 100 200 300 400 500 600 700 800 900 1,000 1,100 1,200 1,300 1,400 1,500

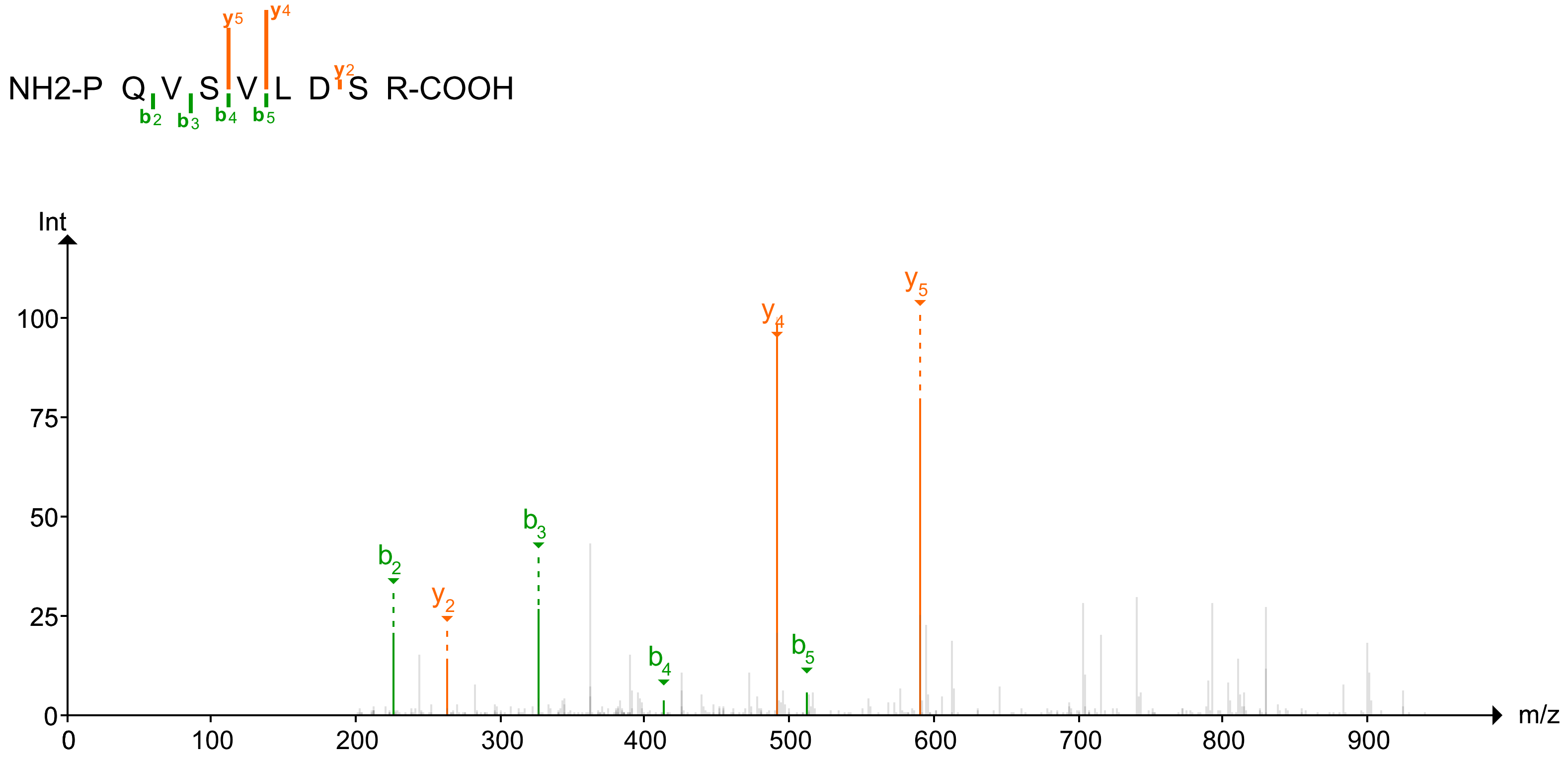

NH2-P Q V S V L D S R-COOH
y5 y4 y2
b2 b3 b4 b5
Int
100
75
50
25
0
m/z
0 100 200 300 400 500 600 700 800 900

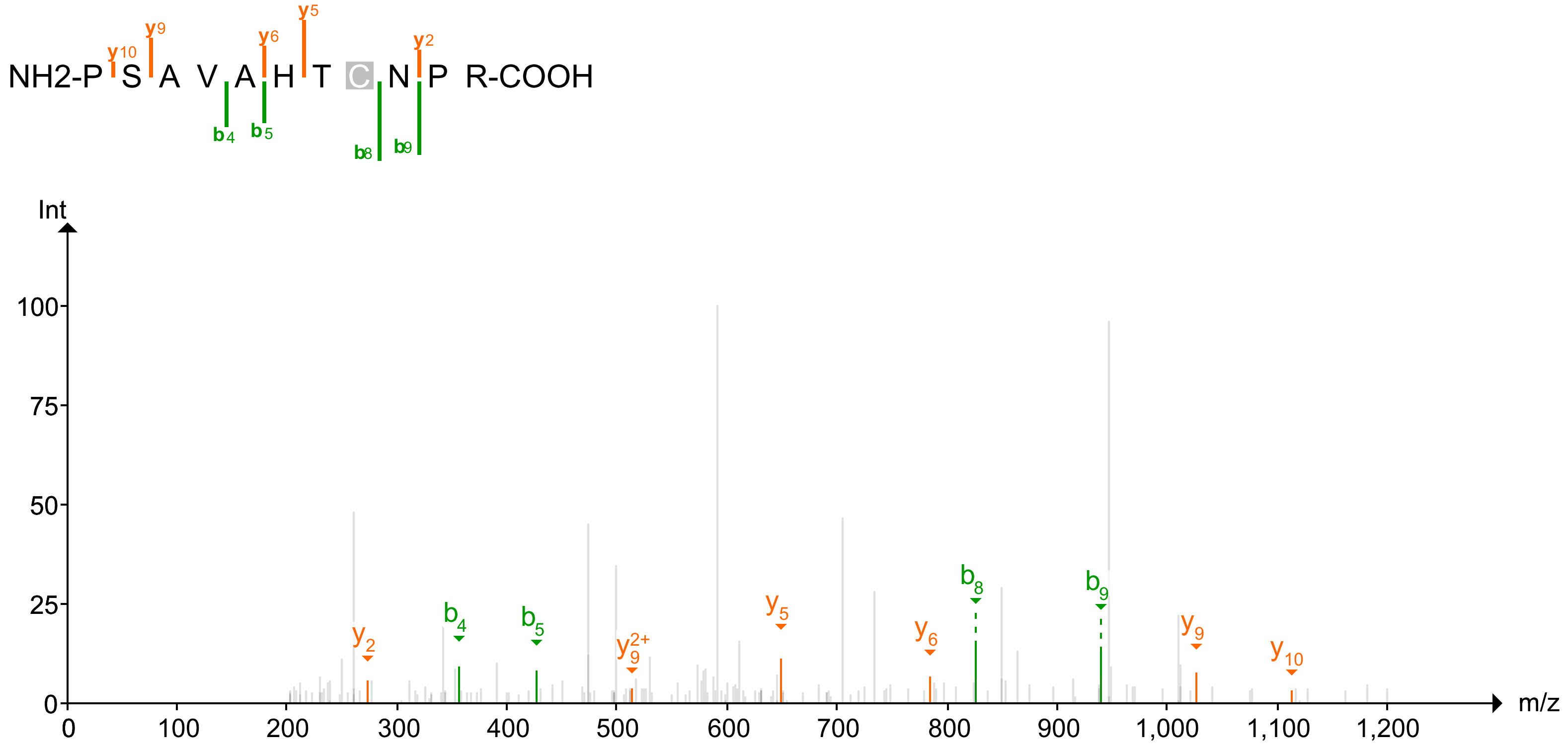

NH2-P S A V A H T C N P R-COOH
y10 y9 y6 y5 y2
b4 b5 b8 b9
Int
100
75
50
25
0
m/z
0 100 200 300 400 500 600 700 800 900 1,000 1,100 1,200
y2 b4 b5 y9 2+ y5 y6 b8 b9 y9 y10

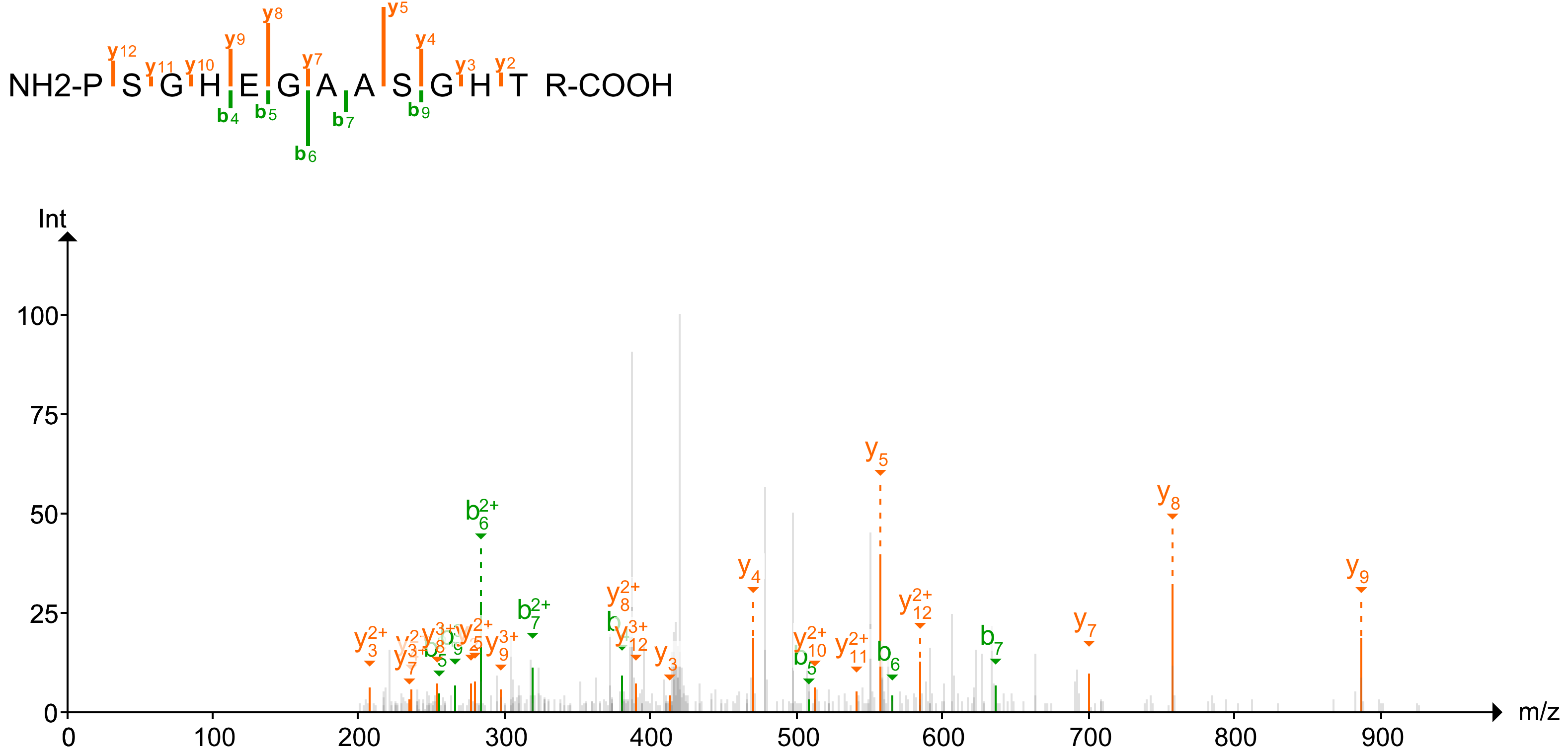

NH2-P S G H E G A A S G H T R-COOH
Int
m/z

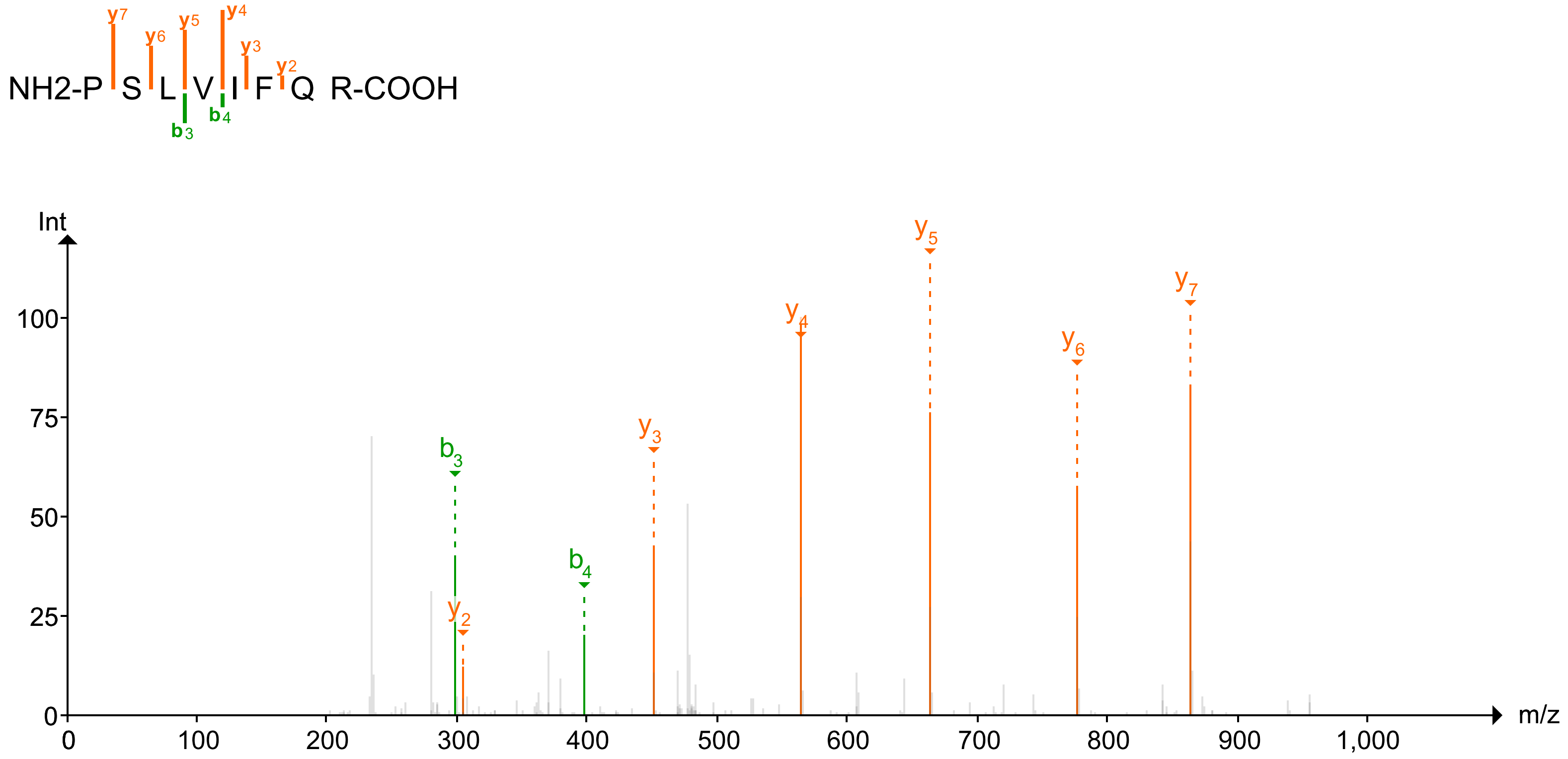

NH2-P S L V I F Q R-COOH
y7 y6 y5 y4 y3 y2
b3 b4
Int
m/z
0 25 50 75 100
0 100 200 300 400 500 600 700 800 900 1,000
y5 y7 y4 y6 y3 b3 b4 y2

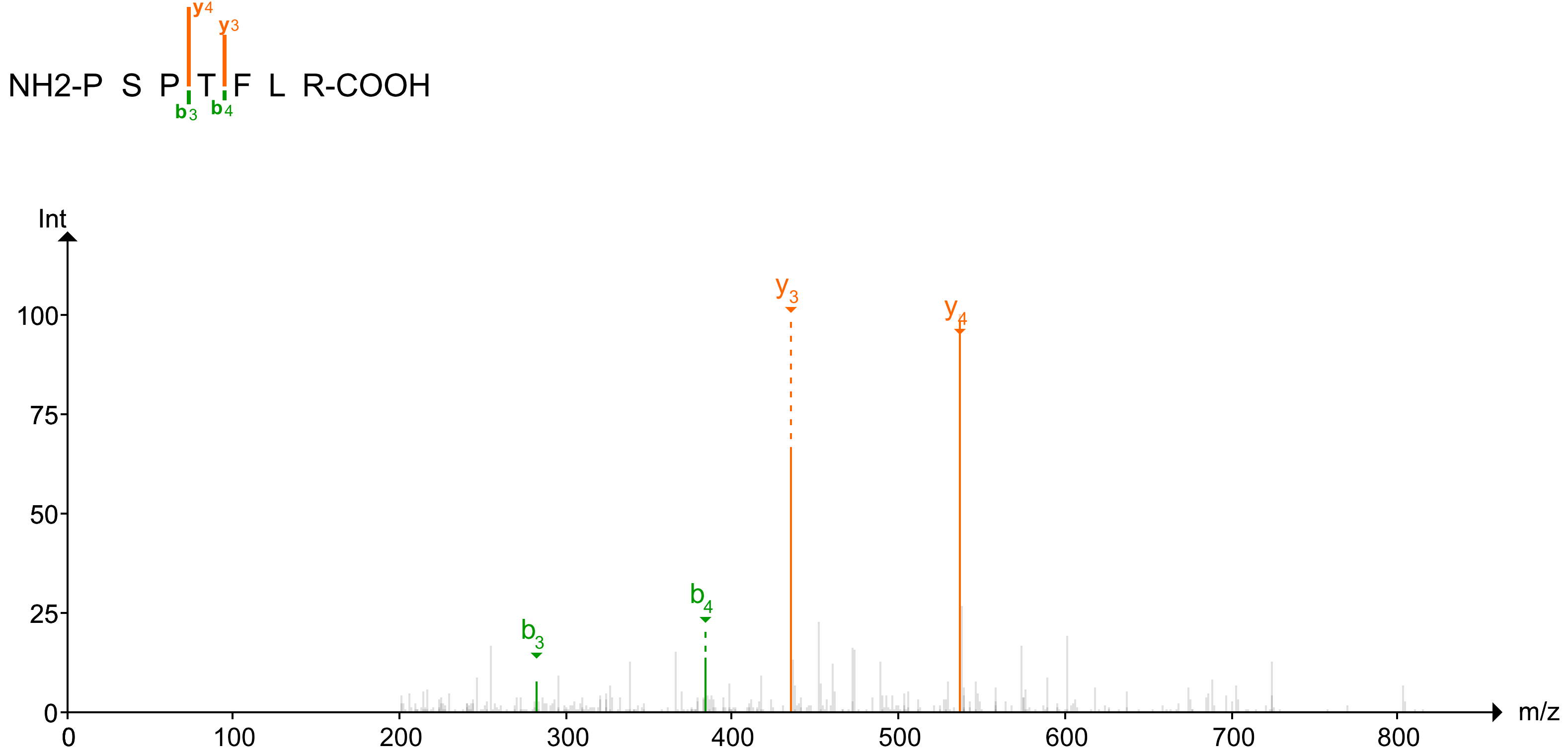

NH2-P S P T F L R-COOH
y4
y3
b3
b4
Int
100
75
50
25
0
0
100
200
300
400
500
600
700
800
m/z

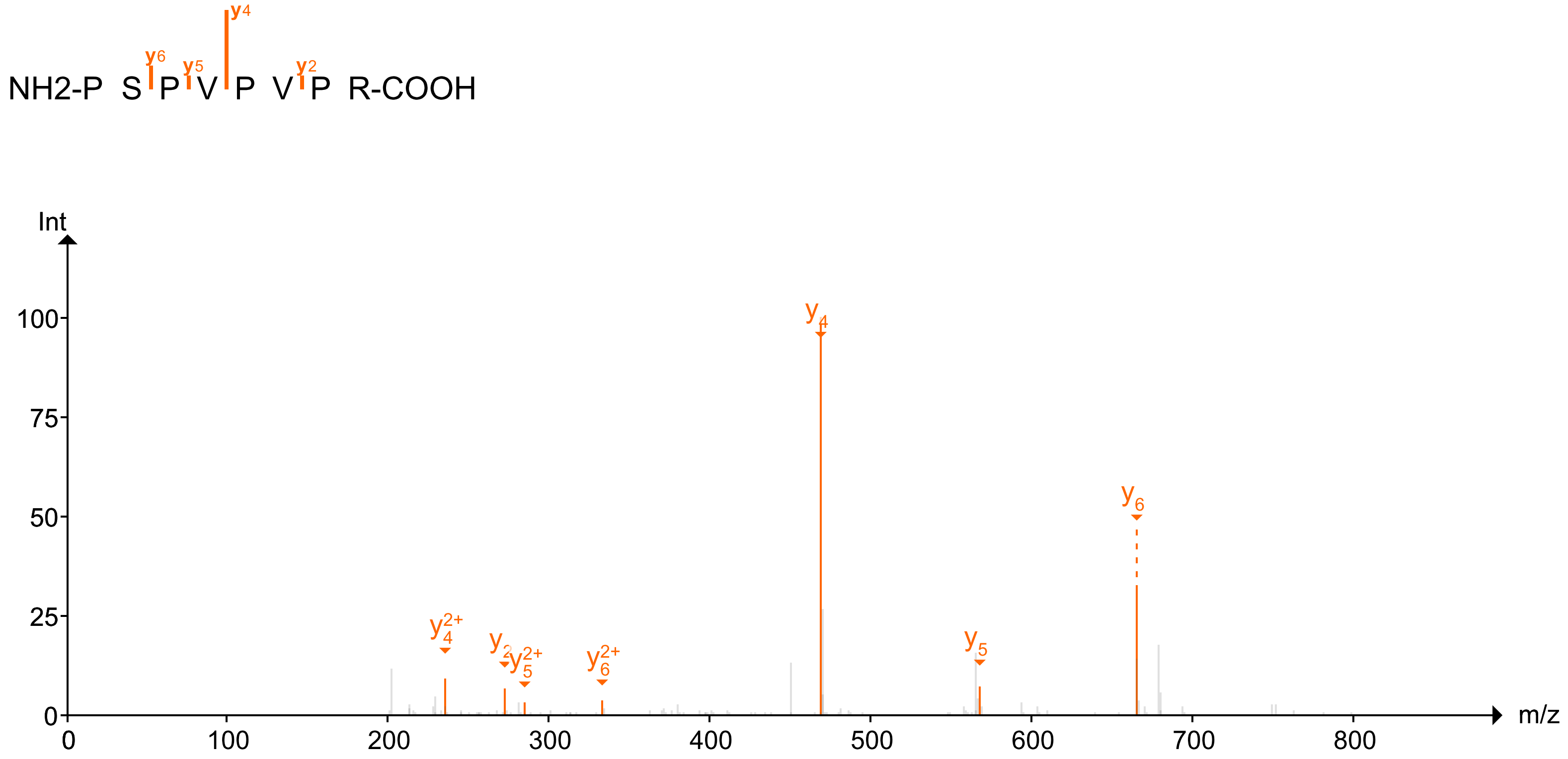

NH2-P S P V P V P R-COOH
y6
y5
y4
y2
Int
100
75
50
25
0
m/z
0
100
200
300
400
500
600
700
800
$y_4^{2+}$
$y_2$
$y_5^{2+}$
$y_6^{2+}$
$y_4$
$y_5$
$y_6$

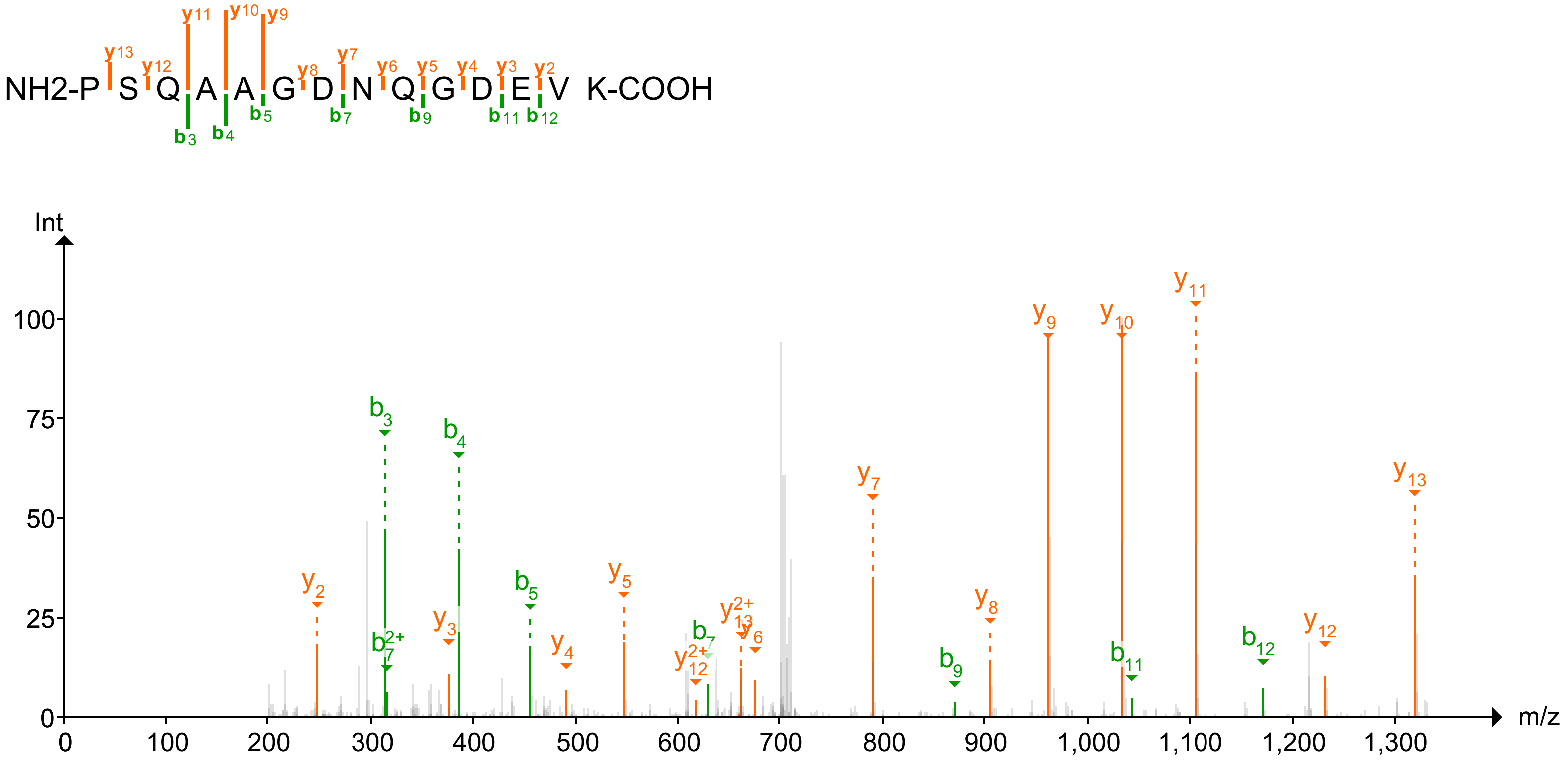

NH2-P S Q A A G D N Q G D E V K-COOH
y13 y12 y11 y10 y9 y8 y7 y6 y5 y4 y3 y2
b3 b4 b5 b7 b9 b11 b12
Int
100
75
50
25
0
m/z
0
100
200
300
400
500
600
700
800
900
1,000
1,100
1,200
1,300

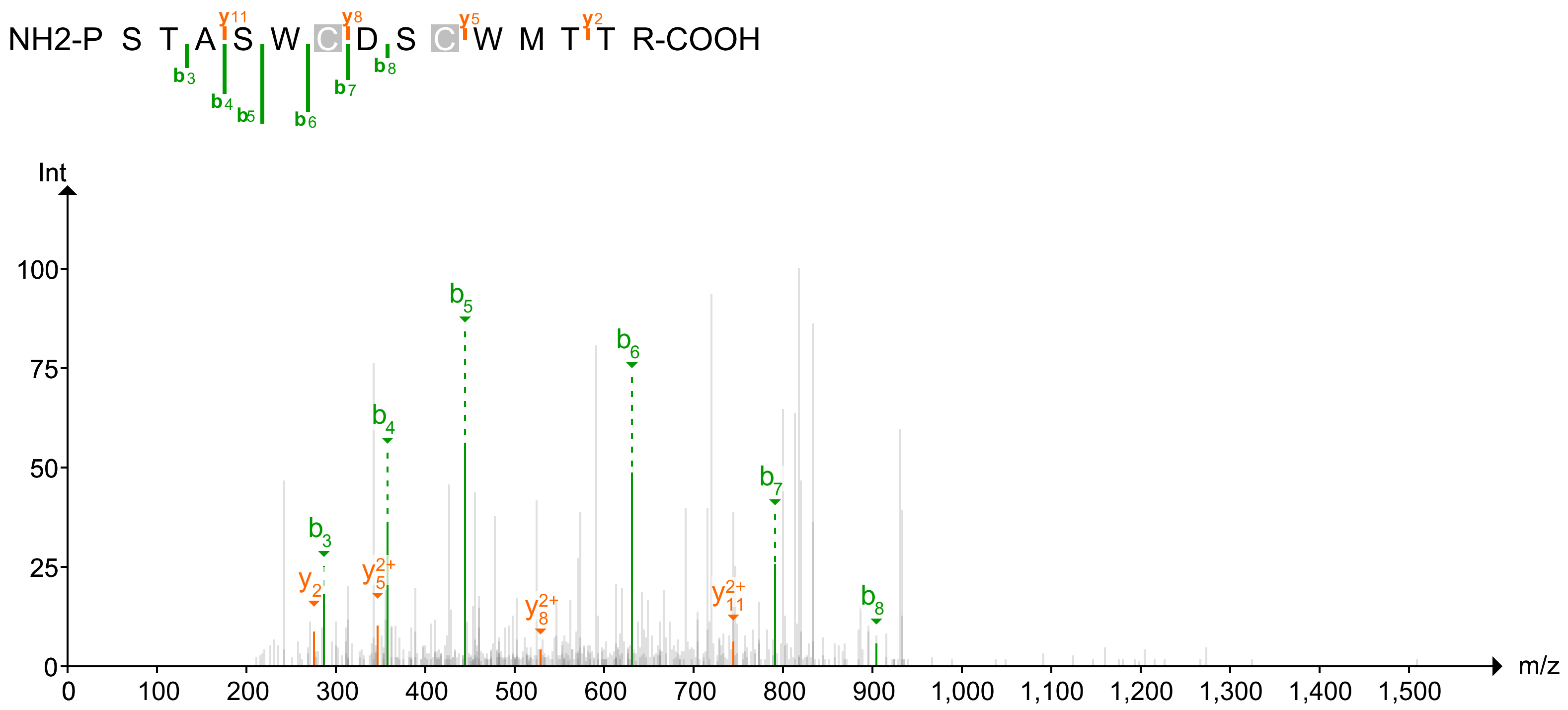
NH2-P S T A S W C D S C W M T T R-COOH
y11 y8 y5 y2
b3 b4 b5 b6 b7 b8
Int
100
75
50
25
0
m/z
0 100 200 300 400 500 600 700 800 900 1,000 1,100 1,200 1,300 1,400 1,500
b5 b6 b4 b7 b3 b8
y2 y5 2+ y8 2+ y11 2+

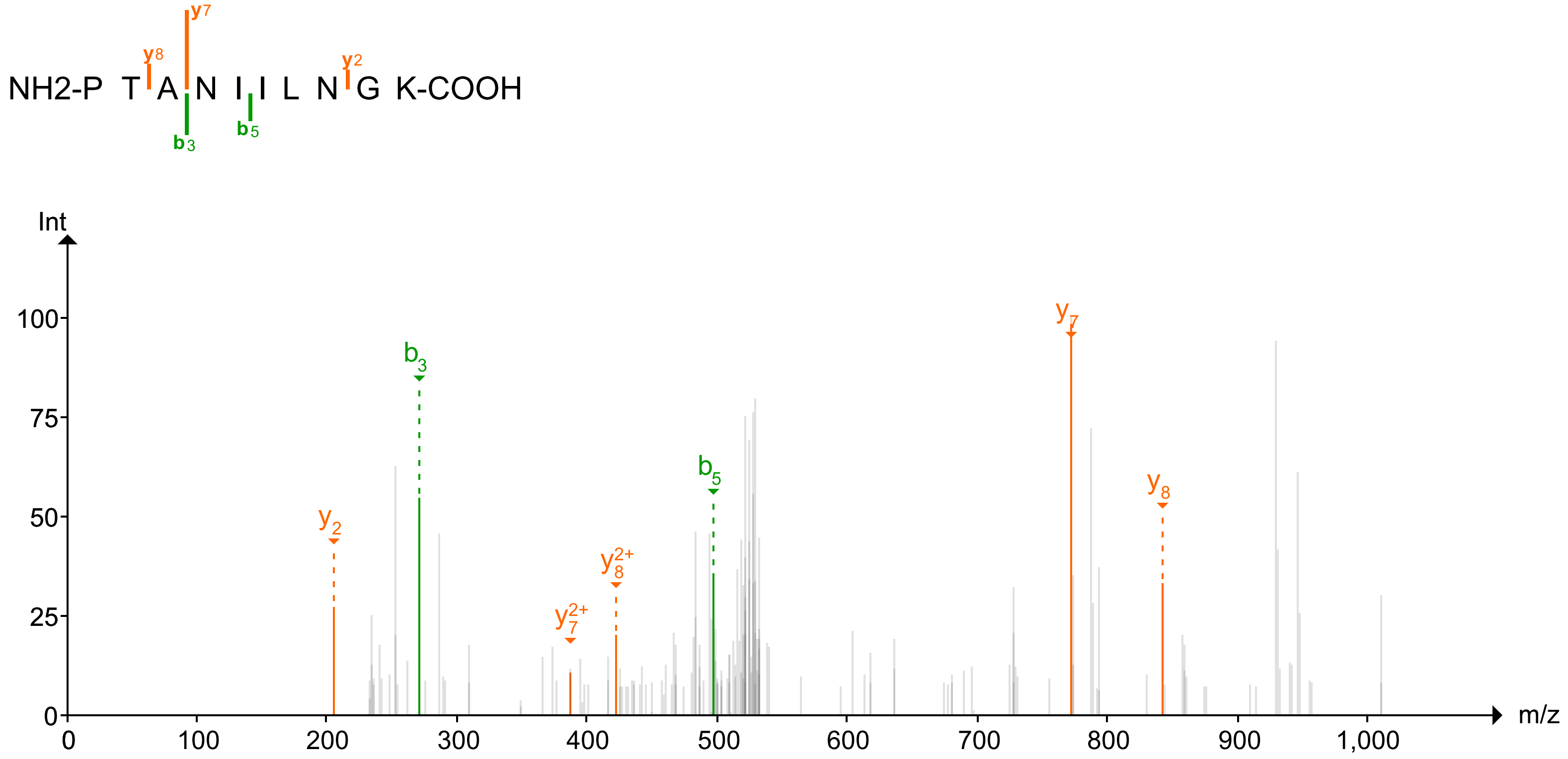

NH2-P T A N I I L N G K-COOH
y8
y7
y2
b3
b5
Int
100
75
50
25
0
0
100
200
300
400
500
600
700
800
900
1,000
m/z
y2
b3
y7 2+
y8 2+
b5
y7
y8

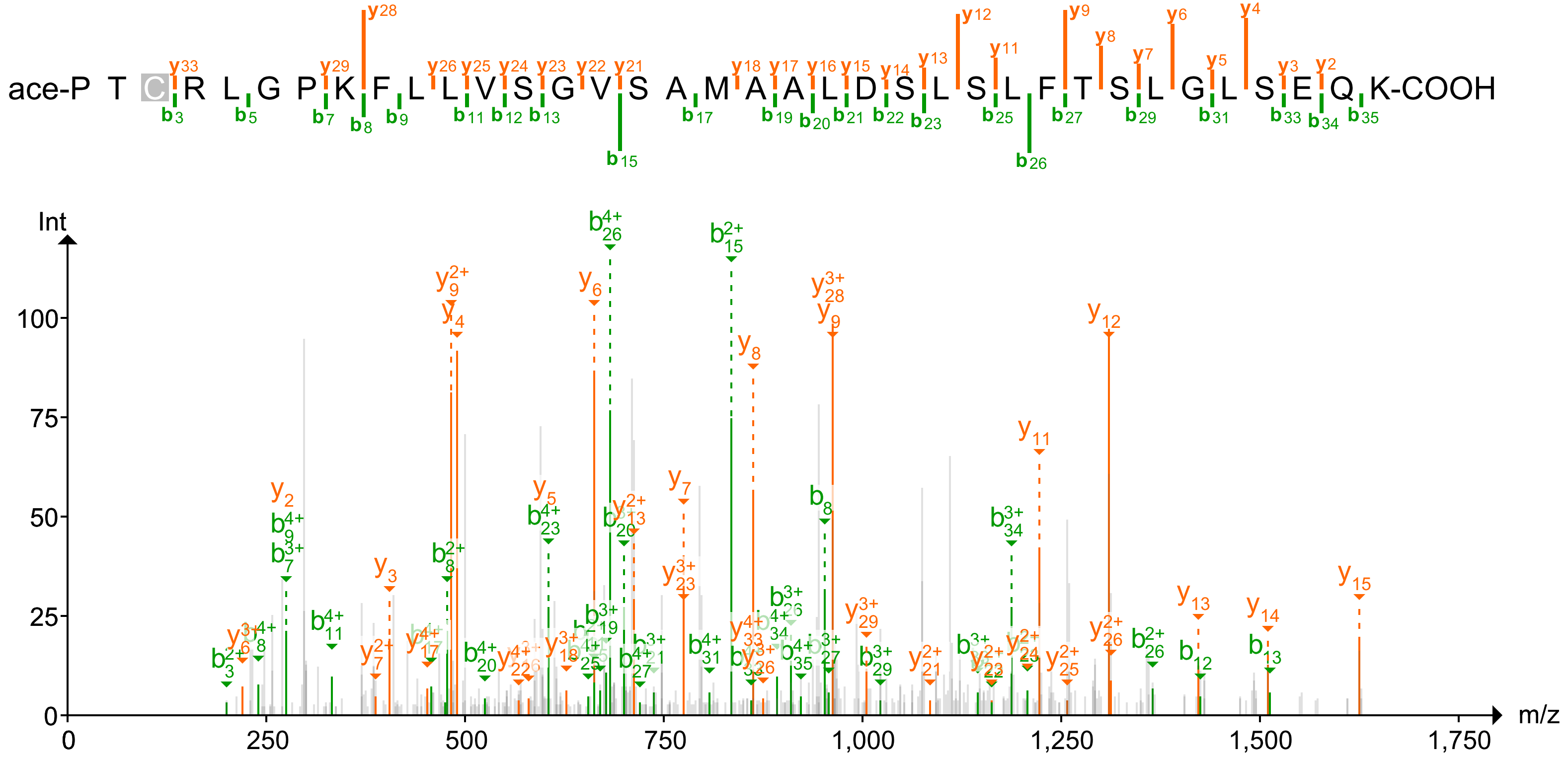

ace-P T C R L G P K F L L V S G V S A M A A L D S L S L F T S L G L S E Q K-COOH
y33 y29 y28 y26 y25 y24 y23 y22 y21 y18 y17 y16 y15 y14 y13 y12 y11 y9 y8 y7 y6 y5 y4 y3 y2
b3 b5 b7 b8 b9 b11 b12 b13 b15 b17 b19 b20 b21 b22 b23 b25 b26 b27 b29 b31 b33 b34 b35
Int
100
75
50
25
0
m/z
0
250
500
750
1,000
1,250
1,500
1,750

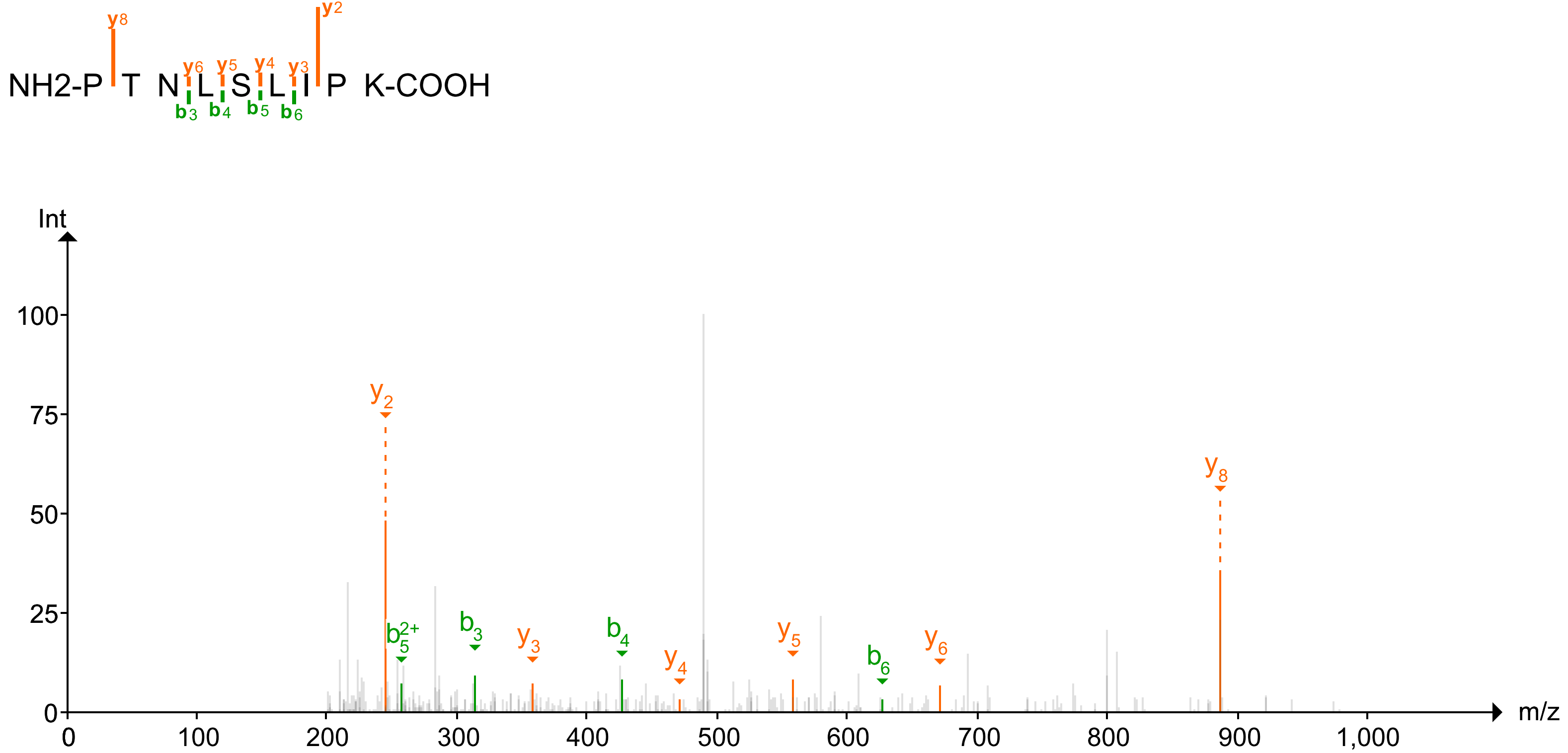

NH2-P T N L S L I P K-COOH
y8 y6 y5 y4 y3 y2
b3 b4 b5 b6
Int
100
75
50
25
0
y2
y8
b5 2+
b3
y3
b4
y4
y5
b6
y6
0
100
200
300
400
500
600
700
800
900
1,000
m/z

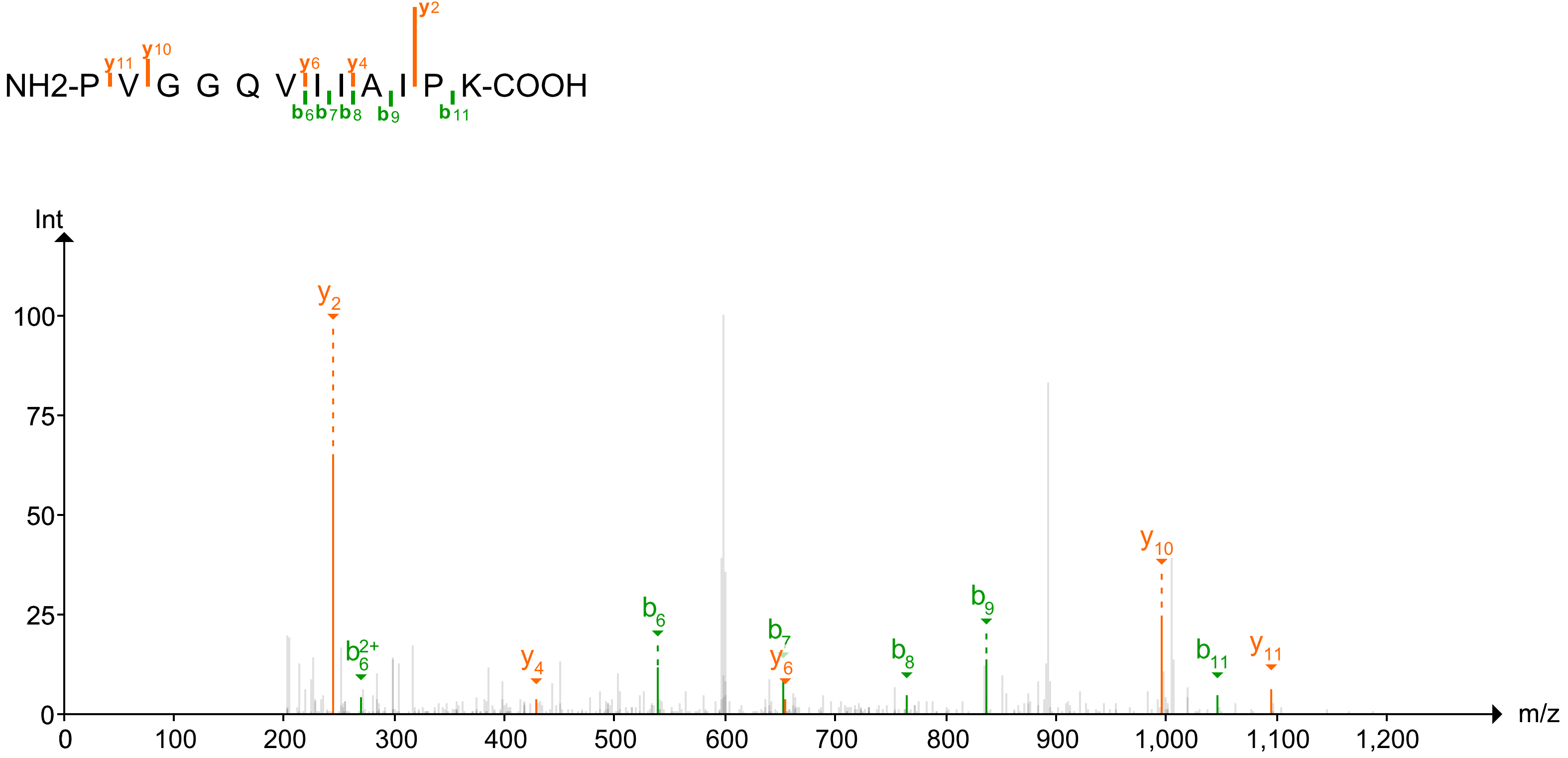

NH2-P V G G Q V I I A I P K-COOH
y11 y10 y6 y4 y2
b6 b7 b8 b9 b11
Int
100
75
50
25
0
y2
b6 2+
y4
b6
b7
y6
b8
b9
y10
b11
y11
0 100 200 300 400 500 600 700 800 900 1,000 1,100 1,200
m/z

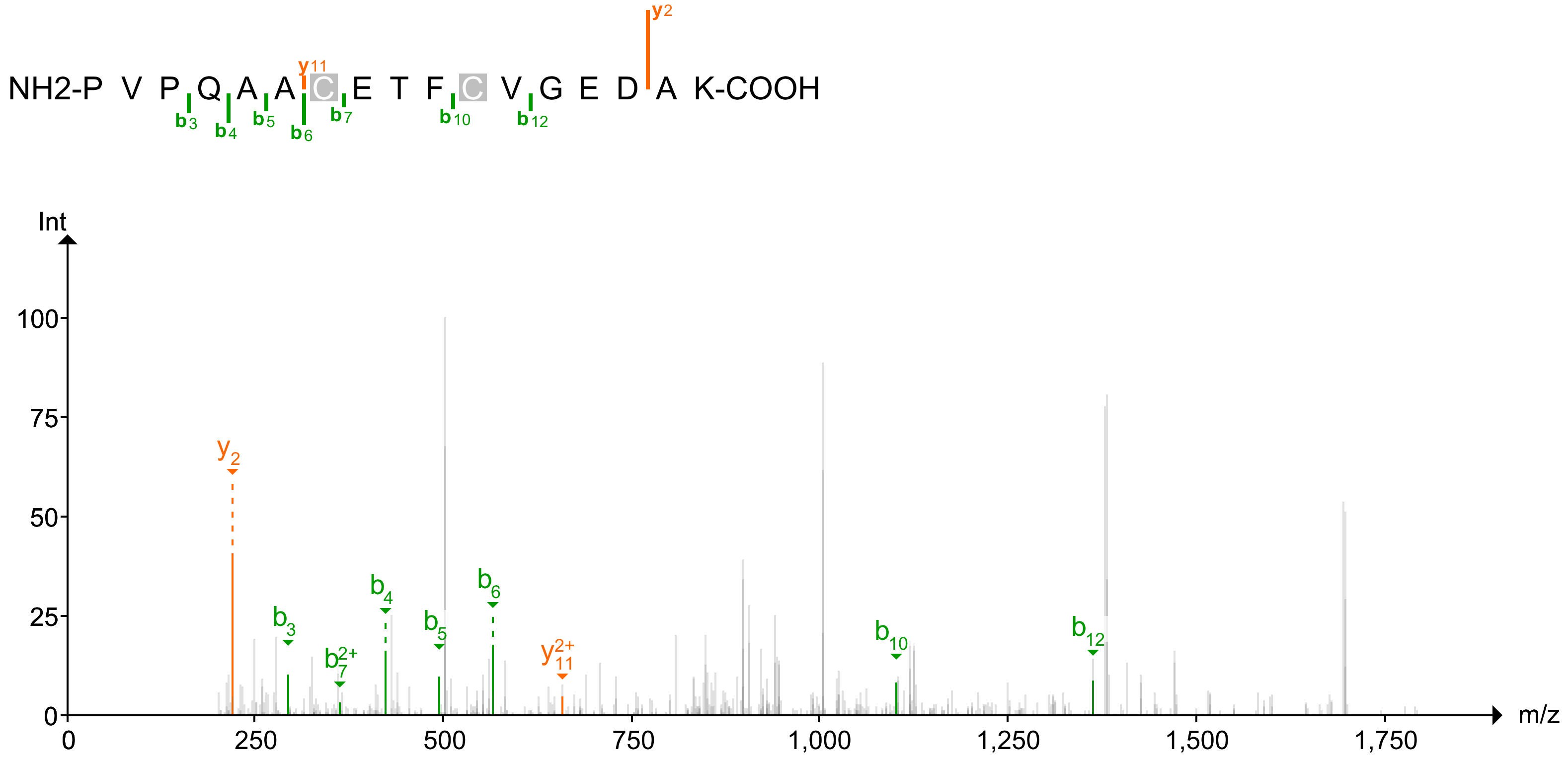

NH2-P V P Q A A C E T F C V G E D A K-COOH
y11
y2
b3
b4
b5
b6
b7
b10
b12
Int
100
75
50
25
0
y2
b3
b7 2+
b4
b5
b6
y11 2+
b10
b12
0
250
500
750
1,000
1,250
1,500
1,750
m/z

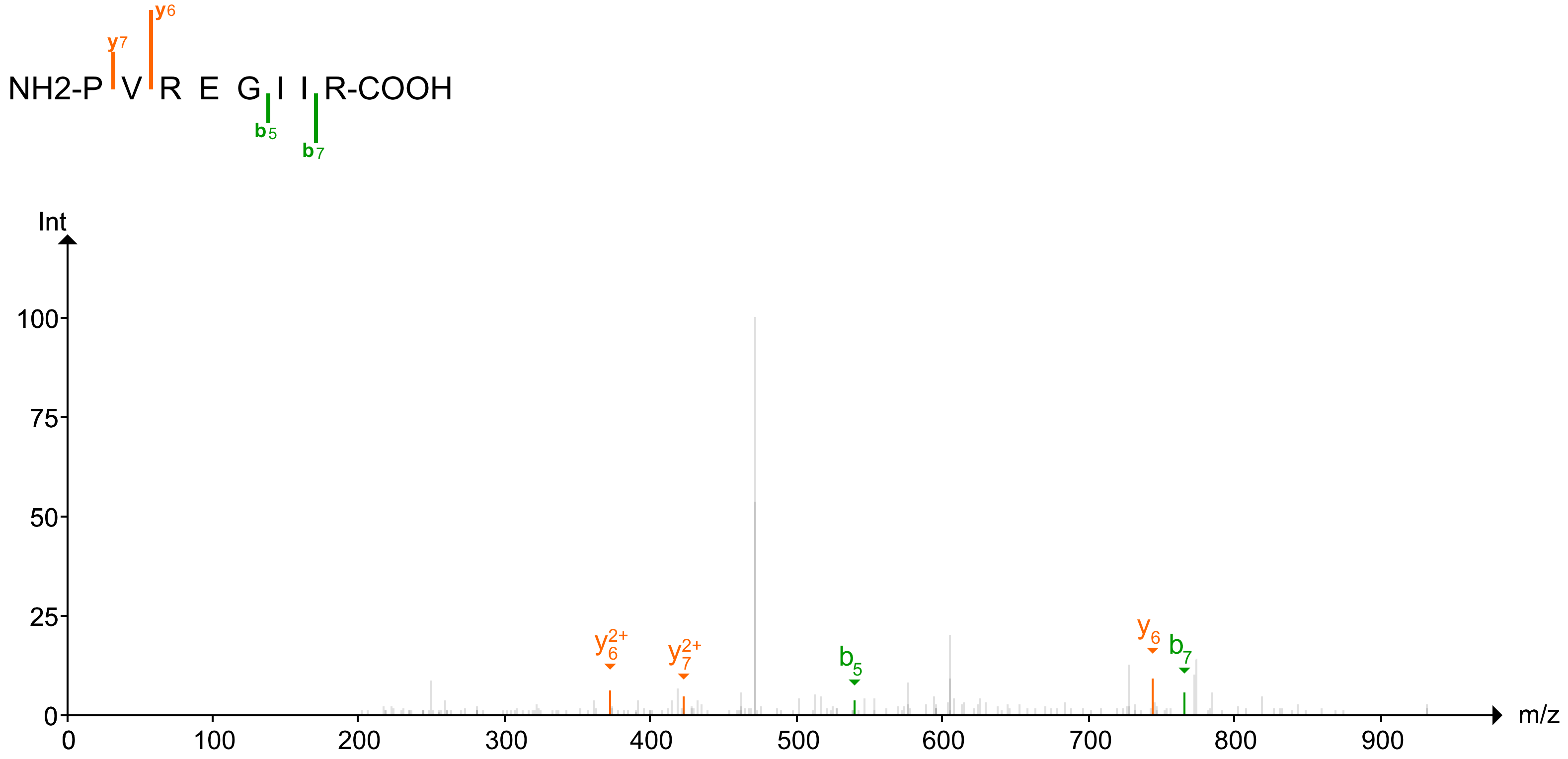

NH2-P V R E G I I R-COOH
y7
y6
b5
b7
Int
100
75
50
25
0
0
100
200
300
400
500
600
700
800
900
m/z
$y_6^{2+}$
$y_7^{2+}$
$b_5$
$y_6$
$b_7$

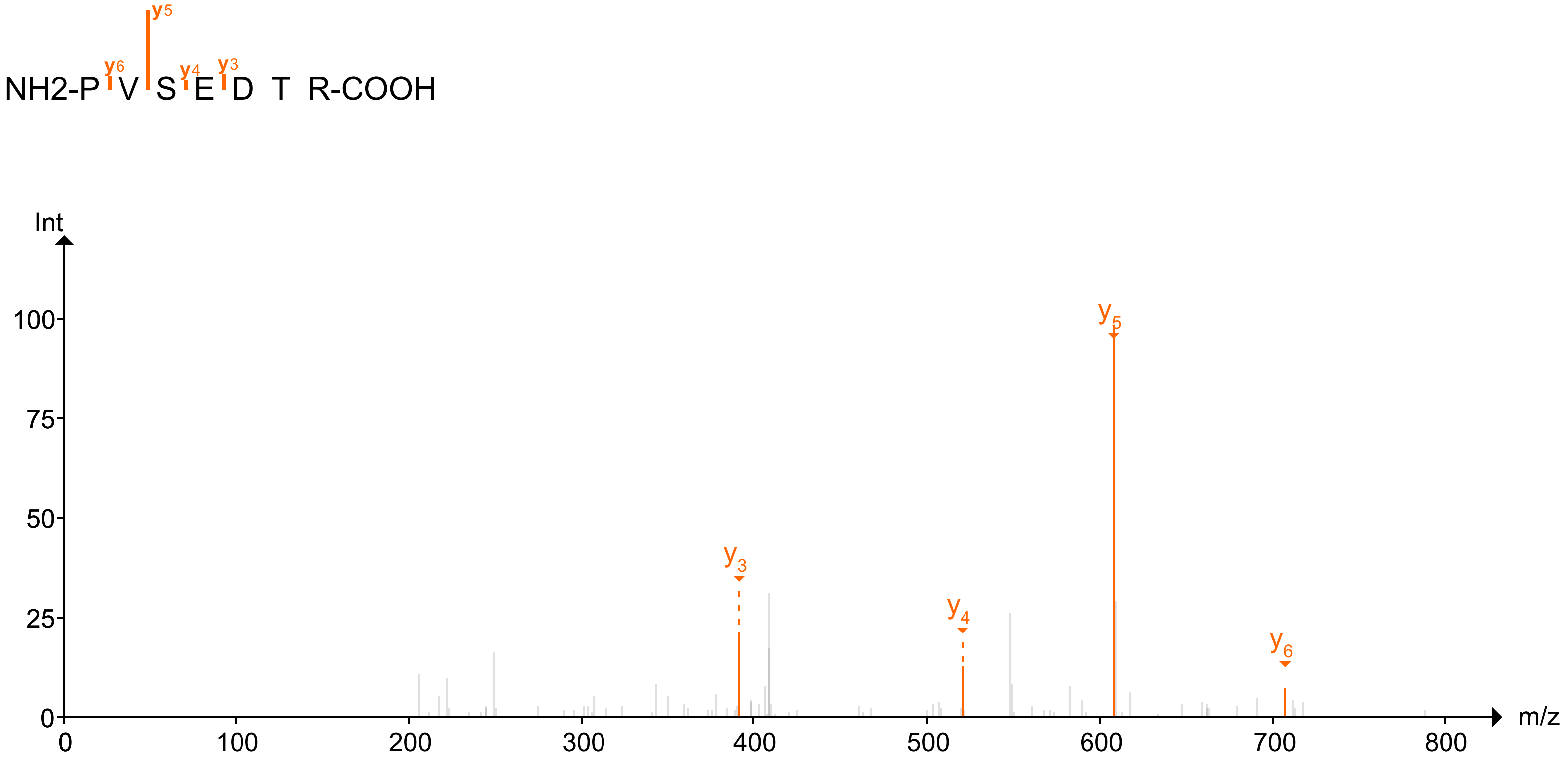

NH2-P V S E D T R-COOH
y5
y6
y4
y3
Int
100
75
50
25
0
m/z
0
100
200
300
400
500
600
700
800

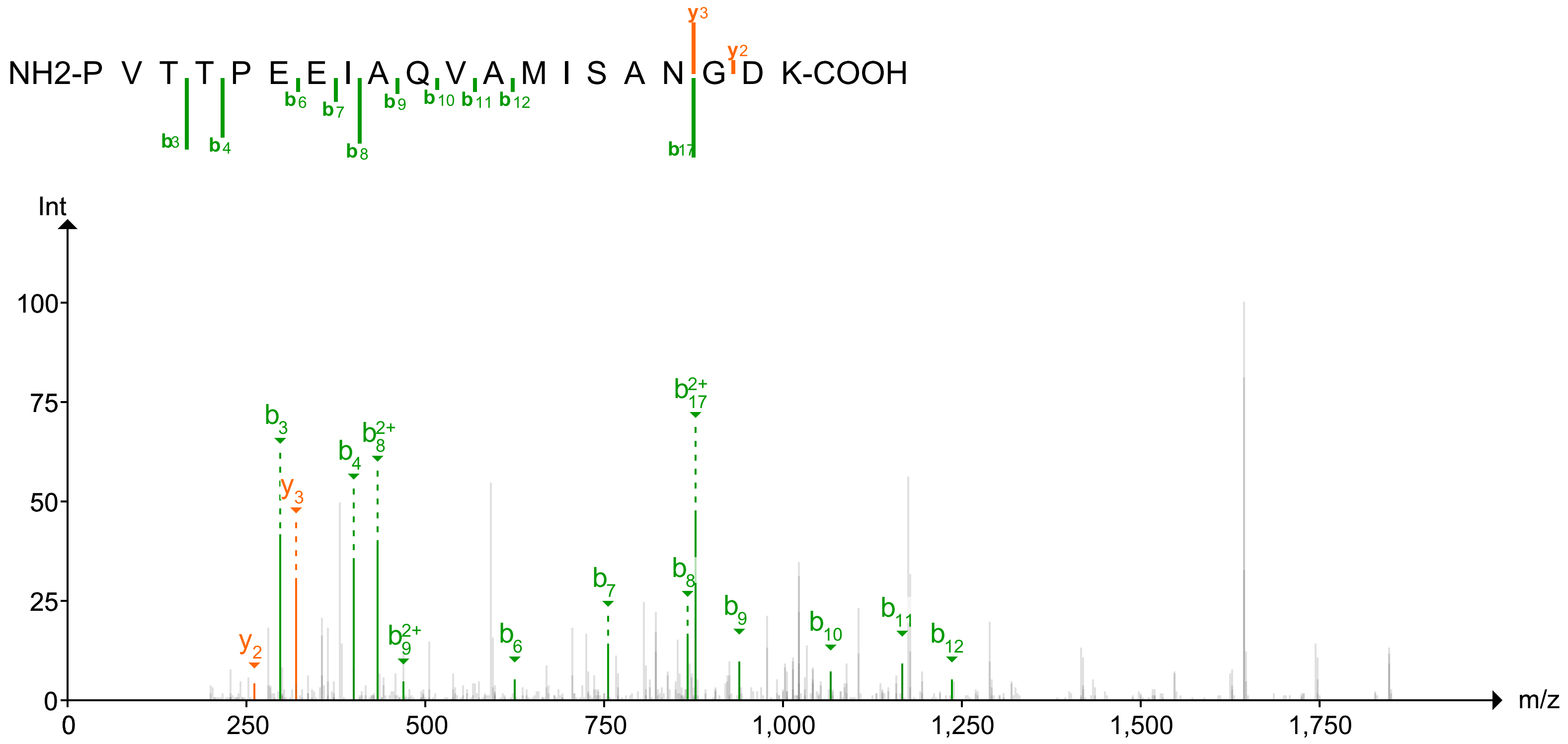

NH2-P V T T P E E I A Q V A M I S A N G D K-COOH
b3 b4 b6 b7 b8 b9 b10 b11 b12 b17
y3 y2
Int
100
75
50
25
0
0
250
500
750
1,000
1,250
1,500
1,750
m/z
b3
b4
b8 2+
b17 2+
y3
y2
b9 2+
b6
b7
b8
b9
b10
b11
b12

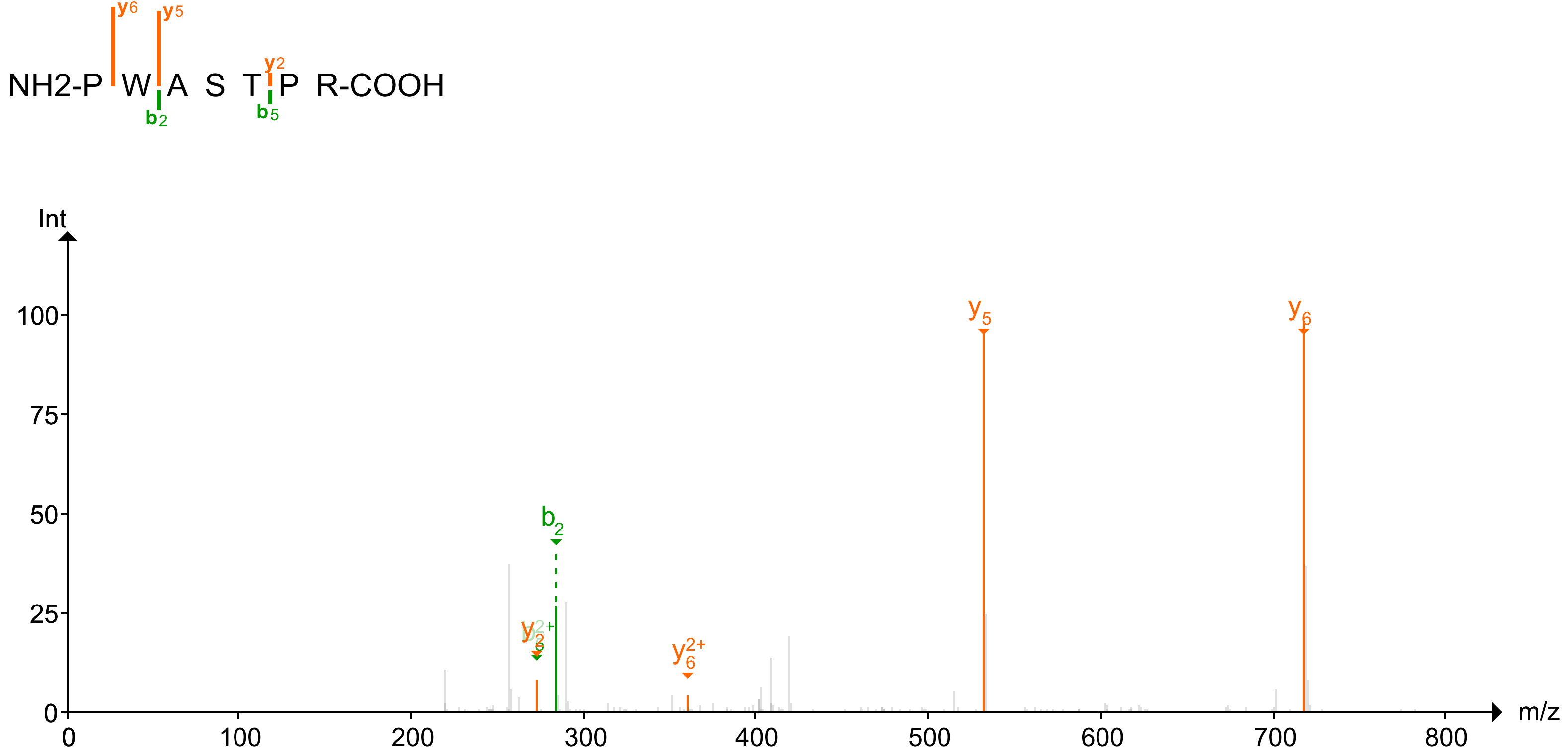

y6
y5
y2
NH2-P W A S T P R-COOH
b2
b5
Int
100
75
50
25
0
y5
y6
b2
y6 2+
0
100
200
300
400
500
600
700
800
m/z

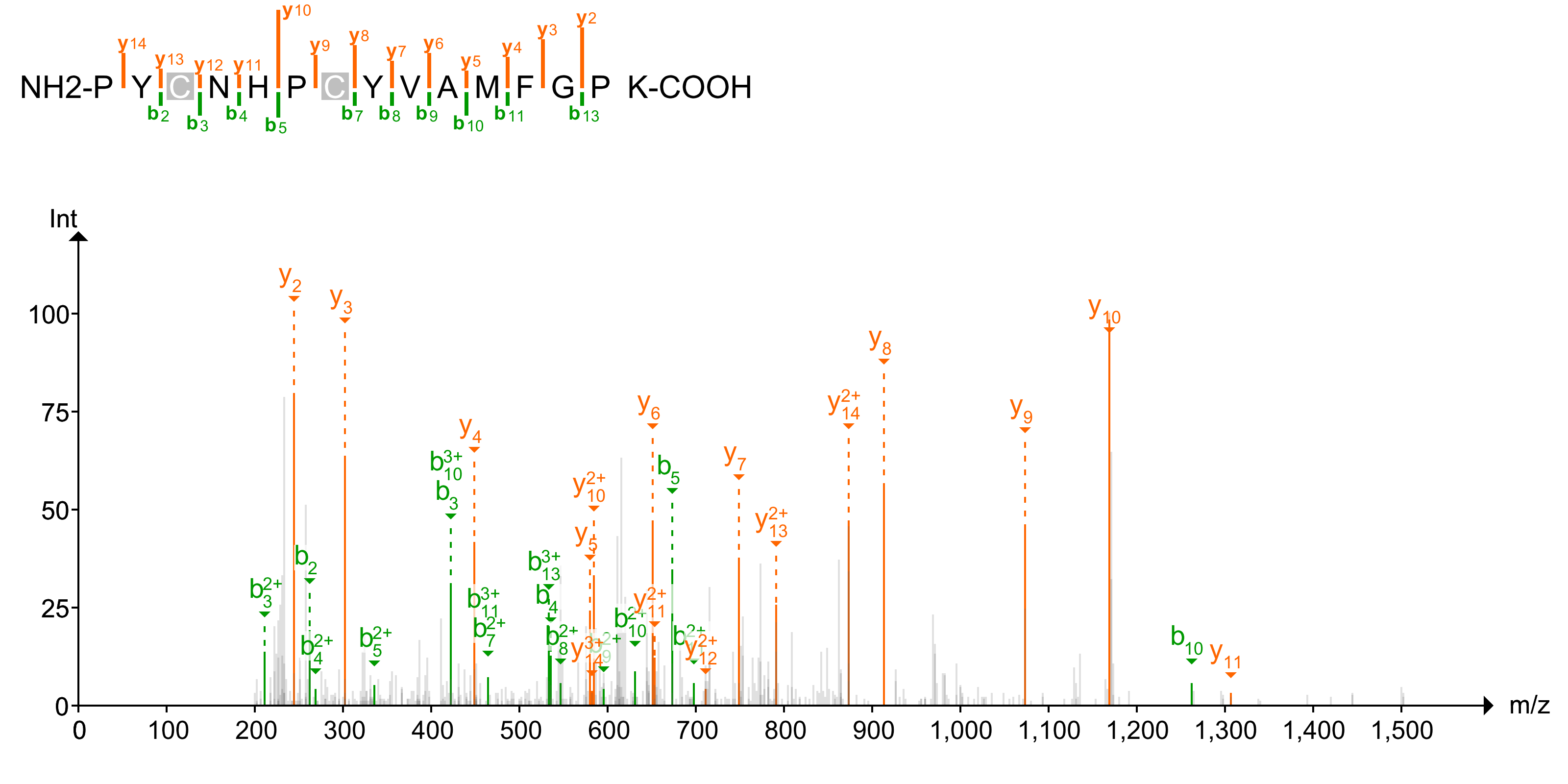

NH2-P Y C N H P C Y V A M F G P K-COOH
y14 y13 y12 y11 y10 y9 y8 y7 y6 y5 y4 y3 y2
b2 b3 b4 b5 b7 b8 b9 b10 b11 b13
Int
m/z
0
25
50
75
100
0 100 200 300 400 500 600 700 800 900 1,000 1,100 1,200 1,300 1,400 1,500
y2
y3
y4
y5
y6
y7
y8
y9
y10
y11
b2
b3
b4
b5
b10

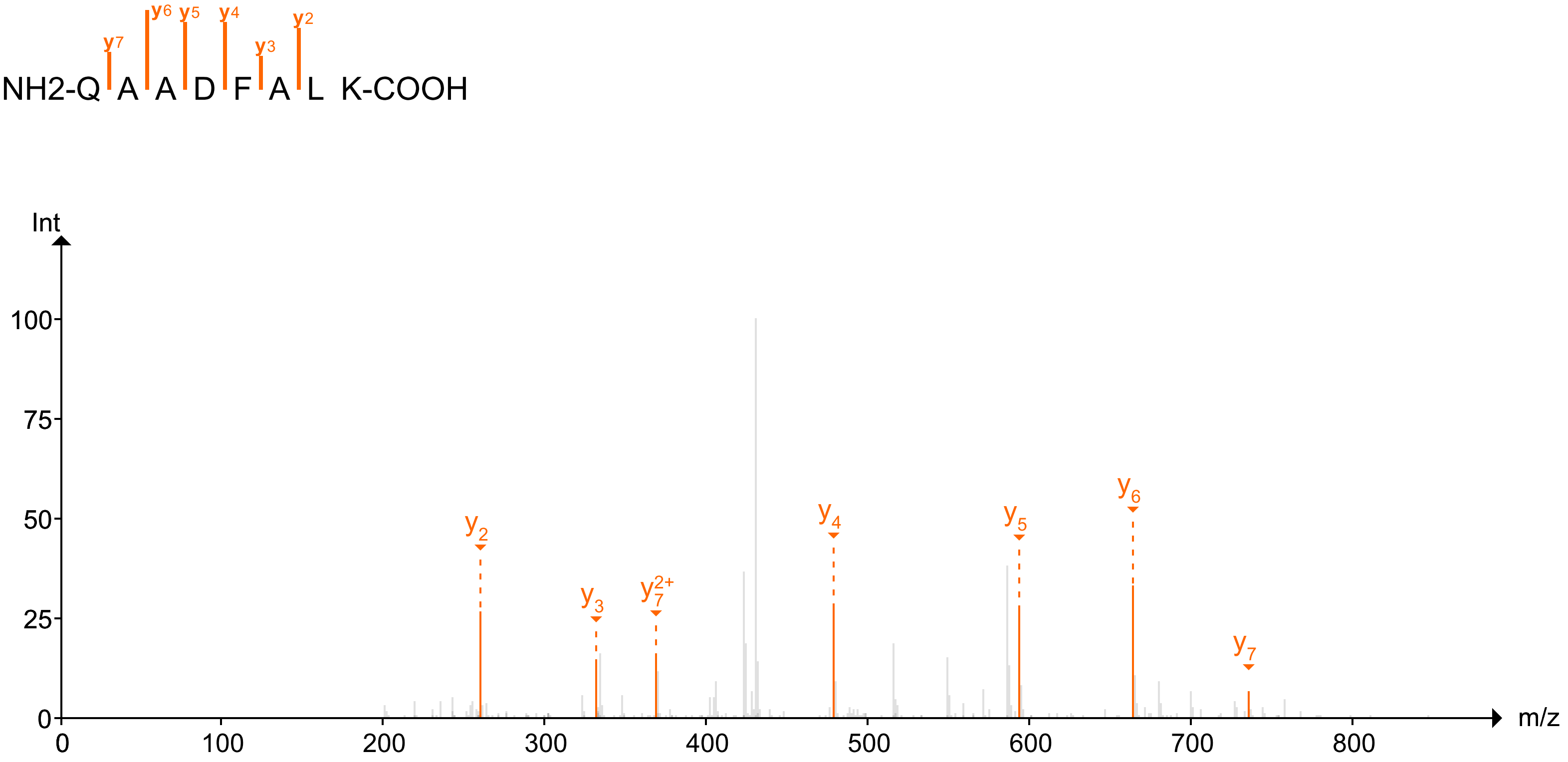

y7
y6
y5
y4
y3
y2
NH2-Q A A D F A L K-COOH
Int
100
75
50
25
0
y2
y3
y7 2+
y4
y5
y6
y7
m/z
0
100
200
300
400
500
600
700
800

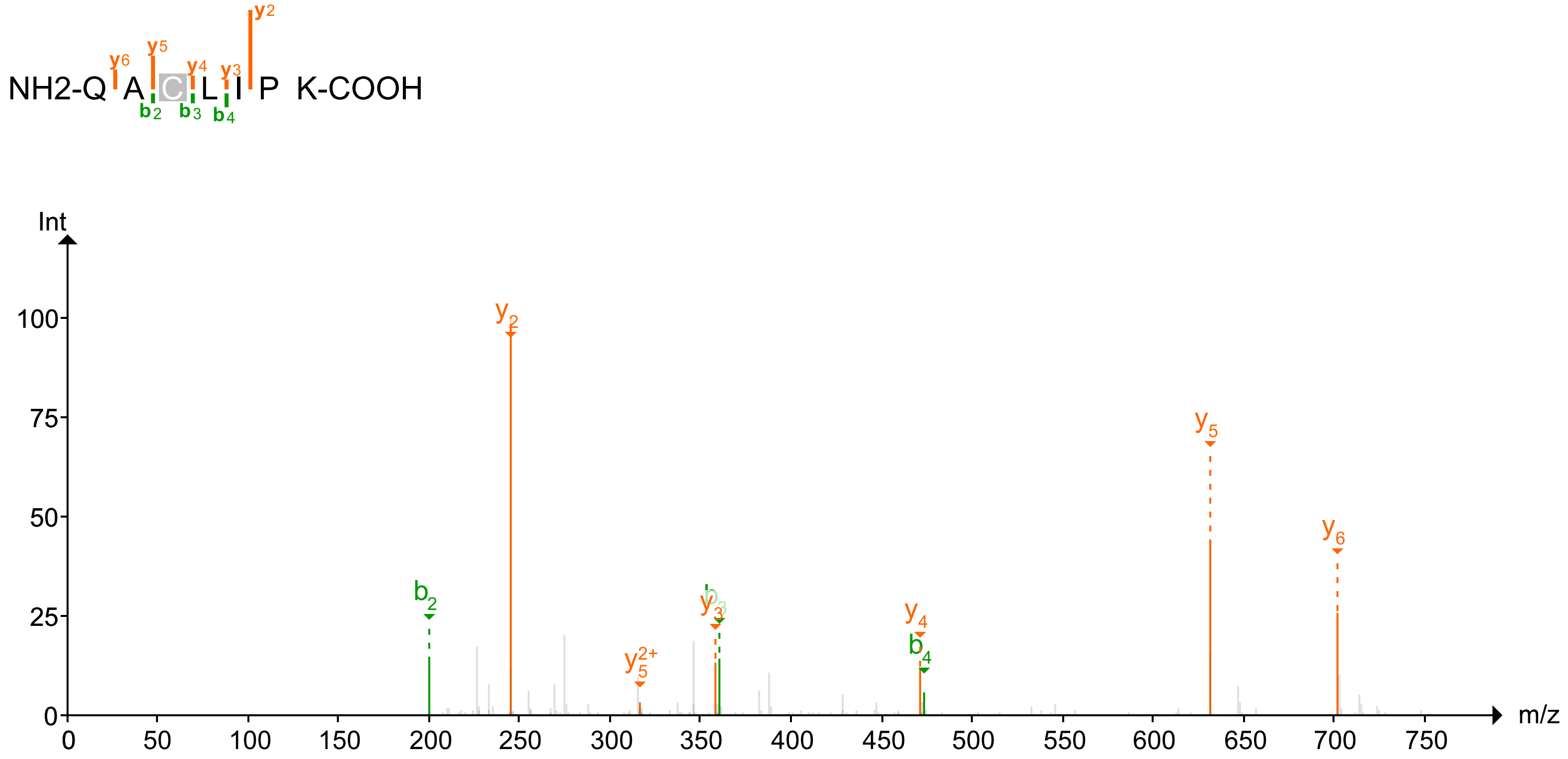

NH2-Q A C L I P K-COOH
y6 y5 y4 y3 y2
b2 b3 b4
Int
m/z
100
75
50
25
0
0 50 100 150 200 250 300 350 400 450 500 550 600 650 700 750
$y_2$
$y_5$
$y_6$
$b_2$
$y_5^{2+}$
$y_3$
$b_3$
$y_4$
$b_4$

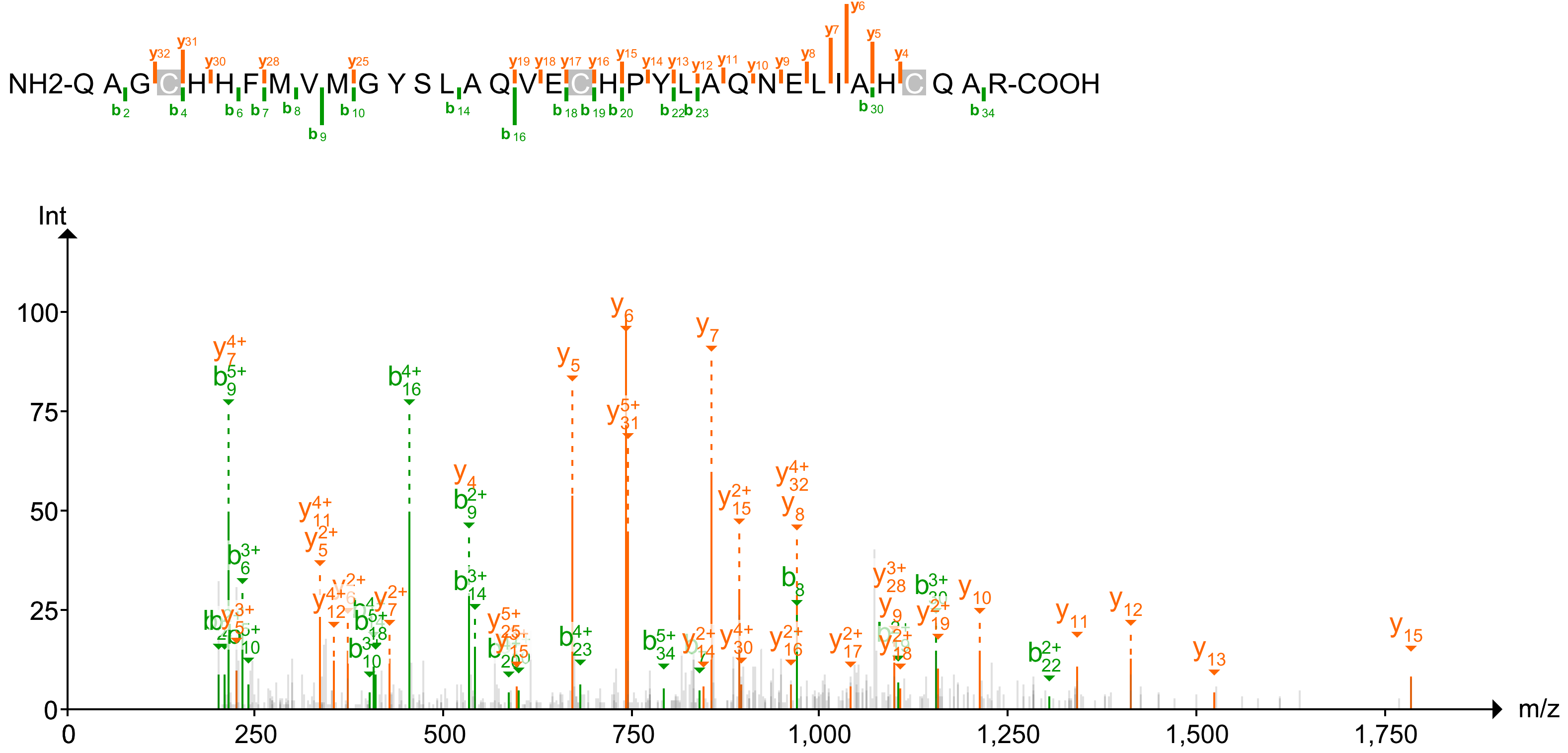
NH2-Q A G C H H F M V M G Y S L A Q V E C H P Y L A Q N E L I A H C Q A R-COOH
Int
100
75
50
25
0
250
500
750
1,000
1,250
1,500
1,750
m/z

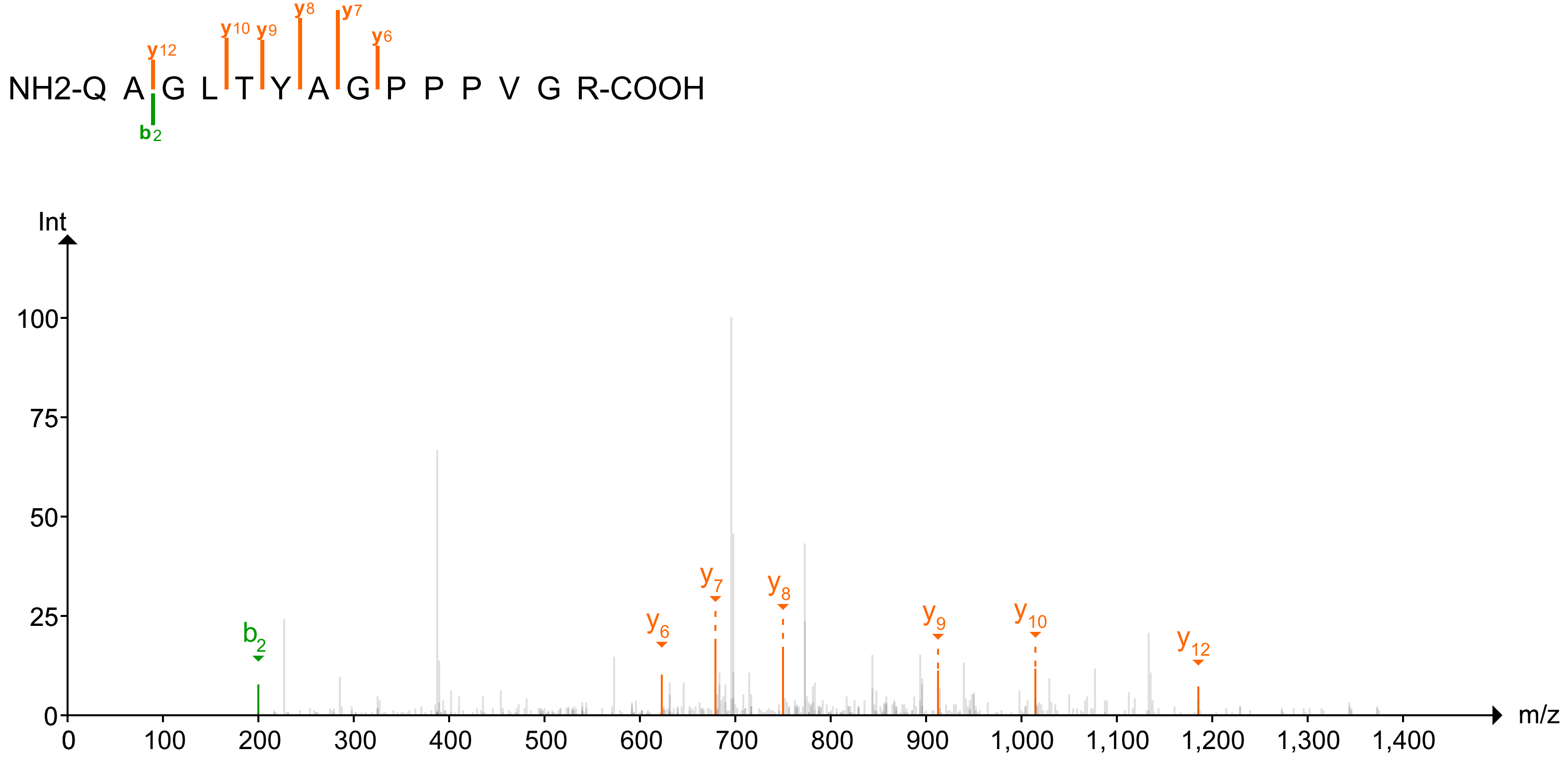

NH2-Q A G L T Y A G P P P V G R-COOH
y12 y10 y9 y8 y7 y6
b2
Int
100
75
50
25
0
b2
y6
y7
y8
y9
y10
y12
0 100 200 300 400 500 600 700 800 900 1,000 1,100 1,200 1,300 1,400
m/z

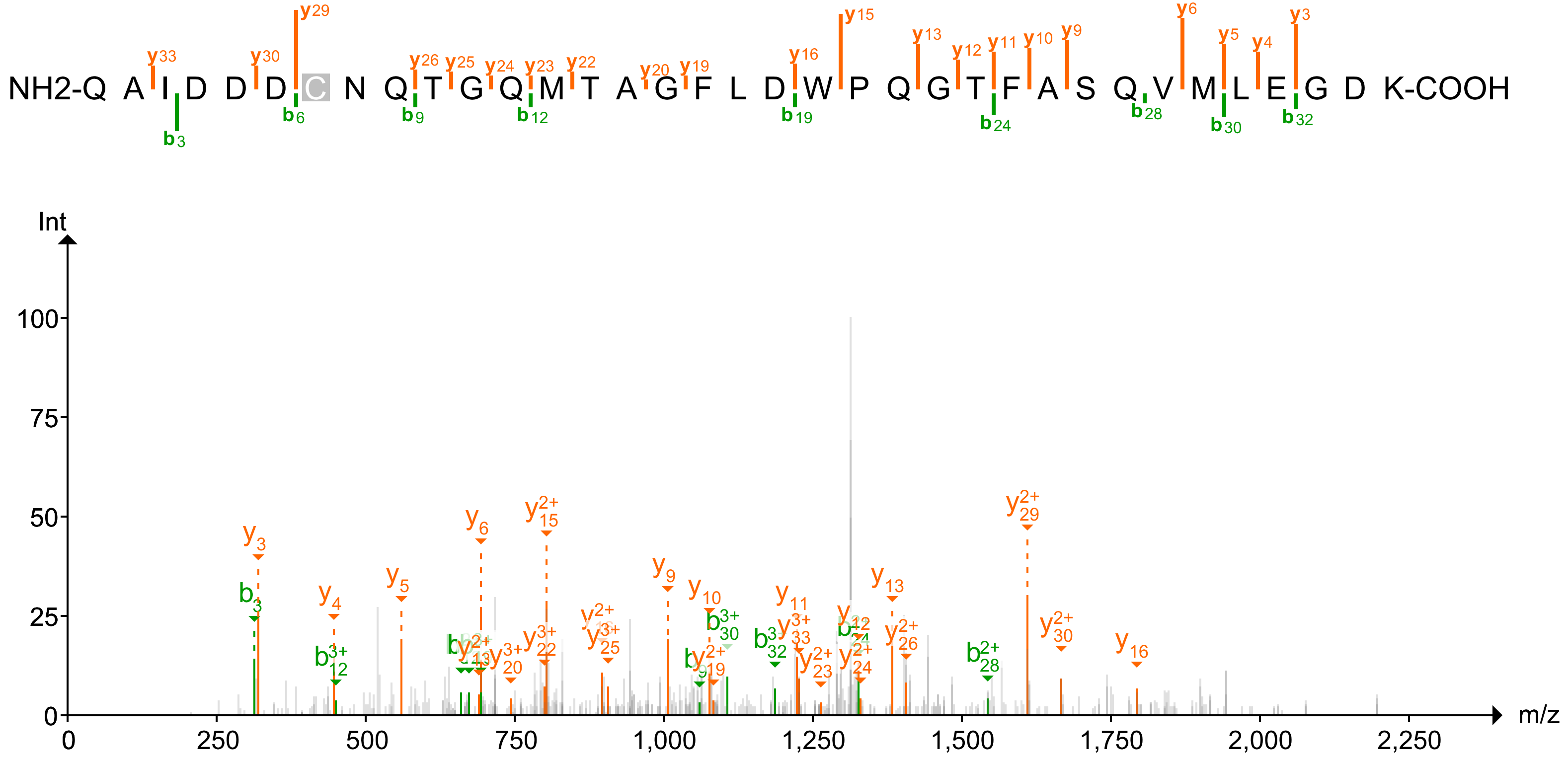
NH2-Q A I D D D C N Q T G Q M T A G F L D W P Q G T F A S Q V M L E G D K-COOH
Int
m/z
0
25
50
75
100
250
500
750
1,000
1,250
1,500
1,750
2,000
2,250

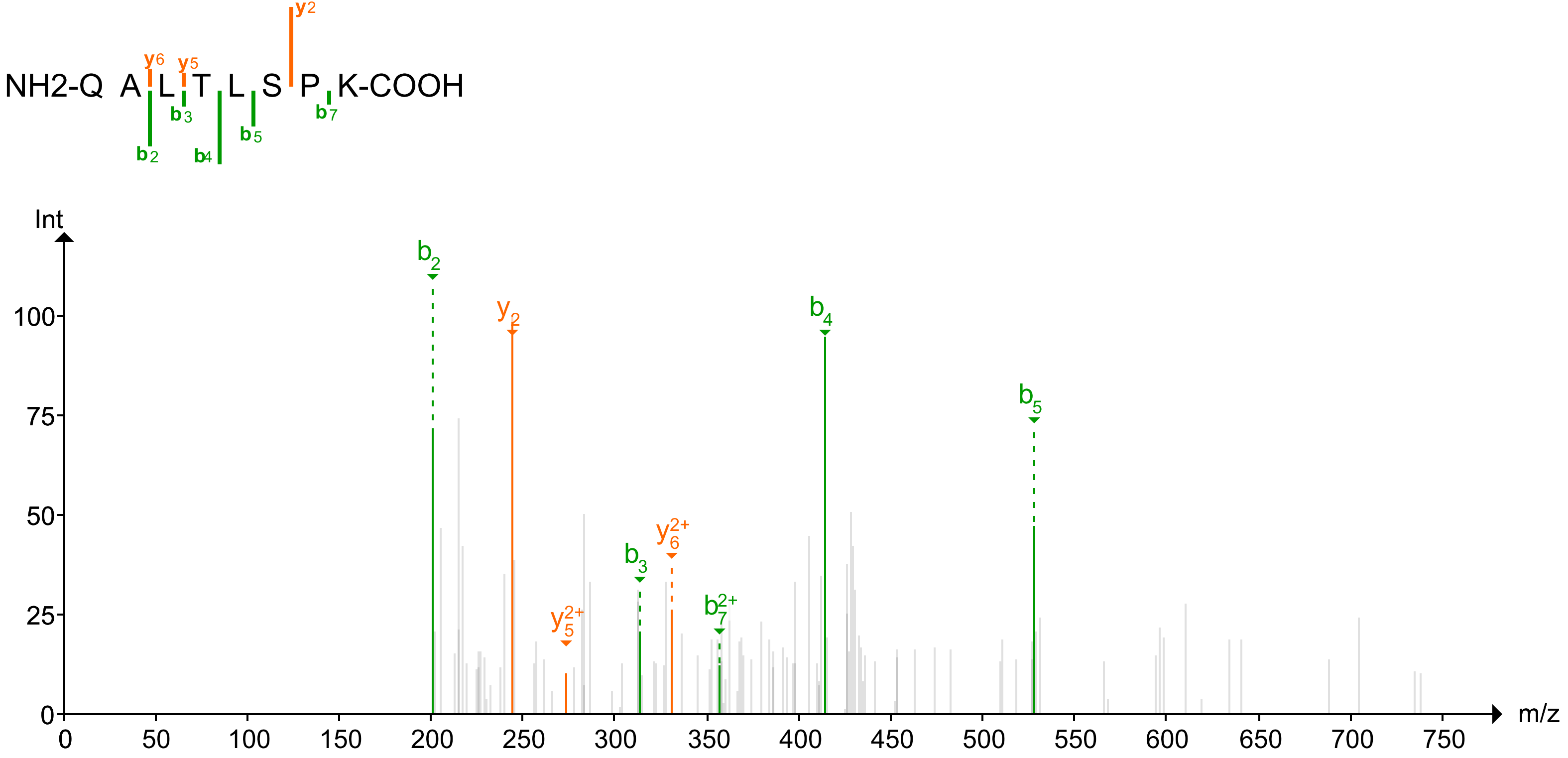

NH2-Q A L T L S P K-COOH
y2
y6
y5
b2
b3
b4
b5
b7
Int
100
75
50
25
0
b2
y2
b4
b5
b3
y6 2+
y5 2+
b7 2+
0
50
100
150
200
250
300
350
400
450
500
550
600
650
700
750
m/z

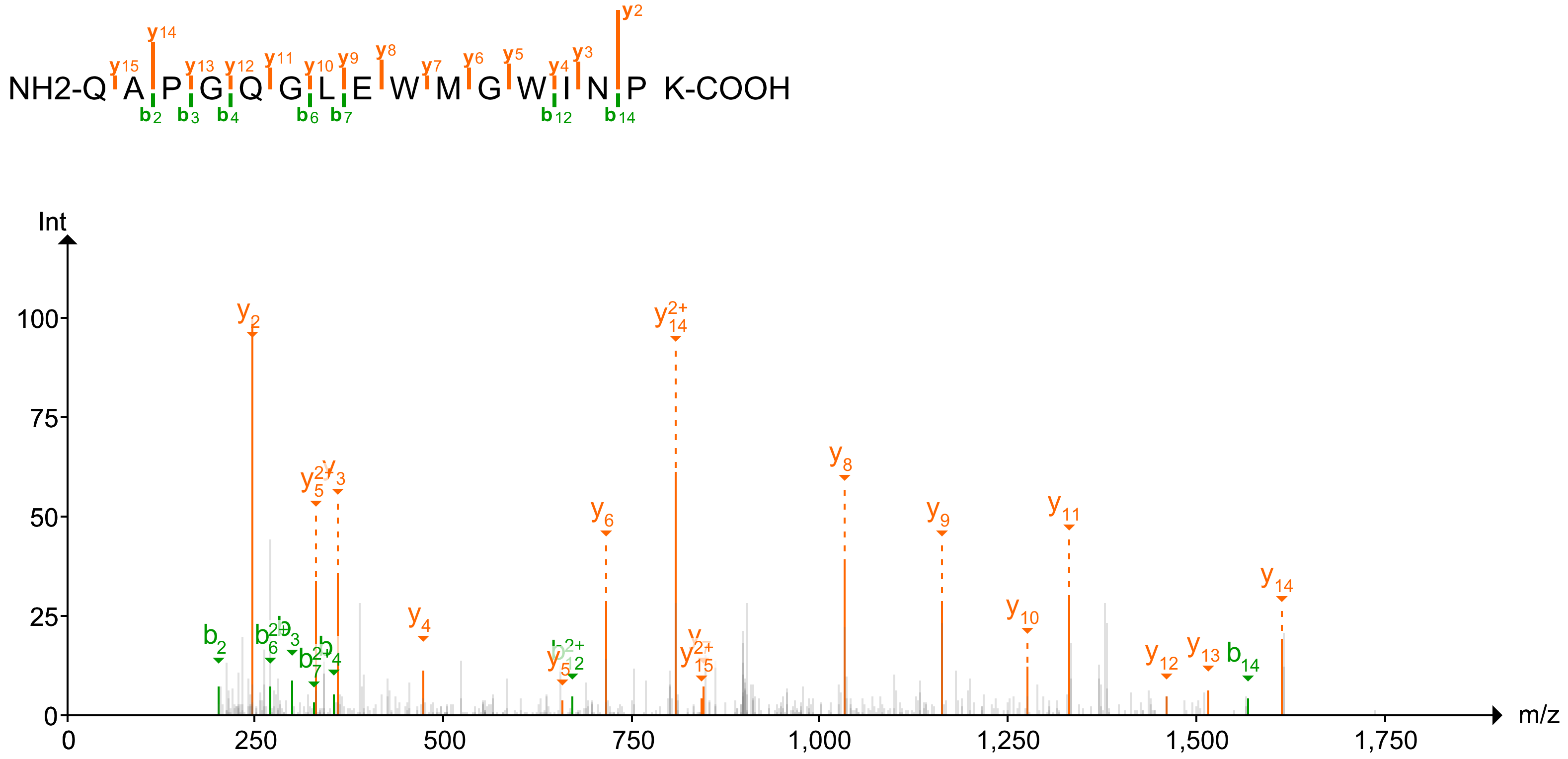

NH2-Q A P G Q G L E W M G W I N P K-COOH
y15 y14 y13 y12 y11 y10 y9 y8 y7 y6 y5 y4 y3 y2
b2 b3 b4 b6 b7 b12 b14
Int
100
75
50
25
0
m/z
0
250
500
750
1,000
1,250
1,500
1,750

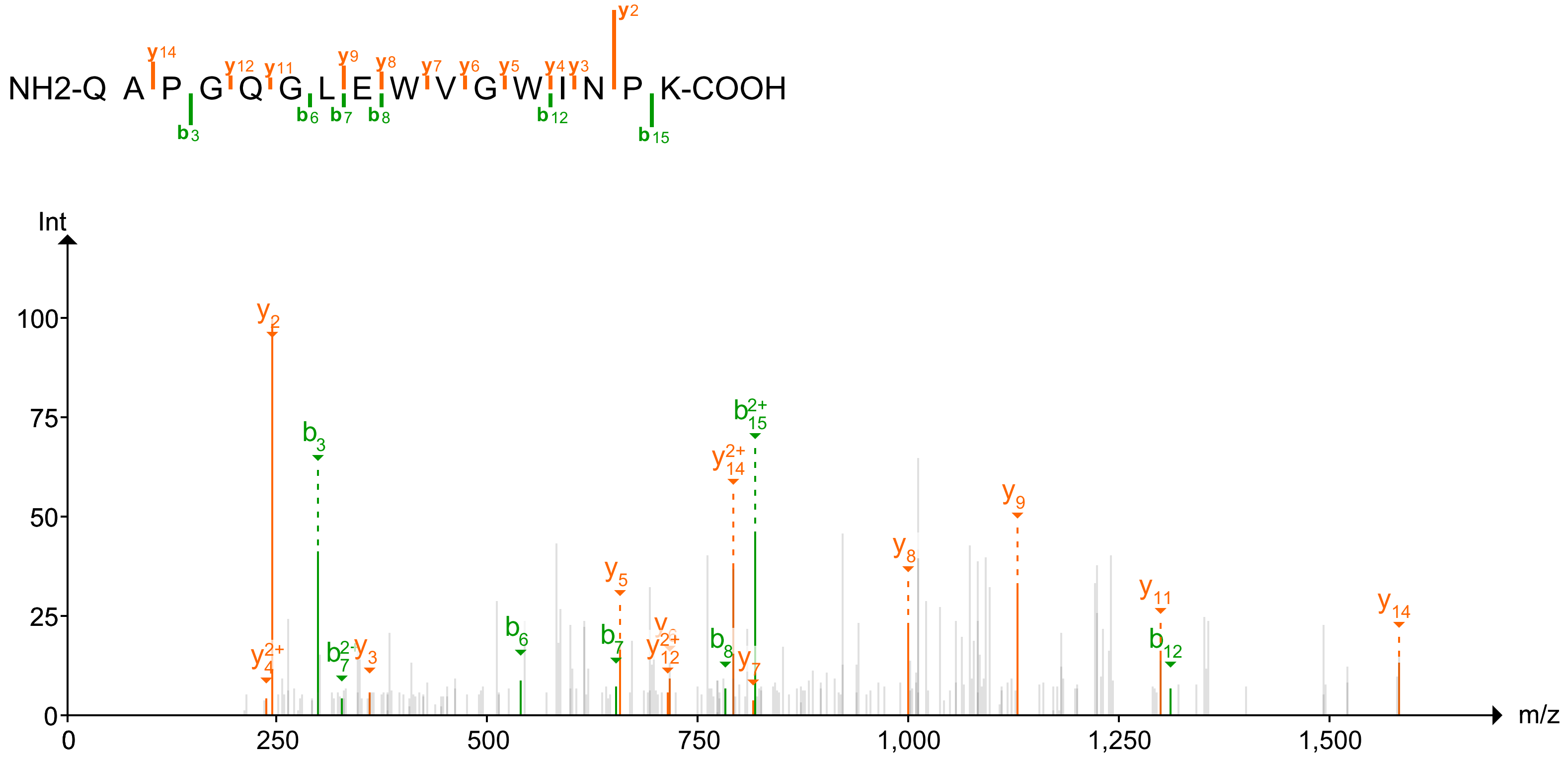

NH2-Q A P G Q G L E W V G W I N P K-COOH
y14 y12 y11 y9 y8 y7 y6 y5 y4 y3 y2
b3 b6 b7 b8 b12 b15
Int
100
75
50
25
0
m/z
0
250
500
750
1,000
1,250
1,500

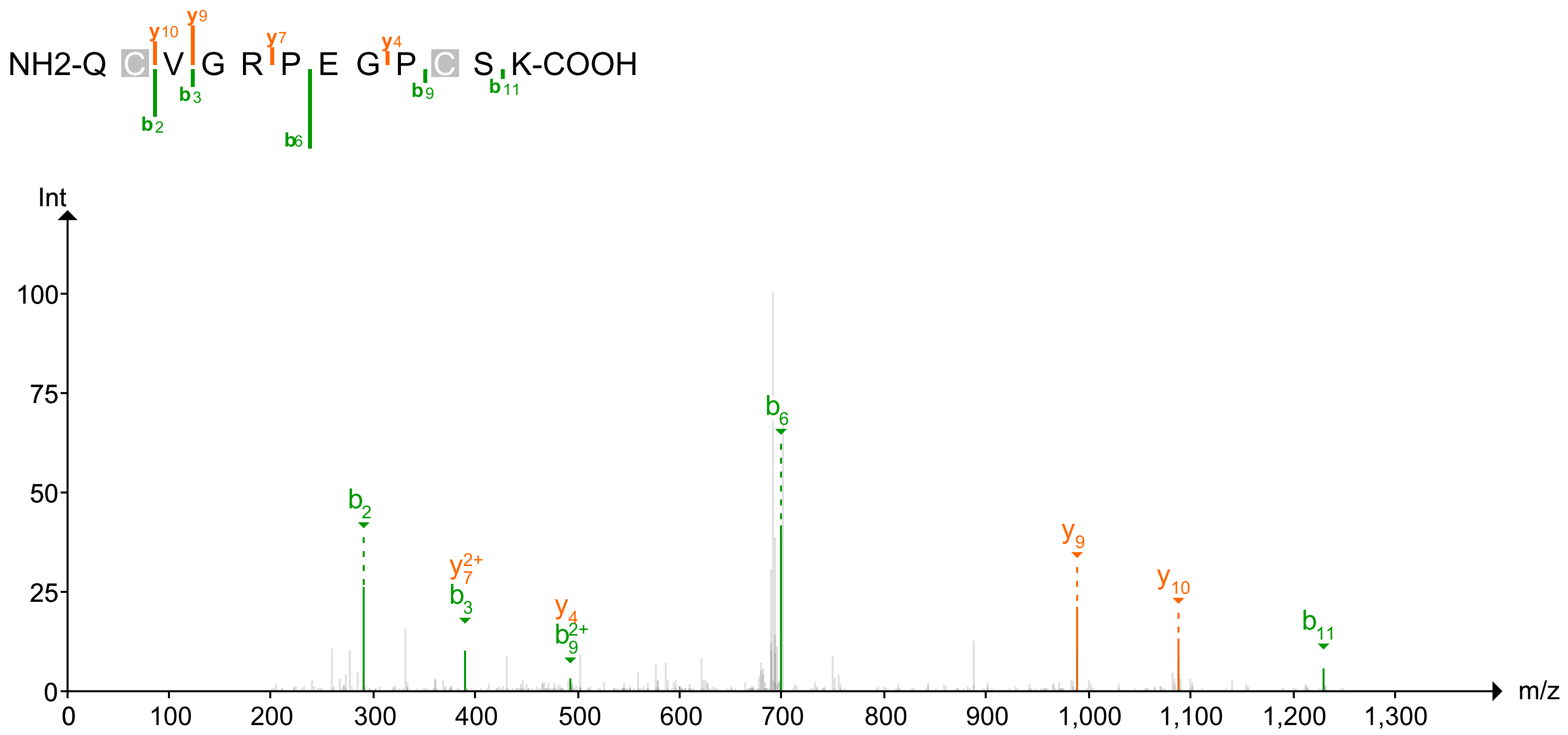

NH2-Q C V G R P E G P C S K-COOH
y10 y9 y7 y4
b2 b3 b6 b9 b11
Int
100
75
50
25
0
0 100 200 300 400 500 600 700 800 900 1,000 1,100 1,200 1,300
m/z
b2
y7 2+
b3
y4
b9 2+
b6
y9
y10
b11

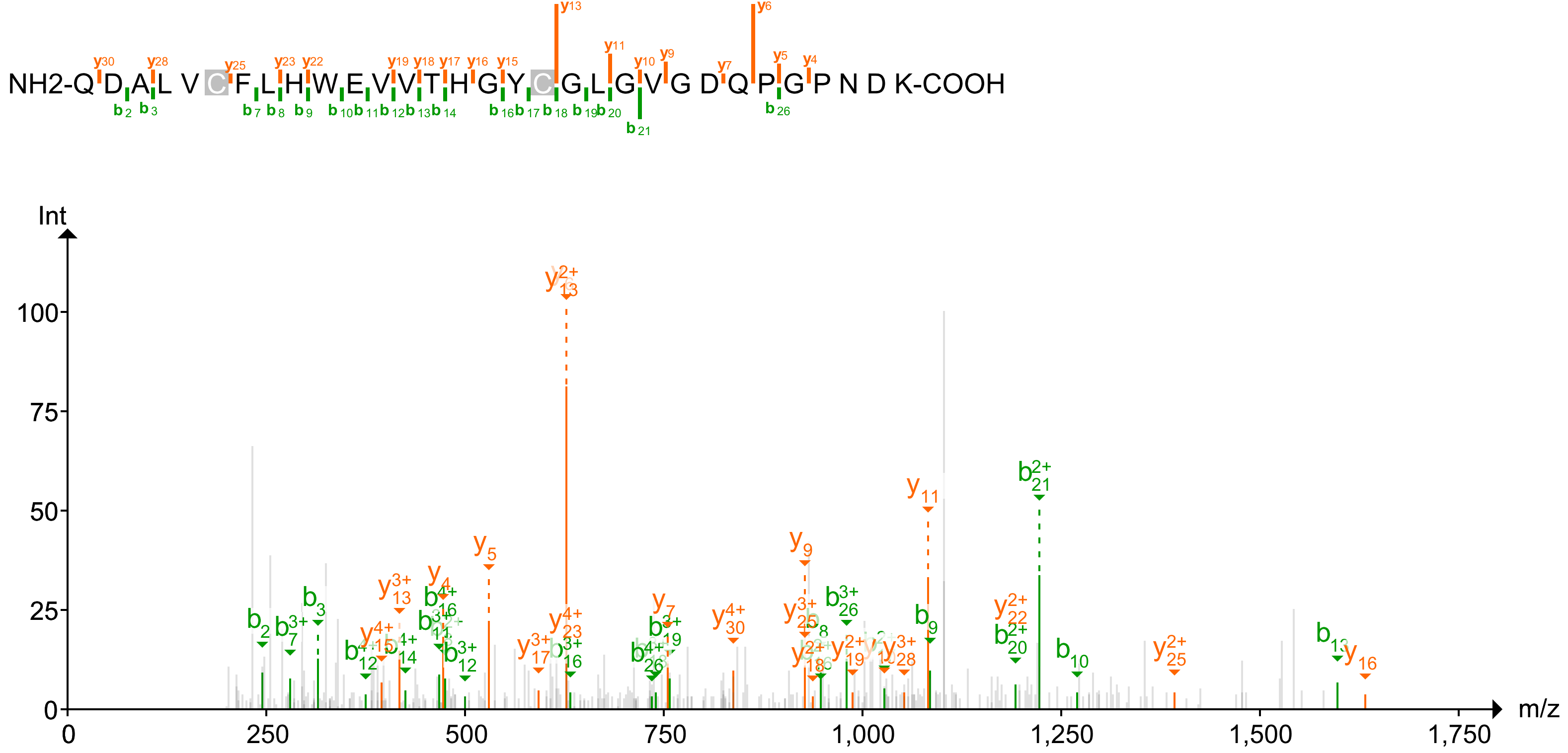

NH2-Q D A L V C F L H W E V V T H G Y C G L G V G D Q P G P N D K-COOH
Int
100
75
50
25
0
m/z
0
250
500
750
1,000
1,250
1,500
1,750

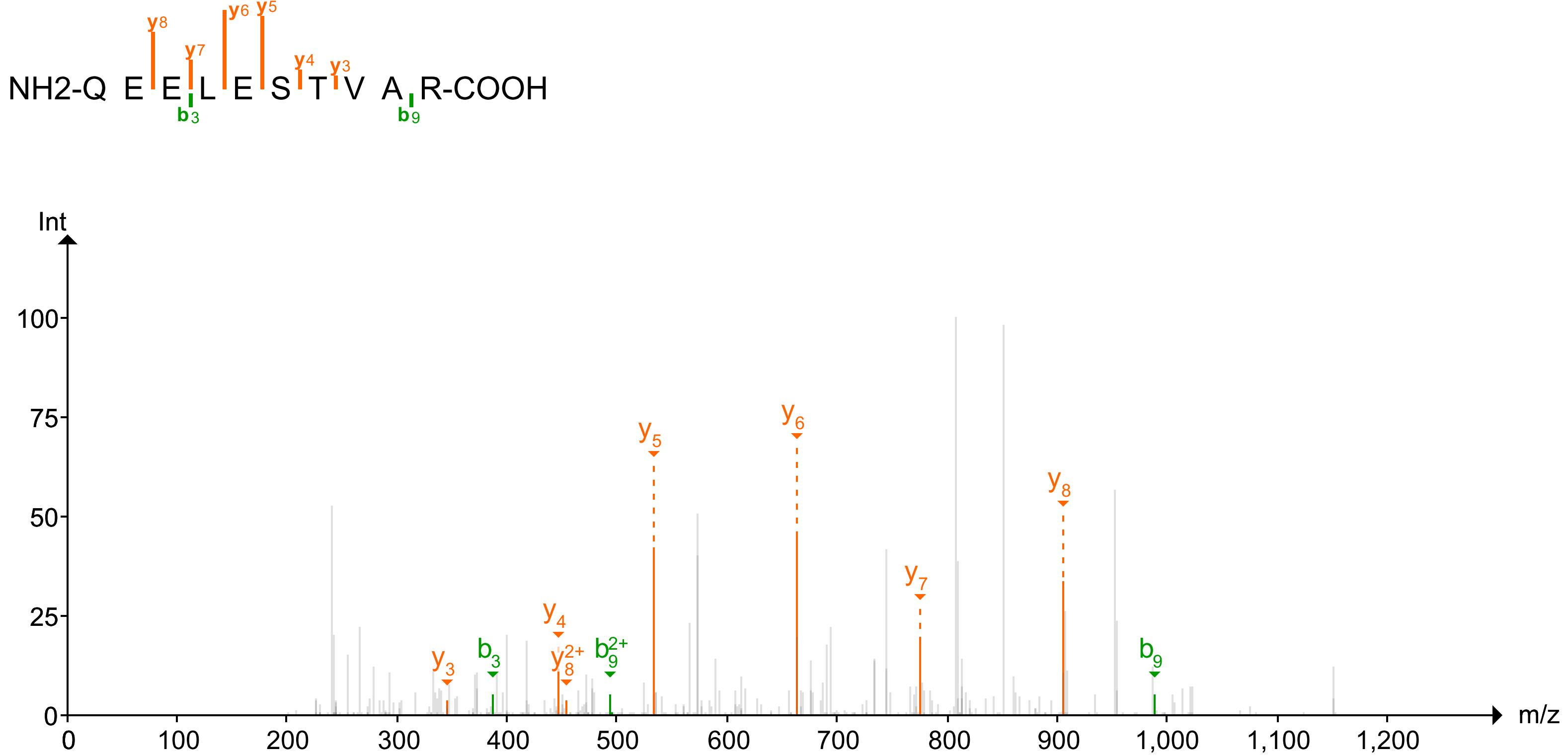

NH2-Q E E L E S T V A R-COOH
y8 y7 y6 y5 y4 y3
b3 b9
Int
100
75
50
25
0
m/z
0
100
200
300
400
500
600
700
800
900
1,000
1,100
1,200
y3
b3
y4
y8 2+
b9 2+
y5
y6
y7
y8
b9

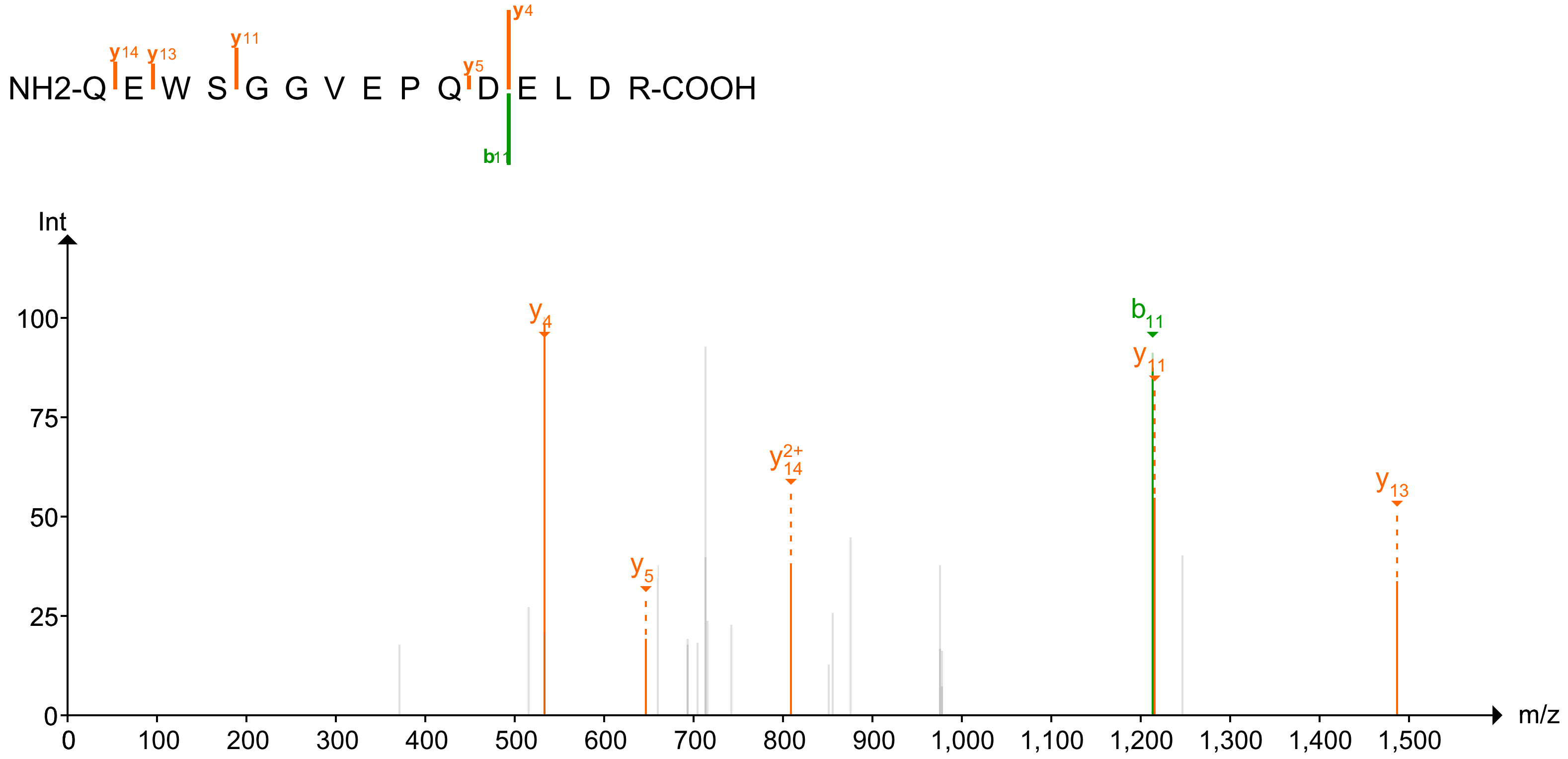

NH2-Q E W S G G V E P Q D E L D R-COOH
y14
y13
y11
y5
y4
b11
Int
100
75
50
25
0
m/z
0
100
200
300
400
500
600
700
800
900
1,000
1,100
1,200
1,300
1,400
1,500
$y_4$
$y_5$
$y_{14}^{2+}$
$b_{11}$
$y_{11}$
$y_{13}$

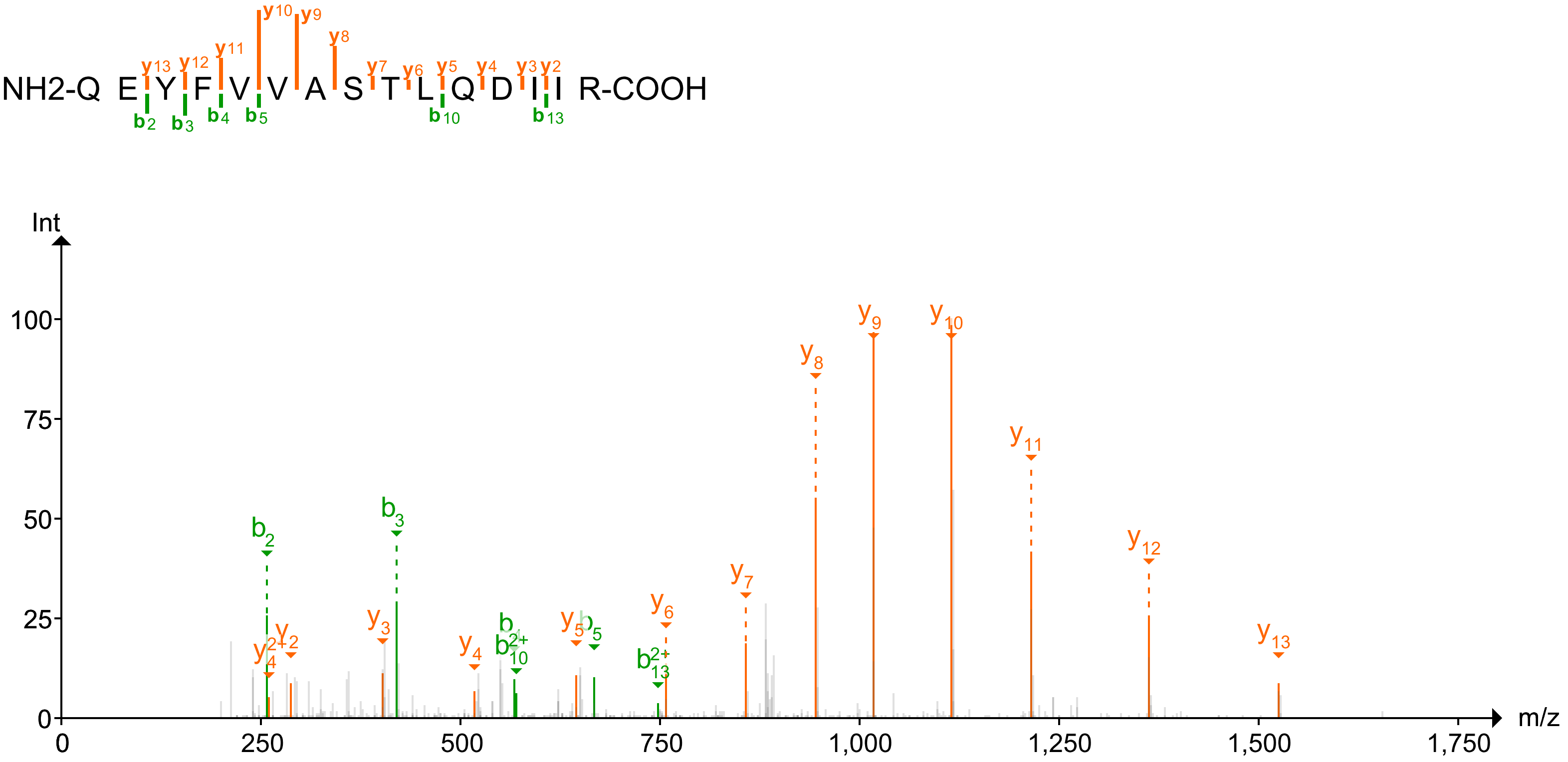
NH2-Q E Y F V V A S T L Q D I I R-COOH
y13 y12 y11 y10 y9 y8 y7 y6 y5 y4 y3 y2
b2 b3 b4 b5 b10 b13
Int
100
75
50
25
0
0
250
500
750
1,000
1,250
1,500
1,750
m/z
b2
b3
y9
y10
y8
y11
y12
y13
y7
y6
y5
b5
y4
y3
y2
y4 2+
b10 2+
b13 2+

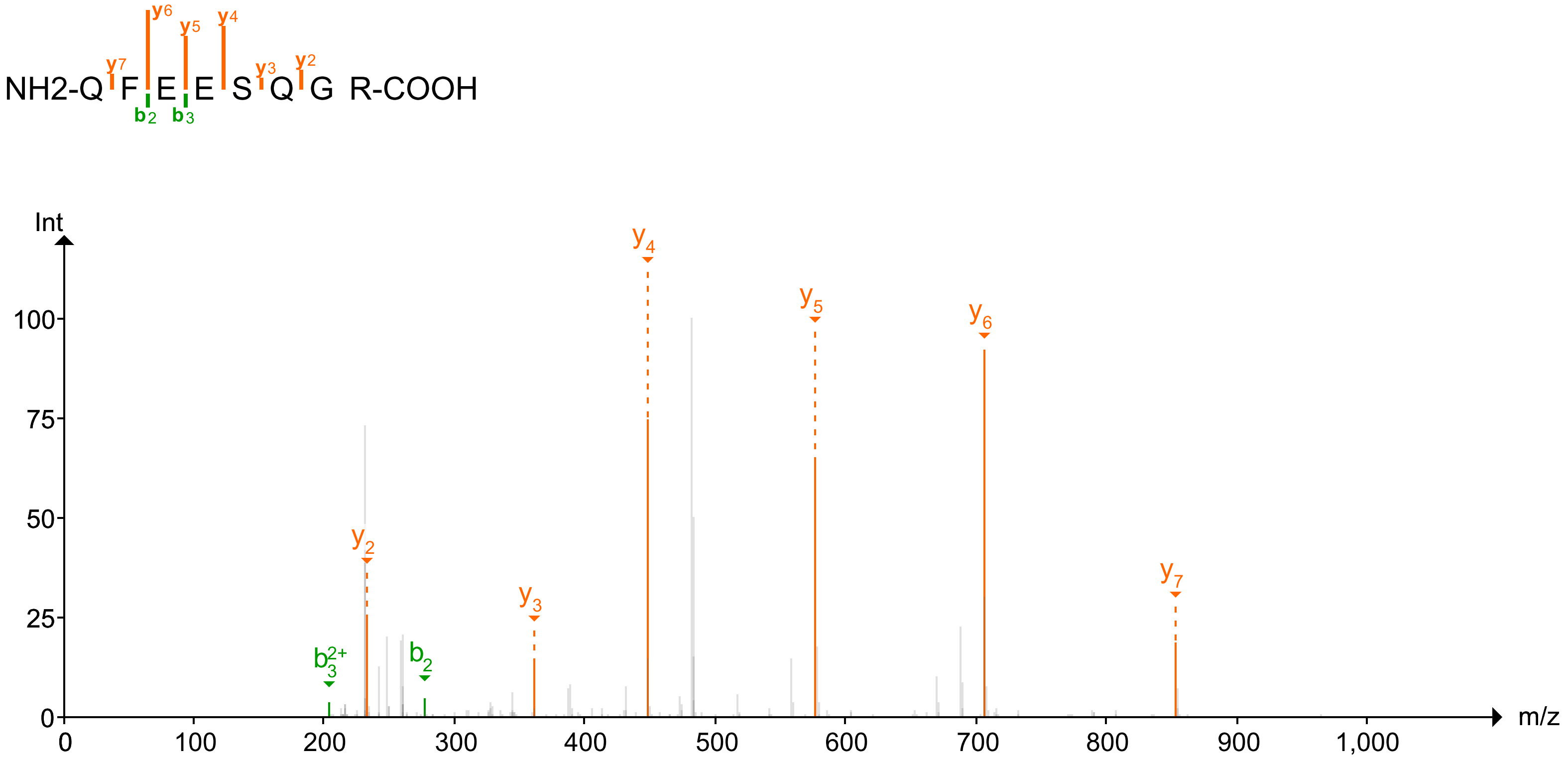

NH2-Q F E E S Q G R-COOH
y7 y6 y5 y4 y3 y2
b2 b3
Int
m/z
0
25
50
75
100
0
100
200
300
400
500
600
700
800
900
1,000
y2
y3
y4
y5
y6
y7
b2
b3 2+

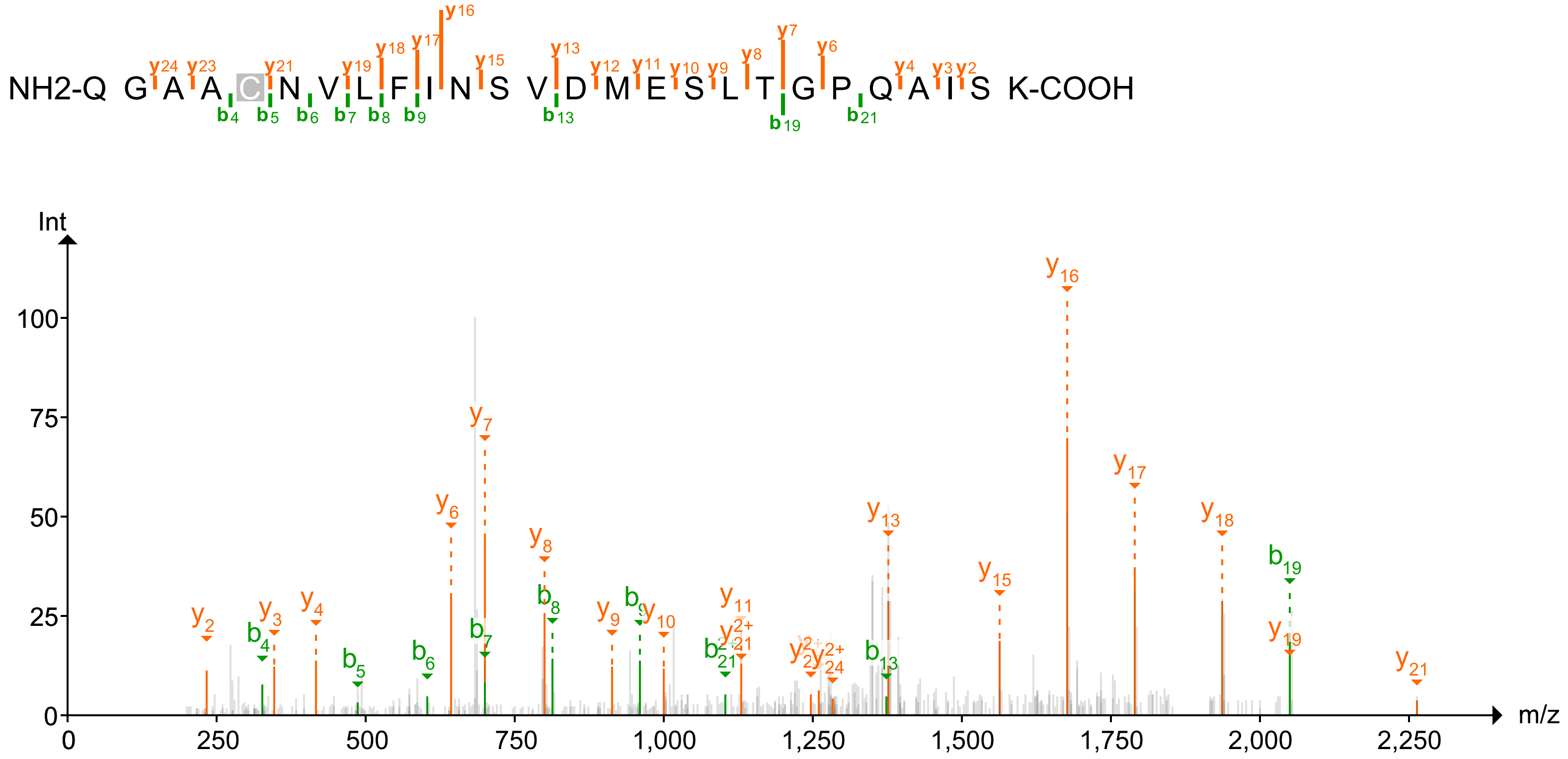

NH2-Q G A A C N V L F I N S V D M E S L T G P Q A I S K-COOH
Int
m/z
0
25
50
75
100
250
500
750
1,000
1,250
1,500
1,750
2,000
2,250

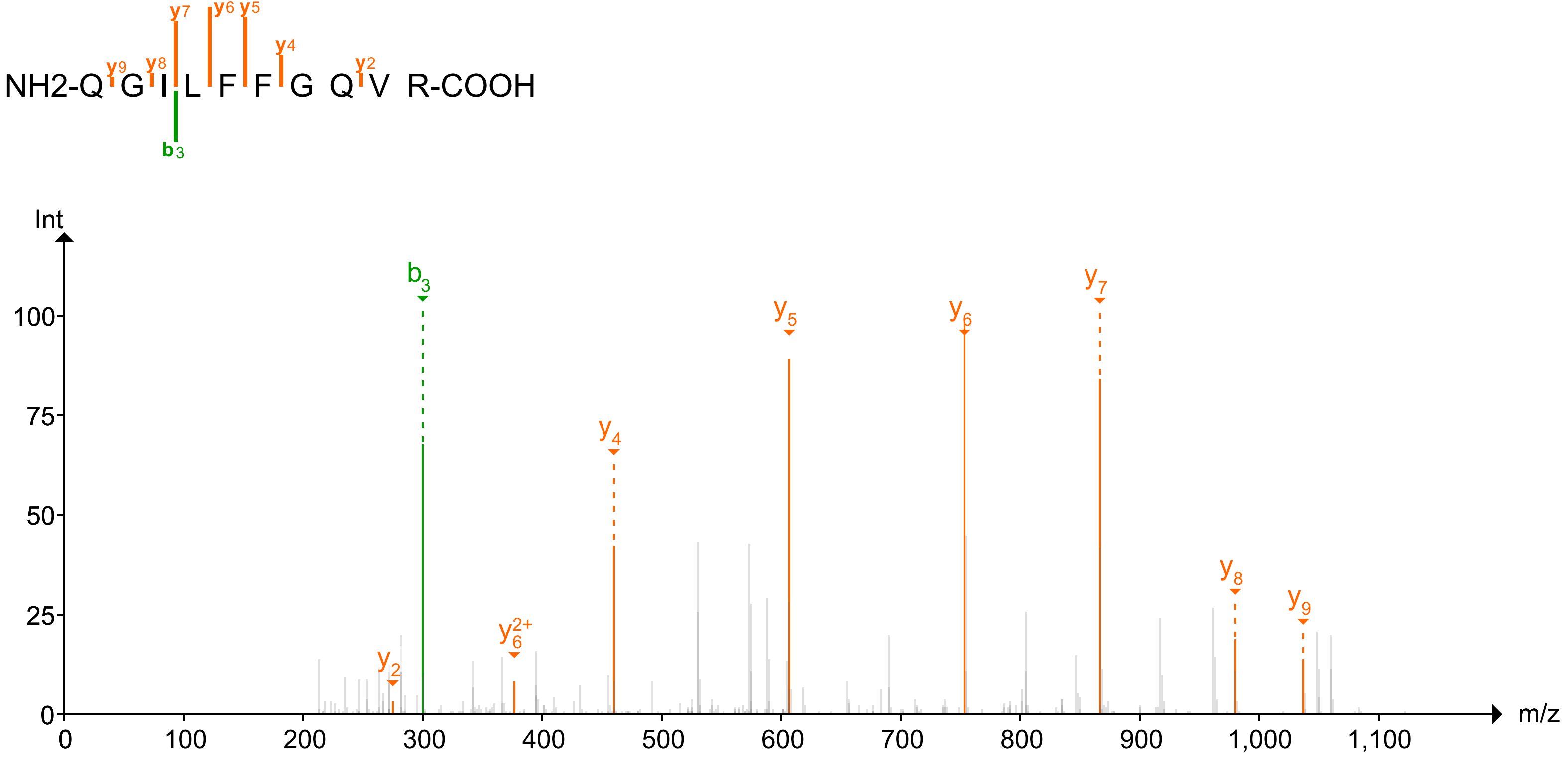

NH2-Q G I L F F G Q V R-COOH
y9 y8 y7 y6 y5 y4 y2
b3
Int
m/z
0
25
50
75
100
0
100
200
300
400
500
600
700
800
900
1,000
1,100
b3
y2
y6 2+
y4
y5
y6
y7
y8
y9

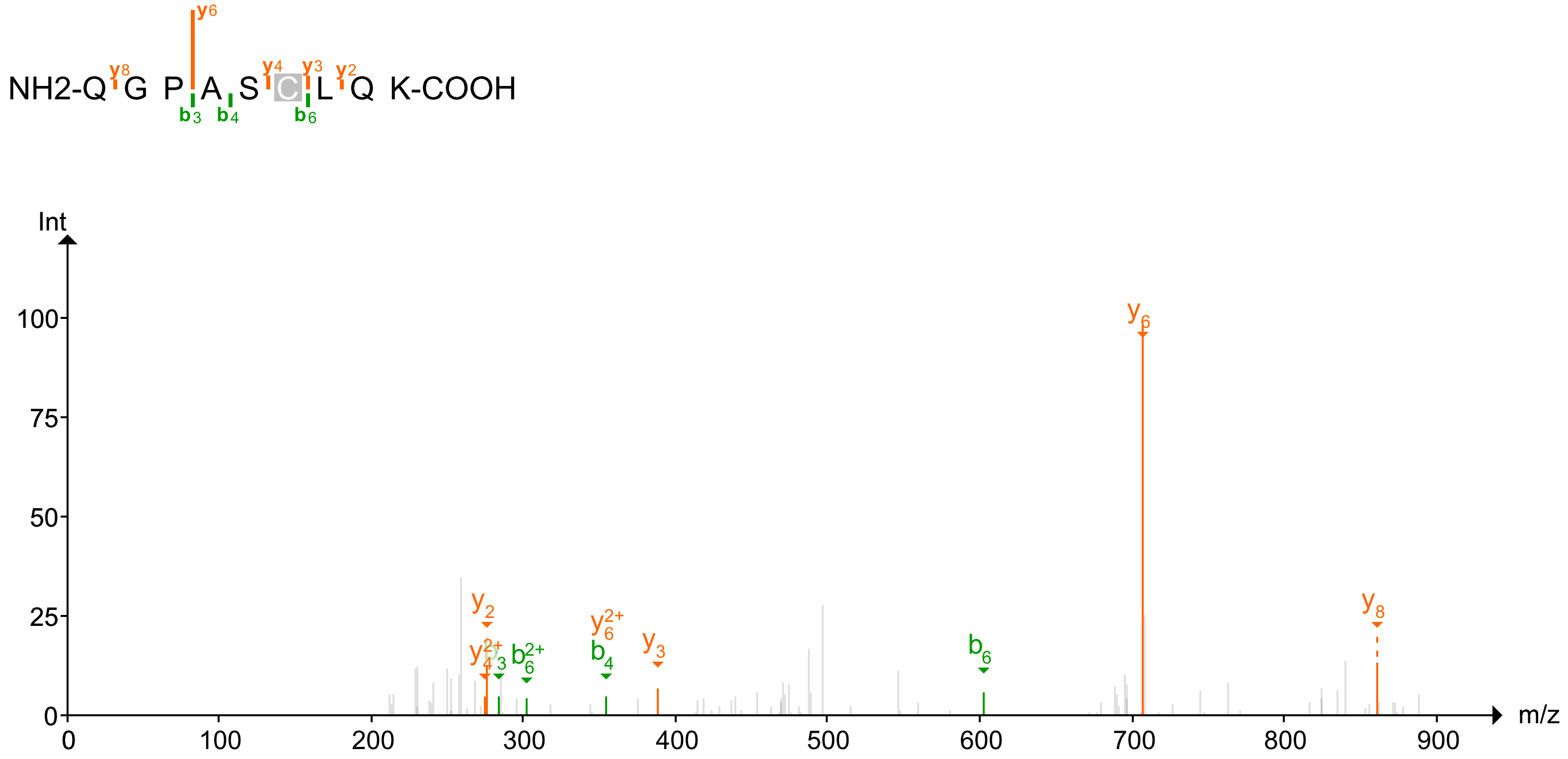
NH2-Q G P A S C L Q K-COOH
y8 y6 y4 y3 y2
b3 b4 b6
Int
100
75
50
25
0
m/z
0
100
200
300
400
500
600
700
800
900
y6
y2
y4 2+
b3
b6 2+
y6 2+
b4
y3
b6
y8

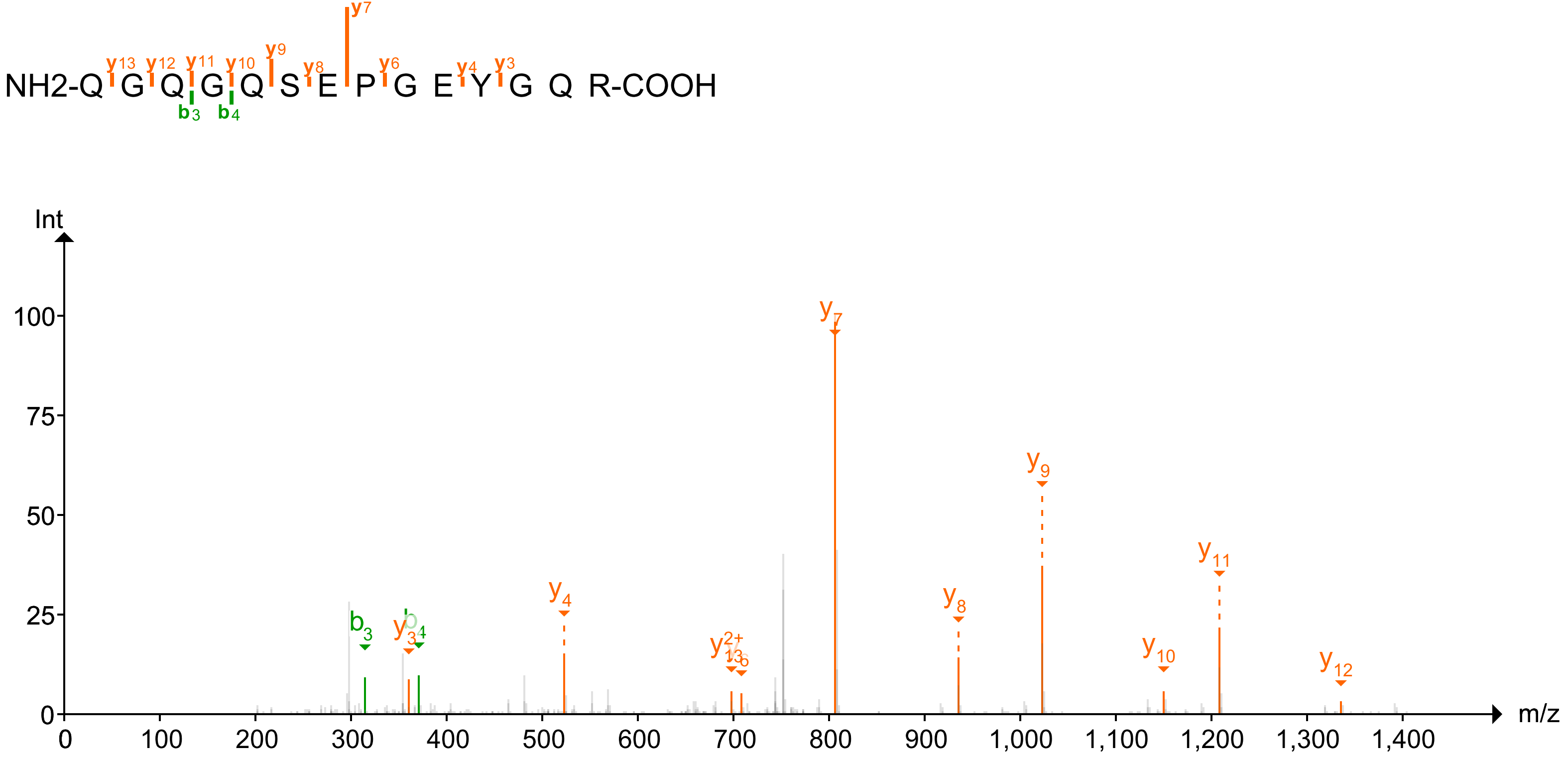

NH2-Q G Q G Q S E P G E Y G Q R-COOH
y13 y12 y11 y10 y9 y8 y7 y6 y4 y3
b3 b4
Int
100
75
50
25
0
m/z
0 100 200 300 400 500 600 700 800 900 1,000 1,100 1,200 1,300 1,400
y7
y9
y11
y4
y8
y10
y12
b3
y3
b4
y13 2+
y6

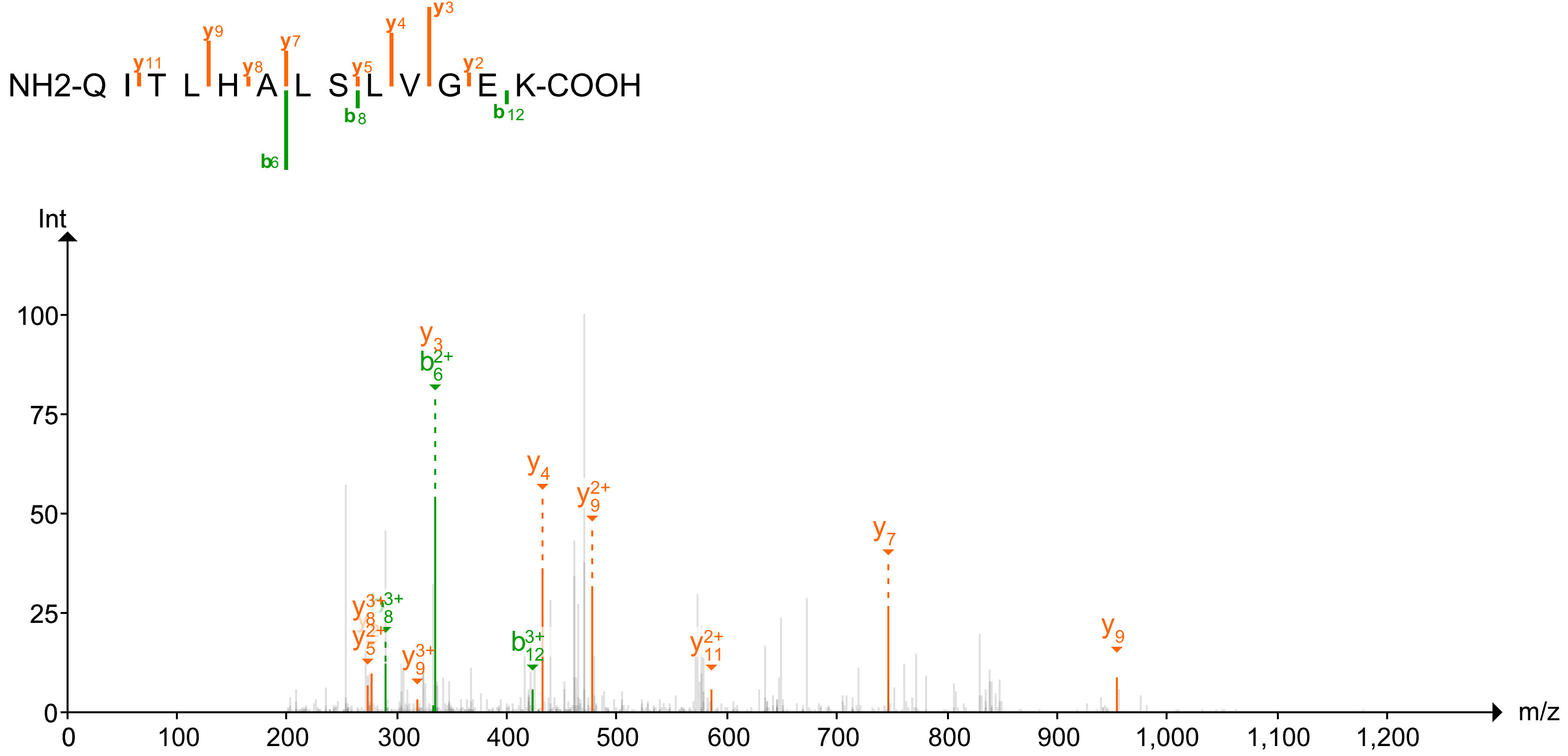

NH2-Q I T L H A L S L V G E K-COOH
y11
y9
y8
y7
y5
y4
y3
y2
b6
b8
b12
Int
100
75
50
25
0
m/z
0
100
200
300
400
500
600
700
800
900
1,000
1,100
1,200
y3
b6 2+
y4
y9 2+
y7
y9
y8 3+
b8 3+
y5 2+
y9 3+
b12 3+
y11 2+

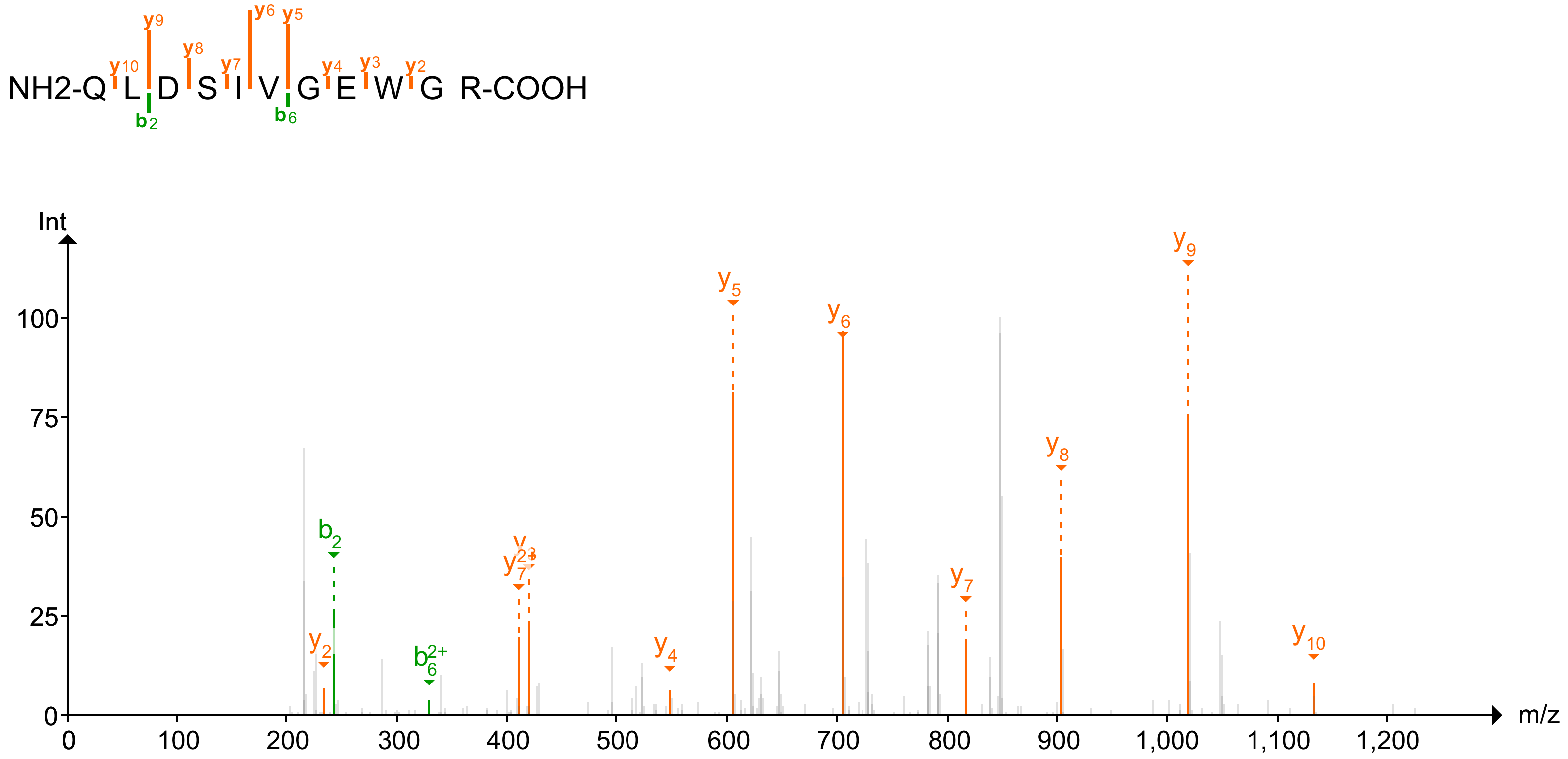

NH2-QLDSIVGEWGR-COOH
y10 y9 y8 y7 y6 y5 y4 y3 y2
b2 b6
Int
m/z
y9 y5 y6 y8 y7 y10 y4 y2 b2 b6 2+ y7 2+
0 25 50 75 100
0 100 200 300 400 500 600 700 800 900 1,000 1,100 1,200

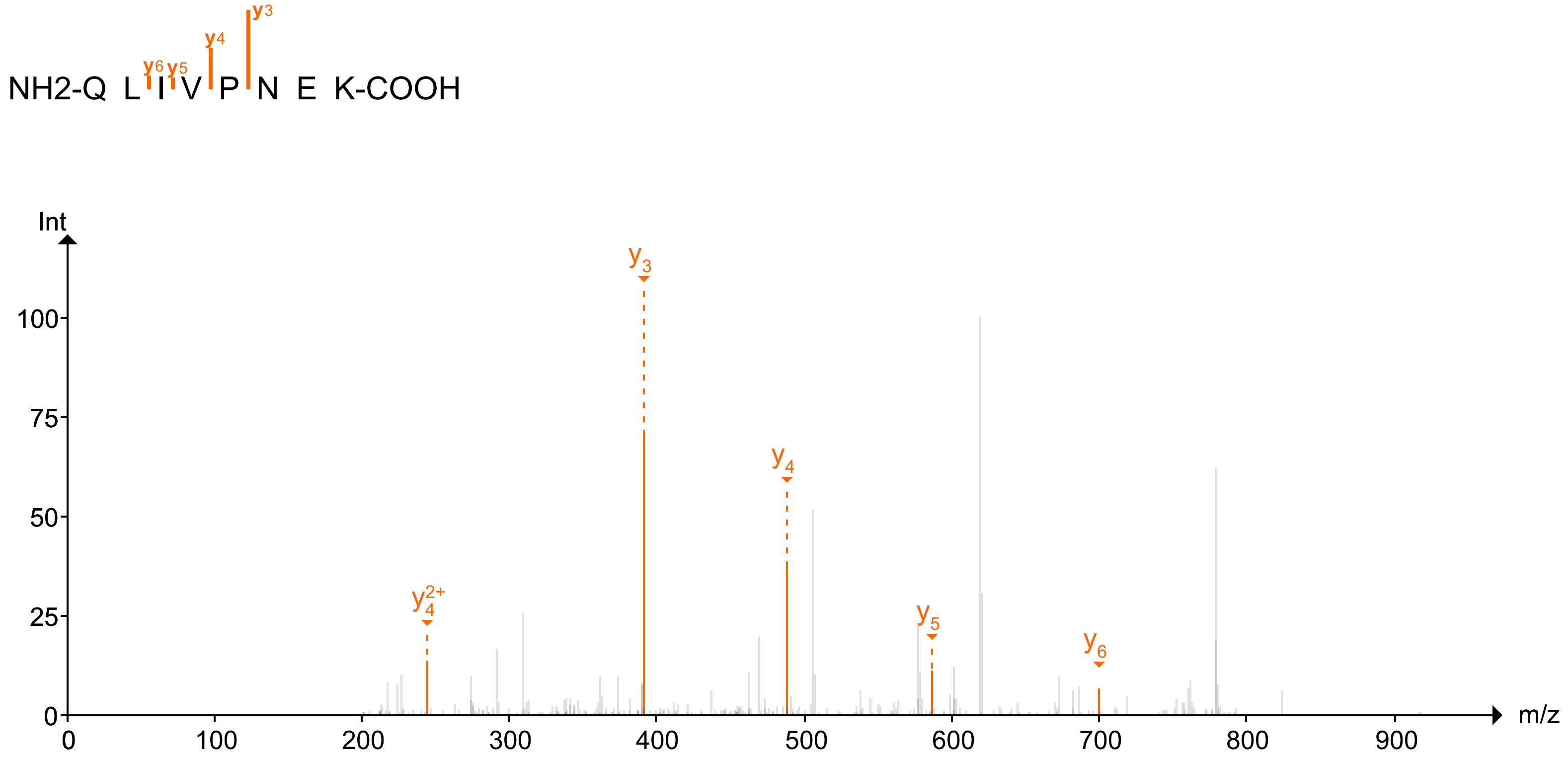

NH2-Q L I V P N E K-COOH
y6 y5 y4 y3
Int
100
75
50
25
0
y3
y4
y4 2+
y5
y6
0
100
200
300
400
500
600
700
800
900
m/z

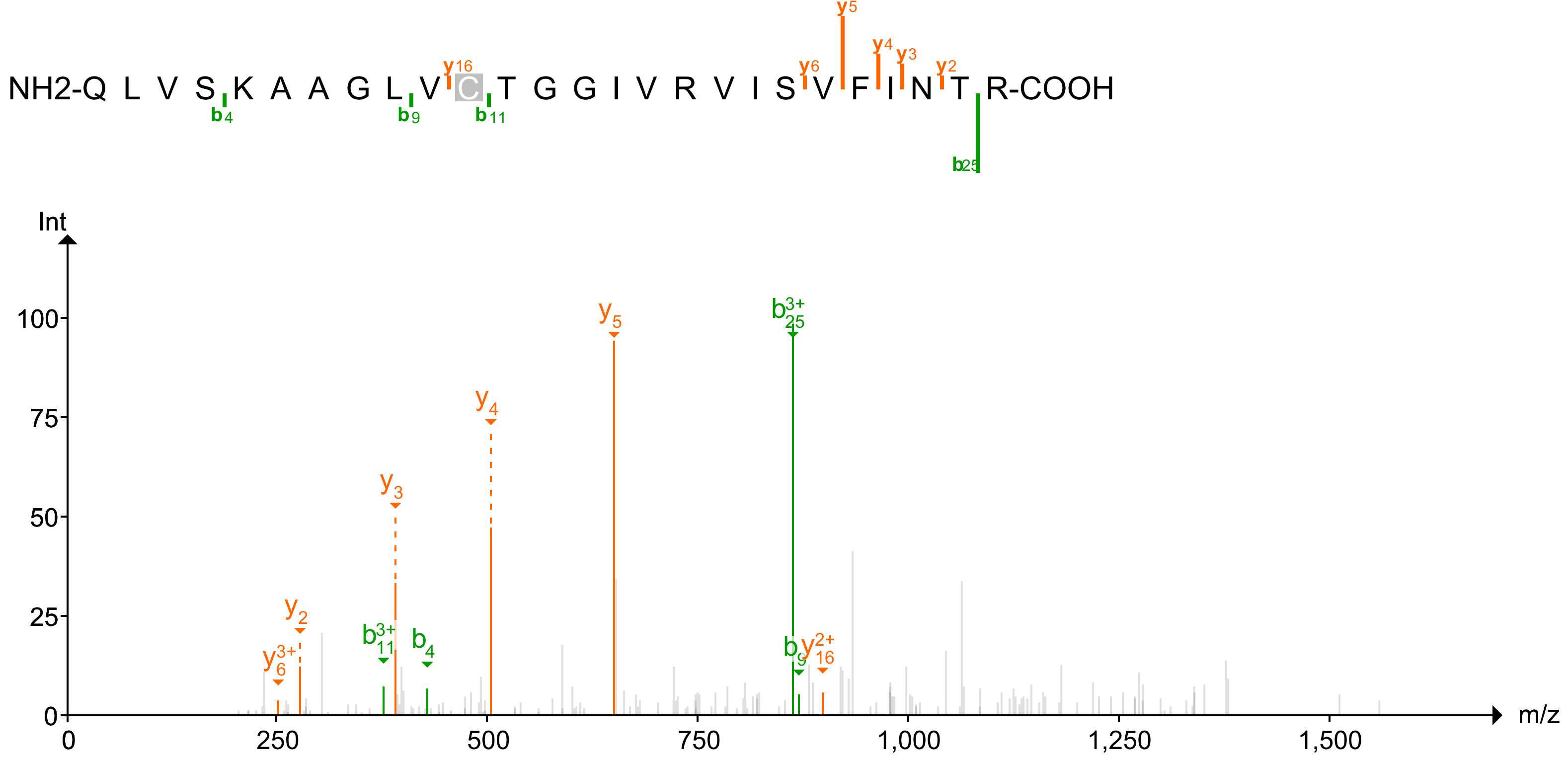

NH2-Q L V S K A A G L V C T G G I V R V I S V F I N T R-COOH
b4
b9
b11
b25
y16
y6
y5
y4
y3
y2
Int
100
75
50
25
0
y5
b25 3+
y4
y3
y2
y6 3+
b11 3+
b4
b9
y16 2+
0
250
500
750
1,000
1,250
1,500
m/z

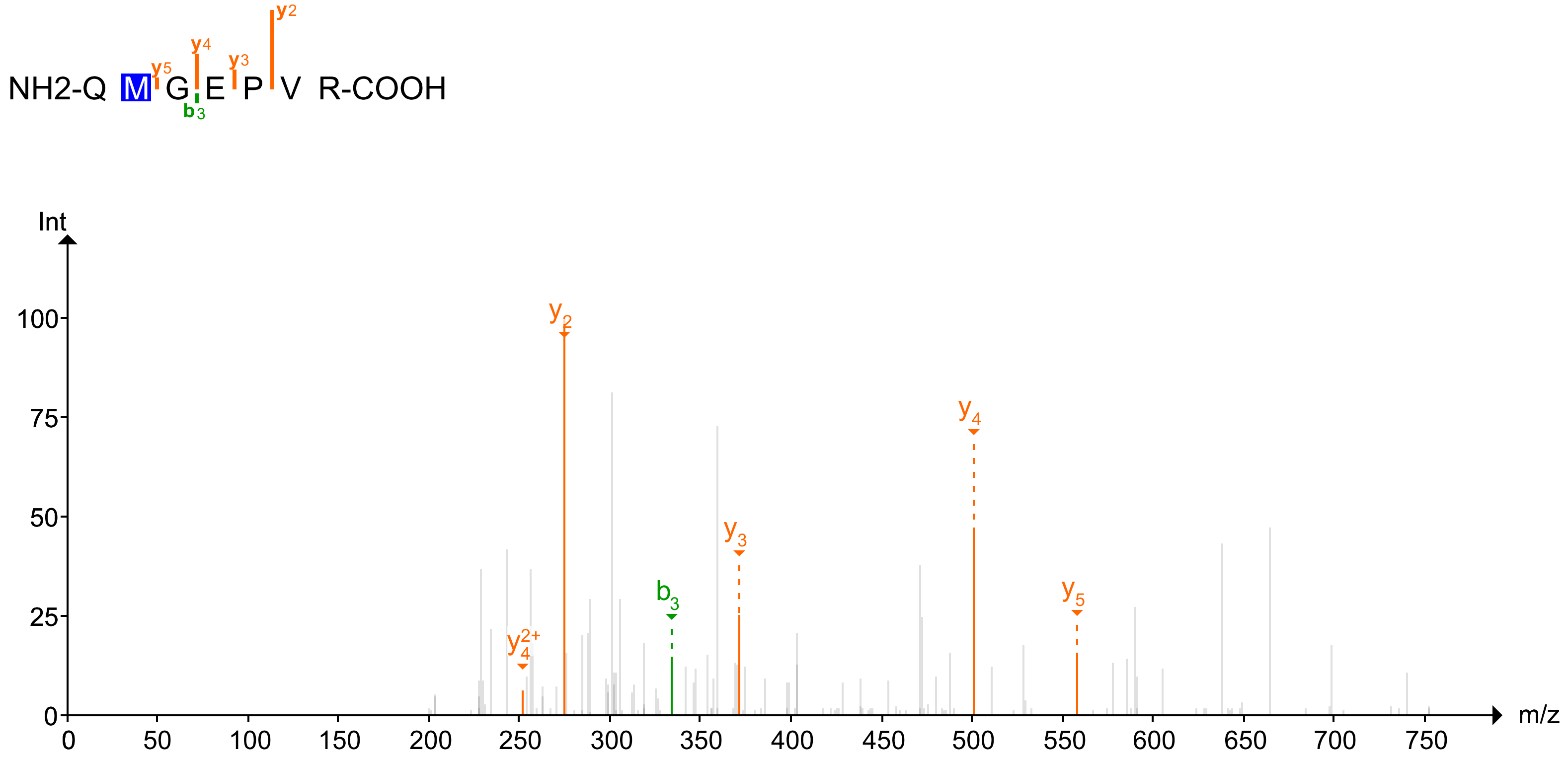
NH2-Q M G E P V R-COOH
y2
y4
y3
y5
b3
Int
100
75
50
25
0
y2
y4
y3
b3
y5
y4 2+
0
50
100
150
200
250
300
350
400
450
500
550
600
650
700
750
m/z

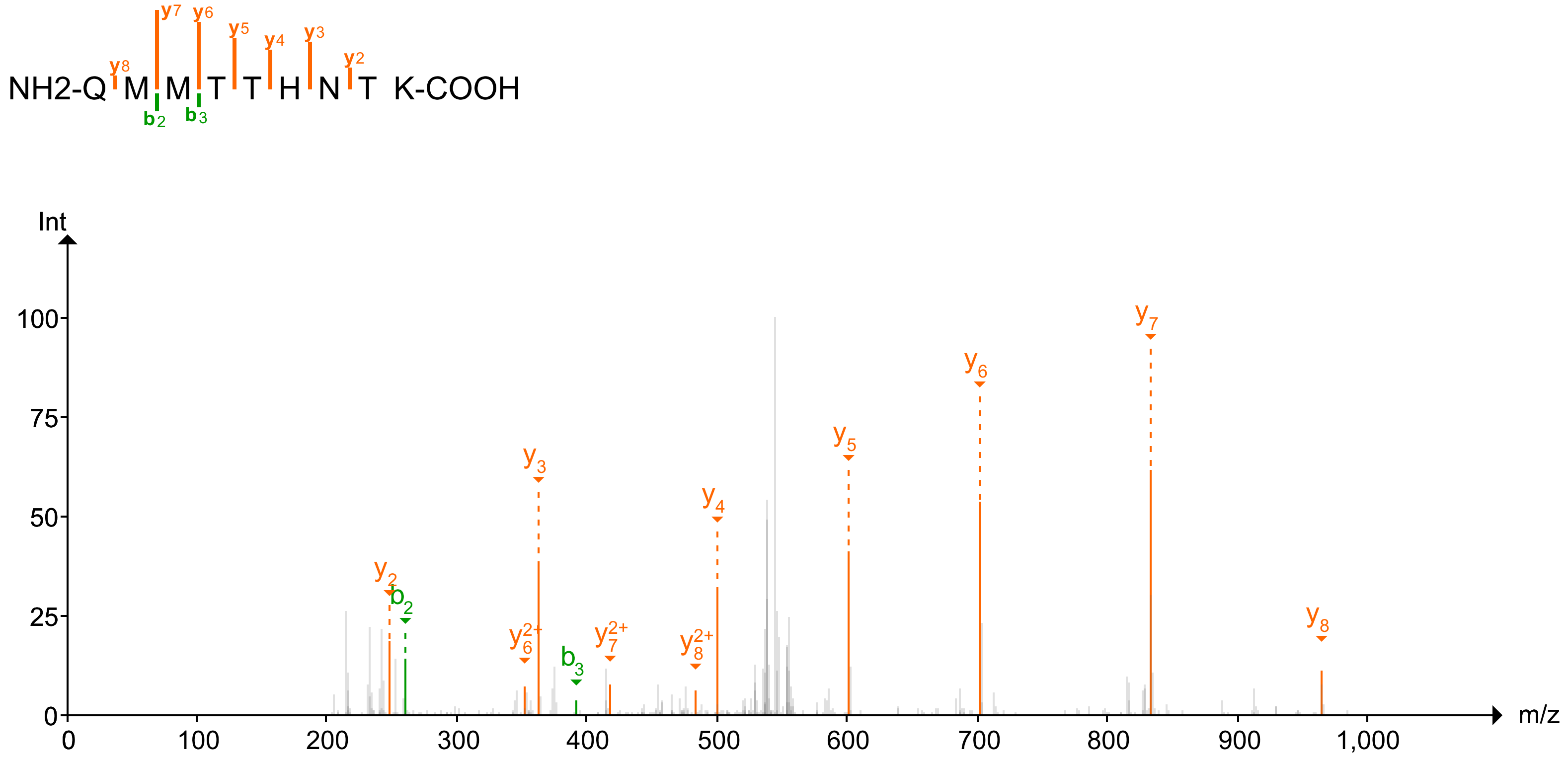

NH2-Q M M T T H N T K-COOH
y8 y7 y6 y5 y4 y3 y2
b2 b3
Int
100
75
50
25
0
0 100 200 300 400 500 600 700 800 900 1,000
m/z
y2 b2 y6 2+ b3 y3 y7 2+ y8 2+ y4 y5 y6 y7 y8

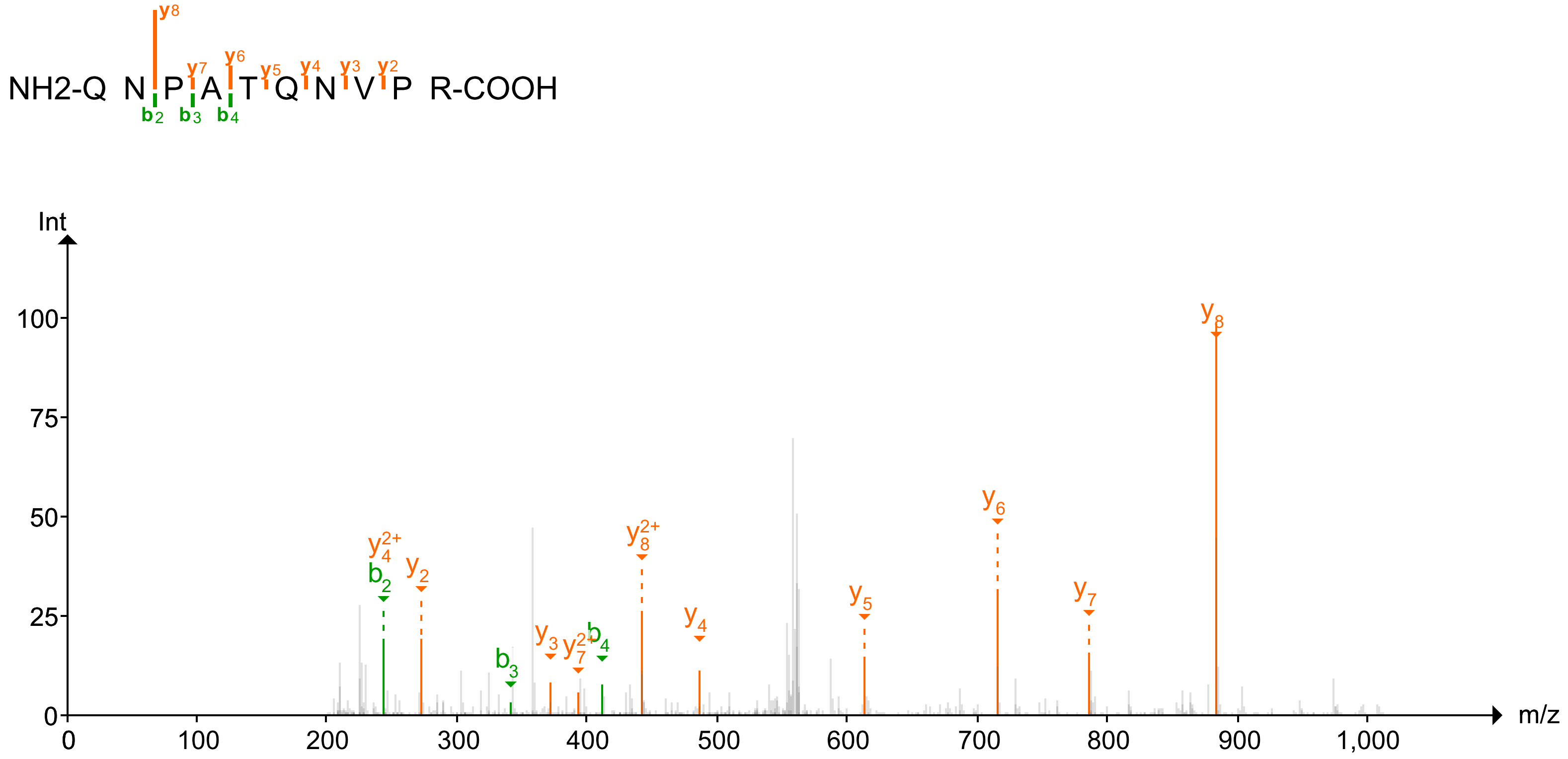

NH2-Q N P A T Q N V P R-COOH
y8 y7 y6 y5 y4 y3 y2
b2 b3 b4
Int
100
75
50
25
0
m/z
0
100
200
300
400
500
600
700
800
900
1,000
y8
y6
y8 2+
y4 2+
b2
y2
y5
y7
y4
b3
y3
y7 2+
b4

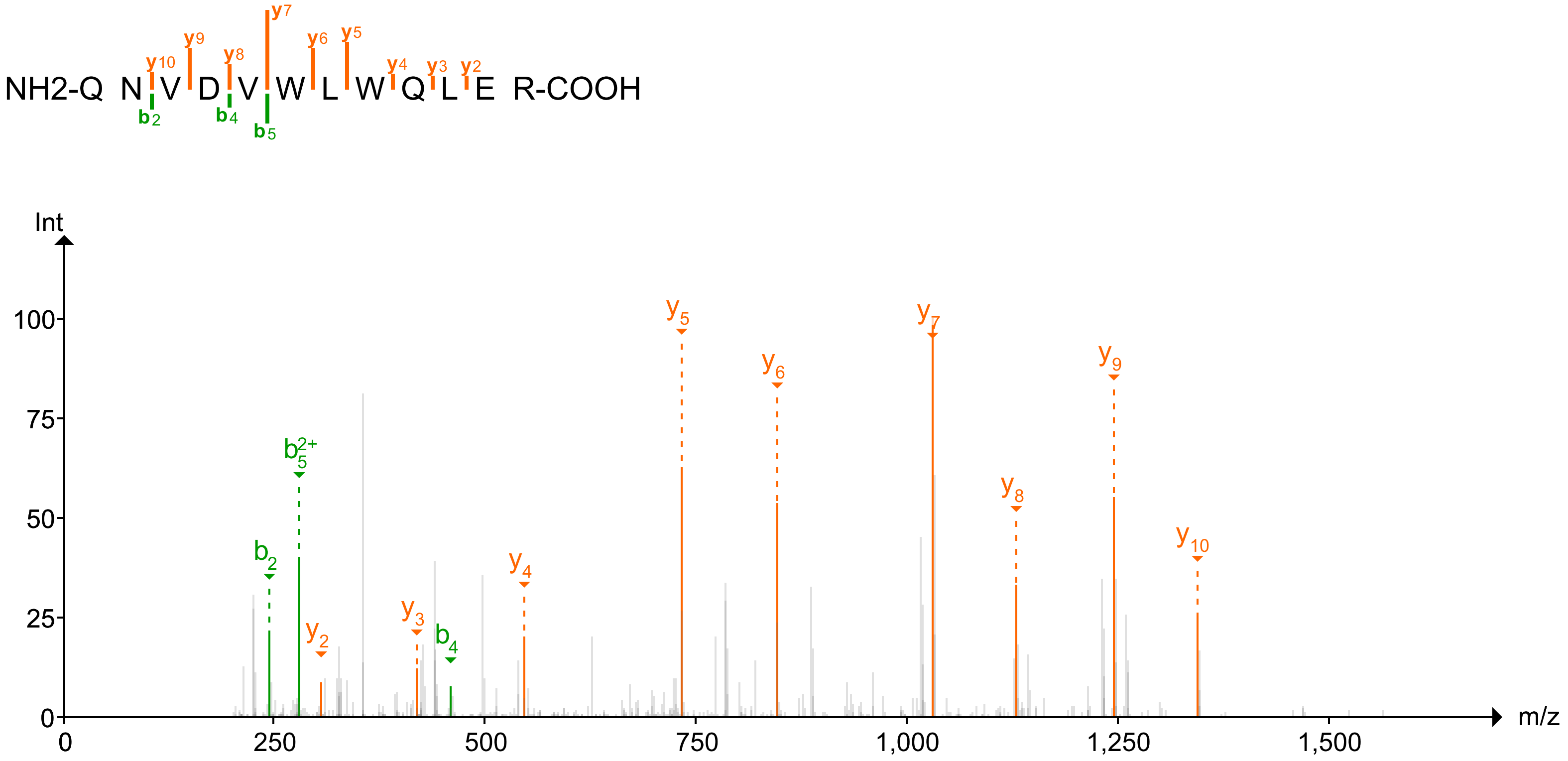

NH2-Q N V D V W L W Q L E R-COOH
y10 y9 y8 y7 y6 y5 y4 y3 y2
b2 b4 b5
Int
100
75
50
25
0
m/z
0
250
500
750
1,000
1,250
1,500
y5
y7
y6
y9
y8
y10
b5 2+
b2
y4
y3
b4
y2

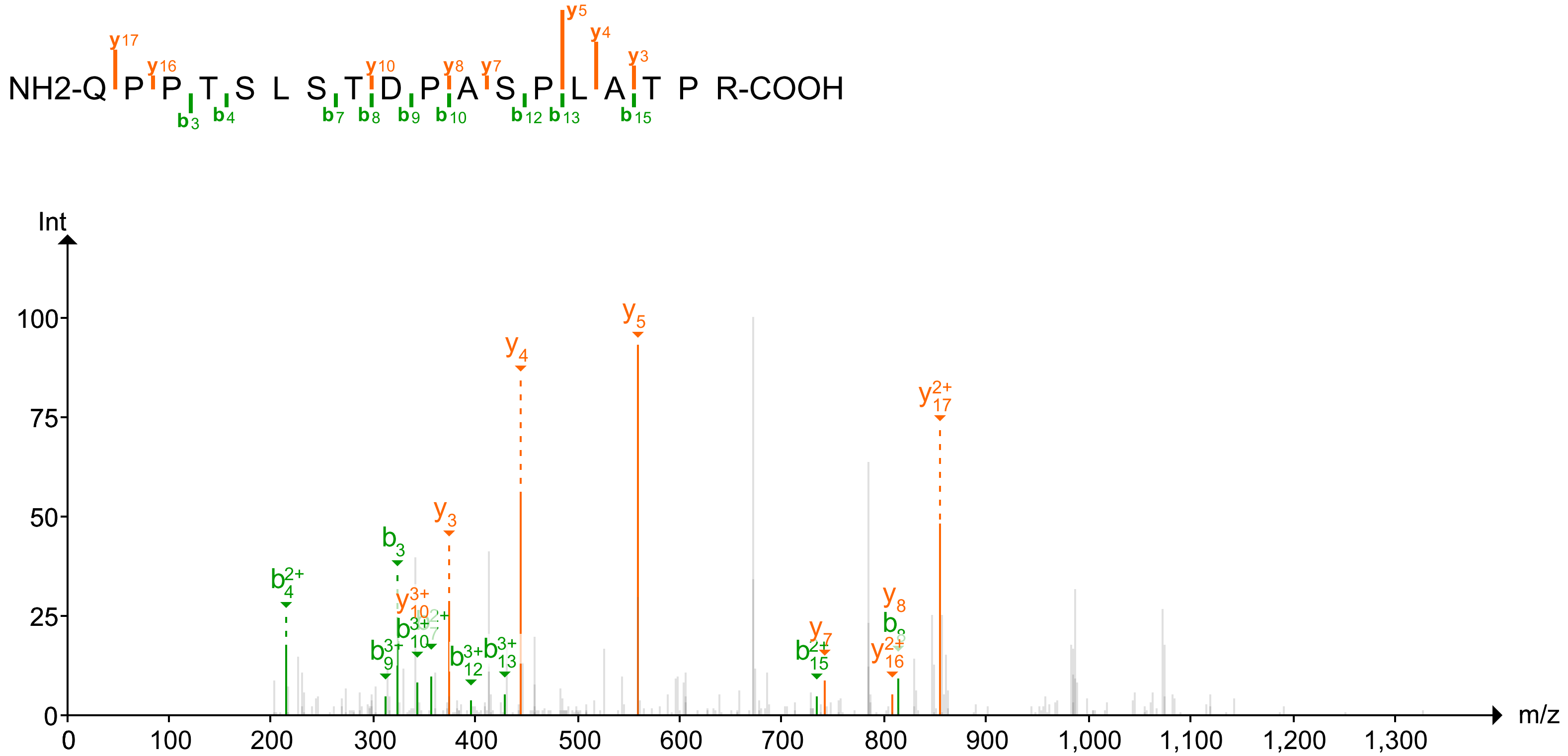

NH2-Q P P T S L S T D P A S P L A T P R-COOH
y17 y16 y10 y8 y7 y5 y4 y3
b3 b4 b7 b8 b9 b10 b12 b13 b15
Int
m/z

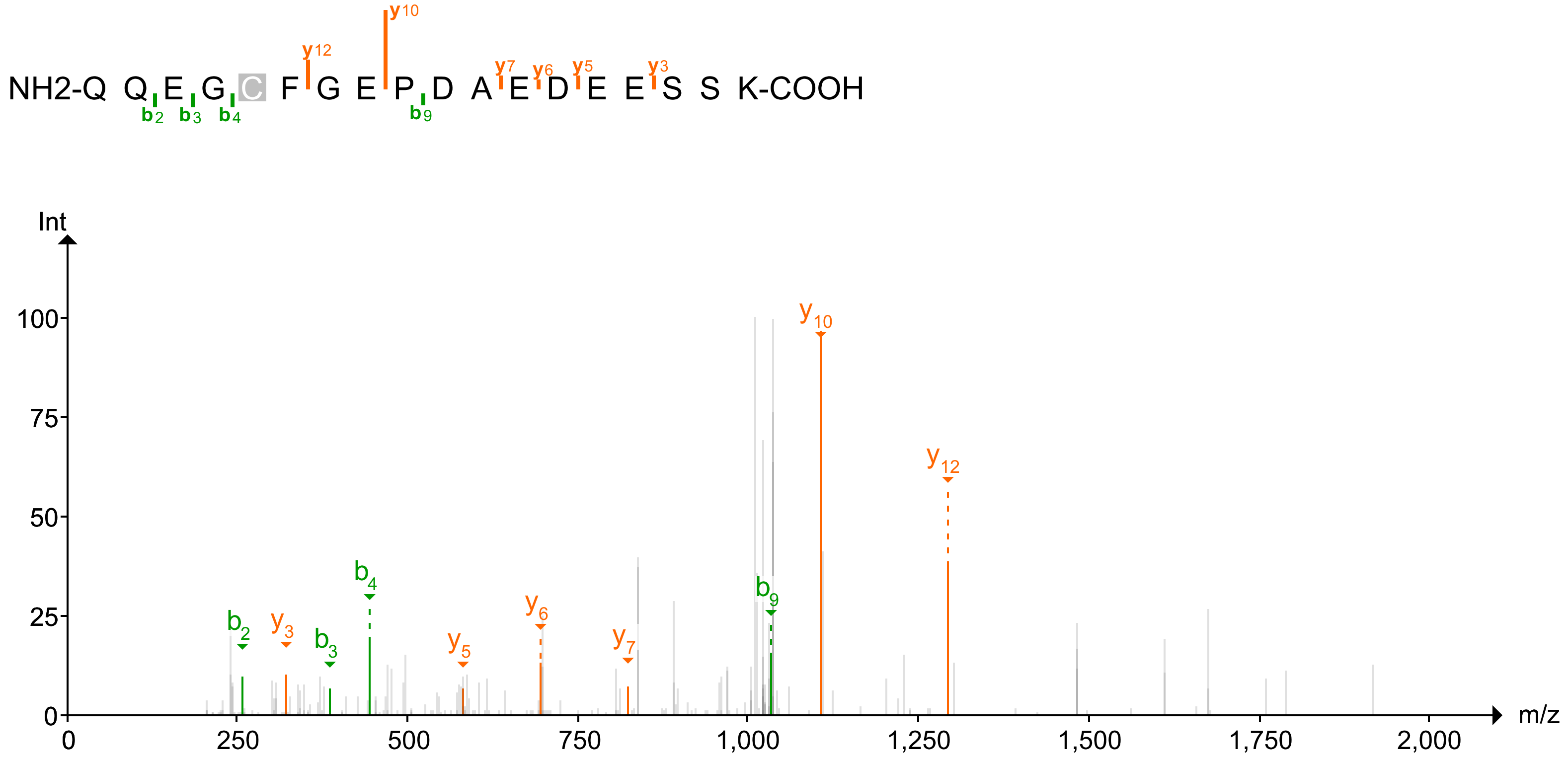
NH2-Q Q E G C F G E P D A E D E E S S K-COOH
y10
y12
y7
y6
y5
y3
b2
b3
b4
b9
Int
100
75
50
25
0
y10
y12
b4
b9
y6
y7
b2
y3
b3
y5
0
250
500
750
1,000
1,250
1,500
1,750
2,000
m/z

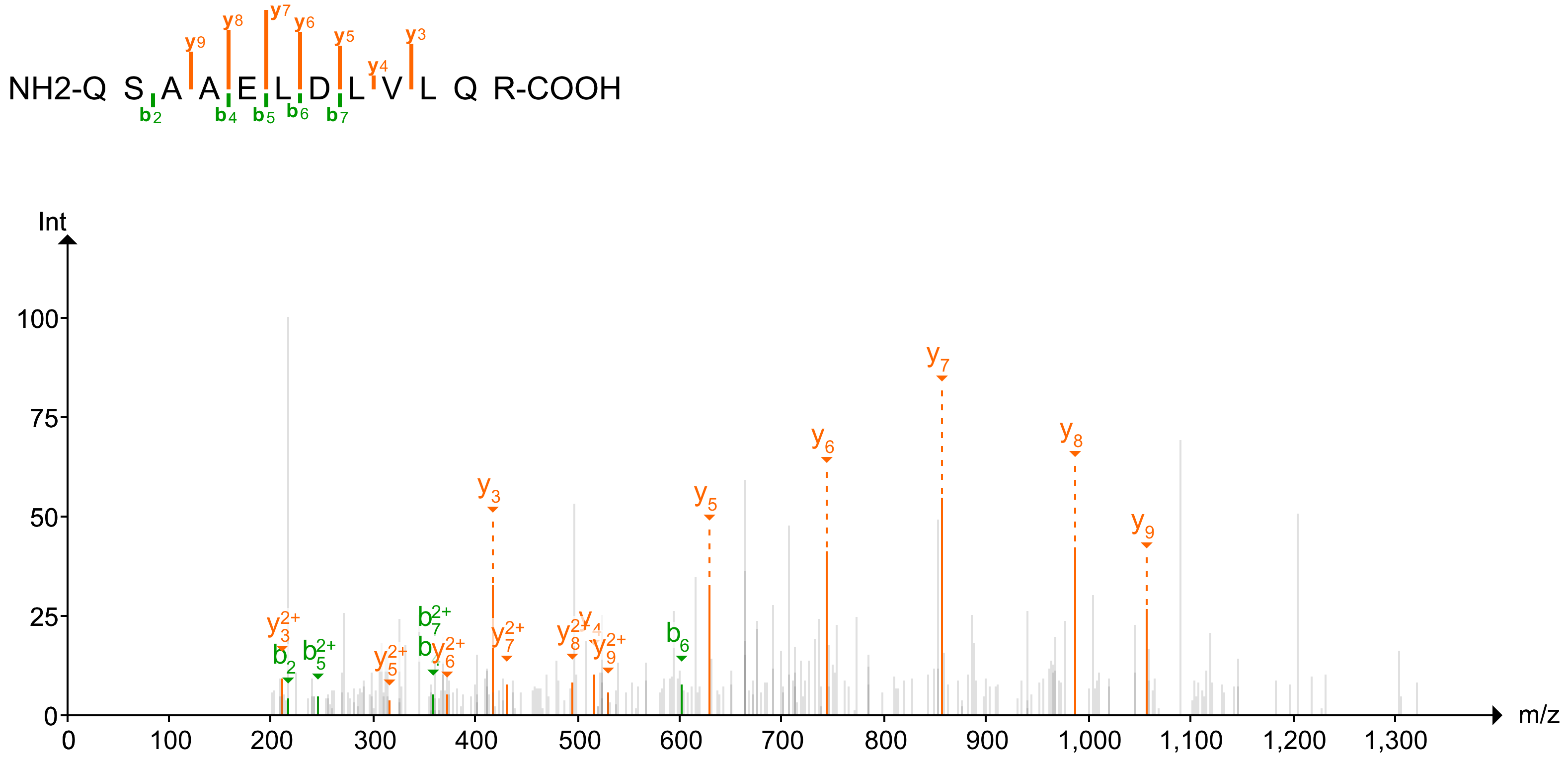
NH2-Q S A A E L D L V L Q R-COOH
y9 y8 y7 y6 y5 y4 y3
b2 b4 b5 b6 b7
Int
100
75
50
25
0
m/z
0 100 200 300 400 500 600 700 800 900 1,000 1,100 1,200 1,300
y7
y6
y8
y3
y5
y9
b6

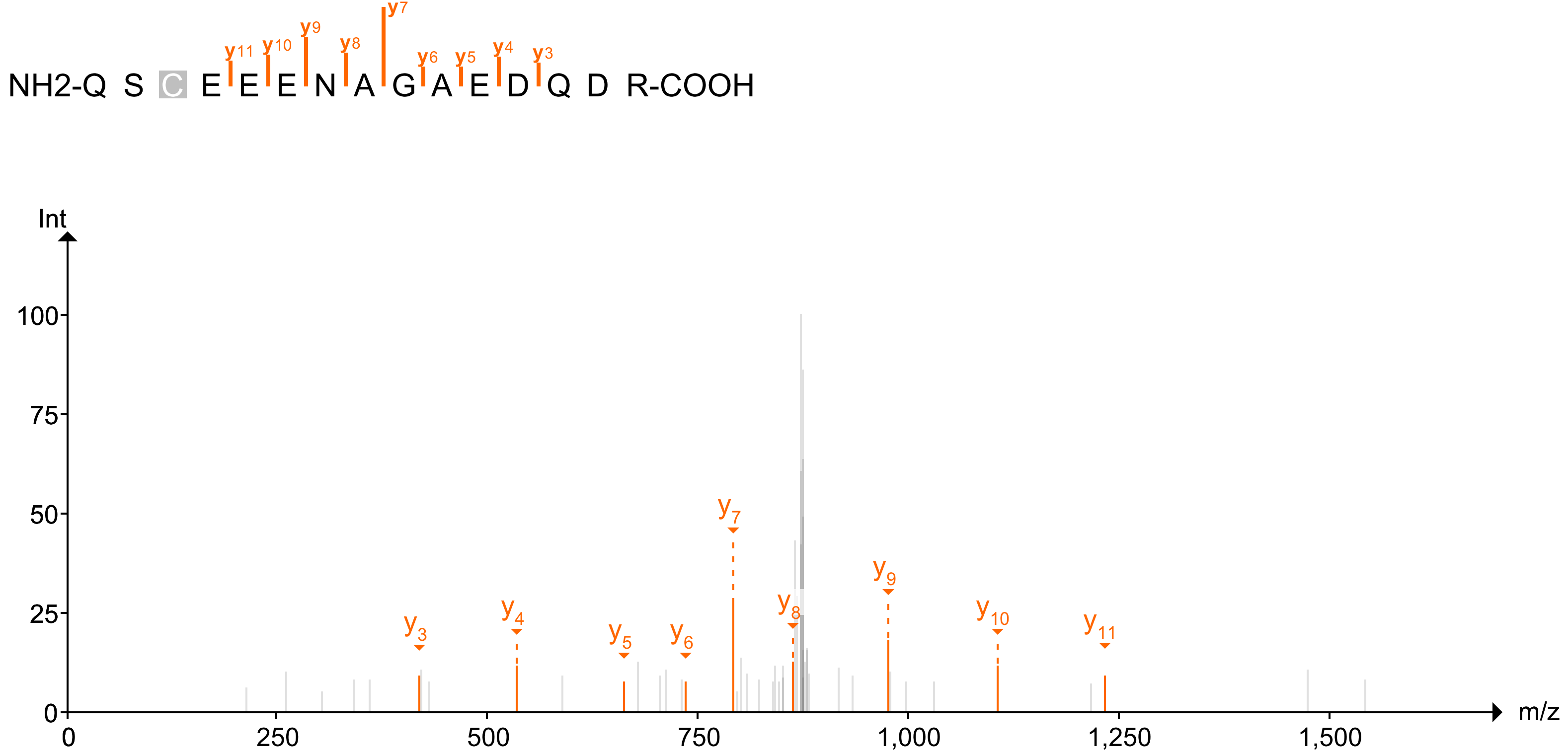

NH2-Q S C E E E N A G A E D Q D R-COOH
y11 y10 y9 y8 y7 y6 y5 y4 y3
Int
100
75
50
25
0
y3
y4
y5
y6
y7
y8
y9
y10
y11
0
250
500
750
1,000
1,250
1,500
m/z

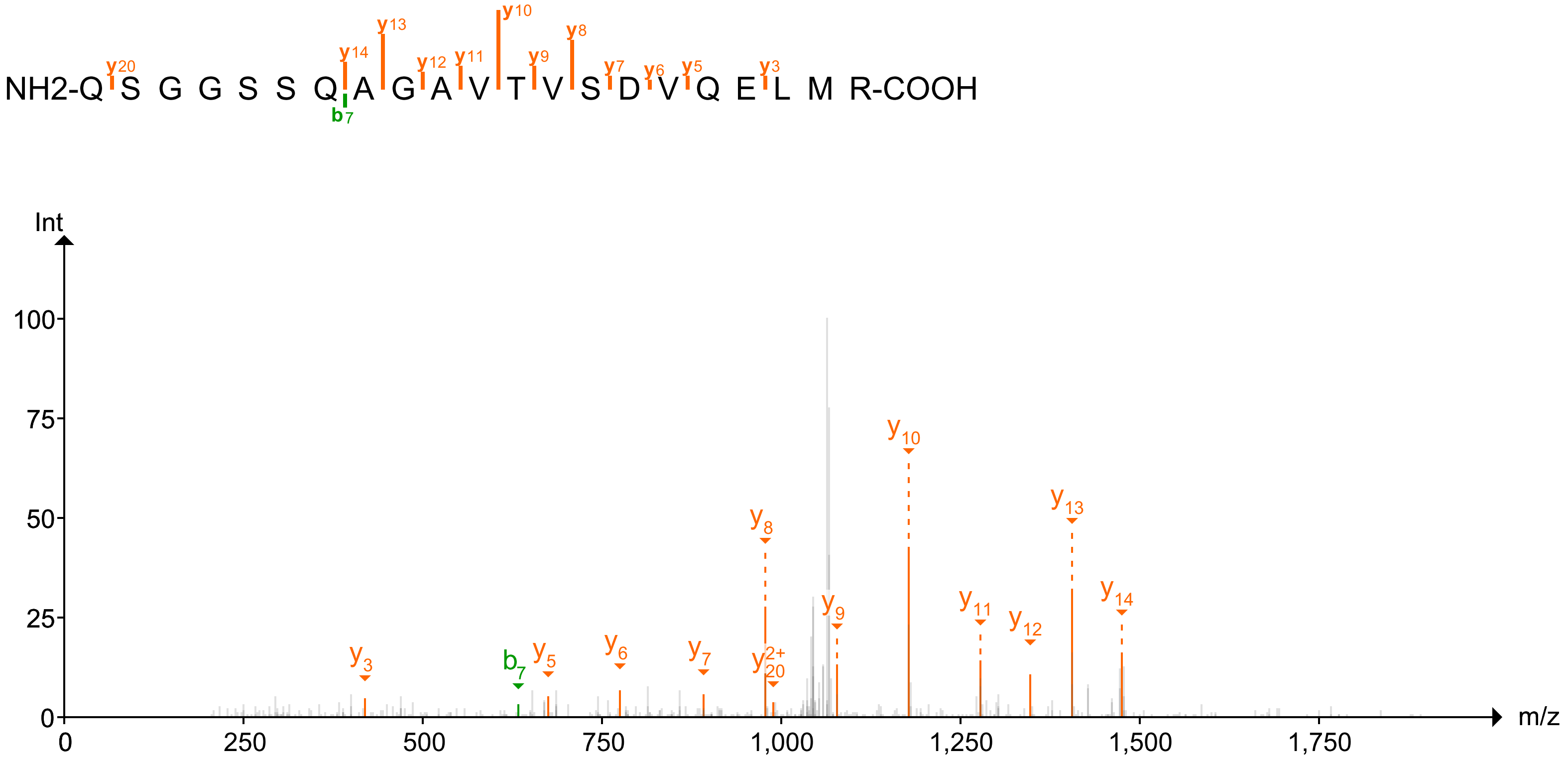

NH2-Q S G G S S Q A G A V T V S D V Q E L M R-COOH
y20 y14 y13 y12 y11 y10 y9 y8 y7 y6 y5 y3
b7
Int
100
75
50
25
0
250
500
750
1,000
1,250
1,500
1,750
m/z
y3 b7 y5 y6 y7 y8 y20 2+ y9 y10 y11 y12 y13 y14

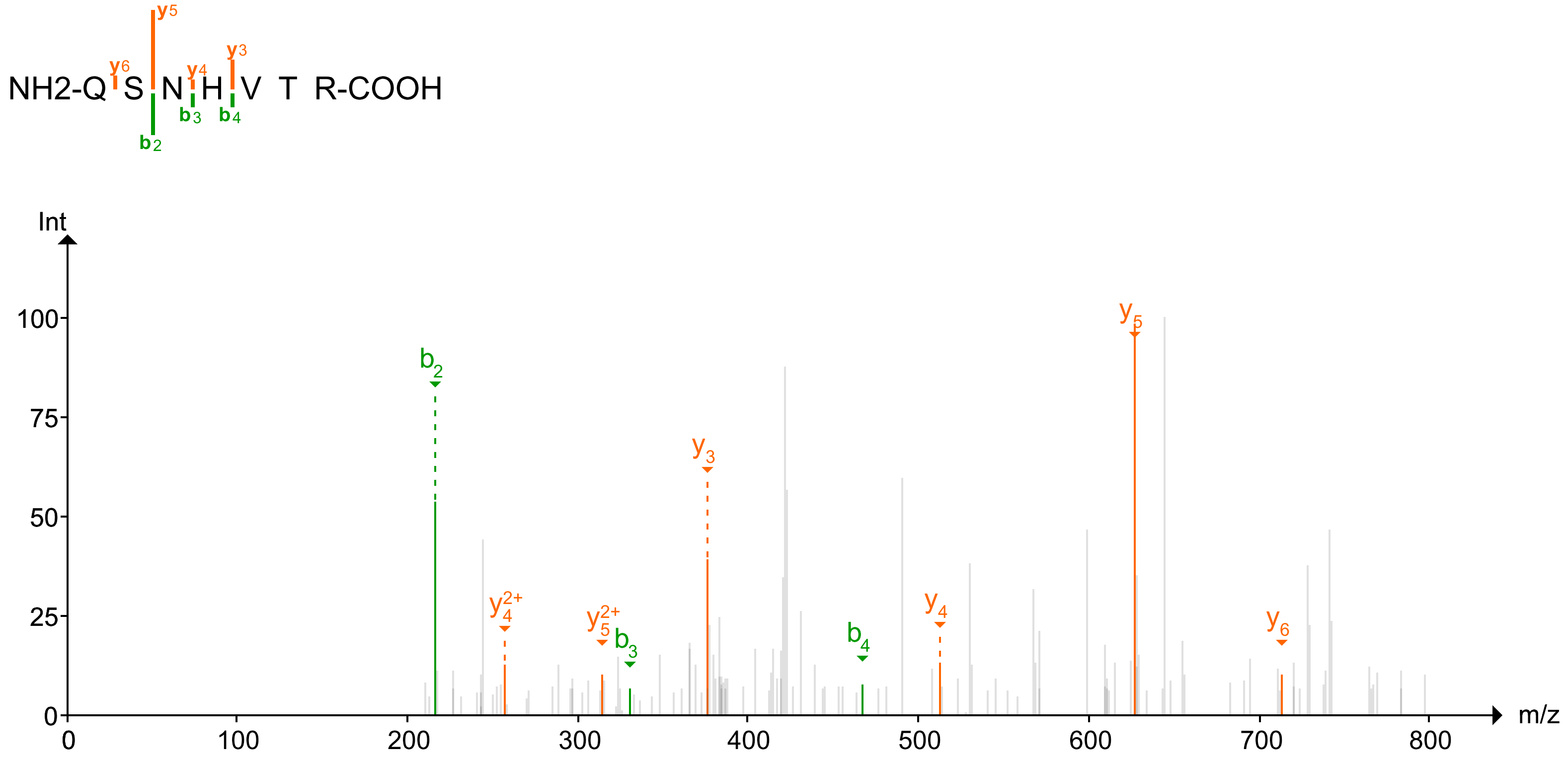

NH2-Q S N H V T R-COOH
y5
y6
y4
y3
b2
b3
b4
Int
100
75
50
25
0
m/z
0
100
200
300
400
500
600
700
800
b2
y4 2+
y5 2+
b3
y3
b4
y4
y5
y6

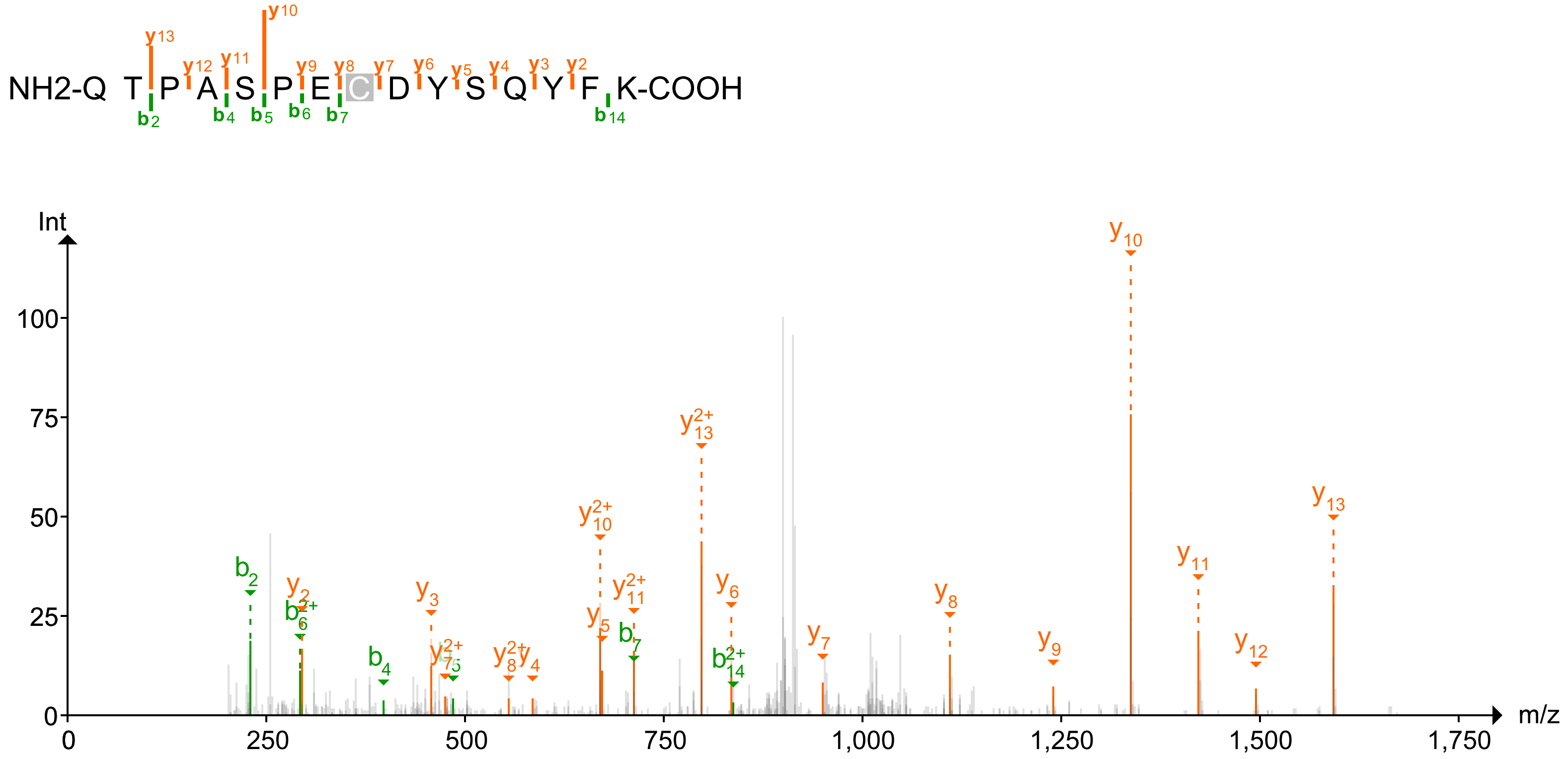
NH2-Q T P A S P E C D Y S Q Y F K-COOH
y13 y12 y11 y10 y9 y8 y7 y6 y5 y4 y3 y2
b2 b4 b5 b6 b7 b14
Int
m/z
0
25
50
75
100
250
500
750
1,000
1,250
1,500
1,750

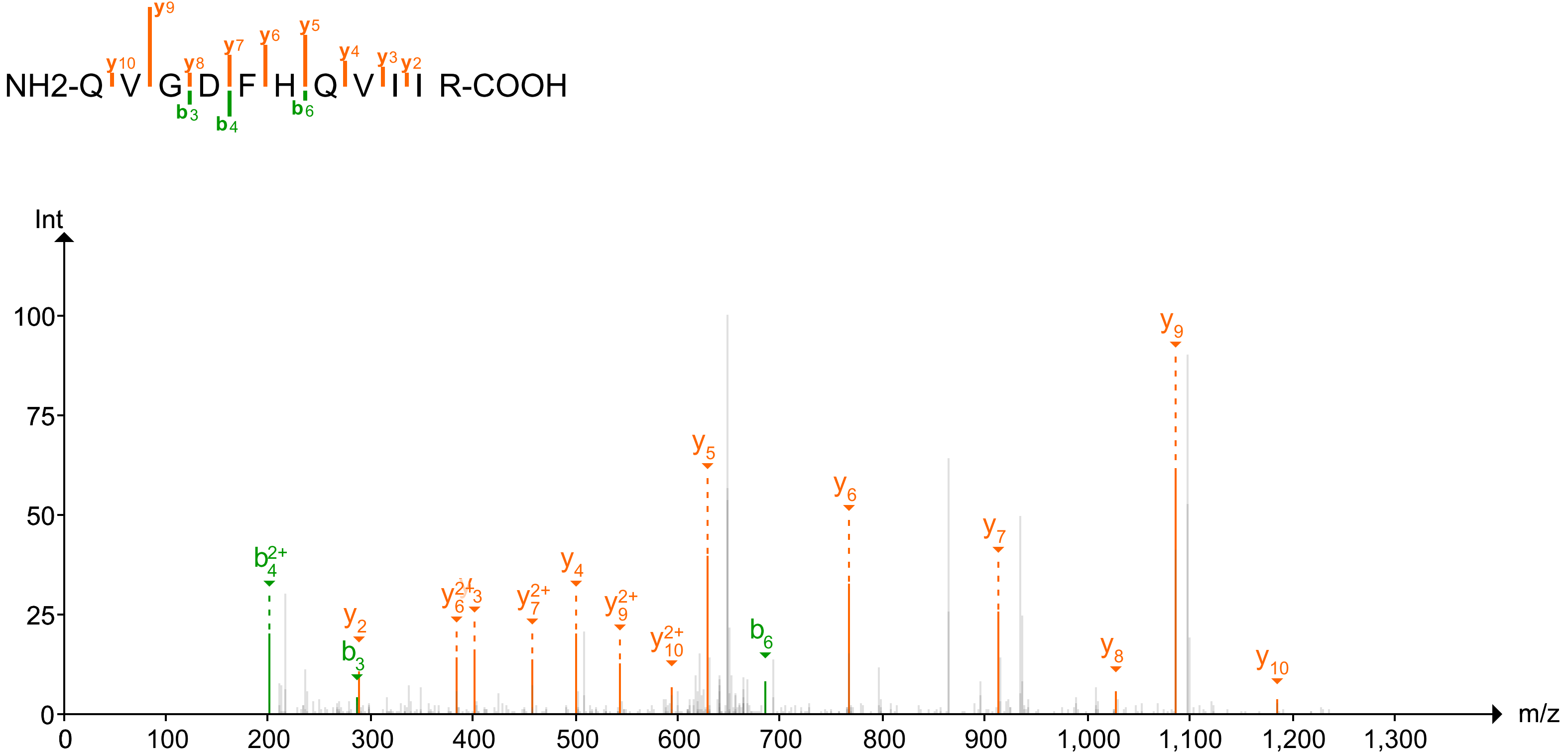

NH2-Q V G D F H Q V I I R-COOH
y10 y9 y8 y7 y6 y5 y4 y3 y2
b3 b4 b6
Int
m/z
0
25
50
75
100
0
100
200
300
400
500
600
700
800
900
1,000
1,100
1,200
1,300

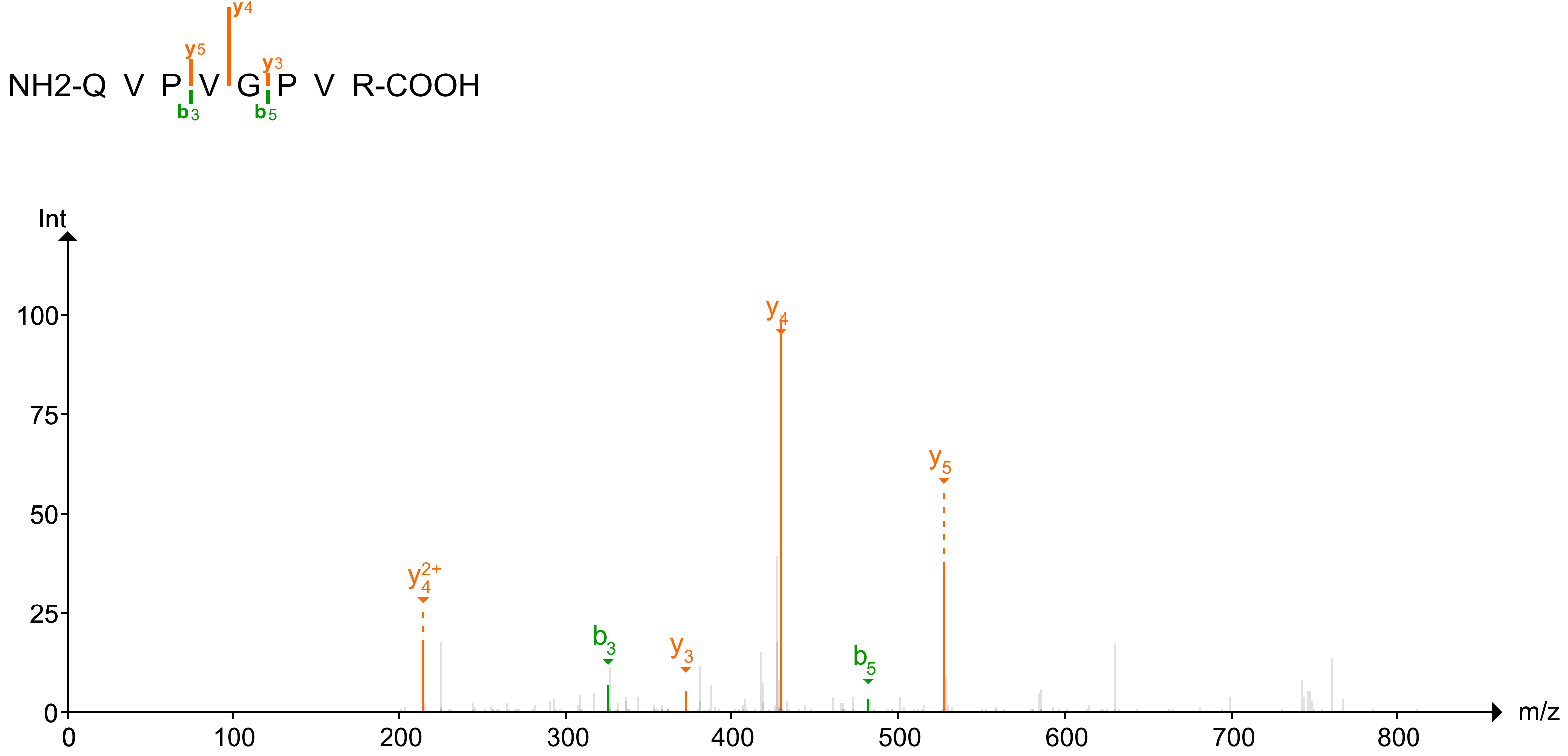

NH2-Q V P V G P V R-COOH
y4
y5
y3
b3
b5
Int
100
75
50
25
0
$y_4^{2+}$
$b_3$
$y_3$
$y_4$
$b_5$
$y_5$
0
100
200
300
400
500
600
700
800
m/z

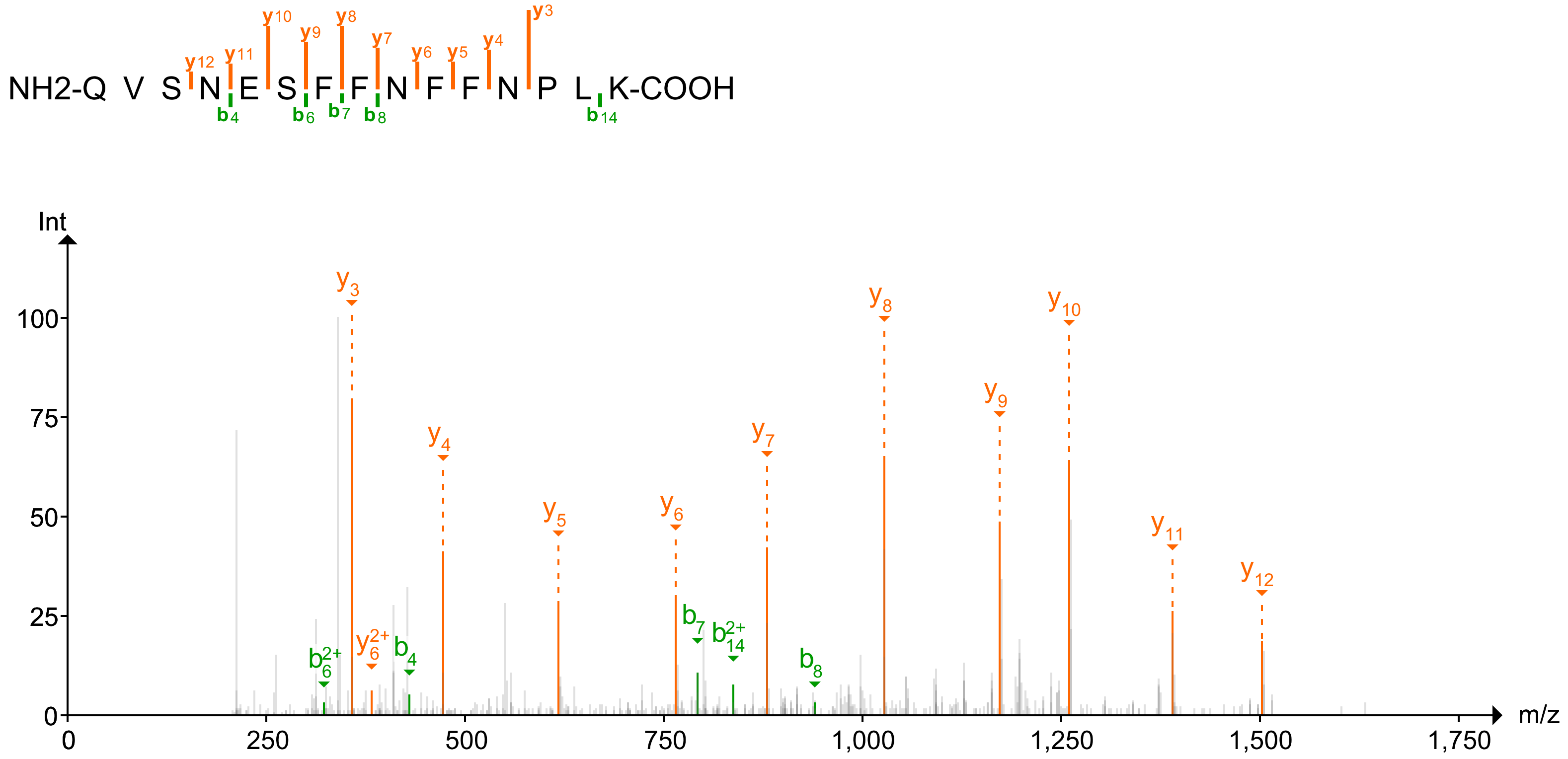

NH2-Q V S N E S F F N F F N P L K-COOH
y12 y11 y10 y9 y8 y7 y6 y5 y4 y3
b4 b6 b7 b8 b14
Int
100
75
50
25
0
m/z
0
250
500
750
1,000
1,250
1,500
1,750
y3 y4 y5 y6 y7 y8 y9 y10 y11 y12
b6 2+ y6 2+ b4 b7 b14 2+ b8

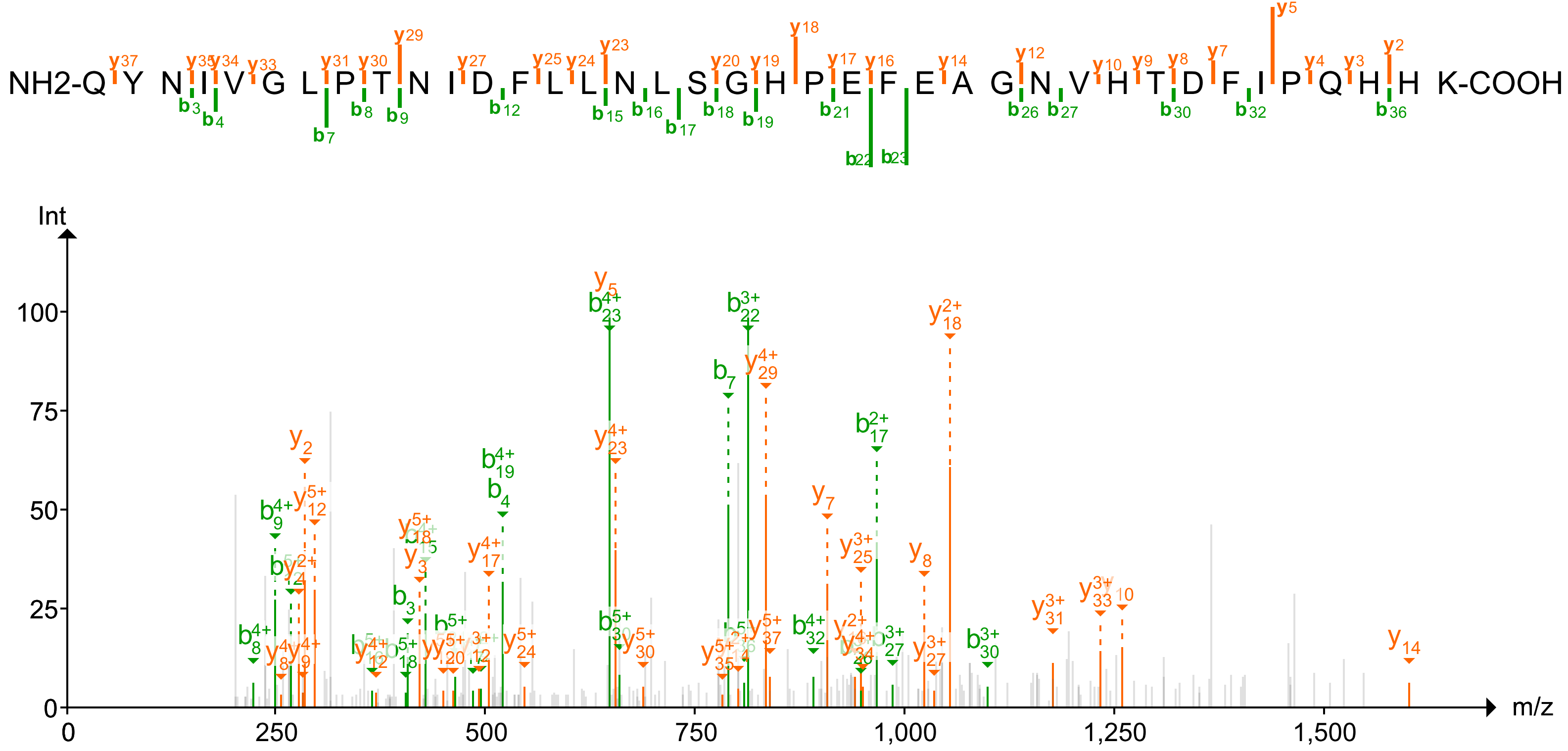

NH2-Q Y N I V G L P T N I D F L L N L S G H P E F E A G N V H T D F I P Q H H K-COOH
Int
m/z
0
25
50
75
100
0
250
500
750
1,000
1,250
1,500

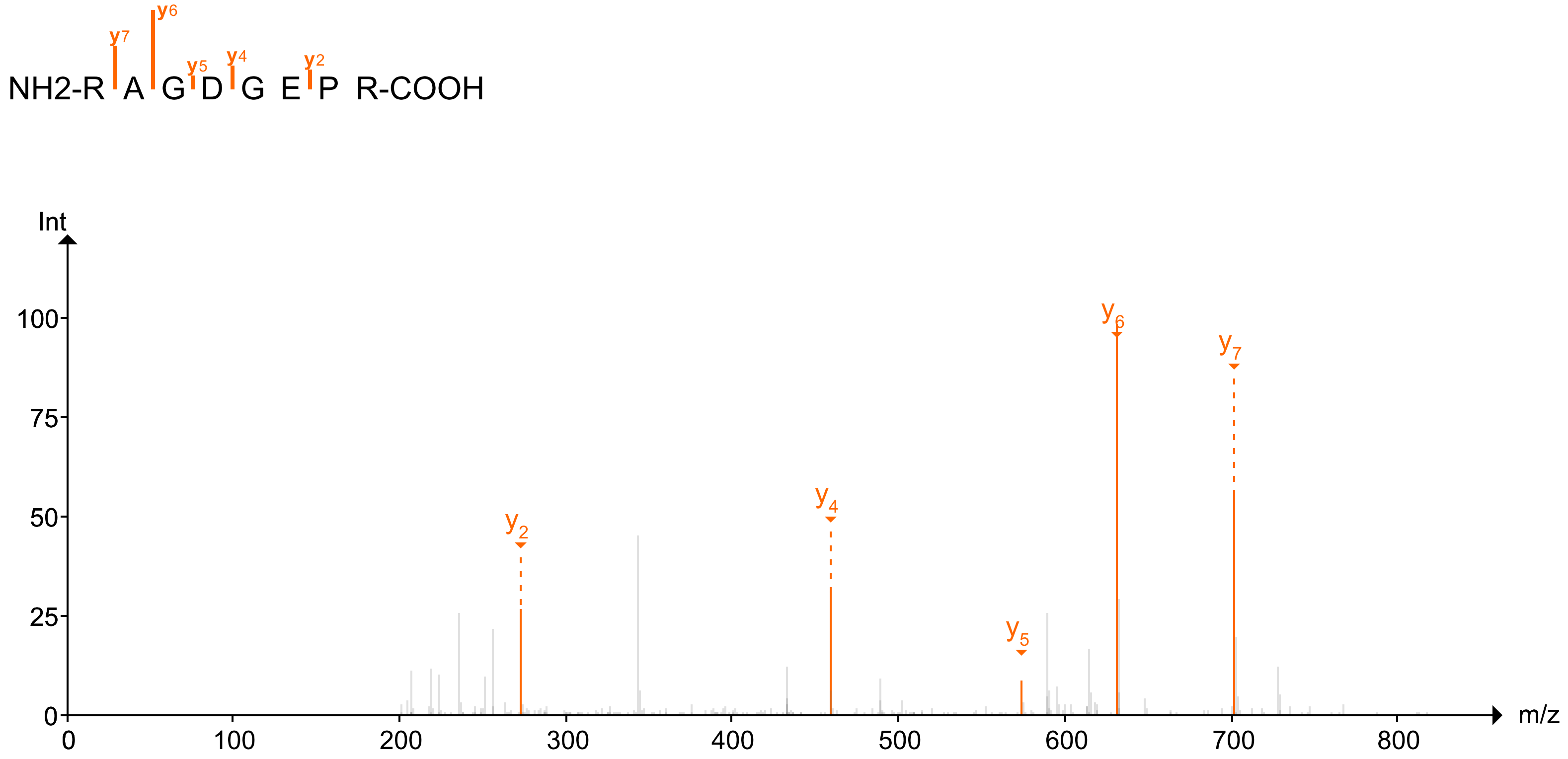

NH2-R A G D G E P R-COOH
y7
y6
y5
y4
y2
Int
100
75
50
25
0
0
100
200
300
400
500
600
700
800
m/z

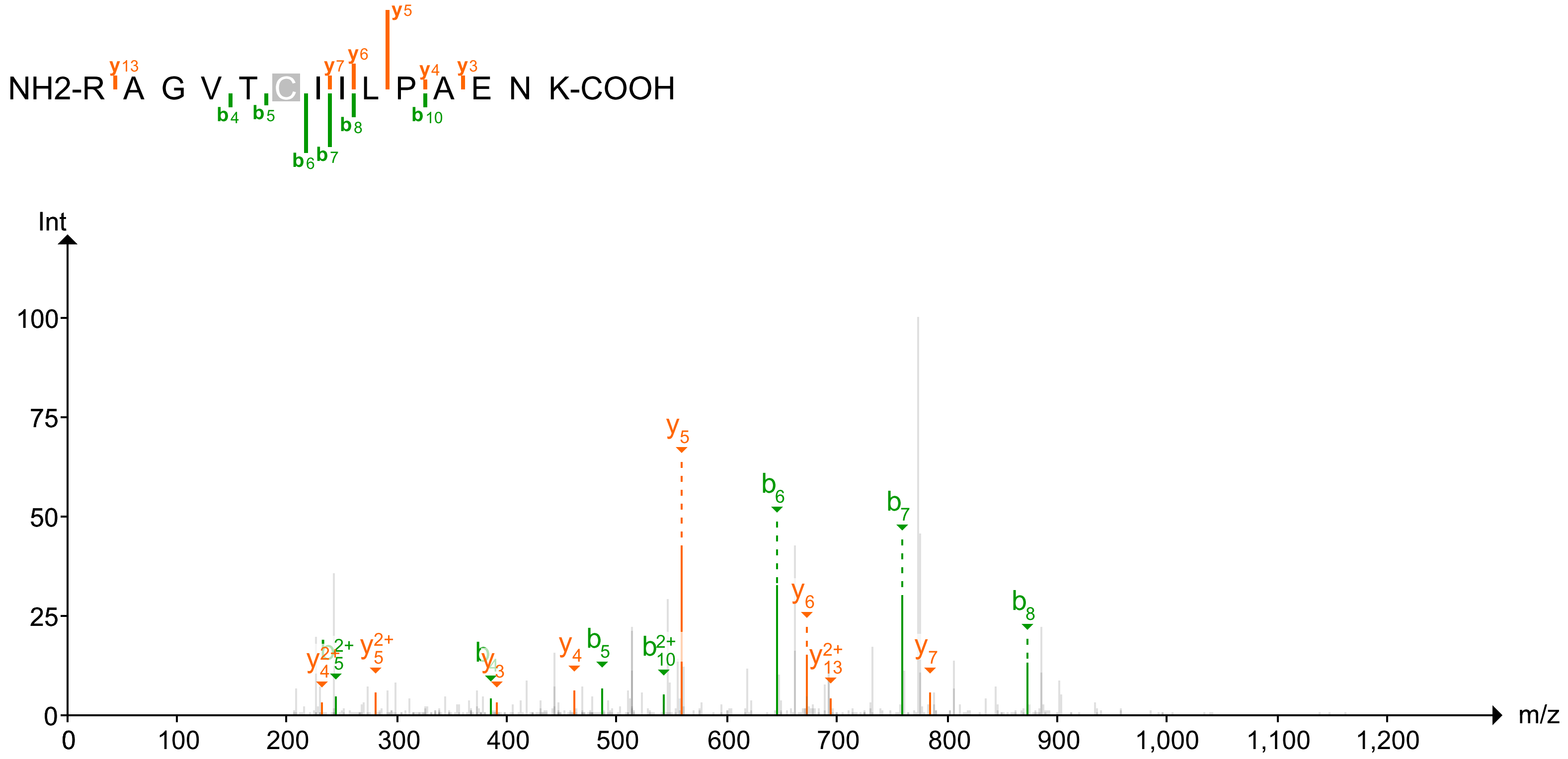

NH2-R A G V T C I I L P A E N K-COOH
y13 y7 y6 y5 y4 y3
b4 b5 b6 b7 b8 b10
Int
m/z
0
25
50
75
100
0
100
200
300
400
500
600
700
800
900
1,000
1,100
1,200
y5
b6
b7
y6
b8
y7
y4
b5
b10 2+
y13 2+
y4 2+
b5 2+
y5 2+
y3
b3

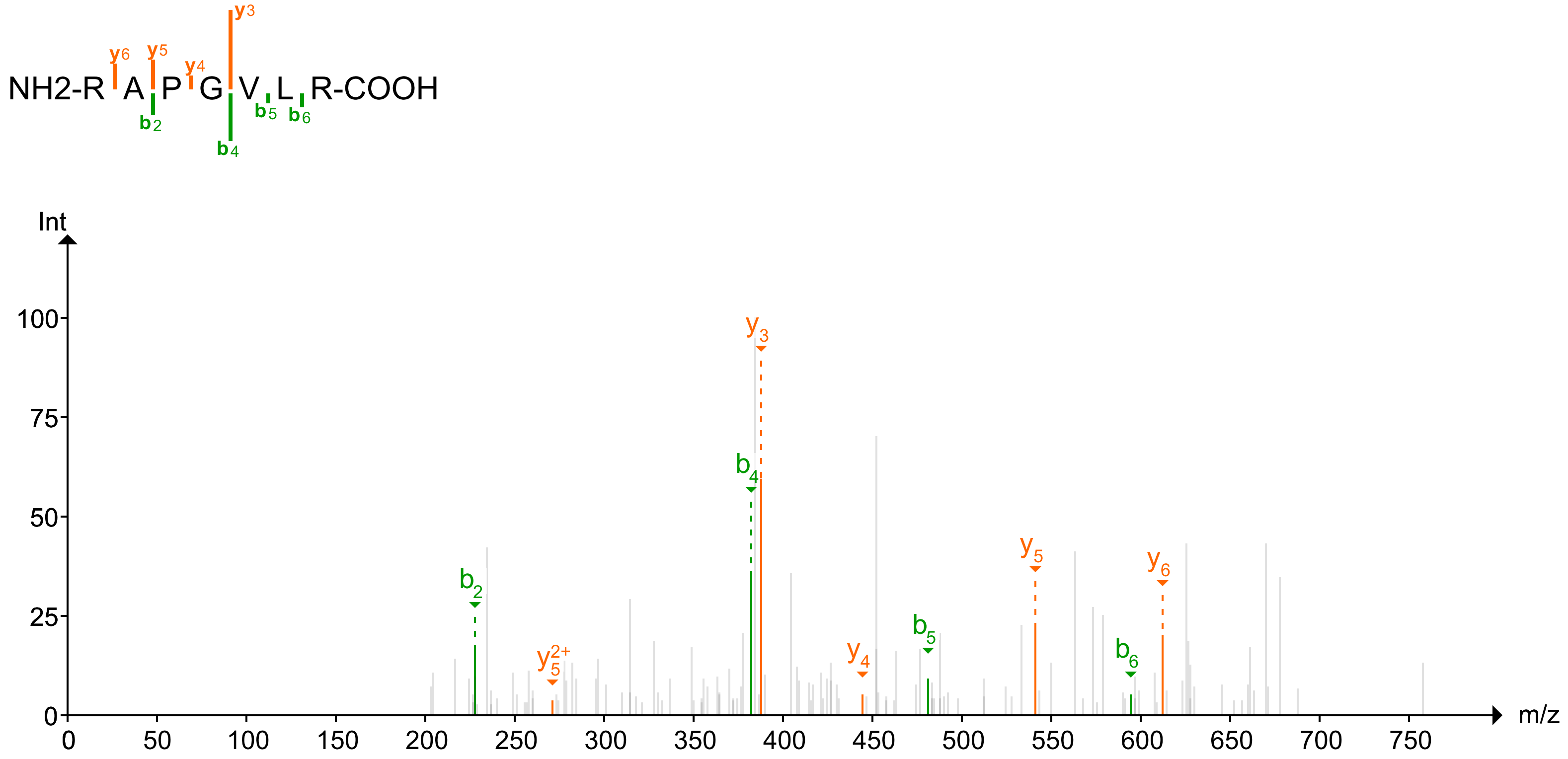

NH2-R A P G V L R-COOH
y6
y5
y4
y3
b2
b4
b5
b6
Int
m/z
0
25
50
75
100
0
50
100
150
200
250
300
350
400
450
500
550
600
650
700
750
$b_2$
$y_5^{2+}$
$b_4$
$y_3$
$y_4$
$b_5$
$y_5$
$b_6$
$y_6$

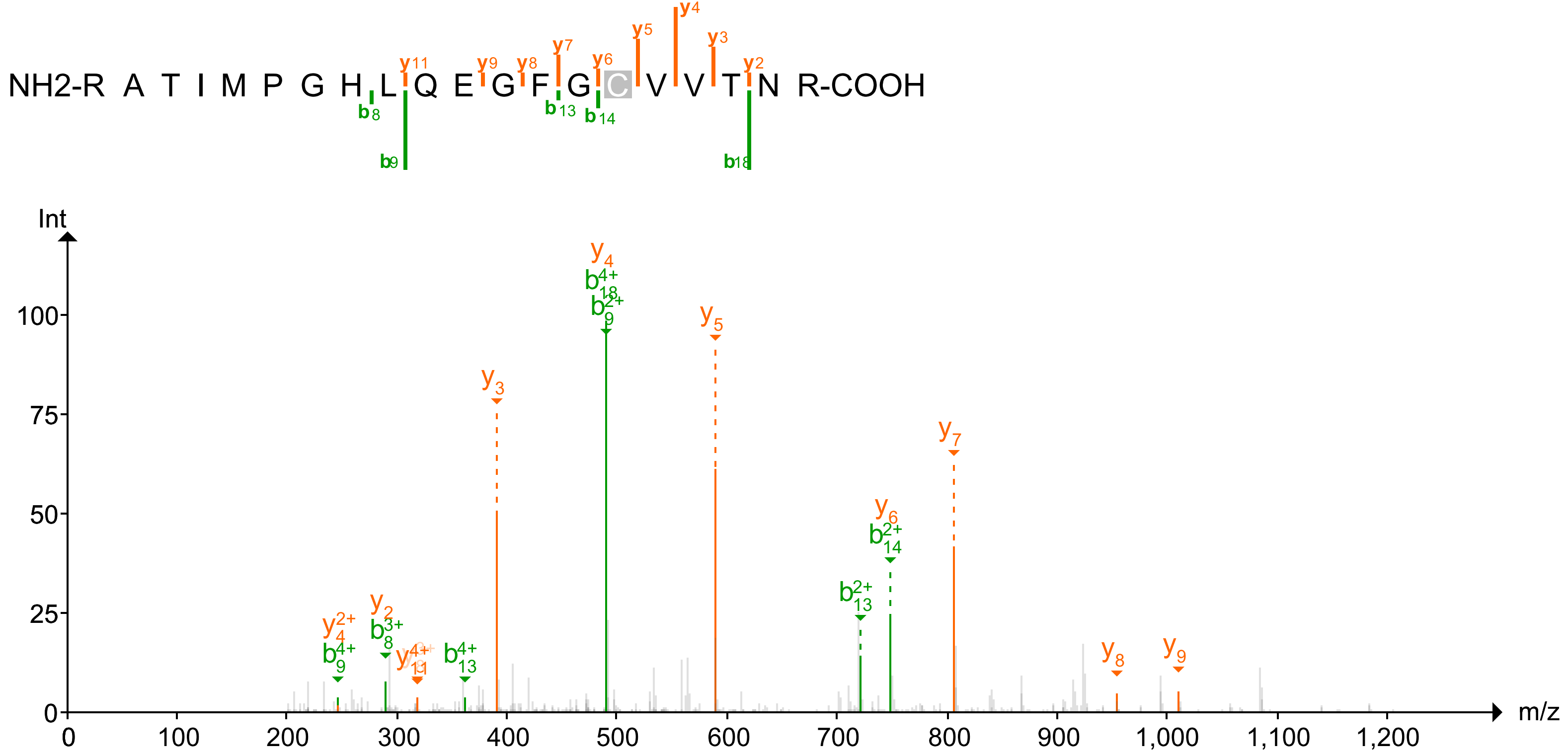

NH2-R A T I M P G H L Q E G F G C V V T N R-COOH
y11 y9 y8 y7 y6 y5 y4 y3 y2
b8 b9 b13 b14 b18
Int
100
75
50
25
0
y4
b4+18
b2+9
y5
y3
y7
y6
b2+14
b2+13
y2+4
b4+9
y2
b3+8
y4+11
b4+13
y8
y9
0
100
200
300
400
500
600
700
800
900
1,000
1,100
1,200
m/z

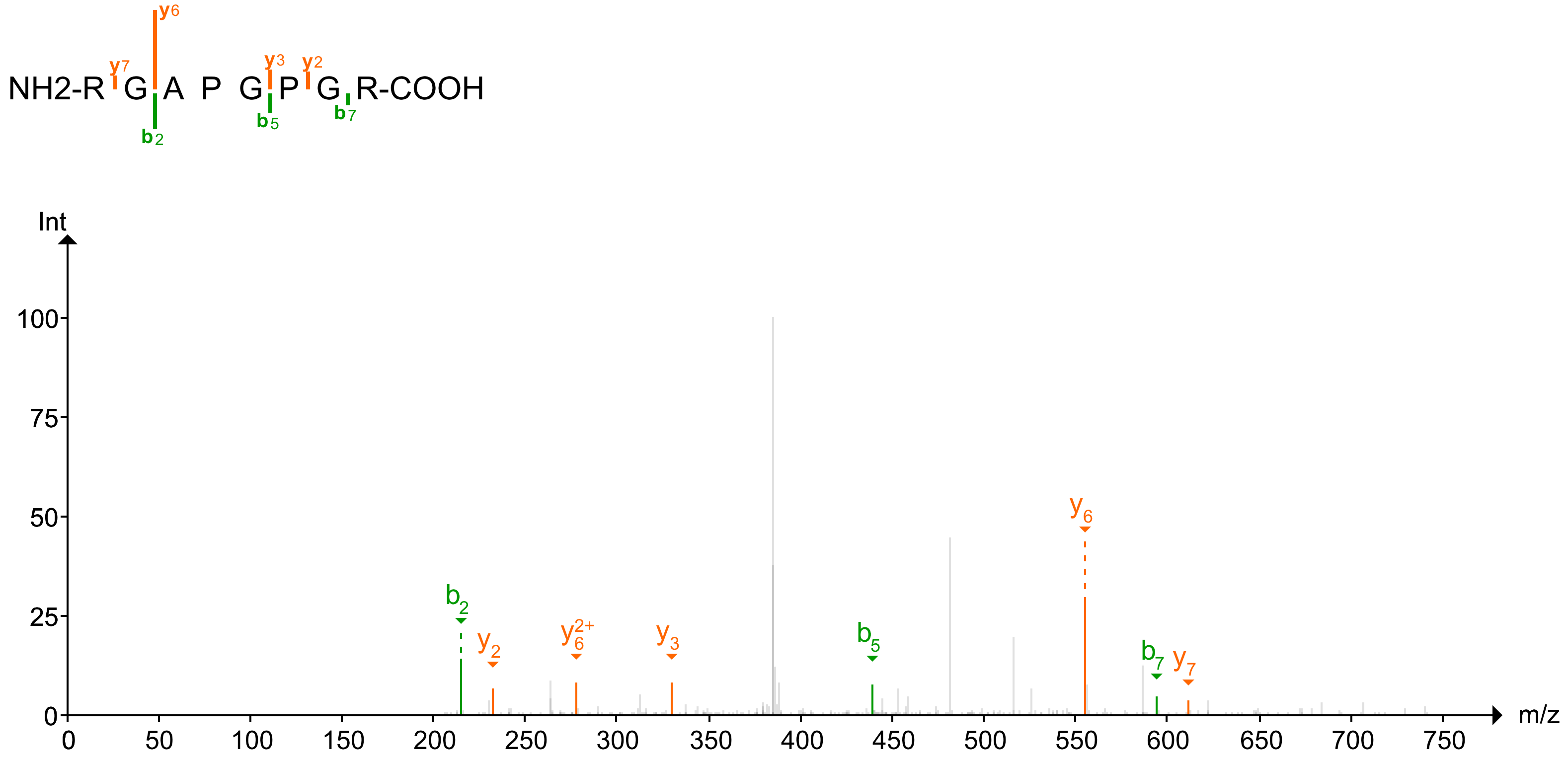
NH2-R G A P G P G R-COOH
y6
y7
y3
y2
b2
b5
b7
Int
100
75
50
25
0
0
50
100
150
200
250
300
350
400
450
500
550
600
650
700
750
m/z
b2
y2
y6 2+
y3
b5
y6
b7
y7

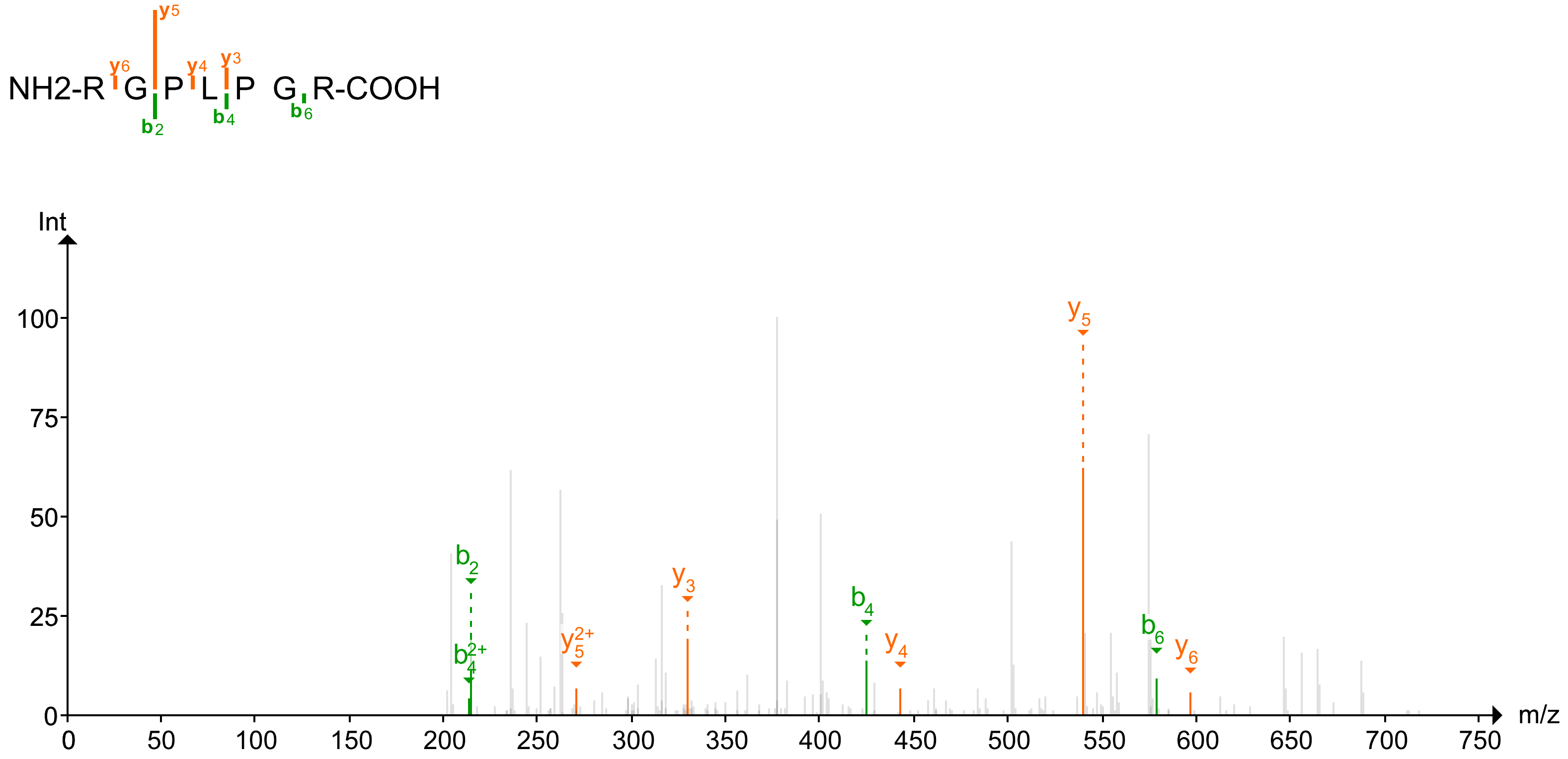

NH2-R G P L P G R-COOH
y6 y5 y4 y3
b2 b4 b6
Int
100
75
50
25
0
m/z
0 50 100 150 200 250 300 350 400 450 500 550 600 650 700 750
b2
b4 2+
y5 2+
y3
b4
y4
y5
b6
y6

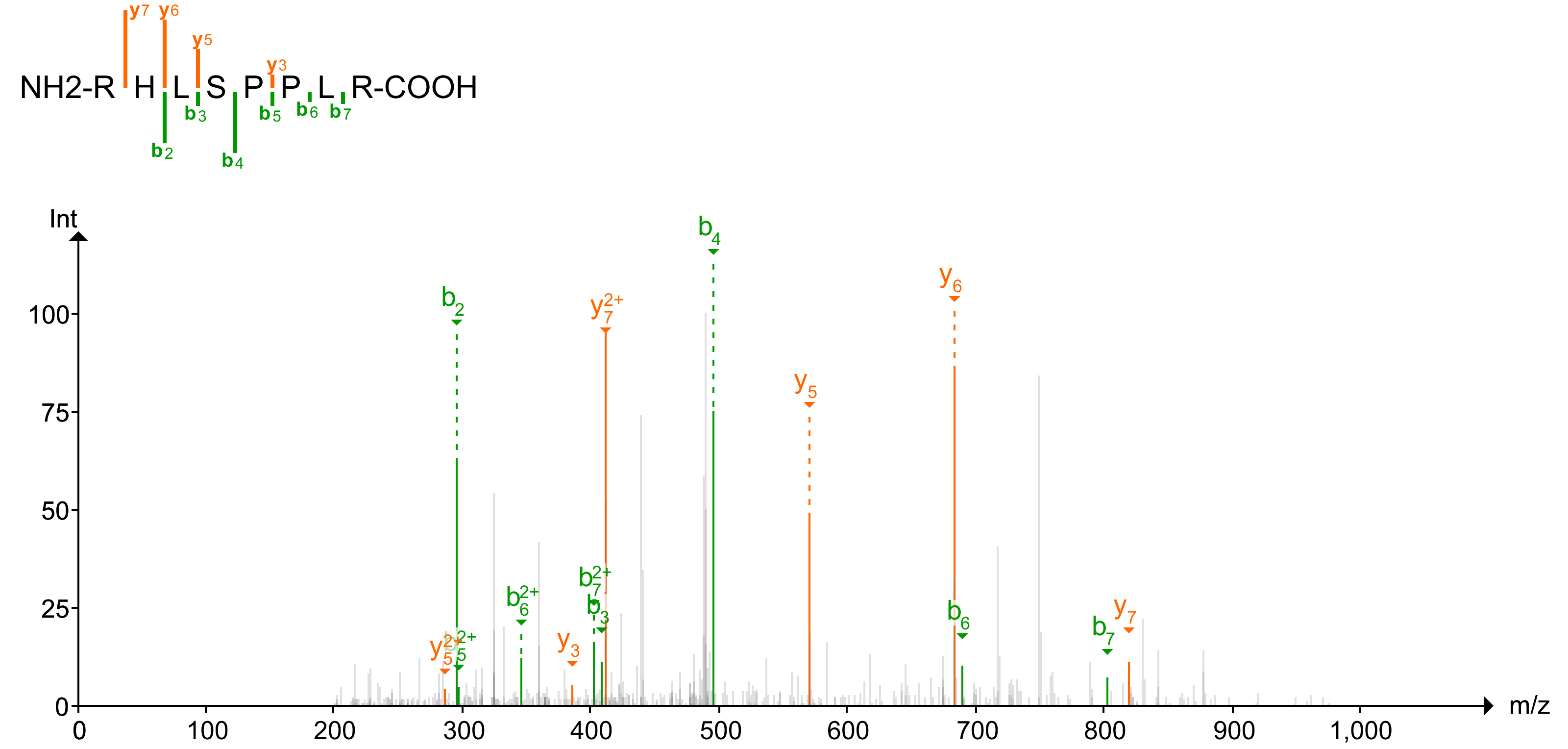

NH2-R H L S P P L R-COOH
y7 y6 y5 y3
b2 b3 b4 b5 b6 b7
Int
m/z
0
25
50
75
100
0
100
200
300
400
500
600
700
800
900
1,000
b4
y6
b2
y7 2+
y5
b7 2+
b6 2+
b3
y3
y5 2+
b5 2+
b6
y7
b7

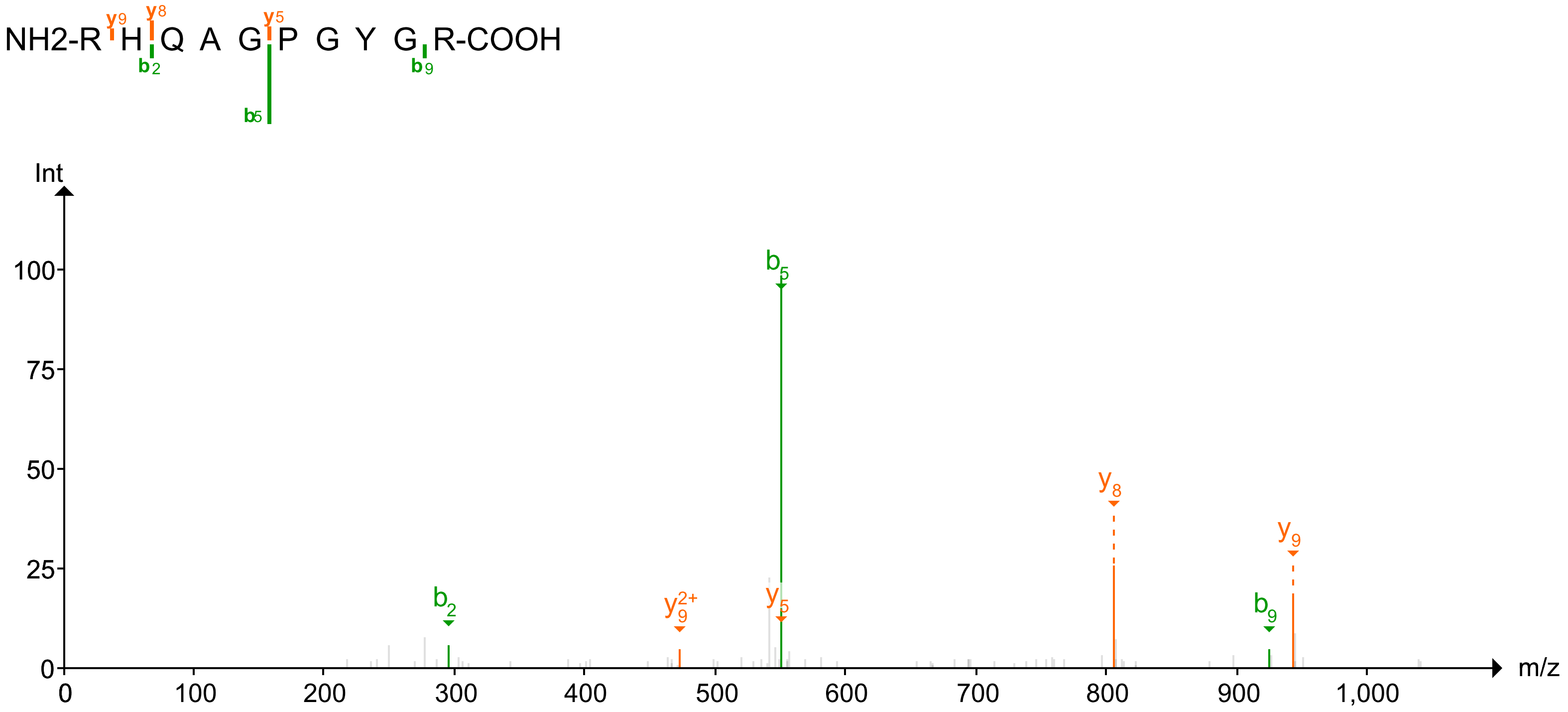

NH2-R H Q A G P G Y G R-COOH
y9
y8
y5
b2
b5
b9
Int
100
75
50
25
0
m/z
0
100
200
300
400
500
600
700
800
900
1,000
b5
y8
y9
b2
y9 2+
y5
b9

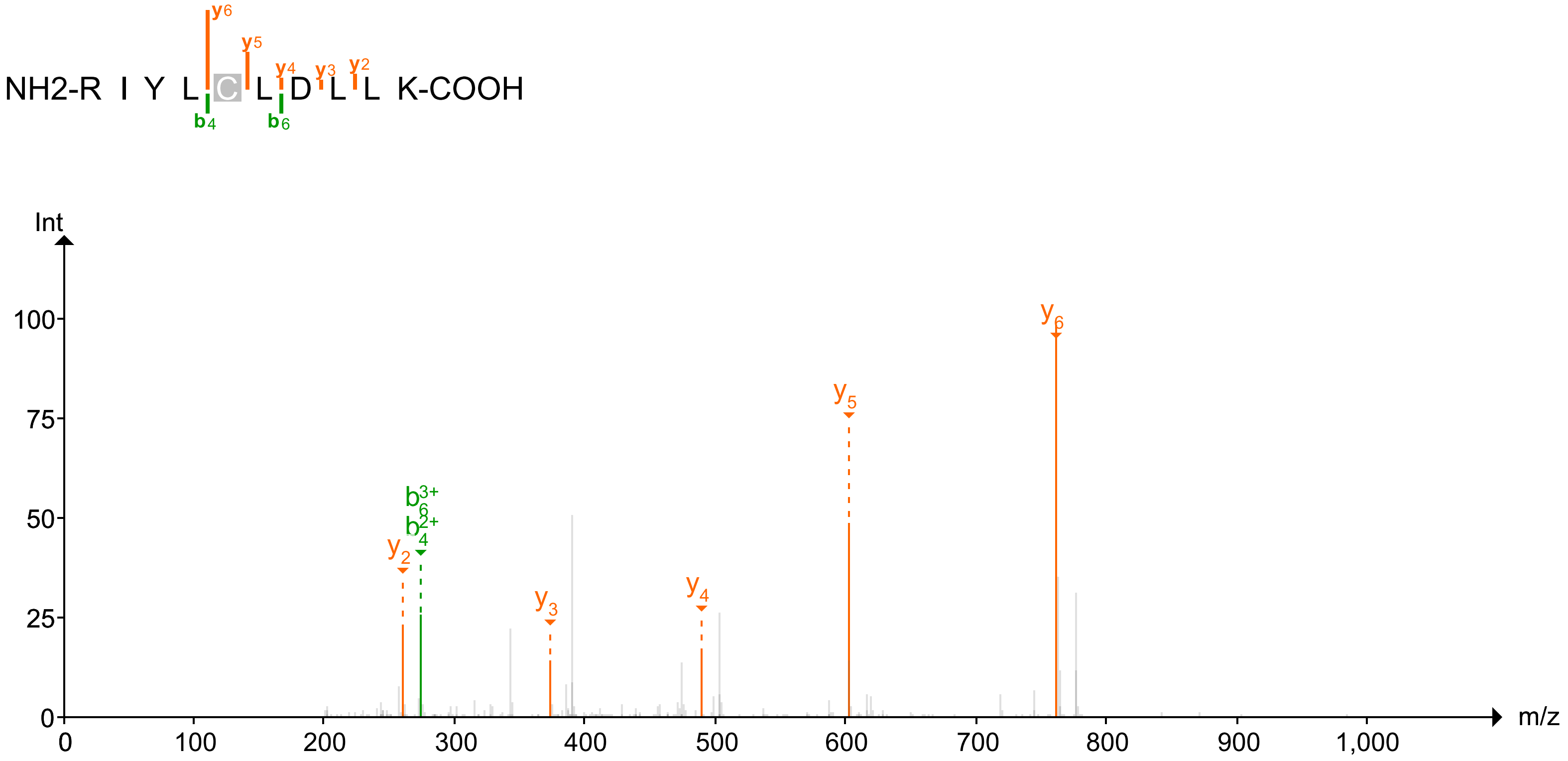

NH2-R I Y L C L D L L K-COOH
y6
y5
y4
y3
y2
b4
b6
Int
100
75
50
25
0
m/z
0
100
200
300
400
500
600
700
800
900
1,000
y6
y5
y4
y3
y2
b6 3+
b4 2+

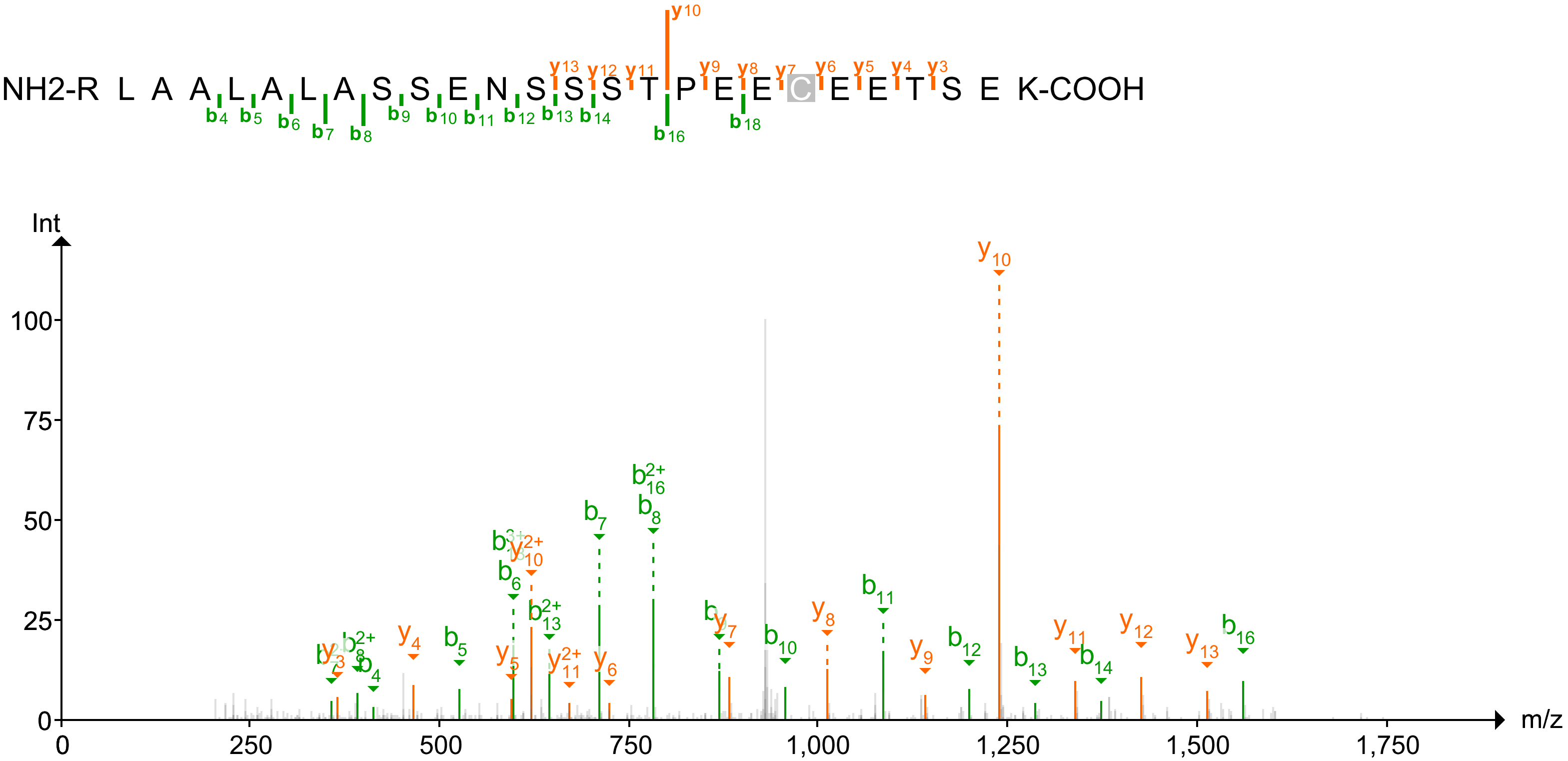

NH2-R L A A L A L A S S E N S S S T P E E C E E T S E K-COOH
y13 y12 y11 y10 y9 y8 y7 y6 y5 y4 y3
b4 b5 b6 b7 b8 b9 b10 b11 b12 b13 b14 b16 b18
Int
100
75
50
25
0
m/z
0
250
500
750
1,000
1,250
1,500
1,750
y10
b16 2+
b8
b7
b6
y10 2+
b13 2+
b11
y8
y7
b10
y9
b12
y11
y12
y13
b16
b13
b14
y4
b5
y5
y11 2+
y6
b4
b8 2+
y3

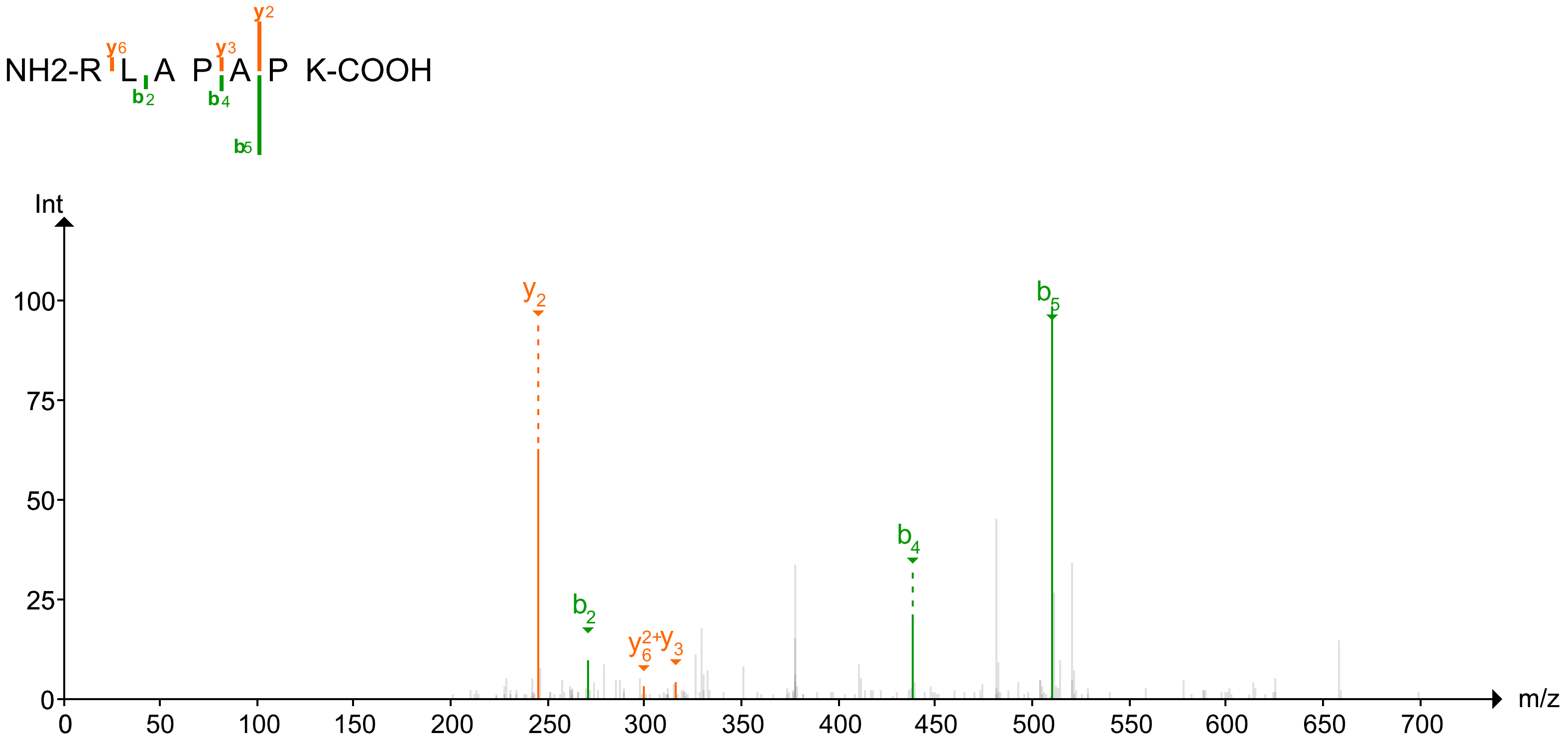

NH2-R L A P A P K-COOH
y6
y3
y2
b2
b4
b5
Int
100
75
50
25
0
y2
b2
y6 2+ y3
b4
b5
0
50
100
150
200
250
300
350
400
450
500
550
600
650
700
m/z

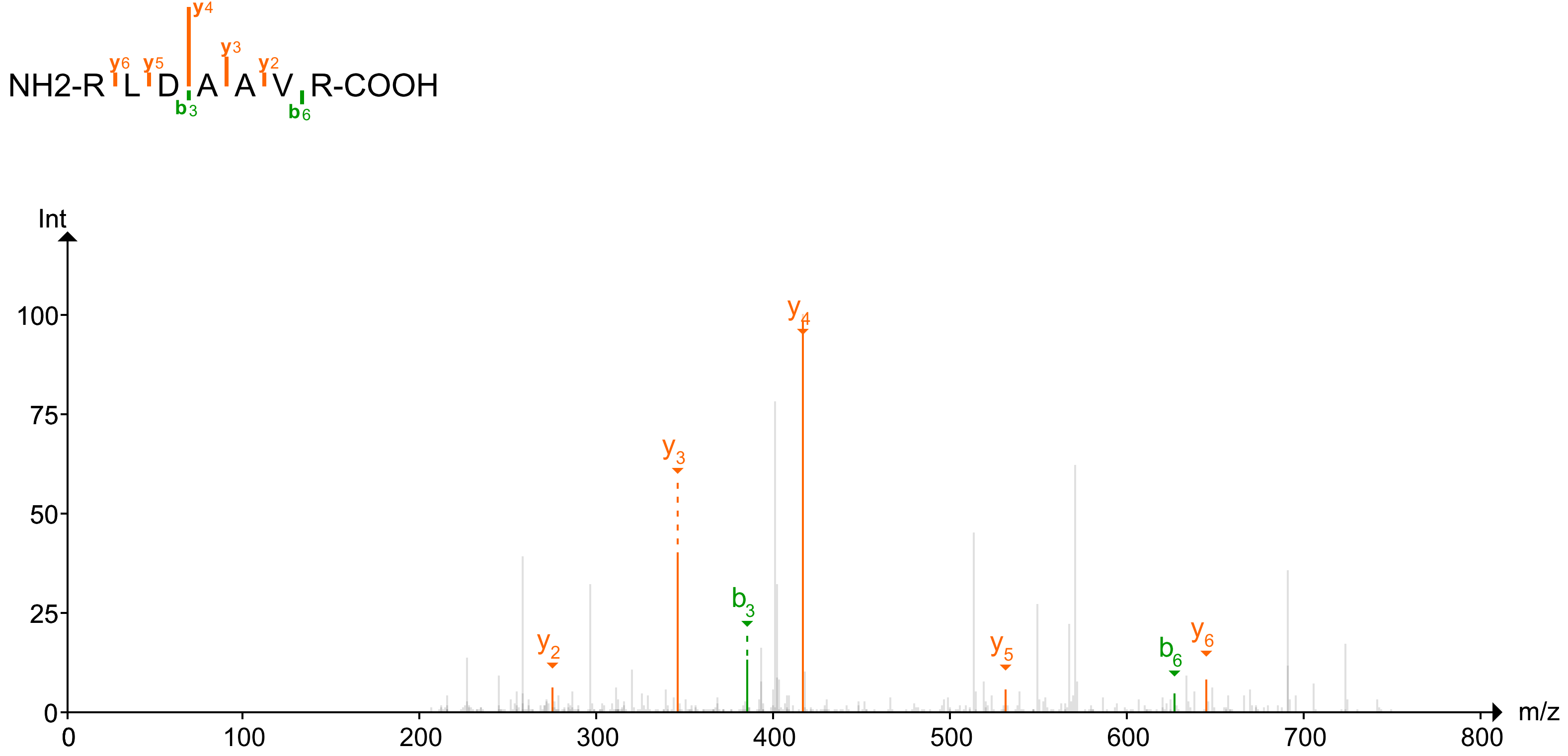

NH2-R L D A A V R-COOH
y6 y5 y4 y3 y2
b3 b6
Int
100
75
50
25
0
m/z
0 100 200 300 400 500 600 700 800
y2 y3 b3 y4 y5 b6 y6

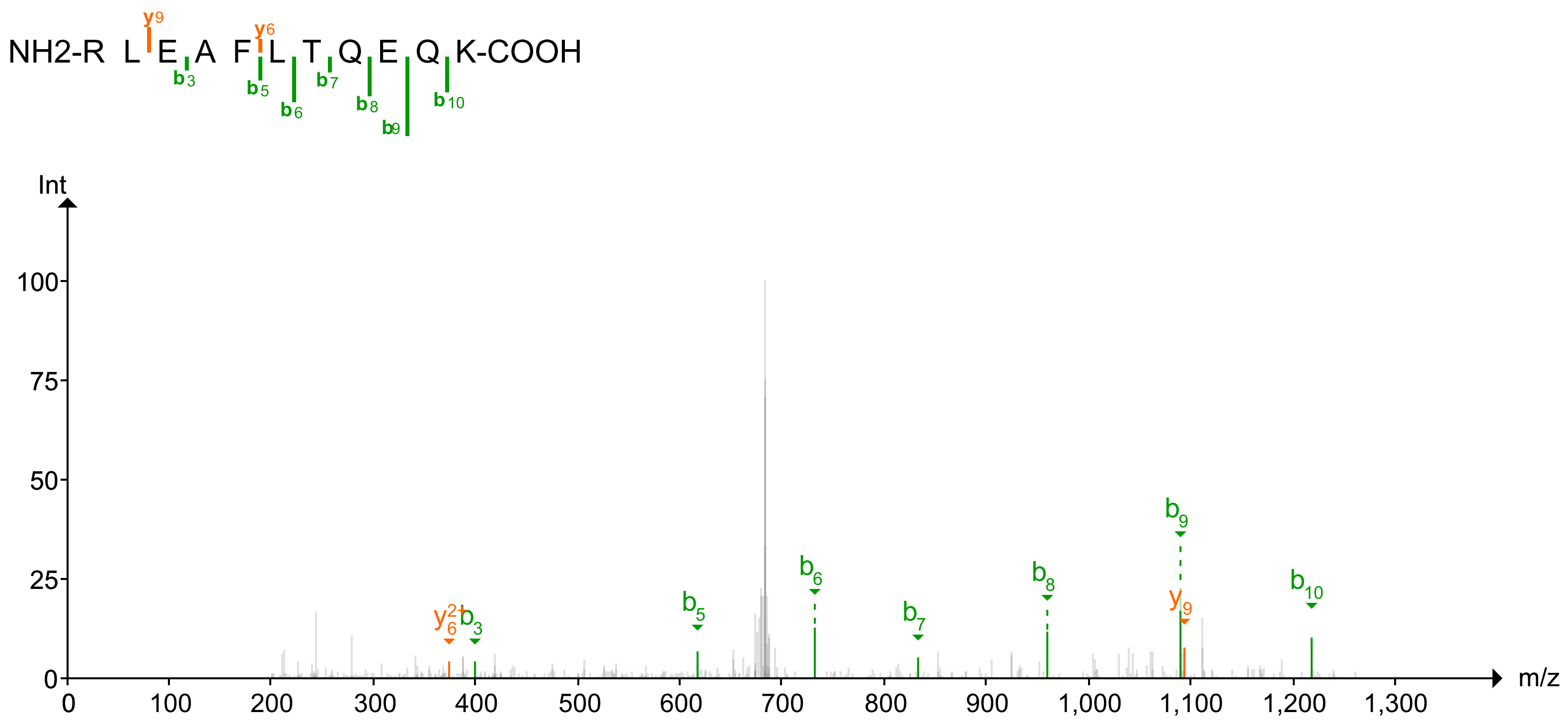

NH2-R L E A F L T Q E Q K-COOH
y9
y6
b3
b5
b6
b7
b8
b9
b10
Int
100
75
50
25
0
m/z
0
100
200
300
400
500
600
700
800
900
1,000
1,100
1,200
1,300
y6 2+
b3
b5
b6
b7
b8
b9
y9
b10

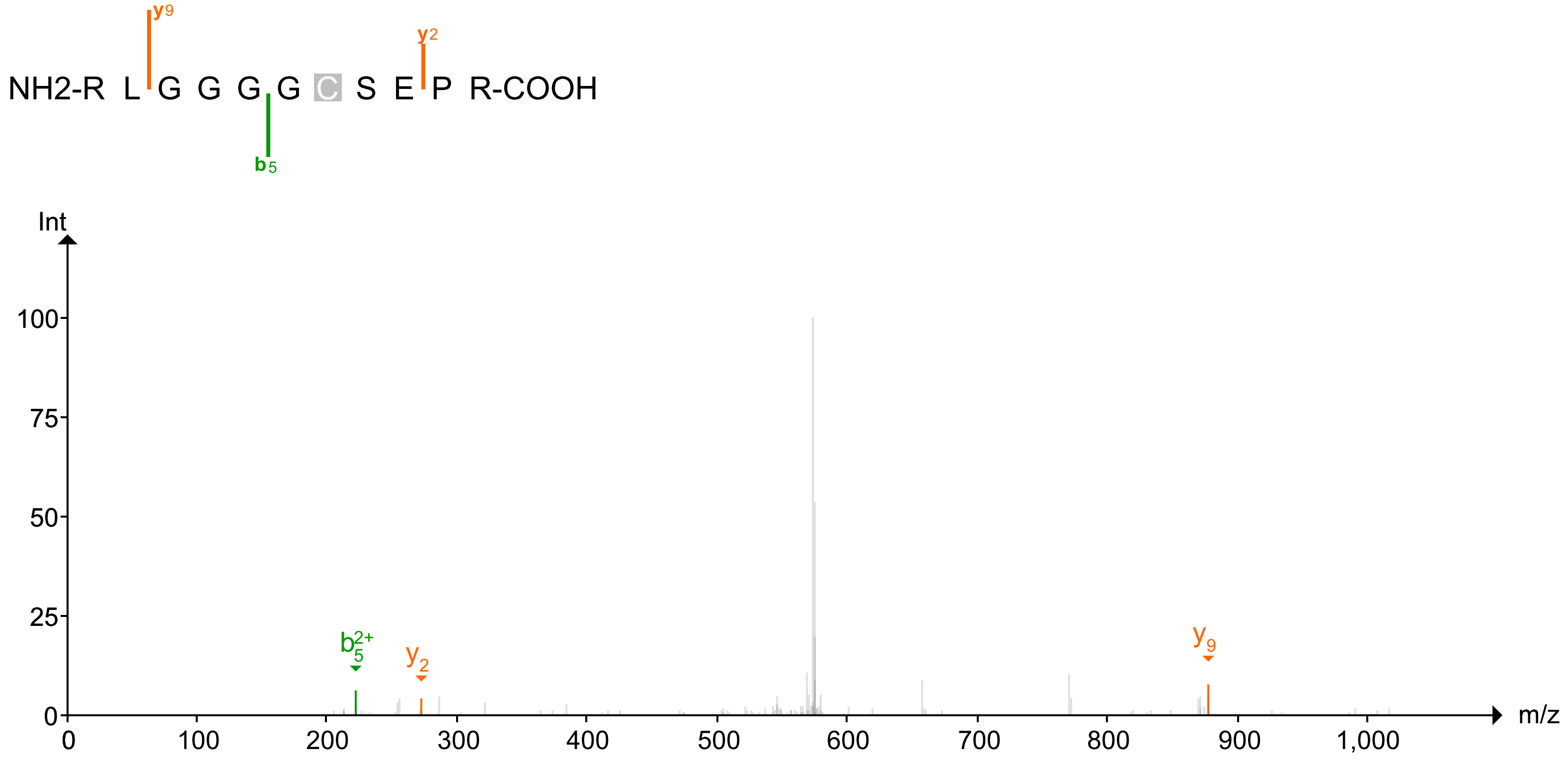

NH2-R L G G G G C S E P R-COOH
y9
y2
b5
Int
100
75
50
25
0
$b_5^{2+}$
$y_2$
$y_9$
0
100
200
300
400
500
600
700
800
900
1,000
m/z

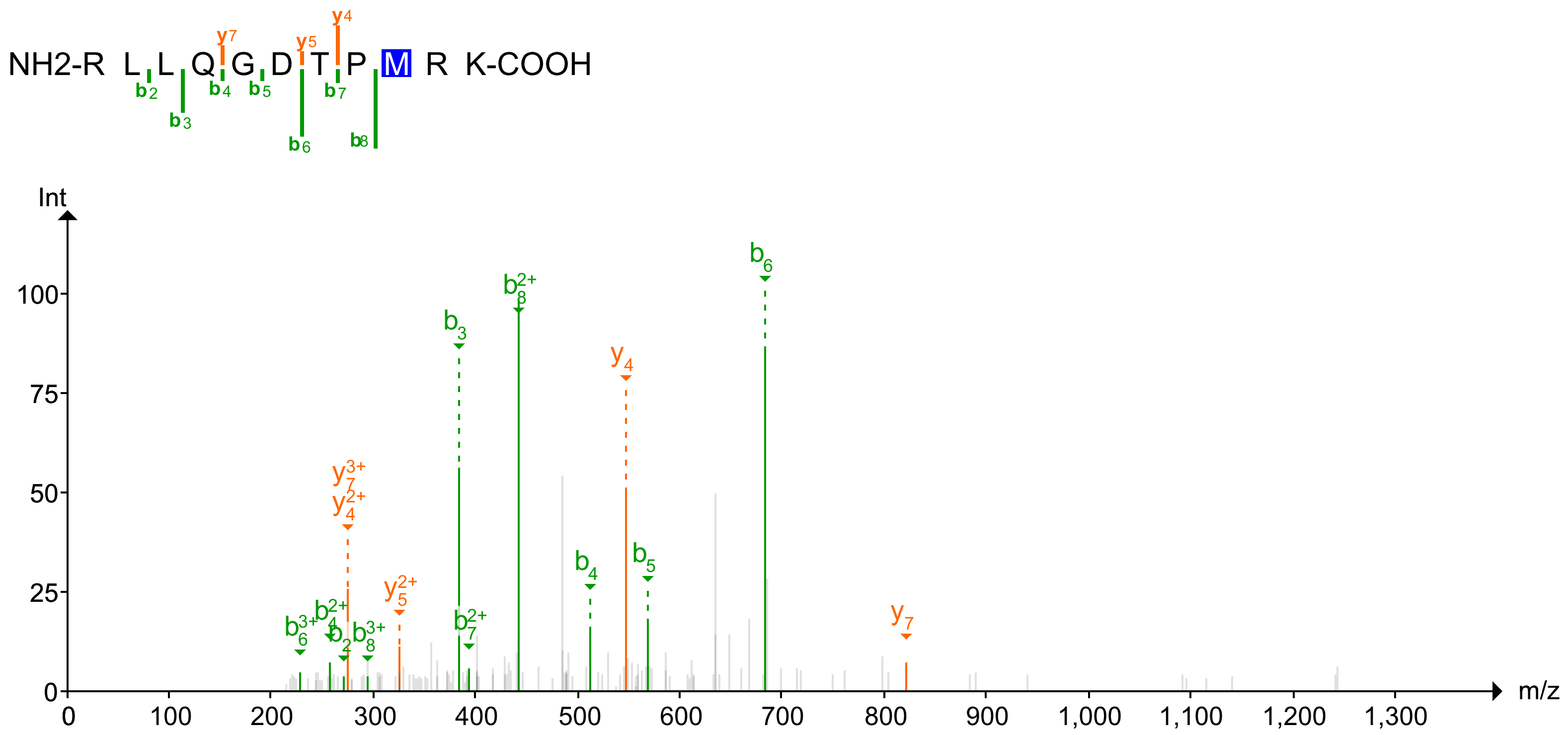

NH2-R L L Q G D T P M R K-COOH
Int
m/z

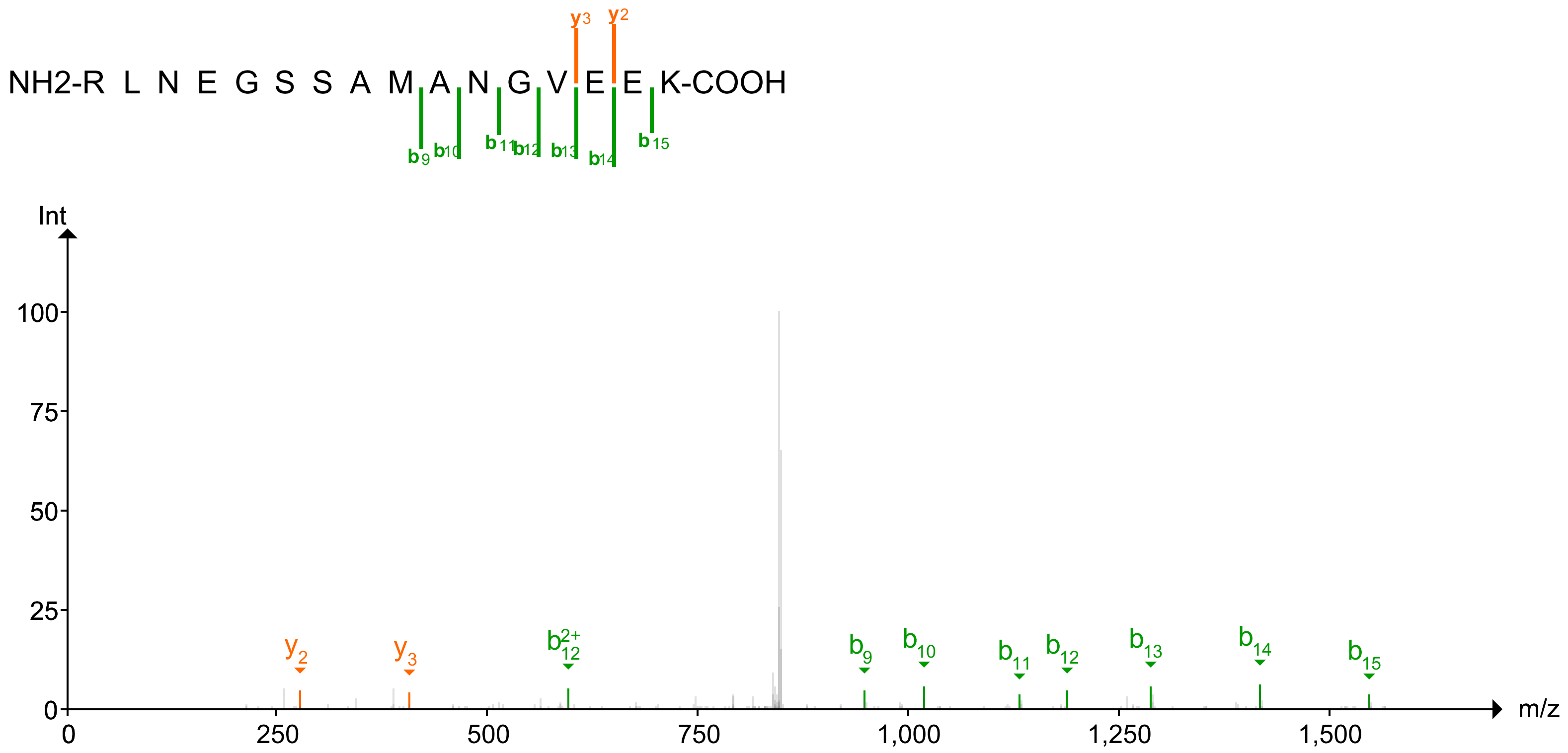

NH2-R L N E G S S A M A N G V E E K-COOH
y3 y2
b9 b10 b11 b12 b13 b14 b15
Int
100
75
50
25
0
y2 y3 b12 2+ b9 b10 b11 b12 b13 b14 b15
0 250 500 750 1,000 1,250 1,500
m/z

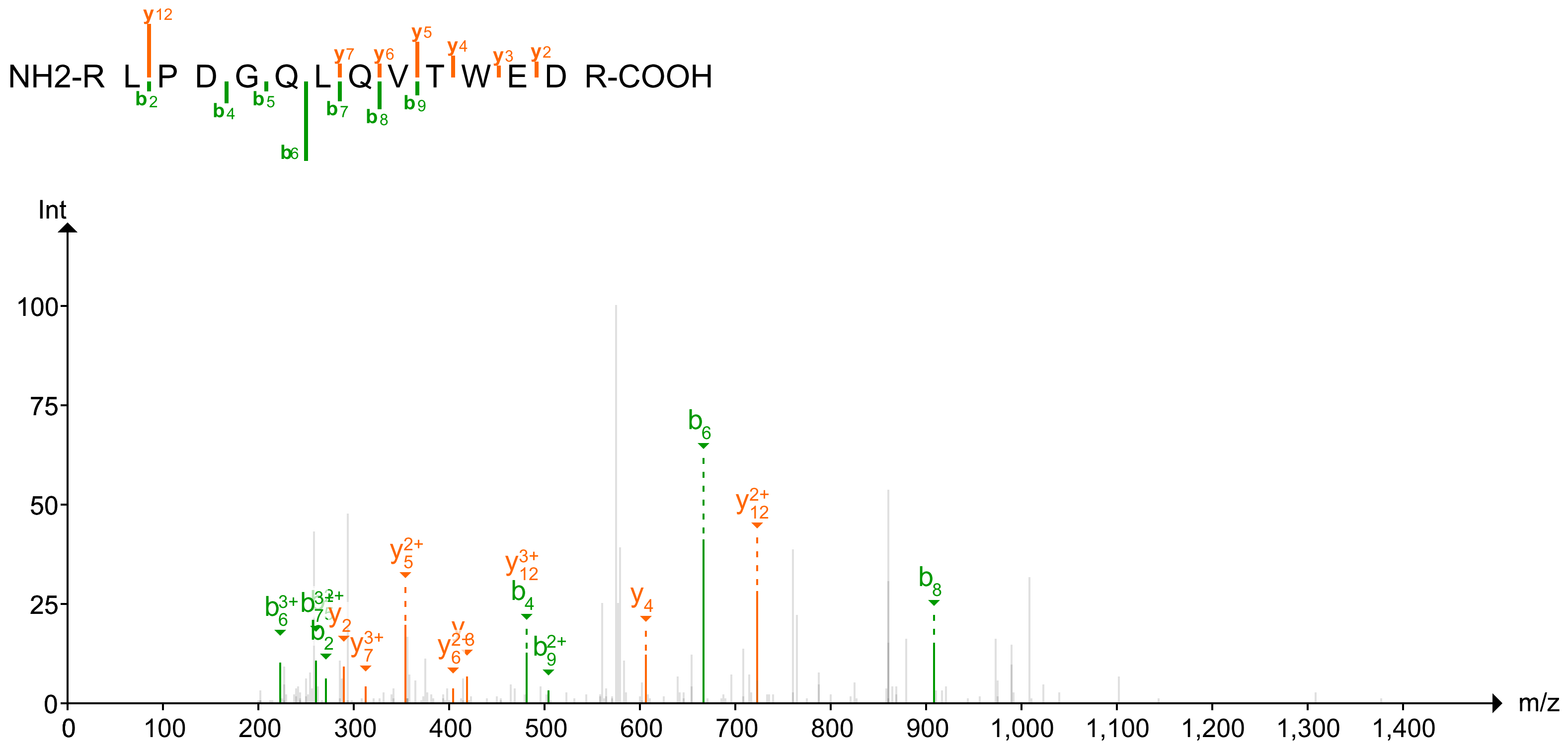
NH2-R L P D G Q L Q V T W E D R-COOH
Int
m/z
0
25
50
75
100
0
100
200
300
400
500
600
700
800
900
1,000
1,100
1,200
1,300
1,400

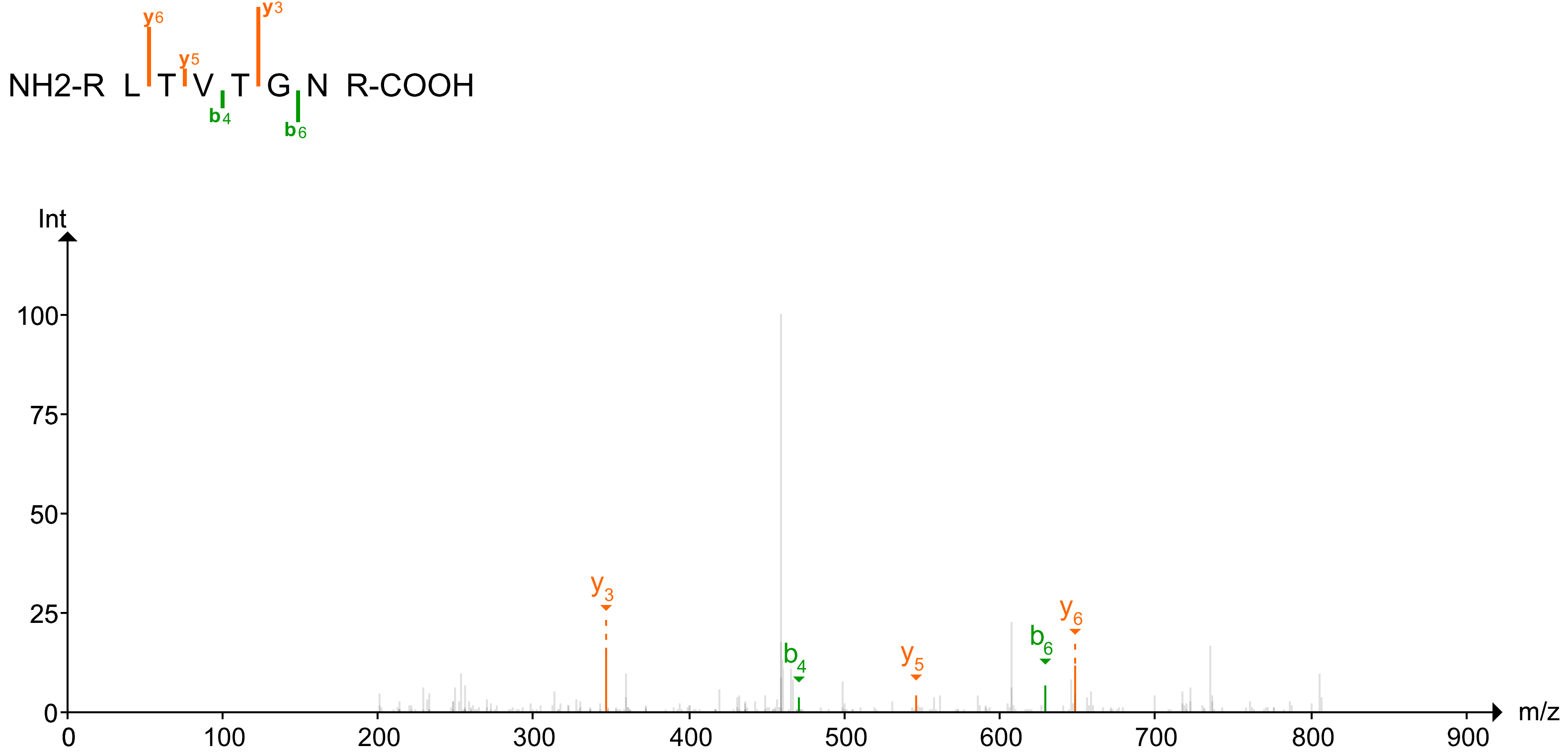

NH2-R L T V T G N R-COOH
y6
y5
y3
b4
b6
Int
100
75
50
25
0
m/z
0
100
200
300
400
500
600
700
800
900
y3
b4
y5
b6
y6

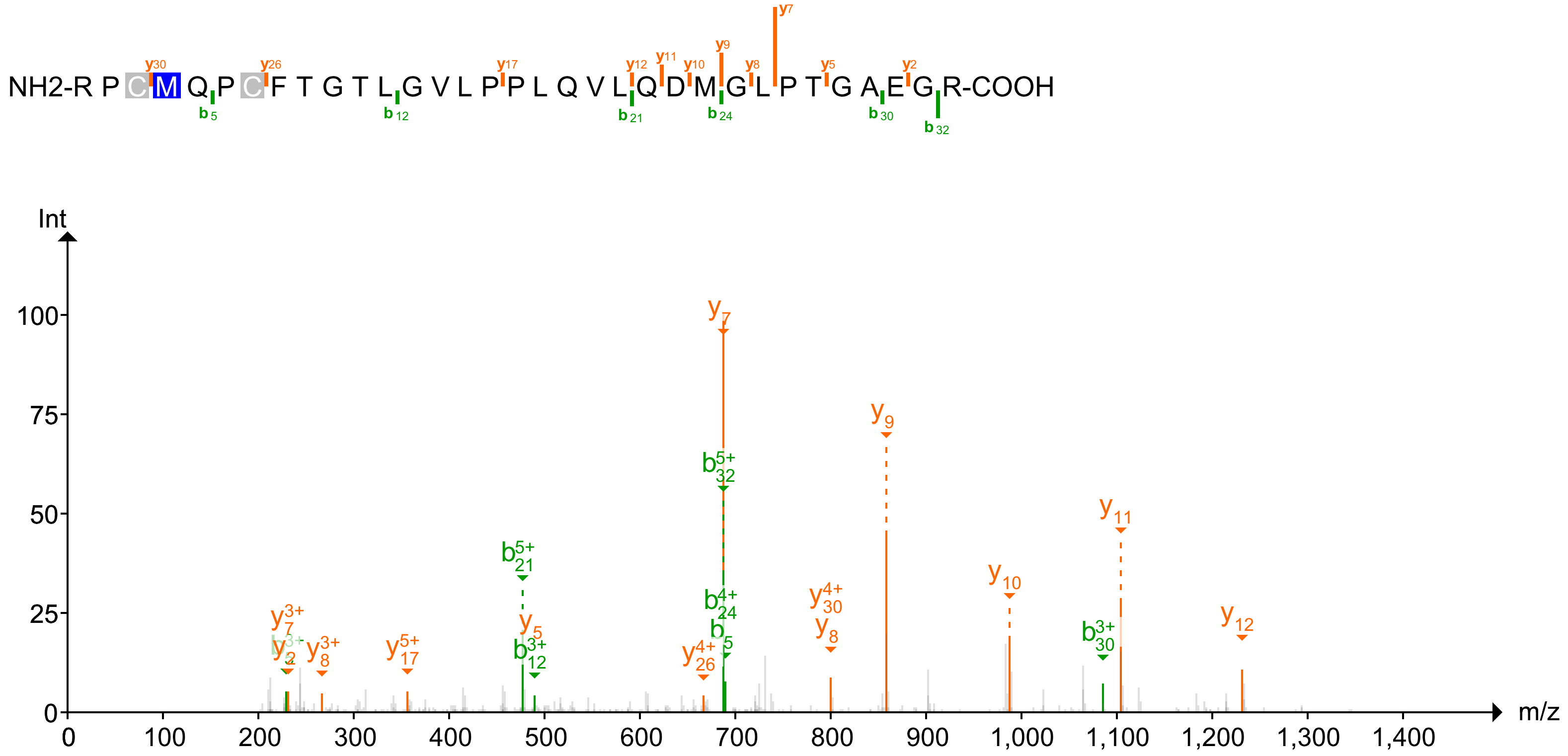
NH2-R P C M Q P C F T G T L G V L P P L Q V L Q D M G L P T G A E G R-COOH
y30 y26 y17 y12 y11 y10 y9 y8 y7 y5 y2
b 5 b 12 b 21 b 24 b 30 b 32
Int
100
75
50
25
0
m/z
0 100 200 300 400 500 600 700 800 900 1,000 1,100 1,200 1,300 1,400
y7
y9
b32 5+
y11
b21 5+
y10
b24 4+
y30 4+
y12
y7 3+
y5
b5
y8
b30 3+
y2 3+
y8 3+
y17 5+
b12 3+
y26 4+

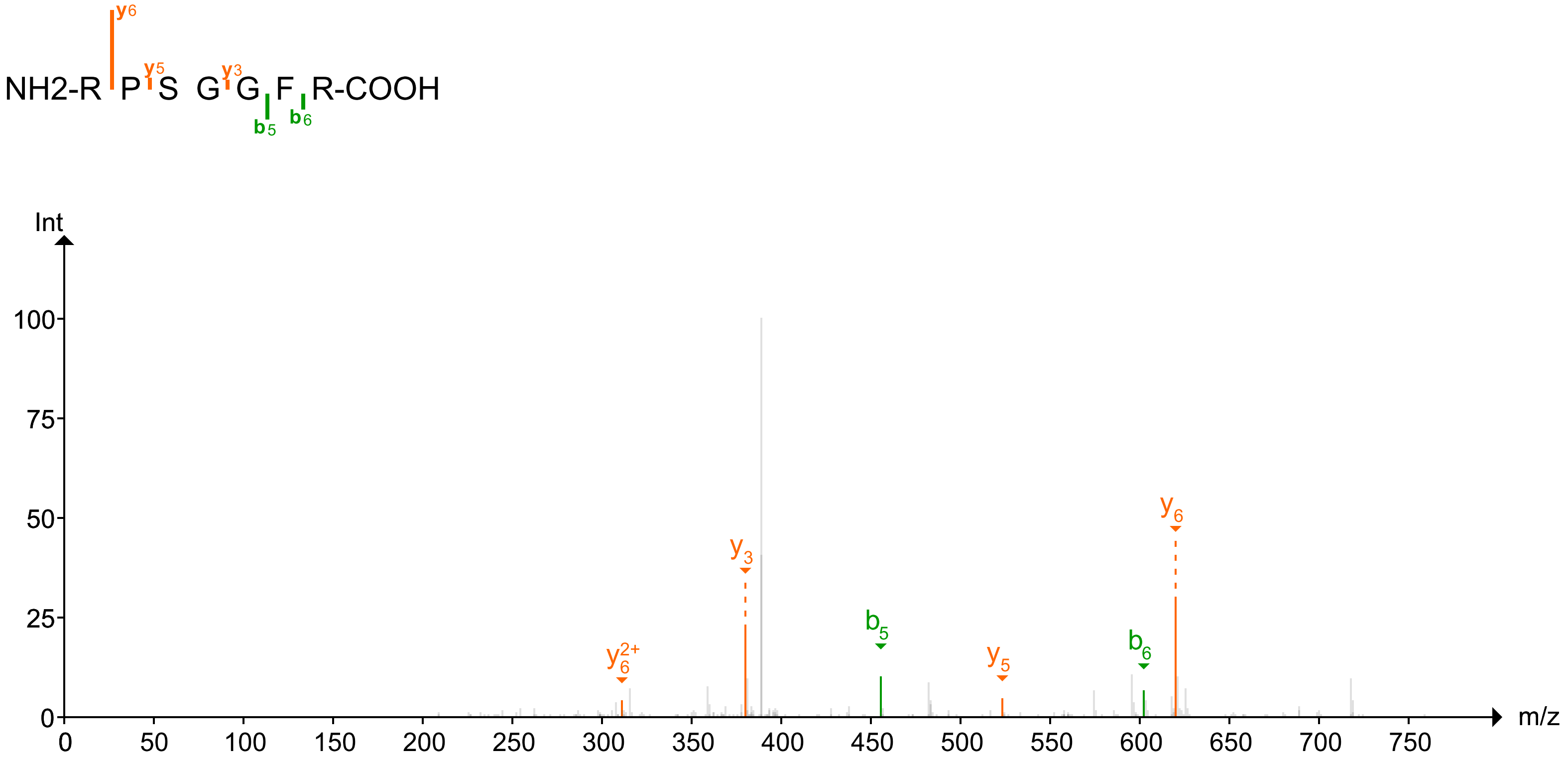

NH2-R P S G G F R-COOH
y6
y5
y3
b5
b6
Int
100
75
50
25
0
m/z
0
50
100
150
200
250
300
350
400
450
500
550
600
650
700
750
y6 2+
y3
b5
y5
b6
y6

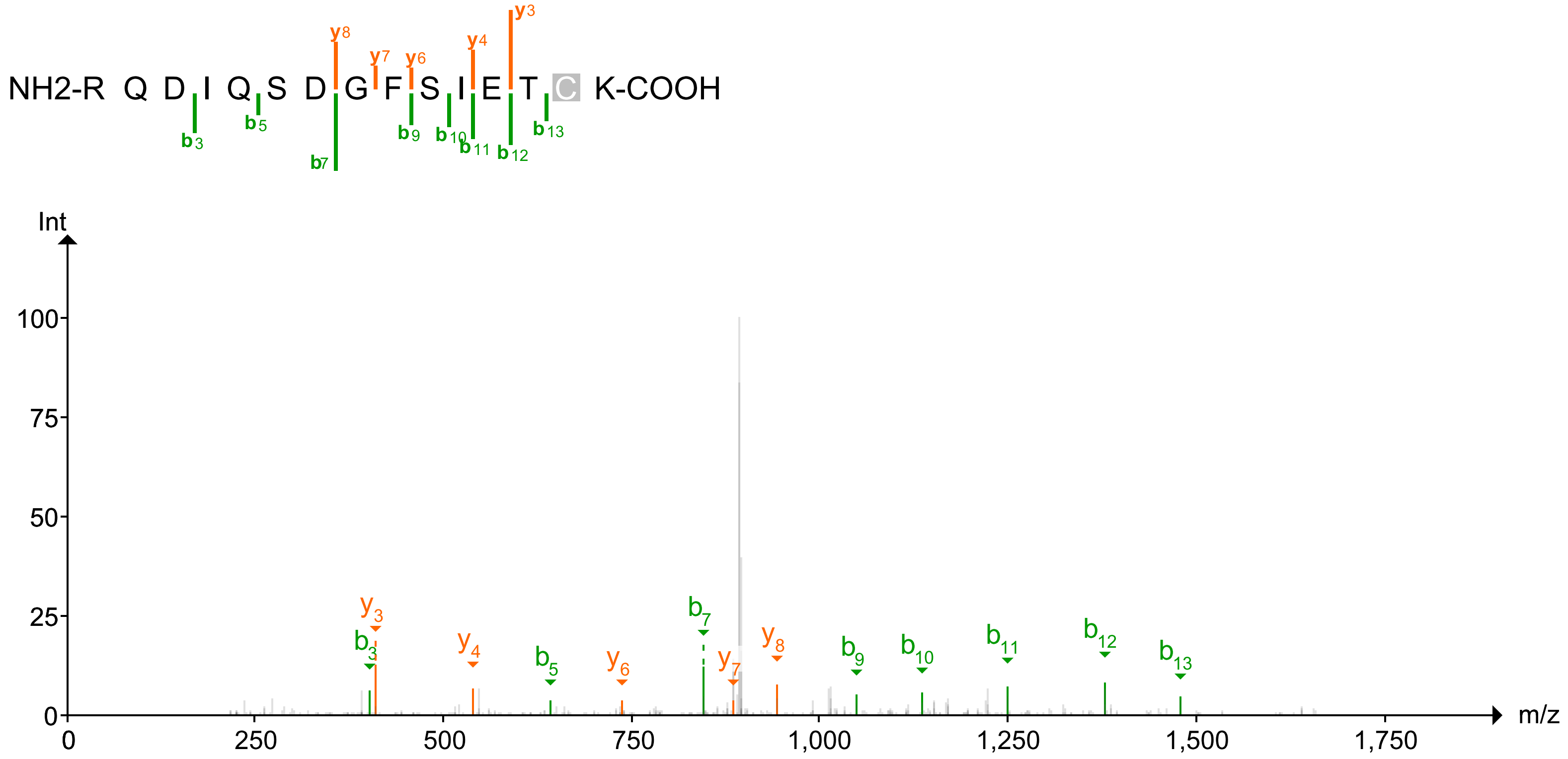

NH2-R Q D I Q S D G F S I E T C K-COOH
y3
y4
y6
y7
y8
b3
b5
b7
b9
b10
b11
b12
b13
Int
100
75
50
25
0
m/z
0
250
500
750
1,000
1,250
1,500
1,750

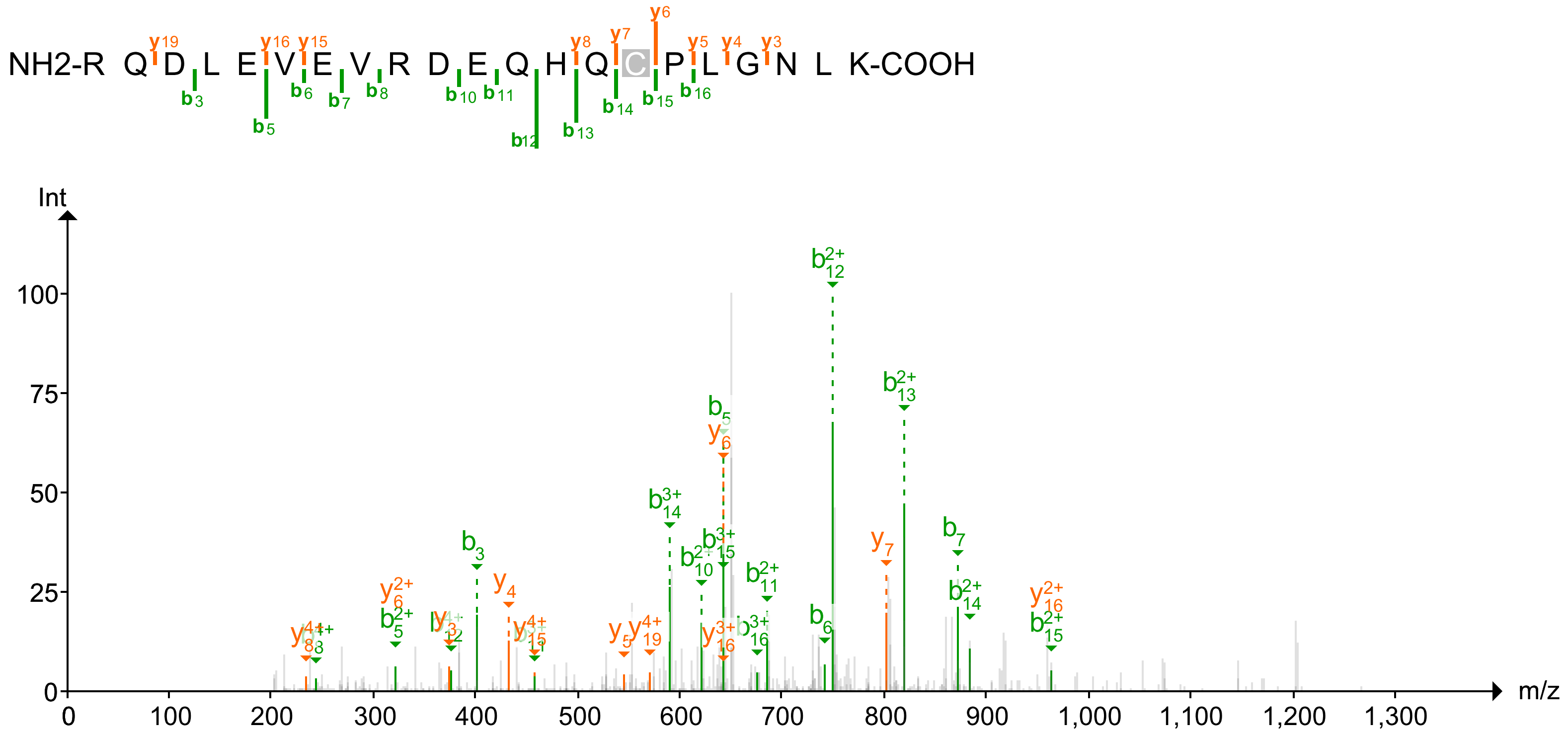

NH2-R Q D L E V E V R D E Q H Q C P L G N L K-COOH
y19 y16 y15 y8 y7 y6 y5 y4 y3
b3 b5 b6 b7 b8 b10 b11 b12 b13 b14 b15 b16
Int
100
75
50
25
0
m/z
0 100 200 300 400 500 600 700 800 900 1,000 1,100 1,200 1,300

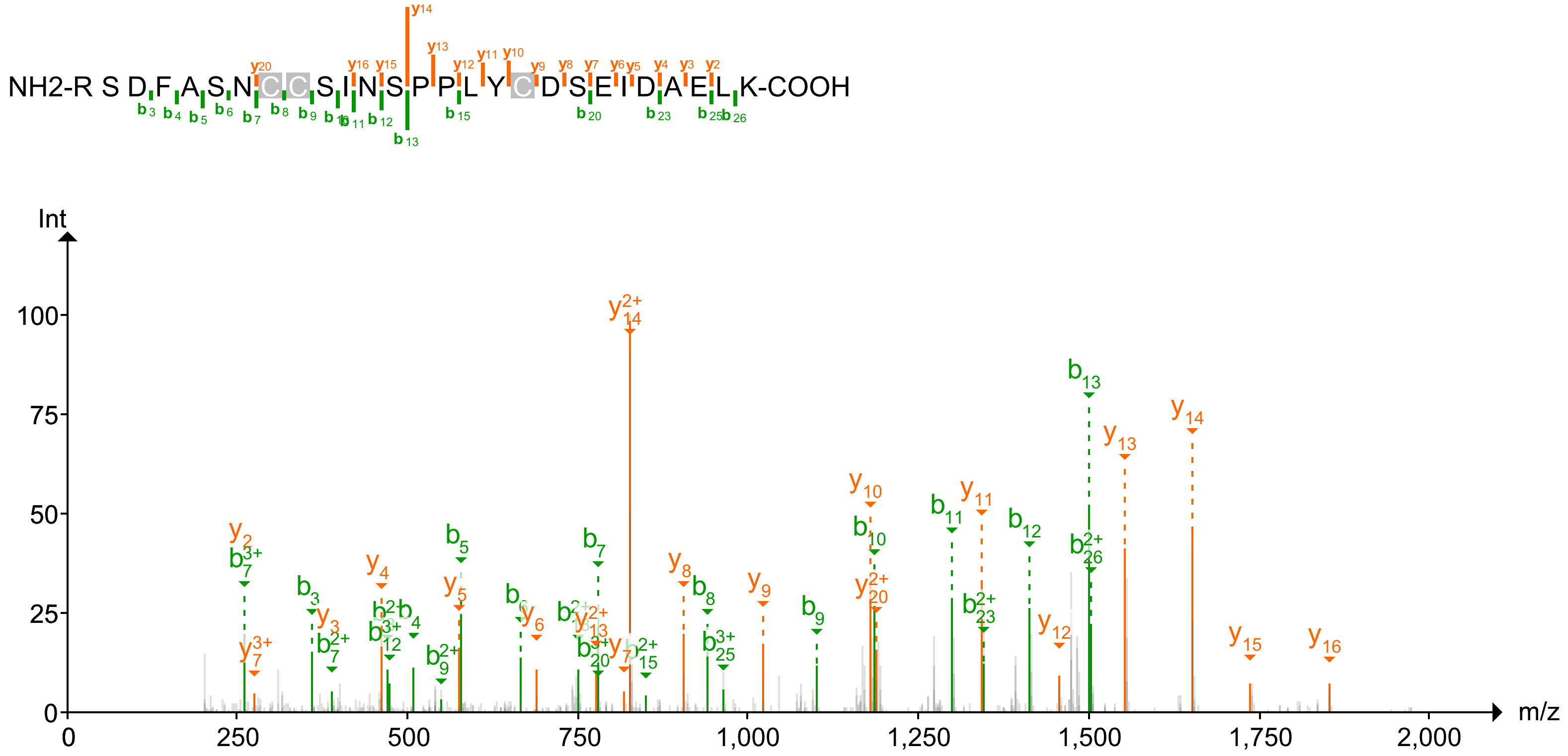
NH2-R S D F A S N C C S I N S P P L Y C D S E I D A E L K-COOH
Int
m/z
0
25
50
75
100
250
500
750
1,000
1,250
1,500
1,750
2,000

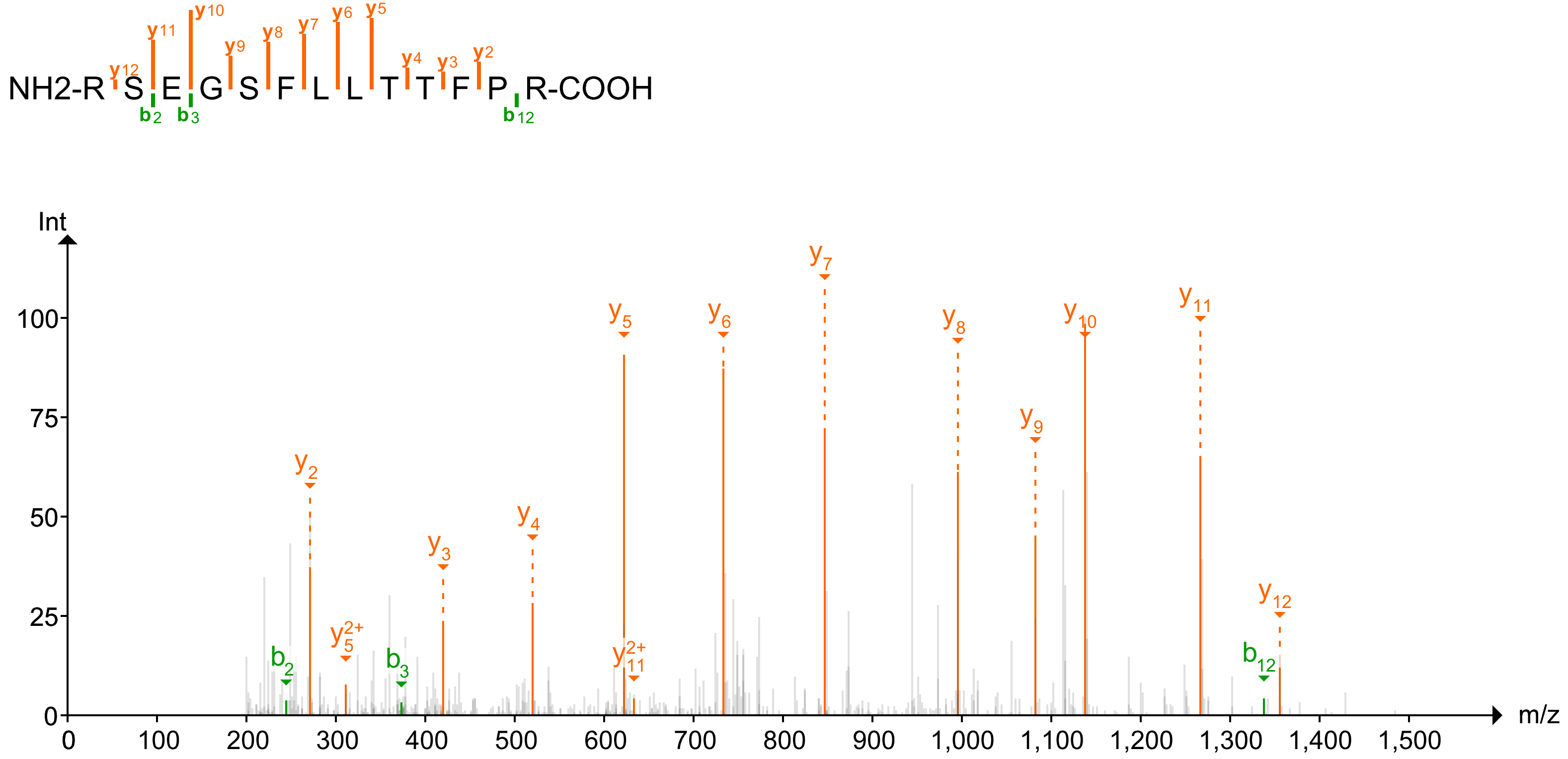

NH2-R S E G S F L L T T F P R-COOH
y12 y11 y10 y9 y8 y7 y6 y5 y4 y3 y2
b2 b3 b12
Int
100
75
50
25
0
m/z
0
100
200
300
400
500
600
700
800
900
1,000
1,100
1,200
1,300
1,400
1,500
y2
y5 2+
b2
b3
y3
y4
y5
y11 2+
y6
y7
y8
y9
y10
y11
y12
b12

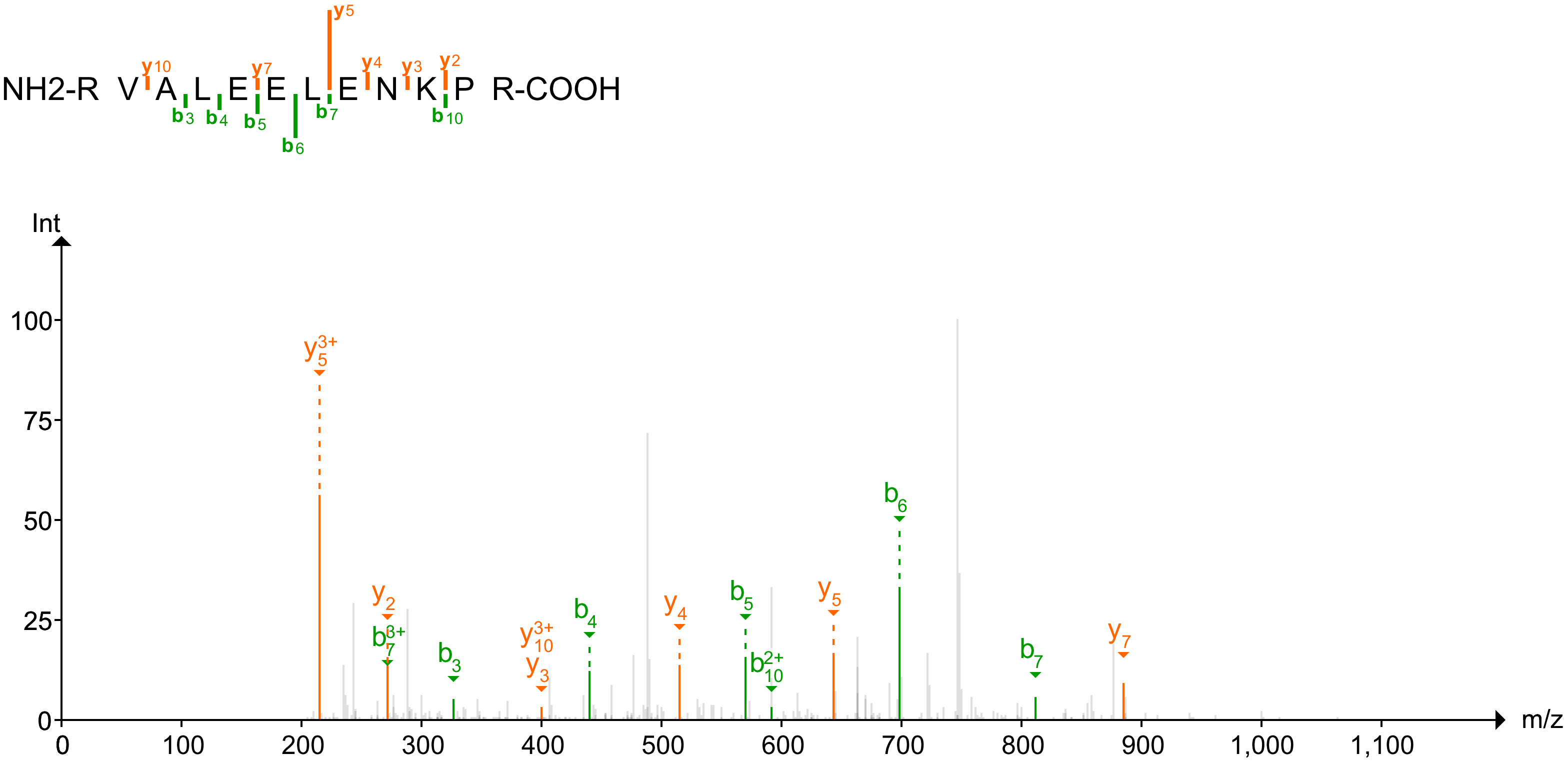

NH2-R V A L E E L E N K P R-COOH
y10 y7 y5 y4 y3 y2
b3 b4 b5 b6 b7 b10
Int
100
75
50
25
0
m/z
0 100 200 300 400 500 600 700 800 900 1,000 1,100
y5 3+
y2
b7 3+
b3
y10 3+
y3
b4
y4
b5
b10 2+
y5
b6
b7
y7

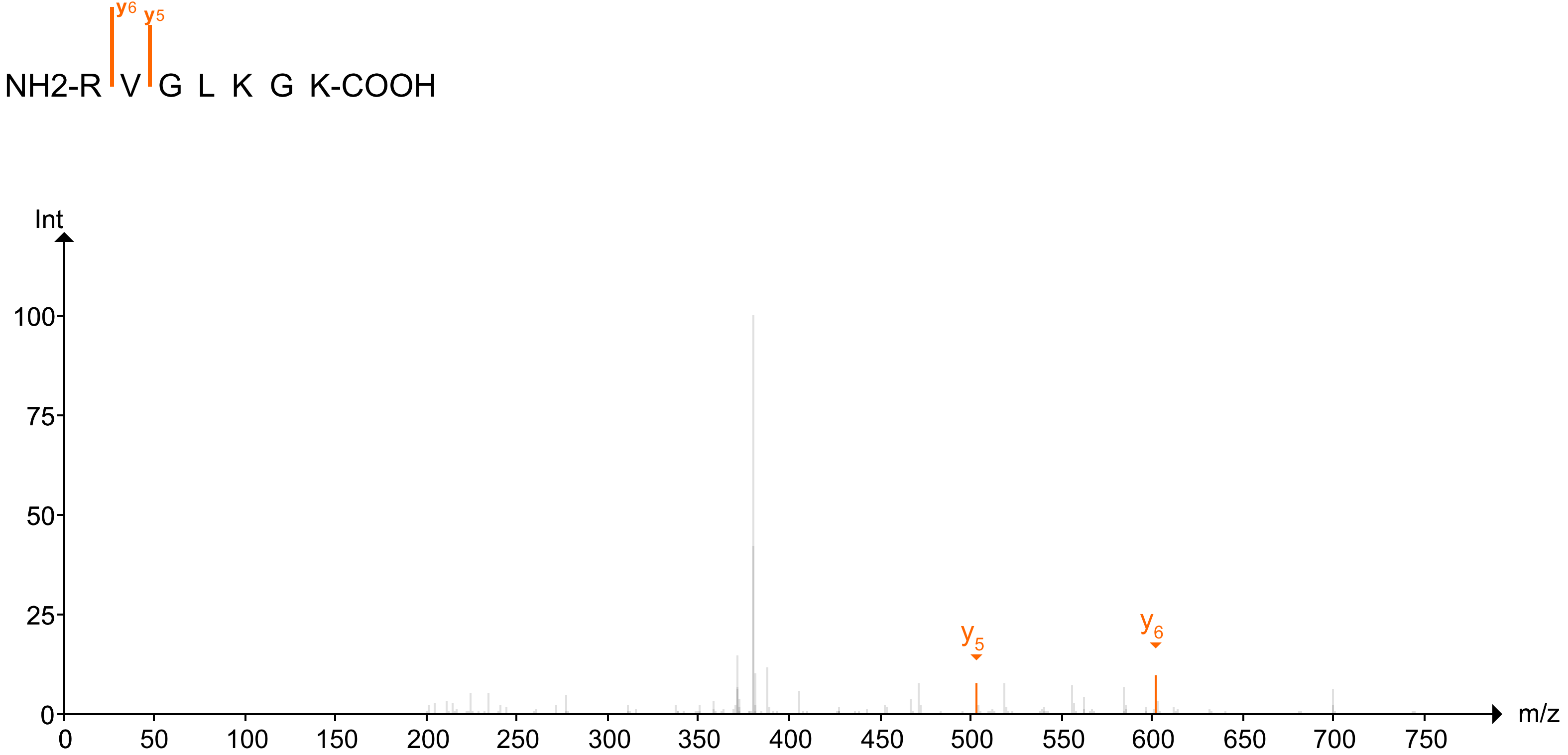
y6
y5
NH2-R V G L K G K-COOH
Int
100
75
50
25
0
y5
y6
0
50
100
150
200
250
300
350
400
450
500
550
600
650
700
750
m/z

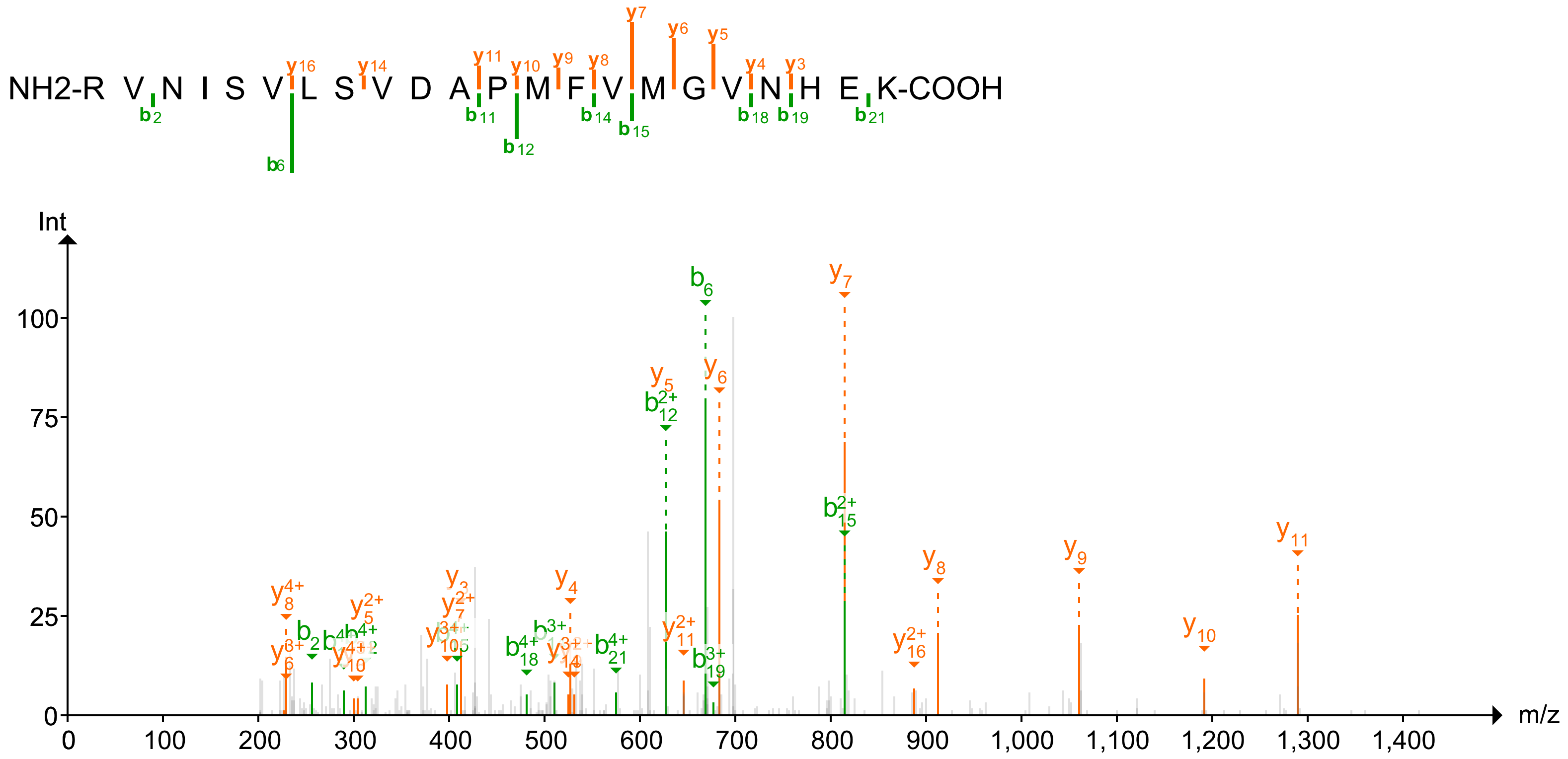

NH2-R V N I S V L S V D A P M F V M G V N H E K-COOH
Int
m/z
0
25
50
75
100
0
100
200
300
400
500
600
700
800
900
1,000
1,100
1,200
1,300
1,400

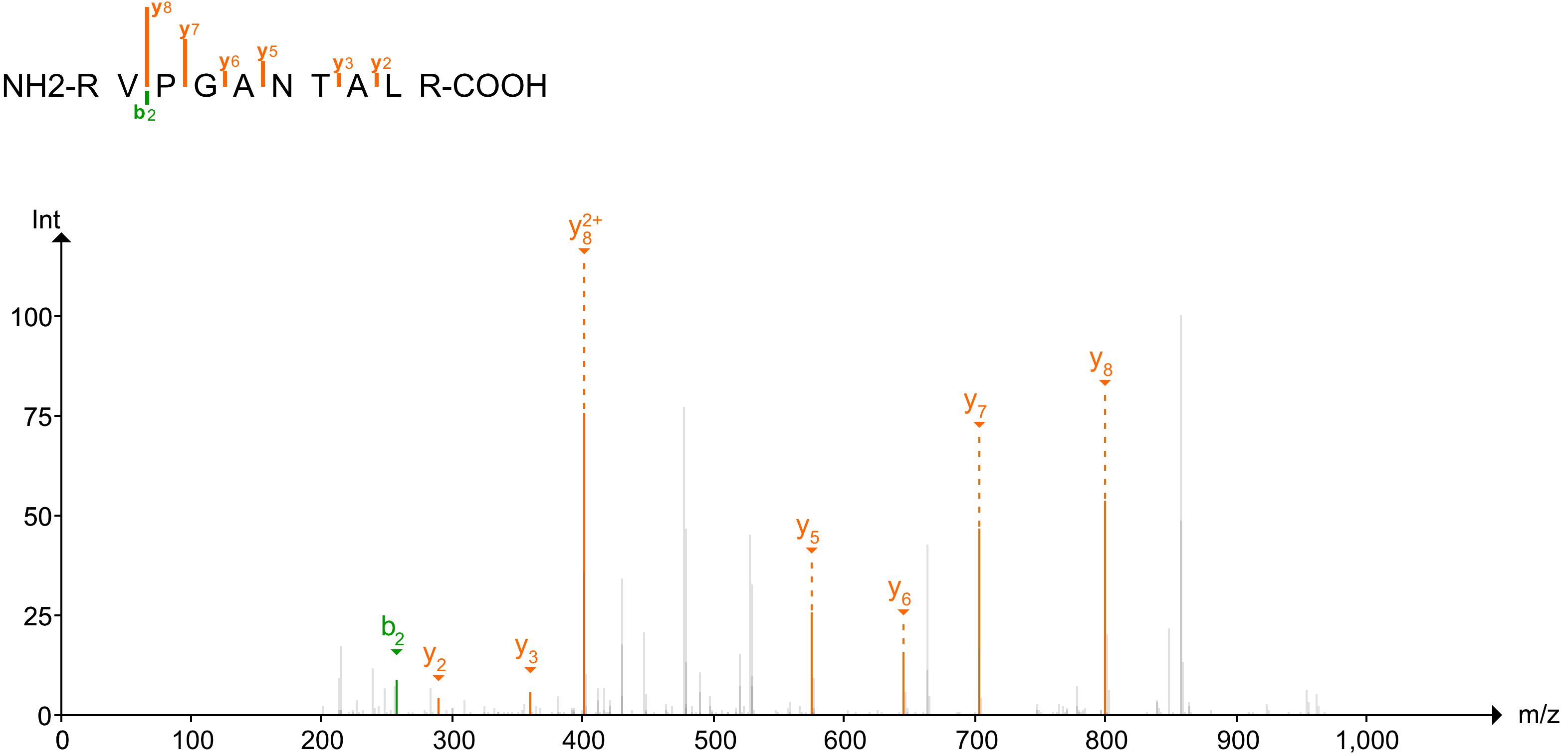

NH2-R V P G A N T A L R-COOH
y8
y7
y6
y5
y3
y2
b2
Int
100
75
50
25
0
m/z
0
100
200
300
400
500
600
700
800
900
1,000
y8 2+
y8
y7
y6
y5
y3
y2
b2

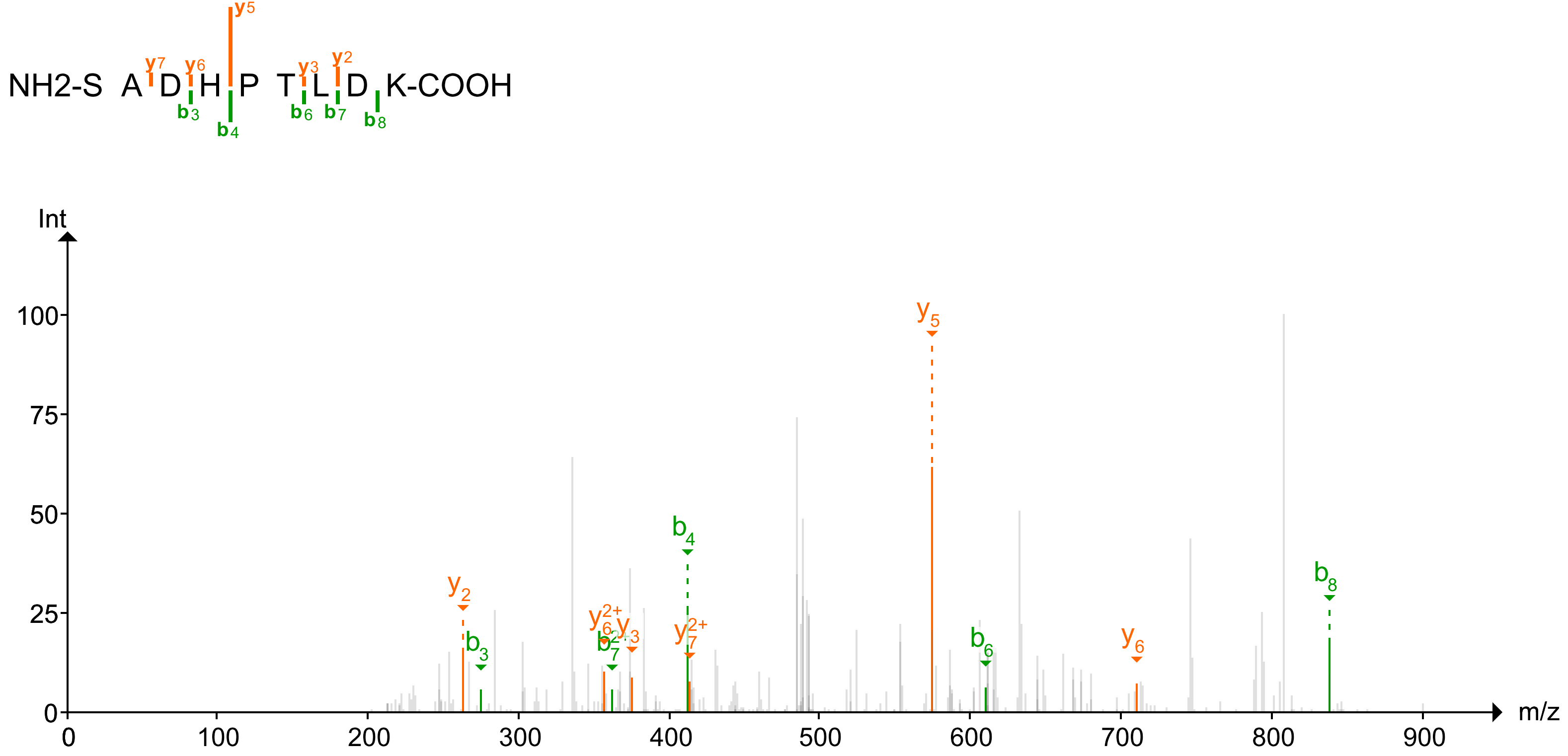

NH2-S A D H P T L D K-COOH
y7 y6 y5 y3 y2
b3 b4 b6 b7 b8
Int
100
75
50
25
0
0 100 200 300 400 500 600 700 800 900
m/z
y5
b4
b8
y2
b3
y6 2+
b7 2+
y3
y7 2+
b6
y6

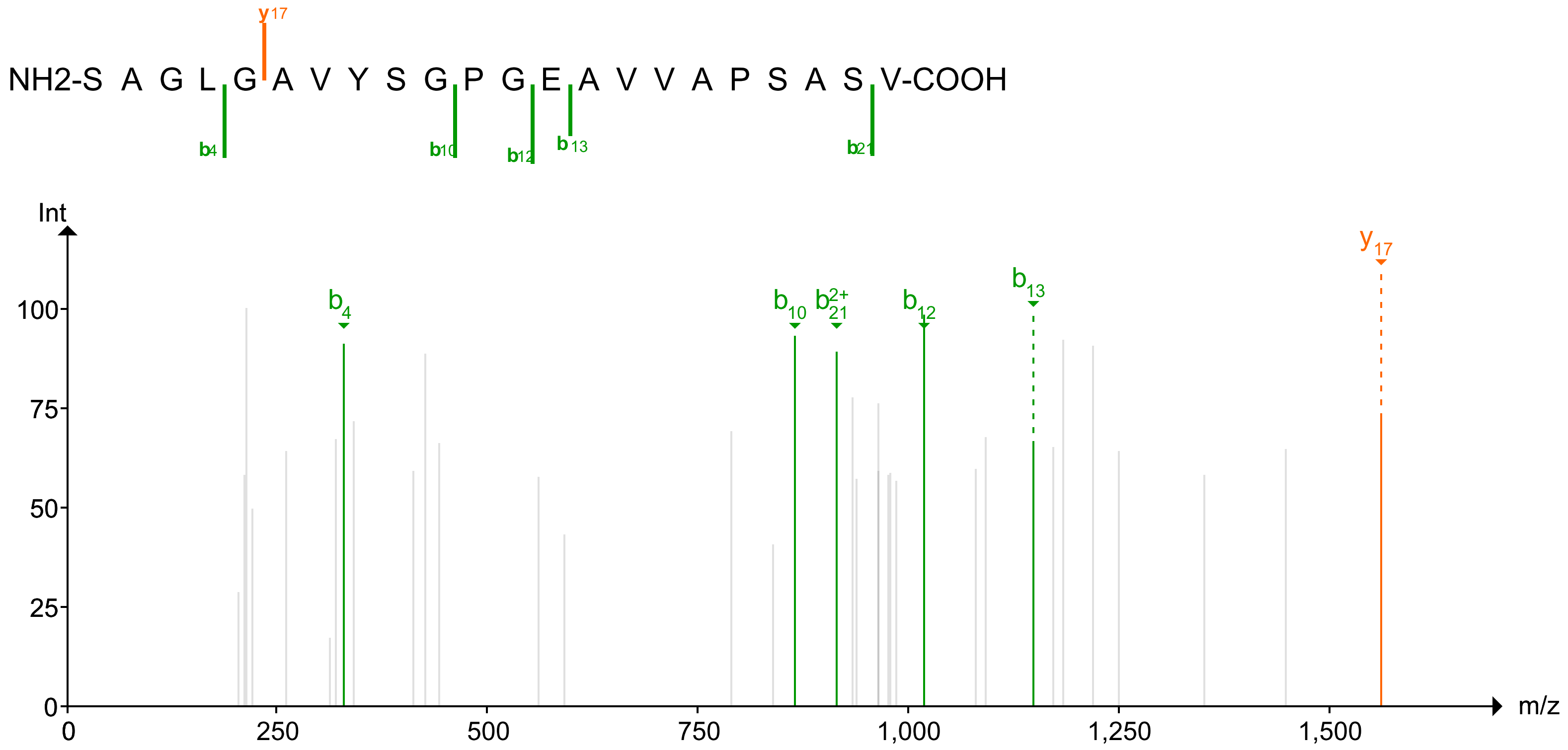
NH2-S A G L G A V Y S G P G E A V V A P S A S V-COOH
y17
b4
b10
b12
b13
b21
Int
m/z
0
25
50
75
100
250
500
750
1,000
1,250
1,500
b4
b10
b21 2+
b12
b13
y17

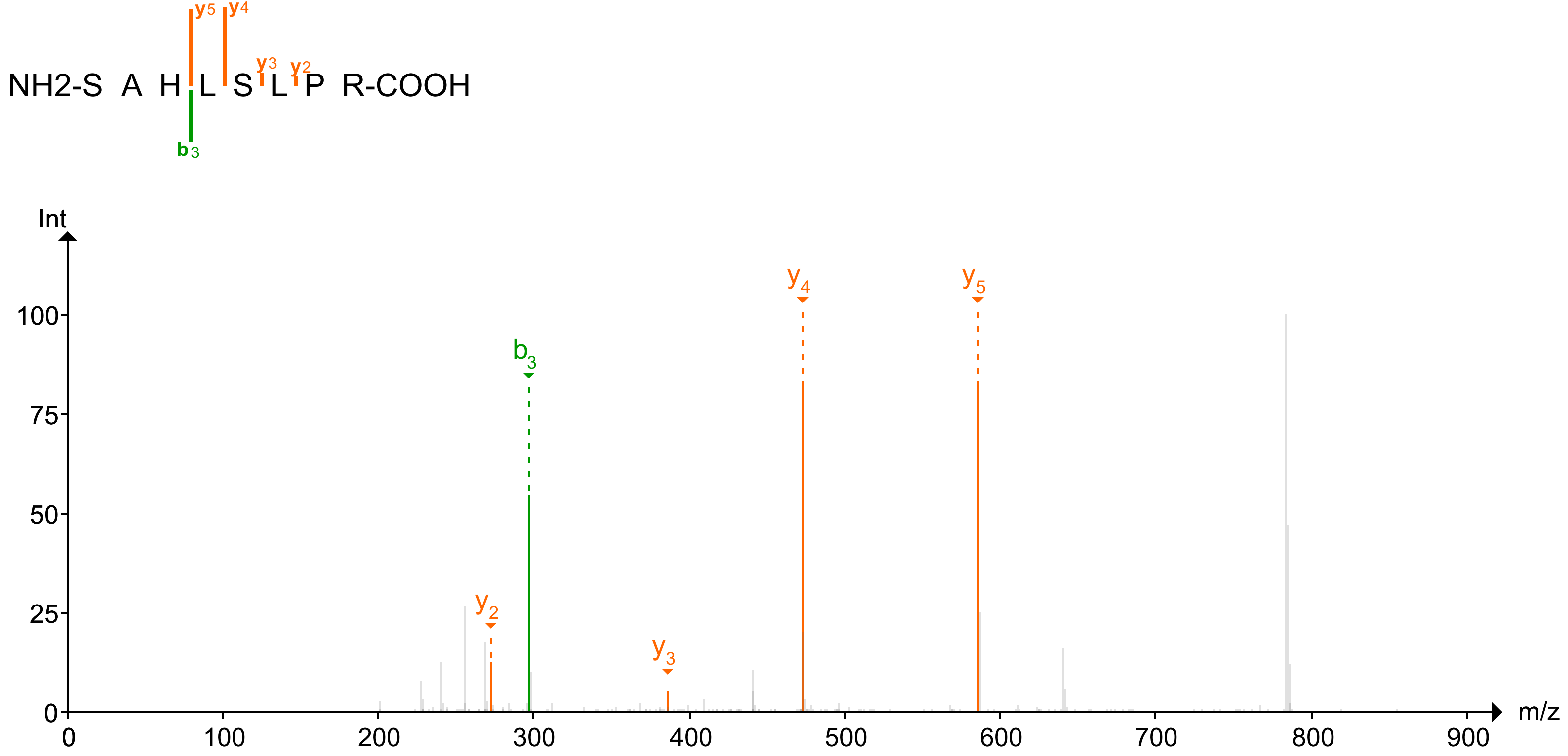

NH2-S A H L S L P R-COOH
y5 y4 y3 y2 b3
Int
100
75
50
25
0
y4
y5
b3
y2
y3
0 100 200 300 400 500 600 700 800 900
m/z

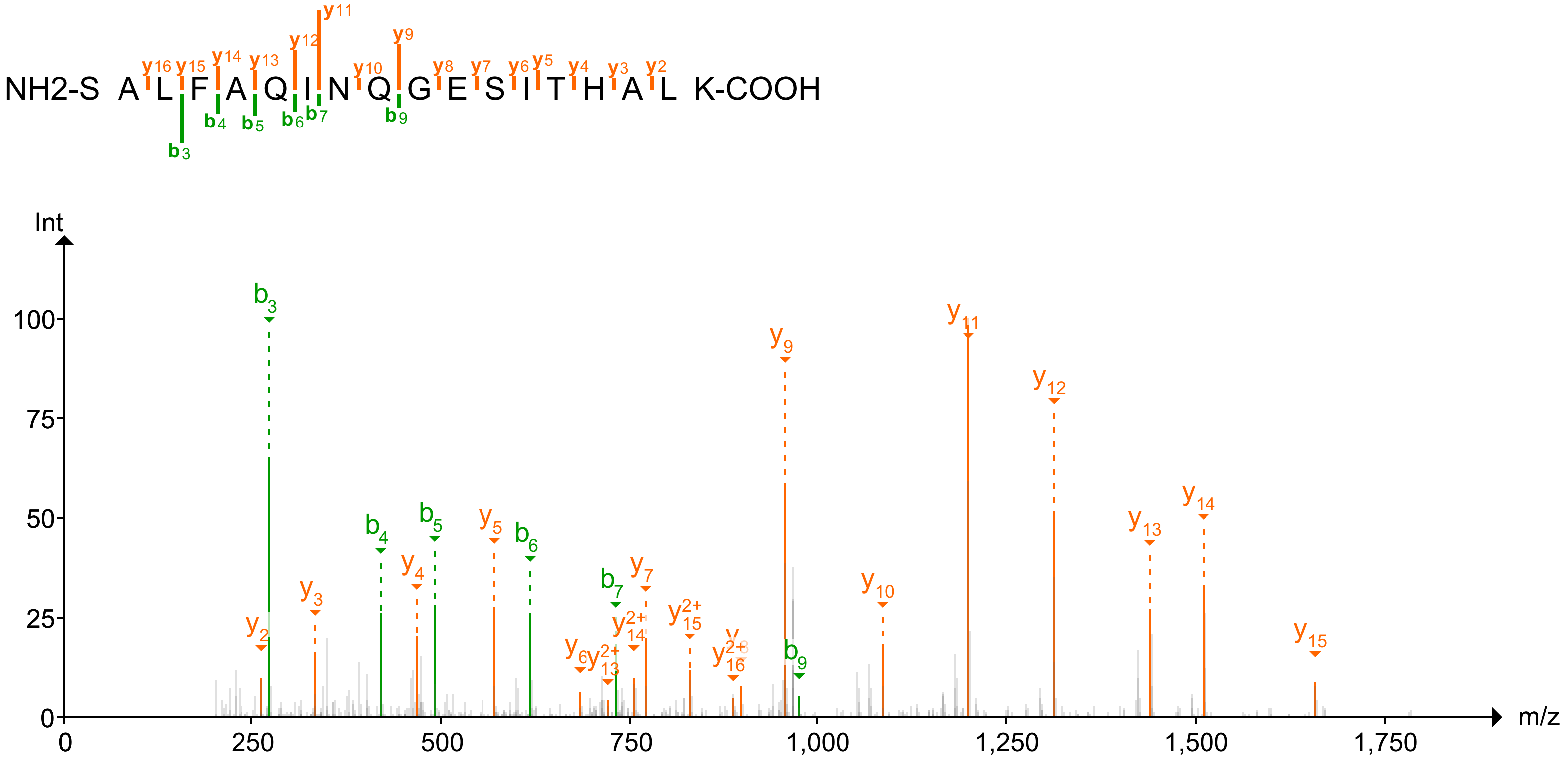

NH2-S A L F A Q I N Q G E S I T H A L K-COOH
Int
m/z

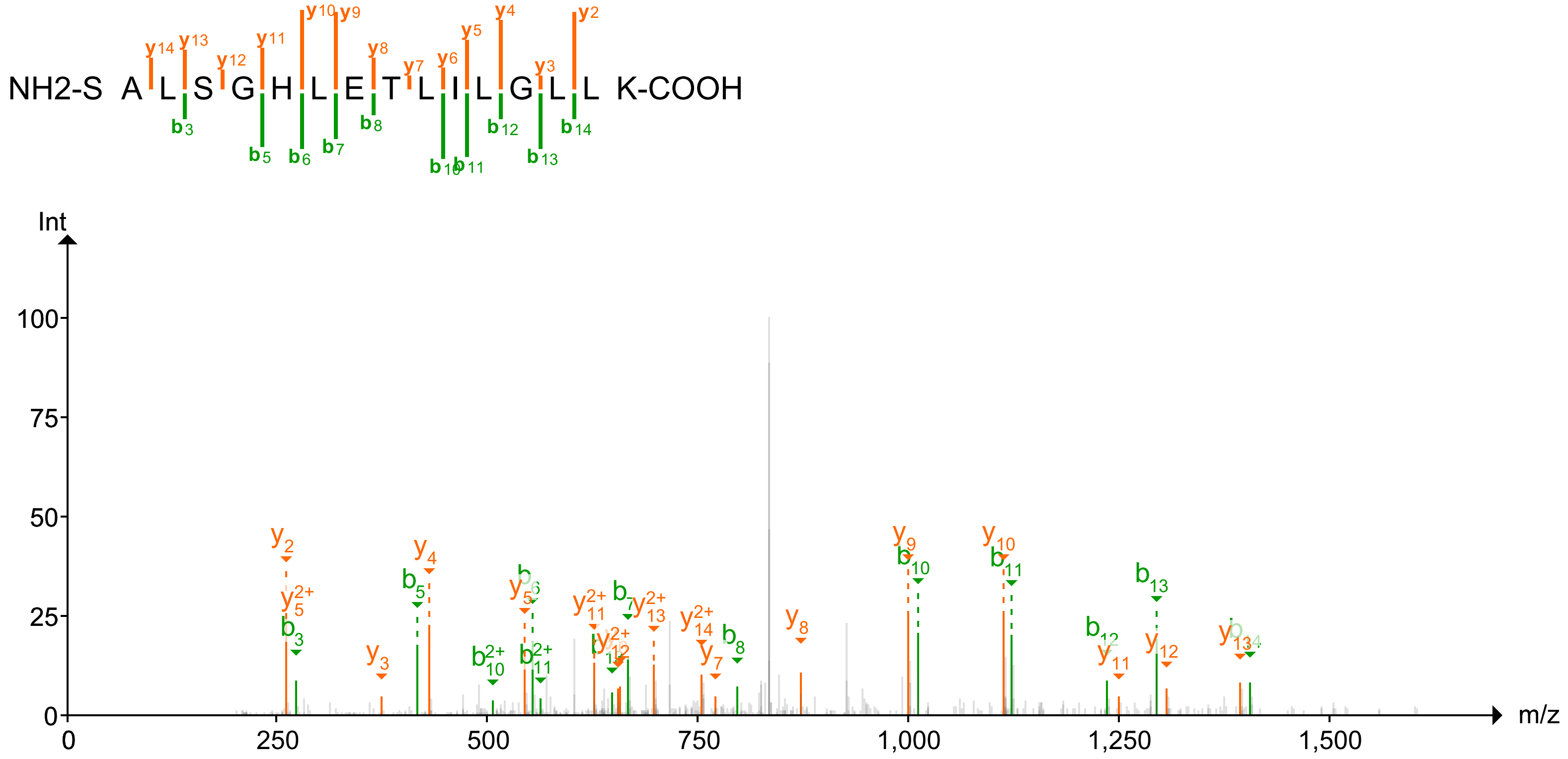

NH2-S A L S G H L E T L I L G L L K-COOH
y14 y13 y12 y11 y10 y9 y8 y7 y6 y5 y4 y3 y2
b3 b5 b6 b7 b8 b10 b11 b12 b13 b14
Int
100
75
50
25
0
0
250
500
750
1,000
1,250
1,500
m/z

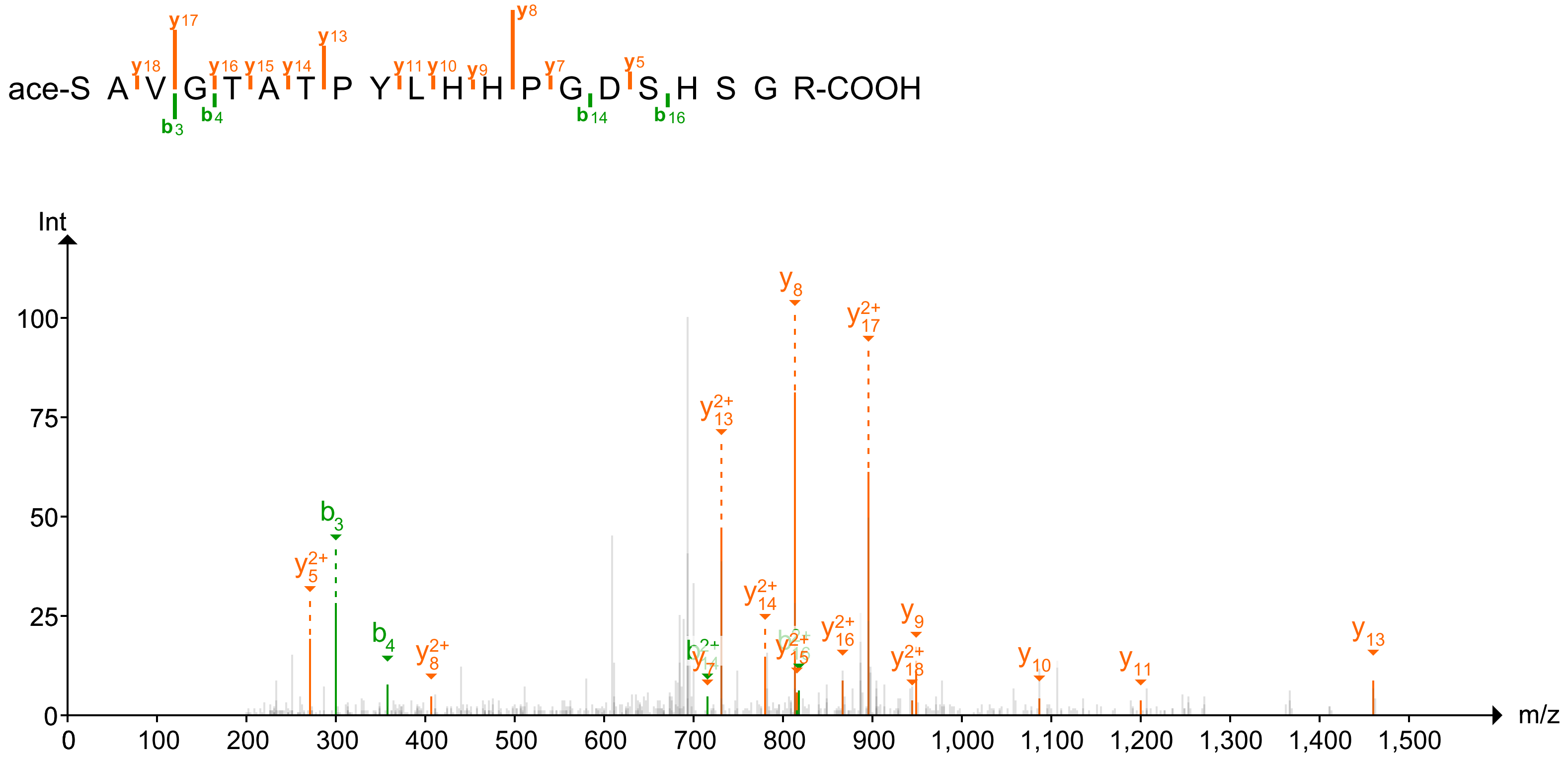

ace-S A V G T A T P Y L H H P G D S H S G R-COOH
y17 y18 y16 y15 y14 y13 y11 y10 y9 y8 y7 y5
b3 b4 b14 b16
Int
m/z
0
25
50
75
100
0 100 200 300 400 500 600 700 800 900 1,000 1,100 1,200 1,300 1,400 1,500
y8
y17 2+
y13 2+
b3
y5 2+
b4
y8 2+
y7
y14 2+
y15 2+
y16 2+
y9
y18 2+
y10
y11
y13

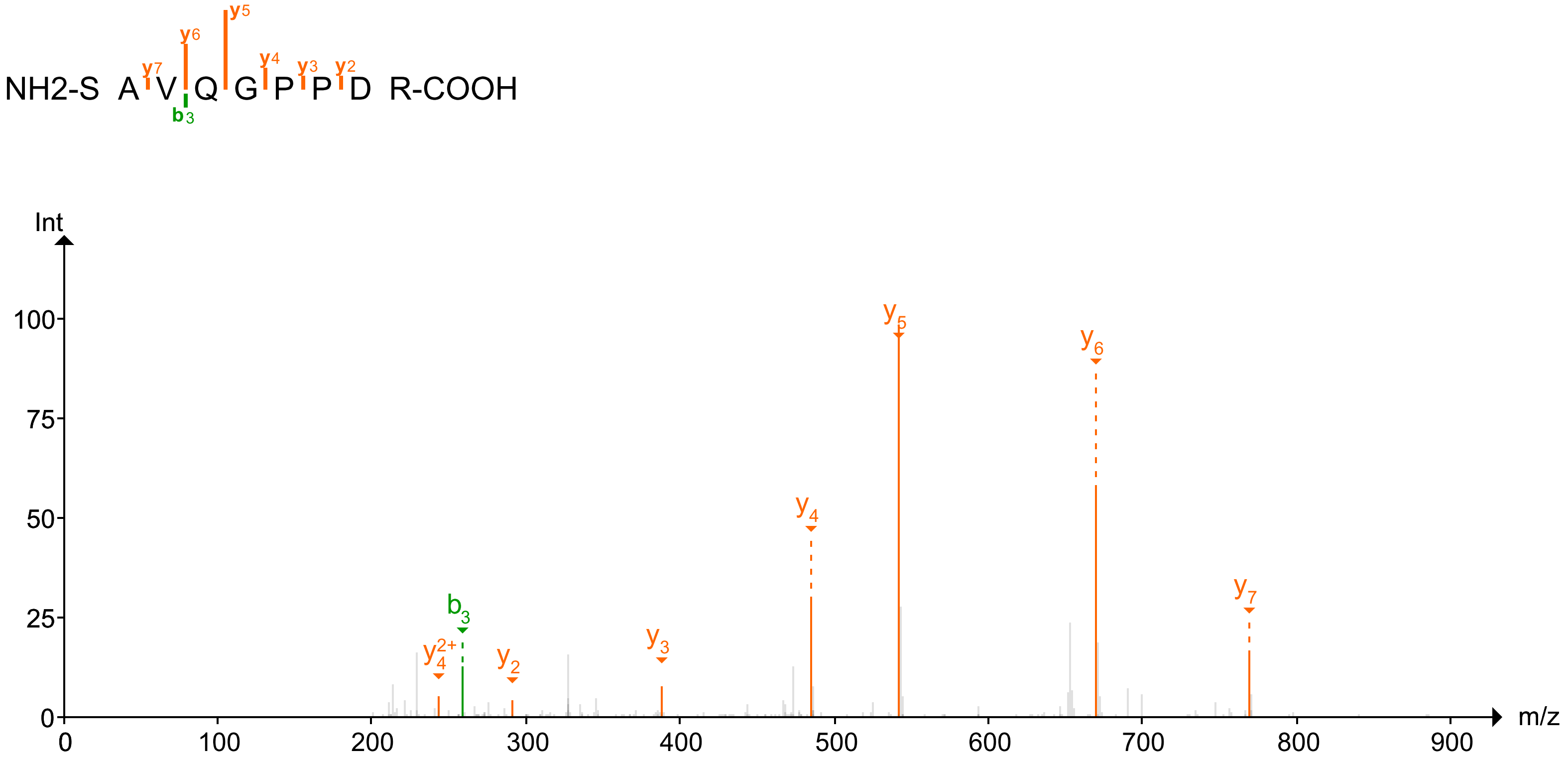

NH2-S A V Q G P P D R-COOH
y7 y6 y5 y4 y3 y2
b3
Int
100
75
50
25
0
m/z
0
100
200
300
400
500
600
700
800
900
y5
y6
y4
y7
b3
y3
y2
y4 2+

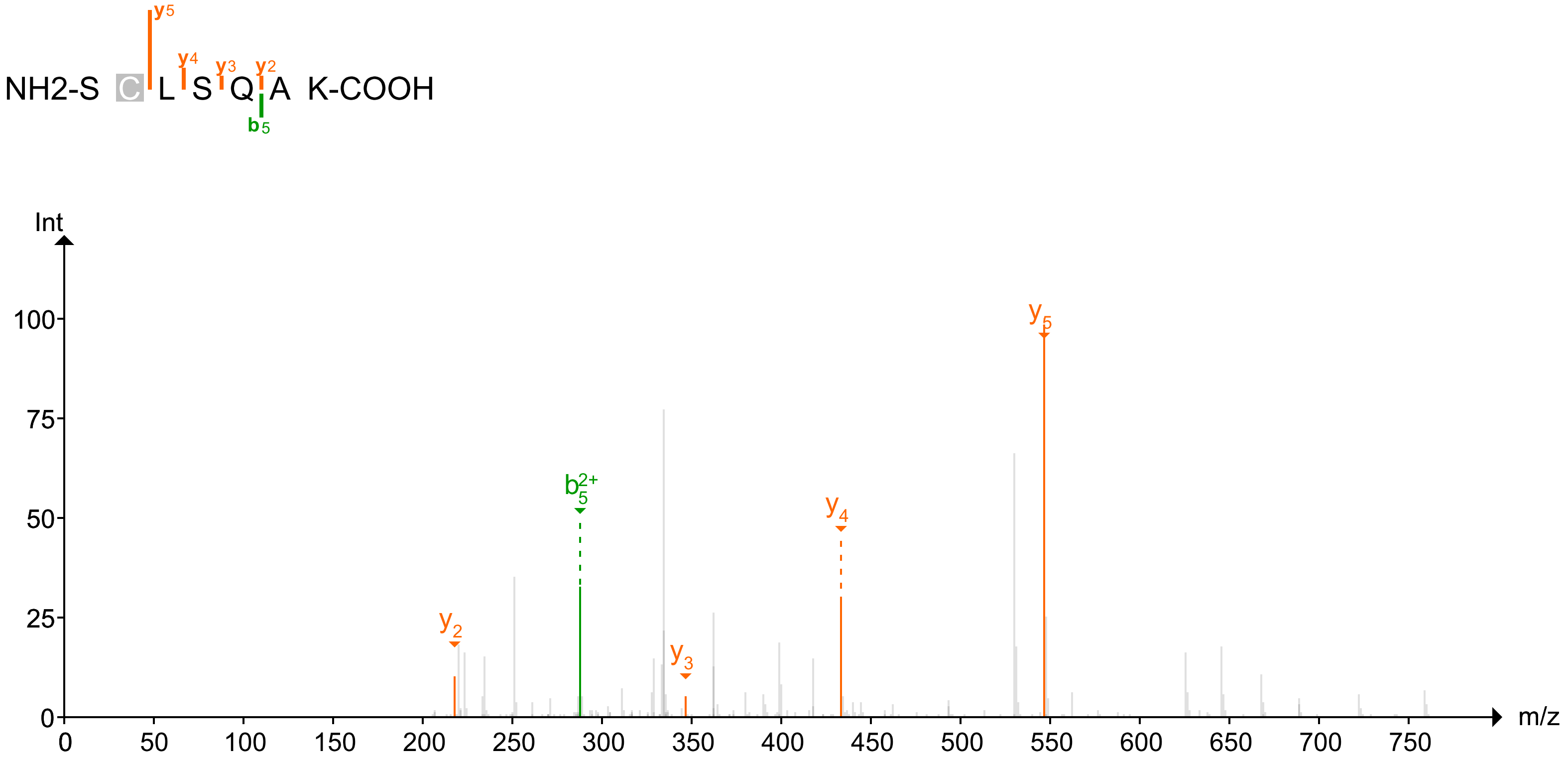

NH2-S C L S Q A K-COOH
y5
y4
y3
y2
b5
Int
100
75
50
25
0
y5
$b_5^{2+}$
y4
y2
y3
0
50
100
150
200
250
300
350
400
450
500
550
600
650
700
750
m/z

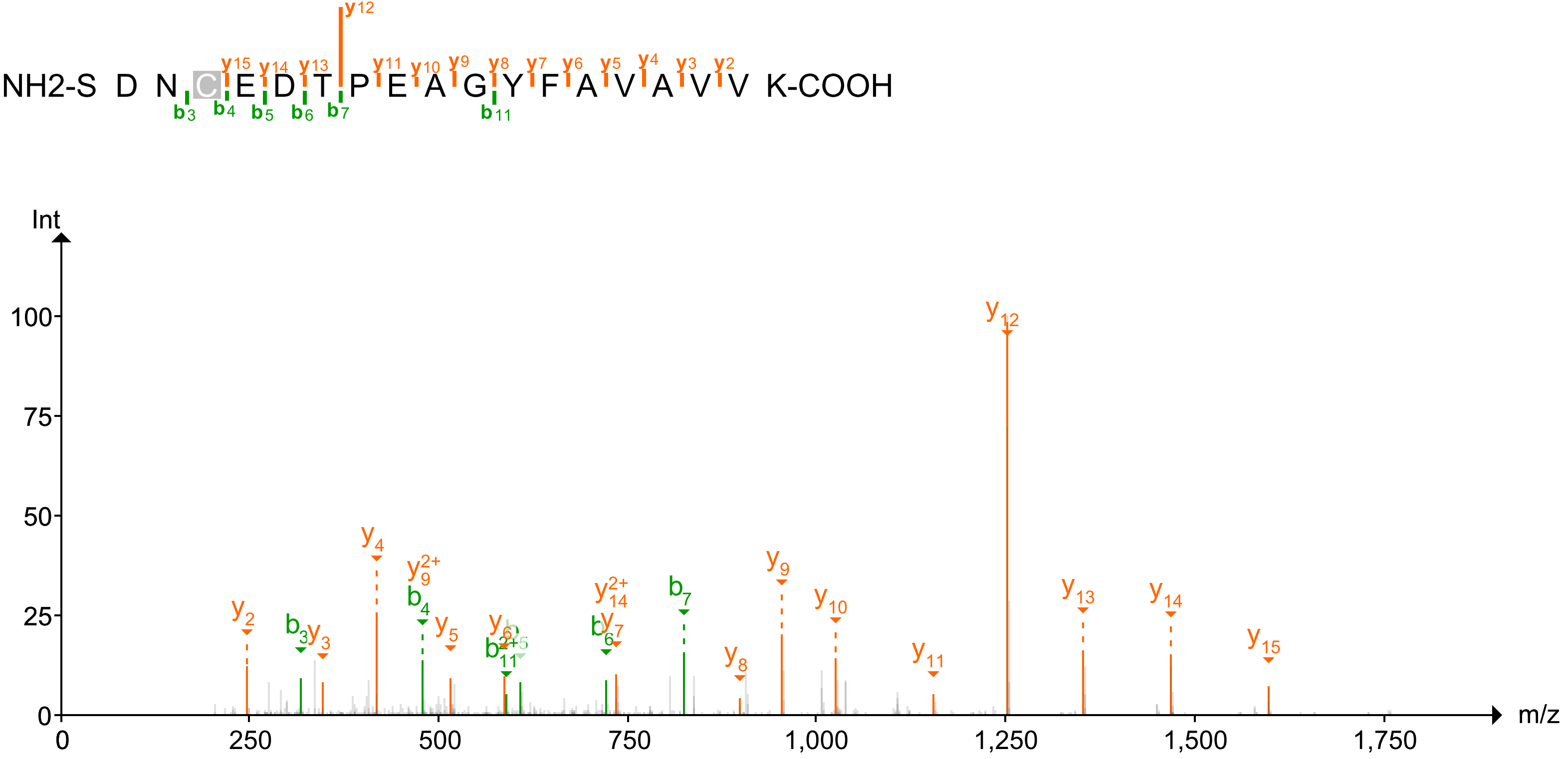

NH2-S D N C E D T P E A G Y F A V A V V K-COOH
y12
y15 y14 y13 y11 y10 y9 y8 y7 y6 y5 y4 y3 y2
b3 b4 b5 b6 b7 b11
Int
100
75
50
25
0
0
250
500
750
1,000
1,250
1,500
1,750
m/z
y12
y4
y9 2+
b4
y14 2+
b7
y9
y10
y13
y14
y2
b3
y3
y5
y6
b6
y7
b11 2+
y8
y11
y15

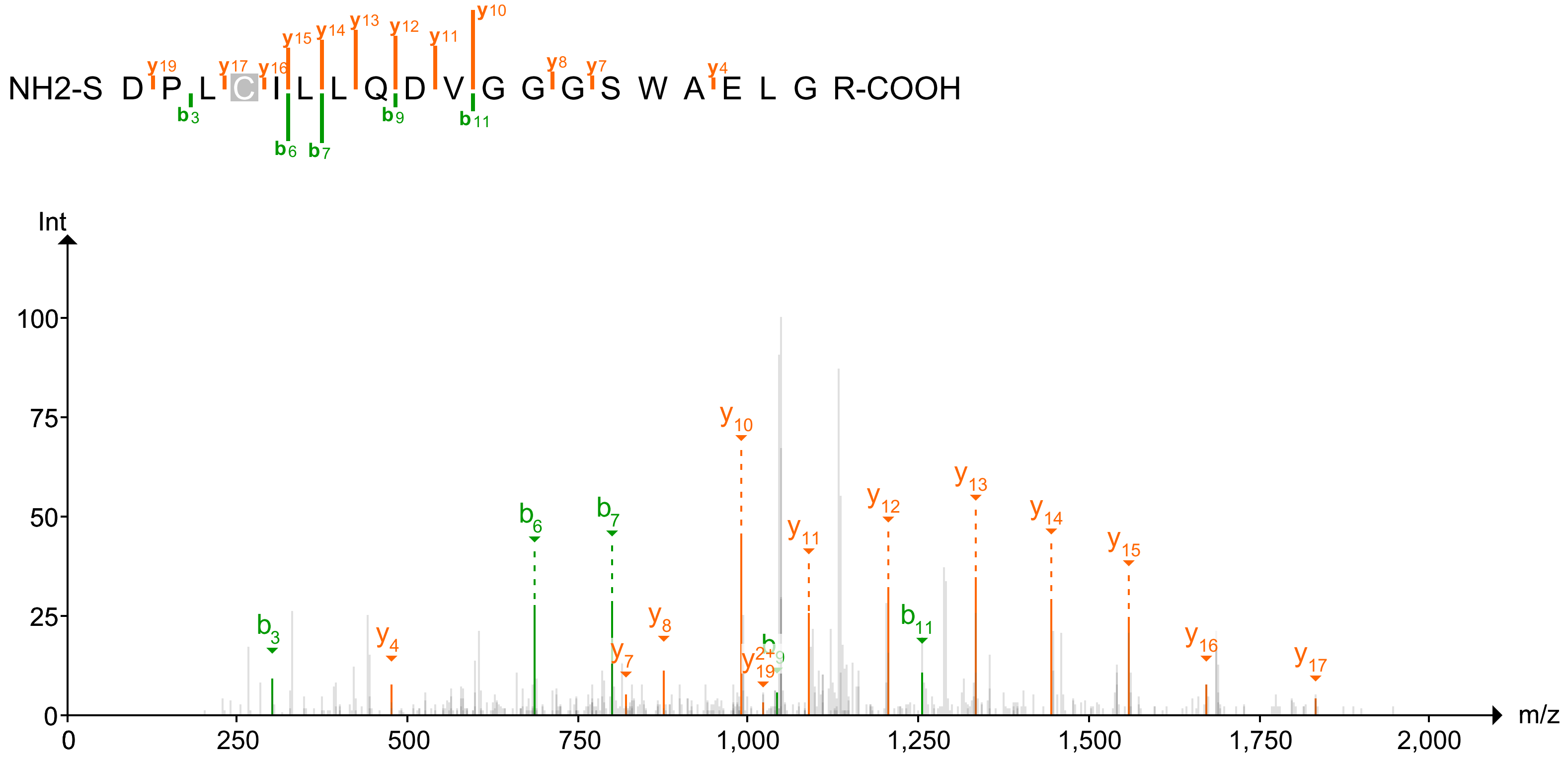

NH2-S D P L C I L L Q D V G G G S W A E L G R-COOH
y19 y17 y16 y15 y14 y13 y12 y11 y10 y8 y7 y4
b3 b6 b7 b9 b11
Int
100
75
50
25
0
m/z
0
250
500
750
1,000
1,250
1,500
1,750
2,000
b3 y4 b6 b7 y7 y8 y10 y11 y12 y13 y14 y15 y16 y17 b11 b9

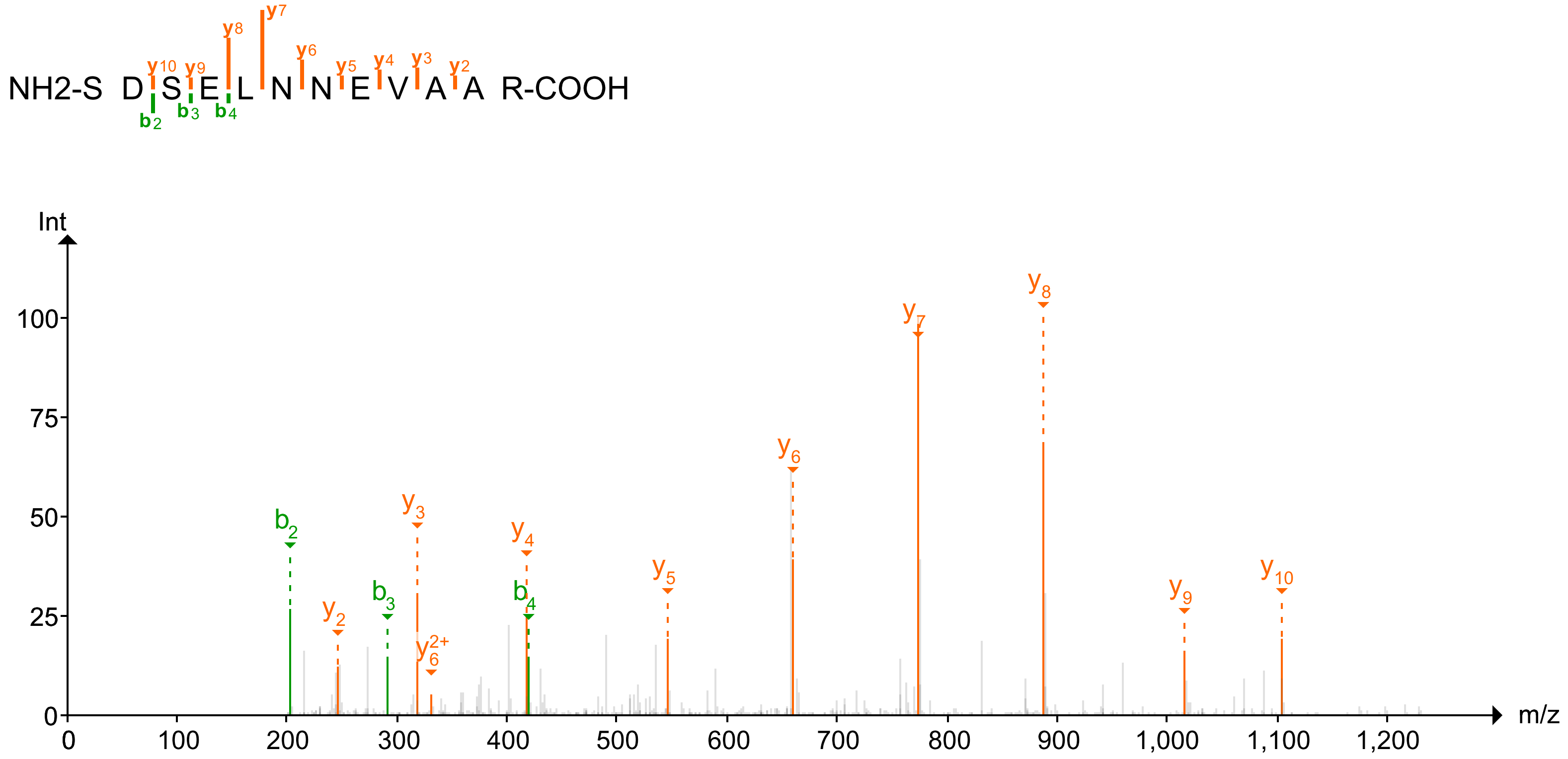

NH2-S D S E L N N E V A A R-COOH
y10 y9 y8 y7 y6 y5 y4 y3 y2
b2 b3 b4
Int
m/z
0
25
50
75
100
100
200
300
400
500
600
700
800
900
1,000
1,100
1,200
b2
y2
b3
y3
y6 2+
y4
b4
y5
y6
y7
y8
y9
y10

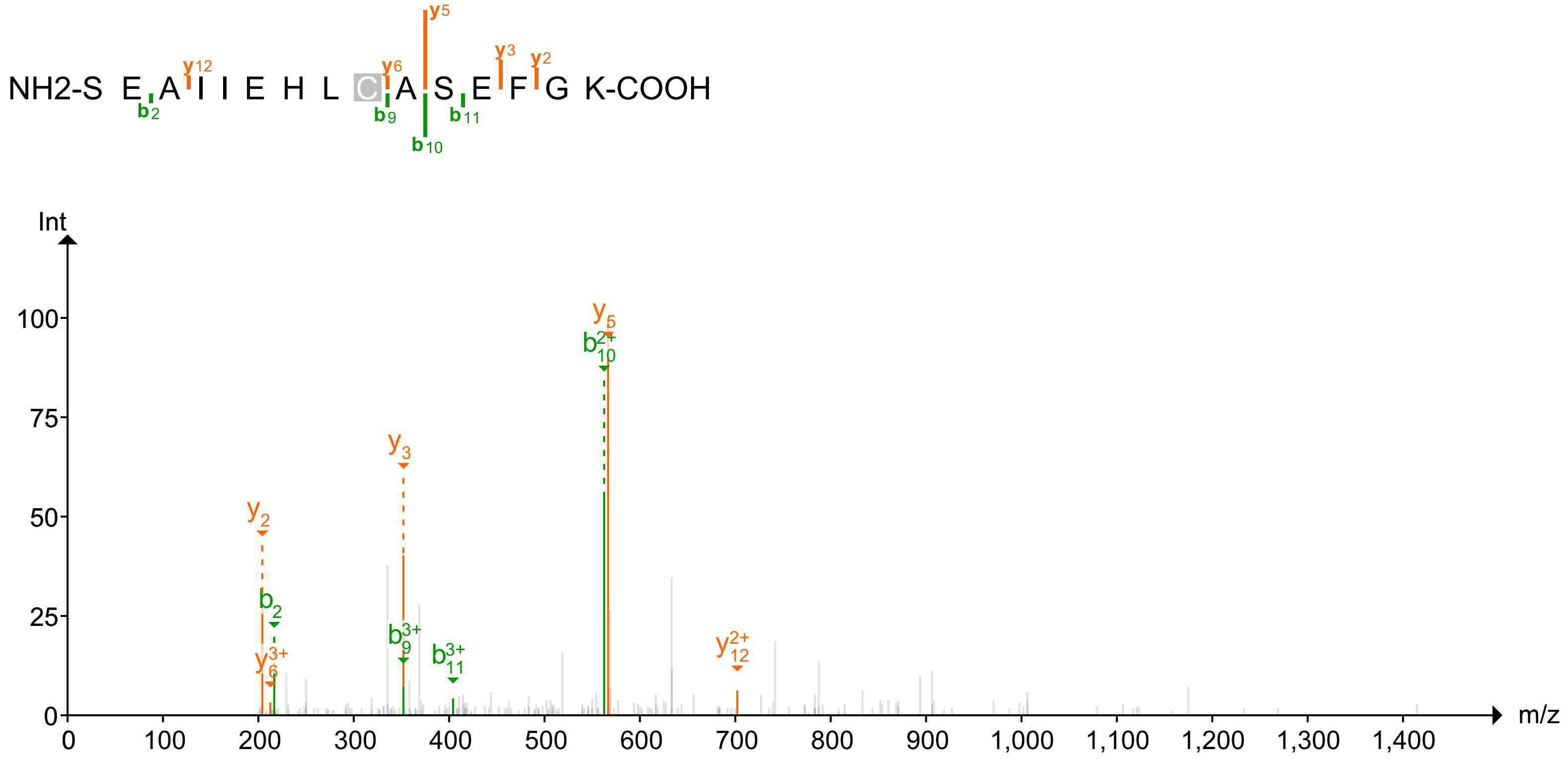

NH2-S E A I I E H L C A S E F G K-COOH
y12 y6 y5 y3 y2
b2 b9 b10 b11
Int
100
75
50
25
0
y5
b2+ 10
y3
y2
b2
y3+ 6
b3+ 9
b3+ 11
y2+ 12
0 100 200 300 400 500 600 700 800 900 1,000 1,100 1,200 1,300 1,400
m/z

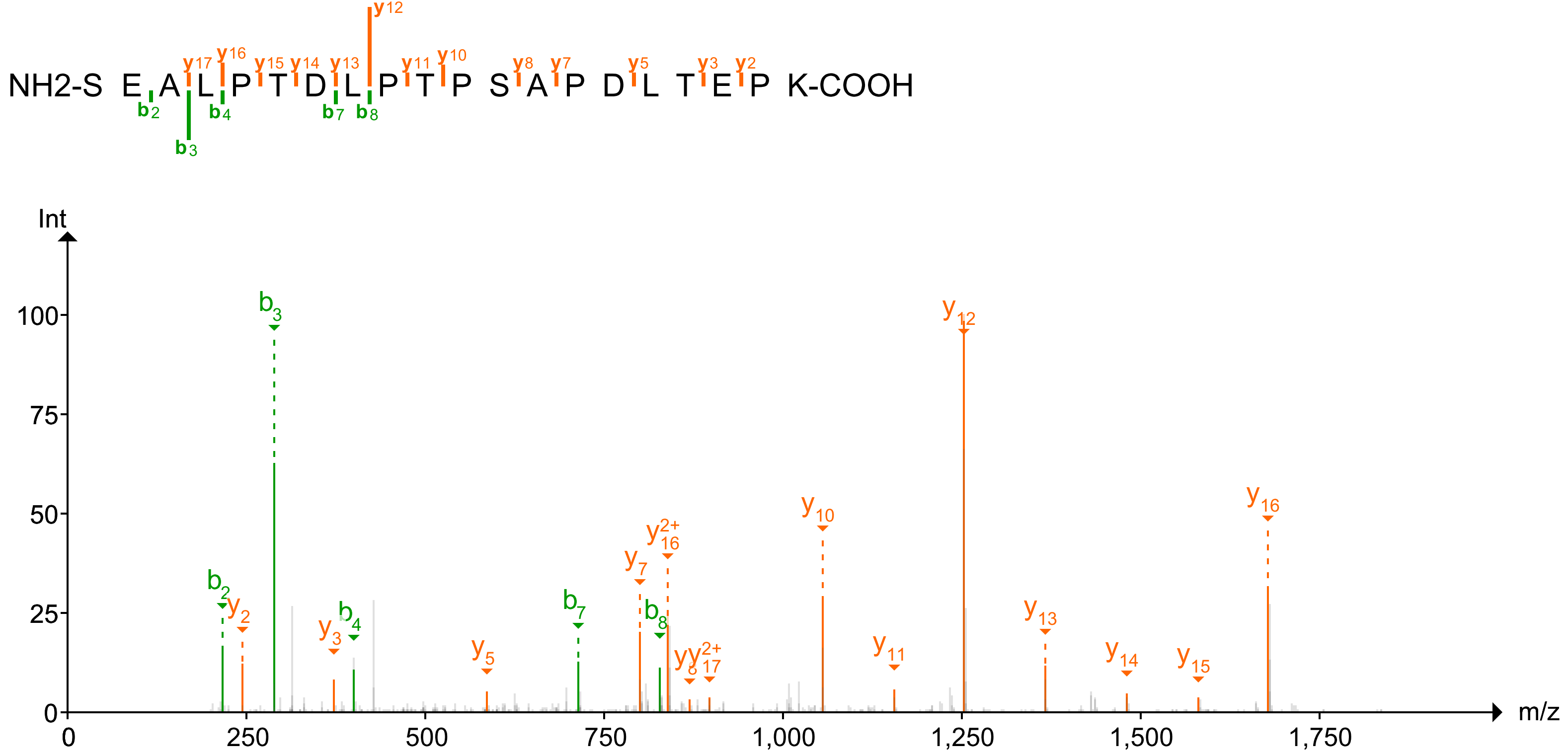

NH2-S E A L P T D L P T P S A P D L T E P K-COOH
y17 y16 y15 y14 y13 y12 y11 y10 y8 y7 y5 y3 y2
b2 b3 b4 b7 b8
Int
100
75
50
25
0
m/z
0
250
500
750
1,000
1,250
1,500
1,750
b3
b2
y2
y3
b4
y5
b7
y7
b8
y16 2+
y8
y17 2+
y10
y11
y12
y13
y14
y15
y16

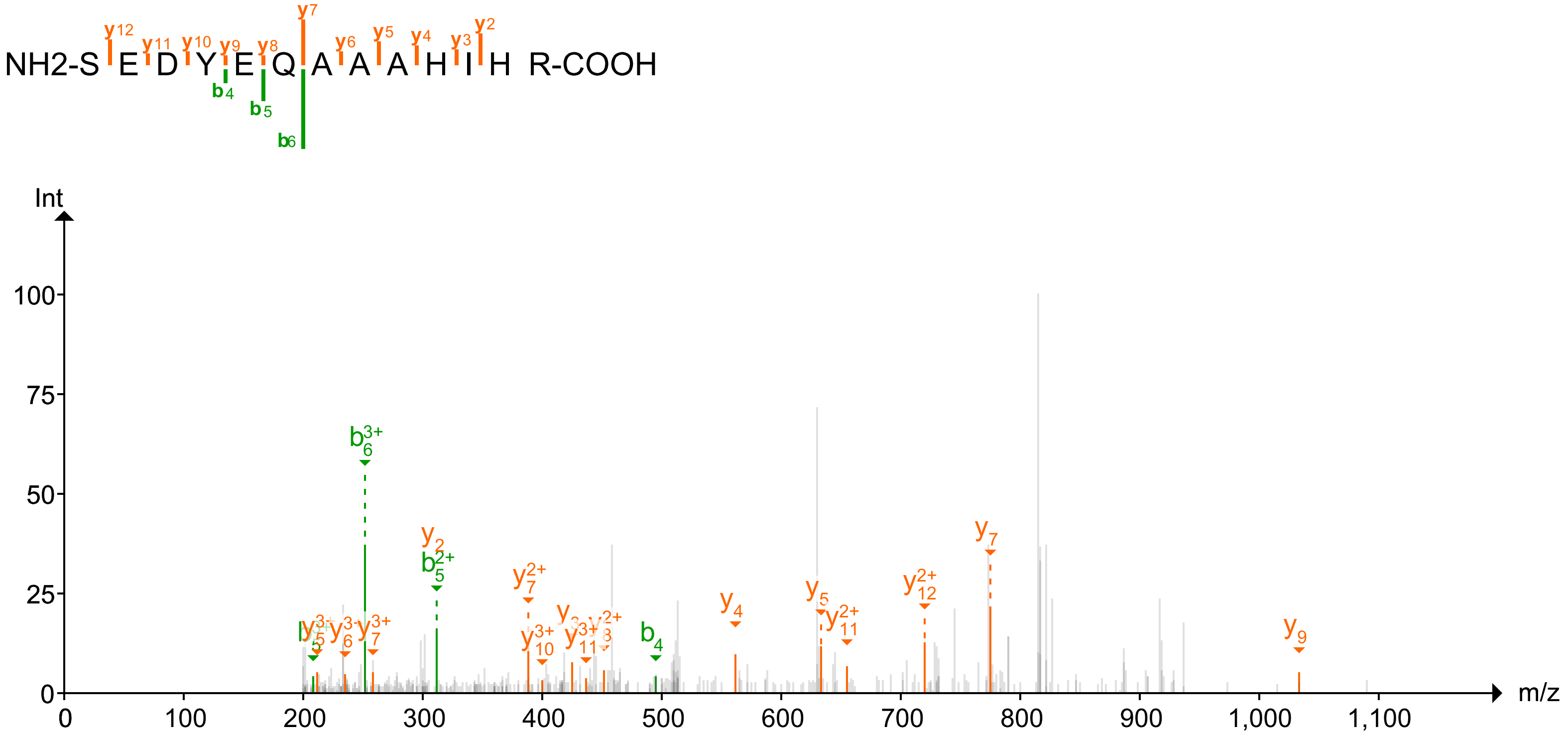

NH2-S E D Y E Q A A A H I H R-COOH
y12 y11 y10 y9 y8 y7 y6 y5 y4 y3 y2
b4 b5 b6
Int
m/z
0
25
50
75
100
0
100
200
300
400
500
600
700
800
900
1,000
1,100

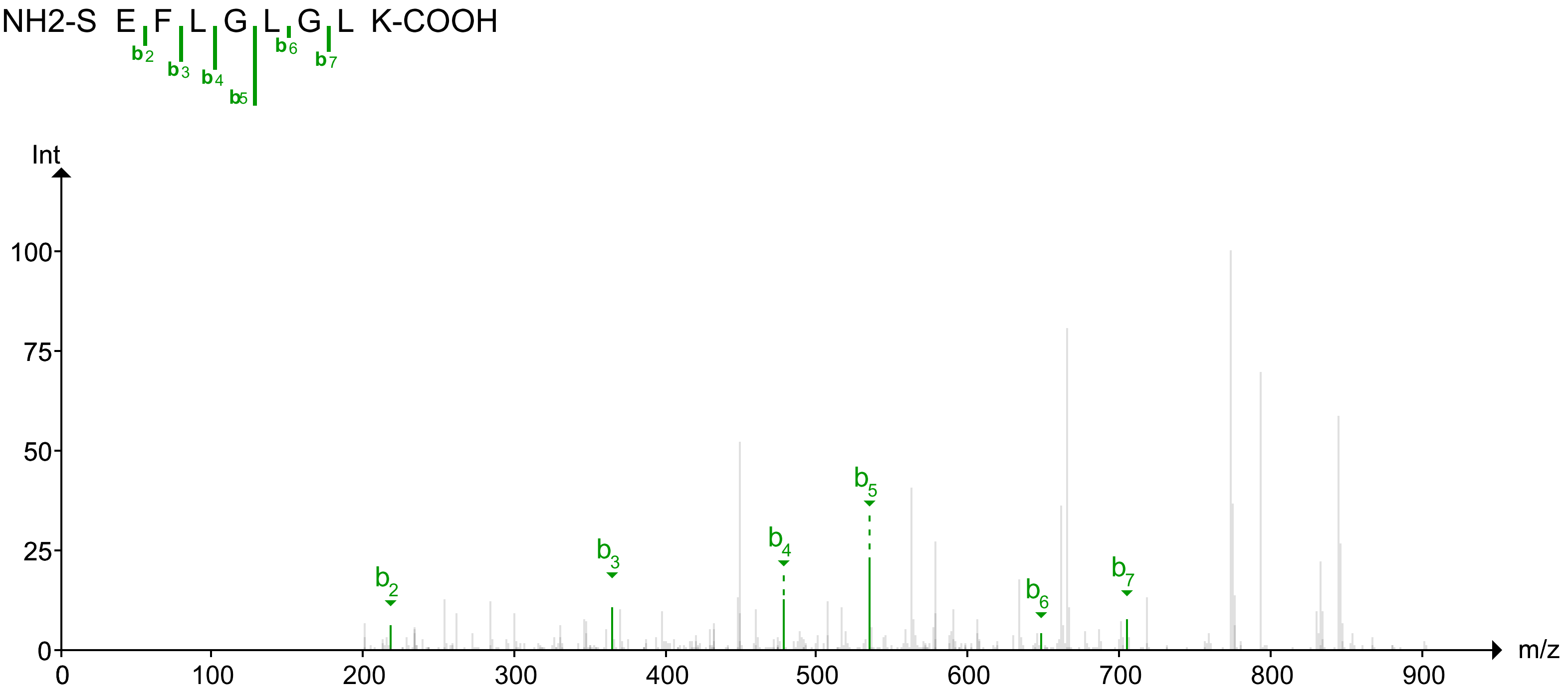
NH2-S E F L G L G L K-COOH
b2
b3
b4
b5
b6
b7
Int
100
75
50
25
0
0
100
200
300
400
500
600
700
800
900
m/z

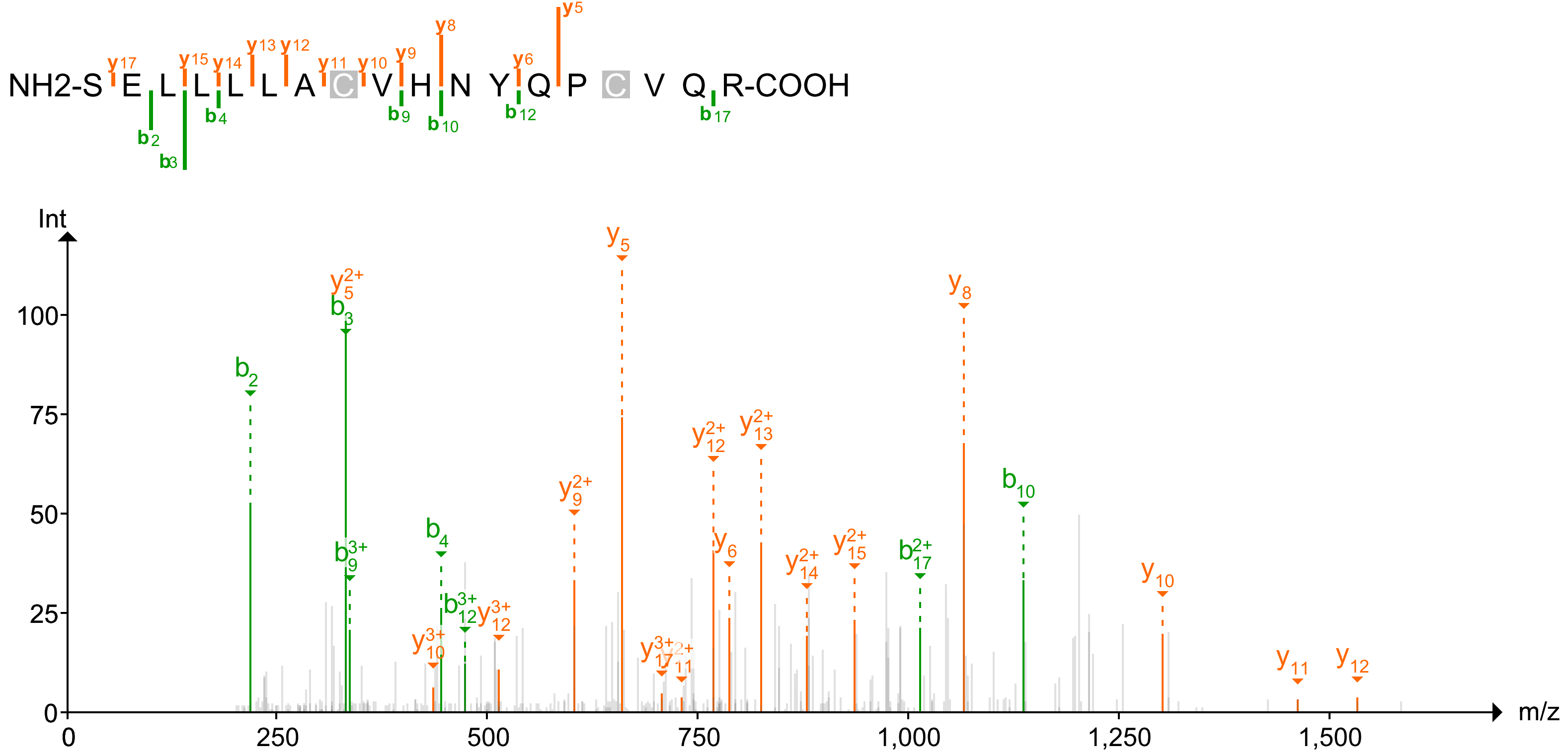

NH2-S E L L L L A C V H N Y Q P C V Q R-COOH
y5
y8
y13 y12
y9
y17
y15 y14
y11 y10
y6
b2
b3
b4
b9
b10
b12
b17
Int
100
75
50
25
0
0
250
500
750
1,000
1,250
1,500
m/z

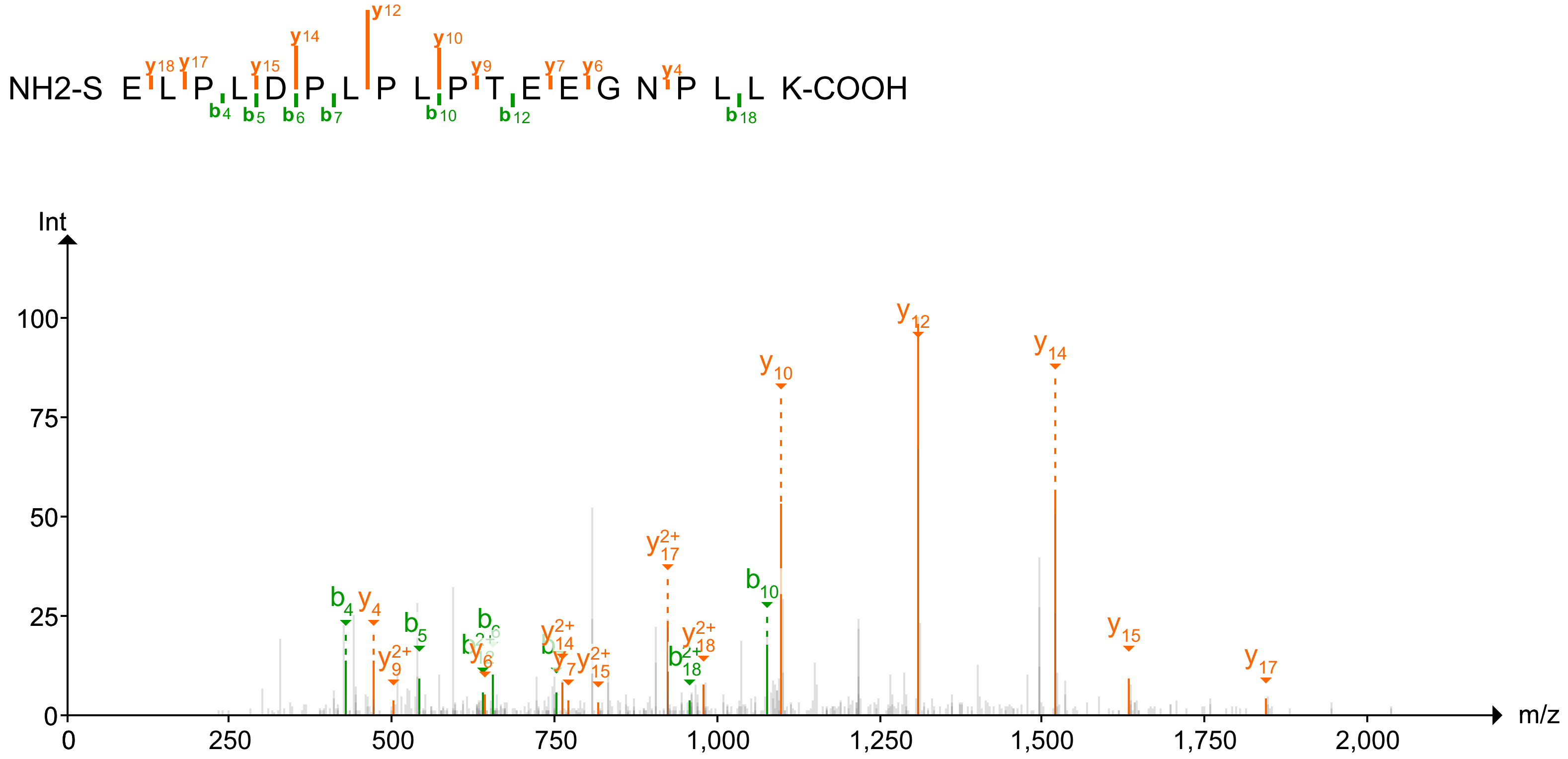
NH2-S E L P L D P L P L P T E E G N P L L K-COOH
Int
m/z
y12
y14
y10
y17
y15
y4
b10
b4
b5
b6
0
25
50
75
100
0
250
500
750
1,000
1,250
1,500
1,750
2,000

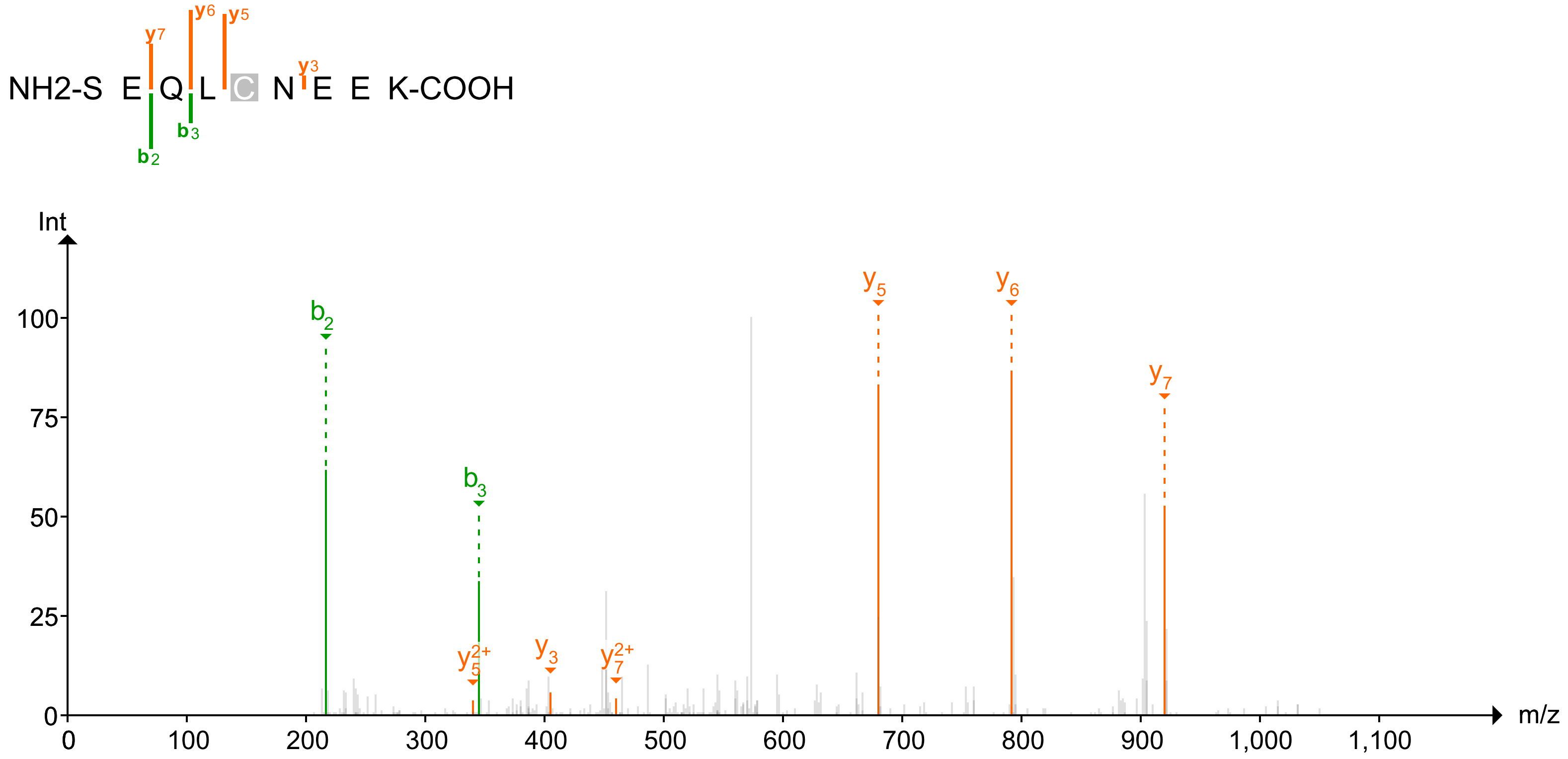

NH2-S E Q L C N E E K-COOH
y7 y6 y5 y3
b2 b3
Int
100
75
50
25
0
m/z
0 100 200 300 400 500 600 700 800 900 1,000 1,100
b2 b3 y5 y6 y7 y3
y5 2+
y7 2+

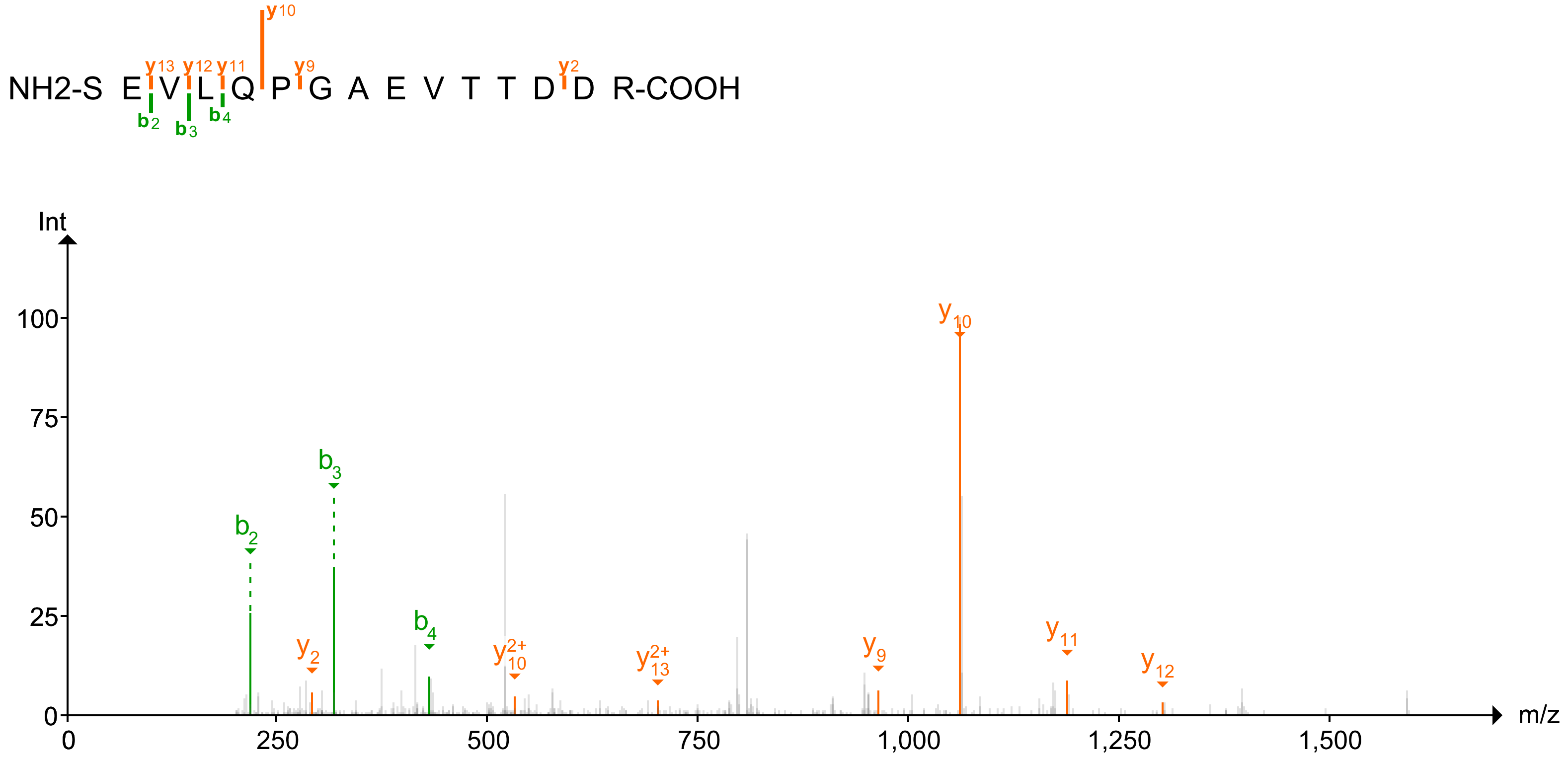

NH2-S E V L Q P G A E V T T D D R-COOH
y13 y12 y11 y10 y9 y2
b2 b3 b4
Int
100
75
50
25
0
b2
b3
b4
y2
y10 2+
y13 2+
y9
y10
y11
y12
0
250
500
750
1,000
1,250
1,500
m/z

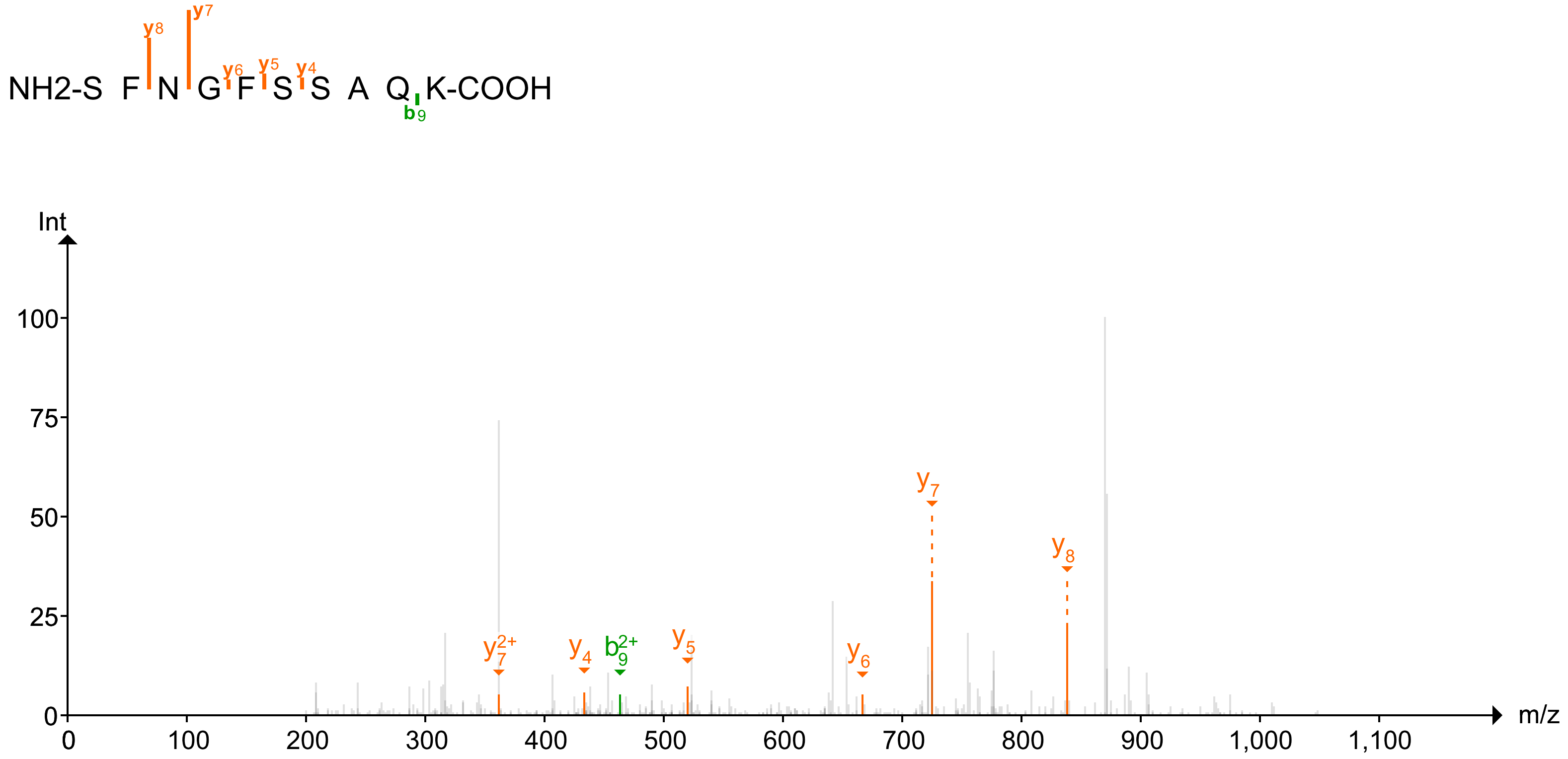

NH2-S F N G F S S A Q K-COOH
y8
y7
y6
y5
y4
b9
Int
100
75
50
25
0
y7
y8
y7 2+
y4
b9 2+
y5
y6
0
100
200
300
400
500
600
700
800
900
1,000
1,100
m/z

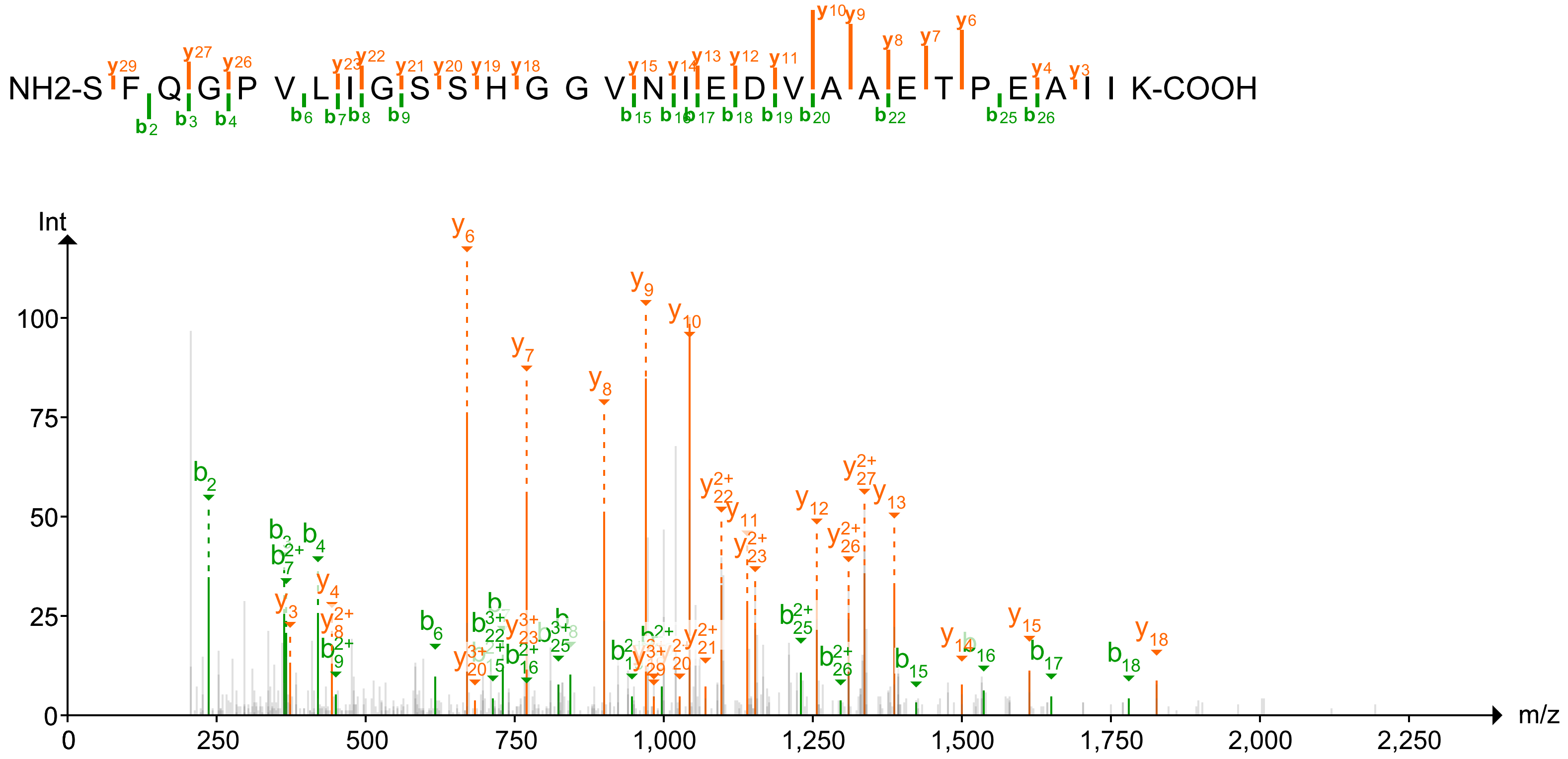

NH2-S F Q G P V L I G S S H G G V N I E D V A A E T P E A I I K-COOH
Int
m/z
0
25
50
75
100
250
500
750
1,000
1,250
1,500
1,750
2,000
2,250

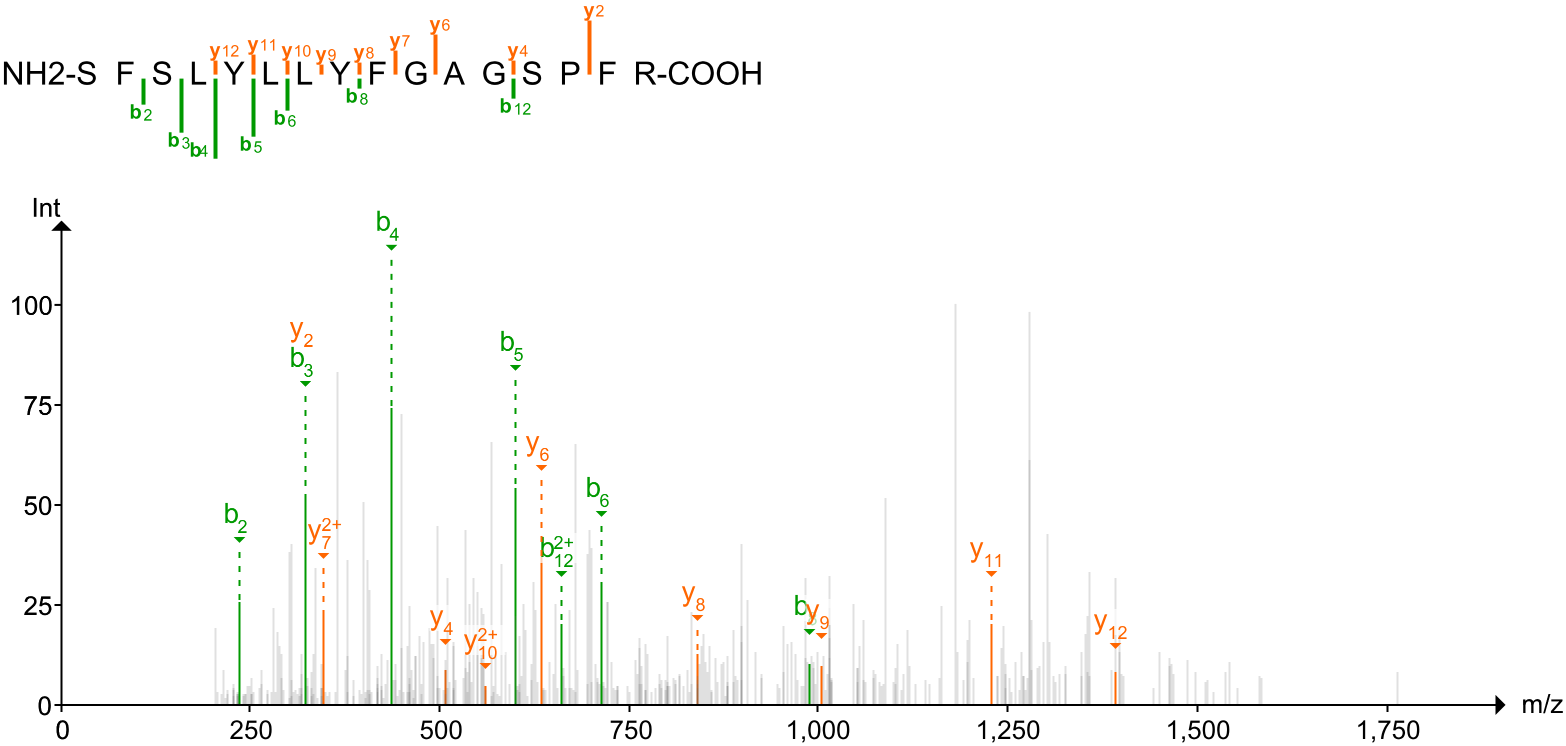

NH2-S F S L Y L L Y F G A G S P F R-COOH
y12 y11 y10 y9 y8 y7 y6 y4 y2
b2 b3 b4 b5 b6 b8 b12
Int
100
75
50
25
0
m/z
0
250
500
750
1,000
1,250
1,500
1,750
b4
y2
b3
b5
y6
b6
b2
y7 2+
b12 2+
y4
y10 2+
y8
b
y9
y11
y12

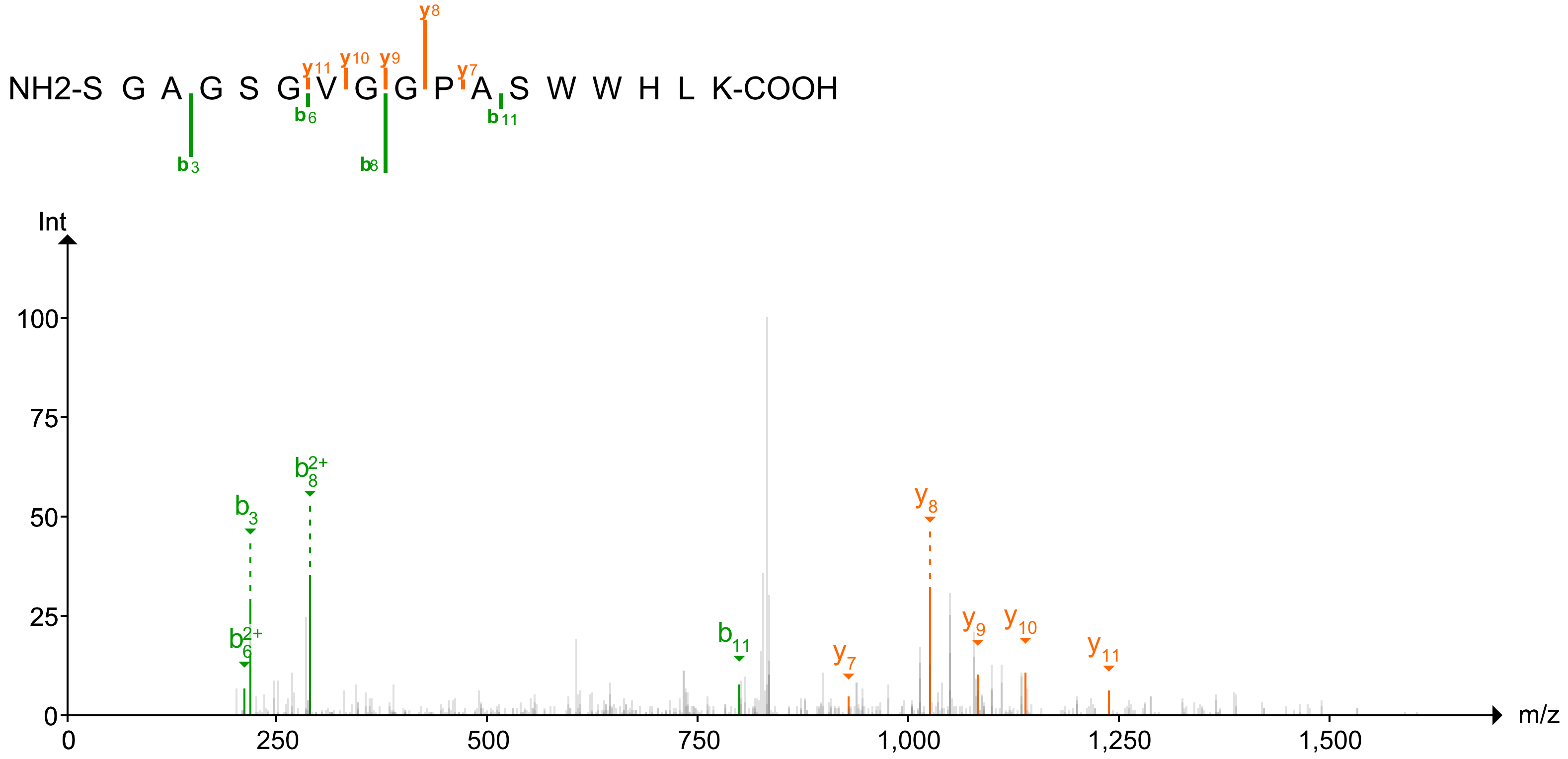

NH2-S G A G S G V G G P A S W W H L K-COOH
y8
y11 y10 y9 y7
b6 b11
b3 b8
Int
100
75
50
25
0
b8 2+
b3
y8
b6 2+
b11
y7
y9
y10
y11
0
250
500
750
1,000
1,250
1,500
m/z

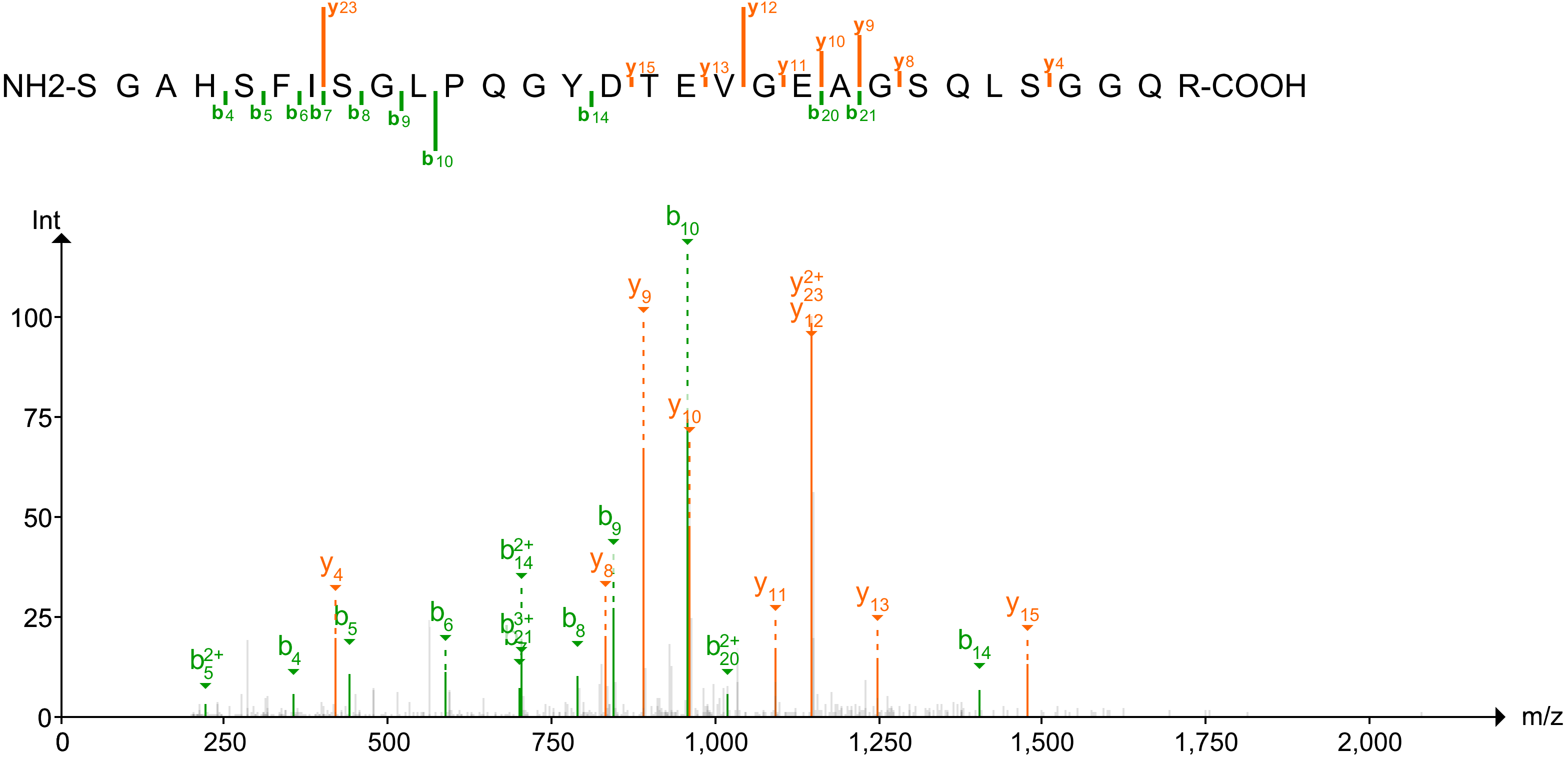
NH2-S G A H S F I S G L P Q G Y D T E V G E A G S Q L S G G Q R-COOH
y23
y15
y13
y12
y11
y10
y9
y8
y4
b4
b5
b6
b7
b8
b9
b10
b14
b20
b21
Int
m/z
0
25
50
75
100
250
500
750
1,000
1,250
1,500
1,750
2,000

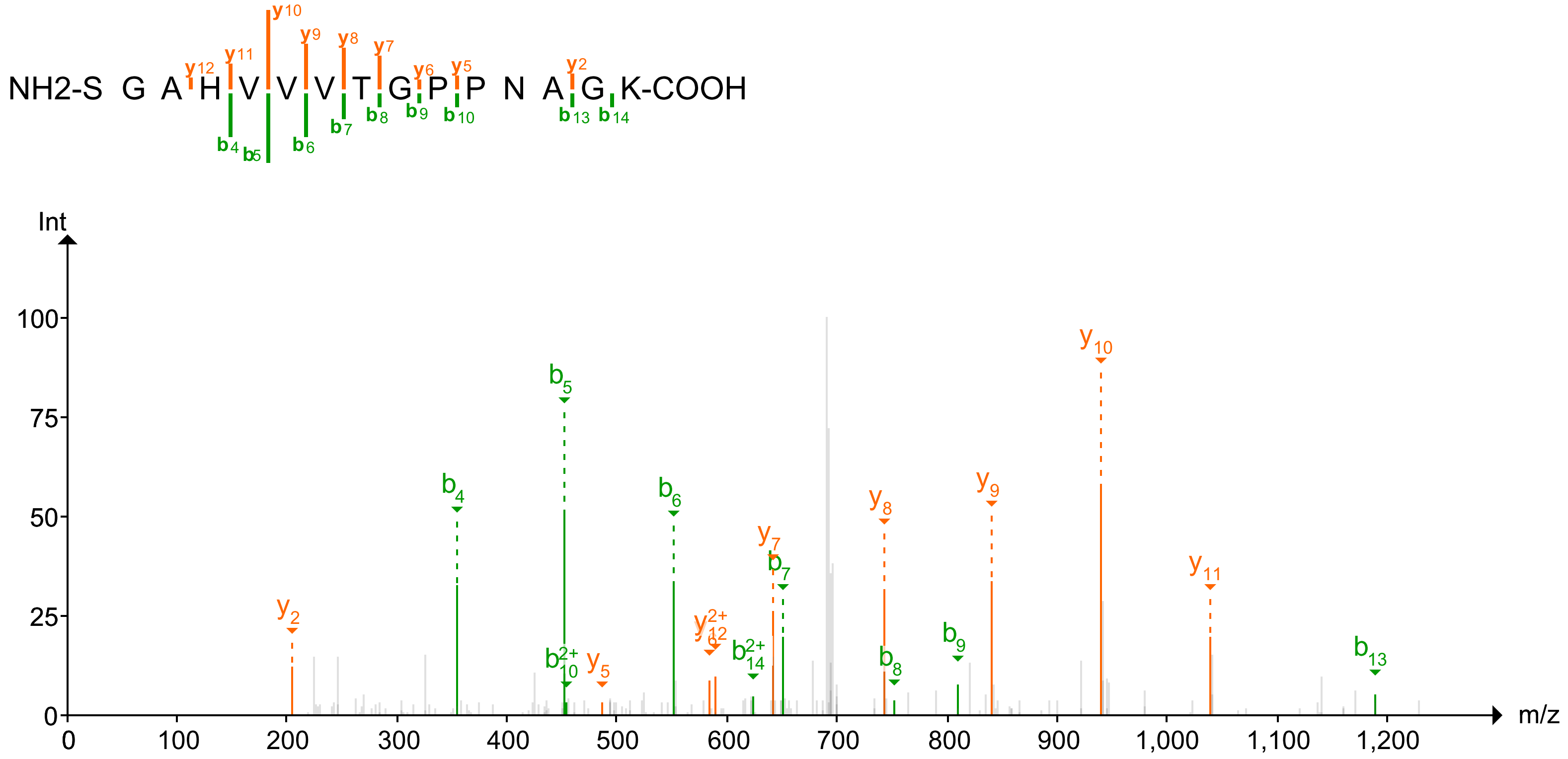

NH2-S G A H V V V T G P P N A G K-COOH
y12 y11 y10 y9 y8 y7 y6 y5 y2
b4 b5 b6 b7 b8 b9 b10 b13 b14
Int
100
75
50
25
0
m/z
0 100 200 300 400 500 600 700 800 900 1,000 1,100 1,200
y2 b4 b5 b10 2+ y5 b6 y12 2+ y6 b14 2+ y7 b7 y8 b8 b9 y9 y10 y11 b13

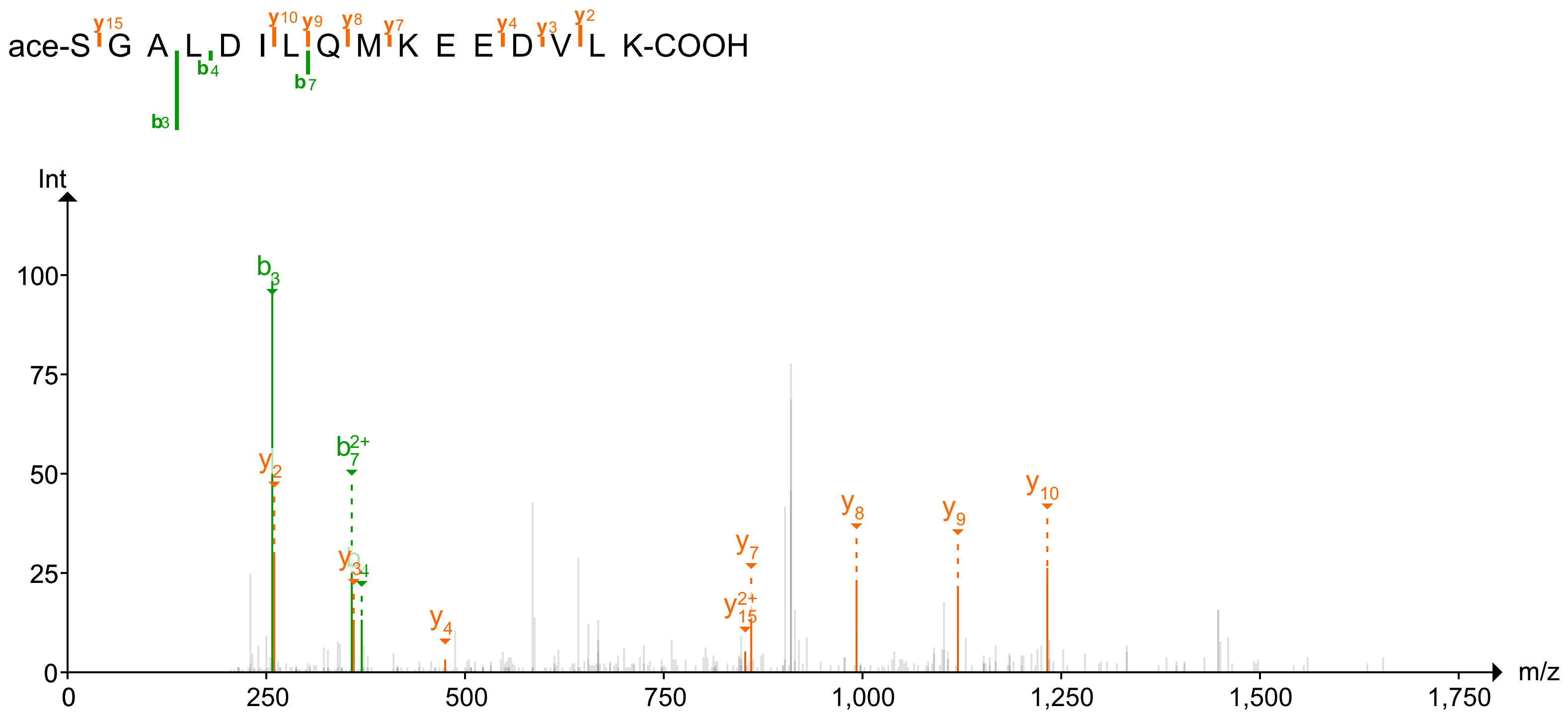

ace-S G A L D I L Q M K E E D V L K-COOH
y15 y10 y9 y8 y7 y4 y3 y2
b3 b4 b7
Int
100
75
50
25
0
b3
y2
b7 2+
y3
b4
y4
y7
y15 2+
y8
y9
y10
0
250
500
750
1,000
1,250
1,500
1,750
m/z

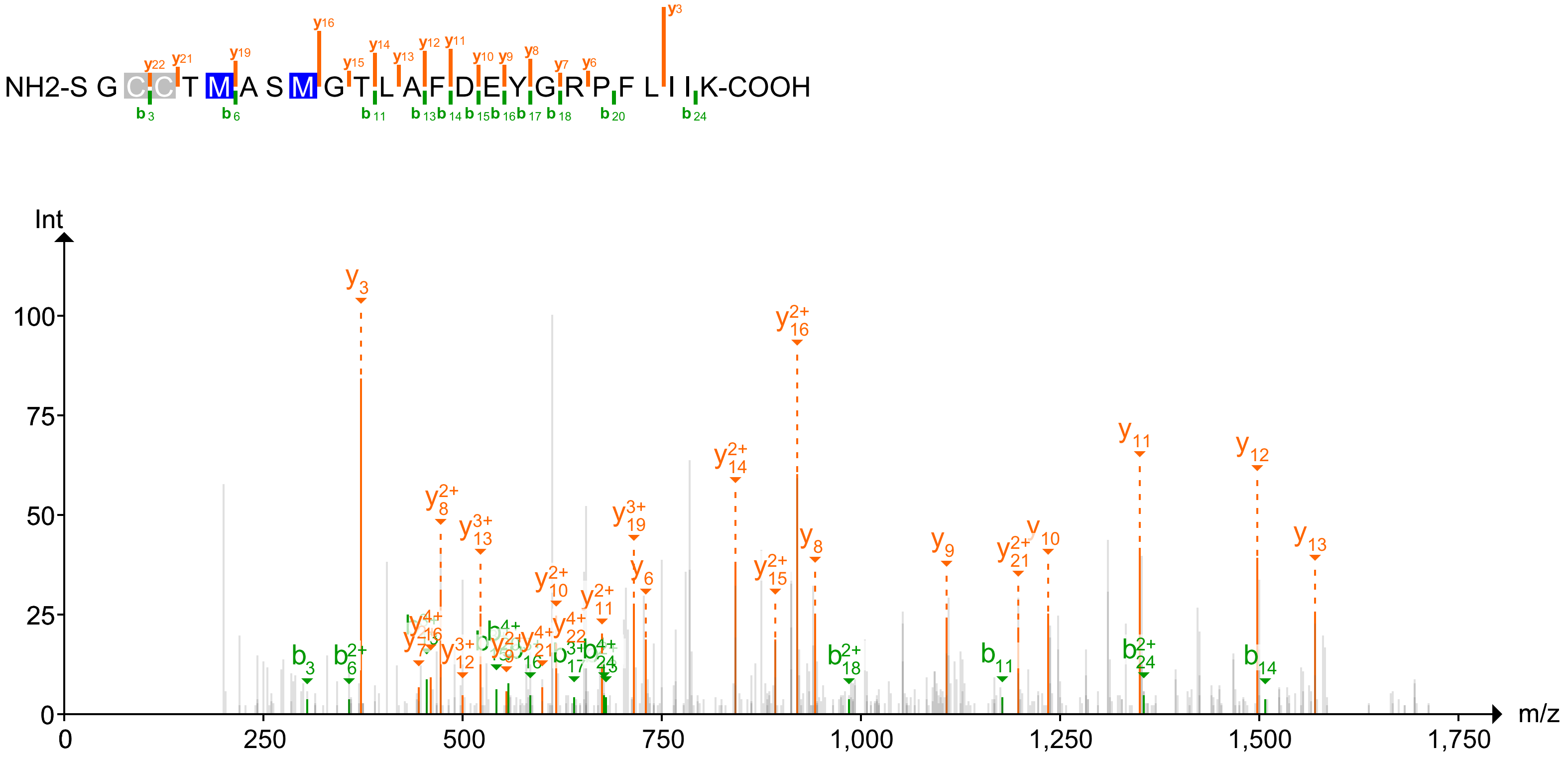

NH2-S G C C T M A S M G T L A F D E Y G R P F L I I K-COOH
Int
m/z
0
25
50
75
100
250
500
750
1,000
1,250
1,500
1,750

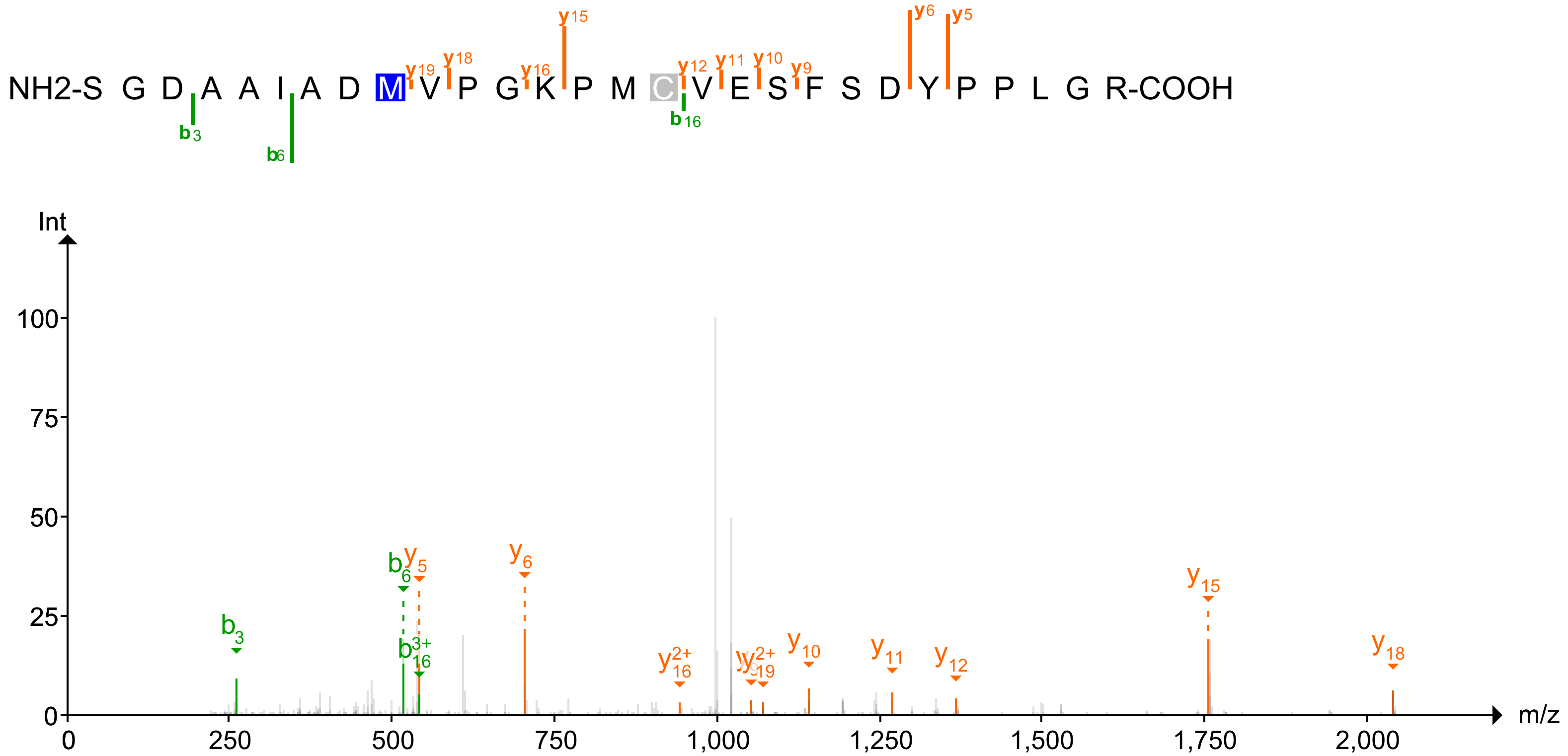

NH2-S G D A A I A D M V P G K P M C V E S F S D Y P P L G R-COOH
b3
b6
b16
y19
y18
y16
y15
y12
y11
y10
y9
y6
y5
Int
100
75
50
25
0
m/z
0
250
500
750
1,000
1,250
1,500
1,750
2,000

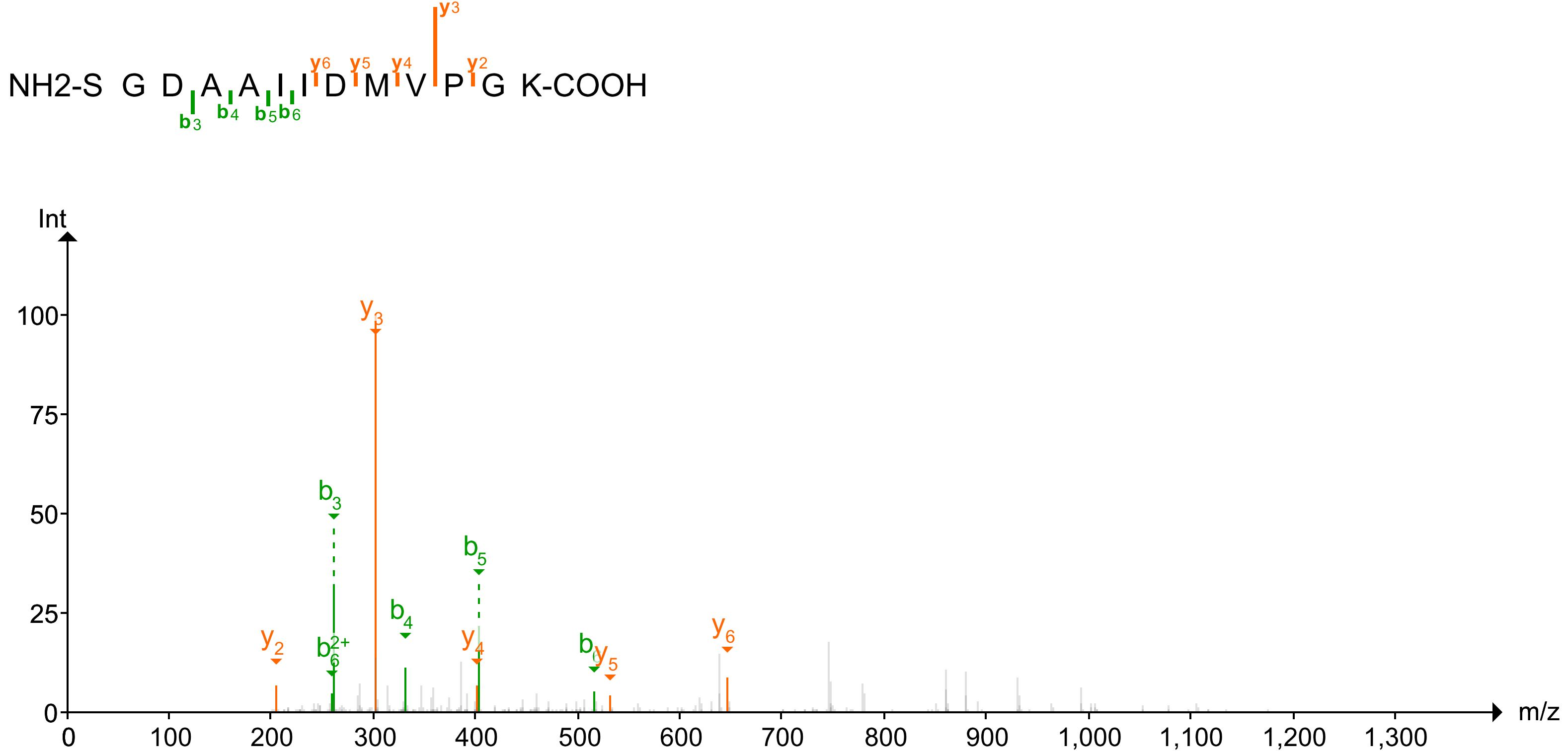

NH2-S G D A A I I D M V P G K-COOH
b3 b4 b5 b6
y6 y5 y4 y3 y2
Int
100
75
50
25
0
m/z
0
100
200
300
400
500
600
700
800
900
1,000
1,100
1,200
1,300
y3
b3
b5
b4
y2
b6 2+
y4
b6
y5
y6

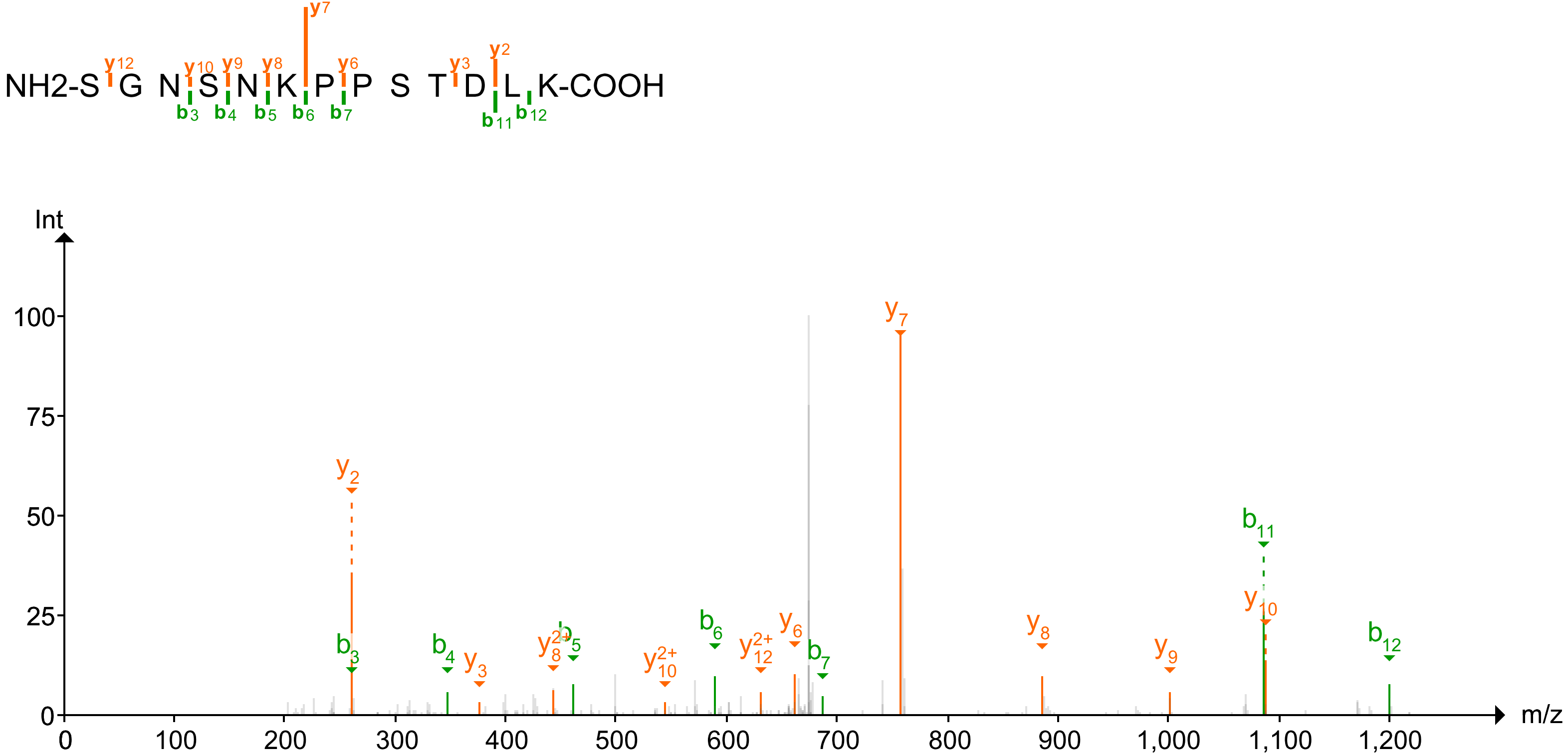

NH2-S G N S N K P P S T D L K-COOH
y12 y10 y9 y8 y7 y6 y3 y2
b3 b4 b5 b6 b7 b11 b12
Int
100
75
50
25
0
y7
y2
b11
y10
b12
y9
y8
y6
b7
b6
y2+ 12
y2+ 10
y2+ 8
b5
y3
b4
b3
0
100
200
300
400
500
600
700
800
900
1,000
1,100
1,200
m/z

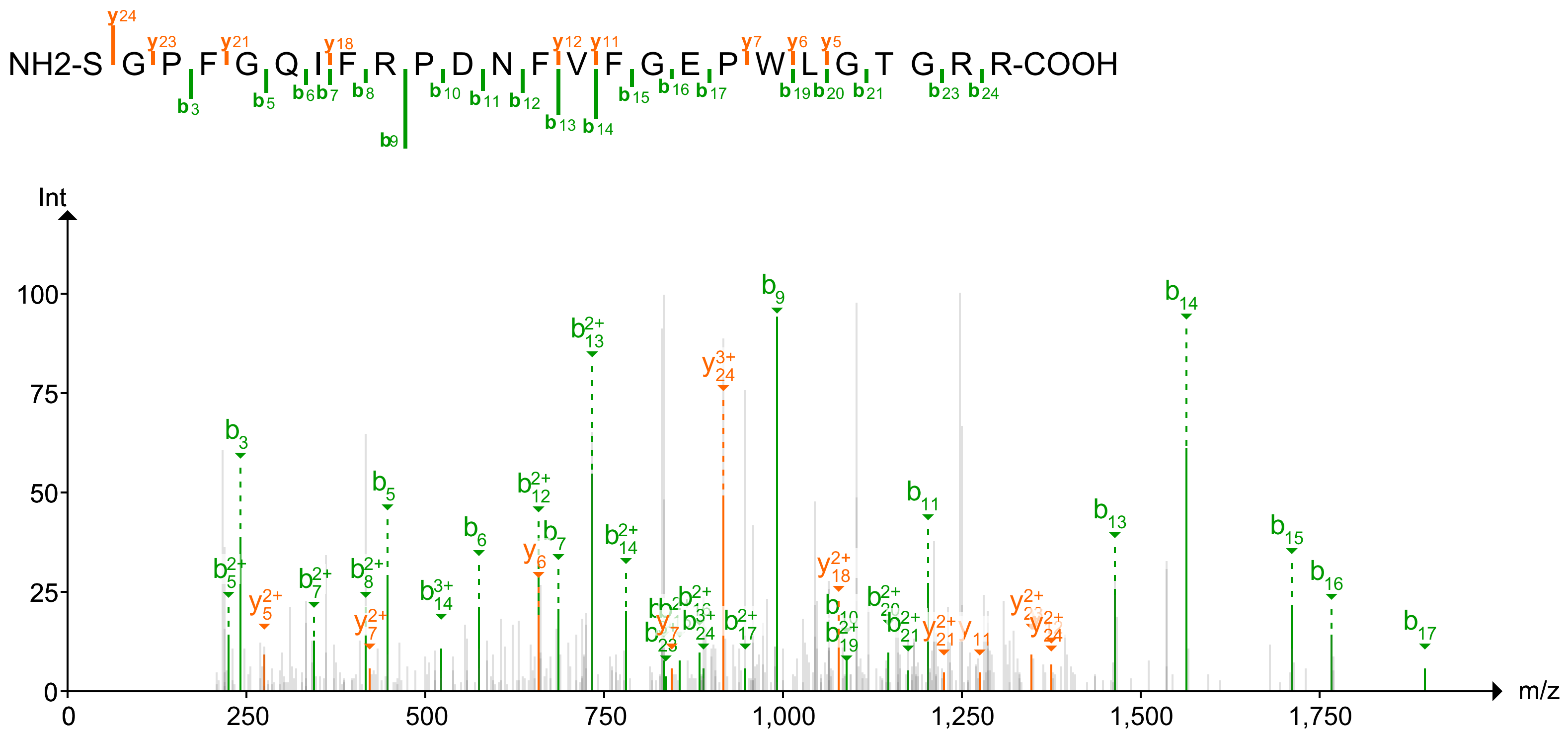

NH2-S G P F G Q I F R P D N F V F G E P W L G T G R R-COOH
Int
m/z
0
25
50
75
100
250
500
750
1,000
1,250
1,500
1,750

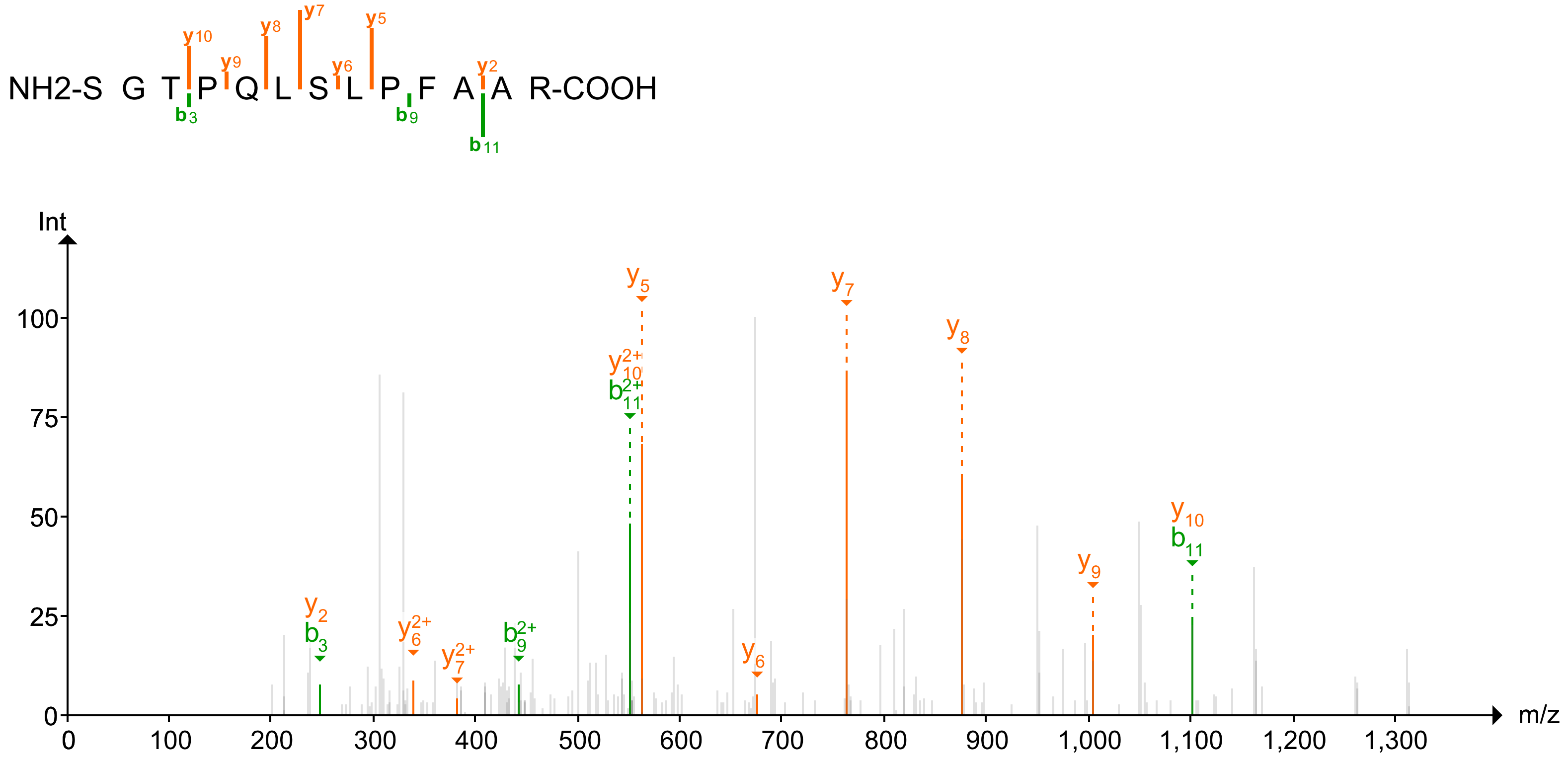

NH2-S G T P Q L S L P F A A R-COOH
y10 y9 y8 y7 y6 y5 y2
b3 b9 b11
Int
m/z
0
25
50
75
100
0 100 200 300 400 500 600 700 800 900 1,000 1,100 1,200 1,300
y5
y7
y8
y10 2+
b11 2+
y10
b11
y9
y6
y2
b3
y6 2+
y7 2+
b9 2+

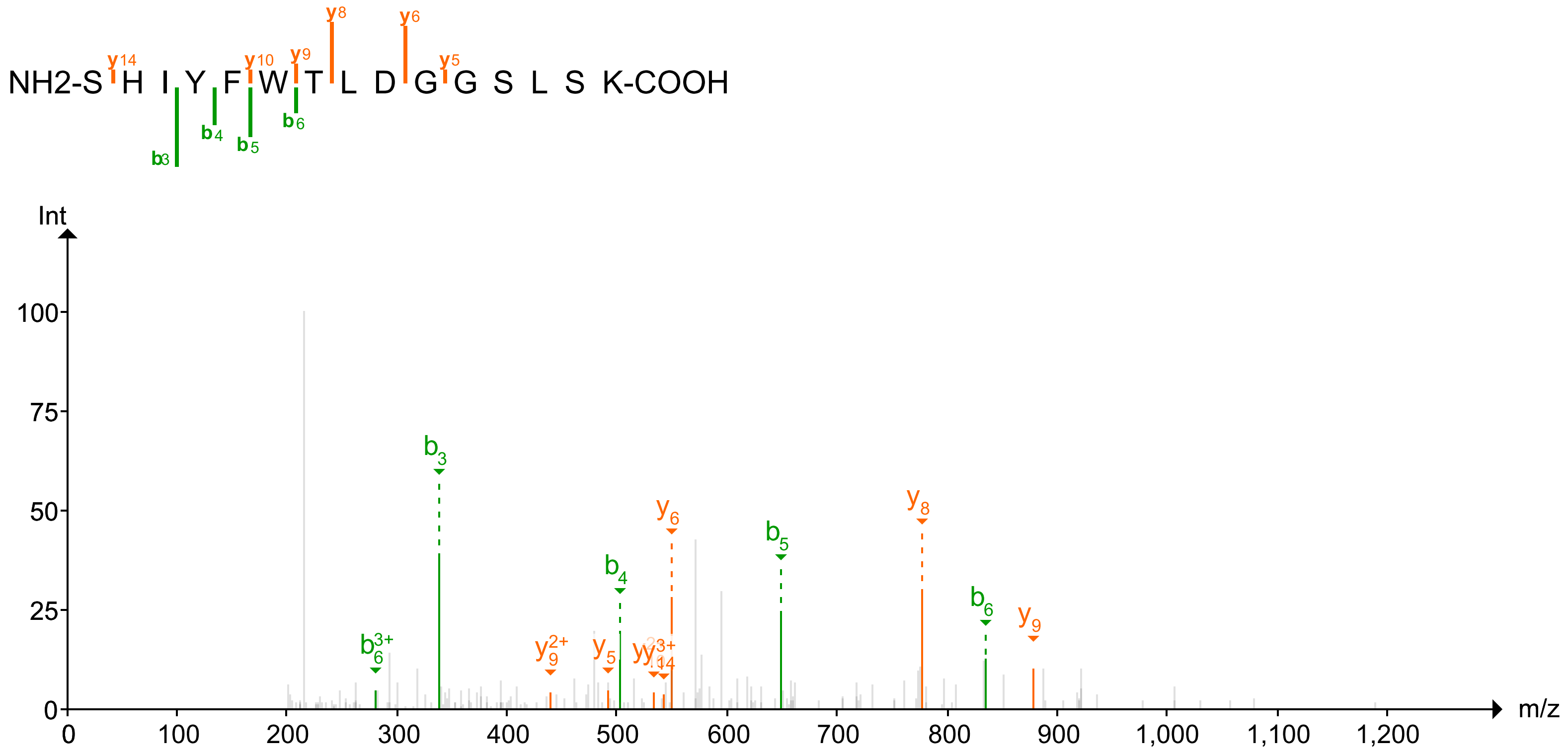
NH2-S H I Y F W T L D G G S L S K-COOH
y14 y10 y9 y8 y6 y5
b3 b4 b5 b6
Int
100
75
50
25
0
b3
b4
b5
b6
$b_6^{3+}$
y6
y8
y9
$y_9^{2+}$
y5
$y_{14}^{3+}$
0 100 200 300 400 500 600 700 800 900 1,000 1,100 1,200
m/z

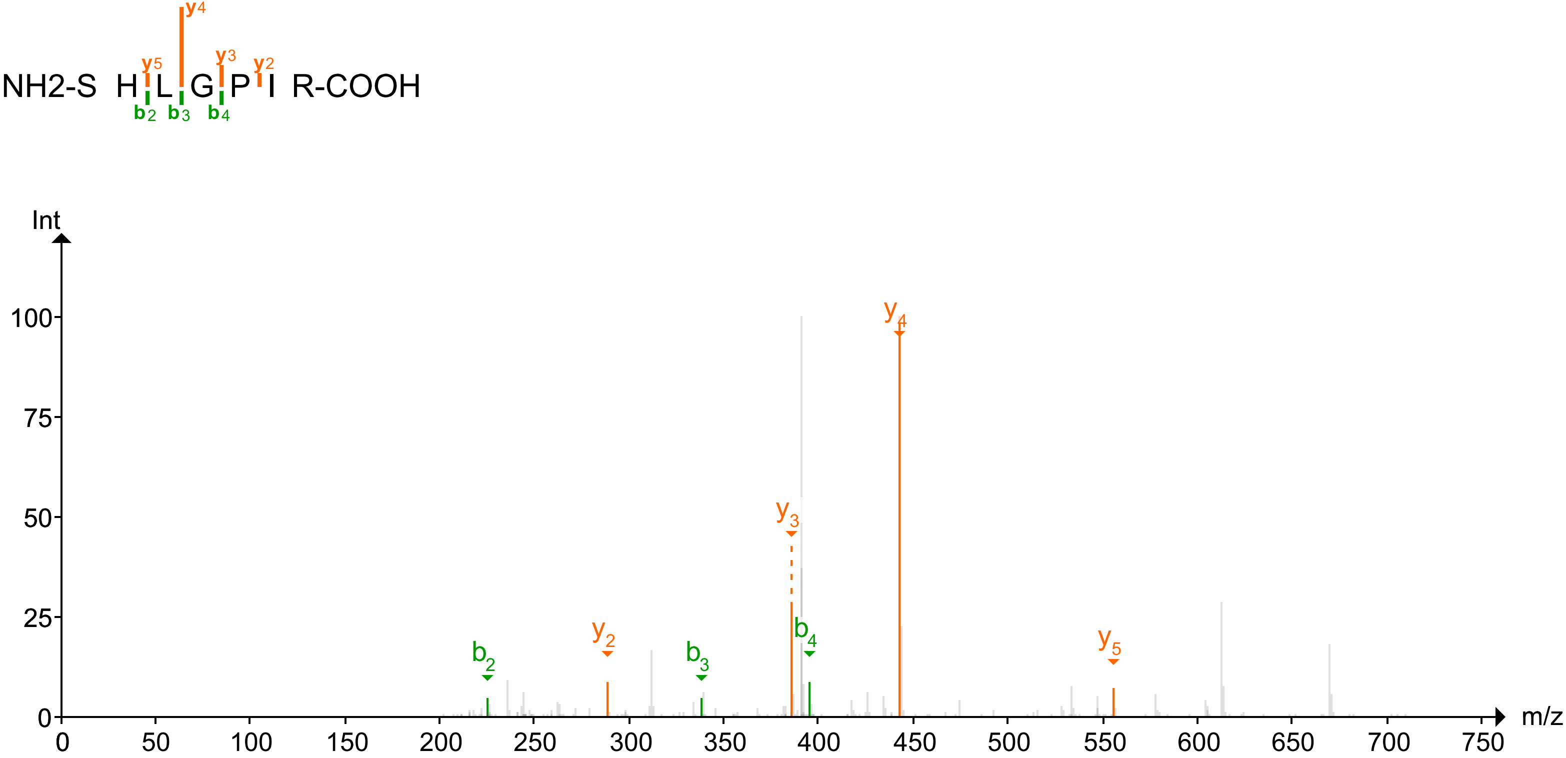
NH2-S H L G P I R-COOH
y4
y5
y3
y2
b2
b3
b4
Int
100
75
50
25
0
m/z
0
50
100
150
200
250
300
350
400
450
500
550
600
650
700
750
b2
y2
b3
y3
b4
y4
y5

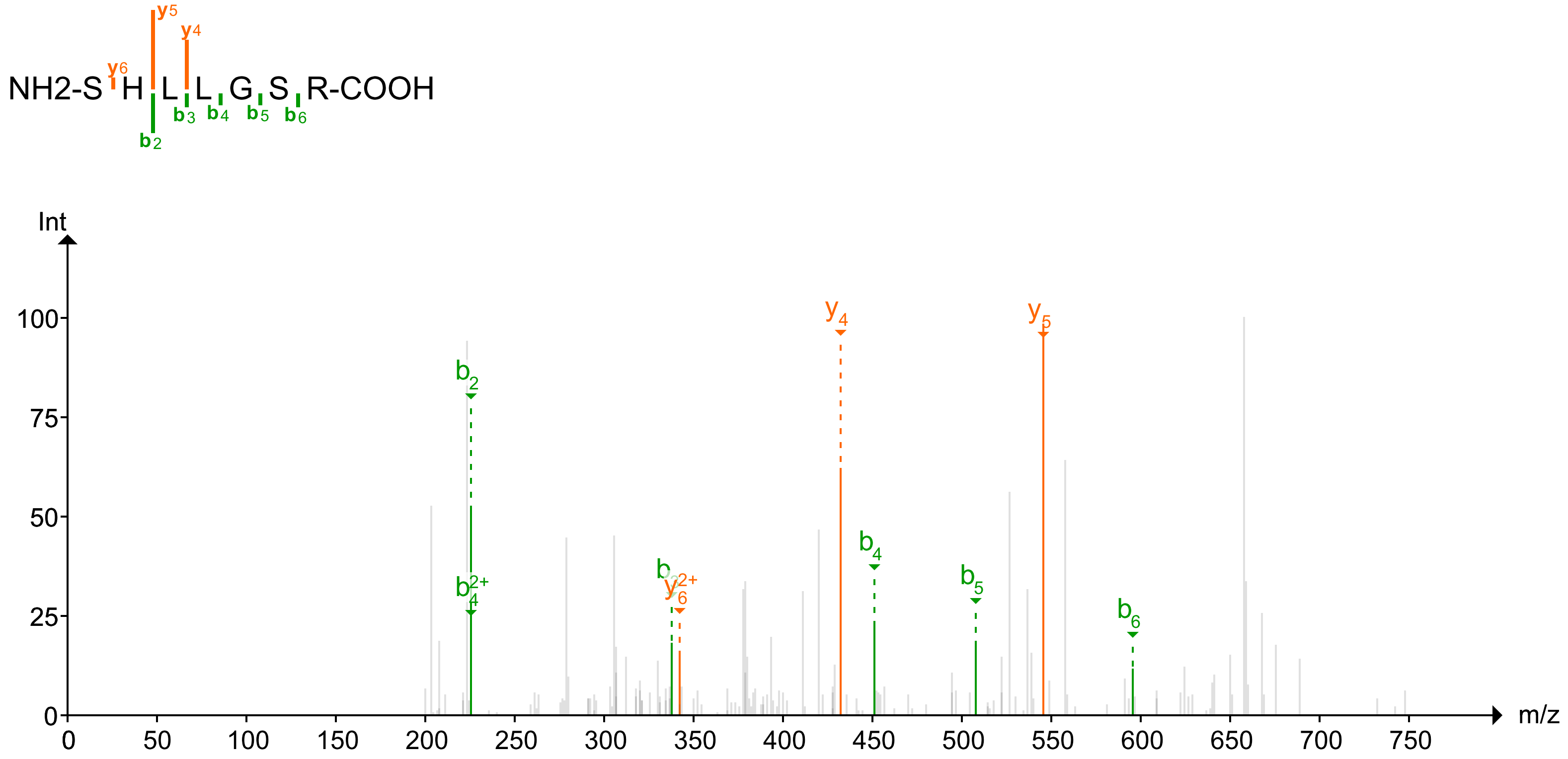

NH2-S H L L G S R-COOH
y6 y5 y4
b2 b3 b4 b5 b6
Int
m/z

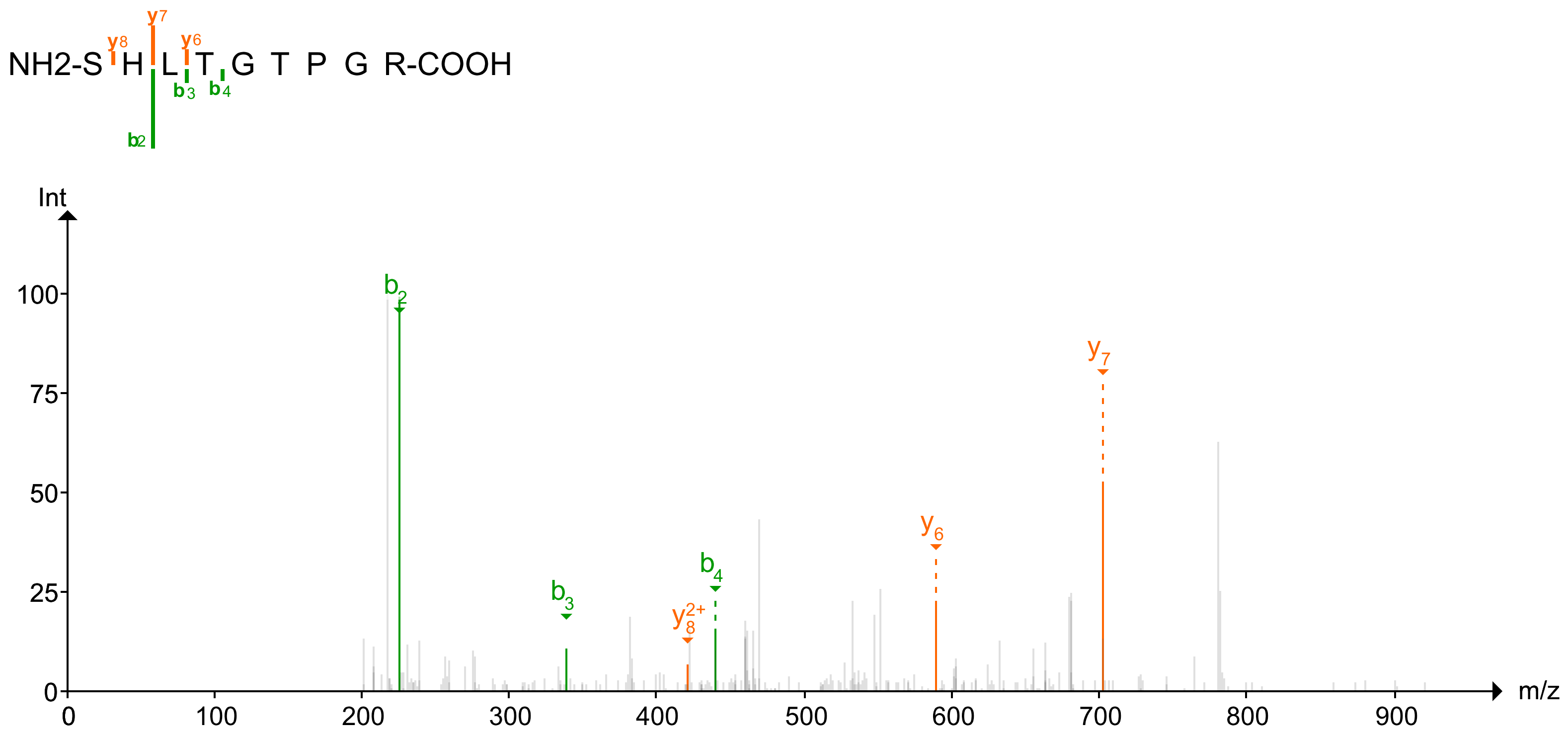

NH2-S H L T G T P G R-COOH
y8 y7 y6
b2 b3 b4
Int
100
75
50
25
0
b2
y7
y6
b4
b3
y8 2+
0 100 200 300 400 500 600 700 800 900
m/z

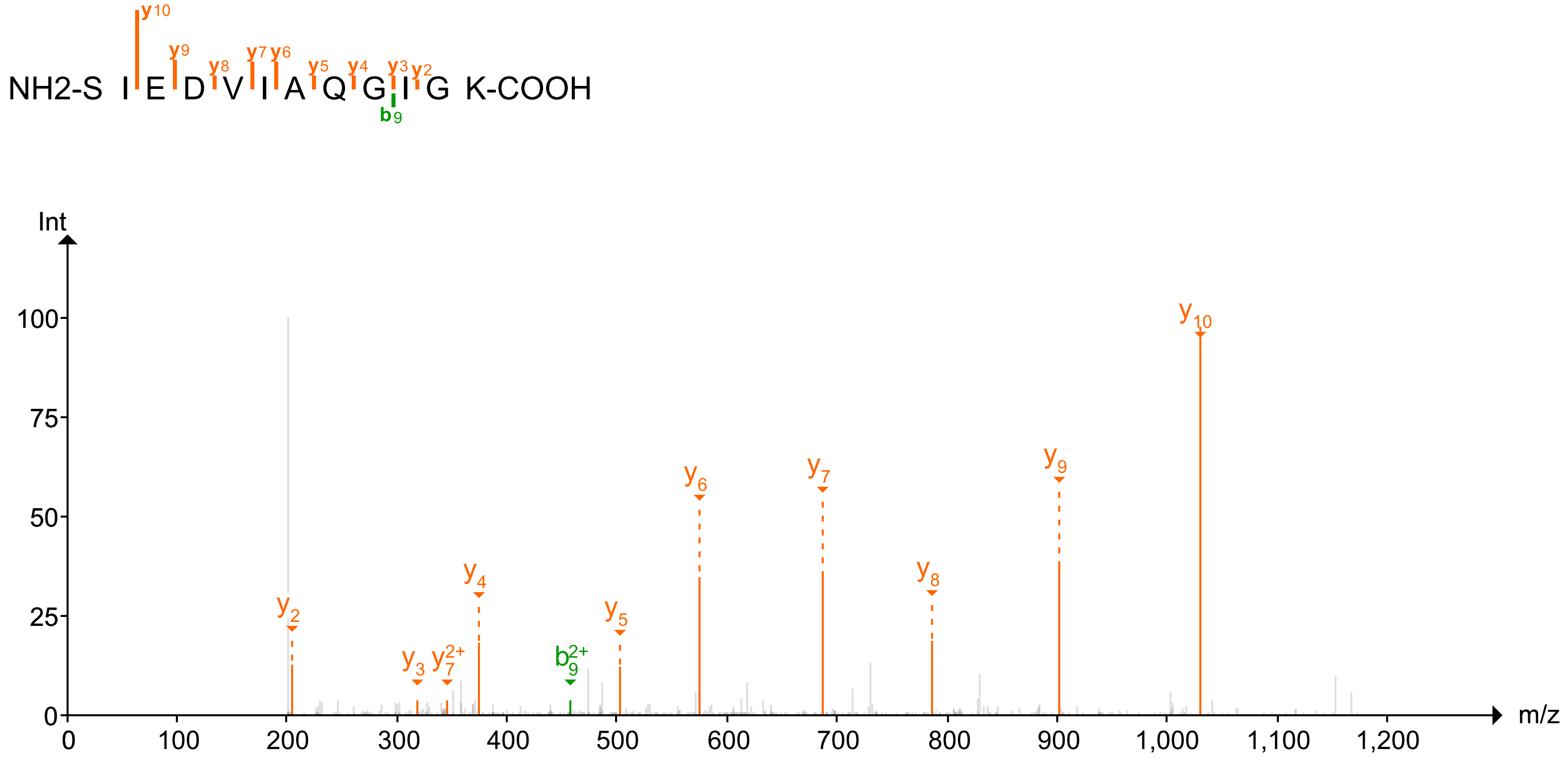

NH2-S I E D V I A Q G I I G K-COOH
y10 y9 y8 y7 y6 y5 y4 y3 y2
b9
Int
100
75
50
25
0
m/z
0 100 200 300 400 500 600 700 800 900 1,000 1,100 1,200
y10
y9
y7
y6
y8
y4
y2
y5
y3
y7 2+
b9 2+

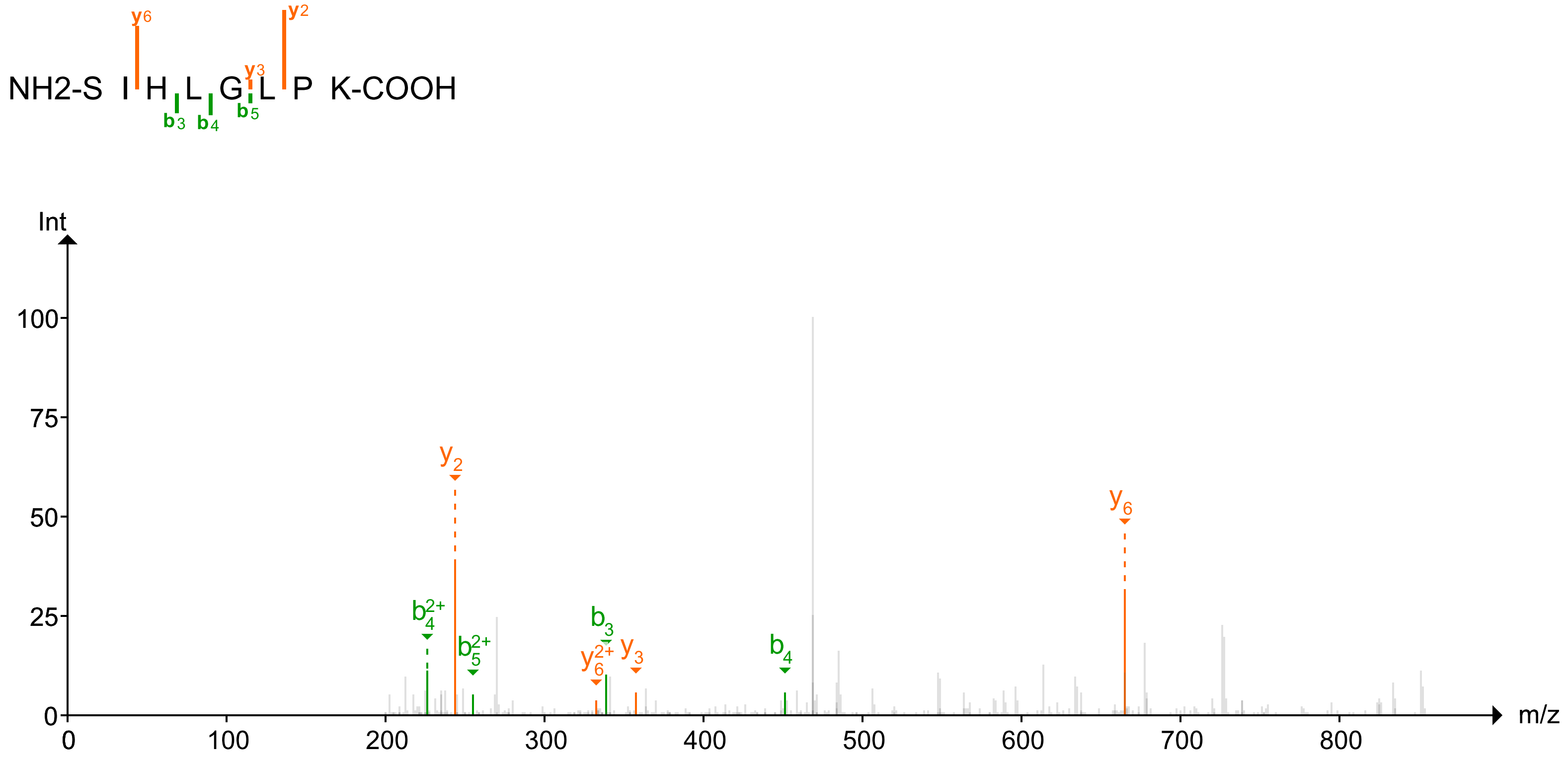

NH2-S I H L G L P K-COOH
y6
y3
y2
b3
b4
b5
Int
100
75
50
25
0
m/z
0
100
200
300
400
500
600
700
800
y2
y6
b4 2+
b5 2+
b3
y6 2+
y3
b4

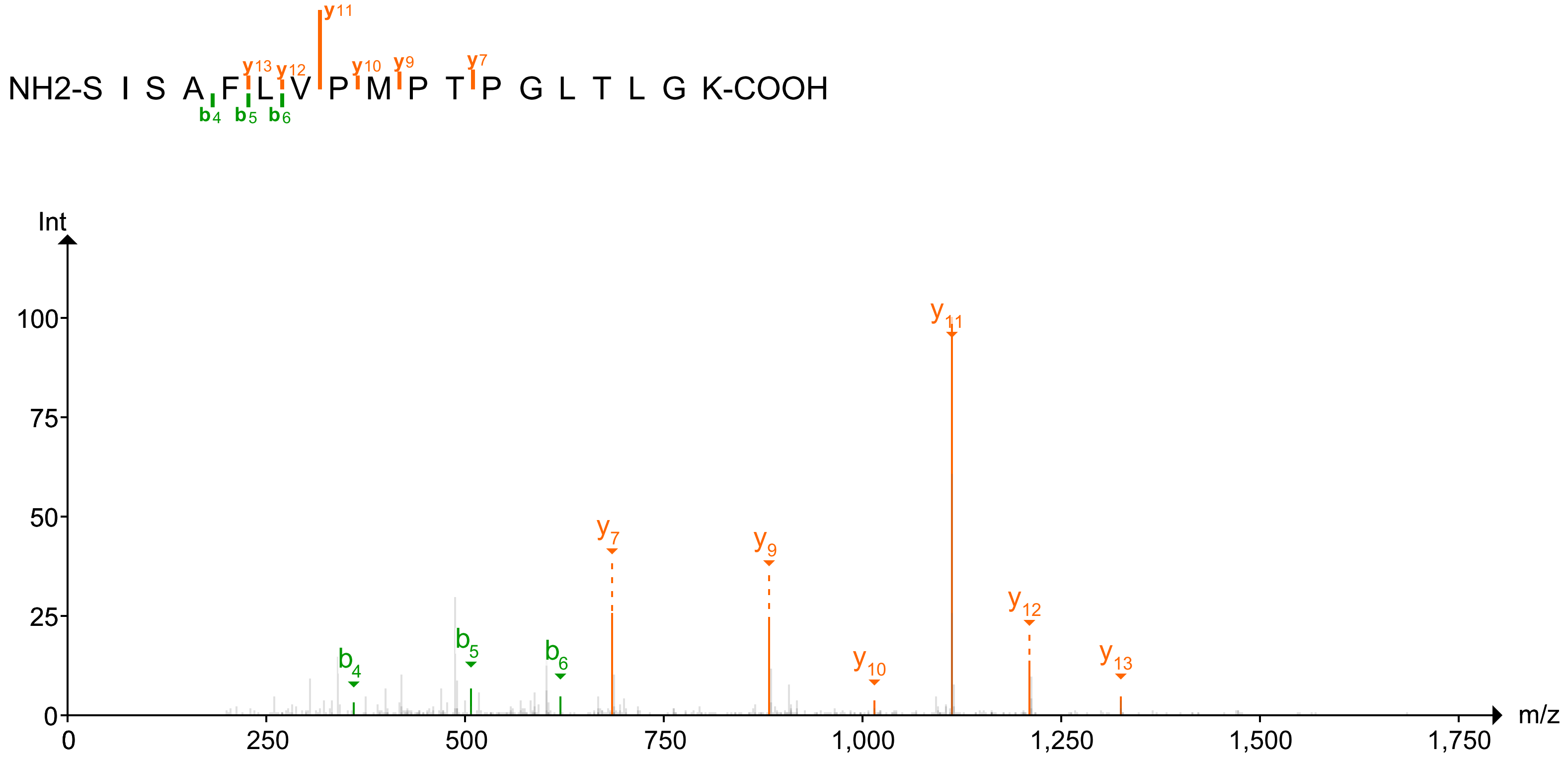

NH2-S I S A F L V P M P T P G L T L G K-COOH
b4 b5 b6
y13 y12 y11 y10 y9 y7
Int
100
75
50
25
0
m/z
0
250
500
750
1,000
1,250
1,500
1,750
b4 b5 b6 y7 y9 y10 y11 y12 y13

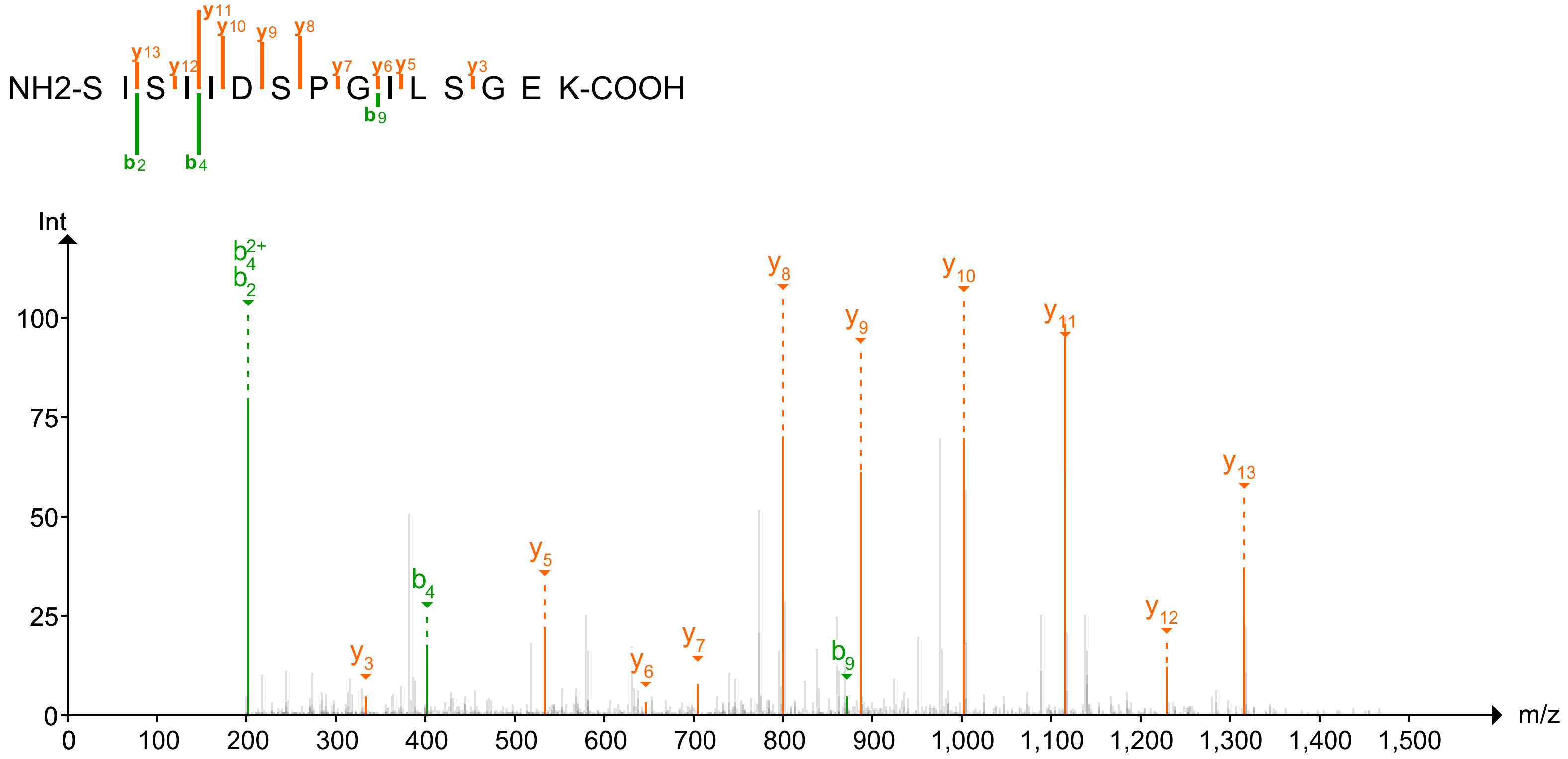

NH2-S I S I I D S P G I L S G E K-COOH
y13
y12
y11
y10
y9
y8
y7
y6
y5
y3
b2
b4
b9
Int
m/z
0
25
50
75
100
0
100
200
300
400
500
600
700
800
900
1,000
1,100
1,200
1,300
1,400
1,500

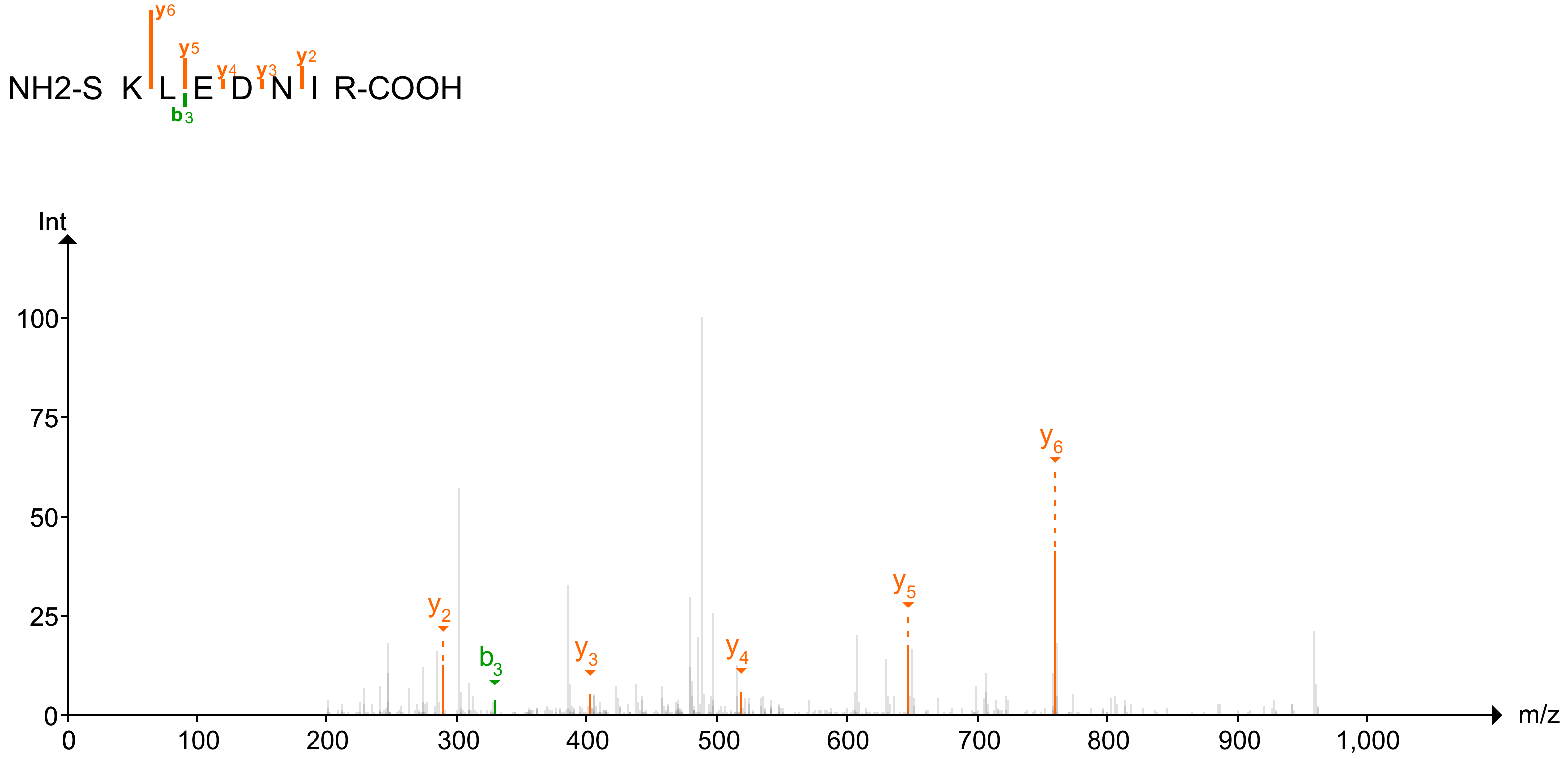

NH2-S K L E D N I R-COOH
y6
y5
y4
y3
y2
b3
Int
100
75
50
25
0
m/z
0
100
200
300
400
500
600
700
800
900
1,000

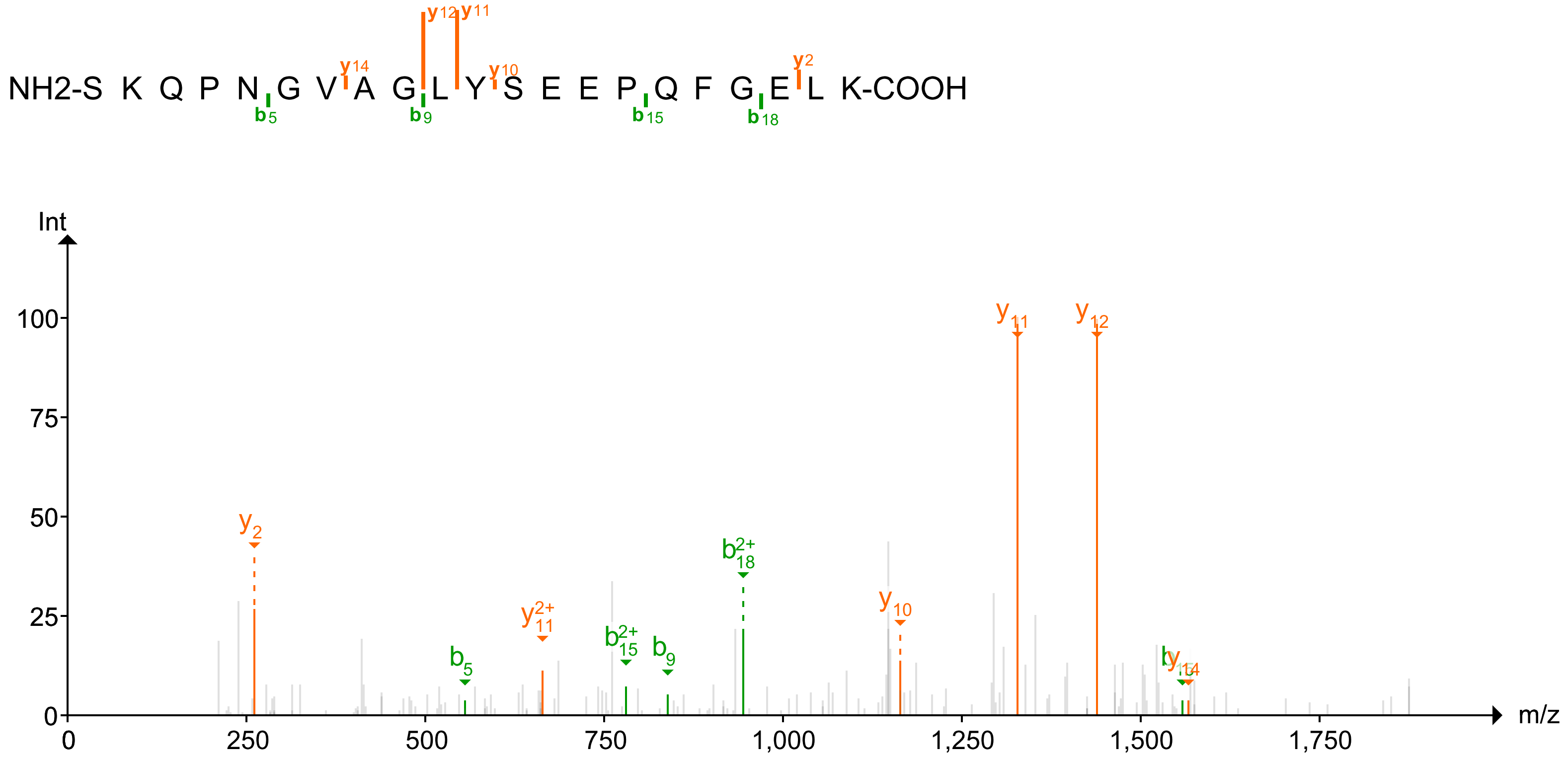

NH2-S K Q P N G V A G L Y S E E P Q F G E L K-COOH
y12 y11 y14 y10 y2
b5 b9 b15 b18
Int
100
75
50
25
0
y2
y2+11
b5
b2+15
b9
b2+18
y10
y11
y12
y14
0
250
500
750
1,000
1,250
1,500
1,750
m/z

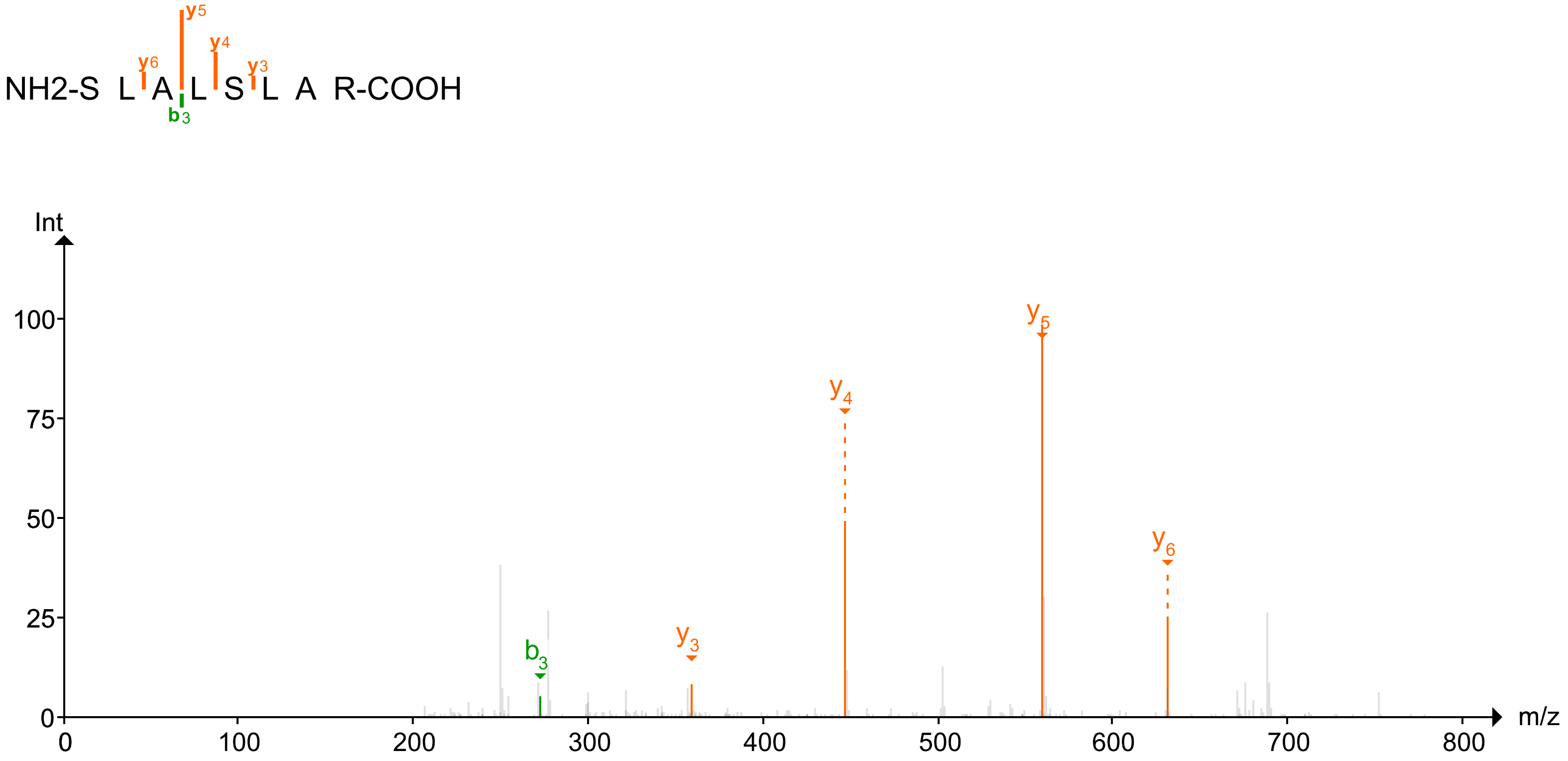

NH2-S L A L S L A R-COOH
y5
y4
y6
y3
b3
Int
100
75
50
25
0
m/z
0
100
200
300
400
500
600
700
800
b3
y3
y4
y5
y6

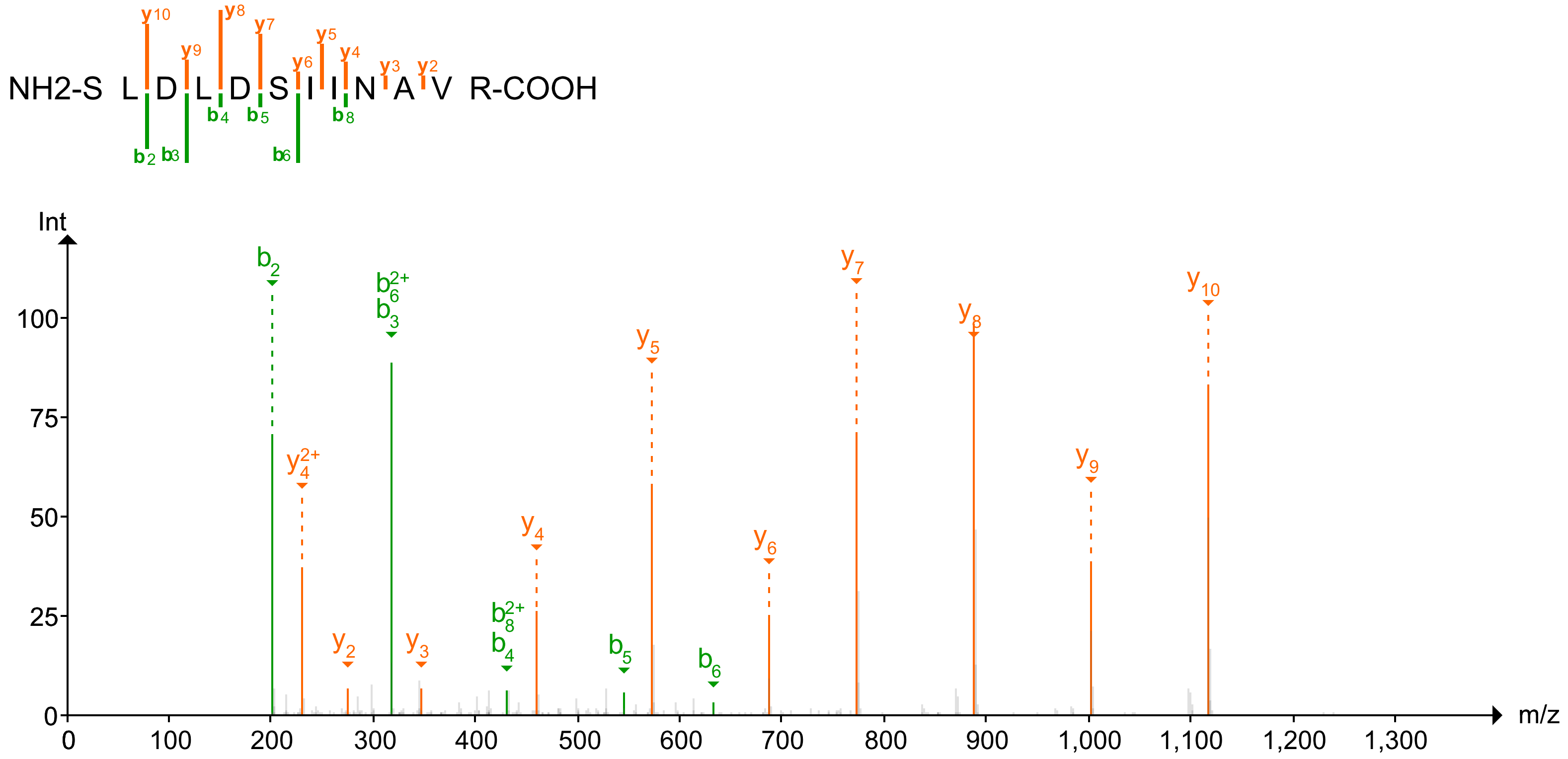

NH2-S L D L D S I I N A V R-COOH
y10 y9 y8 y7 y6 y5 y4 y3 y2
b2 b3 b4 b5 b6 b8
Int
m/z
0
25
50
75
100
0
100
200
300
400
500
600
700
800
900
1,000
1,100
1,200
1,300
b2
$y_4^{2+}$
$y_2$
$b_6^{2+}$
$b_3$
$y_3$
$b_8^{2+}$
$b_4$
$y_4$
$b_5$
$y_5$
$b_6$
$y_6$
$y_7$
$y_8$
$y_9$
$y_{10}$

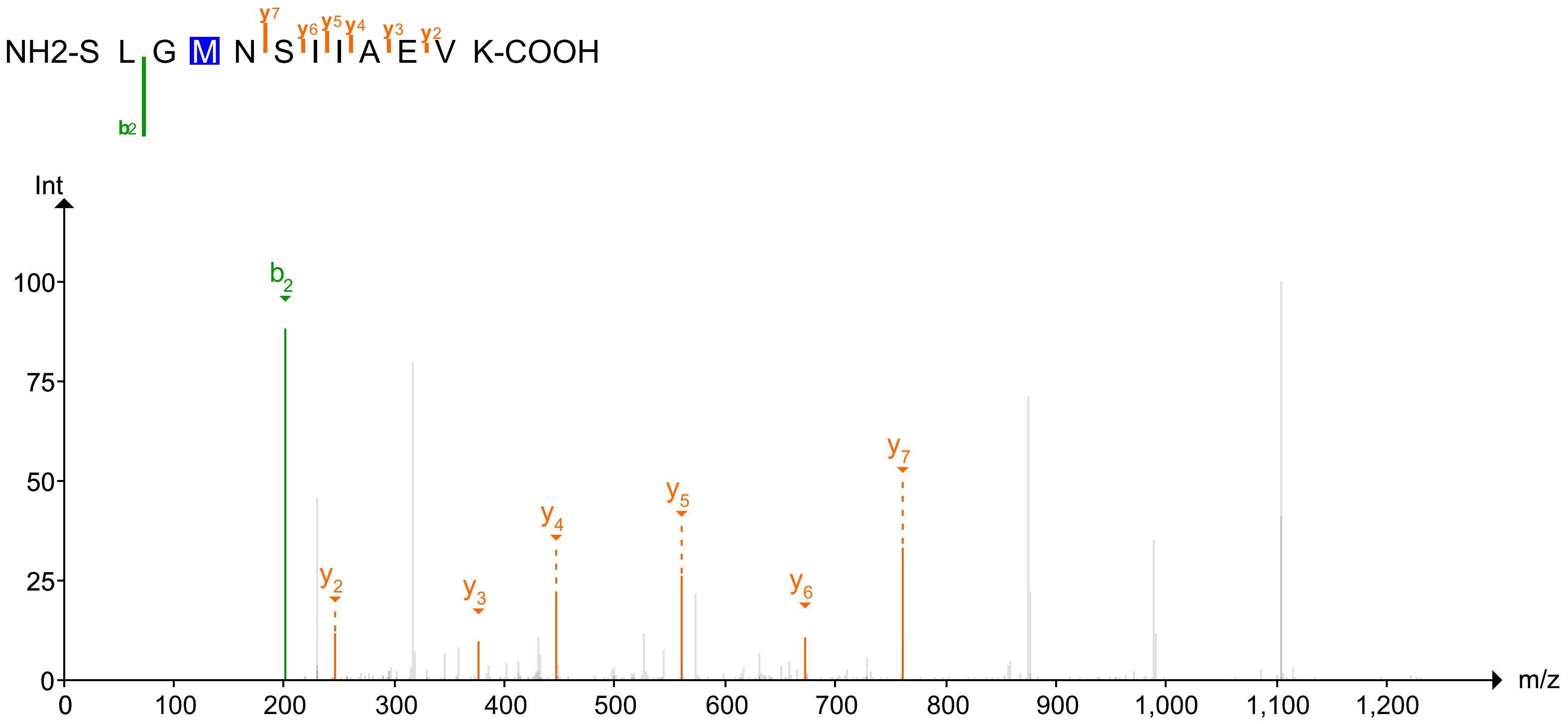

NH2-S L G M N S I I A E V K-COOH
b2
y7 y6 y5 y4 y3 y2
Int
100
75
50
25
0
b2
y2
y3
y4
y5
y6
y7
0 100 200 300 400 500 600 700 800 900 1,000 1,100 1,200
m/z

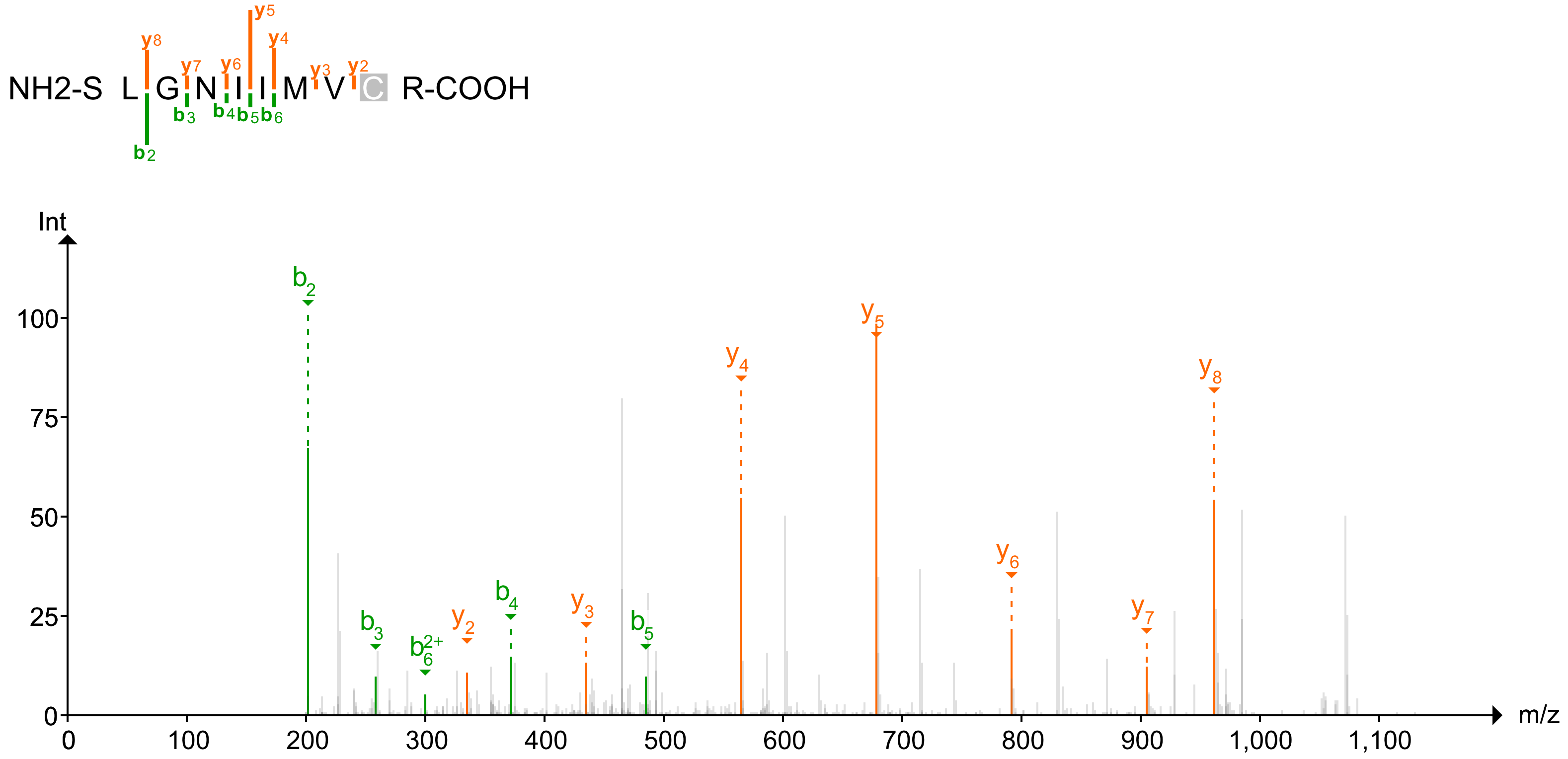

NH2-S L G N I I M V C R-COOH
y8 y7 y6 y5 y4 y3 y2
b2 b3 b4 b5 b6
Int
100
75
50
25
0
b2
b3
b6 2+
y2
b4
y3
b5
y4
y5
y6
y7
y8
0 100 200 300 400 500 600 700 800 900 1,000 1,100
m/z

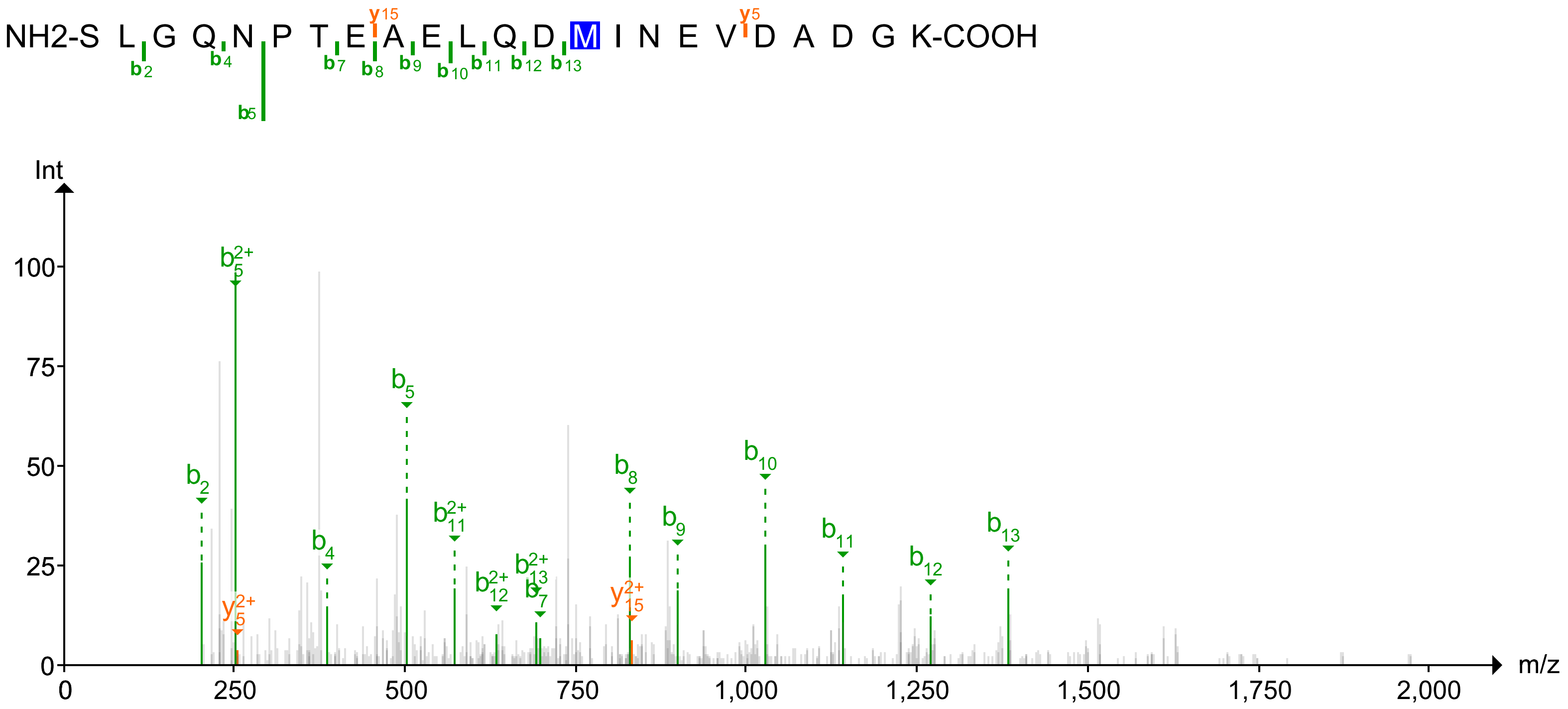

NH2-S L G Q N P T E A E L Q D M I N E V D A D G K-COOH
y15
y5
b2
b4
b5
b7
b8
b9
b10
b11
b12
b13
Int
100
75
50
25
0
m/z
0
250
500
750
1,000
1,250
1,500
1,750
2,000
b5 2+
b5
b2
b4
y5 2+
b11 2+
b12 2+
b13 2+
b7
y15 2+
b8
b9
b10
b11
b12
b13

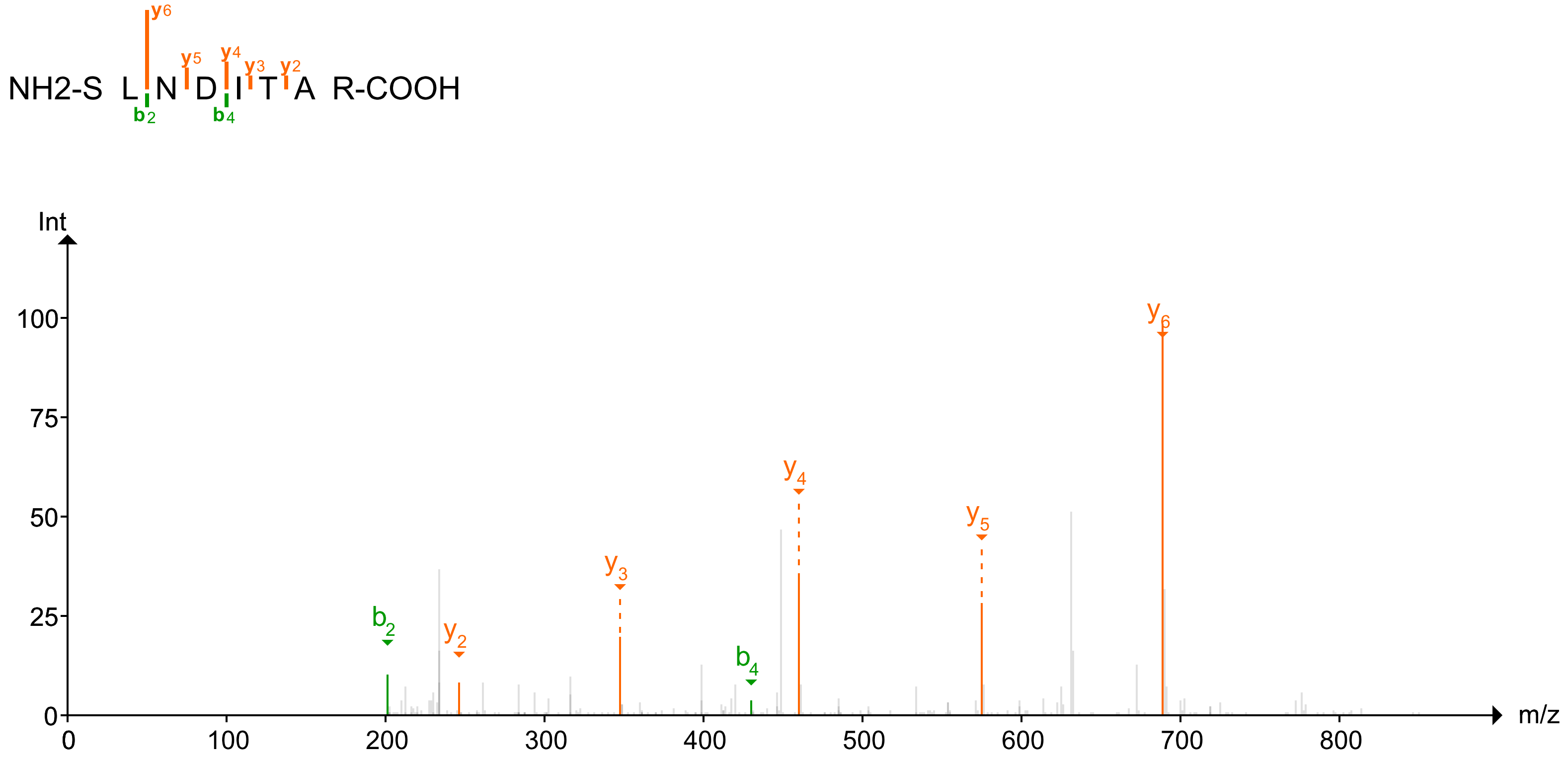

NH2-S L N D I T A R-COOH
y6
y5
y4
y3
y2
b2
b4
Int
100
75
50
25
0
m/z
0
100
200
300
400
500
600
700
800

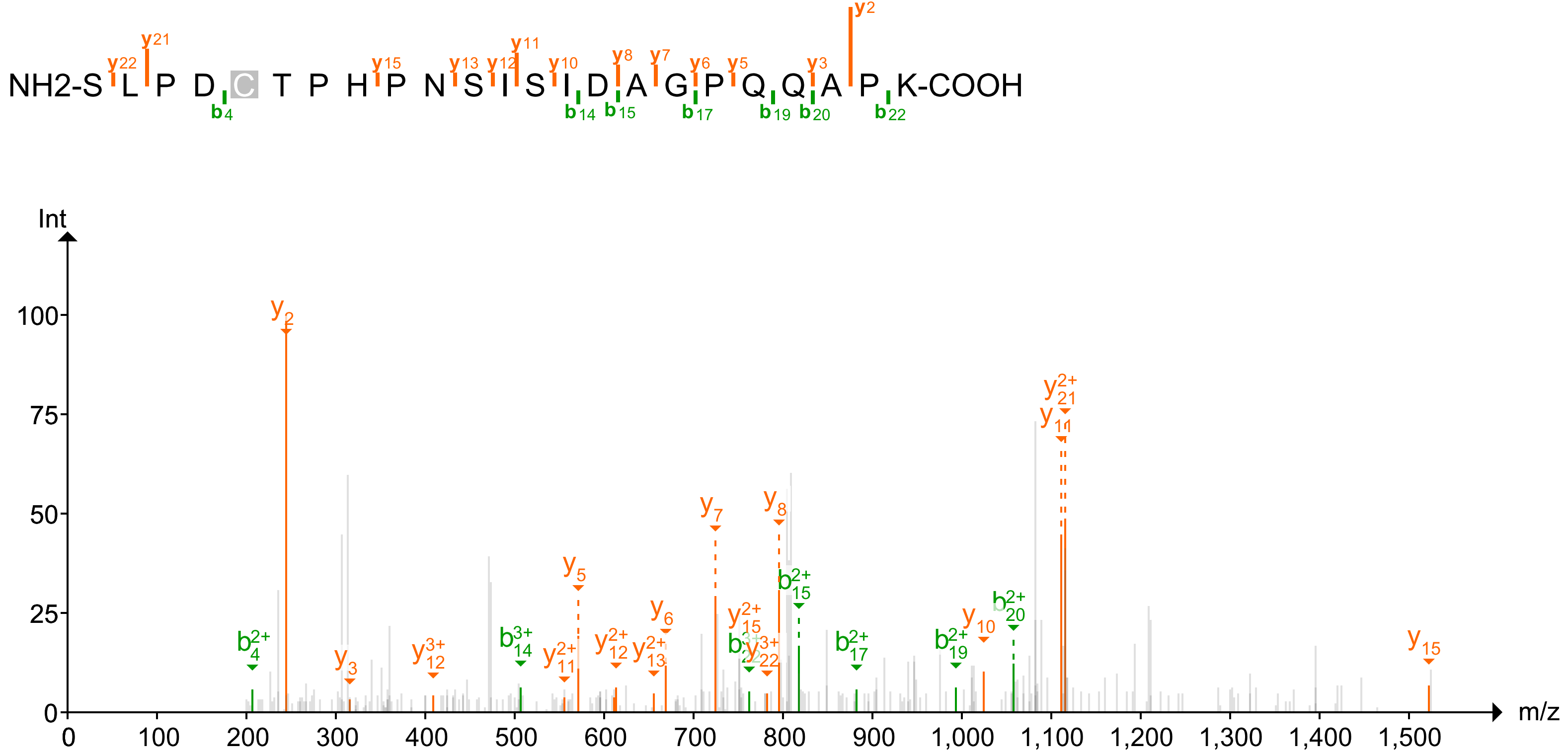

NH2-SLPDCTPHPNSISIDAGPQQAPK-COOH
y2
y3
y5
y6
y7
y8
y10
y11
y12
y13
y15
y21
y22
b4
b14
b15
b17
b19
b20
b22
Int
m/z
0
25
50
75
100
0
100
200
300
400
500
600
700
800
900
1,000
1,100
1,200
1,300
1,400
1,500

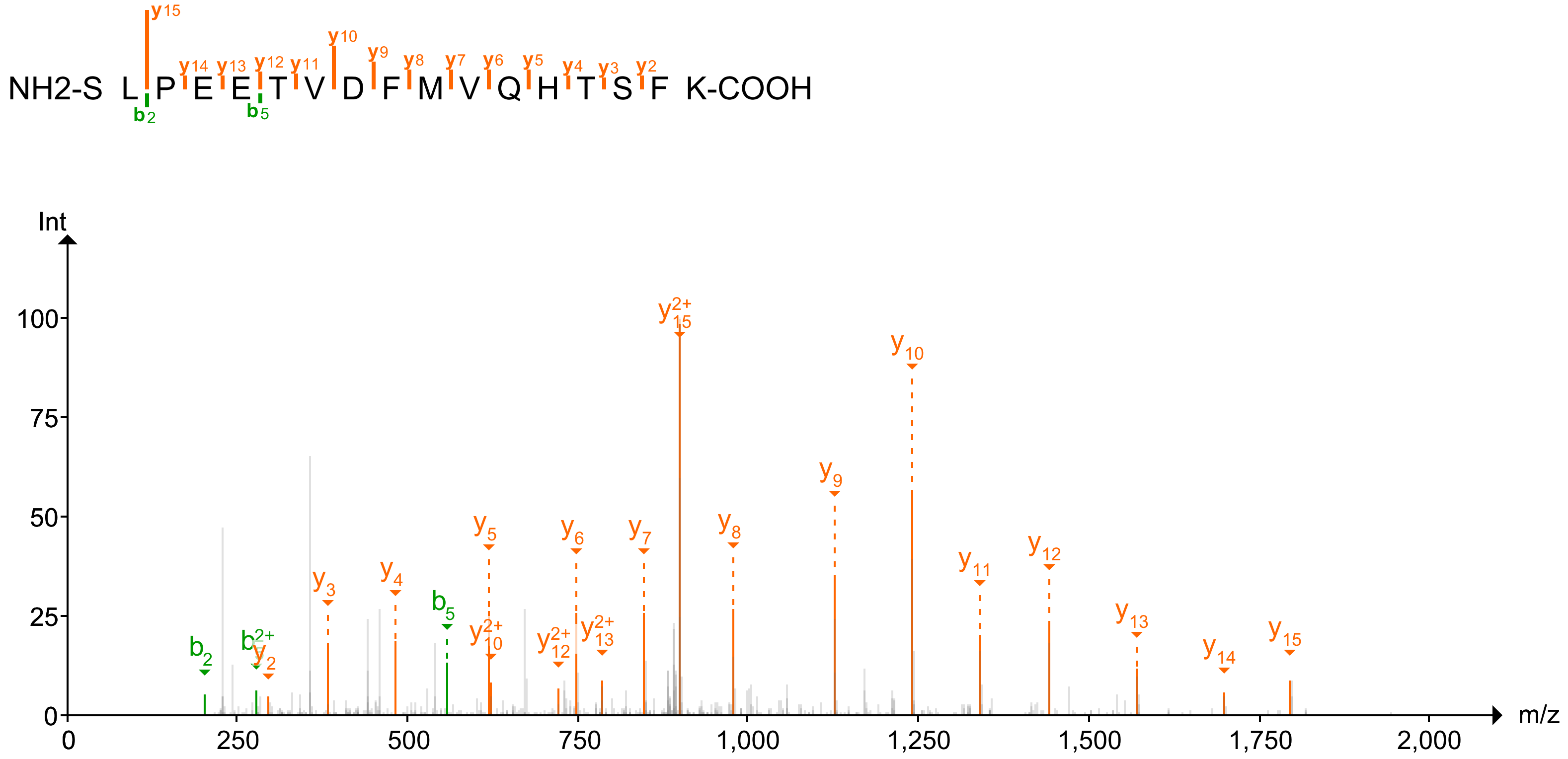

NH2-S L P E E T V D F M V Q H T S F K-COOH
y15 y14 y13 y12 y11 y10 y9 y8 y7 y6 y5 y4 y3 y2
b2 b5
Int
m/z
0
25
50
75
100
250
500
750
1,000
1,250
1,500
1,750
2,000

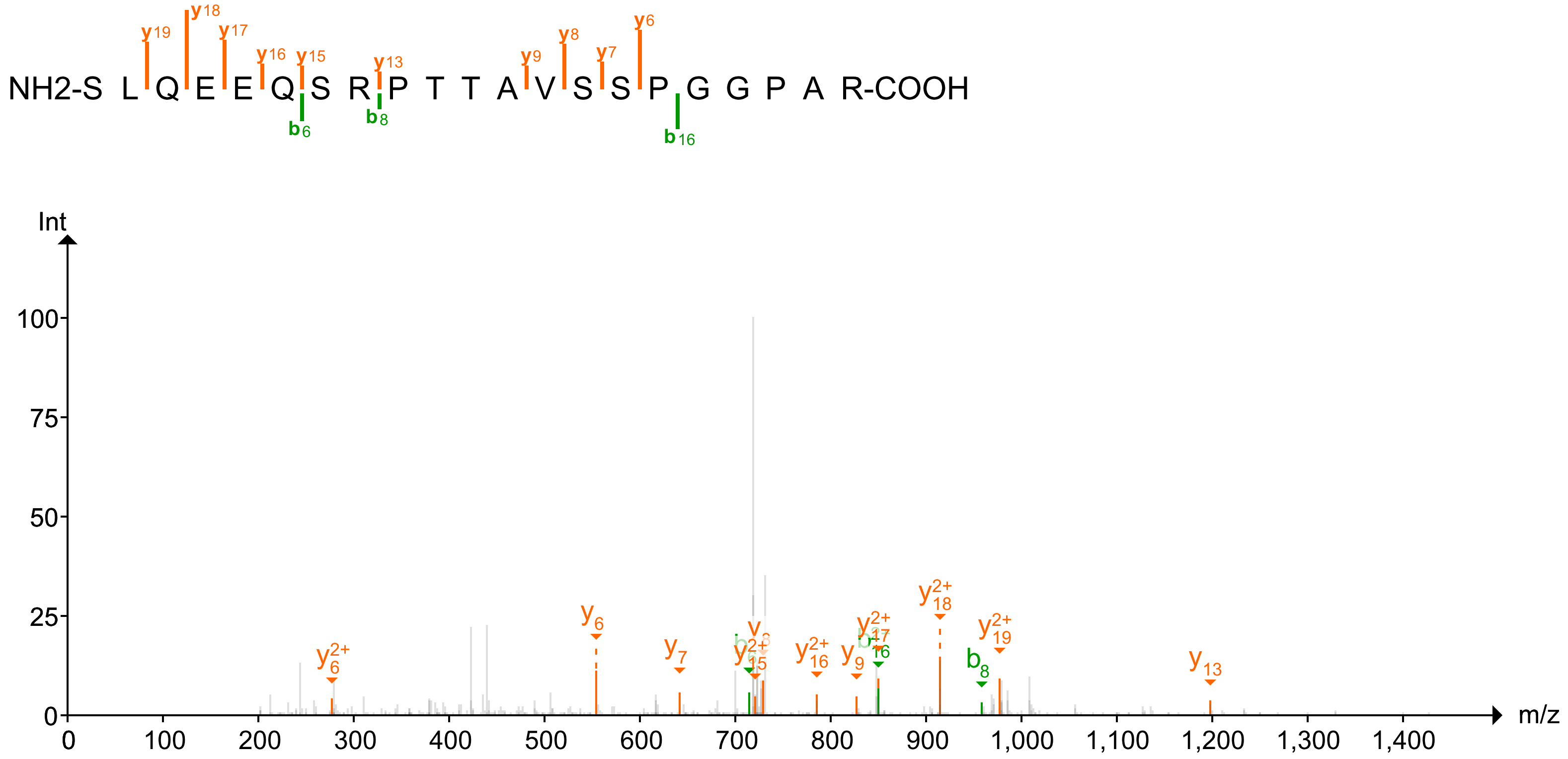

NH2-S L Q E E Q S R P T T A V S S P G G P A R-COOH
y19 y18 y17 y16 y15 y13 y9 y8 y7 y6
b6 b8 b16
Int
100
75
50
25
0
m/z
0 100 200 300 400 500 600 700 800 900 1,000 1,100 1,200 1,300 1,400

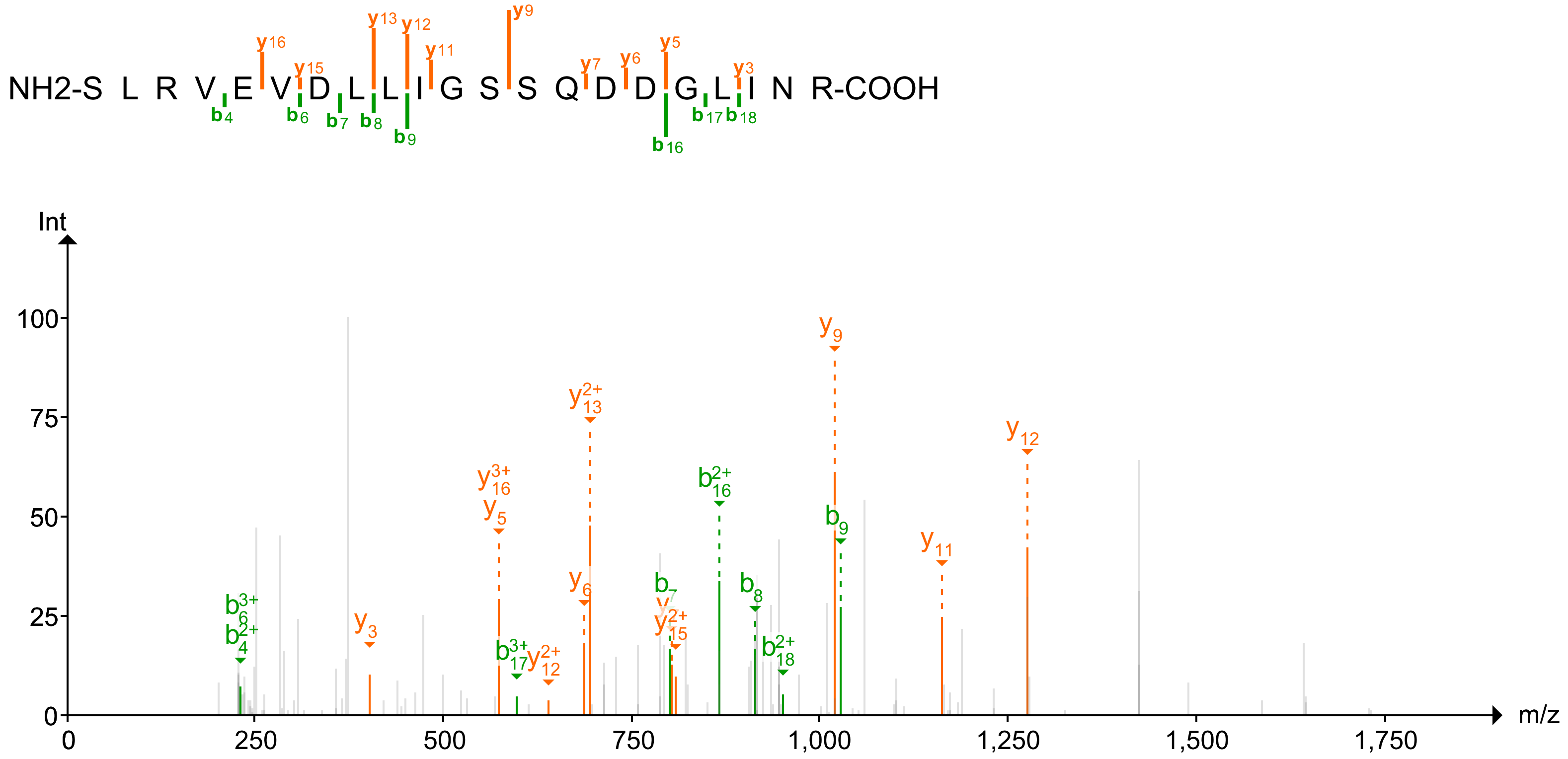

NH2-S L R V E V D L L I G S S Q D D G L I N R-COOH
y16 y15 y13 y12 y11 y9 y7 y6 y5 y3
b4 b6 b7 b8 b9 b16 b17 b18
Int
100
75
50
25
0
m/z
0
250
500
750
1,000
1,250
1,500
1,750
y9
y12
y13 2+
y16 3+
y5
b16 2+
b9
y11
y6
b7
y15 2+
b8
b18 2+
b6 3+
b4 2+
y3
b17 3+
y12 2+

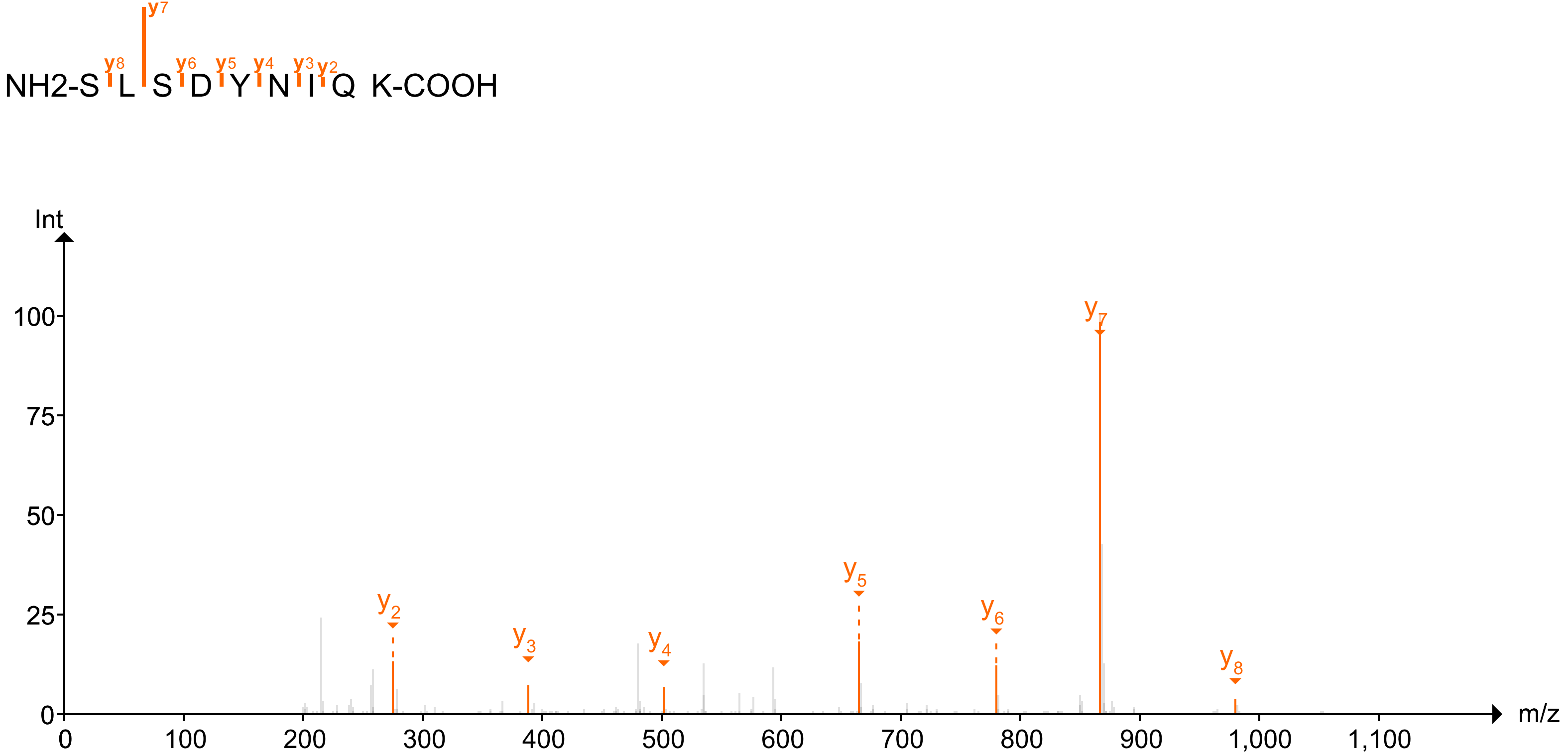

NH2-S L S D Y N I Q K-COOH
y8 y7 y6 y5 y4 y3 y2
Int
100
75
50
25
0
y2
y3
y4
y5
y6
y7
y8
0
100
200
300
400
500
600
700
800
900
1,000
1,100
m/z

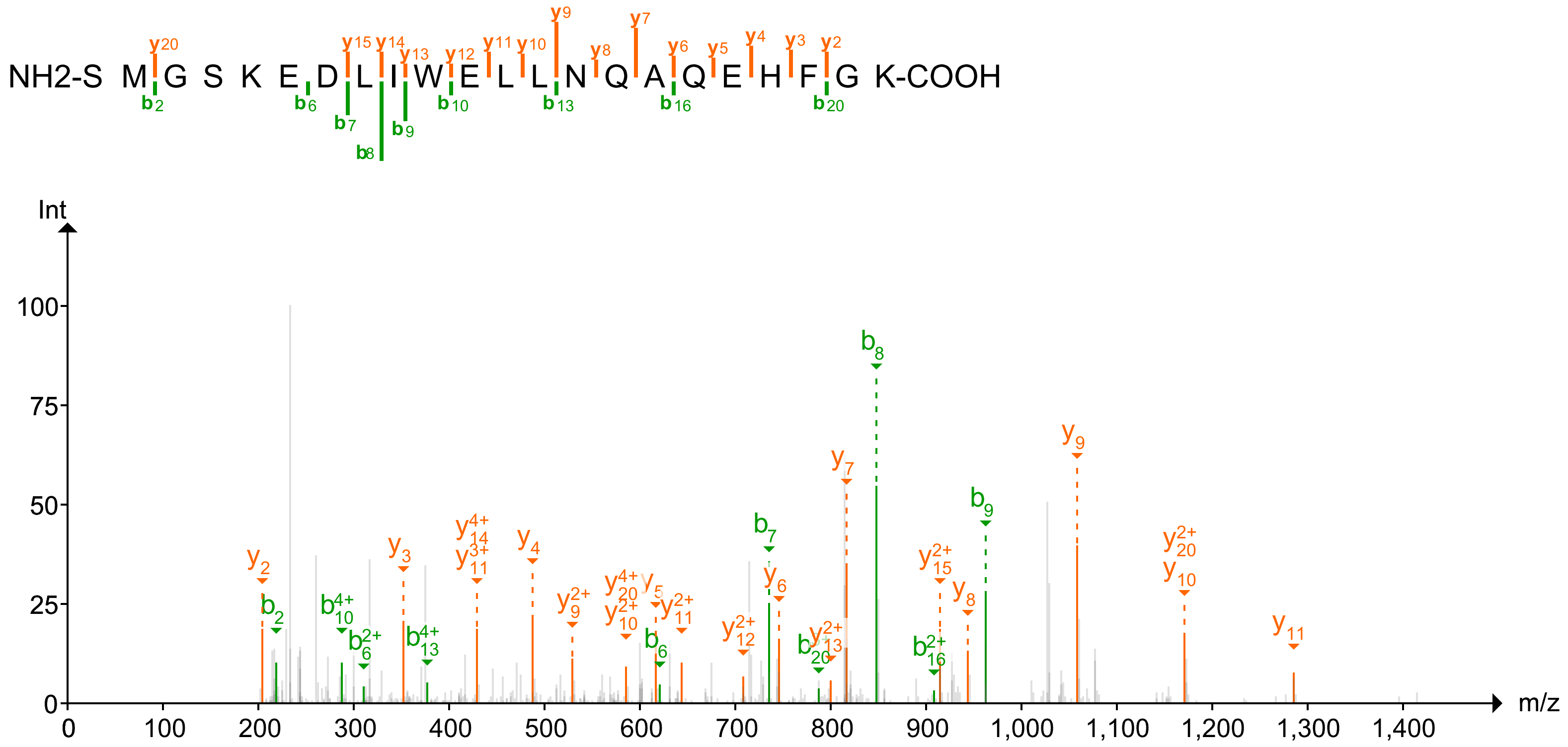
NH2-S M G S K E D L I W E L L N Q A Q E H F G K-COOH
y20 y15 y14 y13 y12 y11 y10 y9 y8 y7 y6 y5 y4 y3 y2
b2 b6 b7 b8 b9 b10 b13 b16 b20
Int
100
75
50
25
0
m/z
0 100 200 300 400 500 600 700 800 900 1,000 1,100 1,200 1,300 1,400

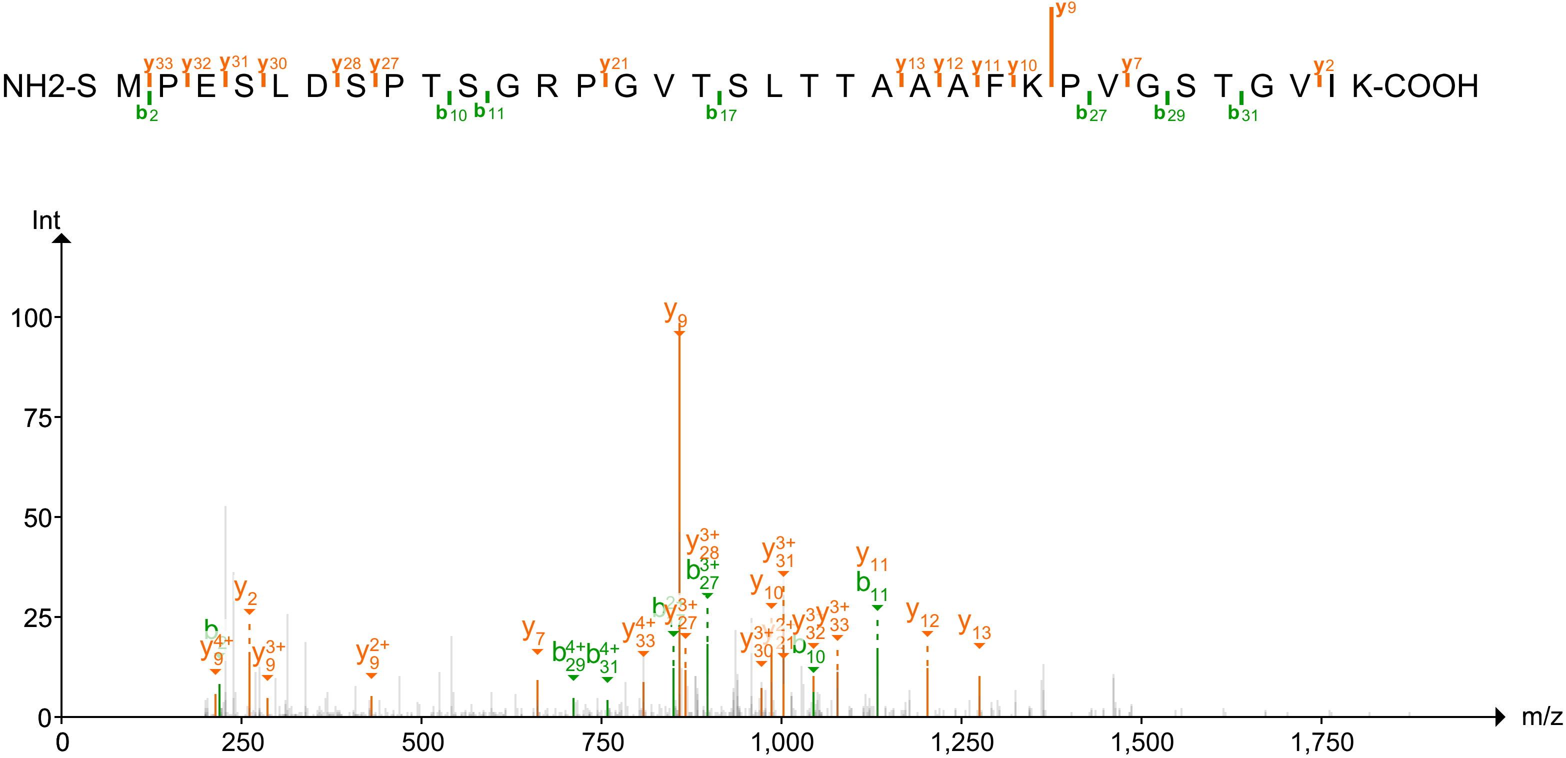

NH2-S M P E S L D S P T S G R P G V T S L T T A A A F K P V G S T G V I K-COOH
y33 y32 y31 y30 y28 y27 y21 y13 y12 y11 y10 y9 y7 y2
b2 b10 b11 b17 b27 b29 b31
Int
100
75
50
25
0
m/z
0
250
500
750
1,000
1,250
1,500
1,750

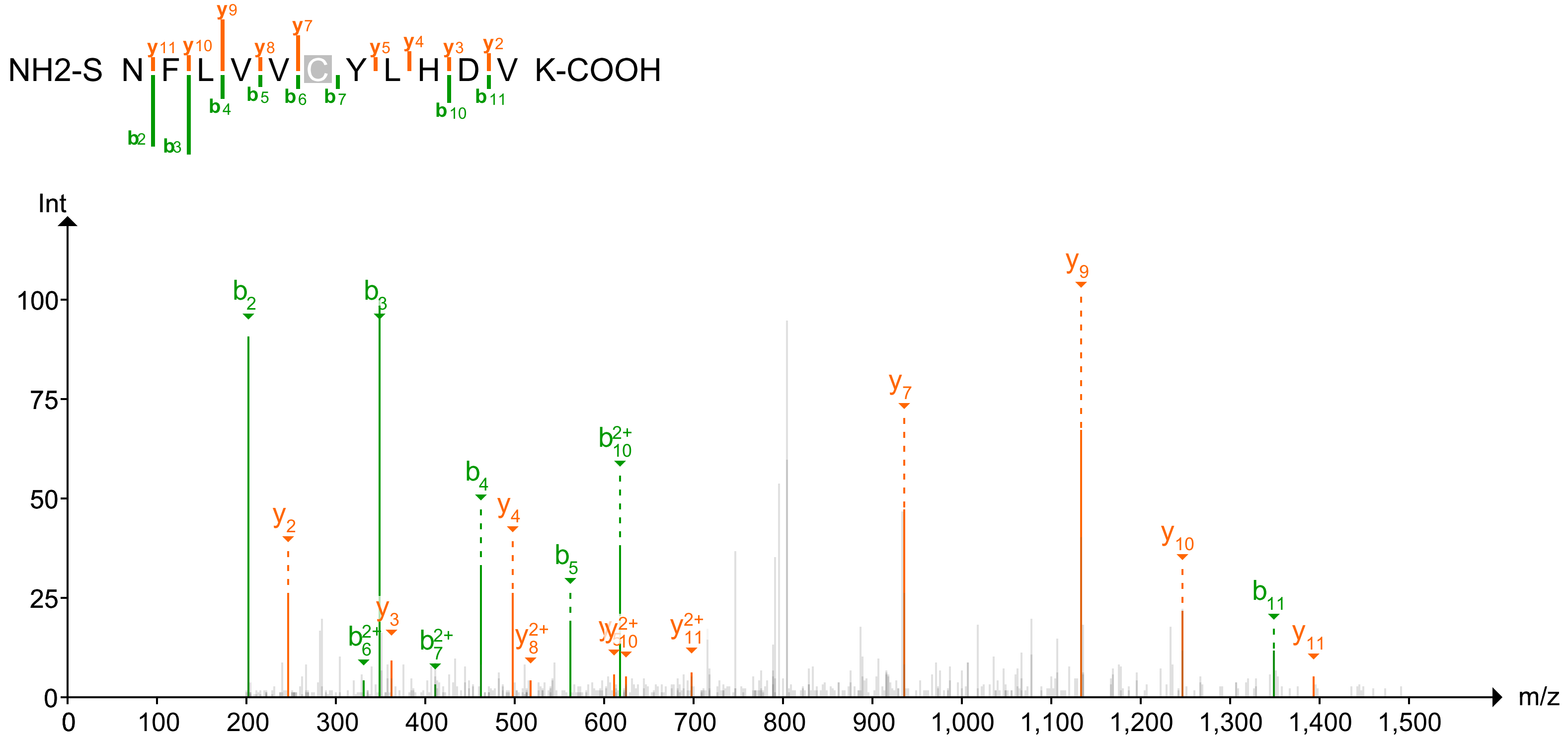

NH2-S N F L V V C Y L H D V K-COOH
Int
m/z
b2
b3
b4
b5
b10
b11
y2
y4
y7
y9
y10
y11

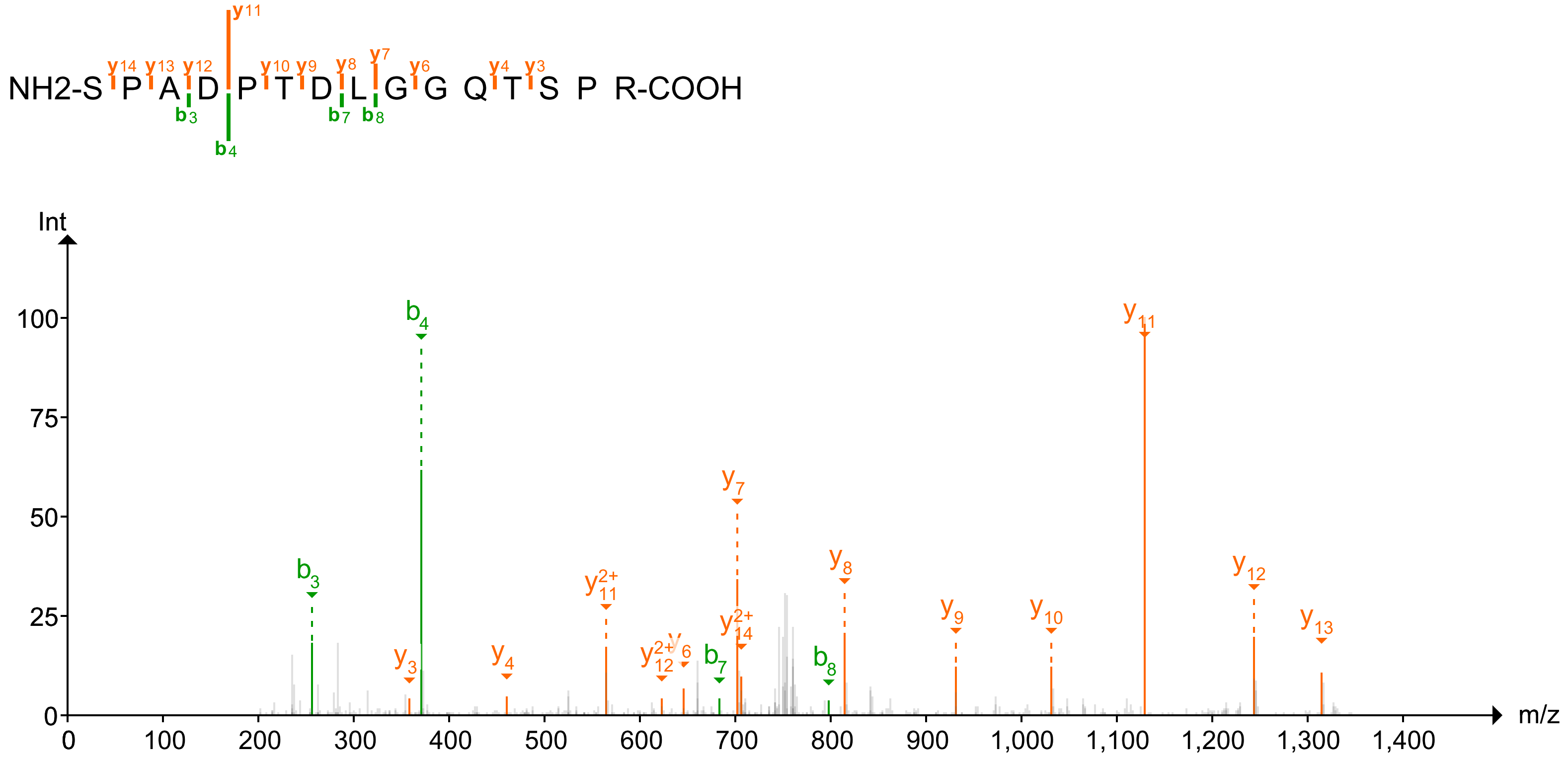

NH2-S P A D P T D L G G Q T S P R-COOH
y14 y13 y12 y11 y10 y9 y8 y7 y6 y4 y3
b3 b4 b7 b8
Int
100
75
50
25
0
m/z
0 100 200 300 400 500 600 700 800 900 1,000 1,100 1,200 1,300 1,400
b3 b4 y3 y4 y11 2+ y12 2+ y6 b7 y7 y14 2+ b8 y8 y9 y10 y11 y12 y13

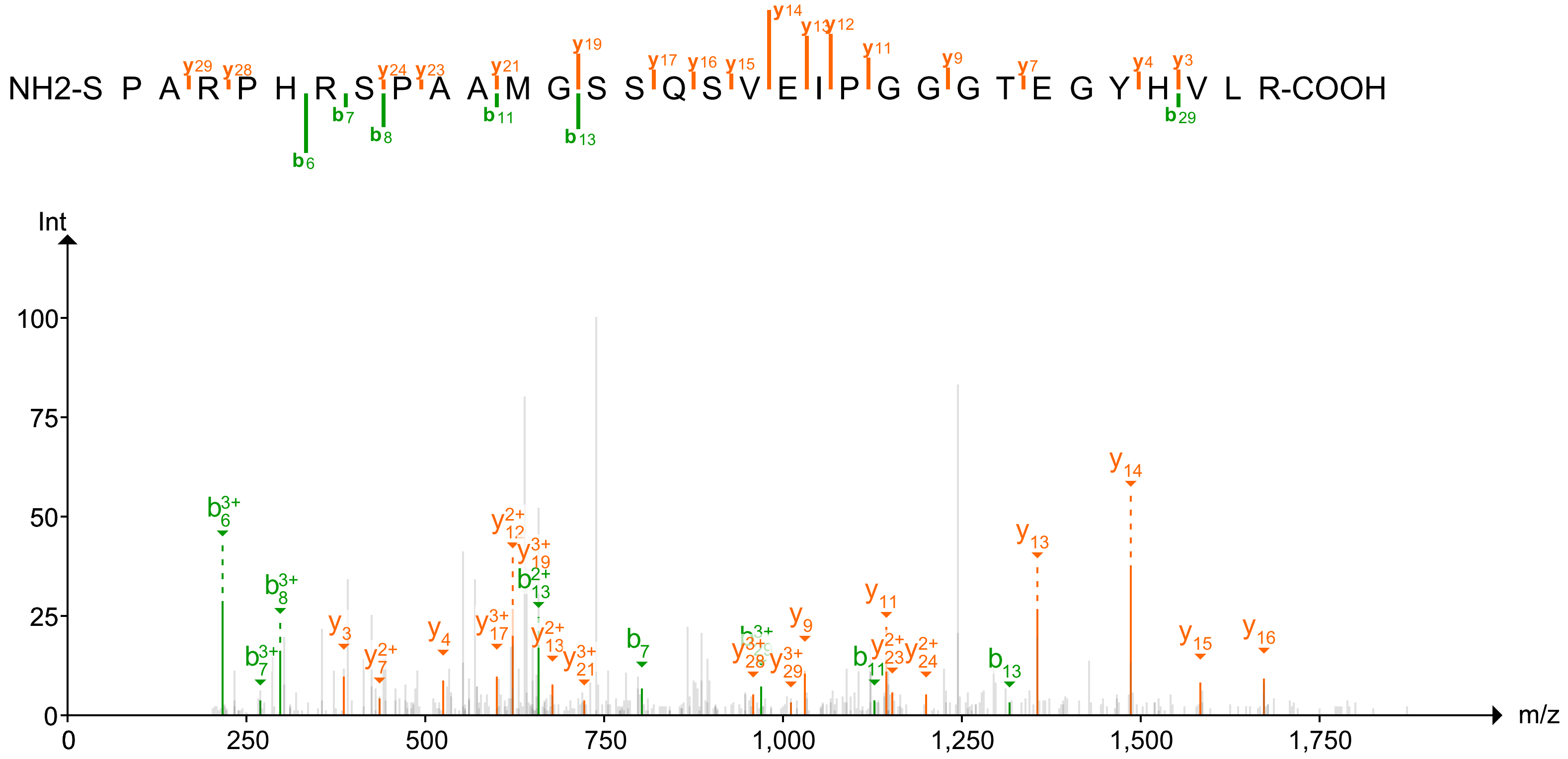

NH2-S P A R P H R S P A A M G S S Q S V E I P G G G T E G Y H V L R-COOH
Int
m/z

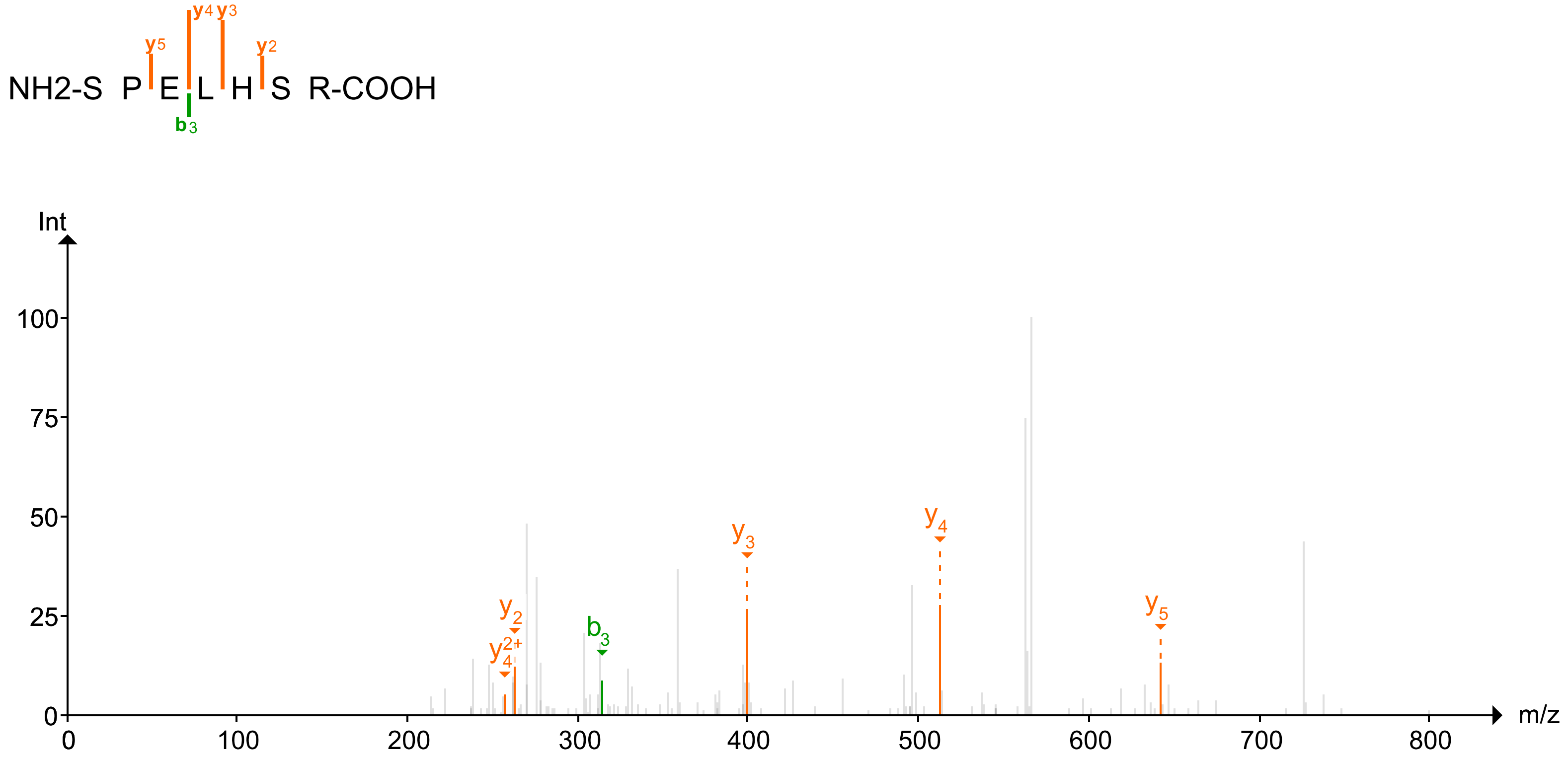

NH2-S P E L H S R-COOH
y5
y4
y3
y2
b3
Int
100
75
50
25
0
0
100
200
300
400
500
600
700
800
m/z
y2
y4 2+
b3
y3
y4
y5

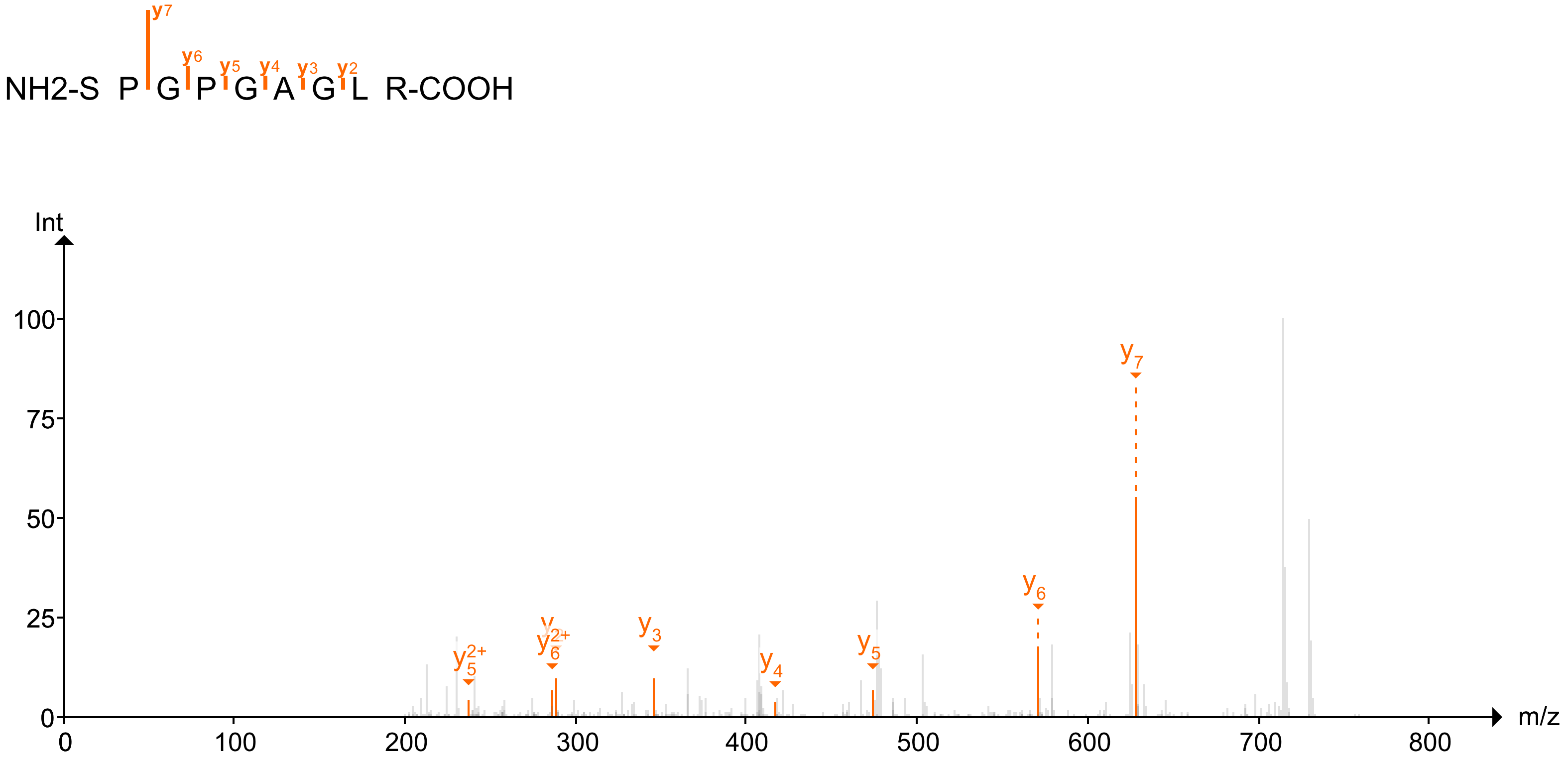

NH2-S P G P G A G L R-COOH
y7 y6 y5 y4 y3 y2
Int
100
75
50
25
0
y7
y6
y3
y4
y5
$y_5^{2+}$
$y_6^{2+}$
0 100 200 300 400 500 600 700 800
m/z

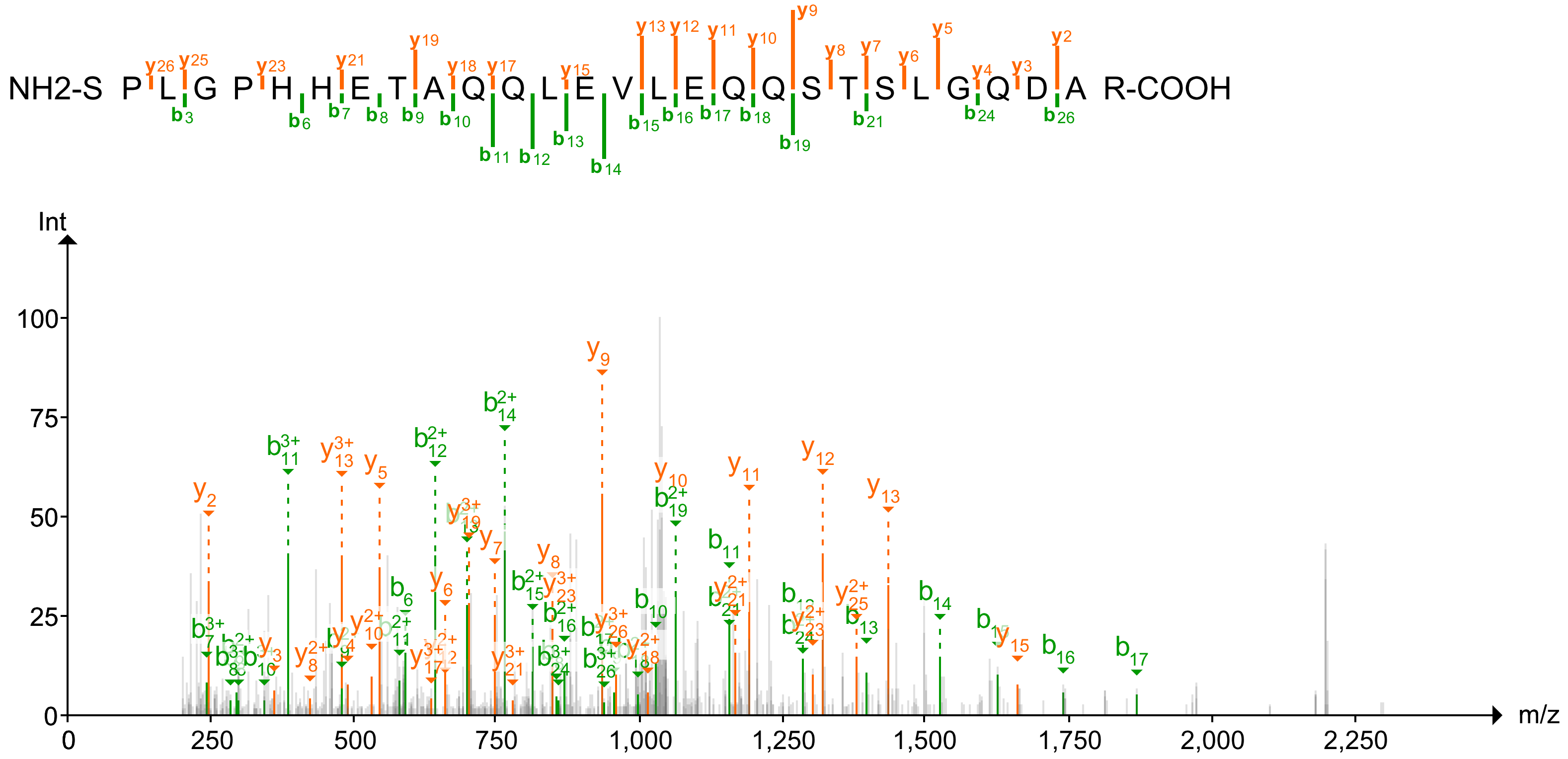

NH2-S P L G P H H E T A Q Q L E V L E Q Q S T S L G Q D A R-COOH
Int
m/z
0
25
50
75
100
250
500
750
1,000
1,250
1,500
1,750
2,000
2,250

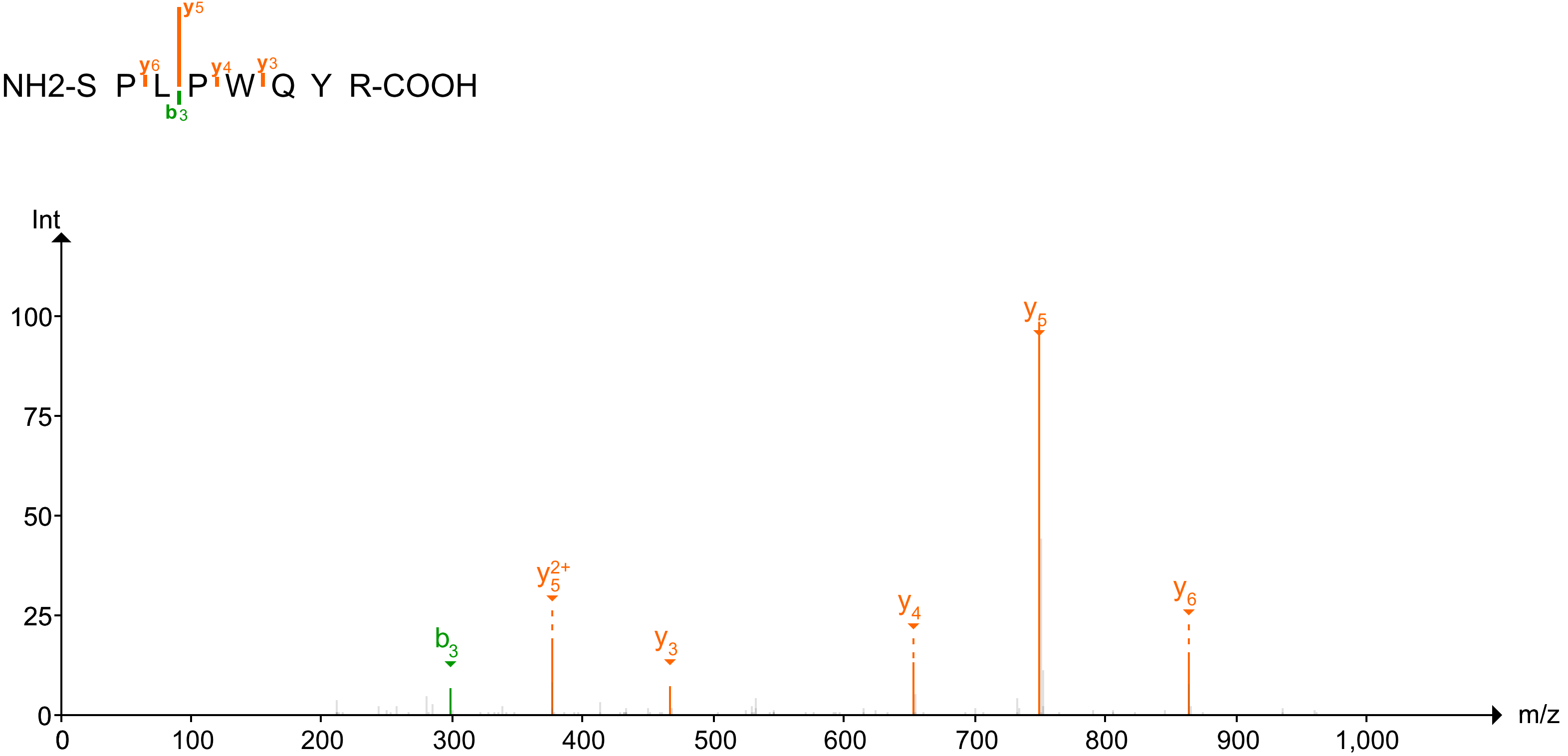

NH2-S P L P W Q Y R-COOH
y5
y6
y4
y3
b3
Int
100
75
50
25
0
y5
y5 2+
b3
y3
y4
y6
0
100
200
300
400
500
600
700
800
900
1,000
m/z

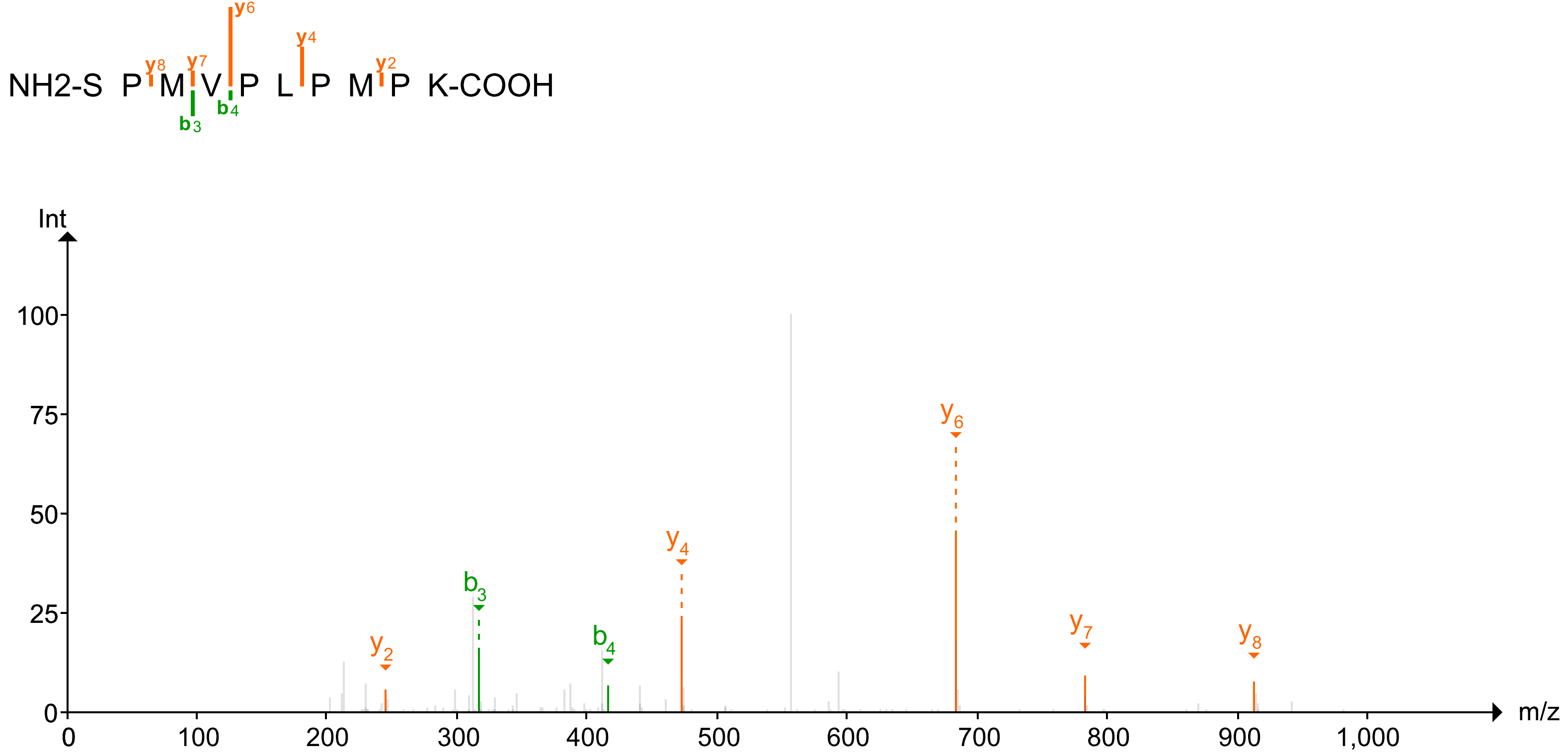
NH2-S P M V P L P M P K-COOH
y8 y7 y6 y4 y2
b3 b4
Int
100
75
50
25
0
m/z
0 100 200 300 400 500 600 700 800 900 1,000
y2 b3 b4 y4 y6 y7 y8

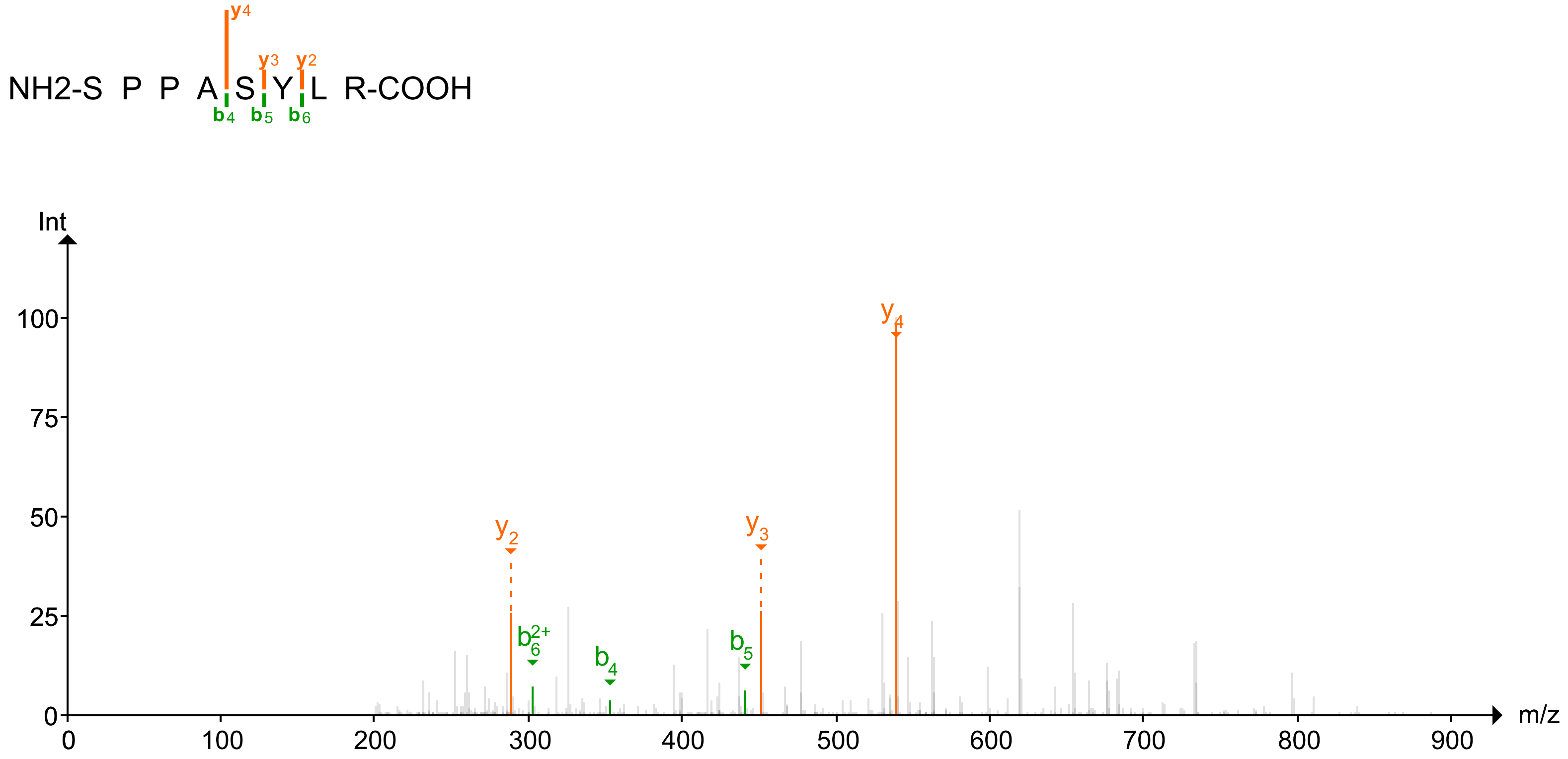

y4
y3
y2
NH2-S P P A S Y L R-COOH
b4
b5
b6
Int
100
75
50
25
0
y4
y2
y3
b6 2+
b4
b5
0
100
200
300
400
500
600
700
800
900
m/z

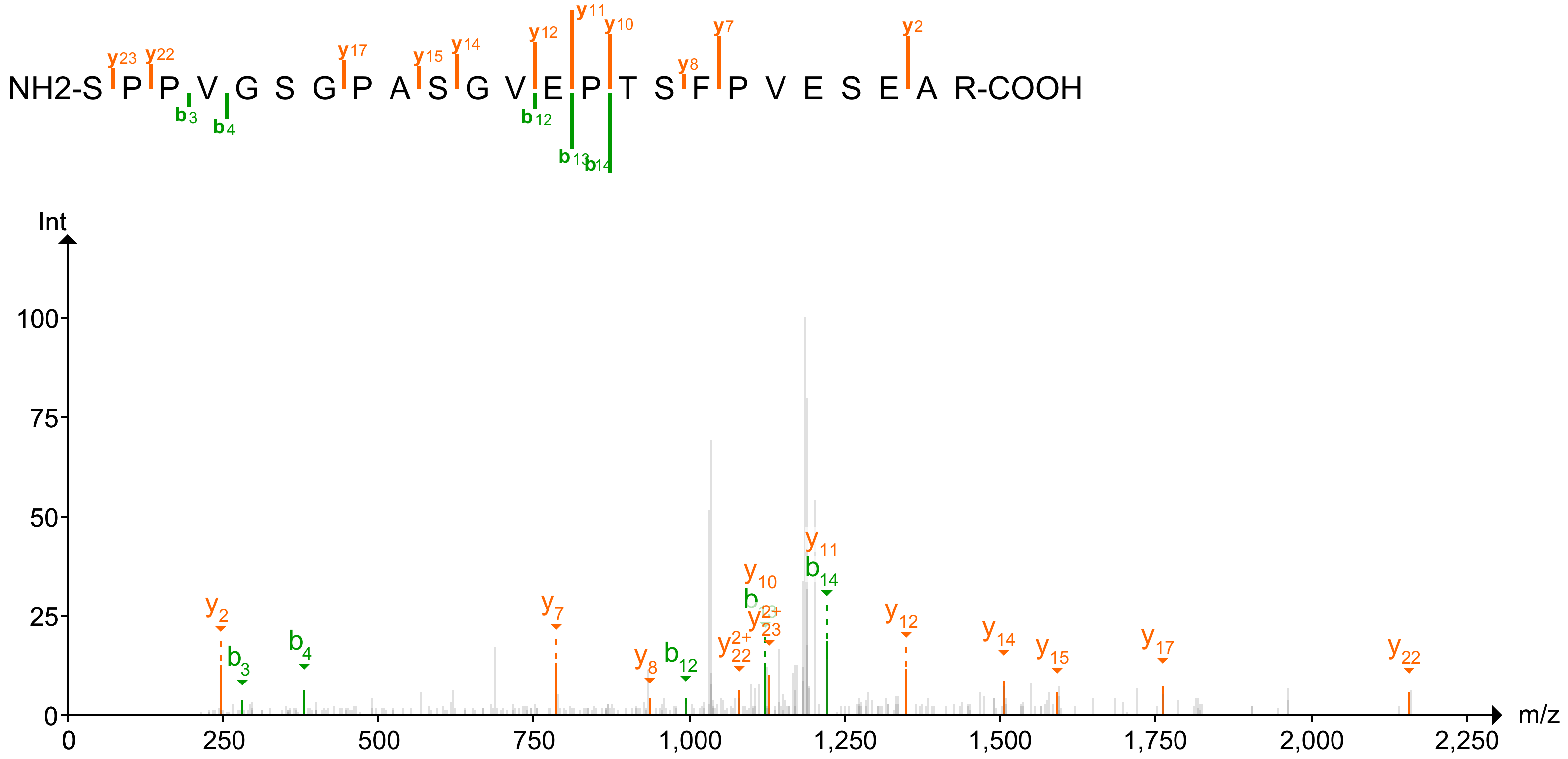
NH2-S P P V G S G P A S G V E P T S F P V E S E A R-COOH
y23 y22 y17 y15 y14 y12 y11 y10 y8 y7 y2
b3 b4 b12 b13 b14
Int
100
75
50
25
0
0
250
500
750
1,000
1,250
1,500
1,750
2,000
2,250
m/z
y2 b3 b4 y7 y8 b12 y22 2+ y23 2+ y10 y11 b14 y12 y14 y15 y17 y22

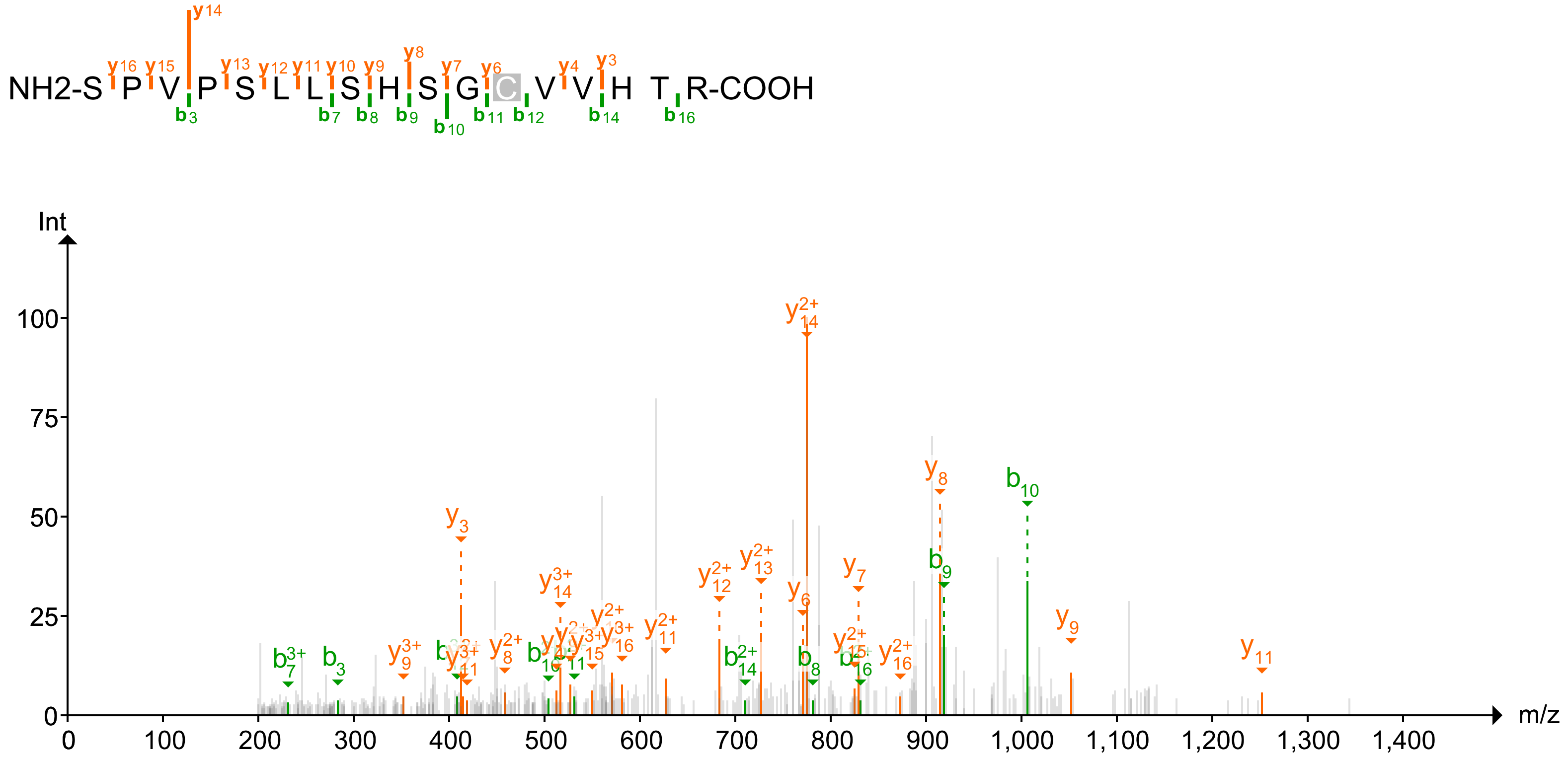

NH2-S P V P S L L S H S G C V V H T R-COOH
Int
m/z
100
75
50
25
0
0
100
200
300
400
500
600
700
800
900
1,000
1,100
1,200
1,300
1,400

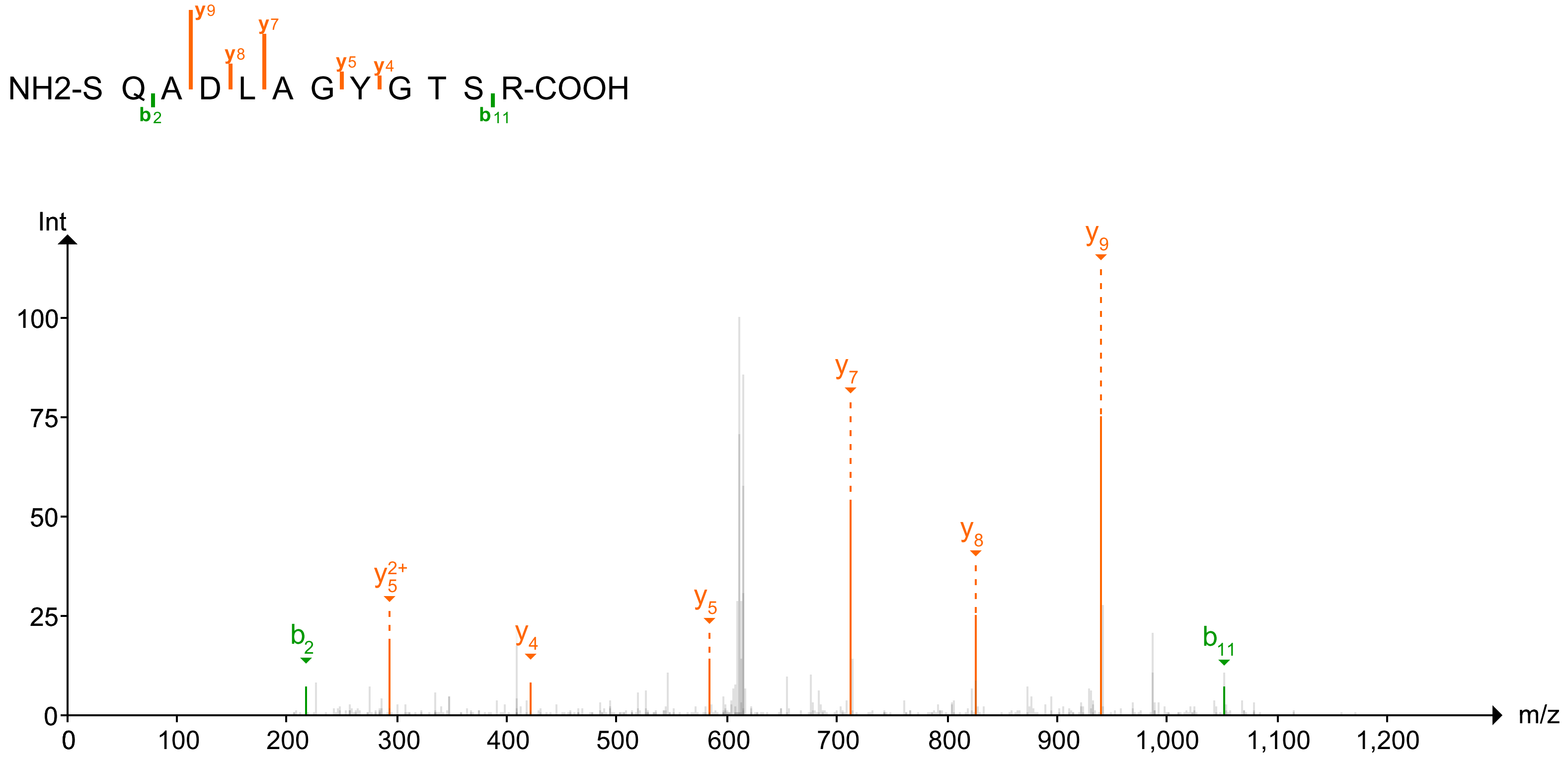

NH2-S Q A D L A G Y G T S R-COOH
y9
y8
y7
y5
y4
b2
b11
Int
100
75
50
25
0
y9
y7
y8
y5 2+
y5
y4
b2
b11
0
100
200
300
400
500
600
700
800
900
1,000
1,100
1,200
m/z

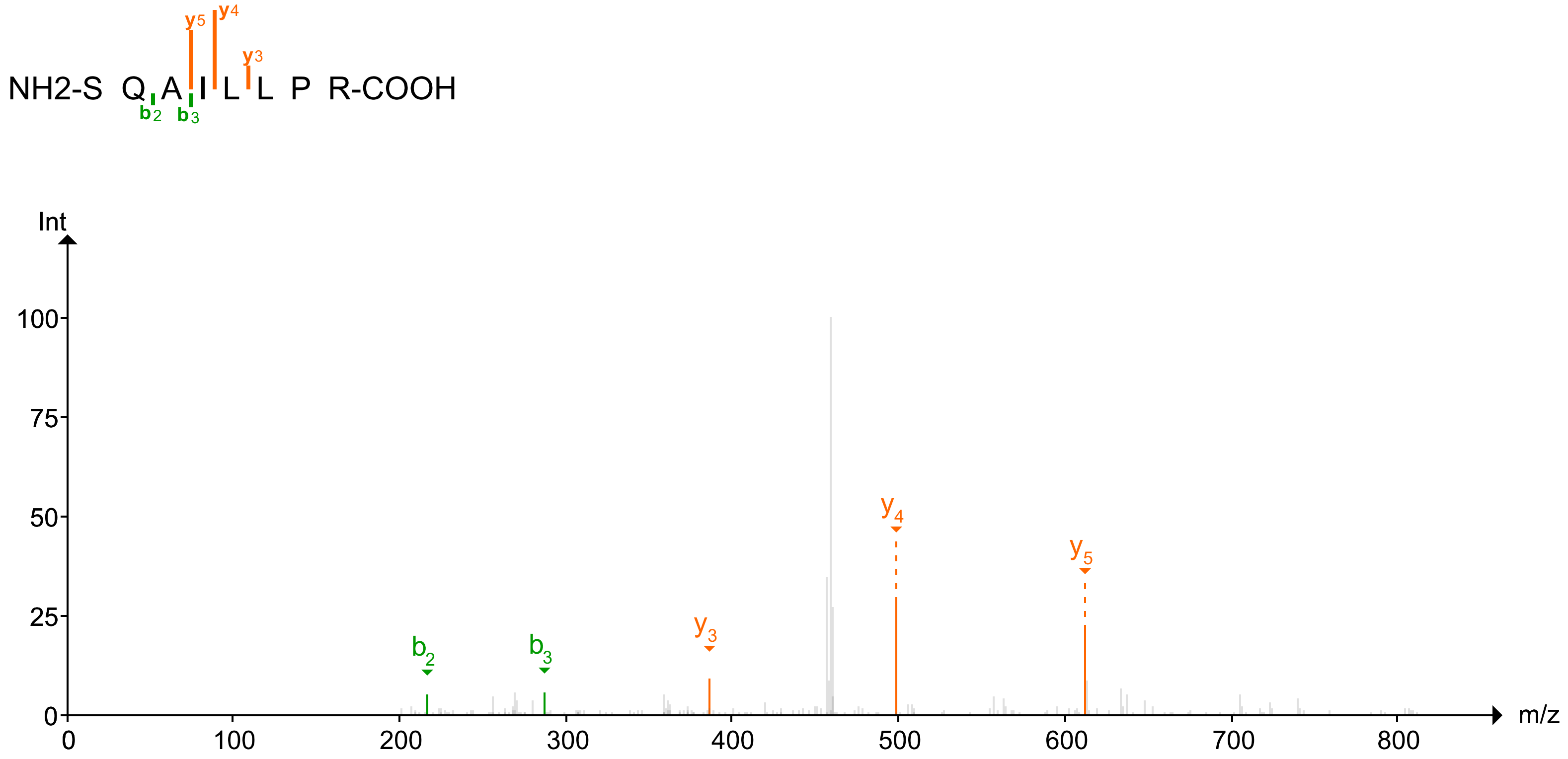

y5
y4
y3
NH2-S Q A I L L P R-COOH
b2
b3
Int
100
75
50
25
0
y4
y5
b2
b3
y3
0
100
200
300
400
500
600
700
800
m/z

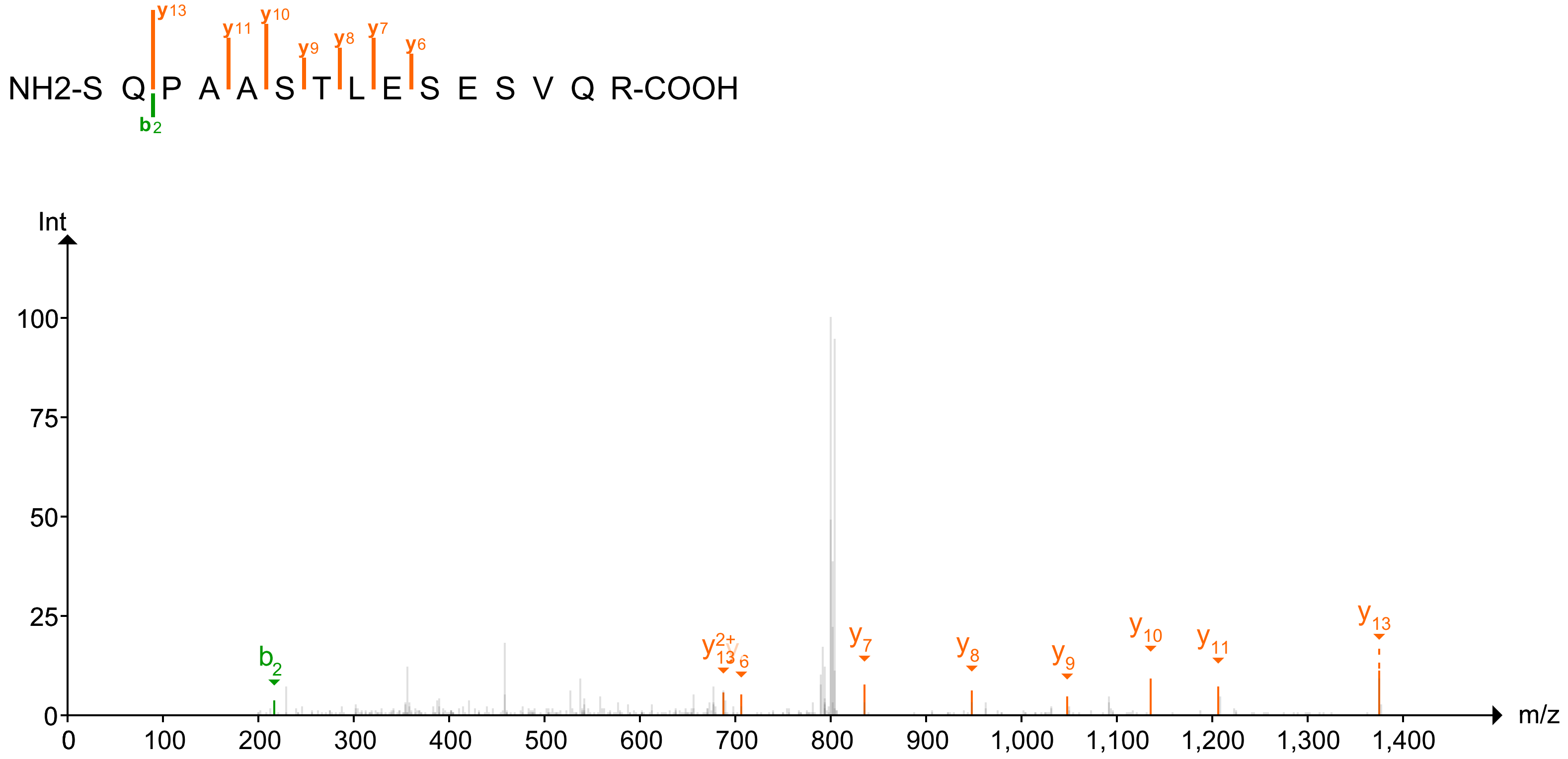

y13 y11 y10 y9 y8 y7 y6
NH2-S Q P A A S T L E S E S V Q R-COOH
b2
Int
100
75
50
25
0
b2
y13 2+
y6
y7
y8
y9
y10
y11
y13
0 100 200 300 400 500 600 700 800 900 1,000 1,100 1,200 1,300 1,400
m/z

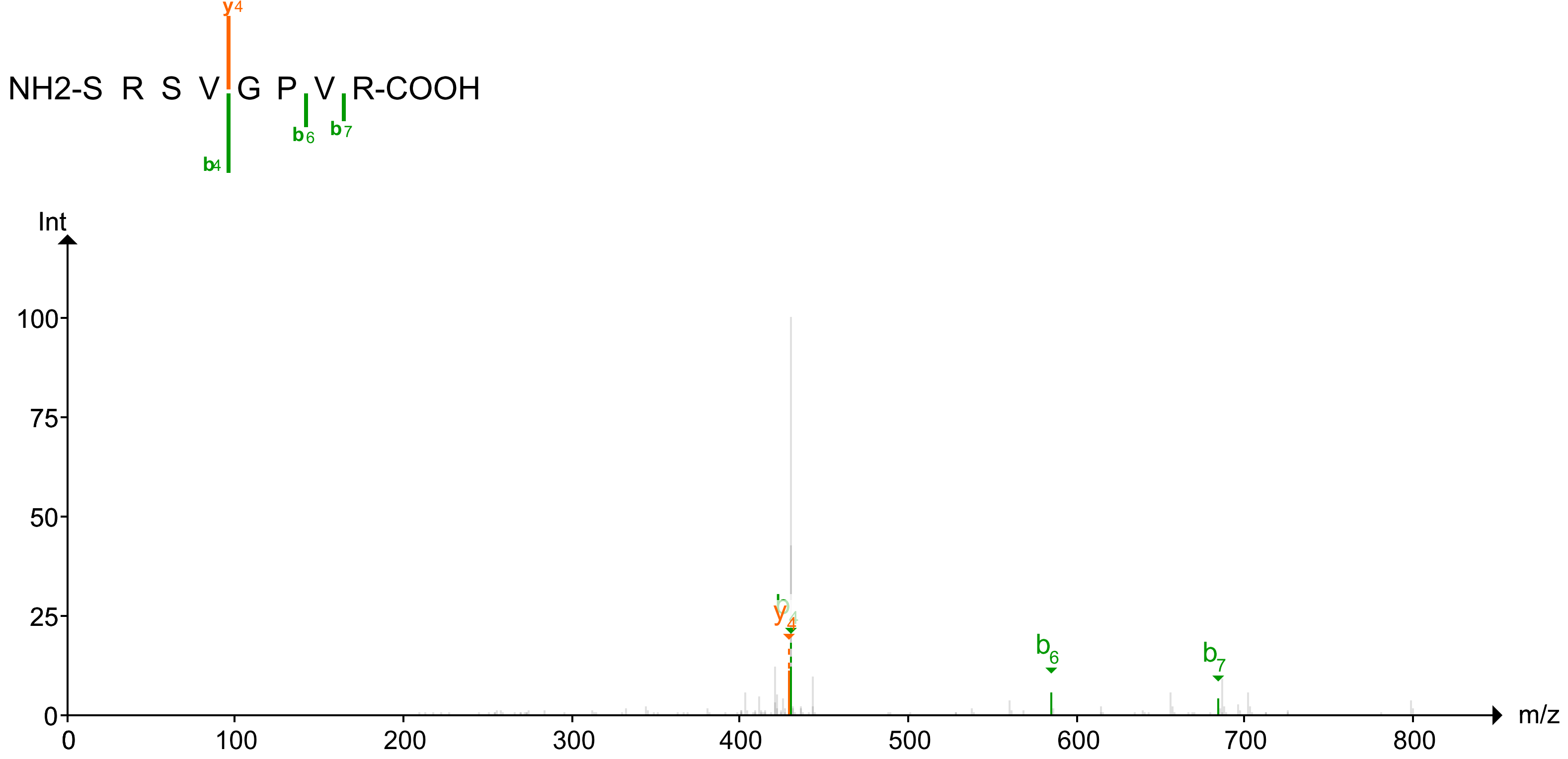

NH2-S R S V G P V R-COOH
y4
b4
b6
b7
Int
100
75
50
25
0
m/z
0
100
200
300
400
500
600
700
800
y4
b4
b6
b7

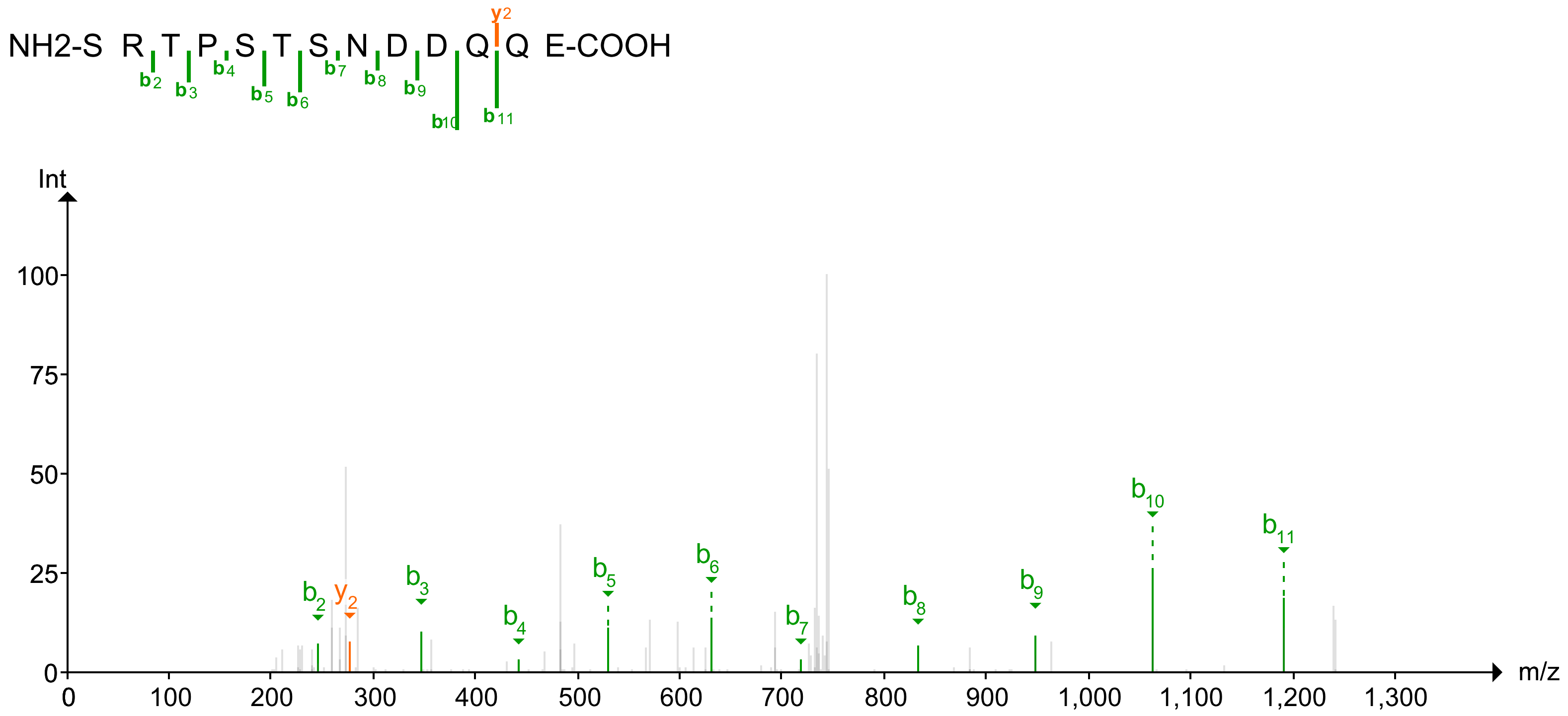
NH2-S R T P S T S N D D Q Q E-COOH
y2
b2 b3 b4 b5 b6 b7 b8 b9 b10 b11
Int
100
75
50
25
0
0 100 200 300 400 500 600 700 800 900 1,000 1,100 1,200 1,300
m/z

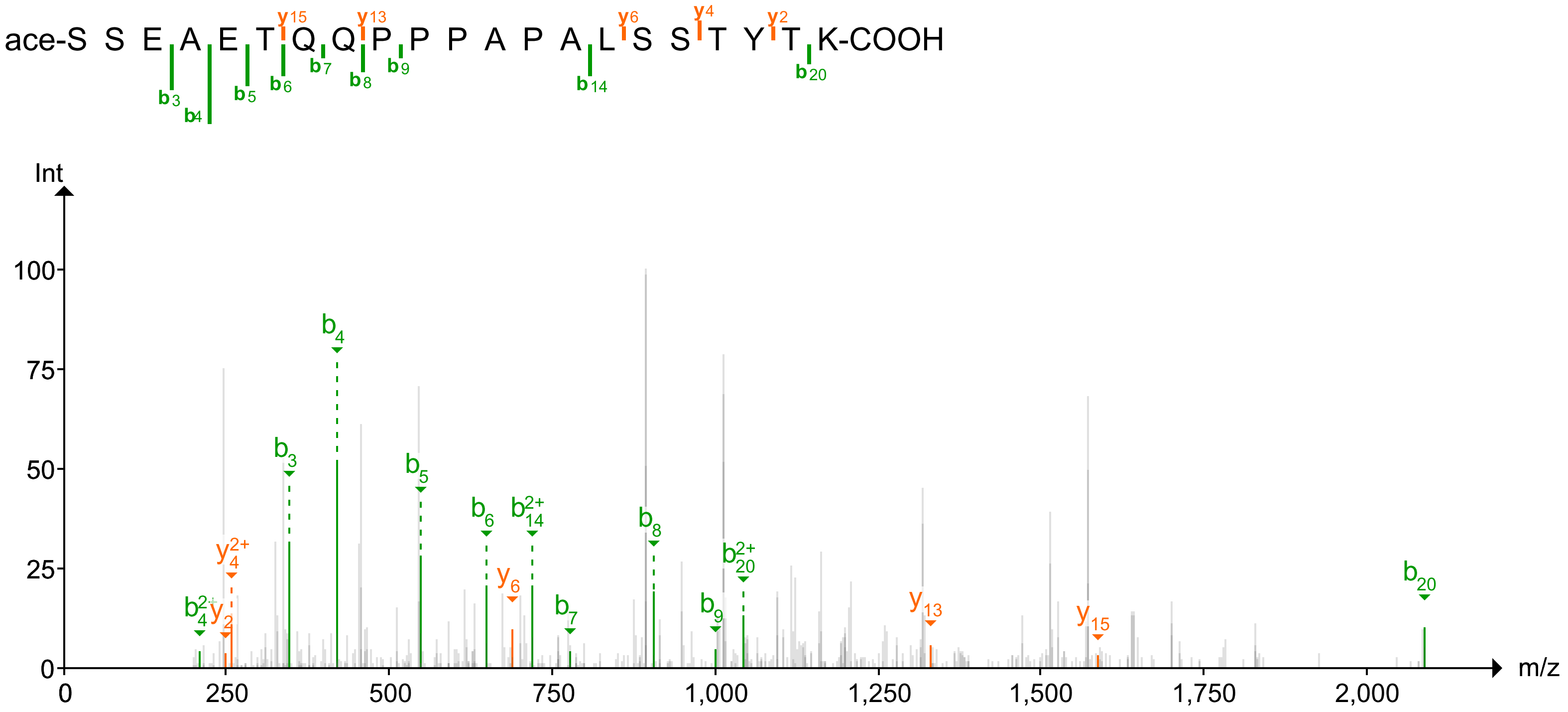

ace-S S E A E T Q Q P P P A P A L S S T Y T K-COOH
y15 y13 y6 y4 y2
b3 b4 b5 b6 b7 b8 b9 b14 b20
Int
100
75
50
25
0
m/z
0
250
500
750
1,000
1,250
1,500
1,750
2,000
b4
b3
b5
b6
b14 2+
b8
b20 2+
b20
y4 2+
b4 2+
y2
y6
b7
b9
y13
y15

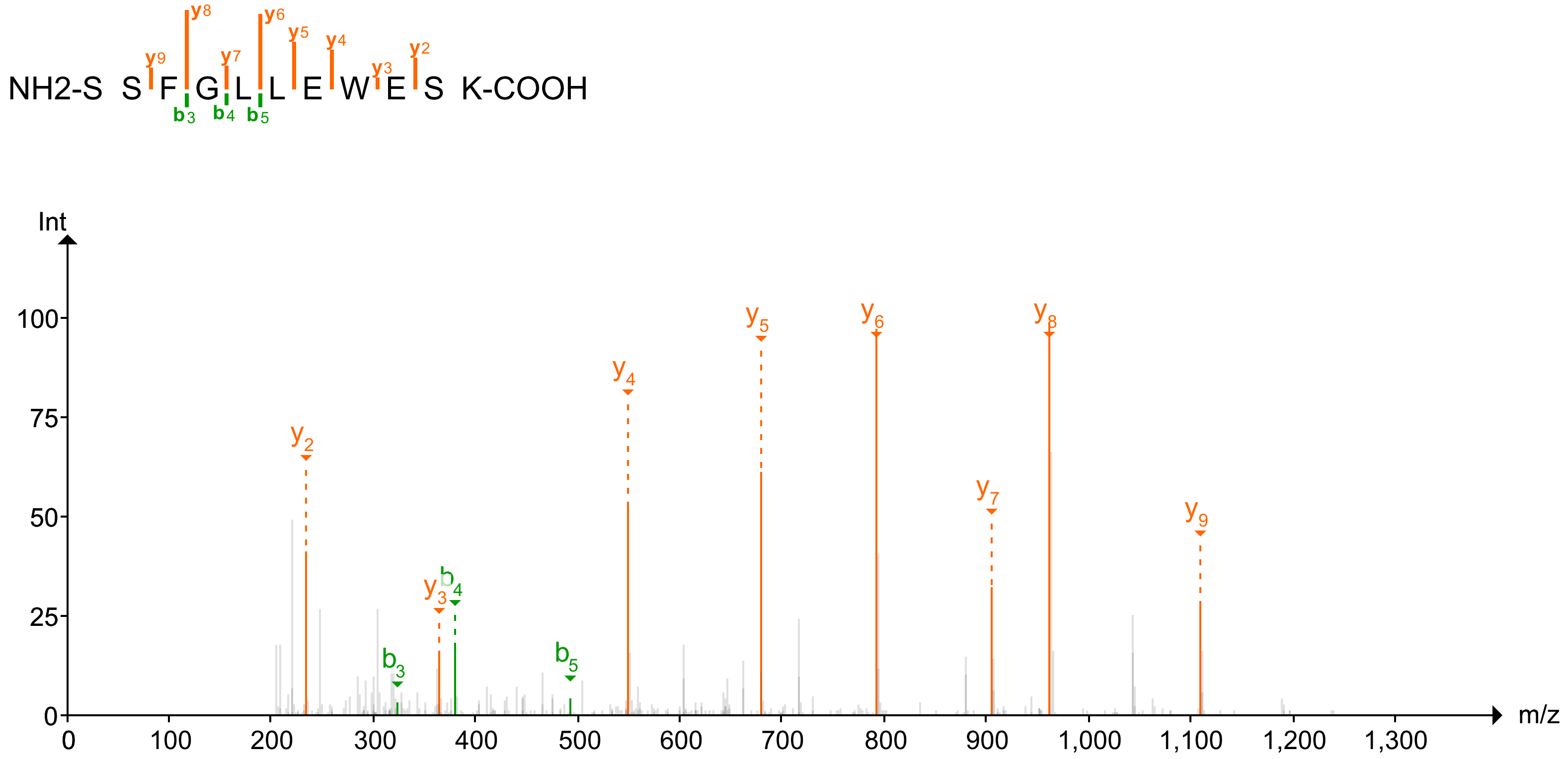

NH2-S S F G L L E W E S K-COOH
y9 y8 y7 y6 y5 y4 y3 y2
b3 b4 b5
Int
100
75
50
25
0
m/z
0 100 200 300 400 500 600 700 800 900 1,000 1,100 1,200 1,300
y2 y3 b4 b3 b5 y4 y5 y6 y7 y8 y9

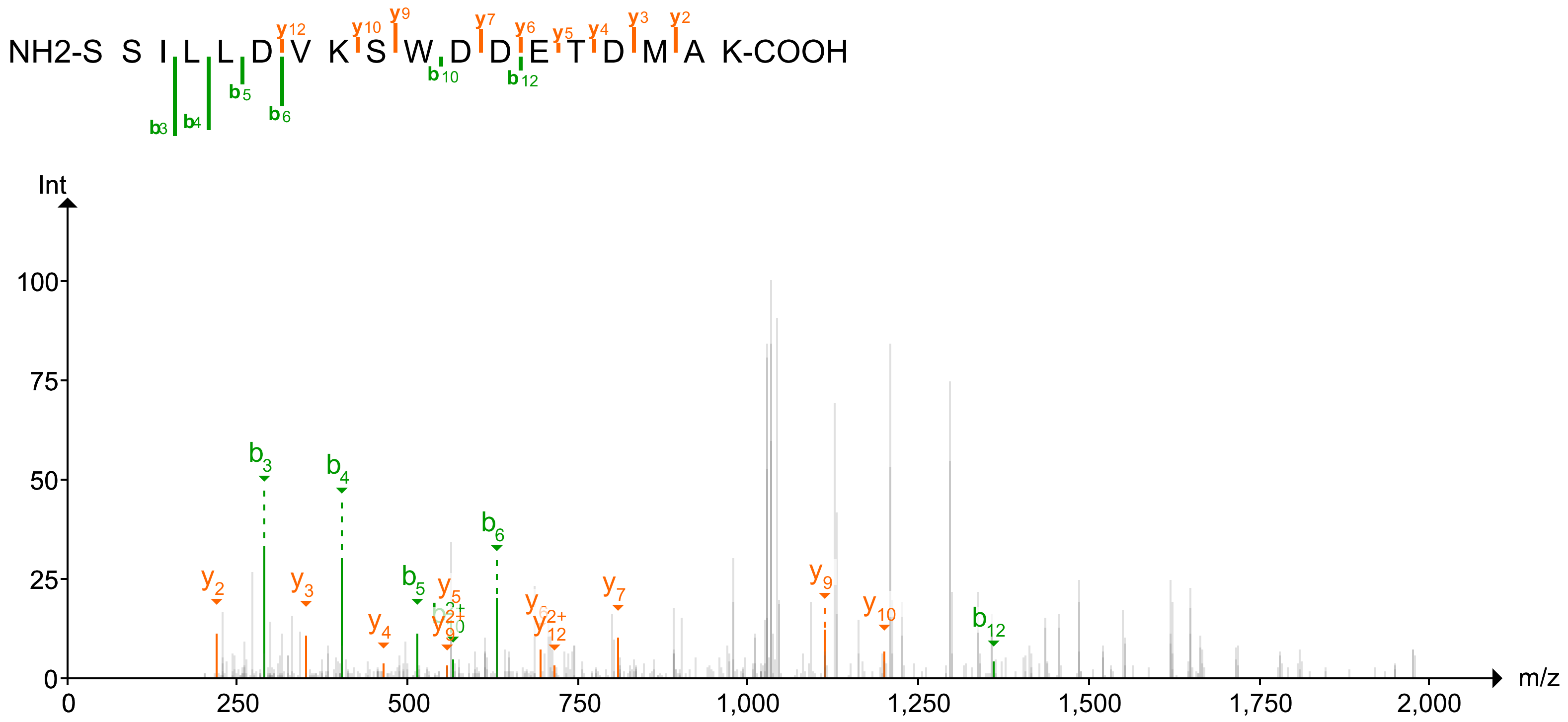

NH2-S S I L L D V K S W D D E T D M A K-COOH
y12 y10 y9 y7 y6 y5 y4 y3 y2
b3 b4 b5 b6 b10 b12
Int
100
75
50
25
0
b3
b4
b6
b5
y2
y3
y4
y5
y7
y9
y10
b12
m/z
0
250
500
750
1,000
1,250
1,500
1,750
2,000

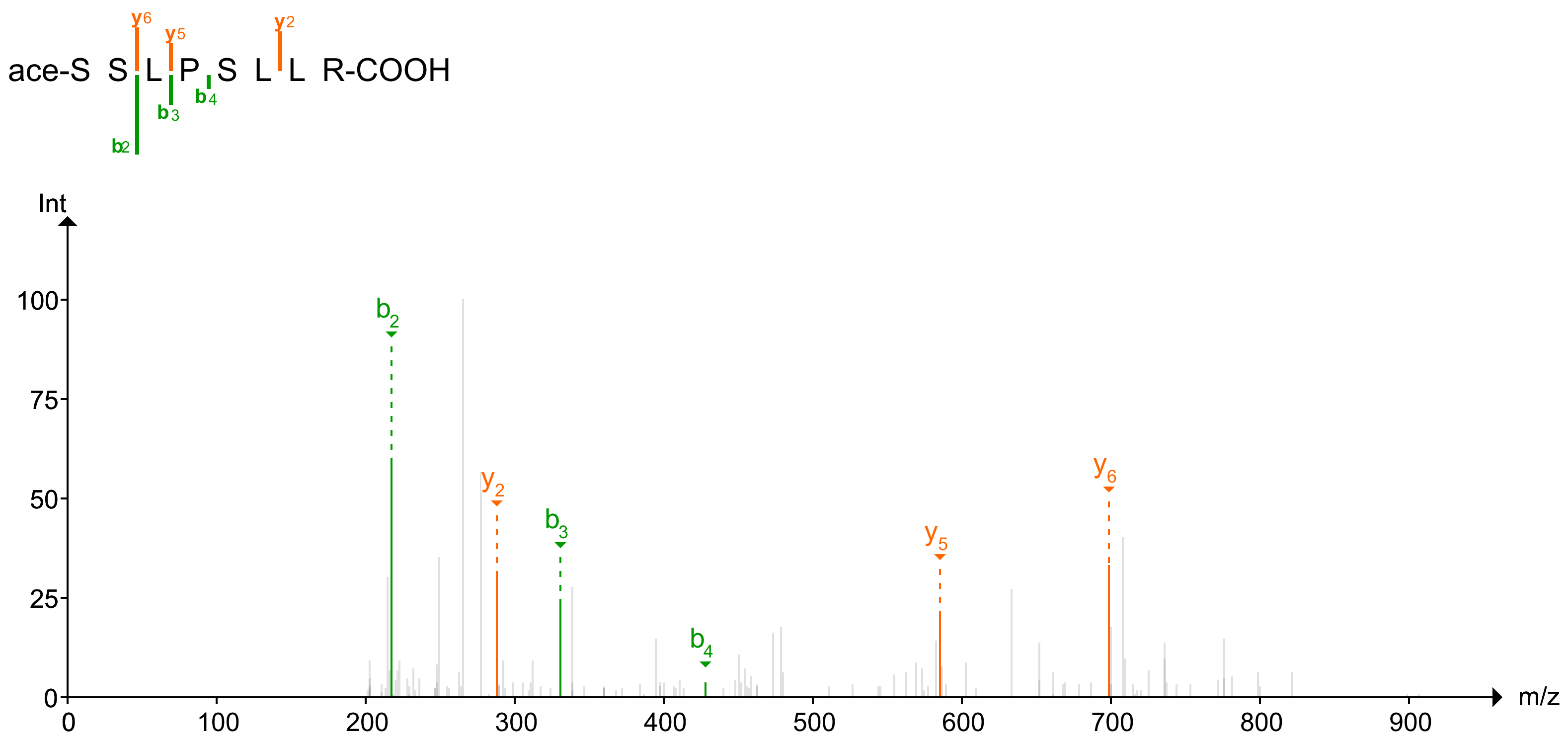
ace-S S L P S L L R-COOH
Int
m/z
100
75
50
25
0
0
100
200
300
400
500
600
700
800
900
b2
b3
b4
y2
y5
y6

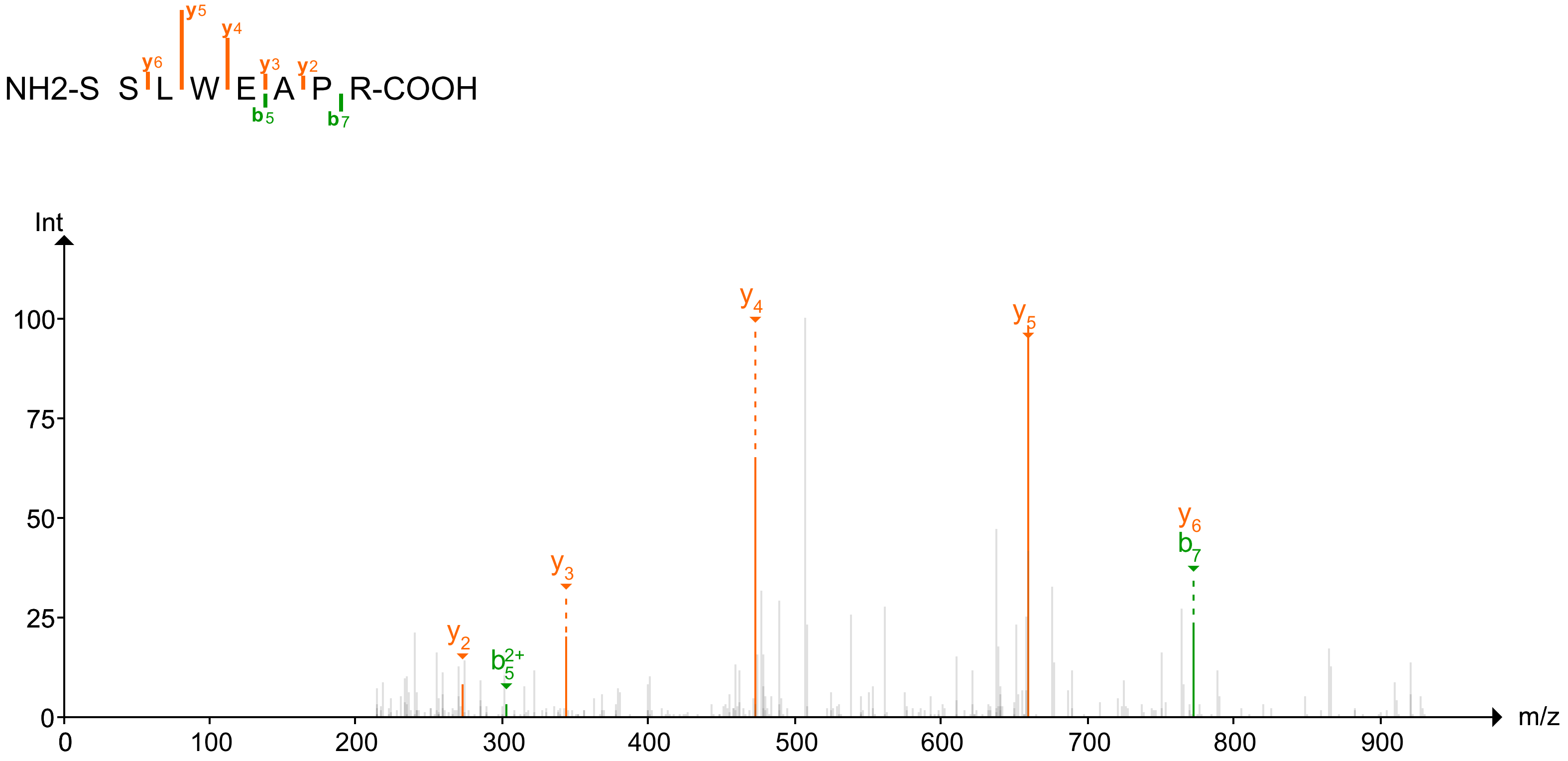

NH2-S S L W E A P R-COOH
y5 y4 y6 y3 y2 b5 b7
Int
m/z
0
25
50
75
100
0
100
200
300
400
500
600
700
800
900
y4
y5
y6
b7
y3
y2
b5 2+

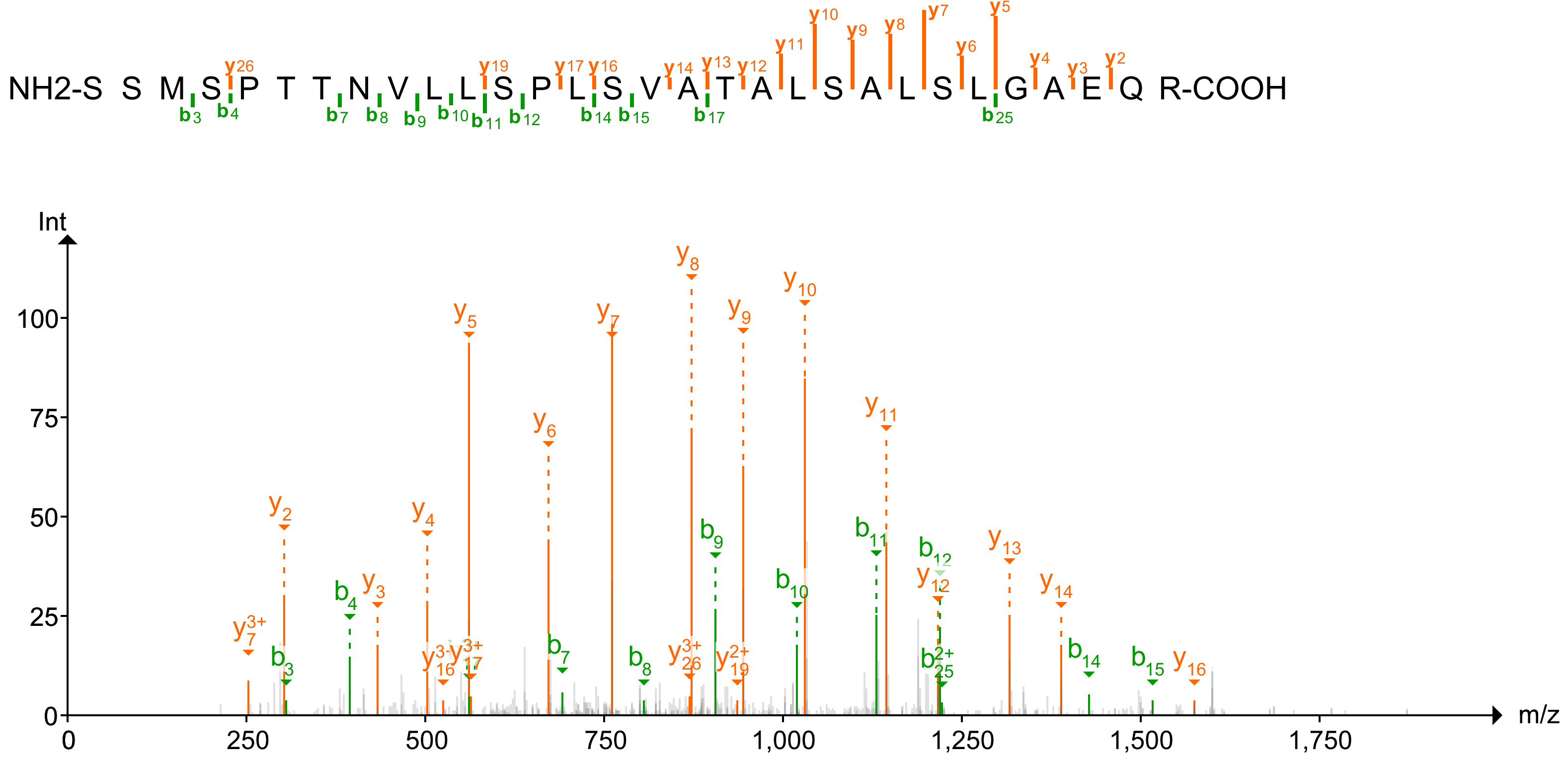

NH2-S S M S P T T N V L L S P L S V A T T A L S A L S L G A E Q R-COOH
y26 y19 y17 y16 y14 y13 y12 y11 y10 y9 y8 y7 y6 y5 y4 y3 y2
b3 b4 b7 b8 b9 b10 b11 b12 b14 b15 b17 b25
Int
m/z
0
25
50
75
100
250
500
750
1,000
1,250
1,500
1,750
y2 y3 y4 y5 y6 y7 y8 y9 y10 y11 y12 y13 y14 y16
y7 3+ y16 3+ y17 3+ y26 3+ y19 2+
b3 b4 b7 b8 b9 b10 b11 b12 b14 b15 b25 2+

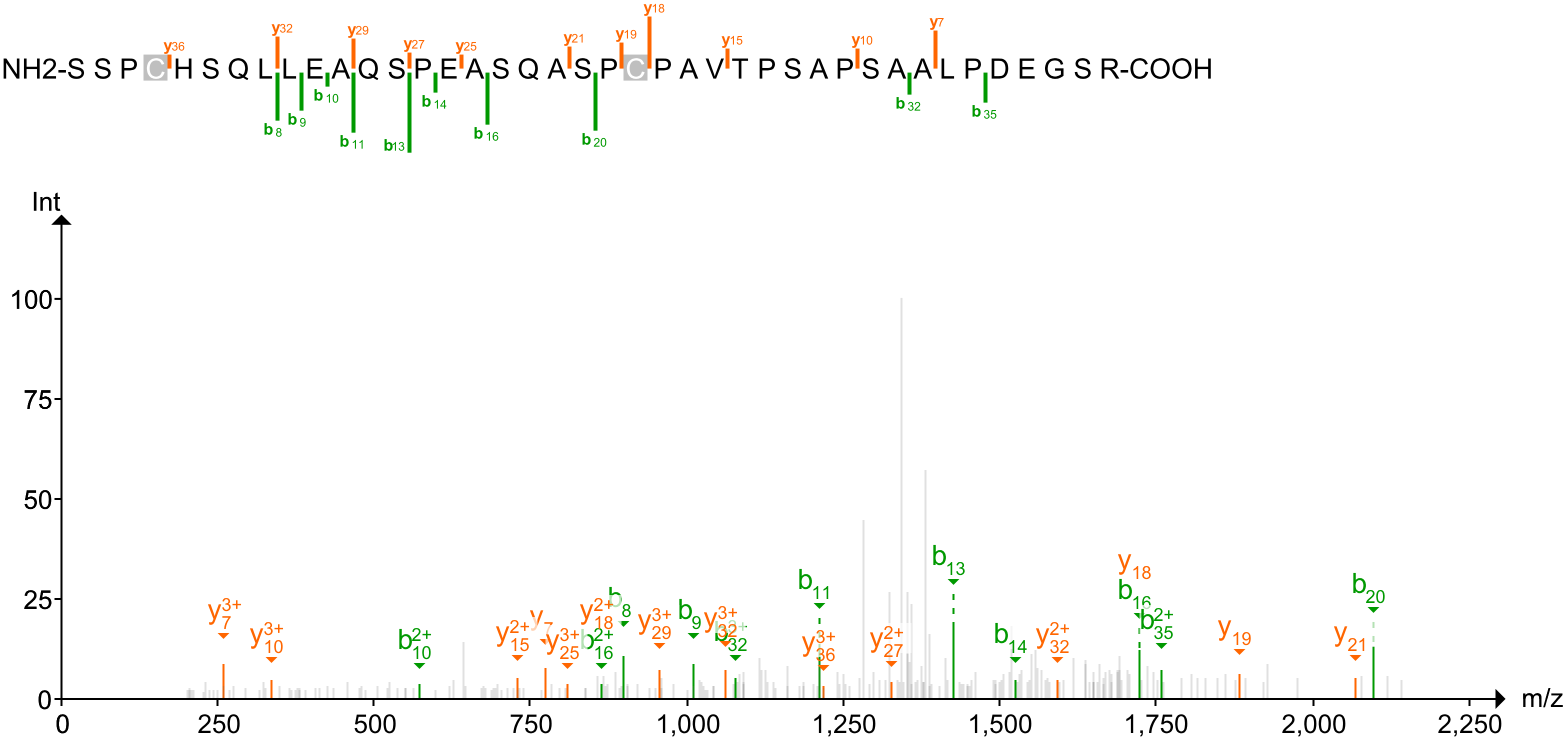

NH2-S S P C H S Q L L E A Q S P E A S Q A S P C P A V T P S A P S A A L P D E G S R-COOH
y36 y32 y29 y27 y25 y21 y19 y18 y15 y10 y7
b 8 b 9 b 10 b 11 b13 b 14 b 16 b 20 b 32 b 35
Int
100
75
50
25
0
m/z
0
250
500
750
1,000
1,250
1,500
1,750
2,000
2,250

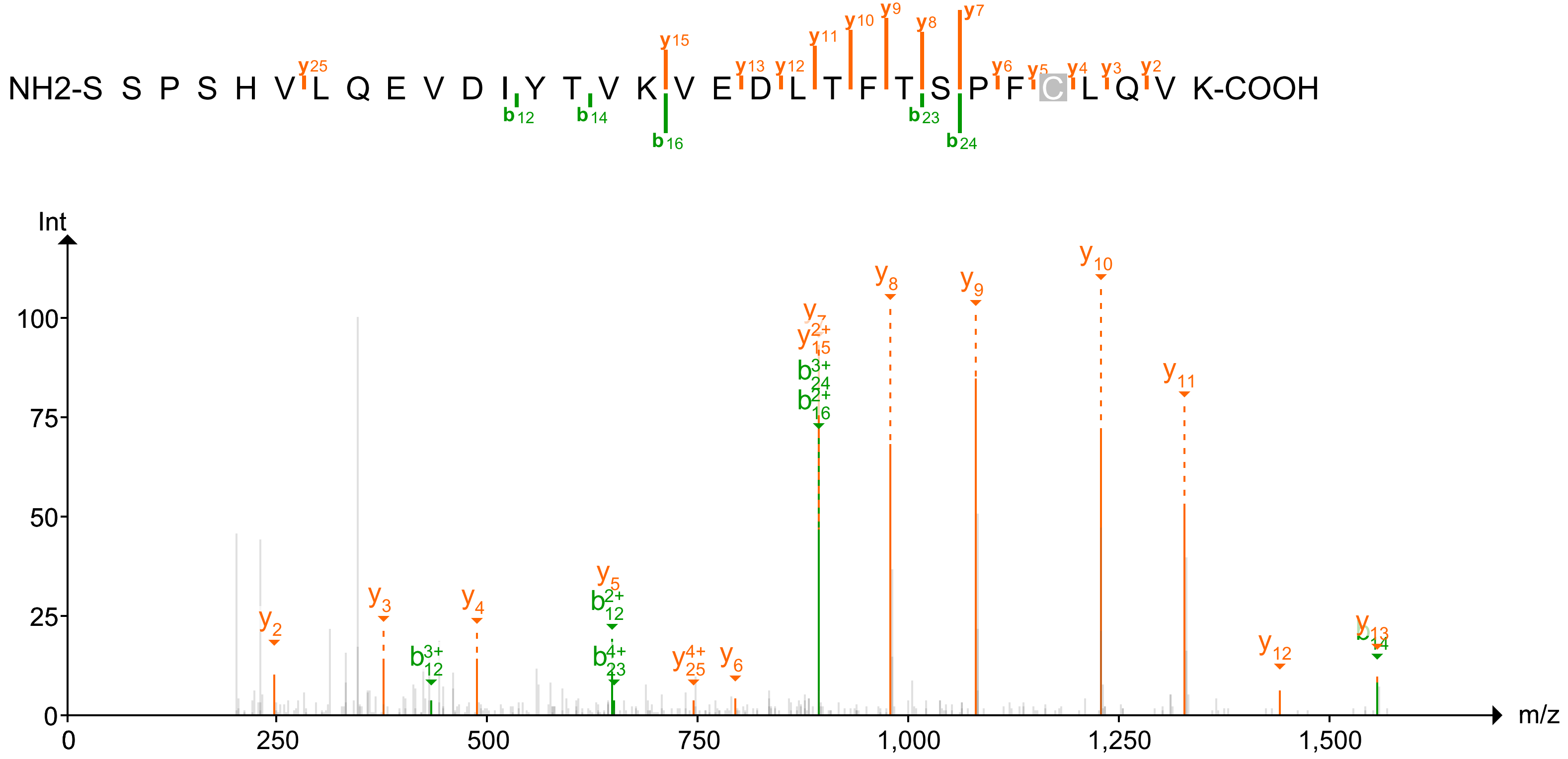

NH2-S S P S H V L Q E V D I Y T V K V E D L T F T S P F C L Q V K-COOH
y25 y15 y13 y12 y11 y10 y9 y8 y7 y6 y5 y4 y3 y2
b12 b14 b16 b23 b24
Int
m/z
0
25
50
75
100
250
500
750
1,000
1,250
1,500

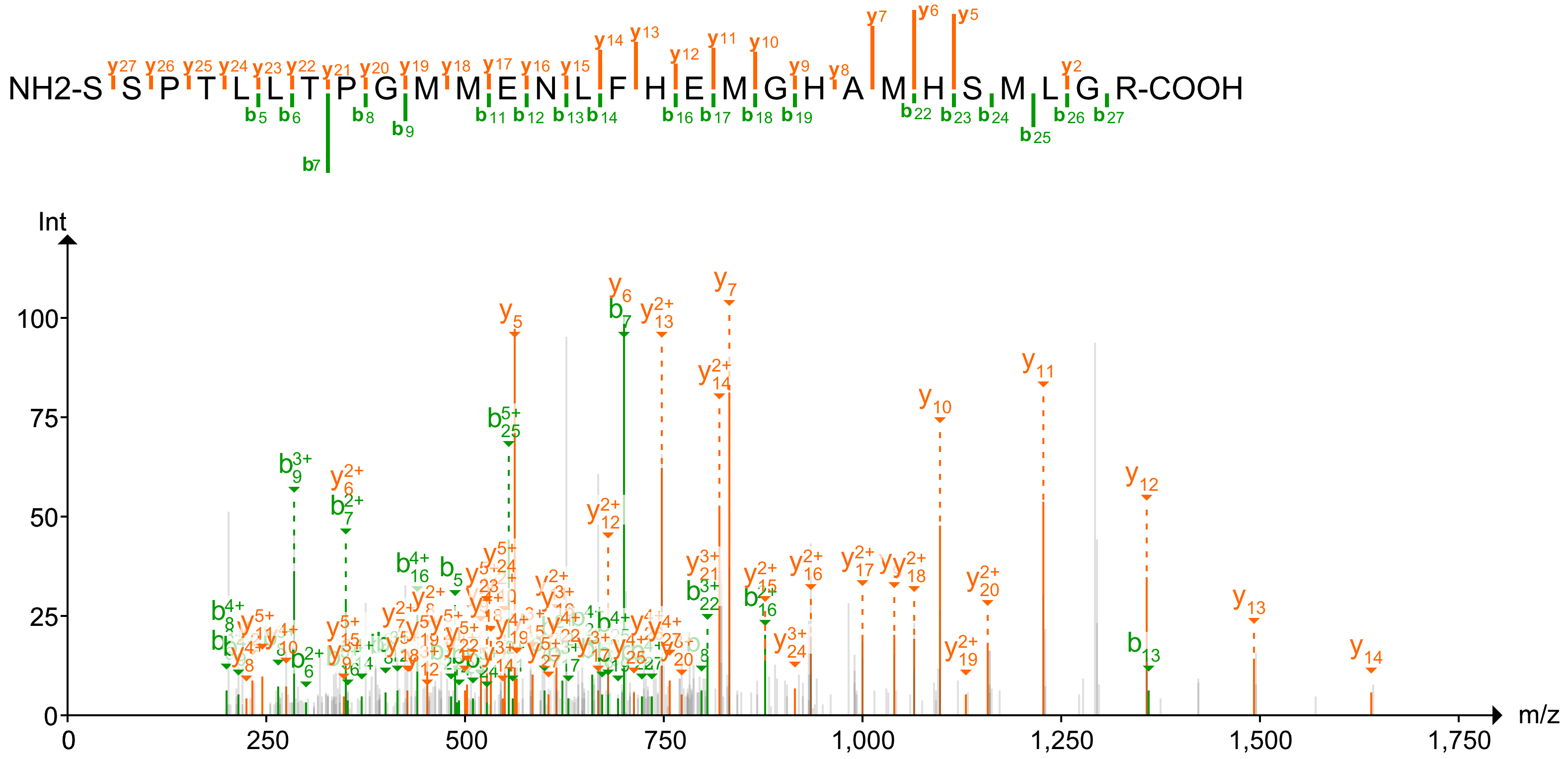

NH2-S S P T L L T P G M M E N L F H E M G H A M H S M L G R-COOH
Int
m/z
0
25
50
75
100
250
500
750
1,000
1,250
1,500
1,750
y5
y6
b7
y7
y13 2+
y14 2+
y10
y11
y12
y13
y14
b13
b25 5+
b9 3+
y6 2+
b7 2+
y12 2+
y21 3+
b22 3+
y15 2+
b16 2+
y16 2+
y17 2+
y18 2+
y20 2+
y19 2+
y24 3+
b16 4+
b5
b6 2+

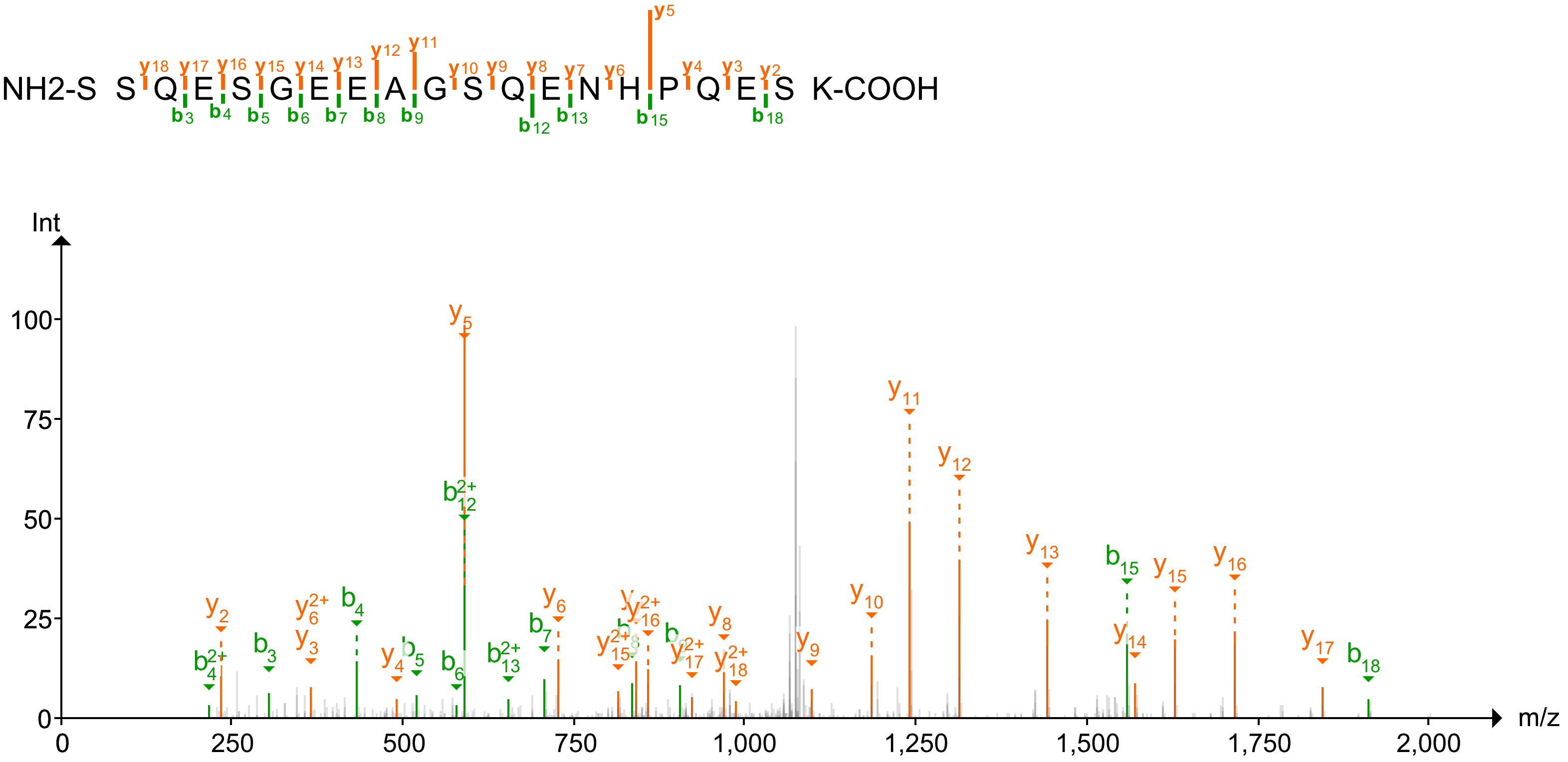

NH2-S S Q E S G E E A G S Q E N H P Q E S K-COOH
y18 y17 y16 y15 y14 y13 y12 y11 y10 y9 y8 y7 y6 y5 y4 y3 y2
b3 b4 b5 b6 b7 b8 b9 b12 b13 b15 b18
Int
100
75
50
25
0
m/z
0
250
500
750
1,000
1,250
1,500
1,750
2,000

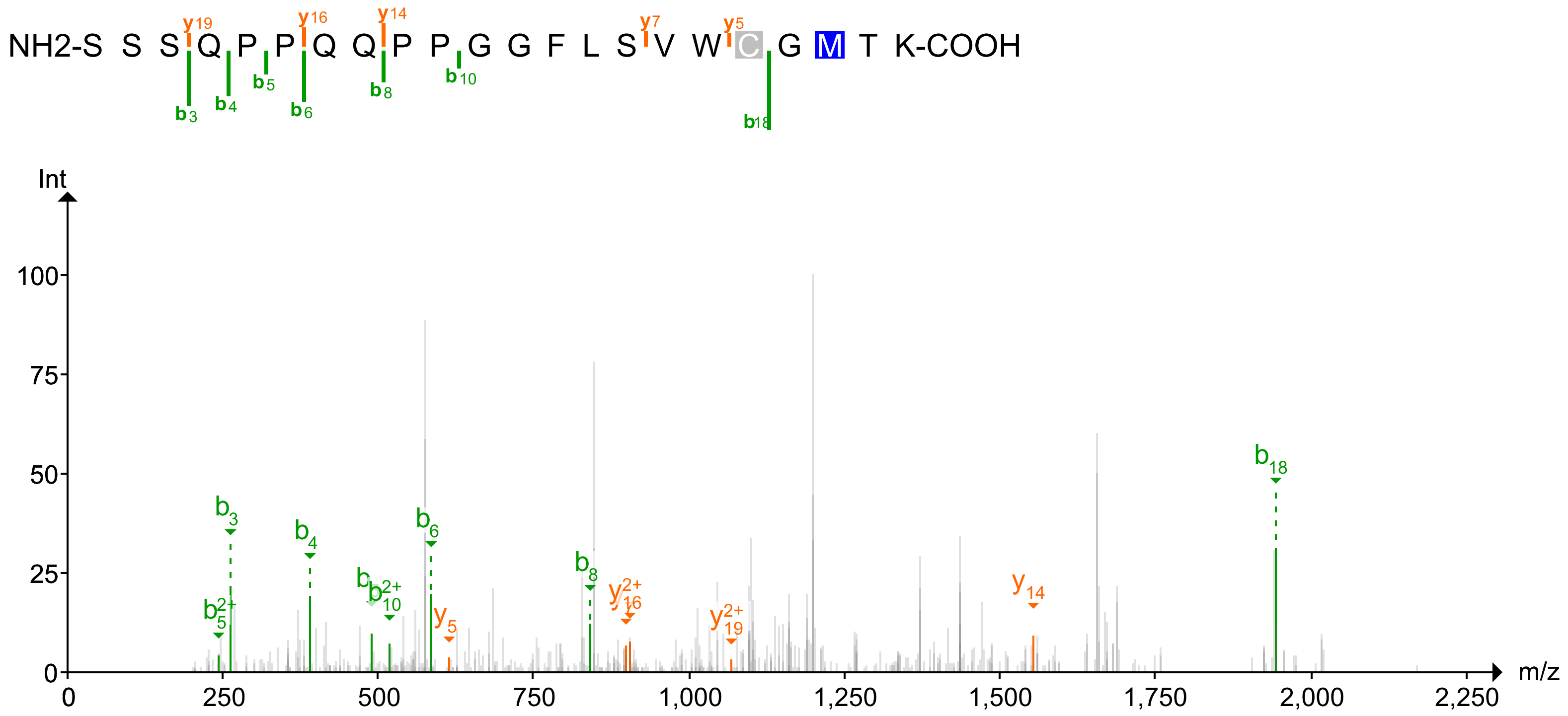

NH2-S S S Q P P Q Q P P G G F L S V W C G M T K-COOH
y19 y16 y14 y7 y5
b3 b4 b5 b6 b8 b10 b18
Int
m/z
0
25
50
75
100
250
500
750
1,000
1,250
1,500
1,750
2,000
2,250
b3
b4
b6
b8
b18
y14

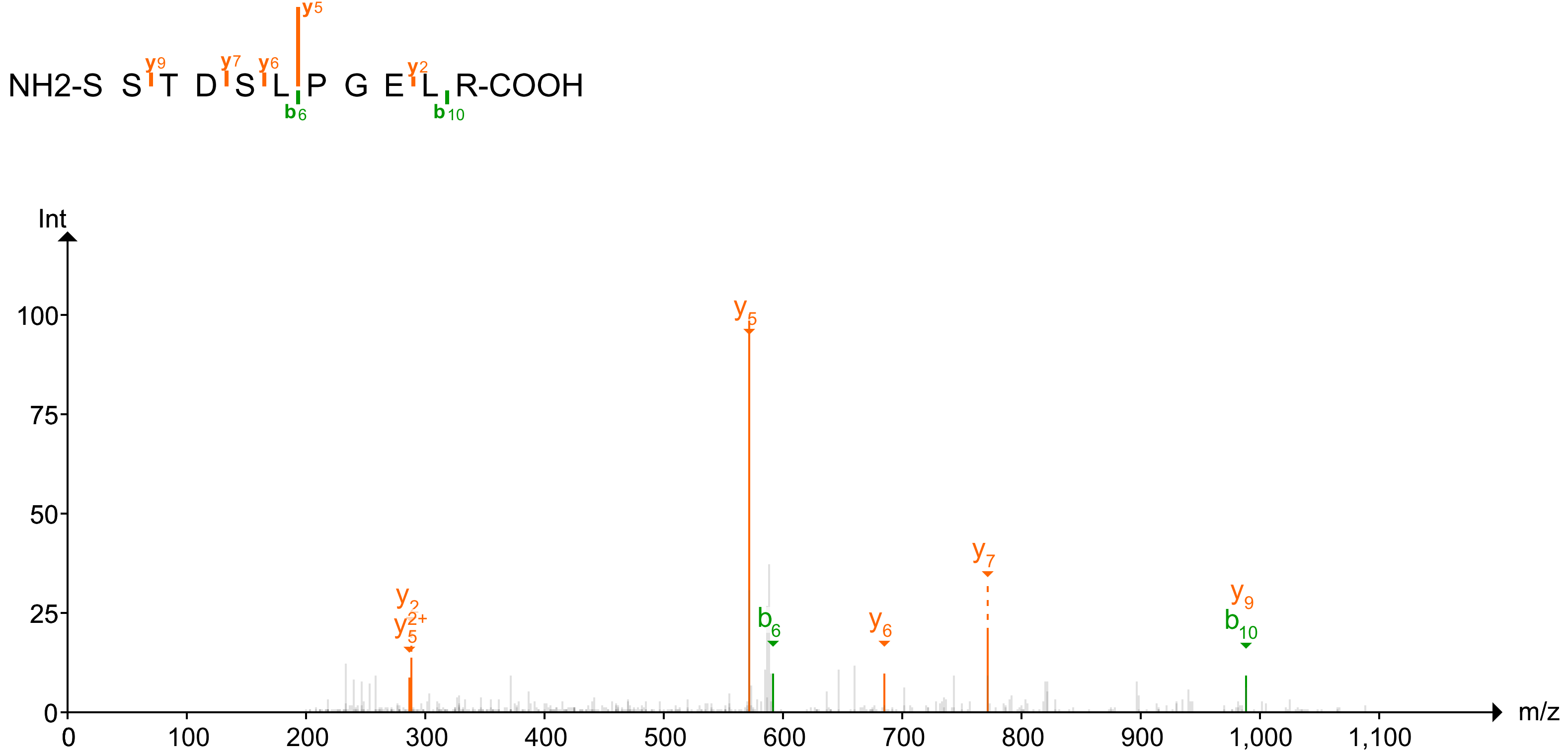
NH2-S S T D S L P G E L R-COOH
y9
y7
y6
y5
y2
b6
b10
Int
100
75
50
25
0
y5
y2
$y_5^{2+}$
b6
y6
y7
y9
b10
0
100
200
300
400
500
600
700
800
900
1,000
1,100
m/z

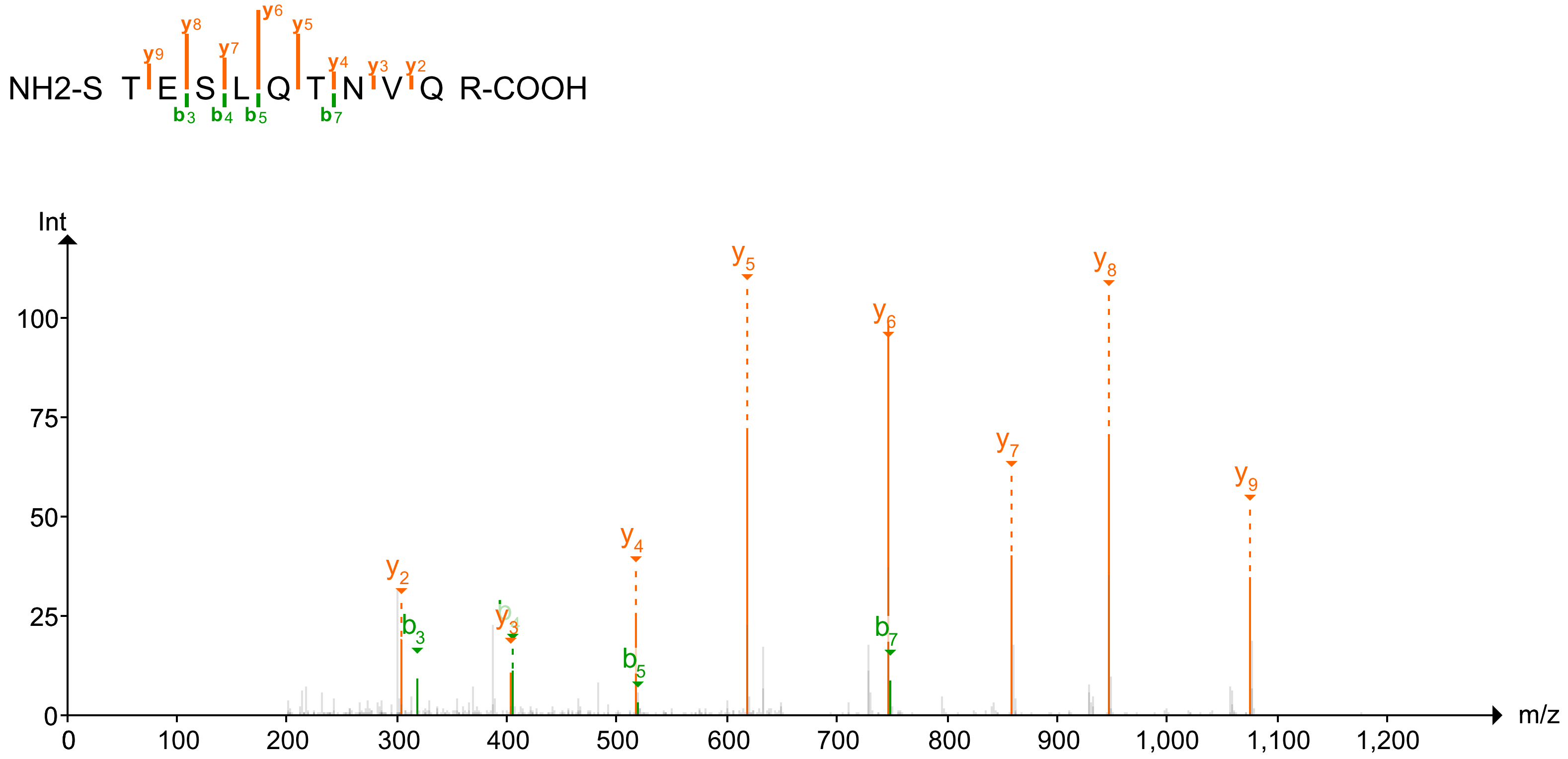

NH2-S T E S L Q T N V Q R-COOH
y9 y8 y7 y6 y5 y4 y3 y2
b3 b4 b5 b7
Int
100
75
50
25
0
y5
y8
y6
y7
y9
y4
y2
b3
y3
b5
b7
0 100 200 300 400 500 600 700 800 900 1,000 1,100 1,200
m/z

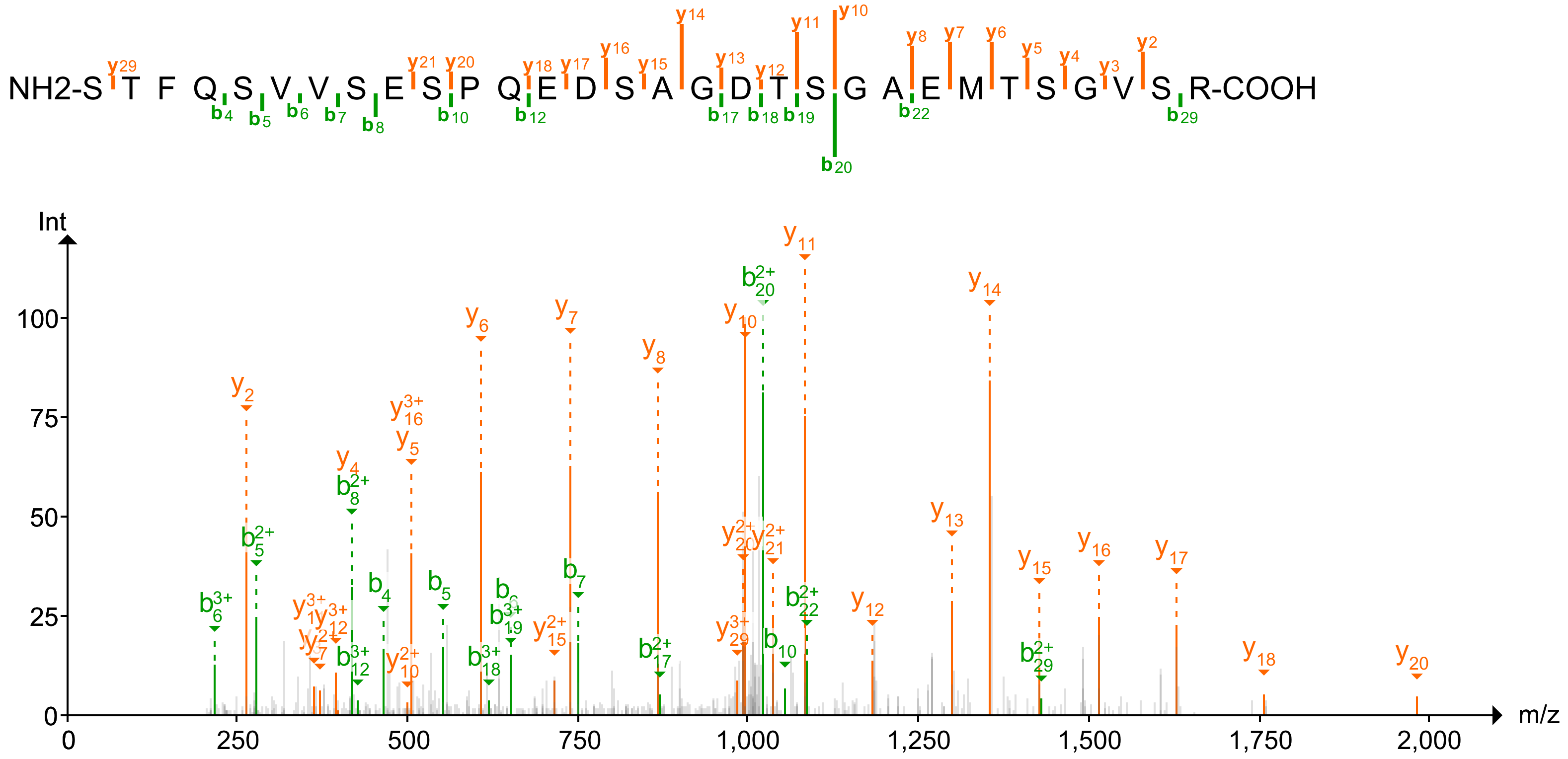

NH2-S T F Q S V V S E S P Q E D S A G D T S G A E M T S G V S R-COOH
Int
m/z
0
25
50
75
100
250
500
750
1,000
1,250
1,500
1,750
2,000

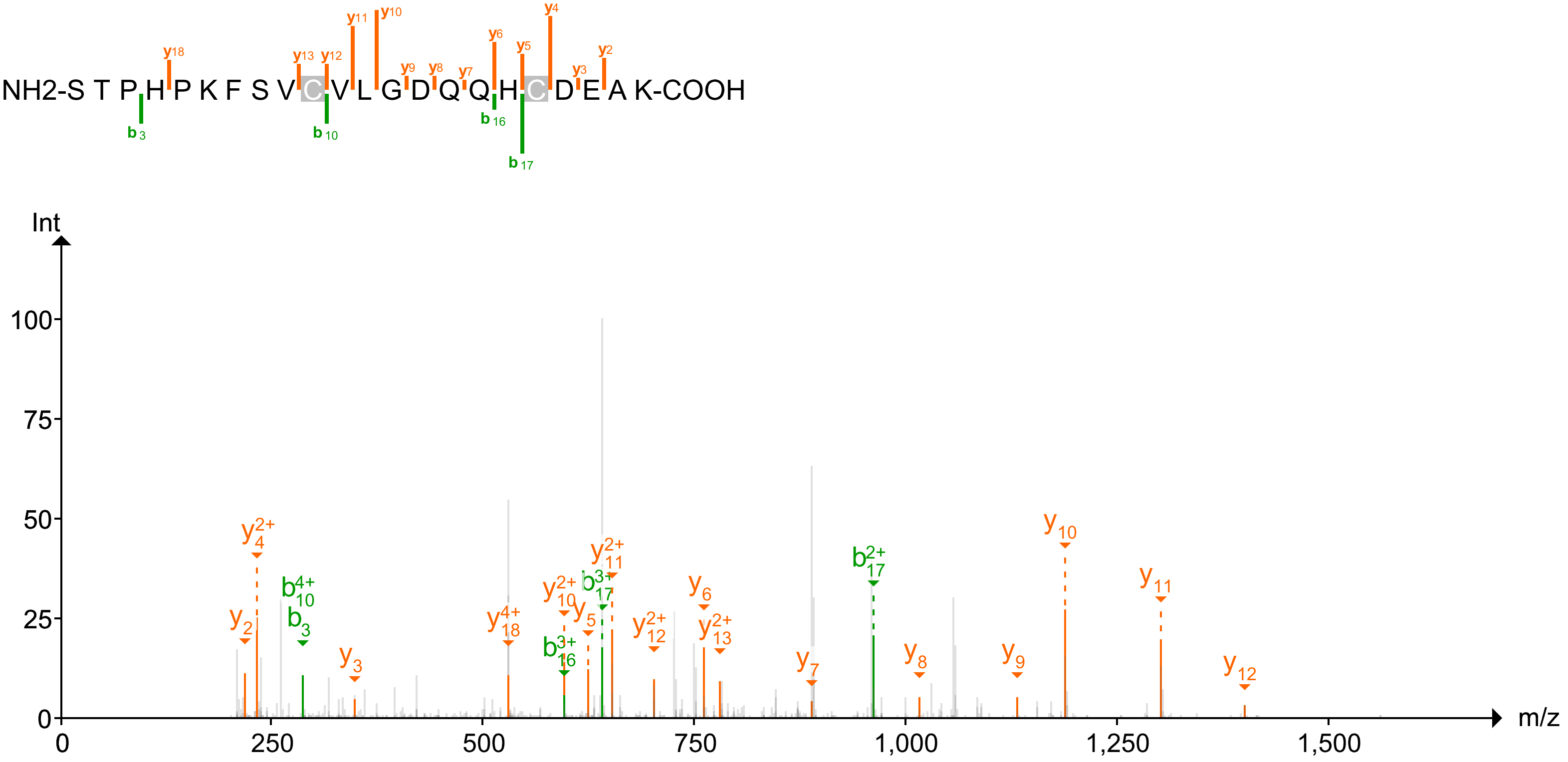

NH2-S T P H P K F S V C V L G D Q Q H C D E A K-COOH
y18 y13 y12 y11 y10 y9 y8 y7 y6 y5 y4 y3 y2
b3 b10 b16 b17
Int
100
75
50
25
0
m/z
0
250
500
750
1,000
1,250
1,500
y4 2+
y2
b10 4+
b3
y3
y18 4+
b16 3+
y10 2+
y5
b17 3+
y11 2+
y12 2+
y6
y13 2+
y7
b17 2+
y8
y9
y10
y11
y12

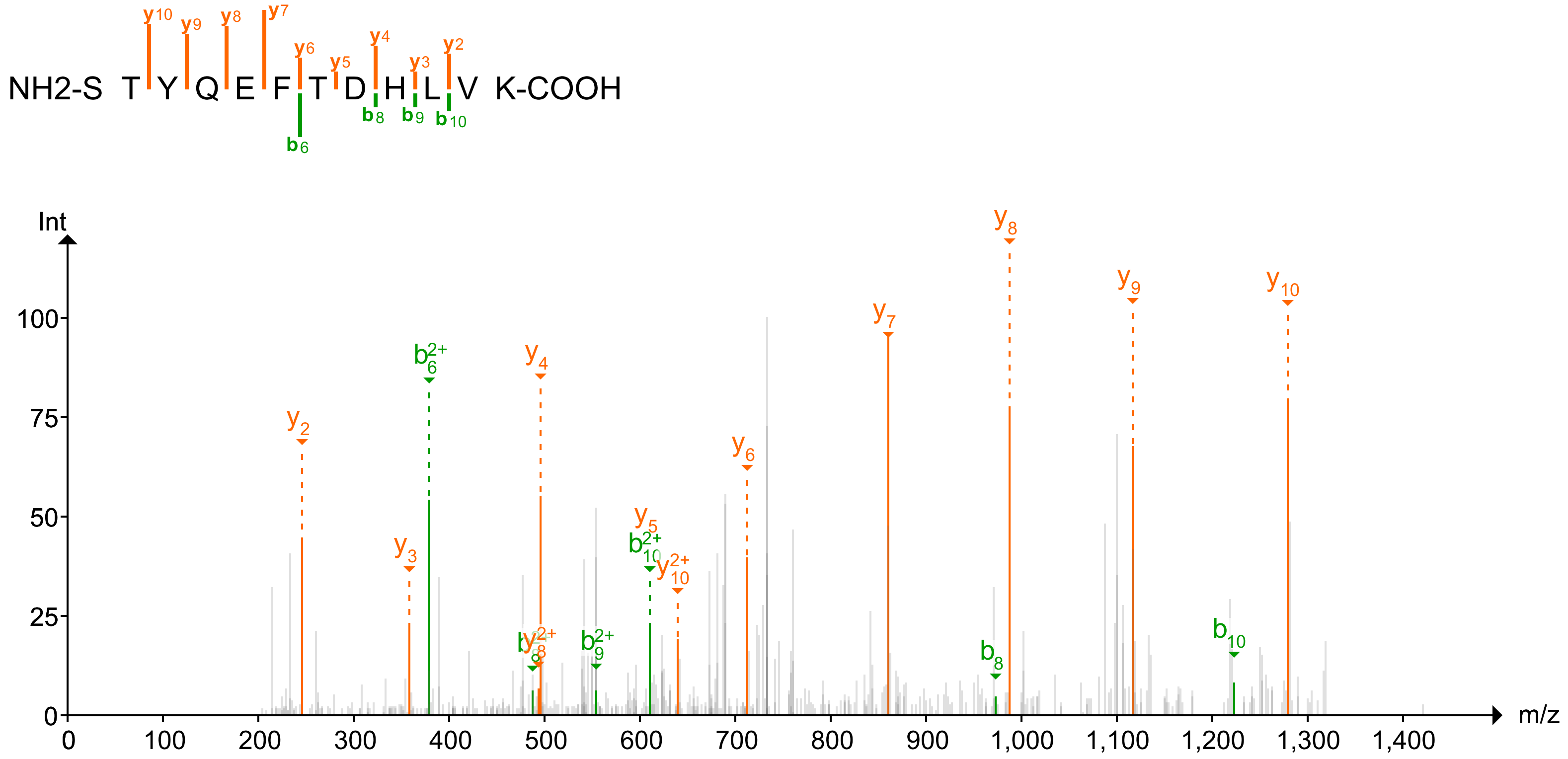

NH2-S T Y Q E F T D H L V K-COOH
y10 y9 y8 y7 y6 y5 y4 y3 y2
b6 b8 b9 b10
Int
m/z
0 25 50 75 100
0 100 200 300 400 500 600 700 800 900 1,000 1,100 1,200 1,300 1,400
y2 y3 b6 2+ y4 b8 2+ y8 2+ b9 2+ y5 b10 2+ y10 2+ y6 y7 b8 y8 y9 b10 y10

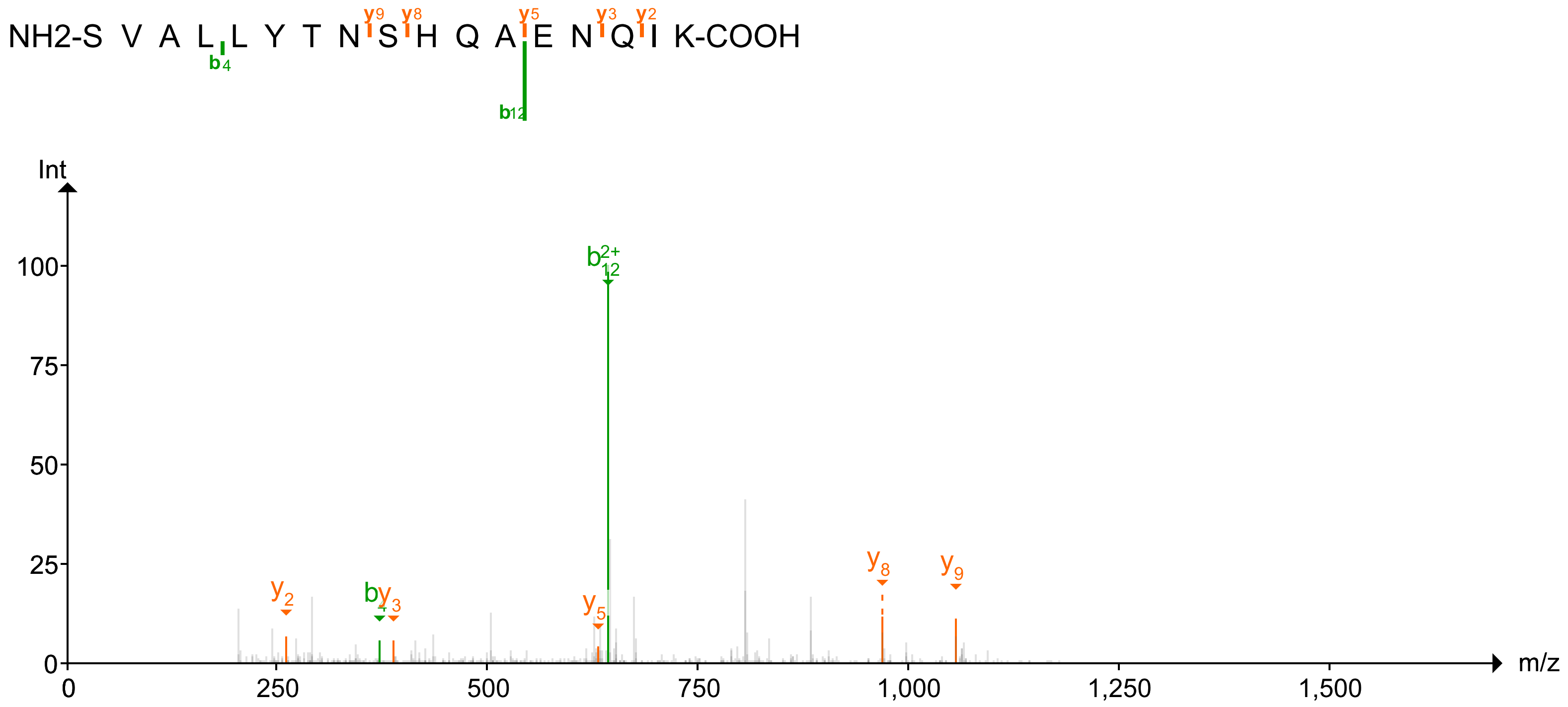
NH2-S V A L L Y T N S H Q A E N Q I K-COOH
y9 y8 y5 y3 y2
b4
b12
Int
100
75
50
25
0
b12 2+
y2
b4
y3
y5
y8
y9
0
250
500
750
1,000
1,250
1,500
m/z

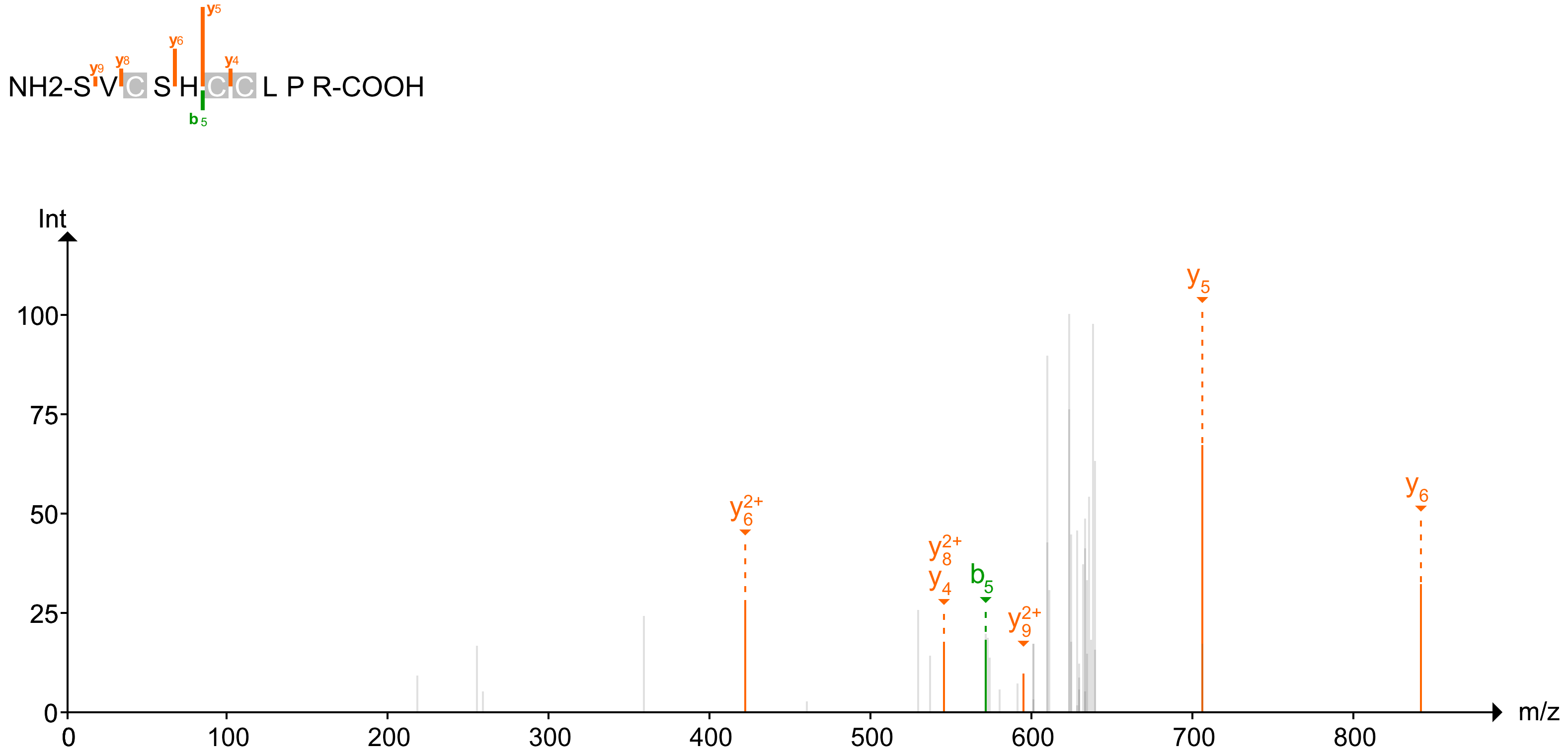
NH2-S V C S H C C L P R-COOH
y9
y8
y6
y5
y4
b5
Int
m/z
$y_5$
$y_6$
$y_6^{2+}$
$y_8^{2+}$
$y_4$
$b_5$
$y_9^{2+}$
0
25
50
75
100
0
100
200
300
400
500
600
700
800

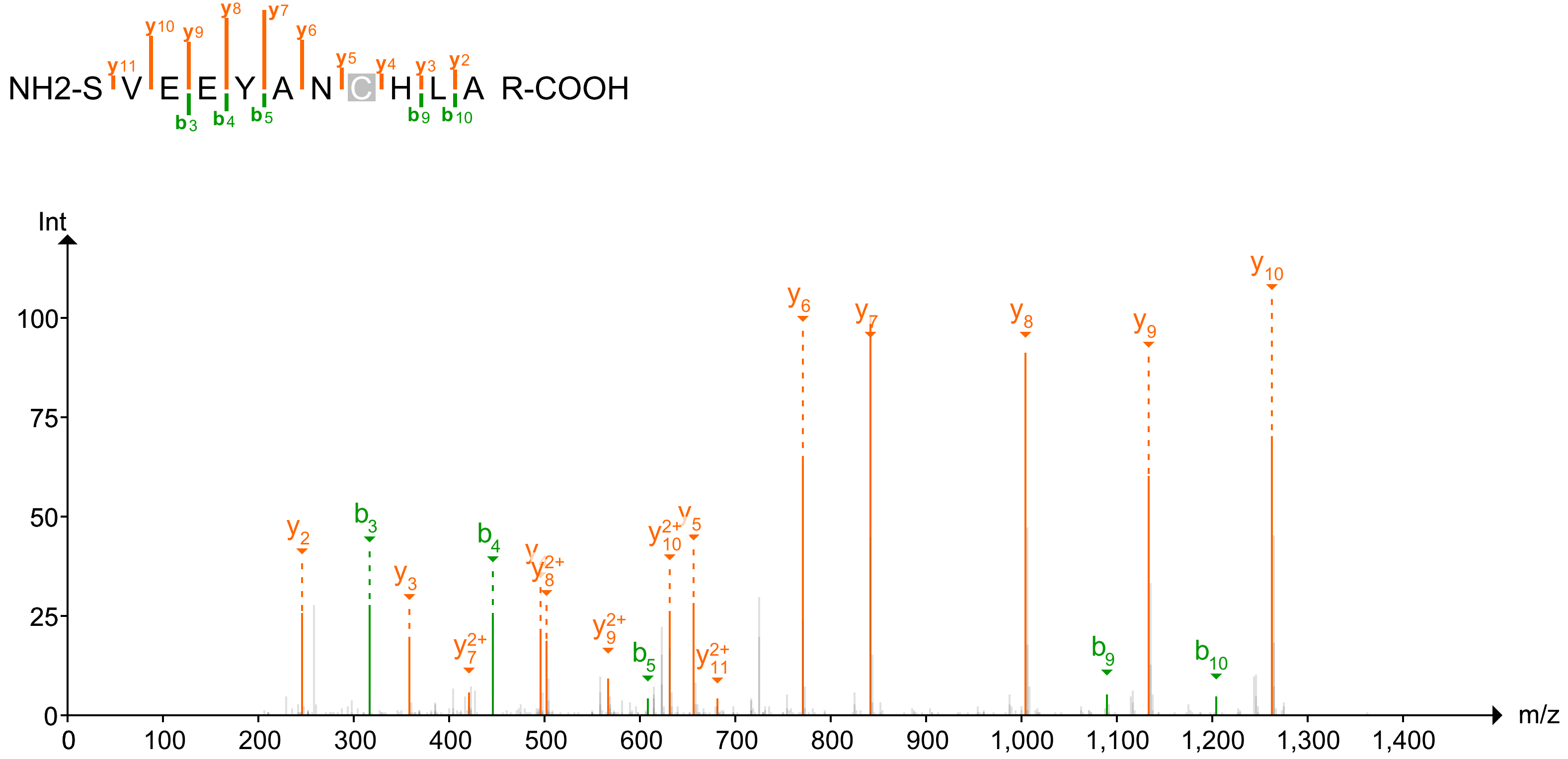

NH2-S V E E Y A N C H L A R-COOH
y11 y10 y9 y8 y7 y6 y5 y4 y3 y2
b3 b4 b5 b9 b10
Int
100
75
50
25
0
y10
y6
y7
y8
y9
y2
b3
b4
y3
y8 2+
y7 2+
y9 2+
b5
y10 2+
y5
y11 2+
b9
b10
0
100
200
300
400
500
600
700
800
900
1,000
1,100
1,200
1,300
1,400
m/z

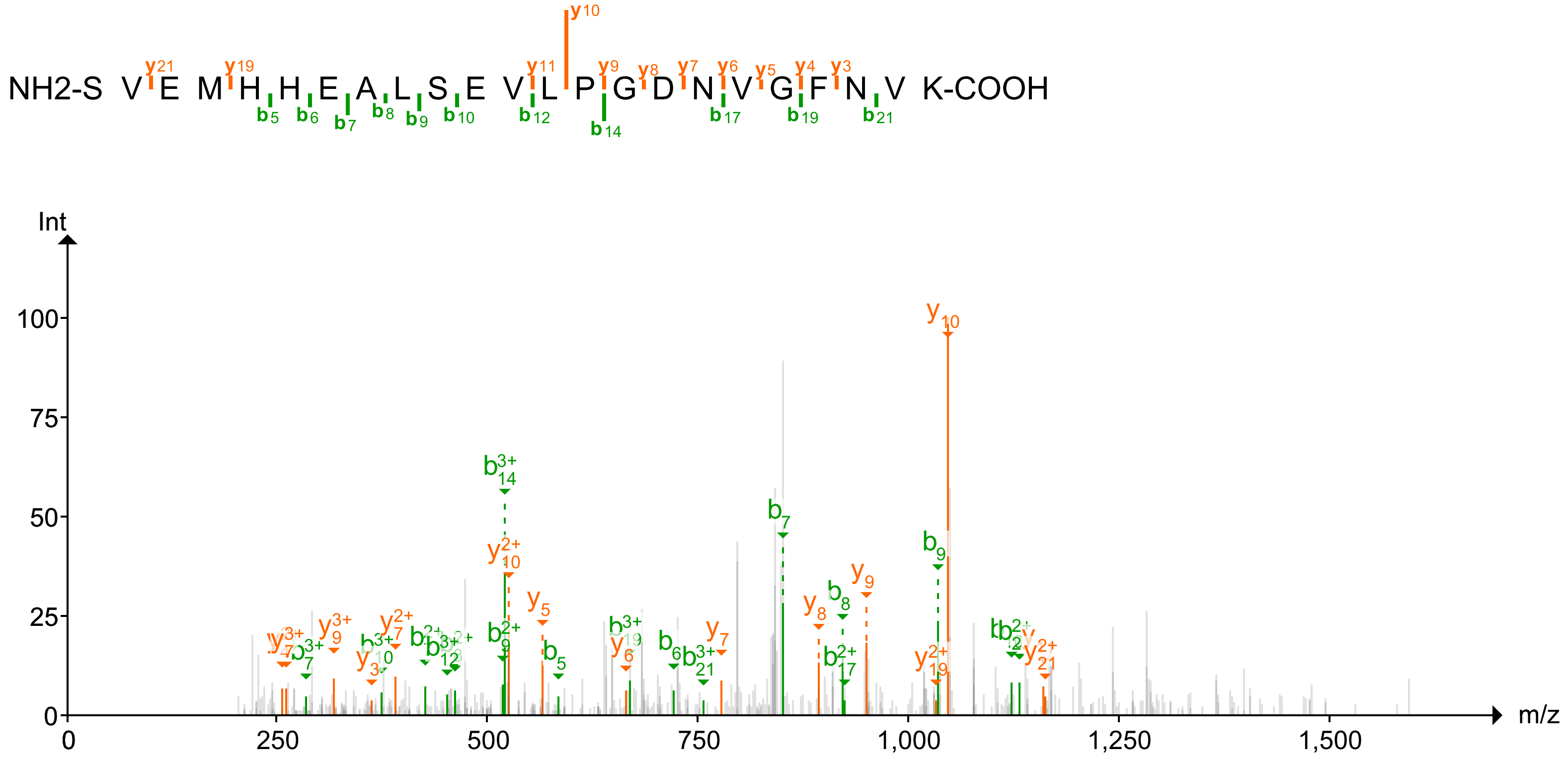

NH2-S V E M H H E A L S E V L P G D N V G F N V K-COOH
y21 y19 y11 y10 y9 y8 y7 y6 y5 y4 y3
b5 b6 b7 b8 b9 b10 b12 b14 b17 b19 b21
Int
100
75
50
25
0
m/z
0
250
500
750
1,000
1,250
1,500
$y_{10}$
$b_{14}^{3+}$
$y_{10}^{2+}$
$b_7$
$b_9$
$y_9$
$b_8$
$y_8$
$y_5$
$b_5$
$b_9^{2+}$
$y_6$
$b_6$
$b_{21}^{3+}$
$y_7$
$b_{17}^{2+}$
$y_{19}^{2+}$
$y_{21}^{2+}$
$y_9^{3+}$
$y_7^{2+}$
$b_7^{3+}$
$b_{19}^{3+}$

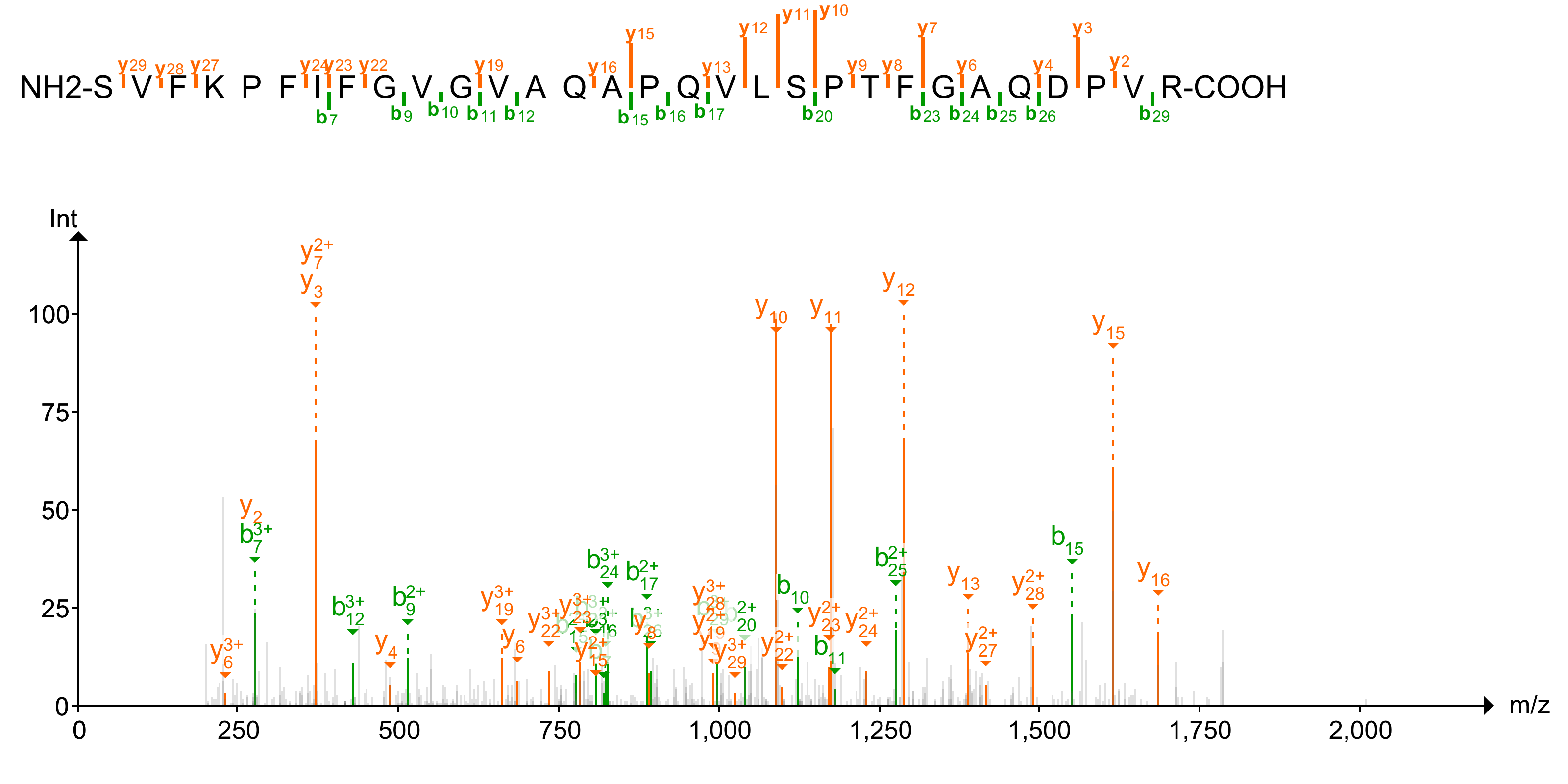

NH2-S V F K P F I F G V G V A Q A P Q V L S P T F G A Q D P V R-COOH
y29 y28 y27 y24 y23 y22 y19 y16 y15 y13 y12 y11 y10 y9 y8 y7 y6 y4 y3 y2
b7 b9 b10 b11 b12 b15 b16 b17 b20 b23 b24 b25 b26 b29
Int
m/z
0
25
50
75
100
250
500
750
1,000
1,250
1,500
1,750
2,000
$y_7^{2+}$
$y_3$
$y_{10}$
$y_{11}$
$y_{12}$
$y_{15}$
$y_2$
$b_7^{3+}$
$y_6^{3+}$
$b_{12}^{3+}$
$y_4$
$b_9^{2+}$
$y_{19}^{3+}$
$y_6$
$y_{22}^{3+}$
$y_{23}^{3+}$
$y_{15}^{2+}$
$b_{24}^{3+}$
$b_{17}^{2+}$
$y_{28}^{3+}$
$y_{19}^{2+}$
$y_{29}^{3+}$
$b_{20}^{2+}$
$b_{10}$
$y_{22}^{2+}$
$y_{23}^{2+}$
$b_{11}$
$y_{24}^{2+}$
$b_{25}^{2+}$
$y_{13}$
$y_{27}^{2+}$
$y_{28}^{2+}$
$b_{15}$
$y_{16}$

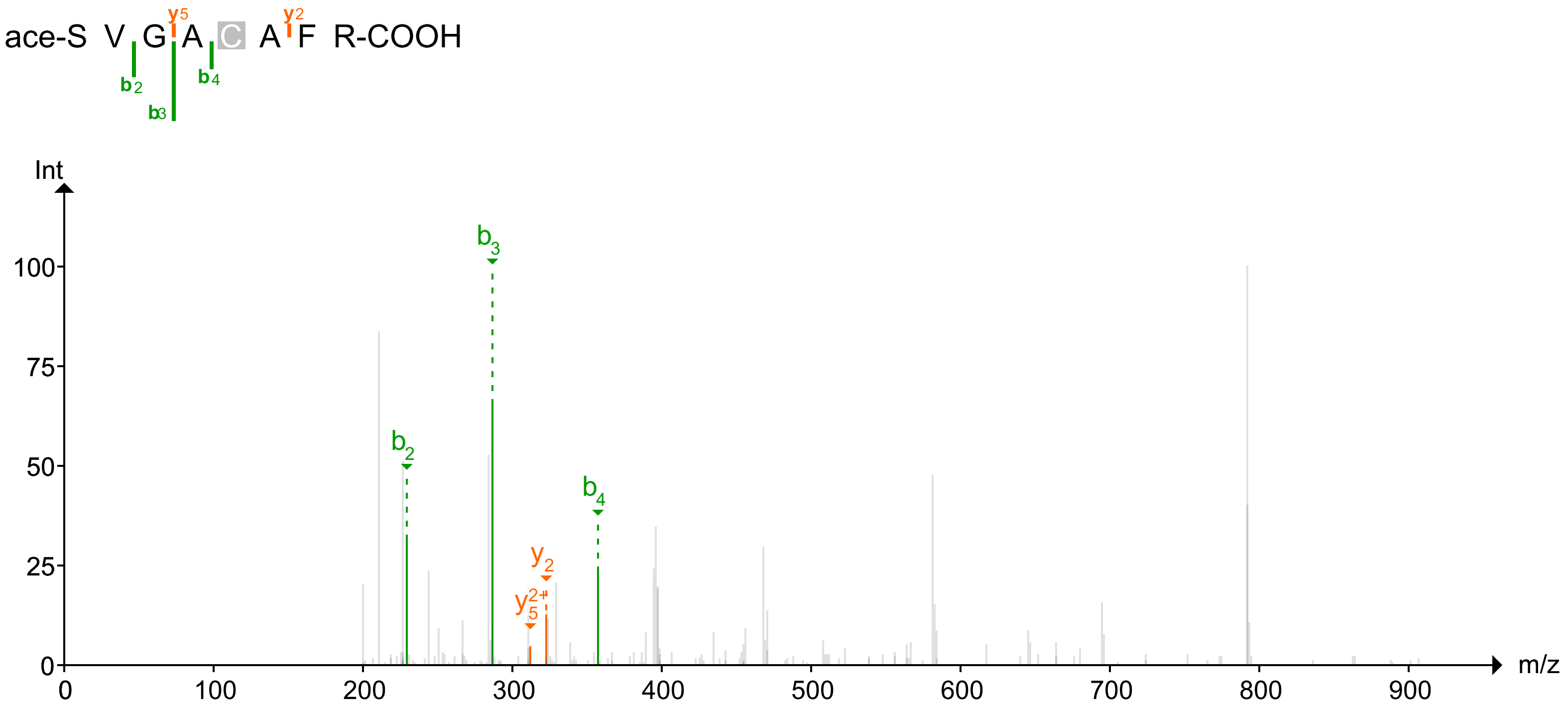

ace-S V G A C A F R-COOH
y5
y2
b2
b3
b4
Int
100
75
50
25
0
b3
b2
b4
y2
y5 2+
m/z
0
100
200
300
400
500
600
700
800
900

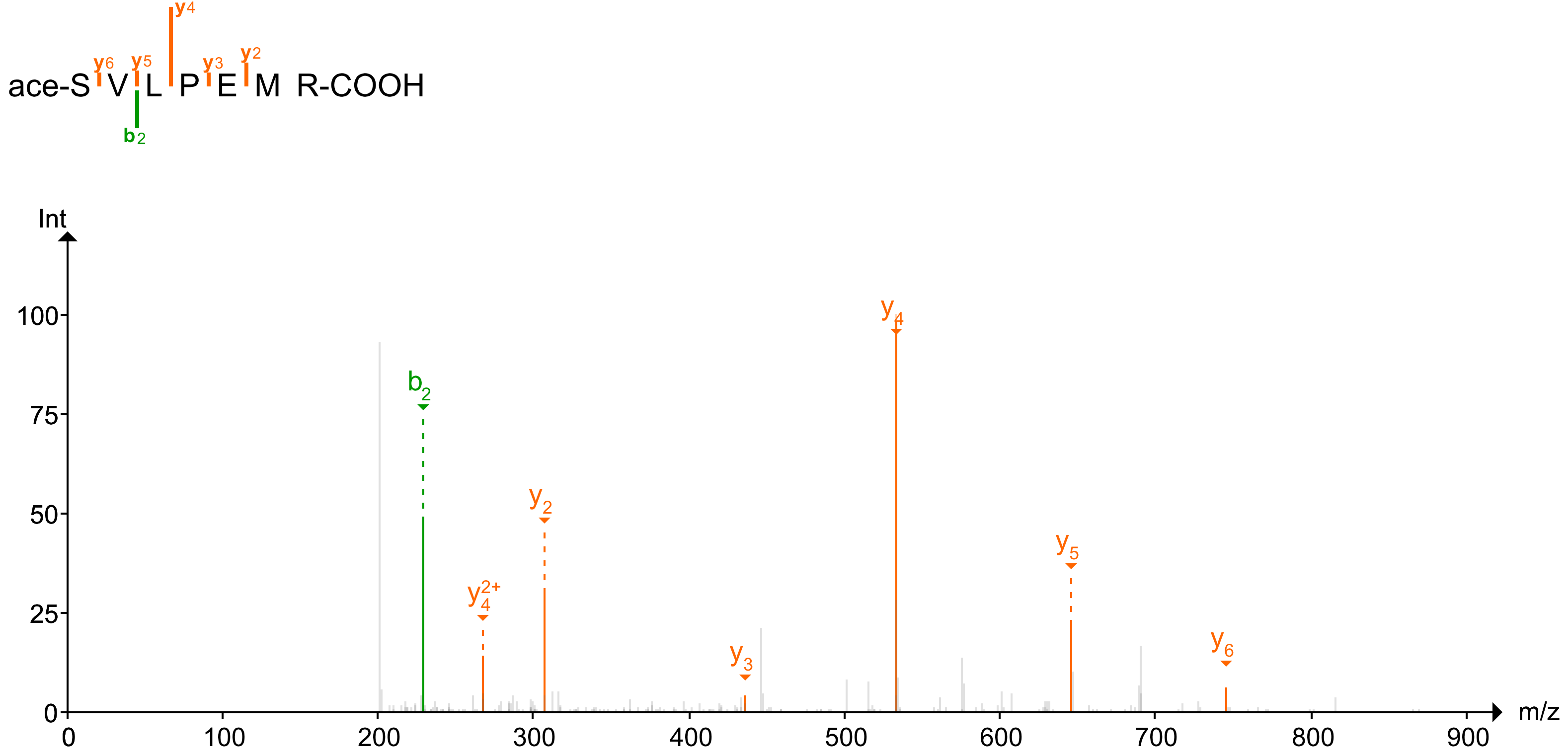

ace-S V L P E M R-COOH
y6 y5 y4 y3 y2
b2
Int
100
75
50
25
0
m/z
0 100 200 300 400 500 600 700 800 900
b2
y4 2+
y2
y3
y4
y5
y6

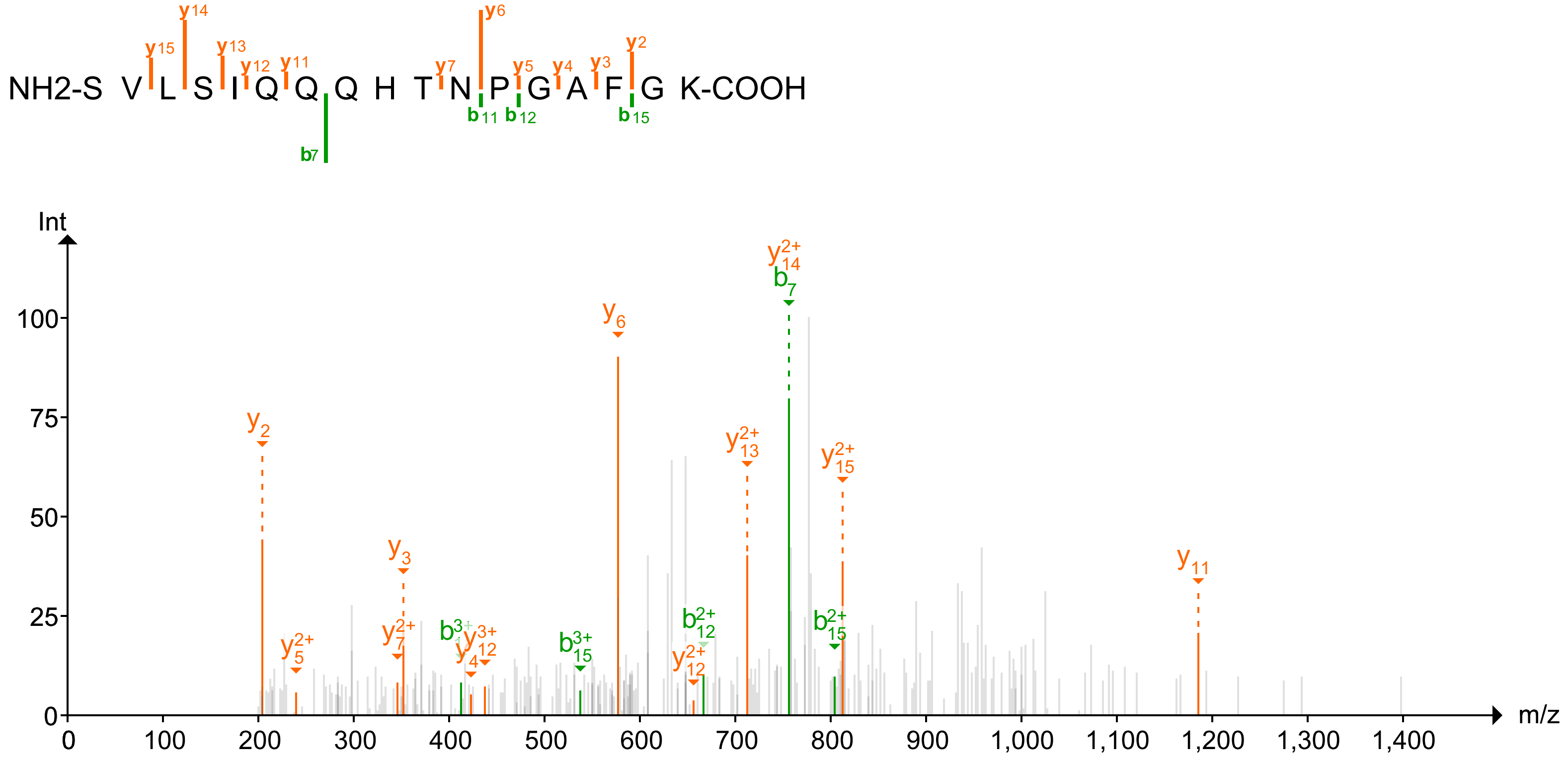

NH2-S V L S I Q Q Q H T N P G A F G K-COOH
y15 y14 y13 y12 y11 y7 y6 y5 y4 y3 y2
b7 b11 b12 b15
Int
100
75
50
25
0
y2
y5 2+
y3
y7 2+
b3+
y4
y12 3+
b15 3+
y6
b12 2+
y12 2+
y13 2+
y14 2+
b7
b15 2+
y15 2+
y11
0 100 200 300 400 500 600 700 800 900 1,000 1,100 1,200 1,300 1,400
m/z

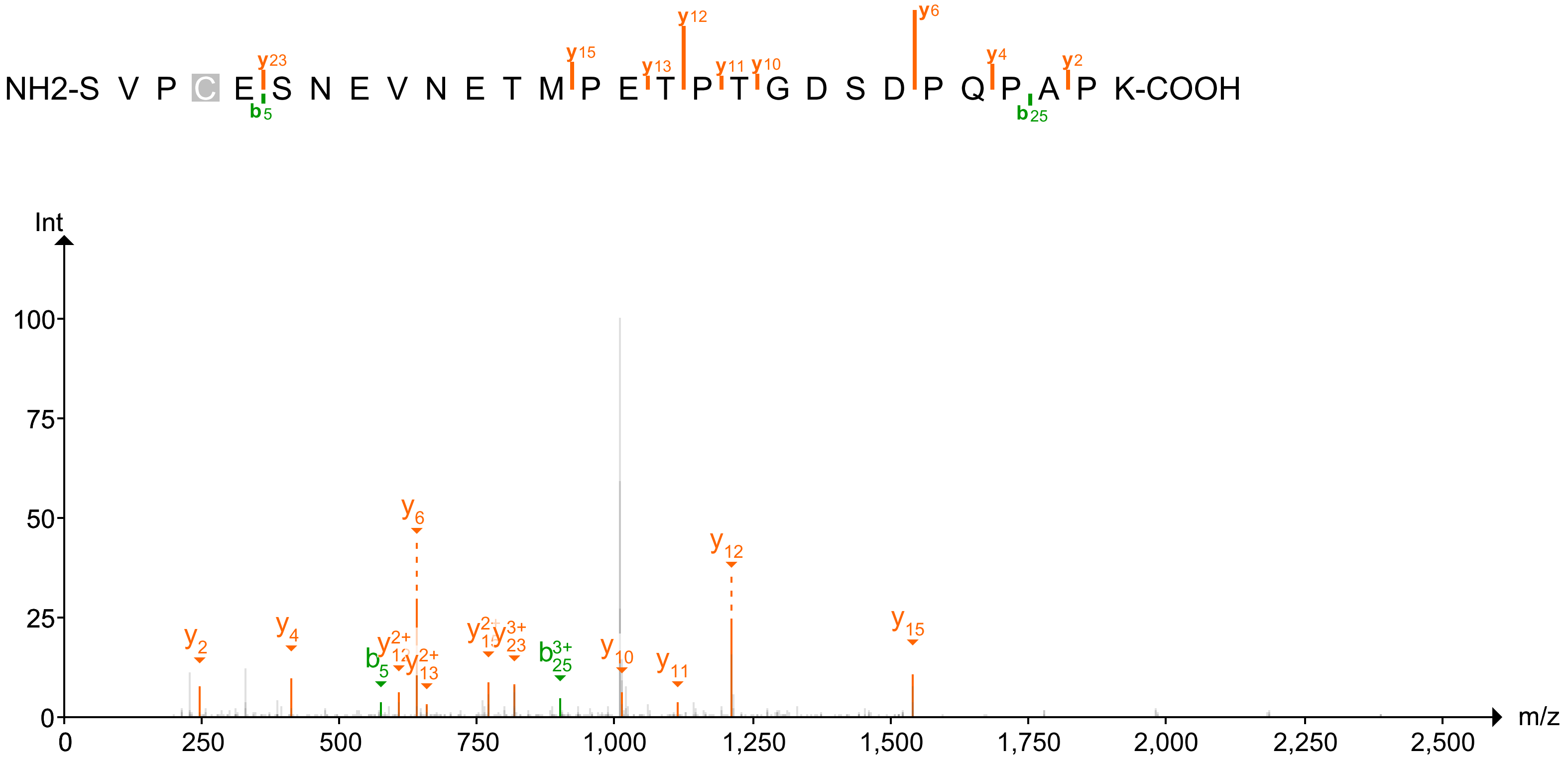
NH2-S V P C E S N E V N E T M P E T P T G D S D P Q P A P K-COOH
y23 y15 y13 y12 y11 y10 y6 y4 y2
b5 b25
Int
100
75
50
25
0
m/z
0
250
500
750
1,000
1,250
1,500
1,750
2,000
2,250
2,500
y2
y4
b5
y6
y12
y15
y10
y11
b25 3+
y23 3+
y13 2+

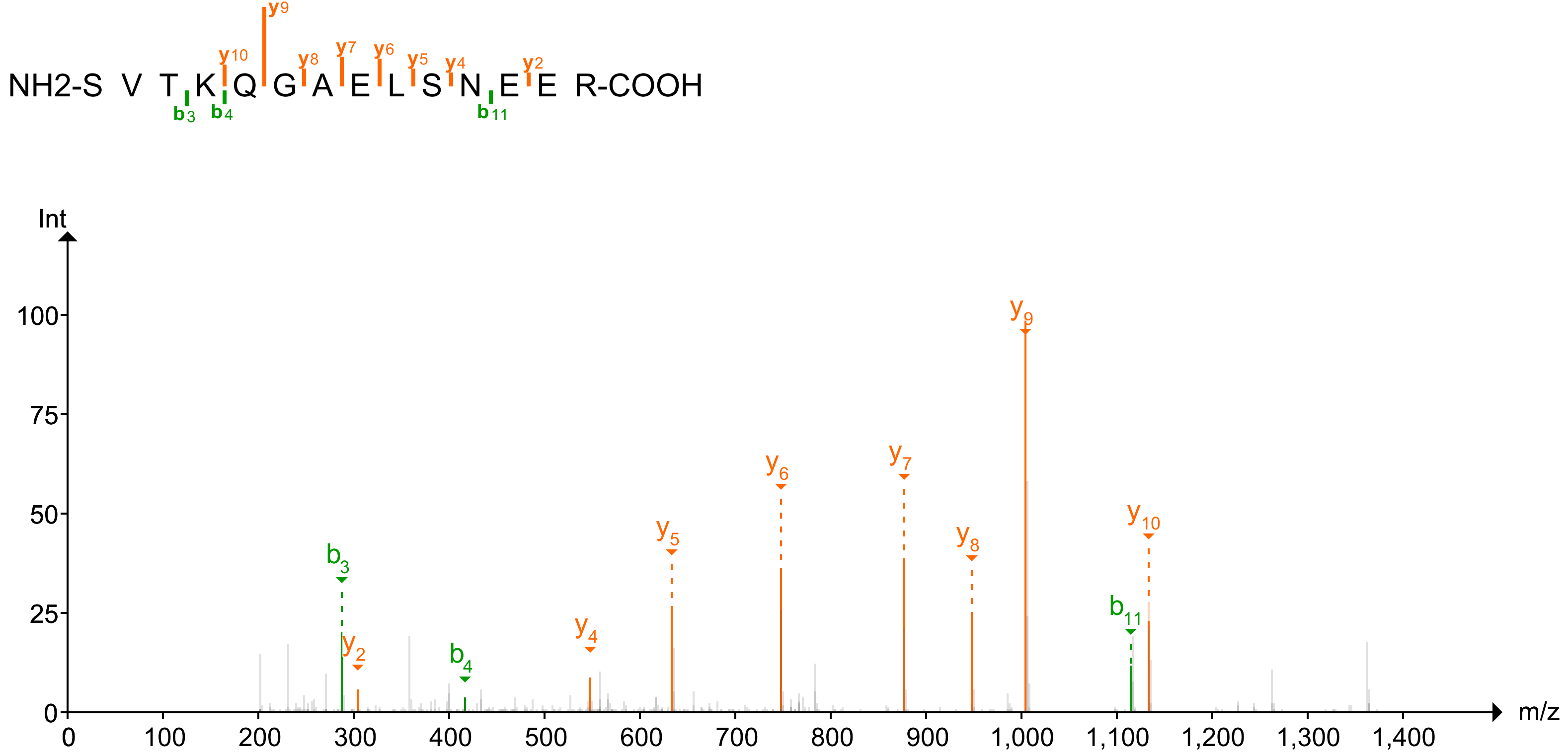

NH2-S V T K Q G A E L S N E E R-COOH
y9 y10 y8 y7 y6 y5 y4 y2
b3 b4 b11
Int
100
75
50
25
0
0 100 200 300 400 500 600 700 800 900 1,000 1,100 1,200 1,300 1,400
m/z
y9 y6 y7 y5 y8 y10 b3 b11 y4 y2 b4

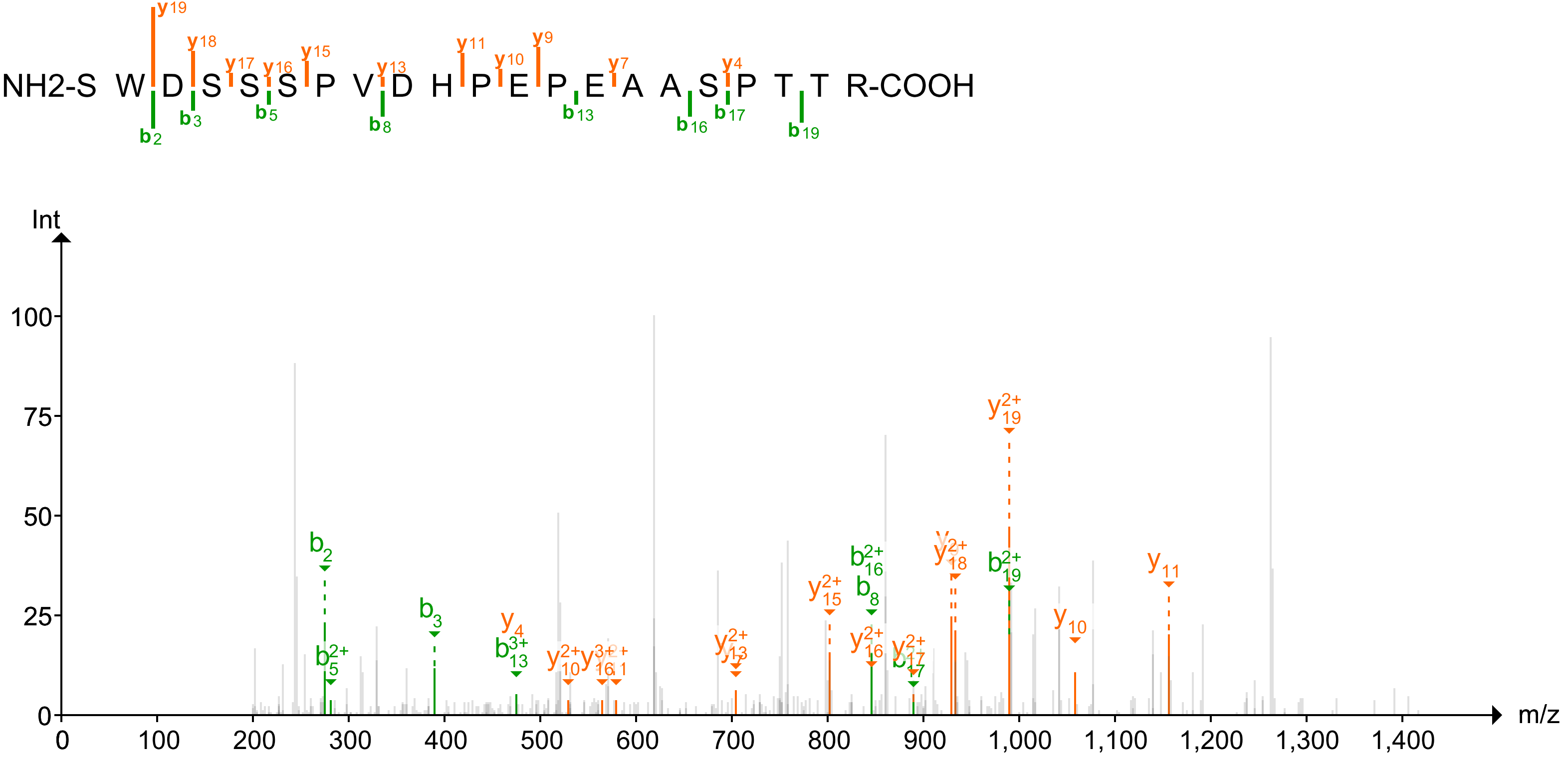

NH2-S W D S S S P V D H P E P E A A S P T T R-COOH
y19 y18 y17 y16 y15 y13 y11 y10 y9 y7 y4
b2 b3 b5 b8 b13 b16 b17 b19
Int
m/z
0
25
50
75
100
0
100
200
300
400
500
600
700
800
900
1,000
1,100
1,200
1,300
1,400
b2
b3
b5 2+
y4
b13 3+
y10 2+
y16 3+
y13 2+
y15 2+
b16 2+
b8
y16 2+
y17 2+
y18 2+
y19 2+
b19 2+
y10
y11

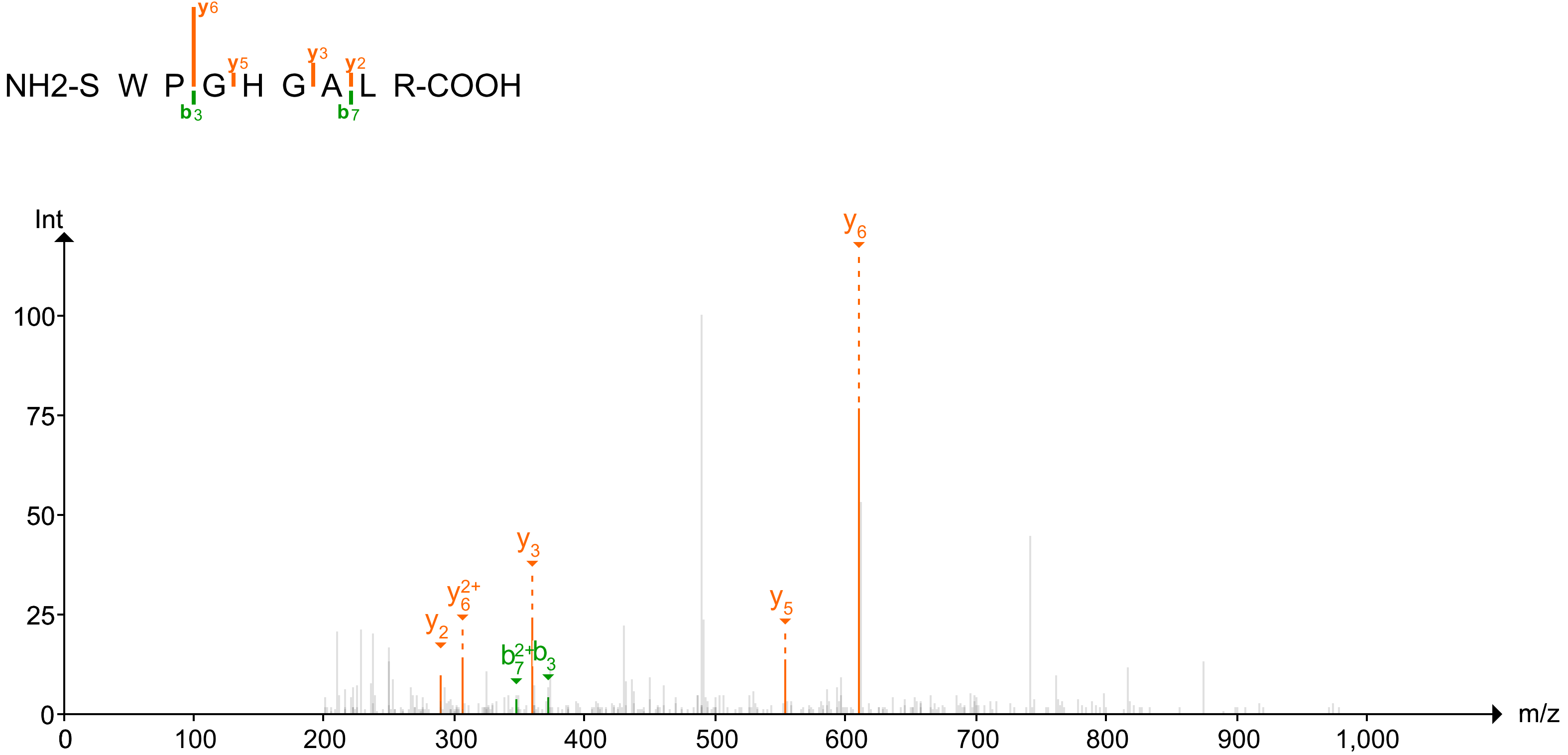
NH2-S W P G H G A L R-COOH
y6
y5
y3
y2
b3
b7
Int
m/z
y6
y3
y6 2+
y2
y5
b7 2+
b3
0
25
50
75
100
0
100
200
300
400
500
600
700
800
900
1,000

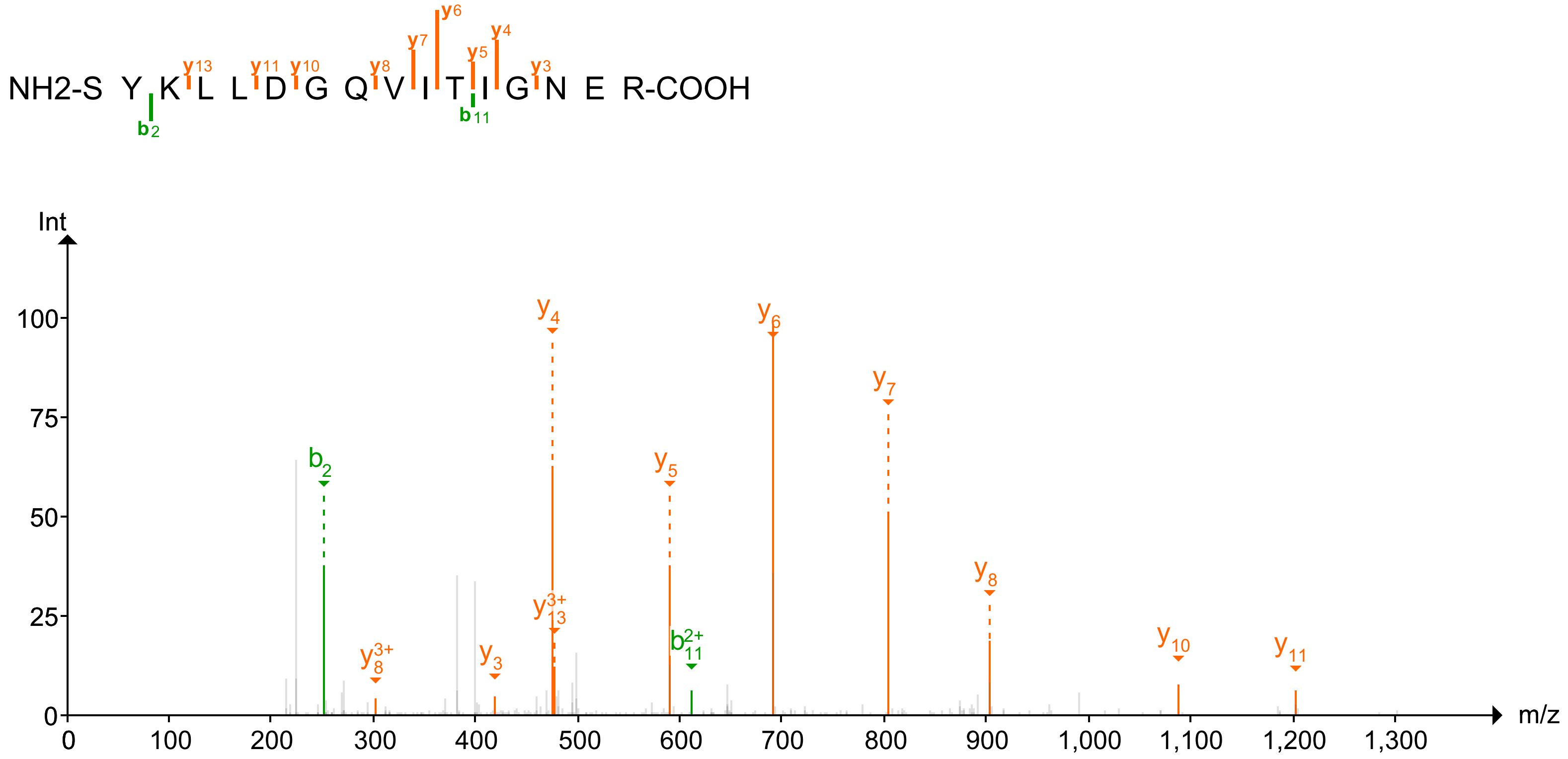

NH2-S Y K L L D G Q V I T I G N E R-COOH
y13 y11 y10 y8 y7 y6 y5 y4 y3
b2 b11
Int
100
75
50
25
0
m/z
0 100 200 300 400 500 600 700 800 900 1,000 1,100 1,200 1,300
b2
y8 3+
y3
y4
y13 3+
y5
b11 2+
y6
y7
y8
y10
y11

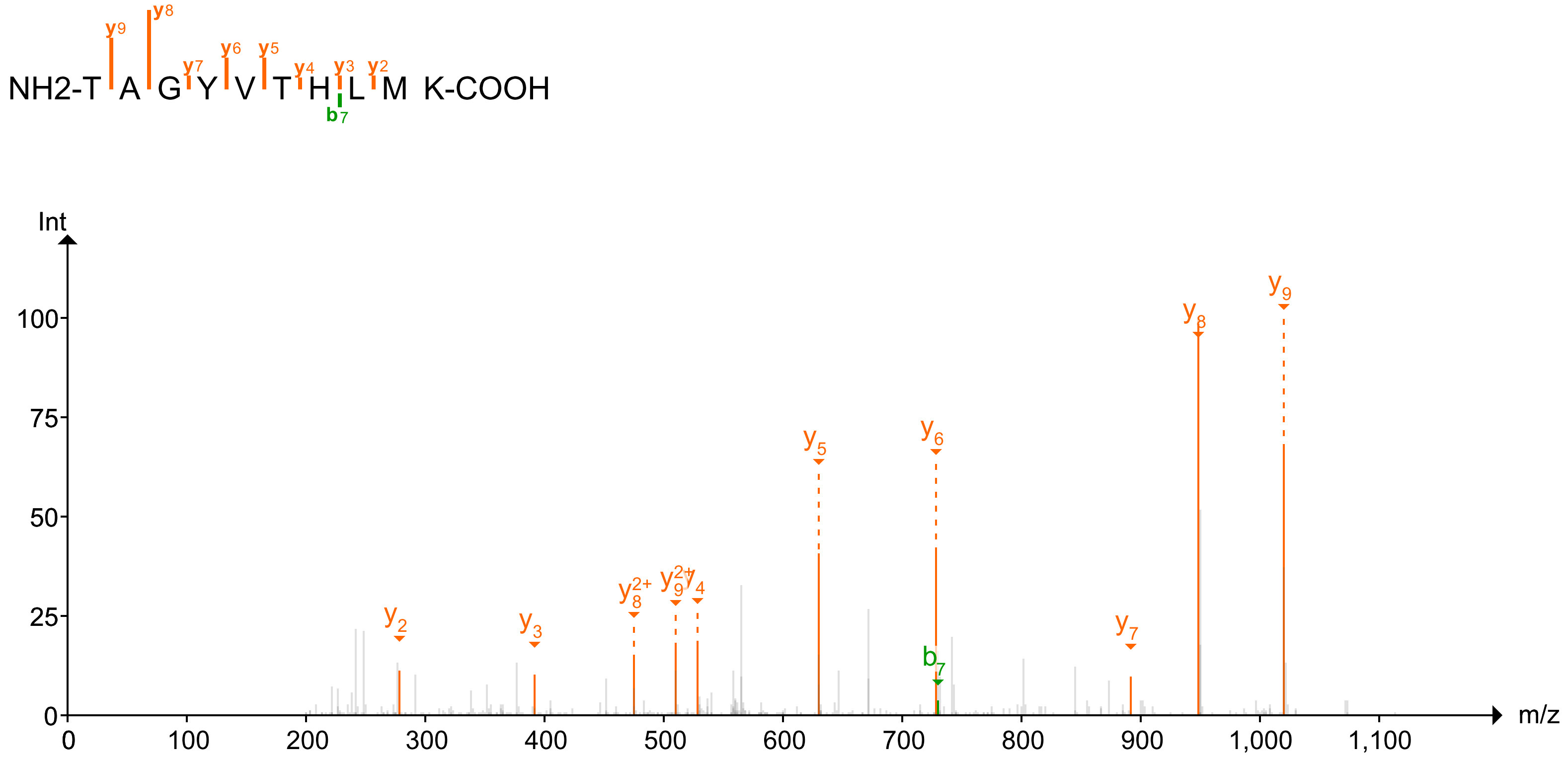

NH2-T A G Y V T H L M K-COOH
y9 y8 y7 y6 y5 y4 y3 y2
b7
Int
100
75
50
25
0
m/z
0
100
200
300
400
500
600
700
800
900
1,000
1,100
y2
y3
y8 2+
y9 2+
y4
y5
y6
b7
y7
y8
y9

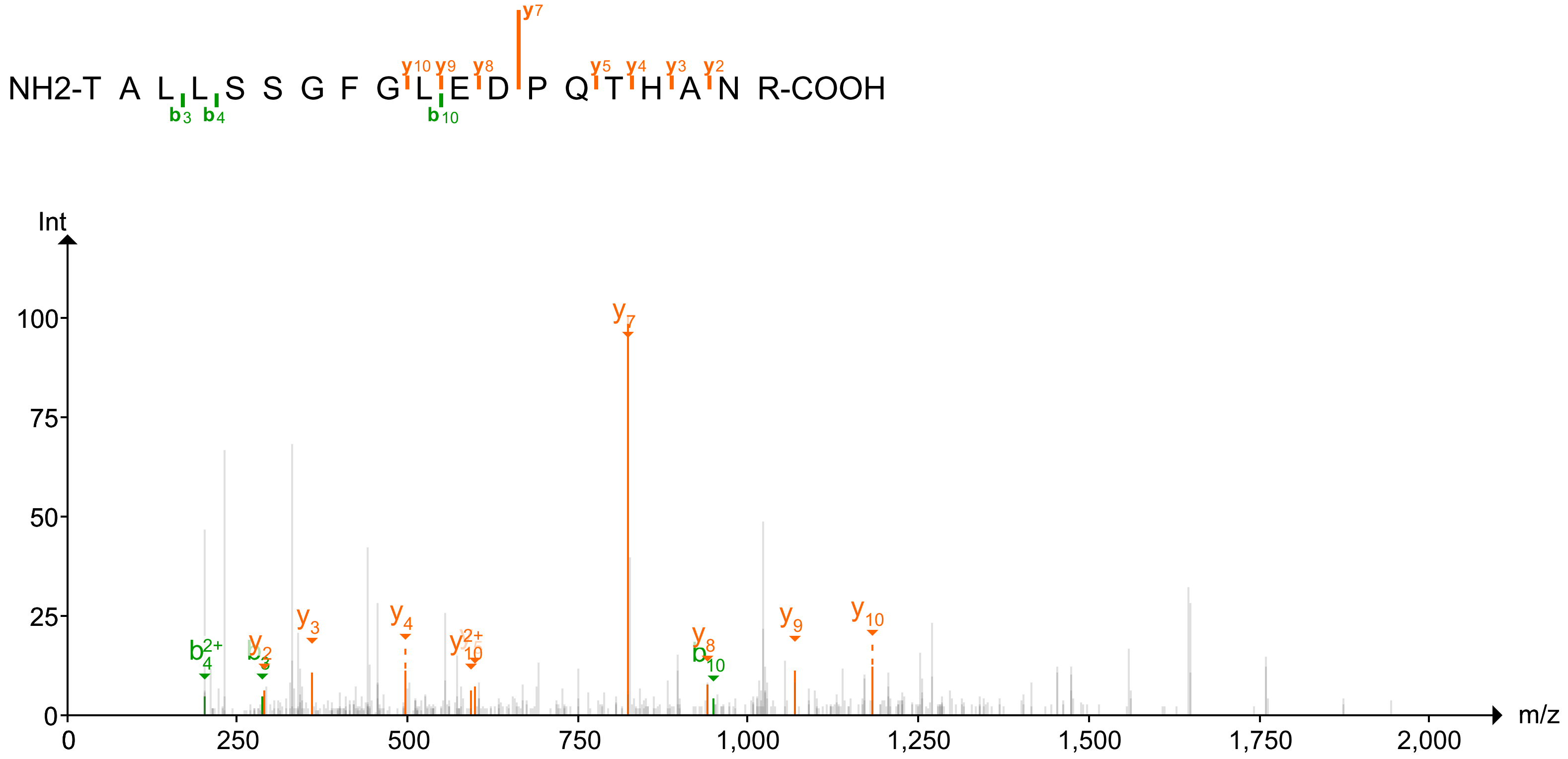

NH2-T A L L S S G F G L E D P Q T H A N R-COOH
y7
y10 y9 y8
y5 y4 y3 y2
b3 b4 b10
Int
100
75
50
25
0
y7
y3
y4
y9
y10
y8
b10
b4 2+
y2
b3
y10 2+
0
250
500
750
1,000
1,250
1,500
1,750
2,000
m/z

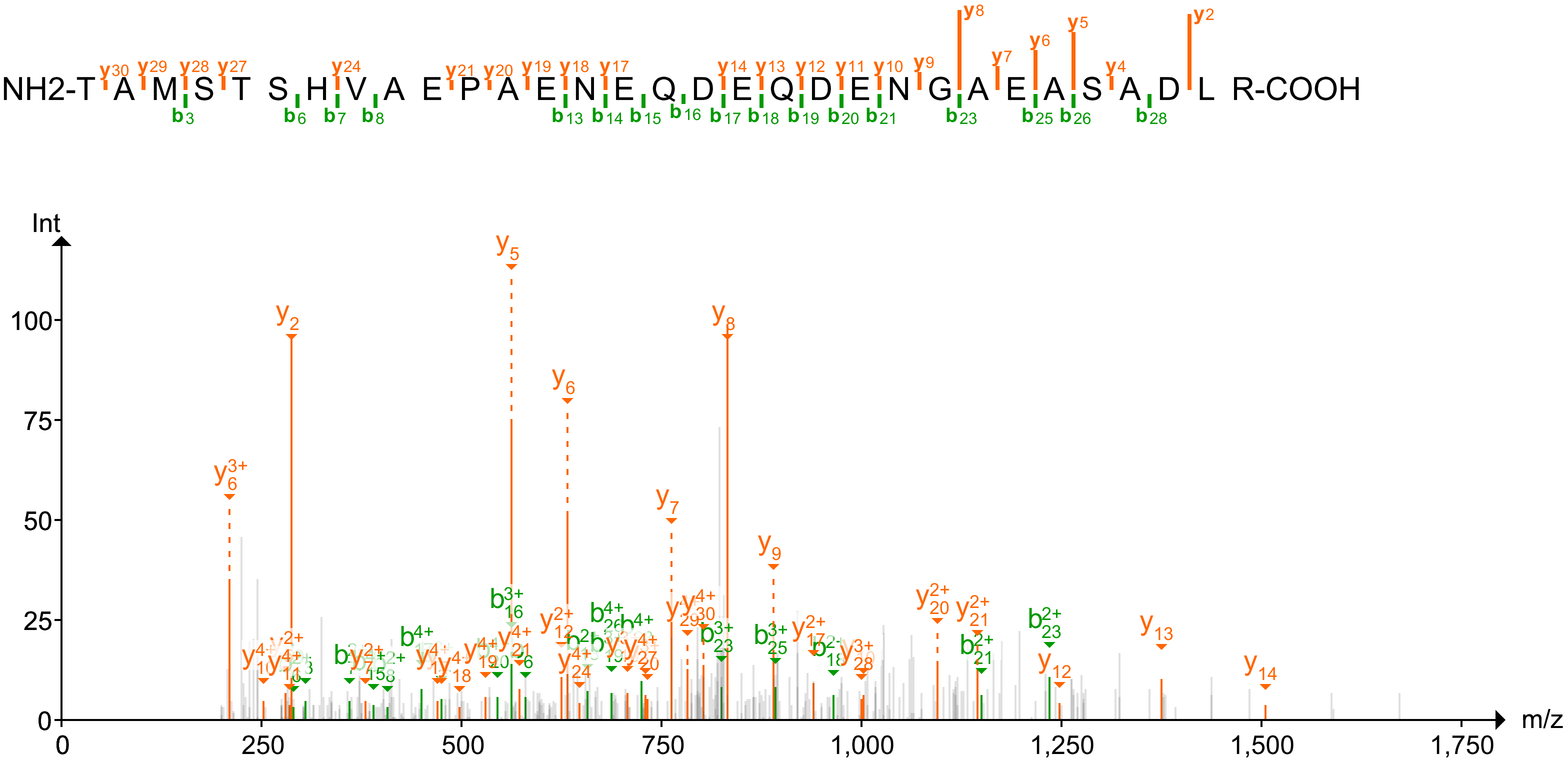

NH2-T A M S T S H V A E P A E N E Q D E Q D E N G A E A S A D L R-COOH
Int
m/z
0
25
50
75
100
250
500
750
1,000
1,250
1,500
1,750
y2
y5
y6
y8
y7
y9
y13
y14
y12

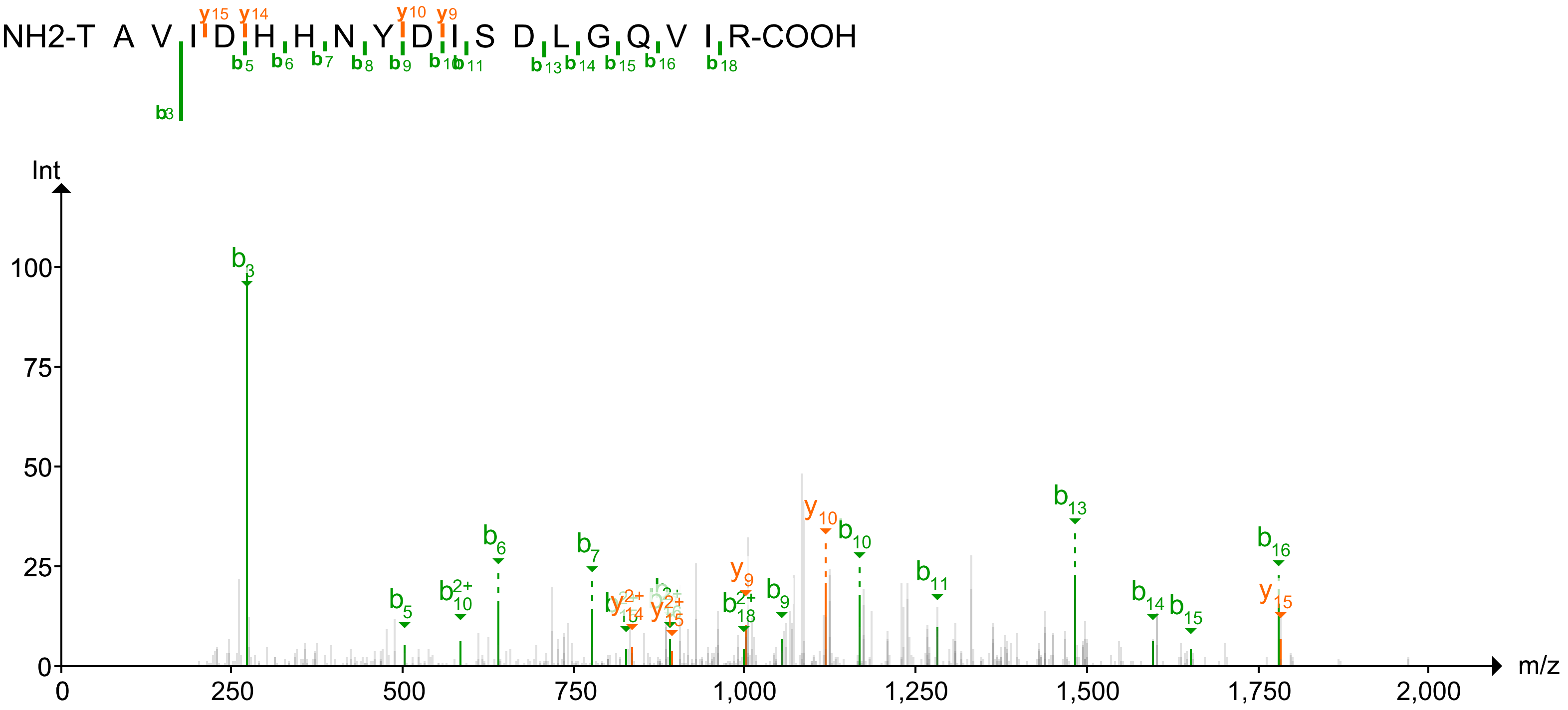
NH2-T A V I D H H N Y D I S D L G Q V I R-COOH
y15 y14 y10 y9
b3 b5 b6 b7 b8 b9 b10 b11 b13 b14 b15 b16 b18
Int
100
75
50
25
0
m/z
0
250
500
750
1,000
1,250
1,500
1,750
2,000
b3
b5
b6
b7
b9
b10
b11
b13
b14
b15
b16
y9
y10
y15
b2+ 10
b2+ 18
y2+ 14
y2+ 15

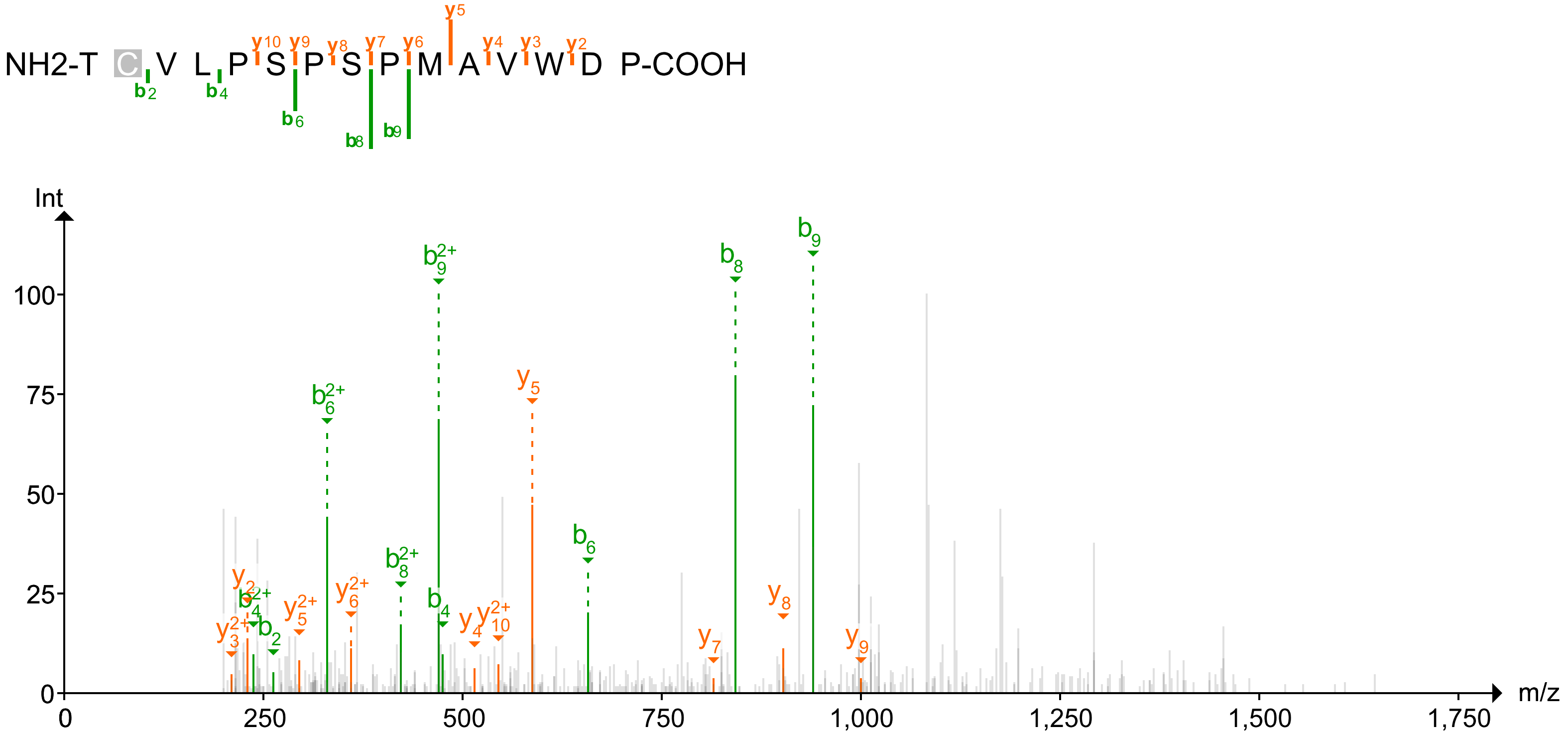
NH2-T C V L P S P S P M A V W D P-COOH
y10 y9 y8 y7 y6 y5 y4 y3 y2
b2 b4 b6 b8 b9
Int
100
75
50
25
0
m/z
0
250
500
750
1,000
1,250
1,500
1,750
b9 2+
b8
b9
b6 2+
y5
b8 2+
b6
y2
b4 2+
b2
y3 2+
y5 2+
y6 2+
b4
y4
y10 2+
y7
y8
y9

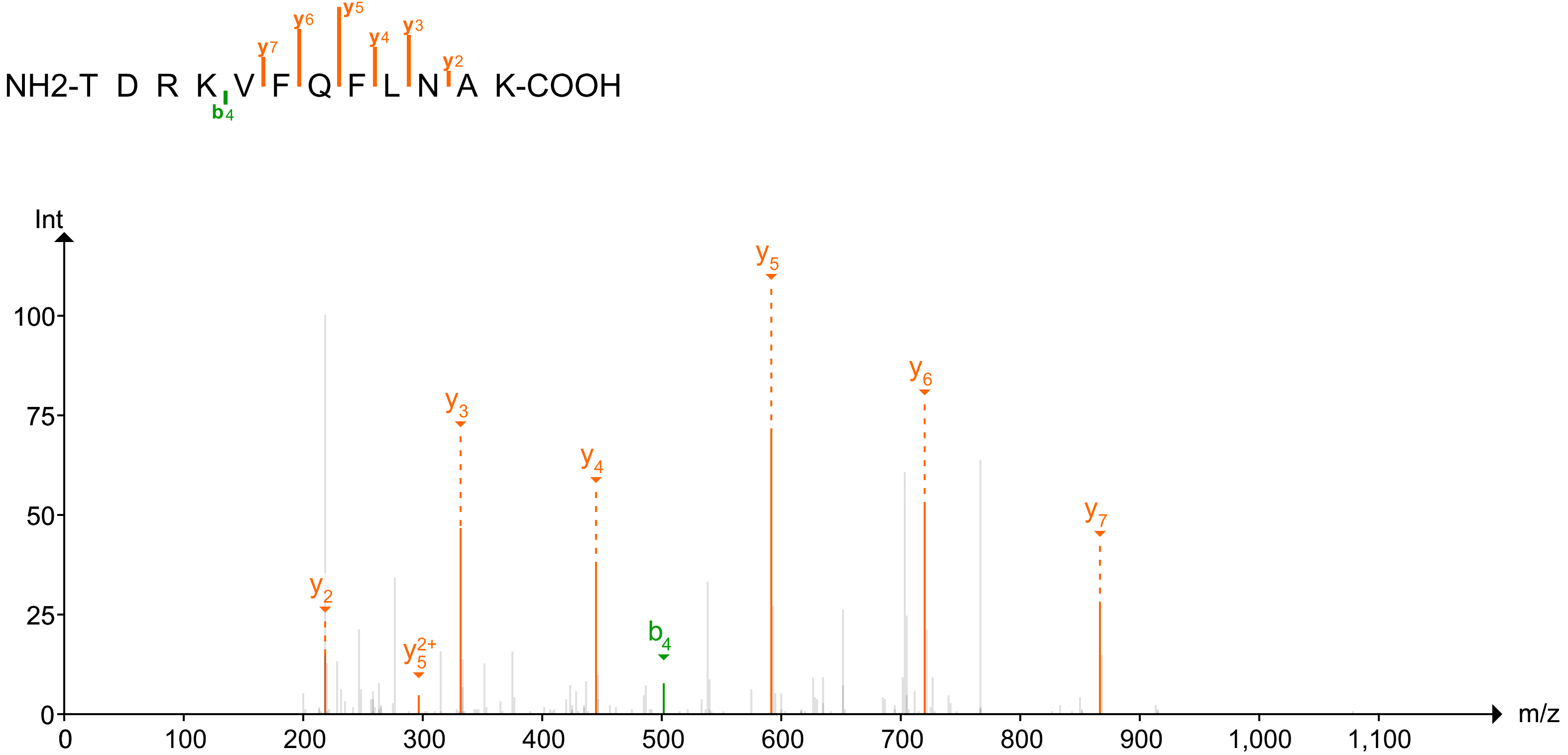

NH2-T D R K V F Q F L N A K-COOH
y7
y6
y5
y4
y3
y2
b4
Int
100
75
50
25
0
$y_2$
$y_5^{2+}$
$y_3$
$y_4$
$b_4$
$y_5$
$y_6$
$y_7$
0
100
200
300
400
500
600
700
800
900
1,000
1,100
m/z

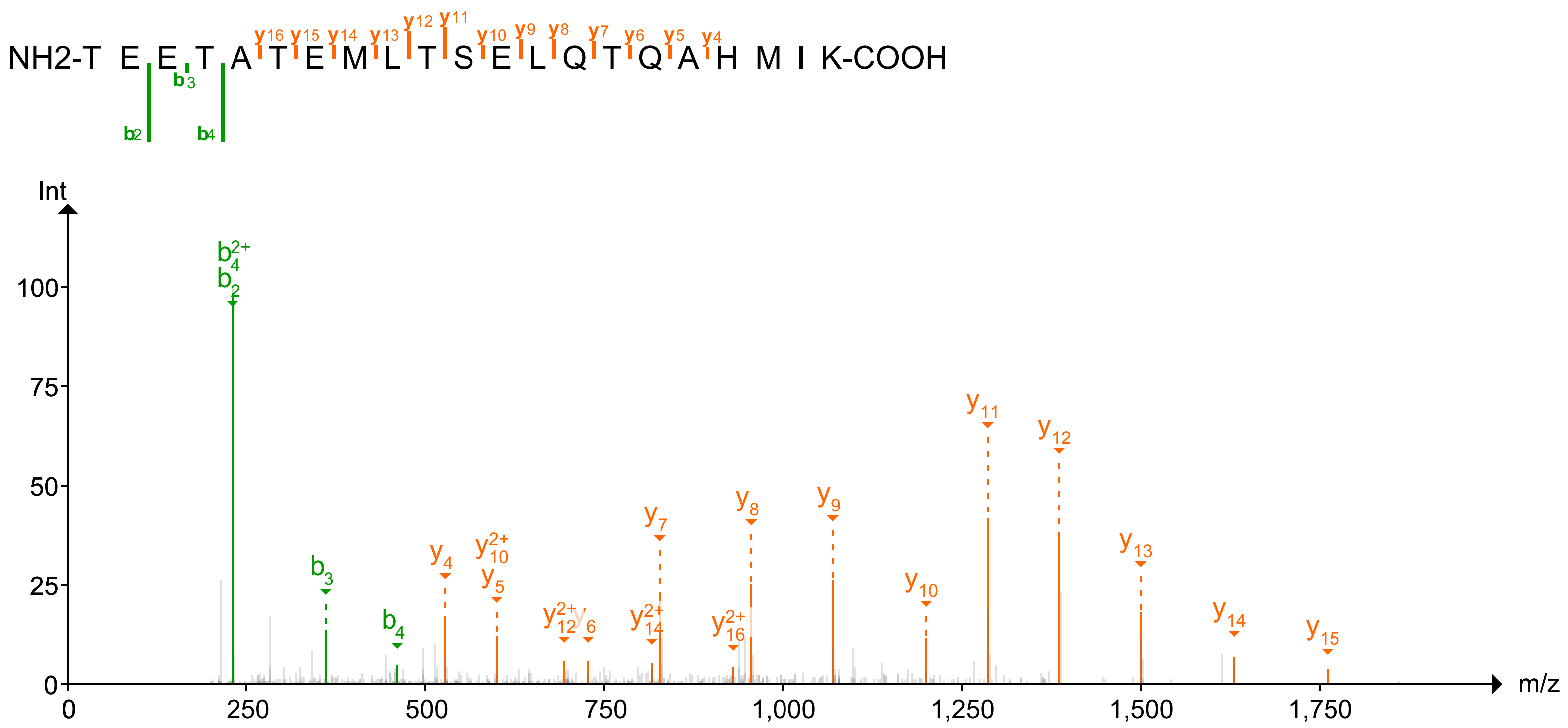
NH2-T E E T A T E M L T S E L Q T Q A H M I K-COOH
b2 b3 b4
y16 y15 y14 y13 y12 y11 y10 y9 y8 y7 y6 y5 y4
Int
m/z
0 25 50 75 100
0 250 500 750 1,000 1,250 1,500 1,750
b4 2+
b2
b3
b4
y4
y10 2+
y5
y12 2+
y6
y14 2+
y7
y16 2+
y8
y9
y10
y11
y12
y13
y14
y15

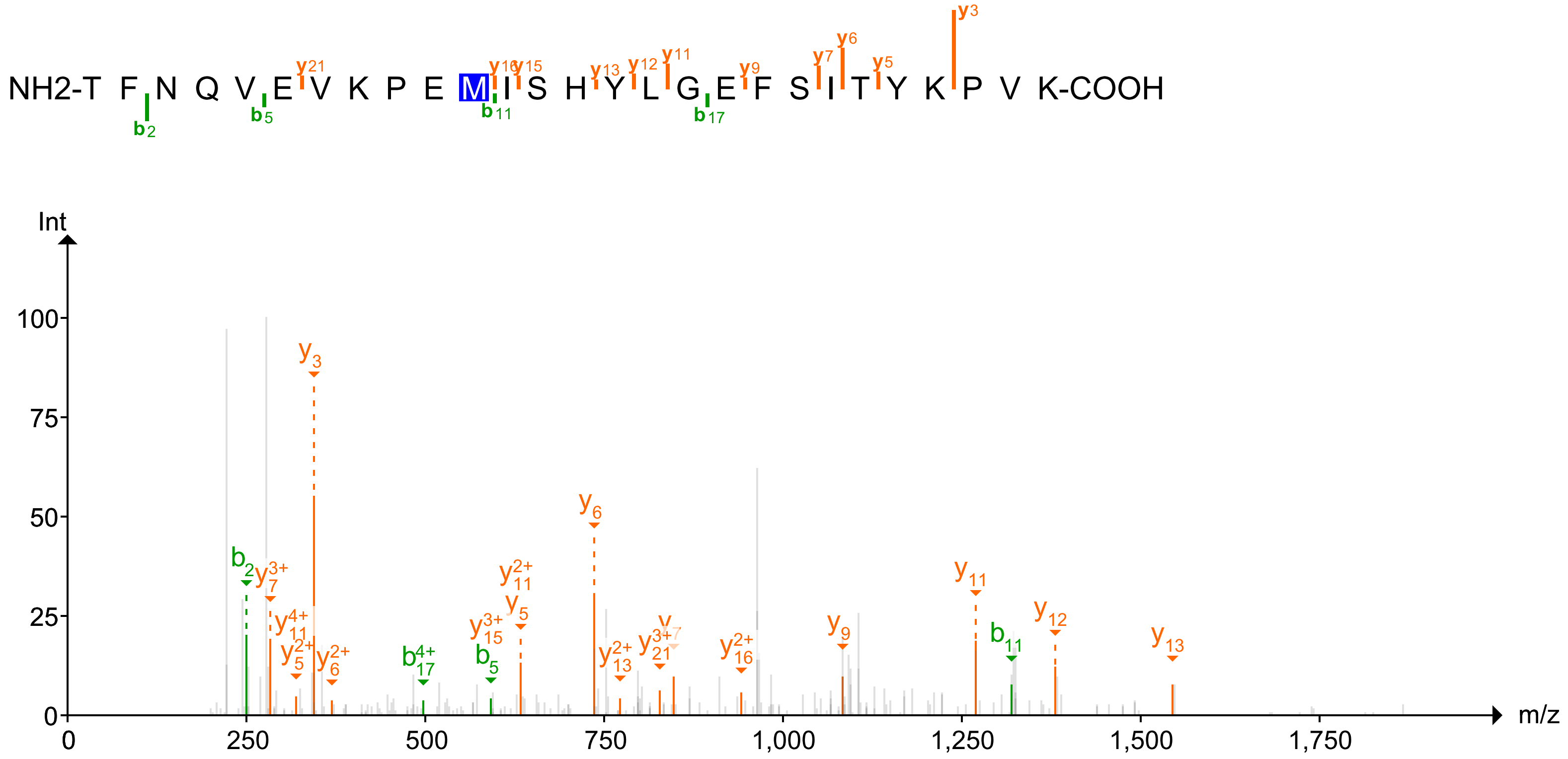

NH2-T F N Q V E V K P E M I S H Y L G E F S I T Y K P V K-COOH
Int
100
75
50
25
0
m/z
0
250
500
750
1,000
1,250
1,500
1,750

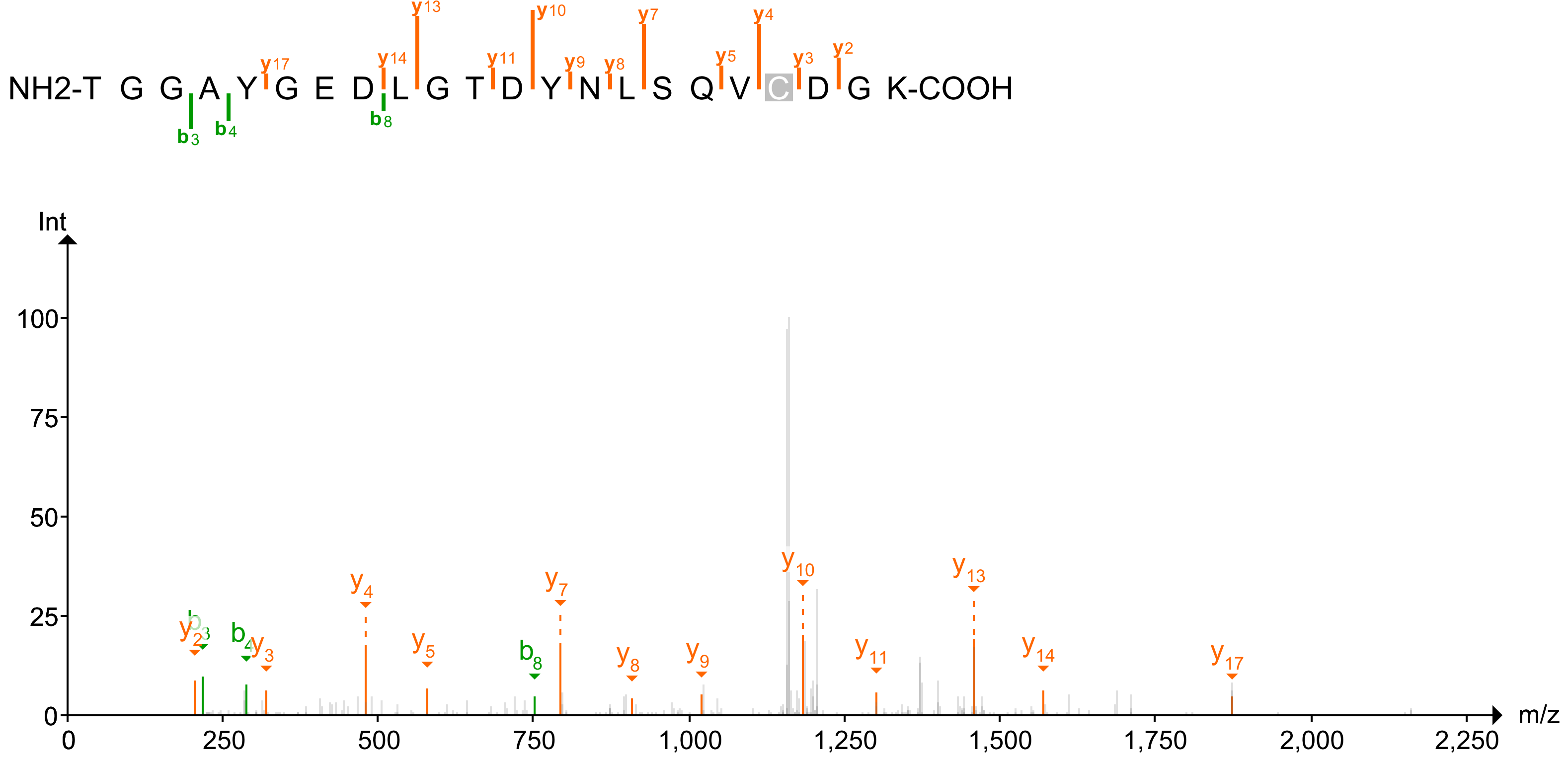
NH2-T G G A Y G E D L G T D Y N L S Q V C D G K-COOH
y17 y14 y13 y11 y10 y9 y8 y7 y5 y4 y3 y2
b3 b4 b8
Int
100
75
50
25
0
m/z
0
250
500
750
1,000
1,250
1,500
1,750
2,000
2,250
y2 b3 b4 y3 y4 y5 b8 y7 y8 y9 y10 y11 y13 y14 y17

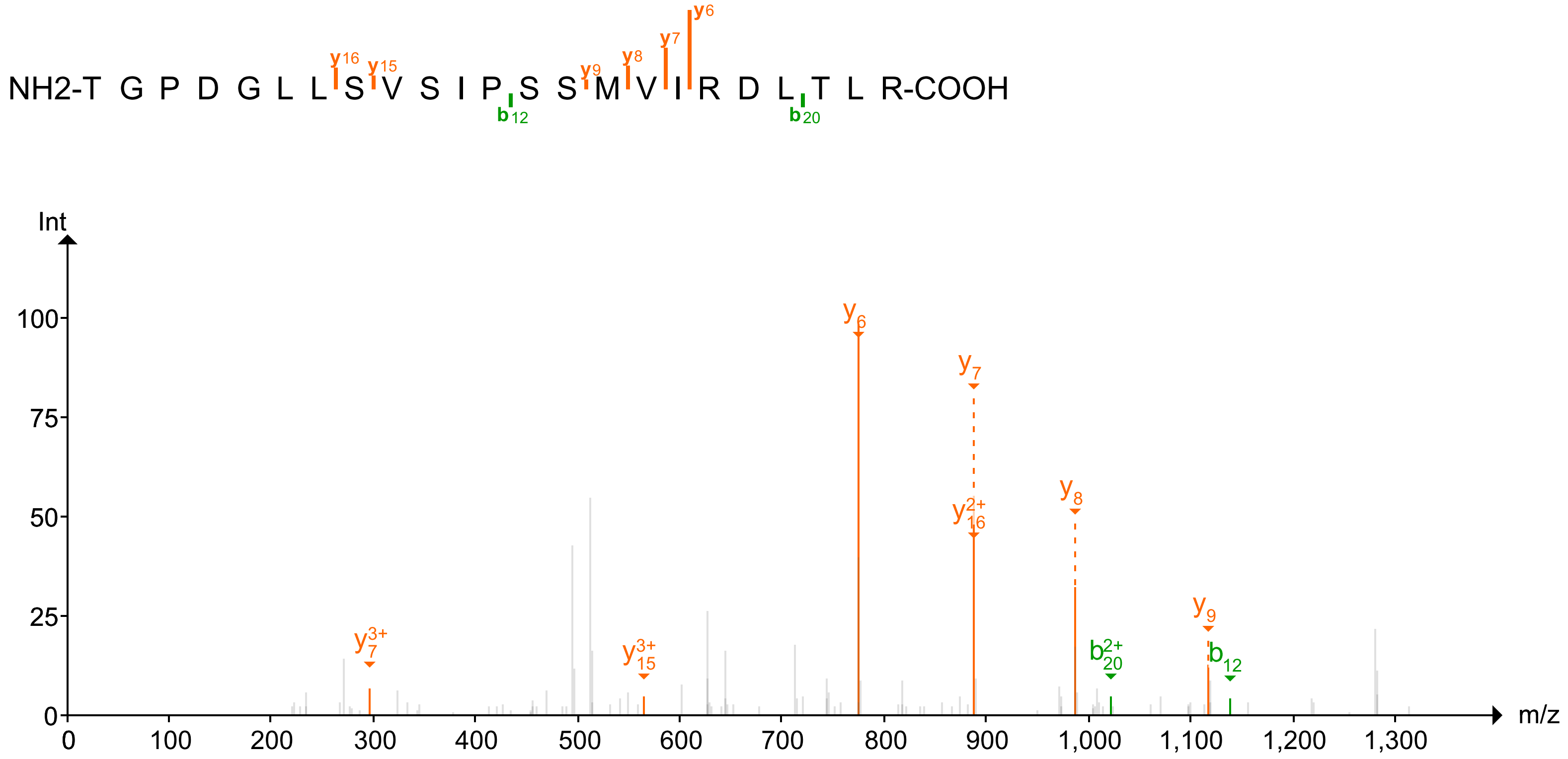

NH2-T G P D G L L S V S I P S S M V I R D L T L R-COOH
y16
y15
y9
y8
y7
y6
b12
b20
Int
100
75
50
25
0
m/z
0
100
200
300
400
500
600
700
800
900
1,000
1,100
1,200
1,300
y6
y7
y16 2+
y8
y9
b20 2+
b12
y7 3+
y15 3+

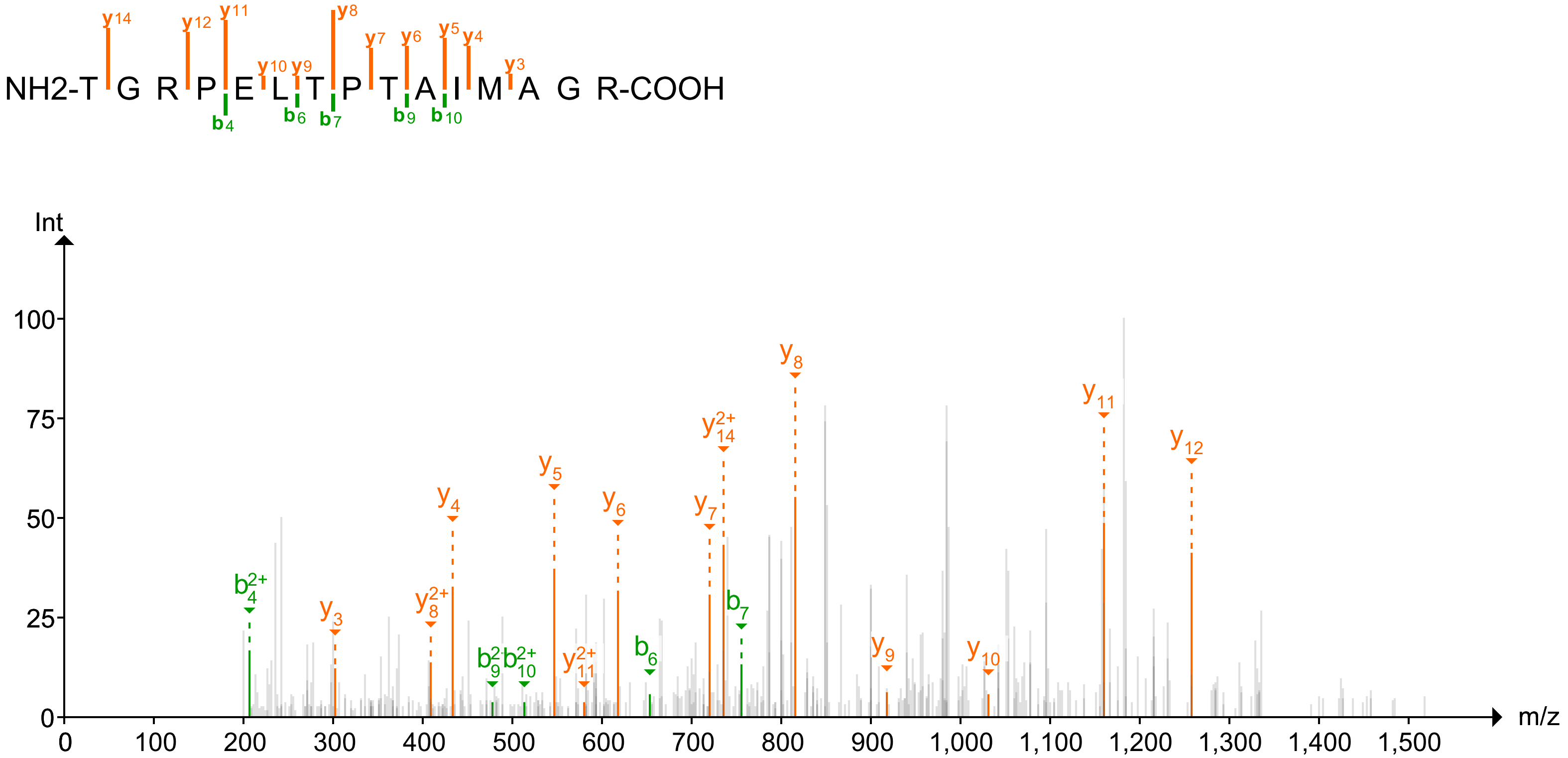

NH2-T G R P E L T P T A I M A G R-COOH
y14 y12 y11 y10 y9 y8 y7 y6 y5 y4 y3
b4 b6 b7 b9 b10
Int
m/z

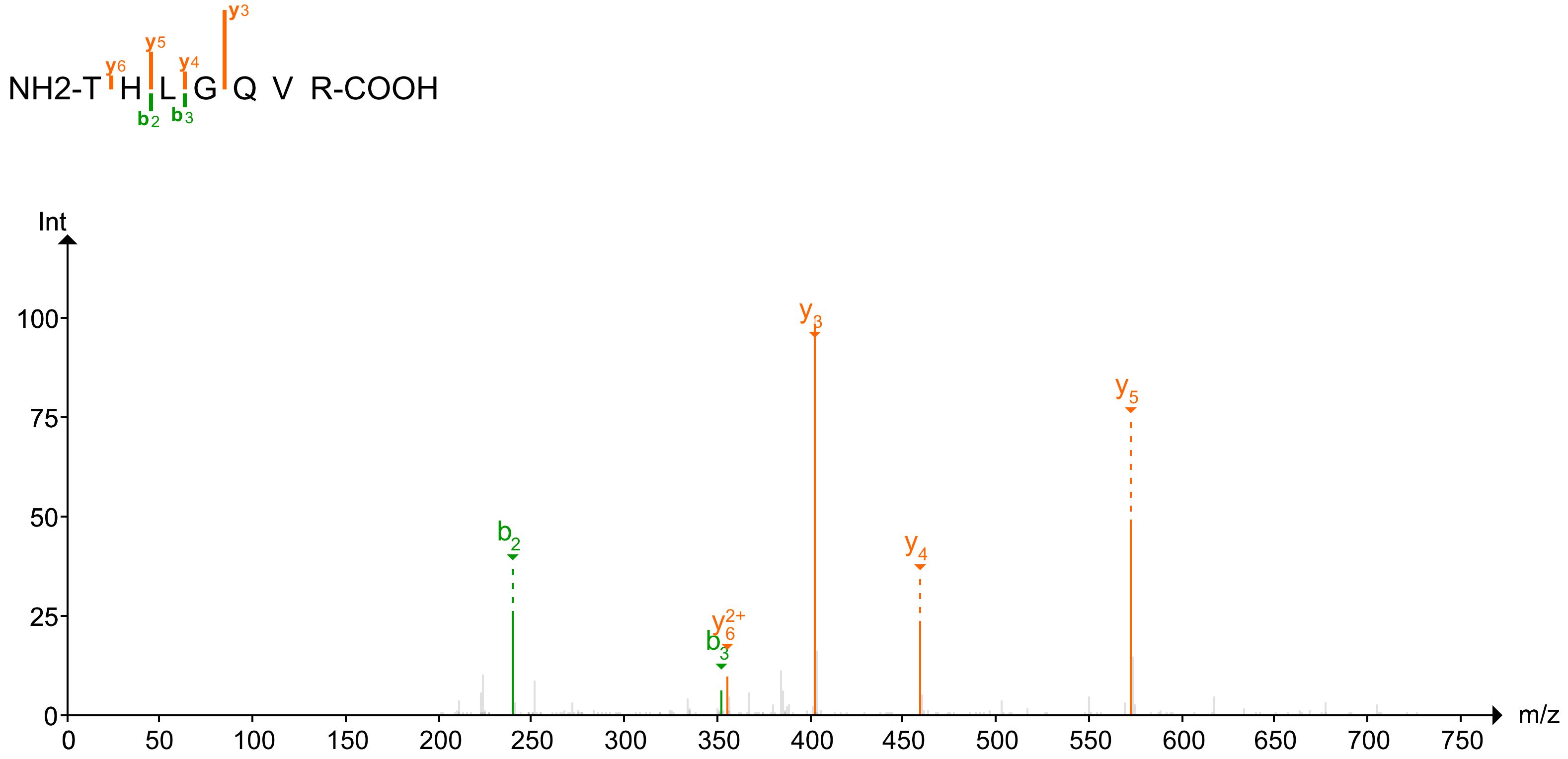

NH2-T H L G Q V R-COOH
y6 y5 y4 y3
b2 b3
Int
100
75
50
25
0
m/z
0 50 100 150 200 250 300 350 400 450 500 550 600 650 700 750
y3
y5
b2
y4
y6 2+
b3

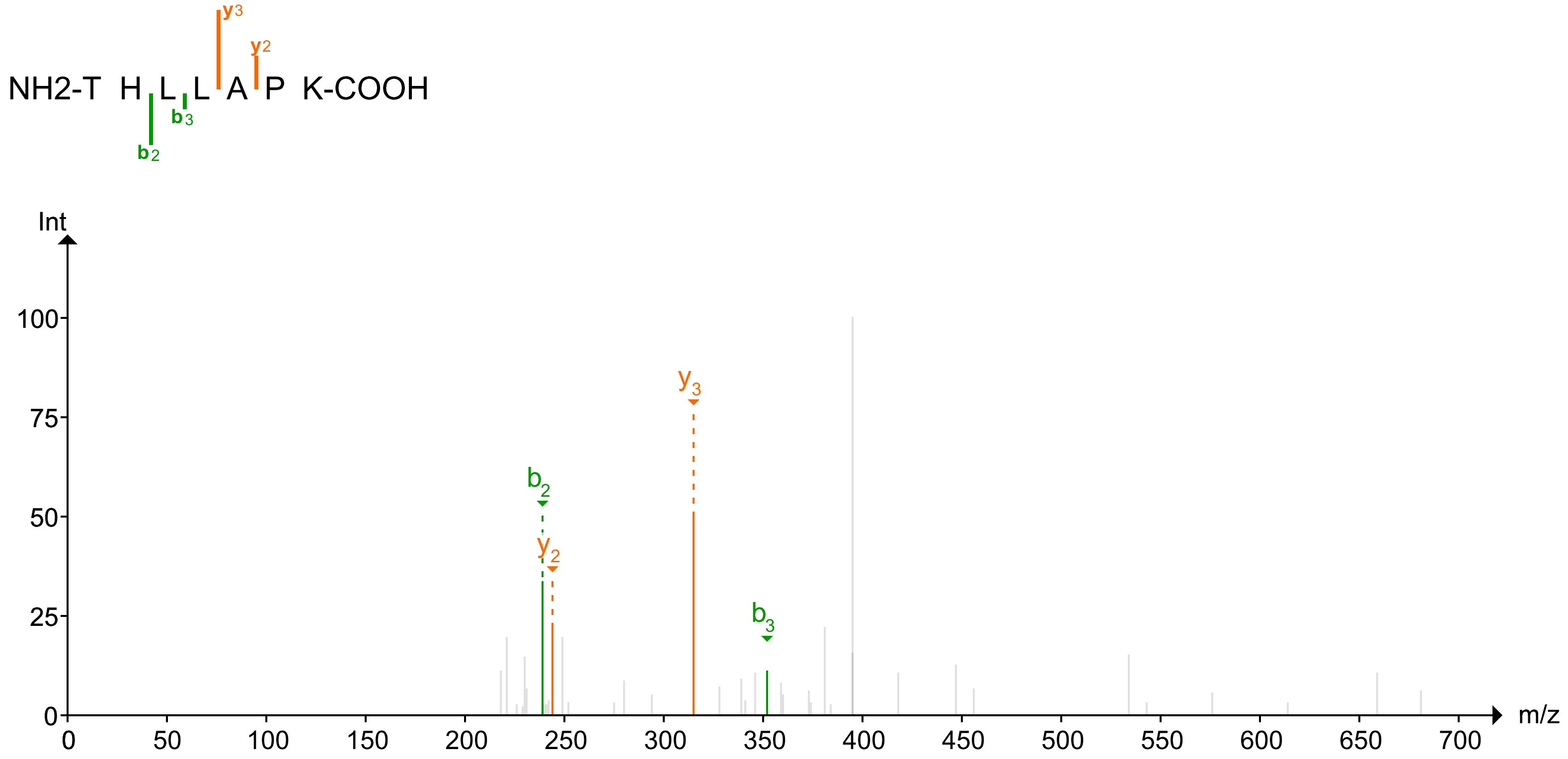
NH2-T H L L A P K-COOH
y3
y2
b2
b3
Int
100
75
50
25
0
m/z
0
50
100
150
200
250
300
350
400
450
500
550
600
650
700

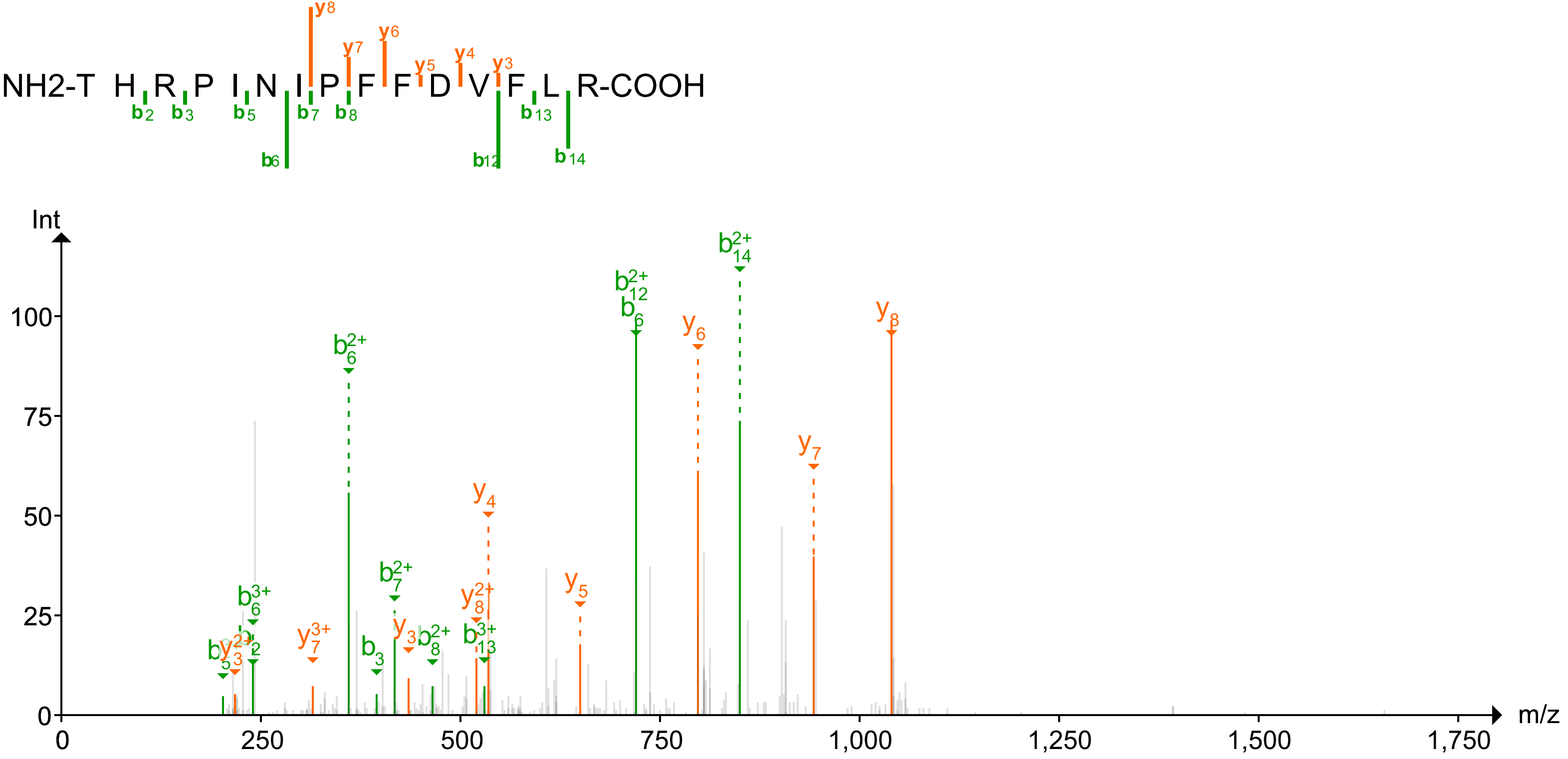

NH2-T H R P I N I P F F D V F L R-COOH
Int
m/z
100
75
50
25
0
250
500
750
1,000
1,250
1,500
1,750

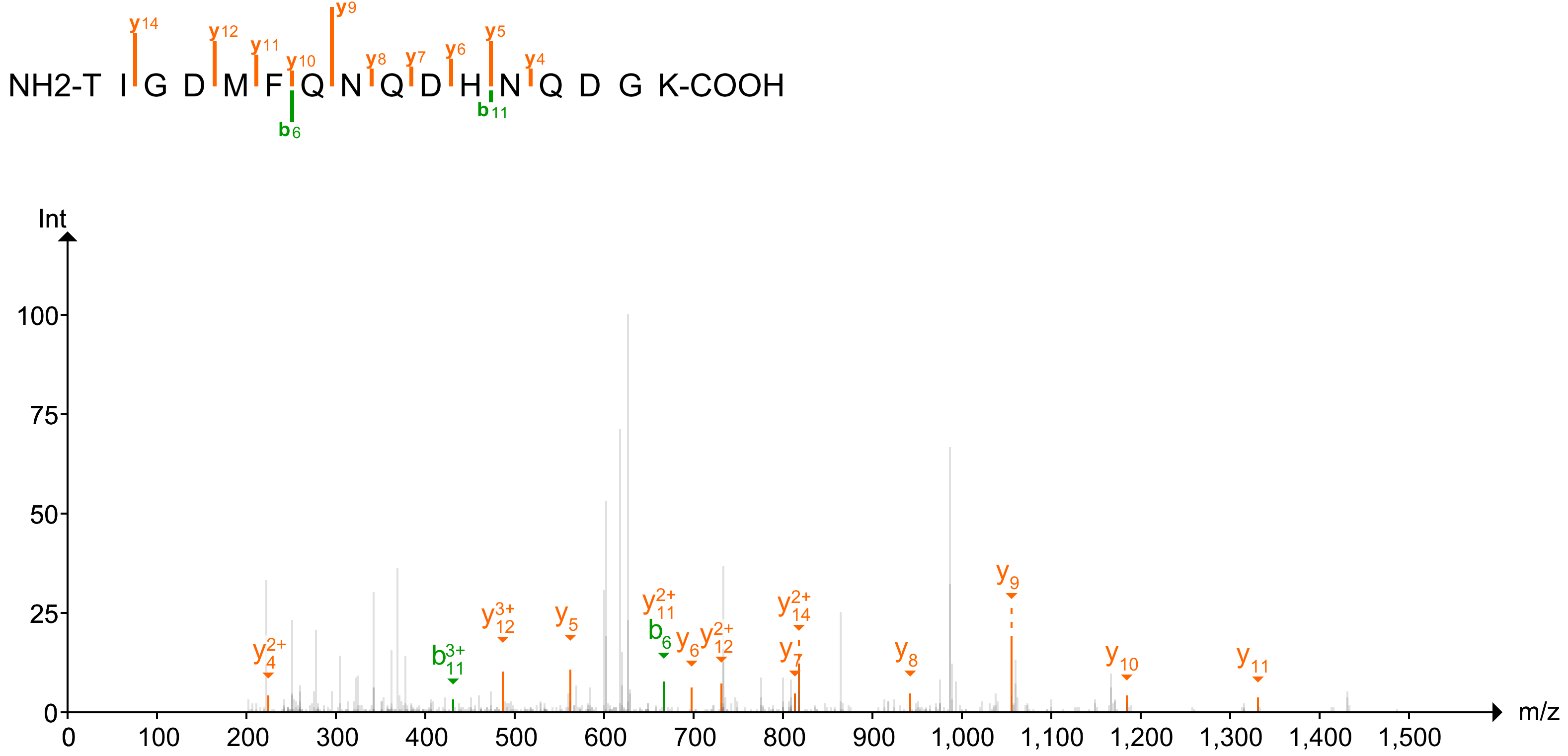

NH2-T I G D M F Q N Q D H N Q D G K-COOH
y14 y12 y11 y10 y9 y8 y7 y6 y5 y4
b6 b11
Int
100
75
50
25
0
m/z
0 100 200 300 400 500 600 700 800 900 1,000 1,100 1,200 1,300 1,400 1,500
y4 2+
b11 3+
y12 3+
y5
y11 2+
b6
y6
y12 2+
y14 2+
y7
y8
y9
y10
y11

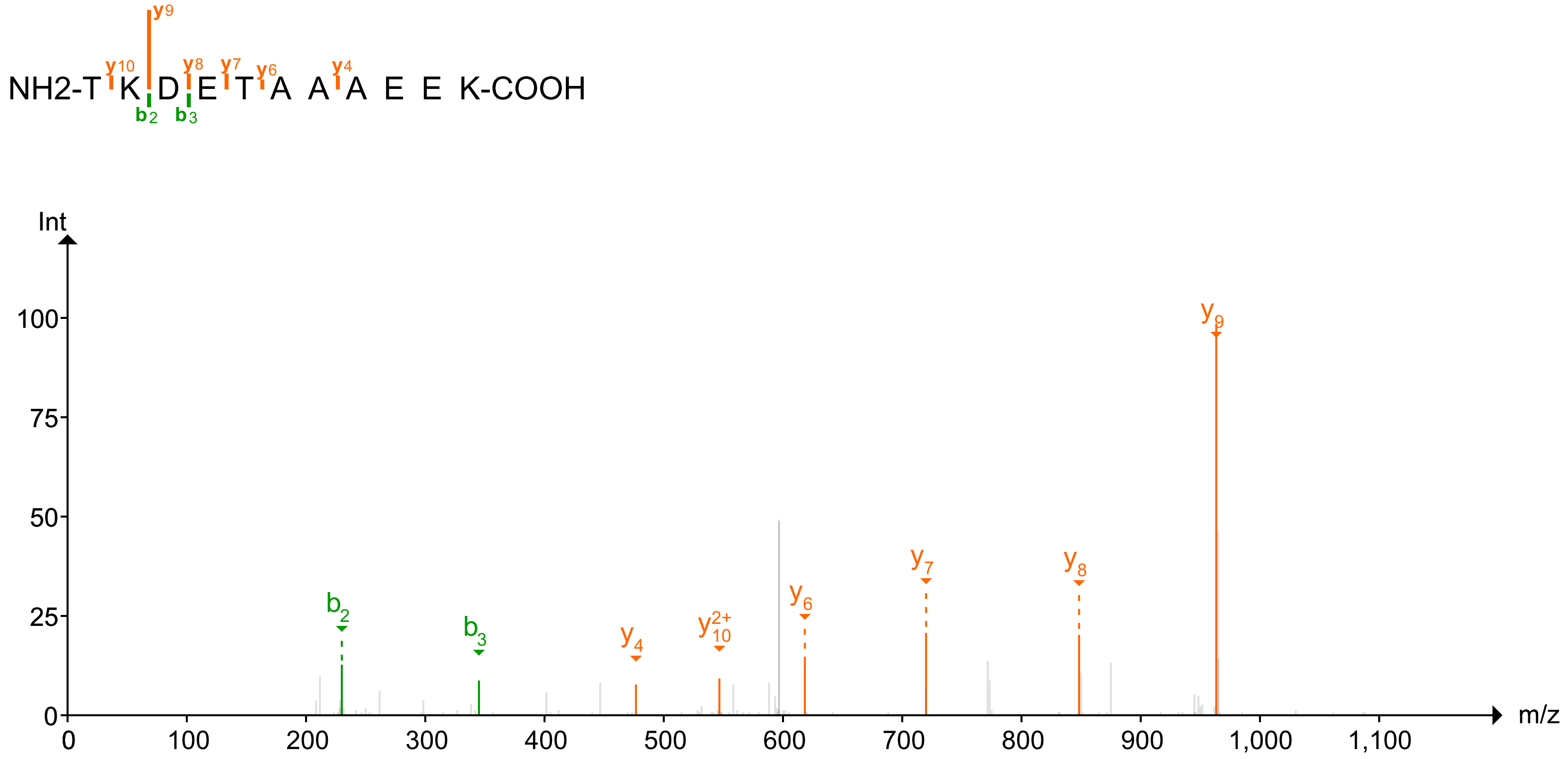

NH2-T K D E T A A A E E K-COOH
y9
y10
y8
y7
y6
y4
b2
b3
Int
m/z
100
75
50
25
0
0
100
200
300
400
500
600
700
800
900
1,000
1,100
y9
y7
y8
y6
y10 2+
y4
b2
b3

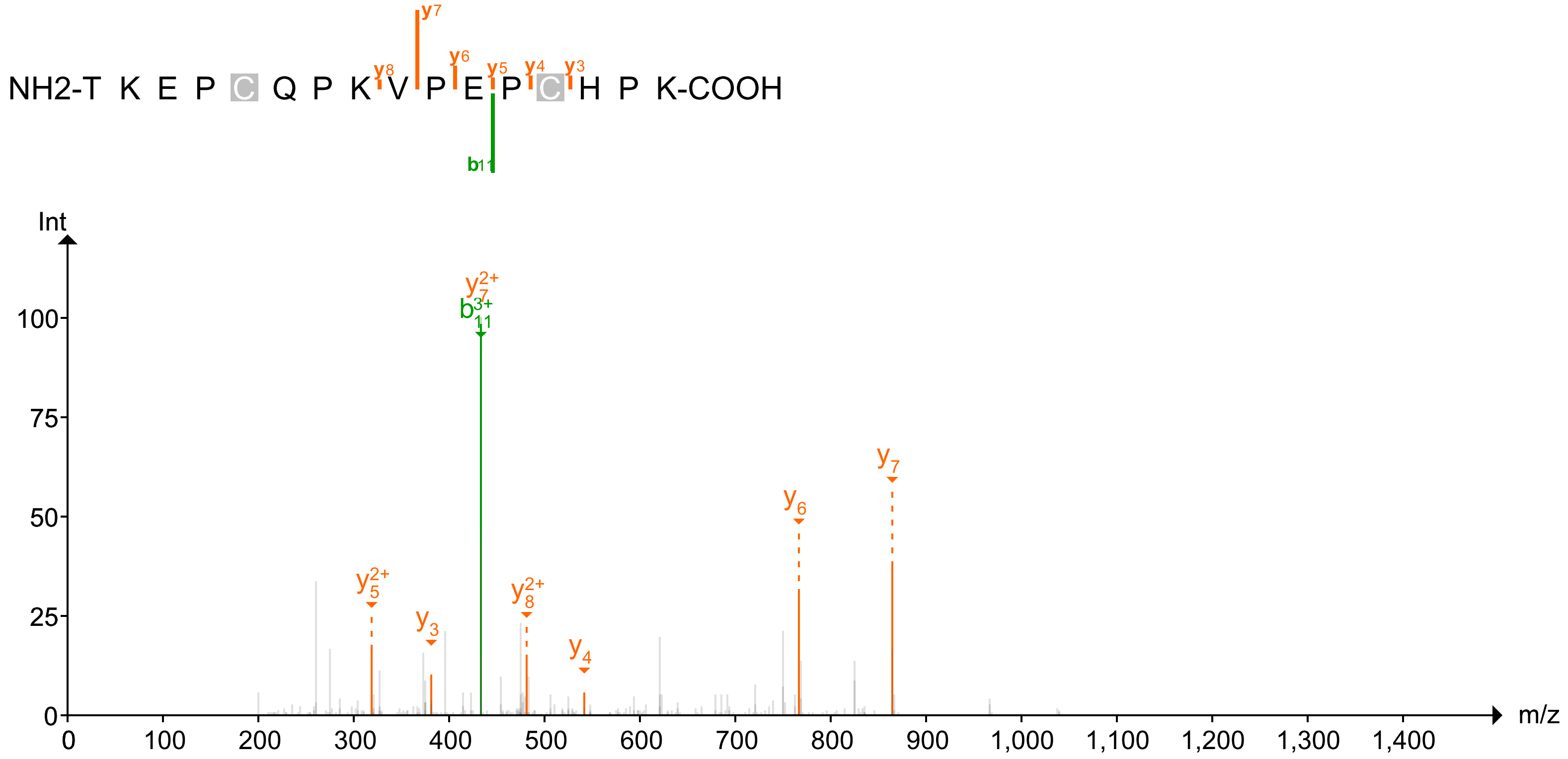

NH2-T K E P C Q P K V P E P C H P K-COOH
y8 y7 y6 y5 y4 y3
b11
Int
100
75
50
25
0
m/z
0 100 200 300 400 500 600 700 800 900 1,000 1,100 1,200 1,300 1,400
y7 2+
b11 3+
y5 2+
y3
y8 2+
y4
y6
y7

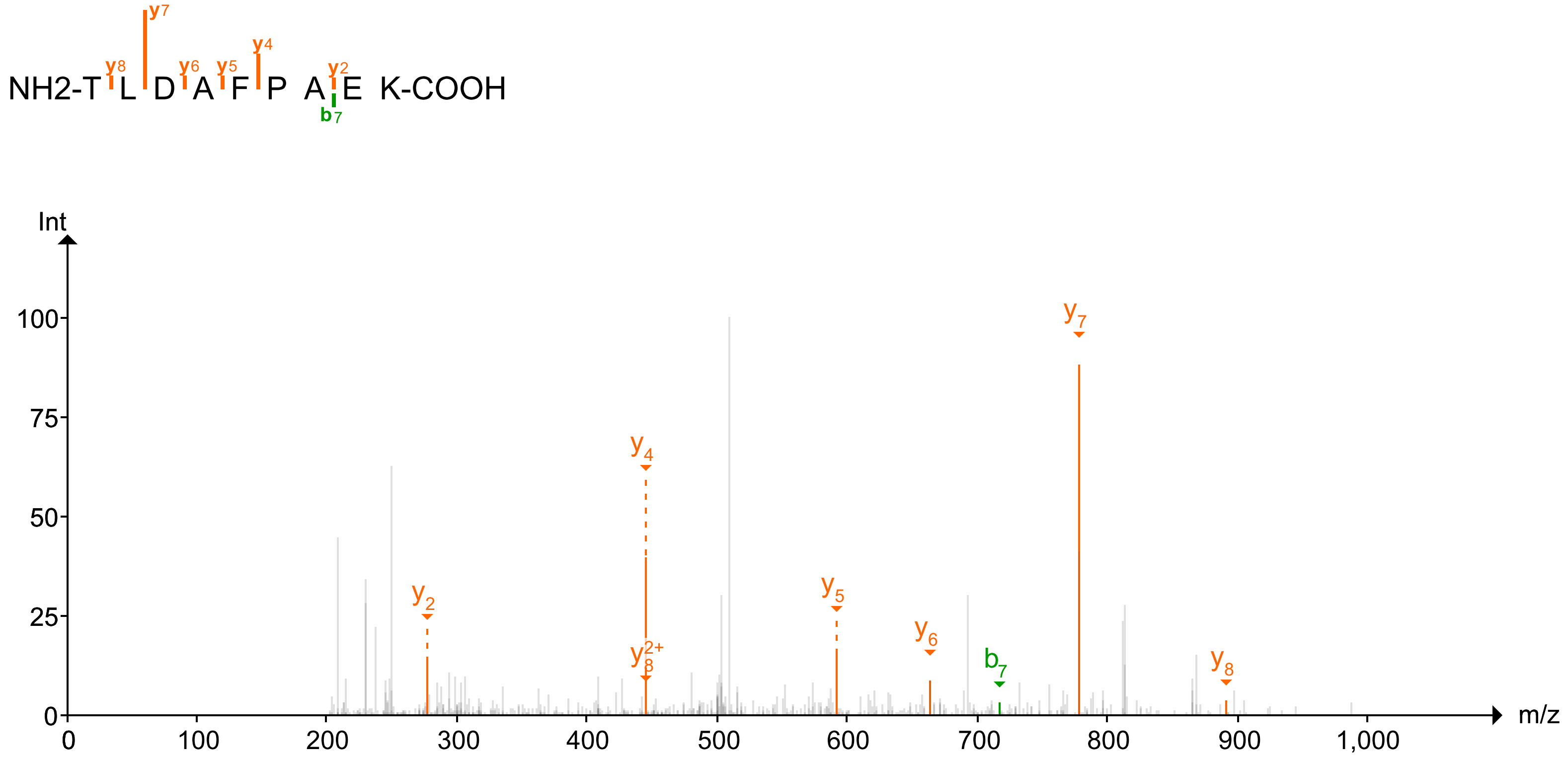

NH2-T L D A F P A E K-COOH
y8 y7 y6 y5 y4 y2
b7
Int
100
75
50
25
0
y7
y4
y2
y5
y6
b7
y8
y8 2+
m/z
0
100
200
300
400
500
600
700
800
900
1,000

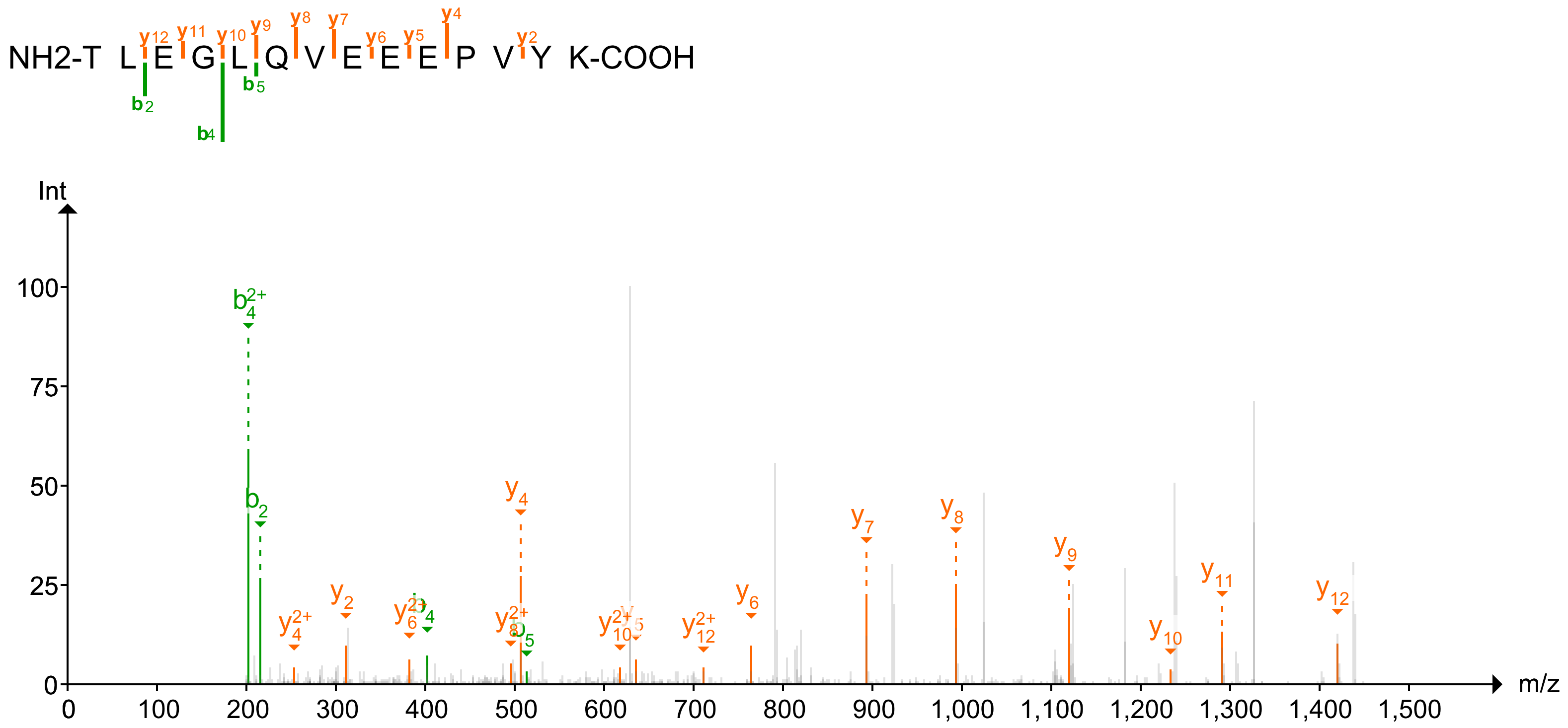

NH2-T L E G L Q V E E E P V Y K-COOH
y12 y11 y10 y9 y8 y7 y6 y5 y4 y2
b2 b4 b5
Int
100
75
50
25
0
m/z
0 100 200 300 400 500 600 700 800 900 1,000 1,100 1,200 1,300 1,400 1,500

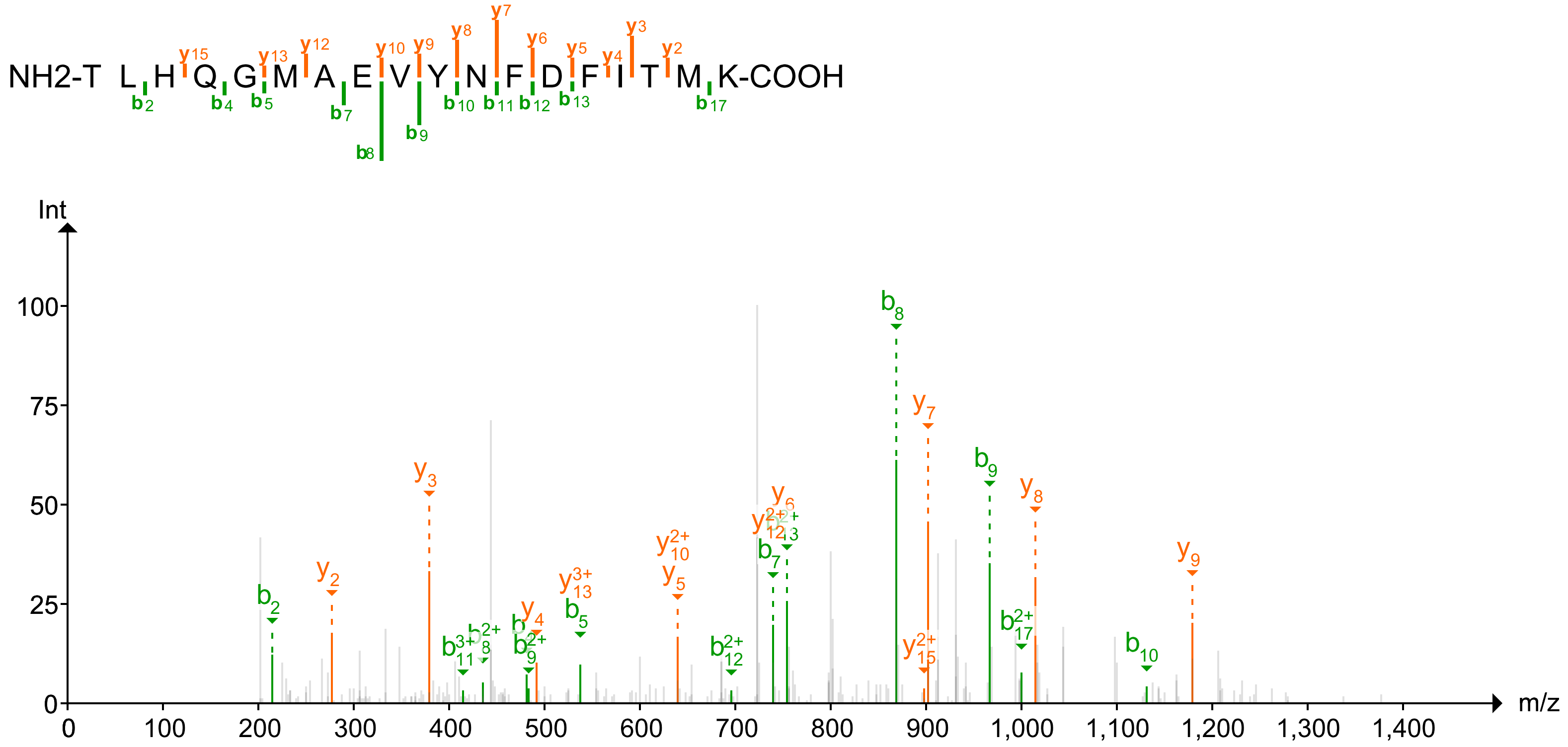

NH2-T L H Q G M A E V Y N F D F I T M K-COOH
y15 y13 y12 y10 y9 y8 y7 y6 y5 y4 y3 y2
b2 b4 b5 b7 b8 b9 b10 b11 b12 b13 b17
Int
m/z
0 25 50 75 100
0 100 200 300 400 500 600 700 800 900 1,000 1,100 1,200 1,300 1,400

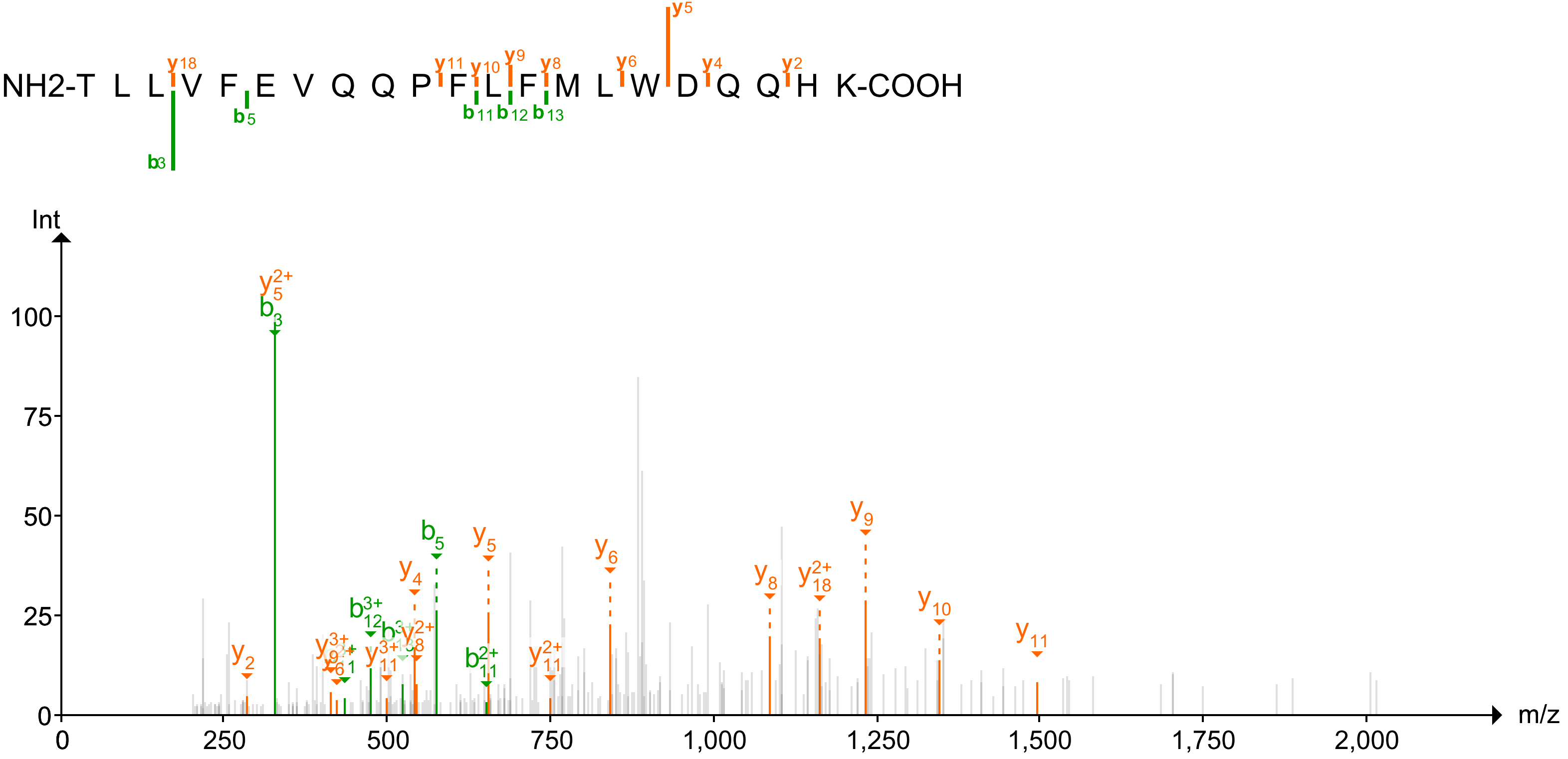

NH2-T L L V F E V Q Q P F L F M L W D Q Q H K-COOH
y18
y11
y10
y9
y8
y6
y5
y4
y2
b3
b5
b11
b12
b13
Int
100
75
50
25
0
m/z
0
250
500
750
1,000
1,250
1,500
1,750
2,000

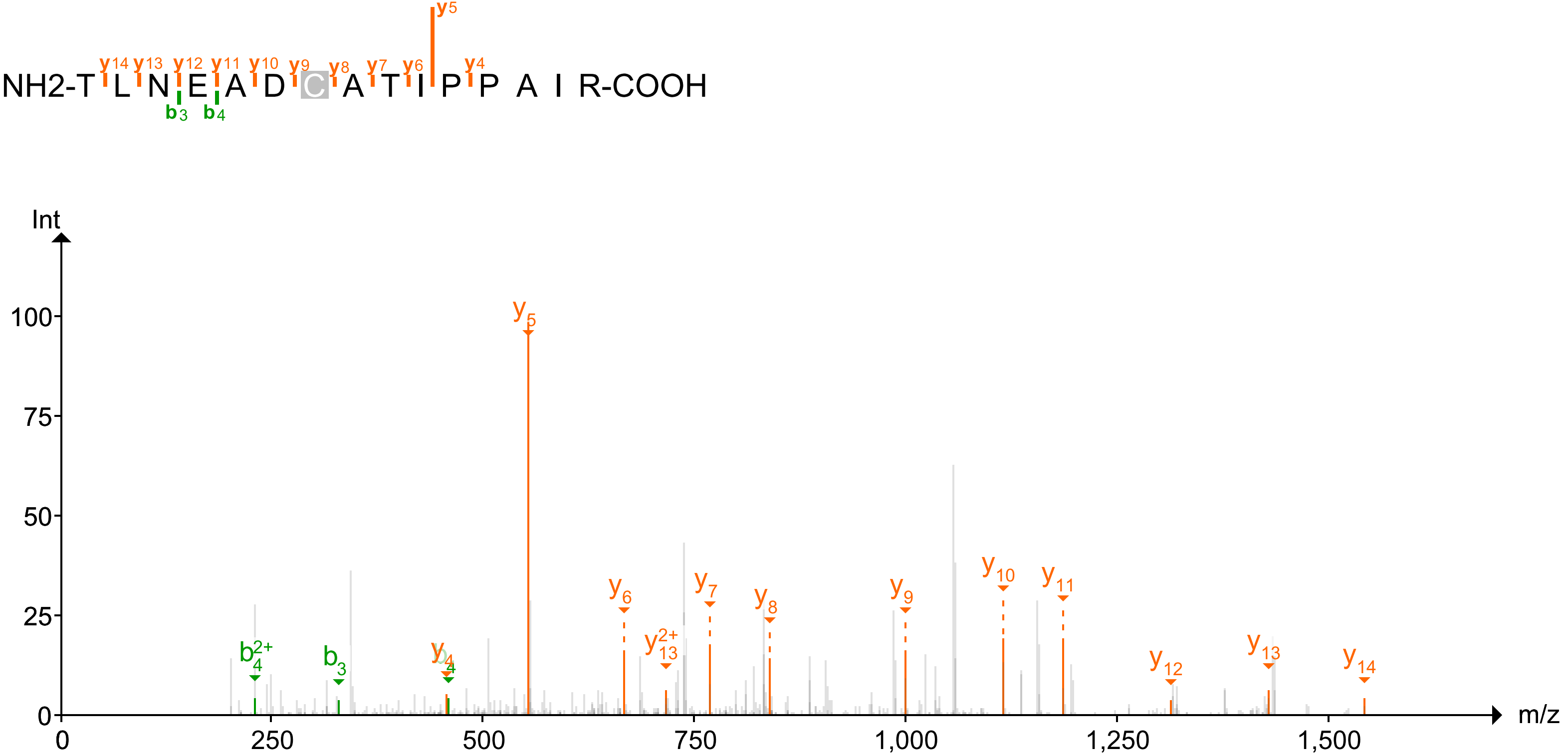

NH2-T L N E A D C A T I P P A I R-COOH
y14 y13 y12 y11 y10 y9 y8 y7 y6 y5 y4
b3 b4
Int
100
75
50
25
0
m/z
0
250
500
750
1,000
1,250
1,500
y5
y6
y7
y8
y9
y10
y11
y12
y13
y14
y13 2+
b4 2+
b3
y4

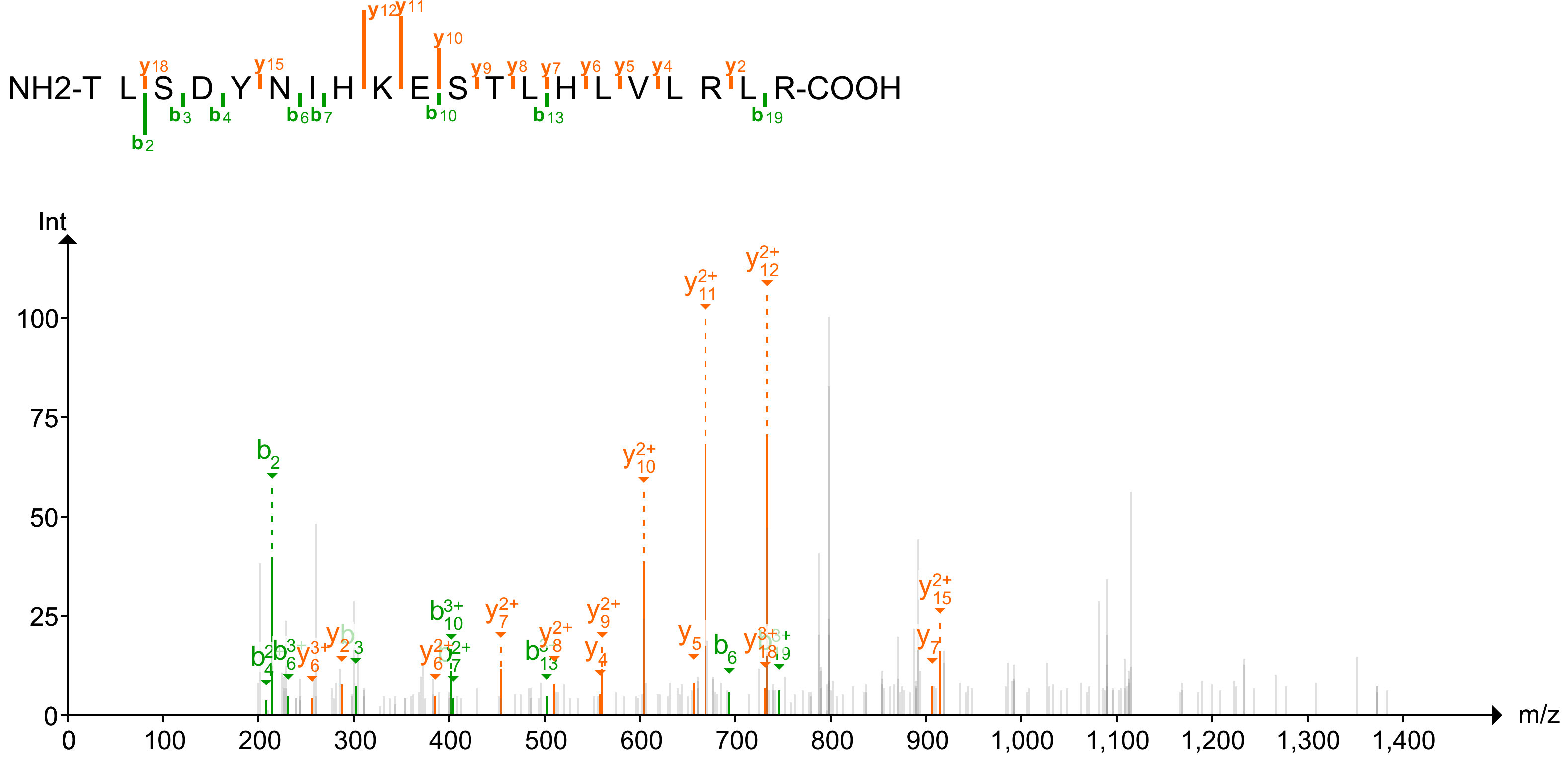

NH2-T L S D Y N I H K E S T L H L V L R L R-COOH
Int
m/z
0
25
50
75
100
0
100
200
300
400
500
600
700
800
900
1,000
1,100
1,200
1,300
1,400

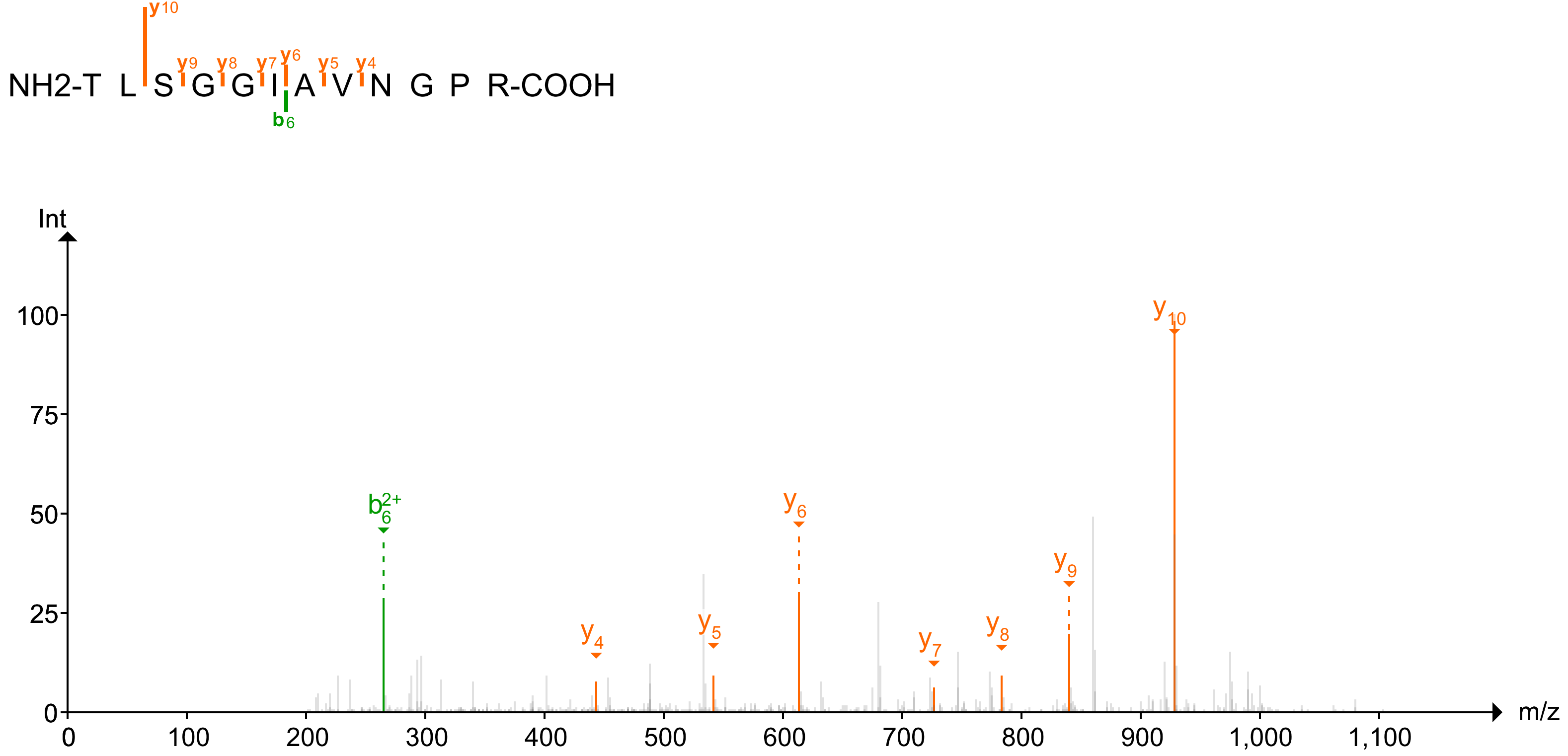

NH2-T L S G G I A V N G P R-COOH
y10
y9
y8
y7
y6
y5
y4
b6
Int
100
75
50
25
0
$b_6^{2+}$
$y_4$
$y_5$
$y_6$
$y_7$
$y_8$
$y_9$
$y_{10}$
0
100
200
300
400
500
600
700
800
900
1,000
1,100
m/z

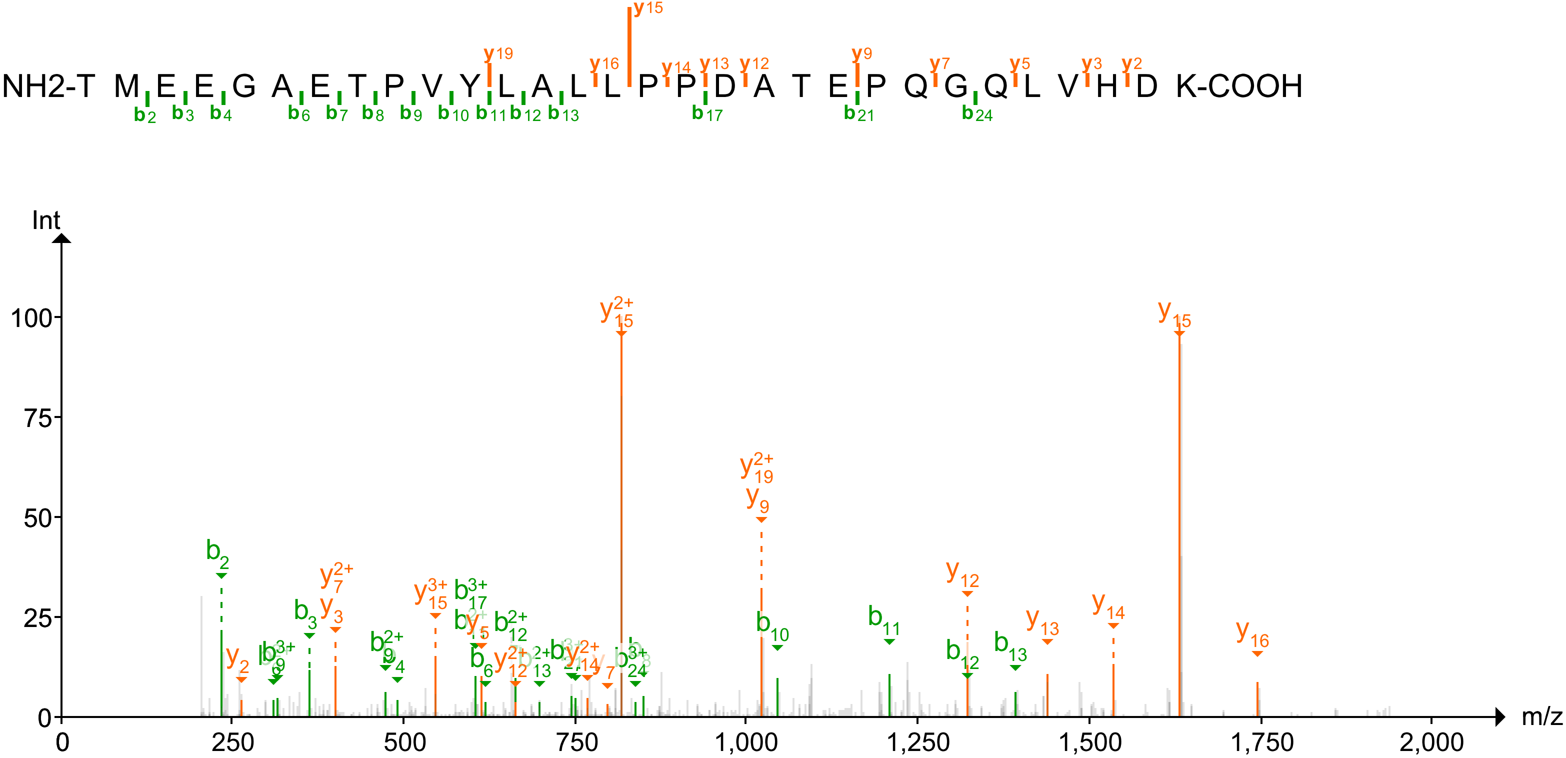

NH2-T M E E G A E T P V Y L A L L P P D A T E P Q G Q L V H D K-COOH
Int
m/z
0
25
50
75
100
250
500
750
1,000
1,250
1,500
1,750
2,000

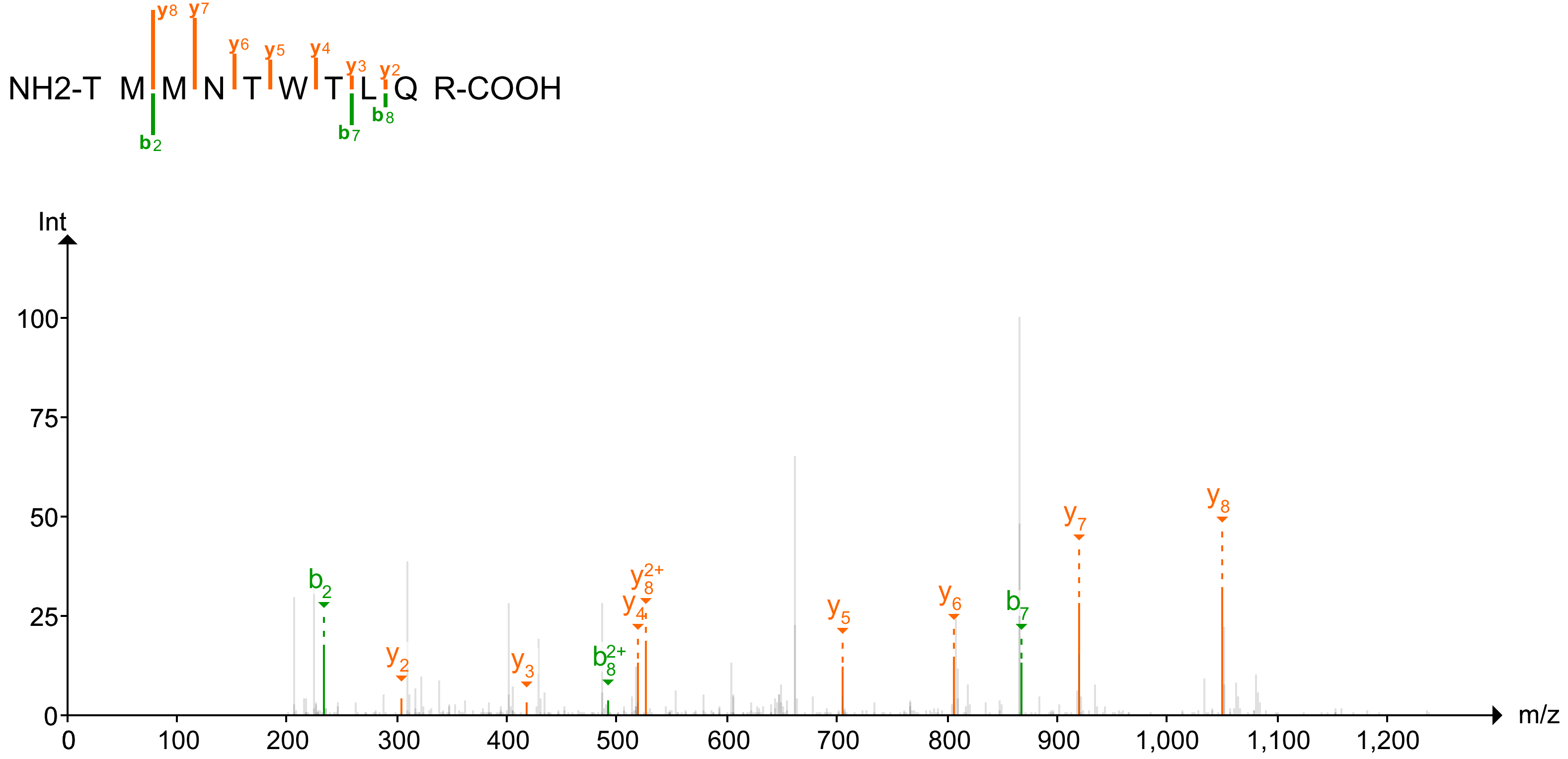

NH2-T M M N T W T L Q R-COOH
y8 y7 y6 y5 y4 y3 y2
b2 b7 b8
Int
100
75
50
25
0
m/z
0
100
200
300
400
500
600
700
800
900
1,000
1,100
1,200
b2
y2
y3
b8 2+
y4
y8 2+
y5
y6
b7
y7
y8

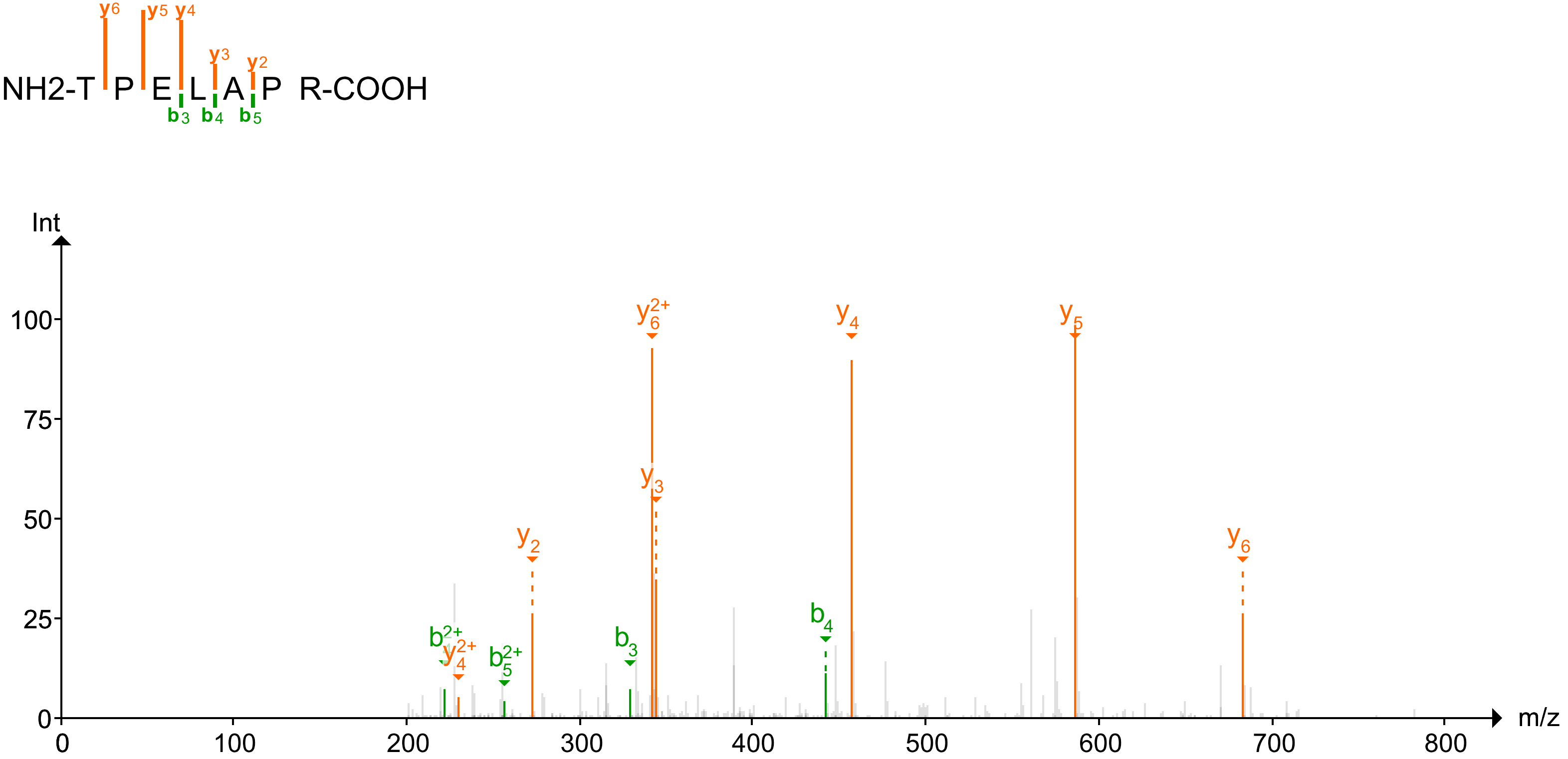

y6 y5 y4 y3 y2
NH2-T P E L A P R-COOH
b3 b4 b5
Int
100
75
50
25
0
m/z
0
100
200
300
400
500
600
700
800
y6 2+
y4
y5
y3
y2
y6
b4
b3
b2+
y4 2+
b5 2+

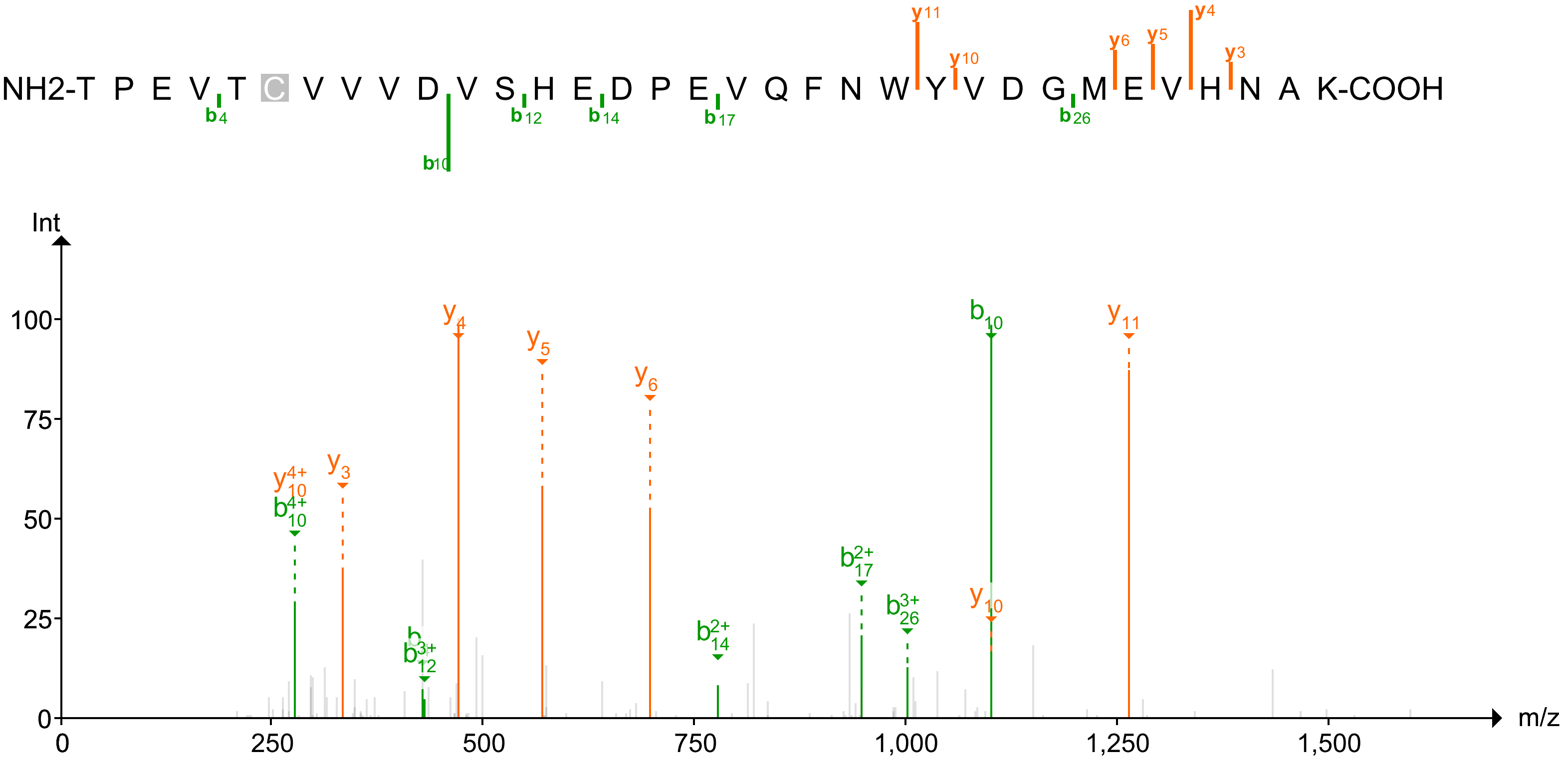

NH2-T P E V T C V V V D V S H E D P E V Q F N W Y V D G M E V H N A K-COOH
b4 b10 b12 b14 b17 b26
y11 y10 y6 y5 y4 y3
Int
100
75
50
25
0
m/z
0
250
500
750
1,000
1,250
1,500
y4
y5
y6
b10
y11
y3
y10 4+
b10 4+
b12 3+
b14 2+
b17 2+
b26 3+
y10

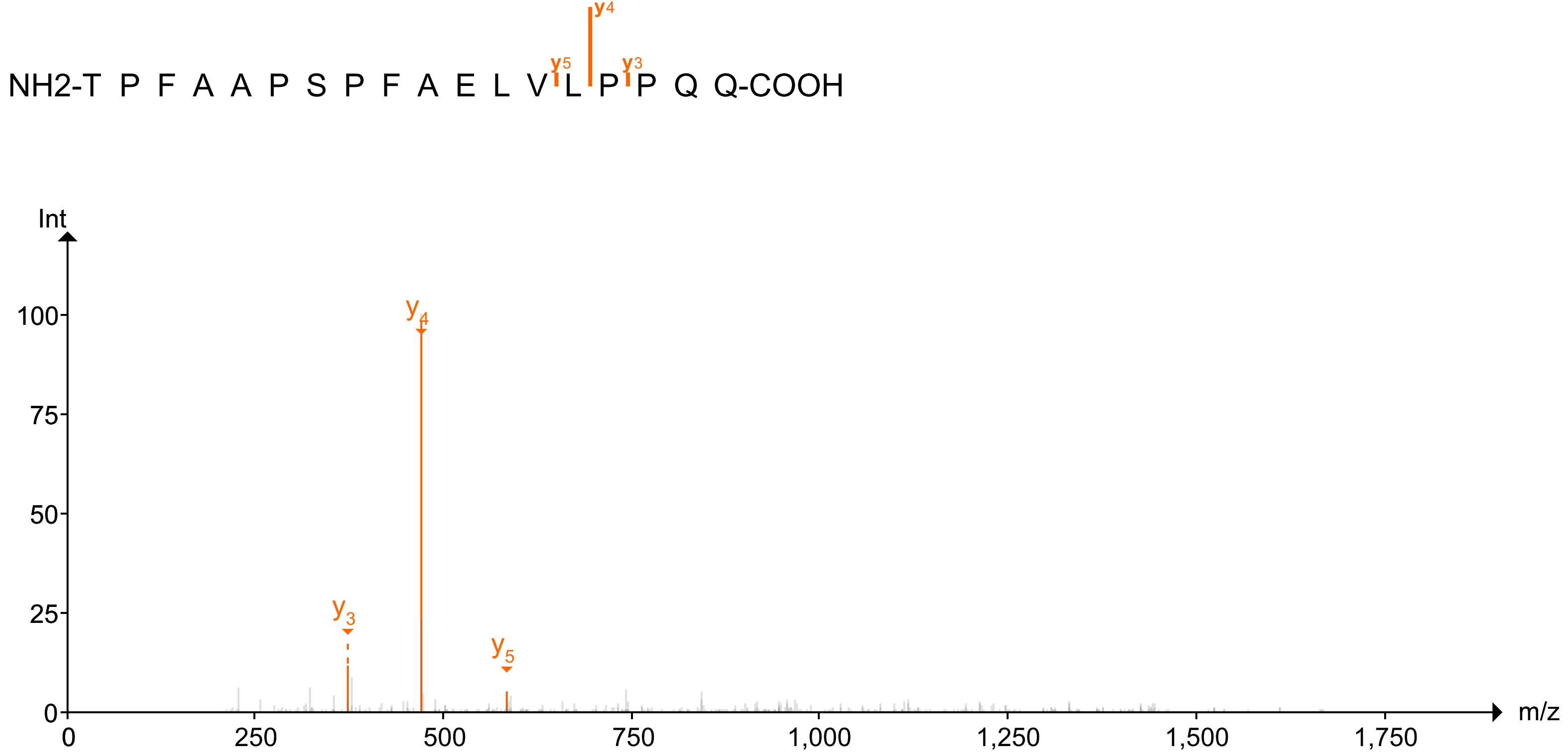

NH2-T P F A A P S P F A E L V L P P Q Q-COOH
y4
y5
y3
Int
100
75
50
25
0
y4
y3
y5
m/z
0
250
500
750
1,000
1,250
1,500
1,750

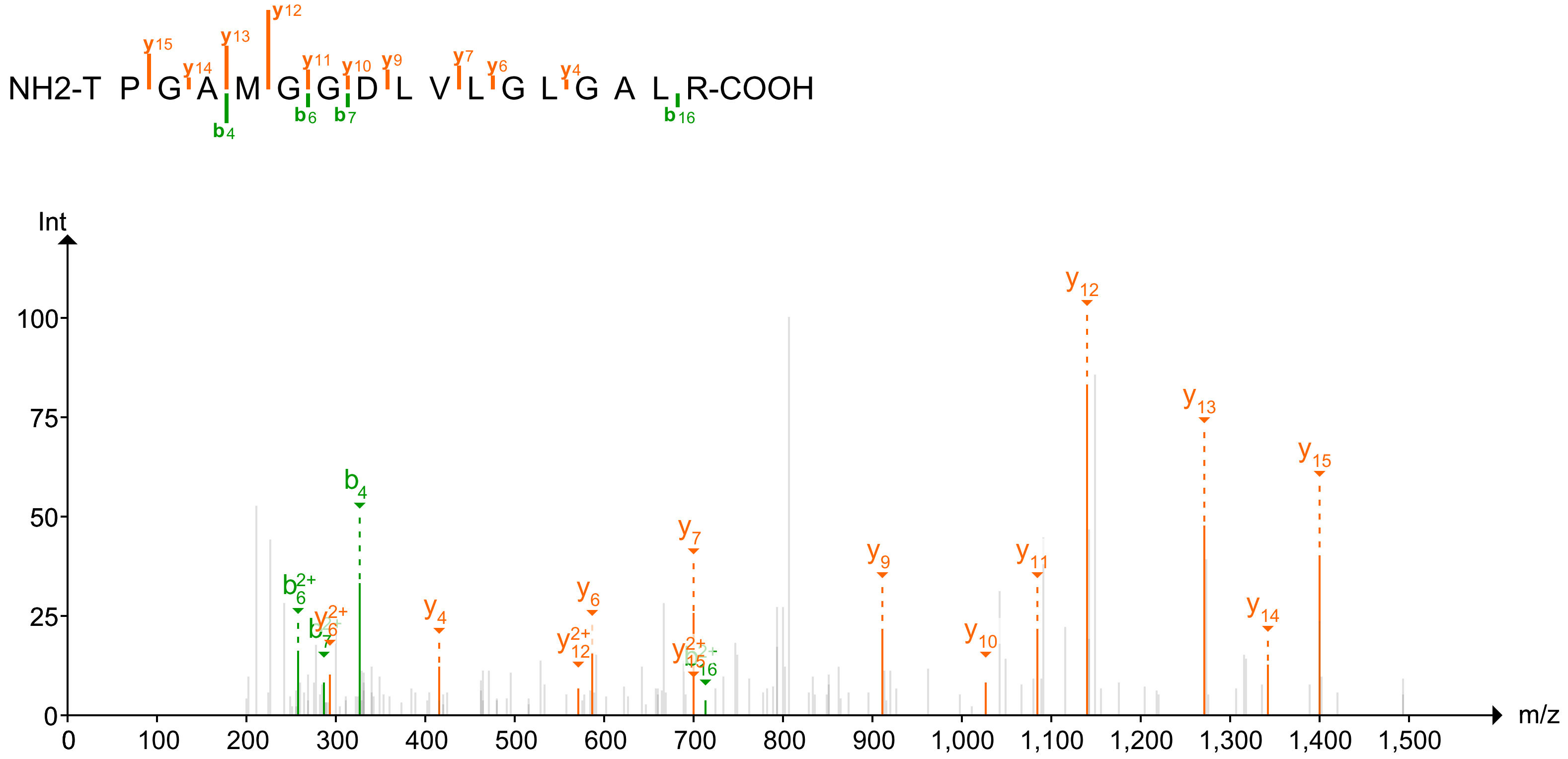

NH2-T P G A M G G D L V L G L G A L R-COOH
y15
y14
y13
y12
y11
y10
y9
y7
y6
y4
b4
b6
b7
b16
Int
m/z
0
25
50
75
100
0
100
200
300
400
500
600
700
800
900
1,000
1,100
1,200
1,300
1,400
1,500

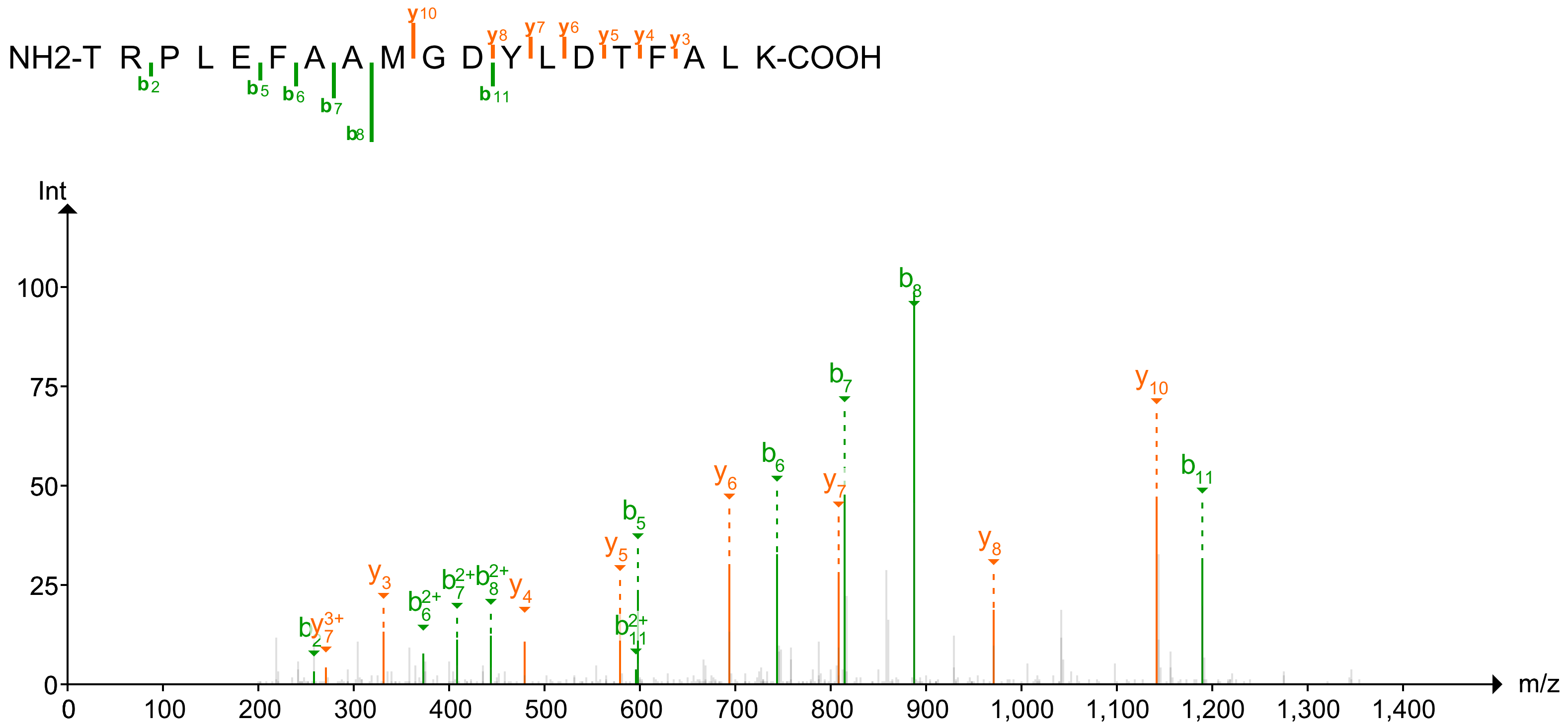

NH2-T R P L E F A A M G D Y L D T F A L K-COOH
b2 b5 b6 b7 b8 b11
y10 y8 y7 y6 y5 y4 y3
Int
100
75
50
25
0
m/z
0
100
200
300
400
500
600
700
800
900
1,000
1,100
1,200
1,300
1,400
b8
b7
y10
b11
b6
y6
y7
b5
y5
y8
y3
y4
b6 2+
b7 2+
b8 2+
b11 2+
b2
y7 3+

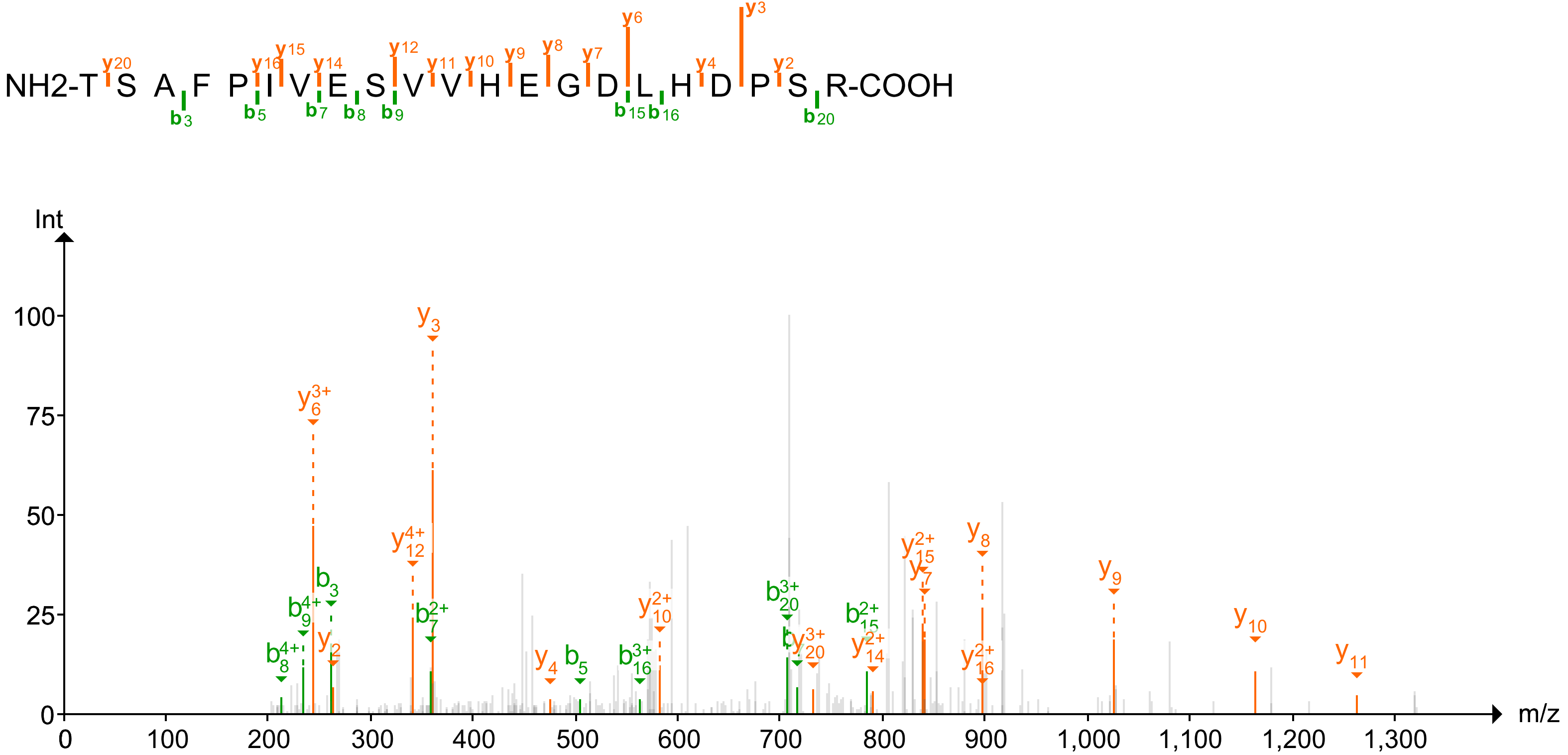

NH2-T S A F P I V E S V V H E G D L H D P S R-COOH
y20 y16 y15 y14 y12 y11 y10 y9 y8 y7 y6 y4 y3 y2
b3 b5 b7 b8 b9 b15 b16 b20
Int
100
75
50
25
0
m/z
0
100
200
300
400
500
600
700
800
900
1,000
1,100
1,200
1,300

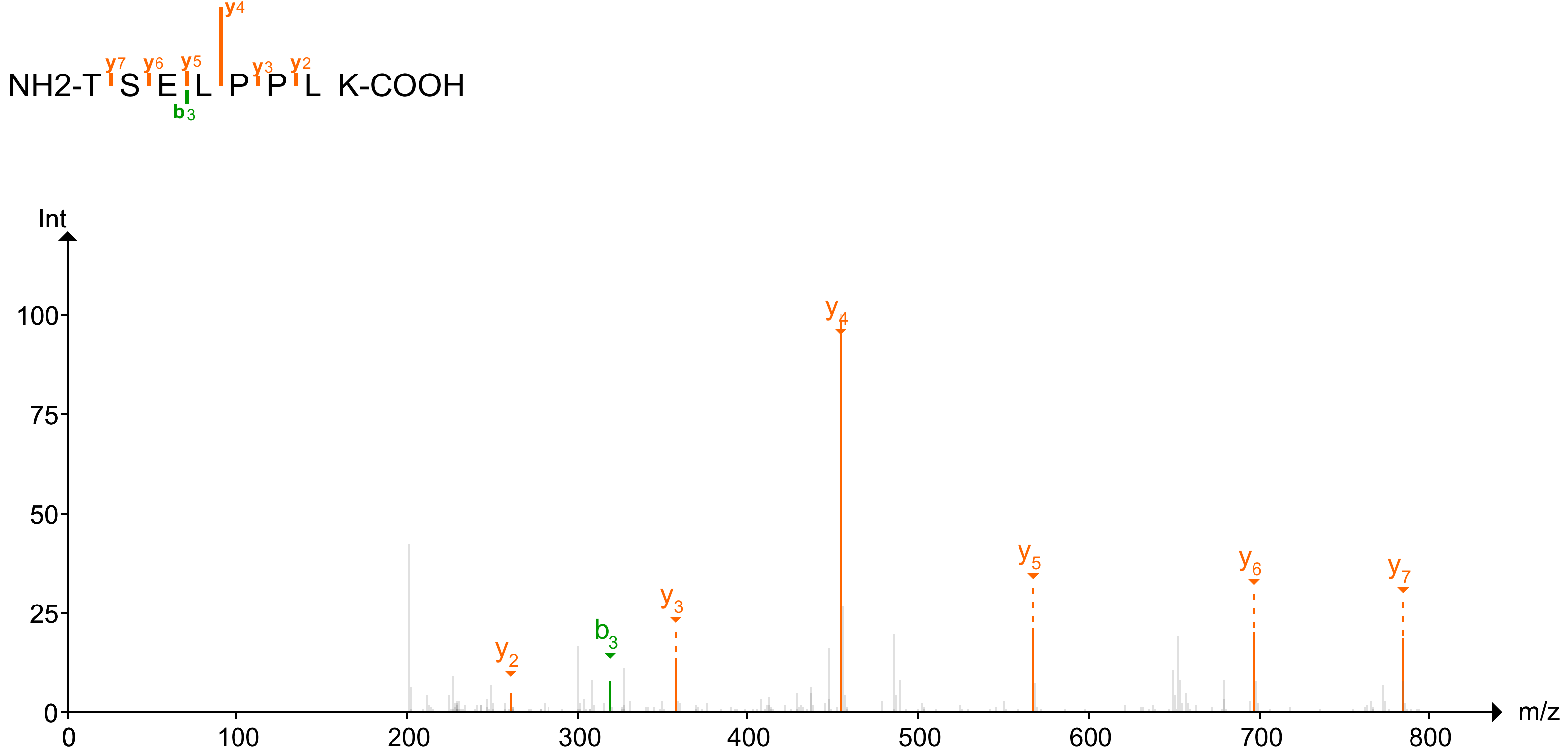
NH2-T S E L P P L K-COOH
y7 y6 y5 y4 y3 y2
b3
Int
100
75
50
25
0
m/z
0
100
200
300
400
500
600
700
800
y2
b3
y3
y4
y5
y6
y7

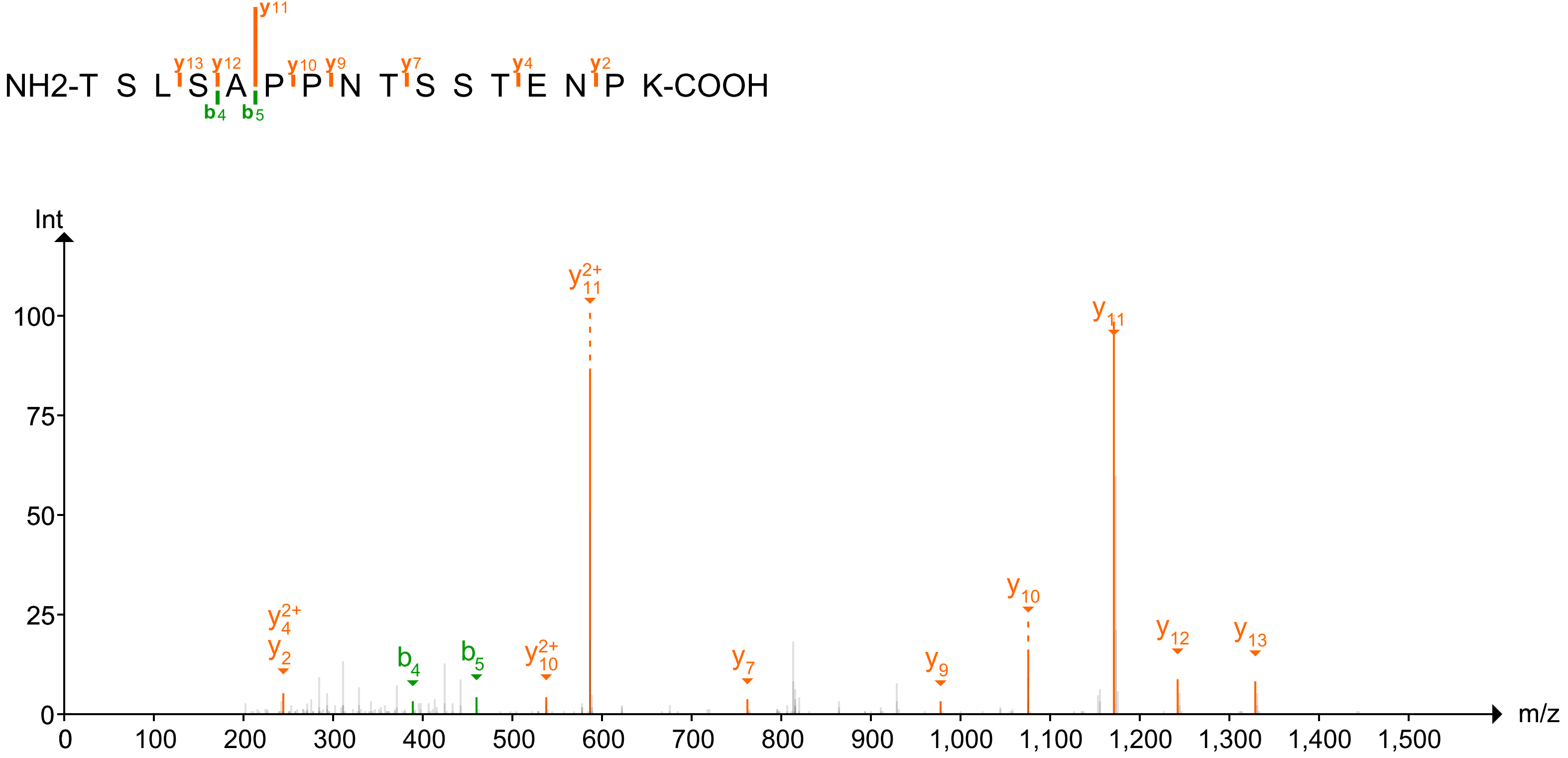

NH2-T S L S A P P N T S S T E N P K-COOH
y13 y12 y11 y10 y9 y7 y4 y2
b4 b5
Int
m/z
y11 2+
y11
y10
y12
y13
y4 2+
y2
b4
b5
y10 2+
y7
y9
0
25
50
75
100
0
100
200
300
400
500
600
700
800
900
1,000
1,100
1,200
1,300
1,400
1,500

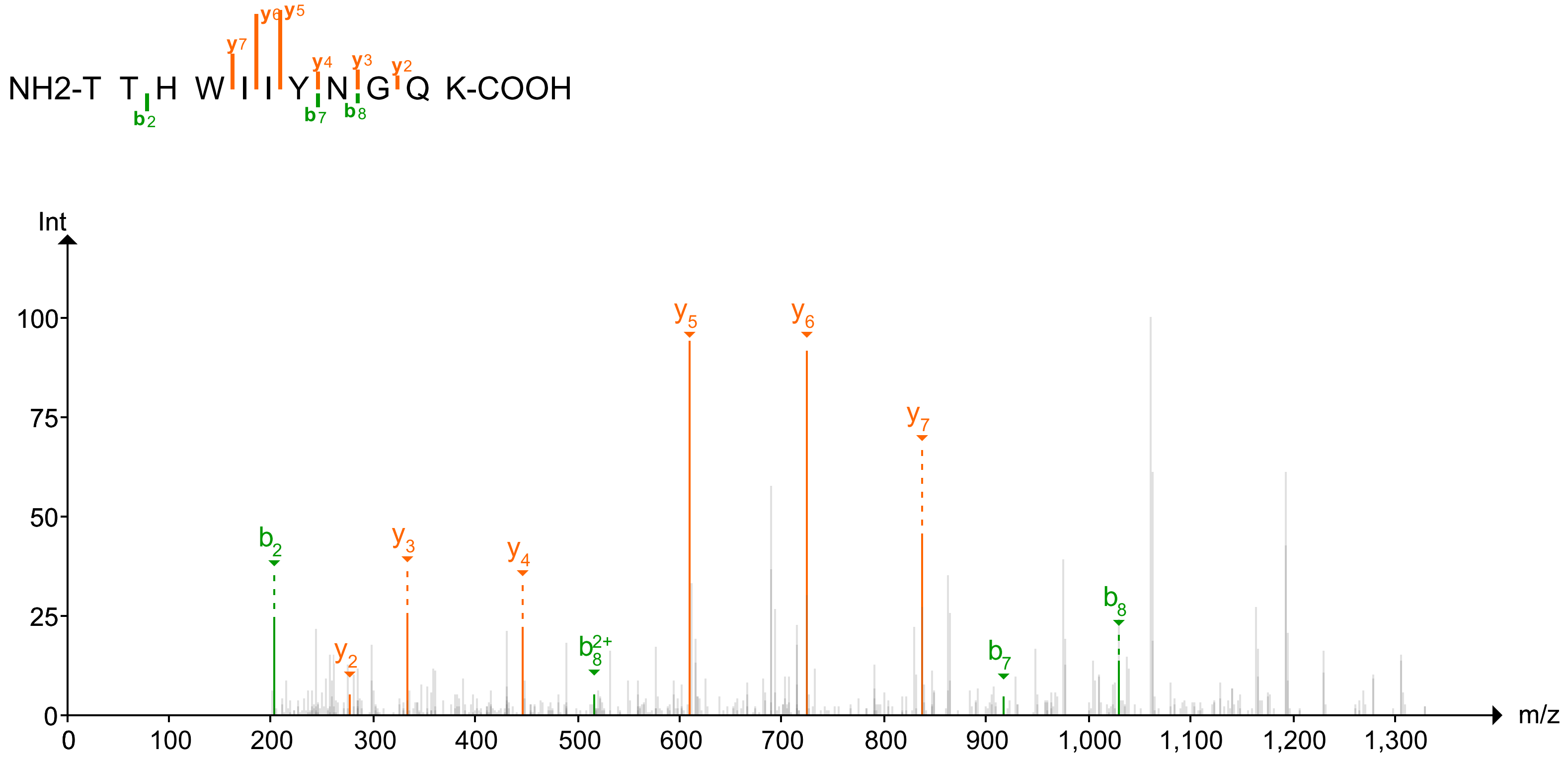
NH2-T T H W I I Y N G Q K-COOH
y7 y6 y5 y4 y3 y2
b2 b7 b8
Int
100
75
50
25
0
0 100 200 300 400 500 600 700 800 900 1,000 1,100 1,200 1,300
m/z
y5 y6 y7 b2 y3 y4 y2 b8 2+ b7 b8

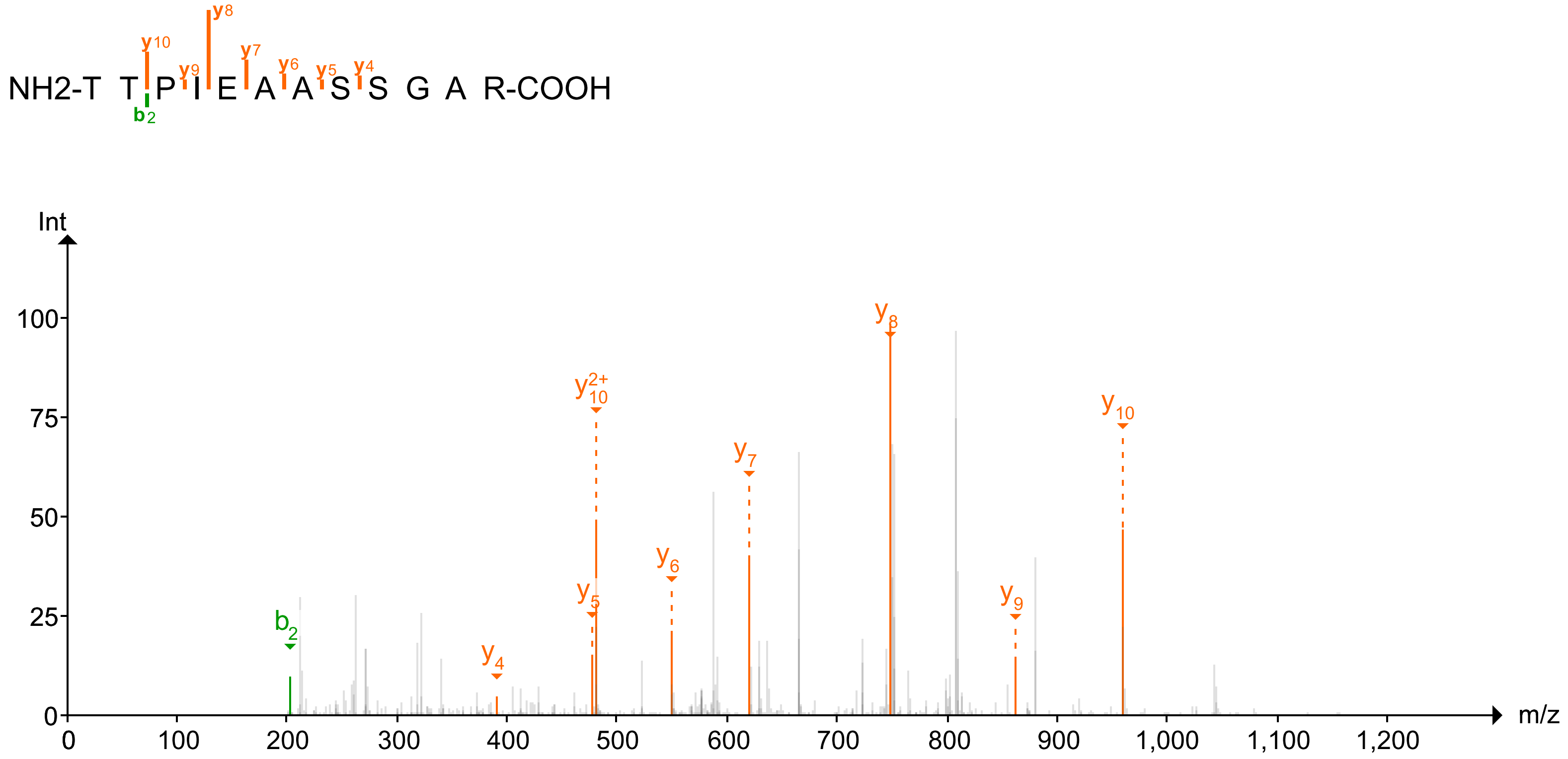

NH2-T T P I E A A S S G A R-COOH
y10
y9
y8
y7
y6
y5
y4
b2
Int
100
75
50
25
0
m/z
0
100
200
300
400
500
600
700
800
900
1,000
1,100
1,200
b2
y4
y5
y10 2+
y6
y7
y8
y9
y10

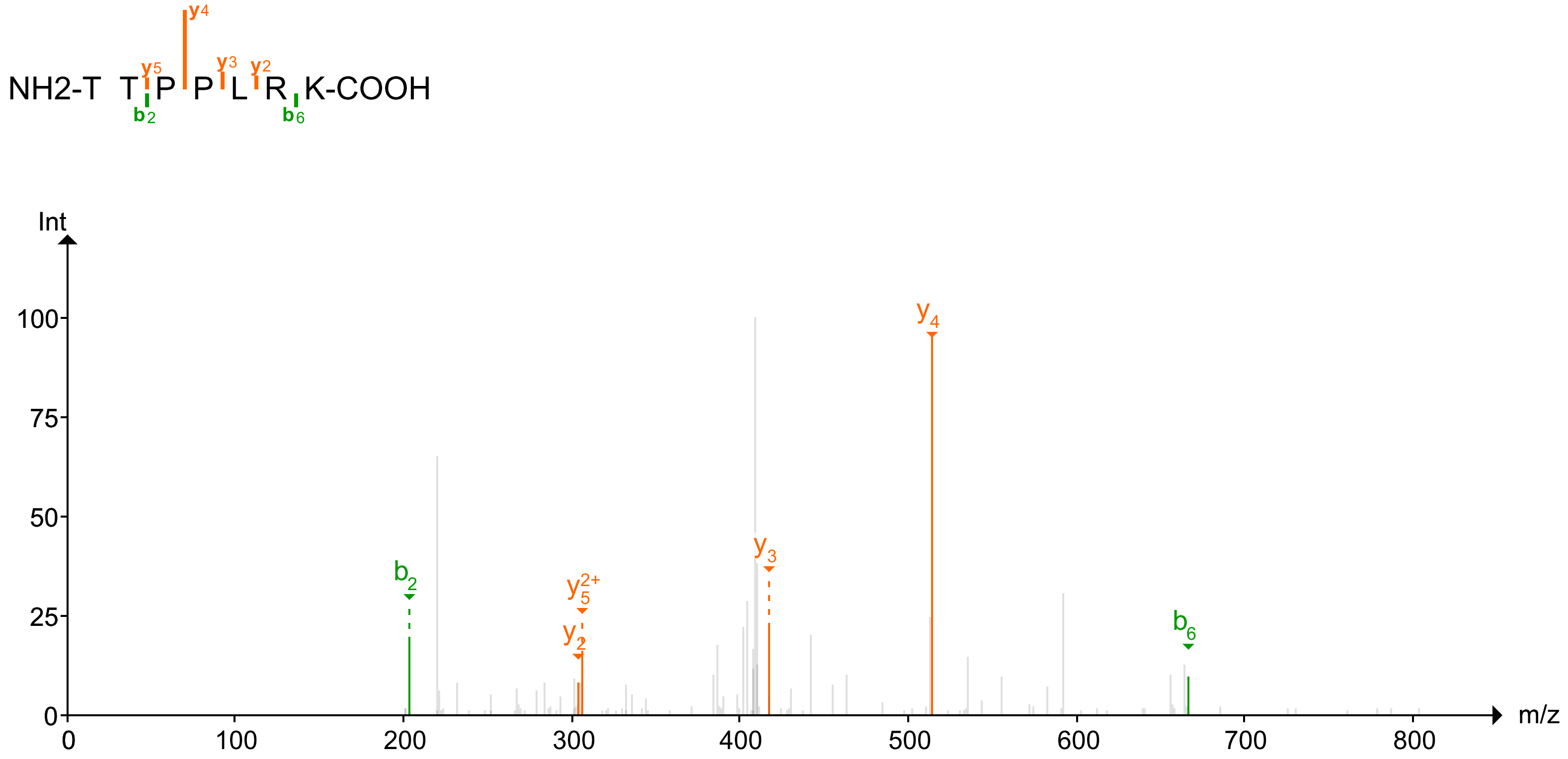

NH2-T T P P L R K-COOH
y4
y5
y3
y2
b2
b6
Int
100
75
50
25
0
m/z
0
100
200
300
400
500
600
700
800
b2
y5 2+
y2
y3
y4
b6

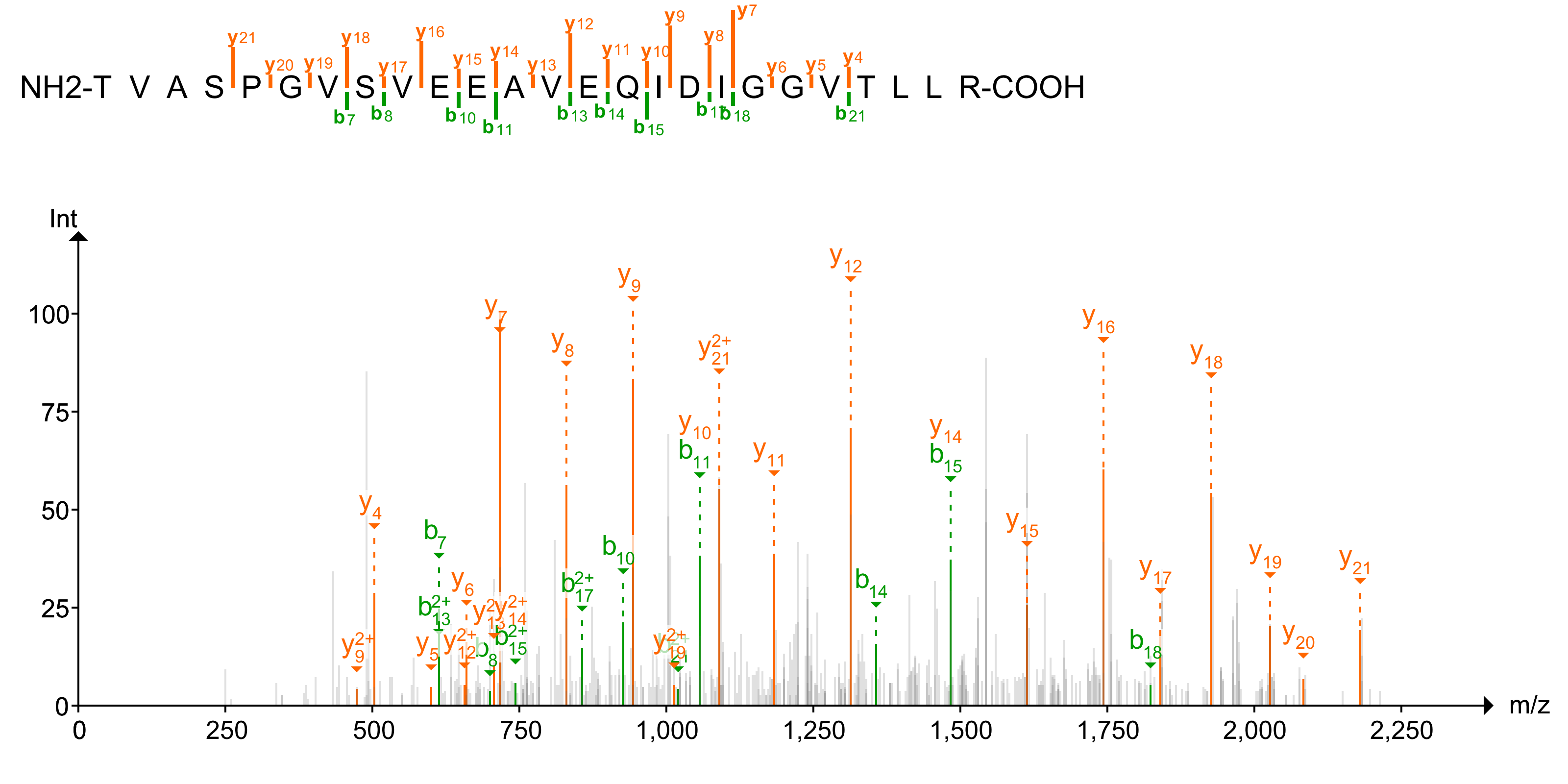

NH2-T V A S P G V S V E E A V E Q I D I G G V T L L R-COOH
Int
m/z
0
25
50
75
100
250
500
750
1,000
1,250
1,500
1,750
2,000
2,250

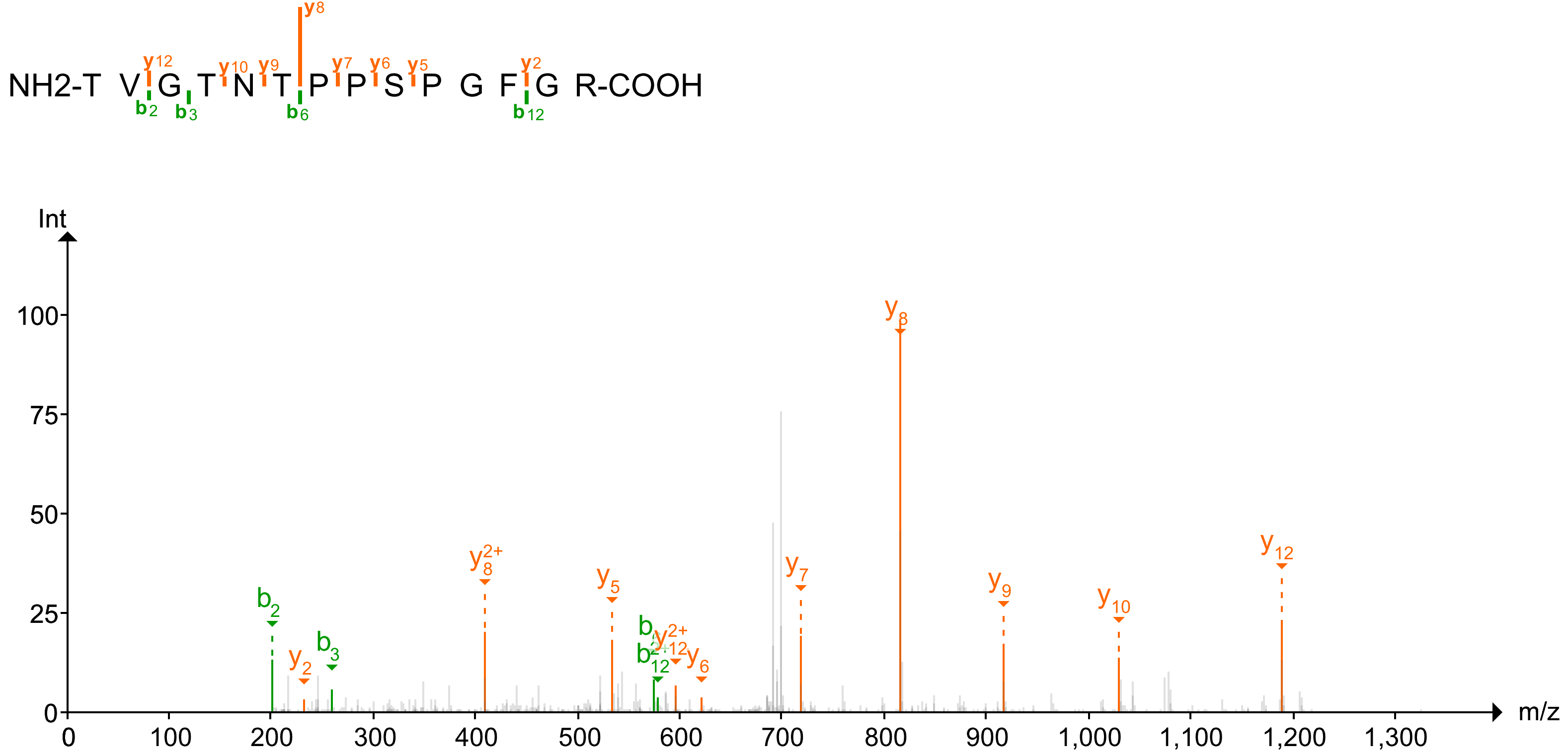
NH2-T V G T N T P P S P G F G R-COOH
y12 y10 y9 y8 y7 y6 y5 y2
b2 b3 b6 b12
Int
100
75
50
25
0
m/z
0 100 200 300 400 500 600 700 800 900 1,000 1,100 1,200 1,300
b2 y2 b3 y8 2+ y5 b y2+ 12 b2+ 12 y6 y7 y8 y9 y10 y12

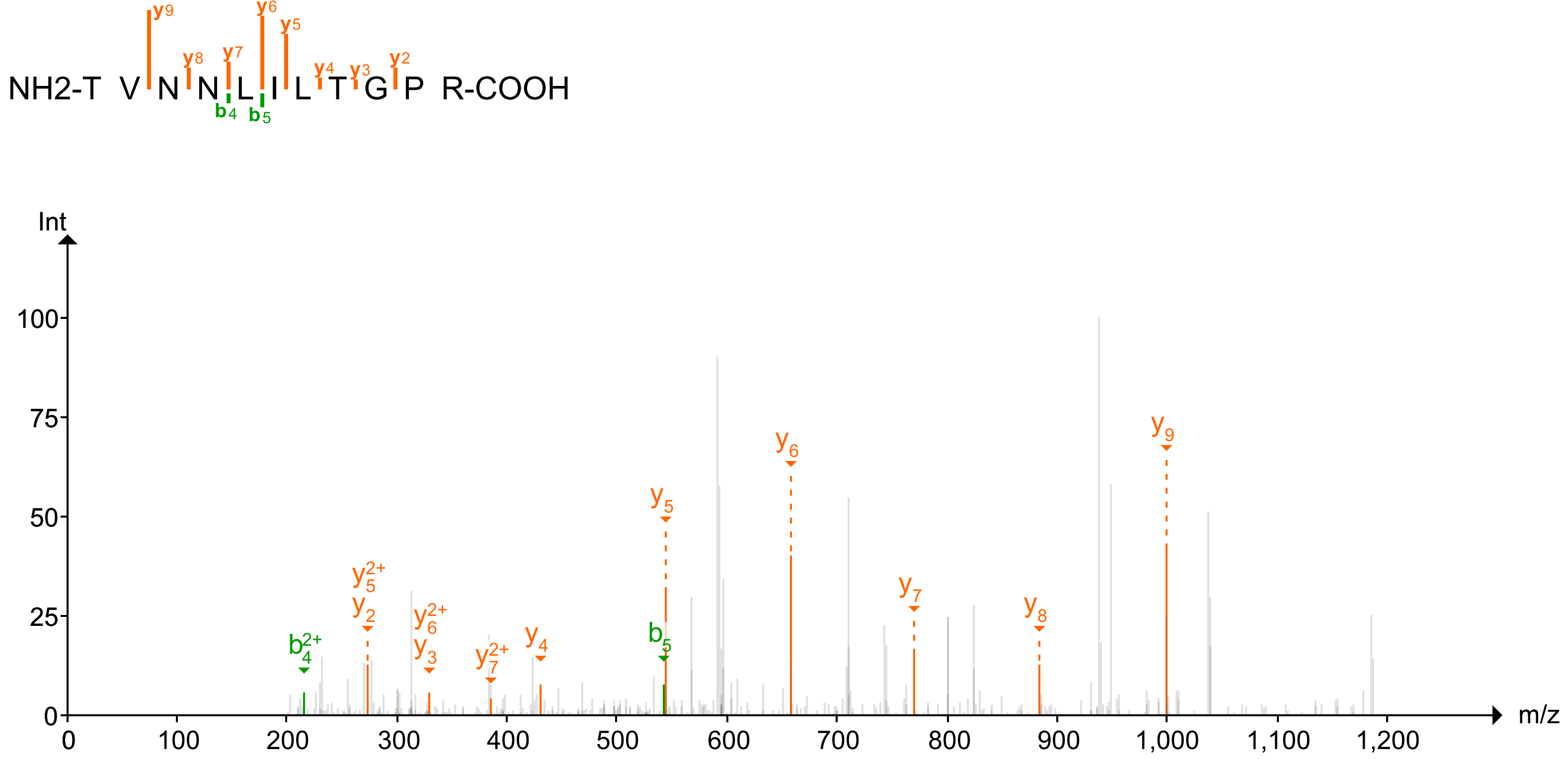
NH2-T V N N L I L T G P R-COOH
y9 y8 y7 y6 y5 y4 y3 y2
b4 b5
Int
100
75
50
25
0
m/z
0 100 200 300 400 500 600 700 800 900 1,000 1,100 1,200
$b_4^{2+}$
$y_5^{2+}$
$y_2$
$y_6^{2+}$
$y_3$
$y_7^{2+}$
$y_4$
$b_5$
$y_5$
$y_6$
$y_7$
$y_8$
$y_9$

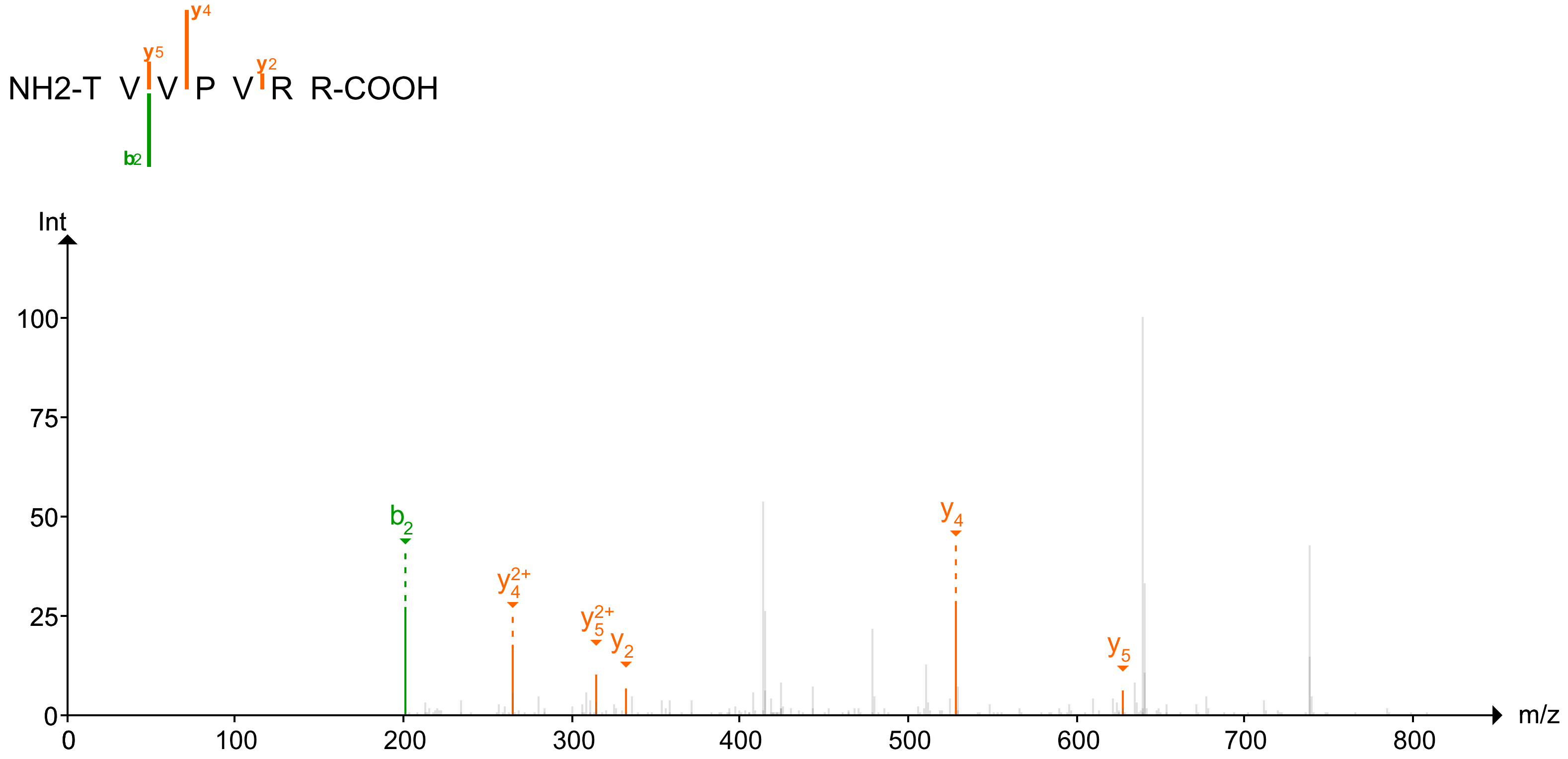

y4
y5
y2
NH2-T V V P V R R-COOH
b2
Int
100
75
50
25
0
b2
y4 2+
y5 2+
y2
y4
y5
0
100
200
300
400
500
600
700
800
m/z

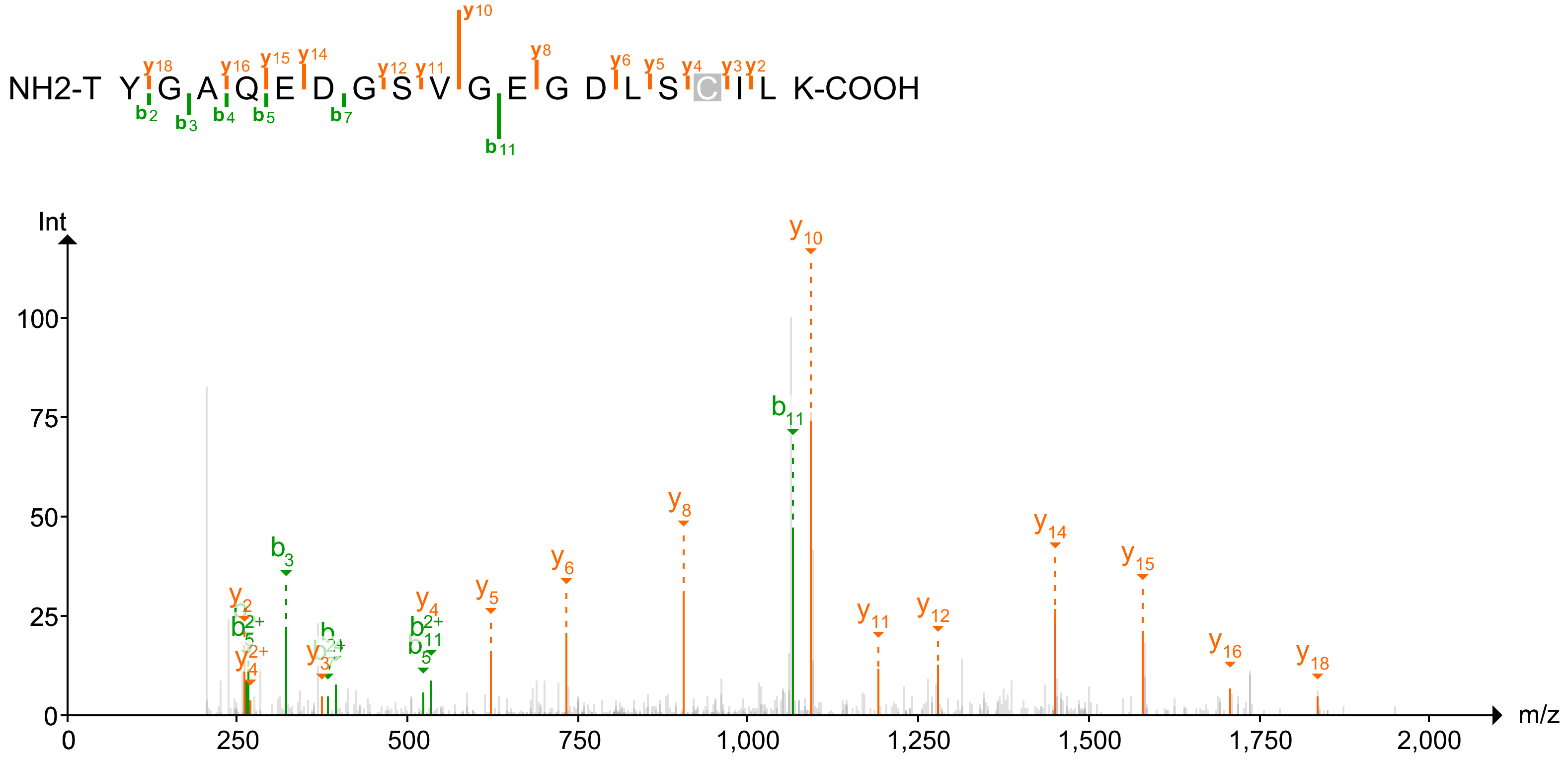
NH2-T Y G A Q E D G S V G E G D L S C I L K-COOH
y18 y16 y15 y14 y12 y11 y10 y8 y6 y5 y4 y3 y2
b2 b3 b4 b5 b7 b11
Int
100
75
50
25
0
m/z
0
250
500
750
1,000
1,250
1,500
1,750
2,000
y10
b11
y8
y14
y15
y6
y5
y4
y16
y18
y11
y12
b3
y2
$b_{11}^{2+}$
$y_4^{2+}$
$b_5^{2+}$
y3
b5

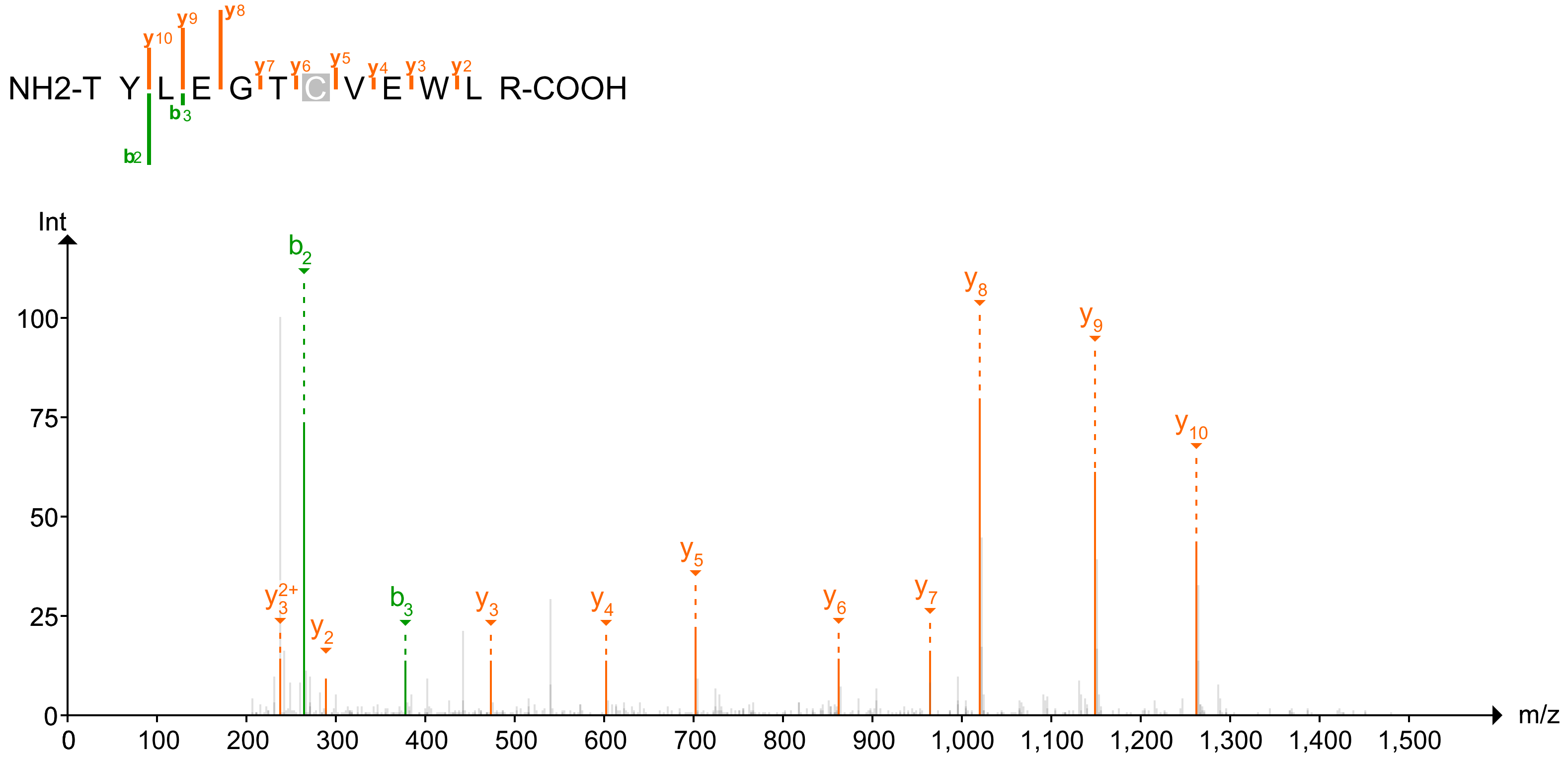

NH2-T Y L E G T C V E W L R-COOH
y10 y9 y8 y7 y6 y5 y4 y3 y2
b2 b3
Int
100
75
50
25
0
b2
y8
y9
y10
y5
y3 2+
y2
b3
y3
y4
y6
y7
0 100 200 300 400 500 600 700 800 900 1,000 1,100 1,200 1,300 1,400 1,500
m/z

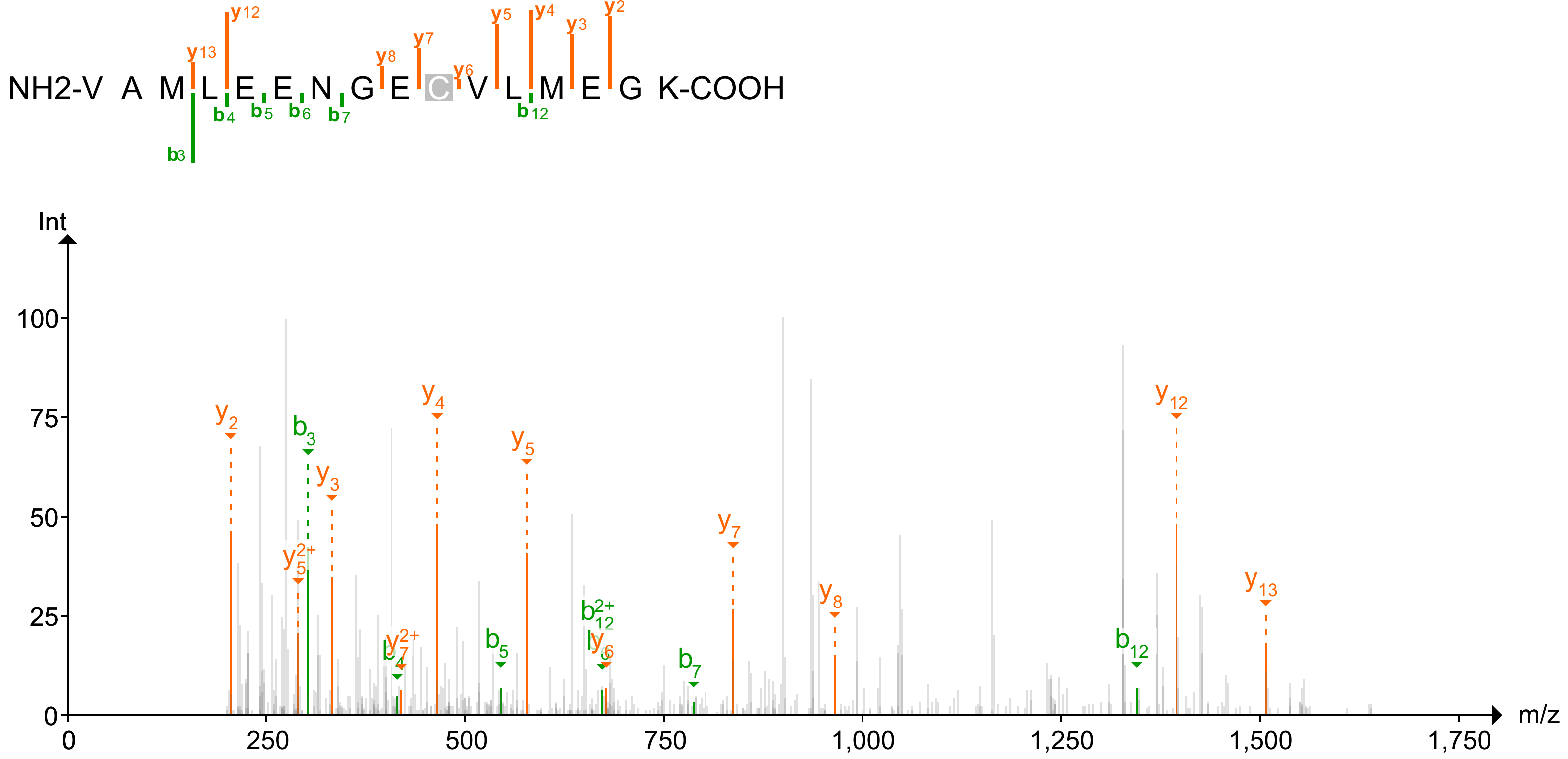

NH2-V A M L E E N G E C V L M E G K-COOH
y13 y12 y8 y7 y6 y5 y4 y3 y2
b3 b4 b5 b6 b7 b12
Int
100
75
50
25
0
m/z
0
250
500
750
1,000
1,250
1,500
1,750

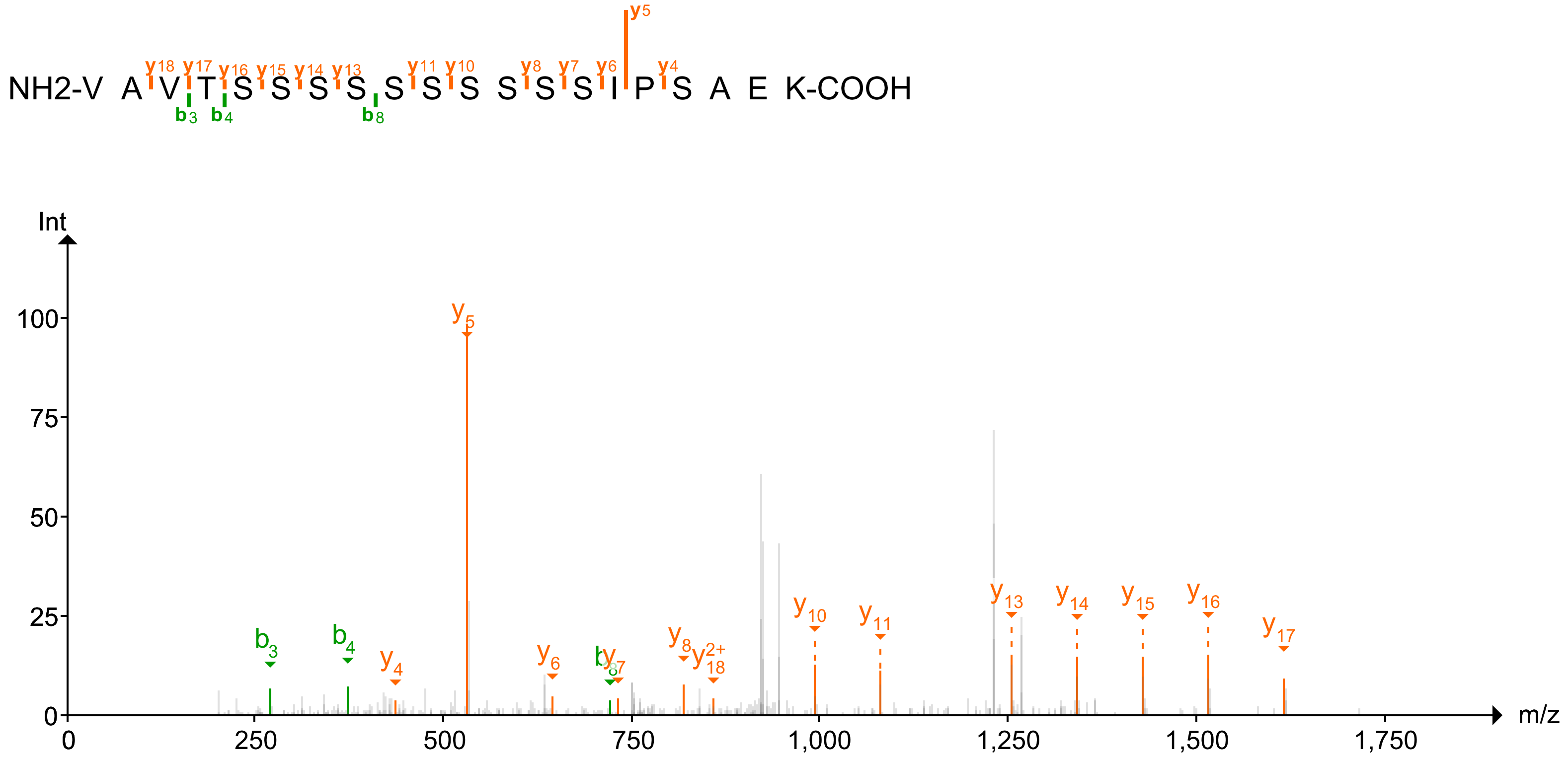

NH2-V A V T S S S S S S S S S S I P S A E K-COOH
y5
y18 y17 y16 y15 y14 y13 y11 y10 y8 y7 y6 y4
b3 b4 b8
Int
100
75
50
25
0
0
250
500
750
1,000
1,250
1,500
1,750
m/z
y5
b3
b4
y4
y6
b8
y7
y8
y2+ 18
y10
y11
y13
y14
y15
y16
y17

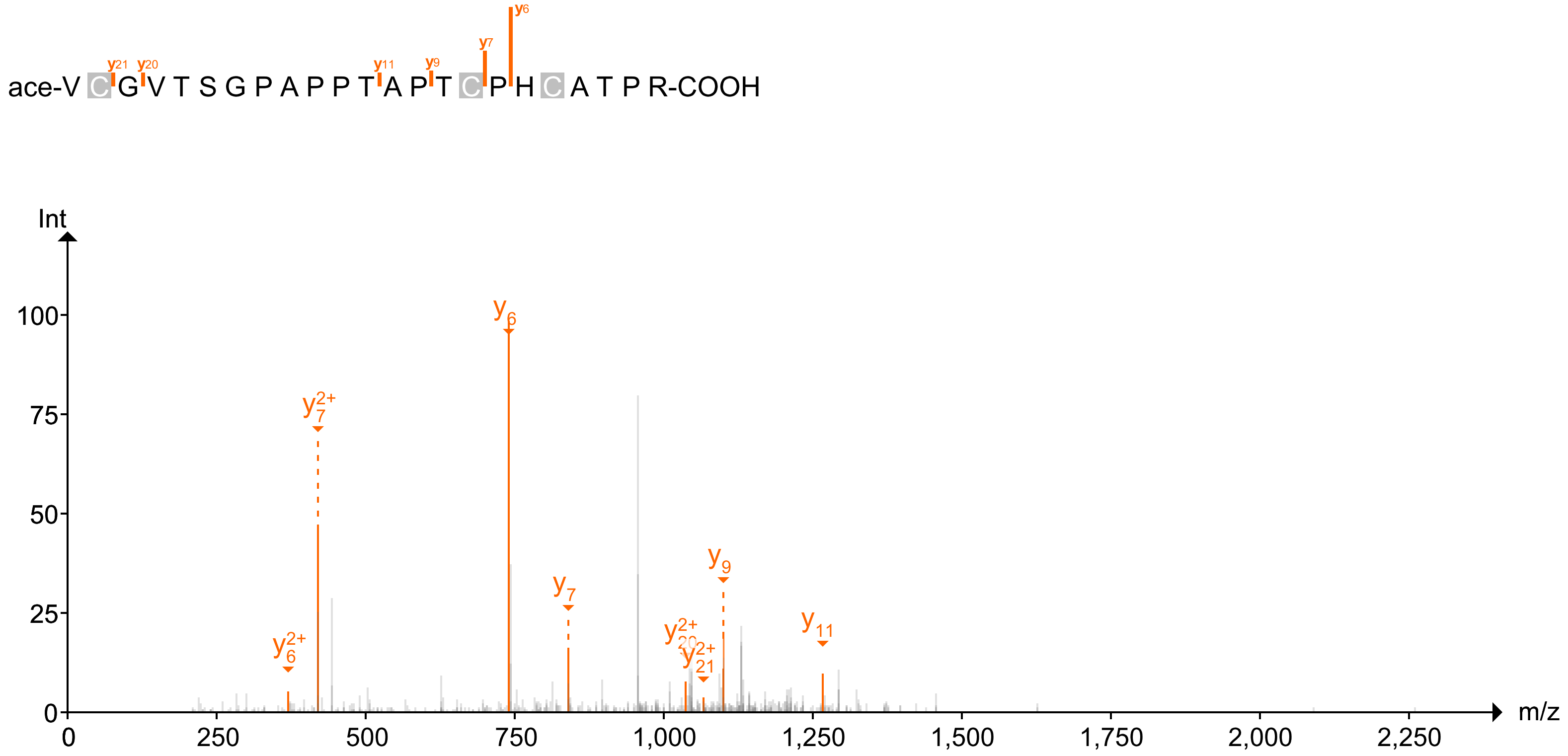

ace-V C G V T S G P A P P T A P T C P H C A T P R-COOH
y21 y20 y11 y9 y7 y6
Int
m/z
100
75
50
25
0
250
500
750
1,000
1,250
1,500
1,750
2,000
2,250

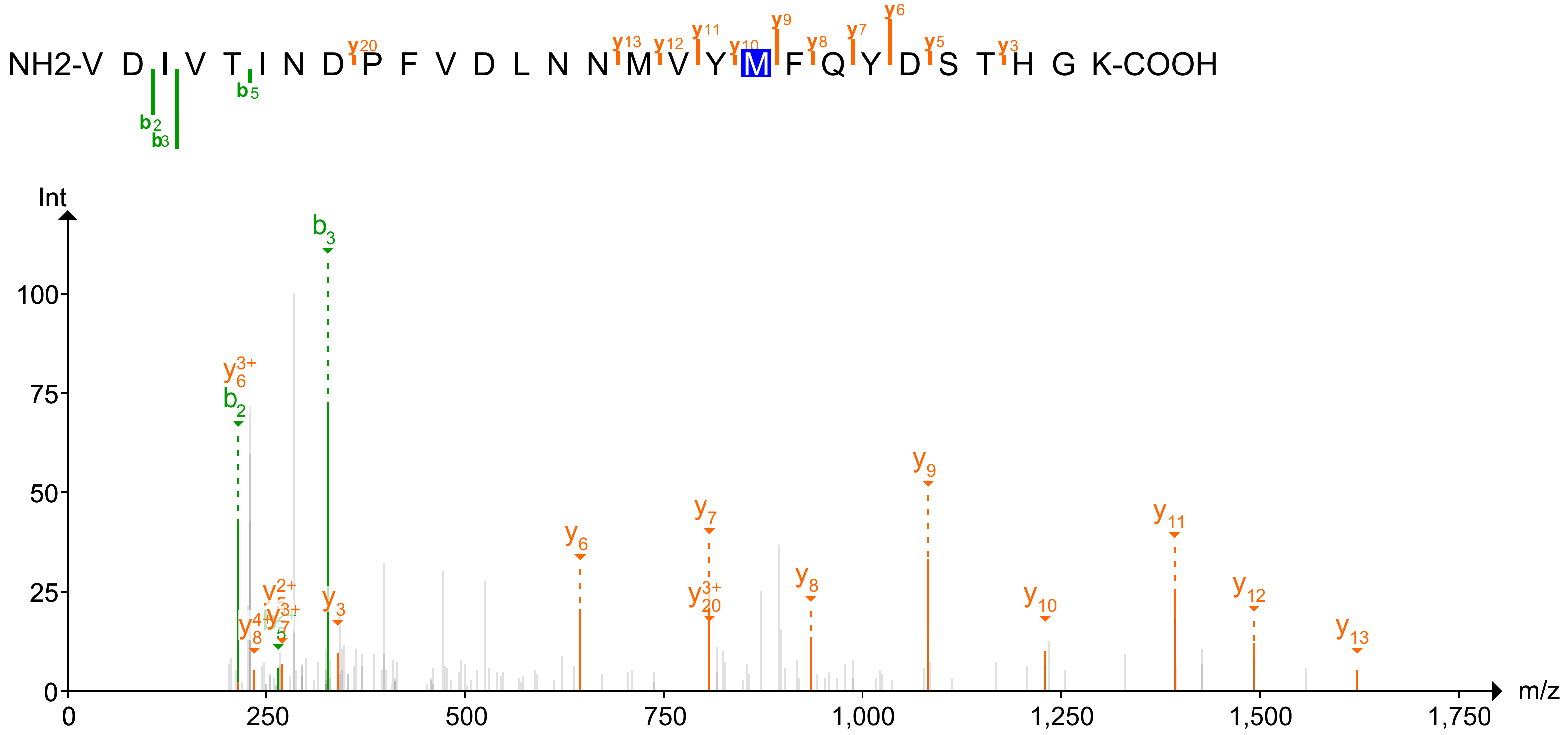
NH2-V D I V T I N D P F V D L N N M V Y M F Q Y D S T H G K-COOH
b2
b3
b5
y20
y13
y12
y11
y10
y9
y8
y7
y6
y5
y3
Int
m/z
0
25
50
75
100
0
250
500
750
1,000
1,250
1,500
1,750

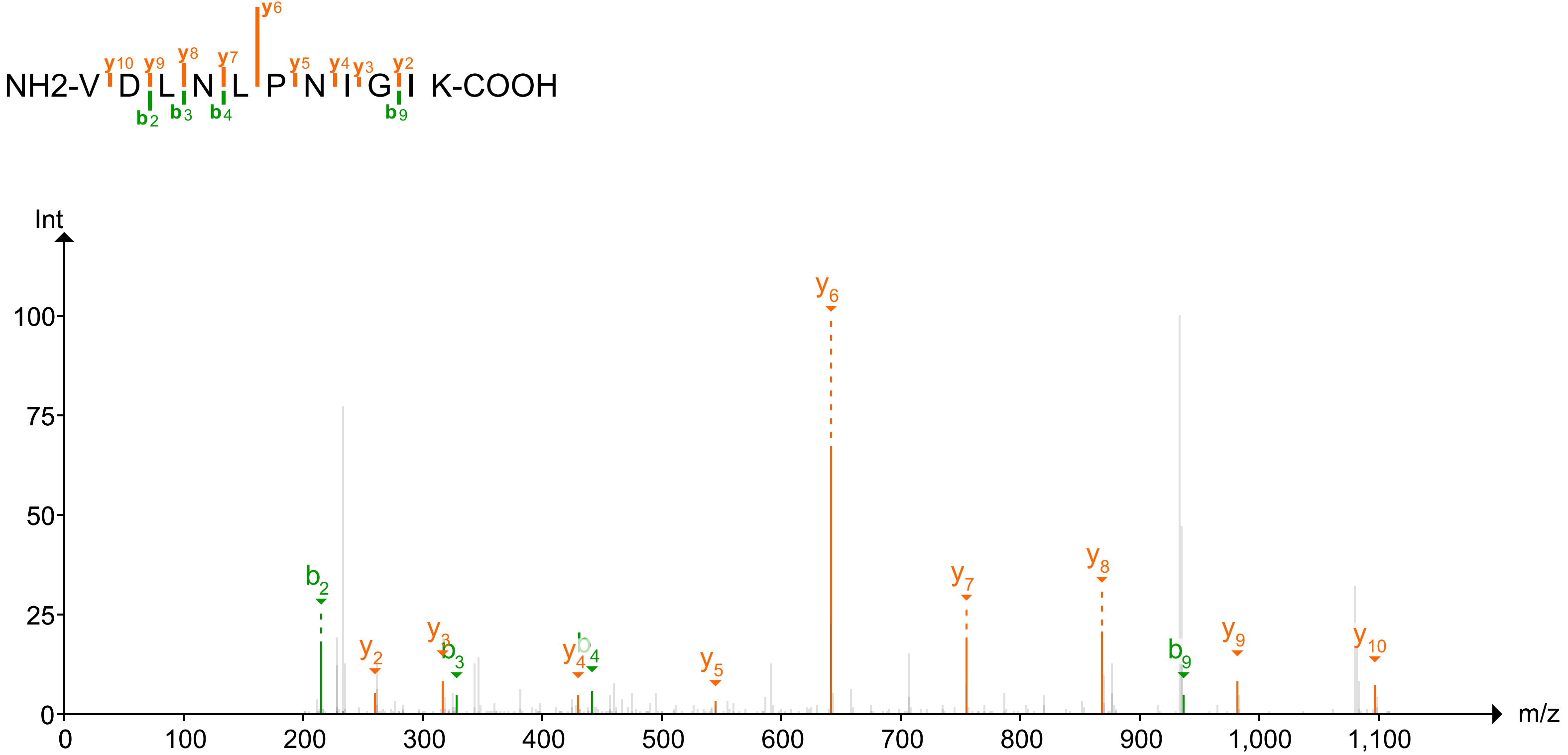
NH2-V D L N L P N I G I K-COOH
y10 y9 y8 y7 y6 y5 y4 y3 y2
b2 b3 b4 b9
Int
100
75
50
25
0
0 100 200 300 400 500 600 700 800 900 1,000 1,100
m/z
y6
y7
y8
b2
y2
y3
b3
y4
b4
y5
b9
y9
y10

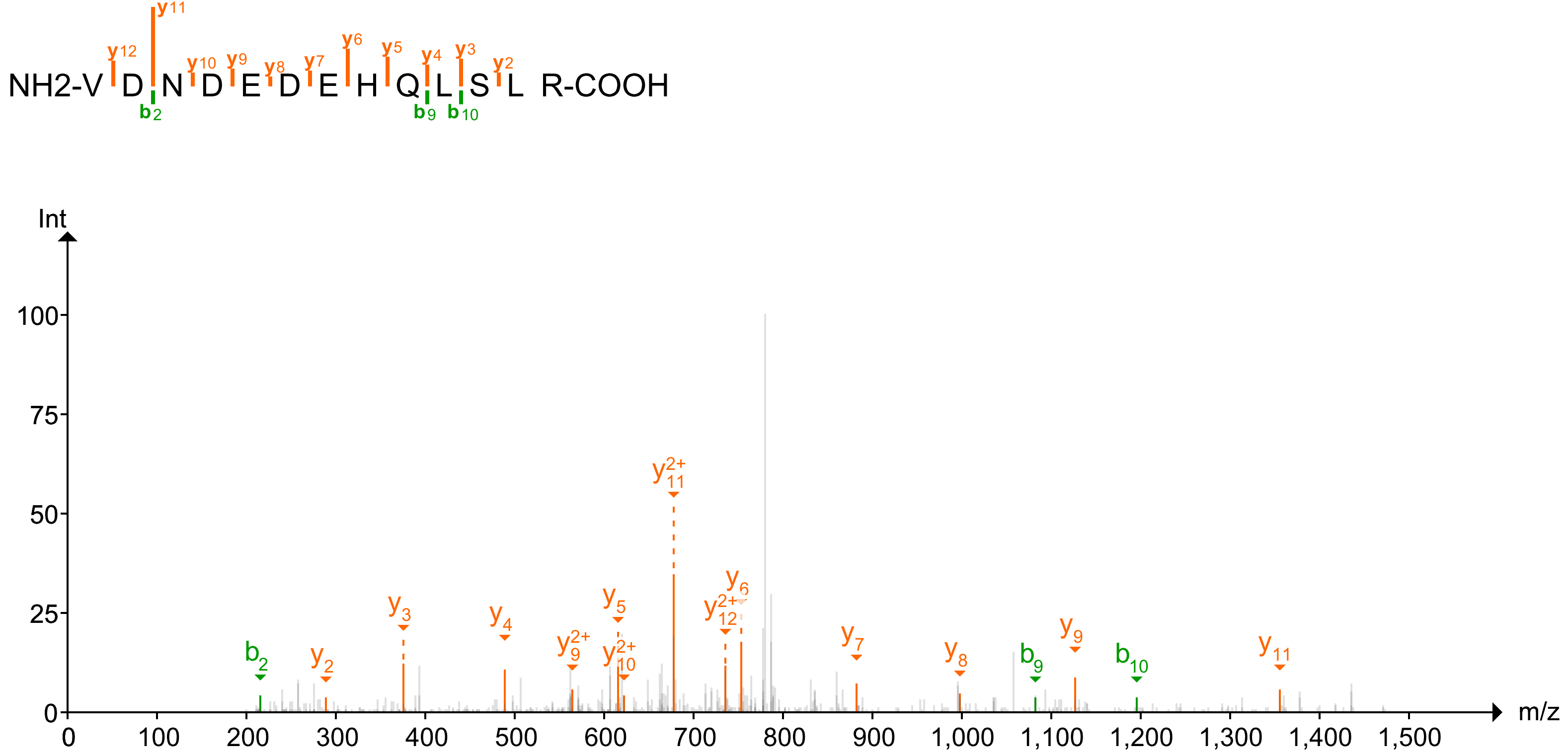

NH2-V D N D E D E H Q L S L R-COOH
y12 y11 y10 y9 y8 y7 y6 y5 y4 y3 y2
b2 b9 b10
Int
100
75
50
25
0
m/z
0 100 200 300 400 500 600 700 800 900 1,000 1,100 1,200 1,300 1,400 1,500
b2 y2 y3 y4 y9 2+ y5 y10 2+ y11 2+ y12 2+ y6 y7 y8 b9 y9 b10 y11

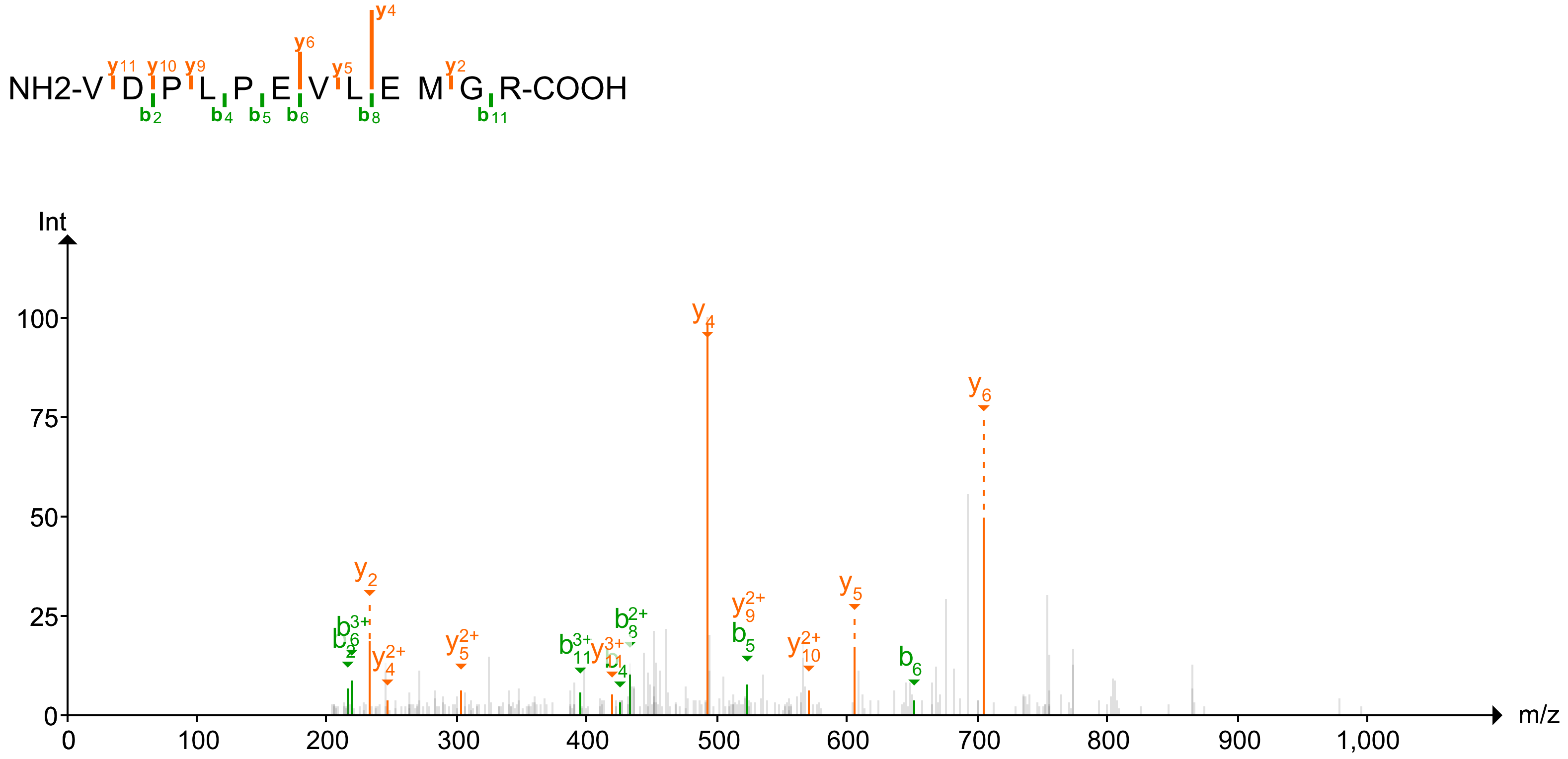

NH2-V D P L P E V L E M G R-COOH
y11 y10 y9 y6 y5 y4 y2
b2 b4 b5 b6 b8 b11
Int
100
75
50
25
0
m/z
0
100
200
300
400
500
600
700
800
900
1,000
y4
y6
y2
y5
b5
b6

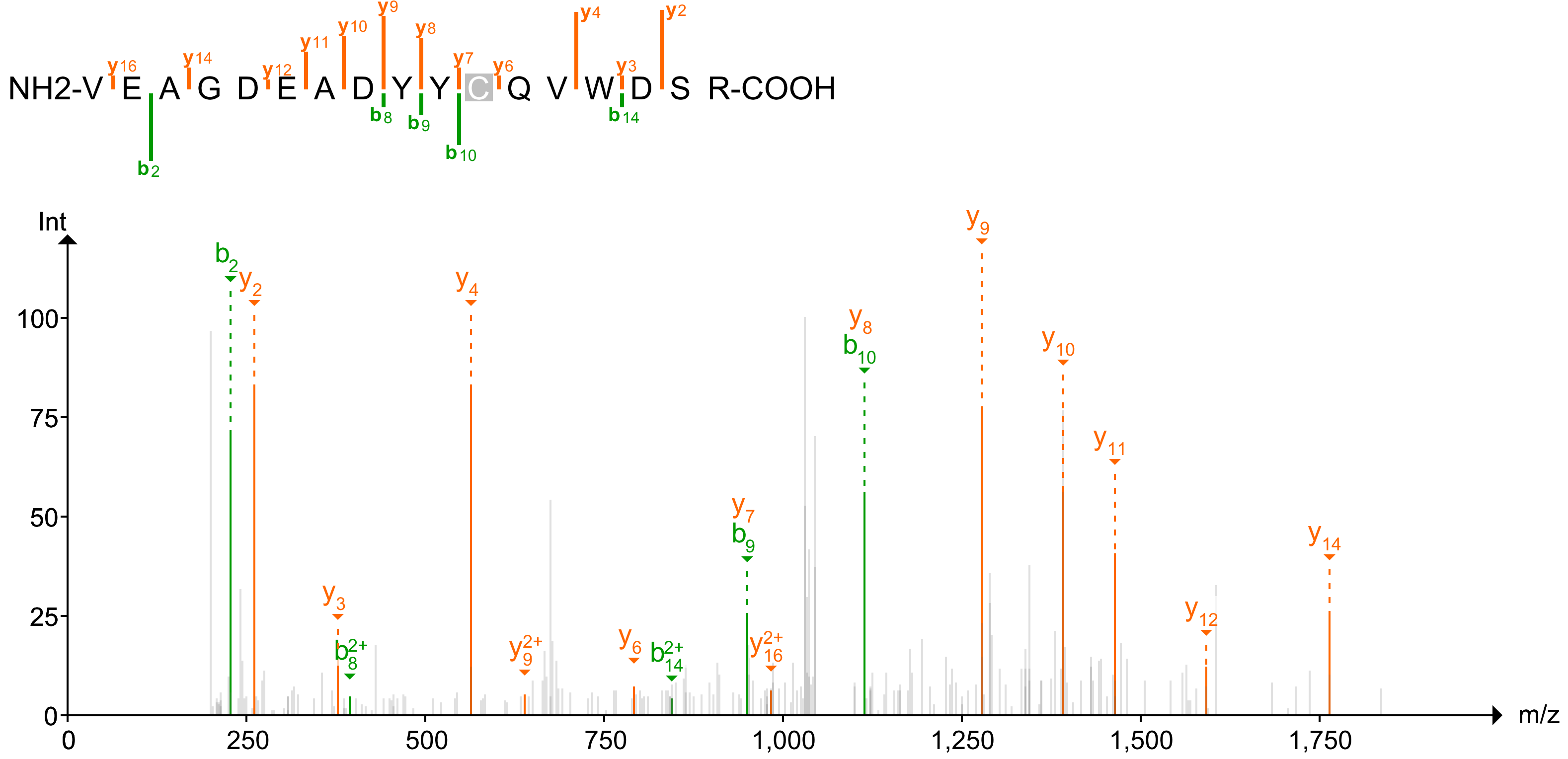

NH2-V E A G D E A D Y Y C Q V W D S R-COOH
y16 y14 y12 y11 y10 y9 y8 y7 y6 y4 y3 y2
b2 b8 b9 b10 b14
Int
m/z
0
25
50
75
100
250
500
750
1,000
1,250
1,500
1,750
b2 y2 y3 b8 2+ y4 y9 2+ y6 b14 2+ y7 b9 y16 2+ y8 b10 y9 y10 y11 y12 y14

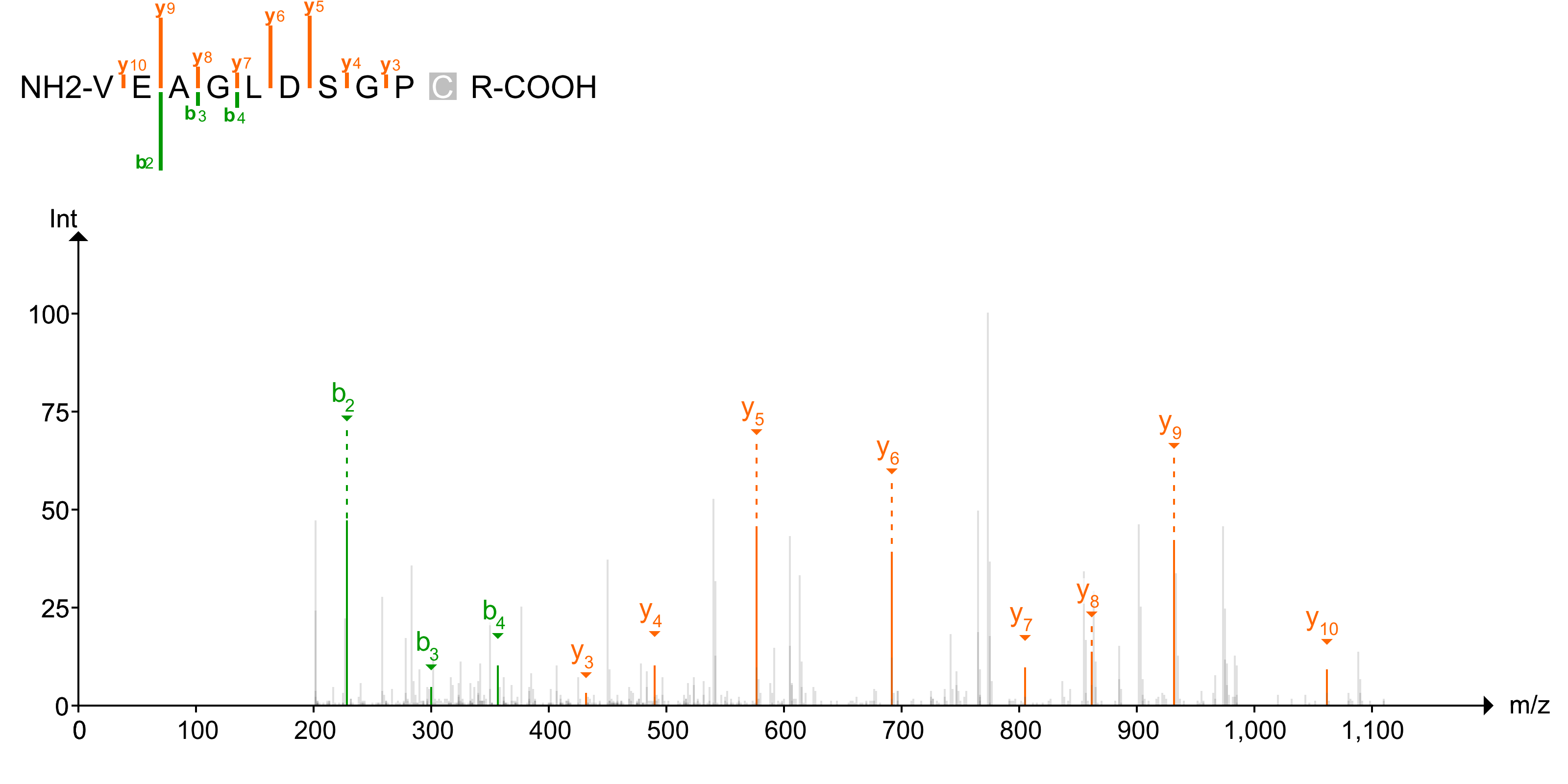

NH2-V E A G L D S G P C R-COOH
y9
y10
y8
y7
y6
y5
y4
y3
b2
b3
b4
Int
100
75
50
25
0
m/z
0
100
200
300
400
500
600
700
800
900
1,000
1,100

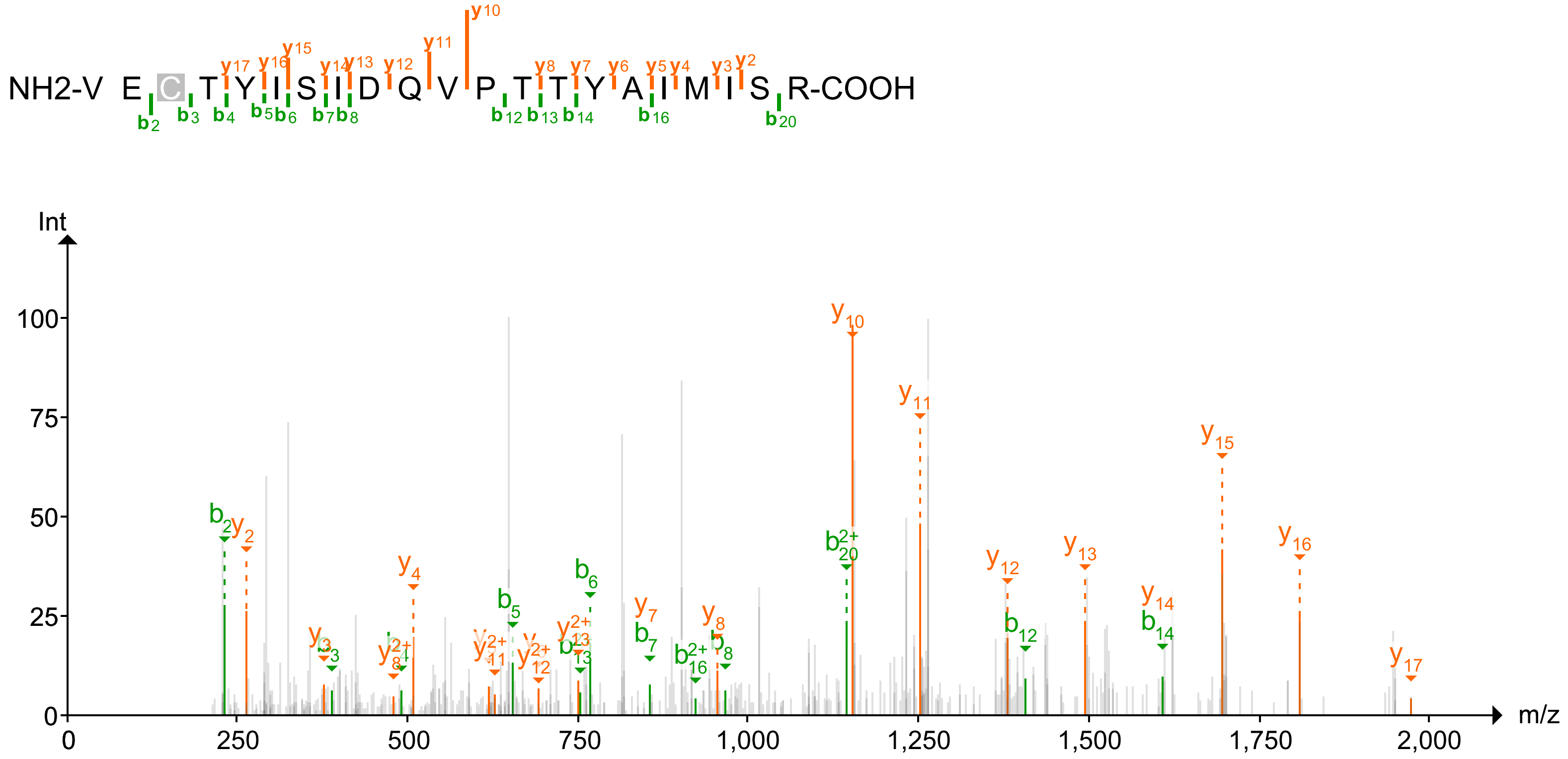

NH2-V E C T Y I I S I I D Q V P T T Y A I M I S R-COOH
Int
m/z
100
75
50
25
0
250
500
750
1,000
1,250
1,500
1,750
2,000

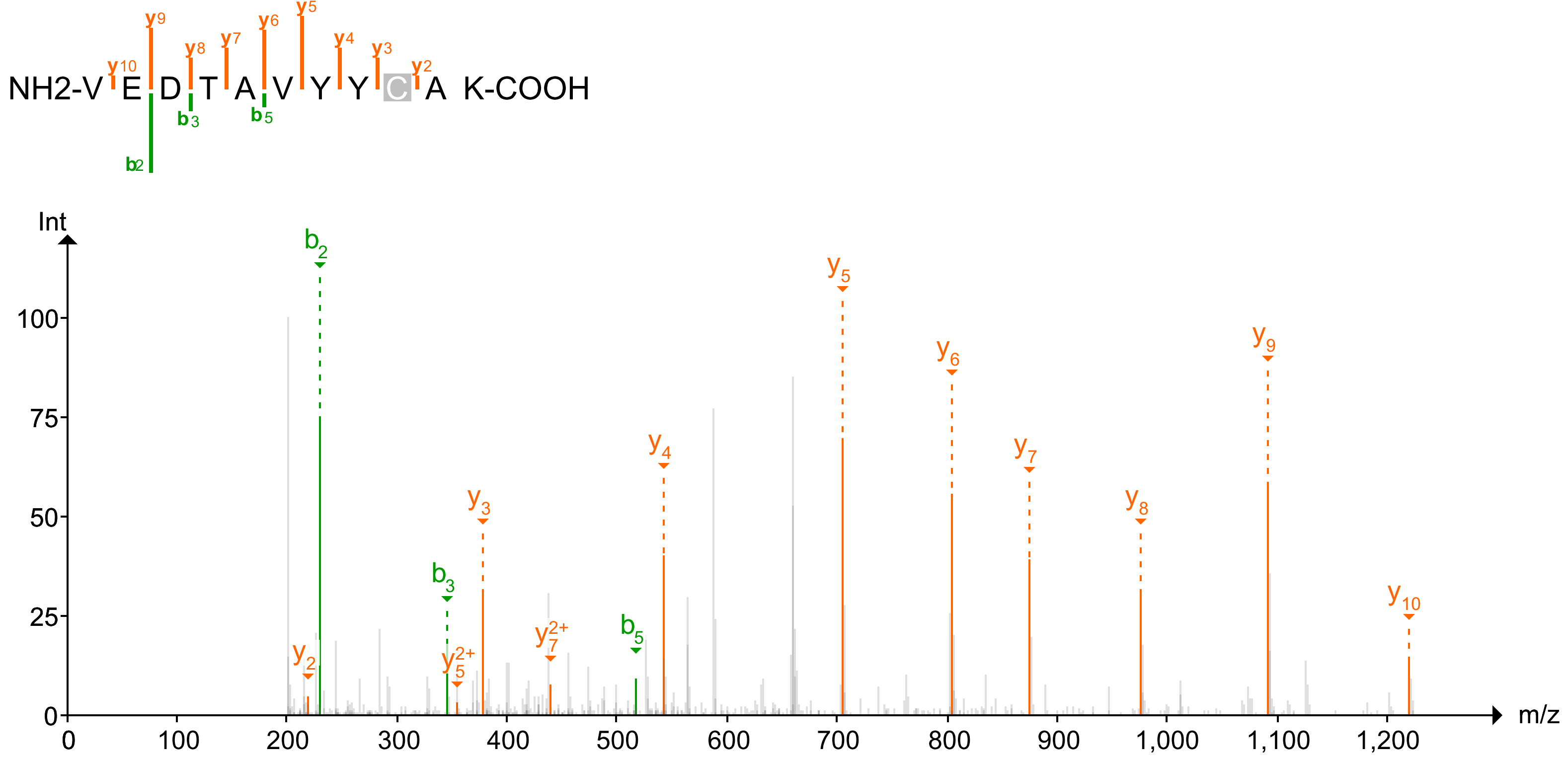
NH2-V E D T A V Y Y C A K-COOH
y10 y9 y8 y7 y6 y5 y4 y3 y2
b2 b3 b5
Int
100
75
50
25
0
m/z
0 100 200 300 400 500 600 700 800 900 1,000 1,100 1,200
b2
b3
b5
y2
y3
y4
y5
y6
y7
y8
y9
y10
y5 2+
y7 2+

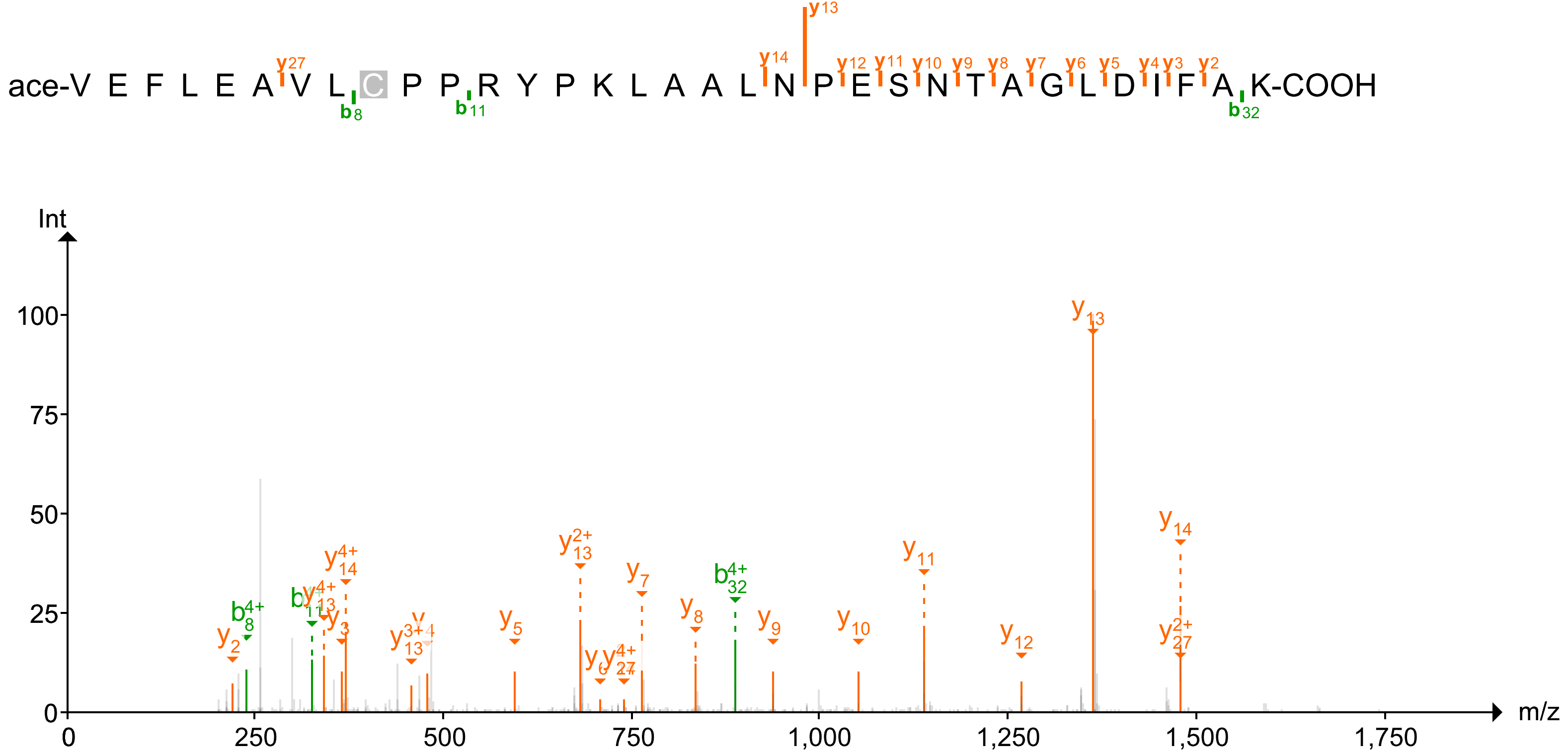

ace-V E F L E A V L C P P R Y P K L A A L N P E S N T A G L D I F A K-COOH
y27 y14 y13 y12 y11 y10 y9 y8 y7 y6 y5 y4 y3 y2
b8 b11 b32
Int
m/z
0 25 50 75 100
0 250 500 750 1,000 1,250 1,500 1,750

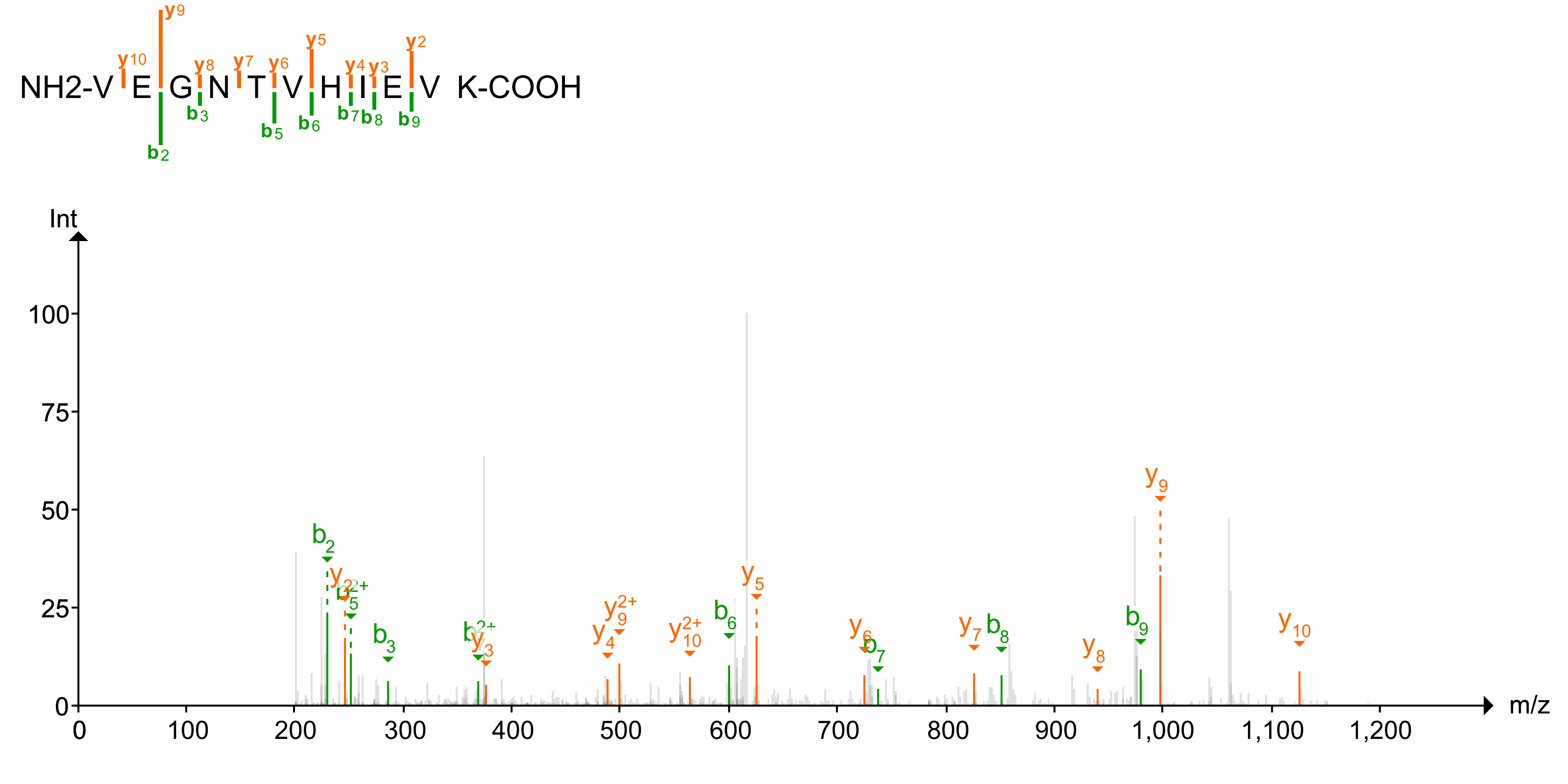

NH2-V E G N T V H I E V K-COOH
Int
m/z

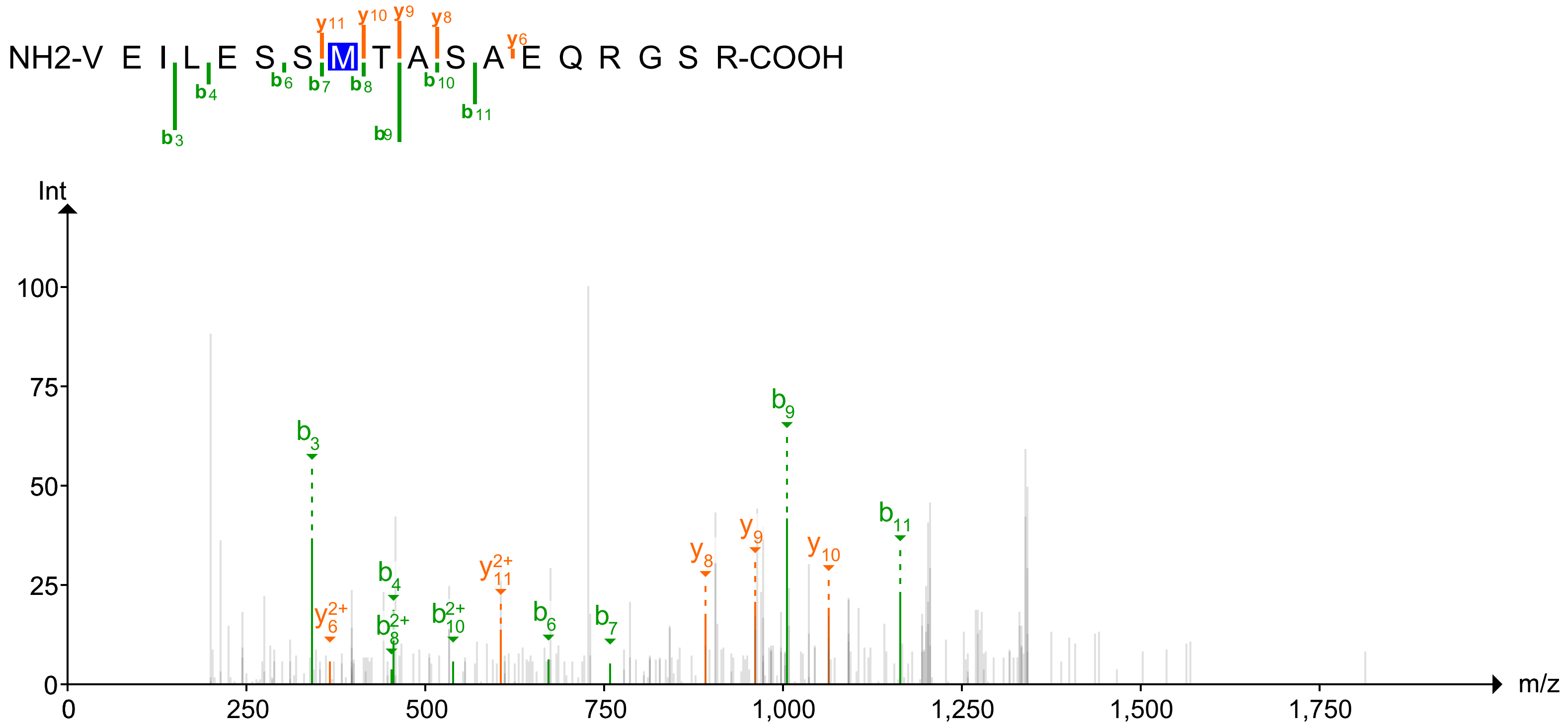

NH2-V E I L E S S M T A S A E Q R G S R-COOH
Int
m/z
100
75
50
25
0
250
500
750
1,000
1,250
1,500
1,750

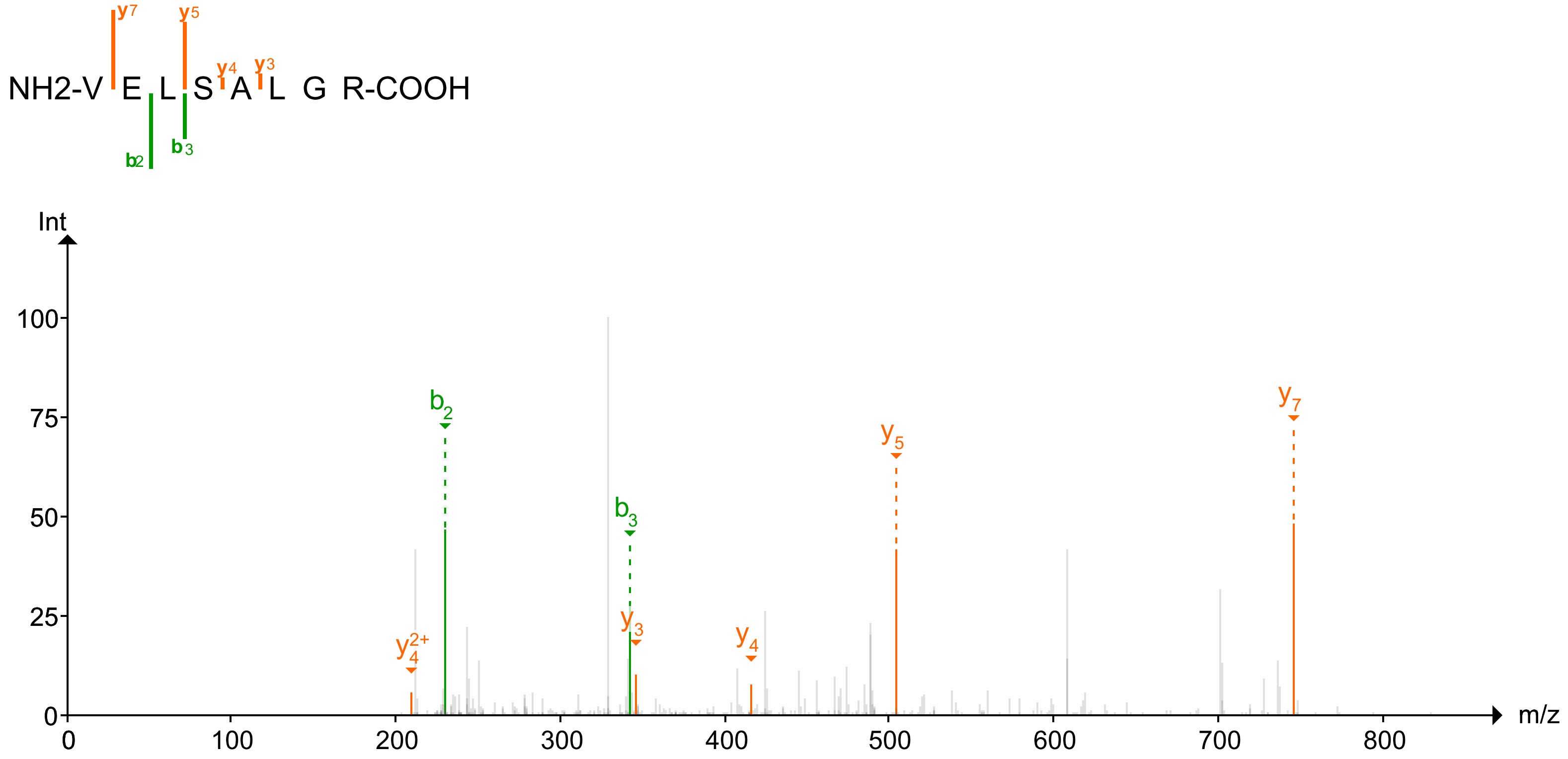

NH2-V E L S A L G R-COOH
y7
y5
y4
y3
b2
b3
Int
100
75
50
25
0
b2
b3
y7
y5
y3
y4
y4 2+
m/z
0
100
200
300
400
500
600
700
800

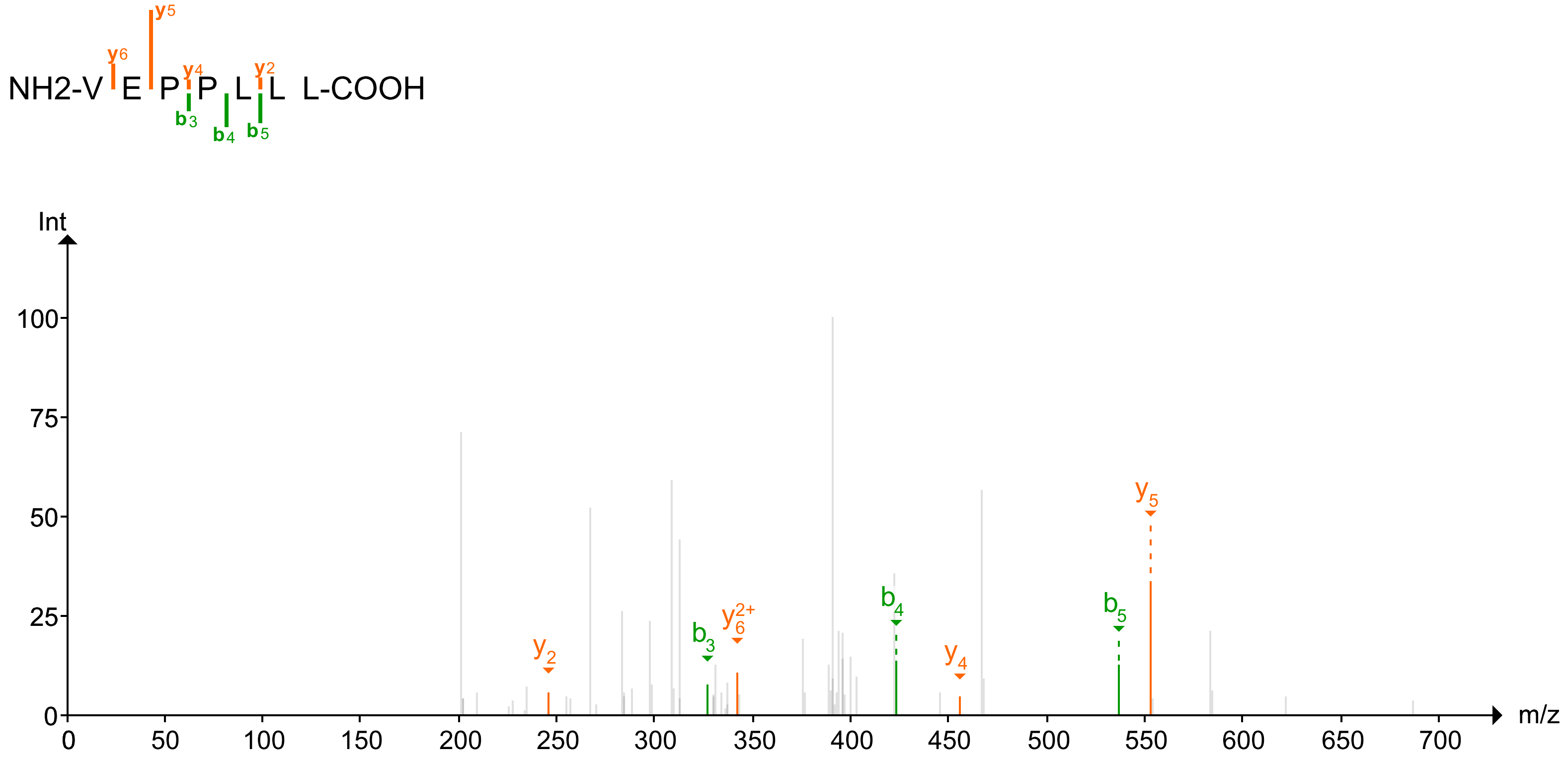

NH2-V E P P L L L-COOH
y6
y5
y4
y2
b3
b4
b5
Int
100
75
50
25
0
m/z
0
50
100
150
200
250
300
350
400
450
500
550
600
650
700
y2
b3
y6 2+
b4
y4
b5
y5

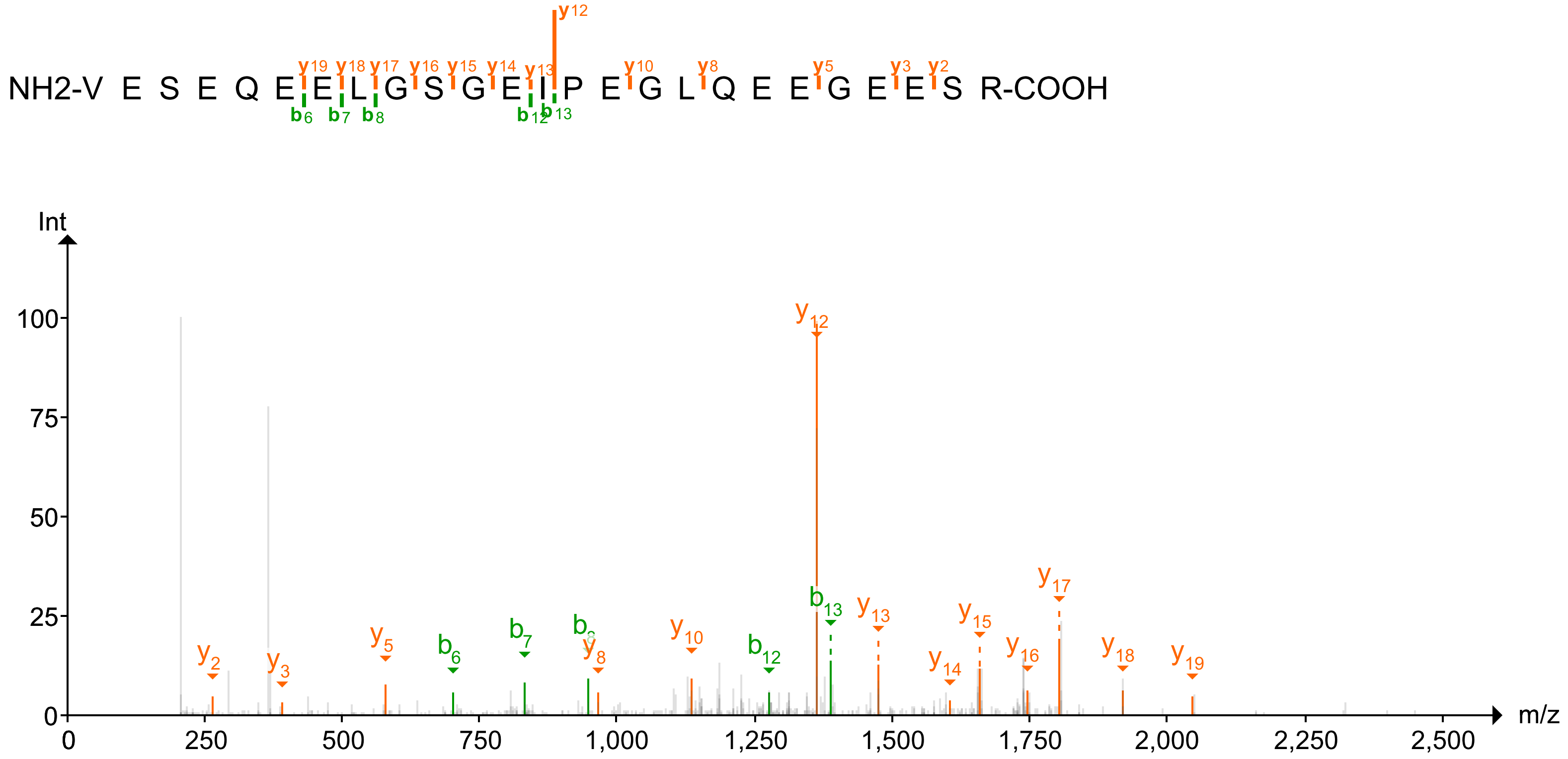

NH2-V E S E Q E E L G S G E I P E G L Q E E G E E S R-COOH
y12 y19 y18 y17 y16 y15 y14 y13 y10 y8 y5 y3 y2
b6 b7 b8 b12 b13
Int
100
75
50
25
0
m/z
0 250 500 750 1,000 1,250 1,500 1,750 2,000 2,250 2,500
y2 y3 y5 b6 b7 b8 y8 y10 b12 y12 b13 y13 y14 y15 y16 y17 y18 y19

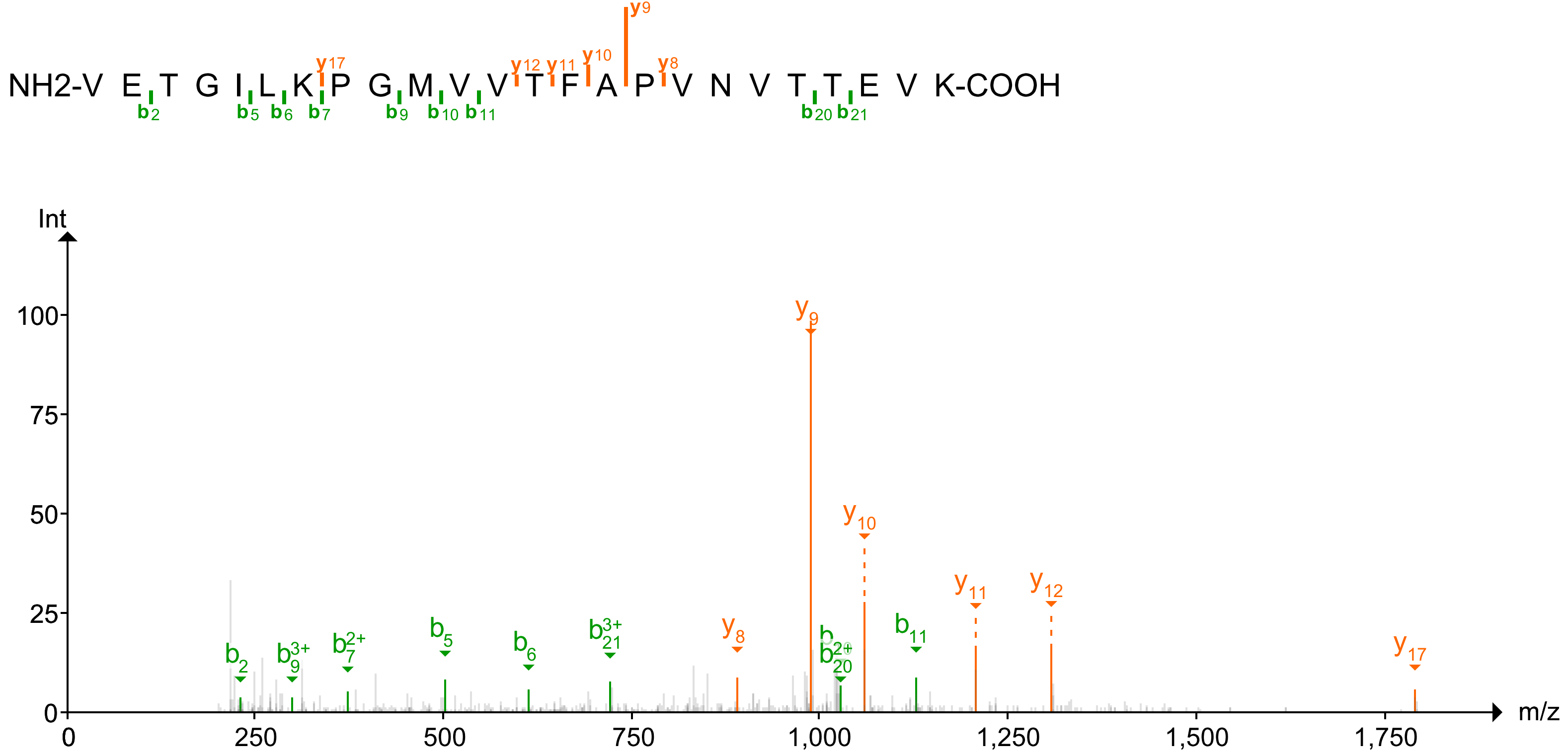

NH2-V E T G I L K P G M V V T F A P V N V T T E V K-COOH
b2 b5 b6 b7 b9 b10 b11 b20 b21
y17 y12 y11 y10 y9 y8
Int
100
75
50
25
0
m/z
0
250
500
750
1,000
1,250
1,500
1,750

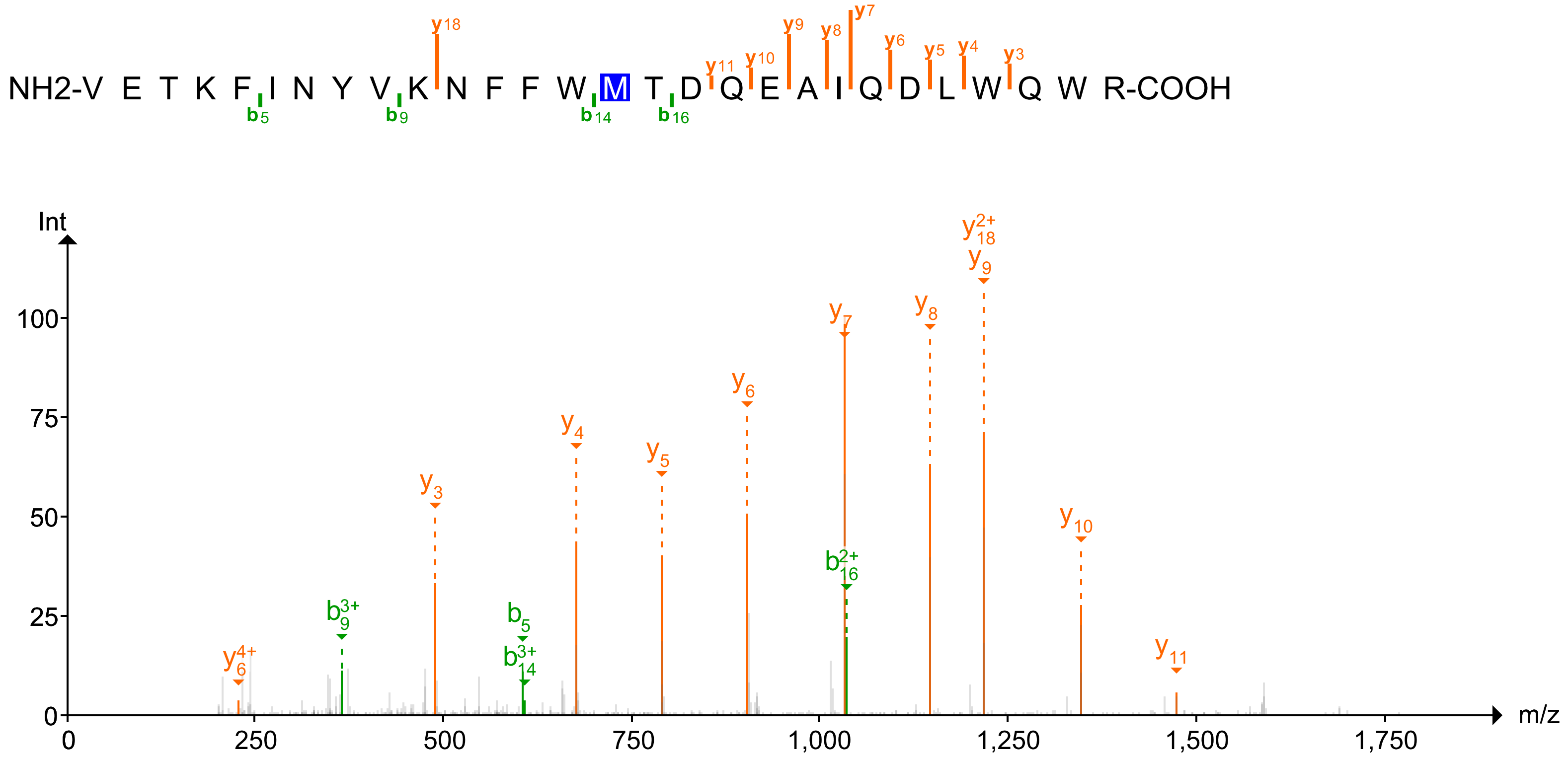
NH2-V E T K F I N Y V K N F F W M T D Q E A I Q D L W Q W R-COOH
b5 b9 b14 b16
y18 y11 y10 y9 y8 y7 y6 y5 y4 y3
Int
100
75
50
25
0
m/z
0 250 500 750 1,000 1,250 1,500 1,750

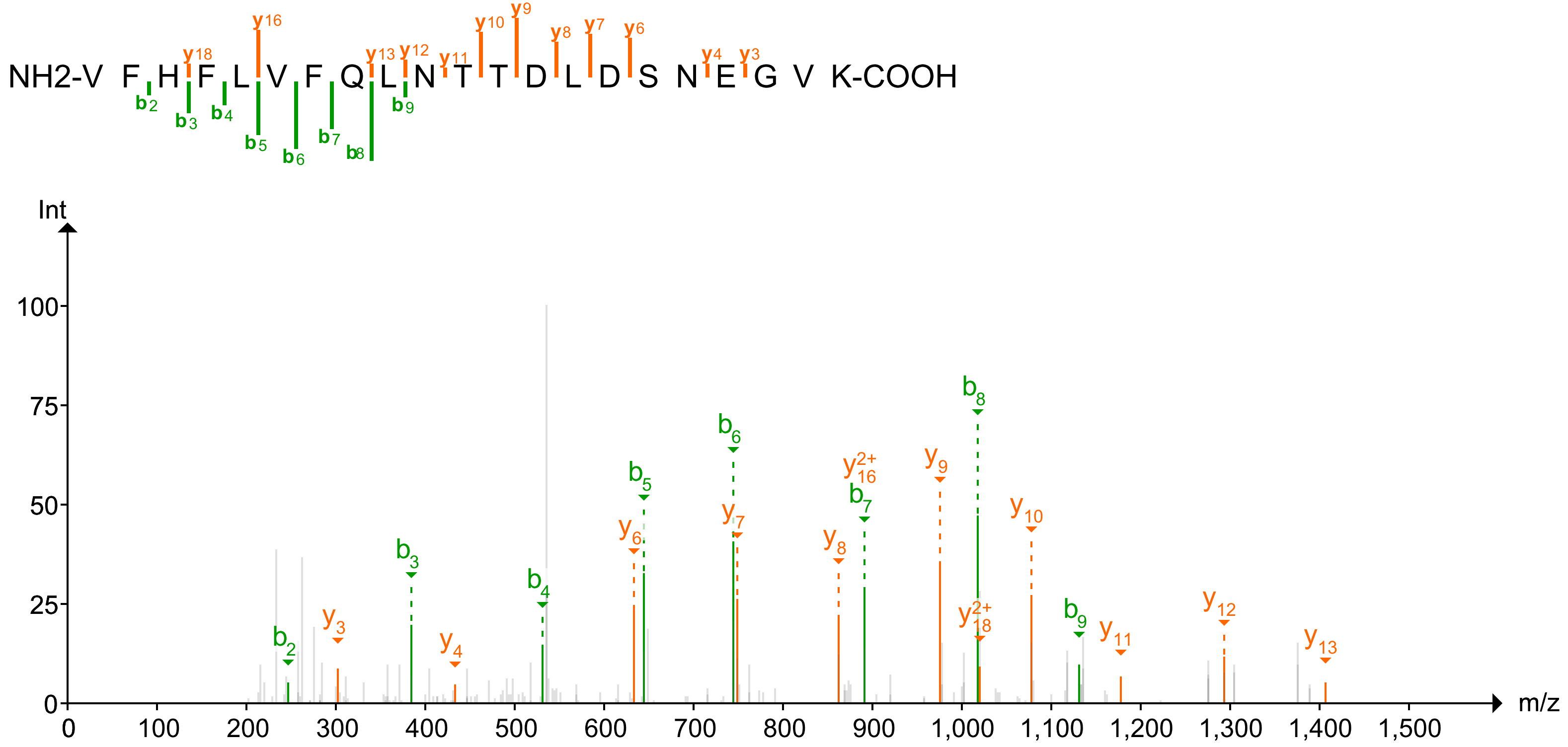
NH2-V F H F L V F Q L N T T D L D S N E G V K-COOH
y18 y16 y13 y12 y11 y10 y9 y8 y7 y6 y4 y3
b2 b3 b4 b5 b6 b7 b8 b9
Int
m/z
0 25 50 75 100
0 100 200 300 400 500 600 700 800 900 1,000 1,100 1,200 1,300 1,400 1,500

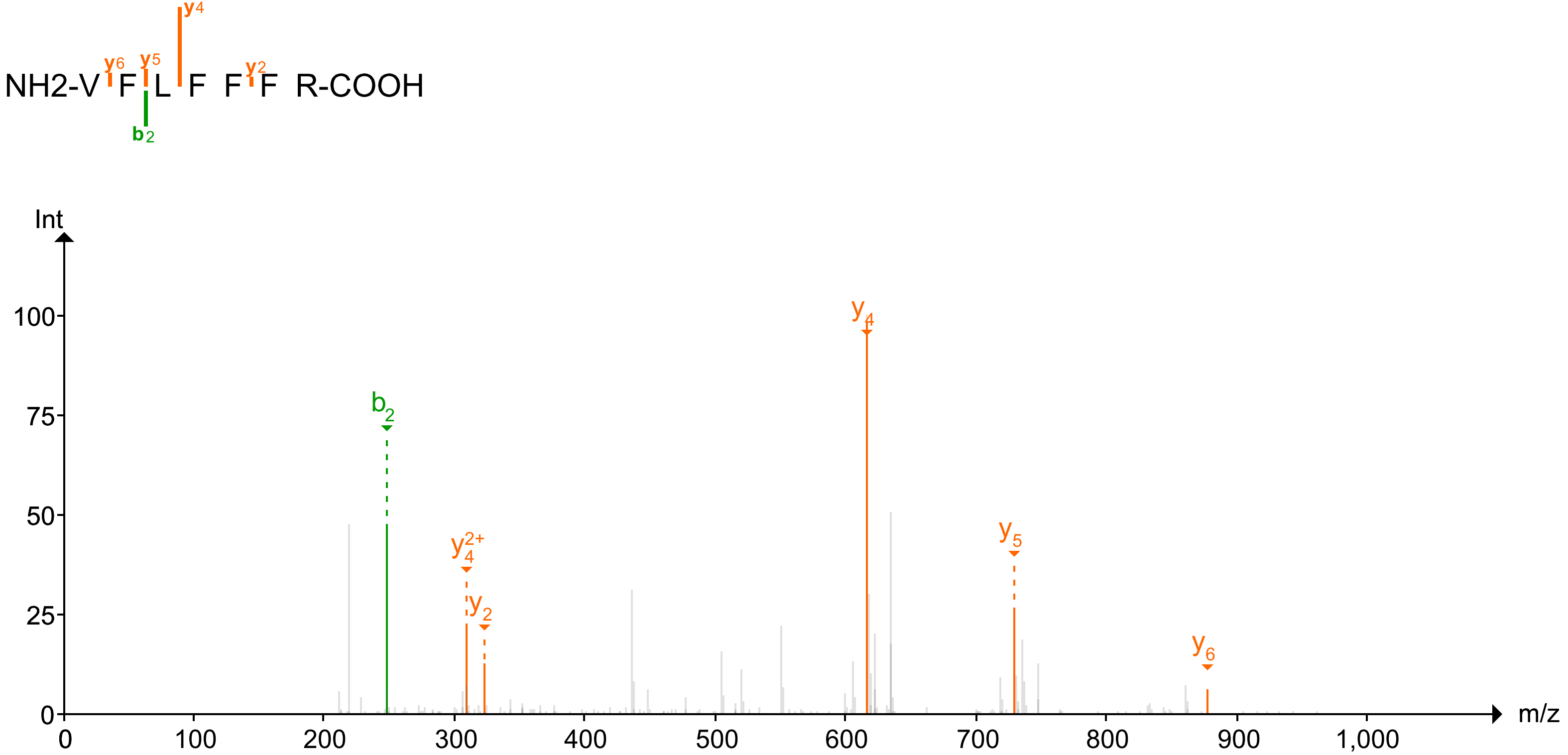

NH2-V F L F F F R-COOH
y4
y6
y5
y2
b2
Int
100
75
50
25
0
y4
b2
y4 2+
y2
y5
y6
0
100
200
300
400
500
600
700
800
900
1,000
m/z

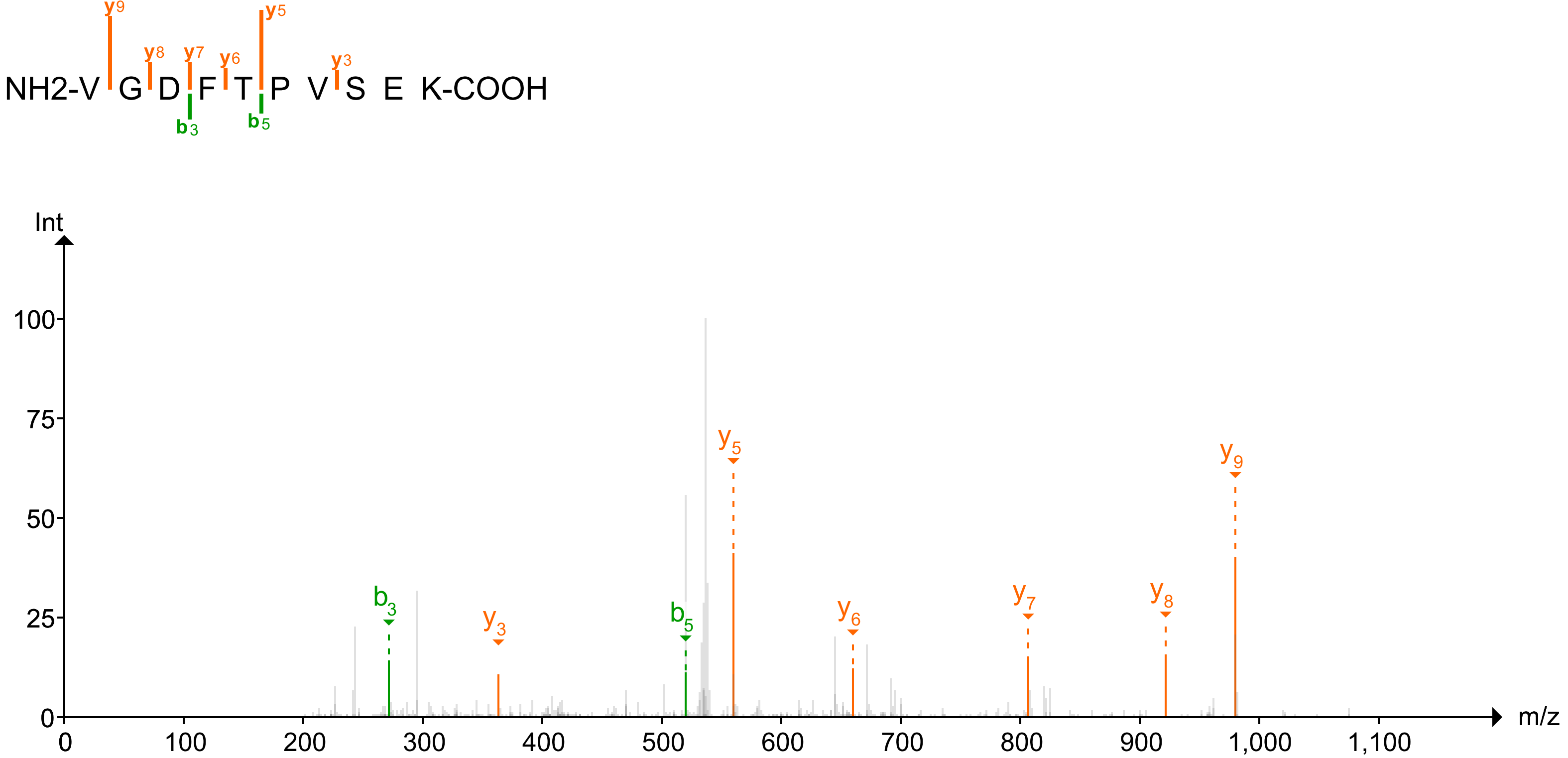

NH2-V G D F T P V S E K-COOH
y9 y8 y7 y6 y5 y3
b3 b5
Int
100
75
50
25
0
m/z
0 100 200 300 400 500 600 700 800 900 1,000 1,100

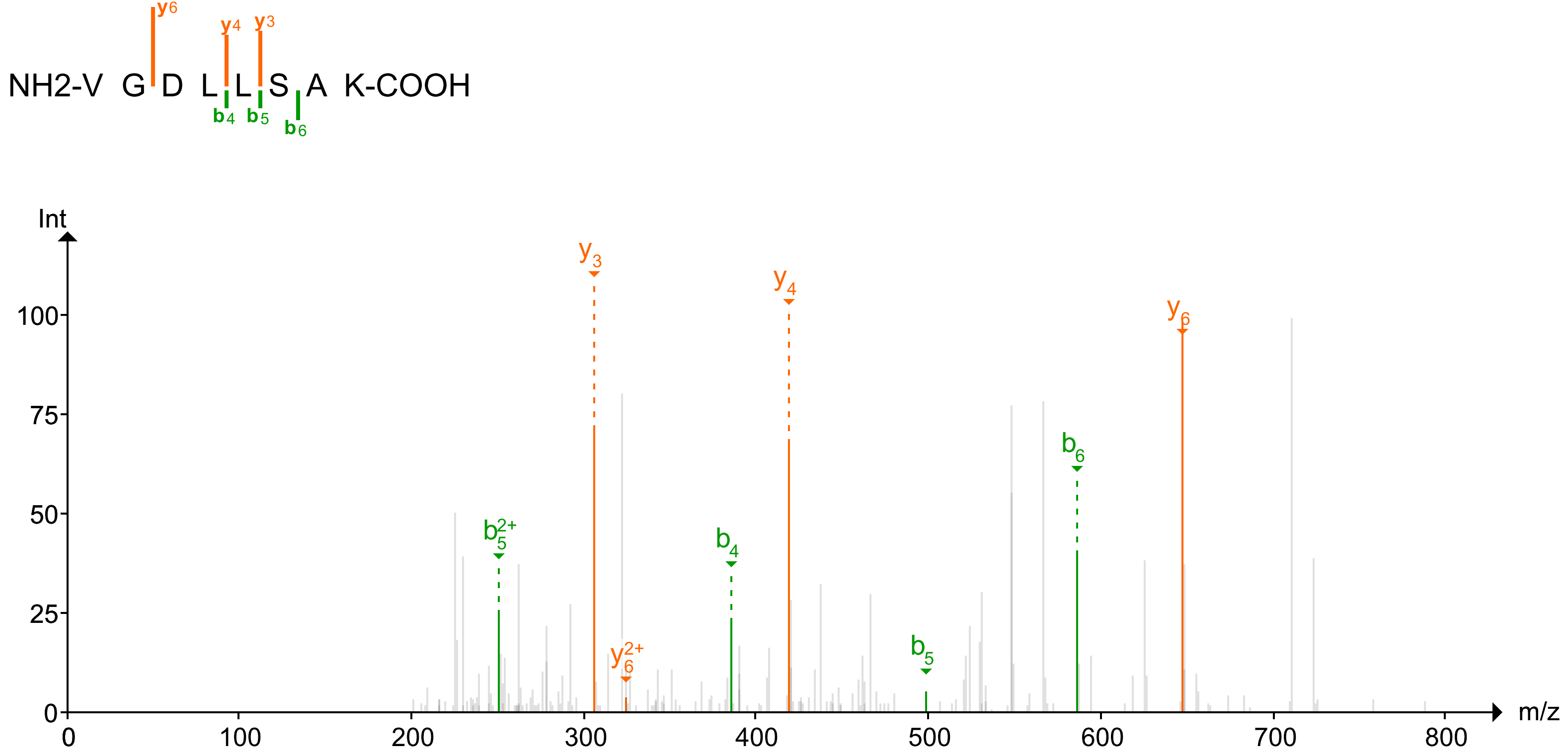

NH2-V G D L L S A K-COOH
y6
y4
y3
b4
b5
b6
Int
m/z
y3
y4
y6
b6
b5 2+
b4
y6 2+
b5
0
25
50
75
100
0
100
200
300
400
500
600
700
800

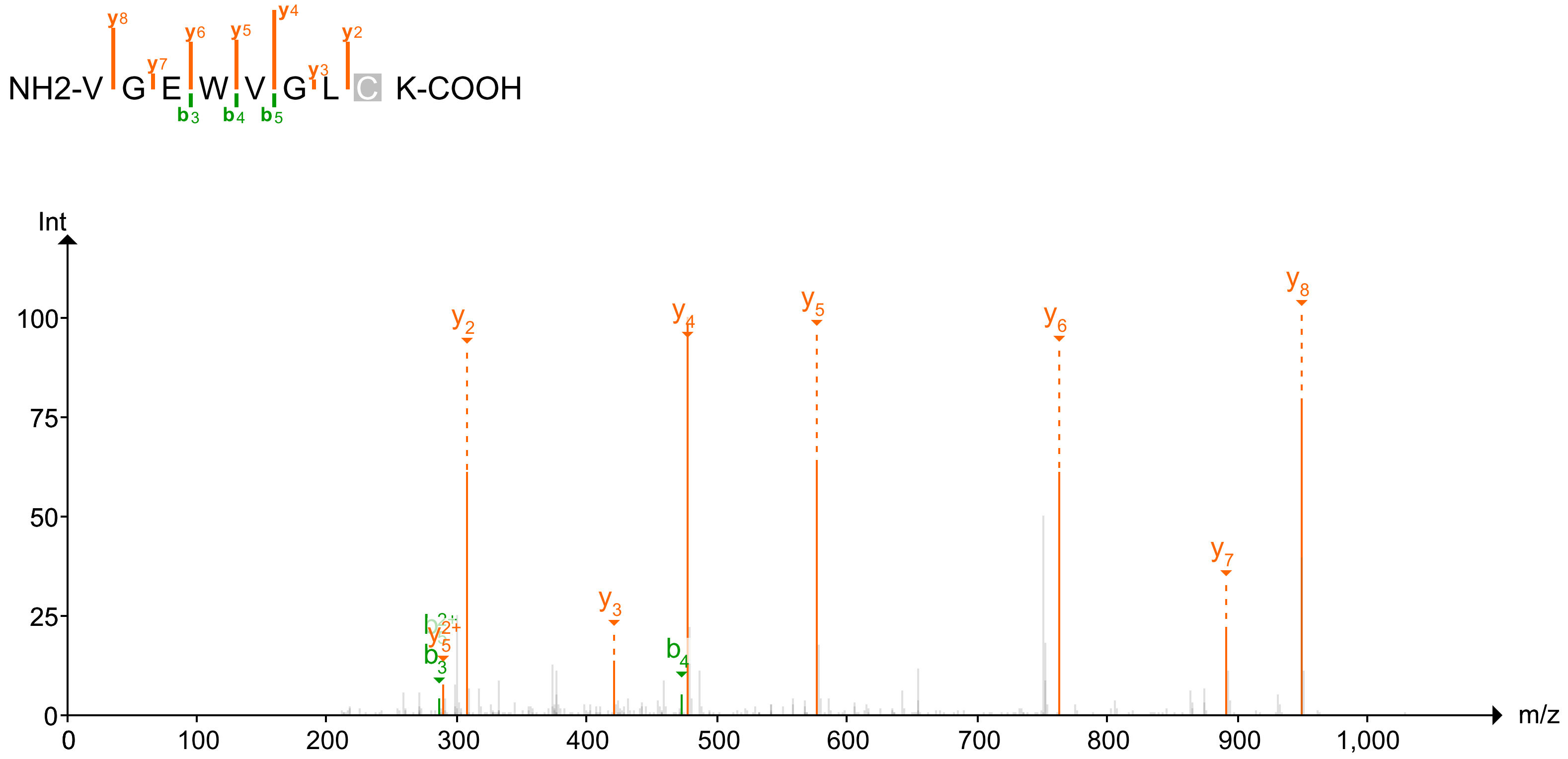
NH2-V G E W V G L C K-COOH
y8 y7 y6 y5 y4 y3 y2
b3 b4 b5
Int
100
75
50
25
0
m/z
0
100
200
300
400
500
600
700
800
900
1,000
y2
y3
y4
y5
y6
y7
y8
b3
b4

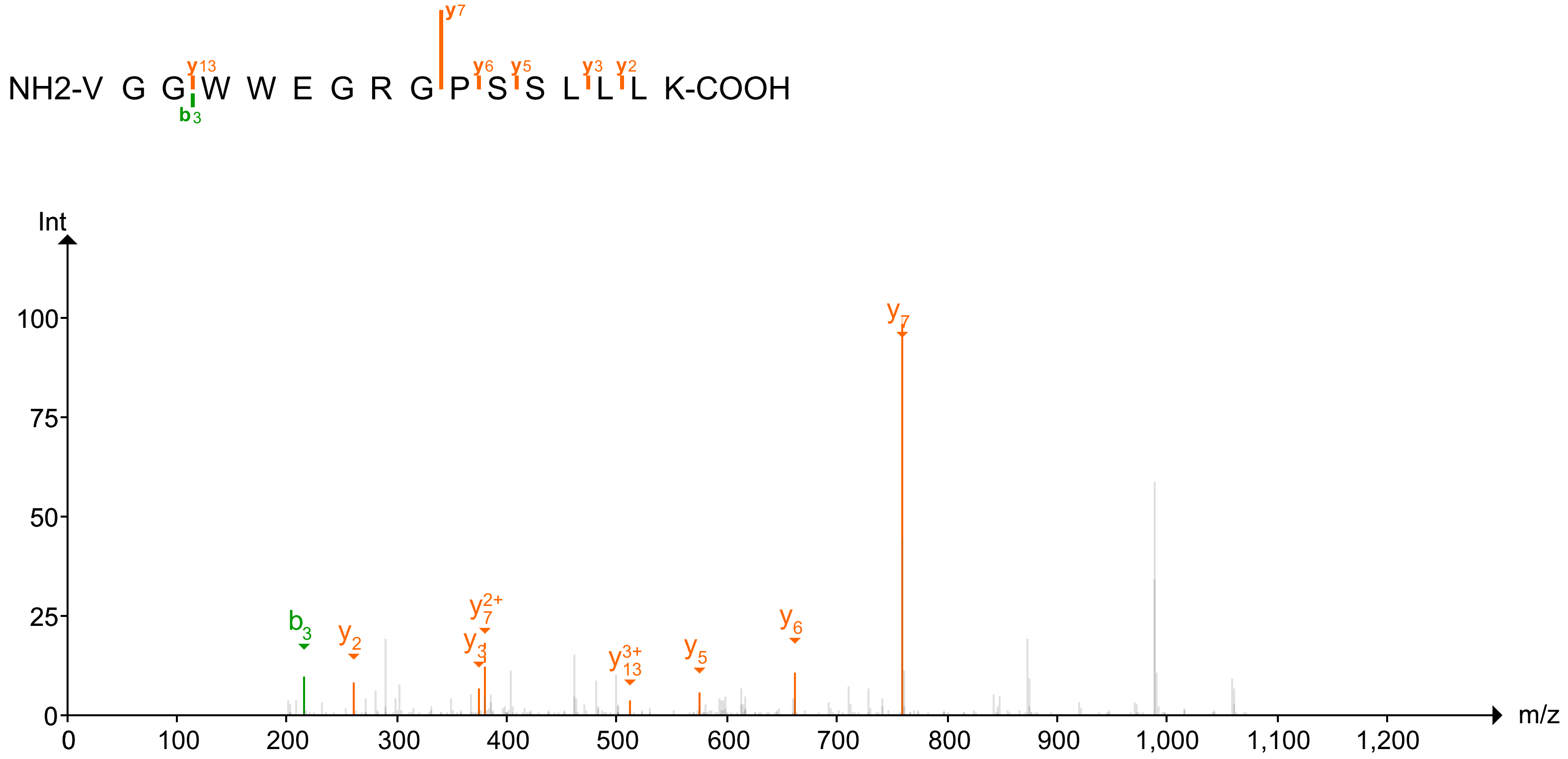

NH2-V G G W W E G R G P S S L L L K-COOH
y7
y13
y6
y5
y3
y2
b3
Int
100
75
50
25
0
m/z
0
100
200
300
400
500
600
700
800
900
1,000
1,100
1,200
b3
y2
y3
y7 2+
y13 3+
y5
y6
y7

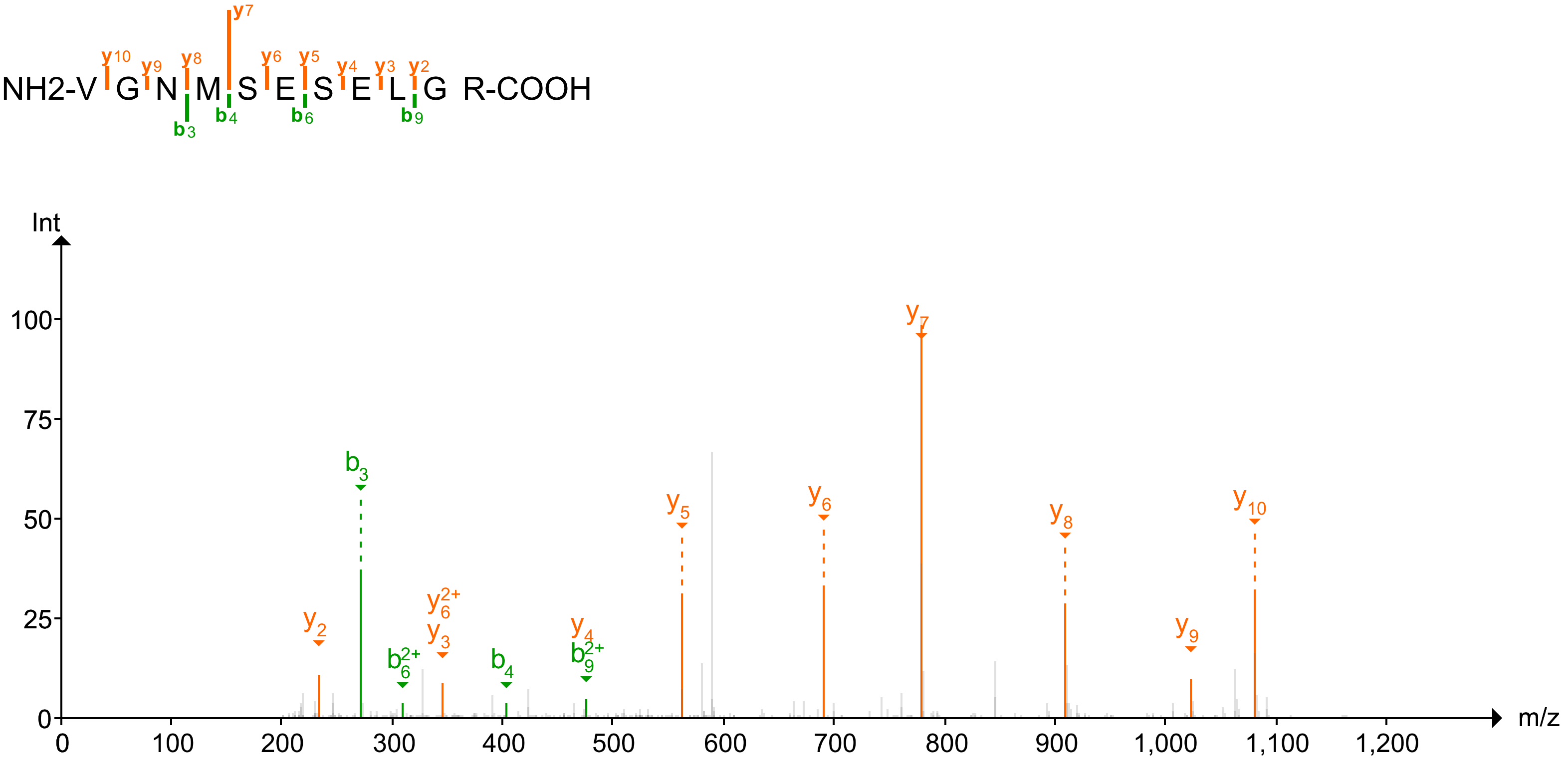
NH2-V G N M S E S E L G R-COOH
y10 y9 y8 y7 y6 y5 y4 y3 y2
b3 b4 b6 b9
Int
100
75
50
25
0
b3
y2
y6 2+
y3
b6 2+
b4
y4
b9 2+
y5
y6
y7
y8
y9
y10
0
100
200
300
400
500
600
700
800
900
1,000
1,100
1,200
m/z

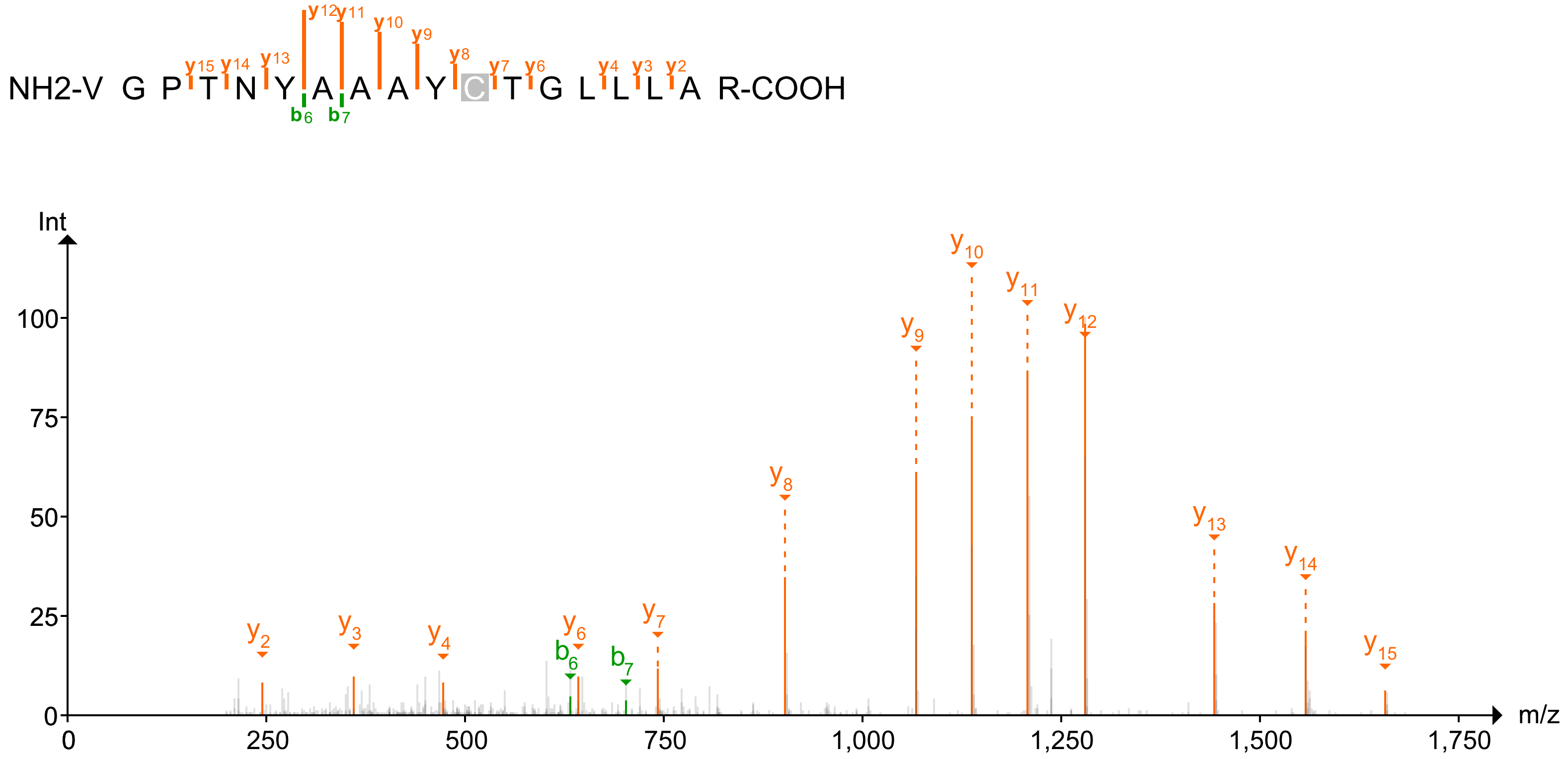

NH2-V G P T N Y A A A Y C T G L L L A R-COOH
y15 y14 y13 y12 y11 y10 y9 y8 y7 y6 y4 y3 y2
b6 b7
Int
100
75
50
25
0
0 250 500 750 1,000 1,250 1,500 1,750
m/z
y2 y3 y4 b6 y6 b7 y7 y8 y9 y10 y11 y12 y13 y14 y15

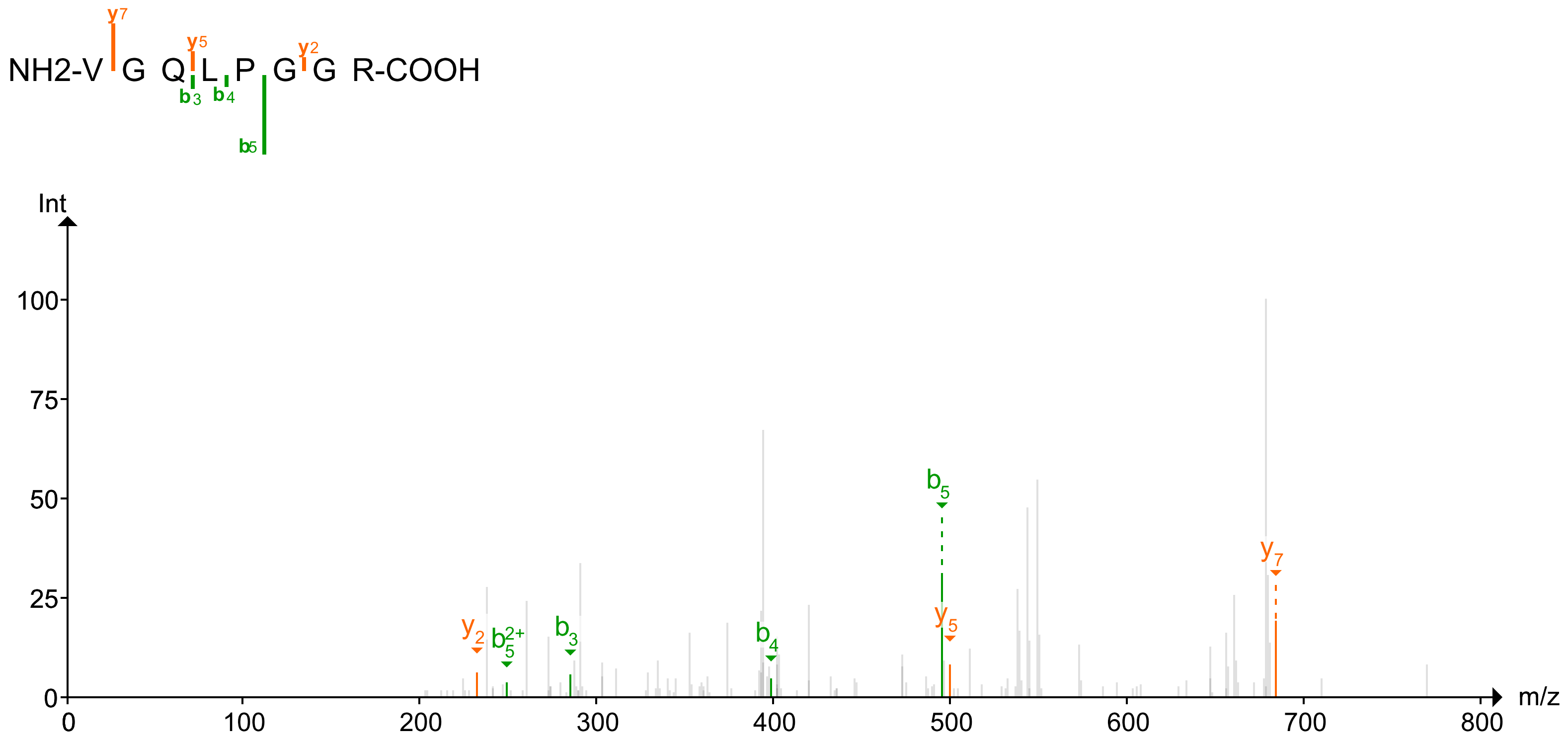

NH2-V G Q L P G G R-COOH
y7
y5
y2
b3
b4
b5
Int
100
75
50
25
0
m/z
0
100
200
300
400
500
600
700
800
y2
b5 2+
b3
b4
b5
y5
y7

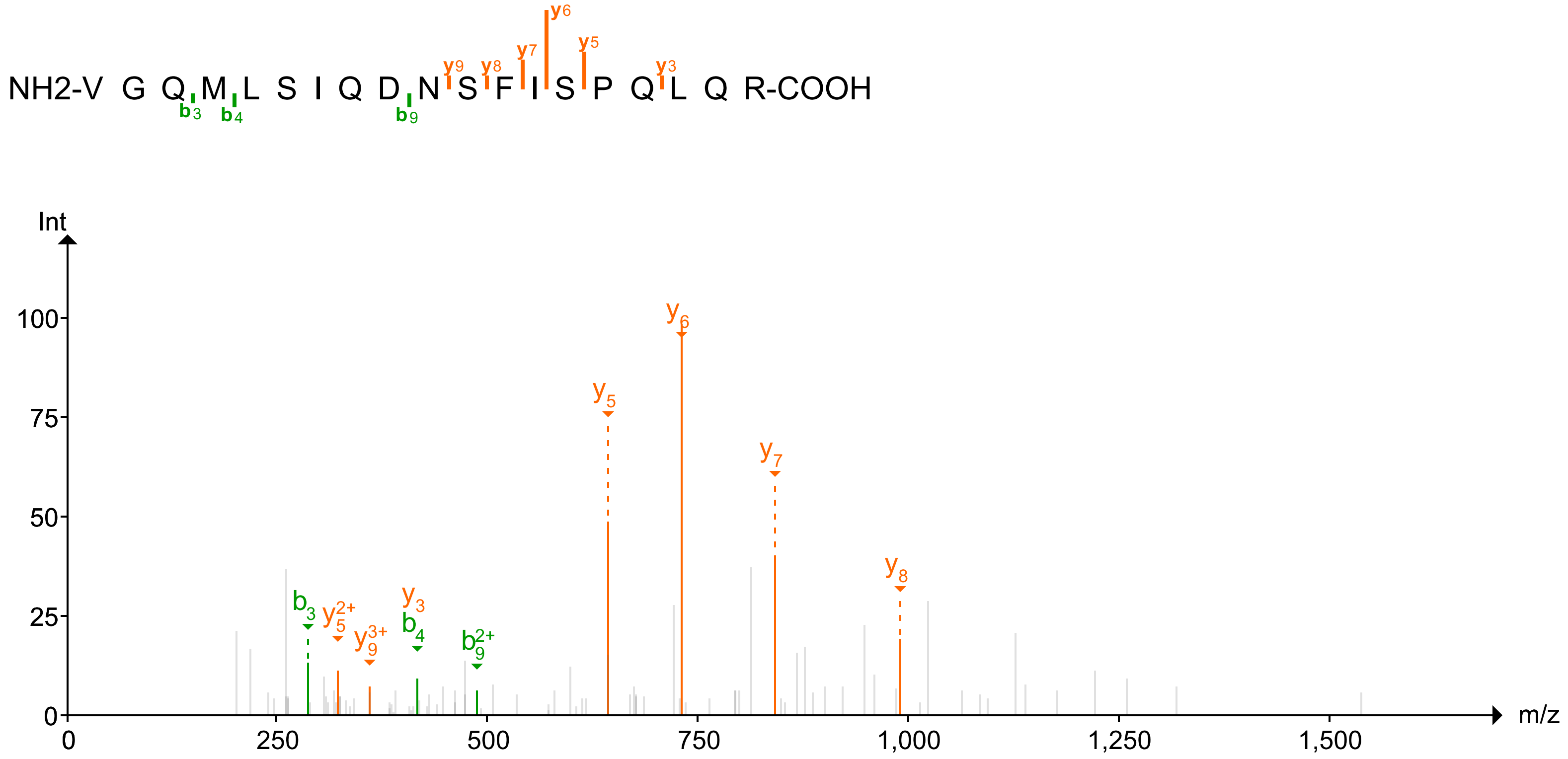

NH2-V G Q M L S I Q D N S F I S P Q L Q R-COOH
b3 b4 b9
y9 y8 y7 y6 y5 y3
Int
m/z
0
25
50
75
100
250
500
750
1,000
1,250
1,500

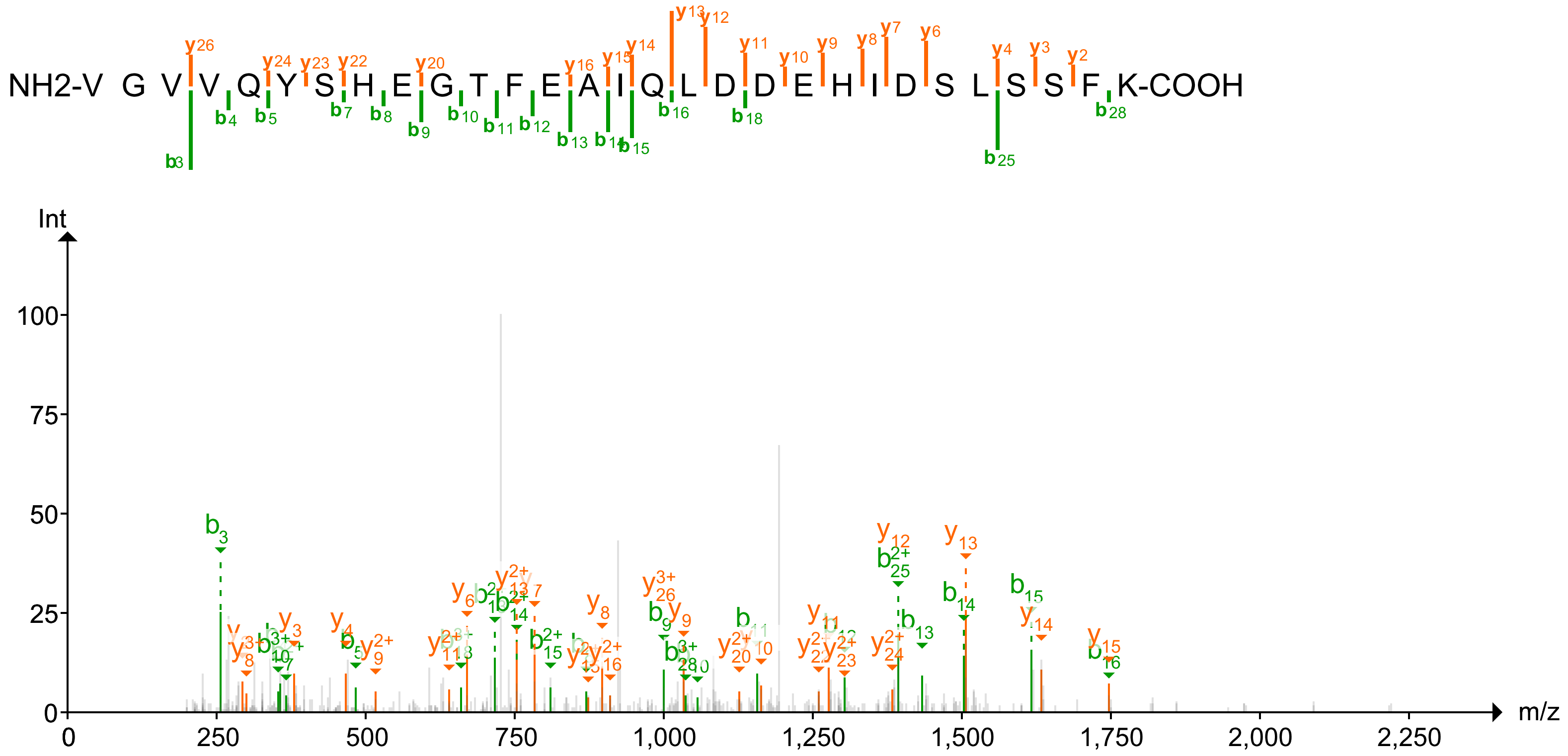
NH2-V G V V Q Y S H E G T F E A I Q L D D E H I D S L S S F K-COOH
Int
100
75
50
25
0
m/z
0
250
500
750
1,000
1,250
1,500
1,750
2,000
2,250

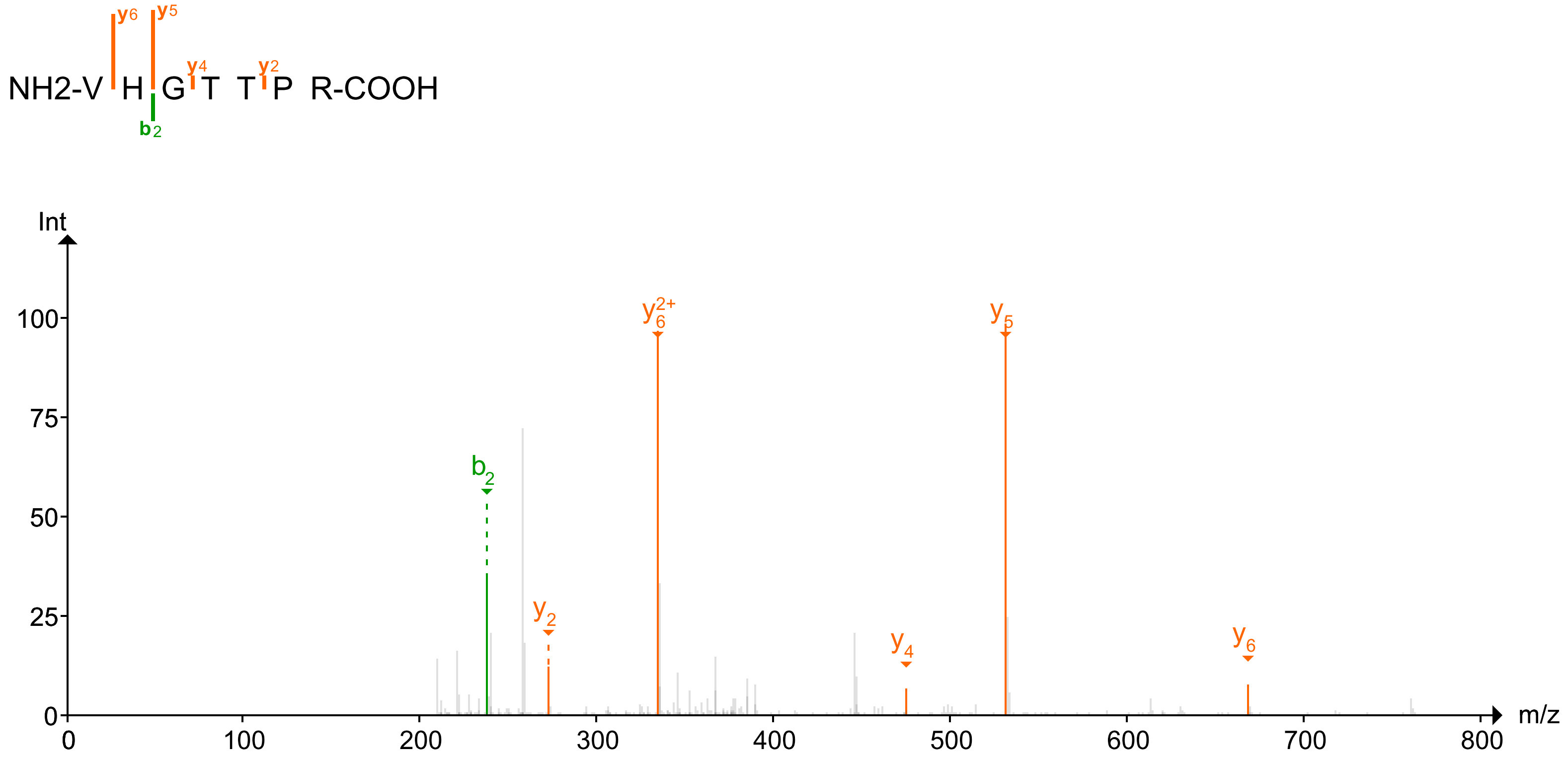
NH2-V H G T T P R-COOH
y6
y5
y4
y2
b2
Int
100
75
50
25
0
b2
y2
y6 2+
y4
y5
y6
0
100
200
300
400
500
600
700
800
m/z

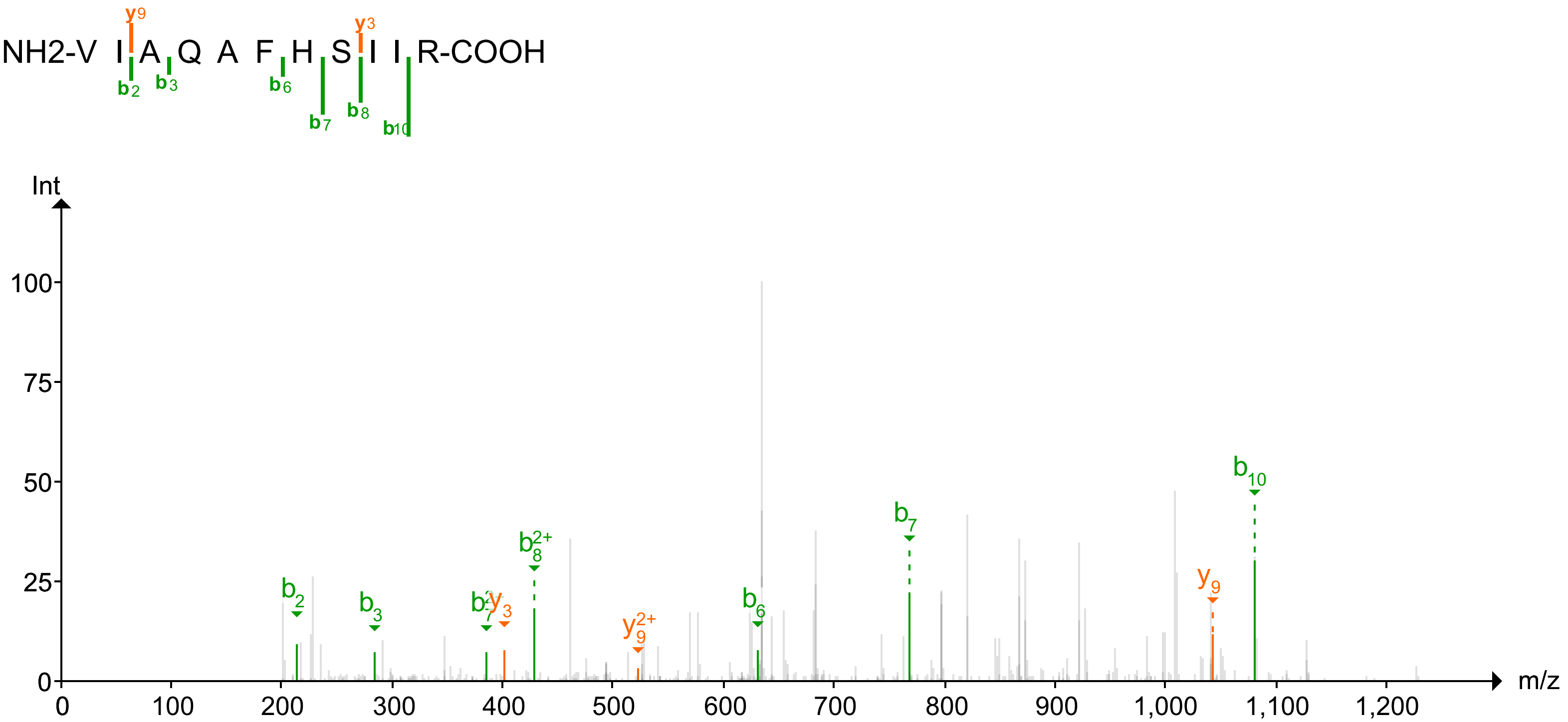
NH2-V I A Q A F H S I I R-COOH
y9
y3
b2
b3
b6
b7
b8
b10
Int
100
75
50
25
0
b2
b3
b7
y3
b8 2+
y9 2+
b6
b7
y9
b10
0
100
200
300
400
500
600
700
800
900
1,000
1,100
1,200
m/z

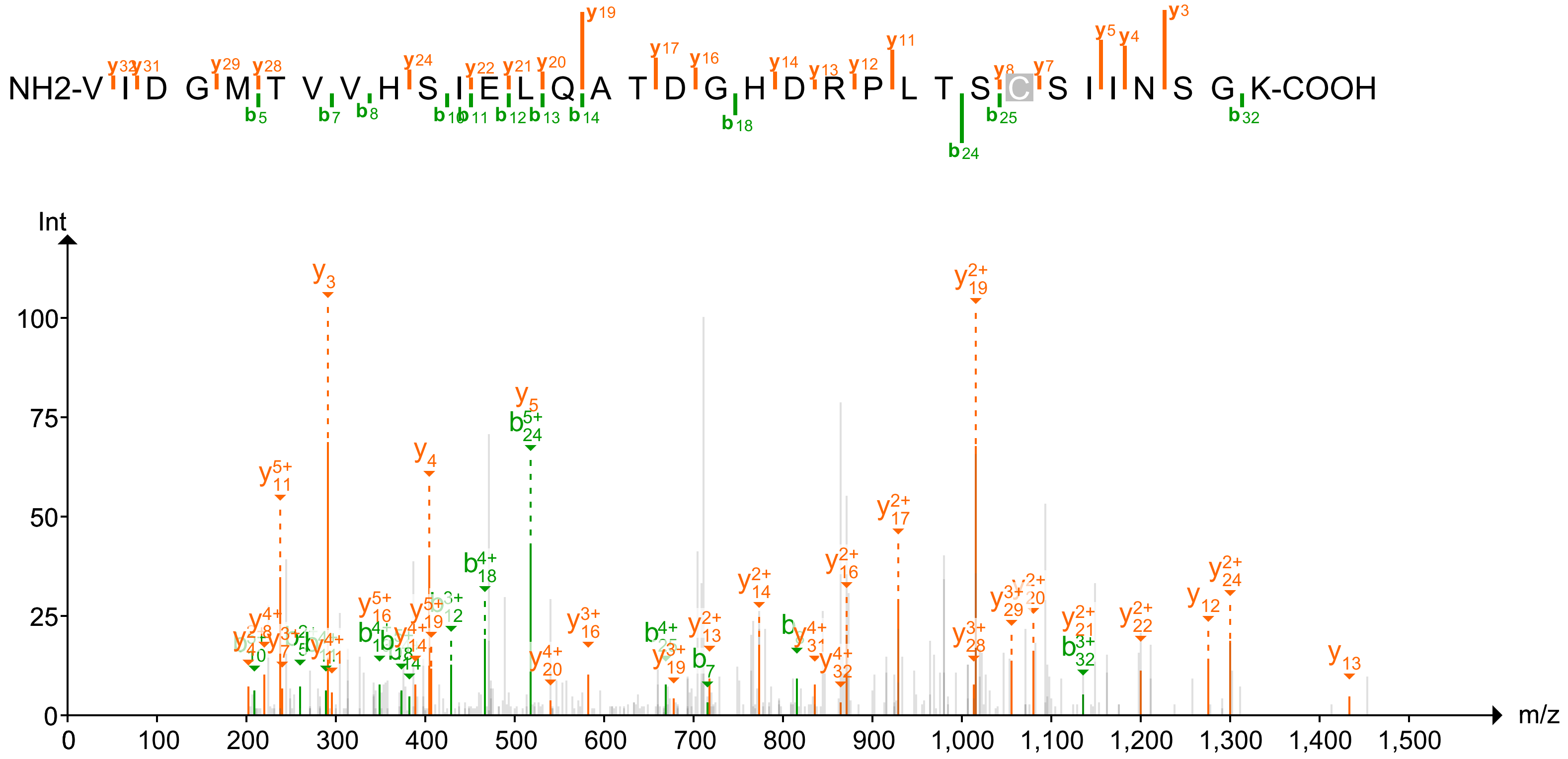

NH2-V I D G M T V V H S I E L Q A T D G H D R P L T S C S I I N S G K-COOH
y19
y3
y5 y4
y17 y16 y14 y13 y12 y11
y24 y22 y21 y20
y29 y28
y32 y31
y8 y7
b5 b7 b8 b10 b11 b12 b13 b14 b18 b24 b25 b32
Int
100
75
50
25
0
m/z
0
100
200
300
400
500
600
700
800
900
1,000
1,100
1,200
1,300
1,400
1,500
$y_3$
$y^{2+}_{19}$
$y_5$
$b^{5+}_{24}$
$y_4$
$y^{5+}_{11}$
$y^{2+}_{17}$
$b^{4+}_{18}$
$y^{2+}_{16}$
$y^{2+}_{14}$
$y^{2+}_{24}$
$y^{2+}_{20}$
$y^{3+}_{29}$
$y_{12}$
$y^{2+}_{22}$
$y^{2+}_{21}$
$b^{3+}_{32}$
$y^{3+}_{28}$
$y_{13}$
$y^{3+}_{16}$
$y^{4+}_{20}$
$y^{2+}_{13}$
$y^{3+}_{19}$
$b_7$
$y^{4+}_{31}$
$y^{4+}_{32}$
$y^{5+}_{16}$
$y^{5+}_{19}$
$y^{4+}_{14}$
$y^{4+}_8$
$y^{3+}_7$
$y^{4+}_{11}$

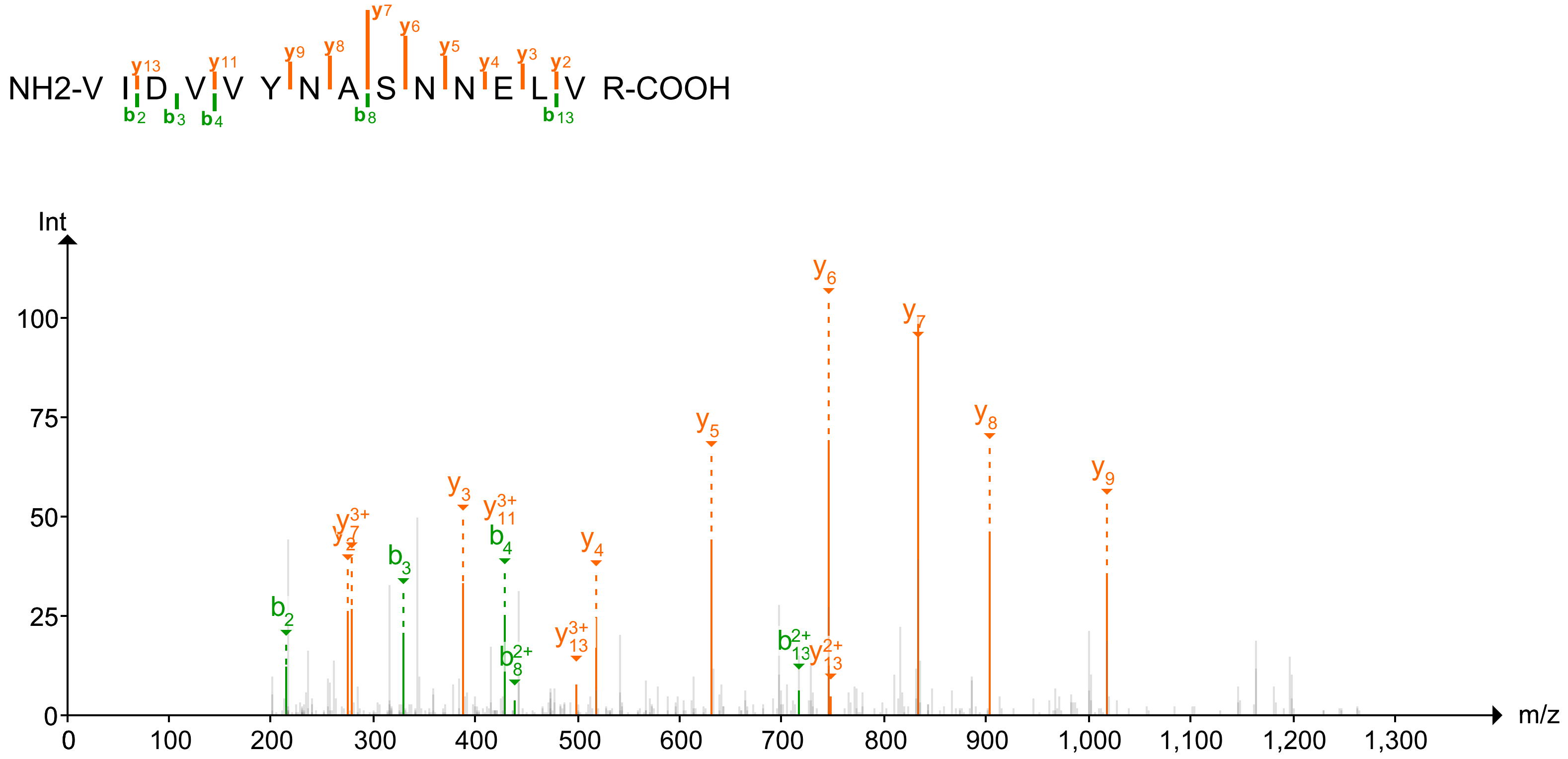
NH2-V I D V V Y N A S N N E L V R-COOH
y13 y11 y9 y8 y7 y6 y5 y4 y3 y2
b2 b3 b4 b8 b13
Int
m/z
0
25
50
75
100
0 100 200 300 400 500 600 700 800 900 1,000 1,100 1,200 1,300
b2 b3 b4 y3 y4 y5 y6 y7 y8 y9

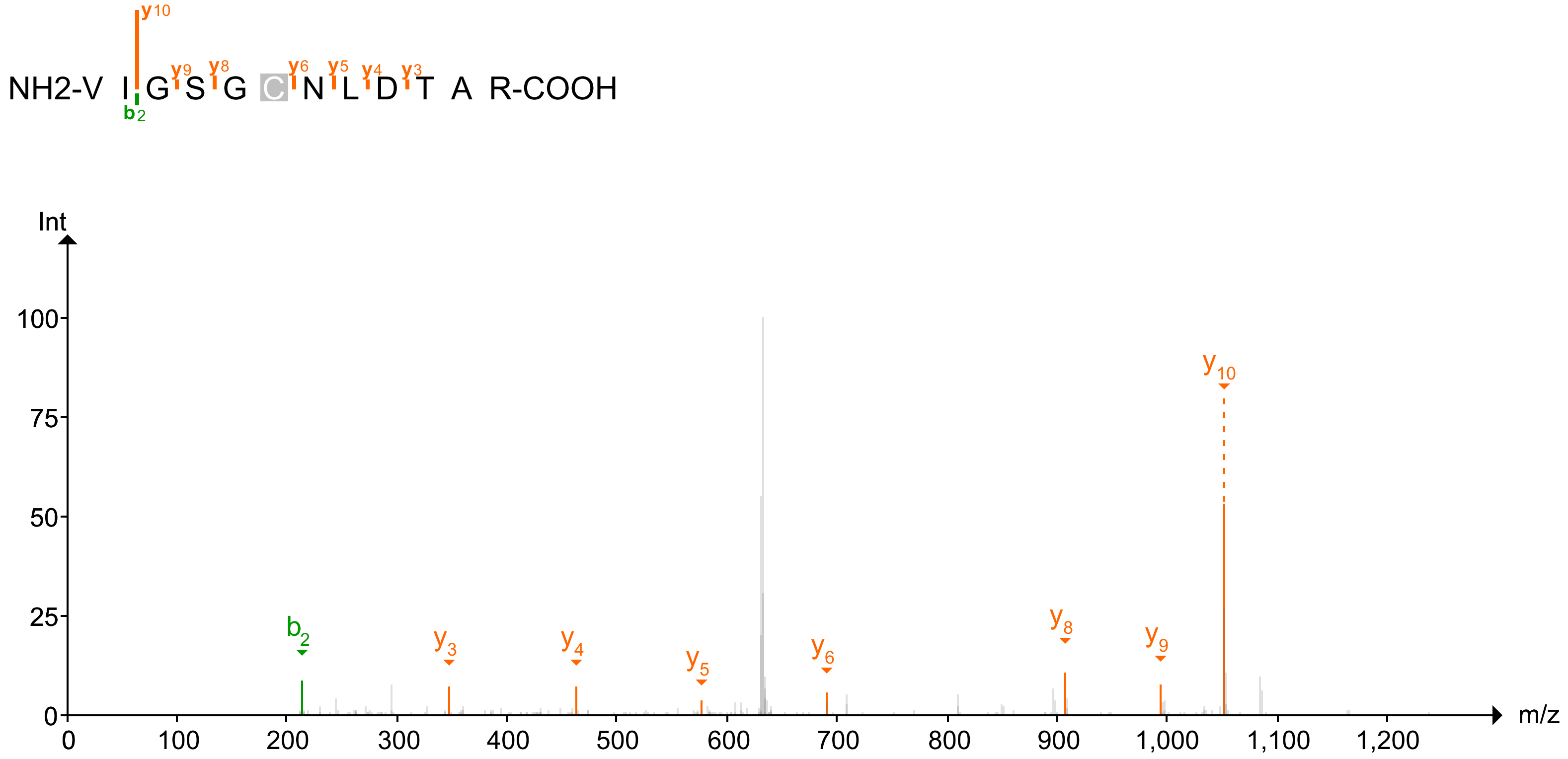
NH2-V I G S G C N L D T A R-COOH
y10
y9
y8
y6
y5
y4
y3
b2
Int
100
75
50
25
0
m/z
0
100
200
300
400
500
600
700
800
900
1,000
1,100
1,200

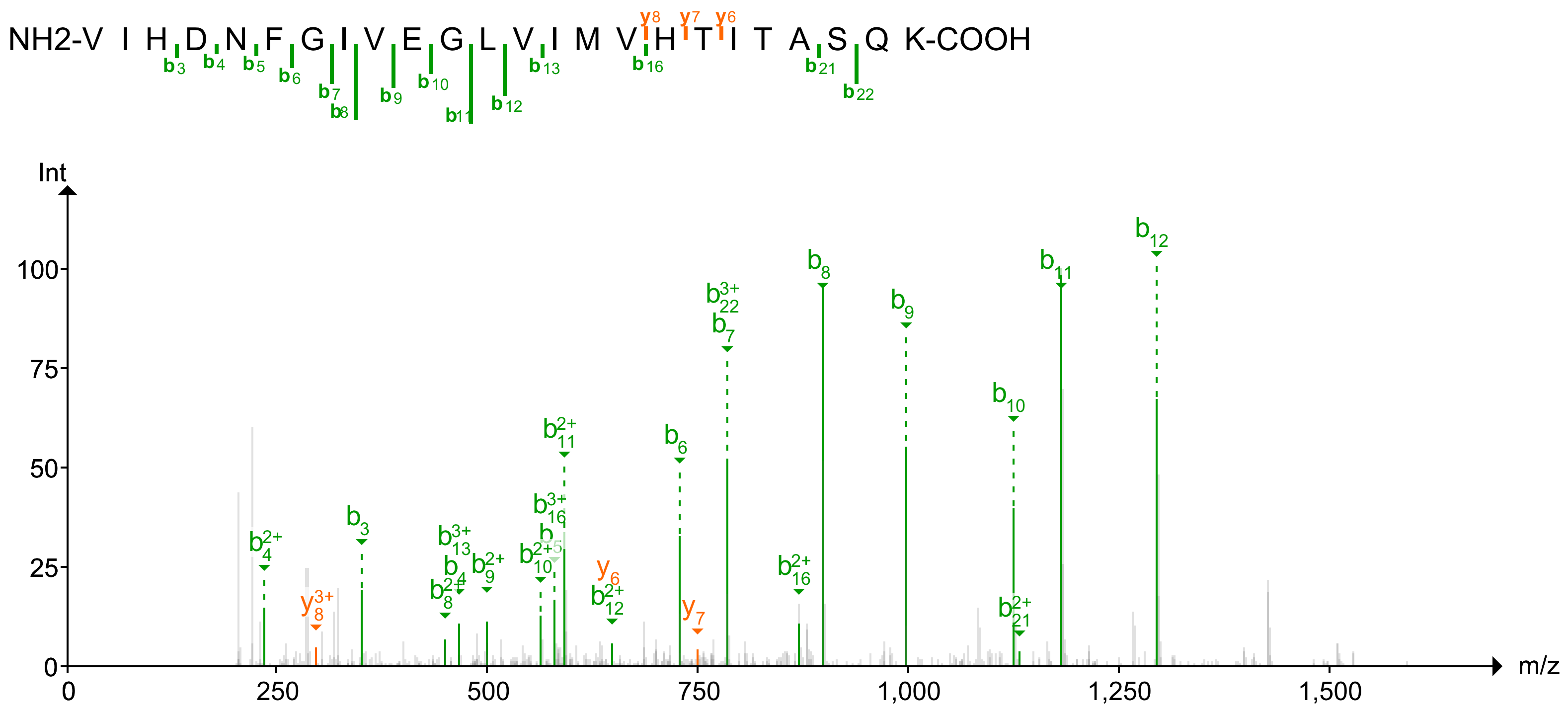

NH2-V I H D N F G I V E G L V I M V H T I T A S Q K-COOH
Int
m/z
0
25
50
75
100
250
500
750
1,000
1,250
1,500

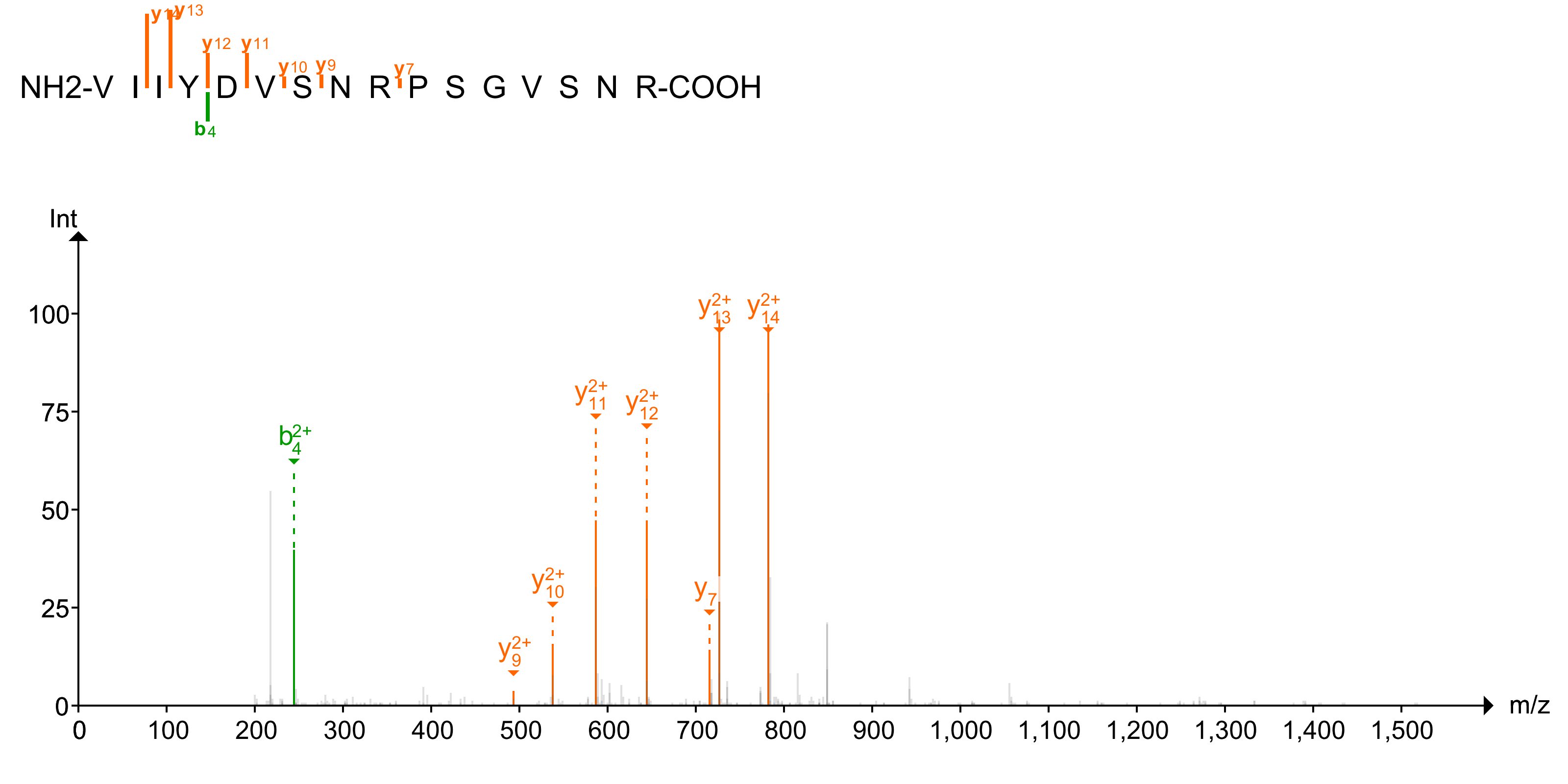
NH2-V I I Y D V S N R P S G V S N R-COOH
y14 y13 y12 y11 y10 y9 y7
b4
Int
m/z
0
25
50
75
100
0 100 200 300 400 500 600 700 800 900 1,000 1,100 1,200 1,300 1,400 1,500
$b_4^{2+}$
$y_9^{2+}$
$y_{10}^{2+}$
$y_{11}^{2+}$
$y_{12}^{2+}$
$y_7$
$y_{13}^{2+}$
$y_{14}^{2+}$

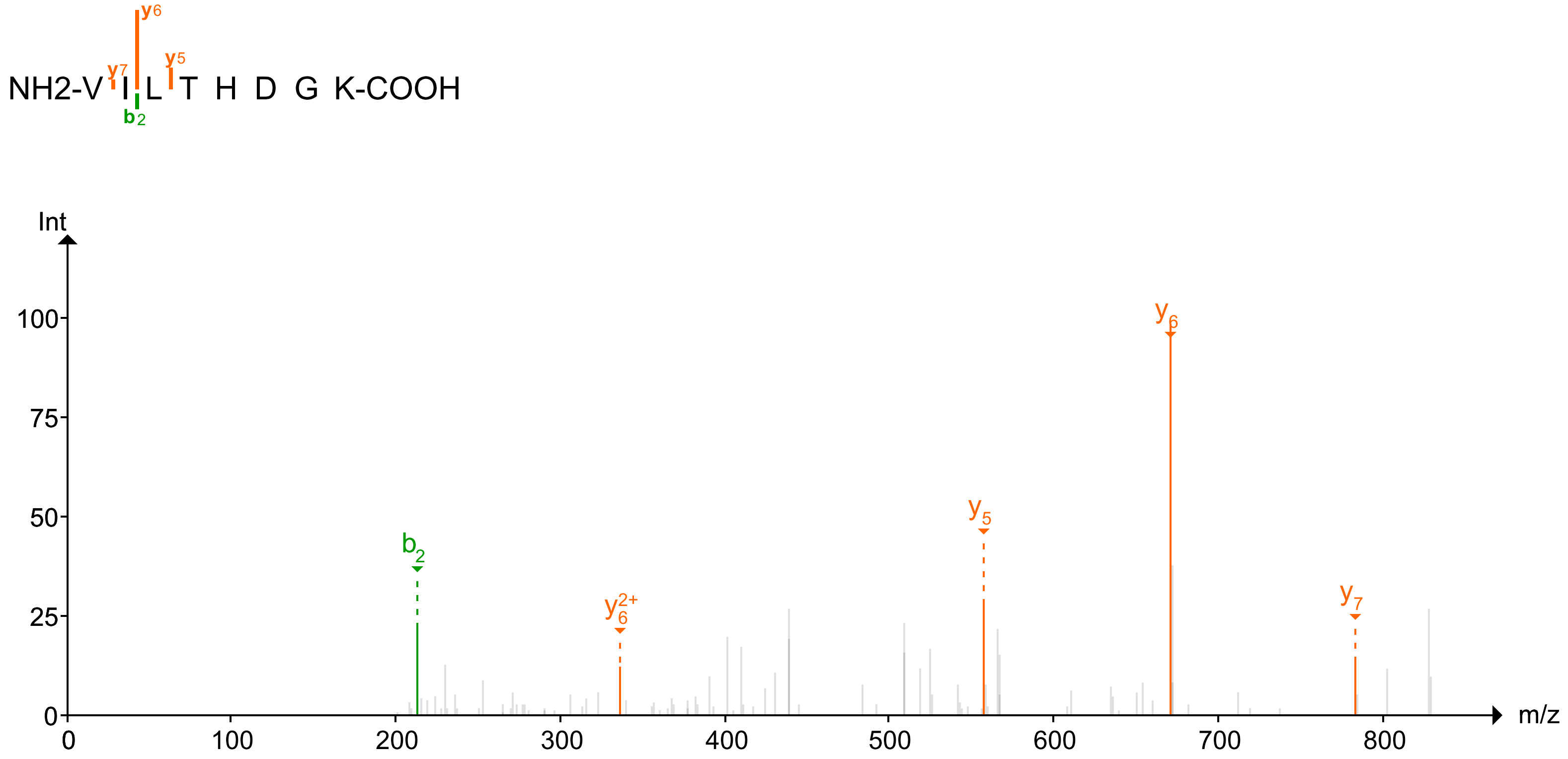

NH2-V I L T H D G K-COOH
y6
y7
y5
b2
Int
100
75
50
25
0
m/z
0
100
200
300
400
500
600
700
800
$b_2$
$y_6^{2+}$
$y_5$
$y_6$
$y_7$

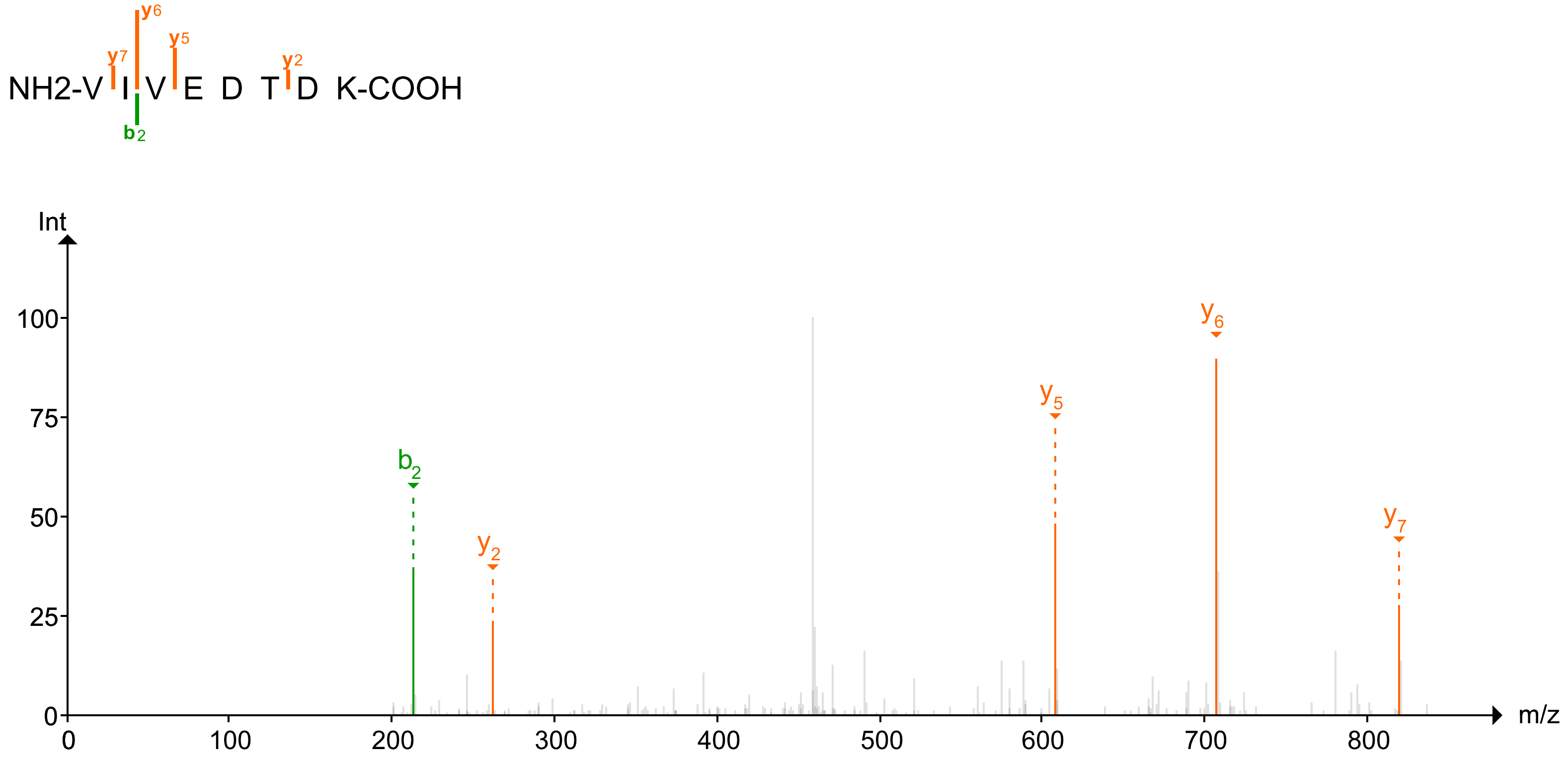

NH2-V I V E D T D K-COOH
y6
y5
y7
y2
b2
Int
100
75
50
25
0
m/z
0
100
200
300
400
500
600
700
800
b2
y2
y5
y6
y7

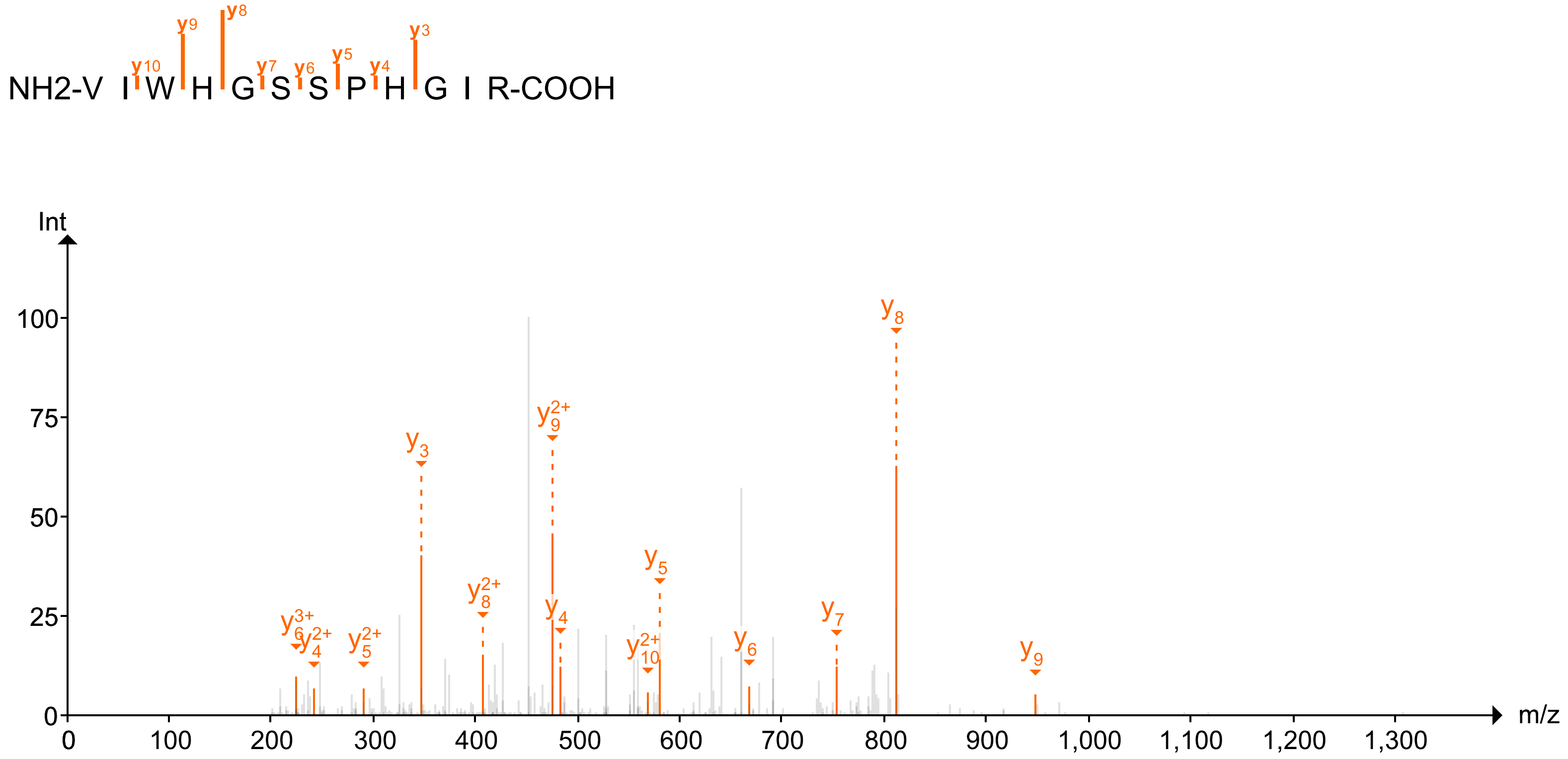

NH2-V I W H G S S P H G I R-COOH
y10
y9
y8
y7
y6
y5
y4
y3
Int
100
75
50
25
0
y8
y9 2+
y3
y8 2+
y5
y4
y6 3+
y4 2+
y5 2+
y10 2+
y6
y7
y9
0
100
200
300
400
500
600
700
800
900
1,000
1,100
1,200
1,300
m/z

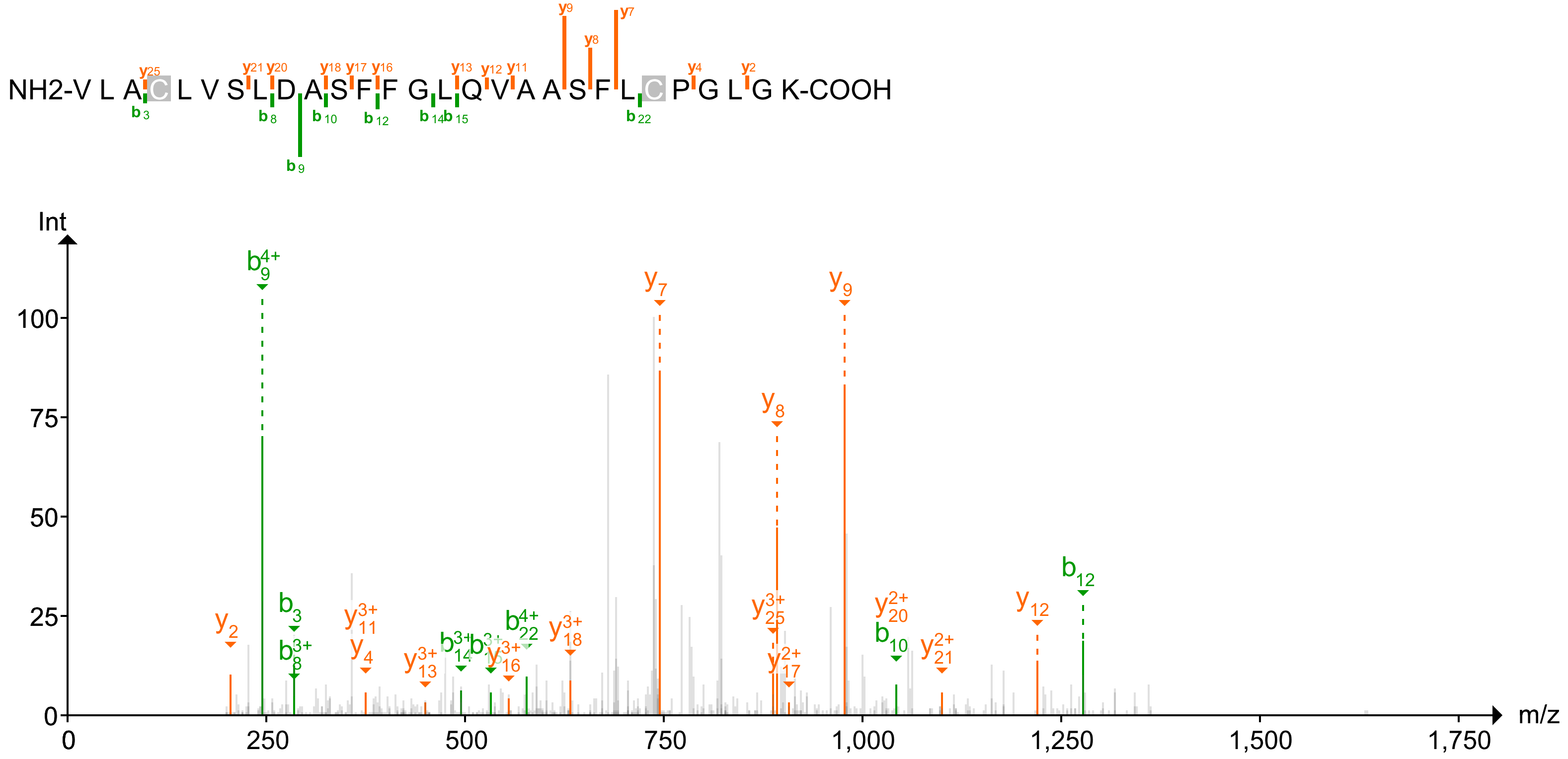

NH2-V L A C L V S L D A S F F G L Q V A A S F L C P G L G K-COOH
Int
m/z
0
25
50
75
100
250
500
750
1,000
1,250
1,500
1,750

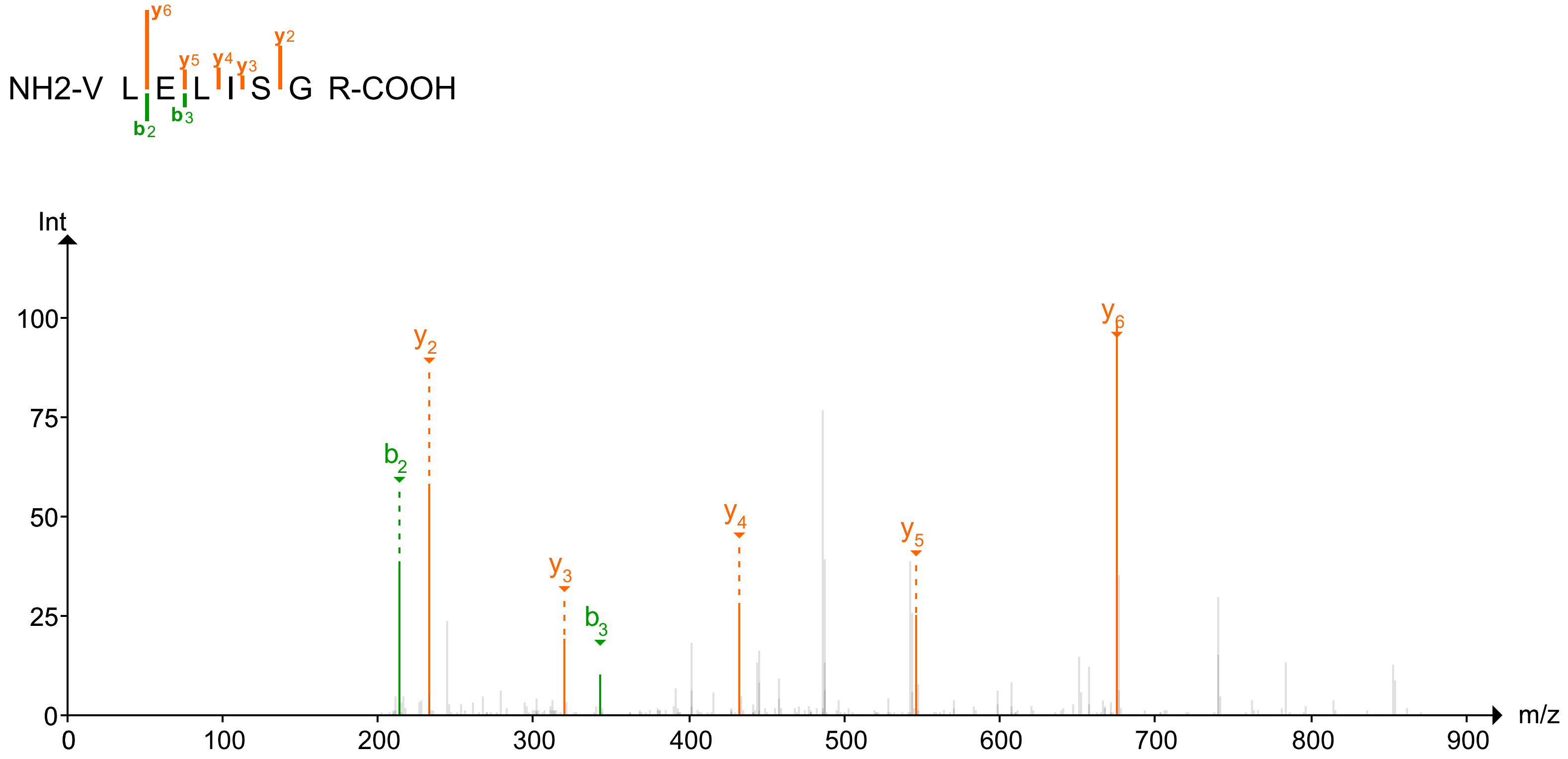

NH2-V L E L I S G R-COOH
y6 y5 y4 y3 y2
b2 b3
Int
m/z
0
25
50
75
100
0
100
200
300
400
500
600
700
800
900
$b_2$
$y_2$
$y_3$
$b_3$
$y_4$
$y_5$
$y_6$

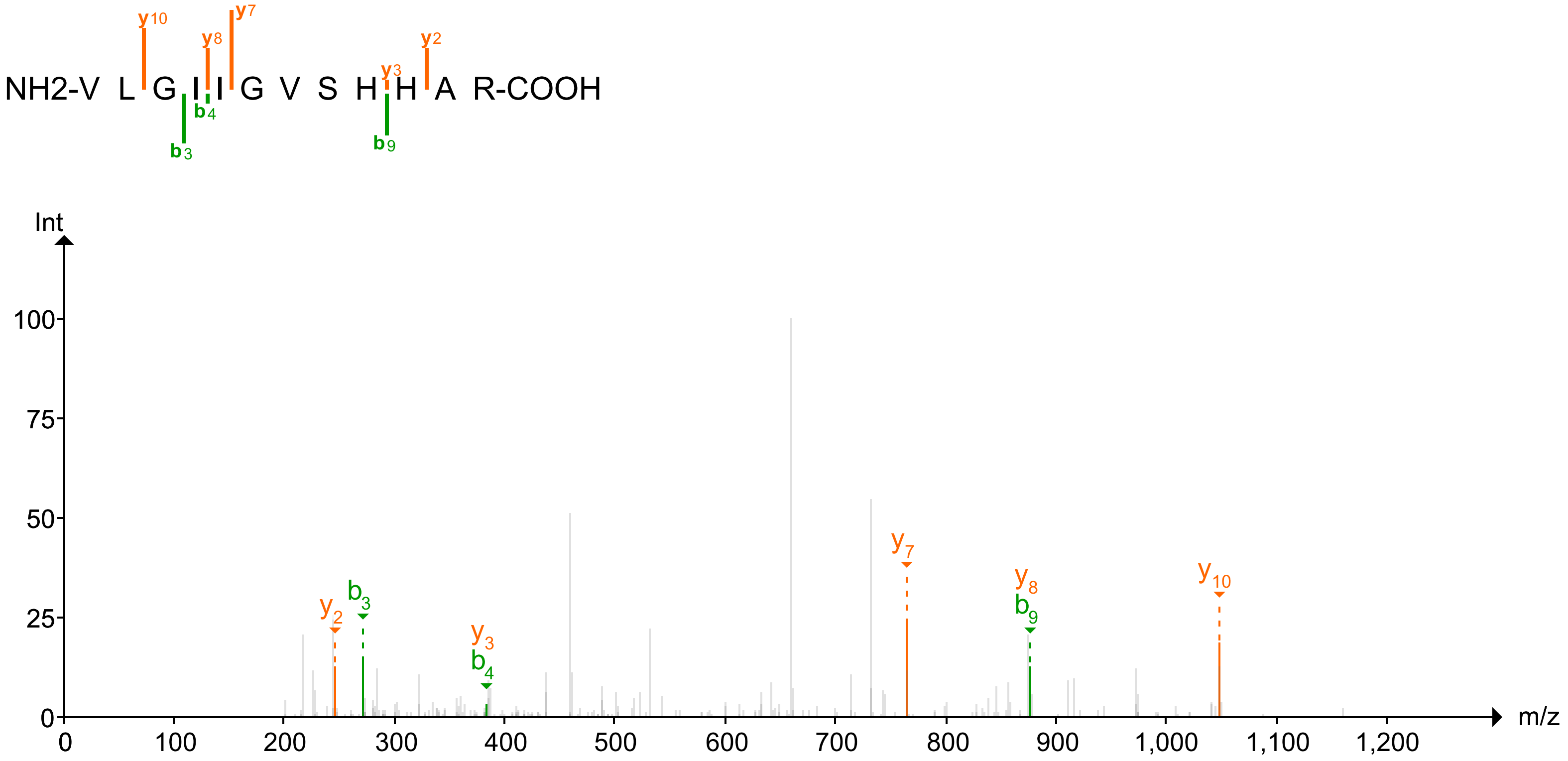

y10
y7
y8
y2
y3
NH2-V L G I I G V S H H A R-COOH
b4
b3
b9
Int
100
75
50
25
0
y2
b3
y3
b4
y7
y8
b9
y10
0
100
200
300
400
500
600
700
800
900
1,000
1,100
1,200
m/z

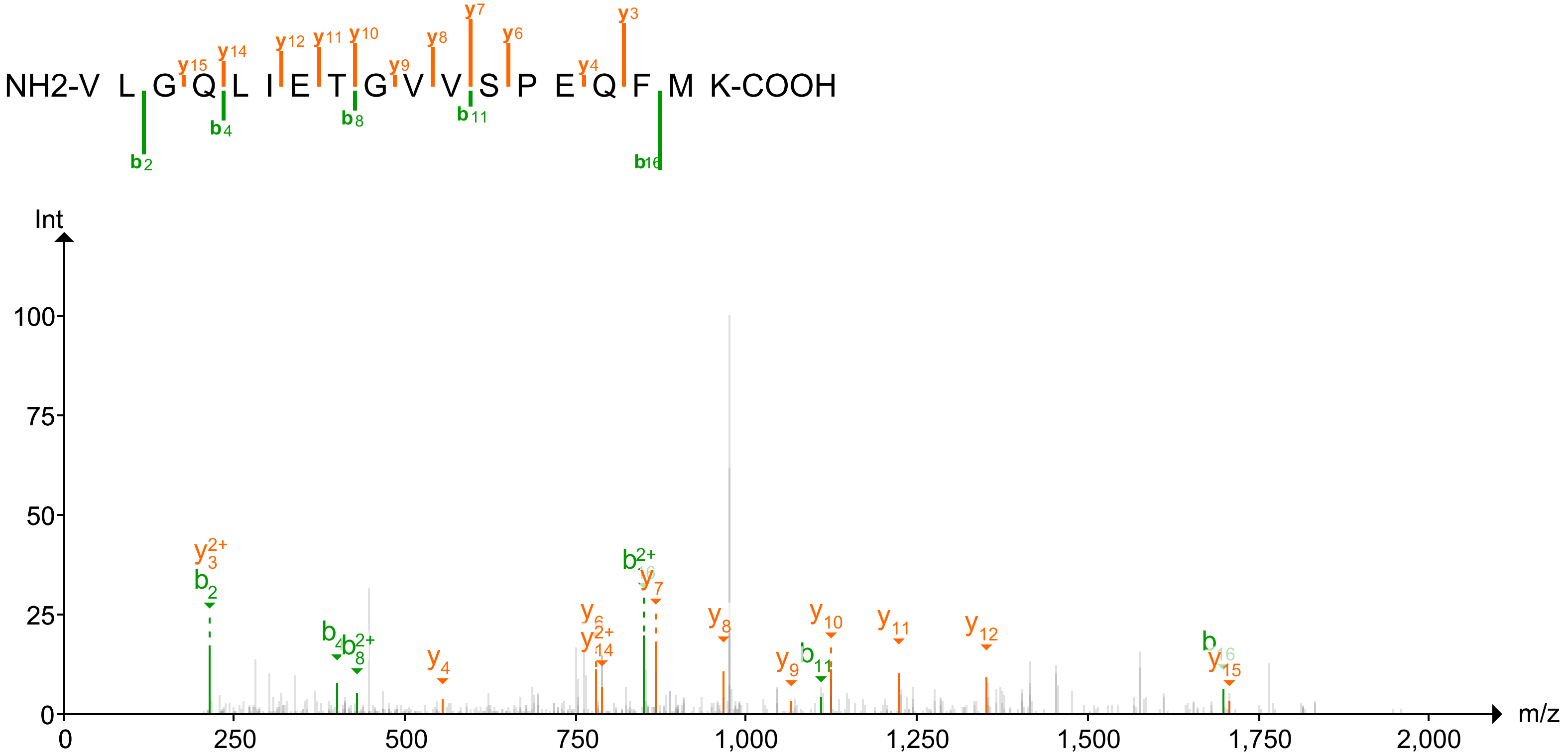

NH2-V L G Q L I E T G V V S P E Q F M K-COOH
y15 y14 y12 y11 y10 y9 y8 y7 y6 y4 y3
b2 b4 b8 b11 b16
Int
100
75
50
25
0
m/z
0
250
500
750
1,000
1,250
1,500
1,750
2,000

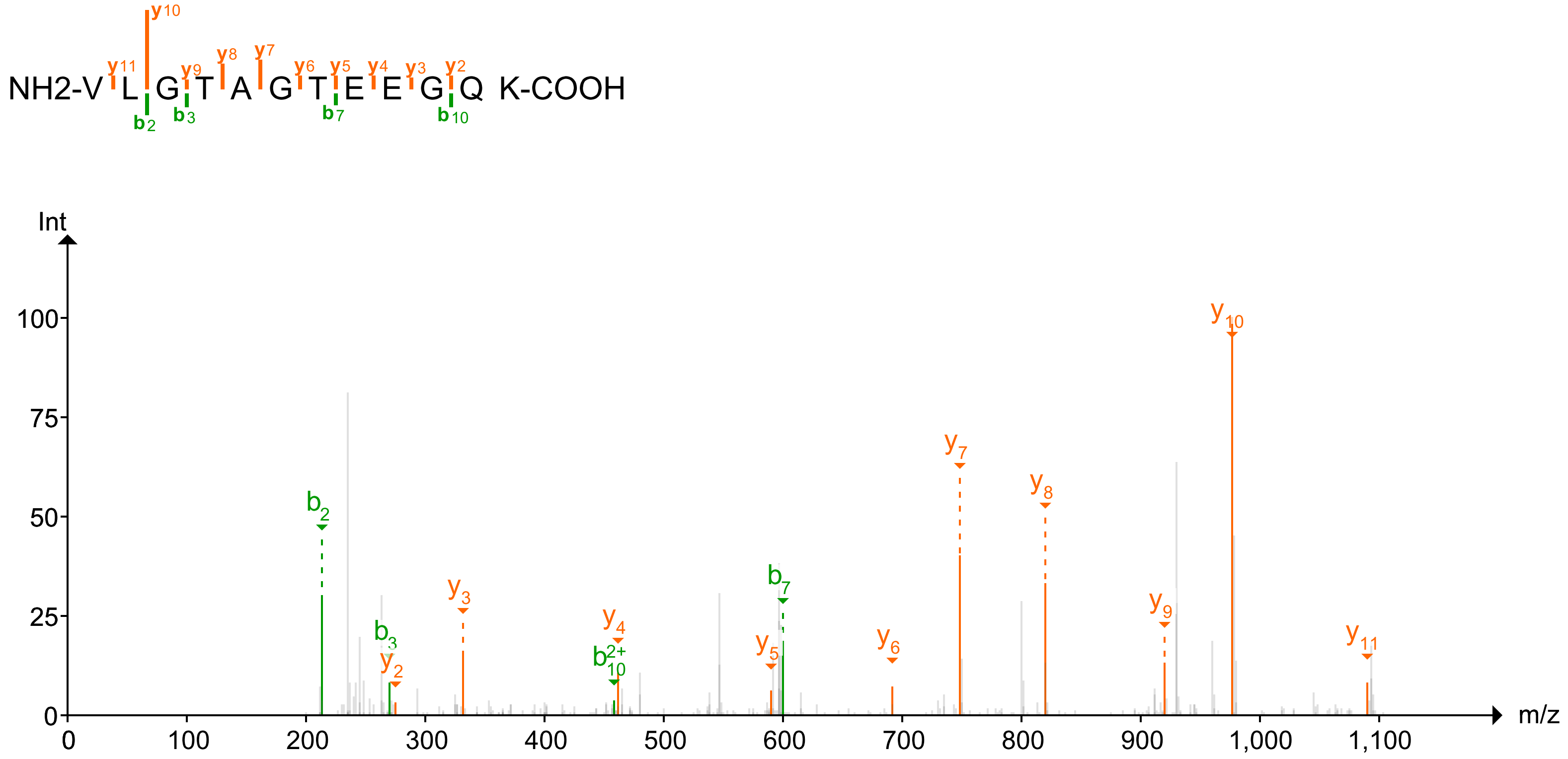

NH2-V L G T A G T E E G Q K-COOH
y10
y11
y9
y8
y7
y6
y5
y4
y3
y2
b2
b3
b7
b10
Int
100
75
50
25
0
m/z
0
100
200
300
400
500
600
700
800
900
1,000
1,100
b2
b3
y2
y3
y4
b10 2+
y5
b7
y6
y7
y8
y9
y10
y11

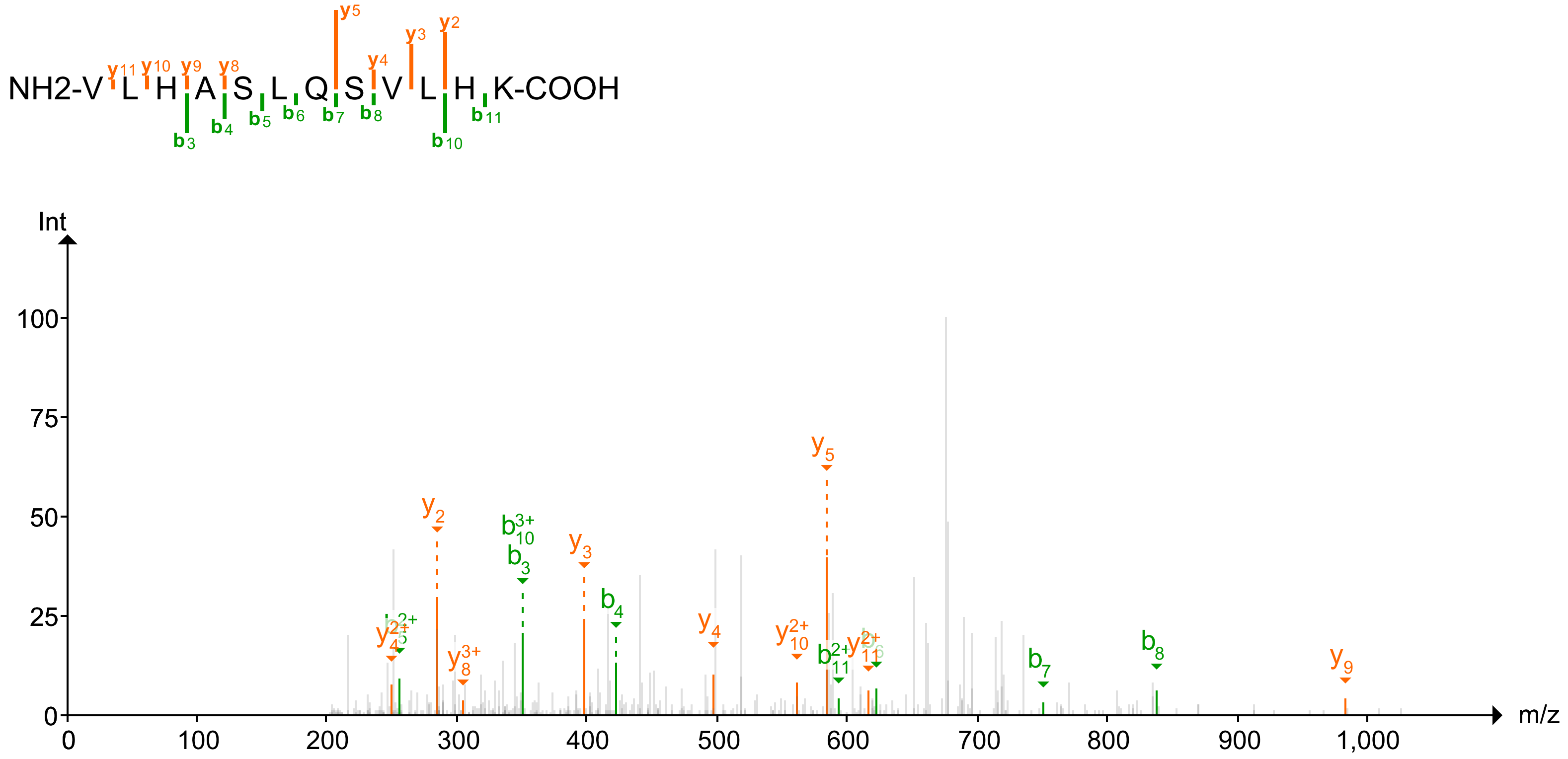

NH2-V L H A S L Q S V L H K-COOH
y11 y10 y9 y8 y5 y4 y3 y2
b3 b4 b5 b6 b7 b8 b10 b11
Int
100
75
50
25
0
0
100
200
300
400
500
600
700
800
900
1,000
m/z
y2
y3
y4
y5
y9
b3
b4
b7
b8

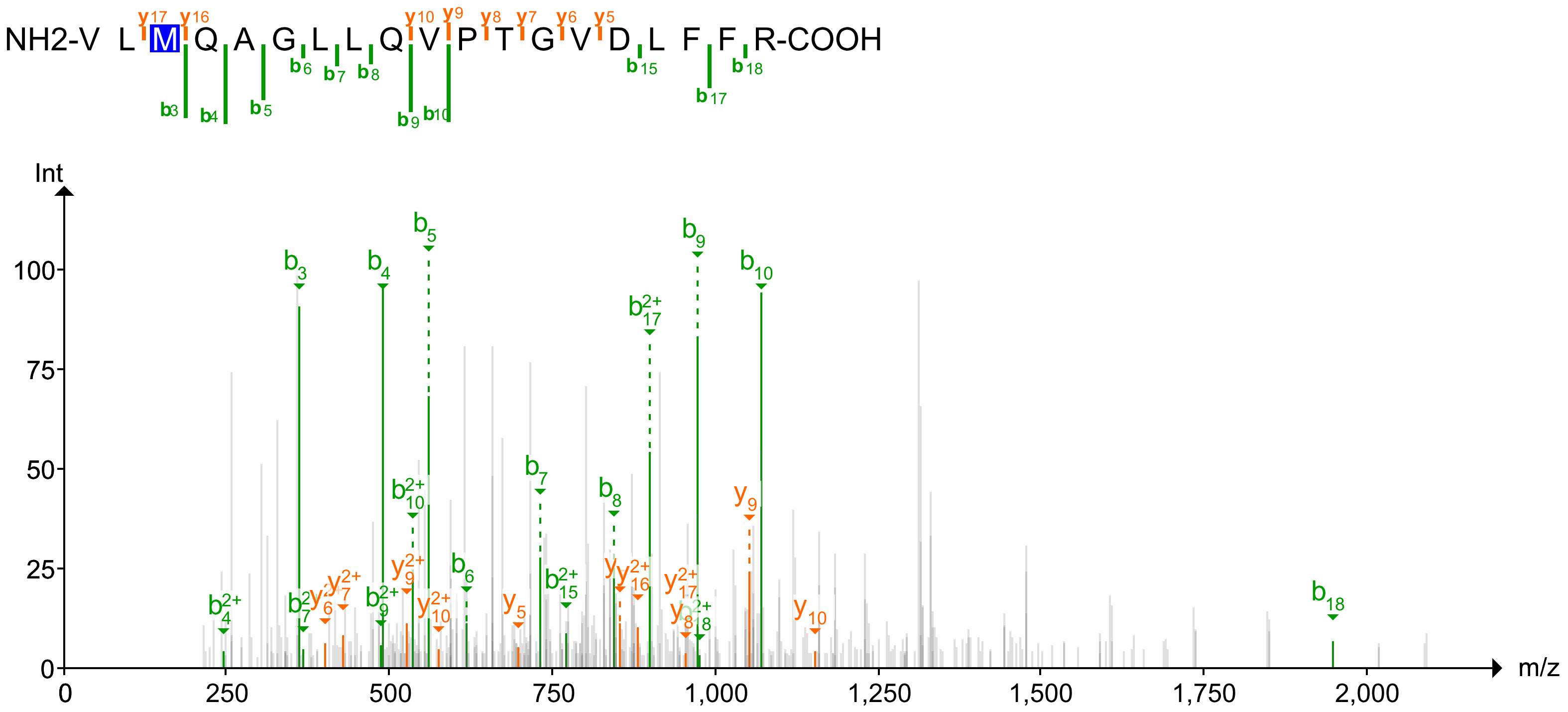

NH2-V L M Q A G L L Q V P T G V D L F F R-COOH
y17 y16 y10 y9 y8 y7 y6 y5
b3 b4 b5 b6 b7 b8 b9 b10 b15 b17 b18
Int
m/z
0
25
50
75
100
250
500
750
1,000
1,250
1,500
1,750
2,000

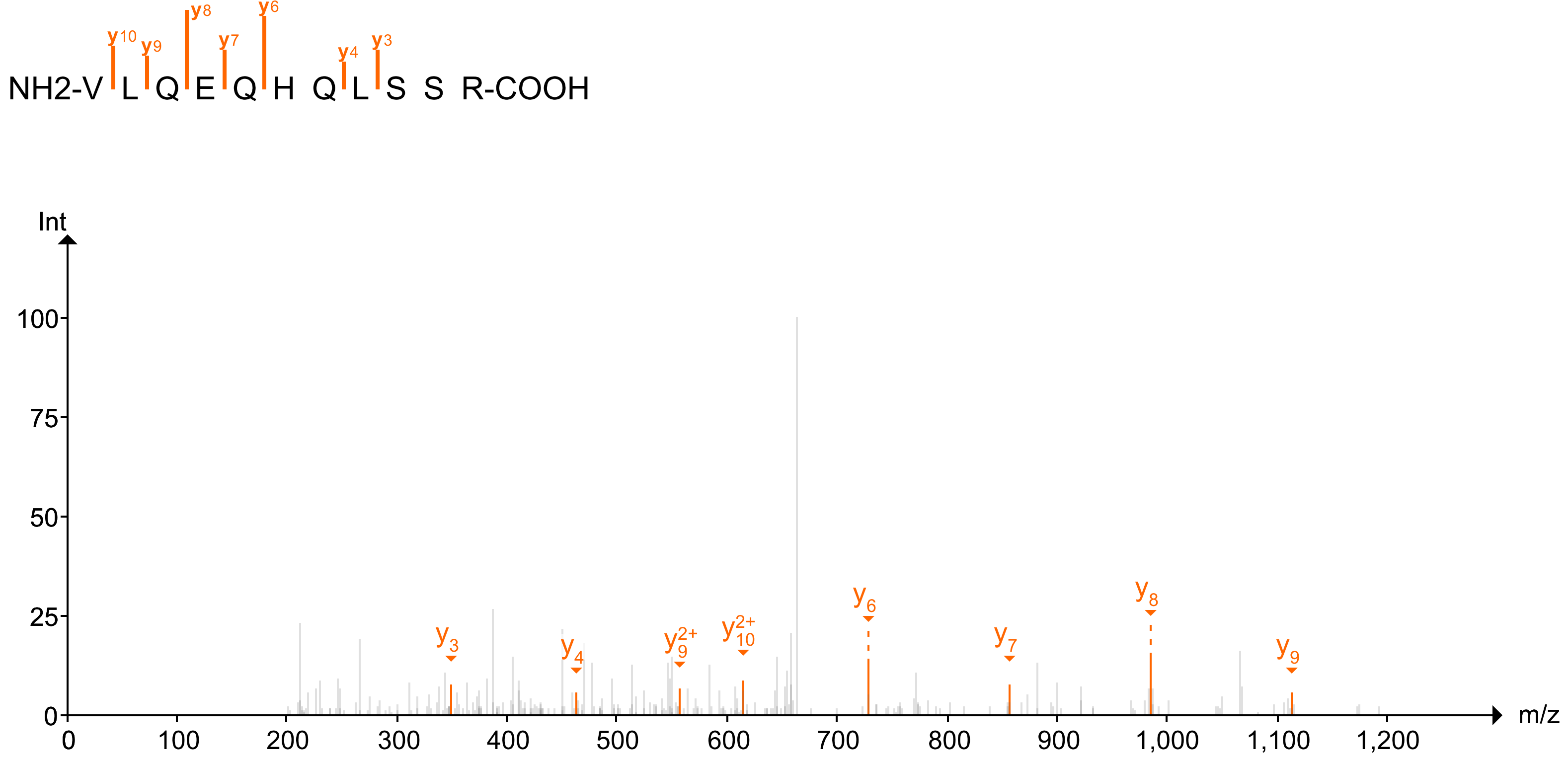

y10 y9 y8 y7 y6 y4 y3
NH2-V L Q E Q H Q L S S R-COOH
Int
100
75
50
25
0
y3
y4
$y_9^{2+}$
$y_{10}^{2+}$
y6
y7
y8
y9
0
100
200
300
400
500
600
700
800
900
1,000
1,100
1,200
m/z

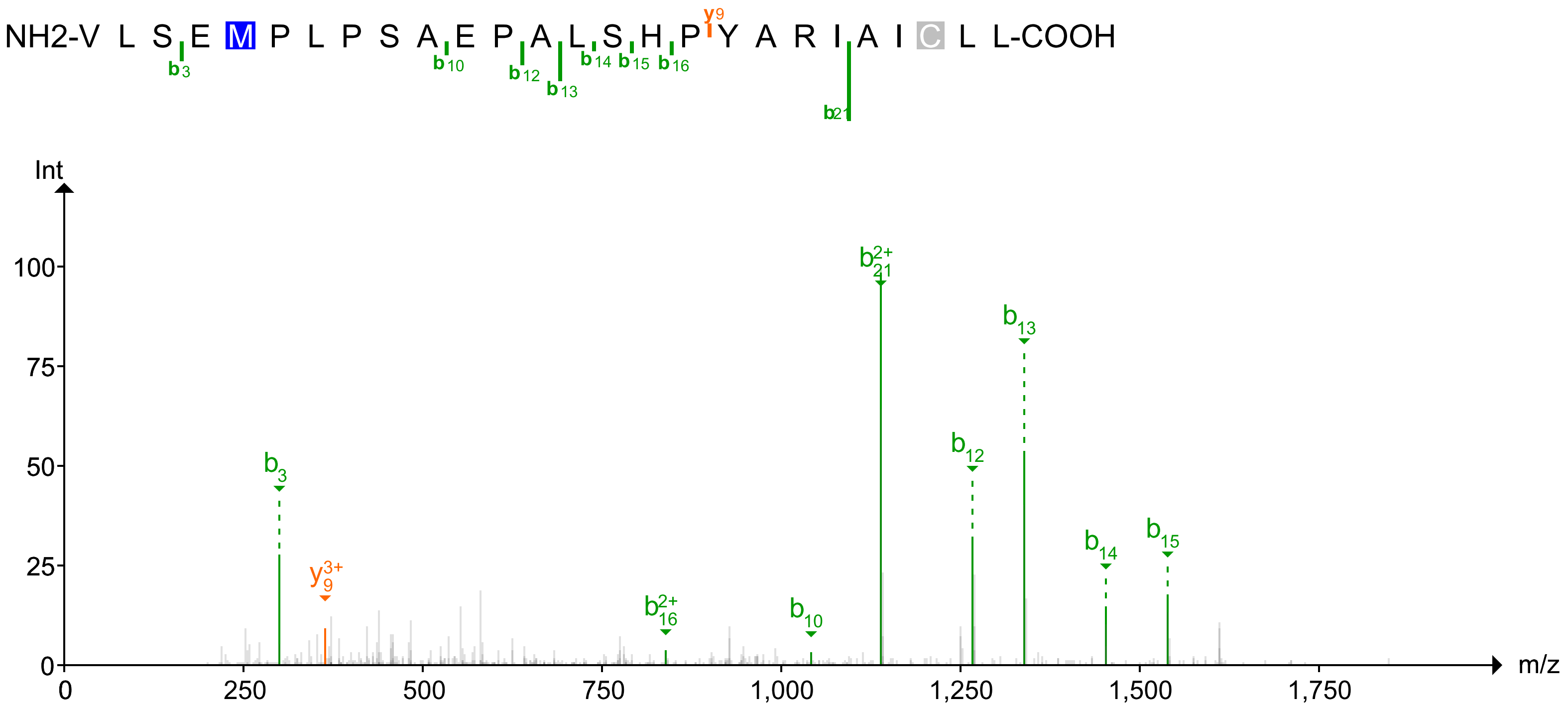

NH2-V L S E M P L P S A E P A L S H P Y A R I A I C L L-COOH
b3
b10
b12
b13
b14
b15
b16
b21
y9
Int
100
75
50
25
0
m/z
0
250
500
750
1,000
1,250
1,500
1,750
b3
y9 3+
b16 2+
b10
b21 2+
b12
b13
b14
b15

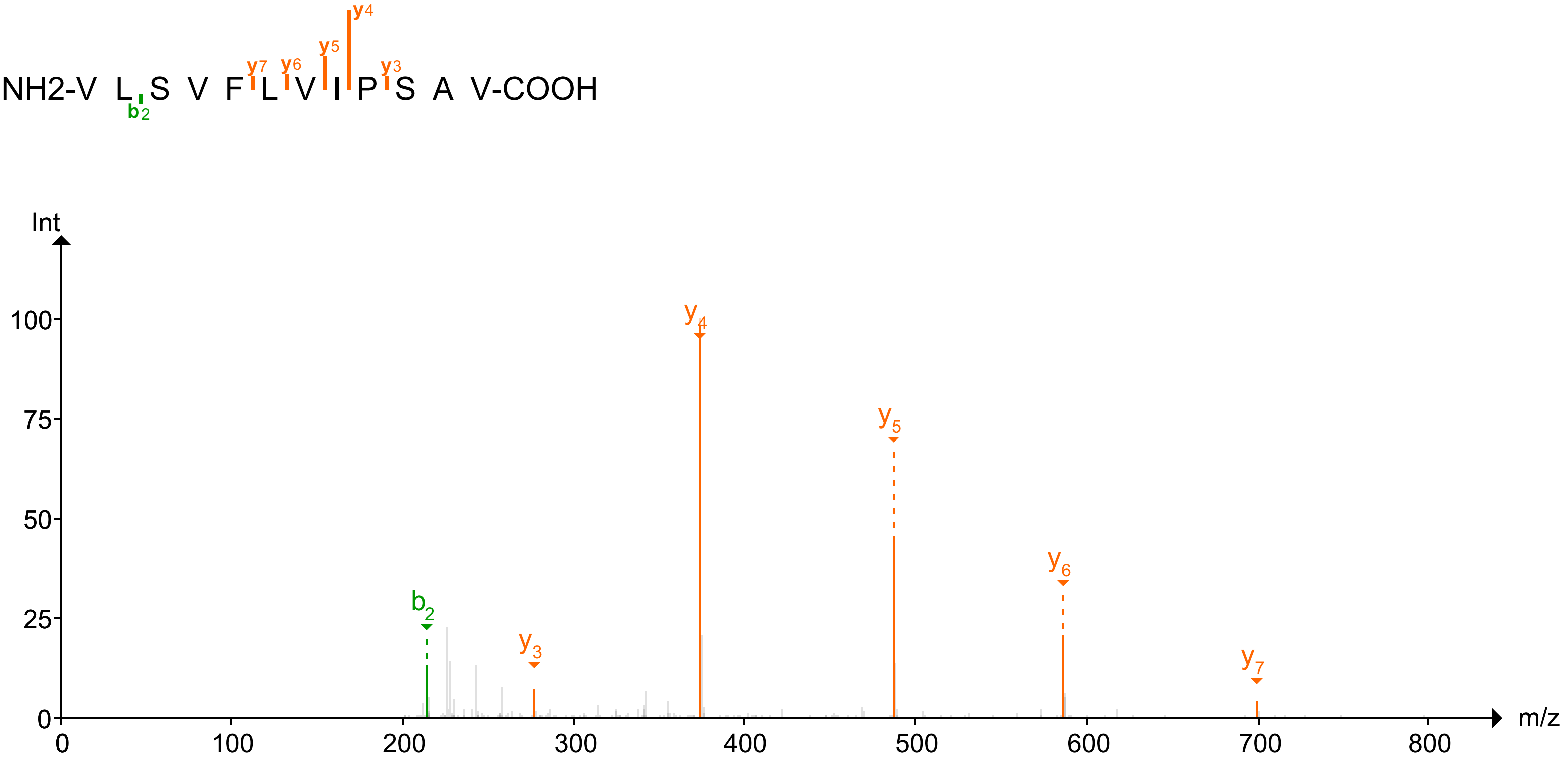

NH2-V L S V F L V I P S A V-COOH
b2
y7
y6
y5
y4
y3
Int
100
75
50
25
0
0
100
200
300
400
500
600
700
800
m/z

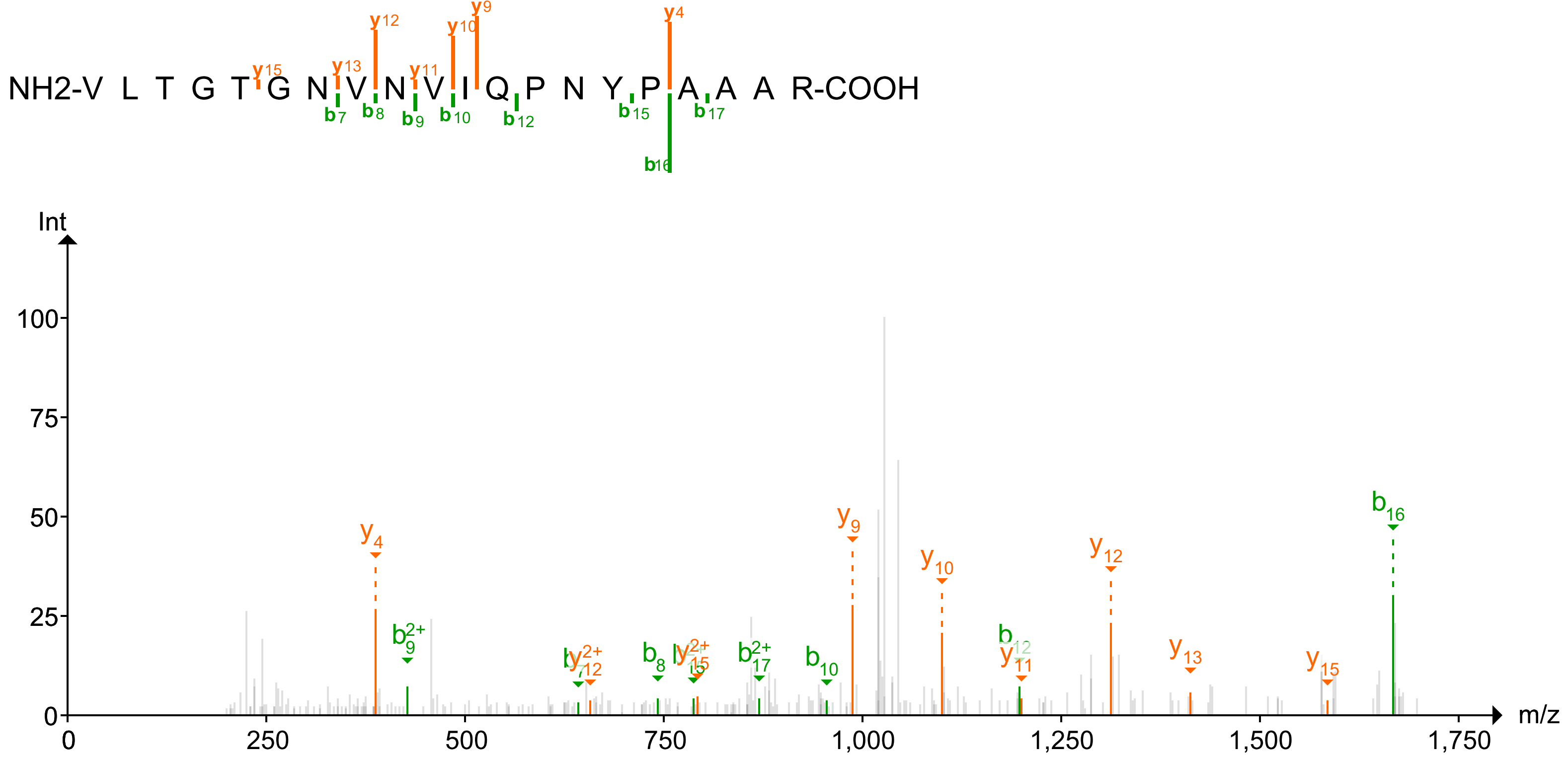

NH2-V L T G T G N V N V I Q P N Y P A A A R-COOH
y15
y13
y12
y11
y10
y9
y4
b7
b8
b9
b10
b12
b15
b16
b17
Int
100
75
50
25
0
m/z
0
250
500
750
1,000
1,250
1,500
1,750
y4
b9 2+
y12 2+
b8
y15 2+
b17 2+
b10
y9
y10
y11
b12
y12
y13
y15
b16

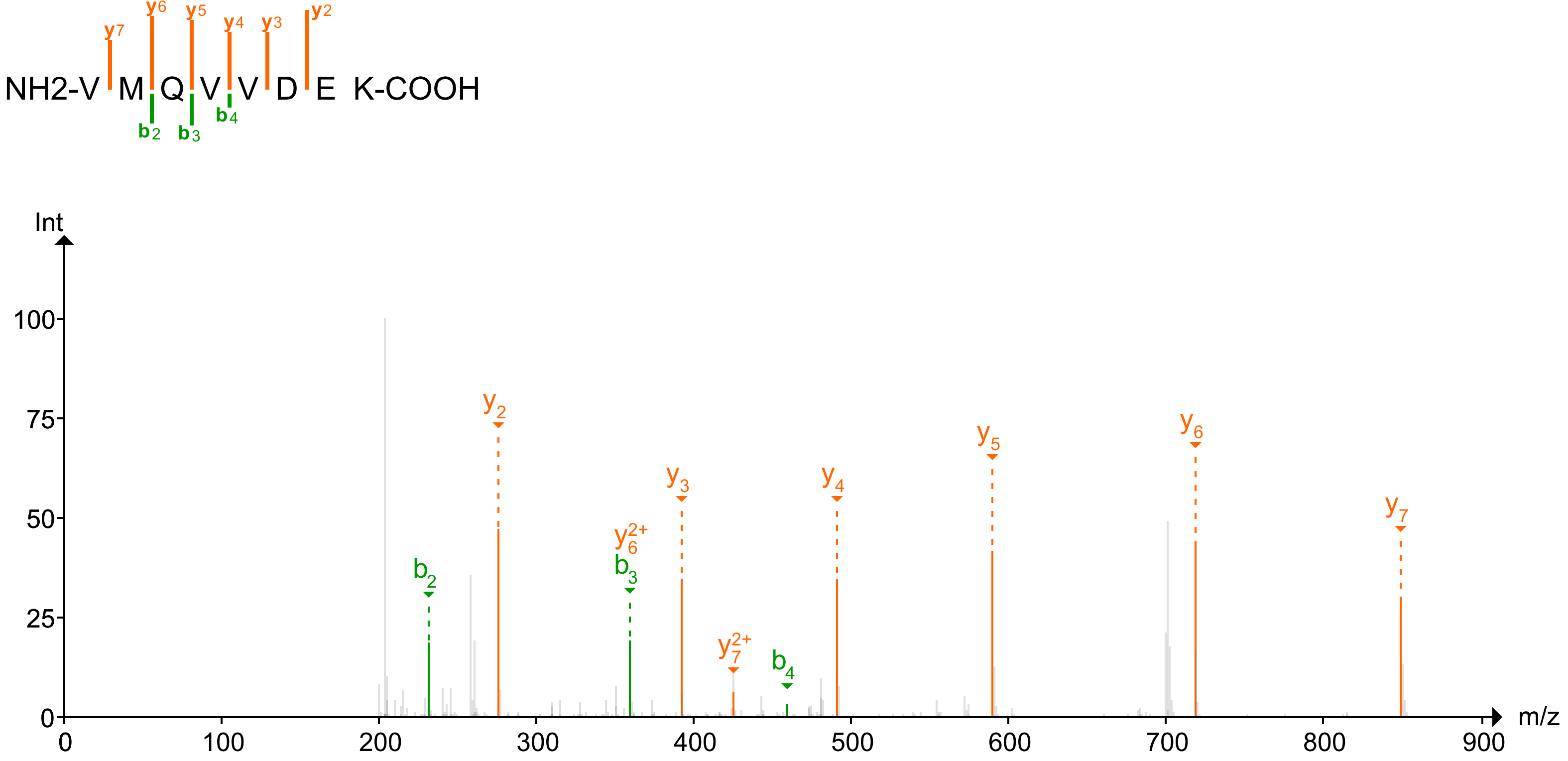
NH2-V M Q V V D E K-COOH
y7 y6 y5 y4 y3 y2
b2 b3 b4
Int
100
75
50
25
0
m/z
0 100 200 300 400 500 600 700 800 900
y2
y3
y4
y5
y6
y7
b2
b3
b4
y6 2+
y7 2+

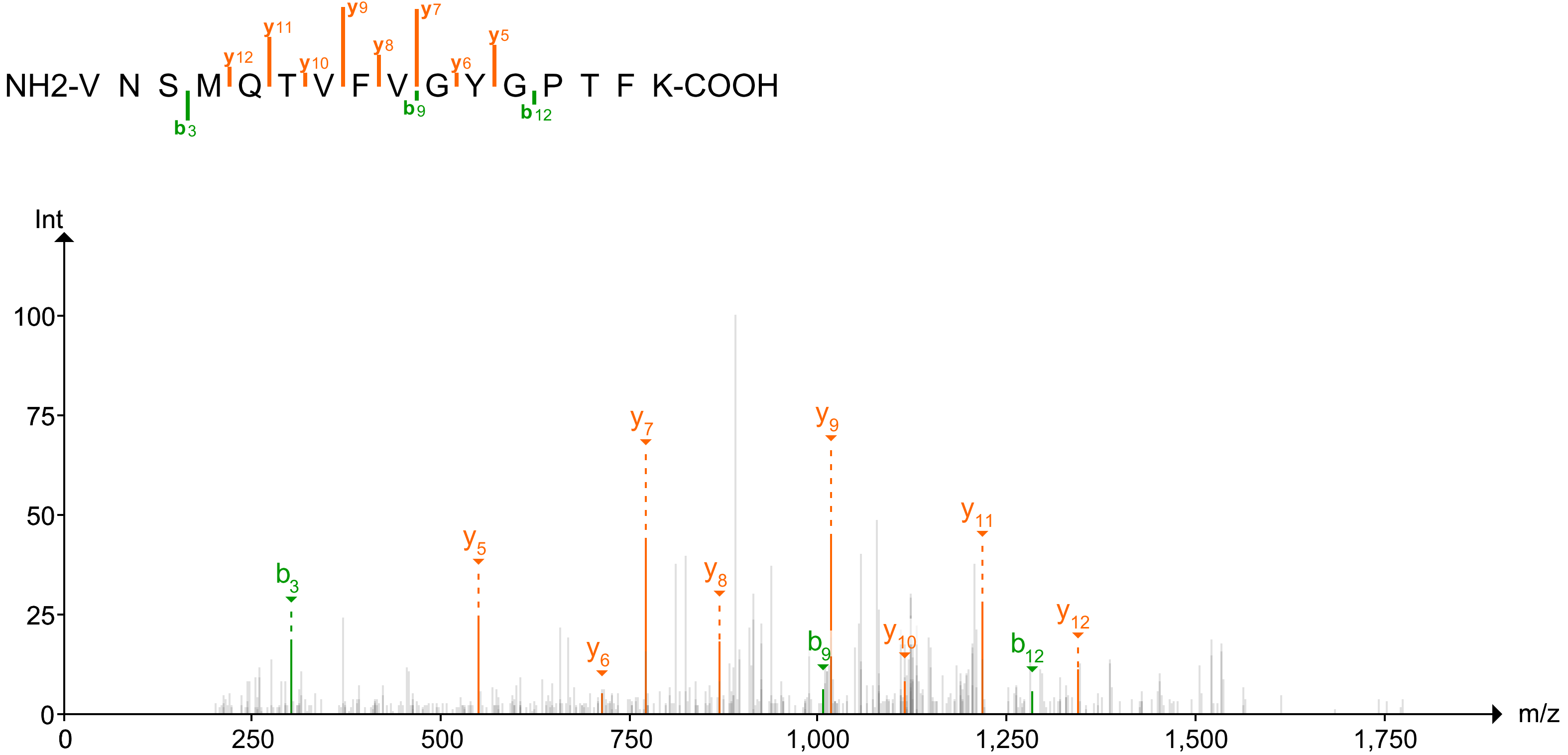

NH2-V N S M Q T V F V G Y G P T F K-COOH
y12 y11 y10 y9 y8 y7 y6 y5
b3 b9 b12
Int
100
75
50
25
0
0 250 500 750 1,000 1,250 1,500 1,750
m/z

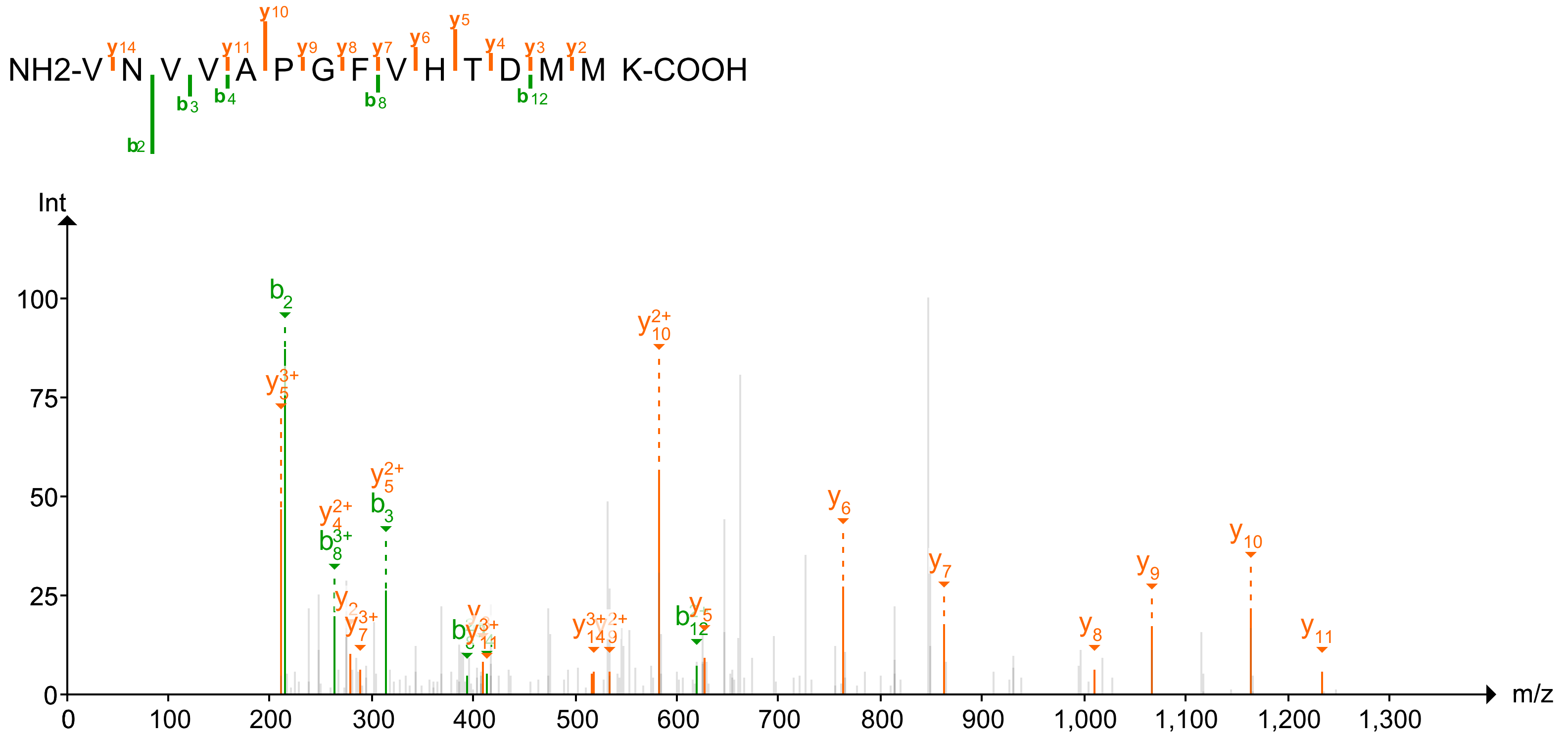

NH2-V N V V A P G F V H T D M M K-COOH
y14 y11 y10 y9 y8 y7 y6 y5 y4 y3 y2
b2 b3 b4 b8 b12
Int
100
75
50
25
0
m/z
0 100 200 300 400 500 600 700 800 900 1,000 1,100 1,200 1,300
b2
y5 3+
y10 2+
y5 2+
b3
y4 2+
b8 3+
y6
y7
y9
y10
y8
y11

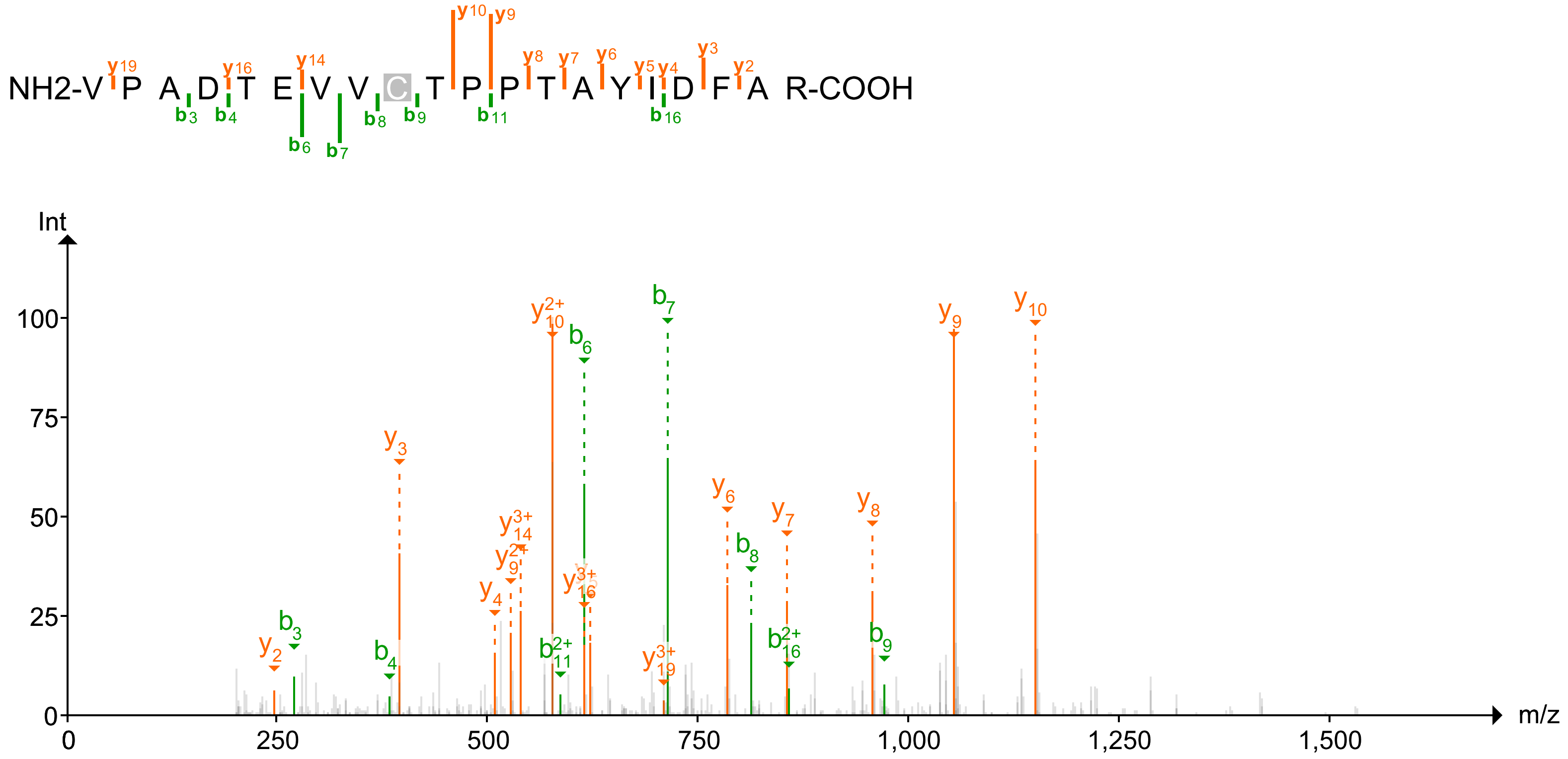

NH2-V P A D T E V V C T P P T A Y I D F A R-COOH
Int
m/z
0
25
50
75
100
250
500
750
1,000
1,250
1,500

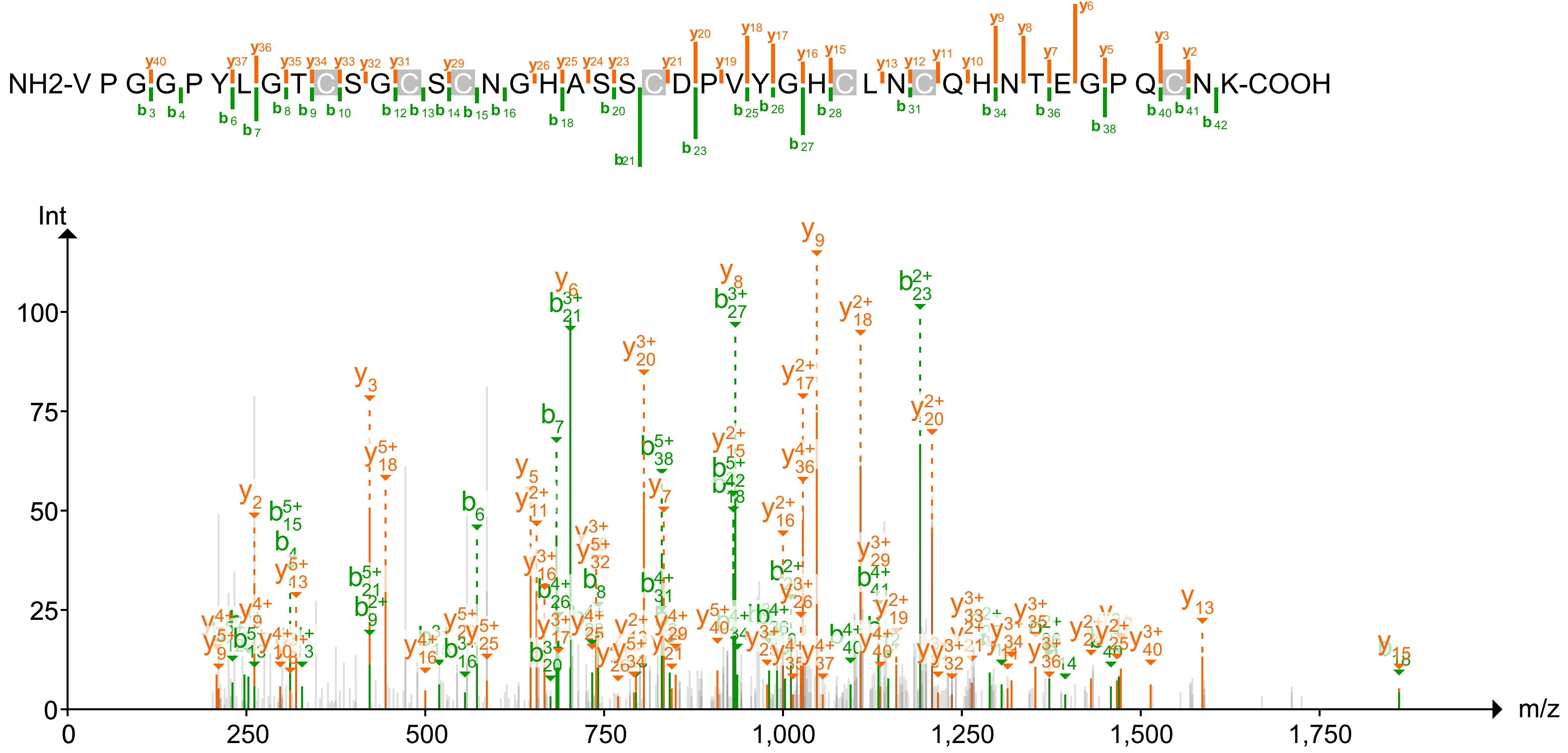

NH2-V P G G P Y L G T C S G C S C N G H A S S C D P V Y G H C L N C Q H N T E G P Q C N K-COOH
Int
100
75
50
25
0
m/z
0
250
500
750
1,000
1,250
1,500
1,750

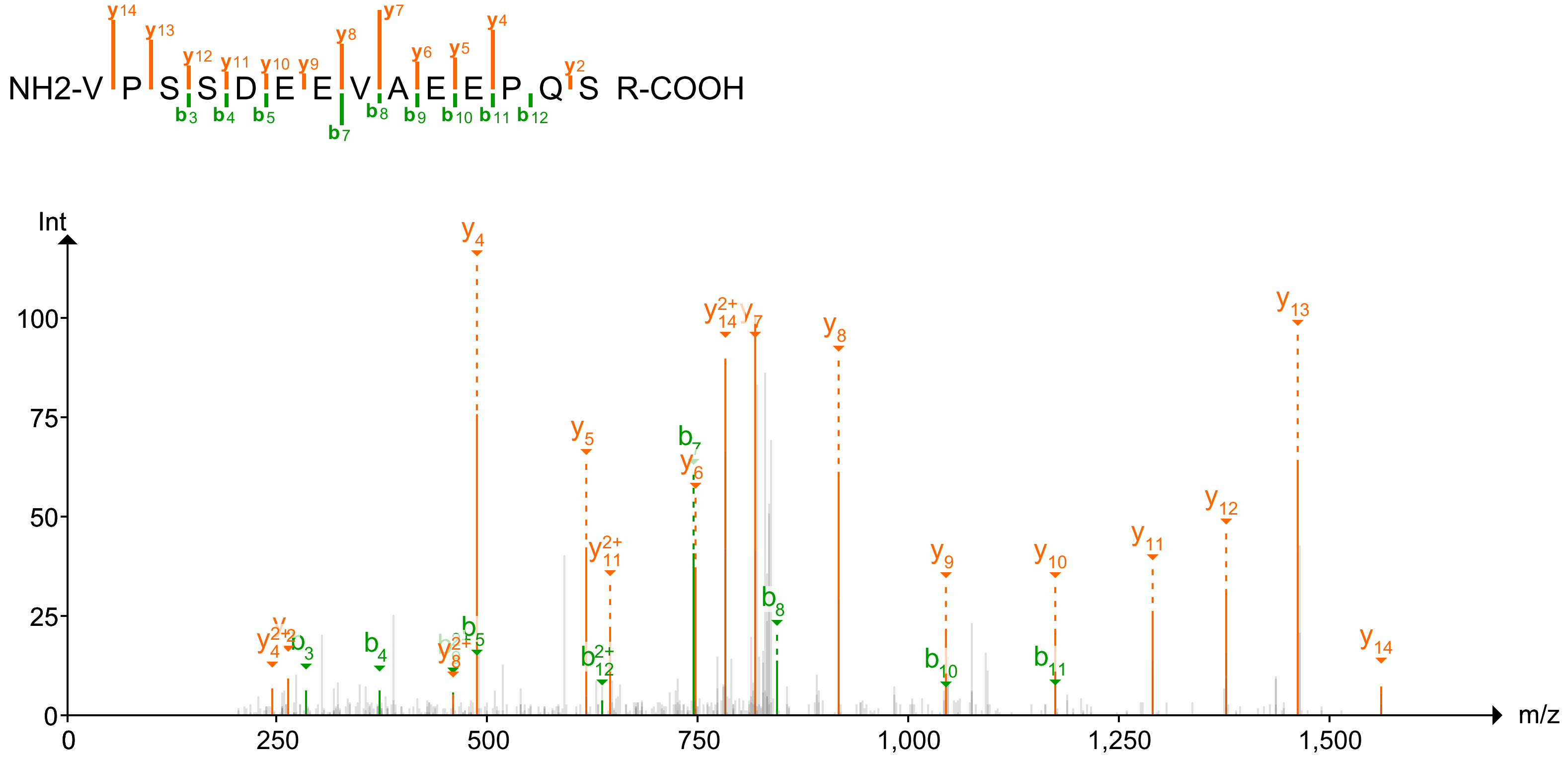

NH2-V P S S D E E V A E E P Q S R-COOH
Int
m/z

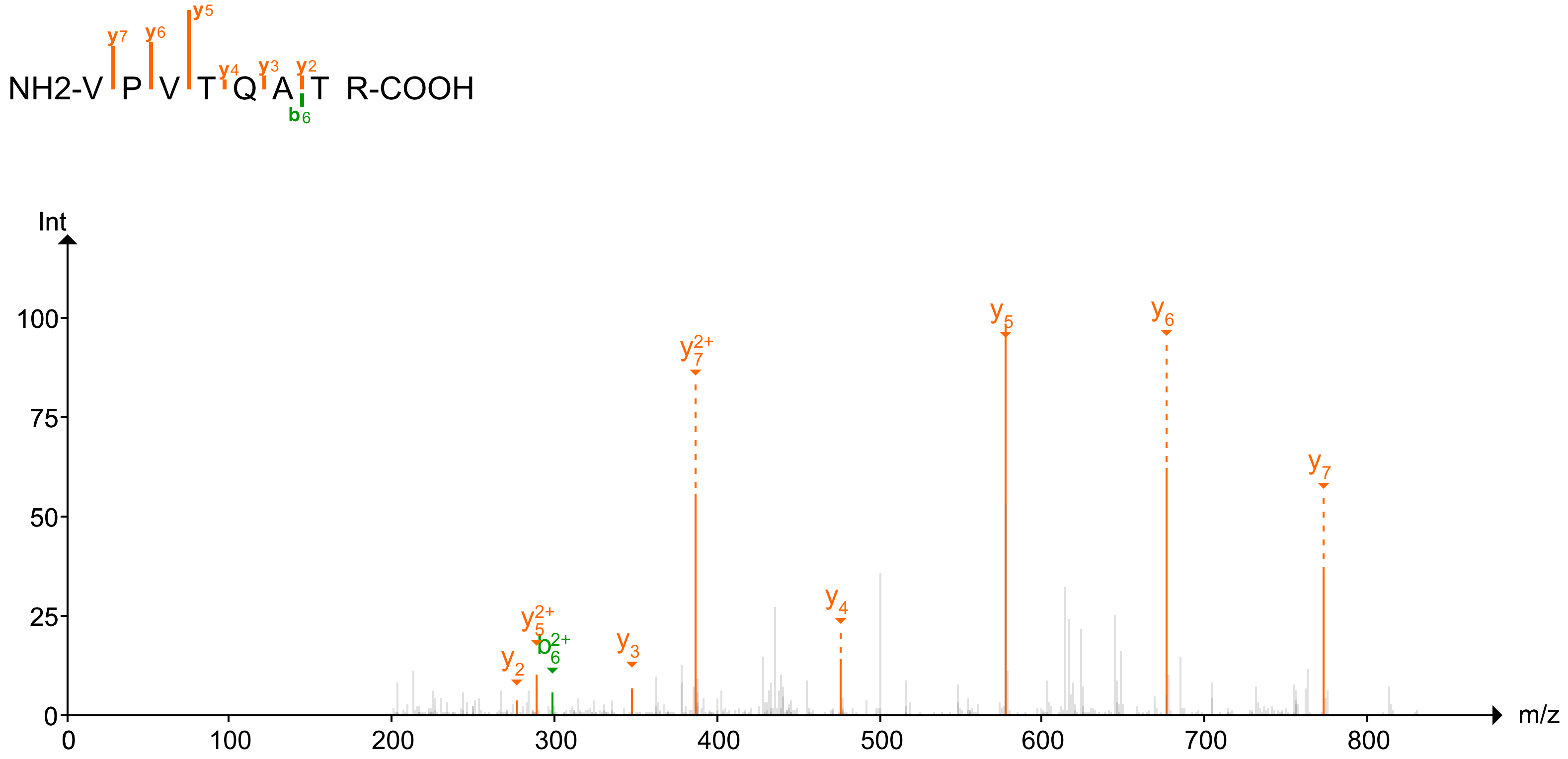

NH2-V P V T Q A T R-COOH
y7 y6 y5 y4 y3 y2
b6
Int
100
75
50
25
0
m/z
0
100
200
300
400
500
600
700
800
y2
y5 2+
b6 2+
y3
y7 2+
y4
y5
y6
y7

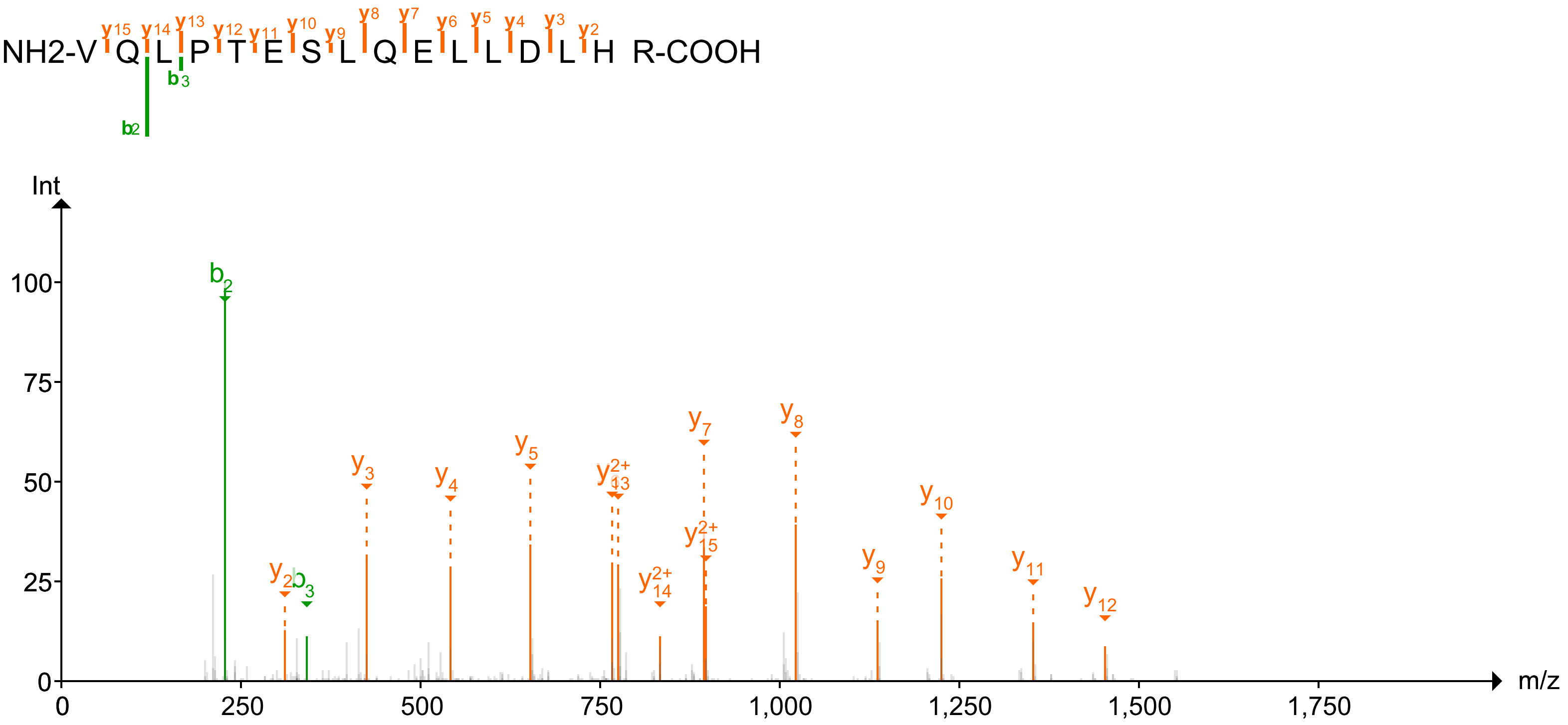

NH2-V Q L P T E S L Q E L L D L H R-COOH
y15 y14 y13 y12 y11 y10 y9 y8 y7 y6 y5 y4 y3 y2
b2 b3
Int
100
75
50
25
0
b2
y2
b3
y3
y4
y5
y2+ 13
y7
y8
y2+ 15
y2+ 14
y9
y10
y11
y12
0
250
500
750
1,000
1,250
1,500
1,750
m/z

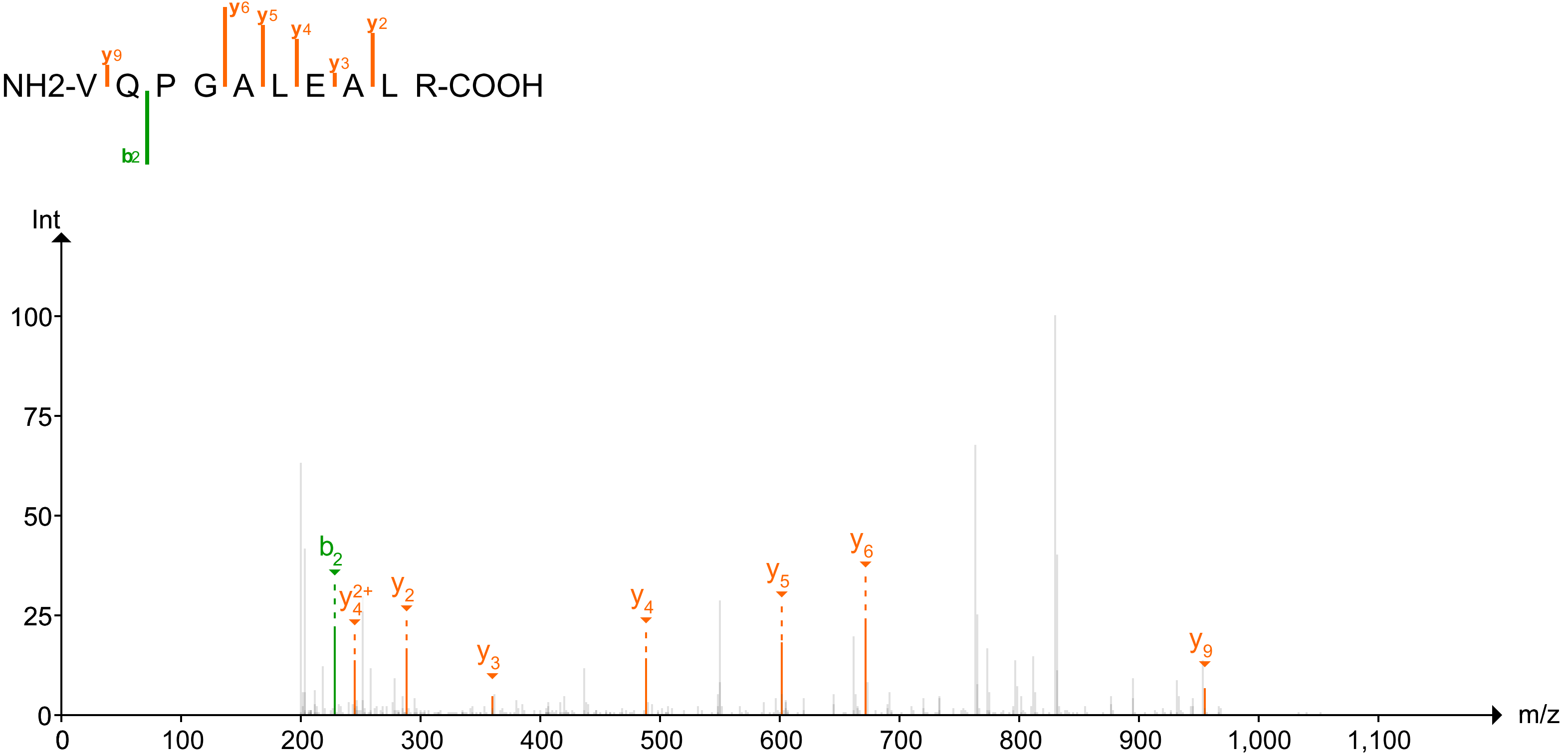

NH2-V Q P G A L E A L R-COOH
y9
y6
y5
y4
y3
y2
b2
Int
100
75
50
25
0
m/z
0
100
200
300
400
500
600
700
800
900
1,000
1,100
$b_2$
$y_4^{2+}$
$y_2$
$y_3$
$y_4$
$y_5$
$y_6$
$y_9$

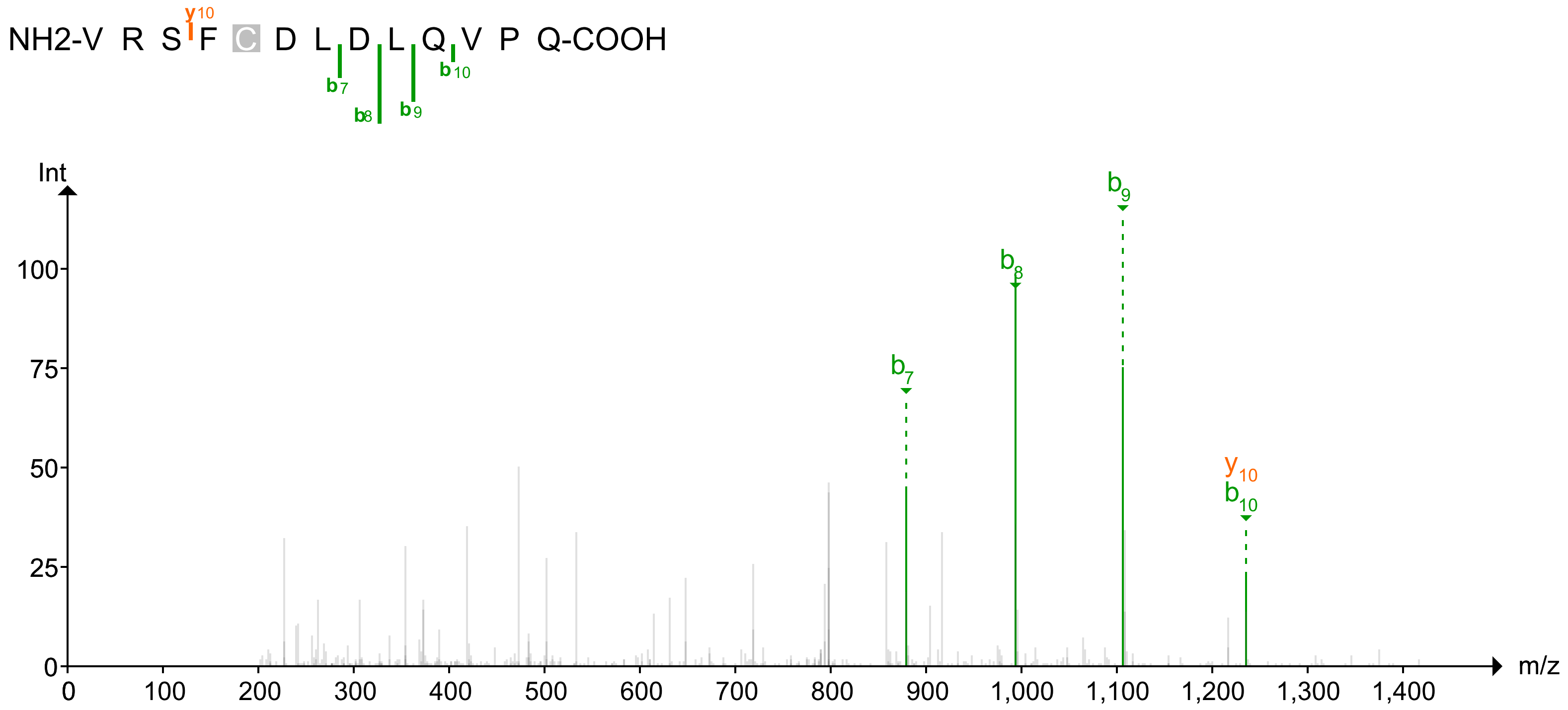

NH2-V R S F C D L D L Q V P Q-COOH
y10
b7
b8
b9
b10
Int
100
75
50
25
0
b9
b8
b7
y10
b10
0
100
200
300
400
500
600
700
800
900
1,000
1,100
1,200
1,300
1,400
m/z

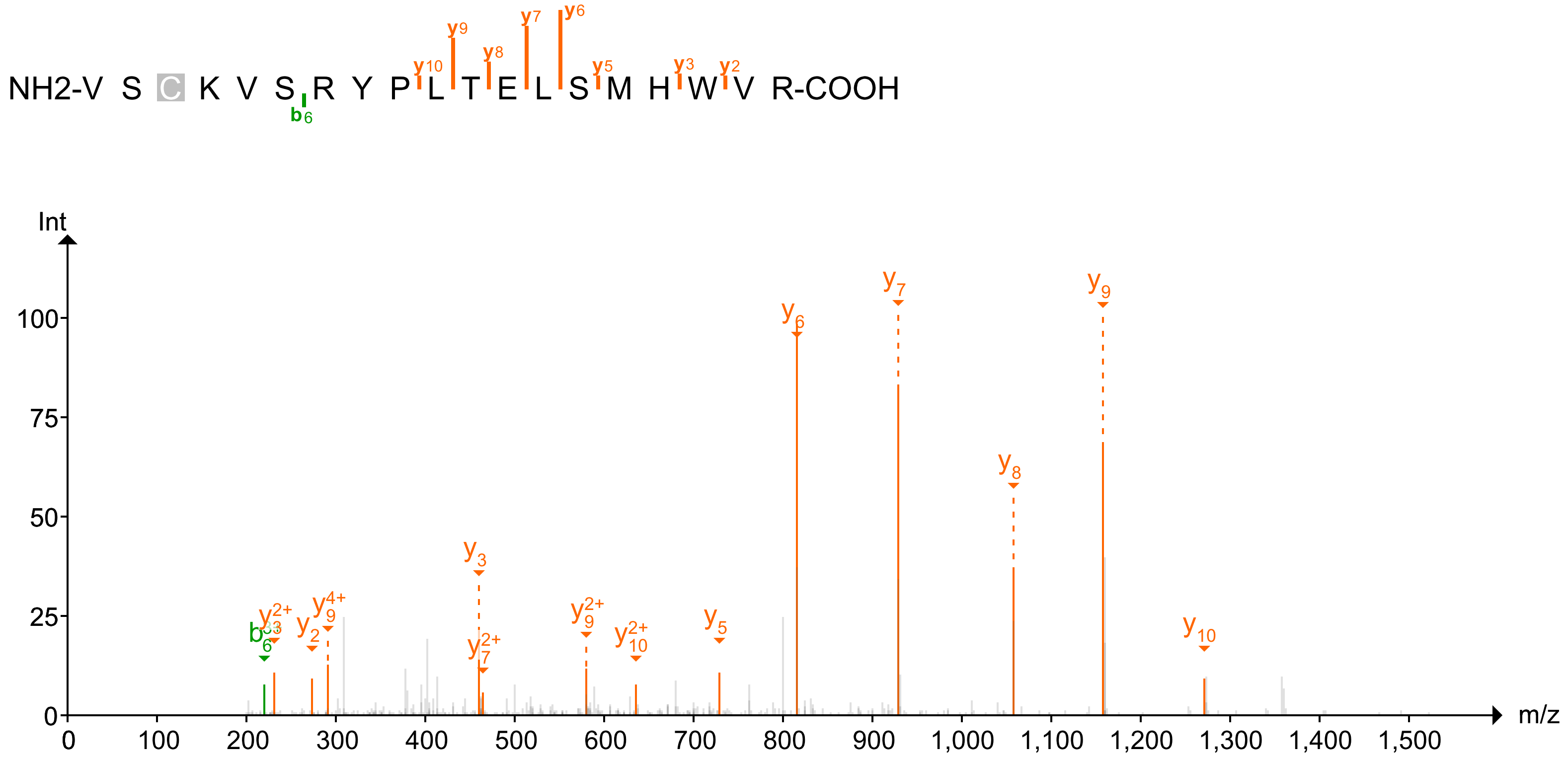
NH2-V S C K V S R Y P L T E L S M H W V R-COOH
b6
y10 y9 y8 y7 y6 y5 y3 y2
Int
100
75
50
25
0
m/z
0 100 200 300 400 500 600 700 800 900 1,000 1,100 1,200 1,300 1,400 1,500

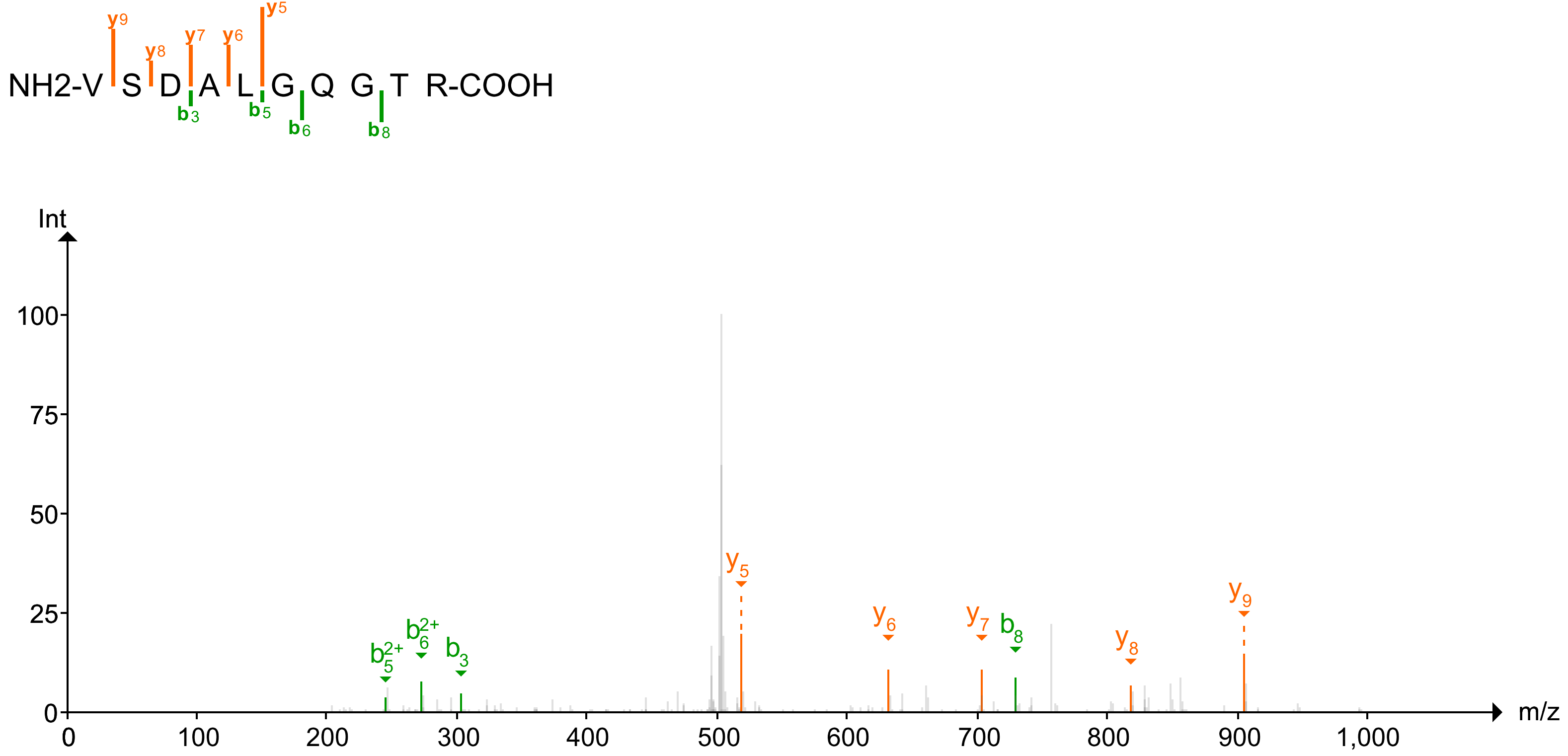

NH2-V S D A L G Q G T R-COOH
y9
y8
y7
y6
y5
b3
b5
b6
b8
Int
100
75
50
25
0
m/z
0
100
200
300
400
500
600
700
800
900
1,000
b5 2+
b6 2+
b3
y5
y6
y7
b8
y8
y9

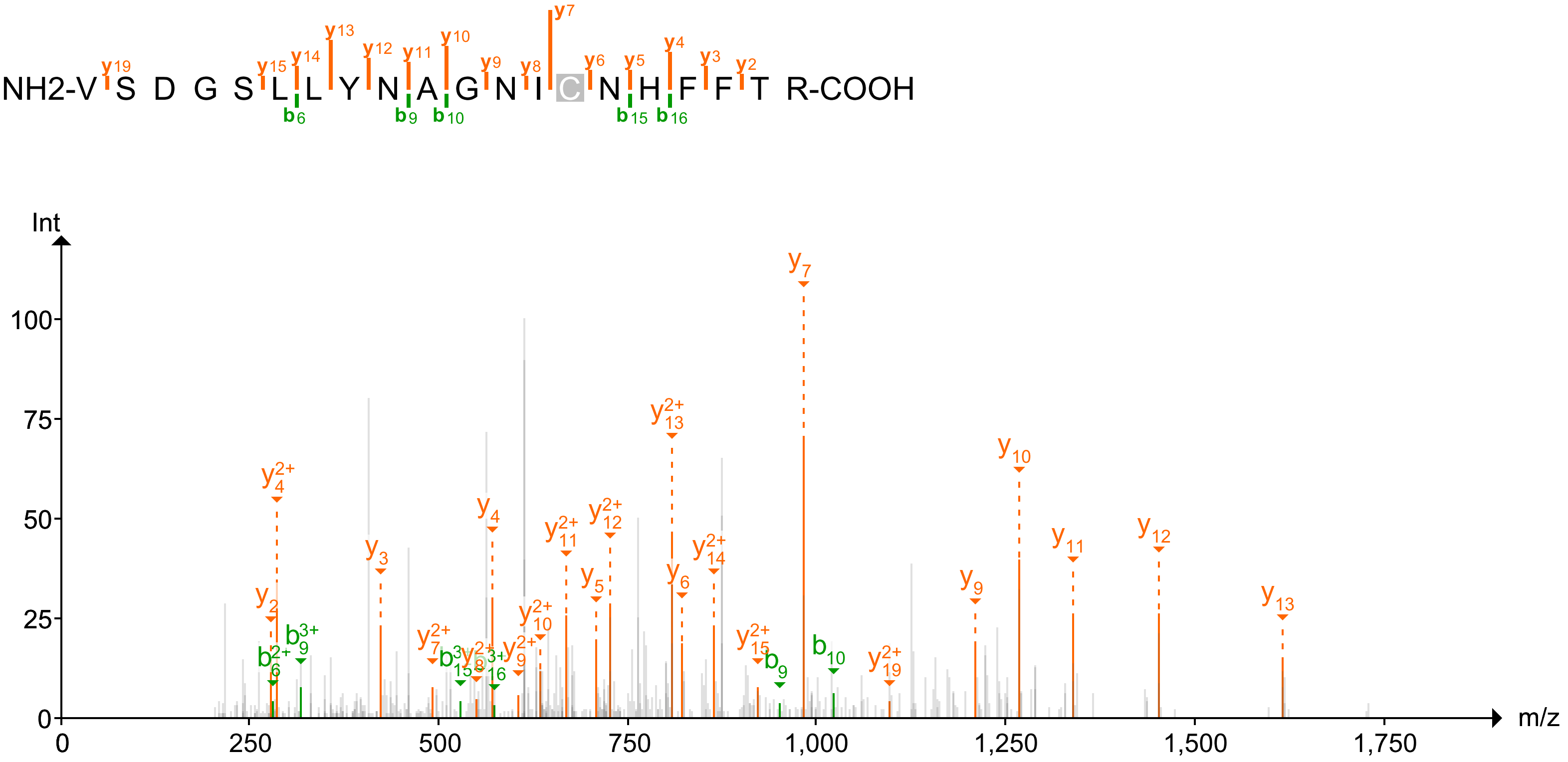

NH2-V S D G S L L Y N A G N I C N H F F T R-COOH
y19 y15 y14 y13 y12 y11 y10 y9 y8 y7 y6 y5 y4 y3 y2
b6 b9 b10 b15 b16
Int
100
75
50
25
0
0
250
500
750
1,000
1,250
1,500
1,750
m/z

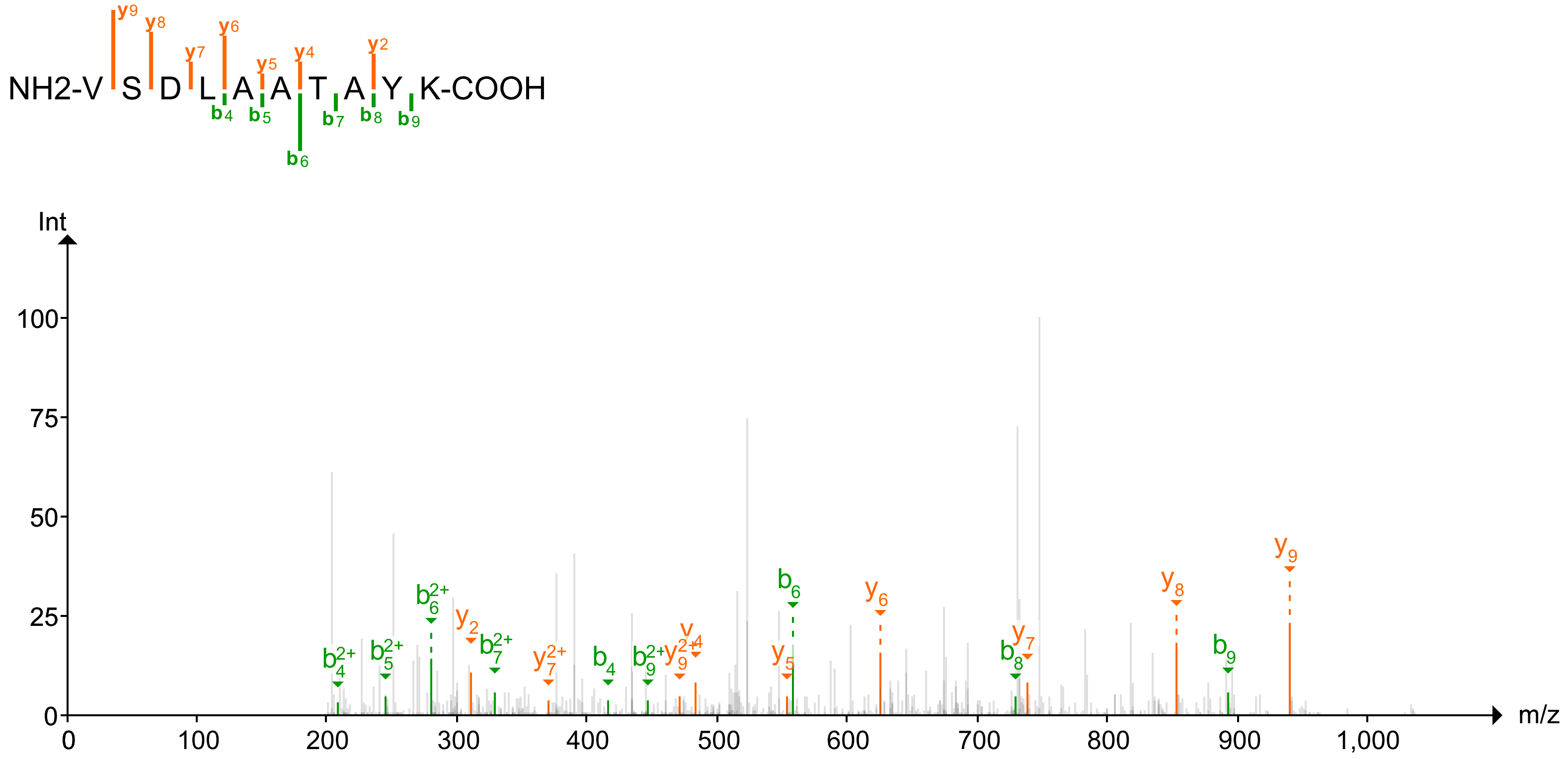

NH2-V S D L A A T A Y K-COOH
y9 y8 y7 y6 y5 y4 y2
b4 b5 b6 b7 b8 b9
Int
m/z

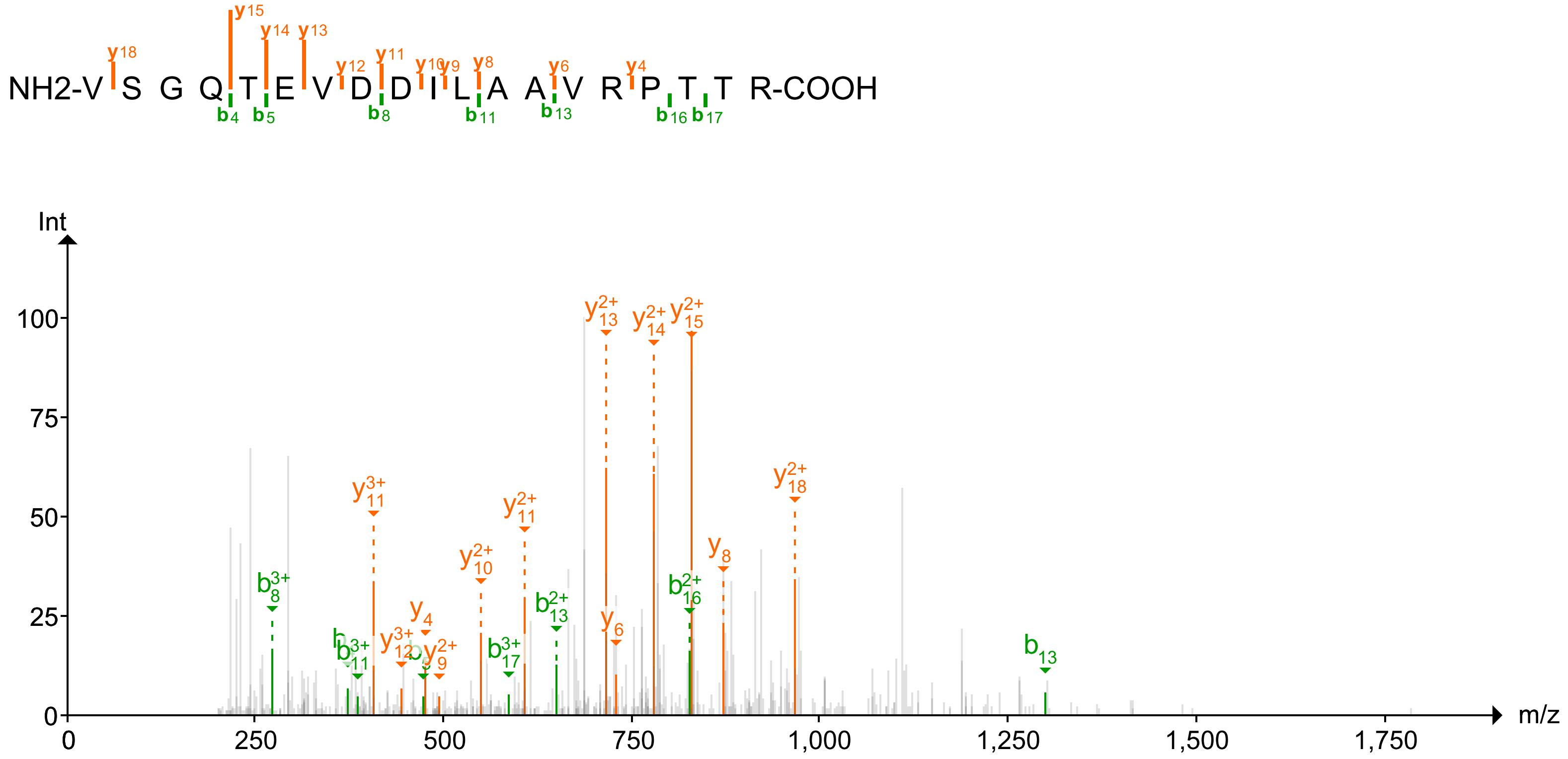

NH2-V S G Q T E V D D I L A A V R P T T R-COOH
y18 y15 y14 y13 y12 y11 y10 y9 y8 y6 y4
b4 b5 b8 b11 b13 b16 b17
Int
100
75
50
25
0
m/z
0
250
500
750
1,000
1,250
1,500
1,750
y2+ 13
y2+ 14
y2+ 15
y2+ 18
y3+ 11
y2+ 11
y2+ 10
y8
b2+ 16
b3+ 8
b2+ 13
y6
y4
b3+ 11
y3+ 12
y2+ 9
b3+ 17
b13

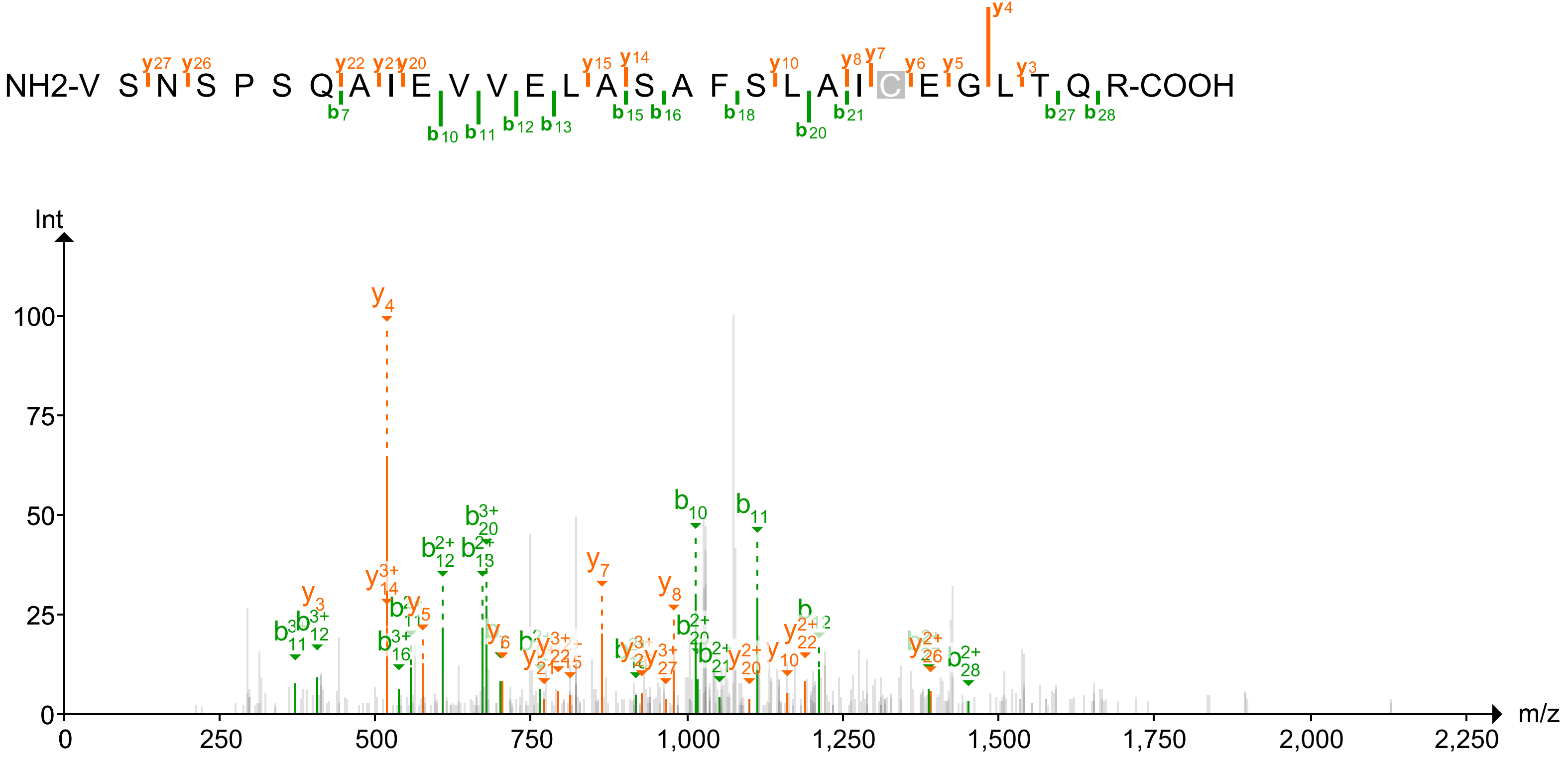
NH2-V S N S P S Q A I E V V E L A S A F S L A I C E G L T Q R-COOH
Int
m/z
100
75
50
25
0
250
500
750
1,000
1,250
1,500
1,750
2,000
2,250
y4
y7
y8
b10
b11
y3
y10

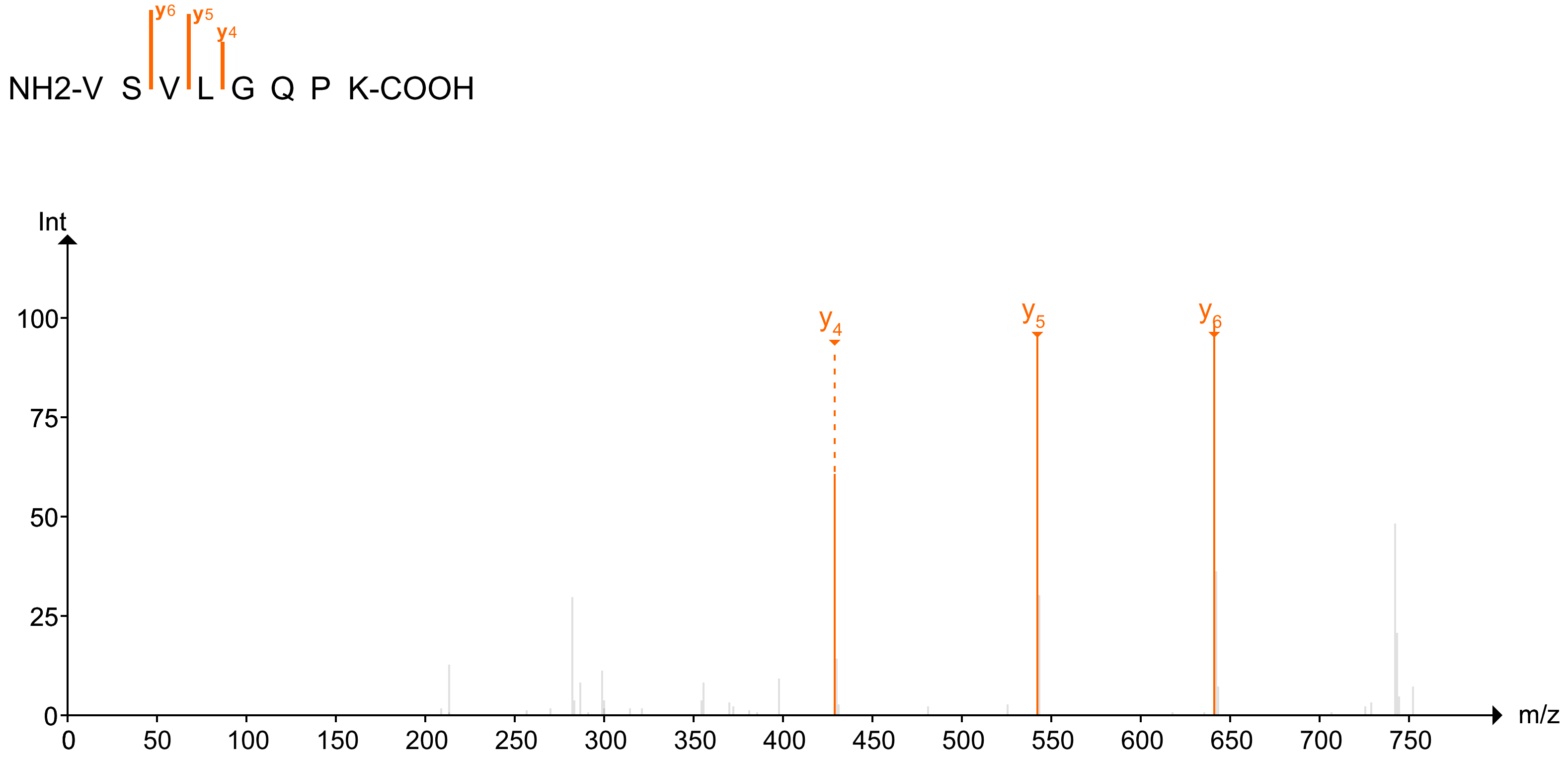

y6
y5
y4
NH2-V S V L G Q P K-COOH
Int
100
75
50
25
0
y4
y5
y6
0
50
100
150
200
250
300
350
400
450
500
550
600
650
700
750
m/z

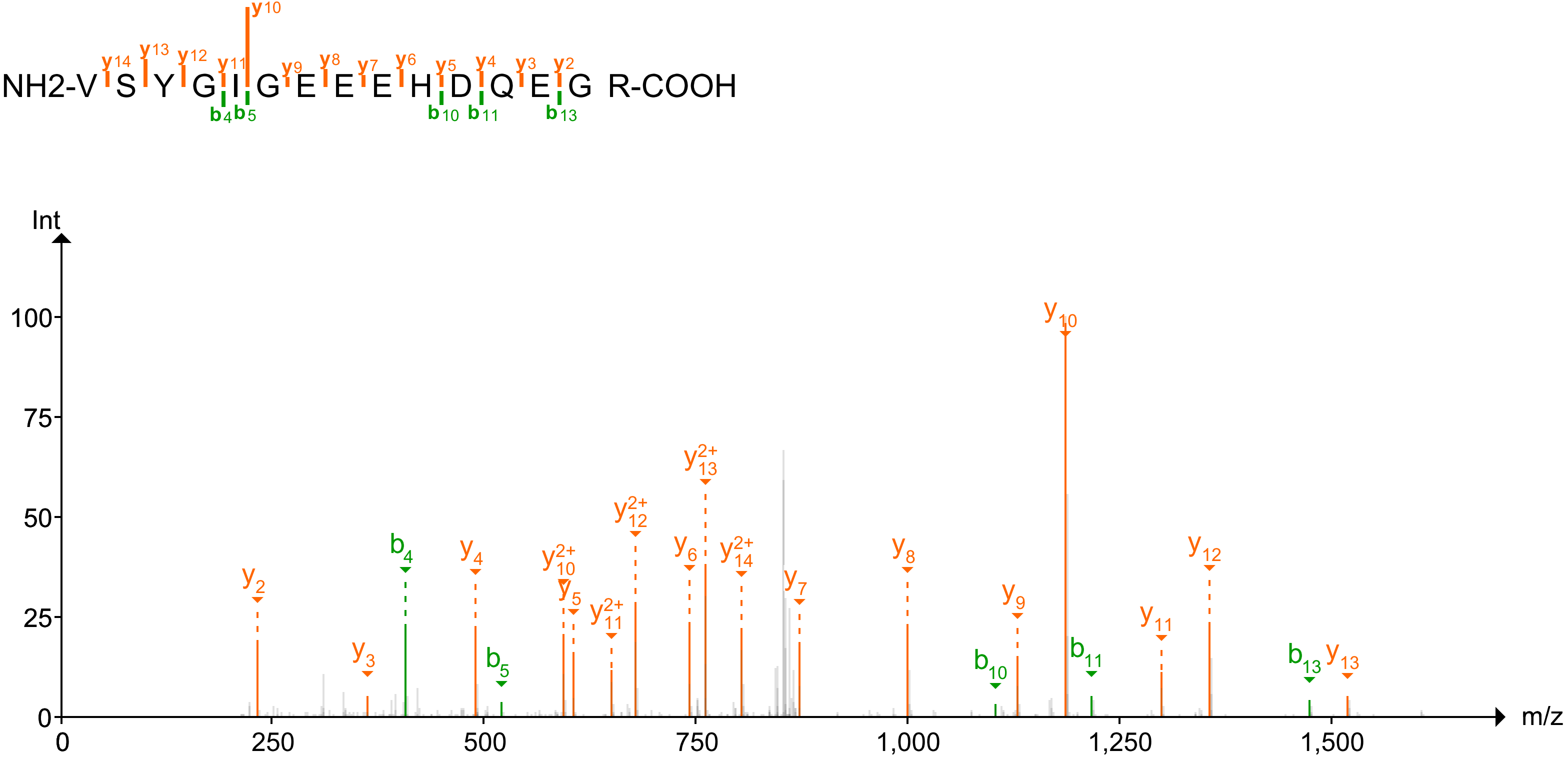

NH2-V S Y G I G E E E H D Q E G R-COOH
y14 y13 y12 y11 y10 y9 y8 y7 y6 y5 y4 y3 y2
b4 b5 b10 b11 b13
Int
100
75
50
25
0
250
500
750
1,000
1,250
1,500
m/z
y10
y13 2+
y12 2+
b4
y4
y10 2+
y6
y14 2+
y8
y12
y2
y5
y11 2+
y7
y9
y11
y3
b5
b10
b11
b13
y13

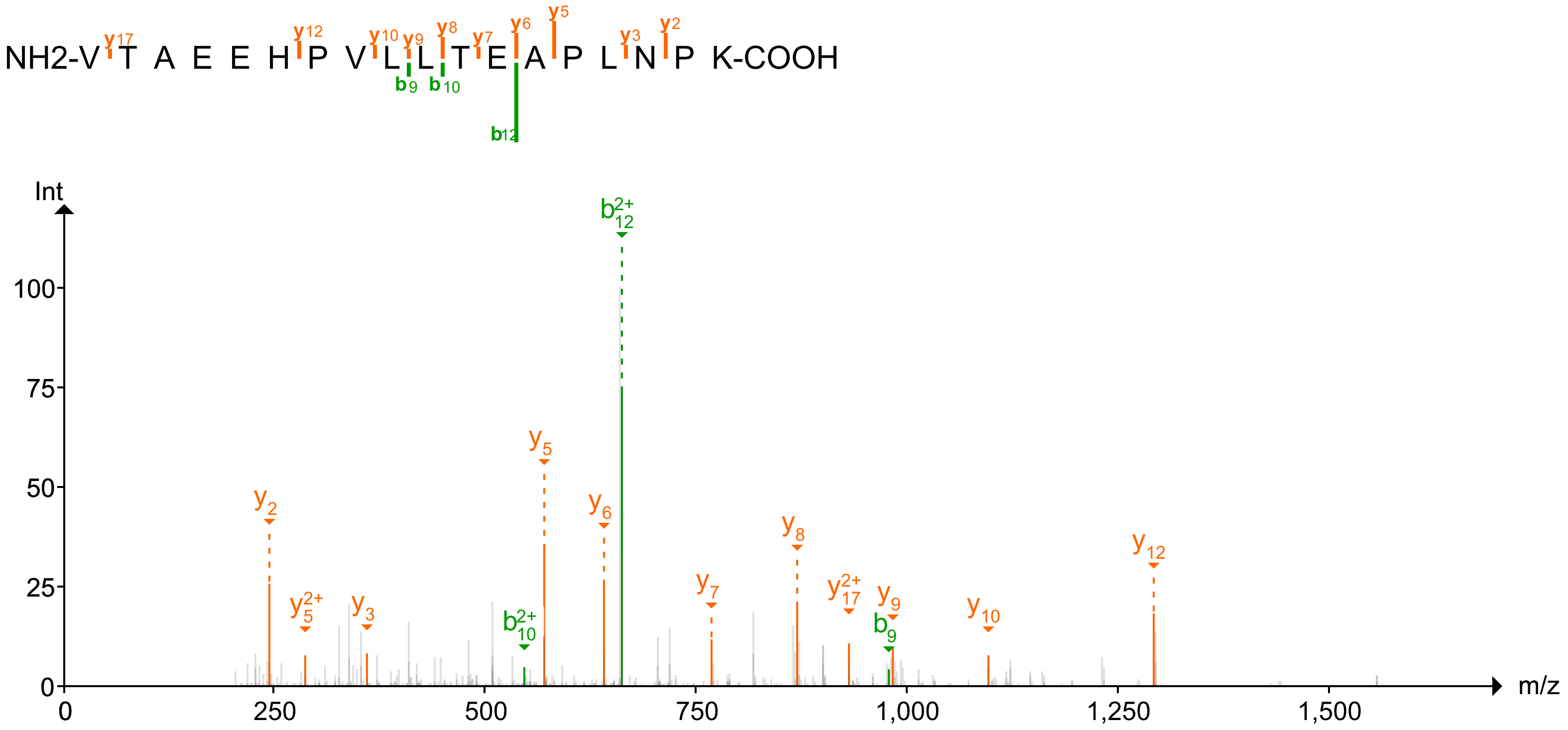

NH2-V T A E E H P V L L T E A P L N P K-COOH
y17 y12 y10 y9 y8 y7 y6 y5 y3 y2
b9 b10 b12
Int
100
75
50
25
0
m/z
0
250
500
750
1,000
1,250
1,500
y2
y5 2+
y3
b10 2+
y5
y6
b12 2+
y7
y8
y17 2+
y9
b9
y10
y12

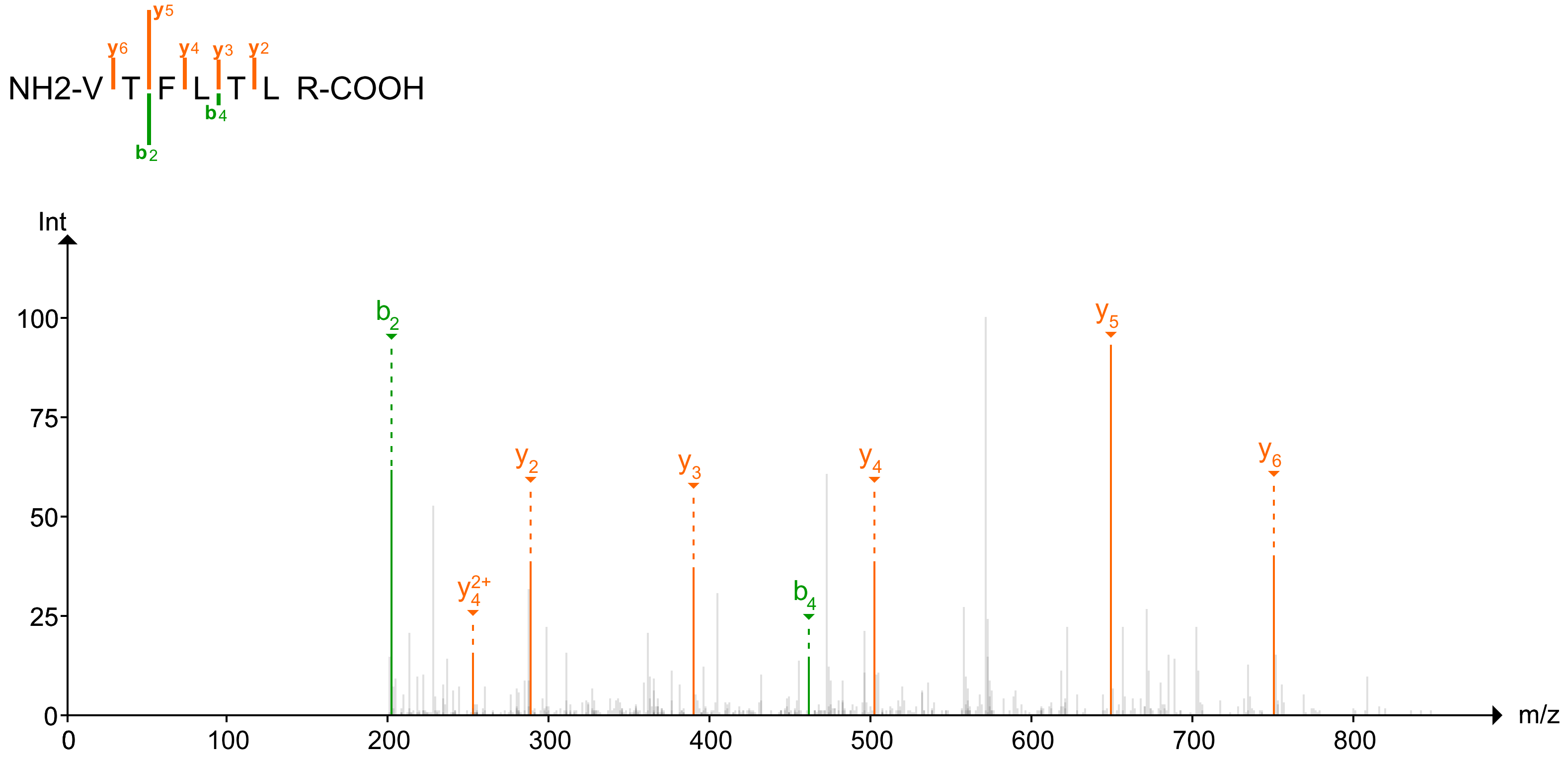

NH2-V T F L T L R-COOH
y6 y5 y4 y3 y2
b2 b4
Int
100
75
50
25
0
b2
y2
y3
y4
y5
y6
y4 2+
b4
0
100
200
300
400
500
600
700
800
m/z

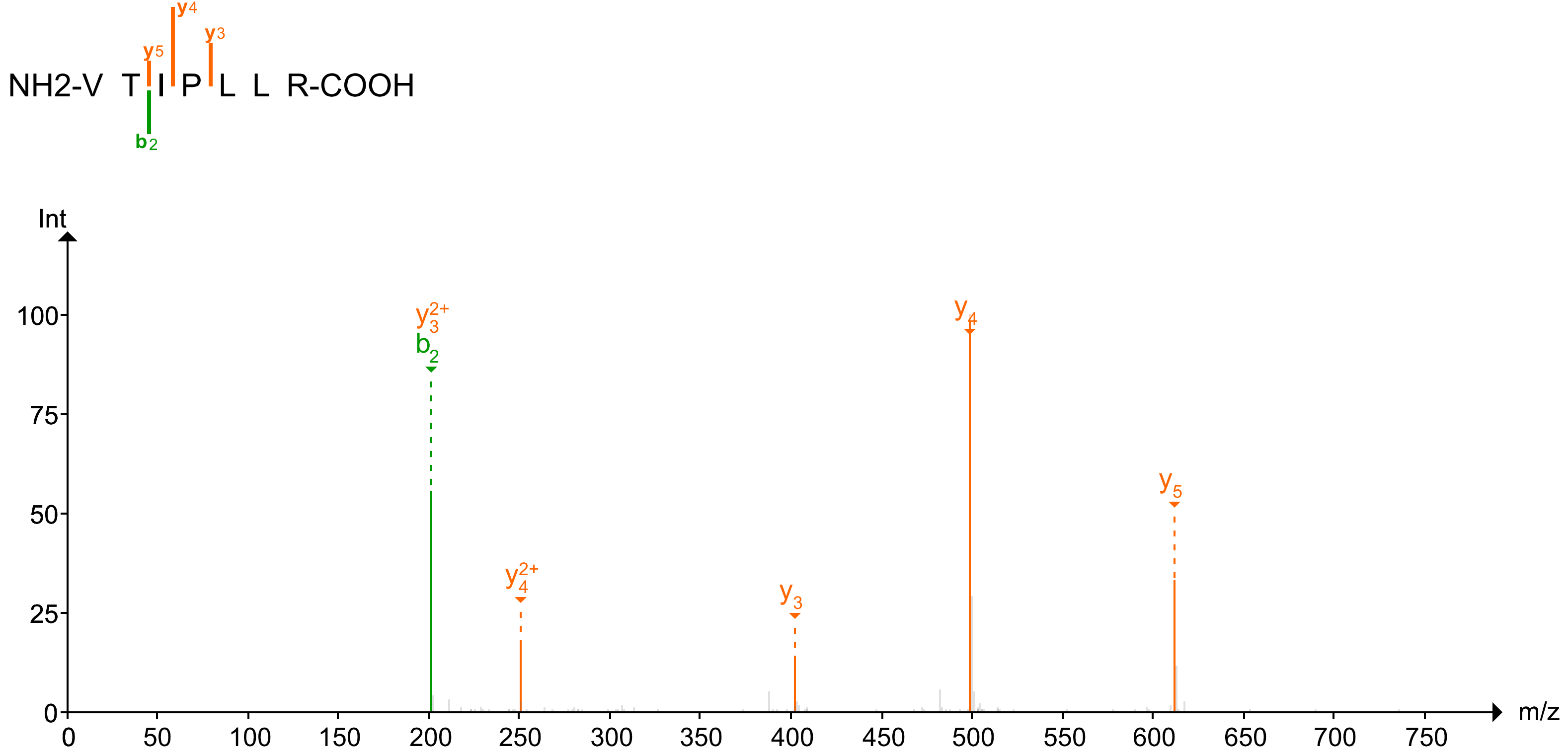

NH2-V T I P L L R-COOH
y5
y4
y3
b2
Int
100
75
50
25
0
m/z
0
50
100
150
200
250
300
350
400
450
500
550
600
650
700
750
$y_3^{2+}$
$b_2$
$y_4^{2+}$
$y_3$
$y_4$
$y_5$

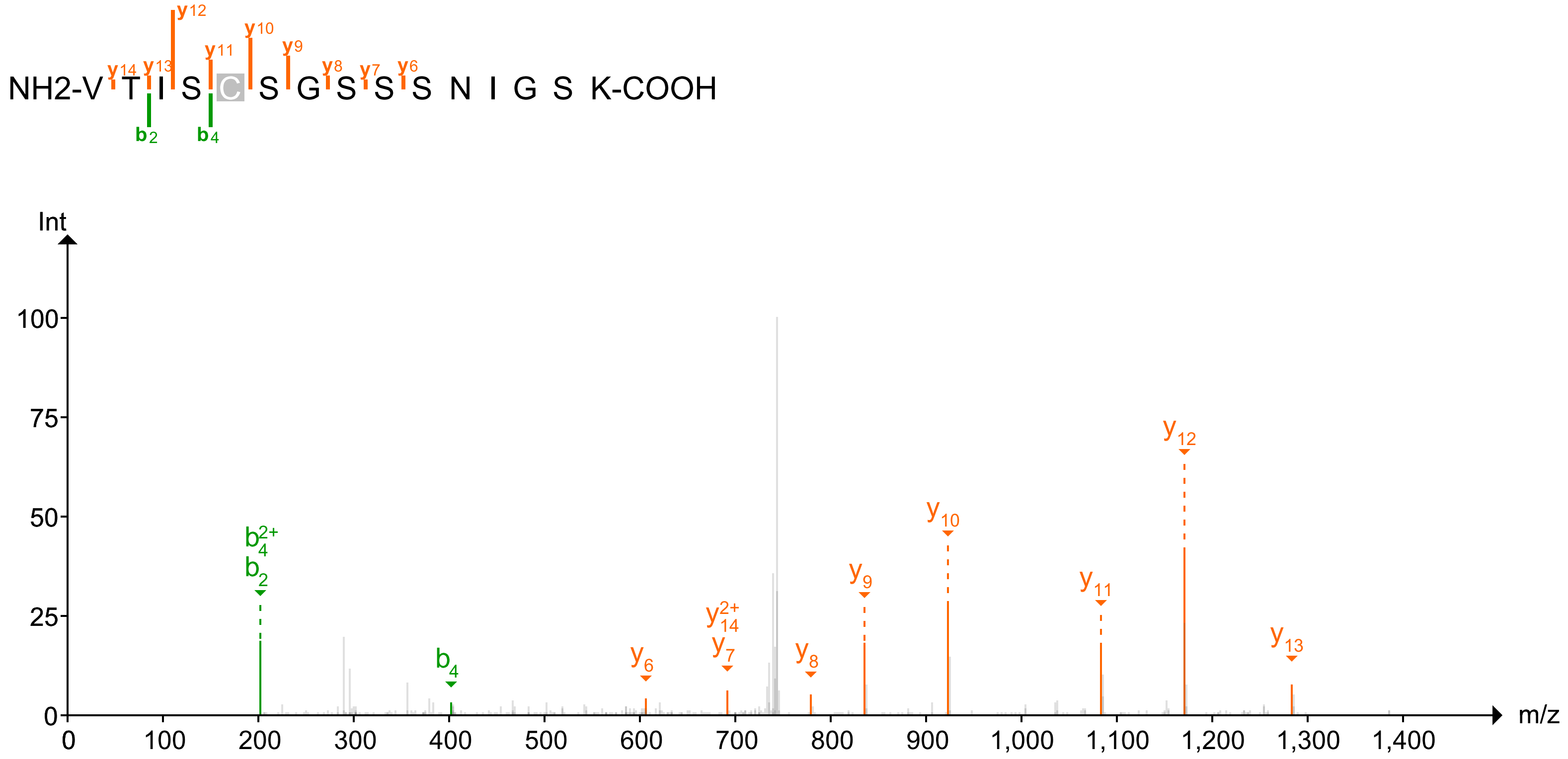

NH2-V T I S C S G S S S N I G S K-COOH
y14 y13 y12 y11 y10 y9 y8 y7 y6
b2 b4
Int
100
75
50
25
0
0 100 200 300 400 500 600 700 800 900 1,000 1,100 1,200 1,300 1,400
m/z
b4 2+
b2
b4
y6
y14 2+
y7
y8
y9
y10
y11
y12
y13

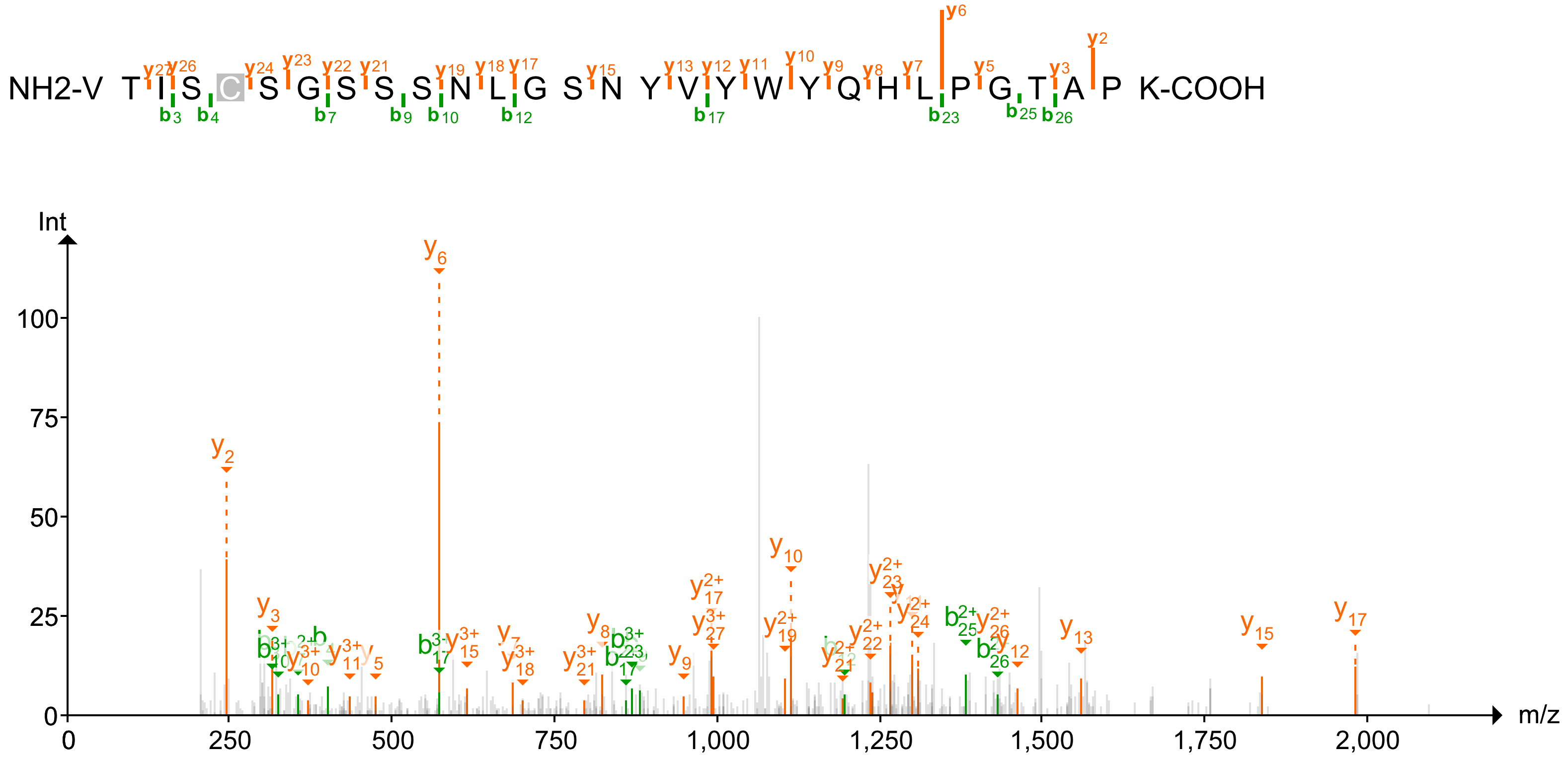

NH2-V T I S C S G S S S N L G S N Y V Y W Y Q H L P G T A P K-COOH
Int
100
75
50
25
0
250
500
750
1,000
1,250
1,500
1,750
2,000
m/z

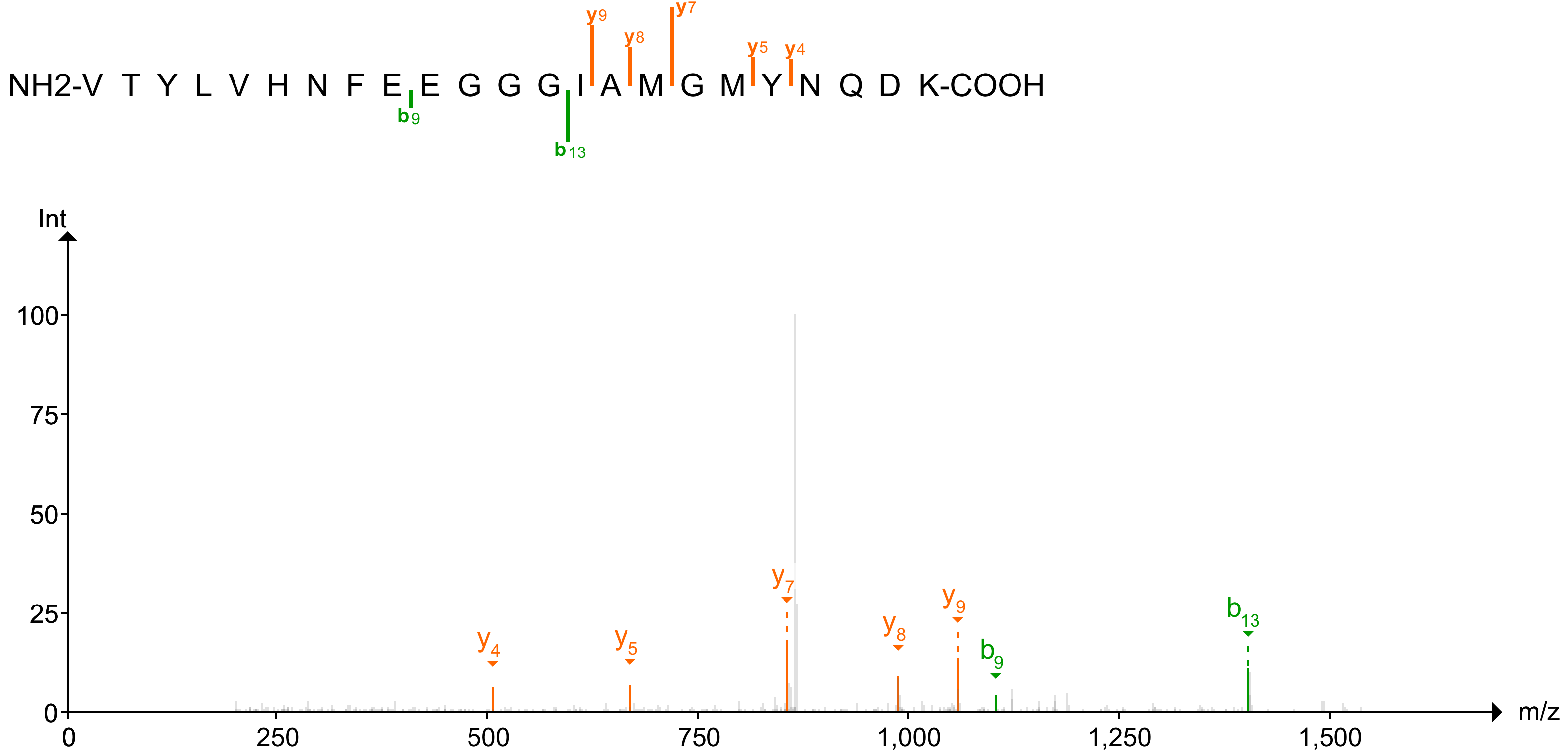

NH2-V T Y L V H N F E E G G G I A M G M Y N Q D K-COOH
y9
y8
y7
y5
y4
b9
b13
Int
100
75
50
25
0
m/z
0
250
500
750
1,000
1,250
1,500

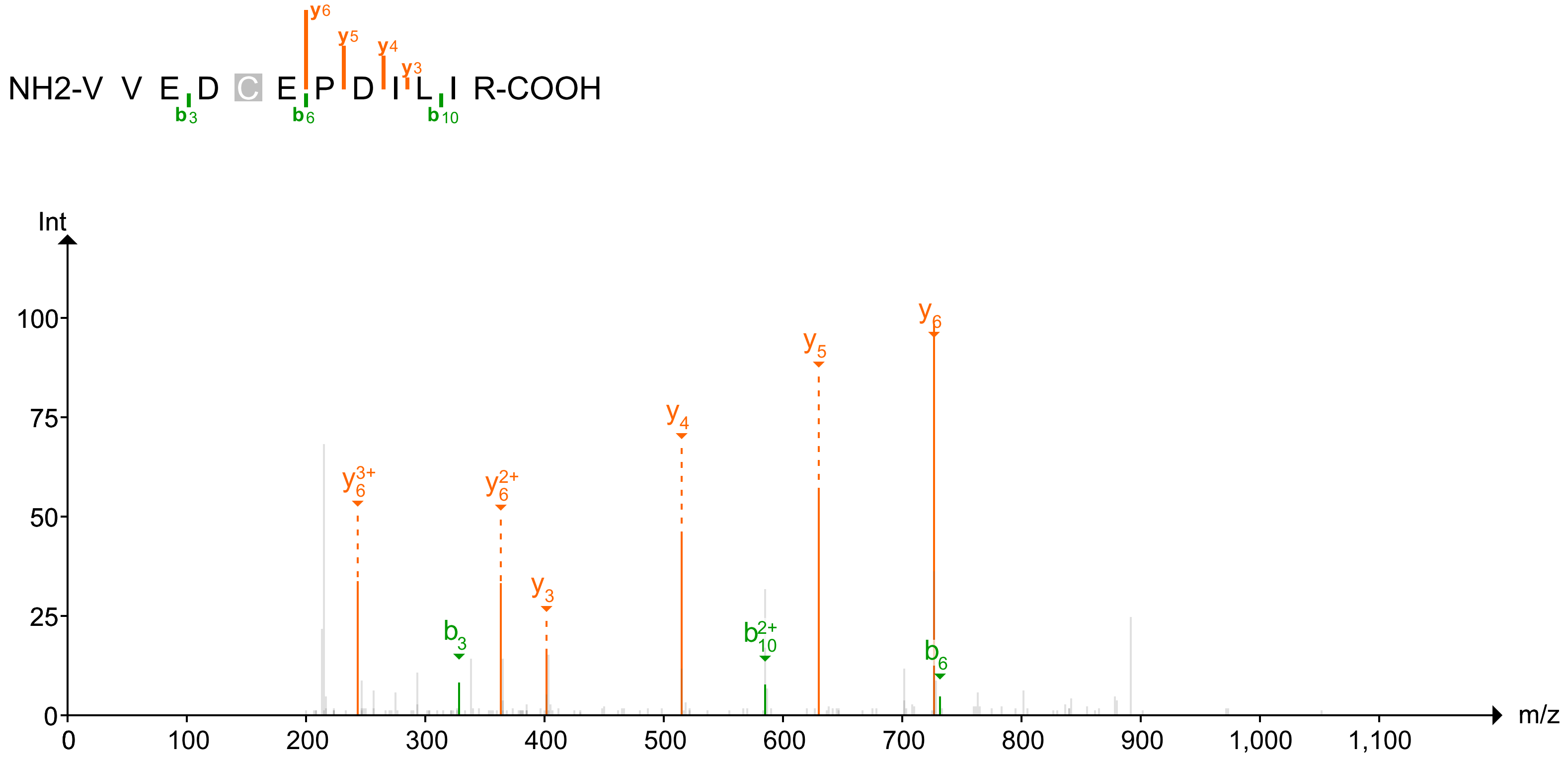

NH2-V V E D C E P D I L I R-COOH
b3
b6
b10
y6
y5
y4
y3
Int
100
75
50
25
0
0
100
200
300
400
500
600
700
800
900
1,000
1,100
m/z
y6 3+
y6 2+
y3
b3
y4
b10 2+
y5
y6
b6

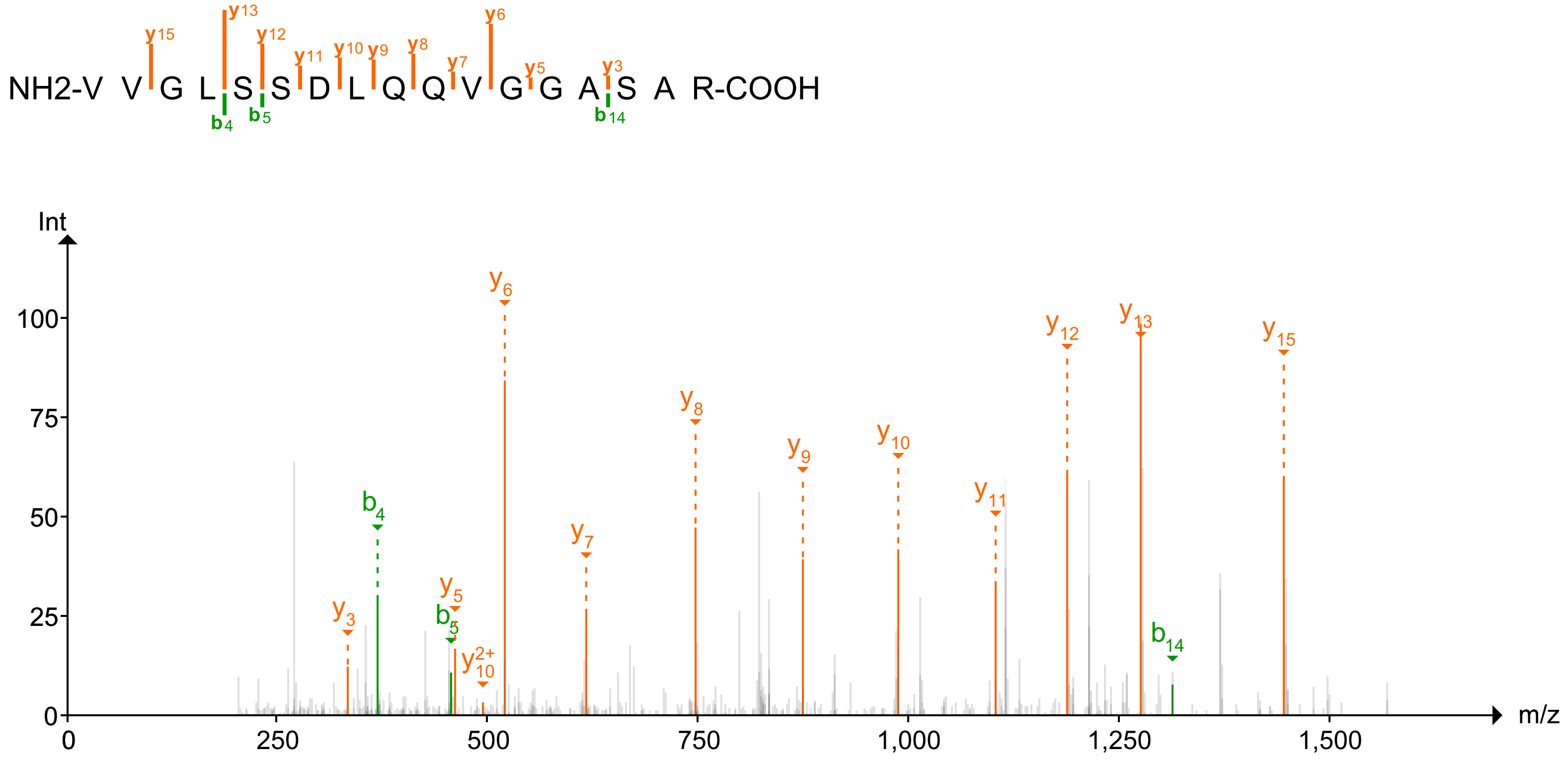

NH2-V V G L S S D L Q Q V G G A S A R-COOH
y15
y13
y12
y11
y10
y9
y8
y7
y6
y5
y3
b4
b5
b14
Int
m/z
0
25
50
75
100
250
500
750
1,000
1,250
1,500
y10 2+

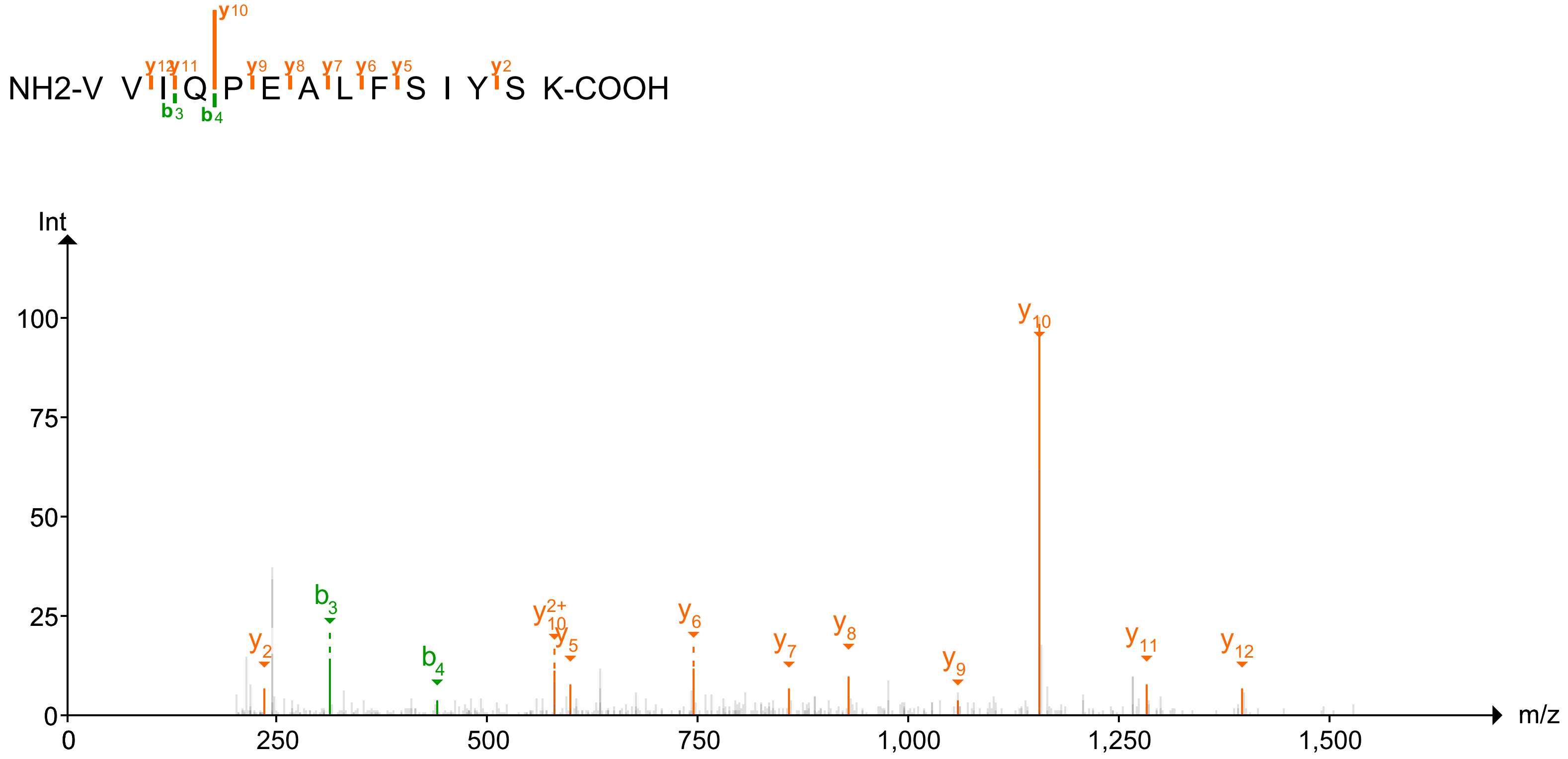

NH2-V V I Q P E A L F S I Y S K-COOH
y12 y11 y10 y9 y8 y7 y6 y5 y2
b3 b4
Int
100
75
50
25
0
m/z
0
250
500
750
1,000
1,250
1,500
y10
b3
y2
b4
y10 2+
y5
y6
y7
y8
y9
y11
y12

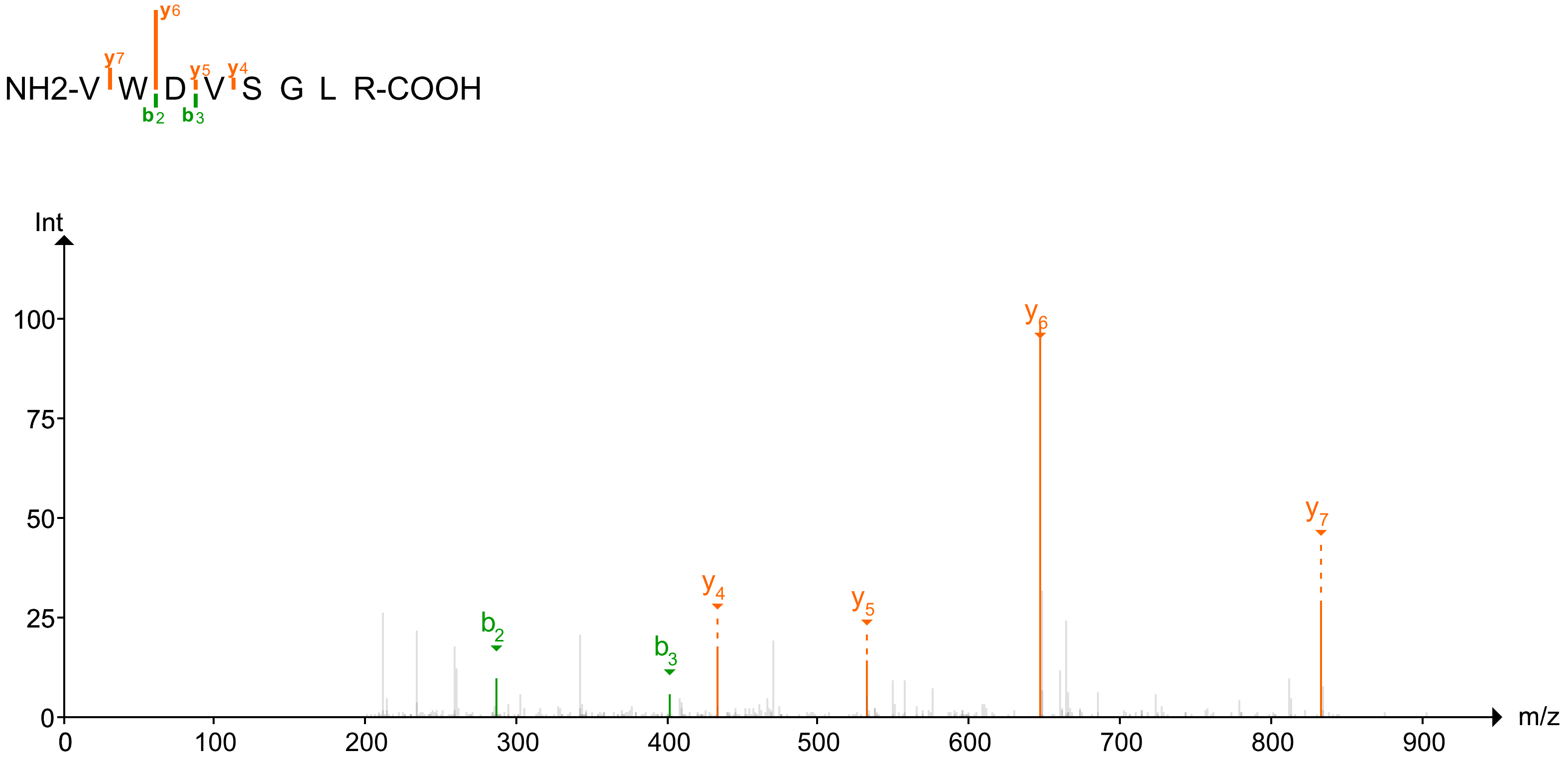

NH2-V W D V S G L R-COOH
y7 y6 y5 y4
b2 b3
Int
100
75
50
25
0
m/z
0
100
200
300
400
500
600
700
800
900
y6
y7
y4
y5
b2
b3

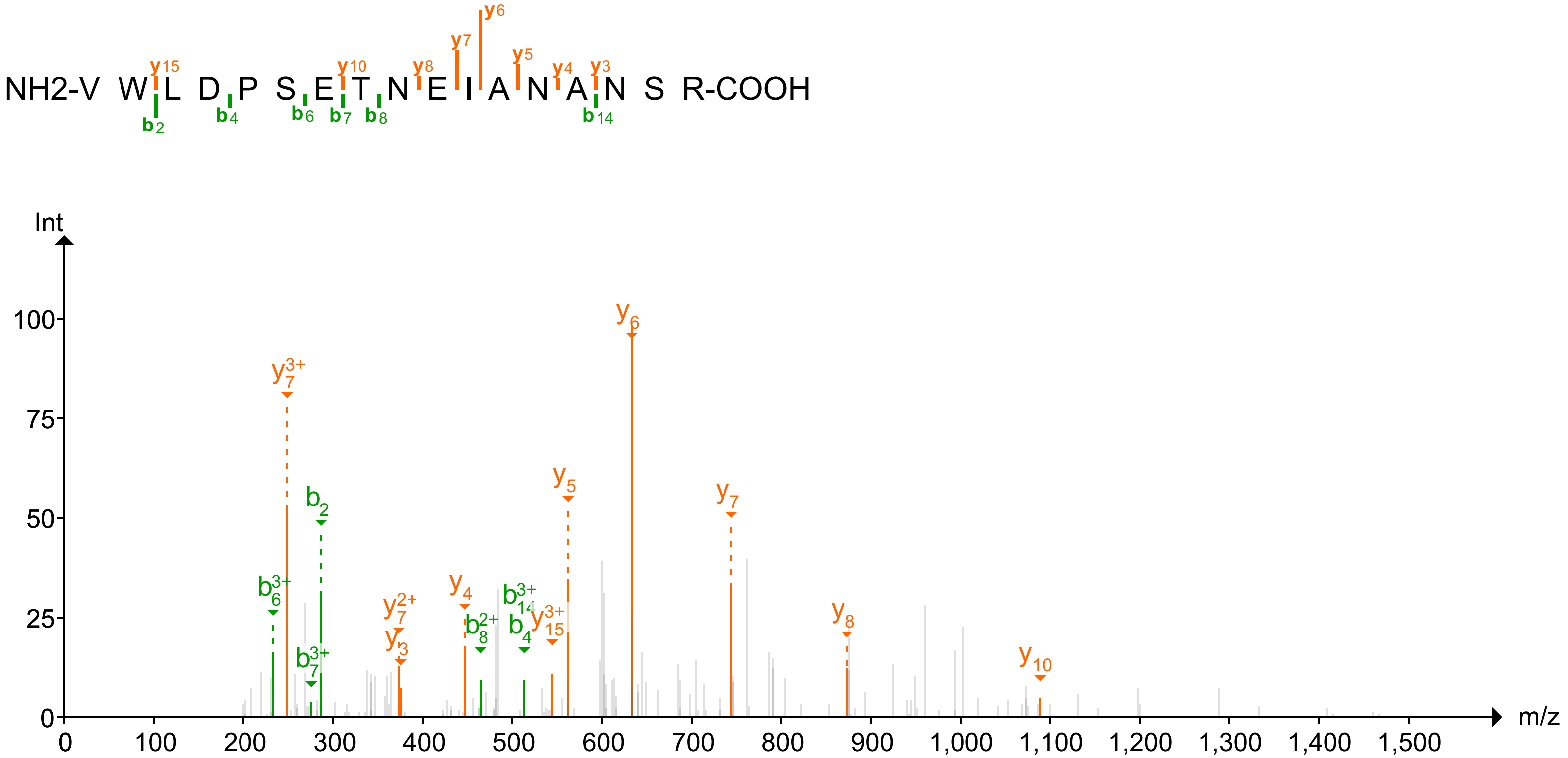
NH2-V W L D P S E T N E I A N A N S R-COOH
y15 y10 y8 y7 y6 y5 y4 y3
b2 b4 b6 b7 b8 b14
Int
m/z
0
25
50
75
100
0 100 200 300 400 500 600 700 800 900 1,000 1,100 1,200 1,300 1,400 1,500

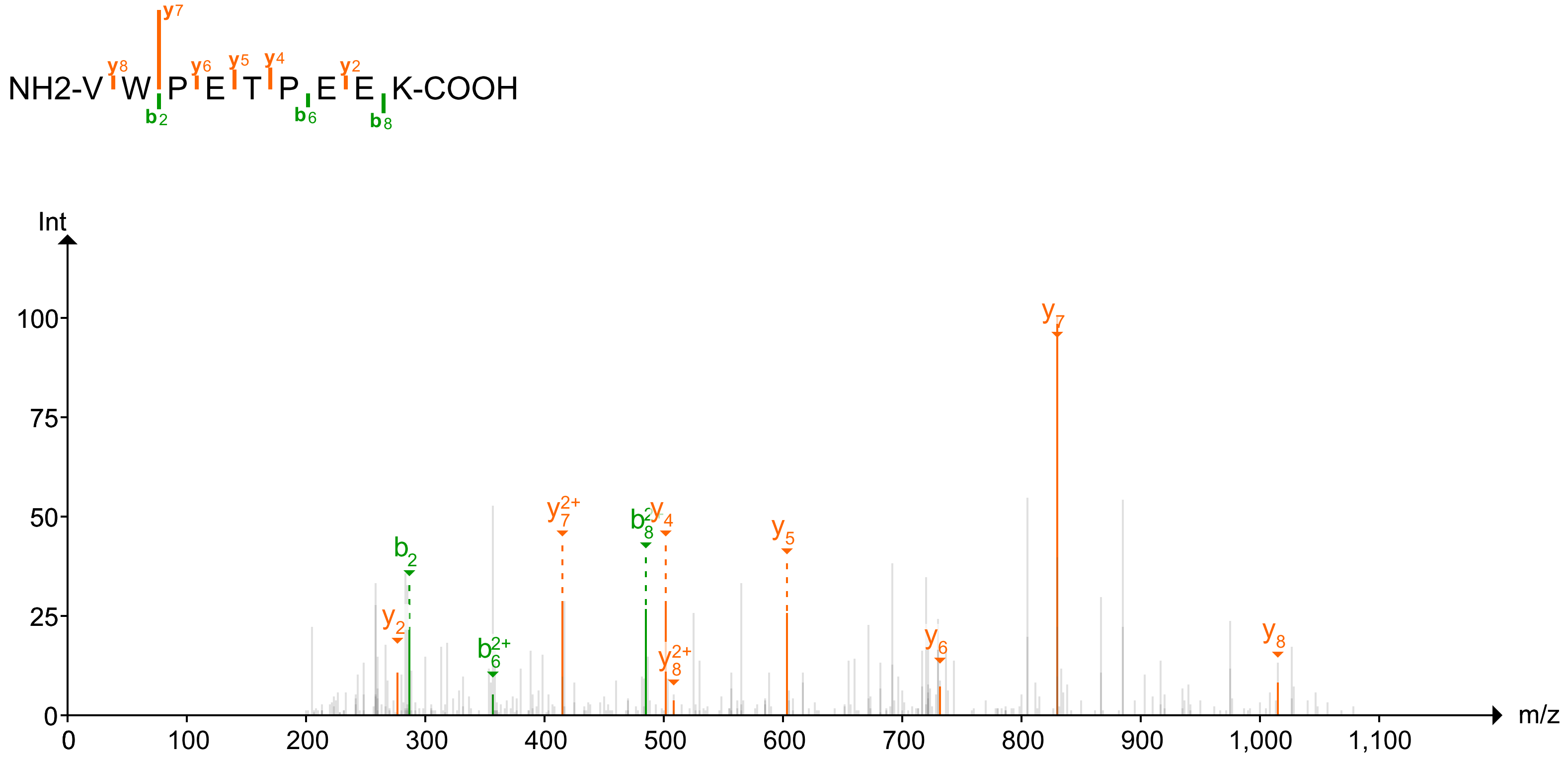

NH2-V W P E T P E E K-COOH
y8 y7 y6 y5 y4 y2
b2 b6 b8
Int
100
75
50
25
0
m/z
0
100
200
300
400
500
600
700
800
900
1,000
1,100
y7
y7 2+
b8 2+
y4
y5
b2
y2
b6 2+
y8 2+
y6
y8

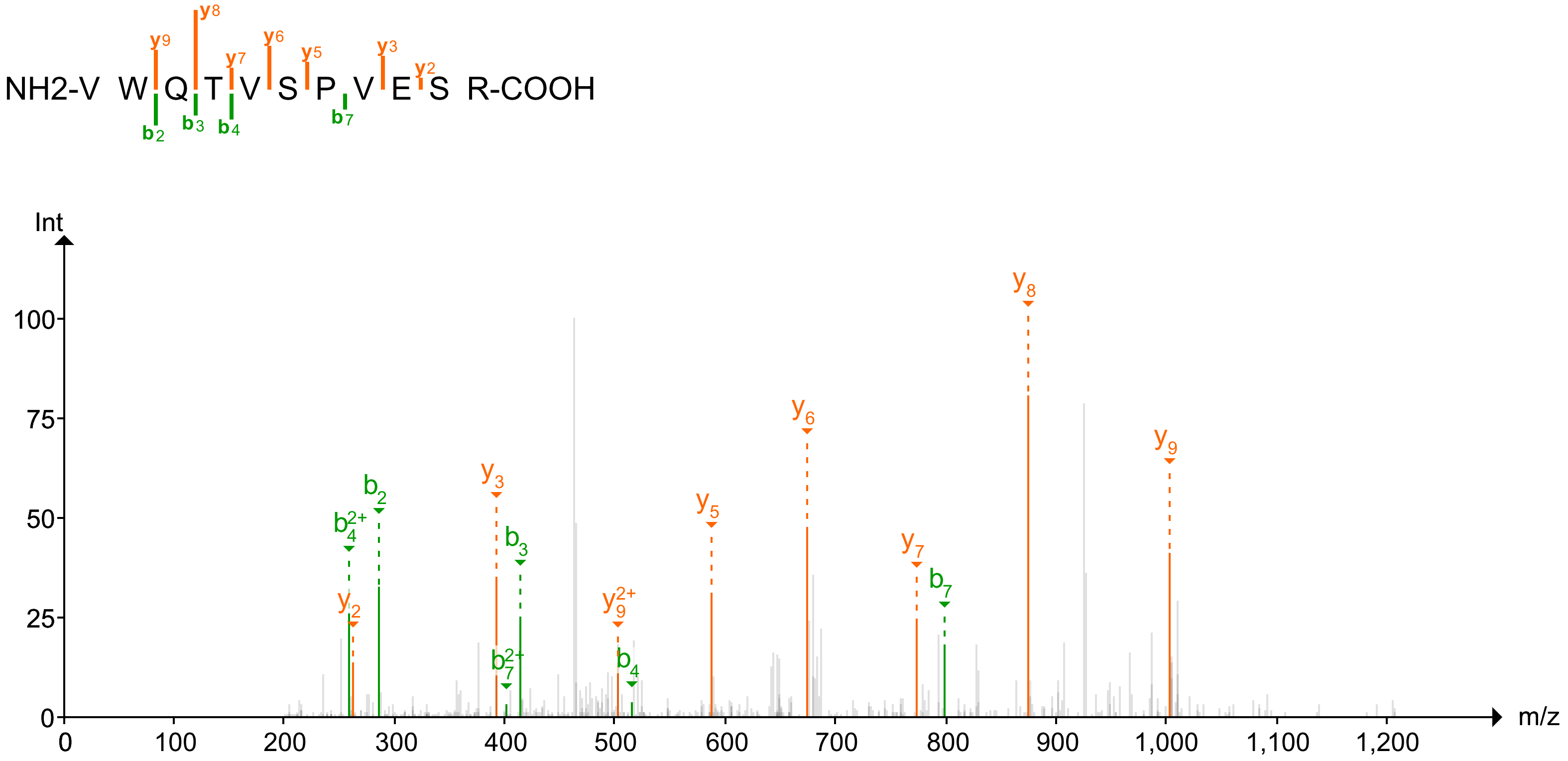

NH2-V W Q T V S P V E S R-COOH
y9 y8 y7 y6 y5 y3 y2
b2 b3 b4 b7
Int
100
75
50
25
0
m/z
0 100 200 300 400 500 600 700 800 900 1,000 1,100 1,200
y8
y6
y9
y3
b2
$b_4^{2+}$
b3
y5
y7
b7
y2
$y_9^{2+}$
$b_7^{2+}$
b4

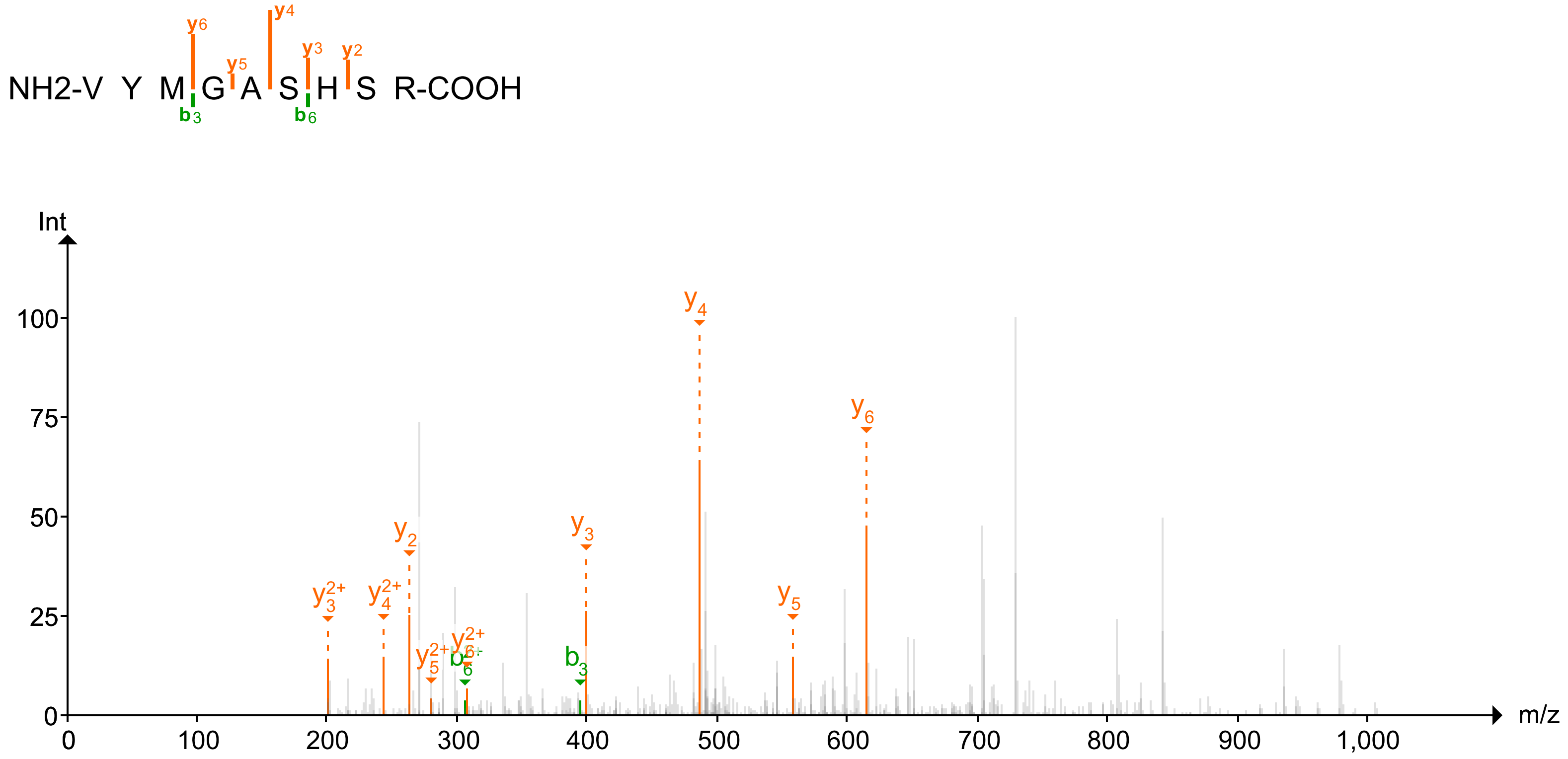

NH2-V Y M G A S H S R-COOH
y6 y5 y4 y3 y2
b3 b6
Int
100
75
50
25
0
m/z
0 100 200 300 400 500 600 700 800 900 1,000

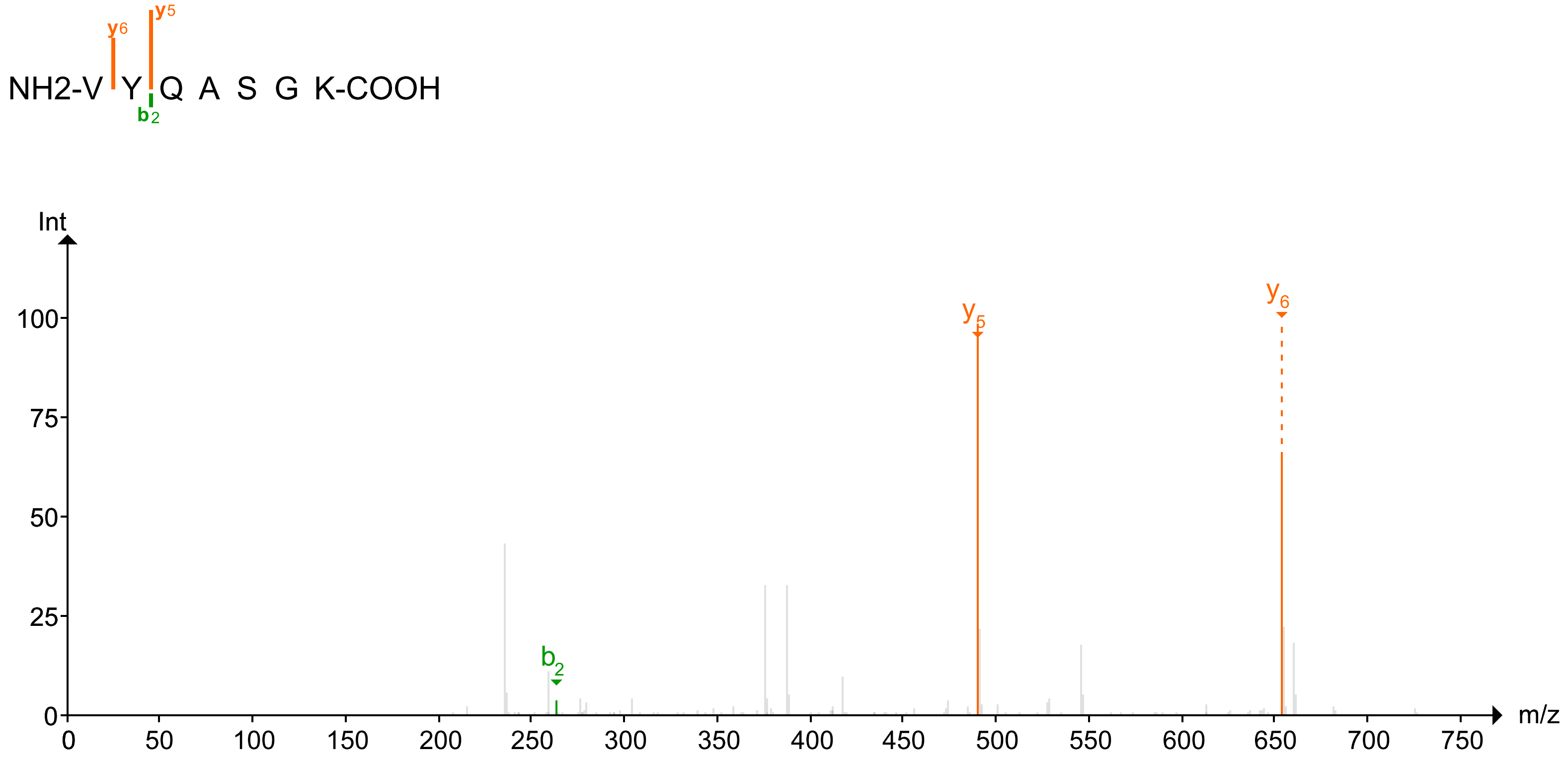

NH2-V Y Q A S G K-COOH
y6
y5
b2
Int
100
75
50
25
0
m/z
0
50
100
150
200
250
300
350
400
450
500
550
600
650
700
750

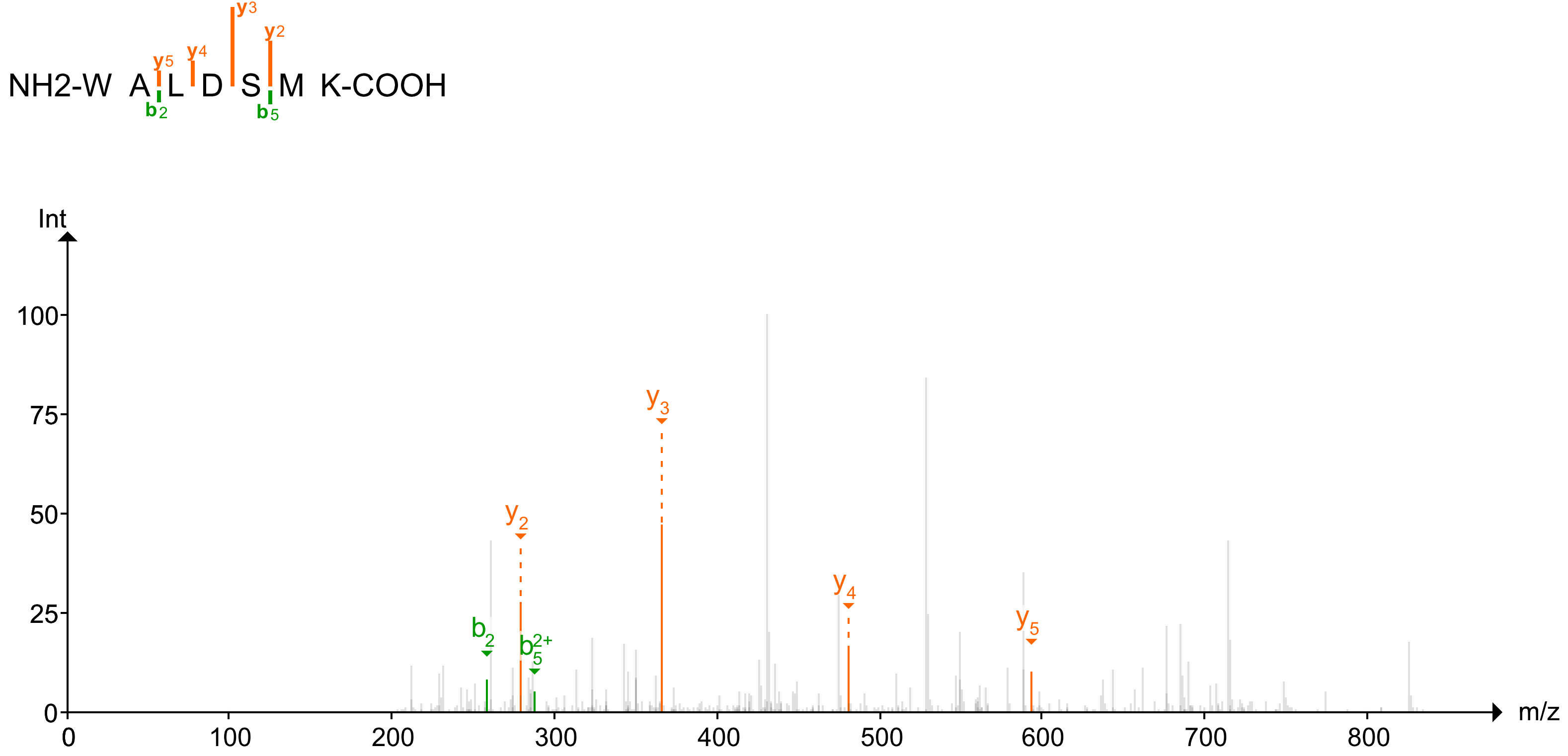

NH2-W A L D S M K-COOH
y5
y4
y3
y2
b2
b5
Int
100
75
50
25
0
y3
y2
y4
y5
b2
b5 2+
0
100
200
300
400
500
600
700
800
m/z

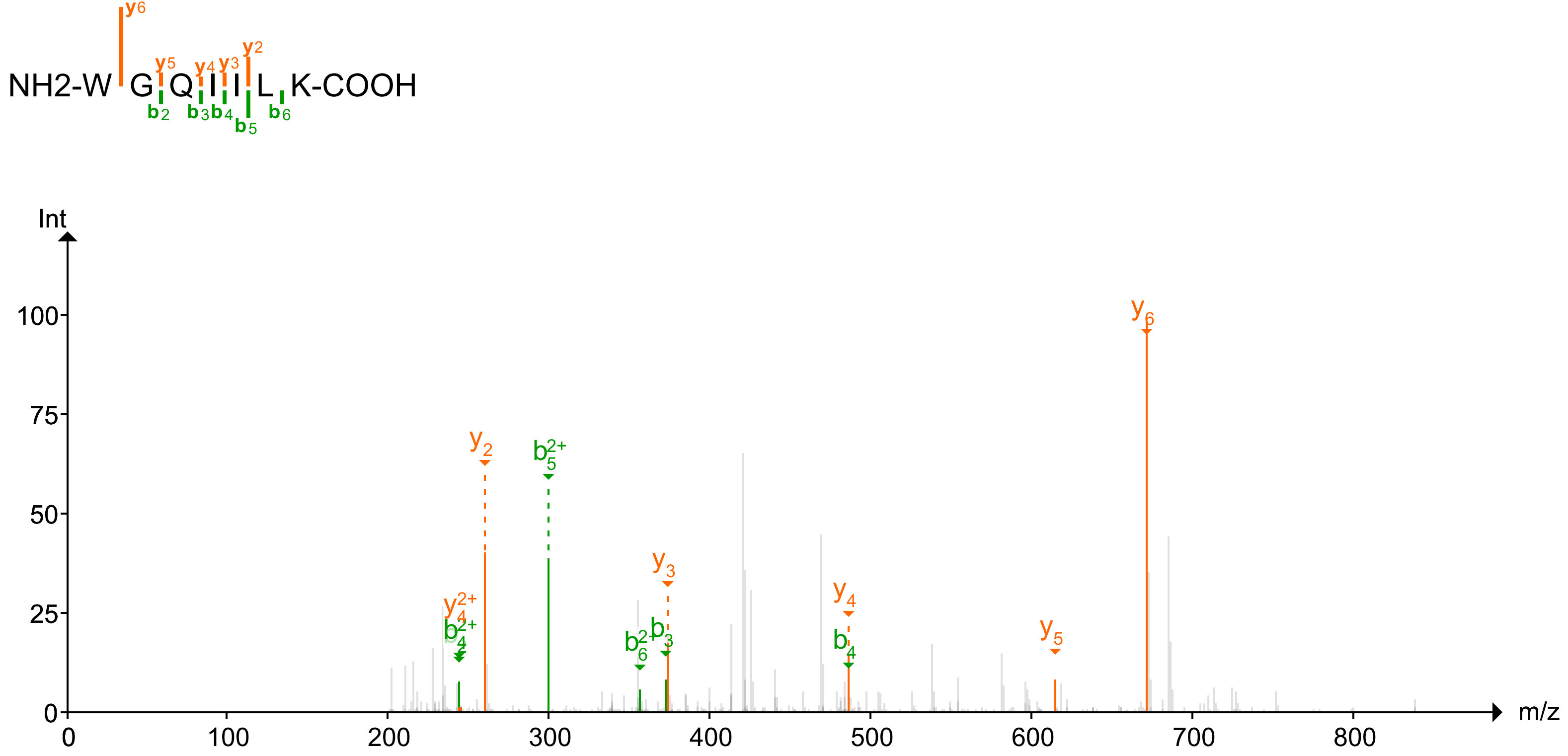

NH2-W G Q I I L K-COOH
y6
y5
y4
y3
y2
b2
b3
b4
b5
b6
Int
100
75
50
25
0
m/z
0
100
200
300
400
500
600
700
800
y2
b5 2+
y3
y4
y5
y6
y4 2+
b4 2+
b6 2+
b3
b4

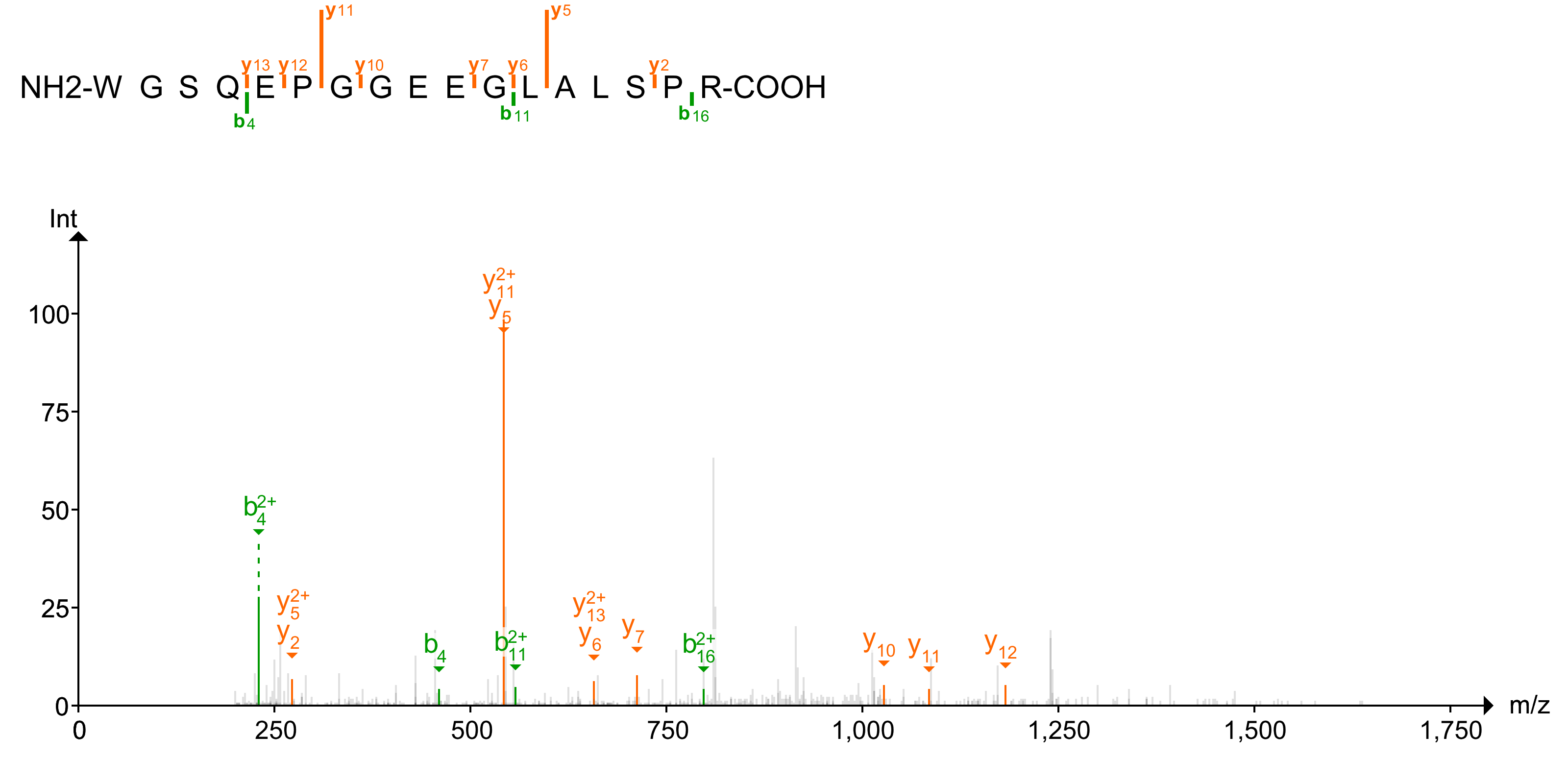

NH2-W G S Q E P G G E E G L A L S P R-COOH
y13 y12 y11 y10 y7 y6 y5 y2
b4 b11 b16
Int
m/z
y$_{11}^{2+}$
y$_5$
b$_4^{2+}$
y$_5^{2+}$
y$_2$
b$_4$
b$_{11}^{2+}$
y$_{13}^{2+}$
y$_6$
y$_7$
b$_{16}^{2+}$
y$_{10}$
y$_{11}$
y$_{12}$

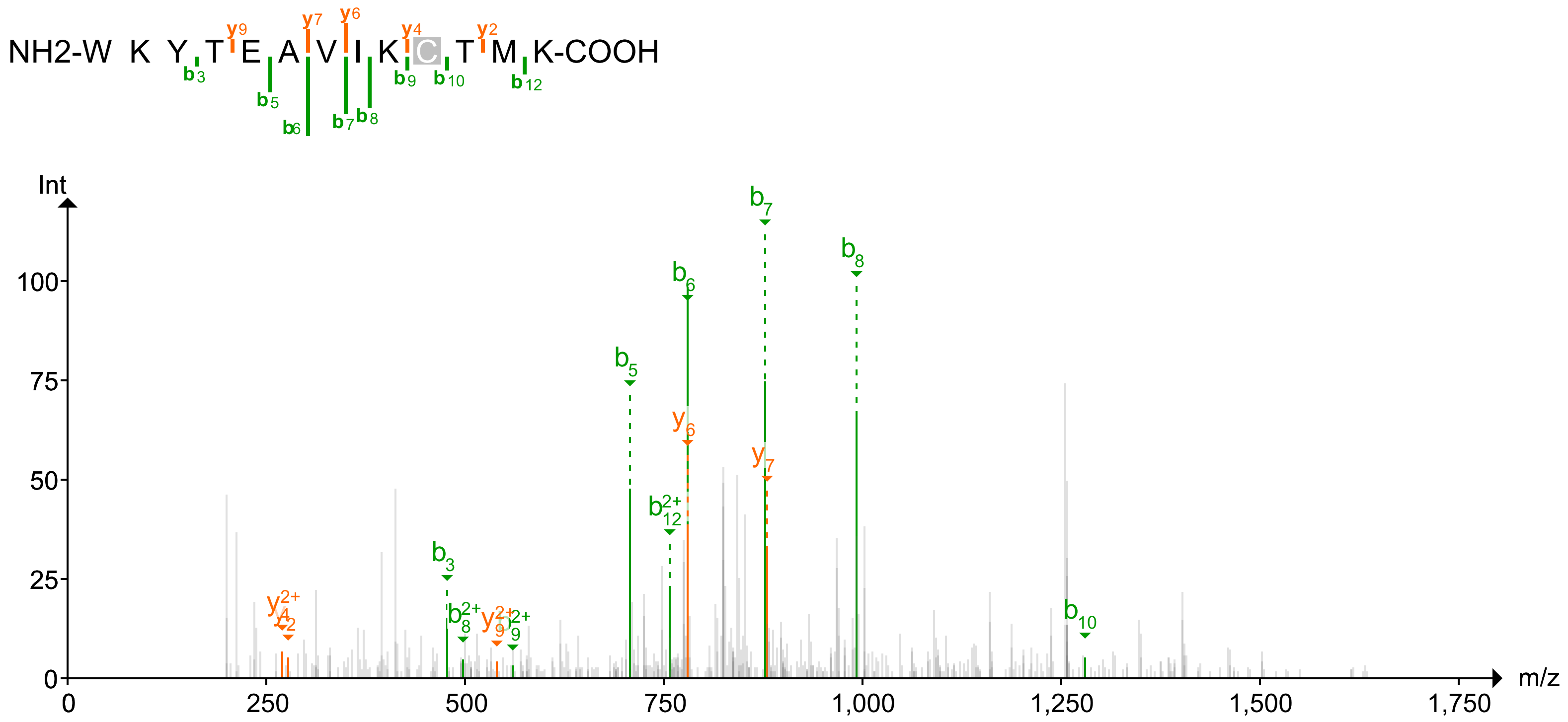
NH2-W K Y T E A V I K C T M K-COOH
y9 y7 y6 y4 y2
b3 b5 b6 b7 b8 b9 b10 b12
Int
100
75
50
25
0
m/z
0
250
500
750
1,000
1,250
1,500
1,750
b7
b8
b6
b5
y6
y7
b12 2+
b3
b10
y4 2+
y2
b8 2+
y9 2+
b9 2+

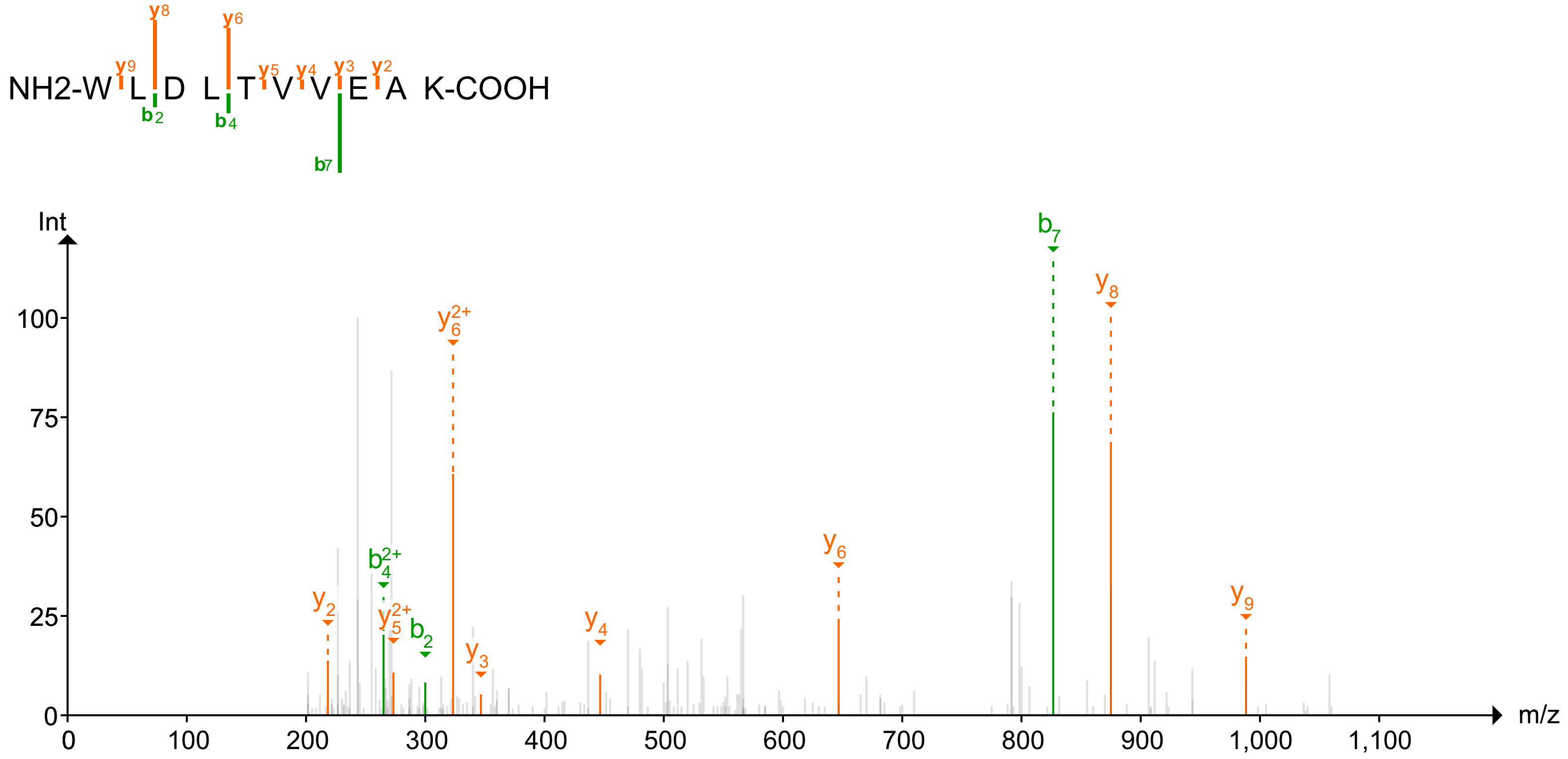

NH2-W L D L T V V E A K-COOH
y9
y8
y6
y5
y4
y3
y2
b2
b4
b7
Int
100
75
50
25
0
m/z
0
100
200
300
400
500
600
700
800
900
1,000
1,100
b7
y8
y6 2+
b4 2+
y2
y5 2+
b2
y3
y4
y6
y9

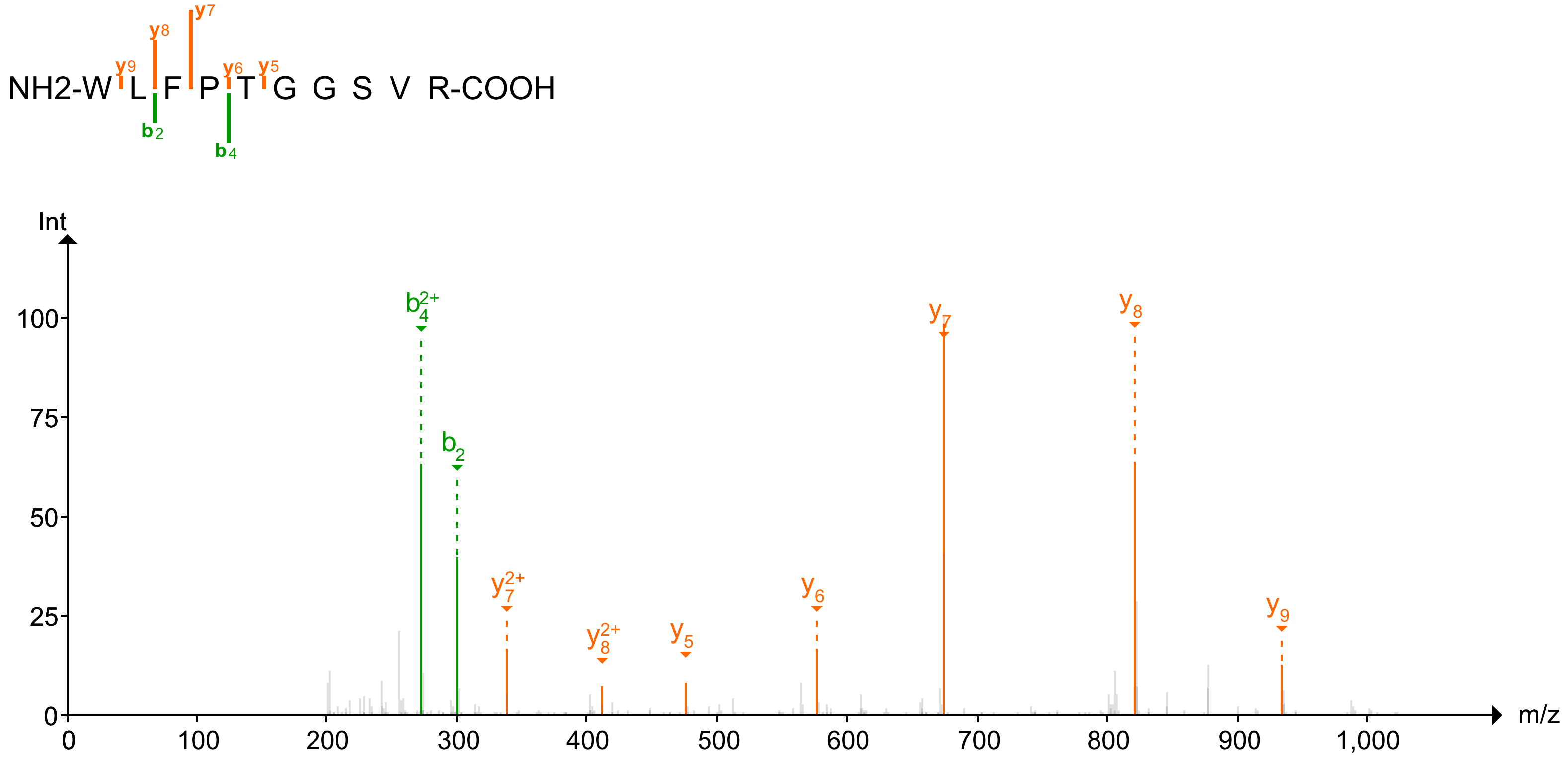

NH2-W L F P T G G S V R-COOH
y9 y8 y7 y6 y5
b2 b4
Int
100
75
50
25
0
$b_4^{2+}$
$b_2$
$y_7^{2+}$
$y_8^{2+}$
$y_5$
$y_6$
$y_7$
$y_8$
$y_9$
0 100 200 300 400 500 600 700 800 900 1,000
m/z

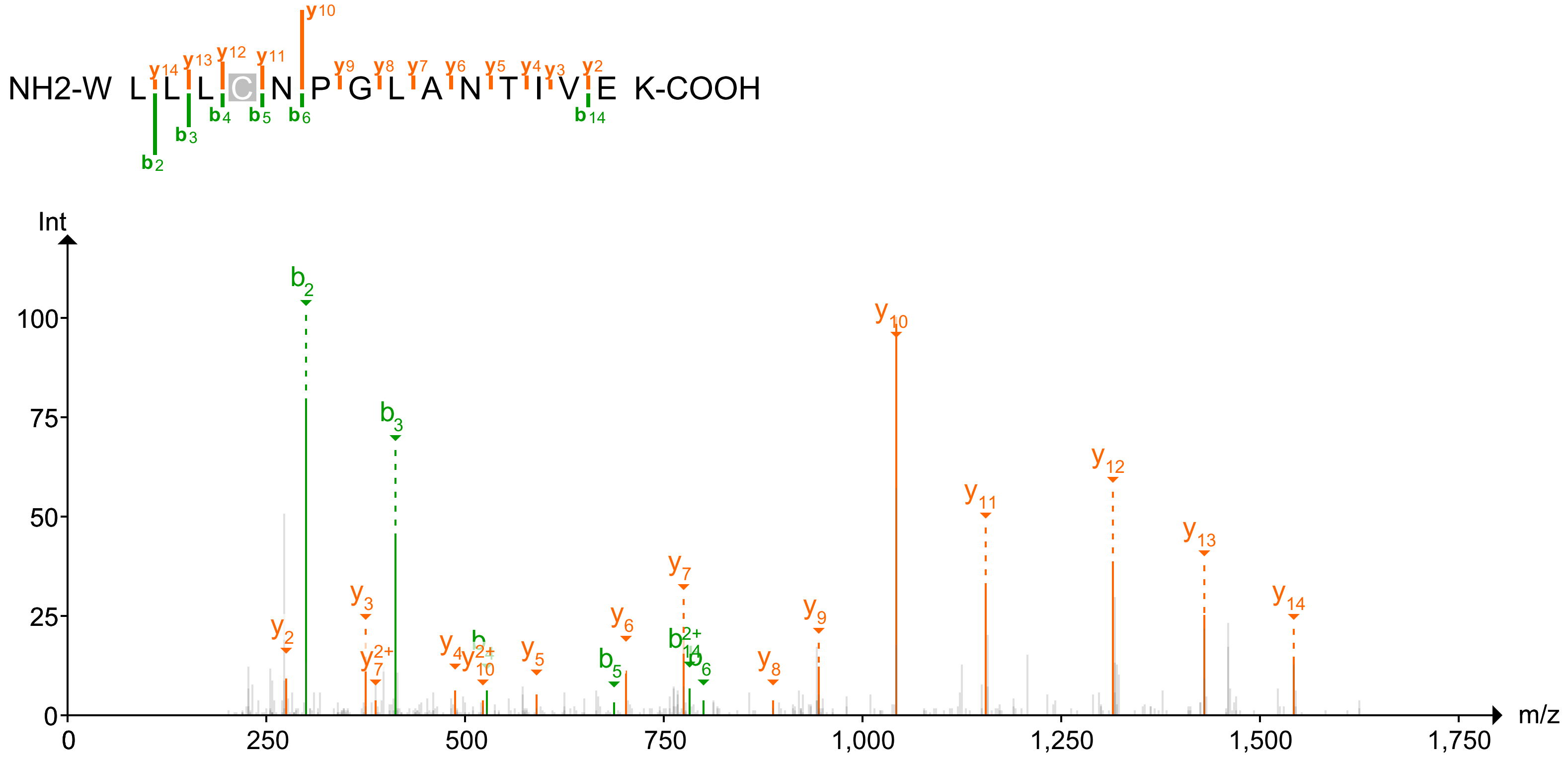

NH2-W L L L C N P G L A N T I V E K-COOH
y14 y13 y12 y11 y10 y9 y8 y7 y6 y5 y4 y3 y2
b2 b3 b4 b5 b6 b14
Int
100
75
50
25
0
m/z
0
250
500
750
1,000
1,250
1,500
1,750
b2
b3
y10
y11
y12
y13
y14
y2
y3
y7
y6
y9
y8
y5
y4
b5
b6

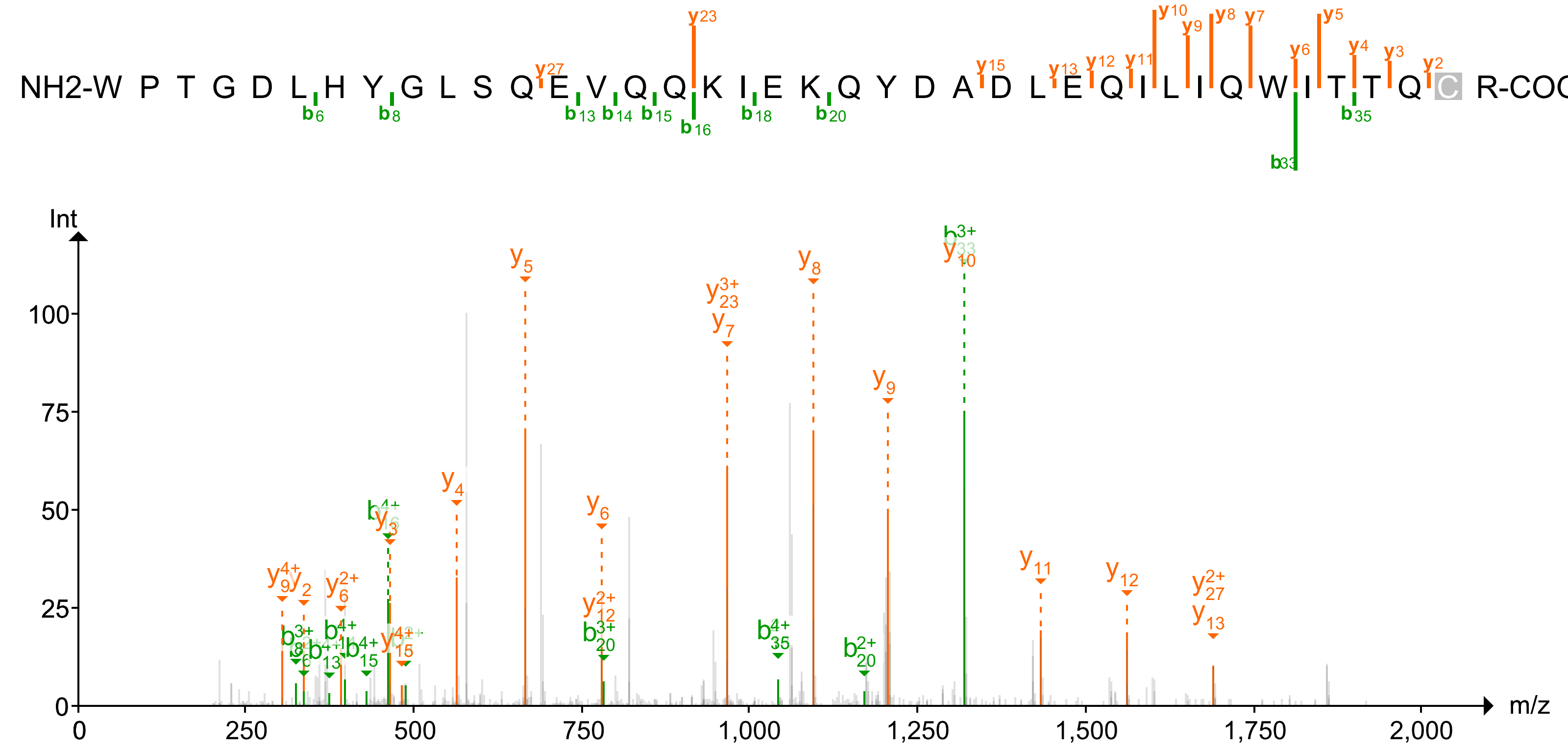

NH2-W P T G D L H Y G L S Q E V Q Q K I E K Q Y D A D L E Q I L I Q W I T T Q C R-COO
Int
m/z
0
25
50
75
100
0
250
500
750
1,000
1,250
1,500
1,750
2,000

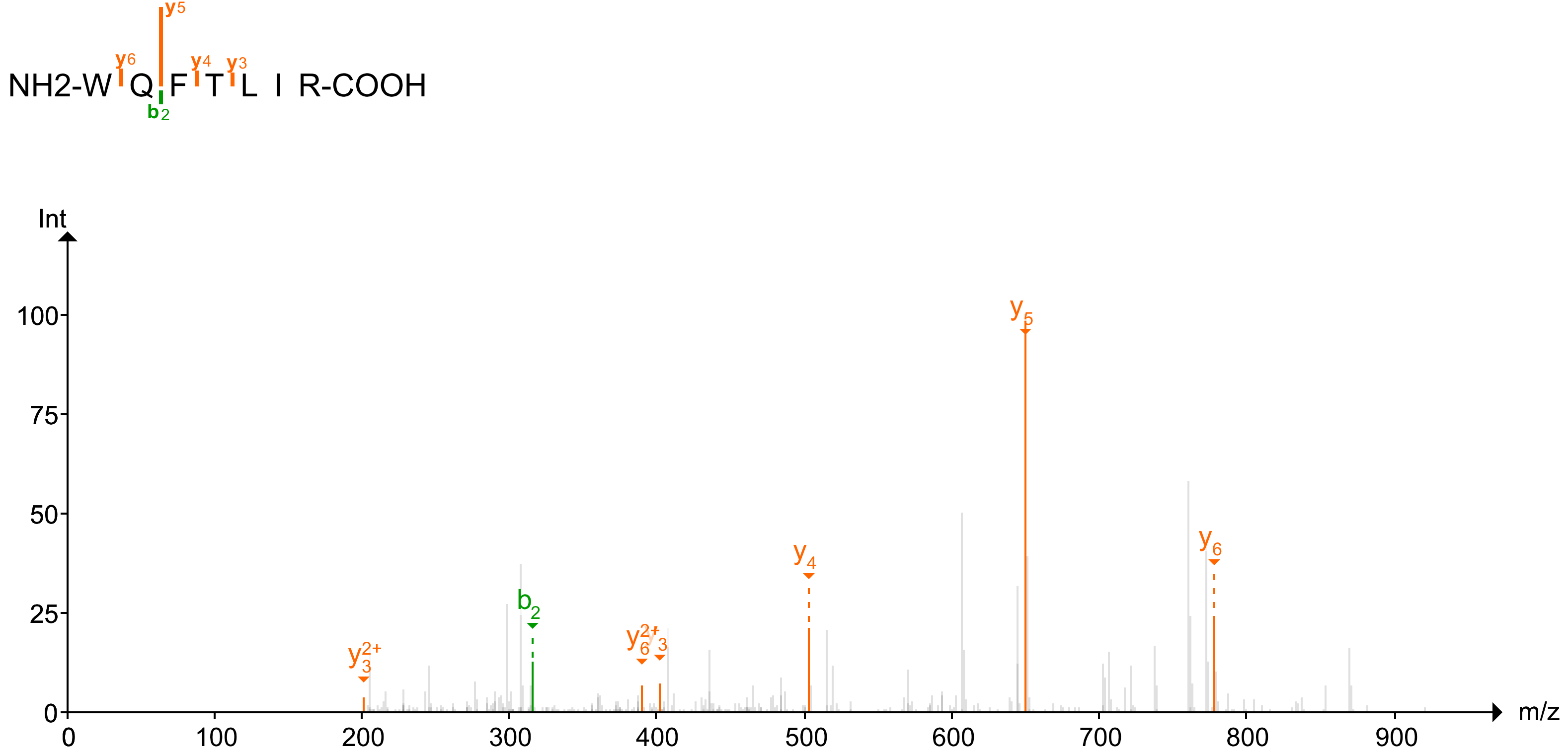

NH2-W Q F T L I R-COOH
y6
y5
y4
y3
b2
Int
m/z
0
25
50
75
100
100
200
300
400
500
600
700
800
900
$y_5$
$y_6$
$y_4$
$b_2$
$y_3^{2+}$
$y_6^{2+}$
$y_3$

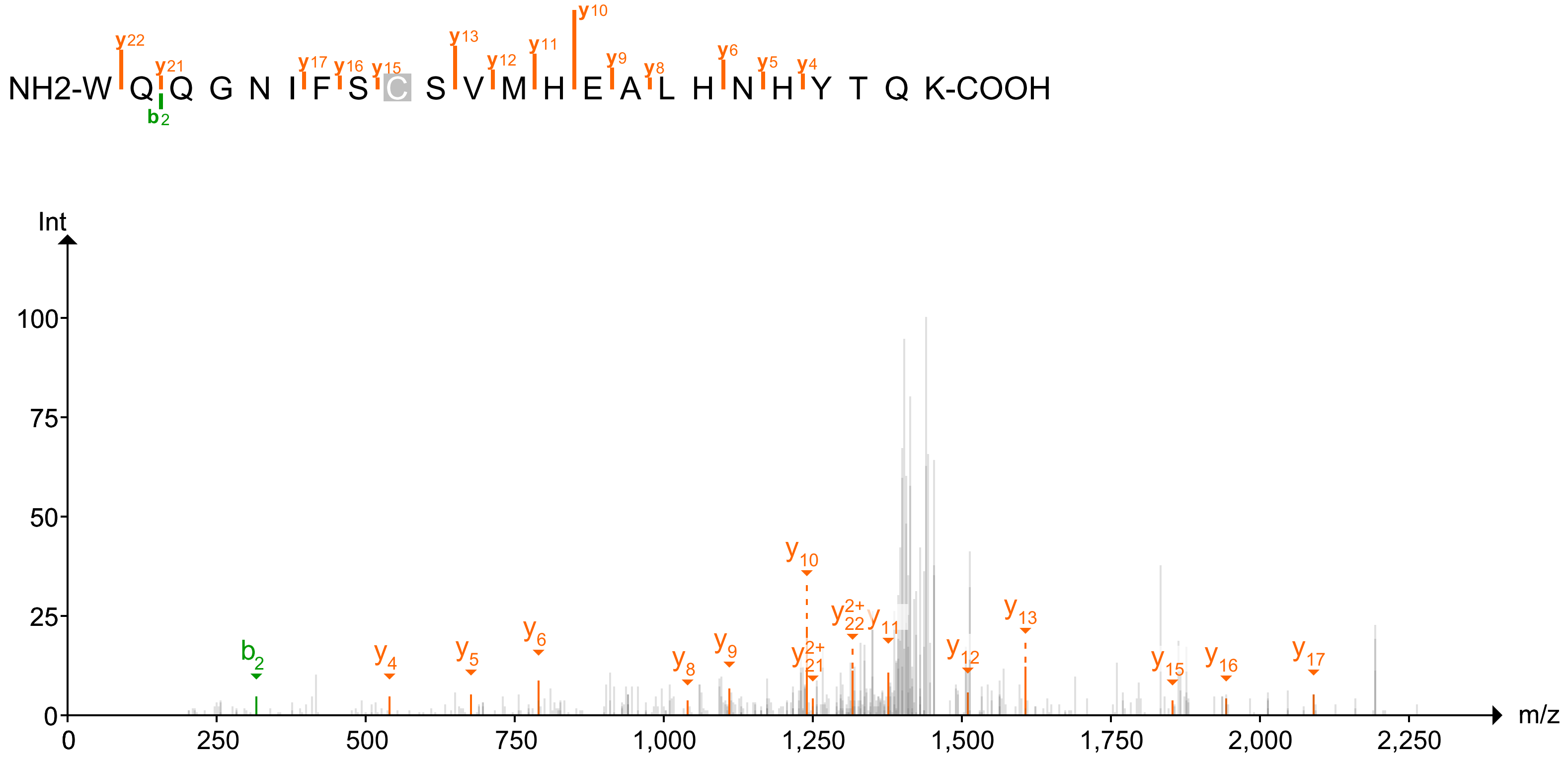

NH2-W Q Q G N I F S C S V M H E A L H N H Y T Q K-COOH
y22 y21 y17 y16 y15 y13 y12 y11 y10 y9 y8 y6 y5 y4
b2
Int
100
75
50
25
0
m/z
0
250
500
750
1,000
1,250
1,500
1,750
2,000
2,250
b2
y4
y5
y6
y8
y9
y10
y21 2+
y22 2+
y11
y12
y13
y15
y16
y17

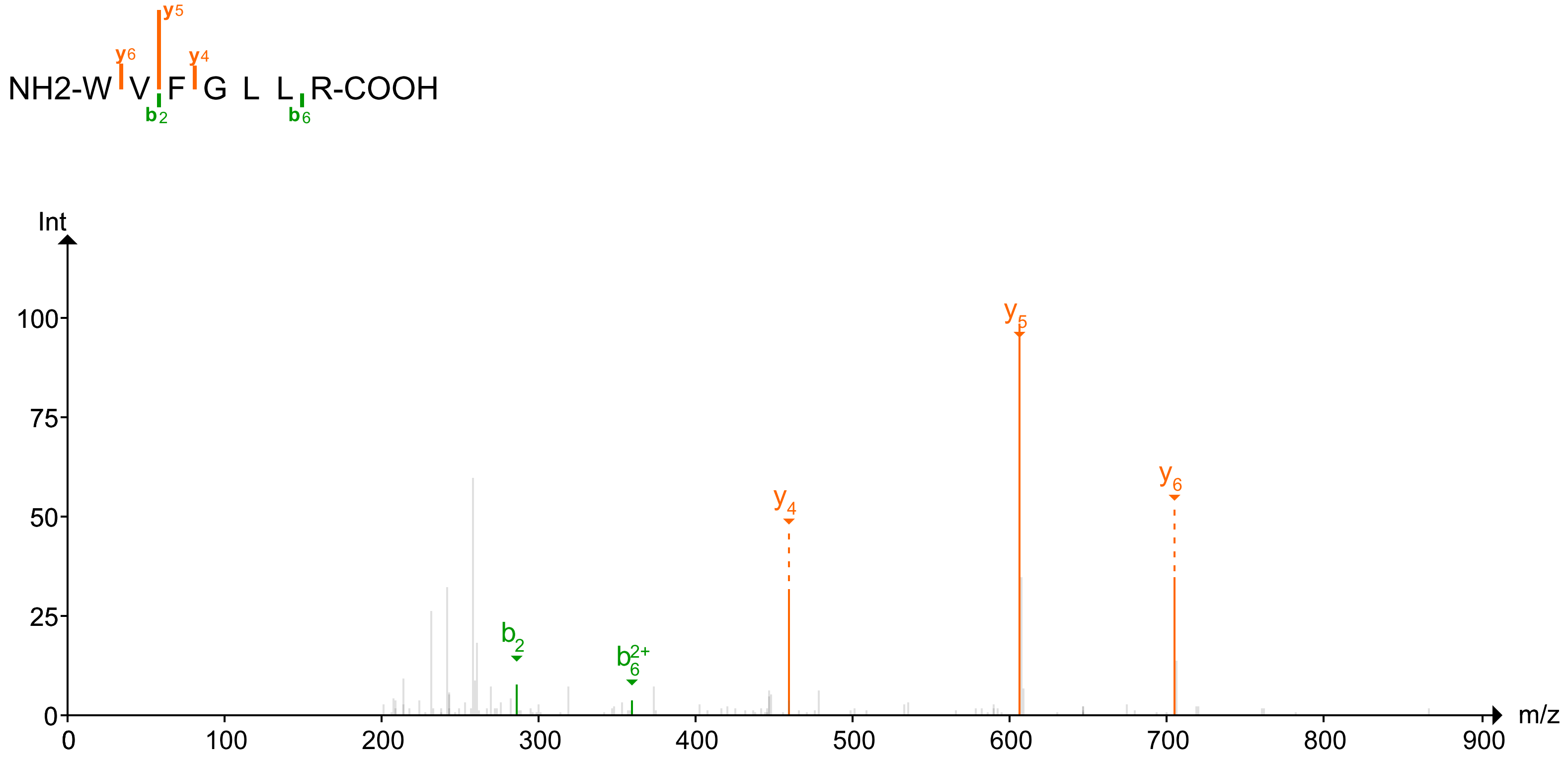

NH2-W V F G L L R-COOH
y6
y5
y4
b2
b6
Int
100
75
50
25
0
y5
y6
y4
b2
b6 2+
m/z
0
100
200
300
400
500
600
700
800
900

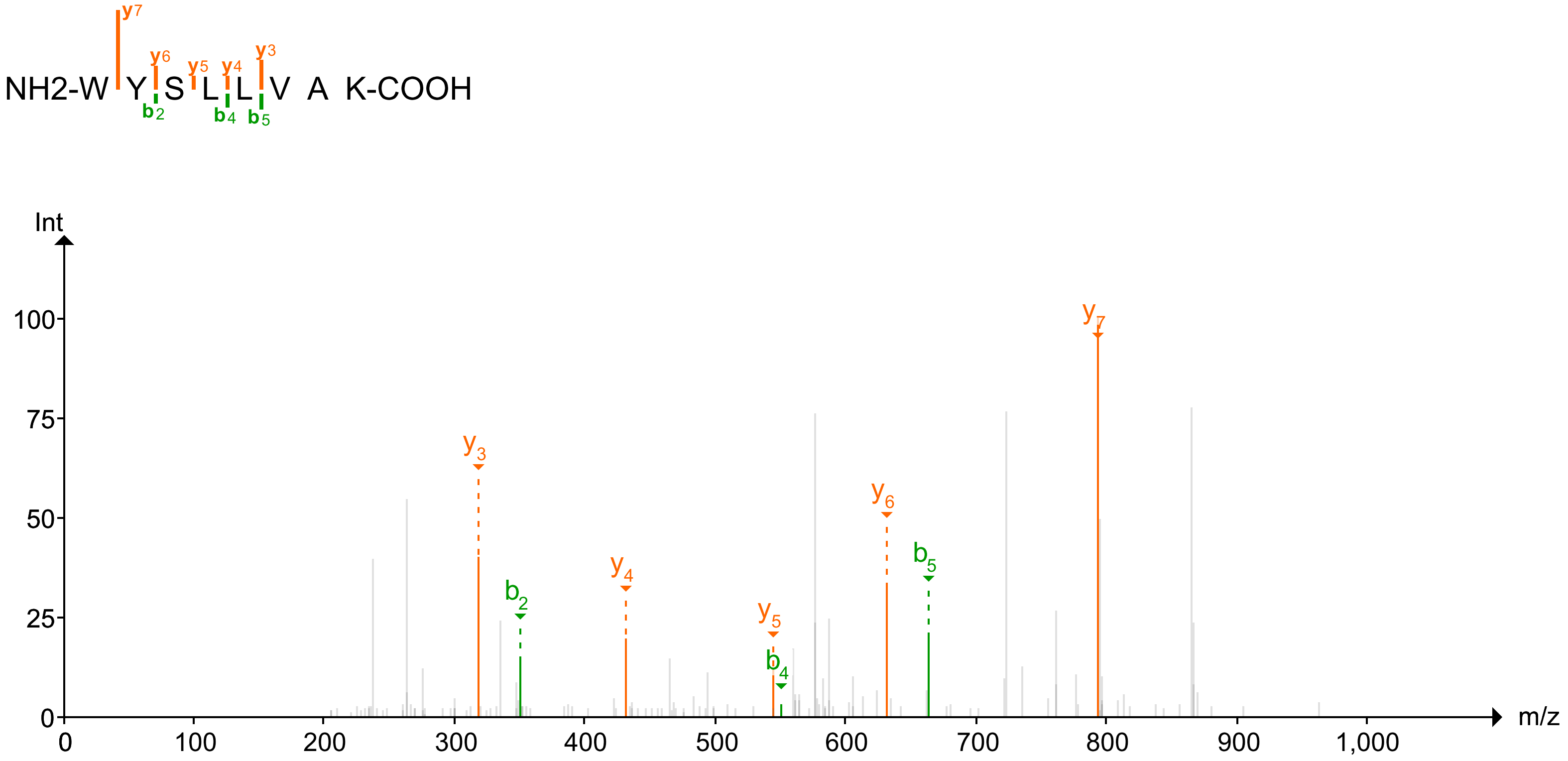

NH2-W Y S L L V A K-COOH
y7
y6
y5
y4
y3
b2
b4
b5
Int
100
75
50
25
0
m/z
0
100
200
300
400
500
600
700
800
900
1,000

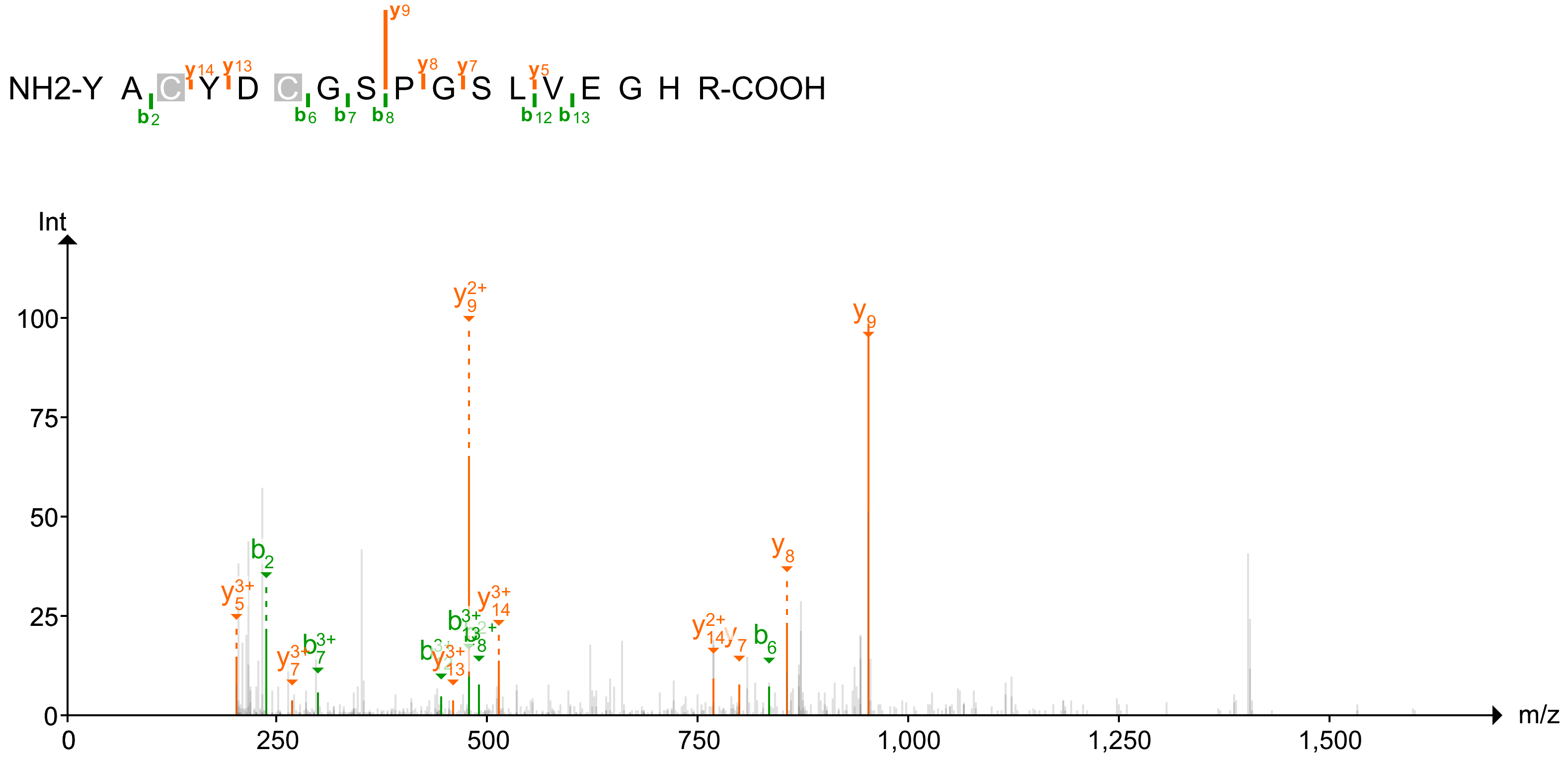
NH2-Y A C Y D C G S P G S L V E G H R-COOH
y14 y13 y9 y8 y7 y5
b2 b6 b7 b8 b12 b13
Int
m/z
0
25
50
75
100
250
500
750
1,000
1,250
1,500
y9
y8
b2
b6

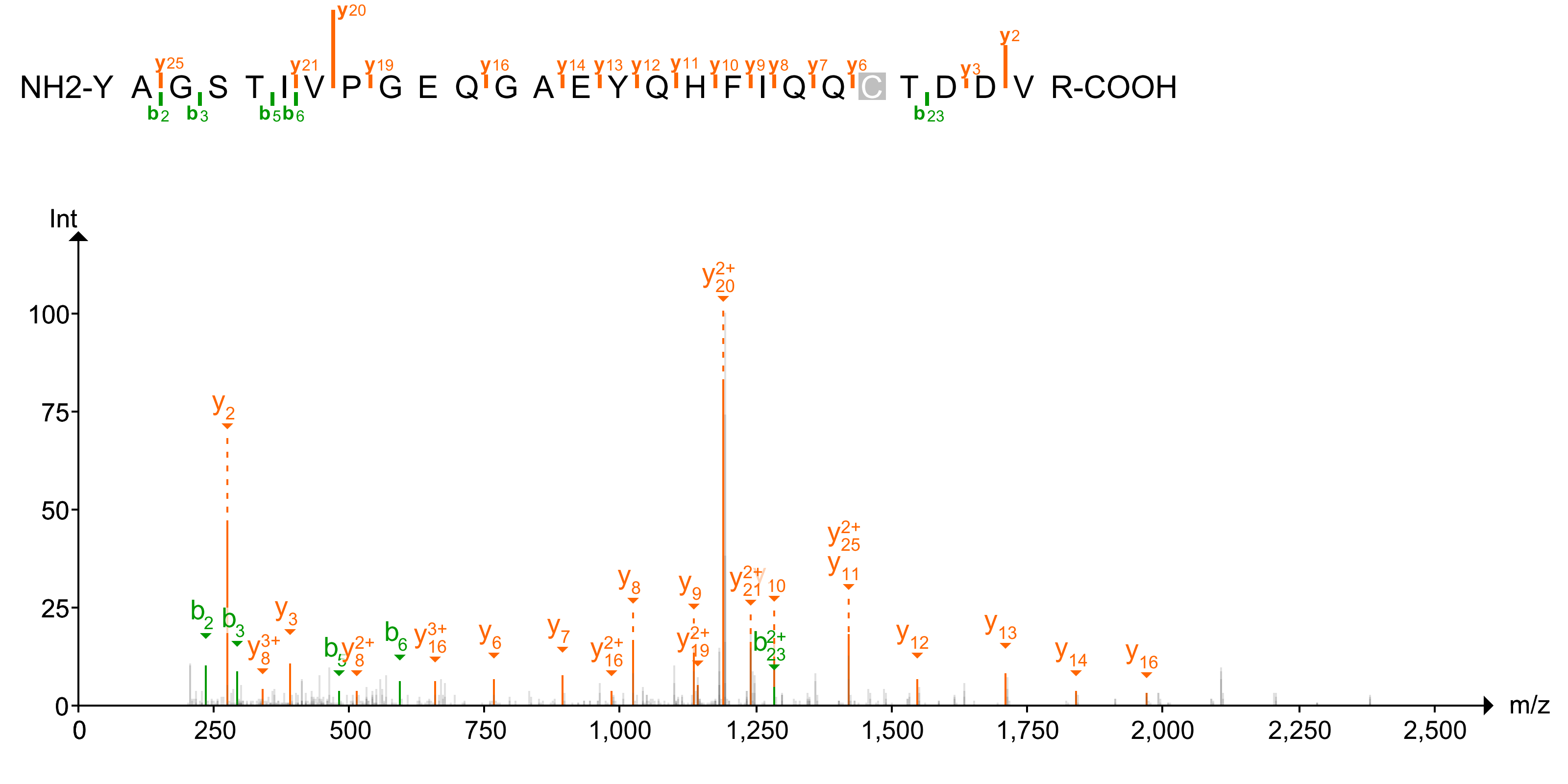
NH2-Y A G S T I V P G E Q G A E Y Q H F I Q Q C T D D V R-COOH
y20 y25 y21 y19 y16 y14 y13 y12 y11 y10 y9 y8 y7 y6 y3 y2
b2 b3 b5 b6 b23
Int
100
75
50
25
0
m/z
0
250
500
750
1,000
1,250
1,500
1,750
2,000
2,250
2,500

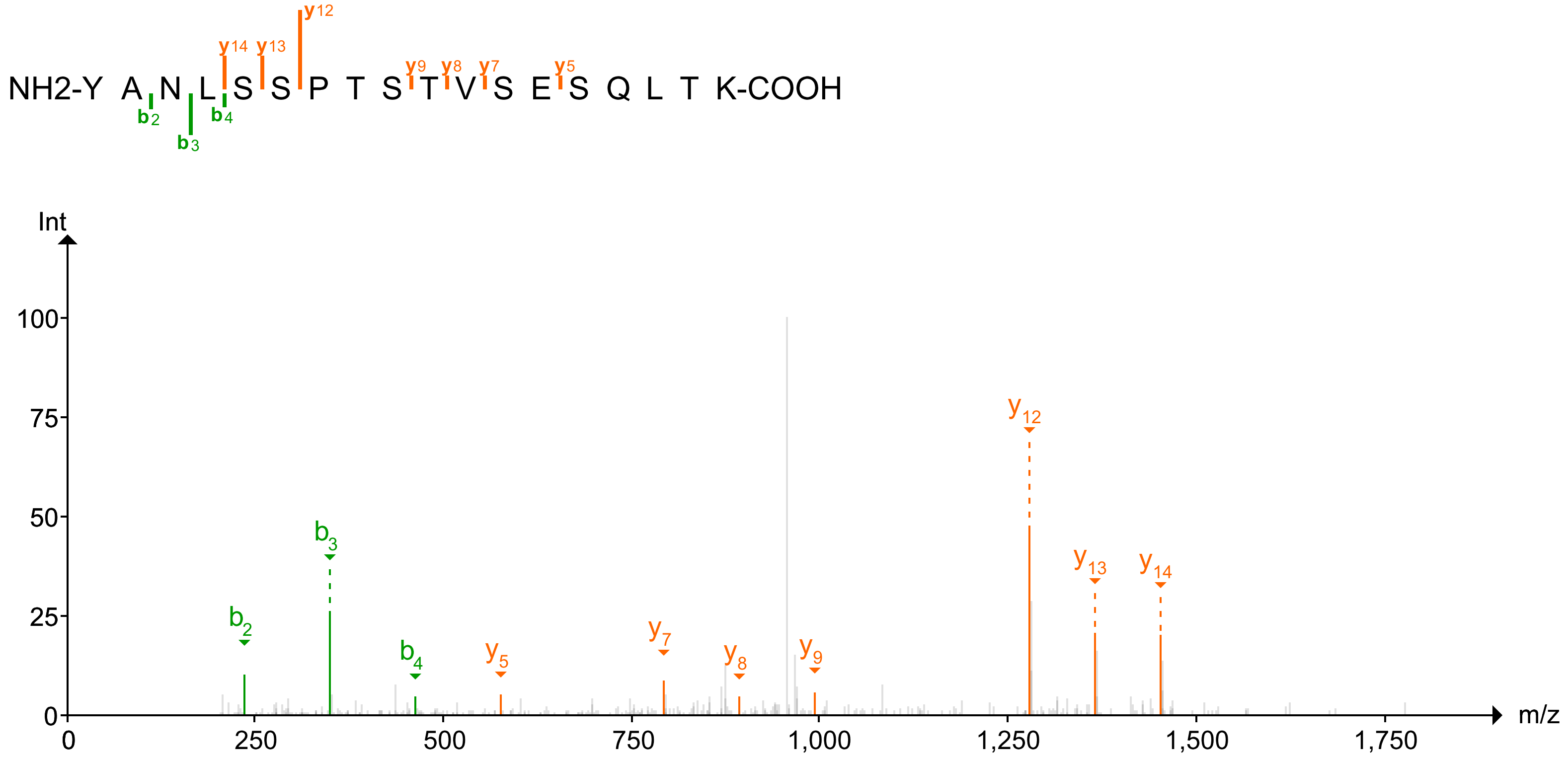

NH2-Y A N L S S P T S T V S E S Q L T K-COOH
b2 b3 b4
y14 y13 y12 y9 y8 y7 y5
Int
100
75
50
25
0
b2
b3
b4
y5
y7
y8
y9
y12
y13
y14
0
250
500
750
1,000
1,250
1,500
1,750
m/z

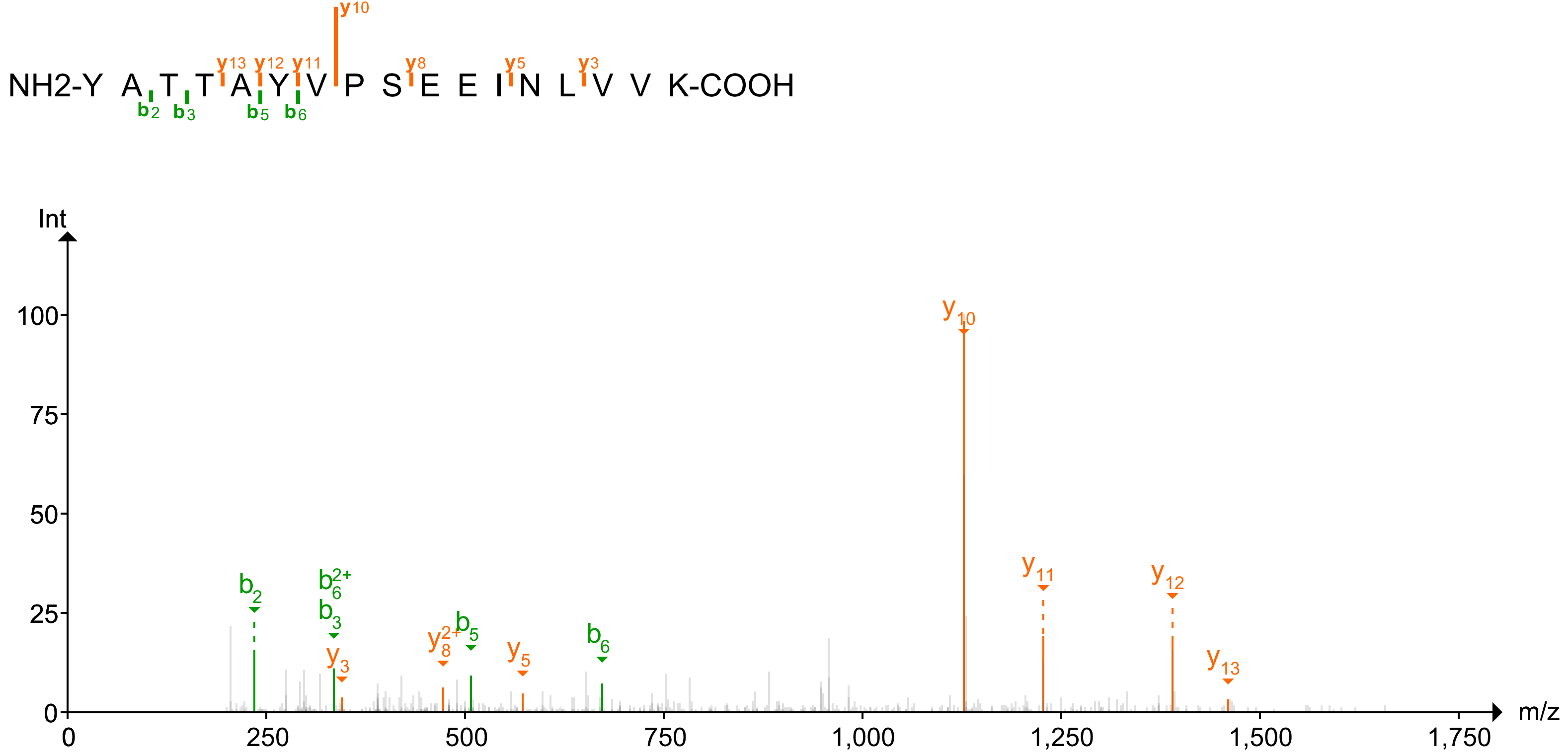

NH2-Y A T T A Y V P S E E I N L V V K-COOH
y10
y13 y12 y11 y8 y5 y3
b2 b3 b5 b6
Int
100
75
50
25
0
0
250
500
750
1,000
1,250
1,500
1,750
m/z
b2
b6 2+
b3
y3
y8 2+
b5
y5
b6
y10
y11
y12
y13

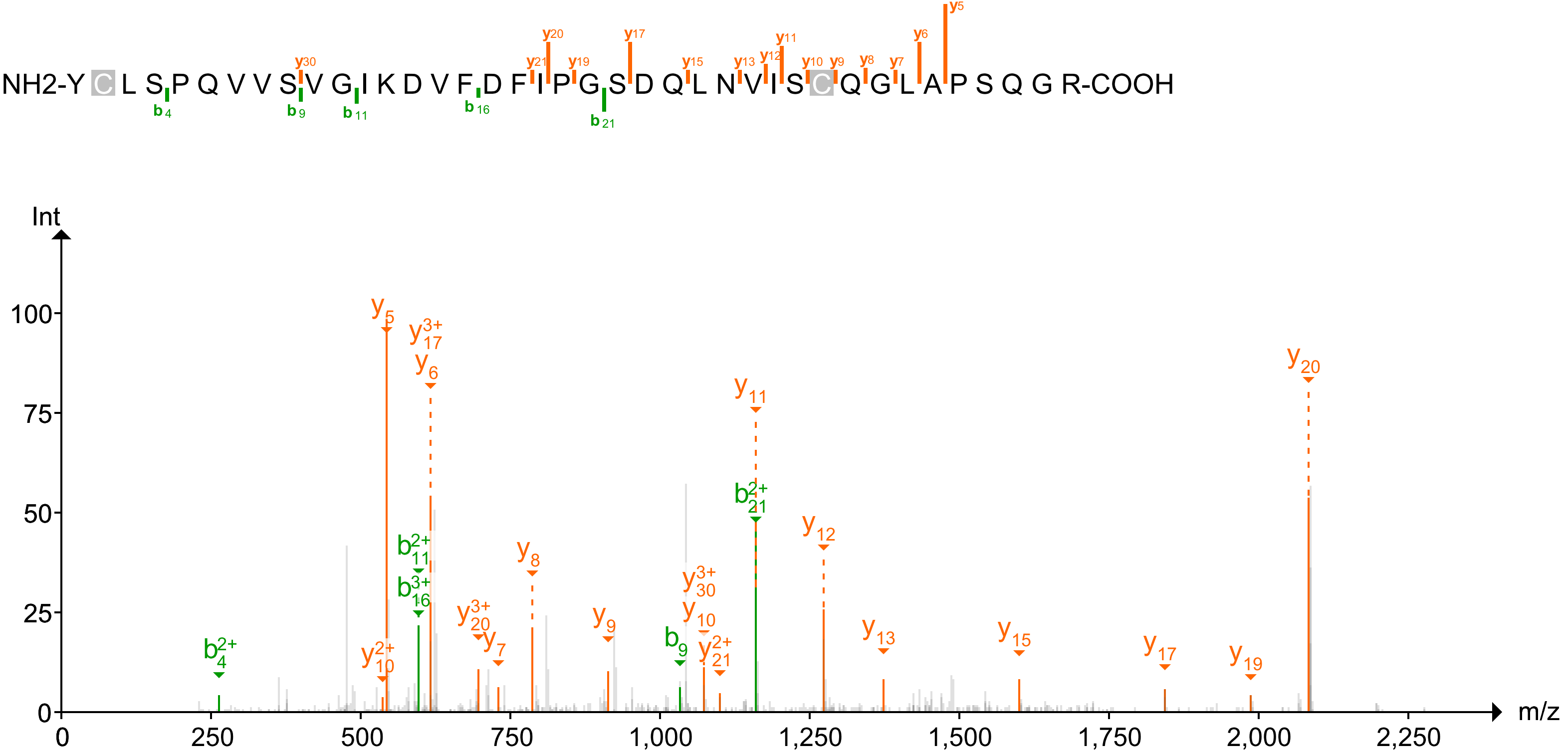

NH2-Y C L S P Q V V S V G I K D V F D F I P G S D Q L N V I S C Q G L A P S Q G R-COOH
y30 y21 y20 y19 y17 y15 y13 y12 y11 y10 y9 y8 y7 y6 y5
b4 b9 b11 b16 b21
Int
100
75
50
25
0
m/z
0
250
500
750
1,000
1,250
1,500
1,750
2,000
2,250
y5
y17 3+
y6
y11
y20
b21 2+
y12
b11 2+
b16 3+
y8
y30 3+
y10
y20 3+
y7
y9
b9
y21 2+
y13
y15
y17
y19
b4 2+
y10 2+

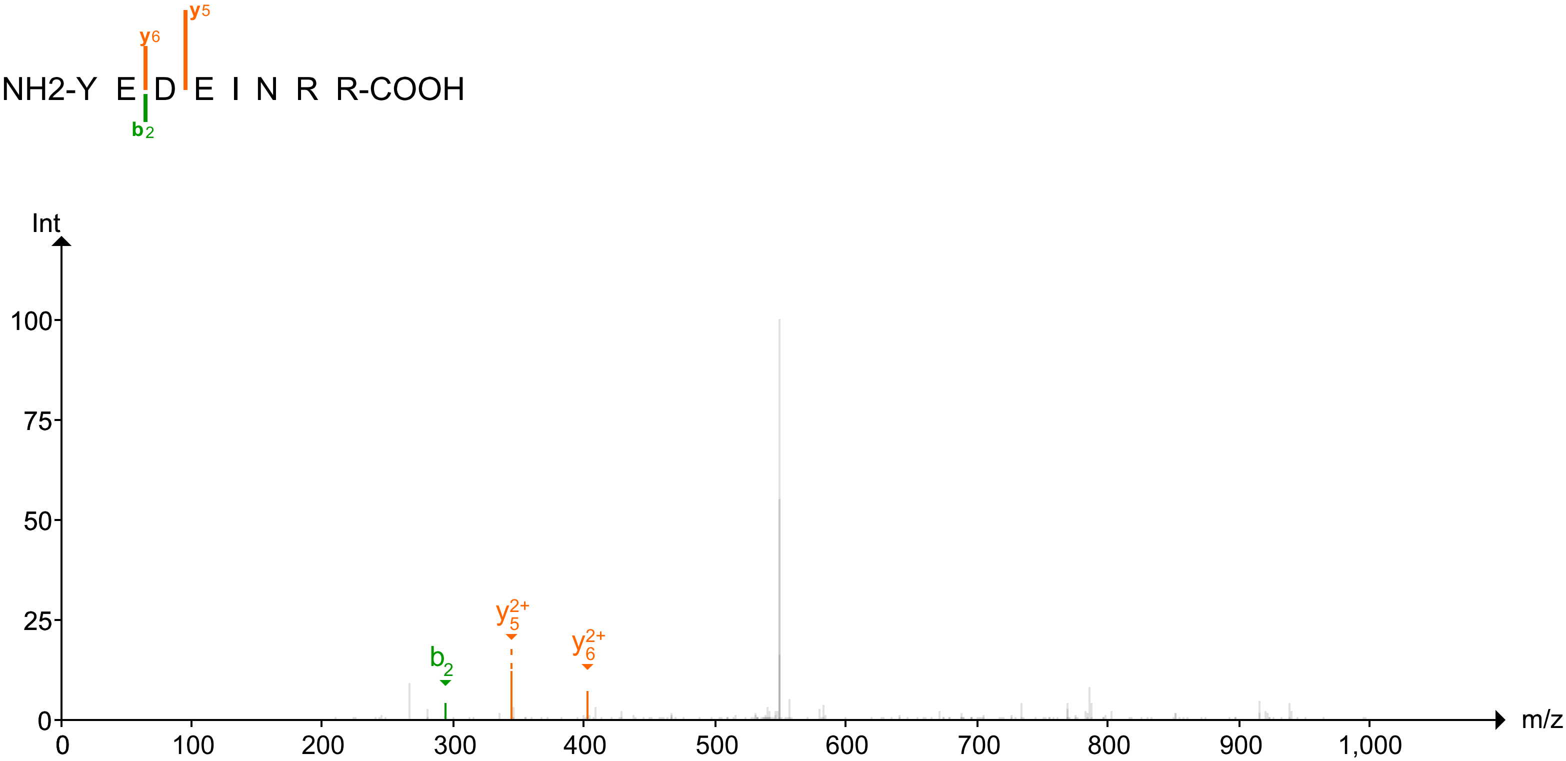

y5
y6
NH2-Y E D E I N R R-COOH
b2
Int
100
75
50
25
0
b2
y5 2+
y6 2+
0
100
200
300
400
500
600
700
800
900
1,000
m/z

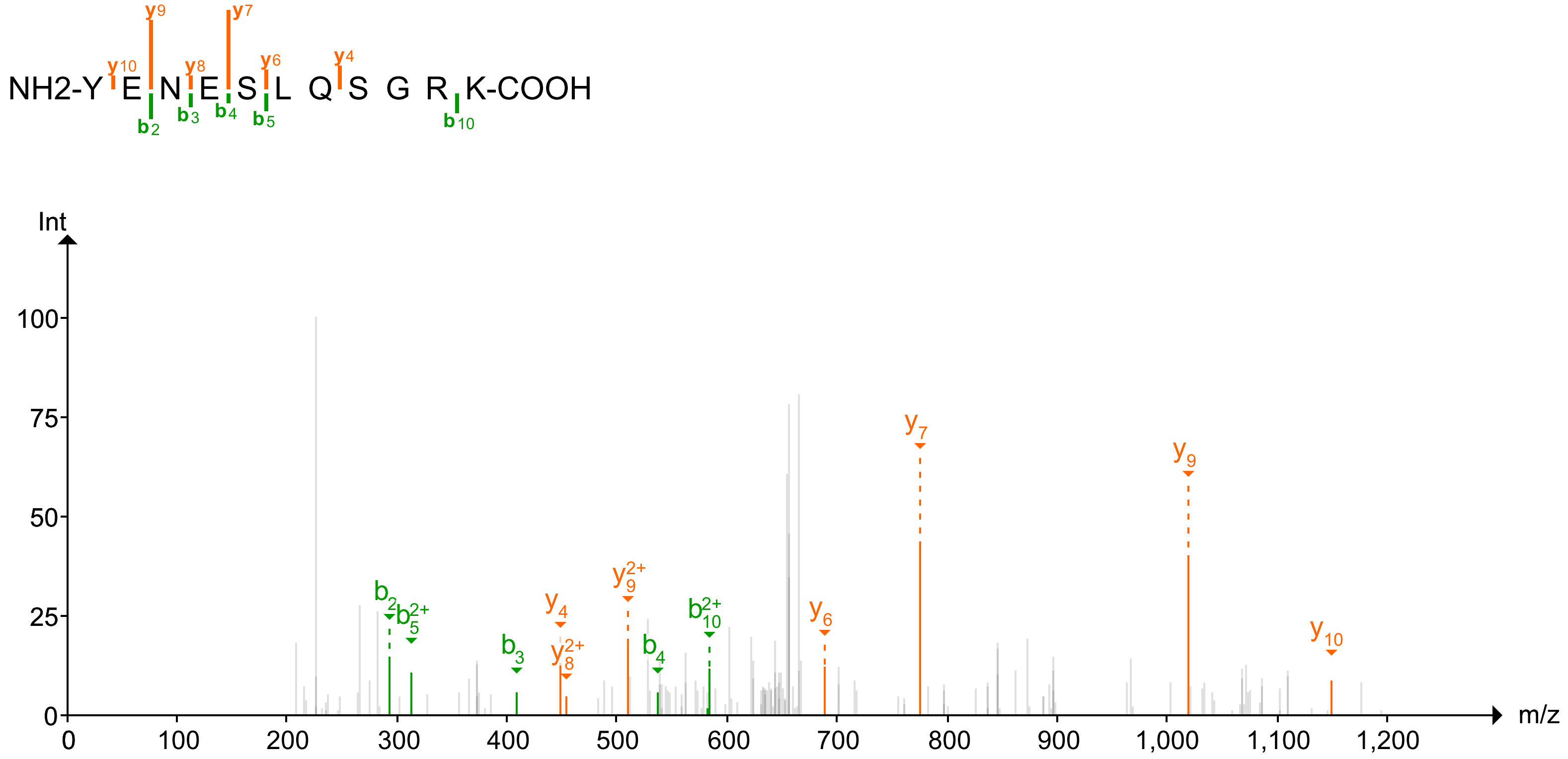
NH2-Y E N E S L Q S G R K-COOH
y10 y9 y8 y7 y6 y4
b2 b3 b4 b5 b10
Int
100
75
50
25
0
m/z
0 100 200 300 400 500 600 700 800 900 1,000 1,100 1,200
y7
y9
y10
y6
b2
b5 2+
b3
y4
y8 2+
y9 2+
b4
b10 2+

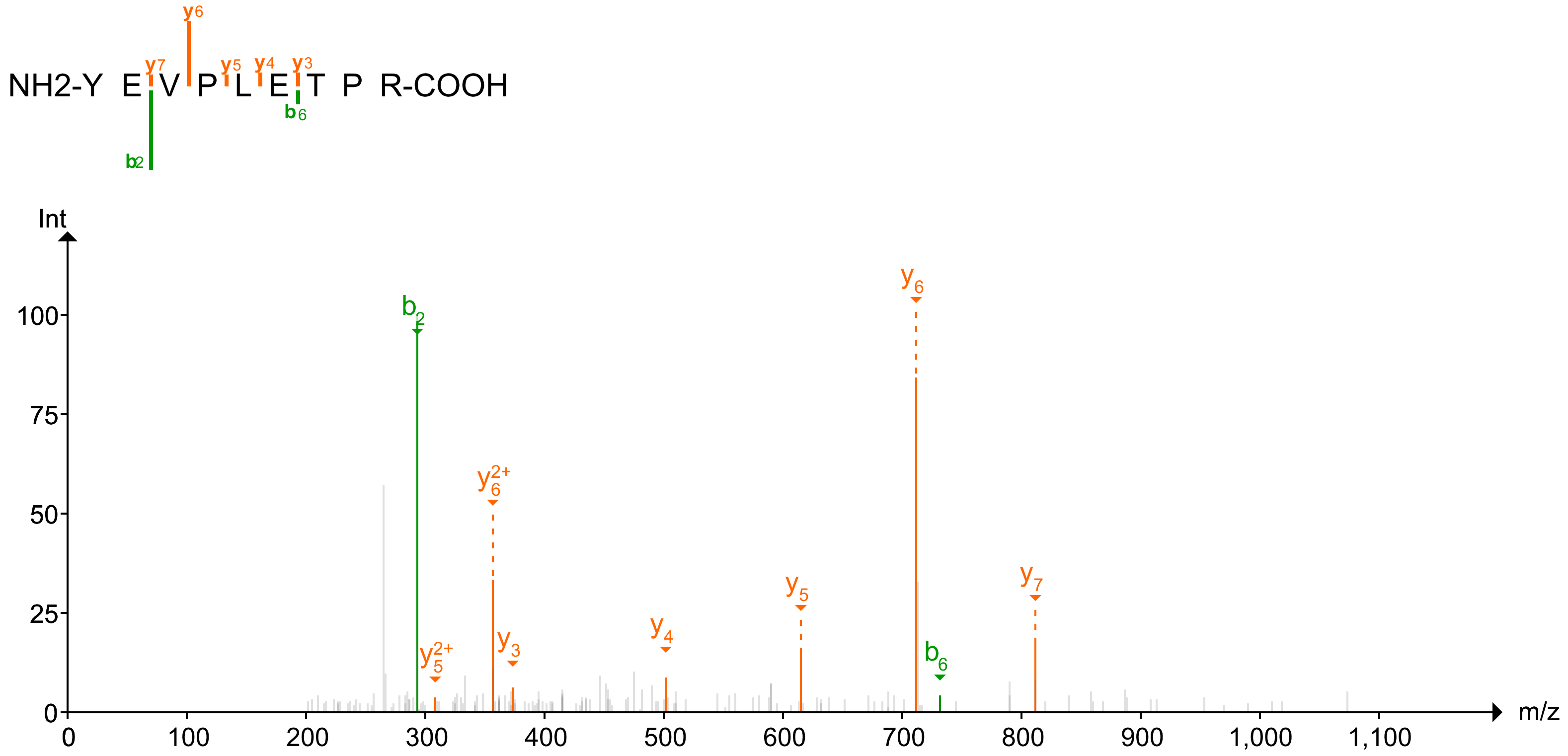

NH2-Y E V P L E T P R-COOH
y6
y7
y5
y4
y3
b2
b6
Int
100
75
50
25
0
m/z
0
100
200
300
400
500
600
700
800
900
1,000
1,100
b2
y6 2+
y5 2+
y3
y4
y5
y6
b6
y7

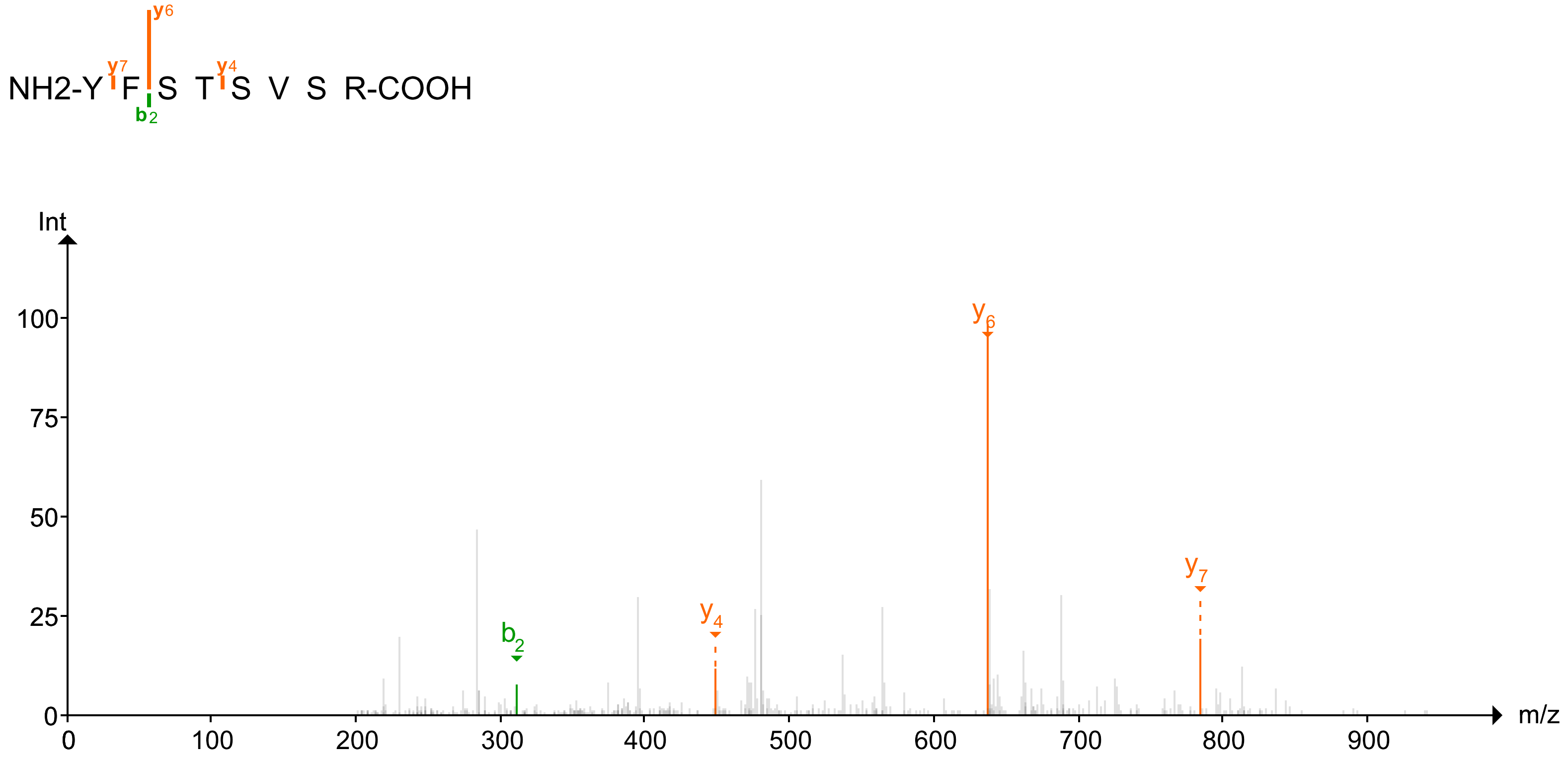

NH2-Y F S T S V S R-COOH
y6
y7
y4
b2
Int
100
75
50
25
0
m/z
0
100
200
300
400
500
600
700
800
900

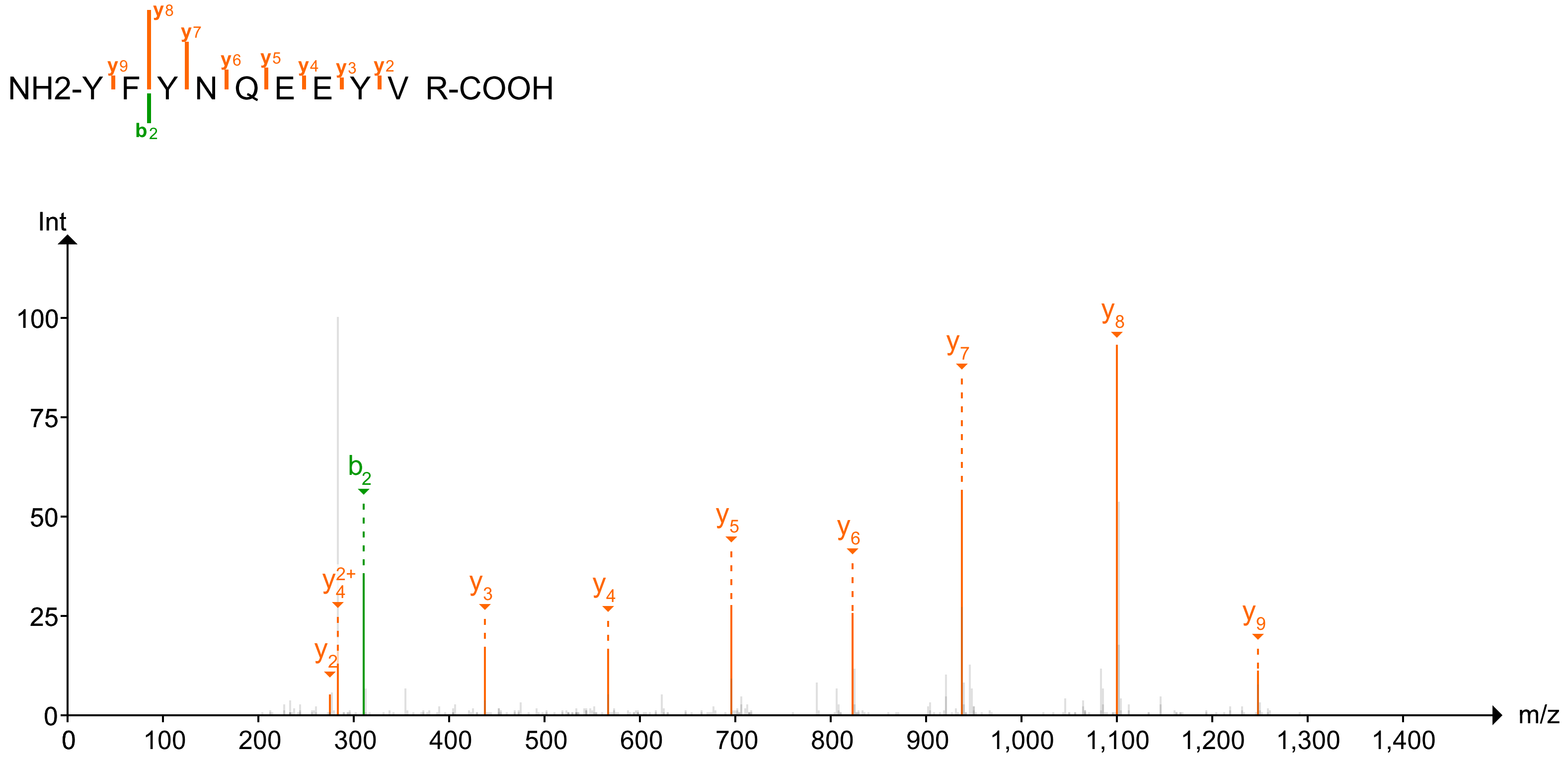

NH2-Y F Y N Q E E Y V R-COOH
y9 y8 y7 y6 y5 y4 y3 y2
b2
Int
100
75
50
25
0
m/z
0 100 200 300 400 500 600 700 800 900 1,000 1,100 1,200 1,300 1,400
y2
y4 2+
b2
y3
y4
y5
y6
y7
y8
y9

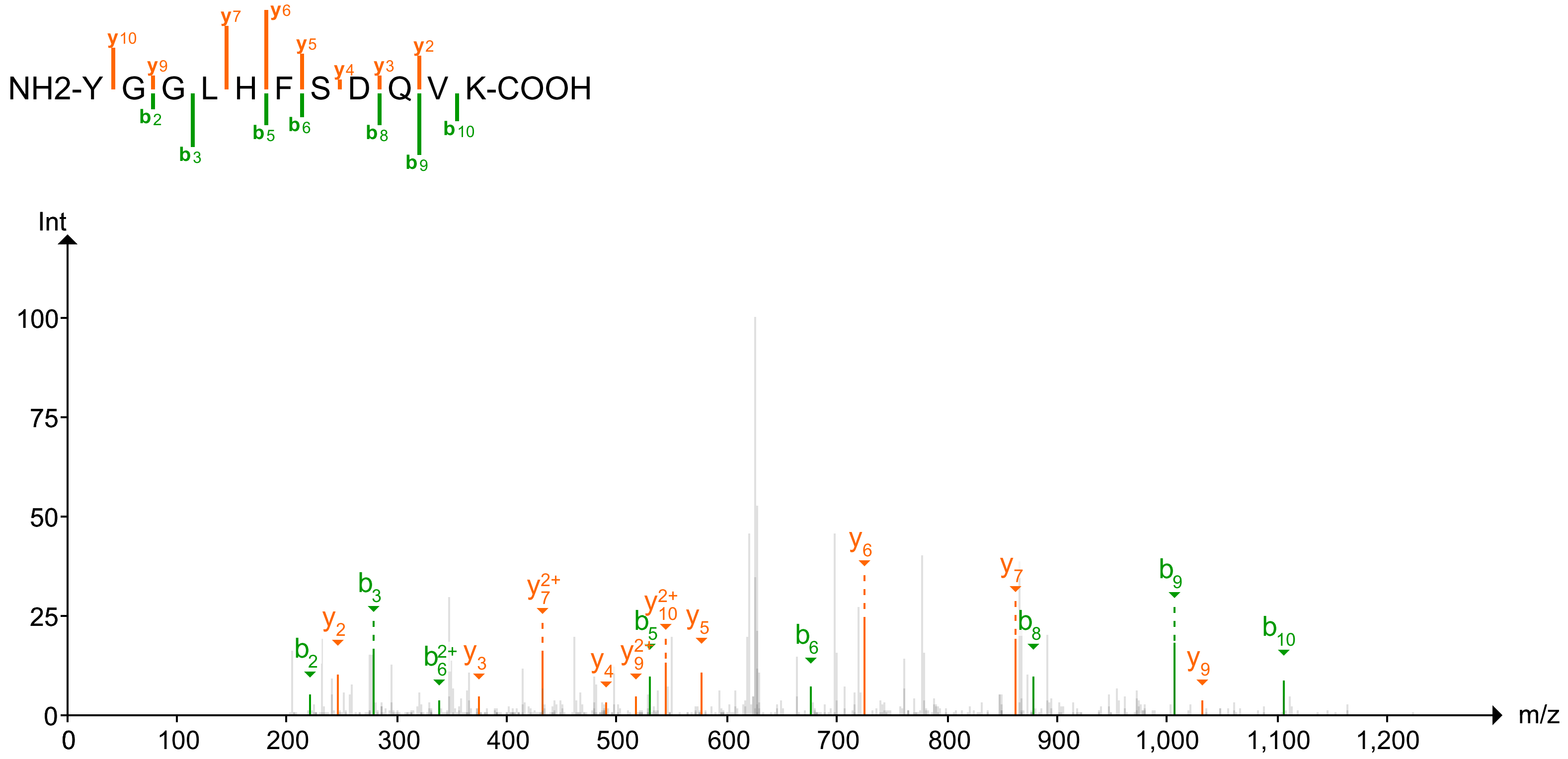

NH2-Y G G L H F S D Q V K-COOH
y10 y9 y7 y6 y5 y4 y3 y2
b2 b3 b5 b6 b8 b9 b10
Int
100
75
50
25
0
m/z
0
100
200
300
400
500
600
700
800
900
1,000
1,100
1,200
b2 y2 b3 b6 2+ y3 y7 2+ y4 y9 2+ b5 y10 2+ y5 b6 y6 y7 b8 b9 y9 b10

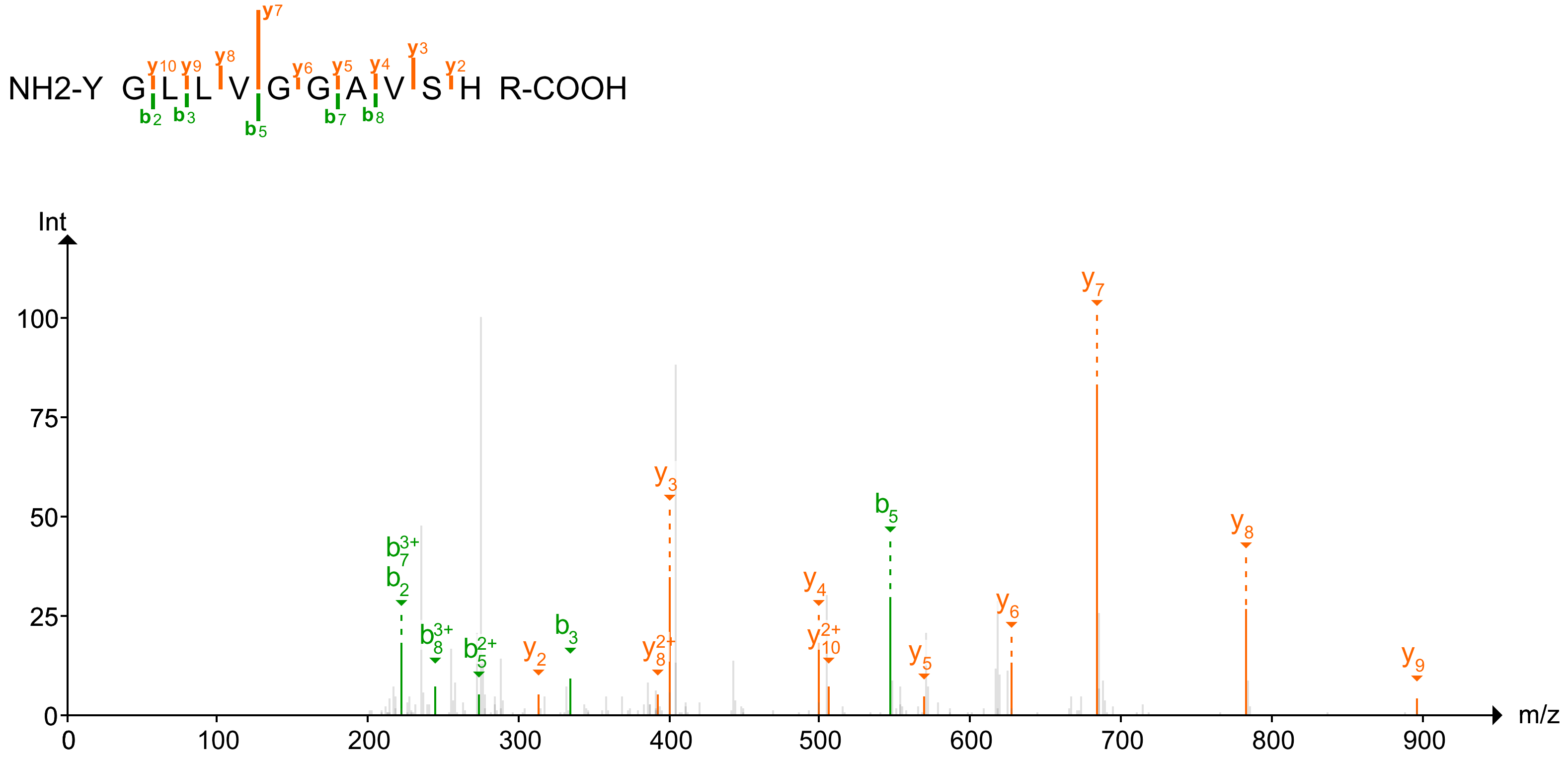

NH2-Y G L L V G G A V S H R-COOH
y10 y9 y8 y7 y6 y5 y4 y3 y2
b2 b3 b5 b7 b8
Int
100
75
50
25
0
m/z
0 100 200 300 400 500 600 700 800 900
y7
y3
b5
y8
y4
y6
b7 3+
b2
b8 3+
b5 2+
y2
b3
y8 2+
y10 2+
y5
y9

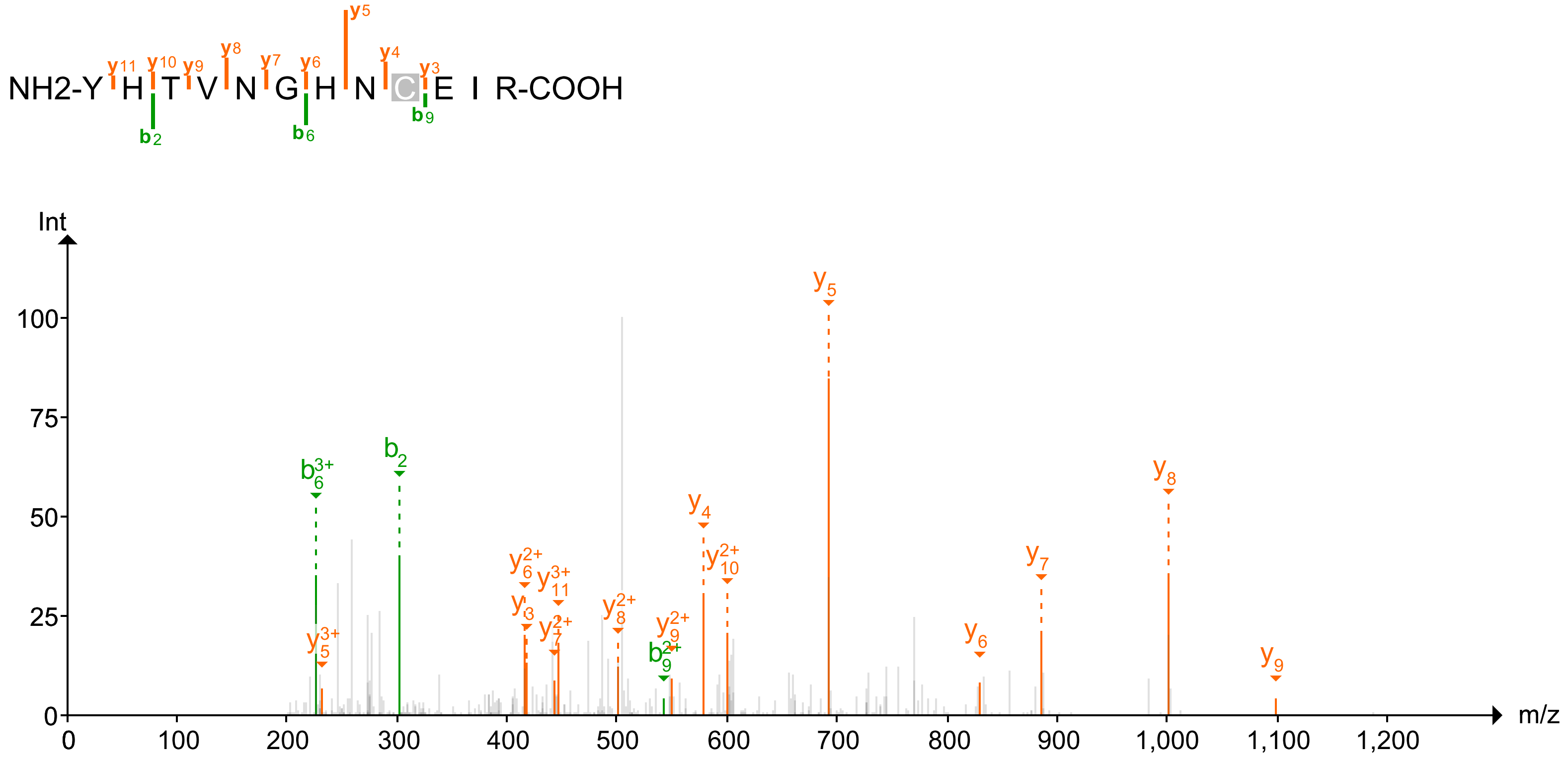

NH2-Y H T V N G H N C E I R-COOH
y11 y10 y9 y8 y7 y6 y5 y4 y3
b2 b6 b9
Int
100
75
50
25
0
m/z
0 100 200 300 400 500 600 700 800 900 1,000 1,100 1,200
y5
b2
b6 3+
y8
y4
y6 2+
y10 2+
y7
y11 3+
y3
y8 2+
y9 2+
y7 2+
b9 2+
y5 3+
y6
y9

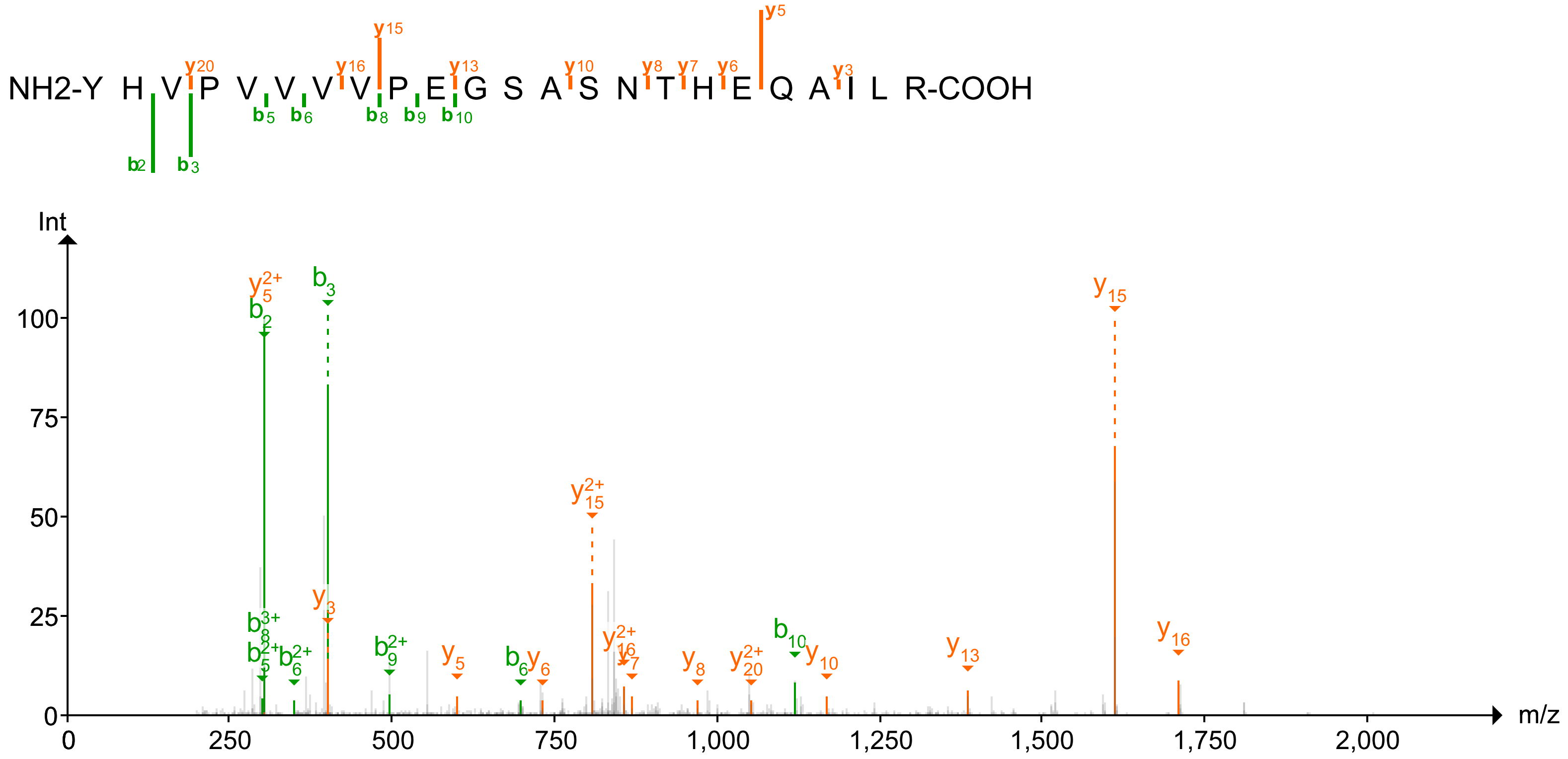
NH2-Y H V P V V V V P E G S A S N T H E Q A I L R-COOH
Int
m/z

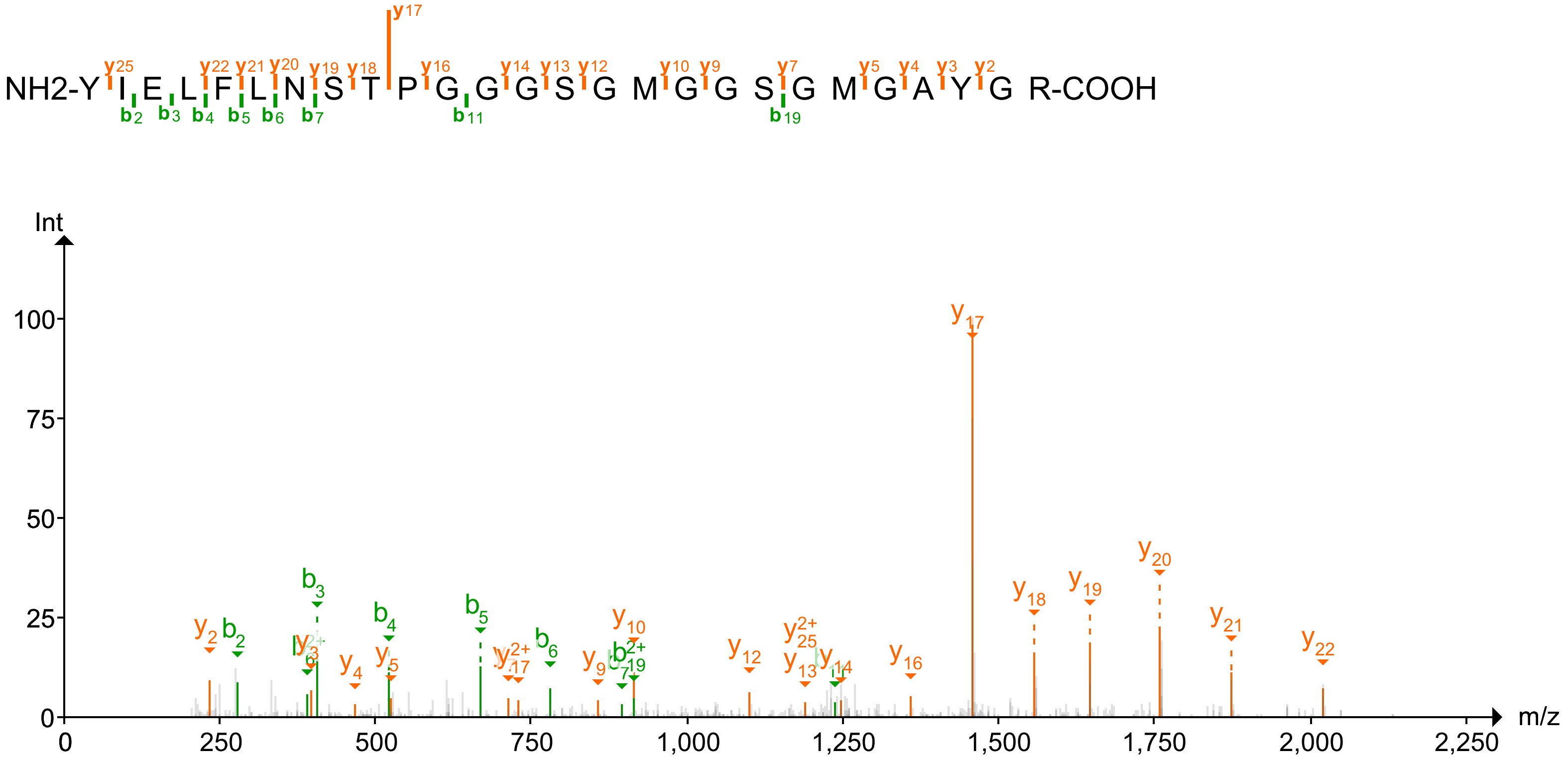

NH2-Y I E L F L N S T P G G G S G M G G S G M G A Y G R-COOH
y25 y22 y21 y20 y19 y18 y17 y16 y14 y13 y12 y10 y9 y7 y5 y4 y3 y2
b2 b3 b4 b5 b6 b7 b11 b19
Int
100
75
50
25
0
m/z
0
250
500
750
1,000
1,250
1,500
1,750
2,000
2,250
y17
y18
y19
y20
y21
y22
y2
b2
b3
b4
b5
b6
y4
y5
y9
y10
y12
y13
y14
y16
y25 2+
b19 2+
y17 2+

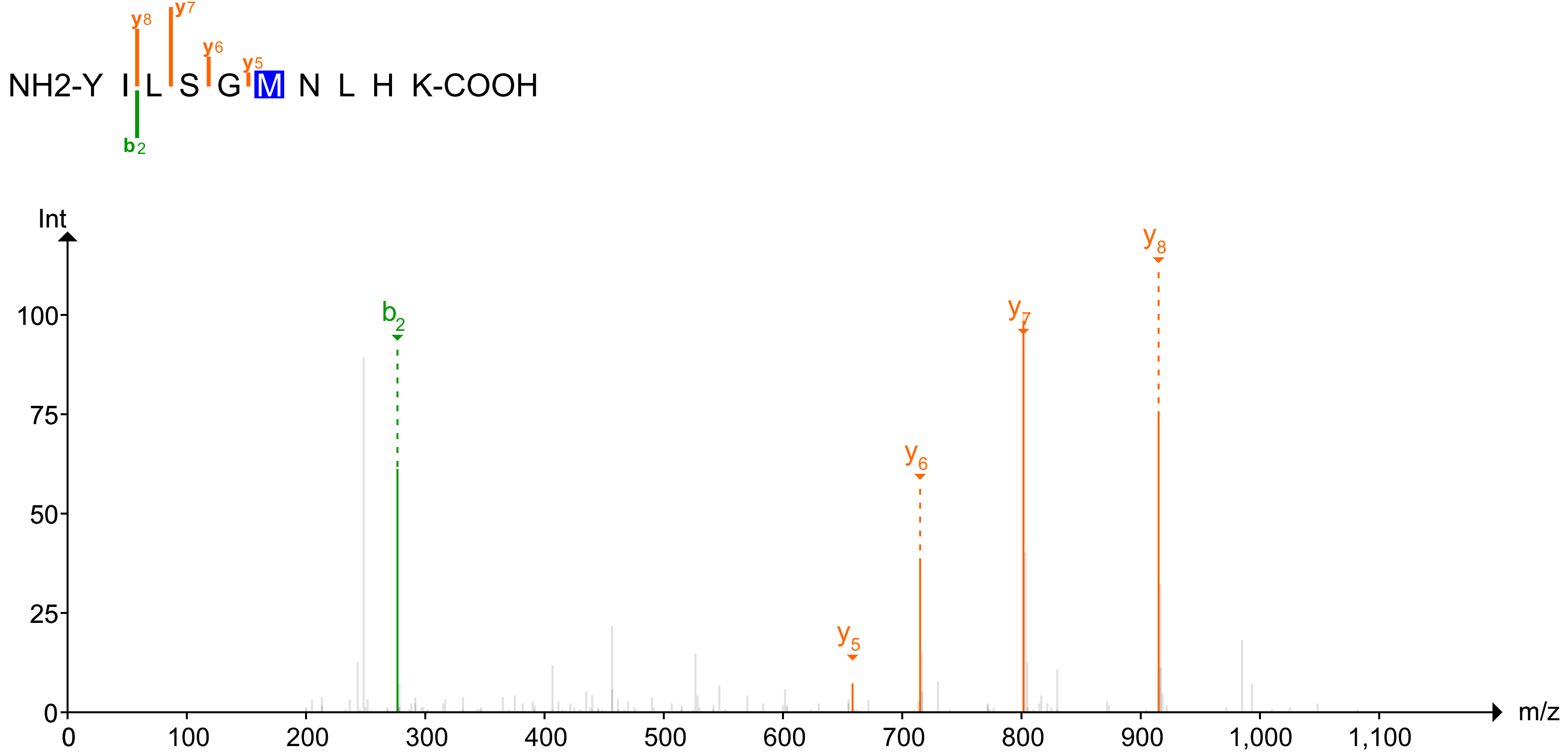

y8
y7
y6
y5
NH2-Y I L S G M N L H K-COOH
b2
Int
100
75
50
25
0
b2
y5
y6
y7
y8
0
100
200
300
400
500
600
700
800
900
1,000
1,100
m/z

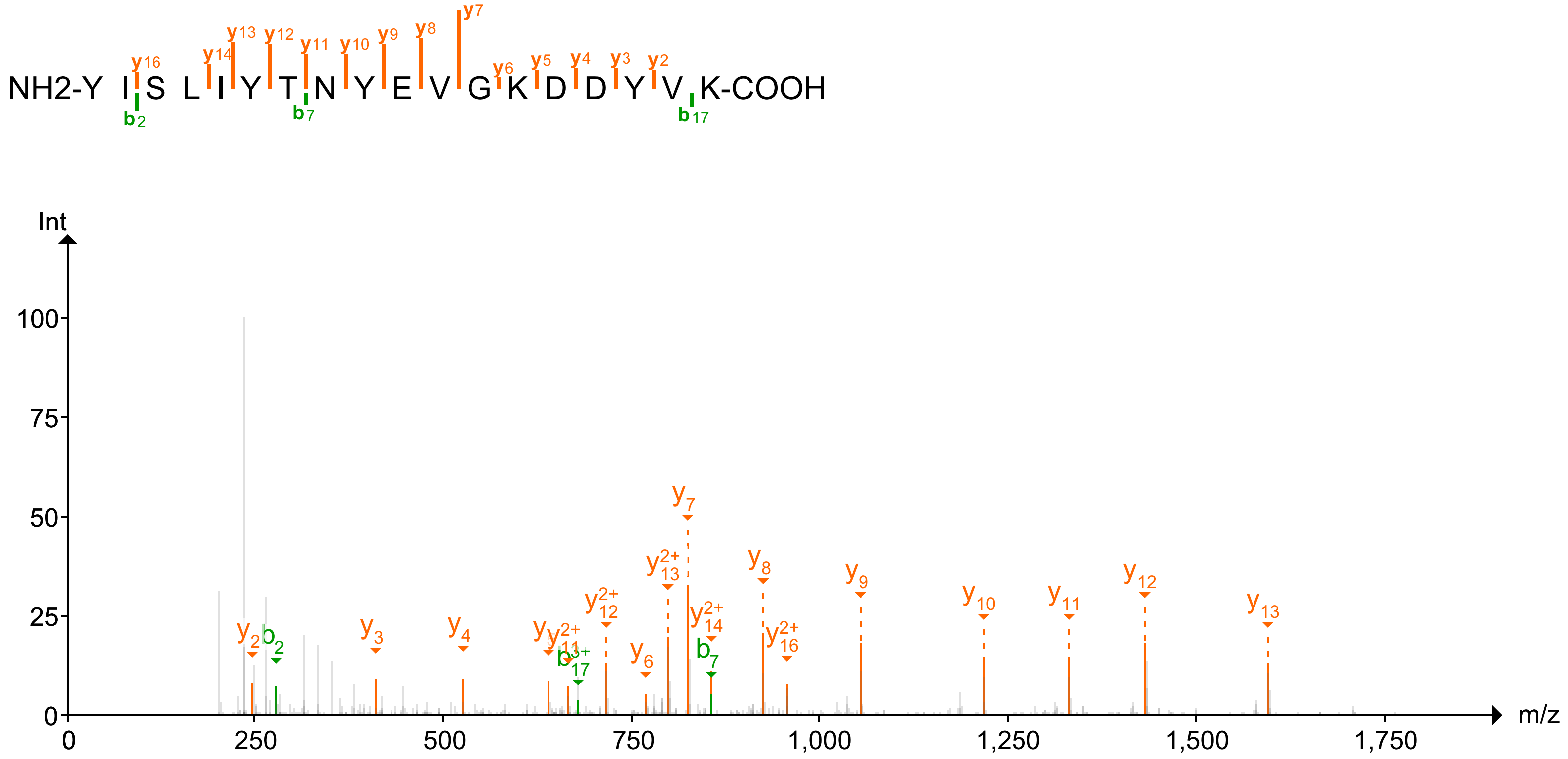

NH2-Y I S L I Y T N Y E V G K D D Y V K-COOH
y16 y14 y13 y12 y11 y10 y9 y8 y7 y6 y5 y4 y3 y2
b2 b7 b17
Int
100
75
50
25
0
m/z
0
250
500
750
1,000
1,250
1,500
1,750
y7
y8
y9
y10
y11
y12
y13
y2
b2
y3
y4
y6
y7
b7

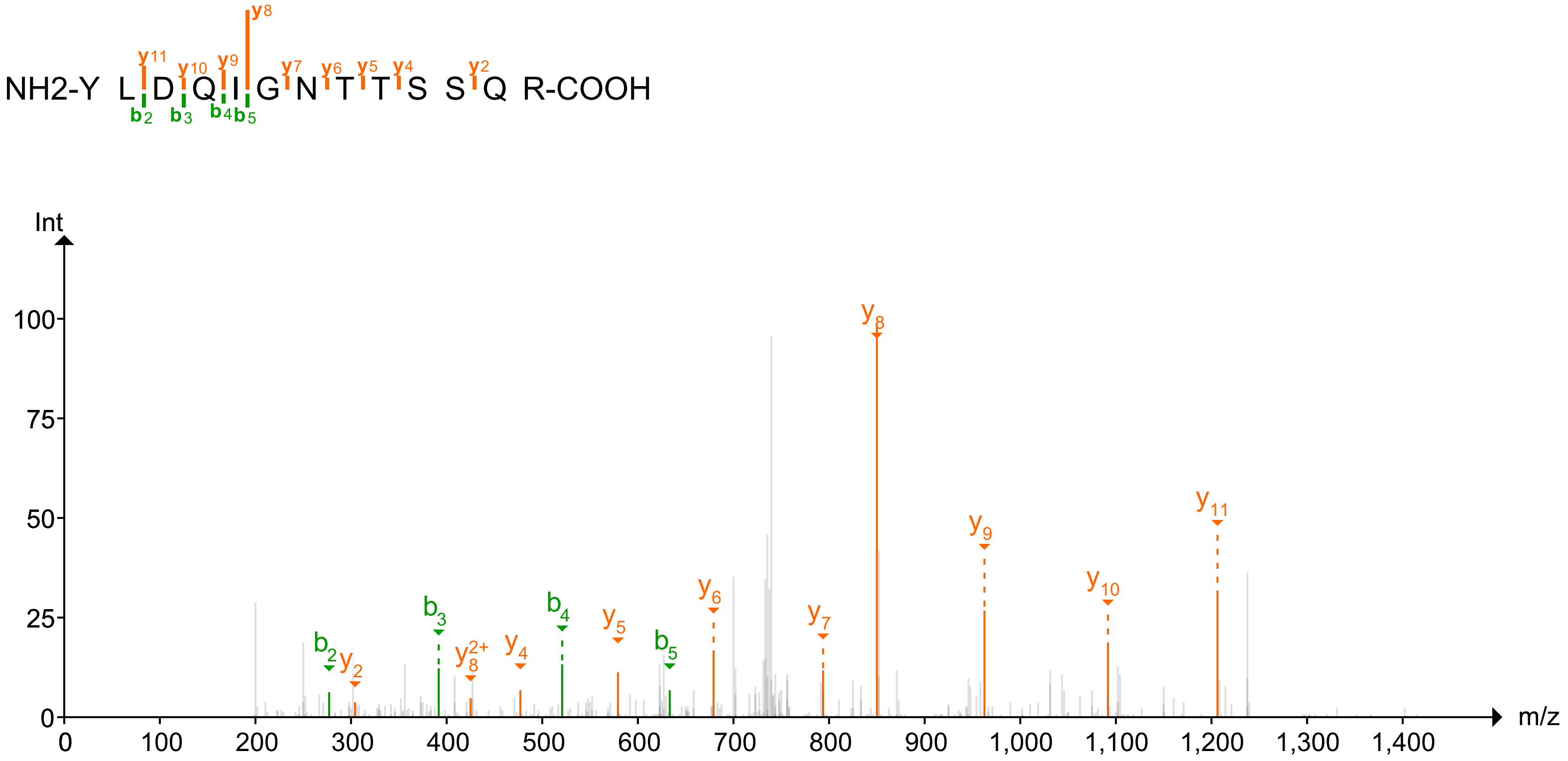

NH2-Y L D Q I G N T T S S Q R-COOH
y11 y10 y9 y8 y7 y6 y5 y4 y2
b2 b3 b4 b5
Int
100
75
50
25
0
m/z
0 100 200 300 400 500 600 700 800 900 1,000 1,100 1,200 1,300 1,400
$y_8$ $y_9$ $y_{11}$ $y_{10}$ $y_6$ $y_7$ $y_5$ $b_5$ $b_4$ $y_4$ $y_8^{2+}$ $b_3$ $b_2$ $y_2$

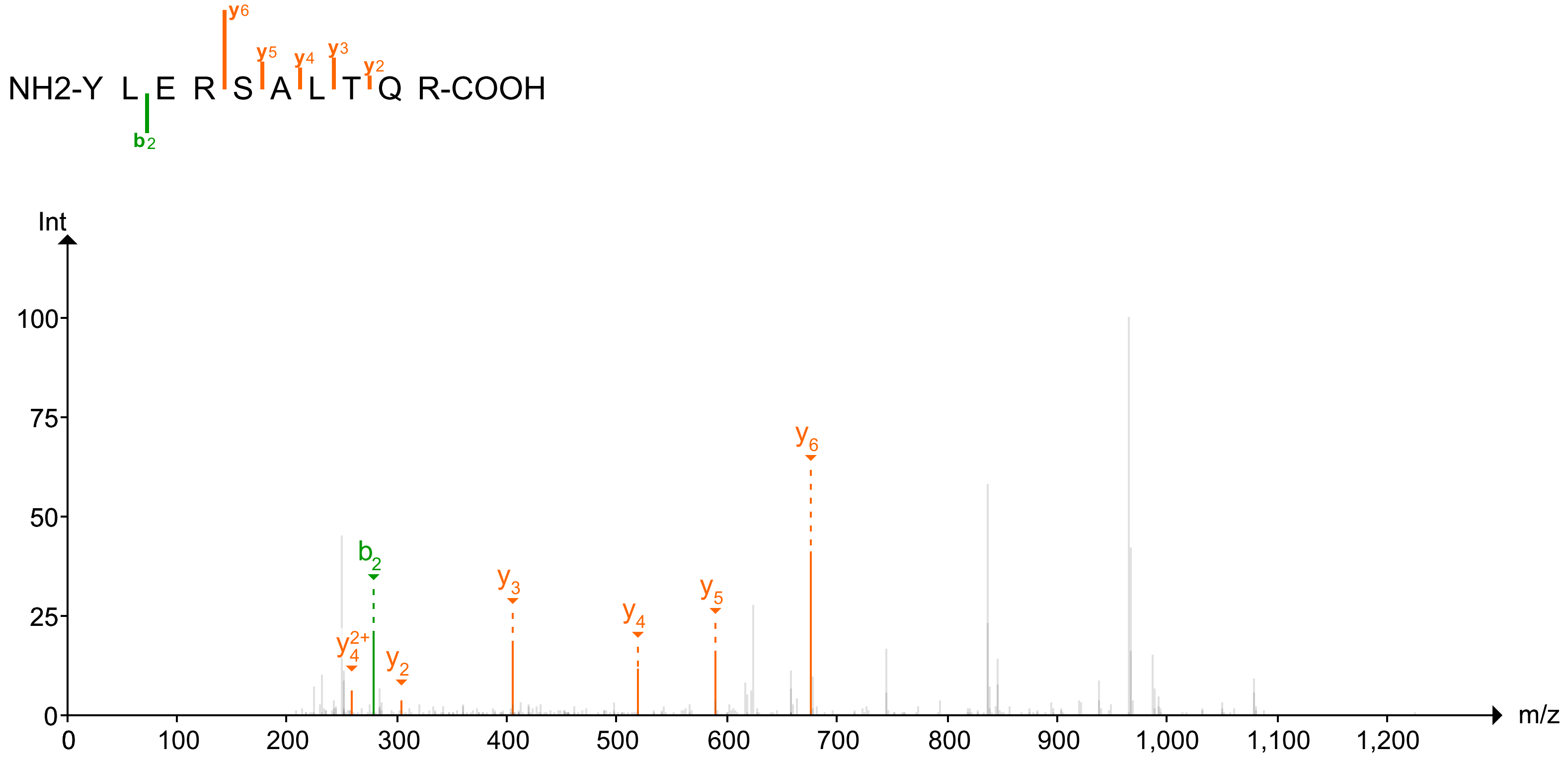

NH2-Y L E R S A L T Q R-COOH
y6
y5
y4
y3
y2
b2
Int
100
75
50
25
0
b2
y3
y4
y5
y6
y4 2+
y2
0
100
200
300
400
500
600
700
800
900
1,000
1,100
1,200
m/z

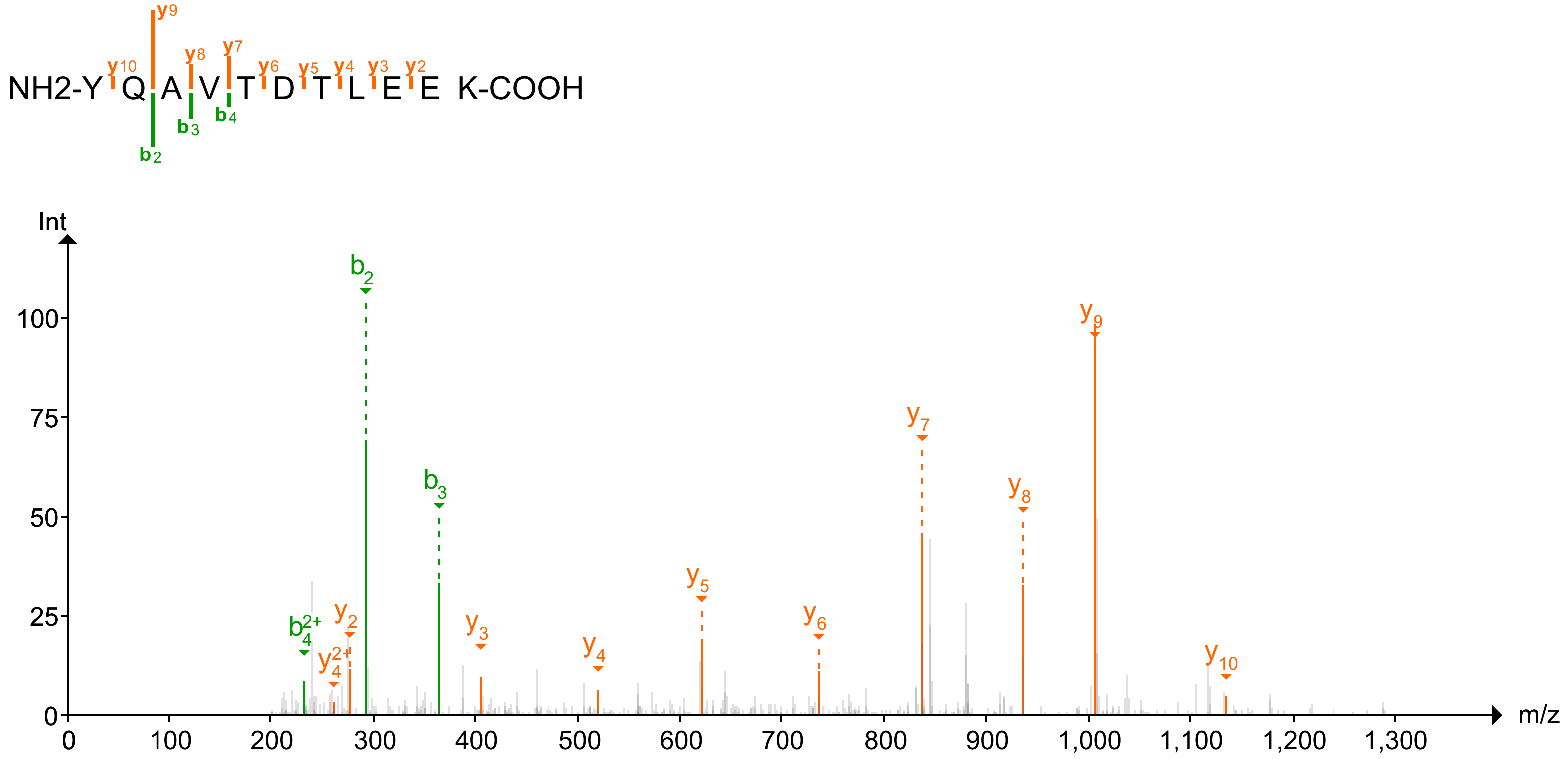

NH2-Y Q A V T D T L E E K-COOH
y9
y10
y8
y7
y6
y5
y4
y3
y2
b2
b3
b4
Int
100
75
50
25
0
b2
b3
y9
y7
y8
y5
y6
y10
y4
y3
y2
b4 2+
y4 2+
0
100
200
300
400
500
600
700
800
900
1,000
1,100
1,200
1,300
m/z

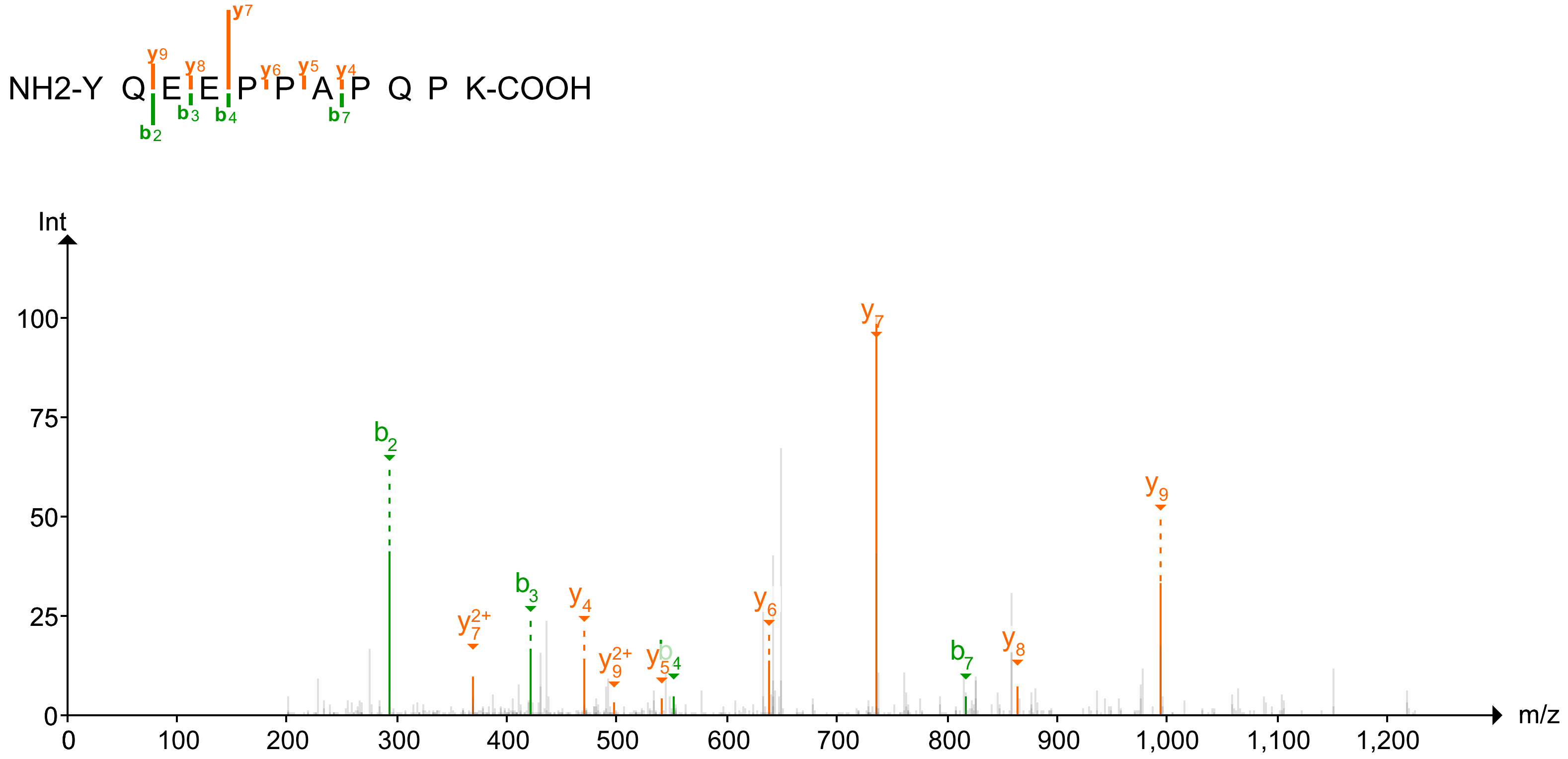
NH2-Y Q E E P P A P Q P K-COOH
y9 y8 y7 y6 y5 y4
b2 b3 b4 b7
Int
100
75
50
25
0
m/z
0
100
200
300
400
500
600
700
800
900
1,000
1,100
1,200
b2
y7 2+
b3
y4
y9 2+
y5
b4
y6
y7
b7
y8
y9

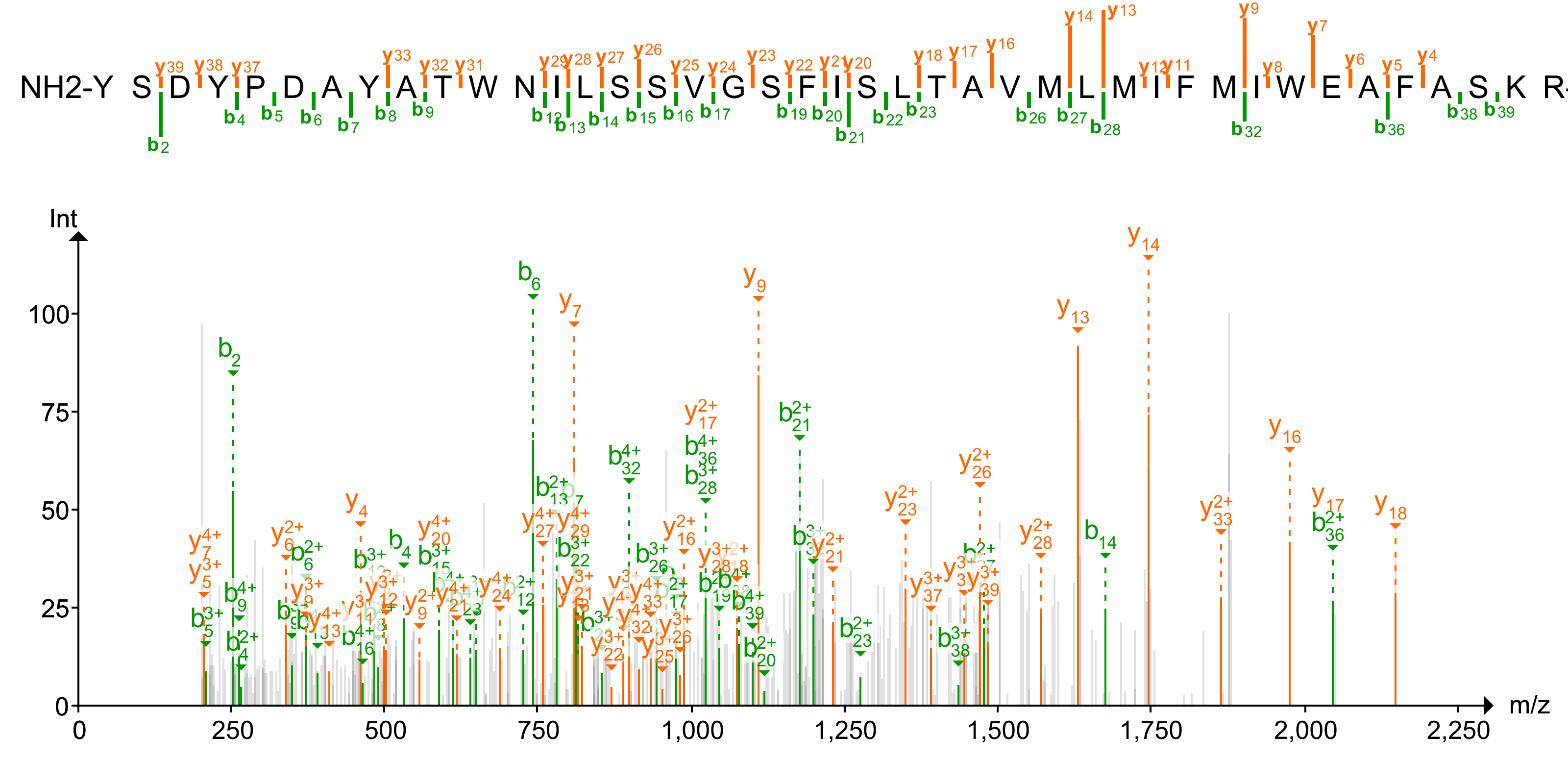

NH2-Y S D Y P D A Y A T W N I L S S V G S F I S L T A V M L M I F M I W E A F A S K R-
Int
100
75
50
25
0
m/z
0
250
500
750
1,000
1,250
1,500
1,750
2,000
2,250
b2
b6
y7
y9
y13
y14
y16
y17
y18
y4
b14

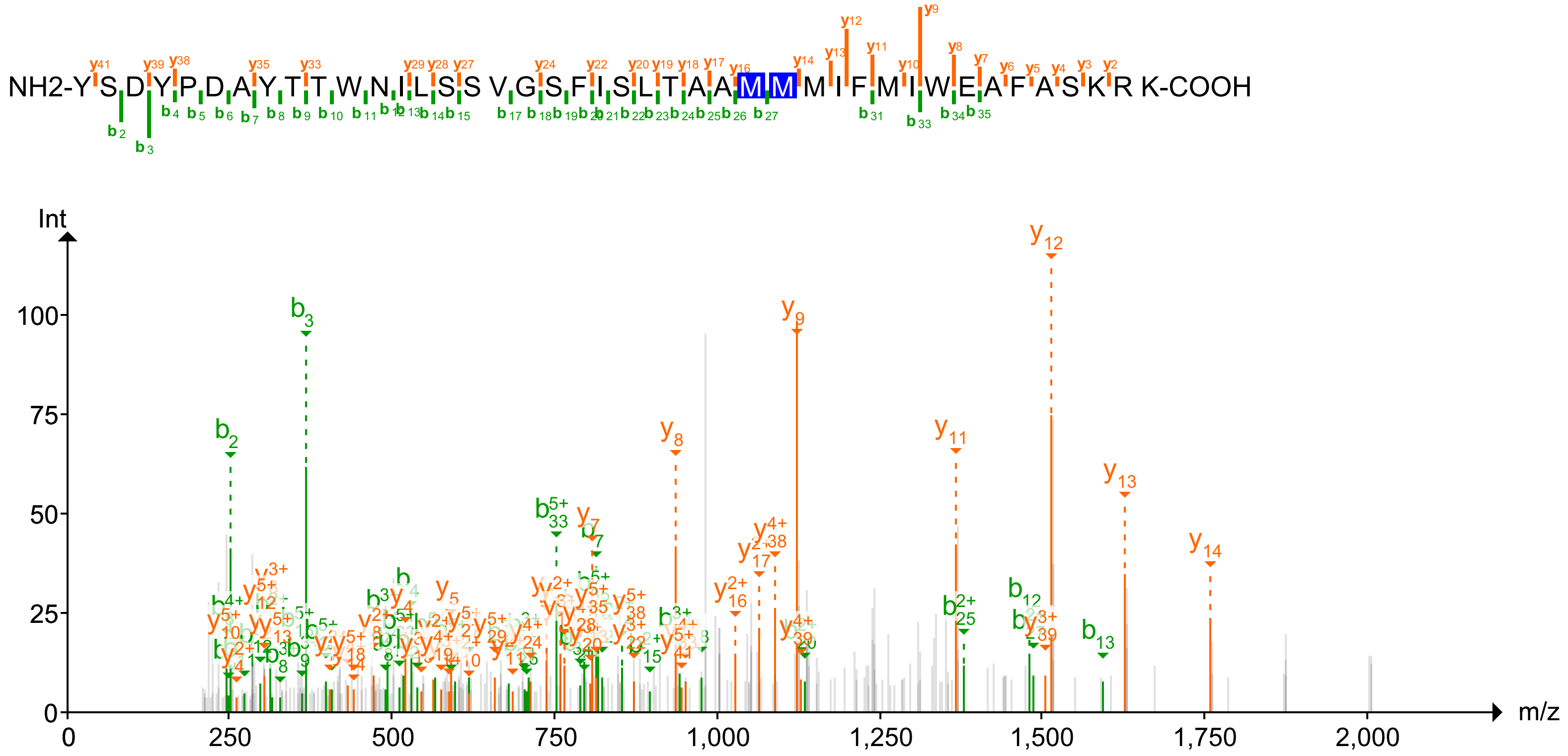

NH2-YSDYPDAYTTWNILSSVGSFISLTAAMMMIFMIWEAFASKRK-COOH
Int
m/z
0
25
50
75
100
250
500
750
1,000
1,250
1,500
1,750
2,000
b2
b3
y8
y9
y11
y12
y13
y14
b12
b13

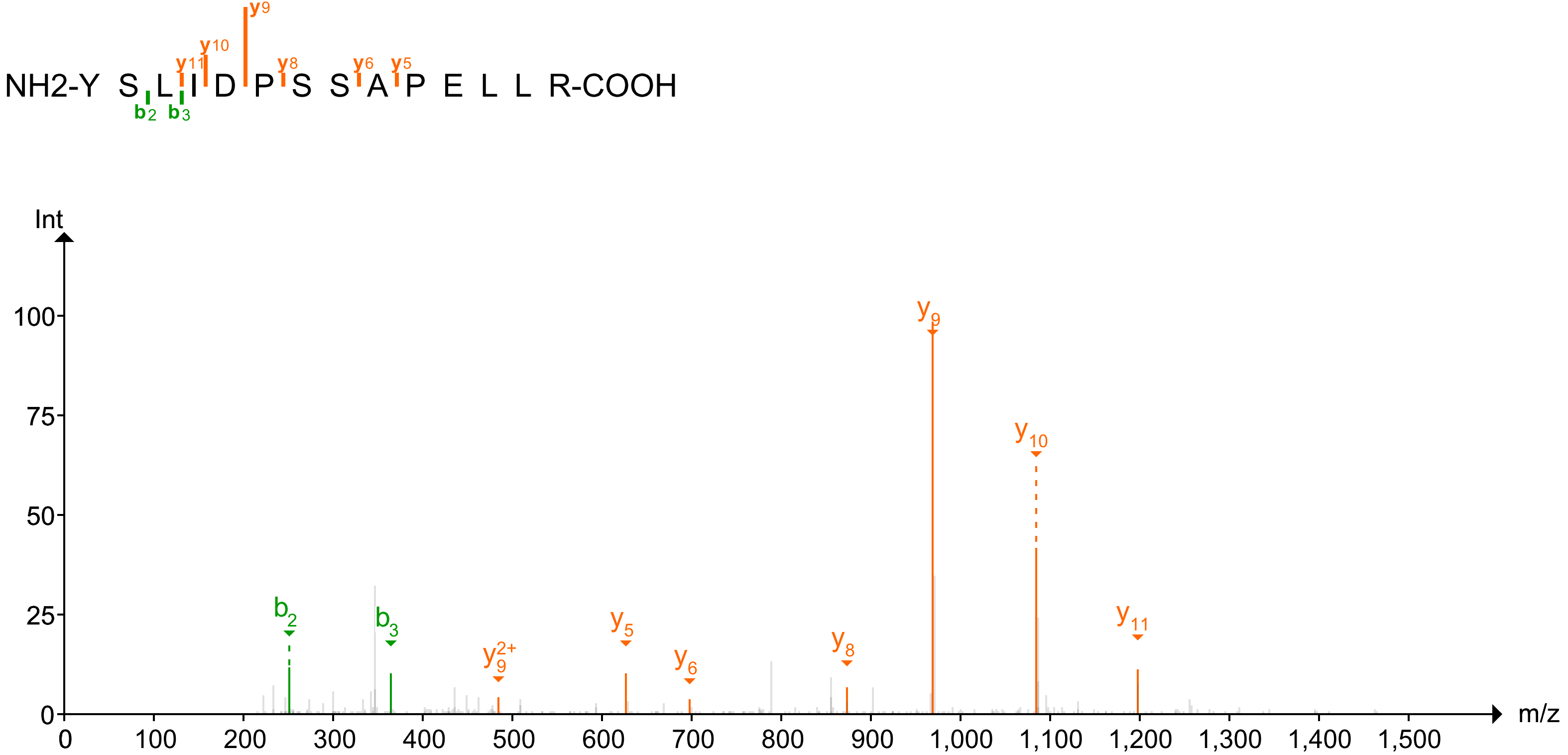

NH2-Y S L I D P S S A P E L L R-COOH
y9
y10
y11
y8
y6
y5
b2
b3
Int
100
75
50
25
0
m/z
0
100
200
300
400
500
600
700
800
900
1,000
1,100
1,200
1,300
1,400
1,500
y9
y10
y11
b2
b3
y9 2+
y5
y6
y8

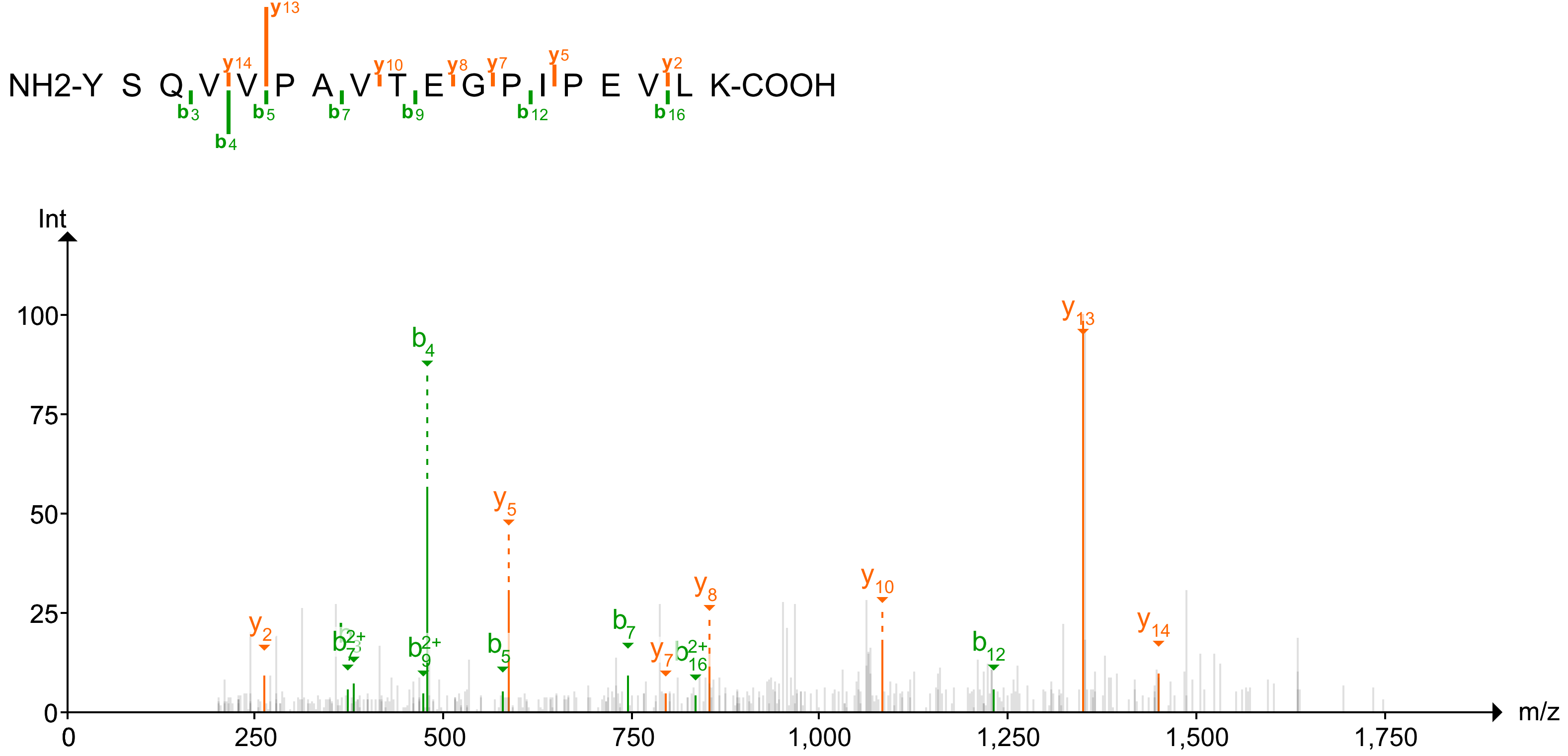

NH2-Y S Q V V P A V T E G P I P E V L K-COOH
y13
y14
y10
y8
y7
y5
y2
b3
b4
b5
b7
b9
b12
b16
Int
100
75
50
25
0
m/z
0
250
500
750
1,000
1,250
1,500
1,750

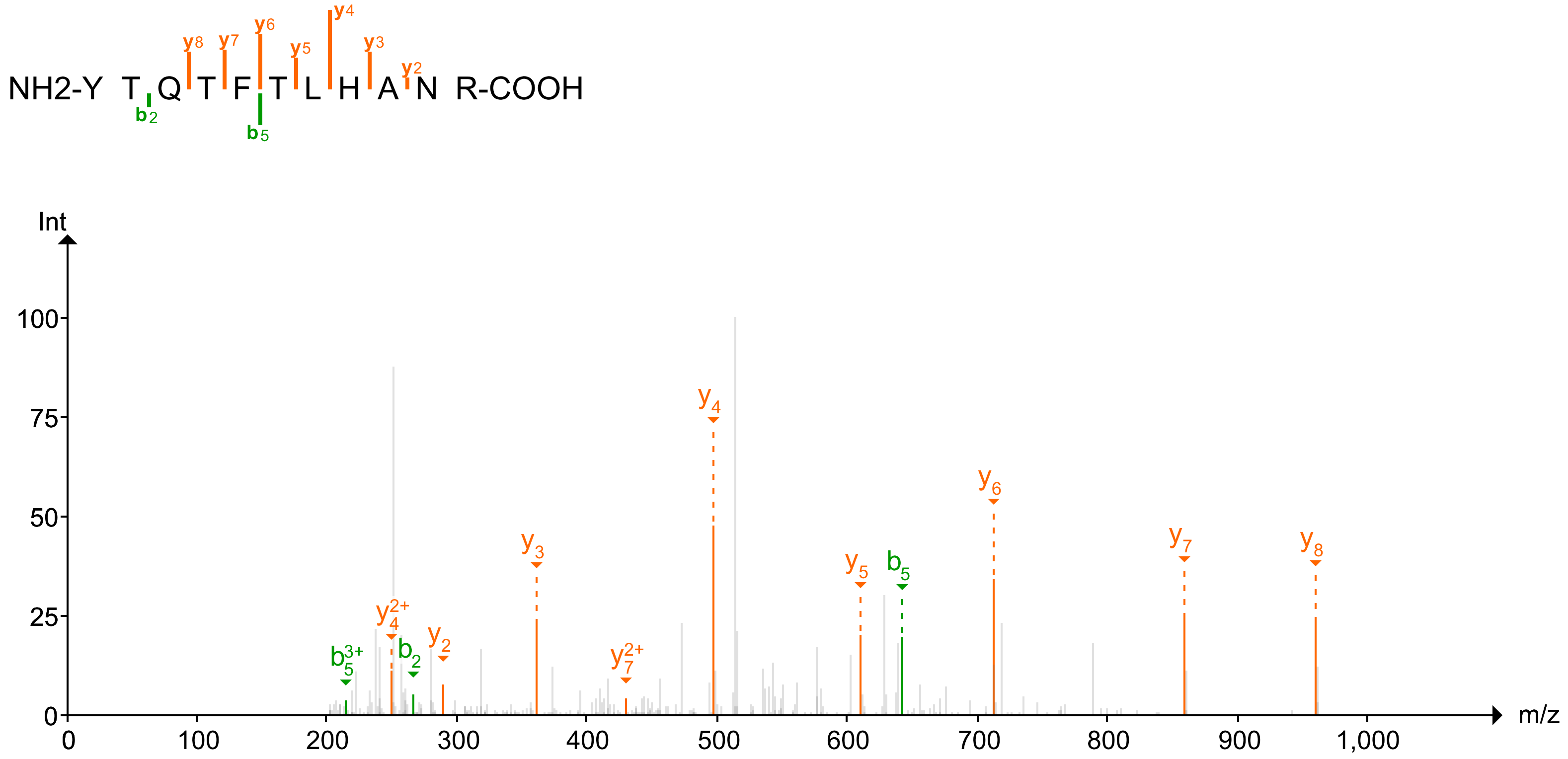

NH2-Y T Q T F T L H A N R-COOH
y8 y7 y6 y5 y4 y3 y2
b2 b5
Int
100
75
50
25
0
0 100 200 300 400 500 600 700 800 900 1,000
m/z
y4
y6
y3
y5
b5
y7
y8
y4 2+
y2
b2
b5 3+
y7 2+

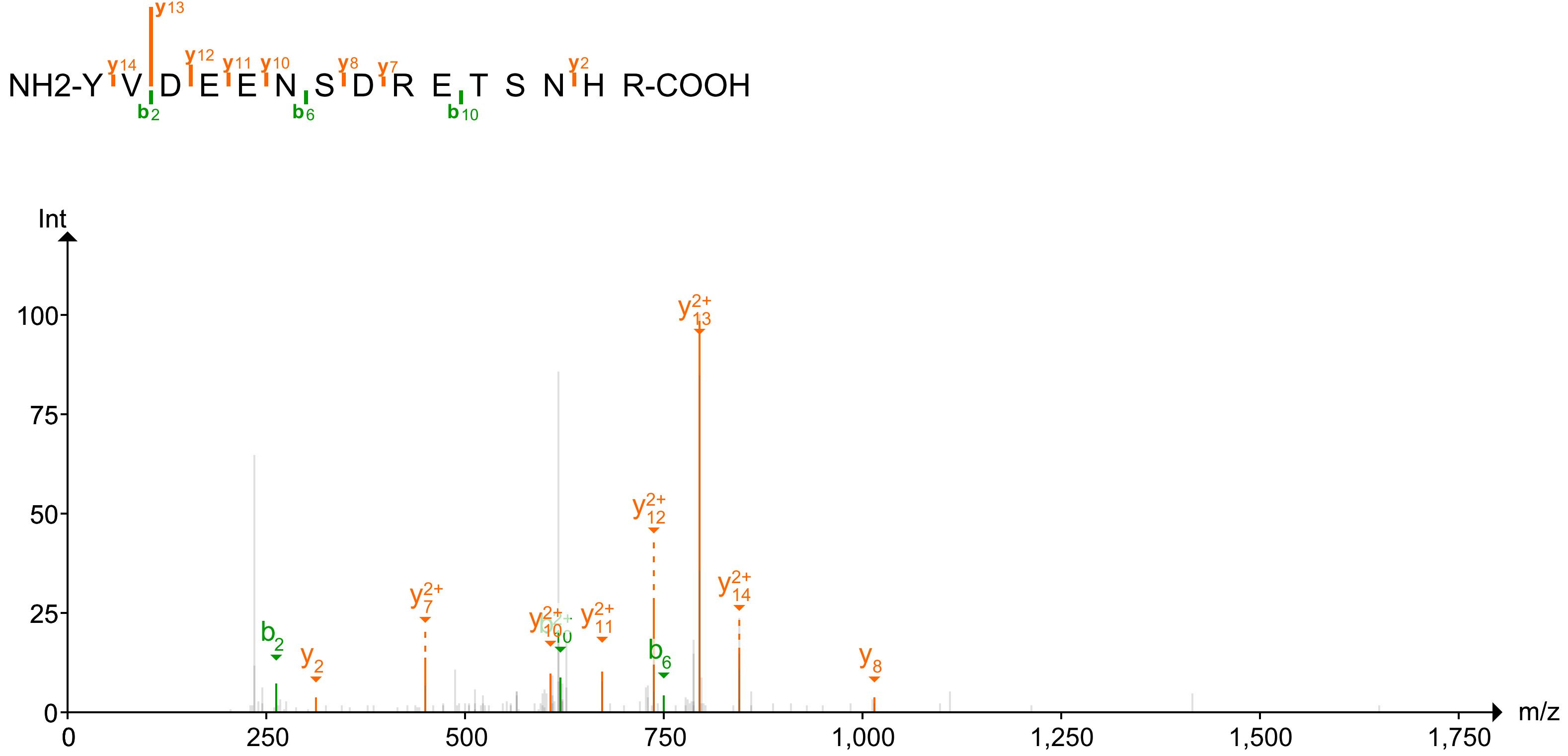

NH2-Y V D E E N S D R E T S N H R-COOH
y13 y14 y12 y11 y10 y8 y7 y2
b2 b6 b10
Int
100
75
50
25
0
m/z
0
250
500
750
1,000
1,250
1,500
1,750
$y_{13}^{2+}$
$y_{12}^{2+}$
$y_{7}^{2+}$
$y_{14}^{2+}$
$y_{11}^{2+}$
$y_{10}^{2+}$
$b_2$
$y_2$
$b_6$
$y_8$

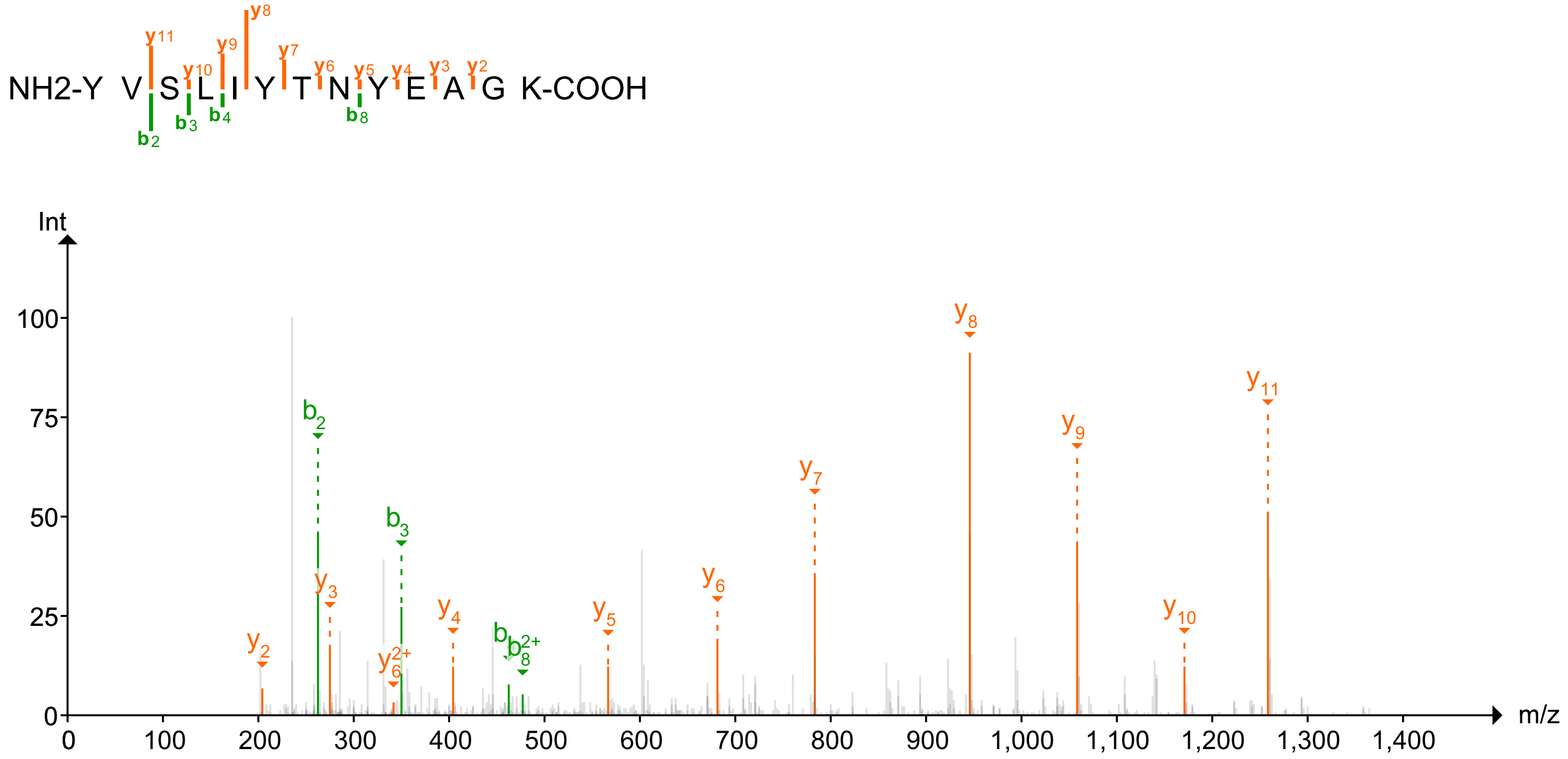
NH2-Y V S L I Y T N Y E A G K-COOH
y11 y10 y9 y8 y7 y6 y5 y4 y3 y2
b2 b3 b4 b8
Int
100
75
50
25
0
m/z
0 100 200 300 400 500 600 700 800 900 1,000 1,100 1,200 1,300 1,400
y2 b2 y3 b3 y6 2+ y4 b8 2+ b8 y5 y6 y7 y8 y9 y10 y11

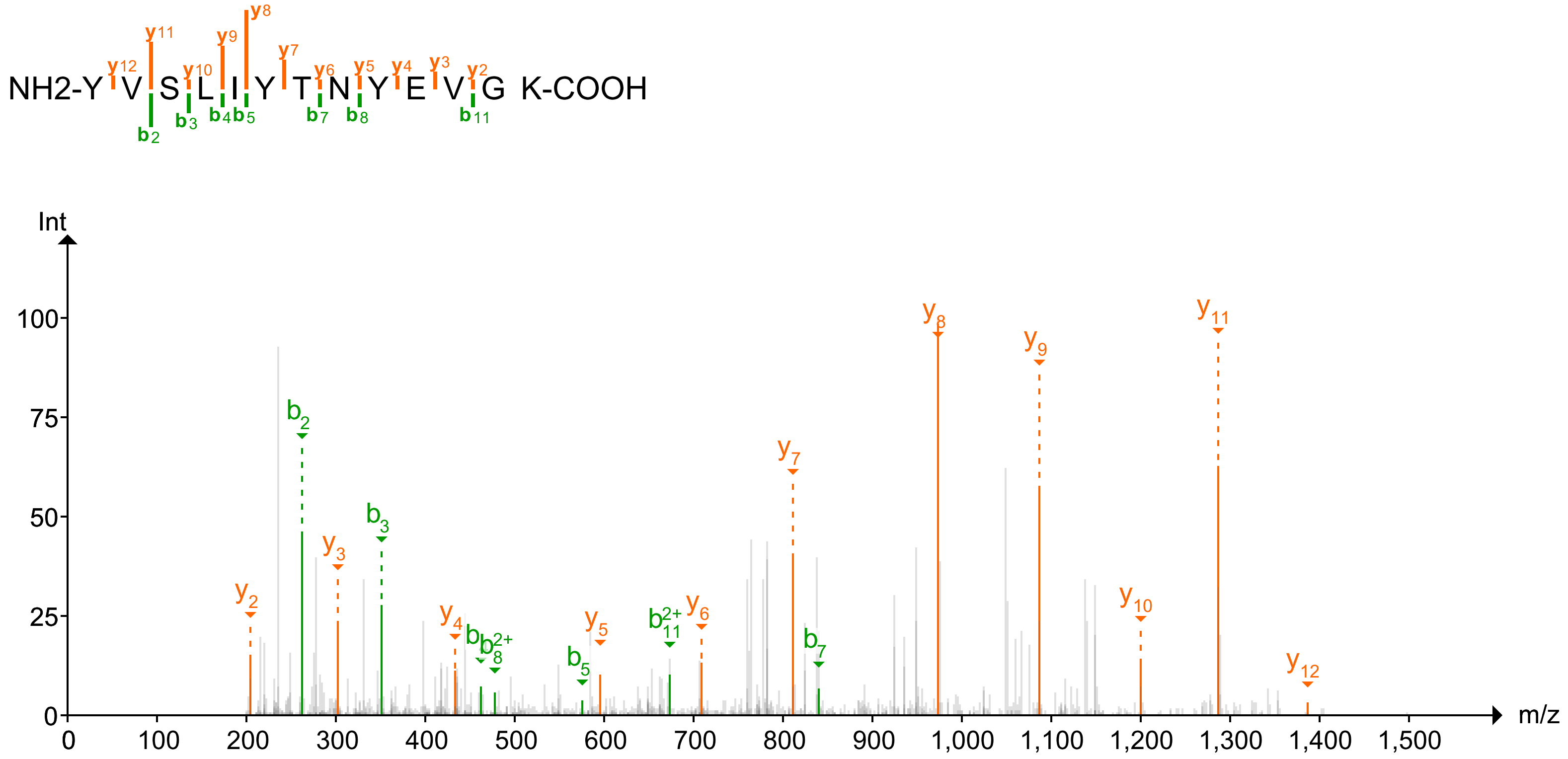

NH2-Y V S L I Y T N Y E V G K-COOH
y12 y11 y10 y9 y8 y7 y6 y5 y4 y3 y2
b2 b3 b4 b5 b7 b8 b11
Int
100
75
50
25
0
m/z
0 100 200 300 400 500 600 700 800 900 1,000 1,100 1,200 1,300 1,400 1,500
y2 b2 y3 b3 y4 b5 y5 y6 y7 b7 y8 y9 y10 y11 y12
b8 2+
b11 2+
